# Employing genome-wide SNP discovery and genotyping strategy to extrapolate the natural allelic diversity and domestication patterns in chickpea

**DOI:** 10.3389/fpls.2015.00162

**Published:** 2015-03-31

**Authors:** Alice Kujur, Deepak Bajaj, Hari D. Upadhyaya, Shouvik Das, Rajeev Ranjan, Tanima Shree, Maneesha S. Saxena, Saurabh Badoni, Vinod Kumar, Shailesh Tripathi, C. L. L. Gowda, Shivali Sharma, Sube Singh, Akhilesh K. Tyagi, Swarup K. Parida

**Affiliations:** ^1^National Institute of Plant Genome Research (NIPGR)New Delhi, India; ^2^International Crops Research Institute for the Semi-Arid Tropics (ICRISAT)Telangana, India; ^3^National Research Centre on Plant Biotechnology (NRCPB)New Delhi, India; ^4^Division of Genetics, Indian Agricultural Research Institute (IARI)New Delhi, India

**Keywords:** *Cicer*, chickpea, *desi*, GBS, *kabuli*, linkage disequilibrium, SNP

## Abstract

The genome-wide discovery and high-throughput genotyping of SNPs in chickpea natural germplasm lines is indispensable to extrapolate their natural allelic diversity, domestication, and linkage disequilibrium (LD) patterns leading to the genetic enhancement of this vital legume crop. We discovered 44,844 high-quality SNPs by sequencing of 93 diverse cultivated *desi, kabuli*, and wild chickpea accessions using reference genome- and *de novo*-based GBS (genotyping-by-sequencing) assays that were physically mapped across eight chromosomes of *desi* and *kabuli*. Of these, 22,542 SNPs were structurally annotated in different coding and non-coding sequence components of genes. Genes with 3296 non-synonymous and 269 regulatory SNPs could functionally differentiate accessions based on their contrasting agronomic traits. A high experimental validation success rate (92%) and reproducibility (100%) along with strong sensitivity (93–96%) and specificity (99%) of GBS-based SNPs was observed. This infers the robustness of GBS as a high-throughput assay for rapid large-scale mining and genotyping of genome-wide SNPs in chickpea with sub-optimal use of resources. With 23,798 genome-wide SNPs, a relatively high intra-specific polymorphic potential (49.5%) and broader molecular diversity (13–89%)/functional allelic diversity (18–77%) was apparent among 93 chickpea accessions, suggesting their tremendous applicability in rapid selection of desirable diverse accessions/inter-specific hybrids in chickpea crossbred varietal improvement program. The genome-wide SNPs revealed complex admixed domestication pattern, extensive LD estimates (0.54–0.68) and extended LD decay (400–500 kb) in a structured population inclusive of 93 accessions. These findings reflect the utility of our identified SNPs for subsequent genome-wide association study (GWAS) and selective sweep-based domestication trait dissection analysis to identify potential genomic loci (gene-associated targets) specifically regulating important complex quantitative agronomic traits in chickpea. The numerous informative genome-wide SNPs, natural allelic diversity-led domestication pattern, and LD-based information generated in our study have got multidimensional applicability with respect to chickpea genomics-assisted breeding.

## Introduction

The second most abundantly grown food legume chickpea (*Cicer arietinum* L.) is a self-pollinated and diploid (2n = 2x = 16) crop species belonging to Fabaceae (Kumar et al., 2011). It is a vital source of human dietary protein enriched with essential amino acids. Chickpea is believed to have diverged from a wild progenitor *C. reticulatum* by single domestication event at a Fertile Crescent (South Eastern Turkey and adjacent Syria) near about 10,000 years ago (Abbo et al., 2003; Berger et al., 2005; Burger et al., 2008; Toker, 2009; Jain et al., 2013; Kujur et al., 2013; Varshney et al., 2013a; Saxena et al., 2014a). A series of four sequential evolutionary bottlenecks coupled with strong adaptation-based selection pressure/selective sweeps during chickpea domestication might have significantly narrowed-down the genetic base of the presently cultivated varieties (Abbo et al., 2003; Berger et al., 2005; Burger et al., 2008; Toker, 2009). The draft genomes of two major chickpea cultivars-*kabuli* (large seeded) and *desi* (small seeded) representing diverse gene pools have been successfully sequenced (Jain et al., 2013; Varshney et al., 2013a). These genome sequencing efforts signified that ~70% of their total sequenced draft genomes are represented by low-complexity regions, which could serve as reference for subsequent resequencing of diverse *desi* and *kabuli* accessions in order to discover and validate informative sequence-based markers at a genome-wide scale by deploying suitable high-throughput genotyping assay.

Single nucleotide polymorphisms (SNPs) are highly preferred in plant genetic and genome analyses because of their excellent genetic attributes, such as wide genomic distribution, co-dominant inheritance, high reproducibility, and chromosome-specific location (Brookes, 1999; Cho et al., 1999; Gupta et al., 2001; Sachidanandam et al., 2001; Rafalski, 2002; Gupta and Rustgi, 2004). For a large chickpea genome (~740 Mb) with a narrow genetic base, these informative SNPs aids in identifying trait-regulatory genes/QTLs (quantitative trait loci) for marker-assisted genetic enhancement. Tremendous efforts have been made in chickpea toward mining a vast number of SNPs *in silico* from the ESTs (expressed sequence tag), transcripts, genes and genome sequences of diverse *desi, kabuli*, and wild accessions using traditional Sanger and high-throughput next-generation sequencing (NGS) approaches (Varshney et al., 2007, 2013a; Rajesh and Muehlbauer, 2008; Nayak et al., 2010; Garg et al., 2011; Gujaria et al., 2011; Hiremath et al., 2011; Agarwal et al., 2012; Azam et al., 2012; Jhanwar et al., 2012; Jain et al., 2013; Kujur et al., 2013). Also a smaller subset of selected SNPs have been successfully validated and genotyped in diverse chickpea accessions by low-throughput allele-specific resequencing, allele-specific PCR (polymerase chain reaction) and CAPS (cleaved amplified polymorphic sequence) assays to be utilized for various marker-based genotyping applications. More recently, the assistance of several array-based SNP genotyping approaches, like Illumina GoldenGate/Infinium (Bead Xpress array) and Competitive Allele Specific PCR (KASPar) assays have pushed ahead the process of large-scale validation and high-throughput genotyping of SNPs in different chickpea accessions. In chickpea, so far about 3000 such validated SNPs have been exploited, specifically in genetic diversity and evolutionary studies (Gaur et al., 2012; Hiremath et al., 2012; Thudi et al., 2012; Roorkiwal et al., 2013; Stephens et al., 2014).

With advent of barcoded multiplexing and restriction enzyme (RE)-based NGS approaches, the simultaneous integration of large-scale discovery, validation and genotyping of SNPs is now possible in numerous diverse crop accessions. These NGS-based methods have evolved plenty of high-throughput and cost-effective SNP genotyping assays and applications for many small diploid and larger polyploid crop genomes. Some of these assays include reduced representation libraries (RRLs) (van Tassell et al., 2008), complexity reduction of polymorphic sequences (CRoPs) (van Orsouw et al., 2007), restriction-site-associated DNA sequencing (RAD-seq) (Miller et al., 2007; Baird et al., 2008), and more recently, genotyping-by-sequencing (GBS) (Elshire et al., 2011) assays. In addition to simultaneous high-throughput genome-wide SNP discovery and genotyping in a larger set of diverse crop accessions, these assays are also particularly helpful for ultra-high density genetic and physical linkage map construction and genome-wide high-resolution trait association and genetic mapping (Huang et al., 2009, 2010; Barchi et al., 2011; Chutimanitsakun et al., 2011; Davey et al., 2011; Pfender et al., 2011; Yu et al., 2011). Quite recently, the significance of RAD-seq has been clearly demonstrated in mining and genotyping of ~4.4 million genome-wide SNPs for understanding the molecular diversity and phylogenies among 90 diverse chickpea accessions (Varshney et al., 2013a).

GBS is a simple, yet fast and reproducible latest RE (restriction enzyme)-based improved NGS method that has gained considerable attention of crop researchers for large-scale discovery and genotyping of SNPs simultaneously in diverse crop germplasm accessions and mapping populations at a genome-wide scale (Schnable et al., 2009; Elshire et al., 2011). The maximal efficiency of GBS in mining and genotyping of SNPs with wider genomic distribution is due to its highest barcoded multiplexing capability (pooling up to 384 barcoded accessions in a single sequencing lane) at methylation-sensitive RE (restriction endonuclease)-sites (*APe*KI) and also complexity reduction (targeting lower copy number of genome) ability, especially in larger complex plant genomes (Schnable et al., 2009; Elshire et al., 2011; Poland et al., 2012a). This assay is now the most preferred and well-established SNP discovery and genotyping approach, thus has got multiple utilities in high-throughput marker-based applications of plant genomics and breeding with optimal use of resources, high genotype multiplexing and rapid SNP genotyping potential.

The GBS method has also been extensively applied in diverse germplasm lines of barley (Mayer et al., 2012), wheat (Poland et al., 2012b), soybean (Sonah et al., 2013), sorghum (Morris et al., 2013; Thurber et al., 2013), and rice (Spindel et al., 2013) for large-scale mining and high-throughput genotyping of SNPs at a genome-wide scale. The significant outcomes of these studies have proven the efficacy of GBS for understanding the molecular diversity and phylogeny in crop plants (Byrne et al., 2013; Crossa et al., 2013; Mascher et al., 2013; Uitdewilligen et al., 2013; Bastien et al., 2014; Huang et al., 2014; Jarquín et al., 2014; Liu et al., 2014; Tardivel et al., 2014; Sonah et al., 2014). Notably, the above assay was recently employed for constructing high-resolution intra- and inter-specific genetic linkage maps and fine mapping of QTLs/genes associated with drought tolerance in chickpea (Deokar et al., 2014; Jaganathan et al., 2014). Nonetheless, as far as molecular diversity analysis in core/mini-core germplasm lines is concerned, the GBS assay is yet to be utilized in chickpea. Thus, considering the successful application of GBS assay in high-throughput genetic analyses in other crop plants (He et al., 2014), its use for mining and validating of genome-wide SNPs in diverse natural populations of chickpea assumes significance. The information acquired from diverse germplasm lines by GBS assay could enable the realistic estimation of natural allelic diversity and domestication pattern in chickpea. Moreover, this will accelerate various large-scale genotyping applications, including construction of high-density integrated genetic, physical and genome maps, comparative genome mapping, genome-wide association study (GWAS), selective sweep-based trait dissection analysis and fine mapping/map-based cloning of QTLs/genes controlling traits of agronomic importance and eventually marker-assisted genetic improvement of chickpea.

Keeping that in view, the present study has utilized GBS approach for the first time to discover, validate and genotype SNPs in 93 diverse [with significant seed yield (g) per plant variability] cultivated (*desi* and *kabuli*) and wild chickpea accessions at a genome-wide scale. The genotyping information of validated SNPs was further utilized to understand their functional significance and determine the molecular diversity pattern, population genetic structure and LD patterns among chickpea accessions.

## Materials and methods

### Chickpea accessions used for genomic DNA extraction

Ninty-three accessions belonging to 92 cultivated *C. arietinum desi* (39 accessions) and *kabuli* (53), and one *C. reticulatum* wild accession (ICC 17160) were utilized (Supplementary Table S1) for genome-wide discovery and genotyping of SNPs employing GBS assay. Ninty-two *desi* and *kabuli* accessions of these, with significant phenotypic [seed yield (g)/plant] and genotypic diversity (>80% diversity of total germplasm lines evaluated) were selected from available chickpea germplasm collections (16,991, including 211 minicore and 300 reference core germplasm lines) (Upadhyaya and Ortiz, 2001; Upadhyaya et al., 2001, 2008) following the methods of Kujur et al. (2014). An additional wild accession (ICC 17160) was included in GBS assay for understanding its molecular diversity and phylogenies with cultivated *desi* and *kabuli* chickpea. The genomic DNA was isolated from the young leaf samples of 93 accessions using a QIAGEN DNeasy 96 Plant Kit (QIAGEN, CA, USA) following the manufacturer's instructions.

### Library preparation, sequencing, and sequence read mapping

The isolated genomic DNA of 96 chickpea accessions (93 accessions along with three accessions as biological replicates) was digested with *Ape*KI and ligated to adapters containing one of 96 unique barcodes to construct 96-plex GBS libraries. These libraries were pooled together (following Elshire et al., 2011; Spindel et al., 2013) and sequenced (100-bp single end) using Illumina HiSeq 2000. The reproducibility of the GBS assay was evaluated using three *desi* and *kabuli* accessions (ICCX-810800, ICCV10, and ICCV95334) as biological replicates (Supplementary Table S1). The FASTQ sequence reads generated from accessions were processed for quality assessment. For this assessment, a sliding window approach implemented in the STACKS v1.0 (Catchen et al., 2013; http://creskolab.uoregon.edu/stacks) was utilized to examine the quality distribution of each sequence read. The reads with an average quality below 90% confidence (a *phred* score of 10; Ewing and Green, 1998) within a sliding window were discarded. Additionally, the sequence reads showing prolonged drops in quality were discarded. The rest of the good quality sequence reads were further analyzed for their quality using FASTQC v0.10.1. The high-quality reads were de-multiplexed based upon their unique barcodes to extract sequence reads of individual accessions. The individual sequence reads of 96 accessions were separately aligned and mapped to reference drafts of *desi* (ICC 4958; Jain et al., 2013) and *kabuli* (CDC Frontier; Varshney et al., 2013a) chickpea genome sequences using Bowtie v2.1.0 with default parameters (Langmead and Salzberg, 2012). The unaligned sequence reads of *desi* and *kabuli* reference genomes were further processed individually using the *de novo* genotyping approach of STACKS.

### Discovery and genotyping of SNPs

The sequence alignment map (SAM) files generated from each *desi* and *kabuli* genome were processed using reference-based GBS pipeline of STACKS to identify accurate SNPs in 96 accessions. The SNP identification process in pstacks is efficient enough for handling gapped alignments properly to accommodate InDels and soft-masked alignment fragments. This prevents the SNP model from wrongly calling polymorphisms due to InDel frameshifts. Stacks algorithm also filters paralog and high-copy loci by read coverage, assuming random coverage across loci. We removed the putative loci with more than twice the standard diversion of coverage depth to filter out repetitive elements and stacks of paralogs loci. A maximum likelihood statistical model was used in STACKS to identify valid and high-quality SNPs (with no sequencing errors and SNP base quality = 20), supported by at least 10 sequence reads. A catalog was built to record all SNP loci identified in 96 accessions by matching each accession to that catalog and detecting accurate alleles (minimum sequence read depth: 10) that are present at every SNP locus in each accession.

Using the *de novo* genotyping approach of STACKS, the sampled SNP loci from each accession were reassembled from the sequenced reads (reads unaligned with reference genome) for creating stacks. A *de novo* method was employed to group all exactly matching sequences into stacks, forming putative loci from high-confidence unique stacks. To detect polymorphism and infer SNP alleles, each putative locus of one-nucleotide position at a time was examined using a maximum likelihood framework (Hohenlohe et al., 2010) implemented in STACKS. We set the parameters in a way that for each stack in the locus there was another stack in the locus, which is at most one nucleotide divergent (−M 1). A minimum of three raw reads that requires to form a stack (−m 3) were specified. The stack information was further used to create a catalog against which the other accessions matched, resulting in a map of all the alleles (minimum sequence read depth: 10 with SNP base quality = 20) in 96 accessions for a set of SNP loci.

### Structural and functional annotation of SNPs

The physical positions (bp) of SNPs identified by reference-based GBS approaches were correlated with the GFF file containing the genome annotation of *desi* (CGAP v1.0; Jain et al., 2013) and *kabuli* (Varshney et al., 2013a) chickpea to determine the distribution of SNPs in various coding and non-coding sequence components of genes and genomes (chromosomes/pseudomolecules and unanchored scaffolds). The customized Perl scripts and a single-nucleotide polymorphism effect predictor (SnpEff v3.1h; http://snpeff.sourceforge.net/) were used to deduce accurate genomic distribution, including the structural and functional annotation of SNPs (synonymous and non-synonymous SNPs). To determine the genomic distribution of identified SNPs across chickpea chromosomes, the SNPs with synonymous and non-synonymous substitutions were plotted individually based on their unique physical positions (bp) on eight chromosomes (pseudomolecules) of *desi* and *kabuli* genomes and visualized using Circos visualization tool (Krzywinski et al., 2009).

The putative functions, including conserved domains in the proteins encoded by SNP-carrying genes were predicted based on available *desi* and *kabuli* genome annotations (Jain et al., 2013; Varshney et al., 2013a) and PFAM database v27.0 (http://pfam.sanger.ac.uk). The SNP-carrying genes were BLAST searched (cut-off *E*-value = 1e-41) against the KOGnitor NCBI database (ftp://ftp.ncbi.nih.gov/pub/COG/KOG) for detailed functional classification. Gene Ontology (GO) enrichment analysis of SNPs-containing genes was performed using BiNGO plugin of Cytoscape V2.6 (Maere et al., 2005). For the GO enrichment significance test (*P* = 0.05), the Benjamini and Hochberg false discovery rate correction was employed.

For estimation of Ka (number of non-synonymous substitutions per non-synonymous site)/Ks (number of synonymous substitutions per synonymous site) substitution ratio, the SNP-carrying coding sequences of genes annotated from the reference *desi* and *kabuli* genomes were compared among accessions and analyzed following the methods of Li et al. (2011) and Devisetty et al. (2014). To further deduce the direction as well as magnitude of natural selection acting on protein-coding *desi* and *kabuli* genes, the significant departure from standard neutral model Ka/Ks = 1 was evaluated using maximum-likelihood ratio method implemented in the CODEML program of PAMLv4.8a (http://abacus.gene.ucl.ac.uk/software/paml.html).

### Large-scale validation of SNPs

For validating SNPs identified through GBS assay, the SNP genotyping information among four chickpea accessions (ICC 4958, ICC 4951, ICC 12968, and ICC 17160) was compared/correlated with an available *in silico* SNP database of same accessions according to their nature/types and bp on the *desi* (ICC 4958) genome (Gaur et al., 2012; Jain et al., 2013). For experimental validation, 384 non-synonymous and upstream regulatory SNPs were selected based on their presence in the transcription factor (TF) genes, potential to reveal polymorphism between a representative set of stress tolerant and sensitive chickpea accessions and localization in the genes underlying the known QTLs for stress tolerance in chickpea. Moreover, 70 *de novo*-based GBS-SNPs screened as per their uniform physical distribution (within ~1 Mb physical distance between SNPs) on *desi* and *kabuli* genomes, were included in our validation study. For these analyses, the primers designed from 300-bp sequences flanking either side of 214 selected SNPs were PCR amplified using the genomic DNA of 93 chickpea accessions. The amplified PCR products were purified and sequenced. The aligned high-quality sequences were used to detect SNPs among accessions following Kujur et al. (2013) and Saxena et al. (2014a,b). Additionally, to validate GBS-based SNPs, a representative set of 240 SNPs were genotyped in the genomic DNA of 93 accessions using 34-plex Sequenom MALDI-TOF (matrix-assisted laser desorption ionization-time of flight) MassARRAY (http://www.sequenom.com) following Saxena et al. (2014a,b). The SNP alleles scanned among accessions were cataloged according to allele-specific differences in mass between extension products. The sensitivity [TP/(TP + FN) × 100] and specificity [TN/(TN + FP) × 100] rate (%) of validated SNPs were determined by estimating the number of true positives (TP), false negatives (FN), true negatives (TN), and false positives (FP).

### Estimation of diversity statistics, population genetic structure, and LD patterns

The SNP genotyping data (minor allele frequency/MAF ≥ 5% with missing data < 10%, Zhao et al., 2011) were analyzed with PowerMarker v3.51 (Liu and Muse, 2005), MEGA v5.0 (Tamura et al., 2011), and TASSEL v5.0 (Bradbury et al., 2007; http://www.maizegenetics.net) to estimate the PIC (polymorphism information content) and for constructing an unrooted neighbor-joining (NJ)-based phylogenetic tree (Nei's genetic distance; Nei et al., 1983 with 1000 bootstrap replicates) among 93 chickpea accessions. Various nucleotide diversity parameters, including θπ (average number of nucleotide substitutions per site between any two DNA sequences selected randomly from chickpea population under study), θω (average estimates of mutation rate derived from a total number of segregating sites that corrected for population size) and Tajima's D (statistical test to infer the evidence of neutral evolution for each defined region within a selected population) were measured using a 100-kb non-overlapping sliding window approach of TASSEL v5.0 and following the methods of Xu et al. (2012) and Varshney et al. (2013a).

For determining the population structure among 93 cultivated and wild chickpea accessions, the SNP genotyping data were analyzed in a model-based program STRUCTURE v2.3.4 (Pritchard et al., 2000) adopting the methods of Kujur et al. (2013, 2014). The optimal value of population number (K) was determined in accordance with *ad-hoc* method of Pritchard et al. (2000) and *delta* K procedure of Evanno et al. (2005). To determine the genome-wide LD patterns (*r*^2^; frequency correlation among pair of alleles across a pair of SNP loci), including LD decay (by plotting average *r*^2^ against 10 and 100 kb uniform physical intervals across eight *desi* and *kabuli* chromosomes) in each *desi* and *kabuli* genomes, the genotyping data of SNPs that were physically mapped on eight chromosomes were analyzed using a command (–r2 −ld-window 99999 −ld-window-r2 0) line interface of PLINK (Purcell et al., 2007) and the full-matrix approach of TASSEL (following Zhao et al., 2011; Kujur et al., 2013, 2014). The ANOVA (analysis of variance)-based significance test of LD estimates by comparing their *r^2^-values* across population groups and chromosomes was performed using SPSS v17.0 (http://www.spss.com/statsistics) and following Saxena et al. (2014a).

## Results

### Genome-wide SNPs discovery using a GBS assay

**Figure 1:**
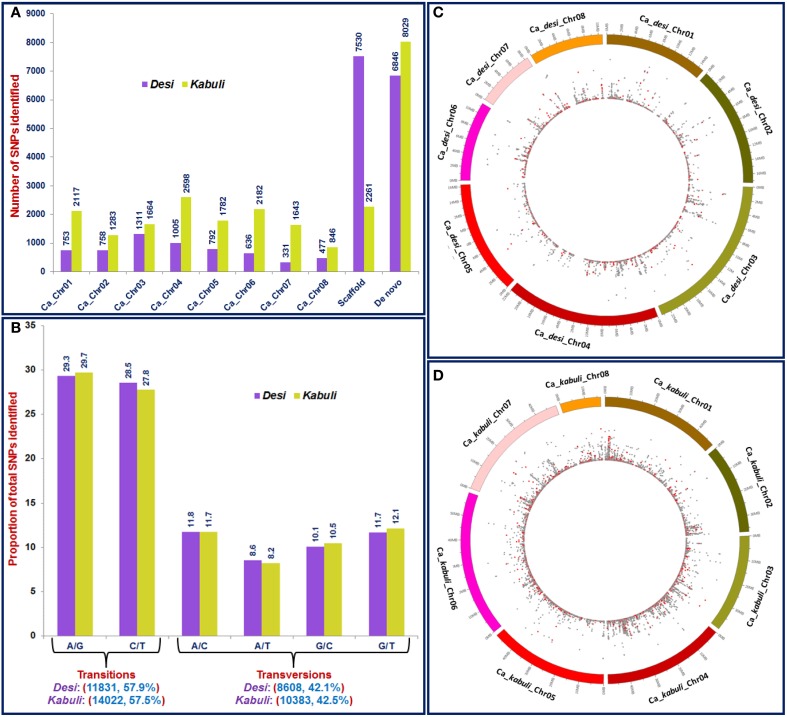
**(A)** Frequency and distribution of 44,844 SNPs identified from *desi* and *kabuli* chickpea using reference genome (eight chromosomes and unanchored scaffolds)- and *de novo*-based GBS assays. **(B)** Percentage distribution of transition and transversion SNPs identified using GBS assay. The relative distribution of 20,178 SNPs physically mapped on eight chromosomes of *desi* **(C)** and *kabuli* **(D)** genomes are depicted in the Circos circular ideogram. The outermost circles represent the eight chickpea chromosomes coded with different colors, whereas the innermost circles display the distribution of SNPs, including non-synonymous SNPs (marked with red dots) identified through GBS assay.

The sequencing of 96-plex *Ape*KI libraries constructed from 96 diverse chickpea accessions (Supplementary Table S1) using a GBS assay generated 246.5 million reads (~30-fold sequencing depth of coverage), with an average of 2.5 million reads (varied from 1.5 to 9.9 million reads) per accession (Supplementary Figure S1). After quality filtering, 207.9 (84.3%) million processed high-quality reads uniformly distributed across 96 accessions were obtained. Of these high-quality sequence reads, 81.2 and 72.6% were mapped to unique physical locations on *desi* and *kabuli* reference genomes, respectively (Supplementary Table S2). The sequencing data generated in our study have been submitted to a free publicly-accessible NCBI-short read archive (SRA) database (www.ncbi.nlm.nih.gov/sra) with accession number SRX845396 (Release date: 31st March 2015). Using both reference and *de novo*-based GBS approaches in 96 accessions, 44,844, including 20,439 and 24,405 high-quality SNPs (with read-depth = 10, SNP base quality = 20, < 10% missing data and ~2% heterozygosity in each accession) were identified from *desi* and *kabuli* genomes, respectively (Table 1, Figures 1A–C, Supplementary Table S3). A high degree of reproducibility (100%) of SNPs identified by GBS assay in three *desi* and *kabuli* chickpea accessions (ICCX-810800, ICCV10, and ICCV 95334) used as biological replicates was observed. The transitions were more frequent than the transversions that composed slightly more than a-half (57.9%; 11,831 SNPs in *desi* and 57.5%; 14,022 SNPs in *kabuli*) of the identified SNPs (Figure 1D). A higher frequency of A/G transitions was evident in both *desi* (29.3%) and *kabuli* (29.7%) chickpea compared to A/C (11.8% in *desi*) and G/T (12.1% in *kabuli*) transversions (Figure 1D).

**Table 1:** **Genomic distribution and nucleotide diversity potential of SNPs physically mapped on eight *desi* and *kabuli* chickpea chromosomes**.

**Chromosomes**	**Size (Mb) of chromosomes (pseudomolecules)**		**Number (%) of SNPs mapped**		**Average map density (kb)**		**Nucleotide diversity**					
	***Desi***	***Kabuli***	***Desi***	***Kabuli***	***Desi***	***Kabuli***	***Desi***			***Kabuli***		
							**Theta Pi (θπ) kb^−1^**	**Theta w (θω) kb^−1^**	**Tajima's D**	**Theta Pi (θπ) kb^−1^**	**Theta w (θω) kb^−1^**	**Tajima's D**
Ca_Chr01	14.79	48.36	753 (12.4)	2117 (15.0)	19.6	22.8	0.89	1.56	−2.54	0.87	1.62	−2.71
Ca_Chr02	17.30	36.63	758 (12.5)	1283 (9.1)	22.8	28.6	0.81	1.45	−2.61	0.96	1.32	−1.99
Ca_Chr03	23.38	39.99	1311 (21.6)	1664 (11.8)	17.8	24.0	0.90	1.54	−2.51	0.78	1.29	−2.43
Ca_Chr04	22.09	49.19	1005 (16.6)	2598 (18.4)	22.0	18.9	1.33	1.69	−2.78	1.31	1.72	−2.91
Ca_Chr05	16.30	48.17	792 (13.1)	1782 (12.6)	20.6	27.0	0.47	1.25	−2.27	0.43	1.01	−2.11
Ca_Chr06	11.48	59.46	636 (10.5)	2182 (15.5)	18.1	27.3	0.74	1.42	−2.73	0.70	1.51	−2.99
Ca_Chr07	8.46	48.96	331 (5.5)	1643 (11.6)	25.6	29.8	1.13	1.53	−1.96	0.95	1.67	−2.58
Ca_Chr08	10.57	16.48	477 (7.9)	846 (6.0)	22.2	19.5	0.98	1.30	−1.88	0.75	1.19	−2.35
Total	124.37	347.24	6063	14115	20.5	24.6	0.99	1.47	−2.31	0.92	1.65	−2.39
Unanchored scaffolds	NA	NA	7530	2261	NA	NA	1.06	1.93	−2.66	1.10	2.28	−2.86
*De novo*	NA	NA	6846	8029	NA	NA	0.93	0.66	−2.99	2.17	0.99	−2.95
Total	NA	NA	20,439	24,405	NA	NA	1.02	1.35	−2.67	1.40	1.64	−2.75

*Ca_Chr: Cicer arietinum Chromosomes*.

*θπ per kb: Average pair-wise nucleotide diversity*.

*θω per kb: Watterson's estimator of segregating sites*.

**Figure 2:**
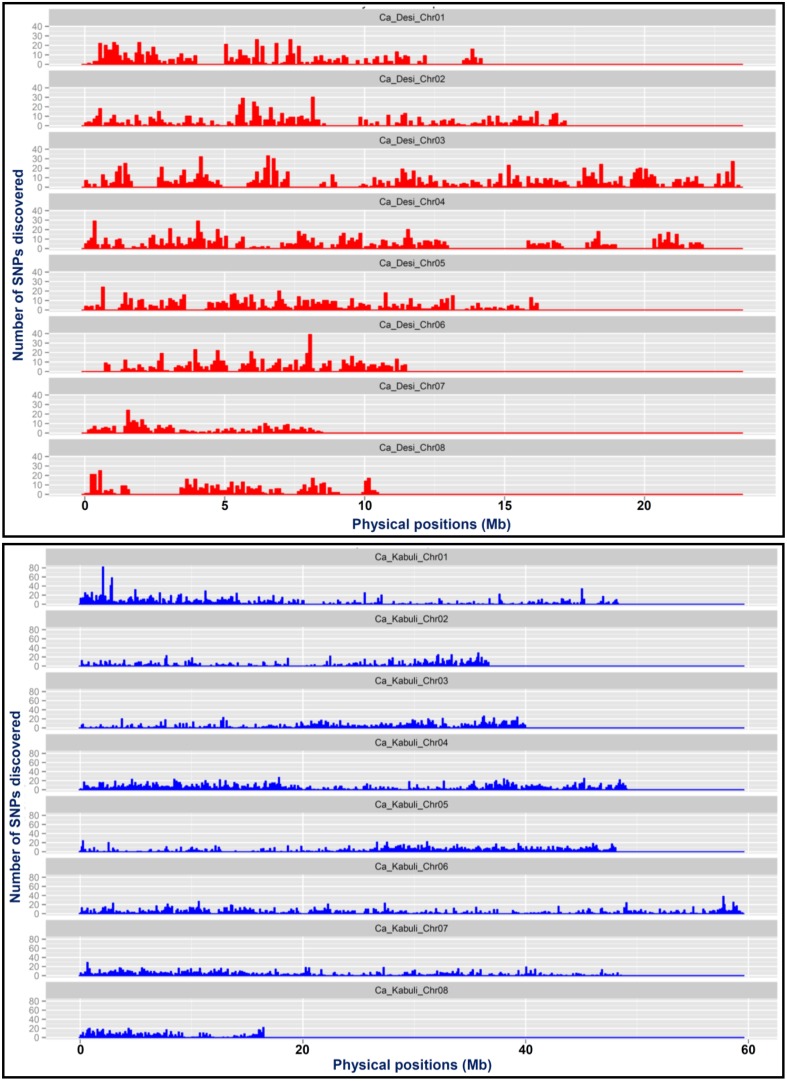
**A genome scan plot depicting the density of SNPs physically mapped across eight chromosomes of *desi* (A) and *kabuli* (B) genomes at 100-kb sliding window**. The SNP density is represented by the number of SNPs mapped within 1-Mb physical intervals across chromosomes.

A total of 20,439 SNPs identified in the *desi* genome includes 13,593 SNPs from reference genome as well as 6846 *de novo* discovered SNPs from its unaligned sequence reads (Figure 1A, Supplementary Table S3). The mined 6063 reference genome-based SNPs were physically mapped across eight *desi* chromosomes with an average map density of 20.5 kb (Table 1, Figures 1B,C). A genome-wide SNP density plot revealed that a maximum proportion of SNPs were physically mapped on *desi* chromosome 3 (21.6%, 1311 SNPs) (Figures 1A, 2A). The average marker density was maximum on chromosome 3 (17.8 kb), whereas it was minimum on chromosome 7 (Table 1, Figure 2A). The remaining 7530 SNPs were physically mapped on unanchored scaffolds of *desi* genome.

A total of 24,405 SNPs discovered from the *kabuli* genome includes 16,376 and 8029 reference genome and *de novo*-based SNPs, respectively (Figure 1A, Supplementary Table S3). The reference genome-based 14,115 SNPs were physically mapped across eight *kabuli* chromosomes with a mean map density of 24.6 kb (Table 1, Figures 1B,C). A genome-wide SNP density plot indicated that a higher proportion (18.4%, 2598 SNPs) of SNPs were physically mapped on *kabuli* chromosome 4 (Figures 1A, 2B). The *kabuli* chromosomes 4 and 7 had maximum (18.9 kb) and minimum (29.8 kb) average marker density, respectively (Table 1, Figure 2B). The rest 2261 SNPs were physically mapped on unanchored scaffolds of *kabuli* genome. In total, 16,736 SNPs identified from the reference *kabuli* genome have been submitted to NCBI dbSNP (http://www.ncbi.nlm.nih.gov/SNP), with SNP submission (SS) accession numbers 974751673–974768048, which will be publicly available in the next NCBI db SNP Build (B142), year 2015 (http://www.ncbi.nlm.nih.gov/SNP/snp_viewTable.cgi?handle=NIPGR).

### Structural and functional annotation of SNPs

**Figure 3:**
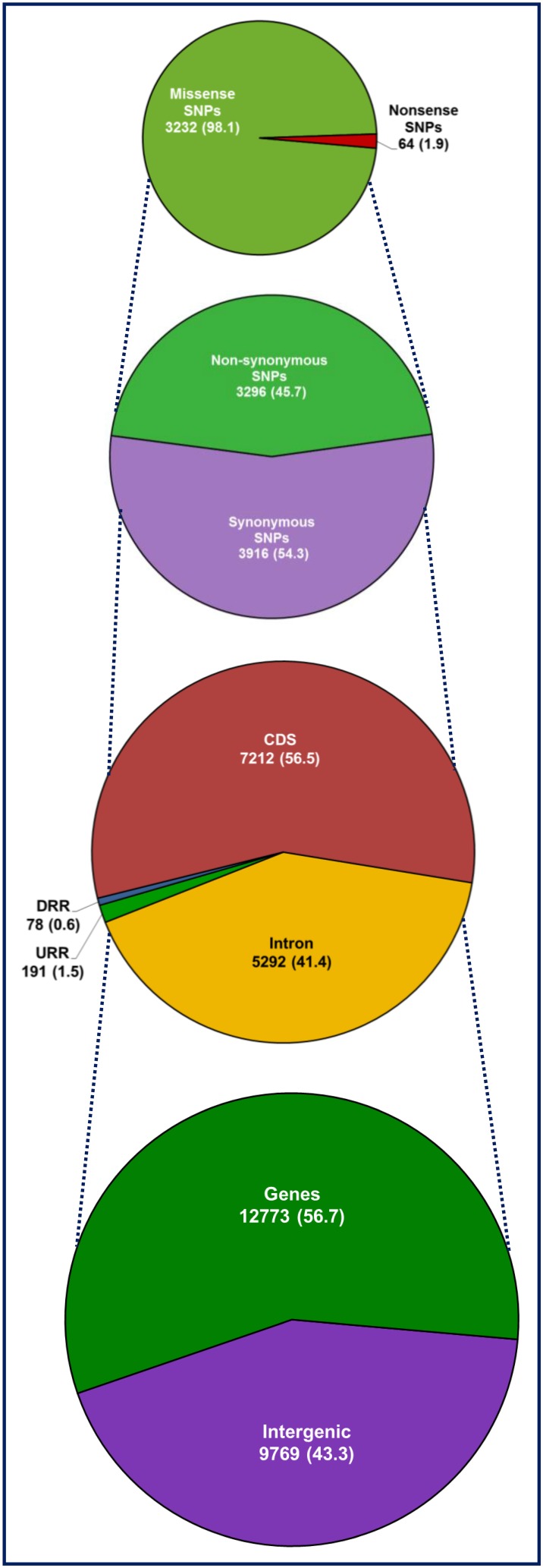
**Relative frequency of 22,542 SNPs identified in the intergenic regions and different coding and non-coding sequence components of 5588 non-redundant genes annotated from *desi* and *kabuli* genomes**. Number (%) of SNPs, including synonymous and non-synonymous (missense and non-sense) SNPs annotated in the coding as well as non-coding intronic and regulatory sequences of chickpea genes and intergenic regions are illustrated. The URR (upstream regulatory region) and DRR (downstream regulatory region) of protein-coding genes were defined based on the available gene annotation information of *desi* and *kabuli* genomes (Jain et al., 2013; Varshney et al., 2013a).

The structural annotation of 13,593 *desi* reference genome-based SNPs showed the presence of 9567 SNPs in 4010 genes and 4026 SNPs in the intergenic regions (Supplementary Table S2). Likewise, the annotation of 16,372 *kabuli* reference genome-based SNPs identified 10,656 and 5720 SNPs in 4643 genes and intergenic regions, respectively. The SNPs-carrying gene sequences, including SNPs with their 100-bp flanking sequences were compared between *desi* and *kabuli* genomes based on sequence homology to filter out the duplicated genes and SNPs observed commonly between the two genomes. This analysis identified 6170 non-redundant SNPs (including 2117 SNPs in 945 genes and 4053 intergenic SNPs) specifically from *desi* genome (Supplementary Table S2). Combining these unique *desi*-specific SNPs data with 16,376 SNPs identified from *kabuli*, the detailed structural annotation of 22,542 SNPs (Supplementary Table S4) in different sequence components of chickpea genes/genomes and the functional annotation of SNP-carrying genes were performed. The maximum percentage (56.7%, 12,773 SNPs) of SNPs were identified in 5588 chickpea genes, whereas the remaining 43.3% (9773) of SNPs were detected in the intergenic regions (Figure 3). The number of SNPs identified in the genes varied from 1 to 34, with an average frequency of 3 SNPs/gene. The annotation of SNPs in various structural components of genes revealed the presence of a greater percentage (56.5%, 7212 SNPs) of SNPs in the exons, followed by introns (41.4%, 5292) and 2000-bp upstream regulatory regions (URRs) (1.5%, 191), and a minimum percentage (0.6%, 78 SNPs) in the 1000-bp downstream regulatory regions (DRRs) (Figure 3). Among the identified coding SNPs, 3916 SNPs within 2510 genes showed synonymous substitutions, whereas 3296 SNPs in 2048 genes resulted in non-synonymous substitutions (Figure 3). The identified non-synonymous SNPs included 3232 missense SNPs in 2025 genes showing amino acid substitutions and 64 non-sense SNPs in 23 genes culminating into premature termination codons introduced by nucleotide replacements (Figure 3). The estimation of average non-synonymous to synonymous substitution rates in 7212 coding SNPs across 4558 chickpea genes revealed that a larger proportion (~97%) of these genes had Ka/Ks = 0.84 (<1.0) and thus under negative/purifying selection pressure. A maximum (1.04) Ka/Ks was obtained between ICC 15264 (*kabuli*) and ICC 17160 (*C. reticulatum*) and minimum (0.47) between ICC 7346 (*kabuli*) and ICC 15512 (*kabuli*). The Tajima's D estimate further inferred that a higher proportion (95%) of SNP-carrying genes under purifying selection with reduced *D* = −2.48 (*D* < −2) and remaining 5% genes had elevated *D* > 2.

**Figure 4:**
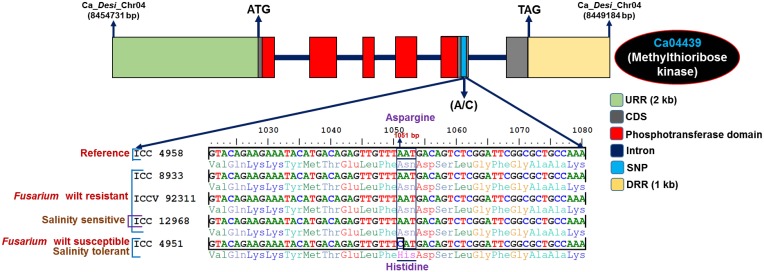
**SNP loci (A–C) revealing missense non-synonymous amino acid substitutions [asparagine (AAT) to histidine (CAT)] in the phosphotransferase domain encoding the methylthioribose kinase gene of *desi* chickpea showed polymorphism between the *Fusarium* wilt resistant (ICC 8933, ICCV 92311, and ICC 12968) and susceptible (ICC 4951) chickpea accessions**. Moreover, this analysis identified non-synonymous SNPs showing polymorphism between salinity tolerant (ICC 4951) and sensitive (ICC 12968) accessions. The *desi* accession ICC 4958 was used as a reference genome for identifying SNPs. The missense non-synonymous SNPs are highlighted.

The functional annotation of 5588 genes with SNPs revealed their maximum correspondence to growth, development and metabolism-related proteins (3073, 55%), followed by TFs (92, 16.3%) and signal transduction proteins (39, 7%), with a minimal correspondence to genes encoding hypothetical proteins (12, 2.2%) (Supplementary Figure S2A). The KOG-based determination of putative functions (excluding unknown and general functions) for SNP-carrying genes indicated their primary roles in signal transduction mechanisms (20.4%), followed by transcription (10.2%) and carbohydrate transport and metabolism (8.8%) (Supplementary Figure S2B). Furthermore, GO enrichment analysis of SNP-carrying genes depicted a significant overrepresentation/enrichment of GO terms in the genes associated with molecular function (nucleic acid binding, 22%, P: 1.9 × 10^−31^), followed by biological process (metabolism, 6.6%, 3.4 × 10^−94^) and cellular component (macromolecular complex, 1.7%, 1.4 × 10^−50^) (Supplementary Figure S3). The detailed functional annotation of genes with SNPs detected a greater percentage of SNPs belonging to growth, development and metabolism-related protein-encoding genes (11.8%, 659 of total identified 5588 SNPs carrying genes), TF-encoding genes (3.7%, 207), and unknown expressed proteins (4.1%, 229) that result in missense and non-sense non-synonymous substitutions. Interestingly, 123 SNPs, including 13 non-synonymous SNPs and 12 upstream regulatory SNPs identified in the 73 chickpea genes showed polymorphism between abiotic stress tolerant (ICC 4958 and IC 296131) and sensitive (ICCV 93954) chickpea accessions (Supplementary Tables S4, S5). For instance, one SNP (C–G) showing missense non-synonymous amino acid substitution [aspartic acid (GAC) to glutamic acid (GAG)] in the plant homeodomain (PHD) zinc finger encoding *kabuli* transcription factor (TF) gene was found to be polymorphic between drought tolerant (ICC 4958 and ICCV 93954) and sensitive (ICCV 93954) chickpea accessions (Supplementary Figure S4). Similarly, 21 SNPs (including three non-synonymous SNPs and four upstream regulatory SNPs) detected in 16 genes showed polymorphism between *Fusarium* wilt resistant (ICC 8933, ICCV 92311, and ICC 12968) and susceptible (ICC 4951) chickpea accessions (Supplementary Tables S4, S5). A total of 242 SNPs (42 non-synonymous SNPs and 10 upstream regulatory SNPs) were detected in 156 genes showing polymorphism between the salinity stress tolerant (ICC 4951) and sensitive (ICC 12968) accessions (Supplementary Tables S4, S5). For example, SNP (A–C) showing missense non-synonymous amino acid substitution [asparagine (AAT) to histidine (CAT)] in the phosphotransferase domain encoding methylthioribose kinase *desi* gene was found to be polymorphic between *Fusarium* wilt resistant (ICC 8933, ICCV 92311, and ICC 12968) and susceptible (ICC 4951) chickpea accessions. In addition, this non-synonymous SNP revealed polymorphism between salinity tolerant (ICC 4951) and sensitive (ICC 12968) chickpea accessions (Figure 4). Notably, SNPs identified in the coding regions of two stress-responsive PHD zinc finger TF and methylthioribose kinase genes had lower mean Ka/Ks = (0.39) (< 1.0) and reduced Tajima's *D* = −2.66 (*D* < −2) compared to that estimated in other genic SNPs (Ka/Ks: 0.84 and Tajima's D: −2.48).

In order to determine the significance of identified genome-wide GBS-based SNPs for subsequent fine-mapping/map-based cloning of QTLs/genes, these SNPs were annotated in the genes underlying the known QTLs reported earlier for drought tolerance, and *Ascochyta* blight and *Fusarium* wilt resistance traits in chickpea (Udupa and Baum, 2003; Sabbavarapu et al., 2013; Varshney et al., 2013b, 2014a,b; Thudi et al., 2014). Based on these analyses, we identified 303 SNPs (78 non-synonymous SNPs) in the 140 genes localized within a 0.03 Mb (10.39–10.42 Mb) major “*QTL-hotspot*” region (mapped on *kabuli* chromosome 4) controlling drought tolerance-component root traits in chickpea (Supplementary Tables S4, S6). Interestingly, 18 SNPs-carrying 11 genes of these, showed polymorphism between drought tolerant (ICC 4958 and IC 296131) and sensitive (ICCV 93954) chickpea accessions. A total of 190 (59 non-synonymous SNPs) and 12 SNPs (1 non-synonymous SNPs) in 64 and 3 genes harboring the two major *Ascochyta* blight (*ar2aQTL*) and *Fusarium* wilt (*FW_Q_APR_6_1*) resistance QTLs mapped on *kabuli* chromosomes 2 (2.39 Mb: 31.58–33.97 Mb) and 6 (0.1 Mb: 29.47–29.57 Mb), respectively were discovered (Supplementary Tables S4, S6).

### Validation and polymorphic potential of SNPs

For validating SNPs identified through GBS assay, 12,719 *desi* (ICC 4958) reference genome-based SNPs (with MAF ≥ 0.05) discovered in 93 accessions were compared and correlated with the available resequencing-based SNP database (31,019 SNPs) of four chickpea accessions (*desi*: ICC 4958, *desi*: ICC 4951, *kabuli*: ICC 12968, and wild *C. reticulatum*: ICC 17160). On the basis of congruent physical positions (bp), the correspondence of 8.8% (777 SNPs) of the SNPs present in our GBS data (8776 SNPs) was observed with that of SNPs mined by genome resequencing of the four accessions (Supplementary Table S7). Among these corresponding SNPs, the maximum percentage of SNPs (9.5%, 593 SNPs) showing polymorphism between ICC 4958 (*desi*) and ICC 17160 (wild) and minimum between ICC 4958 (*desi*) and ICC 4951 (*kabuli*) (7%, 86) were found to be common between past genome sequencing and our present GBS data (6244 SNPs) (Supplementary Table S7). Notably, the sensitivity and specificity of these *in silico* validated SNPs were 96.5% and 99.6%, respectively. Additionally, the PCR amplicon-based Sanger sequencing (214 SNPs) and MALDI-TOF mass array SNP genotyping (240 SNPs) of 454 selected non-synonymous and regulatory SNP loci discovered by reference genome- and *de novo-based* GBS approaches (Supplementary Table S8) successfully validated 418 (92.2%) SNPs in 93 *desi, kabuli*, and wild chickpea accessions. The sensitivity and specificity of these experimentally validated SNPs were estimated to be 93.7% and 99.8%, respectively. Multiple sequence alignment of amplicons resequenced from a representative set of *desi* and *kabuli* accessions exhibited the presence of similar expected SNP alleles in these accessions at specific genomic locations as discovered through our reference genome- and *de novo*-based GBS assay (Supplementary Figure S5). The MALDI-TOF mass array-based genotyping of SNPs in a selected set of *desi* and *kabuli* accessions further enabled to validate similar expected homozygous/heterozygous SNP alleles in these accessions at definite bp as detected through our GBS assay (Supplementary Figure S6).

In total, 23,798 SNPs (including 12,719 and 11,079 SNPs from *desi* and *kabuli*, respectively with MAF ≥ 0.05) showing polymorphism among 93 diverse *desi, kabuli*, and wild chickpea accessions had higher PIC (varied from 0.12 to 0.47, mean 0.42), MAF (mean: 0.37), and nucleotide diversity (mean θπ: 1.30 and mean θω: 1.51) (Supplementary Table S8, Supplementary Figure S7). Remarkably, 11,780 (49.5%) of these SNPs were polymorphic (PIC: 0.38, MAF: 0.34, θπ: 1.15, and θω: 1.26) between at least two accessions belonging to the cultivated *C. arietinum* species. The remaining 50.5% (12,018) of SNPs revealed polymorphism between cultivated *C. arietinum* and wild *C. reticulatum* species. The SNPs detected a higher intra-specific polymorphic potential within 39 *desi* accessions (42.1% polymorphism and mean PIC: 0.35, MAF: 0.31, θπ: 1.20, and θω: 1.33) compared with that of 53 *kabuli* accessions (37.6%, 0.30, 0.27, 0.96, and 1.07) (Supplementary Table S9). The nucleotide diversity estimated in eight *desi* chromosomes (mean θπ: 0.99, θω: 1.47, and Tajima's D: −2.31) was almost comparable to that of *kabuli* chromosomes (0.92, 1.65, and −2.39) (Table 1). Maximum nucleotide diversity was obtained in *desi* (mean θπ: 1.33 and θω: 1.69) and *kabuli* chromosomes 4 (1.31 and 1.72).

### Molecular diversity and population genetic structure among chickpea accessions revealed by genome-wide SNPs

**Figure 5:**
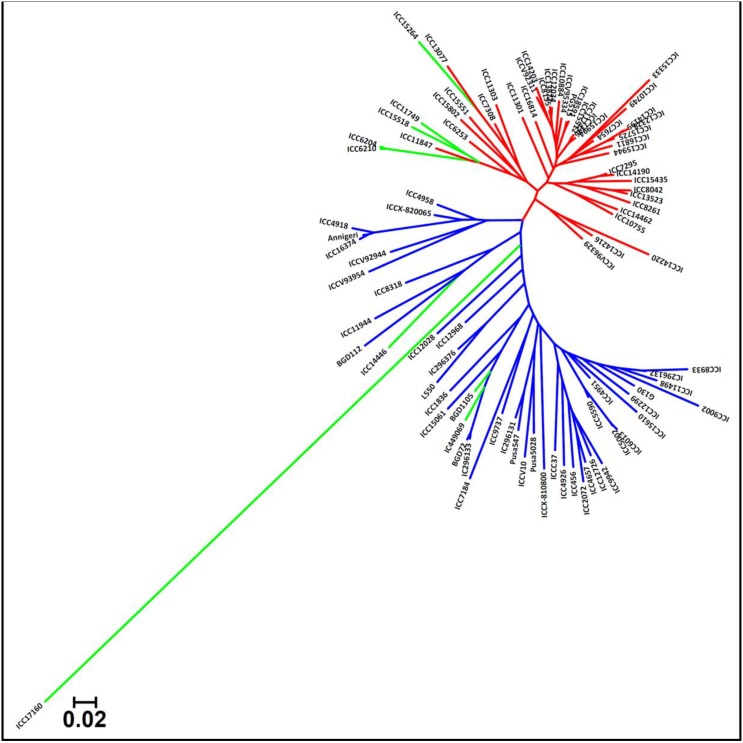
**Unrooted phylogenetic tree depicting the genetic relationships among 93 cultivated and wild chickpea accessions based on Nei's genetic distance using 23,798 high-quality GBS-based SNPs (MAF ≥ 0.05)**. Molecular classification differentiated these accessions into two major clusters/population groups (*kabuli* and *desi*) as expected based on their cultivar/species-specific origination. The wild accession grouped distinctly within a POP III. Three different colors correspond to three populations (POP I, POP II, and POP III) as defined by population genetic structure.

The genetic distance estimation among 93 *desi, kabuli*, and wild chickpea accessions using 23,798 SNPs (with MAF ≥ 0.05) revealed a wider range of genetic distances, from 0.13 (ICC 7346 and ICC 15512) to 0.89 (ICC 15264 and ICC 17160), with an average of 0.56 (Supplementary Table S9). The 11,780 SNPs-based genetic distances among 92 *desi* and *kabuli* accessions within the cultivated (*C. arietinum*) species varied from 0.12 to 0.76, with a mean of 0.45. A relatively less genetic diversity in 53 cultivated *kabuli* accessions (mean genetic distance: 0.38) as compared to the 39 *desi* accessions (0.43) was evident (Supplementary Table S9). A broader range of genetic distances (0.18–0.77, mean: 0.51) and thus, a higher functional allelic diversity level was observed among 93 accessions using 12,802 gene-derived SNPs (MAF ≥ 0.05). The unrooted neighbor-joining phylogenetic tree among 93 accessions using 23,978 genome-wide SNPs reflected a clear differentiation and clustering of accessions into two major population groups (*kabuli* and *desi*) in accordance with their pedigree relationships and parentage (Figure 5). One wild *C. reticulatum* accession ICC 17160 being divergent, got clearly separated and clustered distinctly within *desi* group.

**Figure 6:**
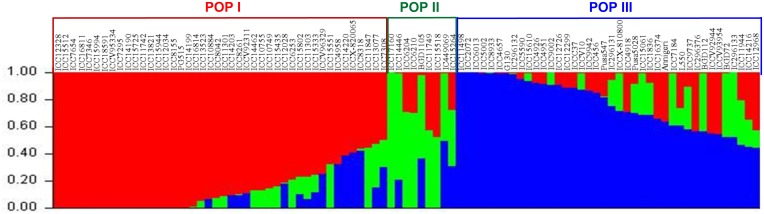
**Population structure with population number *K* = 3 and their inferred best possible population genetic structure for 93 chickpea accessions using 23,798 high-quality GBS-based SNPs (MAF ≥ 0.05)**. These mapped markers assigned accessions into three populations (POP I, POP II, and POP III) that primarily corresponded to their cultivar/species-specific origination. The accessions represented by vertical bars along the horizontal axis were classified into K color segments based on their estimated membership fraction in each K cluster. Three diverse colors represent different populations based on the optimal population number *K* = 3.

The population genetic structure determination among 93 accessions with genotyping data of genome-wide (physically mapped on eight chickpea chromosomes) 96 SSR (simple sequence repeats selected from Kujur et al., 2013) and 23,798 SNP markers classified all the accessions into following three distinct populations at a K (population number) value of 3: POP I (44 *kabuli* accessions), POP II (1 wild, 4 *desi* and 4 *kabuli* accessions), and POP III (40 accessions) (Figure 6). The results of *K*-value with the best replicate were confirmed by accessing the average LnP(D) (log-likelihood) value (showing the greatest apparent inflection) (Supplementary Figure S8A) and using the second order statistics (Supplementary Figure S8B) of ΔK estimation (showing a sharp peak with maximum value of ΔK). The estimation of significance of SNPs to detect polymorphism among the three populations inferred their maximum polymorphic potential in POP II (PIC: 0.10–0.48 with mean: 0.40 and mean MAF: 0.36, θπ: 1.28, and θω: 1.47), followed by POP III and POP I (Supplementary Table S10). A wider level of significant quantitative genetic and population divergence based on pair-wise F_*ST*_ (*P* < 0.001) was observed between POP I and POP III (0.62), followed between POP II and POP III (0.43) and minimum between POP I and POP II (0.30). The F_*ST*_-based population differentiation was highest within POP II (0.21), followed within POP III (0.16), whereas it was least within POP I (0.10). All 93 accessions clearly belonged to a structured population with 78% inferred ancestry derived from one of the model-based population and the remaining 22% had admixed ancestry. Admixed ancestry was maximum in POP II (16%), followed by POP III (11%) and minimum in POP I (5%). Highest admixed ancestry was obtained between POP III and POP II (19%) compared to that between POP III and POP I (13%). The admixture was not detected in 9.7% (nine accessions) of chickpea accessions, whereas 37.6% (35) and 47.3% (44) of accessions showed up to 20 and 10% admixed ancestry, respectively.

### Genome-wide and population-specific LD patterns in chickpea

**Figure 7:**
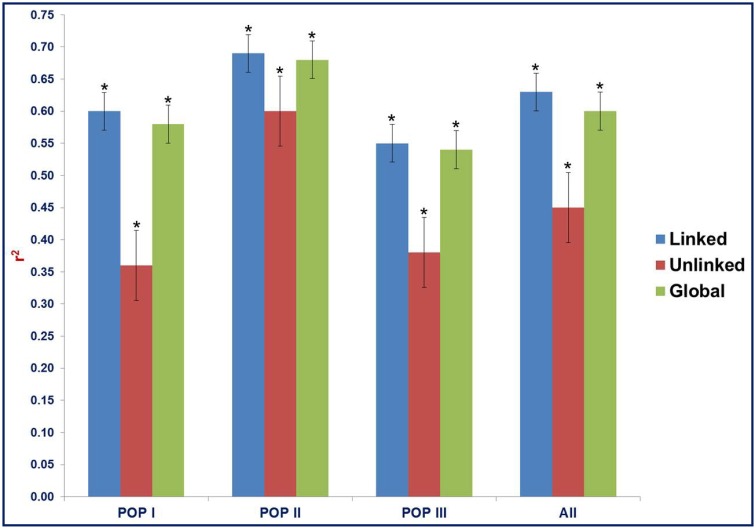
**Estimates of LD (mean *r*^2^) for linked, unlinked, and global markers (3321 *desi* and 8592 *kabuli* SNPs) in three populations as defined by the population genetic structure**. “All” includes the LD estimates and decay across three populations. The bar indicates the standard error. ^*^ANOVA significance at *p* < 0.001.

**Figure 8:**
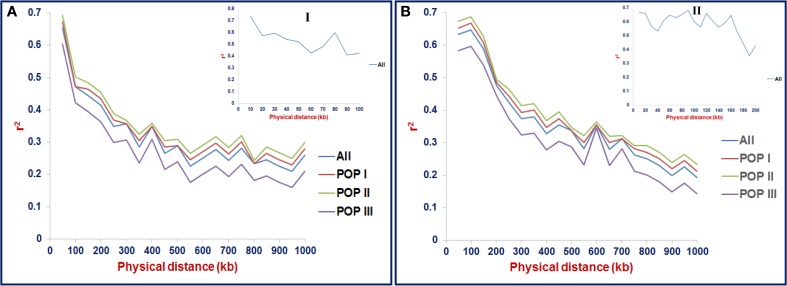
**LD decay (mean *r*^2^) estimated in three populations (as defined by population genetic structure) using 3321 *desi* (A) and 8592 *kabuli* (B) SNPs, respectively**. For LD decay, the *r*^2^-value of the marker physical distance of 0 kb is considered 1. The plotted curved lines indicate the mean *r*^2^-values among markers spaced with uniform 100 kb physical intervals from 0 to 1000 kb. The plotted line in uppermost panel **I** indicate the mean *r*^2^-values among markers spaced with uniform 10 kb physical intervals from 0 to 100 kb. The marked line in panels **I, II** indicate the mean *r*^2^-values among markers spaced with uniform 10 and 20 kb physical intervals from 0 to 100 kb and 0 to 200 kb, respectively. “All” includes the LD decay across entire three populations.

The LD estimates (average *r*^2^) and extent of LD decay using all possible pair-combination of genome-wide 3321 and 8592 *desi* and *kabuli* SNPs (physically mapped on eight chromosomes) were calculated among 93 chickpea accessions constituting three populations (POP I, II, and III) and a diversity panel. In the whole population, 64.3% global marker-pairs exhibited significant LD (*P* < 0.0001), indicating the existence of an extreme LD level in a panel of 93 accessions (Table 2). The LD estimates of global markers ranged from 0.54 in POP III to 0.68 in POP II, with an average of 0.60. The proportion of significant LD and LD estimates for global, linked and unlinked markers was maximum in POP II compared to POP I and POP III (Table 2, Figure 7). The LD estimates of linked markers in all three populations was 0.63, whereas it was 0.45 for unlinked markers. Significant LD (*P* < 0.0001) was observed for 65 and 54.8% of the linked and unlinked marker-pairs, respectively (Table 2). However, the trend of significant LD proportion and LD estimates with linked and unlinked marker-pairs was similar to that observed among three populations using global markers. The linked marker-pairs detected maximum percentage of LD estimates and significant LD proportion on *desi* chromosome 3 (67.3%) and *kabuli* chromosome 4 (58.4%) (Supplementary Table S11, Supplementary Figures S9A, B). The significant LD proportion and LD estimates of linked markers obtained in *desi* (56.9%, 0.56) and *kabuli* (54.6%, 0.52) chromosomes are comparable to each other, whereas these parameters for unlinked markers were higher in the chromosomes of *desi* (43%, 0.53) than *kabuli* (35.9%, 0.33) (Supplementary Table S11). The LD decay of genome-wide 3321 *desi* and 8592 *kabuli* reference genome-based SNPs was determined individually across three populations by pooling the *r*^2^ estimates across eight chromosomes and plotting their average *r*^2^ against the uniform physical distance of 0–100 kb (Figures 8A,B). In all three populations, a non-linear regression curve exhibiting a decreasing trend of LD decay with an increase in the physical distance (kb) was observed. All these populations sustained a significant level of LD up to a physical distance of 1000 kb. No significant decay of LD (below *r*^2^ = 0.1) was observed in any population up to a 1000 kb physical distance for the *desi* and *kabuli* chromosomes (Figures 8A,B). However, the *r*^2^ decreased to half of its maximum value at ~400–500 kb physical distance in both *desi* and *kabuli* chromosomes. The genetically more diverse population (POP III) showed a shorter LD decay (~400 kb in *desi* chromosomes and ~600 kb in *kabuli* chromosomes) than that of the less diverse populations of POP I (~700 kb in both *desi* and *kabuli* chromosomes) and POP II (~750 kb) (Figures 8A,B).

**Table 2:** **LD estimates among linked, unlinked, and global SNP-pairs in three model-based individual populations and entire populations**.

**Populations**	**Global**			**Linked**			**Unlinked**		
	**Number of SNP-pairs (MAF ≥ 0.05) used**	**^a^ Number (%) of significant SNP-pairs in LD**	**Extent of (mean *r*^2^)**	**Number of SNP-pairs (MAF ≥ 0.05) used**	**Number (%) of significant marker-pairs in LD**	**Extent of (mean *r*^2^)**	**Number of SNP-pairs (MAF ≥ 0.05) used**	**Number (%) of significant marker-pairs in LD**	**Extent of LD (mean *r*^2^)**
POP I	119,453	71,319 (59.7)	0.58	111,328	67,642 (60.8)	0.60	8125	3571 (44.0)	0.36
POP II	69,194	60,301 (87.1)	0.68	62,599	54,817 (87.6)	0.69	6594	5484 (83.2)	0.60
POP III	131,749	74,331 (56.4)	0.54	123,199	70,645 (57.3)	0.55	8550	3686 (43.1)	0.38
Total populations	320,396	205,951 (64.3)	0.60	297,126	193,104 (65.0)	0.63	23,269	12,741 (54.8)	0.45

a *Percentage of SNP-pairs in significant (P < 0.0001) LD*.

## Discussion

### GBS assay expedites genome-wide discovery and high-throughput genotyping of SNPs in chickpea

GBS assay is currently considered as the most convenient approach for high-throughput SNP discovery and genotyping in crop plants, including chickpea (Mayer et al., 2012; Poland et al., 2012a,b; Byrne et al., 2013; Crossa et al., 2013; Mascher et al., 2013; Morris et al., 2013; Sonah et al., 2013; Spindel et al., 2013; Thurber et al., 2013; Uitdewilligen et al., 2013; Bastien et al., 2014; Deokar et al., 2014; He et al., 2014; Huang et al., 2014; Jaganathan et al., 2014; Jarquín et al., 2014; Liu et al., 2014; Sonah et al., 2014; Tardivel et al., 2014). Nevertheless, it also suffers from certain technical drawbacks, including non-uniform distribution of short sequence reads in different genotyped accessions and a high percentage of missing and erroneous (heterozygous) SNP genotyping data. This assay is also accompanied with difficulties in storing and handling much low quality SNP genotyping data generated from diverse crop accessions during bioinformatics analyses (Davey et al., 2011; Mir and Varshney, 2012; Poland and Rife, 2012). Recent efforts have been made to address these technical concerns involving generation of high-quality, uniform and accurate SNP genotyping information by physical mapping of short sequence reads (~64-bp tag) on available reference genomes, identifying bi-allelic tags through SNP imputation and use of suitable computational genomics softwares/pipelines (Beissinger et al., 2013). Moreover, the possibility of identifying such valid SNPs from large-scale GBS data can be enriched using the latest optimized *de novo* approaches, as suggested by several early reports in diverse crop plants, including chickpea (Craig et al., 2008; Cronn et al., 2008; Schnable et al., 2009; Poland et al., 2012a,b; Deokar et al., 2014; Jaganathan et al., 2014).

In the present study, an optimized GBS assay protocol was developed by sequencing of 96-plex *Ape*KI GBS libraries (generated from 96 diverse chickpea accessions) and integrating both reference genome- and *de novo*-based GBS pipelines for genome-wide discovery and genotyping of SNPs in chickpea. By implementing these novel approaches, many (44,844) non-erroneous and high-quality SNPs with wider genome coverage were identified in chickpea. In contrast to the conventional TASSEL GBS pipeline (maximum SNP detection limited to 64-bp sequence reads), the use of integrated reference genome (*desi* and *kabuli*)–and *de novo*-based GBS approaches would be of heightened interest for SNP discovery and genotyping. Consequently, our study for the first time identified SNPs in high-quality longer sequence reads (average sequence read length of > 75 bp after removal of barcode and adapter sequences) by involving a broader range of filtration tools (modifying stringency of SNP calling parameters), thereby enriching the chickpea genome with reduced computational time. The advantages of this integrated GBS approach for discovery and genotyping of SNPs at a genome-wide scale is well-documented in many crop plants, including soybean and rice (Sonah et al., 2013, 2014; Spindel et al., 2013). A high (100%) reproducibility of SNPs identified from the 93 accessions with our optimized integrated GBS assay was clearly evident. Their correspondence with an available SNP database *in silico* along with high experimental validation success rate (92%) through amplicon resequencing and MALDI-TOF mass array SNP genotyping assay based on strong sensitivity (93–96%) and specificity (99%) were also apparent. These findings reflect the major strength and reliability of GBS assay in rapid discovery and high-throughput genotyping of accurate SNPs in chickpea genome with sub-optimal use of resources. Henceforth, this highly versatile integrated GBS approach developed in our study could find multidimensional applicability for genome-wide discovery and genotyping of SNPs in small as well as large genome plant species. The identification of a relatively higher frequency of SNPs showing transition substitutions (57%) than transversions is consistent with previous genome-wide SNP discovery studies in crop plants, including chickpea (Agarwal et al., 2012; Parida et al., 2012; Subbaiyan et al., 2012; Jain et al., 2013, 2014; Varshney et al., 2013a).

The physical mapping of a lesser number of SNPs on eight *desi* chromosomes (6063 SNPs) compared with *kabuli* chromosomes (14,115) could be attributed to the greater (~2.8 times) differences in pseudomolecule assemblies/lengths between *desi* (124.37 Mb) and *kabuli* (347.24 Mb) genomes (Jain et al., 2013; Varshney et al., 2013a). This infers a direct correlation between numbers of SNPs physically mapped on chromosomes and length of *desi* and *kabuli* chromosomes. However, the SNPs mined with GBS assay were represented from different portions of the *desi* and *kabuli* genomes, which could be the possible reason for significant differences in physical map density of SNPs between the two genomes. This in turn is due to uneven pseudomolecule assemblies with significant differences in genome coverage (*kabuli*: 39.4% and *desi*: 14.3%) resulting in sequencing of different regions of genomes between *desi* and *kabuli* chickpea (Ruperao et al., 2014). These clues indicate that large-scale mining and genotyping of SNPs at a genome-wide scale can be essentially enriched in chickpea by use of both *desi* and *kabuli* genomes as references along with comparison of their individual SNP outcomes from GBS assay. This strategy appears to be more relevant considering identification of more valid and accurate common as well as unique SNPs covering different fractions of *desi* and *kabuli* genomes, thereby accelerating the large-scale genome-wide SNP discovery in chickpea. The 20,178 SNPs-based physical maps composed of *desi* and *kabuli* chickpea chromosomes generated in our study can serve as references for a faster selection of genome-wide SNP markers for large-scale genotyping applications, including mapping of genomes and genes/QTLs controlling important agronomic traits and comparative genome mapping involving chickpea and legumes. Additionally, 24,666 high-quality and valid GBS-based SNPs (including 14,875 SNPs annotated on unanchored scaffolds and 9791 *de novo* SNPs) identified from *desi* and *kabuli* genomes could prove useful for various marker-based applications of chickpea genetics, genomics, and breeding.

### Functional significance of GBS-based genome-wide SNPs in chickpea

The identified SNPs were structurally annotated in different coding and non-coding (URR, intron, and DRR) sequence components of protein-coding genes individually predicted from *desi* (9567 SNPs in 4010 genes) and *kabuli* (10,656 SNPs in 4643 genes) genomes. Furthermore, the structural and functional annotation of 12,773 SNPs (submitted to NCBI dbSNP) detected in 5588 non-redundant chickpea genes and 3296 non-synonymous and 269 regulatory SNPs in 2225 genes provided a faster mode of selection of functional gene-based SNPs to establish marker-trait linkages and identify a number of genes/QTLs governing qualitative and quantitative agronomic traits in chickpea. The estimated mean ratio of non-synonymous to synonymous SNPs (Ka/Ks: 0.84) in chickpea genes is comparable with that obtained (Ka/Ks < 1) from orthologous chickpea genes/transcripts (Agarwal et al., 2012; Jhanwar et al., 2012; Jain et al., 2013; Varshney et al., 2013a) and some selected stress-responsive rice genes (Parida et al., 2012). Consequently, this finding revealed the superior efficiency of purifying selection over non-synonymous sequence polymorphism in the scanned chickpea genes (Jhanwar et al., 2012; Parida et al., 2012; Jain et al., 2013). The purifying selection pressure on a larger proportion of SNPs-carrying genes is further evident from their reduced mean Tajima's D estimates (*D* < −2). The identification of non-synonymous and regulatory SNPs in protein-coding genes, (e.g., PHD zinc finger and methylthioribose kinase) showing polymorphism between abiotic (drought and salinity)/biotic (*Fusarium* wilt) stress tolerant/resistant and sensitive/susceptible accessions, have documented the evolutionary/adaptive advantages of these non-synonymous SNP loci (Wang et al., 1998; Parida et al., 2012) and probable functional significance of SNP-carrying genes for stress tolerance in chickpea. The adaptive significance of SNPs in two stress-responsive genes was well-supported by their estimated low mean Ka/Ks (0.39) and reduced Tajima's D (D < −2), and thus enduring more purifying selection compared with other genic SNPs. The differential expression and transcriptional regulation of plant homeodomain (PHD) zinc finger-encoding TF genes have confirmed its involvement in improving abiotic (drought) stress tolerance in plant species (Wei et al., 2009; Ray et al., 2011). The potential role of methylthioribose kinase-encoding genes in regulating abiotic and biotic stress responses through methionine homeostasis, ROS-mediated oxidation and polyamine biosynthesis has also been clearly demonstrated in crop plants, including rice and *Arabidopsis* (Sauter et al., 2004; Burstenbinder et al., 2007; Atkinson et al., 2013; Zagorchev et al., 2013). Henceforth, these informative SNPs identified, particularly in diverse coding and non-coding regulatory sequence components of various genes, once validated with a selected set of contrasting stress tolerant and susceptible accessions in a large-scale, can be utilized as potential markers in genetic and association mapping for identifying major trait-regulatory candidate genes/QTLs in chickpea.

### Broader functional molecular diversity, admixed domestication, and high-resolution complex LD patterns in a structured chickpea population

The intra (49.5% and PIC: 0.38)- and inter (50.5% and 0.40)-specific polymorphic potential detected by 23,798 SNPs among 93 cultivated and wild chickpea accessions was much higher than that estimated previously with SNP markers (Nayak et al., 2010; Gujaria et al., 2011; Roorkiwal et al., 2013; Varshney et al., 2013a). However, the nucleotide diversity measured by these SNPs in 39 *desi* (mean θ_π_: 1.20 and θ_w_: 1.33) and 53 *kabuli* (θ_π_: 0.96 and θ_w_: 1.07) accessions was comparable to that obtained using genome-wide SNP markers (Varshney et al., 2013a). A plenty of genome-wide genic SNP markers showing relatively high intra (*desi*: 42.1% and *kabuli*: 37.6%)–and inter-specific polymorphic potential and wider genetic diversity (13–89%, mean: 56%)/functional molecular diversity (18–77%, mean: 51%) among 93 accessions were identified. They could assist us in selecting desirable diverse accessions/inter-specific hybrids in cross-breeding program (introgression breeding) for chickpea varietal improvement.

The classification of 93 chickpea accessions into three major model-based genetically distinct populations; POP I, POP II, and POP III by wider population differentiation (F_ST_: 0.30–0.62, mean: 0.42) was consistent with their expected pedigree relationships and parentage. A significant deviation from accurate population assignment based on pedigree relations was more pronounced in cultivated *desi* and *kabuli* accessions belonging to POP II. The geographical origin and adaptive environment, rather than parentage, of these accessions had a greater effect on their assignment to a specific population group. Higher (22%) admixed ancestry among accessions reflected their multiple domestication at archeological sites of south eastern Turkey ~10,000 years ago, followed by a complex breeding history involving inter-crossing/introgression coupled with strong adaptive selection pressure. The inclusion of diverse non-recurrent *desi* and *kabuli* germplasm lines as common parental lines in crop improvement breeding program to develop/breed most of the selected chickpea accessions in our study for valuable agronomic traits and higher yield might have influenced their population group assignment and domestication pattern, resulting in numerous admixtures among these accessions (Kujur et al., 2013; Varshney et al., 2013a). A more admixed ancestry between POP III and POP II compared with POP III and POP I is quite relevant because of complex domestication patterns during their evolutionary divergence from wild relatives.

A greater LD estimate (mean *r*^2^: 0.63) in three populations of 93 accessions and furthermore, across eight *desi* (0.56) and *kabuli* (0.52) chromosomes using linked SNP-pairs compared with unlinked SNPs, reflected the direct correlation of LD patterning with the physical linkage of SNPs on chromosomes and the density required to cover genomic regions. The extensive LD estimates and extended chromosomal LD decay (~1000 kb) calculated in three populations of self-pollinated chickpea, were much higher than the cross-pollinated (Riedelsheimer et al., 2012; Sakiroglu et al., 2012; Xiao et al., 2012) and selfing (Mather et al., 2007; Atwell et al., 2010; Lam et al., 2010; Branca et al., 2011; Zhao et al., 2011) crop plants. It could be due to extensive contribution of selective sweeps and four sequential bottlenecks during chickpea domestication (Abbo et al., 2003; Berger et al., 2005; Burger et al., 2008; Toker, 2009; Jain et al., 2013; Kujur et al., 2013; Varshney et al., 2013a; Saxena et al., 2014a), reducing its genetic base in contrast to other domesticated selfing plant species. Shorter LD decay (~400–600 kb) in a more diverse population (POP III) compared with POP I and POP II suggests the effect of factors other than marker density, including genetic diversity, population genetic structure, and demographic history on shaping the LD patterns in chickpea. The genome-wide and population-specific LD patterns generated in our study using 3321 *desi* and 8592 *kabuli* SNP genotyping information provided insight regarding the SNP marker density required for GWAS to identify potential genomic loci (gene-associated targets) regulating important agronomic traits in chickpea. Moreover, our studies regarding the molecular diversity, population genetic structure and LD mapping among chickpea accessions (three population groups) using genome-wide SNPs could essentially identify trait-influencing target genomic regions (candidate genes) across chromosomes/genomes that possibly got influenced by strong selection pressure during domestication.

## Conclusions

We identified 44,844 non-erroneous SNPs in chickpea at a genome-wide scale using reference genome- and *de novo*-based GBS assays. The structurally and functionally annotated SNPs in diverse coding and non-coding sequence components of genes could have potential to be utilized for various large-scale marker-based genotyping applications in chickpea (Supplementary Figure S10). High reproducibility (100%) and experimental validation success rate (92%) along with strong specificity (99%) and sensitivity (93–96%) of SNPs identified from 93 accessions using our optimized integrated GBS assay suggest the strength and reliability of GBS assay in rapid genome-wide discovery and high-throughput genotyping of accurate SNPs in chickpea. A wider natural allelic diversity, admixed domestication pattern and differential genome-wide and population-specific LD estimates/decay assayed by mapped SNPs in a structured population of cultivated and wild accessions further implies their efficacy in genomics-assisted breeding applications of chickpea.

### Conflict of interest statement

The authors declare that the research was conducted in the absence of any commercial or financial relationships that could be construed as a potential conflict of interest.
