# Supplementary material for: Employing genome-wide SNP discovery and genotyping strategy to extrapolate the natural allelic diversity and domestication patterns in chickpea
Source: Front Plant Sci. 2015 Mar 31;6:162. doi: 10.3389/fpls.2015.00162 (PMC4379880; doi:10.3389/fpls.2015.00162)
Supplement: Supplementary file 1 [file Image1.PDF]

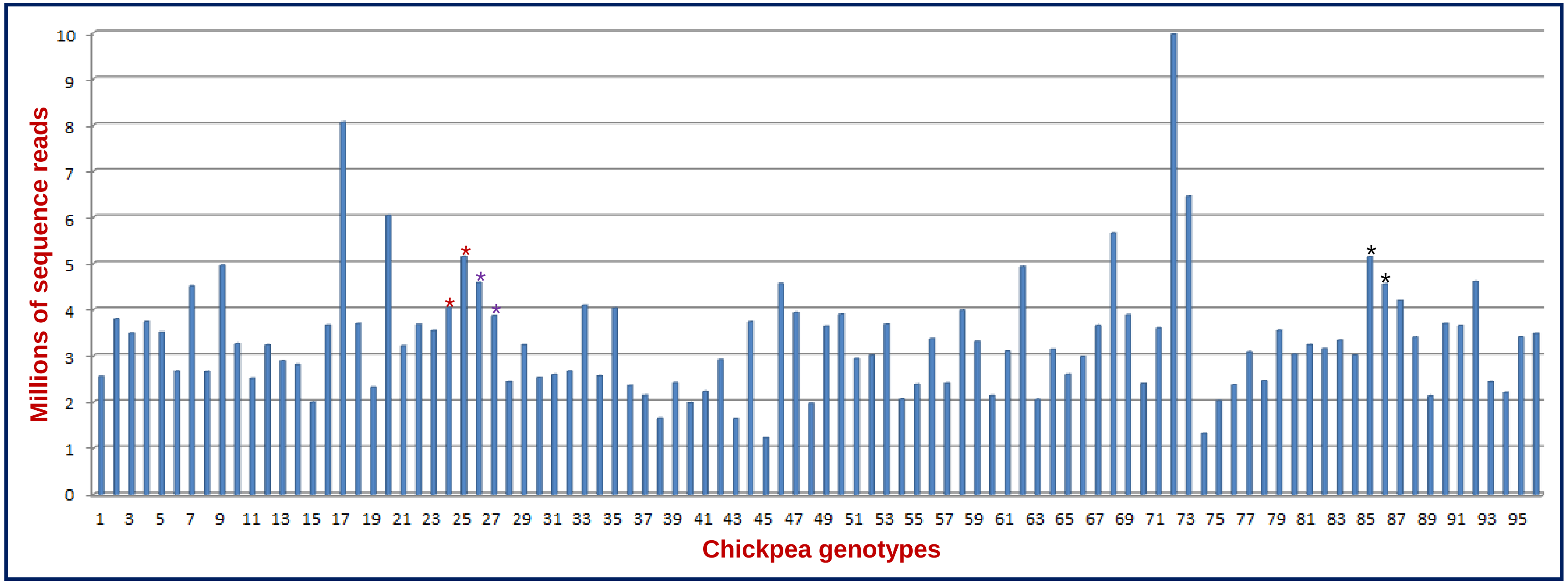

Figure S1: Frequency distribution of million sequence reads generated from each of the 96 chickpea accessions using a 96-plex GBS assay. The three chickpea accessions, *ICCV10 (genotypes number 24 and 25), *ICCX-810800 (26 and 27) and *ICCV95334 (86 and 87) used as biological replicates are indicated.
