# Supplementary material for: Employing genome-wide SNP discovery and genotyping strategy to extrapolate the natural allelic diversity and domestication patterns in chickpea
Source: Front Plant Sci. 2015 Mar 31;6:162. doi: 10.3389/fpls.2015.00162 (PMC4379880; doi:10.3389/fpls.2015.00162)
Supplement: Supplementary file 2 [file Image2.PDF]

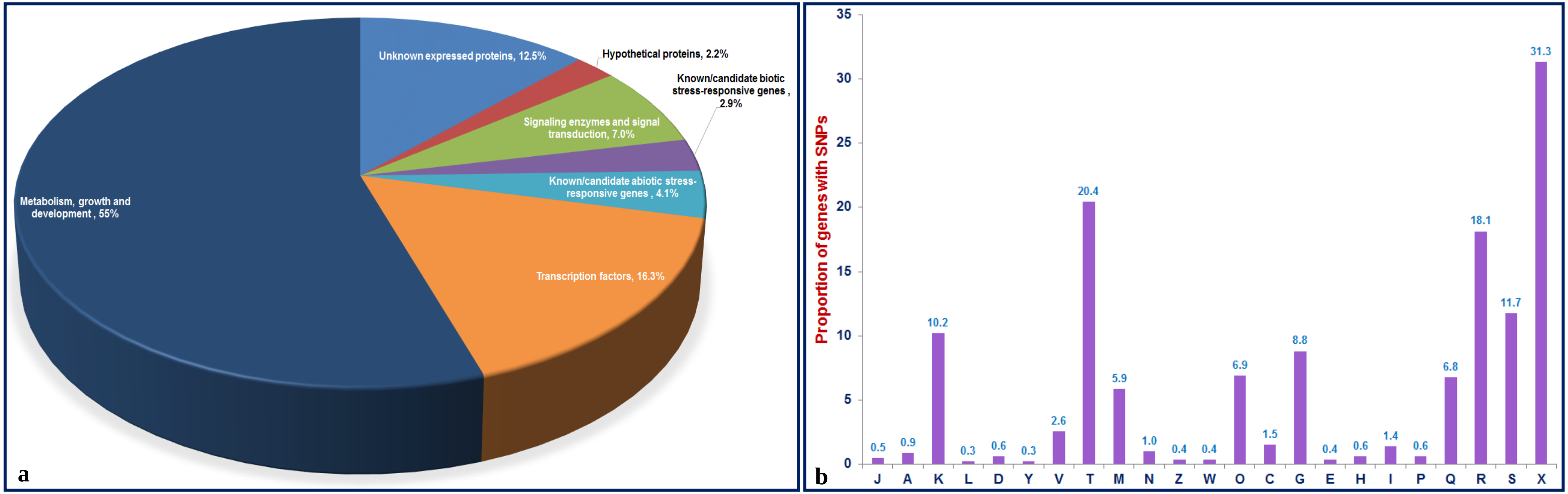

Fig. S2: (a) Functional annotation of SNP-carrying 5588 chickpea genes. (b) KOG-based putative function determination of genes identified with SNPs. The diverse KOG classes are defined as J: Translation, ribosomal structure and biogenesis, A: RNA processing and modification, K: Transcription, L: Replication, recombination and repair, D: Cell cycle control, cell division and chromosome partitioning, Y: Nuclear structure, V: Defence mechanisms, T: Signal transduction mechanisms, M: Cell wall/membrane/envelope biogenesis, N: Cell motility, Z: Cytoskeleton, W: Extracellular structures, O: Post-translational modification, protein turnover and chaperones, C: Energy production and conversion, G: Carbohydrate transport and metabolism, E: Amino acid transport and metabolism, H: Coenzyme transport and metabolism, I: Lipid transport and metabolism, P: Inorganic ion transport and metabolism, Q: Secondary metabolites biosynthesis, transport and catabolism, R: General function prediction only, S: Function unknown and X: Unnamed proteins.
