# Supplementary material for: Employing genome-wide SNP discovery and genotyping strategy to extrapolate the natural allelic diversity and domestication patterns in chickpea
Source: Front Plant Sci. 2015 Mar 31;6:162. doi: 10.3389/fpls.2015.00162 (PMC4379880; doi:10.3389/fpls.2015.00162)
Supplement: Supplementary file 3 [file Image3.PDF]

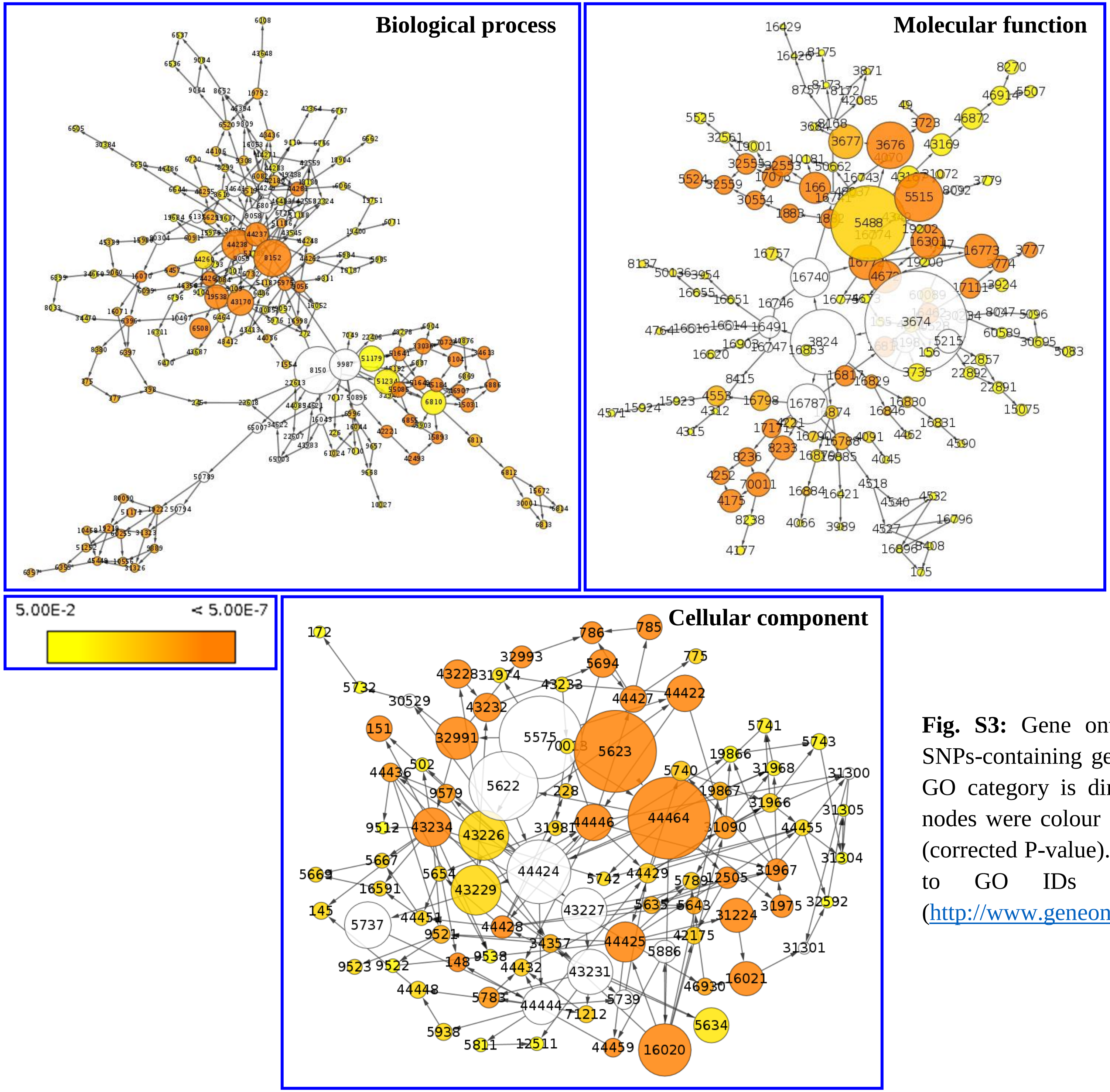


**Fig. S3:** Gene ontology (GO) enrichment analysis of the SNPs-containing genes. The number of genes falling in each GO category is directly proportional to the node size. The nodes were colour shaded according to the significance level (corrected P-value). Numbers denoted in the nodes correspond to GO IDs available in the public database ([http://www.geneontology.org](http://www.geneontology.org)).
