# Supplementary material for: Employing genome-wide SNP discovery and genotyping strategy to extrapolate the natural allelic diversity and domestication patterns in chickpea
Source: Front Plant Sci. 2015 Mar 31;6:162. doi: 10.3389/fpls.2015.00162 (PMC4379880; doi:10.3389/fpls.2015.00162)
Supplement: Supplementary file 4 [file Image4.PDF]

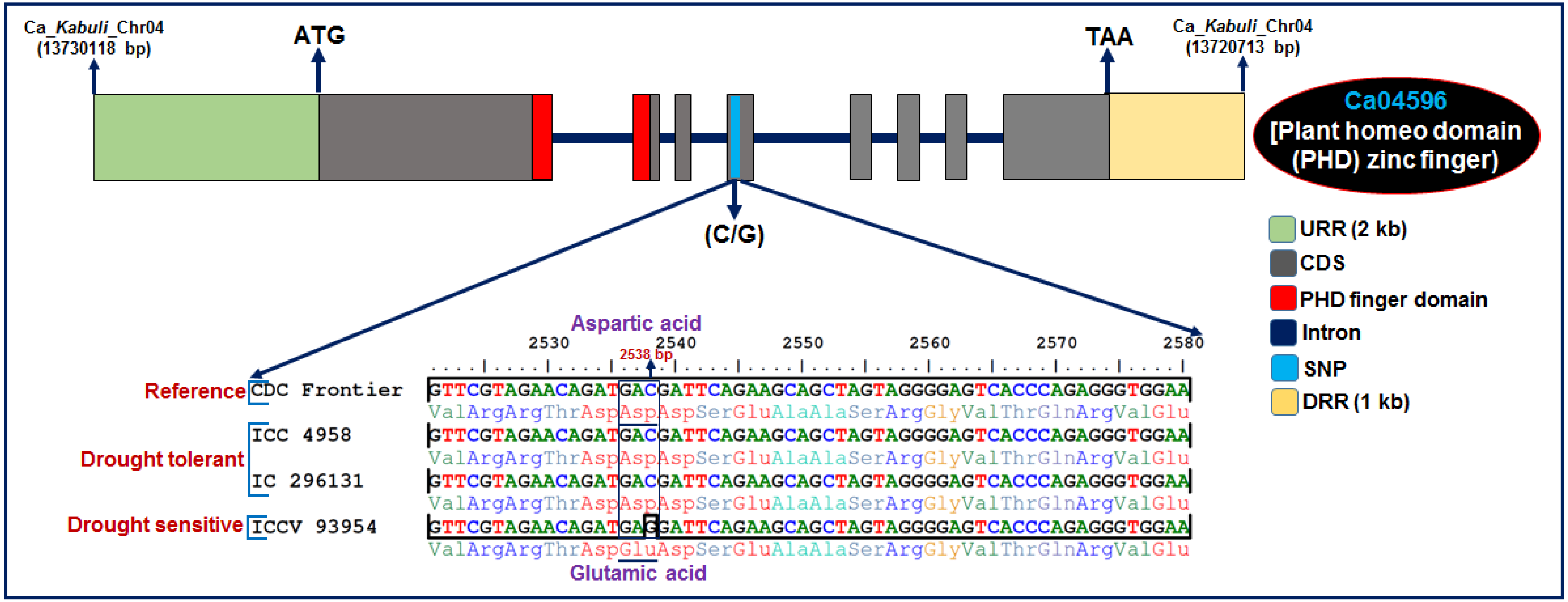

Fig. S4: SNP (C to G) revealing missense non-synonymous amino acid substitution [aspartic acid (GAC) to glutamic acid (GAG)] in the PHD finger functional domain encoding plant homeodomain (PHD) zinc finger of *kabuli* chickpea gene exhibited polymorphism between drought tolerant (ICC 4958 and ICCV 93954) and sensitive (ICCV 93954) chickpea accessions. The *kabuli* accession CDC Frontier was used as reference genome for SNP identification. The missense non-synonymous SNPs are highlighted.
