# Supplementary material for: Employing genome-wide SNP discovery and genotyping strategy to extrapolate the natural allelic diversity and domestication patterns in chickpea
Source: Front Plant Sci. 2015 Mar 31;6:162. doi: 10.3389/fpls.2015.00162 (PMC4379880; doi:10.3389/fpls.2015.00162)
Supplement: Supplementary file 5 [file Image5.PDF]

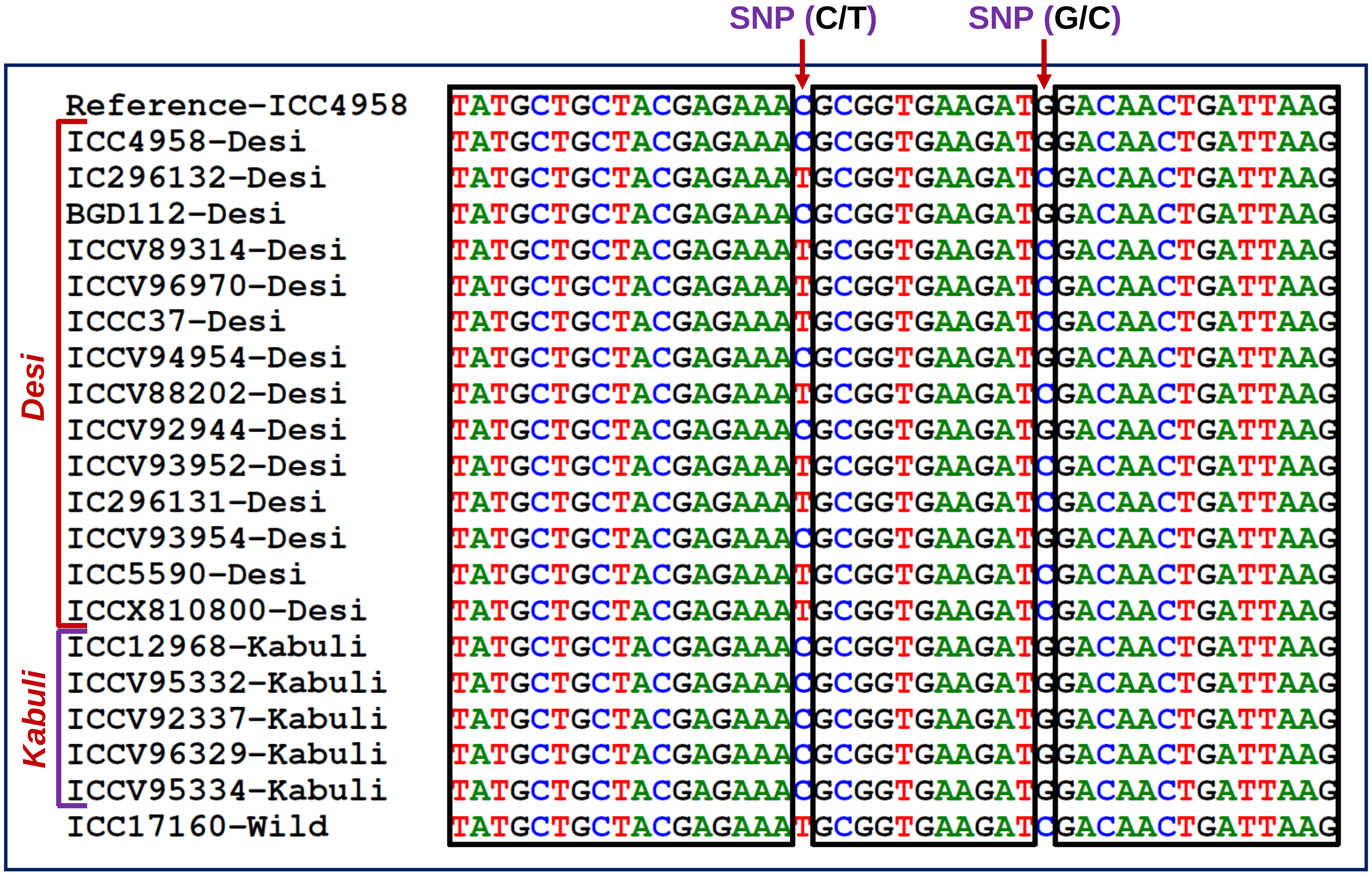

Fig. S5: A representative figure showing the validation of two GBS-based SNP loci (C/T and G/C) by the multiple alignment of amplicon resequences generated from 20 *desi* and *kabuli* chickpea accessions. The two validated SNP sites are highlighted.
