# Supplementary material for: Employing genome-wide SNP discovery and genotyping strategy to extrapolate the natural allelic diversity and domestication patterns in chickpea
Source: Front Plant Sci. 2015 Mar 31;6:162. doi: 10.3389/fpls.2015.00162 (PMC4379880; doi:10.3389/fpls.2015.00162)
Supplement: Supplementary file 6 [file Image6.PDF]

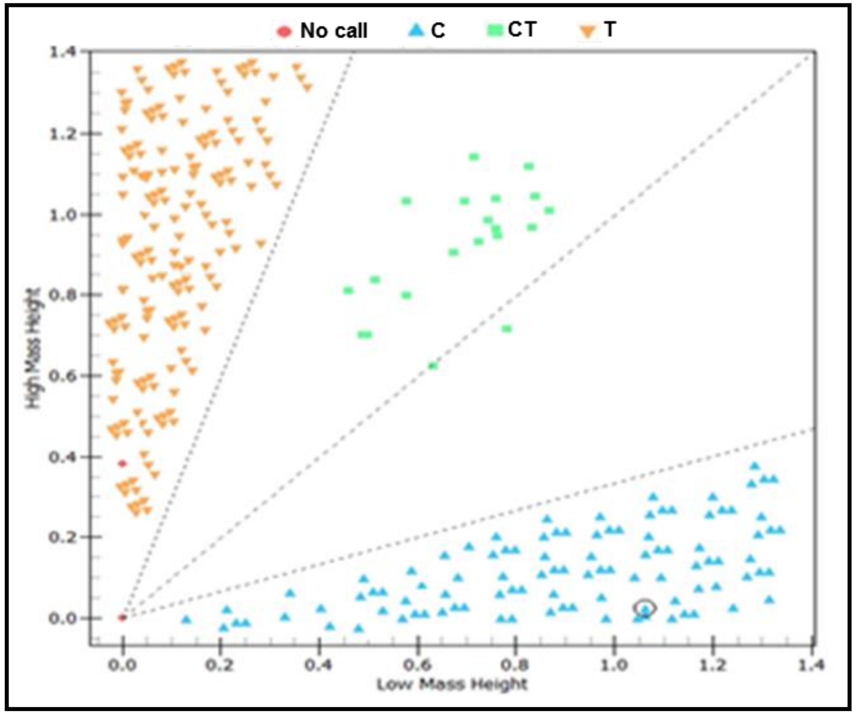

Fig. S6: Call cluster plot for one representative GBS-based SNP locus (C/T) demonstrating its validation and genotyping potential in 93 cultivated *desi*, *kabuli* and wild chickpea accessions assayed using a MALDI-TOF mass array. Distinct differentiation of homozygous and heterozygous SNPs based on the mass differences of corresponding alleles are evident.
