# Supplementary material for: Employing genome-wide SNP discovery and genotyping strategy to extrapolate the natural allelic diversity and domestication patterns in chickpea
Source: Front Plant Sci. 2015 Mar 31;6:162. doi: 10.3389/fpls.2015.00162 (PMC4379880; doi:10.3389/fpls.2015.00162)
Supplement: Supplementary file 7 [file Image7.PDF]

Desi

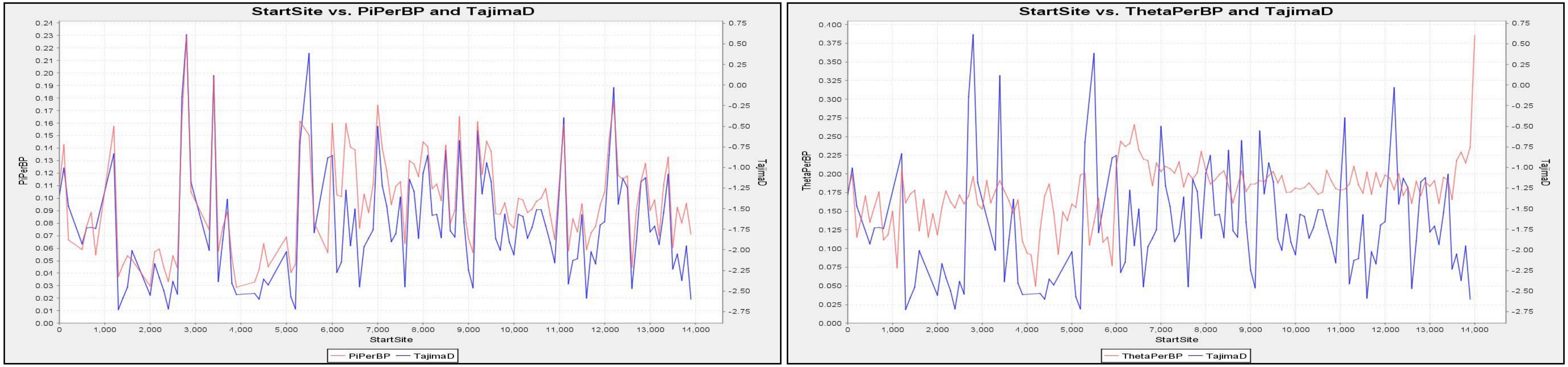

Kabuli

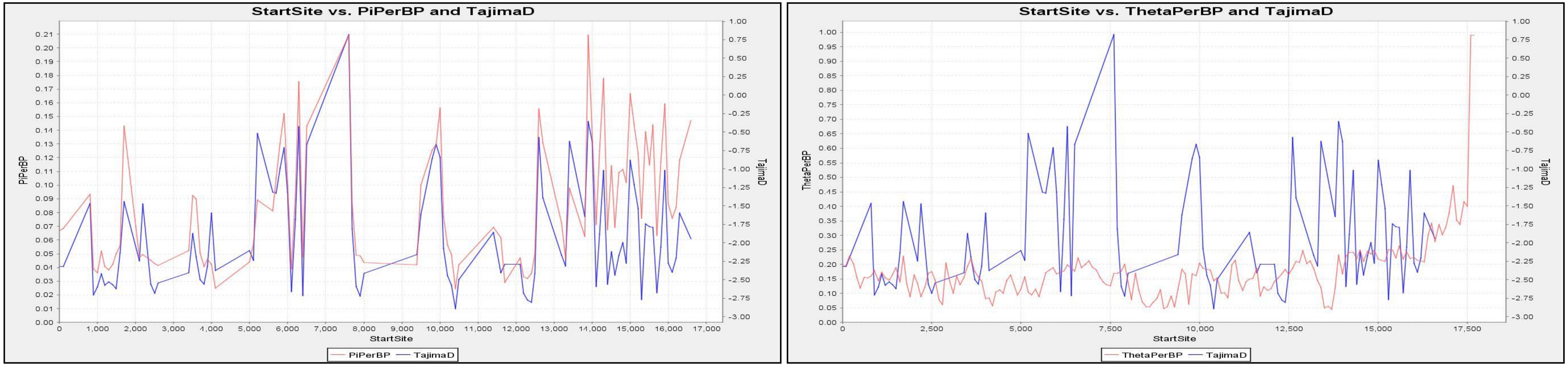

Fig. S7: Plots depicting the estimates of three nucleotide diversity metrics (θπ, θw and Tajima's D) across *desi* and *kabuli* genomes obtained from 93 *desi*, *kabuli* and wild chickpea accessions using 23798 GBS-based SNPs.
