# Supplementary material for: Employing genome-wide SNP discovery and genotyping strategy to extrapolate the natural allelic diversity and domestication patterns in chickpea
Source: Front Plant Sci. 2015 Mar 31;6:162. doi: 10.3389/fpls.2015.00162 (PMC4379880; doi:10.3389/fpls.2015.00162)
Supplement: Supplementary file 8 [file Image8.PDF]

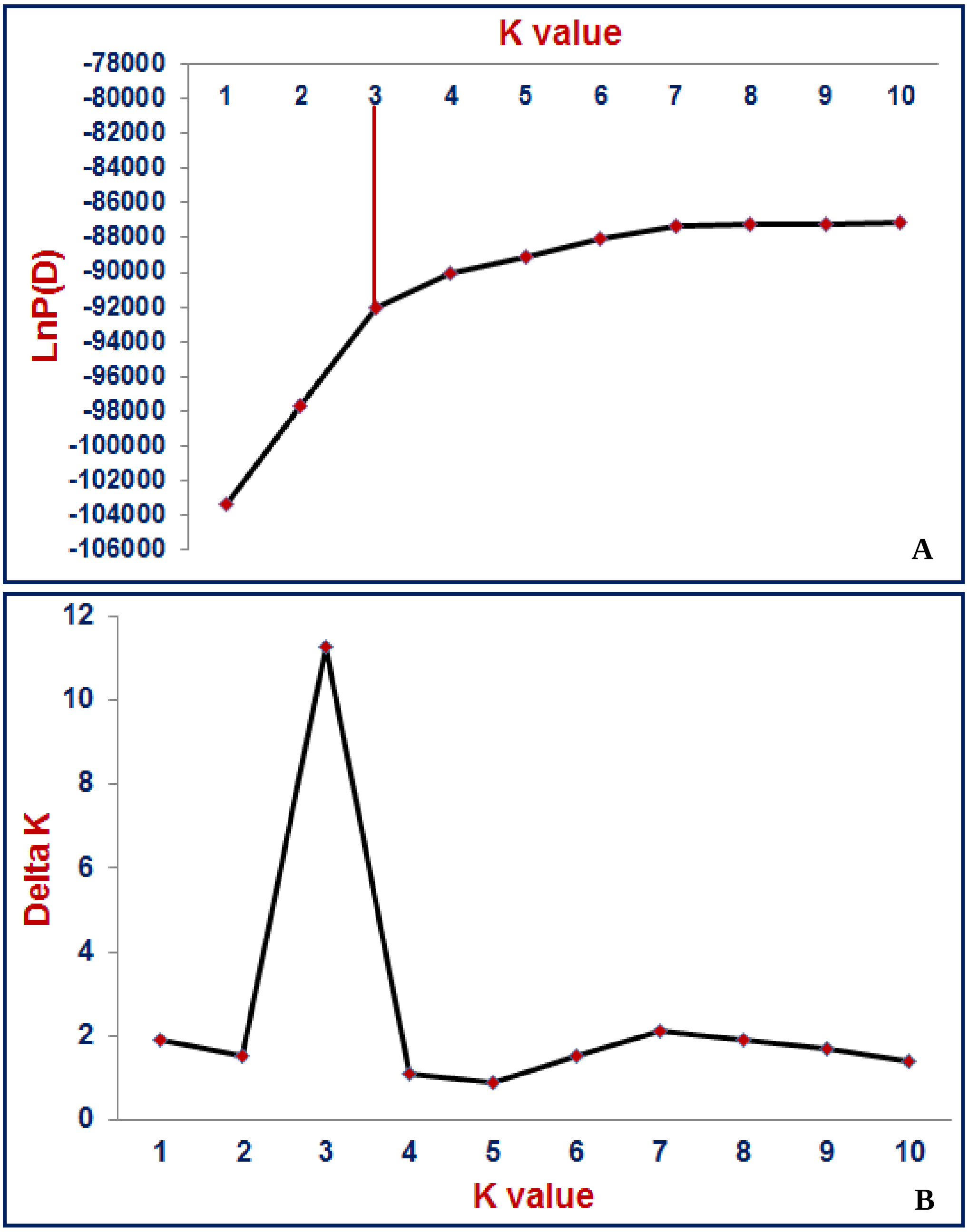

Fig. S8: Optimisation of the number of populations (K value) varying from K= 1 to 10 to determine the best possible population number for 93 chickpea accessions using the *ad hoc* procedure (A) in STRUCTURE (Pritchard et al., 2000) and the second order statistics (*delta K*) (B) of Evanno et al. (2005).
