# Supplementary material for: Employing genome-wide SNP discovery and genotyping strategy to extrapolate the natural allelic diversity and domestication patterns in chickpea
Source: Front Plant Sci. 2015 Mar 31;6:162. doi: 10.3389/fpls.2015.00162 (PMC4379880; doi:10.3389/fpls.2015.00162)
Supplement: Supplementary file 9 [file Image9.PDF]

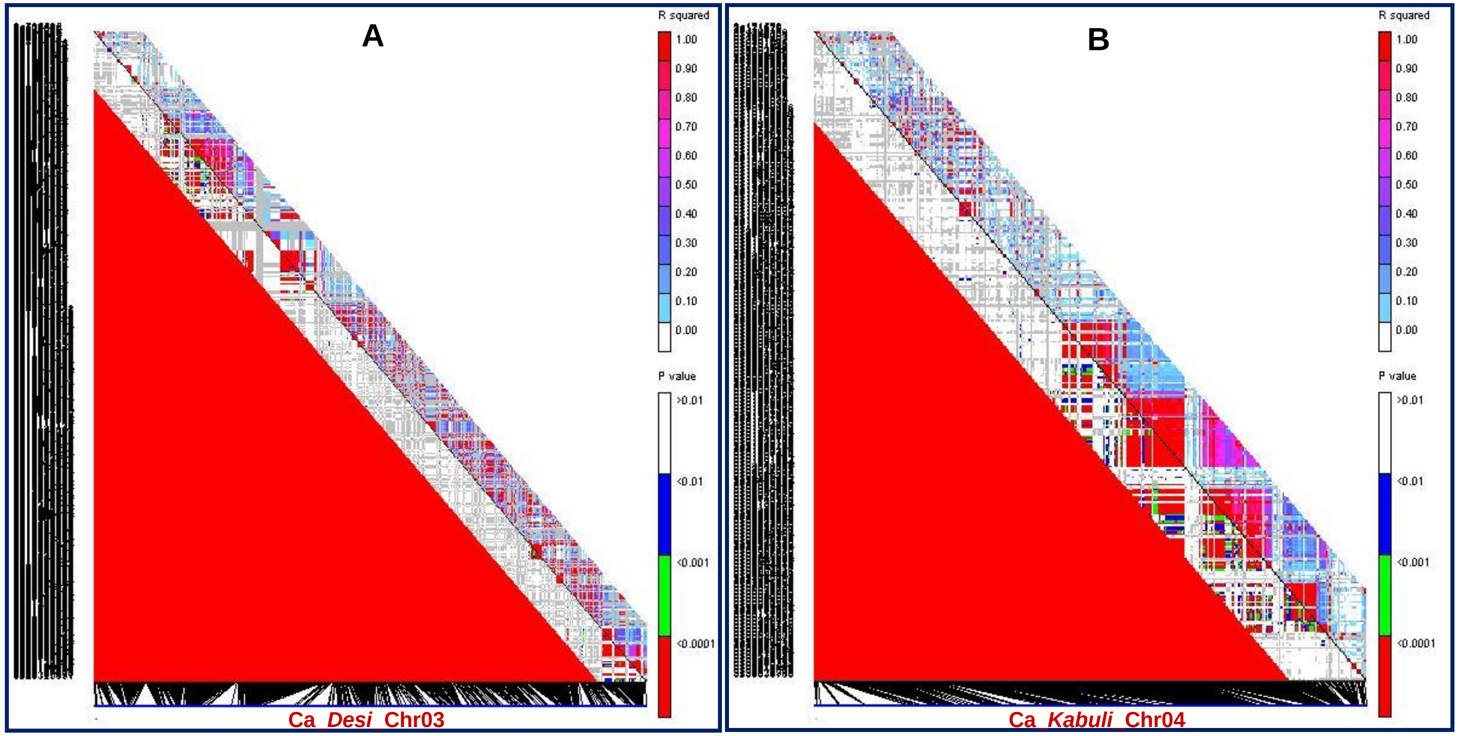

Fig. S9: The LD plots depicting the highest LD estimates (average r^2) and significant LD (%) at P value <0.0001 of linked SNP marker-pairs that are physically mapped on chromosomes 3 (**A**) and 4 (**B**) of *desi* and *kabuli* chickpea genomes, respectively.
