# Supplementary material for: Employing genome-wide SNP discovery and genotyping strategy to extrapolate the natural allelic diversity and domestication patterns in chickpea
Source: Front Plant Sci. 2015 Mar 31;6:162. doi: 10.3389/fpls.2015.00162 (PMC4379880; doi:10.3389/fpls.2015.00162)
Supplement: Supplementary file 10 [file Image10.PDF]

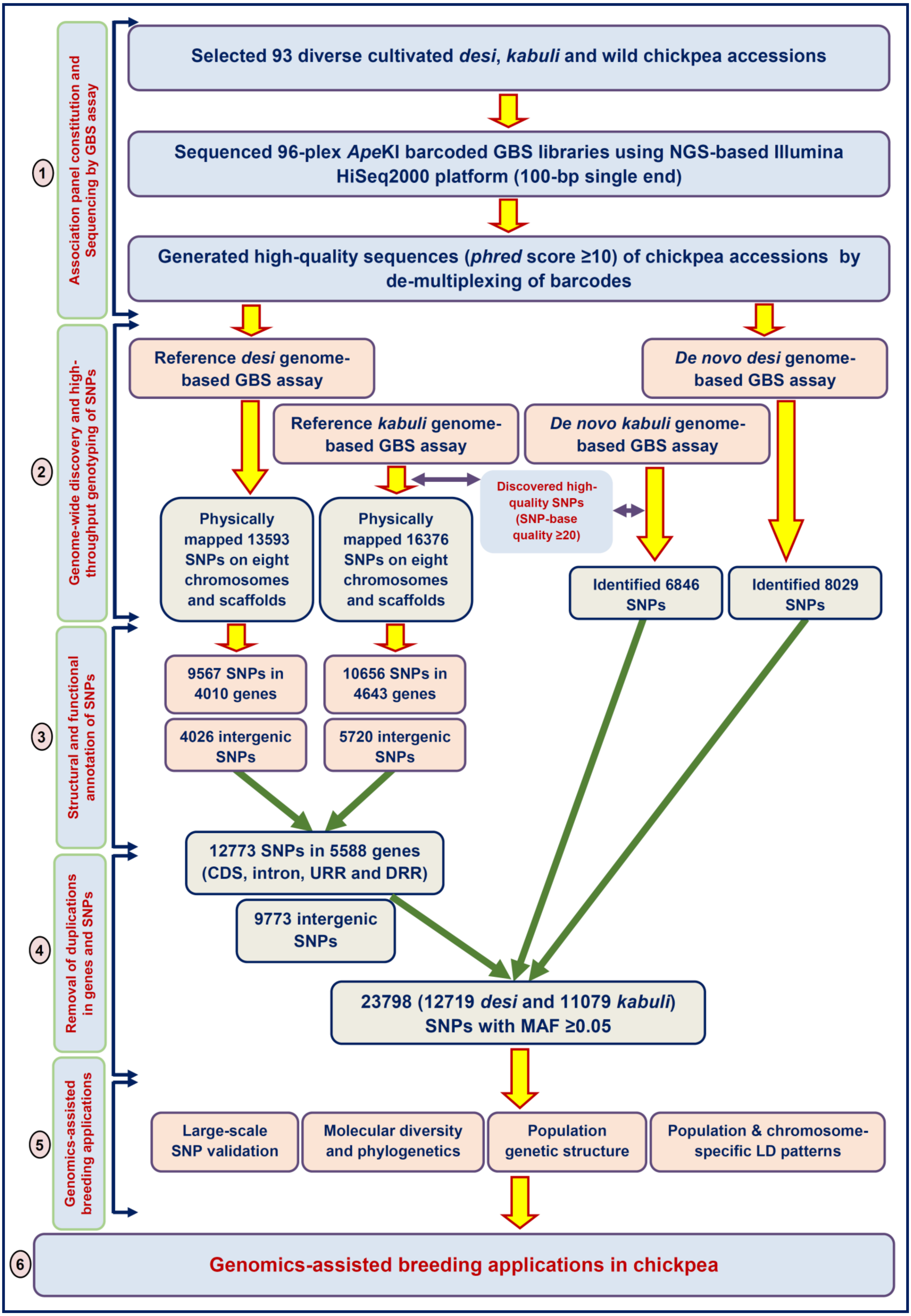

Fig. S10: A combinatorial reference genome- and *de novo*-based GBS approach developed for large-scale validation and high-throughput genotyping of SNPs simultaneously in diverse accessions to expedite multiple genomics-assisted breeding applications in chickpea.
