# Supplementary material for: Employing genome-wide SNP discovery and genotyping strategy to extrapolate the natural allelic diversity and domestication patterns in chickpea
Source: Front Plant Sci. 2015 Mar 31;6:162. doi: 10.3389/fpls.2015.00162 (PMC4379880; doi:10.3389/fpls.2015.00162)
Supplement: Supplementary file 11 [file Table1.PDF]

Table S1: Ninty-three chickpea accessions with diverse seed yield potential selected for discovery and high-throughput genotyping of genome-wide SNPs using NGS-based GBS assay

Sl. No.	Accession Numbers	Cultivar types	Geographical origin	Biological status	Seed yield (g) per plant
1	ICC5590	*Desi*	India	Breeding/Research material	10.3
2	ICC6013	*Desi*	India	Breeding/Research material	10.1
3	ICC5002	*Desi*	India	Breeding/Research material	9.4
4	ICC7184	*Desi*	Turkey	Traditional cultivar/Landrace	12.6
5	ICC4926	*Desi*	India	Breeding/Research material	12.9
6	ICC4657*	*Desi*	India	Traditional cultivar/Landrace	13.5
7	ICC12299	*Desi*	Nepal	Traditional cultivar/Landrace	10.9
8	ICC456	*Desi*	India	Traditional cultivar/Landrace	14.8
9	ICC12726	*Desi*	Ethiopia	Traditional cultivar/Landrace	14.5
10	ICC11944	*Desi*	Nepal	Traditional cultivar/Landrace	12.0
11	ICC11498	*Desi*	India	Traditional cultivar/Landrace	14.0
12	ICC9942	*Desi*	India	Traditional cultivar/Landrace	15.0
13	ICC2072	*Desi*	India	Traditional cultivar/Landrace	15.2
14	ICC9737	*Desi*	Afghanistan	Traditional cultivar/Landrace	9.7
15	ICC9002	*Desi*	Iran	Traditional cultivar/Landrace	13.4
16	ICC16374	*Desi*	Malawi	Breeding material	13.8
17	ICC12028	*Desi*	Mexico	Traditional cultivar/Landrace	12.7
18	ICC8318	*Desi*	India	Traditional cultivar/Landrace	13.1
19	ICC15610	*Desi*	India	Traditional cultivar/Landrace	14.6
20	ICC4918*	*Desi*	India	Advanced cultivar	16.6
21	ICC1836	*Desi*	India	Traditional cultivar/Landrace	15.3
22	ICC15061	*Desi*	India	Traditional cultivar/Landrace	15.8
23	ICC4958	*Desi*	Central India	Traditional cultivar/Landrace/genetic stock	22.3
24	ICCV10*#	*Desi*	India	Released Cultivar	17.6
25	ICCX-810800#	*Desi*	Northern India	Variety	17.1
26	IC296132	*Desi*	Northern India	Variety	23.0
27	IC296131	*Desi*	Northern India	Variety	23.1
28	BGD112	*Desi*	Northern India	Genetic stock	20.3
29	ICC4951*	*Desi*	Central India	Landrace	18.0
30	ICC8933*	*Desi*	Northern India	Genetic stock	22.9
31	ICCV93954	*Desi*	Southern India	Variety	21.5
32	BGD72	*Desi*	Northern India	Genetic stock	17.4
33	IC296133	*Desi*	Northern India	Variety	21.3
34	ICCV92944	*Desi*	Central India	Variety	19.9
35	ICCC37	*Desi*	Southern India	Variety	21.6
36	ICCX-820065	*Desi*	Western India	Variety	22.3
37	Pusa5028	*Desi*	Northern India	Variety	20.5
38	Pusa547	*Desi*	Northern India	Variety	22.0
39	G130	*Desi*	India	Released Cultivar	15.3
40	ICC13523	*Kabuli*	Iran	Traditional cultivar/Landrace	12.0
41	ICC15802	*Kabuli*	Syria	Traditional cultivar/Landrace	13.2
42	ICC13077	*Kabuli*	India	Traditional cultivar/Landrace	10.8
43	ICC15264	*Kabuli*	Iran	Traditional cultivar/Landrace	12.9
44	ICC7308	*Kabuli*	Peru	Traditional cultivar/Landrace	14.4
45	ICC15435	*Kabuli*	Morocco	Traditional cultivar/Landrace	14.4
46	ICC7295	*Kabuli*	Tunisia	Traditional cultivar/Landrace	12.8
47	ICC12328	*Kabuli*	Cyprus	Traditional cultivar/Landrace	17.7
48	ICC14446	*Kabuli*	Italy	Traditional cultivar/Landrace	9.4
49	ICC10755	*Kabuli*	Turkey	Traditional cultivar/Landrace	13.6
50	ICC14190	*Kabuli*	India	Traditional cultivar/Landrace	12.1
51	ICC8042	*Kabuli*	Iran	Traditional cultivar/Landrace	12.9

Sl. No.	Accession Numbers	Cultivar types	Geographical origin	Biological status	Seed yield (g) per plant
52	ICC8261*	*Kabuli*	Turkey	Traditional cultivar/Landrace	13.3
53	ICC15333	*Kabuli*	Iran	Traditional cultivar/Landrace	12.2
54	ICC15512	*Kabuli*	Morocco	Traditional cultivar/Landrace	12.7
55	ICC10884	*Kabuli*	Ethiopia	Traditional cultivar/Landrace	13.3
56	ICC15551	*Kabuli*	Australia	Traditional cultivar/Landrace	11.7
57	ICC15725	*Kabuli*	Syrian Arab Republic	Traditional cultivar/Landrace	12.3
58	ICC6204	*Kabuli*	Spain	Traditional cultivar/Landrace	13.9
59	ICC6210	*Kabuli*	Spain	Traditional cultivar/Landrace	11.9
60	ICC7654	*Kabuli*	Turkey	Traditional cultivar/Landrace	9.5
61	ICC11847	*Kabuli*	Chile	Traditional cultivar/Landrace	11.0
62	ICC11749	*Kabuli*	Chile	Traditional cultivar/Landrace	12.7
63	ICC14199	*Kabuli*	Mexico	Breeding material	15.1
64	ICC15518	*Kabuli*	Morocco	Traditional cultivar/Landrace	14.1
65	ICC16814	*Kabuli*	Portugal	Traditional cultivar/Landrace	14.0
66	ICC16811	*Kabuli*	Portugal	Traditional cultivar/Landrace	10.4
67	ICC6253	*Kabuli*	Morocco	Traditional cultivar/Landrace	13.0
68	ICC11301	*Kabuli*	United States of America	Traditional cultivar/Landrace	15.7
69	ICC10749	*Kabuli*	Turkey	Traditional cultivar/Landrace	13.0
70	ICC14216	*Kabuli*	Mexico	Breeding/Research material	13.3
71	ICC14203	*Kabuli*	Mexico	Traditional cultivar/Landrace	10.7
72	ICC11742	*Kabuli*	Chile	Traditional cultivar/Landrace	10.4
73	ICC13821	*Kabuli*	Ethiopia	Traditional cultivar/Landrace	13.9
74	ICC14462	*Kabuli*	India	Traditional cultivar/Landrace	12.6
75	ICC14220	*Kabuli*	Kenya	Traditional cultivar/Landrace	15.2
76	ICC15944	*Kabuli*	United States of America	Advanced/Improved cultivar	14.0
77	ICC11303	*Kabuli*	Chile	Traditional cultivar/Landrace	12.3
78	ICC12034	*Kabuli*	Mexico	Advanced/Improved cultivar	11.4
79	ICC7346	*Kabuli*	Mexico	Breeding/Research material	16.8
80	ICC15994	*Kabuli*	Spain	Traditional cultivar/Landrace	13.4
81	ICC18591	*Kabuli*	Mexico	Traditional cultivar/Landrace	13.7
82	ICC8155	*Kabuli*	United States of America	Traditional cultivar/Landrace	12.6
83	ICCV92311*	*Kabuli*	India	Released Cultivar/Variety	22.3
84	ICCV95334*#	*Kabuli*	Central India	Variety	20.6
85	PhuleG0515	*Kabuli*	Central India	Variety	18.8
86	ICCV96329	*Kabuli*	Southern India	Variety	21.4
87	IC296376	*Kabuli*	Northern India	Variety	20.1
88	ICC12968*	*Kabuli*	Southern India	Variety	19.2
89	IC449069	*Kabuli*	Northern India	Variety	20.8
90	BGD1105	*Kabuli*	Northern India	Genetic stock	18.1
91	Annigeri	*Kabuli*	India	Released Cultivar	16.1
92	L550	*Kabuli*	India	Released Cultivar	13.6
93	ICC17160*	Wild	Turkey	Wild species	11.8

*Ten chickpea accessions resequenced previously through NGS-based RAD-seq assay [Varshney et al. (2013a)]

#Three chickpea accessions used as biological replicates in GBS assay
