# Supplementary material for: Employing genome-wide SNP discovery and genotyping strategy to extrapolate the natural allelic diversity and domestication patterns in chickpea
Source: Front Plant Sci. 2015 Mar 31;6:162. doi: 10.3389/fpls.2015.00162 (PMC4379880; doi:10.3389/fpls.2015.00162)
Supplement: Supplementary file 12 [file Table2.PDF]

Table S2: Statistics of sequence read generated/mapped and structural annotation of SNPs identified through GBS assay in different sequence components of *desi* and *kabuli* genomes and/or genes

Characteristics	*Desi*	*Kabuli*
Total high-quality sequence reads mapped	168.8 (81.2%)	151 (72.6%)
Total SNPs identified	20439	24405
SNPs identified from reference genomes	13593	16376
SNPs physically mapped on eight chromosomes	6063	14115
SNPs physically mapped on scaffolds	7530	2261
SNPs identified by *de novo* approaches	6846	8029
SNPs in the genes	9567 (4010)*	10656 (4643)
SNPs in the intergenic regions	4026	5720
SNPs in the unique genes	2117 (945)	10656 (4643)
SNPs in the unique intergenic regions	9773	
SNPs in the unique genes of chickpea	12773 (5588)	
SNPs in the exons (CDS)	7212 (4558)	
SNPs in the introns	5292	
SNPs in the URRs	191 (126)	
SNPs in the DRR	78 (51)	
Synonymous SNPs in the CDS	3916 (2510)	
Non-synonymous SNPs in the CDS	3296 (2048)	
Missense non-synonymous SNPs in the CDS	3232 (2025)	
Nonsense non-synonymous SNPs in the CDS	64 (23)	

*value mentioned in the parentheses indicates the number of genes with SNPs
