# Supplementary material for: Employing genome-wide SNP discovery and genotyping strategy to extrapolate the natural allelic diversity and domestication patterns in chickpea
Source: Front Plant Sci. 2015 Mar 31;6:162. doi: 10.3389/fpls.2015.00162 (PMC4379880; doi:10.3389/fpls.2015.00162)
Supplement: Supplementary file 15 [file Table5.PDF]

Table S5: Functional significance of SNPs, including non-synonymous and upstream regulatory SNPs identified through GBS assay

Contrasting Traits	Tolerant/resistant chickpea accessions	Susceptible/sensitive chickpea accessions	Number of SNPs showing polymorphism between tolerant/resistant and susceptible/sensitive accessions	Number of SNPs in chickpea genes showing polymorphism between tolerant/resistant and susceptible/sensitive accessions	Number of non-synonymous SNPs in genes showing polymorphism between tolerant/resistant and susceptible/sensitive accessions	Number of upstream regulatory SNPs in genes showing polymorphism between tolerant/resistant and susceptible/sensitive accessions
**Drought tolerance**[a]	ICC 4958	ICCV 93954	377	180 (104 genes)	21 (20 genes)	12 (12 genes)
	IC 296131	ICCV 93954	994	507 (291 genes)	69 (67 genes)	15 (15 genes)
	ICC 4958 and IC 296131	ICCV 93954	215	123 (73 genes)	13 (12 genes)	12 (12 genes)
**Salt tolerance**[b]	ICC 4951	ICC 12968	448	242 (156 genes)	42 (40 genes)	10 (10 genes)
**Fusarium wilt resistance**[c]	ICC 8933	ICC 4951	214	109 (77 genes)	14 (13 genes)	3 (1 gene)
	ICC 12968	ICC 4951	448	232 (146 genes)	42 (41 genes)	2 (2 genes)
	ICCV 92311	ICC 4951	668	336 (206 genes)	65 (63 genes)	4 (4 genes)
	ICC 8933, ICC 12968 and ICCV 92311	ICC 4951	34	21 (16 genes)	3 (2 genes)	4 (3 genes)

[a]Chickpea accessions with contrasting drought tolerance and sensitive traits reported earlier by Varshney et al. (2013) *Plant Genome* doi:10.3835/plantgenome2013.07.0022 and Varshney et al. (2014) *Theor Appl Genet* 127:445

[b]Chickpea accessions with contrasting salinity tolerance and sensitive traits reported previously by Vadez et al. (2007) *Fields Crop Res* 104:123 and Vadez et al. (2012) *Mol Breed* 30:9

[c]Chickpea accessions with contrasting *Fusarium* wilt resistance and susceptibility traits reported earlier by Haware and Nene (1982) *Plant Disease* 66:809, Gaur et al. (2006) *J SAT Agri Res* 2:1 and Varshney et al. (2014) *Plant Genome* doi:10.3835/plantgenome2013.10.0035
