# Supplementary material for: Employing genome-wide SNP discovery and genotyping strategy to extrapolate the natural allelic diversity and domestication patterns in chickpea
Source: Front Plant Sci. 2015 Mar 31;6:162. doi: 10.3389/fpls.2015.00162 (PMC4379880; doi:10.3389/fpls.2015.00162)
Supplement: Supplementary file 16 [file Table6.PDF]

Table S6: Structural annotation of GBS-based SNPs localized in the genes underlying three known major QTLs reported earlier for stress tolerance in chickpea

**Known QTLs for abiotic and biotic stress tolerance**	**Traits**	**Chromosomes**	**Physical positions (Mb)**	**Number of genes localized at target QTL interval**	**Number of SNPs in the genes**	**Number of coding SNPs**	**Number of intronic SNPs**	**Number of non-synonymous coding SNPs**	**References**
AB-Q-SR-4-1	*Ascochyta* blight	*Ca_Kabuli*_Chr02	31.58-33.97	306	190 (64)	121 (64)	69 (28)	59 (33)	Varshney et al., (2013b, 2014a)
FW_Q_APR_6_1	*Fusarium* wilt	*Ca_Kabuli*_Chr06	29.47-29.57	23	12 (3)	5 (3)	7 (2)	1 (1)	Varshney et al., (2014b)
QTL hotspot	Drought tolerance (root traits)	*Ca_Kabuli*_Chr04	10.39-10.42	654	303 (140)	195 (98)	108 (60)	78 (53)	Sabbavarapu et al., (2013)

*value mentioned in the parentheses indicates the number of genes with SNPs
