# Supplementary material for: Employing genome-wide SNP discovery and genotyping strategy to extrapolate the natural allelic diversity and domestication patterns in chickpea
Source: Front Plant Sci. 2015 Mar 31;6:162. doi: 10.3389/fpls.2015.00162 (PMC4379880; doi:10.3389/fpls.2015.00162)
Supplement: Supplementary file 17 [file Table7.PDF]

Table S7: Validation success rate of genome-wide GBS-based SNPs with previously discovered resequencing-based SNPs among four chickpea accessions

**Genotypes**	**SNPs identified through whole genome resequencing***	**SNPs discovered through GBS assay**	**Number (%) of common SNP loci identified based on congruent physical position (bp) on ICC 4958**
ICC 4958 vs PI 489777	10490	6244	593 (9.5)
ICC 4958 vs ICC 12968	10047	1296	98 (7.6)
ICC 4958 vs ICC 4951	10482	1236	86 (7.0)
**In all three genotype combinations**	**31019**	**8776**	**777 (8.8)**

*Whole genome resequencing-based SNPs identified among four chickpea accessions were obtained from Jain et al. (2013)
