# Supplementary material for: Employing genome-wide SNP discovery and genotyping strategy to extrapolate the natural allelic diversity and domestication patterns in chickpea
Source: Front Plant Sci. 2015 Mar 31;6:162. doi: 10.3389/fpls.2015.00162 (PMC4379880; doi:10.3389/fpls.2015.00162)
Supplement: Supplementary file 18 [file Table8.PDF]

Table S8: Experimental validation of 454 reference genome (*desi* and *kabuli*) and *de novo*-based GBS-SNPs through amplicon resequencing and MALDI-TOF mass array SNP genotyping assay

S.No.	SNP IDs	Chickpea cultivars	Chromosomes	Physical positions (bp)	SNPs	Gene accession IDs	Putative functions	Sequence components of genome
70	CakSNP70	*Kabuli*	Ca_Kabuli_Chr01	644318	[C/T]	Ca_00075	Protein of unknown function DUF639	NON_SYNONYMOUS_CODING
96	CakSNP96	*Kabuli*	Ca_Kabuli_Chr01	750693	[C/T]	Ca_00092	Tubby, C-terminal	NON_SYNONYMOUS_CODING
209	CakSNP209	*Kabuli*	Ca_Kabuli_Chr01	1783923	[C/A]	Ca_00224	Zinc finger, RING-type	NON_SYNONYMOUS_CODING
426	CakSNP426	*Kabuli*	Ca_Kabuli_Chr01	2832939	[G/T]	Ca_00347	Transcriptional factor B3	NON_SYNONYMOUS_CODING
542	CakSNP542	*Kabuli*	Ca_Kabuli_Chr01	4302669	[G/C]	Ca_00512	Zinc finger, CCCH-type	NON_SYNONYMOUS_CODING
555	CakSNP555	*Kabuli*	Ca_Kabuli_Chr01	4429044	[C/T]	Ca_00530	DNA-directed RNA polymerase, subunit 2, domain 6	NON_SYNONYMOUS_CODING
593	CakSNP593	*Kabuli*	Ca_Kabuli_Chr01	4715563	[T/C]	Ca_00555	Zinc finger, RING-type	NON_SYNONYMOUS_CODING
594	CakSNP594	*Kabuli*	Ca_Kabuli_Chr01	4780330	[C/T]	Ca_00562	Zinc finger, RING-type	NON_SYNONYMOUS_CODING
595	CakSNP595	*Kabuli*	Ca_Kabuli_Chr01	4780345	[G/T]	Ca_00562	Zinc finger, RING-type	NON_SYNONYMOUS_CODING
663	CakSNP663	*Kabuli*	Ca_Kabuli_Chr01	5249839	[G/A]	Ca_00613	Protein of unknown function DUF584	NON_SYNONYMOUS_CODING
685	CakSNP685	*Kabuli*	Ca_Kabuli_Chr01	5666217	[C/A]	Ca_00656	DNA-binding WRKY	NON_SYNONYMOUS_CODING
687	CakSNP687	*Kabuli*	Ca_Kabuli_Chr01	5666266	[T/G]	Ca_00656	DNA-binding WRKY	NON_SYNONYMOUS_CODING
689	CakSNP689	*Kabuli*	Ca_Kabuli_Chr01	5666355	[T/G]	Ca_00656	DNA-binding WRKY	NON_SYNONYMOUS_CODING
835	CakSNP835	*Kabuli*	Ca_Kabuli_Chr01	7325164	[T/A]	Ca_07989	Homeobox	NON_SYNONYMOUS_CODING
1070	CakSNP1070	*Kabuli*	Ca_Kabuli_Chr01	10649758	[C/T]	Ca_02701	Protein of unknown function DUF3755	NON_SYNONYMOUS_CODING
1122	CakSNP1122	*Kabuli*	Ca_Kabuli_Chr01	11285291	[C/A]	Ca_02625	SANT domain, DNA binding	NON_SYNONYMOUS_CODING
1133	CakSNP1133	*Kabuli*	Ca_Kabuli_Chr01	11342388	[T/C]	Ca_02619	Protein kinase, catalytic domain	NON_SYNONYMOUS_CODING
1184	CakSNP1184	*Kabuli*	Ca_Kabuli_Chr01	12326318	[C/G]	Ca_02517	AUX/IAA protein	NON_SYNONYMOUS_CODING
1225	CakSNP1225	*Kabuli*	Ca_Kabuli_Chr01	12794147	[G/T]	Ca_02472	Basic-leucine zipper (bZIP) transcription factor	NON_SYNONYMOUS_CODING
1365	CakSNP1365	*Kabuli*	Ca_Kabuli_Chr01	14393313	[C/A]	Ca_07095	Helix-loop-helix DNA-binding domain	NON_SYNONYMOUS_CODING
1476	CakSNP1476	*Kabuli*	Ca_Kabuli_Chr01	17314911	[A/G]	Ca_22780	WD40 repeat	NON_SYNONYMOUS_CODING
1794	CakSNP1794	*Kabuli*	Ca_Kabuli_Chr01	34772813	[T/A]	Ca_21416	SANT domain, DNA binding	NON_SYNONYMOUS_CODING
2172	CakSNP2172	*Kabuli*	Ca_Kabuli_Chr02	1828406	[G/A]	Ca_12602	Protein of unknown function DUF1421	NON_SYNONYMOUS_CODING
2232	CakSNP2232	*Kabuli*	Ca_Kabuli_Chr02	3366023	[C/T]	Ca_10517	ZF-HD homeobox protein, Cys/His-rich dimerisation domain	NON_SYNONYMOUS_CODING
2233	CakSNP2233	*Kabuli*	Ca_Kabuli_Chr02	3410375	[C/T]	Ca_10515	ZF-HD homeobox protein, Cys/His-rich dimerisation domain	NON_SYNONYMOUS_CODING
2251	CakSNP2251	*Kabuli*	Ca_Kabuli_Chr02	3915549	[A/G]	Ca_10464	Zinc finger, PHD-type	NON_SYNONYMOUS_CODING
2492	CakSNP2492	*Kabuli*	Ca_Kabuli_Chr02	10705982	[C/T]	Ca_17569	Zinc finger, C2H2-type	NON_SYNONYMOUS_CODING
2493	CakSNP2493	*Kabuli*	Ca_Kabuli_Chr02	10760939	[G/A]	Ca_17564	Zinc finger, C2H2-type	NON_SYNONYMOUS_CODING
2515	CakSNP2515	*Kabuli*	Ca_Kabuli_Chr02	13079417	[T/G]	Ca_11686	Protein of unknown function DUF3110	NON_SYNONYMOUS_CODING
2710	CakSNP2710	*Kabuli*	Ca_Kabuli_Chr02	24911154	[G/C]	Ca_24902	Zinc finger, B-box	NON_SYNONYMOUS_CODING
2724	CakSNP2724	*Kabuli*	Ca_Kabuli_Chr02	25507637	[C/G]	Ca_14280	Zinc finger, CCCH-type	NON_SYNONYMOUS_CODING
2725	CakSNP2725	*Kabuli*	Ca_Kabuli_Chr02	25507681	[A/G]	Ca_14280	Zinc finger, CCCH-type	NON_SYNONYMOUS_CODING
2727	CakSNP2727	*Kabuli*	Ca_Kabuli_Chr02	25508679	[A/G]	Ca_14280	Zinc finger, CCCH-type	NON_SYNONYMOUS_CODING
2728	CakSNP2728	*Kabuli*	Ca_Kabuli_Chr02	25508660	[G/C]	Ca_14280	Zinc finger, CCCH-type	NON_SYNONYMOUS_CODING
2729	CakSNP2729	*Kabuli*	Ca_Kabuli_Chr02	25508920	[G/A]	Ca_14280	Zinc finger, CCCH-type	NON_SYNONYMOUS_CODING
2731	CakSNP2731	*Kabuli*	Ca_Kabuli_Chr02	25508964	[A/C]	Ca_14280	Zinc finger, CCCH-type	NON_SYNONYMOUS_CODING
2733	CakSNP2733	*Kabuli*	Ca_Kabuli_Chr02	25508935	[A/T]	Ca_14280	Zinc finger, CCCH-type	NON_SYNONYMOUS_CODING

S.No.	SNP IDs	Chickpea cultivars	Chromosomes	Physical positions (bp)	SNPs	Gene accession IDs	Putative functions	Sequence components of genome
2734	CakSNP2734	*Kabuli*	Ca_Kabuli_Chr02	25509087	[C/A]	Ca_14280	Zinc finger, CCCH-type	NON_SYNONYMOUS_CODING
2735	CakSNP2735	*Kabuli*	Ca_Kabuli_Chr02	25509093	[C/G]	Ca_14280	Zinc finger, CCCH-type	NON_SYNONYMOUS_CODING
2738	CakSNP2738	*Kabuli*	Ca_Kabuli_Chr02	25509202	[T/A]	Ca_14280	Zinc finger, CCCH-type	NON_SYNONYMOUS_CODING
2911	CakSNP2911	*Kabuli*	Ca_Kabuli_Chr02	30702121	[T/C]	Ca_12487	Zinc finger, CCCH-type	NON_SYNONYMOUS_CODING
2962	CakSNP2962	*Kabuli*	Ca_Kabuli_Chr02	31205531	[C/T]	Ca_12443	Protein of unknown function DUF827, plant	NON_SYNONYMOUS_CODING
2963	CakSNP2963	*Kabuli*	Ca_Kabuli_Chr02	31427861	[A/G]	Ca_12419	Protein of unknown function DUF827, plant	NON_SYNONYMOUS_CODING
2964	CakSNP2964	*Kabuli*	Ca_Kabuli_Chr02	31427902	[G/C]	Ca_12419	Protein of unknown function DUF827, plant	NON_SYNONYMOUS_CODING
2965	CakSNP2965	*Kabuli*	Ca_Kabuli_Chr02	31427915	[A/G]	Ca_12419	Protein of unknown function DUF827, plant	NON_SYNONYMOUS_CODING
2966	CakSNP2966	*Kabuli*	Ca_Kabuli_Chr02	31428024	[T/G]	Ca_12419	Protein of unknown function DUF827, plant	NON_SYNONYMOUS_CODING
3043	CakSNP3043	*Kabuli*	Ca_Kabuli_Chr02	32203036	[A/T]	Ca_10295	Protein kinase, catalytic domain	NON_SYNONYMOUS_CODING
3057	CakSNP3057	*Kabuli*	Ca_Kabuli_Chr02	32545351	[G/A]	Ca_10268	Protein kinase, catalytic domain	NON_SYNONYMOUS_CODING
3071	CakSNP3071	*Kabuli*	Ca_Kabuli_Chr02	32608900	[A/T]	Ca_10261	Aldehyde oxidase/xanthine dehydrogenase, a/b hammerhead	NON_SYNONYMOUS_CODING
3074	CakSNP3074	*Kabuli*	Ca_Kabuli_Chr02	32611601	[G/T]	Ca_10261	Aldehyde oxidase/xanthine dehydrogenase, a/b hammerhead	NON_SYNONYMOUS_CODING
3076	CakSNP3076	*Kabuli*	Ca_Kabuli_Chr02	32629763	[A/G]	Ca_10259	BTB/POZ-like	NON_SYNONYMOUS_CODING
3079	CakSNP3079	*Kabuli*	Ca_Kabuli_Chr02	32670181	[T/G]	Ca_10254	BTB/POZ-like	NON_SYNONYMOUS_CODING
3082	CakSNP3082	*Kabuli*	Ca_Kabuli_Chr02	32844754	[T/G]	Ca_10238	Late embryogenesis abundant protein, group 2	NON_SYNONYMOUS_CODING
3083	CakSNP3083	*Kabuli*	Ca_Kabuli_Chr02	32844842	[C/G]	Ca_10238	Ethylene insensitive 3	NON_SYNONYMOUS_CODING
3087	CakSNP3087	*Kabuli*	Ca_Kabuli_Chr02	32936855	[A/G]	Ca_10230	Protein of unknown function DM15	NON_SYNONYMOUS_CODING
3088	CakSNP3088	*Kabuli*	Ca_Kabuli_Chr02	32936908	[G/A]	Ca_10230	Basic-leucine zipper (bZIP) transcription factor	NON_SYNONYMOUS_CODING
3101	CakSNP3101	*Kabuli*	Ca_Kabuli_Chr02	33103466	[G/A]	Ca_10211	Ubiquitin-associated/translation elongation factor EF1B	NON_SYNONYMOUS_CODING
3126	CakSNP3126	*Kabuli*	Ca_Kabuli_Chr02	33399010	[C/T]	Ca_10186	Disease resistance protein	NON_SYNONYMOUS_CODING
3127	CakSNP3127	*Kabuli*	Ca_Kabuli_Chr02	33399019	[T/C]	Ca_10186	Disease resistance protein	NON_SYNONYMOUS_CODING
3129	CakSNP3129	*Kabuli*	Ca_Kabuli_Chr02	33399098	[G/A]	Ca_10186	Disease resistance protein	NON_SYNONYMOUS_CODING
3132	CakSNP3132	*Kabuli*	Ca_Kabuli_Chr02	33399404	[C/A]	Ca_10186	Disease resistance protein	NON_SYNONYMOUS_CODING
3133	CakSNP3133	*Kabuli*	Ca_Kabuli_Chr02	33399397	[C/T]	Ca_10186	Disease resistance protein	NON_SYNONYMOUS_CODING
3134	CakSNP3134	*Kabuli*	Ca_Kabuli_Chr02	33399393	[C/A]	Ca_10186	Disease resistance protein	NON_SYNONYMOUS_CODING
3136	CakSNP3136	*Kabuli*	Ca_Kabuli_Chr02	33399342	[A/C]	Ca_10186	Disease resistance protein	NON_SYNONYMOUS_CODING
3137	CakSNP3137	*Kabuli*	Ca_Kabuli_Chr02	33399465	[C/G]	Ca_10186	Disease resistance protein	NON_SYNONYMOUS_CODING
3138	CakSNP3138	*Kabuli*	Ca_Kabuli_Chr02	33399608	[C/A]	Ca_10186	Disease resistance protein	NON_SYNONYMOUS_CODING
3155	CakSNP3155	*Kabuli*	Ca_Kabuli_Chr02	33745281	[A/G]	Ca_10163	ATPase, BadF/BadG/BcrA/BcrD type	NON_SYNONYMOUS_CODING
3159	CakSNP3159	*Kabuli*	Ca_Kabuli_Chr02	33786243	[G/A]	Ca_10157	Amine oxidase	NON_SYNONYMOUS_CODING
3175	CakSNP3175	*Kabuli*	Ca_Kabuli_Chr02	34250266	[T/C]	Ca_15249	Protein kinase, catalytic domain	NON_SYNONYMOUS_CODING
3176	CakSNP3176	*Kabuli*	Ca_Kabuli_Chr02	34266109	[A/G]	Ca_15250	Potassium channel, two pore-domain	NON_SYNONYMOUS_CODING
3180	CakSNP3180	*Kabuli*	Ca_Kabuli_Chr02	34299074	[A/C]	Ca_15255	Domain of unknown function DUF296	NON_SYNONYMOUS_CODING
3182	CakSNP3182	*Kabuli*	Ca_Kabuli_Chr02	34299079	[A/C]	Ca_15255	Domain of unknown function DUF296	NON_SYNONYMOUS_CODING
3201	CakSNP3201	*Kabuli*	Ca_Kabuli_Chr02	34734329	[G/A]	Ca_16897	Heat shock protein Hsp70	NON_SYNONYMOUS_CODING
3287	CakSNP3287	*Kabuli*	Ca_Kabuli_Chr02	35612895	[T/C]	Ca_09731	Protein of unknown function DUF827, plant	NON_SYNONYMOUS_CODING
3288	CakSNP3288	*Kabuli*	Ca_Kabuli_Chr02	35612900	[C/T]	Ca_09731	Protein of unknown function DUF827, plant	NON_SYNONYMOUS_CODING
3293	CakSNP3293	*Kabuli*	Ca_Kabuli_Chr02	35661053	[C/T]	Ca_09735	Protein of unknown function DUF1296	NON_SYNONYMOUS_CODING

S.No.	SNP IDs	Chickpea cultivars	Chromosomes	Physical positions (bp)	SNPs	Gene accession IDs	Putative functions	Sequence components of genome
3354	CakSNP3354	*Kabuli*	Ca_Kabuli_Chr02	36088623	[C/T]	Ca_09786	Transcription factor GRAS	NON_SYNONYMOUS_CODING
3707	CakSNP3707	*Kabuli*	Ca_Kabuli_Chr03	13420758	[A/G]	Ca_22696	Zinc finger, C2H2-type	NON_SYNONYMOUS_CODING
3708	CakSNP3708	*Kabuli*	Ca_Kabuli_Chr03	13420756	[A/G]	Ca_22696	Zinc finger, C2H2-type	NON_SYNONYMOUS_CODING
3776	CakSNP3776	*Kabuli*	Ca_Kabuli_Chr03	18215332	[T/C]	Ca_19612	Protein of unknown function DUF3411	NON_SYNONYMOUS_CODING
3835	CakSNP3835	*Kabuli*	Ca_Kabuli_Chr03	20000967	[C/G]	Ca_09402	Protein of unknown function DUF688	NON_SYNONYMOUS_CODING
3883	CakSNP3883	*Kabuli*	Ca_Kabuli_Chr03	21020182	[A/G]	Ca_09470	Protein of unknown function DUF827, plant	NON_SYNONYMOUS_CODING
4022	CakSNP4022	*Kabuli*	Ca_Kabuli_Chr03	23273069	[C/G]	Ca_06189	Zinc finger, RING-type	NON_SYNONYMOUS_CODING
4197	CakSNP4197	*Kabuli*	Ca_Kabuli_Chr03	27632100	[G/A]	Ca_08144	Myb, DNA-binding	NON_SYNONYMOUS_CODING
4200	CakSNP4200	*Kabuli*	Ca_Kabuli_Chr03	27673727	[C/A]	Ca_08139	Protein of unknown function DUF309	NON_SYNONYMOUS_CODING
4218	CakSNP4218	*Kabuli*	Ca_Kabuli_Chr03	27968979	[T/G]	Ca_08113	Zinc finger, C2H2-type	NON_SYNONYMOUS_CODING
4219	CakSNP4219	*Kabuli*	Ca_Kabuli_Chr03	27969014	[T/C]	Ca_08113	Zinc finger, C2H2-type	NON_SYNONYMOUS_CODING
4220	CakSNP4220	*Kabuli*	Ca_Kabuli_Chr03	27969005	[A/G]	Ca_08113	Zinc finger, C2H2-type	NON_SYNONYMOUS_CODING
4407	CakSNP4407	*Kabuli*	Ca_Kabuli_Chr03	30794046	[A/C]	Ca_07400	Domain of unknown function DUF828	NON_SYNONYMOUS_CODING
4410	CakSNP4410	*Kabuli*	Ca_Kabuli_Chr03	30837265	[T/C]	Ca_12181	Domain of unknown function DUF828	NON_SYNONYMOUS_CODING
4596	CakSNP4596	*Kabuli*	Ca_Kabuli_Chr03	34547141	[A/G]	Ca_00726	Transcription factor, MADS-box	NON_SYNONYMOUS_CODING
4597	CakSNP4597	*Kabuli*	Ca_Kabuli_Chr03	34576769	[G/A]	Ca_00729	Zinc finger, PHD-type	NON_SYNONYMOUS_CODING
4609	CakSNP4609	*Kabuli*	Ca_Kabuli_Chr03	34722992	[T/G]	Ca_00747	Protein of unknown function DUF81	NON_SYNONYMOUS_CODING
4661	CakSNP4661	*Kabuli*	Ca_Kabuli_Chr03	35829834	[A/G]	Ca_00870	WD40 repeat	NON_SYNONYMOUS_CODING
4710	CakSNP4710	*Kabuli*	Ca_Kabuli_Chr03	36212843	[T/A]	Ca_00926	Zinc finger, B-box	NON_SYNONYMOUS_CODING
4730	CakSNP4730	*Kabuli*	Ca_Kabuli_Chr03	36330750	[A/G]	Ca_00942	Homeobox	NON_SYNONYMOUS_CODING
4732	CakSNP4732	*Kabuli*	Ca_Kabuli_Chr03	36345158	[T/C]	Ca_00943	Zinc finger, C2H2-type	NON_SYNONYMOUS_CODING
4799	CakSNP4799	*Kabuli*	Ca_Kabuli_Chr03	37094437	[T/C]	Ca_01036	Transcription factor, SBP-box	NON_SYNONYMOUS_CODING
4801	CakSNP4801	*Kabuli*	Ca_Kabuli_Chr03	37101339	[A/T]	Ca_01036	Transcription factor, SBP-box	NON_SYNONYMOUS_CODING
4925	CakSNP4925	*Kabuli*	Ca_Kabuli_Chr03	38539721	[T/G]	Ca_01207	WD40 repeat	NON_SYNONYMOUS_CODING
4972	CakSNP4972	*Kabuli*	Ca_Kabuli_Chr03	38987916	[A/C]	Ca_01261	SANT domain, DNA binding	NON_SYNONYMOUS_CODING
4983	CakSNP4983	*Kabuli*	Ca_Kabuli_Chr03	39084916	[T/A]	Ca_01271	Domain of unknown function DUF296	STOP_GAINED
5143	CakSNP5143	*Kabuli*	Ca_Kabuli_Chr04	1474922	[G/T]	Ca_07777	DNA-binding WRKY	NON_SYNONYMOUS_CODING
5206	CakSNP5206	*Kabuli*	Ca_Kabuli_Chr04	2143155	[A/G]	Ca_07834	Helix-loop-helix DNA-binding domain	NON_SYNONYMOUS_CODING
5384	CakSNP5384	*Kabuli*	Ca_Kabuli_Chr04	4757416	[G/A]	Ca_03781	SET domain	NON_SYNONYMOUS_CODING
5388	CakSNP5388	*Kabuli*	Ca_Kabuli_Chr04	4799122	[C/T]	Ca_03778	SNF2-related	NON_SYNONYMOUS_CODING
5389	CakSNP5389	*Kabuli*	Ca_Kabuli_Chr04	4799116	[C/T]	Ca_03778	SNF2-related	NON_SYNONYMOUS_CODING
5402	CakSNP5402	*Kabuli*	Ca_Kabuli_Chr04	4920706	[T/C]	Ca_03766	Zinc finger, PMZ-type	NON_SYNONYMOUS_CODING
5403	CakSNP5403	*Kabuli*	Ca_Kabuli_Chr04	4920712	[A/T]	Ca_03766	Zinc finger, PMZ-type	NON_SYNONYMOUS_CODING
5472	CakSNP5472	*Kabuli*	Ca_Kabuli_Chr04	5841462	[A/C]	Ca_03665	Zinc finger, RING-type	NON_SYNONYMOUS_CODING
5545	CakSNP5545	*Kabuli*	Ca_Kabuli_Chr04	6761778	[C/A]	Ca_03565	SANT domain, DNA binding	STOP_GAINED
5547	CakSNP5547	*Kabuli*	Ca_Kabuli_Chr04	6765884	[T/C]	Ca_03564	SANT domain, DNA binding	NON_SYNONYMOUS_CODING
5565	CakSNP5565	*Kabuli*	Ca_Kabuli_Chr04	6919896	[C/T]	Ca_03548	Transcription factor, SBP-box	NON_SYNONYMOUS_CODING
5582	CakSNP5582	*Kabuli*	Ca_Kabuli_Chr04	7155826	[G/A]	Ca_03520	SANT domain, DNA binding	NON_SYNONYMOUS_CODING
5631	CakSNP5631	*Kabuli*	Ca_Kabuli_Chr04	8050770	[T/C]	Ca_03429	Tubby, C-terminal	NON_SYNONYMOUS_CODING

S.No.	SNP IDs	Chickpea cultivars	Chromosomes	Physical positions (bp)	SNPs	Gene accession IDs	Putative functions	Sequence components of genome
5666	CakSNP5666	*Kabuli*	Ca_Kabuli_Chr04	8484976	[G/A]	Ca_08340	WD40 repeat	NON_SYNONYMOUS_CODING
5667	CakSNP5667	*Kabuli*	Ca_Kabuli_Chr04	8484978	[A/G]	Ca_08340	WD40 repeat	NON_SYNONYMOUS_CODING
5704	CakSNP5704	*Kabuli*	Ca_Kabuli_Chr04	8751618	[A/C]	Ca_08371	No apical meristem (NAM) protein	NON_SYNONYMOUS_CODING
5720	CakSNP5720	*Kabuli*	Ca_Kabuli_Chr04	8848653	[T/C]	Ca_08381	Zinc finger, RING-type	NON_SYNONYMOUS_CODING
5727	CakSNP5727	*Kabuli*	Ca_Kabuli_Chr04	8919395	[A/G]	Ca_08388	WD40 repeat	NON_SYNONYMOUS_CODING
5754	CakSNP5754	*Kabuli*	Ca_Kabuli_Chr04	9189976	[T/A]	Ca_08410	Serine/threonine dehydratase, pyridoxal-phosphate-binding site	NON_SYNONYMOUS_CODING
5812	CakSNP5812	*Kabuli*	Ca_Kabuli_Chr04	10151943	[C/T]	Ca_08487	GCN5-related N-acetyltransferase (GNAT) domain	NON_SYNONYMOUS_CODING
5813	CakSNP5813	*Kabuli*	Ca_Kabuli_Chr04	10184428	[G/T]	Ca_08489	GCN5-related N-acetyltransferase (GNAT) domain	NON_SYNONYMOUS_CODING
5815	CakSNP5815	*Kabuli*	Ca_Kabuli_Chr04	10185602	[T/G]	Ca_08489	GCN5-related N-acetyltransferase (GNAT) domain	NON_SYNONYMOUS_CODING
5820	CakSNP5820	*Kabuli*	Ca_Kabuli_Chr04	10293241	[C/G]	Ca_08496	Carbamoyl-phosphate synthase, GATase domain	NON_SYNONYMOUS_CODING
5840	CakSNP5840	*Kabuli*	Ca_Kabuli_Chr04	10642043	[T/G]	Ca_04299	Forkhead-associated (FHA) domain	NON_SYNONYMOUS_CODING
5881	CakSNP5881	*Kabuli*	Ca_Kabuli_Chr04	11230403	[G/T]	Ca_04355	Major intrinsic protein	NON_SYNONYMOUS_CODING
5882	CakSNP5882	*Kabuli*	Ca_Kabuli_Chr04	11230400	[T/A]	Ca_04355	Major intrinsic protein	NON_SYNONYMOUS_CODING
5883	CakSNP5883	*Kabuli*	Ca_Kabuli_Chr04	11230397	[G/C]	Ca_04355	Major intrinsic protein	NON_SYNONYMOUS_CODING
5885	CakSNP5885	*Kabuli*	Ca_Kabuli_Chr04	11231137	[A/G]	Ca_04355	Major intrinsic protein	NON_SYNONYMOUS_CODING
5900	CakSNP5900	*Kabuli*	Ca_Kabuli_Chr04	11277574	[G/C]	Ca_04359	SET domain	NON_SYNONYMOUS_CODING
5918	CakSNP5918	*Kabuli*	Ca_Kabuli_Chr04	11465113	[C/T]	Ca_04383	Tubby, C-terminal	NON_SYNONYMOUS_CODING
5928	CakSNP5928	*Kabuli*	Ca_Kabuli_Chr04	11646453	[G/A]	Ca_04392	Domain of unknown function DUF125, transmembrane	NON_SYNONYMOUS_CODING
5931	CakSNP5931	*Kabuli*	Ca_Kabuli_Chr04	11689611	[C/T]	Ca_04395	Basic-leucine zipper (bZIP) transcription factor	NON_SYNONYMOUS_CODING
5935	CakSNP5935	*Kabuli*	Ca_Kabuli_Chr04	11772023	[G/T]	Ca_04401	ABC transporter, transmembrane domain	NON_SYNONYMOUS_CODING
5944	CakSNP5944	*Kabuli*	Ca_Kabuli_Chr04	12023137	[A/T]	Ca_04428	Protein kinase, catalytic domain	NON_SYNONYMOUS_CODING
5985	CakSNP5985	*Kabuli*	Ca_Kabuli_Chr04	12574650	[G/A]	Ca_04480	DNA mismatch repair protein MutS, C-terminal domain	NON_SYNONYMOUS_CODING
5998	CakSNP5998	*Kabuli*	Ca_Kabuli_Chr04	12869023	[C/T]	Ca_04506	Protein kinase, catalytic domain	NON_SYNONYMOUS_CODING
6007	CakSNP6007	*Kabuli*	Ca_Kabuli_Chr04	12973657	[C/A]	Ca_04519	Alpha-D-phosphohexomutase	NON_SYNONYMOUS_CODING
6027	CakSNP6027	*Kabuli*	Ca_Kabuli_Chr04	13072208	[C/A]	Ca_04530	FAE1/Type III polyketide synthase-like protein	NON_SYNONYMOUS_CODING
6030	CakSNP6030	*Kabuli*	Ca_Kabuli_Chr04	13102348	[G/T]	Ca_04534	Cytochrome P450	NON_SYNONYMOUS_CODING
6052	CakSNP6052	*Kabuli*	Ca_Kabuli_Chr04	13376456	[A/T]	Ca_04560	Protein kinase, catalytic domain	NON_SYNONYMOUS_CODING
6063	CakSNP6063	*Kabuli*	Ca_Kabuli_Chr04	13724666	[G/C]	Ca_04596	Copper amine oxidase	NON_SYNONYMOUS_CODING
6108	CakSNP6108	*Kabuli*	Ca_Kabuli_Chr04	14149589	[G/A]	Ca_04645	Protein kinase, catalytic domain	NON_SYNONYMOUS_CODING
6109	CakSNP6109	*Kabuli*	Ca_Kabuli_Chr04	14149588	[A/G]	Ca_04645	Protein kinase, catalytic domain	NON_SYNONYMOUS_CODING
6113	CakSNP6113	*Kabuli*	Ca_Kabuli_Chr04	14181527	[A/G]	Ca_04651	Protein kinase, catalytic domain	NON_SYNONYMOUS_CODING
6114	CakSNP6114	*Kabuli*	Ca_Kabuli_Chr04	14189356	[A/C]	Ca_04652	Protein kinase, catalytic domain	NON_SYNONYMOUS_CODING
6132	CakSNP6132	*Kabuli*	Ca_Kabuli_Chr04	14699270	[T/C]	Ca_05634	Protein of unknown function DUF620	NON_SYNONYMOUS_CODING
6166	CakSNP6166	*Kabuli*	Ca_Kabuli_Chr04	15240675	[G/A]	Ca_05575	Defects-in-morphology protein 1-like, mitochondrial	NON_SYNONYMOUS_CODING
6172	CakSNP6172	*Kabuli*	Ca_Kabuli_Chr04	15394470	[T/C]	Ca_05556	Double-stranded RNA-binding	NON_SYNONYMOUS_CODING
6223	CakSNP6223	*Kabuli*	Ca_Kabuli_Chr04	16278600	[C/T]	Ca_05487	Zinc finger, B-box	NON_SYNONYMOUS_CODING
6230	CakSNP6230	*Kabuli*	Ca_Kabuli_Chr04	16501291	[T/A]	Ca_05477	Domain of unknown function DUF292, eukaryotic	NON_SYNONYMOUS_CODING
6236	CakSNP6236	*Kabuli*	Ca_Kabuli_Chr04	16501738	[G/A]	Ca_05477	Domain of unknown function DUF292, eukaryotic	NON_SYNONYMOUS_CODING
6361	CakSNP6361	*Kabuli*	Ca_Kabuli_Chr04	17771708	[C/G]	Ca_05347	Protein of unknown function DUF639	NON_SYNONYMOUS_CODING

S.No.	SNP IDs	Chickpea cultivars	Chromosomes	Physical positions (bp)	SNPs	Gene accession IDs	Putative functions	Sequence components of genome
6408	CakSNP6408	*Kabuli*	Ca_Kabuli_Chr04	18117833	[T/C]	Ca_18651	SET domain	NON_SYNONYMOUS_CODING
6409	CakSNP6409	*Kabuli*	Ca_Kabuli_Chr04	18222762	[T/C]	Ca_18659	SET domain	NON_SYNONYMOUS_CODING
6517	CakSNP6517	*Kabuli*	Ca_Kabuli_Chr04	22850596	[C/G]	Ca_14467	Protein of unknown function DUF408	NON_SYNONYMOUS_CODING
6677	CakSNP6677	*Kabuli*	Ca_Kabuli_Chr04	29683718	[A/T]	Ca_14166	Plant peroxidase	NON_SYNONYMOUS_CODING
6799	CakSNP6799	*Kabuli*	Ca_Kabuli_Chr04	35141281	[A/T]	Ca_20010	Glycoside hydrolase, family 9	NON_SYNONYMOUS_CODING
6853	CakSNP6853	*Kabuli*	Ca_Kabuli_Chr04	36153818	[A/G]	Ca_14853	Protein of unknown function DUF827, plant	NON_SYNONYMOUS_CODING
6855	CakSNP6855	*Kabuli*	Ca_Kabuli_Chr04	36154820	[G/A]	Ca_14853	Protein of unknown function DUF827, plant	NON_SYNONYMOUS_CODING
6858	CakSNP6858	*Kabuli*	Ca_Kabuli_Chr04	36277383	[C/T]	Ca_14843	Domain of unknown function DUF1084	NON_SYNONYMOUS_CODING
6940	CakSNP6940	*Kabuli*	Ca_Kabuli_Chr04	37316738	[C/T]	Ca_15144	Zinc finger, RING-type	NON_SYNONYMOUS_CODING
6941	CakSNP6941	*Kabuli*	Ca_Kabuli_Chr04	37316713	[A/T]	Ca_15144	Zinc finger, RING-type	NON_SYNONYMOUS_CODING
6942	CakSNP6942	*Kabuli*	Ca_Kabuli_Chr04	37316700	[C/A]	Ca_15144	Zinc finger, RING-type	NON_SYNONYMOUS_CODING
7022	CakSNP7022	*Kabuli*	Ca_Kabuli_Chr04	38029136	[G/T]	Ca_13142	Protein of unknown function DUF3326	NON_SYNONYMOUS_CODING
7023	CakSNP7023	*Kabuli*	Ca_Kabuli_Chr04	38038865	[C/T]	Ca_13141	Protein of unknown function DUF3326	NON_SYNONYMOUS_CODING
7031	CakSNP7031	*Kabuli*	Ca_Kabuli_Chr04	38039297	[C/T]	Ca_13141	Protein of unknown function DUF3326	NON_SYNONYMOUS_CODING
7032	CakSNP7032	*Kabuli*	Ca_Kabuli_Chr04	38039313	[G/A]	Ca_13141	Protein of unknown function DUF3326	NON_SYNONYMOUS_CODING
7042	CakSNP7042	*Kabuli*	Ca_Kabuli_Chr04	38181292	[G/A]	Ca_13135	Domain of unknown function DUF250	NON_SYNONYMOUS_CODING
7043	CakSNP7043	*Kabuli*	Ca_Kabuli_Chr04	38195411	[T/A]	Ca_13134	Domain of unknown function DUF250	NON_SYNONYMOUS_CODING
7048	CakSNP7048	*Kabuli*	Ca_Kabuli_Chr04	38288877	[C/G]	Ca_13128	Protein of unknown function DUF869, plant	NON_SYNONYMOUS_CODING
7105	CakSNP7105	*Kabuli*	Ca_Kabuli_Chr04	38770370	[G/C]	Ca_13096	Fatty acid desaturase, type 2	NON_SYNONYMOUS_CODING
7207	CakSNP7207	*Kabuli*	Ca_Kabuli_Chr04	40583205	[G/T]	Ca_14868	Zinc finger, RING-type	STOP_GAINED
7208	CakSNP7208	*Kabuli*	Ca_Kabuli_Chr04	40583206	[A/T]	Ca_14868	Zinc finger, RING-type	NON_SYNONYMOUS_CODING
7209	CakSNP7209	*Kabuli*	Ca_Kabuli_Chr04	40583211	[G/T]	Ca_14868	Zinc finger, RING-type	STOP_GAINED
7210	CakSNP7210	*Kabuli*	Ca_Kabuli_Chr04	40583214	[G/A]	Ca_14868	Zinc finger, RING-type	NON_SYNONYMOUS_CODING
7211	CakSNP7211	*Kabuli*	Ca_Kabuli_Chr04	40583216	[C/A]	Ca_14868	Zinc finger, RING-type	NON_SYNONYMOUS_CODING
7212	CakSNP7212	*Kabuli*	Ca_Kabuli_Chr04	40583217	[G/A]	Ca_14868	Zinc finger, RING-type	NON_SYNONYMOUS_CODING
7347	CakSNP7347	*Kabuli*	Ca_Kabuli_Chr04	43729266	[A/G]	Ca_09058	WD40 repeat	NON_SYNONYMOUS_CODING
7367	CakSNP7367	*Kabuli*	Ca_Kabuli_Chr04	44266819	[G/A]	Ca_09101	Protein of unknown function DUF1077, TMEM85	NON_SYNONYMOUS_CODING
7392	CakSNP7392	*Kabuli*	Ca_Kabuli_Chr04	44472278	[A/G]	Ca_09120	Protein of unknown function DUF1350	NON_SYNONYMOUS_CODING
7393	CakSNP7393	*Kabuli*	Ca_Kabuli_Chr04	44472237	[T/A]	Ca_09120	Protein of unknown function DUF1350	NON_SYNONYMOUS_CODING
7449	CakSNP7449	*Kabuli*	Ca_Kabuli_Chr04	45220916	[T/C]	Ca_09184	Protein of unknown function DUF647	NON_SYNONYMOUS_CODING
7452	CakSNP7452	*Kabuli*	Ca_Kabuli_Chr04	45224997	[G/C]	Ca_09185	Protein of unknown function DUF647	NON_SYNONYMOUS_CODING
7496	CakSNP7496	*Kabuli*	Ca_Kabuli_Chr04	46134302	[C/G]	Ca_18358	Zinc finger, RING-type	NON_SYNONYMOUS_CODING
7513	CakSNP7513	*Kabuli*	Ca_Kabuli_Chr04	46612244	[C/T]	Ca_19256	Protein of unknown function DUF827, plant	NON_SYNONYMOUS_CODING
7602	CakSNP7602	*Kabuli*	Ca_Kabuli_Chr04	48464493	[T/C]	Ca_10746	Zinc finger, CCHC-type	NON_SYNONYMOUS_CODING
7672	CakSNP7672	*Kabuli*	Ca_Kabuli_Chr05	166217	[G/C]	Ca_18171	No apical meristem (NAM) protein	NON_SYNONYMOUS_CODING
7674	CakSNP7674	*Kabuli*	Ca_Kabuli_Chr05	166341	[C/A]	Ca_18171	No apical meristem (NAM) protein	NON_SYNONYMOUS_CODING
7721	CakSNP7721	*Kabuli*	Ca_Kabuli_Chr05	1280977	[T/G]	Ca_26279	Transcription factor GRAS	NON_SYNONYMOUS_CODING
7840	CakSNP7840	*Kabuli*	Ca_Kabuli_Chr05	9961380	[C/T]	Ca_20508	DNA-binding WRKY	NON_SYNONYMOUS_CODING
8012	CakSNP8012	*Kabuli*	Ca_Kabuli_Chr05	20644984	[C/A]	Ca_18219	Domain of unknown function DUF231, plant	NON_SYNONYMOUS_CODING

S.No.	SNP IDs	Chickpea cultivars	Chromosomes	Physical positions (bp)	SNPs	Gene accession IDs	Putative functions	Sequence components of genome
8078	CakSNP8078	*Kabuli*	Ca_Kabuli_Chr05	24090533	[C/T]	Ca_17657	SANT domain, DNA binding	NON_SYNONYMOUS_CODING
8079	CakSNP8079	*Kabuli*	Ca_Kabuli_Chr05	24090523	[A/C]	Ca_17657	SANT domain, DNA binding	NON_SYNONYMOUS_CODING
8080	CakSNP8080	*Kabuli*	Ca_Kabuli_Chr05	24090515	[A/C]	Ca_17657	SANT domain, DNA binding	NON_SYNONYMOUS_CODING
8081	CakSNP8081	*Kabuli*	Ca_Kabuli_Chr05	24090749	[A/C]	Ca_17657	Protein of unknown function DUF3437	NON_SYNONYMOUS_CODING
8082	CakSNP8082	*Kabuli*	Ca_Kabuli_Chr05	24097823	[T/C]	Ca_17656	Protein of unknown function DUF3437	NON_SYNONYMOUS_CODING
8083	CakSNP8083	*Kabuli*	Ca_Kabuli_Chr05	24153061	[G/C]	Ca_17653	Protein of unknown function DUF3437	NON_SYNONYMOUS_CODING
8084	CakSNP8084	*Kabuli*	Ca_Kabuli_Chr05	24153205	[T/C]	Ca_17653	Protein of unknown function DUF3437	NON_SYNONYMOUS_CODING
8085	CakSNP8085	*Kabuli*	Ca_Kabuli_Chr05	24153273	[T/C]	Ca_17653	Protein of unknown function DUF3437	NON_SYNONYMOUS_CODING
8086	CakSNP8086	*Kabuli*	Ca_Kabuli_Chr05	24153202	[A/C]	Ca_17653	Protein of unknown function DUF3437	NON_SYNONYMOUS_CODING
8087	CakSNP8087	*Kabuli*	Ca_Kabuli_Chr05	24153199	[C/T]	Ca_17653	Protein of unknown function DUF3437	NON_SYNONYMOUS_CODING
8235	CakSNP8235	*Kabuli*	Ca_Kabuli_Chr05	27694205	[C/A]	Ca_08882	AUX/IAA protein	NON_SYNONYMOUS_CODING
8307	CakSNP8307	*Kabuli*	Ca_Kabuli_Chr05	28746139	[A/G]	Ca_13355	Protein of unknown function DUF827, plant	NON_SYNONYMOUS_CODING
8308	CakSNP8308	*Kabuli*	Ca_Kabuli_Chr05	28757467	[A/T]	Ca_13354	Protein of unknown function DUF827, plant	NON_SYNONYMOUS_CODING
8309	CakSNP8309	*Kabuli*	Ca_Kabuli_Chr05	28757474	[T/C]	Ca_13354	Protein of unknown function DUF827, plant	NON_SYNONYMOUS_CODING
8310	CakSNP8310	*Kabuli*	Ca_Kabuli_Chr05	28757479	[T/C]	Ca_13354	Protein of unknown function DUF827, plant	NON_SYNONYMOUS_CODING
8311	CakSNP8311	*Kabuli*	Ca_Kabuli_Chr05	28757485	[A/C]	Ca_13354	Protein of unknown function DUF827, plant	NON_SYNONYMOUS_CODING
8312	CakSNP8312	*Kabuli*	Ca_Kabuli_Chr05	28757507	[A/T]	Ca_13354	Protein of unknown function DUF827, plant	NON_SYNONYMOUS_CODING
8399	CakSNP8399	*Kabuli*	Ca_Kabuli_Chr05	30086232	[A/C]	Ca_04684	Protein of unknown function DUF300	NON_SYNONYMOUS_CODING
8524	CakSNP8524	*Kabuli*	Ca_Kabuli_Chr05	31758166	[T/C]	Ca_04880	Protein of unknown function DUF668	NON_SYNONYMOUS_CODING
8535	CakSNP8535	*Kabuli*	Ca_Kabuli_Chr05	32015967	[T/C]	Ca_04905	Protein of unknown function DUF726	NON_SYNONYMOUS_CODING
8574	CakSNP8574	*Kabuli*	Ca_Kabuli_Chr05	32840541	[T/G]	Ca_04983	Protein of unknown function DUF3453	NON_SYNONYMOUS_CODING
8597	CakSNP8597	*Kabuli*	Ca_Kabuli_Chr05	33379873	[C/T]	Ca_01919	Homeobox	NON_SYNONYMOUS_CODING
8676	CakSNP8676	*Kabuli*	Ca_Kabuli_Chr05	34482245	[G/T]	Ca_01795	WD40 repeat	NON_SYNONYMOUS_CODING
8678	CakSNP8678	*Kabuli*	Ca_Kabuli_Chr05	34511669	[G/T]	Ca_01790	WD40 repeat	NON_SYNONYMOUS_CODING
8800	CakSNP8800	*Kabuli*	Ca_Kabuli_Chr05	36797187	[G/T]	Ca_01541	Protein of unknown function DUF827, plant	NON_SYNONYMOUS_CODING
9010	CakSNP9010	*Kabuli*	Ca_Kabuli_Chr05	40464608	[A/G]	Ca_07566	Zinc finger, CCHC-type	NON_SYNONYMOUS_CODING
9054	CakSNP9054	*Kabuli*	Ca_Kabuli_Chr05	41283253	[T/C]	Ca_07642	Protein of unknown function DUF1296	NON_SYNONYMOUS_CODING
9055	CakSNP9055	*Kabuli*	Ca_Kabuli_Chr05	41283453	[A/C]	Ca_07642	Protein of unknown function DUF1296	NON_SYNONYMOUS_CODING
9211	CakSNP9211	*Kabuli*	Ca_Kabuli_Chr05	44109260	[G/T]	Ca_12646	SANT domain, DNA binding	STOP_GAINED
9230	CakSNP9230	*Kabuli*	Ca_Kabuli_Chr05	44760347	[G/T]	Ca_03920	Transcription factor GRAS	NON_SYNONYMOUS_CODING
9284	CakSNP9284	*Kabuli*	Ca_Kabuli_Chr05	45649189	[G/A]	Ca_04010	Protein of unknown function DUF791	NON_SYNONYMOUS_CODING
9331	CakSNP9331	*Kabuli*	Ca_Kabuli_Chr05	46176366	[T/C]	Ca_04069	No apical meristem (NAM) protein	NON_SYNONYMOUS_CODING
9357	CakSNP9357	*Kabuli*	Ca_Kabuli_Chr05	46376532	[A/C]	Ca_04094	Protein of unknown function DUF3133	NON_SYNONYMOUS_CODING
9409	CakSNP9409	*Kabuli*	Ca_Kabuli_Chr05	47774516	[A/G]	Ca_04239	Protein of unknown function DUF869, plant	NON_SYNONYMOUS_CODING
9410	CakSNP9410	*Kabuli*	Ca_Kabuli_Chr05	47774559	[C/T]	Ca_04239	Protein of unknown function DUF869, plant	NON_SYNONYMOUS_CODING
9411	CakSNP9411	*Kabuli*	Ca_Kabuli_Chr05	47774718	[C/G]	Ca_04239	Protein of unknown function DUF869, plant	NON_SYNONYMOUS_CODING
9533	CakSNP9533	*Kabuli*	Ca_Kabuli_Chr06	2079137	[T/G]	Ca_10329	Protein of unknown function DUF760	NON_SYNONYMOUS_CODING
9728	CakSNP9728	*Kabuli*	Ca_Kabuli_Chr06	6443828	[C/G]	Ca_16913	SANT domain, DNA binding	NON_SYNONYMOUS_CODING
9742	CakSNP9742	*Kabuli*	Ca_Kabuli_Chr06	6779147	[G/C]	Ca_16938	Protein of unknown function DUF869, plant	NON_SYNONYMOUS_CODING

S.No.	SNP IDs	Chickpea cultivars	Chromosomes	Physical positions (bp)	SNPs	Gene accession IDs	Putative functions	Sequence components of genome
9762	CakSNP9762	*Kabuli*	Ca_Kabuli_Chr06	6913639	[T/C]	Ca_16954	SNF2-related	NON_SYNONYMOUS_CODING
9763	CakSNP9763	*Kabuli*	Ca_Kabuli_Chr06	6913622	[A/C]	Ca_16954	SNF2-related	NON_SYNONYMOUS_CODING
9764	CakSNP9764	*Kabuli*	Ca_Kabuli_Chr06	6913695	[G/A]	Ca_16954	SNF2-related	NON_SYNONYMOUS_CODING
9823	CakSNP9823	*Kabuli*	Ca_Kabuli_Chr06	7831899	[G/A]	Ca_09595	C2 calcium-dependent membrane targeting	NON_SYNONYMOUS_CODING
9834	CakSNP9834	*Kabuli*	Ca_Kabuli_Chr06	7929263	[C/T]	Ca_09581	Acyl-CoA oxidase, C-terminal	NON_SYNONYMOUS_CODING
9883	CakSNP9883	*Kabuli*	Ca_Kabuli_Chr06	8269326	[A/C]	Ca_09543	Protein of unknown function DUF3550/UPF0682	NON_SYNONYMOUS_CODING
9946	CakSNP9946	*Kabuli*	Ca_Kabuli_Chr06	9834198	[C/A]	Ca_08608	WD40 repeat	NON_SYNONYMOUS_CODING
9947	CakSNP9947	*Kabuli*	Ca_Kabuli_Chr06	9834187	[A/T]	Ca_08608	WD40 repeat	NON_SYNONYMOUS_CODING
9949	CakSNP9949	*Kabuli*	Ca_Kabuli_Chr06	9834183	[C/T]	Ca_08608	WD40 repeat	NON_SYNONYMOUS_CODING
9981	CakSNP9981	*Kabuli*	Ca_Kabuli_Chr06	10138984	[C/T]	Ca_08579	Protein of unknown function DUF616	NON_SYNONYMOUS_CODING
10111	CakSNP10111	*Kabuli*	Ca_Kabuli_Chr06	12437275	[A/T]	Ca_05025	AUX/IAA protein	NON_SYNONYMOUS_CODING
10114	CakSNP10114	*Kabuli*	Ca_Kabuli_Chr06	12437319	[C/T]	Ca_05025	Zinc finger, C3HC-like	STOP_GAINED
10123	CakSNP10123	*Kabuli*	Ca_Kabuli_Chr06	12799890	[T/G]	Ca_05052	Protein of unknown function DUF593	NON_SYNONYMOUS_CODING
10157	CakSNP10157	*Kabuli*	Ca_Kabuli_Chr06	13192177	[G/A]	Ca_05091	Zinc finger, CCCH-type	NON_SYNONYMOUS_CODING
10160	CakSNP10160	*Kabuli*	Ca_Kabuli_Chr06	13296128	[G/A]	Ca_05103	Zinc finger, RING-type	NON_SYNONYMOUS_CODING
10248	CakSNP10248	*Kabuli*	Ca_Kabuli_Chr06	14416997	[A/G]	Ca_05213	Domain of unknown function DUF676, hydrolase-like	NON_SYNONYMOUS_CODING
10387	CakSNP10387	*Kabuli*	Ca_Kabuli_Chr06	17258827	[C/A]	Ca_06343	Homeobox	NON_SYNONYMOUS_CODING
10388	CakSNP10388	*Kabuli*	Ca_Kabuli_Chr06	17258886	[T/G]	Ca_06343	Homeobox	NON_SYNONYMOUS_CODING
10416	CakSNP10416	*Kabuli*	Ca_Kabuli_Chr06	18444725	[T/C]	Ca_06458	Helix-loop-helix DNA-binding domain	NON_SYNONYMOUS_CODING
10483	CakSNP10483	*Kabuli*	Ca_Kabuli_Chr06	20766377	[C/A]	Ca_16355	Helix-loop-helix DNA-binding domain	NON_SYNONYMOUS_CODING
10539	CakSNP10539	*Kabuli*	Ca_Kabuli_Chr06	21675734	[T/G]	Ca_11272	Homeobox	NON_SYNONYMOUS_CODING
10540	CakSNP10540	*Kabuli*	Ca_Kabuli_Chr06	21675757	[A/G]	Ca_11272	Homeobox	NON_SYNONYMOUS_CODING
10542	CakSNP10542	*Kabuli*	Ca_Kabuli_Chr06	21675816	[C/G]	Ca_11272	Myb, DNA-binding	NON_SYNONYMOUS_CODING
10589	CakSNP10589	*Kabuli*	Ca_Kabuli_Chr06	22217157	[T/G]	Ca_11216	Zinc finger, CHCC-type	NON_SYNONYMOUS_CODING
10718	CakSNP10718	*Kabuli*	Ca_Kabuli_Chr06	26349363	[C/A]	Ca_16676	Transcription factor GRAS	NON_SYNONYMOUS_CODING
10720	CakSNP10720	*Kabuli*	Ca_Kabuli_Chr06	26349434	[G/A]	Ca_16676	Transcription factor GRAS	NON_SYNONYMOUS_CODING
10843	CakSNP10843	*Kabuli*	Ca_Kabuli_Chr06	29205940	[A/G]	Ca_17457	DNA ligase, ATP-dependent, N-terminal	NON_SYNONYMOUS_CODING
10896	CakSNP10896	*Kabuli*	Ca_Kabuli_Chr06	32328468	[T/C]	Ca_15224	Protein of unknown function DUF936, plant	NON_SYNONYMOUS_CODING
11227	CakSNP11227	*Kabuli*	Ca_Kabuli_Chr06	49565934	[C/T]	Ca_13509	Domain of unknown function DUF632	START_LOST
11368	CakSNP11368	*Kabuli*	Ca_Kabuli_Chr06	55180211	[A/G]	Ca_21215	Homeobox	NON_SYNONYMOUS_CODING
11444	CakSNP11444	*Kabuli*	Ca_Kabuli_Chr06	57634387	[G/T]	Ca_13682	Protein of unknown function DUF936, plant	NON_SYNONYMOUS_CODING
11559	CakSNP11559	*Kabuli*	Ca_Kabuli_Chr06	58650459	[A/T]	Ca_15410	WD40 repeat	NON_SYNONYMOUS_CODING
11560	CakSNP11560	*Kabuli*	Ca_Kabuli_Chr06	58650491	[A/G]	Ca_15410	WD40 repeat	NON_SYNONYMOUS_CODING
11635	CakSNP11635	*Kabuli*	Ca_Kabuli_Chr07	218341	[C/G]	Ca_20223	Transcription factor GRAS	NON_SYNONYMOUS_CODING
11667	CakSNP11667	*Kabuli*	Ca_Kabuli_Chr07	610723	[G/T]	Ca_24417	Transcription factor GRAS	NON_SYNONYMOUS_CODING
11713	CakSNP11713	*Kabuli*	Ca_Kabuli_Chr07	1585622	[T/C]	Ca_03308	WD40 repeat	NON_SYNONYMOUS_CODING
11727	CakSNP11727	*Kabuli*	Ca_Kabuli_Chr07	1787308	[T/A]	Ca_03291	Domain of unknown function DUF828	NON_SYNONYMOUS_CODING
11770	CakSNP11770	*Kabuli*	Ca_Kabuli_Chr07	2757255	[G/C]	Ca_03202	Transcription factor jumonji/aspartyl beta-hydroxylase	NON_SYNONYMOUS_CODING
11771	CakSNP11771	*Kabuli*	Ca_Kabuli_Chr07	2757253	[T/G]	Ca_03202	Transcription factor jumonji/aspartyl beta-hydroxylase	NON_SYNONYMOUS_CODING

S.No.	SNP IDs	Chickpea cultivars	Chromosomes	Physical positions (bp)	SNPs	Gene accession IDs	Putative functions	Sequence components of genome
11810	CakSNP11810	*Kabuli*	Ca_Kabuli_Chr07	3265823	[A/G]	Ca_03142	Domain of unknown function DUF231, plant	NON_SYNONYMOUS_CODING
11816	CakSNP11816	*Kabuli*	Ca_Kabuli_Chr07	3355627	[A/G]	Ca_03128	AUX/IAA protein	NON_SYNONYMOUS_CODING
11966	CakSNP11966	*Kabuli*	Ca_Kabuli_Chr07	5467744	[G/C]	Ca_06820	SNF2-related	NON_SYNONYMOUS_CODING
11967	CakSNP11967	*Kabuli*	Ca_Kabuli_Chr07	5500318	[C/A]	Ca_06816	SNF2-related	NON_SYNONYMOUS_CODING
12073	CakSNP12073	*Kabuli*	Ca_Kabuli_Chr07	6814954	[G/T]	Ca_06681	YABBY protein	NON_SYNONYMOUS_CODING
12116	CakSNP12116	*Kabuli*	Ca_Kabuli_Chr07	7706816	[A/G]	Ca_06587	SNF2-related	NON_SYNONYMOUS_CODING
12117	CakSNP12117	*Kabuli*	Ca_Kabuli_Chr07	7756455	[C/T]	Ca_13243	SNF2-related	NON_SYNONYMOUS_CODING
12118	CakSNP12118	*Kabuli*	Ca_Kabuli_Chr07	7756482	[G/A]	Ca_13243	SNF2-related	NON_SYNONYMOUS_CODING
12149	CakSNP12149	*Kabuli*	Ca_Kabuli_Chr07	8569632	[A/G]	Ca_15361	Zinc finger, C2HC5-type	NON_SYNONYMOUS_CODING
12173	CakSNP12173	*Kabuli*	Ca_Kabuli_Chr07	8894362	[A/T]	Ca_15325	Domain of unknown function DUF629	NON_SYNONYMOUS_CODING
12279	CakSNP12279	*Kabuli*	Ca_Kabuli_Chr07	10485414	[C/T]	Ca_12810	Zinc finger, RING-type	NON_SYNONYMOUS_CODING
12458	CakSNP12458	*Kabuli*	Ca_Kabuli_Chr07	13903980	[T/G]	Ca_16064	Tubby, C-terminal	NON_SYNONYMOUS_CODING
12473	CakSNP12473	*Kabuli*	Ca_Kabuli_Chr07	14233343	[T/G]	Ca_23041	Protein of unknown function DUF248, methyltransferase putative	NON_SYNONYMOUS_CODING
12475	CakSNP12475	*Kabuli*	Ca_Kabuli_Chr07	14281993	[G/T]	Ca_23042	Protein of unknown function DUF702	NON_SYNONYMOUS_CODING
12571	CakSNP12571	*Kabuli*	Ca_Kabuli_Chr07	16936123	[C/G]	Ca_15885	Domain of unknown function DUF231, plant	NON_SYNONYMOUS_CODING
12597	CakSNP12597	*Kabuli*	Ca_Kabuli_Chr07	17597348	[T/C]	Ca_15849	Protein of unknown function DUF1639	NON_SYNONYMOUS_CODING
12599	CakSNP12599	*Kabuli*	Ca_Kabuli_Chr07	17597420	[C/T]	Ca_15849	Protein of unknown function DUF1639	NON_SYNONYMOUS_CODING
12605	CakSNP12605	*Kabuli*	Ca_Kabuli_Chr07	17779414	[A/G]	Ca_15839	Basic-leucine zipper (bZIP) transcription factor	NON_SYNONYMOUS_CODING
12606	CakSNP12606	*Kabuli*	Ca_Kabuli_Chr07	17779594	[T/C]	Ca_15839	Basic-leucine zipper (bZIP) transcription factor	NON_SYNONYMOUS_CODING
12624	CakSNP12624	*Kabuli*	Ca_Kabuli_Chr07	18796748	[T/C]	Ca_12308	ZF-HD homeobox protein, Cys/His-rich dimerisation domain	NON_SYNONYMOUS_CODING
12874	CakSNP12874	*Kabuli*	Ca_Kabuli_Chr07	30085847	[C/T]	Ca_11718	Domain of unknown function DUF828	NON_SYNONYMOUS_CODING
12896	CakSNP12896	*Kabuli*	Ca_Kabuli_Chr07	31612697	[T/A]	Ca_10100	Domain of unknown function DUF250	NON_SYNONYMOUS_CODING
12924	CakSNP12924	*Kabuli*	Ca_Kabuli_Chr07	32522203	[A/G]	Ca_10028	SANT domain, DNA binding	NON_SYNONYMOUS_CODING
12940	CakSNP12940	*Kabuli*	Ca_Kabuli_Chr07	32942199	[A/G]	Ca_10004	Transcription factor GRAS	NON_SYNONYMOUS_CODING
13024	CakSNP13024	*Kabuli*	Ca_Kabuli_Chr07	35181619	[T/G]	Ca_17639	Helix-loop-helix DNA-binding domain	NON_SYNONYMOUS_CODING
13026	CakSNP13026	*Kabuli*	Ca_Kabuli_Chr07	35181660	[G/A]	Ca_17639	Helix-loop-helix DNA-binding domain	NON_SYNONYMOUS_CODING
13027	CakSNP13027	*Kabuli*	Ca_Kabuli_Chr07	35181635	[G/C]	Ca_17639	Helix-loop-helix DNA-binding domain	NON_SYNONYMOUS_CODING
13104	CakSNP13104	*Kabuli*	Ca_Kabuli_Chr07	39339510	[T/G]	Ca_19588	Domain of unknown function DUF296	NON_SYNONYMOUS_CODING
13184	CakSNP13184	*Kabuli*	Ca_Kabuli_Chr07	41387502	[A/C]	Ca_17996	WD40 repeat	NON_SYNONYMOUS_CODING
13185	CakSNP13185	*Kabuli*	Ca_Kabuli_Chr07	41387475	[A/C]	Ca_17996	WD40 repeat	NON_SYNONYMOUS_CODING
13274	CakSNP13274	*Kabuli*	Ca_Kabuli_Chr08	85710	[C/A]	Ca_11933	SNF2-related	NON_SYNONYMOUS_CODING
13287	CakSNP13287	*Kabuli*	Ca_Kabuli_Chr08	281608	[T/A]	Ca_11909	Helix-loop-helix DNA-binding domain	NON_SYNONYMOUS_CODING
13328	CakSNP13328	*Kabuli*	Ca_Kabuli_Chr08	709063	[T/C]	Ca_11856	Protein of unknown function DUF1950	NON_SYNONYMOUS_CODING
13329	CakSNP13329	*Kabuli*	Ca_Kabuli_Chr08	709094	[T/C]	Ca_11856	Protein of unknown function DUF1950	NON_SYNONYMOUS_CODING
13331	CakSNP13331	*Kabuli*	Ca_Kabuli_Chr08	714967	[A/G]	Ca_11855	Protein of unknown function DUF1950	NON_SYNONYMOUS_CODING
13340	CakSNP13340	*Kabuli*	Ca_Kabuli_Chr08	822302	[A/G]	Ca_11839	Zinc finger, PHD-type	NON_SYNONYMOUS_CODING
13341	CakSNP13341	*Kabuli*	Ca_Kabuli_Chr08	822942	[T/G]	Ca_11839	Zinc finger, PHD-type	NON_SYNONYMOUS_CODING
13344	CakSNP13344	*Kabuli*	Ca_Kabuli_Chr08	855332	[G/A]	Ca_11834	SANT domain, DNA binding	NON_SYNONYMOUS_CODING
13371	CakSNP13371	*Kabuli*	Ca_Kabuli_Chr08	1070728	[T/C]	Ca_15078	WD40 repeat	NON_SYNONYMOUS_CODING

S.No.	SNP IDs	Chickpea cultivars	Chromosomes	Physical positions (bp)	SNPs	Gene accession IDs	Putative functions	Sequence components of genome
13388	CakSNP13388	*Kabuli*	Ca_Kabuli_Chr08	1241739	[C/T]	Ca_15053	Zinc finger, CCCH-type	NON_SYNONYMOUS_CODING
13393	CakSNP13393	*Kabuli*	Ca_Kabuli_Chr08	1265537	[G/A]	Ca_15049	Zinc finger, CCCH-type	NON_SYNONYMOUS_CODING
13394	CakSNP13394	*Kabuli*	Ca_Kabuli_Chr08	1265535	[A/G]	Ca_15049	Zinc finger, CCCH-type	NON_SYNONYMOUS_CODING
13399	CakSNP13399	*Kabuli*	Ca_Kabuli_Chr08	1382266	[G/A]	Ca_15035	Zinc finger, PHD-type	NON_SYNONYMOUS_CODING
13400	CakSNP13400	*Kabuli*	Ca_Kabuli_Chr08	1382433	[G/T]	Ca_15035	Zinc finger, PHD-type	NON_SYNONYMOUS_CODING
13428	CakSNP13428	*Kabuli*	Ca_Kabuli_Chr08	1675888	[C/T]	Ca_02440	Protein of unknown function DUF604	NON_SYNONYMOUS_CODING
13443	CakSNP13443	*Kabuli*	Ca_Kabuli_Chr08	1830348	[T/C]	Ca_02423	Transcription factor TFIIE beta subunit, DNA-binding domain	NON_SYNONYMOUS_CODING
13608	CakSNP13608	*Kabuli*	Ca_Kabuli_Chr08	4042424	[G/A]	Ca_02167	SET domain	NON_SYNONYMOUS_CODING
13611	CakSNP13611	*Kabuli*	Ca_Kabuli_Chr08	4073528	[C/T]	Ca_02163	Zinc finger, RING-type	NON_SYNONYMOUS_CODING
13612	CakSNP13612	*Kabuli*	Ca_Kabuli_Chr08	4073603	[A/G]	Ca_02163	Zinc finger, RING-type	NON_SYNONYMOUS_CODING
13630	CakSNP13630	*Kabuli*	Ca_Kabuli_Chr08	4290690	[T/A]	Ca_02137	Protein of unknown function DUF3651, TMEM131	NON_SYNONYMOUS_CODING
13699	CakSNP13699	*Kabuli*	Ca_Kabuli_Chr08	5197004	[G/T]	Ca_02034	Homeobox	NON_SYNONYMOUS_CODING
13708	CakSNP13708	*Kabuli*	Ca_Kabuli_Chr08	5390602	[T/C]	Ca_02008	Protein of unknown function DUF248, methyltransferase putative	NON_SYNONYMOUS_CODING
13709	CakSNP13709	*Kabuli*	Ca_Kabuli_Chr08	5399503	[G/A]	Ca_02005	Protein of unknown function DUF248, methyltransferase putative	STOP_GAINED
13969	CakSNP13969	*Kabuli*	Ca_Kabuli_Chr08	11403754	[G/A]	Ca_16820	SANT domain, DNA binding	NON_SYNONYMOUS_CODING
14045	CakSNP14045	*Kabuli*	Ca_Kabuli_Chr08	16004976	[G/C]	Ca_15507	Glycoside hydrolase, family 17	NON_SYNONYMOUS_CODING
14073	CakSNP14073	*Kabuli*	Ca_Kabuli_Chr08	16145411	[C/G]	Ca_15527	Domain of unknown function DUF292, eukaryotic	NON_SYNONYMOUS_CODING
14157	CakSNP14157	*Kabuli*	Ca_Kabuli_Scaffold_C1116	5778	[T/A]	Ca_28056	Protein of unknown function DUF2930	NON_SYNONYMOUS_CODING
14471	CakSNP14471	*Kabuli*	Ca_Kabuli_Scaffold_1324	262208	[C/G]	Ca_19766	SET domain	NON_SYNONYMOUS_CODING
14473	CakSNP14473	*Kabuli*	Ca_Kabuli_Scaffold_134	218352	[T/G]	Ca_22581	SET domain	NON_SYNONYMOUS_CODING
14474	CakSNP14474	*Kabuli*	Ca_Kabuli_Scaffold_134	218336	[T/G]	Ca_22581	SET domain	NON_SYNONYMOUS_CODING
14494	CakSNP14494	*Kabuli*	Ca_Kabuli_Scaffold_1348_1	535984	[G/T]	Ca_08741	Zinc finger, RING-type	NON_SYNONYMOUS_CODING
14534	CakSNP14534	*Kabuli*	Ca_Kabuli_Scaffold_1348_1	959608	[A/C]	Ca_08787	Transcription factor jumonji/aspartyl beta-hydroxylase	NON_SYNONYMOUS_CODING
14569	CakSNP14569	*Kabuli*	Ca_Kabuli_Scaffold_1351	172110	[T/C]	Ca_21995	Protein of unknown function DUF2419	NON_SYNONYMOUS_CODING
14701	CakSNP14701	*Kabuli*	Ca_Kabuli_Scaffold_157	151081	[G/A]	Ca_21070	Zinc finger, DHHC-type, palmitoyltransferase	NON_SYNONYMOUS_CODING
15082	CakSNP15086	*Kabuli*	Ca_Kabuli_Scaffold_2557	110378	[C/T]	Ca_26665	Domain of unknown function DUF828	NON_SYNONYMOUS_CODING
15127	CakSNP15131	*Kabuli*	Ca_Kabuli_Scaffold_2763	25771	[T/G]	Ca_24394	Zinc finger, RING-type	NON_SYNONYMOUS_CODING
15128	CakSNP15132	*Kabuli*	Ca_Kabuli_Scaffold_2763	43207	[G/A]	Ca_24396	Zinc finger, RING-type	NON_SYNONYMOUS_CODING
15281	CakSNP15285	*Kabuli*	Ca_Kabuli_Scaffold_314	535269	[A/C]	Ca_21812	Zinc finger, CCCH-type	NON_SYNONYMOUS_CODING
15341	CakSNP15345	*Kabuli*	Ca_Kabuli_Scaffold_3337	17963	[C/T]	Ca_28178	Domain of unknown function DUF828	NON_SYNONYMOUS_CODING
15342	CakSNP15346	*Kabuli*	Ca_Kabuli_Scaffold_3337	17999	[T/G]	Ca_28178	Domain of unknown function DUF828	NON_SYNONYMOUS_CODING
15345	CakSNP15349	*Kabuli*	Ca_Kabuli_Scaffold_3337	17959	[A/G]	Ca_28178	Domain of unknown function DUF828	NON_SYNONYMOUS_CODING
15346	CakSNP15350	*Kabuli*	Ca_Kabuli_Scaffold_3337	17956	[G/A]	Ca_28178	Domain of unknown function DUF828	NON_SYNONYMOUS_CODING
15526	CakSNP15530	*Kabuli*	Ca_Kabuli_Scaffold_401	49065	[G/A]	Ca_25591	Protein of unknown function DUF1296	STOP_GAINED
15714	CakSNP15718	*Kabuli*	Ca_Kabuli_Scaffold_50	153631	[C/T]	Ca_24566	Protein of unknown function DUF3527	NON_SYNONYMOUS_CODING
15788	CakSNP15792	*Kabuli*	Ca_Kabuli_Scaffold_545	147568	[T/G]	Ca_23924	WD40 repeat	NON_SYNONYMOUS_CODING
15842	CakSNP15846	*Kabuli*	Ca_Kabuli_Scaffold_562	289080	[C/A]	Ca_23141	Protein of unknown function DUF642	NON_SYNONYMOUS_CODING
15969	CakSNP15973	*Kabuli*	Ca_Kabuli_Scaffold_681	18376	[C/G]	Ca_26833	Transcriptional factor B3	NON_SYNONYMOUS_CODING
16380	CakSNP16380	*Kabuli*	Ca_Kabuli_denovo	2003	(A/G)	-	-	-

S.No.	SNP IDs	Chickpea cultivars	Chromosomes	Physical positions (bp)	SNPs	Gene accession IDs	Putative functions	Sequence components of genome
16386	CadSNP1845	*Desi*	Ca_Desi_Chr03	5804745	[G/C]	Ca_02600	WD repeat-containing protein 3	NON_SYNONYMOUS_CODING
16391	CadSNP3327	*Desi*	Ca_Desi_Chr04	8450693	[T/G]	Ca_04439	Methylthioribose kinase	NON_SYNONYMOUS_CODING
16410	CadSNP4895	*Desi*	Ca_Desi_Chr06	5944054	[G/A]	Ca_06902	U-box domain-containing protein 4	NON_SYNONYMOUS_CODING
16416	CadSNP4906	*Desi*	Ca_Desi_Chr06	6048824	[C/T]	Ca_06916	Acyl-coenzyme A oxidase 2, peroxisomal	NON_SYNONYMOUS_CODING
16476	CadSNP9055	*Desi*	Ca_Desi_Scaffold_935	34654	[T/C]	Ca_14515	Glutamine--tRNA ligase	NON_SYNONYMOUS_CODING
16498	CadSNP11448	*Desi*	Ca_Desi_Scaffold_3429	26838	[T/A]	Ca_18368	U-box domain-containing protein 13	NON_SYNONYMOUS_CODING
16505	CadSNP12847	*Desi*	Ca_Desi_Scaffold_11652	290	[G/C]	Ca_20403	Primary amine oxidase (Fragment)	NON_SYNONYMOUS_CODING
16670	CadSNP681	*Desi*	Ca_Desi_Chr01	11411433	[G/C]	Ca_00882	MYB family transcription factor	NON_SYNONYMOUS_CODING
16837	CadSNP1131	*Desi*	Ca_Desi_Chr02	7082872	[A/C]	Ca_01546	ABSCISIC ACID-INSENSITIVE 5-like protein 2	NON_SYNONYMOUS_CODING
16896	CakSNP16896	*Kabuli*	Ca_Kabuli_denovo	100026	(C/G)	-	-	-
17081	CadSNP1819	*Desi*	Ca_Desi_Chr03	4356981	[C/T]	Ca_02541	WD repeat domain phosphoinositide-interacting protein 3	NON_SYNONYMOUS_CODING
17255	CadSNP2316	*Desi*	Ca_Desi_Chr03	15385992	[G/C]	Ca_03197	Zinc finger CCCH domain-containing protein 5	NON_SYNONYMOUS_CODING
17256	CadSNP2317	*Desi*	Ca_Desi_Chr03	15386093	[A/C]	Ca_03197	Zinc finger CCCH domain-containing protein 5	NON_SYNONYMOUS_CODING
17465	CadSNP3012	*Desi*	Ca_Desi_Chr04	3248872	[G/C]	Ca_04110	Transcriptional corepressor LEUNIG	NON_SYNONYMOUS_CODING
17540	CadSNP3309	*Desi*	Ca_Desi_Chr04	8131383	[G/T]	Ca_04406	Transcriptional corepressor LEUNIG	NON_SYNONYMOUS_CODING
17615	CadSNP3473	*Desi*	Ca_Desi_Chr04	11399103	[C/T]	Ca_04679	NAC domain-containing protein 78	NON_SYNONYMOUS_CODING
17696	CakSNP17696	*Kabuli*	Ca_Kabuli_denovo	575809	(A/G)	-	-	-
17710	CakSNP17710	*Kabuli*	Ca_Kabuli_denovo	1158419	(G/C)	-	-	-
17875	CakSNP17875	*Kabuli*	Ca_Kabuli_denovo	2017269	(C/T)	-	-	-
17898	CadSNP4178	*Desi*	Ca_Desi_Chr05	6320255	[T/C]	Ca_05724	Protein ETHYLENE INSENSITIVE 3	NON_SYNONYMOUS_CODING
17899	CadSNP4179	*Desi*	Ca_Desi_Chr05	6320247	[G/C]	Ca_05724	Protein ETHYLENE INSENSITIVE 3	NON_SYNONYMOUS_CODING
17942	CakSNP17942	*Kabuli*	Ca_Kabuli_denovo	3004600	(C/G)	-	-	-
18011	CakSNP18011	*Kabuli*	Ca_Kabuli_denovo	4058620	(G/C)	-	-	-
18048	CadSNP4543	*Desi*	Ca_Desi_Chr05	13173580	[G/A]	Ca_06336	Protein of unknown function (DUF399 and DUF3411)	NON_SYNONYMOUS_CODING
18074	CakSNP18074	*Kabuli*	Ca_Kabuli_denovo	5021916	(A/T)	-	-	-
18164	CakSNP18164	*Kabuli*	Ca_Kabuli_denovo	6010227	(T/A)	-	-	-
18249	CadSNP5179	*Desi*	Ca_Desi_Chr06	10172037	[G/T]	Ca_07258	Homeobox protein 10	NON_SYNONYMOUS_CODING
18250	CadSNP5180	*Desi*	Ca_Desi_Chr06	10171978	[A/C]	Ca_07258	Homeobox protein 10	NON_SYNONYMOUS_CODING
18330	CakSNP18330	*Kabuli*	Ca_Kabuli_denovo	7000764	(A/T)	-	-	-
18576	CadSNP5964	*Desi*	Ca_Desi_Chr08	8124369	[T/G]	Ca_08579	Transcription factor MYB98	NON_SYNONYMOUS_CODING
18611	CakSNP18611	*Kabuli*	Ca_Kabuli_denovo	8001503	(T/C)	-	-	-
18613	CadSNP6067	*Desi*	Ca_Desi_Scaffold_32	210202	[G/T]	Ca_10618	Probable WRKY transcription factor 19	NON_SYNONYMOUS_CODING
18659	CadSNP6181	*Desi*	Ca_Desi_Scaffold_93	157340	[G/C]	Ca_10810	Protein of unknown function (DUF1644)	URR
18992	CakSNP18992	*Kabuli*	Ca_Kabuli_denovo	9007535	(C/A)	-	-	-
19034	CadSNP7143	*Desi*	Ca_Desi_Scaffold_310	49502	[C/G]	Ca_11984	Zinc finger CCCH domain-containing protein 48	NON_SYNONYMOUS_CODING
19181	CadSNP7678	*Desi*	Ca_Desi_Scaffold_450	42711	[C/G]	Ca_12689	Transcription factor TCP7	NON_SYNONYMOUS_CODING
19208	CadSNP7737	*Desi*	Ca_Desi_Scaffold_473	13637	[G/C]	Ca_12770	SWI/SNF complex subunit SWI3C	NON_SYNONYMOUS_CODING
19404	CadSNP8263	*Desi*	Ca_Desi_Scaffold_624	69711	[A/G]	Ca_13464	WRKY transcription factor 6	NON_SYNONYMOUS_CODING
19419	CakSNP19419	*Kabuli*	Ca_Kabuli_denovo	10003680	(G/T)	-	-	-

S.No.	SNP IDs	Chickpea cultivars	Chromosomes	Physical positions (bp)	SNPs	Gene accession IDs	Putative functions	Sequence components of genome
19458	CadSNP8418	*Desi*	Ca_Desi_Scaffold_680	76297	[G/A]	Ca_13667	Protein of unknown function (DUF604)	NON_SYNONYMOUS_CODING
19459	CadSNP8419	*Desi*	Ca_Desi_Scaffold_680	76290	[G/A]	Ca_13667	Protein of unknown function (DUF604)	NON_SYNONYMOUS_CODING
19803	CakSNP19803	*Kabuli*	Ca_Kabuli_denovo	11000849	(G/C)	-	-	-
19859	CadSNP9459	*Desi*	Ca_Desi_Scaffold_1166	34273	[C/G]	Ca_15118	Dof zinc finger protein DOF5.2	NON_SYNONYMOUS_CODING
20173	CakSNP20173	*Kabuli*	Ca_Kabuli_denovo	12005056	(T/C)	-	-	-
20213	CadSNP10164	*Desi*	Ca_Desi_Scaffold_1667	33602	[T/G]	Ca_16252	Zinc finger CCCH domain-containing protein 53	NON_SYNONYMOUS_CODING
20500	CakSNP20500	*Kabuli*	Ca_Kabuli_denovo	13000283	(A/C)	-	-	-
20501	CadSNP10717	*Desi*	Ca_Desi_Scaffold_2257	32638	[A/G]	Ca_17178	Protein of unknown function, DUF617	URR
20852	CadSNP11361	*Desi*	Ca_Desi_Scaffold_3151	27219	[T/C]	Ca_18179	SET domain-containing protein	NON_SYNONYMOUS_CODING
20867	CakSNP20867	*Kabuli*	Ca_Kabuli_denovo	14013165	(T/C)	-	-	-
21086	CadSNP11790	*Desi*	Ca_Desi_Scaffold_4448	15163	[G/A]	Ca_18943	Protein of unknown function (DUF810)	NON_SYNONYMOUS_CODING
21210	CakSNP21210	*Kabuli*	Ca_Kabuli_denovo	15002955	(T/C)	-	-	-
21324	CadSNP12159	*Desi*	Ca_Desi_Scaffold_5938	6461	[G/C]	Ca_19525	Transcriptional corepressor LEUNIG	NON_SYNONYMOUS_CODING
21583	CadSNP12532	*Desi*	Ca_Desi_Scaffold_8872	3778	[C/T]	Ca_20116	Zinc finger CCCH domain-containing protein	URR
21591	CakSNP21591	*Kabuli*	Ca_Kabuli_denovo	16007356	(G/C)	-	-	-
21957	CakSNP21957	*Kabuli*	Ca_Kabuli_denovo	17004468	(G/A)	-	-	-
22347	CakSNP22347	*Kabuli*	Ca_Kabuli_denovo	18000376	(T/C)	-	-	-
22769	CakSNP22769	*Kabuli*	Ca_Kabuli_denovo	19005025	(A/C)	-	-	-
23147	CakSNP23147	*Kabuli*	Ca_Kabuli_denovo	20000681	(G/A)	-	-	-
23523	CakSNP23523	*Kabuli*	Ca_Kabuli_denovo	21002777	(T/C)	-	-	-
23897	CakSNP23897	*Kabuli*	Ca_Kabuli_denovo	22001325	(C/A)	-	-	-
24180	CakSNP24180	*Kabuli*	Ca_Kabuli_denovo	23389902	(A/G)	-	-	-
24181	CakSNP24181	*Kabuli*	Ca_Kabuli_denovo	26279213	(G/C)	-	-	-
24184	CakSNP24184	*Kabuli*	Ca_Kabuli_denovo	28463588	(A/G)	-	-	-
24186	CakSNP24186	*Kabuli*	Ca_Kabuli_denovo	30521758	(C/T)	-	-	-
24382	CakSNP24382	*Kabuli*	Ca_Kabuli_denovo	39369006	(C/G)	-	-	-
24383	CakSNP24383	*Kabuli*	Ca_Kabuli_denovo	43194868	(T/A)	-	-	-
24395	CakSNP24395	*Kabuli*	Ca_Kabuli_denovo	44029557	(G/C)	-	-	-
38010	CadSNP13605	*Desi*	Ca_Desi_denovo	5897	(T/G)	-	-	-
38494	CadSNP14089	*Desi*	Ca_Desi_denovo	1010473	(G/C)	-	-	-
38517	CadSNP14112	*Desi*	Ca_Desi_denovo	2216494	(A/G)	-	-	-
38597	CadSNP14192	*Desi*	Ca_Desi_denovo	3004633	(G/A)	-	-	-
38663	CadSNP14258	*Desi*	Ca_Desi_denovo	4006808	(C/T)	-	-	-
38754	CadSNP14349	*Desi*	Ca_Desi_denovo	5001267	(C/G)	-	-	-
38835	CadSNP14430	*Desi*	Ca_Desi_denovo	6010338	(T/C)	-	-	-
38903	CadSNP14498	*Desi*	Ca_Desi_denovo	7038125	(C/G)	-	-	-
39047	CadSNP14642	*Desi*	Ca_Desi_denovo	8007420	(C/T)	-	-	-
39384	CadSNP14979	*Desi*	Ca_Desi_denovo	9001802	(G/A)	-	-	-
39748	CadSNP15343	*Desi*	Ca_Desi_denovo	10007242	(T/G)	-	-	-

S.No.	SNP IDs	Chickpea cultivars	Chromosomes	Physical positions (bp)	SNPs	Gene accession IDs	Putative functions	Sequence components of genome
40091	CadSNP15686	*Desi*	Ca_Desi_denovo	11002604	(G/A)	-	-	-
40420	CadSNP16015	*Desi*	Ca_Desi_denovo	12008450	(C/A)	-	-	-
40750	CadSNP16345	*Desi*	Ca_Desi_denovo	13000685	(G/T)	-	-	-
41086	CadSNP16681	*Desi*	Ca_Desi_denovo	14016547	(G/A)	-	-	-
41415	CadSNP17010	*Desi*	Ca_Desi_denovo	15000265	(G/A)	-	-	-
41785	CadSNP17380	*Desi*	Ca_Desi_denovo	16001698	(C/G)	-	-	-
42100	CadSNP17695	*Desi*	Ca_Desi_denovo	17000658	(G/A)	-	-	-
42473	CadSNP18068	*Desi*	Ca_Desi_denovo	18001097	(T/C)	-	-	-
42845	CadSNP18440	*Desi*	Ca_Desi_denovo	19000611	(A/C)	-	-	-
43200	CadSNP18795	*Desi*	Ca_Desi_denovo	20000686	(C/T)	-	-	-
43575	CadSNP19170	*Desi*	Ca_Desi_denovo	21003641	(A/G)	-	-	-
43914	CadSNP19509	*Desi*	Ca_Desi_denovo	22014511	(C/T)	-	-	-
44223	CadSNP19818	*Desi*	Ca_Desi_denovo	23046661	(C/T)	-	-	-
44277	CadSNP19872	*Desi*	Ca_Desi_denovo	24002753	(G/T)	-	-	-
44330	CadSNP19925	*Desi*	Ca_Desi_denovo	25033757	(A/G)	-	-	-
44379	CadSNP19974	*Desi*	Ca_Desi_denovo	26009730	(A/G)	-	-	-
44433	CadSNP20028	*Desi*	Ca_Desi_denovo	27089069	(A/G)	-	-	-
44496	CadSNP20091	*Desi*	Ca_Desi_denovo	28003445	(C/A)	-	-	-
44548	CadSNP20143	*Desi*	Ca_Desi_denovo	29032209	(T/C)	-	-	-
44605	CadSNP20200	*Desi*	Ca_Desi_denovo	30023670	(A/G)	-	-	-
44683	CadSNP20278	*Desi*	Ca_Desi_denovo	31078584	(A/G)	-	-	-
44701	CadSNP20296	*Desi*	Ca_Desi_denovo	32026141	(G/T)	-	-	-
44774	CadSNP20369	*Desi*	Ca_Desi_denovo	33111070	(C/A)	-	-	-
44792	CadSNP20387	*Desi*	Ca_Desi_denovo	34002689	(C/T)	-	-	-
44823	CadSNP20418	*Desi*	Ca_Desi_denovo	39746958	(C/G)	-	-	-
44824	CadSNP20419	*Desi*	Ca_Desi_denovo	43386023	(C/T)	-	-	-
44830	CadSNP20425	*Desi*	Ca_Desi_denovo	44195103	(T/C)	-	-	-

SNPs showing polymorphism between a representaive set of contrasting stress tolerant and sensitive chickpea accessions are marked with blue colour fonts

SNPs annoated in the genes underlying the known QTLs reported earlier for stress tolerance in chickpea are indicated with green colour fonts

SNPs annoated in the transcription factor genes are highlighted with red colour fonts
