# Supplementary material for: Employing genome-wide SNP discovery and genotyping strategy to extrapolate the natural allelic diversity and domestication patterns in chickpea
Source: Front Plant Sci. 2015 Mar 31;6:162. doi: 10.3389/fpls.2015.00162 (PMC4379880; doi:10.3389/fpls.2015.00162)
Supplement: Supplementary file 19 [file Table9.PDF]

Table S9: Polymorphism and molecular diversity potential of GBS-based genome-wide SNPs in 93 chickpea accessions estimated by diverse polymorphism and diversity statistical measures

**Accessions**	**Polymorphism and diversity statistics**									
	**Number (%) of SNP showing polymorphism (MAF ≥ 0.05)**	**Polymorphism information content (PIC)**			**Minor Allele Frequency (MAF)**	**Nucleotide diversity**		**Genetic distance**		
		**Maximum**	**Minimum**	**Mean**		**θπ**	**θω**	**Maximum**	**Minimum**	**Average**
All 93 accessions	23798	0.47	0.12	0.42	0.37	1.30	1.51	0.89 [ICC15264 (*kabuli*) and ICC17160 (wild)]	0.13 [ICC7346 (*kabuli*) and ICC15512 (*kabuli*)]	0.56
92 cultivated accessions	11780 (49.5)	0.42	0.13	0.38	0.34	1.15	1.26	0.76 [ICC15264 (*kabuli*) and ICC9002 (*desi*)]	0.12 [ICC7346 (*kabuli*) and ICC15512 (*kabuli*)]	0.45
39 *desi* accessions	10012 (42.1)	0.40	0.13	0.35	0.31	1.20	1.33	0.67 [ICC7184 (*desi*) and ICCV93954 (*desi*)]	0.10 [ICC5002 (*desi*) and ICC6013 (*desi*)]	0.43
53 *kabuli* accessions	8948 (37.6)	0.38	0.12	0.30	0.27	0.96	1.07	0.65 [ICC15512 (*kabuli*) and IC449069 (*kabuli*)]	0.10 [ICC15512 (*kabuli*) and ICC7346 (*kabuli)*]	0.38

θπ: Average pair-wise nucleotide diversity

θω: Watterson's estimator of segregating sites
