# Supplementary material for: Employing genome-wide SNP discovery and genotyping strategy to extrapolate the natural allelic diversity and domestication patterns in chickpea
Source: Front Plant Sci. 2015 Mar 31;6:162. doi: 10.3389/fpls.2015.00162 (PMC4379880; doi:10.3389/fpls.2015.00162)
Supplement: Supplementary file 20 [file Table10.PDF]

**Table S10:** Polymorphic potential (represented by diverse statistical measures) estimated in three model-based populations (inferred by population genetic structure analysis) using GBS-based genome-wide SNPs

| Populations | Number of accessions | PIC (polymorphism information content) | Minor allele frequency (MAF) | Nucleotide diversity | | Genetic distance Minimum-Maximum (mean) |
|---|---|---|---|---|---|---|
| | | | | θπ | θω | |
| **POP I** | 44 | 0.10-0.38 (0.28) | 0.25 | 0.98 | 1.10 | 0.11 [ICC 14199 and ICC 11742] - 0.62 [ICC 7308 and ICC 12028] (0.36) |
| **POP II** | 9 | 0.10-0.48 (0.40) | 0.36 | 1.28 | 1.47 | 0.08 [ICC 6204 and ICC 6210]- 0.91 [ICC 14446 and ICC 6253] (0.53) |
| **POP III** | 40 | 0.10-0.42 (0.36) | 0.30 | 1.22 | 1.35 | 0.10 [Annigeri and ICC 16374]- 0.65 [ICCV 93954 and IC 296131] (0.42) |
