# Supplementary material for: Employing genome-wide SNP discovery and genotyping strategy to extrapolate the natural allelic diversity and domestication patterns in chickpea
Source: Front Plant Sci. 2015 Mar 31;6:162. doi: 10.3389/fpls.2015.00162 (PMC4379880; doi:10.3389/fpls.2015.00162)
Supplement: Supplementary file 21 [file Table11.PDF]

Table S11: LD estimates among SNP-pairs physically mapped across eight chromosomes of *desi* and *kabuli* chickpea genomes

**Chromosomes**	**Linked**			**Unlinked**		
	**Number of SNP-pairs used**	**[a]Number of SNP-pairs (%) in significant LD**	**Extent of LD (mean r^2)**	**Number of SNP-pairs used**	**[a]Number of SNP-pairs (%) in significant LD**	**Extent of LD (mean r^2)**
Ca_*desi*_chr01	9692	5069 (52.3)	0.59	0	0	0
Ca_*desi*_chr02	8687	4763 (54.8)	0.60	665	327 (49.2)	0.77
Ca_*desi*_chr03	16673	11228 (67.3)	0.59	618	338 (54.7)	0.63
Ca_*desi*_chr04	17965	9658 (53.8)	0.60	1166	345 (29.6)	0.40
Ca_*desi*_chr05	5456	3505 (64.2)	0.59	451	274 (60.8)	0.52
Ca_*desi*_chr06	6678	3639 (54.5)	0.60	474	267 (56.3)	0.56
Ca_*desi*_chr07	3797	1749 (46.1)	0.43	484	245 (50.6)	0.52
Ca_*desi*_chr08	5741	2911 (50.7)	0.51	796	203 (25.5)	0.33
**Total *desi* chromosomes**	**74689**	**42522 (56.9)**	**0.56**	**4654**	**1999 (43.0)**	**0.53**
Ca_*kabuli*_chr01	19294	10462 (54.2)	0.53	0	0	0
Ca_*kabuli*_chr02	12157	6533 (53.7)	0.53	518	213 (41.1)	0.36
Ca_*kabuli*_chr03	12991	7530 (57.8)	0.55	893	245 (27.4)	0.45
Ca_*kabuli*_chr04	36231	21165 (58.4)	0.54	965	317 (32.8)	0.25
Ca_*kabuli*_chr05	6840	3408 (49.8)	0.48	1038	442 (42.6)	0.20
Ca_*kabuli*_chr06	16061	8722 (54.3)	0.54	715	226 (31.6)	0.36
Ca_*kabuli*_chr07	14301	7017 (49.1)	0.48	833	290 (34.8)	0.33
Ca_*kabuli*_chr08	5310	2463 (46.4)	0.49	656	282 (43.0)	0.37
**Total *kabuli* chromosomes**	**123185**	**67300 (54.6)**	**0.52**	**5618**	**2015 (35.9)**	**0.33**

[a]SNP-pairs in significant (P <0.0001) LD
