# Supplementary material for: Employing genome-wide SNP discovery and genotyping strategy to extrapolate the natural allelic diversity and domestication patterns in chickpea
Source: Front Plant Sci. 2015 Mar 31;6:162. doi: 10.3389/fpls.2015.00162 (PMC4379880; doi:10.3389/fpls.2015.00162)
Supplement: Supplementary file 13 [file Table3.PDF]

**Table S3:** 44844 genome-wide SNPs identified using reference genome (*desi* and *kabuli*)- and *de novo* -based GBS assay in chickpea					
**S.N.**	**SNP IDs**	**Cultivars**	**Chromosomes/scaffolds**	**Physical positions (bp)**	**SNPs**
1	CakSNP1	*Kabuli*	Ca_Kabuli_Chr01	523	(G/C)
2	CakSNP2	*Kabuli*	Ca_Kabuli_Chr01	462	(G/C)
3	CakSNP3	*Kabuli*	Ca_Kabuli_Chr01	548	(G/C)
4	CakSNP4	*Kabuli*	Ca_Kabuli_Chr01	578	(G/A)
5	CakSNP5	*Kabuli*	Ca_Kabuli_Chr01	579	(T/C)
6	CakSNP6	*Kabuli*	Ca_Kabuli_Chr01	589	(A/C)
7	CakSNP7	*Kabuli*	Ca_Kabuli_Chr01	627	(C/A)
8	CakSNP8	*Kabuli*	Ca_Kabuli_Chr01	839	(A/T)
9	CakSNP9	*Kabuli*	Ca_Kabuli_Chr01	903	(G/T)
10	CakSNP10	*Kabuli*	Ca_Kabuli_Chr01	927	(C/A)
11	CakSNP11	*Kabuli*	Ca_Kabuli_Chr01	98930	(T/C)
12	CakSNP12	*Kabuli*	Ca_Kabuli_Chr01	98992	(G/T)
13	CakSNP13	*Kabuli*	Ca_Kabuli_Chr01	99242	(G/A)
14	CakSNP14	*Kabuli*	Ca_Kabuli_Chr01	108882	(C/A)
15	CakSNP15	*Kabuli*	Ca_Kabuli_Chr01	108984	(G/A)
16	CakSNP16	*Kabuli*	Ca_Kabuli_Chr01	108979	(T/G)
17	CakSNP17	*Kabuli*	Ca_Kabuli_Chr01	129394	(A/G)
18	CakSNP18	*Kabuli*	Ca_Kabuli_Chr01	129401	(A/G)
19	CakSNP19	*Kabuli*	Ca_Kabuli_Chr01	173669	(C/T)
20	CakSNP20	*Kabuli*	Ca_Kabuli_Chr01	202954	(G/T)
21	CakSNP21	*Kabuli*	Ca_Kabuli_Chr01	208244	(G/A)
22	CakSNP22	*Kabuli*	Ca_Kabuli_Chr01	214979	(T/A)
23	CakSNP23	*Kabuli*	Ca_Kabuli_Chr01	215066	(G/T)
24	CakSNP24	*Kabuli*	Ca_Kabuli_Chr01	215058	(T/C)
25	CakSNP25	*Kabuli*	Ca_Kabuli_Chr01	215053	(G/T)
26	CakSNP26	*Kabuli*	Ca_Kabuli_Chr01	215048	(C/T)
27	CakSNP27	*Kabuli*	Ca_Kabuli_Chr01	215029	(C/A)
28	CakSNP28	*Kabuli*	Ca_Kabuli_Chr01	215027	(T/C)
29	CakSNP29	*Kabuli*	Ca_Kabuli_Chr01	215006	(C/T)
30	CakSNP30	*Kabuli*	Ca_Kabuli_Chr01	215239	(C/G)
31	CakSNP31	*Kabuli*	Ca_Kabuli_Chr01	292962	(T/C)
32	CakSNP32	*Kabuli*	Ca_Kabuli_Chr01	292972	(G/A)
33	CakSNP33	*Kabuli*	Ca_Kabuli_Chr01	292998	(T/C)
34	CakSNP34	*Kabuli*	Ca_Kabuli_Chr01	347620	(A/C)
35	CakSNP35	*Kabuli*	Ca_Kabuli_Chr01	372530	(A/G)
36	CakSNP36	*Kabuli*	Ca_Kabuli_Chr01	406570	(A/G)
37	CakSNP37	*Kabuli*	Ca_Kabuli_Chr01	425149	(T/C)

S.N.	SNP IDs	Cultivars	Chromosomes/scaffolds	Physical positions (bp)	SNPs
38	CakSNP38	*Kabuli*	Ca_Kabuli_Chr01	425076	(A/G)
39	CakSNP39	*Kabuli*	Ca_Kabuli_Chr01	435259	(A/G)
40	CakSNP40	*Kabuli*	Ca_Kabuli_Chr01	435274	(G/T)
41	CakSNP41	*Kabuli*	Ca_Kabuli_Chr01	435280	(T/C)
42	CakSNP42	*Kabuli*	Ca_Kabuli_Chr01	435297	(G/A)
43	CakSNP43	*Kabuli*	Ca_Kabuli_Chr01	435304	(G/T)
44	CakSNP44	*Kabuli*	Ca_Kabuli_Chr01	435343	(C/A)
45	CakSNP45	*Kabuli*	Ca_Kabuli_Chr01	435338	(T/C)
46	CakSNP46	*Kabuli*	Ca_Kabuli_Chr01	435264	(G/T)
47	CakSNP47	*Kabuli*	Ca_Kabuli_Chr01	441283	(G/A)
48	CakSNP48	*Kabuli*	Ca_Kabuli_Chr01	441286	(C/T)
49	CakSNP49	*Kabuli*	Ca_Kabuli_Chr01	441288	(T/A)
50	CakSNP50	*Kabuli*	Ca_Kabuli_Chr01	441343	(T/A)
51	CakSNP51	*Kabuli*	Ca_Kabuli_Chr01	446409	(T/C)
52	CakSNP52	*Kabuli*	Ca_Kabuli_Chr01	446443	(A/G)
53	CakSNP53	*Kabuli*	Ca_Kabuli_Chr01	446484	(C/T)
54	CakSNP54	*Kabuli*	Ca_Kabuli_Chr01	446558	(G/T)
55	CakSNP55	*Kabuli*	Ca_Kabuli_Chr01	459968	(T/G)
56	CakSNP56	*Kabuli*	Ca_Kabuli_Chr01	464039	(A/C)
57	CakSNP57	*Kabuli*	Ca_Kabuli_Chr01	464040	(G/C)
58	CakSNP58	*Kabuli*	Ca_Kabuli_Chr01	464043	(C/G)
59	CakSNP59	*Kabuli*	Ca_Kabuli_Chr01	471007	(C/T)
60	CakSNP60	*Kabuli*	Ca_Kabuli_Chr01	471037	(C/T)
61	CakSNP61	*Kabuli*	Ca_Kabuli_Chr01	527271	(T/G)
62	CakSNP62	*Kabuli*	Ca_Kabuli_Chr01	527321	(C/T)
63	CakSNP63	*Kabuli*	Ca_Kabuli_Chr01	554581	(G/T)
64	CakSNP64	*Kabuli*	Ca_Kabuli_Chr01	554677	(G/A)
65	CakSNP65	*Kabuli*	Ca_Kabuli_Chr01	571020	(T/G)
66	CakSNP66	*Kabuli*	Ca_Kabuli_Chr01	591609	(T/C)
67	CakSNP67	*Kabuli*	Ca_Kabuli_Chr01	609709	(A/G)
68	CakSNP68	*Kabuli*	Ca_Kabuli_Chr01	609671	(C/T)
69	CakSNP69	*Kabuli*	Ca_Kabuli_Chr01	609669	(C/G)
70	CakSNP70	*Kabuli*	Ca_Kabuli_Chr01	644318	(C/T)
71	CakSNP71	*Kabuli*	Ca_Kabuli_Chr01	644353	(G/A)
72	CakSNP72	*Kabuli*	Ca_Kabuli_Chr01	644359	(T/A)
73	CakSNP73	*Kabuli*	Ca_Kabuli_Chr01	644440	(T/C)
74	CakSNP74	*Kabuli*	Ca_Kabuli_Chr01	644425	(G/A)
75	CakSNP75	*Kabuli*	Ca_Kabuli_Chr01	661882	(A/G)
76	CakSNP76	*Kabuli*	Ca_Kabuli_Chr01	662341	(A/C)

S.N.	SNP IDs	Cultivars	Chromosomes/scaffolds	Physical positions (bp)	SNPs
77	CakSNP77	*Kabuli*	Ca_Kabuli_Chr01	662489	(T/A)
78	CakSNP78	*Kabuli*	Ca_Kabuli_Chr01	671420	(C/A)
79	CakSNP79	*Kabuli*	Ca_Kabuli_Chr01	671410	(T/C)
80	CakSNP80	*Kabuli*	Ca_Kabuli_Chr01	671391	(T/C)
81	CakSNP81	*Kabuli*	Ca_Kabuli_Chr01	671389	(A/G)
82	CakSNP82	*Kabuli*	Ca_Kabuli_Chr01	671388	(A/T)
83	CakSNP83	*Kabuli*	Ca_Kabuli_Chr01	690156	(C/A)
84	CakSNP84	*Kabuli*	Ca_Kabuli_Chr01	690431	(G/A)
85	CakSNP85	*Kabuli*	Ca_Kabuli_Chr01	691102	(A/T)
86	CakSNP86	*Kabuli*	Ca_Kabuli_Chr01	695962	(A/C)
87	CakSNP87	*Kabuli*	Ca_Kabuli_Chr01	712110	(T/C)
88	CakSNP88	*Kabuli*	Ca_Kabuli_Chr01	712073	(C/T)
89	CakSNP89	*Kabuli*	Ca_Kabuli_Chr01	741514	(G/T)
90	CakSNP90	*Kabuli*	Ca_Kabuli_Chr01	741535	(C/T)
91	CakSNP91	*Kabuli*	Ca_Kabuli_Chr01	741565	(T/C)
92	CakSNP92	*Kabuli*	Ca_Kabuli_Chr01	741549	(C/T)
93	CakSNP93	*Kabuli*	Ca_Kabuli_Chr01	741980	(C/G)
94	CakSNP94	*Kabuli*	Ca_Kabuli_Chr01	742043	(G/A)
95	CakSNP95	*Kabuli*	Ca_Kabuli_Chr01	742026	(T/G)
96	CakSNP96	*Kabuli*	Ca_Kabuli_Chr01	750693	(C/T)
97	CakSNP97	*Kabuli*	Ca_Kabuli_Chr01	777273	(C/T)
98	CakSNP98	*Kabuli*	Ca_Kabuli_Chr01	777240	(G/A)
99	CakSNP99	*Kabuli*	Ca_Kabuli_Chr01	790004	(A/C)
100	CakSNP100	*Kabuli*	Ca_Kabuli_Chr01	790000	(C/T)
101	CakSNP101	*Kabuli*	Ca_Kabuli_Chr01	790102	(G/T)
102	CakSNP102	*Kabuli*	Ca_Kabuli_Chr01	864205	(T/C)
103	CakSNP103	*Kabuli*	Ca_Kabuli_Chr01	864382	(T/C)
104	CakSNP104	*Kabuli*	Ca_Kabuli_Chr01	864381	(G/A)
105	CakSNP105	*Kabuli*	Ca_Kabuli_Chr01	864351	(T/C)
106	CakSNP106	*Kabuli*	Ca_Kabuli_Chr01	866872	(T/C)
107	CakSNP107	*Kabuli*	Ca_Kabuli_Chr01	872443	(G/T)
108	CakSNP108	*Kabuli*	Ca_Kabuli_Chr01	872452	(C/T)
109	CakSNP109	*Kabuli*	Ca_Kabuli_Chr01	876616	(T/C)
110	CakSNP110	*Kabuli*	Ca_Kabuli_Chr01	876633	(T/A)
111	CakSNP111	*Kabuli*	Ca_Kabuli_Chr01	881543	(C/A)
112	CakSNP112	*Kabuli*	Ca_Kabuli_Chr01	881673	(G/A)
113	CakSNP113	*Kabuli*	Ca_Kabuli_Chr01	881703	(G/T)
114	CakSNP114	*Kabuli*	Ca_Kabuli_Chr01	890949	(G/A)
115	CakSNP115	*Kabuli*	Ca_Kabuli_Chr01	890948	(C/T)

S.N.	SNP IDs	Cultivars	Chromosomes/scaffolds	Physical positions (bp)	SNPs
116	CakSNP116	*Kabuli*	Ca_Kabuli_Chr01	910486	(T/A)
117	CakSNP117	*Kabuli*	Ca_Kabuli_Chr01	970315	(A/T)
118	CakSNP118	*Kabuli*	Ca_Kabuli_Chr01	996439	(T/C)
119	CakSNP119	*Kabuli*	Ca_Kabuli_Chr01	1007698	(A/G)
120	CakSNP120	*Kabuli*	Ca_Kabuli_Chr01	1008895	(A/G)
121	CakSNP121	*Kabuli*	Ca_Kabuli_Chr01	1008971	(C/G)
122	CakSNP122	*Kabuli*	Ca_Kabuli_Chr01	1009058	(A/C)
123	CakSNP123	*Kabuli*	Ca_Kabuli_Chr01	1018966	(C/G)
124	CakSNP124	*Kabuli*	Ca_Kabuli_Chr01	1020800	(A/G)
125	CakSNP125	*Kabuli*	Ca_Kabuli_Chr01	1023550	(C/T)
126	CakSNP126	*Kabuli*	Ca_Kabuli_Chr01	1023538	(T/C)
127	CakSNP127	*Kabuli*	Ca_Kabuli_Chr01	1032734	(A/T)
128	CakSNP128	*Kabuli*	Ca_Kabuli_Chr01	1059209	(A/G)
129	CakSNP129	*Kabuli*	Ca_Kabuli_Chr01	1059311	(A/G)
130	CakSNP130	*Kabuli*	Ca_Kabuli_Chr01	1083274	(T/C)
131	CakSNP131	*Kabuli*	Ca_Kabuli_Chr01	1083388	(G/A)
132	CakSNP132	*Kabuli*	Ca_Kabuli_Chr01	1083403	(C/T)
133	CakSNP133	*Kabuli*	Ca_Kabuli_Chr01	1085228	(A/C)
134	CakSNP134	*Kabuli*	Ca_Kabuli_Chr01	1085230	(T/G)
135	CakSNP135	*Kabuli*	Ca_Kabuli_Chr01	1085238	(A/G)
136	CakSNP136	*Kabuli*	Ca_Kabuli_Chr01	1085254	(A/C)
137	CakSNP137	*Kabuli*	Ca_Kabuli_Chr01	1085263	(A/C)
138	CakSNP138	*Kabuli*	Ca_Kabuli_Chr01	1085293	(G/A)
139	CakSNP139	*Kabuli*	Ca_Kabuli_Chr01	1085348	(G/A)
140	CakSNP140	*Kabuli*	Ca_Kabuli_Chr01	1091548	(T/A)
141	CakSNP141	*Kabuli*	Ca_Kabuli_Chr01	1091534	(A/C)
142	CakSNP142	*Kabuli*	Ca_Kabuli_Chr01	1091504	(C/T)
143	CakSNP143	*Kabuli*	Ca_Kabuli_Chr01	1098703	(C/G)
144	CakSNP144	*Kabuli*	Ca_Kabuli_Chr01	1098789	(A/G)
145	CakSNP145	*Kabuli*	Ca_Kabuli_Chr01	1115668	(C/T)
146	CakSNP146	*Kabuli*	Ca_Kabuli_Chr01	1115809	(A/G)
147	CakSNP147	*Kabuli*	Ca_Kabuli_Chr01	1116043	(C/T)
148	CakSNP148	*Kabuli*	Ca_Kabuli_Chr01	1140313	(A/C)
149	CakSNP149	*Kabuli*	Ca_Kabuli_Chr01	1140405	(A/T)
150	CakSNP150	*Kabuli*	Ca_Kabuli_Chr01	1150973	(C/T)
151	CakSNP151	*Kabuli*	Ca_Kabuli_Chr01	1159383	(C/A)
152	CakSNP152	*Kabuli*	Ca_Kabuli_Chr01	1159411	(T/G)
153	CakSNP153	*Kabuli*	Ca_Kabuli_Chr01	1215024	(T/C)
154	CakSNP154	*Kabuli*	Ca_Kabuli_Chr01	1269212	(C/G)

S.N.	SNP IDs	Cultivars	Chromosomes/scaffolds	Physical positions (bp)	SNPs
155	CakSNP155	*Kabuli*	Ca_Kabuli_Chr01	1269180	(C/A)
156	CakSNP156	*Kabuli*	Ca_Kabuli_Chr01	1278884	(G/T)
157	CakSNP157	*Kabuli*	Ca_Kabuli_Chr01	1278997	(T/A)
158	CakSNP158	*Kabuli*	Ca_Kabuli_Chr01	1295527	(T/G)
159	CakSNP159	*Kabuli*	Ca_Kabuli_Chr01	1335518	(A/C)
160	CakSNP160	*Kabuli*	Ca_Kabuli_Chr01	1385553	(C/A)
161	CakSNP161	*Kabuli*	Ca_Kabuli_Chr01	1385554	(G/T)
162	CakSNP162	*Kabuli*	Ca_Kabuli_Chr01	1390073	(A/G)
163	CakSNP163	*Kabuli*	Ca_Kabuli_Chr01	1411348	(C/T)
164	CakSNP164	*Kabuli*	Ca_Kabuli_Chr01	1411351	(A/C)
165	CakSNP165	*Kabuli*	Ca_Kabuli_Chr01	1411501	(C/T)
166	CakSNP166	*Kabuli*	Ca_Kabuli_Chr01	1411498	(T/A)
167	CakSNP167	*Kabuli*	Ca_Kabuli_Chr01	1411495	(A/G)
168	CakSNP168	*Kabuli*	Ca_Kabuli_Chr01	1411490	(T/C)
169	CakSNP169	*Kabuli*	Ca_Kabuli_Chr01	1428222	(C/G)
170	CakSNP170	*Kabuli*	Ca_Kabuli_Chr01	1428251	(T/G)
171	CakSNP171	*Kabuli*	Ca_Kabuli_Chr01	1428264	(T/G)
172	CakSNP172	*Kabuli*	Ca_Kabuli_Chr01	1434121	(A/G)
173	CakSNP173	*Kabuli*	Ca_Kabuli_Chr01	1434883	(A/G)
174	CakSNP174	*Kabuli*	Ca_Kabuli_Chr01	1434875	(A/G)
175	CakSNP175	*Kabuli*	Ca_Kabuli_Chr01	1449848	(G/A)
176	CakSNP176	*Kabuli*	Ca_Kabuli_Chr01	1449913	(T/G)
177	CakSNP177	*Kabuli*	Ca_Kabuli_Chr01	1449949	(G/A)
178	CakSNP178	*Kabuli*	Ca_Kabuli_Chr01	1449980	(A/G)
179	CakSNP179	*Kabuli*	Ca_Kabuli_Chr01	1461574	(T/A)
180	CakSNP180	*Kabuli*	Ca_Kabuli_Chr01	1461600	(T/C)
181	CakSNP181	*Kabuli*	Ca_Kabuli_Chr01	1467056	(A/G)
182	CakSNP182	*Kabuli*	Ca_Kabuli_Chr01	1527513	(A/G)
183	CakSNP183	*Kabuli*	Ca_Kabuli_Chr01	1528325	(G/A)
184	CakSNP184	*Kabuli*	Ca_Kabuli_Chr01	1533723	(G/A)
185	CakSNP185	*Kabuli*	Ca_Kabuli_Chr01	1546679	(G/C)
186	CakSNP186	*Kabuli*	Ca_Kabuli_Chr01	1599821	(A/G)
187	CakSNP187	*Kabuli*	Ca_Kabuli_Chr01	1654823	(C/T)
188	CakSNP188	*Kabuli*	Ca_Kabuli_Chr01	1654855	(A/C)
189	CakSNP189	*Kabuli*	Ca_Kabuli_Chr01	1655018	(G/A)
190	CakSNP190	*Kabuli*	Ca_Kabuli_Chr01	1655012	(G/T)
191	CakSNP191	*Kabuli*	Ca_Kabuli_Chr01	1693010	(T/A)
192	CakSNP192	*Kabuli*	Ca_Kabuli_Chr01	1693013	(C/T)
193	CakSNP193	*Kabuli*	Ca_Kabuli_Chr01	1693022	(T/C)

S.N.	SNP IDs	Cultivars	Chromosomes/scaffolds	Physical positions (bp)	SNPs
194	CakSNP194	*Kabuli*	Ca_Kabuli_Chr01	1693023	(T/C)
195	CakSNP195	*Kabuli*	Ca_Kabuli_Chr01	1693031	(C/A)
196	CakSNP196	*Kabuli*	Ca_Kabuli_Chr01	1738179	(G/C)
197	CakSNP197	*Kabuli*	Ca_Kabuli_Chr01	1772813	(T/C)
198	CakSNP198	*Kabuli*	Ca_Kabuli_Chr01	1782760	(G/A)
199	CakSNP199	*Kabuli*	Ca_Kabuli_Chr01	1782793	(G/T)
200	CakSNP200	*Kabuli*	Ca_Kabuli_Chr01	1782808	(G/C)
201	CakSNP201	*Kabuli*	Ca_Kabuli_Chr01	1782953	(G/A)
202	CakSNP202	*Kabuli*	Ca_Kabuli_Chr01	1782948	(T/C)
203	CakSNP203	*Kabuli*	Ca_Kabuli_Chr01	1782930	(G/A)
204	CakSNP204	*Kabuli*	Ca_Kabuli_Chr01	1782929	(C/T)
205	CakSNP205	*Kabuli*	Ca_Kabuli_Chr01	1783207	(G/A)
206	CakSNP206	*Kabuli*	Ca_Kabuli_Chr01	1783277	(G/A)
207	CakSNP207	*Kabuli*	Ca_Kabuli_Chr01	1783280	(A/C)
208	CakSNP208	*Kabuli*	Ca_Kabuli_Chr01	1783342	(A/T)
209	CakSNP209	*Kabuli*	Ca_Kabuli_Chr01	1783923	(C/A)
210	CakSNP210	*Kabuli*	Ca_Kabuli_Chr01	1784006	(T/C)
211	CakSNP211	*Kabuli*	Ca_Kabuli_Chr01	1788958	(T/A)
212	CakSNP212	*Kabuli*	Ca_Kabuli_Chr01	1789087	(T/C)
213	CakSNP213	*Kabuli*	Ca_Kabuli_Chr01	1802992	(C/G)
214	CakSNP214	*Kabuli*	Ca_Kabuli_Chr01	1858073	(C/T)
215	CakSNP215	*Kabuli*	Ca_Kabuli_Chr01	1858078	(T/C)
216	CakSNP216	*Kabuli*	Ca_Kabuli_Chr01	1858084	(C/T)
217	CakSNP217	*Kabuli*	Ca_Kabuli_Chr01	1858132	(T/A)
218	CakSNP218	*Kabuli*	Ca_Kabuli_Chr01	1858156	(C/T)
219	CakSNP219	*Kabuli*	Ca_Kabuli_Chr01	1858177	(T/C)
220	CakSNP220	*Kabuli*	Ca_Kabuli_Chr01	1858159	(A/G)
221	CakSNP221	*Kabuli*	Ca_Kabuli_Chr01	1858509	(C/T)
222	CakSNP222	*Kabuli*	Ca_Kabuli_Chr01	1858608	(T/A)
223	CakSNP223	*Kabuli*	Ca_Kabuli_Chr01	1858600	(G/A)
224	CakSNP224	*Kabuli*	Ca_Kabuli_Chr01	1859789	(C/T)
225	CakSNP225	*Kabuli*	Ca_Kabuli_Chr01	1859857	(A/G)
226	CakSNP226	*Kabuli*	Ca_Kabuli_Chr01	1859914	(G/A)
227	CakSNP227	*Kabuli*	Ca_Kabuli_Chr01	1859902	(G/A)
228	CakSNP228	*Kabuli*	Ca_Kabuli_Chr01	1861696	(C/T)
229	CakSNP229	*Kabuli*	Ca_Kabuli_Chr01	1861707	(T/G)
230	CakSNP230	*Kabuli*	Ca_Kabuli_Chr01	1861722	(T/A)
231	CakSNP231	*Kabuli*	Ca_Kabuli_Chr01	1861729	(T/C)
232	CakSNP232	*Kabuli*	Ca_Kabuli_Chr01	1861837	(T/G)

S.N.	SNP IDs	Cultivars	Chromosomes/scaffolds	Physical positions (bp)	SNPs
233	CakSNP233	*Kabuli*	Ca_Kabuli_Chr01	1904233	(T/C)
234	CakSNP234	*Kabuli*	Ca_Kabuli_Chr01	1904230	(G/T)
235	CakSNP235	*Kabuli*	Ca_Kabuli_Chr01	1904365	(G/A)
236	CakSNP236	*Kabuli*	Ca_Kabuli_Chr01	1904391	(A/G)
237	CakSNP237	*Kabuli*	Ca_Kabuli_Chr01	1935729	(A/G)
238	CakSNP238	*Kabuli*	Ca_Kabuli_Chr01	1962876	(A/G)
239	CakSNP239	*Kabuli*	Ca_Kabuli_Chr01	1968828	(G/A)
240	CakSNP240	*Kabuli*	Ca_Kabuli_Chr01	2002844	(G/A)
241	CakSNP241	*Kabuli*	Ca_Kabuli_Chr01	2002922	(G/T)
242	CakSNP242	*Kabuli*	Ca_Kabuli_Chr01	2040943	(C/T)
243	CakSNP243	*Kabuli*	Ca_Kabuli_Chr01	2040946	(G/T)
244	CakSNP244	*Kabuli*	Ca_Kabuli_Chr01	2041027	(C/G)
245	CakSNP245	*Kabuli*	Ca_Kabuli_Chr01	2041190	(A/C)
246	CakSNP246	*Kabuli*	Ca_Kabuli_Chr01	2041180	(T/C)
247	CakSNP247	*Kabuli*	Ca_Kabuli_Chr01	2041178	(A/C)
248	CakSNP248	*Kabuli*	Ca_Kabuli_Chr01	2041174	(C/T)
249	CakSNP249	*Kabuli*	Ca_Kabuli_Chr01	2041154	(G/C)
250	CakSNP250	*Kabuli*	Ca_Kabuli_Chr01	2041132	(C/T)
251	CakSNP251	*Kabuli*	Ca_Kabuli_Chr01	2042071	(C/G)
252	CakSNP252	*Kabuli*	Ca_Kabuli_Chr01	2042185	(T/C)
253	CakSNP253	*Kabuli*	Ca_Kabuli_Chr01	2042152	(G/C)
254	CakSNP254	*Kabuli*	Ca_Kabuli_Chr01	2042149	(G/T)
255	CakSNP255	*Kabuli*	Ca_Kabuli_Chr01	2044338	(G/A)
256	CakSNP256	*Kabuli*	Ca_Kabuli_Chr01	2044304	(A/C)
257	CakSNP257	*Kabuli*	Ca_Kabuli_Chr01	2044289	(A/G)
258	CakSNP258	*Kabuli*	Ca_Kabuli_Chr01	2044263	(A/C)
259	CakSNP259	*Kabuli*	Ca_Kabuli_Chr01	2044359	(C/T)
260	CakSNP260	*Kabuli*	Ca_Kabuli_Chr01	2044374	(C/G)
261	CakSNP261	*Kabuli*	Ca_Kabuli_Chr01	2044380	(G/C)
262	CakSNP262	*Kabuli*	Ca_Kabuli_Chr01	2044408	(A/C)
263	CakSNP263	*Kabuli*	Ca_Kabuli_Chr01	2044414	(G/A)
264	CakSNP264	*Kabuli*	Ca_Kabuli_Chr01	2044474	(A/G)
265	CakSNP265	*Kabuli*	Ca_Kabuli_Chr01	2050232	(A/T)
266	CakSNP266	*Kabuli*	Ca_Kabuli_Chr01	2050187	(C/G)
267	CakSNP267	*Kabuli*	Ca_Kabuli_Chr01	2050479	(A/C)
268	CakSNP268	*Kabuli*	Ca_Kabuli_Chr01	2050469	(T/C)
269	CakSNP269	*Kabuli*	Ca_Kabuli_Chr01	2050467	(A/C)
270	CakSNP270	*Kabuli*	Ca_Kabuli_Chr01	2050465	(A/G)
271	CakSNP271	*Kabuli*	Ca_Kabuli_Chr01	2050463	(C/T)

S.N.	SNP IDs	Cultivars	Chromosomes/scaffolds	Physical positions (bp)	SNPs
272	CakSNP272	*Kabuli*	Ca_Kabuli_Chr01	2050443	(G/C)
273	CakSNP273	*Kabuli*	Ca_Kabuli_Chr01	2050421	(C/T)
274	CakSNP274	*Kabuli*	Ca_Kabuli_Chr01	2051360	(A/G)
275	CakSNP275	*Kabuli*	Ca_Kabuli_Chr01	2051375	(G/T)
276	CakSNP276	*Kabuli*	Ca_Kabuli_Chr01	2051379	(C/T)
277	CakSNP277	*Kabuli*	Ca_Kabuli_Chr01	2053732	(C/T)
278	CakSNP278	*Kabuli*	Ca_Kabuli_Chr01	2053744	(C/A)
279	CakSNP279	*Kabuli*	Ca_Kabuli_Chr01	2053749	(T/A)
280	CakSNP280	*Kabuli*	Ca_Kabuli_Chr01	2053756	(G/T)
281	CakSNP281	*Kabuli*	Ca_Kabuli_Chr01	2053757	(C/T)
282	CakSNP282	*Kabuli*	Ca_Kabuli_Chr01	2053758	(A/G)
283	CakSNP283	*Kabuli*	Ca_Kabuli_Chr01	2053784	(C/A)
284	CakSNP284	*Kabuli*	Ca_Kabuli_Chr01	2053788	(A/G)
285	CakSNP285	*Kabuli*	Ca_Kabuli_Chr01	2053790	(G/A)
286	CakSNP286	*Kabuli*	Ca_Kabuli_Chr01	2053808	(G/T)
287	CakSNP287	*Kabuli*	Ca_Kabuli_Chr01	2053856	(T/G)
288	CakSNP288	*Kabuli*	Ca_Kabuli_Chr01	2053843	(T/A)
289	CakSNP289	*Kabuli*	Ca_Kabuli_Chr01	2053841	(C/A)
290	CakSNP290	*Kabuli*	Ca_Kabuli_Chr01	2053840	(A/C)
291	CakSNP291	*Kabuli*	Ca_Kabuli_Chr01	2053833	(T/G)
292	CakSNP292	*Kabuli*	Ca_Kabuli_Chr01	2053830	(A/G)
293	CakSNP293	*Kabuli*	Ca_Kabuli_Chr01	2053819	(C/A)
294	CakSNP294	*Kabuli*	Ca_Kabuli_Chr01	2053818	(G/C)
295	CakSNP295	*Kabuli*	Ca_Kabuli_Chr01	2071456	(T/A)
296	CakSNP296	*Kabuli*	Ca_Kabuli_Chr01	2071448	(A/T)
297	CakSNP297	*Kabuli*	Ca_Kabuli_Chr01	2071424	(T/A)
298	CakSNP298	*Kabuli*	Ca_Kabuli_Chr01	2071708	(C/G)
299	CakSNP299	*Kabuli*	Ca_Kabuli_Chr01	2071703	(A/C)
300	CakSNP300	*Kabuli*	Ca_Kabuli_Chr01	2071699	(T/C)
301	CakSNP301	*Kabuli*	Ca_Kabuli_Chr01	2071693	(T/C)
302	CakSNP302	*Kabuli*	Ca_Kabuli_Chr01	2071691	(A/C)
303	CakSNP303	*Kabuli*	Ca_Kabuli_Chr01	2071689	(A/G)
304	CakSNP304	*Kabuli*	Ca_Kabuli_Chr01	2071687	(C/T)
305	CakSNP305	*Kabuli*	Ca_Kabuli_Chr01	2071667	(G/C)
306	CakSNP306	*Kabuli*	Ca_Kabuli_Chr01	2071645	(C/T)
307	CakSNP307	*Kabuli*	Ca_Kabuli_Chr01	2072613	(G/A)
308	CakSNP308	*Kabuli*	Ca_Kabuli_Chr01	2072627	(A/T)
309	CakSNP309	*Kabuli*	Ca_Kabuli_Chr01	2074998	(A/C)
310	CakSNP310	*Kabuli*	Ca_Kabuli_Chr01	2075087	(A/T)

S.N.	SNP IDs	Cultivars	Chromosomes/scaffolds	Physical positions (bp)	SNPs
311	CakSNP311	*Kabuli*	Ca_Kabuli_Chr01	2075057	(G/T)
312	CakSNP312	*Kabuli*	Ca_Kabuli_Chr01	2075045	(C/A)
313	CakSNP313	*Kabuli*	Ca_Kabuli_Chr01	2075044	(A/G)
314	CakSNP314	*Kabuli*	Ca_Kabuli_Chr01	2075043	(C/A)
315	CakSNP315	*Kabuli*	Ca_Kabuli_Chr01	2075015	(G/A)
316	CakSNP316	*Kabuli*	Ca_Kabuli_Chr01	2075013	(A/G)
317	CakSNP317	*Kabuli*	Ca_Kabuli_Chr01	2075009	(C/A)
318	CakSNP318	*Kabuli*	Ca_Kabuli_Chr01	2082760	(C/G)
319	CakSNP319	*Kabuli*	Ca_Kabuli_Chr01	2082801	(G/A)
320	CakSNP320	*Kabuli*	Ca_Kabuli_Chr01	2083003	(G/C)
321	CakSNP321	*Kabuli*	Ca_Kabuli_Chr01	2083059	(A/T)
322	CakSNP322	*Kabuli*	Ca_Kabuli_Chr01	2126267	(G/A)
323	CakSNP323	*Kabuli*	Ca_Kabuli_Chr01	2148583	(T/G)
324	CakSNP324	*Kabuli*	Ca_Kabuli_Chr01	2170090	(G/C)
325	CakSNP325	*Kabuli*	Ca_Kabuli_Chr01	2170935	(A/C)
326	CakSNP326	*Kabuli*	Ca_Kabuli_Chr01	2172076	(T/C)
327	CakSNP327	*Kabuli*	Ca_Kabuli_Chr01	2172113	(G/A)
328	CakSNP328	*Kabuli*	Ca_Kabuli_Chr01	2172143	(T/G)
329	CakSNP329	*Kabuli*	Ca_Kabuli_Chr01	2172149	(G/A)
330	CakSNP330	*Kabuli*	Ca_Kabuli_Chr01	2183544	(A/G)
331	CakSNP331	*Kabuli*	Ca_Kabuli_Chr01	2188472	(T/C)
332	CakSNP332	*Kabuli*	Ca_Kabuli_Chr01	2232724	(A/G)
333	CakSNP333	*Kabuli*	Ca_Kabuli_Chr01	2232764	(C/T)
334	CakSNP334	*Kabuli*	Ca_Kabuli_Chr01	2232779	(A/G)
335	CakSNP335	*Kabuli*	Ca_Kabuli_Chr01	2252270	(C/T)
336	CakSNP336	*Kabuli*	Ca_Kabuli_Chr01	2252320	(G/T)
337	CakSNP337	*Kabuli*	Ca_Kabuli_Chr01	2252328	(C/A)
338	CakSNP338	*Kabuli*	Ca_Kabuli_Chr01	2252342	(C/T)
339	CakSNP339	*Kabuli*	Ca_Kabuli_Chr01	2252343	(G/T)
340	CakSNP340	*Kabuli*	Ca_Kabuli_Chr01	2252463	(T/C)
341	CakSNP341	*Kabuli*	Ca_Kabuli_Chr01	2252401	(A/G)
342	CakSNP342	*Kabuli*	Ca_Kabuli_Chr01	2252496	(C/A)
343	CakSNP343	*Kabuli*	Ca_Kabuli_Chr01	2252513	(C/T)
344	CakSNP344	*Kabuli*	Ca_Kabuli_Chr01	2269319	(T/G)
345	CakSNP345	*Kabuli*	Ca_Kabuli_Chr01	2269489	(A/C)
346	CakSNP346	*Kabuli*	Ca_Kabuli_Chr01	2277459	(G/A)
347	CakSNP347	*Kabuli*	Ca_Kabuli_Chr01	2285879	(G/A)
348	CakSNP348	*Kabuli*	Ca_Kabuli_Chr01	2287408	(A/T)
349	CakSNP349	*Kabuli*	Ca_Kabuli_Chr01	2287478	(A/T)

S.N.	SNP IDs	Cultivars	Chromosomes/scaffolds	Physical positions (bp)	SNPs
350	CakSNP350	*Kabuli*	Ca_Kabuli_Chr01	2366413	(T/C)
351	CakSNP351	*Kabuli*	Ca_Kabuli_Chr01	2366409	(T/A)
352	CakSNP352	*Kabuli*	Ca_Kabuli_Chr01	2373166	(A/G)
353	CakSNP353	*Kabuli*	Ca_Kabuli_Chr01	2373862	(G/A)
354	CakSNP354	*Kabuli*	Ca_Kabuli_Chr01	2373910	(C/T)
355	CakSNP355	*Kabuli*	Ca_Kabuli_Chr01	2373954	(G/T)
356	CakSNP356	*Kabuli*	Ca_Kabuli_Chr01	2395842	(A/C)
357	CakSNP357	*Kabuli*	Ca_Kabuli_Chr01	2400799	(T/G)
358	CakSNP358	*Kabuli*	Ca_Kabuli_Chr01	2400892	(C/T)
359	CakSNP359	*Kabuli*	Ca_Kabuli_Chr01	2428531	(A/G)
360	CakSNP360	*Kabuli*	Ca_Kabuli_Chr01	2439915	(A/G)
361	CakSNP361	*Kabuli*	Ca_Kabuli_Chr01	2439917	(G/A)
362	CakSNP362	*Kabuli*	Ca_Kabuli_Chr01	2473309	(T/C)
363	CakSNP363	*Kabuli*	Ca_Kabuli_Chr01	2513476	(T/A)
364	CakSNP364	*Kabuli*	Ca_Kabuli_Chr01	2513516	(T/A)
365	CakSNP365	*Kabuli*	Ca_Kabuli_Chr01	2513535	(T/C)
366	CakSNP366	*Kabuli*	Ca_Kabuli_Chr01	2513737	(T/C)
367	CakSNP367	*Kabuli*	Ca_Kabuli_Chr01	2513860	(G/A)
368	CakSNP368	*Kabuli*	Ca_Kabuli_Chr01	2513854	(G/A)
369	CakSNP369	*Kabuli*	Ca_Kabuli_Chr01	2513792	(A/G)
370	CakSNP370	*Kabuli*	Ca_Kabuli_Chr01	2513914	(T/C)
371	CakSNP371	*Kabuli*	Ca_Kabuli_Chr01	2542652	(G/A)
372	CakSNP372	*Kabuli*	Ca_Kabuli_Chr01	2649822	(G/C)
373	CakSNP373	*Kabuli*	Ca_Kabuli_Chr01	2649864	(C/A)
374	CakSNP374	*Kabuli*	Ca_Kabuli_Chr01	2649921	(T/C)
375	CakSNP375	*Kabuli*	Ca_Kabuli_Chr01	2649906	(C/T)
376	CakSNP376	*Kabuli*	Ca_Kabuli_Chr01	2649974	(A/C)
377	CakSNP377	*Kabuli*	Ca_Kabuli_Chr01	2671403	(G/T)
378	CakSNP378	*Kabuli*	Ca_Kabuli_Chr01	2694584	(T/C)
379	CakSNP379	*Kabuli*	Ca_Kabuli_Chr01	2745499	(C/A)
380	CakSNP380	*Kabuli*	Ca_Kabuli_Chr01	2745514	(A/C)
381	CakSNP381	*Kabuli*	Ca_Kabuli_Chr01	2745519	(G/C)
382	CakSNP382	*Kabuli*	Ca_Kabuli_Chr01	2745486	(C/T)
383	CakSNP383	*Kabuli*	Ca_Kabuli_Chr01	2754998	(C/T)
384	CakSNP384	*Kabuli*	Ca_Kabuli_Chr01	2755012	(C/T)
385	CakSNP385	*Kabuli*	Ca_Kabuli_Chr01	2755013	(T/A)
386	CakSNP386	*Kabuli*	Ca_Kabuli_Chr01	2755015	(T/C)
387	CakSNP387	*Kabuli*	Ca_Kabuli_Chr01	2755443	(T/A)
388	CakSNP388	*Kabuli*	Ca_Kabuli_Chr01	2755455	(G/A)

S.N.	SNP IDs	Cultivars	Chromosomes/scaffolds	Physical positions (bp)	SNPs
389	CakSNP389	*Kabuli*	Ca_Kabuli_Chr01	2755457	(C/T)
390	CakSNP390	*Kabuli*	Ca_Kabuli_Chr01	2755459	(T/G)
391	CakSNP391	*Kabuli*	Ca_Kabuli_Chr01	2755465	(T/A)
392	CakSNP392	*Kabuli*	Ca_Kabuli_Chr01	2755466	(C/G)
393	CakSNP393	*Kabuli*	Ca_Kabuli_Chr01	2755470	(A/T)
394	CakSNP394	*Kabuli*	Ca_Kabuli_Chr01	2755471	(C/A)
395	CakSNP395	*Kabuli*	Ca_Kabuli_Chr01	2755489	(G/A)
396	CakSNP396	*Kabuli*	Ca_Kabuli_Chr01	2755494	(T/C)
397	CakSNP397	*Kabuli*	Ca_Kabuli_Chr01	2755496	(A/G)
398	CakSNP398	*Kabuli*	Ca_Kabuli_Chr01	2755613	(G/T)
399	CakSNP399	*Kabuli*	Ca_Kabuli_Chr01	2755604	(A/C)
400	CakSNP400	*Kabuli*	Ca_Kabuli_Chr01	2755596	(C/A)
401	CakSNP401	*Kabuli*	Ca_Kabuli_Chr01	2757711	(T/G)
402	CakSNP402	*Kabuli*	Ca_Kabuli_Chr01	2757696	(A/G)
403	CakSNP403	*Kabuli*	Ca_Kabuli_Chr01	2757693	(C/T)
404	CakSNP404	*Kabuli*	Ca_Kabuli_Chr01	2757678	(A/G)
405	CakSNP405	*Kabuli*	Ca_Kabuli_Chr01	2758781	(G/C)
406	CakSNP406	*Kabuli*	Ca_Kabuli_Chr01	2758759	(G/C)
407	CakSNP407	*Kabuli*	Ca_Kabuli_Chr01	2758750	(A/G)
408	CakSNP408	*Kabuli*	Ca_Kabuli_Chr01	2758746	(G/A)
409	CakSNP409	*Kabuli*	Ca_Kabuli_Chr01	2758739	(A/T)
410	CakSNP410	*Kabuli*	Ca_Kabuli_Chr01	2758738	(T/G)
411	CakSNP411	*Kabuli*	Ca_Kabuli_Chr01	2758859	(A/T)
412	CakSNP412	*Kabuli*	Ca_Kabuli_Chr01	2758835	(T/A)
413	CakSNP413	*Kabuli*	Ca_Kabuli_Chr01	2758827	(A/T)
414	CakSNP414	*Kabuli*	Ca_Kabuli_Chr01	2787006	(C/T)
415	CakSNP415	*Kabuli*	Ca_Kabuli_Chr01	2786994	(A/C)
416	CakSNP416	*Kabuli*	Ca_Kabuli_Chr01	2786989	(T/C)
417	CakSNP417	*Kabuli*	Ca_Kabuli_Chr01	2786976	(A/C)
418	CakSNP418	*Kabuli*	Ca_Kabuli_Chr01	2786974	(T/C)
419	CakSNP419	*Kabuli*	Ca_Kabuli_Chr01	2786970	(T/A)
420	CakSNP420	*Kabuli*	Ca_Kabuli_Chr01	2807605	(G/C)
421	CakSNP421	*Kabuli*	Ca_Kabuli_Chr01	2807616	(G/A)
422	CakSNP422	*Kabuli*	Ca_Kabuli_Chr01	2808197	(T/C)
423	CakSNP423	*Kabuli*	Ca_Kabuli_Chr01	2808170	(T/G)
424	CakSNP424	*Kabuli*	Ca_Kabuli_Chr01	2832792	(G/A)
425	CakSNP425	*Kabuli*	Ca_Kabuli_Chr01	2832969	(G/A)
426	CakSNP426	*Kabuli*	Ca_Kabuli_Chr01	2832939	(G/T)
427	CakSNP427	*Kabuli*	Ca_Kabuli_Chr01	2833448	(C/A)

S.N.	SNP IDs	Cultivars	Chromosomes/scaffolds	Physical positions (bp)	SNPs
428	CakSNP428	*Kabuli*	Ca_Kabuli_Chr01	2855919	(T/G)
429	CakSNP429	*Kabuli*	Ca_Kabuli_Chr01	2855926	(T/A)
430	CakSNP430	*Kabuli*	Ca_Kabuli_Chr01	2855927	(T/A)
431	CakSNP431	*Kabuli*	Ca_Kabuli_Chr01	2855941	(T/G)
432	CakSNP432	*Kabuli*	Ca_Kabuli_Chr01	2855962	(A/G)
433	CakSNP433	*Kabuli*	Ca_Kabuli_Chr01	2855969	(C/T)
434	CakSNP434	*Kabuli*	Ca_Kabuli_Chr01	2856003	(T/C)
435	CakSNP435	*Kabuli*	Ca_Kabuli_Chr01	2856045	(C/T)
436	CakSNP436	*Kabuli*	Ca_Kabuli_Chr01	2856079	(T/G)
437	CakSNP437	*Kabuli*	Ca_Kabuli_Chr01	2856094	(T/G)
438	CakSNP438	*Kabuli*	Ca_Kabuli_Chr01	2856120	(T/G)
439	CakSNP439	*Kabuli*	Ca_Kabuli_Chr01	2856534	(T/A)
440	CakSNP440	*Kabuli*	Ca_Kabuli_Chr01	2858263	(A/G)
441	CakSNP441	*Kabuli*	Ca_Kabuli_Chr01	2858278	(T/A)
442	CakSNP442	*Kabuli*	Ca_Kabuli_Chr01	2858287	(G/T)
443	CakSNP443	*Kabuli*	Ca_Kabuli_Chr01	2858299	(C/A)
444	CakSNP444	*Kabuli*	Ca_Kabuli_Chr01	2868942	(G/A)
445	CakSNP445	*Kabuli*	Ca_Kabuli_Chr01	2881315	(T/C)
446	CakSNP446	*Kabuli*	Ca_Kabuli_Chr01	2881321	(C/A)
447	CakSNP447	*Kabuli*	Ca_Kabuli_Chr01	2881323	(A/T)
448	CakSNP448	*Kabuli*	Ca_Kabuli_Chr01	2881327	(C/T)
449	CakSNP449	*Kabuli*	Ca_Kabuli_Chr01	2881356	(C/A)
450	CakSNP450	*Kabuli*	Ca_Kabuli_Chr01	2881357	(A/T)
451	CakSNP451	*Kabuli*	Ca_Kabuli_Chr01	2881422	(T/C)
452	CakSNP452	*Kabuli*	Ca_Kabuli_Chr01	2881389	(G/C)
453	CakSNP453	*Kabuli*	Ca_Kabuli_Chr01	2881386	(G/T)
454	CakSNP454	*Kabuli*	Ca_Kabuli_Chr01	2883625	(T/A)
455	CakSNP455	*Kabuli*	Ca_Kabuli_Chr01	2883634	(T/A)
456	CakSNP456	*Kabuli*	Ca_Kabuli_Chr01	2883635	(A/G)
457	CakSNP457	*Kabuli*	Ca_Kabuli_Chr01	2883660	(C/G)
458	CakSNP458	*Kabuli*	Ca_Kabuli_Chr01	2883666	(G/A)
459	CakSNP459	*Kabuli*	Ca_Kabuli_Chr01	2883684	(G/T)
460	CakSNP460	*Kabuli*	Ca_Kabuli_Chr01	2883717	(C/A)
461	CakSNP461	*Kabuli*	Ca_Kabuli_Chr01	2883716	(A/C)
462	CakSNP462	*Kabuli*	Ca_Kabuli_Chr01	2883709	(A/G)
463	CakSNP463	*Kabuli*	Ca_Kabuli_Chr01	2885508	(T/C)
464	CakSNP464	*Kabuli*	Ca_Kabuli_Chr01	2885525	(T/C)
465	CakSNP465	*Kabuli*	Ca_Kabuli_Chr01	2885531	(C/T)
466	CakSNP466	*Kabuli*	Ca_Kabuli_Chr01	2885626	(A/G)

S.N.	SNP IDs	Cultivars	Chromosomes/scaffolds	Physical positions (bp)	SNPs
467	CakSNP467	*Kabuli*	Ca_Kabuli_Chr01	2885673	(G/C)
468	CakSNP468	*Kabuli*	Ca_Kabuli_Chr01	2885892	(T/A)
469	CakSNP469	*Kabuli*	Ca_Kabuli_Chr01	2885906	(C/T)
470	CakSNP470	*Kabuli*	Ca_Kabuli_Chr01	2885923	(C/G)
471	CakSNP471	*Kabuli*	Ca_Kabuli_Chr01	2886025	(A/G)
472	CakSNP472	*Kabuli*	Ca_Kabuli_Chr01	2886020	(G/T)
473	CakSNP473	*Kabuli*	Ca_Kabuli_Chr01	2886015	(G/A)
474	CakSNP474	*Kabuli*	Ca_Kabuli_Chr01	2886005	(G/A)
475	CakSNP475	*Kabuli*	Ca_Kabuli_Chr01	2885997	(G/A)
476	CakSNP476	*Kabuli*	Ca_Kabuli_Chr01	2885988	(C/G)
477	CakSNP477	*Kabuli*	Ca_Kabuli_Chr01	2885987	(T/A)
478	CakSNP478	*Kabuli*	Ca_Kabuli_Chr01	2948211	(C/T)
479	CakSNP479	*Kabuli*	Ca_Kabuli_Chr01	3009417	(C/T)
480	CakSNP480	*Kabuli*	Ca_Kabuli_Chr01	3032061	(C/A)
481	CakSNP481	*Kabuli*	Ca_Kabuli_Chr01	3081723	(G/T)
482	CakSNP482	*Kabuli*	Ca_Kabuli_Chr01	3081851	(T/G)
483	CakSNP483	*Kabuli*	Ca_Kabuli_Chr01	3081850	(C/A)
484	CakSNP484	*Kabuli*	Ca_Kabuli_Chr01	3082280	(G/A)
485	CakSNP485	*Kabuli*	Ca_Kabuli_Chr01	3151007	(G/A)
486	CakSNP486	*Kabuli*	Ca_Kabuli_Chr01	3150986	(T/C)
487	CakSNP487	*Kabuli*	Ca_Kabuli_Chr01	3150964	(C/G)
488	CakSNP488	*Kabuli*	Ca_Kabuli_Chr01	3259114	(G/A)
489	CakSNP489	*Kabuli*	Ca_Kabuli_Chr01	3259117	(G/C)
490	CakSNP490	*Kabuli*	Ca_Kabuli_Chr01	3259177	(G/C)
491	CakSNP491	*Kabuli*	Ca_Kabuli_Chr01	3298327	(C/G)
492	CakSNP492	*Kabuli*	Ca_Kabuli_Chr01	3298783	(T/G)
493	CakSNP493	*Kabuli*	Ca_Kabuli_Chr01	3359148	(C/A)
494	CakSNP494	*Kabuli*	Ca_Kabuli_Chr01	3364952	(A/C)
495	CakSNP495	*Kabuli*	Ca_Kabuli_Chr01	3374375	(G/A)
496	CakSNP496	*Kabuli*	Ca_Kabuli_Chr01	3427555	(A/C)
497	CakSNP497	*Kabuli*	Ca_Kabuli_Chr01	3427649	(A/G)
498	CakSNP498	*Kabuli*	Ca_Kabuli_Chr01	3503059	(C/A)
499	CakSNP499	*Kabuli*	Ca_Kabuli_Chr01	3503153	(G/A)
500	CakSNP500	*Kabuli*	Ca_Kabuli_Chr01	3542263	(A/C)
501	CakSNP501	*Kabuli*	Ca_Kabuli_Chr01	3544948	(A/T)
502	CakSNP502	*Kabuli*	Ca_Kabuli_Chr01	3545011	(G/A)
503	CakSNP503	*Kabuli*	Ca_Kabuli_Chr01	3564342	(G/T)
504	CakSNP504	*Kabuli*	Ca_Kabuli_Chr01	3596241	(C/T)
505	CakSNP505	*Kabuli*	Ca_Kabuli_Chr01	3597842	(G/A)

S.N.	SNP IDs	Cultivars	Chromosomes/scaffolds	Physical positions (bp)	SNPs
506	CakSNP506	*Kabuli*	Ca_Kabuli_Chr01	3605582	(G/A)
507	CakSNP507	*Kabuli*	Ca_Kabuli_Chr01	3605583	(C/T)
508	CakSNP508	*Kabuli*	Ca_Kabuli_Chr01	3609580	(T/C)
509	CakSNP509	*Kabuli*	Ca_Kabuli_Chr01	3611231	(C/A)
510	CakSNP510	*Kabuli*	Ca_Kabuli_Chr01	3614966	(A/G)
511	CakSNP511	*Kabuli*	Ca_Kabuli_Chr01	3615037	(G/T)
512	CakSNP512	*Kabuli*	Ca_Kabuli_Chr01	3615064	(T/C)
513	CakSNP513	*Kabuli*	Ca_Kabuli_Chr01	3615052	(C/A)
514	CakSNP514	*Kabuli*	Ca_Kabuli_Chr01	3622757	(C/T)
515	CakSNP515	*Kabuli*	Ca_Kabuli_Chr01	3711498	(T/G)
516	CakSNP516	*Kabuli*	Ca_Kabuli_Chr01	3711525	(T/G)
517	CakSNP517	*Kabuli*	Ca_Kabuli_Chr01	3751865	(G/T)
518	CakSNP518	*Kabuli*	Ca_Kabuli_Chr01	3777223	(C/T)
519	CakSNP519	*Kabuli*	Ca_Kabuli_Chr01	3810931	(T/C)
520	CakSNP520	*Kabuli*	Ca_Kabuli_Chr01	3852035	(A/C)
521	CakSNP521	*Kabuli*	Ca_Kabuli_Chr01	3852476	(A/C)
522	CakSNP522	*Kabuli*	Ca_Kabuli_Chr01	3853408	(T/C)
523	CakSNP523	*Kabuli*	Ca_Kabuli_Chr01	3855229	(G/A)
524	CakSNP524	*Kabuli*	Ca_Kabuli_Chr01	3891319	(T/C)
525	CakSNP525	*Kabuli*	Ca_Kabuli_Chr01	3911547	(G/A)
526	CakSNP526	*Kabuli*	Ca_Kabuli_Chr01	3941250	(C/T)
527	CakSNP527	*Kabuli*	Ca_Kabuli_Chr01	3941399	(T/C)
528	CakSNP528	*Kabuli*	Ca_Kabuli_Chr01	3952819	(G/A)
529	CakSNP529	*Kabuli*	Ca_Kabuli_Chr01	3956031	(T/C)
530	CakSNP530	*Kabuli*	Ca_Kabuli_Chr01	3956028	(C/T)
531	CakSNP531	*Kabuli*	Ca_Kabuli_Chr01	3972671	(G/T)
532	CakSNP532	*Kabuli*	Ca_Kabuli_Chr01	4048854	(G/A)
533	CakSNP533	*Kabuli*	Ca_Kabuli_Chr01	4048875	(A/T)
534	CakSNP534	*Kabuli*	Ca_Kabuli_Chr01	4073941	(A/C)
535	CakSNP535	*Kabuli*	Ca_Kabuli_Chr01	4112929	(A/T)
536	CakSNP536	*Kabuli*	Ca_Kabuli_Chr01	4189333	(C/T)
537	CakSNP537	*Kabuli*	Ca_Kabuli_Chr01	4189297	(T/C)
538	CakSNP538	*Kabuli*	Ca_Kabuli_Chr01	4245058	(C/G)
539	CakSNP539	*Kabuli*	Ca_Kabuli_Chr01	4245073	(C/G)
540	CakSNP540	*Kabuli*	Ca_Kabuli_Chr01	4245176	(G/T)
541	CakSNP541	*Kabuli*	Ca_Kabuli_Chr01	4292981	(G/C)
542	CakSNP542	*Kabuli*	Ca_Kabuli_Chr01	4302669	(G/C)
543	CakSNP543	*Kabuli*	Ca_Kabuli_Chr01	4302770	(A/C)
544	CakSNP544	*Kabuli*	Ca_Kabuli_Chr01	4302751	(T/C)

S.N.	SNP IDs	Cultivars	Chromosomes/scaffolds	Physical positions (bp)	SNPs
545	CakSNP545	*Kabuli*	Ca_Kabuli_Chr01	4334299	(A/T)
546	CakSNP546	*Kabuli*	Ca_Kabuli_Chr01	4334336	(A/G)
547	CakSNP547	*Kabuli*	Ca_Kabuli_Chr01	4372257	(A/G)
548	CakSNP548	*Kabuli*	Ca_Kabuli_Chr01	4375710	(T/C)
549	CakSNP549	*Kabuli*	Ca_Kabuli_Chr01	4385002	(C/T)
550	CakSNP550	*Kabuli*	Ca_Kabuli_Chr01	4386114	(T/G)
551	CakSNP551	*Kabuli*	Ca_Kabuli_Chr01	4386111	(T/C)
552	CakSNP552	*Kabuli*	Ca_Kabuli_Chr01	4392680	(A/G)
553	CakSNP553	*Kabuli*	Ca_Kabuli_Chr01	4414410	(A/T)
554	CakSNP554	*Kabuli*	Ca_Kabuli_Chr01	4423984	(A/G)
555	CakSNP555	*Kabuli*	Ca_Kabuli_Chr01	4429044	(C/T)
556	CakSNP556	*Kabuli*	Ca_Kabuli_Chr01	4432569	(A/G)
557	CakSNP557	*Kabuli*	Ca_Kabuli_Chr01	4432549	(C/T)
558	CakSNP558	*Kabuli*	Ca_Kabuli_Chr01	4432545	(C/A)
559	CakSNP559	*Kabuli*	Ca_Kabuli_Chr01	4432541	(C/A)
560	CakSNP560	*Kabuli*	Ca_Kabuli_Chr01	4456662	(G/A)
561	CakSNP561	*Kabuli*	Ca_Kabuli_Chr01	4456654	(A/C)
562	CakSNP562	*Kabuli*	Ca_Kabuli_Chr01	4456648	(T/C)
563	CakSNP563	*Kabuli*	Ca_Kabuli_Chr01	4457588	(A/G)
564	CakSNP564	*Kabuli*	Ca_Kabuli_Chr01	4460112	(A/T)
565	CakSNP565	*Kabuli*	Ca_Kabuli_Chr01	4494270	(C/T)
566	CakSNP566	*Kabuli*	Ca_Kabuli_Chr01	4494467	(A/G)
567	CakSNP567	*Kabuli*	Ca_Kabuli_Chr01	4512566	(T/C)
568	CakSNP568	*Kabuli*	Ca_Kabuli_Chr01	4528117	(A/G)
569	CakSNP569	*Kabuli*	Ca_Kabuli_Chr01	4528184	(C/T)
570	CakSNP570	*Kabuli*	Ca_Kabuli_Chr01	4530304	(T/C)
571	CakSNP571	*Kabuli*	Ca_Kabuli_Chr01	4560792	(C/T)
572	CakSNP572	*Kabuli*	Ca_Kabuli_Chr01	4560920	(G/A)
573	CakSNP573	*Kabuli*	Ca_Kabuli_Chr01	4569669	(T/A)
574	CakSNP574	*Kabuli*	Ca_Kabuli_Chr01	4569670	(T/A)
575	CakSNP575	*Kabuli*	Ca_Kabuli_Chr01	4569690	(A/G)
576	CakSNP576	*Kabuli*	Ca_Kabuli_Chr01	4571615	(T/C)
577	CakSNP577	*Kabuli*	Ca_Kabuli_Chr01	4571540	(C/G)
578	CakSNP578	*Kabuli*	Ca_Kabuli_Chr01	4571539	(G/A)
579	CakSNP579	*Kabuli*	Ca_Kabuli_Chr01	4576888	(A/G)
580	CakSNP580	*Kabuli*	Ca_Kabuli_Chr01	4578967	(T/G)
581	CakSNP581	*Kabuli*	Ca_Kabuli_Chr01	4578999	(T/A)
582	CakSNP582	*Kabuli*	Ca_Kabuli_Chr01	4582189	(C/T)
583	CakSNP583	*Kabuli*	Ca_Kabuli_Chr01	4626099	(T/G)

S.N.	SNP IDs	Cultivars	Chromosomes/scaffolds	Physical positions (bp)	SNPs
584	CakSNP584	*Kabuli*	Ca_Kabuli_Chr01	4626098	(C/G)
585	CakSNP585	*Kabuli*	Ca_Kabuli_Chr01	4631467	(G/A)
586	CakSNP586	*Kabuli*	Ca_Kabuli_Chr01	4632188	(G/A)
587	CakSNP587	*Kabuli*	Ca_Kabuli_Chr01	4632965	(G/A)
588	CakSNP588	*Kabuli*	Ca_Kabuli_Chr01	4634413	(C/T)
589	CakSNP589	*Kabuli*	Ca_Kabuli_Chr01	4635347	(G/A)
590	CakSNP590	*Kabuli*	Ca_Kabuli_Chr01	4635302	(A/C)
591	CakSNP591	*Kabuli*	Ca_Kabuli_Chr01	4662564	(A/G)
592	CakSNP592	*Kabuli*	Ca_Kabuli_Chr01	4672439	(G/A)
593	CakSNP593	*Kabuli*	Ca_Kabuli_Chr01	4715563	(T/C)
594	CakSNP594	*Kabuli*	Ca_Kabuli_Chr01	4780330	(C/T)
595	CakSNP595	*Kabuli*	Ca_Kabuli_Chr01	4780345	(G/T)
596	CakSNP596	*Kabuli*	Ca_Kabuli_Chr01	4781362	(C/T)
597	CakSNP597	*Kabuli*	Ca_Kabuli_Chr01	4781416	(G/A)
598	CakSNP598	*Kabuli*	Ca_Kabuli_Chr01	4781704	(G/A)
599	CakSNP599	*Kabuli*	Ca_Kabuli_Chr01	4808910	(A/T)
600	CakSNP600	*Kabuli*	Ca_Kabuli_Chr01	4878683	(T/C)
601	CakSNP601	*Kabuli*	Ca_Kabuli_Chr01	4878691	(A/C)
602	CakSNP602	*Kabuli*	Ca_Kabuli_Chr01	4878695	(T/C)
603	CakSNP603	*Kabuli*	Ca_Kabuli_Chr01	4878823	(A/G)
604	CakSNP604	*Kabuli*	Ca_Kabuli_Chr01	4903185	(T/A)
605	CakSNP605	*Kabuli*	Ca_Kabuli_Chr01	4903203	(A/G)
606	CakSNP606	*Kabuli*	Ca_Kabuli_Chr01	4903239	(G/A)
607	CakSNP607	*Kabuli*	Ca_Kabuli_Chr01	4906759	(G/C)
608	CakSNP608	*Kabuli*	Ca_Kabuli_Chr01	4906775	(A/T)
609	CakSNP609	*Kabuli*	Ca_Kabuli_Chr01	4906777	(G/T)
610	CakSNP610	*Kabuli*	Ca_Kabuli_Chr01	4906778	(T/G)
611	CakSNP611	*Kabuli*	Ca_Kabuli_Chr01	4906800	(T/G)
612	CakSNP612	*Kabuli*	Ca_Kabuli_Chr01	4906816	(A/G)
613	CakSNP613	*Kabuli*	Ca_Kabuli_Chr01	4906868	(G/C)
614	CakSNP614	*Kabuli*	Ca_Kabuli_Chr01	4906859	(T/C)
615	CakSNP615	*Kabuli*	Ca_Kabuli_Chr01	4906854	(A/G)
616	CakSNP616	*Kabuli*	Ca_Kabuli_Chr01	4909155	(T/C)
617	CakSNP617	*Kabuli*	Ca_Kabuli_Chr01	4909286	(T/G)
618	CakSNP618	*Kabuli*	Ca_Kabuli_Chr01	4909280	(T/G)
619	CakSNP619	*Kabuli*	Ca_Kabuli_Chr01	4909271	(A/G)
620	CakSNP620	*Kabuli*	Ca_Kabuli_Chr01	4909268	(C/T)
621	CakSNP621	*Kabuli*	Ca_Kabuli_Chr01	4909261	(G/T)
622	CakSNP622	*Kabuli*	Ca_Kabuli_Chr01	4909255	(G/A)

S.N.	SNP IDs	Cultivars	Chromosomes/scaffolds	Physical positions (bp)	SNPs
623	CakSNP623	*Kabuli*	Ca_Kabuli_Chr01	4909253	(A/G)
624	CakSNP624	*Kabuli*	Ca_Kabuli_Chr01	4910497	(A/G)
625	CakSNP625	*Kabuli*	Ca_Kabuli_Chr01	4910494	(C/A)
626	CakSNP626	*Kabuli*	Ca_Kabuli_Chr01	4948740	(A/T)
627	CakSNP627	*Kabuli*	Ca_Kabuli_Chr01	4949578	(A/T)
628	CakSNP628	*Kabuli*	Ca_Kabuli_Chr01	4950457	(G/A)
629	CakSNP629	*Kabuli*	Ca_Kabuli_Chr01	4950726	(G/T)
630	CakSNP630	*Kabuli*	Ca_Kabuli_Chr01	4959562	(A/C)
631	CakSNP631	*Kabuli*	Ca_Kabuli_Chr01	4995240	(T/A)
632	CakSNP632	*Kabuli*	Ca_Kabuli_Chr01	4996404	(A/C)
633	CakSNP633	*Kabuli*	Ca_Kabuli_Chr01	4996459	(T/C)
634	CakSNP634	*Kabuli*	Ca_Kabuli_Chr01	4996476	(G/A)
635	CakSNP635	*Kabuli*	Ca_Kabuli_Chr01	4996536	(C/T)
636	CakSNP636	*Kabuli*	Ca_Kabuli_Chr01	5071210	(A/G)
637	CakSNP637	*Kabuli*	Ca_Kabuli_Chr01	5071195	(T/A)
638	CakSNP638	*Kabuli*	Ca_Kabuli_Chr01	5071177	(A/G)
639	CakSNP639	*Kabuli*	Ca_Kabuli_Chr01	5071158	(G/A)
640	CakSNP640	*Kabuli*	Ca_Kabuli_Chr01	5072022	(C/A)
641	CakSNP641	*Kabuli*	Ca_Kabuli_Chr01	5080342	(A/T)
642	CakSNP642	*Kabuli*	Ca_Kabuli_Chr01	5080358	(G/A)
643	CakSNP643	*Kabuli*	Ca_Kabuli_Chr01	5087428	(T/C)
644	CakSNP644	*Kabuli*	Ca_Kabuli_Chr01	5096676	(A/T)
645	CakSNP645	*Kabuli*	Ca_Kabuli_Chr01	5096650	(C/T)
646	CakSNP646	*Kabuli*	Ca_Kabuli_Chr01	5122663	(A/G)
647	CakSNP647	*Kabuli*	Ca_Kabuli_Chr01	5129940	(G/A)
648	CakSNP648	*Kabuli*	Ca_Kabuli_Chr01	5138013	(A/C)
649	CakSNP649	*Kabuli*	Ca_Kabuli_Chr01	5140801	(C/A)
650	CakSNP650	*Kabuli*	Ca_Kabuli_Chr01	5140837	(A/T)
651	CakSNP651	*Kabuli*	Ca_Kabuli_Chr01	5141009	(G/C)
652	CakSNP652	*Kabuli*	Ca_Kabuli_Chr01	5140944	(C/G)
653	CakSNP653	*Kabuli*	Ca_Kabuli_Chr01	5146658	(C/A)
654	CakSNP654	*Kabuli*	Ca_Kabuli_Chr01	5146667	(T/G)
655	CakSNP655	*Kabuli*	Ca_Kabuli_Chr01	5146696	(T/A)
656	CakSNP656	*Kabuli*	Ca_Kabuli_Chr01	5164127	(A/G)
657	CakSNP657	*Kabuli*	Ca_Kabuli_Chr01	5164136	(C/T)
658	CakSNP658	*Kabuli*	Ca_Kabuli_Chr01	5164198	(C/A)
659	CakSNP659	*Kabuli*	Ca_Kabuli_Chr01	5197461	(C/T)
660	CakSNP660	*Kabuli*	Ca_Kabuli_Chr01	5197475	(T/A)
661	CakSNP661	*Kabuli*	Ca_Kabuli_Chr01	5200250	(T/G)

S.N.	SNP IDs	Cultivars	Chromosomes/scaffolds	Physical positions (bp)	SNPs
662	CakSNP662	*Kabuli*	Ca_Kabuli_Chr01	5200289	(G/T)
663	CakSNP663	*Kabuli*	Ca_Kabuli_Chr01	5249839	(G/A)
664	CakSNP664	*Kabuli*	Ca_Kabuli_Chr01	5249846	(A/C)
665	CakSNP665	*Kabuli*	Ca_Kabuli_Chr01	5249868	(G/A)
666	CakSNP666	*Kabuli*	Ca_Kabuli_Chr01	5288427	(T/C)
667	CakSNP667	*Kabuli*	Ca_Kabuli_Chr01	5288421	(A/C)
668	CakSNP668	*Kabuli*	Ca_Kabuli_Chr01	5304576	(A/C)
669	CakSNP669	*Kabuli*	Ca_Kabuli_Chr01	5321398	(A/G)
670	CakSNP670	*Kabuli*	Ca_Kabuli_Chr01	5430700	(A/C)
671	CakSNP671	*Kabuli*	Ca_Kabuli_Chr01	5430703	(T/G)
672	CakSNP672	*Kabuli*	Ca_Kabuli_Chr01	5430809	(G/A)
673	CakSNP673	*Kabuli*	Ca_Kabuli_Chr01	5444130	(A/C)
674	CakSNP674	*Kabuli*	Ca_Kabuli_Chr01	5444142	(A/T)
675	CakSNP675	*Kabuli*	Ca_Kabuli_Chr01	5474604	(G/A)
676	CakSNP676	*Kabuli*	Ca_Kabuli_Chr01	5477196	(T/C)
677	CakSNP677	*Kabuli*	Ca_Kabuli_Chr01	5487535	(A/G)
678	CakSNP678	*Kabuli*	Ca_Kabuli_Chr01	5489806	(C/G)
679	CakSNP679	*Kabuli*	Ca_Kabuli_Chr01	5577080	(T/C)
680	CakSNP680	*Kabuli*	Ca_Kabuli_Chr01	5629027	(G/A)
681	CakSNP681	*Kabuli*	Ca_Kabuli_Chr01	5637388	(T/A)
682	CakSNP682	*Kabuli*	Ca_Kabuli_Chr01	5637442	(C/T)
683	CakSNP683	*Kabuli*	Ca_Kabuli_Chr01	5643313	(G/C)
684	CakSNP684	*Kabuli*	Ca_Kabuli_Chr01	5666178	(A/C)
685	CakSNP685	*Kabuli*	Ca_Kabuli_Chr01	5666217	(C/A)
686	CakSNP686	*Kabuli*	Ca_Kabuli_Chr01	5666260	(T/G)
687	CakSNP687	*Kabuli*	Ca_Kabuli_Chr01	5666266	(T/G)
688	CakSNP688	*Kabuli*	Ca_Kabuli_Chr01	5666263	(T/G)
689	CakSNP689	*Kabuli*	Ca_Kabuli_Chr01	5666355	(T/G)
690	CakSNP690	*Kabuli*	Ca_Kabuli_Chr01	5667605	(G/A)
691	CakSNP691	*Kabuli*	Ca_Kabuli_Chr01	5702638	(C/T)
692	CakSNP692	*Kabuli*	Ca_Kabuli_Chr01	5706858	(A/T)
693	CakSNP693	*Kabuli*	Ca_Kabuli_Chr01	5706961	(G/T)
694	CakSNP694	*Kabuli*	Ca_Kabuli_Chr01	5706948	(A/T)
695	CakSNP695	*Kabuli*	Ca_Kabuli_Chr01	5706944	(T/G)
696	CakSNP696	*Kabuli*	Ca_Kabuli_Chr01	5706920	(G/A)
697	CakSNP697	*Kabuli*	Ca_Kabuli_Chr01	5721616	(A/T)
698	CakSNP698	*Kabuli*	Ca_Kabuli_Chr01	5749456	(C/T)
699	CakSNP699	*Kabuli*	Ca_Kabuli_Chr01	5791118	(T/G)
700	CakSNP700	*Kabuli*	Ca_Kabuli_Chr01	5796146	(C/T)

S.N.	SNP IDs	Cultivars	Chromosomes/scaffolds	Physical positions (bp)	SNPs
701	CakSNP701	*Kabuli*	Ca_Kabuli_Chr01	5796886	(A/G)
702	CakSNP702	*Kabuli*	Ca_Kabuli_Chr01	5807492	(T/C)
703	CakSNP703	*Kabuli*	Ca_Kabuli_Chr01	5812283	(G/T)
704	CakSNP704	*Kabuli*	Ca_Kabuli_Chr01	5812298	(G/A)
705	CakSNP705	*Kabuli*	Ca_Kabuli_Chr01	5862565	(C/T)
706	CakSNP706	*Kabuli*	Ca_Kabuli_Chr01	5960264	(T/C)
707	CakSNP707	*Kabuli*	Ca_Kabuli_Chr01	5978146	(G/A)
708	CakSNP708	*Kabuli*	Ca_Kabuli_Chr01	5978289	(C/T)
709	CakSNP709	*Kabuli*	Ca_Kabuli_Chr01	5981127	(C/T)
710	CakSNP710	*Kabuli*	Ca_Kabuli_Chr01	5981178	(A/G)
711	CakSNP711	*Kabuli*	Ca_Kabuli_Chr01	6011727	(G/T)
712	CakSNP712	*Kabuli*	Ca_Kabuli_Chr01	6011697	(T/G)
713	CakSNP713	*Kabuli*	Ca_Kabuli_Chr01	6011689	(G/A)
714	CakSNP714	*Kabuli*	Ca_Kabuli_Chr01	6011657	(T/C)
715	CakSNP715	*Kabuli*	Ca_Kabuli_Chr01	6011761	(C/T)
716	CakSNP716	*Kabuli*	Ca_Kabuli_Chr01	6017854	(A/C)
717	CakSNP717	*Kabuli*	Ca_Kabuli_Chr01	6066972	(C/T)
718	CakSNP718	*Kabuli*	Ca_Kabuli_Chr01	6066936	(A/G)
719	CakSNP719	*Kabuli*	Ca_Kabuli_Chr01	6066933	(C/T)
720	CakSNP720	*Kabuli*	Ca_Kabuli_Chr01	6067846	(T/C)
721	CakSNP721	*Kabuli*	Ca_Kabuli_Chr01	6088283	(C/T)
722	CakSNP722	*Kabuli*	Ca_Kabuli_Chr01	6101205	(C/T)
723	CakSNP723	*Kabuli*	Ca_Kabuli_Chr01	6126761	(T/A)
724	CakSNP724	*Kabuli*	Ca_Kabuli_Chr01	6126772	(G/A)
725	CakSNP725	*Kabuli*	Ca_Kabuli_Chr01	6155341	(A/G)
726	CakSNP726	*Kabuli*	Ca_Kabuli_Chr01	6224010	(C/G)
727	CakSNP727	*Kabuli*	Ca_Kabuli_Chr01	6224015	(C/T)
728	CakSNP728	*Kabuli*	Ca_Kabuli_Chr01	6249062	(T/C)
729	CakSNP729	*Kabuli*	Ca_Kabuli_Chr01	6249035	(T/C)
730	CakSNP730	*Kabuli*	Ca_Kabuli_Chr01	6257653	(C/G)
731	CakSNP731	*Kabuli*	Ca_Kabuli_Chr01	6257712	(A/T)
732	CakSNP732	*Kabuli*	Ca_Kabuli_Chr01	6259458	(T/C)
733	CakSNP733	*Kabuli*	Ca_Kabuli_Chr01	6262461	(A/G)
734	CakSNP734	*Kabuli*	Ca_Kabuli_Chr01	6262529	(T/G)
735	CakSNP735	*Kabuli*	Ca_Kabuli_Chr01	6262577	(A/G)
736	CakSNP736	*Kabuli*	Ca_Kabuli_Chr01	6278572	(T/C)
737	CakSNP737	*Kabuli*	Ca_Kabuli_Chr01	6278625	(T/C)
738	CakSNP738	*Kabuli*	Ca_Kabuli_Chr01	6279644	(G/T)
739	CakSNP739	*Kabuli*	Ca_Kabuli_Chr01	6296858	(G/A)

S.N.	SNP IDs	Cultivars	Chromosomes/scaffolds	Physical positions (bp)	SNPs
740	CakSNP740	*Kabuli*	Ca_Kabuli_Chr01	6296986	(C/A)
741	CakSNP741	*Kabuli*	Ca_Kabuli_Chr01	6296929	(C/T)
742	CakSNP742	*Kabuli*	Ca_Kabuli_Chr01	6315337	(G/T)
743	CakSNP743	*Kabuli*	Ca_Kabuli_Chr01	6315402	(C/T)
744	CakSNP744	*Kabuli*	Ca_Kabuli_Chr01	6315399	(T/G)
745	CakSNP745	*Kabuli*	Ca_Kabuli_Chr01	6315397	(C/G)
746	CakSNP746	*Kabuli*	Ca_Kabuli_Chr01	6328801	(T/A)
747	CakSNP747	*Kabuli*	Ca_Kabuli_Chr01	6328957	(C/T)
748	CakSNP748	*Kabuli*	Ca_Kabuli_Chr01	6328963	(A/G)
749	CakSNP749	*Kabuli*	Ca_Kabuli_Chr01	6329805	(A/G)
750	CakSNP750	*Kabuli*	Ca_Kabuli_Chr01	6329814	(T/A)
751	CakSNP751	*Kabuli*	Ca_Kabuli_Chr01	6329844	(C/T)
752	CakSNP752	*Kabuli*	Ca_Kabuli_Chr01	6329847	(A/G)
753	CakSNP753	*Kabuli*	Ca_Kabuli_Chr01	6332229	(T/C)
754	CakSNP754	*Kabuli*	Ca_Kabuli_Chr01	6347626	(C/T)
755	CakSNP755	*Kabuli*	Ca_Kabuli_Chr01	6347720	(G/A)
756	CakSNP756	*Kabuli*	Ca_Kabuli_Chr01	6381114	(T/G)
757	CakSNP757	*Kabuli*	Ca_Kabuli_Chr01	6381191	(T/C)
758	CakSNP758	*Kabuli*	Ca_Kabuli_Chr01	6390541	(T/A)
759	CakSNP759	*Kabuli*	Ca_Kabuli_Chr01	6390849	(G/A)
760	CakSNP760	*Kabuli*	Ca_Kabuli_Chr01	6391017	(T/C)
761	CakSNP761	*Kabuli*	Ca_Kabuli_Chr01	6433592	(T/G)
762	CakSNP762	*Kabuli*	Ca_Kabuli_Chr01	6433826	(G/A)
763	CakSNP763	*Kabuli*	Ca_Kabuli_Chr01	6433838	(C/T)
764	CakSNP764	*Kabuli*	Ca_Kabuli_Chr01	6433841	(C/T)
765	CakSNP765	*Kabuli*	Ca_Kabuli_Chr01	6433883	(A/G)
766	CakSNP766	*Kabuli*	Ca_Kabuli_Chr01	6433934	(T/A)
767	CakSNP767	*Kabuli*	Ca_Kabuli_Chr01	6433925	(C/T)
768	CakSNP768	*Kabuli*	Ca_Kabuli_Chr01	6478736	(A/G)
769	CakSNP769	*Kabuli*	Ca_Kabuli_Chr01	6478758	(A/G)
770	CakSNP770	*Kabuli*	Ca_Kabuli_Chr01	6486446	(G/C)
771	CakSNP771	*Kabuli*	Ca_Kabuli_Chr01	6490978	(A/T)
772	CakSNP772	*Kabuli*	Ca_Kabuli_Chr01	6491008	(A/T)
773	CakSNP773	*Kabuli*	Ca_Kabuli_Chr01	6491030	(T/G)
774	CakSNP774	*Kabuli*	Ca_Kabuli_Chr01	6491017	(T/C)
775	CakSNP775	*Kabuli*	Ca_Kabuli_Chr01	6521670	(C/T)
776	CakSNP776	*Kabuli*	Ca_Kabuli_Chr01	6521673	(G/A)
777	CakSNP777	*Kabuli*	Ca_Kabuli_Chr01	6521856	(A/G)
778	CakSNP778	*Kabuli*	Ca_Kabuli_Chr01	6521859	(T/A)

S.N.	SNP IDs	Cultivars	Chromosomes/scaffolds	Physical positions (bp)	SNPs
779	CakSNP779	*Kabuli*	Ca_Kabuli_Chr01	6521970	(C/T)
780	CakSNP780	*Kabuli*	Ca_Kabuli_Chr01	6540056	(C/T)
781	CakSNP781	*Kabuli*	Ca_Kabuli_Chr01	6540170	(G/A)
782	CakSNP782	*Kabuli*	Ca_Kabuli_Chr01	6553107	(A/C)
783	CakSNP783	*Kabuli*	Ca_Kabuli_Chr01	6564716	(C/T)
784	CakSNP784	*Kabuli*	Ca_Kabuli_Chr01	6564789	(C/T)
785	CakSNP785	*Kabuli*	Ca_Kabuli_Chr01	6591506	(G/C)
786	CakSNP786	*Kabuli*	Ca_Kabuli_Chr01	6591518	(G/T)
787	CakSNP787	*Kabuli*	Ca_Kabuli_Chr01	6599917	(C/A)
788	CakSNP788	*Kabuli*	Ca_Kabuli_Chr01	6634482	(G/C)
789	CakSNP789	*Kabuli*	Ca_Kabuli_Chr01	6635404	(C/T)
790	CakSNP790	*Kabuli*	Ca_Kabuli_Chr01	6635401	(T/C)
791	CakSNP791	*Kabuli*	Ca_Kabuli_Chr01	6635366	(T/C)
792	CakSNP792	*Kabuli*	Ca_Kabuli_Chr01	6679253	(A/G)
793	CakSNP793	*Kabuli*	Ca_Kabuli_Chr01	6679329	(A/G)
794	CakSNP794	*Kabuli*	Ca_Kabuli_Chr01	6803364	(G/A)
795	CakSNP795	*Kabuli*	Ca_Kabuli_Chr01	6804031	(C/T)
796	CakSNP796	*Kabuli*	Ca_Kabuli_Chr01	6804110	(A/T)
797	CakSNP797	*Kabuli*	Ca_Kabuli_Chr01	6849437	(C/T)
798	CakSNP798	*Kabuli*	Ca_Kabuli_Chr01	6849512	(G/C)
799	CakSNP799	*Kabuli*	Ca_Kabuli_Chr01	6879110	(G/C)
800	CakSNP800	*Kabuli*	Ca_Kabuli_Chr01	6880666	(A/G)
801	CakSNP801	*Kabuli*	Ca_Kabuli_Chr01	6880837	(A/G)
802	CakSNP802	*Kabuli*	Ca_Kabuli_Chr01	6904278	(A/C)
803	CakSNP803	*Kabuli*	Ca_Kabuli_Chr01	6904313	(A/C)
804	CakSNP804	*Kabuli*	Ca_Kabuli_Chr01	6904338	(C/T)
805	CakSNP805	*Kabuli*	Ca_Kabuli_Chr01	6952382	(G/T)
806	CakSNP806	*Kabuli*	Ca_Kabuli_Chr01	7050825	(G/A)
807	CakSNP807	*Kabuli*	Ca_Kabuli_Chr01	7050958	(C/T)
808	CakSNP808	*Kabuli*	Ca_Kabuli_Chr01	7050953	(C/T)
809	CakSNP809	*Kabuli*	Ca_Kabuli_Chr01	7104985	(G/T)
810	CakSNP810	*Kabuli*	Ca_Kabuli_Chr01	7127843	(A/G)
811	CakSNP811	*Kabuli*	Ca_Kabuli_Chr01	7136677	(T/C)
812	CakSNP812	*Kabuli*	Ca_Kabuli_Chr01	7147651	(T/A)
813	CakSNP813	*Kabuli*	Ca_Kabuli_Chr01	7147643	(A/G)
814	CakSNP814	*Kabuli*	Ca_Kabuli_Chr01	7155344	(A/G)
815	CakSNP815	*Kabuli*	Ca_Kabuli_Chr01	7155302	(A/G)
816	CakSNP816	*Kabuli*	Ca_Kabuli_Chr01	7156354	(G/T)
817	CakSNP817	*Kabuli*	Ca_Kabuli_Chr01	7156355	(A/T)

S.N.	SNP IDs	Cultivars	Chromosomes/scaffolds	Physical positions (bp)	SNPs
818	CakSNP818	*Kabuli*	Ca_Kabuli_Chr01	7157057	(A/C)
819	CakSNP819	*Kabuli*	Ca_Kabuli_Chr01	7157112	(T/C)
820	CakSNP820	*Kabuli*	Ca_Kabuli_Chr01	7160649	(T/C)
821	CakSNP821	*Kabuli*	Ca_Kabuli_Chr01	7160648	(A/T)
822	CakSNP822	*Kabuli*	Ca_Kabuli_Chr01	7160695	(G/A)
823	CakSNP823	*Kabuli*	Ca_Kabuli_Chr01	7160698	(A/C)
824	CakSNP824	*Kabuli*	Ca_Kabuli_Chr01	7160754	(A/T)
825	CakSNP825	*Kabuli*	Ca_Kabuli_Chr01	7160781	(A/C)
826	CakSNP826	*Kabuli*	Ca_Kabuli_Chr01	7160773	(G/A)
827	CakSNP827	*Kabuli*	Ca_Kabuli_Chr01	7161122	(A/G)
828	CakSNP828	*Kabuli*	Ca_Kabuli_Chr01	7184813	(A/C)
829	CakSNP829	*Kabuli*	Ca_Kabuli_Chr01	7184773	(G/A)
830	CakSNP830	*Kabuli*	Ca_Kabuli_Chr01	7187934	(A/G)
831	CakSNP831	*Kabuli*	Ca_Kabuli_Chr01	7187969	(C/G)
832	CakSNP832	*Kabuli*	Ca_Kabuli_Chr01	7196570	(T/C)
833	CakSNP833	*Kabuli*	Ca_Kabuli_Chr01	7196601	(T/C)
834	CakSNP834	*Kabuli*	Ca_Kabuli_Chr01	7231750	(A/C)
835	CakSNP835	*Kabuli*	Ca_Kabuli_Chr01	7325164	(T/A)
836	CakSNP836	*Kabuli*	Ca_Kabuli_Chr01	7417717	(G/A)
837	CakSNP837	*Kabuli*	Ca_Kabuli_Chr01	7417699	(G/A)
838	CakSNP838	*Kabuli*	Ca_Kabuli_Chr01	7417788	(G/A)
839	CakSNP839	*Kabuli*	Ca_Kabuli_Chr01	7439421	(C/T)
840	CakSNP840	*Kabuli*	Ca_Kabuli_Chr01	7467280	(A/G)
841	CakSNP841	*Kabuli*	Ca_Kabuli_Chr01	7478022	(G/A)
842	CakSNP842	*Kabuli*	Ca_Kabuli_Chr01	7478046	(T/G)
843	CakSNP843	*Kabuli*	Ca_Kabuli_Chr01	7486102	(C/T)
844	CakSNP844	*Kabuli*	Ca_Kabuli_Chr01	7486273	(T/C)
845	CakSNP845	*Kabuli*	Ca_Kabuli_Chr01	7486282	(G/A)
846	CakSNP846	*Kabuli*	Ca_Kabuli_Chr01	7497334	(C/A)
847	CakSNP847	*Kabuli*	Ca_Kabuli_Chr01	7497362	(T/C)
848	CakSNP848	*Kabuli*	Ca_Kabuli_Chr01	7500494	(T/G)
849	CakSNP849	*Kabuli*	Ca_Kabuli_Chr01	7500524	(T/C)
850	CakSNP850	*Kabuli*	Ca_Kabuli_Chr01	7500665	(C/A)
851	CakSNP851	*Kabuli*	Ca_Kabuli_Chr01	7505779	(A/T)
852	CakSNP852	*Kabuli*	Ca_Kabuli_Chr01	7505792	(A/T)
853	CakSNP853	*Kabuli*	Ca_Kabuli_Chr01	7515002	(T/C)
854	CakSNP854	*Kabuli*	Ca_Kabuli_Chr01	7515605	(T/C)
855	CakSNP855	*Kabuli*	Ca_Kabuli_Chr01	7515578	(A/C)
856	CakSNP856	*Kabuli*	Ca_Kabuli_Chr01	7557751	(T/G)

S.N.	SNP IDs	Cultivars	Chromosomes/scaffolds	Physical positions (bp)	SNPs
857	CakSNP857	*Kabuli*	Ca_Kabuli_Chr01	7557811	(A/T)
858	CakSNP858	*Kabuli*	Ca_Kabuli_Chr01	7690613	(T/G)
859	CakSNP859	*Kabuli*	Ca_Kabuli_Chr01	7690620	(T/G)
860	CakSNP860	*Kabuli*	Ca_Kabuli_Chr01	7690625	(A/C)
861	CakSNP861	*Kabuli*	Ca_Kabuli_Chr01	7695844	(T/C)
862	CakSNP862	*Kabuli*	Ca_Kabuli_Chr01	7811388	(C/T)
863	CakSNP863	*Kabuli*	Ca_Kabuli_Chr01	7925666	(A/C)
864	CakSNP864	*Kabuli*	Ca_Kabuli_Chr01	7925801	(C/T)
865	CakSNP865	*Kabuli*	Ca_Kabuli_Chr01	7925774	(C/T)
866	CakSNP866	*Kabuli*	Ca_Kabuli_Chr01	7925738	(C/T)
867	CakSNP867	*Kabuli*	Ca_Kabuli_Chr01	7969202	(A/C)
868	CakSNP868	*Kabuli*	Ca_Kabuli_Chr01	7969694	(A/G)
869	CakSNP869	*Kabuli*	Ca_Kabuli_Chr01	7969647	(G/A)
870	CakSNP870	*Kabuli*	Ca_Kabuli_Chr01	8036666	(C/T)
871	CakSNP871	*Kabuli*	Ca_Kabuli_Chr01	8036706	(A/G)
872	CakSNP872	*Kabuli*	Ca_Kabuli_Chr01	8036917	(T/G)
873	CakSNP873	*Kabuli*	Ca_Kabuli_Chr01	8036903	(A/T)
874	CakSNP874	*Kabuli*	Ca_Kabuli_Chr01	8075631	(A/T)
875	CakSNP875	*Kabuli*	Ca_Kabuli_Chr01	8103090	(G/T)
876	CakSNP876	*Kabuli*	Ca_Kabuli_Chr01	8103339	(T/C)
877	CakSNP877	*Kabuli*	Ca_Kabuli_Chr01	8103401	(T/A)
878	CakSNP878	*Kabuli*	Ca_Kabuli_Chr01	8107967	(A/C)
879	CakSNP879	*Kabuli*	Ca_Kabuli_Chr01	8108014	(C/T)
880	CakSNP880	*Kabuli*	Ca_Kabuli_Chr01	8111588	(G/A)
881	CakSNP881	*Kabuli*	Ca_Kabuli_Chr01	8112343	(C/T)
882	CakSNP882	*Kabuli*	Ca_Kabuli_Chr01	8113235	(G/T)
883	CakSNP883	*Kabuli*	Ca_Kabuli_Chr01	8113383	(T/G)
884	CakSNP884	*Kabuli*	Ca_Kabuli_Chr01	8113449	(T/A)
885	CakSNP885	*Kabuli*	Ca_Kabuli_Chr01	8113403	(A/T)
886	CakSNP886	*Kabuli*	Ca_Kabuli_Chr01	8113398	(C/G)
887	CakSNP887	*Kabuli*	Ca_Kabuli_Chr01	8123033	(A/G)
888	CakSNP888	*Kabuli*	Ca_Kabuli_Chr01	8123257	(A/G)
889	CakSNP889	*Kabuli*	Ca_Kabuli_Chr01	8123481	(A/G)
890	CakSNP890	*Kabuli*	Ca_Kabuli_Chr01	8134368	(T/C)
891	CakSNP891	*Kabuli*	Ca_Kabuli_Chr01	8178109	(T/C)
892	CakSNP892	*Kabuli*	Ca_Kabuli_Chr01	8178146	(G/A)
893	CakSNP893	*Kabuli*	Ca_Kabuli_Chr01	8192580	(T/C)
894	CakSNP894	*Kabuli*	Ca_Kabuli_Chr01	8220996	(T/A)
895	CakSNP895	*Kabuli*	Ca_Kabuli_Chr01	8220968	(A/G)

S.N.	SNP IDs	Cultivars	Chromosomes/scaffolds	Physical positions (bp)	SNPs
896	CakSNP896	*Kabuli*	Ca_Kabuli_Chr01	8343356	(G/A)
897	CakSNP897	*Kabuli*	Ca_Kabuli_Chr01	8389242	(G/A)
898	CakSNP898	*Kabuli*	Ca_Kabuli_Chr01	8464621	(C/G)
899	CakSNP899	*Kabuli*	Ca_Kabuli_Chr01	8582341	(T/C)
900	CakSNP900	*Kabuli*	Ca_Kabuli_Chr01	8607820	(T/C)
901	CakSNP901	*Kabuli*	Ca_Kabuli_Chr01	8607919	(A/C)
902	CakSNP902	*Kabuli*	Ca_Kabuli_Chr01	8633598	(G/A)
903	CakSNP903	*Kabuli*	Ca_Kabuli_Chr01	8665705	(T/G)
904	CakSNP904	*Kabuli*	Ca_Kabuli_Chr01	8697177	(G/T)
905	CakSNP905	*Kabuli*	Ca_Kabuli_Chr01	8698059	(T/C)
906	CakSNP906	*Kabuli*	Ca_Kabuli_Chr01	8735740	(G/T)
907	CakSNP907	*Kabuli*	Ca_Kabuli_Chr01	8735727	(G/C)
908	CakSNP908	*Kabuli*	Ca_Kabuli_Chr01	8736733	(G/A)
909	CakSNP909	*Kabuli*	Ca_Kabuli_Chr01	8736711	(G/T)
910	CakSNP910	*Kabuli*	Ca_Kabuli_Chr01	8737380	(A/G)
911	CakSNP911	*Kabuli*	Ca_Kabuli_Chr01	8738691	(T/G)
912	CakSNP912	*Kabuli*	Ca_Kabuli_Chr01	8738695	(G/C)
913	CakSNP913	*Kabuli*	Ca_Kabuli_Chr01	8767122	(T/A)
914	CakSNP914	*Kabuli*	Ca_Kabuli_Chr01	8767121	(A/T)
915	CakSNP915	*Kabuli*	Ca_Kabuli_Chr01	8799685	(T/G)
916	CakSNP916	*Kabuli*	Ca_Kabuli_Chr01	8799701	(T/C)
917	CakSNP917	*Kabuli*	Ca_Kabuli_Chr01	8799708	(T/C)
918	CakSNP918	*Kabuli*	Ca_Kabuli_Chr01	8799758	(C/G)
919	CakSNP919	*Kabuli*	Ca_Kabuli_Chr01	8809188	(T/A)
920	CakSNP920	*Kabuli*	Ca_Kabuli_Chr01	8809191	(C/T)
921	CakSNP921	*Kabuli*	Ca_Kabuli_Chr01	8809295	(A/T)
922	CakSNP922	*Kabuli*	Ca_Kabuli_Chr01	8809517	(C/G)
923	CakSNP923	*Kabuli*	Ca_Kabuli_Chr01	8822121	(G/T)
924	CakSNP924	*Kabuli*	Ca_Kabuli_Chr01	8822375	(G/A)
925	CakSNP925	*Kabuli*	Ca_Kabuli_Chr01	8822366	(T/A)
926	CakSNP926	*Kabuli*	Ca_Kabuli_Chr01	8824856	(T/C)
927	CakSNP927	*Kabuli*	Ca_Kabuli_Chr01	8852863	(G/T)
928	CakSNP928	*Kabuli*	Ca_Kabuli_Chr01	8852928	(T/A)
929	CakSNP929	*Kabuli*	Ca_Kabuli_Chr01	8852981	(C/A)
930	CakSNP930	*Kabuli*	Ca_Kabuli_Chr01	8852957	(C/A)
931	CakSNP931	*Kabuli*	Ca_Kabuli_Chr01	8852943	(C/A)
932	CakSNP932	*Kabuli*	Ca_Kabuli_Chr01	8853010	(A/G)
933	CakSNP933	*Kabuli*	Ca_Kabuli_Chr01	8854211	(G/T)
934	CakSNP934	*Kabuli*	Ca_Kabuli_Chr01	8891438	(T/G)

S.N.	SNP IDs	Cultivars	Chromosomes/scaffolds	Physical positions (bp)	SNPs
935	CakSNP935	*Kabuli*	Ca_Kabuli_Chr01	8891485	(T/G)
936	CakSNP936	*Kabuli*	Ca_Kabuli_Chr01	8891562	(C/G)
937	CakSNP937	*Kabuli*	Ca_Kabuli_Chr01	8891561	(G/A)
938	CakSNP938	*Kabuli*	Ca_Kabuli_Chr01	8893985	(G/A)
939	CakSNP939	*Kabuli*	Ca_Kabuli_Chr01	8901769	(C/A)
940	CakSNP940	*Kabuli*	Ca_Kabuli_Chr01	8901842	(T/A)
941	CakSNP941	*Kabuli*	Ca_Kabuli_Chr01	8941004	(A/C)
942	CakSNP942	*Kabuli*	Ca_Kabuli_Chr01	8941029	(G/A)
943	CakSNP943	*Kabuli*	Ca_Kabuli_Chr01	8941031	(G/C)
944	CakSNP944	*Kabuli*	Ca_Kabuli_Chr01	8942137	(T/C)
945	CakSNP945	*Kabuli*	Ca_Kabuli_Chr01	8942131	(T/C)
946	CakSNP946	*Kabuli*	Ca_Kabuli_Chr01	8942198	(A/G)
947	CakSNP947	*Kabuli*	Ca_Kabuli_Chr01	8942179	(C/G)
948	CakSNP948	*Kabuli*	Ca_Kabuli_Chr01	8949723	(G/T)
949	CakSNP949	*Kabuli*	Ca_Kabuli_Chr01	8960630	(T/C)
950	CakSNP950	*Kabuli*	Ca_Kabuli_Chr01	8969321	(C/T)
951	CakSNP951	*Kabuli*	Ca_Kabuli_Chr01	8971718	(C/G)
952	CakSNP952	*Kabuli*	Ca_Kabuli_Chr01	8971776	(A/G)
953	CakSNP953	*Kabuli*	Ca_Kabuli_Chr01	8976169	(A/T)
954	CakSNP954	*Kabuli*	Ca_Kabuli_Chr01	8980112	(C/A)
955	CakSNP955	*Kabuli*	Ca_Kabuli_Chr01	8993300	(T/C)
956	CakSNP956	*Kabuli*	Ca_Kabuli_Chr01	8993329	(A/C)
957	CakSNP957	*Kabuli*	Ca_Kabuli_Chr01	9018113	(A/C)
958	CakSNP958	*Kabuli*	Ca_Kabuli_Chr01	9031417	(G/T)
959	CakSNP959	*Kabuli*	Ca_Kabuli_Chr01	9031825	(G/T)
960	CakSNP960	*Kabuli*	Ca_Kabuli_Chr01	9032126	(G/A)
961	CakSNP961	*Kabuli*	Ca_Kabuli_Chr01	9035744	(G/A)
962	CakSNP962	*Kabuli*	Ca_Kabuli_Chr01	9040419	(T/A)
963	CakSNP963	*Kabuli*	Ca_Kabuli_Chr01	9048675	(A/T)
964	CakSNP964	*Kabuli*	Ca_Kabuli_Chr01	9048682	(T/G)
965	CakSNP965	*Kabuli*	Ca_Kabuli_Chr01	9054490	(G/A)
966	CakSNP966	*Kabuli*	Ca_Kabuli_Chr01	9065933	(T/C)
967	CakSNP967	*Kabuli*	Ca_Kabuli_Chr01	9093812	(C/G)
968	CakSNP968	*Kabuli*	Ca_Kabuli_Chr01	9093964	(T/A)
969	CakSNP969	*Kabuli*	Ca_Kabuli_Chr01	9097885	(A/G)
970	CakSNP970	*Kabuli*	Ca_Kabuli_Chr01	9097881	(C/G)
971	CakSNP971	*Kabuli*	Ca_Kabuli_Chr01	9097879	(C/A)
972	CakSNP972	*Kabuli*	Ca_Kabuli_Chr01	9097878	(A/G)
973	CakSNP973	*Kabuli*	Ca_Kabuli_Chr01	9097877	(C/A)

S.N.	SNP IDs	Cultivars	Chromosomes/scaffolds	Physical positions (bp)	SNPs
974	CakSNP974	*Kabuli*	Ca_Kabuli_Chr01	9097869	(G/T)
975	CakSNP975	*Kabuli*	Ca_Kabuli_Chr01	9097947	(C/A)
976	CakSNP976	*Kabuli*	Ca_Kabuli_Chr01	9097949	(A/C)
977	CakSNP977	*Kabuli*	Ca_Kabuli_Chr01	9098003	(C/T)
978	CakSNP978	*Kabuli*	Ca_Kabuli_Chr01	9098051	(A/G)
979	CakSNP979	*Kabuli*	Ca_Kabuli_Chr01	9105432	(C/T)
980	CakSNP980	*Kabuli*	Ca_Kabuli_Chr01	9195312	(G/A)
981	CakSNP981	*Kabuli*	Ca_Kabuli_Chr01	9237766	(T/G)
982	CakSNP982	*Kabuli*	Ca_Kabuli_Chr01	9237813	(G/T)
983	CakSNP983	*Kabuli*	Ca_Kabuli_Chr01	9269888	(T/A)
984	CakSNP984	*Kabuli*	Ca_Kabuli_Chr01	9468041	(C/T)
985	CakSNP985	*Kabuli*	Ca_Kabuli_Chr01	9468895	(C/A)
986	CakSNP986	*Kabuli*	Ca_Kabuli_Chr01	9476207	(A/C)
987	CakSNP987	*Kabuli*	Ca_Kabuli_Chr01	9476388	(A/G)
988	CakSNP988	*Kabuli*	Ca_Kabuli_Chr01	9476385	(A/G)
989	CakSNP989	*Kabuli*	Ca_Kabuli_Chr01	9535616	(C/T)
990	CakSNP990	*Kabuli*	Ca_Kabuli_Chr01	9547223	(A/G)
991	CakSNP991	*Kabuli*	Ca_Kabuli_Chr01	9547310	(A/G)
992	CakSNP992	*Kabuli*	Ca_Kabuli_Chr01	9547854	(G/A)
993	CakSNP993	*Kabuli*	Ca_Kabuli_Chr01	9547834	(C/T)
994	CakSNP994	*Kabuli*	Ca_Kabuli_Chr01	9547819	(T/C)
995	CakSNP995	*Kabuli*	Ca_Kabuli_Chr01	9556418	(C/G)
996	CakSNP996	*Kabuli*	Ca_Kabuli_Chr01	9556486	(T/G)
997	CakSNP997	*Kabuli*	Ca_Kabuli_Chr01	9556487	(T/A)
998	CakSNP998	*Kabuli*	Ca_Kabuli_Chr01	9556488	(G/A)
999	CakSNP999	*Kabuli*	Ca_Kabuli_Chr01	9556588	(G/T)
1000	CakSNP1000	*Kabuli*	Ca_Kabuli_Chr01	9556604	(G/C)
1001	CakSNP1001	*Kabuli*	Ca_Kabuli_Chr01	9556628	(G/A)
1002	CakSNP1002	*Kabuli*	Ca_Kabuli_Chr01	9556735	(G/A)
1003	CakSNP1003	*Kabuli*	Ca_Kabuli_Chr01	9556662	(C/T)
1004	CakSNP1004	*Kabuli*	Ca_Kabuli_Chr01	9567049	(C/G)
1005	CakSNP1005	*Kabuli*	Ca_Kabuli_Chr01	9603896	(T/A)
1006	CakSNP1006	*Kabuli*	Ca_Kabuli_Chr01	9634823	(C/T)
1007	CakSNP1007	*Kabuli*	Ca_Kabuli_Chr01	9647861	(G/C)
1008	CakSNP1008	*Kabuli*	Ca_Kabuli_Chr01	9659316	(C/T)
1009	CakSNP1009	*Kabuli*	Ca_Kabuli_Chr01	9705162	(A/C)
1010	CakSNP1010	*Kabuli*	Ca_Kabuli_Chr01	9706910	(C/T)
1011	CakSNP1011	*Kabuli*	Ca_Kabuli_Chr01	9737032	(C/T)
1012	CakSNP1012	*Kabuli*	Ca_Kabuli_Chr01	9737067	(T/C)

S.N.	SNP IDs	Cultivars	Chromosomes/scaffolds	Physical positions (bp)	SNPs
1013	CakSNP1013	*Kabuli*	Ca_Kabuli_Chr01	9757006	(G/A)
1014	CakSNP1014	*Kabuli*	Ca_Kabuli_Chr01	9779398	(G/A)
1015	CakSNP1015	*Kabuli*	Ca_Kabuli_Chr01	9797342	(G/T)
1016	CakSNP1016	*Kabuli*	Ca_Kabuli_Chr01	9810275	(T/C)
1017	CakSNP1017	*Kabuli*	Ca_Kabuli_Chr01	9815448	(A/T)
1018	CakSNP1018	*Kabuli*	Ca_Kabuli_Chr01	9815508	(G/C)
1019	CakSNP1019	*Kabuli*	Ca_Kabuli_Chr01	9836198	(A/C)
1020	CakSNP1020	*Kabuli*	Ca_Kabuli_Chr01	9896511	(C/T)
1021	CakSNP1021	*Kabuli*	Ca_Kabuli_Chr01	9920951	(G/A)
1022	CakSNP1022	*Kabuli*	Ca_Kabuli_Chr01	9920986	(A/C)
1023	CakSNP1023	*Kabuli*	Ca_Kabuli_Chr01	9932690	(C/G)
1024	CakSNP1024	*Kabuli*	Ca_Kabuli_Chr01	10038486	(T/G)
1025	CakSNP1025	*Kabuli*	Ca_Kabuli_Chr01	10049364	(A/G)
1026	CakSNP1026	*Kabuli*	Ca_Kabuli_Chr01	10049469	(A/G)
1027	CakSNP1027	*Kabuli*	Ca_Kabuli_Chr01	10142688	(A/G)
1028	CakSNP1028	*Kabuli*	Ca_Kabuli_Chr01	10155496	(T/G)
1029	CakSNP1029	*Kabuli*	Ca_Kabuli_Chr01	10155584	(T/C)
1030	CakSNP1030	*Kabuli*	Ca_Kabuli_Chr01	10191578	(A/C)
1031	CakSNP1031	*Kabuli*	Ca_Kabuli_Chr01	10201074	(C/G)
1032	CakSNP1032	*Kabuli*	Ca_Kabuli_Chr01	10212925	(G/C)
1033	CakSNP1033	*Kabuli*	Ca_Kabuli_Chr01	10212926	(A/C)
1034	CakSNP1034	*Kabuli*	Ca_Kabuli_Chr01	10212948	(T/C)
1035	CakSNP1035	*Kabuli*	Ca_Kabuli_Chr01	10212976	(T/A)
1036	CakSNP1036	*Kabuli*	Ca_Kabuli_Chr01	10213111	(T/C)
1037	CakSNP1037	*Kabuli*	Ca_Kabuli_Chr01	10266774	(C/T)
1038	CakSNP1038	*Kabuli*	Ca_Kabuli_Chr01	10289781	(G/A)
1039	CakSNP1039	*Kabuli*	Ca_Kabuli_Chr01	10340870	(G/C)
1040	CakSNP1040	*Kabuli*	Ca_Kabuli_Chr01	10364986	(C/G)
1041	CakSNP1041	*Kabuli*	Ca_Kabuli_Chr01	10365024	(G/A)
1042	CakSNP1042	*Kabuli*	Ca_Kabuli_Chr01	10365029	(T/A)
1043	CakSNP1043	*Kabuli*	Ca_Kabuli_Chr01	10366383	(G/A)
1044	CakSNP1044	*Kabuli*	Ca_Kabuli_Chr01	10377700	(G/C)
1045	CakSNP1045	*Kabuli*	Ca_Kabuli_Chr01	10377698	(T/C)
1046	CakSNP1046	*Kabuli*	Ca_Kabuli_Chr01	10377691	(T/C)
1047	CakSNP1047	*Kabuli*	Ca_Kabuli_Chr01	10377860	(T/A)
1048	CakSNP1048	*Kabuli*	Ca_Kabuli_Chr01	10408322	(A/G)
1049	CakSNP1049	*Kabuli*	Ca_Kabuli_Chr01	10408349	(G/T)
1050	CakSNP1050	*Kabuli*	Ca_Kabuli_Chr01	10410479	(T/C)
1051	CakSNP1051	*Kabuli*	Ca_Kabuli_Chr01	10439153	(C/G)

S.N.	SNP IDs	Cultivars	Chromosomes/scaffolds	Physical positions (bp)	SNPs
1052	CakSNP1052	*Kabuli*	Ca_Kabuli_Chr01	10439177	(T/C)
1053	CakSNP1053	*Kabuli*	Ca_Kabuli_Chr01	10439190	(C/A)
1054	CakSNP1054	*Kabuli*	Ca_Kabuli_Chr01	10440393	(C/T)
1055	CakSNP1055	*Kabuli*	Ca_Kabuli_Chr01	10440430	(G/T)
1056	CakSNP1056	*Kabuli*	Ca_Kabuli_Chr01	10440533	(C/A)
1057	CakSNP1057	*Kabuli*	Ca_Kabuli_Chr01	10440479	(C/T)
1058	CakSNP1058	*Kabuli*	Ca_Kabuli_Chr01	10489197	(A/G)
1059	CakSNP1059	*Kabuli*	Ca_Kabuli_Chr01	10527733	(A/G)
1060	CakSNP1060	*Kabuli*	Ca_Kabuli_Chr01	10551440	(T/C)
1061	CakSNP1061	*Kabuli*	Ca_Kabuli_Chr01	10556610	(T/G)
1062	CakSNP1062	*Kabuli*	Ca_Kabuli_Chr01	10560693	(A/G)
1063	CakSNP1063	*Kabuli*	Ca_Kabuli_Chr01	10560690	(T/G)
1064	CakSNP1064	*Kabuli*	Ca_Kabuli_Chr01	10567303	(A/C)
1065	CakSNP1065	*Kabuli*	Ca_Kabuli_Chr01	10571949	(G/A)
1066	CakSNP1066	*Kabuli*	Ca_Kabuli_Chr01	10601318	(T/G)
1067	CakSNP1067	*Kabuli*	Ca_Kabuli_Chr01	10605977	(G/A)
1068	CakSNP1068	*Kabuli*	Ca_Kabuli_Chr01	10623359	(T/G)
1069	CakSNP1069	*Kabuli*	Ca_Kabuli_Chr01	10630658	(A/T)
1070	CakSNP1070	*Kabuli*	Ca_Kabuli_Chr01	10649758	(C/T)
1071	CakSNP1071	*Kabuli*	Ca_Kabuli_Chr01	10655633	(G/T)
1072	CakSNP1072	*Kabuli*	Ca_Kabuli_Chr01	10657464	(T/A)
1073	CakSNP1073	*Kabuli*	Ca_Kabuli_Chr01	10697972	(C/G)
1074	CakSNP1074	*Kabuli*	Ca_Kabuli_Chr01	10710672	(T/G)
1075	CakSNP1075	*Kabuli*	Ca_Kabuli_Chr01	10712235	(G/A)
1076	CakSNP1076	*Kabuli*	Ca_Kabuli_Chr01	10713001	(T/A)
1077	CakSNP1077	*Kabuli*	Ca_Kabuli_Chr01	10734511	(G/A)
1078	CakSNP1078	*Kabuli*	Ca_Kabuli_Chr01	10734570	(G/A)
1079	CakSNP1079	*Kabuli*	Ca_Kabuli_Chr01	10739374	(T/A)
1080	CakSNP1080	*Kabuli*	Ca_Kabuli_Chr01	10761609	(G/T)
1081	CakSNP1081	*Kabuli*	Ca_Kabuli_Chr01	10761648	(C/T)
1082	CakSNP1082	*Kabuli*	Ca_Kabuli_Chr01	10814166	(T/C)
1083	CakSNP1083	*Kabuli*	Ca_Kabuli_Chr01	10837203	(C/T)
1084	CakSNP1084	*Kabuli*	Ca_Kabuli_Chr01	10861334	(A/G)
1085	CakSNP1085	*Kabuli*	Ca_Kabuli_Chr01	10861332	(C/G)
1086	CakSNP1086	*Kabuli*	Ca_Kabuli_Chr01	10861326	(T/G)
1087	CakSNP1087	*Kabuli*	Ca_Kabuli_Chr01	10869338	(A/T)
1088	CakSNP1088	*Kabuli*	Ca_Kabuli_Chr01	10869336	(G/A)
1089	CakSNP1089	*Kabuli*	Ca_Kabuli_Chr01	10887927	(C/A)
1090	CakSNP1090	*Kabuli*	Ca_Kabuli_Chr01	10939428	(T/C)

S.N.	SNP IDs	Cultivars	Chromosomes/scaffolds	Physical positions (bp)	SNPs
1091	CakSNP1091	*Kabuli*	Ca_Kabuli_Chr01	10973356	(C/T)
1092	CakSNP1092	*Kabuli*	Ca_Kabuli_Chr01	11065411	(T/A)
1093	CakSNP1093	*Kabuli*	Ca_Kabuli_Chr01	11103176	(T/C)
1094	CakSNP1094	*Kabuli*	Ca_Kabuli_Chr01	11121162	(G/A)
1095	CakSNP1095	*Kabuli*	Ca_Kabuli_Chr01	11122665	(G/T)
1096	CakSNP1096	*Kabuli*	Ca_Kabuli_Chr01	11191023	(G/A)
1097	CakSNP1097	*Kabuli*	Ca_Kabuli_Chr01	11193334	(G/C)
1098	CakSNP1098	*Kabuli*	Ca_Kabuli_Chr01	11225828	(T/C)
1099	CakSNP1099	*Kabuli*	Ca_Kabuli_Chr01	11227614	(T/C)
1100	CakSNP1100	*Kabuli*	Ca_Kabuli_Chr01	11230086	(A/G)
1101	CakSNP1101	*Kabuli*	Ca_Kabuli_Chr01	11230109	(T/C)
1102	CakSNP1102	*Kabuli*	Ca_Kabuli_Chr01	11230115	(G/C)
1103	CakSNP1103	*Kabuli*	Ca_Kabuli_Chr01	11230116	(T/C)
1104	CakSNP1104	*Kabuli*	Ca_Kabuli_Chr01	11230118	(A/T)
1105	CakSNP1105	*Kabuli*	Ca_Kabuli_Chr01	11230125	(A/C)
1106	CakSNP1106	*Kabuli*	Ca_Kabuli_Chr01	11230126	(G/T)
1107	CakSNP1107	*Kabuli*	Ca_Kabuli_Chr01	11230127	(G/T)
1108	CakSNP1108	*Kabuli*	Ca_Kabuli_Chr01	11230128	(T/G)
1109	CakSNP1109	*Kabuli*	Ca_Kabuli_Chr01	11230133	(G/A)
1110	CakSNP1110	*Kabuli*	Ca_Kabuli_Chr01	11230972	(C/T)
1111	CakSNP1111	*Kabuli*	Ca_Kabuli_Chr01	11230998	(A/T)
1112	CakSNP1112	*Kabuli*	Ca_Kabuli_Chr01	11231052	(A/G)
1113	CakSNP1113	*Kabuli*	Ca_Kabuli_Chr01	11231064	(A/G)
1114	CakSNP1114	*Kabuli*	Ca_Kabuli_Chr01	11231077	(T/C)
1115	CakSNP1115	*Kabuli*	Ca_Kabuli_Chr01	11235211	(C/T)
1116	CakSNP1116	*Kabuli*	Ca_Kabuli_Chr01	11265367	(T/C)
1117	CakSNP1117	*Kabuli*	Ca_Kabuli_Chr01	11277703	(T/A)
1118	CakSNP1118	*Kabuli*	Ca_Kabuli_Chr01	11277964	(C/T)
1119	CakSNP1119	*Kabuli*	Ca_Kabuli_Chr01	11284183	(T/C)
1120	CakSNP1120	*Kabuli*	Ca_Kabuli_Chr01	11285192	(G/A)
1121	CakSNP1121	*Kabuli*	Ca_Kabuli_Chr01	11285247	(T/C)
1122	CakSNP1122	*Kabuli*	Ca_Kabuli_Chr01	11285291	(C/A)
1123	CakSNP1123	*Kabuli*	Ca_Kabuli_Chr01	11291516	(G/A)
1124	CakSNP1124	*Kabuli*	Ca_Kabuli_Chr01	11291741	(A/C)
1125	CakSNP1125	*Kabuli*	Ca_Kabuli_Chr01	11291740	(T/A)
1126	CakSNP1126	*Kabuli*	Ca_Kabuli_Chr01	11291737	(C/A)
1127	CakSNP1127	*Kabuli*	Ca_Kabuli_Chr01	11319264	(A/C)
1128	CakSNP1128	*Kabuli*	Ca_Kabuli_Chr01	11321670	(G/C)
1129	CakSNP1129	*Kabuli*	Ca_Kabuli_Chr01	11324018	(C/A)

S.N.	SNP IDs	Cultivars	Chromosomes/scaffolds	Physical positions (bp)	SNPs
1130	CakSNP1130	*Kabuli*	Ca_Kabuli_Chr01	11324023	(C/T)
1131	CakSNP1131	*Kabuli*	Ca_Kabuli_Chr01	11324035	(C/T)
1132	CakSNP1132	*Kabuli*	Ca_Kabuli_Chr01	11324050	(G/T)
1133	CakSNP1133	*Kabuli*	Ca_Kabuli_Chr01	11342388	(T/C)
1134	CakSNP1134	*Kabuli*	Ca_Kabuli_Chr01	11361646	(G/A)
1135	CakSNP1135	*Kabuli*	Ca_Kabuli_Chr01	11361588	(T/G)
1136	CakSNP1136	*Kabuli*	Ca_Kabuli_Chr01	11361583	(T/C)
1137	CakSNP1137	*Kabuli*	Ca_Kabuli_Chr01	11374707	(G/A)
1138	CakSNP1138	*Kabuli*	Ca_Kabuli_Chr01	11405015	(A/G)
1139	CakSNP1139	*Kabuli*	Ca_Kabuli_Chr01	11405118	(G/A)
1140	CakSNP1140	*Kabuli*	Ca_Kabuli_Chr01	11423677	(C/A)
1141	CakSNP1141	*Kabuli*	Ca_Kabuli_Chr01	11487689	(A/G)
1142	CakSNP1142	*Kabuli*	Ca_Kabuli_Chr01	11487630	(C/A)
1143	CakSNP1143	*Kabuli*	Ca_Kabuli_Chr01	11487752	(G/T)
1144	CakSNP1144	*Kabuli*	Ca_Kabuli_Chr01	11502148	(C/A)
1145	CakSNP1145	*Kabuli*	Ca_Kabuli_Chr01	11514888	(T/A)
1146	CakSNP1146	*Kabuli*	Ca_Kabuli_Chr01	11514898	(T/C)
1147	CakSNP1147	*Kabuli*	Ca_Kabuli_Chr01	11514903	(A/G)
1148	CakSNP1148	*Kabuli*	Ca_Kabuli_Chr01	11515055	(G/A)
1149	CakSNP1149	*Kabuli*	Ca_Kabuli_Chr01	11515130	(T/G)
1150	CakSNP1150	*Kabuli*	Ca_Kabuli_Chr01	11522656	(G/T)
1151	CakSNP1151	*Kabuli*	Ca_Kabuli_Chr01	11522743	(T/C)
1152	CakSNP1152	*Kabuli*	Ca_Kabuli_Chr01	11522875	(C/T)
1153	CakSNP1153	*Kabuli*	Ca_Kabuli_Chr01	11601347	(T/C)
1154	CakSNP1154	*Kabuli*	Ca_Kabuli_Chr01	11624419	(C/G)
1155	CakSNP1155	*Kabuli*	Ca_Kabuli_Chr01	11624464	(C/T)
1156	CakSNP1156	*Kabuli*	Ca_Kabuli_Chr01	11669794	(T/G)
1157	CakSNP1157	*Kabuli*	Ca_Kabuli_Chr01	11673478	(A/C)
1158	CakSNP1158	*Kabuli*	Ca_Kabuli_Chr01	11677097	(C/A)
1159	CakSNP1159	*Kabuli*	Ca_Kabuli_Chr01	11685769	(A/G)
1160	CakSNP1160	*Kabuli*	Ca_Kabuli_Chr01	11685790	(T/C)
1161	CakSNP1161	*Kabuli*	Ca_Kabuli_Chr01	11706451	(C/T)
1162	CakSNP1162	*Kabuli*	Ca_Kabuli_Chr01	11706494	(G/C)
1163	CakSNP1163	*Kabuli*	Ca_Kabuli_Chr01	11706583	(A/C)
1164	CakSNP1164	*Kabuli*	Ca_Kabuli_Chr01	11706545	(C/G)
1165	CakSNP1165	*Kabuli*	Ca_Kabuli_Chr01	11741448	(T/A)
1166	CakSNP1166	*Kabuli*	Ca_Kabuli_Chr01	11793443	(C/A)
1167	CakSNP1167	*Kabuli*	Ca_Kabuli_Chr01	11822248	(A/T)
1168	CakSNP1168	*Kabuli*	Ca_Kabuli_Chr01	11822305	(G/T)

S.N.	SNP IDs	Cultivars	Chromosomes/scaffolds	Physical positions (bp)	SNPs
1169	CakSNP1169	*Kabuli*	Ca_Kabuli_Chr01	11822309	(C/A)
1170	CakSNP1170	*Kabuli*	Ca_Kabuli_Chr01	11870401	(A/G)
1171	CakSNP1171	*Kabuli*	Ca_Kabuli_Chr01	11935333	(A/T)
1172	CakSNP1172	*Kabuli*	Ca_Kabuli_Chr01	11955896	(G/T)
1173	CakSNP1173	*Kabuli*	Ca_Kabuli_Chr01	11955866	(G/A)
1174	CakSNP1174	*Kabuli*	Ca_Kabuli_Chr01	11955853	(A/T)
1175	CakSNP1175	*Kabuli*	Ca_Kabuli_Chr01	12054809	(C/T)
1176	CakSNP1176	*Kabuli*	Ca_Kabuli_Chr01	12189822	(T/G)
1177	CakSNP1177	*Kabuli*	Ca_Kabuli_Chr01	12189825	(T/G)
1178	CakSNP1178	*Kabuli*	Ca_Kabuli_Chr01	12193933	(T/G)
1179	CakSNP1179	*Kabuli*	Ca_Kabuli_Chr01	12199691	(C/G)
1180	CakSNP1180	*Kabuli*	Ca_Kabuli_Chr01	12236387	(T/A)
1181	CakSNP1181	*Kabuli*	Ca_Kabuli_Chr01	12264697	(T/G)
1182	CakSNP1182	*Kabuli*	Ca_Kabuli_Chr01	12326230	(A/G)
1183	CakSNP1183	*Kabuli*	Ca_Kabuli_Chr01	12326234	(T/A)
1184	CakSNP1184	*Kabuli*	Ca_Kabuli_Chr01	12326318	(C/G)
1185	CakSNP1185	*Kabuli*	Ca_Kabuli_Chr01	12334259	(C/T)
1186	CakSNP1186	*Kabuli*	Ca_Kabuli_Chr01	12340884	(C/T)
1187	CakSNP1187	*Kabuli*	Ca_Kabuli_Chr01	12390756	(G/T)
1188	CakSNP1188	*Kabuli*	Ca_Kabuli_Chr01	12390750	(T/C)
1189	CakSNP1189	*Kabuli*	Ca_Kabuli_Chr01	12391116	(G/C)
1190	CakSNP1190	*Kabuli*	Ca_Kabuli_Chr01	12391074	(G/A)
1191	CakSNP1191	*Kabuli*	Ca_Kabuli_Chr01	12391067	(C/A)
1192	CakSNP1192	*Kabuli*	Ca_Kabuli_Chr01	12413012	(G/T)
1193	CakSNP1193	*Kabuli*	Ca_Kabuli_Chr01	12413016	(T/A)
1194	CakSNP1194	*Kabuli*	Ca_Kabuli_Chr01	12413025	(G/C)
1195	CakSNP1195	*Kabuli*	Ca_Kabuli_Chr01	12413116	(G/A)
1196	CakSNP1196	*Kabuli*	Ca_Kabuli_Chr01	12414040	(G/A)
1197	CakSNP1197	*Kabuli*	Ca_Kabuli_Chr01	12481879	(C/A)
1198	CakSNP1198	*Kabuli*	Ca_Kabuli_Chr01	12525445	(A/G)
1199	CakSNP1199	*Kabuli*	Ca_Kabuli_Chr01	12525640	(G/C)
1200	CakSNP1200	*Kabuli*	Ca_Kabuli_Chr01	12566565	(C/T)
1201	CakSNP1201	*Kabuli*	Ca_Kabuli_Chr01	12566579	(C/T)
1202	CakSNP1202	*Kabuli*	Ca_Kabuli_Chr01	12566607	(T/C)
1203	CakSNP1203	*Kabuli*	Ca_Kabuli_Chr01	12566615	(C/T)
1204	CakSNP1204	*Kabuli*	Ca_Kabuli_Chr01	12566641	(T/C)
1205	CakSNP1205	*Kabuli*	Ca_Kabuli_Chr01	12588525	(A/G)
1206	CakSNP1206	*Kabuli*	Ca_Kabuli_Chr01	12597939	(A/T)
1207	CakSNP1207	*Kabuli*	Ca_Kabuli_Chr01	12639552	(T/A)

S.N.	SNP IDs	Cultivars	Chromosomes/scaffolds	Physical positions (bp)	SNPs
1208	CakSNP1208	*Kabuli*	Ca_Kabuli_Chr01	12639584	(T/C)
1209	CakSNP1209	*Kabuli*	Ca_Kabuli_Chr01	12687506	(C/T)
1210	CakSNP1210	*Kabuli*	Ca_Kabuli_Chr01	12688424	(A/G)
1211	CakSNP1211	*Kabuli*	Ca_Kabuli_Chr01	12688435	(A/C)
1212	CakSNP1212	*Kabuli*	Ca_Kabuli_Chr01	12704406	(G/T)
1213	CakSNP1213	*Kabuli*	Ca_Kabuli_Chr01	12705665	(C/T)
1214	CakSNP1214	*Kabuli*	Ca_Kabuli_Chr01	12705731	(G/A)
1215	CakSNP1215	*Kabuli*	Ca_Kabuli_Chr01	12715427	(C/T)
1216	CakSNP1216	*Kabuli*	Ca_Kabuli_Chr01	12719734	(T/A)
1217	CakSNP1217	*Kabuli*	Ca_Kabuli_Chr01	12719735	(G/T)
1218	CakSNP1218	*Kabuli*	Ca_Kabuli_Chr01	12719752	(G/C)
1219	CakSNP1219	*Kabuli*	Ca_Kabuli_Chr01	12736298	(A/G)
1220	CakSNP1220	*Kabuli*	Ca_Kabuli_Chr01	12736353	(A/C)
1221	CakSNP1221	*Kabuli*	Ca_Kabuli_Chr01	12736622	(T/G)
1222	CakSNP1222	*Kabuli*	Ca_Kabuli_Chr01	12738564	(C/T)
1223	CakSNP1223	*Kabuli*	Ca_Kabuli_Chr01	12754422	(G/T)
1224	CakSNP1224	*Kabuli*	Ca_Kabuli_Chr01	12794075	(C/A)
1225	CakSNP1225	*Kabuli*	Ca_Kabuli_Chr01	12794147	(G/T)
1226	CakSNP1226	*Kabuli*	Ca_Kabuli_Chr01	12794251	(A/C)
1227	CakSNP1227	*Kabuli*	Ca_Kabuli_Chr01	12800638	(T/C)
1228	CakSNP1228	*Kabuli*	Ca_Kabuli_Chr01	12800610	(A/C)
1229	CakSNP1229	*Kabuli*	Ca_Kabuli_Chr01	12800607	(T/A)
1230	CakSNP1230	*Kabuli*	Ca_Kabuli_Chr01	12800601	(T/A)
1231	CakSNP1231	*Kabuli*	Ca_Kabuli_Chr01	12800597	(G/T)
1232	CakSNP1232	*Kabuli*	Ca_Kabuli_Chr01	12831717	(G/T)
1233	CakSNP1233	*Kabuli*	Ca_Kabuli_Chr01	12845445	(A/C)
1234	CakSNP1234	*Kabuli*	Ca_Kabuli_Chr01	12875506	(G/A)
1235	CakSNP1235	*Kabuli*	Ca_Kabuli_Chr01	12880408	(G/A)
1236	CakSNP1236	*Kabuli*	Ca_Kabuli_Chr01	12912621	(C/T)
1237	CakSNP1237	*Kabuli*	Ca_Kabuli_Chr01	12912625	(T/C)
1238	CakSNP1238	*Kabuli*	Ca_Kabuli_Chr01	12912670	(C/T)
1239	CakSNP1239	*Kabuli*	Ca_Kabuli_Chr01	12947377	(C/T)
1240	CakSNP1240	*Kabuli*	Ca_Kabuli_Chr01	12947376	(C/T)
1241	CakSNP1241	*Kabuli*	Ca_Kabuli_Chr01	12947358	(A/T)
1242	CakSNP1242	*Kabuli*	Ca_Kabuli_Chr01	12947347	(A/G)
1243	CakSNP1243	*Kabuli*	Ca_Kabuli_Chr01	12992445	(A/C)
1244	CakSNP1244	*Kabuli*	Ca_Kabuli_Chr01	12992423	(A/C)
1245	CakSNP1245	*Kabuli*	Ca_Kabuli_Chr01	12992569	(A/G)
1246	CakSNP1246	*Kabuli*	Ca_Kabuli_Chr01	13028543	(T/C)

S.N.	SNP IDs	Cultivars	Chromosomes/scaffolds	Physical positions (bp)	SNPs
1247	CakSNP1247	*Kabuli*	Ca_Kabuli_Chr01	13028517	(G/A)
1248	CakSNP1248	*Kabuli*	Ca_Kabuli_Chr01	13028505	(A/G)
1249	CakSNP1249	*Kabuli*	Ca_Kabuli_Chr01	13034708	(T/G)
1250	CakSNP1250	*Kabuli*	Ca_Kabuli_Chr01	13034810	(G/A)
1251	CakSNP1251	*Kabuli*	Ca_Kabuli_Chr01	13040664	(T/C)
1252	CakSNP1252	*Kabuli*	Ca_Kabuli_Chr01	13066954	(C/T)
1253	CakSNP1253	*Kabuli*	Ca_Kabuli_Chr01	13066968	(C/G)
1254	CakSNP1254	*Kabuli*	Ca_Kabuli_Chr01	13076647	(T/A)
1255	CakSNP1255	*Kabuli*	Ca_Kabuli_Chr01	13076695	(G/A)
1256	CakSNP1256	*Kabuli*	Ca_Kabuli_Chr01	13156473	(A/C)
1257	CakSNP1257	*Kabuli*	Ca_Kabuli_Chr01	13158244	(C/T)
1258	CakSNP1258	*Kabuli*	Ca_Kabuli_Chr01	13158201	(C/T)
1259	CakSNP1259	*Kabuli*	Ca_Kabuli_Chr01	13158192	(T/C)
1260	CakSNP1260	*Kabuli*	Ca_Kabuli_Chr01	13199761	(T/A)
1261	CakSNP1261	*Kabuli*	Ca_Kabuli_Chr01	13202986	(T/G)
1262	CakSNP1262	*Kabuli*	Ca_Kabuli_Chr01	13202991	(T/G)
1263	CakSNP1263	*Kabuli*	Ca_Kabuli_Chr01	13216776	(G/T)
1264	CakSNP1264	*Kabuli*	Ca_Kabuli_Chr01	13216741	(T/A)
1265	CakSNP1265	*Kabuli*	Ca_Kabuli_Chr01	13216710	(G/A)
1266	CakSNP1266	*Kabuli*	Ca_Kabuli_Chr01	13267896	(T/C)
1267	CakSNP1267	*Kabuli*	Ca_Kabuli_Chr01	13282556	(A/G)
1268	CakSNP1268	*Kabuli*	Ca_Kabuli_Chr01	13282552	(T/C)
1269	CakSNP1269	*Kabuli*	Ca_Kabuli_Chr01	13316992	(C/A)
1270	CakSNP1270	*Kabuli*	Ca_Kabuli_Chr01	13331955	(T/A)
1271	CakSNP1271	*Kabuli*	Ca_Kabuli_Chr01	13331941	(C/T)
1272	CakSNP1272	*Kabuli*	Ca_Kabuli_Chr01	13332015	(C/A)
1273	CakSNP1273	*Kabuli*	Ca_Kabuli_Chr01	13332025	(T/G)
1274	CakSNP1274	*Kabuli*	Ca_Kabuli_Chr01	13365594	(T/A)
1275	CakSNP1275	*Kabuli*	Ca_Kabuli_Chr01	13365625	(C/G)
1276	CakSNP1276	*Kabuli*	Ca_Kabuli_Chr01	13368341	(G/A)
1277	CakSNP1277	*Kabuli*	Ca_Kabuli_Chr01	13368556	(T/A)
1278	CakSNP1278	*Kabuli*	Ca_Kabuli_Chr01	13369730	(G/A)
1279	CakSNP1279	*Kabuli*	Ca_Kabuli_Chr01	13369986	(C/A)
1280	CakSNP1280	*Kabuli*	Ca_Kabuli_Chr01	13497572	(G/T)
1281	CakSNP1281	*Kabuli*	Ca_Kabuli_Chr01	13549635	(T/C)
1282	CakSNP1282	*Kabuli*	Ca_Kabuli_Chr01	13550600	(G/A)
1283	CakSNP1283	*Kabuli*	Ca_Kabuli_Chr01	13560364	(T/G)
1284	CakSNP1284	*Kabuli*	Ca_Kabuli_Chr01	13590903	(G/T)
1285	CakSNP1285	*Kabuli*	Ca_Kabuli_Chr01	13591177	(G/A)

S.N.	SNP IDs	Cultivars	Chromosomes/scaffolds	Physical positions (bp)	SNPs
1286	CakSNP1286	*Kabuli*	Ca_Kabuli_Chr01	13591203	(C/G)
1287	CakSNP1287	*Kabuli*	Ca_Kabuli_Chr01	13591314	(C/T)
1288	CakSNP1288	*Kabuli*	Ca_Kabuli_Chr01	13593931	(T/A)
1289	CakSNP1289	*Kabuli*	Ca_Kabuli_Chr01	13596411	(G/T)
1290	CakSNP1290	*Kabuli*	Ca_Kabuli_Chr01	13596528	(T/A)
1291	CakSNP1291	*Kabuli*	Ca_Kabuli_Chr01	13596539	(A/G)
1292	CakSNP1292	*Kabuli*	Ca_Kabuli_Chr01	13596666	(T/A)
1293	CakSNP1293	*Kabuli*	Ca_Kabuli_Chr01	13596659	(T/A)
1294	CakSNP1294	*Kabuli*	Ca_Kabuli_Chr01	13596631	(C/T)
1295	CakSNP1295	*Kabuli*	Ca_Kabuli_Chr01	13596621	(C/T)
1296	CakSNP1296	*Kabuli*	Ca_Kabuli_Chr01	13596741	(C/T)
1297	CakSNP1297	*Kabuli*	Ca_Kabuli_Chr01	13596771	(C/A)
1298	CakSNP1298	*Kabuli*	Ca_Kabuli_Chr01	13607486	(A/C)
1299	CakSNP1299	*Kabuli*	Ca_Kabuli_Chr01	13608414	(T/C)
1300	CakSNP1300	*Kabuli*	Ca_Kabuli_Chr01	13637705	(G/T)
1301	CakSNP1301	*Kabuli*	Ca_Kabuli_Chr01	13637688	(A/G)
1302	CakSNP1302	*Kabuli*	Ca_Kabuli_Chr01	13637687	(C/T)
1303	CakSNP1303	*Kabuli*	Ca_Kabuli_Chr01	13637684	(C/T)
1304	CakSNP1304	*Kabuli*	Ca_Kabuli_Chr01	13639157	(T/C)
1305	CakSNP1305	*Kabuli*	Ca_Kabuli_Chr01	13641517	(A/G)
1306	CakSNP1306	*Kabuli*	Ca_Kabuli_Chr01	13641644	(G/A)
1307	CakSNP1307	*Kabuli*	Ca_Kabuli_Chr01	13641615	(A/G)
1308	CakSNP1308	*Kabuli*	Ca_Kabuli_Chr01	13641608	(G/T)
1309	CakSNP1309	*Kabuli*	Ca_Kabuli_Chr01	13641574	(A/C)
1310	CakSNP1310	*Kabuli*	Ca_Kabuli_Chr01	13641564	(G/A)
1311	CakSNP1311	*Kabuli*	Ca_Kabuli_Chr01	13668759	(T/A)
1312	CakSNP1312	*Kabuli*	Ca_Kabuli_Chr01	13680002	(G/T)
1313	CakSNP1313	*Kabuli*	Ca_Kabuli_Chr01	13680008	(G/C)
1314	CakSNP1314	*Kabuli*	Ca_Kabuli_Chr01	13762410	(C/A)
1315	CakSNP1315	*Kabuli*	Ca_Kabuli_Chr01	13769805	(C/T)
1316	CakSNP1316	*Kabuli*	Ca_Kabuli_Chr01	13769771	(T/C)
1317	CakSNP1317	*Kabuli*	Ca_Kabuli_Chr01	13782049	(C/T)
1318	CakSNP1318	*Kabuli*	Ca_Kabuli_Chr01	13814790	(T/C)
1319	CakSNP1319	*Kabuli*	Ca_Kabuli_Chr01	13828703	(C/T)
1320	CakSNP1320	*Kabuli*	Ca_Kabuli_Chr01	13828659	(G/A)
1321	CakSNP1321	*Kabuli*	Ca_Kabuli_Chr01	13828657	(G/A)
1322	CakSNP1322	*Kabuli*	Ca_Kabuli_Chr01	13828744	(A/T)
1323	CakSNP1323	*Kabuli*	Ca_Kabuli_Chr01	13991356	(T/G)
1324	CakSNP1324	*Kabuli*	Ca_Kabuli_Chr01	14014566	(C/A)

S.N.	SNP IDs	Cultivars	Chromosomes/scaffolds	Physical positions (bp)	SNPs
1325	CakSNP1325	*Kabuli*	Ca_Kabuli_Chr01	14015757	(T/C)
1326	CakSNP1326	*Kabuli*	Ca_Kabuli_Chr01	14015781	(G/C)
1327	CakSNP1327	*Kabuli*	Ca_Kabuli_Chr01	14015818	(C/T)
1328	CakSNP1328	*Kabuli*	Ca_Kabuli_Chr01	14022892	(C/A)
1329	CakSNP1329	*Kabuli*	Ca_Kabuli_Chr01	14022890	(A/T)
1330	CakSNP1330	*Kabuli*	Ca_Kabuli_Chr01	14023097	(A/C)
1331	CakSNP1331	*Kabuli*	Ca_Kabuli_Chr01	14023095	(T/A)
1332	CakSNP1332	*Kabuli*	Ca_Kabuli_Chr01	14023078	(T/A)
1333	CakSNP1333	*Kabuli*	Ca_Kabuli_Chr01	14025015	(G/A)
1334	CakSNP1334	*Kabuli*	Ca_Kabuli_Chr01	14025075	(A/G)
1335	CakSNP1335	*Kabuli*	Ca_Kabuli_Chr01	14026347	(C/G)
1336	CakSNP1336	*Kabuli*	Ca_Kabuli_Chr01	14026391	(T/C)
1337	CakSNP1337	*Kabuli*	Ca_Kabuli_Chr01	14026467	(C/T)
1338	CakSNP1338	*Kabuli*	Ca_Kabuli_Chr01	14026444	(G/T)
1339	CakSNP1339	*Kabuli*	Ca_Kabuli_Chr01	14026437	(C/A)
1340	CakSNP1340	*Kabuli*	Ca_Kabuli_Chr01	14033226	(C/T)
1341	CakSNP1341	*Kabuli*	Ca_Kabuli_Chr01	14033285	(C/T)
1342	CakSNP1342	*Kabuli*	Ca_Kabuli_Chr01	14033277	(G/C)
1343	CakSNP1343	*Kabuli*	Ca_Kabuli_Chr01	14033951	(G/A)
1344	CakSNP1344	*Kabuli*	Ca_Kabuli_Chr01	14034011	(A/G)
1345	CakSNP1345	*Kabuli*	Ca_Kabuli_Chr01	14045110	(T/C)
1346	CakSNP1346	*Kabuli*	Ca_Kabuli_Chr01	14045239	(A/G)
1347	CakSNP1347	*Kabuli*	Ca_Kabuli_Chr01	14076924	(T/C)
1348	CakSNP1348	*Kabuli*	Ca_Kabuli_Chr01	14101608	(C/T)
1349	CakSNP1349	*Kabuli*	Ca_Kabuli_Chr01	14101621	(T/C)
1350	CakSNP1350	*Kabuli*	Ca_Kabuli_Chr01	14101624	(C/T)
1351	CakSNP1351	*Kabuli*	Ca_Kabuli_Chr01	14101644	(A/G)
1352	CakSNP1352	*Kabuli*	Ca_Kabuli_Chr01	14163638	(C/T)
1353	CakSNP1353	*Kabuli*	Ca_Kabuli_Chr01	14163721	(T/C)
1354	CakSNP1354	*Kabuli*	Ca_Kabuli_Chr01	14163836	(A/C)
1355	CakSNP1355	*Kabuli*	Ca_Kabuli_Chr01	14225210	(G/T)
1356	CakSNP1356	*Kabuli*	Ca_Kabuli_Chr01	14237163	(T/G)
1357	CakSNP1357	*Kabuli*	Ca_Kabuli_Chr01	14237164	(T/A)
1358	CakSNP1358	*Kabuli*	Ca_Kabuli_Chr01	14237273	(A/T)
1359	CakSNP1359	*Kabuli*	Ca_Kabuli_Chr01	14240571	(C/T)
1360	CakSNP1360	*Kabuli*	Ca_Kabuli_Chr01	14259435	(G/C)
1361	CakSNP1361	*Kabuli*	Ca_Kabuli_Chr01	14259455	(A/G)
1362	CakSNP1362	*Kabuli*	Ca_Kabuli_Chr01	14281643	(T/C)
1363	CakSNP1363	*Kabuli*	Ca_Kabuli_Chr01	14348207	(C/T)

S.N.	SNP IDs	Cultivars	Chromosomes/scaffolds	Physical positions (bp)	SNPs
1364	CakSNP1364	*Kabuli*	Ca_Kabuli_Chr01	14393255	(A/G)
1365	CakSNP1365	*Kabuli*	Ca_Kabuli_Chr01	14393313	(C/A)
1366	CakSNP1366	*Kabuli*	Ca_Kabuli_Chr01	14459908	(T/C)
1367	CakSNP1367	*Kabuli*	Ca_Kabuli_Chr01	14576525	(C/T)
1368	CakSNP1368	*Kabuli*	Ca_Kabuli_Chr01	14623271	(T/C)
1369	CakSNP1369	*Kabuli*	Ca_Kabuli_Chr01	14698461	(A/T)
1370	CakSNP1370	*Kabuli*	Ca_Kabuli_Chr01	14801406	(A/G)
1371	CakSNP1371	*Kabuli*	Ca_Kabuli_Chr01	14884940	(G/T)
1372	CakSNP1372	*Kabuli*	Ca_Kabuli_Chr01	14916633	(G/C)
1373	CakSNP1373	*Kabuli*	Ca_Kabuli_Chr01	15126244	(T/C)
1374	CakSNP1374	*Kabuli*	Ca_Kabuli_Chr01	15137319	(C/T)
1375	CakSNP1375	*Kabuli*	Ca_Kabuli_Chr01	15146123	(A/G)
1376	CakSNP1376	*Kabuli*	Ca_Kabuli_Chr01	15146149	(G/A)
1377	CakSNP1377	*Kabuli*	Ca_Kabuli_Chr01	15167641	(T/C)
1378	CakSNP1378	*Kabuli*	Ca_Kabuli_Chr01	15167643	(G/A)
1379	CakSNP1379	*Kabuli*	Ca_Kabuli_Chr01	15196154	(T/A)
1380	CakSNP1380	*Kabuli*	Ca_Kabuli_Chr01	15236202	(A/G)
1381	CakSNP1381	*Kabuli*	Ca_Kabuli_Chr01	15253283	(G/A)
1382	CakSNP1382	*Kabuli*	Ca_Kabuli_Chr01	15253285	(A/G)
1383	CakSNP1383	*Kabuli*	Ca_Kabuli_Chr01	15291748	(C/T)
1384	CakSNP1384	*Kabuli*	Ca_Kabuli_Chr01	15319181	(T/C)
1385	CakSNP1385	*Kabuli*	Ca_Kabuli_Chr01	15611784	(T/C)
1386	CakSNP1386	*Kabuli*	Ca_Kabuli_Chr01	15613071	(A/C)
1387	CakSNP1387	*Kabuli*	Ca_Kabuli_Chr01	15683091	(G/A)
1388	CakSNP1388	*Kabuli*	Ca_Kabuli_Chr01	15683101	(A/C)
1389	CakSNP1389	*Kabuli*	Ca_Kabuli_Chr01	15683106	(C/A)
1390	CakSNP1390	*Kabuli*	Ca_Kabuli_Chr01	15683117	(G/T)
1391	CakSNP1391	*Kabuli*	Ca_Kabuli_Chr01	15683138	(A/G)
1392	CakSNP1392	*Kabuli*	Ca_Kabuli_Chr01	15760638	(C/A)
1393	CakSNP1393	*Kabuli*	Ca_Kabuli_Chr01	15784021	(C/T)
1394	CakSNP1394	*Kabuli*	Ca_Kabuli_Chr01	15802393	(G/A)
1395	CakSNP1395	*Kabuli*	Ca_Kabuli_Chr01	15862493	(C/T)
1396	CakSNP1396	*Kabuli*	Ca_Kabuli_Chr01	15960425	(A/C)
1397	CakSNP1397	*Kabuli*	Ca_Kabuli_Chr01	15965278	(G/A)
1398	CakSNP1398	*Kabuli*	Ca_Kabuli_Chr01	16061242	(A/G)
1399	CakSNP1399	*Kabuli*	Ca_Kabuli_Chr01	16061293	(C/G)
1400	CakSNP1400	*Kabuli*	Ca_Kabuli_Chr01	16061294	(T/A)
1401	CakSNP1401	*Kabuli*	Ca_Kabuli_Chr01	16061295	(A/G)
1402	CakSNP1402	*Kabuli*	Ca_Kabuli_Chr01	16061300	(A/G)

S.N.	SNP IDs	Cultivars	Chromosomes/scaffolds	Physical positions (bp)	SNPs
1403	CakSNP1403	*Kabuli*	Ca_Kabuli_Chr01	16061385	(A/G)
1404	CakSNP1404	*Kabuli*	Ca_Kabuli_Chr01	16061486	(G/A)
1405	CakSNP1405	*Kabuli*	Ca_Kabuli_Chr01	16061541	(G/A)
1406	CakSNP1406	*Kabuli*	Ca_Kabuli_Chr01	16061536	(A/C)
1407	CakSNP1407	*Kabuli*	Ca_Kabuli_Chr01	16081438	(T/C)
1408	CakSNP1408	*Kabuli*	Ca_Kabuli_Chr01	16099437	(T/G)
1409	CakSNP1409	*Kabuli*	Ca_Kabuli_Chr01	16099438	(G/A)
1410	CakSNP1410	*Kabuli*	Ca_Kabuli_Chr01	16099468	(G/A)
1411	CakSNP1411	*Kabuli*	Ca_Kabuli_Chr01	16099444	(T/C)
1412	CakSNP1412	*Kabuli*	Ca_Kabuli_Chr01	16099459	(T/C)
1413	CakSNP1413	*Kabuli*	Ca_Kabuli_Chr01	16099457	(T/C)
1414	CakSNP1414	*Kabuli*	Ca_Kabuli_Chr01	16104255	(G/T)
1415	CakSNP1415	*Kabuli*	Ca_Kabuli_Chr01	16104351	(G/A)
1416	CakSNP1416	*Kabuli*	Ca_Kabuli_Chr01	16104334	(T/G)
1417	CakSNP1417	*Kabuli*	Ca_Kabuli_Chr01	16104298	(C/T)
1418	CakSNP1418	*Kabuli*	Ca_Kabuli_Chr01	16114607	(C/A)
1419	CakSNP1419	*Kabuli*	Ca_Kabuli_Chr01	16196930	(A/G)
1420	CakSNP1420	*Kabuli*	Ca_Kabuli_Chr01	16228049	(C/A)
1421	CakSNP1421	*Kabuli*	Ca_Kabuli_Chr01	16228039	(C/A)
1422	CakSNP1422	*Kabuli*	Ca_Kabuli_Chr01	16289397	(G/A)
1423	CakSNP1423	*Kabuli*	Ca_Kabuli_Chr01	16329161	(G/A)
1424	CakSNP1424	*Kabuli*	Ca_Kabuli_Chr01	16376254	(G/T)
1425	CakSNP1425	*Kabuli*	Ca_Kabuli_Chr01	16379239	(A/G)
1426	CakSNP1426	*Kabuli*	Ca_Kabuli_Chr01	16379258	(C/T)
1427	CakSNP1427	*Kabuli*	Ca_Kabuli_Chr01	16410186	(C/T)
1428	CakSNP1428	*Kabuli*	Ca_Kabuli_Chr01	16419469	(C/T)
1429	CakSNP1429	*Kabuli*	Ca_Kabuli_Chr01	16454432	(C/T)
1430	CakSNP1430	*Kabuli*	Ca_Kabuli_Chr01	16454487	(G/C)
1431	CakSNP1431	*Kabuli*	Ca_Kabuli_Chr01	16493894	(A/G)
1432	CakSNP1432	*Kabuli*	Ca_Kabuli_Chr01	16512011	(G/T)
1433	CakSNP1433	*Kabuli*	Ca_Kabuli_Chr01	16512026	(C/T)
1434	CakSNP1434	*Kabuli*	Ca_Kabuli_Chr01	16512171	(G/A)
1435	CakSNP1435	*Kabuli*	Ca_Kabuli_Chr01	16512195	(G/A)
1436	CakSNP1436	*Kabuli*	Ca_Kabuli_Chr01	16515112	(T/C)
1437	CakSNP1437	*Kabuli*	Ca_Kabuli_Chr01	16527347	(T/A)
1438	CakSNP1438	*Kabuli*	Ca_Kabuli_Chr01	16527366	(G/A)
1439	CakSNP1439	*Kabuli*	Ca_Kabuli_Chr01	16575167	(A/G)
1440	CakSNP1440	*Kabuli*	Ca_Kabuli_Chr01	16636587	(G/A)
1441	CakSNP1441	*Kabuli*	Ca_Kabuli_Chr01	16743569	(C/T)

S.N.	SNP IDs	Cultivars	Chromosomes/scaffolds	Physical positions (bp)	SNPs
1442	CakSNP1442	*Kabuli*	Ca_Kabuli_Chr01	16799112	(T/C)
1443	CakSNP1443	*Kabuli*	Ca_Kabuli_Chr01	16799142	(C/T)
1444	CakSNP1444	*Kabuli*	Ca_Kabuli_Chr01	16813600	(T/A)
1445	CakSNP1445	*Kabuli*	Ca_Kabuli_Chr01	16813603	(T/G)
1446	CakSNP1446	*Kabuli*	Ca_Kabuli_Chr01	16813604	(T/C)
1447	CakSNP1447	*Kabuli*	Ca_Kabuli_Chr01	16813657	(A/T)
1448	CakSNP1448	*Kabuli*	Ca_Kabuli_Chr01	16813616	(T/A)
1449	CakSNP1449	*Kabuli*	Ca_Kabuli_Chr01	16873356	(C/T)
1450	CakSNP1450	*Kabuli*	Ca_Kabuli_Chr01	16877812	(C/A)
1451	CakSNP1451	*Kabuli*	Ca_Kabuli_Chr01	16877829	(C/A)
1452	CakSNP1452	*Kabuli*	Ca_Kabuli_Chr01	16883153	(C/T)
1453	CakSNP1453	*Kabuli*	Ca_Kabuli_Chr01	16901798	(C/A)
1454	CakSNP1454	*Kabuli*	Ca_Kabuli_Chr01	16902129	(G/A)
1455	CakSNP1455	*Kabuli*	Ca_Kabuli_Chr01	16908636	(G/A)
1456	CakSNP1456	*Kabuli*	Ca_Kabuli_Chr01	16933423	(T/G)
1457	CakSNP1457	*Kabuli*	Ca_Kabuli_Chr01	16933431	(T/G)
1458	CakSNP1458	*Kabuli*	Ca_Kabuli_Chr01	17006391	(T/C)
1459	CakSNP1459	*Kabuli*	Ca_Kabuli_Chr01	17023111	(G/C)
1460	CakSNP1460	*Kabuli*	Ca_Kabuli_Chr01	17024083	(A/C)
1461	CakSNP1461	*Kabuli*	Ca_Kabuli_Chr01	17068798	(A/C)
1462	CakSNP1462	*Kabuli*	Ca_Kabuli_Chr01	17092204	(C/T)
1463	CakSNP1463	*Kabuli*	Ca_Kabuli_Chr01	17092404	(G/A)
1464	CakSNP1464	*Kabuli*	Ca_Kabuli_Chr01	17092413	(C/T)
1465	CakSNP1465	*Kabuli*	Ca_Kabuli_Chr01	17093640	(T/C)
1466	CakSNP1466	*Kabuli*	Ca_Kabuli_Chr01	17167019	(G/A)
1467	CakSNP1467	*Kabuli*	Ca_Kabuli_Chr01	17170443	(A/T)
1468	CakSNP1468	*Kabuli*	Ca_Kabuli_Chr01	17170727	(T/C)
1469	CakSNP1469	*Kabuli*	Ca_Kabuli_Chr01	17182711	(C/T)
1470	CakSNP1470	*Kabuli*	Ca_Kabuli_Chr01	17182697	(A/T)
1471	CakSNP1471	*Kabuli*	Ca_Kabuli_Chr01	17182867	(G/A)
1472	CakSNP1472	*Kabuli*	Ca_Kabuli_Chr01	17194835	(C/T)
1473	CakSNP1473	*Kabuli*	Ca_Kabuli_Chr01	17272338	(A/C)
1474	CakSNP1474	*Kabuli*	Ca_Kabuli_Chr01	17272401	(C/T)
1475	CakSNP1475	*Kabuli*	Ca_Kabuli_Chr01	17314880	(A/C)
1476	CakSNP1476	*Kabuli*	Ca_Kabuli_Chr01	17314911	(A/G)
1477	CakSNP1477	*Kabuli*	Ca_Kabuli_Chr01	17314945	(T/C)
1478	CakSNP1478	*Kabuli*	Ca_Kabuli_Chr01	17315046	(G/A)
1479	CakSNP1479	*Kabuli*	Ca_Kabuli_Chr01	17328721	(G/A)
1480	CakSNP1480	*Kabuli*	Ca_Kabuli_Chr01	17328808	(G/A)

S.N.	SNP IDs	Cultivars	Chromosomes/scaffolds	Physical positions (bp)	SNPs
1481	CakSNP1481	*Kabuli*	Ca_Kabuli_Chr01	17332981	(C/T)
1482	CakSNP1482	*Kabuli*	Ca_Kabuli_Chr01	17333056	(C/G)
1483	CakSNP1483	*Kabuli*	Ca_Kabuli_Chr01	17333049	(C/T)
1484	CakSNP1484	*Kabuli*	Ca_Kabuli_Chr01	17340321	(T/C)
1485	CakSNP1485	*Kabuli*	Ca_Kabuli_Chr01	17340350	(G/T)
1486	CakSNP1486	*Kabuli*	Ca_Kabuli_Chr01	17340398	(G/T)
1487	CakSNP1487	*Kabuli*	Ca_Kabuli_Chr01	17359712	(A/G)
1488	CakSNP1488	*Kabuli*	Ca_Kabuli_Chr01	17359688	(A/C)
1489	CakSNP1489	*Kabuli*	Ca_Kabuli_Chr01	17359686	(C/T)
1490	CakSNP1490	*Kabuli*	Ca_Kabuli_Chr01	17359682	(A/C)
1491	CakSNP1491	*Kabuli*	Ca_Kabuli_Chr01	17386549	(C/T)
1492	CakSNP1492	*Kabuli*	Ca_Kabuli_Chr01	17386603	(A/T)
1493	CakSNP1493	*Kabuli*	Ca_Kabuli_Chr01	17395543	(C/A)
1494	CakSNP1494	*Kabuli*	Ca_Kabuli_Chr01	17437336	(G/C)
1495	CakSNP1495	*Kabuli*	Ca_Kabuli_Chr01	17558885	(T/C)
1496	CakSNP1496	*Kabuli*	Ca_Kabuli_Chr01	17558940	(A/G)
1497	CakSNP1497	*Kabuli*	Ca_Kabuli_Chr01	17558975	(A/G)
1498	CakSNP1498	*Kabuli*	Ca_Kabuli_Chr01	17576793	(T/G)
1499	CakSNP1499	*Kabuli*	Ca_Kabuli_Chr01	17761244	(T/C)
1500	CakSNP1500	*Kabuli*	Ca_Kabuli_Chr01	17763949	(G/A)
1501	CakSNP1501	*Kabuli*	Ca_Kabuli_Chr01	17819374	(A/C)
1502	CakSNP1502	*Kabuli*	Ca_Kabuli_Chr01	17856892	(A/G)
1503	CakSNP1503	*Kabuli*	Ca_Kabuli_Chr01	17857089	(T/A)
1504	CakSNP1504	*Kabuli*	Ca_Kabuli_Chr01	17880143	(T/C)
1505	CakSNP1505	*Kabuli*	Ca_Kabuli_Chr01	17880185	(T/C)
1506	CakSNP1506	*Kabuli*	Ca_Kabuli_Chr01	17961779	(T/C)
1507	CakSNP1507	*Kabuli*	Ca_Kabuli_Chr01	17983301	(T/G)
1508	CakSNP1508	*Kabuli*	Ca_Kabuli_Chr01	17983310	(T/G)
1509	CakSNP1509	*Kabuli*	Ca_Kabuli_Chr01	18009665	(C/G)
1510	CakSNP1510	*Kabuli*	Ca_Kabuli_Chr01	18050590	(G/T)
1511	CakSNP1511	*Kabuli*	Ca_Kabuli_Chr01	18064930	(C/T)
1512	CakSNP1512	*Kabuli*	Ca_Kabuli_Chr01	18065239	(T/C)
1513	CakSNP1513	*Kabuli*	Ca_Kabuli_Chr01	18092704	(T/G)
1514	CakSNP1514	*Kabuli*	Ca_Kabuli_Chr01	18092761	(C/T)
1515	CakSNP1515	*Kabuli*	Ca_Kabuli_Chr01	18098516	(C/T)
1516	CakSNP1516	*Kabuli*	Ca_Kabuli_Chr01	18099331	(C/T)
1517	CakSNP1517	*Kabuli*	Ca_Kabuli_Chr01	18099334	(T/A)
1518	CakSNP1518	*Kabuli*	Ca_Kabuli_Chr01	18112318	(A/G)
1519	CakSNP1519	*Kabuli*	Ca_Kabuli_Chr01	18112353	(T/C)

S.N.	SNP IDs	Cultivars	Chromosomes/scaffolds	Physical positions (bp)	SNPs
1520	CakSNP1520	*Kabuli*	Ca_Kabuli_Chr01	18131516	(T/G)
1521	CakSNP1521	*Kabuli*	Ca_Kabuli_Chr01	18131545	(A/T)
1522	CakSNP1522	*Kabuli*	Ca_Kabuli_Chr01	18131898	(G/A)
1523	CakSNP1523	*Kabuli*	Ca_Kabuli_Chr01	18141990	(C/A)
1524	CakSNP1524	*Kabuli*	Ca_Kabuli_Chr01	18144107	(C/G)
1525	CakSNP1525	*Kabuli*	Ca_Kabuli_Chr01	18144137	(T/A)
1526	CakSNP1526	*Kabuli*	Ca_Kabuli_Chr01	18147011	(C/G)
1527	CakSNP1527	*Kabuli*	Ca_Kabuli_Chr01	18150151	(G/A)
1528	CakSNP1528	*Kabuli*	Ca_Kabuli_Chr01	18153870	(T/G)
1529	CakSNP1529	*Kabuli*	Ca_Kabuli_Chr01	18154083	(A/G)
1530	CakSNP1530	*Kabuli*	Ca_Kabuli_Chr01	18156634	(A/G)
1531	CakSNP1531	*Kabuli*	Ca_Kabuli_Chr01	18172762	(G/T)
1532	CakSNP1532	*Kabuli*	Ca_Kabuli_Chr01	18198444	(C/T)
1533	CakSNP1533	*Kabuli*	Ca_Kabuli_Chr01	18488172	(G/A)
1534	CakSNP1534	*Kabuli*	Ca_Kabuli_Chr01	18746045	(T/G)
1535	CakSNP1535	*Kabuli*	Ca_Kabuli_Chr01	18763916	(G/A)
1536	CakSNP1536	*Kabuli*	Ca_Kabuli_Chr01	18763911	(C/A)
1537	CakSNP1537	*Kabuli*	Ca_Kabuli_Chr01	18764225	(C/T)
1538	CakSNP1538	*Kabuli*	Ca_Kabuli_Chr01	18768563	(C/A)
1539	CakSNP1539	*Kabuli*	Ca_Kabuli_Chr01	18797330	(T/C)
1540	CakSNP1540	*Kabuli*	Ca_Kabuli_Chr01	18890559	(A/G)
1541	CakSNP1541	*Kabuli*	Ca_Kabuli_Chr01	18890616	(G/A)
1542	CakSNP1542	*Kabuli*	Ca_Kabuli_Chr01	18890833	(G/A)
1543	CakSNP1543	*Kabuli*	Ca_Kabuli_Chr01	18910213	(C/T)
1544	CakSNP1544	*Kabuli*	Ca_Kabuli_Chr01	18910202	(G/A)
1545	CakSNP1545	*Kabuli*	Ca_Kabuli_Chr01	18910173	(T/C)
1546	CakSNP1546	*Kabuli*	Ca_Kabuli_Chr01	19000445	(T/C)
1547	CakSNP1547	*Kabuli*	Ca_Kabuli_Chr01	19055085	(C/T)
1548	CakSNP1548	*Kabuli*	Ca_Kabuli_Chr01	19130894	(T/C)
1549	CakSNP1549	*Kabuli*	Ca_Kabuli_Chr01	19130884	(C/A)
1550	CakSNP1550	*Kabuli*	Ca_Kabuli_Chr01	19130883	(C/A)
1551	CakSNP1551	*Kabuli*	Ca_Kabuli_Chr01	19130872	(G/A)
1552	CakSNP1552	*Kabuli*	Ca_Kabuli_Chr01	19130833	(G/T)
1553	CakSNP1553	*Kabuli*	Ca_Kabuli_Chr01	19130831	(A/G)
1554	CakSNP1554	*Kabuli*	Ca_Kabuli_Chr01	19130848	(C/T)
1555	CakSNP1555	*Kabuli*	Ca_Kabuli_Chr01	19130853	(G/A)
1556	CakSNP1556	*Kabuli*	Ca_Kabuli_Chr01	19164976	(T/C)
1557	CakSNP1557	*Kabuli*	Ca_Kabuli_Chr01	19166597	(A/G)
1558	CakSNP1558	*Kabuli*	Ca_Kabuli_Chr01	19218067	(G/A)

S.N.	SNP IDs	Cultivars	Chromosomes/scaffolds	Physical positions (bp)	SNPs
1559	CakSNP1559	*Kabuli*	Ca_Kabuli_Chr01	19218093	(T/G)
1560	CakSNP1560	*Kabuli*	Ca_Kabuli_Chr01	19252952	(G/T)
1561	CakSNP1561	*Kabuli*	Ca_Kabuli_Chr01	19252938	(G/A)
1562	CakSNP1562	*Kabuli*	Ca_Kabuli_Chr01	19253655	(C/T)
1563	CakSNP1563	*Kabuli*	Ca_Kabuli_Chr01	19254161	(G/A)
1564	CakSNP1564	*Kabuli*	Ca_Kabuli_Chr01	19276489	(A/G)
1565	CakSNP1565	*Kabuli*	Ca_Kabuli_Chr01	19276697	(C/G)
1566	CakSNP1566	*Kabuli*	Ca_Kabuli_Chr01	19359812	(A/C)
1567	CakSNP1567	*Kabuli*	Ca_Kabuli_Chr01	19359844	(C/A)
1568	CakSNP1568	*Kabuli*	Ca_Kabuli_Chr01	19399112	(C/T)
1569	CakSNP1569	*Kabuli*	Ca_Kabuli_Chr01	19526320	(T/C)
1570	CakSNP1570	*Kabuli*	Ca_Kabuli_Chr01	19526531	(C/G)
1571	CakSNP1571	*Kabuli*	Ca_Kabuli_Chr01	19526548	(G/A)
1572	CakSNP1572	*Kabuli*	Ca_Kabuli_Chr01	19526561	(T/C)
1573	CakSNP1573	*Kabuli*	Ca_Kabuli_Chr01	19526592	(T/A)
1574	CakSNP1574	*Kabuli*	Ca_Kabuli_Chr01	19526652	(G/T)
1575	CakSNP1575	*Kabuli*	Ca_Kabuli_Chr01	19526632	(C/T)
1576	CakSNP1576	*Kabuli*	Ca_Kabuli_Chr01	19572408	(C/T)
1577	CakSNP1577	*Kabuli*	Ca_Kabuli_Chr01	19572494	(G/C)
1578	CakSNP1578	*Kabuli*	Ca_Kabuli_Chr01	19591671	(A/G)
1579	CakSNP1579	*Kabuli*	Ca_Kabuli_Chr01	19877654	(A/T)
1580	CakSNP1580	*Kabuli*	Ca_Kabuli_Chr01	19902735	(T/A)
1581	CakSNP1581	*Kabuli*	Ca_Kabuli_Chr01	19903665	(A/G)
1582	CakSNP1582	*Kabuli*	Ca_Kabuli_Chr01	19903683	(G/A)
1583	CakSNP1583	*Kabuli*	Ca_Kabuli_Chr01	19903686	(T/A)
1584	CakSNP1584	*Kabuli*	Ca_Kabuli_Chr01	19903690	(A/G)
1585	CakSNP1585	*Kabuli*	Ca_Kabuli_Chr01	19903693	(G/T)
1586	CakSNP1586	*Kabuli*	Ca_Kabuli_Chr01	19903701	(G/A)
1587	CakSNP1587	*Kabuli*	Ca_Kabuli_Chr01	19903704	(G/A)
1588	CakSNP1588	*Kabuli*	Ca_Kabuli_Chr01	20004655	(A/G)
1589	CakSNP1589	*Kabuli*	Ca_Kabuli_Chr01	20005747	(C/T)
1590	CakSNP1590	*Kabuli*	Ca_Kabuli_Chr01	20016525	(C/T)
1591	CakSNP1591	*Kabuli*	Ca_Kabuli_Chr01	20070045	(A/G)
1592	CakSNP1592	*Kabuli*	Ca_Kabuli_Chr01	20070197	(T/C)
1593	CakSNP1593	*Kabuli*	Ca_Kabuli_Chr01	20091291	(T/G)
1594	CakSNP1594	*Kabuli*	Ca_Kabuli_Chr01	20092571	(C/G)
1595	CakSNP1595	*Kabuli*	Ca_Kabuli_Chr01	20092677	(C/A)
1596	CakSNP1596	*Kabuli*	Ca_Kabuli_Chr01	21822975	(A/G)
1597	CakSNP1597	*Kabuli*	Ca_Kabuli_Chr01	21881133	(G/A)

S.N.	SNP IDs	Cultivars	Chromosomes/scaffolds	Physical positions (bp)	SNPs
1598	CakSNP1598	*Kabuli*	Ca_Kabuli_Chr01	21881112	(C/T)
1599	CakSNP1599	*Kabuli*	Ca_Kabuli_Chr01	21881103	(G/A)
1600	CakSNP1600	*Kabuli*	Ca_Kabuli_Chr01	21881094	(G/A)
1601	CakSNP1601	*Kabuli*	Ca_Kabuli_Chr01	21918747	(G/A)
1602	CakSNP1602	*Kabuli*	Ca_Kabuli_Chr01	22344003	(G/A)
1603	CakSNP1603	*Kabuli*	Ca_Kabuli_Chr01	22437033	(C/T)
1604	CakSNP1604	*Kabuli*	Ca_Kabuli_Chr01	22465555	(C/T)
1605	CakSNP1605	*Kabuli*	Ca_Kabuli_Chr01	22465692	(A/G)
1606	CakSNP1606	*Kabuli*	Ca_Kabuli_Chr01	22719581	(T/G)
1607	CakSNP1607	*Kabuli*	Ca_Kabuli_Chr01	22743361	(T/G)
1608	CakSNP1608	*Kabuli*	Ca_Kabuli_Chr01	23122951	(A/G)
1609	CakSNP1609	*Kabuli*	Ca_Kabuli_Chr01	23128921	(T/A)
1610	CakSNP1610	*Kabuli*	Ca_Kabuli_Chr01	23128979	(G/T)
1611	CakSNP1611	*Kabuli*	Ca_Kabuli_Chr01	23129094	(C/T)
1612	CakSNP1612	*Kabuli*	Ca_Kabuli_Chr01	23131178	(A/C)
1613	CakSNP1613	*Kabuli*	Ca_Kabuli_Chr01	23131914	(T/C)
1614	CakSNP1614	*Kabuli*	Ca_Kabuli_Chr01	23148736	(A/C)
1615	CakSNP1615	*Kabuli*	Ca_Kabuli_Chr01	23149053	(A/G)
1616	CakSNP1616	*Kabuli*	Ca_Kabuli_Chr01	23149096	(A/C)
1617	CakSNP1617	*Kabuli*	Ca_Kabuli_Chr01	23341499	(C/G)
1618	CakSNP1618	*Kabuli*	Ca_Kabuli_Chr01	23382483	(T/A)
1619	CakSNP1619	*Kabuli*	Ca_Kabuli_Chr01	23382454	(G/T)
1620	CakSNP1620	*Kabuli*	Ca_Kabuli_Chr01	23382664	(C/T)
1621	CakSNP1621	*Kabuli*	Ca_Kabuli_Chr01	23382645	(C/G)
1622	CakSNP1622	*Kabuli*	Ca_Kabuli_Chr01	23915127	(C/G)
1623	CakSNP1623	*Kabuli*	Ca_Kabuli_Chr01	23941438	(G/A)
1624	CakSNP1624	*Kabuli*	Ca_Kabuli_Chr01	23941518	(C/T)
1625	CakSNP1625	*Kabuli*	Ca_Kabuli_Chr01	24070908	(C/T)
1626	CakSNP1626	*Kabuli*	Ca_Kabuli_Chr01	24070968	(C/T)
1627	CakSNP1627	*Kabuli*	Ca_Kabuli_Chr01	24071007	(C/A)
1628	CakSNP1628	*Kabuli*	Ca_Kabuli_Chr01	24071014	(T/G)
1629	CakSNP1629	*Kabuli*	Ca_Kabuli_Chr01	24173273	(A/G)
1630	CakSNP1630	*Kabuli*	Ca_Kabuli_Chr01	24450415	(T/C)
1631	CakSNP1631	*Kabuli*	Ca_Kabuli_Chr01	24450511	(A/G)
1632	CakSNP1632	*Kabuli*	Ca_Kabuli_Chr01	24450483	(T/G)
1633	CakSNP1633	*Kabuli*	Ca_Kabuli_Chr01	24693506	(C/T)
1634	CakSNP1634	*Kabuli*	Ca_Kabuli_Chr01	24693602	(A/G)
1635	CakSNP1635	*Kabuli*	Ca_Kabuli_Chr01	24693601	(C/T)
1636	CakSNP1636	*Kabuli*	Ca_Kabuli_Chr01	24693586	(C/T)

S.N.	SNP IDs	Cultivars	Chromosomes/scaffolds	Physical positions (bp)	SNPs
1637	CakSNP1637	*Kabuli*	Ca_Kabuli_Chr01	24693571	(G/A)
1638	CakSNP1638	*Kabuli*	Ca_Kabuli_Chr01	24693550	(G/A)
1639	CakSNP1639	*Kabuli*	Ca_Kabuli_Chr01	24693533	(G/A)
1640	CakSNP1640	*Kabuli*	Ca_Kabuli_Chr01	24693521	(G/A)
1641	CakSNP1641	*Kabuli*	Ca_Kabuli_Chr01	24828169	(C/T)
1642	CakSNP1642	*Kabuli*	Ca_Kabuli_Chr01	24828245	(A/C)
1643	CakSNP1643	*Kabuli*	Ca_Kabuli_Chr01	24845148	(A/C)
1644	CakSNP1644	*Kabuli*	Ca_Kabuli_Chr01	24983908	(A/G)
1645	CakSNP1645	*Kabuli*	Ca_Kabuli_Chr01	25039836	(T/C)
1646	CakSNP1646	*Kabuli*	Ca_Kabuli_Chr01	25083482	(A/C)
1647	CakSNP1647	*Kabuli*	Ca_Kabuli_Chr01	25083534	(A/C)
1648	CakSNP1648	*Kabuli*	Ca_Kabuli_Chr01	25175124	(C/T)
1649	CakSNP1649	*Kabuli*	Ca_Kabuli_Chr01	25176162	(C/A)
1650	CakSNP1650	*Kabuli*	Ca_Kabuli_Chr01	25192363	(T/C)
1651	CakSNP1651	*Kabuli*	Ca_Kabuli_Chr01	25524382	(C/T)
1652	CakSNP1652	*Kabuli*	Ca_Kabuli_Chr01	25524425	(C/T)
1653	CakSNP1653	*Kabuli*	Ca_Kabuli_Chr01	25524441	(T/C)
1654	CakSNP1654	*Kabuli*	Ca_Kabuli_Chr01	25524459	(C/G)
1655	CakSNP1655	*Kabuli*	Ca_Kabuli_Chr01	25524532	(T/C)
1656	CakSNP1656	*Kabuli*	Ca_Kabuli_Chr01	25524519	(C/T)
1657	CakSNP1657	*Kabuli*	Ca_Kabuli_Chr01	25524481	(T/A)
1658	CakSNP1658	*Kabuli*	Ca_Kabuli_Chr01	25524480	(G/A)
1659	CakSNP1659	*Kabuli*	Ca_Kabuli_Chr01	25524473	(C/T)
1660	CakSNP1660	*Kabuli*	Ca_Kabuli_Chr01	25524465	(A/G)
1661	CakSNP1661	*Kabuli*	Ca_Kabuli_Chr01	25524460	(G/A)
1662	CakSNP1662	*Kabuli*	Ca_Kabuli_Chr01	25524458	(G/A)
1663	CakSNP1663	*Kabuli*	Ca_Kabuli_Chr01	25524456	(C/T)
1664	CakSNP1664	*Kabuli*	Ca_Kabuli_Chr01	25524455	(G/A)
1665	CakSNP1665	*Kabuli*	Ca_Kabuli_Chr01	25524453	(C/T)
1666	CakSNP1666	*Kabuli*	Ca_Kabuli_Chr01	25524451	(A/T)
1667	CakSNP1667	*Kabuli*	Ca_Kabuli_Chr01	25524450	(C/T)
1668	CakSNP1668	*Kabuli*	Ca_Kabuli_Chr01	25524464	(G/A)
1669	CakSNP1669	*Kabuli*	Ca_Kabuli_Chr01	25524478	(C/A)
1670	CakSNP1670	*Kabuli*	Ca_Kabuli_Chr01	25524488	(G/T)
1671	CakSNP1671	*Kabuli*	Ca_Kabuli_Chr01	25524498	(G/C)
1672	CakSNP1672	*Kabuli*	Ca_Kabuli_Chr01	25524505	(A/G)
1673	CakSNP1673	*Kabuli*	Ca_Kabuli_Chr01	25524520	(C/T)
1674	CakSNP1674	*Kabuli*	Ca_Kabuli_Chr01	25524522	(G/A)
1675	CakSNP1675	*Kabuli*	Ca_Kabuli_Chr01	25524524	(C/T)

S.N.	SNP IDs	Cultivars	Chromosomes/scaffolds	Physical positions (bp)	SNPs
1676	CakSNP1676	*Kabuli*	Ca_Kabuli_Chr01	25732004	(T/C)
1677	CakSNP1677	*Kabuli*	Ca_Kabuli_Chr01	25887539	(G/A)
1678	CakSNP1678	*Kabuli*	Ca_Kabuli_Chr01	25987201	(A/C)
1679	CakSNP1679	*Kabuli*	Ca_Kabuli_Chr01	26026817	(C/T)
1680	CakSNP1680	*Kabuli*	Ca_Kabuli_Chr01	26190096	(G/A)
1681	CakSNP1681	*Kabuli*	Ca_Kabuli_Chr01	26190050	(G/A)
1682	CakSNP1682	*Kabuli*	Ca_Kabuli_Chr01	26481730	(C/T)
1683	CakSNP1683	*Kabuli*	Ca_Kabuli_Chr01	26481747	(T/G)
1684	CakSNP1684	*Kabuli*	Ca_Kabuli_Chr01	26484868	(G/C)
1685	CakSNP1685	*Kabuli*	Ca_Kabuli_Chr01	26574098	(C/T)
1686	CakSNP1686	*Kabuli*	Ca_Kabuli_Chr01	26574146	(C/T)
1687	CakSNP1687	*Kabuli*	Ca_Kabuli_Chr01	26579621	(A/C)
1688	CakSNP1688	*Kabuli*	Ca_Kabuli_Chr01	26746094	(A/T)
1689	CakSNP1689	*Kabuli*	Ca_Kabuli_Chr01	26767522	(A/G)
1690	CakSNP1690	*Kabuli*	Ca_Kabuli_Chr01	26767514	(A/G)
1691	CakSNP1691	*Kabuli*	Ca_Kabuli_Chr01	26784404	(G/A)
1692	CakSNP1692	*Kabuli*	Ca_Kabuli_Chr01	26784421	(G/T)
1693	CakSNP1693	*Kabuli*	Ca_Kabuli_Chr01	26784422	(C/T)
1694	CakSNP1694	*Kabuli*	Ca_Kabuli_Chr01	26784464	(C/T)
1695	CakSNP1695	*Kabuli*	Ca_Kabuli_Chr01	26784548	(C/A)
1696	CakSNP1696	*Kabuli*	Ca_Kabuli_Chr01	26784570	(A/G)
1697	CakSNP1697	*Kabuli*	Ca_Kabuli_Chr01	26784592	(A/G)
1698	CakSNP1698	*Kabuli*	Ca_Kabuli_Chr01	26784618	(G/A)
1699	CakSNP1699	*Kabuli*	Ca_Kabuli_Chr01	26784732	(C/G)
1700	CakSNP1700	*Kabuli*	Ca_Kabuli_Chr01	26785566	(T/G)
1701	CakSNP1701	*Kabuli*	Ca_Kabuli_Chr01	26807401	(A/G)
1702	CakSNP1702	*Kabuli*	Ca_Kabuli_Chr01	26807395	(T/G)
1703	CakSNP1703	*Kabuli*	Ca_Kabuli_Chr01	26856541	(A/T)
1704	CakSNP1704	*Kabuli*	Ca_Kabuli_Chr01	26856691	(G/A)
1705	CakSNP1705	*Kabuli*	Ca_Kabuli_Chr01	26921471	(C/T)
1706	CakSNP1706	*Kabuli*	Ca_Kabuli_Chr01	26921487	(C/A)
1707	CakSNP1707	*Kabuli*	Ca_Kabuli_Chr01	26922011	(T/G)
1708	CakSNP1708	*Kabuli*	Ca_Kabuli_Chr01	26922631	(C/T)
1709	CakSNP1709	*Kabuli*	Ca_Kabuli_Chr01	26953535	(C/T)
1710	CakSNP1710	*Kabuli*	Ca_Kabuli_Chr01	27012495	(C/T)
1711	CakSNP1711	*Kabuli*	Ca_Kabuli_Chr01	27012660	(G/C)
1712	CakSNP1712	*Kabuli*	Ca_Kabuli_Chr01	27014895	(T/G)
1713	CakSNP1713	*Kabuli*	Ca_Kabuli_Chr01	27014981	(A/G)
1714	CakSNP1714	*Kabuli*	Ca_Kabuli_Chr01	27014951	(A/C)

S.N.	SNP IDs	Cultivars	Chromosomes/scaffolds	Physical positions (bp)	SNPs
1715	CakSNP1715	*Kabuli*	Ca_Kabuli_Chr01	27016101	(G/A)
1716	CakSNP1716	*Kabuli*	Ca_Kabuli_Chr01	27016206	(T/A)
1717	CakSNP1717	*Kabuli*	Ca_Kabuli_Chr01	27017559	(G/A)
1718	CakSNP1718	*Kabuli*	Ca_Kabuli_Chr01	27017560	(G/A)
1719	CakSNP1719	*Kabuli*	Ca_Kabuli_Chr01	27017570	(A/G)
1720	CakSNP1720	*Kabuli*	Ca_Kabuli_Chr01	27017604	(T/G)
1721	CakSNP1721	*Kabuli*	Ca_Kabuli_Chr01	27017606	(C/T)
1722	CakSNP1722	*Kabuli*	Ca_Kabuli_Chr01	27017617	(G/A)
1723	CakSNP1723	*Kabuli*	Ca_Kabuli_Chr01	27017626	(A/C)
1724	CakSNP1724	*Kabuli*	Ca_Kabuli_Chr01	27017628	(C/A)
1725	CakSNP1725	*Kabuli*	Ca_Kabuli_Chr01	27017734	(T/C)
1726	CakSNP1726	*Kabuli*	Ca_Kabuli_Chr01	27017765	(A/T)
1727	CakSNP1727	*Kabuli*	Ca_Kabuli_Chr01	27017771	(G/A)
1728	CakSNP1728	*Kabuli*	Ca_Kabuli_Chr01	27020193	(T/C)
1729	CakSNP1729	*Kabuli*	Ca_Kabuli_Chr01	27020354	(C/A)
1730	CakSNP1730	*Kabuli*	Ca_Kabuli_Chr01	28088501	(T/C)
1731	CakSNP1731	*Kabuli*	Ca_Kabuli_Chr01	28106756	(G/A)
1732	CakSNP1732	*Kabuli*	Ca_Kabuli_Chr01	28133847	(A/G)
1733	CakSNP1733	*Kabuli*	Ca_Kabuli_Chr01	28133830	(A/T)
1734	CakSNP1734	*Kabuli*	Ca_Kabuli_Chr01	28133814	(C/T)
1735	CakSNP1735	*Kabuli*	Ca_Kabuli_Chr01	28143955	(G/A)
1736	CakSNP1736	*Kabuli*	Ca_Kabuli_Chr01	28152260	(A/G)
1737	CakSNP1737	*Kabuli*	Ca_Kabuli_Chr01	28656197	(C/A)
1738	CakSNP1738	*Kabuli*	Ca_Kabuli_Chr01	28832810	(C/A)
1739	CakSNP1739	*Kabuli*	Ca_Kabuli_Chr01	28907617	(G/A)
1740	CakSNP1740	*Kabuli*	Ca_Kabuli_Chr01	28909059	(T/A)
1741	CakSNP1741	*Kabuli*	Ca_Kabuli_Chr01	29020220	(T/G)
1742	CakSNP1742	*Kabuli*	Ca_Kabuli_Chr01	29116430	(T/C)
1743	CakSNP1743	*Kabuli*	Ca_Kabuli_Chr01	29460440	(C/A)
1744	CakSNP1744	*Kabuli*	Ca_Kabuli_Chr01	29634320	(G/T)
1745	CakSNP1745	*Kabuli*	Ca_Kabuli_Chr01	29637509	(A/C)
1746	CakSNP1746	*Kabuli*	Ca_Kabuli_Chr01	29637526	(C/T)
1747	CakSNP1747	*Kabuli*	Ca_Kabuli_Chr01	29637572	(T/G)
1748	CakSNP1748	*Kabuli*	Ca_Kabuli_Chr01	29637571	(T/G)
1749	CakSNP1749	*Kabuli*	Ca_Kabuli_Chr01	30262124	(A/G)
1750	CakSNP1750	*Kabuli*	Ca_Kabuli_Chr01	31080673	(T/G)
1751	CakSNP1751	*Kabuli*	Ca_Kabuli_Chr01	31080792	(T/C)
1752	CakSNP1752	*Kabuli*	Ca_Kabuli_Chr01	31240370	(A/G)
1753	CakSNP1753	*Kabuli*	Ca_Kabuli_Chr01	31359861	(C/A)

S.N.	SNP IDs	Cultivars	Chromosomes/scaffolds	Physical positions (bp)	SNPs
1754	CakSNP1754	*Kabuli*	Ca_Kabuli_Chr01	31369599	(T/C)
1755	CakSNP1755	*Kabuli*	Ca_Kabuli_Chr01	31590263	(C/G)
1756	CakSNP1756	*Kabuli*	Ca_Kabuli_Chr01	31590510	(G/A)
1757	CakSNP1757	*Kabuli*	Ca_Kabuli_Chr01	31590548	(C/T)
1758	CakSNP1758	*Kabuli*	Ca_Kabuli_Chr01	31838239	(G/A)
1759	CakSNP1759	*Kabuli*	Ca_Kabuli_Chr01	32286538	(C/T)
1760	CakSNP1760	*Kabuli*	Ca_Kabuli_Chr01	32286534	(G/C)
1761	CakSNP1761	*Kabuli*	Ca_Kabuli_Chr01	32286518	(C/A)
1762	CakSNP1762	*Kabuli*	Ca_Kabuli_Chr01	32286517	(G/A)
1763	CakSNP1763	*Kabuli*	Ca_Kabuli_Chr01	32286469	(G/A)
1764	CakSNP1764	*Kabuli*	Ca_Kabuli_Chr01	32286466	(A/G)
1765	CakSNP1765	*Kabuli*	Ca_Kabuli_Chr01	32286568	(A/C)
1766	CakSNP1766	*Kabuli*	Ca_Kabuli_Chr01	32286558	(A/G)
1767	CakSNP1767	*Kabuli*	Ca_Kabuli_Chr01	32286550	(G/A)
1768	CakSNP1768	*Kabuli*	Ca_Kabuli_Chr01	32286536	(G/A)
1769	CakSNP1769	*Kabuli*	Ca_Kabuli_Chr01	32286551	(C/T)
1770	CakSNP1770	*Kabuli*	Ca_Kabuli_Chr01	32286567	(C/T)
1771	CakSNP1771	*Kabuli*	Ca_Kabuli_Chr01	32467929	(G/A)
1772	CakSNP1772	*Kabuli*	Ca_Kabuli_Chr01	32467954	(A/C)
1773	CakSNP1773	*Kabuli*	Ca_Kabuli_Chr01	32468053	(A/C)
1774	CakSNP1774	*Kabuli*	Ca_Kabuli_Chr01	32468080	(A/T)
1775	CakSNP1775	*Kabuli*	Ca_Kabuli_Chr01	32468105	(C/G)
1776	CakSNP1776	*Kabuli*	Ca_Kabuli_Chr01	32468116	(A/G)
1777	CakSNP1777	*Kabuli*	Ca_Kabuli_Chr01	32468119	(T/C)
1778	CakSNP1778	*Kabuli*	Ca_Kabuli_Chr01	33058743	(T/C)
1779	CakSNP1779	*Kabuli*	Ca_Kabuli_Chr01	33063207	(C/T)
1780	CakSNP1780	*Kabuli*	Ca_Kabuli_Chr01	33194600	(T/C)
1781	CakSNP1781	*Kabuli*	Ca_Kabuli_Chr01	33804152	(C/T)
1782	CakSNP1782	*Kabuli*	Ca_Kabuli_Chr01	33804153	(C/T)
1783	CakSNP1783	*Kabuli*	Ca_Kabuli_Chr01	33804189	(C/T)
1784	CakSNP1784	*Kabuli*	Ca_Kabuli_Chr01	33804201	(C/T)
1785	CakSNP1785	*Kabuli*	Ca_Kabuli_Chr01	33804232	(T/G)
1786	CakSNP1786	*Kabuli*	Ca_Kabuli_Chr01	33804247	(A/G)
1787	CakSNP1787	*Kabuli*	Ca_Kabuli_Chr01	33804262	(C/G)
1788	CakSNP1788	*Kabuli*	Ca_Kabuli_Chr01	34161921	(G/A)
1789	CakSNP1789	*Kabuli*	Ca_Kabuli_Chr01	34261166	(C/A)
1790	CakSNP1790	*Kabuli*	Ca_Kabuli_Chr01	34261158	(C/A)
1791	CakSNP1791	*Kabuli*	Ca_Kabuli_Chr01	34311145	(T/G)
1792	CakSNP1792	*Kabuli*	Ca_Kabuli_Chr01	34437114	(C/T)

S.N.	SNP IDs	Cultivars	Chromosomes/scaffolds	Physical positions (bp)	SNPs
1793	CakSNP1793	*Kabuli*	Ca_Kabuli_Chr01	34727251	(T/G)
1794	CakSNP1794	*Kabuli*	Ca_Kabuli_Chr01	34772813	(T/A)
1795	CakSNP1795	*Kabuli*	Ca_Kabuli_Chr01	34866709	(C/A)
1796	CakSNP1796	*Kabuli*	Ca_Kabuli_Chr01	34905580	(A/G)
1797	CakSNP1797	*Kabuli*	Ca_Kabuli_Chr01	34911065	(T/A)
1798	CakSNP1798	*Kabuli*	Ca_Kabuli_Chr01	34948934	(A/G)
1799	CakSNP1799	*Kabuli*	Ca_Kabuli_Chr01	35041241	(T/G)
1800	CakSNP1800	*Kabuli*	Ca_Kabuli_Chr01	35041324	(G/T)
1801	CakSNP1801	*Kabuli*	Ca_Kabuli_Chr01	35313277	(A/G)
1802	CakSNP1802	*Kabuli*	Ca_Kabuli_Chr01	35315381	(T/A)
1803	CakSNP1803	*Kabuli*	Ca_Kabuli_Chr01	35315379	(A/T)
1804	CakSNP1804	*Kabuli*	Ca_Kabuli_Chr01	35315347	(C/T)
1805	CakSNP1805	*Kabuli*	Ca_Kabuli_Chr01	35315327	(T/A)
1806	CakSNP1806	*Kabuli*	Ca_Kabuli_Chr01	35315436	(G/A)
1807	CakSNP1807	*Kabuli*	Ca_Kabuli_Chr01	35708841	(C/T)
1808	CakSNP1808	*Kabuli*	Ca_Kabuli_Chr01	36126090	(C/T)
1809	CakSNP1809	*Kabuli*	Ca_Kabuli_Chr01	36794731	(A/G)
1810	CakSNP1810	*Kabuli*	Ca_Kabuli_Chr01	36794797	(G/A)
1811	CakSNP1811	*Kabuli*	Ca_Kabuli_Chr01	36794800	(G/T)
1812	CakSNP1812	*Kabuli*	Ca_Kabuli_Chr01	36794806	(G/T)
1813	CakSNP1813	*Kabuli*	Ca_Kabuli_Chr01	36794813	(C/T)
1814	CakSNP1814	*Kabuli*	Ca_Kabuli_Chr01	36794887	(G/A)
1815	CakSNP1815	*Kabuli*	Ca_Kabuli_Chr01	36794870	(C/T)
1816	CakSNP1816	*Kabuli*	Ca_Kabuli_Chr01	36794900	(C/T)
1817	CakSNP1817	*Kabuli*	Ca_Kabuli_Chr01	36794906	(A/C)
1818	CakSNP1818	*Kabuli*	Ca_Kabuli_Chr01	36794912	(G/T)
1819	CakSNP1819	*Kabuli*	Ca_Kabuli_Chr01	36794913	(A/T)
1820	CakSNP1820	*Kabuli*	Ca_Kabuli_Chr01	36794915	(G/A)
1821	CakSNP1821	*Kabuli*	Ca_Kabuli_Chr01	36892533	(C/T)
1822	CakSNP1822	*Kabuli*	Ca_Kabuli_Chr01	37026302	(C/T)
1823	CakSNP1823	*Kabuli*	Ca_Kabuli_Chr01	37608030	(G/A)
1824	CakSNP1824	*Kabuli*	Ca_Kabuli_Chr01	37608050	(T/G)
1825	CakSNP1825	*Kabuli*	Ca_Kabuli_Chr01	37642546	(G/A)
1826	CakSNP1826	*Kabuli*	Ca_Kabuli_Chr01	37643213	(T/C)
1827	CakSNP1827	*Kabuli*	Ca_Kabuli_Chr01	37647602	(C/G)
1828	CakSNP1828	*Kabuli*	Ca_Kabuli_Chr01	37651679	(G/A)
1829	CakSNP1829	*Kabuli*	Ca_Kabuli_Chr01	37651695	(G/A)
1830	CakSNP1830	*Kabuli*	Ca_Kabuli_Chr01	37651764	(A/C)
1831	CakSNP1831	*Kabuli*	Ca_Kabuli_Chr01	37651711	(G/T)

S.N.	SNP IDs	Cultivars	Chromosomes/scaffolds	Physical positions (bp)	SNPs
1832	CakSNP1832	*Kabuli*	Ca_Kabuli_Chr01	37651800	(T/A)
1833	CakSNP1833	*Kabuli*	Ca_Kabuli_Chr01	37651823	(A/G)
1834	CakSNP1834	*Kabuli*	Ca_Kabuli_Chr01	37651827	(C/T)
1835	CakSNP1835	*Kabuli*	Ca_Kabuli_Chr01	37651843	(T/C)
1836	CakSNP1836	*Kabuli*	Ca_Kabuli_Chr01	37654039	(C/G)
1837	CakSNP1837	*Kabuli*	Ca_Kabuli_Chr01	37654136	(C/G)
1838	CakSNP1838	*Kabuli*	Ca_Kabuli_Chr01	37654926	(C/T)
1839	CakSNP1839	*Kabuli*	Ca_Kabuli_Chr01	37690416	(G/A)
1840	CakSNP1840	*Kabuli*	Ca_Kabuli_Chr01	37690465	(T/C)
1841	CakSNP1841	*Kabuli*	Ca_Kabuli_Chr01	37690498	(C/A)
1842	CakSNP1842	*Kabuli*	Ca_Kabuli_Chr01	37690497	(A/T)
1843	CakSNP1843	*Kabuli*	Ca_Kabuli_Chr01	37699039	(T/G)
1844	CakSNP1844	*Kabuli*	Ca_Kabuli_Chr01	37699036	(T/C)
1845	CakSNP1845	*Kabuli*	Ca_Kabuli_Chr01	37722095	(G/A)
1846	CakSNP1846	*Kabuli*	Ca_Kabuli_Chr01	37722143	(G/C)
1847	CakSNP1847	*Kabuli*	Ca_Kabuli_Chr01	37768179	(T/A)
1848	CakSNP1848	*Kabuli*	Ca_Kabuli_Chr01	37768206	(A/T)
1849	CakSNP1849	*Kabuli*	Ca_Kabuli_Chr01	37768238	(A/G)
1850	CakSNP1850	*Kabuli*	Ca_Kabuli_Chr01	37768333	(C/T)
1851	CakSNP1851	*Kabuli*	Ca_Kabuli_Chr01	37768299	(A/G)
1852	CakSNP1852	*Kabuli*	Ca_Kabuli_Chr01	37768276	(G/C)
1853	CakSNP1853	*Kabuli*	Ca_Kabuli_Chr01	37768264	(A/G)
1854	CakSNP1854	*Kabuli*	Ca_Kabuli_Chr01	37862342	(T/G)
1855	CakSNP1855	*Kabuli*	Ca_Kabuli_Chr01	37880995	(T/C)
1856	CakSNP1856	*Kabuli*	Ca_Kabuli_Chr01	37892088	(A/C)
1857	CakSNP1857	*Kabuli*	Ca_Kabuli_Chr01	37892089	(C/T)
1858	CakSNP1858	*Kabuli*	Ca_Kabuli_Chr01	37892095	(A/G)
1859	CakSNP1859	*Kabuli*	Ca_Kabuli_Chr01	37892139	(A/T)
1860	CakSNP1860	*Kabuli*	Ca_Kabuli_Chr01	38049721	(C/T)
1861	CakSNP1861	*Kabuli*	Ca_Kabuli_Chr01	38678712	(T/C)
1862	CakSNP1862	*Kabuli*	Ca_Kabuli_Chr01	38877670	(T/C)
1863	CakSNP1863	*Kabuli*	Ca_Kabuli_Chr01	38877692	(G/T)
1864	CakSNP1864	*Kabuli*	Ca_Kabuli_Chr01	39436291	(T/C)
1865	CakSNP1865	*Kabuli*	Ca_Kabuli_Chr01	39601982	(T/G)
1866	CakSNP1866	*Kabuli*	Ca_Kabuli_Chr01	39602029	(G/T)
1867	CakSNP1867	*Kabuli*	Ca_Kabuli_Chr01	39616139	(A/C)
1868	CakSNP1868	*Kabuli*	Ca_Kabuli_Chr01	39663432	(C/G)
1869	CakSNP1869	*Kabuli*	Ca_Kabuli_Chr01	40224835	(T/A)
1870	CakSNP1870	*Kabuli*	Ca_Kabuli_Chr01	40224834	(C/G)

S.N.	SNP IDs	Cultivars	Chromosomes/scaffolds	Physical positions (bp)	SNPs
1871	CakSNP1871	*Kabuli*	Ca_Kabuli_Chr01	40224804	(A/G)
1872	CakSNP1872	*Kabuli*	Ca_Kabuli_Chr01	40343216	(G/T)
1873	CakSNP1873	*Kabuli*	Ca_Kabuli_Chr01	40346280	(A/C)
1874	CakSNP1874	*Kabuli*	Ca_Kabuli_Chr01	40358693	(C/T)
1875	CakSNP1875	*Kabuli*	Ca_Kabuli_Chr01	40358747	(T/G)
1876	CakSNP1876	*Kabuli*	Ca_Kabuli_Chr01	40367940	(G/A)
1877	CakSNP1877	*Kabuli*	Ca_Kabuli_Chr01	40368845	(C/T)
1878	CakSNP1878	*Kabuli*	Ca_Kabuli_Chr01	40502077	(T/C)
1879	CakSNP1879	*Kabuli*	Ca_Kabuli_Chr01	40570811	(G/T)
1880	CakSNP1880	*Kabuli*	Ca_Kabuli_Chr01	40585026	(T/C)
1881	CakSNP1881	*Kabuli*	Ca_Kabuli_Chr01	40630215	(C/T)
1882	CakSNP1882	*Kabuli*	Ca_Kabuli_Chr01	40630213	(G/T)
1883	CakSNP1883	*Kabuli*	Ca_Kabuli_Chr01	40630158	(T/A)
1884	CakSNP1884	*Kabuli*	Ca_Kabuli_Chr01	40660069	(T/G)
1885	CakSNP1885	*Kabuli*	Ca_Kabuli_Chr01	40660116	(G/A)
1886	CakSNP1886	*Kabuli*	Ca_Kabuli_Chr01	40745421	(G/T)
1887	CakSNP1887	*Kabuli*	Ca_Kabuli_Chr01	40745452	(T/C)
1888	CakSNP1888	*Kabuli*	Ca_Kabuli_Chr01	40745492	(A/G)
1889	CakSNP1889	*Kabuli*	Ca_Kabuli_Chr01	40745846	(T/C)
1890	CakSNP1890	*Kabuli*	Ca_Kabuli_Chr01	41029732	(C/T)
1891	CakSNP1891	*Kabuli*	Ca_Kabuli_Chr01	41065797	(G/C)
1892	CakSNP1892	*Kabuli*	Ca_Kabuli_Chr01	41125493	(T/C)
1893	CakSNP1893	*Kabuli*	Ca_Kabuli_Chr01	41169218	(G/T)
1894	CakSNP1894	*Kabuli*	Ca_Kabuli_Chr01	41169430	(T/G)
1895	CakSNP1895	*Kabuli*	Ca_Kabuli_Chr01	41169372	(T/A)
1896	CakSNP1896	*Kabuli*	Ca_Kabuli_Chr01	41178168	(G/A)
1897	CakSNP1897	*Kabuli*	Ca_Kabuli_Chr01	41178183	(C/A)
1898	CakSNP1898	*Kabuli*	Ca_Kabuli_Chr01	41179660	(G/T)
1899	CakSNP1899	*Kabuli*	Ca_Kabuli_Chr01	41179650	(A/T)
1900	CakSNP1900	*Kabuli*	Ca_Kabuli_Chr01	41185512	(T/G)
1901	CakSNP1901	*Kabuli*	Ca_Kabuli_Chr01	41202173	(T/G)
1902	CakSNP1902	*Kabuli*	Ca_Kabuli_Chr01	41202206	(A/G)
1903	CakSNP1903	*Kabuli*	Ca_Kabuli_Chr01	41310401	(T/G)
1904	CakSNP1904	*Kabuli*	Ca_Kabuli_Chr01	41339408	(T/A)
1905	CakSNP1905	*Kabuli*	Ca_Kabuli_Chr01	41339445	(C/A)
1906	CakSNP1906	*Kabuli*	Ca_Kabuli_Chr01	41380061	(G/T)
1907	CakSNP1907	*Kabuli*	Ca_Kabuli_Chr01	41437222	(A/G)
1908	CakSNP1908	*Kabuli*	Ca_Kabuli_Chr01	41437366	(T/C)
1909	CakSNP1909	*Kabuli*	Ca_Kabuli_Chr01	41437453	(T/G)

S.N.	SNP IDs	Cultivars	Chromosomes/scaffolds	Physical positions (bp)	SNPs
1910	CakSNP1910	*Kabuli*	Ca_Kabuli_Chr01	41437483	(G/A)
1911	CakSNP1911	*Kabuli*	Ca_Kabuli_Chr01	41439539	(A/G)
1912	CakSNP1912	*Kabuli*	Ca_Kabuli_Chr01	41439593	(A/G)
1913	CakSNP1913	*Kabuli*	Ca_Kabuli_Chr01	41451910	(C/T)
1914	CakSNP1914	*Kabuli*	Ca_Kabuli_Chr01	41451977	(C/T)
1915	CakSNP1915	*Kabuli*	Ca_Kabuli_Chr01	41475442	(T/C)
1916	CakSNP1916	*Kabuli*	Ca_Kabuli_Chr01	41484033	(T/C)
1917	CakSNP1917	*Kabuli*	Ca_Kabuli_Chr01	41484085	(C/T)
1918	CakSNP1918	*Kabuli*	Ca_Kabuli_Chr01	41564809	(T/C)
1919	CakSNP1919	*Kabuli*	Ca_Kabuli_Chr01	41795343	(G/A)
1920	CakSNP1920	*Kabuli*	Ca_Kabuli_Chr01	41795367	(C/T)
1921	CakSNP1921	*Kabuli*	Ca_Kabuli_Chr01	41822419	(C/T)
1922	CakSNP1922	*Kabuli*	Ca_Kabuli_Chr01	41827388	(T/C)
1923	CakSNP1923	*Kabuli*	Ca_Kabuli_Chr01	41867035	(C/T)
1924	CakSNP1924	*Kabuli*	Ca_Kabuli_Chr01	41897216	(C/A)
1925	CakSNP1925	*Kabuli*	Ca_Kabuli_Chr01	41946005	(A/G)
1926	CakSNP1926	*Kabuli*	Ca_Kabuli_Chr01	41981244	(G/A)
1927	CakSNP1927	*Kabuli*	Ca_Kabuli_Chr01	41981259	(G/A)
1928	CakSNP1928	*Kabuli*	Ca_Kabuli_Chr01	42189380	(A/G)
1929	CakSNP1929	*Kabuli*	Ca_Kabuli_Chr01	42452801	(G/A)
1930	CakSNP1930	*Kabuli*	Ca_Kabuli_Chr01	42452939	(G/T)
1931	CakSNP1931	*Kabuli*	Ca_Kabuli_Chr01	42515406	(C/T)
1932	CakSNP1932	*Kabuli*	Ca_Kabuli_Chr01	42551574	(T/G)
1933	CakSNP1933	*Kabuli*	Ca_Kabuli_Chr01	42551585	(C/A)
1934	CakSNP1934	*Kabuli*	Ca_Kabuli_Chr01	42552658	(A/C)
1935	CakSNP1935	*Kabuli*	Ca_Kabuli_Chr01	42605239	(T/G)
1936	CakSNP1936	*Kabuli*	Ca_Kabuli_Chr01	42610115	(A/C)
1937	CakSNP1937	*Kabuli*	Ca_Kabuli_Chr01	42613060	(T/G)
1938	CakSNP1938	*Kabuli*	Ca_Kabuli_Chr01	42894518	(T/C)
1939	CakSNP1939	*Kabuli*	Ca_Kabuli_Chr01	42894525	(T/C)
1940	CakSNP1940	*Kabuli*	Ca_Kabuli_Chr01	42894536	(G/T)
1941	CakSNP1941	*Kabuli*	Ca_Kabuli_Chr01	43053424	(G/C)
1942	CakSNP1942	*Kabuli*	Ca_Kabuli_Chr01	43273910	(G/T)
1943	CakSNP1943	*Kabuli*	Ca_Kabuli_Chr01	43273922	(T/C)
1944	CakSNP1944	*Kabuli*	Ca_Kabuli_Chr01	43273925	(A/G)
1945	CakSNP1945	*Kabuli*	Ca_Kabuli_Chr01	43273931	(G/A)
1946	CakSNP1946	*Kabuli*	Ca_Kabuli_Chr01	43273941	(C/G)
1947	CakSNP1947	*Kabuli*	Ca_Kabuli_Chr01	43273943	(A/G)
1948	CakSNP1948	*Kabuli*	Ca_Kabuli_Chr01	43273961	(C/T)

S.N.	SNP IDs	Cultivars	Chromosomes/scaffolds	Physical positions (bp)	SNPs
1949	CakSNP1949	*Kabuli*	Ca_Kabuli_Chr01	43273962	(A/G)
1950	CakSNP1950	*Kabuli*	Ca_Kabuli_Chr01	43273968	(T/G)
1951	CakSNP1951	*Kabuli*	Ca_Kabuli_Chr01	43273972	(C/T)
1952	CakSNP1952	*Kabuli*	Ca_Kabuli_Chr01	43273981	(G/T)
1953	CakSNP1953	*Kabuli*	Ca_Kabuli_Chr01	43273985	(G/A)
1954	CakSNP1954	*Kabuli*	Ca_Kabuli_Chr01	43304584	(A/C)
1955	CakSNP1955	*Kabuli*	Ca_Kabuli_Chr01	43304573	(C/T)
1956	CakSNP1956	*Kabuli*	Ca_Kabuli_Chr01	43305858	(T/C)
1957	CakSNP1957	*Kabuli*	Ca_Kabuli_Chr01	43305870	(G/A)
1958	CakSNP1958	*Kabuli*	Ca_Kabuli_Chr01	43305886	(T/C)
1959	CakSNP1959	*Kabuli*	Ca_Kabuli_Chr01	43305904	(C/A)
1960	CakSNP1960	*Kabuli*	Ca_Kabuli_Chr01	43306048	(G/A)
1961	CakSNP1961	*Kabuli*	Ca_Kabuli_Chr01	43306045	(C/A)
1962	CakSNP1962	*Kabuli*	Ca_Kabuli_Chr01	43306036	(G/A)
1963	CakSNP1963	*Kabuli*	Ca_Kabuli_Chr01	43306012	(G/A)
1964	CakSNP1964	*Kabuli*	Ca_Kabuli_Chr01	43306221	(A/G)
1965	CakSNP1965	*Kabuli*	Ca_Kabuli_Chr01	43509159	(A/G)
1966	CakSNP1966	*Kabuli*	Ca_Kabuli_Chr01	43509409	(T/C)
1967	CakSNP1967	*Kabuli*	Ca_Kabuli_Chr01	43576059	(A/G)
1968	CakSNP1968	*Kabuli*	Ca_Kabuli_Chr01	43585657	(T/G)
1969	CakSNP1969	*Kabuli*	Ca_Kabuli_Chr01	43590106	(C/T)
1970	CakSNP1970	*Kabuli*	Ca_Kabuli_Chr01	43590258	(T/C)
1971	CakSNP1971	*Kabuli*	Ca_Kabuli_Chr01	43724824	(C/T)
1972	CakSNP1972	*Kabuli*	Ca_Kabuli_Chr01	43724839	(C/T)
1973	CakSNP1973	*Kabuli*	Ca_Kabuli_Chr01	43724963	(C/T)
1974	CakSNP1974	*Kabuli*	Ca_Kabuli_Chr01	43725059	(C/T)
1975	CakSNP1975	*Kabuli*	Ca_Kabuli_Chr01	43725104	(T/C)
1976	CakSNP1976	*Kabuli*	Ca_Kabuli_Chr01	43727804	(C/T)
1977	CakSNP1977	*Kabuli*	Ca_Kabuli_Chr01	43792565	(G/A)
1978	CakSNP1978	*Kabuli*	Ca_Kabuli_Chr01	43800367	(C/T)
1979	CakSNP1979	*Kabuli*	Ca_Kabuli_Chr01	43930143	(A/T)
1980	CakSNP1980	*Kabuli*	Ca_Kabuli_Chr01	43930138	(T/C)
1981	CakSNP1981	*Kabuli*	Ca_Kabuli_Chr01	43933881	(G/C)
1982	CakSNP1982	*Kabuli*	Ca_Kabuli_Chr01	43933904	(A/G)
1983	CakSNP1983	*Kabuli*	Ca_Kabuli_Chr01	43934024	(G/T)
1984	CakSNP1984	*Kabuli*	Ca_Kabuli_Chr01	43934120	(A/G)
1985	CakSNP1985	*Kabuli*	Ca_Kabuli_Chr01	43934117	(G/T)
1986	CakSNP1986	*Kabuli*	Ca_Kabuli_Chr01	43934081	(C/T)
1987	CakSNP1987	*Kabuli*	Ca_Kabuli_Chr01	43943035	(G/T)

S.N.	SNP IDs	Cultivars	Chromosomes/scaffolds	Physical positions (bp)	SNPs
1988	CakSNP1988	*Kabuli*	Ca_Kabuli_Chr01	43954743	(C/G)
1989	CakSNP1989	*Kabuli*	Ca_Kabuli_Chr01	44043281	(G/A)
1990	CakSNP1990	*Kabuli*	Ca_Kabuli_Chr01	44085069	(C/T)
1991	CakSNP1991	*Kabuli*	Ca_Kabuli_Chr01	44348947	(C/T)
1992	CakSNP1992	*Kabuli*	Ca_Kabuli_Chr01	44349004	(T/C)
1993	CakSNP1993	*Kabuli*	Ca_Kabuli_Chr01	44350953	(G/T)
1994	CakSNP1994	*Kabuli*	Ca_Kabuli_Chr01	44635939	(T/C)
1995	CakSNP1995	*Kabuli*	Ca_Kabuli_Chr01	44635926	(A/G)
1996	CakSNP1996	*Kabuli*	Ca_Kabuli_Chr01	44635893	(A/G)
1997	CakSNP1997	*Kabuli*	Ca_Kabuli_Chr01	44976738	(T/A)
1998	CakSNP1998	*Kabuli*	Ca_Kabuli_Chr01	44976735	(C/T)
1999	CakSNP1999	*Kabuli*	Ca_Kabuli_Chr01	44988344	(A/G)
2000	CakSNP2000	*Kabuli*	Ca_Kabuli_Chr01	44988387	(C/A)
2001	CakSNP2001	*Kabuli*	Ca_Kabuli_Chr01	45003190	(G/A)
2002	CakSNP2002	*Kabuli*	Ca_Kabuli_Chr01	45003514	(G/A)
2003	CakSNP2003	*Kabuli*	Ca_Kabuli_Chr01	45003509	(C/A)
2004	CakSNP2004	*Kabuli*	Ca_Kabuli_Chr01	45003505	(A/C)
2005	CakSNP2005	*Kabuli*	Ca_Kabuli_Chr01	45003938	(A/G)
2006	CakSNP2006	*Kabuli*	Ca_Kabuli_Chr01	45003939	(T/A)
2007	CakSNP2007	*Kabuli*	Ca_Kabuli_Chr01	45003940	(T/G)
2008	CakSNP2008	*Kabuli*	Ca_Kabuli_Chr01	45003944	(C/T)
2009	CakSNP2009	*Kabuli*	Ca_Kabuli_Chr01	45003945	(T/A)
2010	CakSNP2010	*Kabuli*	Ca_Kabuli_Chr01	45003946	(T/G)
2011	CakSNP2011	*Kabuli*	Ca_Kabuli_Chr01	45003947	(C/A)
2012	CakSNP2012	*Kabuli*	Ca_Kabuli_Chr01	45003973	(T/C)
2013	CakSNP2013	*Kabuli*	Ca_Kabuli_Chr01	45003985	(G/T)
2014	CakSNP2014	*Kabuli*	Ca_Kabuli_Chr01	45003998	(G/A)
2015	CakSNP2015	*Kabuli*	Ca_Kabuli_Chr01	45004001	(C/T)
2016	CakSNP2016	*Kabuli*	Ca_Kabuli_Chr01	45007032	(A/C)
2017	CakSNP2017	*Kabuli*	Ca_Kabuli_Chr01	45007227	(T/C)
2018	CakSNP2018	*Kabuli*	Ca_Kabuli_Chr01	45007259	(A/G)
2019	CakSNP2019	*Kabuli*	Ca_Kabuli_Chr01	45007331	(C/T)
2020	CakSNP2020	*Kabuli*	Ca_Kabuli_Chr01	45008076	(G/A)
2021	CakSNP2021	*Kabuli*	Ca_Kabuli_Chr01	45008075	(T/C)
2022	CakSNP2022	*Kabuli*	Ca_Kabuli_Chr01	45008073	(T/G)
2023	CakSNP2023	*Kabuli*	Ca_Kabuli_Chr01	45008071	(G/C)
2024	CakSNP2024	*Kabuli*	Ca_Kabuli_Chr01	45008050	(C/G)
2025	CakSNP2025	*Kabuli*	Ca_Kabuli_Chr01	45008044	(T/C)
2026	CakSNP2026	*Kabuli*	Ca_Kabuli_Chr01	45008041	(C/G)

S.N.	SNP IDs	Cultivars	Chromosomes/scaffolds	Physical positions (bp)	SNPs
2027	CakSNP2027	*Kabuli*	Ca_Kabuli_Chr01	45008040	(G/C)
2028	CakSNP2028	*Kabuli*	Ca_Kabuli_Chr01	45008039	(C/T)
2029	CakSNP2029	*Kabuli*	Ca_Kabuli_Chr01	45008016	(T/A)
2030	CakSNP2030	*Kabuli*	Ca_Kabuli_Chr01	45008015	(G/A)
2031	CakSNP2031	*Kabuli*	Ca_Kabuli_Chr01	45008009	(C/T)
2032	CakSNP2032	*Kabuli*	Ca_Kabuli_Chr01	45008006	(C/A)
2033	CakSNP2033	*Kabuli*	Ca_Kabuli_Chr01	45008004	(C/T)
2034	CakSNP2034	*Kabuli*	Ca_Kabuli_Chr01	45008074	(C/T)
2035	CakSNP2035	*Kabuli*	Ca_Kabuli_Chr01	45250874	(G/A)
2036	CakSNP2036	*Kabuli*	Ca_Kabuli_Chr01	45250872	(G/T)
2037	CakSNP2037	*Kabuli*	Ca_Kabuli_Chr01	45250869	(G/A)
2038	CakSNP2038	*Kabuli*	Ca_Kabuli_Chr01	45250863	(C/T)
2039	CakSNP2039	*Kabuli*	Ca_Kabuli_Chr01	45250862	(A/T)
2040	CakSNP2040	*Kabuli*	Ca_Kabuli_Chr01	45250834	(A/G)
2041	CakSNP2041	*Kabuli*	Ca_Kabuli_Chr01	45250828	(G/C)
2042	CakSNP2042	*Kabuli*	Ca_Kabuli_Chr01	45250807	(C/G)
2043	CakSNP2043	*Kabuli*	Ca_Kabuli_Chr01	45250805	(A/C)
2044	CakSNP2044	*Kabuli*	Ca_Kabuli_Chr01	45250803	(A/G)
2045	CakSNP2045	*Kabuli*	Ca_Kabuli_Chr01	45250802	(C/T)
2046	CakSNP2046	*Kabuli*	Ca_Kabuli_Chr01	46437023	(T/G)
2047	CakSNP2047	*Kabuli*	Ca_Kabuli_Chr01	46523221	(C/T)
2048	CakSNP2048	*Kabuli*	Ca_Kabuli_Chr01	46597131	(T/G)
2049	CakSNP2049	*Kabuli*	Ca_Kabuli_Chr01	46745628	(C/A)
2050	CakSNP2050	*Kabuli*	Ca_Kabuli_Chr01	46778814	(A/C)
2051	CakSNP2051	*Kabuli*	Ca_Kabuli_Chr01	46791939	(T/G)
2052	CakSNP2052	*Kabuli*	Ca_Kabuli_Chr01	46793244	(G/A)
2053	CakSNP2053	*Kabuli*	Ca_Kabuli_Chr01	46793357	(T/C)
2054	CakSNP2054	*Kabuli*	Ca_Kabuli_Chr01	46793331	(C/G)
2055	CakSNP2055	*Kabuli*	Ca_Kabuli_Chr01	46816041	(A/T)
2056	CakSNP2056	*Kabuli*	Ca_Kabuli_Chr01	46825211	(G/A)
2057	CakSNP2057	*Kabuli*	Ca_Kabuli_Chr01	46829662	(C/T)
2058	CakSNP2058	*Kabuli*	Ca_Kabuli_Chr01	46832306	(T/G)
2059	CakSNP2059	*Kabuli*	Ca_Kabuli_Chr01	46832395	(A/G)
2060	CakSNP2060	*Kabuli*	Ca_Kabuli_Chr01	46832383	(T/C)
2061	CakSNP2061	*Kabuli*	Ca_Kabuli_Chr01	46920524	(C/T)
2062	CakSNP2062	*Kabuli*	Ca_Kabuli_Chr01	46920554	(T/C)
2063	CakSNP2063	*Kabuli*	Ca_Kabuli_Chr01	46936196	(C/A)
2064	CakSNP2064	*Kabuli*	Ca_Kabuli_Chr01	46936201	(C/T)
2065	CakSNP2065	*Kabuli*	Ca_Kabuli_Chr01	46936207	(C/A)

S.N.	SNP IDs	Cultivars	Chromosomes/scaffolds	Physical positions (bp)	SNPs
2066	CakSNP2066	*Kabuli*	Ca_Kabuli_Chr01	46936209	(C/T)
2067	CakSNP2067	*Kabuli*	Ca_Kabuli_Chr01	46936329	(C/A)
2068	CakSNP2068	*Kabuli*	Ca_Kabuli_Chr01	46936297	(A/G)
2069	CakSNP2069	*Kabuli*	Ca_Kabuli_Chr01	46936428	(T/C)
2070	CakSNP2070	*Kabuli*	Ca_Kabuli_Chr01	46936460	(A/C)
2071	CakSNP2071	*Kabuli*	Ca_Kabuli_Chr01	46936456	(T/C)
2072	CakSNP2072	*Kabuli*	Ca_Kabuli_Chr01	46936448	(A/C)
2073	CakSNP2073	*Kabuli*	Ca_Kabuli_Chr01	46936447	(G/A)
2074	CakSNP2074	*Kabuli*	Ca_Kabuli_Chr01	46941875	(T/C)
2075	CakSNP2075	*Kabuli*	Ca_Kabuli_Chr01	46968740	(T/C)
2076	CakSNP2076	*Kabuli*	Ca_Kabuli_Chr01	46969221	(T/A)
2077	CakSNP2077	*Kabuli*	Ca_Kabuli_Chr01	46976508	(A/G)
2078	CakSNP2078	*Kabuli*	Ca_Kabuli_Chr01	47042798	(T/A)
2079	CakSNP2079	*Kabuli*	Ca_Kabuli_Chr01	47203123	(A/C)
2080	CakSNP2080	*Kabuli*	Ca_Kabuli_Chr01	47263589	(C/T)
2081	CakSNP2081	*Kabuli*	Ca_Kabuli_Chr01	47278505	(T/C)
2082	CakSNP2082	*Kabuli*	Ca_Kabuli_Chr01	47331082	(T/C)
2083	CakSNP2083	*Kabuli*	Ca_Kabuli_Chr01	47331080	(T/C)
2084	CakSNP2084	*Kabuli*	Ca_Kabuli_Chr01	47423715	(A/T)
2085	CakSNP2085	*Kabuli*	Ca_Kabuli_Chr01	47449818	(A/G)
2086	CakSNP2086	*Kabuli*	Ca_Kabuli_Chr01	47449950	(T/C)
2087	CakSNP2087	*Kabuli*	Ca_Kabuli_Chr01	47449891	(T/C)
2088	CakSNP2088	*Kabuli*	Ca_Kabuli_Chr01	47449882	(A/C)
2089	CakSNP2089	*Kabuli*	Ca_Kabuli_Chr01	47801491	(A/G)
2090	CakSNP2090	*Kabuli*	Ca_Kabuli_Chr01	47890026	(C/T)
2091	CakSNP2091	*Kabuli*	Ca_Kabuli_Chr01	47897914	(C/T)
2092	CakSNP2092	*Kabuli*	Ca_Kabuli_Chr01	47936748	(G/A)
2093	CakSNP2093	*Kabuli*	Ca_Kabuli_Chr01	47936841	(A/G)
2094	CakSNP2094	*Kabuli*	Ca_Kabuli_Chr01	47938884	(T/G)
2095	CakSNP2095	*Kabuli*	Ca_Kabuli_Chr01	47947009	(T/C)
2096	CakSNP2096	*Kabuli*	Ca_Kabuli_Chr01	47947281	(A/C)
2097	CakSNP2097	*Kabuli*	Ca_Kabuli_Chr01	48026664	(G/T)
2098	CakSNP2098	*Kabuli*	Ca_Kabuli_Chr01	48026701	(C/T)
2099	CakSNP2099	*Kabuli*	Ca_Kabuli_Chr01	48026716	(T/G)
2100	CakSNP2100	*Kabuli*	Ca_Kabuli_Chr01	48026727	(A/C)
2101	CakSNP2101	*Kabuli*	Ca_Kabuli_Chr01	48027714	(C/A)
2102	CakSNP2102	*Kabuli*	Ca_Kabuli_Chr01	48027869	(T/C)
2103	CakSNP2103	*Kabuli*	Ca_Kabuli_Chr01	48028094	(G/A)
2104	CakSNP2104	*Kabuli*	Ca_Kabuli_Chr01	48049293	(C/T)

S.N.	SNP IDs	Cultivars	Chromosomes/scaffolds	Physical positions (bp)	SNPs
2105	CakSNP2105	*Kabuli*	Ca_Kabuli_Chr01	48132217	(G/A)
2106	CakSNP2106	*Kabuli*	Ca_Kabuli_Chr01	48132521	(A/C)
2107	CakSNP2107	*Kabuli*	Ca_Kabuli_Chr01	48154321	(A/C)
2108	CakSNP2108	*Kabuli*	Ca_Kabuli_Chr01	48191460	(T/A)
2109	CakSNP2109	*Kabuli*	Ca_Kabuli_Chr01	48195174	(T/A)
2110	CakSNP2110	*Kabuli*	Ca_Kabuli_Chr01	48197895	(A/T)
2111	CakSNP2111	*Kabuli*	Ca_Kabuli_Chr01	48197911	(T/G)
2112	CakSNP2112	*Kabuli*	Ca_Kabuli_Chr01	48197979	(A/G)
2113	CakSNP2113	*Kabuli*	Ca_Kabuli_Chr01	48197969	(G/A)
2114	CakSNP2114	*Kabuli*	Ca_Kabuli_Chr01	48198932	(A/T)
2115	CakSNP2115	*Kabuli*	Ca_Kabuli_Chr01	48265590	(T/G)
2116	CakSNP2116	*Kabuli*	Ca_Kabuli_Chr01	48273097	(G/C)
2117	CakSNP2117	*Kabuli*	Ca_Kabuli_Chr01	48273307	(C/T)
2118	CakSNP2118	*Kabuli*	Ca_Kabuli_Chr02	113330	(C/T)
2119	CakSNP2119	*Kabuli*	Ca_Kabuli_Chr02	149250	(C/A)
2120	CakSNP2120	*Kabuli*	Ca_Kabuli_Chr02	149251	(A/T)
2121	CakSNP2121	*Kabuli*	Ca_Kabuli_Chr02	149259	(A/C)
2122	CakSNP2122	*Kabuli*	Ca_Kabuli_Chr02	149269	(C/T)
2123	CakSNP2123	*Kabuli*	Ca_Kabuli_Chr02	149278	(G/T)
2124	CakSNP2124	*Kabuli*	Ca_Kabuli_Chr02	149315	(C/A)
2125	CakSNP2125	*Kabuli*	Ca_Kabuli_Chr02	149322	(C/A)
2126	CakSNP2126	*Kabuli*	Ca_Kabuli_Chr02	149402	(C/T)
2127	CakSNP2127	*Kabuli*	Ca_Kabuli_Chr02	149395	(T/C)
2128	CakSNP2128	*Kabuli*	Ca_Kabuli_Chr02	189287	(A/G)
2129	CakSNP2129	*Kabuli*	Ca_Kabuli_Chr02	189784	(C/A)
2130	CakSNP2130	*Kabuli*	Ca_Kabuli_Chr02	221903	(A/T)
2131	CakSNP2131	*Kabuli*	Ca_Kabuli_Chr02	221955	(A/G)
2132	CakSNP2132	*Kabuli*	Ca_Kabuli_Chr02	233589	(C/T)
2133	CakSNP2133	*Kabuli*	Ca_Kabuli_Chr02	270139	(G/A)
2134	CakSNP2134	*Kabuli*	Ca_Kabuli_Chr02	354795	(T/C)
2135	CakSNP2135	*Kabuli*	Ca_Kabuli_Chr02	355905	(T/C)
2136	CakSNP2136	*Kabuli*	Ca_Kabuli_Chr02	507857	(G/T)
2137	CakSNP2137	*Kabuli*	Ca_Kabuli_Chr02	522943	(A/G)
2138	CakSNP2138	*Kabuli*	Ca_Kabuli_Chr02	543360	(G/T)
2139	CakSNP2139	*Kabuli*	Ca_Kabuli_Chr02	616251	(C/T)
2140	CakSNP2140	*Kabuli*	Ca_Kabuli_Chr02	616410	(C/G)
2141	CakSNP2141	*Kabuli*	Ca_Kabuli_Chr02	669304	(A/T)
2142	CakSNP2142	*Kabuli*	Ca_Kabuli_Chr02	728957	(T/C)
2143	CakSNP2143	*Kabuli*	Ca_Kabuli_Chr02	737183	(T/G)

S.N.	SNP IDs	Cultivars	Chromosomes/scaffolds	Physical positions (bp)	SNPs
2144	CakSNP2144	*Kabuli*	Ca_Kabuli_Chr02	770164	(A/C)
2145	CakSNP2145	*Kabuli*	Ca_Kabuli_Chr02	770183	(C/T)
2146	CakSNP2146	*Kabuli*	Ca_Kabuli_Chr02	770234	(C/T)
2147	CakSNP2147	*Kabuli*	Ca_Kabuli_Chr02	770237	(C/T)
2148	CakSNP2148	*Kabuli*	Ca_Kabuli_Chr02	770225	(C/T)
2149	CakSNP2149	*Kabuli*	Ca_Kabuli_Chr02	770260	(C/T)
2150	CakSNP2150	*Kabuli*	Ca_Kabuli_Chr02	770266	(C/A)
2151	CakSNP2151	*Kabuli*	Ca_Kabuli_Chr02	1231486	(A/G)
2152	CakSNP2152	*Kabuli*	Ca_Kabuli_Chr02	1231500	(A/G)
2153	CakSNP2153	*Kabuli*	Ca_Kabuli_Chr02	1231519	(T/C)
2154	CakSNP2154	*Kabuli*	Ca_Kabuli_Chr02	1231618	(G/A)
2155	CakSNP2155	*Kabuli*	Ca_Kabuli_Chr02	1234932	(C/T)
2156	CakSNP2156	*Kabuli*	Ca_Kabuli_Chr02	1235018	(G/A)
2157	CakSNP2157	*Kabuli*	Ca_Kabuli_Chr02	1234983	(T/C)
2158	CakSNP2158	*Kabuli*	Ca_Kabuli_Chr02	1314367	(G/A)
2159	CakSNP2159	*Kabuli*	Ca_Kabuli_Chr02	1368911	(A/G)
2160	CakSNP2160	*Kabuli*	Ca_Kabuli_Chr02	1389661	(A/G)
2161	CakSNP2161	*Kabuli*	Ca_Kabuli_Chr02	1397779	(C/T)
2162	CakSNP2162	*Kabuli*	Ca_Kabuli_Chr02	1429186	(T/C)
2163	CakSNP2163	*Kabuli*	Ca_Kabuli_Chr02	1429471	(G/C)
2164	CakSNP2164	*Kabuli*	Ca_Kabuli_Chr02	1431368	(A/C)
2165	CakSNP2165	*Kabuli*	Ca_Kabuli_Chr02	1493971	(A/G)
2166	CakSNP2166	*Kabuli*	Ca_Kabuli_Chr02	1610477	(G/T)
2167	CakSNP2167	*Kabuli*	Ca_Kabuli_Chr02	1668116	(A/G)
2168	CakSNP2168	*Kabuli*	Ca_Kabuli_Chr02	1713343	(T/C)
2169	CakSNP2169	*Kabuli*	Ca_Kabuli_Chr02	1797399	(A/G)
2170	CakSNP2170	*Kabuli*	Ca_Kabuli_Chr02	1801341	(C/T)
2171	CakSNP2171	*Kabuli*	Ca_Kabuli_Chr02	1828318	(T/C)
2172	CakSNP2172	*Kabuli*	Ca_Kabuli_Chr02	1828406	(G/A)
2173	CakSNP2173	*Kabuli*	Ca_Kabuli_Chr02	1829772	(C/T)
2174	CakSNP2174	*Kabuli*	Ca_Kabuli_Chr02	1835055	(G/A)
2175	CakSNP2175	*Kabuli*	Ca_Kabuli_Chr02	1841290	(A/T)
2176	CakSNP2176	*Kabuli*	Ca_Kabuli_Chr02	1841267	(C/T)
2177	CakSNP2177	*Kabuli*	Ca_Kabuli_Chr02	1867041	(C/A)
2178	CakSNP2178	*Kabuli*	Ca_Kabuli_Chr02	1882691	(C/T)
2179	CakSNP2179	*Kabuli*	Ca_Kabuli_Chr02	1884583	(C/T)
2180	CakSNP2180	*Kabuli*	Ca_Kabuli_Chr02	1884704	(A/G)
2181	CakSNP2181	*Kabuli*	Ca_Kabuli_Chr02	1888657	(C/G)
2182	CakSNP2182	*Kabuli*	Ca_Kabuli_Chr02	1953614	(T/C)

S.N.	SNP IDs	Cultivars	Chromosomes/scaffolds	Physical positions (bp)	SNPs
2183	CakSNP2183	*Kabuli*	Ca_Kabuli_Chr02	1997914	(C/T)
2184	CakSNP2184	*Kabuli*	Ca_Kabuli_Chr02	1998003	(C/T)
2185	CakSNP2185	*Kabuli*	Ca_Kabuli_Chr02	2086763	(T/G)
2186	CakSNP2186	*Kabuli*	Ca_Kabuli_Chr02	2220871	(C/T)
2187	CakSNP2187	*Kabuli*	Ca_Kabuli_Chr02	2227118	(A/G)
2188	CakSNP2188	*Kabuli*	Ca_Kabuli_Chr02	2227074	(T/C)
2189	CakSNP2189	*Kabuli*	Ca_Kabuli_Chr02	2227465	(G/A)
2190	CakSNP2190	*Kabuli*	Ca_Kabuli_Chr02	2230089	(G/A)
2191	CakSNP2191	*Kabuli*	Ca_Kabuli_Chr02	2230056	(T/C)
2192	CakSNP2192	*Kabuli*	Ca_Kabuli_Chr02	2230984	(T/G)
2193	CakSNP2193	*Kabuli*	Ca_Kabuli_Chr02	2230980	(C/A)
2194	CakSNP2194	*Kabuli*	Ca_Kabuli_Chr02	2230976	(T/G)
2195	CakSNP2195	*Kabuli*	Ca_Kabuli_Chr02	2246678	(T/C)
2196	CakSNP2196	*Kabuli*	Ca_Kabuli_Chr02	2311915	(C/A)
2197	CakSNP2197	*Kabuli*	Ca_Kabuli_Chr02	2311917	(A/C)
2198	CakSNP2198	*Kabuli*	Ca_Kabuli_Chr02	2312104	(C/T)
2199	CakSNP2199	*Kabuli*	Ca_Kabuli_Chr02	2312037	(T/G)
2200	CakSNP2200	*Kabuli*	Ca_Kabuli_Chr02	2344777	(G/A)
2201	CakSNP2201	*Kabuli*	Ca_Kabuli_Chr02	2344778	(A/G)
2202	CakSNP2202	*Kabuli*	Ca_Kabuli_Chr02	2344786	(G/A)
2203	CakSNP2203	*Kabuli*	Ca_Kabuli_Chr02	2344835	(G/T)
2204	CakSNP2204	*Kabuli*	Ca_Kabuli_Chr02	2414265	(A/G)
2205	CakSNP2205	*Kabuli*	Ca_Kabuli_Chr02	2416422	(C/T)
2206	CakSNP2206	*Kabuli*	Ca_Kabuli_Chr02	2441571	(G/A)
2207	CakSNP2207	*Kabuli*	Ca_Kabuli_Chr02	2602831	(A/C)
2208	CakSNP2208	*Kabuli*	Ca_Kabuli_Chr02	2650375	(T/C)
2209	CakSNP2209	*Kabuli*	Ca_Kabuli_Chr02	2650373	(A/T)
2210	CakSNP2210	*Kabuli*	Ca_Kabuli_Chr02	2652189	(A/T)
2211	CakSNP2211	*Kabuli*	Ca_Kabuli_Chr02	2682235	(T/C)
2212	CakSNP2212	*Kabuli*	Ca_Kabuli_Chr02	2682293	(T/C)
2213	CakSNP2213	*Kabuli*	Ca_Kabuli_Chr02	2682321	(G/A)
2214	CakSNP2214	*Kabuli*	Ca_Kabuli_Chr02	2682375	(A/G)
2215	CakSNP2215	*Kabuli*	Ca_Kabuli_Chr02	2682408	(G/A)
2216	CakSNP2216	*Kabuli*	Ca_Kabuli_Chr02	2682471	(A/G)
2217	CakSNP2217	*Kabuli*	Ca_Kabuli_Chr02	2685299	(T/C)
2218	CakSNP2218	*Kabuli*	Ca_Kabuli_Chr02	2697688	(G/C)
2219	CakSNP2219	*Kabuli*	Ca_Kabuli_Chr02	2708805	(G/C)
2220	CakSNP2220	*Kabuli*	Ca_Kabuli_Chr02	2780283	(C/T)
2221	CakSNP2221	*Kabuli*	Ca_Kabuli_Chr02	2783727	(G/A)

S.N.	SNP IDs	Cultivars	Chromosomes/scaffolds	Physical positions (bp)	SNPs
2222	CakSNP2222	*Kabuli*	Ca_Kabuli_Chr02	2846667	(T/C)
2223	CakSNP2223	*Kabuli*	Ca_Kabuli_Chr02	2846774	(C/A)
2224	CakSNP2224	*Kabuli*	Ca_Kabuli_Chr02	2965867	(G/A)
2225	CakSNP2225	*Kabuli*	Ca_Kabuli_Chr02	3099959	(C/A)
2226	CakSNP2226	*Kabuli*	Ca_Kabuli_Chr02	3099997	(A/T)
2227	CakSNP2227	*Kabuli*	Ca_Kabuli_Chr02	3100116	(A/G)
2228	CakSNP2228	*Kabuli*	Ca_Kabuli_Chr02	3113560	(G/A)
2229	CakSNP2229	*Kabuli*	Ca_Kabuli_Chr02	3141128	(A/C)
2230	CakSNP2230	*Kabuli*	Ca_Kabuli_Chr02	3164404	(A/G)
2231	CakSNP2231	*Kabuli*	Ca_Kabuli_Chr02	3164355	(G/A)
2232	CakSNP2232	*Kabuli*	Ca_Kabuli_Chr02	3366023	(C/T)
2233	CakSNP2233	*Kabuli*	Ca_Kabuli_Chr02	3410375	(C/T)
2234	CakSNP2234	*Kabuli*	Ca_Kabuli_Chr02	3450904	(C/A)
2235	CakSNP2235	*Kabuli*	Ca_Kabuli_Chr02	3503763	(T/G)
2236	CakSNP2236	*Kabuli*	Ca_Kabuli_Chr02	3503759	(T/A)
2237	CakSNP2237	*Kabuli*	Ca_Kabuli_Chr02	3503753	(C/G)
2238	CakSNP2238	*Kabuli*	Ca_Kabuli_Chr02	3503750	(C/G)
2239	CakSNP2239	*Kabuli*	Ca_Kabuli_Chr02	3789808	(G/T)
2240	CakSNP2240	*Kabuli*	Ca_Kabuli_Chr02	3843814	(C/T)
2241	CakSNP2241	*Kabuli*	Ca_Kabuli_Chr02	3843826	(T/C)
2242	CakSNP2242	*Kabuli*	Ca_Kabuli_Chr02	3843836	(G/A)
2243	CakSNP2243	*Kabuli*	Ca_Kabuli_Chr02	3852203	(C/A)
2244	CakSNP2244	*Kabuli*	Ca_Kabuli_Chr02	3894041	(G/T)
2245	CakSNP2245	*Kabuli*	Ca_Kabuli_Chr02	3904757	(G/A)
2246	CakSNP2246	*Kabuli*	Ca_Kabuli_Chr02	3904761	(A/G)
2247	CakSNP2247	*Kabuli*	Ca_Kabuli_Chr02	3908928	(A/G)
2248	CakSNP2248	*Kabuli*	Ca_Kabuli_Chr02	3908955	(G/A)
2249	CakSNP2249	*Kabuli*	Ca_Kabuli_Chr02	3909068	(G/A)
2250	CakSNP2250	*Kabuli*	Ca_Kabuli_Chr02	3909062	(C/T)
2251	CakSNP2251	*Kabuli*	Ca_Kabuli_Chr02	3915549	(A/G)
2252	CakSNP2252	*Kabuli*	Ca_Kabuli_Chr02	3930211	(A/G)
2253	CakSNP2253	*Kabuli*	Ca_Kabuli_Chr02	3930182	(A/G)
2254	CakSNP2254	*Kabuli*	Ca_Kabuli_Chr02	3941397	(A/T)
2255	CakSNP2255	*Kabuli*	Ca_Kabuli_Chr02	3994150	(A/C)
2256	CakSNP2256	*Kabuli*	Ca_Kabuli_Chr02	3998491	(A/C)
2257	CakSNP2257	*Kabuli*	Ca_Kabuli_Chr02	3999615	(C/T)
2258	CakSNP2258	*Kabuli*	Ca_Kabuli_Chr02	4039745	(T/C)
2259	CakSNP2259	*Kabuli*	Ca_Kabuli_Chr02	4039854	(G/A)
2260	CakSNP2260	*Kabuli*	Ca_Kabuli_Chr02	4325658	(A/C)

S.N.	SNP IDs	Cultivars	Chromosomes/scaffolds	Physical positions (bp)	SNPs
2261	CakSNP2261	*Kabuli*	Ca_Kabuli_Chr02	4325684	(C/A)
2262	CakSNP2262	*Kabuli*	Ca_Kabuli_Chr02	4404813	(C/T)
2263	CakSNP2263	*Kabuli*	Ca_Kabuli_Chr02	4404797	(G/A)
2264	CakSNP2264	*Kabuli*	Ca_Kabuli_Chr02	4966216	(C/T)
2265	CakSNP2265	*Kabuli*	Ca_Kabuli_Chr02	4966168	(G/C)
2266	CakSNP2266	*Kabuli*	Ca_Kabuli_Chr02	4981626	(A/G)
2267	CakSNP2267	*Kabuli*	Ca_Kabuli_Chr02	4981698	(C/T)
2268	CakSNP2268	*Kabuli*	Ca_Kabuli_Chr02	4981686	(T/C)
2269	CakSNP2269	*Kabuli*	Ca_Kabuli_Chr02	4981666	(G/C)
2270	CakSNP2270	*Kabuli*	Ca_Kabuli_Chr02	5017291	(T/A)
2271	CakSNP2271	*Kabuli*	Ca_Kabuli_Chr02	5017330	(G/C)
2272	CakSNP2272	*Kabuli*	Ca_Kabuli_Chr02	5078323	(T/C)
2273	CakSNP2273	*Kabuli*	Ca_Kabuli_Chr02	5079639	(C/T)
2274	CakSNP2274	*Kabuli*	Ca_Kabuli_Chr02	5085761	(A/C)
2275	CakSNP2275	*Kabuli*	Ca_Kabuli_Chr02	5085836	(G/C)
2276	CakSNP2276	*Kabuli*	Ca_Kabuli_Chr02	5100017	(T/A)
2277	CakSNP2277	*Kabuli*	Ca_Kabuli_Chr02	5099985	(A/G)
2278	CakSNP2278	*Kabuli*	Ca_Kabuli_Chr02	5118370	(A/T)
2279	CakSNP2279	*Kabuli*	Ca_Kabuli_Chr02	5118364	(C/T)
2280	CakSNP2280	*Kabuli*	Ca_Kabuli_Chr02	5141075	(C/T)
2281	CakSNP2281	*Kabuli*	Ca_Kabuli_Chr02	5141081	(A/T)
2282	CakSNP2282	*Kabuli*	Ca_Kabuli_Chr02	5247305	(T/C)
2283	CakSNP2283	*Kabuli*	Ca_Kabuli_Chr02	5247548	(G/T)
2284	CakSNP2284	*Kabuli*	Ca_Kabuli_Chr02	5247547	(A/G)
2285	CakSNP2285	*Kabuli*	Ca_Kabuli_Chr02	5247540	(T/C)
2286	CakSNP2286	*Kabuli*	Ca_Kabuli_Chr02	5247481	(T/A)
2287	CakSNP2287	*Kabuli*	Ca_Kabuli_Chr02	5247557	(A/C)
2288	CakSNP2288	*Kabuli*	Ca_Kabuli_Chr02	5247558	(G/T)
2289	CakSNP2289	*Kabuli*	Ca_Kabuli_Chr02	5259254	(T/C)
2290	CakSNP2290	*Kabuli*	Ca_Kabuli_Chr02	5276208	(T/C)
2291	CakSNP2291	*Kabuli*	Ca_Kabuli_Chr02	5342864	(C/A)
2292	CakSNP2292	*Kabuli*	Ca_Kabuli_Chr02	5342923	(A/G)
2293	CakSNP2293	*Kabuli*	Ca_Kabuli_Chr02	5359177	(C/A)
2294	CakSNP2294	*Kabuli*	Ca_Kabuli_Chr02	5449886	(C/T)
2295	CakSNP2295	*Kabuli*	Ca_Kabuli_Chr02	5449978	(T/C)
2296	CakSNP2296	*Kabuli*	Ca_Kabuli_Chr02	5449907	(G/A)
2297	CakSNP2297	*Kabuli*	Ca_Kabuli_Chr02	5462902	(A/T)
2298	CakSNP2298	*Kabuli*	Ca_Kabuli_Chr02	5468575	(A/T)
2299	CakSNP2299	*Kabuli*	Ca_Kabuli_Chr02	5475329	(C/A)

S.N.	SNP IDs	Cultivars	Chromosomes/scaffolds	Physical positions (bp)	SNPs
2300	CakSNP2300	*Kabuli*	Ca_Kabuli_Chr02	5475344	(A/G)
2301	CakSNP2301	*Kabuli*	Ca_Kabuli_Chr02	5475365	(T/C)
2302	CakSNP2302	*Kabuli*	Ca_Kabuli_Chr02	5475407	(C/T)
2303	CakSNP2303	*Kabuli*	Ca_Kabuli_Chr02	5504300	(T/G)
2304	CakSNP2304	*Kabuli*	Ca_Kabuli_Chr02	5601092	(G/C)
2305	CakSNP2305	*Kabuli*	Ca_Kabuli_Chr02	5787862	(G/A)
2306	CakSNP2306	*Kabuli*	Ca_Kabuli_Chr02	5787887	(A/C)
2307	CakSNP2307	*Kabuli*	Ca_Kabuli_Chr02	5877688	(A/G)
2308	CakSNP2308	*Kabuli*	Ca_Kabuli_Chr02	5881503	(A/G)
2309	CakSNP2309	*Kabuli*	Ca_Kabuli_Chr02	5897314	(T/C)
2310	CakSNP2310	*Kabuli*	Ca_Kabuli_Chr02	5904368	(T/C)
2311	CakSNP2311	*Kabuli*	Ca_Kabuli_Chr02	5936796	(C/A)
2312	CakSNP2312	*Kabuli*	Ca_Kabuli_Chr02	5936902	(A/T)
2313	CakSNP2313	*Kabuli*	Ca_Kabuli_Chr02	6042131	(G/A)
2314	CakSNP2314	*Kabuli*	Ca_Kabuli_Chr02	6042139	(C/A)
2315	CakSNP2315	*Kabuli*	Ca_Kabuli_Chr02	6042210	(C/A)
2316	CakSNP2316	*Kabuli*	Ca_Kabuli_Chr02	6060259	(G/C)
2317	CakSNP2317	*Kabuli*	Ca_Kabuli_Chr02	6130301	(G/C)
2318	CakSNP2318	*Kabuli*	Ca_Kabuli_Chr02	6130257	(C/A)
2319	CakSNP2319	*Kabuli*	Ca_Kabuli_Chr02	6130247	(G/C)
2320	CakSNP2320	*Kabuli*	Ca_Kabuli_Chr02	6162378	(T/G)
2321	CakSNP2321	*Kabuli*	Ca_Kabuli_Chr02	6162406	(G/C)
2322	CakSNP2322	*Kabuli*	Ca_Kabuli_Chr02	6213582	(T/A)
2323	CakSNP2323	*Kabuli*	Ca_Kabuli_Chr02	6213580	(C/A)
2324	CakSNP2324	*Kabuli*	Ca_Kabuli_Chr02	6213566	(C/T)
2325	CakSNP2325	*Kabuli*	Ca_Kabuli_Chr02	6213530	(C/A)
2326	CakSNP2326	*Kabuli*	Ca_Kabuli_Chr02	6213520	(G/T)
2327	CakSNP2327	*Kabuli*	Ca_Kabuli_Chr02	6349208	(T/G)
2328	CakSNP2328	*Kabuli*	Ca_Kabuli_Chr02	6367649	(A/G)
2329	CakSNP2329	*Kabuli*	Ca_Kabuli_Chr02	6367704	(A/G)
2330	CakSNP2330	*Kabuli*	Ca_Kabuli_Chr02	6391819	(T/C)
2331	CakSNP2331	*Kabuli*	Ca_Kabuli_Chr02	6392158	(A/T)
2332	CakSNP2332	*Kabuli*	Ca_Kabuli_Chr02	6392209	(G/A)
2333	CakSNP2333	*Kabuli*	Ca_Kabuli_Chr02	6392272	(T/C)
2334	CakSNP2334	*Kabuli*	Ca_Kabuli_Chr02	6392254	(T/C)
2335	CakSNP2335	*Kabuli*	Ca_Kabuli_Chr02	6392449	(A/G)
2336	CakSNP2336	*Kabuli*	Ca_Kabuli_Chr02	6397551	(A/G)
2337	CakSNP2337	*Kabuli*	Ca_Kabuli_Chr02	6397597	(A/C)
2338	CakSNP2338	*Kabuli*	Ca_Kabuli_Chr02	6408406	(C/A)

S.N.	SNP IDs	Cultivars	Chromosomes/scaffolds	Physical positions (bp)	SNPs
2339	CakSNP2339	*Kabuli*	Ca_Kabuli_Chr02	6534776	(C/A)
2340	CakSNP2340	*Kabuli*	Ca_Kabuli_Chr02	6537296	(G/T)
2341	CakSNP2341	*Kabuli*	Ca_Kabuli_Chr02	6537300	(G/T)
2342	CakSNP2342	*Kabuli*	Ca_Kabuli_Chr02	6727932	(G/C)
2343	CakSNP2343	*Kabuli*	Ca_Kabuli_Chr02	6727905	(G/C)
2344	CakSNP2344	*Kabuli*	Ca_Kabuli_Chr02	6727900	(G/C)
2345	CakSNP2345	*Kabuli*	Ca_Kabuli_Chr02	6728716	(A/T)
2346	CakSNP2346	*Kabuli*	Ca_Kabuli_Chr02	6728719	(C/T)
2347	CakSNP2347	*Kabuli*	Ca_Kabuli_Chr02	6728763	(T/C)
2348	CakSNP2348	*Kabuli*	Ca_Kabuli_Chr02	6989950	(A/G)
2349	CakSNP2349	*Kabuli*	Ca_Kabuli_Chr02	6990083	(G/A)
2350	CakSNP2350	*Kabuli*	Ca_Kabuli_Chr02	7007701	(G/C)
2351	CakSNP2351	*Kabuli*	Ca_Kabuli_Chr02	7027093	(G/A)
2352	CakSNP2352	*Kabuli*	Ca_Kabuli_Chr02	7027146	(T/C)
2353	CakSNP2353	*Kabuli*	Ca_Kabuli_Chr02	7047195	(C/A)
2354	CakSNP2354	*Kabuli*	Ca_Kabuli_Chr02	7047196	(T/C)
2355	CakSNP2355	*Kabuli*	Ca_Kabuli_Chr02	7047197	(G/C)
2356	CakSNP2356	*Kabuli*	Ca_Kabuli_Chr02	7051451	(G/A)
2357	CakSNP2357	*Kabuli*	Ca_Kabuli_Chr02	7125992	(T/G)
2358	CakSNP2358	*Kabuli*	Ca_Kabuli_Chr02	7126018	(T/G)
2359	CakSNP2359	*Kabuli*	Ca_Kabuli_Chr02	7507259	(C/A)
2360	CakSNP2360	*Kabuli*	Ca_Kabuli_Chr02	7633520	(G/A)
2361	CakSNP2361	*Kabuli*	Ca_Kabuli_Chr02	7633547	(C/T)
2362	CakSNP2362	*Kabuli*	Ca_Kabuli_Chr02	7633564	(G/T)
2363	CakSNP2363	*Kabuli*	Ca_Kabuli_Chr02	7633572	(T/C)
2364	CakSNP2364	*Kabuli*	Ca_Kabuli_Chr02	7633605	(T/A)
2365	CakSNP2365	*Kabuli*	Ca_Kabuli_Chr02	7633592	(G/A)
2366	CakSNP2366	*Kabuli*	Ca_Kabuli_Chr02	7633585	(G/A)
2367	CakSNP2367	*Kabuli*	Ca_Kabuli_Chr02	7633869	(G/A)
2368	CakSNP2368	*Kabuli*	Ca_Kabuli_Chr02	7633826	(G/A)
2369	CakSNP2369	*Kabuli*	Ca_Kabuli_Chr02	7633818	(G/A)
2370	CakSNP2370	*Kabuli*	Ca_Kabuli_Chr02	7633816	(G/A)
2371	CakSNP2371	*Kabuli*	Ca_Kabuli_Chr02	7633813	(G/T)
2372	CakSNP2372	*Kabuli*	Ca_Kabuli_Chr02	7633793	(C/T)
2373	CakSNP2373	*Kabuli*	Ca_Kabuli_Chr02	7634041	(G/A)
2374	CakSNP2374	*Kabuli*	Ca_Kabuli_Chr02	7679627	(A/T)
2375	CakSNP2375	*Kabuli*	Ca_Kabuli_Chr02	7679693	(C/G)
2376	CakSNP2376	*Kabuli*	Ca_Kabuli_Chr02	7701189	(T/G)
2377	CakSNP2377	*Kabuli*	Ca_Kabuli_Chr02	7701199	(T/G)

S.N.	SNP IDs	Cultivars	Chromosomes/scaffolds	Physical positions (bp)	SNPs
2378	CakSNP2378	*Kabuli*	Ca_Kabuli_Chr02	7701216	(T/G)
2379	CakSNP2379	*Kabuli*	Ca_Kabuli_Chr02	7701230	(T/G)
2380	CakSNP2380	*Kabuli*	Ca_Kabuli_Chr02	7702468	(A/T)
2381	CakSNP2381	*Kabuli*	Ca_Kabuli_Chr02	7708668	(C/T)
2382	CakSNP2382	*Kabuli*	Ca_Kabuli_Chr02	7708663	(T/C)
2383	CakSNP2383	*Kabuli*	Ca_Kabuli_Chr02	7708660	(G/A)
2384	CakSNP2384	*Kabuli*	Ca_Kabuli_Chr02	7708630	(C/A)
2385	CakSNP2385	*Kabuli*	Ca_Kabuli_Chr02	7708617	(G/T)
2386	CakSNP2386	*Kabuli*	Ca_Kabuli_Chr02	7708603	(C/T)
2387	CakSNP2387	*Kabuli*	Ca_Kabuli_Chr02	7708598	(C/T)
2388	CakSNP2388	*Kabuli*	Ca_Kabuli_Chr02	7708596	(T/C)
2389	CakSNP2389	*Kabuli*	Ca_Kabuli_Chr02	7708590	(A/T)
2390	CakSNP2390	*Kabuli*	Ca_Kabuli_Chr02	7708678	(C/T)
2391	CakSNP2391	*Kabuli*	Ca_Kabuli_Chr02	7712181	(C/T)
2392	CakSNP2392	*Kabuli*	Ca_Kabuli_Chr02	7712155	(G/T)
2393	CakSNP2393	*Kabuli*	Ca_Kabuli_Chr02	7714392	(G/A)
2394	CakSNP2394	*Kabuli*	Ca_Kabuli_Chr02	7714454	(A/G)
2395	CakSNP2395	*Kabuli*	Ca_Kabuli_Chr02	7714463	(T/C)
2396	CakSNP2396	*Kabuli*	Ca_Kabuli_Chr02	7753419	(G/C)
2397	CakSNP2397	*Kabuli*	Ca_Kabuli_Chr02	7753425	(T/C)
2398	CakSNP2398	*Kabuli*	Ca_Kabuli_Chr02	7753440	(C/T)
2399	CakSNP2399	*Kabuli*	Ca_Kabuli_Chr02	7819281	(T/C)
2400	CakSNP2400	*Kabuli*	Ca_Kabuli_Chr02	7870919	(G/T)
2401	CakSNP2401	*Kabuli*	Ca_Kabuli_Chr02	7870943	(T/G)
2402	CakSNP2402	*Kabuli*	Ca_Kabuli_Chr02	7938890	(T/C)
2403	CakSNP2403	*Kabuli*	Ca_Kabuli_Chr02	8337517	(C/T)
2404	CakSNP2404	*Kabuli*	Ca_Kabuli_Chr02	8484840	(A/T)
2405	CakSNP2405	*Kabuli*	Ca_Kabuli_Chr02	8487331	(T/A)
2406	CakSNP2406	*Kabuli*	Ca_Kabuli_Chr02	8487343	(C/T)
2407	CakSNP2407	*Kabuli*	Ca_Kabuli_Chr02	8489241	(C/A)
2408	CakSNP2408	*Kabuli*	Ca_Kabuli_Chr02	8489237	(G/A)
2409	CakSNP2409	*Kabuli*	Ca_Kabuli_Chr02	8489172	(C/A)
2410	CakSNP2410	*Kabuli*	Ca_Kabuli_Chr02	8600256	(C/G)
2411	CakSNP2411	*Kabuli*	Ca_Kabuli_Chr02	8600298	(A/G)
2412	CakSNP2412	*Kabuli*	Ca_Kabuli_Chr02	8600309	(T/C)
2413	CakSNP2413	*Kabuli*	Ca_Kabuli_Chr02	8600450	(C/T)
2414	CakSNP2414	*Kabuli*	Ca_Kabuli_Chr02	8600666	(T/C)
2415	CakSNP2415	*Kabuli*	Ca_Kabuli_Chr02	8685674	(G/A)
2416	CakSNP2416	*Kabuli*	Ca_Kabuli_Chr02	8719725	(T/A)

S.N.	SNP IDs	Cultivars	Chromosomes/scaffolds	Physical positions (bp)	SNPs
2417	CakSNP2417	*Kabuli*	Ca_Kabuli_Chr02	8734136	(G/A)
2418	CakSNP2418	*Kabuli*	Ca_Kabuli_Chr02	8802884	(A/G)
2419	CakSNP2419	*Kabuli*	Ca_Kabuli_Chr02	9458933	(C/T)
2420	CakSNP2420	*Kabuli*	Ca_Kabuli_Chr02	9458985	(A/G)
2421	CakSNP2421	*Kabuli*	Ca_Kabuli_Chr02	9473339	(T/C)
2422	CakSNP2422	*Kabuli*	Ca_Kabuli_Chr02	9473353	(C/A)
2423	CakSNP2423	*Kabuli*	Ca_Kabuli_Chr02	9473362	(G/A)
2424	CakSNP2424	*Kabuli*	Ca_Kabuli_Chr02	9473380	(A/G)
2425	CakSNP2425	*Kabuli*	Ca_Kabuli_Chr02	9473393	(G/A)
2426	CakSNP2426	*Kabuli*	Ca_Kabuli_Chr02	9473474	(T/A)
2427	CakSNP2427	*Kabuli*	Ca_Kabuli_Chr02	9473452	(A/G)
2428	CakSNP2428	*Kabuli*	Ca_Kabuli_Chr02	9473430	(A/G)
2429	CakSNP2429	*Kabuli*	Ca_Kabuli_Chr02	9621953	(A/C)
2430	CakSNP2430	*Kabuli*	Ca_Kabuli_Chr02	9740018	(G/A)
2431	CakSNP2431	*Kabuli*	Ca_Kabuli_Chr02	9740017	(C/T)
2432	CakSNP2432	*Kabuli*	Ca_Kabuli_Chr02	9740010	(A/G)
2433	CakSNP2433	*Kabuli*	Ca_Kabuli_Chr02	9740007	(A/G)
2434	CakSNP2434	*Kabuli*	Ca_Kabuli_Chr02	9739980	(G/C)
2435	CakSNP2435	*Kabuli*	Ca_Kabuli_Chr02	9739974	(T/C)
2436	CakSNP2436	*Kabuli*	Ca_Kabuli_Chr02	9739959	(T/C)
2437	CakSNP2437	*Kabuli*	Ca_Kabuli_Chr02	9739965	(A/G)
2438	CakSNP2438	*Kabuli*	Ca_Kabuli_Chr02	9756955	(G/A)
2439	CakSNP2439	*Kabuli*	Ca_Kabuli_Chr02	9756946	(C/T)
2440	CakSNP2440	*Kabuli*	Ca_Kabuli_Chr02	9756910	(G/A)
2441	CakSNP2441	*Kabuli*	Ca_Kabuli_Chr02	9756887	(G/T)
2442	CakSNP2442	*Kabuli*	Ca_Kabuli_Chr02	9823224	(G/A)
2443	CakSNP2443	*Kabuli*	Ca_Kabuli_Chr02	9823165	(T/C)
2444	CakSNP2444	*Kabuli*	Ca_Kabuli_Chr02	9825526	(C/G)
2445	CakSNP2445	*Kabuli*	Ca_Kabuli_Chr02	9841625	(A/G)
2446	CakSNP2446	*Kabuli*	Ca_Kabuli_Chr02	9862794	(A/G)
2447	CakSNP2447	*Kabuli*	Ca_Kabuli_Chr02	9906042	(T/G)
2448	CakSNP2448	*Kabuli*	Ca_Kabuli_Chr02	9906077	(T/A)
2449	CakSNP2449	*Kabuli*	Ca_Kabuli_Chr02	9918536	(T/A)
2450	CakSNP2450	*Kabuli*	Ca_Kabuli_Chr02	9943370	(A/G)
2451	CakSNP2451	*Kabuli*	Ca_Kabuli_Chr02	9943374	(C/T)
2452	CakSNP2452	*Kabuli*	Ca_Kabuli_Chr02	9943404	(T/G)
2453	CakSNP2453	*Kabuli*	Ca_Kabuli_Chr02	9986639	(C/T)
2454	CakSNP2454	*Kabuli*	Ca_Kabuli_Chr02	9986683	(C/T)
2455	CakSNP2455	*Kabuli*	Ca_Kabuli_Chr02	10004694	(G/A)

S.N.	SNP IDs	Cultivars	Chromosomes/scaffolds	Physical positions (bp)	SNPs
2456	CakSNP2456	*Kabuli*	Ca_Kabuli_Chr02	10025451	(G/A)
2457	CakSNP2457	*Kabuli*	Ca_Kabuli_Chr02	10033991	(T/C)
2458	CakSNP2458	*Kabuli*	Ca_Kabuli_Chr02	10060196	(G/T)
2459	CakSNP2459	*Kabuli*	Ca_Kabuli_Chr02	10060200	(G/T)
2460	CakSNP2460	*Kabuli*	Ca_Kabuli_Chr02	10060207	(C/T)
2461	CakSNP2461	*Kabuli*	Ca_Kabuli_Chr02	10067093	(G/C)
2462	CakSNP2462	*Kabuli*	Ca_Kabuli_Chr02	10067078	(T/G)
2463	CakSNP2463	*Kabuli*	Ca_Kabuli_Chr02	10067061	(T/G)
2464	CakSNP2464	*Kabuli*	Ca_Kabuli_Chr02	10067195	(T/A)
2465	CakSNP2465	*Kabuli*	Ca_Kabuli_Chr02	10067262	(A/C)
2466	CakSNP2466	*Kabuli*	Ca_Kabuli_Chr02	10067278	(G/T)
2467	CakSNP2467	*Kabuli*	Ca_Kabuli_Chr02	10067285	(T/G)
2468	CakSNP2468	*Kabuli*	Ca_Kabuli_Chr02	10067286	(A/T)
2469	CakSNP2469	*Kabuli*	Ca_Kabuli_Chr02	10067451	(A/T)
2470	CakSNP2470	*Kabuli*	Ca_Kabuli_Chr02	10067516	(G/A)
2471	CakSNP2471	*Kabuli*	Ca_Kabuli_Chr02	10067538	(C/T)
2472	CakSNP2472	*Kabuli*	Ca_Kabuli_Chr02	10068214	(A/G)
2473	CakSNP2473	*Kabuli*	Ca_Kabuli_Chr02	10220396	(T/C)
2474	CakSNP2474	*Kabuli*	Ca_Kabuli_Chr02	10270474	(C/A)
2475	CakSNP2475	*Kabuli*	Ca_Kabuli_Chr02	10284426	(C/G)
2476	CakSNP2476	*Kabuli*	Ca_Kabuli_Chr02	10294698	(A/G)
2477	CakSNP2477	*Kabuli*	Ca_Kabuli_Chr02	10294635	(T/C)
2478	CakSNP2478	*Kabuli*	Ca_Kabuli_Chr02	10322943	(T/C)
2479	CakSNP2479	*Kabuli*	Ca_Kabuli_Chr02	10352705	(T/A)
2480	CakSNP2480	*Kabuli*	Ca_Kabuli_Chr02	10358484	(C/T)
2481	CakSNP2481	*Kabuli*	Ca_Kabuli_Chr02	10358496	(G/T)
2482	CakSNP2482	*Kabuli*	Ca_Kabuli_Chr02	10377695	(G/A)
2483	CakSNP2483	*Kabuli*	Ca_Kabuli_Chr02	10434727	(T/C)
2484	CakSNP2484	*Kabuli*	Ca_Kabuli_Chr02	10434726	(G/C)
2485	CakSNP2485	*Kabuli*	Ca_Kabuli_Chr02	10434714	(C/G)
2486	CakSNP2486	*Kabuli*	Ca_Kabuli_Chr02	10540735	(C/T)
2487	CakSNP2487	*Kabuli*	Ca_Kabuli_Chr02	10637631	(C/A)
2488	CakSNP2488	*Kabuli*	Ca_Kabuli_Chr02	10656518	(A/T)
2489	CakSNP2489	*Kabuli*	Ca_Kabuli_Chr02	10705902	(C/A)
2490	CakSNP2490	*Kabuli*	Ca_Kabuli_Chr02	10705923	(T/A)
2491	CakSNP2491	*Kabuli*	Ca_Kabuli_Chr02	10705979	(C/A)
2492	CakSNP2492	*Kabuli*	Ca_Kabuli_Chr02	10705982	(C/T)
2493	CakSNP2493	*Kabuli*	Ca_Kabuli_Chr02	10760939	(G/A)
2494	CakSNP2494	*Kabuli*	Ca_Kabuli_Chr02	10761036	(C/G)

S.N.	SNP IDs	Cultivars	Chromosomes/scaffolds	Physical positions (bp)	SNPs
2495	CakSNP2495	*Kabuli*	Ca_Kabuli_Chr02	11799472	(G/A)
2496	CakSNP2496	*Kabuli*	Ca_Kabuli_Chr02	11799462	(G/A)
2497	CakSNP2497	*Kabuli*	Ca_Kabuli_Chr02	12252285	(T/G)
2498	CakSNP2498	*Kabuli*	Ca_Kabuli_Chr02	12252370	(A/G)
2499	CakSNP2499	*Kabuli*	Ca_Kabuli_Chr02	12278583	(G/A)
2500	CakSNP2500	*Kabuli*	Ca_Kabuli_Chr02	12278584	(T/C)
2501	CakSNP2501	*Kabuli*	Ca_Kabuli_Chr02	12278606	(T/C)
2502	CakSNP2502	*Kabuli*	Ca_Kabuli_Chr02	12281599	(A/C)
2503	CakSNP2503	*Kabuli*	Ca_Kabuli_Chr02	12357732	(A/G)
2504	CakSNP2504	*Kabuli*	Ca_Kabuli_Chr02	12389166	(G/T)
2505	CakSNP2505	*Kabuli*	Ca_Kabuli_Chr02	12389112	(G/A)
2506	CakSNP2506	*Kabuli*	Ca_Kabuli_Chr02	12515595	(A/G)
2507	CakSNP2507	*Kabuli*	Ca_Kabuli_Chr02	12528825	(C/G)
2508	CakSNP2508	*Kabuli*	Ca_Kabuli_Chr02	12710137	(G/A)
2509	CakSNP2509	*Kabuli*	Ca_Kabuli_Chr02	12737814	(T/C)
2510	CakSNP2510	*Kabuli*	Ca_Kabuli_Chr02	12794438	(G/A)
2511	CakSNP2511	*Kabuli*	Ca_Kabuli_Chr02	12854610	(A/G)
2512	CakSNP2512	*Kabuli*	Ca_Kabuli_Chr02	12854591	(T/A)
2513	CakSNP2513	*Kabuli*	Ca_Kabuli_Chr02	12854586	(G/A)
2514	CakSNP2514	*Kabuli*	Ca_Kabuli_Chr02	12862414	(G/C)
2515	CakSNP2515	*Kabuli*	Ca_Kabuli_Chr02	13079417	(T/G)
2516	CakSNP2516	*Kabuli*	Ca_Kabuli_Chr02	13079445	(T/G)
2517	CakSNP2517	*Kabuli*	Ca_Kabuli_Chr02	13217781	(T/C)
2518	CakSNP2518	*Kabuli*	Ca_Kabuli_Chr02	13355018	(T/G)
2519	CakSNP2519	*Kabuli*	Ca_Kabuli_Chr02	13355022	(G/T)
2520	CakSNP2520	*Kabuli*	Ca_Kabuli_Chr02	13355033	(A/T)
2521	CakSNP2521	*Kabuli*	Ca_Kabuli_Chr02	13355615	(T/G)
2522	CakSNP2522	*Kabuli*	Ca_Kabuli_Chr02	13425568	(T/A)
2523	CakSNP2523	*Kabuli*	Ca_Kabuli_Chr02	13434904	(G/A)
2524	CakSNP2524	*Kabuli*	Ca_Kabuli_Chr02	13434884	(T/A)
2525	CakSNP2525	*Kabuli*	Ca_Kabuli_Chr02	13435043	(C/T)
2526	CakSNP2526	*Kabuli*	Ca_Kabuli_Chr02	13435030	(G/C)
2527	CakSNP2527	*Kabuli*	Ca_Kabuli_Chr02	13434995	(A/C)
2528	CakSNP2528	*Kabuli*	Ca_Kabuli_Chr02	13437317	(A/G)
2529	CakSNP2529	*Kabuli*	Ca_Kabuli_Chr02	13529057	(G/A)
2530	CakSNP2530	*Kabuli*	Ca_Kabuli_Chr02	13552505	(T/A)
2531	CakSNP2531	*Kabuli*	Ca_Kabuli_Chr02	13554038	(T/C)
2532	CakSNP2532	*Kabuli*	Ca_Kabuli_Chr02	13554045	(T/C)
2533	CakSNP2533	*Kabuli*	Ca_Kabuli_Chr02	13554047	(A/T)

S.N.	SNP IDs	Cultivars	Chromosomes/scaffolds	Physical positions (bp)	SNPs
2534	CakSNP2534	*Kabuli*	Ca_Kabuli_Chr02	13703083	(G/A)
2535	CakSNP2535	*Kabuli*	Ca_Kabuli_Chr02	13782906	(C/A)
2536	CakSNP2536	*Kabuli*	Ca_Kabuli_Chr02	14101913	(T/A)
2537	CakSNP2537	*Kabuli*	Ca_Kabuli_Chr02	14134413	(A/C)
2538	CakSNP2538	*Kabuli*	Ca_Kabuli_Chr02	14134676	(T/C)
2539	CakSNP2539	*Kabuli*	Ca_Kabuli_Chr02	14136062	(C/A)
2540	CakSNP2540	*Kabuli*	Ca_Kabuli_Chr02	14136019	(A/G)
2541	CakSNP2541	*Kabuli*	Ca_Kabuli_Chr02	14136013	(T/G)
2542	CakSNP2542	*Kabuli*	Ca_Kabuli_Chr02	14136223	(A/C)
2543	CakSNP2543	*Kabuli*	Ca_Kabuli_Chr02	15160301	(T/C)
2544	CakSNP2544	*Kabuli*	Ca_Kabuli_Chr02	15178565	(G/A)
2545	CakSNP2545	*Kabuli*	Ca_Kabuli_Chr02	15178564	(A/G)
2546	CakSNP2546	*Kabuli*	Ca_Kabuli_Chr02	15178540	(A/G)
2547	CakSNP2547	*Kabuli*	Ca_Kabuli_Chr02	15181635	(G/T)
2548	CakSNP2548	*Kabuli*	Ca_Kabuli_Chr02	15234951	(C/A)
2549	CakSNP2549	*Kabuli*	Ca_Kabuli_Chr02	15389412	(G/A)
2550	CakSNP2550	*Kabuli*	Ca_Kabuli_Chr02	15390673	(A/G)
2551	CakSNP2551	*Kabuli*	Ca_Kabuli_Chr02	15390731	(G/A)
2552	CakSNP2552	*Kabuli*	Ca_Kabuli_Chr02	15390684	(A/G)
2553	CakSNP2553	*Kabuli*	Ca_Kabuli_Chr02	15688657	(G/T)
2554	CakSNP2554	*Kabuli*	Ca_Kabuli_Chr02	15817688	(T/C)
2555	CakSNP2555	*Kabuli*	Ca_Kabuli_Chr02	16460174	(G/A)
2556	CakSNP2556	*Kabuli*	Ca_Kabuli_Chr02	16460142	(A/G)
2557	CakSNP2557	*Kabuli*	Ca_Kabuli_Chr02	16460097	(C/A)
2558	CakSNP2558	*Kabuli*	Ca_Kabuli_Chr02	16460106	(C/T)
2559	CakSNP2559	*Kabuli*	Ca_Kabuli_Chr02	16460175	(C/A)
2560	CakSNP2560	*Kabuli*	Ca_Kabuli_Chr02	16462095	(A/G)
2561	CakSNP2561	*Kabuli*	Ca_Kabuli_Chr02	16462089	(C/T)
2562	CakSNP2562	*Kabuli*	Ca_Kabuli_Chr02	16462056	(A/C)
2563	CakSNP2563	*Kabuli*	Ca_Kabuli_Chr02	16462038	(G/T)
2564	CakSNP2564	*Kabuli*	Ca_Kabuli_Chr02	16555733	(C/T)
2565	CakSNP2565	*Kabuli*	Ca_Kabuli_Chr02	16555790	(C/G)
2566	CakSNP2566	*Kabuli*	Ca_Kabuli_Chr02	16563117	(A/T)
2567	CakSNP2567	*Kabuli*	Ca_Kabuli_Chr02	16563121	(A/T)
2568	CakSNP2568	*Kabuli*	Ca_Kabuli_Chr02	16563123	(C/T)
2569	CakSNP2569	*Kabuli*	Ca_Kabuli_Chr02	16563125	(A/T)
2570	CakSNP2570	*Kabuli*	Ca_Kabuli_Chr02	16563133	(A/T)
2571	CakSNP2571	*Kabuli*	Ca_Kabuli_Chr02	16705119	(C/T)
2572	CakSNP2572	*Kabuli*	Ca_Kabuli_Chr02	17450780	(C/T)

S.N.	SNP IDs	Cultivars	Chromosomes/scaffolds	Physical positions (bp)	SNPs
2573	CakSNP2573	*Kabuli*	Ca_Kabuli_Chr02	17578623	(G/A)
2574	CakSNP2574	*Kabuli*	Ca_Kabuli_Chr02	17578633	(C/G)
2575	CakSNP2575	*Kabuli*	Ca_Kabuli_Chr02	17578659	(A/G)
2576	CakSNP2576	*Kabuli*	Ca_Kabuli_Chr02	17578689	(G/A)
2577	CakSNP2577	*Kabuli*	Ca_Kabuli_Chr02	17578663	(C/A)
2578	CakSNP2578	*Kabuli*	Ca_Kabuli_Chr02	17578676	(T/C)
2579	CakSNP2579	*Kabuli*	Ca_Kabuli_Chr02	17578701	(A/C)
2580	CakSNP2580	*Kabuli*	Ca_Kabuli_Chr02	17578706	(A/T)
2581	CakSNP2581	*Kabuli*	Ca_Kabuli_Chr02	17578726	(A/T)
2582	CakSNP2582	*Kabuli*	Ca_Kabuli_Chr02	17578740	(A/G)
2583	CakSNP2583	*Kabuli*	Ca_Kabuli_Chr02	17681915	(C/T)
2584	CakSNP2584	*Kabuli*	Ca_Kabuli_Chr02	17716022	(A/T)
2585	CakSNP2585	*Kabuli*	Ca_Kabuli_Chr02	17727340	(G/A)
2586	CakSNP2586	*Kabuli*	Ca_Kabuli_Chr02	17727362	(G/C)
2587	CakSNP2587	*Kabuli*	Ca_Kabuli_Chr02	17727403	(C/T)
2588	CakSNP2588	*Kabuli*	Ca_Kabuli_Chr02	17839209	(T/A)
2589	CakSNP2589	*Kabuli*	Ca_Kabuli_Chr02	17839297	(T/C)
2590	CakSNP2590	*Kabuli*	Ca_Kabuli_Chr02	17910111	(C/G)
2591	CakSNP2591	*Kabuli*	Ca_Kabuli_Chr02	17910145	(C/T)
2592	CakSNP2592	*Kabuli*	Ca_Kabuli_Chr02	17910783	(A/T)
2593	CakSNP2593	*Kabuli*	Ca_Kabuli_Chr02	18312200	(T/C)
2594	CakSNP2594	*Kabuli*	Ca_Kabuli_Chr02	18395345	(A/C)
2595	CakSNP2595	*Kabuli*	Ca_Kabuli_Chr02	18395410	(T/C)
2596	CakSNP2596	*Kabuli*	Ca_Kabuli_Chr02	18395378	(A/G)
2597	CakSNP2597	*Kabuli*	Ca_Kabuli_Chr02	18546345	(T/C)
2598	CakSNP2598	*Kabuli*	Ca_Kabuli_Chr02	18546366	(A/T)
2599	CakSNP2599	*Kabuli*	Ca_Kabuli_Chr02	18631367	(T/A)
2600	CakSNP2600	*Kabuli*	Ca_Kabuli_Chr02	18631383	(A/G)
2601	CakSNP2601	*Kabuli*	Ca_Kabuli_Chr02	18631404	(T/G)
2602	CakSNP2602	*Kabuli*	Ca_Kabuli_Chr02	18631412	(T/G)
2603	CakSNP2603	*Kabuli*	Ca_Kabuli_Chr02	18631522	(A/C)
2604	CakSNP2604	*Kabuli*	Ca_Kabuli_Chr02	18671692	(C/A)
2605	CakSNP2605	*Kabuli*	Ca_Kabuli_Chr02	18671672	(T/A)
2606	CakSNP2606	*Kabuli*	Ca_Kabuli_Chr02	18671666	(C/T)
2607	CakSNP2607	*Kabuli*	Ca_Kabuli_Chr02	18671637	(G/A)
2608	CakSNP2608	*Kabuli*	Ca_Kabuli_Chr02	18671618	(T/G)
2609	CakSNP2609	*Kabuli*	Ca_Kabuli_Chr02	18671723	(C/G)
2610	CakSNP2610	*Kabuli*	Ca_Kabuli_Chr02	18671746	(C/T)
2611	CakSNP2611	*Kabuli*	Ca_Kabuli_Chr02	18671756	(T/A)

S.N.	SNP IDs	Cultivars	Chromosomes/scaffolds	Physical positions (bp)	SNPs
2612	CakSNP2612	*Kabuli*	Ca_Kabuli_Chr02	18671765	(C/A)
2613	CakSNP2613	*Kabuli*	Ca_Kabuli_Chr02	18671767	(G/C)
2614	CakSNP2614	*Kabuli*	Ca_Kabuli_Chr02	18671785	(G/A)
2615	CakSNP2615	*Kabuli*	Ca_Kabuli_Chr02	18671824	(C/T)
2616	CakSNP2616	*Kabuli*	Ca_Kabuli_Chr02	20392224	(A/G)
2617	CakSNP2617	*Kabuli*	Ca_Kabuli_Chr02	20762187	(A/G)
2618	CakSNP2618	*Kabuli*	Ca_Kabuli_Chr02	20762205	(A/T)
2619	CakSNP2619	*Kabuli*	Ca_Kabuli_Chr02	21000509	(C/T)
2620	CakSNP2620	*Kabuli*	Ca_Kabuli_Chr02	21000466	(C/T)
2621	CakSNP2621	*Kabuli*	Ca_Kabuli_Chr02	21000463	(C/T)
2622	CakSNP2622	*Kabuli*	Ca_Kabuli_Chr02	21849874	(A/T)
2623	CakSNP2623	*Kabuli*	Ca_Kabuli_Chr02	21906952	(A/G)
2624	CakSNP2624	*Kabuli*	Ca_Kabuli_Chr02	21965365	(T/C)
2625	CakSNP2625	*Kabuli*	Ca_Kabuli_Chr02	22158593	(C/T)
2626	CakSNP2626	*Kabuli*	Ca_Kabuli_Chr02	22158602	(C/T)
2627	CakSNP2627	*Kabuli*	Ca_Kabuli_Chr02	22190700	(C/T)
2628	CakSNP2628	*Kabuli*	Ca_Kabuli_Chr02	22190743	(C/T)
2629	CakSNP2629	*Kabuli*	Ca_Kabuli_Chr02	22190748	(G/T)
2630	CakSNP2630	*Kabuli*	Ca_Kabuli_Chr02	22190751	(C/T)
2631	CakSNP2631	*Kabuli*	Ca_Kabuli_Chr02	22190756	(C/A)
2632	CakSNP2632	*Kabuli*	Ca_Kabuli_Chr02	22190753	(C/T)
2633	CakSNP2633	*Kabuli*	Ca_Kabuli_Chr02	22190790	(G/A)
2634	CakSNP2634	*Kabuli*	Ca_Kabuli_Chr02	22190815	(C/T)
2635	CakSNP2635	*Kabuli*	Ca_Kabuli_Chr02	22205024	(G/C)
2636	CakSNP2636	*Kabuli*	Ca_Kabuli_Chr02	22347405	(C/T)
2637	CakSNP2637	*Kabuli*	Ca_Kabuli_Chr02	22415930	(C/T)
2638	CakSNP2638	*Kabuli*	Ca_Kabuli_Chr02	22416899	(A/C)
2639	CakSNP2639	*Kabuli*	Ca_Kabuli_Chr02	22419501	(C/G)
2640	CakSNP2640	*Kabuli*	Ca_Kabuli_Chr02	22419513	(T/C)
2641	CakSNP2641	*Kabuli*	Ca_Kabuli_Chr02	22419514	(C/T)
2642	CakSNP2642	*Kabuli*	Ca_Kabuli_Chr02	22419529	(T/C)
2643	CakSNP2643	*Kabuli*	Ca_Kabuli_Chr02	22419625	(C/T)
2644	CakSNP2644	*Kabuli*	Ca_Kabuli_Chr02	22419633	(C/T)
2645	CakSNP2645	*Kabuli*	Ca_Kabuli_Chr02	22419634	(G/C)
2646	CakSNP2646	*Kabuli*	Ca_Kabuli_Chr02	22419679	(G/A)
2647	CakSNP2647	*Kabuli*	Ca_Kabuli_Chr02	22457234	(G/A)
2648	CakSNP2648	*Kabuli*	Ca_Kabuli_Chr02	22457221	(T/A)
2649	CakSNP2649	*Kabuli*	Ca_Kabuli_Chr02	22457215	(G/A)
2650	CakSNP2650	*Kabuli*	Ca_Kabuli_Chr02	22457181	(G/T)

S.N.	SNP IDs	Cultivars	Chromosomes/scaffolds	Physical positions (bp)	SNPs
2651	CakSNP2651	*Kabuli*	Ca_Kabuli_Chr02	22457233	(C/T)
2652	CakSNP2652	*Kabuli*	Ca_Kabuli_Chr02	22457255	(C/T)
2653	CakSNP2653	*Kabuli*	Ca_Kabuli_Chr02	22457263	(G/A)
2654	CakSNP2654	*Kabuli*	Ca_Kabuli_Chr02	22457257	(G/A)
2655	CakSNP2655	*Kabuli*	Ca_Kabuli_Chr02	22483072	(C/T)
2656	CakSNP2656	*Kabuli*	Ca_Kabuli_Chr02	22483067	(G/A)
2657	CakSNP2657	*Kabuli*	Ca_Kabuli_Chr02	22483052	(C/T)
2658	CakSNP2658	*Kabuli*	Ca_Kabuli_Chr02	22483019	(A/T)
2659	CakSNP2659	*Kabuli*	Ca_Kabuli_Chr02	22703091	(A/C)
2660	CakSNP2660	*Kabuli*	Ca_Kabuli_Chr02	22820616	(C/G)
2661	CakSNP2661	*Kabuli*	Ca_Kabuli_Chr02	22820588	(A/C)
2662	CakSNP2662	*Kabuli*	Ca_Kabuli_Chr02	22897694	(G/A)
2663	CakSNP2663	*Kabuli*	Ca_Kabuli_Chr02	22897647	(T/C)
2664	CakSNP2664	*Kabuli*	Ca_Kabuli_Chr02	23123734	(C/T)
2665	CakSNP2665	*Kabuli*	Ca_Kabuli_Chr02	23141564	(C/T)
2666	CakSNP2666	*Kabuli*	Ca_Kabuli_Chr02	23181353	(C/T)
2667	CakSNP2667	*Kabuli*	Ca_Kabuli_Chr02	23247801	(C/T)
2668	CakSNP2668	*Kabuli*	Ca_Kabuli_Chr02	23282374	(T/C)
2669	CakSNP2669	*Kabuli*	Ca_Kabuli_Chr02	23297412	(G/A)
2670	CakSNP2670	*Kabuli*	Ca_Kabuli_Chr02	23329325	(G/T)
2671	CakSNP2671	*Kabuli*	Ca_Kabuli_Chr02	23329424	(C/T)
2672	CakSNP2672	*Kabuli*	Ca_Kabuli_Chr02	23329440	(G/A)
2673	CakSNP2673	*Kabuli*	Ca_Kabuli_Chr02	23417518	(T/G)
2674	CakSNP2674	*Kabuli*	Ca_Kabuli_Chr02	23435939	(A/G)
2675	CakSNP2675	*Kabuli*	Ca_Kabuli_Chr02	23439966	(T/C)
2676	CakSNP2676	*Kabuli*	Ca_Kabuli_Chr02	23441740	(A/G)
2677	CakSNP2677	*Kabuli*	Ca_Kabuli_Chr02	23910254	(T/C)
2678	CakSNP2678	*Kabuli*	Ca_Kabuli_Chr02	23961003	(G/A)
2679	CakSNP2679	*Kabuli*	Ca_Kabuli_Chr02	24048087	(A/C)
2680	CakSNP2680	*Kabuli*	Ca_Kabuli_Chr02	24098243	(T/C)
2681	CakSNP2681	*Kabuli*	Ca_Kabuli_Chr02	24101371	(C/T)
2682	CakSNP2682	*Kabuli*	Ca_Kabuli_Chr02	24164335	(A/T)
2683	CakSNP2683	*Kabuli*	Ca_Kabuli_Chr02	24164840	(T/A)
2684	CakSNP2684	*Kabuli*	Ca_Kabuli_Chr02	24164903	(T/C)
2685	CakSNP2685	*Kabuli*	Ca_Kabuli_Chr02	24164900	(A/T)
2686	CakSNP2686	*Kabuli*	Ca_Kabuli_Chr02	24221498	(G/T)
2687	CakSNP2687	*Kabuli*	Ca_Kabuli_Chr02	24291665	(T/C)
2688	CakSNP2688	*Kabuli*	Ca_Kabuli_Chr02	24353303	(A/G)
2689	CakSNP2689	*Kabuli*	Ca_Kabuli_Chr02	24353243	(A/T)

S.N.	SNP IDs	Cultivars	Chromosomes/scaffolds	Physical positions (bp)	SNPs
2690	CakSNP2690	*Kabuli*	Ca_Kabuli_Chr02	24511053	(G/A)
2691	CakSNP2691	*Kabuli*	Ca_Kabuli_Chr02	24511114	(G/A)
2692	CakSNP2692	*Kabuli*	Ca_Kabuli_Chr02	24511121	(G/A)
2693	CakSNP2693	*Kabuli*	Ca_Kabuli_Chr02	24511119	(T/G)
2694	CakSNP2694	*Kabuli*	Ca_Kabuli_Chr02	24511116	(C/A)
2695	CakSNP2695	*Kabuli*	Ca_Kabuli_Chr02	24586360	(T/C)
2696	CakSNP2696	*Kabuli*	Ca_Kabuli_Chr02	24709014	(C/G)
2697	CakSNP2697	*Kabuli*	Ca_Kabuli_Chr02	24709103	(G/A)
2698	CakSNP2698	*Kabuli*	Ca_Kabuli_Chr02	24709139	(A/G)
2699	CakSNP2699	*Kabuli*	Ca_Kabuli_Chr02	24709159	(T/A)
2700	CakSNP2700	*Kabuli*	Ca_Kabuli_Chr02	24709295	(G/A)
2701	CakSNP2701	*Kabuli*	Ca_Kabuli_Chr02	24741195	(C/T)
2702	CakSNP2702	*Kabuli*	Ca_Kabuli_Chr02	24744726	(A/T)
2703	CakSNP2703	*Kabuli*	Ca_Kabuli_Chr02	24817957	(G/A)
2704	CakSNP2704	*Kabuli*	Ca_Kabuli_Chr02	24817945	(C/T)
2705	CakSNP2705	*Kabuli*	Ca_Kabuli_Chr02	24817940	(G/A)
2706	CakSNP2706	*Kabuli*	Ca_Kabuli_Chr02	24817934	(A/G)
2707	CakSNP2707	*Kabuli*	Ca_Kabuli_Chr02	24817921	(G/A)
2708	CakSNP2708	*Kabuli*	Ca_Kabuli_Chr02	24876355	(T/G)
2709	CakSNP2709	*Kabuli*	Ca_Kabuli_Chr02	24911080	(T/C)
2710	CakSNP2710	*Kabuli*	Ca_Kabuli_Chr02	24911154	(G/C)
2711	CakSNP2711	*Kabuli*	Ca_Kabuli_Chr02	25022971	(A/G)
2712	CakSNP2712	*Kabuli*	Ca_Kabuli_Chr02	25104319	(G/T)
2713	CakSNP2713	*Kabuli*	Ca_Kabuli_Chr02	25104291	(A/T)
2714	CakSNP2714	*Kabuli*	Ca_Kabuli_Chr02	25108249	(T/C)
2715	CakSNP2715	*Kabuli*	Ca_Kabuli_Chr02	25126798	(C/T)
2716	CakSNP2716	*Kabuli*	Ca_Kabuli_Chr02	25126804	(A/G)
2717	CakSNP2717	*Kabuli*	Ca_Kabuli_Chr02	25126837	(G/T)
2718	CakSNP2718	*Kabuli*	Ca_Kabuli_Chr02	25126910	(G/C)
2719	CakSNP2719	*Kabuli*	Ca_Kabuli_Chr02	25163062	(C/G)
2720	CakSNP2720	*Kabuli*	Ca_Kabuli_Chr02	25194583	(G/A)
2721	CakSNP2721	*Kabuli*	Ca_Kabuli_Chr02	25317391	(A/G)
2722	CakSNP2722	*Kabuli*	Ca_Kabuli_Chr02	25321993	(C/A)
2723	CakSNP2723	*Kabuli*	Ca_Kabuli_Chr02	25493973	(T/G)
2724	CakSNP2724	*Kabuli*	Ca_Kabuli_Chr02	25507637	(C/G)
2725	CakSNP2725	*Kabuli*	Ca_Kabuli_Chr02	25507681	(A/G)
2726	CakSNP2726	*Kabuli*	Ca_Kabuli_Chr02	25508579	(C/A)
2727	CakSNP2727	*Kabuli*	Ca_Kabuli_Chr02	25508679	(A/G)
2728	CakSNP2728	*Kabuli*	Ca_Kabuli_Chr02	25508660	(G/C)

S.N.	SNP IDs	Cultivars	Chromosomes/scaffolds	Physical positions (bp)	SNPs
2729	CakSNP2729	*Kabuli*	Ca_Kabuli_Chr02	25508920	(G/A)
2730	CakSNP2730	*Kabuli*	Ca_Kabuli_Chr02	25508969	(T/C)
2731	CakSNP2731	*Kabuli*	Ca_Kabuli_Chr02	25508964	(A/C)
2732	CakSNP2732	*Kabuli*	Ca_Kabuli_Chr02	25508963	(A/G)
2733	CakSNP2733	*Kabuli*	Ca_Kabuli_Chr02	25508935	(A/T)
2734	CakSNP2734	*Kabuli*	Ca_Kabuli_Chr02	25509087	(C/A)
2735	CakSNP2735	*Kabuli*	Ca_Kabuli_Chr02	25509093	(C/G)
2736	CakSNP2736	*Kabuli*	Ca_Kabuli_Chr02	25509095	(G/A)
2737	CakSNP2737	*Kabuli*	Ca_Kabuli_Chr02	25509203	(T/C)
2738	CakSNP2738	*Kabuli*	Ca_Kabuli_Chr02	25509202	(T/A)
2739	CakSNP2739	*Kabuli*	Ca_Kabuli_Chr02	25667417	(G/C)
2740	CakSNP2740	*Kabuli*	Ca_Kabuli_Chr02	25667427	(T/C)
2741	CakSNP2741	*Kabuli*	Ca_Kabuli_Chr02	25788124	(T/A)
2742	CakSNP2742	*Kabuli*	Ca_Kabuli_Chr02	25788139	(T/C)
2743	CakSNP2743	*Kabuli*	Ca_Kabuli_Chr02	25880318	(T/C)
2744	CakSNP2744	*Kabuli*	Ca_Kabuli_Chr02	25898931	(T/C)
2745	CakSNP2745	*Kabuli*	Ca_Kabuli_Chr02	25930490	(G/A)
2746	CakSNP2746	*Kabuli*	Ca_Kabuli_Chr02	26051667	(T/G)
2747	CakSNP2747	*Kabuli*	Ca_Kabuli_Chr02	26092881	(C/G)
2748	CakSNP2748	*Kabuli*	Ca_Kabuli_Chr02	26092913	(C/A)
2749	CakSNP2749	*Kabuli*	Ca_Kabuli_Chr02	26092928	(T/G)
2750	CakSNP2750	*Kabuli*	Ca_Kabuli_Chr02	26092991	(T/C)
2751	CakSNP2751	*Kabuli*	Ca_Kabuli_Chr02	26121495	(A/C)
2752	CakSNP2752	*Kabuli*	Ca_Kabuli_Chr02	26121491	(T/G)
2753	CakSNP2753	*Kabuli*	Ca_Kabuli_Chr02	26417311	(T/A)
2754	CakSNP2754	*Kabuli*	Ca_Kabuli_Chr02	26417281	(A/G)
2755	CakSNP2755	*Kabuli*	Ca_Kabuli_Chr02	26417256	(T/C)
2756	CakSNP2756	*Kabuli*	Ca_Kabuli_Chr02	26431061	(T/C)
2757	CakSNP2757	*Kabuli*	Ca_Kabuli_Chr02	26431058	(G/C)
2758	CakSNP2758	*Kabuli*	Ca_Kabuli_Chr02	26431052	(T/C)
2759	CakSNP2759	*Kabuli*	Ca_Kabuli_Chr02	26461867	(C/G)
2760	CakSNP2760	*Kabuli*	Ca_Kabuli_Chr02	26461986	(A/C)
2761	CakSNP2761	*Kabuli*	Ca_Kabuli_Chr02	26461960	(T/A)
2762	CakSNP2762	*Kabuli*	Ca_Kabuli_Chr02	26486049	(G/A)
2763	CakSNP2763	*Kabuli*	Ca_Kabuli_Chr02	26486065	(C/T)
2764	CakSNP2764	*Kabuli*	Ca_Kabuli_Chr02	26486109	(T/C)
2765	CakSNP2765	*Kabuli*	Ca_Kabuli_Chr02	26502153	(T/C)
2766	CakSNP2766	*Kabuli*	Ca_Kabuli_Chr02	26503262	(C/A)
2767	CakSNP2767	*Kabuli*	Ca_Kabuli_Chr02	26567088	(T/C)

S.N.	SNP IDs	Cultivars	Chromosomes/scaffolds	Physical positions (bp)	SNPs
2768	CakSNP2768	*Kabuli*	Ca_Kabuli_Chr02	26585842	(T/A)
2769	CakSNP2769	*Kabuli*	Ca_Kabuli_Chr02	26601337	(C/A)
2770	CakSNP2770	*Kabuli*	Ca_Kabuli_Chr02	26601331	(G/C)
2771	CakSNP2771	*Kabuli*	Ca_Kabuli_Chr02	26706849	(A/G)
2772	CakSNP2772	*Kabuli*	Ca_Kabuli_Chr02	27057582	(C/T)
2773	CakSNP2773	*Kabuli*	Ca_Kabuli_Chr02	27063749	(C/A)
2774	CakSNP2774	*Kabuli*	Ca_Kabuli_Chr02	27134574	(A/G)
2775	CakSNP2775	*Kabuli*	Ca_Kabuli_Chr02	27134560	(G/T)
2776	CakSNP2776	*Kabuli*	Ca_Kabuli_Chr02	27182855	(T/C)
2777	CakSNP2777	*Kabuli*	Ca_Kabuli_Chr02	27317272	(A/T)
2778	CakSNP2778	*Kabuli*	Ca_Kabuli_Chr02	27374064	(A/T)
2779	CakSNP2779	*Kabuli*	Ca_Kabuli_Chr02	27374094	(C/A)
2780	CakSNP2780	*Kabuli*	Ca_Kabuli_Chr02	27377809	(T/G)
2781	CakSNP2781	*Kabuli*	Ca_Kabuli_Chr02	27623258	(A/G)
2782	CakSNP2782	*Kabuli*	Ca_Kabuli_Chr02	27703356	(G/A)
2783	CakSNP2783	*Kabuli*	Ca_Kabuli_Chr02	27724425	(G/T)
2784	CakSNP2784	*Kabuli*	Ca_Kabuli_Chr02	27757490	(T/C)
2785	CakSNP2785	*Kabuli*	Ca_Kabuli_Chr02	27757824	(A/G)
2786	CakSNP2786	*Kabuli*	Ca_Kabuli_Chr02	27757804	(A/G)
2787	CakSNP2787	*Kabuli*	Ca_Kabuli_Chr02	27757795	(T/G)
2788	CakSNP2788	*Kabuli*	Ca_Kabuli_Chr02	27758057	(C/T)
2789	CakSNP2789	*Kabuli*	Ca_Kabuli_Chr02	27758058	(A/G)
2790	CakSNP2790	*Kabuli*	Ca_Kabuli_Chr02	27758065	(C/G)
2791	CakSNP2791	*Kabuli*	Ca_Kabuli_Chr02	27758093	(C/T)
2792	CakSNP2792	*Kabuli*	Ca_Kabuli_Chr02	27766561	(T/A)
2793	CakSNP2793	*Kabuli*	Ca_Kabuli_Chr02	27766623	(T/C)
2794	CakSNP2794	*Kabuli*	Ca_Kabuli_Chr02	27766605	(A/C)
2795	CakSNP2795	*Kabuli*	Ca_Kabuli_Chr02	27766594	(C/A)
2796	CakSNP2796	*Kabuli*	Ca_Kabuli_Chr02	27807994	(A/G)
2797	CakSNP2797	*Kabuli*	Ca_Kabuli_Chr02	27808070	(G/A)
2798	CakSNP2798	*Kabuli*	Ca_Kabuli_Chr02	27824962	(G/A)
2799	CakSNP2799	*Kabuli*	Ca_Kabuli_Chr02	27831340	(A/C)
2800	CakSNP2800	*Kabuli*	Ca_Kabuli_Chr02	27831398	(A/G)
2801	CakSNP2801	*Kabuli*	Ca_Kabuli_Chr02	27835871	(G/A)
2802	CakSNP2802	*Kabuli*	Ca_Kabuli_Chr02	27835850	(G/A)
2803	CakSNP2803	*Kabuli*	Ca_Kabuli_Chr02	27835843	(C/A)
2804	CakSNP2804	*Kabuli*	Ca_Kabuli_Chr02	27835837	(C/T)
2805	CakSNP2805	*Kabuli*	Ca_Kabuli_Chr02	27835832	(C/T)
2806	CakSNP2806	*Kabuli*	Ca_Kabuli_Chr02	27847670	(A/C)

S.N.	SNP IDs	Cultivars	Chromosomes/scaffolds	Physical positions (bp)	SNPs
2807	CakSNP2807	*Kabuli*	Ca_Kabuli_Chr02	28017163	(A/G)
2808	CakSNP2808	*Kabuli*	Ca_Kabuli_Chr02	28018158	(T/C)
2809	CakSNP2809	*Kabuli*	Ca_Kabuli_Chr02	28060867	(T/G)
2810	CakSNP2810	*Kabuli*	Ca_Kabuli_Chr02	28060850	(A/G)
2811	CakSNP2811	*Kabuli*	Ca_Kabuli_Chr02	28060879	(C/T)
2812	CakSNP2812	*Kabuli*	Ca_Kabuli_Chr02	28060896	(T/C)
2813	CakSNP2813	*Kabuli*	Ca_Kabuli_Chr02	28060921	(C/T)
2814	CakSNP2814	*Kabuli*	Ca_Kabuli_Chr02	28060922	(A/G)
2815	CakSNP2815	*Kabuli*	Ca_Kabuli_Chr02	28060928	(C/T)
2816	CakSNP2816	*Kabuli*	Ca_Kabuli_Chr02	28060930	(T/G)
2817	CakSNP2817	*Kabuli*	Ca_Kabuli_Chr02	28060940	(C/T)
2818	CakSNP2818	*Kabuli*	Ca_Kabuli_Chr02	28061049	(G/T)
2819	CakSNP2819	*Kabuli*	Ca_Kabuli_Chr02	28076664	(C/T)
2820	CakSNP2820	*Kabuli*	Ca_Kabuli_Chr02	28076834	(C/T)
2821	CakSNP2821	*Kabuli*	Ca_Kabuli_Chr02	28076851	(G/A)
2822	CakSNP2822	*Kabuli*	Ca_Kabuli_Chr02	28084910	(C/T)
2823	CakSNP2823	*Kabuli*	Ca_Kabuli_Chr02	28084913	(C/A)
2824	CakSNP2824	*Kabuli*	Ca_Kabuli_Chr02	28084953	(C/T)
2825	CakSNP2825	*Kabuli*	Ca_Kabuli_Chr02	28229031	(G/A)
2826	CakSNP2826	*Kabuli*	Ca_Kabuli_Chr02	28229046	(G/A)
2827	CakSNP2827	*Kabuli*	Ca_Kabuli_Chr02	28324577	(T/A)
2828	CakSNP2828	*Kabuli*	Ca_Kabuli_Chr02	28352533	(C/A)
2829	CakSNP2829	*Kabuli*	Ca_Kabuli_Chr02	28407316	(T/C)
2830	CakSNP2830	*Kabuli*	Ca_Kabuli_Chr02	28407324	(C/T)
2831	CakSNP2831	*Kabuli*	Ca_Kabuli_Chr02	28534235	(T/C)
2832	CakSNP2832	*Kabuli*	Ca_Kabuli_Chr02	28699149	(C/T)
2833	CakSNP2833	*Kabuli*	Ca_Kabuli_Chr02	28699178	(T/C)
2834	CakSNP2834	*Kabuli*	Ca_Kabuli_Chr02	28699185	(C/A)
2835	CakSNP2835	*Kabuli*	Ca_Kabuli_Chr02	28699219	(T/G)
2836	CakSNP2836	*Kabuli*	Ca_Kabuli_Chr02	28699229	(C/T)
2837	CakSNP2837	*Kabuli*	Ca_Kabuli_Chr02	28699276	(T/C)
2838	CakSNP2838	*Kabuli*	Ca_Kabuli_Chr02	28699383	(A/T)
2839	CakSNP2839	*Kabuli*	Ca_Kabuli_Chr02	28699403	(C/T)
2840	CakSNP2840	*Kabuli*	Ca_Kabuli_Chr02	28699414	(C/T)
2841	CakSNP2841	*Kabuli*	Ca_Kabuli_Chr02	28699579	(A/C)
2842	CakSNP2842	*Kabuli*	Ca_Kabuli_Chr02	28701724	(A/G)
2843	CakSNP2843	*Kabuli*	Ca_Kabuli_Chr02	28741764	(T/C)
2844	CakSNP2844	*Kabuli*	Ca_Kabuli_Chr02	28931402	(T/C)
2845	CakSNP2845	*Kabuli*	Ca_Kabuli_Chr02	28931340	(T/C)

S.N.	SNP IDs	Cultivars	Chromosomes/scaffolds	Physical positions (bp)	SNPs
2846	CakSNP2846	*Kabuli*	Ca_Kabuli_Chr02	28944712	(T/C)
2847	CakSNP2847	*Kabuli*	Ca_Kabuli_Chr02	28944707	(A/C)
2848	CakSNP2848	*Kabuli*	Ca_Kabuli_Chr02	28949900	(C/T)
2849	CakSNP2849	*Kabuli*	Ca_Kabuli_Chr02	28949909	(G/A)
2850	CakSNP2850	*Kabuli*	Ca_Kabuli_Chr02	28949911	(A/C)
2851	CakSNP2851	*Kabuli*	Ca_Kabuli_Chr02	28949919	(A/T)
2852	CakSNP2852	*Kabuli*	Ca_Kabuli_Chr02	28949954	(T/A)
2853	CakSNP2853	*Kabuli*	Ca_Kabuli_Chr02	28949948	(A/G)
2854	CakSNP2854	*Kabuli*	Ca_Kabuli_Chr02	28970621	(A/G)
2855	CakSNP2855	*Kabuli*	Ca_Kabuli_Chr02	28970618	(A/G)
2856	CakSNP2856	*Kabuli*	Ca_Kabuli_Chr02	29103993	(T/C)
2857	CakSNP2857	*Kabuli*	Ca_Kabuli_Chr02	29103970	(C/T)
2858	CakSNP2858	*Kabuli*	Ca_Kabuli_Chr02	29122581	(G/A)
2859	CakSNP2859	*Kabuli*	Ca_Kabuli_Chr02	29122589	(T/G)
2860	CakSNP2860	*Kabuli*	Ca_Kabuli_Chr02	29592666	(G/A)
2861	CakSNP2861	*Kabuli*	Ca_Kabuli_Chr02	29592694	(T/C)
2862	CakSNP2862	*Kabuli*	Ca_Kabuli_Chr02	29601458	(C/T)
2863	CakSNP2863	*Kabuli*	Ca_Kabuli_Chr02	29601463	(C/T)
2864	CakSNP2864	*Kabuli*	Ca_Kabuli_Chr02	29614370	(C/T)
2865	CakSNP2865	*Kabuli*	Ca_Kabuli_Chr02	29614375	(C/T)
2866	CakSNP2866	*Kabuli*	Ca_Kabuli_Chr02	29693107	(A/G)
2867	CakSNP2867	*Kabuli*	Ca_Kabuli_Chr02	29948356	(G/A)
2868	CakSNP2868	*Kabuli*	Ca_Kabuli_Chr02	29960049	(G/A)
2869	CakSNP2869	*Kabuli*	Ca_Kabuli_Chr02	29960054	(G/A)
2870	CakSNP2870	*Kabuli*	Ca_Kabuli_Chr02	29960130	(C/T)
2871	CakSNP2871	*Kabuli*	Ca_Kabuli_Chr02	30042255	(A/C)
2872	CakSNP2872	*Kabuli*	Ca_Kabuli_Chr02	30042309	(T/G)
2873	CakSNP2873	*Kabuli*	Ca_Kabuli_Chr02	30042302	(A/T)
2874	CakSNP2874	*Kabuli*	Ca_Kabuli_Chr02	30042297	(C/T)
2875	CakSNP2875	*Kabuli*	Ca_Kabuli_Chr02	30042267	(C/T)
2876	CakSNP2876	*Kabuli*	Ca_Kabuli_Chr02	30045338	(C/G)
2877	CakSNP2877	*Kabuli*	Ca_Kabuli_Chr02	30091313	(G/A)
2878	CakSNP2878	*Kabuli*	Ca_Kabuli_Chr02	30091311	(C/T)
2879	CakSNP2879	*Kabuli*	Ca_Kabuli_Chr02	30091308	(A/T)
2880	CakSNP2880	*Kabuli*	Ca_Kabuli_Chr02	30091307	(C/T)
2881	CakSNP2881	*Kabuli*	Ca_Kabuli_Chr02	30091295	(T/A)
2882	CakSNP2882	*Kabuli*	Ca_Kabuli_Chr02	30091290	(T/C)
2883	CakSNP2883	*Kabuli*	Ca_Kabuli_Chr02	30091286	(G/A)
2884	CakSNP2884	*Kabuli*	Ca_Kabuli_Chr02	30091284	(A/C)

S.N.	SNP IDs	Cultivars	Chromosomes/scaffolds	Physical positions (bp)	SNPs
2885	CakSNP2885	*Kabuli*	Ca_Kabuli_Chr02	30091280	(C/G)
2886	CakSNP2886	*Kabuli*	Ca_Kabuli_Chr02	30133162	(G/A)
2887	CakSNP2887	*Kabuli*	Ca_Kabuli_Chr02	30133189	(G/A)
2888	CakSNP2888	*Kabuli*	Ca_Kabuli_Chr02	30133215	(G/T)
2889	CakSNP2889	*Kabuli*	Ca_Kabuli_Chr02	30133221	(A/T)
2890	CakSNP2890	*Kabuli*	Ca_Kabuli_Chr02	30335160	(T/C)
2891	CakSNP2891	*Kabuli*	Ca_Kabuli_Chr02	30335212	(T/A)
2892	CakSNP2892	*Kabuli*	Ca_Kabuli_Chr02	30335194	(A/G)
2893	CakSNP2893	*Kabuli*	Ca_Kabuli_Chr02	30363957	(C/A)
2894	CakSNP2894	*Kabuli*	Ca_Kabuli_Chr02	30364073	(T/A)
2895	CakSNP2895	*Kabuli*	Ca_Kabuli_Chr02	30364042	(A/G)
2896	CakSNP2896	*Kabuli*	Ca_Kabuli_Chr02	30364025	(T/C)
2897	CakSNP2897	*Kabuli*	Ca_Kabuli_Chr02	30370013	(C/A)
2898	CakSNP2898	*Kabuli*	Ca_Kabuli_Chr02	30370003	(A/C)
2899	CakSNP2899	*Kabuli*	Ca_Kabuli_Chr02	30370411	(T/C)
2900	CakSNP2900	*Kabuli*	Ca_Kabuli_Chr02	30467552	(C/T)
2901	CakSNP2901	*Kabuli*	Ca_Kabuli_Chr02	30467551	(A/T)
2902	CakSNP2902	*Kabuli*	Ca_Kabuli_Chr02	30508454	(T/C)
2903	CakSNP2903	*Kabuli*	Ca_Kabuli_Chr02	30530642	(A/T)
2904	CakSNP2904	*Kabuli*	Ca_Kabuli_Chr02	30537667	(A/T)
2905	CakSNP2905	*Kabuli*	Ca_Kabuli_Chr02	30537673	(A/G)
2906	CakSNP2906	*Kabuli*	Ca_Kabuli_Chr02	30537687	(T/C)
2907	CakSNP2907	*Kabuli*	Ca_Kabuli_Chr02	30537701	(T/A)
2908	CakSNP2908	*Kabuli*	Ca_Kabuli_Chr02	30537953	(T/C)
2909	CakSNP2909	*Kabuli*	Ca_Kabuli_Chr02	30614086	(C/T)
2910	CakSNP2910	*Kabuli*	Ca_Kabuli_Chr02	30686920	(C/T)
2911	CakSNP2911	*Kabuli*	Ca_Kabuli_Chr02	30702121	(T/C)
2912	CakSNP2912	*Kabuli*	Ca_Kabuli_Chr02	30702090	(G/A)
2913	CakSNP2913	*Kabuli*	Ca_Kabuli_Chr02	30867547	(G/A)
2914	CakSNP2914	*Kabuli*	Ca_Kabuli_Chr02	30880148	(A/G)
2915	CakSNP2915	*Kabuli*	Ca_Kabuli_Chr02	30880243	(G/C)
2916	CakSNP2916	*Kabuli*	Ca_Kabuli_Chr02	30880246	(G/A)
2917	CakSNP2917	*Kabuli*	Ca_Kabuli_Chr02	30880303	(T/A)
2918	CakSNP2918	*Kabuli*	Ca_Kabuli_Chr02	30880302	(T/C)
2919	CakSNP2919	*Kabuli*	Ca_Kabuli_Chr02	30880282	(A/G)
2920	CakSNP2920	*Kabuli*	Ca_Kabuli_Chr02	30921092	(C/A)
2921	CakSNP2921	*Kabuli*	Ca_Kabuli_Chr02	30921758	(C/T)
2922	CakSNP2922	*Kabuli*	Ca_Kabuli_Chr02	30921888	(T/C)
2923	CakSNP2923	*Kabuli*	Ca_Kabuli_Chr02	30921882	(C/G)

S.N.	SNP IDs	Cultivars	Chromosomes/scaffolds	Physical positions (bp)	SNPs
2924	CakSNP2924	*Kabuli*	Ca_Kabuli_Chr02	30921917	(C/A)
2925	CakSNP2925	*Kabuli*	Ca_Kabuli_Chr02	30921923	(A/G)
2926	CakSNP2926	*Kabuli*	Ca_Kabuli_Chr02	30954317	(G/A)
2927	CakSNP2927	*Kabuli*	Ca_Kabuli_Chr02	30954305	(T/G)
2928	CakSNP2928	*Kabuli*	Ca_Kabuli_Chr02	30977572	(A/G)
2929	CakSNP2929	*Kabuli*	Ca_Kabuli_Chr02	30977528	(C/T)
2930	CakSNP2930	*Kabuli*	Ca_Kabuli_Chr02	30977522	(A/T)
2931	CakSNP2931	*Kabuli*	Ca_Kabuli_Chr02	30977508	(A/T)
2932	CakSNP2932	*Kabuli*	Ca_Kabuli_Chr02	30997769	(T/A)
2933	CakSNP2933	*Kabuli*	Ca_Kabuli_Chr02	30997780	(A/C)
2934	CakSNP2934	*Kabuli*	Ca_Kabuli_Chr02	30997784	(T/A)
2935	CakSNP2935	*Kabuli*	Ca_Kabuli_Chr02	31005847	(T/G)
2936	CakSNP2936	*Kabuli*	Ca_Kabuli_Chr02	31005845	(C/T)
2937	CakSNP2937	*Kabuli*	Ca_Kabuli_Chr02	31014295	(A/T)
2938	CakSNP2938	*Kabuli*	Ca_Kabuli_Chr02	31055083	(C/T)
2939	CakSNP2939	*Kabuli*	Ca_Kabuli_Chr02	31055007	(G/A)
2940	CakSNP2940	*Kabuli*	Ca_Kabuli_Chr02	31088681	(G/T)
2941	CakSNP2941	*Kabuli*	Ca_Kabuli_Chr02	31088676	(G/C)
2942	CakSNP2942	*Kabuli*	Ca_Kabuli_Chr02	31088670	(C/T)
2943	CakSNP2943	*Kabuli*	Ca_Kabuli_Chr02	31088648	(A/G)
2944	CakSNP2944	*Kabuli*	Ca_Kabuli_Chr02	31088645	(A/C)
2945	CakSNP2945	*Kabuli*	Ca_Kabuli_Chr02	31088778	(A/T)
2946	CakSNP2946	*Kabuli*	Ca_Kabuli_Chr02	31088800	(T/C)
2947	CakSNP2947	*Kabuli*	Ca_Kabuli_Chr02	31088882	(A/G)
2948	CakSNP2948	*Kabuli*	Ca_Kabuli_Chr02	31109311	(G/T)
2949	CakSNP2949	*Kabuli*	Ca_Kabuli_Chr02	31109358	(C/T)
2950	CakSNP2950	*Kabuli*	Ca_Kabuli_Chr02	31109481	(G/A)
2951	CakSNP2951	*Kabuli*	Ca_Kabuli_Chr02	31109441	(C/G)
2952	CakSNP2952	*Kabuli*	Ca_Kabuli_Chr02	31109435	(G/A)
2953	CakSNP2953	*Kabuli*	Ca_Kabuli_Chr02	31109427	(C/T)
2954	CakSNP2954	*Kabuli*	Ca_Kabuli_Chr02	31141019	(G/A)
2955	CakSNP2955	*Kabuli*	Ca_Kabuli_Chr02	31167603	(G/A)
2956	CakSNP2956	*Kabuli*	Ca_Kabuli_Chr02	31167594	(A/G)
2957	CakSNP2957	*Kabuli*	Ca_Kabuli_Chr02	31188355	(A/G)
2958	CakSNP2958	*Kabuli*	Ca_Kabuli_Chr02	31201872	(T/C)
2959	CakSNP2959	*Kabuli*	Ca_Kabuli_Chr02	31201859	(T/A)
2960	CakSNP2960	*Kabuli*	Ca_Kabuli_Chr02	31201795	(A/G)
2961	CakSNP2961	*Kabuli*	Ca_Kabuli_Chr02	31203154	(G/A)
2962	CakSNP2962	*Kabuli*	Ca_Kabuli_Chr02	31205531	(C/T)

S.N.	SNP IDs	Cultivars	Chromosomes/scaffolds	Physical positions (bp)	SNPs
2963	CakSNP2963	*Kabuli*	Ca_Kabuli_Chr02	31427861	(A/G)
2964	CakSNP2964	*Kabuli*	Ca_Kabuli_Chr02	31427902	(G/C)
2965	CakSNP2965	*Kabuli*	Ca_Kabuli_Chr02	31427915	(A/G)
2966	CakSNP2966	*Kabuli*	Ca_Kabuli_Chr02	31428024	(T/G)
2967	CakSNP2967	*Kabuli*	Ca_Kabuli_Chr02	31428132	(G/A)
2968	CakSNP2968	*Kabuli*	Ca_Kabuli_Chr02	31428405	(T/C)
2969	CakSNP2969	*Kabuli*	Ca_Kabuli_Chr02	31428429	(T/G)
2970	CakSNP2970	*Kabuli*	Ca_Kabuli_Chr02	31534265	(A/C)
2971	CakSNP2971	*Kabuli*	Ca_Kabuli_Chr02	31534283	(A/G)
2972	CakSNP2972	*Kabuli*	Ca_Kabuli_Chr02	31534455	(T/C)
2973	CakSNP2973	*Kabuli*	Ca_Kabuli_Chr02	31686714	(C/T)
2974	CakSNP2974	*Kabuli*	Ca_Kabuli_Chr02	31686716	(C/T)
2975	CakSNP2975	*Kabuli*	Ca_Kabuli_Chr02	31686742	(A/C)
2976	CakSNP2976	*Kabuli*	Ca_Kabuli_Chr02	31686751	(A/T)
2977	CakSNP2977	*Kabuli*	Ca_Kabuli_Chr02	31686758	(C/A)
2978	CakSNP2978	*Kabuli*	Ca_Kabuli_Chr02	31686761	(A/C)
2979	CakSNP2979	*Kabuli*	Ca_Kabuli_Chr02	31686767	(C/T)
2980	CakSNP2980	*Kabuli*	Ca_Kabuli_Chr02	31686779	(G/A)
2981	CakSNP2981	*Kabuli*	Ca_Kabuli_Chr02	31686820	(T/C)
2982	CakSNP2982	*Kabuli*	Ca_Kabuli_Chr02	31756777	(G/T)
2983	CakSNP2983	*Kabuli*	Ca_Kabuli_Chr02	31847033	(A/C)
2984	CakSNP2984	*Kabuli*	Ca_Kabuli_Chr02	31881918	(G/T)
2985	CakSNP2985	*Kabuli*	Ca_Kabuli_Chr02	31885934	(A/G)
2986	CakSNP2986	*Kabuli*	Ca_Kabuli_Chr02	31908694	(G/A)
2987	CakSNP2987	*Kabuli*	Ca_Kabuli_Chr02	31908742	(G/A)
2988	CakSNP2988	*Kabuli*	Ca_Kabuli_Chr02	31908803	(C/T)
2989	CakSNP2989	*Kabuli*	Ca_Kabuli_Chr02	31908787	(G/A)
2990	CakSNP2990	*Kabuli*	Ca_Kabuli_Chr02	31908845	(T/A)
2991	CakSNP2991	*Kabuli*	Ca_Kabuli_Chr02	31908857	(C/T)
2992	CakSNP2992	*Kabuli*	Ca_Kabuli_Chr02	31941463	(T/C)
2993	CakSNP2993	*Kabuli*	Ca_Kabuli_Chr02	31941554	(A/T)
2994	CakSNP2994	*Kabuli*	Ca_Kabuli_Chr02	31975269	(C/T)
2995	CakSNP2995	*Kabuli*	Ca_Kabuli_Chr02	32003536	(G/T)
2996	CakSNP2996	*Kabuli*	Ca_Kabuli_Chr02	32017361	(G/A)
2997	CakSNP2997	*Kabuli*	Ca_Kabuli_Chr02	32049523	(C/A)
2998	CakSNP2998	*Kabuli*	Ca_Kabuli_Chr02	32049525	(A/C)
2999	CakSNP2999	*Kabuli*	Ca_Kabuli_Chr02	32058591	(T/C)
3000	CakSNP3000	*Kabuli*	Ca_Kabuli_Chr02	32061053	(C/T)
3001	CakSNP3001	*Kabuli*	Ca_Kabuli_Chr02	32061054	(T/C)

S.N.	SNP IDs	Cultivars	Chromosomes/scaffolds	Physical positions (bp)	SNPs
3002	CakSNP3002	*Kabuli*	Ca_Kabuli_Chr02	32062010	(A/C)
3003	CakSNP3003	*Kabuli*	Ca_Kabuli_Chr02	32071107	(C/G)
3004	CakSNP3004	*Kabuli*	Ca_Kabuli_Chr02	32080002	(T/C)
3005	CakSNP3005	*Kabuli*	Ca_Kabuli_Chr02	32080007	(T/C)
3006	CakSNP3006	*Kabuli*	Ca_Kabuli_Chr02	32080010	(G/C)
3007	CakSNP3007	*Kabuli*	Ca_Kabuli_Chr02	32080016	(A/T)
3008	CakSNP3008	*Kabuli*	Ca_Kabuli_Chr02	32080022	(C/A)
3009	CakSNP3009	*Kabuli*	Ca_Kabuli_Chr02	32080037	(A/G)
3010	CakSNP3010	*Kabuli*	Ca_Kabuli_Chr02	32096192	(A/C)
3011	CakSNP3011	*Kabuli*	Ca_Kabuli_Chr02	32096186	(T/A)
3012	CakSNP3012	*Kabuli*	Ca_Kabuli_Chr02	32096180	(C/G)
3013	CakSNP3013	*Kabuli*	Ca_Kabuli_Chr02	32096177	(C/T)
3014	CakSNP3014	*Kabuli*	Ca_Kabuli_Chr02	32096172	(C/T)
3015	CakSNP3015	*Kabuli*	Ca_Kabuli_Chr02	32099757	(C/T)
3016	CakSNP3016	*Kabuli*	Ca_Kabuli_Chr02	32099907	(T/C)
3017	CakSNP3017	*Kabuli*	Ca_Kabuli_Chr02	32109805	(G/A)
3018	CakSNP3018	*Kabuli*	Ca_Kabuli_Chr02	32109811	(G/A)
3019	CakSNP3019	*Kabuli*	Ca_Kabuli_Chr02	32109813	(C/T)
3020	CakSNP3020	*Kabuli*	Ca_Kabuli_Chr02	32109821	(G/A)
3021	CakSNP3021	*Kabuli*	Ca_Kabuli_Chr02	32109827	(T/C)
3022	CakSNP3022	*Kabuli*	Ca_Kabuli_Chr02	32109859	(A/C)
3023	CakSNP3023	*Kabuli*	Ca_Kabuli_Chr02	32109904	(T/A)
3024	CakSNP3024	*Kabuli*	Ca_Kabuli_Chr02	32109895	(A/G)
3025	CakSNP3025	*Kabuli*	Ca_Kabuli_Chr02	32109892	(T/A)
3026	CakSNP3026	*Kabuli*	Ca_Kabuli_Chr02	32119094	(A/T)
3027	CakSNP3027	*Kabuli*	Ca_Kabuli_Chr02	32119268	(T/A)
3028	CakSNP3028	*Kabuli*	Ca_Kabuli_Chr02	32119274	(T/C)
3029	CakSNP3029	*Kabuli*	Ca_Kabuli_Chr02	32119331	(A/T)
3030	CakSNP3030	*Kabuli*	Ca_Kabuli_Chr02	32129272	(T/C)
3031	CakSNP3031	*Kabuli*	Ca_Kabuli_Chr02	32129395	(C/G)
3032	CakSNP3032	*Kabuli*	Ca_Kabuli_Chr02	32140939	(A/T)
3033	CakSNP3033	*Kabuli*	Ca_Kabuli_Chr02	32140948	(A/C)
3034	CakSNP3034	*Kabuli*	Ca_Kabuli_Chr02	32140987	(A/G)
3035	CakSNP3035	*Kabuli*	Ca_Kabuli_Chr02	32141047	(A/G)
3036	CakSNP3036	*Kabuli*	Ca_Kabuli_Chr02	32141038	(T/C)
3037	CakSNP3037	*Kabuli*	Ca_Kabuli_Chr02	32141019	(G/A)
3038	CakSNP3038	*Kabuli*	Ca_Kabuli_Chr02	32141013	(G/C)
3039	CakSNP3039	*Kabuli*	Ca_Kabuli_Chr02	32141002	(A/T)
3040	CakSNP3040	*Kabuli*	Ca_Kabuli_Chr02	32140998	(G/T)

S.N.	SNP IDs	Cultivars	Chromosomes/scaffolds	Physical positions (bp)	SNPs
3041	CakSNP3041	*Kabuli*	Ca_Kabuli_Chr02	32140990	(G/A)
3042	CakSNP3042	*Kabuli*	Ca_Kabuli_Chr02	32203032	(A/G)
3043	CakSNP3043	*Kabuli*	Ca_Kabuli_Chr02	32203036	(A/T)
3044	CakSNP3044	*Kabuli*	Ca_Kabuli_Chr02	32242818	(T/G)
3045	CakSNP3045	*Kabuli*	Ca_Kabuli_Chr02	32348054	(T/A)
3046	CakSNP3046	*Kabuli*	Ca_Kabuli_Chr02	32348087	(A/C)
3047	CakSNP3047	*Kabuli*	Ca_Kabuli_Chr02	32348119	(G/T)
3048	CakSNP3048	*Kabuli*	Ca_Kabuli_Chr02	32348793	(G/A)
3049	CakSNP3049	*Kabuli*	Ca_Kabuli_Chr02	32348870	(T/C)
3050	CakSNP3050	*Kabuli*	Ca_Kabuli_Chr02	32411994	(G/T)
3051	CakSNP3051	*Kabuli*	Ca_Kabuli_Chr02	32412000	(G/T)
3052	CakSNP3052	*Kabuli*	Ca_Kabuli_Chr02	32481969	(C/T)
3053	CakSNP3053	*Kabuli*	Ca_Kabuli_Chr02	32481983	(G/T)
3054	CakSNP3054	*Kabuli*	Ca_Kabuli_Chr02	32484223	(C/T)
3055	CakSNP3055	*Kabuli*	Ca_Kabuli_Chr02	32545320	(A/G)
3056	CakSNP3056	*Kabuli*	Ca_Kabuli_Chr02	32545329	(T/C)
3057	CakSNP3057	*Kabuli*	Ca_Kabuli_Chr02	32545351	(G/A)
3058	CakSNP3058	*Kabuli*	Ca_Kabuli_Chr02	32556992	(T/A)
3059	CakSNP3059	*Kabuli*	Ca_Kabuli_Chr02	32564427	(T/C)
3060	CakSNP3060	*Kabuli*	Ca_Kabuli_Chr02	32564480	(G/A)
3061	CakSNP3061	*Kabuli*	Ca_Kabuli_Chr02	32564770	(G/T)
3062	CakSNP3062	*Kabuli*	Ca_Kabuli_Chr02	32564796	(A/T)
3063	CakSNP3063	*Kabuli*	Ca_Kabuli_Chr02	32570394	(G/A)
3064	CakSNP3064	*Kabuli*	Ca_Kabuli_Chr02	32570479	(T/G)
3065	CakSNP3065	*Kabuli*	Ca_Kabuli_Chr02	32570429	(G/A)
3066	CakSNP3066	*Kabuli*	Ca_Kabuli_Chr02	32570504	(G/C)
3067	CakSNP3067	*Kabuli*	Ca_Kabuli_Chr02	32577274	(T/A)
3068	CakSNP3068	*Kabuli*	Ca_Kabuli_Chr02	32577593	(A/G)
3069	CakSNP3069	*Kabuli*	Ca_Kabuli_Chr02	32579849	(T/C)
3070	CakSNP3070	*Kabuli*	Ca_Kabuli_Chr02	32579900	(C/G)
3071	CakSNP3071	*Kabuli*	Ca_Kabuli_Chr02	32608900	(A/T)
3072	CakSNP3072	*Kabuli*	Ca_Kabuli_Chr02	32611624	(C/T)
3073	CakSNP3073	*Kabuli*	Ca_Kabuli_Chr02	32611609	(A/G)
3074	CakSNP3074	*Kabuli*	Ca_Kabuli_Chr02	32611601	(G/T)
3075	CakSNP3075	*Kabuli*	Ca_Kabuli_Chr02	32627727	(C/G)
3076	CakSNP3076	*Kabuli*	Ca_Kabuli_Chr02	32629763	(A/G)
3077	CakSNP3077	*Kabuli*	Ca_Kabuli_Chr02	32670054	(C/T)
3078	CakSNP3078	*Kabuli*	Ca_Kabuli_Chr02	32670099	(C/T)
3079	CakSNP3079	*Kabuli*	Ca_Kabuli_Chr02	32670181	(T/G)

S.N.	SNP IDs	Cultivars	Chromosomes/scaffolds	Physical positions (bp)	SNPs
3080	CakSNP3080	*Kabuli*	Ca_Kabuli_Chr02	32671354	(T/A)
3081	CakSNP3081	*Kabuli*	Ca_Kabuli_Chr02	32781880	(A/G)
3082	CakSNP3082	*Kabuli*	Ca_Kabuli_Chr02	32844754	(T/G)
3083	CakSNP3083	*Kabuli*	Ca_Kabuli_Chr02	32844842	(C/G)
3084	CakSNP3084	*Kabuli*	Ca_Kabuli_Chr02	32867125	(A/G)
3085	CakSNP3085	*Kabuli*	Ca_Kabuli_Chr02	32934646	(C/T)
3086	CakSNP3086	*Kabuli*	Ca_Kabuli_Chr02	32934633	(G/C)
3087	CakSNP3087	*Kabuli*	Ca_Kabuli_Chr02	32936855	(A/G)
3088	CakSNP3088	*Kabuli*	Ca_Kabuli_Chr02	32936908	(G/A)
3089	CakSNP3089	*Kabuli*	Ca_Kabuli_Chr02	32957515	(A/G)
3090	CakSNP3090	*Kabuli*	Ca_Kabuli_Chr02	32957523	(T/C)
3091	CakSNP3091	*Kabuli*	Ca_Kabuli_Chr02	32957538	(A/C)
3092	CakSNP3092	*Kabuli*	Ca_Kabuli_Chr02	32957572	(A/C)
3093	CakSNP3093	*Kabuli*	Ca_Kabuli_Chr02	32957958	(A/C)
3094	CakSNP3094	*Kabuli*	Ca_Kabuli_Chr02	32958034	(C/G)
3095	CakSNP3095	*Kabuli*	Ca_Kabuli_Chr02	32989134	(G/T)
3096	CakSNP3096	*Kabuli*	Ca_Kabuli_Chr02	32989205	(G/T)
3097	CakSNP3097	*Kabuli*	Ca_Kabuli_Chr02	33083903	(T/A)
3098	CakSNP3098	*Kabuli*	Ca_Kabuli_Chr02	33084095	(T/G)
3099	CakSNP3099	*Kabuli*	Ca_Kabuli_Chr02	33084132	(T/A)
3100	CakSNP3100	*Kabuli*	Ca_Kabuli_Chr02	33103318	(G/A)
3101	CakSNP3101	*Kabuli*	Ca_Kabuli_Chr02	33103466	(G/A)
3102	CakSNP3102	*Kabuli*	Ca_Kabuli_Chr02	33103750	(A/G)
3103	CakSNP3103	*Kabuli*	Ca_Kabuli_Chr02	33103766	(G/A)
3104	CakSNP3104	*Kabuli*	Ca_Kabuli_Chr02	33172936	(C/T)
3105	CakSNP3105	*Kabuli*	Ca_Kabuli_Chr02	33172971	(T/G)
3106	CakSNP3106	*Kabuli*	Ca_Kabuli_Chr02	33178391	(G/A)
3107	CakSNP3107	*Kabuli*	Ca_Kabuli_Chr02	33178407	(C/T)
3108	CakSNP3108	*Kabuli*	Ca_Kabuli_Chr02	33178419	(C/A)
3109	CakSNP3109	*Kabuli*	Ca_Kabuli_Chr02	33178420	(A/C)
3110	CakSNP3110	*Kabuli*	Ca_Kabuli_Chr02	33178506	(T/C)
3111	CakSNP3111	*Kabuli*	Ca_Kabuli_Chr02	33184262	(T/C)
3112	CakSNP3112	*Kabuli*	Ca_Kabuli_Chr02	33194198	(A/G)
3113	CakSNP3113	*Kabuli*	Ca_Kabuli_Chr02	33204253	(C/T)
3114	CakSNP3114	*Kabuli*	Ca_Kabuli_Chr02	33326392	(T/G)
3115	CakSNP3115	*Kabuli*	Ca_Kabuli_Chr02	33326398	(C/T)
3116	CakSNP3116	*Kabuli*	Ca_Kabuli_Chr02	33326444	(G/A)
3117	CakSNP3117	*Kabuli*	Ca_Kabuli_Chr02	33326522	(G/A)
3118	CakSNP3118	*Kabuli*	Ca_Kabuli_Chr02	33326520	(T/C)

S.N.	SNP IDs	Cultivars	Chromosomes/scaffolds	Physical positions (bp)	SNPs
3119	CakSNP3119	*Kabuli*	Ca_Kabuli_Chr02	33326516	(G/A)
3120	CakSNP3120	*Kabuli*	Ca_Kabuli_Chr02	33326510	(C/G)
3121	CakSNP3121	*Kabuli*	Ca_Kabuli_Chr02	33393209	(T/C)
3122	CakSNP3122	*Kabuli*	Ca_Kabuli_Chr02	33398561	(C/T)
3123	CakSNP3123	*Kabuli*	Ca_Kabuli_Chr02	33398550	(G/A)
3124	CakSNP3124	*Kabuli*	Ca_Kabuli_Chr02	33398549	(T/C)
3125	CakSNP3125	*Kabuli*	Ca_Kabuli_Chr02	33398527	(T/G)
3126	CakSNP3126	*Kabuli*	Ca_Kabuli_Chr02	33399010	(C/T)
3127	CakSNP3127	*Kabuli*	Ca_Kabuli_Chr02	33399019	(T/C)
3128	CakSNP3128	*Kabuli*	Ca_Kabuli_Chr02	33399045	(A/C)
3129	CakSNP3129	*Kabuli*	Ca_Kabuli_Chr02	33399098	(G/A)
3130	CakSNP3130	*Kabuli*	Ca_Kabuli_Chr02	33399063	(C/T)
3131	CakSNP3131	*Kabuli*	Ca_Kabuli_Chr02	33399405	(C/T)
3132	CakSNP3132	*Kabuli*	Ca_Kabuli_Chr02	33399404	(C/A)
3133	CakSNP3133	*Kabuli*	Ca_Kabuli_Chr02	33399397	(C/T)
3134	CakSNP3134	*Kabuli*	Ca_Kabuli_Chr02	33399393	(C/A)
3135	CakSNP3135	*Kabuli*	Ca_Kabuli_Chr02	33399381	(C/T)
3136	CakSNP3136	*Kabuli*	Ca_Kabuli_Chr02	33399342	(A/C)
3137	CakSNP3137	*Kabuli*	Ca_Kabuli_Chr02	33399465	(C/G)
3138	CakSNP3138	*Kabuli*	Ca_Kabuli_Chr02	33399608	(C/A)
3139	CakSNP3139	*Kabuli*	Ca_Kabuli_Chr02	33401608	(A/G)
3140	CakSNP3140	*Kabuli*	Ca_Kabuli_Chr02	33490058	(C/A)
3141	CakSNP3141	*Kabuli*	Ca_Kabuli_Chr02	33489989	(T/C)
3142	CakSNP3142	*Kabuli*	Ca_Kabuli_Chr02	33575286	(A/C)
3143	CakSNP3143	*Kabuli*	Ca_Kabuli_Chr02	33582948	(G/A)
3144	CakSNP3144	*Kabuli*	Ca_Kabuli_Chr02	33582900	(G/A)
3145	CakSNP3145	*Kabuli*	Ca_Kabuli_Chr02	33650447	(A/T)
3146	CakSNP3146	*Kabuli*	Ca_Kabuli_Chr02	33651426	(G/A)
3147	CakSNP3147	*Kabuli*	Ca_Kabuli_Chr02	33651408	(A/G)
3148	CakSNP3148	*Kabuli*	Ca_Kabuli_Chr02	33661177	(C/T)
3149	CakSNP3149	*Kabuli*	Ca_Kabuli_Chr02	33661316	(T/C)
3150	CakSNP3150	*Kabuli*	Ca_Kabuli_Chr02	33686794	(G/A)
3151	CakSNP3151	*Kabuli*	Ca_Kabuli_Chr02	33692368	(C/A)
3152	CakSNP3152	*Kabuli*	Ca_Kabuli_Chr02	33713018	(G/A)
3153	CakSNP3153	*Kabuli*	Ca_Kabuli_Chr02	33721515	(T/A)
3154	CakSNP3154	*Kabuli*	Ca_Kabuli_Chr02	33733893	(C/T)
3155	CakSNP3155	*Kabuli*	Ca_Kabuli_Chr02	33745281	(A/G)
3156	CakSNP3156	*Kabuli*	Ca_Kabuli_Chr02	33764634	(G/A)
3157	CakSNP3157	*Kabuli*	Ca_Kabuli_Chr02	33783871	(C/T)

S.N.	SNP IDs	Cultivars	Chromosomes/scaffolds	Physical positions (bp)	SNPs
3158	CakSNP3158	*Kabuli*	Ca_Kabuli_Chr02	33785203	(G/A)
3159	CakSNP3159	*Kabuli*	Ca_Kabuli_Chr02	33786243	(G/A)
3160	CakSNP3160	*Kabuli*	Ca_Kabuli_Chr02	33788913	(A/C)
3161	CakSNP3161	*Kabuli*	Ca_Kabuli_Chr02	33798717	(T/C)
3162	CakSNP3162	*Kabuli*	Ca_Kabuli_Chr02	33798748	(T/C)
3163	CakSNP3163	*Kabuli*	Ca_Kabuli_Chr02	34152352	(T/C)
3164	CakSNP3164	*Kabuli*	Ca_Kabuli_Chr02	34152384	(T/C)
3165	CakSNP3165	*Kabuli*	Ca_Kabuli_Chr02	34152476	(C/T)
3166	CakSNP3166	*Kabuli*	Ca_Kabuli_Chr02	34155090	(A/G)
3167	CakSNP3167	*Kabuli*	Ca_Kabuli_Chr02	34155179	(A/G)
3168	CakSNP3168	*Kabuli*	Ca_Kabuli_Chr02	34178534	(G/A)
3169	CakSNP3169	*Kabuli*	Ca_Kabuli_Chr02	34203162	(T/C)
3170	CakSNP3170	*Kabuli*	Ca_Kabuli_Chr02	34203444	(T/C)
3171	CakSNP3171	*Kabuli*	Ca_Kabuli_Chr02	34222585	(G/A)
3172	CakSNP3172	*Kabuli*	Ca_Kabuli_Chr02	34249987	(C/T)
3173	CakSNP3173	*Kabuli*	Ca_Kabuli_Chr02	34249953	(G/T)
3174	CakSNP3174	*Kabuli*	Ca_Kabuli_Chr02	34250042	(C/T)
3175	CakSNP3175	*Kabuli*	Ca_Kabuli_Chr02	34250266	(T/C)
3176	CakSNP3176	*Kabuli*	Ca_Kabuli_Chr02	34266109	(A/G)
3177	CakSNP3177	*Kabuli*	Ca_Kabuli_Chr02	34288116	(G/C)
3178	CakSNP3178	*Kabuli*	Ca_Kabuli_Chr02	34299096	(G/A)
3179	CakSNP3179	*Kabuli*	Ca_Kabuli_Chr02	34299075	(C/T)
3180	CakSNP3180	*Kabuli*	Ca_Kabuli_Chr02	34299074	(A/C)
3181	CakSNP3181	*Kabuli*	Ca_Kabuli_Chr02	34299063	(G/A)
3182	CakSNP3182	*Kabuli*	Ca_Kabuli_Chr02	34299079	(A/C)
3183	CakSNP3183	*Kabuli*	Ca_Kabuli_Chr02	34299846	(C/T)
3184	CakSNP3184	*Kabuli*	Ca_Kabuli_Chr02	34300059	(A/G)
3185	CakSNP3185	*Kabuli*	Ca_Kabuli_Chr02	34300057	(A/G)
3186	CakSNP3186	*Kabuli*	Ca_Kabuli_Chr02	34343478	(C/G)
3187	CakSNP3187	*Kabuli*	Ca_Kabuli_Chr02	34343705	(G/A)
3188	CakSNP3188	*Kabuli*	Ca_Kabuli_Chr02	34346849	(C/T)
3189	CakSNP3189	*Kabuli*	Ca_Kabuli_Chr02	34346842	(T/C)
3190	CakSNP3190	*Kabuli*	Ca_Kabuli_Chr02	34372503	(T/G)
3191	CakSNP3191	*Kabuli*	Ca_Kabuli_Chr02	34428978	(C/A)
3192	CakSNP3192	*Kabuli*	Ca_Kabuli_Chr02	34428979	(G/C)
3193	CakSNP3193	*Kabuli*	Ca_Kabuli_Chr02	34429029	(G/T)
3194	CakSNP3194	*Kabuli*	Ca_Kabuli_Chr02	34429030	(A/T)
3195	CakSNP3195	*Kabuli*	Ca_Kabuli_Chr02	34551849	(A/C)
3196	CakSNP3196	*Kabuli*	Ca_Kabuli_Chr02	34554164	(A/C)

S.N.	SNP IDs	Cultivars	Chromosomes/scaffolds	Physical positions (bp)	SNPs
3197	CakSNP3197	*Kabuli*	Ca_Kabuli_Chr02	34601444	(C/A)
3198	CakSNP3198	*Kabuli*	Ca_Kabuli_Chr02	34634128	(C/T)
3199	CakSNP3199	*Kabuli*	Ca_Kabuli_Chr02	34638960	(A/C)
3200	CakSNP3200	*Kabuli*	Ca_Kabuli_Chr02	34638955	(T/A)
3201	CakSNP3201	*Kabuli*	Ca_Kabuli_Chr02	34734329	(G/A)
3202	CakSNP3202	*Kabuli*	Ca_Kabuli_Chr02	34734379	(G/A)
3203	CakSNP3203	*Kabuli*	Ca_Kabuli_Chr02	34734439	(G/A)
3204	CakSNP3204	*Kabuli*	Ca_Kabuli_Chr02	34734472	(A/G)
3205	CakSNP3205	*Kabuli*	Ca_Kabuli_Chr02	34736649	(T/C)
3206	CakSNP3206	*Kabuli*	Ca_Kabuli_Chr02	34736658	(A/C)
3207	CakSNP3207	*Kabuli*	Ca_Kabuli_Chr02	34736753	(C/T)
3208	CakSNP3208	*Kabuli*	Ca_Kabuli_Chr02	34754363	(T/C)
3209	CakSNP3209	*Kabuli*	Ca_Kabuli_Chr02	34754507	(C/T)
3210	CakSNP3210	*Kabuli*	Ca_Kabuli_Chr02	34820075	(C/T)
3211	CakSNP3211	*Kabuli*	Ca_Kabuli_Chr02	34830995	(A/G)
3212	CakSNP3212	*Kabuli*	Ca_Kabuli_Chr02	34847681	(C/A)
3213	CakSNP3213	*Kabuli*	Ca_Kabuli_Chr02	34848053	(G/A)
3214	CakSNP3214	*Kabuli*	Ca_Kabuli_Chr02	34848480	(T/C)
3215	CakSNP3215	*Kabuli*	Ca_Kabuli_Chr02	34862129	(G/T)
3216	CakSNP3216	*Kabuli*	Ca_Kabuli_Chr02	34862135	(G/A)
3217	CakSNP3217	*Kabuli*	Ca_Kabuli_Chr02	34862139	(A/G)
3218	CakSNP3218	*Kabuli*	Ca_Kabuli_Chr02	34862150	(C/T)
3219	CakSNP3219	*Kabuli*	Ca_Kabuli_Chr02	34862276	(C/G)
3220	CakSNP3220	*Kabuli*	Ca_Kabuli_Chr02	34888414	(T/C)
3221	CakSNP3221	*Kabuli*	Ca_Kabuli_Chr02	35013088	(G/C)
3222	CakSNP3222	*Kabuli*	Ca_Kabuli_Chr02	35013084	(T/C)
3223	CakSNP3223	*Kabuli*	Ca_Kabuli_Chr02	35052930	(C/A)
3224	CakSNP3224	*Kabuli*	Ca_Kabuli_Chr02	35054244	(C/A)
3225	CakSNP3225	*Kabuli*	Ca_Kabuli_Chr02	35059446	(A/T)
3226	CakSNP3226	*Kabuli*	Ca_Kabuli_Chr02	35059434	(T/C)
3227	CakSNP3227	*Kabuli*	Ca_Kabuli_Chr02	35059440	(A/C)
3228	CakSNP3228	*Kabuli*	Ca_Kabuli_Chr02	35059451	(C/A)
3229	CakSNP3229	*Kabuli*	Ca_Kabuli_Chr02	35060171	(A/G)
3230	CakSNP3230	*Kabuli*	Ca_Kabuli_Chr02	35060178	(A/T)
3231	CakSNP3231	*Kabuli*	Ca_Kabuli_Chr02	35060228	(T/C)
3232	CakSNP3232	*Kabuli*	Ca_Kabuli_Chr02	35060273	(G/A)
3233	CakSNP3233	*Kabuli*	Ca_Kabuli_Chr02	35060264	(A/T)
3234	CakSNP3234	*Kabuli*	Ca_Kabuli_Chr02	35066138	(G/A)
3235	CakSNP3235	*Kabuli*	Ca_Kabuli_Chr02	35095010	(A/G)

S.N.	SNP IDs	Cultivars	Chromosomes/scaffolds	Physical positions (bp)	SNPs
3236	CakSNP3236	*Kabuli*	Ca_Kabuli_Chr02	35096648	(A/T)
3237	CakSNP3237	*Kabuli*	Ca_Kabuli_Chr02	35096649	(A/T)
3238	CakSNP3238	*Kabuli*	Ca_Kabuli_Chr02	35120761	(G/A)
3239	CakSNP3239	*Kabuli*	Ca_Kabuli_Chr02	35122109	(C/G)
3240	CakSNP3240	*Kabuli*	Ca_Kabuli_Chr02	35154629	(C/A)
3241	CakSNP3241	*Kabuli*	Ca_Kabuli_Chr02	35154603	(G/A)
3242	CakSNP3242	*Kabuli*	Ca_Kabuli_Chr02	35171882	(T/G)
3243	CakSNP3243	*Kabuli*	Ca_Kabuli_Chr02	35189471	(C/T)
3244	CakSNP3244	*Kabuli*	Ca_Kabuli_Chr02	35195303	(T/C)
3245	CakSNP3245	*Kabuli*	Ca_Kabuli_Chr02	35198352	(T/C)
3246	CakSNP3246	*Kabuli*	Ca_Kabuli_Chr02	35209940	(C/T)
3247	CakSNP3247	*Kabuli*	Ca_Kabuli_Chr02	35362587	(A/G)
3248	CakSNP3248	*Kabuli*	Ca_Kabuli_Chr02	35362642	(A/G)
3249	CakSNP3249	*Kabuli*	Ca_Kabuli_Chr02	35362657	(C/A)
3250	CakSNP3250	*Kabuli*	Ca_Kabuli_Chr02	35362729	(A/G)
3251	CakSNP3251	*Kabuli*	Ca_Kabuli_Chr02	35363488	(T/C)
3252	CakSNP3252	*Kabuli*	Ca_Kabuli_Chr02	35363501	(T/C)
3253	CakSNP3253	*Kabuli*	Ca_Kabuli_Chr02	35365029	(C/T)
3254	CakSNP3254	*Kabuli*	Ca_Kabuli_Chr02	35367161	(G/A)
3255	CakSNP3255	*Kabuli*	Ca_Kabuli_Chr02	35413400	(T/A)
3256	CakSNP3256	*Kabuli*	Ca_Kabuli_Chr02	35441184	(C/T)
3257	CakSNP3257	*Kabuli*	Ca_Kabuli_Chr02	35441180	(A/G)
3258	CakSNP3258	*Kabuli*	Ca_Kabuli_Chr02	35441174	(T/G)
3259	CakSNP3259	*Kabuli*	Ca_Kabuli_Chr02	35447976	(A/G)
3260	CakSNP3260	*Kabuli*	Ca_Kabuli_Chr02	35448018	(C/G)
3261	CakSNP3261	*Kabuli*	Ca_Kabuli_Chr02	35448126	(T/A)
3262	CakSNP3262	*Kabuli*	Ca_Kabuli_Chr02	35449653	(G/A)
3263	CakSNP3263	*Kabuli*	Ca_Kabuli_Chr02	35473250	(C/T)
3264	CakSNP3264	*Kabuli*	Ca_Kabuli_Chr02	35505791	(C/G)
3265	CakSNP3265	*Kabuli*	Ca_Kabuli_Chr02	35505874	(T/G)
3266	CakSNP3266	*Kabuli*	Ca_Kabuli_Chr02	35533246	(G/A)
3267	CakSNP3267	*Kabuli*	Ca_Kabuli_Chr02	35593439	(T/C)
3268	CakSNP3268	*Kabuli*	Ca_Kabuli_Chr02	35593797	(G/T)
3269	CakSNP3269	*Kabuli*	Ca_Kabuli_Chr02	35593824	(A/C)
3270	CakSNP3270	*Kabuli*	Ca_Kabuli_Chr02	35593827	(A/G)
3271	CakSNP3271	*Kabuli*	Ca_Kabuli_Chr02	35594018	(A/T)
3272	CakSNP3272	*Kabuli*	Ca_Kabuli_Chr02	35594013	(C/T)
3273	CakSNP3273	*Kabuli*	Ca_Kabuli_Chr02	35593985	(A/G)
3274	CakSNP3274	*Kabuli*	Ca_Kabuli_Chr02	35593965	(C/T)

S.N.	SNP IDs	Cultivars	Chromosomes/scaffolds	Physical positions (bp)	SNPs
3275	CakSNP3275	*Kabuli*	Ca_Kabuli_Chr02	35593956	(A/G)
3276	CakSNP3276	*Kabuli*	Ca_Kabuli_Chr02	35601566	(T/C)
3277	CakSNP3277	*Kabuli*	Ca_Kabuli_Chr02	35601571	(A/C)
3278	CakSNP3278	*Kabuli*	Ca_Kabuli_Chr02	35601578	(C/T)
3279	CakSNP3279	*Kabuli*	Ca_Kabuli_Chr02	35601605	(G/A)
3280	CakSNP3280	*Kabuli*	Ca_Kabuli_Chr02	35601691	(A/G)
3281	CakSNP3281	*Kabuli*	Ca_Kabuli_Chr02	35601657	(T/C)
3282	CakSNP3282	*Kabuli*	Ca_Kabuli_Chr02	35601632	(G/T)
3283	CakSNP3283	*Kabuli*	Ca_Kabuli_Chr02	35612587	(C/A)
3284	CakSNP3284	*Kabuli*	Ca_Kabuli_Chr02	35612590	(G/A)
3285	CakSNP3285	*Kabuli*	Ca_Kabuli_Chr02	35612644	(G/A)
3286	CakSNP3286	*Kabuli*	Ca_Kabuli_Chr02	35612788	(G/A)
3287	CakSNP3287	*Kabuli*	Ca_Kabuli_Chr02	35612895	(T/C)
3288	CakSNP3288	*Kabuli*	Ca_Kabuli_Chr02	35612900	(C/T)
3289	CakSNP3289	*Kabuli*	Ca_Kabuli_Chr02	35613016	(G/A)
3290	CakSNP3290	*Kabuli*	Ca_Kabuli_Chr02	35613135	(T/C)
3291	CakSNP3291	*Kabuli*	Ca_Kabuli_Chr02	35658426	(C/T)
3292	CakSNP3292	*Kabuli*	Ca_Kabuli_Chr02	35658537	(T/C)
3293	CakSNP3293	*Kabuli*	Ca_Kabuli_Chr02	35661053	(C/T)
3294	CakSNP3294	*Kabuli*	Ca_Kabuli_Chr02	35670478	(A/G)
3295	CakSNP3295	*Kabuli*	Ca_Kabuli_Chr02	35704018	(A/G)
3296	CakSNP3296	*Kabuli*	Ca_Kabuli_Chr02	35704069	(G/A)
3297	CakSNP3297	*Kabuli*	Ca_Kabuli_Chr02	35704228	(C/A)
3298	CakSNP3298	*Kabuli*	Ca_Kabuli_Chr02	35704213	(A/G)
3299	CakSNP3299	*Kabuli*	Ca_Kabuli_Chr02	35704204	(T/C)
3300	CakSNP3300	*Kabuli*	Ca_Kabuli_Chr02	35704200	(T/C)
3301	CakSNP3301	*Kabuli*	Ca_Kabuli_Chr02	35704180	(A/G)
3302	CakSNP3302	*Kabuli*	Ca_Kabuli_Chr02	35704178	(G/A)
3303	CakSNP3303	*Kabuli*	Ca_Kabuli_Chr02	35728285	(T/A)
3304	CakSNP3304	*Kabuli*	Ca_Kabuli_Chr02	35754617	(G/T)
3305	CakSNP3305	*Kabuli*	Ca_Kabuli_Chr02	35754557	(C/T)
3306	CakSNP3306	*Kabuli*	Ca_Kabuli_Chr02	35754680	(C/A)
3307	CakSNP3307	*Kabuli*	Ca_Kabuli_Chr02	35754682	(A/G)
3308	CakSNP3308	*Kabuli*	Ca_Kabuli_Chr02	35754806	(G/C)
3309	CakSNP3309	*Kabuli*	Ca_Kabuli_Chr02	35754757	(G/C)
3310	CakSNP3310	*Kabuli*	Ca_Kabuli_Chr02	35759932	(A/T)
3311	CakSNP3311	*Kabuli*	Ca_Kabuli_Chr02	35759940	(T/C)
3312	CakSNP3312	*Kabuli*	Ca_Kabuli_Chr02	35759981	(G/A)
3313	CakSNP3313	*Kabuli*	Ca_Kabuli_Chr02	35760062	(G/C)

S.N.	SNP IDs	Cultivars	Chromosomes/scaffolds	Physical positions (bp)	SNPs
3314	CakSNP3314	*Kabuli*	Ca_Kabuli_Chr02	35760073	(A/T)
3315	CakSNP3315	*Kabuli*	Ca_Kabuli_Chr02	35760170	(A/G)
3316	CakSNP3316	*Kabuli*	Ca_Kabuli_Chr02	35760155	(G/A)
3317	CakSNP3317	*Kabuli*	Ca_Kabuli_Chr02	35770569	(A/G)
3318	CakSNP3318	*Kabuli*	Ca_Kabuli_Chr02	35795446	(G/A)
3319	CakSNP3319	*Kabuli*	Ca_Kabuli_Chr02	35795395	(A/G)
3320	CakSNP3320	*Kabuli*	Ca_Kabuli_Chr02	35795494	(G/A)
3321	CakSNP3321	*Kabuli*	Ca_Kabuli_Chr02	35795496	(G/T)
3322	CakSNP3322	*Kabuli*	Ca_Kabuli_Chr02	35795497	(G/T)
3323	CakSNP3323	*Kabuli*	Ca_Kabuli_Chr02	35795521	(T/C)
3324	CakSNP3324	*Kabuli*	Ca_Kabuli_Chr02	35828553	(G/A)
3325	CakSNP3325	*Kabuli*	Ca_Kabuli_Chr02	35829782	(A/G)
3326	CakSNP3326	*Kabuli*	Ca_Kabuli_Chr02	35844790	(A/G)
3327	CakSNP3327	*Kabuli*	Ca_Kabuli_Chr02	35844762	(A/G)
3328	CakSNP3328	*Kabuli*	Ca_Kabuli_Chr02	35862852	(C/T)
3329	CakSNP3329	*Kabuli*	Ca_Kabuli_Chr02	35862878	(T/G)
3330	CakSNP3330	*Kabuli*	Ca_Kabuli_Chr02	35875666	(A/G)
3331	CakSNP3331	*Kabuli*	Ca_Kabuli_Chr02	35875653	(C/T)
3332	CakSNP3332	*Kabuli*	Ca_Kabuli_Chr02	35930699	(T/G)
3333	CakSNP3333	*Kabuli*	Ca_Kabuli_Chr02	35993062	(C/T)
3334	CakSNP3334	*Kabuli*	Ca_Kabuli_Chr02	35993082	(T/G)
3335	CakSNP3335	*Kabuli*	Ca_Kabuli_Chr02	35993089	(C/T)
3336	CakSNP3336	*Kabuli*	Ca_Kabuli_Chr02	35993092	(A/T)
3337	CakSNP3337	*Kabuli*	Ca_Kabuli_Chr02	35993178	(G/A)
3338	CakSNP3338	*Kabuli*	Ca_Kabuli_Chr02	36013809	(A/G)
3339	CakSNP3339	*Kabuli*	Ca_Kabuli_Chr02	36039428	(C/T)
3340	CakSNP3340	*Kabuli*	Ca_Kabuli_Chr02	36039377	(C/T)
3341	CakSNP3341	*Kabuli*	Ca_Kabuli_Chr02	36039364	(G/T)
3342	CakSNP3342	*Kabuli*	Ca_Kabuli_Chr02	36046593	(G/T)
3343	CakSNP3343	*Kabuli*	Ca_Kabuli_Chr02	36046595	(T/G)
3344	CakSNP3344	*Kabuli*	Ca_Kabuli_Chr02	36046741	(G/A)
3345	CakSNP3345	*Kabuli*	Ca_Kabuli_Chr02	36046720	(T/A)
3346	CakSNP3346	*Kabuli*	Ca_Kabuli_Chr02	36046767	(T/A)
3347	CakSNP3347	*Kabuli*	Ca_Kabuli_Chr02	36046799	(C/A)
3348	CakSNP3348	*Kabuli*	Ca_Kabuli_Chr02	36046820	(T/A)
3349	CakSNP3349	*Kabuli*	Ca_Kabuli_Chr02	36046844	(T/G)
3350	CakSNP3350	*Kabuli*	Ca_Kabuli_Chr02	36069340	(T/C)
3351	CakSNP3351	*Kabuli*	Ca_Kabuli_Chr02	36072247	(C/A)
3352	CakSNP3352	*Kabuli*	Ca_Kabuli_Chr02	36074147	(T/C)

S.N.	SNP IDs	Cultivars	Chromosomes/scaffolds	Physical positions (bp)	SNPs
3353	CakSNP3353	*Kabuli*	Ca_Kabuli_Chr02	36088577	(A/T)
3354	CakSNP3354	*Kabuli*	Ca_Kabuli_Chr02	36088623	(C/T)
3355	CakSNP3355	*Kabuli*	Ca_Kabuli_Chr02	36088676	(T/C)
3356	CakSNP3356	*Kabuli*	Ca_Kabuli_Chr02	36088853	(G/A)
3357	CakSNP3357	*Kabuli*	Ca_Kabuli_Chr02	36101358	(A/G)
3358	CakSNP3358	*Kabuli*	Ca_Kabuli_Chr02	36101379	(A/G)
3359	CakSNP3359	*Kabuli*	Ca_Kabuli_Chr02	36101421	(C/T)
3360	CakSNP3360	*Kabuli*	Ca_Kabuli_Chr02	36117695	(T/A)
3361	CakSNP3361	*Kabuli*	Ca_Kabuli_Chr02	36117647	(G/A)
3362	CakSNP3362	*Kabuli*	Ca_Kabuli_Chr02	36253581	(C/T)
3363	CakSNP3363	*Kabuli*	Ca_Kabuli_Chr02	36276791	(G/A)
3364	CakSNP3364	*Kabuli*	Ca_Kabuli_Chr02	36323575	(G/T)
3365	CakSNP3365	*Kabuli*	Ca_Kabuli_Chr02	36326361	(A/C)
3366	CakSNP3366	*Kabuli*	Ca_Kabuli_Chr02	36335252	(A/C)
3367	CakSNP3367	*Kabuli*	Ca_Kabuli_Chr02	36343900	(C/T)
3368	CakSNP3368	*Kabuli*	Ca_Kabuli_Chr02	36343933	(T/C)
3369	CakSNP3369	*Kabuli*	Ca_Kabuli_Chr02	36346336	(A/G)
3370	CakSNP3370	*Kabuli*	Ca_Kabuli_Chr02	36375224	(A/G)
3371	CakSNP3371	*Kabuli*	Ca_Kabuli_Chr02	36375638	(A/G)
3372	CakSNP3372	*Kabuli*	Ca_Kabuli_Chr02	36387189	(G/C)
3373	CakSNP3373	*Kabuli*	Ca_Kabuli_Chr02	36387245	(T/C)
3374	CakSNP3374	*Kabuli*	Ca_Kabuli_Chr02	36389041	(G/A)
3375	CakSNP3375	*Kabuli*	Ca_Kabuli_Chr02	36389096	(C/T)
3376	CakSNP3376	*Kabuli*	Ca_Kabuli_Chr02	36403788	(A/G)
3377	CakSNP3377	*Kabuli*	Ca_Kabuli_Chr02	36427841	(C/T)
3378	CakSNP3378	*Kabuli*	Ca_Kabuli_Chr02	36429512	(T/C)
3379	CakSNP3379	*Kabuli*	Ca_Kabuli_Chr02	36438939	(T/G)
3380	CakSNP3380	*Kabuli*	Ca_Kabuli_Chr02	36453691	(A/G)
3381	CakSNP3381	*Kabuli*	Ca_Kabuli_Chr02	36453685	(G/A)
3382	CakSNP3382	*Kabuli*	Ca_Kabuli_Chr02	36466837	(C/T)
3383	CakSNP3383	*Kabuli*	Ca_Kabuli_Chr02	36466998	(A/G)
3384	CakSNP3384	*Kabuli*	Ca_Kabuli_Chr02	36467100	(A/C)
3385	CakSNP3385	*Kabuli*	Ca_Kabuli_Chr02	36468390	(A/G)
3386	CakSNP3386	*Kabuli*	Ca_Kabuli_Chr02	36468505	(C/A)
3387	CakSNP3387	*Kabuli*	Ca_Kabuli_Chr02	36469921	(A/G)
3388	CakSNP3388	*Kabuli*	Ca_Kabuli_Chr02	36505316	(C/A)
3389	CakSNP3389	*Kabuli*	Ca_Kabuli_Chr02	36521200	(T/C)
3390	CakSNP3390	*Kabuli*	Ca_Kabuli_Chr02	36533821	(A/C)
3391	CakSNP3391	*Kabuli*	Ca_Kabuli_Chr02	36533910	(G/A)

S.N.	SNP IDs	Cultivars	Chromosomes/scaffolds	Physical positions (bp)	SNPs
3392	CakSNP3392	*Kabuli*	Ca_Kabuli_Chr02	36554430	(G/A)
3393	CakSNP3393	*Kabuli*	Ca_Kabuli_Chr02	36609730	(T/A)
3394	CakSNP3394	*Kabuli*	Ca_Kabuli_Chr02	36609743	(C/T)
3395	CakSNP3395	*Kabuli*	Ca_Kabuli_Chr02	36609744	(G/C)
3396	CakSNP3396	*Kabuli*	Ca_Kabuli_Chr02	36609858	(C/G)
3397	CakSNP3397	*Kabuli*	Ca_Kabuli_Chr02	36609950	(C/T)
3398	CakSNP3398	*Kabuli*	Ca_Kabuli_Chr02	36609968	(C/T)
3399	CakSNP3399	*Kabuli*	Ca_Kabuli_Chr02	36634234	(T/C)
3400	CakSNP3400	*Kabuli*	Ca_Kabuli_Chr02	36634297	(C/G)
3401	CakSNP3401	*Kabuli*	Ca_Kabuli_Chr03	171409	(A/T)
3402	CakSNP3402	*Kabuli*	Ca_Kabuli_Chr03	171468	(A/G)
3403	CakSNP3403	*Kabuli*	Ca_Kabuli_Chr03	171574	(C/A)
3404	CakSNP3404	*Kabuli*	Ca_Kabuli_Chr03	171576	(G/T)
3405	CakSNP3405	*Kabuli*	Ca_Kabuli_Chr03	171579	(C/T)
3406	CakSNP3406	*Kabuli*	Ca_Kabuli_Chr03	171675	(T/A)
3407	CakSNP3407	*Kabuli*	Ca_Kabuli_Chr03	208233	(C/A)
3408	CakSNP3408	*Kabuli*	Ca_Kabuli_Chr03	220564	(A/G)
3409	CakSNP3409	*Kabuli*	Ca_Kabuli_Chr03	220568	(A/T)
3410	CakSNP3410	*Kabuli*	Ca_Kabuli_Chr03	220572	(T/C)
3411	CakSNP3411	*Kabuli*	Ca_Kabuli_Chr03	220573	(G/T)
3412	CakSNP3412	*Kabuli*	Ca_Kabuli_Chr03	223943	(G/A)
3413	CakSNP3413	*Kabuli*	Ca_Kabuli_Chr03	223976	(G/C)
3414	CakSNP3414	*Kabuli*	Ca_Kabuli_Chr03	286425	(G/T)
3415	CakSNP3415	*Kabuli*	Ca_Kabuli_Chr03	419715	(T/G)
3416	CakSNP3416	*Kabuli*	Ca_Kabuli_Chr03	988477	(G/A)
3417	CakSNP3417	*Kabuli*	Ca_Kabuli_Chr03	988471	(T/C)
3418	CakSNP3418	*Kabuli*	Ca_Kabuli_Chr03	1499038	(G/A)
3419	CakSNP3419	*Kabuli*	Ca_Kabuli_Chr03	1499006	(A/G)
3420	CakSNP3420	*Kabuli*	Ca_Kabuli_Chr03	1746388	(C/A)
3421	CakSNP3421	*Kabuli*	Ca_Kabuli_Chr03	1746389	(A/G)
3422	CakSNP3422	*Kabuli*	Ca_Kabuli_Chr03	1829412	(T/G)
3423	CakSNP3423	*Kabuli*	Ca_Kabuli_Chr03	1829358	(T/C)
3424	CakSNP3424	*Kabuli*	Ca_Kabuli_Chr03	1829352	(C/T)
3425	CakSNP3425	*Kabuli*	Ca_Kabuli_Chr03	1831920	(T/G)
3426	CakSNP3426	*Kabuli*	Ca_Kabuli_Chr03	1831866	(T/C)
3427	CakSNP3427	*Kabuli*	Ca_Kabuli_Chr03	1831860	(C/T)
3428	CakSNP3428	*Kabuli*	Ca_Kabuli_Chr03	1831931	(A/C)
3429	CakSNP3429	*Kabuli*	Ca_Kabuli_Chr03	2097376	(T/G)
3430	CakSNP3430	*Kabuli*	Ca_Kabuli_Chr03	2097413	(A/G)

S.N.	SNP IDs	Cultivars	Chromosomes/scaffolds	Physical positions (bp)	SNPs
3431	CakSNP3431	*Kabuli*	Ca_Kabuli_Chr03	2391515	(T/G)
3432	CakSNP3432	*Kabuli*	Ca_Kabuli_Chr03	2423760	(T/C)
3433	CakSNP3433	*Kabuli*	Ca_Kabuli_Chr03	2708703	(C/T)
3434	CakSNP3434	*Kabuli*	Ca_Kabuli_Chr03	2825546	(G/T)
3435	CakSNP3435	*Kabuli*	Ca_Kabuli_Chr03	2927148	(T/A)
3436	CakSNP3436	*Kabuli*	Ca_Kabuli_Chr03	2927150	(A/T)
3437	CakSNP3437	*Kabuli*	Ca_Kabuli_Chr03	2931027	(G/C)
3438	CakSNP3438	*Kabuli*	Ca_Kabuli_Chr03	2931046	(C/T)
3439	CakSNP3439	*Kabuli*	Ca_Kabuli_Chr03	2975179	(T/C)
3440	CakSNP3440	*Kabuli*	Ca_Kabuli_Chr03	3280478	(C/T)
3441	CakSNP3441	*Kabuli*	Ca_Kabuli_Chr03	3280500	(C/T)
3442	CakSNP3442	*Kabuli*	Ca_Kabuli_Chr03	3280530	(G/A)
3443	CakSNP3443	*Kabuli*	Ca_Kabuli_Chr03	3280595	(G/A)
3444	CakSNP3444	*Kabuli*	Ca_Kabuli_Chr03	3617555	(G/A)
3445	CakSNP3445	*Kabuli*	Ca_Kabuli_Chr03	3701394	(A/T)
3446	CakSNP3446	*Kabuli*	Ca_Kabuli_Chr03	3701396	(C/T)
3447	CakSNP3447	*Kabuli*	Ca_Kabuli_Chr03	3701398	(A/T)
3448	CakSNP3448	*Kabuli*	Ca_Kabuli_Chr03	3701401	(A/T)
3449	CakSNP3449	*Kabuli*	Ca_Kabuli_Chr03	3701403	(A/T)
3450	CakSNP3450	*Kabuli*	Ca_Kabuli_Chr03	3701405	(G/T)
3451	CakSNP3451	*Kabuli*	Ca_Kabuli_Chr03	3701415	(C/T)
3452	CakSNP3452	*Kabuli*	Ca_Kabuli_Chr03	3701422	(C/T)
3453	CakSNP3453	*Kabuli*	Ca_Kabuli_Chr03	3701426	(T/C)
3454	CakSNP3454	*Kabuli*	Ca_Kabuli_Chr03	3701435	(A/T)
3455	CakSNP3455	*Kabuli*	Ca_Kabuli_Chr03	3701436	(C/T)
3456	CakSNP3456	*Kabuli*	Ca_Kabuli_Chr03	3701437	(A/T)
3457	CakSNP3457	*Kabuli*	Ca_Kabuli_Chr03	3701440	(G/T)
3458	CakSNP3458	*Kabuli*	Ca_Kabuli_Chr03	3701450	(C/T)
3459	CakSNP3459	*Kabuli*	Ca_Kabuli_Chr03	3701375	(G/A)
3460	CakSNP3460	*Kabuli*	Ca_Kabuli_Chr03	3701370	(G/A)
3461	CakSNP3461	*Kabuli*	Ca_Kabuli_Chr03	3744908	(G/A)
3462	CakSNP3462	*Kabuli*	Ca_Kabuli_Chr03	3764420	(T/A)
3463	CakSNP3463	*Kabuli*	Ca_Kabuli_Chr03	3764508	(T/C)
3464	CakSNP3464	*Kabuli*	Ca_Kabuli_Chr03	3764545	(C/T)
3465	CakSNP3465	*Kabuli*	Ca_Kabuli_Chr03	4145743	(G/A)
3466	CakSNP3466	*Kabuli*	Ca_Kabuli_Chr03	4178154	(C/T)
3467	CakSNP3467	*Kabuli*	Ca_Kabuli_Chr03	4266739	(G/A)
3468	CakSNP3468	*Kabuli*	Ca_Kabuli_Chr03	4266700	(T/G)
3469	CakSNP3469	*Kabuli*	Ca_Kabuli_Chr03	4266691	(C/T)

S.N.	SNP IDs	Cultivars	Chromosomes/scaffolds	Physical positions (bp)	SNPs
3470	CakSNP3470	*Kabuli*	Ca_Kabuli_Chr03	4266686	(C/T)
3471	CakSNP3471	*Kabuli*	Ca_Kabuli_Chr03	4266668	(G/A)
3472	CakSNP3472	*Kabuli*	Ca_Kabuli_Chr03	4555124	(G/A)
3473	CakSNP3473	*Kabuli*	Ca_Kabuli_Chr03	4555142	(T/A)
3474	CakSNP3474	*Kabuli*	Ca_Kabuli_Chr03	4555167	(C/G)
3475	CakSNP3475	*Kabuli*	Ca_Kabuli_Chr03	4555177	(C/T)
3476	CakSNP3476	*Kabuli*	Ca_Kabuli_Chr03	4596815	(C/T)
3477	CakSNP3477	*Kabuli*	Ca_Kabuli_Chr03	4596782	(A/G)
3478	CakSNP3478	*Kabuli*	Ca_Kabuli_Chr03	4604649	(A/G)
3479	CakSNP3479	*Kabuli*	Ca_Kabuli_Chr03	4606129	(T/A)
3480	CakSNP3480	*Kabuli*	Ca_Kabuli_Chr03	4606128	(A/G)
3481	CakSNP3481	*Kabuli*	Ca_Kabuli_Chr03	4629742	(A/T)
3482	CakSNP3482	*Kabuli*	Ca_Kabuli_Chr03	4629747	(A/C)
3483	CakSNP3483	*Kabuli*	Ca_Kabuli_Chr03	4654906	(C/T)
3484	CakSNP3484	*Kabuli*	Ca_Kabuli_Chr03	4654898	(G/T)
3485	CakSNP3485	*Kabuli*	Ca_Kabuli_Chr03	4654896	(T/C)
3486	CakSNP3486	*Kabuli*	Ca_Kabuli_Chr03	4823828	(T/C)
3487	CakSNP3487	*Kabuli*	Ca_Kabuli_Chr03	5224781	(A/G)
3488	CakSNP3488	*Kabuli*	Ca_Kabuli_Chr03	5225052	(A/G)
3489	CakSNP3489	*Kabuli*	Ca_Kabuli_Chr03	5225063	(G/T)
3490	CakSNP3490	*Kabuli*	Ca_Kabuli_Chr03	5225108	(T/G)
3491	CakSNP3491	*Kabuli*	Ca_Kabuli_Chr03	5225079	(G/A)
3492	CakSNP3492	*Kabuli*	Ca_Kabuli_Chr03	5415782	(G/A)
3493	CakSNP3493	*Kabuli*	Ca_Kabuli_Chr03	5436267	(A/G)
3494	CakSNP3494	*Kabuli*	Ca_Kabuli_Chr03	5436272	(T/C)
3495	CakSNP3495	*Kabuli*	Ca_Kabuli_Chr03	5436277	(C/T)
3496	CakSNP3496	*Kabuli*	Ca_Kabuli_Chr03	5436288	(G/T)
3497	CakSNP3497	*Kabuli*	Ca_Kabuli_Chr03	5436295	(G/A)
3498	CakSNP3498	*Kabuli*	Ca_Kabuli_Chr03	5436296	(C/T)
3499	CakSNP3499	*Kabuli*	Ca_Kabuli_Chr03	5436299	(A/C)
3500	CakSNP3500	*Kabuli*	Ca_Kabuli_Chr03	5436331	(C/A)
3501	CakSNP3501	*Kabuli*	Ca_Kabuli_Chr03	5514176	(A/G)
3502	CakSNP3502	*Kabuli*	Ca_Kabuli_Chr03	5514287	(C/A)
3503	CakSNP3503	*Kabuli*	Ca_Kabuli_Chr03	6169456	(G/T)
3504	CakSNP3504	*Kabuli*	Ca_Kabuli_Chr03	6169539	(T/C)
3505	CakSNP3505	*Kabuli*	Ca_Kabuli_Chr03	6169510	(A/C)
3506	CakSNP3506	*Kabuli*	Ca_Kabuli_Chr03	6278104	(G/A)
3507	CakSNP3507	*Kabuli*	Ca_Kabuli_Chr03	6467014	(G/A)
3508	CakSNP3508	*Kabuli*	Ca_Kabuli_Chr03	6683646	(A/G)

S.N.	SNP IDs	Cultivars	Chromosomes/scaffolds	Physical positions (bp)	SNPs
3509	CakSNP3509	*Kabuli*	Ca_Kabuli_Chr03	6683587	(T/A)
3510	CakSNP3510	*Kabuli*	Ca_Kabuli_Chr03	6828181	(A/T)
3511	CakSNP3511	*Kabuli*	Ca_Kabuli_Chr03	6840644	(C/T)
3512	CakSNP3512	*Kabuli*	Ca_Kabuli_Chr03	6894987	(C/A)
3513	CakSNP3513	*Kabuli*	Ca_Kabuli_Chr03	6895085	(T/C)
3514	CakSNP3514	*Kabuli*	Ca_Kabuli_Chr03	6899667	(T/C)
3515	CakSNP3515	*Kabuli*	Ca_Kabuli_Chr03	7013492	(G/A)
3516	CakSNP3516	*Kabuli*	Ca_Kabuli_Chr03	7015213	(T/G)
3517	CakSNP3517	*Kabuli*	Ca_Kabuli_Chr03	7024620	(G/A)
3518	CakSNP3518	*Kabuli*	Ca_Kabuli_Chr03	7204053	(G/A)
3519	CakSNP3519	*Kabuli*	Ca_Kabuli_Chr03	7204071	(T/C)
3520	CakSNP3520	*Kabuli*	Ca_Kabuli_Chr03	7204087	(C/T)
3521	CakSNP3521	*Kabuli*	Ca_Kabuli_Chr03	7204088	(G/C)
3522	CakSNP3522	*Kabuli*	Ca_Kabuli_Chr03	7204098	(G/A)
3523	CakSNP3523	*Kabuli*	Ca_Kabuli_Chr03	7204099	(C/T)
3524	CakSNP3524	*Kabuli*	Ca_Kabuli_Chr03	7204111	(G/A)
3525	CakSNP3525	*Kabuli*	Ca_Kabuli_Chr03	7204112	(G/A)
3526	CakSNP3526	*Kabuli*	Ca_Kabuli_Chr03	7204133	(G/T)
3527	CakSNP3527	*Kabuli*	Ca_Kabuli_Chr03	7226689	(T/C)
3528	CakSNP3528	*Kabuli*	Ca_Kabuli_Chr03	7226661	(G/T)
3529	CakSNP3529	*Kabuli*	Ca_Kabuli_Chr03	7226632	(G/T)
3530	CakSNP3530	*Kabuli*	Ca_Kabuli_Chr03	7246049	(T/G)
3531	CakSNP3531	*Kabuli*	Ca_Kabuli_Chr03	7420705	(G/A)
3532	CakSNP3532	*Kabuli*	Ca_Kabuli_Chr03	7433747	(C/A)
3533	CakSNP3533	*Kabuli*	Ca_Kabuli_Chr03	7461833	(A/G)
3534	CakSNP3534	*Kabuli*	Ca_Kabuli_Chr03	7482871	(C/T)
3535	CakSNP3535	*Kabuli*	Ca_Kabuli_Chr03	7606843	(A/G)
3536	CakSNP3536	*Kabuli*	Ca_Kabuli_Chr03	7606901	(T/C)
3537	CakSNP3537	*Kabuli*	Ca_Kabuli_Chr03	7606905	(C/G)
3538	CakSNP3538	*Kabuli*	Ca_Kabuli_Chr03	7608100	(A/G)
3539	CakSNP3539	*Kabuli*	Ca_Kabuli_Chr03	7665123	(G/A)
3540	CakSNP3540	*Kabuli*	Ca_Kabuli_Chr03	7665112	(C/T)
3541	CakSNP3541	*Kabuli*	Ca_Kabuli_Chr03	7665100	(G/T)
3542	CakSNP3542	*Kabuli*	Ca_Kabuli_Chr03	7665098	(C/T)
3543	CakSNP3543	*Kabuli*	Ca_Kabuli_Chr03	7665090	(C/T)
3544	CakSNP3544	*Kabuli*	Ca_Kabuli_Chr03	7665078	(G/A)
3545	CakSNP3545	*Kabuli*	Ca_Kabuli_Chr03	7665075	(G/A)
3546	CakSNP3546	*Kabuli*	Ca_Kabuli_Chr03	7665156	(G/A)
3547	CakSNP3547	*Kabuli*	Ca_Kabuli_Chr03	7665149	(T/G)

S.N.	SNP IDs	Cultivars	Chromosomes/scaffolds	Physical positions (bp)	SNPs
3548	CakSNP3548	*Kabuli*	Ca_Kabuli_Chr03	7665142	(G/A)
3549	CakSNP3549	*Kabuli*	Ca_Kabuli_Chr03	7665140	(G/T)
3550	CakSNP3550	*Kabuli*	Ca_Kabuli_Chr03	7665157	(C/T)
3551	CakSNP3551	*Kabuli*	Ca_Kabuli_Chr03	7694198	(C/T)
3552	CakSNP3552	*Kabuli*	Ca_Kabuli_Chr03	7694297	(T/C)
3553	CakSNP3553	*Kabuli*	Ca_Kabuli_Chr03	8536213	(T/C)
3554	CakSNP3554	*Kabuli*	Ca_Kabuli_Chr03	8536275	(C/T)
3555	CakSNP3555	*Kabuli*	Ca_Kabuli_Chr03	8536295	(A/T)
3556	CakSNP3556	*Kabuli*	Ca_Kabuli_Chr03	8536301	(G/T)
3557	CakSNP3557	*Kabuli*	Ca_Kabuli_Chr03	8536425	(C/A)
3558	CakSNP3558	*Kabuli*	Ca_Kabuli_Chr03	8536498	(T/A)
3559	CakSNP3559	*Kabuli*	Ca_Kabuli_Chr03	8536559	(T/C)
3560	CakSNP3560	*Kabuli*	Ca_Kabuli_Chr03	8668628	(A/C)
3561	CakSNP3561	*Kabuli*	Ca_Kabuli_Chr03	8669004	(A/T)
3562	CakSNP3562	*Kabuli*	Ca_Kabuli_Chr03	8669132	(T/A)
3563	CakSNP3563	*Kabuli*	Ca_Kabuli_Chr03	8697186	(A/G)
3564	CakSNP3564	*Kabuli*	Ca_Kabuli_Chr03	8712381	(A/C)
3565	CakSNP3565	*Kabuli*	Ca_Kabuli_Chr03	8933037	(T/C)
3566	CakSNP3566	*Kabuli*	Ca_Kabuli_Chr03	8933084	(G/A)
3567	CakSNP3567	*Kabuli*	Ca_Kabuli_Chr03	8933086	(C/A)
3568	CakSNP3568	*Kabuli*	Ca_Kabuli_Chr03	8933221	(T/A)
3569	CakSNP3569	*Kabuli*	Ca_Kabuli_Chr03	8933183	(A/G)
3570	CakSNP3570	*Kabuli*	Ca_Kabuli_Chr03	8933255	(A/T)
3571	CakSNP3571	*Kabuli*	Ca_Kabuli_Chr03	8933266	(G/T)
3572	CakSNP3572	*Kabuli*	Ca_Kabuli_Chr03	8933409	(A/C)
3573	CakSNP3573	*Kabuli*	Ca_Kabuli_Chr03	8933370	(A/G)
3574	CakSNP3574	*Kabuli*	Ca_Kabuli_Chr03	8933355	(A/T)
3575	CakSNP3575	*Kabuli*	Ca_Kabuli_Chr03	9221403	(G/A)
3576	CakSNP3576	*Kabuli*	Ca_Kabuli_Chr03	9221528	(C/T)
3577	CakSNP3577	*Kabuli*	Ca_Kabuli_Chr03	9221579	(G/C)
3578	CakSNP3578	*Kabuli*	Ca_Kabuli_Chr03	9221580	(T/G)
3579	CakSNP3579	*Kabuli*	Ca_Kabuli_Chr03	9223444	(G/C)
3580	CakSNP3580	*Kabuli*	Ca_Kabuli_Chr03	9223875	(G/A)
3581	CakSNP3581	*Kabuli*	Ca_Kabuli_Chr03	9224033	(C/A)
3582	CakSNP3582	*Kabuli*	Ca_Kabuli_Chr03	9224011	(G/A)
3583	CakSNP3583	*Kabuli*	Ca_Kabuli_Chr03	9296469	(T/G)
3584	CakSNP3584	*Kabuli*	Ca_Kabuli_Chr03	9448525	(T/C)
3585	CakSNP3585	*Kabuli*	Ca_Kabuli_Chr03	9577402	(G/A)
3586	CakSNP3586	*Kabuli*	Ca_Kabuli_Chr03	9579805	(A/T)

S.N.	SNP IDs	Cultivars	Chromosomes/scaffolds	Physical positions (bp)	SNPs
3587	CakSNP3587	*Kabuli*	Ca_Kabuli_Chr03	9579808	(C/T)
3588	CakSNP3588	*Kabuli*	Ca_Kabuli_Chr03	9579811	(G/C)
3589	CakSNP3589	*Kabuli*	Ca_Kabuli_Chr03	9579817	(G/A)
3590	CakSNP3590	*Kabuli*	Ca_Kabuli_Chr03	9579818	(G/T)
3591	CakSNP3591	*Kabuli*	Ca_Kabuli_Chr03	9579824	(A/G)
3592	CakSNP3592	*Kabuli*	Ca_Kabuli_Chr03	9579830	(T/A)
3593	CakSNP3593	*Kabuli*	Ca_Kabuli_Chr03	9579831	(C/G)
3594	CakSNP3594	*Kabuli*	Ca_Kabuli_Chr03	9579839	(G/A)
3595	CakSNP3595	*Kabuli*	Ca_Kabuli_Chr03	9894316	(C/T)
3596	CakSNP3596	*Kabuli*	Ca_Kabuli_Chr03	9894310	(C/A)
3597	CakSNP3597	*Kabuli*	Ca_Kabuli_Chr03	10039539	(T/A)
3598	CakSNP3598	*Kabuli*	Ca_Kabuli_Chr03	10105733	(A/G)
3599	CakSNP3599	*Kabuli*	Ca_Kabuli_Chr03	10142152	(C/A)
3600	CakSNP3600	*Kabuli*	Ca_Kabuli_Chr03	10159836	(A/C)
3601	CakSNP3601	*Kabuli*	Ca_Kabuli_Chr03	10159859	(G/A)
3602	CakSNP3602	*Kabuli*	Ca_Kabuli_Chr03	10159941	(C/A)
3603	CakSNP3603	*Kabuli*	Ca_Kabuli_Chr03	10159943	(G/A)
3604	CakSNP3604	*Kabuli*	Ca_Kabuli_Chr03	10159944	(T/C)
3605	CakSNP3605	*Kabuli*	Ca_Kabuli_Chr03	10186893	(G/C)
3606	CakSNP3606	*Kabuli*	Ca_Kabuli_Chr03	10186861	(G/T)
3607	CakSNP3607	*Kabuli*	Ca_Kabuli_Chr03	10186857	(A/G)
3608	CakSNP3608	*Kabuli*	Ca_Kabuli_Chr03	10186880	(A/G)
3609	CakSNP3609	*Kabuli*	Ca_Kabuli_Chr03	10186889	(G/A)
3610	CakSNP3610	*Kabuli*	Ca_Kabuli_Chr03	10186891	(C/G)
3611	CakSNP3611	*Kabuli*	Ca_Kabuli_Chr03	10201361	(G/T)
3612	CakSNP3612	*Kabuli*	Ca_Kabuli_Chr03	10722780	(G/T)
3613	CakSNP3613	*Kabuli*	Ca_Kabuli_Chr03	10722849	(G/C)
3614	CakSNP3614	*Kabuli*	Ca_Kabuli_Chr03	10722859	(G/A)
3615	CakSNP3615	*Kabuli*	Ca_Kabuli_Chr03	11143751	(G/A)
3616	CakSNP3616	*Kabuli*	Ca_Kabuli_Chr03	11143818	(G/A)
3617	CakSNP3617	*Kabuli*	Ca_Kabuli_Chr03	11143816	(C/T)
3618	CakSNP3618	*Kabuli*	Ca_Kabuli_Chr03	11376752	(C/A)
3619	CakSNP3619	*Kabuli*	Ca_Kabuli_Chr03	11378141	(C/T)
3620	CakSNP3620	*Kabuli*	Ca_Kabuli_Chr03	11378425	(G/T)
3621	CakSNP3621	*Kabuli*	Ca_Kabuli_Chr03	11590767	(C/T)
3622	CakSNP3622	*Kabuli*	Ca_Kabuli_Chr03	11590793	(C/T)
3623	CakSNP3623	*Kabuli*	Ca_Kabuli_Chr03	11590817	(C/G)
3624	CakSNP3624	*Kabuli*	Ca_Kabuli_Chr03	11590874	(A/G)
3625	CakSNP3625	*Kabuli*	Ca_Kabuli_Chr03	11590860	(G/A)

S.N.	SNP IDs	Cultivars	Chromosomes/scaffolds	Physical positions (bp)	SNPs
3626	CakSNP3626	*Kabuli*	Ca_Kabuli_Chr03	11590832	(G/A)
3627	CakSNP3627	*Kabuli*	Ca_Kabuli_Chr03	11590814	(T/C)
3628	CakSNP3628	*Kabuli*	Ca_Kabuli_Chr03	11610500	(A/G)
3629	CakSNP3629	*Kabuli*	Ca_Kabuli_Chr03	11610494	(C/T)
3630	CakSNP3630	*Kabuli*	Ca_Kabuli_Chr03	11610460	(A/G)
3631	CakSNP3631	*Kabuli*	Ca_Kabuli_Chr03	11610441	(G/T)
3632	CakSNP3632	*Kabuli*	Ca_Kabuli_Chr03	11610481	(C/T)
3633	CakSNP3633	*Kabuli*	Ca_Kabuli_Chr03	11610451	(C/T)
3634	CakSNP3634	*Kabuli*	Ca_Kabuli_Chr03	11678625	(G/A)
3635	CakSNP3635	*Kabuli*	Ca_Kabuli_Chr03	11684552	(G/A)
3636	CakSNP3636	*Kabuli*	Ca_Kabuli_Chr03	11709777	(G/T)
3637	CakSNP3637	*Kabuli*	Ca_Kabuli_Chr03	11811685	(A/G)
3638	CakSNP3638	*Kabuli*	Ca_Kabuli_Chr03	12051826	(A/C)
3639	CakSNP3639	*Kabuli*	Ca_Kabuli_Chr03	12058576	(T/C)
3640	CakSNP3640	*Kabuli*	Ca_Kabuli_Chr03	12058756	(A/G)
3641	CakSNP3641	*Kabuli*	Ca_Kabuli_Chr03	12058748	(A/T)
3642	CakSNP3642	*Kabuli*	Ca_Kabuli_Chr03	12067458	(A/C)
3643	CakSNP3643	*Kabuli*	Ca_Kabuli_Chr03	12069092	(T/G)
3644	CakSNP3644	*Kabuli*	Ca_Kabuli_Chr03	12095571	(T/G)
3645	CakSNP3645	*Kabuli*	Ca_Kabuli_Chr03	12124099	(G/A)
3646	CakSNP3646	*Kabuli*	Ca_Kabuli_Chr03	12394742	(G/A)
3647	CakSNP3647	*Kabuli*	Ca_Kabuli_Chr03	12599496	(A/C)
3648	CakSNP3648	*Kabuli*	Ca_Kabuli_Chr03	12622503	(A/G)
3649	CakSNP3649	*Kabuli*	Ca_Kabuli_Chr03	12622504	(G/T)
3650	CakSNP3650	*Kabuli*	Ca_Kabuli_Chr03	12622518	(T/G)
3651	CakSNP3651	*Kabuli*	Ca_Kabuli_Chr03	12622534	(C/T)
3652	CakSNP3652	*Kabuli*	Ca_Kabuli_Chr03	12622550	(A/T)
3653	CakSNP3653	*Kabuli*	Ca_Kabuli_Chr03	12622578	(G/A)
3654	CakSNP3654	*Kabuli*	Ca_Kabuli_Chr03	12622567	(A/C)
3655	CakSNP3655	*Kabuli*	Ca_Kabuli_Chr03	12622543	(G/A)
3656	CakSNP3656	*Kabuli*	Ca_Kabuli_Chr03	12627120	(C/T)
3657	CakSNP3657	*Kabuli*	Ca_Kabuli_Chr03	12627125	(T/C)
3658	CakSNP3658	*Kabuli*	Ca_Kabuli_Chr03	12627131	(G/A)
3659	CakSNP3659	*Kabuli*	Ca_Kabuli_Chr03	12627144	(G/A)
3660	CakSNP3660	*Kabuli*	Ca_Kabuli_Chr03	12627165	(T/C)
3661	CakSNP3661	*Kabuli*	Ca_Kabuli_Chr03	12627143	(T/C)
3662	CakSNP3662	*Kabuli*	Ca_Kabuli_Chr03	12627164	(G/A)
3663	CakSNP3663	*Kabuli*	Ca_Kabuli_Chr03	12627170	(C/G)
3664	CakSNP3664	*Kabuli*	Ca_Kabuli_Chr03	12809390	(A/G)

S.N.	SNP IDs	Cultivars	Chromosomes/scaffolds	Physical positions (bp)	SNPs
3665	CakSNP3665	*Kabuli*	Ca_Kabuli_Chr03	12809794	(T/C)
3666	CakSNP3666	*Kabuli*	Ca_Kabuli_Chr03	12814631	(C/T)
3667	CakSNP3667	*Kabuli*	Ca_Kabuli_Chr03	12814603	(T/C)
3668	CakSNP3668	*Kabuli*	Ca_Kabuli_Chr03	12814601	(A/C)
3669	CakSNP3669	*Kabuli*	Ca_Kabuli_Chr03	12814600	(A/G)
3670	CakSNP3670	*Kabuli*	Ca_Kabuli_Chr03	12814599	(A/T)
3671	CakSNP3671	*Kabuli*	Ca_Kabuli_Chr03	12814597	(T/A)
3672	CakSNP3672	*Kabuli*	Ca_Kabuli_Chr03	12814571	(C/A)
3673	CakSNP3673	*Kabuli*	Ca_Kabuli_Chr03	12814558	(A/G)
3674	CakSNP3674	*Kabuli*	Ca_Kabuli_Chr03	12897820	(C/T)
3675	CakSNP3675	*Kabuli*	Ca_Kabuli_Chr03	12897831	(C/A)
3676	CakSNP3676	*Kabuli*	Ca_Kabuli_Chr03	12897839	(G/A)
3677	CakSNP3677	*Kabuli*	Ca_Kabuli_Chr03	12897841	(C/A)
3678	CakSNP3678	*Kabuli*	Ca_Kabuli_Chr03	12897876	(C/T)
3679	CakSNP3679	*Kabuli*	Ca_Kabuli_Chr03	12897881	(C/T)
3680	CakSNP3680	*Kabuli*	Ca_Kabuli_Chr03	12897882	(T/G)
3681	CakSNP3681	*Kabuli*	Ca_Kabuli_Chr03	12897926	(A/C)
3682	CakSNP3682	*Kabuli*	Ca_Kabuli_Chr03	12897906	(C/A)
3683	CakSNP3683	*Kabuli*	Ca_Kabuli_Chr03	12897887	(T/C)
3684	CakSNP3684	*Kabuli*	Ca_Kabuli_Chr03	12897884	(C/T)
3685	CakSNP3685	*Kabuli*	Ca_Kabuli_Chr03	12897883	(G/T)
3686	CakSNP3686	*Kabuli*	Ca_Kabuli_Chr03	12897886	(G/A)
3687	CakSNP3687	*Kabuli*	Ca_Kabuli_Chr03	13185541	(G/A)
3688	CakSNP3688	*Kabuli*	Ca_Kabuli_Chr03	13185530	(C/T)
3689	CakSNP3689	*Kabuli*	Ca_Kabuli_Chr03	13185496	(G/A)
3690	CakSNP3690	*Kabuli*	Ca_Kabuli_Chr03	13185490	(G/A)
3691	CakSNP3691	*Kabuli*	Ca_Kabuli_Chr03	13185493	(G/A)
3692	CakSNP3692	*Kabuli*	Ca_Kabuli_Chr03	13185508	(C/T)
3693	CakSNP3693	*Kabuli*	Ca_Kabuli_Chr03	13185516	(C/T)
3694	CakSNP3694	*Kabuli*	Ca_Kabuli_Chr03	13185583	(T/C)
3695	CakSNP3695	*Kabuli*	Ca_Kabuli_Chr03	13185581	(T/A)
3696	CakSNP3696	*Kabuli*	Ca_Kabuli_Chr03	13185574	(G/A)
3697	CakSNP3697	*Kabuli*	Ca_Kabuli_Chr03	13185560	(G/A)
3698	CakSNP3698	*Kabuli*	Ca_Kabuli_Chr03	13185575	(C/T)
3699	CakSNP3699	*Kabuli*	Ca_Kabuli_Chr03	13185582	(A/G)
3700	CakSNP3700	*Kabuli*	Ca_Kabuli_Chr03	13185590	(C/A)
3701	CakSNP3701	*Kabuli*	Ca_Kabuli_Chr03	13185591	(C/T)
3702	CakSNP3702	*Kabuli*	Ca_Kabuli_Chr03	13185592	(A/C)
3703	CakSNP3703	*Kabuli*	Ca_Kabuli_Chr03	13202989	(C/T)

S.N.	SNP IDs	Cultivars	Chromosomes/scaffolds	Physical positions (bp)	SNPs
3704	CakSNP3704	*Kabuli*	Ca_Kabuli_Chr03	13396925	(A/T)
3705	CakSNP3705	*Kabuli*	Ca_Kabuli_Chr03	13397207	(A/C)
3706	CakSNP3706	*Kabuli*	Ca_Kabuli_Chr03	13420776	(T/C)
3707	CakSNP3707	*Kabuli*	Ca_Kabuli_Chr03	13420758	(A/G)
3708	CakSNP3708	*Kabuli*	Ca_Kabuli_Chr03	13420756	(A/G)
3709	CakSNP3709	*Kabuli*	Ca_Kabuli_Chr03	13617948	(A/C)
3710	CakSNP3710	*Kabuli*	Ca_Kabuli_Chr03	14200381	(A/C)
3711	CakSNP3711	*Kabuli*	Ca_Kabuli_Chr03	14465236	(T/G)
3712	CakSNP3712	*Kabuli*	Ca_Kabuli_Chr03	14497374	(C/A)
3713	CakSNP3713	*Kabuli*	Ca_Kabuli_Chr03	14653195	(G/T)
3714	CakSNP3714	*Kabuli*	Ca_Kabuli_Chr03	14653194	(C/T)
3715	CakSNP3715	*Kabuli*	Ca_Kabuli_Chr03	14653193	(T/G)
3716	CakSNP3716	*Kabuli*	Ca_Kabuli_Chr03	14653270	(C/A)
3717	CakSNP3717	*Kabuli*	Ca_Kabuli_Chr03	14653271	(G/A)
3718	CakSNP3718	*Kabuli*	Ca_Kabuli_Chr03	14658175	(A/C)
3719	CakSNP3719	*Kabuli*	Ca_Kabuli_Chr03	14812960	(A/C)
3720	CakSNP3720	*Kabuli*	Ca_Kabuli_Chr03	14982667	(C/T)
3721	CakSNP3721	*Kabuli*	Ca_Kabuli_Chr03	14982739	(A/T)
3722	CakSNP3722	*Kabuli*	Ca_Kabuli_Chr03	15744906	(T/A)
3723	CakSNP3723	*Kabuli*	Ca_Kabuli_Chr03	15745010	(C/T)
3724	CakSNP3724	*Kabuli*	Ca_Kabuli_Chr03	15745017	(A/G)
3725	CakSNP3725	*Kabuli*	Ca_Kabuli_Chr03	15745264	(G/C)
3726	CakSNP3726	*Kabuli*	Ca_Kabuli_Chr03	15745311	(G/A)
3727	CakSNP3727	*Kabuli*	Ca_Kabuli_Chr03	15757804	(T/C)
3728	CakSNP3728	*Kabuli*	Ca_Kabuli_Chr03	15757829	(C/T)
3729	CakSNP3729	*Kabuli*	Ca_Kabuli_Chr03	15757852	(A/C)
3730	CakSNP3730	*Kabuli*	Ca_Kabuli_Chr03	16071411	(G/C)
3731	CakSNP3731	*Kabuli*	Ca_Kabuli_Chr03	16396662	(C/G)
3732	CakSNP3732	*Kabuli*	Ca_Kabuli_Chr03	16396673	(A/C)
3733	CakSNP3733	*Kabuli*	Ca_Kabuli_Chr03	16436286	(T/A)
3734	CakSNP3734	*Kabuli*	Ca_Kabuli_Chr03	16458123	(G/T)
3735	CakSNP3735	*Kabuli*	Ca_Kabuli_Chr03	16458413	(T/A)
3736	CakSNP3736	*Kabuli*	Ca_Kabuli_Chr03	16540328	(T/G)
3737	CakSNP3737	*Kabuli*	Ca_Kabuli_Chr03	16568567	(A/G)
3738	CakSNP3738	*Kabuli*	Ca_Kabuli_Chr03	16621498	(G/A)
3739	CakSNP3739	*Kabuli*	Ca_Kabuli_Chr03	16621624	(G/T)
3740	CakSNP3740	*Kabuli*	Ca_Kabuli_Chr03	16768867	(A/G)
3741	CakSNP3741	*Kabuli*	Ca_Kabuli_Chr03	16768936	(A/G)
3742	CakSNP3742	*Kabuli*	Ca_Kabuli_Chr03	16768917	(C/T)

S.N.	SNP IDs	Cultivars	Chromosomes/scaffolds	Physical positions (bp)	SNPs
3743	CakSNP3743	*Kabuli*	Ca_Kabuli_Chr03	16948510	(G/T)
3744	CakSNP3744	*Kabuli*	Ca_Kabuli_Chr03	16948854	(A/T)
3745	CakSNP3745	*Kabuli*	Ca_Kabuli_Chr03	16995243	(C/T)
3746	CakSNP3746	*Kabuli*	Ca_Kabuli_Chr03	16997328	(G/A)
3747	CakSNP3747	*Kabuli*	Ca_Kabuli_Chr03	16997331	(C/T)
3748	CakSNP3748	*Kabuli*	Ca_Kabuli_Chr03	16997400	(T/C)
3749	CakSNP3749	*Kabuli*	Ca_Kabuli_Chr03	17136071	(G/C)
3750	CakSNP3750	*Kabuli*	Ca_Kabuli_Chr03	17136097	(T/C)
3751	CakSNP3751	*Kabuli*	Ca_Kabuli_Chr03	17145639	(A/T)
3752	CakSNP3752	*Kabuli*	Ca_Kabuli_Chr03	17145643	(G/T)
3753	CakSNP3753	*Kabuli*	Ca_Kabuli_Chr03	17181213	(A/G)
3754	CakSNP3754	*Kabuli*	Ca_Kabuli_Chr03	17185682	(A/G)
3755	CakSNP3755	*Kabuli*	Ca_Kabuli_Chr03	17334924	(G/T)
3756	CakSNP3756	*Kabuli*	Ca_Kabuli_Chr03	17335851	(A/C)
3757	CakSNP3757	*Kabuli*	Ca_Kabuli_Chr03	17351553	(A/T)
3758	CakSNP3758	*Kabuli*	Ca_Kabuli_Chr03	17351526	(A/C)
3759	CakSNP3759	*Kabuli*	Ca_Kabuli_Chr03	17459986	(T/A)
3760	CakSNP3760	*Kabuli*	Ca_Kabuli_Chr03	17460002	(G/A)
3761	CakSNP3761	*Kabuli*	Ca_Kabuli_Chr03	17460012	(C/A)
3762	CakSNP3762	*Kabuli*	Ca_Kabuli_Chr03	17574247	(C/T)
3763	CakSNP3763	*Kabuli*	Ca_Kabuli_Chr03	17635591	(T/A)
3764	CakSNP3764	*Kabuli*	Ca_Kabuli_Chr03	17635531	(T/C)
3765	CakSNP3765	*Kabuli*	Ca_Kabuli_Chr03	17679409	(C/T)
3766	CakSNP3766	*Kabuli*	Ca_Kabuli_Chr03	17679404	(C/T)
3767	CakSNP3767	*Kabuli*	Ca_Kabuli_Chr03	17914677	(T/G)
3768	CakSNP3768	*Kabuli*	Ca_Kabuli_Chr03	17936321	(T/C)
3769	CakSNP3769	*Kabuli*	Ca_Kabuli_Chr03	17936729	(G/A)
3770	CakSNP3770	*Kabuli*	Ca_Kabuli_Chr03	17936842	(A/G)
3771	CakSNP3771	*Kabuli*	Ca_Kabuli_Chr03	17984978	(T/G)
3772	CakSNP3772	*Kabuli*	Ca_Kabuli_Chr03	17986521	(G/C)
3773	CakSNP3773	*Kabuli*	Ca_Kabuli_Chr03	17986525	(G/T)
3774	CakSNP3774	*Kabuli*	Ca_Kabuli_Chr03	17986740	(T/C)
3775	CakSNP3775	*Kabuli*	Ca_Kabuli_Chr03	18215321	(G/C)
3776	CakSNP3776	*Kabuli*	Ca_Kabuli_Chr03	18215332	(T/C)
3777	CakSNP3777	*Kabuli*	Ca_Kabuli_Chr03	18355427	(T/C)
3778	CakSNP3778	*Kabuli*	Ca_Kabuli_Chr03	18355445	(T/C)
3779	CakSNP3779	*Kabuli*	Ca_Kabuli_Chr03	18375547	(T/C)
3780	CakSNP3780	*Kabuli*	Ca_Kabuli_Chr03	18375581	(G/C)
3781	CakSNP3781	*Kabuli*	Ca_Kabuli_Chr03	18382404	(T/C)

S.N.	SNP IDs	Cultivars	Chromosomes/scaffolds	Physical positions (bp)	SNPs
3782	CakSNP3782	*Kabuli*	Ca_Kabuli_Chr03	18382438	(G/C)
3783	CakSNP3783	*Kabuli*	Ca_Kabuli_Chr03	18383884	(T/C)
3784	CakSNP3784	*Kabuli*	Ca_Kabuli_Chr03	18476006	(A/G)
3785	CakSNP3785	*Kabuli*	Ca_Kabuli_Chr03	18476065	(C/A)
3786	CakSNP3786	*Kabuli*	Ca_Kabuli_Chr03	18497633	(T/C)
3787	CakSNP3787	*Kabuli*	Ca_Kabuli_Chr03	18573701	(T/A)
3788	CakSNP3788	*Kabuli*	Ca_Kabuli_Chr03	18645114	(T/C)
3789	CakSNP3789	*Kabuli*	Ca_Kabuli_Chr03	18687994	(T/A)
3790	CakSNP3790	*Kabuli*	Ca_Kabuli_Chr03	18709398	(G/A)
3791	CakSNP3791	*Kabuli*	Ca_Kabuli_Chr03	18731098	(T/C)
3792	CakSNP3792	*Kabuli*	Ca_Kabuli_Chr03	18825708	(T/C)
3793	CakSNP3793	*Kabuli*	Ca_Kabuli_Chr03	18825687	(T/C)
3794	CakSNP3794	*Kabuli*	Ca_Kabuli_Chr03	18939425	(C/G)
3795	CakSNP3795	*Kabuli*	Ca_Kabuli_Chr03	19372722	(G/T)
3796	CakSNP3796	*Kabuli*	Ca_Kabuli_Chr03	19372739	(C/T)
3797	CakSNP3797	*Kabuli*	Ca_Kabuli_Chr03	19400104	(T/C)
3798	CakSNP3798	*Kabuli*	Ca_Kabuli_Chr03	19400121	(G/T)
3799	CakSNP3799	*Kabuli*	Ca_Kabuli_Chr03	19400276	(C/T)
3800	CakSNP3800	*Kabuli*	Ca_Kabuli_Chr03	19442839	(G/A)
3801	CakSNP3801	*Kabuli*	Ca_Kabuli_Chr03	19442884	(G/C)
3802	CakSNP3802	*Kabuli*	Ca_Kabuli_Chr03	19442941	(G/A)
3803	CakSNP3803	*Kabuli*	Ca_Kabuli_Chr03	19445925	(A/G)
3804	CakSNP3804	*Kabuli*	Ca_Kabuli_Chr03	19445920	(T/A)
3805	CakSNP3805	*Kabuli*	Ca_Kabuli_Chr03	19448140	(A/G)
3806	CakSNP3806	*Kabuli*	Ca_Kabuli_Chr03	19475095	(G/T)
3807	CakSNP3807	*Kabuli*	Ca_Kabuli_Chr03	19571396	(C/A)
3808	CakSNP3808	*Kabuli*	Ca_Kabuli_Chr03	19571382	(G/T)
3809	CakSNP3809	*Kabuli*	Ca_Kabuli_Chr03	19571371	(A/G)
3810	CakSNP3810	*Kabuli*	Ca_Kabuli_Chr03	19742600	(G/A)
3811	CakSNP3811	*Kabuli*	Ca_Kabuli_Chr03	19742530	(C/T)
3812	CakSNP3812	*Kabuli*	Ca_Kabuli_Chr03	19788512	(G/A)
3813	CakSNP3813	*Kabuli*	Ca_Kabuli_Chr03	19802763	(C/T)
3814	CakSNP3814	*Kabuli*	Ca_Kabuli_Chr03	19804985	(T/C)
3815	CakSNP3815	*Kabuli*	Ca_Kabuli_Chr03	19805063	(A/C)
3816	CakSNP3816	*Kabuli*	Ca_Kabuli_Chr03	19829510	(G/T)
3817	CakSNP3817	*Kabuli*	Ca_Kabuli_Chr03	19850532	(T/C)
3818	CakSNP3818	*Kabuli*	Ca_Kabuli_Chr03	19850589	(C/T)
3819	CakSNP3819	*Kabuli*	Ca_Kabuli_Chr03	19855217	(T/C)
3820	CakSNP3820	*Kabuli*	Ca_Kabuli_Chr03	19857613	(T/A)

S.N.	SNP IDs	Cultivars	Chromosomes/scaffolds	Physical positions (bp)	SNPs
3821	CakSNP3821	*Kabuli*	Ca_Kabuli_Chr03	19883898	(T/C)
3822	CakSNP3822	*Kabuli*	Ca_Kabuli_Chr03	19883927	(A/G)
3823	CakSNP3823	*Kabuli*	Ca_Kabuli_Chr03	19883936	(T/G)
3824	CakSNP3824	*Kabuli*	Ca_Kabuli_Chr03	19896800	(C/T)
3825	CakSNP3825	*Kabuli*	Ca_Kabuli_Chr03	19896849	(G/A)
3826	CakSNP3826	*Kabuli*	Ca_Kabuli_Chr03	19907774	(T/C)
3827	CakSNP3827	*Kabuli*	Ca_Kabuli_Chr03	19956343	(C/T)
3828	CakSNP3828	*Kabuli*	Ca_Kabuli_Chr03	19956391	(C/T)
3829	CakSNP3829	*Kabuli*	Ca_Kabuli_Chr03	19978343	(A/C)
3830	CakSNP3830	*Kabuli*	Ca_Kabuli_Chr03	19978334	(A/C)
3831	CakSNP3831	*Kabuli*	Ca_Kabuli_Chr03	19978407	(C/T)
3832	CakSNP3832	*Kabuli*	Ca_Kabuli_Chr03	19979288	(A/T)
3833	CakSNP3833	*Kabuli*	Ca_Kabuli_Chr03	19979318	(C/A)
3834	CakSNP3834	*Kabuli*	Ca_Kabuli_Chr03	19979390	(A/T)
3835	CakSNP3835	*Kabuli*	Ca_Kabuli_Chr03	20000967	(C/G)
3836	CakSNP3836	*Kabuli*	Ca_Kabuli_Chr03	20030262	(T/C)
3837	CakSNP3837	*Kabuli*	Ca_Kabuli_Chr03	20030298	(G/A)
3838	CakSNP3838	*Kabuli*	Ca_Kabuli_Chr03	20030484	(A/G)
3839	CakSNP3839	*Kabuli*	Ca_Kabuli_Chr03	20103039	(G/A)
3840	CakSNP3840	*Kabuli*	Ca_Kabuli_Chr03	20103060	(C/T)
3841	CakSNP3841	*Kabuli*	Ca_Kabuli_Chr03	20103126	(A/C)
3842	CakSNP3842	*Kabuli*	Ca_Kabuli_Chr03	20172492	(C/T)
3843	CakSNP3843	*Kabuli*	Ca_Kabuli_Chr03	20240118	(T/G)
3844	CakSNP3844	*Kabuli*	Ca_Kabuli_Chr03	20242663	(C/T)
3845	CakSNP3845	*Kabuli*	Ca_Kabuli_Chr03	20331659	(G/A)
3846	CakSNP3846	*Kabuli*	Ca_Kabuli_Chr03	20365442	(A/C)
3847	CakSNP3847	*Kabuli*	Ca_Kabuli_Chr03	20387399	(T/A)
3848	CakSNP3848	*Kabuli*	Ca_Kabuli_Chr03	20387471	(A/G)
3849	CakSNP3849	*Kabuli*	Ca_Kabuli_Chr03	20415660	(C/G)
3850	CakSNP3850	*Kabuli*	Ca_Kabuli_Chr03	20415615	(A/G)
3851	CakSNP3851	*Kabuli*	Ca_Kabuli_Chr03	20462715	(T/A)
3852	CakSNP3852	*Kabuli*	Ca_Kabuli_Chr03	20490488	(T/C)
3853	CakSNP3853	*Kabuli*	Ca_Kabuli_Chr03	20495810	(G/A)
3854	CakSNP3854	*Kabuli*	Ca_Kabuli_Chr03	20503939	(G/T)
3855	CakSNP3855	*Kabuli*	Ca_Kabuli_Chr03	20506663	(C/G)
3856	CakSNP3856	*Kabuli*	Ca_Kabuli_Chr03	20552836	(T/G)
3857	CakSNP3857	*Kabuli*	Ca_Kabuli_Chr03	20555942	(G/A)
3858	CakSNP3858	*Kabuli*	Ca_Kabuli_Chr03	20690207	(T/G)
3859	CakSNP3859	*Kabuli*	Ca_Kabuli_Chr03	20690185	(A/T)

S.N.	SNP IDs	Cultivars	Chromosomes/scaffolds	Physical positions (bp)	SNPs
3860	CakSNP3860	*Kabuli*	Ca_Kabuli_Chr03	20794236	(A/T)
3861	CakSNP3861	*Kabuli*	Ca_Kabuli_Chr03	20794206	(A/T)
3862	CakSNP3862	*Kabuli*	Ca_Kabuli_Chr03	20794984	(C/T)
3863	CakSNP3863	*Kabuli*	Ca_Kabuli_Chr03	20795019	(C/T)
3864	CakSNP3864	*Kabuli*	Ca_Kabuli_Chr03	20795014	(C/T)
3865	CakSNP3865	*Kabuli*	Ca_Kabuli_Chr03	20794976	(A/C)
3866	CakSNP3866	*Kabuli*	Ca_Kabuli_Chr03	20794993	(A/G)
3867	CakSNP3867	*Kabuli*	Ca_Kabuli_Chr03	20794990	(G/A)
3868	CakSNP3868	*Kabuli*	Ca_Kabuli_Chr03	20799767	(A/G)
3869	CakSNP3869	*Kabuli*	Ca_Kabuli_Chr03	20799778	(C/T)
3870	CakSNP3870	*Kabuli*	Ca_Kabuli_Chr03	20799811	(C/T)
3871	CakSNP3871	*Kabuli*	Ca_Kabuli_Chr03	20799860	(A/G)
3872	CakSNP3872	*Kabuli*	Ca_Kabuli_Chr03	20799841	(C/T)
3873	CakSNP3873	*Kabuli*	Ca_Kabuli_Chr03	20799819	(C/T)
3874	CakSNP3874	*Kabuli*	Ca_Kabuli_Chr03	20799820	(A/G)
3875	CakSNP3875	*Kabuli*	Ca_Kabuli_Chr03	20822676	(T/C)
3876	CakSNP3876	*Kabuli*	Ca_Kabuli_Chr03	20823146	(C/T)
3877	CakSNP3877	*Kabuli*	Ca_Kabuli_Chr03	20828272	(G/T)
3878	CakSNP3878	*Kabuli*	Ca_Kabuli_Chr03	20829399	(A/G)
3879	CakSNP3879	*Kabuli*	Ca_Kabuli_Chr03	20829407	(T/G)
3880	CakSNP3880	*Kabuli*	Ca_Kabuli_Chr03	20841298	(T/G)
3881	CakSNP3881	*Kabuli*	Ca_Kabuli_Chr03	20841352	(C/T)
3882	CakSNP3882	*Kabuli*	Ca_Kabuli_Chr03	20880294	(T/A)
3883	CakSNP3883	*Kabuli*	Ca_Kabuli_Chr03	21020182	(A/G)
3884	CakSNP3884	*Kabuli*	Ca_Kabuli_Chr03	21020191	(C/T)
3885	CakSNP3885	*Kabuli*	Ca_Kabuli_Chr03	21069672	(A/G)
3886	CakSNP3886	*Kabuli*	Ca_Kabuli_Chr03	21069714	(G/A)
3887	CakSNP3887	*Kabuli*	Ca_Kabuli_Chr03	21069916	(C/T)
3888	CakSNP3888	*Kabuli*	Ca_Kabuli_Chr03	21069917	(G/A)
3889	CakSNP3889	*Kabuli*	Ca_Kabuli_Chr03	21069938	(C/A)
3890	CakSNP3890	*Kabuli*	Ca_Kabuli_Chr03	21069959	(G/A)
3891	CakSNP3891	*Kabuli*	Ca_Kabuli_Chr03	21069986	(A/C)
3892	CakSNP3892	*Kabuli*	Ca_Kabuli_Chr03	21127162	(C/G)
3893	CakSNP3893	*Kabuli*	Ca_Kabuli_Chr03	21127156	(T/G)
3894	CakSNP3894	*Kabuli*	Ca_Kabuli_Chr03	21198607	(A/T)
3895	CakSNP3895	*Kabuli*	Ca_Kabuli_Chr03	21210002	(T/A)
3896	CakSNP3896	*Kabuli*	Ca_Kabuli_Chr03	21210005	(C/A)
3897	CakSNP3897	*Kabuli*	Ca_Kabuli_Chr03	21210008	(T/C)
3898	CakSNP3898	*Kabuli*	Ca_Kabuli_Chr03	21210049	(A/G)

S.N.	SNP IDs	Cultivars	Chromosomes/scaffolds	Physical positions (bp)	SNPs
3899	CakSNP3899	*Kabuli*	Ca_Kabuli_Chr03	21273368	(A/G)
3900	CakSNP3900	*Kabuli*	Ca_Kabuli_Chr03	21288626	(C/A)
3901	CakSNP3901	*Kabuli*	Ca_Kabuli_Chr03	21288652	(G/C)
3902	CakSNP3902	*Kabuli*	Ca_Kabuli_Chr03	21288695	(G/A)
3903	CakSNP3903	*Kabuli*	Ca_Kabuli_Chr03	21335892	(C/T)
3904	CakSNP3904	*Kabuli*	Ca_Kabuli_Chr03	21382790	(G/A)
3905	CakSNP3905	*Kabuli*	Ca_Kabuli_Chr03	21421082	(A/G)
3906	CakSNP3906	*Kabuli*	Ca_Kabuli_Chr03	21421085	(G/A)
3907	CakSNP3907	*Kabuli*	Ca_Kabuli_Chr03	21421146	(C/A)
3908	CakSNP3908	*Kabuli*	Ca_Kabuli_Chr03	21421162	(G/A)
3909	CakSNP3909	*Kabuli*	Ca_Kabuli_Chr03	21421358	(G/A)
3910	CakSNP3910	*Kabuli*	Ca_Kabuli_Chr03	21421362	(T/A)
3911	CakSNP3911	*Kabuli*	Ca_Kabuli_Chr03	21421369	(G/A)
3912	CakSNP3912	*Kabuli*	Ca_Kabuli_Chr03	21421424	(G/A)
3913	CakSNP3913	*Kabuli*	Ca_Kabuli_Chr03	21421440	(C/T)
3914	CakSNP3914	*Kabuli*	Ca_Kabuli_Chr03	21450650	(A/G)
3915	CakSNP3915	*Kabuli*	Ca_Kabuli_Chr03	21452017	(G/A)
3916	CakSNP3916	*Kabuli*	Ca_Kabuli_Chr03	21468634	(G/A)
3917	CakSNP3917	*Kabuli*	Ca_Kabuli_Chr03	21468692	(A/C)
3918	CakSNP3918	*Kabuli*	Ca_Kabuli_Chr03	21470784	(C/T)
3919	CakSNP3919	*Kabuli*	Ca_Kabuli_Chr03	21500178	(G/A)
3920	CakSNP3920	*Kabuli*	Ca_Kabuli_Chr03	21500179	(A/T)
3921	CakSNP3921	*Kabuli*	Ca_Kabuli_Chr03	21507422	(T/C)
3922	CakSNP3922	*Kabuli*	Ca_Kabuli_Chr03	21524084	(T/C)
3923	CakSNP3923	*Kabuli*	Ca_Kabuli_Chr03	21524192	(T/C)
3924	CakSNP3924	*Kabuli*	Ca_Kabuli_Chr03	21524296	(G/T)
3925	CakSNP3925	*Kabuli*	Ca_Kabuli_Chr03	21524379	(A/G)
3926	CakSNP3926	*Kabuli*	Ca_Kabuli_Chr03	21525184	(C/T)
3927	CakSNP3927	*Kabuli*	Ca_Kabuli_Chr03	21525177	(G/A)
3928	CakSNP3928	*Kabuli*	Ca_Kabuli_Chr03	21558388	(A/G)
3929	CakSNP3929	*Kabuli*	Ca_Kabuli_Chr03	21558357	(T/G)
3930	CakSNP3930	*Kabuli*	Ca_Kabuli_Chr03	21627610	(T/C)
3931	CakSNP3931	*Kabuli*	Ca_Kabuli_Chr03	21634275	(A/C)
3932	CakSNP3932	*Kabuli*	Ca_Kabuli_Chr03	21634271	(A/G)
3933	CakSNP3933	*Kabuli*	Ca_Kabuli_Chr03	21646863	(C/T)
3934	CakSNP3934	*Kabuli*	Ca_Kabuli_Chr03	21646872	(T/C)
3935	CakSNP3935	*Kabuli*	Ca_Kabuli_Chr03	21646893	(C/A)
3936	CakSNP3936	*Kabuli*	Ca_Kabuli_Chr03	21646965	(C/T)
3937	CakSNP3937	*Kabuli*	Ca_Kabuli_Chr03	21646962	(A/G)

S.N.	SNP IDs	Cultivars	Chromosomes/scaffolds	Physical positions (bp)	SNPs
3938	CakSNP3938	*Kabuli*	Ca_Kabuli_Chr03	21654592	(T/A)
3939	CakSNP3939	*Kabuli*	Ca_Kabuli_Chr03	21686470	(C/T)
3940	CakSNP3940	*Kabuli*	Ca_Kabuli_Chr03	21687439	(G/T)
3941	CakSNP3941	*Kabuli*	Ca_Kabuli_Chr03	21687966	(C/A)
3942	CakSNP3942	*Kabuli*	Ca_Kabuli_Chr03	21687961	(C/A)
3943	CakSNP3943	*Kabuli*	Ca_Kabuli_Chr03	21687960	(G/A)
3944	CakSNP3944	*Kabuli*	Ca_Kabuli_Chr03	21687959	(T/C)
3945	CakSNP3945	*Kabuli*	Ca_Kabuli_Chr03	21687956	(C/T)
3946	CakSNP3946	*Kabuli*	Ca_Kabuli_Chr03	21687953	(C/T)
3947	CakSNP3947	*Kabuli*	Ca_Kabuli_Chr03	21741914	(C/A)
3948	CakSNP3948	*Kabuli*	Ca_Kabuli_Chr03	21809395	(G/A)
3949	CakSNP3949	*Kabuli*	Ca_Kabuli_Chr03	21809456	(T/C)
3950	CakSNP3950	*Kabuli*	Ca_Kabuli_Chr03	21809457	(G/A)
3951	CakSNP3951	*Kabuli*	Ca_Kabuli_Chr03	21809546	(T/A)
3952	CakSNP3952	*Kabuli*	Ca_Kabuli_Chr03	21809505	(A/G)
3953	CakSNP3953	*Kabuli*	Ca_Kabuli_Chr03	21809503	(T/C)
3954	CakSNP3954	*Kabuli*	Ca_Kabuli_Chr03	21950053	(C/G)
3955	CakSNP3955	*Kabuli*	Ca_Kabuli_Chr03	21950186	(A/G)
3956	CakSNP3956	*Kabuli*	Ca_Kabuli_Chr03	21950164	(A/G)
3957	CakSNP3957	*Kabuli*	Ca_Kabuli_Chr03	22008087	(G/A)
3958	CakSNP3958	*Kabuli*	Ca_Kabuli_Chr03	22026111	(A/G)
3959	CakSNP3959	*Kabuli*	Ca_Kabuli_Chr03	22026647	(A/G)
3960	CakSNP3960	*Kabuli*	Ca_Kabuli_Chr03	22060199	(G/T)
3961	CakSNP3961	*Kabuli*	Ca_Kabuli_Chr03	22061236	(A/G)
3962	CakSNP3962	*Kabuli*	Ca_Kabuli_Chr03	22062029	(G/T)
3963	CakSNP3963	*Kabuli*	Ca_Kabuli_Chr03	22062958	(T/C)
3964	CakSNP3964	*Kabuli*	Ca_Kabuli_Chr03	22062965	(C/T)
3965	CakSNP3965	*Kabuli*	Ca_Kabuli_Chr03	22063826	(G/A)
3966	CakSNP3966	*Kabuli*	Ca_Kabuli_Chr03	22063829	(G/A)
3967	CakSNP3967	*Kabuli*	Ca_Kabuli_Chr03	22063835	(C/T)
3968	CakSNP3968	*Kabuli*	Ca_Kabuli_Chr03	22073381	(C/T)
3969	CakSNP3969	*Kabuli*	Ca_Kabuli_Chr03	22073447	(T/A)
3970	CakSNP3970	*Kabuli*	Ca_Kabuli_Chr03	22075779	(C/G)
3971	CakSNP3971	*Kabuli*	Ca_Kabuli_Chr03	22075990	(T/C)
3972	CakSNP3972	*Kabuli*	Ca_Kabuli_Chr03	22169816	(T/C)
3973	CakSNP3973	*Kabuli*	Ca_Kabuli_Chr03	22188124	(T/C)
3974	CakSNP3974	*Kabuli*	Ca_Kabuli_Chr03	22188169	(T/A)
3975	CakSNP3975	*Kabuli*	Ca_Kabuli_Chr03	22200927	(T/C)
3976	CakSNP3976	*Kabuli*	Ca_Kabuli_Chr03	22201045	(T/A)

S.N.	SNP IDs	Cultivars	Chromosomes/scaffolds	Physical positions (bp)	SNPs
3977	CakSNP3977	*Kabuli*	Ca_Kabuli_Chr03	22202317	(C/T)
3978	CakSNP3978	*Kabuli*	Ca_Kabuli_Chr03	22202819	(T/C)
3979	CakSNP3979	*Kabuli*	Ca_Kabuli_Chr03	22203460	(A/G)
3980	CakSNP3980	*Kabuli*	Ca_Kabuli_Chr03	22225186	(C/A)
3981	CakSNP3981	*Kabuli*	Ca_Kabuli_Chr03	22225154	(C/G)
3982	CakSNP3982	*Kabuli*	Ca_Kabuli_Chr03	22225129	(C/G)
3983	CakSNP3983	*Kabuli*	Ca_Kabuli_Chr03	22233428	(G/A)
3984	CakSNP3984	*Kabuli*	Ca_Kabuli_Chr03	22233409	(T/C)
3985	CakSNP3985	*Kabuli*	Ca_Kabuli_Chr03	22235801	(G/A)
3986	CakSNP3986	*Kabuli*	Ca_Kabuli_Chr03	22253842	(T/C)
3987	CakSNP3987	*Kabuli*	Ca_Kabuli_Chr03	22331057	(C/T)
3988	CakSNP3988	*Kabuli*	Ca_Kabuli_Chr03	22346294	(T/C)
3989	CakSNP3989	*Kabuli*	Ca_Kabuli_Chr03	22404660	(G/T)
3990	CakSNP3990	*Kabuli*	Ca_Kabuli_Chr03	22527529	(A/C)
3991	CakSNP3991	*Kabuli*	Ca_Kabuli_Chr03	22539384	(G/A)
3992	CakSNP3992	*Kabuli*	Ca_Kabuli_Chr03	22539702	(C/T)
3993	CakSNP3993	*Kabuli*	Ca_Kabuli_Chr03	22539885	(C/T)
3994	CakSNP3994	*Kabuli*	Ca_Kabuli_Chr03	22539952	(G/A)
3995	CakSNP3995	*Kabuli*	Ca_Kabuli_Chr03	22636201	(T/C)
3996	CakSNP3996	*Kabuli*	Ca_Kabuli_Chr03	22646897	(T/C)
3997	CakSNP3997	*Kabuli*	Ca_Kabuli_Chr03	22659924	(G/A)
3998	CakSNP3998	*Kabuli*	Ca_Kabuli_Chr03	22681658	(G/A)
3999	CakSNP3999	*Kabuli*	Ca_Kabuli_Chr03	22719470	(T/C)
4000	CakSNP4000	*Kabuli*	Ca_Kabuli_Chr03	22719487	(T/C)
4001	CakSNP4001	*Kabuli*	Ca_Kabuli_Chr03	22719494	(C/T)
4002	CakSNP4002	*Kabuli*	Ca_Kabuli_Chr03	22719519	(G/A)
4003	CakSNP4003	*Kabuli*	Ca_Kabuli_Chr03	22727393	(G/A)
4004	CakSNP4004	*Kabuli*	Ca_Kabuli_Chr03	22727439	(A/C)
4005	CakSNP4005	*Kabuli*	Ca_Kabuli_Chr03	22765806	(G/A)
4006	CakSNP4006	*Kabuli*	Ca_Kabuli_Chr03	22765862	(T/C)
4007	CakSNP4007	*Kabuli*	Ca_Kabuli_Chr03	22765987	(G/T)
4008	CakSNP4008	*Kabuli*	Ca_Kabuli_Chr03	22798281	(T/G)
4009	CakSNP4009	*Kabuli*	Ca_Kabuli_Chr03	22812717	(A/T)
4010	CakSNP4010	*Kabuli*	Ca_Kabuli_Chr03	22846051	(G/A)
4011	CakSNP4011	*Kabuli*	Ca_Kabuli_Chr03	22847885	(G/T)
4012	CakSNP4012	*Kabuli*	Ca_Kabuli_Chr03	22847984	(A/G)
4013	CakSNP4013	*Kabuli*	Ca_Kabuli_Chr03	22990523	(C/T)
4014	CakSNP4014	*Kabuli*	Ca_Kabuli_Chr03	23028855	(A/G)
4015	CakSNP4015	*Kabuli*	Ca_Kabuli_Chr03	23033373	(A/C)

S.N.	SNP IDs	Cultivars	Chromosomes/scaffolds	Physical positions (bp)	SNPs
4016	CakSNP4016	*Kabuli*	Ca_Kabuli_Chr03	23042249	(A/C)
4017	CakSNP4017	*Kabuli*	Ca_Kabuli_Chr03	23053008	(C/G)
4018	CakSNP4018	*Kabuli*	Ca_Kabuli_Chr03	23053005	(G/C)
4019	CakSNP4019	*Kabuli*	Ca_Kabuli_Chr03	23053066	(A/C)
4020	CakSNP4020	*Kabuli*	Ca_Kabuli_Chr03	23095498	(C/T)
4021	CakSNP4021	*Kabuli*	Ca_Kabuli_Chr03	23273057	(T/A)
4022	CakSNP4022	*Kabuli*	Ca_Kabuli_Chr03	23273069	(C/G)
4023	CakSNP4023	*Kabuli*	Ca_Kabuli_Chr03	23327648	(C/T)
4024	CakSNP4024	*Kabuli*	Ca_Kabuli_Chr03	23327700	(G/C)
4025	CakSNP4025	*Kabuli*	Ca_Kabuli_Chr03	23331530	(T/G)
4026	CakSNP4026	*Kabuli*	Ca_Kabuli_Chr03	23335788	(A/T)
4027	CakSNP4027	*Kabuli*	Ca_Kabuli_Chr03	23335789	(T/G)
4028	CakSNP4028	*Kabuli*	Ca_Kabuli_Chr03	23340171	(A/G)
4029	CakSNP4029	*Kabuli*	Ca_Kabuli_Chr03	23340134	(G/A)
4030	CakSNP4030	*Kabuli*	Ca_Kabuli_Chr03	23340133	(T/C)
4031	CakSNP4031	*Kabuli*	Ca_Kabuli_Chr03	23341384	(T/G)
4032	CakSNP4032	*Kabuli*	Ca_Kabuli_Chr03	23342297	(T/C)
4033	CakSNP4033	*Kabuli*	Ca_Kabuli_Chr03	23342305	(T/C)
4034	CakSNP4034	*Kabuli*	Ca_Kabuli_Chr03	23342338	(G/T)
4035	CakSNP4035	*Kabuli*	Ca_Kabuli_Chr03	23342347	(G/A)
4036	CakSNP4036	*Kabuli*	Ca_Kabuli_Chr03	23342350	(A/G)
4037	CakSNP4037	*Kabuli*	Ca_Kabuli_Chr03	23342351	(T/C)
4038	CakSNP4038	*Kabuli*	Ca_Kabuli_Chr03	23360857	(C/T)
4039	CakSNP4039	*Kabuli*	Ca_Kabuli_Chr03	23400864	(A/C)
4040	CakSNP4040	*Kabuli*	Ca_Kabuli_Chr03	23455008	(A/G)
4041	CakSNP4041	*Kabuli*	Ca_Kabuli_Chr03	23455128	(A/G)
4042	CakSNP4042	*Kabuli*	Ca_Kabuli_Chr03	23461370	(C/A)
4043	CakSNP4043	*Kabuli*	Ca_Kabuli_Chr03	23555586	(C/A)
4044	CakSNP4044	*Kabuli*	Ca_Kabuli_Chr03	23593288	(G/A)
4045	CakSNP4045	*Kabuli*	Ca_Kabuli_Chr03	23593294	(T/C)
4046	CakSNP4046	*Kabuli*	Ca_Kabuli_Chr03	23593309	(T/C)
4047	CakSNP4047	*Kabuli*	Ca_Kabuli_Chr03	23593269	(T/C)
4048	CakSNP4048	*Kabuli*	Ca_Kabuli_Chr03	23714158	(T/A)
4049	CakSNP4049	*Kabuli*	Ca_Kabuli_Chr03	23714129	(C/A)
4050	CakSNP4050	*Kabuli*	Ca_Kabuli_Chr03	23745324	(C/T)
4051	CakSNP4051	*Kabuli*	Ca_Kabuli_Chr03	23792975	(A/T)
4052	CakSNP4052	*Kabuli*	Ca_Kabuli_Chr03	23833683	(G/A)
4053	CakSNP4053	*Kabuli*	Ca_Kabuli_Chr03	23833680	(G/C)
4054	CakSNP4054	*Kabuli*	Ca_Kabuli_Chr03	23833668	(G/A)

S.N.	SNP IDs	Cultivars	Chromosomes/scaffolds	Physical positions (bp)	SNPs
4055	CakSNP4055	*Kabuli*	Ca_Kabuli_Chr03	23886582	(A/G)
4056	CakSNP4056	*Kabuli*	Ca_Kabuli_Chr03	23918105	(T/C)
4057	CakSNP4057	*Kabuli*	Ca_Kabuli_Chr03	23966110	(G/A)
4058	CakSNP4058	*Kabuli*	Ca_Kabuli_Chr03	23997287	(A/T)
4059	CakSNP4059	*Kabuli*	Ca_Kabuli_Chr03	24000847	(G/T)
4060	CakSNP4060	*Kabuli*	Ca_Kabuli_Chr03	24000818	(T/C)
4061	CakSNP4061	*Kabuli*	Ca_Kabuli_Chr03	24024560	(T/C)
4062	CakSNP4062	*Kabuli*	Ca_Kabuli_Chr03	24024513	(A/C)
4063	CakSNP4063	*Kabuli*	Ca_Kabuli_Chr03	24025900	(G/C)
4064	CakSNP4064	*Kabuli*	Ca_Kabuli_Chr03	24025924	(T/G)
4065	CakSNP4065	*Kabuli*	Ca_Kabuli_Chr03	24026428	(C/T)
4066	CakSNP4066	*Kabuli*	Ca_Kabuli_Chr03	24041806	(C/T)
4067	CakSNP4067	*Kabuli*	Ca_Kabuli_Chr03	24095268	(T/C)
4068	CakSNP4068	*Kabuli*	Ca_Kabuli_Chr03	24099191	(C/G)
4069	CakSNP4069	*Kabuli*	Ca_Kabuli_Chr03	24127222	(C/T)
4070	CakSNP4070	*Kabuli*	Ca_Kabuli_Chr03	24127201	(A/C)
4071	CakSNP4071	*Kabuli*	Ca_Kabuli_Chr03	24162153	(C/T)
4072	CakSNP4072	*Kabuli*	Ca_Kabuli_Chr03	24194574	(C/T)
4073	CakSNP4073	*Kabuli*	Ca_Kabuli_Chr03	24247161	(G/C)
4074	CakSNP4074	*Kabuli*	Ca_Kabuli_Chr03	24297842	(T/G)
4075	CakSNP4075	*Kabuli*	Ca_Kabuli_Chr03	24297894	(C/A)
4076	CakSNP4076	*Kabuli*	Ca_Kabuli_Chr03	24417477	(T/G)
4077	CakSNP4077	*Kabuli*	Ca_Kabuli_Chr03	24417507	(A/T)
4078	CakSNP4078	*Kabuli*	Ca_Kabuli_Chr03	24418063	(G/T)
4079	CakSNP4079	*Kabuli*	Ca_Kabuli_Chr03	24418111	(A/G)
4080	CakSNP4080	*Kabuli*	Ca_Kabuli_Chr03	24418130	(T/A)
4081	CakSNP4081	*Kabuli*	Ca_Kabuli_Chr03	24459669	(G/A)
4082	CakSNP4082	*Kabuli*	Ca_Kabuli_Chr03	24515489	(T/G)
4083	CakSNP4083	*Kabuli*	Ca_Kabuli_Chr03	24538726	(A/C)
4084	CakSNP4084	*Kabuli*	Ca_Kabuli_Chr03	24540199	(A/G)
4085	CakSNP4085	*Kabuli*	Ca_Kabuli_Chr03	24540304	(T/C)
4086	CakSNP4086	*Kabuli*	Ca_Kabuli_Chr03	24637834	(G/T)
4087	CakSNP4087	*Kabuli*	Ca_Kabuli_Chr03	24637837	(A/C)
4088	CakSNP4088	*Kabuli*	Ca_Kabuli_Chr03	24664582	(G/T)
4089	CakSNP4089	*Kabuli*	Ca_Kabuli_Chr03	24664550	(T/G)
4090	CakSNP4090	*Kabuli*	Ca_Kabuli_Chr03	24664651	(G/T)
4091	CakSNP4091	*Kabuli*	Ca_Kabuli_Chr03	24666594	(T/C)
4092	CakSNP4092	*Kabuli*	Ca_Kabuli_Chr03	24763730	(A/G)
4093	CakSNP4093	*Kabuli*	Ca_Kabuli_Chr03	24933940	(T/C)

S.N.	SNP IDs	Cultivars	Chromosomes/scaffolds	Physical positions (bp)	SNPs
4094	CakSNP4094	*Kabuli*	Ca_Kabuli_Chr03	24933901	(C/A)
4095	CakSNP4095	*Kabuli*	Ca_Kabuli_Chr03	24933899	(G/T)
4096	CakSNP4096	*Kabuli*	Ca_Kabuli_Chr03	24998316	(C/G)
4097	CakSNP4097	*Kabuli*	Ca_Kabuli_Chr03	24998300	(C/T)
4098	CakSNP4098	*Kabuli*	Ca_Kabuli_Chr03	25037746	(G/A)
4099	CakSNP4099	*Kabuli*	Ca_Kabuli_Chr03	25046423	(C/G)
4100	CakSNP4100	*Kabuli*	Ca_Kabuli_Chr03	25046421	(T/C)
4101	CakSNP4101	*Kabuli*	Ca_Kabuli_Chr03	25055049	(C/T)
4102	CakSNP4102	*Kabuli*	Ca_Kabuli_Chr03	25062250	(C/A)
4103	CakSNP4103	*Kabuli*	Ca_Kabuli_Chr03	25110037	(T/G)
4104	CakSNP4104	*Kabuli*	Ca_Kabuli_Chr03	25137192	(T/A)
4105	CakSNP4105	*Kabuli*	Ca_Kabuli_Chr03	25147096	(G/A)
4106	CakSNP4106	*Kabuli*	Ca_Kabuli_Chr03	25205548	(C/A)
4107	CakSNP4107	*Kabuli*	Ca_Kabuli_Chr03	25241115	(G/A)
4108	CakSNP4108	*Kabuli*	Ca_Kabuli_Chr03	25244194	(A/C)
4109	CakSNP4109	*Kabuli*	Ca_Kabuli_Chr03	25270592	(C/G)
4110	CakSNP4110	*Kabuli*	Ca_Kabuli_Chr03	25399911	(C/G)
4111	CakSNP4111	*Kabuli*	Ca_Kabuli_Chr03	25457474	(T/A)
4112	CakSNP4112	*Kabuli*	Ca_Kabuli_Chr03	25457494	(A/T)
4113	CakSNP4113	*Kabuli*	Ca_Kabuli_Chr03	25457514	(C/A)
4114	CakSNP4114	*Kabuli*	Ca_Kabuli_Chr03	25462227	(A/C)
4115	CakSNP4115	*Kabuli*	Ca_Kabuli_Chr03	25504708	(A/G)
4116	CakSNP4116	*Kabuli*	Ca_Kabuli_Chr03	25505438	(C/T)
4117	CakSNP4117	*Kabuli*	Ca_Kabuli_Chr03	25505420	(G/T)
4118	CakSNP4118	*Kabuli*	Ca_Kabuli_Chr03	25534044	(C/T)
4119	CakSNP4119	*Kabuli*	Ca_Kabuli_Chr03	25538867	(T/C)
4120	CakSNP4120	*Kabuli*	Ca_Kabuli_Chr03	25580072	(A/T)
4121	CakSNP4121	*Kabuli*	Ca_Kabuli_Chr03	25614515	(T/G)
4122	CakSNP4122	*Kabuli*	Ca_Kabuli_Chr03	25641434	(C/A)
4123	CakSNP4123	*Kabuli*	Ca_Kabuli_Chr03	25641433	(G/A)
4124	CakSNP4124	*Kabuli*	Ca_Kabuli_Chr03	25688341	(G/A)
4125	CakSNP4125	*Kabuli*	Ca_Kabuli_Chr03	25702238	(G/A)
4126	CakSNP4126	*Kabuli*	Ca_Kabuli_Chr03	25718829	(C/A)
4127	CakSNP4127	*Kabuli*	Ca_Kabuli_Chr03	25718827	(T/A)
4128	CakSNP4128	*Kabuli*	Ca_Kabuli_Chr03	25757693	(C/T)
4129	CakSNP4129	*Kabuli*	Ca_Kabuli_Chr03	25987920	(T/G)
4130	CakSNP4130	*Kabuli*	Ca_Kabuli_Chr03	26119425	(T/G)
4131	CakSNP4131	*Kabuli*	Ca_Kabuli_Chr03	26119461	(C/T)
4132	CakSNP4132	*Kabuli*	Ca_Kabuli_Chr03	26163449	(G/A)

**S.N.**	**SNP IDs**	**Cultivars**	**Chromosomes/scaffolds**	**Physical positions (bp)**	**SNPs**
4133	CakSNP4133	*Kabuli*	Ca_Kabuli_Chr03	26178592	(G/A)
4134	CakSNP4134	*Kabuli*	Ca_Kabuli_Chr03	26198368	(G/A)
4135	CakSNP4135	*Kabuli*	Ca_Kabuli_Chr03	26198372	(G/A)
4136	CakSNP4136	*Kabuli*	Ca_Kabuli_Chr03	26293846	(A/G)
4137	CakSNP4137	*Kabuli*	Ca_Kabuli_Chr03	26335889	(T/A)
4138	CakSNP4138	*Kabuli*	Ca_Kabuli_Chr03	26339286	(T/C)
4139	CakSNP4139	*Kabuli*	Ca_Kabuli_Chr03	26339449	(G/T)
4140	CakSNP4140	*Kabuli*	Ca_Kabuli_Chr03	26562004	(A/G)
4141	CakSNP4141	*Kabuli*	Ca_Kabuli_Chr03	26573902	(A/G)
4142	CakSNP4142	*Kabuli*	Ca_Kabuli_Chr03	26574056	(C/T)
4143	CakSNP4143	*Kabuli*	Ca_Kabuli_Chr03	26574068	(T/G)
4144	CakSNP4144	*Kabuli*	Ca_Kabuli_Chr03	26574831	(A/G)
4145	CakSNP4145	*Kabuli*	Ca_Kabuli_Chr03	26589678	(T/A)
4146	CakSNP4146	*Kabuli*	Ca_Kabuli_Chr03	26627150	(T/G)
4147	CakSNP4147	*Kabuli*	Ca_Kabuli_Chr03	26636553	(A/G)
4148	CakSNP4148	*Kabuli*	Ca_Kabuli_Chr03	26646152	(C/T)
4149	CakSNP4149	*Kabuli*	Ca_Kabuli_Chr03	26678343	(T/C)
4150	CakSNP4150	*Kabuli*	Ca_Kabuli_Chr03	26678433	(T/G)
4151	CakSNP4151	*Kabuli*	Ca_Kabuli_Chr03	26726897	(C/A)
4152	CakSNP4152	*Kabuli*	Ca_Kabuli_Chr03	26758452	(C/A)
4153	CakSNP4153	*Kabuli*	Ca_Kabuli_Chr03	26848870	(G/C)
4154	CakSNP4154	*Kabuli*	Ca_Kabuli_Chr03	26849288	(T/A)
4155	CakSNP4155	*Kabuli*	Ca_Kabuli_Chr03	26878028	(A/T)
4156	CakSNP4156	*Kabuli*	Ca_Kabuli_Chr03	26900271	(T/A)
4157	CakSNP4157	*Kabuli*	Ca_Kabuli_Chr03	26911443	(A/T)
4158	CakSNP4158	*Kabuli*	Ca_Kabuli_Chr03	26911514	(A/G)
4159	CakSNP4159	*Kabuli*	Ca_Kabuli_Chr03	26911723	(T/A)
4160	CakSNP4160	*Kabuli*	Ca_Kabuli_Chr03	26911792	(A/G)
4161	CakSNP4161	*Kabuli*	Ca_Kabuli_Chr03	26976249	(A/G)
4162	CakSNP4162	*Kabuli*	Ca_Kabuli_Chr03	26976323	(G/A)
4163	CakSNP4163	*Kabuli*	Ca_Kabuli_Chr03	27024215	(T/C)
4164	CakSNP4164	*Kabuli*	Ca_Kabuli_Chr03	27047840	(A/G)
4165	CakSNP4165	*Kabuli*	Ca_Kabuli_Chr03	27047847	(A/C)
4166	CakSNP4166	*Kabuli*	Ca_Kabuli_Chr03	27063150	(A/C)
4167	CakSNP4167	*Kabuli*	Ca_Kabuli_Chr03	27074842	(C/A)
4168	CakSNP4168	*Kabuli*	Ca_Kabuli_Chr03	27074896	(A/T)
4169	CakSNP4169	*Kabuli*	Ca_Kabuli_Chr03	27074931	(A/T)
4170	CakSNP4170	*Kabuli*	Ca_Kabuli_Chr03	27074930	(A/C)
4171	CakSNP4171	*Kabuli*	Ca_Kabuli_Chr03	27076716	(T/C)

S.N.	SNP IDs	Cultivars	Chromosomes/scaffolds	Physical positions (bp)	SNPs
4172	CakSNP4172	*Kabuli*	Ca_Kabuli_Chr03	27076735	(T/G)
4173	CakSNP4173	*Kabuli*	Ca_Kabuli_Chr03	27076744	(C/A)
4174	CakSNP4174	*Kabuli*	Ca_Kabuli_Chr03	27105637	(C/T)
4175	CakSNP4175	*Kabuli*	Ca_Kabuli_Chr03	27157840	(T/A)
4176	CakSNP4176	*Kabuli*	Ca_Kabuli_Chr03	27157848	(C/T)
4177	CakSNP4177	*Kabuli*	Ca_Kabuli_Chr03	27161813	(T/G)
4178	CakSNP4178	*Kabuli*	Ca_Kabuli_Chr03	27161801	(A/G)
4179	CakSNP4179	*Kabuli*	Ca_Kabuli_Chr03	27194033	(C/T)
4180	CakSNP4180	*Kabuli*	Ca_Kabuli_Chr03	27194069	(T/C)
4181	CakSNP4181	*Kabuli*	Ca_Kabuli_Chr03	27338521	(T/C)
4182	CakSNP4182	*Kabuli*	Ca_Kabuli_Chr03	27342919	(G/A)
4183	CakSNP4183	*Kabuli*	Ca_Kabuli_Chr03	27391199	(G/A)
4184	CakSNP4184	*Kabuli*	Ca_Kabuli_Chr03	27391354	(G/A)
4185	CakSNP4185	*Kabuli*	Ca_Kabuli_Chr03	27391321	(A/G)
4186	CakSNP4186	*Kabuli*	Ca_Kabuli_Chr03	27417561	(A/T)
4187	CakSNP4187	*Kabuli*	Ca_Kabuli_Chr03	27420276	(T/C)
4188	CakSNP4188	*Kabuli*	Ca_Kabuli_Chr03	27420330	(C/T)
4189	CakSNP4189	*Kabuli*	Ca_Kabuli_Chr03	27435210	(T/C)
4190	CakSNP4190	*Kabuli*	Ca_Kabuli_Chr03	27435209	(G/C)
4191	CakSNP4191	*Kabuli*	Ca_Kabuli_Chr03	27435198	(T/A)
4192	CakSNP4192	*Kabuli*	Ca_Kabuli_Chr03	27447685	(C/A)
4193	CakSNP4193	*Kabuli*	Ca_Kabuli_Chr03	27470443	(A/C)
4194	CakSNP4194	*Kabuli*	Ca_Kabuli_Chr03	27470439	(G/T)
4195	CakSNP4195	*Kabuli*	Ca_Kabuli_Chr03	27597331	(A/C)
4196	CakSNP4196	*Kabuli*	Ca_Kabuli_Chr03	27597409	(A/G)
4197	CakSNP4197	*Kabuli*	Ca_Kabuli_Chr03	27632100	(G/A)
4198	CakSNP4198	*Kabuli*	Ca_Kabuli_Chr03	27632169	(T/C)
4199	CakSNP4199	*Kabuli*	Ca_Kabuli_Chr03	27654798	(A/C)
4200	CakSNP4200	*Kabuli*	Ca_Kabuli_Chr03	27673727	(C/A)
4201	CakSNP4201	*Kabuli*	Ca_Kabuli_Chr03	27681551	(T/C)
4202	CakSNP4202	*Kabuli*	Ca_Kabuli_Chr03	27716396	(C/T)
4203	CakSNP4203	*Kabuli*	Ca_Kabuli_Chr03	27763236	(C/T)
4204	CakSNP4204	*Kabuli*	Ca_Kabuli_Chr03	27812571	(A/T)
4205	CakSNP4205	*Kabuli*	Ca_Kabuli_Chr03	27876405	(A/G)
4206	CakSNP4206	*Kabuli*	Ca_Kabuli_Chr03	27877442	(A/T)
4207	CakSNP4207	*Kabuli*	Ca_Kabuli_Chr03	27877479	(C/T)
4208	CakSNP4208	*Kabuli*	Ca_Kabuli_Chr03	27877480	(G/A)
4209	CakSNP4209	*Kabuli*	Ca_Kabuli_Chr03	27877510	(G/A)
4210	CakSNP4210	*Kabuli*	Ca_Kabuli_Chr03	27888817	(G/C)

S.N.	SNP IDs	Cultivars	Chromosomes/scaffolds	Physical positions (bp)	SNPs
4211	CakSNP4211	*Kabuli*	Ca_Kabuli_Chr03	27889107	(A/G)
4212	CakSNP4212	*Kabuli*	Ca_Kabuli_Chr03	27890849	(C/T)
4213	CakSNP4213	*Kabuli*	Ca_Kabuli_Chr03	27905944	(T/C)
4214	CakSNP4214	*Kabuli*	Ca_Kabuli_Chr03	27905989	(G/A)
4215	CakSNP4215	*Kabuli*	Ca_Kabuli_Chr03	27905995	(C/T)
4216	CakSNP4216	*Kabuli*	Ca_Kabuli_Chr03	27906069	(C/A)
4217	CakSNP4217	*Kabuli*	Ca_Kabuli_Chr03	27914409	(C/T)
4218	CakSNP4218	*Kabuli*	Ca_Kabuli_Chr03	27968979	(T/G)
4219	CakSNP4219	*Kabuli*	Ca_Kabuli_Chr03	27969014	(T/C)
4220	CakSNP4220	*Kabuli*	Ca_Kabuli_Chr03	27969005	(A/G)
4221	CakSNP4221	*Kabuli*	Ca_Kabuli_Chr03	27969207	(C/T)
4222	CakSNP4222	*Kabuli*	Ca_Kabuli_Chr03	28044035	(A/T)
4223	CakSNP4223	*Kabuli*	Ca_Kabuli_Chr03	28176380	(A/C)
4224	CakSNP4224	*Kabuli*	Ca_Kabuli_Chr03	28176333	(C/A)
4225	CakSNP4225	*Kabuli*	Ca_Kabuli_Chr03	28277991	(C/G)
4226	CakSNP4226	*Kabuli*	Ca_Kabuli_Chr03	28278018	(C/G)
4227	CakSNP4227	*Kabuli*	Ca_Kabuli_Chr03	28374024	(C/T)
4228	CakSNP4228	*Kabuli*	Ca_Kabuli_Chr03	28375082	(G/A)
4229	CakSNP4229	*Kabuli*	Ca_Kabuli_Chr03	28409001	(G/A)
4230	CakSNP4230	*Kabuli*	Ca_Kabuli_Chr03	28420948	(C/T)
4231	CakSNP4231	*Kabuli*	Ca_Kabuli_Chr03	28478044	(C/T)
4232	CakSNP4232	*Kabuli*	Ca_Kabuli_Chr03	28478539	(C/G)
4233	CakSNP4233	*Kabuli*	Ca_Kabuli_Chr03	28478569	(C/A)
4234	CakSNP4234	*Kabuli*	Ca_Kabuli_Chr03	28602055	(C/A)
4235	CakSNP4235	*Kabuli*	Ca_Kabuli_Chr03	28603892	(T/C)
4236	CakSNP4236	*Kabuli*	Ca_Kabuli_Chr03	28613067	(A/G)
4237	CakSNP4237	*Kabuli*	Ca_Kabuli_Chr03	28614784	(A/G)
4238	CakSNP4238	*Kabuli*	Ca_Kabuli_Chr03	28614919	(A/T)
4239	CakSNP4239	*Kabuli*	Ca_Kabuli_Chr03	28680858	(C/T)
4240	CakSNP4240	*Kabuli*	Ca_Kabuli_Chr03	28706106	(T/A)
4241	CakSNP4241	*Kabuli*	Ca_Kabuli_Chr03	28706292	(G/C)
4242	CakSNP4242	*Kabuli*	Ca_Kabuli_Chr03	28712993	(T/C)
4243	CakSNP4243	*Kabuli*	Ca_Kabuli_Chr03	28716003	(C/T)
4244	CakSNP4244	*Kabuli*	Ca_Kabuli_Chr03	28716027	(A/T)
4245	CakSNP4245	*Kabuli*	Ca_Kabuli_Chr03	28716028	(A/T)
4246	CakSNP4246	*Kabuli*	Ca_Kabuli_Chr03	28716029	(A/G)
4247	CakSNP4247	*Kabuli*	Ca_Kabuli_Chr03	28716038	(T/G)
4248	CakSNP4248	*Kabuli*	Ca_Kabuli_Chr03	28716047	(G/A)
4249	CakSNP4249	*Kabuli*	Ca_Kabuli_Chr03	28755111	(A/G)

S.N.	SNP IDs	Cultivars	Chromosomes/scaffolds	Physical positions (bp)	SNPs
4250	CakSNP4250	*Kabuli*	Ca_Kabuli_Chr03	28755149	(G/A)
4251	CakSNP4251	*Kabuli*	Ca_Kabuli_Chr03	28845958	(A/C)
4252	CakSNP4252	*Kabuli*	Ca_Kabuli_Chr03	28926094	(T/A)
4253	CakSNP4253	*Kabuli*	Ca_Kabuli_Chr03	28926290	(T/C)
4254	CakSNP4254	*Kabuli*	Ca_Kabuli_Chr03	28934196	(C/T)
4255	CakSNP4255	*Kabuli*	Ca_Kabuli_Chr03	28943956	(C/T)
4256	CakSNP4256	*Kabuli*	Ca_Kabuli_Chr03	28943989	(G/T)
4257	CakSNP4257	*Kabuli*	Ca_Kabuli_Chr03	29056451	(T/C)
4258	CakSNP4258	*Kabuli*	Ca_Kabuli_Chr03	29056528	(G/T)
4259	CakSNP4259	*Kabuli*	Ca_Kabuli_Chr03	29056768	(T/C)
4260	CakSNP4260	*Kabuli*	Ca_Kabuli_Chr03	29107776	(A/G)
4261	CakSNP4261	*Kabuli*	Ca_Kabuli_Chr03	29107947	(C/A)
4262	CakSNP4262	*Kabuli*	Ca_Kabuli_Chr03	29155741	(C/G)
4263	CakSNP4263	*Kabuli*	Ca_Kabuli_Chr03	29177885	(A/C)
4264	CakSNP4264	*Kabuli*	Ca_Kabuli_Chr03	29188300	(T/G)
4265	CakSNP4265	*Kabuli*	Ca_Kabuli_Chr03	29188308	(G/A)
4266	CakSNP4266	*Kabuli*	Ca_Kabuli_Chr03	29207338	(G/A)
4267	CakSNP4267	*Kabuli*	Ca_Kabuli_Chr03	29293770	(G/T)
4268	CakSNP4268	*Kabuli*	Ca_Kabuli_Chr03	29293783	(G/A)
4269	CakSNP4269	*Kabuli*	Ca_Kabuli_Chr03	29293840	(C/A)
4270	CakSNP4270	*Kabuli*	Ca_Kabuli_Chr03	29302251	(C/T)
4271	CakSNP4271	*Kabuli*	Ca_Kabuli_Chr03	29302448	(A/C)
4272	CakSNP4272	*Kabuli*	Ca_Kabuli_Chr03	29303144	(A/C)
4273	CakSNP4273	*Kabuli*	Ca_Kabuli_Chr03	29304116	(A/G)
4274	CakSNP4274	*Kabuli*	Ca_Kabuli_Chr03	29304161	(C/T)
4275	CakSNP4275	*Kabuli*	Ca_Kabuli_Chr03	29311672	(C/A)
4276	CakSNP4276	*Kabuli*	Ca_Kabuli_Chr03	29348267	(C/T)
4277	CakSNP4277	*Kabuli*	Ca_Kabuli_Chr03	29355268	(T/A)
4278	CakSNP4278	*Kabuli*	Ca_Kabuli_Chr03	29376025	(A/G)
4279	CakSNP4279	*Kabuli*	Ca_Kabuli_Chr03	29376008	(G/A)
4280	CakSNP4280	*Kabuli*	Ca_Kabuli_Chr03	29439505	(A/G)
4281	CakSNP4281	*Kabuli*	Ca_Kabuli_Chr03	29485590	(T/C)
4282	CakSNP4282	*Kabuli*	Ca_Kabuli_Chr03	29485566	(T/A)
4283	CakSNP4283	*Kabuli*	Ca_Kabuli_Chr03	29508831	(C/T)
4284	CakSNP4284	*Kabuli*	Ca_Kabuli_Chr03	29509323	(G/A)
4285	CakSNP4285	*Kabuli*	Ca_Kabuli_Chr03	29509310	(G/T)
4286	CakSNP4286	*Kabuli*	Ca_Kabuli_Chr03	29547528	(G/A)
4287	CakSNP4287	*Kabuli*	Ca_Kabuli_Chr03	29588940	(C/T)
4288	CakSNP4288	*Kabuli*	Ca_Kabuli_Chr03	29588934	(T/C)

S.N.	SNP IDs	Cultivars	Chromosomes/scaffolds	Physical positions (bp)	SNPs
4289	CakSNP4289	*Kabuli*	Ca_Kabuli_Chr03	29588930	(T/C)
4290	CakSNP4290	*Kabuli*	Ca_Kabuli_Chr03	29591575	(T/G)
4291	CakSNP4291	*Kabuli*	Ca_Kabuli_Chr03	29591613	(G/T)
4292	CakSNP4292	*Kabuli*	Ca_Kabuli_Chr03	29603720	(T/C)
4293	CakSNP4293	*Kabuli*	Ca_Kabuli_Chr03	29640075	(A/C)
4294	CakSNP4294	*Kabuli*	Ca_Kabuli_Chr03	29640071	(T/C)
4295	CakSNP4295	*Kabuli*	Ca_Kabuli_Chr03	29666633	(C/T)
4296	CakSNP4296	*Kabuli*	Ca_Kabuli_Chr03	29690732	(C/A)
4297	CakSNP4297	*Kabuli*	Ca_Kabuli_Chr03	29698166	(G/T)
4298	CakSNP4298	*Kabuli*	Ca_Kabuli_Chr03	29698245	(T/A)
4299	CakSNP4299	*Kabuli*	Ca_Kabuli_Chr03	29698289	(G/A)
4300	CakSNP4300	*Kabuli*	Ca_Kabuli_Chr03	29698273	(A/C)
4301	CakSNP4301	*Kabuli*	Ca_Kabuli_Chr03	29730019	(G/T)
4302	CakSNP4302	*Kabuli*	Ca_Kabuli_Chr03	29744878	(T/G)
4303	CakSNP4303	*Kabuli*	Ca_Kabuli_Chr03	29744890	(A/C)
4304	CakSNP4304	*Kabuli*	Ca_Kabuli_Chr03	29803866	(C/T)
4305	CakSNP4305	*Kabuli*	Ca_Kabuli_Chr03	29803962	(A/G)
4306	CakSNP4306	*Kabuli*	Ca_Kabuli_Chr03	29807091	(C/G)
4307	CakSNP4307	*Kabuli*	Ca_Kabuli_Chr03	29807248	(A/G)
4308	CakSNP4308	*Kabuli*	Ca_Kabuli_Chr03	29836337	(C/A)
4309	CakSNP4309	*Kabuli*	Ca_Kabuli_Chr03	29840029	(C/G)
4310	CakSNP4310	*Kabuli*	Ca_Kabuli_Chr03	29840028	(T/C)
4311	CakSNP4311	*Kabuli*	Ca_Kabuli_Chr03	29840022	(C/A)
4312	CakSNP4312	*Kabuli*	Ca_Kabuli_Chr03	29840012	(A/T)
4313	CakSNP4313	*Kabuli*	Ca_Kabuli_Chr03	29844509	(T/A)
4314	CakSNP4314	*Kabuli*	Ca_Kabuli_Chr03	29869190	(G/T)
4315	CakSNP4315	*Kabuli*	Ca_Kabuli_Chr03	29869199	(T/C)
4316	CakSNP4316	*Kabuli*	Ca_Kabuli_Chr03	29869428	(G/T)
4317	CakSNP4317	*Kabuli*	Ca_Kabuli_Chr03	29869381	(G/A)
4318	CakSNP4318	*Kabuli*	Ca_Kabuli_Chr03	29869380	(G/A)
4319	CakSNP4319	*Kabuli*	Ca_Kabuli_Chr03	29872643	(A/G)
4320	CakSNP4320	*Kabuli*	Ca_Kabuli_Chr03	29872682	(G/A)
4321	CakSNP4321	*Kabuli*	Ca_Kabuli_Chr03	29898396	(A/G)
4322	CakSNP4322	*Kabuli*	Ca_Kabuli_Chr03	29912488	(A/G)
4323	CakSNP4323	*Kabuli*	Ca_Kabuli_Chr03	29915510	(G/A)
4324	CakSNP4324	*Kabuli*	Ca_Kabuli_Chr03	29932378	(C/G)
4325	CakSNP4325	*Kabuli*	Ca_Kabuli_Chr03	29932504	(T/A)
4326	CakSNP4326	*Kabuli*	Ca_Kabuli_Chr03	29932502	(G/C)
4327	CakSNP4327	*Kabuli*	Ca_Kabuli_Chr03	29954498	(G/A)

S.N.	SNP IDs	Cultivars	Chromosomes/scaffolds	Physical positions (bp)	SNPs
4328	CakSNP4328	*Kabuli*	Ca_Kabuli_Chr03	29954533	(A/G)
4329	CakSNP4329	*Kabuli*	Ca_Kabuli_Chr03	29954648	(A/T)
4330	CakSNP4330	*Kabuli*	Ca_Kabuli_Chr03	29954612	(T/G)
4331	CakSNP4331	*Kabuli*	Ca_Kabuli_Chr03	29974532	(A/G)
4332	CakSNP4332	*Kabuli*	Ca_Kabuli_Chr03	29974535	(A/G)
4333	CakSNP4333	*Kabuli*	Ca_Kabuli_Chr03	29974595	(C/T)
4334	CakSNP4334	*Kabuli*	Ca_Kabuli_Chr03	29982638	(A/G)
4335	CakSNP4335	*Kabuli*	Ca_Kabuli_Chr03	29982644	(T/C)
4336	CakSNP4336	*Kabuli*	Ca_Kabuli_Chr03	29982718	(C/A)
4337	CakSNP4337	*Kabuli*	Ca_Kabuli_Chr03	30004352	(T/A)
4338	CakSNP4338	*Kabuli*	Ca_Kabuli_Chr03	30004311	(T/G)
4339	CakSNP4339	*Kabuli*	Ca_Kabuli_Chr03	30004295	(A/G)
4340	CakSNP4340	*Kabuli*	Ca_Kabuli_Chr03	30004279	(G/T)
4341	CakSNP4341	*Kabuli*	Ca_Kabuli_Chr03	30009698	(C/T)
4342	CakSNP4342	*Kabuli*	Ca_Kabuli_Chr03	30009751	(A/C)
4343	CakSNP4343	*Kabuli*	Ca_Kabuli_Chr03	30011584	(T/C)
4344	CakSNP4344	*Kabuli*	Ca_Kabuli_Chr03	30011669	(A/G)
4345	CakSNP4345	*Kabuli*	Ca_Kabuli_Chr03	30011642	(T/A)
4346	CakSNP4346	*Kabuli*	Ca_Kabuli_Chr03	30011621	(C/T)
4347	CakSNP4347	*Kabuli*	Ca_Kabuli_Chr03	30029874	(C/T)
4348	CakSNP4348	*Kabuli*	Ca_Kabuli_Chr03	30029858	(T/A)
4349	CakSNP4349	*Kabuli*	Ca_Kabuli_Chr03	30029842	(G/C)
4350	CakSNP4350	*Kabuli*	Ca_Kabuli_Chr03	30029826	(T/C)
4351	CakSNP4351	*Kabuli*	Ca_Kabuli_Chr03	30029817	(T/G)
4352	CakSNP4352	*Kabuli*	Ca_Kabuli_Chr03	30084154	(T/C)
4353	CakSNP4353	*Kabuli*	Ca_Kabuli_Chr03	30101412	(T/C)
4354	CakSNP4354	*Kabuli*	Ca_Kabuli_Chr03	30101476	(A/C)
4355	CakSNP4355	*Kabuli*	Ca_Kabuli_Chr03	30111154	(A/C)
4356	CakSNP4356	*Kabuli*	Ca_Kabuli_Chr03	30111146	(G/C)
4357	CakSNP4357	*Kabuli*	Ca_Kabuli_Chr03	30148907	(T/C)
4358	CakSNP4358	*Kabuli*	Ca_Kabuli_Chr03	30194810	(C/T)
4359	CakSNP4359	*Kabuli*	Ca_Kabuli_Chr03	30206121	(T/A)
4360	CakSNP4360	*Kabuli*	Ca_Kabuli_Chr03	30206266	(G/A)
4361	CakSNP4361	*Kabuli*	Ca_Kabuli_Chr03	30206281	(A/T)
4362	CakSNP4362	*Kabuli*	Ca_Kabuli_Chr03	30206436	(G/A)
4363	CakSNP4363	*Kabuli*	Ca_Kabuli_Chr03	30267761	(T/G)
4364	CakSNP4364	*Kabuli*	Ca_Kabuli_Chr03	30271022	(G/T)
4365	CakSNP4365	*Kabuli*	Ca_Kabuli_Chr03	30271028	(G/A)
4366	CakSNP4366	*Kabuli*	Ca_Kabuli_Chr03	30271034	(G/A)

S.N.	SNP IDs	Cultivars	Chromosomes/scaffolds	Physical positions (bp)	SNPs
4367	CakSNP4367	*Kabuli*	Ca_Kabuli_Chr03	30309625	(G/C)
4368	CakSNP4368	*Kabuli*	Ca_Kabuli_Chr03	30309624	(A/T)
4369	CakSNP4369	*Kabuli*	Ca_Kabuli_Chr03	30321177	(C/G)
4370	CakSNP4370	*Kabuli*	Ca_Kabuli_Chr03	30321245	(C/T)
4371	CakSNP4371	*Kabuli*	Ca_Kabuli_Chr03	30321272	(C/A)
4372	CakSNP4372	*Kabuli*	Ca_Kabuli_Chr03	30323585	(G/A)
4373	CakSNP4373	*Kabuli*	Ca_Kabuli_Chr03	30376319	(G/A)
4374	CakSNP4374	*Kabuli*	Ca_Kabuli_Chr03	30385144	(G/A)
4375	CakSNP4375	*Kabuli*	Ca_Kabuli_Chr03	30385153	(T/A)
4376	CakSNP4376	*Kabuli*	Ca_Kabuli_Chr03	30390663	(C/T)
4377	CakSNP4377	*Kabuli*	Ca_Kabuli_Chr03	30397262	(A/T)
4378	CakSNP4378	*Kabuli*	Ca_Kabuli_Chr03	30425496	(G/T)
4379	CakSNP4379	*Kabuli*	Ca_Kabuli_Chr03	30510392	(T/A)
4380	CakSNP4380	*Kabuli*	Ca_Kabuli_Chr03	30510333	(C/T)
4381	CakSNP4381	*Kabuli*	Ca_Kabuli_Chr03	30522442	(A/C)
4382	CakSNP4382	*Kabuli*	Ca_Kabuli_Chr03	30522485	(C/T)
4383	CakSNP4383	*Kabuli*	Ca_Kabuli_Chr03	30522498	(T/C)
4384	CakSNP4384	*Kabuli*	Ca_Kabuli_Chr03	30650598	(C/A)
4385	CakSNP4385	*Kabuli*	Ca_Kabuli_Chr03	30650634	(T/C)
4386	CakSNP4386	*Kabuli*	Ca_Kabuli_Chr03	30650659	(C/T)
4387	CakSNP4387	*Kabuli*	Ca_Kabuli_Chr03	30659438	(T/A)
4388	CakSNP4388	*Kabuli*	Ca_Kabuli_Chr03	30675468	(T/G)
4389	CakSNP4389	*Kabuli*	Ca_Kabuli_Chr03	30681031	(A/G)
4390	CakSNP4390	*Kabuli*	Ca_Kabuli_Chr03	30681032	(A/G)
4391	CakSNP4391	*Kabuli*	Ca_Kabuli_Chr03	30688336	(T/G)
4392	CakSNP4392	*Kabuli*	Ca_Kabuli_Chr03	30688354	(G/A)
4393	CakSNP4393	*Kabuli*	Ca_Kabuli_Chr03	30688369	(T/C)
4394	CakSNP4394	*Kabuli*	Ca_Kabuli_Chr03	30688418	(A/C)
4395	CakSNP4395	*Kabuli*	Ca_Kabuli_Chr03	30688511	(G/C)
4396	CakSNP4396	*Kabuli*	Ca_Kabuli_Chr03	30688485	(C/A)
4397	CakSNP4397	*Kabuli*	Ca_Kabuli_Chr03	30688484	(A/C)
4398	CakSNP4398	*Kabuli*	Ca_Kabuli_Chr03	30688454	(A/G)
4399	CakSNP4399	*Kabuli*	Ca_Kabuli_Chr03	30688451	(T/C)
4400	CakSNP4400	*Kabuli*	Ca_Kabuli_Chr03	30718334	(G/T)
4401	CakSNP4401	*Kabuli*	Ca_Kabuli_Chr03	30751893	(A/C)
4402	CakSNP4402	*Kabuli*	Ca_Kabuli_Chr03	30751949	(A/T)
4403	CakSNP4403	*Kabuli*	Ca_Kabuli_Chr03	30791169	(A/T)
4404	CakSNP4404	*Kabuli*	Ca_Kabuli_Chr03	30791229	(G/A)
4405	CakSNP4405	*Kabuli*	Ca_Kabuli_Chr03	30791693	(C/T)

S.N.	SNP IDs	Cultivars	Chromosomes/scaffolds	Physical positions (bp)	SNPs
4406	CakSNP4406	*Kabuli*	Ca_Kabuli_Chr03	30794040	(C/A)
4407	CakSNP4407	*Kabuli*	Ca_Kabuli_Chr03	30794046	(A/C)
4408	CakSNP4408	*Kabuli*	Ca_Kabuli_Chr03	30837147	(A/G)
4409	CakSNP4409	*Kabuli*	Ca_Kabuli_Chr03	30837284	(C/T)
4410	CakSNP4410	*Kabuli*	Ca_Kabuli_Chr03	30837265	(T/C)
4411	CakSNP4411	*Kabuli*	Ca_Kabuli_Chr03	30880452	(T/C)
4412	CakSNP4412	*Kabuli*	Ca_Kabuli_Chr03	30880610	(A/G)
4413	CakSNP4413	*Kabuli*	Ca_Kabuli_Chr03	31020357	(C/A)
4414	CakSNP4414	*Kabuli*	Ca_Kabuli_Chr03	31020358	(C/G)
4415	CakSNP4415	*Kabuli*	Ca_Kabuli_Chr03	31073396	(T/C)
4416	CakSNP4416	*Kabuli*	Ca_Kabuli_Chr03	31073425	(G/C)
4417	CakSNP4417	*Kabuli*	Ca_Kabuli_Chr03	31073451	(A/G)
4418	CakSNP4418	*Kabuli*	Ca_Kabuli_Chr03	31091875	(T/G)
4419	CakSNP4419	*Kabuli*	Ca_Kabuli_Chr03	31267639	(T/A)
4420	CakSNP4420	*Kabuli*	Ca_Kabuli_Chr03	31267675	(A/G)
4421	CakSNP4421	*Kabuli*	Ca_Kabuli_Chr03	31267681	(G/A)
4422	CakSNP4422	*Kabuli*	Ca_Kabuli_Chr03	31267828	(A/G)
4423	CakSNP4423	*Kabuli*	Ca_Kabuli_Chr03	31267927	(T/G)
4424	CakSNP4424	*Kabuli*	Ca_Kabuli_Chr03	31268143	(A/G)
4425	CakSNP4425	*Kabuli*	Ca_Kabuli_Chr03	31279502	(C/T)
4426	CakSNP4426	*Kabuli*	Ca_Kabuli_Chr03	31279541	(A/G)
4427	CakSNP4427	*Kabuli*	Ca_Kabuli_Chr03	31279639	(C/T)
4428	CakSNP4428	*Kabuli*	Ca_Kabuli_Chr03	31279629	(C/G)
4429	CakSNP4429	*Kabuli*	Ca_Kabuli_Chr03	31279628	(T/C)
4430	CakSNP4430	*Kabuli*	Ca_Kabuli_Chr03	31279616	(T/A)
4431	CakSNP4431	*Kabuli*	Ca_Kabuli_Chr03	31279606	(G/A)
4432	CakSNP4432	*Kabuli*	Ca_Kabuli_Chr03	31279605	(G/A)
4433	CakSNP4433	*Kabuli*	Ca_Kabuli_Chr03	31279604	(C/G)
4434	CakSNP4434	*Kabuli*	Ca_Kabuli_Chr03	31279600	(T/C)
4435	CakSNP4435	*Kabuli*	Ca_Kabuli_Chr03	31279586	(A/G)
4436	CakSNP4436	*Kabuli*	Ca_Kabuli_Chr03	31287618	(C/A)
4437	CakSNP4437	*Kabuli*	Ca_Kabuli_Chr03	31287909	(A/G)
4438	CakSNP4438	*Kabuli*	Ca_Kabuli_Chr03	31288143	(T/C)
4439	CakSNP4439	*Kabuli*	Ca_Kabuli_Chr03	31301723	(G/A)
4440	CakSNP4440	*Kabuli*	Ca_Kabuli_Chr03	31301783	(A/G)
4441	CakSNP4441	*Kabuli*	Ca_Kabuli_Chr03	31302060	(T/C)
4442	CakSNP4442	*Kabuli*	Ca_Kabuli_Chr03	31302117	(C/T)
4443	CakSNP4443	*Kabuli*	Ca_Kabuli_Chr03	31313021	(G/T)
4444	CakSNP4444	*Kabuli*	Ca_Kabuli_Chr03	31317521	(T/G)

S.N.	SNP IDs	Cultivars	Chromosomes/scaffolds	Physical positions (bp)	SNPs
4445	CakSNP4445	*Kabuli*	Ca_Kabuli_Chr03	31321725	(A/G)
4446	CakSNP4446	*Kabuli*	Ca_Kabuli_Chr03	31321778	(G/A)
4447	CakSNP4447	*Kabuli*	Ca_Kabuli_Chr03	31321850	(A/G)
4448	CakSNP4448	*Kabuli*	Ca_Kabuli_Chr03	31341335	(C/T)
4449	CakSNP4449	*Kabuli*	Ca_Kabuli_Chr03	31341323	(T/C)
4450	CakSNP4450	*Kabuli*	Ca_Kabuli_Chr03	31342119	(A/T)
4451	CakSNP4451	*Kabuli*	Ca_Kabuli_Chr03	31354988	(G/C)
4452	CakSNP4452	*Kabuli*	Ca_Kabuli_Chr03	31355001	(C/T)
4453	CakSNP4453	*Kabuli*	Ca_Kabuli_Chr03	31355026	(T/C)
4454	CakSNP4454	*Kabuli*	Ca_Kabuli_Chr03	31355066	(A/T)
4455	CakSNP4455	*Kabuli*	Ca_Kabuli_Chr03	31357787	(A/T)
4456	CakSNP4456	*Kabuli*	Ca_Kabuli_Chr03	31431216	(C/T)
4457	CakSNP4457	*Kabuli*	Ca_Kabuli_Chr03	31478804	(T/C)
4458	CakSNP4458	*Kabuli*	Ca_Kabuli_Chr03	31478882	(C/T)
4459	CakSNP4459	*Kabuli*	Ca_Kabuli_Chr03	31480603	(T/C)
4460	CakSNP4460	*Kabuli*	Ca_Kabuli_Chr03	31516662	(G/T)
4461	CakSNP4461	*Kabuli*	Ca_Kabuli_Chr03	31516766	(T/C)
4462	CakSNP4462	*Kabuli*	Ca_Kabuli_Chr03	31520119	(C/T)
4463	CakSNP4463	*Kabuli*	Ca_Kabuli_Chr03	31520225	(A/T)
4464	CakSNP4464	*Kabuli*	Ca_Kabuli_Chr03	31520198	(G/A)
4465	CakSNP4465	*Kabuli*	Ca_Kabuli_Chr03	31520190	(T/C)
4466	CakSNP4466	*Kabuli*	Ca_Kabuli_Chr03	31520832	(G/A)
4467	CakSNP4467	*Kabuli*	Ca_Kabuli_Chr03	31521142	(T/C)
4468	CakSNP4468	*Kabuli*	Ca_Kabuli_Chr03	31521155	(T/A)
4469	CakSNP4469	*Kabuli*	Ca_Kabuli_Chr03	31589355	(A/G)
4470	CakSNP4470	*Kabuli*	Ca_Kabuli_Chr03	31589329	(C/T)
4471	CakSNP4471	*Kabuli*	Ca_Kabuli_Chr03	31591065	(G/A)
4472	CakSNP4472	*Kabuli*	Ca_Kabuli_Chr03	31606359	(C/T)
4473	CakSNP4473	*Kabuli*	Ca_Kabuli_Chr03	31630175	(T/A)
4474	CakSNP4474	*Kabuli*	Ca_Kabuli_Chr03	31630178	(G/T)
4475	CakSNP4475	*Kabuli*	Ca_Kabuli_Chr03	31630195	(T/C)
4476	CakSNP4476	*Kabuli*	Ca_Kabuli_Chr03	31631451	(A/C)
4477	CakSNP4477	*Kabuli*	Ca_Kabuli_Chr03	31631444	(T/G)
4478	CakSNP4478	*Kabuli*	Ca_Kabuli_Chr03	31631561	(G/A)
4479	CakSNP4479	*Kabuli*	Ca_Kabuli_Chr03	31631534	(A/G)
4480	CakSNP4480	*Kabuli*	Ca_Kabuli_Chr03	31631622	(G/A)
4481	CakSNP4481	*Kabuli*	Ca_Kabuli_Chr03	31631644	(A/C)
4482	CakSNP4482	*Kabuli*	Ca_Kabuli_Chr03	31646735	(C/T)
4483	CakSNP4483	*Kabuli*	Ca_Kabuli_Chr03	31646817	(C/T)

S.N.	SNP IDs	Cultivars	Chromosomes/scaffolds	Physical positions (bp)	SNPs
4484	CakSNP4484	*Kabuli*	Ca_Kabuli_Chr03	31646802	(G/A)
4485	CakSNP4485	*Kabuli*	Ca_Kabuli_Chr03	31683906	(A/G)
4486	CakSNP4486	*Kabuli*	Ca_Kabuli_Chr03	31689128	(C/T)
4487	CakSNP4487	*Kabuli*	Ca_Kabuli_Chr03	31698493	(A/C)
4488	CakSNP4488	*Kabuli*	Ca_Kabuli_Chr03	31766363	(G/A)
4489	CakSNP4489	*Kabuli*	Ca_Kabuli_Chr03	31770365	(A/C)
4490	CakSNP4490	*Kabuli*	Ca_Kabuli_Chr03	31771314	(C/A)
4491	CakSNP4491	*Kabuli*	Ca_Kabuli_Chr03	31790189	(C/T)
4492	CakSNP4492	*Kabuli*	Ca_Kabuli_Chr03	31790216	(G/A)
4493	CakSNP4493	*Kabuli*	Ca_Kabuli_Chr03	31790239	(G/T)
4494	CakSNP4494	*Kabuli*	Ca_Kabuli_Chr03	31790352	(G/A)
4495	CakSNP4495	*Kabuli*	Ca_Kabuli_Chr03	31814300	(A/G)
4496	CakSNP4496	*Kabuli*	Ca_Kabuli_Chr03	31852191	(T/C)
4497	CakSNP4497	*Kabuli*	Ca_Kabuli_Chr03	31857877	(C/T)
4498	CakSNP4498	*Kabuli*	Ca_Kabuli_Chr03	31859152	(C/G)
4499	CakSNP4499	*Kabuli*	Ca_Kabuli_Chr03	31859251	(T/C)
4500	CakSNP4500	*Kabuli*	Ca_Kabuli_Chr03	31926694	(C/G)
4501	CakSNP4501	*Kabuli*	Ca_Kabuli_Chr03	31931014	(A/C)
4502	CakSNP4502	*Kabuli*	Ca_Kabuli_Chr03	31945504	(C/T)
4503	CakSNP4503	*Kabuli*	Ca_Kabuli_Chr03	31945634	(A/G)
4504	CakSNP4504	*Kabuli*	Ca_Kabuli_Chr03	31951994	(G/A)
4505	CakSNP4505	*Kabuli*	Ca_Kabuli_Chr03	31953466	(G/A)
4506	CakSNP4506	*Kabuli*	Ca_Kabuli_Chr03	31953549	(C/T)
4507	CakSNP4507	*Kabuli*	Ca_Kabuli_Chr03	32351052	(C/T)
4508	CakSNP4508	*Kabuli*	Ca_Kabuli_Chr03	32351031	(G/C)
4509	CakSNP4509	*Kabuli*	Ca_Kabuli_Chr03	32357599	(C/T)
4510	CakSNP4510	*Kabuli*	Ca_Kabuli_Chr03	32357578	(G/C)
4511	CakSNP4511	*Kabuli*	Ca_Kabuli_Chr03	32448959	(T/C)
4512	CakSNP4512	*Kabuli*	Ca_Kabuli_Chr03	32449654	(T/G)
4513	CakSNP4513	*Kabuli*	Ca_Kabuli_Chr03	32449700	(C/T)
4514	CakSNP4514	*Kabuli*	Ca_Kabuli_Chr03	32510033	(T/G)
4515	CakSNP4515	*Kabuli*	Ca_Kabuli_Chr03	32510090	(A/G)
4516	CakSNP4516	*Kabuli*	Ca_Kabuli_Chr03	32510130	(A/G)
4517	CakSNP4517	*Kabuli*	Ca_Kabuli_Chr03	32510124	(C/T)
4518	CakSNP4518	*Kabuli*	Ca_Kabuli_Chr03	32510111	(C/T)
4519	CakSNP4519	*Kabuli*	Ca_Kabuli_Chr03	32510071	(G/T)
4520	CakSNP4520	*Kabuli*	Ca_Kabuli_Chr03	32510081	(C/T)
4521	CakSNP4521	*Kabuli*	Ca_Kabuli_Chr03	32514908	(A/G)
4522	CakSNP4522	*Kabuli*	Ca_Kabuli_Chr03	32514890	(A/G)

S.N.	SNP IDs	Cultivars	Chromosomes/scaffolds	Physical positions (bp)	SNPs
4523	CakSNP4523	*Kabuli*	Ca_Kabuli_Chr03	32514889	(C/T)
4524	CakSNP4524	*Kabuli*	Ca_Kabuli_Chr03	32514869	(C/T)
4525	CakSNP4525	*Kabuli*	Ca_Kabuli_Chr03	32514868	(A/G)
4526	CakSNP4526	*Kabuli*	Ca_Kabuli_Chr03	32514867	(C/T)
4527	CakSNP4527	*Kabuli*	Ca_Kabuli_Chr03	32514863	(G/C)
4528	CakSNP4528	*Kabuli*	Ca_Kabuli_Chr03	32514859	(C/T)
4529	CakSNP4529	*Kabuli*	Ca_Kabuli_Chr03	32514916	(G/C)
4530	CakSNP4530	*Kabuli*	Ca_Kabuli_Chr03	32525128	(G/T)
4531	CakSNP4531	*Kabuli*	Ca_Kabuli_Chr03	32557850	(C/T)
4532	CakSNP4532	*Kabuli*	Ca_Kabuli_Chr03	32571237	(G/A)
4533	CakSNP4533	*Kabuli*	Ca_Kabuli_Chr03	32571291	(A/G)
4534	CakSNP4534	*Kabuli*	Ca_Kabuli_Chr03	32571275	(T/C)
4535	CakSNP4535	*Kabuli*	Ca_Kabuli_Chr03	32600003	(A/G)
4536	CakSNP4536	*Kabuli*	Ca_Kabuli_Chr03	32601732	(T/C)
4537	CakSNP4537	*Kabuli*	Ca_Kabuli_Chr03	32608947	(A/G)
4538	CakSNP4538	*Kabuli*	Ca_Kabuli_Chr03	32662861	(A/G)
4539	CakSNP4539	*Kabuli*	Ca_Kabuli_Chr03	32662858	(A/G)
4540	CakSNP4540	*Kabuli*	Ca_Kabuli_Chr03	32663015	(C/T)
4541	CakSNP4541	*Kabuli*	Ca_Kabuli_Chr03	32663059	(A/C)
4542	CakSNP4542	*Kabuli*	Ca_Kabuli_Chr03	32694509	(T/C)
4543	CakSNP4543	*Kabuli*	Ca_Kabuli_Chr03	32694454	(T/C)
4544	CakSNP4544	*Kabuli*	Ca_Kabuli_Chr03	32704734	(G/A)
4545	CakSNP4545	*Kabuli*	Ca_Kabuli_Chr03	32704868	(G/T)
4546	CakSNP4546	*Kabuli*	Ca_Kabuli_Chr03	32704953	(G/A)
4547	CakSNP4547	*Kabuli*	Ca_Kabuli_Chr03	32706200	(C/T)
4548	CakSNP4548	*Kabuli*	Ca_Kabuli_Chr03	32725039	(C/G)
4549	CakSNP4549	*Kabuli*	Ca_Kabuli_Chr03	32725096	(A/T)
4550	CakSNP4550	*Kabuli*	Ca_Kabuli_Chr03	32758259	(C/A)
4551	CakSNP4551	*Kabuli*	Ca_Kabuli_Chr03	32758293	(T/C)
4552	CakSNP4552	*Kabuli*	Ca_Kabuli_Chr03	32785228	(A/G)
4553	CakSNP4553	*Kabuli*	Ca_Kabuli_Chr03	32827067	(C/G)
4554	CakSNP4554	*Kabuli*	Ca_Kabuli_Chr03	32827065	(T/G)
4555	CakSNP4555	*Kabuli*	Ca_Kabuli_Chr03	32845361	(A/G)
4556	CakSNP4556	*Kabuli*	Ca_Kabuli_Chr03	32845395	(C/A)
4557	CakSNP4557	*Kabuli*	Ca_Kabuli_Chr03	32847120	(T/A)
4558	CakSNP4558	*Kabuli*	Ca_Kabuli_Chr03	32847216	(A/G)
4559	CakSNP4559	*Kabuli*	Ca_Kabuli_Chr03	32865549	(C/T)
4560	CakSNP4560	*Kabuli*	Ca_Kabuli_Chr03	32865530	(C/G)
4561	CakSNP4561	*Kabuli*	Ca_Kabuli_Chr03	32954428	(T/C)

S.N.	SNP IDs	Cultivars	Chromosomes/scaffolds	Physical positions (bp)	SNPs
4562	CakSNP4562	*Kabuli*	Ca_Kabuli_Chr03	33033245	(T/C)
4563	CakSNP4563	*Kabuli*	Ca_Kabuli_Chr03	33091737	(A/C)
4564	CakSNP4564	*Kabuli*	Ca_Kabuli_Chr03	33120619	(G/A)
4565	CakSNP4565	*Kabuli*	Ca_Kabuli_Chr03	33120825	(G/A)
4566	CakSNP4566	*Kabuli*	Ca_Kabuli_Chr03	33520615	(A/C)
4567	CakSNP4567	*Kabuli*	Ca_Kabuli_Chr03	33520887	(G/A)
4568	CakSNP4568	*Kabuli*	Ca_Kabuli_Chr03	33522974	(G/A)
4569	CakSNP4569	*Kabuli*	Ca_Kabuli_Chr03	33533273	(G/A)
4570	CakSNP4570	*Kabuli*	Ca_Kabuli_Chr03	33627826	(A/C)
4571	CakSNP4571	*Kabuli*	Ca_Kabuli_Chr03	33715215	(G/T)
4572	CakSNP4572	*Kabuli*	Ca_Kabuli_Chr03	33898250	(C/T)
4573	CakSNP4573	*Kabuli*	Ca_Kabuli_Chr03	33900522	(A/G)
4574	CakSNP4574	*Kabuli*	Ca_Kabuli_Chr03	33900533	(A/G)
4575	CakSNP4575	*Kabuli*	Ca_Kabuli_Chr03	33904394	(T/A)
4576	CakSNP4576	*Kabuli*	Ca_Kabuli_Chr03	34247736	(T/G)
4577	CakSNP4577	*Kabuli*	Ca_Kabuli_Chr03	34247831	(G/A)
4578	CakSNP4578	*Kabuli*	Ca_Kabuli_Chr03	34247828	(T/C)
4579	CakSNP4579	*Kabuli*	Ca_Kabuli_Chr03	34247893	(A/G)
4580	CakSNP4580	*Kabuli*	Ca_Kabuli_Chr03	34268356	(A/T)
4581	CakSNP4581	*Kabuli*	Ca_Kabuli_Chr03	34282419	(C/T)
4582	CakSNP4582	*Kabuli*	Ca_Kabuli_Chr03	34290129	(T/C)
4583	CakSNP4583	*Kabuli*	Ca_Kabuli_Chr03	34290135	(G/A)
4584	CakSNP4584	*Kabuli*	Ca_Kabuli_Chr03	34290173	(C/T)
4585	CakSNP4585	*Kabuli*	Ca_Kabuli_Chr03	34352385	(A/C)
4586	CakSNP4586	*Kabuli*	Ca_Kabuli_Chr03	34356844	(A/C)
4587	CakSNP4587	*Kabuli*	Ca_Kabuli_Chr03	34357171	(T/G)
4588	CakSNP4588	*Kabuli*	Ca_Kabuli_Chr03	34357296	(C/G)
4589	CakSNP4589	*Kabuli*	Ca_Kabuli_Chr03	34496982	(G/C)
4590	CakSNP4590	*Kabuli*	Ca_Kabuli_Chr03	34524656	(C/A)
4591	CakSNP4591	*Kabuli*	Ca_Kabuli_Chr03	34542046	(T/G)
4592	CakSNP4592	*Kabuli*	Ca_Kabuli_Chr03	34543039	(C/T)
4593	CakSNP4593	*Kabuli*	Ca_Kabuli_Chr03	34543111	(A/G)
4594	CakSNP4594	*Kabuli*	Ca_Kabuli_Chr03	34547052	(A/T)
4595	CakSNP4595	*Kabuli*	Ca_Kabuli_Chr03	34547069	(G/T)
4596	CakSNP4596	*Kabuli*	Ca_Kabuli_Chr03	34547141	(A/G)
4597	CakSNP4597	*Kabuli*	Ca_Kabuli_Chr03	34576769	(G/A)
4598	CakSNP4598	*Kabuli*	Ca_Kabuli_Chr03	34595487	(A/G)
4599	CakSNP4599	*Kabuli*	Ca_Kabuli_Chr03	34601038	(T/C)
4600	CakSNP4600	*Kabuli*	Ca_Kabuli_Chr03	34603669	(T/A)

S.N.	SNP IDs	Cultivars	Chromosomes/scaffolds	Physical positions (bp)	SNPs
4601	CakSNP4601	*Kabuli*	Ca_Kabuli_Chr03	34650308	(G/A)
4602	CakSNP4602	*Kabuli*	Ca_Kabuli_Chr03	34650245	(G/A)
4603	CakSNP4603	*Kabuli*	Ca_Kabuli_Chr03	34662736	(T/G)
4604	CakSNP4604	*Kabuli*	Ca_Kabuli_Chr03	34699114	(C/A)
4605	CakSNP4605	*Kabuli*	Ca_Kabuli_Chr03	34699076	(T/C)
4606	CakSNP4606	*Kabuli*	Ca_Kabuli_Chr03	34722508	(A/T)
4607	CakSNP4607	*Kabuli*	Ca_Kabuli_Chr03	34722931	(A/G)
4608	CakSNP4608	*Kabuli*	Ca_Kabuli_Chr03	34722979	(T/C)
4609	CakSNP4609	*Kabuli*	Ca_Kabuli_Chr03	34722992	(T/G)
4610	CakSNP4610	*Kabuli*	Ca_Kabuli_Chr03	34730463	(T/C)
4611	CakSNP4611	*Kabuli*	Ca_Kabuli_Chr03	34730954	(G/A)
4612	CakSNP4612	*Kabuli*	Ca_Kabuli_Chr03	34800277	(G/A)
4613	CakSNP4613	*Kabuli*	Ca_Kabuli_Chr03	34820472	(T/A)
4614	CakSNP4614	*Kabuli*	Ca_Kabuli_Chr03	34820532	(T/A)
4615	CakSNP4615	*Kabuli*	Ca_Kabuli_Chr03	34852030	(G/A)
4616	CakSNP4616	*Kabuli*	Ca_Kabuli_Chr03	34852070	(T/G)
4617	CakSNP4617	*Kabuli*	Ca_Kabuli_Chr03	34852047	(T/A)
4618	CakSNP4618	*Kabuli*	Ca_Kabuli_Chr03	34889316	(C/G)
4619	CakSNP4619	*Kabuli*	Ca_Kabuli_Chr03	34889361	(T/C)
4620	CakSNP4620	*Kabuli*	Ca_Kabuli_Chr03	34911621	(G/A)
4621	CakSNP4621	*Kabuli*	Ca_Kabuli_Chr03	35071821	(A/G)
4622	CakSNP4622	*Kabuli*	Ca_Kabuli_Chr03	35071852	(T/G)
4623	CakSNP4623	*Kabuli*	Ca_Kabuli_Chr03	35085559	(T/A)
4624	CakSNP4624	*Kabuli*	Ca_Kabuli_Chr03	35085658	(C/A)
4625	CakSNP4625	*Kabuli*	Ca_Kabuli_Chr03	35126649	(C/G)
4626	CakSNP4626	*Kabuli*	Ca_Kabuli_Chr03	35126766	(G/A)
4627	CakSNP4627	*Kabuli*	Ca_Kabuli_Chr03	35126760	(G/A)
4628	CakSNP4628	*Kabuli*	Ca_Kabuli_Chr03	35167312	(T/G)
4629	CakSNP4629	*Kabuli*	Ca_Kabuli_Chr03	35167359	(T/G)
4630	CakSNP4630	*Kabuli*	Ca_Kabuli_Chr03	35185001	(G/T)
4631	CakSNP4631	*Kabuli*	Ca_Kabuli_Chr03	35274992	(A/G)
4632	CakSNP4632	*Kabuli*	Ca_Kabuli_Chr03	35275199	(T/C)
4633	CakSNP4633	*Kabuli*	Ca_Kabuli_Chr03	35332088	(T/A)
4634	CakSNP4634	*Kabuli*	Ca_Kabuli_Chr03	35332091	(T/C)
4635	CakSNP4635	*Kabuli*	Ca_Kabuli_Chr03	35383318	(C/T)
4636	CakSNP4636	*Kabuli*	Ca_Kabuli_Chr03	35394684	(G/T)
4637	CakSNP4637	*Kabuli*	Ca_Kabuli_Chr03	35403074	(A/G)
4638	CakSNP4638	*Kabuli*	Ca_Kabuli_Chr03	35420061	(A/G)
4639	CakSNP4639	*Kabuli*	Ca_Kabuli_Chr03	35420108	(T/A)

S.N.	SNP IDs	Cultivars	Chromosomes/scaffolds	Physical positions (bp)	SNPs
4640	CakSNP4640	*Kabuli*	Ca_Kabuli_Chr03	35424994	(T/C)
4641	CakSNP4641	*Kabuli*	Ca_Kabuli_Chr03	35483932	(T/C)
4642	CakSNP4642	*Kabuli*	Ca_Kabuli_Chr03	35483960	(T/C)
4643	CakSNP4643	*Kabuli*	Ca_Kabuli_Chr03	35483995	(T/C)
4644	CakSNP4644	*Kabuli*	Ca_Kabuli_Chr03	35483999	(C/T)
4645	CakSNP4645	*Kabuli*	Ca_Kabuli_Chr03	35484034	(G/C)
4646	CakSNP4646	*Kabuli*	Ca_Kabuli_Chr03	35487736	(T/C)
4647	CakSNP4647	*Kabuli*	Ca_Kabuli_Chr03	35488503	(A/C)
4648	CakSNP4648	*Kabuli*	Ca_Kabuli_Chr03	35488597	(T/C)
4649	CakSNP4649	*Kabuli*	Ca_Kabuli_Chr03	35488570	(G/T)
4650	CakSNP4650	*Kabuli*	Ca_Kabuli_Chr03	35490991	(A/G)
4651	CakSNP4651	*Kabuli*	Ca_Kabuli_Chr03	35490951	(C/T)
4652	CakSNP4652	*Kabuli*	Ca_Kabuli_Chr03	35543614	(C/T)
4653	CakSNP4653	*Kabuli*	Ca_Kabuli_Chr03	35631679	(C/G)
4654	CakSNP4654	*Kabuli*	Ca_Kabuli_Chr03	35631689	(C/A)
4655	CakSNP4655	*Kabuli*	Ca_Kabuli_Chr03	35631723	(A/G)
4656	CakSNP4656	*Kabuli*	Ca_Kabuli_Chr03	35647986	(C/G)
4657	CakSNP4657	*Kabuli*	Ca_Kabuli_Chr03	35747394	(C/G)
4658	CakSNP4658	*Kabuli*	Ca_Kabuli_Chr03	35807222	(A/G)
4659	CakSNP4659	*Kabuli*	Ca_Kabuli_Chr03	35807265	(T/C)
4660	CakSNP4660	*Kabuli*	Ca_Kabuli_Chr03	35807407	(A/T)
4661	CakSNP4661	*Kabuli*	Ca_Kabuli_Chr03	35829834	(A/G)
4662	CakSNP4662	*Kabuli*	Ca_Kabuli_Chr03	35898320	(G/C)
4663	CakSNP4663	*Kabuli*	Ca_Kabuli_Chr03	35898374	(G/A)
4664	CakSNP4664	*Kabuli*	Ca_Kabuli_Chr03	35898581	(C/T)
4665	CakSNP4665	*Kabuli*	Ca_Kabuli_Chr03	35920684	(A/C)
4666	CakSNP4666	*Kabuli*	Ca_Kabuli_Chr03	35920860	(C/A)
4667	CakSNP4667	*Kabuli*	Ca_Kabuli_Chr03	35948487	(G/A)
4668	CakSNP4668	*Kabuli*	Ca_Kabuli_Chr03	35979412	(G/T)
4669	CakSNP4669	*Kabuli*	Ca_Kabuli_Chr03	36003699	(G/A)
4670	CakSNP4670	*Kabuli*	Ca_Kabuli_Chr03	36003682	(G/T)
4671	CakSNP4671	*Kabuli*	Ca_Kabuli_Chr03	36004638	(C/T)
4672	CakSNP4672	*Kabuli*	Ca_Kabuli_Chr03	36004663	(T/C)
4673	CakSNP4673	*Kabuli*	Ca_Kabuli_Chr03	36004668	(A/G)
4674	CakSNP4674	*Kabuli*	Ca_Kabuli_Chr03	36046759	(G/A)
4675	CakSNP4675	*Kabuli*	Ca_Kabuli_Chr03	36046938	(C/T)
4676	CakSNP4676	*Kabuli*	Ca_Kabuli_Chr03	36046956	(A/C)
4677	CakSNP4677	*Kabuli*	Ca_Kabuli_Chr03	36050259	(T/G)
4678	CakSNP4678	*Kabuli*	Ca_Kabuli_Chr03	36050319	(T/C)

S.N.	SNP IDs	Cultivars	Chromosomes/scaffolds	Physical positions (bp)	SNPs
4679	CakSNP4679	*Kabuli*	Ca_Kabuli_Chr03	36071033	(A/G)
4680	CakSNP4680	*Kabuli*	Ca_Kabuli_Chr03	36085966	(G/A)
4681	CakSNP4681	*Kabuli*	Ca_Kabuli_Chr03	36117521	(A/G)
4682	CakSNP4682	*Kabuli*	Ca_Kabuli_Chr03	36118120	(T/C)
4683	CakSNP4683	*Kabuli*	Ca_Kabuli_Chr03	36118156	(C/T)
4684	CakSNP4684	*Kabuli*	Ca_Kabuli_Chr03	36119592	(T/C)
4685	CakSNP4685	*Kabuli*	Ca_Kabuli_Chr03	36119602	(C/A)
4686	CakSNP4686	*Kabuli*	Ca_Kabuli_Chr03	36130561	(T/A)
4687	CakSNP4687	*Kabuli*	Ca_Kabuli_Chr03	36130610	(C/A)
4688	CakSNP4688	*Kabuli*	Ca_Kabuli_Chr03	36132440	(T/C)
4689	CakSNP4689	*Kabuli*	Ca_Kabuli_Chr03	36132437	(C/T)
4690	CakSNP4690	*Kabuli*	Ca_Kabuli_Chr03	36132401	(C/T)
4691	CakSNP4691	*Kabuli*	Ca_Kabuli_Chr03	36132480	(C/T)
4692	CakSNP4692	*Kabuli*	Ca_Kabuli_Chr03	36132539	(C/T)
4693	CakSNP4693	*Kabuli*	Ca_Kabuli_Chr03	36132586	(T/G)
4694	CakSNP4694	*Kabuli*	Ca_Kabuli_Chr03	36132570	(G/A)
4695	CakSNP4695	*Kabuli*	Ca_Kabuli_Chr03	36132554	(A/T)
4696	CakSNP4696	*Kabuli*	Ca_Kabuli_Chr03	36150722	(G/A)
4697	CakSNP4697	*Kabuli*	Ca_Kabuli_Chr03	36150717	(T/C)
4698	CakSNP4698	*Kabuli*	Ca_Kabuli_Chr03	36164863	(T/G)
4699	CakSNP4699	*Kabuli*	Ca_Kabuli_Chr03	36164866	(T/C)
4700	CakSNP4700	*Kabuli*	Ca_Kabuli_Chr03	36165997	(C/G)
4701	CakSNP4701	*Kabuli*	Ca_Kabuli_Chr03	36167136	(G/T)
4702	CakSNP4702	*Kabuli*	Ca_Kabuli_Chr03	36175164	(G/A)
4703	CakSNP4703	*Kabuli*	Ca_Kabuli_Chr03	36177201	(T/C)
4704	CakSNP4704	*Kabuli*	Ca_Kabuli_Chr03	36205806	(T/G)
4705	CakSNP4705	*Kabuli*	Ca_Kabuli_Chr03	36205819	(A/G)
4706	CakSNP4706	*Kabuli*	Ca_Kabuli_Chr03	36205845	(A/G)
4707	CakSNP4707	*Kabuli*	Ca_Kabuli_Chr03	36206615	(A/C)
4708	CakSNP4708	*Kabuli*	Ca_Kabuli_Chr03	36212799	(A/T)
4709	CakSNP4709	*Kabuli*	Ca_Kabuli_Chr03	36212809	(G/A)
4710	CakSNP4710	*Kabuli*	Ca_Kabuli_Chr03	36212843	(T/A)
4711	CakSNP4711	*Kabuli*	Ca_Kabuli_Chr03	36217686	(G/A)
4712	CakSNP4712	*Kabuli*	Ca_Kabuli_Chr03	36227565	(A/T)
4713	CakSNP4713	*Kabuli*	Ca_Kabuli_Chr03	36227566	(A/T)
4714	CakSNP4714	*Kabuli*	Ca_Kabuli_Chr03	36227948	(A/G)
4715	CakSNP4715	*Kabuli*	Ca_Kabuli_Chr03	36227949	(C/T)
4716	CakSNP4716	*Kabuli*	Ca_Kabuli_Chr03	36228078	(G/A)
4717	CakSNP4717	*Kabuli*	Ca_Kabuli_Chr03	36228032	(G/T)

S.N.	SNP IDs	Cultivars	Chromosomes/scaffolds	Physical positions (bp)	SNPs
4718	CakSNP4718	*Kabuli*	Ca_Kabuli_Chr03	36228121	(T/A)
4719	CakSNP4719	*Kabuli*	Ca_Kabuli_Chr03	36228126	(T/A)
4720	CakSNP4720	*Kabuli*	Ca_Kabuli_Chr03	36242506	(C/T)
4721	CakSNP4721	*Kabuli*	Ca_Kabuli_Chr03	36242504	(C/T)
4722	CakSNP4722	*Kabuli*	Ca_Kabuli_Chr03	36242490	(G/T)
4723	CakSNP4723	*Kabuli*	Ca_Kabuli_Chr03	36242480	(G/C)
4724	CakSNP4724	*Kabuli*	Ca_Kabuli_Chr03	36247792	(T/C)
4725	CakSNP4725	*Kabuli*	Ca_Kabuli_Chr03	36260865	(C/A)
4726	CakSNP4726	*Kabuli*	Ca_Kabuli_Chr03	36282091	(T/G)
4727	CakSNP4727	*Kabuli*	Ca_Kabuli_Chr03	36293589	(A/G)
4728	CakSNP4728	*Kabuli*	Ca_Kabuli_Chr03	36293625	(C/T)
4729	CakSNP4729	*Kabuli*	Ca_Kabuli_Chr03	36293637	(C/T)
4730	CakSNP4730	*Kabuli*	Ca_Kabuli_Chr03	36330750	(A/G)
4731	CakSNP4731	*Kabuli*	Ca_Kabuli_Chr03	36330815	(T/C)
4732	CakSNP4732	*Kabuli*	Ca_Kabuli_Chr03	36345158	(T/C)
4733	CakSNP4733	*Kabuli*	Ca_Kabuli_Chr03	36347203	(T/A)
4734	CakSNP4734	*Kabuli*	Ca_Kabuli_Chr03	36361339	(C/T)
4735	CakSNP4735	*Kabuli*	Ca_Kabuli_Chr03	36378294	(G/T)
4736	CakSNP4736	*Kabuli*	Ca_Kabuli_Chr03	36378299	(C/G)
4737	CakSNP4737	*Kabuli*	Ca_Kabuli_Chr03	36378358	(G/A)
4738	CakSNP4738	*Kabuli*	Ca_Kabuli_Chr03	36387615	(A/T)
4739	CakSNP4739	*Kabuli*	Ca_Kabuli_Chr03	36387732	(G/A)
4740	CakSNP4740	*Kabuli*	Ca_Kabuli_Chr03	36387718	(C/T)
4741	CakSNP4741	*Kabuli*	Ca_Kabuli_Chr03	36396559	(C/A)
4742	CakSNP4742	*Kabuli*	Ca_Kabuli_Chr03	36420902	(T/C)
4743	CakSNP4743	*Kabuli*	Ca_Kabuli_Chr03	36432389	(A/G)
4744	CakSNP4744	*Kabuli*	Ca_Kabuli_Chr03	36447274	(G/A)
4745	CakSNP4745	*Kabuli*	Ca_Kabuli_Chr03	36447364	(T/A)
4746	CakSNP4746	*Kabuli*	Ca_Kabuli_Chr03	36447520	(T/A)
4747	CakSNP4747	*Kabuli*	Ca_Kabuli_Chr03	36447525	(C/T)
4748	CakSNP4748	*Kabuli*	Ca_Kabuli_Chr03	36488722	(T/A)
4749	CakSNP4749	*Kabuli*	Ca_Kabuli_Chr03	36488736	(T/C)
4750	CakSNP4750	*Kabuli*	Ca_Kabuli_Chr03	36488853	(A/G)
4751	CakSNP4751	*Kabuli*	Ca_Kabuli_Chr03	36488890	(A/G)
4752	CakSNP4752	*Kabuli*	Ca_Kabuli_Chr03	36503906	(C/T)
4753	CakSNP4753	*Kabuli*	Ca_Kabuli_Chr03	36503912	(T/C)
4754	CakSNP4754	*Kabuli*	Ca_Kabuli_Chr03	36524598	(G/C)
4755	CakSNP4755	*Kabuli*	Ca_Kabuli_Chr03	36524635	(A/G)
4756	CakSNP4756	*Kabuli*	Ca_Kabuli_Chr03	36524659	(T/C)

S.N.	SNP IDs	Cultivars	Chromosomes/scaffolds	Physical positions (bp)	SNPs
4757	CakSNP4757	*Kabuli*	Ca_Kabuli_Chr03	36531151	(C/A)
4758	CakSNP4758	*Kabuli*	Ca_Kabuli_Chr03	36588856	(C/T)
4759	CakSNP4759	*Kabuli*	Ca_Kabuli_Chr03	36588896	(C/G)
4760	CakSNP4760	*Kabuli*	Ca_Kabuli_Chr03	36606527	(G/A)
4761	CakSNP4761	*Kabuli*	Ca_Kabuli_Chr03	36609043	(G/A)
4762	CakSNP4762	*Kabuli*	Ca_Kabuli_Chr03	36616855	(T/C)
4763	CakSNP4763	*Kabuli*	Ca_Kabuli_Chr03	36644117	(A/T)
4764	CakSNP4764	*Kabuli*	Ca_Kabuli_Chr03	36733097	(G/A)
4765	CakSNP4765	*Kabuli*	Ca_Kabuli_Chr03	36733157	(A/G)
4766	CakSNP4766	*Kabuli*	Ca_Kabuli_Chr03	36733211	(A/G)
4767	CakSNP4767	*Kabuli*	Ca_Kabuli_Chr03	36749775	(T/A)
4768	CakSNP4768	*Kabuli*	Ca_Kabuli_Chr03	36749826	(G/A)
4769	CakSNP4769	*Kabuli*	Ca_Kabuli_Chr03	36749829	(T/C)
4770	CakSNP4770	*Kabuli*	Ca_Kabuli_Chr03	36749831	(T/C)
4771	CakSNP4771	*Kabuli*	Ca_Kabuli_Chr03	36749871	(T/C)
4772	CakSNP4772	*Kabuli*	Ca_Kabuli_Chr03	36749904	(C/T)
4773	CakSNP4773	*Kabuli*	Ca_Kabuli_Chr03	36749916	(C/A)
4774	CakSNP4774	*Kabuli*	Ca_Kabuli_Chr03	36752689	(T/G)
4775	CakSNP4775	*Kabuli*	Ca_Kabuli_Chr03	36769775	(G/T)
4776	CakSNP4776	*Kabuli*	Ca_Kabuli_Chr03	36783494	(T/A)
4777	CakSNP4777	*Kabuli*	Ca_Kabuli_Chr03	36825463	(T/C)
4778	CakSNP4778	*Kabuli*	Ca_Kabuli_Chr03	36860134	(A/G)
4779	CakSNP4779	*Kabuli*	Ca_Kabuli_Chr03	36860246	(T/C)
4780	CakSNP4780	*Kabuli*	Ca_Kabuli_Chr03	36873720	(T/C)
4781	CakSNP4781	*Kabuli*	Ca_Kabuli_Chr03	36883268	(T/C)
4782	CakSNP4782	*Kabuli*	Ca_Kabuli_Chr03	36883333	(A/T)
4783	CakSNP4783	*Kabuli*	Ca_Kabuli_Chr03	36895362	(T/C)
4784	CakSNP4784	*Kabuli*	Ca_Kabuli_Chr03	36938148	(A/G)
4785	CakSNP4785	*Kabuli*	Ca_Kabuli_Chr03	36938271	(G/C)
4786	CakSNP4786	*Kabuli*	Ca_Kabuli_Chr03	36943522	(A/G)
4787	CakSNP4787	*Kabuli*	Ca_Kabuli_Chr03	36991718	(C/A)
4788	CakSNP4788	*Kabuli*	Ca_Kabuli_Chr03	37003976	(C/T)
4789	CakSNP4789	*Kabuli*	Ca_Kabuli_Chr03	37004026	(A/G)
4790	CakSNP4790	*Kabuli*	Ca_Kabuli_Chr03	37022354	(A/G)
4791	CakSNP4791	*Kabuli*	Ca_Kabuli_Chr03	37034618	(G/A)
4792	CakSNP4792	*Kabuli*	Ca_Kabuli_Chr03	37067095	(T/G)
4793	CakSNP4793	*Kabuli*	Ca_Kabuli_Chr03	37072289	(A/G)
4794	CakSNP4794	*Kabuli*	Ca_Kabuli_Chr03	37072276	(T/G)
4795	CakSNP4795	*Kabuli*	Ca_Kabuli_Chr03	37072312	(T/C)

S.N.	SNP IDs	Cultivars	Chromosomes/scaffolds	Physical positions (bp)	SNPs
4796	CakSNP4796	*Kabuli*	Ca_Kabuli_Chr03	37077087	(G/T)
4797	CakSNP4797	*Kabuli*	Ca_Kabuli_Chr03	37090968	(T/C)
4798	CakSNP4798	*Kabuli*	Ca_Kabuli_Chr03	37091004	(A/G)
4799	CakSNP4799	*Kabuli*	Ca_Kabuli_Chr03	37094437	(T/C)
4800	CakSNP4800	*Kabuli*	Ca_Kabuli_Chr03	37100897	(G/T)
4801	CakSNP4801	*Kabuli*	Ca_Kabuli_Chr03	37101339	(A/T)
4802	CakSNP4802	*Kabuli*	Ca_Kabuli_Chr03	37101433	(T/G)
4803	CakSNP4803	*Kabuli*	Ca_Kabuli_Chr03	37101409	(A/G)
4804	CakSNP4804	*Kabuli*	Ca_Kabuli_Chr03	37108762	(A/G)
4805	CakSNP4805	*Kabuli*	Ca_Kabuli_Chr03	37108908	(A/G)
4806	CakSNP4806	*Kabuli*	Ca_Kabuli_Chr03	37108904	(A/T)
4807	CakSNP4807	*Kabuli*	Ca_Kabuli_Chr03	37108876	(C/A)
4808	CakSNP4808	*Kabuli*	Ca_Kabuli_Chr03	37110070	(A/G)
4809	CakSNP4809	*Kabuli*	Ca_Kabuli_Chr03	37110124	(A/T)
4810	CakSNP4810	*Kabuli*	Ca_Kabuli_Chr03	37146766	(C/G)
4811	CakSNP4811	*Kabuli*	Ca_Kabuli_Chr03	37146840	(A/G)
4812	CakSNP4812	*Kabuli*	Ca_Kabuli_Chr03	37154761	(T/G)
4813	CakSNP4813	*Kabuli*	Ca_Kabuli_Chr03	37154857	(T/C)
4814	CakSNP4814	*Kabuli*	Ca_Kabuli_Chr03	37154938	(A/C)
4815	CakSNP4815	*Kabuli*	Ca_Kabuli_Chr03	37154957	(C/A)
4816	CakSNP4816	*Kabuli*	Ca_Kabuli_Chr03	37155925	(G/A)
4817	CakSNP4817	*Kabuli*	Ca_Kabuli_Chr03	37156951	(A/G)
4818	CakSNP4818	*Kabuli*	Ca_Kabuli_Chr03	37160822	(G/T)
4819	CakSNP4819	*Kabuli*	Ca_Kabuli_Chr03	37160939	(A/G)
4820	CakSNP4820	*Kabuli*	Ca_Kabuli_Chr03	37164633	(T/C)
4821	CakSNP4821	*Kabuli*	Ca_Kabuli_Chr03	37170249	(C/T)
4822	CakSNP4822	*Kabuli*	Ca_Kabuli_Chr03	37271869	(T/C)
4823	CakSNP4823	*Kabuli*	Ca_Kabuli_Chr03	37300206	(T/G)
4824	CakSNP4824	*Kabuli*	Ca_Kabuli_Chr03	37303478	(G/A)
4825	CakSNP4825	*Kabuli*	Ca_Kabuli_Chr03	37308614	(T/C)
4826	CakSNP4826	*Kabuli*	Ca_Kabuli_Chr03	37309420	(A/C)
4827	CakSNP4827	*Kabuli*	Ca_Kabuli_Chr03	37316736	(T/C)
4828	CakSNP4828	*Kabuli*	Ca_Kabuli_Chr03	37316737	(C/T)
4829	CakSNP4829	*Kabuli*	Ca_Kabuli_Chr03	37316782	(G/A)
4830	CakSNP4830	*Kabuli*	Ca_Kabuli_Chr03	37355013	(A/G)
4831	CakSNP4831	*Kabuli*	Ca_Kabuli_Chr03	37444451	(C/A)
4832	CakSNP4832	*Kabuli*	Ca_Kabuli_Chr03	37566557	(C/A)
4833	CakSNP4833	*Kabuli*	Ca_Kabuli_Chr03	37599142	(T/C)
4834	CakSNP4834	*Kabuli*	Ca_Kabuli_Chr03	37604672	(T/A)

S.N.	SNP IDs	Cultivars	Chromosomes/scaffolds	Physical positions (bp)	SNPs
4835	CakSNP4835	*Kabuli*	Ca_Kabuli_Chr03	37630502	(T/C)
4836	CakSNP4836	*Kabuli*	Ca_Kabuli_Chr03	37635909	(G/A)
4837	CakSNP4837	*Kabuli*	Ca_Kabuli_Chr03	37635961	(C/T)
4838	CakSNP4838	*Kabuli*	Ca_Kabuli_Chr03	37639300	(A/G)
4839	CakSNP4839	*Kabuli*	Ca_Kabuli_Chr03	37682816	(T/C)
4840	CakSNP4840	*Kabuli*	Ca_Kabuli_Chr03	37682824	(G/A)
4841	CakSNP4841	*Kabuli*	Ca_Kabuli_Chr03	37723959	(C/T)
4842	CakSNP4842	*Kabuli*	Ca_Kabuli_Chr03	37759329	(G/A)
4843	CakSNP4843	*Kabuli*	Ca_Kabuli_Chr03	37765767	(A/C)
4844	CakSNP4844	*Kabuli*	Ca_Kabuli_Chr03	37806690	(G/A)
4845	CakSNP4845	*Kabuli*	Ca_Kabuli_Chr03	37814955	(A/T)
4846	CakSNP4846	*Kabuli*	Ca_Kabuli_Chr03	37814954	(T/A)
4847	CakSNP4847	*Kabuli*	Ca_Kabuli_Chr03	37816895	(T/A)
4848	CakSNP4848	*Kabuli*	Ca_Kabuli_Chr03	37859943	(T/C)
4849	CakSNP4849	*Kabuli*	Ca_Kabuli_Chr03	37864390	(C/T)
4850	CakSNP4850	*Kabuli*	Ca_Kabuli_Chr03	37866258	(A/G)
4851	CakSNP4851	*Kabuli*	Ca_Kabuli_Chr03	37886983	(G/T)
4852	CakSNP4852	*Kabuli*	Ca_Kabuli_Chr03	37886971	(A/G)
4853	CakSNP4853	*Kabuli*	Ca_Kabuli_Chr03	37886942	(A/C)
4854	CakSNP4854	*Kabuli*	Ca_Kabuli_Chr03	37908317	(T/C)
4855	CakSNP4855	*Kabuli*	Ca_Kabuli_Chr03	37908492	(A/C)
4856	CakSNP4856	*Kabuli*	Ca_Kabuli_Chr03	37910881	(A/G)
4857	CakSNP4857	*Kabuli*	Ca_Kabuli_Chr03	37910899	(T/C)
4858	CakSNP4858	*Kabuli*	Ca_Kabuli_Chr03	37910911	(C/T)
4859	CakSNP4859	*Kabuli*	Ca_Kabuli_Chr03	37913266	(C/T)
4860	CakSNP4860	*Kabuli*	Ca_Kabuli_Chr03	37919880	(C/T)
4861	CakSNP4861	*Kabuli*	Ca_Kabuli_Chr03	37942807	(A/G)
4862	CakSNP4862	*Kabuli*	Ca_Kabuli_Chr03	37942962	(T/C)
4863	CakSNP4863	*Kabuli*	Ca_Kabuli_Chr03	37985612	(A/C)
4864	CakSNP4864	*Kabuli*	Ca_Kabuli_Chr03	37985652	(C/T)
4865	CakSNP4865	*Kabuli*	Ca_Kabuli_Chr03	37985776	(T/A)
4866	CakSNP4866	*Kabuli*	Ca_Kabuli_Chr03	37985977	(A/G)
4867	CakSNP4867	*Kabuli*	Ca_Kabuli_Chr03	37985947	(A/T)
4868	CakSNP4868	*Kabuli*	Ca_Kabuli_Chr03	38026470	(G/C)
4869	CakSNP4869	*Kabuli*	Ca_Kabuli_Chr03	38026539	(A/G)
4870	CakSNP4870	*Kabuli*	Ca_Kabuli_Chr03	38026734	(G/C)
4871	CakSNP4871	*Kabuli*	Ca_Kabuli_Chr03	38026725	(G/A)
4872	CakSNP4872	*Kabuli*	Ca_Kabuli_Chr03	38029095	(G/C)
4873	CakSNP4873	*Kabuli*	Ca_Kabuli_Chr03	38033965	(G/A)

S.N.	SNP IDs	Cultivars	Chromosomes/scaffolds	Physical positions (bp)	SNPs
4874	CakSNP4874	*Kabuli*	Ca_Kabuli_Chr03	38033969	(A/G)
4875	CakSNP4875	*Kabuli*	Ca_Kabuli_Chr03	38046510	(T/C)
4876	CakSNP4876	*Kabuli*	Ca_Kabuli_Chr03	38046575	(A/C)
4877	CakSNP4877	*Kabuli*	Ca_Kabuli_Chr03	38049996	(A/G)
4878	CakSNP4878	*Kabuli*	Ca_Kabuli_Chr03	38059303	(T/C)
4879	CakSNP4879	*Kabuli*	Ca_Kabuli_Chr03	38059311	(T/A)
4880	CakSNP4880	*Kabuli*	Ca_Kabuli_Chr03	38060814	(A/G)
4881	CakSNP4881	*Kabuli*	Ca_Kabuli_Chr03	38060755	(T/C)
4882	CakSNP4882	*Kabuli*	Ca_Kabuli_Chr03	38060742	(C/T)
4883	CakSNP4883	*Kabuli*	Ca_Kabuli_Chr03	38074259	(C/A)
4884	CakSNP4884	*Kabuli*	Ca_Kabuli_Chr03	38103375	(T/C)
4885	CakSNP4885	*Kabuli*	Ca_Kabuli_Chr03	38103378	(C/T)
4886	CakSNP4886	*Kabuli*	Ca_Kabuli_Chr03	38103381	(C/T)
4887	CakSNP4887	*Kabuli*	Ca_Kabuli_Chr03	38103385	(C/T)
4888	CakSNP4888	*Kabuli*	Ca_Kabuli_Chr03	38103524	(C/T)
4889	CakSNP4889	*Kabuli*	Ca_Kabuli_Chr03	38112801	(C/T)
4890	CakSNP4890	*Kabuli*	Ca_Kabuli_Chr03	38112802	(T/C)
4891	CakSNP4891	*Kabuli*	Ca_Kabuli_Chr03	38112971	(A/G)
4892	CakSNP4892	*Kabuli*	Ca_Kabuli_Chr03	38117169	(T/C)
4893	CakSNP4893	*Kabuli*	Ca_Kabuli_Chr03	38130415	(T/C)
4894	CakSNP4894	*Kabuli*	Ca_Kabuli_Chr03	38130410	(A/G)
4895	CakSNP4895	*Kabuli*	Ca_Kabuli_Chr03	38145115	(C/T)
4896	CakSNP4896	*Kabuli*	Ca_Kabuli_Chr03	38157519	(T/C)
4897	CakSNP4897	*Kabuli*	Ca_Kabuli_Chr03	38166074	(A/G)
4898	CakSNP4898	*Kabuli*	Ca_Kabuli_Chr03	38166881	(C/T)
4899	CakSNP4899	*Kabuli*	Ca_Kabuli_Chr03	38202223	(C/T)
4900	CakSNP4900	*Kabuli*	Ca_Kabuli_Chr03	38220910	(T/C)
4901	CakSNP4901	*Kabuli*	Ca_Kabuli_Chr03	38220949	(A/T)
4902	CakSNP4902	*Kabuli*	Ca_Kabuli_Chr03	38220958	(A/G)
4903	CakSNP4903	*Kabuli*	Ca_Kabuli_Chr03	38222750	(A/C)
4904	CakSNP4904	*Kabuli*	Ca_Kabuli_Chr03	38222747	(G/A)
4905	CakSNP4905	*Kabuli*	Ca_Kabuli_Chr03	38222733	(C/G)
4906	CakSNP4906	*Kabuli*	Ca_Kabuli_Chr03	38228118	(G/T)
4907	CakSNP4907	*Kabuli*	Ca_Kabuli_Chr03	38237246	(T/G)
4908	CakSNP4908	*Kabuli*	Ca_Kabuli_Chr03	38251660	(C/A)
4909	CakSNP4909	*Kabuli*	Ca_Kabuli_Chr03	38253654	(T/C)
4910	CakSNP4910	*Kabuli*	Ca_Kabuli_Chr03	38258645	(C/A)
4911	CakSNP4911	*Kabuli*	Ca_Kabuli_Chr03	38264250	(C/T)
4912	CakSNP4912	*Kabuli*	Ca_Kabuli_Chr03	38294286	(C/A)

S.N.	SNP IDs	Cultivars	Chromosomes/scaffolds	Physical positions (bp)	SNPs
4913	CakSNP4913	*Kabuli*	Ca_Kabuli_Chr03	38296573	(A/G)
4914	CakSNP4914	*Kabuli*	Ca_Kabuli_Chr03	38315560	(G/T)
4915	CakSNP4915	*Kabuli*	Ca_Kabuli_Chr03	38321159	(T/C)
4916	CakSNP4916	*Kabuli*	Ca_Kabuli_Chr03	38321153	(G/T)
4917	CakSNP4917	*Kabuli*	Ca_Kabuli_Chr03	38386392	(T/C)
4918	CakSNP4918	*Kabuli*	Ca_Kabuli_Chr03	38424238	(A/T)
4919	CakSNP4919	*Kabuli*	Ca_Kabuli_Chr03	38452060	(A/C)
4920	CakSNP4920	*Kabuli*	Ca_Kabuli_Chr03	38465366	(G/A)
4921	CakSNP4921	*Kabuli*	Ca_Kabuli_Chr03	38465335	(G/A)
4922	CakSNP4922	*Kabuli*	Ca_Kabuli_Chr03	38465440	(G/A)
4923	CakSNP4923	*Kabuli*	Ca_Kabuli_Chr03	38496236	(A/G)
4924	CakSNP4924	*Kabuli*	Ca_Kabuli_Chr03	38501705	(T/C)
4925	CakSNP4925	*Kabuli*	Ca_Kabuli_Chr03	38539721	(T/G)
4926	CakSNP4926	*Kabuli*	Ca_Kabuli_Chr03	38551142	(C/T)
4927	CakSNP4927	*Kabuli*	Ca_Kabuli_Chr03	38555941	(G/A)
4928	CakSNP4928	*Kabuli*	Ca_Kabuli_Chr03	38555944	(A/G)
4929	CakSNP4929	*Kabuli*	Ca_Kabuli_Chr03	38592077	(G/A)
4930	CakSNP4930	*Kabuli*	Ca_Kabuli_Chr03	38624786	(C/T)
4931	CakSNP4931	*Kabuli*	Ca_Kabuli_Chr03	38624934	(T/C)
4932	CakSNP4932	*Kabuli*	Ca_Kabuli_Chr03	38672863	(C/A)
4933	CakSNP4933	*Kabuli*	Ca_Kabuli_Chr03	38704356	(C/T)
4934	CakSNP4934	*Kabuli*	Ca_Kabuli_Chr03	38728528	(C/T)
4935	CakSNP4935	*Kabuli*	Ca_Kabuli_Chr03	38728679	(T/C)
4936	CakSNP4936	*Kabuli*	Ca_Kabuli_Chr03	38731315	(C/T)
4937	CakSNP4937	*Kabuli*	Ca_Kabuli_Chr03	38731466	(T/G)
4938	CakSNP4938	*Kabuli*	Ca_Kabuli_Chr03	38764372	(G/A)
4939	CakSNP4939	*Kabuli*	Ca_Kabuli_Chr03	38764390	(A/C)
4940	CakSNP4940	*Kabuli*	Ca_Kabuli_Chr03	38766156	(T/A)
4941	CakSNP4941	*Kabuli*	Ca_Kabuli_Chr03	38766174	(A/G)
4942	CakSNP4942	*Kabuli*	Ca_Kabuli_Chr03	38766311	(A/C)
4943	CakSNP4943	*Kabuli*	Ca_Kabuli_Chr03	38771817	(A/G)
4944	CakSNP4944	*Kabuli*	Ca_Kabuli_Chr03	38776366	(T/G)
4945	CakSNP4945	*Kabuli*	Ca_Kabuli_Chr03	38785843	(C/G)
4946	CakSNP4946	*Kabuli*	Ca_Kabuli_Chr03	38785845	(C/T)
4947	CakSNP4947	*Kabuli*	Ca_Kabuli_Chr03	38793194	(T/C)
4948	CakSNP4948	*Kabuli*	Ca_Kabuli_Chr03	38805822	(T/A)
4949	CakSNP4949	*Kabuli*	Ca_Kabuli_Chr03	38805831	(A/C)
4950	CakSNP4950	*Kabuli*	Ca_Kabuli_Chr03	38809132	(G/A)
4951	CakSNP4951	*Kabuli*	Ca_Kabuli_Chr03	38810765	(A/T)

S.N.	SNP IDs	Cultivars	Chromosomes/scaffolds	Physical positions (bp)	SNPs
4952	CakSNP4952	*Kabuli*	Ca_Kabuli_Chr03	38818340	(A/G)
4953	CakSNP4953	*Kabuli*	Ca_Kabuli_Chr03	38821990	(A/G)
4954	CakSNP4954	*Kabuli*	Ca_Kabuli_Chr03	38831533	(T/C)
4955	CakSNP4955	*Kabuli*	Ca_Kabuli_Chr03	38848358	(C/T)
4956	CakSNP4956	*Kabuli*	Ca_Kabuli_Chr03	38848412	(C/G)
4957	CakSNP4957	*Kabuli*	Ca_Kabuli_Chr03	38878577	(C/G)
4958	CakSNP4958	*Kabuli*	Ca_Kabuli_Chr03	38878664	(G/C)
4959	CakSNP4959	*Kabuli*	Ca_Kabuli_Chr03	38878649	(T/C)
4960	CakSNP4960	*Kabuli*	Ca_Kabuli_Chr03	38878736	(G/C)
4961	CakSNP4961	*Kabuli*	Ca_Kabuli_Chr03	38887640	(C/T)
4962	CakSNP4962	*Kabuli*	Ca_Kabuli_Chr03	38891567	(T/C)
4963	CakSNP4963	*Kabuli*	Ca_Kabuli_Chr03	38896013	(A/G)
4964	CakSNP4964	*Kabuli*	Ca_Kabuli_Chr03	38913449	(T/C)
4965	CakSNP4965	*Kabuli*	Ca_Kabuli_Chr03	38914528	(A/G)
4966	CakSNP4966	*Kabuli*	Ca_Kabuli_Chr03	38923321	(C/T)
4967	CakSNP4967	*Kabuli*	Ca_Kabuli_Chr03	38951428	(G/A)
4968	CakSNP4968	*Kabuli*	Ca_Kabuli_Chr03	38951698	(A/C)
4969	CakSNP4969	*Kabuli*	Ca_Kabuli_Chr03	38965610	(T/A)
4970	CakSNP4970	*Kabuli*	Ca_Kabuli_Chr03	38982481	(A/G)
4971	CakSNP4971	*Kabuli*	Ca_Kabuli_Chr03	38987810	(G/A)
4972	CakSNP4972	*Kabuli*	Ca_Kabuli_Chr03	38987916	(A/C)
4973	CakSNP4973	*Kabuli*	Ca_Kabuli_Chr03	39003771	(G/A)
4974	CakSNP4974	*Kabuli*	Ca_Kabuli_Chr03	39013988	(T/G)
4975	CakSNP4975	*Kabuli*	Ca_Kabuli_Chr03	39029251	(A/C)
4976	CakSNP4976	*Kabuli*	Ca_Kabuli_Chr03	39034246	(C/T)
4977	CakSNP4977	*Kabuli*	Ca_Kabuli_Chr03	39034433	(A/G)
4978	CakSNP4978	*Kabuli*	Ca_Kabuli_Chr03	39036130	(T/C)
4979	CakSNP4979	*Kabuli*	Ca_Kabuli_Chr03	39052024	(T/C)
4980	CakSNP4980	*Kabuli*	Ca_Kabuli_Chr03	39055529	(C/T)
4981	CakSNP4981	*Kabuli*	Ca_Kabuli_Chr03	39055455	(T/C)
4982	CakSNP4982	*Kabuli*	Ca_Kabuli_Chr03	39081040	(A/G)
4983	CakSNP4983	*Kabuli*	Ca_Kabuli_Chr03	39084916	(T/A)
4984	CakSNP4984	*Kabuli*	Ca_Kabuli_Chr03	39084979	(A/C)
4985	CakSNP4985	*Kabuli*	Ca_Kabuli_Chr03	39093242	(G/A)
4986	CakSNP4986	*Kabuli*	Ca_Kabuli_Chr03	39093243	(C/A)
4987	CakSNP4987	*Kabuli*	Ca_Kabuli_Chr03	39093245	(T/A)
4988	CakSNP4988	*Kabuli*	Ca_Kabuli_Chr03	39093215	(G/A)
4989	CakSNP4989	*Kabuli*	Ca_Kabuli_Chr03	39115274	(A/C)
4990	CakSNP4990	*Kabuli*	Ca_Kabuli_Chr03	39115986	(C/G)

S.N.	SNP IDs	Cultivars	Chromosomes/scaffolds	Physical positions (bp)	SNPs
4991	CakSNP4991	*Kabuli*	Ca_Kabuli_Chr03	39115979	(A/G)
4992	CakSNP4992	*Kabuli*	Ca_Kabuli_Chr03	39115972	(T/A)
4993	CakSNP4993	*Kabuli*	Ca_Kabuli_Chr03	39115970	(T/G)
4994	CakSNP4994	*Kabuli*	Ca_Kabuli_Chr03	39118510	(C/T)
4995	CakSNP4995	*Kabuli*	Ca_Kabuli_Chr03	39200823	(C/A)
4996	CakSNP4996	*Kabuli*	Ca_Kabuli_Chr03	39200847	(A/C)
4997	CakSNP4997	*Kabuli*	Ca_Kabuli_Chr03	39202479	(G/C)
4998	CakSNP4998	*Kabuli*	Ca_Kabuli_Chr03	39202481	(A/T)
4999	CakSNP4999	*Kabuli*	Ca_Kabuli_Chr03	39207112	(A/C)
5000	CakSNP5000	*Kabuli*	Ca_Kabuli_Chr03	39215327	(C/T)
5001	CakSNP5001	*Kabuli*	Ca_Kabuli_Chr03	39215476	(G/A)
5002	CakSNP5002	*Kabuli*	Ca_Kabuli_Chr03	39215558	(C/T)
5003	CakSNP5003	*Kabuli*	Ca_Kabuli_Chr03	39233774	(A/T)
5004	CakSNP5004	*Kabuli*	Ca_Kabuli_Chr03	39233852	(A/C)
5005	CakSNP5005	*Kabuli*	Ca_Kabuli_Chr03	39233885	(A/T)
5006	CakSNP5006	*Kabuli*	Ca_Kabuli_Chr03	39234161	(A/G)
5007	CakSNP5007	*Kabuli*	Ca_Kabuli_Chr03	39254527	(T/G)
5008	CakSNP5008	*Kabuli*	Ca_Kabuli_Chr03	39254532	(C/G)
5009	CakSNP5009	*Kabuli*	Ca_Kabuli_Chr03	39255721	(A/C)
5010	CakSNP5010	*Kabuli*	Ca_Kabuli_Chr03	39255715	(G/A)
5011	CakSNP5011	*Kabuli*	Ca_Kabuli_Chr03	39255713	(C/A)
5012	CakSNP5012	*Kabuli*	Ca_Kabuli_Chr03	39255709	(A/T)
5013	CakSNP5013	*Kabuli*	Ca_Kabuli_Chr03	39255729	(C/T)
5014	CakSNP5014	*Kabuli*	Ca_Kabuli_Chr03	39255792	(C/T)
5015	CakSNP5015	*Kabuli*	Ca_Kabuli_Chr03	39257226	(A/G)
5016	CakSNP5016	*Kabuli*	Ca_Kabuli_Chr03	39258671	(A/C)
5017	CakSNP5017	*Kabuli*	Ca_Kabuli_Chr03	39265412	(A/C)
5018	CakSNP5018	*Kabuli*	Ca_Kabuli_Chr03	39289840	(T/A)
5019	CakSNP5019	*Kabuli*	Ca_Kabuli_Chr03	39303386	(C/G)
5020	CakSNP5020	*Kabuli*	Ca_Kabuli_Chr03	39303416	(G/T)
5021	CakSNP5021	*Kabuli*	Ca_Kabuli_Chr03	39303407	(C/A)
5022	CakSNP5022	*Kabuli*	Ca_Kabuli_Chr03	39323779	(A/G)
5023	CakSNP5023	*Kabuli*	Ca_Kabuli_Chr03	39326387	(C/T)
5024	CakSNP5024	*Kabuli*	Ca_Kabuli_Chr03	39405252	(C/A)
5025	CakSNP5025	*Kabuli*	Ca_Kabuli_Chr03	39405873	(C/G)
5026	CakSNP5026	*Kabuli*	Ca_Kabuli_Chr03	39472291	(G/C)
5027	CakSNP5027	*Kabuli*	Ca_Kabuli_Chr03	39481371	(A/C)
5028	CakSNP5028	*Kabuli*	Ca_Kabuli_Chr03	39485182	(G/A)
5029	CakSNP5029	*Kabuli*	Ca_Kabuli_Chr03	39504242	(A/G)

S.N.	SNP IDs	Cultivars	Chromosomes/scaffolds	Physical positions (bp)	SNPs
5030	CakSNP5030	*Kabuli*	Ca_Kabuli_Chr03	39515531	(A/G)
5031	CakSNP5031	*Kabuli*	Ca_Kabuli_Chr03	39515710	(T/A)
5032	CakSNP5032	*Kabuli*	Ca_Kabuli_Chr03	39519640	(A/G)
5033	CakSNP5033	*Kabuli*	Ca_Kabuli_Chr03	39556535	(T/A)
5034	CakSNP5034	*Kabuli*	Ca_Kabuli_Chr03	39587620	(T/G)
5035	CakSNP5035	*Kabuli*	Ca_Kabuli_Chr03	39592340	(C/T)
5036	CakSNP5036	*Kabuli*	Ca_Kabuli_Chr03	39612466	(T/C)
5037	CakSNP5037	*Kabuli*	Ca_Kabuli_Chr03	39612458	(C/A)
5038	CakSNP5038	*Kabuli*	Ca_Kabuli_Chr03	39612447	(C/A)
5039	CakSNP5039	*Kabuli*	Ca_Kabuli_Chr03	39612568	(A/T)
5040	CakSNP5040	*Kabuli*	Ca_Kabuli_Chr03	39628916	(G/A)
5041	CakSNP5041	*Kabuli*	Ca_Kabuli_Chr03	39698060	(T/C)
5042	CakSNP5042	*Kabuli*	Ca_Kabuli_Chr03	39701532	(G/A)
5043	CakSNP5043	*Kabuli*	Ca_Kabuli_Chr03	39722314	(C/T)
5044	CakSNP5044	*Kabuli*	Ca_Kabuli_Chr03	39731437	(A/C)
5045	CakSNP5045	*Kabuli*	Ca_Kabuli_Chr03	39787513	(A/C)
5046	CakSNP5046	*Kabuli*	Ca_Kabuli_Chr03	39787615	(C/T)
5047	CakSNP5047	*Kabuli*	Ca_Kabuli_Chr03	39787716	(A/C)
5048	CakSNP5048	*Kabuli*	Ca_Kabuli_Chr03	39791717	(C/T)
5049	CakSNP5049	*Kabuli*	Ca_Kabuli_Chr03	39793539	(G/A)
5050	CakSNP5050	*Kabuli*	Ca_Kabuli_Chr03	39794672	(C/T)
5051	CakSNP5051	*Kabuli*	Ca_Kabuli_Chr03	39794639	(C/G)
5052	CakSNP5052	*Kabuli*	Ca_Kabuli_Chr03	39801213	(A/G)
5053	CakSNP5053	*Kabuli*	Ca_Kabuli_Chr03	39801242	(C/T)
5054	CakSNP5054	*Kabuli*	Ca_Kabuli_Chr03	39817122	(G/A)
5055	CakSNP5055	*Kabuli*	Ca_Kabuli_Chr03	39839763	(T/G)
5056	CakSNP5056	*Kabuli*	Ca_Kabuli_Chr03	39891075	(G/A)
5057	CakSNP5057	*Kabuli*	Ca_Kabuli_Chr03	39890991	(A/T)
5058	CakSNP5058	*Kabuli*	Ca_Kabuli_Chr03	39896395	(A/G)
5059	CakSNP5059	*Kabuli*	Ca_Kabuli_Chr03	39908983	(C/G)
5060	CakSNP5060	*Kabuli*	Ca_Kabuli_Chr03	39909824	(C/G)
5061	CakSNP5061	*Kabuli*	Ca_Kabuli_Chr03	39909839	(T/C)
5062	CakSNP5062	*Kabuli*	Ca_Kabuli_Chr03	39914344	(T/G)
5063	CakSNP5063	*Kabuli*	Ca_Kabuli_Chr03	39941101	(T/G)
5064	CakSNP5064	*Kabuli*	Ca_Kabuli_Chr03	39941090	(G/A)
5065	CakSNP5065	*Kabuli*	Ca_Kabuli_Chr04	20297	(A/G)
5066	CakSNP5066	*Kabuli*	Ca_Kabuli_Chr04	20495	(G/C)
5067	CakSNP5067	*Kabuli*	Ca_Kabuli_Chr04	167229	(T/G)
5068	CakSNP5068	*Kabuli*	Ca_Kabuli_Chr04	228864	(G/A)

S.N.	SNP IDs	Cultivars	Chromosomes/scaffolds	Physical positions (bp)	SNPs
5069	CakSNP5069	*Kabuli*	Ca_Kabuli_Chr04	305558	(C/T)
5070	CakSNP5070	*Kabuli*	Ca_Kabuli_Chr04	335941	(C/T)
5071	CakSNP5071	*Kabuli*	Ca_Kabuli_Chr04	336055	(G/A)
5072	CakSNP5072	*Kabuli*	Ca_Kabuli_Chr04	336050	(T/C)
5073	CakSNP5073	*Kabuli*	Ca_Kabuli_Chr04	336029	(G/A)
5074	CakSNP5074	*Kabuli*	Ca_Kabuli_Chr04	338641	(A/G)
5075	CakSNP5075	*Kabuli*	Ca_Kabuli_Chr04	338721	(C/T)
5076	CakSNP5076	*Kabuli*	Ca_Kabuli_Chr04	338707	(G/T)
5077	CakSNP5077	*Kabuli*	Ca_Kabuli_Chr04	349309	(C/T)
5078	CakSNP5078	*Kabuli*	Ca_Kabuli_Chr04	353069	(C/A)
5079	CakSNP5079	*Kabuli*	Ca_Kabuli_Chr04	353079	(C/T)
5080	CakSNP5080	*Kabuli*	Ca_Kabuli_Chr04	353119	(G/A)
5081	CakSNP5081	*Kabuli*	Ca_Kabuli_Chr04	353120	(C/G)
5082	CakSNP5082	*Kabuli*	Ca_Kabuli_Chr04	353124	(A/G)
5083	CakSNP5083	*Kabuli*	Ca_Kabuli_Chr04	353126	(T/G)
5084	CakSNP5084	*Kabuli*	Ca_Kabuli_Chr04	369258	(C/T)
5085	CakSNP5085	*Kabuli*	Ca_Kabuli_Chr04	394661	(C/G)
5086	CakSNP5086	*Kabuli*	Ca_Kabuli_Chr04	411208	(A/C)
5087	CakSNP5087	*Kabuli*	Ca_Kabuli_Chr04	412017	(C/A)
5088	CakSNP5088	*Kabuli*	Ca_Kabuli_Chr04	455361	(A/G)
5089	CakSNP5089	*Kabuli*	Ca_Kabuli_Chr04	459360	(T/C)
5090	CakSNP5090	*Kabuli*	Ca_Kabuli_Chr04	480138	(C/A)
5091	CakSNP5091	*Kabuli*	Ca_Kabuli_Chr04	481556	(A/G)
5092	CakSNP5092	*Kabuli*	Ca_Kabuli_Chr04	481687	(T/G)
5093	CakSNP5093	*Kabuli*	Ca_Kabuli_Chr04	504928	(C/A)
5094	CakSNP5094	*Kabuli*	Ca_Kabuli_Chr04	586273	(A/G)
5095	CakSNP5095	*Kabuli*	Ca_Kabuli_Chr04	586703	(T/C)
5096	CakSNP5096	*Kabuli*	Ca_Kabuli_Chr04	589140	(T/C)
5097	CakSNP5097	*Kabuli*	Ca_Kabuli_Chr04	589191	(A/G)
5098	CakSNP5098	*Kabuli*	Ca_Kabuli_Chr04	589204	(G/A)
5099	CakSNP5099	*Kabuli*	Ca_Kabuli_Chr04	589220	(A/C)
5100	CakSNP5100	*Kabuli*	Ca_Kabuli_Chr04	589224	(T/C)
5101	CakSNP5101	*Kabuli*	Ca_Kabuli_Chr04	595982	(C/G)
5102	CakSNP5102	*Kabuli*	Ca_Kabuli_Chr04	613150	(G/A)
5103	CakSNP5103	*Kabuli*	Ca_Kabuli_Chr04	613166	(A/G)
5104	CakSNP5104	*Kabuli*	Ca_Kabuli_Chr04	645103	(T/A)
5105	CakSNP5105	*Kabuli*	Ca_Kabuli_Chr04	645075	(T/A)
5106	CakSNP5106	*Kabuli*	Ca_Kabuli_Chr04	646567	(C/T)
5107	CakSNP5107	*Kabuli*	Ca_Kabuli_Chr04	646576	(A/G)

S.N.	SNP IDs	Cultivars	Chromosomes/scaffolds	Physical positions (bp)	SNPs
5108	CakSNP5108	*Kabuli*	Ca_Kabuli_Chr04	651040	(C/T)
5109	CakSNP5109	*Kabuli*	Ca_Kabuli_Chr04	660034	(A/C)
5110	CakSNP5110	*Kabuli*	Ca_Kabuli_Chr04	660029	(T/G)
5111	CakSNP5111	*Kabuli*	Ca_Kabuli_Chr04	703978	(A/T)
5112	CakSNP5112	*Kabuli*	Ca_Kabuli_Chr04	703975	(G/T)
5113	CakSNP5113	*Kabuli*	Ca_Kabuli_Chr04	704009	(G/A)
5114	CakSNP5114	*Kabuli*	Ca_Kabuli_Chr04	705336	(T/G)
5115	CakSNP5115	*Kabuli*	Ca_Kabuli_Chr04	783302	(C/T)
5116	CakSNP5116	*Kabuli*	Ca_Kabuli_Chr04	783382	(G/T)
5117	CakSNP5117	*Kabuli*	Ca_Kabuli_Chr04	827630	(T/C)
5118	CakSNP5118	*Kabuli*	Ca_Kabuli_Chr04	848192	(G/A)
5119	CakSNP5119	*Kabuli*	Ca_Kabuli_Chr04	913689	(A/G)
5120	CakSNP5120	*Kabuli*	Ca_Kabuli_Chr04	951389	(T/G)
5121	CakSNP5121	*Kabuli*	Ca_Kabuli_Chr04	951390	(G/A)
5122	CakSNP5122	*Kabuli*	Ca_Kabuli_Chr04	1035328	(G/C)
5123	CakSNP5123	*Kabuli*	Ca_Kabuli_Chr04	1047639	(G/T)
5124	CakSNP5124	*Kabuli*	Ca_Kabuli_Chr04	1065107	(C/T)
5125	CakSNP5125	*Kabuli*	Ca_Kabuli_Chr04	1065245	(A/G)
5126	CakSNP5126	*Kabuli*	Ca_Kabuli_Chr04	1189997	(C/T)
5127	CakSNP5127	*Kabuli*	Ca_Kabuli_Chr04	1190007	(C/G)
5128	CakSNP5128	*Kabuli*	Ca_Kabuli_Chr04	1190038	(G/T)
5129	CakSNP5129	*Kabuli*	Ca_Kabuli_Chr04	1190208	(T/C)
5130	CakSNP5130	*Kabuli*	Ca_Kabuli_Chr04	1190184	(A/C)
5131	CakSNP5131	*Kabuli*	Ca_Kabuli_Chr04	1190158	(T/G)
5132	CakSNP5132	*Kabuli*	Ca_Kabuli_Chr04	1259917	(G/T)
5133	CakSNP5133	*Kabuli*	Ca_Kabuli_Chr04	1259915	(T/G)
5134	CakSNP5134	*Kabuli*	Ca_Kabuli_Chr04	1264361	(T/C)
5135	CakSNP5135	*Kabuli*	Ca_Kabuli_Chr04	1266175	(A/G)
5136	CakSNP5136	*Kabuli*	Ca_Kabuli_Chr04	1266174	(C/T)
5137	CakSNP5137	*Kabuli*	Ca_Kabuli_Chr04	1283369	(A/G)
5138	CakSNP5138	*Kabuli*	Ca_Kabuli_Chr04	1344151	(T/C)
5139	CakSNP5139	*Kabuli*	Ca_Kabuli_Chr04	1396281	(G/C)
5140	CakSNP5140	*Kabuli*	Ca_Kabuli_Chr04	1396425	(C/A)
5141	CakSNP5141	*Kabuli*	Ca_Kabuli_Chr04	1474771	(T/C)
5142	CakSNP5142	*Kabuli*	Ca_Kabuli_Chr04	1474898	(A/G)
5143	CakSNP5143	*Kabuli*	Ca_Kabuli_Chr04	1474922	(G/T)
5144	CakSNP5144	*Kabuli*	Ca_Kabuli_Chr04	1493927	(T/C)
5145	CakSNP5145	*Kabuli*	Ca_Kabuli_Chr04	1513664	(T/A)
5146	CakSNP5146	*Kabuli*	Ca_Kabuli_Chr04	1526270	(T/C)

S.N.	SNP IDs	Cultivars	Chromosomes/scaffolds	Physical positions (bp)	SNPs
5147	CakSNP5147	*Kabuli*	Ca_Kabuli_Chr04	1526731	(T/G)
5148	CakSNP5148	*Kabuli*	Ca_Kabuli_Chr04	1613153	(G/A)
5149	CakSNP5149	*Kabuli*	Ca_Kabuli_Chr04	1613193	(G/A)
5150	CakSNP5150	*Kabuli*	Ca_Kabuli_Chr04	1613303	(G/A)
5151	CakSNP5151	*Kabuli*	Ca_Kabuli_Chr04	1687346	(A/G)
5152	CakSNP5152	*Kabuli*	Ca_Kabuli_Chr04	1691555	(T/G)
5153	CakSNP5153	*Kabuli*	Ca_Kabuli_Chr04	1708370	(C/T)
5154	CakSNP5154	*Kabuli*	Ca_Kabuli_Chr04	1711864	(A/C)
5155	CakSNP5155	*Kabuli*	Ca_Kabuli_Chr04	1711865	(G/A)
5156	CakSNP5156	*Kabuli*	Ca_Kabuli_Chr04	1720544	(A/C)
5157	CakSNP5157	*Kabuli*	Ca_Kabuli_Chr04	1720658	(A/G)
5158	CakSNP5158	*Kabuli*	Ca_Kabuli_Chr04	1720642	(A/G)
5159	CakSNP5159	*Kabuli*	Ca_Kabuli_Chr04	1720619	(A/C)
5160	CakSNP5160	*Kabuli*	Ca_Kabuli_Chr04	1742468	(C/T)
5161	CakSNP5161	*Kabuli*	Ca_Kabuli_Chr04	1747181	(A/T)
5162	CakSNP5162	*Kabuli*	Ca_Kabuli_Chr04	1754555	(C/T)
5163	CakSNP5163	*Kabuli*	Ca_Kabuli_Chr04	1755066	(G/C)
5164	CakSNP5164	*Kabuli*	Ca_Kabuli_Chr04	1773886	(C/G)
5165	CakSNP5165	*Kabuli*	Ca_Kabuli_Chr04	1773939	(C/T)
5166	CakSNP5166	*Kabuli*	Ca_Kabuli_Chr04	1773951	(C/G)
5167	CakSNP5167	*Kabuli*	Ca_Kabuli_Chr04	1785705	(G/C)
5168	CakSNP5168	*Kabuli*	Ca_Kabuli_Chr04	1785680	(C/T)
5169	CakSNP5169	*Kabuli*	Ca_Kabuli_Chr04	1807484	(G/A)
5170	CakSNP5170	*Kabuli*	Ca_Kabuli_Chr04	1817779	(G/C)
5171	CakSNP5171	*Kabuli*	Ca_Kabuli_Chr04	1833570	(T/C)
5172	CakSNP5172	*Kabuli*	Ca_Kabuli_Chr04	1833678	(A/G)
5173	CakSNP5173	*Kabuli*	Ca_Kabuli_Chr04	1833725	(C/T)
5174	CakSNP5174	*Kabuli*	Ca_Kabuli_Chr04	1845301	(G/A)
5175	CakSNP5175	*Kabuli*	Ca_Kabuli_Chr04	1867921	(G/A)
5176	CakSNP5176	*Kabuli*	Ca_Kabuli_Chr04	1869577	(C/T)
5177	CakSNP5177	*Kabuli*	Ca_Kabuli_Chr04	1870333	(A/G)
5178	CakSNP5178	*Kabuli*	Ca_Kabuli_Chr04	1870317	(T/C)
5179	CakSNP5179	*Kabuli*	Ca_Kabuli_Chr04	1870666	(G/C)
5180	CakSNP5180	*Kabuli*	Ca_Kabuli_Chr04	1870806	(C/A)
5181	CakSNP5181	*Kabuli*	Ca_Kabuli_Chr04	1870802	(C/T)
5182	CakSNP5182	*Kabuli*	Ca_Kabuli_Chr04	1873733	(T/C)
5183	CakSNP5183	*Kabuli*	Ca_Kabuli_Chr04	1890220	(G/C)
5184	CakSNP5184	*Kabuli*	Ca_Kabuli_Chr04	1941001	(T/G)
5185	CakSNP5185	*Kabuli*	Ca_Kabuli_Chr04	1941089	(C/T)

S.N.	SNP IDs	Cultivars	Chromosomes/scaffolds	Physical positions (bp)	SNPs
5186	CakSNP5186	*Kabuli*	Ca_Kabuli_Chr04	1970471	(A/C)
5187	CakSNP5187	*Kabuli*	Ca_Kabuli_Chr04	1973375	(A/C)
5188	CakSNP5188	*Kabuli*	Ca_Kabuli_Chr04	2009707	(A/G)
5189	CakSNP5189	*Kabuli*	Ca_Kabuli_Chr04	2025214	(A/C)
5190	CakSNP5190	*Kabuli*	Ca_Kabuli_Chr04	2027063	(G/A)
5191	CakSNP5191	*Kabuli*	Ca_Kabuli_Chr04	2027101	(T/A)
5192	CakSNP5192	*Kabuli*	Ca_Kabuli_Chr04	2034429	(C/A)
5193	CakSNP5193	*Kabuli*	Ca_Kabuli_Chr04	2034398	(C/T)
5194	CakSNP5194	*Kabuli*	Ca_Kabuli_Chr04	2034396	(A/G)
5195	CakSNP5195	*Kabuli*	Ca_Kabuli_Chr04	2034739	(A/C)
5196	CakSNP5196	*Kabuli*	Ca_Kabuli_Chr04	2035797	(G/A)
5197	CakSNP5197	*Kabuli*	Ca_Kabuli_Chr04	2035801	(G/A)
5198	CakSNP5198	*Kabuli*	Ca_Kabuli_Chr04	2035841	(C/T)
5199	CakSNP5199	*Kabuli*	Ca_Kabuli_Chr04	2035894	(C/A)
5200	CakSNP5200	*Kabuli*	Ca_Kabuli_Chr04	2051684	(T/C)
5201	CakSNP5201	*Kabuli*	Ca_Kabuli_Chr04	2051642	(G/C)
5202	CakSNP5202	*Kabuli*	Ca_Kabuli_Chr04	2060244	(G/A)
5203	CakSNP5203	*Kabuli*	Ca_Kabuli_Chr04	2060262	(C/T)
5204	CakSNP5204	*Kabuli*	Ca_Kabuli_Chr04	2113564	(A/G)
5205	CakSNP5205	*Kabuli*	Ca_Kabuli_Chr04	2125774	(G/A)
5206	CakSNP5206	*Kabuli*	Ca_Kabuli_Chr04	2143155	(A/G)
5207	CakSNP5207	*Kabuli*	Ca_Kabuli_Chr04	2213710	(G/A)
5208	CakSNP5208	*Kabuli*	Ca_Kabuli_Chr04	2238480	(A/T)
5209	CakSNP5209	*Kabuli*	Ca_Kabuli_Chr04	2270360	(T/C)
5210	CakSNP5210	*Kabuli*	Ca_Kabuli_Chr04	2277421	(A/T)
5211	CakSNP5211	*Kabuli*	Ca_Kabuli_Chr04	2311977	(A/G)
5212	CakSNP5212	*Kabuli*	Ca_Kabuli_Chr04	2313969	(G/A)
5213	CakSNP5213	*Kabuli*	Ca_Kabuli_Chr04	2361088	(T/A)
5214	CakSNP5214	*Kabuli*	Ca_Kabuli_Chr04	2380206	(A/G)
5215	CakSNP5215	*Kabuli*	Ca_Kabuli_Chr04	2393033	(T/G)
5216	CakSNP5216	*Kabuli*	Ca_Kabuli_Chr04	2396883	(A/G)
5217	CakSNP5217	*Kabuli*	Ca_Kabuli_Chr04	2411925	(G/T)
5218	CakSNP5218	*Kabuli*	Ca_Kabuli_Chr04	2422087	(A/G)
5219	CakSNP5219	*Kabuli*	Ca_Kabuli_Chr04	2422092	(G/A)
5220	CakSNP5220	*Kabuli*	Ca_Kabuli_Chr04	2422949	(C/T)
5221	CakSNP5221	*Kabuli*	Ca_Kabuli_Chr04	2463064	(A/G)
5222	CakSNP5222	*Kabuli*	Ca_Kabuli_Chr04	2463038	(T/C)
5223	CakSNP5223	*Kabuli*	Ca_Kabuli_Chr04	2492726	(A/G)
5224	CakSNP5224	*Kabuli*	Ca_Kabuli_Chr04	2493195	(T/C)

S.N.	SNP IDs	Cultivars	Chromosomes/scaffolds	Physical positions (bp)	SNPs
5225	CakSNP5225	*Kabuli*	Ca_Kabuli_Chr04	2494101	(C/A)
5226	CakSNP5226	*Kabuli*	Ca_Kabuli_Chr04	2494140	(C/A)
5227	CakSNP5227	*Kabuli*	Ca_Kabuli_Chr04	2499257	(A/C)
5228	CakSNP5228	*Kabuli*	Ca_Kabuli_Chr04	2562541	(G/C)
5229	CakSNP5229	*Kabuli*	Ca_Kabuli_Chr04	2564500	(T/A)
5230	CakSNP5230	*Kabuli*	Ca_Kabuli_Chr04	2609945	(C/T)
5231	CakSNP5231	*Kabuli*	Ca_Kabuli_Chr04	2676174	(C/T)
5232	CakSNP5232	*Kabuli*	Ca_Kabuli_Chr04	2740041	(T/G)
5233	CakSNP5233	*Kabuli*	Ca_Kabuli_Chr04	2740068	(A/T)
5234	CakSNP5234	*Kabuli*	Ca_Kabuli_Chr04	2740065	(A/G)
5235	CakSNP5235	*Kabuli*	Ca_Kabuli_Chr04	2752538	(G/A)
5236	CakSNP5236	*Kabuli*	Ca_Kabuli_Chr04	2752568	(C/T)
5237	CakSNP5237	*Kabuli*	Ca_Kabuli_Chr04	2886775	(T/G)
5238	CakSNP5238	*Kabuli*	Ca_Kabuli_Chr04	2886863	(C/T)
5239	CakSNP5239	*Kabuli*	Ca_Kabuli_Chr04	2978875	(T/C)
5240	CakSNP5240	*Kabuli*	Ca_Kabuli_Chr04	2978876	(T/G)
5241	CakSNP5241	*Kabuli*	Ca_Kabuli_Chr04	2978882	(G/T)
5242	CakSNP5242	*Kabuli*	Ca_Kabuli_Chr04	2978932	(A/C)
5243	CakSNP5243	*Kabuli*	Ca_Kabuli_Chr04	2978931	(A/T)
5244	CakSNP5244	*Kabuli*	Ca_Kabuli_Chr04	3002755	(A/G)
5245	CakSNP5245	*Kabuli*	Ca_Kabuli_Chr04	3002810	(T/C)
5246	CakSNP5246	*Kabuli*	Ca_Kabuli_Chr04	3037899	(A/C)
5247	CakSNP5247	*Kabuli*	Ca_Kabuli_Chr04	3037986	(T/A)
5248	CakSNP5248	*Kabuli*	Ca_Kabuli_Chr04	3037929	(T/G)
5249	CakSNP5249	*Kabuli*	Ca_Kabuli_Chr04	3038022	(C/T)
5250	CakSNP5250	*Kabuli*	Ca_Kabuli_Chr04	3046920	(A/G)
5251	CakSNP5251	*Kabuli*	Ca_Kabuli_Chr04	3144454	(T/C)
5252	CakSNP5252	*Kabuli*	Ca_Kabuli_Chr04	3158321	(A/G)
5253	CakSNP5253	*Kabuli*	Ca_Kabuli_Chr04	3158323	(T/G)
5254	CakSNP5254	*Kabuli*	Ca_Kabuli_Chr04	3158328	(G/T)
5255	CakSNP5255	*Kabuli*	Ca_Kabuli_Chr04	3158331	(A/T)
5256	CakSNP5256	*Kabuli*	Ca_Kabuli_Chr04	3158426	(G/A)
5257	CakSNP5257	*Kabuli*	Ca_Kabuli_Chr04	3158504	(A/T)
5258	CakSNP5258	*Kabuli*	Ca_Kabuli_Chr04	3158506	(T/C)
5259	CakSNP5259	*Kabuli*	Ca_Kabuli_Chr04	3158549	(A/G)
5260	CakSNP5260	*Kabuli*	Ca_Kabuli_Chr04	3164115	(G/A)
5261	CakSNP5261	*Kabuli*	Ca_Kabuli_Chr04	3164083	(G/A)
5262	CakSNP5262	*Kabuli*	Ca_Kabuli_Chr04	3166657	(C/T)
5263	CakSNP5263	*Kabuli*	Ca_Kabuli_Chr04	3166663	(C/T)

S.N.	SNP IDs	Cultivars	Chromosomes/scaffolds	Physical positions (bp)	SNPs
5264	CakSNP5264	*Kabuli*	Ca_Kabuli_Chr04	3166674	(G/T)
5265	CakSNP5265	*Kabuli*	Ca_Kabuli_Chr04	3171162	(G/A)
5266	CakSNP5266	*Kabuli*	Ca_Kabuli_Chr04	3171384	(T/G)
5267	CakSNP5267	*Kabuli*	Ca_Kabuli_Chr04	3171385	(A/G)
5268	CakSNP5268	*Kabuli*	Ca_Kabuli_Chr04	3171428	(T/C)
5269	CakSNP5269	*Kabuli*	Ca_Kabuli_Chr04	3171551	(C/T)
5270	CakSNP5270	*Kabuli*	Ca_Kabuli_Chr04	3264351	(T/C)
5271	CakSNP5271	*Kabuli*	Ca_Kabuli_Chr04	3280147	(G/A)
5272	CakSNP5272	*Kabuli*	Ca_Kabuli_Chr04	3280217	(A/G)
5273	CakSNP5273	*Kabuli*	Ca_Kabuli_Chr04	3281050	(T/G)
5274	CakSNP5274	*Kabuli*	Ca_Kabuli_Chr04	3290298	(T/G)
5275	CakSNP5275	*Kabuli*	Ca_Kabuli_Chr04	3353106	(T/C)
5276	CakSNP5276	*Kabuli*	Ca_Kabuli_Chr04	3356252	(C/G)
5277	CakSNP5277	*Kabuli*	Ca_Kabuli_Chr04	3356242	(C/G)
5278	CakSNP5278	*Kabuli*	Ca_Kabuli_Chr04	3400828	(G/C)
5279	CakSNP5279	*Kabuli*	Ca_Kabuli_Chr04	3427396	(A/G)
5280	CakSNP5280	*Kabuli*	Ca_Kabuli_Chr04	3429035	(G/A)
5281	CakSNP5281	*Kabuli*	Ca_Kabuli_Chr04	3429059	(C/A)
5282	CakSNP5282	*Kabuli*	Ca_Kabuli_Chr04	3455029	(T/C)
5283	CakSNP5283	*Kabuli*	Ca_Kabuli_Chr04	3455047	(G/A)
5284	CakSNP5284	*Kabuli*	Ca_Kabuli_Chr04	3456450	(G/T)
5285	CakSNP5285	*Kabuli*	Ca_Kabuli_Chr04	3456501	(C/T)
5286	CakSNP5286	*Kabuli*	Ca_Kabuli_Chr04	3483687	(A/C)
5287	CakSNP5287	*Kabuli*	Ca_Kabuli_Chr04	3627810	(G/T)
5288	CakSNP5288	*Kabuli*	Ca_Kabuli_Chr04	3642051	(A/T)
5289	CakSNP5289	*Kabuli*	Ca_Kabuli_Chr04	3642070	(T/C)
5290	CakSNP5290	*Kabuli*	Ca_Kabuli_Chr04	3642116	(A/T)
5291	CakSNP5291	*Kabuli*	Ca_Kabuli_Chr04	3689840	(T/C)
5292	CakSNP5292	*Kabuli*	Ca_Kabuli_Chr04	3689871	(G/A)
5293	CakSNP5293	*Kabuli*	Ca_Kabuli_Chr04	3739386	(C/A)
5294	CakSNP5294	*Kabuli*	Ca_Kabuli_Chr04	3741522	(T/G)
5295	CakSNP5295	*Kabuli*	Ca_Kabuli_Chr04	3741539	(T/G)
5296	CakSNP5296	*Kabuli*	Ca_Kabuli_Chr04	3752141	(C/G)
5297	CakSNP5297	*Kabuli*	Ca_Kabuli_Chr04	3762559	(T/C)
5298	CakSNP5298	*Kabuli*	Ca_Kabuli_Chr04	3762561	(C/A)
5299	CakSNP5299	*Kabuli*	Ca_Kabuli_Chr04	3762585	(G/T)
5300	CakSNP5300	*Kabuli*	Ca_Kabuli_Chr04	3794764	(G/A)
5301	CakSNP5301	*Kabuli*	Ca_Kabuli_Chr04	3794885	(C/A)
5302	CakSNP5302	*Kabuli*	Ca_Kabuli_Chr04	3885444	(G/C)

S.N.	SNP IDs	Cultivars	Chromosomes/scaffolds	Physical positions (bp)	SNPs
5303	CakSNP5303	*Kabuli*	Ca_Kabuli_Chr04	3888010	(G/C)
5304	CakSNP5304	*Kabuli*	Ca_Kabuli_Chr04	3888053	(C/A)
5305	CakSNP5305	*Kabuli*	Ca_Kabuli_Chr04	3888164	(T/A)
5306	CakSNP5306	*Kabuli*	Ca_Kabuli_Chr04	3888185	(A/G)
5307	CakSNP5307	*Kabuli*	Ca_Kabuli_Chr04	3888236	(A/G)
5308	CakSNP5308	*Kabuli*	Ca_Kabuli_Chr04	3888328	(C/T)
5309	CakSNP5309	*Kabuli*	Ca_Kabuli_Chr04	3888462	(T/C)
5310	CakSNP5310	*Kabuli*	Ca_Kabuli_Chr04	3888510	(G/A)
5311	CakSNP5311	*Kabuli*	Ca_Kabuli_Chr04	3888514	(G/A)
5312	CakSNP5312	*Kabuli*	Ca_Kabuli_Chr04	3888529	(T/G)
5313	CakSNP5313	*Kabuli*	Ca_Kabuli_Chr04	3890556	(A/G)
5314	CakSNP5314	*Kabuli*	Ca_Kabuli_Chr04	3890709	(A/G)
5315	CakSNP5315	*Kabuli*	Ca_Kabuli_Chr04	3990810	(C/G)
5316	CakSNP5316	*Kabuli*	Ca_Kabuli_Chr04	3990809	(G/A)
5317	CakSNP5317	*Kabuli*	Ca_Kabuli_Chr04	4002917	(T/C)
5318	CakSNP5318	*Kabuli*	Ca_Kabuli_Chr04	4006213	(A/C)
5319	CakSNP5319	*Kabuli*	Ca_Kabuli_Chr04	4027829	(A/T)
5320	CakSNP5320	*Kabuli*	Ca_Kabuli_Chr04	4027872	(A/C)
5321	CakSNP5321	*Kabuli*	Ca_Kabuli_Chr04	4097989	(G/A)
5322	CakSNP5322	*Kabuli*	Ca_Kabuli_Chr04	4097935	(T/C)
5323	CakSNP5323	*Kabuli*	Ca_Kabuli_Chr04	4116386	(G/C)
5324	CakSNP5324	*Kabuli*	Ca_Kabuli_Chr04	4148061	(T/C)
5325	CakSNP5325	*Kabuli*	Ca_Kabuli_Chr04	4148250	(A/G)
5326	CakSNP5326	*Kabuli*	Ca_Kabuli_Chr04	4154417	(A/C)
5327	CakSNP5327	*Kabuli*	Ca_Kabuli_Chr04	4154507	(G/T)
5328	CakSNP5328	*Kabuli*	Ca_Kabuli_Chr04	4229295	(T/A)
5329	CakSNP5329	*Kabuli*	Ca_Kabuli_Chr04	4229345	(C/T)
5330	CakSNP5330	*Kabuli*	Ca_Kabuli_Chr04	4229381	(T/C)
5331	CakSNP5331	*Kabuli*	Ca_Kabuli_Chr04	4229371	(C/T)
5332	CakSNP5332	*Kabuli*	Ca_Kabuli_Chr04	4255863	(T/G)
5333	CakSNP5333	*Kabuli*	Ca_Kabuli_Chr04	4256023	(C/T)
5334	CakSNP5334	*Kabuli*	Ca_Kabuli_Chr04	4258002	(A/G)
5335	CakSNP5335	*Kabuli*	Ca_Kabuli_Chr04	4258149	(A/G)
5336	CakSNP5336	*Kabuli*	Ca_Kabuli_Chr04	4305891	(A/C)
5337	CakSNP5337	*Kabuli*	Ca_Kabuli_Chr04	4320615	(C/T)
5338	CakSNP5338	*Kabuli*	Ca_Kabuli_Chr04	4320743	(A/G)
5339	CakSNP5339	*Kabuli*	Ca_Kabuli_Chr04	4327268	(A/G)
5340	CakSNP5340	*Kabuli*	Ca_Kabuli_Chr04	4334307	(T/G)
5341	CakSNP5341	*Kabuli*	Ca_Kabuli_Chr04	4343586	(C/T)

S.N.	SNP IDs	Cultivars	Chromosomes/scaffolds	Physical positions (bp)	SNPs
5342	CakSNP5342	*Kabuli*	Ca_Kabuli_Chr04	4343540	(C/A)
5343	CakSNP5343	*Kabuli*	Ca_Kabuli_Chr04	4410351	(T/C)
5344	CakSNP5344	*Kabuli*	Ca_Kabuli_Chr04	4426659	(C/T)
5345	CakSNP5345	*Kabuli*	Ca_Kabuli_Chr04	4426760	(T/C)
5346	CakSNP5346	*Kabuli*	Ca_Kabuli_Chr04	4435621	(T/C)
5347	CakSNP5347	*Kabuli*	Ca_Kabuli_Chr04	4451928	(T/C)
5348	CakSNP5348	*Kabuli*	Ca_Kabuli_Chr04	4452018	(T/A)
5349	CakSNP5349	*Kabuli*	Ca_Kabuli_Chr04	4452380	(C/G)
5350	CakSNP5350	*Kabuli*	Ca_Kabuli_Chr04	4454305	(A/C)
5351	CakSNP5351	*Kabuli*	Ca_Kabuli_Chr04	4454464	(A/G)
5352	CakSNP5352	*Kabuli*	Ca_Kabuli_Chr04	4461562	(G/C)
5353	CakSNP5353	*Kabuli*	Ca_Kabuli_Chr04	4468394	(C/T)
5354	CakSNP5354	*Kabuli*	Ca_Kabuli_Chr04	4536310	(A/G)
5355	CakSNP5355	*Kabuli*	Ca_Kabuli_Chr04	4536286	(G/C)
5356	CakSNP5356	*Kabuli*	Ca_Kabuli_Chr04	4578836	(C/T)
5357	CakSNP5357	*Kabuli*	Ca_Kabuli_Chr04	4579274	(C/T)
5358	CakSNP5358	*Kabuli*	Ca_Kabuli_Chr04	4605340	(A/C)
5359	CakSNP5359	*Kabuli*	Ca_Kabuli_Chr04	4622932	(T/G)
5360	CakSNP5360	*Kabuli*	Ca_Kabuli_Chr04	4622913	(C/T)
5361	CakSNP5361	*Kabuli*	Ca_Kabuli_Chr04	4628221	(T/G)
5362	CakSNP5362	*Kabuli*	Ca_Kabuli_Chr04	4628232	(G/A)
5363	CakSNP5363	*Kabuli*	Ca_Kabuli_Chr04	4628240	(C/T)
5364	CakSNP5364	*Kabuli*	Ca_Kabuli_Chr04	4628243	(G/A)
5365	CakSNP5365	*Kabuli*	Ca_Kabuli_Chr04	4628309	(C/T)
5366	CakSNP5366	*Kabuli*	Ca_Kabuli_Chr04	4630407	(G/A)
5367	CakSNP5367	*Kabuli*	Ca_Kabuli_Chr04	4630419	(A/C)
5368	CakSNP5368	*Kabuli*	Ca_Kabuli_Chr04	4630514	(T/C)
5369	CakSNP5369	*Kabuli*	Ca_Kabuli_Chr04	4633913	(T/G)
5370	CakSNP5370	*Kabuli*	Ca_Kabuli_Chr04	4634542	(A/T)
5371	CakSNP5371	*Kabuli*	Ca_Kabuli_Chr04	4660220	(A/G)
5372	CakSNP5372	*Kabuli*	Ca_Kabuli_Chr04	4670913	(A/G)
5373	CakSNP5373	*Kabuli*	Ca_Kabuli_Chr04	4670975	(C/A)
5374	CakSNP5374	*Kabuli*	Ca_Kabuli_Chr04	4675182	(T/G)
5375	CakSNP5375	*Kabuli*	Ca_Kabuli_Chr04	4675177	(T/A)
5376	CakSNP5376	*Kabuli*	Ca_Kabuli_Chr04	4675176	(T/G)
5377	CakSNP5377	*Kabuli*	Ca_Kabuli_Chr04	4675172	(T/A)
5378	CakSNP5378	*Kabuli*	Ca_Kabuli_Chr04	4675171	(T/A)
5379	CakSNP5379	*Kabuli*	Ca_Kabuli_Chr04	4675166	(A/T)
5380	CakSNP5380	*Kabuli*	Ca_Kabuli_Chr04	4679236	(A/G)

S.N.	SNP IDs	Cultivars	Chromosomes/scaffolds	Physical positions (bp)	SNPs
5381	CakSNP5381	*Kabuli*	Ca_Kabuli_Chr04	4707682	(A/G)
5382	CakSNP5382	*Kabuli*	Ca_Kabuli_Chr04	4722348	(G/A)
5383	CakSNP5383	*Kabuli*	Ca_Kabuli_Chr04	4757357	(T/C)
5384	CakSNP5384	*Kabuli*	Ca_Kabuli_Chr04	4757416	(G/A)
5385	CakSNP5385	*Kabuli*	Ca_Kabuli_Chr04	4757498	(C/G)
5386	CakSNP5386	*Kabuli*	Ca_Kabuli_Chr04	4794122	(G/A)
5387	CakSNP5387	*Kabuli*	Ca_Kabuli_Chr04	4794192	(G/C)
5388	CakSNP5388	*Kabuli*	Ca_Kabuli_Chr04	4799122	(C/T)
5389	CakSNP5389	*Kabuli*	Ca_Kabuli_Chr04	4799116	(C/T)
5390	CakSNP5390	*Kabuli*	Ca_Kabuli_Chr04	4803168	(T/A)
5391	CakSNP5391	*Kabuli*	Ca_Kabuli_Chr04	4803213	(C/T)
5392	CakSNP5392	*Kabuli*	Ca_Kabuli_Chr04	4874780	(A/T)
5393	CakSNP5393	*Kabuli*	Ca_Kabuli_Chr04	4874782	(C/A)
5394	CakSNP5394	*Kabuli*	Ca_Kabuli_Chr04	4874836	(C/T)
5395	CakSNP5395	*Kabuli*	Ca_Kabuli_Chr04	4882813	(C/G)
5396	CakSNP5396	*Kabuli*	Ca_Kabuli_Chr04	4897934	(C/G)
5397	CakSNP5397	*Kabuli*	Ca_Kabuli_Chr04	4907987	(G/A)
5398	CakSNP5398	*Kabuli*	Ca_Kabuli_Chr04	4911083	(T/C)
5399	CakSNP5399	*Kabuli*	Ca_Kabuli_Chr04	4911130	(T/C)
5400	CakSNP5400	*Kabuli*	Ca_Kabuli_Chr04	4911209	(A/G)
5401	CakSNP5401	*Kabuli*	Ca_Kabuli_Chr04	4911217	(A/C)
5402	CakSNP5402	*Kabuli*	Ca_Kabuli_Chr04	4920706	(T/C)
5403	CakSNP5403	*Kabuli*	Ca_Kabuli_Chr04	4920712	(A/T)
5404	CakSNP5404	*Kabuli*	Ca_Kabuli_Chr04	4920719	(A/G)
5405	CakSNP5405	*Kabuli*	Ca_Kabuli_Chr04	4922290	(A/C)
5406	CakSNP5406	*Kabuli*	Ca_Kabuli_Chr04	4928764	(T/C)
5407	CakSNP5407	*Kabuli*	Ca_Kabuli_Chr04	4928890	(A/G)
5408	CakSNP5408	*Kabuli*	Ca_Kabuli_Chr04	4929387	(T/C)
5409	CakSNP5409	*Kabuli*	Ca_Kabuli_Chr04	4953629	(C/A)
5410	CakSNP5410	*Kabuli*	Ca_Kabuli_Chr04	4967336	(T/C)
5411	CakSNP5411	*Kabuli*	Ca_Kabuli_Chr04	4967342	(G/A)
5412	CakSNP5412	*Kabuli*	Ca_Kabuli_Chr04	4967347	(A/G)
5413	CakSNP5413	*Kabuli*	Ca_Kabuli_Chr04	5018591	(G/A)
5414	CakSNP5414	*Kabuli*	Ca_Kabuli_Chr04	5038300	(G/C)
5415	CakSNP5415	*Kabuli*	Ca_Kabuli_Chr04	5051617	(A/G)
5416	CakSNP5416	*Kabuli*	Ca_Kabuli_Chr04	5051574	(T/G)
5417	CakSNP5417	*Kabuli*	Ca_Kabuli_Chr04	5051573	(A/C)
5418	CakSNP5418	*Kabuli*	Ca_Kabuli_Chr04	5086705	(T/C)
5419	CakSNP5419	*Kabuli*	Ca_Kabuli_Chr04	5120207	(C/T)

S.N.	SNP IDs	Cultivars	Chromosomes/scaffolds	Physical positions (bp)	SNPs
5420	CakSNP5420	*Kabuli*	Ca_Kabuli_Chr04	5186401	(A/G)
5421	CakSNP5421	*Kabuli*	Ca_Kabuli_Chr04	5187402	(G/C)
5422	CakSNP5422	*Kabuli*	Ca_Kabuli_Chr04	5187344	(C/G)
5423	CakSNP5423	*Kabuli*	Ca_Kabuli_Chr04	5188084	(G/A)
5424	CakSNP5424	*Kabuli*	Ca_Kabuli_Chr04	5188040	(A/G)
5425	CakSNP5425	*Kabuli*	Ca_Kabuli_Chr04	5197824	(T/C)
5426	CakSNP5426	*Kabuli*	Ca_Kabuli_Chr04	5199907	(C/T)
5427	CakSNP5427	*Kabuli*	Ca_Kabuli_Chr04	5222669	(A/C)
5428	CakSNP5428	*Kabuli*	Ca_Kabuli_Chr04	5222805	(G/C)
5429	CakSNP5429	*Kabuli*	Ca_Kabuli_Chr04	5222851	(C/T)
5430	CakSNP5430	*Kabuli*	Ca_Kabuli_Chr04	5223100	(G/A)
5431	CakSNP5431	*Kabuli*	Ca_Kabuli_Chr04	5223206	(A/T)
5432	CakSNP5432	*Kabuli*	Ca_Kabuli_Chr04	5228950	(G/T)
5433	CakSNP5433	*Kabuli*	Ca_Kabuli_Chr04	5232364	(T/C)
5434	CakSNP5434	*Kabuli*	Ca_Kabuli_Chr04	5263199	(A/G)
5435	CakSNP5435	*Kabuli*	Ca_Kabuli_Chr04	5268995	(A/C)
5436	CakSNP5436	*Kabuli*	Ca_Kabuli_Chr04	5282131	(T/C)
5437	CakSNP5437	*Kabuli*	Ca_Kabuli_Chr04	5310873	(A/G)
5438	CakSNP5438	*Kabuli*	Ca_Kabuli_Chr04	5345520	(T/G)
5439	CakSNP5439	*Kabuli*	Ca_Kabuli_Chr04	5377067	(A/C)
5440	CakSNP5440	*Kabuli*	Ca_Kabuli_Chr04	5377400	(C/T)
5441	CakSNP5441	*Kabuli*	Ca_Kabuli_Chr04	5390236	(C/A)
5442	CakSNP5442	*Kabuli*	Ca_Kabuli_Chr04	5390317	(G/A)
5443	CakSNP5443	*Kabuli*	Ca_Kabuli_Chr04	5453008	(A/C)
5444	CakSNP5444	*Kabuli*	Ca_Kabuli_Chr04	5473174	(C/T)
5445	CakSNP5445	*Kabuli*	Ca_Kabuli_Chr04	5484259	(G/T)
5446	CakSNP5446	*Kabuli*	Ca_Kabuli_Chr04	5484257	(G/C)
5447	CakSNP5447	*Kabuli*	Ca_Kabuli_Chr04	5484238	(G/A)
5448	CakSNP5448	*Kabuli*	Ca_Kabuli_Chr04	5484236	(C/T)
5449	CakSNP5449	*Kabuli*	Ca_Kabuli_Chr04	5484220	(A/G)
5450	CakSNP5450	*Kabuli*	Ca_Kabuli_Chr04	5485052	(G/T)
5451	CakSNP5451	*Kabuli*	Ca_Kabuli_Chr04	5485050	(G/C)
5452	CakSNP5452	*Kabuli*	Ca_Kabuli_Chr04	5485031	(G/A)
5453	CakSNP5453	*Kabuli*	Ca_Kabuli_Chr04	5485029	(C/T)
5454	CakSNP5454	*Kabuli*	Ca_Kabuli_Chr04	5485013	(A/G)
5455	CakSNP5455	*Kabuli*	Ca_Kabuli_Chr04	5528421	(T/G)
5456	CakSNP5456	*Kabuli*	Ca_Kabuli_Chr04	5530612	(A/G)
5457	CakSNP5457	*Kabuli*	Ca_Kabuli_Chr04	5535661	(A/G)
5458	CakSNP5458	*Kabuli*	Ca_Kabuli_Chr04	5575846	(G/C)

S.N.	SNP IDs	Cultivars	Chromosomes/scaffolds	Physical positions (bp)	SNPs
5459	CakSNP5459	*Kabuli*	Ca_Kabuli_Chr04	5595079	(T/C)
5460	CakSNP5460	*Kabuli*	Ca_Kabuli_Chr04	5595077	(T/A)
5461	CakSNP5461	*Kabuli*	Ca_Kabuli_Chr04	5623571	(A/G)
5462	CakSNP5462	*Kabuli*	Ca_Kabuli_Chr04	5627096	(C/T)
5463	CakSNP5463	*Kabuli*	Ca_Kabuli_Chr04	5701873	(A/C)
5464	CakSNP5464	*Kabuli*	Ca_Kabuli_Chr04	5701900	(G/T)
5465	CakSNP5465	*Kabuli*	Ca_Kabuli_Chr04	5701902	(C/T)
5466	CakSNP5466	*Kabuli*	Ca_Kabuli_Chr04	5701907	(G/T)
5467	CakSNP5467	*Kabuli*	Ca_Kabuli_Chr04	5779698	(A/G)
5468	CakSNP5468	*Kabuli*	Ca_Kabuli_Chr04	5779765	(A/T)
5469	CakSNP5469	*Kabuli*	Ca_Kabuli_Chr04	5787727	(G/A)
5470	CakSNP5470	*Kabuli*	Ca_Kabuli_Chr04	5787722	(G/A)
5471	CakSNP5471	*Kabuli*	Ca_Kabuli_Chr04	5841444	(C/G)
5472	CakSNP5472	*Kabuli*	Ca_Kabuli_Chr04	5841462	(A/C)
5473	CakSNP5473	*Kabuli*	Ca_Kabuli_Chr04	5853789	(C/T)
5474	CakSNP5474	*Kabuli*	Ca_Kabuli_Chr04	5853798	(T/G)
5475	CakSNP5475	*Kabuli*	Ca_Kabuli_Chr04	5900194	(C/A)
5476	CakSNP5476	*Kabuli*	Ca_Kabuli_Chr04	5900290	(T/C)
5477	CakSNP5477	*Kabuli*	Ca_Kabuli_Chr04	5905252	(C/T)
5478	CakSNP5478	*Kabuli*	Ca_Kabuli_Chr04	5905276	(C/T)
5479	CakSNP5479	*Kabuli*	Ca_Kabuli_Chr04	5905303	(A/C)
5480	CakSNP5480	*Kabuli*	Ca_Kabuli_Chr04	5905385	(G/A)
5481	CakSNP5481	*Kabuli*	Ca_Kabuli_Chr04	5907409	(T/G)
5482	CakSNP5482	*Kabuli*	Ca_Kabuli_Chr04	5907421	(T/G)
5483	CakSNP5483	*Kabuli*	Ca_Kabuli_Chr04	5907437	(C/A)
5484	CakSNP5484	*Kabuli*	Ca_Kabuli_Chr04	5907465	(C/A)
5485	CakSNP5485	*Kabuli*	Ca_Kabuli_Chr04	5940176	(C/T)
5486	CakSNP5486	*Kabuli*	Ca_Kabuli_Chr04	5999964	(T/C)
5487	CakSNP5487	*Kabuli*	Ca_Kabuli_Chr04	6000010	(T/A)
5488	CakSNP5488	*Kabuli*	Ca_Kabuli_Chr04	6000013	(A/C)
5489	CakSNP5489	*Kabuli*	Ca_Kabuli_Chr04	6000037	(T/G)
5490	CakSNP5490	*Kabuli*	Ca_Kabuli_Chr04	6006849	(T/G)
5491	CakSNP5491	*Kabuli*	Ca_Kabuli_Chr04	6030948	(T/G)
5492	CakSNP5492	*Kabuli*	Ca_Kabuli_Chr04	6030966	(A/G)
5493	CakSNP5493	*Kabuli*	Ca_Kabuli_Chr04	6041134	(A/C)
5494	CakSNP5494	*Kabuli*	Ca_Kabuli_Chr04	6041130	(C/A)
5495	CakSNP5495	*Kabuli*	Ca_Kabuli_Chr04	6041126	(A/G)
5496	CakSNP5496	*Kabuli*	Ca_Kabuli_Chr04	6041120	(G/T)
5497	CakSNP5497	*Kabuli*	Ca_Kabuli_Chr04	6041117	(C/T)

S.N.	SNP IDs	Cultivars	Chromosomes/scaffolds	Physical positions (bp)	SNPs
5498	CakSNP5498	*Kabuli*	Ca_Kabuli_Chr04	6076530	(T/C)
5499	CakSNP5499	*Kabuli*	Ca_Kabuli_Chr04	6076594	(G/C)
5500	CakSNP5500	*Kabuli*	Ca_Kabuli_Chr04	6076614	(A/G)
5501	CakSNP5501	*Kabuli*	Ca_Kabuli_Chr04	6077103	(G/A)
5502	CakSNP5502	*Kabuli*	Ca_Kabuli_Chr04	6077113	(G/T)
5503	CakSNP5503	*Kabuli*	Ca_Kabuli_Chr04	6077156	(G/T)
5504	CakSNP5504	*Kabuli*	Ca_Kabuli_Chr04	6077168	(T/C)
5505	CakSNP5505	*Kabuli*	Ca_Kabuli_Chr04	6157625	(T/C)
5506	CakSNP5506	*Kabuli*	Ca_Kabuli_Chr04	6201137	(T/C)
5507	CakSNP5507	*Kabuli*	Ca_Kabuli_Chr04	6238354	(G/A)
5508	CakSNP5508	*Kabuli*	Ca_Kabuli_Chr04	6238367	(T/A)
5509	CakSNP5509	*Kabuli*	Ca_Kabuli_Chr04	6238376	(A/G)
5510	CakSNP5510	*Kabuli*	Ca_Kabuli_Chr04	6238414	(T/A)
5511	CakSNP5511	*Kabuli*	Ca_Kabuli_Chr04	6277398	(C/T)
5512	CakSNP5512	*Kabuli*	Ca_Kabuli_Chr04	6281228	(A/C)
5513	CakSNP5513	*Kabuli*	Ca_Kabuli_Chr04	6296089	(G/T)
5514	CakSNP5514	*Kabuli*	Ca_Kabuli_Chr04	6387938	(T/G)
5515	CakSNP5515	*Kabuli*	Ca_Kabuli_Chr04	6403910	(A/C)
5516	CakSNP5516	*Kabuli*	Ca_Kabuli_Chr04	6408543	(T/G)
5517	CakSNP5517	*Kabuli*	Ca_Kabuli_Chr04	6415058	(A/T)
5518	CakSNP5518	*Kabuli*	Ca_Kabuli_Chr04	6462228	(C/T)
5519	CakSNP5519	*Kabuli*	Ca_Kabuli_Chr04	6507540	(G/A)
5520	CakSNP5520	*Kabuli*	Ca_Kabuli_Chr04	6509701	(T/A)
5521	CakSNP5521	*Kabuli*	Ca_Kabuli_Chr04	6509761	(C/A)
5522	CakSNP5522	*Kabuli*	Ca_Kabuli_Chr04	6511977	(C/T)
5523	CakSNP5523	*Kabuli*	Ca_Kabuli_Chr04	6512060	(A/T)
5524	CakSNP5524	*Kabuli*	Ca_Kabuli_Chr04	6513321	(A/T)
5525	CakSNP5525	*Kabuli*	Ca_Kabuli_Chr04	6513493	(C/T)
5526	CakSNP5526	*Kabuli*	Ca_Kabuli_Chr04	6513576	(A/T)
5527	CakSNP5527	*Kabuli*	Ca_Kabuli_Chr04	6544385	(G/A)
5528	CakSNP5528	*Kabuli*	Ca_Kabuli_Chr04	6544342	(A/C)
5529	CakSNP5529	*Kabuli*	Ca_Kabuli_Chr04	6544337	(A/C)
5530	CakSNP5530	*Kabuli*	Ca_Kabuli_Chr04	6552776	(A/G)
5531	CakSNP5531	*Kabuli*	Ca_Kabuli_Chr04	6562373	(T/A)
5532	CakSNP5532	*Kabuli*	Ca_Kabuli_Chr04	6562391	(A/C)
5533	CakSNP5533	*Kabuli*	Ca_Kabuli_Chr04	6562402	(G/C)
5534	CakSNP5534	*Kabuli*	Ca_Kabuli_Chr04	6611820	(G/A)
5535	CakSNP5535	*Kabuli*	Ca_Kabuli_Chr04	6616016	(C/T)
5536	CakSNP5536	*Kabuli*	Ca_Kabuli_Chr04	6616071	(C/T)

S.N.	SNP IDs	Cultivars	Chromosomes/scaffolds	Physical positions (bp)	SNPs
5537	CakSNP5537	*Kabuli*	Ca_Kabuli_Chr04	6688218	(G/A)
5538	CakSNP5538	*Kabuli*	Ca_Kabuli_Chr04	6715326	(C/T)
5539	CakSNP5539	*Kabuli*	Ca_Kabuli_Chr04	6723103	(G/T)
5540	CakSNP5540	*Kabuli*	Ca_Kabuli_Chr04	6749334	(A/G)
5541	CakSNP5541	*Kabuli*	Ca_Kabuli_Chr04	6749481	(C/T)
5542	CakSNP5542	*Kabuli*	Ca_Kabuli_Chr04	6749550	(G/A)
5543	CakSNP5543	*Kabuli*	Ca_Kabuli_Chr04	6749522	(T/C)
5544	CakSNP5544	*Kabuli*	Ca_Kabuli_Chr04	6751549	(A/T)
5545	CakSNP5545	*Kabuli*	Ca_Kabuli_Chr04	6761778	(C/A)
5546	CakSNP5546	*Kabuli*	Ca_Kabuli_Chr04	6765922	(T/G)
5547	CakSNP5547	*Kabuli*	Ca_Kabuli_Chr04	6765884	(T/C)
5548	CakSNP5548	*Kabuli*	Ca_Kabuli_Chr04	6792858	(T/G)
5549	CakSNP5549	*Kabuli*	Ca_Kabuli_Chr04	6801632	(C/A)
5550	CakSNP5550	*Kabuli*	Ca_Kabuli_Chr04	6841854	(G/A)
5551	CakSNP5551	*Kabuli*	Ca_Kabuli_Chr04	6841856	(G/A)
5552	CakSNP5552	*Kabuli*	Ca_Kabuli_Chr04	6841858	(G/A)
5553	CakSNP5553	*Kabuli*	Ca_Kabuli_Chr04	6841859	(A/C)
5554	CakSNP5554	*Kabuli*	Ca_Kabuli_Chr04	6841860	(G/A)
5555	CakSNP5555	*Kabuli*	Ca_Kabuli_Chr04	6841861	(A/C)
5556	CakSNP5556	*Kabuli*	Ca_Kabuli_Chr04	6843659	(T/C)
5557	CakSNP5557	*Kabuli*	Ca_Kabuli_Chr04	6852908	(T/C)
5558	CakSNP5558	*Kabuli*	Ca_Kabuli_Chr04	6889964	(T/G)
5559	CakSNP5559	*Kabuli*	Ca_Kabuli_Chr04	6889974	(T/G)
5560	CakSNP5560	*Kabuli*	Ca_Kabuli_Chr04	6890000	(A/G)
5561	CakSNP5561	*Kabuli*	Ca_Kabuli_Chr04	6890005	(C/T)
5562	CakSNP5562	*Kabuli*	Ca_Kabuli_Chr04	6890014	(G/T)
5563	CakSNP5563	*Kabuli*	Ca_Kabuli_Chr04	6889994	(T/G)
5564	CakSNP5564	*Kabuli*	Ca_Kabuli_Chr04	6895804	(G/A)
5565	CakSNP5565	*Kabuli*	Ca_Kabuli_Chr04	6919896	(C/T)
5566	CakSNP5566	*Kabuli*	Ca_Kabuli_Chr04	6920068	(G/A)
5567	CakSNP5567	*Kabuli*	Ca_Kabuli_Chr04	6931668	(T/A)
5568	CakSNP5568	*Kabuli*	Ca_Kabuli_Chr04	6960336	(G/A)
5569	CakSNP5569	*Kabuli*	Ca_Kabuli_Chr04	6969771	(G/T)
5570	CakSNP5570	*Kabuli*	Ca_Kabuli_Chr04	6969799	(T/C)
5571	CakSNP5571	*Kabuli*	Ca_Kabuli_Chr04	6970808	(A/G)
5572	CakSNP5572	*Kabuli*	Ca_Kabuli_Chr04	6970800	(A/G)
5573	CakSNP5573	*Kabuli*	Ca_Kabuli_Chr04	6970792	(T/A)
5574	CakSNP5574	*Kabuli*	Ca_Kabuli_Chr04	6970781	(G/A)
5575	CakSNP5575	*Kabuli*	Ca_Kabuli_Chr04	6970856	(T/C)

S.N.	SNP IDs	Cultivars	Chromosomes/scaffolds	Physical positions (bp)	SNPs
5576	CakSNP5576	*Kabuli*	Ca_Kabuli_Chr04	7013498	(A/G)
5577	CakSNP5577	*Kabuli*	Ca_Kabuli_Chr04	7040229	(A/T)
5578	CakSNP5578	*Kabuli*	Ca_Kabuli_Chr04	7142498	(G/A)
5579	CakSNP5579	*Kabuli*	Ca_Kabuli_Chr04	7143207	(G/T)
5580	CakSNP5580	*Kabuli*	Ca_Kabuli_Chr04	7143214	(T/C)
5581	CakSNP5581	*Kabuli*	Ca_Kabuli_Chr04	7143238	(C/T)
5582	CakSNP5582	*Kabuli*	Ca_Kabuli_Chr04	7155826	(G/A)
5583	CakSNP5583	*Kabuli*	Ca_Kabuli_Chr04	7185974	(G/A)
5584	CakSNP5584	*Kabuli*	Ca_Kabuli_Chr04	7231299	(C/T)
5585	CakSNP5585	*Kabuli*	Ca_Kabuli_Chr04	7239917	(T/G)
5586	CakSNP5586	*Kabuli*	Ca_Kabuli_Chr04	7244301	(G/A)
5587	CakSNP5587	*Kabuli*	Ca_Kabuli_Chr04	7335587	(C/T)
5588	CakSNP5588	*Kabuli*	Ca_Kabuli_Chr04	7349958	(T/C)
5589	CakSNP5589	*Kabuli*	Ca_Kabuli_Chr04	7349948	(T/C)
5590	CakSNP5590	*Kabuli*	Ca_Kabuli_Chr04	7349900	(G/A)
5591	CakSNP5591	*Kabuli*	Ca_Kabuli_Chr04	7349885	(G/A)
5592	CakSNP5592	*Kabuli*	Ca_Kabuli_Chr04	7350192	(G/T)
5593	CakSNP5593	*Kabuli*	Ca_Kabuli_Chr04	7371667	(G/A)
5594	CakSNP5594	*Kabuli*	Ca_Kabuli_Chr04	7371708	(T/C)
5595	CakSNP5595	*Kabuli*	Ca_Kabuli_Chr04	7416949	(C/T)
5596	CakSNP5596	*Kabuli*	Ca_Kabuli_Chr04	7496298	(A/G)
5597	CakSNP5597	*Kabuli*	Ca_Kabuli_Chr04	7520182	(A/T)
5598	CakSNP5598	*Kabuli*	Ca_Kabuli_Chr04	7520184	(A/C)
5599	CakSNP5599	*Kabuli*	Ca_Kabuli_Chr04	7522047	(C/T)
5600	CakSNP5600	*Kabuli*	Ca_Kabuli_Chr04	7522033	(A/G)
5601	CakSNP5601	*Kabuli*	Ca_Kabuli_Chr04	7587317	(A/G)
5602	CakSNP5602	*Kabuli*	Ca_Kabuli_Chr04	7587516	(T/C)
5603	CakSNP5603	*Kabuli*	Ca_Kabuli_Chr04	7598474	(G/C)
5604	CakSNP5604	*Kabuli*	Ca_Kabuli_Chr04	7699429	(C/T)
5605	CakSNP5605	*Kabuli*	Ca_Kabuli_Chr04	7717770	(A/G)
5606	CakSNP5606	*Kabuli*	Ca_Kabuli_Chr04	7717935	(T/G)
5607	CakSNP5607	*Kabuli*	Ca_Kabuli_Chr04	7717978	(G/A)
5608	CakSNP5608	*Kabuli*	Ca_Kabuli_Chr04	7718270	(T/C)
5609	CakSNP5609	*Kabuli*	Ca_Kabuli_Chr04	7718277	(G/A)
5610	CakSNP5610	*Kabuli*	Ca_Kabuli_Chr04	7739202	(C/T)
5611	CakSNP5611	*Kabuli*	Ca_Kabuli_Chr04	7746230	(A/C)
5612	CakSNP5612	*Kabuli*	Ca_Kabuli_Chr04	7746242	(C/G)
5613	CakSNP5613	*Kabuli*	Ca_Kabuli_Chr04	7812468	(C/T)
5614	CakSNP5614	*Kabuli*	Ca_Kabuli_Chr04	7812491	(T/A)

S.N.	SNP IDs	Cultivars	Chromosomes/scaffolds	Physical positions (bp)	SNPs
5615	CakSNP5615	*Kabuli*	Ca_Kabuli_Chr04	7812521	(A/G)
5616	CakSNP5616	*Kabuli*	Ca_Kabuli_Chr04	7819865	(T/C)
5617	CakSNP5617	*Kabuli*	Ca_Kabuli_Chr04	7821269	(G/A)
5618	CakSNP5618	*Kabuli*	Ca_Kabuli_Chr04	7868480	(A/T)
5619	CakSNP5619	*Kabuli*	Ca_Kabuli_Chr04	7883707	(T/A)
5620	CakSNP5620	*Kabuli*	Ca_Kabuli_Chr04	7892596	(A/C)
5621	CakSNP5621	*Kabuli*	Ca_Kabuli_Chr04	7975577	(C/G)
5622	CakSNP5622	*Kabuli*	Ca_Kabuli_Chr04	7975586	(T/G)
5623	CakSNP5623	*Kabuli*	Ca_Kabuli_Chr04	7988367	(A/G)
5624	CakSNP5624	*Kabuli*	Ca_Kabuli_Chr04	7988396	(C/T)
5625	CakSNP5625	*Kabuli*	Ca_Kabuli_Chr04	7988399	(G/A)
5626	CakSNP5626	*Kabuli*	Ca_Kabuli_Chr04	7988422	(T/G)
5627	CakSNP5627	*Kabuli*	Ca_Kabuli_Chr04	7988444	(T/G)
5628	CakSNP5628	*Kabuli*	Ca_Kabuli_Chr04	8025363	(T/C)
5629	CakSNP5629	*Kabuli*	Ca_Kabuli_Chr04	8025348	(A/T)
5630	CakSNP5630	*Kabuli*	Ca_Kabuli_Chr04	8025530	(A/G)
5631	CakSNP5631	*Kabuli*	Ca_Kabuli_Chr04	8050770	(T/C)
5632	CakSNP5632	*Kabuli*	Ca_Kabuli_Chr04	8051306	(T/C)
5633	CakSNP5633	*Kabuli*	Ca_Kabuli_Chr04	8117401	(G/A)
5634	CakSNP5634	*Kabuli*	Ca_Kabuli_Chr04	8178043	(G/A)
5635	CakSNP5635	*Kabuli*	Ca_Kabuli_Chr04	8198702	(T/G)
5636	CakSNP5636	*Kabuli*	Ca_Kabuli_Chr04	8203586	(C/T)
5637	CakSNP5637	*Kabuli*	Ca_Kabuli_Chr04	8236449	(C/A)
5638	CakSNP5638	*Kabuli*	Ca_Kabuli_Chr04	8237145	(C/T)
5639	CakSNP5639	*Kabuli*	Ca_Kabuli_Chr04	8269099	(T/G)
5640	CakSNP5640	*Kabuli*	Ca_Kabuli_Chr04	8269152	(C/T)
5641	CakSNP5641	*Kabuli*	Ca_Kabuli_Chr04	8269347	(C/G)
5642	CakSNP5642	*Kabuli*	Ca_Kabuli_Chr04	8302198	(A/G)
5643	CakSNP5643	*Kabuli*	Ca_Kabuli_Chr04	8383890	(G/A)
5644	CakSNP5644	*Kabuli*	Ca_Kabuli_Chr04	8396861	(T/A)
5645	CakSNP5645	*Kabuli*	Ca_Kabuli_Chr04	8396837	(T/G)
5646	CakSNP5646	*Kabuli*	Ca_Kabuli_Chr04	8401109	(A/G)
5647	CakSNP5647	*Kabuli*	Ca_Kabuli_Chr04	8401126	(G/T)
5648	CakSNP5648	*Kabuli*	Ca_Kabuli_Chr04	8401143	(A/T)
5649	CakSNP5649	*Kabuli*	Ca_Kabuli_Chr04	8401248	(T/A)
5650	CakSNP5650	*Kabuli*	Ca_Kabuli_Chr04	8401230	(A/G)
5651	CakSNP5651	*Kabuli*	Ca_Kabuli_Chr04	8404108	(T/C)
5652	CakSNP5652	*Kabuli*	Ca_Kabuli_Chr04	8404165	(C/A)
5653	CakSNP5653	*Kabuli*	Ca_Kabuli_Chr04	8404244	(C/G)

S.N.	SNP IDs	Cultivars	Chromosomes/scaffolds	Physical positions (bp)	SNPs
5654	CakSNP5654	*Kabuli*	Ca_Kabuli_Chr04	8471029	(G/A)
5655	CakSNP5655	*Kabuli*	Ca_Kabuli_Chr04	8473120	(G/A)
5656	CakSNP5656	*Kabuli*	Ca_Kabuli_Chr04	8473127	(G/A)
5657	CakSNP5657	*Kabuli*	Ca_Kabuli_Chr04	8473294	(G/A)
5658	CakSNP5658	*Kabuli*	Ca_Kabuli_Chr04	8473288	(A/G)
5659	CakSNP5659	*Kabuli*	Ca_Kabuli_Chr04	8473246	(T/C)
5660	CakSNP5660	*Kabuli*	Ca_Kabuli_Chr04	8474442	(C/T)
5661	CakSNP5661	*Kabuli*	Ca_Kabuli_Chr04	8474441	(G/A)
5662	CakSNP5662	*Kabuli*	Ca_Kabuli_Chr04	8474401	(T/A)
5663	CakSNP5663	*Kabuli*	Ca_Kabuli_Chr04	8475634	(A/C)
5664	CakSNP5664	*Kabuli*	Ca_Kabuli_Chr04	8483482	(T/C)
5665	CakSNP5665	*Kabuli*	Ca_Kabuli_Chr04	8483494	(T/C)
5666	CakSNP5666	*Kabuli*	Ca_Kabuli_Chr04	8484976	(G/A)
5667	CakSNP5667	*Kabuli*	Ca_Kabuli_Chr04	8484978	(A/G)
5668	CakSNP5668	*Kabuli*	Ca_Kabuli_Chr04	8496641	(C/A)
5669	CakSNP5669	*Kabuli*	Ca_Kabuli_Chr04	8510011	(A/C)
5670	CakSNP5670	*Kabuli*	Ca_Kabuli_Chr04	8512095	(G/A)
5671	CakSNP5671	*Kabuli*	Ca_Kabuli_Chr04	8512280	(C/T)
5672	CakSNP5672	*Kabuli*	Ca_Kabuli_Chr04	8518979	(G/A)
5673	CakSNP5673	*Kabuli*	Ca_Kabuli_Chr04	8534120	(C/T)
5674	CakSNP5674	*Kabuli*	Ca_Kabuli_Chr04	8567380	(A/T)
5675	CakSNP5675	*Kabuli*	Ca_Kabuli_Chr04	8567431	(C/T)
5676	CakSNP5676	*Kabuli*	Ca_Kabuli_Chr04	8567490	(C/A)
5677	CakSNP5677	*Kabuli*	Ca_Kabuli_Chr04	8567478	(A/C)
5678	CakSNP5678	*Kabuli*	Ca_Kabuli_Chr04	8567477	(T/C)
5679	CakSNP5679	*Kabuli*	Ca_Kabuli_Chr04	8567465	(C/T)
5680	CakSNP5680	*Kabuli*	Ca_Kabuli_Chr04	8570458	(T/G)
5681	CakSNP5681	*Kabuli*	Ca_Kabuli_Chr04	8570501	(G/C)
5682	CakSNP5682	*Kabuli*	Ca_Kabuli_Chr04	8595427	(C/T)
5683	CakSNP5683	*Kabuli*	Ca_Kabuli_Chr04	8601364	(T/G)
5684	CakSNP5684	*Kabuli*	Ca_Kabuli_Chr04	8659774	(G/A)
5685	CakSNP5685	*Kabuli*	Ca_Kabuli_Chr04	8659935	(T/A)
5686	CakSNP5686	*Kabuli*	Ca_Kabuli_Chr04	8659946	(C/T)
5687	CakSNP5687	*Kabuli*	Ca_Kabuli_Chr04	8659985	(T/C)
5688	CakSNP5688	*Kabuli*	Ca_Kabuli_Chr04	8660115	(A/T)
5689	CakSNP5689	*Kabuli*	Ca_Kabuli_Chr04	8660136	(G/A)
5690	CakSNP5690	*Kabuli*	Ca_Kabuli_Chr04	8660608	(G/T)
5691	CakSNP5691	*Kabuli*	Ca_Kabuli_Chr04	8660599	(A/G)
5692	CakSNP5692	*Kabuli*	Ca_Kabuli_Chr04	8668648	(G/A)

**S.N.**	**SNP IDs**	**Cultivars**	**Chromosomes/scaffolds**	**Physical positions (bp)**	**SNPs**
5693	CakSNP5693	*Kabuli*	Ca_Kabuli_Chr04	8668753	(A/G)
5694	CakSNP5694	*Kabuli*	Ca_Kabuli_Chr04	8668945	(T/C)
5695	CakSNP5695	*Kabuli*	Ca_Kabuli_Chr04	8669014	(C/G)
5696	CakSNP5696	*Kabuli*	Ca_Kabuli_Chr04	8669104	(T/C)
5697	CakSNP5697	*Kabuli*	Ca_Kabuli_Chr04	8669515	(C/T)
5698	CakSNP5698	*Kabuli*	Ca_Kabuli_Chr04	8669513	(G/A)
5699	CakSNP5699	*Kabuli*	Ca_Kabuli_Chr04	8669505	(G/T)
5700	CakSNP5700	*Kabuli*	Ca_Kabuli_Chr04	8681949	(G/A)
5701	CakSNP5701	*Kabuli*	Ca_Kabuli_Chr04	8695562	(C/T)
5702	CakSNP5702	*Kabuli*	Ca_Kabuli_Chr04	8751638	(A/C)
5703	CakSNP5703	*Kabuli*	Ca_Kabuli_Chr04	8751629	(T/C)
5704	CakSNP5704	*Kabuli*	Ca_Kabuli_Chr04	8751618	(A/C)
5705	CakSNP5705	*Kabuli*	Ca_Kabuli_Chr04	8751591	(A/C)
5706	CakSNP5706	*Kabuli*	Ca_Kabuli_Chr04	8772215	(A/C)
5707	CakSNP5707	*Kabuli*	Ca_Kabuli_Chr04	8782879	(G/C)
5708	CakSNP5708	*Kabuli*	Ca_Kabuli_Chr04	8791654	(A/G)
5709	CakSNP5709	*Kabuli*	Ca_Kabuli_Chr04	8805504	(A/G)
5710	CakSNP5710	*Kabuli*	Ca_Kabuli_Chr04	8805578	(A/C)
5711	CakSNP5711	*Kabuli*	Ca_Kabuli_Chr04	8805667	(C/T)
5712	CakSNP5712	*Kabuli*	Ca_Kabuli_Chr04	8805867	(A/C)
5713	CakSNP5713	*Kabuli*	Ca_Kabuli_Chr04	8805839	(G/A)
5714	CakSNP5714	*Kabuli*	Ca_Kabuli_Chr04	8809032	(C/T)
5715	CakSNP5715	*Kabuli*	Ca_Kabuli_Chr04	8809064	(T/C)
5716	CakSNP5716	*Kabuli*	Ca_Kabuli_Chr04	8809010	(A/G)
5717	CakSNP5717	*Kabuli*	Ca_Kabuli_Chr04	8809456	(A/C)
5718	CakSNP5718	*Kabuli*	Ca_Kabuli_Chr04	8844846	(A/G)
5719	CakSNP5719	*Kabuli*	Ca_Kabuli_Chr04	8848654	(C/T)
5720	CakSNP5720	*Kabuli*	Ca_Kabuli_Chr04	8848653	(T/C)
5721	CakSNP5721	*Kabuli*	Ca_Kabuli_Chr04	8852334	(T/A)
5722	CakSNP5722	*Kabuli*	Ca_Kabuli_Chr04	8852420	(T/C)
5723	CakSNP5723	*Kabuli*	Ca_Kabuli_Chr04	8873494	(G/T)
5724	CakSNP5724	*Kabuli*	Ca_Kabuli_Chr04	8873509	(G/C)
5725	CakSNP5725	*Kabuli*	Ca_Kabuli_Chr04	8919264	(C/T)
5726	CakSNP5726	*Kabuli*	Ca_Kabuli_Chr04	8919300	(C/T)
5727	CakSNP5727	*Kabuli*	Ca_Kabuli_Chr04	8919395	(A/G)
5728	CakSNP5728	*Kabuli*	Ca_Kabuli_Chr04	8952236	(A/G)
5729	CakSNP5729	*Kabuli*	Ca_Kabuli_Chr04	8985949	(C/T)
5730	CakSNP5730	*Kabuli*	Ca_Kabuli_Chr04	8985961	(C/T)
5731	CakSNP5731	*Kabuli*	Ca_Kabuli_Chr04	8986031	(T/A)

S.N.	SNP IDs	Cultivars	Chromosomes/scaffolds	Physical positions (bp)	SNPs
5732	CakSNP5732	*Kabuli*	Ca_Kabuli_Chr04	8986026	(T/A)
5733	CakSNP5733	*Kabuli*	Ca_Kabuli_Chr04	8986024	(T/A)
5734	CakSNP5734	*Kabuli*	Ca_Kabuli_Chr04	8986018	(G/A)
5735	CakSNP5735	*Kabuli*	Ca_Kabuli_Chr04	8986008	(T/A)
5736	CakSNP5736	*Kabuli*	Ca_Kabuli_Chr04	8986002	(T/A)
5737	CakSNP5737	*Kabuli*	Ca_Kabuli_Chr04	8985998	(T/A)
5738	CakSNP5738	*Kabuli*	Ca_Kabuli_Chr04	8985994	(C/A)
5739	CakSNP5739	*Kabuli*	Ca_Kabuli_Chr04	8986207	(G/C)
5740	CakSNP5740	*Kabuli*	Ca_Kabuli_Chr04	8986217	(T/A)
5741	CakSNP5741	*Kabuli*	Ca_Kabuli_Chr04	9075398	(G/A)
5742	CakSNP5742	*Kabuli*	Ca_Kabuli_Chr04	9075395	(A/G)
5743	CakSNP5743	*Kabuli*	Ca_Kabuli_Chr04	9075459	(C/T)
5744	CakSNP5744	*Kabuli*	Ca_Kabuli_Chr04	9075520	(C/T)
5745	CakSNP5745	*Kabuli*	Ca_Kabuli_Chr04	9075565	(A/G)
5746	CakSNP5746	*Kabuli*	Ca_Kabuli_Chr04	9075543	(T/C)
5747	CakSNP5747	*Kabuli*	Ca_Kabuli_Chr04	9075671	(A/G)
5748	CakSNP5748	*Kabuli*	Ca_Kabuli_Chr04	9075677	(T/C)
5749	CakSNP5749	*Kabuli*	Ca_Kabuli_Chr04	9075682	(G/A)
5750	CakSNP5750	*Kabuli*	Ca_Kabuli_Chr04	9075737	(G/A)
5751	CakSNP5751	*Kabuli*	Ca_Kabuli_Chr04	9075744	(T/G)
5752	CakSNP5752	*Kabuli*	Ca_Kabuli_Chr04	9187887	(G/C)
5753	CakSNP5753	*Kabuli*	Ca_Kabuli_Chr04	9187973	(T/G)
5754	CakSNP5754	*Kabuli*	Ca_Kabuli_Chr04	9189976	(T/A)
5755	CakSNP5755	*Kabuli*	Ca_Kabuli_Chr04	9211570	(A/G)
5756	CakSNP5756	*Kabuli*	Ca_Kabuli_Chr04	9257131	(G/A)
5757	CakSNP5757	*Kabuli*	Ca_Kabuli_Chr04	9331949	(C/T)
5758	CakSNP5758	*Kabuli*	Ca_Kabuli_Chr04	9392373	(A/T)
5759	CakSNP5759	*Kabuli*	Ca_Kabuli_Chr04	9392455	(A/G)
5760	CakSNP5760	*Kabuli*	Ca_Kabuli_Chr04	9392427	(G/A)
5761	CakSNP5761	*Kabuli*	Ca_Kabuli_Chr04	9427890	(T/G)
5762	CakSNP5762	*Kabuli*	Ca_Kabuli_Chr04	9427892	(G/T)
5763	CakSNP5763	*Kabuli*	Ca_Kabuli_Chr04	9427894	(A/C)
5764	CakSNP5764	*Kabuli*	Ca_Kabuli_Chr04	9427964	(C/A)
5765	CakSNP5765	*Kabuli*	Ca_Kabuli_Chr04	9451895	(T/A)
5766	CakSNP5766	*Kabuli*	Ca_Kabuli_Chr04	9451850	(T/G)
5767	CakSNP5767	*Kabuli*	Ca_Kabuli_Chr04	9491741	(A/T)
5768	CakSNP5768	*Kabuli*	Ca_Kabuli_Chr04	9533658	(A/G)
5769	CakSNP5769	*Kabuli*	Ca_Kabuli_Chr04	9533688	(T/G)
5770	CakSNP5770	*Kabuli*	Ca_Kabuli_Chr04	9579383	(C/A)

S.N.	SNP IDs	Cultivars	Chromosomes/scaffolds	Physical positions (bp)	SNPs
5771	CakSNP5771	*Kabuli*	Ca_Kabuli_Chr04	9579393	(G/A)
5772	CakSNP5772	*Kabuli*	Ca_Kabuli_Chr04	9579401	(G/T)
5773	CakSNP5773	*Kabuli*	Ca_Kabuli_Chr04	9579519	(A/T)
5774	CakSNP5774	*Kabuli*	Ca_Kabuli_Chr04	9579517	(T/C)
5775	CakSNP5775	*Kabuli*	Ca_Kabuli_Chr04	9580341	(T/A)
5776	CakSNP5776	*Kabuli*	Ca_Kabuli_Chr04	9580339	(T/G)
5777	CakSNP5777	*Kabuli*	Ca_Kabuli_Chr04	9614897	(G/A)
5778	CakSNP5778	*Kabuli*	Ca_Kabuli_Chr04	9614905	(C/A)
5779	CakSNP5779	*Kabuli*	Ca_Kabuli_Chr04	9661685	(G/A)
5780	CakSNP5780	*Kabuli*	Ca_Kabuli_Chr04	9670905	(C/T)
5781	CakSNP5781	*Kabuli*	Ca_Kabuli_Chr04	9670996	(G/T)
5782	CakSNP5782	*Kabuli*	Ca_Kabuli_Chr04	9671007	(G/T)
5783	CakSNP5783	*Kabuli*	Ca_Kabuli_Chr04	9671089	(T/G)
5784	CakSNP5784	*Kabuli*	Ca_Kabuli_Chr04	9706453	(T/G)
5785	CakSNP5785	*Kabuli*	Ca_Kabuli_Chr04	9706480	(T/A)
5786	CakSNP5786	*Kabuli*	Ca_Kabuli_Chr04	9706481	(A/C)
5787	CakSNP5787	*Kabuli*	Ca_Kabuli_Chr04	9740099	(A/G)
5788	CakSNP5788	*Kabuli*	Ca_Kabuli_Chr04	9800865	(C/T)
5789	CakSNP5789	*Kabuli*	Ca_Kabuli_Chr04	9802603	(T/C)
5790	CakSNP5790	*Kabuli*	Ca_Kabuli_Chr04	9836559	(G/T)
5791	CakSNP5791	*Kabuli*	Ca_Kabuli_Chr04	9840763	(C/A)
5792	CakSNP5792	*Kabuli*	Ca_Kabuli_Chr04	9840906	(A/C)
5793	CakSNP5793	*Kabuli*	Ca_Kabuli_Chr04	9846321	(T/A)
5794	CakSNP5794	*Kabuli*	Ca_Kabuli_Chr04	9947077	(A/G)
5795	CakSNP5795	*Kabuli*	Ca_Kabuli_Chr04	9973728	(C/G)
5796	CakSNP5796	*Kabuli*	Ca_Kabuli_Chr04	9976039	(C/T)
5797	CakSNP5797	*Kabuli*	Ca_Kabuli_Chr04	9999907	(A/T)
5798	CakSNP5798	*Kabuli*	Ca_Kabuli_Chr04	10007069	(A/G)
5799	CakSNP5799	*Kabuli*	Ca_Kabuli_Chr04	10007151	(C/T)
5800	CakSNP5800	*Kabuli*	Ca_Kabuli_Chr04	10007193	(T/A)
5801	CakSNP5801	*Kabuli*	Ca_Kabuli_Chr04	10007332	(T/C)
5802	CakSNP5802	*Kabuli*	Ca_Kabuli_Chr04	10007286	(C/T)
5803	CakSNP5803	*Kabuli*	Ca_Kabuli_Chr04	10007372	(C/A)
5804	CakSNP5804	*Kabuli*	Ca_Kabuli_Chr04	10009839	(G/A)
5805	CakSNP5805	*Kabuli*	Ca_Kabuli_Chr04	10013120	(G/A)
5806	CakSNP5806	*Kabuli*	Ca_Kabuli_Chr04	10013148	(G/T)
5807	CakSNP5807	*Kabuli*	Ca_Kabuli_Chr04	10017467	(A/C)
5808	CakSNP5808	*Kabuli*	Ca_Kabuli_Chr04	10024226	(G/A)
5809	CakSNP5809	*Kabuli*	Ca_Kabuli_Chr04	10030134	(A/G)

**S.N.**	**SNP IDs**	**Cultivars**	**Chromosomes/scaffolds**	**Physical positions (bp)**	**SNPs**
5810	CakSNP5810	*Kabuli*	Ca_Kabuli_Chr04	10116516	(T/G)
5811	CakSNP5811	*Kabuli*	Ca_Kabuli_Chr04	10151711	(A/C)
5812	CakSNP5812	*Kabuli*	Ca_Kabuli_Chr04	10151943	(C/T)
5813	CakSNP5813	*Kabuli*	Ca_Kabuli_Chr04	10184428	(G/T)
5814	CakSNP5814	*Kabuli*	Ca_Kabuli_Chr04	10184427	(C/A)
5815	CakSNP5815	*Kabuli*	Ca_Kabuli_Chr04	10185602	(T/G)
5816	CakSNP5816	*Kabuli*	Ca_Kabuli_Chr04	10185625	(A/G)
5817	CakSNP5817	*Kabuli*	Ca_Kabuli_Chr04	10205246	(C/T)
5818	CakSNP5818	*Kabuli*	Ca_Kabuli_Chr04	10290706	(G/T)
5819	CakSNP5819	*Kabuli*	Ca_Kabuli_Chr04	10293203	(T/C)
5820	CakSNP5820	*Kabuli*	Ca_Kabuli_Chr04	10293241	(C/G)
5821	CakSNP5821	*Kabuli*	Ca_Kabuli_Chr04	10294380	(A/G)
5822	CakSNP5822	*Kabuli*	Ca_Kabuli_Chr04	10305276	(G/A)
5823	CakSNP5823	*Kabuli*	Ca_Kabuli_Chr04	10305235	(A/G)
5824	CakSNP5824	*Kabuli*	Ca_Kabuli_Chr04	10305285	(C/A)
5825	CakSNP5825	*Kabuli*	Ca_Kabuli_Chr04	10411809	(G/A)
5826	CakSNP5826	*Kabuli*	Ca_Kabuli_Chr04	10422142	(A/T)
5827	CakSNP5827	*Kabuli*	Ca_Kabuli_Chr04	10422469	(C/A)
5828	CakSNP5828	*Kabuli*	Ca_Kabuli_Chr04	10430622	(A/C)
5829	CakSNP5829	*Kabuli*	Ca_Kabuli_Chr04	10430617	(A/C)
5830	CakSNP5830	*Kabuli*	Ca_Kabuli_Chr04	10430613	(C/A)
5831	CakSNP5831	*Kabuli*	Ca_Kabuli_Chr04	10430603	(G/A)
5832	CakSNP5832	*Kabuli*	Ca_Kabuli_Chr04	10430595	(A/C)
5833	CakSNP5833	*Kabuli*	Ca_Kabuli_Chr04	10430588	(T/C)
5834	CakSNP5834	*Kabuli*	Ca_Kabuli_Chr04	10430574	(A/T)
5835	CakSNP5835	*Kabuli*	Ca_Kabuli_Chr04	10464927	(T/G)
5836	CakSNP5836	*Kabuli*	Ca_Kabuli_Chr04	10465328	(T/C)
5837	CakSNP5837	*Kabuli*	Ca_Kabuli_Chr04	10547575	(G/A)
5838	CakSNP5838	*Kabuli*	Ca_Kabuli_Chr04	10547561	(T/C)
5839	CakSNP5839	*Kabuli*	Ca_Kabuli_Chr04	10642002	(A/G)
5840	CakSNP5840	*Kabuli*	Ca_Kabuli_Chr04	10642043	(T/G)
5841	CakSNP5841	*Kabuli*	Ca_Kabuli_Chr04	10642044	(A/T)
5842	CakSNP5842	*Kabuli*	Ca_Kabuli_Chr04	10643339	(T/A)
5843	CakSNP5843	*Kabuli*	Ca_Kabuli_Chr04	10643331	(T/A)
5844	CakSNP5844	*Kabuli*	Ca_Kabuli_Chr04	10644245	(G/A)
5845	CakSNP5845	*Kabuli*	Ca_Kabuli_Chr04	10670664	(C/A)
5846	CakSNP5846	*Kabuli*	Ca_Kabuli_Chr04	10699624	(A/T)
5847	CakSNP5847	*Kabuli*	Ca_Kabuli_Chr04	10780990	(A/G)
5848	CakSNP5848	*Kabuli*	Ca_Kabuli_Chr04	10807993	(T/C)

S.N.	SNP IDs	Cultivars	Chromosomes/scaffolds	Physical positions (bp)	SNPs
5849	CakSNP5849	*Kabuli*	Ca_Kabuli_Chr04	10827485	(G/A)
5850	CakSNP5850	*Kabuli*	Ca_Kabuli_Chr04	10827515	(C/G)
5851	CakSNP5851	*Kabuli*	Ca_Kabuli_Chr04	10901282	(T/C)
5852	CakSNP5852	*Kabuli*	Ca_Kabuli_Chr04	10917723	(T/C)
5853	CakSNP5853	*Kabuli*	Ca_Kabuli_Chr04	10917717	(C/G)
5854	CakSNP5854	*Kabuli*	Ca_Kabuli_Chr04	10925490	(G/A)
5855	CakSNP5855	*Kabuli*	Ca_Kabuli_Chr04	10928923	(T/C)
5856	CakSNP5856	*Kabuli*	Ca_Kabuli_Chr04	10950823	(C/T)
5857	CakSNP5857	*Kabuli*	Ca_Kabuli_Chr04	10971039	(C/A)
5858	CakSNP5858	*Kabuli*	Ca_Kabuli_Chr04	10990000	(T/C)
5859	CakSNP5859	*Kabuli*	Ca_Kabuli_Chr04	10990008	(A/T)
5860	CakSNP5860	*Kabuli*	Ca_Kabuli_Chr04	10990049	(G/T)
5861	CakSNP5861	*Kabuli*	Ca_Kabuli_Chr04	10990058	(T/C)
5862	CakSNP5862	*Kabuli*	Ca_Kabuli_Chr04	10990265	(T/A)
5863	CakSNP5863	*Kabuli*	Ca_Kabuli_Chr04	10990261	(A/G)
5864	CakSNP5864	*Kabuli*	Ca_Kabuli_Chr04	10991842	(G/A)
5865	CakSNP5865	*Kabuli*	Ca_Kabuli_Chr04	10991910	(C/T)
5866	CakSNP5866	*Kabuli*	Ca_Kabuli_Chr04	11040108	(C/T)
5867	CakSNP5867	*Kabuli*	Ca_Kabuli_Chr04	11051335	(C/T)
5868	CakSNP5868	*Kabuli*	Ca_Kabuli_Chr04	11059403	(T/G)
5869	CakSNP5869	*Kabuli*	Ca_Kabuli_Chr04	11059404	(T/G)
5870	CakSNP5870	*Kabuli*	Ca_Kabuli_Chr04	11059414	(T/G)
5871	CakSNP5871	*Kabuli*	Ca_Kabuli_Chr04	11059572	(G/A)
5872	CakSNP5872	*Kabuli*	Ca_Kabuli_Chr04	11059540	(T/C)
5873	CakSNP5873	*Kabuli*	Ca_Kabuli_Chr04	11096124	(A/G)
5874	CakSNP5874	*Kabuli*	Ca_Kabuli_Chr04	11096225	(A/G)
5875	CakSNP5875	*Kabuli*	Ca_Kabuli_Chr04	11096290	(G/A)
5876	CakSNP5876	*Kabuli*	Ca_Kabuli_Chr04	11113277	(A/C)
5877	CakSNP5877	*Kabuli*	Ca_Kabuli_Chr04	11114238	(T/C)
5878	CakSNP5878	*Kabuli*	Ca_Kabuli_Chr04	11142202	(A/G)
5879	CakSNP5879	*Kabuli*	Ca_Kabuli_Chr04	11142227	(C/T)
5880	CakSNP5880	*Kabuli*	Ca_Kabuli_Chr04	11196803	(T/G)
5881	CakSNP5881	*Kabuli*	Ca_Kabuli_Chr04	11230403	(G/T)
5882	CakSNP5882	*Kabuli*	Ca_Kabuli_Chr04	11230400	(T/A)
5883	CakSNP5883	*Kabuli*	Ca_Kabuli_Chr04	11230397	(G/C)
5884	CakSNP5884	*Kabuli*	Ca_Kabuli_Chr04	11231142	(A/G)
5885	CakSNP5885	*Kabuli*	Ca_Kabuli_Chr04	11231137	(A/G)
5886	CakSNP5886	*Kabuli*	Ca_Kabuli_Chr04	11231135	(C/T)
5887	CakSNP5887	*Kabuli*	Ca_Kabuli_Chr04	11244334	(T/G)

S.N.	SNP IDs	Cultivars	Chromosomes/scaffolds	Physical positions (bp)	SNPs
5888	CakSNP5888	*Kabuli*	Ca_Kabuli_Chr04	11244395	(G/T)
5889	CakSNP5889	*Kabuli*	Ca_Kabuli_Chr04	11246093	(G/A)
5890	CakSNP5890	*Kabuli*	Ca_Kabuli_Chr04	11246173	(C/T)
5891	CakSNP5891	*Kabuli*	Ca_Kabuli_Chr04	11246164	(G/A)
5892	CakSNP5892	*Kabuli*	Ca_Kabuli_Chr04	11273328	(T/C)
5893	CakSNP5893	*Kabuli*	Ca_Kabuli_Chr04	11273405	(A/T)
5894	CakSNP5894	*Kabuli*	Ca_Kabuli_Chr04	11274281	(T/C)
5895	CakSNP5895	*Kabuli*	Ca_Kabuli_Chr04	11275171	(T/C)
5896	CakSNP5896	*Kabuli*	Ca_Kabuli_Chr04	11276413	(C/G)
5897	CakSNP5897	*Kabuli*	Ca_Kabuli_Chr04	11276484	(T/C)
5898	CakSNP5898	*Kabuli*	Ca_Kabuli_Chr04	11277138	(A/G)
5899	CakSNP5899	*Kabuli*	Ca_Kabuli_Chr04	11277297	(G/A)
5900	CakSNP5900	*Kabuli*	Ca_Kabuli_Chr04	11277574	(G/C)
5901	CakSNP5901	*Kabuli*	Ca_Kabuli_Chr04	11334350	(A/G)
5902	CakSNP5902	*Kabuli*	Ca_Kabuli_Chr04	11334343	(A/T)
5903	CakSNP5903	*Kabuli*	Ca_Kabuli_Chr04	11343257	(T/C)
5904	CakSNP5904	*Kabuli*	Ca_Kabuli_Chr04	11343230	(G/C)
5905	CakSNP5905	*Kabuli*	Ca_Kabuli_Chr04	11379077	(T/C)
5906	CakSNP5906	*Kabuli*	Ca_Kabuli_Chr04	11398699	(A/G)
5907	CakSNP5907	*Kabuli*	Ca_Kabuli_Chr04	11398682	(G/A)
5908	CakSNP5908	*Kabuli*	Ca_Kabuli_Chr04	11398889	(A/G)
5909	CakSNP5909	*Kabuli*	Ca_Kabuli_Chr04	11414506	(C/T)
5910	CakSNP5910	*Kabuli*	Ca_Kabuli_Chr04	11435651	(A/G)
5911	CakSNP5911	*Kabuli*	Ca_Kabuli_Chr04	11441604	(T/A)
5912	CakSNP5912	*Kabuli*	Ca_Kabuli_Chr04	11441615	(T/C)
5913	CakSNP5913	*Kabuli*	Ca_Kabuli_Chr04	11441619	(G/A)
5914	CakSNP5914	*Kabuli*	Ca_Kabuli_Chr04	11441655	(T/A)
5915	CakSNP5915	*Kabuli*	Ca_Kabuli_Chr04	11441707	(A/C)
5916	CakSNP5916	*Kabuli*	Ca_Kabuli_Chr04	11465057	(C/G)
5917	CakSNP5917	*Kabuli*	Ca_Kabuli_Chr04	11465075	(T/C)
5918	CakSNP5918	*Kabuli*	Ca_Kabuli_Chr04	11465113	(C/T)
5919	CakSNP5919	*Kabuli*	Ca_Kabuli_Chr04	11490100	(C/T)
5920	CakSNP5920	*Kabuli*	Ca_Kabuli_Chr04	11490154	(A/T)
5921	CakSNP5921	*Kabuli*	Ca_Kabuli_Chr04	11490125	(G/T)
5922	CakSNP5922	*Kabuli*	Ca_Kabuli_Chr04	11490496	(G/A)
5923	CakSNP5923	*Kabuli*	Ca_Kabuli_Chr04	11517582	(C/T)
5924	CakSNP5924	*Kabuli*	Ca_Kabuli_Chr04	11517517	(C/A)
5925	CakSNP5925	*Kabuli*	Ca_Kabuli_Chr04	11572051	(C/T)
5926	CakSNP5926	*Kabuli*	Ca_Kabuli_Chr04	11645738	(A/G)

S.N.	SNP IDs	Cultivars	Chromosomes/scaffolds	Physical positions (bp)	SNPs
5927	CakSNP5927	*Kabuli*	Ca_Kabuli_Chr04	11646347	(C/G)
5928	CakSNP5928	*Kabuli*	Ca_Kabuli_Chr04	11646453	(G/A)
5929	CakSNP5929	*Kabuli*	Ca_Kabuli_Chr04	11658323	(A/G)
5930	CakSNP5930	*Kabuli*	Ca_Kabuli_Chr04	11658314	(A/G)
5931	CakSNP5931	*Kabuli*	Ca_Kabuli_Chr04	11689611	(C/T)
5932	CakSNP5932	*Kabuli*	Ca_Kabuli_Chr04	11689647	(T/C)
5933	CakSNP5933	*Kabuli*	Ca_Kabuli_Chr04	11752862	(A/G)
5934	CakSNP5934	*Kabuli*	Ca_Kabuli_Chr04	11752855	(A/G)
5935	CakSNP5935	*Kabuli*	Ca_Kabuli_Chr04	11772023	(G/T)
5936	CakSNP5936	*Kabuli*	Ca_Kabuli_Chr04	11838005	(C/T)
5937	CakSNP5937	*Kabuli*	Ca_Kabuli_Chr04	11852291	(A/G)
5938	CakSNP5938	*Kabuli*	Ca_Kabuli_Chr04	11926352	(G/C)
5939	CakSNP5939	*Kabuli*	Ca_Kabuli_Chr04	11985628	(G/A)
5940	CakSNP5940	*Kabuli*	Ca_Kabuli_Chr04	11985604	(C/T)
5941	CakSNP5941	*Kabuli*	Ca_Kabuli_Chr04	12004082	(A/T)
5942	CakSNP5942	*Kabuli*	Ca_Kabuli_Chr04	12004209	(C/T)
5943	CakSNP5943	*Kabuli*	Ca_Kabuli_Chr04	12004205	(A/C)
5944	CakSNP5944	*Kabuli*	Ca_Kabuli_Chr04	12023137	(A/T)
5945	CakSNP5945	*Kabuli*	Ca_Kabuli_Chr04	12023215	(G/A)
5946	CakSNP5946	*Kabuli*	Ca_Kabuli_Chr04	12045899	(T/A)
5947	CakSNP5947	*Kabuli*	Ca_Kabuli_Chr04	12045903	(C/A)
5948	CakSNP5948	*Kabuli*	Ca_Kabuli_Chr04	12045906	(C/A)
5949	CakSNP5949	*Kabuli*	Ca_Kabuli_Chr04	12045910	(G/A)
5950	CakSNP5950	*Kabuli*	Ca_Kabuli_Chr04	12070990	(T/A)
5951	CakSNP5951	*Kabuli*	Ca_Kabuli_Chr04	12070994	(C/A)
5952	CakSNP5952	*Kabuli*	Ca_Kabuli_Chr04	12070997	(C/A)
5953	CakSNP5953	*Kabuli*	Ca_Kabuli_Chr04	12071001	(G/A)
5954	CakSNP5954	*Kabuli*	Ca_Kabuli_Chr04	12167328	(A/G)
5955	CakSNP5955	*Kabuli*	Ca_Kabuli_Chr04	12167568	(G/A)
5956	CakSNP5956	*Kabuli*	Ca_Kabuli_Chr04	12212917	(G/A)
5957	CakSNP5957	*Kabuli*	Ca_Kabuli_Chr04	12213071	(C/A)
5958	CakSNP5958	*Kabuli*	Ca_Kabuli_Chr04	12213046	(T/C)
5959	CakSNP5959	*Kabuli*	Ca_Kabuli_Chr04	12234358	(C/A)
5960	CakSNP5960	*Kabuli*	Ca_Kabuli_Chr04	12249494	(T/C)
5961	CakSNP5961	*Kabuli*	Ca_Kabuli_Chr04	12251184	(G/A)
5962	CakSNP5962	*Kabuli*	Ca_Kabuli_Chr04	12274290	(A/T)
5963	CakSNP5963	*Kabuli*	Ca_Kabuli_Chr04	12316847	(T/C)
5964	CakSNP5964	*Kabuli*	Ca_Kabuli_Chr04	12431895	(C/T)
5965	CakSNP5965	*Kabuli*	Ca_Kabuli_Chr04	12431915	(A/G)

S.N.	SNP IDs	Cultivars	Chromosomes/scaffolds	Physical positions (bp)	SNPs
5966	CakSNP5966	*Kabuli*	Ca_Kabuli_Chr04	12484911	(A/T)
5967	CakSNP5967	*Kabuli*	Ca_Kabuli_Chr04	12506440	(A/G)
5968	CakSNP5968	*Kabuli*	Ca_Kabuli_Chr04	12506450	(T/A)
5969	CakSNP5969	*Kabuli*	Ca_Kabuli_Chr04	12514728	(G/A)
5970	CakSNP5970	*Kabuli*	Ca_Kabuli_Chr04	12514726	(C/T)
5971	CakSNP5971	*Kabuli*	Ca_Kabuli_Chr04	12514724	(T/C)
5972	CakSNP5972	*Kabuli*	Ca_Kabuli_Chr04	12514706	(G/A)
5973	CakSNP5973	*Kabuli*	Ca_Kabuli_Chr04	12525328	(A/G)
5974	CakSNP5974	*Kabuli*	Ca_Kabuli_Chr04	12525358	(C/G)
5975	CakSNP5975	*Kabuli*	Ca_Kabuli_Chr04	12525639	(T/A)
5976	CakSNP5976	*Kabuli*	Ca_Kabuli_Chr04	12534847	(T/C)
5977	CakSNP5977	*Kabuli*	Ca_Kabuli_Chr04	12558541	(T/C)
5978	CakSNP5978	*Kabuli*	Ca_Kabuli_Chr04	12558680	(C/T)
5979	CakSNP5979	*Kabuli*	Ca_Kabuli_Chr04	12568707	(G/A)
5980	CakSNP5980	*Kabuli*	Ca_Kabuli_Chr04	12568845	(G/T)
5981	CakSNP5981	*Kabuli*	Ca_Kabuli_Chr04	12568905	(G/A)
5982	CakSNP5982	*Kabuli*	Ca_Kabuli_Chr04	12568944	(A/G)
5983	CakSNP5983	*Kabuli*	Ca_Kabuli_Chr04	12568995	(G/A)
5984	CakSNP5984	*Kabuli*	Ca_Kabuli_Chr04	12569133	(G/A)
5985	CakSNP5985	*Kabuli*	Ca_Kabuli_Chr04	12574650	(G/A)
5986	CakSNP5986	*Kabuli*	Ca_Kabuli_Chr04	12578259	(A/G)
5987	CakSNP5987	*Kabuli*	Ca_Kabuli_Chr04	12578350	(A/G)
5988	CakSNP5988	*Kabuli*	Ca_Kabuli_Chr04	12581371	(G/T)
5989	CakSNP5989	*Kabuli*	Ca_Kabuli_Chr04	12692925	(C/T)
5990	CakSNP5990	*Kabuli*	Ca_Kabuli_Chr04	12692958	(G/C)
5991	CakSNP5991	*Kabuli*	Ca_Kabuli_Chr04	12693000	(T/C)
5992	CakSNP5992	*Kabuli*	Ca_Kabuli_Chr04	12740330	(A/G)
5993	CakSNP5993	*Kabuli*	Ca_Kabuli_Chr04	12740351	(T/G)
5994	CakSNP5994	*Kabuli*	Ca_Kabuli_Chr04	12779146	(A/C)
5995	CakSNP5995	*Kabuli*	Ca_Kabuli_Chr04	12780274	(C/T)
5996	CakSNP5996	*Kabuli*	Ca_Kabuli_Chr04	12795036	(G/A)
5997	CakSNP5997	*Kabuli*	Ca_Kabuli_Chr04	12795037	(G/A)
5998	CakSNP5998	*Kabuli*	Ca_Kabuli_Chr04	12869023	(C/T)
5999	CakSNP5999	*Kabuli*	Ca_Kabuli_Chr04	12869049	(C/T)
6000	CakSNP6000	*Kabuli*	Ca_Kabuli_Chr04	12907910	(C/A)
6001	CakSNP6001	*Kabuli*	Ca_Kabuli_Chr04	12908080	(C/T)
6002	CakSNP6002	*Kabuli*	Ca_Kabuli_Chr04	12911860	(A/G)
6003	CakSNP6003	*Kabuli*	Ca_Kabuli_Chr04	12911874	(T/C)
6004	CakSNP6004	*Kabuli*	Ca_Kabuli_Chr04	12911888	(G/C)

S.N.	SNP IDs	Cultivars	Chromosomes/scaffolds	Physical positions (bp)	SNPs
6005	CakSNP6005	*Kabuli*	Ca_Kabuli_Chr04	12912015	(G/A)
6006	CakSNP6006	*Kabuli*	Ca_Kabuli_Chr04	12955130	(G/A)
6007	CakSNP6007	*Kabuli*	Ca_Kabuli_Chr04	12973657	(C/A)
6008	CakSNP6008	*Kabuli*	Ca_Kabuli_Chr04	12982420	(G/A)
6009	CakSNP6009	*Kabuli*	Ca_Kabuli_Chr04	13018788	(C/G)
6010	CakSNP6010	*Kabuli*	Ca_Kabuli_Chr04	13020891	(A/G)
6011	CakSNP6011	*Kabuli*	Ca_Kabuli_Chr04	13050470	(T/C)
6012	CakSNP6012	*Kabuli*	Ca_Kabuli_Chr04	13050516	(A/G)
6013	CakSNP6013	*Kabuli*	Ca_Kabuli_Chr04	13050527	(C/T)
6014	CakSNP6014	*Kabuli*	Ca_Kabuli_Chr04	13050585	(C/T)
6015	CakSNP6015	*Kabuli*	Ca_Kabuli_Chr04	13062744	(T/A)
6016	CakSNP6016	*Kabuli*	Ca_Kabuli_Chr04	13062761	(A/T)
6017	CakSNP6017	*Kabuli*	Ca_Kabuli_Chr04	13062805	(C/G)
6018	CakSNP6018	*Kabuli*	Ca_Kabuli_Chr04	13062972	(A/T)
6019	CakSNP6019	*Kabuli*	Ca_Kabuli_Chr04	13063689	(T/A)
6020	CakSNP6020	*Kabuli*	Ca_Kabuli_Chr04	13063699	(G/T)
6021	CakSNP6021	*Kabuli*	Ca_Kabuli_Chr04	13069574	(T/C)
6022	CakSNP6022	*Kabuli*	Ca_Kabuli_Chr04	13071169	(T/A)
6023	CakSNP6023	*Kabuli*	Ca_Kabuli_Chr04	13071199	(A/T)
6024	CakSNP6024	*Kabuli*	Ca_Kabuli_Chr04	13071230	(T/G)
6025	CakSNP6025	*Kabuli*	Ca_Kabuli_Chr04	13072114	(T/A)
6026	CakSNP6026	*Kabuli*	Ca_Kabuli_Chr04	13072118	(T/G)
6027	CakSNP6027	*Kabuli*	Ca_Kabuli_Chr04	13072208	(C/A)
6028	CakSNP6028	*Kabuli*	Ca_Kabuli_Chr04	13082999	(A/G)
6029	CakSNP6029	*Kabuli*	Ca_Kabuli_Chr04	13102381	(G/A)
6030	CakSNP6030	*Kabuli*	Ca_Kabuli_Chr04	13102348	(G/T)
6031	CakSNP6031	*Kabuli*	Ca_Kabuli_Chr04	13147948	(A/G)
6032	CakSNP6032	*Kabuli*	Ca_Kabuli_Chr04	13147951	(C/T)
6033	CakSNP6033	*Kabuli*	Ca_Kabuli_Chr04	13147960	(T/C)
6034	CakSNP6034	*Kabuli*	Ca_Kabuli_Chr04	13147965	(A/G)
6035	CakSNP6035	*Kabuli*	Ca_Kabuli_Chr04	13148016	(G/A)
6036	CakSNP6036	*Kabuli*	Ca_Kabuli_Chr04	13179829	(T/C)
6037	CakSNP6037	*Kabuli*	Ca_Kabuli_Chr04	13203008	(A/T)
6038	CakSNP6038	*Kabuli*	Ca_Kabuli_Chr04	13229035	(C/T)
6039	CakSNP6039	*Kabuli*	Ca_Kabuli_Chr04	13252015	(A/T)
6040	CakSNP6040	*Kabuli*	Ca_Kabuli_Chr04	13252077	(C/G)
6041	CakSNP6041	*Kabuli*	Ca_Kabuli_Chr04	13252126	(T/C)
6042	CakSNP6042	*Kabuli*	Ca_Kabuli_Chr04	13252212	(G/A)
6043	CakSNP6043	*Kabuli*	Ca_Kabuli_Chr04	13272282	(T/C)

S.N.	SNP IDs	Cultivars	Chromosomes/scaffolds	Physical positions (bp)	SNPs
6044	CakSNP6044	*Kabuli*	Ca_Kabuli_Chr04	13282984	(A/C)
6045	CakSNP6045	*Kabuli*	Ca_Kabuli_Chr04	13296221	(A/T)
6046	CakSNP6046	*Kabuli*	Ca_Kabuli_Chr04	13296217	(G/T)
6047	CakSNP6047	*Kabuli*	Ca_Kabuli_Chr04	13310700	(T/C)
6048	CakSNP6048	*Kabuli*	Ca_Kabuli_Chr04	13318811	(C/T)
6049	CakSNP6049	*Kabuli*	Ca_Kabuli_Chr04	13331280	(A/G)
6050	CakSNP6050	*Kabuli*	Ca_Kabuli_Chr04	13332384	(G/A)
6051	CakSNP6051	*Kabuli*	Ca_Kabuli_Chr04	13332452	(T/C)
6052	CakSNP6052	*Kabuli*	Ca_Kabuli_Chr04	13376456	(A/T)
6053	CakSNP6053	*Kabuli*	Ca_Kabuli_Chr04	13463481	(C/T)
6054	CakSNP6054	*Kabuli*	Ca_Kabuli_Chr04	13500679	(A/C)
6055	CakSNP6055	*Kabuli*	Ca_Kabuli_Chr04	13500693	(G/A)
6056	CakSNP6056	*Kabuli*	Ca_Kabuli_Chr04	13588956	(C/T)
6057	CakSNP6057	*Kabuli*	Ca_Kabuli_Chr04	13588928	(T/C)
6058	CakSNP6058	*Kabuli*	Ca_Kabuli_Chr04	13646571	(T/C)
6059	CakSNP6059	*Kabuli*	Ca_Kabuli_Chr04	13646628	(C/T)
6060	CakSNP6060	*Kabuli*	Ca_Kabuli_Chr04	13687456	(G/A)
6061	CakSNP6061	*Kabuli*	Ca_Kabuli_Chr04	13713380	(G/T)
6062	CakSNP6062	*Kabuli*	Ca_Kabuli_Chr04	13713367	(G/C)
6063	CakSNP6063	*Kabuli*	Ca_Kabuli_Chr04	13724666	(G/C)
6064	CakSNP6064	*Kabuli*	Ca_Kabuli_Chr04	13787448	(A/C)
6065	CakSNP6065	*Kabuli*	Ca_Kabuli_Chr04	13787649	(T/C)
6066	CakSNP6066	*Kabuli*	Ca_Kabuli_Chr04	13787720	(G/C)
6067	CakSNP6067	*Kabuli*	Ca_Kabuli_Chr04	13823667	(T/A)
6068	CakSNP6068	*Kabuli*	Ca_Kabuli_Chr04	13823672	(G/T)
6069	CakSNP6069	*Kabuli*	Ca_Kabuli_Chr04	13823695	(T/C)
6070	CakSNP6070	*Kabuli*	Ca_Kabuli_Chr04	13838241	(G/T)
6071	CakSNP6071	*Kabuli*	Ca_Kabuli_Chr04	13838796	(A/G)
6072	CakSNP6072	*Kabuli*	Ca_Kabuli_Chr04	13839294	(A/G)
6073	CakSNP6073	*Kabuli*	Ca_Kabuli_Chr04	13839288	(G/A)
6074	CakSNP6074	*Kabuli*	Ca_Kabuli_Chr04	13840191	(T/A)
6075	CakSNP6075	*Kabuli*	Ca_Kabuli_Chr04	13840227	(C/A)
6076	CakSNP6076	*Kabuli*	Ca_Kabuli_Chr04	13840251	(A/G)
6077	CakSNP6077	*Kabuli*	Ca_Kabuli_Chr04	13840484	(T/C)
6078	CakSNP6078	*Kabuli*	Ca_Kabuli_Chr04	13841340	(G/T)
6079	CakSNP6079	*Kabuli*	Ca_Kabuli_Chr04	13841401	(A/G)
6080	CakSNP6080	*Kabuli*	Ca_Kabuli_Chr04	13845581	(A/T)
6081	CakSNP6081	*Kabuli*	Ca_Kabuli_Chr04	13845639	(A/C)
6082	CakSNP6082	*Kabuli*	Ca_Kabuli_Chr04	13845675	(T/A)

S.N.	SNP IDs	Cultivars	Chromosomes/scaffolds	Physical positions (bp)	SNPs
6083	CakSNP6083	*Kabuli*	Ca_Kabuli_Chr04	13900013	(A/G)
6084	CakSNP6084	*Kabuli*	Ca_Kabuli_Chr04	13923366	(T/C)
6085	CakSNP6085	*Kabuli*	Ca_Kabuli_Chr04	13949513	(A/C)
6086	CakSNP6086	*Kabuli*	Ca_Kabuli_Chr04	13949580	(C/G)
6087	CakSNP6087	*Kabuli*	Ca_Kabuli_Chr04	13954832	(G/A)
6088	CakSNP6088	*Kabuli*	Ca_Kabuli_Chr04	13968291	(C/T)
6089	CakSNP6089	*Kabuli*	Ca_Kabuli_Chr04	13968397	(A/G)
6090	CakSNP6090	*Kabuli*	Ca_Kabuli_Chr04	13968354	(A/G)
6091	CakSNP6091	*Kabuli*	Ca_Kabuli_Chr04	13982970	(G/A)
6092	CakSNP6092	*Kabuli*	Ca_Kabuli_Chr04	14031548	(A/G)
6093	CakSNP6093	*Kabuli*	Ca_Kabuli_Chr04	14056908	(A/G)
6094	CakSNP6094	*Kabuli*	Ca_Kabuli_Chr04	14073059	(C/T)
6095	CakSNP6095	*Kabuli*	Ca_Kabuli_Chr04	14073056	(C/T)
6096	CakSNP6096	*Kabuli*	Ca_Kabuli_Chr04	14073045	(G/A)
6097	CakSNP6097	*Kabuli*	Ca_Kabuli_Chr04	14073044	(C/T)
6098	CakSNP6098	*Kabuli*	Ca_Kabuli_Chr04	14073004	(C/A)
6099	CakSNP6099	*Kabuli*	Ca_Kabuli_Chr04	14082157	(C/A)
6100	CakSNP6100	*Kabuli*	Ca_Kabuli_Chr04	14082158	(T/G)
6101	CakSNP6101	*Kabuli*	Ca_Kabuli_Chr04	14082186	(G/A)
6102	CakSNP6102	*Kabuli*	Ca_Kabuli_Chr04	14082210	(T/C)
6103	CakSNP6103	*Kabuli*	Ca_Kabuli_Chr04	14082285	(C/G)
6104	CakSNP6104	*Kabuli*	Ca_Kabuli_Chr04	14093027	(A/G)
6105	CakSNP6105	*Kabuli*	Ca_Kabuli_Chr04	14104096	(A/C)
6106	CakSNP6106	*Kabuli*	Ca_Kabuli_Chr04	14112911	(C/A)
6107	CakSNP6107	*Kabuli*	Ca_Kabuli_Chr04	14132659	(A/C)
6108	CakSNP6108	*Kabuli*	Ca_Kabuli_Chr04	14149589	(G/A)
6109	CakSNP6109	*Kabuli*	Ca_Kabuli_Chr04	14149588	(A/G)
6110	CakSNP6110	*Kabuli*	Ca_Kabuli_Chr04	14155978	(A/G)
6111	CakSNP6111	*Kabuli*	Ca_Kabuli_Chr04	14156014	(C/T)
6112	CakSNP6112	*Kabuli*	Ca_Kabuli_Chr04	14163060	(C/T)
6113	CakSNP6113	*Kabuli*	Ca_Kabuli_Chr04	14181527	(A/G)
6114	CakSNP6114	*Kabuli*	Ca_Kabuli_Chr04	14189356	(A/C)
6115	CakSNP6115	*Kabuli*	Ca_Kabuli_Chr04	14199191	(G/C)
6116	CakSNP6116	*Kabuli*	Ca_Kabuli_Chr04	14225284	(A/G)
6117	CakSNP6117	*Kabuli*	Ca_Kabuli_Chr04	14270811	(G/A)
6118	CakSNP6118	*Kabuli*	Ca_Kabuli_Chr04	14270763	(G/A)
6119	CakSNP6119	*Kabuli*	Ca_Kabuli_Chr04	14453942	(T/C)
6120	CakSNP6120	*Kabuli*	Ca_Kabuli_Chr04	14515543	(T/A)
6121	CakSNP6121	*Kabuli*	Ca_Kabuli_Chr04	14515531	(T/A)

S.N.	SNP IDs	Cultivars	Chromosomes/scaffolds	Physical positions (bp)	SNPs
6122	CakSNP6122	*Kabuli*	Ca_Kabuli_Chr04	14517424	(C/T)
6123	CakSNP6123	*Kabuli*	Ca_Kabuli_Chr04	14529007	(A/G)
6124	CakSNP6124	*Kabuli*	Ca_Kabuli_Chr04	14565948	(A/C)
6125	CakSNP6125	*Kabuli*	Ca_Kabuli_Chr04	14566064	(G/A)
6126	CakSNP6126	*Kabuli*	Ca_Kabuli_Chr04	14566074	(T/A)
6127	CakSNP6127	*Kabuli*	Ca_Kabuli_Chr04	14566081	(A/G)
6128	CakSNP6128	*Kabuli*	Ca_Kabuli_Chr04	14611177	(A/G)
6129	CakSNP6129	*Kabuli*	Ca_Kabuli_Chr04	14657743	(C/T)
6130	CakSNP6130	*Kabuli*	Ca_Kabuli_Chr04	14696775	(T/C)
6131	CakSNP6131	*Kabuli*	Ca_Kabuli_Chr04	14698825	(A/T)
6132	CakSNP6132	*Kabuli*	Ca_Kabuli_Chr04	14699270	(T/C)
6133	CakSNP6133	*Kabuli*	Ca_Kabuli_Chr04	14748051	(A/C)
6134	CakSNP6134	*Kabuli*	Ca_Kabuli_Chr04	14770484	(C/T)
6135	CakSNP6135	*Kabuli*	Ca_Kabuli_Chr04	14770524	(G/C)
6136	CakSNP6136	*Kabuli*	Ca_Kabuli_Chr04	14770525	(G/A)
6137	CakSNP6137	*Kabuli*	Ca_Kabuli_Chr04	14770532	(C/T)
6138	CakSNP6138	*Kabuli*	Ca_Kabuli_Chr04	14785530	(G/A)
6139	CakSNP6139	*Kabuli*	Ca_Kabuli_Chr04	14785557	(A/C)
6140	CakSNP6140	*Kabuli*	Ca_Kabuli_Chr04	14786093	(C/G)
6141	CakSNP6141	*Kabuli*	Ca_Kabuli_Chr04	14831204	(A/T)
6142	CakSNP6142	*Kabuli*	Ca_Kabuli_Chr04	14831298	(A/T)
6143	CakSNP6143	*Kabuli*	Ca_Kabuli_Chr04	14855406	(T/C)
6144	CakSNP6144	*Kabuli*	Ca_Kabuli_Chr04	14855417	(T/A)
6145	CakSNP6145	*Kabuli*	Ca_Kabuli_Chr04	14870658	(T/G)
6146	CakSNP6146	*Kabuli*	Ca_Kabuli_Chr04	14870641	(G/A)
6147	CakSNP6147	*Kabuli*	Ca_Kabuli_Chr04	14989557	(C/T)
6148	CakSNP6148	*Kabuli*	Ca_Kabuli_Chr04	14989544	(C/T)
6149	CakSNP6149	*Kabuli*	Ca_Kabuli_Chr04	14989526	(G/A)
6150	CakSNP6150	*Kabuli*	Ca_Kabuli_Chr04	15025765	(T/A)
6151	CakSNP6151	*Kabuli*	Ca_Kabuli_Chr04	15025785	(T/G)
6152	CakSNP6152	*Kabuli*	Ca_Kabuli_Chr04	15026058	(C/T)
6153	CakSNP6153	*Kabuli*	Ca_Kabuli_Chr04	15036362	(A/G)
6154	CakSNP6154	*Kabuli*	Ca_Kabuli_Chr04	15039897	(C/G)
6155	CakSNP6155	*Kabuli*	Ca_Kabuli_Chr04	15039905	(T/C)
6156	CakSNP6156	*Kabuli*	Ca_Kabuli_Chr04	15039948	(G/C)
6157	CakSNP6157	*Kabuli*	Ca_Kabuli_Chr04	15039996	(C/T)
6158	CakSNP6158	*Kabuli*	Ca_Kabuli_Chr04	15040072	(A/C)
6159	CakSNP6159	*Kabuli*	Ca_Kabuli_Chr04	15053643	(T/C)
6160	CakSNP6160	*Kabuli*	Ca_Kabuli_Chr04	15075489	(T/G)

S.N.	SNP IDs	Cultivars	Chromosomes/scaffolds	Physical positions (bp)	SNPs
6161	CakSNP6161	*Kabuli*	Ca_Kabuli_Chr04	15075502	(G/T)
6162	CakSNP6162	*Kabuli*	Ca_Kabuli_Chr04	15159143	(A/G)
6163	CakSNP6163	*Kabuli*	Ca_Kabuli_Chr04	15190387	(T/G)
6164	CakSNP6164	*Kabuli*	Ca_Kabuli_Chr04	15190423	(T/A)
6165	CakSNP6165	*Kabuli*	Ca_Kabuli_Chr04	15212787	(G/A)
6166	CakSNP6166	*Kabuli*	Ca_Kabuli_Chr04	15240675	(G/A)
6167	CakSNP6167	*Kabuli*	Ca_Kabuli_Chr04	15240678	(T/C)
6168	CakSNP6168	*Kabuli*	Ca_Kabuli_Chr04	15299232	(G/A)
6169	CakSNP6169	*Kabuli*	Ca_Kabuli_Chr04	15320777	(G/A)
6170	CakSNP6170	*Kabuli*	Ca_Kabuli_Chr04	15357651	(C/T)
6171	CakSNP6171	*Kabuli*	Ca_Kabuli_Chr04	15357695	(A/G)
6172	CakSNP6172	*Kabuli*	Ca_Kabuli_Chr04	15394470	(T/C)
6173	CakSNP6173	*Kabuli*	Ca_Kabuli_Chr04	15448826	(G/T)
6174	CakSNP6174	*Kabuli*	Ca_Kabuli_Chr04	15449843	(A/G)
6175	CakSNP6175	*Kabuli*	Ca_Kabuli_Chr04	15542543	(T/C)
6176	CakSNP6176	*Kabuli*	Ca_Kabuli_Chr04	15542597	(A/G)
6177	CakSNP6177	*Kabuli*	Ca_Kabuli_Chr04	15552690	(C/T)
6178	CakSNP6178	*Kabuli*	Ca_Kabuli_Chr04	15552683	(C/T)
6179	CakSNP6179	*Kabuli*	Ca_Kabuli_Chr04	15697128	(A/G)
6180	CakSNP6180	*Kabuli*	Ca_Kabuli_Chr04	15697303	(G/T)
6181	CakSNP6181	*Kabuli*	Ca_Kabuli_Chr04	15698212	(G/T)
6182	CakSNP6182	*Kabuli*	Ca_Kabuli_Chr04	15698179	(G/A)
6183	CakSNP6183	*Kabuli*	Ca_Kabuli_Chr04	15698233	(A/C)
6184	CakSNP6184	*Kabuli*	Ca_Kabuli_Chr04	15698305	(T/C)
6185	CakSNP6185	*Kabuli*	Ca_Kabuli_Chr04	15698405	(G/A)
6186	CakSNP6186	*Kabuli*	Ca_Kabuli_Chr04	15698486	(T/C)
6187	CakSNP6187	*Kabuli*	Ca_Kabuli_Chr04	15698456	(T/C)
6188	CakSNP6188	*Kabuli*	Ca_Kabuli_Chr04	15698444	(C/T)
6189	CakSNP6189	*Kabuli*	Ca_Kabuli_Chr04	15698420	(G/C)
6190	CakSNP6190	*Kabuli*	Ca_Kabuli_Chr04	15700603	(A/T)
6191	CakSNP6191	*Kabuli*	Ca_Kabuli_Chr04	15700630	(G/A)
6192	CakSNP6192	*Kabuli*	Ca_Kabuli_Chr04	15713955	(G/A)
6193	CakSNP6193	*Kabuli*	Ca_Kabuli_Chr04	15717281	(T/C)
6194	CakSNP6194	*Kabuli*	Ca_Kabuli_Chr04	15735993	(T/C)
6195	CakSNP6195	*Kabuli*	Ca_Kabuli_Chr04	15736015	(C/T)
6196	CakSNP6196	*Kabuli*	Ca_Kabuli_Chr04	15736562	(A/T)
6197	CakSNP6197	*Kabuli*	Ca_Kabuli_Chr04	15736619	(T/G)
6198	CakSNP6198	*Kabuli*	Ca_Kabuli_Chr04	15736627	(T/C)
6199	CakSNP6199	*Kabuli*	Ca_Kabuli_Chr04	15744703	(T/G)

S.N.	SNP IDs	Cultivars	Chromosomes/scaffolds	Physical positions (bp)	SNPs
6200	CakSNP6200	*Kabuli*	Ca_Kabuli_Chr04	15752567	(G/C)
6201	CakSNP6201	*Kabuli*	Ca_Kabuli_Chr04	15766341	(A/G)
6202	CakSNP6202	*Kabuli*	Ca_Kabuli_Chr04	15766360	(G/A)
6203	CakSNP6203	*Kabuli*	Ca_Kabuli_Chr04	15772235	(C/A)
6204	CakSNP6204	*Kabuli*	Ca_Kabuli_Chr04	15772275	(A/G)
6205	CakSNP6205	*Kabuli*	Ca_Kabuli_Chr04	15821282	(G/A)
6206	CakSNP6206	*Kabuli*	Ca_Kabuli_Chr04	15821311	(T/A)
6207	CakSNP6207	*Kabuli*	Ca_Kabuli_Chr04	15825621	(A/T)
6208	CakSNP6208	*Kabuli*	Ca_Kabuli_Chr04	15860388	(A/G)
6209	CakSNP6209	*Kabuli*	Ca_Kabuli_Chr04	15860359	(C/A)
6210	CakSNP6210	*Kabuli*	Ca_Kabuli_Chr04	15900602	(G/A)
6211	CakSNP6211	*Kabuli*	Ca_Kabuli_Chr04	15925936	(T/G)
6212	CakSNP6212	*Kabuli*	Ca_Kabuli_Chr04	15926160	(A/G)
6213	CakSNP6213	*Kabuli*	Ca_Kabuli_Chr04	15934607	(A/T)
6214	CakSNP6214	*Kabuli*	Ca_Kabuli_Chr04	15934901	(T/G)
6215	CakSNP6215	*Kabuli*	Ca_Kabuli_Chr04	15957070	(T/A)
6216	CakSNP6216	*Kabuli*	Ca_Kabuli_Chr04	16027272	(G/A)
6217	CakSNP6217	*Kabuli*	Ca_Kabuli_Chr04	16027292	(A/C)
6218	CakSNP6218	*Kabuli*	Ca_Kabuli_Chr04	16027368	(G/T)
6219	CakSNP6219	*Kabuli*	Ca_Kabuli_Chr04	16027299	(T/A)
6220	CakSNP6220	*Kabuli*	Ca_Kabuli_Chr04	16045694	(T/G)
6221	CakSNP6221	*Kabuli*	Ca_Kabuli_Chr04	16045870	(G/A)
6222	CakSNP6222	*Kabuli*	Ca_Kabuli_Chr04	16046928	(T/C)
6223	CakSNP6223	*Kabuli*	Ca_Kabuli_Chr04	16278600	(C/T)
6224	CakSNP6224	*Kabuli*	Ca_Kabuli_Chr04	16278671	(T/A)
6225	CakSNP6225	*Kabuli*	Ca_Kabuli_Chr04	16446305	(T/C)
6226	CakSNP6226	*Kabuli*	Ca_Kabuli_Chr04	16446341	(T/G)
6227	CakSNP6227	*Kabuli*	Ca_Kabuli_Chr04	16446336	(C/G)
6228	CakSNP6228	*Kabuli*	Ca_Kabuli_Chr04	16446956	(T/C)
6229	CakSNP6229	*Kabuli*	Ca_Kabuli_Chr04	16457940	(G/A)
6230	CakSNP6230	*Kabuli*	Ca_Kabuli_Chr04	16501291	(T/A)
6231	CakSNP6231	*Kabuli*	Ca_Kabuli_Chr04	16501306	(T/A)
6232	CakSNP6232	*Kabuli*	Ca_Kabuli_Chr04	16501354	(C/T)
6233	CakSNP6233	*Kabuli*	Ca_Kabuli_Chr04	16501782	(G/A)
6234	CakSNP6234	*Kabuli*	Ca_Kabuli_Chr04	16501781	(C/A)
6235	CakSNP6235	*Kabuli*	Ca_Kabuli_Chr04	16501769	(C/T)
6236	CakSNP6236	*Kabuli*	Ca_Kabuli_Chr04	16501738	(G/A)
6237	CakSNP6237	*Kabuli*	Ca_Kabuli_Chr04	16501716	(T/C)
6238	CakSNP6238	*Kabuli*	Ca_Kabuli_Chr04	16505159	(A/G)

S.N.	SNP IDs	Cultivars	Chromosomes/scaffolds	Physical positions (bp)	SNPs
6239	CakSNP6239	*Kabuli*	Ca_Kabuli_Chr04	16536754	(C/T)
6240	CakSNP6240	*Kabuli*	Ca_Kabuli_Chr04	16571288	(T/C)
6241	CakSNP6241	*Kabuli*	Ca_Kabuli_Chr04	16586863	(G/C)
6242	CakSNP6242	*Kabuli*	Ca_Kabuli_Chr04	16586913	(G/C)
6243	CakSNP6243	*Kabuli*	Ca_Kabuli_Chr04	16586929	(G/C)
6244	CakSNP6244	*Kabuli*	Ca_Kabuli_Chr04	16619312	(T/C)
6245	CakSNP6245	*Kabuli*	Ca_Kabuli_Chr04	16622514	(C/A)
6246	CakSNP6246	*Kabuli*	Ca_Kabuli_Chr04	16625148	(T/G)
6247	CakSNP6247	*Kabuli*	Ca_Kabuli_Chr04	16625270	(T/G)
6248	CakSNP6248	*Kabuli*	Ca_Kabuli_Chr04	16625221	(G/A)
6249	CakSNP6249	*Kabuli*	Ca_Kabuli_Chr04	16628559	(T/G)
6250	CakSNP6250	*Kabuli*	Ca_Kabuli_Chr04	16628591	(T/G)
6251	CakSNP6251	*Kabuli*	Ca_Kabuli_Chr04	16628594	(T/G)
6252	CakSNP6252	*Kabuli*	Ca_Kabuli_Chr04	16628542	(G/A)
6253	CakSNP6253	*Kabuli*	Ca_Kabuli_Chr04	16628540	(G/A)
6254	CakSNP6254	*Kabuli*	Ca_Kabuli_Chr04	16628536	(T/A)
6255	CakSNP6255	*Kabuli*	Ca_Kabuli_Chr04	16628529	(C/A)
6256	CakSNP6256	*Kabuli*	Ca_Kabuli_Chr04	16628527	(G/A)
6257	CakSNP6257	*Kabuli*	Ca_Kabuli_Chr04	16628523	(T/C)
6258	CakSNP6258	*Kabuli*	Ca_Kabuli_Chr04	16629560	(G/T)
6259	CakSNP6259	*Kabuli*	Ca_Kabuli_Chr04	16629559	(T/G)
6260	CakSNP6260	*Kabuli*	Ca_Kabuli_Chr04	16629638	(C/A)
6261	CakSNP6261	*Kabuli*	Ca_Kabuli_Chr04	16630385	(G/T)
6262	CakSNP6262	*Kabuli*	Ca_Kabuli_Chr04	16669916	(T/C)
6263	CakSNP6263	*Kabuli*	Ca_Kabuli_Chr04	16749740	(A/G)
6264	CakSNP6264	*Kabuli*	Ca_Kabuli_Chr04	16750416	(A/G)
6265	CakSNP6265	*Kabuli*	Ca_Kabuli_Chr04	16751205	(T/C)
6266	CakSNP6266	*Kabuli*	Ca_Kabuli_Chr04	16789476	(G/A)
6267	CakSNP6267	*Kabuli*	Ca_Kabuli_Chr04	16789532	(A/C)
6268	CakSNP6268	*Kabuli*	Ca_Kabuli_Chr04	16820726	(C/A)
6269	CakSNP6269	*Kabuli*	Ca_Kabuli_Chr04	16837336	(T/A)
6270	CakSNP6270	*Kabuli*	Ca_Kabuli_Chr04	16839081	(T/A)
6271	CakSNP6271	*Kabuli*	Ca_Kabuli_Chr04	16845299	(G/A)
6272	CakSNP6272	*Kabuli*	Ca_Kabuli_Chr04	16889650	(A/T)
6273	CakSNP6273	*Kabuli*	Ca_Kabuli_Chr04	16926545	(T/C)
6274	CakSNP6274	*Kabuli*	Ca_Kabuli_Chr04	16926527	(T/A)
6275	CakSNP6275	*Kabuli*	Ca_Kabuli_Chr04	16926614	(A/T)
6276	CakSNP6276	*Kabuli*	Ca_Kabuli_Chr04	17028303	(A/G)
6277	CakSNP6277	*Kabuli*	Ca_Kabuli_Chr04	17028368	(G/A)

S.N.	SNP IDs	Cultivars	Chromosomes/scaffolds	Physical positions (bp)	SNPs
6278	CakSNP6278	*Kabuli*	Ca_Kabuli_Chr04	17028523	(C/T)
6279	CakSNP6279	*Kabuli*	Ca_Kabuli_Chr04	17028495	(T/A)
6280	CakSNP6280	*Kabuli*	Ca_Kabuli_Chr04	17032676	(T/C)
6281	CakSNP6281	*Kabuli*	Ca_Kabuli_Chr04	17032673	(C/T)
6282	CakSNP6282	*Kabuli*	Ca_Kabuli_Chr04	17059830	(G/A)
6283	CakSNP6283	*Kabuli*	Ca_Kabuli_Chr04	17059833	(A/T)
6284	CakSNP6284	*Kabuli*	Ca_Kabuli_Chr04	17059852	(T/C)
6285	CakSNP6285	*Kabuli*	Ca_Kabuli_Chr04	17059866	(G/A)
6286	CakSNP6286	*Kabuli*	Ca_Kabuli_Chr04	17059872	(A/G)
6287	CakSNP6287	*Kabuli*	Ca_Kabuli_Chr04	17059887	(G/A)
6288	CakSNP6288	*Kabuli*	Ca_Kabuli_Chr04	17093090	(C/G)
6289	CakSNP6289	*Kabuli*	Ca_Kabuli_Chr04	17147875	(C/T)
6290	CakSNP6290	*Kabuli*	Ca_Kabuli_Chr04	17147926	(T/C)
6291	CakSNP6291	*Kabuli*	Ca_Kabuli_Chr04	17187560	(C/G)
6292	CakSNP6292	*Kabuli*	Ca_Kabuli_Chr04	17191562	(T/A)
6293	CakSNP6293	*Kabuli*	Ca_Kabuli_Chr04	17199316	(T/C)
6294	CakSNP6294	*Kabuli*	Ca_Kabuli_Chr04	17199477	(G/A)
6295	CakSNP6295	*Kabuli*	Ca_Kabuli_Chr04	17199432	(G/A)
6296	CakSNP6296	*Kabuli*	Ca_Kabuli_Chr04	17202770	(A/G)
6297	CakSNP6297	*Kabuli*	Ca_Kabuli_Chr04	17225334	(G/A)
6298	CakSNP6298	*Kabuli*	Ca_Kabuli_Chr04	17264248	(C/A)
6299	CakSNP6299	*Kabuli*	Ca_Kabuli_Chr04	17264592	(G/A)
6300	CakSNP6300	*Kabuli*	Ca_Kabuli_Chr04	17264621	(C/G)
6301	CakSNP6301	*Kabuli*	Ca_Kabuli_Chr04	17264637	(C/T)
6302	CakSNP6302	*Kabuli*	Ca_Kabuli_Chr04	17282119	(T/C)
6303	CakSNP6303	*Kabuli*	Ca_Kabuli_Chr04	17282139	(G/C)
6304	CakSNP6304	*Kabuli*	Ca_Kabuli_Chr04	17282148	(G/C)
6305	CakSNP6305	*Kabuli*	Ca_Kabuli_Chr04	17282336	(G/T)
6306	CakSNP6306	*Kabuli*	Ca_Kabuli_Chr04	17282345	(G/C)
6307	CakSNP6307	*Kabuli*	Ca_Kabuli_Chr04	17289594	(G/C)
6308	CakSNP6308	*Kabuli*	Ca_Kabuli_Chr04	17297498	(G/T)
6309	CakSNP6309	*Kabuli*	Ca_Kabuli_Chr04	17297567	(G/A)
6310	CakSNP6310	*Kabuli*	Ca_Kabuli_Chr04	17297563	(A/G)
6311	CakSNP6311	*Kabuli*	Ca_Kabuli_Chr04	17309656	(A/C)
6312	CakSNP6312	*Kabuli*	Ca_Kabuli_Chr04	17345431	(T/C)
6313	CakSNP6313	*Kabuli*	Ca_Kabuli_Chr04	17345436	(G/T)
6314	CakSNP6314	*Kabuli*	Ca_Kabuli_Chr04	17345439	(G/A)
6315	CakSNP6315	*Kabuli*	Ca_Kabuli_Chr04	17345448	(T/G)
6316	CakSNP6316	*Kabuli*	Ca_Kabuli_Chr04	17345500	(G/A)

S.N.	SNP IDs	Cultivars	Chromosomes/scaffolds	Physical positions (bp)	SNPs
6317	CakSNP6317	*Kabuli*	Ca_Kabuli_Chr04	17345483	(C/A)
6318	CakSNP6318	*Kabuli*	Ca_Kabuli_Chr04	17345454	(A/G)
6319	CakSNP6319	*Kabuli*	Ca_Kabuli_Chr04	17358736	(C/T)
6320	CakSNP6320	*Kabuli*	Ca_Kabuli_Chr04	17361143	(T/A)
6321	CakSNP6321	*Kabuli*	Ca_Kabuli_Chr04	17396325	(A/G)
6322	CakSNP6322	*Kabuli*	Ca_Kabuli_Chr04	17396349	(T/G)
6323	CakSNP6323	*Kabuli*	Ca_Kabuli_Chr04	17396352	(T/G)
6324	CakSNP6324	*Kabuli*	Ca_Kabuli_Chr04	17429456	(A/T)
6325	CakSNP6325	*Kabuli*	Ca_Kabuli_Chr04	17429457	(A/T)
6326	CakSNP6326	*Kabuli*	Ca_Kabuli_Chr04	17429468	(T/C)
6327	CakSNP6327	*Kabuli*	Ca_Kabuli_Chr04	17429747	(G/A)
6328	CakSNP6328	*Kabuli*	Ca_Kabuli_Chr04	17429742	(A/G)
6329	CakSNP6329	*Kabuli*	Ca_Kabuli_Chr04	17429735	(A/G)
6330	CakSNP6330	*Kabuli*	Ca_Kabuli_Chr04	17429699	(G/C)
6331	CakSNP6331	*Kabuli*	Ca_Kabuli_Chr04	17433782	(G/A)
6332	CakSNP6332	*Kabuli*	Ca_Kabuli_Chr04	17433914	(G/A)
6333	CakSNP6333	*Kabuli*	Ca_Kabuli_Chr04	17444578	(A/C)
6334	CakSNP6334	*Kabuli*	Ca_Kabuli_Chr04	17472129	(T/A)
6335	CakSNP6335	*Kabuli*	Ca_Kabuli_Chr04	17472992	(T/G)
6336	CakSNP6336	*Kabuli*	Ca_Kabuli_Chr04	17473174	(C/T)
6337	CakSNP6337	*Kabuli*	Ca_Kabuli_Chr04	17473237	(T/C)
6338	CakSNP6338	*Kabuli*	Ca_Kabuli_Chr04	17473234	(C/A)
6339	CakSNP6339	*Kabuli*	Ca_Kabuli_Chr04	17603613	(T/C)
6340	CakSNP6340	*Kabuli*	Ca_Kabuli_Chr04	17603677	(G/C)
6341	CakSNP6341	*Kabuli*	Ca_Kabuli_Chr04	17603689	(T/C)
6342	CakSNP6342	*Kabuli*	Ca_Kabuli_Chr04	17603725	(T/C)
6343	CakSNP6343	*Kabuli*	Ca_Kabuli_Chr04	17609554	(A/T)
6344	CakSNP6344	*Kabuli*	Ca_Kabuli_Chr04	17621035	(G/T)
6345	CakSNP6345	*Kabuli*	Ca_Kabuli_Chr04	17621609	(G/A)
6346	CakSNP6346	*Kabuli*	Ca_Kabuli_Chr04	17647558	(G/A)
6347	CakSNP6347	*Kabuli*	Ca_Kabuli_Chr04	17652735	(T/C)
6348	CakSNP6348	*Kabuli*	Ca_Kabuli_Chr04	17685058	(T/G)
6349	CakSNP6349	*Kabuli*	Ca_Kabuli_Chr04	17685113	(C/A)
6350	CakSNP6350	*Kabuli*	Ca_Kabuli_Chr04	17718540	(C/T)
6351	CakSNP6351	*Kabuli*	Ca_Kabuli_Chr04	17718536	(C/A)
6352	CakSNP6352	*Kabuli*	Ca_Kabuli_Chr04	17718894	(G/A)
6353	CakSNP6353	*Kabuli*	Ca_Kabuli_Chr04	17718922	(T/G)
6354	CakSNP6354	*Kabuli*	Ca_Kabuli_Chr04	17718997	(C/A)
6355	CakSNP6355	*Kabuli*	Ca_Kabuli_Chr04	17729988	(A/C)

S.N.	SNP IDs	Cultivars	Chromosomes/scaffolds	Physical positions (bp)	SNPs
6356	CakSNP6356	*Kabuli*	Ca_Kabuli_Chr04	17748171	(A/T)
6357	CakSNP6357	*Kabuli*	Ca_Kabuli_Chr04	17752013	(C/T)
6358	CakSNP6358	*Kabuli*	Ca_Kabuli_Chr04	17760841	(A/G)
6359	CakSNP6359	*Kabuli*	Ca_Kabuli_Chr04	17760853	(T/G)
6360	CakSNP6360	*Kabuli*	Ca_Kabuli_Chr04	17768507	(T/C)
6361	CakSNP6361	*Kabuli*	Ca_Kabuli_Chr04	17771708	(C/G)
6362	CakSNP6362	*Kabuli*	Ca_Kabuli_Chr04	17784792	(T/A)
6363	CakSNP6363	*Kabuli*	Ca_Kabuli_Chr04	17826472	(G/A)
6364	CakSNP6364	*Kabuli*	Ca_Kabuli_Chr04	17826533	(A/C)
6365	CakSNP6365	*Kabuli*	Ca_Kabuli_Chr04	17826601	(T/C)
6366	CakSNP6366	*Kabuli*	Ca_Kabuli_Chr04	17826580	(C/G)
6367	CakSNP6367	*Kabuli*	Ca_Kabuli_Chr04	17826543	(T/C)
6368	CakSNP6368	*Kabuli*	Ca_Kabuli_Chr04	17843480	(G/T)
6369	CakSNP6369	*Kabuli*	Ca_Kabuli_Chr04	17847737	(G/A)
6370	CakSNP6370	*Kabuli*	Ca_Kabuli_Chr04	17847854	(T/C)
6371	CakSNP6371	*Kabuli*	Ca_Kabuli_Chr04	17856307	(T/C)
6372	CakSNP6372	*Kabuli*	Ca_Kabuli_Chr04	17856311	(T/G)
6373	CakSNP6373	*Kabuli*	Ca_Kabuli_Chr04	17856334	(A/G)
6374	CakSNP6374	*Kabuli*	Ca_Kabuli_Chr04	17857753	(C/T)
6375	CakSNP6375	*Kabuli*	Ca_Kabuli_Chr04	17858122	(T/G)
6376	CakSNP6376	*Kabuli*	Ca_Kabuli_Chr04	17858133	(C/T)
6377	CakSNP6377	*Kabuli*	Ca_Kabuli_Chr04	17858135	(G/A)
6378	CakSNP6378	*Kabuli*	Ca_Kabuli_Chr04	17858140	(A/C)
6379	CakSNP6379	*Kabuli*	Ca_Kabuli_Chr04	17858143	(A/T)
6380	CakSNP6380	*Kabuli*	Ca_Kabuli_Chr04	17858144	(G/C)
6381	CakSNP6381	*Kabuli*	Ca_Kabuli_Chr04	17858152	(A/T)
6382	CakSNP6382	*Kabuli*	Ca_Kabuli_Chr04	17858159	(C/A)
6383	CakSNP6383	*Kabuli*	Ca_Kabuli_Chr04	17858226	(C/T)
6384	CakSNP6384	*Kabuli*	Ca_Kabuli_Chr04	17871769	(C/A)
6385	CakSNP6385	*Kabuli*	Ca_Kabuli_Chr04	17871763	(T/C)
6386	CakSNP6386	*Kabuli*	Ca_Kabuli_Chr04	17871804	(T/C)
6387	CakSNP6387	*Kabuli*	Ca_Kabuli_Chr04	17871828	(A/C)
6388	CakSNP6388	*Kabuli*	Ca_Kabuli_Chr04	17871919	(A/G)
6389	CakSNP6389	*Kabuli*	Ca_Kabuli_Chr04	17871915	(T/A)
6390	CakSNP6390	*Kabuli*	Ca_Kabuli_Chr04	17913731	(A/C)
6391	CakSNP6391	*Kabuli*	Ca_Kabuli_Chr04	17913726	(G/A)
6392	CakSNP6392	*Kabuli*	Ca_Kabuli_Chr04	17913719	(G/C)
6393	CakSNP6393	*Kabuli*	Ca_Kabuli_Chr04	17913698	(C/T)
6394	CakSNP6394	*Kabuli*	Ca_Kabuli_Chr04	17913682	(C/A)

S.N.	SNP IDs	Cultivars	Chromosomes/scaffolds	Physical positions (bp)	SNPs
6395	CakSNP6395	*Kabuli*	Ca_Kabuli_Chr04	17920616	(C/G)
6396	CakSNP6396	*Kabuli*	Ca_Kabuli_Chr04	17931955	(G/A)
6397	CakSNP6397	*Kabuli*	Ca_Kabuli_Chr04	17970218	(T/G)
6398	CakSNP6398	*Kabuli*	Ca_Kabuli_Chr04	17970221	(C/T)
6399	CakSNP6399	*Kabuli*	Ca_Kabuli_Chr04	17970225	(T/C)
6400	CakSNP6400	*Kabuli*	Ca_Kabuli_Chr04	17970232	(A/G)
6401	CakSNP6401	*Kabuli*	Ca_Kabuli_Chr04	17970280	(T/A)
6402	CakSNP6402	*Kabuli*	Ca_Kabuli_Chr04	18053756	(A/G)
6403	CakSNP6403	*Kabuli*	Ca_Kabuli_Chr04	18070293	(G/T)
6404	CakSNP6404	*Kabuli*	Ca_Kabuli_Chr04	18071114	(C/A)
6405	CakSNP6405	*Kabuli*	Ca_Kabuli_Chr04	18071324	(G/A)
6406	CakSNP6406	*Kabuli*	Ca_Kabuli_Chr04	18089423	(G/C)
6407	CakSNP6407	*Kabuli*	Ca_Kabuli_Chr04	18089422	(T/C)
6408	CakSNP6408	*Kabuli*	Ca_Kabuli_Chr04	18117833	(T/C)
6409	CakSNP6409	*Kabuli*	Ca_Kabuli_Chr04	18222762	(T/C)
6410	CakSNP6410	*Kabuli*	Ca_Kabuli_Chr04	18231443	(T/C)
6411	CakSNP6411	*Kabuli*	Ca_Kabuli_Chr04	18231463	(T/C)
6412	CakSNP6412	*Kabuli*	Ca_Kabuli_Chr04	18231466	(T/C)
6413	CakSNP6413	*Kabuli*	Ca_Kabuli_Chr04	18242020	(C/G)
6414	CakSNP6414	*Kabuli*	Ca_Kabuli_Chr04	18241995	(C/G)
6415	CakSNP6415	*Kabuli*	Ca_Kabuli_Chr04	18427385	(T/G)
6416	CakSNP6416	*Kabuli*	Ca_Kabuli_Chr04	18427384	(C/T)
6417	CakSNP6417	*Kabuli*	Ca_Kabuli_Chr04	18429046	(G/T)
6418	CakSNP6418	*Kabuli*	Ca_Kabuli_Chr04	18429139	(C/T)
6419	CakSNP6419	*Kabuli*	Ca_Kabuli_Chr04	18485782	(T/C)
6420	CakSNP6420	*Kabuli*	Ca_Kabuli_Chr04	18489456	(G/C)
6421	CakSNP6421	*Kabuli*	Ca_Kabuli_Chr04	18495186	(A/G)
6422	CakSNP6422	*Kabuli*	Ca_Kabuli_Chr04	18545489	(A/T)
6423	CakSNP6423	*Kabuli*	Ca_Kabuli_Chr04	18546209	(T/G)
6424	CakSNP6424	*Kabuli*	Ca_Kabuli_Chr04	18625387	(G/A)
6425	CakSNP6425	*Kabuli*	Ca_Kabuli_Chr04	18628582	(A/G)
6426	CakSNP6426	*Kabuli*	Ca_Kabuli_Chr04	18710941	(T/G)
6427	CakSNP6427	*Kabuli*	Ca_Kabuli_Chr04	18719417	(A/G)
6428	CakSNP6428	*Kabuli*	Ca_Kabuli_Chr04	18721801	(G/A)
6429	CakSNP6429	*Kabuli*	Ca_Kabuli_Chr04	18779396	(A/C)
6430	CakSNP6430	*Kabuli*	Ca_Kabuli_Chr04	18812610	(T/C)
6431	CakSNP6431	*Kabuli*	Ca_Kabuli_Chr04	18880757	(A/G)
6432	CakSNP6432	*Kabuli*	Ca_Kabuli_Chr04	18904060	(G/A)
6433	CakSNP6433	*Kabuli*	Ca_Kabuli_Chr04	18912038	(G/C)

S.N.	SNP IDs	Cultivars	Chromosomes/scaffolds	Physical positions (bp)	SNPs
6434	CakSNP6434	*Kabuli*	Ca_Kabuli_Chr04	18912137	(T/C)
6435	CakSNP6435	*Kabuli*	Ca_Kabuli_Chr04	18912124	(C/A)
6436	CakSNP6436	*Kabuli*	Ca_Kabuli_Chr04	18912142	(A/G)
6437	CakSNP6437	*Kabuli*	Ca_Kabuli_Chr04	18923641	(G/T)
6438	CakSNP6438	*Kabuli*	Ca_Kabuli_Chr04	18953354	(C/A)
6439	CakSNP6439	*Kabuli*	Ca_Kabuli_Chr04	18953423	(A/C)
6440	CakSNP6440	*Kabuli*	Ca_Kabuli_Chr04	18953511	(C/G)
6441	CakSNP6441	*Kabuli*	Ca_Kabuli_Chr04	18998727	(C/T)
6442	CakSNP6442	*Kabuli*	Ca_Kabuli_Chr04	19005285	(T/G)
6443	CakSNP6443	*Kabuli*	Ca_Kabuli_Chr04	19005416	(T/G)
6444	CakSNP6444	*Kabuli*	Ca_Kabuli_Chr04	19006493	(C/T)
6445	CakSNP6445	*Kabuli*	Ca_Kabuli_Chr04	19024709	(C/T)
6446	CakSNP6446	*Kabuli*	Ca_Kabuli_Chr04	19024699	(T/C)
6447	CakSNP6447	*Kabuli*	Ca_Kabuli_Chr04	19206563	(A/G)
6448	CakSNP6448	*Kabuli*	Ca_Kabuli_Chr04	19206581	(C/T)
6449	CakSNP6449	*Kabuli*	Ca_Kabuli_Chr04	19206611	(T/C)
6450	CakSNP6450	*Kabuli*	Ca_Kabuli_Chr04	19206613	(T/G)
6451	CakSNP6451	*Kabuli*	Ca_Kabuli_Chr04	19206678	(A/T)
6452	CakSNP6452	*Kabuli*	Ca_Kabuli_Chr04	19207121	(A/T)
6453	CakSNP6453	*Kabuli*	Ca_Kabuli_Chr04	19227691	(G/C)
6454	CakSNP6454	*Kabuli*	Ca_Kabuli_Chr04	19263964	(G/C)
6455	CakSNP6455	*Kabuli*	Ca_Kabuli_Chr04	19263967	(T/G)
6456	CakSNP6456	*Kabuli*	Ca_Kabuli_Chr04	19267581	(A/G)
6457	CakSNP6457	*Kabuli*	Ca_Kabuli_Chr04	19481258	(A/G)
6458	CakSNP6458	*Kabuli*	Ca_Kabuli_Chr04	19540632	(A/C)
6459	CakSNP6459	*Kabuli*	Ca_Kabuli_Chr04	19540646	(T/G)
6460	CakSNP6460	*Kabuli*	Ca_Kabuli_Chr04	19579390	(G/T)
6461	CakSNP6461	*Kabuli*	Ca_Kabuli_Chr04	19579401	(C/A)
6462	CakSNP6462	*Kabuli*	Ca_Kabuli_Chr04	19579423	(A/G)
6463	CakSNP6463	*Kabuli*	Ca_Kabuli_Chr04	19579433	(G/A)
6464	CakSNP6464	*Kabuli*	Ca_Kabuli_Chr04	19579435	(A/G)
6465	CakSNP6465	*Kabuli*	Ca_Kabuli_Chr04	19579436	(A/T)
6466	CakSNP6466	*Kabuli*	Ca_Kabuli_Chr04	19579442	(C/A)
6467	CakSNP6467	*Kabuli*	Ca_Kabuli_Chr04	19649499	(A/G)
6468	CakSNP6468	*Kabuli*	Ca_Kabuli_Chr04	19668499	(T/C)
6469	CakSNP6469	*Kabuli*	Ca_Kabuli_Chr04	19668502	(T/G)
6470	CakSNP6470	*Kabuli*	Ca_Kabuli_Chr04	19668595	(T/C)
6471	CakSNP6471	*Kabuli*	Ca_Kabuli_Chr04	19688016	(A/G)
6472	CakSNP6472	*Kabuli*	Ca_Kabuli_Chr04	19712406	(C/T)

S.N.	SNP IDs	Cultivars	Chromosomes/scaffolds	Physical positions (bp)	SNPs
6473	CakSNP6473	*Kabuli*	Ca_Kabuli_Chr04	19713311	(T/G)
6474	CakSNP6474	*Kabuli*	Ca_Kabuli_Chr04	19734940	(G/A)
6475	CakSNP6475	*Kabuli*	Ca_Kabuli_Chr04	19844376	(A/G)
6476	CakSNP6476	*Kabuli*	Ca_Kabuli_Chr04	19869729	(G/T)
6477	CakSNP6477	*Kabuli*	Ca_Kabuli_Chr04	19869722	(T/G)
6478	CakSNP6478	*Kabuli*	Ca_Kabuli_Chr04	20218430	(T/G)
6479	CakSNP6479	*Kabuli*	Ca_Kabuli_Chr04	20308992	(C/A)
6480	CakSNP6480	*Kabuli*	Ca_Kabuli_Chr04	20309151	(C/T)
6481	CakSNP6481	*Kabuli*	Ca_Kabuli_Chr04	20398548	(T/G)
6482	CakSNP6482	*Kabuli*	Ca_Kabuli_Chr04	20398685	(A/C)
6483	CakSNP6483	*Kabuli*	Ca_Kabuli_Chr04	20402361	(A/G)
6484	CakSNP6484	*Kabuli*	Ca_Kabuli_Chr04	20402391	(A/G)
6485	CakSNP6485	*Kabuli*	Ca_Kabuli_Chr04	20412329	(A/T)
6486	CakSNP6486	*Kabuli*	Ca_Kabuli_Chr04	20436136	(G/A)
6487	CakSNP6487	*Kabuli*	Ca_Kabuli_Chr04	20444953	(T/C)
6488	CakSNP6488	*Kabuli*	Ca_Kabuli_Chr04	20599050	(A/G)
6489	CakSNP6489	*Kabuli*	Ca_Kabuli_Chr04	20599058	(T/C)
6490	CakSNP6490	*Kabuli*	Ca_Kabuli_Chr04	20599081	(C/T)
6491	CakSNP6491	*Kabuli*	Ca_Kabuli_Chr04	20599091	(T/G)
6492	CakSNP6492	*Kabuli*	Ca_Kabuli_Chr04	20656170	(T/G)
6493	CakSNP6493	*Kabuli*	Ca_Kabuli_Chr04	20669174	(T/C)
6494	CakSNP6494	*Kabuli*	Ca_Kabuli_Chr04	20669442	(G/A)
6495	CakSNP6495	*Kabuli*	Ca_Kabuli_Chr04	20696913	(A/G)
6496	CakSNP6496	*Kabuli*	Ca_Kabuli_Chr04	20696952	(G/T)
6497	CakSNP6497	*Kabuli*	Ca_Kabuli_Chr04	20696979	(C/T)
6498	CakSNP6498	*Kabuli*	Ca_Kabuli_Chr04	20697338	(T/A)
6499	CakSNP6499	*Kabuli*	Ca_Kabuli_Chr04	20697408	(A/G)
6500	CakSNP6500	*Kabuli*	Ca_Kabuli_Chr04	20774180	(C/T)
6501	CakSNP6501	*Kabuli*	Ca_Kabuli_Chr04	20905105	(G/A)
6502	CakSNP6502	*Kabuli*	Ca_Kabuli_Chr04	21096178	(G/A)
6503	CakSNP6503	*Kabuli*	Ca_Kabuli_Chr04	21385308	(A/T)
6504	CakSNP6504	*Kabuli*	Ca_Kabuli_Chr04	21830534	(C/T)
6505	CakSNP6505	*Kabuli*	Ca_Kabuli_Chr04	21835459	(T/A)
6506	CakSNP6506	*Kabuli*	Ca_Kabuli_Chr04	21835457	(T/A)
6507	CakSNP6507	*Kabuli*	Ca_Kabuli_Chr04	21835454	(C/A)
6508	CakSNP6508	*Kabuli*	Ca_Kabuli_Chr04	21835446	(T/A)
6509	CakSNP6509	*Kabuli*	Ca_Kabuli_Chr04	21931559	(A/G)
6510	CakSNP6510	*Kabuli*	Ca_Kabuli_Chr04	21931558	(G/A)
6511	CakSNP6511	*Kabuli*	Ca_Kabuli_Chr04	21931541	(C/T)

S.N.	SNP IDs	Cultivars	Chromosomes/scaffolds	Physical positions (bp)	SNPs
6512	CakSNP6512	*Kabuli*	Ca_Kabuli_Chr04	22079566	(G/A)
6513	CakSNP6513	*Kabuli*	Ca_Kabuli_Chr04	22237989	(A/T)
6514	CakSNP6514	*Kabuli*	Ca_Kabuli_Chr04	22237971	(C/T)
6515	CakSNP6515	*Kabuli*	Ca_Kabuli_Chr04	22288433	(T/C)
6516	CakSNP6516	*Kabuli*	Ca_Kabuli_Chr04	22293495	(T/C)
6517	CakSNP6517	*Kabuli*	Ca_Kabuli_Chr04	22850596	(C/G)
6518	CakSNP6518	*Kabuli*	Ca_Kabuli_Chr04	22859422	(G/A)
6519	CakSNP6519	*Kabuli*	Ca_Kabuli_Chr04	22860281	(A/C)
6520	CakSNP6520	*Kabuli*	Ca_Kabuli_Chr04	22865436	(A/T)
6521	CakSNP6521	*Kabuli*	Ca_Kabuli_Chr04	22865390	(G/A)
6522	CakSNP6522	*Kabuli*	Ca_Kabuli_Chr04	22865389	(C/T)
6523	CakSNP6523	*Kabuli*	Ca_Kabuli_Chr04	22908360	(A/G)
6524	CakSNP6524	*Kabuli*	Ca_Kabuli_Chr04	22971660	(G/A)
6525	CakSNP6525	*Kabuli*	Ca_Kabuli_Chr04	22971766	(A/G)
6526	CakSNP6526	*Kabuli*	Ca_Kabuli_Chr04	22971746	(G/A)
6527	CakSNP6527	*Kabuli*	Ca_Kabuli_Chr04	22971744	(A/C)
6528	CakSNP6528	*Kabuli*	Ca_Kabuli_Chr04	22971847	(C/A)
6529	CakSNP6529	*Kabuli*	Ca_Kabuli_Chr04	22971853	(C/A)
6530	CakSNP6530	*Kabuli*	Ca_Kabuli_Chr04	22971884	(G/A)
6531	CakSNP6531	*Kabuli*	Ca_Kabuli_Chr04	23157656	(C/G)
6532	CakSNP6532	*Kabuli*	Ca_Kabuli_Chr04	23267842	(T/C)
6533	CakSNP6533	*Kabuli*	Ca_Kabuli_Chr04	23267856	(C/T)
6534	CakSNP6534	*Kabuli*	Ca_Kabuli_Chr04	23267858	(C/T)
6535	CakSNP6535	*Kabuli*	Ca_Kabuli_Chr04	23267859	(T/C)
6536	CakSNP6536	*Kabuli*	Ca_Kabuli_Chr04	23314624	(C/T)
6537	CakSNP6537	*Kabuli*	Ca_Kabuli_Chr04	23314665	(A/C)
6538	CakSNP6538	*Kabuli*	Ca_Kabuli_Chr04	23318875	(T/C)
6539	CakSNP6539	*Kabuli*	Ca_Kabuli_Chr04	23319309	(G/C)
6540	CakSNP6540	*Kabuli*	Ca_Kabuli_Chr04	23333610	(C/T)
6541	CakSNP6541	*Kabuli*	Ca_Kabuli_Chr04	23333554	(T/C)
6542	CakSNP6542	*Kabuli*	Ca_Kabuli_Chr04	23337341	(T/C)
6543	CakSNP6543	*Kabuli*	Ca_Kabuli_Chr04	23337312	(T/G)
6544	CakSNP6544	*Kabuli*	Ca_Kabuli_Chr04	23481919	(G/A)
6545	CakSNP6545	*Kabuli*	Ca_Kabuli_Chr04	23481927	(G/T)
6546	CakSNP6546	*Kabuli*	Ca_Kabuli_Chr04	23481962	(G/A)
6547	CakSNP6547	*Kabuli*	Ca_Kabuli_Chr04	23482033	(A/G)
6548	CakSNP6548	*Kabuli*	Ca_Kabuli_Chr04	23481998	(T/C)
6549	CakSNP6549	*Kabuli*	Ca_Kabuli_Chr04	23486812	(A/G)
6550	CakSNP6550	*Kabuli*	Ca_Kabuli_Chr04	23486859	(A/G)

S.N.	SNP IDs	Cultivars	Chromosomes/scaffolds	Physical positions (bp)	SNPs
6551	CakSNP6551	*Kabuli*	Ca_Kabuli_Chr04	23486824	(T/C)
6552	CakSNP6552	*Kabuli*	Ca_Kabuli_Chr04	23671441	(T/G)
6553	CakSNP6553	*Kabuli*	Ca_Kabuli_Chr04	23671443	(A/T)
6554	CakSNP6554	*Kabuli*	Ca_Kabuli_Chr04	23731572	(C/T)
6555	CakSNP6555	*Kabuli*	Ca_Kabuli_Chr04	23732099	(A/T)
6556	CakSNP6556	*Kabuli*	Ca_Kabuli_Chr04	24050459	(G/A)
6557	CakSNP6557	*Kabuli*	Ca_Kabuli_Chr04	24050464	(G/A)
6558	CakSNP6558	*Kabuli*	Ca_Kabuli_Chr04	24120866	(A/G)
6559	CakSNP6559	*Kabuli*	Ca_Kabuli_Chr04	24544345	(G/A)
6560	CakSNP6560	*Kabuli*	Ca_Kabuli_Chr04	24614582	(G/T)
6561	CakSNP6561	*Kabuli*	Ca_Kabuli_Chr04	24729302	(T/C)
6562	CakSNP6562	*Kabuli*	Ca_Kabuli_Chr04	24732991	(G/T)
6563	CakSNP6563	*Kabuli*	Ca_Kabuli_Chr04	24732990	(T/C)
6564	CakSNP6564	*Kabuli*	Ca_Kabuli_Chr04	24771737	(T/A)
6565	CakSNP6565	*Kabuli*	Ca_Kabuli_Chr04	24782714	(C/A)
6566	CakSNP6566	*Kabuli*	Ca_Kabuli_Chr04	24782715	(A/G)
6567	CakSNP6567	*Kabuli*	Ca_Kabuli_Chr04	24856115	(A/C)
6568	CakSNP6568	*Kabuli*	Ca_Kabuli_Chr04	24857501	(A/G)
6569	CakSNP6569	*Kabuli*	Ca_Kabuli_Chr04	25401714	(T/C)
6570	CakSNP6570	*Kabuli*	Ca_Kabuli_Chr04	25446677	(G/C)
6571	CakSNP6571	*Kabuli*	Ca_Kabuli_Chr04	25446684	(T/C)
6572	CakSNP6572	*Kabuli*	Ca_Kabuli_Chr04	25449897	(T/G)
6573	CakSNP6573	*Kabuli*	Ca_Kabuli_Chr04	25455156	(G/A)
6574	CakSNP6574	*Kabuli*	Ca_Kabuli_Chr04	25455204	(A/G)
6575	CakSNP6575	*Kabuli*	Ca_Kabuli_Chr04	25455225	(G/A)
6576	CakSNP6576	*Kabuli*	Ca_Kabuli_Chr04	25592561	(T/C)
6577	CakSNP6577	*Kabuli*	Ca_Kabuli_Chr04	25592548	(A/G)
6578	CakSNP6578	*Kabuli*	Ca_Kabuli_Chr04	25592528	(A/C)
6579	CakSNP6579	*Kabuli*	Ca_Kabuli_Chr04	25592525	(C/A)
6580	CakSNP6580	*Kabuli*	Ca_Kabuli_Chr04	25592492	(T/C)
6581	CakSNP6581	*Kabuli*	Ca_Kabuli_Chr04	25592487	(A/G)
6582	CakSNP6582	*Kabuli*	Ca_Kabuli_Chr04	25592484	(A/C)
6583	CakSNP6583	*Kabuli*	Ca_Kabuli_Chr04	25592481	(T/C)
6584	CakSNP6584	*Kabuli*	Ca_Kabuli_Chr04	25729905	(C/A)
6585	CakSNP6585	*Kabuli*	Ca_Kabuli_Chr04	25729914	(C/A)
6586	CakSNP6586	*Kabuli*	Ca_Kabuli_Chr04	25729974	(G/C)
6587	CakSNP6587	*Kabuli*	Ca_Kabuli_Chr04	25729977	(C/T)
6588	CakSNP6588	*Kabuli*	Ca_Kabuli_Chr04	25747256	(T/C)
6589	CakSNP6589	*Kabuli*	Ca_Kabuli_Chr04	25747262	(G/A)

S.N.	SNP IDs	Cultivars	Chromosomes/scaffolds	Physical positions (bp)	SNPs
6590	CakSNP6590	*Kabuli*	Ca_Kabuli_Chr04	25747274	(T/G)
6591	CakSNP6591	*Kabuli*	Ca_Kabuli_Chr04	25747275	(G/A)
6592	CakSNP6592	*Kabuli*	Ca_Kabuli_Chr04	25747281	(T/C)
6593	CakSNP6593	*Kabuli*	Ca_Kabuli_Chr04	25747295	(G/A)
6594	CakSNP6594	*Kabuli*	Ca_Kabuli_Chr04	25747296	(T/C)
6595	CakSNP6595	*Kabuli*	Ca_Kabuli_Chr04	25747364	(T/A)
6596	CakSNP6596	*Kabuli*	Ca_Kabuli_Chr04	25747297	(G/A)
6597	CakSNP6597	*Kabuli*	Ca_Kabuli_Chr04	25809665	(T/C)
6598	CakSNP6598	*Kabuli*	Ca_Kabuli_Chr04	25921165	(T/C)
6599	CakSNP6599	*Kabuli*	Ca_Kabuli_Chr04	26000532	(C/T)
6600	CakSNP6600	*Kabuli*	Ca_Kabuli_Chr04	26000483	(T/A)
6601	CakSNP6601	*Kabuli*	Ca_Kabuli_Chr04	26002991	(C/A)
6602	CakSNP6602	*Kabuli*	Ca_Kabuli_Chr04	26008744	(G/A)
6603	CakSNP6603	*Kabuli*	Ca_Kabuli_Chr04	26008745	(G/A)
6604	CakSNP6604	*Kabuli*	Ca_Kabuli_Chr04	26008748	(C/T)
6605	CakSNP6605	*Kabuli*	Ca_Kabuli_Chr04	26008768	(A/G)
6606	CakSNP6606	*Kabuli*	Ca_Kabuli_Chr04	26008816	(C/A)
6607	CakSNP6607	*Kabuli*	Ca_Kabuli_Chr04	26009438	(G/T)
6608	CakSNP6608	*Kabuli*	Ca_Kabuli_Chr04	26009439	(G/T)
6609	CakSNP6609	*Kabuli*	Ca_Kabuli_Chr04	26222180	(A/G)
6610	CakSNP6610	*Kabuli*	Ca_Kabuli_Chr04	26282236	(A/C)
6611	CakSNP6611	*Kabuli*	Ca_Kabuli_Chr04	26324460	(T/C)
6612	CakSNP6612	*Kabuli*	Ca_Kabuli_Chr04	26529606	(C/T)
6613	CakSNP6613	*Kabuli*	Ca_Kabuli_Chr04	26532763	(T/G)
6614	CakSNP6614	*Kabuli*	Ca_Kabuli_Chr04	26536919	(C/G)
6615	CakSNP6615	*Kabuli*	Ca_Kabuli_Chr04	26537053	(C/T)
6616	CakSNP6616	*Kabuli*	Ca_Kabuli_Chr04	26537258	(A/C)
6617	CakSNP6617	*Kabuli*	Ca_Kabuli_Chr04	26869756	(A/G)
6618	CakSNP6618	*Kabuli*	Ca_Kabuli_Chr04	26915602	(G/A)
6619	CakSNP6619	*Kabuli*	Ca_Kabuli_Chr04	26915615	(A/G)
6620	CakSNP6620	*Kabuli*	Ca_Kabuli_Chr04	26915621	(G/A)
6621	CakSNP6621	*Kabuli*	Ca_Kabuli_Chr04	26915626	(C/T)
6622	CakSNP6622	*Kabuli*	Ca_Kabuli_Chr04	26989085	(G/A)
6623	CakSNP6623	*Kabuli*	Ca_Kabuli_Chr04	26989090	(G/T)
6624	CakSNP6624	*Kabuli*	Ca_Kabuli_Chr04	26989204	(A/C)
6625	CakSNP6625	*Kabuli*	Ca_Kabuli_Chr04	27102807	(G/A)
6626	CakSNP6626	*Kabuli*	Ca_Kabuli_Chr04	27102817	(A/C)
6627	CakSNP6627	*Kabuli*	Ca_Kabuli_Chr04	27102833	(G/T)
6628	CakSNP6628	*Kabuli*	Ca_Kabuli_Chr04	27262241	(C/T)

S.N.	SNP IDs	Cultivars	Chromosomes/scaffolds	Physical positions (bp)	SNPs
6629	CakSNP6629	*Kabuli*	Ca_Kabuli_Chr04	27262893	(G/A)
6630	CakSNP6630	*Kabuli*	Ca_Kabuli_Chr04	27262870	(C/T)
6631	CakSNP6631	*Kabuli*	Ca_Kabuli_Chr04	27262830	(T/A)
6632	CakSNP6632	*Kabuli*	Ca_Kabuli_Chr04	27268301	(G/A)
6633	CakSNP6633	*Kabuli*	Ca_Kabuli_Chr04	27268278	(C/T)
6634	CakSNP6634	*Kabuli*	Ca_Kabuli_Chr04	27493010	(G/T)
6635	CakSNP6635	*Kabuli*	Ca_Kabuli_Chr04	27613450	(C/T)
6636	CakSNP6636	*Kabuli*	Ca_Kabuli_Chr04	27617803	(A/G)
6637	CakSNP6637	*Kabuli*	Ca_Kabuli_Chr04	27626559	(G/A)
6638	CakSNP6638	*Kabuli*	Ca_Kabuli_Chr04	27626999	(C/T)
6639	CakSNP6639	*Kabuli*	Ca_Kabuli_Chr04	27661241	(T/G)
6640	CakSNP6640	*Kabuli*	Ca_Kabuli_Chr04	27669829	(C/G)
6641	CakSNP6641	*Kabuli*	Ca_Kabuli_Chr04	27777968	(A/C)
6642	CakSNP6642	*Kabuli*	Ca_Kabuli_Chr04	27786162	(G/C)
6643	CakSNP6643	*Kabuli*	Ca_Kabuli_Chr04	27970989	(T/G)
6644	CakSNP6644	*Kabuli*	Ca_Kabuli_Chr04	28061002	(A/C)
6645	CakSNP6645	*Kabuli*	Ca_Kabuli_Chr04	28092609	(G/A)
6646	CakSNP6646	*Kabuli*	Ca_Kabuli_Chr04	28092727	(T/A)
6647	CakSNP6647	*Kabuli*	Ca_Kabuli_Chr04	28092725	(T/G)
6648	CakSNP6648	*Kabuli*	Ca_Kabuli_Chr04	28092724	(T/A)
6649	CakSNP6649	*Kabuli*	Ca_Kabuli_Chr04	28268788	(G/A)
6650	CakSNP6650	*Kabuli*	Ca_Kabuli_Chr04	28289900	(C/A)
6651	CakSNP6651	*Kabuli*	Ca_Kabuli_Chr04	28299238	(G/A)
6652	CakSNP6652	*Kabuli*	Ca_Kabuli_Chr04	28301853	(T/C)
6653	CakSNP6653	*Kabuli*	Ca_Kabuli_Chr04	28423079	(T/C)
6654	CakSNP6654	*Kabuli*	Ca_Kabuli_Chr04	28423140	(A/G)
6655	CakSNP6655	*Kabuli*	Ca_Kabuli_Chr04	28431706	(G/A)
6656	CakSNP6656	*Kabuli*	Ca_Kabuli_Chr04	28790085	(A/G)
6657	CakSNP6657	*Kabuli*	Ca_Kabuli_Chr04	29582296	(C/T)
6658	CakSNP6658	*Kabuli*	Ca_Kabuli_Chr04	29582289	(G/A)
6659	CakSNP6659	*Kabuli*	Ca_Kabuli_Chr04	29582285	(C/A)
6660	CakSNP6660	*Kabuli*	Ca_Kabuli_Chr04	29582282	(C/A)
6661	CakSNP6661	*Kabuli*	Ca_Kabuli_Chr04	29582273	(G/A)
6662	CakSNP6662	*Kabuli*	Ca_Kabuli_Chr04	29582268	(C/G)
6663	CakSNP6663	*Kabuli*	Ca_Kabuli_Chr04	29582257	(G/A)
6664	CakSNP6664	*Kabuli*	Ca_Kabuli_Chr04	29582255	(C/T)
6665	CakSNP6665	*Kabuli*	Ca_Kabuli_Chr04	29582252	(G/A)
6666	CakSNP6666	*Kabuli*	Ca_Kabuli_Chr04	29582249	(C/T)
6667	CakSNP6667	*Kabuli*	Ca_Kabuli_Chr04	29582235	(T/A)

S.N.	SNP IDs	Cultivars	Chromosomes/scaffolds	Physical positions (bp)	SNPs
6668	CakSNP6668	*Kabuli*	Ca_Kabuli_Chr04	29582232	(C/T)
6669	CakSNP6669	*Kabuli*	Ca_Kabuli_Chr04	29582227	(G/A)
6670	CakSNP6670	*Kabuli*	Ca_Kabuli_Chr04	29582219	(A/C)
6671	CakSNP6671	*Kabuli*	Ca_Kabuli_Chr04	29582271	(C/T)
6672	CakSNP6672	*Kabuli*	Ca_Kabuli_Chr04	29582286	(T/A)
6673	CakSNP6673	*Kabuli*	Ca_Kabuli_Chr04	29582294	(G/A)
6674	CakSNP6674	*Kabuli*	Ca_Kabuli_Chr04	29582301	(G/T)
6675	CakSNP6675	*Kabuli*	Ca_Kabuli_Chr04	29671563	(G/A)
6676	CakSNP6676	*Kabuli*	Ca_Kabuli_Chr04	29671606	(A/G)
6677	CakSNP6677	*Kabuli*	Ca_Kabuli_Chr04	29683718	(A/T)
6678	CakSNP6678	*Kabuli*	Ca_Kabuli_Chr04	29923380	(G/A)
6679	CakSNP6679	*Kabuli*	Ca_Kabuli_Chr04	29958122	(T/G)
6680	CakSNP6680	*Kabuli*	Ca_Kabuli_Chr04	29958113	(G/A)
6681	CakSNP6681	*Kabuli*	Ca_Kabuli_Chr04	29972268	(T/G)
6682	CakSNP6682	*Kabuli*	Ca_Kabuli_Chr04	30066903	(G/A)
6683	CakSNP6683	*Kabuli*	Ca_Kabuli_Chr04	30153390	(G/A)
6684	CakSNP6684	*Kabuli*	Ca_Kabuli_Chr04	30153448	(T/C)
6685	CakSNP6685	*Kabuli*	Ca_Kabuli_Chr04	30153505	(C/G)
6686	CakSNP6686	*Kabuli*	Ca_Kabuli_Chr04	30258044	(A/T)
6687	CakSNP6687	*Kabuli*	Ca_Kabuli_Chr04	30258045	(A/T)
6688	CakSNP6688	*Kabuli*	Ca_Kabuli_Chr04	30258056	(A/G)
6689	CakSNP6689	*Kabuli*	Ca_Kabuli_Chr04	30258484	(G/C)
6690	CakSNP6690	*Kabuli*	Ca_Kabuli_Chr04	30258613	(C/A)
6691	CakSNP6691	*Kabuli*	Ca_Kabuli_Chr04	30258607	(C/A)
6692	CakSNP6692	*Kabuli*	Ca_Kabuli_Chr04	30258604	(C/A)
6693	CakSNP6693	*Kabuli*	Ca_Kabuli_Chr04	30258596	(G/C)
6694	CakSNP6694	*Kabuli*	Ca_Kabuli_Chr04	30258567	(C/A)
6695	CakSNP6695	*Kabuli*	Ca_Kabuli_Chr04	30260485	(C/A)
6696	CakSNP6696	*Kabuli*	Ca_Kabuli_Chr04	30281991	(A/G)
6697	CakSNP6697	*Kabuli*	Ca_Kabuli_Chr04	30301002	(T/G)
6698	CakSNP6698	*Kabuli*	Ca_Kabuli_Chr04	30308719	(A/C)
6699	CakSNP6699	*Kabuli*	Ca_Kabuli_Chr04	30308812	(G/A)
6700	CakSNP6700	*Kabuli*	Ca_Kabuli_Chr04	30308788	(T/C)
6701	CakSNP6701	*Kabuli*	Ca_Kabuli_Chr04	30463063	(T/C)
6702	CakSNP6702	*Kabuli*	Ca_Kabuli_Chr04	30463117	(C/A)
6703	CakSNP6703	*Kabuli*	Ca_Kabuli_Chr04	30599096	(C/A)
6704	CakSNP6704	*Kabuli*	Ca_Kabuli_Chr04	30599188	(C/T)
6705	CakSNP6705	*Kabuli*	Ca_Kabuli_Chr04	30667916	(G/A)
6706	CakSNP6706	*Kabuli*	Ca_Kabuli_Chr04	30668893	(A/C)

S.N.	SNP IDs	Cultivars	Chromosomes/scaffolds	Physical positions (bp)	SNPs
6707	CakSNP6707	*Kabuli*	Ca_Kabuli_Chr04	30851027	(A/T)
6708	CakSNP6708	*Kabuli*	Ca_Kabuli_Chr04	30851411	(T/G)
6709	CakSNP6709	*Kabuli*	Ca_Kabuli_Chr04	30851840	(A/T)
6710	CakSNP6710	*Kabuli*	Ca_Kabuli_Chr04	30931586	(G/T)
6711	CakSNP6711	*Kabuli*	Ca_Kabuli_Chr04	31218647	(T/C)
6712	CakSNP6712	*Kabuli*	Ca_Kabuli_Chr04	31221264	(T/G)
6713	CakSNP6713	*Kabuli*	Ca_Kabuli_Chr04	31221253	(G/A)
6714	CakSNP6714	*Kabuli*	Ca_Kabuli_Chr04	31221226	(T/A)
6715	CakSNP6715	*Kabuli*	Ca_Kabuli_Chr04	31224001	(G/A)
6716	CakSNP6716	*Kabuli*	Ca_Kabuli_Chr04	31262596	(T/A)
6717	CakSNP6717	*Kabuli*	Ca_Kabuli_Chr04	31263205	(T/A)
6718	CakSNP6718	*Kabuli*	Ca_Kabuli_Chr04	31594126	(A/C)
6719	CakSNP6719	*Kabuli*	Ca_Kabuli_Chr04	31618949	(T/A)
6720	CakSNP6720	*Kabuli*	Ca_Kabuli_Chr04	31875713	(A/G)
6721	CakSNP6721	*Kabuli*	Ca_Kabuli_Chr04	31987890	(A/C)
6722	CakSNP6722	*Kabuli*	Ca_Kabuli_Chr04	32042488	(T/G)
6723	CakSNP6723	*Kabuli*	Ca_Kabuli_Chr04	32042527	(C/T)
6724	CakSNP6724	*Kabuli*	Ca_Kabuli_Chr04	32068961	(A/G)
6725	CakSNP6725	*Kabuli*	Ca_Kabuli_Chr04	32073552	(T/C)
6726	CakSNP6726	*Kabuli*	Ca_Kabuli_Chr04	32073550	(C/G)
6727	CakSNP6727	*Kabuli*	Ca_Kabuli_Chr04	32073549	(G/C)
6728	CakSNP6728	*Kabuli*	Ca_Kabuli_Chr04	32166559	(C/G)
6729	CakSNP6729	*Kabuli*	Ca_Kabuli_Chr04	32166557	(T/A)
6730	CakSNP6730	*Kabuli*	Ca_Kabuli_Chr04	32183307	(C/G)
6731	CakSNP6731	*Kabuli*	Ca_Kabuli_Chr04	32184628	(A/G)
6732	CakSNP6732	*Kabuli*	Ca_Kabuli_Chr04	32212899	(G/A)
6733	CakSNP6733	*Kabuli*	Ca_Kabuli_Chr04	32297780	(C/A)
6734	CakSNP6734	*Kabuli*	Ca_Kabuli_Chr04	32297758	(G/A)
6735	CakSNP6735	*Kabuli*	Ca_Kabuli_Chr04	32297734	(A/G)
6736	CakSNP6736	*Kabuli*	Ca_Kabuli_Chr04	32303394	(C/T)
6737	CakSNP6737	*Kabuli*	Ca_Kabuli_Chr04	32429228	(A/T)
6738	CakSNP6738	*Kabuli*	Ca_Kabuli_Chr04	32429222	(A/C)
6739	CakSNP6739	*Kabuli*	Ca_Kabuli_Chr04	32521346	(T/C)
6740	CakSNP6740	*Kabuli*	Ca_Kabuli_Chr04	32602658	(C/T)
6741	CakSNP6741	*Kabuli*	Ca_Kabuli_Chr04	32604261	(G/A)
6742	CakSNP6742	*Kabuli*	Ca_Kabuli_Chr04	32604538	(T/C)
6743	CakSNP6743	*Kabuli*	Ca_Kabuli_Chr04	32604510	(A/C)
6744	CakSNP6744	*Kabuli*	Ca_Kabuli_Chr04	32636315	(T/C)
6745	CakSNP6745	*Kabuli*	Ca_Kabuli_Chr04	32636276	(T/C)

S.N.	SNP IDs	Cultivars	Chromosomes/scaffolds	Physical positions (bp)	SNPs
6746	CakSNP6746	*Kabuli*	Ca_Kabuli_Chr04	32636428	(C/A)
6747	CakSNP6747	*Kabuli*	Ca_Kabuli_Chr04	32636459	(A/G)
6748	CakSNP6748	*Kabuli*	Ca_Kabuli_Chr04	32636690	(C/T)
6749	CakSNP6749	*Kabuli*	Ca_Kabuli_Chr04	32636953	(A/G)
6750	CakSNP6750	*Kabuli*	Ca_Kabuli_Chr04	32639121	(C/T)
6751	CakSNP6751	*Kabuli*	Ca_Kabuli_Chr04	32639188	(C/G)
6752	CakSNP6752	*Kabuli*	Ca_Kabuli_Chr04	32639118	(A/C)
6753	CakSNP6753	*Kabuli*	Ca_Kabuli_Chr04	32639262	(A/G)
6754	CakSNP6754	*Kabuli*	Ca_Kabuli_Chr04	32639315	(A/G)
6755	CakSNP6755	*Kabuli*	Ca_Kabuli_Chr04	32639654	(T/C)
6756	CakSNP6756	*Kabuli*	Ca_Kabuli_Chr04	32639681	(C/T)
6757	CakSNP6757	*Kabuli*	Ca_Kabuli_Chr04	32639770	(C/G)
6758	CakSNP6758	*Kabuli*	Ca_Kabuli_Chr04	32640911	(A/G)
6759	CakSNP6759	*Kabuli*	Ca_Kabuli_Chr04	32853834	(T/C)
6760	CakSNP6760	*Kabuli*	Ca_Kabuli_Chr04	32853838	(G/T)
6761	CakSNP6761	*Kabuli*	Ca_Kabuli_Chr04	33068276	(G/A)
6762	CakSNP6762	*Kabuli*	Ca_Kabuli_Chr04	33221361	(T/C)
6763	CakSNP6763	*Kabuli*	Ca_Kabuli_Chr04	33221371	(T/C)
6764	CakSNP6764	*Kabuli*	Ca_Kabuli_Chr04	33221397	(T/G)
6765	CakSNP6765	*Kabuli*	Ca_Kabuli_Chr04	33509759	(A/C)
6766	CakSNP6766	*Kabuli*	Ca_Kabuli_Chr04	33509762	(G/A)
6767	CakSNP6767	*Kabuli*	Ca_Kabuli_Chr04	33509804	(A/G)
6768	CakSNP6768	*Kabuli*	Ca_Kabuli_Chr04	33641757	(C/T)
6769	CakSNP6769	*Kabuli*	Ca_Kabuli_Chr04	33820657	(A/C)
6770	CakSNP6770	*Kabuli*	Ca_Kabuli_Chr04	33874346	(T/C)
6771	CakSNP6771	*Kabuli*	Ca_Kabuli_Chr04	33874327	(T/C)
6772	CakSNP6772	*Kabuli*	Ca_Kabuli_Chr04	33874313	(G/A)
6773	CakSNP6773	*Kabuli*	Ca_Kabuli_Chr04	33929598	(A/C)
6774	CakSNP6774	*Kabuli*	Ca_Kabuli_Chr04	33929777	(C/T)
6775	CakSNP6775	*Kabuli*	Ca_Kabuli_Chr04	34032176	(C/T)
6776	CakSNP6776	*Kabuli*	Ca_Kabuli_Chr04	34155135	(A/C)
6777	CakSNP6777	*Kabuli*	Ca_Kabuli_Chr04	34155131	(T/A)
6778	CakSNP6778	*Kabuli*	Ca_Kabuli_Chr04	34155128	(C/T)
6779	CakSNP6779	*Kabuli*	Ca_Kabuli_Chr04	34155127	(G/A)
6780	CakSNP6780	*Kabuli*	Ca_Kabuli_Chr04	34155124	(G/A)
6781	CakSNP6781	*Kabuli*	Ca_Kabuli_Chr04	34155084	(C/G)
6782	CakSNP6782	*Kabuli*	Ca_Kabuli_Chr04	34155083	(C/T)
6783	CakSNP6783	*Kabuli*	Ca_Kabuli_Chr04	34222792	(G/C)
6784	CakSNP6784	*Kabuli*	Ca_Kabuli_Chr04	34354519	(G/T)

S.N.	SNP IDs	Cultivars	Chromosomes/scaffolds	Physical positions (bp)	SNPs
6785	CakSNP6785	*Kabuli*	Ca_Kabuli_Chr04	34487064	(T/G)
6786	CakSNP6786	*Kabuli*	Ca_Kabuli_Chr04	34744344	(T/G)
6787	CakSNP6787	*Kabuli*	Ca_Kabuli_Chr04	34744398	(T/A)
6788	CakSNP6788	*Kabuli*	Ca_Kabuli_Chr04	34784285	(G/A)
6789	CakSNP6789	*Kabuli*	Ca_Kabuli_Chr04	34798597	(C/A)
6790	CakSNP6790	*Kabuli*	Ca_Kabuli_Chr04	34916032	(C/T)
6791	CakSNP6791	*Kabuli*	Ca_Kabuli_Chr04	34924575	(C/T)
6792	CakSNP6792	*Kabuli*	Ca_Kabuli_Chr04	34955953	(A/G)
6793	CakSNP6793	*Kabuli*	Ca_Kabuli_Chr04	34955971	(A/T)
6794	CakSNP6794	*Kabuli*	Ca_Kabuli_Chr04	34956006	(C/T)
6795	CakSNP6795	*Kabuli*	Ca_Kabuli_Chr04	34956019	(G/A)
6796	CakSNP6796	*Kabuli*	Ca_Kabuli_Chr04	34956035	(C/T)
6797	CakSNP6797	*Kabuli*	Ca_Kabuli_Chr04	34956111	(G/T)
6798	CakSNP6798	*Kabuli*	Ca_Kabuli_Chr04	34956031	(G/A)
6799	CakSNP6799	*Kabuli*	Ca_Kabuli_Chr04	35141281	(A/T)
6800	CakSNP6800	*Kabuli*	Ca_Kabuli_Chr04	35141309	(A/C)
6801	CakSNP6801	*Kabuli*	Ca_Kabuli_Chr04	35141340	(G/A)
6802	CakSNP6802	*Kabuli*	Ca_Kabuli_Chr04	35141353	(C/G)
6803	CakSNP6803	*Kabuli*	Ca_Kabuli_Chr04	35141359	(C/G)
6804	CakSNP6804	*Kabuli*	Ca_Kabuli_Chr04	35141375	(A/G)
6805	CakSNP6805	*Kabuli*	Ca_Kabuli_Chr04	35141403	(C/T)
6806	CakSNP6806	*Kabuli*	Ca_Kabuli_Chr04	35141425	(A/T)
6807	CakSNP6807	*Kabuli*	Ca_Kabuli_Chr04	35141421	(T/A)
6808	CakSNP6808	*Kabuli*	Ca_Kabuli_Chr04	35141408	(A/G)
6809	CakSNP6809	*Kabuli*	Ca_Kabuli_Chr04	35168915	(C/T)
6810	CakSNP6810	*Kabuli*	Ca_Kabuli_Chr04	35168939	(C/T)
6811	CakSNP6811	*Kabuli*	Ca_Kabuli_Chr04	35229966	(C/T)
6812	CakSNP6812	*Kabuli*	Ca_Kabuli_Chr04	35230144	(A/G)
6813	CakSNP6813	*Kabuli*	Ca_Kabuli_Chr04	35231891	(C/T)
6814	CakSNP6814	*Kabuli*	Ca_Kabuli_Chr04	35304102	(C/T)
6815	CakSNP6815	*Kabuli*	Ca_Kabuli_Chr04	35304108	(C/A)
6816	CakSNP6816	*Kabuli*	Ca_Kabuli_Chr04	35309709	(A/G)
6817	CakSNP6817	*Kabuli*	Ca_Kabuli_Chr04	35311815	(A/C)
6818	CakSNP6818	*Kabuli*	Ca_Kabuli_Chr04	35337282	(A/G)
6819	CakSNP6819	*Kabuli*	Ca_Kabuli_Chr04	35344356	(A/G)
6820	CakSNP6820	*Kabuli*	Ca_Kabuli_Chr04	35344353	(T/C)
6821	CakSNP6821	*Kabuli*	Ca_Kabuli_Chr04	35344323	(C/T)
6822	CakSNP6822	*Kabuli*	Ca_Kabuli_Chr04	35346415	(G/A)
6823	CakSNP6823	*Kabuli*	Ca_Kabuli_Chr04	35346417	(C/T)

S.N.	SNP IDs	Cultivars	Chromosomes/scaffolds	Physical positions (bp)	SNPs
6824	CakSNP6824	*Kabuli*	Ca_Kabuli_Chr04	35346495	(C/T)
6825	CakSNP6825	*Kabuli*	Ca_Kabuli_Chr04	35346465	(G/T)
6826	CakSNP6826	*Kabuli*	Ca_Kabuli_Chr04	35346558	(G/T)
6827	CakSNP6827	*Kabuli*	Ca_Kabuli_Chr04	35346563	(G/T)
6828	CakSNP6828	*Kabuli*	Ca_Kabuli_Chr04	35346573	(A/G)
6829	CakSNP6829	*Kabuli*	Ca_Kabuli_Chr04	35346576	(C/G)
6830	CakSNP6830	*Kabuli*	Ca_Kabuli_Chr04	35346624	(G/A)
6831	CakSNP6831	*Kabuli*	Ca_Kabuli_Chr04	35393505	(G/T)
6832	CakSNP6832	*Kabuli*	Ca_Kabuli_Chr04	35393643	(G/A)
6833	CakSNP6833	*Kabuli*	Ca_Kabuli_Chr04	35455095	(A/C)
6834	CakSNP6834	*Kabuli*	Ca_Kabuli_Chr04	35455062	(A/T)
6835	CakSNP6835	*Kabuli*	Ca_Kabuli_Chr04	35455708	(G/A)
6836	CakSNP6836	*Kabuli*	Ca_Kabuli_Chr04	35469308	(T/G)
6837	CakSNP6837	*Kabuli*	Ca_Kabuli_Chr04	35469304	(G/A)
6838	CakSNP6838	*Kabuli*	Ca_Kabuli_Chr04	35487504	(G/C)
6839	CakSNP6839	*Kabuli*	Ca_Kabuli_Chr04	35487496	(C/A)
6840	CakSNP6840	*Kabuli*	Ca_Kabuli_Chr04	35487471	(A/C)
6841	CakSNP6841	*Kabuli*	Ca_Kabuli_Chr04	35528758	(C/A)
6842	CakSNP6842	*Kabuli*	Ca_Kabuli_Chr04	35534777	(G/C)
6843	CakSNP6843	*Kabuli*	Ca_Kabuli_Chr04	35534877	(T/C)
6844	CakSNP6844	*Kabuli*	Ca_Kabuli_Chr04	35534891	(C/T)
6845	CakSNP6845	*Kabuli*	Ca_Kabuli_Chr04	35574379	(C/A)
6846	CakSNP6846	*Kabuli*	Ca_Kabuli_Chr04	35634712	(A/G)
6847	CakSNP6847	*Kabuli*	Ca_Kabuli_Chr04	35634751	(A/G)
6848	CakSNP6848	*Kabuli*	Ca_Kabuli_Chr04	35634873	(G/T)
6849	CakSNP6849	*Kabuli*	Ca_Kabuli_Chr04	35961593	(A/C)
6850	CakSNP6850	*Kabuli*	Ca_Kabuli_Chr04	35977422	(G/A)
6851	CakSNP6851	*Kabuli*	Ca_Kabuli_Chr04	36005847	(T/G)
6852	CakSNP6852	*Kabuli*	Ca_Kabuli_Chr04	36010756	(T/G)
6853	CakSNP6853	*Kabuli*	Ca_Kabuli_Chr04	36153818	(A/G)
6854	CakSNP6854	*Kabuli*	Ca_Kabuli_Chr04	36154771	(T/C)
6855	CakSNP6855	*Kabuli*	Ca_Kabuli_Chr04	36154820	(G/A)
6856	CakSNP6856	*Kabuli*	Ca_Kabuli_Chr04	36154822	(T/G)
6857	CakSNP6857	*Kabuli*	Ca_Kabuli_Chr04	36184969	(A/G)
6858	CakSNP6858	*Kabuli*	Ca_Kabuli_Chr04	36277383	(C/T)
6859	CakSNP6859	*Kabuli*	Ca_Kabuli_Chr04	36368713	(T/C)
6860	CakSNP6860	*Kabuli*	Ca_Kabuli_Chr04	36445345	(G/A)
6861	CakSNP6861	*Kabuli*	Ca_Kabuli_Chr04	36445367	(A/G)
6862	CakSNP6862	*Kabuli*	Ca_Kabuli_Chr04	36449101	(C/T)

S.N.	SNP IDs	Cultivars	Chromosomes/scaffolds	Physical positions (bp)	SNPs
6863	CakSNP6863	*Kabuli*	Ca_Kabuli_Chr04	36461836	(A/G)
6864	CakSNP6864	*Kabuli*	Ca_Kabuli_Chr04	36467928	(C/T)
6865	CakSNP6865	*Kabuli*	Ca_Kabuli_Chr04	36467943	(A/G)
6866	CakSNP6866	*Kabuli*	Ca_Kabuli_Chr04	36467964	(G/A)
6867	CakSNP6867	*Kabuli*	Ca_Kabuli_Chr04	36467967	(T/C)
6868	CakSNP6868	*Kabuli*	Ca_Kabuli_Chr04	36467980	(C/A)
6869	CakSNP6869	*Kabuli*	Ca_Kabuli_Chr04	36467988	(C/T)
6870	CakSNP6870	*Kabuli*	Ca_Kabuli_Chr04	36467990	(T/G)
6871	CakSNP6871	*Kabuli*	Ca_Kabuli_Chr04	36468003	(T/A)
6872	CakSNP6872	*Kabuli*	Ca_Kabuli_Chr04	36468053	(A/G)
6873	CakSNP6873	*Kabuli*	Ca_Kabuli_Chr04	36488966	(A/G)
6874	CakSNP6874	*Kabuli*	Ca_Kabuli_Chr04	36537118	(C/T)
6875	CakSNP6875	*Kabuli*	Ca_Kabuli_Chr04	36537124	(C/T)
6876	CakSNP6876	*Kabuli*	Ca_Kabuli_Chr04	36537170	(C/T)
6877	CakSNP6877	*Kabuli*	Ca_Kabuli_Chr04	36541088	(G/T)
6878	CakSNP6878	*Kabuli*	Ca_Kabuli_Chr04	36541087	(T/C)
6879	CakSNP6879	*Kabuli*	Ca_Kabuli_Chr04	36541083	(A/C)
6880	CakSNP6880	*Kabuli*	Ca_Kabuli_Chr04	36639333	(A/T)
6881	CakSNP6881	*Kabuli*	Ca_Kabuli_Chr04	36639382	(T/C)
6882	CakSNP6882	*Kabuli*	Ca_Kabuli_Chr04	36639474	(G/C)
6883	CakSNP6883	*Kabuli*	Ca_Kabuli_Chr04	36673677	(C/A)
6884	CakSNP6884	*Kabuli*	Ca_Kabuli_Chr04	36682215	(G/A)
6885	CakSNP6885	*Kabuli*	Ca_Kabuli_Chr04	36682213	(C/T)
6886	CakSNP6886	*Kabuli*	Ca_Kabuli_Chr04	36682211	(G/A)
6887	CakSNP6887	*Kabuli*	Ca_Kabuli_Chr04	36682253	(T/G)
6888	CakSNP6888	*Kabuli*	Ca_Kabuli_Chr04	36682231	(G/T)
6889	CakSNP6889	*Kabuli*	Ca_Kabuli_Chr04	36709731	(G/A)
6890	CakSNP6890	*Kabuli*	Ca_Kabuli_Chr04	36754121	(T/C)
6891	CakSNP6891	*Kabuli*	Ca_Kabuli_Chr04	36754149	(G/A)
6892	CakSNP6892	*Kabuli*	Ca_Kabuli_Chr04	36754290	(T/A)
6893	CakSNP6893	*Kabuli*	Ca_Kabuli_Chr04	36754378	(C/T)
6894	CakSNP6894	*Kabuli*	Ca_Kabuli_Chr04	36754377	(T/A)
6895	CakSNP6895	*Kabuli*	Ca_Kabuli_Chr04	36754348	(C/T)
6896	CakSNP6896	*Kabuli*	Ca_Kabuli_Chr04	36754343	(A/C)
6897	CakSNP6897	*Kabuli*	Ca_Kabuli_Chr04	36754327	(G/A)
6898	CakSNP6898	*Kabuli*	Ca_Kabuli_Chr04	36754468	(G/A)
6899	CakSNP6899	*Kabuli*	Ca_Kabuli_Chr04	36754453	(A/G)
6900	CakSNP6900	*Kabuli*	Ca_Kabuli_Chr04	36799551	(C/T)
6901	CakSNP6901	*Kabuli*	Ca_Kabuli_Chr04	36831035	(A/G)

S.N.	SNP IDs	Cultivars	Chromosomes/scaffolds	Physical positions (bp)	SNPs
6902	CakSNP6902	*Kabuli*	Ca_Kabuli_Chr04	36831199	(G/A)
6903	CakSNP6903	*Kabuli*	Ca_Kabuli_Chr04	36831267	(A/C)
6904	CakSNP6904	*Kabuli*	Ca_Kabuli_Chr04	36955706	(G/T)
6905	CakSNP6905	*Kabuli*	Ca_Kabuli_Chr04	36977192	(C/T)
6906	CakSNP6906	*Kabuli*	Ca_Kabuli_Chr04	36977194	(G/A)
6907	CakSNP6907	*Kabuli*	Ca_Kabuli_Chr04	36979866	(T/G)
6908	CakSNP6908	*Kabuli*	Ca_Kabuli_Chr04	36979926	(G/T)
6909	CakSNP6909	*Kabuli*	Ca_Kabuli_Chr04	36979879	(C/T)
6910	CakSNP6910	*Kabuli*	Ca_Kabuli_Chr04	36982955	(A/G)
6911	CakSNP6911	*Kabuli*	Ca_Kabuli_Chr04	36984727	(C/G)
6912	CakSNP6912	*Kabuli*	Ca_Kabuli_Chr04	36984829	(C/T)
6913	CakSNP6913	*Kabuli*	Ca_Kabuli_Chr04	36984795	(T/A)
6914	CakSNP6914	*Kabuli*	Ca_Kabuli_Chr04	36998201	(A/C)
6915	CakSNP6915	*Kabuli*	Ca_Kabuli_Chr04	36998209	(C/T)
6916	CakSNP6916	*Kabuli*	Ca_Kabuli_Chr04	37064588	(A/G)
6917	CakSNP6917	*Kabuli*	Ca_Kabuli_Chr04	37064630	(G/A)
6918	CakSNP6918	*Kabuli*	Ca_Kabuli_Chr04	37185576	(T/A)
6919	CakSNP6919	*Kabuli*	Ca_Kabuli_Chr04	37185577	(T/A)
6920	CakSNP6920	*Kabuli*	Ca_Kabuli_Chr04	37185578	(T/A)
6921	CakSNP6921	*Kabuli*	Ca_Kabuli_Chr04	37237211	(G/A)
6922	CakSNP6922	*Kabuli*	Ca_Kabuli_Chr04	37237276	(T/C)
6923	CakSNP6923	*Kabuli*	Ca_Kabuli_Chr04	37241235	(A/G)
6924	CakSNP6924	*Kabuli*	Ca_Kabuli_Chr04	37241262	(G/A)
6925	CakSNP6925	*Kabuli*	Ca_Kabuli_Chr04	37260697	(T/C)
6926	CakSNP6926	*Kabuli*	Ca_Kabuli_Chr04	37260873	(A/G)
6927	CakSNP6927	*Kabuli*	Ca_Kabuli_Chr04	37260841	(T/A)
6928	CakSNP6928	*Kabuli*	Ca_Kabuli_Chr04	37264628	(G/A)
6929	CakSNP6929	*Kabuli*	Ca_Kabuli_Chr04	37286591	(T/A)
6930	CakSNP6930	*Kabuli*	Ca_Kabuli_Chr04	37286625	(C/A)
6931	CakSNP6931	*Kabuli*	Ca_Kabuli_Chr04	37286632	(G/A)
6932	CakSNP6932	*Kabuli*	Ca_Kabuli_Chr04	37286663	(A/G)
6933	CakSNP6933	*Kabuli*	Ca_Kabuli_Chr04	37293752	(G/A)
6934	CakSNP6934	*Kabuli*	Ca_Kabuli_Chr04	37293780	(T/A)
6935	CakSNP6935	*Kabuli*	Ca_Kabuli_Chr04	37293773	(A/T)
6936	CakSNP6936	*Kabuli*	Ca_Kabuli_Chr04	37293769	(C/A)
6937	CakSNP6937	*Kabuli*	Ca_Kabuli_Chr04	37294720	(A/G)
6938	CakSNP6938	*Kabuli*	Ca_Kabuli_Chr04	37316574	(T/A)
6939	CakSNP6939	*Kabuli*	Ca_Kabuli_Chr04	37316748	(T/A)
6940	CakSNP6940	*Kabuli*	Ca_Kabuli_Chr04	37316738	(C/T)

S.N.	SNP IDs	Cultivars	Chromosomes/scaffolds	Physical positions (bp)	SNPs
6941	CakSNP6941	*Kabuli*	Ca_Kabuli_Chr04	37316713	(A/T)
6942	CakSNP6942	*Kabuli*	Ca_Kabuli_Chr04	37316700	(C/A)
6943	CakSNP6943	*Kabuli*	Ca_Kabuli_Chr04	37349071	(T/C)
6944	CakSNP6944	*Kabuli*	Ca_Kabuli_Chr04	37349212	(A/C)
6945	CakSNP6945	*Kabuli*	Ca_Kabuli_Chr04	37349239	(C/G)
6946	CakSNP6946	*Kabuli*	Ca_Kabuli_Chr04	37349304	(T/C)
6947	CakSNP6947	*Kabuli*	Ca_Kabuli_Chr04	37349331	(C/T)
6948	CakSNP6948	*Kabuli*	Ca_Kabuli_Chr04	37349321	(T/C)
6949	CakSNP6949	*Kabuli*	Ca_Kabuli_Chr04	37356865	(C/A)
6950	CakSNP6950	*Kabuli*	Ca_Kabuli_Chr04	37356889	(C/T)
6951	CakSNP6951	*Kabuli*	Ca_Kabuli_Chr04	37356892	(C/T)
6952	CakSNP6952	*Kabuli*	Ca_Kabuli_Chr04	37356919	(A/T)
6953	CakSNP6953	*Kabuli*	Ca_Kabuli_Chr04	37356951	(C/T)
6954	CakSNP6954	*Kabuli*	Ca_Kabuli_Chr04	37364980	(G/A)
6955	CakSNP6955	*Kabuli*	Ca_Kabuli_Chr04	37365020	(C/T)
6956	CakSNP6956	*Kabuli*	Ca_Kabuli_Chr04	37371262	(G/A)
6957	CakSNP6957	*Kabuli*	Ca_Kabuli_Chr04	37371263	(G/A)
6958	CakSNP6958	*Kabuli*	Ca_Kabuli_Chr04	37433459	(G/T)
6959	CakSNP6959	*Kabuli*	Ca_Kabuli_Chr04	37450206	(A/C)
6960	CakSNP6960	*Kabuli*	Ca_Kabuli_Chr04	37450326	(A/T)
6961	CakSNP6961	*Kabuli*	Ca_Kabuli_Chr04	37450308	(C/T)
6962	CakSNP6962	*Kabuli*	Ca_Kabuli_Chr04	37450294	(T/C)
6963	CakSNP6963	*Kabuli*	Ca_Kabuli_Chr04	37453497	(C/G)
6964	CakSNP6964	*Kabuli*	Ca_Kabuli_Chr04	37453449	(A/G)
6965	CakSNP6965	*Kabuli*	Ca_Kabuli_Chr04	37453590	(C/A)
6966	CakSNP6966	*Kabuli*	Ca_Kabuli_Chr04	37453586	(C/A)
6967	CakSNP6967	*Kabuli*	Ca_Kabuli_Chr04	37454296	(A/G)
6968	CakSNP6968	*Kabuli*	Ca_Kabuli_Chr04	37492422	(C/A)
6969	CakSNP6969	*Kabuli*	Ca_Kabuli_Chr04	37549883	(T/C)
6970	CakSNP6970	*Kabuli*	Ca_Kabuli_Chr04	37549871	(A/T)
6971	CakSNP6971	*Kabuli*	Ca_Kabuli_Chr04	37550122	(G/A)
6972	CakSNP6972	*Kabuli*	Ca_Kabuli_Chr04	37552973	(C/T)
6973	CakSNP6973	*Kabuli*	Ca_Kabuli_Chr04	37558880	(C/T)
6974	CakSNP6974	*Kabuli*	Ca_Kabuli_Chr04	37558881	(G/T)
6975	CakSNP6975	*Kabuli*	Ca_Kabuli_Chr04	37575636	(T/C)
6976	CakSNP6976	*Kabuli*	Ca_Kabuli_Chr04	37575670	(C/T)
6977	CakSNP6977	*Kabuli*	Ca_Kabuli_Chr04	37575709	(C/T)
6978	CakSNP6978	*Kabuli*	Ca_Kabuli_Chr04	37612154	(A/G)
6979	CakSNP6979	*Kabuli*	Ca_Kabuli_Chr04	37630404	(A/G)

S.N.	SNP IDs	Cultivars	Chromosomes/scaffolds	Physical positions (bp)	SNPs
6980	CakSNP6980	*Kabuli*	Ca_Kabuli_Chr04	37630445	(A/G)
6981	CakSNP6981	*Kabuli*	Ca_Kabuli_Chr04	37630527	(G/A)
6982	CakSNP6982	*Kabuli*	Ca_Kabuli_Chr04	37630520	(G/C)
6983	CakSNP6983	*Kabuli*	Ca_Kabuli_Chr04	37634624	(C/T)
6984	CakSNP6984	*Kabuli*	Ca_Kabuli_Chr04	37635300	(T/A)
6985	CakSNP6985	*Kabuli*	Ca_Kabuli_Chr04	37685824	(T/C)
6986	CakSNP6986	*Kabuli*	Ca_Kabuli_Chr04	37685842	(T/G)
6987	CakSNP6987	*Kabuli*	Ca_Kabuli_Chr04	37699176	(G/A)
6988	CakSNP6988	*Kabuli*	Ca_Kabuli_Chr04	37699141	(T/G)
6989	CakSNP6989	*Kabuli*	Ca_Kabuli_Chr04	37699140	(C/T)
6990	CakSNP6990	*Kabuli*	Ca_Kabuli_Chr04	37703656	(G/T)
6991	CakSNP6991	*Kabuli*	Ca_Kabuli_Chr04	37703653	(A/C)
6992	CakSNP6992	*Kabuli*	Ca_Kabuli_Chr04	37703651	(T/C)
6993	CakSNP6993	*Kabuli*	Ca_Kabuli_Chr04	37703649	(A/C)
6994	CakSNP6994	*Kabuli*	Ca_Kabuli_Chr04	37703648	(C/T)
6995	CakSNP6995	*Kabuli*	Ca_Kabuli_Chr04	37707115	(G/A)
6996	CakSNP6996	*Kabuli*	Ca_Kabuli_Chr04	37708260	(A/G)
6997	CakSNP6997	*Kabuli*	Ca_Kabuli_Chr04	37708348	(T/G)
6998	CakSNP6998	*Kabuli*	Ca_Kabuli_Chr04	37708334	(T/A)
6999	CakSNP6999	*Kabuli*	Ca_Kabuli_Chr04	37753479	(C/T)
7000	CakSNP7000	*Kabuli*	Ca_Kabuli_Chr04	37806689	(T/C)
7001	CakSNP7001	*Kabuli*	Ca_Kabuli_Chr04	37812917	(G/C)
7002	CakSNP7002	*Kabuli*	Ca_Kabuli_Chr04	37859069	(T/G)
7003	CakSNP7003	*Kabuli*	Ca_Kabuli_Chr04	37897261	(G/T)
7004	CakSNP7004	*Kabuli*	Ca_Kabuli_Chr04	37897266	(A/T)
7005	CakSNP7005	*Kabuli*	Ca_Kabuli_Chr04	37897351	(C/A)
7006	CakSNP7006	*Kabuli*	Ca_Kabuli_Chr04	37898404	(A/G)
7007	CakSNP7007	*Kabuli*	Ca_Kabuli_Chr04	37899597	(C/T)
7008	CakSNP7008	*Kabuli*	Ca_Kabuli_Chr04	37901584	(C/T)
7009	CakSNP7009	*Kabuli*	Ca_Kabuli_Chr04	37901575	(G/A)
7010	CakSNP7010	*Kabuli*	Ca_Kabuli_Chr04	38008062	(T/A)
7011	CakSNP7011	*Kabuli*	Ca_Kabuli_Chr04	38008082	(T/C)
7012	CakSNP7012	*Kabuli*	Ca_Kabuli_Chr04	38008243	(C/T)
7013	CakSNP7013	*Kabuli*	Ca_Kabuli_Chr04	38008227	(G/T)
7014	CakSNP7014	*Kabuli*	Ca_Kabuli_Chr04	38008213	(C/T)
7015	CakSNP7015	*Kabuli*	Ca_Kabuli_Chr04	38008205	(A/G)
7016	CakSNP7016	*Kabuli*	Ca_Kabuli_Chr04	38008204	(G/A)
7017	CakSNP7017	*Kabuli*	Ca_Kabuli_Chr04	38008186	(A/C)
7018	CakSNP7018	*Kabuli*	Ca_Kabuli_Chr04	38008174	(A/G)

S.N.	SNP IDs	Cultivars	Chromosomes/scaffolds	Physical positions (bp)	SNPs
7019	CakSNP7019	*Kabuli*	Ca_Kabuli_Chr04	38027560	(G/A)
7020	CakSNP7020	*Kabuli*	Ca_Kabuli_Chr04	38029012	(T/C)
7021	CakSNP7021	*Kabuli*	Ca_Kabuli_Chr04	38029050	(G/A)
7022	CakSNP7022	*Kabuli*	Ca_Kabuli_Chr04	38029136	(G/T)
7023	CakSNP7023	*Kabuli*	Ca_Kabuli_Chr04	38038865	(C/T)
7024	CakSNP7024	*Kabuli*	Ca_Kabuli_Chr04	38038959	(A/G)
7025	CakSNP7025	*Kabuli*	Ca_Kabuli_Chr04	38039037	(T/C)
7026	CakSNP7026	*Kabuli*	Ca_Kabuli_Chr04	38039046	(C/T)
7027	CakSNP7027	*Kabuli*	Ca_Kabuli_Chr04	38039076	(A/G)
7028	CakSNP7028	*Kabuli*	Ca_Kabuli_Chr04	38039088	(T/C)
7029	CakSNP7029	*Kabuli*	Ca_Kabuli_Chr04	38039251	(G/C)
7030	CakSNP7030	*Kabuli*	Ca_Kabuli_Chr04	38039289	(G/A)
7031	CakSNP7031	*Kabuli*	Ca_Kabuli_Chr04	38039297	(C/T)
7032	CakSNP7032	*Kabuli*	Ca_Kabuli_Chr04	38039313	(G/A)
7033	CakSNP7033	*Kabuli*	Ca_Kabuli_Chr04	38039371	(T/C)
7034	CakSNP7034	*Kabuli*	Ca_Kabuli_Chr04	38153183	(C/A)
7035	CakSNP7035	*Kabuli*	Ca_Kabuli_Chr04	38153195	(T/C)
7036	CakSNP7036	*Kabuli*	Ca_Kabuli_Chr04	38153216	(T/C)
7037	CakSNP7037	*Kabuli*	Ca_Kabuli_Chr04	38153277	(A/G)
7038	CakSNP7038	*Kabuli*	Ca_Kabuli_Chr04	38153275	(C/A)
7039	CakSNP7039	*Kabuli*	Ca_Kabuli_Chr04	38153269	(A/C)
7040	CakSNP7040	*Kabuli*	Ca_Kabuli_Chr04	38179583	(A/C)
7041	CakSNP7041	*Kabuli*	Ca_Kabuli_Chr04	38179614	(G/C)
7042	CakSNP7042	*Kabuli*	Ca_Kabuli_Chr04	38181292	(G/A)
7043	CakSNP7043	*Kabuli*	Ca_Kabuli_Chr04	38195411	(T/A)
7044	CakSNP7044	*Kabuli*	Ca_Kabuli_Chr04	38195438	(C/T)
7045	CakSNP7045	*Kabuli*	Ca_Kabuli_Chr04	38215267	(G/A)
7046	CakSNP7046	*Kabuli*	Ca_Kabuli_Chr04	38221009	(A/G)
7047	CakSNP7047	*Kabuli*	Ca_Kabuli_Chr04	38288823	(G/T)
7048	CakSNP7048	*Kabuli*	Ca_Kabuli_Chr04	38288877	(C/G)
7049	CakSNP7049	*Kabuli*	Ca_Kabuli_Chr04	38289066	(G/A)
7050	CakSNP7050	*Kabuli*	Ca_Kabuli_Chr04	38289052	(G/A)
7051	CakSNP7051	*Kabuli*	Ca_Kabuli_Chr04	38288997	(T/A)
7052	CakSNP7052	*Kabuli*	Ca_Kabuli_Chr04	38343633	(A/T)
7053	CakSNP7053	*Kabuli*	Ca_Kabuli_Chr04	38343750	(C/T)
7054	CakSNP7054	*Kabuli*	Ca_Kabuli_Chr04	38343687	(C/T)
7055	CakSNP7055	*Kabuli*	Ca_Kabuli_Chr04	38358067	(A/G)
7056	CakSNP7056	*Kabuli*	Ca_Kabuli_Chr04	38358398	(A/G)
7057	CakSNP7057	*Kabuli*	Ca_Kabuli_Chr04	38358677	(G/A)

S.N.	SNP IDs	Cultivars	Chromosomes/scaffolds	Physical positions (bp)	SNPs
7058	CakSNP7058	*Kabuli*	Ca_Kabuli_Chr04	38358887	(G/A)
7059	CakSNP7059	*Kabuli*	Ca_Kabuli_Chr04	38358869	(G/A)
7060	CakSNP7060	*Kabuli*	Ca_Kabuli_Chr04	38362132	(T/A)
7061	CakSNP7061	*Kabuli*	Ca_Kabuli_Chr04	38362139	(T/A)
7062	CakSNP7062	*Kabuli*	Ca_Kabuli_Chr04	38364369	(C/T)
7063	CakSNP7063	*Kabuli*	Ca_Kabuli_Chr04	38370848	(A/G)
7064	CakSNP7064	*Kabuli*	Ca_Kabuli_Chr04	38370864	(G/A)
7065	CakSNP7065	*Kabuli*	Ca_Kabuli_Chr04	38370941	(A/G)
7066	CakSNP7066	*Kabuli*	Ca_Kabuli_Chr04	38370930	(C/T)
7067	CakSNP7067	*Kabuli*	Ca_Kabuli_Chr04	38370926	(T/C)
7068	CakSNP7068	*Kabuli*	Ca_Kabuli_Chr04	38370925	(G/A)
7069	CakSNP7069	*Kabuli*	Ca_Kabuli_Chr04	38370922	(G/A)
7070	CakSNP7070	*Kabuli*	Ca_Kabuli_Chr04	38370921	(T/G)
7071	CakSNP7071	*Kabuli*	Ca_Kabuli_Chr04	38382809	(T/C)
7072	CakSNP7072	*Kabuli*	Ca_Kabuli_Chr04	38382890	(C/G)
7073	CakSNP7073	*Kabuli*	Ca_Kabuli_Chr04	38421798	(A/C)
7074	CakSNP7074	*Kabuli*	Ca_Kabuli_Chr04	38422037	(C/T)
7075	CakSNP7075	*Kabuli*	Ca_Kabuli_Chr04	38421984	(C/T)
7076	CakSNP7076	*Kabuli*	Ca_Kabuli_Chr04	38425675	(A/G)
7077	CakSNP7077	*Kabuli*	Ca_Kabuli_Chr04	38425720	(T/A)
7078	CakSNP7078	*Kabuli*	Ca_Kabuli_Chr04	38473540	(A/G)
7079	CakSNP7079	*Kabuli*	Ca_Kabuli_Chr04	38473653	(T/C)
7080	CakSNP7080	*Kabuli*	Ca_Kabuli_Chr04	38474123	(A/T)
7081	CakSNP7081	*Kabuli*	Ca_Kabuli_Chr04	38474108	(T/C)
7082	CakSNP7082	*Kabuli*	Ca_Kabuli_Chr04	38474192	(T/C)
7083	CakSNP7083	*Kabuli*	Ca_Kabuli_Chr04	38475310	(A/C)
7084	CakSNP7084	*Kabuli*	Ca_Kabuli_Chr04	38475425	(A/G)
7085	CakSNP7085	*Kabuli*	Ca_Kabuli_Chr04	38475449	(T/A)
7086	CakSNP7086	*Kabuli*	Ca_Kabuli_Chr04	38475442	(A/G)
7087	CakSNP7087	*Kabuli*	Ca_Kabuli_Chr04	38475424	(C/G)
7088	CakSNP7088	*Kabuli*	Ca_Kabuli_Chr04	38475547	(C/T)
7089	CakSNP7089	*Kabuli*	Ca_Kabuli_Chr04	38540201	(C/T)
7090	CakSNP7090	*Kabuli*	Ca_Kabuli_Chr04	38540236	(C/T)
7091	CakSNP7091	*Kabuli*	Ca_Kabuli_Chr04	38540247	(G/A)
7092	CakSNP7092	*Kabuli*	Ca_Kabuli_Chr04	38620579	(C/T)
7093	CakSNP7093	*Kabuli*	Ca_Kabuli_Chr04	38620620	(A/T)
7094	CakSNP7094	*Kabuli*	Ca_Kabuli_Chr04	38659377	(T/C)
7095	CakSNP7095	*Kabuli*	Ca_Kabuli_Chr04	38659398	(G/A)
7096	CakSNP7096	*Kabuli*	Ca_Kabuli_Chr04	38659534	(T/A)

S.N.	SNP IDs	Cultivars	Chromosomes/scaffolds	Physical positions (bp)	SNPs
7097	CakSNP7097	*Kabuli*	Ca_Kabuli_Chr04	38679987	(A/G)
7098	CakSNP7098	*Kabuli*	Ca_Kabuli_Chr04	38721154	(A/G)
7099	CakSNP7099	*Kabuli*	Ca_Kabuli_Chr04	38721160	(A/G)
7100	CakSNP7100	*Kabuli*	Ca_Kabuli_Chr04	38721207	(A/C)
7101	CakSNP7101	*Kabuli*	Ca_Kabuli_Chr04	38756507	(T/C)
7102	CakSNP7102	*Kabuli*	Ca_Kabuli_Chr04	38756510	(C/G)
7103	CakSNP7103	*Kabuli*	Ca_Kabuli_Chr04	38759640	(A/C)
7104	CakSNP7104	*Kabuli*	Ca_Kabuli_Chr04	38770348	(T/A)
7105	CakSNP7105	*Kabuli*	Ca_Kabuli_Chr04	38770370	(G/C)
7106	CakSNP7106	*Kabuli*	Ca_Kabuli_Chr04	38770450	(C/G)
7107	CakSNP7107	*Kabuli*	Ca_Kabuli_Chr04	38770510	(G/A)
7108	CakSNP7108	*Kabuli*	Ca_Kabuli_Chr04	38770606	(G/A)
7109	CakSNP7109	*Kabuli*	Ca_Kabuli_Chr04	38770582	(G/A)
7110	CakSNP7110	*Kabuli*	Ca_Kabuli_Chr04	38785535	(G/A)
7111	CakSNP7111	*Kabuli*	Ca_Kabuli_Chr04	38829959	(T/G)
7112	CakSNP7112	*Kabuli*	Ca_Kabuli_Chr04	38833304	(C/A)
7113	CakSNP7113	*Kabuli*	Ca_Kabuli_Chr04	38833280	(C/T)
7114	CakSNP7114	*Kabuli*	Ca_Kabuli_Chr04	38833333	(A/C)
7115	CakSNP7115	*Kabuli*	Ca_Kabuli_Chr04	38833414	(A/C)
7116	CakSNP7116	*Kabuli*	Ca_Kabuli_Chr04	38834383	(G/A)
7117	CakSNP7117	*Kabuli*	Ca_Kabuli_Chr04	38834405	(G/A)
7118	CakSNP7118	*Kabuli*	Ca_Kabuli_Chr04	38834423	(G/C)
7119	CakSNP7119	*Kabuli*	Ca_Kabuli_Chr04	38834431	(G/A)
7120	CakSNP7120	*Kabuli*	Ca_Kabuli_Chr04	38834448	(A/T)
7121	CakSNP7121	*Kabuli*	Ca_Kabuli_Chr04	38834477	(A/G)
7122	CakSNP7122	*Kabuli*	Ca_Kabuli_Chr04	38834474	(G/A)
7123	CakSNP7123	*Kabuli*	Ca_Kabuli_Chr04	38834453	(C/T)
7124	CakSNP7124	*Kabuli*	Ca_Kabuli_Chr04	38888779	(C/T)
7125	CakSNP7125	*Kabuli*	Ca_Kabuli_Chr04	38888791	(G/C)
7126	CakSNP7126	*Kabuli*	Ca_Kabuli_Chr04	38889023	(C/T)
7127	CakSNP7127	*Kabuli*	Ca_Kabuli_Chr04	38918394	(A/T)
7128	CakSNP7128	*Kabuli*	Ca_Kabuli_Chr04	38918424	(G/A)
7129	CakSNP7129	*Kabuli*	Ca_Kabuli_Chr04	38918430	(C/A)
7130	CakSNP7130	*Kabuli*	Ca_Kabuli_Chr04	38918628	(C/T)
7131	CakSNP7131	*Kabuli*	Ca_Kabuli_Chr04	38918547	(T/C)
7132	CakSNP7132	*Kabuli*	Ca_Kabuli_Chr04	38918649	(G/C)
7133	CakSNP7133	*Kabuli*	Ca_Kabuli_Chr04	38929681	(A/G)
7134	CakSNP7134	*Kabuli*	Ca_Kabuli_Chr04	38948414	(C/A)
7135	CakSNP7135	*Kabuli*	Ca_Kabuli_Chr04	38961521	(G/A)

S.N.	SNP IDs	Cultivars	Chromosomes/scaffolds	Physical positions (bp)	SNPs
7136	CakSNP7136	*Kabuli*	Ca_Kabuli_Chr04	38978462	(A/G)
7137	CakSNP7137	*Kabuli*	Ca_Kabuli_Chr04	39031801	(C/T)
7138	CakSNP7138	*Kabuli*	Ca_Kabuli_Chr04	39031823	(G/A)
7139	CakSNP7139	*Kabuli*	Ca_Kabuli_Chr04	39042938	(T/G)
7140	CakSNP7140	*Kabuli*	Ca_Kabuli_Chr04	39042921	(T/C)
7141	CakSNP7141	*Kabuli*	Ca_Kabuli_Chr04	39057991	(T/C)
7142	CakSNP7142	*Kabuli*	Ca_Kabuli_Chr04	39058011	(G/C)
7143	CakSNP7143	*Kabuli*	Ca_Kabuli_Chr04	39081951	(A/G)
7144	CakSNP7144	*Kabuli*	Ca_Kabuli_Chr04	39110408	(T/C)
7145	CakSNP7145	*Kabuli*	Ca_Kabuli_Chr04	39110448	(A/G)
7146	CakSNP7146	*Kabuli*	Ca_Kabuli_Chr04	39110735	(A/G)
7147	CakSNP7147	*Kabuli*	Ca_Kabuli_Chr04	39113309	(T/C)
7148	CakSNP7148	*Kabuli*	Ca_Kabuli_Chr04	39113327	(G/A)
7149	CakSNP7149	*Kabuli*	Ca_Kabuli_Chr04	39113330	(G/A)
7150	CakSNP7150	*Kabuli*	Ca_Kabuli_Chr04	39113401	(A/T)
7151	CakSNP7151	*Kabuli*	Ca_Kabuli_Chr04	39114000	(A/C)
7152	CakSNP7152	*Kabuli*	Ca_Kabuli_Chr04	39136826	(C/T)
7153	CakSNP7153	*Kabuli*	Ca_Kabuli_Chr04	39189012	(A/G)
7154	CakSNP7154	*Kabuli*	Ca_Kabuli_Chr04	39189016	(G/A)
7155	CakSNP7155	*Kabuli*	Ca_Kabuli_Chr04	39268505	(T/C)
7156	CakSNP7156	*Kabuli*	Ca_Kabuli_Chr04	39306900	(G/A)
7157	CakSNP7157	*Kabuli*	Ca_Kabuli_Chr04	39635746	(T/A)
7158	CakSNP7158	*Kabuli*	Ca_Kabuli_Chr04	39635721	(A/G)
7159	CakSNP7159	*Kabuli*	Ca_Kabuli_Chr04	39635720	(G/A)
7160	CakSNP7160	*Kabuli*	Ca_Kabuli_Chr04	39635718	(C/T)
7161	CakSNP7161	*Kabuli*	Ca_Kabuli_Chr04	39650872	(T/C)
7162	CakSNP7162	*Kabuli*	Ca_Kabuli_Chr04	39659757	(C/T)
7163	CakSNP7163	*Kabuli*	Ca_Kabuli_Chr04	39659750	(G/A)
7164	CakSNP7164	*Kabuli*	Ca_Kabuli_Chr04	39659832	(C/T)
7165	CakSNP7165	*Kabuli*	Ca_Kabuli_Chr04	39715087	(A/G)
7166	CakSNP7166	*Kabuli*	Ca_Kabuli_Chr04	39719606	(C/G)
7167	CakSNP7167	*Kabuli*	Ca_Kabuli_Chr04	39719747	(G/T)
7168	CakSNP7168	*Kabuli*	Ca_Kabuli_Chr04	39779796	(G/A)
7169	CakSNP7169	*Kabuli*	Ca_Kabuli_Chr04	39803305	(T/C)
7170	CakSNP7170	*Kabuli*	Ca_Kabuli_Chr04	39849612	(G/T)
7171	CakSNP7171	*Kabuli*	Ca_Kabuli_Chr04	39849657	(G/A)
7172	CakSNP7172	*Kabuli*	Ca_Kabuli_Chr04	39964930	(T/C)
7173	CakSNP7173	*Kabuli*	Ca_Kabuli_Chr04	40010119	(A/C)
7174	CakSNP7174	*Kabuli*	Ca_Kabuli_Chr04	40010201	(A/G)

S.N.	SNP IDs	Cultivars	Chromosomes/scaffolds	Physical positions (bp)	SNPs
7175	CakSNP7175	*Kabuli*	Ca_Kabuli_Chr04	40010177	(G/A)
7176	CakSNP7176	*Kabuli*	Ca_Kabuli_Chr04	40010165	(A/C)
7177	CakSNP7177	*Kabuli*	Ca_Kabuli_Chr04	40059880	(C/T)
7178	CakSNP7178	*Kabuli*	Ca_Kabuli_Chr04	40060022	(C/T)
7179	CakSNP7179	*Kabuli*	Ca_Kabuli_Chr04	40060063	(T/G)
7180	CakSNP7180	*Kabuli*	Ca_Kabuli_Chr04	40061195	(T/C)
7181	CakSNP7181	*Kabuli*	Ca_Kabuli_Chr04	40061262	(A/C)
7182	CakSNP7182	*Kabuli*	Ca_Kabuli_Chr04	40065505	(T/A)
7183	CakSNP7183	*Kabuli*	Ca_Kabuli_Chr04	40134460	(A/G)
7184	CakSNP7184	*Kabuli*	Ca_Kabuli_Chr04	40134518	(T/C)
7185	CakSNP7185	*Kabuli*	Ca_Kabuli_Chr04	40149064	(C/T)
7186	CakSNP7186	*Kabuli*	Ca_Kabuli_Chr04	40149132	(G/C)
7187	CakSNP7187	*Kabuli*	Ca_Kabuli_Chr04	40149324	(G/C)
7188	CakSNP7188	*Kabuli*	Ca_Kabuli_Chr04	40169176	(T/G)
7189	CakSNP7189	*Kabuli*	Ca_Kabuli_Chr04	40185606	(A/G)
7190	CakSNP7190	*Kabuli*	Ca_Kabuli_Chr04	40199692	(A/C)
7191	CakSNP7191	*Kabuli*	Ca_Kabuli_Chr04	40218375	(G/T)
7192	CakSNP7192	*Kabuli*	Ca_Kabuli_Chr04	40218410	(G/A)
7193	CakSNP7193	*Kabuli*	Ca_Kabuli_Chr04	40267713	(G/T)
7194	CakSNP7194	*Kabuli*	Ca_Kabuli_Chr04	40267719	(G/A)
7195	CakSNP7195	*Kabuli*	Ca_Kabuli_Chr04	40279513	(G/T)
7196	CakSNP7196	*Kabuli*	Ca_Kabuli_Chr04	40313721	(A/G)
7197	CakSNP7197	*Kabuli*	Ca_Kabuli_Chr04	40349170	(C/T)
7198	CakSNP7198	*Kabuli*	Ca_Kabuli_Chr04	40368028	(C/T)
7199	CakSNP7199	*Kabuli*	Ca_Kabuli_Chr04	40368278	(T/C)
7200	CakSNP7200	*Kabuli*	Ca_Kabuli_Chr04	40368765	(G/A)
7201	CakSNP7201	*Kabuli*	Ca_Kabuli_Chr04	40375386	(C/T)
7202	CakSNP7202	*Kabuli*	Ca_Kabuli_Chr04	40375361	(A/G)
7203	CakSNP7203	*Kabuli*	Ca_Kabuli_Chr04	40429387	(G/A)
7204	CakSNP7204	*Kabuli*	Ca_Kabuli_Chr04	40471412	(G/C)
7205	CakSNP7205	*Kabuli*	Ca_Kabuli_Chr04	40472097	(A/G)
7206	CakSNP7206	*Kabuli*	Ca_Kabuli_Chr04	40513733	(C/T)
7207	CakSNP7207	*Kabuli*	Ca_Kabuli_Chr04	40583205	(G/T)
7208	CakSNP7208	*Kabuli*	Ca_Kabuli_Chr04	40583206	(A/T)
7209	CakSNP7209	*Kabuli*	Ca_Kabuli_Chr04	40583211	(G/T)
7210	CakSNP7210	*Kabuli*	Ca_Kabuli_Chr04	40583214	(G/A)
7211	CakSNP7211	*Kabuli*	Ca_Kabuli_Chr04	40583216	(C/A)
7212	CakSNP7212	*Kabuli*	Ca_Kabuli_Chr04	40583217	(G/A)
7213	CakSNP7213	*Kabuli*	Ca_Kabuli_Chr04	40583219	(G/A)

S.N.	SNP IDs	Cultivars	Chromosomes/scaffolds	Physical positions (bp)	SNPs
7214	CakSNP7214	*Kabuli*	Ca_Kabuli_Chr04	40597192	(G/A)
7215	CakSNP7215	*Kabuli*	Ca_Kabuli_Chr04	40597534	(A/G)
7216	CakSNP7216	*Kabuli*	Ca_Kabuli_Chr04	40597464	(G/A)
7217	CakSNP7217	*Kabuli*	Ca_Kabuli_Chr04	40723208	(C/G)
7218	CakSNP7218	*Kabuli*	Ca_Kabuli_Chr04	40723212	(G/A)
7219	CakSNP7219	*Kabuli*	Ca_Kabuli_Chr04	40734572	(T/G)
7220	CakSNP7220	*Kabuli*	Ca_Kabuli_Chr04	40734765	(G/A)
7221	CakSNP7221	*Kabuli*	Ca_Kabuli_Chr04	40738927	(C/T)
7222	CakSNP7222	*Kabuli*	Ca_Kabuli_Chr04	40795060	(G/A)
7223	CakSNP7223	*Kabuli*	Ca_Kabuli_Chr04	40795085	(C/G)
7224	CakSNP7224	*Kabuli*	Ca_Kabuli_Chr04	40795137	(T/G)
7225	CakSNP7225	*Kabuli*	Ca_Kabuli_Chr04	40801783	(T/C)
7226	CakSNP7226	*Kabuli*	Ca_Kabuli_Chr04	40801791	(C/G)
7227	CakSNP7227	*Kabuli*	Ca_Kabuli_Chr04	40805740	(C/A)
7228	CakSNP7228	*Kabuli*	Ca_Kabuli_Chr04	40806993	(A/G)
7229	CakSNP7229	*Kabuli*	Ca_Kabuli_Chr04	40839967	(G/A)
7230	CakSNP7230	*Kabuli*	Ca_Kabuli_Chr04	40839949	(G/A)
7231	CakSNP7231	*Kabuli*	Ca_Kabuli_Chr04	40852381	(A/C)
7232	CakSNP7232	*Kabuli*	Ca_Kabuli_Chr04	40852475	(T/C)
7233	CakSNP7233	*Kabuli*	Ca_Kabuli_Chr04	40912677	(A/G)
7234	CakSNP7234	*Kabuli*	Ca_Kabuli_Chr04	40912681	(A/G)
7235	CakSNP7235	*Kabuli*	Ca_Kabuli_Chr04	40912714	(C/G)
7236	CakSNP7236	*Kabuli*	Ca_Kabuli_Chr04	40912731	(C/T)
7237	CakSNP7237	*Kabuli*	Ca_Kabuli_Chr04	40917776	(C/A)
7238	CakSNP7238	*Kabuli*	Ca_Kabuli_Chr04	40917777	(T/A)
7239	CakSNP7239	*Kabuli*	Ca_Kabuli_Chr04	40917807	(C/T)
7240	CakSNP7240	*Kabuli*	Ca_Kabuli_Chr04	40941283	(A/G)
7241	CakSNP7241	*Kabuli*	Ca_Kabuli_Chr04	41032564	(A/G)
7242	CakSNP7242	*Kabuli*	Ca_Kabuli_Chr04	41032590	(C/A)
7243	CakSNP7243	*Kabuli*	Ca_Kabuli_Chr04	41032599	(T/C)
7244	CakSNP7244	*Kabuli*	Ca_Kabuli_Chr04	41032608	(T/C)
7245	CakSNP7245	*Kabuli*	Ca_Kabuli_Chr04	41070048	(C/G)
7246	CakSNP7246	*Kabuli*	Ca_Kabuli_Chr04	41070017	(T/A)
7247	CakSNP7247	*Kabuli*	Ca_Kabuli_Chr04	41094082	(T/C)
7248	CakSNP7248	*Kabuli*	Ca_Kabuli_Chr04	41094554	(A/C)
7249	CakSNP7249	*Kabuli*	Ca_Kabuli_Chr04	41094685	(G/C)
7250	CakSNP7250	*Kabuli*	Ca_Kabuli_Chr04	41117822	(C/A)
7251	CakSNP7251	*Kabuli*	Ca_Kabuli_Chr04	41141086	(G/T)
7252	CakSNP7252	*Kabuli*	Ca_Kabuli_Chr04	41142299	(T/C)

S.N.	SNP IDs	Cultivars	Chromosomes/scaffolds	Physical positions (bp)	SNPs
7253	CakSNP7253	*Kabuli*	Ca_Kabuli_Chr04	41142290	(A/G)
7254	CakSNP7254	*Kabuli*	Ca_Kabuli_Chr04	41142288	(T/C)
7255	CakSNP7255	*Kabuli*	Ca_Kabuli_Chr04	41144277	(T/A)
7256	CakSNP7256	*Kabuli*	Ca_Kabuli_Chr04	41145767	(C/T)
7257	CakSNP7257	*Kabuli*	Ca_Kabuli_Chr04	41145875	(G/A)
7258	CakSNP7258	*Kabuli*	Ca_Kabuli_Chr04	41207328	(C/T)
7259	CakSNP7259	*Kabuli*	Ca_Kabuli_Chr04	41207356	(C/T)
7260	CakSNP7260	*Kabuli*	Ca_Kabuli_Chr04	41207392	(T/G)
7261	CakSNP7261	*Kabuli*	Ca_Kabuli_Chr04	41207354	(A/C)
7262	CakSNP7262	*Kabuli*	Ca_Kabuli_Chr04	41209521	(T/G)
7263	CakSNP7263	*Kabuli*	Ca_Kabuli_Chr04	41209711	(C/A)
7264	CakSNP7264	*Kabuli*	Ca_Kabuli_Chr04	41215850	(A/C)
7265	CakSNP7265	*Kabuli*	Ca_Kabuli_Chr04	41228918	(T/C)
7266	CakSNP7266	*Kabuli*	Ca_Kabuli_Chr04	41229605	(C/T)
7267	CakSNP7267	*Kabuli*	Ca_Kabuli_Chr04	41277786	(G/A)
7268	CakSNP7268	*Kabuli*	Ca_Kabuli_Chr04	41277799	(T/G)
7269	CakSNP7269	*Kabuli*	Ca_Kabuli_Chr04	41292152	(T/C)
7270	CakSNP7270	*Kabuli*	Ca_Kabuli_Chr04	41319598	(T/C)
7271	CakSNP7271	*Kabuli*	Ca_Kabuli_Chr04	41319610	(G/A)
7272	CakSNP7272	*Kabuli*	Ca_Kabuli_Chr04	41344739	(A/T)
7273	CakSNP7273	*Kabuli*	Ca_Kabuli_Chr04	41427081	(A/T)
7274	CakSNP7274	*Kabuli*	Ca_Kabuli_Chr04	41427086	(C/A)
7275	CakSNP7275	*Kabuli*	Ca_Kabuli_Chr04	41427093	(T/G)
7276	CakSNP7276	*Kabuli*	Ca_Kabuli_Chr04	41445171	(T/C)
7277	CakSNP7277	*Kabuli*	Ca_Kabuli_Chr04	41445212	(A/G)
7278	CakSNP7278	*Kabuli*	Ca_Kabuli_Chr04	41445225	(A/T)
7279	CakSNP7279	*Kabuli*	Ca_Kabuli_Chr04	41445242	(C/T)
7280	CakSNP7280	*Kabuli*	Ca_Kabuli_Chr04	41458426	(A/G)
7281	CakSNP7281	*Kabuli*	Ca_Kabuli_Chr04	41500284	(A/C)
7282	CakSNP7282	*Kabuli*	Ca_Kabuli_Chr04	41500286	(A/G)
7283	CakSNP7283	*Kabuli*	Ca_Kabuli_Chr04	41512840	(G/A)
7284	CakSNP7284	*Kabuli*	Ca_Kabuli_Chr04	41568682	(A/C)
7285	CakSNP7285	*Kabuli*	Ca_Kabuli_Chr04	41629852	(T/A)
7286	CakSNP7286	*Kabuli*	Ca_Kabuli_Chr04	41648927	(C/T)
7287	CakSNP7287	*Kabuli*	Ca_Kabuli_Chr04	41648998	(A/G)
7288	CakSNP7288	*Kabuli*	Ca_Kabuli_Chr04	41675299	(A/T)
7289	CakSNP7289	*Kabuli*	Ca_Kabuli_Chr04	41698291	(A/G)
7290	CakSNP7290	*Kabuli*	Ca_Kabuli_Chr04	41698386	(A/G)
7291	CakSNP7291	*Kabuli*	Ca_Kabuli_Chr04	41700712	(C/A)

S.N.	SNP IDs	Cultivars	Chromosomes/scaffolds	Physical positions (bp)	SNPs
7292	CakSNP7292	*Kabuli*	Ca_Kabuli_Chr04	41721289	(G/A)
7293	CakSNP7293	*Kabuli*	Ca_Kabuli_Chr04	41772690	(C/T)
7294	CakSNP7294	*Kabuli*	Ca_Kabuli_Chr04	41772823	(G/A)
7295	CakSNP7295	*Kabuli*	Ca_Kabuli_Chr04	41772796	(T/A)
7296	CakSNP7296	*Kabuli*	Ca_Kabuli_Chr04	41772794	(T/C)
7297	CakSNP7297	*Kabuli*	Ca_Kabuli_Chr04	41864826	(G/A)
7298	CakSNP7298	*Kabuli*	Ca_Kabuli_Chr04	41864846	(C/G)
7299	CakSNP7299	*Kabuli*	Ca_Kabuli_Chr04	41938784	(A/G)
7300	CakSNP7300	*Kabuli*	Ca_Kabuli_Chr04	41938812	(T/G)
7301	CakSNP7301	*Kabuli*	Ca_Kabuli_Chr04	41956050	(C/G)
7302	CakSNP7302	*Kabuli*	Ca_Kabuli_Chr04	41956085	(T/C)
7303	CakSNP7303	*Kabuli*	Ca_Kabuli_Chr04	41987193	(A/G)
7304	CakSNP7304	*Kabuli*	Ca_Kabuli_Chr04	42056479	(G/A)
7305	CakSNP7305	*Kabuli*	Ca_Kabuli_Chr04	42092379	(T/G)
7306	CakSNP7306	*Kabuli*	Ca_Kabuli_Chr04	42092381	(A/G)
7307	CakSNP7307	*Kabuli*	Ca_Kabuli_Chr04	42092385	(G/A)
7308	CakSNP7308	*Kabuli*	Ca_Kabuli_Chr04	42105737	(C/T)
7309	CakSNP7309	*Kabuli*	Ca_Kabuli_Chr04	42105701	(A/T)
7310	CakSNP7310	*Kabuli*	Ca_Kabuli_Chr04	42108980	(C/A)
7311	CakSNP7311	*Kabuli*	Ca_Kabuli_Chr04	42246499	(C/T)
7312	CakSNP7312	*Kabuli*	Ca_Kabuli_Chr04	42246527	(C/A)
7313	CakSNP7313	*Kabuli*	Ca_Kabuli_Chr04	42246565	(T/G)
7314	CakSNP7314	*Kabuli*	Ca_Kabuli_Chr04	42246566	(T/G)
7315	CakSNP7315	*Kabuli*	Ca_Kabuli_Chr04	42246572	(T/G)
7316	CakSNP7316	*Kabuli*	Ca_Kabuli_Chr04	42246653	(C/G)
7317	CakSNP7317	*Kabuli*	Ca_Kabuli_Chr04	42273252	(G/A)
7318	CakSNP7318	*Kabuli*	Ca_Kabuli_Chr04	42353949	(C/T)
7319	CakSNP7319	*Kabuli*	Ca_Kabuli_Chr04	42499699	(A/T)
7320	CakSNP7320	*Kabuli*	Ca_Kabuli_Chr04	42603642	(T/C)
7321	CakSNP7321	*Kabuli*	Ca_Kabuli_Chr04	42768142	(T/G)
7322	CakSNP7322	*Kabuli*	Ca_Kabuli_Chr04	42807483	(G/A)
7323	CakSNP7323	*Kabuli*	Ca_Kabuli_Chr04	42807518	(A/G)
7324	CakSNP7324	*Kabuli*	Ca_Kabuli_Chr04	42807509	(C/T)
7325	CakSNP7325	*Kabuli*	Ca_Kabuli_Chr04	43319846	(A/G)
7326	CakSNP7326	*Kabuli*	Ca_Kabuli_Chr04	43324547	(G/C)
7327	CakSNP7327	*Kabuli*	Ca_Kabuli_Chr04	43431770	(C/T)
7328	CakSNP7328	*Kabuli*	Ca_Kabuli_Chr04	43431772	(T/A)
7329	CakSNP7329	*Kabuli*	Ca_Kabuli_Chr04	43431815	(G/T)
7330	CakSNP7330	*Kabuli*	Ca_Kabuli_Chr04	43446753	(T/A)

S.N.	SNP IDs	Cultivars	Chromosomes/scaffolds	Physical positions (bp)	SNPs
7331	CakSNP7331	*Kabuli*	Ca_Kabuli_Chr04	43447262	(A/T)
7332	CakSNP7332	*Kabuli*	Ca_Kabuli_Chr04	43476193	(T/C)
7333	CakSNP7333	*Kabuli*	Ca_Kabuli_Chr04	43653225	(A/G)
7334	CakSNP7334	*Kabuli*	Ca_Kabuli_Chr04	43653226	(C/T)
7335	CakSNP7335	*Kabuli*	Ca_Kabuli_Chr04	43662224	(A/C)
7336	CakSNP7336	*Kabuli*	Ca_Kabuli_Chr04	43677006	(T/G)
7337	CakSNP7337	*Kabuli*	Ca_Kabuli_Chr04	43677222	(C/T)
7338	CakSNP7338	*Kabuli*	Ca_Kabuli_Chr04	43722728	(T/C)
7339	CakSNP7339	*Kabuli*	Ca_Kabuli_Chr04	43722727	(A/G)
7340	CakSNP7340	*Kabuli*	Ca_Kabuli_Chr04	43722704	(C/G)
7341	CakSNP7341	*Kabuli*	Ca_Kabuli_Chr04	43723219	(T/G)
7342	CakSNP7342	*Kabuli*	Ca_Kabuli_Chr04	43723224	(C/A)
7343	CakSNP7343	*Kabuli*	Ca_Kabuli_Chr04	43723248	(T/C)
7344	CakSNP7344	*Kabuli*	Ca_Kabuli_Chr04	43726406	(C/T)
7345	CakSNP7345	*Kabuli*	Ca_Kabuli_Chr04	43729063	(G/T)
7346	CakSNP7346	*Kabuli*	Ca_Kabuli_Chr04	43729261	(C/G)
7347	CakSNP7347	*Kabuli*	Ca_Kabuli_Chr04	43729266	(A/G)
7348	CakSNP7348	*Kabuli*	Ca_Kabuli_Chr04	43807399	(C/T)
7349	CakSNP7349	*Kabuli*	Ca_Kabuli_Chr04	43807460	(C/T)
7350	CakSNP7350	*Kabuli*	Ca_Kabuli_Chr04	43840666	(C/G)
7351	CakSNP7351	*Kabuli*	Ca_Kabuli_Chr04	43840650	(C/T)
7352	CakSNP7352	*Kabuli*	Ca_Kabuli_Chr04	43979065	(A/G)
7353	CakSNP7353	*Kabuli*	Ca_Kabuli_Chr04	44008835	(T/C)
7354	CakSNP7354	*Kabuli*	Ca_Kabuli_Chr04	44059060	(C/T)
7355	CakSNP7355	*Kabuli*	Ca_Kabuli_Chr04	44059061	(G/A)
7356	CakSNP7356	*Kabuli*	Ca_Kabuli_Chr04	44067780	(G/A)
7357	CakSNP7357	*Kabuli*	Ca_Kabuli_Chr04	44090929	(T/G)
7358	CakSNP7358	*Kabuli*	Ca_Kabuli_Chr04	44186434	(C/G)
7359	CakSNP7359	*Kabuli*	Ca_Kabuli_Chr04	44239783	(A/C)
7360	CakSNP7360	*Kabuli*	Ca_Kabuli_Chr04	44239883	(A/T)
7361	CakSNP7361	*Kabuli*	Ca_Kabuli_Chr04	44250159	(C/T)
7362	CakSNP7362	*Kabuli*	Ca_Kabuli_Chr04	44261184	(A/C)
7363	CakSNP7363	*Kabuli*	Ca_Kabuli_Chr04	44261909	(T/A)
7364	CakSNP7364	*Kabuli*	Ca_Kabuli_Chr04	44266692	(T/A)
7365	CakSNP7365	*Kabuli*	Ca_Kabuli_Chr04	44266695	(G/A)
7366	CakSNP7366	*Kabuli*	Ca_Kabuli_Chr04	44266758	(C/T)
7367	CakSNP7367	*Kabuli*	Ca_Kabuli_Chr04	44266819	(G/A)
7368	CakSNP7368	*Kabuli*	Ca_Kabuli_Chr04	44353354	(C/T)
7369	CakSNP7369	*Kabuli*	Ca_Kabuli_Chr04	44353343	(G/A)

S.N.	SNP IDs	Cultivars	Chromosomes/scaffolds	Physical positions (bp)	SNPs
7370	CakSNP7370	*Kabuli*	Ca_Kabuli_Chr04	44375761	(T/C)
7371	CakSNP7371	*Kabuli*	Ca_Kabuli_Chr04	44375831	(T/C)
7372	CakSNP7372	*Kabuli*	Ca_Kabuli_Chr04	44382515	(A/G)
7373	CakSNP7373	*Kabuli*	Ca_Kabuli_Chr04	44382677	(C/T)
7374	CakSNP7374	*Kabuli*	Ca_Kabuli_Chr04	44382671	(C/G)
7375	CakSNP7375	*Kabuli*	Ca_Kabuli_Chr04	44382614	(C/A)
7376	CakSNP7376	*Kabuli*	Ca_Kabuli_Chr04	44382602	(A/G)
7377	CakSNP7377	*Kabuli*	Ca_Kabuli_Chr04	44386579	(A/G)
7378	CakSNP7378	*Kabuli*	Ca_Kabuli_Chr04	44431319	(A/C)
7379	CakSNP7379	*Kabuli*	Ca_Kabuli_Chr04	44431353	(G/A)
7380	CakSNP7380	*Kabuli*	Ca_Kabuli_Chr04	44439372	(C/T)
7381	CakSNP7381	*Kabuli*	Ca_Kabuli_Chr04	44439503	(C/A)
7382	CakSNP7382	*Kabuli*	Ca_Kabuli_Chr04	44439495	(G/A)
7383	CakSNP7383	*Kabuli*	Ca_Kabuli_Chr04	44439493	(A/G)
7384	CakSNP7384	*Kabuli*	Ca_Kabuli_Chr04	44439492	(G/T)
7385	CakSNP7385	*Kabuli*	Ca_Kabuli_Chr04	44439448	(T/C)
7386	CakSNP7386	*Kabuli*	Ca_Kabuli_Chr04	44446468	(G/A)
7387	CakSNP7387	*Kabuli*	Ca_Kabuli_Chr04	44446575	(G/A)
7388	CakSNP7388	*Kabuli*	Ca_Kabuli_Chr04	44446506	(C/T)
7389	CakSNP7389	*Kabuli*	Ca_Kabuli_Chr04	44446503	(T/G)
7390	CakSNP7390	*Kabuli*	Ca_Kabuli_Chr04	44446497	(C/T)
7391	CakSNP7391	*Kabuli*	Ca_Kabuli_Chr04	44472282	(G/A)
7392	CakSNP7392	*Kabuli*	Ca_Kabuli_Chr04	44472278	(A/G)
7393	CakSNP7393	*Kabuli*	Ca_Kabuli_Chr04	44472237	(T/A)
7394	CakSNP7394	*Kabuli*	Ca_Kabuli_Chr04	44590652	(T/C)
7395	CakSNP7395	*Kabuli*	Ca_Kabuli_Chr04	44590678	(G/A)
7396	CakSNP7396	*Kabuli*	Ca_Kabuli_Chr04	44590697	(A/C)
7397	CakSNP7397	*Kabuli*	Ca_Kabuli_Chr04	44591405	(G/T)
7398	CakSNP7398	*Kabuli*	Ca_Kabuli_Chr04	44754355	(C/T)
7399	CakSNP7399	*Kabuli*	Ca_Kabuli_Chr04	44802374	(G/A)
7400	CakSNP7400	*Kabuli*	Ca_Kabuli_Chr04	44802364	(A/C)
7401	CakSNP7401	*Kabuli*	Ca_Kabuli_Chr04	44803694	(T/A)
7402	CakSNP7402	*Kabuli*	Ca_Kabuli_Chr04	44811519	(G/A)
7403	CakSNP7403	*Kabuli*	Ca_Kabuli_Chr04	44814857	(A/G)
7404	CakSNP7404	*Kabuli*	Ca_Kabuli_Chr04	44814981	(T/C)
7405	CakSNP7405	*Kabuli*	Ca_Kabuli_Chr04	44814982	(A/G)
7406	CakSNP7406	*Kabuli*	Ca_Kabuli_Chr04	44824103	(G/T)
7407	CakSNP7407	*Kabuli*	Ca_Kabuli_Chr04	44824105	(T/G)
7408	CakSNP7408	*Kabuli*	Ca_Kabuli_Chr04	44824178	(C/G)

S.N.	SNP IDs	Cultivars	Chromosomes/scaffolds	Physical positions (bp)	SNPs
7409	CakSNP7409	*Kabuli*	Ca_Kabuli_Chr04	44824258	(A/G)
7410	CakSNP7410	*Kabuli*	Ca_Kabuli_Chr04	44832121	(G/C)
7411	CakSNP7411	*Kabuli*	Ca_Kabuli_Chr04	44832144	(T/C)
7412	CakSNP7412	*Kabuli*	Ca_Kabuli_Chr04	44906128	(C/A)
7413	CakSNP7413	*Kabuli*	Ca_Kabuli_Chr04	44906255	(A/G)
7414	CakSNP7414	*Kabuli*	Ca_Kabuli_Chr04	44979108	(G/A)
7415	CakSNP7415	*Kabuli*	Ca_Kabuli_Chr04	45019764	(T/C)
7416	CakSNP7416	*Kabuli*	Ca_Kabuli_Chr04	45019778	(T/C)
7417	CakSNP7417	*Kabuli*	Ca_Kabuli_Chr04	45019787	(G/C)
7418	CakSNP7418	*Kabuli*	Ca_Kabuli_Chr04	45019805	(C/A)
7419	CakSNP7419	*Kabuli*	Ca_Kabuli_Chr04	45019807	(C/T)
7420	CakSNP7420	*Kabuli*	Ca_Kabuli_Chr04	45019814	(C/A)
7421	CakSNP7421	*Kabuli*	Ca_Kabuli_Chr04	45019820	(C/T)
7422	CakSNP7422	*Kabuli*	Ca_Kabuli_Chr04	45019824	(A/C)
7423	CakSNP7423	*Kabuli*	Ca_Kabuli_Chr04	45019867	(C/G)
7424	CakSNP7424	*Kabuli*	Ca_Kabuli_Chr04	45019858	(G/T)
7425	CakSNP7425	*Kabuli*	Ca_Kabuli_Chr04	45019848	(C/T)
7426	CakSNP7426	*Kabuli*	Ca_Kabuli_Chr04	45035589	(G/T)
7427	CakSNP7427	*Kabuli*	Ca_Kabuli_Chr04	45035629	(A/C)
7428	CakSNP7428	*Kabuli*	Ca_Kabuli_Chr04	45035745	(A/G)
7429	CakSNP7429	*Kabuli*	Ca_Kabuli_Chr04	45067534	(C/T)
7430	CakSNP7430	*Kabuli*	Ca_Kabuli_Chr04	45104767	(G/A)
7431	CakSNP7431	*Kabuli*	Ca_Kabuli_Chr04	45104779	(C/A)
7432	CakSNP7432	*Kabuli*	Ca_Kabuli_Chr04	45104805	(A/G)
7433	CakSNP7433	*Kabuli*	Ca_Kabuli_Chr04	45141976	(C/A)
7434	CakSNP7434	*Kabuli*	Ca_Kabuli_Chr04	45141990	(A/G)
7435	CakSNP7435	*Kabuli*	Ca_Kabuli_Chr04	45142020	(T/C)
7436	CakSNP7436	*Kabuli*	Ca_Kabuli_Chr04	45142009	(C/T)
7437	CakSNP7437	*Kabuli*	Ca_Kabuli_Chr04	45142003	(A/G)
7438	CakSNP7438	*Kabuli*	Ca_Kabuli_Chr04	45142077	(C/A)
7439	CakSNP7439	*Kabuli*	Ca_Kabuli_Chr04	45142087	(T/A)
7440	CakSNP7440	*Kabuli*	Ca_Kabuli_Chr04	45142104	(T/C)
7441	CakSNP7441	*Kabuli*	Ca_Kabuli_Chr04	45165191	(T/C)
7442	CakSNP7442	*Kabuli*	Ca_Kabuli_Chr04	45165246	(C/G)
7443	CakSNP7443	*Kabuli*	Ca_Kabuli_Chr04	45165240	(T/C)
7444	CakSNP7444	*Kabuli*	Ca_Kabuli_Chr04	45165231	(A/C)
7445	CakSNP7445	*Kabuli*	Ca_Kabuli_Chr04	45206203	(G/T)
7446	CakSNP7446	*Kabuli*	Ca_Kabuli_Chr04	45206209	(G/C)
7447	CakSNP7447	*Kabuli*	Ca_Kabuli_Chr04	45220084	(G/T)

S.N.	SNP IDs	Cultivars	Chromosomes/scaffolds	Physical positions (bp)	SNPs
7448	CakSNP7448	*Kabuli*	Ca_Kabuli_Chr04	45220642	(C/T)
7449	CakSNP7449	*Kabuli*	Ca_Kabuli_Chr04	45220916	(T/C)
7450	CakSNP7450	*Kabuli*	Ca_Kabuli_Chr04	45225063	(T/C)
7451	CakSNP7451	*Kabuli*	Ca_Kabuli_Chr04	45225059	(G/A)
7452	CakSNP7452	*Kabuli*	Ca_Kabuli_Chr04	45224997	(G/C)
7453	CakSNP7453	*Kabuli*	Ca_Kabuli_Chr04	45225077	(A/T)
7454	CakSNP7454	*Kabuli*	Ca_Kabuli_Chr04	45225093	(G/A)
7455	CakSNP7455	*Kabuli*	Ca_Kabuli_Chr04	45225145	(T/A)
7456	CakSNP7456	*Kabuli*	Ca_Kabuli_Chr04	45225154	(T/A)
7457	CakSNP7457	*Kabuli*	Ca_Kabuli_Chr04	45225155	(C/A)
7458	CakSNP7458	*Kabuli*	Ca_Kabuli_Chr04	45225210	(G/T)
7459	CakSNP7459	*Kabuli*	Ca_Kabuli_Chr04	45225193	(A/G)
7460	CakSNP7460	*Kabuli*	Ca_Kabuli_Chr04	45225165	(T/C)
7461	CakSNP7461	*Kabuli*	Ca_Kabuli_Chr04	45231839	(T/C)
7462	CakSNP7462	*Kabuli*	Ca_Kabuli_Chr04	45231867	(A/T)
7463	CakSNP7463	*Kabuli*	Ca_Kabuli_Chr04	45231868	(C/A)
7464	CakSNP7464	*Kabuli*	Ca_Kabuli_Chr04	45231893	(G/T)
7465	CakSNP7465	*Kabuli*	Ca_Kabuli_Chr04	45292852	(G/T)
7466	CakSNP7466	*Kabuli*	Ca_Kabuli_Chr04	45292918	(T/G)
7467	CakSNP7467	*Kabuli*	Ca_Kabuli_Chr04	45292981	(G/T)
7468	CakSNP7468	*Kabuli*	Ca_Kabuli_Chr04	45292969	(T/A)
7469	CakSNP7469	*Kabuli*	Ca_Kabuli_Chr04	45292960	(A/T)
7470	CakSNP7470	*Kabuli*	Ca_Kabuli_Chr04	45552675	(C/A)
7471	CakSNP7471	*Kabuli*	Ca_Kabuli_Chr04	45595233	(G/A)
7472	CakSNP7472	*Kabuli*	Ca_Kabuli_Chr04	45612178	(T/G)
7473	CakSNP7473	*Kabuli*	Ca_Kabuli_Chr04	45615465	(C/T)
7474	CakSNP7474	*Kabuli*	Ca_Kabuli_Chr04	45615457	(C/T)
7475	CakSNP7475	*Kabuli*	Ca_Kabuli_Chr04	45615452	(A/C)
7476	CakSNP7476	*Kabuli*	Ca_Kabuli_Chr04	45615401	(T/G)
7477	CakSNP7477	*Kabuli*	Ca_Kabuli_Chr04	45646003	(A/G)
7478	CakSNP7478	*Kabuli*	Ca_Kabuli_Chr04	45645996	(A/G)
7479	CakSNP7479	*Kabuli*	Ca_Kabuli_Chr04	45645957	(A/C)
7480	CakSNP7480	*Kabuli*	Ca_Kabuli_Chr04	45708895	(C/A)
7481	CakSNP7481	*Kabuli*	Ca_Kabuli_Chr04	45735754	(G/C)
7482	CakSNP7482	*Kabuli*	Ca_Kabuli_Chr04	45735819	(T/C)
7483	CakSNP7483	*Kabuli*	Ca_Kabuli_Chr04	45751623	(G/A)
7484	CakSNP7484	*Kabuli*	Ca_Kabuli_Chr04	45751618	(C/T)
7485	CakSNP7485	*Kabuli*	Ca_Kabuli_Chr04	45752844	(G/A)
7486	CakSNP7486	*Kabuli*	Ca_Kabuli_Chr04	45752869	(G/T)

S.N.	SNP IDs	Cultivars	Chromosomes/scaffolds	Physical positions (bp)	SNPs
7487	CakSNP7487	*Kabuli*	Ca_Kabuli_Chr04	45752874	(A/T)
7488	CakSNP7488	*Kabuli*	Ca_Kabuli_Chr04	45782453	(A/T)
7489	CakSNP7489	*Kabuli*	Ca_Kabuli_Chr04	45870087	(T/C)
7490	CakSNP7490	*Kabuli*	Ca_Kabuli_Chr04	45870042	(C/T)
7491	CakSNP7491	*Kabuli*	Ca_Kabuli_Chr04	45918351	(A/C)
7492	CakSNP7492	*Kabuli*	Ca_Kabuli_Chr04	45935594	(A/C)
7493	CakSNP7493	*Kabuli*	Ca_Kabuli_Chr04	45942252	(G/A)
7494	CakSNP7494	*Kabuli*	Ca_Kabuli_Chr04	45942341	(T/C)
7495	CakSNP7495	*Kabuli*	Ca_Kabuli_Chr04	45942729	(G/A)
7496	CakSNP7496	*Kabuli*	Ca_Kabuli_Chr04	46134302	(C/G)
7497	CakSNP7497	*Kabuli*	Ca_Kabuli_Chr04	46140729	(A/T)
7498	CakSNP7498	*Kabuli*	Ca_Kabuli_Chr04	46148985	(A/T)
7499	CakSNP7499	*Kabuli*	Ca_Kabuli_Chr04	46149013	(A/G)
7500	CakSNP7500	*Kabuli*	Ca_Kabuli_Chr04	46150670	(T/C)
7501	CakSNP7501	*Kabuli*	Ca_Kabuli_Chr04	46202862	(A/G)
7502	CakSNP7502	*Kabuli*	Ca_Kabuli_Chr04	46344790	(G/A)
7503	CakSNP7503	*Kabuli*	Ca_Kabuli_Chr04	46344826	(A/G)
7504	CakSNP7504	*Kabuli*	Ca_Kabuli_Chr04	46345042	(A/G)
7505	CakSNP7505	*Kabuli*	Ca_Kabuli_Chr04	46402035	(G/A)
7506	CakSNP7506	*Kabuli*	Ca_Kabuli_Chr04	46430326	(C/T)
7507	CakSNP7507	*Kabuli*	Ca_Kabuli_Chr04	46430311	(C/A)
7508	CakSNP7508	*Kabuli*	Ca_Kabuli_Chr04	46430288	(G/C)
7509	CakSNP7509	*Kabuli*	Ca_Kabuli_Chr04	46605167	(A/G)
7510	CakSNP7510	*Kabuli*	Ca_Kabuli_Chr04	46605131	(T/A)
7511	CakSNP7511	*Kabuli*	Ca_Kabuli_Chr04	46612175	(G/A)
7512	CakSNP7512	*Kabuli*	Ca_Kabuli_Chr04	46612197	(G/A)
7513	CakSNP7513	*Kabuli*	Ca_Kabuli_Chr04	46612244	(C/T)
7514	CakSNP7514	*Kabuli*	Ca_Kabuli_Chr04	46613068	(T/G)
7515	CakSNP7515	*Kabuli*	Ca_Kabuli_Chr04	46694919	(T/A)
7516	CakSNP7516	*Kabuli*	Ca_Kabuli_Chr04	46695043	(A/C)
7517	CakSNP7517	*Kabuli*	Ca_Kabuli_Chr04	46695008	(T/C)
7518	CakSNP7518	*Kabuli*	Ca_Kabuli_Chr04	46705181	(G/T)
7519	CakSNP7519	*Kabuli*	Ca_Kabuli_Chr04	46705178	(A/T)
7520	CakSNP7520	*Kabuli*	Ca_Kabuli_Chr04	46705225	(A/C)
7521	CakSNP7521	*Kabuli*	Ca_Kabuli_Chr04	46713972	(C/T)
7522	CakSNP7522	*Kabuli*	Ca_Kabuli_Chr04	46715350	(T/C)
7523	CakSNP7523	*Kabuli*	Ca_Kabuli_Chr04	46715518	(G/C)
7524	CakSNP7524	*Kabuli*	Ca_Kabuli_Chr04	46717155	(T/C)
7525	CakSNP7525	*Kabuli*	Ca_Kabuli_Chr04	46717153	(C/T)

S.N.	SNP IDs	Cultivars	Chromosomes/scaffolds	Physical positions (bp)	SNPs
7526	CakSNP7526	*Kabuli*	Ca_Kabuli_Chr04	46717200	(C/T)
7527	CakSNP7527	*Kabuli*	Ca_Kabuli_Chr04	46717508	(G/A)
7528	CakSNP7528	*Kabuli*	Ca_Kabuli_Chr04	46763540	(C/T)
7529	CakSNP7529	*Kabuli*	Ca_Kabuli_Chr04	46763555	(C/T)
7530	CakSNP7530	*Kabuli*	Ca_Kabuli_Chr04	46763868	(T/C)
7531	CakSNP7531	*Kabuli*	Ca_Kabuli_Chr04	46763841	(A/C)
7532	CakSNP7532	*Kabuli*	Ca_Kabuli_Chr04	47223016	(T/C)
7533	CakSNP7533	*Kabuli*	Ca_Kabuli_Chr04	47223047	(A/G)
7534	CakSNP7534	*Kabuli*	Ca_Kabuli_Chr04	47223635	(G/A)
7535	CakSNP7535	*Kabuli*	Ca_Kabuli_Chr04	47223668	(T/G)
7536	CakSNP7536	*Kabuli*	Ca_Kabuli_Chr04	47296400	(C/T)
7537	CakSNP7537	*Kabuli*	Ca_Kabuli_Chr04	47379992	(C/T)
7538	CakSNP7538	*Kabuli*	Ca_Kabuli_Chr04	47405685	(T/A)
7539	CakSNP7539	*Kabuli*	Ca_Kabuli_Chr04	47419514	(A/G)
7540	CakSNP7540	*Kabuli*	Ca_Kabuli_Chr04	47419517	(C/T)
7541	CakSNP7541	*Kabuli*	Ca_Kabuli_Chr04	47428580	(C/T)
7542	CakSNP7542	*Kabuli*	Ca_Kabuli_Chr04	47428599	(C/T)
7543	CakSNP7543	*Kabuli*	Ca_Kabuli_Chr04	47428631	(A/G)
7544	CakSNP7544	*Kabuli*	Ca_Kabuli_Chr04	47428733	(A/G)
7545	CakSNP7545	*Kabuli*	Ca_Kabuli_Chr04	47440207	(G/A)
7546	CakSNP7546	*Kabuli*	Ca_Kabuli_Chr04	47450409	(C/T)
7547	CakSNP7547	*Kabuli*	Ca_Kabuli_Chr04	47450479	(C/G)
7548	CakSNP7548	*Kabuli*	Ca_Kabuli_Chr04	47562991	(A/T)
7549	CakSNP7549	*Kabuli*	Ca_Kabuli_Chr04	47572228	(G/A)
7550	CakSNP7550	*Kabuli*	Ca_Kabuli_Chr04	47572276	(C/T)
7551	CakSNP7551	*Kabuli*	Ca_Kabuli_Chr04	47578508	(C/T)
7552	CakSNP7552	*Kabuli*	Ca_Kabuli_Chr04	47578556	(T/G)
7553	CakSNP7553	*Kabuli*	Ca_Kabuli_Chr04	47578655	(C/T)
7554	CakSNP7554	*Kabuli*	Ca_Kabuli_Chr04	47578596	(C/T)
7555	CakSNP7555	*Kabuli*	Ca_Kabuli_Chr04	47588760	(G/A)
7556	CakSNP7556	*Kabuli*	Ca_Kabuli_Chr04	47612395	(C/A)
7557	CakSNP7557	*Kabuli*	Ca_Kabuli_Chr04	47612396	(G/T)
7558	CakSNP7558	*Kabuli*	Ca_Kabuli_Chr04	47615800	(G/C)
7559	CakSNP7559	*Kabuli*	Ca_Kabuli_Chr04	47616120	(C/T)
7560	CakSNP7560	*Kabuli*	Ca_Kabuli_Chr04	47616216	(A/G)
7561	CakSNP7561	*Kabuli*	Ca_Kabuli_Chr04	47622112	(G/T)
7562	CakSNP7562	*Kabuli*	Ca_Kabuli_Chr04	47675446	(A/G)
7563	CakSNP7563	*Kabuli*	Ca_Kabuli_Chr04	47675758	(A/T)
7564	CakSNP7564	*Kabuli*	Ca_Kabuli_Chr04	47675860	(G/A)

S.N.	SNP IDs	Cultivars	Chromosomes/scaffolds	Physical positions (bp)	SNPs
7565	CakSNP7565	*Kabuli*	Ca_Kabuli_Chr04	47697239	(C/T)
7566	CakSNP7566	*Kabuli*	Ca_Kabuli_Chr04	47798580	(A/G)
7567	CakSNP7567	*Kabuli*	Ca_Kabuli_Chr04	47798622	(G/A)
7568	CakSNP7568	*Kabuli*	Ca_Kabuli_Chr04	47819943	(T/C)
7569	CakSNP7569	*Kabuli*	Ca_Kabuli_Chr04	48008987	(T/C)
7570	CakSNP7570	*Kabuli*	Ca_Kabuli_Chr04	48009201	(C/T)
7571	CakSNP7571	*Kabuli*	Ca_Kabuli_Chr04	48011446	(A/G)
7572	CakSNP7572	*Kabuli*	Ca_Kabuli_Chr04	48098570	(G/A)
7573	CakSNP7573	*Kabuli*	Ca_Kabuli_Chr04	48098562	(G/A)
7574	CakSNP7574	*Kabuli*	Ca_Kabuli_Chr04	48103202	(G/T)
7575	CakSNP7575	*Kabuli*	Ca_Kabuli_Chr04	48103196	(G/A)
7576	CakSNP7576	*Kabuli*	Ca_Kabuli_Chr04	48103159	(G/A)
7577	CakSNP7577	*Kabuli*	Ca_Kabuli_Chr04	48103153	(A/C)
7578	CakSNP7578	*Kabuli*	Ca_Kabuli_Chr04	48150277	(C/T)
7579	CakSNP7579	*Kabuli*	Ca_Kabuli_Chr04	48150481	(T/C)
7580	CakSNP7580	*Kabuli*	Ca_Kabuli_Chr04	48150480	(C/A)
7581	CakSNP7581	*Kabuli*	Ca_Kabuli_Chr04	48150553	(A/G)
7582	CakSNP7582	*Kabuli*	Ca_Kabuli_Chr04	48150641	(G/T)
7583	CakSNP7583	*Kabuli*	Ca_Kabuli_Chr04	48238919	(C/T)
7584	CakSNP7584	*Kabuli*	Ca_Kabuli_Chr04	48239048	(A/G)
7585	CakSNP7585	*Kabuli*	Ca_Kabuli_Chr04	48263603	(G/A)
7586	CakSNP7586	*Kabuli*	Ca_Kabuli_Chr04	48269138	(G/C)
7587	CakSNP7587	*Kabuli*	Ca_Kabuli_Chr04	48275016	(C/T)
7588	CakSNP7588	*Kabuli*	Ca_Kabuli_Chr04	48322502	(C/T)
7589	CakSNP7589	*Kabuli*	Ca_Kabuli_Chr04	48322507	(C/T)
7590	CakSNP7590	*Kabuli*	Ca_Kabuli_Chr04	48322633	(T/C)
7591	CakSNP7591	*Kabuli*	Ca_Kabuli_Chr04	48331069	(C/A)
7592	CakSNP7592	*Kabuli*	Ca_Kabuli_Chr04	48345604	(A/G)
7593	CakSNP7593	*Kabuli*	Ca_Kabuli_Chr04	48345608	(G/T)
7594	CakSNP7594	*Kabuli*	Ca_Kabuli_Chr04	48345655	(A/C)
7595	CakSNP7595	*Kabuli*	Ca_Kabuli_Chr04	48345697	(C/G)
7596	CakSNP7596	*Kabuli*	Ca_Kabuli_Chr04	48355358	(A/T)
7597	CakSNP7597	*Kabuli*	Ca_Kabuli_Chr04	48355468	(G/T)
7598	CakSNP7598	*Kabuli*	Ca_Kabuli_Chr04	48364170	(G/A)
7599	CakSNP7599	*Kabuli*	Ca_Kabuli_Chr04	48365208	(C/A)
7600	CakSNP7600	*Kabuli*	Ca_Kabuli_Chr04	48365388	(G/A)
7601	CakSNP7601	*Kabuli*	Ca_Kabuli_Chr04	48382666	(G/C)
7602	CakSNP7602	*Kabuli*	Ca_Kabuli_Chr04	48464493	(T/C)
7603	CakSNP7603	*Kabuli*	Ca_Kabuli_Chr04	48464434	(G/C)

S.N.	SNP IDs	Cultivars	Chromosomes/scaffolds	Physical positions (bp)	SNPs
7604	CakSNP7604	*Kabuli*	Ca_Kabuli_Chr04	48470477	(A/G)
7605	CakSNP7605	*Kabuli*	Ca_Kabuli_Chr04	48475447	(T/C)
7606	CakSNP7606	*Kabuli*	Ca_Kabuli_Chr04	48475461	(G/A)
7607	CakSNP7607	*Kabuli*	Ca_Kabuli_Chr04	48478018	(A/G)
7608	CakSNP7608	*Kabuli*	Ca_Kabuli_Chr04	48478058	(T/C)
7609	CakSNP7609	*Kabuli*	Ca_Kabuli_Chr04	48478303	(G/A)
7610	CakSNP7610	*Kabuli*	Ca_Kabuli_Chr04	48478524	(C/T)
7611	CakSNP7611	*Kabuli*	Ca_Kabuli_Chr04	48484453	(A/T)
7612	CakSNP7612	*Kabuli*	Ca_Kabuli_Chr04	48497731	(A/C)
7613	CakSNP7613	*Kabuli*	Ca_Kabuli_Chr04	48497725	(A/C)
7614	CakSNP7614	*Kabuli*	Ca_Kabuli_Chr04	48497756	(A/G)
7615	CakSNP7615	*Kabuli*	Ca_Kabuli_Chr04	48497765	(G/A)
7616	CakSNP7616	*Kabuli*	Ca_Kabuli_Chr04	48497839	(G/A)
7617	CakSNP7617	*Kabuli*	Ca_Kabuli_Chr04	48498133	(C/T)
7618	CakSNP7618	*Kabuli*	Ca_Kabuli_Chr04	48498181	(G/C)
7619	CakSNP7619	*Kabuli*	Ca_Kabuli_Chr04	48498166	(A/G)
7620	CakSNP7620	*Kabuli*	Ca_Kabuli_Chr04	48498368	(C/T)
7621	CakSNP7621	*Kabuli*	Ca_Kabuli_Chr04	48498334	(T/G)
7622	CakSNP7622	*Kabuli*	Ca_Kabuli_Chr04	48498331	(T/A)
7623	CakSNP7623	*Kabuli*	Ca_Kabuli_Chr04	48498312	(T/C)
7624	CakSNP7624	*Kabuli*	Ca_Kabuli_Chr04	48501567	(G/T)
7625	CakSNP7625	*Kabuli*	Ca_Kabuli_Chr04	48501618	(C/A)
7626	CakSNP7626	*Kabuli*	Ca_Kabuli_Chr04	48501662	(A/T)
7627	CakSNP7627	*Kabuli*	Ca_Kabuli_Chr04	48502554	(G/T)
7628	CakSNP7628	*Kabuli*	Ca_Kabuli_Chr04	48502718	(G/C)
7629	CakSNP7629	*Kabuli*	Ca_Kabuli_Chr04	48502714	(T/C)
7630	CakSNP7630	*Kabuli*	Ca_Kabuli_Chr04	48502687	(G/A)
7631	CakSNP7631	*Kabuli*	Ca_Kabuli_Chr04	48591482	(T/G)
7632	CakSNP7632	*Kabuli*	Ca_Kabuli_Chr04	48591539	(T/C)
7633	CakSNP7633	*Kabuli*	Ca_Kabuli_Chr04	48591578	(T/C)
7634	CakSNP7634	*Kabuli*	Ca_Kabuli_Chr04	48591625	(G/T)
7635	CakSNP7635	*Kabuli*	Ca_Kabuli_Chr04	48678278	(T/C)
7636	CakSNP7636	*Kabuli*	Ca_Kabuli_Chr04	48678692	(T/C)
7637	CakSNP7637	*Kabuli*	Ca_Kabuli_Chr04	48688766	(A/G)
7638	CakSNP7638	*Kabuli*	Ca_Kabuli_Chr04	48705815	(C/T)
7639	CakSNP7639	*Kabuli*	Ca_Kabuli_Chr04	48705856	(A/T)
7640	CakSNP7640	*Kabuli*	Ca_Kabuli_Chr04	48705957	(C/T)
7641	CakSNP7641	*Kabuli*	Ca_Kabuli_Chr04	48714912	(G/A)
7642	CakSNP7642	*Kabuli*	Ca_Kabuli_Chr04	48715028	(A/G)

S.N.	SNP IDs	Cultivars	Chromosomes/scaffolds	Physical positions (bp)	SNPs
7643	CakSNP7643	*Kabuli*	Ca_Kabuli_Chr04	48720330	(C/T)
7644	CakSNP7644	*Kabuli*	Ca_Kabuli_Chr04	48720266	(C/T)
7645	CakSNP7645	*Kabuli*	Ca_Kabuli_Chr04	48784151	(T/G)
7646	CakSNP7646	*Kabuli*	Ca_Kabuli_Chr04	48784176	(T/C)
7647	CakSNP7647	*Kabuli*	Ca_Kabuli_Chr04	48784178	(C/G)
7648	CakSNP7648	*Kabuli*	Ca_Kabuli_Chr04	48784179	(G/A)
7649	CakSNP7649	*Kabuli*	Ca_Kabuli_Chr04	48784189	(A/T)
7650	CakSNP7650	*Kabuli*	Ca_Kabuli_Chr04	48784202	(A/C)
7651	CakSNP7651	*Kabuli*	Ca_Kabuli_Chr04	48784204	(G/C)
7652	CakSNP7652	*Kabuli*	Ca_Kabuli_Chr04	48931112	(T/G)
7653	CakSNP7653	*Kabuli*	Ca_Kabuli_Chr04	48931109	(A/G)
7654	CakSNP7654	*Kabuli*	Ca_Kabuli_Chr04	48936643	(G/A)
7655	CakSNP7655	*Kabuli*	Ca_Kabuli_Chr04	48936669	(G/A)
7656	CakSNP7656	*Kabuli*	Ca_Kabuli_Chr04	48936710	(A/G)
7657	CakSNP7657	*Kabuli*	Ca_Kabuli_Chr04	48936819	(C/T)
7658	CakSNP7658	*Kabuli*	Ca_Kabuli_Chr04	48964200	(T/C)
7659	CakSNP7659	*Kabuli*	Ca_Kabuli_Chr04	48964260	(G/C)
7660	CakSNP7660	*Kabuli*	Ca_Kabuli_Chr04	48964248	(C/T)
7661	CakSNP7661	*Kabuli*	Ca_Kabuli_Chr04	49012733	(T/A)
7662	CakSNP7662	*Kabuli*	Ca_Kabuli_Chr04	49012756	(C/T)
7663	CakSNP7663	*Kabuli*	Ca_Kabuli_Chr05	95087	(G/A)
7664	CakSNP7664	*Kabuli*	Ca_Kabuli_Chr05	95253	(A/G)
7665	CakSNP7665	*Kabuli*	Ca_Kabuli_Chr05	131558	(A/C)
7666	CakSNP7666	*Kabuli*	Ca_Kabuli_Chr05	154084	(T/G)
7667	CakSNP7667	*Kabuli*	Ca_Kabuli_Chr05	154095	(C/G)
7668	CakSNP7668	*Kabuli*	Ca_Kabuli_Chr05	154097	(A/T)
7669	CakSNP7669	*Kabuli*	Ca_Kabuli_Chr05	154162	(C/T)
7670	CakSNP7670	*Kabuli*	Ca_Kabuli_Chr05	154128	(G/T)
7671	CakSNP7671	*Kabuli*	Ca_Kabuli_Chr05	166222	(T/G)
7672	CakSNP7672	*Kabuli*	Ca_Kabuli_Chr05	166217	(G/C)
7673	CakSNP7673	*Kabuli*	Ca_Kabuli_Chr05	166216	(T/G)
7674	CakSNP7674	*Kabuli*	Ca_Kabuli_Chr05	166341	(C/A)
7675	CakSNP7675	*Kabuli*	Ca_Kabuli_Chr05	166381	(A/G)
7676	CakSNP7676	*Kabuli*	Ca_Kabuli_Chr05	210625	(T/C)
7677	CakSNP7677	*Kabuli*	Ca_Kabuli_Chr05	210649	(C/A)
7678	CakSNP7678	*Kabuli*	Ca_Kabuli_Chr05	222651	(A/C)
7679	CakSNP7679	*Kabuli*	Ca_Kabuli_Chr05	222630	(A/G)
7680	CakSNP7680	*Kabuli*	Ca_Kabuli_Chr05	222641	(C/T)
7681	CakSNP7681	*Kabuli*	Ca_Kabuli_Chr05	222653	(A/C)

S.N.	SNP IDs	Cultivars	Chromosomes/scaffolds	Physical positions (bp)	SNPs
7682	CakSNP7682	*Kabuli*	Ca_Kabuli_Chr05	222657	(G/A)
7683	CakSNP7683	*Kabuli*	Ca_Kabuli_Chr05	223384	(C/G)
7684	CakSNP7684	*Kabuli*	Ca_Kabuli_Chr05	234247	(T/C)
7685	CakSNP7685	*Kabuli*	Ca_Kabuli_Chr05	234319	(T/A)
7686	CakSNP7686	*Kabuli*	Ca_Kabuli_Chr05	234311	(T/A)
7687	CakSNP7687	*Kabuli*	Ca_Kabuli_Chr05	234310	(G/A)
7688	CakSNP7688	*Kabuli*	Ca_Kabuli_Chr05	234305	(T/A)
7689	CakSNP7689	*Kabuli*	Ca_Kabuli_Chr05	234303	(C/A)
7690	CakSNP7690	*Kabuli*	Ca_Kabuli_Chr05	234410	(A/C)
7691	CakSNP7691	*Kabuli*	Ca_Kabuli_Chr05	245850	(T/G)
7692	CakSNP7692	*Kabuli*	Ca_Kabuli_Chr05	245863	(A/T)
7693	CakSNP7693	*Kabuli*	Ca_Kabuli_Chr05	245896	(A/T)
7694	CakSNP7694	*Kabuli*	Ca_Kabuli_Chr05	246466	(A/T)
7695	CakSNP7695	*Kabuli*	Ca_Kabuli_Chr05	246654	(A/G)
7696	CakSNP7696	*Kabuli*	Ca_Kabuli_Chr05	256745	(G/A)
7697	CakSNP7697	*Kabuli*	Ca_Kabuli_Chr05	272972	(T/A)
7698	CakSNP7698	*Kabuli*	Ca_Kabuli_Chr05	277040	(G/A)
7699	CakSNP7699	*Kabuli*	Ca_Kabuli_Chr05	277316	(T/A)
7700	CakSNP7700	*Kabuli*	Ca_Kabuli_Chr05	343517	(C/T)
7701	CakSNP7701	*Kabuli*	Ca_Kabuli_Chr05	353792	(T/A)
7702	CakSNP7702	*Kabuli*	Ca_Kabuli_Chr05	438448	(C/A)
7703	CakSNP7703	*Kabuli*	Ca_Kabuli_Chr05	438445	(C/A)
7704	CakSNP7704	*Kabuli*	Ca_Kabuli_Chr05	438442	(T/C)
7705	CakSNP7705	*Kabuli*	Ca_Kabuli_Chr05	443309	(A/T)
7706	CakSNP7706	*Kabuli*	Ca_Kabuli_Chr05	469397	(G/A)
7707	CakSNP7707	*Kabuli*	Ca_Kabuli_Chr05	805859	(C/T)
7708	CakSNP7708	*Kabuli*	Ca_Kabuli_Chr05	805882	(A/T)
7709	CakSNP7709	*Kabuli*	Ca_Kabuli_Chr05	805883	(G/T)
7710	CakSNP7710	*Kabuli*	Ca_Kabuli_Chr05	805910	(G/A)
7711	CakSNP7711	*Kabuli*	Ca_Kabuli_Chr05	870867	(A/G)
7712	CakSNP7712	*Kabuli*	Ca_Kabuli_Chr05	870909	(G/A)
7713	CakSNP7713	*Kabuli*	Ca_Kabuli_Chr05	925554	(T/C)
7714	CakSNP7714	*Kabuli*	Ca_Kabuli_Chr05	925493	(T/C)
7715	CakSNP7715	*Kabuli*	Ca_Kabuli_Chr05	1141755	(C/T)
7716	CakSNP7716	*Kabuli*	Ca_Kabuli_Chr05	1220867	(A/G)
7717	CakSNP7717	*Kabuli*	Ca_Kabuli_Chr05	1252394	(G/A)
7718	CakSNP7718	*Kabuli*	Ca_Kabuli_Chr05	1252388	(A/T)
7719	CakSNP7719	*Kabuli*	Ca_Kabuli_Chr05	1270830	(T/C)
7720	CakSNP7720	*Kabuli*	Ca_Kabuli_Chr05	1272581	(T/C)

S.N.	SNP IDs	Cultivars	Chromosomes/scaffolds	Physical positions (bp)	SNPs
7721	CakSNP7721	*Kabuli*	Ca_Kabuli_Chr05	1280977	(T/G)
7722	CakSNP7722	*Kabuli*	Ca_Kabuli_Chr05	1281006	(A/C)
7723	CakSNP7723	*Kabuli*	Ca_Kabuli_Chr05	1281017	(G/A)
7724	CakSNP7724	*Kabuli*	Ca_Kabuli_Chr05	1281021	(C/T)
7725	CakSNP7725	*Kabuli*	Ca_Kabuli_Chr05	1534904	(C/T)
7726	CakSNP7726	*Kabuli*	Ca_Kabuli_Chr05	1534901	(C/T)
7727	CakSNP7727	*Kabuli*	Ca_Kabuli_Chr05	1534888	(T/C)
7728	CakSNP7728	*Kabuli*	Ca_Kabuli_Chr05	2166586	(A/G)
7729	CakSNP7729	*Kabuli*	Ca_Kabuli_Chr05	2519977	(C/T)
7730	CakSNP7730	*Kabuli*	Ca_Kabuli_Chr05	2519949	(C/T)
7731	CakSNP7731	*Kabuli*	Ca_Kabuli_Chr05	2519961	(G/A)
7732	CakSNP7732	*Kabuli*	Ca_Kabuli_Chr05	2519976	(G/A)
7733	CakSNP7733	*Kabuli*	Ca_Kabuli_Chr05	2520035	(G/A)
7734	CakSNP7734	*Kabuli*	Ca_Kabuli_Chr05	2520058	(C/T)
7735	CakSNP7735	*Kabuli*	Ca_Kabuli_Chr05	2520015	(C/G)
7736	CakSNP7736	*Kabuli*	Ca_Kabuli_Chr05	2520049	(C/T)
7737	CakSNP7737	*Kabuli*	Ca_Kabuli_Chr05	2520050	(G/C)
7738	CakSNP7738	*Kabuli*	Ca_Kabuli_Chr05	2520051	(G/T)
7739	CakSNP7739	*Kabuli*	Ca_Kabuli_Chr05	2520055	(C/T)
7740	CakSNP7740	*Kabuli*	Ca_Kabuli_Chr05	2520068	(T/A)
7741	CakSNP7741	*Kabuli*	Ca_Kabuli_Chr05	2520069	(C/T)
7742	CakSNP7742	*Kabuli*	Ca_Kabuli_Chr05	2520094	(G/A)
7743	CakSNP7743	*Kabuli*	Ca_Kabuli_Chr05	2520061	(C/T)
7744	CakSNP7744	*Kabuli*	Ca_Kabuli_Chr05	2520093	(G/A)
7745	CakSNP7745	*Kabuli*	Ca_Kabuli_Chr05	2520123	(A/G)
7746	CakSNP7746	*Kabuli*	Ca_Kabuli_Chr05	2520114	(G/A)
7747	CakSNP7747	*Kabuli*	Ca_Kabuli_Chr05	2520109	(G/A)
7748	CakSNP7748	*Kabuli*	Ca_Kabuli_Chr05	2520101	(C/T)
7749	CakSNP7749	*Kabuli*	Ca_Kabuli_Chr05	2613423	(T/G)
7750	CakSNP7750	*Kabuli*	Ca_Kabuli_Chr05	2948530	(G/T)
7751	CakSNP7751	*Kabuli*	Ca_Kabuli_Chr05	2948531	(T/C)
7752	CakSNP7752	*Kabuli*	Ca_Kabuli_Chr05	2948549	(C/A)
7753	CakSNP7753	*Kabuli*	Ca_Kabuli_Chr05	2948592	(T/C)
7754	CakSNP7754	*Kabuli*	Ca_Kabuli_Chr05	2948596	(C/T)
7755	CakSNP7755	*Kabuli*	Ca_Kabuli_Chr05	2948616	(G/A)
7756	CakSNP7756	*Kabuli*	Ca_Kabuli_Chr05	2948698	(G/A)
7757	CakSNP7757	*Kabuli*	Ca_Kabuli_Chr05	2948977	(G/A)
7758	CakSNP7758	*Kabuli*	Ca_Kabuli_Chr05	3007213	(A/G)
7759	CakSNP7759	*Kabuli*	Ca_Kabuli_Chr05	3007316	(T/C)

S.N.	SNP IDs	Cultivars	Chromosomes/scaffolds	Physical positions (bp)	SNPs
7760	CakSNP7760	*Kabuli*	Ca_Kabuli_Chr05	3040264	(G/T)
7761	CakSNP7761	*Kabuli*	Ca_Kabuli_Chr05	3040948	(G/C)
7762	CakSNP7762	*Kabuli*	Ca_Kabuli_Chr05	3159061	(G/A)
7763	CakSNP7763	*Kabuli*	Ca_Kabuli_Chr05	3348291	(G/A)
7764	CakSNP7764	*Kabuli*	Ca_Kabuli_Chr05	3348310	(A/G)
7765	CakSNP7765	*Kabuli*	Ca_Kabuli_Chr05	3348366	(G/C)
7766	CakSNP7766	*Kabuli*	Ca_Kabuli_Chr05	3353052	(A/G)
7767	CakSNP7767	*Kabuli*	Ca_Kabuli_Chr05	4122395	(T/C)
7768	CakSNP7768	*Kabuli*	Ca_Kabuli_Chr05	4122342	(T/C)
7769	CakSNP7769	*Kabuli*	Ca_Kabuli_Chr05	5133644	(A/G)
7770	CakSNP7770	*Kabuli*	Ca_Kabuli_Chr05	5205261	(T/G)
7771	CakSNP7771	*Kabuli*	Ca_Kabuli_Chr05	5404261	(G/A)
7772	CakSNP7772	*Kabuli*	Ca_Kabuli_Chr05	6190494	(T/C)
7773	CakSNP7773	*Kabuli*	Ca_Kabuli_Chr05	6225426	(T/A)
7774	CakSNP7774	*Kabuli*	Ca_Kabuli_Chr05	6225415	(G/C)
7775	CakSNP7775	*Kabuli*	Ca_Kabuli_Chr05	6225410	(T/A)
7776	CakSNP7776	*Kabuli*	Ca_Kabuli_Chr05	6290453	(G/A)
7777	CakSNP7777	*Kabuli*	Ca_Kabuli_Chr05	6300337	(C/A)
7778	CakSNP7778	*Kabuli*	Ca_Kabuli_Chr05	6300360	(C/A)
7779	CakSNP7779	*Kabuli*	Ca_Kabuli_Chr05	6300502	(C/A)
7780	CakSNP7780	*Kabuli*	Ca_Kabuli_Chr05	6316787	(C/T)
7781	CakSNP7781	*Kabuli*	Ca_Kabuli_Chr05	6317014	(C/T)
7782	CakSNP7782	*Kabuli*	Ca_Kabuli_Chr05	6931865	(A/G)
7783	CakSNP7783	*Kabuli*	Ca_Kabuli_Chr05	7082295	(G/A)
7784	CakSNP7784	*Kabuli*	Ca_Kabuli_Chr05	7082296	(T/G)
7785	CakSNP7785	*Kabuli*	Ca_Kabuli_Chr05	7082301	(G/T)
7786	CakSNP7786	*Kabuli*	Ca_Kabuli_Chr05	7278953	(C/T)
7787	CakSNP7787	*Kabuli*	Ca_Kabuli_Chr05	7398246	(A/C)
7788	CakSNP7788	*Kabuli*	Ca_Kabuli_Chr05	7425116	(C/T)
7789	CakSNP7789	*Kabuli*	Ca_Kabuli_Chr05	7617914	(T/A)
7790	CakSNP7790	*Kabuli*	Ca_Kabuli_Chr05	7628097	(C/A)
7791	CakSNP7791	*Kabuli*	Ca_Kabuli_Chr05	7641993	(T/C)
7792	CakSNP7792	*Kabuli*	Ca_Kabuli_Chr05	7641925	(G/T)
7793	CakSNP7793	*Kabuli*	Ca_Kabuli_Chr05	7641923	(A/G)
7794	CakSNP7794	*Kabuli*	Ca_Kabuli_Chr05	7664421	(T/C)
7795	CakSNP7795	*Kabuli*	Ca_Kabuli_Chr05	7775286	(C/A)
7796	CakSNP7796	*Kabuli*	Ca_Kabuli_Chr05	7775290	(A/T)
7797	CakSNP7797	*Kabuli*	Ca_Kabuli_Chr05	7775298	(A/T)
7798	CakSNP7798	*Kabuli*	Ca_Kabuli_Chr05	7775329	(A/G)

S.N.	SNP IDs	Cultivars	Chromosomes/scaffolds	Physical positions (bp)	SNPs
7799	CakSNP7799	*Kabuli*	Ca_Kabuli_Chr05	7775516	(A/G)
7800	CakSNP7800	*Kabuli*	Ca_Kabuli_Chr05	7775497	(G/C)
7801	CakSNP7801	*Kabuli*	Ca_Kabuli_Chr05	7775472	(G/A)
7802	CakSNP7802	*Kabuli*	Ca_Kabuli_Chr05	7775456	(T/C)
7803	CakSNP7803	*Kabuli*	Ca_Kabuli_Chr05	7775918	(C/T)
7804	CakSNP7804	*Kabuli*	Ca_Kabuli_Chr05	7789021	(G/T)
7805	CakSNP7805	*Kabuli*	Ca_Kabuli_Chr05	7789065	(A/G)
7806	CakSNP7806	*Kabuli*	Ca_Kabuli_Chr05	7826336	(G/T)
7807	CakSNP7807	*Kabuli*	Ca_Kabuli_Chr05	7826338	(T/G)
7808	CakSNP7808	*Kabuli*	Ca_Kabuli_Chr05	7826362	(C/T)
7809	CakSNP7809	*Kabuli*	Ca_Kabuli_Chr05	7826366	(A/G)
7810	CakSNP7810	*Kabuli*	Ca_Kabuli_Chr05	7925356	(A/C)
7811	CakSNP7811	*Kabuli*	Ca_Kabuli_Chr05	8263483	(A/G)
7812	CakSNP7812	*Kabuli*	Ca_Kabuli_Chr05	8653283	(G/A)
7813	CakSNP7813	*Kabuli*	Ca_Kabuli_Chr05	8676750	(T/G)
7814	CakSNP7814	*Kabuli*	Ca_Kabuli_Chr05	8676807	(G/A)
7815	CakSNP7815	*Kabuli*	Ca_Kabuli_Chr05	8676789	(T/C)
7816	CakSNP7816	*Kabuli*	Ca_Kabuli_Chr05	8676771	(G/A)
7817	CakSNP7817	*Kabuli*	Ca_Kabuli_Chr05	8946218	(G/A)
7818	CakSNP7818	*Kabuli*	Ca_Kabuli_Chr05	9034100	(C/G)
7819	CakSNP7819	*Kabuli*	Ca_Kabuli_Chr05	9076709	(C/T)
7820	CakSNP7820	*Kabuli*	Ca_Kabuli_Chr05	9076712	(G/A)
7821	CakSNP7821	*Kabuli*	Ca_Kabuli_Chr05	9076737	(C/A)
7822	CakSNP7822	*Kabuli*	Ca_Kabuli_Chr05	9076738	(G/C)
7823	CakSNP7823	*Kabuli*	Ca_Kabuli_Chr05	9076767	(C/A)
7824	CakSNP7824	*Kabuli*	Ca_Kabuli_Chr05	9076824	(G/A)
7825	CakSNP7825	*Kabuli*	Ca_Kabuli_Chr05	9076821	(G/T)
7826	CakSNP7826	*Kabuli*	Ca_Kabuli_Chr05	9076809	(T/A)
7827	CakSNP7827	*Kabuli*	Ca_Kabuli_Chr05	9077489	(T/C)
7828	CakSNP7828	*Kabuli*	Ca_Kabuli_Chr05	9519382	(C/A)
7829	CakSNP7829	*Kabuli*	Ca_Kabuli_Chr05	9519390	(G/A)
7830	CakSNP7830	*Kabuli*	Ca_Kabuli_Chr05	9609055	(T/C)
7831	CakSNP7831	*Kabuli*	Ca_Kabuli_Chr05	9620998	(A/C)
7832	CakSNP7832	*Kabuli*	Ca_Kabuli_Chr05	9728498	(A/T)
7833	CakSNP7833	*Kabuli*	Ca_Kabuli_Chr05	9728609	(T/A)
7834	CakSNP7834	*Kabuli*	Ca_Kabuli_Chr05	9728591	(A/G)
7835	CakSNP7835	*Kabuli*	Ca_Kabuli_Chr05	9786213	(C/A)
7836	CakSNP7836	*Kabuli*	Ca_Kabuli_Chr05	9868796	(T/C)
7837	CakSNP7837	*Kabuli*	Ca_Kabuli_Chr05	9869005	(T/G)

S.N.	SNP IDs	Cultivars	Chromosomes/scaffolds	Physical positions (bp)	SNPs
7838	CakSNP7838	*Kabuli*	Ca_Kabuli_Chr05	9961273	(T/C)
7839	CakSNP7839	*Kabuli*	Ca_Kabuli_Chr05	9961396	(T/C)
7840	CakSNP7840	*Kabuli*	Ca_Kabuli_Chr05	9961380	(C/T)
7841	CakSNP7841	*Kabuli*	Ca_Kabuli_Chr05	9965880	(G/C)
7842	CakSNP7842	*Kabuli*	Ca_Kabuli_Chr05	9965890	(T/C)
7843	CakSNP7843	*Kabuli*	Ca_Kabuli_Chr05	9965913	(A/C)
7844	CakSNP7844	*Kabuli*	Ca_Kabuli_Chr05	9965848	(T/C)
7845	CakSNP7845	*Kabuli*	Ca_Kabuli_Chr05	9965846	(A/C)
7846	CakSNP7846	*Kabuli*	Ca_Kabuli_Chr05	10004386	(G/T)
7847	CakSNP7847	*Kabuli*	Ca_Kabuli_Chr05	10004427	(T/G)
7848	CakSNP7848	*Kabuli*	Ca_Kabuli_Chr05	10004430	(T/G)
7849	CakSNP7849	*Kabuli*	Ca_Kabuli_Chr05	10004451	(T/G)
7850	CakSNP7850	*Kabuli*	Ca_Kabuli_Chr05	10054140	(G/C)
7851	CakSNP7851	*Kabuli*	Ca_Kabuli_Chr05	10054146	(A/G)
7852	CakSNP7852	*Kabuli*	Ca_Kabuli_Chr05	10054266	(T/C)
7853	CakSNP7853	*Kabuli*	Ca_Kabuli_Chr05	10054213	(A/C)
7854	CakSNP7854	*Kabuli*	Ca_Kabuli_Chr05	10072358	(G/A)
7855	CakSNP7855	*Kabuli*	Ca_Kabuli_Chr05	10072403	(G/C)
7856	CakSNP7856	*Kabuli*	Ca_Kabuli_Chr05	10154016	(A/G)
7857	CakSNP7857	*Kabuli*	Ca_Kabuli_Chr05	10247223	(T/C)
7858	CakSNP7858	*Kabuli*	Ca_Kabuli_Chr05	10247224	(T/A)
7859	CakSNP7859	*Kabuli*	Ca_Kabuli_Chr05	10247233	(C/T)
7860	CakSNP7860	*Kabuli*	Ca_Kabuli_Chr05	10247244	(C/T)
7861	CakSNP7861	*Kabuli*	Ca_Kabuli_Chr05	10390882	(C/T)
7862	CakSNP7862	*Kabuli*	Ca_Kabuli_Chr05	10406562	(C/A)
7863	CakSNP7863	*Kabuli*	Ca_Kabuli_Chr05	10406602	(C/T)
7864	CakSNP7864	*Kabuli*	Ca_Kabuli_Chr05	10406613	(C/T)
7865	CakSNP7865	*Kabuli*	Ca_Kabuli_Chr05	10406636	(A/G)
7866	CakSNP7866	*Kabuli*	Ca_Kabuli_Chr05	10406649	(A/C)
7867	CakSNP7867	*Kabuli*	Ca_Kabuli_Chr05	10406650	(A/G)
7868	CakSNP7868	*Kabuli*	Ca_Kabuli_Chr05	10406754	(G/T)
7869	CakSNP7869	*Kabuli*	Ca_Kabuli_Chr05	10510166	(G/A)
7870	CakSNP7870	*Kabuli*	Ca_Kabuli_Chr05	10544823	(A/G)
7871	CakSNP7871	*Kabuli*	Ca_Kabuli_Chr05	10546944	(A/T)
7872	CakSNP7872	*Kabuli*	Ca_Kabuli_Chr05	10675252	(A/G)
7873	CakSNP7873	*Kabuli*	Ca_Kabuli_Chr05	10675279	(C/G)
7874	CakSNP7874	*Kabuli*	Ca_Kabuli_Chr05	10675294	(A/G)
7875	CakSNP7875	*Kabuli*	Ca_Kabuli_Chr05	10769765	(C/T)
7876	CakSNP7876	*Kabuli*	Ca_Kabuli_Chr05	10832275	(T/C)

S.N.	SNP IDs	Cultivars	Chromosomes/scaffolds	Physical positions (bp)	SNPs
7877	CakSNP7877	*Kabuli*	Ca_Kabuli_Chr05	10858490	(C/T)
7878	CakSNP7878	*Kabuli*	Ca_Kabuli_Chr05	10918368	(A/G)
7879	CakSNP7879	*Kabuli*	Ca_Kabuli_Chr05	10919246	(C/G)
7880	CakSNP7880	*Kabuli*	Ca_Kabuli_Chr05	11230617	(C/G)
7881	CakSNP7881	*Kabuli*	Ca_Kabuli_Chr05	11230627	(A/G)
7882	CakSNP7882	*Kabuli*	Ca_Kabuli_Chr05	11230648	(G/A)
7883	CakSNP7883	*Kabuli*	Ca_Kabuli_Chr05	11230671	(T/C)
7884	CakSNP7884	*Kabuli*	Ca_Kabuli_Chr05	11263571	(C/G)
7885	CakSNP7885	*Kabuli*	Ca_Kabuli_Chr05	11263574	(C/A)
7886	CakSNP7886	*Kabuli*	Ca_Kabuli_Chr05	11317487	(C/T)
7887	CakSNP7887	*Kabuli*	Ca_Kabuli_Chr05	11317502	(C/A)
7888	CakSNP7888	*Kabuli*	Ca_Kabuli_Chr05	11317534	(C/A)
7889	CakSNP7889	*Kabuli*	Ca_Kabuli_Chr05	11385780	(A/C)
7890	CakSNP7890	*Kabuli*	Ca_Kabuli_Chr05	11385874	(A/G)
7891	CakSNP7891	*Kabuli*	Ca_Kabuli_Chr05	11765841	(C/T)
7892	CakSNP7892	*Kabuli*	Ca_Kabuli_Chr05	11777112	(C/T)
7893	CakSNP7893	*Kabuli*	Ca_Kabuli_Chr05	11807822	(G/T)
7894	CakSNP7894	*Kabuli*	Ca_Kabuli_Chr05	11807842	(T/C)
7895	CakSNP7895	*Kabuli*	Ca_Kabuli_Chr05	11845515	(C/T)
7896	CakSNP7896	*Kabuli*	Ca_Kabuli_Chr05	11853090	(C/T)
7897	CakSNP7897	*Kabuli*	Ca_Kabuli_Chr05	11864959	(T/C)
7898	CakSNP7898	*Kabuli*	Ca_Kabuli_Chr05	12044714	(A/G)
7899	CakSNP7899	*Kabuli*	Ca_Kabuli_Chr05	12157678	(A/G)
7900	CakSNP7900	*Kabuli*	Ca_Kabuli_Chr05	12157683	(T/C)
7901	CakSNP7901	*Kabuli*	Ca_Kabuli_Chr05	12157688	(C/T)
7902	CakSNP7902	*Kabuli*	Ca_Kabuli_Chr05	12157706	(G/A)
7903	CakSNP7903	*Kabuli*	Ca_Kabuli_Chr05	12157707	(C/T)
7904	CakSNP7904	*Kabuli*	Ca_Kabuli_Chr05	12157710	(A/C)
7905	CakSNP7905	*Kabuli*	Ca_Kabuli_Chr05	12157742	(C/A)
7906	CakSNP7906	*Kabuli*	Ca_Kabuli_Chr05	12157699	(G/T)
7907	CakSNP7907	*Kabuli*	Ca_Kabuli_Chr05	12162108	(T/G)
7908	CakSNP7908	*Kabuli*	Ca_Kabuli_Chr05	12162156	(T/C)
7909	CakSNP7909	*Kabuli*	Ca_Kabuli_Chr05	12162160	(G/T)
7910	CakSNP7910	*Kabuli*	Ca_Kabuli_Chr05	12162164	(C/G)
7911	CakSNP7911	*Kabuli*	Ca_Kabuli_Chr05	12198014	(G/A)
7912	CakSNP7912	*Kabuli*	Ca_Kabuli_Chr05	12204521	(T/A)
7913	CakSNP7913	*Kabuli*	Ca_Kabuli_Chr05	12204588	(A/G)
7914	CakSNP7914	*Kabuli*	Ca_Kabuli_Chr05	12427931	(C/T)
7915	CakSNP7915	*Kabuli*	Ca_Kabuli_Chr05	12427940	(T/A)

S.N.	SNP IDs	Cultivars	Chromosomes/scaffolds	Physical positions (bp)	SNPs
7916	CakSNP7916	*Kabuli*	Ca_Kabuli_Chr05	12427970	(T/C)
7917	CakSNP7917	*Kabuli*	Ca_Kabuli_Chr05	12427969	(T/C)
7918	CakSNP7918	*Kabuli*	Ca_Kabuli_Chr05	12427966	(T/C)
7919	CakSNP7919	*Kabuli*	Ca_Kabuli_Chr05	12428027	(G/A)
7920	CakSNP7920	*Kabuli*	Ca_Kabuli_Chr05	12428121	(A/T)
7921	CakSNP7921	*Kabuli*	Ca_Kabuli_Chr05	12428120	(G/A)
7922	CakSNP7922	*Kabuli*	Ca_Kabuli_Chr05	12428102	(A/G)
7923	CakSNP7923	*Kabuli*	Ca_Kabuli_Chr05	12480948	(T/G)
7924	CakSNP7924	*Kabuli*	Ca_Kabuli_Chr05	12553469	(A/C)
7925	CakSNP7925	*Kabuli*	Ca_Kabuli_Chr05	12553470	(G/C)
7926	CakSNP7926	*Kabuli*	Ca_Kabuli_Chr05	12590330	(A/G)
7927	CakSNP7927	*Kabuli*	Ca_Kabuli_Chr05	12590361	(T/C)
7928	CakSNP7928	*Kabuli*	Ca_Kabuli_Chr05	12590478	(T/C)
7929	CakSNP7929	*Kabuli*	Ca_Kabuli_Chr05	12662138	(G/T)
7930	CakSNP7930	*Kabuli*	Ca_Kabuli_Chr05	13982000	(T/G)
7931	CakSNP7931	*Kabuli*	Ca_Kabuli_Chr05	13982041	(C/T)
7932	CakSNP7932	*Kabuli*	Ca_Kabuli_Chr05	15401230	(C/T)
7933	CakSNP7933	*Kabuli*	Ca_Kabuli_Chr05	15500782	(G/C)
7934	CakSNP7934	*Kabuli*	Ca_Kabuli_Chr05	15548719	(G/A)
7935	CakSNP7935	*Kabuli*	Ca_Kabuli_Chr05	15952395	(C/T)
7936	CakSNP7936	*Kabuli*	Ca_Kabuli_Chr05	16227319	(G/T)
7937	CakSNP7937	*Kabuli*	Ca_Kabuli_Chr05	16227321	(C/T)
7938	CakSNP7938	*Kabuli*	Ca_Kabuli_Chr05	16227353	(G/A)
7939	CakSNP7939	*Kabuli*	Ca_Kabuli_Chr05	16227395	(G/A)
7940	CakSNP7940	*Kabuli*	Ca_Kabuli_Chr05	16227401	(G/A)
7941	CakSNP7941	*Kabuli*	Ca_Kabuli_Chr05	16227408	(C/T)
7942	CakSNP7942	*Kabuli*	Ca_Kabuli_Chr05	16426578	(G/A)
7943	CakSNP7943	*Kabuli*	Ca_Kabuli_Chr05	16426575	(G/A)
7944	CakSNP7944	*Kabuli*	Ca_Kabuli_Chr05	16426549	(G/A)
7945	CakSNP7945	*Kabuli*	Ca_Kabuli_Chr05	16426662	(C/T)
7946	CakSNP7946	*Kabuli*	Ca_Kabuli_Chr05	16426657	(C/T)
7947	CakSNP7947	*Kabuli*	Ca_Kabuli_Chr05	16426656	(A/G)
7948	CakSNP7948	*Kabuli*	Ca_Kabuli_Chr05	16426642	(A/G)
7949	CakSNP7949	*Kabuli*	Ca_Kabuli_Chr05	16593880	(A/G)
7950	CakSNP7950	*Kabuli*	Ca_Kabuli_Chr05	16751536	(C/T)
7951	CakSNP7951	*Kabuli*	Ca_Kabuli_Chr05	16767134	(C/T)
7952	CakSNP7952	*Kabuli*	Ca_Kabuli_Chr05	16821406	(A/G)
7953	CakSNP7953	*Kabuli*	Ca_Kabuli_Chr05	16929903	(C/G)
7954	CakSNP7954	*Kabuli*	Ca_Kabuli_Chr05	16929897	(A/G)

S.N.	SNP IDs	Cultivars	Chromosomes/scaffolds	Physical positions (bp)	SNPs
7955	CakSNP7955	*Kabuli*	Ca_Kabuli_Chr05	17037918	(G/T)
7956	CakSNP7956	*Kabuli*	Ca_Kabuli_Chr05	17044340	(G/A)
7957	CakSNP7957	*Kabuli*	Ca_Kabuli_Chr05	17044338	(G/A)
7958	CakSNP7958	*Kabuli*	Ca_Kabuli_Chr05	17044332	(A/G)
7959	CakSNP7959	*Kabuli*	Ca_Kabuli_Chr05	17045235	(T/C)
7960	CakSNP7960	*Kabuli*	Ca_Kabuli_Chr05	17045244	(A/G)
7961	CakSNP7961	*Kabuli*	Ca_Kabuli_Chr05	17045485	(T/G)
7962	CakSNP7962	*Kabuli*	Ca_Kabuli_Chr05	17239612	(T/C)
7963	CakSNP7963	*Kabuli*	Ca_Kabuli_Chr05	17494714	(A/G)
7964	CakSNP7964	*Kabuli*	Ca_Kabuli_Chr05	17494702	(G/A)
7965	CakSNP7965	*Kabuli*	Ca_Kabuli_Chr05	17494700	(T/C)
7966	CakSNP7966	*Kabuli*	Ca_Kabuli_Chr05	17494697	(C/T)
7967	CakSNP7967	*Kabuli*	Ca_Kabuli_Chr05	17494672	(C/T)
7968	CakSNP7968	*Kabuli*	Ca_Kabuli_Chr05	17494699	(C/A)
7969	CakSNP7969	*Kabuli*	Ca_Kabuli_Chr05	17494705	(A/T)
7970	CakSNP7970	*Kabuli*	Ca_Kabuli_Chr05	17494710	(C/T)
7971	CakSNP7971	*Kabuli*	Ca_Kabuli_Chr05	17494731	(A/T)
7972	CakSNP7972	*Kabuli*	Ca_Kabuli_Chr05	18044808	(C/G)
7973	CakSNP7973	*Kabuli*	Ca_Kabuli_Chr05	18664706	(C/T)
7974	CakSNP7974	*Kabuli*	Ca_Kabuli_Chr05	18667782	(T/C)
7975	CakSNP7975	*Kabuli*	Ca_Kabuli_Chr05	18667775	(T/C)
7976	CakSNP7976	*Kabuli*	Ca_Kabuli_Chr05	18682788	(A/C)
7977	CakSNP7977	*Kabuli*	Ca_Kabuli_Chr05	18682792	(T/C)
7978	CakSNP7978	*Kabuli*	Ca_Kabuli_Chr05	18682793	(A/G)
7979	CakSNP7979	*Kabuli*	Ca_Kabuli_Chr05	18682794	(A/G)
7980	CakSNP7980	*Kabuli*	Ca_Kabuli_Chr05	18682812	(A/G)
7981	CakSNP7981	*Kabuli*	Ca_Kabuli_Chr05	18682814	(T/A)
7982	CakSNP7982	*Kabuli*	Ca_Kabuli_Chr05	18682822	(A/G)
7983	CakSNP7983	*Kabuli*	Ca_Kabuli_Chr05	18682827	(C/G)
7984	CakSNP7984	*Kabuli*	Ca_Kabuli_Chr05	18682830	(G/A)
7985	CakSNP7985	*Kabuli*	Ca_Kabuli_Chr05	18684334	(C/T)
7986	CakSNP7986	*Kabuli*	Ca_Kabuli_Chr05	18971085	(G/A)
7987	CakSNP7987	*Kabuli*	Ca_Kabuli_Chr05	18971088	(C/A)
7988	CakSNP7988	*Kabuli*	Ca_Kabuli_Chr05	19421820	(C/T)
7989	CakSNP7989	*Kabuli*	Ca_Kabuli_Chr05	19421868	(C/T)
7990	CakSNP7990	*Kabuli*	Ca_Kabuli_Chr05	19423387	(G/A)
7991	CakSNP7991	*Kabuli*	Ca_Kabuli_Chr05	19423357	(C/T)
7992	CakSNP7992	*Kabuli*	Ca_Kabuli_Chr05	19828124	(G/A)
7993	CakSNP7993	*Kabuli*	Ca_Kabuli_Chr05	19828151	(C/A)

S.N.	SNP IDs	Cultivars	Chromosomes/scaffolds	Physical positions (bp)	SNPs
7994	CakSNP7994	*Kabuli*	Ca_Kabuli_Chr05	19828163	(C/T)
7995	CakSNP7995	*Kabuli*	Ca_Kabuli_Chr05	19828166	(C/T)
7996	CakSNP7996	*Kabuli*	Ca_Kabuli_Chr05	19828167	(A/C)
7997	CakSNP7997	*Kabuli*	Ca_Kabuli_Chr05	19828226	(G/A)
7998	CakSNP7998	*Kabuli*	Ca_Kabuli_Chr05	19828190	(T/C)
7999	CakSNP7999	*Kabuli*	Ca_Kabuli_Chr05	19828184	(C/A)
8000	CakSNP8000	*Kabuli*	Ca_Kabuli_Chr05	19828172	(T/G)
8001	CakSNP8001	*Kabuli*	Ca_Kabuli_Chr05	19828144	(A/G)
8002	CakSNP8002	*Kabuli*	Ca_Kabuli_Chr05	19884132	(C/T)
8003	CakSNP8003	*Kabuli*	Ca_Kabuli_Chr05	19884124	(A/G)
8004	CakSNP8004	*Kabuli*	Ca_Kabuli_Chr05	20008545	(C/T)
8005	CakSNP8005	*Kabuli*	Ca_Kabuli_Chr05	20031711	(A/G)
8006	CakSNP8006	*Kabuli*	Ca_Kabuli_Chr05	20354352	(T/C)
8007	CakSNP8007	*Kabuli*	Ca_Kabuli_Chr05	20359674	(G/A)
8008	CakSNP8008	*Kabuli*	Ca_Kabuli_Chr05	20460857	(G/A)
8009	CakSNP8009	*Kabuli*	Ca_Kabuli_Chr05	20460859	(T/G)
8010	CakSNP8010	*Kabuli*	Ca_Kabuli_Chr05	20546402	(T/C)
8011	CakSNP8011	*Kabuli*	Ca_Kabuli_Chr05	20546385	(T/G)
8012	CakSNP8012	*Kabuli*	Ca_Kabuli_Chr05	20644984	(C/A)
8013	CakSNP8013	*Kabuli*	Ca_Kabuli_Chr05	20834519	(A/G)
8014	CakSNP8014	*Kabuli*	Ca_Kabuli_Chr05	21163371	(G/A)
8015	CakSNP8015	*Kabuli*	Ca_Kabuli_Chr05	21385948	(T/C)
8016	CakSNP8016	*Kabuli*	Ca_Kabuli_Chr05	21495583	(C/A)
8017	CakSNP8017	*Kabuli*	Ca_Kabuli_Chr05	21495631	(C/T)
8018	CakSNP8018	*Kabuli*	Ca_Kabuli_Chr05	21495672	(G/A)
8019	CakSNP8019	*Kabuli*	Ca_Kabuli_Chr05	21495707	(A/G)
8020	CakSNP8020	*Kabuli*	Ca_Kabuli_Chr05	21495679	(G/A)
8021	CakSNP8021	*Kabuli*	Ca_Kabuli_Chr05	21495674	(G/A)
8022	CakSNP8022	*Kabuli*	Ca_Kabuli_Chr05	21769327	(G/A)
8023	CakSNP8023	*Kabuli*	Ca_Kabuli_Chr05	21769419	(C/G)
8024	CakSNP8024	*Kabuli*	Ca_Kabuli_Chr05	21769794	(A/C)
8025	CakSNP8025	*Kabuli*	Ca_Kabuli_Chr05	22027215	(T/C)
8026	CakSNP8026	*Kabuli*	Ca_Kabuli_Chr05	22027257	(G/T)
8027	CakSNP8027	*Kabuli*	Ca_Kabuli_Chr05	22027265	(A/T)
8028	CakSNP8028	*Kabuli*	Ca_Kabuli_Chr05	22029684	(C/A)
8029	CakSNP8029	*Kabuli*	Ca_Kabuli_Chr05	22029686	(G/A)
8030	CakSNP8030	*Kabuli*	Ca_Kabuli_Chr05	22029761	(C/T)
8031	CakSNP8031	*Kabuli*	Ca_Kabuli_Chr05	22065518	(C/T)
8032	CakSNP8032	*Kabuli*	Ca_Kabuli_Chr05	22065859	(G/A)

S.N.	SNP IDs	Cultivars	Chromosomes/scaffolds	Physical positions (bp)	SNPs
8033	CakSNP8033	*Kabuli*	Ca_Kabuli_Chr05	22066034	(A/T)
8034	CakSNP8034	*Kabuli*	Ca_Kabuli_Chr05	22116961	(T/G)
8035	CakSNP8035	*Kabuli*	Ca_Kabuli_Chr05	22188341	(C/T)
8036	CakSNP8036	*Kabuli*	Ca_Kabuli_Chr05	22188318	(C/T)
8037	CakSNP8037	*Kabuli*	Ca_Kabuli_Chr05	22296731	(G/A)
8038	CakSNP8038	*Kabuli*	Ca_Kabuli_Chr05	22296728	(G/T)
8039	CakSNP8039	*Kabuli*	Ca_Kabuli_Chr05	22302350	(A/C)
8040	CakSNP8040	*Kabuli*	Ca_Kabuli_Chr05	22344868	(A/G)
8041	CakSNP8041	*Kabuli*	Ca_Kabuli_Chr05	22445954	(T/C)
8042	CakSNP8042	*Kabuli*	Ca_Kabuli_Chr05	22679380	(C/A)
8043	CakSNP8043	*Kabuli*	Ca_Kabuli_Chr05	23039312	(C/T)
8044	CakSNP8044	*Kabuli*	Ca_Kabuli_Chr05	23049683	(C/A)
8045	CakSNP8045	*Kabuli*	Ca_Kabuli_Chr05	23157899	(A/G)
8046	CakSNP8046	*Kabuli*	Ca_Kabuli_Chr05	23161492	(A/T)
8047	CakSNP8047	*Kabuli*	Ca_Kabuli_Chr05	23261964	(A/G)
8048	CakSNP8048	*Kabuli*	Ca_Kabuli_Chr05	23262114	(C/A)
8049	CakSNP8049	*Kabuli*	Ca_Kabuli_Chr05	23262264	(C/A)
8050	CakSNP8050	*Kabuli*	Ca_Kabuli_Chr05	23323538	(G/A)
8051	CakSNP8051	*Kabuli*	Ca_Kabuli_Chr05	23323520	(C/A)
8052	CakSNP8052	*Kabuli*	Ca_Kabuli_Chr05	23323514	(C/A)
8053	CakSNP8053	*Kabuli*	Ca_Kabuli_Chr05	23323479	(A/T)
8054	CakSNP8054	*Kabuli*	Ca_Kabuli_Chr05	23323478	(A/T)
8055	CakSNP8055	*Kabuli*	Ca_Kabuli_Chr05	23440293	(C/T)
8056	CakSNP8056	*Kabuli*	Ca_Kabuli_Chr05	23449048	(T/C)
8057	CakSNP8057	*Kabuli*	Ca_Kabuli_Chr05	23449134	(A/C)
8058	CakSNP8058	*Kabuli*	Ca_Kabuli_Chr05	23449127	(C/A)
8059	CakSNP8059	*Kabuli*	Ca_Kabuli_Chr05	23562595	(G/A)
8060	CakSNP8060	*Kabuli*	Ca_Kabuli_Chr05	23562827	(G/A)
8061	CakSNP8061	*Kabuli*	Ca_Kabuli_Chr05	23674814	(C/G)
8062	CakSNP8062	*Kabuli*	Ca_Kabuli_Chr05	23674815	(C/T)
8063	CakSNP8063	*Kabuli*	Ca_Kabuli_Chr05	23674845	(G/C)
8064	CakSNP8064	*Kabuli*	Ca_Kabuli_Chr05	23783140	(T/C)
8065	CakSNP8065	*Kabuli*	Ca_Kabuli_Chr05	23783156	(C/A)
8066	CakSNP8066	*Kabuli*	Ca_Kabuli_Chr05	23783157	(G/C)
8067	CakSNP8067	*Kabuli*	Ca_Kabuli_Chr05	23783188	(G/A)
8068	CakSNP8068	*Kabuli*	Ca_Kabuli_Chr05	23783195	(C/G)
8069	CakSNP8069	*Kabuli*	Ca_Kabuli_Chr05	23783254	(A/G)
8070	CakSNP8070	*Kabuli*	Ca_Kabuli_Chr05	23783252	(T/A)
8071	CakSNP8071	*Kabuli*	Ca_Kabuli_Chr05	23884909	(C/T)

S.N.	SNP IDs	Cultivars	Chromosomes/scaffolds	Physical positions (bp)	SNPs
8072	CakSNP8072	*Kabuli*	Ca_Kabuli_Chr05	23884936	(G/A)
8073	CakSNP8073	*Kabuli*	Ca_Kabuli_Chr05	23930933	(C/T)
8074	CakSNP8074	*Kabuli*	Ca_Kabuli_Chr05	23930988	(G/A)
8075	CakSNP8075	*Kabuli*	Ca_Kabuli_Chr05	23932354	(C/T)
8076	CakSNP8076	*Kabuli*	Ca_Kabuli_Chr05	23934198	(G/T)
8077	CakSNP8077	*Kabuli*	Ca_Kabuli_Chr05	24059182	(C/G)
8078	CakSNP8078	*Kabuli*	Ca_Kabuli_Chr05	24090533	(C/T)
8079	CakSNP8079	*Kabuli*	Ca_Kabuli_Chr05	24090523	(A/C)
8080	CakSNP8080	*Kabuli*	Ca_Kabuli_Chr05	24090515	(A/C)
8081	CakSNP8081	*Kabuli*	Ca_Kabuli_Chr05	24090749	(A/C)
8082	CakSNP8082	*Kabuli*	Ca_Kabuli_Chr05	24097823	(T/C)
8083	CakSNP8083	*Kabuli*	Ca_Kabuli_Chr05	24153061	(G/C)
8084	CakSNP8084	*Kabuli*	Ca_Kabuli_Chr05	24153205	(T/C)
8085	CakSNP8085	*Kabuli*	Ca_Kabuli_Chr05	24153273	(T/C)
8086	CakSNP8086	*Kabuli*	Ca_Kabuli_Chr05	24153202	(A/C)
8087	CakSNP8087	*Kabuli*	Ca_Kabuli_Chr05	24153199	(C/T)
8088	CakSNP8088	*Kabuli*	Ca_Kabuli_Chr05	24166103	(C/T)
8089	CakSNP8089	*Kabuli*	Ca_Kabuli_Chr05	24166095	(G/A)
8090	CakSNP8090	*Kabuli*	Ca_Kabuli_Chr05	24166093	(A/G)
8091	CakSNP8091	*Kabuli*	Ca_Kabuli_Chr05	24166046	(G/A)
8092	CakSNP8092	*Kabuli*	Ca_Kabuli_Chr05	24166047	(C/T)
8093	CakSNP8093	*Kabuli*	Ca_Kabuli_Chr05	24166082	(G/A)
8094	CakSNP8094	*Kabuli*	Ca_Kabuli_Chr05	24181232	(C/A)
8095	CakSNP8095	*Kabuli*	Ca_Kabuli_Chr05	24181228	(C/A)
8096	CakSNP8096	*Kabuli*	Ca_Kabuli_Chr05	24245312	(C/A)
8097	CakSNP8097	*Kabuli*	Ca_Kabuli_Chr05	24245308	(C/A)
8098	CakSNP8098	*Kabuli*	Ca_Kabuli_Chr05	24346350	(A/T)
8099	CakSNP8099	*Kabuli*	Ca_Kabuli_Chr05	24576960	(C/T)
8100	CakSNP8100	*Kabuli*	Ca_Kabuli_Chr05	24576970	(A/G)
8101	CakSNP8101	*Kabuli*	Ca_Kabuli_Chr05	24576993	(T/G)
8102	CakSNP8102	*Kabuli*	Ca_Kabuli_Chr05	24596227	(T/C)
8103	CakSNP8103	*Kabuli*	Ca_Kabuli_Chr05	24596257	(C/T)
8104	CakSNP8104	*Kabuli*	Ca_Kabuli_Chr05	24596269	(G/A)
8105	CakSNP8105	*Kabuli*	Ca_Kabuli_Chr05	24605438	(A/C)
8106	CakSNP8106	*Kabuli*	Ca_Kabuli_Chr05	24843015	(G/T)
8107	CakSNP8107	*Kabuli*	Ca_Kabuli_Chr05	25003171	(C/T)
8108	CakSNP8108	*Kabuli*	Ca_Kabuli_Chr05	25006110	(A/C)
8109	CakSNP8109	*Kabuli*	Ca_Kabuli_Chr05	25006123	(C/T)
8110	CakSNP8110	*Kabuli*	Ca_Kabuli_Chr05	25184089	(T/C)

S.N.	SNP IDs	Cultivars	Chromosomes/scaffolds	Physical positions (bp)	SNPs
8111	CakSNP8111	*Kabuli*	Ca_Kabuli_Chr05	25184133	(G/A)
8112	CakSNP8112	*Kabuli*	Ca_Kabuli_Chr05	25399115	(T/G)
8113	CakSNP8113	*Kabuli*	Ca_Kabuli_Chr05	25400604	(A/G)
8114	CakSNP8114	*Kabuli*	Ca_Kabuli_Chr05	25474110	(A/C)
8115	CakSNP8115	*Kabuli*	Ca_Kabuli_Chr05	25474105	(C/T)
8116	CakSNP8116	*Kabuli*	Ca_Kabuli_Chr05	25474093	(G/C)
8117	CakSNP8117	*Kabuli*	Ca_Kabuli_Chr05	25474089	(C/A)
8118	CakSNP8118	*Kabuli*	Ca_Kabuli_Chr05	25597077	(A/G)
8119	CakSNP8119	*Kabuli*	Ca_Kabuli_Chr05	25678975	(G/T)
8120	CakSNP8120	*Kabuli*	Ca_Kabuli_Chr05	25697487	(C/G)
8121	CakSNP8121	*Kabuli*	Ca_Kabuli_Chr05	25805020	(A/G)
8122	CakSNP8122	*Kabuli*	Ca_Kabuli_Chr05	26025243	(C/T)
8123	CakSNP8123	*Kabuli*	Ca_Kabuli_Chr05	26025282	(A/G)
8124	CakSNP8124	*Kabuli*	Ca_Kabuli_Chr05	26025356	(A/T)
8125	CakSNP8125	*Kabuli*	Ca_Kabuli_Chr05	26027313	(C/A)
8126	CakSNP8126	*Kabuli*	Ca_Kabuli_Chr05	26095084	(T/G)
8127	CakSNP8127	*Kabuli*	Ca_Kabuli_Chr05	26124240	(T/C)
8128	CakSNP8128	*Kabuli*	Ca_Kabuli_Chr05	26191896	(T/C)
8129	CakSNP8129	*Kabuli*	Ca_Kabuli_Chr05	26194579	(A/G)
8130	CakSNP8130	*Kabuli*	Ca_Kabuli_Chr05	26238764	(T/G)
8131	CakSNP8131	*Kabuli*	Ca_Kabuli_Chr05	26238752	(A/T)
8132	CakSNP8132	*Kabuli*	Ca_Kabuli_Chr05	26238748	(A/T)
8133	CakSNP8133	*Kabuli*	Ca_Kabuli_Chr05	26238741	(G/T)
8134	CakSNP8134	*Kabuli*	Ca_Kabuli_Chr05	26238739	(C/T)
8135	CakSNP8135	*Kabuli*	Ca_Kabuli_Chr05	26384636	(C/G)
8136	CakSNP8136	*Kabuli*	Ca_Kabuli_Chr05	26467869	(A/C)
8137	CakSNP8137	*Kabuli*	Ca_Kabuli_Chr05	26474910	(A/C)
8138	CakSNP8138	*Kabuli*	Ca_Kabuli_Chr05	26475067	(T/C)
8139	CakSNP8139	*Kabuli*	Ca_Kabuli_Chr05	26477751	(A/T)
8140	CakSNP8140	*Kabuli*	Ca_Kabuli_Chr05	26494631	(A/G)
8141	CakSNP8141	*Kabuli*	Ca_Kabuli_Chr05	26494778	(T/C)
8142	CakSNP8142	*Kabuli*	Ca_Kabuli_Chr05	26494769	(T/C)
8143	CakSNP8143	*Kabuli*	Ca_Kabuli_Chr05	26496197	(A/G)
8144	CakSNP8144	*Kabuli*	Ca_Kabuli_Chr05	26558406	(A/G)
8145	CakSNP8145	*Kabuli*	Ca_Kabuli_Chr05	26558366	(A/G)
8146	CakSNP8146	*Kabuli*	Ca_Kabuli_Chr05	26558363	(C/G)
8147	CakSNP8147	*Kabuli*	Ca_Kabuli_Chr05	26558357	(G/A)
8148	CakSNP8148	*Kabuli*	Ca_Kabuli_Chr05	26558356	(G/T)
8149	CakSNP8149	*Kabuli*	Ca_Kabuli_Chr05	26569400	(G/C)

S.N.	SNP IDs	Cultivars	Chromosomes/scaffolds	Physical positions (bp)	SNPs
8150	CakSNP8150	*Kabuli*	Ca_Kabuli_Chr05	26609014	(T/C)
8151	CakSNP8151	*Kabuli*	Ca_Kabuli_Chr05	26608993	(A/G)
8152	CakSNP8152	*Kabuli*	Ca_Kabuli_Chr05	26621093	(C/G)
8153	CakSNP8153	*Kabuli*	Ca_Kabuli_Chr05	26626861	(A/G)
8154	CakSNP8154	*Kabuli*	Ca_Kabuli_Chr05	26626855	(T/G)
8155	CakSNP8155	*Kabuli*	Ca_Kabuli_Chr05	26638041	(G/A)
8156	CakSNP8156	*Kabuli*	Ca_Kabuli_Chr05	26638042	(C/T)
8157	CakSNP8157	*Kabuli*	Ca_Kabuli_Chr05	26638080	(A/G)
8158	CakSNP8158	*Kabuli*	Ca_Kabuli_Chr05	26638126	(C/T)
8159	CakSNP8159	*Kabuli*	Ca_Kabuli_Chr05	26638118	(C/T)
8160	CakSNP8160	*Kabuli*	Ca_Kabuli_Chr05	26679836	(C/T)
8161	CakSNP8161	*Kabuli*	Ca_Kabuli_Chr05	26696390	(G/A)
8162	CakSNP8162	*Kabuli*	Ca_Kabuli_Chr05	26696412	(C/A)
8163	CakSNP8163	*Kabuli*	Ca_Kabuli_Chr05	26696455	(G/A)
8164	CakSNP8164	*Kabuli*	Ca_Kabuli_Chr05	26696547	(C/T)
8165	CakSNP8165	*Kabuli*	Ca_Kabuli_Chr05	26696533	(C/T)
8166	CakSNP8166	*Kabuli*	Ca_Kabuli_Chr05	26696494	(G/A)
8167	CakSNP8167	*Kabuli*	Ca_Kabuli_Chr05	26696578	(G/A)
8168	CakSNP8168	*Kabuli*	Ca_Kabuli_Chr05	26696602	(C/T)
8169	CakSNP8169	*Kabuli*	Ca_Kabuli_Chr05	26696620	(C/T)
8170	CakSNP8170	*Kabuli*	Ca_Kabuli_Chr05	26696624	(G/A)
8171	CakSNP8171	*Kabuli*	Ca_Kabuli_Chr05	26785140	(G/A)
8172	CakSNP8172	*Kabuli*	Ca_Kabuli_Chr05	26785226	(C/A)
8173	CakSNP8173	*Kabuli*	Ca_Kabuli_Chr05	26997335	(T/G)
8174	CakSNP8174	*Kabuli*	Ca_Kabuli_Chr05	26999793	(C/T)
8175	CakSNP8175	*Kabuli*	Ca_Kabuli_Chr05	27000127	(C/G)
8176	CakSNP8176	*Kabuli*	Ca_Kabuli_Chr05	27000104	(C/A)
8177	CakSNP8177	*Kabuli*	Ca_Kabuli_Chr05	27000278	(T/A)
8178	CakSNP8178	*Kabuli*	Ca_Kabuli_Chr05	27000280	(T/G)
8179	CakSNP8179	*Kabuli*	Ca_Kabuli_Chr05	27025766	(A/G)
8180	CakSNP8180	*Kabuli*	Ca_Kabuli_Chr05	27025752	(A/C)
8181	CakSNP8181	*Kabuli*	Ca_Kabuli_Chr05	27042679	(G/A)
8182	CakSNP8182	*Kabuli*	Ca_Kabuli_Chr05	27117953	(A/G)
8183	CakSNP8183	*Kabuli*	Ca_Kabuli_Chr05	27150550	(A/G)
8184	CakSNP8184	*Kabuli*	Ca_Kabuli_Chr05	27230515	(T/A)
8185	CakSNP8185	*Kabuli*	Ca_Kabuli_Chr05	27230514	(A/G)
8186	CakSNP8186	*Kabuli*	Ca_Kabuli_Chr05	27231394	(T/C)
8187	CakSNP8187	*Kabuli*	Ca_Kabuli_Chr05	27231641	(T/A)
8188	CakSNP8188	*Kabuli*	Ca_Kabuli_Chr05	27232304	(T/A)

S.N.	SNP IDs	Cultivars	Chromosomes/scaffolds	Physical positions (bp)	SNPs
8189	CakSNP8189	*Kabuli*	Ca_Kabuli_Chr05	27234806	(G/A)
8190	CakSNP8190	*Kabuli*	Ca_Kabuli_Chr05	27235002	(T/G)
8191	CakSNP8191	*Kabuli*	Ca_Kabuli_Chr05	27328552	(G/A)
8192	CakSNP8192	*Kabuli*	Ca_Kabuli_Chr05	27328737	(G/T)
8193	CakSNP8193	*Kabuli*	Ca_Kabuli_Chr05	27334056	(A/C)
8194	CakSNP8194	*Kabuli*	Ca_Kabuli_Chr05	27334057	(G/C)
8195	CakSNP8195	*Kabuli*	Ca_Kabuli_Chr05	27334061	(G/C)
8196	CakSNP8196	*Kabuli*	Ca_Kabuli_Chr05	27349210	(T/A)
8197	CakSNP8197	*Kabuli*	Ca_Kabuli_Chr05	27349237	(C/T)
8198	CakSNP8198	*Kabuli*	Ca_Kabuli_Chr05	27361515	(C/T)
8199	CakSNP8199	*Kabuli*	Ca_Kabuli_Chr05	27361579	(T/G)
8200	CakSNP8200	*Kabuli*	Ca_Kabuli_Chr05	27362517	(C/G)
8201	CakSNP8201	*Kabuli*	Ca_Kabuli_Chr05	27362570	(G/T)
8202	CakSNP8202	*Kabuli*	Ca_Kabuli_Chr05	27362573	(A/G)
8203	CakSNP8203	*Kabuli*	Ca_Kabuli_Chr05	27362584	(T/G)
8204	CakSNP8204	*Kabuli*	Ca_Kabuli_Chr05	27452484	(C/G)
8205	CakSNP8205	*Kabuli*	Ca_Kabuli_Chr05	27516054	(T/A)
8206	CakSNP8206	*Kabuli*	Ca_Kabuli_Chr05	27552106	(A/C)
8207	CakSNP8207	*Kabuli*	Ca_Kabuli_Chr05	27555637	(T/G)
8208	CakSNP8208	*Kabuli*	Ca_Kabuli_Chr05	27568101	(T/G)
8209	CakSNP8209	*Kabuli*	Ca_Kabuli_Chr05	27568095	(C/A)
8210	CakSNP8210	*Kabuli*	Ca_Kabuli_Chr05	27568064	(A/C)
8211	CakSNP8211	*Kabuli*	Ca_Kabuli_Chr05	27568059	(C/T)
8212	CakSNP8212	*Kabuli*	Ca_Kabuli_Chr05	27568028	(A/T)
8213	CakSNP8213	*Kabuli*	Ca_Kabuli_Chr05	27568033	(G/A)
8214	CakSNP8214	*Kabuli*	Ca_Kabuli_Chr05	27568082	(G/A)
8215	CakSNP8215	*Kabuli*	Ca_Kabuli_Chr05	27568097	(G/A)
8216	CakSNP8216	*Kabuli*	Ca_Kabuli_Chr05	27568098	(C/T)
8217	CakSNP8217	*Kabuli*	Ca_Kabuli_Chr05	27568118	(A/G)
8218	CakSNP8218	*Kabuli*	Ca_Kabuli_Chr05	27568133	(G/T)
8219	CakSNP8219	*Kabuli*	Ca_Kabuli_Chr05	27568081	(G/C)
8220	CakSNP8220	*Kabuli*	Ca_Kabuli_Chr05	27568107	(C/G)
8221	CakSNP8221	*Kabuli*	Ca_Kabuli_Chr05	27568156	(C/G)
8222	CakSNP8222	*Kabuli*	Ca_Kabuli_Chr05	27568141	(C/T)
8223	CakSNP8223	*Kabuli*	Ca_Kabuli_Chr05	27568180	(C/T)
8224	CakSNP8224	*Kabuli*	Ca_Kabuli_Chr05	27568176	(T/C)
8225	CakSNP8225	*Kabuli*	Ca_Kabuli_Chr05	27579347	(C/T)
8226	CakSNP8226	*Kabuli*	Ca_Kabuli_Chr05	27605037	(A/T)
8227	CakSNP8227	*Kabuli*	Ca_Kabuli_Chr05	27605063	(T/A)

S.N.	SNP IDs	Cultivars	Chromosomes/scaffolds	Physical positions (bp)	SNPs
8228	CakSNP8228	*Kabuli*	Ca_Kabuli_Chr05	27605081	(C/T)
8229	CakSNP8229	*Kabuli*	Ca_Kabuli_Chr05	27605095	(G/C)
8230	CakSNP8230	*Kabuli*	Ca_Kabuli_Chr05	27692816	(C/T)
8231	CakSNP8231	*Kabuli*	Ca_Kabuli_Chr05	27692818	(C/T)
8232	CakSNP8232	*Kabuli*	Ca_Kabuli_Chr05	27692822	(C/T)
8233	CakSNP8233	*Kabuli*	Ca_Kabuli_Chr05	27692823	(A/T)
8234	CakSNP8234	*Kabuli*	Ca_Kabuli_Chr05	27692831	(A/T)
8235	CakSNP8235	*Kabuli*	Ca_Kabuli_Chr05	27694205	(C/A)
8236	CakSNP8236	*Kabuli*	Ca_Kabuli_Chr05	27750155	(C/T)
8237	CakSNP8237	*Kabuli*	Ca_Kabuli_Chr05	27786025	(T/C)
8238	CakSNP8238	*Kabuli*	Ca_Kabuli_Chr05	27838003	(T/C)
8239	CakSNP8239	*Kabuli*	Ca_Kabuli_Chr05	27850760	(T/C)
8240	CakSNP8240	*Kabuli*	Ca_Kabuli_Chr05	27850735	(C/A)
8241	CakSNP8241	*Kabuli*	Ca_Kabuli_Chr05	27853444	(C/T)
8242	CakSNP8242	*Kabuli*	Ca_Kabuli_Chr05	27872968	(C/T)
8243	CakSNP8243	*Kabuli*	Ca_Kabuli_Chr05	27873001	(T/C)
8244	CakSNP8244	*Kabuli*	Ca_Kabuli_Chr05	27874414	(C/A)
8245	CakSNP8245	*Kabuli*	Ca_Kabuli_Chr05	27936521	(A/G)
8246	CakSNP8246	*Kabuli*	Ca_Kabuli_Chr05	27936524	(C/T)
8247	CakSNP8247	*Kabuli*	Ca_Kabuli_Chr05	27939162	(G/A)
8248	CakSNP8248	*Kabuli*	Ca_Kabuli_Chr05	27939135	(G/C)
8249	CakSNP8249	*Kabuli*	Ca_Kabuli_Chr05	27939134	(A/T)
8250	CakSNP8250	*Kabuli*	Ca_Kabuli_Chr05	27939128	(A/G)
8251	CakSNP8251	*Kabuli*	Ca_Kabuli_Chr05	27974691	(G/T)
8252	CakSNP8252	*Kabuli*	Ca_Kabuli_Chr05	28016628	(C/T)
8253	CakSNP8253	*Kabuli*	Ca_Kabuli_Chr05	28016669	(A/G)
8254	CakSNP8254	*Kabuli*	Ca_Kabuli_Chr05	28071467	(A/G)
8255	CakSNP8255	*Kabuli*	Ca_Kabuli_Chr05	28116818	(A/G)
8256	CakSNP8256	*Kabuli*	Ca_Kabuli_Chr05	28116988	(A/T)
8257	CakSNP8257	*Kabuli*	Ca_Kabuli_Chr05	28117011	(C/T)
8258	CakSNP8258	*Kabuli*	Ca_Kabuli_Chr05	28164985	(G/C)
8259	CakSNP8259	*Kabuli*	Ca_Kabuli_Chr05	28168910	(A/G)
8260	CakSNP8260	*Kabuli*	Ca_Kabuli_Chr05	28169475	(T/C)
8261	CakSNP8261	*Kabuli*	Ca_Kabuli_Chr05	28175039	(A/G)
8262	CakSNP8262	*Kabuli*	Ca_Kabuli_Chr05	28266046	(T/G)
8263	CakSNP8263	*Kabuli*	Ca_Kabuli_Chr05	28271638	(G/C)
8264	CakSNP8264	*Kabuli*	Ca_Kabuli_Chr05	28271732	(C/T)
8265	CakSNP8265	*Kabuli*	Ca_Kabuli_Chr05	28271733	(G/A)
8266	CakSNP8266	*Kabuli*	Ca_Kabuli_Chr05	28271742	(G/A)

S.N.	SNP IDs	Cultivars	Chromosomes/scaffolds	Physical positions (bp)	SNPs
8267	CakSNP8267	*Kabuli*	Ca_Kabuli_Chr05	28271767	(G/C)
8268	CakSNP8268	*Kabuli*	Ca_Kabuli_Chr05	28284640	(T/C)
8269	CakSNP8269	*Kabuli*	Ca_Kabuli_Chr05	28327832	(T/G)
8270	CakSNP8270	*Kabuli*	Ca_Kabuli_Chr05	28327883	(G/A)
8271	CakSNP8271	*Kabuli*	Ca_Kabuli_Chr05	28327870	(T/C)
8272	CakSNP8272	*Kabuli*	Ca_Kabuli_Chr05	28327868	(G/A)
8273	CakSNP8273	*Kabuli*	Ca_Kabuli_Chr05	28327856	(T/C)
8274	CakSNP8274	*Kabuli*	Ca_Kabuli_Chr05	28335455	(A/C)
8275	CakSNP8275	*Kabuli*	Ca_Kabuli_Chr05	28346497	(C/T)
8276	CakSNP8276	*Kabuli*	Ca_Kabuli_Chr05	28360495	(C/T)
8277	CakSNP8277	*Kabuli*	Ca_Kabuli_Chr05	28360492	(C/T)
8278	CakSNP8278	*Kabuli*	Ca_Kabuli_Chr05	28360585	(A/G)
8279	CakSNP8279	*Kabuli*	Ca_Kabuli_Chr05	28383374	(G/T)
8280	CakSNP8280	*Kabuli*	Ca_Kabuli_Chr05	28491113	(G/A)
8281	CakSNP8281	*Kabuli*	Ca_Kabuli_Chr05	28491131	(G/A)
8282	CakSNP8282	*Kabuli*	Ca_Kabuli_Chr05	28509048	(G/A)
8283	CakSNP8283	*Kabuli*	Ca_Kabuli_Chr05	28509066	(G/A)
8284	CakSNP8284	*Kabuli*	Ca_Kabuli_Chr05	28562734	(G/A)
8285	CakSNP8285	*Kabuli*	Ca_Kabuli_Chr05	28581814	(A/G)
8286	CakSNP8286	*Kabuli*	Ca_Kabuli_Chr05	28581815	(C/G)
8287	CakSNP8287	*Kabuli*	Ca_Kabuli_Chr05	28581827	(T/C)
8288	CakSNP8288	*Kabuli*	Ca_Kabuli_Chr05	28581866	(T/C)
8289	CakSNP8289	*Kabuli*	Ca_Kabuli_Chr05	28581926	(C/A)
8290	CakSNP8290	*Kabuli*	Ca_Kabuli_Chr05	28582525	(C/T)
8291	CakSNP8291	*Kabuli*	Ca_Kabuli_Chr05	28582806	(T/C)
8292	CakSNP8292	*Kabuli*	Ca_Kabuli_Chr05	28582763	(A/G)
8293	CakSNP8293	*Kabuli*	Ca_Kabuli_Chr05	28583402	(G/A)
8294	CakSNP8294	*Kabuli*	Ca_Kabuli_Chr05	28583375	(C/G)
8295	CakSNP8295	*Kabuli*	Ca_Kabuli_Chr05	28682973	(A/C)
8296	CakSNP8296	*Kabuli*	Ca_Kabuli_Chr05	28682995	(G/A)
8297	CakSNP8297	*Kabuli*	Ca_Kabuli_Chr05	28683084	(A/C)
8298	CakSNP8298	*Kabuli*	Ca_Kabuli_Chr05	28683070	(T/A)
8299	CakSNP8299	*Kabuli*	Ca_Kabuli_Chr05	28683069	(A/T)
8300	CakSNP8300	*Kabuli*	Ca_Kabuli_Chr05	28714658	(C/T)
8301	CakSNP8301	*Kabuli*	Ca_Kabuli_Chr05	28717362	(C/T)
8302	CakSNP8302	*Kabuli*	Ca_Kabuli_Chr05	28717384	(C/T)
8303	CakSNP8303	*Kabuli*	Ca_Kabuli_Chr05	28721236	(C/T)
8304	CakSNP8304	*Kabuli*	Ca_Kabuli_Chr05	28721237	(A/G)
8305	CakSNP8305	*Kabuli*	Ca_Kabuli_Chr05	28721386	(T/C)

S.N.	SNP IDs	Cultivars	Chromosomes/scaffolds	Physical positions (bp)	SNPs
8306	CakSNP8306	*Kabuli*	Ca_Kabuli_Chr05	28721383	(A/C)
8307	CakSNP8307	*Kabuli*	Ca_Kabuli_Chr05	28746139	(A/G)
8308	CakSNP8308	*Kabuli*	Ca_Kabuli_Chr05	28757467	(A/T)
8309	CakSNP8309	*Kabuli*	Ca_Kabuli_Chr05	28757474	(T/C)
8310	CakSNP8310	*Kabuli*	Ca_Kabuli_Chr05	28757479	(T/C)
8311	CakSNP8311	*Kabuli*	Ca_Kabuli_Chr05	28757485	(A/C)
8312	CakSNP8312	*Kabuli*	Ca_Kabuli_Chr05	28757507	(A/T)
8313	CakSNP8313	*Kabuli*	Ca_Kabuli_Chr05	28758723	(A/G)
8314	CakSNP8314	*Kabuli*	Ca_Kabuli_Chr05	28817832	(A/G)
8315	CakSNP8315	*Kabuli*	Ca_Kabuli_Chr05	28827467	(A/G)
8316	CakSNP8316	*Kabuli*	Ca_Kabuli_Chr05	28843854	(G/A)
8317	CakSNP8317	*Kabuli*	Ca_Kabuli_Chr05	28843801	(A/G)
8318	CakSNP8318	*Kabuli*	Ca_Kabuli_Chr05	28843795	(A/T)
8319	CakSNP8319	*Kabuli*	Ca_Kabuli_Chr05	28843782	(C/G)
8320	CakSNP8320	*Kabuli*	Ca_Kabuli_Chr05	28843775	(C/T)
8321	CakSNP8321	*Kabuli*	Ca_Kabuli_Chr05	28854486	(C/T)
8322	CakSNP8322	*Kabuli*	Ca_Kabuli_Chr05	28905203	(A/C)
8323	CakSNP8323	*Kabuli*	Ca_Kabuli_Chr05	29007895	(T/C)
8324	CakSNP8324	*Kabuli*	Ca_Kabuli_Chr05	29007886	(C/A)
8325	CakSNP8325	*Kabuli*	Ca_Kabuli_Chr05	29007884	(A/C)
8326	CakSNP8326	*Kabuli*	Ca_Kabuli_Chr05	29007882	(A/C)
8327	CakSNP8327	*Kabuli*	Ca_Kabuli_Chr05	29007879	(T/C)
8328	CakSNP8328	*Kabuli*	Ca_Kabuli_Chr05	29007847	(A/C)
8329	CakSNP8329	*Kabuli*	Ca_Kabuli_Chr05	29007844	(T/C)
8330	CakSNP8330	*Kabuli*	Ca_Kabuli_Chr05	29061056	(T/A)
8331	CakSNP8331	*Kabuli*	Ca_Kabuli_Chr05	29061025	(A/C)
8332	CakSNP8332	*Kabuli*	Ca_Kabuli_Chr05	29060992	(C/T)
8333	CakSNP8333	*Kabuli*	Ca_Kabuli_Chr05	29062696	(G/A)
8334	CakSNP8334	*Kabuli*	Ca_Kabuli_Chr05	29062738	(A/G)
8335	CakSNP8335	*Kabuli*	Ca_Kabuli_Chr05	29079931	(G/A)
8336	CakSNP8336	*Kabuli*	Ca_Kabuli_Chr05	29079962	(A/G)
8337	CakSNP8337	*Kabuli*	Ca_Kabuli_Chr05	29080066	(G/C)
8338	CakSNP8338	*Kabuli*	Ca_Kabuli_Chr05	29080060	(C/T)
8339	CakSNP8339	*Kabuli*	Ca_Kabuli_Chr05	29111292	(T/G)
8340	CakSNP8340	*Kabuli*	Ca_Kabuli_Chr05	29111293	(T/C)
8341	CakSNP8341	*Kabuli*	Ca_Kabuli_Chr05	29186179	(T/G)
8342	CakSNP8342	*Kabuli*	Ca_Kabuli_Chr05	29220980	(T/A)
8343	CakSNP8343	*Kabuli*	Ca_Kabuli_Chr05	29221003	(T/C)
8344	CakSNP8344	*Kabuli*	Ca_Kabuli_Chr05	29221919	(T/A)

S.N.	SNP IDs	Cultivars	Chromosomes/scaffolds	Physical positions (bp)	SNPs
8345	CakSNP8345	*Kabuli*	Ca_Kabuli_Chr05	29272117	(C/G)
8346	CakSNP8346	*Kabuli*	Ca_Kabuli_Chr05	29272125	(C/T)
8347	CakSNP8347	*Kabuli*	Ca_Kabuli_Chr05	29354269	(A/T)
8348	CakSNP8348	*Kabuli*	Ca_Kabuli_Chr05	29422029	(T/A)
8349	CakSNP8349	*Kabuli*	Ca_Kabuli_Chr05	29422066	(A/T)
8350	CakSNP8350	*Kabuli*	Ca_Kabuli_Chr05	29422091	(C/T)
8351	CakSNP8351	*Kabuli*	Ca_Kabuli_Chr05	29437139	(C/T)
8352	CakSNP8352	*Kabuli*	Ca_Kabuli_Chr05	29437146	(C/A)
8353	CakSNP8353	*Kabuli*	Ca_Kabuli_Chr05	29437159	(C/T)
8354	CakSNP8354	*Kabuli*	Ca_Kabuli_Chr05	29437162	(A/C)
8355	CakSNP8355	*Kabuli*	Ca_Kabuli_Chr05	29437171	(A/T)
8356	CakSNP8356	*Kabuli*	Ca_Kabuli_Chr05	29437181	(A/C)
8357	CakSNP8357	*Kabuli*	Ca_Kabuli_Chr05	29437189	(C/T)
8358	CakSNP8358	*Kabuli*	Ca_Kabuli_Chr05	29437193	(A/C)
8359	CakSNP8359	*Kabuli*	Ca_Kabuli_Chr05	29437182	(C/A)
8360	CakSNP8360	*Kabuli*	Ca_Kabuli_Chr05	29528859	(A/T)
8361	CakSNP8361	*Kabuli*	Ca_Kabuli_Chr05	29528890	(T/A)
8362	CakSNP8362	*Kabuli*	Ca_Kabuli_Chr05	29528904	(T/G)
8363	CakSNP8363	*Kabuli*	Ca_Kabuli_Chr05	29530996	(G/A)
8364	CakSNP8364	*Kabuli*	Ca_Kabuli_Chr05	29531122	(A/T)
8365	CakSNP8365	*Kabuli*	Ca_Kabuli_Chr05	29531113	(G/A)
8366	CakSNP8366	*Kabuli*	Ca_Kabuli_Chr05	29531218	(A/T)
8367	CakSNP8367	*Kabuli*	Ca_Kabuli_Chr05	29531416	(A/G)
8368	CakSNP8368	*Kabuli*	Ca_Kabuli_Chr05	29567676	(T/C)
8369	CakSNP8369	*Kabuli*	Ca_Kabuli_Chr05	29567820	(T/C)
8370	CakSNP8370	*Kabuli*	Ca_Kabuli_Chr05	29576690	(A/T)
8371	CakSNP8371	*Kabuli*	Ca_Kabuli_Chr05	29576689	(G/A)
8372	CakSNP8372	*Kabuli*	Ca_Kabuli_Chr05	29576716	(A/C)
8373	CakSNP8373	*Kabuli*	Ca_Kabuli_Chr05	29608657	(T/C)
8374	CakSNP8374	*Kabuli*	Ca_Kabuli_Chr05	29641659	(T/C)
8375	CakSNP8375	*Kabuli*	Ca_Kabuli_Chr05	29658258	(G/C)
8376	CakSNP8376	*Kabuli*	Ca_Kabuli_Chr05	29658280	(A/C)
8377	CakSNP8377	*Kabuli*	Ca_Kabuli_Chr05	29667787	(C/G)
8378	CakSNP8378	*Kabuli*	Ca_Kabuli_Chr05	29667783	(C/T)
8379	CakSNP8379	*Kabuli*	Ca_Kabuli_Chr05	29667774	(G/C)
8380	CakSNP8380	*Kabuli*	Ca_Kabuli_Chr05	29809862	(A/C)
8381	CakSNP8381	*Kabuli*	Ca_Kabuli_Chr05	29816799	(T/C)
8382	CakSNP8382	*Kabuli*	Ca_Kabuli_Chr05	29816800	(T/A)
8383	CakSNP8383	*Kabuli*	Ca_Kabuli_Chr05	29824218	(T/G)

S.N.	SNP IDs	Cultivars	Chromosomes/scaffolds	Physical positions (bp)	SNPs
8384	CakSNP8384	*Kabuli*	Ca_Kabuli_Chr05	29852130	(T/C)
8385	CakSNP8385	*Kabuli*	Ca_Kabuli_Chr05	29852105	(T/G)
8386	CakSNP8386	*Kabuli*	Ca_Kabuli_Chr05	29886086	(T/A)
8387	CakSNP8387	*Kabuli*	Ca_Kabuli_Chr05	29981826	(A/G)
8388	CakSNP8388	*Kabuli*	Ca_Kabuli_Chr05	29999414	(C/T)
8389	CakSNP8389	*Kabuli*	Ca_Kabuli_Chr05	29999427	(G/A)
8390	CakSNP8390	*Kabuli*	Ca_Kabuli_Chr05	29999433	(C/A)
8391	CakSNP8391	*Kabuli*	Ca_Kabuli_Chr05	29999465	(T/G)
8392	CakSNP8392	*Kabuli*	Ca_Kabuli_Chr05	29999476	(G/A)
8393	CakSNP8393	*Kabuli*	Ca_Kabuli_Chr05	29999523	(A/T)
8394	CakSNP8394	*Kabuli*	Ca_Kabuli_Chr05	30035763	(A/T)
8395	CakSNP8395	*Kabuli*	Ca_Kabuli_Chr05	30049265	(G/A)
8396	CakSNP8396	*Kabuli*	Ca_Kabuli_Chr05	30053540	(C/G)
8397	CakSNP8397	*Kabuli*	Ca_Kabuli_Chr05	30060160	(C/T)
8398	CakSNP8398	*Kabuli*	Ca_Kabuli_Chr05	30086073	(A/G)
8399	CakSNP8399	*Kabuli*	Ca_Kabuli_Chr05	30086232	(A/C)
8400	CakSNP8400	*Kabuli*	Ca_Kabuli_Chr05	30090906	(A/G)
8401	CakSNP8401	*Kabuli*	Ca_Kabuli_Chr05	30091063	(G/A)
8402	CakSNP8402	*Kabuli*	Ca_Kabuli_Chr05	30116978	(C/T)
8403	CakSNP8403	*Kabuli*	Ca_Kabuli_Chr05	30116992	(T/G)
8404	CakSNP8404	*Kabuli*	Ca_Kabuli_Chr05	30159226	(C/T)
8405	CakSNP8405	*Kabuli*	Ca_Kabuli_Chr05	30159173	(G/A)
8406	CakSNP8406	*Kabuli*	Ca_Kabuli_Chr05	30159161	(G/A)
8407	CakSNP8407	*Kabuli*	Ca_Kabuli_Chr05	30191389	(A/C)
8408	CakSNP8408	*Kabuli*	Ca_Kabuli_Chr05	30248447	(C/A)
8409	CakSNP8409	*Kabuli*	Ca_Kabuli_Chr05	30258178	(T/G)
8410	CakSNP8410	*Kabuli*	Ca_Kabuli_Chr05	30287853	(C/T)
8411	CakSNP8411	*Kabuli*	Ca_Kabuli_Chr05	30304076	(A/T)
8412	CakSNP8412	*Kabuli*	Ca_Kabuli_Chr05	30319316	(A/G)
8413	CakSNP8413	*Kabuli*	Ca_Kabuli_Chr05	30359047	(T/C)
8414	CakSNP8414	*Kabuli*	Ca_Kabuli_Chr05	30359022	(G/A)
8415	CakSNP8415	*Kabuli*	Ca_Kabuli_Chr05	30360185	(T/C)
8416	CakSNP8416	*Kabuli*	Ca_Kabuli_Chr05	30360348	(T/C)
8417	CakSNP8417	*Kabuli*	Ca_Kabuli_Chr05	30367349	(G/T)
8418	CakSNP8418	*Kabuli*	Ca_Kabuli_Chr05	30373542	(A/G)
8419	CakSNP8419	*Kabuli*	Ca_Kabuli_Chr05	30373783	(T/A)
8420	CakSNP8420	*Kabuli*	Ca_Kabuli_Chr05	30376661	(C/G)
8421	CakSNP8421	*Kabuli*	Ca_Kabuli_Chr05	30376722	(T/C)
8422	CakSNP8422	*Kabuli*	Ca_Kabuli_Chr05	30376723	(T/C)

S.N.	SNP IDs	Cultivars	Chromosomes/scaffolds	Physical positions (bp)	SNPs
8423	CakSNP8423	*Kabuli*	Ca_Kabuli_Chr05	30378114	(A/G)
8424	CakSNP8424	*Kabuli*	Ca_Kabuli_Chr05	30378184	(A/G)
8425	CakSNP8425	*Kabuli*	Ca_Kabuli_Chr05	30407725	(C/G)
8426	CakSNP8426	*Kabuli*	Ca_Kabuli_Chr05	30408006	(C/T)
8427	CakSNP8427	*Kabuli*	Ca_Kabuli_Chr05	30438913	(G/A)
8428	CakSNP8428	*Kabuli*	Ca_Kabuli_Chr05	30443858	(A/T)
8429	CakSNP8429	*Kabuli*	Ca_Kabuli_Chr05	30562430	(C/G)
8430	CakSNP8430	*Kabuli*	Ca_Kabuli_Chr05	30562524	(G/T)
8431	CakSNP8431	*Kabuli*	Ca_Kabuli_Chr05	30601932	(T/C)
8432	CakSNP8432	*Kabuli*	Ca_Kabuli_Chr05	30601926	(T/C)
8433	CakSNP8433	*Kabuli*	Ca_Kabuli_Chr05	30601920	(T/C)
8434	CakSNP8434	*Kabuli*	Ca_Kabuli_Chr05	30627937	(G/T)
8435	CakSNP8435	*Kabuli*	Ca_Kabuli_Chr05	30632697	(A/G)
8436	CakSNP8436	*Kabuli*	Ca_Kabuli_Chr05	30655941	(T/C)
8437	CakSNP8437	*Kabuli*	Ca_Kabuli_Chr05	30656058	(A/T)
8438	CakSNP8438	*Kabuli*	Ca_Kabuli_Chr05	30690891	(T/G)
8439	CakSNP8439	*Kabuli*	Ca_Kabuli_Chr05	30690852	(G/A)
8440	CakSNP8440	*Kabuli*	Ca_Kabuli_Chr05	30690885	(A/G)
8441	CakSNP8441	*Kabuli*	Ca_Kabuli_Chr05	30691510	(A/G)
8442	CakSNP8442	*Kabuli*	Ca_Kabuli_Chr05	30691515	(G/A)
8443	CakSNP8443	*Kabuli*	Ca_Kabuli_Chr05	30691543	(A/G)
8444	CakSNP8444	*Kabuli*	Ca_Kabuli_Chr05	30697511	(G/A)
8445	CakSNP8445	*Kabuli*	Ca_Kabuli_Chr05	30745571	(G/T)
8446	CakSNP8446	*Kabuli*	Ca_Kabuli_Chr05	30749553	(A/T)
8447	CakSNP8447	*Kabuli*	Ca_Kabuli_Chr05	30749696	(A/T)
8448	CakSNP8448	*Kabuli*	Ca_Kabuli_Chr05	30755644	(A/G)
8449	CakSNP8449	*Kabuli*	Ca_Kabuli_Chr05	30755584	(G/A)
8450	CakSNP8450	*Kabuli*	Ca_Kabuli_Chr05	30811250	(A/T)
8451	CakSNP8451	*Kabuli*	Ca_Kabuli_Chr05	30864010	(T/C)
8452	CakSNP8452	*Kabuli*	Ca_Kabuli_Chr05	30868770	(G/C)
8453	CakSNP8453	*Kabuli*	Ca_Kabuli_Chr05	30868695	(G/C)
8454	CakSNP8454	*Kabuli*	Ca_Kabuli_Chr05	30878047	(C/T)
8455	CakSNP8455	*Kabuli*	Ca_Kabuli_Chr05	30878092	(G/T)
8456	CakSNP8456	*Kabuli*	Ca_Kabuli_Chr05	30878100	(T/A)
8457	CakSNP8457	*Kabuli*	Ca_Kabuli_Chr05	30890480	(G/C)
8458	CakSNP8458	*Kabuli*	Ca_Kabuli_Chr05	30962640	(A/T)
8459	CakSNP8459	*Kabuli*	Ca_Kabuli_Chr05	30962641	(C/A)
8460	CakSNP8460	*Kabuli*	Ca_Kabuli_Chr05	30962650	(G/A)
8461	CakSNP8461	*Kabuli*	Ca_Kabuli_Chr05	30964628	(A/G)

S.N.	SNP IDs	Cultivars	Chromosomes/scaffolds	Physical positions (bp)	SNPs
8462	CakSNP8462	*Kabuli*	Ca_Kabuli_Chr05	31024408	(T/C)
8463	CakSNP8463	*Kabuli*	Ca_Kabuli_Chr05	31025764	(G/T)
8464	CakSNP8464	*Kabuli*	Ca_Kabuli_Chr05	31034500	(T/C)
8465	CakSNP8465	*Kabuli*	Ca_Kabuli_Chr05	31034898	(A/G)
8466	CakSNP8466	*Kabuli*	Ca_Kabuli_Chr05	31064173	(C/G)
8467	CakSNP8467	*Kabuli*	Ca_Kabuli_Chr05	31072213	(T/C)
8468	CakSNP8468	*Kabuli*	Ca_Kabuli_Chr05	31072276	(G/A)
8469	CakSNP8469	*Kabuli*	Ca_Kabuli_Chr05	31084839	(A/C)
8470	CakSNP8470	*Kabuli*	Ca_Kabuli_Chr05	31088698	(T/G)
8471	CakSNP8471	*Kabuli*	Ca_Kabuli_Chr05	31126125	(C/T)
8472	CakSNP8472	*Kabuli*	Ca_Kabuli_Chr05	31131830	(T/C)
8473	CakSNP8473	*Kabuli*	Ca_Kabuli_Chr05	31132568	(A/T)
8474	CakSNP8474	*Kabuli*	Ca_Kabuli_Chr05	31138650	(G/A)
8475	CakSNP8475	*Kabuli*	Ca_Kabuli_Chr05	31140531	(T/C)
8476	CakSNP8476	*Kabuli*	Ca_Kabuli_Chr05	31162533	(G/A)
8477	CakSNP8477	*Kabuli*	Ca_Kabuli_Chr05	31164387	(G/C)
8478	CakSNP8478	*Kabuli*	Ca_Kabuli_Chr05	31164539	(T/C)
8479	CakSNP8479	*Kabuli*	Ca_Kabuli_Chr05	31165048	(T/C)
8480	CakSNP8480	*Kabuli*	Ca_Kabuli_Chr05	31165039	(T/C)
8481	CakSNP8481	*Kabuli*	Ca_Kabuli_Chr05	31165035	(T/C)
8482	CakSNP8482	*Kabuli*	Ca_Kabuli_Chr05	31165002	(A/C)
8483	CakSNP8483	*Kabuli*	Ca_Kabuli_Chr05	31171555	(G/A)
8484	CakSNP8484	*Kabuli*	Ca_Kabuli_Chr05	31176722	(T/G)
8485	CakSNP8485	*Kabuli*	Ca_Kabuli_Chr05	31176810	(C/T)
8486	CakSNP8486	*Kabuli*	Ca_Kabuli_Chr05	31176844	(G/A)
8487	CakSNP8487	*Kabuli*	Ca_Kabuli_Chr05	31192415	(C/T)
8488	CakSNP8488	*Kabuli*	Ca_Kabuli_Chr05	31192413	(G/A)
8489	CakSNP8489	*Kabuli*	Ca_Kabuli_Chr05	31194845	(G/T)
8490	CakSNP8490	*Kabuli*	Ca_Kabuli_Chr05	31194974	(T/C)
8491	CakSNP8491	*Kabuli*	Ca_Kabuli_Chr05	31194988	(G/T)
8492	CakSNP8492	*Kabuli*	Ca_Kabuli_Chr05	31194989	(A/C)
8493	CakSNP8493	*Kabuli*	Ca_Kabuli_Chr05	31215109	(C/T)
8494	CakSNP8494	*Kabuli*	Ca_Kabuli_Chr05	31310698	(C/T)
8495	CakSNP8495	*Kabuli*	Ca_Kabuli_Chr05	31310702	(C/G)
8496	CakSNP8496	*Kabuli*	Ca_Kabuli_Chr05	31310703	(A/G)
8497	CakSNP8497	*Kabuli*	Ca_Kabuli_Chr05	31310741	(A/G)
8498	CakSNP8498	*Kabuli*	Ca_Kabuli_Chr05	31314012	(A/C)
8499	CakSNP8499	*Kabuli*	Ca_Kabuli_Chr05	31314056	(A/C)
8500	CakSNP8500	*Kabuli*	Ca_Kabuli_Chr05	31344725	(G/A)

S.N.	SNP IDs	Cultivars	Chromosomes/scaffolds	Physical positions (bp)	SNPs
8501	CakSNP8501	*Kabuli*	Ca_Kabuli_Chr05	31377103	(C/T)
8502	CakSNP8502	*Kabuli*	Ca_Kabuli_Chr05	31377122	(C/T)
8503	CakSNP8503	*Kabuli*	Ca_Kabuli_Chr05	31377134	(A/T)
8504	CakSNP8504	*Kabuli*	Ca_Kabuli_Chr05	31390221	(A/G)
8505	CakSNP8505	*Kabuli*	Ca_Kabuli_Chr05	31390319	(C/T)
8506	CakSNP8506	*Kabuli*	Ca_Kabuli_Chr05	31522389	(T/G)
8507	CakSNP8507	*Kabuli*	Ca_Kabuli_Chr05	31548555	(G/A)
8508	CakSNP8508	*Kabuli*	Ca_Kabuli_Chr05	31651335	(G/T)
8509	CakSNP8509	*Kabuli*	Ca_Kabuli_Chr05	31651336	(T/G)
8510	CakSNP8510	*Kabuli*	Ca_Kabuli_Chr05	31653418	(T/C)
8511	CakSNP8511	*Kabuli*	Ca_Kabuli_Chr05	31653423	(T/C)
8512	CakSNP8512	*Kabuli*	Ca_Kabuli_Chr05	31653432	(A/G)
8513	CakSNP8513	*Kabuli*	Ca_Kabuli_Chr05	31653437	(C/T)
8514	CakSNP8514	*Kabuli*	Ca_Kabuli_Chr05	31653452	(C/A)
8515	CakSNP8515	*Kabuli*	Ca_Kabuli_Chr05	31687881	(C/T)
8516	CakSNP8516	*Kabuli*	Ca_Kabuli_Chr05	31687891	(A/C)
8517	CakSNP8517	*Kabuli*	Ca_Kabuli_Chr05	31696236	(T/C)
8518	CakSNP8518	*Kabuli*	Ca_Kabuli_Chr05	31710670	(G/A)
8519	CakSNP8519	*Kabuli*	Ca_Kabuli_Chr05	31724217	(G/A)
8520	CakSNP8520	*Kabuli*	Ca_Kabuli_Chr05	31724272	(C/T)
8521	CakSNP8521	*Kabuli*	Ca_Kabuli_Chr05	31724332	(G/A)
8522	CakSNP8522	*Kabuli*	Ca_Kabuli_Chr05	31750150	(T/G)
8523	CakSNP8523	*Kabuli*	Ca_Kabuli_Chr05	31750242	(A/G)
8524	CakSNP8524	*Kabuli*	Ca_Kabuli_Chr05	31758166	(T/C)
8525	CakSNP8525	*Kabuli*	Ca_Kabuli_Chr05	31802684	(G/T)
8526	CakSNP8526	*Kabuli*	Ca_Kabuli_Chr05	31802727	(G/A)
8527	CakSNP8527	*Kabuli*	Ca_Kabuli_Chr05	31816202	(A/C)
8528	CakSNP8528	*Kabuli*	Ca_Kabuli_Chr05	31816228	(A/G)
8529	CakSNP8529	*Kabuli*	Ca_Kabuli_Chr05	31935674	(T/C)
8530	CakSNP8530	*Kabuli*	Ca_Kabuli_Chr05	31951925	(G/A)
8531	CakSNP8531	*Kabuli*	Ca_Kabuli_Chr05	31951944	(C/T)
8532	CakSNP8532	*Kabuli*	Ca_Kabuli_Chr05	31985390	(G/C)
8533	CakSNP8533	*Kabuli*	Ca_Kabuli_Chr05	32011781	(G/T)
8534	CakSNP8534	*Kabuli*	Ca_Kabuli_Chr05	32011792	(T/G)
8535	CakSNP8535	*Kabuli*	Ca_Kabuli_Chr05	32015967	(T/C)
8536	CakSNP8536	*Kabuli*	Ca_Kabuli_Chr05	32019864	(G/A)
8537	CakSNP8537	*Kabuli*	Ca_Kabuli_Chr05	32023925	(T/C)
8538	CakSNP8538	*Kabuli*	Ca_Kabuli_Chr05	32090295	(C/A)
8539	CakSNP8539	*Kabuli*	Ca_Kabuli_Chr05	32144081	(G/C)

S.N.	SNP IDs	Cultivars	Chromosomes/scaffolds	Physical positions (bp)	SNPs
8540	CakSNP8540	*Kabuli*	Ca_Kabuli_Chr05	32144108	(A/C)
8541	CakSNP8541	*Kabuli*	Ca_Kabuli_Chr05	32144120	(C/T)
8542	CakSNP8542	*Kabuli*	Ca_Kabuli_Chr05	32147522	(C/T)
8543	CakSNP8543	*Kabuli*	Ca_Kabuli_Chr05	32171632	(G/A)
8544	CakSNP8544	*Kabuli*	Ca_Kabuli_Chr05	32171652	(T/A)
8545	CakSNP8545	*Kabuli*	Ca_Kabuli_Chr05	32209547	(C/G)
8546	CakSNP8546	*Kabuli*	Ca_Kabuli_Chr05	32209750	(T/C)
8547	CakSNP8547	*Kabuli*	Ca_Kabuli_Chr05	32229485	(A/T)
8548	CakSNP8548	*Kabuli*	Ca_Kabuli_Chr05	32291158	(C/T)
8549	CakSNP8549	*Kabuli*	Ca_Kabuli_Chr05	32291118	(G/A)
8550	CakSNP8550	*Kabuli*	Ca_Kabuli_Chr05	32328110	(T/C)
8551	CakSNP8551	*Kabuli*	Ca_Kabuli_Chr05	32346655	(A/C)
8552	CakSNP8552	*Kabuli*	Ca_Kabuli_Chr05	32357511	(G/A)
8553	CakSNP8553	*Kabuli*	Ca_Kabuli_Chr05	32368485	(G/C)
8554	CakSNP8554	*Kabuli*	Ca_Kabuli_Chr05	32369197	(C/A)
8555	CakSNP8555	*Kabuli*	Ca_Kabuli_Chr05	32399391	(T/A)
8556	CakSNP8556	*Kabuli*	Ca_Kabuli_Chr05	32476432	(A/G)
8557	CakSNP8557	*Kabuli*	Ca_Kabuli_Chr05	32476423	(A/G)
8558	CakSNP8558	*Kabuli*	Ca_Kabuli_Chr05	32516773	(T/A)
8559	CakSNP8559	*Kabuli*	Ca_Kabuli_Chr05	32524646	(T/C)
8560	CakSNP8560	*Kabuli*	Ca_Kabuli_Chr05	32566131	(C/G)
8561	CakSNP8561	*Kabuli*	Ca_Kabuli_Chr05	32598221	(C/A)
8562	CakSNP8562	*Kabuli*	Ca_Kabuli_Chr05	32623507	(T/G)
8563	CakSNP8563	*Kabuli*	Ca_Kabuli_Chr05	32631930	(C/A)
8564	CakSNP8564	*Kabuli*	Ca_Kabuli_Chr05	32643759	(A/G)
8565	CakSNP8565	*Kabuli*	Ca_Kabuli_Chr05	32732862	(A/T)
8566	CakSNP8566	*Kabuli*	Ca_Kabuli_Chr05	32732913	(A/T)
8567	CakSNP8567	*Kabuli*	Ca_Kabuli_Chr05	32759420	(T/A)
8568	CakSNP8568	*Kabuli*	Ca_Kabuli_Chr05	32761420	(G/A)
8569	CakSNP8569	*Kabuli*	Ca_Kabuli_Chr05	32794288	(G/A)
8570	CakSNP8570	*Kabuli*	Ca_Kabuli_Chr05	32816626	(G/T)
8571	CakSNP8571	*Kabuli*	Ca_Kabuli_Chr05	32839347	(C/A)
8572	CakSNP8572	*Kabuli*	Ca_Kabuli_Chr05	32840512	(A/T)
8573	CakSNP8573	*Kabuli*	Ca_Kabuli_Chr05	32840522	(T/C)
8574	CakSNP8574	*Kabuli*	Ca_Kabuli_Chr05	32840541	(T/G)
8575	CakSNP8575	*Kabuli*	Ca_Kabuli_Chr05	32859483	(T/A)
8576	CakSNP8576	*Kabuli*	Ca_Kabuli_Chr05	32859504	(T/G)
8577	CakSNP8577	*Kabuli*	Ca_Kabuli_Chr05	32859515	(T/G)
8578	CakSNP8578	*Kabuli*	Ca_Kabuli_Chr05	32860087	(C/T)

S.N.	SNP IDs	Cultivars	Chromosomes/scaffolds	Physical positions (bp)	SNPs
8579	CakSNP8579	*Kabuli*	Ca_Kabuli_Chr05	32978975	(A/T)
8580	CakSNP8580	*Kabuli*	Ca_Kabuli_Chr05	33052329	(C/G)
8581	CakSNP8581	*Kabuli*	Ca_Kabuli_Chr05	33143038	(A/T)
8582	CakSNP8582	*Kabuli*	Ca_Kabuli_Chr05	33189733	(A/G)
8583	CakSNP8583	*Kabuli*	Ca_Kabuli_Chr05	33189791	(G/A)
8584	CakSNP8584	*Kabuli*	Ca_Kabuli_Chr05	33189808	(A/G)
8585	CakSNP8585	*Kabuli*	Ca_Kabuli_Chr05	33255823	(T/G)
8586	CakSNP8586	*Kabuli*	Ca_Kabuli_Chr05	33255971	(G/T)
8587	CakSNP8587	*Kabuli*	Ca_Kabuli_Chr05	33256053	(C/G)
8588	CakSNP8588	*Kabuli*	Ca_Kabuli_Chr05	33256089	(T/C)
8589	CakSNP8589	*Kabuli*	Ca_Kabuli_Chr05	33256245	(T/C)
8590	CakSNP8590	*Kabuli*	Ca_Kabuli_Chr05	33256412	(A/T)
8591	CakSNP8591	*Kabuli*	Ca_Kabuli_Chr05	33256388	(T/A)
8592	CakSNP8592	*Kabuli*	Ca_Kabuli_Chr05	33336127	(C/G)
8593	CakSNP8593	*Kabuli*	Ca_Kabuli_Chr05	33336135	(G/T)
8594	CakSNP8594	*Kabuli*	Ca_Kabuli_Chr05	33349598	(C/T)
8595	CakSNP8595	*Kabuli*	Ca_Kabuli_Chr05	33349700	(A/G)
8596	CakSNP8596	*Kabuli*	Ca_Kabuli_Chr05	33379904	(A/G)
8597	CakSNP8597	*Kabuli*	Ca_Kabuli_Chr05	33379873	(C/T)
8598	CakSNP8598	*Kabuli*	Ca_Kabuli_Chr05	33381774	(C/T)
8599	CakSNP8599	*Kabuli*	Ca_Kabuli_Chr05	33422813	(C/T)
8600	CakSNP8600	*Kabuli*	Ca_Kabuli_Chr05	33422780	(A/G)
8601	CakSNP8601	*Kabuli*	Ca_Kabuli_Chr05	33422779	(C/T)
8602	CakSNP8602	*Kabuli*	Ca_Kabuli_Chr05	33422767	(T/C)
8603	CakSNP8603	*Kabuli*	Ca_Kabuli_Chr05	33431897	(T/C)
8604	CakSNP8604	*Kabuli*	Ca_Kabuli_Chr05	33431996	(T/C)
8605	CakSNP8605	*Kabuli*	Ca_Kabuli_Chr05	33523676	(T/C)
8606	CakSNP8606	*Kabuli*	Ca_Kabuli_Chr05	33523686	(G/A)
8607	CakSNP8607	*Kabuli*	Ca_Kabuli_Chr05	33523691	(T/C)
8608	CakSNP8608	*Kabuli*	Ca_Kabuli_Chr05	33532807	(A/G)
8609	CakSNP8609	*Kabuli*	Ca_Kabuli_Chr05	33532804	(A/T)
8610	CakSNP8610	*Kabuli*	Ca_Kabuli_Chr05	33532798	(A/T)
8611	CakSNP8611	*Kabuli*	Ca_Kabuli_Chr05	33574726	(T/C)
8612	CakSNP8612	*Kabuli*	Ca_Kabuli_Chr05	33578061	(T/A)
8613	CakSNP8613	*Kabuli*	Ca_Kabuli_Chr05	33578167	(A/T)
8614	CakSNP8614	*Kabuli*	Ca_Kabuli_Chr05	33578438	(T/C)
8615	CakSNP8615	*Kabuli*	Ca_Kabuli_Chr05	33578477	(A/G)
8616	CakSNP8616	*Kabuli*	Ca_Kabuli_Chr05	33581040	(A/G)
8617	CakSNP8617	*Kabuli*	Ca_Kabuli_Chr05	33626841	(C/T)

S.N.	SNP IDs	Cultivars	Chromosomes/scaffolds	Physical positions (bp)	SNPs
8618	CakSNP8618	*Kabuli*	Ca_Kabuli_Chr05	33627132	(T/C)
8619	CakSNP8619	*Kabuli*	Ca_Kabuli_Chr05	33631108	(A/G)
8620	CakSNP8620	*Kabuli*	Ca_Kabuli_Chr05	33631266	(T/A)
8621	CakSNP8621	*Kabuli*	Ca_Kabuli_Chr05	33656560	(C/G)
8622	CakSNP8622	*Kabuli*	Ca_Kabuli_Chr05	33673894	(T/C)
8623	CakSNP8623	*Kabuli*	Ca_Kabuli_Chr05	33700181	(G/C)
8624	CakSNP8624	*Kabuli*	Ca_Kabuli_Chr05	33700196	(A/T)
8625	CakSNP8625	*Kabuli*	Ca_Kabuli_Chr05	33721487	(C/T)
8626	CakSNP8626	*Kabuli*	Ca_Kabuli_Chr05	33721544	(A/G)
8627	CakSNP8627	*Kabuli*	Ca_Kabuli_Chr05	33771359	(T/C)
8628	CakSNP8628	*Kabuli*	Ca_Kabuli_Chr05	33812108	(T/C)
8629	CakSNP8629	*Kabuli*	Ca_Kabuli_Chr05	33812213	(A/C)
8630	CakSNP8630	*Kabuli*	Ca_Kabuli_Chr05	33812363	(T/A)
8631	CakSNP8631	*Kabuli*	Ca_Kabuli_Chr05	33818742	(T/A)
8632	CakSNP8632	*Kabuli*	Ca_Kabuli_Chr05	33818789	(C/G)
8633	CakSNP8633	*Kabuli*	Ca_Kabuli_Chr05	33818823	(A/G)
8634	CakSNP8634	*Kabuli*	Ca_Kabuli_Chr05	33824516	(G/T)
8635	CakSNP8635	*Kabuli*	Ca_Kabuli_Chr05	33824513	(G/A)
8636	CakSNP8636	*Kabuli*	Ca_Kabuli_Chr05	33824503	(G/A)
8637	CakSNP8637	*Kabuli*	Ca_Kabuli_Chr05	33835816	(T/C)
8638	CakSNP8638	*Kabuli*	Ca_Kabuli_Chr05	33841544	(T/G)
8639	CakSNP8639	*Kabuli*	Ca_Kabuli_Chr05	33844056	(A/T)
8640	CakSNP8640	*Kabuli*	Ca_Kabuli_Chr05	33844059	(G/C)
8641	CakSNP8641	*Kabuli*	Ca_Kabuli_Chr05	33844222	(T/C)
8642	CakSNP8642	*Kabuli*	Ca_Kabuli_Chr05	33844436	(T/A)
8643	CakSNP8643	*Kabuli*	Ca_Kabuli_Chr05	33892596	(A/C)
8644	CakSNP8644	*Kabuli*	Ca_Kabuli_Chr05	33937307	(G/A)
8645	CakSNP8645	*Kabuli*	Ca_Kabuli_Chr05	33937316	(G/A)
8646	CakSNP8646	*Kabuli*	Ca_Kabuli_Chr05	33963525	(G/C)
8647	CakSNP8647	*Kabuli*	Ca_Kabuli_Chr05	33985819	(A/G)
8648	CakSNP8648	*Kabuli*	Ca_Kabuli_Chr05	33986234	(C/T)
8649	CakSNP8649	*Kabuli*	Ca_Kabuli_Chr05	33986253	(A/C)
8650	CakSNP8650	*Kabuli*	Ca_Kabuli_Chr05	34022362	(G/T)
8651	CakSNP8651	*Kabuli*	Ca_Kabuli_Chr05	34040479	(A/G)
8652	CakSNP8652	*Kabuli*	Ca_Kabuli_Chr05	34040508	(A/C)
8653	CakSNP8653	*Kabuli*	Ca_Kabuli_Chr05	34040535	(G/A)
8654	CakSNP8654	*Kabuli*	Ca_Kabuli_Chr05	34041705	(G/T)
8655	CakSNP8655	*Kabuli*	Ca_Kabuli_Chr05	34073378	(A/T)
8656	CakSNP8656	*Kabuli*	Ca_Kabuli_Chr05	34073395	(G/A)

S.N.	SNP IDs	Cultivars	Chromosomes/scaffolds	Physical positions (bp)	SNPs
8657	CakSNP8657	*Kabuli*	Ca_Kabuli_Chr05	34073445	(A/T)
8658	CakSNP8658	*Kabuli*	Ca_Kabuli_Chr05	34201989	(C/G)
8659	CakSNP8659	*Kabuli*	Ca_Kabuli_Chr05	34202000	(T/C)
8660	CakSNP8660	*Kabuli*	Ca_Kabuli_Chr05	34202075	(T/G)
8661	CakSNP8661	*Kabuli*	Ca_Kabuli_Chr05	34202222	(G/A)
8662	CakSNP8662	*Kabuli*	Ca_Kabuli_Chr05	34241269	(C/G)
8663	CakSNP8663	*Kabuli*	Ca_Kabuli_Chr05	34244499	(C/T)
8664	CakSNP8664	*Kabuli*	Ca_Kabuli_Chr05	34244681	(G/A)
8665	CakSNP8665	*Kabuli*	Ca_Kabuli_Chr05	34244670	(A/G)
8666	CakSNP8666	*Kabuli*	Ca_Kabuli_Chr05	34244662	(C/G)
8667	CakSNP8667	*Kabuli*	Ca_Kabuli_Chr05	34244754	(T/A)
8668	CakSNP8668	*Kabuli*	Ca_Kabuli_Chr05	34313779	(A/G)
8669	CakSNP8669	*Kabuli*	Ca_Kabuli_Chr05	34313758	(T/G)
8670	CakSNP8670	*Kabuli*	Ca_Kabuli_Chr05	34334980	(A/G)
8671	CakSNP8671	*Kabuli*	Ca_Kabuli_Chr05	34334993	(A/C)
8672	CakSNP8672	*Kabuli*	Ca_Kabuli_Chr05	34335252	(T/A)
8673	CakSNP8673	*Kabuli*	Ca_Kabuli_Chr05	34418798	(G/C)
8674	CakSNP8674	*Kabuli*	Ca_Kabuli_Chr05	34441581	(A/C)
8675	CakSNP8675	*Kabuli*	Ca_Kabuli_Chr05	34461159	(T/C)
8676	CakSNP8676	*Kabuli*	Ca_Kabuli_Chr05	34482245	(G/T)
8677	CakSNP8677	*Kabuli*	Ca_Kabuli_Chr05	34482217	(G/T)
8678	CakSNP8678	*Kabuli*	Ca_Kabuli_Chr05	34511669	(G/T)
8679	CakSNP8679	*Kabuli*	Ca_Kabuli_Chr05	34511678	(T/C)
8680	CakSNP8680	*Kabuli*	Ca_Kabuli_Chr05	34511693	(C/T)
8681	CakSNP8681	*Kabuli*	Ca_Kabuli_Chr05	34511705	(G/T)
8682	CakSNP8682	*Kabuli*	Ca_Kabuli_Chr05	34521655	(T/C)
8683	CakSNP8683	*Kabuli*	Ca_Kabuli_Chr05	34531222	(A/G)
8684	CakSNP8684	*Kabuli*	Ca_Kabuli_Chr05	34535758	(T/C)
8685	CakSNP8685	*Kabuli*	Ca_Kabuli_Chr05	34535893	(G/T)
8686	CakSNP8686	*Kabuli*	Ca_Kabuli_Chr05	34535878	(G/A)
8687	CakSNP8687	*Kabuli*	Ca_Kabuli_Chr05	34629028	(A/G)
8688	CakSNP8688	*Kabuli*	Ca_Kabuli_Chr05	34673784	(T/A)
8689	CakSNP8689	*Kabuli*	Ca_Kabuli_Chr05	34691234	(A/C)
8690	CakSNP8690	*Kabuli*	Ca_Kabuli_Chr05	34691302	(T/C)
8691	CakSNP8691	*Kabuli*	Ca_Kabuli_Chr05	34691453	(G/A)
8692	CakSNP8692	*Kabuli*	Ca_Kabuli_Chr05	34807071	(C/T)
8693	CakSNP8693	*Kabuli*	Ca_Kabuli_Chr05	34814216	(T/C)
8694	CakSNP8694	*Kabuli*	Ca_Kabuli_Chr05	34824647	(C/G)
8695	CakSNP8695	*Kabuli*	Ca_Kabuli_Chr05	34824749	(A/T)

S.N.	SNP IDs	Cultivars	Chromosomes/scaffolds	Physical positions (bp)	SNPs
8696	CakSNP8696	*Kabuli*	Ca_Kabuli_Chr05	34845398	(T/G)
8697	CakSNP8697	*Kabuli*	Ca_Kabuli_Chr05	34879731	(A/G)
8698	CakSNP8698	*Kabuli*	Ca_Kabuli_Chr05	34974615	(T/A)
8699	CakSNP8699	*Kabuli*	Ca_Kabuli_Chr05	34974703	(C/T)
8700	CakSNP8700	*Kabuli*	Ca_Kabuli_Chr05	34974795	(A/G)
8701	CakSNP8701	*Kabuli*	Ca_Kabuli_Chr05	34975712	(T/A)
8702	CakSNP8702	*Kabuli*	Ca_Kabuli_Chr05	34998725	(G/A)
8703	CakSNP8703	*Kabuli*	Ca_Kabuli_Chr05	35126544	(A/C)
8704	CakSNP8704	*Kabuli*	Ca_Kabuli_Chr05	35164295	(C/T)
8705	CakSNP8705	*Kabuli*	Ca_Kabuli_Chr05	35191371	(C/G)
8706	CakSNP8706	*Kabuli*	Ca_Kabuli_Chr05	35209511	(T/C)
8707	CakSNP8707	*Kabuli*	Ca_Kabuli_Chr05	35213601	(C/T)
8708	CakSNP8708	*Kabuli*	Ca_Kabuli_Chr05	35226049	(G/C)
8709	CakSNP8709	*Kabuli*	Ca_Kabuli_Chr05	35290452	(A/G)
8710	CakSNP8710	*Kabuli*	Ca_Kabuli_Chr05	35296135	(A/C)
8711	CakSNP8711	*Kabuli*	Ca_Kabuli_Chr05	35330946	(G/A)
8712	CakSNP8712	*Kabuli*	Ca_Kabuli_Chr05	35330995	(A/C)
8713	CakSNP8713	*Kabuli*	Ca_Kabuli_Chr05	35340068	(A/G)
8714	CakSNP8714	*Kabuli*	Ca_Kabuli_Chr05	35340065	(G/A)
8715	CakSNP8715	*Kabuli*	Ca_Kabuli_Chr05	35374184	(G/A)
8716	CakSNP8716	*Kabuli*	Ca_Kabuli_Chr05	35438327	(G/T)
8717	CakSNP8717	*Kabuli*	Ca_Kabuli_Chr05	35495900	(T/A)
8718	CakSNP8718	*Kabuli*	Ca_Kabuli_Chr05	35500751	(T/C)
8719	CakSNP8719	*Kabuli*	Ca_Kabuli_Chr05	35534923	(T/A)
8720	CakSNP8720	*Kabuli*	Ca_Kabuli_Chr05	35534897	(G/A)
8721	CakSNP8721	*Kabuli*	Ca_Kabuli_Chr05	35540372	(T/C)
8722	CakSNP8722	*Kabuli*	Ca_Kabuli_Chr05	35543648	(T/C)
8723	CakSNP8723	*Kabuli*	Ca_Kabuli_Chr05	35554534	(C/A)
8724	CakSNP8724	*Kabuli*	Ca_Kabuli_Chr05	35554593	(C/T)
8725	CakSNP8725	*Kabuli*	Ca_Kabuli_Chr05	35555184	(A/G)
8726	CakSNP8726	*Kabuli*	Ca_Kabuli_Chr05	35576495	(A/G)
8727	CakSNP8727	*Kabuli*	Ca_Kabuli_Chr05	35617798	(A/C)
8728	CakSNP8728	*Kabuli*	Ca_Kabuli_Chr05	35688558	(T/C)
8729	CakSNP8729	*Kabuli*	Ca_Kabuli_Chr05	35690996	(G/A)
8730	CakSNP8730	*Kabuli*	Ca_Kabuli_Chr05	35691581	(T/C)
8731	CakSNP8731	*Kabuli*	Ca_Kabuli_Chr05	35715495	(C/T)
8732	CakSNP8732	*Kabuli*	Ca_Kabuli_Chr05	35715550	(G/C)
8733	CakSNP8733	*Kabuli*	Ca_Kabuli_Chr05	35715571	(A/C)
8734	CakSNP8734	*Kabuli*	Ca_Kabuli_Chr05	35715563	(G/A)

S.N.	SNP IDs	Cultivars	Chromosomes/scaffolds	Physical positions (bp)	SNPs
8735	CakSNP8735	*Kabuli*	Ca_Kabuli_Chr05	35715548	(T/C)
8736	CakSNP8736	*Kabuli*	Ca_Kabuli_Chr05	35773040	(C/A)
8737	CakSNP8737	*Kabuli*	Ca_Kabuli_Chr05	35773044	(A/G)
8738	CakSNP8738	*Kabuli*	Ca_Kabuli_Chr05	35775200	(T/C)
8739	CakSNP8739	*Kabuli*	Ca_Kabuli_Chr05	35775191	(T/G)
8740	CakSNP8740	*Kabuli*	Ca_Kabuli_Chr05	35775266	(G/A)
8741	CakSNP8741	*Kabuli*	Ca_Kabuli_Chr05	35789373	(T/A)
8742	CakSNP8742	*Kabuli*	Ca_Kabuli_Chr05	35789775	(A/G)
8743	CakSNP8743	*Kabuli*	Ca_Kabuli_Chr05	35839230	(T/C)
8744	CakSNP8744	*Kabuli*	Ca_Kabuli_Chr05	35839233	(G/A)
8745	CakSNP8745	*Kabuli*	Ca_Kabuli_Chr05	35844295	(G/T)
8746	CakSNP8746	*Kabuli*	Ca_Kabuli_Chr05	35845094	(G/A)
8747	CakSNP8747	*Kabuli*	Ca_Kabuli_Chr05	35845780	(T/C)
8748	CakSNP8748	*Kabuli*	Ca_Kabuli_Chr05	35845801	(T/C)
8749	CakSNP8749	*Kabuli*	Ca_Kabuli_Chr05	35846404	(A/G)
8750	CakSNP8750	*Kabuli*	Ca_Kabuli_Chr05	35868865	(G/A)
8751	CakSNP8751	*Kabuli*	Ca_Kabuli_Chr05	35877505	(T/C)
8752	CakSNP8752	*Kabuli*	Ca_Kabuli_Chr05	35926105	(T/C)
8753	CakSNP8753	*Kabuli*	Ca_Kabuli_Chr05	35937904	(C/T)
8754	CakSNP8754	*Kabuli*	Ca_Kabuli_Chr05	35937842	(G/T)
8755	CakSNP8755	*Kabuli*	Ca_Kabuli_Chr05	35947574	(G/A)
8756	CakSNP8756	*Kabuli*	Ca_Kabuli_Chr05	35954656	(T/C)
8757	CakSNP8757	*Kabuli*	Ca_Kabuli_Chr05	35956534	(C/T)
8758	CakSNP8758	*Kabuli*	Ca_Kabuli_Chr05	36012698	(C/G)
8759	CakSNP8759	*Kabuli*	Ca_Kabuli_Chr05	36039365	(A/C)
8760	CakSNP8760	*Kabuli*	Ca_Kabuli_Chr05	36128655	(G/T)
8761	CakSNP8761	*Kabuli*	Ca_Kabuli_Chr05	36131156	(A/C)
8762	CakSNP8762	*Kabuli*	Ca_Kabuli_Chr05	36217803	(C/T)
8763	CakSNP8763	*Kabuli*	Ca_Kabuli_Chr05	36238656	(G/A)
8764	CakSNP8764	*Kabuli*	Ca_Kabuli_Chr05	36238655	(G/C)
8765	CakSNP8765	*Kabuli*	Ca_Kabuli_Chr05	36248568	(C/T)
8766	CakSNP8766	*Kabuli*	Ca_Kabuli_Chr05	36292611	(C/G)
8767	CakSNP8767	*Kabuli*	Ca_Kabuli_Chr05	36334569	(C/A)
8768	CakSNP8768	*Kabuli*	Ca_Kabuli_Chr05	36334592	(C/T)
8769	CakSNP8769	*Kabuli*	Ca_Kabuli_Chr05	36334625	(A/G)
8770	CakSNP8770	*Kabuli*	Ca_Kabuli_Chr05	36338598	(A/C)
8771	CakSNP8771	*Kabuli*	Ca_Kabuli_Chr05	36338658	(A/G)
8772	CakSNP8772	*Kabuli*	Ca_Kabuli_Chr05	36397489	(A/C)
8773	CakSNP8773	*Kabuli*	Ca_Kabuli_Chr05	36400943	(C/T)

S.N.	SNP IDs	Cultivars	Chromosomes/scaffolds	Physical positions (bp)	SNPs
8774	CakSNP8774	*Kabuli*	Ca_Kabuli_Chr05	36408701	(G/A)
8775	CakSNP8775	*Kabuli*	Ca_Kabuli_Chr05	36408719	(G/A)
8776	CakSNP8776	*Kabuli*	Ca_Kabuli_Chr05	36408724	(T/C)
8777	CakSNP8777	*Kabuli*	Ca_Kabuli_Chr05	36408736	(C/A)
8778	CakSNP8778	*Kabuli*	Ca_Kabuli_Chr05	36430677	(A/C)
8779	CakSNP8779	*Kabuli*	Ca_Kabuli_Chr05	36441063	(C/T)
8780	CakSNP8780	*Kabuli*	Ca_Kabuli_Chr05	36533996	(T/A)
8781	CakSNP8781	*Kabuli*	Ca_Kabuli_Chr05	36534056	(G/C)
8782	CakSNP8782	*Kabuli*	Ca_Kabuli_Chr05	36534057	(T/G)
8783	CakSNP8783	*Kabuli*	Ca_Kabuli_Chr05	36534060	(G/A)
8784	CakSNP8784	*Kabuli*	Ca_Kabuli_Chr05	36534063	(C/T)
8785	CakSNP8785	*Kabuli*	Ca_Kabuli_Chr05	36534126	(A/G)
8786	CakSNP8786	*Kabuli*	Ca_Kabuli_Chr05	36534110	(T/C)
8787	CakSNP8787	*Kabuli*	Ca_Kabuli_Chr05	36566175	(G/A)
8788	CakSNP8788	*Kabuli*	Ca_Kabuli_Chr05	36613610	(T/C)
8789	CakSNP8789	*Kabuli*	Ca_Kabuli_Chr05	36619837	(A/G)
8790	CakSNP8790	*Kabuli*	Ca_Kabuli_Chr05	36621872	(A/T)
8791	CakSNP8791	*Kabuli*	Ca_Kabuli_Chr05	36672147	(C/T)
8792	CakSNP8792	*Kabuli*	Ca_Kabuli_Chr05	36672164	(A/G)
8793	CakSNP8793	*Kabuli*	Ca_Kabuli_Chr05	36673950	(C/A)
8794	CakSNP8794	*Kabuli*	Ca_Kabuli_Chr05	36731961	(T/C)
8795	CakSNP8795	*Kabuli*	Ca_Kabuli_Chr05	36735459	(A/T)
8796	CakSNP8796	*Kabuli*	Ca_Kabuli_Chr05	36751625	(C/T)
8797	CakSNP8797	*Kabuli*	Ca_Kabuli_Chr05	36751851	(T/A)
8798	CakSNP8798	*Kabuli*	Ca_Kabuli_Chr05	36757408	(G/A)
8799	CakSNP8799	*Kabuli*	Ca_Kabuli_Chr05	36797173	(A/G)
8800	CakSNP8800	*Kabuli*	Ca_Kabuli_Chr05	36797187	(G/T)
8801	CakSNP8801	*Kabuli*	Ca_Kabuli_Chr05	36800497	(G/T)
8802	CakSNP8802	*Kabuli*	Ca_Kabuli_Chr05	36842784	(G/T)
8803	CakSNP8803	*Kabuli*	Ca_Kabuli_Chr05	36855028	(G/A)
8804	CakSNP8804	*Kabuli*	Ca_Kabuli_Chr05	36916560	(T/C)
8805	CakSNP8805	*Kabuli*	Ca_Kabuli_Chr05	36999074	(G/C)
8806	CakSNP8806	*Kabuli*	Ca_Kabuli_Chr05	37016704	(C/G)
8807	CakSNP8807	*Kabuli*	Ca_Kabuli_Chr05	37039246	(A/T)
8808	CakSNP8808	*Kabuli*	Ca_Kabuli_Chr05	37039300	(A/G)
8809	CakSNP8809	*Kabuli*	Ca_Kabuli_Chr05	37040045	(G/A)
8810	CakSNP8810	*Kabuli*	Ca_Kabuli_Chr05	37067293	(A/G)
8811	CakSNP8811	*Kabuli*	Ca_Kabuli_Chr05	37067371	(T/C)
8812	CakSNP8812	*Kabuli*	Ca_Kabuli_Chr05	37067384	(G/C)

S.N.	SNP IDs	Cultivars	Chromosomes/scaffolds	Physical positions (bp)	SNPs
8813	CakSNP8813	*Kabuli*	Ca_Kabuli_Chr05	37083276	(T/G)
8814	CakSNP8814	*Kabuli*	Ca_Kabuli_Chr05	37083275	(G/T)
8815	CakSNP8815	*Kabuli*	Ca_Kabuli_Chr05	37114574	(A/G)
8816	CakSNP8816	*Kabuli*	Ca_Kabuli_Chr05	37126371	(A/G)
8817	CakSNP8817	*Kabuli*	Ca_Kabuli_Chr05	37126354	(T/C)
8818	CakSNP8818	*Kabuli*	Ca_Kabuli_Chr05	37147572	(C/T)
8819	CakSNP8819	*Kabuli*	Ca_Kabuli_Chr05	37167061	(A/G)
8820	CakSNP8820	*Kabuli*	Ca_Kabuli_Chr05	37167114	(A/T)
8821	CakSNP8821	*Kabuli*	Ca_Kabuli_Chr05	37167108	(A/T)
8822	CakSNP8822	*Kabuli*	Ca_Kabuli_Chr05	37194939	(G/A)
8823	CakSNP8823	*Kabuli*	Ca_Kabuli_Chr05	37194917	(G/A)
8824	CakSNP8824	*Kabuli*	Ca_Kabuli_Chr05	37197321	(C/A)
8825	CakSNP8825	*Kabuli*	Ca_Kabuli_Chr05	37207821	(A/T)
8826	CakSNP8826	*Kabuli*	Ca_Kabuli_Chr05	37207837	(G/T)
8827	CakSNP8827	*Kabuli*	Ca_Kabuli_Chr05	37211645	(T/G)
8828	CakSNP8828	*Kabuli*	Ca_Kabuli_Chr05	37213853	(C/T)
8829	CakSNP8829	*Kabuli*	Ca_Kabuli_Chr05	37232307	(T/C)
8830	CakSNP8830	*Kabuli*	Ca_Kabuli_Chr05	37254596	(A/G)
8831	CakSNP8831	*Kabuli*	Ca_Kabuli_Chr05	37260601	(T/C)
8832	CakSNP8832	*Kabuli*	Ca_Kabuli_Chr05	37274572	(C/T)
8833	CakSNP8833	*Kabuli*	Ca_Kabuli_Chr05	37274583	(C/T)
8834	CakSNP8834	*Kabuli*	Ca_Kabuli_Chr05	37274616	(C/G)
8835	CakSNP8835	*Kabuli*	Ca_Kabuli_Chr05	37309917	(T/G)
8836	CakSNP8836	*Kabuli*	Ca_Kabuli_Chr05	37309935	(T/G)
8837	CakSNP8837	*Kabuli*	Ca_Kabuli_Chr05	37332101	(G/T)
8838	CakSNP8838	*Kabuli*	Ca_Kabuli_Chr05	37332198	(G/A)
8839	CakSNP8839	*Kabuli*	Ca_Kabuli_Chr05	37332304	(G/A)
8840	CakSNP8840	*Kabuli*	Ca_Kabuli_Chr05	37332303	(A/C)
8841	CakSNP8841	*Kabuli*	Ca_Kabuli_Chr05	37332370	(T/C)
8842	CakSNP8842	*Kabuli*	Ca_Kabuli_Chr05	37332441	(T/C)
8843	CakSNP8843	*Kabuli*	Ca_Kabuli_Chr05	37332501	(C/T)
8844	CakSNP8844	*Kabuli*	Ca_Kabuli_Chr05	37339022	(T/G)
8845	CakSNP8845	*Kabuli*	Ca_Kabuli_Chr05	37339358	(T/G)
8846	CakSNP8846	*Kabuli*	Ca_Kabuli_Chr05	37339327	(A/G)
8847	CakSNP8847	*Kabuli*	Ca_Kabuli_Chr05	37339300	(A/G)
8848	CakSNP8848	*Kabuli*	Ca_Kabuli_Chr05	37342358	(G/T)
8849	CakSNP8849	*Kabuli*	Ca_Kabuli_Chr05	37363626	(C/T)
8850	CakSNP8850	*Kabuli*	Ca_Kabuli_Chr05	37375575	(C/G)
8851	CakSNP8851	*Kabuli*	Ca_Kabuli_Chr05	37375620	(C/T)

S.N.	SNP IDs	Cultivars	Chromosomes/scaffolds	Physical positions (bp)	SNPs
8852	CakSNP8852	*Kabuli*	Ca_Kabuli_Chr05	37445226	(A/G)
8853	CakSNP8853	*Kabuli*	Ca_Kabuli_Chr05	37460980	(A/G)
8854	CakSNP8854	*Kabuli*	Ca_Kabuli_Chr05	37468727	(T/C)
8855	CakSNP8855	*Kabuli*	Ca_Kabuli_Chr05	37468755	(G/A)
8856	CakSNP8856	*Kabuli*	Ca_Kabuli_Chr05	37555816	(T/A)
8857	CakSNP8857	*Kabuli*	Ca_Kabuli_Chr05	37555846	(T/A)
8858	CakSNP8858	*Kabuli*	Ca_Kabuli_Chr05	37578999	(T/G)
8859	CakSNP8859	*Kabuli*	Ca_Kabuli_Chr05	37591782	(A/T)
8860	CakSNP8860	*Kabuli*	Ca_Kabuli_Chr05	37593444	(A/T)
8861	CakSNP8861	*Kabuli*	Ca_Kabuli_Chr05	37594347	(T/C)
8862	CakSNP8862	*Kabuli*	Ca_Kabuli_Chr05	37604030	(T/C)
8863	CakSNP8863	*Kabuli*	Ca_Kabuli_Chr05	37604090	(G/T)
8864	CakSNP8864	*Kabuli*	Ca_Kabuli_Chr05	37604780	(C/T)
8865	CakSNP8865	*Kabuli*	Ca_Kabuli_Chr05	37796337	(T/G)
8866	CakSNP8866	*Kabuli*	Ca_Kabuli_Chr05	37798313	(T/G)
8867	CakSNP8867	*Kabuli*	Ca_Kabuli_Chr05	37868250	(C/T)
8868	CakSNP8868	*Kabuli*	Ca_Kabuli_Chr05	37868256	(T/A)
8869	CakSNP8869	*Kabuli*	Ca_Kabuli_Chr05	37869744	(T/C)
8870	CakSNP8870	*Kabuli*	Ca_Kabuli_Chr05	37872223	(C/G)
8871	CakSNP8871	*Kabuli*	Ca_Kabuli_Chr05	37874564	(T/A)
8872	CakSNP8872	*Kabuli*	Ca_Kabuli_Chr05	37874725	(A/C)
8873	CakSNP8873	*Kabuli*	Ca_Kabuli_Chr05	37874694	(C/T)
8874	CakSNP8874	*Kabuli*	Ca_Kabuli_Chr05	37886417	(T/G)
8875	CakSNP8875	*Kabuli*	Ca_Kabuli_Chr05	37899098	(T/C)
8876	CakSNP8876	*Kabuli*	Ca_Kabuli_Chr05	37899238	(T/C)
8877	CakSNP8877	*Kabuli*	Ca_Kabuli_Chr05	37908818	(A/C)
8878	CakSNP8878	*Kabuli*	Ca_Kabuli_Chr05	37908807	(A/T)
8879	CakSNP8879	*Kabuli*	Ca_Kabuli_Chr05	37911813	(A/C)
8880	CakSNP8880	*Kabuli*	Ca_Kabuli_Chr05	38018552	(A/T)
8881	CakSNP8881	*Kabuli*	Ca_Kabuli_Chr05	38018558	(G/C)
8882	CakSNP8882	*Kabuli*	Ca_Kabuli_Chr05	38057582	(C/T)
8883	CakSNP8883	*Kabuli*	Ca_Kabuli_Chr05	38080226	(A/G)
8884	CakSNP8884	*Kabuli*	Ca_Kabuli_Chr05	38080349	(C/T)
8885	CakSNP8885	*Kabuli*	Ca_Kabuli_Chr05	38092606	(G/A)
8886	CakSNP8886	*Kabuli*	Ca_Kabuli_Chr05	38092619	(T/C)
8887	CakSNP8887	*Kabuli*	Ca_Kabuli_Chr05	38092732	(C/T)
8888	CakSNP8888	*Kabuli*	Ca_Kabuli_Chr05	38092735	(C/T)
8889	CakSNP8889	*Kabuli*	Ca_Kabuli_Chr05	38092793	(C/T)
8890	CakSNP8890	*Kabuli*	Ca_Kabuli_Chr05	38102941	(T/G)

S.N.	SNP IDs	Cultivars	Chromosomes/scaffolds	Physical positions (bp)	SNPs
8891	CakSNP8891	*Kabuli*	Ca_Kabuli_Chr05	38108396	(G/A)
8892	CakSNP8892	*Kabuli*	Ca_Kabuli_Chr05	38110219	(T/G)
8893	CakSNP8893	*Kabuli*	Ca_Kabuli_Chr05	38220843	(G/A)
8894	CakSNP8894	*Kabuli*	Ca_Kabuli_Chr05	38220908	(G/T)
8895	CakSNP8895	*Kabuli*	Ca_Kabuli_Chr05	38248895	(A/C)
8896	CakSNP8896	*Kabuli*	Ca_Kabuli_Chr05	38249134	(T/C)
8897	CakSNP8897	*Kabuli*	Ca_Kabuli_Chr05	38249132	(A/C)
8898	CakSNP8898	*Kabuli*	Ca_Kabuli_Chr05	38266153	(G/A)
8899	CakSNP8899	*Kabuli*	Ca_Kabuli_Chr05	38307036	(G/A)
8900	CakSNP8900	*Kabuli*	Ca_Kabuli_Chr05	38333718	(C/T)
8901	CakSNP8901	*Kabuli*	Ca_Kabuli_Chr05	38333746	(G/A)
8902	CakSNP8902	*Kabuli*	Ca_Kabuli_Chr05	38333774	(A/G)
8903	CakSNP8903	*Kabuli*	Ca_Kabuli_Chr05	38347201	(A/C)
8904	CakSNP8904	*Kabuli*	Ca_Kabuli_Chr05	38455563	(A/C)
8905	CakSNP8905	*Kabuli*	Ca_Kabuli_Chr05	38509657	(A/C)
8906	CakSNP8906	*Kabuli*	Ca_Kabuli_Chr05	38538904	(A/G)
8907	CakSNP8907	*Kabuli*	Ca_Kabuli_Chr05	38551438	(C/T)
8908	CakSNP8908	*Kabuli*	Ca_Kabuli_Chr05	38551381	(G/A)
8909	CakSNP8909	*Kabuli*	Ca_Kabuli_Chr05	38647040	(A/C)
8910	CakSNP8910	*Kabuli*	Ca_Kabuli_Chr05	38656599	(C/T)
8911	CakSNP8911	*Kabuli*	Ca_Kabuli_Chr05	38783604	(C/A)
8912	CakSNP8912	*Kabuli*	Ca_Kabuli_Chr05	38784078	(G/A)
8913	CakSNP8913	*Kabuli*	Ca_Kabuli_Chr05	38784074	(A/G)
8914	CakSNP8914	*Kabuli*	Ca_Kabuli_Chr05	38784150	(T/A)
8915	CakSNP8915	*Kabuli*	Ca_Kabuli_Chr05	38784129	(G/C)
8916	CakSNP8916	*Kabuli*	Ca_Kabuli_Chr05	38857904	(T/C)
8917	CakSNP8917	*Kabuli*	Ca_Kabuli_Chr05	38872143	(A/G)
8918	CakSNP8918	*Kabuli*	Ca_Kabuli_Chr05	38892604	(A/C)
8919	CakSNP8919	*Kabuli*	Ca_Kabuli_Chr05	38898971	(G/A)
8920	CakSNP8920	*Kabuli*	Ca_Kabuli_Chr05	38909701	(C/T)
8921	CakSNP8921	*Kabuli*	Ca_Kabuli_Chr05	38937304	(C/G)
8922	CakSNP8922	*Kabuli*	Ca_Kabuli_Chr05	38937398	(C/A)
8923	CakSNP8923	*Kabuli*	Ca_Kabuli_Chr05	38939578	(C/A)
8924	CakSNP8924	*Kabuli*	Ca_Kabuli_Chr05	38984762	(A/G)
8925	CakSNP8925	*Kabuli*	Ca_Kabuli_Chr05	38984769	(A/C)
8926	CakSNP8926	*Kabuli*	Ca_Kabuli_Chr05	38984776	(A/G)
8927	CakSNP8927	*Kabuli*	Ca_Kabuli_Chr05	39008980	(G/A)
8928	CakSNP8928	*Kabuli*	Ca_Kabuli_Chr05	39137170	(A/G)
8929	CakSNP8929	*Kabuli*	Ca_Kabuli_Chr05	39175007	(A/T)

S.N.	SNP IDs	Cultivars	Chromosomes/scaffolds	Physical positions (bp)	SNPs
8930	CakSNP8930	*Kabuli*	Ca_Kabuli_Chr05	39176391	(T/A)
8931	CakSNP8931	*Kabuli*	Ca_Kabuli_Chr05	39266333	(T/C)
8932	CakSNP8932	*Kabuli*	Ca_Kabuli_Chr05	39277581	(T/C)
8933	CakSNP8933	*Kabuli*	Ca_Kabuli_Chr05	39283856	(G/A)
8934	CakSNP8934	*Kabuli*	Ca_Kabuli_Chr05	39326933	(G/T)
8935	CakSNP8935	*Kabuli*	Ca_Kabuli_Chr05	39353755	(T/G)
8936	CakSNP8936	*Kabuli*	Ca_Kabuli_Chr05	39364522	(T/C)
8937	CakSNP8937	*Kabuli*	Ca_Kabuli_Chr05	39364569	(G/A)
8938	CakSNP8938	*Kabuli*	Ca_Kabuli_Chr05	39364571	(C/A)
8939	CakSNP8939	*Kabuli*	Ca_Kabuli_Chr05	39364720	(A/T)
8940	CakSNP8940	*Kabuli*	Ca_Kabuli_Chr05	39364700	(C/A)
8941	CakSNP8941	*Kabuli*	Ca_Kabuli_Chr05	39364695	(A/C)
8942	CakSNP8942	*Kabuli*	Ca_Kabuli_Chr05	39364667	(T/A)
8943	CakSNP8943	*Kabuli*	Ca_Kabuli_Chr05	39364741	(A/T)
8944	CakSNP8944	*Kabuli*	Ca_Kabuli_Chr05	39364752	(G/T)
8945	CakSNP8945	*Kabuli*	Ca_Kabuli_Chr05	39364895	(A/C)
8946	CakSNP8946	*Kabuli*	Ca_Kabuli_Chr05	39364856	(A/G)
8947	CakSNP8947	*Kabuli*	Ca_Kabuli_Chr05	39364848	(C/T)
8948	CakSNP8948	*Kabuli*	Ca_Kabuli_Chr05	39364841	(A/T)
8949	CakSNP8949	*Kabuli*	Ca_Kabuli_Chr05	39366353	(C/G)
8950	CakSNP8950	*Kabuli*	Ca_Kabuli_Chr05	39385603	(T/G)
8951	CakSNP8951	*Kabuli*	Ca_Kabuli_Chr05	39385630	(C/T)
8952	CakSNP8952	*Kabuli*	Ca_Kabuli_Chr05	39410377	(T/G)
8953	CakSNP8953	*Kabuli*	Ca_Kabuli_Chr05	39410329	(T/G)
8954	CakSNP8954	*Kabuli*	Ca_Kabuli_Chr05	39460567	(A/C)
8955	CakSNP8955	*Kabuli*	Ca_Kabuli_Chr05	39475960	(T/C)
8956	CakSNP8956	*Kabuli*	Ca_Kabuli_Chr05	39476040	(T/G)
8957	CakSNP8957	*Kabuli*	Ca_Kabuli_Chr05	39498665	(G/A)
8958	CakSNP8958	*Kabuli*	Ca_Kabuli_Chr05	39513563	(C/A)
8959	CakSNP8959	*Kabuli*	Ca_Kabuli_Chr05	39543929	(C/T)
8960	CakSNP8960	*Kabuli*	Ca_Kabuli_Chr05	39576823	(C/T)
8961	CakSNP8961	*Kabuli*	Ca_Kabuli_Chr05	39576836	(T/A)
8962	CakSNP8962	*Kabuli*	Ca_Kabuli_Chr05	39576839	(C/T)
8963	CakSNP8963	*Kabuli*	Ca_Kabuli_Chr05	39576864	(C/A)
8964	CakSNP8964	*Kabuli*	Ca_Kabuli_Chr05	39576870	(C/T)
8965	CakSNP8965	*Kabuli*	Ca_Kabuli_Chr05	39576874	(G/T)
8966	CakSNP8966	*Kabuli*	Ca_Kabuli_Chr05	39576841	(G/A)
8967	CakSNP8967	*Kabuli*	Ca_Kabuli_Chr05	39592439	(A/G)
8968	CakSNP8968	*Kabuli*	Ca_Kabuli_Chr05	39597485	(C/T)

S.N.	SNP IDs	Cultivars	Chromosomes/scaffolds	Physical positions (bp)	SNPs
8969	CakSNP8969	*Kabuli*	Ca_Kabuli_Chr05	39597459	(T/C)
8970	CakSNP8970	*Kabuli*	Ca_Kabuli_Chr05	39608204	(A/G)
8971	CakSNP8971	*Kabuli*	Ca_Kabuli_Chr05	39608209	(G/T)
8972	CakSNP8972	*Kabuli*	Ca_Kabuli_Chr05	39684480	(A/G)
8973	CakSNP8973	*Kabuli*	Ca_Kabuli_Chr05	39684592	(A/T)
8974	CakSNP8974	*Kabuli*	Ca_Kabuli_Chr05	39713021	(G/A)
8975	CakSNP8975	*Kabuli*	Ca_Kabuli_Chr05	39776794	(G/A)
8976	CakSNP8976	*Kabuli*	Ca_Kabuli_Chr05	39776795	(G/A)
8977	CakSNP8977	*Kabuli*	Ca_Kabuli_Chr05	39776818	(C/T)
8978	CakSNP8978	*Kabuli*	Ca_Kabuli_Chr05	39776850	(G/A)
8979	CakSNP8979	*Kabuli*	Ca_Kabuli_Chr05	39776869	(C/T)
8980	CakSNP8980	*Kabuli*	Ca_Kabuli_Chr05	39794718	(C/T)
8981	CakSNP8981	*Kabuli*	Ca_Kabuli_Chr05	39851570	(T/G)
8982	CakSNP8982	*Kabuli*	Ca_Kabuli_Chr05	39869398	(C/T)
8983	CakSNP8983	*Kabuli*	Ca_Kabuli_Chr05	39943298	(C/T)
8984	CakSNP8984	*Kabuli*	Ca_Kabuli_Chr05	39985042	(G/A)
8985	CakSNP8985	*Kabuli*	Ca_Kabuli_Chr05	39985332	(A/G)
8986	CakSNP8986	*Kabuli*	Ca_Kabuli_Chr05	40018264	(A/C)
8987	CakSNP8987	*Kabuli*	Ca_Kabuli_Chr05	40020062	(C/T)
8988	CakSNP8988	*Kabuli*	Ca_Kabuli_Chr05	40020073	(C/G)
8989	CakSNP8989	*Kabuli*	Ca_Kabuli_Chr05	40025250	(G/A)
8990	CakSNP8990	*Kabuli*	Ca_Kabuli_Chr05	40025319	(T/C)
8991	CakSNP8991	*Kabuli*	Ca_Kabuli_Chr05	40025326	(C/T)
8992	CakSNP8992	*Kabuli*	Ca_Kabuli_Chr05	40025376	(A/G)
8993	CakSNP8993	*Kabuli*	Ca_Kabuli_Chr05	40025377	(T/A)
8994	CakSNP8994	*Kabuli*	Ca_Kabuli_Chr05	40027707	(A/G)
8995	CakSNP8995	*Kabuli*	Ca_Kabuli_Chr05	40078085	(C/G)
8996	CakSNP8996	*Kabuli*	Ca_Kabuli_Chr05	40078077	(A/G)
8997	CakSNP8997	*Kabuli*	Ca_Kabuli_Chr05	40159260	(C/A)
8998	CakSNP8998	*Kabuli*	Ca_Kabuli_Chr05	40159348	(G/A)
8999	CakSNP8999	*Kabuli*	Ca_Kabuli_Chr05	40162755	(T/C)
9000	CakSNP9000	*Kabuli*	Ca_Kabuli_Chr05	40162995	(C/T)
9001	CakSNP9001	*Kabuli*	Ca_Kabuli_Chr05	40164176	(C/G)
9002	CakSNP9002	*Kabuli*	Ca_Kabuli_Chr05	40239397	(G/C)
9003	CakSNP9003	*Kabuli*	Ca_Kabuli_Chr05	40290082	(G/A)
9004	CakSNP9004	*Kabuli*	Ca_Kabuli_Chr05	40290066	(A/G)
9005	CakSNP9005	*Kabuli*	Ca_Kabuli_Chr05	40404136	(C/T)
9006	CakSNP9006	*Kabuli*	Ca_Kabuli_Chr05	40404105	(G/C)
9007	CakSNP9007	*Kabuli*	Ca_Kabuli_Chr05	40410891	(C/A)

S.N.	SNP IDs	Cultivars	Chromosomes/scaffolds	Physical positions (bp)	SNPs
9008	CakSNP9008	*Kabuli*	Ca_Kabuli_Chr05	40410895	(G/T)
9009	CakSNP9009	*Kabuli*	Ca_Kabuli_Chr05	40411325	(G/C)
9010	CakSNP9010	*Kabuli*	Ca_Kabuli_Chr05	40464608	(A/G)
9011	CakSNP9011	*Kabuli*	Ca_Kabuli_Chr05	40505498	(T/G)
9012	CakSNP9012	*Kabuli*	Ca_Kabuli_Chr05	40507018	(T/A)
9013	CakSNP9013	*Kabuli*	Ca_Kabuli_Chr05	40507107	(A/G)
9014	CakSNP9014	*Kabuli*	Ca_Kabuli_Chr05	40507116	(A/G)
9015	CakSNP9015	*Kabuli*	Ca_Kabuli_Chr05	40507158	(A/G)
9016	CakSNP9016	*Kabuli*	Ca_Kabuli_Chr05	40507653	(G/C)
9017	CakSNP9017	*Kabuli*	Ca_Kabuli_Chr05	40507662	(C/T)
9018	CakSNP9018	*Kabuli*	Ca_Kabuli_Chr05	40507683	(G/T)
9019	CakSNP9019	*Kabuli*	Ca_Kabuli_Chr05	40532435	(A/G)
9020	CakSNP9020	*Kabuli*	Ca_Kabuli_Chr05	40543989	(G/T)
9021	CakSNP9021	*Kabuli*	Ca_Kabuli_Chr05	40553279	(C/G)
9022	CakSNP9022	*Kabuli*	Ca_Kabuli_Chr05	40631069	(G/C)
9023	CakSNP9023	*Kabuli*	Ca_Kabuli_Chr05	40666160	(T/A)
9024	CakSNP9024	*Kabuli*	Ca_Kabuli_Chr05	40666171	(T/C)
9025	CakSNP9025	*Kabuli*	Ca_Kabuli_Chr05	40666174	(C/T)
9026	CakSNP9026	*Kabuli*	Ca_Kabuli_Chr05	40673241	(A/G)
9027	CakSNP9027	*Kabuli*	Ca_Kabuli_Chr05	40700278	(C/G)
9028	CakSNP9028	*Kabuli*	Ca_Kabuli_Chr05	40700260	(T/C)
9029	CakSNP9029	*Kabuli*	Ca_Kabuli_Chr05	40700318	(C/A)
9030	CakSNP9030	*Kabuli*	Ca_Kabuli_Chr05	40700772	(C/T)
9031	CakSNP9031	*Kabuli*	Ca_Kabuli_Chr05	40736291	(C/G)
9032	CakSNP9032	*Kabuli*	Ca_Kabuli_Chr05	40828566	(A/C)
9033	CakSNP9033	*Kabuli*	Ca_Kabuli_Chr05	40828567	(G/C)
9034	CakSNP9034	*Kabuli*	Ca_Kabuli_Chr05	40841690	(C/T)
9035	CakSNP9035	*Kabuli*	Ca_Kabuli_Chr05	40845320	(C/T)
9036	CakSNP9036	*Kabuli*	Ca_Kabuli_Chr05	40883438	(C/A)
9037	CakSNP9037	*Kabuli*	Ca_Kabuli_Chr05	40955055	(A/T)
9038	CakSNP9038	*Kabuli*	Ca_Kabuli_Chr05	40966058	(G/A)
9039	CakSNP9039	*Kabuli*	Ca_Kabuli_Chr05	41024079	(A/G)
9040	CakSNP9040	*Kabuli*	Ca_Kabuli_Chr05	41024135	(C/G)
9041	CakSNP9041	*Kabuli*	Ca_Kabuli_Chr05	41024201	(G/A)
9042	CakSNP9042	*Kabuli*	Ca_Kabuli_Chr05	41054578	(A/G)
9043	CakSNP9043	*Kabuli*	Ca_Kabuli_Chr05	41055111	(T/A)
9044	CakSNP9044	*Kabuli*	Ca_Kabuli_Chr05	41063657	(C/T)
9045	CakSNP9045	*Kabuli*	Ca_Kabuli_Chr05	41066644	(C/G)
9046	CakSNP9046	*Kabuli*	Ca_Kabuli_Chr05	41066664	(T/C)

S.N.	SNP IDs	Cultivars	Chromosomes/scaffolds	Physical positions (bp)	SNPs
9047	CakSNP9047	*Kabuli*	Ca_Kabuli_Chr05	41066667	(C/A)
9048	CakSNP9048	*Kabuli*	Ca_Kabuli_Chr05	41080035	(C/T)
9049	CakSNP9049	*Kabuli*	Ca_Kabuli_Chr05	41191566	(A/T)
9050	CakSNP9050	*Kabuli*	Ca_Kabuli_Chr05	41191676	(G/C)
9051	CakSNP9051	*Kabuli*	Ca_Kabuli_Chr05	41197909	(A/T)
9052	CakSNP9052	*Kabuli*	Ca_Kabuli_Chr05	41203594	(C/T)
9053	CakSNP9053	*Kabuli*	Ca_Kabuli_Chr05	41253742	(A/G)
9054	CakSNP9054	*Kabuli*	Ca_Kabuli_Chr05	41283253	(T/C)
9055	CakSNP9055	*Kabuli*	Ca_Kabuli_Chr05	41283453	(A/C)
9056	CakSNP9056	*Kabuli*	Ca_Kabuli_Chr05	41285223	(A/C)
9057	CakSNP9057	*Kabuli*	Ca_Kabuli_Chr05	41302544	(C/G)
9058	CakSNP9058	*Kabuli*	Ca_Kabuli_Chr05	41302601	(G/A)
9059	CakSNP9059	*Kabuli*	Ca_Kabuli_Chr05	41302733	(T/C)
9060	CakSNP9060	*Kabuli*	Ca_Kabuli_Chr05	41302804	(A/G)
9061	CakSNP9061	*Kabuli*	Ca_Kabuli_Chr05	41302859	(C/A)
9062	CakSNP9062	*Kabuli*	Ca_Kabuli_Chr05	41302901	(C/A)
9063	CakSNP9063	*Kabuli*	Ca_Kabuli_Chr05	41302900	(T/A)
9064	CakSNP9064	*Kabuli*	Ca_Kabuli_Chr05	41302895	(G/A)
9065	CakSNP9065	*Kabuli*	Ca_Kabuli_Chr05	41304103	(G/A)
9066	CakSNP9066	*Kabuli*	Ca_Kabuli_Chr05	41399241	(C/T)
9067	CakSNP9067	*Kabuli*	Ca_Kabuli_Chr05	41407077	(A/C)
9068	CakSNP9068	*Kabuli*	Ca_Kabuli_Chr05	41407253	(T/C)
9069	CakSNP9069	*Kabuli*	Ca_Kabuli_Chr05	41519141	(C/T)
9070	CakSNP9070	*Kabuli*	Ca_Kabuli_Chr05	41521854	(T/G)
9071	CakSNP9071	*Kabuli*	Ca_Kabuli_Chr05	41526026	(T/G)
9072	CakSNP9072	*Kabuli*	Ca_Kabuli_Chr05	41635262	(C/T)
9073	CakSNP9073	*Kabuli*	Ca_Kabuli_Chr05	41678144	(C/T)
9074	CakSNP9074	*Kabuli*	Ca_Kabuli_Chr05	41696426	(T/C)
9075	CakSNP9075	*Kabuli*	Ca_Kabuli_Chr05	41696496	(G/C)
9076	CakSNP9076	*Kabuli*	Ca_Kabuli_Chr05	41701479	(T/C)
9077	CakSNP9077	*Kabuli*	Ca_Kabuli_Chr05	41772759	(G/A)
9078	CakSNP9078	*Kabuli*	Ca_Kabuli_Chr05	41887214	(A/T)
9079	CakSNP9079	*Kabuli*	Ca_Kabuli_Chr05	41902419	(A/C)
9080	CakSNP9080	*Kabuli*	Ca_Kabuli_Chr05	41945316	(A/G)
9081	CakSNP9081	*Kabuli*	Ca_Kabuli_Chr05	41945373	(C/T)
9082	CakSNP9082	*Kabuli*	Ca_Kabuli_Chr05	41945308	(G/A)
9083	CakSNP9083	*Kabuli*	Ca_Kabuli_Chr05	41974690	(T/C)
9084	CakSNP9084	*Kabuli*	Ca_Kabuli_Chr05	42004600	(T/G)
9085	CakSNP9085	*Kabuli*	Ca_Kabuli_Chr05	42004602	(A/G)

S.N.	SNP IDs	Cultivars	Chromosomes/scaffolds	Physical positions (bp)	SNPs
9086	CakSNP9086	*Kabuli*	Ca_Kabuli_Chr05	42004734	(G/A)
9087	CakSNP9087	*Kabuli*	Ca_Kabuli_Chr05	42004742	(G/A)
9088	CakSNP9088	*Kabuli*	Ca_Kabuli_Chr05	42004751	(G/A)
9089	CakSNP9089	*Kabuli*	Ca_Kabuli_Chr05	42004753	(C/A)
9090	CakSNP9090	*Kabuli*	Ca_Kabuli_Chr05	42008626	(A/C)
9091	CakSNP9091	*Kabuli*	Ca_Kabuli_Chr05	42012972	(C/A)
9092	CakSNP9092	*Kabuli*	Ca_Kabuli_Chr05	42012983	(G/A)
9093	CakSNP9093	*Kabuli*	Ca_Kabuli_Chr05	42012998	(C/T)
9094	CakSNP9094	*Kabuli*	Ca_Kabuli_Chr05	42020435	(A/G)
9095	CakSNP9095	*Kabuli*	Ca_Kabuli_Chr05	42100070	(A/G)
9096	CakSNP9096	*Kabuli*	Ca_Kabuli_Chr05	42117516	(C/T)
9097	CakSNP9097	*Kabuli*	Ca_Kabuli_Chr05	42120698	(T/C)
9098	CakSNP9098	*Kabuli*	Ca_Kabuli_Chr05	42120735	(G/C)
9099	CakSNP9099	*Kabuli*	Ca_Kabuli_Chr05	42121472	(C/A)
9100	CakSNP9100	*Kabuli*	Ca_Kabuli_Chr05	42225634	(G/A)
9101	CakSNP9101	*Kabuli*	Ca_Kabuli_Chr05	42275523	(G/A)
9102	CakSNP9102	*Kabuli*	Ca_Kabuli_Chr05	42282297	(G/A)
9103	CakSNP9103	*Kabuli*	Ca_Kabuli_Chr05	42413430	(A/C)
9104	CakSNP9104	*Kabuli*	Ca_Kabuli_Chr05	42422867	(C/A)
9105	CakSNP9105	*Kabuli*	Ca_Kabuli_Chr05	42422845	(T/C)
9106	CakSNP9106	*Kabuli*	Ca_Kabuli_Chr05	42422837	(G/A)
9107	CakSNP9107	*Kabuli*	Ca_Kabuli_Chr05	42422808	(A/G)
9108	CakSNP9108	*Kabuli*	Ca_Kabuli_Chr05	42422932	(C/A)
9109	CakSNP9109	*Kabuli*	Ca_Kabuli_Chr05	42435263	(C/T)
9110	CakSNP9110	*Kabuli*	Ca_Kabuli_Chr05	42436972	(A/G)
9111	CakSNP9111	*Kabuli*	Ca_Kabuli_Chr05	42436976	(A/T)
9112	CakSNP9112	*Kabuli*	Ca_Kabuli_Chr05	42443786	(A/G)
9113	CakSNP9113	*Kabuli*	Ca_Kabuli_Chr05	42477712	(G/A)
9114	CakSNP9114	*Kabuli*	Ca_Kabuli_Chr05	42485130	(T/C)
9115	CakSNP9115	*Kabuli*	Ca_Kabuli_Chr05	42528744	(T/G)
9116	CakSNP9116	*Kabuli*	Ca_Kabuli_Chr05	42528934	(A/G)
9117	CakSNP9117	*Kabuli*	Ca_Kabuli_Chr05	42540675	(A/T)
9118	CakSNP9118	*Kabuli*	Ca_Kabuli_Chr05	42582262	(T/A)
9119	CakSNP9119	*Kabuli*	Ca_Kabuli_Chr05	42655824	(A/G)
9120	CakSNP9120	*Kabuli*	Ca_Kabuli_Chr05	42749499	(C/T)
9121	CakSNP9121	*Kabuli*	Ca_Kabuli_Chr05	42792635	(G/A)
9122	CakSNP9122	*Kabuli*	Ca_Kabuli_Chr05	42814621	(C/T)
9123	CakSNP9123	*Kabuli*	Ca_Kabuli_Chr05	42814623	(A/G)
9124	CakSNP9124	*Kabuli*	Ca_Kabuli_Chr05	42857977	(G/C)

S.N.	SNP IDs	Cultivars	Chromosomes/scaffolds	Physical positions (bp)	SNPs
9125	CakSNP9125	*Kabuli*	Ca_Kabuli_Chr05	42863366	(C/T)
9126	CakSNP9126	*Kabuli*	Ca_Kabuli_Chr05	42872074	(A/C)
9127	CakSNP9127	*Kabuli*	Ca_Kabuli_Chr05	42872091	(A/G)
9128	CakSNP9128	*Kabuli*	Ca_Kabuli_Chr05	42881185	(C/T)
9129	CakSNP9129	*Kabuli*	Ca_Kabuli_Chr05	42882945	(C/T)
9130	CakSNP9130	*Kabuli*	Ca_Kabuli_Chr05	42882965	(C/T)
9131	CakSNP9131	*Kabuli*	Ca_Kabuli_Chr05	42883163	(T/C)
9132	CakSNP9132	*Kabuli*	Ca_Kabuli_Chr05	42910653	(A/G)
9133	CakSNP9133	*Kabuli*	Ca_Kabuli_Chr05	42910661	(A/G)
9134	CakSNP9134	*Kabuli*	Ca_Kabuli_Chr05	42910732	(A/T)
9135	CakSNP9135	*Kabuli*	Ca_Kabuli_Chr05	42924113	(C/T)
9136	CakSNP9136	*Kabuli*	Ca_Kabuli_Chr05	42928986	(T/G)
9137	CakSNP9137	*Kabuli*	Ca_Kabuli_Chr05	42929087	(A/C)
9138	CakSNP9138	*Kabuli*	Ca_Kabuli_Chr05	42946580	(T/C)
9139	CakSNP9139	*Kabuli*	Ca_Kabuli_Chr05	42946716	(A/T)
9140	CakSNP9140	*Kabuli*	Ca_Kabuli_Chr05	42999704	(C/T)
9141	CakSNP9141	*Kabuli*	Ca_Kabuli_Chr05	43000210	(A/G)
9142	CakSNP9142	*Kabuli*	Ca_Kabuli_Chr05	43034618	(T/A)
9143	CakSNP9143	*Kabuli*	Ca_Kabuli_Chr05	43037863	(G/A)
9144	CakSNP9144	*Kabuli*	Ca_Kabuli_Chr05	43060091	(C/T)
9145	CakSNP9145	*Kabuli*	Ca_Kabuli_Chr05	43067894	(T/C)
9146	CakSNP9146	*Kabuli*	Ca_Kabuli_Chr05	43067891	(G/C)
9147	CakSNP9147	*Kabuli*	Ca_Kabuli_Chr05	43067905	(A/C)
9148	CakSNP9148	*Kabuli*	Ca_Kabuli_Chr05	43080493	(G/A)
9149	CakSNP9149	*Kabuli*	Ca_Kabuli_Chr05	43081783	(G/A)
9150	CakSNP9150	*Kabuli*	Ca_Kabuli_Chr05	43110660	(G/A)
9151	CakSNP9151	*Kabuli*	Ca_Kabuli_Chr05	43110669	(A/T)
9152	CakSNP9152	*Kabuli*	Ca_Kabuli_Chr05	43110726	(T/G)
9153	CakSNP9153	*Kabuli*	Ca_Kabuli_Chr05	43110737	(A/G)
9154	CakSNP9154	*Kabuli*	Ca_Kabuli_Chr05	43175781	(G/A)
9155	CakSNP9155	*Kabuli*	Ca_Kabuli_Chr05	43188824	(T/G)
9156	CakSNP9156	*Kabuli*	Ca_Kabuli_Chr05	43188853	(C/A)
9157	CakSNP9157	*Kabuli*	Ca_Kabuli_Chr05	43210793	(T/C)
9158	CakSNP9158	*Kabuli*	Ca_Kabuli_Chr05	43212083	(G/A)
9159	CakSNP9159	*Kabuli*	Ca_Kabuli_Chr05	43212066	(C/T)
9160	CakSNP9160	*Kabuli*	Ca_Kabuli_Chr05	43221125	(T/C)
9161	CakSNP9161	*Kabuli*	Ca_Kabuli_Chr05	43231220	(T/C)
9162	CakSNP9162	*Kabuli*	Ca_Kabuli_Chr05	43231265	(C/T)
9163	CakSNP9163	*Kabuli*	Ca_Kabuli_Chr05	43253586	(C/A)

S.N.	SNP IDs	Cultivars	Chromosomes/scaffolds	Physical positions (bp)	SNPs
9164	CakSNP9164	*Kabuli*	Ca_Kabuli_Chr05	43275472	(T/C)
9165	CakSNP9165	*Kabuli*	Ca_Kabuli_Chr05	43334903	(T/C)
9166	CakSNP9166	*Kabuli*	Ca_Kabuli_Chr05	43341646	(A/C)
9167	CakSNP9167	*Kabuli*	Ca_Kabuli_Chr05	43402230	(C/T)
9168	CakSNP9168	*Kabuli*	Ca_Kabuli_Chr05	43410326	(T/G)
9169	CakSNP9169	*Kabuli*	Ca_Kabuli_Chr05	43423593	(G/C)
9170	CakSNP9170	*Kabuli*	Ca_Kabuli_Chr05	43521774	(A/T)
9171	CakSNP9171	*Kabuli*	Ca_Kabuli_Chr05	43521789	(G/A)
9172	CakSNP9172	*Kabuli*	Ca_Kabuli_Chr05	43521830	(T/C)
9173	CakSNP9173	*Kabuli*	Ca_Kabuli_Chr05	43521851	(G/A)
9174	CakSNP9174	*Kabuli*	Ca_Kabuli_Chr05	43523560	(A/G)
9175	CakSNP9175	*Kabuli*	Ca_Kabuli_Chr05	43530567	(C/T)
9176	CakSNP9176	*Kabuli*	Ca_Kabuli_Chr05	43565712	(T/C)
9177	CakSNP9177	*Kabuli*	Ca_Kabuli_Chr05	43632249	(C/A)
9178	CakSNP9178	*Kabuli*	Ca_Kabuli_Chr05	43632281	(C/G)
9179	CakSNP9179	*Kabuli*	Ca_Kabuli_Chr05	43632349	(T/A)
9180	CakSNP9180	*Kabuli*	Ca_Kabuli_Chr05	43632400	(A/G)
9181	CakSNP9181	*Kabuli*	Ca_Kabuli_Chr05	43632377	(C/T)
9182	CakSNP9182	*Kabuli*	Ca_Kabuli_Chr05	43632481	(G/T)
9183	CakSNP9183	*Kabuli*	Ca_Kabuli_Chr05	43632508	(A/G)
9184	CakSNP9184	*Kabuli*	Ca_Kabuli_Chr05	43710359	(T/G)
9185	CakSNP9185	*Kabuli*	Ca_Kabuli_Chr05	43710362	(A/T)
9186	CakSNP9186	*Kabuli*	Ca_Kabuli_Chr05	43710366	(G/T)
9187	CakSNP9187	*Kabuli*	Ca_Kabuli_Chr05	43710391	(A/G)
9188	CakSNP9188	*Kabuli*	Ca_Kabuli_Chr05	43742448	(C/T)
9189	CakSNP9189	*Kabuli*	Ca_Kabuli_Chr05	43742456	(G/A)
9190	CakSNP9190	*Kabuli*	Ca_Kabuli_Chr05	43755074	(A/G)
9191	CakSNP9191	*Kabuli*	Ca_Kabuli_Chr05	43755756	(A/G)
9192	CakSNP9192	*Kabuli*	Ca_Kabuli_Chr05	43759059	(A/C)
9193	CakSNP9193	*Kabuli*	Ca_Kabuli_Chr05	43786883	(A/G)
9194	CakSNP9194	*Kabuli*	Ca_Kabuli_Chr05	43789875	(C/G)
9195	CakSNP9195	*Kabuli*	Ca_Kabuli_Chr05	43790108	(G/A)
9196	CakSNP9196	*Kabuli*	Ca_Kabuli_Chr05	43812119	(G/A)
9197	CakSNP9197	*Kabuli*	Ca_Kabuli_Chr05	43812213	(C/T)
9198	CakSNP9198	*Kabuli*	Ca_Kabuli_Chr05	43859301	(G/A)
9199	CakSNP9199	*Kabuli*	Ca_Kabuli_Chr05	43904489	(T/A)
9200	CakSNP9200	*Kabuli*	Ca_Kabuli_Chr05	43919009	(C/T)
9201	CakSNP9201	*Kabuli*	Ca_Kabuli_Chr05	43919017	(T/G)
9202	CakSNP9202	*Kabuli*	Ca_Kabuli_Chr05	43919022	(G/C)

S.N.	SNP IDs	Cultivars	Chromosomes/scaffolds	Physical positions (bp)	SNPs
9203	CakSNP9203	*Kabuli*	Ca_Kabuli_Chr05	43919028	(A/G)
9204	CakSNP9204	*Kabuli*	Ca_Kabuli_Chr05	43982994	(C/T)
9205	CakSNP9205	*Kabuli*	Ca_Kabuli_Chr05	43995843	(T/C)
9206	CakSNP9206	*Kabuli*	Ca_Kabuli_Chr05	44002824	(T/G)
9207	CakSNP9207	*Kabuli*	Ca_Kabuli_Chr05	44002862	(C/A)
9208	CakSNP9208	*Kabuli*	Ca_Kabuli_Chr05	44038229	(T/C)
9209	CakSNP9209	*Kabuli*	Ca_Kabuli_Chr05	44064495	(G/A)
9210	CakSNP9210	*Kabuli*	Ca_Kabuli_Chr05	44080450	(G/C)
9211	CakSNP9211	*Kabuli*	Ca_Kabuli_Chr05	44109260	(G/T)
9212	CakSNP9212	*Kabuli*	Ca_Kabuli_Chr05	44154671	(A/G)
9213	CakSNP9213	*Kabuli*	Ca_Kabuli_Chr05	44159237	(C/G)
9214	CakSNP9214	*Kabuli*	Ca_Kabuli_Chr05	44289612	(T/C)
9215	CakSNP9215	*Kabuli*	Ca_Kabuli_Chr05	44334286	(C/G)
9216	CakSNP9216	*Kabuli*	Ca_Kabuli_Chr05	44334318	(C/T)
9217	CakSNP9217	*Kabuli*	Ca_Kabuli_Chr05	44364134	(C/T)
9218	CakSNP9218	*Kabuli*	Ca_Kabuli_Chr05	44373968	(G/T)
9219	CakSNP9219	*Kabuli*	Ca_Kabuli_Chr05	44378178	(C/A)
9220	CakSNP9220	*Kabuli*	Ca_Kabuli_Chr05	44428965	(G/A)
9221	CakSNP9221	*Kabuli*	Ca_Kabuli_Chr05	44461250	(A/G)
9222	CakSNP9222	*Kabuli*	Ca_Kabuli_Chr05	44491489	(G/T)
9223	CakSNP9223	*Kabuli*	Ca_Kabuli_Chr05	44555718	(A/C)
9224	CakSNP9224	*Kabuli*	Ca_Kabuli_Chr05	44561095	(T/C)
9225	CakSNP9225	*Kabuli*	Ca_Kabuli_Chr05	44656921	(G/A)
9226	CakSNP9226	*Kabuli*	Ca_Kabuli_Chr05	44667285	(C/T)
9227	CakSNP9227	*Kabuli*	Ca_Kabuli_Chr05	44667297	(C/T)
9228	CakSNP9228	*Kabuli*	Ca_Kabuli_Chr05	44667359	(C/A)
9229	CakSNP9229	*Kabuli*	Ca_Kabuli_Chr05	44760403	(A/G)
9230	CakSNP9230	*Kabuli*	Ca_Kabuli_Chr05	44760347	(G/T)
9231	CakSNP9231	*Kabuli*	Ca_Kabuli_Chr05	44760469	(C/T)
9232	CakSNP9232	*Kabuli*	Ca_Kabuli_Chr05	44760490	(C/A)
9233	CakSNP9233	*Kabuli*	Ca_Kabuli_Chr05	44772326	(G/A)
9234	CakSNP9234	*Kabuli*	Ca_Kabuli_Chr05	44884779	(G/C)
9235	CakSNP9235	*Kabuli*	Ca_Kabuli_Chr05	44887105	(T/G)
9236	CakSNP9236	*Kabuli*	Ca_Kabuli_Chr05	44887241	(T/C)
9237	CakSNP9237	*Kabuli*	Ca_Kabuli_Chr05	44887234	(A/C)
9238	CakSNP9238	*Kabuli*	Ca_Kabuli_Chr05	44891426	(G/C)
9239	CakSNP9239	*Kabuli*	Ca_Kabuli_Chr05	44911842	(C/T)
9240	CakSNP9240	*Kabuli*	Ca_Kabuli_Chr05	44911868	(C/G)
9241	CakSNP9241	*Kabuli*	Ca_Kabuli_Chr05	44911881	(G/A)

S.N.	SNP IDs	Cultivars	Chromosomes/scaffolds	Physical positions (bp)	SNPs
9242	CakSNP9242	*Kabuli*	Ca_Kabuli_Chr05	44911906	(C/T)
9243	CakSNP9243	*Kabuli*	Ca_Kabuli_Chr05	44918947	(C/A)
9244	CakSNP9244	*Kabuli*	Ca_Kabuli_Chr05	44938814	(A/T)
9245	CakSNP9245	*Kabuli*	Ca_Kabuli_Chr05	44938832	(A/G)
9246	CakSNP9246	*Kabuli*	Ca_Kabuli_Chr05	44939058	(C/G)
9247	CakSNP9247	*Kabuli*	Ca_Kabuli_Chr05	44939033	(G/A)
9248	CakSNP9248	*Kabuli*	Ca_Kabuli_Chr05	45087170	(G/A)
9249	CakSNP9249	*Kabuli*	Ca_Kabuli_Chr05	45087156	(A/G)
9250	CakSNP9250	*Kabuli*	Ca_Kabuli_Chr05	45087146	(C/G)
9251	CakSNP9251	*Kabuli*	Ca_Kabuli_Chr05	45087140	(C/A)
9252	CakSNP9252	*Kabuli*	Ca_Kabuli_Chr05	45087202	(G/C)
9253	CakSNP9253	*Kabuli*	Ca_Kabuli_Chr05	45087267	(A/G)
9254	CakSNP9254	*Kabuli*	Ca_Kabuli_Chr05	45087228	(A/C)
9255	CakSNP9255	*Kabuli*	Ca_Kabuli_Chr05	45087215	(G/A)
9256	CakSNP9256	*Kabuli*	Ca_Kabuli_Chr05	45215617	(G/A)
9257	CakSNP9257	*Kabuli*	Ca_Kabuli_Chr05	45219125	(C/T)
9258	CakSNP9258	*Kabuli*	Ca_Kabuli_Chr05	45219258	(G/T)
9259	CakSNP9259	*Kabuli*	Ca_Kabuli_Chr05	45219355	(T/G)
9260	CakSNP9260	*Kabuli*	Ca_Kabuli_Chr05	45254102	(C/T)
9261	CakSNP9261	*Kabuli*	Ca_Kabuli_Chr05	45254112	(T/G)
9262	CakSNP9262	*Kabuli*	Ca_Kabuli_Chr05	45301772	(T/G)
9263	CakSNP9263	*Kabuli*	Ca_Kabuli_Chr05	45337274	(C/G)
9264	CakSNP9264	*Kabuli*	Ca_Kabuli_Chr05	45337307	(C/T)
9265	CakSNP9265	*Kabuli*	Ca_Kabuli_Chr05	45406853	(C/T)
9266	CakSNP9266	*Kabuli*	Ca_Kabuli_Chr05	45407063	(G/A)
9267	CakSNP9267	*Kabuli*	Ca_Kabuli_Chr05	45407044	(A/C)
9268	CakSNP9268	*Kabuli*	Ca_Kabuli_Chr05	45414089	(C/G)
9269	CakSNP9269	*Kabuli*	Ca_Kabuli_Chr05	45428229	(A/G)
9270	CakSNP9270	*Kabuli*	Ca_Kabuli_Chr05	45429513	(A/G)
9271	CakSNP9271	*Kabuli*	Ca_Kabuli_Chr05	45429518	(T/G)
9272	CakSNP9272	*Kabuli*	Ca_Kabuli_Chr05	45441223	(C/T)
9273	CakSNP9273	*Kabuli*	Ca_Kabuli_Chr05	45444675	(G/T)
9274	CakSNP9274	*Kabuli*	Ca_Kabuli_Chr05	45444924	(G/T)
9275	CakSNP9275	*Kabuli*	Ca_Kabuli_Chr05	45480175	(T/G)
9276	CakSNP9276	*Kabuli*	Ca_Kabuli_Chr05	45501379	(C/T)
9277	CakSNP9277	*Kabuli*	Ca_Kabuli_Chr05	45540473	(G/A)
9278	CakSNP9278	*Kabuli*	Ca_Kabuli_Chr05	45559874	(C/A)
9279	CakSNP9279	*Kabuli*	Ca_Kabuli_Chr05	45601196	(A/G)
9280	CakSNP9280	*Kabuli*	Ca_Kabuli_Chr05	45601313	(G/A)

S.N.	SNP IDs	Cultivars	Chromosomes/scaffolds	Physical positions (bp)	SNPs
9281	CakSNP9281	*Kabuli*	Ca_Kabuli_Chr05	45642344	(C/A)
9282	CakSNP9282	*Kabuli*	Ca_Kabuli_Chr05	45646235	(A/G)
9283	CakSNP9283	*Kabuli*	Ca_Kabuli_Chr05	45649180	(G/A)
9284	CakSNP9284	*Kabuli*	Ca_Kabuli_Chr05	45649189	(G/A)
9285	CakSNP9285	*Kabuli*	Ca_Kabuli_Chr05	45661109	(T/C)
9286	CakSNP9286	*Kabuli*	Ca_Kabuli_Chr05	45694453	(T/G)
9287	CakSNP9287	*Kabuli*	Ca_Kabuli_Chr05	45710509	(G/A)
9288	CakSNP9288	*Kabuli*	Ca_Kabuli_Chr05	45710476	(A/G)
9289	CakSNP9289	*Kabuli*	Ca_Kabuli_Chr05	45726539	(T/A)
9290	CakSNP9290	*Kabuli*	Ca_Kabuli_Chr05	45726995	(C/T)
9291	CakSNP9291	*Kabuli*	Ca_Kabuli_Chr05	45727022	(T/C)
9292	CakSNP9292	*Kabuli*	Ca_Kabuli_Chr05	45750121	(A/T)
9293	CakSNP9293	*Kabuli*	Ca_Kabuli_Chr05	45798850	(C/A)
9294	CakSNP9294	*Kabuli*	Ca_Kabuli_Chr05	45798966	(T/G)
9295	CakSNP9295	*Kabuli*	Ca_Kabuli_Chr05	45798950	(C/T)
9296	CakSNP9296	*Kabuli*	Ca_Kabuli_Chr05	45798919	(C/A)
9297	CakSNP9297	*Kabuli*	Ca_Kabuli_Chr05	45808905	(G/A)
9298	CakSNP9298	*Kabuli*	Ca_Kabuli_Chr05	45832009	(T/G)
9299	CakSNP9299	*Kabuli*	Ca_Kabuli_Chr05	45832048	(C/G)
9300	CakSNP9300	*Kabuli*	Ca_Kabuli_Chr05	45832070	(A/T)
9301	CakSNP9301	*Kabuli*	Ca_Kabuli_Chr05	45842438	(A/C)
9302	CakSNP9302	*Kabuli*	Ca_Kabuli_Chr05	45845081	(A/C)
9303	CakSNP9303	*Kabuli*	Ca_Kabuli_Chr05	45848030	(A/G)
9304	CakSNP9304	*Kabuli*	Ca_Kabuli_Chr05	45848087	(T/G)
9305	CakSNP9305	*Kabuli*	Ca_Kabuli_Chr05	45952107	(A/G)
9306	CakSNP9306	*Kabuli*	Ca_Kabuli_Chr05	45952842	(A/C)
9307	CakSNP9307	*Kabuli*	Ca_Kabuli_Chr05	45971560	(G/A)
9308	CakSNP9308	*Kabuli*	Ca_Kabuli_Chr05	45978758	(A/G)
9309	CakSNP9309	*Kabuli*	Ca_Kabuli_Chr05	46002514	(C/T)
9310	CakSNP9310	*Kabuli*	Ca_Kabuli_Chr05	46002497	(G/A)
9311	CakSNP9311	*Kabuli*	Ca_Kabuli_Chr05	46018939	(C/T)
9312	CakSNP9312	*Kabuli*	Ca_Kabuli_Chr05	46019052	(T/A)
9313	CakSNP9313	*Kabuli*	Ca_Kabuli_Chr05	46022566	(C/T)
9314	CakSNP9314	*Kabuli*	Ca_Kabuli_Chr05	46022598	(T/C)
9315	CakSNP9315	*Kabuli*	Ca_Kabuli_Chr05	46022551	(G/A)
9316	CakSNP9316	*Kabuli*	Ca_Kabuli_Chr05	46022549	(G/A)
9317	CakSNP9317	*Kabuli*	Ca_Kabuli_Chr05	46022547	(G/A)
9318	CakSNP9318	*Kabuli*	Ca_Kabuli_Chr05	46022544	(A/G)
9319	CakSNP9319	*Kabuli*	Ca_Kabuli_Chr05	46022531	(T/A)

**S.N.**	**SNP IDs**	**Cultivars**	**Chromosomes/scaffolds**	**Physical positions (bp)**	**SNPs**
9320	CakSNP9320	*Kabuli*	Ca_Kabuli_Chr05	46049587	(A/G)
9321	CakSNP9321	*Kabuli*	Ca_Kabuli_Chr05	46050168	(A/T)
9322	CakSNP9322	*Kabuli*	Ca_Kabuli_Chr05	46077190	(T/C)
9323	CakSNP9323	*Kabuli*	Ca_Kabuli_Chr05	46077302	(G/A)
9324	CakSNP9324	*Kabuli*	Ca_Kabuli_Chr05	46083283	(G/A)
9325	CakSNP9325	*Kabuli*	Ca_Kabuli_Chr05	46083343	(T/G)
9326	CakSNP9326	*Kabuli*	Ca_Kabuli_Chr05	46083414	(T/G)
9327	CakSNP9327	*Kabuli*	Ca_Kabuli_Chr05	46119122	(G/A)
9328	CakSNP9328	*Kabuli*	Ca_Kabuli_Chr05	46119182	(A/G)
9329	CakSNP9329	*Kabuli*	Ca_Kabuli_Chr05	46130289	(A/C)
9330	CakSNP9330	*Kabuli*	Ca_Kabuli_Chr05	46155262	(C/T)
9331	CakSNP9331	*Kabuli*	Ca_Kabuli_Chr05	46176366	(T/C)
9332	CakSNP9332	*Kabuli*	Ca_Kabuli_Chr05	46176443	(G/A)
9333	CakSNP9333	*Kabuli*	Ca_Kabuli_Chr05	46214229	(G/T)
9334	CakSNP9334	*Kabuli*	Ca_Kabuli_Chr05	46214233	(C/T)
9335	CakSNP9335	*Kabuli*	Ca_Kabuli_Chr05	46230010	(G/C)
9336	CakSNP9336	*Kabuli*	Ca_Kabuli_Chr05	46230011	(G/A)
9337	CakSNP9337	*Kabuli*	Ca_Kabuli_Chr05	46230109	(T/G)
9338	CakSNP9338	*Kabuli*	Ca_Kabuli_Chr05	46246419	(G/C)
9339	CakSNP9339	*Kabuli*	Ca_Kabuli_Chr05	46252617	(C/T)
9340	CakSNP9340	*Kabuli*	Ca_Kabuli_Chr05	46252643	(T/A)
9341	CakSNP9341	*Kabuli*	Ca_Kabuli_Chr05	46252605	(C/A)
9342	CakSNP9342	*Kabuli*	Ca_Kabuli_Chr05	46260822	(C/T)
9343	CakSNP9343	*Kabuli*	Ca_Kabuli_Chr05	46294000	(G/A)
9344	CakSNP9344	*Kabuli*	Ca_Kabuli_Chr05	46300789	(G/A)
9345	CakSNP9345	*Kabuli*	Ca_Kabuli_Chr05	46301639	(A/G)
9346	CakSNP9346	*Kabuli*	Ca_Kabuli_Chr05	46321992	(C/T)
9347	CakSNP9347	*Kabuli*	Ca_Kabuli_Chr05	46328589	(C/A)
9348	CakSNP9348	*Kabuli*	Ca_Kabuli_Chr05	46328627	(A/G)
9349	CakSNP9349	*Kabuli*	Ca_Kabuli_Chr05	46328895	(T/C)
9350	CakSNP9350	*Kabuli*	Ca_Kabuli_Chr05	46328902	(C/T)
9351	CakSNP9351	*Kabuli*	Ca_Kabuli_Chr05	46344109	(G/T)
9352	CakSNP9352	*Kabuli*	Ca_Kabuli_Chr05	46344121	(G/A)
9353	CakSNP9353	*Kabuli*	Ca_Kabuli_Chr05	46356764	(G/A)
9354	CakSNP9354	*Kabuli*	Ca_Kabuli_Chr05	46366598	(G/T)
9355	CakSNP9355	*Kabuli*	Ca_Kabuli_Chr05	46368606	(G/T)
9356	CakSNP9356	*Kabuli*	Ca_Kabuli_Chr05	46376496	(G/T)
9357	CakSNP9357	*Kabuli*	Ca_Kabuli_Chr05	46376532	(A/C)
9358	CakSNP9358	*Kabuli*	Ca_Kabuli_Chr05	46403922	(G/T)

S.N.	SNP IDs	Cultivars	Chromosomes/scaffolds	Physical positions (bp)	SNPs
9359	CakSNP9359	*Kabuli*	Ca_Kabuli_Chr05	46422788	(C/G)
9360	CakSNP9360	*Kabuli*	Ca_Kabuli_Chr05	46422869	(A/T)
9361	CakSNP9361	*Kabuli*	Ca_Kabuli_Chr05	46445611	(A/T)
9362	CakSNP9362	*Kabuli*	Ca_Kabuli_Chr05	46445651	(G/T)
9363	CakSNP9363	*Kabuli*	Ca_Kabuli_Chr05	46491761	(C/G)
9364	CakSNP9364	*Kabuli*	Ca_Kabuli_Chr05	46526963	(A/C)
9365	CakSNP9365	*Kabuli*	Ca_Kabuli_Chr05	46621351	(G/A)
9366	CakSNP9366	*Kabuli*	Ca_Kabuli_Chr05	46621455	(C/T)
9367	CakSNP9367	*Kabuli*	Ca_Kabuli_Chr05	46668462	(T/C)
9368	CakSNP9368	*Kabuli*	Ca_Kabuli_Chr05	46792131	(C/T)
9369	CakSNP9369	*Kabuli*	Ca_Kabuli_Chr05	46945916	(G/A)
9370	CakSNP9370	*Kabuli*	Ca_Kabuli_Chr05	46956474	(T/C)
9371	CakSNP9371	*Kabuli*	Ca_Kabuli_Chr05	47061531	(C/T)
9372	CakSNP9372	*Kabuli*	Ca_Kabuli_Chr05	47073011	(A/T)
9373	CakSNP9373	*Kabuli*	Ca_Kabuli_Chr05	47073016	(G/C)
9374	CakSNP9374	*Kabuli*	Ca_Kabuli_Chr05	47091499	(C/T)
9375	CakSNP9375	*Kabuli*	Ca_Kabuli_Chr05	47151660	(C/G)
9376	CakSNP9376	*Kabuli*	Ca_Kabuli_Chr05	47151840	(T/G)
9377	CakSNP9377	*Kabuli*	Ca_Kabuli_Chr05	47165299	(A/C)
9378	CakSNP9378	*Kabuli*	Ca_Kabuli_Chr05	47256002	(G/T)
9379	CakSNP9379	*Kabuli*	Ca_Kabuli_Chr05	47256038	(A/T)
9380	CakSNP9380	*Kabuli*	Ca_Kabuli_Chr05	47289004	(A/C)
9381	CakSNP9381	*Kabuli*	Ca_Kabuli_Chr05	47298345	(A/G)
9382	CakSNP9382	*Kabuli*	Ca_Kabuli_Chr05	47329979	(T/G)
9383	CakSNP9383	*Kabuli*	Ca_Kabuli_Chr05	47362666	(T/G)
9384	CakSNP9384	*Kabuli*	Ca_Kabuli_Chr05	47362814	(C/T)
9385	CakSNP9385	*Kabuli*	Ca_Kabuli_Chr05	47375789	(C/T)
9386	CakSNP9386	*Kabuli*	Ca_Kabuli_Chr05	47422536	(G/A)
9387	CakSNP9387	*Kabuli*	Ca_Kabuli_Chr05	47518533	(G/A)
9388	CakSNP9388	*Kabuli*	Ca_Kabuli_Chr05	47539345	(T/C)
9389	CakSNP9389	*Kabuli*	Ca_Kabuli_Chr05	47539505	(G/T)
9390	CakSNP9390	*Kabuli*	Ca_Kabuli_Chr05	47570939	(T/C)
9391	CakSNP9391	*Kabuli*	Ca_Kabuli_Chr05	47591327	(G/T)
9392	CakSNP9392	*Kabuli*	Ca_Kabuli_Chr05	47594471	(T/C)
9393	CakSNP9393	*Kabuli*	Ca_Kabuli_Chr05	47641224	(A/C)
9394	CakSNP9394	*Kabuli*	Ca_Kabuli_Chr05	47641472	(T/C)
9395	CakSNP9395	*Kabuli*	Ca_Kabuli_Chr05	47641628	(T/C)
9396	CakSNP9396	*Kabuli*	Ca_Kabuli_Chr05	47641567	(A/G)
9397	CakSNP9397	*Kabuli*	Ca_Kabuli_Chr05	47643992	(G/T)

S.N.	SNP IDs	Cultivars	Chromosomes/scaffolds	Physical positions (bp)	SNPs
9398	CakSNP9398	*Kabuli*	Ca_Kabuli_Chr05	47669124	(A/G)
9399	CakSNP9399	*Kabuli*	Ca_Kabuli_Chr05	47670649	(G/A)
9400	CakSNP9400	*Kabuli*	Ca_Kabuli_Chr05	47706780	(G/A)
9401	CakSNP9401	*Kabuli*	Ca_Kabuli_Chr05	47718830	(A/G)
9402	CakSNP9402	*Kabuli*	Ca_Kabuli_Chr05	47718839	(A/G)
9403	CakSNP9403	*Kabuli*	Ca_Kabuli_Chr05	47733645	(A/G)
9404	CakSNP9404	*Kabuli*	Ca_Kabuli_Chr05	47759169	(T/G)
9405	CakSNP9405	*Kabuli*	Ca_Kabuli_Chr05	47759745	(G/A)
9406	CakSNP9406	*Kabuli*	Ca_Kabuli_Chr05	47761348	(A/G)
9407	CakSNP9407	*Kabuli*	Ca_Kabuli_Chr05	47761322	(C/A)
9408	CakSNP9408	*Kabuli*	Ca_Kabuli_Chr05	47770909	(T/G)
9409	CakSNP9409	*Kabuli*	Ca_Kabuli_Chr05	47774516	(A/G)
9410	CakSNP9410	*Kabuli*	Ca_Kabuli_Chr05	47774559	(C/T)
9411	CakSNP9411	*Kabuli*	Ca_Kabuli_Chr05	47774718	(C/G)
9412	CakSNP9412	*Kabuli*	Ca_Kabuli_Chr05	47775175	(C/T)
9413	CakSNP9413	*Kabuli*	Ca_Kabuli_Chr05	47782605	(G/A)
9414	CakSNP9414	*Kabuli*	Ca_Kabuli_Chr05	47782606	(C/T)
9415	CakSNP9415	*Kabuli*	Ca_Kabuli_Chr05	47782697	(T/C)
9416	CakSNP9416	*Kabuli*	Ca_Kabuli_Chr05	47782843	(T/C)
9417	CakSNP9417	*Kabuli*	Ca_Kabuli_Chr05	47803636	(C/A)
9418	CakSNP9418	*Kabuli*	Ca_Kabuli_Chr05	47812041	(C/T)
9419	CakSNP9419	*Kabuli*	Ca_Kabuli_Chr05	47813712	(T/C)
9420	CakSNP9420	*Kabuli*	Ca_Kabuli_Chr05	47813767	(A/G)
9421	CakSNP9421	*Kabuli*	Ca_Kabuli_Chr05	47829261	(T/C)
9422	CakSNP9422	*Kabuli*	Ca_Kabuli_Chr05	47829331	(T/A)
9423	CakSNP9423	*Kabuli*	Ca_Kabuli_Chr05	47871094	(C/T)
9424	CakSNP9424	*Kabuli*	Ca_Kabuli_Chr05	47871137	(G/A)
9425	CakSNP9425	*Kabuli*	Ca_Kabuli_Chr05	47871248	(G/T)
9426	CakSNP9426	*Kabuli*	Ca_Kabuli_Chr05	47871234	(A/G)
9427	CakSNP9427	*Kabuli*	Ca_Kabuli_Chr05	47871194	(T/C)
9428	CakSNP9428	*Kabuli*	Ca_Kabuli_Chr05	47878997	(C/T)
9429	CakSNP9429	*Kabuli*	Ca_Kabuli_Chr05	47901017	(G/T)
9430	CakSNP9430	*Kabuli*	Ca_Kabuli_Chr05	47931566	(T/C)
9431	CakSNP9431	*Kabuli*	Ca_Kabuli_Chr05	47941783	(C/T)
9432	CakSNP9432	*Kabuli*	Ca_Kabuli_Chr05	47943743	(G/A)
9433	CakSNP9433	*Kabuli*	Ca_Kabuli_Chr05	47965279	(G/A)
9434	CakSNP9434	*Kabuli*	Ca_Kabuli_Chr05	47994780	(A/C)
9435	CakSNP9435	*Kabuli*	Ca_Kabuli_Chr05	47997751	(A/T)
9436	CakSNP9436	*Kabuli*	Ca_Kabuli_Chr05	48005123	(A/C)

S.N.	SNP IDs	Cultivars	Chromosomes/scaffolds	Physical positions (bp)	SNPs
9437	CakSNP9437	*Kabuli*	Ca_Kabuli_Chr05	48005073	(G/A)
9438	CakSNP9438	*Kabuli*	Ca_Kabuli_Chr05	48042132	(A/C)
9439	CakSNP9439	*Kabuli*	Ca_Kabuli_Chr05	48063028	(A/T)
9440	CakSNP9440	*Kabuli*	Ca_Kabuli_Chr05	48069744	(G/A)
9441	CakSNP9441	*Kabuli*	Ca_Kabuli_Chr05	48069811	(G/C)
9442	CakSNP9442	*Kabuli*	Ca_Kabuli_Chr05	48069859	(A/T)
9443	CakSNP9443	*Kabuli*	Ca_Kabuli_Chr05	48069844	(T/C)
9444	CakSNP9444	*Kabuli*	Ca_Kabuli_Chr05	48069833	(G/A)
9445	CakSNP9445	*Kabuli*	Ca_Kabuli_Chr06	1916	(T/C)
9446	CakSNP9446	*Kabuli*	Ca_Kabuli_Chr06	1847	(G/T)
9447	CakSNP9447	*Kabuli*	Ca_Kabuli_Chr06	140189	(A/G)
9448	CakSNP9448	*Kabuli*	Ca_Kabuli_Chr06	140279	(T/C)
9449	CakSNP9449	*Kabuli*	Ca_Kabuli_Chr06	140320	(G/A)
9450	CakSNP9450	*Kabuli*	Ca_Kabuli_Chr06	152513	(C/T)
9451	CakSNP9451	*Kabuli*	Ca_Kabuli_Chr06	152556	(T/C)
9452	CakSNP9452	*Kabuli*	Ca_Kabuli_Chr06	166823	(G/A)
9453	CakSNP9453	*Kabuli*	Ca_Kabuli_Chr06	166806	(C/T)
9454	CakSNP9454	*Kabuli*	Ca_Kabuli_Chr06	166959	(G/A)
9455	CakSNP9455	*Kabuli*	Ca_Kabuli_Chr06	166943	(G/A)
9456	CakSNP9456	*Kabuli*	Ca_Kabuli_Chr06	166938	(G/T)
9457	CakSNP9457	*Kabuli*	Ca_Kabuli_Chr06	166934	(G/A)
9458	CakSNP9458	*Kabuli*	Ca_Kabuli_Chr06	166932	(G/A)
9459	CakSNP9459	*Kabuli*	Ca_Kabuli_Chr06	166911	(A/G)
9460	CakSNP9460	*Kabuli*	Ca_Kabuli_Chr06	204368	(G/A)
9461	CakSNP9461	*Kabuli*	Ca_Kabuli_Chr06	204412	(G/C)
9462	CakSNP9462	*Kabuli*	Ca_Kabuli_Chr06	204438	(T/C)
9463	CakSNP9463	*Kabuli*	Ca_Kabuli_Chr06	267151	(A/G)
9464	CakSNP9464	*Kabuli*	Ca_Kabuli_Chr06	269269	(T/G)
9465	CakSNP9465	*Kabuli*	Ca_Kabuli_Chr06	324301	(C/T)
9466	CakSNP9466	*Kabuli*	Ca_Kabuli_Chr06	348283	(C/T)
9467	CakSNP9467	*Kabuli*	Ca_Kabuli_Chr06	348295	(G/C)
9468	CakSNP9468	*Kabuli*	Ca_Kabuli_Chr06	375684	(T/G)
9469	CakSNP9469	*Kabuli*	Ca_Kabuli_Chr06	386250	(A/G)
9470	CakSNP9470	*Kabuli*	Ca_Kabuli_Chr06	474489	(C/A)
9471	CakSNP9471	*Kabuli*	Ca_Kabuli_Chr06	505875	(T/C)
9472	CakSNP9472	*Kabuli*	Ca_Kabuli_Chr06	515221	(T/G)
9473	CakSNP9473	*Kabuli*	Ca_Kabuli_Chr06	548416	(A/T)
9474	CakSNP9474	*Kabuli*	Ca_Kabuli_Chr06	551130	(T/G)
9475	CakSNP9475	*Kabuli*	Ca_Kabuli_Chr06	561562	(A/G)

S.N.	SNP IDs	Cultivars	Chromosomes/scaffolds	Physical positions (bp)	SNPs
9476	CakSNP9476	*Kabuli*	Ca_Kabuli_Chr06	580616	(G/C)
9477	CakSNP9477	*Kabuli*	Ca_Kabuli_Chr06	588592	(C/A)
9478	CakSNP9478	*Kabuli*	Ca_Kabuli_Chr06	638983	(T/A)
9479	CakSNP9479	*Kabuli*	Ca_Kabuli_Chr06	645755	(G/T)
9480	CakSNP9480	*Kabuli*	Ca_Kabuli_Chr06	645746	(G/T)
9481	CakSNP9481	*Kabuli*	Ca_Kabuli_Chr06	647322	(A/C)
9482	CakSNP9482	*Kabuli*	Ca_Kabuli_Chr06	697187	(A/G)
9483	CakSNP9483	*Kabuli*	Ca_Kabuli_Chr06	697169	(A/G)
9484	CakSNP9484	*Kabuli*	Ca_Kabuli_Chr06	697168	(C/T)
9485	CakSNP9485	*Kabuli*	Ca_Kabuli_Chr06	697162	(A/G)
9486	CakSNP9486	*Kabuli*	Ca_Kabuli_Chr06	697158	(G/A)
9487	CakSNP9487	*Kabuli*	Ca_Kabuli_Chr06	697148	(C/T)
9488	CakSNP9488	*Kabuli*	Ca_Kabuli_Chr06	697147	(A/G)
9489	CakSNP9489	*Kabuli*	Ca_Kabuli_Chr06	697142	(G/C)
9490	CakSNP9490	*Kabuli*	Ca_Kabuli_Chr06	894921	(A/G)
9491	CakSNP9491	*Kabuli*	Ca_Kabuli_Chr06	902403	(G/A)
9492	CakSNP9492	*Kabuli*	Ca_Kabuli_Chr06	902402	(T/C)
9493	CakSNP9493	*Kabuli*	Ca_Kabuli_Chr06	949263	(C/A)
9494	CakSNP9494	*Kabuli*	Ca_Kabuli_Chr06	961652	(C/T)
9495	CakSNP9495	*Kabuli*	Ca_Kabuli_Chr06	961805	(C/T)
9496	CakSNP9496	*Kabuli*	Ca_Kabuli_Chr06	961733	(T/A)
9497	CakSNP9497	*Kabuli*	Ca_Kabuli_Chr06	997997	(T/A)
9498	CakSNP9498	*Kabuli*	Ca_Kabuli_Chr06	1130293	(C/T)
9499	CakSNP9499	*Kabuli*	Ca_Kabuli_Chr06	1212889	(G/A)
9500	CakSNP9500	*Kabuli*	Ca_Kabuli_Chr06	1220425	(C/T)
9501	CakSNP9501	*Kabuli*	Ca_Kabuli_Chr06	1220460	(G/C)
9502	CakSNP9502	*Kabuli*	Ca_Kabuli_Chr06	1220465	(G/A)
9503	CakSNP9503	*Kabuli*	Ca_Kabuli_Chr06	1220510	(A/T)
9504	CakSNP9504	*Kabuli*	Ca_Kabuli_Chr06	1220499	(C/G)
9505	CakSNP9505	*Kabuli*	Ca_Kabuli_Chr06	1287168	(C/T)
9506	CakSNP9506	*Kabuli*	Ca_Kabuli_Chr06	1290413	(C/G)
9507	CakSNP9507	*Kabuli*	Ca_Kabuli_Chr06	1290538	(T/G)
9508	CakSNP9508	*Kabuli*	Ca_Kabuli_Chr06	1295748	(T/C)
9509	CakSNP9509	*Kabuli*	Ca_Kabuli_Chr06	1312881	(T/C)
9510	CakSNP9510	*Kabuli*	Ca_Kabuli_Chr06	1312932	(G/A)
9511	CakSNP9511	*Kabuli*	Ca_Kabuli_Chr06	1312982	(C/A)
9512	CakSNP9512	*Kabuli*	Ca_Kabuli_Chr06	1315583	(G/C)
9513	CakSNP9513	*Kabuli*	Ca_Kabuli_Chr06	1626723	(T/C)
9514	CakSNP9514	*Kabuli*	Ca_Kabuli_Chr06	1626712	(C/T)

S.N.	SNP IDs	Cultivars	Chromosomes/scaffolds	Physical positions (bp)	SNPs
9515	CakSNP9515	*Kabuli*	Ca_Kabuli_Chr06	1817599	(C/T)
9516	CakSNP9516	*Kabuli*	Ca_Kabuli_Chr06	1817602	(A/T)
9517	CakSNP9517	*Kabuli*	Ca_Kabuli_Chr06	1817607	(T/C)
9518	CakSNP9518	*Kabuli*	Ca_Kabuli_Chr06	1817628	(A/T)
9519	CakSNP9519	*Kabuli*	Ca_Kabuli_Chr06	1932114	(C/G)
9520	CakSNP9520	*Kabuli*	Ca_Kabuli_Chr06	1932175	(G/A)
9521	CakSNP9521	*Kabuli*	Ca_Kabuli_Chr06	1966137	(T/C)
9522	CakSNP9522	*Kabuli*	Ca_Kabuli_Chr06	1978781	(C/A)
9523	CakSNP9523	*Kabuli*	Ca_Kabuli_Chr06	1978864	(A/C)
9524	CakSNP9524	*Kabuli*	Ca_Kabuli_Chr06	1978867	(T/A)
9525	CakSNP9525	*Kabuli*	Ca_Kabuli_Chr06	2037575	(G/T)
9526	CakSNP9526	*Kabuli*	Ca_Kabuli_Chr06	2037599	(A/G)
9527	CakSNP9527	*Kabuli*	Ca_Kabuli_Chr06	2037620	(T/G)
9528	CakSNP9528	*Kabuli*	Ca_Kabuli_Chr06	2037623	(T/G)
9529	CakSNP9529	*Kabuli*	Ca_Kabuli_Chr06	2037626	(C/T)
9530	CakSNP9530	*Kabuli*	Ca_Kabuli_Chr06	2050907	(A/G)
9531	CakSNP9531	*Kabuli*	Ca_Kabuli_Chr06	2061459	(A/G)
9532	CakSNP9532	*Kabuli*	Ca_Kabuli_Chr06	2078416	(T/A)
9533	CakSNP9533	*Kabuli*	Ca_Kabuli_Chr06	2079137	(T/G)
9534	CakSNP9534	*Kabuli*	Ca_Kabuli_Chr06	2079186	(G/T)
9535	CakSNP9535	*Kabuli*	Ca_Kabuli_Chr06	2083784	(A/T)
9536	CakSNP9536	*Kabuli*	Ca_Kabuli_Chr06	2083805	(G/A)
9537	CakSNP9537	*Kabuli*	Ca_Kabuli_Chr06	2083813	(T/C)
9538	CakSNP9538	*Kabuli*	Ca_Kabuli_Chr06	2126667	(C/T)
9539	CakSNP9539	*Kabuli*	Ca_Kabuli_Chr06	2126655	(G/A)
9540	CakSNP9540	*Kabuli*	Ca_Kabuli_Chr06	2140189	(G/T)
9541	CakSNP9541	*Kabuli*	Ca_Kabuli_Chr06	2140406	(G/A)
9542	CakSNP9542	*Kabuli*	Ca_Kabuli_Chr06	2193748	(T/A)
9543	CakSNP9543	*Kabuli*	Ca_Kabuli_Chr06	2193763	(A/C)
9544	CakSNP9544	*Kabuli*	Ca_Kabuli_Chr06	2193803	(G/A)
9545	CakSNP9545	*Kabuli*	Ca_Kabuli_Chr06	2214131	(T/A)
9546	CakSNP9546	*Kabuli*	Ca_Kabuli_Chr06	2214178	(T/C)
9547	CakSNP9547	*Kabuli*	Ca_Kabuli_Chr06	2214247	(A/G)
9548	CakSNP9548	*Kabuli*	Ca_Kabuli_Chr06	2223544	(A/T)
9549	CakSNP9549	*Kabuli*	Ca_Kabuli_Chr06	2223578	(T/G)
9550	CakSNP9550	*Kabuli*	Ca_Kabuli_Chr06	2223664	(A/C)
9551	CakSNP9551	*Kabuli*	Ca_Kabuli_Chr06	2244901	(A/C)
9552	CakSNP9552	*Kabuli*	Ca_Kabuli_Chr06	2275505	(A/G)
9553	CakSNP9553	*Kabuli*	Ca_Kabuli_Chr06	2275524	(G/A)

S.N.	SNP IDs	Cultivars	Chromosomes/scaffolds	Physical positions (bp)	SNPs
9554	CakSNP9554	*Kabuli*	Ca_Kabuli_Chr06	2275553	(A/G)
9555	CakSNP9555	*Kabuli*	Ca_Kabuli_Chr06	2280734	(T/A)
9556	CakSNP9556	*Kabuli*	Ca_Kabuli_Chr06	2280752	(A/G)
9557	CakSNP9557	*Kabuli*	Ca_Kabuli_Chr06	2286205	(A/G)
9558	CakSNP9558	*Kabuli*	Ca_Kabuli_Chr06	2286237	(C/T)
9559	CakSNP9559	*Kabuli*	Ca_Kabuli_Chr06	2294911	(T/G)
9560	CakSNP9560	*Kabuli*	Ca_Kabuli_Chr06	2303123	(T/G)
9561	CakSNP9561	*Kabuli*	Ca_Kabuli_Chr06	2313683	(A/C)
9562	CakSNP9562	*Kabuli*	Ca_Kabuli_Chr06	2313828	(G/A)
9563	CakSNP9563	*Kabuli*	Ca_Kabuli_Chr06	2314029	(T/C)
9564	CakSNP9564	*Kabuli*	Ca_Kabuli_Chr06	2327842	(A/G)
9565	CakSNP9565	*Kabuli*	Ca_Kabuli_Chr06	2462481	(C/T)
9566	CakSNP9566	*Kabuli*	Ca_Kabuli_Chr06	2502485	(A/G)
9567	CakSNP9567	*Kabuli*	Ca_Kabuli_Chr06	2527768	(A/G)
9568	CakSNP9568	*Kabuli*	Ca_Kabuli_Chr06	2542942	(A/T)
9569	CakSNP9569	*Kabuli*	Ca_Kabuli_Chr06	2542981	(T/C)
9570	CakSNP9570	*Kabuli*	Ca_Kabuli_Chr06	2543096	(C/T)
9571	CakSNP9571	*Kabuli*	Ca_Kabuli_Chr06	2543062	(A/G)
9572	CakSNP9572	*Kabuli*	Ca_Kabuli_Chr06	2548412	(A/G)
9573	CakSNP9573	*Kabuli*	Ca_Kabuli_Chr06	2549997	(C/T)
9574	CakSNP9574	*Kabuli*	Ca_Kabuli_Chr06	2549991	(T/C)
9575	CakSNP9575	*Kabuli*	Ca_Kabuli_Chr06	2550154	(A/C)
9576	CakSNP9576	*Kabuli*	Ca_Kabuli_Chr06	2623370	(T/C)
9577	CakSNP9577	*Kabuli*	Ca_Kabuli_Chr06	2625793	(C/G)
9578	CakSNP9578	*Kabuli*	Ca_Kabuli_Chr06	2633941	(A/T)
9579	CakSNP9579	*Kabuli*	Ca_Kabuli_Chr06	2639558	(T/A)
9580	CakSNP9580	*Kabuli*	Ca_Kabuli_Chr06	2639606	(A/G)
9581	CakSNP9581	*Kabuli*	Ca_Kabuli_Chr06	2639607	(T/C)
9582	CakSNP9582	*Kabuli*	Ca_Kabuli_Chr06	2676594	(C/T)
9583	CakSNP9583	*Kabuli*	Ca_Kabuli_Chr06	2714894	(T/C)
9584	CakSNP9584	*Kabuli*	Ca_Kabuli_Chr06	2714897	(C/A)
9585	CakSNP9585	*Kabuli*	Ca_Kabuli_Chr06	2714918	(T/G)
9586	CakSNP9586	*Kabuli*	Ca_Kabuli_Chr06	2717150	(G/A)
9587	CakSNP9587	*Kabuli*	Ca_Kabuli_Chr06	2740630	(A/G)
9588	CakSNP9588	*Kabuli*	Ca_Kabuli_Chr06	2740765	(A/G)
9589	CakSNP9589	*Kabuli*	Ca_Kabuli_Chr06	2740784	(T/C)
9590	CakSNP9590	*Kabuli*	Ca_Kabuli_Chr06	2741185	(G/A)
9591	CakSNP9591	*Kabuli*	Ca_Kabuli_Chr06	2777609	(A/G)
9592	CakSNP9592	*Kabuli*	Ca_Kabuli_Chr06	2802942	(C/A)

S.N.	SNP IDs	Cultivars	Chromosomes/scaffolds	Physical positions (bp)	SNPs
9593	CakSNP9593	*Kabuli*	Ca_Kabuli_Chr06	2811345	(A/G)
9594	CakSNP9594	*Kabuli*	Ca_Kabuli_Chr06	2812794	(A/C)
9595	CakSNP9595	*Kabuli*	Ca_Kabuli_Chr06	2812719	(G/A)
9596	CakSNP9596	*Kabuli*	Ca_Kabuli_Chr06	2813244	(G/A)
9597	CakSNP9597	*Kabuli*	Ca_Kabuli_Chr06	2821836	(G/T)
9598	CakSNP9598	*Kabuli*	Ca_Kabuli_Chr06	2846051	(C/T)
9599	CakSNP9599	*Kabuli*	Ca_Kabuli_Chr06	2846018	(T/C)
9600	CakSNP9600	*Kabuli*	Ca_Kabuli_Chr06	2855691	(C/G)
9601	CakSNP9601	*Kabuli*	Ca_Kabuli_Chr06	2864713	(G/A)
9602	CakSNP9602	*Kabuli*	Ca_Kabuli_Chr06	2919355	(A/C)
9603	CakSNP9603	*Kabuli*	Ca_Kabuli_Chr06	2925908	(A/G)
9604	CakSNP9604	*Kabuli*	Ca_Kabuli_Chr06	2925962	(G/C)
9605	CakSNP9605	*Kabuli*	Ca_Kabuli_Chr06	2926006	(T/C)
9606	CakSNP9606	*Kabuli*	Ca_Kabuli_Chr06	2927224	(G/A)
9607	CakSNP9607	*Kabuli*	Ca_Kabuli_Chr06	2927255	(C/A)
9608	CakSNP9608	*Kabuli*	Ca_Kabuli_Chr06	2927271	(G/A)
9609	CakSNP9609	*Kabuli*	Ca_Kabuli_Chr06	2927386	(C/G)
9610	CakSNP9610	*Kabuli*	Ca_Kabuli_Chr06	2927387	(T/C)
9611	CakSNP9611	*Kabuli*	Ca_Kabuli_Chr06	2929355	(C/T)
9612	CakSNP9612	*Kabuli*	Ca_Kabuli_Chr06	2930453	(T/C)
9613	CakSNP9613	*Kabuli*	Ca_Kabuli_Chr06	2930459	(C/T)
9614	CakSNP9614	*Kabuli*	Ca_Kabuli_Chr06	2930504	(T/C)
9615	CakSNP9615	*Kabuli*	Ca_Kabuli_Chr06	2932029	(T/C)
9616	CakSNP9616	*Kabuli*	Ca_Kabuli_Chr06	2932179	(T/G)
9617	CakSNP9617	*Kabuli*	Ca_Kabuli_Chr06	2932168	(A/C)
9618	CakSNP9618	*Kabuli*	Ca_Kabuli_Chr06	2932143	(C/A)
9619	CakSNP9619	*Kabuli*	Ca_Kabuli_Chr06	2932130	(T/G)
9620	CakSNP9620	*Kabuli*	Ca_Kabuli_Chr06	2932777	(A/G)
9621	CakSNP9621	*Kabuli*	Ca_Kabuli_Chr06	2972319	(C/T)
9622	CakSNP9622	*Kabuli*	Ca_Kabuli_Chr06	2972301	(T/C)
9623	CakSNP9623	*Kabuli*	Ca_Kabuli_Chr06	2973741	(A/G)
9624	CakSNP9624	*Kabuli*	Ca_Kabuli_Chr06	2973756	(A/C)
9625	CakSNP9625	*Kabuli*	Ca_Kabuli_Chr06	3060508	(G/A)
9626	CakSNP9626	*Kabuli*	Ca_Kabuli_Chr06	3060502	(G/A)
9627	CakSNP9627	*Kabuli*	Ca_Kabuli_Chr06	3288481	(C/A)
9628	CakSNP9628	*Kabuli*	Ca_Kabuli_Chr06	3288444	(G/A)
9629	CakSNP9629	*Kabuli*	Ca_Kabuli_Chr06	3288441	(A/G)
9630	CakSNP9630	*Kabuli*	Ca_Kabuli_Chr06	3288505	(G/T)
9631	CakSNP9631	*Kabuli*	Ca_Kabuli_Chr06	3297086	(G/A)

S.N.	SNP IDs	Cultivars	Chromosomes/scaffolds	Physical positions (bp)	SNPs
9632	CakSNP9632	*Kabuli*	Ca_Kabuli_Chr06	3298952	(A/T)
9633	CakSNP9633	*Kabuli*	Ca_Kabuli_Chr06	3395024	(A/G)
9634	CakSNP9634	*Kabuli*	Ca_Kabuli_Chr06	3395253	(G/A)
9635	CakSNP9635	*Kabuli*	Ca_Kabuli_Chr06	3395370	(C/T)
9636	CakSNP9636	*Kabuli*	Ca_Kabuli_Chr06	3397650	(A/G)
9637	CakSNP9637	*Kabuli*	Ca_Kabuli_Chr06	3401260	(A/G)
9638	CakSNP9638	*Kabuli*	Ca_Kabuli_Chr06	3401272	(G/A)
9639	CakSNP9639	*Kabuli*	Ca_Kabuli_Chr06	3401277	(C/T)
9640	CakSNP9640	*Kabuli*	Ca_Kabuli_Chr06	3482411	(A/G)
9641	CakSNP9641	*Kabuli*	Ca_Kabuli_Chr06	3505662	(C/A)
9642	CakSNP9642	*Kabuli*	Ca_Kabuli_Chr06	3530094	(T/C)
9643	CakSNP9643	*Kabuli*	Ca_Kabuli_Chr06	3530137	(A/G)
9644	CakSNP9644	*Kabuli*	Ca_Kabuli_Chr06	3548121	(C/A)
9645	CakSNP9645	*Kabuli*	Ca_Kabuli_Chr06	3548114	(A/C)
9646	CakSNP9646	*Kabuli*	Ca_Kabuli_Chr06	3786843	(A/G)
9647	CakSNP9647	*Kabuli*	Ca_Kabuli_Chr06	3787239	(C/A)
9648	CakSNP9648	*Kabuli*	Ca_Kabuli_Chr06	3829070	(C/A)
9649	CakSNP9649	*Kabuli*	Ca_Kabuli_Chr06	3962516	(A/C)
9650	CakSNP9650	*Kabuli*	Ca_Kabuli_Chr06	3985519	(G/A)
9651	CakSNP9651	*Kabuli*	Ca_Kabuli_Chr06	3992419	(C/T)
9652	CakSNP9652	*Kabuli*	Ca_Kabuli_Chr06	4112085	(T/C)
9653	CakSNP9653	*Kabuli*	Ca_Kabuli_Chr06	4226957	(G/A)
9654	CakSNP9654	*Kabuli*	Ca_Kabuli_Chr06	4226947	(A/T)
9655	CakSNP9655	*Kabuli*	Ca_Kabuli_Chr06	4226924	(G/T)
9656	CakSNP9656	*Kabuli*	Ca_Kabuli_Chr06	4226951	(C/A)
9657	CakSNP9657	*Kabuli*	Ca_Kabuli_Chr06	4228728	(T/C)
9658	CakSNP9658	*Kabuli*	Ca_Kabuli_Chr06	4228759	(C/G)
9659	CakSNP9659	*Kabuli*	Ca_Kabuli_Chr06	4228884	(T/C)
9660	CakSNP9660	*Kabuli*	Ca_Kabuli_Chr06	4344604	(T/C)
9661	CakSNP9661	*Kabuli*	Ca_Kabuli_Chr06	4350822	(A/T)
9662	CakSNP9662	*Kabuli*	Ca_Kabuli_Chr06	4350867	(A/C)
9663	CakSNP9663	*Kabuli*	Ca_Kabuli_Chr06	4350889	(A/C)
9664	CakSNP9664	*Kabuli*	Ca_Kabuli_Chr06	4440034	(T/G)
9665	CakSNP9665	*Kabuli*	Ca_Kabuli_Chr06	4562263	(C/A)
9666	CakSNP9666	*Kabuli*	Ca_Kabuli_Chr06	4562264	(C/G)
9667	CakSNP9667	*Kabuli*	Ca_Kabuli_Chr06	4601303	(A/C)
9668	CakSNP9668	*Kabuli*	Ca_Kabuli_Chr06	4678523	(A/C)
9669	CakSNP9669	*Kabuli*	Ca_Kabuli_Chr06	4681686	(C/A)
9670	CakSNP9670	*Kabuli*	Ca_Kabuli_Chr06	4743563	(C/G)

S.N.	SNP IDs	Cultivars	Chromosomes/scaffolds	Physical positions (bp)	SNPs
9671	CakSNP9671	*Kabuli*	Ca_Kabuli_Chr06	4743608	(C/T)
9672	CakSNP9672	*Kabuli*	Ca_Kabuli_Chr06	4807908	(G/C)
9673	CakSNP9673	*Kabuli*	Ca_Kabuli_Chr06	4858109	(T/C)
9674	CakSNP9674	*Kabuli*	Ca_Kabuli_Chr06	4861184	(T/G)
9675	CakSNP9675	*Kabuli*	Ca_Kabuli_Chr06	4981078	(G/T)
9676	CakSNP9676	*Kabuli*	Ca_Kabuli_Chr06	5185380	(A/G)
9677	CakSNP9677	*Kabuli*	Ca_Kabuli_Chr06	5309445	(G/A)
9678	CakSNP9678	*Kabuli*	Ca_Kabuli_Chr06	5338761	(T/G)
9679	CakSNP9679	*Kabuli*	Ca_Kabuli_Chr06	5341510	(C/A)
9680	CakSNP9680	*Kabuli*	Ca_Kabuli_Chr06	5366094	(A/G)
9681	CakSNP9681	*Kabuli*	Ca_Kabuli_Chr06	5368455	(T/G)
9682	CakSNP9682	*Kabuli*	Ca_Kabuli_Chr06	5392161	(G/A)
9683	CakSNP9683	*Kabuli*	Ca_Kabuli_Chr06	5392185	(T/C)
9684	CakSNP9684	*Kabuli*	Ca_Kabuli_Chr06	5396888	(T/G)
9685	CakSNP9685	*Kabuli*	Ca_Kabuli_Chr06	5396890	(T/G)
9686	CakSNP9686	*Kabuli*	Ca_Kabuli_Chr06	5396897	(T/G)
9687	CakSNP9687	*Kabuli*	Ca_Kabuli_Chr06	5396913	(C/T)
9688	CakSNP9688	*Kabuli*	Ca_Kabuli_Chr06	5396916	(C/T)
9689	CakSNP9689	*Kabuli*	Ca_Kabuli_Chr06	5401307	(T/C)
9690	CakSNP9690	*Kabuli*	Ca_Kabuli_Chr06	5453512	(A/G)
9691	CakSNP9691	*Kabuli*	Ca_Kabuli_Chr06	5498898	(C/T)
9692	CakSNP9692	*Kabuli*	Ca_Kabuli_Chr06	5500791	(C/T)
9693	CakSNP9693	*Kabuli*	Ca_Kabuli_Chr06	5501363	(T/C)
9694	CakSNP9694	*Kabuli*	Ca_Kabuli_Chr06	5541264	(A/G)
9695	CakSNP9695	*Kabuli*	Ca_Kabuli_Chr06	5567967	(T/C)
9696	CakSNP9696	*Kabuli*	Ca_Kabuli_Chr06	5589373	(T/C)
9697	CakSNP9697	*Kabuli*	Ca_Kabuli_Chr06	5616372	(C/T)
9698	CakSNP9698	*Kabuli*	Ca_Kabuli_Chr06	5616482	(A/G)
9699	CakSNP9699	*Kabuli*	Ca_Kabuli_Chr06	5616454	(T/A)
9700	CakSNP9700	*Kabuli*	Ca_Kabuli_Chr06	5628948	(C/A)
9701	CakSNP9701	*Kabuli*	Ca_Kabuli_Chr06	5628956	(T/G)
9702	CakSNP9702	*Kabuli*	Ca_Kabuli_Chr06	5692120	(C/A)
9703	CakSNP9703	*Kabuli*	Ca_Kabuli_Chr06	5694397	(G/T)
9704	CakSNP9704	*Kabuli*	Ca_Kabuli_Chr06	5701004	(A/G)
9705	CakSNP9705	*Kabuli*	Ca_Kabuli_Chr06	5704845	(T/C)
9706	CakSNP9706	*Kabuli*	Ca_Kabuli_Chr06	5704809	(T/A)
9707	CakSNP9707	*Kabuli*	Ca_Kabuli_Chr06	5706649	(C/T)
9708	CakSNP9708	*Kabuli*	Ca_Kabuli_Chr06	5841443	(G/C)
9709	CakSNP9709	*Kabuli*	Ca_Kabuli_Chr06	5871537	(G/A)

S.N.	SNP IDs	Cultivars	Chromosomes/scaffolds	Physical positions (bp)	SNPs
9710	CakSNP9710	*Kabuli*	Ca_Kabuli_Chr06	5886393	(A/G)
9711	CakSNP9711	*Kabuli*	Ca_Kabuli_Chr06	5888423	(A/G)
9712	CakSNP9712	*Kabuli*	Ca_Kabuli_Chr06	5924967	(G/T)
9713	CakSNP9713	*Kabuli*	Ca_Kabuli_Chr06	5925028	(G/T)
9714	CakSNP9714	*Kabuli*	Ca_Kabuli_Chr06	6110805	(T/C)
9715	CakSNP9715	*Kabuli*	Ca_Kabuli_Chr06	6119644	(C/A)
9716	CakSNP9716	*Kabuli*	Ca_Kabuli_Chr06	6131564	(G/A)
9717	CakSNP9717	*Kabuli*	Ca_Kabuli_Chr06	6131518	(G/T)
9718	CakSNP9718	*Kabuli*	Ca_Kabuli_Chr06	6131514	(G/T)
9719	CakSNP9719	*Kabuli*	Ca_Kabuli_Chr06	6169291	(A/G)
9720	CakSNP9720	*Kabuli*	Ca_Kabuli_Chr06	6192895	(A/T)
9721	CakSNP9721	*Kabuli*	Ca_Kabuli_Chr06	6193105	(G/C)
9722	CakSNP9722	*Kabuli*	Ca_Kabuli_Chr06	6216766	(T/G)
9723	CakSNP9723	*Kabuli*	Ca_Kabuli_Chr06	6216746	(G/C)
9724	CakSNP9724	*Kabuli*	Ca_Kabuli_Chr06	6227887	(A/G)
9725	CakSNP9725	*Kabuli*	Ca_Kabuli_Chr06	6248260	(T/C)
9726	CakSNP9726	*Kabuli*	Ca_Kabuli_Chr06	6248203	(A/G)
9727	CakSNP9727	*Kabuli*	Ca_Kabuli_Chr06	6415818	(A/G)
9728	CakSNP9728	*Kabuli*	Ca_Kabuli_Chr06	6443828	(C/G)
9729	CakSNP9729	*Kabuli*	Ca_Kabuli_Chr06	6444169	(A/G)
9730	CakSNP9730	*Kabuli*	Ca_Kabuli_Chr06	6444226	(T/G)
9731	CakSNP9731	*Kabuli*	Ca_Kabuli_Chr06	6444243	(G/A)
9732	CakSNP9732	*Kabuli*	Ca_Kabuli_Chr06	6444220	(G/A)
9733	CakSNP9733	*Kabuli*	Ca_Kabuli_Chr06	6517785	(T/C)
9734	CakSNP9734	*Kabuli*	Ca_Kabuli_Chr06	6517849	(C/T)
9735	CakSNP9735	*Kabuli*	Ca_Kabuli_Chr06	6538236	(C/T)
9736	CakSNP9736	*Kabuli*	Ca_Kabuli_Chr06	6604713	(G/A)
9737	CakSNP9737	*Kabuli*	Ca_Kabuli_Chr06	6604766	(C/A)
9738	CakSNP9738	*Kabuli*	Ca_Kabuli_Chr06	6607773	(A/G)
9739	CakSNP9739	*Kabuli*	Ca_Kabuli_Chr06	6631216	(C/T)
9740	CakSNP9740	*Kabuli*	Ca_Kabuli_Chr06	6654917	(T/C)
9741	CakSNP9741	*Kabuli*	Ca_Kabuli_Chr06	6764292	(G/A)
9742	CakSNP9742	*Kabuli*	Ca_Kabuli_Chr06	6779147	(G/C)
9743	CakSNP9743	*Kabuli*	Ca_Kabuli_Chr06	6779982	(C/T)
9744	CakSNP9744	*Kabuli*	Ca_Kabuli_Chr06	6792012	(C/T)
9745	CakSNP9745	*Kabuli*	Ca_Kabuli_Chr06	6800935	(A/T)
9746	CakSNP9746	*Kabuli*	Ca_Kabuli_Chr06	6800927	(G/T)
9747	CakSNP9747	*Kabuli*	Ca_Kabuli_Chr06	6800877	(T/A)
9748	CakSNP9748	*Kabuli*	Ca_Kabuli_Chr06	6802259	(C/T)

S.N.	SNP IDs	Cultivars	Chromosomes/scaffolds	Physical positions (bp)	SNPs
9749	CakSNP9749	*Kabuli*	Ca_Kabuli_Chr06	6802651	(A/G)
9750	CakSNP9750	*Kabuli*	Ca_Kabuli_Chr06	6803433	(A/G)
9751	CakSNP9751	*Kabuli*	Ca_Kabuli_Chr06	6815594	(G/A)
9752	CakSNP9752	*Kabuli*	Ca_Kabuli_Chr06	6840803	(T/C)
9753	CakSNP9753	*Kabuli*	Ca_Kabuli_Chr06	6853983	(G/A)
9754	CakSNP9754	*Kabuli*	Ca_Kabuli_Chr06	6871801	(A/G)
9755	CakSNP9755	*Kabuli*	Ca_Kabuli_Chr06	6877020	(C/G)
9756	CakSNP9756	*Kabuli*	Ca_Kabuli_Chr06	6877088	(C/A)
9757	CakSNP9757	*Kabuli*	Ca_Kabuli_Chr06	6877163	(T/C)
9758	CakSNP9758	*Kabuli*	Ca_Kabuli_Chr06	6877246	(C/A)
9759	CakSNP9759	*Kabuli*	Ca_Kabuli_Chr06	6877284	(C/T)
9760	CakSNP9760	*Kabuli*	Ca_Kabuli_Chr06	6899977	(G/A)
9761	CakSNP9761	*Kabuli*	Ca_Kabuli_Chr06	6899949	(T/G)
9762	CakSNP9762	*Kabuli*	Ca_Kabuli_Chr06	6913639	(T/C)
9763	CakSNP9763	*Kabuli*	Ca_Kabuli_Chr06	6913622	(A/C)
9764	CakSNP9764	*Kabuli*	Ca_Kabuli_Chr06	6913695	(G/A)
9765	CakSNP9765	*Kabuli*	Ca_Kabuli_Chr06	6913825	(G/A)
9766	CakSNP9766	*Kabuli*	Ca_Kabuli_Chr06	6913821	(A/T)
9767	CakSNP9767	*Kabuli*	Ca_Kabuli_Chr06	6954468	(A/G)
9768	CakSNP9768	*Kabuli*	Ca_Kabuli_Chr06	6954480	(A/G)
9769	CakSNP9769	*Kabuli*	Ca_Kabuli_Chr06	7026495	(G/A)
9770	CakSNP9770	*Kabuli*	Ca_Kabuli_Chr06	7026481	(G/C)
9771	CakSNP9771	*Kabuli*	Ca_Kabuli_Chr06	7039578	(C/T)
9772	CakSNP9772	*Kabuli*	Ca_Kabuli_Chr06	7040231	(A/C)
9773	CakSNP9773	*Kabuli*	Ca_Kabuli_Chr06	7138381	(G/A)
9774	CakSNP9774	*Kabuli*	Ca_Kabuli_Chr06	7259533	(T/A)
9775	CakSNP9775	*Kabuli*	Ca_Kabuli_Chr06	7271634	(C/T)
9776	CakSNP9776	*Kabuli*	Ca_Kabuli_Chr06	7332579	(T/C)
9777	CakSNP9777	*Kabuli*	Ca_Kabuli_Chr06	7332585	(T/C)
9778	CakSNP9778	*Kabuli*	Ca_Kabuli_Chr06	7332762	(C/T)
9779	CakSNP9779	*Kabuli*	Ca_Kabuli_Chr06	7349517	(A/C)
9780	CakSNP9780	*Kabuli*	Ca_Kabuli_Chr06	7377229	(G/A)
9781	CakSNP9781	*Kabuli*	Ca_Kabuli_Chr06	7377276	(G/A)
9782	CakSNP9782	*Kabuli*	Ca_Kabuli_Chr06	7406219	(A/G)
9783	CakSNP9783	*Kabuli*	Ca_Kabuli_Chr06	7406233	(G/A)
9784	CakSNP9784	*Kabuli*	Ca_Kabuli_Chr06	7406301	(T/C)
9785	CakSNP9785	*Kabuli*	Ca_Kabuli_Chr06	7531353	(G/A)
9786	CakSNP9786	*Kabuli*	Ca_Kabuli_Chr06	7531445	(G/A)
9787	CakSNP9787	*Kabuli*	Ca_Kabuli_Chr06	7531456	(T/A)

S.N.	SNP IDs	Cultivars	Chromosomes/scaffolds	Physical positions (bp)	SNPs
9788	CakSNP9788	*Kabuli*	Ca_Kabuli_Chr06	7534360	(G/T)
9789	CakSNP9789	*Kabuli*	Ca_Kabuli_Chr06	7543580	(C/T)
9790	CakSNP9790	*Kabuli*	Ca_Kabuli_Chr06	7554094	(T/A)
9791	CakSNP9791	*Kabuli*	Ca_Kabuli_Chr06	7571950	(T/C)
9792	CakSNP9792	*Kabuli*	Ca_Kabuli_Chr06	7582726	(G/T)
9793	CakSNP9793	*Kabuli*	Ca_Kabuli_Chr06	7600817	(G/A)
9794	CakSNP9794	*Kabuli*	Ca_Kabuli_Chr06	7603195	(G/A)
9795	CakSNP9795	*Kabuli*	Ca_Kabuli_Chr06	7603178	(A/C)
9796	CakSNP9796	*Kabuli*	Ca_Kabuli_Chr06	7609814	(C/T)
9797	CakSNP9797	*Kabuli*	Ca_Kabuli_Chr06	7647907	(T/G)
9798	CakSNP9798	*Kabuli*	Ca_Kabuli_Chr06	7647931	(G/A)
9799	CakSNP9799	*Kabuli*	Ca_Kabuli_Chr06	7661930	(T/G)
9800	CakSNP9800	*Kabuli*	Ca_Kabuli_Chr06	7661924	(T/C)
9801	CakSNP9801	*Kabuli*	Ca_Kabuli_Chr06	7667301	(T/C)
9802	CakSNP9802	*Kabuli*	Ca_Kabuli_Chr06	7667328	(A/T)
9803	CakSNP9803	*Kabuli*	Ca_Kabuli_Chr06	7673403	(C/T)
9804	CakSNP9804	*Kabuli*	Ca_Kabuli_Chr06	7694609	(A/T)
9805	CakSNP9805	*Kabuli*	Ca_Kabuli_Chr06	7694625	(G/T)
9806	CakSNP9806	*Kabuli*	Ca_Kabuli_Chr06	7694696	(T/C)
9807	CakSNP9807	*Kabuli*	Ca_Kabuli_Chr06	7744730	(A/G)
9808	CakSNP9808	*Kabuli*	Ca_Kabuli_Chr06	7744752	(T/C)
9809	CakSNP9809	*Kabuli*	Ca_Kabuli_Chr06	7745641	(A/G)
9810	CakSNP9810	*Kabuli*	Ca_Kabuli_Chr06	7747254	(G/A)
9811	CakSNP9811	*Kabuli*	Ca_Kabuli_Chr06	7764968	(A/G)
9812	CakSNP9812	*Kabuli*	Ca_Kabuli_Chr06	7770357	(C/T)
9813	CakSNP9813	*Kabuli*	Ca_Kabuli_Chr06	7805886	(G/T)
9814	CakSNP9814	*Kabuli*	Ca_Kabuli_Chr06	7821850	(A/G)
9815	CakSNP9815	*Kabuli*	Ca_Kabuli_Chr06	7822666	(C/A)
9816	CakSNP9816	*Kabuli*	Ca_Kabuli_Chr06	7829485	(G/A)
9817	CakSNP9817	*Kabuli*	Ca_Kabuli_Chr06	7829627	(T/G)
9818	CakSNP9818	*Kabuli*	Ca_Kabuli_Chr06	7831214	(C/T)
9819	CakSNP9819	*Kabuli*	Ca_Kabuli_Chr06	7831409	(C/T)
9820	CakSNP9820	*Kabuli*	Ca_Kabuli_Chr06	7831585	(G/A)
9821	CakSNP9821	*Kabuli*	Ca_Kabuli_Chr06	7831874	(A/G)
9822	CakSNP9822	*Kabuli*	Ca_Kabuli_Chr06	7831898	(G/A)
9823	CakSNP9823	*Kabuli*	Ca_Kabuli_Chr06	7831899	(G/A)
9824	CakSNP9824	*Kabuli*	Ca_Kabuli_Chr06	7832029	(C/T)
9825	CakSNP9825	*Kabuli*	Ca_Kabuli_Chr06	7832023	(C/T)
9826	CakSNP9826	*Kabuli*	Ca_Kabuli_Chr06	7834595	(C/T)

S.N.	SNP IDs	Cultivars	Chromosomes/scaffolds	Physical positions (bp)	SNPs
9827	CakSNP9827	*Kabuli*	Ca_Kabuli_Chr06	7834922	(G/A)
9828	CakSNP9828	*Kabuli*	Ca_Kabuli_Chr06	7834895	(G/T)
9829	CakSNP9829	*Kabuli*	Ca_Kabuli_Chr06	7834976	(G/A)
9830	CakSNP9830	*Kabuli*	Ca_Kabuli_Chr06	7835427	(G/A)
9831	CakSNP9831	*Kabuli*	Ca_Kabuli_Chr06	7835738	(C/T)
9832	CakSNP9832	*Kabuli*	Ca_Kabuli_Chr06	7835984	(T/A)
9833	CakSNP9833	*Kabuli*	Ca_Kabuli_Chr06	7851000	(G/C)
9834	CakSNP9834	*Kabuli*	Ca_Kabuli_Chr06	7929263	(C/T)
9835	CakSNP9835	*Kabuli*	Ca_Kabuli_Chr06	7929338	(T/C)
9836	CakSNP9836	*Kabuli*	Ca_Kabuli_Chr06	7929339	(G/A)
9837	CakSNP9837	*Kabuli*	Ca_Kabuli_Chr06	7929348	(C/T)
9838	CakSNP9838	*Kabuli*	Ca_Kabuli_Chr06	7929384	(T/C)
9839	CakSNP9839	*Kabuli*	Ca_Kabuli_Chr06	7929493	(A/G)
9840	CakSNP9840	*Kabuli*	Ca_Kabuli_Chr06	7929629	(G/A)
9841	CakSNP9841	*Kabuli*	Ca_Kabuli_Chr06	7929628	(C/T)
9842	CakSNP9842	*Kabuli*	Ca_Kabuli_Chr06	7929607	(A/C)
9843	CakSNP9843	*Kabuli*	Ca_Kabuli_Chr06	7939277	(T/A)
9844	CakSNP9844	*Kabuli*	Ca_Kabuli_Chr06	7939281	(T/G)
9845	CakSNP9845	*Kabuli*	Ca_Kabuli_Chr06	7939465	(A/G)
9846	CakSNP9846	*Kabuli*	Ca_Kabuli_Chr06	7943546	(C/G)
9847	CakSNP9847	*Kabuli*	Ca_Kabuli_Chr06	7994732	(G/T)
9848	CakSNP9848	*Kabuli*	Ca_Kabuli_Chr06	8010016	(T/C)
9849	CakSNP9849	*Kabuli*	Ca_Kabuli_Chr06	8011685	(G/T)
9850	CakSNP9850	*Kabuli*	Ca_Kabuli_Chr06	8029226	(C/T)
9851	CakSNP9851	*Kabuli*	Ca_Kabuli_Chr06	8041582	(A/G)
9852	CakSNP9852	*Kabuli*	Ca_Kabuli_Chr06	8052987	(G/C)
9853	CakSNP9853	*Kabuli*	Ca_Kabuli_Chr06	8076394	(T/C)
9854	CakSNP9854	*Kabuli*	Ca_Kabuli_Chr06	8076446	(G/A)
9855	CakSNP9855	*Kabuli*	Ca_Kabuli_Chr06	8081800	(C/G)
9856	CakSNP9856	*Kabuli*	Ca_Kabuli_Chr06	8084950	(T/A)
9857	CakSNP9857	*Kabuli*	Ca_Kabuli_Chr06	8090739	(T/A)
9858	CakSNP9858	*Kabuli*	Ca_Kabuli_Chr06	8090980	(A/G)
9859	CakSNP9859	*Kabuli*	Ca_Kabuli_Chr06	8091050	(A/G)
9860	CakSNP9860	*Kabuli*	Ca_Kabuli_Chr06	8139434	(G/A)
9861	CakSNP9861	*Kabuli*	Ca_Kabuli_Chr06	8139446	(C/A)
9862	CakSNP9862	*Kabuli*	Ca_Kabuli_Chr06	8166317	(T/C)
9863	CakSNP9863	*Kabuli*	Ca_Kabuli_Chr06	8166339	(T/C)
9864	CakSNP9864	*Kabuli*	Ca_Kabuli_Chr06	8166435	(A/G)
9865	CakSNP9865	*Kabuli*	Ca_Kabuli_Chr06	8166462	(A/T)

S.N.	SNP IDs	Cultivars	Chromosomes/scaffolds	Physical positions (bp)	SNPs
9866	CakSNP9866	*Kabuli*	Ca_Kabuli_Chr06	8170583	(C/T)
9867	CakSNP9867	*Kabuli*	Ca_Kabuli_Chr06	8170633	(A/G)
9868	CakSNP9868	*Kabuli*	Ca_Kabuli_Chr06	8179815	(T/C)
9869	CakSNP9869	*Kabuli*	Ca_Kabuli_Chr06	8179797	(C/T)
9870	CakSNP9870	*Kabuli*	Ca_Kabuli_Chr06	8221204	(C/A)
9871	CakSNP9871	*Kabuli*	Ca_Kabuli_Chr06	8222666	(T/C)
9872	CakSNP9872	*Kabuli*	Ca_Kabuli_Chr06	8222685	(A/T)
9873	CakSNP9873	*Kabuli*	Ca_Kabuli_Chr06	8223986	(C/G)
9874	CakSNP9874	*Kabuli*	Ca_Kabuli_Chr06	8237286	(A/G)
9875	CakSNP9875	*Kabuli*	Ca_Kabuli_Chr06	8243936	(A/T)
9876	CakSNP9876	*Kabuli*	Ca_Kabuli_Chr06	8243947	(T/C)
9877	CakSNP9877	*Kabuli*	Ca_Kabuli_Chr06	8244152	(C/T)
9878	CakSNP9878	*Kabuli*	Ca_Kabuli_Chr06	8244249	(C/T)
9879	CakSNP9879	*Kabuli*	Ca_Kabuli_Chr06	8246037	(T/C)
9880	CakSNP9880	*Kabuli*	Ca_Kabuli_Chr06	8246187	(A/C)
9881	CakSNP9881	*Kabuli*	Ca_Kabuli_Chr06	8248202	(A/C)
9882	CakSNP9882	*Kabuli*	Ca_Kabuli_Chr06	8251365	(T/C)
9883	CakSNP9883	*Kabuli*	Ca_Kabuli_Chr06	8269326	(A/C)
9884	CakSNP9884	*Kabuli*	Ca_Kabuli_Chr06	8285170	(G/A)
9885	CakSNP9885	*Kabuli*	Ca_Kabuli_Chr06	8285147	(A/G)
9886	CakSNP9886	*Kabuli*	Ca_Kabuli_Chr06	8633231	(C/A)
9887	CakSNP9887	*Kabuli*	Ca_Kabuli_Chr06	8633215	(C/A)
9888	CakSNP9888	*Kabuli*	Ca_Kabuli_Chr06	8633187	(A/C)
9889	CakSNP9889	*Kabuli*	Ca_Kabuli_Chr06	8634513	(G/A)
9890	CakSNP9890	*Kabuli*	Ca_Kabuli_Chr06	8634494	(T/C)
9891	CakSNP9891	*Kabuli*	Ca_Kabuli_Chr06	8643286	(T/C)
9892	CakSNP9892	*Kabuli*	Ca_Kabuli_Chr06	8645079	(A/G)
9893	CakSNP9893	*Kabuli*	Ca_Kabuli_Chr06	8710064	(A/T)
9894	CakSNP9894	*Kabuli*	Ca_Kabuli_Chr06	8787556	(C/T)
9895	CakSNP9895	*Kabuli*	Ca_Kabuli_Chr06	8787557	(T/C)
9896	CakSNP9896	*Kabuli*	Ca_Kabuli_Chr06	8787562	(C/T)
9897	CakSNP9897	*Kabuli*	Ca_Kabuli_Chr06	8787564	(G/T)
9898	CakSNP9898	*Kabuli*	Ca_Kabuli_Chr06	8787568	(G/T)
9899	CakSNP9899	*Kabuli*	Ca_Kabuli_Chr06	8787572	(T/A)
9900	CakSNP9900	*Kabuli*	Ca_Kabuli_Chr06	8787573	(G/A)
9901	CakSNP9901	*Kabuli*	Ca_Kabuli_Chr06	8794430	(A/G)
9902	CakSNP9902	*Kabuli*	Ca_Kabuli_Chr06	8794552	(C/G)
9903	CakSNP9903	*Kabuli*	Ca_Kabuli_Chr06	8890548	(A/G)
9904	CakSNP9904	*Kabuli*	Ca_Kabuli_Chr06	8890552	(C/G)

S.N.	SNP IDs	Cultivars	Chromosomes/scaffolds	Physical positions (bp)	SNPs
9905	CakSNP9905	*Kabuli*	Ca_Kabuli_Chr06	8893216	(A/G)
9906	CakSNP9906	*Kabuli*	Ca_Kabuli_Chr06	8968068	(G/A)
9907	CakSNP9907	*Kabuli*	Ca_Kabuli_Chr06	8969711	(C/T)
9908	CakSNP9908	*Kabuli*	Ca_Kabuli_Chr06	8969725	(C/A)
9909	CakSNP9909	*Kabuli*	Ca_Kabuli_Chr06	9053279	(A/G)
9910	CakSNP9910	*Kabuli*	Ca_Kabuli_Chr06	9054596	(T/C)
9911	CakSNP9911	*Kabuli*	Ca_Kabuli_Chr06	9088121	(T/C)
9912	CakSNP9912	*Kabuli*	Ca_Kabuli_Chr06	9088151	(G/A)
9913	CakSNP9913	*Kabuli*	Ca_Kabuli_Chr06	9088163	(A/G)
9914	CakSNP9914	*Kabuli*	Ca_Kabuli_Chr06	9088184	(G/A)
9915	CakSNP9915	*Kabuli*	Ca_Kabuli_Chr06	9088234	(A/C)
9916	CakSNP9916	*Kabuli*	Ca_Kabuli_Chr06	9190620	(G/T)
9917	CakSNP9917	*Kabuli*	Ca_Kabuli_Chr06	9221201	(A/G)
9918	CakSNP9918	*Kabuli*	Ca_Kabuli_Chr06	9227767	(C/A)
9919	CakSNP9919	*Kabuli*	Ca_Kabuli_Chr06	9228004	(C/A)
9920	CakSNP9920	*Kabuli*	Ca_Kabuli_Chr06	9323775	(A/G)
9921	CakSNP9921	*Kabuli*	Ca_Kabuli_Chr06	9324122	(A/C)
9922	CakSNP9922	*Kabuli*	Ca_Kabuli_Chr06	9373240	(C/A)
9923	CakSNP9923	*Kabuli*	Ca_Kabuli_Chr06	9373383	(G/C)
9924	CakSNP9924	*Kabuli*	Ca_Kabuli_Chr06	9378508	(A/T)
9925	CakSNP9925	*Kabuli*	Ca_Kabuli_Chr06	9378557	(G/C)
9926	CakSNP9926	*Kabuli*	Ca_Kabuli_Chr06	9382091	(C/T)
9927	CakSNP9927	*Kabuli*	Ca_Kabuli_Chr06	9423222	(T/C)
9928	CakSNP9928	*Kabuli*	Ca_Kabuli_Chr06	9424317	(T/C)
9929	CakSNP9929	*Kabuli*	Ca_Kabuli_Chr06	9428522	(C/T)
9930	CakSNP9930	*Kabuli*	Ca_Kabuli_Chr06	9428517	(G/A)
9931	CakSNP9931	*Kabuli*	Ca_Kabuli_Chr06	9523228	(T/C)
9932	CakSNP9932	*Kabuli*	Ca_Kabuli_Chr06	9544233	(T/C)
9933	CakSNP9933	*Kabuli*	Ca_Kabuli_Chr06	9565932	(A/G)
9934	CakSNP9934	*Kabuli*	Ca_Kabuli_Chr06	9568243	(A/C)
9935	CakSNP9935	*Kabuli*	Ca_Kabuli_Chr06	9568247	(G/A)
9936	CakSNP9936	*Kabuli*	Ca_Kabuli_Chr06	9568289	(A/C)
9937	CakSNP9937	*Kabuli*	Ca_Kabuli_Chr06	9569170	(A/G)
9938	CakSNP9938	*Kabuli*	Ca_Kabuli_Chr06	9570753	(C/T)
9939	CakSNP9939	*Kabuli*	Ca_Kabuli_Chr06	9617218	(A/G)
9940	CakSNP9940	*Kabuli*	Ca_Kabuli_Chr06	9617233	(A/G)
9941	CakSNP9941	*Kabuli*	Ca_Kabuli_Chr06	9714240	(T/C)
9942	CakSNP9942	*Kabuli*	Ca_Kabuli_Chr06	9728195	(G/T)
9943	CakSNP9943	*Kabuli*	Ca_Kabuli_Chr06	9728181	(A/G)

S.N.	SNP IDs	Cultivars	Chromosomes/scaffolds	Physical positions (bp)	SNPs
9944	CakSNP9944	*Kabuli*	Ca_Kabuli_Chr06	9814378	(G/T)
9945	CakSNP9945	*Kabuli*	Ca_Kabuli_Chr06	9834199	(T/C)
9946	CakSNP9946	*Kabuli*	Ca_Kabuli_Chr06	9834198	(C/A)
9947	CakSNP9947	*Kabuli*	Ca_Kabuli_Chr06	9834187	(A/T)
9948	CakSNP9948	*Kabuli*	Ca_Kabuli_Chr06	9834184	(A/T)
9949	CakSNP9949	*Kabuli*	Ca_Kabuli_Chr06	9834183	(C/T)
9950	CakSNP9950	*Kabuli*	Ca_Kabuli_Chr06	9841836	(G/A)
9951	CakSNP9951	*Kabuli*	Ca_Kabuli_Chr06	9841890	(G/A)
9952	CakSNP9952	*Kabuli*	Ca_Kabuli_Chr06	9845070	(C/T)
9953	CakSNP9953	*Kabuli*	Ca_Kabuli_Chr06	9845076	(T/C)
9954	CakSNP9954	*Kabuli*	Ca_Kabuli_Chr06	9872557	(G/A)
9955	CakSNP9955	*Kabuli*	Ca_Kabuli_Chr06	9872657	(C/T)
9956	CakSNP9956	*Kabuli*	Ca_Kabuli_Chr06	9872988	(A/T)
9957	CakSNP9957	*Kabuli*	Ca_Kabuli_Chr06	9888237	(G/A)
9958	CakSNP9958	*Kabuli*	Ca_Kabuli_Chr06	9938833	(T/C)
9959	CakSNP9959	*Kabuli*	Ca_Kabuli_Chr06	9939017	(G/A)
9960	CakSNP9960	*Kabuli*	Ca_Kabuli_Chr06	9959686	(T/C)
9961	CakSNP9961	*Kabuli*	Ca_Kabuli_Chr06	10020177	(C/G)
9962	CakSNP9962	*Kabuli*	Ca_Kabuli_Chr06	10020187	(A/G)
9963	CakSNP9963	*Kabuli*	Ca_Kabuli_Chr06	10025115	(A/C)
9964	CakSNP9964	*Kabuli*	Ca_Kabuli_Chr06	10025113	(C/G)
9965	CakSNP9965	*Kabuli*	Ca_Kabuli_Chr06	10027019	(G/A)
9966	CakSNP9966	*Kabuli*	Ca_Kabuli_Chr06	10027015	(G/C)
9967	CakSNP9967	*Kabuli*	Ca_Kabuli_Chr06	10028504	(A/G)
9968	CakSNP9968	*Kabuli*	Ca_Kabuli_Chr06	10028508	(G/C)
9969	CakSNP9969	*Kabuli*	Ca_Kabuli_Chr06	10028515	(A/G)
9970	CakSNP9970	*Kabuli*	Ca_Kabuli_Chr06	10045644	(A/C)
9971	CakSNP9971	*Kabuli*	Ca_Kabuli_Chr06	10045844	(A/G)
9972	CakSNP9972	*Kabuli*	Ca_Kabuli_Chr06	10088963	(G/T)
9973	CakSNP9973	*Kabuli*	Ca_Kabuli_Chr06	10088984	(A/G)
9974	CakSNP9974	*Kabuli*	Ca_Kabuli_Chr06	10100863	(C/G)
9975	CakSNP9975	*Kabuli*	Ca_Kabuli_Chr06	10111488	(C/G)
9976	CakSNP9976	*Kabuli*	Ca_Kabuli_Chr06	10111520	(G/C)
9977	CakSNP9977	*Kabuli*	Ca_Kabuli_Chr06	10111870	(A/G)
9978	CakSNP9978	*Kabuli*	Ca_Kabuli_Chr06	10111938	(G/T)
9979	CakSNP9979	*Kabuli*	Ca_Kabuli_Chr06	10116403	(A/T)
9980	CakSNP9980	*Kabuli*	Ca_Kabuli_Chr06	10116475	(C/A)
9981	CakSNP9981	*Kabuli*	Ca_Kabuli_Chr06	10138984	(C/T)
9982	CakSNP9982	*Kabuli*	Ca_Kabuli_Chr06	10151973	(A/G)

S.N.	SNP IDs	Cultivars	Chromosomes/scaffolds	Physical positions (bp)	SNPs
9983	CakSNP9983	*Kabuli*	Ca_Kabuli_Chr06	10151974	(A/G)
9984	CakSNP9984	*Kabuli*	Ca_Kabuli_Chr06	10158850	(T/C)
9985	CakSNP9985	*Kabuli*	Ca_Kabuli_Chr06	10188400	(G/T)
9986	CakSNP9986	*Kabuli*	Ca_Kabuli_Chr06	10191941	(A/G)
9987	CakSNP9987	*Kabuli*	Ca_Kabuli_Chr06	10215262	(T/G)
9988	CakSNP9988	*Kabuli*	Ca_Kabuli_Chr06	10230617	(A/G)
9989	CakSNP9989	*Kabuli*	Ca_Kabuli_Chr06	10230657	(A/G)
9990	CakSNP9990	*Kabuli*	Ca_Kabuli_Chr06	10231092	(T/G)
9991	CakSNP9991	*Kabuli*	Ca_Kabuli_Chr06	10231095	(G/T)
9992	CakSNP9992	*Kabuli*	Ca_Kabuli_Chr06	10234241	(A/G)
9993	CakSNP9993	*Kabuli*	Ca_Kabuli_Chr06	10259614	(C/T)
9994	CakSNP9994	*Kabuli*	Ca_Kabuli_Chr06	10260274	(C/T)
9995	CakSNP9995	*Kabuli*	Ca_Kabuli_Chr06	10314993	(C/G)
9996	CakSNP9996	*Kabuli*	Ca_Kabuli_Chr06	10395411	(G/A)
9997	CakSNP9997	*Kabuli*	Ca_Kabuli_Chr06	10395379	(T/C)
9998	CakSNP9998	*Kabuli*	Ca_Kabuli_Chr06	10395356	(T/A)
9999	CakSNP9999	*Kabuli*	Ca_Kabuli_Chr06	10441736	(A/C)
10000	CakSNP10000	*Kabuli*	Ca_Kabuli_Chr06	10441705	(T/C)
10001	CakSNP10001	*Kabuli*	Ca_Kabuli_Chr06	10442148	(A/C)
10002	CakSNP10002	*Kabuli*	Ca_Kabuli_Chr06	10445185	(C/T)
10003	CakSNP10003	*Kabuli*	Ca_Kabuli_Chr06	10445274	(G/A)
10004	CakSNP10004	*Kabuli*	Ca_Kabuli_Chr06	10456774	(A/G)
10005	CakSNP10005	*Kabuli*	Ca_Kabuli_Chr06	10456854	(G/T)
10006	CakSNP10006	*Kabuli*	Ca_Kabuli_Chr06	10494134	(C/T)
10007	CakSNP10007	*Kabuli*	Ca_Kabuli_Chr06	10494057	(C/A)
10008	CakSNP10008	*Kabuli*	Ca_Kabuli_Chr06	10494055	(T/A)
10009	CakSNP10009	*Kabuli*	Ca_Kabuli_Chr06	10495747	(G/T)
10010	CakSNP10010	*Kabuli*	Ca_Kabuli_Chr06	10495804	(C/T)
10011	CakSNP10011	*Kabuli*	Ca_Kabuli_Chr06	10502381	(A/G)
10012	CakSNP10012	*Kabuli*	Ca_Kabuli_Chr06	10502387	(C/A)
10013	CakSNP10013	*Kabuli*	Ca_Kabuli_Chr06	10510996	(T/C)
10014	CakSNP10014	*Kabuli*	Ca_Kabuli_Chr06	10520958	(C/T)
10015	CakSNP10015	*Kabuli*	Ca_Kabuli_Chr06	10521741	(A/T)
10016	CakSNP10016	*Kabuli*	Ca_Kabuli_Chr06	10531311	(A/G)
10017	CakSNP10017	*Kabuli*	Ca_Kabuli_Chr06	10531399	(C/T)
10018	CakSNP10018	*Kabuli*	Ca_Kabuli_Chr06	10532182	(A/T)
10019	CakSNP10019	*Kabuli*	Ca_Kabuli_Chr06	10532739	(A/G)
10020	CakSNP10020	*Kabuli*	Ca_Kabuli_Chr06	10539918	(A/C)
10021	CakSNP10021	*Kabuli*	Ca_Kabuli_Chr06	10539977	(C/T)

S.N.	SNP IDs	Cultivars	Chromosomes/scaffolds	Physical positions (bp)	SNPs
10022	CakSNP10022	*Kabuli*	Ca_Kabuli_Chr06	10539994	(A/C)
10023	CakSNP10023	*Kabuli*	Ca_Kabuli_Chr06	10567518	(A/G)
10024	CakSNP10024	*Kabuli*	Ca_Kabuli_Chr06	10588030	(C/T)
10025	CakSNP10025	*Kabuli*	Ca_Kabuli_Chr06	10613546	(A/G)
10026	CakSNP10026	*Kabuli*	Ca_Kabuli_Chr06	10613612	(A/G)
10027	CakSNP10027	*Kabuli*	Ca_Kabuli_Chr06	10652739	(C/T)
10028	CakSNP10028	*Kabuli*	Ca_Kabuli_Chr06	10667504	(A/T)
10029	CakSNP10029	*Kabuli*	Ca_Kabuli_Chr06	10667559	(T/C)
10030	CakSNP10030	*Kabuli*	Ca_Kabuli_Chr06	10667640	(T/G)
10031	CakSNP10031	*Kabuli*	Ca_Kabuli_Chr06	10670343	(G/A)
10032	CakSNP10032	*Kabuli*	Ca_Kabuli_Chr06	10670368	(G/A)
10033	CakSNP10033	*Kabuli*	Ca_Kabuli_Chr06	10670409	(G/A)
10034	CakSNP10034	*Kabuli*	Ca_Kabuli_Chr06	10670412	(A/C)
10035	CakSNP10035	*Kabuli*	Ca_Kabuli_Chr06	10670482	(C/T)
10036	CakSNP10036	*Kabuli*	Ca_Kabuli_Chr06	10670582	(G/A)
10037	CakSNP10037	*Kabuli*	Ca_Kabuli_Chr06	10670675	(C/T)
10038	CakSNP10038	*Kabuli*	Ca_Kabuli_Chr06	10670694	(G/A)
10039	CakSNP10039	*Kabuli*	Ca_Kabuli_Chr06	10670773	(A/C)
10040	CakSNP10040	*Kabuli*	Ca_Kabuli_Chr06	10670959	(T/C)
10041	CakSNP10041	*Kabuli*	Ca_Kabuli_Chr06	10671458	(T/C)
10042	CakSNP10042	*Kabuli*	Ca_Kabuli_Chr06	10671444	(A/C)
10043	CakSNP10043	*Kabuli*	Ca_Kabuli_Chr06	10672468	(C/T)
10044	CakSNP10044	*Kabuli*	Ca_Kabuli_Chr06	10677469	(A/G)
10045	CakSNP10045	*Kabuli*	Ca_Kabuli_Chr06	10677501	(C/T)
10046	CakSNP10046	*Kabuli*	Ca_Kabuli_Chr06	10682221	(G/T)
10047	CakSNP10047	*Kabuli*	Ca_Kabuli_Chr06	10682201	(C/G)
10048	CakSNP10048	*Kabuli*	Ca_Kabuli_Chr06	10683193	(C/T)
10049	CakSNP10049	*Kabuli*	Ca_Kabuli_Chr06	10696986	(A/T)
10050	CakSNP10050	*Kabuli*	Ca_Kabuli_Chr06	10696990	(T/C)
10051	CakSNP10051	*Kabuli*	Ca_Kabuli_Chr06	10697064	(T/A)
10052	CakSNP10052	*Kabuli*	Ca_Kabuli_Chr06	10716986	(G/A)
10053	CakSNP10053	*Kabuli*	Ca_Kabuli_Chr06	10716968	(G/A)
10054	CakSNP10054	*Kabuli*	Ca_Kabuli_Chr06	10744029	(C/A)
10055	CakSNP10055	*Kabuli*	Ca_Kabuli_Chr06	10743996	(A/T)
10056	CakSNP10056	*Kabuli*	Ca_Kabuli_Chr06	10748965	(G/T)
10057	CakSNP10057	*Kabuli*	Ca_Kabuli_Chr06	10779368	(G/C)
10058	CakSNP10058	*Kabuli*	Ca_Kabuli_Chr06	10779465	(A/T)
10059	CakSNP10059	*Kabuli*	Ca_Kabuli_Chr06	10779547	(T/G)
10060	CakSNP10060	*Kabuli*	Ca_Kabuli_Chr06	10779636	(C/A)

S.N.	SNP IDs	Cultivars	Chromosomes/scaffolds	Physical positions (bp)	SNPs
10061	CakSNP10061	*Kabuli*	Ca_Kabuli_Chr06	10785480	(T/G)
10062	CakSNP10062	*Kabuli*	Ca_Kabuli_Chr06	10870187	(T/G)
10063	CakSNP10063	*Kabuli*	Ca_Kabuli_Chr06	10870534	(A/G)
10064	CakSNP10064	*Kabuli*	Ca_Kabuli_Chr06	10880791	(C/T)
10065	CakSNP10065	*Kabuli*	Ca_Kabuli_Chr06	10880819	(T/G)
10066	CakSNP10066	*Kabuli*	Ca_Kabuli_Chr06	10881002	(A/G)
10067	CakSNP10067	*Kabuli*	Ca_Kabuli_Chr06	10880985	(C/T)
10068	CakSNP10068	*Kabuli*	Ca_Kabuli_Chr06	10880962	(G/T)
10069	CakSNP10069	*Kabuli*	Ca_Kabuli_Chr06	10991652	(C/A)
10070	CakSNP10070	*Kabuli*	Ca_Kabuli_Chr06	10991717	(T/A)
10071	CakSNP10071	*Kabuli*	Ca_Kabuli_Chr06	10991737	(A/C)
10072	CakSNP10072	*Kabuli*	Ca_Kabuli_Chr06	11011847	(C/G)
10073	CakSNP10073	*Kabuli*	Ca_Kabuli_Chr06	11128158	(A/G)
10074	CakSNP10074	*Kabuli*	Ca_Kabuli_Chr06	11128170	(T/C)
10075	CakSNP10075	*Kabuli*	Ca_Kabuli_Chr06	11128204	(G/A)
10076	CakSNP10076	*Kabuli*	Ca_Kabuli_Chr06	11131010	(T/A)
10077	CakSNP10077	*Kabuli*	Ca_Kabuli_Chr06	11131464	(G/A)
10078	CakSNP10078	*Kabuli*	Ca_Kabuli_Chr06	11131462	(G/A)
10079	CakSNP10079	*Kabuli*	Ca_Kabuli_Chr06	11131436	(T/G)
10080	CakSNP10080	*Kabuli*	Ca_Kabuli_Chr06	11163819	(T/G)
10081	CakSNP10081	*Kabuli*	Ca_Kabuli_Chr06	11163822	(C/T)
10082	CakSNP10082	*Kabuli*	Ca_Kabuli_Chr06	11163836	(C/T)
10083	CakSNP10083	*Kabuli*	Ca_Kabuli_Chr06	11188803	(G/A)
10084	CakSNP10084	*Kabuli*	Ca_Kabuli_Chr06	11189051	(A/G)
10085	CakSNP10085	*Kabuli*	Ca_Kabuli_Chr06	11229102	(C/T)
10086	CakSNP10086	*Kabuli*	Ca_Kabuli_Chr06	11229143	(A/C)
10087	CakSNP10087	*Kabuli*	Ca_Kabuli_Chr06	11229302	(A/G)
10088	CakSNP10088	*Kabuli*	Ca_Kabuli_Chr06	11229388	(C/T)
10089	CakSNP10089	*Kabuli*	Ca_Kabuli_Chr06	11285884	(G/A)
10090	CakSNP10090	*Kabuli*	Ca_Kabuli_Chr06	11300597	(C/T)
10091	CakSNP10091	*Kabuli*	Ca_Kabuli_Chr06	11300549	(A/C)
10092	CakSNP10092	*Kabuli*	Ca_Kabuli_Chr06	11302037	(A/C)
10093	CakSNP10093	*Kabuli*	Ca_Kabuli_Chr06	11302033	(T/C)
10094	CakSNP10094	*Kabuli*	Ca_Kabuli_Chr06	11314581	(T/C)
10095	CakSNP10095	*Kabuli*	Ca_Kabuli_Chr06	11394963	(A/G)
10096	CakSNP10096	*Kabuli*	Ca_Kabuli_Chr06	11395330	(C/T)
10097	CakSNP10097	*Kabuli*	Ca_Kabuli_Chr06	11508828	(C/T)
10098	CakSNP10098	*Kabuli*	Ca_Kabuli_Chr06	11508824	(T/G)
10099	CakSNP10099	*Kabuli*	Ca_Kabuli_Chr06	11508817	(G/A)

S.N.	SNP IDs	Cultivars	Chromosomes/scaffolds	Physical positions (bp)	SNPs
10100	CakSNP10100	*Kabuli*	Ca_Kabuli_Chr06	11508816	(A/C)
10101	CakSNP10101	*Kabuli*	Ca_Kabuli_Chr06	11514926	(T/C)
10102	CakSNP10102	*Kabuli*	Ca_Kabuli_Chr06	11514883	(C/T)
10103	CakSNP10103	*Kabuli*	Ca_Kabuli_Chr06	11514935	(G/A)
10104	CakSNP10104	*Kabuli*	Ca_Kabuli_Chr06	11543615	(G/C)
10105	CakSNP10105	*Kabuli*	Ca_Kabuli_Chr06	11543618	(T/G)
10106	CakSNP10106	*Kabuli*	Ca_Kabuli_Chr06	11665233	(G/A)
10107	CakSNP10107	*Kabuli*	Ca_Kabuli_Chr06	11665338	(T/G)
10108	CakSNP10108	*Kabuli*	Ca_Kabuli_Chr06	12408955	(A/G)
10109	CakSNP10109	*Kabuli*	Ca_Kabuli_Chr06	12408964	(G/C)
10110	CakSNP10110	*Kabuli*	Ca_Kabuli_Chr06	12434183	(G/T)
10111	CakSNP10111	*Kabuli*	Ca_Kabuli_Chr06	12437275	(A/T)
10112	CakSNP10112	*Kabuli*	Ca_Kabuli_Chr06	12437285	(A/G)
10113	CakSNP10113	*Kabuli*	Ca_Kabuli_Chr06	12437288	(G/A)
10114	CakSNP10114	*Kabuli*	Ca_Kabuli_Chr06	12437319	(C/T)
10115	CakSNP10115	*Kabuli*	Ca_Kabuli_Chr06	12494824	(G/T)
10116	CakSNP10116	*Kabuli*	Ca_Kabuli_Chr06	12494972	(A/G)
10117	CakSNP10117	*Kabuli*	Ca_Kabuli_Chr06	12532947	(T/A)
10118	CakSNP10118	*Kabuli*	Ca_Kabuli_Chr06	12535733	(C/G)
10119	CakSNP10119	*Kabuli*	Ca_Kabuli_Chr06	12619757	(C/T)
10120	CakSNP10120	*Kabuli*	Ca_Kabuli_Chr06	12619786	(C/A)
10121	CakSNP10121	*Kabuli*	Ca_Kabuli_Chr06	12619829	(A/T)
10122	CakSNP10122	*Kabuli*	Ca_Kabuli_Chr06	12647539	(G/A)
10123	CakSNP10123	*Kabuli*	Ca_Kabuli_Chr06	12799890	(T/G)
10124	CakSNP10124	*Kabuli*	Ca_Kabuli_Chr06	12844524	(G/A)
10125	CakSNP10125	*Kabuli*	Ca_Kabuli_Chr06	12893335	(C/A)
10126	CakSNP10126	*Kabuli*	Ca_Kabuli_Chr06	12893424	(C/T)
10127	CakSNP10127	*Kabuli*	Ca_Kabuli_Chr06	12906599	(G/A)
10128	CakSNP10128	*Kabuli*	Ca_Kabuli_Chr06	12906680	(T/C)
10129	CakSNP10129	*Kabuli*	Ca_Kabuli_Chr06	12906669	(G/A)
10130	CakSNP10130	*Kabuli*	Ca_Kabuli_Chr06	12973765	(A/G)
10131	CakSNP10131	*Kabuli*	Ca_Kabuli_Chr06	12976279	(T/A)
10132	CakSNP10132	*Kabuli*	Ca_Kabuli_Chr06	12977129	(A/G)
10133	CakSNP10133	*Kabuli*	Ca_Kabuli_Chr06	12977196	(A/G)
10134	CakSNP10134	*Kabuli*	Ca_Kabuli_Chr06	12977204	(T/G)
10135	CakSNP10135	*Kabuli*	Ca_Kabuli_Chr06	12985896	(A/C)
10136	CakSNP10136	*Kabuli*	Ca_Kabuli_Chr06	12985908	(C/G)
10137	CakSNP10137	*Kabuli*	Ca_Kabuli_Chr06	12985910	(C/T)
10138	CakSNP10138	*Kabuli*	Ca_Kabuli_Chr06	12986776	(A/G)

S.N.	SNP IDs	Cultivars	Chromosomes/scaffolds	Physical positions (bp)	SNPs
10139	CakSNP10139	*Kabuli*	Ca_Kabuli_Chr06	12986833	(A/C)
10140	CakSNP10140	*Kabuli*	Ca_Kabuli_Chr06	13008333	(A/T)
10141	CakSNP10141	*Kabuli*	Ca_Kabuli_Chr06	13008360	(C/T)
10142	CakSNP10142	*Kabuli*	Ca_Kabuli_Chr06	13008465	(C/T)
10143	CakSNP10143	*Kabuli*	Ca_Kabuli_Chr06	13099658	(G/T)
10144	CakSNP10144	*Kabuli*	Ca_Kabuli_Chr06	13099656	(G/T)
10145	CakSNP10145	*Kabuli*	Ca_Kabuli_Chr06	13107887	(C/T)
10146	CakSNP10146	*Kabuli*	Ca_Kabuli_Chr06	13117820	(A/T)
10147	CakSNP10147	*Kabuli*	Ca_Kabuli_Chr06	13131682	(C/G)
10148	CakSNP10148	*Kabuli*	Ca_Kabuli_Chr06	13136852	(C/T)
10149	CakSNP10149	*Kabuli*	Ca_Kabuli_Chr06	13136835	(C/T)
10150	CakSNP10150	*Kabuli*	Ca_Kabuli_Chr06	13136976	(A/G)
10151	CakSNP10151	*Kabuli*	Ca_Kabuli_Chr06	13136955	(G/A)
10152	CakSNP10152	*Kabuli*	Ca_Kabuli_Chr06	13187706	(A/G)
10153	CakSNP10153	*Kabuli*	Ca_Kabuli_Chr06	13187709	(T/C)
10154	CakSNP10154	*Kabuli*	Ca_Kabuli_Chr06	13188124	(G/C)
10155	CakSNP10155	*Kabuli*	Ca_Kabuli_Chr06	13188119	(A/C)
10156	CakSNP10156	*Kabuli*	Ca_Kabuli_Chr06	13188480	(T/C)
10157	CakSNP10157	*Kabuli*	Ca_Kabuli_Chr06	13192177	(G/A)
10158	CakSNP10158	*Kabuli*	Ca_Kabuli_Chr06	13243282	(A/G)
10159	CakSNP10159	*Kabuli*	Ca_Kabuli_Chr06	13252152	(C/T)
10160	CakSNP10160	*Kabuli*	Ca_Kabuli_Chr06	13296128	(G/A)
10161	CakSNP10161	*Kabuli*	Ca_Kabuli_Chr06	13326969	(C/G)
10162	CakSNP10162	*Kabuli*	Ca_Kabuli_Chr06	13340012	(A/G)
10163	CakSNP10163	*Kabuli*	Ca_Kabuli_Chr06	13340757	(T/C)
10164	CakSNP10164	*Kabuli*	Ca_Kabuli_Chr06	13340762	(G/A)
10165	CakSNP10165	*Kabuli*	Ca_Kabuli_Chr06	13340782	(G/A)
10166	CakSNP10166	*Kabuli*	Ca_Kabuli_Chr06	13413848	(C/G)
10167	CakSNP10167	*Kabuli*	Ca_Kabuli_Chr06	13483841	(T/G)
10168	CakSNP10168	*Kabuli*	Ca_Kabuli_Chr06	13565854	(T/C)
10169	CakSNP10169	*Kabuli*	Ca_Kabuli_Chr06	13565990	(C/T)
10170	CakSNP10170	*Kabuli*	Ca_Kabuli_Chr06	13566007	(C/T)
10171	CakSNP10171	*Kabuli*	Ca_Kabuli_Chr06	13566012	(T/G)
10172	CakSNP10172	*Kabuli*	Ca_Kabuli_Chr06	13566041	(C/T)
10173	CakSNP10173	*Kabuli*	Ca_Kabuli_Chr06	13566110	(A/G)
10174	CakSNP10174	*Kabuli*	Ca_Kabuli_Chr06	13566136	(A/C)
10175	CakSNP10175	*Kabuli*	Ca_Kabuli_Chr06	13566130	(G/T)
10176	CakSNP10176	*Kabuli*	Ca_Kabuli_Chr06	13572236	(A/T)
10177	CakSNP10177	*Kabuli*	Ca_Kabuli_Chr06	13572221	(A/C)

S.N.	SNP IDs	Cultivars	Chromosomes/scaffolds	Physical positions (bp)	SNPs
10178	CakSNP10178	*Kabuli*	Ca_Kabuli_Chr06	13576963	(T/A)
10179	CakSNP10179	*Kabuli*	Ca_Kabuli_Chr06	13591590	(T/G)
10180	CakSNP10180	*Kabuli*	Ca_Kabuli_Chr06	13593544	(A/G)
10181	CakSNP10181	*Kabuli*	Ca_Kabuli_Chr06	13593564	(C/A)
10182	CakSNP10182	*Kabuli*	Ca_Kabuli_Chr06	13593600	(T/C)
10183	CakSNP10183	*Kabuli*	Ca_Kabuli_Chr06	13593685	(G/A)
10184	CakSNP10184	*Kabuli*	Ca_Kabuli_Chr06	13593668	(A/G)
10185	CakSNP10185	*Kabuli*	Ca_Kabuli_Chr06	13593661	(T/C)
10186	CakSNP10186	*Kabuli*	Ca_Kabuli_Chr06	13593654	(T/C)
10187	CakSNP10187	*Kabuli*	Ca_Kabuli_Chr06	13619538	(C/T)
10188	CakSNP10188	*Kabuli*	Ca_Kabuli_Chr06	13619555	(C/T)
10189	CakSNP10189	*Kabuli*	Ca_Kabuli_Chr06	13619560	(T/G)
10190	CakSNP10190	*Kabuli*	Ca_Kabuli_Chr06	13619589	(C/T)
10191	CakSNP10191	*Kabuli*	Ca_Kabuli_Chr06	13619658	(A/G)
10192	CakSNP10192	*Kabuli*	Ca_Kabuli_Chr06	13619684	(A/C)
10193	CakSNP10193	*Kabuli*	Ca_Kabuli_Chr06	13622292	(C/T)
10194	CakSNP10194	*Kabuli*	Ca_Kabuli_Chr06	13625388	(A/G)
10195	CakSNP10195	*Kabuli*	Ca_Kabuli_Chr06	13637387	(T/G)
10196	CakSNP10196	*Kabuli*	Ca_Kabuli_Chr06	13637347	(C/T)
10197	CakSNP10197	*Kabuli*	Ca_Kabuli_Chr06	13668916	(T/A)
10198	CakSNP10198	*Kabuli*	Ca_Kabuli_Chr06	13668995	(A/G)
10199	CakSNP10199	*Kabuli*	Ca_Kabuli_Chr06	13669052	(G/C)
10200	CakSNP10200	*Kabuli*	Ca_Kabuli_Chr06	13669049	(C/T)
10201	CakSNP10201	*Kabuli*	Ca_Kabuli_Chr06	13669048	(T/A)
10202	CakSNP10202	*Kabuli*	Ca_Kabuli_Chr06	13669042	(A/G)
10203	CakSNP10203	*Kabuli*	Ca_Kabuli_Chr06	13669018	(G/A)
10204	CakSNP10204	*Kabuli*	Ca_Kabuli_Chr06	13669006	(G/C)
10205	CakSNP10205	*Kabuli*	Ca_Kabuli_Chr06	13714897	(A/T)
10206	CakSNP10206	*Kabuli*	Ca_Kabuli_Chr06	13764980	(T/A)
10207	CakSNP10207	*Kabuli*	Ca_Kabuli_Chr06	13827484	(C/T)
10208	CakSNP10208	*Kabuli*	Ca_Kabuli_Chr06	13827452	(C/G)
10209	CakSNP10209	*Kabuli*	Ca_Kabuli_Chr06	13827444	(G/A)
10210	CakSNP10210	*Kabuli*	Ca_Kabuli_Chr06	13913539	(A/G)
10211	CakSNP10211	*Kabuli*	Ca_Kabuli_Chr06	13917076	(T/C)
10212	CakSNP10212	*Kabuli*	Ca_Kabuli_Chr06	13917135	(T/A)
10213	CakSNP10213	*Kabuli*	Ca_Kabuli_Chr06	13921954	(A/T)
10214	CakSNP10214	*Kabuli*	Ca_Kabuli_Chr06	13941701	(A/T)
10215	CakSNP10215	*Kabuli*	Ca_Kabuli_Chr06	13942961	(A/G)
10216	CakSNP10216	*Kabuli*	Ca_Kabuli_Chr06	13948732	(G/T)

S.N.	SNP IDs	Cultivars	Chromosomes/scaffolds	Physical positions (bp)	SNPs
10217	CakSNP10217	*Kabuli*	Ca_Kabuli_Chr06	13948751	(T/G)
10218	CakSNP10218	*Kabuli*	Ca_Kabuli_Chr06	13948778	(A/C)
10219	CakSNP10219	*Kabuli*	Ca_Kabuli_Chr06	13986559	(A/G)
10220	CakSNP10220	*Kabuli*	Ca_Kabuli_Chr06	14042859	(A/T)
10221	CakSNP10221	*Kabuli*	Ca_Kabuli_Chr06	14067312	(A/C)
10222	CakSNP10222	*Kabuli*	Ca_Kabuli_Chr06	14067326	(C/T)
10223	CakSNP10223	*Kabuli*	Ca_Kabuli_Chr06	14112542	(A/G)
10224	CakSNP10224	*Kabuli*	Ca_Kabuli_Chr06	14148771	(A/G)
10225	CakSNP10225	*Kabuli*	Ca_Kabuli_Chr06	14155543	(C/T)
10226	CakSNP10226	*Kabuli*	Ca_Kabuli_Chr06	14155662	(T/C)
10227	CakSNP10227	*Kabuli*	Ca_Kabuli_Chr06	14155643	(A/C)
10228	CakSNP10228	*Kabuli*	Ca_Kabuli_Chr06	14161084	(G/C)
10229	CakSNP10229	*Kabuli*	Ca_Kabuli_Chr06	14201001	(T/C)
10230	CakSNP10230	*Kabuli*	Ca_Kabuli_Chr06	14200997	(G/A)
10231	CakSNP10231	*Kabuli*	Ca_Kabuli_Chr06	14204400	(T/C)
10232	CakSNP10232	*Kabuli*	Ca_Kabuli_Chr06	14204469	(G/A)
10233	CakSNP10233	*Kabuli*	Ca_Kabuli_Chr06	14254394	(A/G)
10234	CakSNP10234	*Kabuli*	Ca_Kabuli_Chr06	14309042	(C/T)
10235	CakSNP10235	*Kabuli*	Ca_Kabuli_Chr06	14330533	(T/C)
10236	CakSNP10236	*Kabuli*	Ca_Kabuli_Chr06	14332018	(A/C)
10237	CakSNP10237	*Kabuli*	Ca_Kabuli_Chr06	14332404	(A/G)
10238	CakSNP10238	*Kabuli*	Ca_Kabuli_Chr06	14353598	(C/T)
10239	CakSNP10239	*Kabuli*	Ca_Kabuli_Chr06	14353624	(G/A)
10240	CakSNP10240	*Kabuli*	Ca_Kabuli_Chr06	14371493	(C/T)
10241	CakSNP10241	*Kabuli*	Ca_Kabuli_Chr06	14371530	(C/T)
10242	CakSNP10242	*Kabuli*	Ca_Kabuli_Chr06	14375135	(G/A)
10243	CakSNP10243	*Kabuli*	Ca_Kabuli_Chr06	14375141	(G/C)
10244	CakSNP10244	*Kabuli*	Ca_Kabuli_Chr06	14375172	(A/G)
10245	CakSNP10245	*Kabuli*	Ca_Kabuli_Chr06	14375196	(G/A)
10246	CakSNP10246	*Kabuli*	Ca_Kabuli_Chr06	14375207	(C/T)
10247	CakSNP10247	*Kabuli*	Ca_Kabuli_Chr06	14401990	(G/A)
10248	CakSNP10248	*Kabuli*	Ca_Kabuli_Chr06	14416997	(A/G)
10249	CakSNP10249	*Kabuli*	Ca_Kabuli_Chr06	14416981	(A/G)
10250	CakSNP10250	*Kabuli*	Ca_Kabuli_Chr06	14419832	(C/T)
10251	CakSNP10251	*Kabuli*	Ca_Kabuli_Chr06	14526540	(A/T)
10252	CakSNP10252	*Kabuli*	Ca_Kabuli_Chr06	14589478	(T/G)
10253	CakSNP10253	*Kabuli*	Ca_Kabuli_Chr06	14589480	(T/C)
10254	CakSNP10254	*Kabuli*	Ca_Kabuli_Chr06	14589520	(G/C)
10255	CakSNP10255	*Kabuli*	Ca_Kabuli_Chr06	14589744	(C/T)

S.N.	SNP IDs	Cultivars	Chromosomes/scaffolds	Physical positions (bp)	SNPs
10256	CakSNP10256	*Kabuli*	Ca_Kabuli_Chr06	14589807	(T/G)
10257	CakSNP10257	*Kabuli*	Ca_Kabuli_Chr06	14589873	(T/C)
10258	CakSNP10258	*Kabuli*	Ca_Kabuli_Chr06	14606892	(A/C)
10259	CakSNP10259	*Kabuli*	Ca_Kabuli_Chr06	14628213	(A/T)
10260	CakSNP10260	*Kabuli*	Ca_Kabuli_Chr06	14628215	(T/C)
10261	CakSNP10261	*Kabuli*	Ca_Kabuli_Chr06	14628216	(A/C)
10262	CakSNP10262	*Kabuli*	Ca_Kabuli_Chr06	14628284	(G/A)
10263	CakSNP10263	*Kabuli*	Ca_Kabuli_Chr06	14719793	(T/C)
10264	CakSNP10264	*Kabuli*	Ca_Kabuli_Chr06	14719840	(G/T)
10265	CakSNP10265	*Kabuli*	Ca_Kabuli_Chr06	14747608	(T/C)
10266	CakSNP10266	*Kabuli*	Ca_Kabuli_Chr06	14753191	(C/T)
10267	CakSNP10267	*Kabuli*	Ca_Kabuli_Chr06	14753190	(G/A)
10268	CakSNP10268	*Kabuli*	Ca_Kabuli_Chr06	14753158	(A/G)
10269	CakSNP10269	*Kabuli*	Ca_Kabuli_Chr06	14753113	(C/A)
10270	CakSNP10270	*Kabuli*	Ca_Kabuli_Chr06	14753145	(C/A)
10271	CakSNP10271	*Kabuli*	Ca_Kabuli_Chr06	14753150	(G/A)
10272	CakSNP10272	*Kabuli*	Ca_Kabuli_Chr06	14762493	(A/G)
10273	CakSNP10273	*Kabuli*	Ca_Kabuli_Chr06	14771564	(G/T)
10274	CakSNP10274	*Kabuli*	Ca_Kabuli_Chr06	14771709	(G/A)
10275	CakSNP10275	*Kabuli*	Ca_Kabuli_Chr06	14786015	(T/G)
10276	CakSNP10276	*Kabuli*	Ca_Kabuli_Chr06	14789964	(A/C)
10277	CakSNP10277	*Kabuli*	Ca_Kabuli_Chr06	14846794	(C/T)
10278	CakSNP10278	*Kabuli*	Ca_Kabuli_Chr06	14857722	(C/A)
10279	CakSNP10279	*Kabuli*	Ca_Kabuli_Chr06	14857709	(A/G)
10280	CakSNP10280	*Kabuli*	Ca_Kabuli_Chr06	14857680	(G/A)
10281	CakSNP10281	*Kabuli*	Ca_Kabuli_Chr06	14861487	(T/A)
10282	CakSNP10282	*Kabuli*	Ca_Kabuli_Chr06	14884604	(T/G)
10283	CakSNP10283	*Kabuli*	Ca_Kabuli_Chr06	14884606	(T/C)
10284	CakSNP10284	*Kabuli*	Ca_Kabuli_Chr06	14891588	(T/C)
10285	CakSNP10285	*Kabuli*	Ca_Kabuli_Chr06	14891533	(T/C)
10286	CakSNP10286	*Kabuli*	Ca_Kabuli_Chr06	14891597	(C/T)
10287	CakSNP10287	*Kabuli*	Ca_Kabuli_Chr06	14891626	(C/A)
10288	CakSNP10288	*Kabuli*	Ca_Kabuli_Chr06	14941348	(C/T)
10289	CakSNP10289	*Kabuli*	Ca_Kabuli_Chr06	14951877	(C/T)
10290	CakSNP10290	*Kabuli*	Ca_Kabuli_Chr06	14951860	(G/A)
10291	CakSNP10291	*Kabuli*	Ca_Kabuli_Chr06	14982998	(T/C)
10292	CakSNP10292	*Kabuli*	Ca_Kabuli_Chr06	14983391	(C/T)
10293	CakSNP10293	*Kabuli*	Ca_Kabuli_Chr06	15024904	(C/A)
10294	CakSNP10294	*Kabuli*	Ca_Kabuli_Chr06	15024881	(C/A)

S.N.	SNP IDs	Cultivars	Chromosomes/scaffolds	Physical positions (bp)	SNPs
10295	CakSNP10295	*Kabuli*	Ca_Kabuli_Chr06	15024979	(T/G)
10296	CakSNP10296	*Kabuli*	Ca_Kabuli_Chr06	15024999	(T/A)
10297	CakSNP10297	*Kabuli*	Ca_Kabuli_Chr06	15025010	(T/C)
10298	CakSNP10298	*Kabuli*	Ca_Kabuli_Chr06	15025012	(A/C)
10299	CakSNP10299	*Kabuli*	Ca_Kabuli_Chr06	15051094	(T/C)
10300	CakSNP10300	*Kabuli*	Ca_Kabuli_Chr06	15061717	(G/A)
10301	CakSNP10301	*Kabuli*	Ca_Kabuli_Chr06	15123739	(C/T)
10302	CakSNP10302	*Kabuli*	Ca_Kabuli_Chr06	15123771	(T/C)
10303	CakSNP10303	*Kabuli*	Ca_Kabuli_Chr06	15123827	(T/G)
10304	CakSNP10304	*Kabuli*	Ca_Kabuli_Chr06	15123947	(G/A)
10305	CakSNP10305	*Kabuli*	Ca_Kabuli_Chr06	15173607	(G/A)
10306	CakSNP10306	*Kabuli*	Ca_Kabuli_Chr06	15185723	(T/C)
10307	CakSNP10307	*Kabuli*	Ca_Kabuli_Chr06	15204957	(G/C)
10308	CakSNP10308	*Kabuli*	Ca_Kabuli_Chr06	15235575	(A/G)
10309	CakSNP10309	*Kabuli*	Ca_Kabuli_Chr06	15235576	(C/T)
10310	CakSNP10310	*Kabuli*	Ca_Kabuli_Chr06	15235615	(C/T)
10311	CakSNP10311	*Kabuli*	Ca_Kabuli_Chr06	15243538	(A/T)
10312	CakSNP10312	*Kabuli*	Ca_Kabuli_Chr06	15243590	(A/G)
10313	CakSNP10313	*Kabuli*	Ca_Kabuli_Chr06	15243630	(C/T)
10314	CakSNP10314	*Kabuli*	Ca_Kabuli_Chr06	15243674	(T/A)
10315	CakSNP10315	*Kabuli*	Ca_Kabuli_Chr06	15268491	(T/G)
10316	CakSNP10316	*Kabuli*	Ca_Kabuli_Chr06	15294332	(T/G)
10317	CakSNP10317	*Kabuli*	Ca_Kabuli_Chr06	15294331	(C/T)
10318	CakSNP10318	*Kabuli*	Ca_Kabuli_Chr06	15294329	(A/C)
10319	CakSNP10319	*Kabuli*	Ca_Kabuli_Chr06	15301137	(C/T)
10320	CakSNP10320	*Kabuli*	Ca_Kabuli_Chr06	15313655	(G/T)
10321	CakSNP10321	*Kabuli*	Ca_Kabuli_Chr06	15328759	(C/A)
10322	CakSNP10322	*Kabuli*	Ca_Kabuli_Chr06	15328805	(A/T)
10323	CakSNP10323	*Kabuli*	Ca_Kabuli_Chr06	15328948	(A/C)
10324	CakSNP10324	*Kabuli*	Ca_Kabuli_Chr06	15347707	(C/T)
10325	CakSNP10325	*Kabuli*	Ca_Kabuli_Chr06	15393321	(A/T)
10326	CakSNP10326	*Kabuli*	Ca_Kabuli_Chr06	15393349	(T/C)
10327	CakSNP10327	*Kabuli*	Ca_Kabuli_Chr06	15463326	(C/G)
10328	CakSNP10328	*Kabuli*	Ca_Kabuli_Chr06	15475948	(A/G)
10329	CakSNP10329	*Kabuli*	Ca_Kabuli_Chr06	15488265	(C/T)
10330	CakSNP10330	*Kabuli*	Ca_Kabuli_Chr06	15488314	(A/T)
10331	CakSNP10331	*Kabuli*	Ca_Kabuli_Chr06	15488309	(G/A)
10332	CakSNP10332	*Kabuli*	Ca_Kabuli_Chr06	15510433	(A/T)
10333	CakSNP10333	*Kabuli*	Ca_Kabuli_Chr06	15510397	(G/A)

S.N.	SNP IDs	Cultivars	Chromosomes/scaffolds	Physical positions (bp)	SNPs
10334	CakSNP10334	*Kabuli*	Ca_Kabuli_Chr06	15537908	(A/C)
10335	CakSNP10335	*Kabuli*	Ca_Kabuli_Chr06	15543295	(C/G)
10336	CakSNP10336	*Kabuli*	Ca_Kabuli_Chr06	15543293	(T/G)
10337	CakSNP10337	*Kabuli*	Ca_Kabuli_Chr06	15543286	(G/A)
10338	CakSNP10338	*Kabuli*	Ca_Kabuli_Chr06	15550044	(C/T)
10339	CakSNP10339	*Kabuli*	Ca_Kabuli_Chr06	15589539	(A/T)
10340	CakSNP10340	*Kabuli*	Ca_Kabuli_Chr06	15589885	(G/T)
10341	CakSNP10341	*Kabuli*	Ca_Kabuli_Chr06	15635126	(A/T)
10342	CakSNP10342	*Kabuli*	Ca_Kabuli_Chr06	15644412	(A/T)
10343	CakSNP10343	*Kabuli*	Ca_Kabuli_Chr06	15644796	(A/T)
10344	CakSNP10344	*Kabuli*	Ca_Kabuli_Chr06	15857728	(T/C)
10345	CakSNP10345	*Kabuli*	Ca_Kabuli_Chr06	15861278	(G/A)
10346	CakSNP10346	*Kabuli*	Ca_Kabuli_Chr06	16113523	(T/C)
10347	CakSNP10347	*Kabuli*	Ca_Kabuli_Chr06	16113522	(A/T)
10348	CakSNP10348	*Kabuli*	Ca_Kabuli_Chr06	16113521	(G/T)
10349	CakSNP10349	*Kabuli*	Ca_Kabuli_Chr06	16115933	(T/C)
10350	CakSNP10350	*Kabuli*	Ca_Kabuli_Chr06	16147469	(A/G)
10351	CakSNP10351	*Kabuli*	Ca_Kabuli_Chr06	16148277	(A/C)
10352	CakSNP10352	*Kabuli*	Ca_Kabuli_Chr06	16189692	(C/A)
10353	CakSNP10353	*Kabuli*	Ca_Kabuli_Chr06	16189693	(G/T)
10354	CakSNP10354	*Kabuli*	Ca_Kabuli_Chr06	16195748	(T/C)
10355	CakSNP10355	*Kabuli*	Ca_Kabuli_Chr06	16196582	(G/C)
10356	CakSNP10356	*Kabuli*	Ca_Kabuli_Chr06	16315552	(T/A)
10357	CakSNP10357	*Kabuli*	Ca_Kabuli_Chr06	16332377	(G/A)
10358	CakSNP10358	*Kabuli*	Ca_Kabuli_Chr06	16353698	(G/A)
10359	CakSNP10359	*Kabuli*	Ca_Kabuli_Chr06	16450107	(C/T)
10360	CakSNP10360	*Kabuli*	Ca_Kabuli_Chr06	16460495	(A/C)
10361	CakSNP10361	*Kabuli*	Ca_Kabuli_Chr06	16561334	(C/T)
10362	CakSNP10362	*Kabuli*	Ca_Kabuli_Chr06	16635061	(C/T)
10363	CakSNP10363	*Kabuli*	Ca_Kabuli_Chr06	16646769	(A/T)
10364	CakSNP10364	*Kabuli*	Ca_Kabuli_Chr06	16646713	(T/G)
10365	CakSNP10365	*Kabuli*	Ca_Kabuli_Chr06	16665385	(G/C)
10366	CakSNP10366	*Kabuli*	Ca_Kabuli_Chr06	16665477	(T/C)
10367	CakSNP10367	*Kabuli*	Ca_Kabuli_Chr06	16665829	(G/C)
10368	CakSNP10368	*Kabuli*	Ca_Kabuli_Chr06	16677320	(C/A)
10369	CakSNP10369	*Kabuli*	Ca_Kabuli_Chr06	16717984	(G/A)
10370	CakSNP10370	*Kabuli*	Ca_Kabuli_Chr06	16718201	(G/A)
10371	CakSNP10371	*Kabuli*	Ca_Kabuli_Chr06	16719918	(C/A)
10372	CakSNP10372	*Kabuli*	Ca_Kabuli_Chr06	16719919	(T/G)

S.N.	SNP IDs	Cultivars	Chromosomes/scaffolds	Physical positions (bp)	SNPs
10373	CakSNP10373	*Kabuli*	Ca_Kabuli_Chr06	16741672	(C/T)
10374	CakSNP10374	*Kabuli*	Ca_Kabuli_Chr06	16748675	(A/C)
10375	CakSNP10375	*Kabuli*	Ca_Kabuli_Chr06	16768782	(G/A)
10376	CakSNP10376	*Kabuli*	Ca_Kabuli_Chr06	16823954	(A/G)
10377	CakSNP10377	*Kabuli*	Ca_Kabuli_Chr06	16932014	(C/G)
10378	CakSNP10378	*Kabuli*	Ca_Kabuli_Chr06	17048493	(A/C)
10379	CakSNP10379	*Kabuli*	Ca_Kabuli_Chr06	17096214	(C/T)
10380	CakSNP10380	*Kabuli*	Ca_Kabuli_Chr06	17096215	(G/A)
10381	CakSNP10381	*Kabuli*	Ca_Kabuli_Chr06	17096224	(C/T)
10382	CakSNP10382	*Kabuli*	Ca_Kabuli_Chr06	17129574	(A/G)
10383	CakSNP10383	*Kabuli*	Ca_Kabuli_Chr06	17148605	(G/A)
10384	CakSNP10384	*Kabuli*	Ca_Kabuli_Chr06	17148773	(A/G)
10385	CakSNP10385	*Kabuli*	Ca_Kabuli_Chr06	17175334	(C/A)
10386	CakSNP10386	*Kabuli*	Ca_Kabuli_Chr06	17175446	(G/A)
10387	CakSNP10387	*Kabuli*	Ca_Kabuli_Chr06	17258827	(C/A)
10388	CakSNP10388	*Kabuli*	Ca_Kabuli_Chr06	17258886	(T/G)
10389	CakSNP10389	*Kabuli*	Ca_Kabuli_Chr06	17262283	(C/T)
10390	CakSNP10390	*Kabuli*	Ca_Kabuli_Chr06	17263126	(G/A)
10391	CakSNP10391	*Kabuli*	Ca_Kabuli_Chr06	17369613	(A/G)
10392	CakSNP10392	*Kabuli*	Ca_Kabuli_Chr06	17445945	(T/G)
10393	CakSNP10393	*Kabuli*	Ca_Kabuli_Chr06	17477741	(A/G)
10394	CakSNP10394	*Kabuli*	Ca_Kabuli_Chr06	17478220	(G/A)
10395	CakSNP10395	*Kabuli*	Ca_Kabuli_Chr06	17480486	(G/C)
10396	CakSNP10396	*Kabuli*	Ca_Kabuli_Chr06	17481288	(T/C)
10397	CakSNP10397	*Kabuli*	Ca_Kabuli_Chr06	17481307	(C/A)
10398	CakSNP10398	*Kabuli*	Ca_Kabuli_Chr06	17481403	(T/G)
10399	CakSNP10399	*Kabuli*	Ca_Kabuli_Chr06	17481392	(A/G)
10400	CakSNP10400	*Kabuli*	Ca_Kabuli_Chr06	17482626	(A/G)
10401	CakSNP10401	*Kabuli*	Ca_Kabuli_Chr06	17482640	(C/T)
10402	CakSNP10402	*Kabuli*	Ca_Kabuli_Chr06	17482649	(A/G)
10403	CakSNP10403	*Kabuli*	Ca_Kabuli_Chr06	17482718	(G/A)
10404	CakSNP10404	*Kabuli*	Ca_Kabuli_Chr06	17482697	(A/G)
10405	CakSNP10405	*Kabuli*	Ca_Kabuli_Chr06	17573309	(A/G)
10406	CakSNP10406	*Kabuli*	Ca_Kabuli_Chr06	17659067	(A/C)
10407	CakSNP10407	*Kabuli*	Ca_Kabuli_Chr06	17741078	(G/A)
10408	CakSNP10408	*Kabuli*	Ca_Kabuli_Chr06	18027391	(G/A)
10409	CakSNP10409	*Kabuli*	Ca_Kabuli_Chr06	18094691	(C/A)
10410	CakSNP10410	*Kabuli*	Ca_Kabuli_Chr06	18125613	(C/A)
10411	CakSNP10411	*Kabuli*	Ca_Kabuli_Chr06	18232696	(G/A)

S.N.	SNP IDs	Cultivars	Chromosomes/scaffolds	Physical positions (bp)	SNPs
10412	CakSNP10412	*Kabuli*	Ca_Kabuli_Chr06	18268960	(G/A)
10413	CakSNP10413	*Kabuli*	Ca_Kabuli_Chr06	18268982	(A/G)
10414	CakSNP10414	*Kabuli*	Ca_Kabuli_Chr06	18389704	(T/G)
10415	CakSNP10415	*Kabuli*	Ca_Kabuli_Chr06	18389661	(A/C)
10416	CakSNP10416	*Kabuli*	Ca_Kabuli_Chr06	18444725	(T/C)
10417	CakSNP10417	*Kabuli*	Ca_Kabuli_Chr06	18549073	(T/C)
10418	CakSNP10418	*Kabuli*	Ca_Kabuli_Chr06	18562366	(C/A)
10419	CakSNP10419	*Kabuli*	Ca_Kabuli_Chr06	18664114	(A/T)
10420	CakSNP10420	*Kabuli*	Ca_Kabuli_Chr06	18747207	(G/A)
10421	CakSNP10421	*Kabuli*	Ca_Kabuli_Chr06	18752554	(G/T)
10422	CakSNP10422	*Kabuli*	Ca_Kabuli_Chr06	18767357	(G/A)
10423	CakSNP10423	*Kabuli*	Ca_Kabuli_Chr06	18771093	(T/A)
10424	CakSNP10424	*Kabuli*	Ca_Kabuli_Chr06	18798747	(A/C)
10425	CakSNP10425	*Kabuli*	Ca_Kabuli_Chr06	18852806	(T/C)
10426	CakSNP10426	*Kabuli*	Ca_Kabuli_Chr06	18852854	(T/C)
10427	CakSNP10427	*Kabuli*	Ca_Kabuli_Chr06	18876221	(T/C)
10428	CakSNP10428	*Kabuli*	Ca_Kabuli_Chr06	18876213	(A/G)
10429	CakSNP10429	*Kabuli*	Ca_Kabuli_Chr06	18899989	(C/A)
10430	CakSNP10430	*Kabuli*	Ca_Kabuli_Chr06	18900031	(C/T)
10431	CakSNP10431	*Kabuli*	Ca_Kabuli_Chr06	18900034	(G/A)
10432	CakSNP10432	*Kabuli*	Ca_Kabuli_Chr06	18916626	(G/T)
10433	CakSNP10433	*Kabuli*	Ca_Kabuli_Chr06	18916619	(C/T)
10434	CakSNP10434	*Kabuli*	Ca_Kabuli_Chr06	18925002	(A/G)
10435	CakSNP10435	*Kabuli*	Ca_Kabuli_Chr06	18938479	(C/T)
10436	CakSNP10436	*Kabuli*	Ca_Kabuli_Chr06	18938512	(G/A)
10437	CakSNP10437	*Kabuli*	Ca_Kabuli_Chr06	18956194	(G/A)
10438	CakSNP10438	*Kabuli*	Ca_Kabuli_Chr06	18987220	(T/G)
10439	CakSNP10439	*Kabuli*	Ca_Kabuli_Chr06	18999175	(C/T)
10440	CakSNP10440	*Kabuli*	Ca_Kabuli_Chr06	19033068	(A/C)
10441	CakSNP10441	*Kabuli*	Ca_Kabuli_Chr06	19033309	(G/A)
10442	CakSNP10442	*Kabuli*	Ca_Kabuli_Chr06	19034129	(A/G)
10443	CakSNP10443	*Kabuli*	Ca_Kabuli_Chr06	19125254	(G/A)
10444	CakSNP10444	*Kabuli*	Ca_Kabuli_Chr06	19125222	(T/C)
10445	CakSNP10445	*Kabuli*	Ca_Kabuli_Chr06	19129651	(T/C)
10446	CakSNP10446	*Kabuli*	Ca_Kabuli_Chr06	19169687	(T/C)
10447	CakSNP10447	*Kabuli*	Ca_Kabuli_Chr06	19195656	(A/T)
10448	CakSNP10448	*Kabuli*	Ca_Kabuli_Chr06	19195659	(A/C)
10449	CakSNP10449	*Kabuli*	Ca_Kabuli_Chr06	19198386	(G/A)
10450	CakSNP10450	*Kabuli*	Ca_Kabuli_Chr06	19217820	(G/A)

S.N.	SNP IDs	Cultivars	Chromosomes/scaffolds	Physical positions (bp)	SNPs
10451	CakSNP10451	*Kabuli*	Ca_Kabuli_Chr06	19304834	(A/G)
10452	CakSNP10452	*Kabuli*	Ca_Kabuli_Chr06	19440988	(T/C)
10453	CakSNP10453	*Kabuli*	Ca_Kabuli_Chr06	19441372	(T/C)
10454	CakSNP10454	*Kabuli*	Ca_Kabuli_Chr06	19450845	(T/A)
10455	CakSNP10455	*Kabuli*	Ca_Kabuli_Chr06	19458843	(C/T)
10456	CakSNP10456	*Kabuli*	Ca_Kabuli_Chr06	19458898	(T/A)
10457	CakSNP10457	*Kabuli*	Ca_Kabuli_Chr06	19458908	(A/T)
10458	CakSNP10458	*Kabuli*	Ca_Kabuli_Chr06	19476915	(T/C)
10459	CakSNP10459	*Kabuli*	Ca_Kabuli_Chr06	19476916	(A/G)
10460	CakSNP10460	*Kabuli*	Ca_Kabuli_Chr06	19477006	(T/C)
10461	CakSNP10461	*Kabuli*	Ca_Kabuli_Chr06	19478493	(T/A)
10462	CakSNP10462	*Kabuli*	Ca_Kabuli_Chr06	19494588	(G/T)
10463	CakSNP10463	*Kabuli*	Ca_Kabuli_Chr06	19494584	(C/A)
10464	CakSNP10464	*Kabuli*	Ca_Kabuli_Chr06	19551459	(G/A)
10465	CakSNP10465	*Kabuli*	Ca_Kabuli_Chr06	19551504	(G/C)
10466	CakSNP10466	*Kabuli*	Ca_Kabuli_Chr06	19556904	(G/A)
10467	CakSNP10467	*Kabuli*	Ca_Kabuli_Chr06	19557045	(C/T)
10468	CakSNP10468	*Kabuli*	Ca_Kabuli_Chr06	19578972	(T/C)
10469	CakSNP10469	*Kabuli*	Ca_Kabuli_Chr06	19725007	(T/A)
10470	CakSNP10470	*Kabuli*	Ca_Kabuli_Chr06	20505641	(A/T)
10471	CakSNP10471	*Kabuli*	Ca_Kabuli_Chr06	20558142	(C/T)
10472	CakSNP10472	*Kabuli*	Ca_Kabuli_Chr06	20558138	(G/A)
10473	CakSNP10473	*Kabuli*	Ca_Kabuli_Chr06	20660779	(T/C)
10474	CakSNP10474	*Kabuli*	Ca_Kabuli_Chr06	20660782	(T/C)
10475	CakSNP10475	*Kabuli*	Ca_Kabuli_Chr06	20660819	(T/C)
10476	CakSNP10476	*Kabuli*	Ca_Kabuli_Chr06	20660820	(T/A)
10477	CakSNP10477	*Kabuli*	Ca_Kabuli_Chr06	20660841	(A/G)
10478	CakSNP10478	*Kabuli*	Ca_Kabuli_Chr06	20660870	(G/C)
10479	CakSNP10479	*Kabuli*	Ca_Kabuli_Chr06	20671043	(T/C)
10480	CakSNP10480	*Kabuli*	Ca_Kabuli_Chr06	20739258	(A/T)
10481	CakSNP10481	*Kabuli*	Ca_Kabuli_Chr06	20739340	(A/G)
10482	CakSNP10482	*Kabuli*	Ca_Kabuli_Chr06	20766342	(T/A)
10483	CakSNP10483	*Kabuli*	Ca_Kabuli_Chr06	20766377	(C/A)
10484	CakSNP10484	*Kabuli*	Ca_Kabuli_Chr06	20766397	(G/T)
10485	CakSNP10485	*Kabuli*	Ca_Kabuli_Chr06	20770728	(C/T)
10486	CakSNP10486	*Kabuli*	Ca_Kabuli_Chr06	20776473	(A/G)
10487	CakSNP10487	*Kabuli*	Ca_Kabuli_Chr06	20786079	(T/A)
10488	CakSNP10488	*Kabuli*	Ca_Kabuli_Chr06	20802747	(G/C)
10489	CakSNP10489	*Kabuli*	Ca_Kabuli_Chr06	20830353	(T/G)

S.N.	SNP IDs	Cultivars	Chromosomes/scaffolds	Physical positions (bp)	SNPs
10490	CakSNP10490	*Kabuli*	Ca_Kabuli_Chr06	20830634	(T/A)
10491	CakSNP10491	*Kabuli*	Ca_Kabuli_Chr06	20890714	(A/G)
10492	CakSNP10492	*Kabuli*	Ca_Kabuli_Chr06	20917713	(A/C)
10493	CakSNP10493	*Kabuli*	Ca_Kabuli_Chr06	20917694	(A/T)
10494	CakSNP10494	*Kabuli*	Ca_Kabuli_Chr06	21025495	(T/A)
10495	CakSNP10495	*Kabuli*	Ca_Kabuli_Chr06	21025494	(C/T)
10496	CakSNP10496	*Kabuli*	Ca_Kabuli_Chr06	21025540	(C/T)
10497	CakSNP10497	*Kabuli*	Ca_Kabuli_Chr06	21025677	(T/A)
10498	CakSNP10498	*Kabuli*	Ca_Kabuli_Chr06	21025742	(T/G)
10499	CakSNP10499	*Kabuli*	Ca_Kabuli_Chr06	21025706	(C/T)
10500	CakSNP10500	*Kabuli*	Ca_Kabuli_Chr06	21025673	(C/T)
10501	CakSNP10501	*Kabuli*	Ca_Kabuli_Chr06	21025680	(C/T)
10502	CakSNP10502	*Kabuli*	Ca_Kabuli_Chr06	21025682	(C/T)
10503	CakSNP10503	*Kabuli*	Ca_Kabuli_Chr06	21025712	(C/T)
10504	CakSNP10504	*Kabuli*	Ca_Kabuli_Chr06	21025718	(C/A)
10505	CakSNP10505	*Kabuli*	Ca_Kabuli_Chr06	21025894	(G/T)
10506	CakSNP10506	*Kabuli*	Ca_Kabuli_Chr06	21054758	(T/C)
10507	CakSNP10507	*Kabuli*	Ca_Kabuli_Chr06	21058286	(G/T)
10508	CakSNP10508	*Kabuli*	Ca_Kabuli_Chr06	21135764	(C/T)
10509	CakSNP10509	*Kabuli*	Ca_Kabuli_Chr06	21135996	(C/T)
10510	CakSNP10510	*Kabuli*	Ca_Kabuli_Chr06	21139038	(C/G)
10511	CakSNP10511	*Kabuli*	Ca_Kabuli_Chr06	21165963	(A/C)
10512	CakSNP10512	*Kabuli*	Ca_Kabuli_Chr06	21165999	(G/T)
10513	CakSNP10513	*Kabuli*	Ca_Kabuli_Chr06	21170382	(G/A)
10514	CakSNP10514	*Kabuli*	Ca_Kabuli_Chr06	21170401	(C/A)
10515	CakSNP10515	*Kabuli*	Ca_Kabuli_Chr06	21177330	(C/A)
10516	CakSNP10516	*Kabuli*	Ca_Kabuli_Chr06	21238178	(A/G)
10517	CakSNP10517	*Kabuli*	Ca_Kabuli_Chr06	21238711	(C/T)
10518	CakSNP10518	*Kabuli*	Ca_Kabuli_Chr06	21238707	(G/T)
10519	CakSNP10519	*Kabuli*	Ca_Kabuli_Chr06	21276479	(G/T)
10520	CakSNP10520	*Kabuli*	Ca_Kabuli_Chr06	21276551	(C/G)
10521	CakSNP10521	*Kabuli*	Ca_Kabuli_Chr06	21323438	(T/C)
10522	CakSNP10522	*Kabuli*	Ca_Kabuli_Chr06	21323390	(C/G)
10523	CakSNP10523	*Kabuli*	Ca_Kabuli_Chr06	21367869	(A/G)
10524	CakSNP10524	*Kabuli*	Ca_Kabuli_Chr06	21367830	(T/A)
10525	CakSNP10525	*Kabuli*	Ca_Kabuli_Chr06	21368163	(C/T)
10526	CakSNP10526	*Kabuli*	Ca_Kabuli_Chr06	21385915	(C/A)
10527	CakSNP10527	*Kabuli*	Ca_Kabuli_Chr06	21478820	(G/T)
10528	CakSNP10528	*Kabuli*	Ca_Kabuli_Chr06	21478816	(C/T)

S.N.	SNP IDs	Cultivars	Chromosomes/scaffolds	Physical positions (bp)	SNPs
10529	CakSNP10529	*Kabuli*	Ca_Kabuli_Chr06	21483425	(T/G)
10530	CakSNP10530	*Kabuli*	Ca_Kabuli_Chr06	21483429	(C/T)
10531	CakSNP10531	*Kabuli*	Ca_Kabuli_Chr06	21483494	(T/G)
10532	CakSNP10532	*Kabuli*	Ca_Kabuli_Chr06	21489498	(A/G)
10533	CakSNP10533	*Kabuli*	Ca_Kabuli_Chr06	21506224	(A/C)
10534	CakSNP10534	*Kabuli*	Ca_Kabuli_Chr06	21540338	(A/G)
10535	CakSNP10535	*Kabuli*	Ca_Kabuli_Chr06	21540382	(T/G)
10536	CakSNP10536	*Kabuli*	Ca_Kabuli_Chr06	21576745	(G/A)
10537	CakSNP10537	*Kabuli*	Ca_Kabuli_Chr06	21602874	(C/T)
10538	CakSNP10538	*Kabuli*	Ca_Kabuli_Chr06	21603102	(G/C)
10539	CakSNP10539	*Kabuli*	Ca_Kabuli_Chr06	21675734	(T/G)
10540	CakSNP10540	*Kabuli*	Ca_Kabuli_Chr06	21675757	(A/G)
10541	CakSNP10541	*Kabuli*	Ca_Kabuli_Chr06	21675762	(G/T)
10542	CakSNP10542	*Kabuli*	Ca_Kabuli_Chr06	21675816	(C/G)
10543	CakSNP10543	*Kabuli*	Ca_Kabuli_Chr06	21718674	(G/T)
10544	CakSNP10544	*Kabuli*	Ca_Kabuli_Chr06	21756666	(A/T)
10545	CakSNP10545	*Kabuli*	Ca_Kabuli_Chr06	21756706	(C/T)
10546	CakSNP10546	*Kabuli*	Ca_Kabuli_Chr06	21763468	(G/A)
10547	CakSNP10547	*Kabuli*	Ca_Kabuli_Chr06	21763611	(C/T)
10548	CakSNP10548	*Kabuli*	Ca_Kabuli_Chr06	21763582	(C/T)
10549	CakSNP10549	*Kabuli*	Ca_Kabuli_Chr06	21804333	(T/A)
10550	CakSNP10550	*Kabuli*	Ca_Kabuli_Chr06	21804337	(G/C)
10551	CakSNP10551	*Kabuli*	Ca_Kabuli_Chr06	21834111	(C/G)
10552	CakSNP10552	*Kabuli*	Ca_Kabuli_Chr06	21861700	(T/A)
10553	CakSNP10553	*Kabuli*	Ca_Kabuli_Chr06	21914387	(T/C)
10554	CakSNP10554	*Kabuli*	Ca_Kabuli_Chr06	21914380	(C/T)
10555	CakSNP10555	*Kabuli*	Ca_Kabuli_Chr06	21916465	(G/A)
10556	CakSNP10556	*Kabuli*	Ca_Kabuli_Chr06	21916476	(T/C)
10557	CakSNP10557	*Kabuli*	Ca_Kabuli_Chr06	21940793	(T/C)
10558	CakSNP10558	*Kabuli*	Ca_Kabuli_Chr06	21979846	(G/A)
10559	CakSNP10559	*Kabuli*	Ca_Kabuli_Chr06	21979860	(C/T)
10560	CakSNP10560	*Kabuli*	Ca_Kabuli_Chr06	21979873	(A/G)
10561	CakSNP10561	*Kabuli*	Ca_Kabuli_Chr06	21979887	(G/A)
10562	CakSNP10562	*Kabuli*	Ca_Kabuli_Chr06	21979889	(A/G)
10563	CakSNP10563	*Kabuli*	Ca_Kabuli_Chr06	21979893	(C/T)
10564	CakSNP10564	*Kabuli*	Ca_Kabuli_Chr06	21979903	(T/C)
10565	CakSNP10565	*Kabuli*	Ca_Kabuli_Chr06	22015915	(G/T)
10566	CakSNP10566	*Kabuli*	Ca_Kabuli_Chr06	22016086	(A/G)
10567	CakSNP10567	*Kabuli*	Ca_Kabuli_Chr06	22016767	(C/T)

S.N.	SNP IDs	Cultivars	Chromosomes/scaffolds	Physical positions (bp)	SNPs
10568	CakSNP10568	*Kabuli*	Ca_Kabuli_Chr06	22016839	(A/G)
10569	CakSNP10569	*Kabuli*	Ca_Kabuli_Chr06	22038857	(A/G)
10570	CakSNP10570	*Kabuli*	Ca_Kabuli_Chr06	22038906	(A/T)
10571	CakSNP10571	*Kabuli*	Ca_Kabuli_Chr06	22074809	(T/C)
10572	CakSNP10572	*Kabuli*	Ca_Kabuli_Chr06	22075447	(C/G)
10573	CakSNP10573	*Kabuli*	Ca_Kabuli_Chr06	22079148	(A/G)
10574	CakSNP10574	*Kabuli*	Ca_Kabuli_Chr06	22137477	(A/T)
10575	CakSNP10575	*Kabuli*	Ca_Kabuli_Chr06	22137464	(G/A)
10576	CakSNP10576	*Kabuli*	Ca_Kabuli_Chr06	22137569	(G/T)
10577	CakSNP10577	*Kabuli*	Ca_Kabuli_Chr06	22175547	(A/G)
10578	CakSNP10578	*Kabuli*	Ca_Kabuli_Chr06	22192503	(G/A)
10579	CakSNP10579	*Kabuli*	Ca_Kabuli_Chr06	22192498	(A/C)
10580	CakSNP10580	*Kabuli*	Ca_Kabuli_Chr06	22194387	(C/A)
10581	CakSNP10581	*Kabuli*	Ca_Kabuli_Chr06	22201688	(C/T)
10582	CakSNP10582	*Kabuli*	Ca_Kabuli_Chr06	22206341	(G/T)
10583	CakSNP10583	*Kabuli*	Ca_Kabuli_Chr06	22208130	(G/C)
10584	CakSNP10584	*Kabuli*	Ca_Kabuli_Chr06	22208125	(G/A)
10585	CakSNP10585	*Kabuli*	Ca_Kabuli_Chr06	22208093	(G/A)
10586	CakSNP10586	*Kabuli*	Ca_Kabuli_Chr06	22211721	(C/T)
10587	CakSNP10587	*Kabuli*	Ca_Kabuli_Chr06	22211823	(T/C)
10588	CakSNP10588	*Kabuli*	Ca_Kabuli_Chr06	22217158	(A/G)
10589	CakSNP10589	*Kabuli*	Ca_Kabuli_Chr06	22217157	(T/G)
10590	CakSNP10590	*Kabuli*	Ca_Kabuli_Chr06	22217107	(G/A)
10591	CakSNP10591	*Kabuli*	Ca_Kabuli_Chr06	22225288	(T/G)
10592	CakSNP10592	*Kabuli*	Ca_Kabuli_Chr06	22235449	(G/A)
10593	CakSNP10593	*Kabuli*	Ca_Kabuli_Chr06	22235421	(T/A)
10594	CakSNP10594	*Kabuli*	Ca_Kabuli_Chr06	22236475	(T/A)
10595	CakSNP10595	*Kabuli*	Ca_Kabuli_Chr06	22252149	(G/A)
10596	CakSNP10596	*Kabuli*	Ca_Kabuli_Chr06	22262999	(A/C)
10597	CakSNP10597	*Kabuli*	Ca_Kabuli_Chr06	22263011	(T/C)
10598	CakSNP10598	*Kabuli*	Ca_Kabuli_Chr06	22263388	(A/G)
10599	CakSNP10599	*Kabuli*	Ca_Kabuli_Chr06	22263452	(A/T)
10600	CakSNP10600	*Kabuli*	Ca_Kabuli_Chr06	22279906	(T/G)
10601	CakSNP10601	*Kabuli*	Ca_Kabuli_Chr06	22287922	(C/T)
10602	CakSNP10602	*Kabuli*	Ca_Kabuli_Chr06	22334733	(C/T)
10603	CakSNP10603	*Kabuli*	Ca_Kabuli_Chr06	22352976	(T/C)
10604	CakSNP10604	*Kabuli*	Ca_Kabuli_Chr06	22370242	(C/T)
10605	CakSNP10605	*Kabuli*	Ca_Kabuli_Chr06	22371908	(A/C)
10606	CakSNP10606	*Kabuli*	Ca_Kabuli_Chr06	22446629	(G/A)

S.N.	SNP IDs	Cultivars	Chromosomes/scaffolds	Physical positions (bp)	SNPs
10607	CakSNP10607	*Kabuli*	Ca_Kabuli_Chr06	22446621	(A/C)
10608	CakSNP10608	*Kabuli*	Ca_Kabuli_Chr06	22446610	(G/A)
10609	CakSNP10609	*Kabuli*	Ca_Kabuli_Chr06	22446570	(C/T)
10610	CakSNP10610	*Kabuli*	Ca_Kabuli_Chr06	22446649	(C/T)
10611	CakSNP10611	*Kabuli*	Ca_Kabuli_Chr06	22446644	(C/T)
10612	CakSNP10612	*Kabuli*	Ca_Kabuli_Chr06	22482366	(A/C)
10613	CakSNP10613	*Kabuli*	Ca_Kabuli_Chr06	22498553	(T/C)
10614	CakSNP10614	*Kabuli*	Ca_Kabuli_Chr06	22498867	(T/C)
10615	CakSNP10615	*Kabuli*	Ca_Kabuli_Chr06	22518747	(G/T)
10616	CakSNP10616	*Kabuli*	Ca_Kabuli_Chr06	22879982	(C/T)
10617	CakSNP10617	*Kabuli*	Ca_Kabuli_Chr06	22881176	(A/T)
10618	CakSNP10618	*Kabuli*	Ca_Kabuli_Chr06	22933938	(T/A)
10619	CakSNP10619	*Kabuli*	Ca_Kabuli_Chr06	22952185	(G/A)
10620	CakSNP10620	*Kabuli*	Ca_Kabuli_Chr06	22952253	(C/T)
10621	CakSNP10621	*Kabuli*	Ca_Kabuli_Chr06	22966001	(C/A)
10622	CakSNP10622	*Kabuli*	Ca_Kabuli_Chr06	22986891	(T/C)
10623	CakSNP10623	*Kabuli*	Ca_Kabuli_Chr06	22986869	(G/C)
10624	CakSNP10624	*Kabuli*	Ca_Kabuli_Chr06	23014724	(T/A)
10625	CakSNP10625	*Kabuli*	Ca_Kabuli_Chr06	23039165	(T/C)
10626	CakSNP10626	*Kabuli*	Ca_Kabuli_Chr06	23039147	(G/A)
10627	CakSNP10627	*Kabuli*	Ca_Kabuli_Chr06	23039145	(A/G)
10628	CakSNP10628	*Kabuli*	Ca_Kabuli_Chr06	23039125	(G/A)
10629	CakSNP10629	*Kabuli*	Ca_Kabuli_Chr06	23039097	(C/T)
10630	CakSNP10630	*Kabuli*	Ca_Kabuli_Chr06	23046076	(C/T)
10631	CakSNP10631	*Kabuli*	Ca_Kabuli_Chr06	23113706	(A/G)
10632	CakSNP10632	*Kabuli*	Ca_Kabuli_Chr06	23129516	(A/G)
10633	CakSNP10633	*Kabuli*	Ca_Kabuli_Chr06	23129504	(C/G)
10634	CakSNP10634	*Kabuli*	Ca_Kabuli_Chr06	23145532	(T/A)
10635	CakSNP10635	*Kabuli*	Ca_Kabuli_Chr06	23166355	(A/C)
10636	CakSNP10636	*Kabuli*	Ca_Kabuli_Chr06	23253687	(G/A)
10637	CakSNP10637	*Kabuli*	Ca_Kabuli_Chr06	23253973	(G/C)
10638	CakSNP10638	*Kabuli*	Ca_Kabuli_Chr06	23254642	(A/G)
10639	CakSNP10639	*Kabuli*	Ca_Kabuli_Chr06	23256848	(C/G)
10640	CakSNP10640	*Kabuli*	Ca_Kabuli_Chr06	23256891	(T/G)
10641	CakSNP10641	*Kabuli*	Ca_Kabuli_Chr06	23380746	(G/A)
10642	CakSNP10642	*Kabuli*	Ca_Kabuli_Chr06	23393426	(T/C)
10643	CakSNP10643	*Kabuli*	Ca_Kabuli_Chr06	23393446	(A/G)
10644	CakSNP10644	*Kabuli*	Ca_Kabuli_Chr06	23393469	(T/C)
10645	CakSNP10645	*Kabuli*	Ca_Kabuli_Chr06	23393491	(T/G)

S.N.	SNP IDs	Cultivars	Chromosomes/scaffolds	Physical positions (bp)	SNPs
10646	CakSNP10646	*Kabuli*	Ca_Kabuli_Chr06	23399103	(A/G)
10647	CakSNP10647	*Kabuli*	Ca_Kabuli_Chr06	23424061	(T/G)
10648	CakSNP10648	*Kabuli*	Ca_Kabuli_Chr06	23549220	(A/T)
10649	CakSNP10649	*Kabuli*	Ca_Kabuli_Chr06	23638882	(A/G)
10650	CakSNP10650	*Kabuli*	Ca_Kabuli_Chr06	23663204	(C/A)
10651	CakSNP10651	*Kabuli*	Ca_Kabuli_Chr06	23663201	(T/C)
10652	CakSNP10652	*Kabuli*	Ca_Kabuli_Chr06	23663180	(G/T)
10653	CakSNP10653	*Kabuli*	Ca_Kabuli_Chr06	23688740	(C/T)
10654	CakSNP10654	*Kabuli*	Ca_Kabuli_Chr06	23688723	(A/G)
10655	CakSNP10655	*Kabuli*	Ca_Kabuli_Chr06	23691440	(A/C)
10656	CakSNP10656	*Kabuli*	Ca_Kabuli_Chr06	23766943	(C/T)
10657	CakSNP10657	*Kabuli*	Ca_Kabuli_Chr06	23768142	(A/T)
10658	CakSNP10658	*Kabuli*	Ca_Kabuli_Chr06	23809120	(G/A)
10659	CakSNP10659	*Kabuli*	Ca_Kabuli_Chr06	23890836	(A/G)
10660	CakSNP10660	*Kabuli*	Ca_Kabuli_Chr06	23891065	(G/A)
10661	CakSNP10661	*Kabuli*	Ca_Kabuli_Chr06	23916260	(A/G)
10662	CakSNP10662	*Kabuli*	Ca_Kabuli_Chr06	23963812	(T/C)
10663	CakSNP10663	*Kabuli*	Ca_Kabuli_Chr06	23973410	(G/A)
10664	CakSNP10664	*Kabuli*	Ca_Kabuli_Chr06	24007185	(C/T)
10665	CakSNP10665	*Kabuli*	Ca_Kabuli_Chr06	24007863	(T/C)
10666	CakSNP10666	*Kabuli*	Ca_Kabuli_Chr06	24061578	(G/T)
10667	CakSNP10667	*Kabuli*	Ca_Kabuli_Chr06	24085747	(G/T)
10668	CakSNP10668	*Kabuli*	Ca_Kabuli_Chr06	24085795	(C/T)
10669	CakSNP10669	*Kabuli*	Ca_Kabuli_Chr06	24085807	(A/T)
10670	CakSNP10670	*Kabuli*	Ca_Kabuli_Chr06	24138357	(A/G)
10671	CakSNP10671	*Kabuli*	Ca_Kabuli_Chr06	24138388	(T/C)
10672	CakSNP10672	*Kabuli*	Ca_Kabuli_Chr06	24174940	(C/T)
10673	CakSNP10673	*Kabuli*	Ca_Kabuli_Chr06	24260527	(T/C)
10674	CakSNP10674	*Kabuli*	Ca_Kabuli_Chr06	24301508	(T/A)
10675	CakSNP10675	*Kabuli*	Ca_Kabuli_Chr06	24302205	(A/G)
10676	CakSNP10676	*Kabuli*	Ca_Kabuli_Chr06	24381949	(A/G)
10677	CakSNP10677	*Kabuli*	Ca_Kabuli_Chr06	24381937	(G/A)
10678	CakSNP10678	*Kabuli*	Ca_Kabuli_Chr06	24403316	(G/T)
10679	CakSNP10679	*Kabuli*	Ca_Kabuli_Chr06	24413675	(T/C)
10680	CakSNP10680	*Kabuli*	Ca_Kabuli_Chr06	24413714	(G/A)
10681	CakSNP10681	*Kabuli*	Ca_Kabuli_Chr06	24414512	(G/C)
10682	CakSNP10682	*Kabuli*	Ca_Kabuli_Chr06	24454583	(C/T)
10683	CakSNP10683	*Kabuli*	Ca_Kabuli_Chr06	24454557	(T/C)
10684	CakSNP10684	*Kabuli*	Ca_Kabuli_Chr06	24516523	(A/T)

S.N.	SNP IDs	Cultivars	Chromosomes/scaffolds	Physical positions (bp)	SNPs
10685	CakSNP10685	*Kabuli*	Ca_Kabuli_Chr06	24516528	(A/T)
10686	CakSNP10686	*Kabuli*	Ca_Kabuli_Chr06	24516532	(C/T)
10687	CakSNP10687	*Kabuli*	Ca_Kabuli_Chr06	24547086	(T/G)
10688	CakSNP10688	*Kabuli*	Ca_Kabuli_Chr06	24644924	(T/G)
10689	CakSNP10689	*Kabuli*	Ca_Kabuli_Chr06	24661895	(G/A)
10690	CakSNP10690	*Kabuli*	Ca_Kabuli_Chr06	24661916	(C/T)
10691	CakSNP10691	*Kabuli*	Ca_Kabuli_Chr06	24661937	(C/T)
10692	CakSNP10692	*Kabuli*	Ca_Kabuli_Chr06	24661945	(T/A)
10693	CakSNP10693	*Kabuli*	Ca_Kabuli_Chr06	24661986	(G/A)
10694	CakSNP10694	*Kabuli*	Ca_Kabuli_Chr06	25575097	(T/C)
10695	CakSNP10695	*Kabuli*	Ca_Kabuli_Chr06	25575078	(C/T)
10696	CakSNP10696	*Kabuli*	Ca_Kabuli_Chr06	25575062	(G/A)
10697	CakSNP10697	*Kabuli*	Ca_Kabuli_Chr06	25575045	(C/A)
10698	CakSNP10698	*Kabuli*	Ca_Kabuli_Chr06	25604942	(C/A)
10699	CakSNP10699	*Kabuli*	Ca_Kabuli_Chr06	25604936	(C/T)
10700	CakSNP10700	*Kabuli*	Ca_Kabuli_Chr06	25604931	(C/A)
10701	CakSNP10701	*Kabuli*	Ca_Kabuli_Chr06	25604922	(A/G)
10702	CakSNP10702	*Kabuli*	Ca_Kabuli_Chr06	25604954	(C/T)
10703	CakSNP10703	*Kabuli*	Ca_Kabuli_Chr06	25604962	(G/A)
10704	CakSNP10704	*Kabuli*	Ca_Kabuli_Chr06	25604994	(T/C)
10705	CakSNP10705	*Kabuli*	Ca_Kabuli_Chr06	25605006	(C/A)
10706	CakSNP10706	*Kabuli*	Ca_Kabuli_Chr06	25605023	(C/A)
10707	CakSNP10707	*Kabuli*	Ca_Kabuli_Chr06	25605022	(T/C)
10708	CakSNP10708	*Kabuli*	Ca_Kabuli_Chr06	25850717	(C/T)
10709	CakSNP10709	*Kabuli*	Ca_Kabuli_Chr06	25850725	(T/G)
10710	CakSNP10710	*Kabuli*	Ca_Kabuli_Chr06	25850736	(C/T)
10711	CakSNP10711	*Kabuli*	Ca_Kabuli_Chr06	25850760	(T/A)
10712	CakSNP10712	*Kabuli*	Ca_Kabuli_Chr06	25850759	(C/A)
10713	CakSNP10713	*Kabuli*	Ca_Kabuli_Chr06	25850783	(G/A)
10714	CakSNP10714	*Kabuli*	Ca_Kabuli_Chr06	25850816	(C/A)
10715	CakSNP10715	*Kabuli*	Ca_Kabuli_Chr06	25850813	(C/T)
10716	CakSNP10716	*Kabuli*	Ca_Kabuli_Chr06	25850866	(C/G)
10717	CakSNP10717	*Kabuli*	Ca_Kabuli_Chr06	25850827	(C/T)
10718	CakSNP10718	*Kabuli*	Ca_Kabuli_Chr06	26349363	(C/A)
10719	CakSNP10719	*Kabuli*	Ca_Kabuli_Chr06	26349321	(C/T)
10720	CakSNP10720	*Kabuli*	Ca_Kabuli_Chr06	26349434	(G/A)
10721	CakSNP10721	*Kabuli*	Ca_Kabuli_Chr06	26349451	(T/C)
10722	CakSNP10722	*Kabuli*	Ca_Kabuli_Chr06	26353512	(C/T)
10723	CakSNP10723	*Kabuli*	Ca_Kabuli_Chr06	26353740	(G/C)

S.N.	SNP IDs	Cultivars	Chromosomes/scaffolds	Physical positions (bp)	SNPs
10724	CakSNP10724	*Kabuli*	Ca_Kabuli_Chr06	26357149	(C/G)
10725	CakSNP10725	*Kabuli*	Ca_Kabuli_Chr06	26360367	(C/A)
10726	CakSNP10726	*Kabuli*	Ca_Kabuli_Chr06	26385448	(A/G)
10727	CakSNP10727	*Kabuli*	Ca_Kabuli_Chr06	26385444	(C/T)
10728	CakSNP10728	*Kabuli*	Ca_Kabuli_Chr06	26385432	(T/C)
10729	CakSNP10729	*Kabuli*	Ca_Kabuli_Chr06	26426239	(C/A)
10730	CakSNP10730	*Kabuli*	Ca_Kabuli_Chr06	26426252	(A/G)
10731	CakSNP10731	*Kabuli*	Ca_Kabuli_Chr06	26426283	(G/A)
10732	CakSNP10732	*Kabuli*	Ca_Kabuli_Chr06	26527086	(T/C)
10733	CakSNP10733	*Kabuli*	Ca_Kabuli_Chr06	26527111	(G/T)
10734	CakSNP10734	*Kabuli*	Ca_Kabuli_Chr06	26527161	(C/G)
10735	CakSNP10735	*Kabuli*	Ca_Kabuli_Chr06	26527416	(C/T)
10736	CakSNP10736	*Kabuli*	Ca_Kabuli_Chr06	26549168	(T/G)
10737	CakSNP10737	*Kabuli*	Ca_Kabuli_Chr06	26549181	(C/T)
10738	CakSNP10738	*Kabuli*	Ca_Kabuli_Chr06	26551390	(C/T)
10739	CakSNP10739	*Kabuli*	Ca_Kabuli_Chr06	26551703	(T/G)
10740	CakSNP10740	*Kabuli*	Ca_Kabuli_Chr06	26567360	(A/G)
10741	CakSNP10741	*Kabuli*	Ca_Kabuli_Chr06	26656016	(T/C)
10742	CakSNP10742	*Kabuli*	Ca_Kabuli_Chr06	26698265	(A/C)
10743	CakSNP10743	*Kabuli*	Ca_Kabuli_Chr06	26703130	(A/G)
10744	CakSNP10744	*Kabuli*	Ca_Kabuli_Chr06	26703132	(T/C)
10745	CakSNP10745	*Kabuli*	Ca_Kabuli_Chr06	26767389	(T/C)
10746	CakSNP10746	*Kabuli*	Ca_Kabuli_Chr06	26769087	(C/A)
10747	CakSNP10747	*Kabuli*	Ca_Kabuli_Chr06	26928255	(T/C)
10748	CakSNP10748	*Kabuli*	Ca_Kabuli_Chr06	27053217	(A/G)
10749	CakSNP10749	*Kabuli*	Ca_Kabuli_Chr06	27053288	(G/C)
10750	CakSNP10750	*Kabuli*	Ca_Kabuli_Chr06	27139791	(T/C)
10751	CakSNP10751	*Kabuli*	Ca_Kabuli_Chr06	27139816	(T/C)
10752	CakSNP10752	*Kabuli*	Ca_Kabuli_Chr06	27190671	(G/A)
10753	CakSNP10753	*Kabuli*	Ca_Kabuli_Chr06	27192557	(C/T)
10754	CakSNP10754	*Kabuli*	Ca_Kabuli_Chr06	27310342	(A/T)
10755	CakSNP10755	*Kabuli*	Ca_Kabuli_Chr06	27310341	(G/T)
10756	CakSNP10756	*Kabuli*	Ca_Kabuli_Chr06	27310336	(A/T)
10757	CakSNP10757	*Kabuli*	Ca_Kabuli_Chr06	27310333	(C/T)
10758	CakSNP10758	*Kabuli*	Ca_Kabuli_Chr06	27310328	(T/A)
10759	CakSNP10759	*Kabuli*	Ca_Kabuli_Chr06	27310327	(A/T)
10760	CakSNP10760	*Kabuli*	Ca_Kabuli_Chr06	27310325	(G/T)
10761	CakSNP10761	*Kabuli*	Ca_Kabuli_Chr06	27310315	(C/T)
10762	CakSNP10762	*Kabuli*	Ca_Kabuli_Chr06	27326420	(G/A)

S.N.	SNP IDs	Cultivars	Chromosomes/scaffolds	Physical positions (bp)	SNPs
10763	CakSNP10763	*Kabuli*	Ca_Kabuli_Chr06	27326978	(C/T)
10764	CakSNP10764	*Kabuli*	Ca_Kabuli_Chr06	27330059	(T/C)
10765	CakSNP10765	*Kabuli*	Ca_Kabuli_Chr06	27330159	(C/T)
10766	CakSNP10766	*Kabuli*	Ca_Kabuli_Chr06	27331165	(T/C)
10767	CakSNP10767	*Kabuli*	Ca_Kabuli_Chr06	27331318	(A/C)
10768	CakSNP10768	*Kabuli*	Ca_Kabuli_Chr06	27364381	(A/G)
10769	CakSNP10769	*Kabuli*	Ca_Kabuli_Chr06	27364395	(C/T)
10770	CakSNP10770	*Kabuli*	Ca_Kabuli_Chr06	27364402	(T/A)
10771	CakSNP10771	*Kabuli*	Ca_Kabuli_Chr06	27364428	(G/T)
10772	CakSNP10772	*Kabuli*	Ca_Kabuli_Chr06	27364452	(A/T)
10773	CakSNP10773	*Kabuli*	Ca_Kabuli_Chr06	27383746	(A/T)
10774	CakSNP10774	*Kabuli*	Ca_Kabuli_Chr06	27383745	(G/T)
10775	CakSNP10775	*Kabuli*	Ca_Kabuli_Chr06	27383744	(G/T)
10776	CakSNP10776	*Kabuli*	Ca_Kabuli_Chr06	27383718	(C/T)
10777	CakSNP10777	*Kabuli*	Ca_Kabuli_Chr06	27515184	(C/T)
10778	CakSNP10778	*Kabuli*	Ca_Kabuli_Chr06	27515293	(G/A)
10779	CakSNP10779	*Kabuli*	Ca_Kabuli_Chr06	27537433	(A/G)
10780	CakSNP10780	*Kabuli*	Ca_Kabuli_Chr06	27558154	(A/G)
10781	CakSNP10781	*Kabuli*	Ca_Kabuli_Chr06	27578968	(A/G)
10782	CakSNP10782	*Kabuli*	Ca_Kabuli_Chr06	27592117	(G/T)
10783	CakSNP10783	*Kabuli*	Ca_Kabuli_Chr06	27592070	(A/G)
10784	CakSNP10784	*Kabuli*	Ca_Kabuli_Chr06	27606193	(A/C)
10785	CakSNP10785	*Kabuli*	Ca_Kabuli_Chr06	27606210	(C/T)
10786	CakSNP10786	*Kabuli*	Ca_Kabuli_Chr06	27622037	(A/C)
10787	CakSNP10787	*Kabuli*	Ca_Kabuli_Chr06	27622045	(A/G)
10788	CakSNP10788	*Kabuli*	Ca_Kabuli_Chr06	27622053	(A/G)
10789	CakSNP10789	*Kabuli*	Ca_Kabuli_Chr06	27636256	(A/G)
10790	CakSNP10790	*Kabuli*	Ca_Kabuli_Chr06	27655605	(A/G)
10791	CakSNP10791	*Kabuli*	Ca_Kabuli_Chr06	27660980	(C/T)
10792	CakSNP10792	*Kabuli*	Ca_Kabuli_Chr06	27660971	(T/C)
10793	CakSNP10793	*Kabuli*	Ca_Kabuli_Chr06	27661951	(C/T)
10794	CakSNP10794	*Kabuli*	Ca_Kabuli_Chr06	27662026	(C/T)
10795	CakSNP10795	*Kabuli*	Ca_Kabuli_Chr06	27710531	(T/G)
10796	CakSNP10796	*Kabuli*	Ca_Kabuli_Chr06	27710547	(A/T)
10797	CakSNP10797	*Kabuli*	Ca_Kabuli_Chr06	27842297	(G/A)
10798	CakSNP10798	*Kabuli*	Ca_Kabuli_Chr06	27865048	(C/T)
10799	CakSNP10799	*Kabuli*	Ca_Kabuli_Chr06	27865046	(G/A)
10800	CakSNP10800	*Kabuli*	Ca_Kabuli_Chr06	27864997	(C/A)
10801	CakSNP10801	*Kabuli*	Ca_Kabuli_Chr06	27986719	(A/G)

S.N.	SNP IDs	Cultivars	Chromosomes/scaffolds	Physical positions (bp)	SNPs
10802	CakSNP10802	*Kabuli*	Ca_Kabuli_Chr06	28021899	(C/T)
10803	CakSNP10803	*Kabuli*	Ca_Kabuli_Chr06	28044830	(C/A)
10804	CakSNP10804	*Kabuli*	Ca_Kabuli_Chr06	28052463	(T/G)
10805	CakSNP10805	*Kabuli*	Ca_Kabuli_Chr06	28074393	(T/C)
10806	CakSNP10806	*Kabuli*	Ca_Kabuli_Chr06	28076323	(T/A)
10807	CakSNP10807	*Kabuli*	Ca_Kabuli_Chr06	28076364	(T/G)
10808	CakSNP10808	*Kabuli*	Ca_Kabuli_Chr06	28076436	(A/T)
10809	CakSNP10809	*Kabuli*	Ca_Kabuli_Chr06	28177397	(A/C)
10810	CakSNP10810	*Kabuli*	Ca_Kabuli_Chr06	28325392	(C/T)
10811	CakSNP10811	*Kabuli*	Ca_Kabuli_Chr06	28372130	(C/T)
10812	CakSNP10812	*Kabuli*	Ca_Kabuli_Chr06	28574699	(T/C)
10813	CakSNP10813	*Kabuli*	Ca_Kabuli_Chr06	28575509	(T/C)
10814	CakSNP10814	*Kabuli*	Ca_Kabuli_Chr06	28575491	(G/A)
10815	CakSNP10815	*Kabuli*	Ca_Kabuli_Chr06	28615404	(G/T)
10816	CakSNP10816	*Kabuli*	Ca_Kabuli_Chr06	28615475	(A/G)
10817	CakSNP10817	*Kabuli*	Ca_Kabuli_Chr06	28650056	(A/G)
10818	CakSNP10818	*Kabuli*	Ca_Kabuli_Chr06	28727478	(T/C)
10819	CakSNP10819	*Kabuli*	Ca_Kabuli_Chr06	28728293	(A/C)
10820	CakSNP10820	*Kabuli*	Ca_Kabuli_Chr06	28755298	(T/A)
10821	CakSNP10821	*Kabuli*	Ca_Kabuli_Chr06	28757208	(C/A)
10822	CakSNP10822	*Kabuli*	Ca_Kabuli_Chr06	28757204	(G/A)
10823	CakSNP10823	*Kabuli*	Ca_Kabuli_Chr06	28757139	(C/A)
10824	CakSNP10824	*Kabuli*	Ca_Kabuli_Chr06	28887176	(G/A)
10825	CakSNP10825	*Kabuli*	Ca_Kabuli_Chr06	28887188	(G/T)
10826	CakSNP10826	*Kabuli*	Ca_Kabuli_Chr06	28887201	(A/G)
10827	CakSNP10827	*Kabuli*	Ca_Kabuli_Chr06	28887212	(C/T)
10828	CakSNP10828	*Kabuli*	Ca_Kabuli_Chr06	28892743	(G/C)
10829	CakSNP10829	*Kabuli*	Ca_Kabuli_Chr06	28893411	(C/T)
10830	CakSNP10830	*Kabuli*	Ca_Kabuli_Chr06	28917705	(T/A)
10831	CakSNP10831	*Kabuli*	Ca_Kabuli_Chr06	28947529	(A/C)
10832	CakSNP10832	*Kabuli*	Ca_Kabuli_Chr06	28990518	(A/G)
10833	CakSNP10833	*Kabuli*	Ca_Kabuli_Chr06	29085558	(T/C)
10834	CakSNP10834	*Kabuli*	Ca_Kabuli_Chr06	29163667	(C/A)
10835	CakSNP10835	*Kabuli*	Ca_Kabuli_Chr06	29163714	(G/C)
10836	CakSNP10836	*Kabuli*	Ca_Kabuli_Chr06	29163699	(T/A)
10837	CakSNP10837	*Kabuli*	Ca_Kabuli_Chr06	29163642	(G/A)
10838	CakSNP10838	*Kabuli*	Ca_Kabuli_Chr06	29163640	(A/C)
10839	CakSNP10839	*Kabuli*	Ca_Kabuli_Chr06	29203129	(C/G)
10840	CakSNP10840	*Kabuli*	Ca_Kabuli_Chr06	29203201	(G/C)

S.N.	SNP IDs	Cultivars	Chromosomes/scaffolds	Physical positions (bp)	SNPs
10841	CakSNP10841	*Kabuli*	Ca_Kabuli_Chr06	29203210	(T/C)
10842	CakSNP10842	*Kabuli*	Ca_Kabuli_Chr06	29205260	(A/G)
10843	CakSNP10843	*Kabuli*	Ca_Kabuli_Chr06	29205940	(A/G)
10844	CakSNP10844	*Kabuli*	Ca_Kabuli_Chr06	29206008	(A/G)
10845	CakSNP10845	*Kabuli*	Ca_Kabuli_Chr06	29307254	(A/G)
10846	CakSNP10846	*Kabuli*	Ca_Kabuli_Chr06	29495856	(C/T)
10847	CakSNP10847	*Kabuli*	Ca_Kabuli_Chr06	29934610	(A/T)
10848	CakSNP10848	*Kabuli*	Ca_Kabuli_Chr06	30127756	(T/G)
10849	CakSNP10849	*Kabuli*	Ca_Kabuli_Chr06	30257217	(C/T)
10850	CakSNP10850	*Kabuli*	Ca_Kabuli_Chr06	30257284	(A/C)
10851	CakSNP10851	*Kabuli*	Ca_Kabuli_Chr06	30567073	(G/A)
10852	CakSNP10852	*Kabuli*	Ca_Kabuli_Chr06	30676031	(G/T)
10853	CakSNP10853	*Kabuli*	Ca_Kabuli_Chr06	30676320	(T/C)
10854	CakSNP10854	*Kabuli*	Ca_Kabuli_Chr06	30676248	(C/T)
10855	CakSNP10855	*Kabuli*	Ca_Kabuli_Chr06	30761620	(C/T)
10856	CakSNP10856	*Kabuli*	Ca_Kabuli_Chr06	30761641	(G/T)
10857	CakSNP10857	*Kabuli*	Ca_Kabuli_Chr06	30913932	(T/C)
10858	CakSNP10858	*Kabuli*	Ca_Kabuli_Chr06	30926084	(G/A)
10859	CakSNP10859	*Kabuli*	Ca_Kabuli_Chr06	30927221	(C/G)
10860	CakSNP10860	*Kabuli*	Ca_Kabuli_Chr06	31011743	(A/G)
10861	CakSNP10861	*Kabuli*	Ca_Kabuli_Chr06	31024728	(A/T)
10862	CakSNP10862	*Kabuli*	Ca_Kabuli_Chr06	31025560	(C/G)
10863	CakSNP10863	*Kabuli*	Ca_Kabuli_Chr06	31038808	(C/T)
10864	CakSNP10864	*Kabuli*	Ca_Kabuli_Chr06	31048949	(T/C)
10865	CakSNP10865	*Kabuli*	Ca_Kabuli_Chr06	31057806	(C/T)
10866	CakSNP10866	*Kabuli*	Ca_Kabuli_Chr06	31068678	(G/A)
10867	CakSNP10867	*Kabuli*	Ca_Kabuli_Chr06	31324962	(A/C)
10868	CakSNP10868	*Kabuli*	Ca_Kabuli_Chr06	31324976	(A/G)
10869	CakSNP10869	*Kabuli*	Ca_Kabuli_Chr06	31408895	(G/T)
10870	CakSNP10870	*Kabuli*	Ca_Kabuli_Chr06	31408969	(C/T)
10871	CakSNP10871	*Kabuli*	Ca_Kabuli_Chr06	31495897	(C/A)
10872	CakSNP10872	*Kabuli*	Ca_Kabuli_Chr06	31495903	(A/G)
10873	CakSNP10873	*Kabuli*	Ca_Kabuli_Chr06	31592707	(A/C)
10874	CakSNP10874	*Kabuli*	Ca_Kabuli_Chr06	31595142	(A/C)
10875	CakSNP10875	*Kabuli*	Ca_Kabuli_Chr06	31595136	(A/T)
10876	CakSNP10876	*Kabuli*	Ca_Kabuli_Chr06	31895214	(G/C)
10877	CakSNP10877	*Kabuli*	Ca_Kabuli_Chr06	31895264	(C/T)
10878	CakSNP10878	*Kabuli*	Ca_Kabuli_Chr06	31900781	(T/G)
10879	CakSNP10879	*Kabuli*	Ca_Kabuli_Chr06	31900785	(T/G)

S.N.	SNP IDs	Cultivars	Chromosomes/scaffolds	Physical positions (bp)	SNPs
10880	CakSNP10880	*Kabuli*	Ca_Kabuli_Chr06	31900790	(A/C)
10881	CakSNP10881	*Kabuli*	Ca_Kabuli_Chr06	31966982	(C/T)
10882	CakSNP10882	*Kabuli*	Ca_Kabuli_Chr06	31967089	(T/G)
10883	CakSNP10883	*Kabuli*	Ca_Kabuli_Chr06	31967106	(G/A)
10884	CakSNP10884	*Kabuli*	Ca_Kabuli_Chr06	31967109	(T/C)
10885	CakSNP10885	*Kabuli*	Ca_Kabuli_Chr06	31967144	(T/C)
10886	CakSNP10886	*Kabuli*	Ca_Kabuli_Chr06	31967201	(C/T)
10887	CakSNP10887	*Kabuli*	Ca_Kabuli_Chr06	31967195	(G/C)
10888	CakSNP10888	*Kabuli*	Ca_Kabuli_Chr06	31992907	(A/G)
10889	CakSNP10889	*Kabuli*	Ca_Kabuli_Chr06	32035882	(C/T)
10890	CakSNP10890	*Kabuli*	Ca_Kabuli_Chr06	32036033	(G/A)
10891	CakSNP10891	*Kabuli*	Ca_Kabuli_Chr06	32035976	(T/C)
10892	CakSNP10892	*Kabuli*	Ca_Kabuli_Chr06	32140428	(C/A)
10893	CakSNP10893	*Kabuli*	Ca_Kabuli_Chr06	32140429	(G/T)
10894	CakSNP10894	*Kabuli*	Ca_Kabuli_Chr06	32262807	(T/C)
10895	CakSNP10895	*Kabuli*	Ca_Kabuli_Chr06	32262855	(C/T)
10896	CakSNP10896	*Kabuli*	Ca_Kabuli_Chr06	32328468	(T/C)
10897	CakSNP10897	*Kabuli*	Ca_Kabuli_Chr06	32717450	(G/A)
10898	CakSNP10898	*Kabuli*	Ca_Kabuli_Chr06	32717665	(C/A)
10899	CakSNP10899	*Kabuli*	Ca_Kabuli_Chr06	32825567	(C/A)
10900	CakSNP10900	*Kabuli*	Ca_Kabuli_Chr06	32825561	(C/T)
10901	CakSNP10901	*Kabuli*	Ca_Kabuli_Chr06	32825547	(A/G)
10902	CakSNP10902	*Kabuli*	Ca_Kabuli_Chr06	32825579	(C/T)
10903	CakSNP10903	*Kabuli*	Ca_Kabuli_Chr06	32825587	(G/A)
10904	CakSNP10904	*Kabuli*	Ca_Kabuli_Chr06	32825612	(G/A)
10905	CakSNP10905	*Kabuli*	Ca_Kabuli_Chr06	32825619	(T/C)
10906	CakSNP10906	*Kabuli*	Ca_Kabuli_Chr06	32825631	(C/A)
10907	CakSNP10907	*Kabuli*	Ca_Kabuli_Chr06	32914927	(C/G)
10908	CakSNP10908	*Kabuli*	Ca_Kabuli_Chr06	33502994	(C/A)
10909	CakSNP10909	*Kabuli*	Ca_Kabuli_Chr06	33502997	(T/A)
10910	CakSNP10910	*Kabuli*	Ca_Kabuli_Chr06	33503007	(T/C)
10911	CakSNP10911	*Kabuli*	Ca_Kabuli_Chr06	33503014	(G/T)
10912	CakSNP10912	*Kabuli*	Ca_Kabuli_Chr06	33503020	(G/A)
10913	CakSNP10913	*Kabuli*	Ca_Kabuli_Chr06	33503032	(A/C)
10914	CakSNP10914	*Kabuli*	Ca_Kabuli_Chr06	33503037	(A/T)
10915	CakSNP10915	*Kabuli*	Ca_Kabuli_Chr06	33503051	(G/C)
10916	CakSNP10916	*Kabuli*	Ca_Kabuli_Chr06	33503063	(C/G)
10917	CakSNP10917	*Kabuli*	Ca_Kabuli_Chr06	33503065	(C/A)
10918	CakSNP10918	*Kabuli*	Ca_Kabuli_Chr06	33503071	(A/G)

S.N.	SNP IDs	Cultivars	Chromosomes/scaffolds	Physical positions (bp)	SNPs
10919	CakSNP10919	*Kabuli*	Ca_Kabuli_Chr06	33598926	(G/A)
10920	CakSNP10920	*Kabuli*	Ca_Kabuli_Chr06	33704792	(C/T)
10921	CakSNP10921	*Kabuli*	Ca_Kabuli_Chr06	33704807	(C/A)
10922	CakSNP10922	*Kabuli*	Ca_Kabuli_Chr06	33704839	(C/T)
10923	CakSNP10923	*Kabuli*	Ca_Kabuli_Chr06	33704782	(C/T)
10924	CakSNP10924	*Kabuli*	Ca_Kabuli_Chr06	33733259	(A/G)
10925	CakSNP10925	*Kabuli*	Ca_Kabuli_Chr06	34235650	(T/C)
10926	CakSNP10926	*Kabuli*	Ca_Kabuli_Chr06	34407309	(T/C)
10927	CakSNP10927	*Kabuli*	Ca_Kabuli_Chr06	34481266	(A/G)
10928	CakSNP10928	*Kabuli*	Ca_Kabuli_Chr06	34481256	(C/T)
10929	CakSNP10929	*Kabuli*	Ca_Kabuli_Chr06	34535695	(T/C)
10930	CakSNP10930	*Kabuli*	Ca_Kabuli_Chr06	34535983	(T/C)
10931	CakSNP10931	*Kabuli*	Ca_Kabuli_Chr06	34596792	(A/G)
10932	CakSNP10932	*Kabuli*	Ca_Kabuli_Chr06	34634028	(T/A)
10933	CakSNP10933	*Kabuli*	Ca_Kabuli_Chr06	34744433	(A/G)
10934	CakSNP10934	*Kabuli*	Ca_Kabuli_Chr06	34744438	(T/G)
10935	CakSNP10935	*Kabuli*	Ca_Kabuli_Chr06	34744681	(T/G)
10936	CakSNP10936	*Kabuli*	Ca_Kabuli_Chr06	34744665	(G/A)
10937	CakSNP10937	*Kabuli*	Ca_Kabuli_Chr06	34773476	(T/A)
10938	CakSNP10938	*Kabuli*	Ca_Kabuli_Chr06	34793312	(T/G)
10939	CakSNP10939	*Kabuli*	Ca_Kabuli_Chr06	34974503	(C/G)
10940	CakSNP10940	*Kabuli*	Ca_Kabuli_Chr06	35126227	(C/T)
10941	CakSNP10941	*Kabuli*	Ca_Kabuli_Chr06	35126273	(T/C)
10942	CakSNP10942	*Kabuli*	Ca_Kabuli_Chr06	35126389	(G/T)
10943	CakSNP10943	*Kabuli*	Ca_Kabuli_Chr06	35129588	(G/A)
10944	CakSNP10944	*Kabuli*	Ca_Kabuli_Chr06	35596037	(C/A)
10945	CakSNP10945	*Kabuli*	Ca_Kabuli_Chr06	35596048	(G/A)
10946	CakSNP10946	*Kabuli*	Ca_Kabuli_Chr06	35596064	(T/C)
10947	CakSNP10947	*Kabuli*	Ca_Kabuli_Chr06	35596070	(G/A)
10948	CakSNP10948	*Kabuli*	Ca_Kabuli_Chr06	35596161	(C/T)
10949	CakSNP10949	*Kabuli*	Ca_Kabuli_Chr06	35596174	(G/A)
10950	CakSNP10950	*Kabuli*	Ca_Kabuli_Chr06	35596184	(A/G)
10951	CakSNP10951	*Kabuli*	Ca_Kabuli_Chr06	35596195	(G/C)
10952	CakSNP10952	*Kabuli*	Ca_Kabuli_Chr06	35596197	(G/A)
10953	CakSNP10953	*Kabuli*	Ca_Kabuli_Chr06	35596207	(G/T)
10954	CakSNP10954	*Kabuli*	Ca_Kabuli_Chr06	35596218	(T/C)
10955	CakSNP10955	*Kabuli*	Ca_Kabuli_Chr06	35596220	(G/A)
10956	CakSNP10956	*Kabuli*	Ca_Kabuli_Chr06	35596230	(T/A)
10957	CakSNP10957	*Kabuli*	Ca_Kabuli_Chr06	35596300	(G/A)

S.N.	SNP IDs	Cultivars	Chromosomes/scaffolds	Physical positions (bp)	SNPs
10958	CakSNP10958	*Kabuli*	Ca_Kabuli_Chr06	35628131	(T/C)
10959	CakSNP10959	*Kabuli*	Ca_Kabuli_Chr06	35719930	(A/C)
10960	CakSNP10960	*Kabuli*	Ca_Kabuli_Chr06	35743792	(A/G)
10961	CakSNP10961	*Kabuli*	Ca_Kabuli_Chr06	35962309	(C/T)
10962	CakSNP10962	*Kabuli*	Ca_Kabuli_Chr06	35962320	(T/C)
10963	CakSNP10963	*Kabuli*	Ca_Kabuli_Chr06	35962360	(T/C)
10964	CakSNP10964	*Kabuli*	Ca_Kabuli_Chr06	35962364	(T/G)
10965	CakSNP10965	*Kabuli*	Ca_Kabuli_Chr06	35962365	(T/C)
10966	CakSNP10966	*Kabuli*	Ca_Kabuli_Chr06	35962366	(G/A)
10967	CakSNP10967	*Kabuli*	Ca_Kabuli_Chr06	36094855	(T/G)
10968	CakSNP10968	*Kabuli*	Ca_Kabuli_Chr06	36371775	(A/G)
10969	CakSNP10969	*Kabuli*	Ca_Kabuli_Chr06	36374561	(C/T)
10970	CakSNP10970	*Kabuli*	Ca_Kabuli_Chr06	36574836	(A/G)
10971	CakSNP10971	*Kabuli*	Ca_Kabuli_Chr06	36790467	(G/A)
10972	CakSNP10972	*Kabuli*	Ca_Kabuli_Chr06	36790458	(G/A)
10973	CakSNP10973	*Kabuli*	Ca_Kabuli_Chr06	36790428	(G/A)
10974	CakSNP10974	*Kabuli*	Ca_Kabuli_Chr06	36790455	(C/T)
10975	CakSNP10975	*Kabuli*	Ca_Kabuli_Chr06	36790460	(A/G)
10976	CakSNP10976	*Kabuli*	Ca_Kabuli_Chr06	37139425	(C/T)
10977	CakSNP10977	*Kabuli*	Ca_Kabuli_Chr06	37139466	(G/C)
10978	CakSNP10978	*Kabuli*	Ca_Kabuli_Chr06	37139484	(T/C)
10979	CakSNP10979	*Kabuli*	Ca_Kabuli_Chr06	37139486	(G/A)
10980	CakSNP10980	*Kabuli*	Ca_Kabuli_Chr06	37139533	(T/A)
10981	CakSNP10981	*Kabuli*	Ca_Kabuli_Chr06	37139514	(C/T)
10982	CakSNP10982	*Kabuli*	Ca_Kabuli_Chr06	37139499	(C/G)
10983	CakSNP10983	*Kabuli*	Ca_Kabuli_Chr06	37139610	(T/G)
10984	CakSNP10984	*Kabuli*	Ca_Kabuli_Chr06	37139604	(G/T)
10985	CakSNP10985	*Kabuli*	Ca_Kabuli_Chr06	37321783	(A/T)
10986	CakSNP10986	*Kabuli*	Ca_Kabuli_Chr06	37476426	(C/T)
10987	CakSNP10987	*Kabuli*	Ca_Kabuli_Chr06	37476437	(A/C)
10988	CakSNP10988	*Kabuli*	Ca_Kabuli_Chr06	37476516	(G/T)
10989	CakSNP10989	*Kabuli*	Ca_Kabuli_Chr06	37476465	(G/A)
10990	CakSNP10990	*Kabuli*	Ca_Kabuli_Chr06	37484467	(C/T)
10991	CakSNP10991	*Kabuli*	Ca_Kabuli_Chr06	37484468	(G/T)
10992	CakSNP10992	*Kabuli*	Ca_Kabuli_Chr06	37484470	(C/A)
10993	CakSNP10993	*Kabuli*	Ca_Kabuli_Chr06	37484489	(C/G)
10994	CakSNP10994	*Kabuli*	Ca_Kabuli_Chr06	37484513	(T/C)
10995	CakSNP10995	*Kabuli*	Ca_Kabuli_Chr06	38816345	(C/T)
10996	CakSNP10996	*Kabuli*	Ca_Kabuli_Chr06	38838320	(A/G)

S.N.	SNP IDs	Cultivars	Chromosomes/scaffolds	Physical positions (bp)	SNPs
10997	CakSNP10997	*Kabuli*	Ca_Kabuli_Chr06	38840279	(G/A)
10998	CakSNP10998	*Kabuli*	Ca_Kabuli_Chr06	38840315	(G/A)
10999	CakSNP10999	*Kabuli*	Ca_Kabuli_Chr06	38840273	(G/A)
11000	CakSNP11000	*Kabuli*	Ca_Kabuli_Chr06	38881909	(C/A)
11001	CakSNP11001	*Kabuli*	Ca_Kabuli_Chr06	38886735	(C/A)
11002	CakSNP11002	*Kabuli*	Ca_Kabuli_Chr06	38994879	(A/G)
11003	CakSNP11003	*Kabuli*	Ca_Kabuli_Chr06	38994925	(G/T)
11004	CakSNP11004	*Kabuli*	Ca_Kabuli_Chr06	38994954	(A/G)
11005	CakSNP11005	*Kabuli*	Ca_Kabuli_Chr06	38994962	(G/A)
11006	CakSNP11006	*Kabuli*	Ca_Kabuli_Chr06	39028564	(T/G)
11007	CakSNP11007	*Kabuli*	Ca_Kabuli_Chr06	39107187	(C/T)
11008	CakSNP11008	*Kabuli*	Ca_Kabuli_Chr06	39107149	(A/C)
11009	CakSNP11009	*Kabuli*	Ca_Kabuli_Chr06	39107188	(A/C)
11010	CakSNP11010	*Kabuli*	Ca_Kabuli_Chr06	39317016	(T/G)
11011	CakSNP11011	*Kabuli*	Ca_Kabuli_Chr06	39380625	(A/C)
11012	CakSNP11012	*Kabuli*	Ca_Kabuli_Chr06	39908314	(T/G)
11013	CakSNP11013	*Kabuli*	Ca_Kabuli_Chr06	40047823	(A/G)
11014	CakSNP11014	*Kabuli*	Ca_Kabuli_Chr06	40128644	(T/C)
11015	CakSNP11015	*Kabuli*	Ca_Kabuli_Chr06	40468356	(T/A)
11016	CakSNP11016	*Kabuli*	Ca_Kabuli_Chr06	40477285	(G/A)
11017	CakSNP11017	*Kabuli*	Ca_Kabuli_Chr06	40657695	(A/C)
11018	CakSNP11018	*Kabuli*	Ca_Kabuli_Chr06	40735186	(C/A)
11019	CakSNP11019	*Kabuli*	Ca_Kabuli_Chr06	40767566	(A/G)
11020	CakSNP11020	*Kabuli*	Ca_Kabuli_Chr06	40821354	(C/T)
11021	CakSNP11021	*Kabuli*	Ca_Kabuli_Chr06	40935441	(A/T)
11022	CakSNP11022	*Kabuli*	Ca_Kabuli_Chr06	41121337	(C/T)
11023	CakSNP11023	*Kabuli*	Ca_Kabuli_Chr06	41121330	(G/A)
11024	CakSNP11024	*Kabuli*	Ca_Kabuli_Chr06	41363546	(G/A)
11025	CakSNP11025	*Kabuli*	Ca_Kabuli_Chr06	41420918	(T/A)
11026	CakSNP11026	*Kabuli*	Ca_Kabuli_Chr06	41420917	(A/G)
11027	CakSNP11027	*Kabuli*	Ca_Kabuli_Chr06	41449028	(C/T)
11028	CakSNP11028	*Kabuli*	Ca_Kabuli_Chr06	41682983	(T/C)
11029	CakSNP11029	*Kabuli*	Ca_Kabuli_Chr06	41682966	(C/T)
11030	CakSNP11030	*Kabuli*	Ca_Kabuli_Chr06	41682943	(T/C)
11031	CakSNP11031	*Kabuli*	Ca_Kabuli_Chr06	41697812	(T/G)
11032	CakSNP11032	*Kabuli*	Ca_Kabuli_Chr06	41708309	(G/A)
11033	CakSNP11033	*Kabuli*	Ca_Kabuli_Chr06	41708306	(G/A)
11034	CakSNP11034	*Kabuli*	Ca_Kabuli_Chr06	41708523	(T/G)
11035	CakSNP11035	*Kabuli*	Ca_Kabuli_Chr06	41708474	(G/A)

S.N.	SNP IDs	Cultivars	Chromosomes/scaffolds	Physical positions (bp)	SNPs
11036	CakSNP11036	*Kabuli*	Ca_Kabuli_Chr06	41708530	(C/A)
11037	CakSNP11037	*Kabuli*	Ca_Kabuli_Chr06	41907459	(C/G)
11038	CakSNP11038	*Kabuli*	Ca_Kabuli_Chr06	42004851	(A/G)
11039	CakSNP11039	*Kabuli*	Ca_Kabuli_Chr06	42004829	(T/G)
11040	CakSNP11040	*Kabuli*	Ca_Kabuli_Chr06	42004818	(C/G)
11041	CakSNP11041	*Kabuli*	Ca_Kabuli_Chr06	42004813	(C/T)
11042	CakSNP11042	*Kabuli*	Ca_Kabuli_Chr06	42161851	(A/G)
11043	CakSNP11043	*Kabuli*	Ca_Kabuli_Chr06	42161857	(C/G)
11044	CakSNP11044	*Kabuli*	Ca_Kabuli_Chr06	42161866	(T/G)
11045	CakSNP11045	*Kabuli*	Ca_Kabuli_Chr06	42521162	(G/T)
11046	CakSNP11046	*Kabuli*	Ca_Kabuli_Chr06	42855091	(T/G)
11047	CakSNP11047	*Kabuli*	Ca_Kabuli_Chr06	42904998	(C/G)
11048	CakSNP11048	*Kabuli*	Ca_Kabuli_Chr06	42905012	(G/A)
11049	CakSNP11049	*Kabuli*	Ca_Kabuli_Chr06	42905039	(C/T)
11050	CakSNP11050	*Kabuli*	Ca_Kabuli_Chr06	42905035	(T/A)
11051	CakSNP11051	*Kabuli*	Ca_Kabuli_Chr06	42905111	(T/C)
11052	CakSNP11052	*Kabuli*	Ca_Kabuli_Chr06	42905094	(C/T)
11053	CakSNP11053	*Kabuli*	Ca_Kabuli_Chr06	42905090	(G/A)
11054	CakSNP11054	*Kabuli*	Ca_Kabuli_Chr06	42905086	(T/C)
11055	CakSNP11055	*Kabuli*	Ca_Kabuli_Chr06	42905058	(C/G)
11056	CakSNP11056	*Kabuli*	Ca_Kabuli_Chr06	42905059	(A/G)
11057	CakSNP11057	*Kabuli*	Ca_Kabuli_Chr06	42905072	(T/C)
11058	CakSNP11058	*Kabuli*	Ca_Kabuli_Chr06	42905115	(G/T)
11059	CakSNP11059	*Kabuli*	Ca_Kabuli_Chr06	42905116	(A/T)
11060	CakSNP11060	*Kabuli*	Ca_Kabuli_Chr06	42919899	(G/A)
11061	CakSNP11061	*Kabuli*	Ca_Kabuli_Chr06	42923969	(A/C)
11062	CakSNP11062	*Kabuli*	Ca_Kabuli_Chr06	42925974	(C/T)
11063	CakSNP11063	*Kabuli*	Ca_Kabuli_Chr06	43148829	(C/T)
11064	CakSNP11064	*Kabuli*	Ca_Kabuli_Chr06	43148824	(C/A)
11065	CakSNP11065	*Kabuli*	Ca_Kabuli_Chr06	43148784	(C/G)
11066	CakSNP11066	*Kabuli*	Ca_Kabuli_Chr06	43905509	(A/T)
11067	CakSNP11067	*Kabuli*	Ca_Kabuli_Chr06	43907445	(T/C)
11068	CakSNP11068	*Kabuli*	Ca_Kabuli_Chr06	44530888	(C/A)
11069	CakSNP11069	*Kabuli*	Ca_Kabuli_Chr06	44530889	(A/G)
11070	CakSNP11070	*Kabuli*	Ca_Kabuli_Chr06	44707313	(T/A)
11071	CakSNP11071	*Kabuli*	Ca_Kabuli_Chr06	44711166	(A/G)
11072	CakSNP11072	*Kabuli*	Ca_Kabuli_Chr06	44792535	(C/T)
11073	CakSNP11073	*Kabuli*	Ca_Kabuli_Chr06	44792529	(G/C)
11074	CakSNP11074	*Kabuli*	Ca_Kabuli_Chr06	44792493	(C/T)

S.N.	SNP IDs	Cultivars	Chromosomes/scaffolds	Physical positions (bp)	SNPs
11075	CakSNP11075	*Kabuli*	Ca_Kabuli_Chr06	44792486	(G/A)
11076	CakSNP11076	*Kabuli*	Ca_Kabuli_Chr06	44937666	(C/T)
11077	CakSNP11077	*Kabuli*	Ca_Kabuli_Chr06	44944917	(G/A)
11078	CakSNP11078	*Kabuli*	Ca_Kabuli_Chr06	45053093	(A/G)
11079	CakSNP11079	*Kabuli*	Ca_Kabuli_Chr06	45053214	(C/T)
11080	CakSNP11080	*Kabuli*	Ca_Kabuli_Chr06	45131295	(C/T)
11081	CakSNP11081	*Kabuli*	Ca_Kabuli_Chr06	45132545	(A/G)
11082	CakSNP11082	*Kabuli*	Ca_Kabuli_Chr06	45178060	(G/A)
11083	CakSNP11083	*Kabuli*	Ca_Kabuli_Chr06	45178056	(A/G)
11084	CakSNP11084	*Kabuli*	Ca_Kabuli_Chr06	45178045	(G/T)
11085	CakSNP11085	*Kabuli*	Ca_Kabuli_Chr06	45178044	(G/A)
11086	CakSNP11086	*Kabuli*	Ca_Kabuli_Chr06	45178033	(C/A)
11087	CakSNP11087	*Kabuli*	Ca_Kabuli_Chr06	45178061	(C/T)
11088	CakSNP11088	*Kabuli*	Ca_Kabuli_Chr06	45201783	(C/T)
11089	CakSNP11089	*Kabuli*	Ca_Kabuli_Chr06	45293271	(T/C)
11090	CakSNP11090	*Kabuli*	Ca_Kabuli_Chr06	45500961	(T/A)
11091	CakSNP11091	*Kabuli*	Ca_Kabuli_Chr06	45501099	(C/A)
11092	CakSNP11092	*Kabuli*	Ca_Kabuli_Chr06	45505942	(A/G)
11093	CakSNP11093	*Kabuli*	Ca_Kabuli_Chr06	45550031	(C/A)
11094	CakSNP11094	*Kabuli*	Ca_Kabuli_Chr06	45554040	(G/A)
11095	CakSNP11095	*Kabuli*	Ca_Kabuli_Chr06	45557656	(T/C)
11096	CakSNP11096	*Kabuli*	Ca_Kabuli_Chr06	45614017	(T/C)
11097	CakSNP11097	*Kabuli*	Ca_Kabuli_Chr06	45637271	(T/G)
11098	CakSNP11098	*Kabuli*	Ca_Kabuli_Chr06	45637292	(C/A)
11099	CakSNP11099	*Kabuli*	Ca_Kabuli_Chr06	45637293	(T/C)
11100	CakSNP11100	*Kabuli*	Ca_Kabuli_Chr06	45637322	(C/A)
11101	CakSNP11101	*Kabuli*	Ca_Kabuli_Chr06	45637309	(G/A)
11102	CakSNP11102	*Kabuli*	Ca_Kabuli_Chr06	45637308	(G/T)
11103	CakSNP11103	*Kabuli*	Ca_Kabuli_Chr06	45693275	(G/A)
11104	CakSNP11104	*Kabuli*	Ca_Kabuli_Chr06	45695051	(T/G)
11105	CakSNP11105	*Kabuli*	Ca_Kabuli_Chr06	45695043	(C/T)
11106	CakSNP11106	*Kabuli*	Ca_Kabuli_Chr06	45695035	(G/A)
11107	CakSNP11107	*Kabuli*	Ca_Kabuli_Chr06	45709318	(T/C)
11108	CakSNP11108	*Kabuli*	Ca_Kabuli_Chr06	45773318	(C/T)
11109	CakSNP11109	*Kabuli*	Ca_Kabuli_Chr06	45773309	(A/G)
11110	CakSNP11110	*Kabuli*	Ca_Kabuli_Chr06	45773298	(C/G)
11111	CakSNP11111	*Kabuli*	Ca_Kabuli_Chr06	45773409	(T/A)
11112	CakSNP11112	*Kabuli*	Ca_Kabuli_Chr06	45773408	(T/A)
11113	CakSNP11113	*Kabuli*	Ca_Kabuli_Chr06	45773373	(G/T)

S.N.	SNP IDs	Cultivars	Chromosomes/scaffolds	Physical positions (bp)	SNPs
11114	CakSNP11114	*Kabuli*	Ca_Kabuli_Chr06	45773367	(G/T)
11115	CakSNP11115	*Kabuli*	Ca_Kabuli_Chr06	45773349	(C/T)
11116	CakSNP11116	*Kabuli*	Ca_Kabuli_Chr06	45812152	(G/A)
11117	CakSNP11117	*Kabuli*	Ca_Kabuli_Chr06	45976962	(G/A)
11118	CakSNP11118	*Kabuli*	Ca_Kabuli_Chr06	45978206	(T/C)
11119	CakSNP11119	*Kabuli*	Ca_Kabuli_Chr06	45978263	(G/A)
11120	CakSNP11120	*Kabuli*	Ca_Kabuli_Chr06	46158211	(C/T)
11121	CakSNP11121	*Kabuli*	Ca_Kabuli_Chr06	46734706	(C/G)
11122	CakSNP11122	*Kabuli*	Ca_Kabuli_Chr06	46734725	(G/A)
11123	CakSNP11123	*Kabuli*	Ca_Kabuli_Chr06	46734726	(T/C)
11124	CakSNP11124	*Kabuli*	Ca_Kabuli_Chr06	46765659	(G/A)
11125	CakSNP11125	*Kabuli*	Ca_Kabuli_Chr06	46765661	(A/T)
11126	CakSNP11126	*Kabuli*	Ca_Kabuli_Chr06	46765673	(G/A)
11127	CakSNP11127	*Kabuli*	Ca_Kabuli_Chr06	46765688	(A/C)
11128	CakSNP11128	*Kabuli*	Ca_Kabuli_Chr06	46765725	(C/T)
11129	CakSNP11129	*Kabuli*	Ca_Kabuli_Chr06	46765778	(C/A)
11130	CakSNP11130	*Kabuli*	Ca_Kabuli_Chr06	46765696	(C/T)
11131	CakSNP11131	*Kabuli*	Ca_Kabuli_Chr06	46830497	(A/T)
11132	CakSNP11132	*Kabuli*	Ca_Kabuli_Chr06	46896903	(C/T)
11133	CakSNP11133	*Kabuli*	Ca_Kabuli_Chr06	46896910	(A/C)
11134	CakSNP11134	*Kabuli*	Ca_Kabuli_Chr06	46898225	(A/C)
11135	CakSNP11135	*Kabuli*	Ca_Kabuli_Chr06	46898305	(T/G)
11136	CakSNP11136	*Kabuli*	Ca_Kabuli_Chr06	46898316	(T/G)
11137	CakSNP11137	*Kabuli*	Ca_Kabuli_Chr06	46898317	(C/T)
11138	CakSNP11138	*Kabuli*	Ca_Kabuli_Chr06	46898363	(C/T)
11139	CakSNP11139	*Kabuli*	Ca_Kabuli_Chr06	46898329	(T/A)
11140	CakSNP11140	*Kabuli*	Ca_Kabuli_Chr06	46898747	(G/A)
11141	CakSNP11141	*Kabuli*	Ca_Kabuli_Chr06	46898743	(A/G)
11142	CakSNP11142	*Kabuli*	Ca_Kabuli_Chr06	46899229	(G/A)
11143	CakSNP11143	*Kabuli*	Ca_Kabuli_Chr06	46979240	(C/T)
11144	CakSNP11144	*Kabuli*	Ca_Kabuli_Chr06	46979267	(C/A)
11145	CakSNP11145	*Kabuli*	Ca_Kabuli_Chr06	46998948	(G/A)
11146	CakSNP11146	*Kabuli*	Ca_Kabuli_Chr06	46999012	(C/T)
11147	CakSNP11147	*Kabuli*	Ca_Kabuli_Chr06	47104478	(T/G)
11148	CakSNP11148	*Kabuli*	Ca_Kabuli_Chr06	47104505	(C/T)
11149	CakSNP11149	*Kabuli*	Ca_Kabuli_Chr06	47106174	(A/G)
11150	CakSNP11150	*Kabuli*	Ca_Kabuli_Chr06	47106238	(C/A)
11151	CakSNP11151	*Kabuli*	Ca_Kabuli_Chr06	47232630	(C/T)
11152	CakSNP11152	*Kabuli*	Ca_Kabuli_Chr06	47232631	(C/G)

S.N.	SNP IDs	Cultivars	Chromosomes/scaffolds	Physical positions (bp)	SNPs
11153	CakSNP11153	*Kabuli*	Ca_Kabuli_Chr06	47232685	(T/A)
11154	CakSNP11154	*Kabuli*	Ca_Kabuli_Chr06	47440468	(A/T)
11155	CakSNP11155	*Kabuli*	Ca_Kabuli_Chr06	47575655	(G/T)
11156	CakSNP11156	*Kabuli*	Ca_Kabuli_Chr06	47575659	(C/G)
11157	CakSNP11157	*Kabuli*	Ca_Kabuli_Chr06	47906683	(G/A)
11158	CakSNP11158	*Kabuli*	Ca_Kabuli_Chr06	47906680	(C/T)
11159	CakSNP11159	*Kabuli*	Ca_Kabuli_Chr06	47906629	(T/G)
11160	CakSNP11160	*Kabuli*	Ca_Kabuli_Chr06	48088550	(G/A)
11161	CakSNP11161	*Kabuli*	Ca_Kabuli_Chr06	48088592	(C/T)
11162	CakSNP11162	*Kabuli*	Ca_Kabuli_Chr06	48255392	(G/A)
11163	CakSNP11163	*Kabuli*	Ca_Kabuli_Chr06	48255538	(A/G)
11164	CakSNP11164	*Kabuli*	Ca_Kabuli_Chr06	48255558	(C/T)
11165	CakSNP11165	*Kabuli*	Ca_Kabuli_Chr06	48255559	(A/G)
11166	CakSNP11166	*Kabuli*	Ca_Kabuli_Chr06	48255568	(A/G)
11167	CakSNP11167	*Kabuli*	Ca_Kabuli_Chr06	48255599	(C/A)
11168	CakSNP11168	*Kabuli*	Ca_Kabuli_Chr06	48314052	(T/A)
11169	CakSNP11169	*Kabuli*	Ca_Kabuli_Chr06	48314788	(T/C)
11170	CakSNP11170	*Kabuli*	Ca_Kabuli_Chr06	48315194	(C/A)
11171	CakSNP11171	*Kabuli*	Ca_Kabuli_Chr06	48315340	(C/A)
11172	CakSNP11172	*Kabuli*	Ca_Kabuli_Chr06	48315343	(G/T)
11173	CakSNP11173	*Kabuli*	Ca_Kabuli_Chr06	48810611	(A/T)
11174	CakSNP11174	*Kabuli*	Ca_Kabuli_Chr06	48810608	(C/T)
11175	CakSNP11175	*Kabuli*	Ca_Kabuli_Chr06	48864622	(T/C)
11176	CakSNP11176	*Kabuli*	Ca_Kabuli_Chr06	48864625	(G/A)
11177	CakSNP11177	*Kabuli*	Ca_Kabuli_Chr06	48864639	(A/C)
11178	CakSNP11178	*Kabuli*	Ca_Kabuli_Chr06	48864699	(T/C)
11179	CakSNP11179	*Kabuli*	Ca_Kabuli_Chr06	48869462	(C/T)
11180	CakSNP11180	*Kabuli*	Ca_Kabuli_Chr06	48869473	(C/A)
11181	CakSNP11181	*Kabuli*	Ca_Kabuli_Chr06	48869484	(G/A)
11182	CakSNP11182	*Kabuli*	Ca_Kabuli_Chr06	48869577	(C/T)
11183	CakSNP11183	*Kabuli*	Ca_Kabuli_Chr06	48869539	(A/G)
11184	CakSNP11184	*Kabuli*	Ca_Kabuli_Chr06	48869529	(T/C)
11185	CakSNP11185	*Kabuli*	Ca_Kabuli_Chr06	48869512	(G/C)
11186	CakSNP11186	*Kabuli*	Ca_Kabuli_Chr06	48940386	(A/C)
11187	CakSNP11187	*Kabuli*	Ca_Kabuli_Chr06	49044772	(A/C)
11188	CakSNP11188	*Kabuli*	Ca_Kabuli_Chr06	49044785	(C/T)
11189	CakSNP11189	*Kabuli*	Ca_Kabuli_Chr06	49044794	(T/A)
11190	CakSNP11190	*Kabuli*	Ca_Kabuli_Chr06	49044802	(T/C)
11191	CakSNP11191	*Kabuli*	Ca_Kabuli_Chr06	49044805	(A/G)

S.N.	SNP IDs	Cultivars	Chromosomes/scaffolds	Physical positions (bp)	SNPs
11192	CakSNP11192	*Kabuli*	Ca_Kabuli_Chr06	49044814	(G/T)
11193	CakSNP11193	*Kabuli*	Ca_Kabuli_Chr06	49044824	(C/T)
11194	CakSNP11194	*Kabuli*	Ca_Kabuli_Chr06	49044839	(T/A)
11195	CakSNP11195	*Kabuli*	Ca_Kabuli_Chr06	49044850	(A/C)
11196	CakSNP11196	*Kabuli*	Ca_Kabuli_Chr06	49044847	(G/A)
11197	CakSNP11197	*Kabuli*	Ca_Kabuli_Chr06	49044842	(G/A)
11198	CakSNP11198	*Kabuli*	Ca_Kabuli_Chr06	49044838	(C/A)
11199	CakSNP11199	*Kabuli*	Ca_Kabuli_Chr06	49044835	(C/A)
11200	CakSNP11200	*Kabuli*	Ca_Kabuli_Chr06	49044826	(G/A)
11201	CakSNP11201	*Kabuli*	Ca_Kabuli_Chr06	49044821	(C/G)
11202	CakSNP11202	*Kabuli*	Ca_Kabuli_Chr06	49044810	(G/A)
11203	CakSNP11203	*Kabuli*	Ca_Kabuli_Chr06	49044808	(C/T)
11204	CakSNP11204	*Kabuli*	Ca_Kabuli_Chr06	49044788	(T/A)
11205	CakSNP11205	*Kabuli*	Ca_Kabuli_Chr06	49044780	(G/A)
11206	CakSNP11206	*Kabuli*	Ca_Kabuli_Chr06	49044915	(A/G)
11207	CakSNP11207	*Kabuli*	Ca_Kabuli_Chr06	49044928	(C/A)
11208	CakSNP11208	*Kabuli*	Ca_Kabuli_Chr06	49044936	(C/T)
11209	CakSNP11209	*Kabuli*	Ca_Kabuli_Chr06	49044937	(A/G)
11210	CakSNP11210	*Kabuli*	Ca_Kabuli_Chr06	49044944	(A/G)
11211	CakSNP11211	*Kabuli*	Ca_Kabuli_Chr06	49101829	(G/C)
11212	CakSNP11212	*Kabuli*	Ca_Kabuli_Chr06	49167341	(T/G)
11213	CakSNP11213	*Kabuli*	Ca_Kabuli_Chr06	49222291	(G/A)
11214	CakSNP11214	*Kabuli*	Ca_Kabuli_Chr06	49222290	(C/T)
11215	CakSNP11215	*Kabuli*	Ca_Kabuli_Chr06	49222275	(A/C)
11216	CakSNP11216	*Kabuli*	Ca_Kabuli_Chr06	49222268	(T/A)
11217	CakSNP11217	*Kabuli*	Ca_Kabuli_Chr06	49222251	(G/A)
11218	CakSNP11218	*Kabuli*	Ca_Kabuli_Chr06	49222235	(G/C)
11219	CakSNP11219	*Kabuli*	Ca_Kabuli_Chr06	49377275	(G/A)
11220	CakSNP11220	*Kabuli*	Ca_Kabuli_Chr06	49403430	(G/C)
11221	CakSNP11221	*Kabuli*	Ca_Kabuli_Chr06	49406604	(G/A)
11222	CakSNP11222	*Kabuli*	Ca_Kabuli_Chr06	49406599	(C/A)
11223	CakSNP11223	*Kabuli*	Ca_Kabuli_Chr06	49466066	(A/G)
11224	CakSNP11224	*Kabuli*	Ca_Kabuli_Chr06	49475716	(G/A)
11225	CakSNP11225	*Kabuli*	Ca_Kabuli_Chr06	49475715	(A/G)
11226	CakSNP11226	*Kabuli*	Ca_Kabuli_Chr06	49524179	(A/T)
11227	CakSNP11227	*Kabuli*	Ca_Kabuli_Chr06	49565934	(C/T)
11228	CakSNP11228	*Kabuli*	Ca_Kabuli_Chr06	49664132	(C/G)
11229	CakSNP11229	*Kabuli*	Ca_Kabuli_Chr06	49724448	(C/T)
11230	CakSNP11230	*Kabuli*	Ca_Kabuli_Chr06	49724515	(C/T)

S.N.	SNP IDs	Cultivars	Chromosomes/scaffolds	Physical positions (bp)	SNPs
11231	CakSNP11231	*Kabuli*	Ca_Kabuli_Chr06	49726734	(C/G)
11232	CakSNP11232	*Kabuli*	Ca_Kabuli_Chr06	49726732	(T/C)
11233	CakSNP11233	*Kabuli*	Ca_Kabuli_Chr06	49726729	(G/A)
11234	CakSNP11234	*Kabuli*	Ca_Kabuli_Chr06	49726711	(C/T)
11235	CakSNP11235	*Kabuli*	Ca_Kabuli_Chr06	49726723	(G/A)
11236	CakSNP11236	*Kabuli*	Ca_Kabuli_Chr06	49830299	(A/G)
11237	CakSNP11237	*Kabuli*	Ca_Kabuli_Chr06	49830384	(G/A)
11238	CakSNP11238	*Kabuli*	Ca_Kabuli_Chr06	49833934	(C/T)
11239	CakSNP11239	*Kabuli*	Ca_Kabuli_Chr06	49843667	(G/A)
11240	CakSNP11240	*Kabuli*	Ca_Kabuli_Chr06	49846257	(A/T)
11241	CakSNP11241	*Kabuli*	Ca_Kabuli_Chr06	49846303	(T/G)
11242	CakSNP11242	*Kabuli*	Ca_Kabuli_Chr06	49931821	(C/T)
11243	CakSNP11243	*Kabuli*	Ca_Kabuli_Chr06	49931879	(T/C)
11244	CakSNP11244	*Kabuli*	Ca_Kabuli_Chr06	49942397	(A/G)
11245	CakSNP11245	*Kabuli*	Ca_Kabuli_Chr06	50086781	(A/G)
11246	CakSNP11246	*Kabuli*	Ca_Kabuli_Chr06	50152053	(C/T)
11247	CakSNP11247	*Kabuli*	Ca_Kabuli_Chr06	50152129	(C/T)
11248	CakSNP11248	*Kabuli*	Ca_Kabuli_Chr06	50152124	(C/T)
11249	CakSNP11249	*Kabuli*	Ca_Kabuli_Chr06	50152119	(T/C)
11250	CakSNP11250	*Kabuli*	Ca_Kabuli_Chr06	50152083	(C/T)
11251	CakSNP11251	*Kabuli*	Ca_Kabuli_Chr06	50217734	(G/A)
11252	CakSNP11252	*Kabuli*	Ca_Kabuli_Chr06	50356239	(A/G)
11253	CakSNP11253	*Kabuli*	Ca_Kabuli_Chr06	50356229	(C/T)
11254	CakSNP11254	*Kabuli*	Ca_Kabuli_Chr06	50356222	(C/A)
11255	CakSNP11255	*Kabuli*	Ca_Kabuli_Chr06	50356217	(T/C)
11256	CakSNP11256	*Kabuli*	Ca_Kabuli_Chr06	50356206	(G/T)
11257	CakSNP11257	*Kabuli*	Ca_Kabuli_Chr06	50356201	(T/C)
11258	CakSNP11258	*Kabuli*	Ca_Kabuli_Chr06	50382452	(C/T)
11259	CakSNP11259	*Kabuli*	Ca_Kabuli_Chr06	50384101	(A/C)
11260	CakSNP11260	*Kabuli*	Ca_Kabuli_Chr06	50390752	(A/G)
11261	CakSNP11261	*Kabuli*	Ca_Kabuli_Chr06	50476721	(T/C)
11262	CakSNP11262	*Kabuli*	Ca_Kabuli_Chr06	50476775	(G/A)
11263	CakSNP11263	*Kabuli*	Ca_Kabuli_Chr06	50477672	(A/C)
11264	CakSNP11264	*Kabuli*	Ca_Kabuli_Chr06	50581295	(T/G)
11265	CakSNP11265	*Kabuli*	Ca_Kabuli_Chr06	50617926	(G/C)
11266	CakSNP11266	*Kabuli*	Ca_Kabuli_Chr06	50617932	(G/A)
11267	CakSNP11267	*Kabuli*	Ca_Kabuli_Chr06	50723595	(T/C)
11268	CakSNP11268	*Kabuli*	Ca_Kabuli_Chr06	50723630	(C/T)
11269	CakSNP11269	*Kabuli*	Ca_Kabuli_Chr06	50723916	(T/C)

S.N.	SNP IDs	Cultivars	Chromosomes/scaffolds	Physical positions (bp)	SNPs
11270	CakSNP11270	*Kabuli*	Ca_Kabuli_Chr06	50723950	(A/G)
11271	CakSNP11271	*Kabuli*	Ca_Kabuli_Chr06	50723980	(A/G)
11272	CakSNP11272	*Kabuli*	Ca_Kabuli_Chr06	50723988	(C/A)
11273	CakSNP11273	*Kabuli*	Ca_Kabuli_Chr06	51157961	(C/T)
11274	CakSNP11274	*Kabuli*	Ca_Kabuli_Chr06	51157939	(A/C)
11275	CakSNP11275	*Kabuli*	Ca_Kabuli_Chr06	51162065	(T/C)
11276	CakSNP11276	*Kabuli*	Ca_Kabuli_Chr06	51281235	(G/A)
11277	CakSNP11277	*Kabuli*	Ca_Kabuli_Chr06	51281178	(C/G)
11278	CakSNP11278	*Kabuli*	Ca_Kabuli_Chr06	51345460	(G/C)
11279	CakSNP11279	*Kabuli*	Ca_Kabuli_Chr06	51392609	(T/C)
11280	CakSNP11280	*Kabuli*	Ca_Kabuli_Chr06	51471573	(T/C)
11281	CakSNP11281	*Kabuli*	Ca_Kabuli_Chr06	52007211	(T/C)
11282	CakSNP11282	*Kabuli*	Ca_Kabuli_Chr06	52007217	(G/A)
11283	CakSNP11283	*Kabuli*	Ca_Kabuli_Chr06	52007471	(C/T)
11284	CakSNP11284	*Kabuli*	Ca_Kabuli_Chr06	52007464	(C/G)
11285	CakSNP11285	*Kabuli*	Ca_Kabuli_Chr06	52007440	(C/T)
11286	CakSNP11286	*Kabuli*	Ca_Kabuli_Chr06	52033279	(G/A)
11287	CakSNP11287	*Kabuli*	Ca_Kabuli_Chr06	52033244	(A/T)
11288	CakSNP11288	*Kabuli*	Ca_Kabuli_Chr06	52059750	(A/G)
11289	CakSNP11289	*Kabuli*	Ca_Kabuli_Chr06	52065393	(C/A)
11290	CakSNP11290	*Kabuli*	Ca_Kabuli_Chr06	52065527	(T/A)
11291	CakSNP11291	*Kabuli*	Ca_Kabuli_Chr06	52164571	(C/A)
11292	CakSNP11292	*Kabuli*	Ca_Kabuli_Chr06	52227277	(T/C)
11293	CakSNP11293	*Kabuli*	Ca_Kabuli_Chr06	52304094	(C/T)
11294	CakSNP11294	*Kabuli*	Ca_Kabuli_Chr06	52304112	(A/G)
11295	CakSNP11295	*Kabuli*	Ca_Kabuli_Chr06	52363121	(C/T)
11296	CakSNP11296	*Kabuli*	Ca_Kabuli_Chr06	52416366	(T/A)
11297	CakSNP11297	*Kabuli*	Ca_Kabuli_Chr06	52417684	(C/T)
11298	CakSNP11298	*Kabuli*	Ca_Kabuli_Chr06	52417717	(T/G)
11299	CakSNP11299	*Kabuli*	Ca_Kabuli_Chr06	52417720	(A/T)
11300	CakSNP11300	*Kabuli*	Ca_Kabuli_Chr06	52417736	(T/A)
11301	CakSNP11301	*Kabuli*	Ca_Kabuli_Chr06	52417747	(G/T)
11302	CakSNP11302	*Kabuli*	Ca_Kabuli_Chr06	52567474	(A/C)
11303	CakSNP11303	*Kabuli*	Ca_Kabuli_Chr06	52596602	(T/A)
11304	CakSNP11304	*Kabuli*	Ca_Kabuli_Chr06	52596601	(A/T)
11305	CakSNP11305	*Kabuli*	Ca_Kabuli_Chr06	52858359	(A/G)
11306	CakSNP11306	*Kabuli*	Ca_Kabuli_Chr06	52993231	(A/G)
11307	CakSNP11307	*Kabuli*	Ca_Kabuli_Chr06	53068176	(C/T)
11308	CakSNP11308	*Kabuli*	Ca_Kabuli_Chr06	53068201	(G/T)

S.N.	SNP IDs	Cultivars	Chromosomes/scaffolds	Physical positions (bp)	SNPs
11309	CakSNP11309	*Kabuli*	Ca_Kabuli_Chr06	53097833	(G/C)
11310	CakSNP11310	*Kabuli*	Ca_Kabuli_Chr06	53097803	(A/G)
11311	CakSNP11311	*Kabuli*	Ca_Kabuli_Chr06	53097766	(G/A)
11312	CakSNP11312	*Kabuli*	Ca_Kabuli_Chr06	53124182	(G/T)
11313	CakSNP11313	*Kabuli*	Ca_Kabuli_Chr06	53168715	(T/G)
11314	CakSNP11314	*Kabuli*	Ca_Kabuli_Chr06	53348993	(C/A)
11315	CakSNP11315	*Kabuli*	Ca_Kabuli_Chr06	53349034	(G/A)
11316	CakSNP11316	*Kabuli*	Ca_Kabuli_Chr06	53390065	(A/G)
11317	CakSNP11317	*Kabuli*	Ca_Kabuli_Chr06	53461929	(C/A)
11318	CakSNP11318	*Kabuli*	Ca_Kabuli_Chr06	53461883	(A/G)
11319	CakSNP11319	*Kabuli*	Ca_Kabuli_Chr06	53541031	(G/A)
11320	CakSNP11320	*Kabuli*	Ca_Kabuli_Chr06	53541094	(C/G)
11321	CakSNP11321	*Kabuli*	Ca_Kabuli_Chr06	53541111	(T/G)
11322	CakSNP11322	*Kabuli*	Ca_Kabuli_Chr06	53551878	(C/A)
11323	CakSNP11323	*Kabuli*	Ca_Kabuli_Chr06	53558430	(A/C)
11324	CakSNP11324	*Kabuli*	Ca_Kabuli_Chr06	53566496	(G/A)
11325	CakSNP11325	*Kabuli*	Ca_Kabuli_Chr06	53566506	(C/T)
11326	CakSNP11326	*Kabuli*	Ca_Kabuli_Chr06	53625264	(A/C)
11327	CakSNP11327	*Kabuli*	Ca_Kabuli_Chr06	53683753	(T/C)
11328	CakSNP11328	*Kabuli*	Ca_Kabuli_Chr06	53683891	(C/A)
11329	CakSNP11329	*Kabuli*	Ca_Kabuli_Chr06	53683885	(C/T)
11330	CakSNP11330	*Kabuli*	Ca_Kabuli_Chr06	53683879	(C/T)
11331	CakSNP11331	*Kabuli*	Ca_Kabuli_Chr06	53683876	(C/T)
11332	CakSNP11332	*Kabuli*	Ca_Kabuli_Chr06	53692784	(G/A)
11333	CakSNP11333	*Kabuli*	Ca_Kabuli_Chr06	53769044	(A/C)
11334	CakSNP11334	*Kabuli*	Ca_Kabuli_Chr06	53769160	(C/T)
11335	CakSNP11335	*Kabuli*	Ca_Kabuli_Chr06	53769240	(T/A)
11336	CakSNP11336	*Kabuli*	Ca_Kabuli_Chr06	53769241	(A/T)
11337	CakSNP11337	*Kabuli*	Ca_Kabuli_Chr06	54091997	(A/C)
11338	CakSNP11338	*Kabuli*	Ca_Kabuli_Chr06	54151902	(T/G)
11339	CakSNP11339	*Kabuli*	Ca_Kabuli_Chr06	54151966	(G/C)
11340	CakSNP11340	*Kabuli*	Ca_Kabuli_Chr06	54151969	(T/G)
11341	CakSNP11341	*Kabuli*	Ca_Kabuli_Chr06	54203356	(G/A)
11342	CakSNP11342	*Kabuli*	Ca_Kabuli_Chr06	54203400	(C/T)
11343	CakSNP11343	*Kabuli*	Ca_Kabuli_Chr06	54226451	(A/T)
11344	CakSNP11344	*Kabuli*	Ca_Kabuli_Chr06	54226432	(A/C)
11345	CakSNP11345	*Kabuli*	Ca_Kabuli_Chr06	54229449	(G/T)
11346	CakSNP11346	*Kabuli*	Ca_Kabuli_Chr06	54237764	(A/C)
11347	CakSNP11347	*Kabuli*	Ca_Kabuli_Chr06	54237815	(C/T)

S.N.	SNP IDs	Cultivars	Chromosomes/scaffolds	Physical positions (bp)	SNPs
11348	CakSNP11348	*Kabuli*	Ca_Kabuli_Chr06	54250702	(C/T)
11349	CakSNP11349	*Kabuli*	Ca_Kabuli_Chr06	54368267	(G/A)
11350	CakSNP11350	*Kabuli*	Ca_Kabuli_Chr06	54869636	(C/T)
11351	CakSNP11351	*Kabuli*	Ca_Kabuli_Chr06	54869666	(G/A)
11352	CakSNP11352	*Kabuli*	Ca_Kabuli_Chr06	54997337	(A/T)
11353	CakSNP11353	*Kabuli*	Ca_Kabuli_Chr06	55024071	(C/T)
11354	CakSNP11354	*Kabuli*	Ca_Kabuli_Chr06	55024091	(G/A)
11355	CakSNP11355	*Kabuli*	Ca_Kabuli_Chr06	55024146	(C/T)
11356	CakSNP11356	*Kabuli*	Ca_Kabuli_Chr06	55024140	(G/A)
11357	CakSNP11357	*Kabuli*	Ca_Kabuli_Chr06	55024130	(T/A)
11358	CakSNP11358	*Kabuli*	Ca_Kabuli_Chr06	55024112	(C/G)
11359	CakSNP11359	*Kabuli*	Ca_Kabuli_Chr06	55024093	(G/C)
11360	CakSNP11360	*Kabuli*	Ca_Kabuli_Chr06	55024108	(C/T)
11361	CakSNP11361	*Kabuli*	Ca_Kabuli_Chr06	55024136	(C/T)
11362	CakSNP11362	*Kabuli*	Ca_Kabuli_Chr06	55024159	(T/A)
11363	CakSNP11363	*Kabuli*	Ca_Kabuli_Chr06	55024198	(T/G)
11364	CakSNP11364	*Kabuli*	Ca_Kabuli_Chr06	55028980	(G/A)
11365	CakSNP11365	*Kabuli*	Ca_Kabuli_Chr06	55074979	(T/A)
11366	CakSNP11366	*Kabuli*	Ca_Kabuli_Chr06	55083311	(T/A)
11367	CakSNP11367	*Kabuli*	Ca_Kabuli_Chr06	55083721	(T/A)
11368	CakSNP11368	*Kabuli*	Ca_Kabuli_Chr06	55180211	(A/G)
11369	CakSNP11369	*Kabuli*	Ca_Kabuli_Chr06	55332515	(G/A)
11370	CakSNP11370	*Kabuli*	Ca_Kabuli_Chr06	55914455	(T/C)
11371	CakSNP11371	*Kabuli*	Ca_Kabuli_Chr06	55914451	(A/G)
11372	CakSNP11372	*Kabuli*	Ca_Kabuli_Chr06	55914443	(T/C)
11373	CakSNP11373	*Kabuli*	Ca_Kabuli_Chr06	55914434	(C/T)
11374	CakSNP11374	*Kabuli*	Ca_Kabuli_Chr06	55914431	(G/A)
11375	CakSNP11375	*Kabuli*	Ca_Kabuli_Chr06	55922843	(T/A)
11376	CakSNP11376	*Kabuli*	Ca_Kabuli_Chr06	55922850	(C/T)
11377	CakSNP11377	*Kabuli*	Ca_Kabuli_Chr06	55942105	(A/G)
11378	CakSNP11378	*Kabuli*	Ca_Kabuli_Chr06	55953320	(T/A)
11379	CakSNP11379	*Kabuli*	Ca_Kabuli_Chr06	55953327	(C/T)
11380	CakSNP11380	*Kabuli*	Ca_Kabuli_Chr06	56025902	(A/G)
11381	CakSNP11381	*Kabuli*	Ca_Kabuli_Chr06	56025888	(G/A)
11382	CakSNP11382	*Kabuli*	Ca_Kabuli_Chr06	56025884	(A/G)
11383	CakSNP11383	*Kabuli*	Ca_Kabuli_Chr06	56029491	(T/A)
11384	CakSNP11384	*Kabuli*	Ca_Kabuli_Chr06	56029544	(A/T)
11385	CakSNP11385	*Kabuli*	Ca_Kabuli_Chr06	56029549	(C/T)
11386	CakSNP11386	*Kabuli*	Ca_Kabuli_Chr06	56137878	(T/C)

S.N.	SNP IDs	Cultivars	Chromosomes/scaffolds	Physical positions (bp)	SNPs
11387	CakSNP11387	*Kabuli*	Ca_Kabuli_Chr06	56553930	(G/A)
11388	CakSNP11388	*Kabuli*	Ca_Kabuli_Chr06	56564249	(G/C)
11389	CakSNP11389	*Kabuli*	Ca_Kabuli_Chr06	56587094	(C/G)
11390	CakSNP11390	*Kabuli*	Ca_Kabuli_Chr06	56593148	(A/G)
11391	CakSNP11391	*Kabuli*	Ca_Kabuli_Chr06	56593121	(T/G)
11392	CakSNP11392	*Kabuli*	Ca_Kabuli_Chr06	56624316	(A/T)
11393	CakSNP11393	*Kabuli*	Ca_Kabuli_Chr06	56624674	(T/C)
11394	CakSNP11394	*Kabuli*	Ca_Kabuli_Chr06	56624718	(C/A)
11395	CakSNP11395	*Kabuli*	Ca_Kabuli_Chr06	56694898	(T/C)
11396	CakSNP11396	*Kabuli*	Ca_Kabuli_Chr06	56765524	(C/A)
11397	CakSNP11397	*Kabuli*	Ca_Kabuli_Chr06	56793077	(G/A)
11398	CakSNP11398	*Kabuli*	Ca_Kabuli_Chr06	56793037	(A/T)
11399	CakSNP11399	*Kabuli*	Ca_Kabuli_Chr06	56793021	(G/T)
11400	CakSNP11400	*Kabuli*	Ca_Kabuli_Chr06	56793122	(C/T)
11401	CakSNP11401	*Kabuli*	Ca_Kabuli_Chr06	56822084	(G/T)
11402	CakSNP11402	*Kabuli*	Ca_Kabuli_Chr06	56822060	(G/A)
11403	CakSNP11403	*Kabuli*	Ca_Kabuli_Chr06	56840030	(A/C)
11404	CakSNP11404	*Kabuli*	Ca_Kabuli_Chr06	56840084	(A/T)
11405	CakSNP11405	*Kabuli*	Ca_Kabuli_Chr06	56840063	(T/A)
11406	CakSNP11406	*Kabuli*	Ca_Kabuli_Chr06	56840038	(A/T)
11407	CakSNP11407	*Kabuli*	Ca_Kabuli_Chr06	56854753	(T/C)
11408	CakSNP11408	*Kabuli*	Ca_Kabuli_Chr06	56946415	(A/G)
11409	CakSNP11409	*Kabuli*	Ca_Kabuli_Chr06	56946431	(T/C)
11410	CakSNP11410	*Kabuli*	Ca_Kabuli_Chr06	56950487	(G/T)
11411	CakSNP11411	*Kabuli*	Ca_Kabuli_Chr06	56980313	(T/C)
11412	CakSNP11412	*Kabuli*	Ca_Kabuli_Chr06	56980286	(T/C)
11413	CakSNP11413	*Kabuli*	Ca_Kabuli_Chr06	56980321	(T/C)
11414	CakSNP11414	*Kabuli*	Ca_Kabuli_Chr06	57147008	(A/G)
11415	CakSNP11415	*Kabuli*	Ca_Kabuli_Chr06	57198837	(G/A)
11416	CakSNP11416	*Kabuli*	Ca_Kabuli_Chr06	57259982	(C/A)
11417	CakSNP11417	*Kabuli*	Ca_Kabuli_Chr06	57260020	(A/G)
11418	CakSNP11418	*Kabuli*	Ca_Kabuli_Chr06	57260022	(T/C)
11419	CakSNP11419	*Kabuli*	Ca_Kabuli_Chr06	57272604	(A/G)
11420	CakSNP11420	*Kabuli*	Ca_Kabuli_Chr06	57272607	(G/A)
11421	CakSNP11421	*Kabuli*	Ca_Kabuli_Chr06	57272608	(C/A)
11422	CakSNP11422	*Kabuli*	Ca_Kabuli_Chr06	57277430	(A/G)
11423	CakSNP11423	*Kabuli*	Ca_Kabuli_Chr06	57277440	(C/T)
11424	CakSNP11424	*Kabuli*	Ca_Kabuli_Chr06	57344963	(G/T)
11425	CakSNP11425	*Kabuli*	Ca_Kabuli_Chr06	57382220	(G/A)

S.N.	SNP IDs	Cultivars	Chromosomes/scaffolds	Physical positions (bp)	SNPs
11426	CakSNP11426	*Kabuli*	Ca_Kabuli_Chr06	57433674	(T/A)
11427	CakSNP11427	*Kabuli*	Ca_Kabuli_Chr06	57436112	(A/T)
11428	CakSNP11428	*Kabuli*	Ca_Kabuli_Chr06	57436119	(T/C)
11429	CakSNP11429	*Kabuli*	Ca_Kabuli_Chr06	57436149	(T/A)
11430	CakSNP11430	*Kabuli*	Ca_Kabuli_Chr06	57436320	(T/G)
11431	CakSNP11431	*Kabuli*	Ca_Kabuli_Chr06	57437904	(A/G)
11432	CakSNP11432	*Kabuli*	Ca_Kabuli_Chr06	57449892	(A/G)
11433	CakSNP11433	*Kabuli*	Ca_Kabuli_Chr06	57475853	(G/T)
11434	CakSNP11434	*Kabuli*	Ca_Kabuli_Chr06	57475892	(A/G)
11435	CakSNP11435	*Kabuli*	Ca_Kabuli_Chr06	57475982	(A/G)
11436	CakSNP11436	*Kabuli*	Ca_Kabuli_Chr06	57533087	(G/A)
11437	CakSNP11437	*Kabuli*	Ca_Kabuli_Chr06	57579770	(G/T)
11438	CakSNP11438	*Kabuli*	Ca_Kabuli_Chr06	57579768	(C/T)
11439	CakSNP11439	*Kabuli*	Ca_Kabuli_Chr06	57580569	(C/T)
11440	CakSNP11440	*Kabuli*	Ca_Kabuli_Chr06	57603257	(T/A)
11441	CakSNP11441	*Kabuli*	Ca_Kabuli_Chr06	57625134	(G/A)
11442	CakSNP11442	*Kabuli*	Ca_Kabuli_Chr06	57625320	(G/A)
11443	CakSNP11443	*Kabuli*	Ca_Kabuli_Chr06	57632486	(C/T)
11444	CakSNP11444	*Kabuli*	Ca_Kabuli_Chr06	57634387	(G/T)
11445	CakSNP11445	*Kabuli*	Ca_Kabuli_Chr06	57634677	(G/A)
11446	CakSNP11446	*Kabuli*	Ca_Kabuli_Chr06	57637091	(G/A)
11447	CakSNP11447	*Kabuli*	Ca_Kabuli_Chr06	57637083	(T/G)
11448	CakSNP11448	*Kabuli*	Ca_Kabuli_Chr06	57637075	(C/T)
11449	CakSNP11449	*Kabuli*	Ca_Kabuli_Chr06	57662858	(T/A)
11450	CakSNP11450	*Kabuli*	Ca_Kabuli_Chr06	57667515	(A/T)
11451	CakSNP11451	*Kabuli*	Ca_Kabuli_Chr06	57688937	(C/T)
11452	CakSNP11452	*Kabuli*	Ca_Kabuli_Chr06	57704057	(C/T)
11453	CakSNP11453	*Kabuli*	Ca_Kabuli_Chr06	57704802	(T/C)
11454	CakSNP11454	*Kabuli*	Ca_Kabuli_Chr06	57705011	(G/A)
11455	CakSNP11455	*Kabuli*	Ca_Kabuli_Chr06	57707716	(C/T)
11456	CakSNP11456	*Kabuli*	Ca_Kabuli_Chr06	57707724	(A/C)
11457	CakSNP11457	*Kabuli*	Ca_Kabuli_Chr06	57707727	(T/G)
11458	CakSNP11458	*Kabuli*	Ca_Kabuli_Chr06	57707848	(A/G)
11459	CakSNP11459	*Kabuli*	Ca_Kabuli_Chr06	57707846	(G/A)
11460	CakSNP11460	*Kabuli*	Ca_Kabuli_Chr06	57707912	(G/C)
11461	CakSNP11461	*Kabuli*	Ca_Kabuli_Chr06	57708019	(A/G)
11462	CakSNP11462	*Kabuli*	Ca_Kabuli_Chr06	57708130	(T/C)
11463	CakSNP11463	*Kabuli*	Ca_Kabuli_Chr06	57708101	(T/G)
11464	CakSNP11464	*Kabuli*	Ca_Kabuli_Chr06	57708198	(C/T)

S.N.	SNP IDs	Cultivars	Chromosomes/scaffolds	Physical positions (bp)	SNPs
11465	CakSNP11465	*Kabuli*	Ca_Kabuli_Chr06	57710133	(C/G)
11466	CakSNP11466	*Kabuli*	Ca_Kabuli_Chr06	57720446	(T/C)
11467	CakSNP11467	*Kabuli*	Ca_Kabuli_Chr06	57723992	(A/C)
11468	CakSNP11468	*Kabuli*	Ca_Kabuli_Chr06	57725372	(A/C)
11469	CakSNP11469	*Kabuli*	Ca_Kabuli_Chr06	57746989	(C/T)
11470	CakSNP11470	*Kabuli*	Ca_Kabuli_Chr06	57747101	(C/T)
11471	CakSNP11471	*Kabuli*	Ca_Kabuli_Chr06	57748565	(A/G)
11472	CakSNP11472	*Kabuli*	Ca_Kabuli_Chr06	57748602	(A/T)
11473	CakSNP11473	*Kabuli*	Ca_Kabuli_Chr06	57753076	(T/C)
11474	CakSNP11474	*Kabuli*	Ca_Kabuli_Chr06	57756699	(G/A)
11475	CakSNP11475	*Kabuli*	Ca_Kabuli_Chr06	57760109	(T/G)
11476	CakSNP11476	*Kabuli*	Ca_Kabuli_Chr06	57760176	(C/T)
11477	CakSNP11477	*Kabuli*	Ca_Kabuli_Chr06	57760227	(C/T)
11478	CakSNP11478	*Kabuli*	Ca_Kabuli_Chr06	57760290	(A/T)
11479	CakSNP11479	*Kabuli*	Ca_Kabuli_Chr06	57760291	(G/T)
11480	CakSNP11480	*Kabuli*	Ca_Kabuli_Chr06	57760360	(A/G)
11481	CakSNP11481	*Kabuli*	Ca_Kabuli_Chr06	57761758	(G/A)
11482	CakSNP11482	*Kabuli*	Ca_Kabuli_Chr06	57761722	(T/C)
11483	CakSNP11483	*Kabuli*	Ca_Kabuli_Chr06	57766444	(C/T)
11484	CakSNP11484	*Kabuli*	Ca_Kabuli_Chr06	57766484	(T/A)
11485	CakSNP11485	*Kabuli*	Ca_Kabuli_Chr06	57766483	(C/A)
11486	CakSNP11486	*Kabuli*	Ca_Kabuli_Chr06	57766570	(C/T)
11487	CakSNP11487	*Kabuli*	Ca_Kabuli_Chr06	57766588	(G/A)
11488	CakSNP11488	*Kabuli*	Ca_Kabuli_Chr06	57766598	(G/A)
11489	CakSNP11489	*Kabuli*	Ca_Kabuli_Chr06	57768314	(T/C)
11490	CakSNP11490	*Kabuli*	Ca_Kabuli_Chr06	57801985	(A/C)
11491	CakSNP11491	*Kabuli*	Ca_Kabuli_Chr06	57831050	(G/T)
11492	CakSNP11492	*Kabuli*	Ca_Kabuli_Chr06	57831049	(C/T)
11493	CakSNP11493	*Kabuli*	Ca_Kabuli_Chr06	57831040	(T/C)
11494	CakSNP11494	*Kabuli*	Ca_Kabuli_Chr06	57835093	(T/A)
11495	CakSNP11495	*Kabuli*	Ca_Kabuli_Chr06	57835120	(G/A)
11496	CakSNP11496	*Kabuli*	Ca_Kabuli_Chr06	57835917	(G/C)
11497	CakSNP11497	*Kabuli*	Ca_Kabuli_Chr06	57835899	(C/T)
11498	CakSNP11498	*Kabuli*	Ca_Kabuli_Chr06	57836024	(G/A)
11499	CakSNP11499	*Kabuli*	Ca_Kabuli_Chr06	57836237	(A/G)
11500	CakSNP11500	*Kabuli*	Ca_Kabuli_Chr06	57836284	(G/A)
11501	CakSNP11501	*Kabuli*	Ca_Kabuli_Chr06	57836314	(T/A)
11502	CakSNP11502	*Kabuli*	Ca_Kabuli_Chr06	57836316	(G/A)
11503	CakSNP11503	*Kabuli*	Ca_Kabuli_Chr06	57870893	(C/A)

S.N.	SNP IDs	Cultivars	Chromosomes/scaffolds	Physical positions (bp)	SNPs
11504	CakSNP11504	*Kabuli*	Ca_Kabuli_Chr06	57877446	(C/A)
11505	CakSNP11505	*Kabuli*	Ca_Kabuli_Chr06	57882364	(C/T)
11506	CakSNP11506	*Kabuli*	Ca_Kabuli_Chr06	57894377	(A/G)
11507	CakSNP11507	*Kabuli*	Ca_Kabuli_Chr06	57894495	(C/G)
11508	CakSNP11508	*Kabuli*	Ca_Kabuli_Chr06	57894590	(G/C)
11509	CakSNP11509	*Kabuli*	Ca_Kabuli_Chr06	57894585	(T/G)
11510	CakSNP11510	*Kabuli*	Ca_Kabuli_Chr06	57904599	(T/C)
11511	CakSNP11511	*Kabuli*	Ca_Kabuli_Chr06	57921373	(C/T)
11512	CakSNP11512	*Kabuli*	Ca_Kabuli_Chr06	57943585	(A/T)
11513	CakSNP11513	*Kabuli*	Ca_Kabuli_Chr06	57943626	(G/A)
11514	CakSNP11514	*Kabuli*	Ca_Kabuli_Chr06	57943628	(A/C)
11515	CakSNP11515	*Kabuli*	Ca_Kabuli_Chr06	57944439	(C/T)
11516	CakSNP11516	*Kabuli*	Ca_Kabuli_Chr06	57968924	(A/T)
11517	CakSNP11517	*Kabuli*	Ca_Kabuli_Chr06	58036471	(C/T)
11518	CakSNP11518	*Kabuli*	Ca_Kabuli_Chr06	58174702	(T/C)
11519	CakSNP11519	*Kabuli*	Ca_Kabuli_Chr06	58187822	(T/G)
11520	CakSNP11520	*Kabuli*	Ca_Kabuli_Chr06	58187859	(T/A)
11521	CakSNP11521	*Kabuli*	Ca_Kabuli_Chr06	58187957	(A/G)
11522	CakSNP11522	*Kabuli*	Ca_Kabuli_Chr06	58191050	(T/C)
11523	CakSNP11523	*Kabuli*	Ca_Kabuli_Chr06	58224023	(G/T)
11524	CakSNP11524	*Kabuli*	Ca_Kabuli_Chr06	58226908	(T/G)
11525	CakSNP11525	*Kabuli*	Ca_Kabuli_Chr06	58227006	(T/C)
11526	CakSNP11526	*Kabuli*	Ca_Kabuli_Chr06	58237503	(A/T)
11527	CakSNP11527	*Kabuli*	Ca_Kabuli_Chr06	58238371	(T/C)
11528	CakSNP11528	*Kabuli*	Ca_Kabuli_Chr06	58238397	(A/G)
11529	CakSNP11529	*Kabuli*	Ca_Kabuli_Chr06	58243505	(G/A)
11530	CakSNP11530	*Kabuli*	Ca_Kabuli_Chr06	58326344	(A/T)
11531	CakSNP11531	*Kabuli*	Ca_Kabuli_Chr06	58380367	(T/C)
11532	CakSNP11532	*Kabuli*	Ca_Kabuli_Chr06	58380394	(G/A)
11533	CakSNP11533	*Kabuli*	Ca_Kabuli_Chr06	58380563	(G/A)
11534	CakSNP11534	*Kabuli*	Ca_Kabuli_Chr06	58381203	(G/C)
11535	CakSNP11535	*Kabuli*	Ca_Kabuli_Chr06	58381259	(A/G)
11536	CakSNP11536	*Kabuli*	Ca_Kabuli_Chr06	58434217	(C/G)
11537	CakSNP11537	*Kabuli*	Ca_Kabuli_Chr06	58452843	(A/C)
11538	CakSNP11538	*Kabuli*	Ca_Kabuli_Chr06	58569576	(G/T)
11539	CakSNP11539	*Kabuli*	Ca_Kabuli_Chr06	58569569	(T/C)
11540	CakSNP11540	*Kabuli*	Ca_Kabuli_Chr06	58571660	(T/C)
11541	CakSNP11541	*Kabuli*	Ca_Kabuli_Chr06	58596821	(T/G)
11542	CakSNP11542	*Kabuli*	Ca_Kabuli_Chr06	58596878	(C/A)

S.N.	SNP IDs	Cultivars	Chromosomes/scaffolds	Physical positions (bp)	SNPs
11543	CakSNP11543	*Kabuli*	Ca_Kabuli_Chr06	58596968	(T/C)
11544	CakSNP11544	*Kabuli*	Ca_Kabuli_Chr06	58596918	(A/G)
11545	CakSNP11545	*Kabuli*	Ca_Kabuli_Chr06	58600930	(C/A)
11546	CakSNP11546	*Kabuli*	Ca_Kabuli_Chr06	58600952	(A/G)
11547	CakSNP11547	*Kabuli*	Ca_Kabuli_Chr06	58600974	(A/G)
11548	CakSNP11548	*Kabuli*	Ca_Kabuli_Chr06	58623095	(A/G)
11549	CakSNP11549	*Kabuli*	Ca_Kabuli_Chr06	58623096	(A/T)
11550	CakSNP11550	*Kabuli*	Ca_Kabuli_Chr06	58623220	(T/C)
11551	CakSNP11551	*Kabuli*	Ca_Kabuli_Chr06	58624183	(G/A)
11552	CakSNP11552	*Kabuli*	Ca_Kabuli_Chr06	58624233	(C/A)
11553	CakSNP11553	*Kabuli*	Ca_Kabuli_Chr06	58624299	(A/G)
11554	CakSNP11554	*Kabuli*	Ca_Kabuli_Chr06	58624281	(A/G)
11555	CakSNP11555	*Kabuli*	Ca_Kabuli_Chr06	58624251	(C/T)
11556	CakSNP11556	*Kabuli*	Ca_Kabuli_Chr06	58650354	(A/G)
11557	CakSNP11557	*Kabuli*	Ca_Kabuli_Chr06	58650330	(G/A)
11558	CakSNP11558	*Kabuli*	Ca_Kabuli_Chr06	58650318	(A/C)
11559	CakSNP11559	*Kabuli*	Ca_Kabuli_Chr06	58650459	(A/T)
11560	CakSNP11560	*Kabuli*	Ca_Kabuli_Chr06	58650491	(A/G)
11561	CakSNP11561	*Kabuli*	Ca_Kabuli_Chr06	58652881	(A/G)
11562	CakSNP11562	*Kabuli*	Ca_Kabuli_Chr06	58672317	(A/G)
11563	CakSNP11563	*Kabuli*	Ca_Kabuli_Chr06	58675788	(T/C)
11564	CakSNP11564	*Kabuli*	Ca_Kabuli_Chr06	58675749	(T/C)
11565	CakSNP11565	*Kabuli*	Ca_Kabuli_Chr06	58676507	(A/G)
11566	CakSNP11566	*Kabuli*	Ca_Kabuli_Chr06	58676517	(A/C)
11567	CakSNP11567	*Kabuli*	Ca_Kabuli_Chr06	58682427	(G/T)
11568	CakSNP11568	*Kabuli*	Ca_Kabuli_Chr06	58682397	(G/A)
11569	CakSNP11569	*Kabuli*	Ca_Kabuli_Chr06	58682387	(T/A)
11570	CakSNP11570	*Kabuli*	Ca_Kabuli_Chr06	58730925	(T/C)
11571	CakSNP11571	*Kabuli*	Ca_Kabuli_Chr06	58730969	(C/A)
11572	CakSNP11572	*Kabuli*	Ca_Kabuli_Chr06	58730967	(T/G)
11573	CakSNP11573	*Kabuli*	Ca_Kabuli_Chr06	58732424	(G/T)
11574	CakSNP11574	*Kabuli*	Ca_Kabuli_Chr06	58732423	(C/T)
11575	CakSNP11575	*Kabuli*	Ca_Kabuli_Chr06	58732514	(G/A)
11576	CakSNP11576	*Kabuli*	Ca_Kabuli_Chr06	58732451	(T/C)
11577	CakSNP11577	*Kabuli*	Ca_Kabuli_Chr06	58732475	(G/A)
11578	CakSNP11578	*Kabuli*	Ca_Kabuli_Chr06	58745356	(A/G)
11579	CakSNP11579	*Kabuli*	Ca_Kabuli_Chr06	58745473	(A/G)
11580	CakSNP11580	*Kabuli*	Ca_Kabuli_Chr06	58745426	(T/C)
11581	CakSNP11581	*Kabuli*	Ca_Kabuli_Chr06	58745583	(C/T)

S.N.	SNP IDs	Cultivars	Chromosomes/scaffolds	Physical positions (bp)	SNPs
11582	CakSNP11582	*Kabuli*	Ca_Kabuli_Chr06	58761879	(G/A)
11583	CakSNP11583	*Kabuli*	Ca_Kabuli_Chr06	58779344	(A/G)
11584	CakSNP11584	*Kabuli*	Ca_Kabuli_Chr06	58779389	(T/G)
11585	CakSNP11585	*Kabuli*	Ca_Kabuli_Chr06	58820632	(A/C)
11586	CakSNP11586	*Kabuli*	Ca_Kabuli_Chr06	58844717	(T/C)
11587	CakSNP11587	*Kabuli*	Ca_Kabuli_Chr06	58844744	(A/T)
11588	CakSNP11588	*Kabuli*	Ca_Kabuli_Chr06	58844776	(C/G)
11589	CakSNP11589	*Kabuli*	Ca_Kabuli_Chr06	58861614	(G/A)
11590	CakSNP11590	*Kabuli*	Ca_Kabuli_Chr06	58878604	(C/G)
11591	CakSNP11591	*Kabuli*	Ca_Kabuli_Chr06	58879570	(T/C)
11592	CakSNP11592	*Kabuli*	Ca_Kabuli_Chr06	58903346	(G/A)
11593	CakSNP11593	*Kabuli*	Ca_Kabuli_Chr06	58903381	(G/A)
11594	CakSNP11594	*Kabuli*	Ca_Kabuli_Chr06	58903395	(A/G)
11595	CakSNP11595	*Kabuli*	Ca_Kabuli_Chr06	58903416	(T/G)
11596	CakSNP11596	*Kabuli*	Ca_Kabuli_Chr06	58903497	(G/C)
11597	CakSNP11597	*Kabuli*	Ca_Kabuli_Chr06	58903538	(G/A)
11598	CakSNP11598	*Kabuli*	Ca_Kabuli_Chr06	58908024	(G/A)
11599	CakSNP11599	*Kabuli*	Ca_Kabuli_Chr06	58908045	(A/G)
11600	CakSNP11600	*Kabuli*	Ca_Kabuli_Chr06	58908069	(G/A)
11601	CakSNP11601	*Kabuli*	Ca_Kabuli_Chr06	58911548	(T/C)
11602	CakSNP11602	*Kabuli*	Ca_Kabuli_Chr06	58951079	(T/C)
11603	CakSNP11603	*Kabuli*	Ca_Kabuli_Chr06	58951298	(A/G)
11604	CakSNP11604	*Kabuli*	Ca_Kabuli_Chr06	58951583	(C/A)
11605	CakSNP11605	*Kabuli*	Ca_Kabuli_Chr06	58953219	(A/G)
11606	CakSNP11606	*Kabuli*	Ca_Kabuli_Chr06	58994014	(C/T)
11607	CakSNP11607	*Kabuli*	Ca_Kabuli_Chr06	58994083	(A/G)
11608	CakSNP11608	*Kabuli*	Ca_Kabuli_Chr06	58994281	(G/A)
11609	CakSNP11609	*Kabuli*	Ca_Kabuli_Chr06	59001443	(C/T)
11610	CakSNP11610	*Kabuli*	Ca_Kabuli_Chr06	59003792	(A/C)
11611	CakSNP11611	*Kabuli*	Ca_Kabuli_Chr06	59064798	(G/T)
11612	CakSNP11612	*Kabuli*	Ca_Kabuli_Chr06	59064815	(C/T)
11613	CakSNP11613	*Kabuli*	Ca_Kabuli_Chr06	59081169	(C/T)
11614	CakSNP11614	*Kabuli*	Ca_Kabuli_Chr06	59107027	(A/T)
11615	CakSNP11615	*Kabuli*	Ca_Kabuli_Chr06	59107007	(G/T)
11616	CakSNP11616	*Kabuli*	Ca_Kabuli_Chr06	59135944	(A/C)
11617	CakSNP11617	*Kabuli*	Ca_Kabuli_Chr06	59194015	(C/G)
11618	CakSNP11618	*Kabuli*	Ca_Kabuli_Chr06	59193994	(G/T)
11619	CakSNP11619	*Kabuli*	Ca_Kabuli_Chr06	59194081	(T/A)
11620	CakSNP11620	*Kabuli*	Ca_Kabuli_Chr06	59202825	(T/G)

S.N.	SNP IDs	Cultivars	Chromosomes/scaffolds	Physical positions (bp)	SNPs
11621	CakSNP11621	*Kabuli*	Ca_Kabuli_Chr06	59219099	(T/C)
11622	CakSNP11622	*Kabuli*	Ca_Kabuli_Chr06	59222848	(C/T)
11623	CakSNP11623	*Kabuli*	Ca_Kabuli_Chr06	59296368	(C/T)
11624	CakSNP11624	*Kabuli*	Ca_Kabuli_Chr06	59296460	(G/A)
11625	CakSNP11625	*Kabuli*	Ca_Kabuli_Chr06	59407761	(G/A)
11626	CakSNP11626	*Kabuli*	Ca_Kabuli_Chr06	59417984	(G/A)
11627	CakSNP11627	*Kabuli*	Ca_Kabuli_Chr07	16760	(T/G)
11628	CakSNP11628	*Kabuli*	Ca_Kabuli_Chr07	16850	(C/T)
11629	CakSNP11629	*Kabuli*	Ca_Kabuli_Chr07	16880	(G/A)
11630	CakSNP11630	*Kabuli*	Ca_Kabuli_Chr07	16881	(A/G)
11631	CakSNP11631	*Kabuli*	Ca_Kabuli_Chr07	41841	(G/A)
11632	CakSNP11632	*Kabuli*	Ca_Kabuli_Chr07	55131	(T/C)
11633	CakSNP11633	*Kabuli*	Ca_Kabuli_Chr07	208824	(A/G)
11634	CakSNP11634	*Kabuli*	Ca_Kabuli_Chr07	218346	(C/A)
11635	CakSNP11635	*Kabuli*	Ca_Kabuli_Chr07	218341	(C/G)
11636	CakSNP11636	*Kabuli*	Ca_Kabuli_Chr07	419301	(C/T)
11637	CakSNP11637	*Kabuli*	Ca_Kabuli_Chr07	419316	(A/G)
11638	CakSNP11638	*Kabuli*	Ca_Kabuli_Chr07	419334	(A/C)
11639	CakSNP11639	*Kabuli*	Ca_Kabuli_Chr07	419385	(T/G)
11640	CakSNP11640	*Kabuli*	Ca_Kabuli_Chr07	419328	(C/T)
11641	CakSNP11641	*Kabuli*	Ca_Kabuli_Chr07	419679	(C/T)
11642	CakSNP11642	*Kabuli*	Ca_Kabuli_Chr07	419638	(C/T)
11643	CakSNP11643	*Kabuli*	Ca_Kabuli_Chr07	419628	(A/C)
11644	CakSNP11644	*Kabuli*	Ca_Kabuli_Chr07	528521	(G/A)
11645	CakSNP11645	*Kabuli*	Ca_Kabuli_Chr07	601563	(T/A)
11646	CakSNP11646	*Kabuli*	Ca_Kabuli_Chr07	601551	(T/C)
11647	CakSNP11647	*Kabuli*	Ca_Kabuli_Chr07	601545	(C/T)
11648	CakSNP11648	*Kabuli*	Ca_Kabuli_Chr07	601538	(C/A)
11649	CakSNP11649	*Kabuli*	Ca_Kabuli_Chr07	601521	(G/A)
11650	CakSNP11650	*Kabuli*	Ca_Kabuli_Chr07	601516	(G/A)
11651	CakSNP11651	*Kabuli*	Ca_Kabuli_Chr07	601513	(C/G)
11652	CakSNP11652	*Kabuli*	Ca_Kabuli_Chr07	601502	(G/T)
11653	CakSNP11653	*Kabuli*	Ca_Kabuli_Chr07	601496	(C/T)
11654	CakSNP11654	*Kabuli*	Ca_Kabuli_Chr07	601586	(C/T)
11655	CakSNP11655	*Kabuli*	Ca_Kabuli_Chr07	601601	(C/T)
11656	CakSNP11656	*Kabuli*	Ca_Kabuli_Chr07	601612	(A/G)
11657	CakSNP11657	*Kabuli*	Ca_Kabuli_Chr07	601643	(G/T)
11658	CakSNP11658	*Kabuli*	Ca_Kabuli_Chr07	601622	(G/C)
11659	CakSNP11659	*Kabuli*	Ca_Kabuli_Chr07	601657	(C/T)

S.N.	SNP IDs	Cultivars	Chromosomes/scaffolds	Physical positions (bp)	SNPs
11660	CakSNP11660	*Kabuli*	Ca_Kabuli_Chr07	601719	(G/T)
11661	CakSNP11661	*Kabuli*	Ca_Kabuli_Chr07	601718	(G/C)
11662	CakSNP11662	*Kabuli*	Ca_Kabuli_Chr07	601714	(A/T)
11663	CakSNP11663	*Kabuli*	Ca_Kabuli_Chr07	601709	(A/C)
11664	CakSNP11664	*Kabuli*	Ca_Kabuli_Chr07	601683	(G/A)
11665	CakSNP11665	*Kabuli*	Ca_Kabuli_Chr07	601647	(C/T)
11666	CakSNP11666	*Kabuli*	Ca_Kabuli_Chr07	610737	(C/A)
11667	CakSNP11667	*Kabuli*	Ca_Kabuli_Chr07	610723	(G/T)
11668	CakSNP11668	*Kabuli*	Ca_Kabuli_Chr07	667597	(C/T)
11669	CakSNP11669	*Kabuli*	Ca_Kabuli_Chr07	674791	(A/T)
11670	CakSNP11670	*Kabuli*	Ca_Kabuli_Chr07	676785	(G/T)
11671	CakSNP11671	*Kabuli*	Ca_Kabuli_Chr07	676786	(G/A)
11672	CakSNP11672	*Kabuli*	Ca_Kabuli_Chr07	676790	(T/C)
11673	CakSNP11673	*Kabuli*	Ca_Kabuli_Chr07	683245	(T/C)
11674	CakSNP11674	*Kabuli*	Ca_Kabuli_Chr07	761710	(A/G)
11675	CakSNP11675	*Kabuli*	Ca_Kabuli_Chr07	767708	(G/A)
11676	CakSNP11676	*Kabuli*	Ca_Kabuli_Chr07	781377	(G/T)
11677	CakSNP11677	*Kabuli*	Ca_Kabuli_Chr07	781409	(A/G)
11678	CakSNP11678	*Kabuli*	Ca_Kabuli_Chr07	807095	(A/T)
11679	CakSNP11679	*Kabuli*	Ca_Kabuli_Chr07	807110	(T/C)
11680	CakSNP11680	*Kabuli*	Ca_Kabuli_Chr07	807133	(T/A)
11681	CakSNP11681	*Kabuli*	Ca_Kabuli_Chr07	815477	(A/T)
11682	CakSNP11682	*Kabuli*	Ca_Kabuli_Chr07	815526	(C/A)
11683	CakSNP11683	*Kabuli*	Ca_Kabuli_Chr07	838581	(C/A)
11684	CakSNP11684	*Kabuli*	Ca_Kabuli_Chr07	838651	(A/C)
11685	CakSNP11685	*Kabuli*	Ca_Kabuli_Chr07	841988	(T/C)
11686	CakSNP11686	*Kabuli*	Ca_Kabuli_Chr07	844248	(G/A)
11687	CakSNP11687	*Kabuli*	Ca_Kabuli_Chr07	845488	(A/G)
11688	CakSNP11688	*Kabuli*	Ca_Kabuli_Chr07	865007	(C/T)
11689	CakSNP11689	*Kabuli*	Ca_Kabuli_Chr07	865170	(C/T)
11690	CakSNP11690	*Kabuli*	Ca_Kabuli_Chr07	873207	(A/G)
11691	CakSNP11691	*Kabuli*	Ca_Kabuli_Chr07	888900	(G/A)
11692	CakSNP11692	*Kabuli*	Ca_Kabuli_Chr07	888987	(A/G)
11693	CakSNP11693	*Kabuli*	Ca_Kabuli_Chr07	975236	(T/A)
11694	CakSNP11694	*Kabuli*	Ca_Kabuli_Chr07	993031	(G/T)
11695	CakSNP11695	*Kabuli*	Ca_Kabuli_Chr07	994976	(G/A)
11696	CakSNP11696	*Kabuli*	Ca_Kabuli_Chr07	995691	(C/T)
11697	CakSNP11697	*Kabuli*	Ca_Kabuli_Chr07	995674	(T/C)
11698	CakSNP11698	*Kabuli*	Ca_Kabuli_Chr07	1022946	(C/T)

S.N.	SNP IDs	Cultivars	Chromosomes/scaffolds	Physical positions (bp)	SNPs
11699	CakSNP11699	*Kabuli*	Ca_Kabuli_Chr07	1045113	(A/C)
11700	CakSNP11700	*Kabuli*	Ca_Kabuli_Chr07	1045127	(T/A)
11701	CakSNP11701	*Kabuli*	Ca_Kabuli_Chr07	1096379	(C/T)
11702	CakSNP11702	*Kabuli*	Ca_Kabuli_Chr07	1153267	(A/C)
11703	CakSNP11703	*Kabuli*	Ca_Kabuli_Chr07	1174188	(C/T)
11704	CakSNP11704	*Kabuli*	Ca_Kabuli_Chr07	1293601	(C/G)
11705	CakSNP11705	*Kabuli*	Ca_Kabuli_Chr07	1350605	(C/T)
11706	CakSNP11706	*Kabuli*	Ca_Kabuli_Chr07	1350856	(G/T)
11707	CakSNP11707	*Kabuli*	Ca_Kabuli_Chr07	1350846	(A/T)
11708	CakSNP11708	*Kabuli*	Ca_Kabuli_Chr07	1350947	(T/G)
11709	CakSNP11709	*Kabuli*	Ca_Kabuli_Chr07	1414206	(C/A)
11710	CakSNP11710	*Kabuli*	Ca_Kabuli_Chr07	1505646	(G/A)
11711	CakSNP11711	*Kabuli*	Ca_Kabuli_Chr07	1509156	(G/A)
11712	CakSNP11712	*Kabuli*	Ca_Kabuli_Chr07	1511252	(C/T)
11713	CakSNP11713	*Kabuli*	Ca_Kabuli_Chr07	1585622	(T/C)
11714	CakSNP11714	*Kabuli*	Ca_Kabuli_Chr07	1635519	(G/A)
11715	CakSNP11715	*Kabuli*	Ca_Kabuli_Chr07	1635471	(G/T)
11716	CakSNP11716	*Kabuli*	Ca_Kabuli_Chr07	1702033	(G/T)
11717	CakSNP11717	*Kabuli*	Ca_Kabuli_Chr07	1702063	(A/G)
11718	CakSNP11718	*Kabuli*	Ca_Kabuli_Chr07	1702077	(G/C)
11719	CakSNP11719	*Kabuli*	Ca_Kabuli_Chr07	1704780	(T/G)
11720	CakSNP11720	*Kabuli*	Ca_Kabuli_Chr07	1704802	(A/G)
11721	CakSNP11721	*Kabuli*	Ca_Kabuli_Chr07	1704892	(A/G)
11722	CakSNP11722	*Kabuli*	Ca_Kabuli_Chr07	1704947	(C/T)
11723	CakSNP11723	*Kabuli*	Ca_Kabuli_Chr07	1705197	(A/C)
11724	CakSNP11724	*Kabuli*	Ca_Kabuli_Chr07	1707283	(G/T)
11725	CakSNP11725	*Kabuli*	Ca_Kabuli_Chr07	1707375	(C/G)
11726	CakSNP11726	*Kabuli*	Ca_Kabuli_Chr07	1739369	(C/A)
11727	CakSNP11727	*Kabuli*	Ca_Kabuli_Chr07	1787308	(T/A)
11728	CakSNP11728	*Kabuli*	Ca_Kabuli_Chr07	1787417	(G/A)
11729	CakSNP11729	*Kabuli*	Ca_Kabuli_Chr07	1946020	(G/A)
11730	CakSNP11730	*Kabuli*	Ca_Kabuli_Chr07	1963659	(T/A)
11731	CakSNP11731	*Kabuli*	Ca_Kabuli_Chr07	1963757	(A/G)
11732	CakSNP11732	*Kabuli*	Ca_Kabuli_Chr07	1991642	(G/T)
11733	CakSNP11733	*Kabuli*	Ca_Kabuli_Chr07	2012854	(T/A)
11734	CakSNP11734	*Kabuli*	Ca_Kabuli_Chr07	2103626	(C/T)
11735	CakSNP11735	*Kabuli*	Ca_Kabuli_Chr07	2106876	(C/A)
11736	CakSNP11736	*Kabuli*	Ca_Kabuli_Chr07	2106917	(A/T)
11737	CakSNP11737	*Kabuli*	Ca_Kabuli_Chr07	2146554	(G/A)

S.N.	SNP IDs	Cultivars	Chromosomes/scaffolds	Physical positions (bp)	SNPs
11738	CakSNP11738	*Kabuli*	Ca_Kabuli_Chr07	2167517	(A/C)
11739	CakSNP11739	*Kabuli*	Ca_Kabuli_Chr07	2167540	(G/C)
11740	CakSNP11740	*Kabuli*	Ca_Kabuli_Chr07	2211897	(G/A)
11741	CakSNP11741	*Kabuli*	Ca_Kabuli_Chr07	2219764	(C/T)
11742	CakSNP11742	*Kabuli*	Ca_Kabuli_Chr07	2230434	(A/G)
11743	CakSNP11743	*Kabuli*	Ca_Kabuli_Chr07	2262749	(C/G)
11744	CakSNP11744	*Kabuli*	Ca_Kabuli_Chr07	2262865	(A/G)
11745	CakSNP11745	*Kabuli*	Ca_Kabuli_Chr07	2262843	(T/A)
11746	CakSNP11746	*Kabuli*	Ca_Kabuli_Chr07	2361064	(T/G)
11747	CakSNP11747	*Kabuli*	Ca_Kabuli_Chr07	2387109	(C/T)
11748	CakSNP11748	*Kabuli*	Ca_Kabuli_Chr07	2391392	(G/A)
11749	CakSNP11749	*Kabuli*	Ca_Kabuli_Chr07	2401235	(T/C)
11750	CakSNP11750	*Kabuli*	Ca_Kabuli_Chr07	2493033	(A/G)
11751	CakSNP11751	*Kabuli*	Ca_Kabuli_Chr07	2538167	(C/G)
11752	CakSNP11752	*Kabuli*	Ca_Kabuli_Chr07	2541501	(A/G)
11753	CakSNP11753	*Kabuli*	Ca_Kabuli_Chr07	2541551	(G/A)
11754	CakSNP11754	*Kabuli*	Ca_Kabuli_Chr07	2541653	(C/G)
11755	CakSNP11755	*Kabuli*	Ca_Kabuli_Chr07	2545613	(T/C)
11756	CakSNP11756	*Kabuli*	Ca_Kabuli_Chr07	2552454	(G/T)
11757	CakSNP11757	*Kabuli*	Ca_Kabuli_Chr07	2603595	(C/T)
11758	CakSNP11758	*Kabuli*	Ca_Kabuli_Chr07	2603633	(C/T)
11759	CakSNP11759	*Kabuli*	Ca_Kabuli_Chr07	2603649	(T/C)
11760	CakSNP11760	*Kabuli*	Ca_Kabuli_Chr07	2609831	(C/A)
11761	CakSNP11761	*Kabuli*	Ca_Kabuli_Chr07	2618478	(C/T)
11762	CakSNP11762	*Kabuli*	Ca_Kabuli_Chr07	2654442	(T/G)
11763	CakSNP11763	*Kabuli*	Ca_Kabuli_Chr07	2654478	(C/T)
11764	CakSNP11764	*Kabuli*	Ca_Kabuli_Chr07	2684213	(A/G)
11765	CakSNP11765	*Kabuli*	Ca_Kabuli_Chr07	2734680	(A/G)
11766	CakSNP11766	*Kabuli*	Ca_Kabuli_Chr07	2734625	(C/T)
11767	CakSNP11767	*Kabuli*	Ca_Kabuli_Chr07	2745665	(A/C)
11768	CakSNP11768	*Kabuli*	Ca_Kabuli_Chr07	2745656	(A/C)
11769	CakSNP11769	*Kabuli*	Ca_Kabuli_Chr07	2757260	(C/T)
11770	CakSNP11770	*Kabuli*	Ca_Kabuli_Chr07	2757255	(G/C)
11771	CakSNP11771	*Kabuli*	Ca_Kabuli_Chr07	2757253	(T/G)
11772	CakSNP11772	*Kabuli*	Ca_Kabuli_Chr07	2762396	(G/A)
11773	CakSNP11773	*Kabuli*	Ca_Kabuli_Chr07	2762406	(C/T)
11774	CakSNP11774	*Kabuli*	Ca_Kabuli_Chr07	2762423	(G/A)
11775	CakSNP11775	*Kabuli*	Ca_Kabuli_Chr07	2832369	(C/T)
11776	CakSNP11776	*Kabuli*	Ca_Kabuli_Chr07	2857630	(T/G)

S.N.	SNP IDs	Cultivars	Chromosomes/scaffolds	Physical positions (bp)	SNPs
11777	CakSNP11777	*Kabuli*	Ca_Kabuli_Chr07	2857818	(T/A)
11778	CakSNP11778	*Kabuli*	Ca_Kabuli_Chr07	2857844	(C/T)
11779	CakSNP11779	*Kabuli*	Ca_Kabuli_Chr07	2909641	(A/G)
11780	CakSNP11780	*Kabuli*	Ca_Kabuli_Chr07	2915375	(C/G)
11781	CakSNP11781	*Kabuli*	Ca_Kabuli_Chr07	2932309	(C/G)
11782	CakSNP11782	*Kabuli*	Ca_Kabuli_Chr07	2932301	(C/A)
11783	CakSNP11783	*Kabuli*	Ca_Kabuli_Chr07	2932377	(C/T)
11784	CakSNP11784	*Kabuli*	Ca_Kabuli_Chr07	2956665	(C/T)
11785	CakSNP11785	*Kabuli*	Ca_Kabuli_Chr07	2956767	(A/G)
11786	CakSNP11786	*Kabuli*	Ca_Kabuli_Chr07	2969653	(A/G)
11787	CakSNP11787	*Kabuli*	Ca_Kabuli_Chr07	2997261	(A/G)
11788	CakSNP11788	*Kabuli*	Ca_Kabuli_Chr07	3023182	(T/C)
11789	CakSNP11789	*Kabuli*	Ca_Kabuli_Chr07	3023154	(T/C)
11790	CakSNP11790	*Kabuli*	Ca_Kabuli_Chr07	3070531	(A/C)
11791	CakSNP11791	*Kabuli*	Ca_Kabuli_Chr07	3090898	(G/A)
11792	CakSNP11792	*Kabuli*	Ca_Kabuli_Chr07	3093884	(T/A)
11793	CakSNP11793	*Kabuli*	Ca_Kabuli_Chr07	3093953	(T/G)
11794	CakSNP11794	*Kabuli*	Ca_Kabuli_Chr07	3094090	(G/A)
11795	CakSNP11795	*Kabuli*	Ca_Kabuli_Chr07	3094087	(A/G)
11796	CakSNP11796	*Kabuli*	Ca_Kabuli_Chr07	3107197	(C/T)
11797	CakSNP11797	*Kabuli*	Ca_Kabuli_Chr07	3107239	(T/A)
11798	CakSNP11798	*Kabuli*	Ca_Kabuli_Chr07	3109250	(T/C)
11799	CakSNP11799	*Kabuli*	Ca_Kabuli_Chr07	3109352	(T/C)
11800	CakSNP11800	*Kabuli*	Ca_Kabuli_Chr07	3110316	(A/G)
11801	CakSNP11801	*Kabuli*	Ca_Kabuli_Chr07	3130070	(A/G)
11802	CakSNP11802	*Kabuli*	Ca_Kabuli_Chr07	3130069	(A/G)
11803	CakSNP11803	*Kabuli*	Ca_Kabuli_Chr07	3143409	(C/T)
11804	CakSNP11804	*Kabuli*	Ca_Kabuli_Chr07	3186341	(C/T)
11805	CakSNP11805	*Kabuli*	Ca_Kabuli_Chr07	3186262	(T/G)
11806	CakSNP11806	*Kabuli*	Ca_Kabuli_Chr07	3198197	(C/A)
11807	CakSNP11807	*Kabuli*	Ca_Kabuli_Chr07	3198263	(T/C)
11808	CakSNP11808	*Kabuli*	Ca_Kabuli_Chr07	3217916	(T/G)
11809	CakSNP11809	*Kabuli*	Ca_Kabuli_Chr07	3236150	(A/C)
11810	CakSNP11810	*Kabuli*	Ca_Kabuli_Chr07	3265823	(A/G)
11811	CakSNP11811	*Kabuli*	Ca_Kabuli_Chr07	3297560	(G/A)
11812	CakSNP11812	*Kabuli*	Ca_Kabuli_Chr07	3297677	(G/T)
11813	CakSNP11813	*Kabuli*	Ca_Kabuli_Chr07	3315157	(A/G)
11814	CakSNP11814	*Kabuli*	Ca_Kabuli_Chr07	3351037	(G/T)
11815	CakSNP11815	*Kabuli*	Ca_Kabuli_Chr07	3353095	(C/T)

S.N.	SNP IDs	Cultivars	Chromosomes/scaffolds	Physical positions (bp)	SNPs
11816	CakSNP11816	*Kabuli*	Ca_Kabuli_Chr07	3355627	(A/G)
11817	CakSNP11817	*Kabuli*	Ca_Kabuli_Chr07	3355763	(A/G)
11818	CakSNP11818	*Kabuli*	Ca_Kabuli_Chr07	3356399	(G/A)
11819	CakSNP11819	*Kabuli*	Ca_Kabuli_Chr07	3357452	(G/A)
11820	CakSNP11820	*Kabuli*	Ca_Kabuli_Chr07	3430492	(A/T)
11821	CakSNP11821	*Kabuli*	Ca_Kabuli_Chr07	3430479	(G/A)
11822	CakSNP11822	*Kabuli*	Ca_Kabuli_Chr07	3445031	(T/C)
11823	CakSNP11823	*Kabuli*	Ca_Kabuli_Chr07	3476546	(T/C)
11824	CakSNP11824	*Kabuli*	Ca_Kabuli_Chr07	3504900	(G/A)
11825	CakSNP11825	*Kabuli*	Ca_Kabuli_Chr07	3505012	(A/C)
11826	CakSNP11826	*Kabuli*	Ca_Kabuli_Chr07	3518048	(G/A)
11827	CakSNP11827	*Kabuli*	Ca_Kabuli_Chr07	3519510	(A/G)
11828	CakSNP11828	*Kabuli*	Ca_Kabuli_Chr07	3519701	(A/C)
11829	CakSNP11829	*Kabuli*	Ca_Kabuli_Chr07	3521150	(A/G)
11830	CakSNP11830	*Kabuli*	Ca_Kabuli_Chr07	3521257	(G/A)
11831	CakSNP11831	*Kabuli*	Ca_Kabuli_Chr07	3521192	(C/A)
11832	CakSNP11832	*Kabuli*	Ca_Kabuli_Chr07	3536194	(C/T)
11833	CakSNP11833	*Kabuli*	Ca_Kabuli_Chr07	3536233	(T/C)
11834	CakSNP11834	*Kabuli*	Ca_Kabuli_Chr07	3543765	(A/G)
11835	CakSNP11835	*Kabuli*	Ca_Kabuli_Chr07	3543768	(T/C)
11836	CakSNP11836	*Kabuli*	Ca_Kabuli_Chr07	3557570	(C/T)
11837	CakSNP11837	*Kabuli*	Ca_Kabuli_Chr07	3560182	(G/A)
11838	CakSNP11838	*Kabuli*	Ca_Kabuli_Chr07	3560250	(C/T)
11839	CakSNP11839	*Kabuli*	Ca_Kabuli_Chr07	3572963	(T/C)
11840	CakSNP11840	*Kabuli*	Ca_Kabuli_Chr07	3581123	(G/A)
11841	CakSNP11841	*Kabuli*	Ca_Kabuli_Chr07	3600826	(C/T)
11842	CakSNP11842	*Kabuli*	Ca_Kabuli_Chr07	3600833	(T/G)
11843	CakSNP11843	*Kabuli*	Ca_Kabuli_Chr07	3600866	(T/C)
11844	CakSNP11844	*Kabuli*	Ca_Kabuli_Chr07	3600867	(C/T)
11845	CakSNP11845	*Kabuli*	Ca_Kabuli_Chr07	3600987	(A/C)
11846	CakSNP11846	*Kabuli*	Ca_Kabuli_Chr07	3628086	(G/A)
11847	CakSNP11847	*Kabuli*	Ca_Kabuli_Chr07	3635307	(G/A)
11848	CakSNP11848	*Kabuli*	Ca_Kabuli_Chr07	3635309	(C/T)
11849	CakSNP11849	*Kabuli*	Ca_Kabuli_Chr07	3635318	(T/A)
11850	CakSNP11850	*Kabuli*	Ca_Kabuli_Chr07	3635325	(T/C)
11851	CakSNP11851	*Kabuli*	Ca_Kabuli_Chr07	3635369	(G/T)
11852	CakSNP11852	*Kabuli*	Ca_Kabuli_Chr07	3651051	(T/C)
11853	CakSNP11853	*Kabuli*	Ca_Kabuli_Chr07	3655451	(G/T)
11854	CakSNP11854	*Kabuli*	Ca_Kabuli_Chr07	3665651	(G/C)

S.N.	SNP IDs	Cultivars	Chromosomes/scaffolds	Physical positions (bp)	SNPs
11855	CakSNP11855	*Kabuli*	Ca_Kabuli_Chr07	3672192	(T/C)
11856	CakSNP11856	*Kabuli*	Ca_Kabuli_Chr07	3672236	(G/A)
11857	CakSNP11857	*Kabuli*	Ca_Kabuli_Chr07	3734030	(T/A)
11858	CakSNP11858	*Kabuli*	Ca_Kabuli_Chr07	3738769	(T/C)
11859	CakSNP11859	*Kabuli*	Ca_Kabuli_Chr07	3754778	(G/A)
11860	CakSNP11860	*Kabuli*	Ca_Kabuli_Chr07	3754743	(G/C)
11861	CakSNP11861	*Kabuli*	Ca_Kabuli_Chr07	3795825	(T/C)
11862	CakSNP11862	*Kabuli*	Ca_Kabuli_Chr07	3795798	(G/A)
11863	CakSNP11863	*Kabuli*	Ca_Kabuli_Chr07	3795776	(C/A)
11864	CakSNP11864	*Kabuli*	Ca_Kabuli_Chr07	3796826	(A/G)
11865	CakSNP11865	*Kabuli*	Ca_Kabuli_Chr07	3796894	(A/G)
11866	CakSNP11866	*Kabuli*	Ca_Kabuli_Chr07	3802188	(T/A)
11867	CakSNP11867	*Kabuli*	Ca_Kabuli_Chr07	3814791	(A/G)
11868	CakSNP11868	*Kabuli*	Ca_Kabuli_Chr07	3817087	(C/T)
11869	CakSNP11869	*Kabuli*	Ca_Kabuli_Chr07	3817733	(T/G)
11870	CakSNP11870	*Kabuli*	Ca_Kabuli_Chr07	3817740	(G/T)
11871	CakSNP11871	*Kabuli*	Ca_Kabuli_Chr07	3829115	(C/T)
11872	CakSNP11872	*Kabuli*	Ca_Kabuli_Chr07	3843168	(A/T)
11873	CakSNP11873	*Kabuli*	Ca_Kabuli_Chr07	3845953	(A/C)
11874	CakSNP11874	*Kabuli*	Ca_Kabuli_Chr07	3845958	(G/A)
11875	CakSNP11875	*Kabuli*	Ca_Kabuli_Chr07	3845996	(G/A)
11876	CakSNP11876	*Kabuli*	Ca_Kabuli_Chr07	3856308	(C/T)
11877	CakSNP11877	*Kabuli*	Ca_Kabuli_Chr07	3859451	(G/A)
11878	CakSNP11878	*Kabuli*	Ca_Kabuli_Chr07	3916849	(A/C)
11879	CakSNP11879	*Kabuli*	Ca_Kabuli_Chr07	3924920	(T/C)
11880	CakSNP11880	*Kabuli*	Ca_Kabuli_Chr07	3946712	(G/A)
11881	CakSNP11881	*Kabuli*	Ca_Kabuli_Chr07	3982635	(T/G)
11882	CakSNP11882	*Kabuli*	Ca_Kabuli_Chr07	3982622	(G/A)
11883	CakSNP11883	*Kabuli*	Ca_Kabuli_Chr07	4017780	(C/T)
11884	CakSNP11884	*Kabuli*	Ca_Kabuli_Chr07	4017851	(T/C)
11885	CakSNP11885	*Kabuli*	Ca_Kabuli_Chr07	4039347	(C/A)
11886	CakSNP11886	*Kabuli*	Ca_Kabuli_Chr07	4039300	(T/C)
11887	CakSNP11887	*Kabuli*	Ca_Kabuli_Chr07	4039290	(A/G)
11888	CakSNP11888	*Kabuli*	Ca_Kabuli_Chr07	4042123	(T/G)
11889	CakSNP11889	*Kabuli*	Ca_Kabuli_Chr07	4095293	(A/G)
11890	CakSNP11890	*Kabuli*	Ca_Kabuli_Chr07	4168933	(G/T)
11891	CakSNP11891	*Kabuli*	Ca_Kabuli_Chr07	4194809	(T/A)
11892	CakSNP11892	*Kabuli*	Ca_Kabuli_Chr07	4194890	(C/A)
11893	CakSNP11893	*Kabuli*	Ca_Kabuli_Chr07	4194891	(C/G)

S.N.	SNP IDs	Cultivars	Chromosomes/scaffolds	Physical positions (bp)	SNPs
11894	CakSNP11894	*Kabuli*	Ca_Kabuli_Chr07	4198812	(A/C)
11895	CakSNP11895	*Kabuli*	Ca_Kabuli_Chr07	4209425	(C/T)
11896	CakSNP11896	*Kabuli*	Ca_Kabuli_Chr07	4209465	(A/C)
11897	CakSNP11897	*Kabuli*	Ca_Kabuli_Chr07	4225314	(T/C)
11898	CakSNP11898	*Kabuli*	Ca_Kabuli_Chr07	4256529	(G/A)
11899	CakSNP11899	*Kabuli*	Ca_Kabuli_Chr07	4256703	(C/G)
11900	CakSNP11900	*Kabuli*	Ca_Kabuli_Chr07	4257726	(A/T)
11901	CakSNP11901	*Kabuli*	Ca_Kabuli_Chr07	4257727	(T/A)
11902	CakSNP11902	*Kabuli*	Ca_Kabuli_Chr07	4257838	(C/T)
11903	CakSNP11903	*Kabuli*	Ca_Kabuli_Chr07	4257830	(G/T)
11904	CakSNP11904	*Kabuli*	Ca_Kabuli_Chr07	4257799	(G/A)
11905	CakSNP11905	*Kabuli*	Ca_Kabuli_Chr07	4289975	(G/A)
11906	CakSNP11906	*Kabuli*	Ca_Kabuli_Chr07	4300525	(C/T)
11907	CakSNP11907	*Kabuli*	Ca_Kabuli_Chr07	4300650	(T/C)
11908	CakSNP11908	*Kabuli*	Ca_Kabuli_Chr07	4300645	(C/T)
11909	CakSNP11909	*Kabuli*	Ca_Kabuli_Chr07	4300592	(G/A)
11910	CakSNP11910	*Kabuli*	Ca_Kabuli_Chr07	4300584	(C/T)
11911	CakSNP11911	*Kabuli*	Ca_Kabuli_Chr07	4305424	(G/T)
11912	CakSNP11912	*Kabuli*	Ca_Kabuli_Chr07	4431138	(A/G)
11913	CakSNP11913	*Kabuli*	Ca_Kabuli_Chr07	4431253	(T/C)
11914	CakSNP11914	*Kabuli*	Ca_Kabuli_Chr07	4438929	(A/G)
11915	CakSNP11915	*Kabuli*	Ca_Kabuli_Chr07	4455045	(T/C)
11916	CakSNP11916	*Kabuli*	Ca_Kabuli_Chr07	4461990	(A/C)
11917	CakSNP11917	*Kabuli*	Ca_Kabuli_Chr07	4470285	(C/G)
11918	CakSNP11918	*Kabuli*	Ca_Kabuli_Chr07	4490514	(G/A)
11919	CakSNP11919	*Kabuli*	Ca_Kabuli_Chr07	4490964	(G/T)
11920	CakSNP11920	*Kabuli*	Ca_Kabuli_Chr07	4490966	(A/G)
11921	CakSNP11921	*Kabuli*	Ca_Kabuli_Chr07	4497784	(C/T)
11922	CakSNP11922	*Kabuli*	Ca_Kabuli_Chr07	4520606	(T/C)
11923	CakSNP11923	*Kabuli*	Ca_Kabuli_Chr07	4525645	(A/G)
11924	CakSNP11924	*Kabuli*	Ca_Kabuli_Chr07	4552790	(T/C)
11925	CakSNP11925	*Kabuli*	Ca_Kabuli_Chr07	4573236	(G/A)
11926	CakSNP11926	*Kabuli*	Ca_Kabuli_Chr07	4625723	(C/T)
11927	CakSNP11927	*Kabuli*	Ca_Kabuli_Chr07	4648569	(A/C)
11928	CakSNP11928	*Kabuli*	Ca_Kabuli_Chr07	4648697	(T/A)
11929	CakSNP11929	*Kabuli*	Ca_Kabuli_Chr07	4648677	(A/T)
11930	CakSNP11930	*Kabuli*	Ca_Kabuli_Chr07	4648662	(G/A)
11931	CakSNP11931	*Kabuli*	Ca_Kabuli_Chr07	4649287	(A/G)
11932	CakSNP11932	*Kabuli*	Ca_Kabuli_Chr07	4651931	(T/C)

S.N.	SNP IDs	Cultivars	Chromosomes/scaffolds	Physical positions (bp)	SNPs
11933	CakSNP11933	*Kabuli*	Ca_Kabuli_Chr07	4652083	(G/A)
11934	CakSNP11934	*Kabuli*	Ca_Kabuli_Chr07	4653235	(A/C)
11935	CakSNP11935	*Kabuli*	Ca_Kabuli_Chr07	4700570	(C/A)
11936	CakSNP11936	*Kabuli*	Ca_Kabuli_Chr07	4749212	(T/G)
11937	CakSNP11937	*Kabuli*	Ca_Kabuli_Chr07	4752954	(G/C)
11938	CakSNP11938	*Kabuli*	Ca_Kabuli_Chr07	4752887	(T/G)
11939	CakSNP11939	*Kabuli*	Ca_Kabuli_Chr07	4756338	(G/A)
11940	CakSNP11940	*Kabuli*	Ca_Kabuli_Chr07	4832993	(A/G)
11941	CakSNP11941	*Kabuli*	Ca_Kabuli_Chr07	4834177	(G/T)
11942	CakSNP11942	*Kabuli*	Ca_Kabuli_Chr07	4849553	(T/G)
11943	CakSNP11943	*Kabuli*	Ca_Kabuli_Chr07	4849573	(T/C)
11944	CakSNP11944	*Kabuli*	Ca_Kabuli_Chr07	4907315	(C/T)
11945	CakSNP11945	*Kabuli*	Ca_Kabuli_Chr07	4926759	(A/T)
11946	CakSNP11946	*Kabuli*	Ca_Kabuli_Chr07	4942847	(A/G)
11947	CakSNP11947	*Kabuli*	Ca_Kabuli_Chr07	4942902	(G/C)
11948	CakSNP11948	*Kabuli*	Ca_Kabuli_Chr07	4945935	(C/G)
11949	CakSNP11949	*Kabuli*	Ca_Kabuli_Chr07	4981846	(T/C)
11950	CakSNP11950	*Kabuli*	Ca_Kabuli_Chr07	5122536	(T/G)
11951	CakSNP11951	*Kabuli*	Ca_Kabuli_Chr07	5251897	(G/C)
11952	CakSNP11952	*Kabuli*	Ca_Kabuli_Chr07	5361558	(T/C)
11953	CakSNP11953	*Kabuli*	Ca_Kabuli_Chr07	5383564	(G/C)
11954	CakSNP11954	*Kabuli*	Ca_Kabuli_Chr07	5383566	(A/T)
11955	CakSNP11955	*Kabuli*	Ca_Kabuli_Chr07	5383671	(G/T)
11956	CakSNP11956	*Kabuli*	Ca_Kabuli_Chr07	5383695	(T/G)
11957	CakSNP11957	*Kabuli*	Ca_Kabuli_Chr07	5383767	(G/A)
11958	CakSNP11958	*Kabuli*	Ca_Kabuli_Chr07	5384447	(G/C)
11959	CakSNP11959	*Kabuli*	Ca_Kabuli_Chr07	5384467	(C/T)
11960	CakSNP11960	*Kabuli*	Ca_Kabuli_Chr07	5406524	(T/G)
11961	CakSNP11961	*Kabuli*	Ca_Kabuli_Chr07	5416862	(C/G)
11962	CakSNP11962	*Kabuli*	Ca_Kabuli_Chr07	5416865	(A/G)
11963	CakSNP11963	*Kabuli*	Ca_Kabuli_Chr07	5416874	(C/T)
11964	CakSNP11964	*Kabuli*	Ca_Kabuli_Chr07	5464941	(G/A)
11965	CakSNP11965	*Kabuli*	Ca_Kabuli_Chr07	5464942	(A/G)
11966	CakSNP11966	*Kabuli*	Ca_Kabuli_Chr07	5467744	(G/C)
11967	CakSNP11967	*Kabuli*	Ca_Kabuli_Chr07	5500318	(C/A)
11968	CakSNP11968	*Kabuli*	Ca_Kabuli_Chr07	5508162	(A/C)
11969	CakSNP11969	*Kabuli*	Ca_Kabuli_Chr07	5520720	(T/G)
11970	CakSNP11970	*Kabuli*	Ca_Kabuli_Chr07	5520934	(T/C)
11971	CakSNP11971	*Kabuli*	Ca_Kabuli_Chr07	5521596	(T/C)

S.N.	SNP IDs	Cultivars	Chromosomes/scaffolds	Physical positions (bp)	SNPs
11972	CakSNP11972	*Kabuli*	Ca_Kabuli_Chr07	5521659	(G/T)
11973	CakSNP11973	*Kabuli*	Ca_Kabuli_Chr07	5529577	(T/G)
11974	CakSNP11974	*Kabuli*	Ca_Kabuli_Chr07	5529729	(A/G)
11975	CakSNP11975	*Kabuli*	Ca_Kabuli_Chr07	5529888	(A/G)
11976	CakSNP11976	*Kabuli*	Ca_Kabuli_Chr07	5529844	(C/A)
11977	CakSNP11977	*Kabuli*	Ca_Kabuli_Chr07	5537851	(G/C)
11978	CakSNP11978	*Kabuli*	Ca_Kabuli_Chr07	5537880	(G/T)
11979	CakSNP11979	*Kabuli*	Ca_Kabuli_Chr07	5561607	(G/T)
11980	CakSNP11980	*Kabuli*	Ca_Kabuli_Chr07	5568114	(C/A)
11981	CakSNP11981	*Kabuli*	Ca_Kabuli_Chr07	5577467	(G/T)
11982	CakSNP11982	*Kabuli*	Ca_Kabuli_Chr07	5577439	(A/G)
11983	CakSNP11983	*Kabuli*	Ca_Kabuli_Chr07	5581968	(G/C)
11984	CakSNP11984	*Kabuli*	Ca_Kabuli_Chr07	5602106	(T/C)
11985	CakSNP11985	*Kabuli*	Ca_Kabuli_Chr07	5602144	(G/T)
11986	CakSNP11986	*Kabuli*	Ca_Kabuli_Chr07	5605118	(G/A)
11987	CakSNP11987	*Kabuli*	Ca_Kabuli_Chr07	5605106	(G/A)
11988	CakSNP11988	*Kabuli*	Ca_Kabuli_Chr07	5658445	(G/T)
11989	CakSNP11989	*Kabuli*	Ca_Kabuli_Chr07	5671902	(G/T)
11990	CakSNP11990	*Kabuli*	Ca_Kabuli_Chr07	5672009	(G/C)
11991	CakSNP11991	*Kabuli*	Ca_Kabuli_Chr07	5672156	(T/C)
11992	CakSNP11992	*Kabuli*	Ca_Kabuli_Chr07	5683412	(T/A)
11993	CakSNP11993	*Kabuli*	Ca_Kabuli_Chr07	5713977	(T/C)
11994	CakSNP11994	*Kabuli*	Ca_Kabuli_Chr07	5735293	(T/A)
11995	CakSNP11995	*Kabuli*	Ca_Kabuli_Chr07	5735448	(A/T)
11996	CakSNP11996	*Kabuli*	Ca_Kabuli_Chr07	5737341	(C/T)
11997	CakSNP11997	*Kabuli*	Ca_Kabuli_Chr07	5737305	(G/A)
11998	CakSNP11998	*Kabuli*	Ca_Kabuli_Chr07	5756269	(C/T)
11999	CakSNP11999	*Kabuli*	Ca_Kabuli_Chr07	5756832	(A/C)
12000	CakSNP12000	*Kabuli*	Ca_Kabuli_Chr07	5774218	(T/A)
12001	CakSNP12001	*Kabuli*	Ca_Kabuli_Chr07	5774219	(T/A)
12002	CakSNP12002	*Kabuli*	Ca_Kabuli_Chr07	5774220	(A/T)
12003	CakSNP12003	*Kabuli*	Ca_Kabuli_Chr07	5774221	(A/T)
12004	CakSNP12004	*Kabuli*	Ca_Kabuli_Chr07	5774230	(G/A)
12005	CakSNP12005	*Kabuli*	Ca_Kabuli_Chr07	5774257	(C/T)
12006	CakSNP12006	*Kabuli*	Ca_Kabuli_Chr07	5774310	(A/G)
12007	CakSNP12007	*Kabuli*	Ca_Kabuli_Chr07	5811097	(G/A)
12008	CakSNP12008	*Kabuli*	Ca_Kabuli_Chr07	5811060	(T/C)
12009	CakSNP12009	*Kabuli*	Ca_Kabuli_Chr07	5811044	(T/A)
12010	CakSNP12010	*Kabuli*	Ca_Kabuli_Chr07	5836421	(A/T)

S.N.	SNP IDs	Cultivars	Chromosomes/scaffolds	Physical positions (bp)	SNPs
12011	CakSNP12011	*Kabuli*	Ca_Kabuli_Chr07	5846477	(A/G)
12012	CakSNP12012	*Kabuli*	Ca_Kabuli_Chr07	5849936	(C/T)
12013	CakSNP12013	*Kabuli*	Ca_Kabuli_Chr07	5849904	(C/A)
12014	CakSNP12014	*Kabuli*	Ca_Kabuli_Chr07	5850058	(T/C)
12015	CakSNP12015	*Kabuli*	Ca_Kabuli_Chr07	5854598	(C/T)
12016	CakSNP12016	*Kabuli*	Ca_Kabuli_Chr07	5929009	(C/G)
12017	CakSNP12017	*Kabuli*	Ca_Kabuli_Chr07	5946113	(G/A)
12018	CakSNP12018	*Kabuli*	Ca_Kabuli_Chr07	5950352	(T/A)
12019	CakSNP12019	*Kabuli*	Ca_Kabuli_Chr07	5950492	(T/C)
12020	CakSNP12020	*Kabuli*	Ca_Kabuli_Chr07	5957696	(T/C)
12021	CakSNP12021	*Kabuli*	Ca_Kabuli_Chr07	5963611	(C/G)
12022	CakSNP12022	*Kabuli*	Ca_Kabuli_Chr07	5963609	(C/A)
12023	CakSNP12023	*Kabuli*	Ca_Kabuli_Chr07	6012561	(A/C)
12024	CakSNP12024	*Kabuli*	Ca_Kabuli_Chr07	6012611	(A/C)
12025	CakSNP12025	*Kabuli*	Ca_Kabuli_Chr07	6029991	(G/A)
12026	CakSNP12026	*Kabuli*	Ca_Kabuli_Chr07	6029990	(C/G)
12027	CakSNP12027	*Kabuli*	Ca_Kabuli_Chr07	6066322	(G/A)
12028	CakSNP12028	*Kabuli*	Ca_Kabuli_Chr07	6074408	(T/A)
12029	CakSNP12029	*Kabuli*	Ca_Kabuli_Chr07	6075124	(T/G)
12030	CakSNP12030	*Kabuli*	Ca_Kabuli_Chr07	6075218	(C/G)
12031	CakSNP12031	*Kabuli*	Ca_Kabuli_Chr07	6096238	(C/A)
12032	CakSNP12032	*Kabuli*	Ca_Kabuli_Chr07	6096237	(C/G)
12033	CakSNP12033	*Kabuli*	Ca_Kabuli_Chr07	6110019	(T/C)
12034	CakSNP12034	*Kabuli*	Ca_Kabuli_Chr07	6132021	(G/A)
12035	CakSNP12035	*Kabuli*	Ca_Kabuli_Chr07	6232777	(T/C)
12036	CakSNP12036	*Kabuli*	Ca_Kabuli_Chr07	6282979	(C/A)
12037	CakSNP12037	*Kabuli*	Ca_Kabuli_Chr07	6282974	(T/A)
12038	CakSNP12038	*Kabuli*	Ca_Kabuli_Chr07	6282949	(T/A)
12039	CakSNP12039	*Kabuli*	Ca_Kabuli_Chr07	6299248	(A/G)
12040	CakSNP12040	*Kabuli*	Ca_Kabuli_Chr07	6299249	(C/G)
12041	CakSNP12041	*Kabuli*	Ca_Kabuli_Chr07	6330237	(A/T)
12042	CakSNP12042	*Kabuli*	Ca_Kabuli_Chr07	6340051	(A/G)
12043	CakSNP12043	*Kabuli*	Ca_Kabuli_Chr07	6340064	(C/G)
12044	CakSNP12044	*Kabuli*	Ca_Kabuli_Chr07	6340111	(C/G)
12045	CakSNP12045	*Kabuli*	Ca_Kabuli_Chr07	6340113	(G/A)
12046	CakSNP12046	*Kabuli*	Ca_Kabuli_Chr07	6340167	(T/A)
12047	CakSNP12047	*Kabuli*	Ca_Kabuli_Chr07	6340216	(T/C)
12048	CakSNP12048	*Kabuli*	Ca_Kabuli_Chr07	6372037	(A/G)
12049	CakSNP12049	*Kabuli*	Ca_Kabuli_Chr07	6393366	(G/A)

S.N.	SNP IDs	Cultivars	Chromosomes/scaffolds	Physical positions (bp)	SNPs
12050	CakSNP12050	*Kabuli*	Ca_Kabuli_Chr07	6430396	(T/A)
12051	CakSNP12051	*Kabuli*	Ca_Kabuli_Chr07	6433480	(C/G)
12052	CakSNP12052	*Kabuli*	Ca_Kabuli_Chr07	6467903	(C/T)
12053	CakSNP12053	*Kabuli*	Ca_Kabuli_Chr07	6540624	(A/G)
12054	CakSNP12054	*Kabuli*	Ca_Kabuli_Chr07	6540602	(G/A)
12055	CakSNP12055	*Kabuli*	Ca_Kabuli_Chr07	6540601	(G/C)
12056	CakSNP12056	*Kabuli*	Ca_Kabuli_Chr07	6566322	(T/C)
12057	CakSNP12057	*Kabuli*	Ca_Kabuli_Chr07	6654718	(C/T)
12058	CakSNP12058	*Kabuli*	Ca_Kabuli_Chr07	6681050	(C/T)
12059	CakSNP12059	*Kabuli*	Ca_Kabuli_Chr07	6682456	(T/A)
12060	CakSNP12060	*Kabuli*	Ca_Kabuli_Chr07	6682454	(A/G)
12061	CakSNP12061	*Kabuli*	Ca_Kabuli_Chr07	6682453	(G/T)
12062	CakSNP12062	*Kabuli*	Ca_Kabuli_Chr07	6682448	(T/C)
12063	CakSNP12063	*Kabuli*	Ca_Kabuli_Chr07	6682443	(C/T)
12064	CakSNP12064	*Kabuli*	Ca_Kabuli_Chr07	6682439	(T/A)
12065	CakSNP12065	*Kabuli*	Ca_Kabuli_Chr07	6682455	(G/T)
12066	CakSNP12066	*Kabuli*	Ca_Kabuli_Chr07	6703759	(C/T)
12067	CakSNP12067	*Kabuli*	Ca_Kabuli_Chr07	6703849	(G/T)
12068	CakSNP12068	*Kabuli*	Ca_Kabuli_Chr07	6767204	(G/T)
12069	CakSNP12069	*Kabuli*	Ca_Kabuli_Chr07	6769694	(T/G)
12070	CakSNP12070	*Kabuli*	Ca_Kabuli_Chr07	6792352	(T/C)
12071	CakSNP12071	*Kabuli*	Ca_Kabuli_Chr07	6792606	(T/G)
12072	CakSNP12072	*Kabuli*	Ca_Kabuli_Chr07	6807817	(A/C)
12073	CakSNP12073	*Kabuli*	Ca_Kabuli_Chr07	6814954	(G/T)
12074	CakSNP12074	*Kabuli*	Ca_Kabuli_Chr07	6815151	(G/T)
12075	CakSNP12075	*Kabuli*	Ca_Kabuli_Chr07	6815113	(T/C)
12076	CakSNP12076	*Kabuli*	Ca_Kabuli_Chr07	6859511	(C/G)
12077	CakSNP12077	*Kabuli*	Ca_Kabuli_Chr07	6904686	(G/C)
12078	CakSNP12078	*Kabuli*	Ca_Kabuli_Chr07	7026448	(C/T)
12079	CakSNP12079	*Kabuli*	Ca_Kabuli_Chr07	7026516	(T/C)
12080	CakSNP12080	*Kabuli*	Ca_Kabuli_Chr07	7026500	(T/A)
12081	CakSNP12081	*Kabuli*	Ca_Kabuli_Chr07	7072853	(G/A)
12082	CakSNP12082	*Kabuli*	Ca_Kabuli_Chr07	7126974	(T/C)
12083	CakSNP12083	*Kabuli*	Ca_Kabuli_Chr07	7126979	(C/G)
12084	CakSNP12084	*Kabuli*	Ca_Kabuli_Chr07	7127030	(G/A)
12085	CakSNP12085	*Kabuli*	Ca_Kabuli_Chr07	7146159	(C/G)
12086	CakSNP12086	*Kabuli*	Ca_Kabuli_Chr07	7147557	(A/G)
12087	CakSNP12087	*Kabuli*	Ca_Kabuli_Chr07	7234422	(T/G)
12088	CakSNP12088	*Kabuli*	Ca_Kabuli_Chr07	7234507	(T/C)

S.N.	SNP IDs	Cultivars	Chromosomes/scaffolds	Physical positions (bp)	SNPs
12089	CakSNP12089	*Kabuli*	Ca_Kabuli_Chr07	7234484	(G/A)
12090	CakSNP12090	*Kabuli*	Ca_Kabuli_Chr07	7234430	(C/T)
12091	CakSNP12091	*Kabuli*	Ca_Kabuli_Chr07	7261971	(C/T)
12092	CakSNP12092	*Kabuli*	Ca_Kabuli_Chr07	7261941	(T/C)
12093	CakSNP12093	*Kabuli*	Ca_Kabuli_Chr07	7265386	(T/A)
12094	CakSNP12094	*Kabuli*	Ca_Kabuli_Chr07	7277353	(G/A)
12095	CakSNP12095	*Kabuli*	Ca_Kabuli_Chr07	7304614	(T/G)
12096	CakSNP12096	*Kabuli*	Ca_Kabuli_Chr07	7304617	(T/G)
12097	CakSNP12097	*Kabuli*	Ca_Kabuli_Chr07	7423419	(G/A)
12098	CakSNP12098	*Kabuli*	Ca_Kabuli_Chr07	7433827	(A/T)
12099	CakSNP12099	*Kabuli*	Ca_Kabuli_Chr07	7449489	(T/C)
12100	CakSNP12100	*Kabuli*	Ca_Kabuli_Chr07	7451931	(G/C)
12101	CakSNP12101	*Kabuli*	Ca_Kabuli_Chr07	7491960	(G/A)
12102	CakSNP12102	*Kabuli*	Ca_Kabuli_Chr07	7498598	(C/T)
12103	CakSNP12103	*Kabuli*	Ca_Kabuli_Chr07	7544142	(A/G)
12104	CakSNP12104	*Kabuli*	Ca_Kabuli_Chr07	7573889	(C/G)
12105	CakSNP12105	*Kabuli*	Ca_Kabuli_Chr07	7576668	(A/C)
12106	CakSNP12106	*Kabuli*	Ca_Kabuli_Chr07	7580158	(T/C)
12107	CakSNP12107	*Kabuli*	Ca_Kabuli_Chr07	7580147	(C/A)
12108	CakSNP12108	*Kabuli*	Ca_Kabuli_Chr07	7580118	(G/A)
12109	CakSNP12109	*Kabuli*	Ca_Kabuli_Chr07	7612046	(A/T)
12110	CakSNP12110	*Kabuli*	Ca_Kabuli_Chr07	7624697	(G/A)
12111	CakSNP12111	*Kabuli*	Ca_Kabuli_Chr07	7625226	(G/T)
12112	CakSNP12112	*Kabuli*	Ca_Kabuli_Chr07	7625277	(A/T)
12113	CakSNP12113	*Kabuli*	Ca_Kabuli_Chr07	7625901	(A/C)
12114	CakSNP12114	*Kabuli*	Ca_Kabuli_Chr07	7625943	(C/G)
12115	CakSNP12115	*Kabuli*	Ca_Kabuli_Chr07	7663564	(A/G)
12116	CakSNP12116	*Kabuli*	Ca_Kabuli_Chr07	7706816	(A/G)
12117	CakSNP12117	*Kabuli*	Ca_Kabuli_Chr07	7756455	(C/T)
12118	CakSNP12118	*Kabuli*	Ca_Kabuli_Chr07	7756482	(G/A)
12119	CakSNP12119	*Kabuli*	Ca_Kabuli_Chr07	7756566	(G/A)
12120	CakSNP12120	*Kabuli*	Ca_Kabuli_Chr07	7809179	(T/G)
12121	CakSNP12121	*Kabuli*	Ca_Kabuli_Chr07	7809192	(A/C)
12122	CakSNP12122	*Kabuli*	Ca_Kabuli_Chr07	7875189	(C/A)
12123	CakSNP12123	*Kabuli*	Ca_Kabuli_Chr07	7876554	(G/A)
12124	CakSNP12124	*Kabuli*	Ca_Kabuli_Chr07	7876561	(C/T)
12125	CakSNP12125	*Kabuli*	Ca_Kabuli_Chr07	7876570	(C/A)
12126	CakSNP12126	*Kabuli*	Ca_Kabuli_Chr07	7928398	(C/T)
12127	CakSNP12127	*Kabuli*	Ca_Kabuli_Chr07	7982650	(G/T)

S.N.	SNP IDs	Cultivars	Chromosomes/scaffolds	Physical positions (bp)	SNPs
12128	CakSNP12128	*Kabuli*	Ca_Kabuli_Chr07	7982871	(T/C)
12129	CakSNP12129	*Kabuli*	Ca_Kabuli_Chr07	7982890	(T/A)
12130	CakSNP12130	*Kabuli*	Ca_Kabuli_Chr07	7983519	(C/T)
12131	CakSNP12131	*Kabuli*	Ca_Kabuli_Chr07	8041465	(T/G)
12132	CakSNP12132	*Kabuli*	Ca_Kabuli_Chr07	8054489	(G/A)
12133	CakSNP12133	*Kabuli*	Ca_Kabuli_Chr07	8061808	(A/G)
12134	CakSNP12134	*Kabuli*	Ca_Kabuli_Chr07	8111735	(T/C)
12135	CakSNP12135	*Kabuli*	Ca_Kabuli_Chr07	8111754	(A/C)
12136	CakSNP12136	*Kabuli*	Ca_Kabuli_Chr07	8111759	(G/T)
12137	CakSNP12137	*Kabuli*	Ca_Kabuli_Chr07	8169857	(A/G)
12138	CakSNP12138	*Kabuli*	Ca_Kabuli_Chr07	8169866	(A/G)
12139	CakSNP12139	*Kabuli*	Ca_Kabuli_Chr07	8169960	(A/T)
12140	CakSNP12140	*Kabuli*	Ca_Kabuli_Chr07	8169947	(T/C)
12141	CakSNP12141	*Kabuli*	Ca_Kabuli_Chr07	8187181	(G/A)
12142	CakSNP12142	*Kabuli*	Ca_Kabuli_Chr07	8188004	(C/G)
12143	CakSNP12143	*Kabuli*	Ca_Kabuli_Chr07	8210963	(G/A)
12144	CakSNP12144	*Kabuli*	Ca_Kabuli_Chr07	8261062	(C/A)
12145	CakSNP12145	*Kabuli*	Ca_Kabuli_Chr07	8286115	(A/T)
12146	CakSNP12146	*Kabuli*	Ca_Kabuli_Chr07	8515092	(C/T)
12147	CakSNP12147	*Kabuli*	Ca_Kabuli_Chr07	8535189	(C/T)
12148	CakSNP12148	*Kabuli*	Ca_Kabuli_Chr07	8553114	(C/T)
12149	CakSNP12149	*Kabuli*	Ca_Kabuli_Chr07	8569632	(A/G)
12150	CakSNP12150	*Kabuli*	Ca_Kabuli_Chr07	8583890	(T/C)
12151	CakSNP12151	*Kabuli*	Ca_Kabuli_Chr07	8609831	(A/T)
12152	CakSNP12152	*Kabuli*	Ca_Kabuli_Chr07	8626840	(A/C)
12153	CakSNP12153	*Kabuli*	Ca_Kabuli_Chr07	8626875	(A/T)
12154	CakSNP12154	*Kabuli*	Ca_Kabuli_Chr07	8626916	(T/C)
12155	CakSNP12155	*Kabuli*	Ca_Kabuli_Chr07	8741098	(T/C)
12156	CakSNP12156	*Kabuli*	Ca_Kabuli_Chr07	8777670	(A/G)
12157	CakSNP12157	*Kabuli*	Ca_Kabuli_Chr07	8777673	(A/G)
12158	CakSNP12158	*Kabuli*	Ca_Kabuli_Chr07	8777690	(G/A)
12159	CakSNP12159	*Kabuli*	Ca_Kabuli_Chr07	8824586	(A/G)
12160	CakSNP12160	*Kabuli*	Ca_Kabuli_Chr07	8824591	(C/T)
12161	CakSNP12161	*Kabuli*	Ca_Kabuli_Chr07	8830772	(A/C)
12162	CakSNP12162	*Kabuli*	Ca_Kabuli_Chr07	8830985	(A/G)
12163	CakSNP12163	*Kabuli*	Ca_Kabuli_Chr07	8831024	(C/T)
12164	CakSNP12164	*Kabuli*	Ca_Kabuli_Chr07	8831096	(G/A)
12165	CakSNP12165	*Kabuli*	Ca_Kabuli_Chr07	8835601	(G/A)
12166	CakSNP12166	*Kabuli*	Ca_Kabuli_Chr07	8835602	(A/G)

S.N.	SNP IDs	Cultivars	Chromosomes/scaffolds	Physical positions (bp)	SNPs
12167	CakSNP12167	*Kabuli*	Ca_Kabuli_Chr07	8835610	(G/A)
12168	CakSNP12168	*Kabuli*	Ca_Kabuli_Chr07	8835659	(G/T)
12169	CakSNP12169	*Kabuli*	Ca_Kabuli_Chr07	8835757	(T/C)
12170	CakSNP12170	*Kabuli*	Ca_Kabuli_Chr07	8835718	(T/G)
12171	CakSNP12171	*Kabuli*	Ca_Kabuli_Chr07	8835705	(T/A)
12172	CakSNP12172	*Kabuli*	Ca_Kabuli_Chr07	8844902	(C/T)
12173	CakSNP12173	*Kabuli*	Ca_Kabuli_Chr07	8894362	(A/T)
12174	CakSNP12174	*Kabuli*	Ca_Kabuli_Chr07	8895087	(T/C)
12175	CakSNP12175	*Kabuli*	Ca_Kabuli_Chr07	8913092	(C/G)
12176	CakSNP12176	*Kabuli*	Ca_Kabuli_Chr07	8937391	(A/G)
12177	CakSNP12177	*Kabuli*	Ca_Kabuli_Chr07	8970387	(A/G)
12178	CakSNP12178	*Kabuli*	Ca_Kabuli_Chr07	9128044	(A/G)
12179	CakSNP12179	*Kabuli*	Ca_Kabuli_Chr07	9128050	(C/A)
12180	CakSNP12180	*Kabuli*	Ca_Kabuli_Chr07	9147367	(A/T)
12181	CakSNP12181	*Kabuli*	Ca_Kabuli_Chr07	9147366	(T/G)
12182	CakSNP12182	*Kabuli*	Ca_Kabuli_Chr07	9147502	(C/T)
12183	CakSNP12183	*Kabuli*	Ca_Kabuli_Chr07	9151709	(T/G)
12184	CakSNP12184	*Kabuli*	Ca_Kabuli_Chr07	9151755	(C/T)
12185	CakSNP12185	*Kabuli*	Ca_Kabuli_Chr07	9153104	(C/A)
12186	CakSNP12186	*Kabuli*	Ca_Kabuli_Chr07	9153062	(G/A)
12187	CakSNP12187	*Kabuli*	Ca_Kabuli_Chr07	9214457	(T/A)
12188	CakSNP12188	*Kabuli*	Ca_Kabuli_Chr07	9214422	(C/T)
12189	CakSNP12189	*Kabuli*	Ca_Kabuli_Chr07	9301825	(G/C)
12190	CakSNP12190	*Kabuli*	Ca_Kabuli_Chr07	9301881	(C/T)
12191	CakSNP12191	*Kabuli*	Ca_Kabuli_Chr07	9327709	(C/T)
12192	CakSNP12192	*Kabuli*	Ca_Kabuli_Chr07	9327687	(G/A)
12193	CakSNP12193	*Kabuli*	Ca_Kabuli_Chr07	9348281	(T/C)
12194	CakSNP12194	*Kabuli*	Ca_Kabuli_Chr07	9350506	(T/G)
12195	CakSNP12195	*Kabuli*	Ca_Kabuli_Chr07	9350535	(A/C)
12196	CakSNP12196	*Kabuli*	Ca_Kabuli_Chr07	9351783	(C/G)
12197	CakSNP12197	*Kabuli*	Ca_Kabuli_Chr07	9351863	(G/A)
12198	CakSNP12198	*Kabuli*	Ca_Kabuli_Chr07	9422842	(T/C)
12199	CakSNP12199	*Kabuli*	Ca_Kabuli_Chr07	9422859	(T/C)
12200	CakSNP12200	*Kabuli*	Ca_Kabuli_Chr07	9422876	(T/C)
12201	CakSNP12201	*Kabuli*	Ca_Kabuli_Chr07	9422946	(G/C)
12202	CakSNP12202	*Kabuli*	Ca_Kabuli_Chr07	9442836	(A/C)
12203	CakSNP12203	*Kabuli*	Ca_Kabuli_Chr07	9475275	(T/G)
12204	CakSNP12204	*Kabuli*	Ca_Kabuli_Chr07	9475817	(C/A)
12205	CakSNP12205	*Kabuli*	Ca_Kabuli_Chr07	9475922	(A/G)

S.N.	SNP IDs	Cultivars	Chromosomes/scaffolds	Physical positions (bp)	SNPs
12206	CakSNP12206	*Kabuli*	Ca_Kabuli_Chr07	9475874	(C/T)
12207	CakSNP12207	*Kabuli*	Ca_Kabuli_Chr07	9523902	(C/T)
12208	CakSNP12208	*Kabuli*	Ca_Kabuli_Chr07	9528324	(A/G)
12209	CakSNP12209	*Kabuli*	Ca_Kabuli_Chr07	9536012	(C/T)
12210	CakSNP12210	*Kabuli*	Ca_Kabuli_Chr07	9550284	(G/C)
12211	CakSNP12211	*Kabuli*	Ca_Kabuli_Chr07	9550230	(C/T)
12212	CakSNP12212	*Kabuli*	Ca_Kabuli_Chr07	9555319	(C/G)
12213	CakSNP12213	*Kabuli*	Ca_Kabuli_Chr07	9577441	(G/A)
12214	CakSNP12214	*Kabuli*	Ca_Kabuli_Chr07	9577424	(G/C)
12215	CakSNP12215	*Kabuli*	Ca_Kabuli_Chr07	9593677	(C/T)
12216	CakSNP12216	*Kabuli*	Ca_Kabuli_Chr07	9612558	(A/C)
12217	CakSNP12217	*Kabuli*	Ca_Kabuli_Chr07	9616335	(G/A)
12218	CakSNP12218	*Kabuli*	Ca_Kabuli_Chr07	9616347	(C/G)
12219	CakSNP12219	*Kabuli*	Ca_Kabuli_Chr07	9616357	(C/T)
12220	CakSNP12220	*Kabuli*	Ca_Kabuli_Chr07	9616363	(A/C)
12221	CakSNP12221	*Kabuli*	Ca_Kabuli_Chr07	9616367	(C/G)
12222	CakSNP12222	*Kabuli*	Ca_Kabuli_Chr07	9616378	(G/A)
12223	CakSNP12223	*Kabuli*	Ca_Kabuli_Chr07	9637456	(C/T)
12224	CakSNP12224	*Kabuli*	Ca_Kabuli_Chr07	9639993	(C/A)
12225	CakSNP12225	*Kabuli*	Ca_Kabuli_Chr07	9661050	(A/C)
12226	CakSNP12226	*Kabuli*	Ca_Kabuli_Chr07	9671210	(T/C)
12227	CakSNP12227	*Kabuli*	Ca_Kabuli_Chr07	9696620	(A/G)
12228	CakSNP12228	*Kabuli*	Ca_Kabuli_Chr07	9738801	(T/C)
12229	CakSNP12229	*Kabuli*	Ca_Kabuli_Chr07	9744436	(G/T)
12230	CakSNP12230	*Kabuli*	Ca_Kabuli_Chr07	9890779	(A/C)
12231	CakSNP12231	*Kabuli*	Ca_Kabuli_Chr07	9890777	(A/G)
12232	CakSNP12232	*Kabuli*	Ca_Kabuli_Chr07	9893688	(A/G)
12233	CakSNP12233	*Kabuli*	Ca_Kabuli_Chr07	9897698	(T/G)
12234	CakSNP12234	*Kabuli*	Ca_Kabuli_Chr07	9898718	(A/T)
12235	CakSNP12235	*Kabuli*	Ca_Kabuli_Chr07	9899348	(C/T)
12236	CakSNP12236	*Kabuli*	Ca_Kabuli_Chr07	9914751	(G/A)
12237	CakSNP12237	*Kabuli*	Ca_Kabuli_Chr07	9914764	(G/A)
12238	CakSNP12238	*Kabuli*	Ca_Kabuli_Chr07	9914770	(T/C)
12239	CakSNP12239	*Kabuli*	Ca_Kabuli_Chr07	9914785	(T/C)
12240	CakSNP12240	*Kabuli*	Ca_Kabuli_Chr07	9914794	(G/A)
12241	CakSNP12241	*Kabuli*	Ca_Kabuli_Chr07	9914737	(C/G)
12242	CakSNP12242	*Kabuli*	Ca_Kabuli_Chr07	9914745	(T/C)
12243	CakSNP12243	*Kabuli*	Ca_Kabuli_Chr07	9914856	(G/A)
12244	CakSNP12244	*Kabuli*	Ca_Kabuli_Chr07	9914845	(A/C)

S.N.	SNP IDs	Cultivars	Chromosomes/scaffolds	Physical positions (bp)	SNPs
12245	CakSNP12245	*Kabuli*	Ca_Kabuli_Chr07	9914832	(C/T)
12246	CakSNP12246	*Kabuli*	Ca_Kabuli_Chr07	9914802	(T/G)
12247	CakSNP12247	*Kabuli*	Ca_Kabuli_Chr07	9932369	(A/G)
12248	CakSNP12248	*Kabuli*	Ca_Kabuli_Chr07	9936661	(C/T)
12249	CakSNP12249	*Kabuli*	Ca_Kabuli_Chr07	9936854	(A/G)
12250	CakSNP12250	*Kabuli*	Ca_Kabuli_Chr07	9938937	(T/C)
12251	CakSNP12251	*Kabuli*	Ca_Kabuli_Chr07	10050020	(A/C)
12252	CakSNP12252	*Kabuli*	Ca_Kabuli_Chr07	10049998	(T/A)
12253	CakSNP12253	*Kabuli*	Ca_Kabuli_Chr07	10049996	(A/G)
12254	CakSNP12254	*Kabuli*	Ca_Kabuli_Chr07	10089428	(T/C)
12255	CakSNP12255	*Kabuli*	Ca_Kabuli_Chr07	10089539	(T/A)
12256	CakSNP12256	*Kabuli*	Ca_Kabuli_Chr07	10106084	(C/G)
12257	CakSNP12257	*Kabuli*	Ca_Kabuli_Chr07	10106065	(A/G)
12258	CakSNP12258	*Kabuli*	Ca_Kabuli_Chr07	10106054	(G/C)
12259	CakSNP12259	*Kabuli*	Ca_Kabuli_Chr07	10137842	(A/G)
12260	CakSNP12260	*Kabuli*	Ca_Kabuli_Chr07	10137890	(A/G)
12261	CakSNP12261	*Kabuli*	Ca_Kabuli_Chr07	10181630	(G/T)
12262	CakSNP12262	*Kabuli*	Ca_Kabuli_Chr07	10208029	(T/C)
12263	CakSNP12263	*Kabuli*	Ca_Kabuli_Chr07	10216813	(G/A)
12264	CakSNP12264	*Kabuli*	Ca_Kabuli_Chr07	10223046	(C/T)
12265	CakSNP12265	*Kabuli*	Ca_Kabuli_Chr07	10223068	(G/T)
12266	CakSNP12266	*Kabuli*	Ca_Kabuli_Chr07	10223069	(G/T)
12267	CakSNP12267	*Kabuli*	Ca_Kabuli_Chr07	10223071	(T/A)
12268	CakSNP12268	*Kabuli*	Ca_Kabuli_Chr07	10223074	(G/T)
12269	CakSNP12269	*Kabuli*	Ca_Kabuli_Chr07	10223075	(C/T)
12270	CakSNP12270	*Kabuli*	Ca_Kabuli_Chr07	10322726	(T/C)
12271	CakSNP12271	*Kabuli*	Ca_Kabuli_Chr07	10340199	(T/C)
12272	CakSNP12272	*Kabuli*	Ca_Kabuli_Chr07	10435983	(C/A)
12273	CakSNP12273	*Kabuli*	Ca_Kabuli_Chr07	10465140	(G/A)
12274	CakSNP12274	*Kabuli*	Ca_Kabuli_Chr07	10480401	(A/C)
12275	CakSNP12275	*Kabuli*	Ca_Kabuli_Chr07	10480462	(A/G)
12276	CakSNP12276	*Kabuli*	Ca_Kabuli_Chr07	10484200	(G/A)
12277	CakSNP12277	*Kabuli*	Ca_Kabuli_Chr07	10484225	(C/A)
12278	CakSNP12278	*Kabuli*	Ca_Kabuli_Chr07	10484196	(C/T)
12279	CakSNP12279	*Kabuli*	Ca_Kabuli_Chr07	10485414	(C/T)
12280	CakSNP12280	*Kabuli*	Ca_Kabuli_Chr07	10521423	(G/T)
12281	CakSNP12281	*Kabuli*	Ca_Kabuli_Chr07	10521449	(C/A)
12282	CakSNP12282	*Kabuli*	Ca_Kabuli_Chr07	10614159	(G/A)
12283	CakSNP12283	*Kabuli*	Ca_Kabuli_Chr07	10658367	(A/T)

S.N.	SNP IDs	Cultivars	Chromosomes/scaffolds	Physical positions (bp)	SNPs
12284	CakSNP12284	*Kabuli*	Ca_Kabuli_Chr07	10658446	(G/T)
12285	CakSNP12285	*Kabuli*	Ca_Kabuli_Chr07	10688189	(T/G)
12286	CakSNP12286	*Kabuli*	Ca_Kabuli_Chr07	10688401	(A/G)
12287	CakSNP12287	*Kabuli*	Ca_Kabuli_Chr07	10688464	(G/A)
12288	CakSNP12288	*Kabuli*	Ca_Kabuli_Chr07	10688434	(T/C)
12289	CakSNP12289	*Kabuli*	Ca_Kabuli_Chr07	10688499	(T/C)
12290	CakSNP12290	*Kabuli*	Ca_Kabuli_Chr07	10703569	(A/T)
12291	CakSNP12291	*Kabuli*	Ca_Kabuli_Chr07	10703612	(A/C)
12292	CakSNP12292	*Kabuli*	Ca_Kabuli_Chr07	10703704	(G/A)
12293	CakSNP12293	*Kabuli*	Ca_Kabuli_Chr07	10703679	(T/C)
12294	CakSNP12294	*Kabuli*	Ca_Kabuli_Chr07	10712202	(T/C)
12295	CakSNP12295	*Kabuli*	Ca_Kabuli_Chr07	10712231	(G/A)
12296	CakSNP12296	*Kabuli*	Ca_Kabuli_Chr07	10758973	(A/T)
12297	CakSNP12297	*Kabuli*	Ca_Kabuli_Chr07	10759142	(T/G)
12298	CakSNP12298	*Kabuli*	Ca_Kabuli_Chr07	10775881	(C/T)
12299	CakSNP12299	*Kabuli*	Ca_Kabuli_Chr07	10775875	(A/G)
12300	CakSNP12300	*Kabuli*	Ca_Kabuli_Chr07	10775851	(C/T)
12301	CakSNP12301	*Kabuli*	Ca_Kabuli_Chr07	10775860	(A/G)
12302	CakSNP12302	*Kabuli*	Ca_Kabuli_Chr07	10780680	(C/G)
12303	CakSNP12303	*Kabuli*	Ca_Kabuli_Chr07	10780682	(G/A)
12304	CakSNP12304	*Kabuli*	Ca_Kabuli_Chr07	10780686	(T/C)
12305	CakSNP12305	*Kabuli*	Ca_Kabuli_Chr07	10797204	(T/C)
12306	CakSNP12306	*Kabuli*	Ca_Kabuli_Chr07	10812617	(G/T)
12307	CakSNP12307	*Kabuli*	Ca_Kabuli_Chr07	10816523	(T/G)
12308	CakSNP12308	*Kabuli*	Ca_Kabuli_Chr07	10816674	(C/T)
12309	CakSNP12309	*Kabuli*	Ca_Kabuli_Chr07	10816662	(T/A)
12310	CakSNP12310	*Kabuli*	Ca_Kabuli_Chr07	10859907	(G/A)
12311	CakSNP12311	*Kabuli*	Ca_Kabuli_Chr07	10859842	(G/T)
12312	CakSNP12312	*Kabuli*	Ca_Kabuli_Chr07	10900910	(T/C)
12313	CakSNP12313	*Kabuli*	Ca_Kabuli_Chr07	11086047	(G/A)
12314	CakSNP12314	*Kabuli*	Ca_Kabuli_Chr07	11086071	(A/T)
12315	CakSNP12315	*Kabuli*	Ca_Kabuli_Chr07	11105847	(C/G)
12316	CakSNP12316	*Kabuli*	Ca_Kabuli_Chr07	11105961	(A/C)
12317	CakSNP12317	*Kabuli*	Ca_Kabuli_Chr07	11192667	(G/A)
12318	CakSNP12318	*Kabuli*	Ca_Kabuli_Chr07	11192712	(C/T)
12319	CakSNP12319	*Kabuli*	Ca_Kabuli_Chr07	11192703	(A/C)
12320	CakSNP12320	*Kabuli*	Ca_Kabuli_Chr07	11261924	(G/A)
12321	CakSNP12321	*Kabuli*	Ca_Kabuli_Chr07	11339511	(C/T)
12322	CakSNP12322	*Kabuli*	Ca_Kabuli_Chr07	11393795	(A/G)

S.N.	SNP IDs	Cultivars	Chromosomes/scaffolds	Physical positions (bp)	SNPs
12323	CakSNP12323	*Kabuli*	Ca_Kabuli_Chr07	11393986	(A/G)
12324	CakSNP12324	*Kabuli*	Ca_Kabuli_Chr07	11473766	(A/G)
12325	CakSNP12325	*Kabuli*	Ca_Kabuli_Chr07	11521694	(G/A)
12326	CakSNP12326	*Kabuli*	Ca_Kabuli_Chr07	11527428	(G/A)
12327	CakSNP12327	*Kabuli*	Ca_Kabuli_Chr07	11545846	(A/G)
12328	CakSNP12328	*Kabuli*	Ca_Kabuli_Chr07	11568963	(T/A)
12329	CakSNP12329	*Kabuli*	Ca_Kabuli_Chr07	11568945	(G/T)
12330	CakSNP12330	*Kabuli*	Ca_Kabuli_Chr07	11590987	(A/C)
12331	CakSNP12331	*Kabuli*	Ca_Kabuli_Chr07	11630509	(A/G)
12332	CakSNP12332	*Kabuli*	Ca_Kabuli_Chr07	11630511	(C/T)
12333	CakSNP12333	*Kabuli*	Ca_Kabuli_Chr07	11638885	(C/A)
12334	CakSNP12334	*Kabuli*	Ca_Kabuli_Chr07	11639117	(C/G)
12335	CakSNP12335	*Kabuli*	Ca_Kabuli_Chr07	11685818	(G/T)
12336	CakSNP12336	*Kabuli*	Ca_Kabuli_Chr07	11685899	(A/G)
12337	CakSNP12337	*Kabuli*	Ca_Kabuli_Chr07	11686238	(C/T)
12338	CakSNP12338	*Kabuli*	Ca_Kabuli_Chr07	11725655	(G/A)
12339	CakSNP12339	*Kabuli*	Ca_Kabuli_Chr07	11730926	(T/C)
12340	CakSNP12340	*Kabuli*	Ca_Kabuli_Chr07	11730946	(A/C)
12341	CakSNP12341	*Kabuli*	Ca_Kabuli_Chr07	11735177	(T/C)
12342	CakSNP12342	*Kabuli*	Ca_Kabuli_Chr07	11735381	(T/C)
12343	CakSNP12343	*Kabuli*	Ca_Kabuli_Chr07	11735368	(G/C)
12344	CakSNP12344	*Kabuli*	Ca_Kabuli_Chr07	11736006	(C/A)
12345	CakSNP12345	*Kabuli*	Ca_Kabuli_Chr07	11792042	(G/A)
12346	CakSNP12346	*Kabuli*	Ca_Kabuli_Chr07	11792126	(T/A)
12347	CakSNP12347	*Kabuli*	Ca_Kabuli_Chr07	11792136	(G/A)
12348	CakSNP12348	*Kabuli*	Ca_Kabuli_Chr07	11803091	(T/G)
12349	CakSNP12349	*Kabuli*	Ca_Kabuli_Chr07	11812410	(G/A)
12350	CakSNP12350	*Kabuli*	Ca_Kabuli_Chr07	11812441	(A/C)
12351	CakSNP12351	*Kabuli*	Ca_Kabuli_Chr07	11858425	(C/T)
12352	CakSNP12352	*Kabuli*	Ca_Kabuli_Chr07	11886954	(A/G)
12353	CakSNP12353	*Kabuli*	Ca_Kabuli_Chr07	11902897	(A/G)
12354	CakSNP12354	*Kabuli*	Ca_Kabuli_Chr07	11902985	(G/T)
12355	CakSNP12355	*Kabuli*	Ca_Kabuli_Chr07	12010432	(G/A)
12356	CakSNP12356	*Kabuli*	Ca_Kabuli_Chr07	12010471	(C/T)
12357	CakSNP12357	*Kabuli*	Ca_Kabuli_Chr07	12010504	(G/A)
12358	CakSNP12358	*Kabuli*	Ca_Kabuli_Chr07	12010499	(C/T)
12359	CakSNP12359	*Kabuli*	Ca_Kabuli_Chr07	12010496	(G/A)
12360	CakSNP12360	*Kabuli*	Ca_Kabuli_Chr07	12010494	(G/A)
12361	CakSNP12361	*Kabuli*	Ca_Kabuli_Chr07	12010491	(G/T)

S.N.	SNP IDs	Cultivars	Chromosomes/scaffolds	Physical positions (bp)	SNPs
12362	CakSNP12362	*Kabuli*	Ca_Kabuli_Chr07	12010483	(C/T)
12363	CakSNP12363	*Kabuli*	Ca_Kabuli_Chr07	12010457	(C/T)
12364	CakSNP12364	*Kabuli*	Ca_Kabuli_Chr07	12010544	(G/A)
12365	CakSNP12365	*Kabuli*	Ca_Kabuli_Chr07	12010489	(C/T)
12366	CakSNP12366	*Kabuli*	Ca_Kabuli_Chr07	12030080	(A/C)
12367	CakSNP12367	*Kabuli*	Ca_Kabuli_Chr07	12071764	(T/C)
12368	CakSNP12368	*Kabuli*	Ca_Kabuli_Chr07	12071773	(A/T)
12369	CakSNP12369	*Kabuli*	Ca_Kabuli_Chr07	12203726	(T/G)
12370	CakSNP12370	*Kabuli*	Ca_Kabuli_Chr07	12203722	(T/C)
12371	CakSNP12371	*Kabuli*	Ca_Kabuli_Chr07	12203706	(T/C)
12372	CakSNP12372	*Kabuli*	Ca_Kabuli_Chr07	12203690	(C/T)
12373	CakSNP12373	*Kabuli*	Ca_Kabuli_Chr07	12244234	(C/T)
12374	CakSNP12374	*Kabuli*	Ca_Kabuli_Chr07	12244259	(G/A)
12375	CakSNP12375	*Kabuli*	Ca_Kabuli_Chr07	12284289	(T/A)
12376	CakSNP12376	*Kabuli*	Ca_Kabuli_Chr07	12284304	(A/G)
12377	CakSNP12377	*Kabuli*	Ca_Kabuli_Chr07	12385785	(C/T)
12378	CakSNP12378	*Kabuli*	Ca_Kabuli_Chr07	12390774	(G/A)
12379	CakSNP12379	*Kabuli*	Ca_Kabuli_Chr07	12481556	(G/C)
12380	CakSNP12380	*Kabuli*	Ca_Kabuli_Chr07	12481627	(A/C)
12381	CakSNP12381	*Kabuli*	Ca_Kabuli_Chr07	12481715	(A/G)
12382	CakSNP12382	*Kabuli*	Ca_Kabuli_Chr07	12481835	(C/T)
12383	CakSNP12383	*Kabuli*	Ca_Kabuli_Chr07	12482851	(G/A)
12384	CakSNP12384	*Kabuli*	Ca_Kabuli_Chr07	12482822	(A/G)
12385	CakSNP12385	*Kabuli*	Ca_Kabuli_Chr07	12482953	(A/C)
12386	CakSNP12386	*Kabuli*	Ca_Kabuli_Chr07	12483290	(A/C)
12387	CakSNP12387	*Kabuli*	Ca_Kabuli_Chr07	12492075	(G/A)
12388	CakSNP12388	*Kabuli*	Ca_Kabuli_Chr07	12492052	(C/T)
12389	CakSNP12389	*Kabuli*	Ca_Kabuli_Chr07	12492046	(A/T)
12390	CakSNP12390	*Kabuli*	Ca_Kabuli_Chr07	12616871	(T/C)
12391	CakSNP12391	*Kabuli*	Ca_Kabuli_Chr07	12616870	(G/A)
12392	CakSNP12392	*Kabuli*	Ca_Kabuli_Chr07	12616957	(C/T)
12393	CakSNP12393	*Kabuli*	Ca_Kabuli_Chr07	12616937	(C/T)
12394	CakSNP12394	*Kabuli*	Ca_Kabuli_Chr07	12623129	(T/C)
12395	CakSNP12395	*Kabuli*	Ca_Kabuli_Chr07	12623128	(G/A)
12396	CakSNP12396	*Kabuli*	Ca_Kabuli_Chr07	12623215	(C/T)
12397	CakSNP12397	*Kabuli*	Ca_Kabuli_Chr07	12623195	(C/T)
12398	CakSNP12398	*Kabuli*	Ca_Kabuli_Chr07	12628148	(A/G)
12399	CakSNP12399	*Kabuli*	Ca_Kabuli_Chr07	12628211	(A/G)
12400	CakSNP12400	*Kabuli*	Ca_Kabuli_Chr07	12628265	(T/A)

S.N.	SNP IDs	Cultivars	Chromosomes/scaffolds	Physical positions (bp)	SNPs
12401	CakSNP12401	*Kabuli*	Ca_Kabuli_Chr07	12629986	(A/G)
12402	CakSNP12402	*Kabuli*	Ca_Kabuli_Chr07	12633590	(A/C)
12403	CakSNP12403	*Kabuli*	Ca_Kabuli_Chr07	12633642	(T/G)
12404	CakSNP12404	*Kabuli*	Ca_Kabuli_Chr07	12633643	(A/T)
12405	CakSNP12405	*Kabuli*	Ca_Kabuli_Chr07	12681440	(T/C)
12406	CakSNP12406	*Kabuli*	Ca_Kabuli_Chr07	12718820	(C/G)
12407	CakSNP12407	*Kabuli*	Ca_Kabuli_Chr07	12718853	(G/A)
12408	CakSNP12408	*Kabuli*	Ca_Kabuli_Chr07	12730600	(C/A)
12409	CakSNP12409	*Kabuli*	Ca_Kabuli_Chr07	12737516	(T/C)
12410	CakSNP12410	*Kabuli*	Ca_Kabuli_Chr07	12893655	(C/T)
12411	CakSNP12411	*Kabuli*	Ca_Kabuli_Chr07	12895182	(A/G)
12412	CakSNP12412	*Kabuli*	Ca_Kabuli_Chr07	12898640	(A/C)
12413	CakSNP12413	*Kabuli*	Ca_Kabuli_Chr07	12898709	(T/A)
12414	CakSNP12414	*Kabuli*	Ca_Kabuli_Chr07	12898707	(G/A)
12415	CakSNP12415	*Kabuli*	Ca_Kabuli_Chr07	12899398	(T/C)
12416	CakSNP12416	*Kabuli*	Ca_Kabuli_Chr07	12913811	(T/A)
12417	CakSNP12417	*Kabuli*	Ca_Kabuli_Chr07	13020788	(C/T)
12418	CakSNP12418	*Kabuli*	Ca_Kabuli_Chr07	13020772	(A/G)
12419	CakSNP12419	*Kabuli*	Ca_Kabuli_Chr07	13021047	(A/G)
12420	CakSNP12420	*Kabuli*	Ca_Kabuli_Chr07	13021030	(A/G)
12421	CakSNP12421	*Kabuli*	Ca_Kabuli_Chr07	13059710	(C/A)
12422	CakSNP12422	*Kabuli*	Ca_Kabuli_Chr07	13059712	(C/A)
12423	CakSNP12423	*Kabuli*	Ca_Kabuli_Chr07	13059717	(T/A)
12424	CakSNP12424	*Kabuli*	Ca_Kabuli_Chr07	13059852	(G/A)
12425	CakSNP12425	*Kabuli*	Ca_Kabuli_Chr07	13172542	(A/G)
12426	CakSNP12426	*Kabuli*	Ca_Kabuli_Chr07	13220439	(G/A)
12427	CakSNP12427	*Kabuli*	Ca_Kabuli_Chr07	13220418	(A/G)
12428	CakSNP12428	*Kabuli*	Ca_Kabuli_Chr07	13262430	(C/T)
12429	CakSNP12429	*Kabuli*	Ca_Kabuli_Chr07	13281255	(T/C)
12430	CakSNP12430	*Kabuli*	Ca_Kabuli_Chr07	13281290	(A/G)
12431	CakSNP12431	*Kabuli*	Ca_Kabuli_Chr07	13281297	(C/G)
12432	CakSNP12432	*Kabuli*	Ca_Kabuli_Chr07	13281307	(C/T)
12433	CakSNP12433	*Kabuli*	Ca_Kabuli_Chr07	13281313	(T/C)
12434	CakSNP12434	*Kabuli*	Ca_Kabuli_Chr07	13281314	(G/A)
12435	CakSNP12435	*Kabuli*	Ca_Kabuli_Chr07	13281374	(G/T)
12436	CakSNP12436	*Kabuli*	Ca_Kabuli_Chr07	13281351	(T/A)
12437	CakSNP12437	*Kabuli*	Ca_Kabuli_Chr07	13288828	(G/A)
12438	CakSNP12438	*Kabuli*	Ca_Kabuli_Chr07	13288813	(G/A)
12439	CakSNP12439	*Kabuli*	Ca_Kabuli_Chr07	13288753	(A/C)

S.N.	SNP IDs	Cultivars	Chromosomes/scaffolds	Physical positions (bp)	SNPs
12440	CakSNP12440	*Kabuli*	Ca_Kabuli_Chr07	13319871	(A/G)
12441	CakSNP12441	*Kabuli*	Ca_Kabuli_Chr07	13514074	(C/T)
12442	CakSNP12442	*Kabuli*	Ca_Kabuli_Chr07	13605200	(A/C)
12443	CakSNP12443	*Kabuli*	Ca_Kabuli_Chr07	13605191	(A/C)
12444	CakSNP12444	*Kabuli*	Ca_Kabuli_Chr07	13605188	(A/C)
12445	CakSNP12445	*Kabuli*	Ca_Kabuli_Chr07	13605578	(C/G)
12446	CakSNP12446	*Kabuli*	Ca_Kabuli_Chr07	13667102	(C/T)
12447	CakSNP12447	*Kabuli*	Ca_Kabuli_Chr07	13667103	(T/G)
12448	CakSNP12448	*Kabuli*	Ca_Kabuli_Chr07	13730350	(A/C)
12449	CakSNP12449	*Kabuli*	Ca_Kabuli_Chr07	13757159	(A/C)
12450	CakSNP12450	*Kabuli*	Ca_Kabuli_Chr07	13776110	(A/C)
12451	CakSNP12451	*Kabuli*	Ca_Kabuli_Chr07	13781815	(T/C)
12452	CakSNP12452	*Kabuli*	Ca_Kabuli_Chr07	13819387	(C/T)
12453	CakSNP12453	*Kabuli*	Ca_Kabuli_Chr07	13881348	(G/A)
12454	CakSNP12454	*Kabuli*	Ca_Kabuli_Chr07	13893199	(T/C)
12455	CakSNP12455	*Kabuli*	Ca_Kabuli_Chr07	13893344	(G/C)
12456	CakSNP12456	*Kabuli*	Ca_Kabuli_Chr07	13893382	(G/T)
12457	CakSNP12457	*Kabuli*	Ca_Kabuli_Chr07	13893383	(C/T)
12458	CakSNP12458	*Kabuli*	Ca_Kabuli_Chr07	13903980	(T/G)
12459	CakSNP12459	*Kabuli*	Ca_Kabuli_Chr07	13904163	(T/C)
12460	CakSNP12460	*Kabuli*	Ca_Kabuli_Chr07	13913735	(A/G)
12461	CakSNP12461	*Kabuli*	Ca_Kabuli_Chr07	13921214	(C/T)
12462	CakSNP12462	*Kabuli*	Ca_Kabuli_Chr07	13921289	(T/C)
12463	CakSNP12463	*Kabuli*	Ca_Kabuli_Chr07	13949259	(A/G)
12464	CakSNP12464	*Kabuli*	Ca_Kabuli_Chr07	13949314	(G/A)
12465	CakSNP12465	*Kabuli*	Ca_Kabuli_Chr07	13951874	(T/C)
12466	CakSNP12466	*Kabuli*	Ca_Kabuli_Chr07	13951856	(T/C)
12467	CakSNP12467	*Kabuli*	Ca_Kabuli_Chr07	14030136	(C/T)
12468	CakSNP12468	*Kabuli*	Ca_Kabuli_Chr07	14051146	(G/T)
12469	CakSNP12469	*Kabuli*	Ca_Kabuli_Chr07	14179629	(T/G)
12470	CakSNP12470	*Kabuli*	Ca_Kabuli_Chr07	14179733	(C/A)
12471	CakSNP12471	*Kabuli*	Ca_Kabuli_Chr07	14212570	(C/A)
12472	CakSNP12472	*Kabuli*	Ca_Kabuli_Chr07	14212597	(C/G)
12473	CakSNP12473	*Kabuli*	Ca_Kabuli_Chr07	14233343	(T/G)
12474	CakSNP12474	*Kabuli*	Ca_Kabuli_Chr07	14233368	(T/C)
12475	CakSNP12475	*Kabuli*	Ca_Kabuli_Chr07	14281993	(G/T)
12476	CakSNP12476	*Kabuli*	Ca_Kabuli_Chr07	14282088	(T/C)
12477	CakSNP12477	*Kabuli*	Ca_Kabuli_Chr07	14320216	(T/C)
12478	CakSNP12478	*Kabuli*	Ca_Kabuli_Chr07	14328655	(T/C)

S.N.	SNP IDs	Cultivars	Chromosomes/scaffolds	Physical positions (bp)	SNPs
12479	CakSNP12479	*Kabuli*	Ca_Kabuli_Chr07	14328649	(T/C)
12480	CakSNP12480	*Kabuli*	Ca_Kabuli_Chr07	14329126	(C/T)
12481	CakSNP12481	*Kabuli*	Ca_Kabuli_Chr07	14329175	(G/A)
12482	CakSNP12482	*Kabuli*	Ca_Kabuli_Chr07	14329196	(G/A)
12483	CakSNP12483	*Kabuli*	Ca_Kabuli_Chr07	14329217	(C/T)
12484	CakSNP12484	*Kabuli*	Ca_Kabuli_Chr07	14329167	(A/T)
12485	CakSNP12485	*Kabuli*	Ca_Kabuli_Chr07	14345827	(T/C)
12486	CakSNP12486	*Kabuli*	Ca_Kabuli_Chr07	14345799	(T/A)
12487	CakSNP12487	*Kabuli*	Ca_Kabuli_Chr07	14470549	(C/G)
12488	CakSNP12488	*Kabuli*	Ca_Kabuli_Chr07	14518292	(A/T)
12489	CakSNP12489	*Kabuli*	Ca_Kabuli_Chr07	14526546	(A/G)
12490	CakSNP12490	*Kabuli*	Ca_Kabuli_Chr07	14526495	(T/C)
12491	CakSNP12491	*Kabuli*	Ca_Kabuli_Chr07	14526493	(C/T)
12492	CakSNP12492	*Kabuli*	Ca_Kabuli_Chr07	14526480	(T/C)
12493	CakSNP12493	*Kabuli*	Ca_Kabuli_Chr07	14526478	(G/C)
12494	CakSNP12494	*Kabuli*	Ca_Kabuli_Chr07	14558741	(C/A)
12495	CakSNP12495	*Kabuli*	Ca_Kabuli_Chr07	14558785	(C/T)
12496	CakSNP12496	*Kabuli*	Ca_Kabuli_Chr07	14559033	(G/A)
12497	CakSNP12497	*Kabuli*	Ca_Kabuli_Chr07	14666535	(T/A)
12498	CakSNP12498	*Kabuli*	Ca_Kabuli_Chr07	14668939	(G/A)
12499	CakSNP12499	*Kabuli*	Ca_Kabuli_Chr07	14668922	(A/C)
12500	CakSNP12500	*Kabuli*	Ca_Kabuli_Chr07	14685011	(G/C)
12501	CakSNP12501	*Kabuli*	Ca_Kabuli_Chr07	14685017	(C/T)
12502	CakSNP12502	*Kabuli*	Ca_Kabuli_Chr07	14685024	(A/G)
12503	CakSNP12503	*Kabuli*	Ca_Kabuli_Chr07	14685034	(T/G)
12504	CakSNP12504	*Kabuli*	Ca_Kabuli_Chr07	14712518	(C/A)
12505	CakSNP12505	*Kabuli*	Ca_Kabuli_Chr07	14712568	(C/A)
12506	CakSNP12506	*Kabuli*	Ca_Kabuli_Chr07	14712596	(T/C)
12507	CakSNP12507	*Kabuli*	Ca_Kabuli_Chr07	14712601	(C/T)
12508	CakSNP12508	*Kabuli*	Ca_Kabuli_Chr07	14748257	(T/A)
12509	CakSNP12509	*Kabuli*	Ca_Kabuli_Chr07	14775135	(C/G)
12510	CakSNP12510	*Kabuli*	Ca_Kabuli_Chr07	14782453	(A/G)
12511	CakSNP12511	*Kabuli*	Ca_Kabuli_Chr07	14899028	(C/A)
12512	CakSNP12512	*Kabuli*	Ca_Kabuli_Chr07	14899101	(T/C)
12513	CakSNP12513	*Kabuli*	Ca_Kabuli_Chr07	14933294	(T/C)
12514	CakSNP12514	*Kabuli*	Ca_Kabuli_Chr07	14933286	(G/A)
12515	CakSNP12515	*Kabuli*	Ca_Kabuli_Chr07	14983364	(T/G)
12516	CakSNP12516	*Kabuli*	Ca_Kabuli_Chr07	14997794	(G/A)
12517	CakSNP12517	*Kabuli*	Ca_Kabuli_Chr07	14997883	(T/C)

S.N.	SNP IDs	Cultivars	Chromosomes/scaffolds	Physical positions (bp)	SNPs
12518	CakSNP12518	*Kabuli*	Ca_Kabuli_Chr07	15028182	(T/G)
12519	CakSNP12519	*Kabuli*	Ca_Kabuli_Chr07	15039606	(T/A)
12520	CakSNP12520	*Kabuli*	Ca_Kabuli_Chr07	15069817	(G/A)
12521	CakSNP12521	*Kabuli*	Ca_Kabuli_Chr07	15070091	(T/A)
12522	CakSNP12522	*Kabuli*	Ca_Kabuli_Chr07	15119205	(T/C)
12523	CakSNP12523	*Kabuli*	Ca_Kabuli_Chr07	15125638	(G/C)
12524	CakSNP12524	*Kabuli*	Ca_Kabuli_Chr07	15125619	(T/G)
12525	CakSNP12525	*Kabuli*	Ca_Kabuli_Chr07	15185603	(A/G)
12526	CakSNP12526	*Kabuli*	Ca_Kabuli_Chr07	15201600	(A/G)
12527	CakSNP12527	*Kabuli*	Ca_Kabuli_Chr07	15399421	(T/C)
12528	CakSNP12528	*Kabuli*	Ca_Kabuli_Chr07	15441316	(A/T)
12529	CakSNP12529	*Kabuli*	Ca_Kabuli_Chr07	15463183	(G/T)
12530	CakSNP12530	*Kabuli*	Ca_Kabuli_Chr07	15463170	(G/A)
12531	CakSNP12531	*Kabuli*	Ca_Kabuli_Chr07	15463169	(G/A)
12532	CakSNP12532	*Kabuli*	Ca_Kabuli_Chr07	15530329	(G/T)
12533	CakSNP12533	*Kabuli*	Ca_Kabuli_Chr07	15530316	(G/A)
12534	CakSNP12534	*Kabuli*	Ca_Kabuli_Chr07	15530315	(G/A)
12535	CakSNP12535	*Kabuli*	Ca_Kabuli_Chr07	15568060	(G/T)
12536	CakSNP12536	*Kabuli*	Ca_Kabuli_Chr07	15568047	(G/A)
12537	CakSNP12537	*Kabuli*	Ca_Kabuli_Chr07	15568046	(G/A)
12538	CakSNP12538	*Kabuli*	Ca_Kabuli_Chr07	15622247	(G/A)
12539	CakSNP12539	*Kabuli*	Ca_Kabuli_Chr07	15622245	(T/A)
12540	CakSNP12540	*Kabuli*	Ca_Kabuli_Chr07	15762068	(A/T)
12541	CakSNP12541	*Kabuli*	Ca_Kabuli_Chr07	15826588	(A/C)
12542	CakSNP12542	*Kabuli*	Ca_Kabuli_Chr07	15856789	(A/G)
12543	CakSNP12543	*Kabuli*	Ca_Kabuli_Chr07	15856955	(T/G)
12544	CakSNP12544	*Kabuli*	Ca_Kabuli_Chr07	15860298	(T/C)
12545	CakSNP12545	*Kabuli*	Ca_Kabuli_Chr07	15887790	(T/C)
12546	CakSNP12546	*Kabuli*	Ca_Kabuli_Chr07	15966680	(A/G)
12547	CakSNP12547	*Kabuli*	Ca_Kabuli_Chr07	15966714	(G/A)
12548	CakSNP12548	*Kabuli*	Ca_Kabuli_Chr07	16008746	(T/C)
12549	CakSNP12549	*Kabuli*	Ca_Kabuli_Chr07	16008733	(A/G)
12550	CakSNP12550	*Kabuli*	Ca_Kabuli_Chr07	16289811	(C/T)
12551	CakSNP12551	*Kabuli*	Ca_Kabuli_Chr07	16289802	(C/T)
12552	CakSNP12552	*Kabuli*	Ca_Kabuli_Chr07	16289794	(G/T)
12553	CakSNP12553	*Kabuli*	Ca_Kabuli_Chr07	16324985	(T/C)
12554	CakSNP12554	*Kabuli*	Ca_Kabuli_Chr07	16331943	(A/G)
12555	CakSNP12555	*Kabuli*	Ca_Kabuli_Chr07	16381854	(T/C)
12556	CakSNP12556	*Kabuli*	Ca_Kabuli_Chr07	16381891	(T/G)

S.N.	SNP IDs	Cultivars	Chromosomes/scaffolds	Physical positions (bp)	SNPs
12557	CakSNP12557	*Kabuli*	Ca_Kabuli_Chr07	16381934	(T/C)
12558	CakSNP12558	*Kabuli*	Ca_Kabuli_Chr07	16466619	(T/C)
12559	CakSNP12559	*Kabuli*	Ca_Kabuli_Chr07	16581695	(C/G)
12560	CakSNP12560	*Kabuli*	Ca_Kabuli_Chr07	16626047	(T/G)
12561	CakSNP12561	*Kabuli*	Ca_Kabuli_Chr07	16626703	(A/G)
12562	CakSNP12562	*Kabuli*	Ca_Kabuli_Chr07	16665577	(A/G)
12563	CakSNP12563	*Kabuli*	Ca_Kabuli_Chr07	16735437	(G/A)
12564	CakSNP12564	*Kabuli*	Ca_Kabuli_Chr07	16735427	(A/G)
12565	CakSNP12565	*Kabuli*	Ca_Kabuli_Chr07	16735423	(G/C)
12566	CakSNP12566	*Kabuli*	Ca_Kabuli_Chr07	16816296	(G/C)
12567	CakSNP12567	*Kabuli*	Ca_Kabuli_Chr07	16821274	(A/C)
12568	CakSNP12568	*Kabuli*	Ca_Kabuli_Chr07	16829081	(C/A)
12569	CakSNP12569	*Kabuli*	Ca_Kabuli_Chr07	16897329	(A/C)
12570	CakSNP12570	*Kabuli*	Ca_Kabuli_Chr07	16897285	(T/C)
12571	CakSNP12571	*Kabuli*	Ca_Kabuli_Chr07	16936123	(C/G)
12572	CakSNP12572	*Kabuli*	Ca_Kabuli_Chr07	17017466	(C/G)
12573	CakSNP12573	*Kabuli*	Ca_Kabuli_Chr07	17017453	(A/G)
12574	CakSNP12574	*Kabuli*	Ca_Kabuli_Chr07	17017447	(A/G)
12575	CakSNP12575	*Kabuli*	Ca_Kabuli_Chr07	17017438	(A/T)
12576	CakSNP12576	*Kabuli*	Ca_Kabuli_Chr07	17017421	(G/A)
12577	CakSNP12577	*Kabuli*	Ca_Kabuli_Chr07	17017418	(T/G)
12578	CakSNP12578	*Kabuli*	Ca_Kabuli_Chr07	17038317	(G/A)
12579	CakSNP12579	*Kabuli*	Ca_Kabuli_Chr07	17064968	(T/C)
12580	CakSNP12580	*Kabuli*	Ca_Kabuli_Chr07	17088603	(A/G)
12581	CakSNP12581	*Kabuli*	Ca_Kabuli_Chr07	17140664	(C/T)
12582	CakSNP12582	*Kabuli*	Ca_Kabuli_Chr07	17178847	(A/C)
12583	CakSNP12583	*Kabuli*	Ca_Kabuli_Chr07	17181160	(G/C)
12584	CakSNP12584	*Kabuli*	Ca_Kabuli_Chr07	17228904	(T/G)
12585	CakSNP12585	*Kabuli*	Ca_Kabuli_Chr07	17318174	(G/T)
12586	CakSNP12586	*Kabuli*	Ca_Kabuli_Chr07	17539110	(G/A)
12587	CakSNP12587	*Kabuli*	Ca_Kabuli_Chr07	17539136	(C/A)
12588	CakSNP12588	*Kabuli*	Ca_Kabuli_Chr07	17539207	(G/A)
12589	CakSNP12589	*Kabuli*	Ca_Kabuli_Chr07	17586154	(C/T)
12590	CakSNP12590	*Kabuli*	Ca_Kabuli_Chr07	17591798	(T/A)
12591	CakSNP12591	*Kabuli*	Ca_Kabuli_Chr07	17591781	(G/A)
12592	CakSNP12592	*Kabuli*	Ca_Kabuli_Chr07	17591843	(G/T)
12593	CakSNP12593	*Kabuli*	Ca_Kabuli_Chr07	17591974	(A/G)
12594	CakSNP12594	*Kabuli*	Ca_Kabuli_Chr07	17591971	(A/G)
12595	CakSNP12595	*Kabuli*	Ca_Kabuli_Chr07	17591924	(T/C)

S.N.	SNP IDs	Cultivars	Chromosomes/scaffolds	Physical positions (bp)	SNPs
12596	CakSNP12596	*Kabuli*	Ca_Kabuli_Chr07	17592701	(C/A)
12597	CakSNP12597	*Kabuli*	Ca_Kabuli_Chr07	17597348	(T/C)
12598	CakSNP12598	*Kabuli*	Ca_Kabuli_Chr07	17597421	(A/G)
12599	CakSNP12599	*Kabuli*	Ca_Kabuli_Chr07	17597420	(C/T)
12600	CakSNP12600	*Kabuli*	Ca_Kabuli_Chr07	17624143	(G/A)
12601	CakSNP12601	*Kabuli*	Ca_Kabuli_Chr07	17624225	(T/C)
12602	CakSNP12602	*Kabuli*	Ca_Kabuli_Chr07	17766520	(T/C)
12603	CakSNP12603	*Kabuli*	Ca_Kabuli_Chr07	17766952	(T/A)
12604	CakSNP12604	*Kabuli*	Ca_Kabuli_Chr07	17766914	(C/A)
12605	CakSNP12605	*Kabuli*	Ca_Kabuli_Chr07	17779414	(A/G)
12606	CakSNP12606	*Kabuli*	Ca_Kabuli_Chr07	17779594	(T/C)
12607	CakSNP12607	*Kabuli*	Ca_Kabuli_Chr07	17779700	(A/T)
12608	CakSNP12608	*Kabuli*	Ca_Kabuli_Chr07	17782558	(C/T)
12609	CakSNP12609	*Kabuli*	Ca_Kabuli_Chr07	18011604	(G/C)
12610	CakSNP12610	*Kabuli*	Ca_Kabuli_Chr07	18090289	(T/A)
12611	CakSNP12611	*Kabuli*	Ca_Kabuli_Chr07	18265803	(A/C)
12612	CakSNP12612	*Kabuli*	Ca_Kabuli_Chr07	18265791	(G/C)
12613	CakSNP12613	*Kabuli*	Ca_Kabuli_Chr07	18265782	(G/C)
12614	CakSNP12614	*Kabuli*	Ca_Kabuli_Chr07	18319049	(T/G)
12615	CakSNP12615	*Kabuli*	Ca_Kabuli_Chr07	18319073	(T/G)
12616	CakSNP12616	*Kabuli*	Ca_Kabuli_Chr07	18365218	(G/A)
12617	CakSNP12617	*Kabuli*	Ca_Kabuli_Chr07	18430468	(C/T)
12618	CakSNP12618	*Kabuli*	Ca_Kabuli_Chr07	18469372	(G/A)
12619	CakSNP12619	*Kabuli*	Ca_Kabuli_Chr07	18469422	(C/T)
12620	CakSNP12620	*Kabuli*	Ca_Kabuli_Chr07	18638565	(A/G)
12621	CakSNP12621	*Kabuli*	Ca_Kabuli_Chr07	18642538	(A/G)
12622	CakSNP12622	*Kabuli*	Ca_Kabuli_Chr07	18658563	(C/G)
12623	CakSNP12623	*Kabuli*	Ca_Kabuli_Chr07	18696645	(G/A)
12624	CakSNP12624	*Kabuli*	Ca_Kabuli_Chr07	18796748	(T/C)
12625	CakSNP12625	*Kabuli*	Ca_Kabuli_Chr07	18796768	(A/G)
12626	CakSNP12626	*Kabuli*	Ca_Kabuli_Chr07	18910468	(A/G)
12627	CakSNP12627	*Kabuli*	Ca_Kabuli_Chr07	19042183	(C/T)
12628	CakSNP12628	*Kabuli*	Ca_Kabuli_Chr07	19189737	(A/C)
12629	CakSNP12629	*Kabuli*	Ca_Kabuli_Chr07	19217501	(A/C)
12630	CakSNP12630	*Kabuli*	Ca_Kabuli_Chr07	19217598	(G/C)
12631	CakSNP12631	*Kabuli*	Ca_Kabuli_Chr07	19232818	(T/A)
12632	CakSNP12632	*Kabuli*	Ca_Kabuli_Chr07	19290521	(G/A)
12633	CakSNP12633	*Kabuli*	Ca_Kabuli_Chr07	19470527	(T/C)
12634	CakSNP12634	*Kabuli*	Ca_Kabuli_Chr07	19472719	(G/A)

S.N.	SNP IDs	Cultivars	Chromosomes/scaffolds	Physical positions (bp)	SNPs
12635	CakSNP12635	*Kabuli*	Ca_Kabuli_Chr07	19472689	(T/A)
12636	CakSNP12636	*Kabuli*	Ca_Kabuli_Chr07	19542901	(A/G)
12637	CakSNP12637	*Kabuli*	Ca_Kabuli_Chr07	19549636	(C/T)
12638	CakSNP12638	*Kabuli*	Ca_Kabuli_Chr07	19707812	(T/A)
12639	CakSNP12639	*Kabuli*	Ca_Kabuli_Chr07	19707842	(G/A)
12640	CakSNP12640	*Kabuli*	Ca_Kabuli_Chr07	19818789	(C/A)
12641	CakSNP12641	*Kabuli*	Ca_Kabuli_Chr07	19818770	(T/C)
12642	CakSNP12642	*Kabuli*	Ca_Kabuli_Chr07	19818759	(G/C)
12643	CakSNP12643	*Kabuli*	Ca_Kabuli_Chr07	19818757	(G/C)
12644	CakSNP12644	*Kabuli*	Ca_Kabuli_Chr07	20031805	(T/C)
12645	CakSNP12645	*Kabuli*	Ca_Kabuli_Chr07	20032205	(A/C)
12646	CakSNP12646	*Kabuli*	Ca_Kabuli_Chr07	20082842	(T/C)
12647	CakSNP12647	*Kabuli*	Ca_Kabuli_Chr07	20083040	(A/T)
12648	CakSNP12648	*Kabuli*	Ca_Kabuli_Chr07	20099384	(C/T)
12649	CakSNP12649	*Kabuli*	Ca_Kabuli_Chr07	20111019	(T/C)
12650	CakSNP12650	*Kabuli*	Ca_Kabuli_Chr07	20111123	(T/C)
12651	CakSNP12651	*Kabuli*	Ca_Kabuli_Chr07	20112897	(G/A)
12652	CakSNP12652	*Kabuli*	Ca_Kabuli_Chr07	20135647	(C/G)
12653	CakSNP12653	*Kabuli*	Ca_Kabuli_Chr07	20168870	(T/A)
12654	CakSNP12654	*Kabuli*	Ca_Kabuli_Chr07	20252133	(T/C)
12655	CakSNP12655	*Kabuli*	Ca_Kabuli_Chr07	20252134	(G/A)
12656	CakSNP12656	*Kabuli*	Ca_Kabuli_Chr07	20252135	(A/G)
12657	CakSNP12657	*Kabuli*	Ca_Kabuli_Chr07	20252144	(G/A)
12658	CakSNP12658	*Kabuli*	Ca_Kabuli_Chr07	20252145	(A/G)
12659	CakSNP12659	*Kabuli*	Ca_Kabuli_Chr07	20252181	(C/T)
12660	CakSNP12660	*Kabuli*	Ca_Kabuli_Chr07	20252192	(G/A)
12661	CakSNP12661	*Kabuli*	Ca_Kabuli_Chr07	20252201	(C/A)
12662	CakSNP12662	*Kabuli*	Ca_Kabuli_Chr07	20252212	(C/T)
12663	CakSNP12663	*Kabuli*	Ca_Kabuli_Chr07	20252214	(C/G)
12664	CakSNP12664	*Kabuli*	Ca_Kabuli_Chr07	20252236	(G/A)
12665	CakSNP12665	*Kabuli*	Ca_Kabuli_Chr07	20252234	(A/C)
12666	CakSNP12666	*Kabuli*	Ca_Kabuli_Chr07	20252233	(C/T)
12667	CakSNP12667	*Kabuli*	Ca_Kabuli_Chr07	20252221	(T/C)
12668	CakSNP12668	*Kabuli*	Ca_Kabuli_Chr07	20252209	(G/A)
12669	CakSNP12669	*Kabuli*	Ca_Kabuli_Chr07	20252309	(C/T)
12670	CakSNP12670	*Kabuli*	Ca_Kabuli_Chr07	20252334	(A/T)
12671	CakSNP12671	*Kabuli*	Ca_Kabuli_Chr07	20252340	(A/G)
12672	CakSNP12672	*Kabuli*	Ca_Kabuli_Chr07	20362883	(G/A)
12673	CakSNP12673	*Kabuli*	Ca_Kabuli_Chr07	20362879	(C/T)

S.N.	SNP IDs	Cultivars	Chromosomes/scaffolds	Physical positions (bp)	SNPs
12674	CakSNP12674	*Kabuli*	Ca_Kabuli_Chr07	20362876	(G/A)
12675	CakSNP12675	*Kabuli*	Ca_Kabuli_Chr07	20362838	(T/C)
12676	CakSNP12676	*Kabuli*	Ca_Kabuli_Chr07	20414653	(G/A)
12677	CakSNP12677	*Kabuli*	Ca_Kabuli_Chr07	20414703	(C/A)
12678	CakSNP12678	*Kabuli*	Ca_Kabuli_Chr07	20486247	(T/G)
12679	CakSNP12679	*Kabuli*	Ca_Kabuli_Chr07	20486280	(G/C)
12680	CakSNP12680	*Kabuli*	Ca_Kabuli_Chr07	20496378	(G/A)
12681	CakSNP12681	*Kabuli*	Ca_Kabuli_Chr07	20496303	(C/T)
12682	CakSNP12682	*Kabuli*	Ca_Kabuli_Chr07	20496438	(C/T)
12683	CakSNP12683	*Kabuli*	Ca_Kabuli_Chr07	20498552	(T/C)
12684	CakSNP12684	*Kabuli*	Ca_Kabuli_Chr07	20498626	(T/G)
12685	CakSNP12685	*Kabuli*	Ca_Kabuli_Chr07	20540780	(C/T)
12686	CakSNP12686	*Kabuli*	Ca_Kabuli_Chr07	20540791	(G/A)
12687	CakSNP12687	*Kabuli*	Ca_Kabuli_Chr07	20543119	(T/C)
12688	CakSNP12688	*Kabuli*	Ca_Kabuli_Chr07	20550563	(G/A)
12689	CakSNP12689	*Kabuli*	Ca_Kabuli_Chr07	20550575	(C/G)
12690	CakSNP12690	*Kabuli*	Ca_Kabuli_Chr07	20552041	(G/A)
12691	CakSNP12691	*Kabuli*	Ca_Kabuli_Chr07	20552053	(C/G)
12692	CakSNP12692	*Kabuli*	Ca_Kabuli_Chr07	20556466	(G/C)
12693	CakSNP12693	*Kabuli*	Ca_Kabuli_Chr07	20556576	(C/T)
12694	CakSNP12694	*Kabuli*	Ca_Kabuli_Chr07	20563931	(G/A)
12695	CakSNP12695	*Kabuli*	Ca_Kabuli_Chr07	20564024	(G/A)
12696	CakSNP12696	*Kabuli*	Ca_Kabuli_Chr07	20564266	(G/A)
12697	CakSNP12697	*Kabuli*	Ca_Kabuli_Chr07	20564269	(C/T)
12698	CakSNP12698	*Kabuli*	Ca_Kabuli_Chr07	20564297	(A/G)
12699	CakSNP12699	*Kabuli*	Ca_Kabuli_Chr07	20579963	(G/T)
12700	CakSNP12700	*Kabuli*	Ca_Kabuli_Chr07	20579974	(A/T)
12701	CakSNP12701	*Kabuli*	Ca_Kabuli_Chr07	20585997	(T/G)
12702	CakSNP12702	*Kabuli*	Ca_Kabuli_Chr07	20605389	(G/A)
12703	CakSNP12703	*Kabuli*	Ca_Kabuli_Chr07	20605384	(C/G)
12704	CakSNP12704	*Kabuli*	Ca_Kabuli_Chr07	20605373	(C/A)
12705	CakSNP12705	*Kabuli*	Ca_Kabuli_Chr07	20633040	(A/G)
12706	CakSNP12706	*Kabuli*	Ca_Kabuli_Chr07	20637152	(G/A)
12707	CakSNP12707	*Kabuli*	Ca_Kabuli_Chr07	20917747	(A/G)
12708	CakSNP12708	*Kabuli*	Ca_Kabuli_Chr07	20917743	(T/G)
12709	CakSNP12709	*Kabuli*	Ca_Kabuli_Chr07	20917740	(A/G)
12710	CakSNP12710	*Kabuli*	Ca_Kabuli_Chr07	21073385	(A/C)
12711	CakSNP12711	*Kabuli*	Ca_Kabuli_Chr07	21081969	(A/G)
12712	CakSNP12712	*Kabuli*	Ca_Kabuli_Chr07	21099782	(G/T)

S.N.	SNP IDs	Cultivars	Chromosomes/scaffolds	Physical positions (bp)	SNPs
12713	CakSNP12713	*Kabuli*	Ca_Kabuli_Chr07	21402075	(A/G)
12714	CakSNP12714	*Kabuli*	Ca_Kabuli_Chr07	21402117	(A/C)
12715	CakSNP12715	*Kabuli*	Ca_Kabuli_Chr07	21595525	(C/T)
12716	CakSNP12716	*Kabuli*	Ca_Kabuli_Chr07	21595527	(T/G)
12717	CakSNP12717	*Kabuli*	Ca_Kabuli_Chr07	21616285	(A/G)
12718	CakSNP12718	*Kabuli*	Ca_Kabuli_Chr07	21616287	(T/G)
12719	CakSNP12719	*Kabuli*	Ca_Kabuli_Chr07	21616294	(T/G)
12720	CakSNP12720	*Kabuli*	Ca_Kabuli_Chr07	21626752	(T/C)
12721	CakSNP12721	*Kabuli*	Ca_Kabuli_Chr07	21633103	(C/G)
12722	CakSNP12722	*Kabuli*	Ca_Kabuli_Chr07	21639234	(G/A)
12723	CakSNP12723	*Kabuli*	Ca_Kabuli_Chr07	21639238	(G/A)
12724	CakSNP12724	*Kabuli*	Ca_Kabuli_Chr07	21639244	(T/G)
12725	CakSNP12725	*Kabuli*	Ca_Kabuli_Chr07	21639251	(T/G)
12726	CakSNP12726	*Kabuli*	Ca_Kabuli_Chr07	21639254	(T/G)
12727	CakSNP12727	*Kabuli*	Ca_Kabuli_Chr07	21639375	(T/G)
12728	CakSNP12728	*Kabuli*	Ca_Kabuli_Chr07	21644279	(C/T)
12729	CakSNP12729	*Kabuli*	Ca_Kabuli_Chr07	21644342	(G/A)
12730	CakSNP12730	*Kabuli*	Ca_Kabuli_Chr07	21713147	(T/G)
12731	CakSNP12731	*Kabuli*	Ca_Kabuli_Chr07	21744514	(T/C)
12732	CakSNP12732	*Kabuli*	Ca_Kabuli_Chr07	21918052	(G/C)
12733	CakSNP12733	*Kabuli*	Ca_Kabuli_Chr07	22707297	(T/C)
12734	CakSNP12734	*Kabuli*	Ca_Kabuli_Chr07	22721356	(A/C)
12735	CakSNP12735	*Kabuli*	Ca_Kabuli_Chr07	22738152	(C/G)
12736	CakSNP12736	*Kabuli*	Ca_Kabuli_Chr07	22738307	(G/C)
12737	CakSNP12737	*Kabuli*	Ca_Kabuli_Chr07	22738482	(A/G)
12738	CakSNP12738	*Kabuli*	Ca_Kabuli_Chr07	22808696	(A/G)
12739	CakSNP12739	*Kabuli*	Ca_Kabuli_Chr07	22808695	(C/T)
12740	CakSNP12740	*Kabuli*	Ca_Kabuli_Chr07	22808687	(C/T)
12741	CakSNP12741	*Kabuli*	Ca_Kabuli_Chr07	22808717	(C/T)
12742	CakSNP12742	*Kabuli*	Ca_Kabuli_Chr07	22808679	(A/C)
12743	CakSNP12743	*Kabuli*	Ca_Kabuli_Chr07	22839776	(G/A)
12744	CakSNP12744	*Kabuli*	Ca_Kabuli_Chr07	22875652	(A/G)
12745	CakSNP12745	*Kabuli*	Ca_Kabuli_Chr07	23087696	(T/C)
12746	CakSNP12746	*Kabuli*	Ca_Kabuli_Chr07	23087732	(T/C)
12747	CakSNP12747	*Kabuli*	Ca_Kabuli_Chr07	23439214	(A/G)
12748	CakSNP12748	*Kabuli*	Ca_Kabuli_Chr07	23439225	(C/A)
12749	CakSNP12749	*Kabuli*	Ca_Kabuli_Chr07	23574823	(C/T)
12750	CakSNP12750	*Kabuli*	Ca_Kabuli_Chr07	23575629	(G/A)
12751	CakSNP12751	*Kabuli*	Ca_Kabuli_Chr07	23616050	(A/G)

S.N.	SNP IDs	Cultivars	Chromosomes/scaffolds	Physical positions (bp)	SNPs
12752	CakSNP12752	*Kabuli*	Ca_Kabuli_Chr07	24021690	(T/A)
12753	CakSNP12753	*Kabuli*	Ca_Kabuli_Chr07	24181321	(G/A)
12754	CakSNP12754	*Kabuli*	Ca_Kabuli_Chr07	24190633	(G/A)
12755	CakSNP12755	*Kabuli*	Ca_Kabuli_Chr07	24190636	(G/A)
12756	CakSNP12756	*Kabuli*	Ca_Kabuli_Chr07	24190645	(A/T)
12757	CakSNP12757	*Kabuli*	Ca_Kabuli_Chr07	24190650	(G/A)
12758	CakSNP12758	*Kabuli*	Ca_Kabuli_Chr07	24190671	(C/T)
12759	CakSNP12759	*Kabuli*	Ca_Kabuli_Chr07	24245096	(C/T)
12760	CakSNP12760	*Kabuli*	Ca_Kabuli_Chr07	24415819	(T/A)
12761	CakSNP12761	*Kabuli*	Ca_Kabuli_Chr07	25218407	(C/T)
12762	CakSNP12762	*Kabuli*	Ca_Kabuli_Chr07	25362272	(T/C)
12763	CakSNP12763	*Kabuli*	Ca_Kabuli_Chr07	25428518	(T/C)
12764	CakSNP12764	*Kabuli*	Ca_Kabuli_Chr07	25428525	(C/G)
12765	CakSNP12765	*Kabuli*	Ca_Kabuli_Chr07	25428548	(G/T)
12766	CakSNP12766	*Kabuli*	Ca_Kabuli_Chr07	25428566	(T/C)
12767	CakSNP12767	*Kabuli*	Ca_Kabuli_Chr07	25428660	(G/A)
12768	CakSNP12768	*Kabuli*	Ca_Kabuli_Chr07	25569665	(T/G)
12769	CakSNP12769	*Kabuli*	Ca_Kabuli_Chr07	25569657	(T/C)
12770	CakSNP12770	*Kabuli*	Ca_Kabuli_Chr07	25740866	(G/T)
12771	CakSNP12771	*Kabuli*	Ca_Kabuli_Chr07	26070513	(G/T)
12772	CakSNP12772	*Kabuli*	Ca_Kabuli_Chr07	26070515	(A/G)
12773	CakSNP12773	*Kabuli*	Ca_Kabuli_Chr07	26094578	(T/C)
12774	CakSNP12774	*Kabuli*	Ca_Kabuli_Chr07	26094572	(C/T)
12775	CakSNP12775	*Kabuli*	Ca_Kabuli_Chr07	26094562	(G/T)
12776	CakSNP12776	*Kabuli*	Ca_Kabuli_Chr07	26094567	(G/A)
12777	CakSNP12777	*Kabuli*	Ca_Kabuli_Chr07	26094609	(C/T)
12778	CakSNP12778	*Kabuli*	Ca_Kabuli_Chr07	26137315	(T/C)
12779	CakSNP12779	*Kabuli*	Ca_Kabuli_Chr07	26146779	(C/T)
12780	CakSNP12780	*Kabuli*	Ca_Kabuli_Chr07	26226146	(G/C)
12781	CakSNP12781	*Kabuli*	Ca_Kabuli_Chr07	26487440	(A/C)
12782	CakSNP12782	*Kabuli*	Ca_Kabuli_Chr07	26692131	(T/G)
12783	CakSNP12783	*Kabuli*	Ca_Kabuli_Chr07	26692130	(C/T)
12784	CakSNP12784	*Kabuli*	Ca_Kabuli_Chr07	26692128	(A/C)
12785	CakSNP12785	*Kabuli*	Ca_Kabuli_Chr07	26692194	(C/A)
12786	CakSNP12786	*Kabuli*	Ca_Kabuli_Chr07	26692195	(G/T)
12787	CakSNP12787	*Kabuli*	Ca_Kabuli_Chr07	26694365	(G/A)
12788	CakSNP12788	*Kabuli*	Ca_Kabuli_Chr07	26694440	(C/A)
12789	CakSNP12789	*Kabuli*	Ca_Kabuli_Chr07	26694441	(G/A)
12790	CakSNP12790	*Kabuli*	Ca_Kabuli_Chr07	26897878	(A/G)

S.N.	SNP IDs	Cultivars	Chromosomes/scaffolds	Physical positions (bp)	SNPs
12791	CakSNP12791	*Kabuli*	Ca_Kabuli_Chr07	26897910	(A/G)
12792	CakSNP12792	*Kabuli*	Ca_Kabuli_Chr07	26897920	(A/G)
12793	CakSNP12793	*Kabuli*	Ca_Kabuli_Chr07	26897923	(G/A)
12794	CakSNP12794	*Kabuli*	Ca_Kabuli_Chr07	26897929	(T/C)
12795	CakSNP12795	*Kabuli*	Ca_Kabuli_Chr07	26897940	(A/G)
12796	CakSNP12796	*Kabuli*	Ca_Kabuli_Chr07	26897948	(A/T)
12797	CakSNP12797	*Kabuli*	Ca_Kabuli_Chr07	26940917	(G/A)
12798	CakSNP12798	*Kabuli*	Ca_Kabuli_Chr07	26940967	(A/G)
12799	CakSNP12799	*Kabuli*	Ca_Kabuli_Chr07	26940979	(A/G)
12800	CakSNP12800	*Kabuli*	Ca_Kabuli_Chr07	26940985	(T/G)
12801	CakSNP12801	*Kabuli*	Ca_Kabuli_Chr07	27050259	(C/G)
12802	CakSNP12802	*Kabuli*	Ca_Kabuli_Chr07	27176481	(C/A)
12803	CakSNP12803	*Kabuli*	Ca_Kabuli_Chr07	27210919	(C/T)
12804	CakSNP12804	*Kabuli*	Ca_Kabuli_Chr07	27210881	(C/T)
12805	CakSNP12805	*Kabuli*	Ca_Kabuli_Chr07	27210871	(C/A)
12806	CakSNP12806	*Kabuli*	Ca_Kabuli_Chr07	27210865	(T/C)
12807	CakSNP12807	*Kabuli*	Ca_Kabuli_Chr07	27210858	(C/T)
12808	CakSNP12808	*Kabuli*	Ca_Kabuli_Chr07	27210853	(A/G)
12809	CakSNP12809	*Kabuli*	Ca_Kabuli_Chr07	27210848	(A/T)
12810	CakSNP12810	*Kabuli*	Ca_Kabuli_Chr07	27210951	(T/C)
12811	CakSNP12811	*Kabuli*	Ca_Kabuli_Chr07	27210945	(C/T)
12812	CakSNP12812	*Kabuli*	Ca_Kabuli_Chr07	27210988	(C/T)
12813	CakSNP12813	*Kabuli*	Ca_Kabuli_Chr07	27210989	(C/T)
12814	CakSNP12814	*Kabuli*	Ca_Kabuli_Chr07	27210992	(C/A)
12815	CakSNP12815	*Kabuli*	Ca_Kabuli_Chr07	27211004	(T/C)
12816	CakSNP12816	*Kabuli*	Ca_Kabuli_Chr07	27211036	(G/A)
12817	CakSNP12817	*Kabuli*	Ca_Kabuli_Chr07	27211051	(C/A)
12818	CakSNP12818	*Kabuli*	Ca_Kabuli_Chr07	27211084	(G/T)
12819	CakSNP12819	*Kabuli*	Ca_Kabuli_Chr07	27211073	(C/T)
12820	CakSNP12820	*Kabuli*	Ca_Kabuli_Chr07	27211060	(C/T)
12821	CakSNP12821	*Kabuli*	Ca_Kabuli_Chr07	27633960	(C/A)
12822	CakSNP12822	*Kabuli*	Ca_Kabuli_Chr07	27735234	(A/G)
12823	CakSNP12823	*Kabuli*	Ca_Kabuli_Chr07	27899782	(T/C)
12824	CakSNP12824	*Kabuli*	Ca_Kabuli_Chr07	27899743	(G/A)
12825	CakSNP12825	*Kabuli*	Ca_Kabuli_Chr07	27971186	(T/G)
12826	CakSNP12826	*Kabuli*	Ca_Kabuli_Chr07	28676139	(T/C)
12827	CakSNP12827	*Kabuli*	Ca_Kabuli_Chr07	28931212	(A/C)
12828	CakSNP12828	*Kabuli*	Ca_Kabuli_Chr07	28931223	(C/A)
12829	CakSNP12829	*Kabuli*	Ca_Kabuli_Chr07	29150927	(A/C)

S.N.	SNP IDs	Cultivars	Chromosomes/scaffolds	Physical positions (bp)	SNPs
12830	CakSNP12830	*Kabuli*	Ca_Kabuli_Chr07	29153994	(T/C)
12831	CakSNP12831	*Kabuli*	Ca_Kabuli_Chr07	29154220	(C/A)
12832	CakSNP12832	*Kabuli*	Ca_Kabuli_Chr07	29157286	(A/G)
12833	CakSNP12833	*Kabuli*	Ca_Kabuli_Chr07	29168982	(G/A)
12834	CakSNP12834	*Kabuli*	Ca_Kabuli_Chr07	29168980	(C/T)
12835	CakSNP12835	*Kabuli*	Ca_Kabuli_Chr07	29190659	(T/G)
12836	CakSNP12836	*Kabuli*	Ca_Kabuli_Chr07	29225385	(T/G)
12837	CakSNP12837	*Kabuli*	Ca_Kabuli_Chr07	29225351	(T/G)
12838	CakSNP12838	*Kabuli*	Ca_Kabuli_Chr07	29225348	(G/C)
12839	CakSNP12839	*Kabuli*	Ca_Kabuli_Chr07	29229965	(A/G)
12840	CakSNP12840	*Kabuli*	Ca_Kabuli_Chr07	29313114	(G/T)
12841	CakSNP12841	*Kabuli*	Ca_Kabuli_Chr07	29313080	(T/C)
12842	CakSNP12842	*Kabuli*	Ca_Kabuli_Chr07	29313067	(G/T)
12843	CakSNP12843	*Kabuli*	Ca_Kabuli_Chr07	29313059	(G/A)
12844	CakSNP12844	*Kabuli*	Ca_Kabuli_Chr07	29313058	(T/C)
12845	CakSNP12845	*Kabuli*	Ca_Kabuli_Chr07	29313051	(T/C)
12846	CakSNP12846	*Kabuli*	Ca_Kabuli_Chr07	29313257	(G/A)
12847	CakSNP12847	*Kabuli*	Ca_Kabuli_Chr07	29434818	(T/C)
12848	CakSNP12848	*Kabuli*	Ca_Kabuli_Chr07	29482556	(G/A)
12849	CakSNP12849	*Kabuli*	Ca_Kabuli_Chr07	29484304	(G/A)
12850	CakSNP12850	*Kabuli*	Ca_Kabuli_Chr07	29607943	(C/T)
12851	CakSNP12851	*Kabuli*	Ca_Kabuli_Chr07	29613699	(C/T)
12852	CakSNP12852	*Kabuli*	Ca_Kabuli_Chr07	29625884	(T/C)
12853	CakSNP12853	*Kabuli*	Ca_Kabuli_Chr07	29705531	(T/G)
12854	CakSNP12854	*Kabuli*	Ca_Kabuli_Chr07	29705499	(T/C)
12855	CakSNP12855	*Kabuli*	Ca_Kabuli_Chr07	29796564	(G/T)
12856	CakSNP12856	*Kabuli*	Ca_Kabuli_Chr07	29801148	(A/G)
12857	CakSNP12857	*Kabuli*	Ca_Kabuli_Chr07	29801154	(T/C)
12858	CakSNP12858	*Kabuli*	Ca_Kabuli_Chr07	29837035	(G/A)
12859	CakSNP12859	*Kabuli*	Ca_Kabuli_Chr07	29838073	(C/T)
12860	CakSNP12860	*Kabuli*	Ca_Kabuli_Chr07	29848248	(T/C)
12861	CakSNP12861	*Kabuli*	Ca_Kabuli_Chr07	29848433	(C/T)
12862	CakSNP12862	*Kabuli*	Ca_Kabuli_Chr07	29849509	(C/T)
12863	CakSNP12863	*Kabuli*	Ca_Kabuli_Chr07	29849544	(A/G)
12864	CakSNP12864	*Kabuli*	Ca_Kabuli_Chr07	29884719	(G/T)
12865	CakSNP12865	*Kabuli*	Ca_Kabuli_Chr07	29884718	(C/A)
12866	CakSNP12866	*Kabuli*	Ca_Kabuli_Chr07	29925008	(G/A)
12867	CakSNP12867	*Kabuli*	Ca_Kabuli_Chr07	30004767	(C/T)
12868	CakSNP12868	*Kabuli*	Ca_Kabuli_Chr07	30004934	(A/C)

S.N.	SNP IDs	Cultivars	Chromosomes/scaffolds	Physical positions (bp)	SNPs
12869	CakSNP12869	*Kabuli*	Ca_Kabuli_Chr07	30026039	(T/G)
12870	CakSNP12870	*Kabuli*	Ca_Kabuli_Chr07	30026017	(C/T)
12871	CakSNP12871	*Kabuli*	Ca_Kabuli_Chr07	30029793	(A/C)
12872	CakSNP12872	*Kabuli*	Ca_Kabuli_Chr07	30029820	(T/C)
12873	CakSNP12873	*Kabuli*	Ca_Kabuli_Chr07	30060299	(C/A)
12874	CakSNP12874	*Kabuli*	Ca_Kabuli_Chr07	30085847	(C/T)
12875	CakSNP12875	*Kabuli*	Ca_Kabuli_Chr07	30085883	(T/G)
12876	CakSNP12876	*Kabuli*	Ca_Kabuli_Chr07	30085906	(C/T)
12877	CakSNP12877	*Kabuli*	Ca_Kabuli_Chr07	30085910	(T/A)
12878	CakSNP12878	*Kabuli*	Ca_Kabuli_Chr07	30099003	(C/T)
12879	CakSNP12879	*Kabuli*	Ca_Kabuli_Chr07	30107340	(A/G)
12880	CakSNP12880	*Kabuli*	Ca_Kabuli_Chr07	30398855	(A/C)
12881	CakSNP12881	*Kabuli*	Ca_Kabuli_Chr07	30403615	(A/G)
12882	CakSNP12882	*Kabuli*	Ca_Kabuli_Chr07	30403618	(G/A)
12883	CakSNP12883	*Kabuli*	Ca_Kabuli_Chr07	30403700	(C/T)
12884	CakSNP12884	*Kabuli*	Ca_Kabuli_Chr07	30403692	(G/A)
12885	CakSNP12885	*Kabuli*	Ca_Kabuli_Chr07	30403621	(G/T)
12886	CakSNP12886	*Kabuli*	Ca_Kabuli_Chr07	30691324	(A/C)
12887	CakSNP12887	*Kabuli*	Ca_Kabuli_Chr07	30691318	(A/C)
12888	CakSNP12888	*Kabuli*	Ca_Kabuli_Chr07	30768315	(G/A)
12889	CakSNP12889	*Kabuli*	Ca_Kabuli_Chr07	30850950	(A/C)
12890	CakSNP12890	*Kabuli*	Ca_Kabuli_Chr07	31190059	(G/A)
12891	CakSNP12891	*Kabuli*	Ca_Kabuli_Chr07	31193786	(T/G)
12892	CakSNP12892	*Kabuli*	Ca_Kabuli_Chr07	31314421	(T/A)
12893	CakSNP12893	*Kabuli*	Ca_Kabuli_Chr07	31324279	(A/G)
12894	CakSNP12894	*Kabuli*	Ca_Kabuli_Chr07	31401807	(T/C)
12895	CakSNP12895	*Kabuli*	Ca_Kabuli_Chr07	31442425	(A/C)
12896	CakSNP12896	*Kabuli*	Ca_Kabuli_Chr07	31612697	(T/A)
12897	CakSNP12897	*Kabuli*	Ca_Kabuli_Chr07	31639701	(C/T)
12898	CakSNP12898	*Kabuli*	Ca_Kabuli_Chr07	31830238	(A/G)
12899	CakSNP12899	*Kabuli*	Ca_Kabuli_Chr07	31851096	(T/A)
12900	CakSNP12900	*Kabuli*	Ca_Kabuli_Chr07	31873079	(G/T)
12901	CakSNP12901	*Kabuli*	Ca_Kabuli_Chr07	31996345	(A/C)
12902	CakSNP12902	*Kabuli*	Ca_Kabuli_Chr07	32017294	(G/A)
12903	CakSNP12903	*Kabuli*	Ca_Kabuli_Chr07	32017288	(G/T)
12904	CakSNP12904	*Kabuli*	Ca_Kabuli_Chr07	32017286	(T/G)
12905	CakSNP12905	*Kabuli*	Ca_Kabuli_Chr07	32017284	(C/T)
12906	CakSNP12906	*Kabuli*	Ca_Kabuli_Chr07	32017253	(G/A)
12907	CakSNP12907	*Kabuli*	Ca_Kabuli_Chr07	32017252	(G/A)

S.N.	SNP IDs	Cultivars	Chromosomes/scaffolds	Physical positions (bp)	SNPs
12908	CakSNP12908	*Kabuli*	Ca_Kabuli_Chr07	32017238	(C/T)
12909	CakSNP12909	*Kabuli*	Ca_Kabuli_Chr07	32017228	(G/A)
12910	CakSNP12910	*Kabuli*	Ca_Kabuli_Chr07	32017221	(C/T)
12911	CakSNP12911	*Kabuli*	Ca_Kabuli_Chr07	32017211	(C/A)
12912	CakSNP12912	*Kabuli*	Ca_Kabuli_Chr07	32127288	(T/A)
12913	CakSNP12913	*Kabuli*	Ca_Kabuli_Chr07	32283585	(T/G)
12914	CakSNP12914	*Kabuli*	Ca_Kabuli_Chr07	32309397	(A/T)
12915	CakSNP12915	*Kabuli*	Ca_Kabuli_Chr07	32309411	(G/T)
12916	CakSNP12916	*Kabuli*	Ca_Kabuli_Chr07	32309415	(C/T)
12917	CakSNP12917	*Kabuli*	Ca_Kabuli_Chr07	32309429	(C/T)
12918	CakSNP12918	*Kabuli*	Ca_Kabuli_Chr07	32309377	(C/T)
12919	CakSNP12919	*Kabuli*	Ca_Kabuli_Chr07	32309376	(C/G)
12920	CakSNP12920	*Kabuli*	Ca_Kabuli_Chr07	32399759	(C/T)
12921	CakSNP12921	*Kabuli*	Ca_Kabuli_Chr07	32404932	(T/C)
12922	CakSNP12922	*Kabuli*	Ca_Kabuli_Chr07	32451377	(A/C)
12923	CakSNP12923	*Kabuli*	Ca_Kabuli_Chr07	32522201	(A/G)
12924	CakSNP12924	*Kabuli*	Ca_Kabuli_Chr07	32522203	(A/G)
12925	CakSNP12925	*Kabuli*	Ca_Kabuli_Chr07	32522207	(T/G)
12926	CakSNP12926	*Kabuli*	Ca_Kabuli_Chr07	32522213	(A/G)
12927	CakSNP12927	*Kabuli*	Ca_Kabuli_Chr07	32522230	(T/G)
12928	CakSNP12928	*Kabuli*	Ca_Kabuli_Chr07	32557599	(G/A)
12929	CakSNP12929	*Kabuli*	Ca_Kabuli_Chr07	32669570	(A/G)
12930	CakSNP12930	*Kabuli*	Ca_Kabuli_Chr07	32754633	(T/G)
12931	CakSNP12931	*Kabuli*	Ca_Kabuli_Chr07	32755647	(A/C)
12932	CakSNP12932	*Kabuli*	Ca_Kabuli_Chr07	32755662	(G/T)
12933	CakSNP12933	*Kabuli*	Ca_Kabuli_Chr07	32784903	(T/G)
12934	CakSNP12934	*Kabuli*	Ca_Kabuli_Chr07	32799037	(A/G)
12935	CakSNP12935	*Kabuli*	Ca_Kabuli_Chr07	32807457	(T/A)
12936	CakSNP12936	*Kabuli*	Ca_Kabuli_Chr07	32807499	(A/G)
12937	CakSNP12937	*Kabuli*	Ca_Kabuli_Chr07	32874847	(C/T)
12938	CakSNP12938	*Kabuli*	Ca_Kabuli_Chr07	32874840	(C/T)
12939	CakSNP12939	*Kabuli*	Ca_Kabuli_Chr07	32875799	(T/A)
12940	CakSNP12940	*Kabuli*	Ca_Kabuli_Chr07	32942199	(A/G)
12941	CakSNP12941	*Kabuli*	Ca_Kabuli_Chr07	32942951	(T/G)
12942	CakSNP12942	*Kabuli*	Ca_Kabuli_Chr07	32989796	(T/A)
12943	CakSNP12943	*Kabuli*	Ca_Kabuli_Chr07	32989838	(C/T)
12944	CakSNP12944	*Kabuli*	Ca_Kabuli_Chr07	32989903	(A/G)
12945	CakSNP12945	*Kabuli*	Ca_Kabuli_Chr07	32989902	(C/A)
12946	CakSNP12946	*Kabuli*	Ca_Kabuli_Chr07	33065611	(G/T)

S.N.	SNP IDs	Cultivars	Chromosomes/scaffolds	Physical positions (bp)	SNPs
12947	CakSNP12947	*Kabuli*	Ca_Kabuli_Chr07	33121674	(G/C)
12948	CakSNP12948	*Kabuli*	Ca_Kabuli_Chr07	33121640	(C/A)
12949	CakSNP12949	*Kabuli*	Ca_Kabuli_Chr07	33122473	(A/C)
12950	CakSNP12950	*Kabuli*	Ca_Kabuli_Chr07	33182932	(G/A)
12951	CakSNP12951	*Kabuli*	Ca_Kabuli_Chr07	33267485	(T/C)
12952	CakSNP12952	*Kabuli*	Ca_Kabuli_Chr07	33267496	(T/A)
12953	CakSNP12953	*Kabuli*	Ca_Kabuli_Chr07	33335978	(C/A)
12954	CakSNP12954	*Kabuli*	Ca_Kabuli_Chr07	33364329	(C/T)
12955	CakSNP12955	*Kabuli*	Ca_Kabuli_Chr07	33364354	(G/A)
12956	CakSNP12956	*Kabuli*	Ca_Kabuli_Chr07	33378427	(C/T)
12957	CakSNP12957	*Kabuli*	Ca_Kabuli_Chr07	33411746	(C/A)
12958	CakSNP12958	*Kabuli*	Ca_Kabuli_Chr07	33465828	(C/A)
12959	CakSNP12959	*Kabuli*	Ca_Kabuli_Chr07	33465818	(G/A)
12960	CakSNP12960	*Kabuli*	Ca_Kabuli_Chr07	33465805	(T/C)
12961	CakSNP12961	*Kabuli*	Ca_Kabuli_Chr07	33465802	(C/A)
12962	CakSNP12962	*Kabuli*	Ca_Kabuli_Chr07	33465800	(T/A)
12963	CakSNP12963	*Kabuli*	Ca_Kabuli_Chr07	33465781	(G/T)
12964	CakSNP12964	*Kabuli*	Ca_Kabuli_Chr07	33465855	(A/C)
12965	CakSNP12965	*Kabuli*	Ca_Kabuli_Chr07	33480940	(C/T)
12966	CakSNP12966	*Kabuli*	Ca_Kabuli_Chr07	33573605	(C/T)
12967	CakSNP12967	*Kabuli*	Ca_Kabuli_Chr07	33573655	(A/C)
12968	CakSNP12968	*Kabuli*	Ca_Kabuli_Chr07	33619268	(G/C)
12969	CakSNP12969	*Kabuli*	Ca_Kabuli_Chr07	33641799	(G/T)
12970	CakSNP12970	*Kabuli*	Ca_Kabuli_Chr07	33641882	(A/T)
12971	CakSNP12971	*Kabuli*	Ca_Kabuli_Chr07	33674454	(T/C)
12972	CakSNP12972	*Kabuli*	Ca_Kabuli_Chr07	33744521	(C/G)
12973	CakSNP12973	*Kabuli*	Ca_Kabuli_Chr07	33753917	(G/A)
12974	CakSNP12974	*Kabuli*	Ca_Kabuli_Chr07	33761944	(G/A)
12975	CakSNP12975	*Kabuli*	Ca_Kabuli_Chr07	33762053	(T/G)
12976	CakSNP12976	*Kabuli*	Ca_Kabuli_Chr07	33821658	(C/T)
12977	CakSNP12977	*Kabuli*	Ca_Kabuli_Chr07	33865463	(T/C)
12978	CakSNP12978	*Kabuli*	Ca_Kabuli_Chr07	33865475	(G/A)
12979	CakSNP12979	*Kabuli*	Ca_Kabuli_Chr07	33867334	(C/T)
12980	CakSNP12980	*Kabuli*	Ca_Kabuli_Chr07	33867491	(C/T)
12981	CakSNP12981	*Kabuli*	Ca_Kabuli_Chr07	33867674	(C/T)
12982	CakSNP12982	*Kabuli*	Ca_Kabuli_Chr07	33869367	(G/A)
12983	CakSNP12983	*Kabuli*	Ca_Kabuli_Chr07	33879559	(C/G)
12984	CakSNP12984	*Kabuli*	Ca_Kabuli_Chr07	33910324	(C/A)
12985	CakSNP12985	*Kabuli*	Ca_Kabuli_Chr07	33910870	(C/A)

S.N.	SNP IDs	Cultivars	Chromosomes/scaffolds	Physical positions (bp)	SNPs
12986	CakSNP12986	*Kabuli*	Ca_Kabuli_Chr07	33912719	(T/G)
12987	CakSNP12987	*Kabuli*	Ca_Kabuli_Chr07	33912766	(G/T)
12988	CakSNP12988	*Kabuli*	Ca_Kabuli_Chr07	33912866	(T/C)
12989	CakSNP12989	*Kabuli*	Ca_Kabuli_Chr07	33912899	(T/A)
12990	CakSNP12990	*Kabuli*	Ca_Kabuli_Chr07	33912902	(A/T)
12991	CakSNP12991	*Kabuli*	Ca_Kabuli_Chr07	33912913	(A/T)
12992	CakSNP12992	*Kabuli*	Ca_Kabuli_Chr07	33912916	(G/T)
12993	CakSNP12993	*Kabuli*	Ca_Kabuli_Chr07	33912921	(T/G)
12994	CakSNP12994	*Kabuli*	Ca_Kabuli_Chr07	33912950	(C/T)
12995	CakSNP12995	*Kabuli*	Ca_Kabuli_Chr07	33912966	(T/C)
12996	CakSNP12996	*Kabuli*	Ca_Kabuli_Chr07	33913094	(A/G)
12997	CakSNP12997	*Kabuli*	Ca_Kabuli_Chr07	33913047	(C/T)
12998	CakSNP12998	*Kabuli*	Ca_Kabuli_Chr07	33913045	(G/T)
12999	CakSNP12999	*Kabuli*	Ca_Kabuli_Chr07	34107603	(C/A)
13000	CakSNP13000	*Kabuli*	Ca_Kabuli_Chr07	34152124	(G/C)
13001	CakSNP13001	*Kabuli*	Ca_Kabuli_Chr07	34271486	(A/G)
13002	CakSNP13002	*Kabuli*	Ca_Kabuli_Chr07	34277014	(G/T)
13003	CakSNP13003	*Kabuli*	Ca_Kabuli_Chr07	34277010	(A/G)
13004	CakSNP13004	*Kabuli*	Ca_Kabuli_Chr07	34279673	(G/A)
13005	CakSNP13005	*Kabuli*	Ca_Kabuli_Chr07	34279725	(A/G)
13006	CakSNP13006	*Kabuli*	Ca_Kabuli_Chr07	34279715	(A/C)
13007	CakSNP13007	*Kabuli*	Ca_Kabuli_Chr07	34318860	(C/T)
13008	CakSNP13008	*Kabuli*	Ca_Kabuli_Chr07	34318862	(A/G)
13009	CakSNP13009	*Kabuli*	Ca_Kabuli_Chr07	34474614	(A/C)
13010	CakSNP13010	*Kabuli*	Ca_Kabuli_Chr07	34801839	(A/T)
13011	CakSNP13011	*Kabuli*	Ca_Kabuli_Chr07	34857693	(C/T)
13012	CakSNP13012	*Kabuli*	Ca_Kabuli_Chr07	34947743	(A/C)
13013	CakSNP13013	*Kabuli*	Ca_Kabuli_Chr07	35016516	(G/C)
13014	CakSNP13014	*Kabuli*	Ca_Kabuli_Chr07	35072425	(A/T)
13015	CakSNP13015	*Kabuli*	Ca_Kabuli_Chr07	35072407	(G/A)
13016	CakSNP13016	*Kabuli*	Ca_Kabuli_Chr07	35072461	(A/T)
13017	CakSNP13017	*Kabuli*	Ca_Kabuli_Chr07	35074345	(T/A)
13018	CakSNP13018	*Kabuli*	Ca_Kabuli_Chr07	35074348	(T/C)
13019	CakSNP13019	*Kabuli*	Ca_Kabuli_Chr07	35074362	(T/C)
13020	CakSNP13020	*Kabuli*	Ca_Kabuli_Chr07	35112785	(T/C)
13021	CakSNP13021	*Kabuli*	Ca_Kabuli_Chr07	35114049	(C/T)
13022	CakSNP13022	*Kabuli*	Ca_Kabuli_Chr07	35134809	(A/C)
13023	CakSNP13023	*Kabuli*	Ca_Kabuli_Chr07	35134863	(T/C)
13024	CakSNP13024	*Kabuli*	Ca_Kabuli_Chr07	35181619	(T/G)

S.N.	SNP IDs	Cultivars	Chromosomes/scaffolds	Physical positions (bp)	SNPs
13025	CakSNP13025	*Kabuli*	Ca_Kabuli_Chr07	35181661	(T/C)
13026	CakSNP13026	*Kabuli*	Ca_Kabuli_Chr07	35181660	(G/A)
13027	CakSNP13027	*Kabuli*	Ca_Kabuli_Chr07	35181635	(G/C)
13028	CakSNP13028	*Kabuli*	Ca_Kabuli_Chr07	35227794	(A/C)
13029	CakSNP13029	*Kabuli*	Ca_Kabuli_Chr07	35258029	(T/G)
13030	CakSNP13030	*Kabuli*	Ca_Kabuli_Chr07	35263711	(A/T)
13031	CakSNP13031	*Kabuli*	Ca_Kabuli_Chr07	35263712	(G/T)
13032	CakSNP13032	*Kabuli*	Ca_Kabuli_Chr07	35263736	(C/G)
13033	CakSNP13033	*Kabuli*	Ca_Kabuli_Chr07	35263758	(A/G)
13034	CakSNP13034	*Kabuli*	Ca_Kabuli_Chr07	35274368	(A/G)
13035	CakSNP13035	*Kabuli*	Ca_Kabuli_Chr07	35274859	(T/G)
13036	CakSNP13036	*Kabuli*	Ca_Kabuli_Chr07	35274858	(G/A)
13037	CakSNP13037	*Kabuli*	Ca_Kabuli_Chr07	35274828	(A/G)
13038	CakSNP13038	*Kabuli*	Ca_Kabuli_Chr07	35279675	(A/G)
13039	CakSNP13039	*Kabuli*	Ca_Kabuli_Chr07	35280189	(G/A)
13040	CakSNP13040	*Kabuli*	Ca_Kabuli_Chr07	35280167	(A/G)
13041	CakSNP13041	*Kabuli*	Ca_Kabuli_Chr07	35576427	(C/A)
13042	CakSNP13042	*Kabuli*	Ca_Kabuli_Chr07	35619530	(G/A)
13043	CakSNP13043	*Kabuli*	Ca_Kabuli_Chr07	35619487	(G/A)
13044	CakSNP13044	*Kabuli*	Ca_Kabuli_Chr07	35619518	(G/C)
13045	CakSNP13045	*Kabuli*	Ca_Kabuli_Chr07	35622168	(A/G)
13046	CakSNP13046	*Kabuli*	Ca_Kabuli_Chr07	35637056	(T/C)
13047	CakSNP13047	*Kabuli*	Ca_Kabuli_Chr07	35680423	(A/C)
13048	CakSNP13048	*Kabuli*	Ca_Kabuli_Chr07	35799096	(A/C)
13049	CakSNP13049	*Kabuli*	Ca_Kabuli_Chr07	35799083	(T/A)
13050	CakSNP13050	*Kabuli*	Ca_Kabuli_Chr07	35799074	(G/C)
13051	CakSNP13051	*Kabuli*	Ca_Kabuli_Chr07	35799073	(T/C)
13052	CakSNP13052	*Kabuli*	Ca_Kabuli_Chr07	35807000	(A/G)
13053	CakSNP13053	*Kabuli*	Ca_Kabuli_Chr07	35806999	(C/G)
13054	CakSNP13054	*Kabuli*	Ca_Kabuli_Chr07	35806990	(A/T)
13055	CakSNP13055	*Kabuli*	Ca_Kabuli_Chr07	35806977	(T/G)
13056	CakSNP13056	*Kabuli*	Ca_Kabuli_Chr07	35911620	(T/G)
13057	CakSNP13057	*Kabuli*	Ca_Kabuli_Chr07	35911617	(A/C)
13058	CakSNP13058	*Kabuli*	Ca_Kabuli_Chr07	35911615	(C/T)
13059	CakSNP13059	*Kabuli*	Ca_Kabuli_Chr07	35911612	(T/G)
13060	CakSNP13060	*Kabuli*	Ca_Kabuli_Chr07	35911606	(T/C)
13061	CakSNP13061	*Kabuli*	Ca_Kabuli_Chr07	35947529	(T/C)
13062	CakSNP13062	*Kabuli*	Ca_Kabuli_Chr07	35947550	(C/T)
13063	CakSNP13063	*Kabuli*	Ca_Kabuli_Chr07	35947551	(A/G)

S.N.	SNP IDs	Cultivars	Chromosomes/scaffolds	Physical positions (bp)	SNPs
13064	CakSNP13064	*Kabuli*	Ca_Kabuli_Chr07	35959805	(C/T)
13065	CakSNP13065	*Kabuli*	Ca_Kabuli_Chr07	35959825	(G/A)
13066	CakSNP13066	*Kabuli*	Ca_Kabuli_Chr07	35959878	(G/A)
13067	CakSNP13067	*Kabuli*	Ca_Kabuli_Chr07	35959917	(C/T)
13068	CakSNP13068	*Kabuli*	Ca_Kabuli_Chr07	35959865	(T/C)
13069	CakSNP13069	*Kabuli*	Ca_Kabuli_Chr07	36711954	(C/A)
13070	CakSNP13070	*Kabuli*	Ca_Kabuli_Chr07	36715249	(T/C)
13071	CakSNP13071	*Kabuli*	Ca_Kabuli_Chr07	36745439	(T/G)
13072	CakSNP13072	*Kabuli*	Ca_Kabuli_Chr07	36916272	(T/G)
13073	CakSNP13073	*Kabuli*	Ca_Kabuli_Chr07	36916273	(T/A)
13074	CakSNP13074	*Kabuli*	Ca_Kabuli_Chr07	36998096	(T/C)
13075	CakSNP13075	*Kabuli*	Ca_Kabuli_Chr07	36998113	(C/T)
13076	CakSNP13076	*Kabuli*	Ca_Kabuli_Chr07	37058979	(C/T)
13077	CakSNP13077	*Kabuli*	Ca_Kabuli_Chr07	37187439	(A/G)
13078	CakSNP13078	*Kabuli*	Ca_Kabuli_Chr07	37626729	(G/T)
13079	CakSNP13079	*Kabuli*	Ca_Kabuli_Chr07	37626653	(A/C)
13080	CakSNP13080	*Kabuli*	Ca_Kabuli_Chr07	37626687	(A/G)
13081	CakSNP13081	*Kabuli*	Ca_Kabuli_Chr07	37626666	(C/T)
13082	CakSNP13082	*Kabuli*	Ca_Kabuli_Chr07	37703967	(G/A)
13083	CakSNP13083	*Kabuli*	Ca_Kabuli_Chr07	37703966	(C/T)
13084	CakSNP13084	*Kabuli*	Ca_Kabuli_Chr07	37703965	(C/T)
13085	CakSNP13085	*Kabuli*	Ca_Kabuli_Chr07	37703921	(G/T)
13086	CakSNP13086	*Kabuli*	Ca_Kabuli_Chr07	37703912	(G/A)
13087	CakSNP13087	*Kabuli*	Ca_Kabuli_Chr07	37703913	(C/G)
13088	CakSNP13088	*Kabuli*	Ca_Kabuli_Chr07	37703936	(C/T)
13089	CakSNP13089	*Kabuli*	Ca_Kabuli_Chr07	37857936	(G/T)
13090	CakSNP13090	*Kabuli*	Ca_Kabuli_Chr07	37857928	(A/T)
13091	CakSNP13091	*Kabuli*	Ca_Kabuli_Chr07	37857917	(G/A)
13092	CakSNP13092	*Kabuli*	Ca_Kabuli_Chr07	37857885	(C/T)
13093	CakSNP13093	*Kabuli*	Ca_Kabuli_Chr07	38047374	(A/G)
13094	CakSNP13094	*Kabuli*	Ca_Kabuli_Chr07	38047378	(C/A)
13095	CakSNP13095	*Kabuli*	Ca_Kabuli_Chr07	38143361	(A/G)
13096	CakSNP13096	*Kabuli*	Ca_Kabuli_Chr07	38143524	(T/A)
13097	CakSNP13097	*Kabuli*	Ca_Kabuli_Chr07	38143452	(G/A)
13098	CakSNP13098	*Kabuli*	Ca_Kabuli_Chr07	38460464	(G/A)
13099	CakSNP13099	*Kabuli*	Ca_Kabuli_Chr07	38460452	(C/T)
13100	CakSNP13100	*Kabuli*	Ca_Kabuli_Chr07	38460425	(G/A)
13101	CakSNP13101	*Kabuli*	Ca_Kabuli_Chr07	38460394	(C/A)
13102	CakSNP13102	*Kabuli*	Ca_Kabuli_Chr07	39181880	(G/A)

S.N.	SNP IDs	Cultivars	Chromosomes/scaffolds	Physical positions (bp)	SNPs
13103	CakSNP13103	*Kabuli*	Ca_Kabuli_Chr07	39182564	(A/T)
13104	CakSNP13104	*Kabuli*	Ca_Kabuli_Chr07	39339510	(T/G)
13105	CakSNP13105	*Kabuli*	Ca_Kabuli_Chr07	39339509	(G/A)
13106	CakSNP13106	*Kabuli*	Ca_Kabuli_Chr07	39339508	(G/A)
13107	CakSNP13107	*Kabuli*	Ca_Kabuli_Chr07	39339503	(C/A)
13108	CakSNP13108	*Kabuli*	Ca_Kabuli_Chr07	39339502	(C/A)
13109	CakSNP13109	*Kabuli*	Ca_Kabuli_Chr07	39339501	(G/C)
13110	CakSNP13110	*Kabuli*	Ca_Kabuli_Chr07	39339500	(G/T)
13111	CakSNP13111	*Kabuli*	Ca_Kabuli_Chr07	39339499	(C/T)
13112	CakSNP13112	*Kabuli*	Ca_Kabuli_Chr07	39339498	(G/T)
13113	CakSNP13113	*Kabuli*	Ca_Kabuli_Chr07	39339495	(G/T)
13114	CakSNP13114	*Kabuli*	Ca_Kabuli_Chr07	39399280	(T/C)
13115	CakSNP13115	*Kabuli*	Ca_Kabuli_Chr07	39680221	(A/C)
13116	CakSNP13116	*Kabuli*	Ca_Kabuli_Chr07	39680222	(G/A)
13117	CakSNP13117	*Kabuli*	Ca_Kabuli_Chr07	39768248	(T/C)
13118	CakSNP13118	*Kabuli*	Ca_Kabuli_Chr07	39944444	(T/C)
13119	CakSNP13119	*Kabuli*	Ca_Kabuli_Chr07	39944937	(T/C)
13120	CakSNP13120	*Kabuli*	Ca_Kabuli_Chr07	39944889	(T/C)
13121	CakSNP13121	*Kabuli*	Ca_Kabuli_Chr07	40092198	(G/A)
13122	CakSNP13122	*Kabuli*	Ca_Kabuli_Chr07	40092181	(C/T)
13123	CakSNP13123	*Kabuli*	Ca_Kabuli_Chr07	40092218	(A/T)
13124	CakSNP13124	*Kabuli*	Ca_Kabuli_Chr07	40092213	(G/A)
13125	CakSNP13125	*Kabuli*	Ca_Kabuli_Chr07	40092163	(T/C)
13126	CakSNP13126	*Kabuli*	Ca_Kabuli_Chr07	40092155	(T/A)
13127	CakSNP13127	*Kabuli*	Ca_Kabuli_Chr07	40092156	(C/A)
13128	CakSNP13128	*Kabuli*	Ca_Kabuli_Chr07	40092168	(G/A)
13129	CakSNP13129	*Kabuli*	Ca_Kabuli_Chr07	40092201	(G/A)
13130	CakSNP13130	*Kabuli*	Ca_Kabuli_Chr07	40092203	(T/G)
13131	CakSNP13131	*Kabuli*	Ca_Kabuli_Chr07	40092211	(C/T)
13132	CakSNP13132	*Kabuli*	Ca_Kabuli_Chr07	40092214	(C/G)
13133	CakSNP13133	*Kabuli*	Ca_Kabuli_Chr07	40092229	(C/A)
13134	CakSNP13134	*Kabuli*	Ca_Kabuli_Chr07	40092237	(C/T)
13135	CakSNP13135	*Kabuli*	Ca_Kabuli_Chr07	40092239	(G/A)
13136	CakSNP13136	*Kabuli*	Ca_Kabuli_Chr07	40092250	(A/C)
13137	CakSNP13137	*Kabuli*	Ca_Kabuli_Chr07	40092253	(C/T)
13138	CakSNP13138	*Kabuli*	Ca_Kabuli_Chr07	40092256	(T/C)
13139	CakSNP13139	*Kabuli*	Ca_Kabuli_Chr07	40092267	(G/C)
13140	CakSNP13140	*Kabuli*	Ca_Kabuli_Chr07	40183449	(C/A)
13141	CakSNP13141	*Kabuli*	Ca_Kabuli_Chr07	40183452	(A/C)

S.N.	SNP IDs	Cultivars	Chromosomes/scaffolds	Physical positions (bp)	SNPs
13142	CakSNP13142	*Kabuli*	Ca_Kabuli_Chr07	40241674	(A/C)
13143	CakSNP13143	*Kabuli*	Ca_Kabuli_Chr07	40242539	(A/G)
13144	CakSNP13144	*Kabuli*	Ca_Kabuli_Chr07	40242765	(A/G)
13145	CakSNP13145	*Kabuli*	Ca_Kabuli_Chr07	40243784	(G/A)
13146	CakSNP13146	*Kabuli*	Ca_Kabuli_Chr07	40323213	(C/A)
13147	CakSNP13147	*Kabuli*	Ca_Kabuli_Chr07	40323251	(T/A)
13148	CakSNP13148	*Kabuli*	Ca_Kabuli_Chr07	40429548	(A/G)
13149	CakSNP13149	*Kabuli*	Ca_Kabuli_Chr07	40429552	(C/T)
13150	CakSNP13150	*Kabuli*	Ca_Kabuli_Chr07	40429564	(G/T)
13151	CakSNP13151	*Kabuli*	Ca_Kabuli_Chr07	40429573	(G/T)
13152	CakSNP13152	*Kabuli*	Ca_Kabuli_Chr07	40429629	(G/T)
13153	CakSNP13153	*Kabuli*	Ca_Kabuli_Chr07	40429621	(G/T)
13154	CakSNP13154	*Kabuli*	Ca_Kabuli_Chr07	40429604	(C/T)
13155	CakSNP13155	*Kabuli*	Ca_Kabuli_Chr07	40429600	(C/T)
13156	CakSNP13156	*Kabuli*	Ca_Kabuli_Chr07	40429596	(G/A)
13157	CakSNP13157	*Kabuli*	Ca_Kabuli_Chr07	40429567	(G/A)
13158	CakSNP13158	*Kabuli*	Ca_Kabuli_Chr07	40456574	(T/C)
13159	CakSNP13159	*Kabuli*	Ca_Kabuli_Chr07	40645278	(A/G)
13160	CakSNP13160	*Kabuli*	Ca_Kabuli_Chr07	40645276	(A/G)
13161	CakSNP13161	*Kabuli*	Ca_Kabuli_Chr07	40645208	(C/G)
13162	CakSNP13162	*Kabuli*	Ca_Kabuli_Chr07	40889596	(A/C)
13163	CakSNP13163	*Kabuli*	Ca_Kabuli_Chr07	40889598	(C/G)
13164	CakSNP13164	*Kabuli*	Ca_Kabuli_Chr07	40889623	(G/A)
13165	CakSNP13165	*Kabuli*	Ca_Kabuli_Chr07	40889632	(C/T)
13166	CakSNP13166	*Kabuli*	Ca_Kabuli_Chr07	40889633	(G/A)
13167	CakSNP13167	*Kabuli*	Ca_Kabuli_Chr07	40889640	(G/A)
13168	CakSNP13168	*Kabuli*	Ca_Kabuli_Chr07	40889641	(C/T)
13169	CakSNP13169	*Kabuli*	Ca_Kabuli_Chr07	40889643	(G/A)
13170	CakSNP13170	*Kabuli*	Ca_Kabuli_Chr07	40889648	(T/A)
13171	CakSNP13171	*Kabuli*	Ca_Kabuli_Chr07	40889649	(G/A)
13172	CakSNP13172	*Kabuli*	Ca_Kabuli_Chr07	40889673	(C/A)
13173	CakSNP13173	*Kabuli*	Ca_Kabuli_Chr07	40889670	(C/T)
13174	CakSNP13174	*Kabuli*	Ca_Kabuli_Chr07	40889723	(C/G)
13175	CakSNP13175	*Kabuli*	Ca_Kabuli_Chr07	40889684	(C/T)
13176	CakSNP13176	*Kabuli*	Ca_Kabuli_Chr07	41282883	(A/T)
13177	CakSNP13177	*Kabuli*	Ca_Kabuli_Chr07	41282961	(G/T)
13178	CakSNP13178	*Kabuli*	Ca_Kabuli_Chr07	41282960	(A/C)
13179	CakSNP13179	*Kabuli*	Ca_Kabuli_Chr07	41282957	(G/A)
13180	CakSNP13180	*Kabuli*	Ca_Kabuli_Chr07	41325886	(A/G)

S.N.	SNP IDs	Cultivars	Chromosomes/scaffolds	Physical positions (bp)	SNPs
13181	CakSNP13181	*Kabuli*	Ca_Kabuli_Chr07	41339748	(C/A)
13182	CakSNP13182	*Kabuli*	Ca_Kabuli_Chr07	41349604	(T/C)
13183	CakSNP13183	*Kabuli*	Ca_Kabuli_Chr07	41387509	(A/C)
13184	CakSNP13184	*Kabuli*	Ca_Kabuli_Chr07	41387502	(A/C)
13185	CakSNP13185	*Kabuli*	Ca_Kabuli_Chr07	41387475	(A/C)
13186	CakSNP13186	*Kabuli*	Ca_Kabuli_Chr07	41657180	(G/T)
13187	CakSNP13187	*Kabuli*	Ca_Kabuli_Chr07	41657241	(T/C)
13188	CakSNP13188	*Kabuli*	Ca_Kabuli_Chr07	41821410	(T/C)
13189	CakSNP13189	*Kabuli*	Ca_Kabuli_Chr07	42166004	(C/T)
13190	CakSNP13190	*Kabuli*	Ca_Kabuli_Chr07	42268628	(A/C)
13191	CakSNP13191	*Kabuli*	Ca_Kabuli_Chr07	42272939	(G/C)
13192	CakSNP13192	*Kabuli*	Ca_Kabuli_Chr07	42355002	(T/G)
13193	CakSNP13193	*Kabuli*	Ca_Kabuli_Chr07	42354958	(G/A)
13194	CakSNP13194	*Kabuli*	Ca_Kabuli_Chr07	42354938	(C/T)
13195	CakSNP13195	*Kabuli*	Ca_Kabuli_Chr07	42354999	(C/T)
13196	CakSNP13196	*Kabuli*	Ca_Kabuli_Chr07	42355011	(G/T)
13197	CakSNP13197	*Kabuli*	Ca_Kabuli_Chr07	42566717	(A/G)
13198	CakSNP13198	*Kabuli*	Ca_Kabuli_Chr07	42846882	(C/T)
13199	CakSNP13199	*Kabuli*	Ca_Kabuli_Chr07	42851182	(T/C)
13200	CakSNP13200	*Kabuli*	Ca_Kabuli_Chr07	43080817	(T/C)
13201	CakSNP13201	*Kabuli*	Ca_Kabuli_Chr07	43118954	(A/C)
13202	CakSNP13202	*Kabuli*	Ca_Kabuli_Chr07	43119266	(C/T)
13203	CakSNP13203	*Kabuli*	Ca_Kabuli_Chr07	43210821	(A/T)
13204	CakSNP13204	*Kabuli*	Ca_Kabuli_Chr07	43646117	(G/A)
13205	CakSNP13205	*Kabuli*	Ca_Kabuli_Chr07	44232239	(G/A)
13206	CakSNP13206	*Kabuli*	Ca_Kabuli_Chr07	44232204	(T/A)
13207	CakSNP13207	*Kabuli*	Ca_Kabuli_Chr07	44232221	(C/T)
13208	CakSNP13208	*Kabuli*	Ca_Kabuli_Chr07	44232266	(C/T)
13209	CakSNP13209	*Kabuli*	Ca_Kabuli_Chr07	44540553	(T/G)
13210	CakSNP13210	*Kabuli*	Ca_Kabuli_Chr07	44540595	(G/A)
13211	CakSNP13211	*Kabuli*	Ca_Kabuli_Chr07	44540596	(C/A)
13212	CakSNP13212	*Kabuli*	Ca_Kabuli_Chr07	44540629	(G/A)
13213	CakSNP13213	*Kabuli*	Ca_Kabuli_Chr07	44540616	(C/A)
13214	CakSNP13214	*Kabuli*	Ca_Kabuli_Chr07	44612651	(C/A)
13215	CakSNP13215	*Kabuli*	Ca_Kabuli_Chr07	44747668	(C/T)
13216	CakSNP13216	*Kabuli*	Ca_Kabuli_Chr07	44765450	(C/T)
13217	CakSNP13217	*Kabuli*	Ca_Kabuli_Chr07	44765467	(G/T)
13218	CakSNP13218	*Kabuli*	Ca_Kabuli_Chr07	44765522	(A/C)
13219	CakSNP13219	*Kabuli*	Ca_Kabuli_Chr07	44877324	(A/C)

S.N.	SNP IDs	Cultivars	Chromosomes/scaffolds	Physical positions (bp)	SNPs
13220	CakSNP13220	*Kabuli*	Ca_Kabuli_Chr07	44926768	(C/T)
13221	CakSNP13221	*Kabuli*	Ca_Kabuli_Chr07	44926783	(C/A)
13222	CakSNP13222	*Kabuli*	Ca_Kabuli_Chr07	44926815	(C/T)
13223	CakSNP13223	*Kabuli*	Ca_Kabuli_Chr07	45147550	(A/G)
13224	CakSNP13224	*Kabuli*	Ca_Kabuli_Chr07	45280659	(T/A)
13225	CakSNP13225	*Kabuli*	Ca_Kabuli_Chr07	45289633	(T/C)
13226	CakSNP13226	*Kabuli*	Ca_Kabuli_Chr07	45936581	(C/A)
13227	CakSNP13227	*Kabuli*	Ca_Kabuli_Chr07	46053109	(G/A)
13228	CakSNP13228	*Kabuli*	Ca_Kabuli_Chr07	46394656	(A/G)
13229	CakSNP13229	*Kabuli*	Ca_Kabuli_Chr07	46485934	(A/G)
13230	CakSNP13230	*Kabuli*	Ca_Kabuli_Chr07	46747255	(A/C)
13231	CakSNP13231	*Kabuli*	Ca_Kabuli_Chr07	46747283	(T/C)
13232	CakSNP13232	*Kabuli*	Ca_Kabuli_Chr07	46747290	(G/T)
13233	CakSNP13233	*Kabuli*	Ca_Kabuli_Chr07	46747295	(G/C)
13234	CakSNP13234	*Kabuli*	Ca_Kabuli_Chr07	46747309	(C/T)
13235	CakSNP13235	*Kabuli*	Ca_Kabuli_Chr07	46747330	(G/A)
13236	CakSNP13236	*Kabuli*	Ca_Kabuli_Chr07	46823471	(T/G)
13237	CakSNP13237	*Kabuli*	Ca_Kabuli_Chr07	46823949	(G/T)
13238	CakSNP13238	*Kabuli*	Ca_Kabuli_Chr07	46826036	(T/A)
13239	CakSNP13239	*Kabuli*	Ca_Kabuli_Chr07	46826030	(T/C)
13240	CakSNP13240	*Kabuli*	Ca_Kabuli_Chr07	46826028	(C/A)
13241	CakSNP13241	*Kabuli*	Ca_Kabuli_Chr07	46826020	(G/A)
13242	CakSNP13242	*Kabuli*	Ca_Kabuli_Chr07	46826015	(G/A)
13243	CakSNP13243	*Kabuli*	Ca_Kabuli_Chr07	46826008	(A/T)
13244	CakSNP13244	*Kabuli*	Ca_Kabuli_Chr07	46825997	(A/G)
13245	CakSNP13245	*Kabuli*	Ca_Kabuli_Chr07	46825992	(T/G)
13246	CakSNP13246	*Kabuli*	Ca_Kabuli_Chr07	46825985	(T/G)
13247	CakSNP13247	*Kabuli*	Ca_Kabuli_Chr07	46825957	(A/T)
13248	CakSNP13248	*Kabuli*	Ca_Kabuli_Chr07	46825952	(A/G)
13249	CakSNP13249	*Kabuli*	Ca_Kabuli_Chr07	47127331	(T/G)
13250	CakSNP13250	*Kabuli*	Ca_Kabuli_Chr07	47127342	(T/G)
13251	CakSNP13251	*Kabuli*	Ca_Kabuli_Chr07	47127343	(C/T)
13252	CakSNP13252	*Kabuli*	Ca_Kabuli_Chr07	47159646	(A/C)
13253	CakSNP13253	*Kabuli*	Ca_Kabuli_Chr07	47335940	(G/A)
13254	CakSNP13254	*Kabuli*	Ca_Kabuli_Chr07	47335916	(C/G)
13255	CakSNP13255	*Kabuli*	Ca_Kabuli_Chr07	47524173	(A/C)
13256	CakSNP13256	*Kabuli*	Ca_Kabuli_Chr07	47554330	(C/G)
13257	CakSNP13257	*Kabuli*	Ca_Kabuli_Chr07	47694706	(A/C)
13258	CakSNP13258	*Kabuli*	Ca_Kabuli_Chr07	47839934	(G/A)

S.N.	SNP IDs	Cultivars	Chromosomes/scaffolds	Physical positions (bp)	SNPs
13259	CakSNP13259	*Kabuli*	Ca_Kabuli_Chr07	47839952	(G/T)
13260	CakSNP13260	*Kabuli*	Ca_Kabuli_Chr07	47840034	(A/G)
13261	CakSNP13261	*Kabuli*	Ca_Kabuli_Chr07	47840092	(A/C)
13262	CakSNP13262	*Kabuli*	Ca_Kabuli_Chr07	48102586	(G/T)
13263	CakSNP13263	*Kabuli*	Ca_Kabuli_Chr07	48200865	(A/G)
13264	CakSNP13264	*Kabuli*	Ca_Kabuli_Chr07	48200852	(G/A)
13265	CakSNP13265	*Kabuli*	Ca_Kabuli_Chr07	48200822	(G/A)
13266	CakSNP13266	*Kabuli*	Ca_Kabuli_Chr07	48200820	(A/C)
13267	CakSNP13267	*Kabuli*	Ca_Kabuli_Chr07	48200823	(G/A)
13268	CakSNP13268	*Kabuli*	Ca_Kabuli_Chr07	48200848	(T/G)
13269	CakSNP13269	*Kabuli*	Ca_Kabuli_Chr07	48509506	(G/T)
13270	CakSNP13270	*Kabuli*	Ca_Kabuli_Chr08	54095	(A/C)
13271	CakSNP13271	*Kabuli*	Ca_Kabuli_Chr08	85693	(T/G)
13272	CakSNP13272	*Kabuli*	Ca_Kabuli_Chr08	85697	(G/A)
13273	CakSNP13273	*Kabuli*	Ca_Kabuli_Chr08	85707	(C/A)
13274	CakSNP13274	*Kabuli*	Ca_Kabuli_Chr08	85710	(C/A)
13275	CakSNP13275	*Kabuli*	Ca_Kabuli_Chr08	158986	(C/G)
13276	CakSNP13276	*Kabuli*	Ca_Kabuli_Chr08	177457	(C/T)
13277	CakSNP13277	*Kabuli*	Ca_Kabuli_Chr08	177436	(A/G)
13278	CakSNP13278	*Kabuli*	Ca_Kabuli_Chr08	177405	(G/A)
13279	CakSNP13279	*Kabuli*	Ca_Kabuli_Chr08	184444	(G/A)
13280	CakSNP13280	*Kabuli*	Ca_Kabuli_Chr08	218813	(C/T)
13281	CakSNP13281	*Kabuli*	Ca_Kabuli_Chr08	218828	(T/G)
13282	CakSNP13282	*Kabuli*	Ca_Kabuli_Chr08	218957	(G/T)
13283	CakSNP13283	*Kabuli*	Ca_Kabuli_Chr08	218928	(A/G)
13284	CakSNP13284	*Kabuli*	Ca_Kabuli_Chr08	249654	(A/C)
13285	CakSNP13285	*Kabuli*	Ca_Kabuli_Chr08	253786	(G/A)
13286	CakSNP13286	*Kabuli*	Ca_Kabuli_Chr08	281622	(G/A)
13287	CakSNP13287	*Kabuli*	Ca_Kabuli_Chr08	281608	(T/A)
13288	CakSNP13288	*Kabuli*	Ca_Kabuli_Chr08	281630	(A/C)
13289	CakSNP13289	*Kabuli*	Ca_Kabuli_Chr08	283147	(C/T)
13290	CakSNP13290	*Kabuli*	Ca_Kabuli_Chr08	285779	(G/A)
13291	CakSNP13291	*Kabuli*	Ca_Kabuli_Chr08	356295	(A/T)
13292	CakSNP13292	*Kabuli*	Ca_Kabuli_Chr08	411967	(G/A)
13293	CakSNP13293	*Kabuli*	Ca_Kabuli_Chr08	499819	(A/C)
13294	CakSNP13294	*Kabuli*	Ca_Kabuli_Chr08	499890	(T/C)
13295	CakSNP13295	*Kabuli*	Ca_Kabuli_Chr08	510824	(G/A)
13296	CakSNP13296	*Kabuli*	Ca_Kabuli_Chr08	560477	(C/T)
13297	CakSNP13297	*Kabuli*	Ca_Kabuli_Chr08	560451	(C/T)

S.N.	SNP IDs	Cultivars	Chromosomes/scaffolds	Physical positions (bp)	SNPs
13298	CakSNP13298	*Kabuli*	Ca_Kabuli_Chr08	560604	(T/G)
13299	CakSNP13299	*Kabuli*	Ca_Kabuli_Chr08	560746	(A/G)
13300	CakSNP13300	*Kabuli*	Ca_Kabuli_Chr08	566112	(A/G)
13301	CakSNP13301	*Kabuli*	Ca_Kabuli_Chr08	566107	(C/T)
13302	CakSNP13302	*Kabuli*	Ca_Kabuli_Chr08	566094	(A/G)
13303	CakSNP13303	*Kabuli*	Ca_Kabuli_Chr08	596171	(C/T)
13304	CakSNP13304	*Kabuli*	Ca_Kabuli_Chr08	614324	(G/A)
13305	CakSNP13305	*Kabuli*	Ca_Kabuli_Chr08	614301	(C/A)
13306	CakSNP13306	*Kabuli*	Ca_Kabuli_Chr08	619605	(T/C)
13307	CakSNP13307	*Kabuli*	Ca_Kabuli_Chr08	634862	(C/T)
13308	CakSNP13308	*Kabuli*	Ca_Kabuli_Chr08	634843	(G/A)
13309	CakSNP13309	*Kabuli*	Ca_Kabuli_Chr08	634818	(G/T)
13310	CakSNP13310	*Kabuli*	Ca_Kabuli_Chr08	634921	(C/G)
13311	CakSNP13311	*Kabuli*	Ca_Kabuli_Chr08	649491	(A/C)
13312	CakSNP13312	*Kabuli*	Ca_Kabuli_Chr08	660610	(T/G)
13313	CakSNP13313	*Kabuli*	Ca_Kabuli_Chr08	666294	(G/A)
13314	CakSNP13314	*Kabuli*	Ca_Kabuli_Chr08	666288	(A/T)
13315	CakSNP13315	*Kabuli*	Ca_Kabuli_Chr08	666284	(T/A)
13316	CakSNP13316	*Kabuli*	Ca_Kabuli_Chr08	666274	(T/G)
13317	CakSNP13317	*Kabuli*	Ca_Kabuli_Chr08	672962	(C/T)
13318	CakSNP13318	*Kabuli*	Ca_Kabuli_Chr08	673820	(A/G)
13319	CakSNP13319	*Kabuli*	Ca_Kabuli_Chr08	674902	(T/G)
13320	CakSNP13320	*Kabuli*	Ca_Kabuli_Chr08	701803	(C/T)
13321	CakSNP13321	*Kabuli*	Ca_Kabuli_Chr08	701811	(T/G)
13322	CakSNP13322	*Kabuli*	Ca_Kabuli_Chr08	701837	(G/A)
13323	CakSNP13323	*Kabuli*	Ca_Kabuli_Chr08	706514	(T/C)
13324	CakSNP13324	*Kabuli*	Ca_Kabuli_Chr08	706482	(G/C)
13325	CakSNP13325	*Kabuli*	Ca_Kabuli_Chr08	706614	(C/T)
13326	CakSNP13326	*Kabuli*	Ca_Kabuli_Chr08	706793	(A/G)
13327	CakSNP13327	*Kabuli*	Ca_Kabuli_Chr08	706800	(G/A)
13328	CakSNP13328	*Kabuli*	Ca_Kabuli_Chr08	709063	(T/C)
13329	CakSNP13329	*Kabuli*	Ca_Kabuli_Chr08	709094	(T/C)
13330	CakSNP13330	*Kabuli*	Ca_Kabuli_Chr08	709124	(A/T)
13331	CakSNP13331	*Kabuli*	Ca_Kabuli_Chr08	714967	(A/G)
13332	CakSNP13332	*Kabuli*	Ca_Kabuli_Chr08	714974	(G/A)
13333	CakSNP13333	*Kabuli*	Ca_Kabuli_Chr08	777760	(A/T)
13334	CakSNP13334	*Kabuli*	Ca_Kabuli_Chr08	777772	(G/A)
13335	CakSNP13335	*Kabuli*	Ca_Kabuli_Chr08	777776	(C/G)
13336	CakSNP13336	*Kabuli*	Ca_Kabuli_Chr08	779589	(C/G)

S.N.	SNP IDs	Cultivars	Chromosomes/scaffolds	Physical positions (bp)	SNPs
13337	CakSNP13337	*Kabuli*	Ca_Kabuli_Chr08	779611	(T/C)
13338	CakSNP13338	*Kabuli*	Ca_Kabuli_Chr08	779640	(T/C)
13339	CakSNP13339	*Kabuli*	Ca_Kabuli_Chr08	817321	(T/G)
13340	CakSNP13340	*Kabuli*	Ca_Kabuli_Chr08	822302	(A/G)
13341	CakSNP13341	*Kabuli*	Ca_Kabuli_Chr08	822942	(T/G)
13342	CakSNP13342	*Kabuli*	Ca_Kabuli_Chr08	822976	(C/T)
13343	CakSNP13343	*Kabuli*	Ca_Kabuli_Chr08	839924	(C/G)
13344	CakSNP13344	*Kabuli*	Ca_Kabuli_Chr08	855332	(G/A)
13345	CakSNP13345	*Kabuli*	Ca_Kabuli_Chr08	866741	(A/C)
13346	CakSNP13346	*Kabuli*	Ca_Kabuli_Chr08	871666	(C/G)
13347	CakSNP13347	*Kabuli*	Ca_Kabuli_Chr08	875236	(T/G)
13348	CakSNP13348	*Kabuli*	Ca_Kabuli_Chr08	894184	(C/A)
13349	CakSNP13349	*Kabuli*	Ca_Kabuli_Chr08	895883	(G/A)
13350	CakSNP13350	*Kabuli*	Ca_Kabuli_Chr08	895947	(C/T)
13351	CakSNP13351	*Kabuli*	Ca_Kabuli_Chr08	897417	(T/C)
13352	CakSNP13352	*Kabuli*	Ca_Kabuli_Chr08	898089	(C/T)
13353	CakSNP13353	*Kabuli*	Ca_Kabuli_Chr08	898150	(C/A)
13354	CakSNP13354	*Kabuli*	Ca_Kabuli_Chr08	898152	(A/G)
13355	CakSNP13355	*Kabuli*	Ca_Kabuli_Chr08	898173	(G/T)
13356	CakSNP13356	*Kabuli*	Ca_Kabuli_Chr08	898164	(G/T)
13357	CakSNP13357	*Kabuli*	Ca_Kabuli_Chr08	898226	(C/G)
13358	CakSNP13358	*Kabuli*	Ca_Kabuli_Chr08	898251	(G/T)
13359	CakSNP13359	*Kabuli*	Ca_Kabuli_Chr08	919690	(T/C)
13360	CakSNP13360	*Kabuli*	Ca_Kabuli_Chr08	923820	(T/C)
13361	CakSNP13361	*Kabuli*	Ca_Kabuli_Chr08	924720	(G/T)
13362	CakSNP13362	*Kabuli*	Ca_Kabuli_Chr08	924681	(C/T)
13363	CakSNP13363	*Kabuli*	Ca_Kabuli_Chr08	924715	(A/G)
13364	CakSNP13364	*Kabuli*	Ca_Kabuli_Chr08	924732	(G/T)
13365	CakSNP13365	*Kabuli*	Ca_Kabuli_Chr08	974170	(T/C)
13366	CakSNP13366	*Kabuli*	Ca_Kabuli_Chr08	974159	(T/G)
13367	CakSNP13367	*Kabuli*	Ca_Kabuli_Chr08	1011333	(C/T)
13368	CakSNP13368	*Kabuli*	Ca_Kabuli_Chr08	1011320	(G/T)
13369	CakSNP13369	*Kabuli*	Ca_Kabuli_Chr08	1011327	(G/A)
13370	CakSNP13370	*Kabuli*	Ca_Kabuli_Chr08	1043905	(T/C)
13371	CakSNP13371	*Kabuli*	Ca_Kabuli_Chr08	1070728	(T/C)
13372	CakSNP13372	*Kabuli*	Ca_Kabuli_Chr08	1077334	(C/T)
13373	CakSNP13373	*Kabuli*	Ca_Kabuli_Chr08	1077430	(C/T)
13374	CakSNP13374	*Kabuli*	Ca_Kabuli_Chr08	1119568	(T/C)
13375	CakSNP13375	*Kabuli*	Ca_Kabuli_Chr08	1119781	(A/G)

S.N.	SNP IDs	Cultivars	Chromosomes/scaffolds	Physical positions (bp)	SNPs
13376	CakSNP13376	*Kabuli*	Ca_Kabuli_Chr08	1119773	(T/G)
13377	CakSNP13377	*Kabuli*	Ca_Kabuli_Chr08	1119761	(G/T)
13378	CakSNP13378	*Kabuli*	Ca_Kabuli_Chr08	1138275	(G/A)
13379	CakSNP13379	*Kabuli*	Ca_Kabuli_Chr08	1138394	(A/G)
13380	CakSNP13380	*Kabuli*	Ca_Kabuli_Chr08	1155597	(C/T)
13381	CakSNP13381	*Kabuli*	Ca_Kabuli_Chr08	1155636	(T/C)
13382	CakSNP13382	*Kabuli*	Ca_Kabuli_Chr08	1155639	(T/A)
13383	CakSNP13383	*Kabuli*	Ca_Kabuli_Chr08	1155641	(C/G)
13384	CakSNP13384	*Kabuli*	Ca_Kabuli_Chr08	1174369	(A/G)
13385	CakSNP13385	*Kabuli*	Ca_Kabuli_Chr08	1193297	(C/T)
13386	CakSNP13386	*Kabuli*	Ca_Kabuli_Chr08	1194886	(G/A)
13387	CakSNP13387	*Kabuli*	Ca_Kabuli_Chr08	1194888	(A/G)
13388	CakSNP13388	*Kabuli*	Ca_Kabuli_Chr08	1241739	(C/T)
13389	CakSNP13389	*Kabuli*	Ca_Kabuli_Chr08	1262576	(T/C)
13390	CakSNP13390	*Kabuli*	Ca_Kabuli_Chr08	1262561	(C/T)
13391	CakSNP13391	*Kabuli*	Ca_Kabuli_Chr08	1262660	(C/A)
13392	CakSNP13392	*Kabuli*	Ca_Kabuli_Chr08	1262686	(T/G)
13393	CakSNP13393	*Kabuli*	Ca_Kabuli_Chr08	1265537	(G/A)
13394	CakSNP13394	*Kabuli*	Ca_Kabuli_Chr08	1265535	(A/G)
13395	CakSNP13395	*Kabuli*	Ca_Kabuli_Chr08	1265509	(T/C)
13396	CakSNP13396	*Kabuli*	Ca_Kabuli_Chr08	1265595	(G/T)
13397	CakSNP13397	*Kabuli*	Ca_Kabuli_Chr08	1265579	(A/G)
13398	CakSNP13398	*Kabuli*	Ca_Kabuli_Chr08	1265577	(T/G)
13399	CakSNP13399	*Kabuli*	Ca_Kabuli_Chr08	1382266	(G/A)
13400	CakSNP13400	*Kabuli*	Ca_Kabuli_Chr08	1382433	(G/T)
13401	CakSNP13401	*Kabuli*	Ca_Kabuli_Chr08	1382454	(A/C)
13402	CakSNP13402	*Kabuli*	Ca_Kabuli_Chr08	1433436	(T/C)
13403	CakSNP13403	*Kabuli*	Ca_Kabuli_Chr08	1477523	(T/C)
13404	CakSNP13404	*Kabuli*	Ca_Kabuli_Chr08	1487202	(G/C)
13405	CakSNP13405	*Kabuli*	Ca_Kabuli_Chr08	1503621	(C/T)
13406	CakSNP13406	*Kabuli*	Ca_Kabuli_Chr08	1503595	(C/T)
13407	CakSNP13407	*Kabuli*	Ca_Kabuli_Chr08	1503588	(T/C)
13408	CakSNP13408	*Kabuli*	Ca_Kabuli_Chr08	1504859	(A/G)
13409	CakSNP13409	*Kabuli*	Ca_Kabuli_Chr08	1507709	(T/G)
13410	CakSNP13410	*Kabuli*	Ca_Kabuli_Chr08	1507782	(G/C)
13411	CakSNP13411	*Kabuli*	Ca_Kabuli_Chr08	1518285	(A/C)
13412	CakSNP13412	*Kabuli*	Ca_Kabuli_Chr08	1518297	(T/G)
13413	CakSNP13413	*Kabuli*	Ca_Kabuli_Chr08	1518355	(G/A)
13414	CakSNP13414	*Kabuli*	Ca_Kabuli_Chr08	1534461	(A/C)

S.N.	SNP IDs	Cultivars	Chromosomes/scaffolds	Physical positions (bp)	SNPs
13415	CakSNP13415	*Kabuli*	Ca_Kabuli_Chr08	1544916	(C/T)
13416	CakSNP13416	*Kabuli*	Ca_Kabuli_Chr08	1552082	(T/A)
13417	CakSNP13417	*Kabuli*	Ca_Kabuli_Chr08	1552143	(A/G)
13418	CakSNP13418	*Kabuli*	Ca_Kabuli_Chr08	1554964	(T/C)
13419	CakSNP13419	*Kabuli*	Ca_Kabuli_Chr08	1555017	(C/A)
13420	CakSNP13420	*Kabuli*	Ca_Kabuli_Chr08	1555122	(A/G)
13421	CakSNP13421	*Kabuli*	Ca_Kabuli_Chr08	1557492	(C/T)
13422	CakSNP13422	*Kabuli*	Ca_Kabuli_Chr08	1573877	(G/T)
13423	CakSNP13423	*Kabuli*	Ca_Kabuli_Chr08	1612107	(G/A)
13424	CakSNP13424	*Kabuli*	Ca_Kabuli_Chr08	1612099	(A/G)
13425	CakSNP13425	*Kabuli*	Ca_Kabuli_Chr08	1612221	(C/G)
13426	CakSNP13426	*Kabuli*	Ca_Kabuli_Chr08	1667832	(T/C)
13427	CakSNP13427	*Kabuli*	Ca_Kabuli_Chr08	1675895	(C/T)
13428	CakSNP13428	*Kabuli*	Ca_Kabuli_Chr08	1675888	(C/T)
13429	CakSNP13429	*Kabuli*	Ca_Kabuli_Chr08	1708743	(C/T)
13430	CakSNP13430	*Kabuli*	Ca_Kabuli_Chr08	1708860	(T/C)
13431	CakSNP13431	*Kabuli*	Ca_Kabuli_Chr08	1718032	(C/T)
13432	CakSNP13432	*Kabuli*	Ca_Kabuli_Chr08	1723593	(G/A)
13433	CakSNP13433	*Kabuli*	Ca_Kabuli_Chr08	1743097	(G/A)
13434	CakSNP13434	*Kabuli*	Ca_Kabuli_Chr08	1747523	(T/A)
13435	CakSNP13435	*Kabuli*	Ca_Kabuli_Chr08	1747522	(T/C)
13436	CakSNP13436	*Kabuli*	Ca_Kabuli_Chr08	1782034	(C/G)
13437	CakSNP13437	*Kabuli*	Ca_Kabuli_Chr08	1794755	(C/A)
13438	CakSNP13438	*Kabuli*	Ca_Kabuli_Chr08	1794756	(G/T)
13439	CakSNP13439	*Kabuli*	Ca_Kabuli_Chr08	1794759	(C/T)
13440	CakSNP13440	*Kabuli*	Ca_Kabuli_Chr08	1794810	(G/A)
13441	CakSNP13441	*Kabuli*	Ca_Kabuli_Chr08	1817388	(T/C)
13442	CakSNP13442	*Kabuli*	Ca_Kabuli_Chr08	1830230	(A/T)
13443	CakSNP13443	*Kabuli*	Ca_Kabuli_Chr08	1830348	(T/C)
13444	CakSNP13444	*Kabuli*	Ca_Kabuli_Chr08	1846478	(A/G)
13445	CakSNP13445	*Kabuli*	Ca_Kabuli_Chr08	1893613	(T/C)
13446	CakSNP13446	*Kabuli*	Ca_Kabuli_Chr08	1932975	(T/C)
13447	CakSNP13447	*Kabuli*	Ca_Kabuli_Chr08	1932981	(T/C)
13448	CakSNP13448	*Kabuli*	Ca_Kabuli_Chr08	1932990	(A/C)
13449	CakSNP13449	*Kabuli*	Ca_Kabuli_Chr08	1932993	(C/T)
13450	CakSNP13450	*Kabuli*	Ca_Kabuli_Chr08	1932997	(G/T)
13451	CakSNP13451	*Kabuli*	Ca_Kabuli_Chr08	1933026	(G/A)
13452	CakSNP13452	*Kabuli*	Ca_Kabuli_Chr08	1933064	(G/C)
13453	CakSNP13453	*Kabuli*	Ca_Kabuli_Chr08	1933053	(G/A)

S.N.	SNP IDs	Cultivars	Chromosomes/scaffolds	Physical positions (bp)	SNPs
13454	CakSNP13454	*Kabuli*	Ca_Kabuli_Chr08	1933052	(T/C)
13455	CakSNP13455	*Kabuli*	Ca_Kabuli_Chr08	1933039	(G/T)
13456	CakSNP13456	*Kabuli*	Ca_Kabuli_Chr08	1933035	(T/A)
13457	CakSNP13457	*Kabuli*	Ca_Kabuli_Chr08	1958433	(G/C)
13458	CakSNP13458	*Kabuli*	Ca_Kabuli_Chr08	1958415	(T/G)
13459	CakSNP13459	*Kabuli*	Ca_Kabuli_Chr08	1978964	(G/C)
13460	CakSNP13460	*Kabuli*	Ca_Kabuli_Chr08	1980189	(T/A)
13461	CakSNP13461	*Kabuli*	Ca_Kabuli_Chr08	2004509	(A/G)
13462	CakSNP13462	*Kabuli*	Ca_Kabuli_Chr08	2004577	(G/C)
13463	CakSNP13463	*Kabuli*	Ca_Kabuli_Chr08	2029405	(C/A)
13464	CakSNP13464	*Kabuli*	Ca_Kabuli_Chr08	2029384	(T/C)
13465	CakSNP13465	*Kabuli*	Ca_Kabuli_Chr08	2052263	(T/G)
13466	CakSNP13466	*Kabuli*	Ca_Kabuli_Chr08	2052286	(T/C)
13467	CakSNP13467	*Kabuli*	Ca_Kabuli_Chr08	2070694	(G/T)
13468	CakSNP13468	*Kabuli*	Ca_Kabuli_Chr08	2073807	(C/G)
13469	CakSNP13469	*Kabuli*	Ca_Kabuli_Chr08	2074619	(T/G)
13470	CakSNP13470	*Kabuli*	Ca_Kabuli_Chr08	2075296	(T/C)
13471	CakSNP13471	*Kabuli*	Ca_Kabuli_Chr08	2075290	(G/T)
13472	CakSNP13472	*Kabuli*	Ca_Kabuli_Chr08	2075278	(T/G)
13473	CakSNP13473	*Kabuli*	Ca_Kabuli_Chr08	2079723	(A/G)
13474	CakSNP13474	*Kabuli*	Ca_Kabuli_Chr08	2081228	(C/T)
13475	CakSNP13475	*Kabuli*	Ca_Kabuli_Chr08	2088903	(T/C)
13476	CakSNP13476	*Kabuli*	Ca_Kabuli_Chr08	2088892	(T/G)
13477	CakSNP13477	*Kabuli*	Ca_Kabuli_Chr08	2090852	(A/G)
13478	CakSNP13478	*Kabuli*	Ca_Kabuli_Chr08	2095607	(A/G)
13479	CakSNP13479	*Kabuli*	Ca_Kabuli_Chr08	2107370	(C/T)
13480	CakSNP13480	*Kabuli*	Ca_Kabuli_Chr08	2113823	(T/G)
13481	CakSNP13481	*Kabuli*	Ca_Kabuli_Chr08	2113816	(T/C)
13482	CakSNP13482	*Kabuli*	Ca_Kabuli_Chr08	2128450	(A/G)
13483	CakSNP13483	*Kabuli*	Ca_Kabuli_Chr08	2194927	(G/A)
13484	CakSNP13484	*Kabuli*	Ca_Kabuli_Chr08	2194898	(A/T)
13485	CakSNP13485	*Kabuli*	Ca_Kabuli_Chr08	2250195	(C/G)
13486	CakSNP13486	*Kabuli*	Ca_Kabuli_Chr08	2250148	(T/C)
13487	CakSNP13487	*Kabuli*	Ca_Kabuli_Chr08	2264108	(G/A)
13488	CakSNP13488	*Kabuli*	Ca_Kabuli_Chr08	2279413	(G/A)
13489	CakSNP13489	*Kabuli*	Ca_Kabuli_Chr08	2309552	(C/G)
13490	CakSNP13490	*Kabuli*	Ca_Kabuli_Chr08	2309549	(A/G)
13491	CakSNP13491	*Kabuli*	Ca_Kabuli_Chr08	2413731	(A/G)
13492	CakSNP13492	*Kabuli*	Ca_Kabuli_Chr08	2456352	(T/C)

S.N.	SNP IDs	Cultivars	Chromosomes/scaffolds	Physical positions (bp)	SNPs
13493	CakSNP13493	*Kabuli*	Ca_Kabuli_Chr08	2482834	(A/G)
13494	CakSNP13494	*Kabuli*	Ca_Kabuli_Chr08	2482849	(T/C)
13495	CakSNP13495	*Kabuli*	Ca_Kabuli_Chr08	2483021	(G/A)
13496	CakSNP13496	*Kabuli*	Ca_Kabuli_Chr08	2488709	(A/G)
13497	CakSNP13497	*Kabuli*	Ca_Kabuli_Chr08	2488769	(C/G)
13498	CakSNP13498	*Kabuli*	Ca_Kabuli_Chr08	2488772	(A/G)
13499	CakSNP13499	*Kabuli*	Ca_Kabuli_Chr08	2515612	(A/G)
13500	CakSNP13500	*Kabuli*	Ca_Kabuli_Chr08	2524188	(A/G)
13501	CakSNP13501	*Kabuli*	Ca_Kabuli_Chr08	2524148	(A/G)
13502	CakSNP13502	*Kabuli*	Ca_Kabuli_Chr08	2539375	(G/A)
13503	CakSNP13503	*Kabuli*	Ca_Kabuli_Chr08	2540644	(G/A)
13504	CakSNP13504	*Kabuli*	Ca_Kabuli_Chr08	2540704	(A/T)
13505	CakSNP13505	*Kabuli*	Ca_Kabuli_Chr08	2543156	(A/G)
13506	CakSNP13506	*Kabuli*	Ca_Kabuli_Chr08	2545070	(T/A)
13507	CakSNP13507	*Kabuli*	Ca_Kabuli_Chr08	2604759	(C/T)
13508	CakSNP13508	*Kabuli*	Ca_Kabuli_Chr08	2605423	(A/T)
13509	CakSNP13509	*Kabuli*	Ca_Kabuli_Chr08	2605460	(C/T)
13510	CakSNP13510	*Kabuli*	Ca_Kabuli_Chr08	2621132	(T/A)
13511	CakSNP13511	*Kabuli*	Ca_Kabuli_Chr08	2622009	(G/T)
13512	CakSNP13512	*Kabuli*	Ca_Kabuli_Chr08	2621987	(C/G)
13513	CakSNP13513	*Kabuli*	Ca_Kabuli_Chr08	2621973	(T/G)
13514	CakSNP13514	*Kabuli*	Ca_Kabuli_Chr08	2651439	(G/T)
13515	CakSNP13515	*Kabuli*	Ca_Kabuli_Chr08	2651425	(C/T)
13516	CakSNP13516	*Kabuli*	Ca_Kabuli_Chr08	2651422	(T/C)
13517	CakSNP13517	*Kabuli*	Ca_Kabuli_Chr08	2651393	(G/T)
13518	CakSNP13518	*Kabuli*	Ca_Kabuli_Chr08	2651373	(G/T)
13519	CakSNP13519	*Kabuli*	Ca_Kabuli_Chr08	2651921	(C/T)
13520	CakSNP13520	*Kabuli*	Ca_Kabuli_Chr08	2664625	(T/C)
13521	CakSNP13521	*Kabuli*	Ca_Kabuli_Chr08	2664711	(T/G)
13522	CakSNP13522	*Kabuli*	Ca_Kabuli_Chr08	2722673	(C/A)
13523	CakSNP13523	*Kabuli*	Ca_Kabuli_Chr08	2735849	(T/C)
13524	CakSNP13524	*Kabuli*	Ca_Kabuli_Chr08	2743075	(T/C)
13525	CakSNP13525	*Kabuli*	Ca_Kabuli_Chr08	2743088	(A/G)
13526	CakSNP13526	*Kabuli*	Ca_Kabuli_Chr08	2743109	(G/C)
13527	CakSNP13527	*Kabuli*	Ca_Kabuli_Chr08	2743139	(A/T)
13528	CakSNP13528	*Kabuli*	Ca_Kabuli_Chr08	2783576	(T/G)
13529	CakSNP13529	*Kabuli*	Ca_Kabuli_Chr08	2783655	(G/C)
13530	CakSNP13530	*Kabuli*	Ca_Kabuli_Chr08	2827822	(C/T)
13531	CakSNP13531	*Kabuli*	Ca_Kabuli_Chr08	2827984	(T/C)

S.N.	SNP IDs	Cultivars	Chromosomes/scaffolds	Physical positions (bp)	SNPs
13532	CakSNP13532	*Kabuli*	Ca_Kabuli_Chr08	2838179	(T/A)
13533	CakSNP13533	*Kabuli*	Ca_Kabuli_Chr08	2871095	(G/A)
13534	CakSNP13534	*Kabuli*	Ca_Kabuli_Chr08	2871064	(G/A)
13535	CakSNP13535	*Kabuli*	Ca_Kabuli_Chr08	2888353	(G/T)
13536	CakSNP13536	*Kabuli*	Ca_Kabuli_Chr08	2888317	(G/A)
13537	CakSNP13537	*Kabuli*	Ca_Kabuli_Chr08	2943631	(G/T)
13538	CakSNP13538	*Kabuli*	Ca_Kabuli_Chr08	2947498	(A/G)
13539	CakSNP13539	*Kabuli*	Ca_Kabuli_Chr08	2974759	(G/C)
13540	CakSNP13540	*Kabuli*	Ca_Kabuli_Chr08	2976709	(C/T)
13541	CakSNP13541	*Kabuli*	Ca_Kabuli_Chr08	2977543	(T/C)
13542	CakSNP13542	*Kabuli*	Ca_Kabuli_Chr08	2977628	(C/T)
13543	CakSNP13543	*Kabuli*	Ca_Kabuli_Chr08	2977603	(T/A)
13544	CakSNP13544	*Kabuli*	Ca_Kabuli_Chr08	3013928	(G/C)
13545	CakSNP13545	*Kabuli*	Ca_Kabuli_Chr08	3013917	(G/A)
13546	CakSNP13546	*Kabuli*	Ca_Kabuli_Chr08	3041947	(A/G)
13547	CakSNP13547	*Kabuli*	Ca_Kabuli_Chr08	3041981	(A/G)
13548	CakSNP13548	*Kabuli*	Ca_Kabuli_Chr08	3050452	(T/C)
13549	CakSNP13549	*Kabuli*	Ca_Kabuli_Chr08	3064477	(G/A)
13550	CakSNP13550	*Kabuli*	Ca_Kabuli_Chr08	3141617	(C/T)
13551	CakSNP13551	*Kabuli*	Ca_Kabuli_Chr08	3171324	(C/T)
13552	CakSNP13552	*Kabuli*	Ca_Kabuli_Chr08	3210879	(C/A)
13553	CakSNP13553	*Kabuli*	Ca_Kabuli_Chr08	3248044	(A/G)
13554	CakSNP13554	*Kabuli*	Ca_Kabuli_Chr08	3248104	(A/G)
13555	CakSNP13555	*Kabuli*	Ca_Kabuli_Chr08	3250707	(C/T)
13556	CakSNP13556	*Kabuli*	Ca_Kabuli_Chr08	3263822	(T/C)
13557	CakSNP13557	*Kabuli*	Ca_Kabuli_Chr08	3269124	(G/A)
13558	CakSNP13558	*Kabuli*	Ca_Kabuli_Chr08	3269118	(A/G)
13559	CakSNP13559	*Kabuli*	Ca_Kabuli_Chr08	3269088	(A/C)
13560	CakSNP13560	*Kabuli*	Ca_Kabuli_Chr08	3269085	(A/C)
13561	CakSNP13561	*Kabuli*	Ca_Kabuli_Chr08	3297410	(T/C)
13562	CakSNP13562	*Kabuli*	Ca_Kabuli_Chr08	3386165	(C/A)
13563	CakSNP13563	*Kabuli*	Ca_Kabuli_Chr08	3386181	(C/A)
13564	CakSNP13564	*Kabuli*	Ca_Kabuli_Chr08	3392929	(A/C)
13565	CakSNP13565	*Kabuli*	Ca_Kabuli_Chr08	3394796	(A/C)
13566	CakSNP13566	*Kabuli*	Ca_Kabuli_Chr08	3394833	(T/C)
13567	CakSNP13567	*Kabuli*	Ca_Kabuli_Chr08	3394931	(T/C)
13568	CakSNP13568	*Kabuli*	Ca_Kabuli_Chr08	3396820	(T/C)
13569	CakSNP13569	*Kabuli*	Ca_Kabuli_Chr08	3438274	(A/T)
13570	CakSNP13570	*Kabuli*	Ca_Kabuli_Chr08	3445568	(G/T)

S.N.	SNP IDs	Cultivars	Chromosomes/scaffolds	Physical positions (bp)	SNPs
13571	CakSNP13571	*Kabuli*	Ca_Kabuli_Chr08	3448194	(C/T)
13572	CakSNP13572	*Kabuli*	Ca_Kabuli_Chr08	3460627	(G/A)
13573	CakSNP13573	*Kabuli*	Ca_Kabuli_Chr08	3499010	(G/A)
13574	CakSNP13574	*Kabuli*	Ca_Kabuli_Chr08	3539690	(A/G)
13575	CakSNP13575	*Kabuli*	Ca_Kabuli_Chr08	3539723	(G/C)
13576	CakSNP13576	*Kabuli*	Ca_Kabuli_Chr08	3539745	(T/C)
13577	CakSNP13577	*Kabuli*	Ca_Kabuli_Chr08	3539851	(T/C)
13578	CakSNP13578	*Kabuli*	Ca_Kabuli_Chr08	3539838	(T/G)
13579	CakSNP13579	*Kabuli*	Ca_Kabuli_Chr08	3539832	(T/C)
13580	CakSNP13580	*Kabuli*	Ca_Kabuli_Chr08	3560271	(G/A)
13581	CakSNP13581	*Kabuli*	Ca_Kabuli_Chr08	3563692	(A/C)
13582	CakSNP13582	*Kabuli*	Ca_Kabuli_Chr08	3563825	(G/T)
13583	CakSNP13583	*Kabuli*	Ca_Kabuli_Chr08	3563824	(T/A)
13584	CakSNP13584	*Kabuli*	Ca_Kabuli_Chr08	3599091	(G/C)
13585	CakSNP13585	*Kabuli*	Ca_Kabuli_Chr08	3601517	(T/C)
13586	CakSNP13586	*Kabuli*	Ca_Kabuli_Chr08	3605513	(T/C)
13587	CakSNP13587	*Kabuli*	Ca_Kabuli_Chr08	3617810	(T/C)
13588	CakSNP13588	*Kabuli*	Ca_Kabuli_Chr08	3617757	(G/C)
13589	CakSNP13589	*Kabuli*	Ca_Kabuli_Chr08	3723085	(C/T)
13590	CakSNP13590	*Kabuli*	Ca_Kabuli_Chr08	3730369	(A/C)
13591	CakSNP13591	*Kabuli*	Ca_Kabuli_Chr08	3775945	(G/A)
13592	CakSNP13592	*Kabuli*	Ca_Kabuli_Chr08	3775960	(T/C)
13593	CakSNP13593	*Kabuli*	Ca_Kabuli_Chr08	3813978	(C/T)
13594	CakSNP13594	*Kabuli*	Ca_Kabuli_Chr08	3829425	(C/G)
13595	CakSNP13595	*Kabuli*	Ca_Kabuli_Chr08	3829576	(A/G)
13596	CakSNP13596	*Kabuli*	Ca_Kabuli_Chr08	3837237	(A/G)
13597	CakSNP13597	*Kabuli*	Ca_Kabuli_Chr08	3837306	(G/C)
13598	CakSNP13598	*Kabuli*	Ca_Kabuli_Chr08	3868511	(A/C)
13599	CakSNP13599	*Kabuli*	Ca_Kabuli_Chr08	3877130	(G/T)
13600	CakSNP13600	*Kabuli*	Ca_Kabuli_Chr08	3877176	(T/G)
13601	CakSNP13601	*Kabuli*	Ca_Kabuli_Chr08	3911930	(A/C)
13602	CakSNP13602	*Kabuli*	Ca_Kabuli_Chr08	3911876	(G/C)
13603	CakSNP13603	*Kabuli*	Ca_Kabuli_Chr08	3951767	(C/A)
13604	CakSNP13604	*Kabuli*	Ca_Kabuli_Chr08	3951768	(A/G)
13605	CakSNP13605	*Kabuli*	Ca_Kabuli_Chr08	3954853	(T/A)
13606	CakSNP13606	*Kabuli*	Ca_Kabuli_Chr08	4042166	(A/C)
13607	CakSNP13607	*Kabuli*	Ca_Kabuli_Chr08	4042233	(G/A)
13608	CakSNP13608	*Kabuli*	Ca_Kabuli_Chr08	4042424	(G/A)
13609	CakSNP13609	*Kabuli*	Ca_Kabuli_Chr08	4052732	(C/T)

S.N.	SNP IDs	Cultivars	Chromosomes/scaffolds	Physical positions (bp)	SNPs
13610	CakSNP13610	*Kabuli*	Ca_Kabuli_Chr08	4052702	(C/T)
13611	CakSNP13611	*Kabuli*	Ca_Kabuli_Chr08	4073528	(C/T)
13612	CakSNP13612	*Kabuli*	Ca_Kabuli_Chr08	4073603	(A/G)
13613	CakSNP13613	*Kabuli*	Ca_Kabuli_Chr08	4074135	(T/C)
13614	CakSNP13614	*Kabuli*	Ca_Kabuli_Chr08	4091946	(T/C)
13615	CakSNP13615	*Kabuli*	Ca_Kabuli_Chr08	4092348	(T/A)
13616	CakSNP13616	*Kabuli*	Ca_Kabuli_Chr08	4092350	(G/C)
13617	CakSNP13617	*Kabuli*	Ca_Kabuli_Chr08	4095131	(A/C)
13618	CakSNP13618	*Kabuli*	Ca_Kabuli_Chr08	4095119	(G/A)
13619	CakSNP13619	*Kabuli*	Ca_Kabuli_Chr08	4106580	(T/C)
13620	CakSNP13620	*Kabuli*	Ca_Kabuli_Chr08	4106583	(T/C)
13621	CakSNP13621	*Kabuli*	Ca_Kabuli_Chr08	4106644	(C/T)
13622	CakSNP13622	*Kabuli*	Ca_Kabuli_Chr08	4172342	(A/G)
13623	CakSNP13623	*Kabuli*	Ca_Kabuli_Chr08	4172368	(T/C)
13624	CakSNP13624	*Kabuli*	Ca_Kabuli_Chr08	4183744	(A/G)
13625	CakSNP13625	*Kabuli*	Ca_Kabuli_Chr08	4183733	(G/A)
13626	CakSNP13626	*Kabuli*	Ca_Kabuli_Chr08	4198214	(A/C)
13627	CakSNP13627	*Kabuli*	Ca_Kabuli_Chr08	4201285	(A/G)
13628	CakSNP13628	*Kabuli*	Ca_Kabuli_Chr08	4237620	(T/A)
13629	CakSNP13629	*Kabuli*	Ca_Kabuli_Chr08	4271620	(T/A)
13630	CakSNP13630	*Kabuli*	Ca_Kabuli_Chr08	4290690	(T/A)
13631	CakSNP13631	*Kabuli*	Ca_Kabuli_Chr08	4322058	(A/G)
13632	CakSNP13632	*Kabuli*	Ca_Kabuli_Chr08	4322202	(C/T)
13633	CakSNP13633	*Kabuli*	Ca_Kabuli_Chr08	4344459	(C/A)
13634	CakSNP13634	*Kabuli*	Ca_Kabuli_Chr08	4346552	(A/G)
13635	CakSNP13635	*Kabuli*	Ca_Kabuli_Chr08	4346749	(A/G)
13636	CakSNP13636	*Kabuli*	Ca_Kabuli_Chr08	4346811	(C/T)
13637	CakSNP13637	*Kabuli*	Ca_Kabuli_Chr08	4346827	(A/C)
13638	CakSNP13638	*Kabuli*	Ca_Kabuli_Chr08	4347535	(A/C)
13639	CakSNP13639	*Kabuli*	Ca_Kabuli_Chr08	4351751	(G/A)
13640	CakSNP13640	*Kabuli*	Ca_Kabuli_Chr08	4353397	(A/C)
13641	CakSNP13641	*Kabuli*	Ca_Kabuli_Chr08	4355388	(G/T)
13642	CakSNP13642	*Kabuli*	Ca_Kabuli_Chr08	4355531	(A/C)
13643	CakSNP13643	*Kabuli*	Ca_Kabuli_Chr08	4368163	(T/C)
13644	CakSNP13644	*Kabuli*	Ca_Kabuli_Chr08	4368184	(T/C)
13645	CakSNP13645	*Kabuli*	Ca_Kabuli_Chr08	4368213	(T/C)
13646	CakSNP13646	*Kabuli*	Ca_Kabuli_Chr08	4368403	(G/A)
13647	CakSNP13647	*Kabuli*	Ca_Kabuli_Chr08	4368463	(A/G)
13648	CakSNP13648	*Kabuli*	Ca_Kabuli_Chr08	4392707	(T/C)

S.N.	SNP IDs	Cultivars	Chromosomes/scaffolds	Physical positions (bp)	SNPs
13649	CakSNP13649	*Kabuli*	Ca_Kabuli_Chr08	4392767	(T/G)
13650	CakSNP13650	*Kabuli*	Ca_Kabuli_Chr08	4392818	(C/A)
13651	CakSNP13651	*Kabuli*	Ca_Kabuli_Chr08	4406956	(C/G)
13652	CakSNP13652	*Kabuli*	Ca_Kabuli_Chr08	4421609	(A/G)
13653	CakSNP13653	*Kabuli*	Ca_Kabuli_Chr08	4421629	(T/A)
13654	CakSNP13654	*Kabuli*	Ca_Kabuli_Chr08	4472423	(A/T)
13655	CakSNP13655	*Kabuli*	Ca_Kabuli_Chr08	4483130	(T/C)
13656	CakSNP13656	*Kabuli*	Ca_Kabuli_Chr08	4483274	(T/C)
13657	CakSNP13657	*Kabuli*	Ca_Kabuli_Chr08	4484606	(T/A)
13658	CakSNP13658	*Kabuli*	Ca_Kabuli_Chr08	4507729	(G/T)
13659	CakSNP13659	*Kabuli*	Ca_Kabuli_Chr08	4510845	(C/T)
13660	CakSNP13660	*Kabuli*	Ca_Kabuli_Chr08	4510810	(A/G)
13661	CakSNP13661	*Kabuli*	Ca_Kabuli_Chr08	4512311	(T/C)
13662	CakSNP13662	*Kabuli*	Ca_Kabuli_Chr08	4512436	(G/A)
13663	CakSNP13663	*Kabuli*	Ca_Kabuli_Chr08	4512492	(T/G)
13664	CakSNP13664	*Kabuli*	Ca_Kabuli_Chr08	4524914	(C/T)
13665	CakSNP13665	*Kabuli*	Ca_Kabuli_Chr08	4557334	(A/G)
13666	CakSNP13666	*Kabuli*	Ca_Kabuli_Chr08	4557328	(T/C)
13667	CakSNP13667	*Kabuli*	Ca_Kabuli_Chr08	4557401	(A/C)
13668	CakSNP13668	*Kabuli*	Ca_Kabuli_Chr08	4557407	(A/C)
13669	CakSNP13669	*Kabuli*	Ca_Kabuli_Chr08	4557440	(A/G)
13670	CakSNP13670	*Kabuli*	Ca_Kabuli_Chr08	4557488	(C/T)
13671	CakSNP13671	*Kabuli*	Ca_Kabuli_Chr08	4580363	(C/A)
13672	CakSNP13672	*Kabuli*	Ca_Kabuli_Chr08	4588869	(T/C)
13673	CakSNP13673	*Kabuli*	Ca_Kabuli_Chr08	4588915	(A/C)
13674	CakSNP13674	*Kabuli*	Ca_Kabuli_Chr08	4611094	(C/T)
13675	CakSNP13675	*Kabuli*	Ca_Kabuli_Chr08	4658502	(A/G)
13676	CakSNP13676	*Kabuli*	Ca_Kabuli_Chr08	4714566	(G/A)
13677	CakSNP13677	*Kabuli*	Ca_Kabuli_Chr08	4744388	(C/T)
13678	CakSNP13678	*Kabuli*	Ca_Kabuli_Chr08	4767567	(A/C)
13679	CakSNP13679	*Kabuli*	Ca_Kabuli_Chr08	4767586	(C/G)
13680	CakSNP13680	*Kabuli*	Ca_Kabuli_Chr08	4773205	(A/C)
13681	CakSNP13681	*Kabuli*	Ca_Kabuli_Chr08	4810954	(A/C)
13682	CakSNP13682	*Kabuli*	Ca_Kabuli_Chr08	4810968	(G/A)
13683	CakSNP13683	*Kabuli*	Ca_Kabuli_Chr08	4810986	(A/C)
13684	CakSNP13684	*Kabuli*	Ca_Kabuli_Chr08	4858262	(C/A)
13685	CakSNP13685	*Kabuli*	Ca_Kabuli_Chr08	4899964	(T/G)
13686	CakSNP13686	*Kabuli*	Ca_Kabuli_Chr08	4899983	(G/A)
13687	CakSNP13687	*Kabuli*	Ca_Kabuli_Chr08	4980949	(A/T)

S.N.	SNP IDs	Cultivars	Chromosomes/scaffolds	Physical positions (bp)	SNPs
13688	CakSNP13688	*Kabuli*	Ca_Kabuli_Chr08	4980916	(C/T)
13689	CakSNP13689	*Kabuli*	Ca_Kabuli_Chr08	5044456	(T/C)
13690	CakSNP13690	*Kabuli*	Ca_Kabuli_Chr08	5044423	(T/A)
13691	CakSNP13691	*Kabuli*	Ca_Kabuli_Chr08	5096194	(T/G)
13692	CakSNP13692	*Kabuli*	Ca_Kabuli_Chr08	5115451	(C/G)
13693	CakSNP13693	*Kabuli*	Ca_Kabuli_Chr08	5115498	(A/T)
13694	CakSNP13694	*Kabuli*	Ca_Kabuli_Chr08	5115512	(T/G)
13695	CakSNP13695	*Kabuli*	Ca_Kabuli_Chr08	5115522	(G/C)
13696	CakSNP13696	*Kabuli*	Ca_Kabuli_Chr08	5134706	(A/C)
13697	CakSNP13697	*Kabuli*	Ca_Kabuli_Chr08	5184227	(C/G)
13698	CakSNP13698	*Kabuli*	Ca_Kabuli_Chr08	5184276	(G/A)
13699	CakSNP13699	*Kabuli*	Ca_Kabuli_Chr08	5197004	(G/T)
13700	CakSNP13700	*Kabuli*	Ca_Kabuli_Chr08	5197254	(G/A)
13701	CakSNP13701	*Kabuli*	Ca_Kabuli_Chr08	5204956	(A/G)
13702	CakSNP13702	*Kabuli*	Ca_Kabuli_Chr08	5313792	(T/C)
13703	CakSNP13703	*Kabuli*	Ca_Kabuli_Chr08	5313782	(T/G)
13704	CakSNP13704	*Kabuli*	Ca_Kabuli_Chr08	5313774	(C/G)
13705	CakSNP13705	*Kabuli*	Ca_Kabuli_Chr08	5313770	(A/T)
13706	CakSNP13706	*Kabuli*	Ca_Kabuli_Chr08	5379556	(G/A)
13707	CakSNP13707	*Kabuli*	Ca_Kabuli_Chr08	5390415	(C/T)
13708	CakSNP13708	*Kabuli*	Ca_Kabuli_Chr08	5390602	(T/C)
13709	CakSNP13709	*Kabuli*	Ca_Kabuli_Chr08	5399503	(G/A)
13710	CakSNP13710	*Kabuli*	Ca_Kabuli_Chr08	5400188	(A/G)
13711	CakSNP13711	*Kabuli*	Ca_Kabuli_Chr08	5412501	(A/G)
13712	CakSNP13712	*Kabuli*	Ca_Kabuli_Chr08	5412489	(T/C)
13713	CakSNP13713	*Kabuli*	Ca_Kabuli_Chr08	5434223	(C/T)
13714	CakSNP13714	*Kabuli*	Ca_Kabuli_Chr08	5434225	(G/C)
13715	CakSNP13715	*Kabuli*	Ca_Kabuli_Chr08	5434232	(A/G)
13716	CakSNP13716	*Kabuli*	Ca_Kabuli_Chr08	5434238	(T/G)
13717	CakSNP13717	*Kabuli*	Ca_Kabuli_Chr08	5434242	(T/A)
13718	CakSNP13718	*Kabuli*	Ca_Kabuli_Chr08	5487769	(G/A)
13719	CakSNP13719	*Kabuli*	Ca_Kabuli_Chr08	5487830	(G/A)
13720	CakSNP13720	*Kabuli*	Ca_Kabuli_Chr08	5536418	(T/G)
13721	CakSNP13721	*Kabuli*	Ca_Kabuli_Chr08	5538696	(A/C)
13722	CakSNP13722	*Kabuli*	Ca_Kabuli_Chr08	5538695	(T/C)
13723	CakSNP13723	*Kabuli*	Ca_Kabuli_Chr08	5538683	(T/C)
13724	CakSNP13724	*Kabuli*	Ca_Kabuli_Chr08	5539044	(C/T)
13725	CakSNP13725	*Kabuli*	Ca_Kabuli_Chr08	5586106	(G/A)
13726	CakSNP13726	*Kabuli*	Ca_Kabuli_Chr08	5591654	(T/C)

S.N.	SNP IDs	Cultivars	Chromosomes/scaffolds	Physical positions (bp)	SNPs
13727	CakSNP13727	*Kabuli*	Ca_Kabuli_Chr08	5591788	(G/C)
13728	CakSNP13728	*Kabuli*	Ca_Kabuli_Chr08	5650080	(C/G)
13729	CakSNP13729	*Kabuli*	Ca_Kabuli_Chr08	5712245	(T/A)
13730	CakSNP13730	*Kabuli*	Ca_Kabuli_Chr08	5811179	(G/C)
13731	CakSNP13731	*Kabuli*	Ca_Kabuli_Chr08	5819008	(G/A)
13732	CakSNP13732	*Kabuli*	Ca_Kabuli_Chr08	5840141	(G/A)
13733	CakSNP13733	*Kabuli*	Ca_Kabuli_Chr08	5870455	(A/G)
13734	CakSNP13734	*Kabuli*	Ca_Kabuli_Chr08	5870664	(T/C)
13735	CakSNP13735	*Kabuli*	Ca_Kabuli_Chr08	5870749	(C/T)
13736	CakSNP13736	*Kabuli*	Ca_Kabuli_Chr08	5871358	(G/C)
13737	CakSNP13737	*Kabuli*	Ca_Kabuli_Chr08	5871361	(T/G)
13738	CakSNP13738	*Kabuli*	Ca_Kabuli_Chr08	5871364	(T/C)
13739	CakSNP13739	*Kabuli*	Ca_Kabuli_Chr08	5871367	(T/A)
13740	CakSNP13740	*Kabuli*	Ca_Kabuli_Chr08	5871421	(A/G)
13741	CakSNP13741	*Kabuli*	Ca_Kabuli_Chr08	5910629	(C/G)
13742	CakSNP13742	*Kabuli*	Ca_Kabuli_Chr08	5927870	(G/T)
13743	CakSNP13743	*Kabuli*	Ca_Kabuli_Chr08	5935435	(A/C)
13744	CakSNP13744	*Kabuli*	Ca_Kabuli_Chr08	5963855	(T/A)
13745	CakSNP13745	*Kabuli*	Ca_Kabuli_Chr08	5992761	(A/G)
13746	CakSNP13746	*Kabuli*	Ca_Kabuli_Chr08	6084594	(G/A)
13747	CakSNP13747	*Kabuli*	Ca_Kabuli_Chr08	6084591	(C/T)
13748	CakSNP13748	*Kabuli*	Ca_Kabuli_Chr08	6115961	(T/G)
13749	CakSNP13749	*Kabuli*	Ca_Kabuli_Chr08	6115965	(T/C)
13750	CakSNP13750	*Kabuli*	Ca_Kabuli_Chr08	6116096	(C/T)
13751	CakSNP13751	*Kabuli*	Ca_Kabuli_Chr08	6116097	(C/A)
13752	CakSNP13752	*Kabuli*	Ca_Kabuli_Chr08	6116101	(G/A)
13753	CakSNP13753	*Kabuli*	Ca_Kabuli_Chr08	6145326	(G/A)
13754	CakSNP13754	*Kabuli*	Ca_Kabuli_Chr08	6149097	(A/C)
13755	CakSNP13755	*Kabuli*	Ca_Kabuli_Chr08	6192944	(T/G)
13756	CakSNP13756	*Kabuli*	Ca_Kabuli_Chr08	6192986	(C/T)
13757	CakSNP13757	*Kabuli*	Ca_Kabuli_Chr08	6193078	(G/A)
13758	CakSNP13758	*Kabuli*	Ca_Kabuli_Chr08	6218250	(G/A)
13759	CakSNP13759	*Kabuli*	Ca_Kabuli_Chr08	6218275	(C/T)
13760	CakSNP13760	*Kabuli*	Ca_Kabuli_Chr08	6218298	(A/G)
13761	CakSNP13761	*Kabuli*	Ca_Kabuli_Chr08	6218299	(T/A)
13762	CakSNP13762	*Kabuli*	Ca_Kabuli_Chr08	6266432	(A/G)
13763	CakSNP13763	*Kabuli*	Ca_Kabuli_Chr08	6372236	(A/T)
13764	CakSNP13764	*Kabuli*	Ca_Kabuli_Chr08	6372230	(G/A)
13765	CakSNP13765	*Kabuli*	Ca_Kabuli_Chr08	6372223	(G/A)

S.N.	SNP IDs	Cultivars	Chromosomes/scaffolds	Physical positions (bp)	SNPs
13766	CakSNP13766	*Kabuli*	Ca_Kabuli_Chr08	6374794	(C/A)
13767	CakSNP13767	*Kabuli*	Ca_Kabuli_Chr08	6437758	(T/G)
13768	CakSNP13768	*Kabuli*	Ca_Kabuli_Chr08	6503723	(T/C)
13769	CakSNP13769	*Kabuli*	Ca_Kabuli_Chr08	6525633	(A/T)
13770	CakSNP13770	*Kabuli*	Ca_Kabuli_Chr08	6526612	(T/C)
13771	CakSNP13771	*Kabuli*	Ca_Kabuli_Chr08	6544248	(G/T)
13772	CakSNP13772	*Kabuli*	Ca_Kabuli_Chr08	6544278	(C/A)
13773	CakSNP13773	*Kabuli*	Ca_Kabuli_Chr08	6624006	(C/T)
13774	CakSNP13774	*Kabuli*	Ca_Kabuli_Chr08	6630663	(C/T)
13775	CakSNP13775	*Kabuli*	Ca_Kabuli_Chr08	6630661	(T/C)
13776	CakSNP13776	*Kabuli*	Ca_Kabuli_Chr08	6664509	(T/A)
13777	CakSNP13777	*Kabuli*	Ca_Kabuli_Chr08	6673855	(C/T)
13778	CakSNP13778	*Kabuli*	Ca_Kabuli_Chr08	6673891	(A/G)
13779	CakSNP13779	*Kabuli*	Ca_Kabuli_Chr08	6674024	(G/A)
13780	CakSNP13780	*Kabuli*	Ca_Kabuli_Chr08	6673956	(G/A)
13781	CakSNP13781	*Kabuli*	Ca_Kabuli_Chr08	6708869	(G/T)
13782	CakSNP13782	*Kabuli*	Ca_Kabuli_Chr08	6708945	(A/G)
13783	CakSNP13783	*Kabuli*	Ca_Kabuli_Chr08	6747091	(T/C)
13784	CakSNP13784	*Kabuli*	Ca_Kabuli_Chr08	6747995	(A/G)
13785	CakSNP13785	*Kabuli*	Ca_Kabuli_Chr08	6748136	(C/T)
13786	CakSNP13786	*Kabuli*	Ca_Kabuli_Chr08	6748294	(T/C)
13787	CakSNP13787	*Kabuli*	Ca_Kabuli_Chr08	6780592	(T/G)
13788	CakSNP13788	*Kabuli*	Ca_Kabuli_Chr08	6940139	(A/G)
13789	CakSNP13789	*Kabuli*	Ca_Kabuli_Chr08	6942651	(G/T)
13790	CakSNP13790	*Kabuli*	Ca_Kabuli_Chr08	6943710	(A/G)
13791	CakSNP13791	*Kabuli*	Ca_Kabuli_Chr08	6968036	(A/G)
13792	CakSNP13792	*Kabuli*	Ca_Kabuli_Chr08	6986090	(G/A)
13793	CakSNP13793	*Kabuli*	Ca_Kabuli_Chr08	6986130	(A/G)
13794	CakSNP13794	*Kabuli*	Ca_Kabuli_Chr08	6987609	(G/T)
13795	CakSNP13795	*Kabuli*	Ca_Kabuli_Chr08	6987615	(A/C)
13796	CakSNP13796	*Kabuli*	Ca_Kabuli_Chr08	6989342	(G/T)
13797	CakSNP13797	*Kabuli*	Ca_Kabuli_Chr08	6989433	(A/G)
13798	CakSNP13798	*Kabuli*	Ca_Kabuli_Chr08	7013049	(G/A)
13799	CakSNP13799	*Kabuli*	Ca_Kabuli_Chr08	7037715	(G/A)
13800	CakSNP13800	*Kabuli*	Ca_Kabuli_Chr08	7041901	(A/C)
13801	CakSNP13801	*Kabuli*	Ca_Kabuli_Chr08	7047509	(T/G)
13802	CakSNP13802	*Kabuli*	Ca_Kabuli_Chr08	7047469	(T/A)
13803	CakSNP13803	*Kabuli*	Ca_Kabuli_Chr08	7099455	(C/T)
13804	CakSNP13804	*Kabuli*	Ca_Kabuli_Chr08	7099569	(T/C)

S.N.	SNP IDs	Cultivars	Chromosomes/scaffolds	Physical positions (bp)	SNPs
13805	CakSNP13805	*Kabuli*	Ca_Kabuli_Chr08	7099530	(C/A)
13806	CakSNP13806	*Kabuli*	Ca_Kabuli_Chr08	7099756	(A/G)
13807	CakSNP13807	*Kabuli*	Ca_Kabuli_Chr08	7162826	(T/G)
13808	CakSNP13808	*Kabuli*	Ca_Kabuli_Chr08	7162976	(A/G)
13809	CakSNP13809	*Kabuli*	Ca_Kabuli_Chr08	7174066	(C/T)
13810	CakSNP13810	*Kabuli*	Ca_Kabuli_Chr08	7175086	(A/C)
13811	CakSNP13811	*Kabuli*	Ca_Kabuli_Chr08	7191825	(A/G)
13812	CakSNP13812	*Kabuli*	Ca_Kabuli_Chr08	7193946	(C/T)
13813	CakSNP13813	*Kabuli*	Ca_Kabuli_Chr08	7193960	(A/T)
13814	CakSNP13814	*Kabuli*	Ca_Kabuli_Chr08	7295151	(T/C)
13815	CakSNP13815	*Kabuli*	Ca_Kabuli_Chr08	7295206	(A/G)
13816	CakSNP13816	*Kabuli*	Ca_Kabuli_Chr08	7295177	(G/T)
13817	CakSNP13817	*Kabuli*	Ca_Kabuli_Chr08	7307737	(G/T)
13818	CakSNP13818	*Kabuli*	Ca_Kabuli_Chr08	7307787	(A/T)
13819	CakSNP13819	*Kabuli*	Ca_Kabuli_Chr08	7357621	(A/G)
13820	CakSNP13820	*Kabuli*	Ca_Kabuli_Chr08	7357583	(T/C)
13821	CakSNP13821	*Kabuli*	Ca_Kabuli_Chr08	7503013	(T/A)
13822	CakSNP13822	*Kabuli*	Ca_Kabuli_Chr08	7503010	(T/C)
13823	CakSNP13823	*Kabuli*	Ca_Kabuli_Chr08	7506004	(A/G)
13824	CakSNP13824	*Kabuli*	Ca_Kabuli_Chr08	7519758	(C/A)
13825	CakSNP13825	*Kabuli*	Ca_Kabuli_Chr08	7519815	(G/A)
13826	CakSNP13826	*Kabuli*	Ca_Kabuli_Chr08	7526956	(A/G)
13827	CakSNP13827	*Kabuli*	Ca_Kabuli_Chr08	7563385	(C/G)
13828	CakSNP13828	*Kabuli*	Ca_Kabuli_Chr08	7563370	(T/C)
13829	CakSNP13829	*Kabuli*	Ca_Kabuli_Chr08	7563373	(T/A)
13830	CakSNP13830	*Kabuli*	Ca_Kabuli_Chr08	7566548	(T/C)
13831	CakSNP13831	*Kabuli*	Ca_Kabuli_Chr08	7614202	(A/C)
13832	CakSNP13832	*Kabuli*	Ca_Kabuli_Chr08	7628690	(T/A)
13833	CakSNP13833	*Kabuli*	Ca_Kabuli_Chr08	7665291	(T/C)
13834	CakSNP13834	*Kabuli*	Ca_Kabuli_Chr08	7665383	(G/T)
13835	CakSNP13835	*Kabuli*	Ca_Kabuli_Chr08	7694747	(A/G)
13836	CakSNP13836	*Kabuli*	Ca_Kabuli_Chr08	7695990	(A/T)
13837	CakSNP13837	*Kabuli*	Ca_Kabuli_Chr08	7695989	(T/A)
13838	CakSNP13838	*Kabuli*	Ca_Kabuli_Chr08	7697886	(G/C)
13839	CakSNP13839	*Kabuli*	Ca_Kabuli_Chr08	7726921	(G/A)
13840	CakSNP13840	*Kabuli*	Ca_Kabuli_Chr08	7726894	(G/T)
13841	CakSNP13841	*Kabuli*	Ca_Kabuli_Chr08	7726879	(A/G)
13842	CakSNP13842	*Kabuli*	Ca_Kabuli_Chr08	7728085	(T/G)
13843	CakSNP13843	*Kabuli*	Ca_Kabuli_Chr08	7728076	(T/C)

S.N.	SNP IDs	Cultivars	Chromosomes/scaffolds	Physical positions (bp)	SNPs
13844	CakSNP13844	*Kabuli*	Ca_Kabuli_Chr08	7729693	(T/C)
13845	CakSNP13845	*Kabuli*	Ca_Kabuli_Chr08	7733508	(C/T)
13846	CakSNP13846	*Kabuli*	Ca_Kabuli_Chr08	7733573	(A/C)
13847	CakSNP13847	*Kabuli*	Ca_Kabuli_Chr08	7733574	(G/A)
13848	CakSNP13848	*Kabuli*	Ca_Kabuli_Chr08	7733670	(C/T)
13849	CakSNP13849	*Kabuli*	Ca_Kabuli_Chr08	7733647	(G/T)
13850	CakSNP13850	*Kabuli*	Ca_Kabuli_Chr08	7776466	(G/A)
13851	CakSNP13851	*Kabuli*	Ca_Kabuli_Chr08	7776592	(T/C)
13852	CakSNP13852	*Kabuli*	Ca_Kabuli_Chr08	7776897	(A/T)
13853	CakSNP13853	*Kabuli*	Ca_Kabuli_Chr08	7776934	(G/T)
13854	CakSNP13854	*Kabuli*	Ca_Kabuli_Chr08	7776970	(T/G)
13855	CakSNP13855	*Kabuli*	Ca_Kabuli_Chr08	7777023	(G/A)
13856	CakSNP13856	*Kabuli*	Ca_Kabuli_Chr08	7807065	(T/C)
13857	CakSNP13857	*Kabuli*	Ca_Kabuli_Chr08	7978269	(C/T)
13858	CakSNP13858	*Kabuli*	Ca_Kabuli_Chr08	8029283	(T/A)
13859	CakSNP13859	*Kabuli*	Ca_Kabuli_Chr08	8039078	(A/T)
13860	CakSNP13860	*Kabuli*	Ca_Kabuli_Chr08	8059732	(A/G)
13861	CakSNP13861	*Kabuli*	Ca_Kabuli_Chr08	8066006	(C/A)
13862	CakSNP13862	*Kabuli*	Ca_Kabuli_Chr08	8066005	(A/G)
13863	CakSNP13863	*Kabuli*	Ca_Kabuli_Chr08	8104804	(G/T)
13864	CakSNP13864	*Kabuli*	Ca_Kabuli_Chr08	8104801	(T/C)
13865	CakSNP13865	*Kabuli*	Ca_Kabuli_Chr08	8104884	(T/A)
13866	CakSNP13866	*Kabuli*	Ca_Kabuli_Chr08	8104892	(C/T)
13867	CakSNP13867	*Kabuli*	Ca_Kabuli_Chr08	8106804	(A/G)
13868	CakSNP13868	*Kabuli*	Ca_Kabuli_Chr08	8106851	(G/A)
13869	CakSNP13869	*Kabuli*	Ca_Kabuli_Chr08	8114316	(A/C)
13870	CakSNP13870	*Kabuli*	Ca_Kabuli_Chr08	8194206	(C/T)
13871	CakSNP13871	*Kabuli*	Ca_Kabuli_Chr08	8194232	(C/A)
13872	CakSNP13872	*Kabuli*	Ca_Kabuli_Chr08	8194234	(C/A)
13873	CakSNP13873	*Kabuli*	Ca_Kabuli_Chr08	8194342	(T/C)
13874	CakSNP13874	*Kabuli*	Ca_Kabuli_Chr08	8194464	(A/G)
13875	CakSNP13875	*Kabuli*	Ca_Kabuli_Chr08	8194436	(T/A)
13876	CakSNP13876	*Kabuli*	Ca_Kabuli_Chr08	8202726	(G/A)
13877	CakSNP13877	*Kabuli*	Ca_Kabuli_Chr08	8270488	(T/C)
13878	CakSNP13878	*Kabuli*	Ca_Kabuli_Chr08	8288041	(A/G)
13879	CakSNP13879	*Kabuli*	Ca_Kabuli_Chr08	8293716	(G/A)
13880	CakSNP13880	*Kabuli*	Ca_Kabuli_Chr08	8293704	(T/C)
13881	CakSNP13881	*Kabuli*	Ca_Kabuli_Chr08	8293693	(T/C)
13882	CakSNP13882	*Kabuli*	Ca_Kabuli_Chr08	8318110	(A/G)

S.N.	SNP IDs	Cultivars	Chromosomes/scaffolds	Physical positions (bp)	SNPs
13883	CakSNP13883	*Kabuli*	Ca_Kabuli_Chr08	8389117	(C/T)
13884	CakSNP13884	*Kabuli*	Ca_Kabuli_Chr08	8458383	(G/A)
13885	CakSNP13885	*Kabuli*	Ca_Kabuli_Chr08	8533479	(G/T)
13886	CakSNP13886	*Kabuli*	Ca_Kabuli_Chr08	8533978	(G/A)
13887	CakSNP13887	*Kabuli*	Ca_Kabuli_Chr08	8560330	(C/A)
13888	CakSNP13888	*Kabuli*	Ca_Kabuli_Chr08	8560331	(C/G)
13889	CakSNP13889	*Kabuli*	Ca_Kabuli_Chr08	8560332	(A/C)
13890	CakSNP13890	*Kabuli*	Ca_Kabuli_Chr08	8560326	(G/T)
13891	CakSNP13891	*Kabuli*	Ca_Kabuli_Chr08	8661327	(T/C)
13892	CakSNP13892	*Kabuli*	Ca_Kabuli_Chr08	8670242	(T/G)
13893	CakSNP13893	*Kabuli*	Ca_Kabuli_Chr08	8772131	(C/G)
13894	CakSNP13894	*Kabuli*	Ca_Kabuli_Chr08	8772124	(G/A)
13895	CakSNP13895	*Kabuli*	Ca_Kabuli_Chr08	8853602	(G/A)
13896	CakSNP13896	*Kabuli*	Ca_Kabuli_Chr08	8853664	(A/G)
13897	CakSNP13897	*Kabuli*	Ca_Kabuli_Chr08	8856046	(C/T)
13898	CakSNP13898	*Kabuli*	Ca_Kabuli_Chr08	8856047	(C/T)
13899	CakSNP13899	*Kabuli*	Ca_Kabuli_Chr08	8856150	(A/G)
13900	CakSNP13900	*Kabuli*	Ca_Kabuli_Chr08	8856143	(G/A)
13901	CakSNP13901	*Kabuli*	Ca_Kabuli_Chr08	8864079	(C/T)
13902	CakSNP13902	*Kabuli*	Ca_Kabuli_Chr08	8864157	(G/A)
13903	CakSNP13903	*Kabuli*	Ca_Kabuli_Chr08	8888412	(A/G)
13904	CakSNP13904	*Kabuli*	Ca_Kabuli_Chr08	8888414	(G/T)
13905	CakSNP13905	*Kabuli*	Ca_Kabuli_Chr08	8888426	(T/C)
13906	CakSNP13906	*Kabuli*	Ca_Kabuli_Chr08	8888476	(A/C)
13907	CakSNP13907	*Kabuli*	Ca_Kabuli_Chr08	8904346	(C/A)
13908	CakSNP13908	*Kabuli*	Ca_Kabuli_Chr08	8904366	(A/G)
13909	CakSNP13909	*Kabuli*	Ca_Kabuli_Chr08	8914018	(A/C)
13910	CakSNP13910	*Kabuli*	Ca_Kabuli_Chr08	9097585	(A/C)
13911	CakSNP13911	*Kabuli*	Ca_Kabuli_Chr08	9099798	(G/T)
13912	CakSNP13912	*Kabuli*	Ca_Kabuli_Chr08	9100111	(C/T)
13913	CakSNP13913	*Kabuli*	Ca_Kabuli_Chr08	9109896	(T/A)
13914	CakSNP13914	*Kabuli*	Ca_Kabuli_Chr08	9109907	(A/T)
13915	CakSNP13915	*Kabuli*	Ca_Kabuli_Chr08	9109920	(C/A)
13916	CakSNP13916	*Kabuli*	Ca_Kabuli_Chr08	9112178	(G/A)
13917	CakSNP13917	*Kabuli*	Ca_Kabuli_Chr08	9112172	(A/C)
13918	CakSNP13918	*Kabuli*	Ca_Kabuli_Chr08	9118582	(C/T)
13919	CakSNP13919	*Kabuli*	Ca_Kabuli_Chr08	9118667	(A/G)
13920	CakSNP13920	*Kabuli*	Ca_Kabuli_Chr08	9143840	(C/A)
13921	CakSNP13921	*Kabuli*	Ca_Kabuli_Chr08	9151722	(G/T)

S.N.	SNP IDs	Cultivars	Chromosomes/scaffolds	Physical positions (bp)	SNPs
13922	CakSNP13922	*Kabuli*	Ca_Kabuli_Chr08	9389347	(G/A)
13923	CakSNP13923	*Kabuli*	Ca_Kabuli_Chr08	10004481	(T/C)
13924	CakSNP13924	*Kabuli*	Ca_Kabuli_Chr08	10155981	(A/C)
13925	CakSNP13925	*Kabuli*	Ca_Kabuli_Chr08	10258892	(T/G)
13926	CakSNP13926	*Kabuli*	Ca_Kabuli_Chr08	10486601	(A/C)
13927	CakSNP13927	*Kabuli*	Ca_Kabuli_Chr08	10556124	(T/C)
13928	CakSNP13928	*Kabuli*	Ca_Kabuli_Chr08	10668422	(A/C)
13929	CakSNP13929	*Kabuli*	Ca_Kabuli_Chr08	10728369	(G/C)
13930	CakSNP13930	*Kabuli*	Ca_Kabuli_Chr08	10733404	(A/G)
13931	CakSNP13931	*Kabuli*	Ca_Kabuli_Chr08	10733503	(C/T)
13932	CakSNP13932	*Kabuli*	Ca_Kabuli_Chr08	10733661	(G/A)
13933	CakSNP13933	*Kabuli*	Ca_Kabuli_Chr08	10733747	(C/T)
13934	CakSNP13934	*Kabuli*	Ca_Kabuli_Chr08	10759078	(G/A)
13935	CakSNP13935	*Kabuli*	Ca_Kabuli_Chr08	10759060	(G/A)
13936	CakSNP13936	*Kabuli*	Ca_Kabuli_Chr08	10759056	(C/T)
13937	CakSNP13937	*Kabuli*	Ca_Kabuli_Chr08	10759102	(C/T)
13938	CakSNP13938	*Kabuli*	Ca_Kabuli_Chr08	10763656	(A/T)
13939	CakSNP13939	*Kabuli*	Ca_Kabuli_Chr08	10953767	(C/T)
13940	CakSNP13940	*Kabuli*	Ca_Kabuli_Chr08	11015680	(G/T)
13941	CakSNP13941	*Kabuli*	Ca_Kabuli_Chr08	11015678	(C/G)
13942	CakSNP13942	*Kabuli*	Ca_Kabuli_Chr08	11015666	(G/C)
13943	CakSNP13943	*Kabuli*	Ca_Kabuli_Chr08	11066644	(C/T)
13944	CakSNP13944	*Kabuli*	Ca_Kabuli_Chr08	11066728	(G/A)
13945	CakSNP13945	*Kabuli*	Ca_Kabuli_Chr08	11087143	(A/G)
13946	CakSNP13946	*Kabuli*	Ca_Kabuli_Chr08	11087169	(C/T)
13947	CakSNP13947	*Kabuli*	Ca_Kabuli_Chr08	11087302	(A/T)
13948	CakSNP13948	*Kabuli*	Ca_Kabuli_Chr08	11087250	(T/C)
13949	CakSNP13949	*Kabuli*	Ca_Kabuli_Chr08	11087247	(T/C)
13950	CakSNP13950	*Kabuli*	Ca_Kabuli_Chr08	11186919	(C/G)
13951	CakSNP13951	*Kabuli*	Ca_Kabuli_Chr08	11187037	(T/C)
13952	CakSNP13952	*Kabuli*	Ca_Kabuli_Chr08	11186986	(A/C)
13953	CakSNP13953	*Kabuli*	Ca_Kabuli_Chr08	11186974	(A/C)
13954	CakSNP13954	*Kabuli*	Ca_Kabuli_Chr08	11231812	(A/G)
13955	CakSNP13955	*Kabuli*	Ca_Kabuli_Chr08	11240258	(C/A)
13956	CakSNP13956	*Kabuli*	Ca_Kabuli_Chr08	11243263	(A/C)
13957	CakSNP13957	*Kabuli*	Ca_Kabuli_Chr08	11243256	(A/C)
13958	CakSNP13958	*Kabuli*	Ca_Kabuli_Chr08	11266447	(G/A)
13959	CakSNP13959	*Kabuli*	Ca_Kabuli_Chr08	11310460	(G/A)
13960	CakSNP13960	*Kabuli*	Ca_Kabuli_Chr08	11310480	(T/A)

**S.N.**	**SNP IDs**	**Cultivars**	**Chromosomes/scaffolds**	**Physical positions (bp)**	**SNPs**
13961	CakSNP13961	*Kabuli*	Ca_Kabuli_Chr08	11332934	(C/T)
13962	CakSNP13962	*Kabuli*	Ca_Kabuli_Chr08	11332940	(T/C)
13963	CakSNP13963	*Kabuli*	Ca_Kabuli_Chr08	11333057	(C/G)
13964	CakSNP13964	*Kabuli*	Ca_Kabuli_Chr08	11334190	(A/T)
13965	CakSNP13965	*Kabuli*	Ca_Kabuli_Chr08	11334210	(C/T)
13966	CakSNP13966	*Kabuli*	Ca_Kabuli_Chr08	11349891	(T/A)
13967	CakSNP13967	*Kabuli*	Ca_Kabuli_Chr08	11349995	(A/G)
13968	CakSNP13968	*Kabuli*	Ca_Kabuli_Chr08	11403805	(G/A)
13969	CakSNP13969	*Kabuli*	Ca_Kabuli_Chr08	11403754	(G/A)
13970	CakSNP13970	*Kabuli*	Ca_Kabuli_Chr08	11562814	(A/C)
13971	CakSNP13971	*Kabuli*	Ca_Kabuli_Chr08	11562833	(A/G)
13972	CakSNP13972	*Kabuli*	Ca_Kabuli_Chr08	11631836	(C/T)
13973	CakSNP13973	*Kabuli*	Ca_Kabuli_Chr08	11656700	(G/T)
13974	CakSNP13974	*Kabuli*	Ca_Kabuli_Chr08	11656687	(A/G)
13975	CakSNP13975	*Kabuli*	Ca_Kabuli_Chr08	11656675	(T/C)
13976	CakSNP13976	*Kabuli*	Ca_Kabuli_Chr08	11656670	(A/G)
13977	CakSNP13977	*Kabuli*	Ca_Kabuli_Chr08	11696021	(G/C)
13978	CakSNP13978	*Kabuli*	Ca_Kabuli_Chr08	11735882	(A/C)
13979	CakSNP13979	*Kabuli*	Ca_Kabuli_Chr08	11962280	(T/C)
13980	CakSNP13980	*Kabuli*	Ca_Kabuli_Chr08	12875217	(A/G)
13981	CakSNP13981	*Kabuli*	Ca_Kabuli_Chr08	13248268	(T/C)
13982	CakSNP13982	*Kabuli*	Ca_Kabuli_Chr08	13745335	(A/G)
13983	CakSNP13983	*Kabuli*	Ca_Kabuli_Chr08	13840865	(A/C)
13984	CakSNP13984	*Kabuli*	Ca_Kabuli_Chr08	13860922	(C/G)
13985	CakSNP13985	*Kabuli*	Ca_Kabuli_Chr08	13864026	(T/G)
13986	CakSNP13986	*Kabuli*	Ca_Kabuli_Chr08	13864077	(T/C)
13987	CakSNP13987	*Kabuli*	Ca_Kabuli_Chr08	13997385	(C/T)
13988	CakSNP13988	*Kabuli*	Ca_Kabuli_Chr08	13998102	(A/G)
13989	CakSNP13989	*Kabuli*	Ca_Kabuli_Chr08	14006709	(G/A)
13990	CakSNP13990	*Kabuli*	Ca_Kabuli_Chr08	14006694	(C/T)
13991	CakSNP13991	*Kabuli*	Ca_Kabuli_Chr08	14007431	(A/G)
13992	CakSNP13992	*Kabuli*	Ca_Kabuli_Chr08	14124588	(C/A)
13993	CakSNP13993	*Kabuli*	Ca_Kabuli_Chr08	14129040	(G/A)
13994	CakSNP13994	*Kabuli*	Ca_Kabuli_Chr08	14325980	(C/A)
13995	CakSNP13995	*Kabuli*	Ca_Kabuli_Chr08	14437006	(C/A)
13996	CakSNP13996	*Kabuli*	Ca_Kabuli_Chr08	14444424	(G/A)
13997	CakSNP13997	*Kabuli*	Ca_Kabuli_Chr08	14444438	(A/C)
13998	CakSNP13998	*Kabuli*	Ca_Kabuli_Chr08	14445205	(C/A)
13999	CakSNP13999	*Kabuli*	Ca_Kabuli_Chr08	14460426	(T/C)

S.N.	SNP IDs	Cultivars	Chromosomes/scaffolds	Physical positions (bp)	SNPs
14000	CakSNP14000	*Kabuli*	Ca_Kabuli_Chr08	14528882	(T/G)
14001	CakSNP14001	*Kabuli*	Ca_Kabuli_Chr08	14528966	(C/G)
14002	CakSNP14002	*Kabuli*	Ca_Kabuli_Chr08	14613302	(G/A)
14003	CakSNP14003	*Kabuli*	Ca_Kabuli_Chr08	14640967	(A/C)
14004	CakSNP14004	*Kabuli*	Ca_Kabuli_Chr08	14715495	(T/G)
14005	CakSNP14005	*Kabuli*	Ca_Kabuli_Chr08	14753763	(T/C)
14006	CakSNP14006	*Kabuli*	Ca_Kabuli_Chr08	14753750	(T/C)
14007	CakSNP14007	*Kabuli*	Ca_Kabuli_Chr08	14754849	(A/T)
14008	CakSNP14008	*Kabuli*	Ca_Kabuli_Chr08	14759784	(G/T)
14009	CakSNP14009	*Kabuli*	Ca_Kabuli_Chr08	14760479	(C/G)
14010	CakSNP14010	*Kabuli*	Ca_Kabuli_Chr08	14760497	(G/A)
14011	CakSNP14011	*Kabuli*	Ca_Kabuli_Chr08	14760503	(A/T)
14012	CakSNP14012	*Kabuli*	Ca_Kabuli_Chr08	14821416	(G/A)
14013	CakSNP14013	*Kabuli*	Ca_Kabuli_Chr08	15000109	(A/G)
14014	CakSNP14014	*Kabuli*	Ca_Kabuli_Chr08	15034465	(T/C)
14015	CakSNP14015	*Kabuli*	Ca_Kabuli_Chr08	15131175	(G/A)
14016	CakSNP14016	*Kabuli*	Ca_Kabuli_Chr08	15192125	(A/G)
14017	CakSNP14017	*Kabuli*	Ca_Kabuli_Chr08	15207944	(C/T)
14018	CakSNP14018	*Kabuli*	Ca_Kabuli_Chr08	15207976	(A/G)
14019	CakSNP14019	*Kabuli*	Ca_Kabuli_Chr08	15220986	(C/G)
14020	CakSNP14020	*Kabuli*	Ca_Kabuli_Chr08	15421764	(T/C)
14021	CakSNP14021	*Kabuli*	Ca_Kabuli_Chr08	15421744	(T/G)
14022	CakSNP14022	*Kabuli*	Ca_Kabuli_Chr08	15421719	(C/G)
14023	CakSNP14023	*Kabuli*	Ca_Kabuli_Chr08	15422163	(A/T)
14024	CakSNP14024	*Kabuli*	Ca_Kabuli_Chr08	15546669	(T/C)
14025	CakSNP14025	*Kabuli*	Ca_Kabuli_Chr08	15546671	(A/C)
14026	CakSNP14026	*Kabuli*	Ca_Kabuli_Chr08	15546696	(C/T)
14027	CakSNP14027	*Kabuli*	Ca_Kabuli_Chr08	15546710	(T/A)
14028	CakSNP14028	*Kabuli*	Ca_Kabuli_Chr08	15546758	(G/C)
14029	CakSNP14029	*Kabuli*	Ca_Kabuli_Chr08	15553251	(A/G)
14030	CakSNP14030	*Kabuli*	Ca_Kabuli_Chr08	15555771	(A/C)
14031	CakSNP14031	*Kabuli*	Ca_Kabuli_Chr08	15699220	(T/A)
14032	CakSNP14032	*Kabuli*	Ca_Kabuli_Chr08	15699180	(T/C)
14033	CakSNP14033	*Kabuli*	Ca_Kabuli_Chr08	15741863	(C/G)
14034	CakSNP14034	*Kabuli*	Ca_Kabuli_Chr08	15764739	(C/T)
14035	CakSNP14035	*Kabuli*	Ca_Kabuli_Chr08	15764748	(G/A)
14036	CakSNP14036	*Kabuli*	Ca_Kabuli_Chr08	15764823	(G/A)
14037	CakSNP14037	*Kabuli*	Ca_Kabuli_Chr08	15764808	(C/A)
14038	CakSNP14038	*Kabuli*	Ca_Kabuli_Chr08	15764874	(G/A)

S.N.	SNP IDs	Cultivars	Chromosomes/scaffolds	Physical positions (bp)	SNPs
14039	CakSNP14039	*Kabuli*	Ca_Kabuli_Chr08	15857176	(G/A)
14040	CakSNP14040	*Kabuli*	Ca_Kabuli_Chr08	15857192	(A/G)
14041	CakSNP14041	*Kabuli*	Ca_Kabuli_Chr08	15857359	(T/G)
14042	CakSNP14042	*Kabuli*	Ca_Kabuli_Chr08	15863509	(T/C)
14043	CakSNP14043	*Kabuli*	Ca_Kabuli_Chr08	15873071	(A/C)
14044	CakSNP14044	*Kabuli*	Ca_Kabuli_Chr08	15873133	(A/G)
14045	CakSNP14045	*Kabuli*	Ca_Kabuli_Chr08	16004976	(G/C)
14046	CakSNP14046	*Kabuli*	Ca_Kabuli_Chr08	16005046	(C/T)
14047	CakSNP14047	*Kabuli*	Ca_Kabuli_Chr08	16005078	(A/G)
14048	CakSNP14048	*Kabuli*	Ca_Kabuli_Chr08	16005235	(A/G)
14049	CakSNP14049	*Kabuli*	Ca_Kabuli_Chr08	16005253	(G/A)
14050	CakSNP14050	*Kabuli*	Ca_Kabuli_Chr08	16020239	(C/A)
14051	CakSNP14051	*Kabuli*	Ca_Kabuli_Chr08	16020277	(T/G)
14052	CakSNP14052	*Kabuli*	Ca_Kabuli_Chr08	16020323	(G/T)
14053	CakSNP14053	*Kabuli*	Ca_Kabuli_Chr08	16020305	(G/A)
14054	CakSNP14054	*Kabuli*	Ca_Kabuli_Chr08	16022432	(C/T)
14055	CakSNP14055	*Kabuli*	Ca_Kabuli_Chr08	16027974	(A/T)
14056	CakSNP14056	*Kabuli*	Ca_Kabuli_Chr08	16028064	(G/T)
14057	CakSNP14057	*Kabuli*	Ca_Kabuli_Chr08	16028062	(G/A)
14058	CakSNP14058	*Kabuli*	Ca_Kabuli_Chr08	16028040	(A/G)
14059	CakSNP14059	*Kabuli*	Ca_Kabuli_Chr08	16033779	(T/G)
14060	CakSNP14060	*Kabuli*	Ca_Kabuli_Chr08	16076161	(C/G)
14061	CakSNP14061	*Kabuli*	Ca_Kabuli_Chr08	16076168	(A/G)
14062	CakSNP14062	*Kabuli*	Ca_Kabuli_Chr08	16102413	(C/T)
14063	CakSNP14063	*Kabuli*	Ca_Kabuli_Chr08	16102395	(G/A)
14064	CakSNP14064	*Kabuli*	Ca_Kabuli_Chr08	16102363	(T/C)
14065	CakSNP14065	*Kabuli*	Ca_Kabuli_Chr08	16102362	(G/T)
14066	CakSNP14066	*Kabuli*	Ca_Kabuli_Chr08	16102348	(A/T)
14067	CakSNP14067	*Kabuli*	Ca_Kabuli_Chr08	16102343	(C/T)
14068	CakSNP14068	*Kabuli*	Ca_Kabuli_Chr08	16102335	(A/T)
14069	CakSNP14069	*Kabuli*	Ca_Kabuli_Chr08	16102414	(G/T)
14070	CakSNP14070	*Kabuli*	Ca_Kabuli_Chr08	16144279	(T/A)
14071	CakSNP14071	*Kabuli*	Ca_Kabuli_Chr08	16145275	(A/G)
14072	CakSNP14072	*Kabuli*	Ca_Kabuli_Chr08	16145419	(A/G)
14073	CakSNP14073	*Kabuli*	Ca_Kabuli_Chr08	16145411	(C/G)
14074	CakSNP14074	*Kabuli*	Ca_Kabuli_Chr08	16145370	(C/A)
14075	CakSNP14075	*Kabuli*	Ca_Kabuli_Chr08	16157015	(C/T)
14076	CakSNP14076	*Kabuli*	Ca_Kabuli_Chr08	16157134	(A/C)
14077	CakSNP14077	*Kabuli*	Ca_Kabuli_Chr08	16158594	(A/C)

S.N.	SNP IDs	Cultivars	Chromosomes/scaffolds	Physical positions (bp)	SNPs
14078	CakSNP14078	*Kabuli*	Ca_Kabuli_Chr08	16264310	(G/T)
14079	CakSNP14079	*Kabuli*	Ca_Kabuli_Chr08	16268878	(T/C)
14080	CakSNP14080	*Kabuli*	Ca_Kabuli_Chr08	16278758	(T/C)
14081	CakSNP14081	*Kabuli*	Ca_Kabuli_Chr08	16283660	(C/T)
14082	CakSNP14082	*Kabuli*	Ca_Kabuli_Chr08	16283661	(A/G)
14083	CakSNP14083	*Kabuli*	Ca_Kabuli_Chr08	16283722	(A/G)
14084	CakSNP14084	*Kabuli*	Ca_Kabuli_Chr08	16283738	(G/C)
14085	CakSNP14085	*Kabuli*	Ca_Kabuli_Chr08	16283859	(T/C)
14086	CakSNP14086	*Kabuli*	Ca_Kabuli_Chr08	16284269	(G/A)
14087	CakSNP14087	*Kabuli*	Ca_Kabuli_Chr08	16284279	(A/C)
14088	CakSNP14088	*Kabuli*	Ca_Kabuli_Chr08	16284306	(C/T)
14089	CakSNP14089	*Kabuli*	Ca_Kabuli_Chr08	16309857	(G/T)
14090	CakSNP14090	*Kabuli*	Ca_Kabuli_Chr08	16321524	(A/G)
14091	CakSNP14091	*Kabuli*	Ca_Kabuli_Chr08	16321545	(G/T)
14092	CakSNP14092	*Kabuli*	Ca_Kabuli_Chr08	16366490	(G/A)
14093	CakSNP14093	*Kabuli*	Ca_Kabuli_Chr08	16381928	(T/G)
14094	CakSNP14094	*Kabuli*	Ca_Kabuli_Chr08	16411550	(A/G)
14095	CakSNP14095	*Kabuli*	Ca_Kabuli_Chr08	16412672	(T/C)
14096	CakSNP14096	*Kabuli*	Ca_Kabuli_Chr08	16421407	(G/A)
14097	CakSNP14097	*Kabuli*	Ca_Kabuli_Chr08	16421441	(C/A)
14098	CakSNP14098	*Kabuli*	Ca_Kabuli_Chr08	16421442	(C/G)
14099	CakSNP14099	*Kabuli*	Ca_Kabuli_Chr08	16421545	(G/T)
14100	CakSNP14100	*Kabuli*	Ca_Kabuli_Chr08	16421497	(C/T)
14101	CakSNP14101	*Kabuli*	Ca_Kabuli_Chr08	16421595	(A/C)
14102	CakSNP14102	*Kabuli*	Ca_Kabuli_Chr08	16432907	(T/G)
14103	CakSNP14103	*Kabuli*	Ca_Kabuli_Chr08	16472919	(T/A)
14104	CakSNP14104	*Kabuli*	Ca_Kabuli_Chr08	16472932	(C/T)
14105	CakSNP14105	*Kabuli*	Ca_Kabuli_Chr08	16472933	(G/C)
14106	CakSNP14106	*Kabuli*	Ca_Kabuli_Chr08	16472999	(A/C)
14107	CakSNP14107	*Kabuli*	Ca_Kabuli_Chr08	16473047	(C/G)
14108	CakSNP14108	*Kabuli*	Ca_Kabuli_Chr08	16473139	(C/T)
14109	CakSNP14109	*Kabuli*	Ca_Kabuli_Chr08	16473154	(T/A)
14110	CakSNP14110	*Kabuli*	Ca_Kabuli_Chr08	16473157	(C/T)
14111	CakSNP14111	*Kabuli*	Ca_Kabuli_Chr08	16473311	(A/G)
14112	CakSNP14112	*Kabuli*	Ca_Kabuli_Chr08	16473290	(A/G)
14113	CakSNP14113	*Kabuli*	Ca_Kabuli_Chr08	16473920	(A/G)
14114	CakSNP14114	*Kabuli*	Ca_Kabuli_Chr08	16473939	(A/G)
14115	CakSNP14115	*Kabuli*	Ca_Kabuli_Chr08	16473987	(C/T)
14116	CakSNP14116	*Kabuli*	Ca_Kabuli_C11058086	341	(G/A)

S.N.	SNP IDs	Cultivars	Chromosomes/scaffolds	Physical positions (bp)	SNPs
14117	CakSNP14117	*Kabuli*	Ca_Kabuli_C11058274	143	(T/A)
14118	CakSNP14118	*Kabuli*	Ca_Kabuli_C11062332	412	(G/A)
14119	CakSNP14119	*Kabuli*	Ca_Kabuli_C11062436	381	(T/A)
14120	CakSNP14120	*Kabuli*	Ca_Kabuli_C11062546	677	(G/T)
14121	CakSNP14121	*Kabuli*	Ca_Kabuli_C11076702	989	(G/A)
14122	CakSNP14122	*Kabuli*	Ca_Kabuli_C11078452	885	(G/A)
14123	CakSNP14123	*Kabuli*	Ca_Kabuli_C11079766	1418	(A/C)
14124	CakSNP14124	*Kabuli*	Ca_Kabuli_C11079766	1422	(G/C)
14125	CakSNP14125	*Kabuli*	Ca_Kabuli_C11079766	1412	(A/T)
14126	CakSNP14126	*Kabuli*	Ca_Kabuli_C11080716	1568	(A/C)
14127	CakSNP14127	*Kabuli*	Ca_Kabuli_C11084624	656	(G/A)
14128	CakSNP14128	*Kabuli*	Ca_Kabuli_C11084992	516	(A/G)
14129	CakSNP14129	*Kabuli*	Ca_Kabuli_C11084992	537	(G/T)
14130	CakSNP14130	*Kabuli*	Ca_Kabuli_C11086066	641	(A/C)
14131	CakSNP14131	*Kabuli*	Ca_Kabuli_C11086066	624	(A/C)
14132	CakSNP14132	*Kabuli*	Ca_Kabuli_C11088694	514	(G/C)
14133	CakSNP14133	*Kabuli*	Ca_Kabuli_C11119498	682	(T/A)
14134	CakSNP14134	*Kabuli*	Ca_Kabuli_C11119498	686	(C/A)
14135	CakSNP14135	*Kabuli*	Ca_Kabuli_C11119498	689	(C/A)
14136	CakSNP14136	*Kabuli*	Ca_Kabuli_C11119498	693	(G/A)
14137	CakSNP14137	*Kabuli*	Ca_Kabuli_C11132768	2177	(G/A)
14138	CakSNP14138	*Kabuli*	Ca_Kabuli_C11142214	2902	(G/T)
14139	CakSNP14139	*Kabuli*	Ca_Kabuli_C11142252	3299	(A/C)
14140	CakSNP14140	*Kabuli*	Ca_Kabuli_C11143608	1389	(A/T)
14141	CakSNP14141	*Kabuli*	Ca_Kabuli_C11147634	3004	(C/A)
14142	CakSNP14142	*Kabuli*	Ca_Kabuli_C11147634	3028	(C/T)
14143	CakSNP14143	*Kabuli*	Ca_Kabuli_C11149092	2181	(A/G)
14144	CakSNP14144	*Kabuli*	Ca_Kabuli_C11149092	2281	(T/C)
14145	CakSNP14145	*Kabuli*	Ca_Kabuli_C11149092	2474	(T/A)
14146	CakSNP14146	*Kabuli*	Ca_Kabuli_C11149092	3190	(T/G)
14147	CakSNP14147	*Kabuli*	Ca_Kabuli_C11149092	3284	(C/G)
14148	CakSNP14148	*Kabuli*	Ca_Kabuli_C11153606	4275	(C/T)
14149	CakSNP14149	*Kabuli*	Ca_Kabuli_C11157320	766	(C/G)
14150	CakSNP14150	*Kabuli*	Ca_Kabuli_C11157320	2169	(T/A)
14151	CakSNP14151	*Kabuli*	Ca_Kabuli_C11157320	2152	(G/C)
14152	CakSNP14152	*Kabuli*	Ca_Kabuli_C11158050	2186	(A/T)
14153	CakSNP14153	*Kabuli*	Ca_Kabuli_C11160518	4522	(A/G)
14154	CakSNP14154	*Kabuli*	Ca_Kabuli_C11162020	3914	(C/T)
14155	CakSNP14155	*Kabuli*	Ca_Kabuli_C11162020	3926	(T/G)

S.N.	SNP IDs	Cultivars	Chromosomes/scaffolds	Physical positions (bp)	SNPs
14156	CakSNP14156	*Kabuli*	Ca_Kabuli_C11162020	5515	(G/A)
14157	CakSNP14157	*Kabuli*	Ca_Kabuli_C11164954	5778	(T/A)
14158	CakSNP14158	*Kabuli*	Ca_Kabuli_C11164954	5784	(G/A)
14159	CakSNP14159	*Kabuli*	Ca_Kabuli_C11165474	6285	(T/A)
14160	CakSNP14160	*Kabuli*	Ca_Kabuli_C11165890	154	(C/G)
14161	CakSNP14161	*Kabuli*	Ca_Kabuli_C11174756	8931	(C/G)
14162	CakSNP14162	*Kabuli*	Ca_Kabuli_C11175430	987	(A/G)
14163	CakSNP14163	*Kabuli*	Ca_Kabuli_C11175430	3929	(T/G)
14164	CakSNP14164	*Kabuli*	Ca_Kabuli_C11175430	3936	(G/T)
14165	CakSNP14165	*Kabuli*	Ca_Kabuli_C11175430	3952	(T/C)
14166	CakSNP14166	*Kabuli*	Ca_Kabuli_C11176688	4197	(G/C)
14167	CakSNP14167	*Kabuli*	Ca_Kabuli_C11177172	2529	(T/G)
14168	CakSNP14168	*Kabuli*	Ca_Kabuli_C11177172	2515	(T/A)
14169	CakSNP14169	*Kabuli*	Ca_Kabuli_C11177172	5324	(G/A)
14170	CakSNP14170	*Kabuli*	Ca_Kabuli_C11177748	1264	(T/A)
14171	CakSNP14171	*Kabuli*	Ca_Kabuli_C11178532	2107	(A/T)
14172	CakSNP14172	*Kabuli*	Ca_Kabuli_C11178532	3524	(A/G)
14173	CakSNP14173	*Kabuli*	Ca_Kabuli_C11181782	6215	(A/G)
14174	CakSNP14174	*Kabuli*	Ca_Kabuli_C11181840	19192	(A/G)
14175	CakSNP14175	*Kabuli*	Ca_Kabuli_C11181840	19202	(T/C)
14176	CakSNP14176	*Kabuli*	Ca_Kabuli_Scaffold1006	40651	(T/A)
14177	CakSNP14177	*Kabuli*	Ca_Kabuli_Scaffold1006	382579	(C/T)
14178	CakSNP14178	*Kabuli*	Ca_Kabuli_Scaffold1006	436923	(T/C)
14179	CakSNP14179	*Kabuli*	Ca_Kabuli_Scaffold1006	644893	(A/C)
14180	CakSNP14180	*Kabuli*	Ca_Kabuli_Scaffold1006	667620	(A/G)
14181	CakSNP14181	*Kabuli*	Ca_Kabuli_Scaffold1006	667713	(G/A)
14182	CakSNP14182	*Kabuli*	Ca_Kabuli_Scaffold1006	674495	(A/G)
14183	CakSNP14183	*Kabuli*	Ca_Kabuli_Scaffold1006	674823	(G/A)
14184	CakSNP14184	*Kabuli*	Ca_Kabuli_Scaffold1006	674826	(C/T)
14185	CakSNP14185	*Kabuli*	Ca_Kabuli_Scaffold1006	674844	(T/C)
14186	CakSNP14186	*Kabuli*	Ca_Kabuli_Scaffold1006	674847	(T/G)
14187	CakSNP14187	*Kabuli*	Ca_Kabuli_Scaffold1006	674862	(G/A)
14188	CakSNP14188	*Kabuli*	Ca_Kabuli_Scaffold1006	674865	(T/C)
14189	CakSNP14189	*Kabuli*	Ca_Kabuli_Scaffold1006	674871	(A/G)
14190	CakSNP14190	*Kabuli*	Ca_Kabuli_Scaffold1006	674881	(G/A)
14191	CakSNP14191	*Kabuli*	Ca_Kabuli_Scaffold1006	674981	(G/A)
14192	CakSNP14192	*Kabuli*	Ca_Kabuli_Scaffold1006	674946	(G/A)
14193	CakSNP14193	*Kabuli*	Ca_Kabuli_Scaffold1010	90092	(G/A)
14194	CakSNP14194	*Kabuli*	Ca_Kabuli_Scaffold1013	56140	(G/A)

S.N.	SNP IDs	Cultivars	Chromosomes/scaffolds	Physical positions (bp)	SNPs
14195	CakSNP14195	*Kabuli*	Ca_Kabuli_Scaffold1013	56116	(C/T)
14196	CakSNP14196	*Kabuli*	Ca_Kabuli_Scaffold1013	56114	(C/A)
14197	CakSNP14197	*Kabuli*	Ca_Kabuli_Scaffold1013	56075	(T/A)
14198	CakSNP14198	*Kabuli*	Ca_Kabuli_Scaffold1013	56074	(C/T)
14199	CakSNP14199	*Kabuli*	Ca_Kabuli_Scaffold1013	56069	(C/T)
14200	CakSNP14200	*Kabuli*	Ca_Kabuli_Scaffold10186	416	(C/T)
14201	CakSNP14201	*Kabuli*	Ca_Kabuli_Scaffold10186	410	(A/C)
14202	CakSNP14202	*Kabuli*	Ca_Kabuli_Scaffold10186	378	(A/T)
14203	CakSNP14203	*Kabuli*	Ca_Kabuli_Scaffold10186	354	(T/A)
14204	CakSNP14204	*Kabuli*	Ca_Kabuli_Scaffold10186	397	(G/A)
14205	CakSNP14205	*Kabuli*	Ca_Kabuli_Scaffold10186	573	(T/G)
14206	CakSNP14206	*Kabuli*	Ca_Kabuli_Scaffold102	82102	(T/C)
14207	CakSNP14207	*Kabuli*	Ca_Kabuli_Scaffold102	82107	(C/A)
14208	CakSNP14208	*Kabuli*	Ca_Kabuli_Scaffold1027	256612	(A/C)
14209	CakSNP14209	*Kabuli*	Ca_Kabuli_Scaffold1034	73684	(G/A)
14210	CakSNP14210	*Kabuli*	Ca_Kabuli_Scaffold1034	73695	(G/T)
14211	CakSNP14211	*Kabuli*	Ca_Kabuli_Scaffold10420	19974	(A/G)
14212	CakSNP14212	*Kabuli*	Ca_Kabuli_Scaffold10420	31513	(C/T)
14213	CakSNP14213	*Kabuli*	Ca_Kabuli_Scaffold10420	31563	(A/G)
14214	CakSNP14214	*Kabuli*	Ca_Kabuli_Scaffold10420	31817	(C/T)
14215	CakSNP14215	*Kabuli*	Ca_Kabuli_Scaffold10420	40193	(G/T)
14216	CakSNP14216	*Kabuli*	Ca_Kabuli_Scaffold1043	16328	(A/T)
14217	CakSNP14217	*Kabuli*	Ca_Kabuli_Scaffold1043	16331	(G/T)
14218	CakSNP14218	*Kabuli*	Ca_Kabuli_Scaffold1043	16333	(A/T)
14219	CakSNP14219	*Kabuli*	Ca_Kabuli_Scaffold1043	16313	(A/G)
14220	CakSNP14220	*Kabuli*	Ca_Kabuli_Scaffold1047	235352	(T/C)
14221	CakSNP14221	*Kabuli*	Ca_Kabuli_Scaffold1047	577874	(T/G)
14222	CakSNP14222	*Kabuli*	Ca_Kabuli_Scaffold1050	3398	(G/A)
14223	CakSNP14223	*Kabuli*	Ca_Kabuli_Scaffold1050	3415	(C/T)
14224	CakSNP14224	*Kabuli*	Ca_Kabuli_Scaffold1050	3463	(C/T)
14225	CakSNP14225	*Kabuli*	Ca_Kabuli_Scaffold1050	14057	(C/A)
14226	CakSNP14226	*Kabuli*	Ca_Kabuli_Scaffold1050	126986	(T/G)
14227	CakSNP14227	*Kabuli*	Ca_Kabuli_Scaffold1050	127022	(G/A)
14228	CakSNP14228	*Kabuli*	Ca_Kabuli_Scaffold1050	127023	(G/T)
14229	CakSNP14229	*Kabuli*	Ca_Kabuli_Scaffold1050	127054	(C/T)
14230	CakSNP14230	*Kabuli*	Ca_Kabuli_Scaffold1050	128763	(A/G)
14231	CakSNP14231	*Kabuli*	Ca_Kabuli_Scaffold1055	344459	(G/A)
14232	CakSNP14232	*Kabuli*	Ca_Kabuli_Scaffold1055	344443	(G/A)
14233	CakSNP14233	*Kabuli*	Ca_Kabuli_Scaffold1055	344399	(C/T)

S.N.	SNP IDs	Cultivars	Chromosomes/scaffolds	Physical positions (bp)	SNPs
14234	CakSNP14234	*Kabuli*	Ca_Kabuli_Scaffold1060	3278	(A/C)
14235	CakSNP14235	*Kabuli*	Ca_Kabuli_Scaffold1060	3310	(G/T)
14236	CakSNP14236	*Kabuli*	Ca_Kabuli_Scaffold1060	394867	(C/T)
14237	CakSNP14237	*Kabuli*	Ca_Kabuli_Scaffold1060	395029	(C/T)
14238	CakSNP14238	*Kabuli*	Ca_Kabuli_Scaffold1060	395042	(A/G)
14239	CakSNP14239	*Kabuli*	Ca_Kabuli_Scaffold1061	63613	(A/G)
14240	CakSNP14240	*Kabuli*	Ca_Kabuli_Scaffold1061	63620	(C/T)
14241	CakSNP14241	*Kabuli*	Ca_Kabuli_Scaffold1065	142678	(A/C)
14242	CakSNP14242	*Kabuli*	Ca_Kabuli_Scaffold1067	38623	(C/A)
14243	CakSNP14243	*Kabuli*	Ca_Kabuli_Scaffold1081	768	(A/C)
14244	CakSNP14244	*Kabuli*	Ca_Kabuli_Scaffold1081	798	(T/C)
14245	CakSNP14245	*Kabuli*	Ca_Kabuli_Scaffold1081	800	(T/C)
14246	CakSNP14246	*Kabuli*	Ca_Kabuli_Scaffold1081	805	(A/C)
14247	CakSNP14247	*Kabuli*	Ca_Kabuli_Scaffold1081	818	(A/C)
14248	CakSNP14248	*Kabuli*	Ca_Kabuli_Scaffold1089	1154	(C/A)
14249	CakSNP14249	*Kabuli*	Ca_Kabuli_Scaffold1089	1240	(T/G)
14250	CakSNP14250	*Kabuli*	Ca_Kabuli_Scaffold1089	1281	(G/A)
14251	CakSNP14251	*Kabuli*	Ca_Kabuli_Scaffold1089	1278	(T/C)
14252	CakSNP14252	*Kabuli*	Ca_Kabuli_Scaffold1089	3160	(C/A)
14253	CakSNP14253	*Kabuli*	Ca_Kabuli_Scaffold1089	3173	(G/T)
14254	CakSNP14254	*Kabuli*	Ca_Kabuli_Scaffold1089	3204	(G/C)
14255	CakSNP14255	*Kabuli*	Ca_Kabuli_Scaffold1089	3236	(T/G)
14256	CakSNP14256	*Kabuli*	Ca_Kabuli_Scaffold1089	3249	(T/G)
14257	CakSNP14257	*Kabuli*	Ca_Kabuli_Scaffold1089	3248	(C/T)
14258	CakSNP14258	*Kabuli*	Ca_Kabuli_Scaffold1089	3240	(G/C)
14259	CakSNP14259	*Kabuli*	Ca_Kabuli_Scaffold1089	3228	(C/T)
14260	CakSNP14260	*Kabuli*	Ca_Kabuli_Scaffold1089	3191	(G/A)
14261	CakSNP14261	*Kabuli*	Ca_Kabuli_Scaffold1089	3274	(T/C)
14262	CakSNP14262	*Kabuli*	Ca_Kabuli_Scaffold1089	137675	(T/C)
14263	CakSNP14263	*Kabuli*	Ca_Kabuli_Scaffold1089	137679	(G/T)
14264	CakSNP14264	*Kabuli*	Ca_Kabuli_Scaffold109_1	156485	(C/T)
14265	CakSNP14265	*Kabuli*	Ca_Kabuli_Scaffold109_1	230701	(G/C)
14266	CakSNP14266	*Kabuli*	Ca_Kabuli_Scaffold109_1	244141	(A/C)
14267	CakSNP14267	*Kabuli*	Ca_Kabuli_Scaffold109_1	244150	(C/T)
14268	CakSNP14268	*Kabuli*	Ca_Kabuli_Scaffold109_1	244156	(A/C)
14269	CakSNP14269	*Kabuli*	Ca_Kabuli_Scaffold109_1	244176	(A/T)
14270	CakSNP14270	*Kabuli*	Ca_Kabuli_Scaffold109_1	328757	(C/T)
14271	CakSNP14271	*Kabuli*	Ca_Kabuli_Scaffold109_1	343415	(G/A)
14272	CakSNP14272	*Kabuli*	Ca_Kabuli_Scaffold109_1	343454	(G/A)

S.N.	SNP IDs	Cultivars	Chromosomes/scaffolds	Physical positions (bp)	SNPs
14273	CakSNP14273	*Kabuli*	Ca_Kabuli_Scaffold109_1	385548	(T/C)
14274	CakSNP14274	*Kabuli*	Ca_Kabuli_Scaffold109_1	385578	(C/G)
14275	CakSNP14275	*Kabuli*	Ca_Kabuli_Scaffold109_1	385602	(C/T)
14276	CakSNP14276	*Kabuli*	Ca_Kabuli_Scaffold109_1	385660	(G/A)
14277	CakSNP14277	*Kabuli*	Ca_Kabuli_Scaffold109_1	385653	(C/T)
14278	CakSNP14278	*Kabuli*	Ca_Kabuli_Scaffold109_1	442149	(A/T)
14279	CakSNP14279	*Kabuli*	Ca_Kabuli_Scaffold109_1	535504	(G/C)
14280	CakSNP14280	*Kabuli*	Ca_Kabuli_Scaffold109_1	586181	(A/C)
14281	CakSNP14281	*Kabuli*	Ca_Kabuli_Scaffold109_1	586286	(C/T)
14282	CakSNP14282	*Kabuli*	Ca_Kabuli_Scaffold109_1	696869	(A/C)
14283	CakSNP14283	*Kabuli*	Ca_Kabuli_Scaffold109_1	734137	(G/A)
14284	CakSNP14284	*Kabuli*	Ca_Kabuli_Scaffold109_1	734141	(C/T)
14285	CakSNP14285	*Kabuli*	Ca_Kabuli_Scaffold109_1	741607	(G/A)
14286	CakSNP14286	*Kabuli*	Ca_Kabuli_Scaffold109_1	763160	(G/A)
14287	CakSNP14287	*Kabuli*	Ca_Kabuli_Scaffold1109	150672	(G/A)
14288	CakSNP14288	*Kabuli*	Ca_Kabuli_Scaffold1109	150720	(G/A)
14289	CakSNP14289	*Kabuli*	Ca_Kabuli_Scaffold1109	150676	(C/T)
14290	CakSNP14290	*Kabuli*	Ca_Kabuli_Scaffold1115	6234	(G/C)
14291	CakSNP14291	*Kabuli*	Ca_Kabuli_Scaffold1115	131039	(G/A)
14292	CakSNP14292	*Kabuli*	Ca_Kabuli_Scaffold1115	153349	(T/C)
14293	CakSNP14293	*Kabuli*	Ca_Kabuli_Scaffold1118	2215	(T/C)
14294	CakSNP14294	*Kabuli*	Ca_Kabuli_Scaffold1118	2224	(T/A)
14295	CakSNP14295	*Kabuli*	Ca_Kabuli_Scaffold1118	2225	(C/T)
14296	CakSNP14296	*Kabuli*	Ca_Kabuli_Scaffold1118	2226	(T/G)
14297	CakSNP14297	*Kabuli*	Ca_Kabuli_Scaffold1118	2315	(T/C)
14298	CakSNP14298	*Kabuli*	Ca_Kabuli_Scaffold1118	9568	(A/G)
14299	CakSNP14299	*Kabuli*	Ca_Kabuli_Scaffold1118	9556	(C/T)
14300	CakSNP14300	*Kabuli*	Ca_Kabuli_Scaffold1128	94196	(T/A)
14301	CakSNP14301	*Kabuli*	Ca_Kabuli_Scaffold1128	112702	(G/A)
14302	CakSNP14302	*Kabuli*	Ca_Kabuli_Scaffold1128	341416	(T/A)
14303	CakSNP14303	*Kabuli*	Ca_Kabuli_Scaffold1128	360567	(T/A)
14304	CakSNP14304	*Kabuli*	Ca_Kabuli_Scaffold1128	360529	(A/C)
14305	CakSNP14305	*Kabuli*	Ca_Kabuli_Scaffold1128	423553	(A/G)
14306	CakSNP14306	*Kabuli*	Ca_Kabuli_Scaffold1132	26497	(C/T)
14307	CakSNP14307	*Kabuli*	Ca_Kabuli_Scaffold1132	26492	(G/A)
14308	CakSNP14308	*Kabuli*	Ca_Kabuli_Scaffold1134	50630	(T/C)
14309	CakSNP14309	*Kabuli*	Ca_Kabuli_Scaffold1151	228312	(C/G)
14310	CakSNP14310	*Kabuli*	Ca_Kabuli_Scaffold1151	228304	(A/T)
14311	CakSNP14311	*Kabuli*	Ca_Kabuli_Scaffold1151	228291	(C/T)

S.N.	SNP IDs	Cultivars	Chromosomes/scaffolds	Physical positions (bp)	SNPs
14312	CakSNP14312	*Kabuli*	Ca_Kabuli_Scaffold1152	9541	(A/G)
14313	CakSNP14313	*Kabuli*	Ca_Kabuli_Scaffold1176_2	52186	(C/T)
14314	CakSNP14314	*Kabuli*	Ca_Kabuli_Scaffold1176_2	52146	(T/C)
14315	CakSNP14315	*Kabuli*	Ca_Kabuli_Scaffold1176_2	52142	(G/T)
14316	CakSNP14316	*Kabuli*	Ca_Kabuli_Scaffold1176_2	52125	(C/T)
14317	CakSNP14317	*Kabuli*	Ca_Kabuli_Scaffold1176_2	52124	(C/T)
14318	CakSNP14318	*Kabuli*	Ca_Kabuli_Scaffold1176_2	330840	(C/A)
14319	CakSNP14319	*Kabuli*	Ca_Kabuli_Scaffold118	206562	(A/G)
14320	CakSNP14320	*Kabuli*	Ca_Kabuli_Scaffold118	303784	(A/C)
14321	CakSNP14321	*Kabuli*	Ca_Kabuli_Scaffold118	332673	(T/C)
14322	CakSNP14322	*Kabuli*	Ca_Kabuli_Scaffold1180	114508	(C/A)
14323	CakSNP14323	*Kabuli*	Ca_Kabuli_Scaffold1185	944	(G/A)
14324	CakSNP14324	*Kabuli*	Ca_Kabuli_Scaffold1185	1110	(G/A)
14325	CakSNP14325	*Kabuli*	Ca_Kabuli_Scaffold1185	1134	(T/A)
14326	CakSNP14326	*Kabuli*	Ca_Kabuli_Scaffold1185	1098	(T/C)
14327	CakSNP14327	*Kabuli*	Ca_Kabuli_Scaffold119	12259	(A/G)
14328	CakSNP14328	*Kabuli*	Ca_Kabuli_Scaffold119	12263	(C/A)
14329	CakSNP14329	*Kabuli*	Ca_Kabuli_Scaffold119	12267	(C/A)
14330	CakSNP14330	*Kabuli*	Ca_Kabuli_Scaffold119	12279	(G/A)
14331	CakSNP14331	*Kabuli*	Ca_Kabuli_Scaffold1196	70506	(G/A)
14332	CakSNP14332	*Kabuli*	Ca_Kabuli_Scaffold1196	70552	(A/C)
14333	CakSNP14333	*Kabuli*	Ca_Kabuli_Scaffold1196	70549	(G/A)
14334	CakSNP14334	*Kabuli*	Ca_Kabuli_Scaffold1196	70547	(G/C)
14335	CakSNP14335	*Kabuli*	Ca_Kabuli_Scaffold1196	70535	(G/A)
14336	CakSNP14336	*Kabuli*	Ca_Kabuli_Scaffold1196	70530	(C/T)
14337	CakSNP14337	*Kabuli*	Ca_Kabuli_Scaffold1196	70526	(T/C)
14338	CakSNP14338	*Kabuli*	Ca_Kabuli_Scaffold1196	70520	(G/A)
14339	CakSNP14339	*Kabuli*	Ca_Kabuli_Scaffold1196	70503	(C/T)
14340	CakSNP14340	*Kabuli*	Ca_Kabuli_Scaffold1196	203001	(G/A)
14341	CakSNP14341	*Kabuli*	Ca_Kabuli_Scaffold1197	219637	(A/T)
14342	CakSNP14342	*Kabuli*	Ca_Kabuli_Scaffold1197	344239	(A/C)
14343	CakSNP14343	*Kabuli*	Ca_Kabuli_Scaffold1197	402506	(T/C)
14344	CakSNP14344	*Kabuli*	Ca_Kabuli_Scaffold1197	407641	(G/A)
14345	CakSNP14345	*Kabuli*	Ca_Kabuli_Scaffold1197	407658	(C/G)
14346	CakSNP14346	*Kabuli*	Ca_Kabuli_Scaffold1197	407691	(T/C)
14347	CakSNP14347	*Kabuli*	Ca_Kabuli_Scaffold1197	407695	(T/C)
14348	CakSNP14348	*Kabuli*	Ca_Kabuli_Scaffold1197	447890	(C/T)
14349	CakSNP14349	*Kabuli*	Ca_Kabuli_Scaffold1197	520164	(T/A)
14350	CakSNP14350	*Kabuli*	Ca_Kabuli_Scaffold1197	520153	(C/G)

S.N.	SNP IDs	Cultivars	Chromosomes/scaffolds	Physical positions (bp)	SNPs
14351	CakSNP14351	*Kabuli*	Ca_Kabuli_Scaffold1197	520148	(A/T)
14352	CakSNP14352	*Kabuli*	Ca_Kabuli_Scaffold12	107756	(G/A)
14353	CakSNP14353	*Kabuli*	Ca_Kabuli_Scaffold12	200573	(G/C)
14354	CakSNP14354	*Kabuli*	Ca_Kabuli_Scaffold12	331877	(G/A)
14355	CakSNP14355	*Kabuli*	Ca_Kabuli_Scaffold12	331890	(A/G)
14356	CakSNP14356	*Kabuli*	Ca_Kabuli_Scaffold12	332909	(C/T)
14357	CakSNP14357	*Kabuli*	Ca_Kabuli_Scaffold12	332921	(G/A)
14358	CakSNP14358	*Kabuli*	Ca_Kabuli_Scaffold12	332963	(C/T)
14359	CakSNP14359	*Kabuli*	Ca_Kabuli_Scaffold1202	46904	(C/T)
14360	CakSNP14360	*Kabuli*	Ca_Kabuli_Scaffold1202	46945	(G/A)
14361	CakSNP14361	*Kabuli*	Ca_Kabuli_Scaffold1215	40741	(G/T)
14362	CakSNP14362	*Kabuli*	Ca_Kabuli_Scaffold1215	40793	(A/G)
14363	CakSNP14363	*Kabuli*	Ca_Kabuli_Scaffold1215	40797	(A/T)
14364	CakSNP14364	*Kabuli*	Ca_Kabuli_Scaffold1215	40817	(G/A)
14365	CakSNP14365	*Kabuli*	Ca_Kabuli_Scaffold1215	40814	(A/T)
14366	CakSNP14366	*Kabuli*	Ca_Kabuli_Scaffold1219	34791	(G/A)
14367	CakSNP14367	*Kabuli*	Ca_Kabuli_Scaffold1219	85015	(A/C)
14368	CakSNP14368	*Kabuli*	Ca_Kabuli_Scaffold1259	71428	(C/T)
14369	CakSNP14369	*Kabuli*	Ca_Kabuli_Scaffold1259	71574	(A/T)
14370	CakSNP14370	*Kabuli*	Ca_Kabuli_Scaffold1259	71649	(C/T)
14371	CakSNP14371	*Kabuli*	Ca_Kabuli_Scaffold127	28809	(A/T)
14372	CakSNP14372	*Kabuli*	Ca_Kabuli_Scaffold127	28780	(G/A)
14373	CakSNP14373	*Kabuli*	Ca_Kabuli_Scaffold127	28778	(C/T)
14374	CakSNP14374	*Kabuli*	Ca_Kabuli_Scaffold127	28776	(T/G)
14375	CakSNP14375	*Kabuli*	Ca_Kabuli_Scaffold127	28772	(G/A)
14376	CakSNP14376	*Kabuli*	Ca_Kabuli_Scaffold1272	57303	(C/A)
14377	CakSNP14377	*Kabuli*	Ca_Kabuli_Scaffold1272	57356	(C/G)
14378	CakSNP14378	*Kabuli*	Ca_Kabuli_Scaffold128	19256	(A/C)
14379	CakSNP14379	*Kabuli*	Ca_Kabuli_Scaffold128	19315	(C/T)
14380	CakSNP14380	*Kabuli*	Ca_Kabuli_Scaffold128	52881	(G/A)
14381	CakSNP14381	*Kabuli*	Ca_Kabuli_Scaffold128	52855	(G/C)
14382	CakSNP14382	*Kabuli*	Ca_Kabuli_Scaffold128	52844	(A/T)
14383	CakSNP14383	*Kabuli*	Ca_Kabuli_Scaffold128	263590	(G/T)
14384	CakSNP14384	*Kabuli*	Ca_Kabuli_Scaffold128	263586	(T/A)
14385	CakSNP14385	*Kabuli*	Ca_Kabuli_Scaffold128	263572	(T/A)
14386	CakSNP14386	*Kabuli*	Ca_Kabuli_Scaffold128	334180	(G/T)
14387	CakSNP14387	*Kabuli*	Ca_Kabuli_Scaffold128	334129	(G/A)
14388	CakSNP14388	*Kabuli*	Ca_Kabuli_Scaffold128	593782	(T/C)
14389	CakSNP14389	*Kabuli*	Ca_Kabuli_Scaffold128	860975	(G/A)

S.N.	SNP IDs	Cultivars	Chromosomes/scaffolds	Physical positions (bp)	SNPs
14390	CakSNP14390	*Kabuli*	Ca_Kabuli_Scaffold128	860981	(T/A)
14391	CakSNP14391	*Kabuli*	Ca_Kabuli_Scaffold128	860983	(A/T)
14392	CakSNP14392	*Kabuli*	Ca_Kabuli_Scaffold128	860987	(C/T)
14393	CakSNP14393	*Kabuli*	Ca_Kabuli_Scaffold128	861003	(C/A)
14394	CakSNP14394	*Kabuli*	Ca_Kabuli_Scaffold128	861010	(C/T)
14395	CakSNP14395	*Kabuli*	Ca_Kabuli_Scaffold128	861022	(T/C)
14396	CakSNP14396	*Kabuli*	Ca_Kabuli_Scaffold128	861028	(T/C)
14397	CakSNP14397	*Kabuli*	Ca_Kabuli_Scaffold128	861072	(G/T)
14398	CakSNP14398	*Kabuli*	Ca_Kabuli_Scaffold128	861053	(G/A)
14399	CakSNP14399	*Kabuli*	Ca_Kabuli_Scaffold128	861036	(G/A)
14400	CakSNP14400	*Kabuli*	Ca_Kabuli_Scaffold128	861015	(C/T)
14401	CakSNP14401	*Kabuli*	Ca_Kabuli_Scaffold1281	211456	(A/T)
14402	CakSNP14402	*Kabuli*	Ca_Kabuli_Scaffold1281	239742	(T/C)
14403	CakSNP14403	*Kabuli*	Ca_Kabuli_Scaffold1281	244153	(A/C)
14404	CakSNP14404	*Kabuli*	Ca_Kabuli_Scaffold1281	258565	(G/A)
14405	CakSNP14405	*Kabuli*	Ca_Kabuli_Scaffold1281	316037	(A/G)
14406	CakSNP14406	*Kabuli*	Ca_Kabuli_Scaffold1281	396888	(G/A)
14407	CakSNP14407	*Kabuli*	Ca_Kabuli_Scaffold1281	396996	(C/T)
14408	CakSNP14408	*Kabuli*	Ca_Kabuli_Scaffold1281	396992	(A/G)
14409	CakSNP14409	*Kabuli*	Ca_Kabuli_Scaffold1281	398693	(G/A)
14410	CakSNP14410	*Kabuli*	Ca_Kabuli_Scaffold1281	398729	(T/G)
14411	CakSNP14411	*Kabuli*	Ca_Kabuli_Scaffold1281	398887	(G/A)
14412	CakSNP14412	*Kabuli*	Ca_Kabuli_Scaffold1281	408214	(C/T)
14413	CakSNP14413	*Kabuli*	Ca_Kabuli_Scaffold1281	408170	(C/T)
14414	CakSNP14414	*Kabuli*	Ca_Kabuli_Scaffold1281	526446	(C/T)
14415	CakSNP14415	*Kabuli*	Ca_Kabuli_Scaffold1281	526442	(T/C)
14416	CakSNP14416	*Kabuli*	Ca_Kabuli_Scaffold1281	527012	(C/T)
14417	CakSNP14417	*Kabuli*	Ca_Kabuli_Scaffold1281	527008	(T/C)
14418	CakSNP14418	*Kabuli*	Ca_Kabuli_Scaffold1285	68330	(G/A)
14419	CakSNP14419	*Kabuli*	Ca_Kabuli_Scaffold1285	68327	(G/T)
14420	CakSNP14420	*Kabuli*	Ca_Kabuli_Scaffold1285	68324	(T/C)
14421	CakSNP14421	*Kabuli*	Ca_Kabuli_Scaffold1285	68315	(T/C)
14422	CakSNP14422	*Kabuli*	Ca_Kabuli_Scaffold1285	68311	(G/A)
14423	CakSNP14423	*Kabuli*	Ca_Kabuli_Scaffold1285	96310	(A/C)
14424	CakSNP14424	*Kabuli*	Ca_Kabuli_Scaffold1285	96358	(T/C)
14425	CakSNP14425	*Kabuli*	Ca_Kabuli_Scaffold1285	96429	(C/T)
14426	CakSNP14426	*Kabuli*	Ca_Kabuli_Scaffold1285	96428	(G/A)
14427	CakSNP14427	*Kabuli*	Ca_Kabuli_Scaffold1285	96414	(C/T)
14428	CakSNP14428	*Kabuli*	Ca_Kabuli_Scaffold1285	96411	(G/A)

S.N.	SNP IDs	Cultivars	Chromosomes/scaffolds	Physical positions (bp)	SNPs
14429	CakSNP14429	*Kabuli*	Ca_Kabuli_Scaffold1285	96409	(C/T)
14430	CakSNP14430	*Kabuli*	Ca_Kabuli_Scaffold1285	96399	(C/T)
14431	CakSNP14431	*Kabuli*	Ca_Kabuli_Scaffold1285	105553	(T/C)
14432	CakSNP14432	*Kabuli*	Ca_Kabuli_Scaffold1285	105507	(A/T)
14433	CakSNP14433	*Kabuli*	Ca_Kabuli_Scaffold1285	105504	(G/A)
14434	CakSNP14434	*Kabuli*	Ca_Kabuli_Scaffold1285	192068	(T/G)
14435	CakSNP14435	*Kabuli*	Ca_Kabuli_Scaffold1285	240762	(C/T)
14436	CakSNP14436	*Kabuli*	Ca_Kabuli_Scaffold1285	240735	(G/A)
14437	CakSNP14437	*Kabuli*	Ca_Kabuli_Scaffold1285	240730	(T/C)
14438	CakSNP14438	*Kabuli*	Ca_Kabuli_Scaffold1285	240723	(C/T)
14439	CakSNP14439	*Kabuli*	Ca_Kabuli_Scaffold1301_1	43433	(T/C)
14440	CakSNP14440	*Kabuli*	Ca_Kabuli_Scaffold1301_1	45565	(A/C)
14441	CakSNP14441	*Kabuli*	Ca_Kabuli_Scaffold1301_1	45566	(G/A)
14442	CakSNP14442	*Kabuli*	Ca_Kabuli_Scaffold1301_1	58243	(A/G)
14443	CakSNP14443	*Kabuli*	Ca_Kabuli_Scaffold1301_1	58337	(C/T)
14444	CakSNP14444	*Kabuli*	Ca_Kabuli_Scaffold1301_1	58460	(A/T)
14445	CakSNP14445	*Kabuli*	Ca_Kabuli_Scaffold1301_1	63148	(T/C)
14446	CakSNP14446	*Kabuli*	Ca_Kabuli_Scaffold1301_1	95495	(G/A)
14447	CakSNP14447	*Kabuli*	Ca_Kabuli_Scaffold1301_1	95497	(G/A)
14448	CakSNP14448	*Kabuli*	Ca_Kabuli_Scaffold1301_1	95602	(T/C)
14449	CakSNP14449	*Kabuli*	Ca_Kabuli_Scaffold1301_1	95533	(C/G)
14450	CakSNP14450	*Kabuli*	Ca_Kabuli_Scaffold1301_1	122496	(T/A)
14451	CakSNP14451	*Kabuli*	Ca_Kabuli_Scaffold1301_1	122563	(T/C)
14452	CakSNP14452	*Kabuli*	Ca_Kabuli_Scaffold1301_1	154342	(A/C)
14453	CakSNP14453	*Kabuli*	Ca_Kabuli_Scaffold1301_1	157390	(A/G)
14454	CakSNP14454	*Kabuli*	Ca_Kabuli_Scaffold1301_1	218538	(G/A)
14455	CakSNP14455	*Kabuli*	Ca_Kabuli_Scaffold1308	44260	(A/G)
14456	CakSNP14456	*Kabuli*	Ca_Kabuli_Scaffold1309	49259	(G/A)
14457	CakSNP14457	*Kabuli*	Ca_Kabuli_Scaffold1312	98400	(T/C)
14458	CakSNP14458	*Kabuli*	Ca_Kabuli_Scaffold1313	18173	(A/G)
14459	CakSNP14459	*Kabuli*	Ca_Kabuli_Scaffold1313	18412	(G/A)
14460	CakSNP14460	*Kabuli*	Ca_Kabuli_Scaffold1313	227732	(G/A)
14461	CakSNP14461	*Kabuli*	Ca_Kabuli_Scaffold1313	227744	(C/T)
14462	CakSNP14462	*Kabuli*	Ca_Kabuli_Scaffold1315	115480	(G/A)
14463	CakSNP14463	*Kabuli*	Ca_Kabuli_Scaffold1315	115476	(A/C)
14464	CakSNP14464	*Kabuli*	Ca_Kabuli_Scaffold1315	115471	(G/A)
14465	CakSNP14465	*Kabuli*	Ca_Kabuli_Scaffold1315	115446	(C/A)
14466	CakSNP14466	*Kabuli*	Ca_Kabuli_Scaffold1315	115481	(G/T)
14467	CakSNP14467	*Kabuli*	Ca_Kabuli_Scaffold132	153364	(A/G)

S.N.	SNP IDs	Cultivars	Chromosomes/scaffolds	Physical positions (bp)	SNPs
14468	CakSNP14468	*Kabuli*	Ca_Kabuli_Scaffold132	153361	(A/G)
14469	CakSNP14469	*Kabuli*	Ca_Kabuli_Scaffold1324	228151	(G/A)
14470	CakSNP14470	*Kabuli*	Ca_Kabuli_Scaffold1324	228159	(A/C)
14471	CakSNP14471	*Kabuli*	Ca_Kabuli_Scaffold1324	262208	(C/G)
14472	CakSNP14472	*Kabuli*	Ca_Kabuli_Scaffold134	218290	(C/T)
14473	CakSNP14473	*Kabuli*	Ca_Kabuli_Scaffold134	218352	(T/G)
14474	CakSNP14474	*Kabuli*	Ca_Kabuli_Scaffold134	218336	(T/G)
14475	CakSNP14475	*Kabuli*	Ca_Kabuli_Scaffold134	223318	(C/T)
14476	CakSNP14476	*Kabuli*	Ca_Kabuli_Scaffold134	223303	(T/C)
14477	CakSNP14477	*Kabuli*	Ca_Kabuli_Scaffold134	233007	(C/T)
14478	CakSNP14478	*Kabuli*	Ca_Kabuli_Scaffold134	275661	(T/A)
14479	CakSNP14479	*Kabuli*	Ca_Kabuli_Scaffold1348_1	84644	(G/C)
14480	CakSNP14480	*Kabuli*	Ca_Kabuli_Scaffold1348_1	134575	(T/C)
14481	CakSNP14481	*Kabuli*	Ca_Kabuli_Scaffold1348_1	134572	(G/A)
14482	CakSNP14482	*Kabuli*	Ca_Kabuli_Scaffold1348_1	294458	(A/G)
14483	CakSNP14483	*Kabuli*	Ca_Kabuli_Scaffold1348_1	294456	(A/G)
14484	CakSNP14484	*Kabuli*	Ca_Kabuli_Scaffold1348_1	380156	(A/T)
14485	CakSNP14485	*Kabuli*	Ca_Kabuli_Scaffold1348_1	380225	(T/G)
14486	CakSNP14486	*Kabuli*	Ca_Kabuli_Scaffold1348_1	380227	(C/G)
14487	CakSNP14487	*Kabuli*	Ca_Kabuli_Scaffold1348_1	380244	(C/G)
14488	CakSNP14488	*Kabuli*	Ca_Kabuli_Scaffold1348_1	380289	(C/T)
14489	CakSNP14489	*Kabuli*	Ca_Kabuli_Scaffold1348_1	380336	(G/C)
14490	CakSNP14490	*Kabuli*	Ca_Kabuli_Scaffold1348_1	381026	(G/T)
14491	CakSNP14491	*Kabuli*	Ca_Kabuli_Scaffold1348_1	452011	(G/T)
14492	CakSNP14492	*Kabuli*	Ca_Kabuli_Scaffold1348_1	454342	(G/T)
14493	CakSNP14493	*Kabuli*	Ca_Kabuli_Scaffold1348_1	515602	(A/C)
14494	CakSNP14494	*Kabuli*	Ca_Kabuli_Scaffold1348_1	535984	(G/T)
14495	CakSNP14495	*Kabuli*	Ca_Kabuli_Scaffold1348_1	552192	(C/A)
14496	CakSNP14496	*Kabuli*	Ca_Kabuli_Scaffold1348_1	552299	(T/C)
14497	CakSNP14497	*Kabuli*	Ca_Kabuli_Scaffold1348_1	552342	(G/A)
14498	CakSNP14498	*Kabuli*	Ca_Kabuli_Scaffold1348_1	552328	(A/G)
14499	CakSNP14499	*Kabuli*	Ca_Kabuli_Scaffold1348_1	553863	(G/T)
14500	CakSNP14500	*Kabuli*	Ca_Kabuli_Scaffold1348_1	553978	(C/T)
14501	CakSNP14501	*Kabuli*	Ca_Kabuli_Scaffold1348_1	554003	(G/C)
14502	CakSNP14502	*Kabuli*	Ca_Kabuli_Scaffold1348_1	554134	(A/G)
14503	CakSNP14503	*Kabuli*	Ca_Kabuli_Scaffold1348_1	554215	(T/G)
14504	CakSNP14504	*Kabuli*	Ca_Kabuli_Scaffold1348_1	651906	(A/T)
14505	CakSNP14505	*Kabuli*	Ca_Kabuli_Scaffold1348_1	652067	(A/G)
14506	CakSNP14506	*Kabuli*	Ca_Kabuli_Scaffold1348_1	667541	(A/C)

S.N.	SNP IDs	Cultivars	Chromosomes/scaffolds	Physical positions (bp)	SNPs
14507	CakSNP14507	*Kabuli*	Ca_Kabuli_Scaffold1348_1	667504	(C/T)
14508	CakSNP14508	*Kabuli*	Ca_Kabuli_Scaffold1348_1	679305	(T/C)
14509	CakSNP14509	*Kabuli*	Ca_Kabuli_Scaffold1348_1	680734	(G/T)
14510	CakSNP14510	*Kabuli*	Ca_Kabuli_Scaffold1348_1	680730	(C/T)
14511	CakSNP14511	*Kabuli*	Ca_Kabuli_Scaffold1348_1	680718	(A/T)
14512	CakSNP14512	*Kabuli*	Ca_Kabuli_Scaffold1348_1	708276	(G/A)
14513	CakSNP14513	*Kabuli*	Ca_Kabuli_Scaffold1348_1	708302	(G/T)
14514	CakSNP14514	*Kabuli*	Ca_Kabuli_Scaffold1348_1	708344	(G/A)
14515	CakSNP14515	*Kabuli*	Ca_Kabuli_Scaffold1348_1	732450	(A/C)
14516	CakSNP14516	*Kabuli*	Ca_Kabuli_Scaffold1348_1	732454	(T/G)
14517	CakSNP14517	*Kabuli*	Ca_Kabuli_Scaffold1348_1	732460	(T/A)
14518	CakSNP14518	*Kabuli*	Ca_Kabuli_Scaffold1348_1	732496	(G/T)
14519	CakSNP14519	*Kabuli*	Ca_Kabuli_Scaffold1348_1	735782	(C/T)
14520	CakSNP14520	*Kabuli*	Ca_Kabuli_Scaffold1348_1	735812	(G/A)
14521	CakSNP14521	*Kabuli*	Ca_Kabuli_Scaffold1348_1	750170	(C/A)
14522	CakSNP14522	*Kabuli*	Ca_Kabuli_Scaffold1348_1	750162	(T/C)
14523	CakSNP14523	*Kabuli*	Ca_Kabuli_Scaffold1348_1	750213	(C/A)
14524	CakSNP14524	*Kabuli*	Ca_Kabuli_Scaffold1348_1	771029	(T/A)
14525	CakSNP14525	*Kabuli*	Ca_Kabuli_Scaffold1348_1	871724	(G/A)
14526	CakSNP14526	*Kabuli*	Ca_Kabuli_Scaffold1348_1	909909	(A/G)
14527	CakSNP14527	*Kabuli*	Ca_Kabuli_Scaffold1348_1	909967	(C/G)
14528	CakSNP14528	*Kabuli*	Ca_Kabuli_Scaffold1348_1	909965	(T/A)
14529	CakSNP14529	*Kabuli*	Ca_Kabuli_Scaffold1348_1	909959	(A/C)
14530	CakSNP14530	*Kabuli*	Ca_Kabuli_Scaffold1348_1	924164	(T/G)
14531	CakSNP14531	*Kabuli*	Ca_Kabuli_Scaffold1348_1	934720	(C/A)
14532	CakSNP14532	*Kabuli*	Ca_Kabuli_Scaffold1348_1	934791	(C/T)
14533	CakSNP14533	*Kabuli*	Ca_Kabuli_Scaffold1348_1	950995	(A/G)
14534	CakSNP14534	*Kabuli*	Ca_Kabuli_Scaffold1348_1	959608	(A/C)
14535	CakSNP14535	*Kabuli*	Ca_Kabuli_Scaffold1348_1	959828	(G/A)
14536	CakSNP14536	*Kabuli*	Ca_Kabuli_Scaffold1348_1	959780	(G/A)
14537	CakSNP14537	*Kabuli*	Ca_Kabuli_Scaffold1348_1	970927	(A/G)
14538	CakSNP14538	*Kabuli*	Ca_Kabuli_Scaffold1348_1	981964	(T/C)
14539	CakSNP14539	*Kabuli*	Ca_Kabuli_Scaffold1348_1	983101	(T/G)
14540	CakSNP14540	*Kabuli*	Ca_Kabuli_Scaffold1348_1	1020258	(C/T)
14541	CakSNP14541	*Kabuli*	Ca_Kabuli_Scaffold1348_1	1023898	(G/A)
14542	CakSNP14542	*Kabuli*	Ca_Kabuli_Scaffold1348_1	1023923	(G/A)
14543	CakSNP14543	*Kabuli*	Ca_Kabuli_Scaffold1348_1	1023954	(G/C)
14544	CakSNP14544	*Kabuli*	Ca_Kabuli_Scaffold1348_1	1066818	(A/C)
14545	CakSNP14545	*Kabuli*	Ca_Kabuli_Scaffold1348_1	1066783	(C/T)

S.N.	SNP IDs	Cultivars	Chromosomes/scaffolds	Physical positions (bp)	SNPs
14546	CakSNP14546	*Kabuli*	Ca_Kabuli_Scaffold1348_1	1066776	(T/C)
14547	CakSNP14547	*Kabuli*	Ca_Kabuli_Scaffold1348_1	1072750	(C/T)
14548	CakSNP14548	*Kabuli*	Ca_Kabuli_Scaffold1348_1	1145082	(A/C)
14549	CakSNP14549	*Kabuli*	Ca_Kabuli_Scaffold1348_1	1145124	(T/C)
14550	CakSNP14550	*Kabuli*	Ca_Kabuli_Scaffold1348_1	1207442	(G/A)
14551	CakSNP14551	*Kabuli*	Ca_Kabuli_Scaffold1348_1	1236956	(A/G)
14552	CakSNP14552	*Kabuli*	Ca_Kabuli_Scaffold1348_1	1263560	(T/G)
14553	CakSNP14553	*Kabuli*	Ca_Kabuli_Scaffold1348_1	1265627	(T/C)
14554	CakSNP14554	*Kabuli*	Ca_Kabuli_Scaffold1348_1	1265635	(T/G)
14555	CakSNP14555	*Kabuli*	Ca_Kabuli_Scaffold1348_1	1273341	(G/A)
14556	CakSNP14556	*Kabuli*	Ca_Kabuli_Scaffold1348_1	1273388	(G/A)
14557	CakSNP14557	*Kabuli*	Ca_Kabuli_Scaffold1348_1	1288448	(A/G)
14558	CakSNP14558	*Kabuli*	Ca_Kabuli_Scaffold1348_1	1294675	(A/T)
14559	CakSNP14559	*Kabuli*	Ca_Kabuli_Scaffold1348_1	1296453	(G/A)
14560	CakSNP14560	*Kabuli*	Ca_Kabuli_Scaffold1348_1	1330256	(A/G)
14561	CakSNP14561	*Kabuli*	Ca_Kabuli_Scaffold1348_1	1330288	(A/G)
14562	CakSNP14562	*Kabuli*	Ca_Kabuli_Scaffold1348_1	1330400	(A/G)
14563	CakSNP14563	*Kabuli*	Ca_Kabuli_Scaffold1348_1	1346914	(G/T)
14564	CakSNP14564	*Kabuli*	Ca_Kabuli_Scaffold1348_1	1346943	(G/A)
14565	CakSNP14565	*Kabuli*	Ca_Kabuli_Scaffold1348_1	1372535	(G/A)
14566	CakSNP14566	*Kabuli*	Ca_Kabuli_Scaffold1348_1	1403452	(G/A)
14567	CakSNP14567	*Kabuli*	Ca_Kabuli_Scaffold1348_1	1403480	(A/G)
14568	CakSNP14568	*Kabuli*	Ca_Kabuli_Scaffold1351	172111	(G/A)
14569	CakSNP14569	*Kabuli*	Ca_Kabuli_Scaffold1351	172110	(T/C)
14570	CakSNP14570	*Kabuli*	Ca_Kabuli_Scaffold1351	177159	(G/A)
14571	CakSNP14571	*Kabuli*	Ca_Kabuli_Scaffold1351	177172	(G/A)
14572	CakSNP14572	*Kabuli*	Ca_Kabuli_Scaffold1351	177178	(T/C)
14573	CakSNP14573	*Kabuli*	Ca_Kabuli_Scaffold1351	177181	(A/G)
14574	CakSNP14574	*Kabuli*	Ca_Kabuli_Scaffold1351	177194	(G/A)
14575	CakSNP14575	*Kabuli*	Ca_Kabuli_Scaffold1351	177202	(G/A)
14576	CakSNP14576	*Kabuli*	Ca_Kabuli_Scaffold1351	177171	(T/G)
14577	CakSNP14577	*Kabuli*	Ca_Kabuli_Scaffold1351	177193	(T/C)
14578	CakSNP14578	*Kabuli*	Ca_Kabuli_Scaffold1351	177256	(T/G)
14579	CakSNP14579	*Kabuli*	Ca_Kabuli_Scaffold1351	177248	(G/T)
14580	CakSNP14580	*Kabuli*	Ca_Kabuli_Scaffold1351	177247	(G/A)
14581	CakSNP14581	*Kabuli*	Ca_Kabuli_Scaffold1351	177242	(G/A)
14582	CakSNP14582	*Kabuli*	Ca_Kabuli_Scaffold1351	177241	(T/G)
14583	CakSNP14583	*Kabuli*	Ca_Kabuli_Scaffold1351	177240	(C/A)
14584	CakSNP14584	*Kabuli*	Ca_Kabuli_Scaffold1351	177235	(G/T)

S.N.	SNP IDs	Cultivars	Chromosomes/scaffolds	Physical positions (bp)	SNPs
14585	CakSNP14585	*Kabuli*	Ca_Kabuli_Scaffold1351	177234	(A/C)
14586	CakSNP14586	*Kabuli*	Ca_Kabuli_Scaffold1351	177210	(T/A)
14587	CakSNP14587	*Kabuli*	Ca_Kabuli_Scaffold1351	177200	(T/G)
14588	CakSNP14588	*Kabuli*	Ca_Kabuli_Scaffold1351	177191	(C/T)
14589	CakSNP14589	*Kabuli*	Ca_Kabuli_Scaffold1351	177188	(G/T)
14590	CakSNP14590	*Kabuli*	Ca_Kabuli_Scaffold1351	204656	(C/A)
14591	CakSNP14591	*Kabuli*	Ca_Kabuli_Scaffold1351	335227	(T/C)
14592	CakSNP14592	*Kabuli*	Ca_Kabuli_Scaffold1351	335239	(G/T)
14593	CakSNP14593	*Kabuli*	Ca_Kabuli_Scaffold1351	367576	(A/G)
14594	CakSNP14594	*Kabuli*	Ca_Kabuli_Scaffold1351	367578	(G/A)
14595	CakSNP14595	*Kabuli*	Ca_Kabuli_Scaffold1351	367745	(G/A)
14596	CakSNP14596	*Kabuli*	Ca_Kabuli_Scaffold1369	8015	(T/A)
14597	CakSNP14597	*Kabuli*	Ca_Kabuli_Scaffold1369	8115	(T/G)
14598	CakSNP14598	*Kabuli*	Ca_Kabuli_Scaffold1369	8154	(T/G)
14599	CakSNP14599	*Kabuli*	Ca_Kabuli_Scaffold1369	8221	(T/G)
14600	CakSNP14600	*Kabuli*	Ca_Kabuli_Scaffold1369	8327	(G/A)
14601	CakSNP14601	*Kabuli*	Ca_Kabuli_Scaffold1369	31000	(A/G)
14602	CakSNP14602	*Kabuli*	Ca_Kabuli_Scaffold1390	98354	(A/G)
14603	CakSNP14603	*Kabuli*	Ca_Kabuli_Scaffold1401	89077	(T/C)
14604	CakSNP14604	*Kabuli*	Ca_Kabuli_Scaffold1401	89074	(T/C)
14605	CakSNP14605	*Kabuli*	Ca_Kabuli_Scaffold1417	4863	(A/G)
14606	CakSNP14606	*Kabuli*	Ca_Kabuli_Scaffold1417	4906	(G/A)
14607	CakSNP14607	*Kabuli*	Ca_Kabuli_Scaffold1417	4917	(G/T)
14608	CakSNP14608	*Kabuli*	Ca_Kabuli_Scaffold1417	76627	(A/C)
14609	CakSNP14609	*Kabuli*	Ca_Kabuli_Scaffold1417	230210	(T/G)
14610	CakSNP14610	*Kabuli*	Ca_Kabuli_Scaffold1419	24845	(C/T)
14611	CakSNP14611	*Kabuli*	Ca_Kabuli_Scaffold143	18492	(A/G)
14612	CakSNP14612	*Kabuli*	Ca_Kabuli_Scaffold1439	242972	(A/G)
14613	CakSNP14613	*Kabuli*	Ca_Kabuli_Scaffold1448	47306	(A/G)
14614	CakSNP14614	*Kabuli*	Ca_Kabuli_Scaffold1448	74436	(C/T)
14615	CakSNP14615	*Kabuli*	Ca_Kabuli_Scaffold1448	74434	(C/T)
14616	CakSNP14616	*Kabuli*	Ca_Kabuli_Scaffold1448	74529	(C/T)
14617	CakSNP14617	*Kabuli*	Ca_Kabuli_Scaffold1448	76809	(G/A)
14618	CakSNP14618	*Kabuli*	Ca_Kabuli_Scaffold1449	55474	(T/G)
14619	CakSNP14619	*Kabuli*	Ca_Kabuli_Scaffold1449	55529	(G/A)
14620	CakSNP14620	*Kabuli*	Ca_Kabuli_Scaffold1449	55532	(G/A)
14621	CakSNP14621	*Kabuli*	Ca_Kabuli_Scaffold1452	3501	(C/A)
14622	CakSNP14622	*Kabuli*	Ca_Kabuli_Scaffold1452	3496	(A/T)
14623	CakSNP14623	*Kabuli*	Ca_Kabuli_Scaffold1452	3495	(C/A)

S.N.	SNP IDs	Cultivars	Chromosomes/scaffolds	Physical positions (bp)	SNPs
14624	CakSNP14624	*Kabuli*	Ca_Kabuli_Scaffold1452	3737	(C/A)
14625	CakSNP14625	*Kabuli*	Ca_Kabuli_Scaffold1462	1556	(T/G)
14626	CakSNP14626	*Kabuli*	Ca_Kabuli_Scaffold1466	90846	(C/A)
14627	CakSNP14627	*Kabuli*	Ca_Kabuli_Scaffold1466	165472	(C/T)
14628	CakSNP14628	*Kabuli*	Ca_Kabuli_Scaffold1466	322287	(G/A)
14629	CakSNP14629	*Kabuli*	Ca_Kabuli_Scaffold1466	322315	(G/T)
14630	CakSNP14630	*Kabuli*	Ca_Kabuli_Scaffold1466	322450	(G/T)
14631	CakSNP14631	*Kabuli*	Ca_Kabuli_Scaffold1466	322448	(T/G)
14632	CakSNP14632	*Kabuli*	Ca_Kabuli_Scaffold1466	322421	(C/T)
14633	CakSNP14633	*Kabuli*	Ca_Kabuli_Scaffold1466	322415	(G/A)
14634	CakSNP14634	*Kabuli*	Ca_Kabuli_Scaffold1466	322414	(G/A)
14635	CakSNP14635	*Kabuli*	Ca_Kabuli_Scaffold1466	322400	(C/T)
14636	CakSNP14636	*Kabuli*	Ca_Kabuli_Scaffold1466	322390	(G/A)
14637	CakSNP14637	*Kabuli*	Ca_Kabuli_Scaffold1466	322383	(C/T)
14638	CakSNP14638	*Kabuli*	Ca_Kabuli_Scaffold1467	205779	(A/G)
14639	CakSNP14639	*Kabuli*	Ca_Kabuli_Scaffold1467	205803	(C/T)
14640	CakSNP14640	*Kabuli*	Ca_Kabuli_Scaffold1467	205833	(G/A)
14641	CakSNP14641	*Kabuli*	Ca_Kabuli_Scaffold1467	205840	(G/A)
14642	CakSNP14642	*Kabuli*	Ca_Kabuli_Scaffold1467	205845	(A/G)
14643	CakSNP14643	*Kabuli*	Ca_Kabuli_Scaffold1467	206121	(G/A)
14644	CakSNP14644	*Kabuli*	Ca_Kabuli_Scaffold1467	206118	(C/T)
14645	CakSNP14645	*Kabuli*	Ca_Kabuli_Scaffold1467	206100	(G/A)
14646	CakSNP14646	*Kabuli*	Ca_Kabuli_Scaffold1467	206094	(T/C)
14647	CakSNP14647	*Kabuli*	Ca_Kabuli_Scaffold1467	206085	(T/C)
14648	CakSNP14648	*Kabuli*	Ca_Kabuli_Scaffold1467	206068	(A/G)
14649	CakSNP14649	*Kabuli*	Ca_Kabuli_Scaffold1467	206067	(T/G)
14650	CakSNP14650	*Kabuli*	Ca_Kabuli_Scaffold1475	4820	(G/A)
14651	CakSNP14651	*Kabuli*	Ca_Kabuli_Scaffold1475	4796	(C/T)
14652	CakSNP14652	*Kabuli*	Ca_Kabuli_Scaffold1475	4794	(C/A)
14653	CakSNP14653	*Kabuli*	Ca_Kabuli_Scaffold1475	4755	(T/A)
14654	CakSNP14654	*Kabuli*	Ca_Kabuli_Scaffold1475	4754	(C/T)
14655	CakSNP14655	*Kabuli*	Ca_Kabuli_Scaffold1475	4749	(C/T)
14656	CakSNP14656	*Kabuli*	Ca_Kabuli_Scaffold1475	4873	(G/T)
14657	CakSNP14657	*Kabuli*	Ca_Kabuli_Scaffold1475	4845	(A/T)
14658	CakSNP14658	*Kabuli*	Ca_Kabuli_Scaffold1475	4833	(G/A)
14659	CakSNP14659	*Kabuli*	Ca_Kabuli_Scaffold1475	4818	(C/T)
14660	CakSNP14660	*Kabuli*	Ca_Kabuli_Scaffold1483	56686	(A/T)
14661	CakSNP14661	*Kabuli*	Ca_Kabuli_Scaffold1483	56721	(G/C)
14662	CakSNP14662	*Kabuli*	Ca_Kabuli_Scaffold1493	81740	(C/T)

S.N.	SNP IDs	Cultivars	Chromosomes/scaffolds	Physical positions (bp)	SNPs
14663	CakSNP14663	*Kabuli*	Ca_Kabuli_Scaffold1493	81784	(C/A)
14664	CakSNP14664	*Kabuli*	Ca_Kabuli_Scaffold1493	81789	(G/A)
14665	CakSNP14665	*Kabuli*	Ca_Kabuli_Scaffold1496	289997	(G/T)
14666	CakSNP14666	*Kabuli*	Ca_Kabuli_Scaffold1496	290018	(C/A)
14667	CakSNP14667	*Kabuli*	Ca_Kabuli_Scaffold1498	3474	(A/C)
14668	CakSNP14668	*Kabuli*	Ca_Kabuli_Scaffold1498	3657	(C/T)
14669	CakSNP14669	*Kabuli*	Ca_Kabuli_Scaffold1498	3641	(T/C)
14670	CakSNP14670	*Kabuli*	Ca_Kabuli_Scaffold1504	160623	(G/T)
14671	CakSNP14671	*Kabuli*	Ca_Kabuli_Scaffold1504	189700	(A/G)
14672	CakSNP14672	*Kabuli*	Ca_Kabuli_Scaffold1504	189699	(C/T)
14673	CakSNP14673	*Kabuli*	Ca_Kabuli_Scaffold1504	189681	(C/T)
14674	CakSNP14674	*Kabuli*	Ca_Kabuli_Scaffold1504	189664	(G/A)
14675	CakSNP14675	*Kabuli*	Ca_Kabuli_Scaffold1504	189663	(G/A)
14676	CakSNP14676	*Kabuli*	Ca_Kabuli_Scaffold1504	197789	(G/A)
14677	CakSNP14677	*Kabuli*	Ca_Kabuli_Scaffold1504	197790	(G/A)
14678	CakSNP14678	*Kabuli*	Ca_Kabuli_Scaffold1504	197819	(T/G)
14679	CakSNP14679	*Kabuli*	Ca_Kabuli_Scaffold1505	33297	(A/C)
14680	CakSNP14680	*Kabuli*	Ca_Kabuli_Scaffold151	267060	(T/G)
14681	CakSNP14681	*Kabuli*	Ca_Kabuli_Scaffold151	275743	(T/A)
14682	CakSNP14682	*Kabuli*	Ca_Kabuli_Scaffold1521	20734	(C/A)
14683	CakSNP14683	*Kabuli*	Ca_Kabuli_Scaffold1521	20779	(T/C)
14684	CakSNP14684	*Kabuli*	Ca_Kabuli_Scaffold1521	20851	(C/T)
14685	CakSNP14685	*Kabuli*	Ca_Kabuli_Scaffold1545	111476	(G/A)
14686	CakSNP14686	*Kabuli*	Ca_Kabuli_Scaffold1545	113148	(T/C)
14687	CakSNP14687	*Kabuli*	Ca_Kabuli_Scaffold1553	5078	(G/A)
14688	CakSNP14688	*Kabuli*	Ca_Kabuli_Scaffold1553	5115	(C/T)
14689	CakSNP14689	*Kabuli*	Ca_Kabuli_Scaffold1553	5125	(A/G)
14690	CakSNP14690	*Kabuli*	Ca_Kabuli_Scaffold157	100228	(A/G)
14691	CakSNP14691	*Kabuli*	Ca_Kabuli_Scaffold157	100529	(T/C)
14692	CakSNP14692	*Kabuli*	Ca_Kabuli_Scaffold157	102779	(C/T)
14693	CakSNP14693	*Kabuli*	Ca_Kabuli_Scaffold157	106331	(G/A)
14694	CakSNP14694	*Kabuli*	Ca_Kabuli_Scaffold157	106349	(C/T)
14695	CakSNP14695	*Kabuli*	Ca_Kabuli_Scaffold157	133602	(A/G)
14696	CakSNP14696	*Kabuli*	Ca_Kabuli_Scaffold157	133583	(T/G)
14697	CakSNP14697	*Kabuli*	Ca_Kabuli_Scaffold157	141880	(A/G)
14698	CakSNP14698	*Kabuli*	Ca_Kabuli_Scaffold157	142444	(A/T)
14699	CakSNP14699	*Kabuli*	Ca_Kabuli_Scaffold157	142455	(T/C)
14700	CakSNP14700	*Kabuli*	Ca_Kabuli_Scaffold157	142470	(G/T)
14701	CakSNP14701	*Kabuli*	Ca_Kabuli_Scaffold157	151081	(G/A)

S.N.	SNP IDs	Cultivars	Chromosomes/scaffolds	Physical positions (bp)	SNPs
14702	CakSNP14702	*Kabuli*	Ca_Kabuli_Scaffold157	151199	(T/A)
14703	CakSNP14703	*Kabuli*	Ca_Kabuli_Scaffold157	151192	(T/C)
14704	CakSNP14704	*Kabuli*	Ca_Kabuli_Scaffold157	151177	(A/G)
14705	CakSNP14705	*Kabuli*	Ca_Kabuli_Scaffold157	151131	(T/A)
14706	CakSNP14706	*Kabuli*	Ca_Kabuli_Scaffold157	213380	(C/G)
14707	CakSNP14707	*Kabuli*	Ca_Kabuli_Scaffold157	213398	(T/G)
14708	CakSNP14708	*Kabuli*	Ca_Kabuli_Scaffold157	213404	(T/G)
14709	CakSNP14709	*Kabuli*	Ca_Kabuli_Scaffold157	213518	(T/C)
14710	CakSNP14710	*Kabuli*	Ca_Kabuli_Scaffold157	213487	(G/C)
14711	CakSNP14711	*Kabuli*	Ca_Kabuli_Scaffold157	213477	(G/A)
14712	CakSNP14712	*Kabuli*	Ca_Kabuli_Scaffold157	229917	(G/A)
14713	CakSNP14713	*Kabuli*	Ca_Kabuli_Scaffold157	229900	(G/A)
14714	CakSNP14714	*Kabuli*	Ca_Kabuli_Scaffold157	321386	(C/T)
14715	CakSNP14715	*Kabuli*	Ca_Kabuli_Scaffold157	326434	(A/G)
14716	CakSNP14716	*Kabuli*	Ca_Kabuli_Scaffold157	326467	(C/T)
14717	CakSNP14717	*Kabuli*	Ca_Kabuli_Scaffold157	326494	(A/G)
14718	CakSNP14718	*Kabuli*	Ca_Kabuli_Scaffold157	326497	(A/G)
14719	CakSNP14719	*Kabuli*	Ca_Kabuli_Scaffold157	352687	(C/T)
14720	CakSNP14720	*Kabuli*	Ca_Kabuli_Scaffold157	352712	(G/A)
14721	CakSNP14721	*Kabuli*	Ca_Kabuli_Scaffold157	352747	(T/C)
14722	CakSNP14722	*Kabuli*	Ca_Kabuli_Scaffold157	355222	(G/A)
14723	CakSNP14723	*Kabuli*	Ca_Kabuli_Scaffold157	469198	(A/C)
14724	CakSNP14724	*Kabuli*	Ca_Kabuli_Scaffold157	535057	(T/G)
14725	CakSNP14725	*Kabuli*	Ca_Kabuli_Scaffold1575	2230	(C/T)
14726	CakSNP14726	*Kabuli*	Ca_Kabuli_Scaffold1580	71861	(G/T)
14727	CakSNP14727	*Kabuli*	Ca_Kabuli_Scaffold1580	119410	(T/C)
14728	CakSNP14728	*Kabuli*	Ca_Kabuli_Scaffold1580	119678	(T/C)
14729	CakSNP14729	*Kabuli*	Ca_Kabuli_Scaffold1580	163148	(A/T)
14730	CakSNP14730	*Kabuli*	Ca_Kabuli_Scaffold1580	163194	(T/C)
14731	CakSNP14731	*Kabuli*	Ca_Kabuli_Scaffold1580	380649	(A/T)
14732	CakSNP14732	*Kabuli*	Ca_Kabuli_Scaffold1585	59010	(C/A)
14733	CakSNP14733	*Kabuli*	Ca_Kabuli_Scaffold1603	11828	(C/G)
14734	CakSNP14734	*Kabuli*	Ca_Kabuli_Scaffold1607	107222	(T/C)
14735	CakSNP14735	*Kabuli*	Ca_Kabuli_Scaffold1613	48917	(T/G)
14736	CakSNP14736	*Kabuli*	Ca_Kabuli_Scaffold1613	107664	(T/A)
14737	CakSNP14737	*Kabuli*	Ca_Kabuli_Scaffold1613	107750	(T/A)
14738	CakSNP14738	*Kabuli*	Ca_Kabuli_Scaffold1631	202418	(A/G)
14739	CakSNP14739	*Kabuli*	Ca_Kabuli_Scaffold1635	83227	(G/C)
14740	CakSNP14740	*Kabuli*	Ca_Kabuli_Scaffold1635	83345	(A/T)

S.N.	SNP IDs	Cultivars	Chromosomes/scaffolds	Physical positions (bp)	SNPs
14741	CakSNP14741	*Kabuli*	Ca_Kabuli_Scaffold1654	26432	(G/A)
14742	CakSNP14742	*Kabuli*	Ca_Kabuli_Scaffold1659	303945	(T/G)
14743	CakSNP14743	*Kabuli*	Ca_Kabuli_Scaffold1661	70434	(A/G)
14744	CakSNP14744	*Kabuli*	Ca_Kabuli_Scaffold1664	94989	(G/A)
14745	CakSNP14745	*Kabuli*	Ca_Kabuli_Scaffold1664	94984	(A/T)
14746	CakSNP14746	*Kabuli*	Ca_Kabuli_Scaffold1664	94930	(G/C)
14747	CakSNP14747	*Kabuli*	Ca_Kabuli_Scaffold167	11018	(T/A)
14748	CakSNP14748	*Kabuli*	Ca_Kabuli_Scaffold167	11009	(A/T)
14749	CakSNP14749	*Kabuli*	Ca_Kabuli_Scaffold167	137869	(C/T)
14750	CakSNP14750	*Kabuli*	Ca_Kabuli_Scaffold167	137848	(A/G)
14751	CakSNP14751	*Kabuli*	Ca_Kabuli_Scaffold167	239687	(G/A)
14752	CakSNP14752	*Kabuli*	Ca_Kabuli_Scaffold167	327829	(T/C)
14753	CakSNP14753	*Kabuli*	Ca_Kabuli_Scaffold1687	82935	(A/C)
14754	CakSNP14754	*Kabuli*	Ca_Kabuli_Scaffold1699	27556	(A/C)
14755	CakSNP14755	*Kabuli*	Ca_Kabuli_Scaffold1699	27607	(T/G)
14756	CakSNP14756	*Kabuli*	Ca_Kabuli_Scaffold1708	69664	(A/T)
14757	CakSNP14757	*Kabuli*	Ca_Kabuli_Scaffold171	96763	(T/C)
14758	CakSNP14758	*Kabuli*	Ca_Kabuli_Scaffold171	183718	(T/C)
14759	CakSNP14759	*Kabuli*	Ca_Kabuli_Scaffold171	183720	(C/T)
14760	CakSNP14760	*Kabuli*	Ca_Kabuli_Scaffold171	183721	(G/A)
14761	CakSNP14761	*Kabuli*	Ca_Kabuli_Scaffold171	183727	(C/T)
14762	CakSNP14762	*Kabuli*	Ca_Kabuli_Scaffold171	183759	(T/C)
14763	CakSNP14763	*Kabuli*	Ca_Kabuli_Scaffold171	186125	(C/A)
14764	CakSNP14764	*Kabuli*	Ca_Kabuli_Scaffold171	208050	(A/G)
14765	CakSNP14765	*Kabuli*	Ca_Kabuli_Scaffold171	209964	(G/T)
14766	CakSNP14766	*Kabuli*	Ca_Kabuli_Scaffold1718	302	(G/A)
14767	CakSNP14767	*Kabuli*	Ca_Kabuli_Scaffold1718	295	(G/A)
14768	CakSNP14768	*Kabuli*	Ca_Kabuli_Scaffold1718	289	(C/T)
14769	CakSNP14769	*Kabuli*	Ca_Kabuli_Scaffold1718	268	(C/G)
14770	CakSNP14770	*Kabuli*	Ca_Kabuli_Scaffold1718	3898	(G/C)
14771	CakSNP14771	*Kabuli*	Ca_Kabuli_Scaffold1718	21521	(A/G)
14772	CakSNP14772	*Kabuli*	Ca_Kabuli_Scaffold1718	40669	(T/C)
14773	CakSNP14773	*Kabuli*	Ca_Kabuli_Scaffold1722	65999	(C/A)
14774	CakSNP14774	*Kabuli*	Ca_Kabuli_Scaffold1722	135589	(C/T)
14775	CakSNP14775	*Kabuli*	Ca_Kabuli_Scaffold1728	33301	(A/C)
14776	CakSNP14776	*Kabuli*	Ca_Kabuli_Scaffold174	17761	(A/C)
14777	CakSNP14777	*Kabuli*	Ca_Kabuli_Scaffold174	32366	(T/C)
14778	CakSNP14778	*Kabuli*	Ca_Kabuli_Scaffold174	44014	(T/G)
14779	CakSNP14779	*Kabuli*	Ca_Kabuli_Scaffold174	61454	(T/C)

S.N.	SNP IDs	Cultivars	Chromosomes/scaffolds	Physical positions (bp)	SNPs
14780	CakSNP14780	*Kabuli*	Ca_Kabuli_Scaffold174	263899	(G/A)
14781	CakSNP14781	*Kabuli*	Ca_Kabuli_Scaffold174	335077	(C/T)
14782	CakSNP14782	*Kabuli*	Ca_Kabuli_Scaffold174	341322	(C/T)
14783	CakSNP14783	*Kabuli*	Ca_Kabuli_Scaffold174	457627	(G/A)
14784	CakSNP14784	*Kabuli*	Ca_Kabuli_Scaffold1750	15510	(C/T)
14785	CakSNP14785	*Kabuli*	Ca_Kabuli_Scaffold1750	137106	(A/C)
14786	CakSNP14786	*Kabuli*	Ca_Kabuli_Scaffold1750	204638	(G/A)
14787	CakSNP14787	*Kabuli*	Ca_Kabuli_Scaffold1751	189367	(T/G)
14788	CakSNP14788	*Kabuli*	Ca_Kabuli_Scaffold1751	192417	(C/A)
14789	CakSNP14789	*Kabuli*	Ca_Kabuli_Scaffold1751	192713	(G/T)
14790	CakSNP14790	*Kabuli*	Ca_Kabuli_Scaffold1751	193026	(T/A)
14791	CakSNP14791	*Kabuli*	Ca_Kabuli_Scaffold1751	193013	(T/G)
14792	CakSNP14792	*Kabuli*	Ca_Kabuli_Scaffold1751	266583	(G/A)
14793	CakSNP14793	*Kabuli*	Ca_Kabuli_Scaffold1751	496626	(G/A)
14794	CakSNP14794	*Kabuli*	Ca_Kabuli_Scaffold1751	528896	(A/C)
14795	CakSNP14795	*Kabuli*	Ca_Kabuli_Scaffold1751	529005	(C/T)
14796	CakSNP14796	*Kabuli*	Ca_Kabuli_Scaffold1751	528979	(T/C)
14797	CakSNP14797	*Kabuli*	Ca_Kabuli_Scaffold1751	528973	(C/G)
14798	CakSNP14798	*Kabuli*	Ca_Kabuli_Scaffold1751	625364	(G/A)
14799	CakSNP14799	*Kabuli*	Ca_Kabuli_Scaffold1751	625362	(C/T)
14800	CakSNP14800	*Kabuli*	Ca_Kabuli_Scaffold1751	625355	(C/T)
14801	CakSNP14801	*Kabuli*	Ca_Kabuli_Scaffold1751	625342	(C/T)
14802	CakSNP14802	*Kabuli*	Ca_Kabuli_Scaffold1751	625335	(G/A)
14803	CakSNP14803	*Kabuli*	Ca_Kabuli_Scaffold1751	625395	(A/G)
14804	CakSNP14804	*Kabuli*	Ca_Kabuli_Scaffold1751	625394	(C/T)
14805	CakSNP14805	*Kabuli*	Ca_Kabuli_Scaffold1751	625389	(A/C)
14806	CakSNP14806	*Kabuli*	Ca_Kabuli_Scaffold1751	625378	(G/A)
14807	CakSNP14807	*Kabuli*	Ca_Kabuli_Scaffold1751	625375	(G/A)
14808	CakSNP14808	*Kabuli*	Ca_Kabuli_Scaffold1751	625366	(T/C)
14809	CakSNP14809	*Kabuli*	Ca_Kabuli_Scaffold1751	625365	(T/C)
14810	CakSNP14810	*Kabuli*	Ca_Kabuli_Scaffold1751	625332	(A/C)
14811	CakSNP14811	*Kabuli*	Ca_Kabuli_Scaffold1751	625396	(G/A)
14812	CakSNP14812	*Kabuli*	Ca_Kabuli_Scaffold1776	740	(C/T)
14813	CakSNP14813	*Kabuli*	Ca_Kabuli_Scaffold1777	164715	(G/A)
14814	CakSNP14814	*Kabuli*	Ca_Kabuli_Scaffold1777	164722	(C/G)
14815	CakSNP14815	*Kabuli*	Ca_Kabuli_Scaffold1777	164747	(G/A)
14816	CakSNP14816	*Kabuli*	Ca_Kabuli_Scaffold1777	164791	(T/G)
14817	CakSNP14817	*Kabuli*	Ca_Kabuli_Scaffold1777	164758	(C/T)
14818	CakSNP14818	*Kabuli*	Ca_Kabuli_Scaffold1777	164753	(A/G)

S.N.	SNP IDs	Cultivars	Chromosomes/scaffolds	Physical positions (bp)	SNPs
14819	CakSNP14819	*Kabuli*	Ca_Kabuli_Scaffold1777	164730	(T/G)
14820	CakSNP14820	*Kabuli*	Ca_Kabuli_Scaffold1777	169037	(G/T)
14821	CakSNP14821	*Kabuli*	Ca_Kabuli_Scaffold1777	343173	(C/T)
14822	CakSNP14822	*Kabuli*	Ca_Kabuli_Scaffold1777	343185	(G/A)
14823	CakSNP14823	*Kabuli*	Ca_Kabuli_Scaffold1777	448166	(A/G)
14824	CakSNP14824	*Kabuli*	Ca_Kabuli_Scaffold1777	448176	(C/T)
14825	CakSNP14825	*Kabuli*	Ca_Kabuli_Scaffold1777	448187	(G/T)
14826	CakSNP14826	*Kabuli*	Ca_Kabuli_Scaffold1777	448216	(C/T)
14827	CakSNP14827	*Kabuli*	Ca_Kabuli_Scaffold1779	957	(G/A)
14828	CakSNP14828	*Kabuli*	Ca_Kabuli_Scaffold1779	896	(A/G)
14829	CakSNP14829	*Kabuli*	Ca_Kabuli_Scaffold1779	890	(G/A)
14830	CakSNP14830	*Kabuli*	Ca_Kabuli_Scaffold1802	17068	(A/G)
14831	CakSNP14831	*Kabuli*	Ca_Kabuli_Scaffold1802	17033	(A/G)
14832	CakSNP14832	*Kabuli*	Ca_Kabuli_Scaffold1802	17111	(C/T)
14833	CakSNP14833	*Kabuli*	Ca_Kabuli_Scaffold1802	17117	(T/C)
14834	CakSNP14834	*Kabuli*	Ca_Kabuli_Scaffold1802	17120	(C/T)
14835	CakSNP14835	*Kabuli*	Ca_Kabuli_Scaffold1802	17123	(G/T)
14836	CakSNP14836	*Kabuli*	Ca_Kabuli_Scaffold1802	17132	(C/T)
14837	CakSNP14837	*Kabuli*	Ca_Kabuli_Scaffold1802	17150	(T/C)
14838	CakSNP14838	*Kabuli*	Ca_Kabuli_Scaffold1802	17151	(T/C)
14839	CakSNP14839	*Kabuli*	Ca_Kabuli_Scaffold1802	17168	(T/C)
14840	CakSNP14840	*Kabuli*	Ca_Kabuli_Scaffold1802	17177	(T/A)
14841	CakSNP14841	*Kabuli*	Ca_Kabuli_Scaffold1835	9398	(C/A)
14842	CakSNP14842	*Kabuli*	Ca_Kabuli_Scaffold1835	10541	(C/G)
14843	CakSNP14843	*Kabuli*	Ca_Kabuli_Scaffold1835	10563	(T/C)
14844	CakSNP14844	*Kabuli*	Ca_Kabuli_Scaffold1844	41899	(C/A)
14845	CakSNP14845	*Kabuli*	Ca_Kabuli_Scaffold1845	16292	(C/G)
14846	CakSNP14846	*Kabuli*	Ca_Kabuli_Scaffold1845	16317	(C/G)
14847	CakSNP14847	*Kabuli*	Ca_Kabuli_Scaffold1845	40886	(T/A)
14848	CakSNP14848	*Kabuli*	Ca_Kabuli_Scaffold1845	40872	(T/G)
14849	CakSNP14849	*Kabuli*	Ca_Kabuli_Scaffold1845	40871	(T/A)
14850	CakSNP14850	*Kabuli*	Ca_Kabuli_Scaffold1845	40842	(T/G)
14851	CakSNP14851	*Kabuli*	Ca_Kabuli_Scaffold1845	40821	(T/G)
14852	CakSNP14852	*Kabuli*	Ca_Kabuli_Scaffold1848	583	(A/G)
14853	CakSNP14853	*Kabuli*	Ca_Kabuli_Scaffold1848	565	(T/C)
14854	CakSNP14854	*Kabuli*	Ca_Kabuli_Scaffold1848	562	(G/C)
14855	CakSNP14855	*Kabuli*	Ca_Kabuli_Scaffold1848	532	(G/C)
14856	CakSNP14856	*Kabuli*	Ca_Kabuli_Scaffold186	11965	(A/G)
14857	CakSNP14857	*Kabuli*	Ca_Kabuli_Scaffold186	279942	(A/G)

S.N.	SNP IDs	Cultivars	Chromosomes/scaffolds	Physical positions (bp)	SNPs
14858	CakSNP14858	*Kabuli*	Ca_Kabuli_Scaffold186	280062	(T/C)
14859	CakSNP14859	*Kabuli*	Ca_Kabuli_Scaffold186	280047	(A/G)
14860	CakSNP14860	*Kabuli*	Ca_Kabuli_Scaffold186	280023	(C/T)
14861	CakSNP14861	*Kabuli*	Ca_Kabuli_Scaffold186	280015	(T/C)
14862	CakSNP14862	*Kabuli*	Ca_Kabuli_Scaffold186	280009	(G/T)
14863	CakSNP14863	*Kabuli*	Ca_Kabuli_Scaffold1866	106318	(T/A)
14864	CakSNP14864	*Kabuli*	Ca_Kabuli_Scaffold1866	106330	(A/T)
14865	CakSNP14865	*Kabuli*	Ca_Kabuli_Scaffold1866	106371	(C/A)
14866	CakSNP14866	*Kabuli*	Ca_Kabuli_Scaffold1866	106374	(A/T)
14867	CakSNP14867	*Kabuli*	Ca_Kabuli_Scaffold1866	106402	(C/T)
14868	CakSNP14868	*Kabuli*	Ca_Kabuli_Scaffold1866	110550	(A/G)
14869	CakSNP14869	*Kabuli*	Ca_Kabuli_Scaffold1866	110570	(G/A)
14870	CakSNP14870	*Kabuli*	Ca_Kabuli_Scaffold1866	110578	(C/A)
14871	CakSNP14871	*Kabuli*	Ca_Kabuli_Scaffold1899	148	(T/G)
14872	CakSNP14872	*Kabuli*	Ca_Kabuli_Scaffold1899	187	(A/G)
14873	CakSNP14873	*Kabuli*	Ca_Kabuli_Scaffold1899	267	(T/A)
14874	CakSNP14874	*Kabuli*	Ca_Kabuli_Scaffold1899	200	(G/A)
14875	CakSNP14875	*Kabuli*	Ca_Kabuli_Scaffold1918	216231	(T/G)
14876	CakSNP14876	*Kabuli*	Ca_Kabuli_Scaffold1918	216213	(T/C)
14877	CakSNP14877	*Kabuli*	Ca_Kabuli_Scaffold1918	216180	(C/A)
14878	CakSNP14878	*Kabuli*	Ca_Kabuli_Scaffold1918	237787	(G/A)
14879	CakSNP14879	*Kabuli*	Ca_Kabuli_Scaffold1918	245929	(T/C)
14880	CakSNP14880	*Kabuli*	Ca_Kabuli_Scaffold1928	103092	(A/G)
14881	CakSNP14881	*Kabuli*	Ca_Kabuli_Scaffold1928	103114	(G/A)
14882	CakSNP14882	*Kabuli*	Ca_Kabuli_Scaffold193	38279	(C/A)
14883	CakSNP14883	*Kabuli*	Ca_Kabuli_Scaffold193	186538	(C/T)
14884	CakSNP14884	*Kabuli*	Ca_Kabuli_Scaffold193	186523	(T/C)
14885	CakSNP14885	*Kabuli*	Ca_Kabuli_Scaffold193	186515	(T/G)
14886	CakSNP14886	*Kabuli*	Ca_Kabuli_Scaffold193	186510	(C/A)
14887	CakSNP14887	*Kabuli*	Ca_Kabuli_Scaffold193	186584	(A/G)
14888	CakSNP14888	*Kabuli*	Ca_Kabuli_Scaffold193	186582	(C/A)
14889	CakSNP14889	*Kabuli*	Ca_Kabuli_Scaffold193	186587	(G/T)
14890	CakSNP14890	*Kabuli*	Ca_Kabuli_Scaffold193	186588	(C/G)
14891	CakSNP14891	*Kabuli*	Ca_Kabuli_Scaffold193	186595	(C/T)
14892	CakSNP14892	*Kabuli*	Ca_Kabuli_Scaffold193	186598	(A/G)
14893	CakSNP14893	*Kabuli*	Ca_Kabuli_Scaffold193	186611	(G/A)
14894	CakSNP14894	*Kabuli*	Ca_Kabuli_Scaffold193	186639	(C/T)
14895	CakSNP14895	*Kabuli*	Ca_Kabuli_Scaffold193	186651	(T/C)
14896	CakSNP14896	*Kabuli*	Ca_Kabuli_Scaffold193	186657	(G/A)

S.N.	SNP IDs	Cultivars	Chromosomes/scaffolds	Physical positions (bp)	SNPs
14897	CakSNP14897	*Kabuli*	Ca_Kabuli_Scaffold193	186661	(T/C)
14898	CakSNP14898	*Kabuli*	Ca_Kabuli_Scaffold193	242763	(T/C)
14899	CakSNP14899	*Kabuli*	Ca_Kabuli_Scaffold193	242733	(G/T)
14900	CakSNP14900	*Kabuli*	Ca_Kabuli_Scaffold193	244735	(T/G)
14901	CakSNP14901	*Kabuli*	Ca_Kabuli_Scaffold1943	163784	(G/A)
14902	CakSNP14902	*Kabuli*	Ca_Kabuli_Scaffold1943	163780	(C/T)
14903	CakSNP14903	*Kabuli*	Ca_Kabuli_Scaffold1959	84160	(G/A)
14904	CakSNP14904	*Kabuli*	Ca_Kabuli_Scaffold1964	54613	(G/A)
14905	CakSNP14905	*Kabuli*	Ca_Kabuli_Scaffold1964	54604	(T/C)
14906	CakSNP14906	*Kabuli*	Ca_Kabuli_Scaffold1964	67416	(G/C)
14907	CakSNP14907	*Kabuli*	Ca_Kabuli_Scaffold198	631160	(G/A)
14908	CakSNP14908	*Kabuli*	Ca_Kabuli_Scaffold198	631174	(C/T)
14909	CakSNP14909	*Kabuli*	Ca_Kabuli_Scaffold198	631185	(G/A)
14910	CakSNP14910	*Kabuli*	Ca_Kabuli_Scaffold198	641266	(C/T)
14911	CakSNP14911	*Kabuli*	Ca_Kabuli_Scaffold198	641252	(A/G)
14912	CakSNP14912	*Kabuli*	Ca_Kabuli_Scaffold198	641243	(A/G)
14913	CakSNP14913	*Kabuli*	Ca_Kabuli_Scaffold1981	265110	(A/C)
14914	CakSNP14914	*Kabuli*	Ca_Kabuli_Scaffold1981	265126	(G/T)
14915	CakSNP14915	*Kabuli*	Ca_Kabuli_Scaffold1981	265216	(A/G)
14916	CakSNP14916	*Kabuli*	Ca_Kabuli_Scaffold1981	265191	(T/G)
14917	CakSNP14917	*Kabuli*	Ca_Kabuli_Scaffold1981	269879	(A/G)
14918	CakSNP14918	*Kabuli*	Ca_Kabuli_Scaffold1981	385683	(T/A)
14919	CakSNP14919	*Kabuli*	Ca_Kabuli_Scaffold1984	212011	(G/A)
14920	CakSNP14920	*Kabuli*	Ca_Kabuli_Scaffold1984	215969	(G/C)
14921	CakSNP14921	*Kabuli*	Ca_Kabuli_Scaffold1984	292507	(A/C)
14922	CakSNP14922	*Kabuli*	Ca_Kabuli_Scaffold1984	292511	(G/C)
14923	CakSNP14923	*Kabuli*	Ca_Kabuli_Scaffold1985	98611	(C/T)
14924	CakSNP14924	*Kabuli*	Ca_Kabuli_Scaffold1985	98659	(C/T)
14925	CakSNP14925	*Kabuli*	Ca_Kabuli_Scaffold1991	62009	(C/A)
14926	CakSNP14926	*Kabuli*	Ca_Kabuli_Scaffold1991	62121	(G/A)
14927	CakSNP14927	*Kabuli*	Ca_Kabuli_Scaffold1991	62085	(A/C)
14928	CakSNP14928	*Kabuli*	Ca_Kabuli_Scaffold1991	62115	(G/A)
14929	CakSNP14929	*Kabuli*	Ca_Kabuli_Scaffold1991	62131	(G/A)
14930	CakSNP14930	*Kabuli*	Ca_Kabuli_Scaffold1991	62154	(T/A)
14931	CakSNP14931	*Kabuli*	Ca_Kabuli_Scaffold1991	62147	(G/A)
14932	CakSNP14932	*Kabuli*	Ca_Kabuli_Scaffold1991	62422	(G/A)
14933	CakSNP14933	*Kabuli*	Ca_Kabuli_Scaffold2	137309	(T/G)
14934	CakSNP14934	*Kabuli*	Ca_Kabuli_Scaffold2	504258	(C/T)
14935	CakSNP14935	*Kabuli*	Ca_Kabuli_Scaffold2	504253	(C/T)

S.N.	SNP IDs	Cultivars	Chromosomes/scaffolds	Physical positions (bp)	SNPs
14936	CakSNP14936	*Kabuli*	Ca_Kabuli_Scaffold2	504248	(G/A)
14937	CakSNP14937	*Kabuli*	Ca_Kabuli_Scaffold202	16084	(T/C)
14938	CakSNP14938	*Kabuli*	Ca_Kabuli_Scaffold202	92532	(C/A)
14939	CakSNP14939	*Kabuli*	Ca_Kabuli_Scaffold202	96456	(T/C)
14940	CakSNP14940	*Kabuli*	Ca_Kabuli_Scaffold202	96444	(G/A)
14941	CakSNP14941	*Kabuli*	Ca_Kabuli_Scaffold202	96532	(G/T)
14942	CakSNP14942	*Kabuli*	Ca_Kabuli_Scaffold202	96589	(A/T)
14943	CakSNP14943	*Kabuli*	Ca_Kabuli_Scaffold202	96580	(T/G)
14944	CakSNP14944	*Kabuli*	Ca_Kabuli_Scaffold202	96576	(A/G)
14945	CakSNP14945	*Kabuli*	Ca_Kabuli_Scaffold202	99123	(C/T)
14946	CakSNP14946	*Kabuli*	Ca_Kabuli_Scaffold2027	100867	(G/C)
14947	CakSNP14947	*Kabuli*	Ca_Kabuli_Scaffold2027	152763	(C/A)
14948	CakSNP14948	*Kabuli*	Ca_Kabuli_Scaffold2030	13855	(G/A)
14949	CakSNP14949	*Kabuli*	Ca_Kabuli_Scaffold2030	13822	(C/T)
14950	CakSNP14950	*Kabuli*	Ca_Kabuli_Scaffold2030	13807	(G/A)
14951	CakSNP14951	*Kabuli*	Ca_Kabuli_Scaffold2030	13889	(C/T)
14952	CakSNP14952	*Kabuli*	Ca_Kabuli_Scaffold2030	13874	(G/T)
14953	CakSNP14953	*Kabuli*	Ca_Kabuli_Scaffold2033	115044	(C/T)
14954	CakSNP14954	*Kabuli*	Ca_Kabuli_Scaffold2033	118686	(C/T)
14955	CakSNP14955	*Kabuli*	Ca_Kabuli_Scaffold2033	118779	(G/T)
14956	CakSNP14956	*Kabuli*	Ca_Kabuli_Scaffold2033	118760	(T/G)
14957	CakSNP14957	*Kabuli*	Ca_Kabuli_Scaffold2033	119057	(T/G)
14958	CakSNP14958	*Kabuli*	Ca_Kabuli_Scaffold2033	119166	(T/C)
14959	CakSNP14959	*Kabuli*	Ca_Kabuli_Scaffold2033	119645	(G/A)
14960	CakSNP14960	*Kabuli*	Ca_Kabuli_Scaffold2033	119648	(G/A)
14961	CakSNP14961	*Kabuli*	Ca_Kabuli_Scaffold2033	119703	(C/T)
14962	CakSNP14962	*Kabuli*	Ca_Kabuli_Scaffold2033	119715	(G/T)
14963	CakSNP14963	*Kabuli*	Ca_Kabuli_Scaffold2033	119728	(G/A)
14964	CakSNP14964	*Kabuli*	Ca_Kabuli_Scaffold2039	116734	(A/G)
14965	CakSNP14965	*Kabuli*	Ca_Kabuli_Scaffold2040	139597	(A/G)
14966	CakSNP14966	*Kabuli*	Ca_Kabuli_Scaffold2040	139585	(A/C)
14967	CakSNP14967	*Kabuli*	Ca_Kabuli_Scaffold2040	139584	(G/A)
14968	CakSNP14968	*Kabuli*	Ca_Kabuli_Scaffold2040	139581	(G/A)
14969	CakSNP14969	*Kabuli*	Ca_Kabuli_Scaffold2040	139560	(G/T)
14970	CakSNP14970	*Kabuli*	Ca_Kabuli_Scaffold2040	139550	(G/A)
14971	CakSNP14971	*Kabuli*	Ca_Kabuli_Scaffold2040	139548	(C/T)
14972	CakSNP14972	*Kabuli*	Ca_Kabuli_Scaffold2040	139544	(C/A)
14973	CakSNP14973	*Kabuli*	Ca_Kabuli_Scaffold2040	139541	(T/C)
14974	CakSNP14974	*Kabuli*	Ca_Kabuli_Scaffold2040	139538	(G/A)

S.N.	SNP IDs	Cultivars	Chromosomes/scaffolds	Physical positions (bp)	SNPs
14975	CakSNP14975	*Kabuli*	Ca_Kabuli_Scaffold2040	139545	(G/A)
14976	CakSNP14976	*Kabuli*	Ca_Kabuli_Scaffold2040	139603	(C/T)
14977	CakSNP14977	*Kabuli*	Ca_Kabuli_Scaffold205	74448	(T/G)
14978	CakSNP14978	*Kabuli*	Ca_Kabuli_Scaffold205	74430	(A/G)
14979	CakSNP14979	*Kabuli*	Ca_Kabuli_Scaffold205	74397	(G/A)
14980	CakSNP14980	*Kabuli*	Ca_Kabuli_Scaffold205	74399	(C/T)
14981	CakSNP14981	*Kabuli*	Ca_Kabuli_Scaffold205	74424	(T/C)
14982	CakSNP14982	*Kabuli*	Ca_Kabuli_Scaffold205	74432	(G/A)
14983	CakSNP14983	*Kabuli*	Ca_Kabuli_Scaffold205	74433	(G/A)
14984	CakSNP14984	*Kabuli*	Ca_Kabuli_Scaffold206	58349	(C/A)
14985	CakSNP14985	*Kabuli*	Ca_Kabuli_Scaffold206	62985	(G/A)
14986	CakSNP14986	*Kabuli*	Ca_Kabuli_Scaffold208	344503	(G/T)
14987	CakSNP14987	*Kabuli*	Ca_Kabuli_Scaffold208	344515	(A/G)
14988	CakSNP14988	*Kabuli*	Ca_Kabuli_Scaffold208	344519	(A/T)
14989	CakSNP14989	*Kabuli*	Ca_Kabuli_Scaffold208	344533	(C/T)
14990	CakSNP14990	*Kabuli*	Ca_Kabuli_Scaffold208	344556	(T/G)
14991	CakSNP14991	*Kabuli*	Ca_Kabuli_Scaffold2097	54210	(A/C)
14992	CakSNP14992	*Kabuli*	Ca_Kabuli_Scaffold210	293799	(G/A)
14993	CakSNP14993	*Kabuli*	Ca_Kabuli_Scaffold210	355284	(G/T)
14994	CakSNP14994	*Kabuli*	Ca_Kabuli_Scaffold210	533483	(G/A)
14995	CakSNP14995	*Kabuli*	Ca_Kabuli_Scaffold210	533416	(G/A)
14996	CakSNP14996	*Kabuli*	Ca_Kabuli_Scaffold210	617431	(T/A)
14997	CakSNP14997	*Kabuli*	Ca_Kabuli_Scaffold2104	61757	(T/C)
14998	CakSNP14998	*Kabuli*	Ca_Kabuli_Scaffold2104	61745	(T/G)
14999	CakSNP14999	*Kabuli*	Ca_Kabuli_Scaffold2104	61734	(T/G)
15000	CakSNP15000	*Kabuli*	Ca_Kabuli_Scaffold2104	61723	(G/A)
15001	CakSNP15001	*Kabuli*	Ca_Kabuli_Scaffold2104	61710	(C/T)
15002	CakSNP15002	*Kabuli*	Ca_Kabuli_Scaffold2104	61705	(C/T)
15003	CakSNP15003	*Kabuli*	Ca_Kabuli_Scaffold2152	372404	(A/T)
15004	CakSNP15004	*Kabuli*	Ca_Kabuli_Scaffold2166	228653	(G/C)
15005	CakSNP15005	*Kabuli*	Ca_Kabuli_Scaffold2166	228710	(G/A)
15006	CakSNP15006	*Kabuli*	Ca_Kabuli_Scaffold2170	4821	(G/A)
15007	CakSNP15007	*Kabuli*	Ca_Kabuli_Scaffold2192	67485	(A/G)
15008	CakSNP15008	*Kabuli*	Ca_Kabuli_Scaffold2192	67589	(A/C)
15009	CakSNP15009	*Kabuli*	Ca_Kabuli_Scaffold221	53841	(T/G)
15010	CakSNP15010	*Kabuli*	Ca_Kabuli_Scaffold2248	5538	(A/T)
15011	CakSNP15011	*Kabuli*	Ca_Kabuli_Scaffold2248	5562	(C/T)
15012	CakSNP15012	*Kabuli*	Ca_Kabuli_Scaffold2248	5570	(T/C)
15013	CakSNP15013	*Kabuli*	Ca_Kabuli_Scaffold2248	5520	(C/A)

S.N.	SNP IDs	Cultivars	Chromosomes/scaffolds	Physical positions (bp)	SNPs
15014	CakSNP15014	*Kabuli*	Ca_Kabuli_Scaffold2249	4446	(A/C)
15015	CakSNP15015	*Kabuli*	Ca_Kabuli_Scaffold2269	58091	(A/T)
15016	CakSNP15016	*Kabuli*	Ca_Kabuli_Scaffold2269	123799	(A/C)
15017	CakSNP15017	*Kabuli*	Ca_Kabuli_Scaffold2269	126013	(A/C)
15018	CakSNP15018	*Kabuli*	Ca_Kabuli_Scaffold2301	70721	(A/G)
15019	CakSNP15019	*Kabuli*	Ca_Kabuli_Scaffold2330	27894	(A/C)
15020	CakSNP15020	*Kabuli*	Ca_Kabuli_Scaffold2330	64157	(T/C)
15021	CakSNP15021	*Kabuli*	Ca_Kabuli_Scaffold2330	65482	(T/G)
15022	CakSNP15022	*Kabuli*	Ca_Kabuli_Scaffold2330	65617	(G/A)
15023	CakSNP15023	*Kabuli*	Ca_Kabuli_Scaffold2330	65650	(C/T)
15024	CakSNP15024	*Kabuli*	Ca_Kabuli_Scaffold2330	83061	(C/T)
15025	CakSNP15025	*Kabuli*	Ca_Kabuli_Scaffold2330	83064	(A/G)
15026	CakSNP15026	*Kabuli*	Ca_Kabuli_Scaffold2330	83077	(G/C)
15027	CakSNP15027	*Kabuli*	Ca_Kabuli_Scaffold2330	83112	(A/G)
15028	CakSNP15028	*Kabuli*	Ca_Kabuli_Scaffold2330	83116	(G/T)
15029	CakSNP15029	*Kabuli*	Ca_Kabuli_Scaffold235	67258	(G/T)
15030	CakSNP15030	*Kabuli*	Ca_Kabuli_Scaffold2371	139149	(A/G)
15031	CakSNP15031	*Kabuli*	Ca_Kabuli_Scaffold2373	3818	(A/G)
15032	CakSNP15032	*Kabuli*	Ca_Kabuli_Scaffold2373	3815	(T/A)
15033	CakSNP15033	*Kabuli*	Ca_Kabuli_Scaffold2373	3811	(C/T)
15034	CakSNP15034	*Kabuli*	Ca_Kabuli_Scaffold2373	3793	(C/T)
15035	CakSNP15035	*Kabuli*	Ca_Kabuli_Scaffold2373	3923	(T/C)
15036	CakSNP15036	*Kabuli*	Ca_Kabuli_Scaffold2373	3871	(A/T)
15037	CakSNP15037	*Kabuli*	Ca_Kabuli_Scaffold2373	3862	(C/T)
15038	CakSNP15038	*Kabuli*	Ca_Kabuli_Scaffold2373	12916	(C/A)
15039	CakSNP15039	*Kabuli*	Ca_Kabuli_Scaffold2373	12969	(G/C)
15040	CakSNP15040	*Kabuli*	Ca_Kabuli_Scaffold2373	12978	(A/T)
15041	CakSNP15041	*Kabuli*	Ca_Kabuli_Scaffold2373	62495	(C/T)
15042	CakSNP15042	*Kabuli*	Ca_Kabuli_Scaffold2373	62494	(A/G)
15043	CakSNP15043	*Kabuli*	Ca_Kabuli_Scaffold2373	62473	(G/A)
15044	CakSNP15044	*Kabuli*	Ca_Kabuli_Scaffold2373	62434	(G/A)
15045	CakSNP15045	*Kabuli*	Ca_Kabuli_Scaffold2373	62433	(A/G)
15046	CakSNP15046	*Kabuli*	Ca_Kabuli_Scaffold2373	127884	(A/C)
15047	CakSNP15047	*Kabuli*	Ca_Kabuli_Scaffold2373	127851	(C/T)
15048	CakSNP15048	*Kabuli*	Ca_Kabuli_Scaffold2373	127905	(C/T)
15049	CakSNP15049	*Kabuli*	Ca_Kabuli_Scaffold2373	127870	(T/G)
15050	CakSNP15050	*Kabuli*	Ca_Kabuli_Scaffold2373	127855	(A/C)
15051	CakSNP15051	*Kabuli*	Ca_Kabuli_Scaffold2392	35402	(A/C)
15052	CakSNP15052	*Kabuli*	Ca_Kabuli_Scaffold2392	198120	(G/T)

S.N.	SNP IDs	Cultivars	Chromosomes/scaffolds	Physical positions (bp)	SNPs
15053	CakSNP15053	*Kabuli*	Ca_Kabuli_Scaffold2392	314304	(T/C)
15054	CakSNP15054	*Kabuli*	Ca_Kabuli_Scaffold2392	314322	(T/C)
15055	CakSNP15055	*Kabuli*	Ca_Kabuli_Scaffold2392	314323	(G/A)
15056	CakSNP15056	*Kabuli*	Ca_Kabuli_Scaffold2392	314343	(G/A)
15057	CakSNP15057	*Kabuli*	Ca_Kabuli_Scaffold2392	314344	(T/C)
15058	CakSNP15058	*Kabuli*	Ca_Kabuli_Scaffold2392	314349	(C/G)
15059	CakSNP15059	*Kabuli*	Ca_Kabuli_Scaffold2392	314353	(G/A)
15060	CakSNP15060	*Kabuli*	Ca_Kabuli_Scaffold2392	314345	(G/A)
15061	CakSNP15061	*Kabuli*	Ca_Kabuli_Scaffold2392	314401	(A/C)
15062	CakSNP15062	*Kabuli*	Ca_Kabuli_Scaffold240	6231	(T/A)
15063	CakSNP15063	*Kabuli*	Ca_Kabuli_Scaffold240	6193	(G/A)
15064	CakSNP15064	*Kabuli*	Ca_Kabuli_Scaffold240	6152	(C/T)
15065	CakSNP15065	*Kabuli*	Ca_Kabuli_Scaffold240	119157	(G/A)
15066	CakSNP15066	*Kabuli*	Ca_Kabuli_Scaffold240	119301	(T/C)
15067	CakSNP15067	*Kabuli*	Ca_Kabuli_Scaffold240	119297	(C/A)
15068	CakSNP15068	*Kabuli*	Ca_Kabuli_Scaffold240	119278	(T/C)
15069	CakSNP15069	*Kabuli*	Ca_Kabuli_Scaffold240	119269	(C/T)
15070	CakSNP15070	*Kabuli*	Ca_Kabuli_Scaffold240	119267	(G/C)
15071	CakSNP15071	*Kabuli*	Ca_Kabuli_Scaffold240	119265	(C/G)
15072	CakSNP15072	*Kabuli*	Ca_Kabuli_Scaffold2415	117840	(A/G)
15073	CakSNP15073	*Kabuli*	Ca_Kabuli_Scaffold2415	117871	(A/C)
15074	CakSNP15074	*Kabuli*	Ca_Kabuli_Scaffold2415	117882	(A/G)
15075	CakSNP15075	*Kabuli*	Ca_Kabuli_Scaffold2415	117970	(G/A)
15076	CakSNP15076	*Kabuli*	Ca_Kabuli_Scaffold242	293872	(A/G)
15077	CakSNP15077	*Kabuli*	Ca_Kabuli_Scaffold250	38270	(A/G)
15078	CakSNP15078	*Kabuli*	Ca_Kabuli_Scaffold2501	287	(C/T)
15079	CakSNP15079	*Kabuli*	Ca_Kabuli_Scaffold2501	282	(C/A)
15080	CakSNP15080	*Kabuli*	Ca_Kabuli_Scaffold2501	251	(T/G)
15081	CakSNP15081	*Kabuli*	Ca_Kabuli_Scaffold2501	242	(C/G)
15082	CakSNP15082	*Kabuli*	Ca_Kabuli_Scaffold2516	58947	(T/C)
15083	CakSNP15083	*Kabuli*	Ca_Kabuli_Scaffold2521	57762	(T/C)
15084	CakSNP15084	*Kabuli*	Ca_Kabuli_Scaffold2521	57725	(G/A)
15085	CakSNP15085	*Kabuli*	Ca_Kabuli_Scaffold2521	57709	(G/T)
15086	CakSNP15086	*Kabuli*	Ca_Kabuli_Scaffold2557	110378	(C/T)
15087	CakSNP15087	*Kabuli*	Ca_Kabuli_Scaffold2557	110414	(T/G)
15088	CakSNP15088	*Kabuli*	Ca_Kabuli_Scaffold2557	110437	(C/T)
15089	CakSNP15089	*Kabuli*	Ca_Kabuli_Scaffold2557	110441	(T/A)
15090	CakSNP15090	*Kabuli*	Ca_Kabuli_Scaffold2569	58152	(T/G)
15091	CakSNP15091	*Kabuli*	Ca_Kabuli_Scaffold2575	101222	(C/T)

S.N.	SNP IDs	Cultivars	Chromosomes/scaffolds	Physical positions (bp)	SNPs
15092	CakSNP15092	*Kabuli*	Ca_Kabuli_Scaffold2617	467848	(C/T)
15093	CakSNP15093	*Kabuli*	Ca_Kabuli_Scaffold263	85006	(G/A)
15094	CakSNP15094	*Kabuli*	Ca_Kabuli_Scaffold263	85003	(C/T)
15095	CakSNP15095	*Kabuli*	Ca_Kabuli_Scaffold263	84998	(G/A)
15096	CakSNP15096	*Kabuli*	Ca_Kabuli_Scaffold263	84976	(A/G)
15097	CakSNP15097	*Kabuli*	Ca_Kabuli_Scaffold263	84970	(T/C)
15098	CakSNP15098	*Kabuli*	Ca_Kabuli_Scaffold263	84952	(T/G)
15099	CakSNP15099	*Kabuli*	Ca_Kabuli_Scaffold2720	30699	(C/T)
15100	CakSNP15100	*Kabuli*	Ca_Kabuli_Scaffold2720	30701	(G/A)
15101	CakSNP15101	*Kabuli*	Ca_Kabuli_Scaffold2720	30760	(T/C)
15102	CakSNP15102	*Kabuli*	Ca_Kabuli_Scaffold2720	177550	(G/T)
15103	CakSNP15103	*Kabuli*	Ca_Kabuli_Scaffold2720	177578	(T/C)
15104	CakSNP15104	*Kabuli*	Ca_Kabuli_Scaffold2722	52416	(G/T)
15105	CakSNP15105	*Kabuli*	Ca_Kabuli_Scaffold2722	52397	(G/T)
15106	CakSNP15106	*Kabuli*	Ca_Kabuli_Scaffold2722	67309	(C/A)
15107	CakSNP15107	*Kabuli*	Ca_Kabuli_Scaffold2722	75609	(A/G)
15108	CakSNP15108	*Kabuli*	Ca_Kabuli_Scaffold2728	1371	(G/A)
15109	CakSNP15109	*Kabuli*	Ca_Kabuli_Scaffold273	202514	(T/G)
15110	CakSNP15110	*Kabuli*	Ca_Kabuli_Scaffold2738	1307	(G/A)
15111	CakSNP15111	*Kabuli*	Ca_Kabuli_Scaffold275	208095	(G/A)
15112	CakSNP15112	*Kabuli*	Ca_Kabuli_Scaffold275	208067	(C/A)
15113	CakSNP15113	*Kabuli*	Ca_Kabuli_Scaffold275	208786	(G/C)
15114	CakSNP15114	*Kabuli*	Ca_Kabuli_Scaffold275	230606	(A/G)
15115	CakSNP15115	*Kabuli*	Ca_Kabuli_Scaffold275	347422	(C/A)
15116	CakSNP15116	*Kabuli*	Ca_Kabuli_Scaffold275	347384	(G/A)
15117	CakSNP15117	*Kabuli*	Ca_Kabuli_Scaffold275	347361	(C/T)
15118	CakSNP15118	*Kabuli*	Ca_Kabuli_Scaffold275	479497	(C/T)
15119	CakSNP15119	*Kabuli*	Ca_Kabuli_Scaffold275	562610	(C/T)
15120	CakSNP15120	*Kabuli*	Ca_Kabuli_Scaffold2763	4688	(A/C)
15121	CakSNP15121	*Kabuli*	Ca_Kabuli_Scaffold2763	4706	(G/T)
15122	CakSNP15122	*Kabuli*	Ca_Kabuli_Scaffold2763	4719	(G/A)
15123	CakSNP15123	*Kabuli*	Ca_Kabuli_Scaffold2763	4732	(C/G)
15124	CakSNP15124	*Kabuli*	Ca_Kabuli_Scaffold2763	4738	(C/G)
15125	CakSNP15125	*Kabuli*	Ca_Kabuli_Scaffold2763	4804	(A/T)
15126	CakSNP15126	*Kabuli*	Ca_Kabuli_Scaffold2763	4800	(T/A)
15127	CakSNP15127	*Kabuli*	Ca_Kabuli_Scaffold2763	4787	(A/G)
15128	CakSNP15128	*Kabuli*	Ca_Kabuli_Scaffold2763	4782	(C/T)
15129	CakSNP15129	*Kabuli*	Ca_Kabuli_Scaffold2763	25756	(T/G)
15130	CakSNP15130	*Kabuli*	Ca_Kabuli_Scaffold2763	25769	(A/G)

S.N.	SNP IDs	Cultivars	Chromosomes/scaffolds	Physical positions (bp)	SNPs
15131	CakSNP15131	*Kabuli*	Ca_Kabuli_Scaffold2763	25771	(T/G)
15132	CakSNP15132	*Kabuli*	Ca_Kabuli_Scaffold2763	43207	(G/A)
15133	CakSNP15133	*Kabuli*	Ca_Kabuli_Scaffold2763	49533	(T/A)
15134	CakSNP15134	*Kabuli*	Ca_Kabuli_Scaffold2763	82193	(C/A)
15135	CakSNP15135	*Kabuli*	Ca_Kabuli_Scaffold2763	82232	(C/T)
15136	CakSNP15136	*Kabuli*	Ca_Kabuli_Scaffold2763	82249	(G/A)
15137	CakSNP15137	*Kabuli*	Ca_Kabuli_Scaffold2763	82250	(T/C)
15138	CakSNP15138	*Kabuli*	Ca_Kabuli_Scaffold2763	82253	(G/C)
15139	CakSNP15139	*Kabuli*	Ca_Kabuli_Scaffold2763	82262	(G/C)
15140	CakSNP15140	*Kabuli*	Ca_Kabuli_Scaffold2763	82261	(C/G)
15141	CakSNP15141	*Kabuli*	Ca_Kabuli_Scaffold2763	82265	(G/T)
15142	CakSNP15142	*Kabuli*	Ca_Kabuli_Scaffold2763	82302	(T/A)
15143	CakSNP15143	*Kabuli*	Ca_Kabuli_Scaffold2763	82319	(T/C)
15144	CakSNP15144	*Kabuli*	Ca_Kabuli_Scaffold2763	82328	(A/G)
15145	CakSNP15145	*Kabuli*	Ca_Kabuli_Scaffold2763	82342	(C/T)
15146	CakSNP15146	*Kabuli*	Ca_Kabuli_Scaffold2763	82369	(G/A)
15147	CakSNP15147	*Kabuli*	Ca_Kabuli_Scaffold2763	82417	(C/T)
15148	CakSNP15148	*Kabuli*	Ca_Kabuli_Scaffold2763	82397	(T/C)
15149	CakSNP15149	*Kabuli*	Ca_Kabuli_Scaffold2763	95823	(G/A)
15150	CakSNP15150	*Kabuli*	Ca_Kabuli_Scaffold2763	99364	(A/G)
15151	CakSNP15151	*Kabuli*	Ca_Kabuli_Scaffold2763	99368	(C/G)
15152	CakSNP15152	*Kabuli*	Ca_Kabuli_Scaffold2763	99549	(C/A)
15153	CakSNP15153	*Kabuli*	Ca_Kabuli_Scaffold2763	104549	(G/C)
15154	CakSNP15154	*Kabuli*	Ca_Kabuli_Scaffold2763	104583	(T/C)
15155	CakSNP15155	*Kabuli*	Ca_Kabuli_Scaffold2763	107087	(A/G)
15156	CakSNP15156	*Kabuli*	Ca_Kabuli_Scaffold2763	107033	(T/A)
15157	CakSNP15157	*Kabuli*	Ca_Kabuli_Scaffold2763	107314	(A/G)
15158	CakSNP15158	*Kabuli*	Ca_Kabuli_Scaffold2763	107437	(T/C)
15159	CakSNP15159	*Kabuli*	Ca_Kabuli_Scaffold2763	107376	(C/T)
15160	CakSNP15160	*Kabuli*	Ca_Kabuli_Scaffold2763	107413	(A/G)
15161	CakSNP15161	*Kabuli*	Ca_Kabuli_Scaffold2763	108560	(T/G)
15162	CakSNP15162	*Kabuli*	Ca_Kabuli_Scaffold2763	108599	(T/A)
15163	CakSNP15163	*Kabuli*	Ca_Kabuli_Scaffold2763	108763	(T/G)
15164	CakSNP15164	*Kabuli*	Ca_Kabuli_Scaffold2763	117440	(C/T)
15165	CakSNP15165	*Kabuli*	Ca_Kabuli_Scaffold2763	117430	(A/C)
15166	CakSNP15166	*Kabuli*	Ca_Kabuli_Scaffold2763	125716	(C/T)
15167	CakSNP15167	*Kabuli*	Ca_Kabuli_Scaffold280	90875	(G/A)
15168	CakSNP15168	*Kabuli*	Ca_Kabuli_Scaffold280	90950	(C/A)
15169	CakSNP15169	*Kabuli*	Ca_Kabuli_Scaffold280	99357	(A/C)

S.N.	SNP IDs	Cultivars	Chromosomes/scaffolds	Physical positions (bp)	SNPs
15170	CakSNP15170	*Kabuli*	Ca_Kabuli_Scaffold280	101752	(G/C)
15171	CakSNP15171	*Kabuli*	Ca_Kabuli_Scaffold280	111160	(C/A)
15172	CakSNP15172	*Kabuli*	Ca_Kabuli_Scaffold2812	56649	(C/T)
15173	CakSNP15173	*Kabuli*	Ca_Kabuli_Scaffold2812	56658	(G/A)
15174	CakSNP15174	*Kabuli*	Ca_Kabuli_Scaffold2812	56672	(C/G)
15175	CakSNP15175	*Kabuli*	Ca_Kabuli_Scaffold2812	56702	(G/A)
15176	CakSNP15176	*Kabuli*	Ca_Kabuli_Scaffold2812	56714	(G/C)
15177	CakSNP15177	*Kabuli*	Ca_Kabuli_Scaffold2812	56648	(C/G)
15178	CakSNP15178	*Kabuli*	Ca_Kabuli_Scaffold2812	89844	(A/C)
15179	CakSNP15179	*Kabuli*	Ca_Kabuli_Scaffold2812	89940	(C/T)
15180	CakSNP15180	*Kabuli*	Ca_Kabuli_Scaffold2812	89888	(T/A)
15181	CakSNP15181	*Kabuli*	Ca_Kabuli_Scaffold2812	89887	(A/T)
15182	CakSNP15182	*Kabuli*	Ca_Kabuli_Scaffold2815	25626	(C/G)
15183	CakSNP15183	*Kabuli*	Ca_Kabuli_Scaffold2815	25655	(T/G)
15184	CakSNP15184	*Kabuli*	Ca_Kabuli_Scaffold2815	25668	(T/G)
15185	CakSNP15185	*Kabuli*	Ca_Kabuli_Scaffold2827	171	(C/T)
15186	CakSNP15186	*Kabuli*	Ca_Kabuli_Scaffold2827	172	(G/A)
15187	CakSNP15187	*Kabuli*	Ca_Kabuli_Scaffold2827	179	(T/G)
15188	CakSNP15188	*Kabuli*	Ca_Kabuli_Scaffold2827	210	(A/G)
15189	CakSNP15189	*Kabuli*	Ca_Kabuli_Scaffold2827	285	(T/G)
15190	CakSNP15190	*Kabuli*	Ca_Kabuli_Scaffold2827	258	(T/C)
15191	CakSNP15191	*Kabuli*	Ca_Kabuli_Scaffold2827	240	(T/G)
15192	CakSNP15192	*Kabuli*	Ca_Kabuli_Scaffold2827	222	(C/T)
15193	CakSNP15193	*Kabuli*	Ca_Kabuli_Scaffold284	242841	(A/T)
15194	CakSNP15194	*Kabuli*	Ca_Kabuli_Scaffold284	242778	(T/C)
15195	CakSNP15195	*Kabuli*	Ca_Kabuli_Scaffold284	522764	(T/G)
15196	CakSNP15196	*Kabuli*	Ca_Kabuli_Scaffold284	613764	(G/A)
15197	CakSNP15197	*Kabuli*	Ca_Kabuli_Scaffold284	613861	(C/T)
15198	CakSNP15198	*Kabuli*	Ca_Kabuli_Scaffold284	613850	(C/T)
15199	CakSNP15199	*Kabuli*	Ca_Kabuli_Scaffold284	613811	(G/A)
15200	CakSNP15200	*Kabuli*	Ca_Kabuli_Scaffold284	826690	(T/C)
15201	CakSNP15201	*Kabuli*	Ca_Kabuli_Scaffold284	826644	(G/A)
15202	CakSNP15202	*Kabuli*	Ca_Kabuli_Scaffold2845	7107	(G/A)
15203	CakSNP15203	*Kabuli*	Ca_Kabuli_Scaffold2848	2403	(C/A)
15204	CakSNP15204	*Kabuli*	Ca_Kabuli_Scaffold2848	2444	(C/A)
15205	CakSNP15205	*Kabuli*	Ca_Kabuli_Scaffold2848	2517	(G/A)
15206	CakSNP15206	*Kabuli*	Ca_Kabuli_Scaffold2848	2464	(A/G)
15207	CakSNP15207	*Kabuli*	Ca_Kabuli_Scaffold2853	14852	(C/G)
15208	CakSNP15208	*Kabuli*	Ca_Kabuli_Scaffold2853	14916	(A/G)

S.N.	SNP IDs	Cultivars	Chromosomes/scaffolds	Physical positions (bp)	SNPs
15209	CakSNP15209	*Kabuli*	Ca_Kabuli_Scaffold2853	14917	(G/A)
15210	CakSNP15210	*Kabuli*	Ca_Kabuli_Scaffold2853	15100	(T/C)
15211	CakSNP15211	*Kabuli*	Ca_Kabuli_Scaffold2853	15086	(G/A)
15212	CakSNP15212	*Kabuli*	Ca_Kabuli_Scaffold287	3224	(G/A)
15213	CakSNP15213	*Kabuli*	Ca_Kabuli_Scaffold287	163600	(G/C)
15214	CakSNP15214	*Kabuli*	Ca_Kabuli_Scaffold287	163591	(C/A)
15215	CakSNP15215	*Kabuli*	Ca_Kabuli_Scaffold287	163576	(G/A)
15216	CakSNP15216	*Kabuli*	Ca_Kabuli_Scaffold290	179998	(T/C)
15217	CakSNP15217	*Kabuli*	Ca_Kabuli_Scaffold290	179993	(C/T)
15218	CakSNP15218	*Kabuli*	Ca_Kabuli_Scaffold290	179984	(G/T)
15219	CakSNP15219	*Kabuli*	Ca_Kabuli_Scaffold290	179940	(G/A)
15220	CakSNP15220	*Kabuli*	Ca_Kabuli_Scaffold290	179937	(C/G)
15221	CakSNP15221	*Kabuli*	Ca_Kabuli_Scaffold290	179969	(A/T)
15222	CakSNP15222	*Kabuli*	Ca_Kabuli_Scaffold290	211634	(A/C)
15223	CakSNP15223	*Kabuli*	Ca_Kabuli_Scaffold290	212536	(A/C)
15224	CakSNP15224	*Kabuli*	Ca_Kabuli_Scaffold290	212514	(C/T)
15225	CakSNP15225	*Kabuli*	Ca_Kabuli_Scaffold290	255161	(G/A)
15226	CakSNP15226	*Kabuli*	Ca_Kabuli_Scaffold290	728010	(T/A)
15227	CakSNP15227	*Kabuli*	Ca_Kabuli_Scaffold290	824906	(C/T)
15228	CakSNP15228	*Kabuli*	Ca_Kabuli_Scaffold2907	31202	(G/A)
15229	CakSNP15229	*Kabuli*	Ca_Kabuli_Scaffold2950	32650	(C/T)
15230	CakSNP15230	*Kabuli*	Ca_Kabuli_Scaffold2950	32703	(A/C)
15231	CakSNP15231	*Kabuli*	Ca_Kabuli_Scaffold2950	32739	(C/A)
15232	CakSNP15232	*Kabuli*	Ca_Kabuli_Scaffold2950	32776	(T/A)
15233	CakSNP15233	*Kabuli*	Ca_Kabuli_Scaffold2950	33081	(A/G)
15234	CakSNP15234	*Kabuli*	Ca_Kabuli_Scaffold2950	33207	(C/T)
15235	CakSNP15235	*Kabuli*	Ca_Kabuli_Scaffold296	41422	(T/G)
15236	CakSNP15236	*Kabuli*	Ca_Kabuli_Scaffold296	59691	(G/A)
15237	CakSNP15237	*Kabuli*	Ca_Kabuli_Scaffold296	59758	(A/G)
15238	CakSNP15238	*Kabuli*	Ca_Kabuli_Scaffold296	59883	(A/C)
15239	CakSNP15239	*Kabuli*	Ca_Kabuli_Scaffold296	361664	(T/C)
15240	CakSNP15240	*Kabuli*	Ca_Kabuli_Scaffold299	199050	(A/T)
15241	CakSNP15241	*Kabuli*	Ca_Kabuli_Scaffold299	199067	(A/T)
15242	CakSNP15242	*Kabuli*	Ca_Kabuli_Scaffold299	199068	(G/T)
15243	CakSNP15243	*Kabuli*	Ca_Kabuli_Scaffold299	199087	(G/A)
15244	CakSNP15244	*Kabuli*	Ca_Kabuli_Scaffold299	199073	(G/A)
15245	CakSNP15245	*Kabuli*	Ca_Kabuli_Scaffold299	204540	(G/A)
15246	CakSNP15246	*Kabuli*	Ca_Kabuli_Scaffold300	111914	(C/T)
15247	CakSNP15247	*Kabuli*	Ca_Kabuli_Scaffold300	111924	(C/T)

S.N.	SNP IDs	Cultivars	Chromosomes/scaffolds	Physical positions (bp)	SNPs
15248	CakSNP15248	*Kabuli*	Ca_Kabuli_Scaffold300	111926	(G/A)
15249	CakSNP15249	*Kabuli*	Ca_Kabuli_Scaffold300	111933	(C/T)
15250	CakSNP15250	*Kabuli*	Ca_Kabuli_Scaffold300	111936	(C/A)
15251	CakSNP15251	*Kabuli*	Ca_Kabuli_Scaffold300	111972	(A/T)
15252	CakSNP15252	*Kabuli*	Ca_Kabuli_Scaffold300	111973	(A/T)
15253	CakSNP15253	*Kabuli*	Ca_Kabuli_Scaffold300	111976	(C/T)
15254	CakSNP15254	*Kabuli*	Ca_Kabuli_Scaffold300	112011	(T/C)
15255	CakSNP15255	*Kabuli*	Ca_Kabuli_Scaffold300	112006	(G/A)
15256	CakSNP15256	*Kabuli*	Ca_Kabuli_Scaffold300	112005	(G/A)
15257	CakSNP15257	*Kabuli*	Ca_Kabuli_Scaffold300	111959	(C/T)
15258	CakSNP15258	*Kabuli*	Ca_Kabuli_Scaffold300	111964	(C/T)
15259	CakSNP15259	*Kabuli*	Ca_Kabuli_Scaffold3016	32790	(C/T)
15260	CakSNP15260	*Kabuli*	Ca_Kabuli_Scaffold303	18213	(G/A)
15261	CakSNP15261	*Kabuli*	Ca_Kabuli_Scaffold305	49263	(G/A)
15262	CakSNP15262	*Kabuli*	Ca_Kabuli_Scaffold305	49266	(T/G)
15263	CakSNP15263	*Kabuli*	Ca_Kabuli_Scaffold305	49278	(C/A)
15264	CakSNP15264	*Kabuli*	Ca_Kabuli_Scaffold305	138929	(G/A)
15265	CakSNP15265	*Kabuli*	Ca_Kabuli_Scaffold306	15514	(C/T)
15266	CakSNP15266	*Kabuli*	Ca_Kabuli_Scaffold306	19215	(C/A)
15267	CakSNP15267	*Kabuli*	Ca_Kabuli_Scaffold306	85993	(T/A)
15268	CakSNP15268	*Kabuli*	Ca_Kabuli_Scaffold306	88389	(T/A)
15269	CakSNP15269	*Kabuli*	Ca_Kabuli_Scaffold3084	4409	(G/A)
15270	CakSNP15270	*Kabuli*	Ca_Kabuli_Scaffold308_2	9421	(G/A)
15271	CakSNP15271	*Kabuli*	Ca_Kabuli_Scaffold308_2	143696	(C/G)
15272	CakSNP15272	*Kabuli*	Ca_Kabuli_Scaffold308_2	143813	(C/G)
15273	CakSNP15273	*Kabuli*	Ca_Kabuli_Scaffold308_2	144354	(C/T)
15274	CakSNP15274	*Kabuli*	Ca_Kabuli_Scaffold308_2	144508	(C/T)
15275	CakSNP15275	*Kabuli*	Ca_Kabuli_Scaffold308_2	145639	(G/T)
15276	CakSNP15276	*Kabuli*	Ca_Kabuli_Scaffold308_2	152546	(A/G)
15277	CakSNP15277	*Kabuli*	Ca_Kabuli_Scaffold308_2	152576	(G/T)
15278	CakSNP15278	*Kabuli*	Ca_Kabuli_Scaffold311	86652	(C/A)
15279	CakSNP15279	*Kabuli*	Ca_Kabuli_Scaffold311	86677	(C/T)
15280	CakSNP15280	*Kabuli*	Ca_Kabuli_Scaffold311	86658	(C/T)
15281	CakSNP15281	*Kabuli*	Ca_Kabuli_Scaffold311	86670	(G/A)
15282	CakSNP15282	*Kabuli*	Ca_Kabuli_Scaffold3116	159	(T/G)
15283	CakSNP15283	*Kabuli*	Ca_Kabuli_Scaffold314	432453	(T/C)
15284	CakSNP15284	*Kabuli*	Ca_Kabuli_Scaffold314	432471	(A/G)
15285	CakSNP15285	*Kabuli*	Ca_Kabuli_Scaffold314	535269	(A/C)
15286	CakSNP15286	*Kabuli*	Ca_Kabuli_Scaffold314	535266	(A/C)

S.N.	SNP IDs	Cultivars	Chromosomes/scaffolds	Physical positions (bp)	SNPs
15287	CakSNP15287	*Kabuli*	Ca_Kabuli_Scaffold314	535263	(T/C)
15288	CakSNP15288	*Kabuli*	Ca_Kabuli_Scaffold314	610535	(C/G)
15289	CakSNP15289	*Kabuli*	Ca_Kabuli_Scaffold314	624361	(C/T)
15290	CakSNP15290	*Kabuli*	Ca_Kabuli_Scaffold3155	7438	(A/C)
15291	CakSNP15291	*Kabuli*	Ca_Kabuli_Scaffold3155	7442	(T/C)
15292	CakSNP15292	*Kabuli*	Ca_Kabuli_Scaffold3155	7443	(A/G)
15293	CakSNP15293	*Kabuli*	Ca_Kabuli_Scaffold3155	7444	(A/G)
15294	CakSNP15294	*Kabuli*	Ca_Kabuli_Scaffold3155	7455	(T/G)
15295	CakSNP15295	*Kabuli*	Ca_Kabuli_Scaffold3155	7462	(A/G)
15296	CakSNP15296	*Kabuli*	Ca_Kabuli_Scaffold3155	7464	(T/A)
15297	CakSNP15297	*Kabuli*	Ca_Kabuli_Scaffold3155	7472	(A/G)
15298	CakSNP15298	*Kabuli*	Ca_Kabuli_Scaffold3155	7477	(C/G)
15299	CakSNP15299	*Kabuli*	Ca_Kabuli_Scaffold3155	7480	(G/A)
15300	CakSNP15300	*Kabuli*	Ca_Kabuli_Scaffold3155	9404	(C/T)
15301	CakSNP15301	*Kabuli*	Ca_Kabuli_Scaffold3155	9746	(A/T)
15302	CakSNP15302	*Kabuli*	Ca_Kabuli_Scaffold3170	56261	(A/G)
15303	CakSNP15303	*Kabuli*	Ca_Kabuli_Scaffold322	55475	(G/A)
15304	CakSNP15304	*Kabuli*	Ca_Kabuli_Scaffold322	55479	(G/T)
15305	CakSNP15305	*Kabuli*	Ca_Kabuli_Scaffold322	55589	(A/T)
15306	CakSNP15306	*Kabuli*	Ca_Kabuli_Scaffold3228	2926	(C/T)
15307	CakSNP15307	*Kabuli*	Ca_Kabuli_Scaffold3228	23138	(A/G)
15308	CakSNP15308	*Kabuli*	Ca_Kabuli_Scaffold3228	23190	(G/C)
15309	CakSNP15309	*Kabuli*	Ca_Kabuli_Scaffold3228	23315	(C/A)
15310	CakSNP15310	*Kabuli*	Ca_Kabuli_Scaffold3228	23270	(A/C)
15311	CakSNP15311	*Kabuli*	Ca_Kabuli_Scaffold324	15569	(A/C)
15312	CakSNP15312	*Kabuli*	Ca_Kabuli_Scaffold324	15574	(G/A)
15313	CakSNP15313	*Kabuli*	Ca_Kabuli_Scaffold324	15575	(G/C)
15314	CakSNP15314	*Kabuli*	Ca_Kabuli_Scaffold324	15581	(G/C)
15315	CakSNP15315	*Kabuli*	Ca_Kabuli_Scaffold324	15590	(G/T)
15316	CakSNP15316	*Kabuli*	Ca_Kabuli_Scaffold324	15593	(G/A)
15317	CakSNP15317	*Kabuli*	Ca_Kabuli_Scaffold324	15594	(C/A)
15318	CakSNP15318	*Kabuli*	Ca_Kabuli_Scaffold324	15614	(C/T)
15319	CakSNP15319	*Kabuli*	Ca_Kabuli_Scaffold324	15619	(A/G)
15320	CakSNP15320	*Kabuli*	Ca_Kabuli_Scaffold324	15636	(G/A)
15321	CakSNP15321	*Kabuli*	Ca_Kabuli_Scaffold3254_2	15616	(A/G)
15322	CakSNP15322	*Kabuli*	Ca_Kabuli_Scaffold3254_2	28089	(T/C)
15323	CakSNP15323	*Kabuli*	Ca_Kabuli_Scaffold3254_2	31210	(T/A)
15324	CakSNP15324	*Kabuli*	Ca_Kabuli_Scaffold3254_2	31217	(C/T)
15325	CakSNP15325	*Kabuli*	Ca_Kabuli_Scaffold3254_2	31219	(A/C)

S.N.	SNP IDs	Cultivars	Chromosomes/scaffolds	Physical positions (bp)	SNPs
15326	CakSNP15326	*Kabuli*	Ca_Kabuli_Scaffold3254_2	31224	(T/G)
15327	CakSNP15327	*Kabuli*	Ca_Kabuli_Scaffold3254_2	31229	(G/T)
15328	CakSNP15328	*Kabuli*	Ca_Kabuli_Scaffold3254_2	31242	(C/A)
15329	CakSNP15329	*Kabuli*	Ca_Kabuli_Scaffold3254_2	31344	(A/G)
15330	CakSNP15330	*Kabuli*	Ca_Kabuli_Scaffold3254_2	31325	(T/A)
15331	CakSNP15331	*Kabuli*	Ca_Kabuli_Scaffold3254_2	31322	(G/A)
15332	CakSNP15332	*Kabuli*	Ca_Kabuli_Scaffold3254_2	31311	(G/A)
15333	CakSNP15333	*Kabuli*	Ca_Kabuli_Scaffold3254_2	31303	(T/C)
15334	CakSNP15334	*Kabuli*	Ca_Kabuli_Scaffold3254_2	39157	(T/A)
15335	CakSNP15335	*Kabuli*	Ca_Kabuli_Scaffold3254_2	46644	(G/A)
15336	CakSNP15336	*Kabuli*	Ca_Kabuli_Scaffold3254_2	186218	(G/A)
15337	CakSNP15337	*Kabuli*	Ca_Kabuli_Scaffold3254_2	186203	(C/G)
15338	CakSNP15338	*Kabuli*	Ca_Kabuli_Scaffold3254_2	186243	(A/C)
15339	CakSNP15339	*Kabuli*	Ca_Kabuli_Scaffold3284	2735	(T/C)
15340	CakSNP15340	*Kabuli*	Ca_Kabuli_Scaffold332	203691	(T/A)
15341	CakSNP15341	*Kabuli*	Ca_Kabuli_Scaffold332	204783	(A/G)
15342	CakSNP15342	*Kabuli*	Ca_Kabuli_Scaffold332	204785	(G/A)
15343	CakSNP15343	*Kabuli*	Ca_Kabuli_Scaffold332	204952	(G/A)
15344	CakSNP15344	*Kabuli*	Ca_Kabuli_Scaffold3321	17935	(C/A)
15345	CakSNP15345	*Kabuli*	Ca_Kabuli_Scaffold3337	17963	(C/T)
15346	CakSNP15346	*Kabuli*	Ca_Kabuli_Scaffold3337	17999	(T/G)
15347	CakSNP15347	*Kabuli*	Ca_Kabuli_Scaffold3337	18022	(C/T)
15348	CakSNP15348	*Kabuli*	Ca_Kabuli_Scaffold3337	18026	(T/A)
15349	CakSNP15349	*Kabuli*	Ca_Kabuli_Scaffold3337	17959	(A/G)
15350	CakSNP15350	*Kabuli*	Ca_Kabuli_Scaffold3337	17956	(G/A)
15351	CakSNP15351	*Kabuli*	Ca_Kabuli_Scaffold334	43160	(G/A)
15352	CakSNP15352	*Kabuli*	Ca_Kabuli_Scaffold335	60951	(T/C)
15353	CakSNP15353	*Kabuli*	Ca_Kabuli_Scaffold335	60952	(T/C)
15354	CakSNP15354	*Kabuli*	Ca_Kabuli_Scaffold335	60956	(T/C)
15355	CakSNP15355	*Kabuli*	Ca_Kabuli_Scaffold335	60957	(T/C)
15356	CakSNP15356	*Kabuli*	Ca_Kabuli_Scaffold335	60960	(T/C)
15357	CakSNP15357	*Kabuli*	Ca_Kabuli_Scaffold335	60961	(C/T)
15358	CakSNP15358	*Kabuli*	Ca_Kabuli_Scaffold335	60962	(T/G)
15359	CakSNP15359	*Kabuli*	Ca_Kabuli_Scaffold335	60906	(T/G)
15360	CakSNP15360	*Kabuli*	Ca_Kabuli_Scaffold335	135942	(T/A)
15361	CakSNP15361	*Kabuli*	Ca_Kabuli_Scaffold335	136017	(C/G)
15362	CakSNP15362	*Kabuli*	Ca_Kabuli_Scaffold335	135969	(C/T)
15363	CakSNP15363	*Kabuli*	Ca_Kabuli_Scaffold335	135976	(C/T)
15364	CakSNP15364	*Kabuli*	Ca_Kabuli_Scaffold335	136077	(G/C)

S.N.	SNP IDs	Cultivars	Chromosomes/scaffolds	Physical positions (bp)	SNPs
15365	CakSNP15365	*Kabuli*	Ca_Kabuli_Scaffold335	136076	(G/C)
15366	CakSNP15366	*Kabuli*	Ca_Kabuli_Scaffold335	136073	(G/A)
15367	CakSNP15367	*Kabuli*	Ca_Kabuli_Scaffold335	136051	(T/A)
15368	CakSNP15368	*Kabuli*	Ca_Kabuli_Scaffold335	136030	(G/A)
15369	CakSNP15369	*Kabuli*	Ca_Kabuli_Scaffold335	136129	(T/C)
15370	CakSNP15370	*Kabuli*	Ca_Kabuli_Scaffold335	136109	(G/T)
15371	CakSNP15371	*Kabuli*	Ca_Kabuli_Scaffold335	136106	(G/A)
15372	CakSNP15372	*Kabuli*	Ca_Kabuli_Scaffold335	136096	(T/C)
15373	CakSNP15373	*Kabuli*	Ca_Kabuli_Scaffold335	136091	(G/A)
15374	CakSNP15374	*Kabuli*	Ca_Kabuli_Scaffold336	468863	(T/A)
15375	CakSNP15375	*Kabuli*	Ca_Kabuli_Scaffold336	563363	(C/T)
15376	CakSNP15376	*Kabuli*	Ca_Kabuli_Scaffold336	618427	(C/T)
15377	CakSNP15377	*Kabuli*	Ca_Kabuli_Scaffold336	771169	(C/T)
15378	CakSNP15378	*Kabuli*	Ca_Kabuli_Scaffold336	771175	(C/T)
15379	CakSNP15379	*Kabuli*	Ca_Kabuli_Scaffold3362	69878	(C/A)
15380	CakSNP15380	*Kabuli*	Ca_Kabuli_Scaffold338	14666	(T/G)
15381	CakSNP15381	*Kabuli*	Ca_Kabuli_Scaffold3397	45363	(T/C)
15382	CakSNP15382	*Kabuli*	Ca_Kabuli_Scaffold342	311411	(G/A)
15383	CakSNP15383	*Kabuli*	Ca_Kabuli_Scaffold342	311394	(A/C)
15384	CakSNP15384	*Kabuli*	Ca_Kabuli_Scaffold342	359376	(A/C)
15385	CakSNP15385	*Kabuli*	Ca_Kabuli_Scaffold3422	13376	(T/A)
15386	CakSNP15386	*Kabuli*	Ca_Kabuli_Scaffold346_1	67110	(G/T)
15387	CakSNP15387	*Kabuli*	Ca_Kabuli_Scaffold346_1	105461	(A/G)
15388	CakSNP15388	*Kabuli*	Ca_Kabuli_Scaffold346_1	105521	(A/C)
15389	CakSNP15389	*Kabuli*	Ca_Kabuli_Scaffold346_1	105523	(G/A)
15390	CakSNP15390	*Kabuli*	Ca_Kabuli_Scaffold346_1	105546	(C/T)
15391	CakSNP15391	*Kabuli*	Ca_Kabuli_Scaffold349	51381	(A/T)
15392	CakSNP15392	*Kabuli*	Ca_Kabuli_Scaffold349	51396	(G/A)
15393	CakSNP15393	*Kabuli*	Ca_Kabuli_Scaffold349	51414	(C/T)
15394	CakSNP15394	*Kabuli*	Ca_Kabuli_Scaffold349	51435	(C/T)
15395	CakSNP15395	*Kabuli*	Ca_Kabuli_Scaffold349	51548	(T/C)
15396	CakSNP15396	*Kabuli*	Ca_Kabuli_Scaffold349	51637	(T/A)
15397	CakSNP15397	*Kabuli*	Ca_Kabuli_Scaffold349	51624	(G/A)
15398	CakSNP15398	*Kabuli*	Ca_Kabuli_Scaffold349	174027	(G/C)
15399	CakSNP15399	*Kabuli*	Ca_Kabuli_Scaffold349	174039	(A/T)
15400	CakSNP15400	*Kabuli*	Ca_Kabuli_Scaffold3514	42134	(G/A)
15401	CakSNP15401	*Kabuli*	Ca_Kabuli_Scaffold3514	42135	(G/T)
15402	CakSNP15402	*Kabuli*	Ca_Kabuli_Scaffold3514	42156	(G/A)
15403	CakSNP15403	*Kabuli*	Ca_Kabuli_Scaffold3514	42163	(T/C)

S.N.	SNP IDs	Cultivars	Chromosomes/scaffolds	Physical positions (bp)	SNPs
15404	CakSNP15404	*Kabuli*	Ca_Kabuli_Scaffold3514	42164	(A/G)
15405	CakSNP15405	*Kabuli*	Ca_Kabuli_Scaffold3514	42175	(G/A)
15406	CakSNP15406	*Kabuli*	Ca_Kabuli_Scaffold352	22672	(C/T)
15407	CakSNP15407	*Kabuli*	Ca_Kabuli_Scaffold352	22675	(G/A)
15408	CakSNP15408	*Kabuli*	Ca_Kabuli_Scaffold352	22693	(C/T)
15409	CakSNP15409	*Kabuli*	Ca_Kabuli_Scaffold352	22708	(A/G)
15410	CakSNP15410	*Kabuli*	Ca_Kabuli_Scaffold352	22787	(A/G)
15411	CakSNP15411	*Kabuli*	Ca_Kabuli_Scaffold352	22777	(T/G)
15412	CakSNP15412	*Kabuli*	Ca_Kabuli_Scaffold352	22726	(A/C)
15413	CakSNP15413	*Kabuli*	Ca_Kabuli_Scaffold36	167556	(C/T)
15414	CakSNP15414	*Kabuli*	Ca_Kabuli_Scaffold36	226511	(A/G)
15415	CakSNP15415	*Kabuli*	Ca_Kabuli_Scaffold362	84943	(A/G)
15416	CakSNP15416	*Kabuli*	Ca_Kabuli_Scaffold362	84945	(T/C)
15417	CakSNP15417	*Kabuli*	Ca_Kabuli_Scaffold362	84954	(T/C)
15418	CakSNP15418	*Kabuli*	Ca_Kabuli_Scaffold362	84972	(C/A)
15419	CakSNP15419	*Kabuli*	Ca_Kabuli_Scaffold362	84988	(T/G)
15420	CakSNP15420	*Kabuli*	Ca_Kabuli_Scaffold362	84994	(G/A)
15421	CakSNP15421	*Kabuli*	Ca_Kabuli_Scaffold362	164620	(C/T)
15422	CakSNP15422	*Kabuli*	Ca_Kabuli_Scaffold362	164789	(T/C)
15423	CakSNP15423	*Kabuli*	Ca_Kabuli_Scaffold362	164787	(C/T)
15424	CakSNP15424	*Kabuli*	Ca_Kabuli_Scaffold362	196556	(G/A)
15425	CakSNP15425	*Kabuli*	Ca_Kabuli_Scaffold362	219176	(T/C)
15426	CakSNP15426	*Kabuli*	Ca_Kabuli_Scaffold366	61810	(C/T)
15427	CakSNP15427	*Kabuli*	Ca_Kabuli_Scaffold369	162735	(T/G)
15428	CakSNP15428	*Kabuli*	Ca_Kabuli_Scaffold3693	81068	(C/T)
15429	CakSNP15429	*Kabuli*	Ca_Kabuli_Scaffold3693	81080	(C/A)
15430	CakSNP15430	*Kabuli*	Ca_Kabuli_Scaffold3693	81204	(C/T)
15431	CakSNP15431	*Kabuli*	Ca_Kabuli_Scaffold3724	68378	(G/A)
15432	CakSNP15432	*Kabuli*	Ca_Kabuli_Scaffold374	132907	(A/C)
15433	CakSNP15433	*Kabuli*	Ca_Kabuli_Scaffold374	132896	(T/C)
15434	CakSNP15434	*Kabuli*	Ca_Kabuli_Scaffold374	132873	(A/G)
15435	CakSNP15435	*Kabuli*	Ca_Kabuli_Scaffold374	132859	(C/T)
15436	CakSNP15436	*Kabuli*	Ca_Kabuli_Scaffold374	132858	(G/A)
15437	CakSNP15437	*Kabuli*	Ca_Kabuli_Scaffold374	132849	(A/C)
15438	CakSNP15438	*Kabuli*	Ca_Kabuli_Scaffold374	132843	(C/T)
15439	CakSNP15439	*Kabuli*	Ca_Kabuli_Scaffold374	198368	(T/C)
15440	CakSNP15440	*Kabuli*	Ca_Kabuli_Scaffold377	393535	(G/T)
15441	CakSNP15441	*Kabuli*	Ca_Kabuli_Scaffold377	393538	(G/A)
15442	CakSNP15442	*Kabuli*	Ca_Kabuli_Scaffold379	69075	(A/C)

S.N.	SNP IDs	Cultivars	Chromosomes/scaffolds	Physical positions (bp)	SNPs
15443	CakSNP15443	*Kabuli*	Ca_Kabuli_Scaffold379	144442	(G/A)
15444	CakSNP15444	*Kabuli*	Ca_Kabuli_Scaffold379	177306	(T/C)
15445	CakSNP15445	*Kabuli*	Ca_Kabuli_Scaffold379	177354	(G/A)
15446	CakSNP15446	*Kabuli*	Ca_Kabuli_Scaffold379	177340	(C/T)
15447	CakSNP15447	*Kabuli*	Ca_Kabuli_Scaffold379	227608	(A/T)
15448	CakSNP15448	*Kabuli*	Ca_Kabuli_Scaffold379	227604	(C/A)
15449	CakSNP15449	*Kabuli*	Ca_Kabuli_Scaffold379	227597	(T/A)
15450	CakSNP15450	*Kabuli*	Ca_Kabuli_Scaffold38	69499	(A/G)
15451	CakSNP15451	*Kabuli*	Ca_Kabuli_Scaffold38	413691	(G/A)
15452	CakSNP15452	*Kabuli*	Ca_Kabuli_Scaffold38	413713	(A/C)
15453	CakSNP15453	*Kabuli*	Ca_Kabuli_Scaffold38	413719	(G/A)
15454	CakSNP15454	*Kabuli*	Ca_Kabuli_Scaffold38	457392	(C/A)
15455	CakSNP15455	*Kabuli*	Ca_Kabuli_Scaffold38	457596	(T/A)
15456	CakSNP15456	*Kabuli*	Ca_Kabuli_Scaffold38	520372	(T/G)
15457	CakSNP15457	*Kabuli*	Ca_Kabuli_Scaffold38	520311	(G/A)
15458	CakSNP15458	*Kabuli*	Ca_Kabuli_Scaffold38	520377	(T/C)
15459	CakSNP15459	*Kabuli*	Ca_Kabuli_Scaffold38	537059	(A/T)
15460	CakSNP15460	*Kabuli*	Ca_Kabuli_Scaffold38	537063	(C/T)
15461	CakSNP15461	*Kabuli*	Ca_Kabuli_Scaffold3819	450	(T/A)
15462	CakSNP15462	*Kabuli*	Ca_Kabuli_Scaffold382	36686	(A/T)
15463	CakSNP15463	*Kabuli*	Ca_Kabuli_Scaffold382	36703	(A/T)
15464	CakSNP15464	*Kabuli*	Ca_Kabuli_Scaffold382	36704	(G/T)
15465	CakSNP15465	*Kabuli*	Ca_Kabuli_Scaffold382	36709	(G/A)
15466	CakSNP15466	*Kabuli*	Ca_Kabuli_Scaffold382	36674	(G/A)
15467	CakSNP15467	*Kabuli*	Ca_Kabuli_Scaffold3824	26207	(G/A)
15468	CakSNP15468	*Kabuli*	Ca_Kabuli_Scaffold3824	26267	(G/A)
15469	CakSNP15469	*Kabuli*	Ca_Kabuli_Scaffold3824	26327	(G/A)
15470	CakSNP15470	*Kabuli*	Ca_Kabuli_Scaffold3824	26387	(G/A)
15471	CakSNP15471	*Kabuli*	Ca_Kabuli_Scaffold3865	12265	(T/A)
15472	CakSNP15472	*Kabuli*	Ca_Kabuli_Scaffold3865	65093	(T/C)
15473	CakSNP15473	*Kabuli*	Ca_Kabuli_Scaffold3865	65127	(C/A)
15474	CakSNP15474	*Kabuli*	Ca_Kabuli_Scaffold3865	65129	(T/A)
15475	CakSNP15475	*Kabuli*	Ca_Kabuli_Scaffold387	3263	(G/A)
15476	CakSNP15476	*Kabuli*	Ca_Kabuli_Scaffold387	3219	(C/A)
15477	CakSNP15477	*Kabuli*	Ca_Kabuli_Scaffold387	3309	(T/C)
15478	CakSNP15478	*Kabuli*	Ca_Kabuli_Scaffold387	3316	(G/A)
15479	CakSNP15479	*Kabuli*	Ca_Kabuli_Scaffold3945	3367	(G/A)
15480	CakSNP15480	*Kabuli*	Ca_Kabuli_Scaffold3945	3371	(G/A)
15481	CakSNP15481	*Kabuli*	Ca_Kabuli_Scaffold3945	3880	(G/T)

S.N.	SNP IDs	Cultivars	Chromosomes/scaffolds	Physical positions (bp)	SNPs
15482	CakSNP15482	*Kabuli*	Ca_Kabuli_Scaffold3945	3879	(T/C)
15483	CakSNP15483	*Kabuli*	Ca_Kabuli_Scaffold3945	7260	(T/A)
15484	CakSNP15484	*Kabuli*	Ca_Kabuli_Scaffold3945	13468	(C/T)
15485	CakSNP15485	*Kabuli*	Ca_Kabuli_Scaffold3945	13440	(C/T)
15486	CakSNP15486	*Kabuli*	Ca_Kabuli_Scaffold3945	13892	(C/G)
15487	CakSNP15487	*Kabuli*	Ca_Kabuli_Scaffold3945	18891	(A/T)
15488	CakSNP15488	*Kabuli*	Ca_Kabuli_Scaffold3945	18892	(G/C)
15489	CakSNP15489	*Kabuli*	Ca_Kabuli_Scaffold3945	19359	(G/A)
15490	CakSNP15490	*Kabuli*	Ca_Kabuli_Scaffold3945	19366	(G/T)
15491	CakSNP15491	*Kabuli*	Ca_Kabuli_Scaffold3945	19367	(A/T)
15492	CakSNP15492	*Kabuli*	Ca_Kabuli_Scaffold3945	19368	(A/C)
15493	CakSNP15493	*Kabuli*	Ca_Kabuli_Scaffold396	130566	(T/C)
15494	CakSNP15494	*Kabuli*	Ca_Kabuli_Scaffold396	130671	(A/G)
15495	CakSNP15495	*Kabuli*	Ca_Kabuli_Scaffold396	130639	(C/A)
15496	CakSNP15496	*Kabuli*	Ca_Kabuli_Scaffold396	130636	(G/A)
15497	CakSNP15497	*Kabuli*	Ca_Kabuli_Scaffold396	130635	(G/T)
15498	CakSNP15498	*Kabuli*	Ca_Kabuli_Scaffold396	130624	(A/G)
15499	CakSNP15499	*Kabuli*	Ca_Kabuli_Scaffold396	130621	(C/A)
15500	CakSNP15500	*Kabuli*	Ca_Kabuli_Scaffold396	195890	(A/G)
15501	CakSNP15501	*Kabuli*	Ca_Kabuli_Scaffold396	297325	(G/C)
15502	CakSNP15502	*Kabuli*	Ca_Kabuli_Scaffold398	246956	(C/T)
15503	CakSNP15503	*Kabuli*	Ca_Kabuli_Scaffold398	247004	(C/T)
15504	CakSNP15504	*Kabuli*	Ca_Kabuli_Scaffold398	441570	(C/T)
15505	CakSNP15505	*Kabuli*	Ca_Kabuli_Scaffold398	441605	(C/A)
15506	CakSNP15506	*Kabuli*	Ca_Kabuli_Scaffold40	386189	(A/C)
15507	CakSNP15507	*Kabuli*	Ca_Kabuli_Scaffold40	497729	(T/C)
15508	CakSNP15508	*Kabuli*	Ca_Kabuli_Scaffold40	542951	(C/G)
15509	CakSNP15509	*Kabuli*	Ca_Kabuli_Scaffold40	548453	(A/G)
15510	CakSNP15510	*Kabuli*	Ca_Kabuli_Scaffold40	636910	(C/T)
15511	CakSNP15511	*Kabuli*	Ca_Kabuli_Scaffold40	691226	(C/G)
15512	CakSNP15512	*Kabuli*	Ca_Kabuli_Scaffold40	871303	(A/C)
15513	CakSNP15513	*Kabuli*	Ca_Kabuli_Scaffold40	911602	(T/G)
15514	CakSNP15514	*Kabuli*	Ca_Kabuli_Scaffold40	968798	(G/A)
15515	CakSNP15515	*Kabuli*	Ca_Kabuli_Scaffold40	996604	(T/C)
15516	CakSNP15516	*Kabuli*	Ca_Kabuli_Scaffold40	1014875	(G/A)
15517	CakSNP15517	*Kabuli*	Ca_Kabuli_Scaffold40	1014834	(T/A)
15518	CakSNP15518	*Kabuli*	Ca_Kabuli_Scaffold40	1014826	(C/T)
15519	CakSNP15519	*Kabuli*	Ca_Kabuli_Scaffold40	1014805	(C/T)
15520	CakSNP15520	*Kabuli*	Ca_Kabuli_Scaffold40	1039218	(T/C)

S.N.	SNP IDs	Cultivars	Chromosomes/scaffolds	Physical positions (bp)	SNPs
15521	CakSNP15521	*Kabuli*	Ca_Kabuli_Scaffold40	1039233	(A/T)
15522	CakSNP15522	*Kabuli*	Ca_Kabuli_Scaffold40	1049594	(G/A)
15523	CakSNP15523	*Kabuli*	Ca_Kabuli_Scaffold40	1050111	(C/T)
15524	CakSNP15524	*Kabuli*	Ca_Kabuli_Scaffold40	1050164	(C/T)
15525	CakSNP15525	*Kabuli*	Ca_Kabuli_Scaffold40	1050255	(T/C)
15526	CakSNP15526	*Kabuli*	Ca_Kabuli_Scaffold40	1050175	(G/C)
15527	CakSNP15527	*Kabuli*	Ca_Kabuli_Scaffold40	1052597	(T/C)
15528	CakSNP15528	*Kabuli*	Ca_Kabuli_Scaffold40	1052983	(G/A)
15529	CakSNP15529	*Kabuli*	Ca_Kabuli_Scaffold40	1053215	(G/A)
15530	CakSNP15530	*Kabuli*	Ca_Kabuli_Scaffold401	49065	(G/A)
15531	CakSNP15531	*Kabuli*	Ca_Kabuli_Scaffold4011	93334	(T/C)
15532	CakSNP15532	*Kabuli*	Ca_Kabuli_Scaffold4011	93347	(C/T)
15533	CakSNP15533	*Kabuli*	Ca_Kabuli_Scaffold4011	96223	(G/C)
15534	CakSNP15534	*Kabuli*	Ca_Kabuli_Scaffold4011	97602	(T/C)
15535	CakSNP15535	*Kabuli*	Ca_Kabuli_Scaffold4011	130271	(G/A)
15536	CakSNP15536	*Kabuli*	Ca_Kabuli_Scaffold4011	130213	(G/A)
15537	CakSNP15537	*Kabuli*	Ca_Kabuli_Scaffold404	273203	(C/A)
15538	CakSNP15538	*Kabuli*	Ca_Kabuli_Scaffold404	273209	(C/A)
15539	CakSNP15539	*Kabuli*	Ca_Kabuli_Scaffold404	273220	(G/A)
15540	CakSNP15540	*Kabuli*	Ca_Kabuli_Scaffold404	273245	(C/T)
15541	CakSNP15541	*Kabuli*	Ca_Kabuli_Scaffold404	285941	(G/A)
15542	CakSNP15542	*Kabuli*	Ca_Kabuli_Scaffold404	285952	(T/C)
15543	CakSNP15543	*Kabuli*	Ca_Kabuli_Scaffold404	285958	(C/G)
15544	CakSNP15544	*Kabuli*	Ca_Kabuli_Scaffold404	285987	(G/A)
15545	CakSNP15545	*Kabuli*	Ca_Kabuli_Scaffold404	285999	(T/G)
15546	CakSNP15546	*Kabuli*	Ca_Kabuli_Scaffold404	286013	(T/A)
15547	CakSNP15547	*Kabuli*	Ca_Kabuli_Scaffold404	355859	(G/A)
15548	CakSNP15548	*Kabuli*	Ca_Kabuli_Scaffold4057	16503	(C/G)
15549	CakSNP15549	*Kabuli*	Ca_Kabuli_Scaffold41	14657	(C/A)
15550	CakSNP15550	*Kabuli*	Ca_Kabuli_Scaffold41	122583	(G/C)
15551	CakSNP15551	*Kabuli*	Ca_Kabuli_Scaffold4102	1035	(C/G)
15552	CakSNP15552	*Kabuli*	Ca_Kabuli_Scaffold4102	1136	(A/G)
15553	CakSNP15553	*Kabuli*	Ca_Kabuli_Scaffold4102	1160	(T/C)
15554	CakSNP15554	*Kabuli*	Ca_Kabuli_Scaffold4102	1163	(G/T)
15555	CakSNP15555	*Kabuli*	Ca_Kabuli_Scaffold4102	1346	(C/T)
15556	CakSNP15556	*Kabuli*	Ca_Kabuli_Scaffold411	60078	(C/T)
15557	CakSNP15557	*Kabuli*	Ca_Kabuli_Scaffold411	323122	(T/C)
15558	CakSNP15558	*Kabuli*	Ca_Kabuli_Scaffold411	323140	(T/C)
15559	CakSNP15559	*Kabuli*	Ca_Kabuli_Scaffold411	323141	(G/A)

S.N.	SNP IDs	Cultivars	Chromosomes/scaffolds	Physical positions (bp)	SNPs
15560	CakSNP15560	*Kabuli*	Ca_Kabuli_Scaffold411	323147	(T/C)
15561	CakSNP15561	*Kabuli*	Ca_Kabuli_Scaffold411	323161	(G/A)
15562	CakSNP15562	*Kabuli*	Ca_Kabuli_Scaffold411	323162	(T/C)
15563	CakSNP15563	*Kabuli*	Ca_Kabuli_Scaffold411	323167	(C/G)
15564	CakSNP15564	*Kabuli*	Ca_Kabuli_Scaffold411	323179	(T/G)
15565	CakSNP15565	*Kabuli*	Ca_Kabuli_Scaffold411	323114	(C/G)
15566	CakSNP15566	*Kabuli*	Ca_Kabuli_Scaffold411	323215	(T/C)
15567	CakSNP15567	*Kabuli*	Ca_Kabuli_Scaffold415	8479	(A/C)
15568	CakSNP15568	*Kabuli*	Ca_Kabuli_Scaffold415	8529	(G/A)
15569	CakSNP15569	*Kabuli*	Ca_Kabuli_Scaffold418	6092	(C/G)
15570	CakSNP15570	*Kabuli*	Ca_Kabuli_Scaffold418	6094	(T/G)
15571	CakSNP15571	*Kabuli*	Ca_Kabuli_Scaffold418	13769	(T/G)
15572	CakSNP15572	*Kabuli*	Ca_Kabuli_Scaffold418	13804	(T/G)
15573	CakSNP15573	*Kabuli*	Ca_Kabuli_Scaffold418	13808	(A/G)
15574	CakSNP15574	*Kabuli*	Ca_Kabuli_Scaffold418	13811	(G/A)
15575	CakSNP15575	*Kabuli*	Ca_Kabuli_Scaffold418	13812	(C/A)
15576	CakSNP15576	*Kabuli*	Ca_Kabuli_Scaffold418	151140	(G/T)
15577	CakSNP15577	*Kabuli*	Ca_Kabuli_Scaffold418	151205	(C/T)
15578	CakSNP15578	*Kabuli*	Ca_Kabuli_Scaffold418	151256	(G/A)
15579	CakSNP15579	*Kabuli*	Ca_Kabuli_Scaffold419	73537	(T/G)
15580	CakSNP15580	*Kabuli*	Ca_Kabuli_Scaffold420	34145	(T/G)
15581	CakSNP15581	*Kabuli*	Ca_Kabuli_Scaffold420	42532	(G/A)
15582	CakSNP15582	*Kabuli*	Ca_Kabuli_Scaffold420	42739	(C/T)
15583	CakSNP15583	*Kabuli*	Ca_Kabuli_Scaffold420	42811	(C/T)
15584	CakSNP15584	*Kabuli*	Ca_Kabuli_Scaffold420	42877	(T/C)
15585	CakSNP15585	*Kabuli*	Ca_Kabuli_Scaffold420	43372	(C/T)
15586	CakSNP15586	*Kabuli*	Ca_Kabuli_Scaffold420	92756	(C/T)
15587	CakSNP15587	*Kabuli*	Ca_Kabuli_Scaffold420	92759	(A/G)
15588	CakSNP15588	*Kabuli*	Ca_Kabuli_Scaffold420	92784	(C/T)
15589	CakSNP15589	*Kabuli*	Ca_Kabuli_Scaffold420	92813	(G/T)
15590	CakSNP15590	*Kabuli*	Ca_Kabuli_Scaffold420	92865	(G/T)
15591	CakSNP15591	*Kabuli*	Ca_Kabuli_Scaffold420	92846	(C/T)
15592	CakSNP15592	*Kabuli*	Ca_Kabuli_Scaffold420	92801	(A/C)
15593	CakSNP15593	*Kabuli*	Ca_Kabuli_Scaffold420	149744	(C/G)
15594	CakSNP15594	*Kabuli*	Ca_Kabuli_Scaffold420	242536	(G/T)
15595	CakSNP15595	*Kabuli*	Ca_Kabuli_Scaffold420	242503	(C/T)
15596	CakSNP15596	*Kabuli*	Ca_Kabuli_Scaffold420	242487	(A/G)
15597	CakSNP15597	*Kabuli*	Ca_Kabuli_Scaffold420	242922	(A/C)
15598	CakSNP15598	*Kabuli*	Ca_Kabuli_Scaffold420	254661	(T/G)

S.N.	SNP IDs	Cultivars	Chromosomes/scaffolds	Physical positions (bp)	SNPs
15599	CakSNP15599	*Kabuli*	Ca_Kabuli_Scaffold420	341146	(T/A)
15600	CakSNP15600	*Kabuli*	Ca_Kabuli_Scaffold420	341184	(A/G)
15601	CakSNP15601	*Kabuli*	Ca_Kabuli_Scaffold420	341202	(T/G)
15602	CakSNP15602	*Kabuli*	Ca_Kabuli_Scaffold420	341206	(G/A)
15603	CakSNP15603	*Kabuli*	Ca_Kabuli_Scaffold421_1	25389	(G/A)
15604	CakSNP15604	*Kabuli*	Ca_Kabuli_Scaffold421_1	71804	(A/G)
15605	CakSNP15605	*Kabuli*	Ca_Kabuli_Scaffold421_1	71770	(G/T)
15606	CakSNP15606	*Kabuli*	Ca_Kabuli_Scaffold421_2	142859	(G/C)
15607	CakSNP15607	*Kabuli*	Ca_Kabuli_Scaffold421_2	341095	(C/A)
15608	CakSNP15608	*Kabuli*	Ca_Kabuli_Scaffold421_2	341136	(C/A)
15609	CakSNP15609	*Kabuli*	Ca_Kabuli_Scaffold421_2	446961	(G/A)
15610	CakSNP15610	*Kabuli*	Ca_Kabuli_Scaffold421_2	446976	(C/A)
15611	CakSNP15611	*Kabuli*	Ca_Kabuli_Scaffold421_2	446979	(C/T)
15612	CakSNP15612	*Kabuli*	Ca_Kabuli_Scaffold424	32402	(C/G)
15613	CakSNP15613	*Kabuli*	Ca_Kabuli_Scaffold4331	4044	(T/G)
15614	CakSNP15614	*Kabuli*	Ca_Kabuli_Scaffold44	66309	(A/C)
15615	CakSNP15615	*Kabuli*	Ca_Kabuli_Scaffold44	66280	(G/A)
15616	CakSNP15616	*Kabuli*	Ca_Kabuli_Scaffold44	87412	(G/A)
15617	CakSNP15617	*Kabuli*	Ca_Kabuli_Scaffold4414	276	(C/T)
15618	CakSNP15618	*Kabuli*	Ca_Kabuli_Scaffold4452	20003	(G/A)
15619	CakSNP15619	*Kabuli*	Ca_Kabuli_Scaffold4452	20005	(G/A)
15620	CakSNP15620	*Kabuli*	Ca_Kabuli_Scaffold4452	20038	(A/G)
15621	CakSNP15621	*Kabuli*	Ca_Kabuli_Scaffold4452	20010	(G/A)
15622	CakSNP15622	*Kabuli*	Ca_Kabuli_Scaffold4452	29414	(G/T)
15623	CakSNP15623	*Kabuli*	Ca_Kabuli_Scaffold4452	36715	(A/T)
15624	CakSNP15624	*Kabuli*	Ca_Kabuli_Scaffold4452	69950	(A/C)
15625	CakSNP15625	*Kabuli*	Ca_Kabuli_Scaffold4452	70041	(T/C)
15626	CakSNP15626	*Kabuli*	Ca_Kabuli_Scaffold450	132367	(C/T)
15627	CakSNP15627	*Kabuli*	Ca_Kabuli_Scaffold450	132427	(A/T)
15628	CakSNP15628	*Kabuli*	Ca_Kabuli_Scaffold450	197287	(G/A)
15629	CakSNP15629	*Kabuli*	Ca_Kabuli_Scaffold450	197272	(G/A)
15630	CakSNP15630	*Kabuli*	Ca_Kabuli_Scaffold450	197262	(T/G)
15631	CakSNP15631	*Kabuli*	Ca_Kabuli_Scaffold450	197230	(C/A)
15632	CakSNP15632	*Kabuli*	Ca_Kabuli_Scaffold450	197226	(G/A)
15633	CakSNP15633	*Kabuli*	Ca_Kabuli_Scaffold450	250303	(G/T)
15634	CakSNP15634	*Kabuli*	Ca_Kabuli_Scaffold451	103555	(G/C)
15635	CakSNP15635	*Kabuli*	Ca_Kabuli_Scaffold451	103596	(A/G)
15636	CakSNP15636	*Kabuli*	Ca_Kabuli_Scaffold451	117231	(A/G)
15637	CakSNP15637	*Kabuli*	Ca_Kabuli_Scaffold451	117300	(C/A)

S.N.	SNP IDs	Cultivars	Chromosomes/scaffolds	Physical positions (bp)	SNPs
15638	CakSNP15638	*Kabuli*	Ca_Kabuli_Scaffold4511	40072	(C/A)
15639	CakSNP15639	*Kabuli*	Ca_Kabuli_Scaffold4511	40014	(C/T)
15640	CakSNP15640	*Kabuli*	Ca_Kabuli_Scaffold4511	40089	(C/T)
15641	CakSNP15641	*Kabuli*	Ca_Kabuli_Scaffold452	1892	(G/A)
15642	CakSNP15642	*Kabuli*	Ca_Kabuli_Scaffold452	1881	(A/G)
15643	CakSNP15643	*Kabuli*	Ca_Kabuli_Scaffold452	1841	(A/G)
15644	CakSNP15644	*Kabuli*	Ca_Kabuli_Scaffold452	1837	(A/C)
15645	CakSNP15645	*Kabuli*	Ca_Kabuli_Scaffold452	1836	(A/G)
15646	CakSNP15646	*Kabuli*	Ca_Kabuli_Scaffold452	1835	(C/T)
15647	CakSNP15647	*Kabuli*	Ca_Kabuli_Scaffold453	303193	(T/C)
15648	CakSNP15648	*Kabuli*	Ca_Kabuli_Scaffold453	303201	(G/C)
15649	CakSNP15649	*Kabuli*	Ca_Kabuli_Scaffold453	303246	(A/G)
15650	CakSNP15650	*Kabuli*	Ca_Kabuli_Scaffold453	303248	(C/T)
15651	CakSNP15651	*Kabuli*	Ca_Kabuli_Scaffold453	303259	(C/G)
15652	CakSNP15652	*Kabuli*	Ca_Kabuli_Scaffold46	39438	(C/A)
15653	CakSNP15653	*Kabuli*	Ca_Kabuli_Scaffold46	39470	(C/T)
15654	CakSNP15654	*Kabuli*	Ca_Kabuli_Scaffold461	20652	(C/A)
15655	CakSNP15655	*Kabuli*	Ca_Kabuli_Scaffold461	20629	(A/T)
15656	CakSNP15656	*Kabuli*	Ca_Kabuli_Scaffold461	20603	(C/T)
15657	CakSNP15657	*Kabuli*	Ca_Kabuli_Scaffold461	20599	(C/A)
15658	CakSNP15658	*Kabuli*	Ca_Kabuli_Scaffold4620	2699	(T/C)
15659	CakSNP15659	*Kabuli*	Ca_Kabuli_Scaffold4620	23442	(T/C)
15660	CakSNP15660	*Kabuli*	Ca_Kabuli_Scaffold4641	511	(G/A)
15661	CakSNP15661	*Kabuli*	Ca_Kabuli_Scaffold4641	547	(A/C)
15662	CakSNP15662	*Kabuli*	Ca_Kabuli_Scaffold4641	553	(C/A)
15663	CakSNP15663	*Kabuli*	Ca_Kabuli_Scaffold4641	556	(G/C)
15664	CakSNP15664	*Kabuli*	Ca_Kabuli_Scaffold4662	7683	(T/C)
15665	CakSNP15665	*Kabuli*	Ca_Kabuli_Scaffold4662	7651	(T/C)
15666	CakSNP15666	*Kabuli*	Ca_Kabuli_Scaffold4662	7638	(C/T)
15667	CakSNP15667	*Kabuli*	Ca_Kabuli_Scaffold4662	7632	(A/G)
15668	CakSNP15668	*Kabuli*	Ca_Kabuli_Scaffold4695	5685	(A/G)
15669	CakSNP15669	*Kabuli*	Ca_Kabuli_Scaffold470	7175	(A/G)
15670	CakSNP15670	*Kabuli*	Ca_Kabuli_Scaffold473	24996	(G/A)
15671	CakSNP15671	*Kabuli*	Ca_Kabuli_Scaffold473	24984	(T/C)
15672	CakSNP15672	*Kabuli*	Ca_Kabuli_Scaffold473	24973	(T/C)
15673	CakSNP15673	*Kabuli*	Ca_Kabuli_Scaffold475	135286	(C/A)
15674	CakSNP15674	*Kabuli*	Ca_Kabuli_Scaffold475	135290	(G/A)
15675	CakSNP15675	*Kabuli*	Ca_Kabuli_Scaffold477	51898	(C/T)
15676	CakSNP15676	*Kabuli*	Ca_Kabuli_Scaffold4777	10546	(G/T)

S.N.	SNP IDs	Cultivars	Chromosomes/scaffolds	Physical positions (bp)	SNPs
15677	CakSNP15677	*Kabuli*	Ca_Kabuli_Scaffold4777	16224	(A/T)
15678	CakSNP15678	*Kabuli*	Ca_Kabuli_Scaffold4777	16239	(G/T)
15679	CakSNP15679	*Kabuli*	Ca_Kabuli_Scaffold4777	55347	(G/T)
15680	CakSNP15680	*Kabuli*	Ca_Kabuli_Scaffold4777	72624	(G/A)
15681	CakSNP15681	*Kabuli*	Ca_Kabuli_Scaffold4777	76950	(C/A)
15682	CakSNP15682	*Kabuli*	Ca_Kabuli_Scaffold4777	106820	(A/G)
15683	CakSNP15683	*Kabuli*	Ca_Kabuli_Scaffold4777	112193	(A/C)
15684	CakSNP15684	*Kabuli*	Ca_Kabuli_Scaffold48	261719	(A/G)
15685	CakSNP15685	*Kabuli*	Ca_Kabuli_Scaffold48	420925	(A/G)
15686	CakSNP15686	*Kabuli*	Ca_Kabuli_Scaffold480	67375	(T/C)
15687	CakSNP15687	*Kabuli*	Ca_Kabuli_Scaffold4836	5866	(T/A)
15688	CakSNP15688	*Kabuli*	Ca_Kabuli_Scaffold4836	5891	(A/T)
15689	CakSNP15689	*Kabuli*	Ca_Kabuli_Scaffold4836	5909	(G/A)
15690	CakSNP15690	*Kabuli*	Ca_Kabuli_Scaffold4836	5846	(C/T)
15691	CakSNP15691	*Kabuli*	Ca_Kabuli_Scaffold484	203947	(C/T)
15692	CakSNP15692	*Kabuli*	Ca_Kabuli_Scaffold484	209786	(A/C)
15693	CakSNP15693	*Kabuli*	Ca_Kabuli_Scaffold484	243567	(C/T)
15694	CakSNP15694	*Kabuli*	Ca_Kabuli_Scaffold484	243575	(T/A)
15695	CakSNP15695	*Kabuli*	Ca_Kabuli_Scaffold484	647868	(C/T)
15696	CakSNP15696	*Kabuli*	Ca_Kabuli_Scaffold484	647866	(A/G)
15697	CakSNP15697	*Kabuli*	Ca_Kabuli_Scaffold484	647809	(A/T)
15698	CakSNP15698	*Kabuli*	Ca_Kabuli_Scaffold484	660749	(C/G)
15699	CakSNP15699	*Kabuli*	Ca_Kabuli_Scaffold485	103572	(C/T)
15700	CakSNP15700	*Kabuli*	Ca_Kabuli_Scaffold485	103546	(A/G)
15701	CakSNP15701	*Kabuli*	Ca_Kabuli_Scaffold485	103528	(A/C)
15702	CakSNP15702	*Kabuli*	Ca_Kabuli_Scaffold485	123578	(T/C)
15703	CakSNP15703	*Kabuli*	Ca_Kabuli_Scaffold485	123642	(G/A)
15704	CakSNP15704	*Kabuli*	Ca_Kabuli_Scaffold495	128200	(A/T)
15705	CakSNP15705	*Kabuli*	Ca_Kabuli_Scaffold496	53652	(G/A)
15706	CakSNP15706	*Kabuli*	Ca_Kabuli_Scaffold496	53657	(A/T)
15707	CakSNP15707	*Kabuli*	Ca_Kabuli_Scaffold496	53616	(G/T)
15708	CakSNP15708	*Kabuli*	Ca_Kabuli_Scaffold496	53587	(A/G)
15709	CakSNP15709	*Kabuli*	Ca_Kabuli_Scaffold496	175429	(G/A)
15710	CakSNP15710	*Kabuli*	Ca_Kabuli_Scaffold496	175493	(G/A)
15711	CakSNP15711	*Kabuli*	Ca_Kabuli_Scaffold498	146772	(A/C)
15712	CakSNP15712	*Kabuli*	Ca_Kabuli_Scaffold498	148658	(G/T)
15713	CakSNP15713	*Kabuli*	Ca_Kabuli_Scaffold498	164638	(T/C)
15714	CakSNP15714	*Kabuli*	Ca_Kabuli_Scaffold498	164615	(G/C)
15715	CakSNP15715	*Kabuli*	Ca_Kabuli_Scaffold498	164590	(T/A)

S.N.	SNP IDs	Cultivars	Chromosomes/scaffolds	Physical positions (bp)	SNPs
15716	CakSNP15716	*Kabuli*	Ca_Kabuli_Scaffold50	153593	(T/A)
15717	CakSNP15717	*Kabuli*	Ca_Kabuli_Scaffold50	153599	(C/G)
15718	CakSNP15718	*Kabuli*	Ca_Kabuli_Scaffold50	153631	(C/T)
15719	CakSNP15719	*Kabuli*	Ca_Kabuli_Scaffold50	153734	(A/C)
15720	CakSNP15720	*Kabuli*	Ca_Kabuli_Scaffold50	153851	(T/C)
15721	CakSNP15721	*Kabuli*	Ca_Kabuli_Scaffold50	191625	(T/C)
15722	CakSNP15722	*Kabuli*	Ca_Kabuli_Scaffold50	201219	(C/G)
15723	CakSNP15723	*Kabuli*	Ca_Kabuli_Scaffold50	448866	(G/A)
15724	CakSNP15724	*Kabuli*	Ca_Kabuli_Scaffold510	12378	(T/A)
15725	CakSNP15725	*Kabuli*	Ca_Kabuli_Scaffold510	12383	(G/T)
15726	CakSNP15726	*Kabuli*	Ca_Kabuli_Scaffold510	12394	(C/A)
15727	CakSNP15727	*Kabuli*	Ca_Kabuli_Scaffold510	12414	(C/T)
15728	CakSNP15728	*Kabuli*	Ca_Kabuli_Scaffold510	12434	(C/T)
15729	CakSNP15729	*Kabuli*	Ca_Kabuli_Scaffold511	10450	(T/G)
15730	CakSNP15730	*Kabuli*	Ca_Kabuli_Scaffold511	10520	(G/T)
15731	CakSNP15731	*Kabuli*	Ca_Kabuli_Scaffold511	173376	(C/A)
15732	CakSNP15732	*Kabuli*	Ca_Kabuli_Scaffold511	173377	(A/G)
15733	CakSNP15733	*Kabuli*	Ca_Kabuli_Scaffold511	173378	(G/A)
15734	CakSNP15734	*Kabuli*	Ca_Kabuli_Scaffold513	7188	(A/C)
15735	CakSNP15735	*Kabuli*	Ca_Kabuli_Scaffold5163	2301	(A/G)
15736	CakSNP15736	*Kabuli*	Ca_Kabuli_Scaffold5185	1091	(G/A)
15737	CakSNP15737	*Kabuli*	Ca_Kabuli_Scaffold520	143359	(C/T)
15738	CakSNP15738	*Kabuli*	Ca_Kabuli_Scaffold520	143358	(G/A)
15739	CakSNP15739	*Kabuli*	Ca_Kabuli_Scaffold520	143356	(C/G)
15740	CakSNP15740	*Kabuli*	Ca_Kabuli_Scaffold520	143342	(G/A)
15741	CakSNP15741	*Kabuli*	Ca_Kabuli_Scaffold520	143336	(T/C)
15742	CakSNP15742	*Kabuli*	Ca_Kabuli_Scaffold520	143334	(G/A)
15743	CakSNP15743	*Kabuli*	Ca_Kabuli_Scaffold520	143333	(A/T)
15744	CakSNP15744	*Kabuli*	Ca_Kabuli_Scaffold520	143324	(G/A)
15745	CakSNP15745	*Kabuli*	Ca_Kabuli_Scaffold520	143317	(T/A)
15746	CakSNP15746	*Kabuli*	Ca_Kabuli_Scaffold520	143300	(G/A)
15747	CakSNP15747	*Kabuli*	Ca_Kabuli_Scaffold520	143294	(G/A)
15748	CakSNP15748	*Kabuli*	Ca_Kabuli_Scaffold520	143291	(G/A)
15749	CakSNP15749	*Kabuli*	Ca_Kabuli_Scaffold520	143375	(G/A)
15750	CakSNP15750	*Kabuli*	Ca_Kabuli_Scaffold520	143372	(G/A)
15751	CakSNP15751	*Kabuli*	Ca_Kabuli_Scaffold520	143355	(C/T)
15752	CakSNP15752	*Kabuli*	Ca_Kabuli_Scaffold520	143390	(A/G)
15753	CakSNP15753	*Kabuli*	Ca_Kabuli_Scaffold520	143391	(A/C)
15754	CakSNP15754	*Kabuli*	Ca_Kabuli_Scaffold528	191764	(A/G)

S.N.	SNP IDs	Cultivars	Chromosomes/scaffolds	Physical positions (bp)	SNPs
15755	CakSNP15755	*Kabuli*	Ca_Kabuli_Scaffold528	191835	(A/G)
15756	CakSNP15756	*Kabuli*	Ca_Kabuli_Scaffold528	232014	(A/C)
15757	CakSNP15757	*Kabuli*	Ca_Kabuli_Scaffold53	49273	(A/G)
15758	CakSNP15758	*Kabuli*	Ca_Kabuli_Scaffold53	123293	(C/A)
15759	CakSNP15759	*Kabuli*	Ca_Kabuli_Scaffold531	2668	(C/T)
15760	CakSNP15760	*Kabuli*	Ca_Kabuli_Scaffold531	2674	(C/T)
15761	CakSNP15761	*Kabuli*	Ca_Kabuli_Scaffold531	65242	(T/G)
15762	CakSNP15762	*Kabuli*	Ca_Kabuli_Scaffold531	101520	(C/G)
15763	CakSNP15763	*Kabuli*	Ca_Kabuli_Scaffold531	124019	(T/G)
15764	CakSNP15764	*Kabuli*	Ca_Kabuli_Scaffold5328	70	(G/A)
15765	CakSNP15765	*Kabuli*	Ca_Kabuli_Scaffold5328	85	(C/T)
15766	CakSNP15766	*Kabuli*	Ca_Kabuli_Scaffold5328	86	(C/T)
15767	CakSNP15767	*Kabuli*	Ca_Kabuli_Scaffold5328	117	(C/T)
15768	CakSNP15768	*Kabuli*	Ca_Kabuli_Scaffold535	156836	(G/A)
15769	CakSNP15769	*Kabuli*	Ca_Kabuli_Scaffold5358	2561	(C/A)
15770	CakSNP15770	*Kabuli*	Ca_Kabuli_Scaffold5358	3329	(C/A)
15771	CakSNP15771	*Kabuli*	Ca_Kabuli_Scaffold537	216656	(A/G)
15772	CakSNP15772	*Kabuli*	Ca_Kabuli_Scaffold537	216669	(T/A)
15773	CakSNP15773	*Kabuli*	Ca_Kabuli_Scaffold537	216846	(C/T)
15774	CakSNP15774	*Kabuli*	Ca_Kabuli_Scaffold537	216829	(C/G)
15775	CakSNP15775	*Kabuli*	Ca_Kabuli_Scaffold537	216778	(T/A)
15776	CakSNP15776	*Kabuli*	Ca_Kabuli_Scaffold537	223108	(T/C)
15777	CakSNP15777	*Kabuli*	Ca_Kabuli_Scaffold543	20780	(T/G)
15778	CakSNP15778	*Kabuli*	Ca_Kabuli_Scaffold543	20819	(T/C)
15779	CakSNP15779	*Kabuli*	Ca_Kabuli_Scaffold543	20827	(G/T)
15780	CakSNP15780	*Kabuli*	Ca_Kabuli_Scaffold543	143169	(C/T)
15781	CakSNP15781	*Kabuli*	Ca_Kabuli_Scaffold543	143182	(G/A)
15782	CakSNP15782	*Kabuli*	Ca_Kabuli_Scaffold543	143185	(A/G)
15783	CakSNP15783	*Kabuli*	Ca_Kabuli_Scaffold543	143191	(G/A)
15784	CakSNP15784	*Kabuli*	Ca_Kabuli_Scaffold543	266748	(A/C)
15785	CakSNP15785	*Kabuli*	Ca_Kabuli_Scaffold543	266729	(A/C)
15786	CakSNP15786	*Kabuli*	Ca_Kabuli_Scaffold543	266848	(A/C)
15787	CakSNP15787	*Kabuli*	Ca_Kabuli_Scaffold545	17699	(A/C)
15788	CakSNP15788	*Kabuli*	Ca_Kabuli_Scaffold545	138848	(T/C)
15789	CakSNP15789	*Kabuli*	Ca_Kabuli_Scaffold545	139118	(A/T)
15790	CakSNP15790	*Kabuli*	Ca_Kabuli_Scaffold545	146720	(A/G)
15791	CakSNP15791	*Kabuli*	Ca_Kabuli_Scaffold545	147549	(G/A)
15792	CakSNP15792	*Kabuli*	Ca_Kabuli_Scaffold545	147568	(T/G)
15793	CakSNP15793	*Kabuli*	Ca_Kabuli_Scaffold545	226799	(G/A)

S.N.	SNP IDs	Cultivars	Chromosomes/scaffolds	Physical positions (bp)	SNPs
15794	CakSNP15794	*Kabuli*	Ca_Kabuli_Scaffold545	226769	(G/C)
15795	CakSNP15795	*Kabuli*	Ca_Kabuli_Scaffold548	17462	(G/A)
15796	CakSNP15796	*Kabuli*	Ca_Kabuli_Scaffold548	231396	(G/A)
15797	CakSNP15797	*Kabuli*	Ca_Kabuli_Scaffold548	231338	(G/A)
15798	CakSNP15798	*Kabuli*	Ca_Kabuli_Scaffold5511	353	(G/A)
15799	CakSNP15799	*Kabuli*	Ca_Kabuli_Scaffold5511	361	(G/A)
15800	CakSNP15800	*Kabuli*	Ca_Kabuli_Scaffold5511	366	(G/A)
15801	CakSNP15801	*Kabuli*	Ca_Kabuli_Scaffold5511	376	(C/T)
15802	CakSNP15802	*Kabuli*	Ca_Kabuli_Scaffold5511	387	(T/C)
15803	CakSNP15803	*Kabuli*	Ca_Kabuli_Scaffold5511	388	(G/A)
15804	CakSNP15804	*Kabuli*	Ca_Kabuli_Scaffold5511	365	(T/G)
15805	CakSNP15805	*Kabuli*	Ca_Kabuli_Scaffold5511	396	(G/A)
15806	CakSNP15806	*Kabuli*	Ca_Kabuli_Scaffold5511	458	(G/A)
15807	CakSNP15807	*Kabuli*	Ca_Kabuli_Scaffold5511	447	(A/C)
15808	CakSNP15808	*Kabuli*	Ca_Kabuli_Scaffold5511	434	(C/T)
15809	CakSNP15809	*Kabuli*	Ca_Kabuli_Scaffold5511	404	(T/G)
15810	CakSNP15810	*Kabuli*	Ca_Kabuli_Scaffold553	302105	(T/C)
15811	CakSNP15811	*Kabuli*	Ca_Kabuli_Scaffold553	302098	(T/C)
15812	CakSNP15812	*Kabuli*	Ca_Kabuli_Scaffold553	310715	(A/G)
15813	CakSNP15813	*Kabuli*	Ca_Kabuli_Scaffold553	310759	(T/A)
15814	CakSNP15814	*Kabuli*	Ca_Kabuli_Scaffold553	402702	(T/C)
15815	CakSNP15815	*Kabuli*	Ca_Kabuli_Scaffold553	441845	(T/C)
15816	CakSNP15816	*Kabuli*	Ca_Kabuli_Scaffold553	441881	(T/C)
15817	CakSNP15817	*Kabuli*	Ca_Kabuli_Scaffold553	441898	(G/A)
15818	CakSNP15818	*Kabuli*	Ca_Kabuli_Scaffold553	550789	(G/C)
15819	CakSNP15819	*Kabuli*	Ca_Kabuli_Scaffold553	607503	(G/T)
15820	CakSNP15820	*Kabuli*	Ca_Kabuli_Scaffold553	607572	(T/C)
15821	CakSNP15821	*Kabuli*	Ca_Kabuli_Scaffold553	794383	(A/G)
15822	CakSNP15822	*Kabuli*	Ca_Kabuli_Scaffold553	794404	(G/A)
15823	CakSNP15823	*Kabuli*	Ca_Kabuli_Scaffold553	794431	(T/C)
15824	CakSNP15824	*Kabuli*	Ca_Kabuli_Scaffold553	794439	(G/T)
15825	CakSNP15825	*Kabuli*	Ca_Kabuli_Scaffold561	46046	(T/G)
15826	CakSNP15826	*Kabuli*	Ca_Kabuli_Scaffold561	49174	(G/A)
15827	CakSNP15827	*Kabuli*	Ca_Kabuli_Scaffold561	95414	(A/C)
15828	CakSNP15828	*Kabuli*	Ca_Kabuli_Scaffold561	95386	(G/A)
15829	CakSNP15829	*Kabuli*	Ca_Kabuli_Scaffold562	221706	(T/C)
15830	CakSNP15830	*Kabuli*	Ca_Kabuli_Scaffold562	221696	(T/G)
15831	CakSNP15831	*Kabuli*	Ca_Kabuli_Scaffold562	221686	(C/A)
15832	CakSNP15832	*Kabuli*	Ca_Kabuli_Scaffold562	221713	(G/A)

S.N.	SNP IDs	Cultivars	Chromosomes/scaffolds	Physical positions (bp)	SNPs
15833	CakSNP15833	*Kabuli*	Ca_Kabuli_Scaffold562	221714	(C/T)
15834	CakSNP15834	*Kabuli*	Ca_Kabuli_Scaffold562	221735	(G/A)
15835	CakSNP15835	*Kabuli*	Ca_Kabuli_Scaffold562	221795	(C/T)
15836	CakSNP15836	*Kabuli*	Ca_Kabuli_Scaffold562	221748	(C/A)
15837	CakSNP15837	*Kabuli*	Ca_Kabuli_Scaffold562	221758	(G/A)
15838	CakSNP15838	*Kabuli*	Ca_Kabuli_Scaffold562	221769	(T/A)
15839	CakSNP15839	*Kabuli*	Ca_Kabuli_Scaffold562	221778	(G/A)
15840	CakSNP15840	*Kabuli*	Ca_Kabuli_Scaffold562	221779	(G/A)
15841	CakSNP15841	*Kabuli*	Ca_Kabuli_Scaffold562	221788	(G/A)
15842	CakSNP15842	*Kabuli*	Ca_Kabuli_Scaffold562	221828	(T/A)
15843	CakSNP15843	*Kabuli*	Ca_Kabuli_Scaffold562	221792	(C/A)
15844	CakSNP15844	*Kabuli*	Ca_Kabuli_Scaffold562	255362	(G/A)
15845	CakSNP15845	*Kabuli*	Ca_Kabuli_Scaffold562	255295	(G/A)
15846	CakSNP15846	*Kabuli*	Ca_Kabuli_Scaffold562	289080	(C/A)
15847	CakSNP15847	*Kabuli*	Ca_Kabuli_Scaffold562	290071	(C/T)
15848	CakSNP15848	*Kabuli*	Ca_Kabuli_Scaffold5714	24882	(A/C)
15849	CakSNP15849	*Kabuli*	Ca_Kabuli_Scaffold5714	36359	(G/C)
15850	CakSNP15850	*Kabuli*	Ca_Kabuli_Scaffold5730	239	(G/A)
15851	CakSNP15851	*Kabuli*	Ca_Kabuli_Scaffold5730	192	(A/G)
15852	CakSNP15852	*Kabuli*	Ca_Kabuli_Scaffold5730	227	(G/A)
15853	CakSNP15853	*Kabuli*	Ca_Kabuli_Scaffold575	36084	(T/C)
15854	CakSNP15854	*Kabuli*	Ca_Kabuli_Scaffold575	36135	(T/C)
15855	CakSNP15855	*Kabuli*	Ca_Kabuli_Scaffold575	36238	(C/T)
15856	CakSNP15856	*Kabuli*	Ca_Kabuli_Scaffold575	45254	(C/T)
15857	CakSNP15857	*Kabuli*	Ca_Kabuli_Scaffold575	45250	(T/C)
15858	CakSNP15858	*Kabuli*	Ca_Kabuli_Scaffold575	45239	(G/T)
15859	CakSNP15859	*Kabuli*	Ca_Kabuli_Scaffold575	45236	(G/A)
15860	CakSNP15860	*Kabuli*	Ca_Kabuli_Scaffold575	45233	(G/A)
15861	CakSNP15861	*Kabuli*	Ca_Kabuli_Scaffold575	45215	(C/A)
15862	CakSNP15862	*Kabuli*	Ca_Kabuli_Scaffold575	45192	(C/T)
15863	CakSNP15863	*Kabuli*	Ca_Kabuli_Scaffold575	45279	(G/C)
15864	CakSNP15864	*Kabuli*	Ca_Kabuli_Scaffold575	45245	(G/A)
15865	CakSNP15865	*Kabuli*	Ca_Kabuli_Scaffold575	45237	(C/T)
15866	CakSNP15866	*Kabuli*	Ca_Kabuli_Scaffold575	45234	(C/T)
15867	CakSNP15867	*Kabuli*	Ca_Kabuli_Scaffold575	110772	(G/T)
15868	CakSNP15868	*Kabuli*	Ca_Kabuli_Scaffold575	110785	(C/T)
15869	CakSNP15869	*Kabuli*	Ca_Kabuli_Scaffold575	110765	(C/T)
15870	CakSNP15870	*Kabuli*	Ca_Kabuli_Scaffold575	110759	(C/T)
15871	CakSNP15871	*Kabuli*	Ca_Kabuli_Scaffold575	110753	(G/A)

S.N.	SNP IDs	Cultivars	Chromosomes/scaffolds	Physical positions (bp)	SNPs
15872	CakSNP15872	*Kabuli*	Ca_Kabuli_Scaffold575	117287	(T/G)
15873	CakSNP15873	*Kabuli*	Ca_Kabuli_Scaffold5792	65166	(A/C)
15874	CakSNP15874	*Kabuli*	Ca_Kabuli_Scaffold5792	86779	(C/A)
15875	CakSNP15875	*Kabuli*	Ca_Kabuli_Scaffold5792	86716	(T/C)
15876	CakSNP15876	*Kabuli*	Ca_Kabuli_Scaffold584	53203	(G/T)
15877	CakSNP15877	*Kabuli*	Ca_Kabuli_Scaffold589	72891	(T/G)
15878	CakSNP15878	*Kabuli*	Ca_Kabuli_Scaffold590	88429	(A/T)
15879	CakSNP15879	*Kabuli*	Ca_Kabuli_Scaffold590	88420	(T/C)
15880	CakSNP15880	*Kabuli*	Ca_Kabuli_Scaffold598	300825	(A/C)
15881	CakSNP15881	*Kabuli*	Ca_Kabuli_Scaffold598	300940	(C/T)
15882	CakSNP15882	*Kabuli*	Ca_Kabuli_Scaffold598	404254	(G/C)
15883	CakSNP15883	*Kabuli*	Ca_Kabuli_Scaffold598	404275	(G/T)
15884	CakSNP15884	*Kabuli*	Ca_Kabuli_Scaffold599	349605	(T/G)
15885	CakSNP15885	*Kabuli*	Ca_Kabuli_Scaffold5997	742	(G/C)
15886	CakSNP15886	*Kabuli*	Ca_Kabuli_Scaffold5997	679	(C/T)
15887	CakSNP15887	*Kabuli*	Ca_Kabuli_Scaffold601	55483	(A/G)
15888	CakSNP15888	*Kabuli*	Ca_Kabuli_Scaffold601	71998	(T/A)
15889	CakSNP15889	*Kabuli*	Ca_Kabuli_Scaffold605	23769	(A/C)
15890	CakSNP15890	*Kabuli*	Ca_Kabuli_Scaffold605	23773	(T/C)
15891	CakSNP15891	*Kabuli*	Ca_Kabuli_Scaffold605	23774	(A/G)
15892	CakSNP15892	*Kabuli*	Ca_Kabuli_Scaffold605	23775	(A/G)
15893	CakSNP15893	*Kabuli*	Ca_Kabuli_Scaffold605	23793	(A/G)
15894	CakSNP15894	*Kabuli*	Ca_Kabuli_Scaffold605	23795	(T/A)
15895	CakSNP15895	*Kabuli*	Ca_Kabuli_Scaffold605	23803	(A/G)
15896	CakSNP15896	*Kabuli*	Ca_Kabuli_Scaffold605	23808	(C/G)
15897	CakSNP15897	*Kabuli*	Ca_Kabuli_Scaffold605	23811	(G/A)
15898	CakSNP15898	*Kabuli*	Ca_Kabuli_Scaffold605	23816	(T/A)
15899	CakSNP15899	*Kabuli*	Ca_Kabuli_Scaffold605	84761	(A/C)
15900	CakSNP15900	*Kabuli*	Ca_Kabuli_Scaffold605	84765	(T/C)
15901	CakSNP15901	*Kabuli*	Ca_Kabuli_Scaffold605	84766	(A/G)
15902	CakSNP15902	*Kabuli*	Ca_Kabuli_Scaffold605	84767	(A/G)
15903	CakSNP15903	*Kabuli*	Ca_Kabuli_Scaffold605	84785	(A/G)
15904	CakSNP15904	*Kabuli*	Ca_Kabuli_Scaffold605	84787	(T/A)
15905	CakSNP15905	*Kabuli*	Ca_Kabuli_Scaffold605	84795	(A/G)
15906	CakSNP15906	*Kabuli*	Ca_Kabuli_Scaffold605	84800	(C/G)
15907	CakSNP15907	*Kabuli*	Ca_Kabuli_Scaffold605	84803	(G/A)
15908	CakSNP15908	*Kabuli*	Ca_Kabuli_Scaffold605	84807	(A/G)
15909	CakSNP15909	*Kabuli*	Ca_Kabuli_Scaffold62	46079	(C/T)
15910	CakSNP15910	*Kabuli*	Ca_Kabuli_Scaffold62	46085	(C/T)

S.N.	SNP IDs	Cultivars	Chromosomes/scaffolds	Physical positions (bp)	SNPs
15911	CakSNP15911	*Kabuli*	Ca_Kabuli_Scaffold62	46087	(G/A)
15912	CakSNP15912	*Kabuli*	Ca_Kabuli_Scaffold62	46088	(C/T)
15913	CakSNP15913	*Kabuli*	Ca_Kabuli_Scaffold624	254901	(C/T)
15914	CakSNP15914	*Kabuli*	Ca_Kabuli_Scaffold624	254950	(G/A)
15915	CakSNP15915	*Kabuli*	Ca_Kabuli_Scaffold624	254959	(T/C)
15916	CakSNP15916	*Kabuli*	Ca_Kabuli_Scaffold624	254971	(G/A)
15917	CakSNP15917	*Kabuli*	Ca_Kabuli_Scaffold624	254992	(C/T)
15918	CakSNP15918	*Kabuli*	Ca_Kabuli_Scaffold624	254942	(A/T)
15919	CakSNP15919	*Kabuli*	Ca_Kabuli_Scaffold6339	190	(T/G)
15920	CakSNP15920	*Kabuli*	Ca_Kabuli_Scaffold6367	6825	(G/T)
15921	CakSNP15921	*Kabuli*	Ca_Kabuli_Scaffold6367	10856	(T/G)
15922	CakSNP15922	*Kabuli*	Ca_Kabuli_Scaffold6367	10842	(T/A)
15923	CakSNP15923	*Kabuli*	Ca_Kabuli_Scaffold6367	13651	(G/A)
15924	CakSNP15924	*Kabuli*	Ca_Kabuli_Scaffold6367	23667	(G/A)
15925	CakSNP15925	*Kabuli*	Ca_Kabuli_Scaffold6367	25674	(T/C)
15926	CakSNP15926	*Kabuli*	Ca_Kabuli_Scaffold637	107671	(G/C)
15927	CakSNP15927	*Kabuli*	Ca_Kabuli_Scaffold637	107661	(A/C)
15928	CakSNP15928	*Kabuli*	Ca_Kabuli_Scaffold637	107636	(T/C)
15929	CakSNP15929	*Kabuli*	Ca_Kabuli_Scaffold637	107613	(C/G)
15930	CakSNP15930	*Kabuli*	Ca_Kabuli_Scaffold6403	1553	(T/A)
15931	CakSNP15931	*Kabuli*	Ca_Kabuli_Scaffold642	28715	(A/T)
15932	CakSNP15932	*Kabuli*	Ca_Kabuli_Scaffold642	615176	(T/C)
15933	CakSNP15933	*Kabuli*	Ca_Kabuli_Scaffold645	91908	(C/T)
15934	CakSNP15934	*Kabuli*	Ca_Kabuli_Scaffold653	145231	(A/G)
15935	CakSNP15935	*Kabuli*	Ca_Kabuli_Scaffold653	183054	(T/C)
15936	CakSNP15936	*Kabuli*	Ca_Kabuli_Scaffold653	183063	(G/A)
15937	CakSNP15937	*Kabuli*	Ca_Kabuli_Scaffold653	183069	(C/A)
15938	CakSNP15938	*Kabuli*	Ca_Kabuli_Scaffold653	183074	(C/G)
15939	CakSNP15939	*Kabuli*	Ca_Kabuli_Scaffold653	183078	(T/G)
15940	CakSNP15940	*Kabuli*	Ca_Kabuli_Scaffold653	183091	(T/C)
15941	CakSNP15941	*Kabuli*	Ca_Kabuli_Scaffold653	183143	(T/A)
15942	CakSNP15942	*Kabuli*	Ca_Kabuli_Scaffold653	183135	(T/C)
15943	CakSNP15943	*Kabuli*	Ca_Kabuli_Scaffold653	183112	(T/C)
15944	CakSNP15944	*Kabuli*	Ca_Kabuli_Scaffold653	189129	(C/T)
15945	CakSNP15945	*Kabuli*	Ca_Kabuli_Scaffold653	197878	(A/T)
15946	CakSNP15946	*Kabuli*	Ca_Kabuli_Scaffold661	180364	(G/T)
15947	CakSNP15947	*Kabuli*	Ca_Kabuli_Scaffold661	180362	(A/C)
15948	CakSNP15948	*Kabuli*	Ca_Kabuli_Scaffold661	180348	(C/T)
15949	CakSNP15949	*Kabuli*	Ca_Kabuli_Scaffold661	180302	(G/T)

S.N.	SNP IDs	Cultivars	Chromosomes/scaffolds	Physical positions (bp)	SNPs
15950	CakSNP15950	*Kabuli*	Ca_Kabuli_Scaffold661	311222	(G/T)
15951	CakSNP15951	*Kabuli*	Ca_Kabuli_Scaffold661	311182	(C/T)
15952	CakSNP15952	*Kabuli*	Ca_Kabuli_Scaffold663	16068	(G/A)
15953	CakSNP15953	*Kabuli*	Ca_Kabuli_Scaffold663	16042	(C/A)
15954	CakSNP15954	*Kabuli*	Ca_Kabuli_Scaffold663	16029	(G/A)
15955	CakSNP15955	*Kabuli*	Ca_Kabuli_Scaffold663	16056	(C/T)
15956	CakSNP15956	*Kabuli*	Ca_Kabuli_Scaffold663	16059	(G/A)
15957	CakSNP15957	*Kabuli*	Ca_Kabuli_Scaffold663	16061	(A/G)
15958	CakSNP15958	*Kabuli*	Ca_Kabuli_Scaffold663	156613	(G/C)
15959	CakSNP15959	*Kabuli*	Ca_Kabuli_Scaffold674	91979	(T/A)
15960	CakSNP15960	*Kabuli*	Ca_Kabuli_Scaffold674	360388	(C/T)
15961	CakSNP15961	*Kabuli*	Ca_Kabuli_Scaffold674	360401	(A/G)
15962	CakSNP15962	*Kabuli*	Ca_Kabuli_Scaffold674	360421	(G/A)
15963	CakSNP15963	*Kabuli*	Ca_Kabuli_Scaffold674	360406	(C/T)
15964	CakSNP15964	*Kabuli*	Ca_Kabuli_Scaffold674	360349	(G/A)
15965	CakSNP15965	*Kabuli*	Ca_Kabuli_Scaffold674	360464	(G/A)
15966	CakSNP15966	*Kabuli*	Ca_Kabuli_Scaffold674	360457	(G/C)
15967	CakSNP15967	*Kabuli*	Ca_Kabuli_Scaffold674	360411	(G/A)
15968	CakSNP15968	*Kabuli*	Ca_Kabuli_Scaffold674	360408	(G/T)
15969	CakSNP15969	*Kabuli*	Ca_Kabuli_Scaffold674	608623	(T/G)
15970	CakSNP15970	*Kabuli*	Ca_Kabuli_Scaffold674	652382	(G/A)
15971	CakSNP15971	*Kabuli*	Ca_Kabuli_Scaffold674	652373	(A/G)
15972	CakSNP15972	*Kabuli*	Ca_Kabuli_Scaffold674	826136	(T/C)
15973	CakSNP15973	*Kabuli*	Ca_Kabuli_Scaffold681	18376	(C/G)
15974	CakSNP15974	*Kabuli*	Ca_Kabuli_Scaffold682	157238	(G/C)
15975	CakSNP15975	*Kabuli*	Ca_Kabuli_Scaffold682	578200	(T/G)
15976	CakSNP15976	*Kabuli*	Ca_Kabuli_Scaffold684	78894	(G/A)
15977	CakSNP15977	*Kabuli*	Ca_Kabuli_Scaffold684	78883	(T/G)
15978	CakSNP15978	*Kabuli*	Ca_Kabuli_Scaffold684	78871	(C/G)
15979	CakSNP15979	*Kabuli*	Ca_Kabuli_Scaffold684	78842	(G/A)
15980	CakSNP15980	*Kabuli*	Ca_Kabuli_Scaffold684	78844	(A/G)
15981	CakSNP15981	*Kabuli*	Ca_Kabuli_Scaffold684	78864	(C/T)
15982	CakSNP15982	*Kabuli*	Ca_Kabuli_Scaffold684	78893	(C/T)
15983	CakSNP15983	*Kabuli*	Ca_Kabuli_Scaffold686	206111	(G/A)
15984	CakSNP15984	*Kabuli*	Ca_Kabuli_Scaffold686	206044	(T/C)
15985	CakSNP15985	*Kabuli*	Ca_Kabuli_Scaffold686	206042	(A/G)
15986	CakSNP15986	*Kabuli*	Ca_Kabuli_Scaffold702	247644	(G/C)
15987	CakSNP15987	*Kabuli*	Ca_Kabuli_Scaffold711	153694	(C/T)
15988	CakSNP15988	*Kabuli*	Ca_Kabuli_Scaffold711	153680	(C/A)

S.N.	SNP IDs	Cultivars	Chromosomes/scaffolds	Physical positions (bp)	SNPs
15989	CakSNP15989	*Kabuli*	Ca_Kabuli_Scaffold711	153667	(G/A)
15990	CakSNP15990	*Kabuli*	Ca_Kabuli_Scaffold711	153697	(G/A)
15991	CakSNP15991	*Kabuli*	Ca_Kabuli_Scaffold711	153699	(A/G)
15992	CakSNP15992	*Kabuli*	Ca_Kabuli_Scaffold711	153706	(G/A)
15993	CakSNP15993	*Kabuli*	Ca_Kabuli_Scaffold7127	1878	(C/T)
15994	CakSNP15994	*Kabuli*	Ca_Kabuli_Scaffold7144	234	(A/T)
15995	CakSNP15995	*Kabuli*	Ca_Kabuli_Scaffold716	128208	(G/T)
15996	CakSNP15996	*Kabuli*	Ca_Kabuli_Scaffold716	212326	(G/A)
15997	CakSNP15997	*Kabuli*	Ca_Kabuli_Scaffold716	212374	(G/A)
15998	CakSNP15998	*Kabuli*	Ca_Kabuli_Scaffold716	269220	(T/C)
15999	CakSNP15999	*Kabuli*	Ca_Kabuli_Scaffold716	269230	(A/C)
16000	CakSNP16000	*Kabuli*	Ca_Kabuli_Scaffold716	269242	(A/G)
16001	CakSNP16001	*Kabuli*	Ca_Kabuli_Scaffold716	269266	(G/T)
16002	CakSNP16002	*Kabuli*	Ca_Kabuli_Scaffold716	269271	(G/A)
16003	CakSNP16003	*Kabuli*	Ca_Kabuli_Scaffold716	269281	(T/G)
16004	CakSNP16004	*Kabuli*	Ca_Kabuli_Scaffold716	269287	(G/A)
16005	CakSNP16005	*Kabuli*	Ca_Kabuli_Scaffold716	269335	(G/A)
16006	CakSNP16006	*Kabuli*	Ca_Kabuli_Scaffold716	269332	(C/T)
16007	CakSNP16007	*Kabuli*	Ca_Kabuli_Scaffold716	269314	(G/A)
16008	CakSNP16008	*Kabuli*	Ca_Kabuli_Scaffold716	269299	(T/C)
16009	CakSNP16009	*Kabuli*	Ca_Kabuli_Scaffold719	11850	(C/T)
16010	CakSNP16010	*Kabuli*	Ca_Kabuli_Scaffold720	60880	(G/A)
16011	CakSNP16011	*Kabuli*	Ca_Kabuli_Scaffold720	370624	(T/G)
16012	CakSNP16012	*Kabuli*	Ca_Kabuli_Scaffold720	370639	(T/A)
16013	CakSNP16013	*Kabuli*	Ca_Kabuli_Scaffold720	370725	(G/A)
16014	CakSNP16014	*Kabuli*	Ca_Kabuli_Scaffold724	25019	(G/A)
16015	CakSNP16015	*Kabuli*	Ca_Kabuli_Scaffold724	25722	(G/C)
16016	CakSNP16016	*Kabuli*	Ca_Kabuli_Scaffold731	315093	(G/A)
16017	CakSNP16017	*Kabuli*	Ca_Kabuli_Scaffold731	315114	(C/T)
16018	CakSNP16018	*Kabuli*	Ca_Kabuli_Scaffold731	315135	(G/T)
16019	CakSNP16019	*Kabuli*	Ca_Kabuli_Scaffold731	315138	(C/T)
16020	CakSNP16020	*Kabuli*	Ca_Kabuli_Scaffold731	315141	(G/A)
16021	CakSNP16021	*Kabuli*	Ca_Kabuli_Scaffold731	332497	(G/A)
16022	CakSNP16022	*Kabuli*	Ca_Kabuli_Scaffold731	332468	(G/T)
16023	CakSNP16023	*Kabuli*	Ca_Kabuli_Scaffold731	332467	(G/C)
16024	CakSNP16024	*Kabuli*	Ca_Kabuli_Scaffold731	332465	(T/A)
16025	CakSNP16025	*Kabuli*	Ca_Kabuli_Scaffold731	332464	(G/T)
16026	CakSNP16026	*Kabuli*	Ca_Kabuli_Scaffold731	332458	(A/T)
16027	CakSNP16027	*Kabuli*	Ca_Kabuli_Scaffold731	332528	(G/C)

S.N.	SNP IDs	Cultivars	Chromosomes/scaffolds	Physical positions (bp)	SNPs
16028	CakSNP16028	*Kabuli*	Ca_Kabuli_Scaffold731	357674	(G/A)
16029	CakSNP16029	*Kabuli*	Ca_Kabuli_Scaffold731	357645	(G/T)
16030	CakSNP16030	*Kabuli*	Ca_Kabuli_Scaffold731	357644	(G/C)
16031	CakSNP16031	*Kabuli*	Ca_Kabuli_Scaffold731	357642	(T/A)
16032	CakSNP16032	*Kabuli*	Ca_Kabuli_Scaffold731	357641	(G/T)
16033	CakSNP16033	*Kabuli*	Ca_Kabuli_Scaffold731	357635	(A/T)
16034	CakSNP16034	*Kabuli*	Ca_Kabuli_Scaffold731	357715	(A/T)
16035	CakSNP16035	*Kabuli*	Ca_Kabuli_Scaffold731	362123	(G/A)
16036	CakSNP16036	*Kabuli*	Ca_Kabuli_Scaffold731	362118	(C/A)
16037	CakSNP16037	*Kabuli*	Ca_Kabuli_Scaffold731	362114	(A/C)
16038	CakSNP16038	*Kabuli*	Ca_Kabuli_Scaffold731	362548	(T/A)
16039	CakSNP16039	*Kabuli*	Ca_Kabuli_Scaffold731	362553	(C/T)
16040	CakSNP16040	*Kabuli*	Ca_Kabuli_Scaffold731	362554	(T/A)
16041	CakSNP16041	*Kabuli*	Ca_Kabuli_Scaffold731	362555	(T/G)
16042	CakSNP16042	*Kabuli*	Ca_Kabuli_Scaffold731	362556	(C/A)
16043	CakSNP16043	*Kabuli*	Ca_Kabuli_Scaffold731	362574	(T/C)
16044	CakSNP16044	*Kabuli*	Ca_Kabuli_Scaffold731	362581	(A/G)
16045	CakSNP16045	*Kabuli*	Ca_Kabuli_Scaffold731	362582	(T/C)
16046	CakSNP16046	*Kabuli*	Ca_Kabuli_Scaffold731	362594	(G/T)
16047	CakSNP16047	*Kabuli*	Ca_Kabuli_Scaffold731	362610	(C/T)
16048	CakSNP16048	*Kabuli*	Ca_Kabuli_Scaffold731	372255	(G/A)
16049	CakSNP16049	*Kabuli*	Ca_Kabuli_Scaffold731	372253	(G/T)
16050	CakSNP16050	*Kabuli*	Ca_Kabuli_Scaffold731	372250	(G/A)
16051	CakSNP16051	*Kabuli*	Ca_Kabuli_Scaffold731	372244	(C/T)
16052	CakSNP16052	*Kabuli*	Ca_Kabuli_Scaffold731	372243	(A/T)
16053	CakSNP16053	*Kabuli*	Ca_Kabuli_Scaffold731	372215	(A/G)
16054	CakSNP16054	*Kabuli*	Ca_Kabuli_Scaffold731	372209	(G/C)
16055	CakSNP16055	*Kabuli*	Ca_Kabuli_Scaffold731	372183	(C/T)
16056	CakSNP16056	*Kabuli*	Ca_Kabuli_Scaffold731	372959	(A/C)
16057	CakSNP16057	*Kabuli*	Ca_Kabuli_Scaffold7355	1606	(C/G)
16058	CakSNP16058	*Kabuli*	Ca_Kabuli_Scaffold7355	1690	(T/C)
16059	CakSNP16059	*Kabuli*	Ca_Kabuli_Scaffold7355	3608	(A/G)
16060	CakSNP16060	*Kabuli*	Ca_Kabuli_Scaffold7355	3602	(T/C)
16061	CakSNP16061	*Kabuli*	Ca_Kabuli_Scaffold7355	3578	(C/T)
16062	CakSNP16062	*Kabuli*	Ca_Kabuli_Scaffold7355	3572	(A/G)
16063	CakSNP16063	*Kabuli*	Ca_Kabuli_Scaffold7355	3566	(C/T)
16064	CakSNP16064	*Kabuli*	Ca_Kabuli_Scaffold752	382375	(G/A)
16065	CakSNP16065	*Kabuli*	Ca_Kabuli_Scaffold752	497452	(T/A)
16066	CakSNP16066	*Kabuli*	Ca_Kabuli_Scaffold752	497454	(A/G)

S.N.	SNP IDs	Cultivars	Chromosomes/scaffolds	Physical positions (bp)	SNPs
16067	CakSNP16067	*Kabuli*	Ca_Kabuli_Scaffold753	8312	(A/G)
16068	CakSNP16068	*Kabuli*	Ca_Kabuli_Scaffold758	16480	(G/T)
16069	CakSNP16069	*Kabuli*	Ca_Kabuli_Scaffold7650	2686	(C/T)
16070	CakSNP16070	*Kabuli*	Ca_Kabuli_Scaffold7715	3434	(A/T)
16071	CakSNP16071	*Kabuli*	Ca_Kabuli_Scaffold772	53192	(C/T)
16072	CakSNP16072	*Kabuli*	Ca_Kabuli_Scaffold772	53196	(C/T)
16073	CakSNP16073	*Kabuli*	Ca_Kabuli_Scaffold775	89238	(T/C)
16074	CakSNP16074	*Kabuli*	Ca_Kabuli_Scaffold775	89239	(C/T)
16075	CakSNP16075	*Kabuli*	Ca_Kabuli_Scaffold775	279828	(A/G)
16076	CakSNP16076	*Kabuli*	Ca_Kabuli_Scaffold775	356107	(C/A)
16077	CakSNP16077	*Kabuli*	Ca_Kabuli_Scaffold775	448604	(G/A)
16078	CakSNP16078	*Kabuli*	Ca_Kabuli_Scaffold775	448743	(T/C)
16079	CakSNP16079	*Kabuli*	Ca_Kabuli_Scaffold776	188906	(C/T)
16080	CakSNP16080	*Kabuli*	Ca_Kabuli_Scaffold776	188895	(C/T)
16081	CakSNP16081	*Kabuli*	Ca_Kabuli_Scaffold776	188888	(G/A)
16082	CakSNP16082	*Kabuli*	Ca_Kabuli_Scaffold7765	503	(C/T)
16083	CakSNP16083	*Kabuli*	Ca_Kabuli_Scaffold7765	536	(C/A)
16084	CakSNP16084	*Kabuli*	Ca_Kabuli_Scaffold7765	549	(G/A)
16085	CakSNP16085	*Kabuli*	Ca_Kabuli_Scaffold783	201617	(T/C)
16086	CakSNP16086	*Kabuli*	Ca_Kabuli_Scaffold783	244209	(T/C)
16087	CakSNP16087	*Kabuli*	Ca_Kabuli_Scaffold7842	727	(C/T)
16088	CakSNP16088	*Kabuli*	Ca_Kabuli_Scaffold7842	1078	(G/A)
16089	CakSNP16089	*Kabuli*	Ca_Kabuli_Scaffold7842	1075	(C/T)
16090	CakSNP16090	*Kabuli*	Ca_Kabuli_Scaffold7842	1068	(C/A)
16091	CakSNP16091	*Kabuli*	Ca_Kabuli_Scaffold7842	1042	(T/C)
16092	CakSNP16092	*Kabuli*	Ca_Kabuli_Scaffold7842	1024	(T/G)
16093	CakSNP16093	*Kabuli*	Ca_Kabuli_Scaffold787	4363	(A/G)
16094	CakSNP16094	*Kabuli*	Ca_Kabuli_Scaffold794	50197	(T/G)
16095	CakSNP16095	*Kabuli*	Ca_Kabuli_Scaffold794	50201	(T/G)
16096	CakSNP16096	*Kabuli*	Ca_Kabuli_Scaffold794	50225	(C/G)
16097	CakSNP16097	*Kabuli*	Ca_Kabuli_Scaffold794	50239	(G/A)
16098	CakSNP16098	*Kabuli*	Ca_Kabuli_Scaffold794	50240	(C/A)
16099	CakSNP16099	*Kabuli*	Ca_Kabuli_Scaffold794	50236	(A/G)
16100	CakSNP16100	*Kabuli*	Ca_Kabuli_Scaffold799	3956	(G/A)
16101	CakSNP16101	*Kabuli*	Ca_Kabuli_Scaffold799	3930	(A/G)
16102	CakSNP16102	*Kabuli*	Ca_Kabuli_Scaffold799	3928	(C/T)
16103	CakSNP16103	*Kabuli*	Ca_Kabuli_Scaffold801	67115	(C/T)
16104	CakSNP16104	*Kabuli*	Ca_Kabuli_Scaffold806	129263	(A/G)
16105	CakSNP16105	*Kabuli*	Ca_Kabuli_Scaffold806	129265	(G/T)

S.N.	SNP IDs	Cultivars	Chromosomes/scaffolds	Physical positions (bp)	SNPs
16106	CakSNP16106	*Kabuli*	Ca_Kabuli_Scaffold809	48678	(T/C)
16107	CakSNP16107	*Kabuli*	Ca_Kabuli_Scaffold809	48650	(C/A)
16108	CakSNP16108	*Kabuli*	Ca_Kabuli_Scaffold809	53417	(C/G)
16109	CakSNP16109	*Kabuli*	Ca_Kabuli_Scaffold809	53425	(T/C)
16110	CakSNP16110	*Kabuli*	Ca_Kabuli_Scaffold809	53431	(G/A)
16111	CakSNP16111	*Kabuli*	Ca_Kabuli_Scaffold809	53444	(G/A)
16112	CakSNP16112	*Kabuli*	Ca_Kabuli_Scaffold809	53474	(G/A)
16113	CakSNP16113	*Kabuli*	Ca_Kabuli_Scaffold809	53464	(G/A)
16114	CakSNP16114	*Kabuli*	Ca_Kabuli_Scaffold809	53470	(C/G)
16115	CakSNP16115	*Kabuli*	Ca_Kabuli_Scaffold811	81373	(C/T)
16116	CakSNP16116	*Kabuli*	Ca_Kabuli_Scaffold812	34686	(C/T)
16117	CakSNP16117	*Kabuli*	Ca_Kabuli_Scaffold812	246208	(A/C)
16118	CakSNP16118	*Kabuli*	Ca_Kabuli_Scaffold812	332746	(T/C)
16119	CakSNP16119	*Kabuli*	Ca_Kabuli_Scaffold812	332758	(T/C)
16120	CakSNP16120	*Kabuli*	Ca_Kabuli_Scaffold812	528815	(C/G)
16121	CakSNP16121	*Kabuli*	Ca_Kabuli_Scaffold8226	21624	(G/A)
16122	CakSNP16122	*Kabuli*	Ca_Kabuli_Scaffold8226	21592	(T/G)
16123	CakSNP16123	*Kabuli*	Ca_Kabuli_Scaffold8226	21590	(T/C)
16124	CakSNP16124	*Kabuli*	Ca_Kabuli_Scaffold8226	21572	(T/A)
16125	CakSNP16125	*Kabuli*	Ca_Kabuli_Scaffold842	13898	(T/C)
16126	CakSNP16126	*Kabuli*	Ca_Kabuli_Scaffold842	13905	(C/G)
16127	CakSNP16127	*Kabuli*	Ca_Kabuli_Scaffold842	13928	(G/T)
16128	CakSNP16128	*Kabuli*	Ca_Kabuli_Scaffold842	13946	(T/C)
16129	CakSNP16129	*Kabuli*	Ca_Kabuli_Scaffold845	28172	(G/T)
16130	CakSNP16130	*Kabuli*	Ca_Kabuli_Scaffold845	45557	(A/G)
16131	CakSNP16131	*Kabuli*	Ca_Kabuli_Scaffold845	71041	(A/G)
16132	CakSNP16132	*Kabuli*	Ca_Kabuli_Scaffold845	102612	(A/G)
16133	CakSNP16133	*Kabuli*	Ca_Kabuli_Scaffold845	129326	(C/G)
16134	CakSNP16134	*Kabuli*	Ca_Kabuli_Scaffold845	129370	(A/G)
16135	CakSNP16135	*Kabuli*	Ca_Kabuli_Scaffold845	130569	(G/C)
16136	CakSNP16136	*Kabuli*	Ca_Kabuli_Scaffold845	131911	(A/G)
16137	CakSNP16137	*Kabuli*	Ca_Kabuli_Scaffold845	136796	(A/G)
16138	CakSNP16138	*Kabuli*	Ca_Kabuli_Scaffold845	139268	(G/A)
16139	CakSNP16139	*Kabuli*	Ca_Kabuli_Scaffold845	154556	(T/C)
16140	CakSNP16140	*Kabuli*	Ca_Kabuli_Scaffold845	205934	(T/C)
16141	CakSNP16141	*Kabuli*	Ca_Kabuli_Scaffold848	37282	(G/A)
16142	CakSNP16142	*Kabuli*	Ca_Kabuli_Scaffold848	37309	(G/A)
16143	CakSNP16143	*Kabuli*	Ca_Kabuli_Scaffold848	39934	(T/G)
16144	CakSNP16144	*Kabuli*	Ca_Kabuli_Scaffold848	39968	(C/T)

S.N.	SNP IDs	Cultivars	Chromosomes/scaffolds	Physical positions (bp)	SNPs
16145	CakSNP16145	*Kabuli*	Ca_Kabuli_Scaffold848	39984	(G/T)
16146	CakSNP16146	*Kabuli*	Ca_Kabuli_Scaffold848	39985	(C/T)
16147	CakSNP16147	*Kabuli*	Ca_Kabuli_Scaffold848	39990	(A/G)
16148	CakSNP16148	*Kabuli*	Ca_Kabuli_Scaffold848	39994	(C/T)
16149	CakSNP16149	*Kabuli*	Ca_Kabuli_Scaffold848	89976	(C/A)
16150	CakSNP16150	*Kabuli*	Ca_Kabuli_Scaffold848	114078	(T/C)
16151	CakSNP16151	*Kabuli*	Ca_Kabuli_Scaffold848	158548	(T/C)
16152	CakSNP16152	*Kabuli*	Ca_Kabuli_Scaffold848	158557	(G/A)
16153	CakSNP16153	*Kabuli*	Ca_Kabuli_Scaffold848	158569	(C/T)
16154	CakSNP16154	*Kabuli*	Ca_Kabuli_Scaffold848	158570	(A/G)
16155	CakSNP16155	*Kabuli*	Ca_Kabuli_Scaffold848	158611	(C/T)
16156	CakSNP16156	*Kabuli*	Ca_Kabuli_Scaffold848	158638	(G/A)
16157	CakSNP16157	*Kabuli*	Ca_Kabuli_Scaffold848	158626	(G/T)
16158	CakSNP16158	*Kabuli*	Ca_Kabuli_Scaffold848	158594	(A/G)
16159	CakSNP16159	*Kabuli*	Ca_Kabuli_Scaffold857	5227	(G/A)
16160	CakSNP16160	*Kabuli*	Ca_Kabuli_Scaffold8586	4415	(A/G)
16161	CakSNP16161	*Kabuli*	Ca_Kabuli_Scaffold859	9382	(A/G)
16162	CakSNP16162	*Kabuli*	Ca_Kabuli_Scaffold8641	502	(G/A)
16163	CakSNP16163	*Kabuli*	Ca_Kabuli_Scaffold8641	472	(C/T)
16164	CakSNP16164	*Kabuli*	Ca_Kabuli_Scaffold8641	466	(C/T)
16165	CakSNP16165	*Kabuli*	Ca_Kabuli_Scaffold8641	460	(T/C)
16166	CakSNP16166	*Kabuli*	Ca_Kabuli_Scaffold8646	2056	(A/T)
16167	CakSNP16167	*Kabuli*	Ca_Kabuli_Scaffold8646	2073	(G/A)
16168	CakSNP16168	*Kabuli*	Ca_Kabuli_Scaffold8648	3938	(C/T)
16169	CakSNP16169	*Kabuli*	Ca_Kabuli_Scaffold8648	3977	(A/G)
16170	CakSNP16170	*Kabuli*	Ca_Kabuli_Scaffold8648	4058	(C/A)
16171	CakSNP16171	*Kabuli*	Ca_Kabuli_Scaffold8648	4050	(A/G)
16172	CakSNP16172	*Kabuli*	Ca_Kabuli_Scaffold8648	4036	(A/C)
16173	CakSNP16173	*Kabuli*	Ca_Kabuli_Scaffold8648	4013	(G/A)
16174	CakSNP16174	*Kabuli*	Ca_Kabuli_Scaffold8648	3992	(G/A)
16175	CakSNP16175	*Kabuli*	Ca_Kabuli_Scaffold866	27325	(C/T)
16176	CakSNP16176	*Kabuli*	Ca_Kabuli_Scaffold866	27293	(T/G)
16177	CakSNP16177	*Kabuli*	Ca_Kabuli_Scaffold866	27279	(C/T)
16178	CakSNP16178	*Kabuli*	Ca_Kabuli_Scaffold87	182677	(C/G)
16179	CakSNP16179	*Kabuli*	Ca_Kabuli_Scaffold87	182724	(G/A)
16180	CakSNP16180	*Kabuli*	Ca_Kabuli_Scaffold87	182769	(G/C)
16181	CakSNP16181	*Kabuli*	Ca_Kabuli_Scaffold87	182788	(A/C)
16182	CakSNP16182	*Kabuli*	Ca_Kabuli_Scaffold873	265386	(C/T)
16183	CakSNP16183	*Kabuli*	Ca_Kabuli_Scaffold873	265395	(C/A)

S.N.	SNP IDs	Cultivars	Chromosomes/scaffolds	Physical positions (bp)	SNPs
16184	CakSNP16184	*Kabuli*	Ca_Kabuli_Scaffold873	265396	(C/T)
16185	CakSNP16185	*Kabuli*	Ca_Kabuli_Scaffold873	265399	(C/A)
16186	CakSNP16186	*Kabuli*	Ca_Kabuli_Scaffold873	265458	(C/T)
16187	CakSNP16187	*Kabuli*	Ca_Kabuli_Scaffold873	265406	(T/C)
16188	CakSNP16188	*Kabuli*	Ca_Kabuli_Scaffold875	49179	(T/C)
16189	CakSNP16189	*Kabuli*	Ca_Kabuli_Scaffold875	51703	(C/G)
16190	CakSNP16190	*Kabuli*	Ca_Kabuli_Scaffold875	51702	(T/A)
16191	CakSNP16191	*Kabuli*	Ca_Kabuli_Scaffold875	51701	(T/C)
16192	CakSNP16192	*Kabuli*	Ca_Kabuli_Scaffold875	51714	(T/A)
16193	CakSNP16193	*Kabuli*	Ca_Kabuli_Scaffold875	51704	(C/A)
16194	CakSNP16194	*Kabuli*	Ca_Kabuli_Scaffold875	62738	(A/G)
16195	CakSNP16195	*Kabuli*	Ca_Kabuli_Scaffold876	152311	(G/T)
16196	CakSNP16196	*Kabuli*	Ca_Kabuli_Scaffold876	188605	(C/G)
16197	CakSNP16197	*Kabuli*	Ca_Kabuli_Scaffold876	297059	(T/C)
16198	CakSNP16198	*Kabuli*	Ca_Kabuli_Scaffold876	297024	(T/G)
16199	CakSNP16199	*Kabuli*	Ca_Kabuli_Scaffold876	297023	(G/A)
16200	CakSNP16200	*Kabuli*	Ca_Kabuli_Scaffold876	297022	(G/A)
16201	CakSNP16201	*Kabuli*	Ca_Kabuli_Scaffold876	297003	(C/T)
16202	CakSNP16202	*Kabuli*	Ca_Kabuli_Scaffold876	296995	(C/T)
16203	CakSNP16203	*Kabuli*	Ca_Kabuli_Scaffold877	9813	(G/A)
16204	CakSNP16204	*Kabuli*	Ca_Kabuli_Scaffold877	9851	(C/T)
16205	CakSNP16205	*Kabuli*	Ca_Kabuli_Scaffold88	9982	(C/A)
16206	CakSNP16206	*Kabuli*	Ca_Kabuli_Scaffold88	16398	(C/T)
16207	CakSNP16207	*Kabuli*	Ca_Kabuli_Scaffold88	110743	(A/G)
16208	CakSNP16208	*Kabuli*	Ca_Kabuli_Scaffold88	240551	(T/C)
16209	CakSNP16209	*Kabuli*	Ca_Kabuli_Scaffold88	240550	(G/A)
16210	CakSNP16210	*Kabuli*	Ca_Kabuli_Scaffold88	240525	(A/G)
16211	CakSNP16211	*Kabuli*	Ca_Kabuli_Scaffold88	274006	(A/G)
16212	CakSNP16212	*Kabuli*	Ca_Kabuli_Scaffold882	117376	(G/A)
16213	CakSNP16213	*Kabuli*	Ca_Kabuli_Scaffold882	118266	(G/T)
16214	CakSNP16214	*Kabuli*	Ca_Kabuli_Scaffold882	232549	(C/A)
16215	CakSNP16215	*Kabuli*	Ca_Kabuli_Scaffold882	232570	(T/G)
16216	CakSNP16216	*Kabuli*	Ca_Kabuli_Scaffold882	232605	(T/A)
16217	CakSNP16217	*Kabuli*	Ca_Kabuli_Scaffold882	358449	(T/A)
16218	CakSNP16218	*Kabuli*	Ca_Kabuli_Scaffold882	570871	(T/A)
16219	CakSNP16219	*Kabuli*	Ca_Kabuli_Scaffold882	571210	(A/T)
16220	CakSNP16220	*Kabuli*	Ca_Kabuli_Scaffold882	573367	(T/A)
16221	CakSNP16221	*Kabuli*	Ca_Kabuli_Scaffold882	573706	(A/T)
16222	CakSNP16222	*Kabuli*	Ca_Kabuli_Scaffold887_1	26025	(A/C)

S.N.	SNP IDs	Cultivars	Chromosomes/scaffolds	Physical positions (bp)	SNPs
16223	CakSNP16223	*Kabuli*	Ca_Kabuli_Scaffold887_1	26134	(C/A)
16224	CakSNP16224	*Kabuli*	Ca_Kabuli_Scaffold887_1	26121	(A/C)
16225	CakSNP16225	*Kabuli*	Ca_Kabuli_Scaffold887_1	26108	(G/T)
16226	CakSNP16226	*Kabuli*	Ca_Kabuli_Scaffold887_1	139386	(T/C)
16227	CakSNP16227	*Kabuli*	Ca_Kabuli_Scaffold887_1	139396	(G/C)
16228	CakSNP16228	*Kabuli*	Ca_Kabuli_Scaffold887_1	139580	(G/T)
16229	CakSNP16229	*Kabuli*	Ca_Kabuli_Scaffold887_1	144885	(T/C)
16230	CakSNP16230	*Kabuli*	Ca_Kabuli_Scaffold887_1	260488	(A/C)
16231	CakSNP16231	*Kabuli*	Ca_Kabuli_Scaffold892	24349	(T/C)
16232	CakSNP16232	*Kabuli*	Ca_Kabuli_Scaffold892	24323	(A/G)
16233	CakSNP16233	*Kabuli*	Ca_Kabuli_Scaffold892	24317	(G/T)
16234	CakSNP16234	*Kabuli*	Ca_Kabuli_Scaffold892	24316	(T/A)
16235	CakSNP16235	*Kabuli*	Ca_Kabuli_Scaffold892	35527	(T/C)
16236	CakSNP16236	*Kabuli*	Ca_Kabuli_Scaffold892	35501	(A/G)
16237	CakSNP16237	*Kabuli*	Ca_Kabuli_Scaffold892	35495	(G/T)
16238	CakSNP16238	*Kabuli*	Ca_Kabuli_Scaffold892	35494	(T/A)
16239	CakSNP16239	*Kabuli*	Ca_Kabuli_Scaffold892	35489	(A/G)
16240	CakSNP16240	*Kabuli*	Ca_Kabuli_Scaffold892	35514	(G/T)
16241	CakSNP16241	*Kabuli*	Ca_Kabuli_Scaffold895	18621	(C/A)
16242	CakSNP16242	*Kabuli*	Ca_Kabuli_Scaffold895	18693	(G/C)
16243	CakSNP16243	*Kabuli*	Ca_Kabuli_Scaffold895	20978	(T/C)
16244	CakSNP16244	*Kabuli*	Ca_Kabuli_Scaffold895	158250	(A/G)
16245	CakSNP16245	*Kabuli*	Ca_Kabuli_Scaffold901	91817	(C/T)
16246	CakSNP16246	*Kabuli*	Ca_Kabuli_Scaffold901	91849	(T/C)
16247	CakSNP16247	*Kabuli*	Ca_Kabuli_Scaffold908	15845	(C/A)
16248	CakSNP16248	*Kabuli*	Ca_Kabuli_Scaffold908	15841	(G/A)
16249	CakSNP16249	*Kabuli*	Ca_Kabuli_Scaffold908	15840	(G/A)
16250	CakSNP16250	*Kabuli*	Ca_Kabuli_Scaffold908	15816	(C/T)
16251	CakSNP16251	*Kabuli*	Ca_Kabuli_Scaffold908	15813	(G/T)
16252	CakSNP16252	*Kabuli*	Ca_Kabuli_Scaffold908	15810	(A/G)
16253	CakSNP16253	*Kabuli*	Ca_Kabuli_Scaffold908	15799	(C/T)
16254	CakSNP16254	*Kabuli*	Ca_Kabuli_Scaffold908	15786	(G/A)
16255	CakSNP16255	*Kabuli*	Ca_Kabuli_Scaffold908	15785	(G/A)
16256	CakSNP16256	*Kabuli*	Ca_Kabuli_Scaffold908	15822	(C/T)
16257	CakSNP16257	*Kabuli*	Ca_Kabuli_Scaffold908	15834	(C/A)
16258	CakSNP16258	*Kabuli*	Ca_Kabuli_Scaffold913	14282	(A/C)
16259	CakSNP16259	*Kabuli*	Ca_Kabuli_Scaffold913	332494	(G/C)
16260	CakSNP16260	*Kabuli*	Ca_Kabuli_Scaffold913	403513	(G/A)
16261	CakSNP16261	*Kabuli*	Ca_Kabuli_Scaffold913	403572	(C/G)

S.N.	SNP IDs	Cultivars	Chromosomes/scaffolds	Physical positions (bp)	SNPs
16262	CakSNP16262	*Kabuli*	Ca_Kabuli_Scaffold913	403553	(T/C)
16263	CakSNP16263	*Kabuli*	Ca_Kabuli_Scaffold913	403531	(A/C)
16264	CakSNP16264	*Kabuli*	Ca_Kabuli_Scaffold914	18826	(A/G)
16265	CakSNP16265	*Kabuli*	Ca_Kabuli_Scaffold914	18810	(C/A)
16266	CakSNP16266	*Kabuli*	Ca_Kabuli_Scaffold914	18809	(C/T)
16267	CakSNP16267	*Kabuli*	Ca_Kabuli_Scaffold914	18750	(C/A)
16268	CakSNP16268	*Kabuli*	Ca_Kabuli_Scaffold914	38792	(A/G)
16269	CakSNP16269	*Kabuli*	Ca_Kabuli_Scaffold914	38776	(C/A)
16270	CakSNP16270	*Kabuli*	Ca_Kabuli_Scaffold914	38775	(C/T)
16271	CakSNP16271	*Kabuli*	Ca_Kabuli_Scaffold914	38716	(C/A)
16272	CakSNP16272	*Kabuli*	Ca_Kabuli_Scaffold914	53125	(T/A)
16273	CakSNP16273	*Kabuli*	Ca_Kabuli_Scaffold914	53132	(C/T)
16274	CakSNP16274	*Kabuli*	Ca_Kabuli_Scaffold914	53270	(A/G)
16275	CakSNP16275	*Kabuli*	Ca_Kabuli_Scaffold916	1036	(A/G)
16276	CakSNP16276	*Kabuli*	Ca_Kabuli_Scaffold916	275497	(T/C)
16277	CakSNP16277	*Kabuli*	Ca_Kabuli_Scaffold916	275510	(A/T)
16278	CakSNP16278	*Kabuli*	Ca_Kabuli_Scaffold916	275512	(G/T)
16279	CakSNP16279	*Kabuli*	Ca_Kabuli_Scaffold916	275519	(A/C)
16280	CakSNP16280	*Kabuli*	Ca_Kabuli_Scaffold916	275493	(A/T)
16281	CakSNP16281	*Kabuli*	Ca_Kabuli_Scaffold919	119274	(T/C)
16282	CakSNP16282	*Kabuli*	Ca_Kabuli_Scaffold922	111921	(C/A)
16283	CakSNP16283	*Kabuli*	Ca_Kabuli_Scaffold922	121629	(T/C)
16284	CakSNP16284	*Kabuli*	Ca_Kabuli_Scaffold93	205677	(G/A)
16285	CakSNP16285	*Kabuli*	Ca_Kabuli_Scaffold93	506983	(C/A)
16286	CakSNP16286	*Kabuli*	Ca_Kabuli_Scaffold93	578138	(G/A)
16287	CakSNP16287	*Kabuli*	Ca_Kabuli_Scaffold93	578146	(A/G)
16288	CakSNP16288	*Kabuli*	Ca_Kabuli_Scaffold93	578158	(A/G)
16289	CakSNP16289	*Kabuli*	Ca_Kabuli_Scaffold93	640373	(A/C)
16290	CakSNP16290	*Kabuli*	Ca_Kabuli_Scaffold93	640389	(G/T)
16291	CakSNP16291	*Kabuli*	Ca_Kabuli_Scaffold93	640430	(C/T)
16292	CakSNP16292	*Kabuli*	Ca_Kabuli_Scaffold93	640487	(A/C)
16293	CakSNP16293	*Kabuli*	Ca_Kabuli_Scaffold93	640459	(G/T)
16294	CakSNP16294	*Kabuli*	Ca_Kabuli_Scaffold93	640448	(T/C)
16295	CakSNP16295	*Kabuli*	Ca_Kabuli_Scaffold93	640445	(C/T)
16296	CakSNP16296	*Kabuli*	Ca_Kabuli_Scaffold93	640444	(G/T)
16297	CakSNP16297	*Kabuli*	Ca_Kabuli_Scaffold93	645328	(A/C)
16298	CakSNP16298	*Kabuli*	Ca_Kabuli_Scaffold93	645344	(G/T)
16299	CakSNP16299	*Kabuli*	Ca_Kabuli_Scaffold93	645442	(A/C)
16300	CakSNP16300	*Kabuli*	Ca_Kabuli_Scaffold93	645414	(G/T)

S.N.	SNP IDs	Cultivars	Chromosomes/scaffolds	Physical positions (bp)	SNPs
16301	CakSNP16301	*Kabuli*	Ca_Kabuli_Scaffold93	645403	(T/C)
16302	CakSNP16302	*Kabuli*	Ca_Kabuli_Scaffold93	645400	(C/T)
16303	CakSNP16303	*Kabuli*	Ca_Kabuli_Scaffold93	645399	(G/T)
16304	CakSNP16304	*Kabuli*	Ca_Kabuli_Scaffold93	645402	(G/A)
16305	CakSNP16305	*Kabuli*	Ca_Kabuli_Scaffold93	651020	(C/T)
16306	CakSNP16306	*Kabuli*	Ca_Kabuli_Scaffold93	651018	(C/T)
16307	CakSNP16307	*Kabuli*	Ca_Kabuli_Scaffold93	651009	(A/G)
16308	CakSNP16308	*Kabuli*	Ca_Kabuli_Scaffold93	650981	(T/C)
16309	CakSNP16309	*Kabuli*	Ca_Kabuli_Scaffold93	650968	(G/A)
16310	CakSNP16310	*Kabuli*	Ca_Kabuli_Scaffold93	650958	(G/A)
16311	CakSNP16311	*Kabuli*	Ca_Kabuli_Scaffold93	650955	(T/C)
16312	CakSNP16312	*Kabuli*	Ca_Kabuli_Scaffold931	13212	(T/C)
16313	CakSNP16313	*Kabuli*	Ca_Kabuli_Scaffold9323	5263	(A/G)
16314	CakSNP16314	*Kabuli*	Ca_Kabuli_Scaffold937	132087	(C/G)
16315	CakSNP16315	*Kabuli*	Ca_Kabuli_Scaffold937	134580	(C/G)
16316	CakSNP16316	*Kabuli*	Ca_Kabuli_Scaffold937	134707	(G/A)
16317	CakSNP16317	*Kabuli*	Ca_Kabuli_Scaffold948	12188	(C/T)
16318	CakSNP16318	*Kabuli*	Ca_Kabuli_Scaffold948	21152	(C/T)
16319	CakSNP16319	*Kabuli*	Ca_Kabuli_Scaffold953	42329	(T/C)
16320	CakSNP16320	*Kabuli*	Ca_Kabuli_Scaffold953	57413	(A/T)
16321	CakSNP16321	*Kabuli*	Ca_Kabuli_Scaffold956	21441	(C/A)
16322	CakSNP16322	*Kabuli*	Ca_Kabuli_Scaffold956	27612	(A/G)
16323	CakSNP16323	*Kabuli*	Ca_Kabuli_Scaffold956	27710	(T/G)
16324	CakSNP16324	*Kabuli*	Ca_Kabuli_Scaffold956	27693	(T/C)
16325	CakSNP16325	*Kabuli*	Ca_Kabuli_Scaffold956	28015	(A/C)
16326	CakSNP16326	*Kabuli*	Ca_Kabuli_Scaffold956	28014	(T/A)
16327	CakSNP16327	*Kabuli*	Ca_Kabuli_Scaffold956	29736	(T/A)
16328	CakSNP16328	*Kabuli*	Ca_Kabuli_Scaffold959	56173	(C/T)
16329	CakSNP16329	*Kabuli*	Ca_Kabuli_Scaffold959	56159	(G/A)
16330	CakSNP16330	*Kabuli*	Ca_Kabuli_Scaffold959	56156	(G/A)
16331	CakSNP16331	*Kabuli*	Ca_Kabuli_Scaffold959	56117	(G/A)
16332	CakSNP16332	*Kabuli*	Ca_Kabuli_Scaffold962	149524	(C/T)
16333	CakSNP16333	*Kabuli*	Ca_Kabuli_Scaffold962	149545	(C/T)
16334	CakSNP16334	*Kabuli*	Ca_Kabuli_Scaffold962	149580	(G/A)
16335	CakSNP16335	*Kabuli*	Ca_Kabuli_Scaffold962	149585	(A/G)
16336	CakSNP16336	*Kabuli*	Ca_Kabuli_Scaffold962	149645	(G/T)
16337	CakSNP16337	*Kabuli*	Ca_Kabuli_Scaffold962	149631	(G/A)
16338	CakSNP16338	*Kabuli*	Ca_Kabuli_Scaffold962	149589	(G/A)
16339	CakSNP16339	*Kabuli*	Ca_Kabuli_Scaffold962	149583	(G/A)

S.N.	SNP IDs	Cultivars	Chromosomes/scaffolds	Physical positions (bp)	SNPs
16340	CakSNP16340	*Kabuli*	Ca_Kabuli_Scaffold962	149577	(G/T)
16341	CakSNP16341	*Kabuli*	Ca_Kabuli_Scaffold962	149689	(G/A)
16342	CakSNP16342	*Kabuli*	Ca_Kabuli_Scaffold962	149687	(A/T)
16343	CakSNP16343	*Kabuli*	Ca_Kabuli_Scaffold962	149676	(T/C)
16344	CakSNP16344	*Kabuli*	Ca_Kabuli_Scaffold962	149661	(G/A)
16345	CakSNP16345	*Kabuli*	Ca_Kabuli_Scaffold962	149649	(C/T)
16346	CakSNP16346	*Kabuli*	Ca_Kabuli_Scaffold962	149644	(C/T)
16347	CakSNP16347	*Kabuli*	Ca_Kabuli_Scaffold962	149628	(G/A)
16348	CakSNP16348	*Kabuli*	Ca_Kabuli_Scaffold962	149613	(G/T)
16349	CakSNP16349	*Kabuli*	Ca_Kabuli_Scaffold963	77099	(C/T)
16350	CakSNP16350	*Kabuli*	Ca_Kabuli_Scaffold98	287956	(T/A)
16351	CakSNP16351	*Kabuli*	Ca_Kabuli_Scaffold98	287933	(T/C)
16352	CakSNP16352	*Kabuli*	Ca_Kabuli_Scaffold98	388565	(T/C)
16353	CakSNP16353	*Kabuli*	Ca_Kabuli_Scaffold98	434956	(A/G)
16354	CakSNP16354	*Kabuli*	Ca_Kabuli_Scaffold98	440243	(G/C)
16355	CakSNP16355	*Kabuli*	Ca_Kabuli_Scaffold98	447314	(G/C)
16356	CakSNP16356	*Kabuli*	Ca_Kabuli_Scaffold98	447311	(G/A)
16357	CakSNP16357	*Kabuli*	Ca_Kabuli_Scaffold98	485305	(G/C)
16358	CakSNP16358	*Kabuli*	Ca_Kabuli_Scaffold98	485302	(G/A)
16359	CakSNP16359	*Kabuli*	Ca_Kabuli_Scaffold98	533710	(T/C)
16360	CakSNP16360	*Kabuli*	Ca_Kabuli_Scaffold98	533677	(T/A)
16361	CakSNP16361	*Kabuli*	Ca_Kabuli_Scaffold98	533812	(G/C)
16362	CakSNP16362	*Kabuli*	Ca_Kabuli_Scaffold98	562979	(G/A)
16363	CakSNP16363	*Kabuli*	Ca_Kabuli_Scaffold98	563016	(T/C)
16364	CakSNP16364	*Kabuli*	Ca_Kabuli_Scaffold98	563048	(A/C)
16365	CakSNP16365	*Kabuli*	Ca_Kabuli_Scaffold981	84091	(G/A)
16366	CakSNP16366	*Kabuli*	Ca_Kabuli_Scaffold981	84088	(C/G)
16367	CakSNP16367	*Kabuli*	Ca_Kabuli_Scaffold981	84087	(C/A)
16368	CakSNP16368	*Kabuli*	Ca_Kabuli_Scaffold981	84072	(G/A)
16369	CakSNP16369	*Kabuli*	Ca_Kabuli_Scaffold981	84057	(A/G)
16370	CakSNP16370	*Kabuli*	Ca_Kabuli_Scaffold981	84034	(C/T)
16371	CakSNP16371	*Kabuli*	Ca_Kabuli_Scaffold981	84026	(G/C)
16372	CakSNP16372	*Kabuli*	Ca_Kabuli_Scaffold981	84019	(C/T)
16373	CakSNP16373	*Kabuli*	Ca_Kabuli_Scaffold981	84007	(C/G)
16374	CakSNP16374	*Kabuli*	Ca_Kabuli_Scaffold999	77761	(A/T)
16375	CakSNP16375	*Kabuli*	Ca_Kabuli_Scaffold999	77763	(C/T)
16376	CakSNP16376	*Kabuli*	Ca_Kabuli_Scaffold999	77767	(A/T)
16377	CakSNP16377	*Kabuli*	Ca_Kabuli_denovo	1061	(T/A)
16378	CakSNP16378	*Kabuli*	Ca_Kabuli_denovo	1965	(G/T)

S.N.	SNP IDs	Cultivars	Chromosomes/scaffolds	Physical positions (bp)	SNPs
16379	CakSNP16379	*Kabuli*	Ca_Kabuli_denovo	1975	(G/A)
16380	CakSNP16380	*Kabuli*	Ca_Kabuli_denovo	2003	(A/G)
16381	CakSNP16381	*Kabuli*	Ca_Kabuli_denovo	2012	(T/G)
16382	CakSNP16382	*Kabuli*	Ca_Kabuli_denovo	2047	(T/G)
16383	CakSNP16383	*Kabuli*	Ca_Kabuli_denovo	3355	(C/A)
16384	CakSNP16384	*Kabuli*	Ca_Kabuli_denovo	3357	(T/G)
16385	CakSNP16385	*Kabuli*	Ca_Kabuli_denovo	3369	(C/G)
16386	CakSNP16386	*Kabuli*	Ca_Kabuli_denovo	3376	(C/G)
16387	CakSNP16387	*Kabuli*	Ca_Kabuli_denovo	3391	(A/G)
16388	CakSNP16388	*Kabuli*	Ca_Kabuli_denovo	3398	(A/G)
16389	CakSNP16389	*Kabuli*	Ca_Kabuli_denovo	3408	(C/G)
16390	CakSNP16390	*Kabuli*	Ca_Kabuli_denovo	3410	(G/T)
16391	CakSNP16391	*Kabuli*	Ca_Kabuli_denovo	3417	(G/A)
16392	CakSNP16392	*Kabuli*	Ca_Kabuli_denovo	3427	(G/C)
16393	CakSNP16393	*Kabuli*	Ca_Kabuli_denovo	3439	(C/G)
16394	CakSNP16394	*Kabuli*	Ca_Kabuli_denovo	3710	(A/T)
16395	CakSNP16395	*Kabuli*	Ca_Kabuli_denovo	3711	(T/G)
16396	CakSNP16396	*Kabuli*	Ca_Kabuli_denovo	3712	(G/C)
16397	CakSNP16397	*Kabuli*	Ca_Kabuli_denovo	3714	(C/G)
16398	CakSNP16398	*Kabuli*	Ca_Kabuli_denovo	3715	(A/G)
16399	CakSNP16399	*Kabuli*	Ca_Kabuli_denovo	3716	(G/A)
16400	CakSNP16400	*Kabuli*	Ca_Kabuli_denovo	3717	(A/G)
16401	CakSNP16401	*Kabuli*	Ca_Kabuli_denovo	3720	(G/T)
16402	CakSNP16402	*Kabuli*	Ca_Kabuli_denovo	3721	(A/C)
16403	CakSNP16403	*Kabuli*	Ca_Kabuli_denovo	3967	(G/A)
16404	CakSNP16404	*Kabuli*	Ca_Kabuli_denovo	3968	(G/A)
16405	CakSNP16405	*Kabuli*	Ca_Kabuli_denovo	3969	(A/G)
16406	CakSNP16406	*Kabuli*	Ca_Kabuli_denovo	3970	(A/G)
16407	CakSNP16407	*Kabuli*	Ca_Kabuli_denovo	3971	(G/A)
16408	CakSNP16408	*Kabuli*	Ca_Kabuli_denovo	3972	(C/A)
16409	CakSNP16409	*Kabuli*	Ca_Kabuli_denovo	3975	(T/G)
16410	CakSNP16410	*Kabuli*	Ca_Kabuli_denovo	4061	(G/A)
16411	CakSNP16411	*Kabuli*	Ca_Kabuli_denovo	4063	(G/A)
16412	CakSNP16412	*Kabuli*	Ca_Kabuli_denovo	4064	(A/G)
16413	CakSNP16413	*Kabuli*	Ca_Kabuli_denovo	4065	(G/C)
16414	CakSNP16414	*Kabuli*	Ca_Kabuli_denovo	4066	(C/G)
16415	CakSNP16415	*Kabuli*	Ca_Kabuli_denovo	4068	(T/G)
16416	CakSNP16416	*Kabuli*	Ca_Kabuli_denovo	4287	(C/G)
16417	CakSNP16417	*Kabuli*	Ca_Kabuli_denovo	4288	(G/A)

S.N.	SNP IDs	Cultivars	Chromosomes/scaffolds	Physical positions (bp)	SNPs
16418	CakSNP16418	*Kabuli*	Ca_Kabuli_denovo	4295	(A/G)
16419	CakSNP16419	*Kabuli*	Ca_Kabuli_denovo	4299	(T/C)
16420	CakSNP16420	*Kabuli*	Ca_Kabuli_denovo	4724	(A/G)
16421	CakSNP16421	*Kabuli*	Ca_Kabuli_denovo	4725	(G/C)
16422	CakSNP16422	*Kabuli*	Ca_Kabuli_denovo	4727	(G/A)
16423	CakSNP16423	*Kabuli*	Ca_Kabuli_denovo	4728	(A/G)
16424	CakSNP16424	*Kabuli*	Ca_Kabuli_denovo	4731	(C/A)
16425	CakSNP16425	*Kabuli*	Ca_Kabuli_denovo	4732	(C/T)
16426	CakSNP16426	*Kabuli*	Ca_Kabuli_denovo	5106	(C/G)
16427	CakSNP16427	*Kabuli*	Ca_Kabuli_denovo	5108	(T/G)
16428	CakSNP16428	*Kabuli*	Ca_Kabuli_denovo	5349	(T/G)
16429	CakSNP16429	*Kabuli*	Ca_Kabuli_denovo	5394	(C/G)
16430	CakSNP16430	*Kabuli*	Ca_Kabuli_denovo	5650	(C/G)
16431	CakSNP16431	*Kabuli*	Ca_Kabuli_denovo	5652	(G/T)
16432	CakSNP16432	*Kabuli*	Ca_Kabuli_denovo	5654	(C/T)
16433	CakSNP16433	*Kabuli*	Ca_Kabuli_denovo	5655	(A/C)
16434	CakSNP16434	*Kabuli*	Ca_Kabuli_denovo	5746	(G/C)
16435	CakSNP16435	*Kabuli*	Ca_Kabuli_denovo	5747	(C/G)
16436	CakSNP16436	*Kabuli*	Ca_Kabuli_denovo	5749	(G/T)
16437	CakSNP16437	*Kabuli*	Ca_Kabuli_denovo	5751	(C/T)
16438	CakSNP16438	*Kabuli*	Ca_Kabuli_denovo	5752	(A/C)
16439	CakSNP16439	*Kabuli*	Ca_Kabuli_denovo	5851	(C/G)
16440	CakSNP16440	*Kabuli*	Ca_Kabuli_denovo	5853	(G/T)
16441	CakSNP16441	*Kabuli*	Ca_Kabuli_denovo	5855	(C/T)
16442	CakSNP16442	*Kabuli*	Ca_Kabuli_denovo	5856	(A/C)
16443	CakSNP16443	*Kabuli*	Ca_Kabuli_denovo	6702	(T/G)
16444	CakSNP16444	*Kabuli*	Ca_Kabuli_denovo	6708	(C/G)
16445	CakSNP16445	*Kabuli*	Ca_Kabuli_denovo	6727	(A/G)
16446	CakSNP16446	*Kabuli*	Ca_Kabuli_denovo	6728	(C/G)
16447	CakSNP16447	*Kabuli*	Ca_Kabuli_denovo	6739	(T/G)
16448	CakSNP16448	*Kabuli*	Ca_Kabuli_denovo	6761	(T/G)
16449	CakSNP16449	*Kabuli*	Ca_Kabuli_denovo	6773	(T/G)
16450	CakSNP16450	*Kabuli*	Ca_Kabuli_denovo	7246	(C/A)
16451	CakSNP16451	*Kabuli*	Ca_Kabuli_denovo	7247	(G/C)
16452	CakSNP16452	*Kabuli*	Ca_Kabuli_denovo	7248	(A/C)
16453	CakSNP16453	*Kabuli*	Ca_Kabuli_denovo	7251	(C/T)
16454	CakSNP16454	*Kabuli*	Ca_Kabuli_denovo	7253	(T/G)
16455	CakSNP16455	*Kabuli*	Ca_Kabuli_denovo	7254	(C/A)
16456	CakSNP16456	*Kabuli*	Ca_Kabuli_denovo	7255	(G/T)

S.N.	SNP IDs	Cultivars	Chromosomes/scaffolds	Physical positions (bp)	SNPs
16457	CakSNP16457	*Kabuli*	Ca_Kabuli_denovo	7380	(A/T)
16458	CakSNP16458	*Kabuli*	Ca_Kabuli_denovo	7690	(A/G)
16459	CakSNP16459	*Kabuli*	Ca_Kabuli_denovo	7691	(C/G)
16460	CakSNP16460	*Kabuli*	Ca_Kabuli_denovo	7883	(G/T)
16461	CakSNP16461	*Kabuli*	Ca_Kabuli_denovo	7937	(C/G)
16462	CakSNP16462	*Kabuli*	Ca_Kabuli_denovo	7944	(T/A)
16463	CakSNP16463	*Kabuli*	Ca_Kabuli_denovo	7976	(T/G)
16464	CakSNP16464	*Kabuli*	Ca_Kabuli_denovo	7999	(T/C)
16465	CakSNP16465	*Kabuli*	Ca_Kabuli_denovo	8051	(C/A)
16466	CakSNP16466	*Kabuli*	Ca_Kabuli_denovo	8069	(T/G)
16467	CakSNP16467	*Kabuli*	Ca_Kabuli_denovo	8088	(C/A)
16468	CakSNP16468	*Kabuli*	Ca_Kabuli_denovo	8136	(A/G)
16469	CakSNP16469	*Kabuli*	Ca_Kabuli_denovo	8162	(T/G)
16470	CakSNP16470	*Kabuli*	Ca_Kabuli_denovo	8537	(A/G)
16471	CakSNP16471	*Kabuli*	Ca_Kabuli_denovo	8539	(A/C)
16472	CakSNP16472	*Kabuli*	Ca_Kabuli_denovo	8541	(T/G)
16473	CakSNP16473	*Kabuli*	Ca_Kabuli_denovo	9009	(G/A)
16474	CakSNP16474	*Kabuli*	Ca_Kabuli_denovo	9010	(A/G)
16475	CakSNP16475	*Kabuli*	Ca_Kabuli_denovo	9011	(A/G)
16476	CakSNP16476	*Kabuli*	Ca_Kabuli_denovo	9012	(C/A)
16477	CakSNP16477	*Kabuli*	Ca_Kabuli_denovo	11242	(C/G)
16478	CakSNP16478	*Kabuli*	Ca_Kabuli_denovo	11244	(T/G)
16479	CakSNP16479	*Kabuli*	Ca_Kabuli_denovo	11246	(C/T)
16480	CakSNP16480	*Kabuli*	Ca_Kabuli_denovo	11711	(G/T)
16481	CakSNP16481	*Kabuli*	Ca_Kabuli_denovo	12623	(A/G)
16482	CakSNP16482	*Kabuli*	Ca_Kabuli_denovo	12624	(G/C)
16483	CakSNP16483	*Kabuli*	Ca_Kabuli_denovo	12625	(C/G)
16484	CakSNP16484	*Kabuli*	Ca_Kabuli_denovo	12627	(T/G)
16485	CakSNP16485	*Kabuli*	Ca_Kabuli_denovo	12629	(C/T)
16486	CakSNP16486	*Kabuli*	Ca_Kabuli_denovo	12630	(A/C)
16487	CakSNP16487	*Kabuli*	Ca_Kabuli_denovo	13473	(G/A)
16488	CakSNP16488	*Kabuli*	Ca_Kabuli_denovo	13475	(G/A)
16489	CakSNP16489	*Kabuli*	Ca_Kabuli_denovo	13476	(A/G)
16490	CakSNP16490	*Kabuli*	Ca_Kabuli_denovo	14124	(A/C)
16491	CakSNP16491	*Kabuli*	Ca_Kabuli_denovo	14560	(C/A)
16492	CakSNP16492	*Kabuli*	Ca_Kabuli_denovo	14672	(G/A)
16493	CakSNP16493	*Kabuli*	Ca_Kabuli_denovo	14674	(G/A)
16494	CakSNP16494	*Kabuli*	Ca_Kabuli_denovo	15543	(G/A)
16495	CakSNP16495	*Kabuli*	Ca_Kabuli_denovo	15553	(C/A)

S.N.	SNP IDs	Cultivars	Chromosomes/scaffolds	Physical positions (bp)	SNPs
16496	CakSNP16496	*Kabuli*	Ca_Kabuli_denovo	15580	(G/A)
16497	CakSNP16497	*Kabuli*	Ca_Kabuli_denovo	17223	(C/G)
16498	CakSNP16498	*Kabuli*	Ca_Kabuli_denovo	17468	(A/G)
16499	CakSNP16499	*Kabuli*	Ca_Kabuli_denovo	17469	(G/C)
16500	CakSNP16500	*Kabuli*	Ca_Kabuli_denovo	17470	(C/G)
16501	CakSNP16501	*Kabuli*	Ca_Kabuli_denovo	17472	(T/G)
16502	CakSNP16502	*Kabuli*	Ca_Kabuli_denovo	17733	(G/C)
16503	CakSNP16503	*Kabuli*	Ca_Kabuli_denovo	17734	(C/G)
16504	CakSNP16504	*Kabuli*	Ca_Kabuli_denovo	17736	(T/G)
16505	CakSNP16505	*Kabuli*	Ca_Kabuli_denovo	17738	(C/T)
16506	CakSNP16506	*Kabuli*	Ca_Kabuli_denovo	18017	(G/A)
16507	CakSNP16507	*Kabuli*	Ca_Kabuli_denovo	18019	(G/A)
16508	CakSNP16508	*Kabuli*	Ca_Kabuli_denovo	18050	(G/C)
16509	CakSNP16509	*Kabuli*	Ca_Kabuli_denovo	18291	(C/G)
16510	CakSNP16510	*Kabuli*	Ca_Kabuli_denovo	18293	(G/T)
16511	CakSNP16511	*Kabuli*	Ca_Kabuli_denovo	18295	(C/T)
16512	CakSNP16512	*Kabuli*	Ca_Kabuli_denovo	18296	(A/C)
16513	CakSNP16513	*Kabuli*	Ca_Kabuli_denovo	18297	(G/A)
16514	CakSNP16514	*Kabuli*	Ca_Kabuli_denovo	18394	(T/G)
16515	CakSNP16515	*Kabuli*	Ca_Kabuli_denovo	18450	(G/A)
16516	CakSNP16516	*Kabuli*	Ca_Kabuli_denovo	19005	(C/T)
16517	CakSNP16517	*Kabuli*	Ca_Kabuli_denovo	19041	(T/C)
16518	CakSNP16518	*Kabuli*	Ca_Kabuli_denovo	19048	(G/C)
16519	CakSNP16519	*Kabuli*	Ca_Kabuli_denovo	20080	(C/G)
16520	CakSNP16520	*Kabuli*	Ca_Kabuli_denovo	20081	(G/A)
16521	CakSNP16521	*Kabuli*	Ca_Kabuli_denovo	20082	(A/T)
16522	CakSNP16522	*Kabuli*	Ca_Kabuli_denovo	20083	(G/C)
16523	CakSNP16523	*Kabuli*	Ca_Kabuli_denovo	20084	(T/A)
16524	CakSNP16524	*Kabuli*	Ca_Kabuli_denovo	20086	(G/C)
16525	CakSNP16525	*Kabuli*	Ca_Kabuli_denovo	20087	(T/G)
16526	CakSNP16526	*Kabuli*	Ca_Kabuli_denovo	21257	(C/A)
16527	CakSNP16527	*Kabuli*	Ca_Kabuli_denovo	22000	(T/G)
16528	CakSNP16528	*Kabuli*	Ca_Kabuli_denovo	22013	(A/G)
16529	CakSNP16529	*Kabuli*	Ca_Kabuli_denovo	22039	(G/T)
16530	CakSNP16530	*Kabuli*	Ca_Kabuli_denovo	22242	(A/G)
16531	CakSNP16531	*Kabuli*	Ca_Kabuli_denovo	22320	(T/A)
16532	CakSNP16532	*Kabuli*	Ca_Kabuli_denovo	27708	(C/G)
16533	CakSNP16533	*Kabuli*	Ca_Kabuli_denovo	27709	(G/A)
16534	CakSNP16534	*Kabuli*	Ca_Kabuli_denovo	27710	(G/A)

S.N.	SNP IDs	Cultivars	Chromosomes/scaffolds	Physical positions (bp)	SNPs
16535	CakSNP16535	*Kabuli*	Ca_Kabuli_denovo	27711	(A/G)
16536	CakSNP16536	*Kabuli*	Ca_Kabuli_denovo	27712	(C/A)
16537	CakSNP16537	*Kabuli*	Ca_Kabuli_denovo	27714	(G/C)
16538	CakSNP16538	*Kabuli*	Ca_Kabuli_denovo	27715	(A/G)
16539	CakSNP16539	*Kabuli*	Ca_Kabuli_denovo	28816	(G/T)
16540	CakSNP16540	*Kabuli*	Ca_Kabuli_denovo	28818	(C/T)
16541	CakSNP16541	*Kabuli*	Ca_Kabuli_denovo	29384	(A/G)
16542	CakSNP16542	*Kabuli*	Ca_Kabuli_denovo	32328	(G/A)
16543	CakSNP16543	*Kabuli*	Ca_Kabuli_denovo	32330	(G/A)
16544	CakSNP16544	*Kabuli*	Ca_Kabuli_denovo	32331	(A/G)
16545	CakSNP16545	*Kabuli*	Ca_Kabuli_denovo	33189	(G/A)
16546	CakSNP16546	*Kabuli*	Ca_Kabuli_denovo	34679	(G/T)
16547	CakSNP16547	*Kabuli*	Ca_Kabuli_denovo	34680	(C/G)
16548	CakSNP16548	*Kabuli*	Ca_Kabuli_denovo	34682	(C/G)
16549	CakSNP16549	*Kabuli*	Ca_Kabuli_denovo	34683	(G/A)
16550	CakSNP16550	*Kabuli*	Ca_Kabuli_denovo	34684	(A/G)
16551	CakSNP16551	*Kabuli*	Ca_Kabuli_denovo	34685	(G/A)
16552	CakSNP16552	*Kabuli*	Ca_Kabuli_denovo	34858	(G/A)
16553	CakSNP16553	*Kabuli*	Ca_Kabuli_denovo	34860	(G/A)
16554	CakSNP16554	*Kabuli*	Ca_Kabuli_denovo	35627	(T/G)
16555	CakSNP16555	*Kabuli*	Ca_Kabuli_denovo	35634	(A/G)
16556	CakSNP16556	*Kabuli*	Ca_Kabuli_denovo	35645	(C/A)
16557	CakSNP16557	*Kabuli*	Ca_Kabuli_denovo	35661	(G/A)
16558	CakSNP16558	*Kabuli*	Ca_Kabuli_denovo	35679	(C/G)
16559	CakSNP16559	*Kabuli*	Ca_Kabuli_denovo	36295	(C/G)
16560	CakSNP16560	*Kabuli*	Ca_Kabuli_denovo	36358	(G/C)
16561	CakSNP16561	*Kabuli*	Ca_Kabuli_denovo	36359	(T/A)
16562	CakSNP16562	*Kabuli*	Ca_Kabuli_denovo	36361	(G/C)
16563	CakSNP16563	*Kabuli*	Ca_Kabuli_denovo	36362	(T/G)
16564	CakSNP16564	*Kabuli*	Ca_Kabuli_denovo	36544	(T/G)
16565	CakSNP16565	*Kabuli*	Ca_Kabuli_denovo	36545	(G/C)
16566	CakSNP16566	*Kabuli*	Ca_Kabuli_denovo	36547	(G/C)
16567	CakSNP16567	*Kabuli*	Ca_Kabuli_denovo	36548	(A/G)
16568	CakSNP16568	*Kabuli*	Ca_Kabuli_denovo	36549	(G/A)
16569	CakSNP16569	*Kabuli*	Ca_Kabuli_denovo	36550	(A/G)
16570	CakSNP16570	*Kabuli*	Ca_Kabuli_denovo	36620	(G/C)
16571	CakSNP16571	*Kabuli*	Ca_Kabuli_denovo	36621	(C/A)
16572	CakSNP16572	*Kabuli*	Ca_Kabuli_denovo	36622	(A/G)
16573	CakSNP16573	*Kabuli*	Ca_Kabuli_denovo	36624	(A/G)

S.N.	SNP IDs	Cultivars	Chromosomes/scaffolds	Physical positions (bp)	SNPs
16574	CakSNP16574	*Kabuli*	Ca_Kabuli_denovo	36626	(T/A)
16575	CakSNP16575	*Kabuli*	Ca_Kabuli_denovo	36627	(G/T)
16576	CakSNP16576	*Kabuli*	Ca_Kabuli_denovo	36628	(C/G)
16577	CakSNP16577	*Kabuli*	Ca_Kabuli_denovo	36630	(G/C)
16578	CakSNP16578	*Kabuli*	Ca_Kabuli_denovo	37457	(G/C)
16579	CakSNP16579	*Kabuli*	Ca_Kabuli_denovo	37458	(C/A)
16580	CakSNP16580	*Kabuli*	Ca_Kabuli_denovo	37459	(A/G)
16581	CakSNP16581	*Kabuli*	Ca_Kabuli_denovo	37461	(G/A)
16582	CakSNP16582	*Kabuli*	Ca_Kabuli_denovo	37463	(A/T)
16583	CakSNP16583	*Kabuli*	Ca_Kabuli_denovo	37464	(T/G)
16584	CakSNP16584	*Kabuli*	Ca_Kabuli_denovo	37465	(C/G)
16585	CakSNP16585	*Kabuli*	Ca_Kabuli_denovo	37467	(G/C)
16586	CakSNP16586	*Kabuli*	Ca_Kabuli_denovo	37468	(A/G)
16587	CakSNP16587	*Kabuli*	Ca_Kabuli_denovo	37469	(G/A)
16588	CakSNP16588	*Kabuli*	Ca_Kabuli_denovo	38106	(G/A)
16589	CakSNP16589	*Kabuli*	Ca_Kabuli_denovo	38108	(T/G)
16590	CakSNP16590	*Kabuli*	Ca_Kabuli_denovo	38109	(T/A)
16591	CakSNP16591	*Kabuli*	Ca_Kabuli_denovo	38110	(C/A)
16592	CakSNP16592	*Kabuli*	Ca_Kabuli_denovo	38111	(T/A)
16593	CakSNP16593	*Kabuli*	Ca_Kabuli_denovo	38114	(C/A)
16594	CakSNP16594	*Kabuli*	Ca_Kabuli_denovo	38932	(G/A)
16595	CakSNP16595	*Kabuli*	Ca_Kabuli_denovo	38934	(G/A)
16596	CakSNP16596	*Kabuli*	Ca_Kabuli_denovo	40259	(A/G)
16597	CakSNP16597	*Kabuli*	Ca_Kabuli_denovo	40260	(G/C)
16598	CakSNP16598	*Kabuli*	Ca_Kabuli_denovo	40261	(C/A)
16599	CakSNP16599	*Kabuli*	Ca_Kabuli_denovo	40262	(A/G)
16600	CakSNP16600	*Kabuli*	Ca_Kabuli_denovo	40264	(G/A)
16601	CakSNP16601	*Kabuli*	Ca_Kabuli_denovo	40266	(T/A)
16602	CakSNP16602	*Kabuli*	Ca_Kabuli_denovo	40267	(G/T)
16603	CakSNP16603	*Kabuli*	Ca_Kabuli_denovo	40268	(C/G)
16604	CakSNP16604	*Kabuli*	Ca_Kabuli_denovo	40270	(G/C)
16605	CakSNP16605	*Kabuli*	Ca_Kabuli_denovo	41456	(T/C)
16606	CakSNP16606	*Kabuli*	Ca_Kabuli_denovo	41457	(T/A)
16607	CakSNP16607	*Kabuli*	Ca_Kabuli_denovo	41458	(G/C)
16608	CakSNP16608	*Kabuli*	Ca_Kabuli_denovo	41460	(A/G)
16609	CakSNP16609	*Kabuli*	Ca_Kabuli_denovo	41888	(A/T)
16610	CakSNP16610	*Kabuli*	Ca_Kabuli_denovo	41895	(C/A)
16611	CakSNP16611	*Kabuli*	Ca_Kabuli_denovo	44072	(T/G)
16612	CakSNP16612	*Kabuli*	Ca_Kabuli_denovo	44074	(C/T)

S.N.	SNP IDs	Cultivars	Chromosomes/scaffolds	Physical positions (bp)	SNPs
16613	CakSNP16613	*Kabuli*	Ca_Kabuli_denovo	45632	(A/C)
16614	CakSNP16614	*Kabuli*	Ca_Kabuli_denovo	45635	(C/T)
16615	CakSNP16615	*Kabuli*	Ca_Kabuli_denovo	45637	(T/G)
16616	CakSNP16616	*Kabuli*	Ca_Kabuli_denovo	45639	(G/A)
16617	CakSNP16617	*Kabuli*	Ca_Kabuli_denovo	48879	(T/G)
16618	CakSNP16618	*Kabuli*	Ca_Kabuli_denovo	49259	(G/A)
16619	CakSNP16619	*Kabuli*	Ca_Kabuli_denovo	49260	(A/G)
16620	CakSNP16620	*Kabuli*	Ca_Kabuli_denovo	49261	(A/G)
16621	CakSNP16621	*Kabuli*	Ca_Kabuli_denovo	49262	(G/A)
16622	CakSNP16622	*Kabuli*	Ca_Kabuli_denovo	49263	(C/A)
16623	CakSNP16623	*Kabuli*	Ca_Kabuli_denovo	49265	(G/C)
16624	CakSNP16624	*Kabuli*	Ca_Kabuli_denovo	49932	(C/G)
16625	CakSNP16625	*Kabuli*	Ca_Kabuli_denovo	49934	(T/G)
16626	CakSNP16626	*Kabuli*	Ca_Kabuli_denovo	49936	(C/T)
16627	CakSNP16627	*Kabuli*	Ca_Kabuli_denovo	50705	(T/G)
16628	CakSNP16628	*Kabuli*	Ca_Kabuli_denovo	50712	(T/C)
16629	CakSNP16629	*Kabuli*	Ca_Kabuli_denovo	50914	(G/A)
16630	CakSNP16630	*Kabuli*	Ca_Kabuli_denovo	51290	(C/G)
16631	CakSNP16631	*Kabuli*	Ca_Kabuli_denovo	51357	(T/G)
16632	CakSNP16632	*Kabuli*	Ca_Kabuli_denovo	51491	(A/G)
16633	CakSNP16633	*Kabuli*	Ca_Kabuli_denovo	51528	(T/C)
16634	CakSNP16634	*Kabuli*	Ca_Kabuli_denovo	51691	(G/C)
16635	CakSNP16635	*Kabuli*	Ca_Kabuli_denovo	51720	(T/G)
16636	CakSNP16636	*Kabuli*	Ca_Kabuli_denovo	51737	(C/G)
16637	CakSNP16637	*Kabuli*	Ca_Kabuli_denovo	51993	(T/G)
16638	CakSNP16638	*Kabuli*	Ca_Kabuli_denovo	52004	(C/A)
16639	CakSNP16639	*Kabuli*	Ca_Kabuli_denovo	52027	(G/T)
16640	CakSNP16640	*Kabuli*	Ca_Kabuli_denovo	52098	(A/G)
16641	CakSNP16641	*Kabuli*	Ca_Kabuli_denovo	52113	(G/T)
16642	CakSNP16642	*Kabuli*	Ca_Kabuli_denovo	52290	(A/G)
16643	CakSNP16643	*Kabuli*	Ca_Kabuli_denovo	52303	(A/C)
16644	CakSNP16644	*Kabuli*	Ca_Kabuli_denovo	53001	(C/A)
16645	CakSNP16645	*Kabuli*	Ca_Kabuli_denovo	53002	(A/G)
16646	CakSNP16646	*Kabuli*	Ca_Kabuli_denovo	53003	(G/C)
16647	CakSNP16647	*Kabuli*	Ca_Kabuli_denovo	53207	(T/G)
16648	CakSNP16648	*Kabuli*	Ca_Kabuli_denovo	53219	(C/A)
16649	CakSNP16649	*Kabuli*	Ca_Kabuli_denovo	53399	(G/C)
16650	CakSNP16650	*Kabuli*	Ca_Kabuli_denovo	53594	(G/A)
16651	CakSNP16651	*Kabuli*	Ca_Kabuli_denovo	53782	(C/A)

S.N.	SNP IDs	Cultivars	Chromosomes/scaffolds	Physical positions (bp)	SNPs
16652	CakSNP16652	*Kabuli*	Ca_Kabuli_denovo	53972	(G/A)
16653	CakSNP16653	*Kabuli*	Ca_Kabuli_denovo	54229	(T/G)
16654	CakSNP16654	*Kabuli*	Ca_Kabuli_denovo	54262	(A/G)
16655	CakSNP16655	*Kabuli*	Ca_Kabuli_denovo	54267	(T/G)
16656	CakSNP16656	*Kabuli*	Ca_Kabuli_denovo	54513	(C/G)
16657	CakSNP16657	*Kabuli*	Ca_Kabuli_denovo	54540	(A/G)
16658	CakSNP16658	*Kabuli*	Ca_Kabuli_denovo	54870	(A/C)
16659	CakSNP16659	*Kabuli*	Ca_Kabuli_denovo	54877	(A/G)
16660	CakSNP16660	*Kabuli*	Ca_Kabuli_denovo	54880	(T/C)
16661	CakSNP16661	*Kabuli*	Ca_Kabuli_denovo	54888	(C/G)
16662	CakSNP16662	*Kabuli*	Ca_Kabuli_denovo	54895	(T/G)
16663	CakSNP16663	*Kabuli*	Ca_Kabuli_denovo	54900	(C/G)
16664	CakSNP16664	*Kabuli*	Ca_Kabuli_denovo	54909	(T/G)
16665	CakSNP16665	*Kabuli*	Ca_Kabuli_denovo	54910	(C/G)
16666	CakSNP16666	*Kabuli*	Ca_Kabuli_denovo	54911	(A/G)
16667	CakSNP16667	*Kabuli*	Ca_Kabuli_denovo	54915	(T/A)
16668	CakSNP16668	*Kabuli*	Ca_Kabuli_denovo	54932	(T/G)
16669	CakSNP16669	*Kabuli*	Ca_Kabuli_denovo	55406	(T/C)
16670	CakSNP16670	*Kabuli*	Ca_Kabuli_denovo	55407	(T/A)
16671	CakSNP16671	*Kabuli*	Ca_Kabuli_denovo	55408	(G/C)
16672	CakSNP16672	*Kabuli*	Ca_Kabuli_denovo	55409	(C/A)
16673	CakSNP16673	*Kabuli*	Ca_Kabuli_denovo	55504	(G/C)
16674	CakSNP16674	*Kabuli*	Ca_Kabuli_denovo	55505	(G/A)
16675	CakSNP16675	*Kabuli*	Ca_Kabuli_denovo	55506	(A/G)
16676	CakSNP16676	*Kabuli*	Ca_Kabuli_denovo	56193	(T/G)
16677	CakSNP16677	*Kabuli*	Ca_Kabuli_denovo	56223	(T/G)
16678	CakSNP16678	*Kabuli*	Ca_Kabuli_denovo	56260	(G/C)
16679	CakSNP16679	*Kabuli*	Ca_Kabuli_denovo	56261	(G/A)
16680	CakSNP16680	*Kabuli*	Ca_Kabuli_denovo	56262	(A/C)
16681	CakSNP16681	*Kabuli*	Ca_Kabuli_denovo	56263	(T/C)
16682	CakSNP16682	*Kabuli*	Ca_Kabuli_denovo	56264	(C/G)
16683	CakSNP16683	*Kabuli*	Ca_Kabuli_denovo	56266	(C/T)
16684	CakSNP16684	*Kabuli*	Ca_Kabuli_denovo	56344	(G/C)
16685	CakSNP16685	*Kabuli*	Ca_Kabuli_denovo	56345	(A/G)
16686	CakSNP16686	*Kabuli*	Ca_Kabuli_denovo	56442	(C/A)
16687	CakSNP16687	*Kabuli*	Ca_Kabuli_denovo	56443	(G/C)
16688	CakSNP16688	*Kabuli*	Ca_Kabuli_denovo	56444	(A/G)
16689	CakSNP16689	*Kabuli*	Ca_Kabuli_denovo	56620	(C/A)
16690	CakSNP16690	*Kabuli*	Ca_Kabuli_denovo	56621	(A/T)

S.N.	SNP IDs	Cultivars	Chromosomes/scaffolds	Physical positions (bp)	SNPs
16691	CakSNP16691	*Kabuli*	Ca_Kabuli_denovo	56623	(G/C)
16692	CakSNP16692	*Kabuli*	Ca_Kabuli_denovo	56624	(C/A)
16693	CakSNP16693	*Kabuli*	Ca_Kabuli_denovo	56625	(G/A)
16694	CakSNP16694	*Kabuli*	Ca_Kabuli_denovo	56721	(C/A)
16695	CakSNP16695	*Kabuli*	Ca_Kabuli_denovo	56723	(C/G)
16696	CakSNP16696	*Kabuli*	Ca_Kabuli_denovo	56724	(G/A)
16697	CakSNP16697	*Kabuli*	Ca_Kabuli_denovo	56811	(A/T)
16698	CakSNP16698	*Kabuli*	Ca_Kabuli_denovo	56812	(T/G)
16699	CakSNP16699	*Kabuli*	Ca_Kabuli_denovo	56813	(G/C)
16700	CakSNP16700	*Kabuli*	Ca_Kabuli_denovo	56815	(C/G)
16701	CakSNP16701	*Kabuli*	Ca_Kabuli_denovo	56816	(G/A)
16702	CakSNP16702	*Kabuli*	Ca_Kabuli_denovo	56817	(G/A)
16703	CakSNP16703	*Kabuli*	Ca_Kabuli_denovo	56818	(A/G)
16704	CakSNP16704	*Kabuli*	Ca_Kabuli_denovo	56900	(A/G)
16705	CakSNP16705	*Kabuli*	Ca_Kabuli_denovo	56902	(G/A)
16706	CakSNP16706	*Kabuli*	Ca_Kabuli_denovo	56904	(A/T)
16707	CakSNP16707	*Kabuli*	Ca_Kabuli_denovo	56905	(T/G)
16708	CakSNP16708	*Kabuli*	Ca_Kabuli_denovo	56906	(G/C)
16709	CakSNP16709	*Kabuli*	Ca_Kabuli_denovo	56908	(C/G)
16710	CakSNP16710	*Kabuli*	Ca_Kabuli_denovo	56909	(G/A)
16711	CakSNP16711	*Kabuli*	Ca_Kabuli_denovo	56910	(A/G)
16712	CakSNP16712	*Kabuli*	Ca_Kabuli_denovo	56911	(G/A)
16713	CakSNP16713	*Kabuli*	Ca_Kabuli_denovo	56912	(A/C)
16714	CakSNP16714	*Kabuli*	Ca_Kabuli_denovo	56913	(T/C)
16715	CakSNP16715	*Kabuli*	Ca_Kabuli_denovo	56915	(G/C)
16716	CakSNP16716	*Kabuli*	Ca_Kabuli_denovo	56916	(T/A)
16717	CakSNP16717	*Kabuli*	Ca_Kabuli_denovo	57097	(T/G)
16718	CakSNP16718	*Kabuli*	Ca_Kabuli_denovo	57098	(C/A)
16719	CakSNP16719	*Kabuli*	Ca_Kabuli_denovo	57100	(C/T)
16720	CakSNP16720	*Kabuli*	Ca_Kabuli_denovo	57101	(G/C)
16721	CakSNP16721	*Kabuli*	Ca_Kabuli_denovo	57170	(C/G)
16722	CakSNP16722	*Kabuli*	Ca_Kabuli_denovo	57172	(G/T)
16723	CakSNP16723	*Kabuli*	Ca_Kabuli_denovo	57174	(T/C)
16724	CakSNP16724	*Kabuli*	Ca_Kabuli_denovo	57175	(C/A)
16725	CakSNP16725	*Kabuli*	Ca_Kabuli_denovo	57176	(A/G)
16726	CakSNP16726	*Kabuli*	Ca_Kabuli_denovo	57177	(C/G)
16727	CakSNP16727	*Kabuli*	Ca_Kabuli_denovo	57178	(A/C)
16728	CakSNP16728	*Kabuli*	Ca_Kabuli_denovo	57193	(G/T)
16729	CakSNP16729	*Kabuli*	Ca_Kabuli_denovo	57194	(A/C)

S.N.	SNP IDs	Cultivars	Chromosomes/scaffolds	Physical positions (bp)	SNPs
16730	CakSNP16730	*Kabuli*	Ca_Kabuli_denovo	57195	(T/G)
16731	CakSNP16731	*Kabuli*	Ca_Kabuli_denovo	57196	(C/T)
16732	CakSNP16732	*Kabuli*	Ca_Kabuli_denovo	57271	(C/A)
16733	CakSNP16733	*Kabuli*	Ca_Kabuli_denovo	57272	(A/G)
16734	CakSNP16734	*Kabuli*	Ca_Kabuli_denovo	57275	(A/G)
16735	CakSNP16735	*Kabuli*	Ca_Kabuli_denovo	57276	(A/T)
16736	CakSNP16736	*Kabuli*	Ca_Kabuli_denovo	57277	(G/T)
16737	CakSNP16737	*Kabuli*	Ca_Kabuli_denovo	57278	(A/C)
16738	CakSNP16738	*Kabuli*	Ca_Kabuli_denovo	57280	(C/G)
16739	CakSNP16739	*Kabuli*	Ca_Kabuli_denovo	57281	(G/A)
16740	CakSNP16740	*Kabuli*	Ca_Kabuli_denovo	57282	(A/G)
16741	CakSNP16741	*Kabuli*	Ca_Kabuli_denovo	57283	(T/A)
16742	CakSNP16742	*Kabuli*	Ca_Kabuli_denovo	57285	(T/C)
16743	CakSNP16743	*Kabuli*	Ca_Kabuli_denovo	57286	(C/G)
16744	CakSNP16744	*Kabuli*	Ca_Kabuli_denovo	57287	(G/A)
16745	CakSNP16745	*Kabuli*	Ca_Kabuli_denovo	57289	(A/C)
16746	CakSNP16746	*Kabuli*	Ca_Kabuli_denovo	57955	(A/G)
16747	CakSNP16747	*Kabuli*	Ca_Kabuli_denovo	57990	(T/G)
16748	CakSNP16748	*Kabuli*	Ca_Kabuli_denovo	58026	(A/C)
16749	CakSNP16749	*Kabuli*	Ca_Kabuli_denovo	58029	(C/T)
16750	CakSNP16750	*Kabuli*	Ca_Kabuli_denovo	58031	(T/G)
16751	CakSNP16751	*Kabuli*	Ca_Kabuli_denovo	58032	(C/A)
16752	CakSNP16752	*Kabuli*	Ca_Kabuli_denovo	58033	(G/T)
16753	CakSNP16753	*Kabuli*	Ca_Kabuli_denovo	58046	(T/G)
16754	CakSNP16754	*Kabuli*	Ca_Kabuli_denovo	58761	(G/A)
16755	CakSNP16755	*Kabuli*	Ca_Kabuli_denovo	58763	(G/A)
16756	CakSNP16756	*Kabuli*	Ca_Kabuli_denovo	58765	(G/A)
16757	CakSNP16757	*Kabuli*	Ca_Kabuli_denovo	59113	(G/T)
16758	CakSNP16758	*Kabuli*	Ca_Kabuli_denovo	60062	(C/G)
16759	CakSNP16759	*Kabuli*	Ca_Kabuli_denovo	60064	(T/G)
16760	CakSNP16760	*Kabuli*	Ca_Kabuli_denovo	60066	(C/T)
16761	CakSNP16761	*Kabuli*	Ca_Kabuli_denovo	60711	(G/A)
16762	CakSNP16762	*Kabuli*	Ca_Kabuli_denovo	60712	(A/G)
16763	CakSNP16763	*Kabuli*	Ca_Kabuli_denovo	62231	(G/A)
16764	CakSNP16764	*Kabuli*	Ca_Kabuli_denovo	62282	(G/A)
16765	CakSNP16765	*Kabuli*	Ca_Kabuli_denovo	62560	(G/A)
16766	CakSNP16766	*Kabuli*	Ca_Kabuli_denovo	62562	(G/A)
16767	CakSNP16767	*Kabuli*	Ca_Kabuli_denovo	63303	(C/G)
16768	CakSNP16768	*Kabuli*	Ca_Kabuli_denovo	63305	(T/G)

S.N.	SNP IDs	Cultivars	Chromosomes/scaffolds	Physical positions (bp)	SNPs
16769	CakSNP16769	*Kabuli*	Ca_Kabuli_denovo	63307	(C/T)
16770	CakSNP16770	*Kabuli*	Ca_Kabuli_denovo	64427	(T/C)
16771	CakSNP16771	*Kabuli*	Ca_Kabuli_denovo	64428	(T/A)
16772	CakSNP16772	*Kabuli*	Ca_Kabuli_denovo	64429	(G/C)
16773	CakSNP16773	*Kabuli*	Ca_Kabuli_denovo	64525	(C/G)
16774	CakSNP16774	*Kabuli*	Ca_Kabuli_denovo	64526	(G/A)
16775	CakSNP16775	*Kabuli*	Ca_Kabuli_denovo	66001	(G/A)
16776	CakSNP16776	*Kabuli*	Ca_Kabuli_denovo	66003	(G/A)
16777	CakSNP16777	*Kabuli*	Ca_Kabuli_denovo	66373	(G/A)
16778	CakSNP16778	*Kabuli*	Ca_Kabuli_denovo	67505	(A/G)
16779	CakSNP16779	*Kabuli*	Ca_Kabuli_denovo	67506	(C/G)
16780	CakSNP16780	*Kabuli*	Ca_Kabuli_denovo	67912	(G/T)
16781	CakSNP16781	*Kabuli*	Ca_Kabuli_denovo	68709	(G/A)
16782	CakSNP16782	*Kabuli*	Ca_Kabuli_denovo	68710	(C/G)
16783	CakSNP16783	*Kabuli*	Ca_Kabuli_denovo	68711	(A/C)
16784	CakSNP16784	*Kabuli*	Ca_Kabuli_denovo	68712	(A/G)
16785	CakSNP16785	*Kabuli*	Ca_Kabuli_denovo	68714	(G/A)
16786	CakSNP16786	*Kabuli*	Ca_Kabuli_denovo	68716	(A/T)
16787	CakSNP16787	*Kabuli*	Ca_Kabuli_denovo	69930	(C/G)
16788	CakSNP16788	*Kabuli*	Ca_Kabuli_denovo	69932	(G/T)
16789	CakSNP16789	*Kabuli*	Ca_Kabuli_denovo	69934	(C/T)
16790	CakSNP16790	*Kabuli*	Ca_Kabuli_denovo	69935	(A/C)
16791	CakSNP16791	*Kabuli*	Ca_Kabuli_denovo	69936	(G/A)
16792	CakSNP16792	*Kabuli*	Ca_Kabuli_denovo	69937	(C/G)
16793	CakSNP16793	*Kabuli*	Ca_Kabuli_denovo	71409	(G/A)
16794	CakSNP16794	*Kabuli*	Ca_Kabuli_denovo	71411	(G/A)
16795	CakSNP16795	*Kabuli*	Ca_Kabuli_denovo	72745	(G/T)
16796	CakSNP16796	*Kabuli*	Ca_Kabuli_denovo	74388	(G/C)
16797	CakSNP16797	*Kabuli*	Ca_Kabuli_denovo	74389	(C/G)
16798	CakSNP16798	*Kabuli*	Ca_Kabuli_denovo	74391	(T/G)
16799	CakSNP16799	*Kabuli*	Ca_Kabuli_denovo	74393	(C/T)
16800	CakSNP16800	*Kabuli*	Ca_Kabuli_denovo	74394	(A/C)
16801	CakSNP16801	*Kabuli*	Ca_Kabuli_denovo	74952	(C/G)
16802	CakSNP16802	*Kabuli*	Ca_Kabuli_denovo	74954	(G/T)
16803	CakSNP16803	*Kabuli*	Ca_Kabuli_denovo	74956	(C/T)
16804	CakSNP16804	*Kabuli*	Ca_Kabuli_denovo	74957	(A/C)
16805	CakSNP16805	*Kabuli*	Ca_Kabuli_denovo	74958	(G/A)
16806	CakSNP16806	*Kabuli*	Ca_Kabuli_denovo	74959	(C/G)
16807	CakSNP16807	*Kabuli*	Ca_Kabuli_denovo	74972	(A/G)

S.N.	SNP IDs	Cultivars	Chromosomes/scaffolds	Physical positions (bp)	SNPs
16808	CakSNP16808	*Kabuli*	Ca_Kabuli_denovo	75008	(G/A)
16809	CakSNP16809	*Kabuli*	Ca_Kabuli_denovo	76173	(A/G)
16810	CakSNP16810	*Kabuli*	Ca_Kabuli_denovo	76178	(G/A)
16811	CakSNP16811	*Kabuli*	Ca_Kabuli_denovo	76193	(C/G)
16812	CakSNP16812	*Kabuli*	Ca_Kabuli_denovo	76204	(C/G)
16813	CakSNP16813	*Kabuli*	Ca_Kabuli_denovo	76218	(T/G)
16814	CakSNP16814	*Kabuli*	Ca_Kabuli_denovo	76228	(G/C)
16815	CakSNP16815	*Kabuli*	Ca_Kabuli_denovo	76288	(C/T)
16816	CakSNP16816	*Kabuli*	Ca_Kabuli_denovo	76295	(G/T)
16817	CakSNP16817	*Kabuli*	Ca_Kabuli_denovo	76452	(T/A)
16818	CakSNP16818	*Kabuli*	Ca_Kabuli_denovo	76513	(A/G)
16819	CakSNP16819	*Kabuli*	Ca_Kabuli_denovo	76517	(T/C)
16820	CakSNP16820	*Kabuli*	Ca_Kabuli_denovo	76530	(T/G)
16821	CakSNP16821	*Kabuli*	Ca_Kabuli_denovo	76561	(G/T)
16822	CakSNP16822	*Kabuli*	Ca_Kabuli_denovo	76579	(G/A)
16823	CakSNP16823	*Kabuli*	Ca_Kabuli_denovo	77007	(T/A)
16824	CakSNP16824	*Kabuli*	Ca_Kabuli_denovo	77013	(A/T)
16825	CakSNP16825	*Kabuli*	Ca_Kabuli_denovo	77051	(T/A)
16826	CakSNP16826	*Kabuli*	Ca_Kabuli_denovo	77063	(A/G)
16827	CakSNP16827	*Kabuli*	Ca_Kabuli_denovo	78895	(C/T)
16828	CakSNP16828	*Kabuli*	Ca_Kabuli_denovo	78946	(C/T)
16829	CakSNP16829	*Kabuli*	Ca_Kabuli_denovo	79307	(T/A)
16830	CakSNP16830	*Kabuli*	Ca_Kabuli_denovo	79308	(T/G)
16831	CakSNP16831	*Kabuli*	Ca_Kabuli_denovo	79309	(C/G)
16832	CakSNP16832	*Kabuli*	Ca_Kabuli_denovo	79311	(A/G)
16833	CakSNP16833	*Kabuli*	Ca_Kabuli_denovo	79312	(T/C)
16834	CakSNP16834	*Kabuli*	Ca_Kabuli_denovo	79313	(G/A)
16835	CakSNP16835	*Kabuli*	Ca_Kabuli_denovo	83072	(C/T)
16836	CakSNP16836	*Kabuli*	Ca_Kabuli_denovo	83103	(C/G)
16837	CakSNP16837	*Kabuli*	Ca_Kabuli_denovo	83773	(C/G)
16838	CakSNP16838	*Kabuli*	Ca_Kabuli_denovo	83775	(A/G)
16839	CakSNP16839	*Kabuli*	Ca_Kabuli_denovo	83776	(T/C)
16840	CakSNP16840	*Kabuli*	Ca_Kabuli_denovo	83777	(G/A)
16841	CakSNP16841	*Kabuli*	Ca_Kabuli_denovo	83885	(A/C)
16842	CakSNP16842	*Kabuli*	Ca_Kabuli_denovo	84999	(T/G)
16843	CakSNP16843	*Kabuli*	Ca_Kabuli_denovo	85000	(G/A)
16844	CakSNP16844	*Kabuli*	Ca_Kabuli_denovo	85001	(C/G)
16845	CakSNP16845	*Kabuli*	Ca_Kabuli_denovo	85003	(G/C)
16846	CakSNP16846	*Kabuli*	Ca_Kabuli_denovo	89165	(G/A)

S.N.	SNP IDs	Cultivars	Chromosomes/scaffolds	Physical positions (bp)	SNPs
16847	CakSNP16847	*Kabuli*	Ca_Kabuli_denovo	89167	(G/A)
16848	CakSNP16848	*Kabuli*	Ca_Kabuli_denovo	89731	(C/G)
16849	CakSNP16849	*Kabuli*	Ca_Kabuli_denovo	89733	(A/G)
16850	CakSNP16850	*Kabuli*	Ca_Kabuli_denovo	89829	(G/A)
16851	CakSNP16851	*Kabuli*	Ca_Kabuli_denovo	89830	(G/A)
16852	CakSNP16852	*Kabuli*	Ca_Kabuli_denovo	90103	(G/A)
16853	CakSNP16853	*Kabuli*	Ca_Kabuli_denovo	90105	(G/A)
16854	CakSNP16854	*Kabuli*	Ca_Kabuli_denovo	90106	(A/G)
16855	CakSNP16855	*Kabuli*	Ca_Kabuli_denovo	90209	(A/C)
16856	CakSNP16856	*Kabuli*	Ca_Kabuli_denovo	90210	(G/T)
16857	CakSNP16857	*Kabuli*	Ca_Kabuli_denovo	90211	(A/G)
16858	CakSNP16858	*Kabuli*	Ca_Kabuli_denovo	90488	(A/C)
16859	CakSNP16859	*Kabuli*	Ca_Kabuli_denovo	92231	(G/A)
16860	CakSNP16860	*Kabuli*	Ca_Kabuli_denovo	92233	(G/A)
16861	CakSNP16861	*Kabuli*	Ca_Kabuli_denovo	92989	(T/A)
16862	CakSNP16862	*Kabuli*	Ca_Kabuli_denovo	92990	(G/T)
16863	CakSNP16863	*Kabuli*	Ca_Kabuli_denovo	94682	(G/A)
16864	CakSNP16864	*Kabuli*	Ca_Kabuli_denovo	94731	(G/A)
16865	CakSNP16865	*Kabuli*	Ca_Kabuli_denovo	96656	(C/T)
16866	CakSNP16866	*Kabuli*	Ca_Kabuli_denovo	96658	(A/C)
16867	CakSNP16867	*Kabuli*	Ca_Kabuli_denovo	96660	(G/A)
16868	CakSNP16868	*Kabuli*	Ca_Kabuli_denovo	96989	(A/G)
16869	CakSNP16869	*Kabuli*	Ca_Kabuli_denovo	96991	(A/G)
16870	CakSNP16870	*Kabuli*	Ca_Kabuli_denovo	96993	(T/A)
16871	CakSNP16871	*Kabuli*	Ca_Kabuli_denovo	96994	(G/T)
16872	CakSNP16872	*Kabuli*	Ca_Kabuli_denovo	96995	(C/G)
16873	CakSNP16873	*Kabuli*	Ca_Kabuli_denovo	98504	(A/G)
16874	CakSNP16874	*Kabuli*	Ca_Kabuli_denovo	98519	(A/G)
16875	CakSNP16875	*Kabuli*	Ca_Kabuli_denovo	98520	(A/G)
16876	CakSNP16876	*Kabuli*	Ca_Kabuli_denovo	98532	(G/A)
16877	CakSNP16877	*Kabuli*	Ca_Kabuli_denovo	98534	(C/G)
16878	CakSNP16878	*Kabuli*	Ca_Kabuli_denovo	98536	(C/G)
16879	CakSNP16879	*Kabuli*	Ca_Kabuli_denovo	98537	(A/G)
16880	CakSNP16880	*Kabuli*	Ca_Kabuli_denovo	98550	(A/C)
16881	CakSNP16881	*Kabuli*	Ca_Kabuli_denovo	98564	(A/G)
16882	CakSNP16882	*Kabuli*	Ca_Kabuli_denovo	98567	(A/G)
16883	CakSNP16883	*Kabuli*	Ca_Kabuli_denovo	98874	(T/A)
16884	CakSNP16884	*Kabuli*	Ca_Kabuli_denovo	98885	(T/C)
16885	CakSNP16885	*Kabuli*	Ca_Kabuli_denovo	98887	(T/C)

S.N.	SNP IDs	Cultivars	Chromosomes/scaffolds	Physical positions (bp)	SNPs
16886	CakSNP16886	*Kabuli*	Ca_Kabuli_denovo	98913	(G/T)
16887	CakSNP16887	*Kabuli*	Ca_Kabuli_denovo	99120	(T/C)
16888	CakSNP16888	*Kabuli*	Ca_Kabuli_denovo	99137	(A/G)
16889	CakSNP16889	*Kabuli*	Ca_Kabuli_denovo	99210	(C/G)
16890	CakSNP16890	*Kabuli*	Ca_Kabuli_denovo	99212	(T/G)
16891	CakSNP16891	*Kabuli*	Ca_Kabuli_denovo	99214	(C/T)
16892	CakSNP16892	*Kabuli*	Ca_Kabuli_denovo	99215	(A/C)
16893	CakSNP16893	*Kabuli*	Ca_Kabuli_denovo	99985	(A/G)
16894	CakSNP16894	*Kabuli*	Ca_Kabuli_denovo	99996	(A/G)
16895	CakSNP16895	*Kabuli*	Ca_Kabuli_denovo	99997	(A/G)
16896	CakSNP16896	*Kabuli*	Ca_Kabuli_denovo	100026	(C/G)
16897	CakSNP16897	*Kabuli*	Ca_Kabuli_denovo	100032	(A/G)
16898	CakSNP16898	*Kabuli*	Ca_Kabuli_denovo	100035	(A/T)
16899	CakSNP16899	*Kabuli*	Ca_Kabuli_denovo	100038	(C/G)
16900	CakSNP16900	*Kabuli*	Ca_Kabuli_denovo	100039	(C/G)
16901	CakSNP16901	*Kabuli*	Ca_Kabuli_denovo	100051	(A/G)
16902	CakSNP16902	*Kabuli*	Ca_Kabuli_denovo	100054	(A/G)
16903	CakSNP16903	*Kabuli*	Ca_Kabuli_denovo	100063	(A/G)
16904	CakSNP16904	*Kabuli*	Ca_Kabuli_denovo	101348	(T/G)
16905	CakSNP16905	*Kabuli*	Ca_Kabuli_denovo	101357	(A/G)
16906	CakSNP16906	*Kabuli*	Ca_Kabuli_denovo	101358	(A/C)
16907	CakSNP16907	*Kabuli*	Ca_Kabuli_denovo	101359	(T/C)
16908	CakSNP16908	*Kabuli*	Ca_Kabuli_denovo	101361	(A/C)
16909	CakSNP16909	*Kabuli*	Ca_Kabuli_denovo	101362	(G/C)
16910	CakSNP16910	*Kabuli*	Ca_Kabuli_denovo	101363	(A/G)
16911	CakSNP16911	*Kabuli*	Ca_Kabuli_denovo	101364	(C/A)
16912	CakSNP16912	*Kabuli*	Ca_Kabuli_denovo	101367	(A/C)
16913	CakSNP16913	*Kabuli*	Ca_Kabuli_denovo	101371	(C/T)
16914	CakSNP16914	*Kabuli*	Ca_Kabuli_denovo	101919	(C/A)
16915	CakSNP16915	*Kabuli*	Ca_Kabuli_denovo	101920	(C/T)
16916	CakSNP16916	*Kabuli*	Ca_Kabuli_denovo	101921	(G/C)
16917	CakSNP16917	*Kabuli*	Ca_Kabuli_denovo	102039	(A/G)
16918	CakSNP16918	*Kabuli*	Ca_Kabuli_denovo	102047	(G/A)
16919	CakSNP16919	*Kabuli*	Ca_Kabuli_denovo	102091	(A/C)
16920	CakSNP16920	*Kabuli*	Ca_Kabuli_denovo	102099	(C/T)
16921	CakSNP16921	*Kabuli*	Ca_Kabuli_denovo	102171	(G/A)
16922	CakSNP16922	*Kabuli*	Ca_Kabuli_denovo	102173	(G/A)
16923	CakSNP16923	*Kabuli*	Ca_Kabuli_denovo	105285	(C/G)
16924	CakSNP16924	*Kabuli*	Ca_Kabuli_denovo	105303	(C/G)

S.N.	SNP IDs	Cultivars	Chromosomes/scaffolds	Physical positions (bp)	SNPs
16925	CakSNP16925	*Kabuli*	Ca_Kabuli_denovo	105305	(T/C)
16926	CakSNP16926	*Kabuli*	Ca_Kabuli_denovo	105319	(G/T)
16927	CakSNP16927	*Kabuli*	Ca_Kabuli_denovo	105320	(T/G)
16928	CakSNP16928	*Kabuli*	Ca_Kabuli_denovo	105321	(C/A)
16929	CakSNP16929	*Kabuli*	Ca_Kabuli_denovo	105326	(T/G)
16930	CakSNP16930	*Kabuli*	Ca_Kabuli_denovo	105336	(A/G)
16931	CakSNP16931	*Kabuli*	Ca_Kabuli_denovo	105339	(C/G)
16932	CakSNP16932	*Kabuli*	Ca_Kabuli_denovo	105790	(G/T)
16933	CakSNP16933	*Kabuli*	Ca_Kabuli_denovo	105792	(T/C)
16934	CakSNP16934	*Kabuli*	Ca_Kabuli_denovo	105795	(A/C)
16935	CakSNP16935	*Kabuli*	Ca_Kabuli_denovo	105819	(G/T)
16936	CakSNP16936	*Kabuli*	Ca_Kabuli_denovo	106278	(A/G)
16937	CakSNP16937	*Kabuli*	Ca_Kabuli_denovo	106280	(C/G)
16938	CakSNP16938	*Kabuli*	Ca_Kabuli_denovo	106282	(T/G)
16939	CakSNP16939	*Kabuli*	Ca_Kabuli_denovo	106284	(C/T)
16940	CakSNP16940	*Kabuli*	Ca_Kabuli_denovo	107119	(C/G)
16941	CakSNP16941	*Kabuli*	Ca_Kabuli_denovo	107120	(A/C)
16942	CakSNP16942	*Kabuli*	Ca_Kabuli_denovo	107121	(G/C)
16943	CakSNP16943	*Kabuli*	Ca_Kabuli_denovo	107124	(G/A)
16944	CakSNP16944	*Kabuli*	Ca_Kabuli_denovo	107125	(A/T)
16945	CakSNP16945	*Kabuli*	Ca_Kabuli_denovo	110466	(G/A)
16946	CakSNP16946	*Kabuli*	Ca_Kabuli_denovo	110468	(G/A)
16947	CakSNP16947	*Kabuli*	Ca_Kabuli_denovo	111292	(C/G)
16948	CakSNP16948	*Kabuli*	Ca_Kabuli_denovo	111294	(T/G)
16949	CakSNP16949	*Kabuli*	Ca_Kabuli_denovo	111296	(C/T)
16950	CakSNP16950	*Kabuli*	Ca_Kabuli_denovo	115118	(A/C)
16951	CakSNP16951	*Kabuli*	Ca_Kabuli_denovo	115119	(G/C)
16952	CakSNP16952	*Kabuli*	Ca_Kabuli_denovo	115122	(G/A)
16953	CakSNP16953	*Kabuli*	Ca_Kabuli_denovo	115123	(A/T)
16954	CakSNP16954	*Kabuli*	Ca_Kabuli_denovo	115219	(G/C)
16955	CakSNP16955	*Kabuli*	Ca_Kabuli_denovo	115220	(A/G)
16956	CakSNP16956	*Kabuli*	Ca_Kabuli_denovo	115768	(C/A)
16957	CakSNP16957	*Kabuli*	Ca_Kabuli_denovo	115769	(T/A)
16958	CakSNP16958	*Kabuli*	Ca_Kabuli_denovo	115771	(C/G)
16959	CakSNP16959	*Kabuli*	Ca_Kabuli_denovo	116794	(G/A)
16960	CakSNP16960	*Kabuli*	Ca_Kabuli_denovo	116795	(A/T)
16961	CakSNP16961	*Kabuli*	Ca_Kabuli_denovo	116796	(G/A)
16962	CakSNP16962	*Kabuli*	Ca_Kabuli_denovo	116797	(C/T)
16963	CakSNP16963	*Kabuli*	Ca_Kabuli_denovo	116798	(C/G)

S.N.	SNP IDs	Cultivars	Chromosomes/scaffolds	Physical positions (bp)	SNPs
16964	CakSNP16964	*Kabuli*	Ca_Kabuli_denovo	116954	(A/G)
16965	CakSNP16965	*Kabuli*	Ca_Kabuli_denovo	117155	(A/G)
16966	CakSNP16966	*Kabuli*	Ca_Kabuli_denovo	117163	(T/C)
16967	CakSNP16967	*Kabuli*	Ca_Kabuli_denovo	117344	(T/C)
16968	CakSNP16968	*Kabuli*	Ca_Kabuli_denovo	117345	(T/A)
16969	CakSNP16969	*Kabuli*	Ca_Kabuli_denovo	117346	(G/C)
16970	CakSNP16970	*Kabuli*	Ca_Kabuli_denovo	117347	(G/A)
16971	CakSNP16971	*Kabuli*	Ca_Kabuli_denovo	117802	(A/G)
16972	CakSNP16972	*Kabuli*	Ca_Kabuli_denovo	117804	(A/G)
16973	CakSNP16973	*Kabuli*	Ca_Kabuli_denovo	117806	(T/C)
16974	CakSNP16974	*Kabuli*	Ca_Kabuli_denovo	117807	(T/G)
16975	CakSNP16975	*Kabuli*	Ca_Kabuli_denovo	117901	(G/C)
16976	CakSNP16976	*Kabuli*	Ca_Kabuli_denovo	117996	(T/G)
16977	CakSNP16977	*Kabuli*	Ca_Kabuli_denovo	118000	(G/C)
16978	CakSNP16978	*Kabuli*	Ca_Kabuli_denovo	118002	(A/G)
16979	CakSNP16979	*Kabuli*	Ca_Kabuli_denovo	119682	(C/G)
16980	CakSNP16980	*Kabuli*	Ca_Kabuli_denovo	119765	(A/G)
16981	CakSNP16981	*Kabuli*	Ca_Kabuli_denovo	119767	(A/C)
16982	CakSNP16982	*Kabuli*	Ca_Kabuli_denovo	122783	(T/C)
16983	CakSNP16983	*Kabuli*	Ca_Kabuli_denovo	122839	(T/G)
16984	CakSNP16984	*Kabuli*	Ca_Kabuli_denovo	124009	(G/T)
16985	CakSNP16985	*Kabuli*	Ca_Kabuli_denovo	124031	(A/G)
16986	CakSNP16986	*Kabuli*	Ca_Kabuli_denovo	124227	(G/T)
16987	CakSNP16987	*Kabuli*	Ca_Kabuli_denovo	124274	(G/A)
16988	CakSNP16988	*Kabuli*	Ca_Kabuli_denovo	124738	(T/A)
16989	CakSNP16989	*Kabuli*	Ca_Kabuli_denovo	124751	(T/A)
16990	CakSNP16990	*Kabuli*	Ca_Kabuli_denovo	124772	(C/G)
16991	CakSNP16991	*Kabuli*	Ca_Kabuli_denovo	124780	(A/G)
16992	CakSNP16992	*Kabuli*	Ca_Kabuli_denovo	125616	(A/G)
16993	CakSNP16993	*Kabuli*	Ca_Kabuli_denovo	125617	(T/G)
16994	CakSNP16994	*Kabuli*	Ca_Kabuli_denovo	125618	(T/C)
16995	CakSNP16995	*Kabuli*	Ca_Kabuli_denovo	127024	(A/G)
16996	CakSNP16996	*Kabuli*	Ca_Kabuli_denovo	127401	(T/G)
16997	CakSNP16997	*Kabuli*	Ca_Kabuli_denovo	127409	(G/T)
16998	CakSNP16998	*Kabuli*	Ca_Kabuli_denovo	127410	(G/T)
16999	CakSNP16999	*Kabuli*	Ca_Kabuli_denovo	127411	(G/C)
17000	CakSNP17000	*Kabuli*	Ca_Kabuli_denovo	127493	(A/G)
17001	CakSNP17001	*Kabuli*	Ca_Kabuli_denovo	127494	(A/G)
17002	CakSNP17002	*Kabuli*	Ca_Kabuli_denovo	129540	(A/C)

S.N.	SNP IDs	Cultivars	Chromosomes/scaffolds	Physical positions (bp)	SNPs
17003	CakSNP17003	*Kabuli*	Ca_Kabuli_denovo	129899	(T/G)
17004	CakSNP17004	*Kabuli*	Ca_Kabuli_denovo	129902	(A/C)
17005	CakSNP17005	*Kabuli*	Ca_Kabuli_denovo	129903	(G/A)
17006	CakSNP17006	*Kabuli*	Ca_Kabuli_denovo	129904	(C/G)
17007	CakSNP17007	*Kabuli*	Ca_Kabuli_denovo	129905	(C/A)
17008	CakSNP17008	*Kabuli*	Ca_Kabuli_denovo	129906	(A/G)
17009	CakSNP17009	*Kabuli*	Ca_Kabuli_denovo	129908	(G/A)
17010	CakSNP17010	*Kabuli*	Ca_Kabuli_denovo	130839	(A/C)
17011	CakSNP17011	*Kabuli*	Ca_Kabuli_denovo	130840	(T/G)
17012	CakSNP17012	*Kabuli*	Ca_Kabuli_denovo	130841	(G/A)
17013	CakSNP17013	*Kabuli*	Ca_Kabuli_denovo	130842	(C/T)
17014	CakSNP17014	*Kabuli*	Ca_Kabuli_denovo	133633	(A/G)
17015	CakSNP17015	*Kabuli*	Ca_Kabuli_denovo	133634	(A/G)
17016	CakSNP17016	*Kabuli*	Ca_Kabuli_denovo	136132	(G/A)
17017	CakSNP17017	*Kabuli*	Ca_Kabuli_denovo	136134	(G/A)
17018	CakSNP17018	*Kabuli*	Ca_Kabuli_denovo	138972	(A/G)
17019	CakSNP17019	*Kabuli*	Ca_Kabuli_denovo	139763	(T/G)
17020	CakSNP17020	*Kabuli*	Ca_Kabuli_denovo	139764	(G/C)
17021	CakSNP17021	*Kabuli*	Ca_Kabuli_denovo	140441	(G/T)
17022	CakSNP17022	*Kabuli*	Ca_Kabuli_denovo	140457	(T/G)
17023	CakSNP17023	*Kabuli*	Ca_Kabuli_denovo	140481	(A/G)
17024	CakSNP17024	*Kabuli*	Ca_Kabuli_denovo	143491	(C/G)
17025	CakSNP17025	*Kabuli*	Ca_Kabuli_denovo	143493	(T/G)
17026	CakSNP17026	*Kabuli*	Ca_Kabuli_denovo	143495	(C/T)
17027	CakSNP17027	*Kabuli*	Ca_Kabuli_denovo	143496	(A/C)
17028	CakSNP17028	*Kabuli*	Ca_Kabuli_denovo	143497	(G/A)
17029	CakSNP17029	*Kabuli*	Ca_Kabuli_denovo	146274	(G/A)
17030	CakSNP17030	*Kabuli*	Ca_Kabuli_denovo	146364	(A/G)
17031	CakSNP17031	*Kabuli*	Ca_Kabuli_denovo	146747	(A/G)
17032	CakSNP17032	*Kabuli*	Ca_Kabuli_denovo	146749	(G/A)
17033	CakSNP17033	*Kabuli*	Ca_Kabuli_denovo	146751	(T/A)
17034	CakSNP17034	*Kabuli*	Ca_Kabuli_denovo	146753	(C/G)
17035	CakSNP17035	*Kabuli*	Ca_Kabuli_denovo	146755	(G/C)
17036	CakSNP17036	*Kabuli*	Ca_Kabuli_denovo	147027	(A/G)
17037	CakSNP17037	*Kabuli*	Ca_Kabuli_denovo	147029	(A/G)
17038	CakSNP17038	*Kabuli*	Ca_Kabuli_denovo	147031	(A/T)
17039	CakSNP17039	*Kabuli*	Ca_Kabuli_denovo	147032	(G/T)
17040	CakSNP17040	*Kabuli*	Ca_Kabuli_denovo	147033	(C/G)
17041	CakSNP17041	*Kabuli*	Ca_Kabuli_denovo	149439	(A/C)

S.N.	SNP IDs	Cultivars	Chromosomes/scaffolds	Physical positions (bp)	SNPs
17042	CakSNP17042	*Kabuli*	Ca_Kabuli_denovo	149440	(G/T)
17043	CakSNP17043	*Kabuli*	Ca_Kabuli_denovo	149628	(C/G)
17044	CakSNP17044	*Kabuli*	Ca_Kabuli_denovo	149629	(C/A)
17045	CakSNP17045	*Kabuli*	Ca_Kabuli_denovo	149632	(C/G)
17046	CakSNP17046	*Kabuli*	Ca_Kabuli_denovo	149809	(A/G)
17047	CakSNP17047	*Kabuli*	Ca_Kabuli_denovo	149811	(A/G)
17048	CakSNP17048	*Kabuli*	Ca_Kabuli_denovo	149813	(T/A)
17049	CakSNP17049	*Kabuli*	Ca_Kabuli_denovo	150092	(A/G)
17050	CakSNP17050	*Kabuli*	Ca_Kabuli_denovo	150094	(G/A)
17051	CakSNP17051	*Kabuli*	Ca_Kabuli_denovo	150096	(T/A)
17052	CakSNP17052	*Kabuli*	Ca_Kabuli_denovo	150097	(G/T)
17053	CakSNP17053	*Kabuli*	Ca_Kabuli_denovo	150098	(C/G)
17054	CakSNP17054	*Kabuli*	Ca_Kabuli_denovo	150669	(A/G)
17055	CakSNP17055	*Kabuli*	Ca_Kabuli_denovo	150688	(T/G)
17056	CakSNP17056	*Kabuli*	Ca_Kabuli_denovo	150703	(A/G)
17057	CakSNP17057	*Kabuli*	Ca_Kabuli_denovo	150706	(T/A)
17058	CakSNP17058	*Kabuli*	Ca_Kabuli_denovo	150720	(A/G)
17059	CakSNP17059	*Kabuli*	Ca_Kabuli_denovo	150730	(A/G)
17060	CakSNP17060	*Kabuli*	Ca_Kabuli_denovo	150734	(G/A)
17061	CakSNP17061	*Kabuli*	Ca_Kabuli_denovo	153852	(A/G)
17062	CakSNP17062	*Kabuli*	Ca_Kabuli_denovo	154095	(C/G)
17063	CakSNP17063	*Kabuli*	Ca_Kabuli_denovo	154096	(G/A)
17064	CakSNP17064	*Kabuli*	Ca_Kabuli_denovo	154097	(T/A)
17065	CakSNP17065	*Kabuli*	Ca_Kabuli_denovo	154098	(C/T)
17066	CakSNP17066	*Kabuli*	Ca_Kabuli_denovo	154640	(G/A)
17067	CakSNP17067	*Kabuli*	Ca_Kabuli_denovo	155679	(A/G)
17068	CakSNP17068	*Kabuli*	Ca_Kabuli_denovo	155681	(A/G)
17069	CakSNP17069	*Kabuli*	Ca_Kabuli_denovo	155683	(A/T)
17070	CakSNP17070	*Kabuli*	Ca_Kabuli_denovo	156229	(A/G)
17071	CakSNP17071	*Kabuli*	Ca_Kabuli_denovo	156322	(T/G)
17072	CakSNP17072	*Kabuli*	Ca_Kabuli_denovo	156323	(G/A)
17073	CakSNP17073	*Kabuli*	Ca_Kabuli_denovo	156324	(C/G)
17074	CakSNP17074	*Kabuli*	Ca_Kabuli_denovo	156325	(A/C)
17075	CakSNP17075	*Kabuli*	Ca_Kabuli_denovo	156492	(A/G)
17076	CakSNP17076	*Kabuli*	Ca_Kabuli_denovo	156493	(G/A)
17077	CakSNP17077	*Kabuli*	Ca_Kabuli_denovo	156494	(C/G)
17078	CakSNP17078	*Kabuli*	Ca_Kabuli_denovo	157819	(C/G)
17079	CakSNP17079	*Kabuli*	Ca_Kabuli_denovo	157821	(G/A)
17080	CakSNP17080	*Kabuli*	Ca_Kabuli_denovo	157822	(A/G)

S.N.	SNP IDs	Cultivars	Chromosomes/scaffolds	Physical positions (bp)	SNPs
17081	CakSNP17081	*Kabuli*	Ca_Kabuli_denovo	157997	(G/C)
17082	CakSNP17082	*Kabuli*	Ca_Kabuli_denovo	158850	(C/A)
17083	CakSNP17083	*Kabuli*	Ca_Kabuli_denovo	159015	(G/A)
17084	CakSNP17084	*Kabuli*	Ca_Kabuli_denovo	159016	(G/C)
17085	CakSNP17085	*Kabuli*	Ca_Kabuli_denovo	159017	(A/C)
17086	CakSNP17086	*Kabuli*	Ca_Kabuli_denovo	159018	(A/G)
17087	CakSNP17087	*Kabuli*	Ca_Kabuli_denovo	159019	(T/A)
17088	CakSNP17088	*Kabuli*	Ca_Kabuli_denovo	159020	(G/T)
17089	CakSNP17089	*Kabuli*	Ca_Kabuli_denovo	164804	(T/G)
17090	CakSNP17090	*Kabuli*	Ca_Kabuli_denovo	164847	(T/C)
17091	CakSNP17091	*Kabuli*	Ca_Kabuli_denovo	164877	(A/G)
17092	CakSNP17092	*Kabuli*	Ca_Kabuli_denovo	164886	(G/C)
17093	CakSNP17093	*Kabuli*	Ca_Kabuli_denovo	165283	(C/G)
17094	CakSNP17094	*Kabuli*	Ca_Kabuli_denovo	165296	(C/A)
17095	CakSNP17095	*Kabuli*	Ca_Kabuli_denovo	165324	(A/G)
17096	CakSNP17096	*Kabuli*	Ca_Kabuli_denovo	165355	(T/G)
17097	CakSNP17097	*Kabuli*	Ca_Kabuli_denovo	166177	(A/G)
17098	CakSNP17098	*Kabuli*	Ca_Kabuli_denovo	166179	(G/A)
17099	CakSNP17099	*Kabuli*	Ca_Kabuli_denovo	166181	(T/A)
17100	CakSNP17100	*Kabuli*	Ca_Kabuli_denovo	166736	(C/G)
17101	CakSNP17101	*Kabuli*	Ca_Kabuli_denovo	166737	(A/G)
17102	CakSNP17102	*Kabuli*	Ca_Kabuli_denovo	166738	(G/A)
17103	CakSNP17103	*Kabuli*	Ca_Kabuli_denovo	166758	(G/C)
17104	CakSNP17104	*Kabuli*	Ca_Kabuli_denovo	166767	(A/G)
17105	CakSNP17105	*Kabuli*	Ca_Kabuli_denovo	166786	(A/G)
17106	CakSNP17106	*Kabuli*	Ca_Kabuli_denovo	166809	(A/G)
17107	CakSNP17107	*Kabuli*	Ca_Kabuli_denovo	166828	(A/C)
17108	CakSNP17108	*Kabuli*	Ca_Kabuli_denovo	166836	(A/C)
17109	CakSNP17109	*Kabuli*	Ca_Kabuli_denovo	166843	(G/T)
17110	CakSNP17110	*Kabuli*	Ca_Kabuli_denovo	167563	(G/C)
17111	CakSNP17111	*Kabuli*	Ca_Kabuli_denovo	167564	(C/A)
17112	CakSNP17112	*Kabuli*	Ca_Kabuli_denovo	167565	(A/G)
17113	CakSNP17113	*Kabuli*	Ca_Kabuli_denovo	167567	(A/G)
17114	CakSNP17114	*Kabuli*	Ca_Kabuli_denovo	167569	(T/A)
17115	CakSNP17115	*Kabuli*	Ca_Kabuli_denovo	169044	(A/G)
17116	CakSNP17116	*Kabuli*	Ca_Kabuli_denovo	169046	(A/G)
17117	CakSNP17117	*Kabuli*	Ca_Kabuli_denovo	169047	(G/A)
17118	CakSNP17118	*Kabuli*	Ca_Kabuli_denovo	171930	(G/A)
17119	CakSNP17119	*Kabuli*	Ca_Kabuli_denovo	171932	(G/A)

S.N.	SNP IDs	Cultivars	Chromosomes/scaffolds	Physical positions (bp)	SNPs
17120	CakSNP17120	*Kabuli*	Ca_Kabuli_denovo	171933	(A/G)
17121	CakSNP17121	*Kabuli*	Ca_Kabuli_denovo	171983	(T/A)
17122	CakSNP17122	*Kabuli*	Ca_Kabuli_denovo	171999	(G/T)
17123	CakSNP17123	*Kabuli*	Ca_Kabuli_denovo	172017	(A/G)
17124	CakSNP17124	*Kabuli*	Ca_Kabuli_denovo	172035	(T/A)
17125	CakSNP17125	*Kabuli*	Ca_Kabuli_denovo	172044	(A/G)
17126	CakSNP17126	*Kabuli*	Ca_Kabuli_denovo	173451	(A/G)
17127	CakSNP17127	*Kabuli*	Ca_Kabuli_denovo	173462	(C/G)
17128	CakSNP17128	*Kabuli*	Ca_Kabuli_denovo	173472	(C/G)
17129	CakSNP17129	*Kabuli*	Ca_Kabuli_denovo	174632	(C/A)
17130	CakSNP17130	*Kabuli*	Ca_Kabuli_denovo	174633	(A/G)
17131	CakSNP17131	*Kabuli*	Ca_Kabuli_denovo	174635	(A/G)
17132	CakSNP17132	*Kabuli*	Ca_Kabuli_denovo	175138	(C/G)
17133	CakSNP17133	*Kabuli*	Ca_Kabuli_denovo	175160	(A/G)
17134	CakSNP17134	*Kabuli*	Ca_Kabuli_denovo	175170	(T/G)
17135	CakSNP17135	*Kabuli*	Ca_Kabuli_denovo	176799	(T/G)
17136	CakSNP17136	*Kabuli*	Ca_Kabuli_denovo	176814	(T/A)
17137	CakSNP17137	*Kabuli*	Ca_Kabuli_denovo	176822	(T/G)
17138	CakSNP17138	*Kabuli*	Ca_Kabuli_denovo	176824	(C/G)
17139	CakSNP17139	*Kabuli*	Ca_Kabuli_denovo	176831	(T/G)
17140	CakSNP17140	*Kabuli*	Ca_Kabuli_denovo	176835	(T/G)
17141	CakSNP17141	*Kabuli*	Ca_Kabuli_denovo	176837	(A/G)
17142	CakSNP17142	*Kabuli*	Ca_Kabuli_denovo	176839	(T/G)
17143	CakSNP17143	*Kabuli*	Ca_Kabuli_denovo	176874	(T/A)
17144	CakSNP17144	*Kabuli*	Ca_Kabuli_denovo	178198	(T/G)
17145	CakSNP17145	*Kabuli*	Ca_Kabuli_denovo	178200	(T/G)
17146	CakSNP17146	*Kabuli*	Ca_Kabuli_denovo	178205	(T/G)
17147	CakSNP17147	*Kabuli*	Ca_Kabuli_denovo	178212	(C/T)
17148	CakSNP17148	*Kabuli*	Ca_Kabuli_denovo	178217	(T/G)
17149	CakSNP17149	*Kabuli*	Ca_Kabuli_denovo	178238	(C/G)
17150	CakSNP17150	*Kabuli*	Ca_Kabuli_denovo	178243	(A/G)
17151	CakSNP17151	*Kabuli*	Ca_Kabuli_denovo	178249	(T/G)
17152	CakSNP17152	*Kabuli*	Ca_Kabuli_denovo	178251	(T/G)
17153	CakSNP17153	*Kabuli*	Ca_Kabuli_denovo	178276	(T/C)
17154	CakSNP17154	*Kabuli*	Ca_Kabuli_denovo	178736	(G/A)
17155	CakSNP17155	*Kabuli*	Ca_Kabuli_denovo	178737	(C/T)
17156	CakSNP17156	*Kabuli*	Ca_Kabuli_denovo	179759	(C/T)
17157	CakSNP17157	*Kabuli*	Ca_Kabuli_denovo	180307	(C/A)
17158	CakSNP17158	*Kabuli*	Ca_Kabuli_denovo	180309	(T/G)

S.N.	SNP IDs	Cultivars	Chromosomes/scaffolds	Physical positions (bp)	SNPs
17159	CakSNP17159	*Kabuli*	Ca_Kabuli_denovo	180571	(G/A)
17160	CakSNP17160	*Kabuli*	Ca_Kabuli_denovo	180573	(G/A)
17161	CakSNP17161	*Kabuli*	Ca_Kabuli_denovo	180686	(A/G)
17162	CakSNP17162	*Kabuli*	Ca_Kabuli_denovo	180689	(G/C)
17163	CakSNP17163	*Kabuli*	Ca_Kabuli_denovo	181636	(T/A)
17164	CakSNP17164	*Kabuli*	Ca_Kabuli_denovo	181639	(C/G)
17165	CakSNP17165	*Kabuli*	Ca_Kabuli_denovo	181670	(A/G)
17166	CakSNP17166	*Kabuli*	Ca_Kabuli_denovo	181675	(G/A)
17167	CakSNP17167	*Kabuli*	Ca_Kabuli_denovo	181679	(G/A)
17168	CakSNP17168	*Kabuli*	Ca_Kabuli_denovo	181698	(G/A)
17169	CakSNP17169	*Kabuli*	Ca_Kabuli_denovo	181720	(C/G)
17170	CakSNP17170	*Kabuli*	Ca_Kabuli_denovo	182277	(A/G)
17171	CakSNP17171	*Kabuli*	Ca_Kabuli_denovo	182279	(A/T)
17172	CakSNP17172	*Kabuli*	Ca_Kabuli_denovo	182280	(G/T)
17173	CakSNP17173	*Kabuli*	Ca_Kabuli_denovo	182281	(C/G)
17174	CakSNP17174	*Kabuli*	Ca_Kabuli_denovo	182628	(G/T)
17175	CakSNP17175	*Kabuli*	Ca_Kabuli_denovo	183987	(G/C)
17176	CakSNP17176	*Kabuli*	Ca_Kabuli_denovo	183990	(A/G)
17177	CakSNP17177	*Kabuli*	Ca_Kabuli_denovo	184118	(G/C)
17178	CakSNP17178	*Kabuli*	Ca_Kabuli_denovo	184181	(T/G)
17179	CakSNP17179	*Kabuli*	Ca_Kabuli_denovo	184211	(T/G)
17180	CakSNP17180	*Kabuli*	Ca_Kabuli_denovo	184231	(C/A)
17181	CakSNP17181	*Kabuli*	Ca_Kabuli_denovo	184333	(C/G)
17182	CakSNP17182	*Kabuli*	Ca_Kabuli_denovo	184359	(G/T)
17183	CakSNP17183	*Kabuli*	Ca_Kabuli_denovo	184367	(T/G)
17184	CakSNP17184	*Kabuli*	Ca_Kabuli_denovo	184370	(C/G)
17185	CakSNP17185	*Kabuli*	Ca_Kabuli_denovo	184372	(C/G)
17186	CakSNP17186	*Kabuli*	Ca_Kabuli_denovo	184427	(C/G)
17187	CakSNP17187	*Kabuli*	Ca_Kabuli_denovo	184440	(C/G)
17188	CakSNP17188	*Kabuli*	Ca_Kabuli_denovo	184460	(T/G)
17189	CakSNP17189	*Kabuli*	Ca_Kabuli_denovo	184487	(C/A)
17190	CakSNP17190	*Kabuli*	Ca_Kabuli_denovo	184509	(C/T)
17191	CakSNP17191	*Kabuli*	Ca_Kabuli_denovo	184521	(T/A)
17192	CakSNP17192	*Kabuli*	Ca_Kabuli_denovo	184541	(C/G)
17193	CakSNP17193	*Kabuli*	Ca_Kabuli_denovo	184553	(T/G)
17194	CakSNP17194	*Kabuli*	Ca_Kabuli_denovo	184583	(T/G)
17195	CakSNP17195	*Kabuli*	Ca_Kabuli_denovo	185564	(C/G)
17196	CakSNP17196	*Kabuli*	Ca_Kabuli_denovo	185568	(T/G)
17197	CakSNP17197	*Kabuli*	Ca_Kabuli_denovo	185573	(G/A)

S.N.	SNP IDs	Cultivars	Chromosomes/scaffolds	Physical positions (bp)	SNPs
17198	CakSNP17198	*Kabuli*	Ca_Kabuli_denovo	185577	(T/G)
17199	CakSNP17199	*Kabuli*	Ca_Kabuli_denovo	185579	(A/G)
17200	CakSNP17200	*Kabuli*	Ca_Kabuli_denovo	185583	(A/G)
17201	CakSNP17201	*Kabuli*	Ca_Kabuli_denovo	185634	(A/G)
17202	CakSNP17202	*Kabuli*	Ca_Kabuli_denovo	185645	(C/G)
17203	CakSNP17203	*Kabuli*	Ca_Kabuli_denovo	185653	(A/G)
17204	CakSNP17204	*Kabuli*	Ca_Kabuli_denovo	185657	(C/G)
17205	CakSNP17205	*Kabuli*	Ca_Kabuli_denovo	185669	(T/G)
17206	CakSNP17206	*Kabuli*	Ca_Kabuli_denovo	185674	(C/G)
17207	CakSNP17207	*Kabuli*	Ca_Kabuli_denovo	185679	(T/A)
17208	CakSNP17208	*Kabuli*	Ca_Kabuli_denovo	185682	(T/G)
17209	CakSNP17209	*Kabuli*	Ca_Kabuli_denovo	187188	(A/G)
17210	CakSNP17210	*Kabuli*	Ca_Kabuli_denovo	188302	(G/A)
17211	CakSNP17211	*Kabuli*	Ca_Kabuli_denovo	188305	(C/A)
17212	CakSNP17212	*Kabuli*	Ca_Kabuli_denovo	188306	(A/G)
17213	CakSNP17213	*Kabuli*	Ca_Kabuli_denovo	188308	(C/A)
17214	CakSNP17214	*Kabuli*	Ca_Kabuli_denovo	188310	(G/T)
17215	CakSNP17215	*Kabuli*	Ca_Kabuli_denovo	188312	(A/C)
17216	CakSNP17216	*Kabuli*	Ca_Kabuli_denovo	188313	(C/A)
17217	CakSNP17217	*Kabuli*	Ca_Kabuli_denovo	190388	(C/G)
17218	CakSNP17218	*Kabuli*	Ca_Kabuli_denovo	190404	(C/G)
17219	CakSNP17219	*Kabuli*	Ca_Kabuli_denovo	194914	(A/G)
17220	CakSNP17220	*Kabuli*	Ca_Kabuli_denovo	194915	(C/G)
17221	CakSNP17221	*Kabuli*	Ca_Kabuli_denovo	195589	(A/G)
17222	CakSNP17222	*Kabuli*	Ca_Kabuli_denovo	195605	(C/G)
17223	CakSNP17223	*Kabuli*	Ca_Kabuli_denovo	195642	(C/G)
17224	CakSNP17224	*Kabuli*	Ca_Kabuli_denovo	195663	(T/A)
17225	CakSNP17225	*Kabuli*	Ca_Kabuli_denovo	195669	(T/G)
17226	CakSNP17226	*Kabuli*	Ca_Kabuli_denovo	196773	(C/G)
17227	CakSNP17227	*Kabuli*	Ca_Kabuli_denovo	196775	(T/G)
17228	CakSNP17228	*Kabuli*	Ca_Kabuli_denovo	196777	(C/T)
17229	CakSNP17229	*Kabuli*	Ca_Kabuli_denovo	197792	(A/G)
17230	CakSNP17230	*Kabuli*	Ca_Kabuli_denovo	197793	(G/C)
17231	CakSNP17231	*Kabuli*	Ca_Kabuli_denovo	197795	(A/G)
17232	CakSNP17232	*Kabuli*	Ca_Kabuli_denovo	197796	(G/A)
17233	CakSNP17233	*Kabuli*	Ca_Kabuli_denovo	197799	(C/A)
17234	CakSNP17234	*Kabuli*	Ca_Kabuli_denovo	197800	(C/T)
17235	CakSNP17235	*Kabuli*	Ca_Kabuli_denovo	197802	(A/C)
17236	CakSNP17236	*Kabuli*	Ca_Kabuli_denovo	197991	(C/G)

S.N.	SNP IDs	Cultivars	Chromosomes/scaffolds	Physical positions (bp)	SNPs
17237	CakSNP17237	*Kabuli*	Ca_Kabuli_denovo	197992	(G/A)
17238	CakSNP17238	*Kabuli*	Ca_Kabuli_denovo	197993	(A/T)
17239	CakSNP17239	*Kabuli*	Ca_Kabuli_denovo	197994	(C/T)
17240	CakSNP17240	*Kabuli*	Ca_Kabuli_denovo	197995	(T/C)
17241	CakSNP17241	*Kabuli*	Ca_Kabuli_denovo	197996	(C/T)
17242	CakSNP17242	*Kabuli*	Ca_Kabuli_denovo	198162	(T/G)
17243	CakSNP17243	*Kabuli*	Ca_Kabuli_denovo	198166	(C/G)
17244	CakSNP17244	*Kabuli*	Ca_Kabuli_denovo	198168	(A/G)
17245	CakSNP17245	*Kabuli*	Ca_Kabuli_denovo	198170	(G/A)
17246	CakSNP17246	*Kabuli*	Ca_Kabuli_denovo	198171	(C/A)
17247	CakSNP17247	*Kabuli*	Ca_Kabuli_denovo	198358	(T/G)
17248	CakSNP17248	*Kabuli*	Ca_Kabuli_denovo	198365	(A/G)
17249	CakSNP17249	*Kabuli*	Ca_Kabuli_denovo	198366	(C/A)
17250	CakSNP17250	*Kabuli*	Ca_Kabuli_denovo	198367	(C/T)
17251	CakSNP17251	*Kabuli*	Ca_Kabuli_denovo	198370	(C/T)
17252	CakSNP17252	*Kabuli*	Ca_Kabuli_denovo	198431	(G/A)
17253	CakSNP17253	*Kabuli*	Ca_Kabuli_denovo	198433	(A/G)
17254	CakSNP17254	*Kabuli*	Ca_Kabuli_denovo	198434	(G/A)
17255	CakSNP17255	*Kabuli*	Ca_Kabuli_denovo	198435	(A/G)
17256	CakSNP17256	*Kabuli*	Ca_Kabuli_denovo	198436	(G/C)
17257	CakSNP17257	*Kabuli*	Ca_Kabuli_denovo	198437	(C/G)
17258	CakSNP17258	*Kabuli*	Ca_Kabuli_denovo	198439	(G/T)
17259	CakSNP17259	*Kabuli*	Ca_Kabuli_denovo	198441	(T/C)
17260	CakSNP17260	*Kabuli*	Ca_Kabuli_denovo	198442	(C/A)
17261	CakSNP17261	*Kabuli*	Ca_Kabuli_denovo	198443	(A/G)
17262	CakSNP17262	*Kabuli*	Ca_Kabuli_denovo	198444	(G/C)
17263	CakSNP17263	*Kabuli*	Ca_Kabuli_denovo	198445	(C/A)
17264	CakSNP17264	*Kabuli*	Ca_Kabuli_denovo	198446	(A/G)
17265	CakSNP17265	*Kabuli*	Ca_Kabuli_denovo	198448	(G/A)
17266	CakSNP17266	*Kabuli*	Ca_Kabuli_denovo	198450	(A/T)
17267	CakSNP17267	*Kabuli*	Ca_Kabuli_denovo	198451	(T/G)
17268	CakSNP17268	*Kabuli*	Ca_Kabuli_denovo	198452	(G/C)
17269	CakSNP17269	*Kabuli*	Ca_Kabuli_denovo	198454	(C/G)
17270	CakSNP17270	*Kabuli*	Ca_Kabuli_denovo	198455	(G/A)
17271	CakSNP17271	*Kabuli*	Ca_Kabuli_denovo	198456	(A/G)
17272	CakSNP17272	*Kabuli*	Ca_Kabuli_denovo	198457	(G/A)
17273	CakSNP17273	*Kabuli*	Ca_Kabuli_denovo	198458	(A/C)
17274	CakSNP17274	*Kabuli*	Ca_Kabuli_denovo	198842	(T/G)
17275	CakSNP17275	*Kabuli*	Ca_Kabuli_denovo	199140	(C/A)

S.N.	SNP IDs	Cultivars	Chromosomes/scaffolds	Physical positions (bp)	SNPs
17276	CakSNP17276	*Kabuli*	Ca_Kabuli_denovo	199240	(T/C)
17277	CakSNP17277	*Kabuli*	Ca_Kabuli_denovo	199747	(C/G)
17278	CakSNP17278	*Kabuli*	Ca_Kabuli_denovo	200458	(A/G)
17279	CakSNP17279	*Kabuli*	Ca_Kabuli_denovo	200459	(T/G)
17280	CakSNP17280	*Kabuli*	Ca_Kabuli_denovo	200728	(T/C)
17281	CakSNP17281	*Kabuli*	Ca_Kabuli_denovo	200743	(C/G)
17282	CakSNP17282	*Kabuli*	Ca_Kabuli_denovo	200890	(T/A)
17283	CakSNP17283	*Kabuli*	Ca_Kabuli_denovo	200910	(G/A)
17284	CakSNP17284	*Kabuli*	Ca_Kabuli_denovo	201180	(G/T)
17285	CakSNP17285	*Kabuli*	Ca_Kabuli_denovo	201201	(A/G)
17286	CakSNP17286	*Kabuli*	Ca_Kabuli_denovo	201287	(A/C)
17287	CakSNP17287	*Kabuli*	Ca_Kabuli_denovo	201365	(A/T)
17288	CakSNP17288	*Kabuli*	Ca_Kabuli_denovo	201444	(T/G)
17289	CakSNP17289	*Kabuli*	Ca_Kabuli_denovo	201979	(G/A)
17290	CakSNP17290	*Kabuli*	Ca_Kabuli_denovo	201981	(G/A)
17291	CakSNP17291	*Kabuli*	Ca_Kabuli_denovo	201982	(A/G)
17292	CakSNP17292	*Kabuli*	Ca_Kabuli_denovo	202419	(T/G)
17293	CakSNP17293	*Kabuli*	Ca_Kabuli_denovo	202456	(T/G)
17294	CakSNP17294	*Kabuli*	Ca_Kabuli_denovo	202459	(C/T)
17295	CakSNP17295	*Kabuli*	Ca_Kabuli_denovo	202460	(G/C)
17296	CakSNP17296	*Kabuli*	Ca_Kabuli_denovo	202461	(T/G)
17297	CakSNP17297	*Kabuli*	Ca_Kabuli_denovo	202523	(G/A)
17298	CakSNP17298	*Kabuli*	Ca_Kabuli_denovo	202525	(A/G)
17299	CakSNP17299	*Kabuli*	Ca_Kabuli_denovo	202526	(G/A)
17300	CakSNP17300	*Kabuli*	Ca_Kabuli_denovo	202527	(A/G)
17301	CakSNP17301	*Kabuli*	Ca_Kabuli_denovo	202529	(C/G)
17302	CakSNP17302	*Kabuli*	Ca_Kabuli_denovo	202531	(G/T)
17303	CakSNP17303	*Kabuli*	Ca_Kabuli_denovo	202533	(T/C)
17304	CakSNP17304	*Kabuli*	Ca_Kabuli_denovo	202534	(C/A)
17305	CakSNP17305	*Kabuli*	Ca_Kabuli_denovo	202535	(A/G)
17306	CakSNP17306	*Kabuli*	Ca_Kabuli_denovo	202536	(G/C)
17307	CakSNP17307	*Kabuli*	Ca_Kabuli_denovo	202537	(C/A)
17308	CakSNP17308	*Kabuli*	Ca_Kabuli_denovo	202538	(A/G)
17309	CakSNP17309	*Kabuli*	Ca_Kabuli_denovo	202540	(G/A)
17310	CakSNP17310	*Kabuli*	Ca_Kabuli_denovo	202542	(A/T)
17311	CakSNP17311	*Kabuli*	Ca_Kabuli_denovo	202544	(G/C)
17312	CakSNP17312	*Kabuli*	Ca_Kabuli_denovo	202546	(C/G)
17313	CakSNP17313	*Kabuli*	Ca_Kabuli_denovo	202547	(G/A)
17314	CakSNP17314	*Kabuli*	Ca_Kabuli_denovo	202548	(A/G)

S.N.	SNP IDs	Cultivars	Chromosomes/scaffolds	Physical positions (bp)	SNPs
17315	CakSNP17315	*Kabuli*	Ca_Kabuli_denovo	202549	(G/A)
17316	CakSNP17316	*Kabuli*	Ca_Kabuli_denovo	202550	(A/T)
17317	CakSNP17317	*Kabuli*	Ca_Kabuli_denovo	202551	(C/A)
17318	CakSNP17318	*Kabuli*	Ca_Kabuli_denovo	202553	(C/G)
17319	CakSNP17319	*Kabuli*	Ca_Kabuli_denovo	204292	(A/G)
17320	CakSNP17320	*Kabuli*	Ca_Kabuli_denovo	204293	(G/C)
17321	CakSNP17321	*Kabuli*	Ca_Kabuli_denovo	207676	(T/A)
17322	CakSNP17322	*Kabuli*	Ca_Kabuli_denovo	207681	(T/G)
17323	CakSNP17323	*Kabuli*	Ca_Kabuli_denovo	207690	(C/G)
17324	CakSNP17324	*Kabuli*	Ca_Kabuli_denovo	207698	(A/G)
17325	CakSNP17325	*Kabuli*	Ca_Kabuli_denovo	207703	(T/A)
17326	CakSNP17326	*Kabuli*	Ca_Kabuli_denovo	207712	(C/G)
17327	CakSNP17327	*Kabuli*	Ca_Kabuli_denovo	207716	(C/G)
17328	CakSNP17328	*Kabuli*	Ca_Kabuli_denovo	207737	(T/G)
17329	CakSNP17329	*Kabuli*	Ca_Kabuli_denovo	208035	(G/A)
17330	CakSNP17330	*Kabuli*	Ca_Kabuli_denovo	208036	(A/T)
17331	CakSNP17331	*Kabuli*	Ca_Kabuli_denovo	208037	(C/T)
17332	CakSNP17332	*Kabuli*	Ca_Kabuli_denovo	208042	(A/T)
17333	CakSNP17333	*Kabuli*	Ca_Kabuli_denovo	209330	(A/G)
17334	CakSNP17334	*Kabuli*	Ca_Kabuli_denovo	209332	(C/T)
17335	CakSNP17335	*Kabuli*	Ca_Kabuli_denovo	209333	(C/G)
17336	CakSNP17336	*Kabuli*	Ca_Kabuli_denovo	209334	(G/C)
17337	CakSNP17337	*Kabuli*	Ca_Kabuli_denovo	210208	(A/T)
17338	CakSNP17338	*Kabuli*	Ca_Kabuli_denovo	210216	(T/C)
17339	CakSNP17339	*Kabuli*	Ca_Kabuli_denovo	210225	(G/A)
17340	CakSNP17340	*Kabuli*	Ca_Kabuli_denovo	210237	(A/T)
17341	CakSNP17341	*Kabuli*	Ca_Kabuli_denovo	215170	(G/A)
17342	CakSNP17342	*Kabuli*	Ca_Kabuli_denovo	215678	(T/G)
17343	CakSNP17343	*Kabuli*	Ca_Kabuli_denovo	215689	(C/A)
17344	CakSNP17344	*Kabuli*	Ca_Kabuli_denovo	215699	(A/G)
17345	CakSNP17345	*Kabuli*	Ca_Kabuli_denovo	215705	(T/G)
17346	CakSNP17346	*Kabuli*	Ca_Kabuli_denovo	215723	(A/G)
17347	CakSNP17347	*Kabuli*	Ca_Kabuli_denovo	215731	(C/G)
17348	CakSNP17348	*Kabuli*	Ca_Kabuli_denovo	215752	(G/A)
17349	CakSNP17349	*Kabuli*	Ca_Kabuli_denovo	215757	(G/T)
17350	CakSNP17350	*Kabuli*	Ca_Kabuli_denovo	217228	(C/A)
17351	CakSNP17351	*Kabuli*	Ca_Kabuli_denovo	217229	(A/T)
17352	CakSNP17352	*Kabuli*	Ca_Kabuli_denovo	217232	(C/A)
17353	CakSNP17353	*Kabuli*	Ca_Kabuli_denovo	220674	(C/G)

S.N.	SNP IDs	Cultivars	Chromosomes/scaffolds	Physical positions (bp)	SNPs
17354	CakSNP17354	*Kabuli*	Ca_Kabuli_denovo	220676	(T/G)
17355	CakSNP17355	*Kabuli*	Ca_Kabuli_denovo	220678	(C/T)
17356	CakSNP17356	*Kabuli*	Ca_Kabuli_denovo	220679	(A/C)
17357	CakSNP17357	*Kabuli*	Ca_Kabuli_denovo	222258	(C/G)
17358	CakSNP17358	*Kabuli*	Ca_Kabuli_denovo	222260	(T/G)
17359	CakSNP17359	*Kabuli*	Ca_Kabuli_denovo	222262	(C/T)
17360	CakSNP17360	*Kabuli*	Ca_Kabuli_denovo	223208	(C/A)
17361	CakSNP17361	*Kabuli*	Ca_Kabuli_denovo	223209	(T/A)
17362	CakSNP17362	*Kabuli*	Ca_Kabuli_denovo	223211	(C/G)
17363	CakSNP17363	*Kabuli*	Ca_Kabuli_denovo	223224	(G/A)
17364	CakSNP17364	*Kabuli*	Ca_Kabuli_denovo	223241	(T/G)
17365	CakSNP17365	*Kabuli*	Ca_Kabuli_denovo	223243	(A/G)
17366	CakSNP17366	*Kabuli*	Ca_Kabuli_denovo	223248	(A/C)
17367	CakSNP17367	*Kabuli*	Ca_Kabuli_denovo	223251	(T/G)
17368	CakSNP17368	*Kabuli*	Ca_Kabuli_denovo	223258	(A/G)
17369	CakSNP17369	*Kabuli*	Ca_Kabuli_denovo	223261	(G/A)
17370	CakSNP17370	*Kabuli*	Ca_Kabuli_denovo	224544	(T/C)
17371	CakSNP17371	*Kabuli*	Ca_Kabuli_denovo	224555	(G/A)
17372	CakSNP17372	*Kabuli*	Ca_Kabuli_denovo	224557	(T/C)
17373	CakSNP17373	*Kabuli*	Ca_Kabuli_denovo	224865	(C/A)
17374	CakSNP17374	*Kabuli*	Ca_Kabuli_denovo	224867	(C/G)
17375	CakSNP17375	*Kabuli*	Ca_Kabuli_denovo	224868	(G/A)
17376	CakSNP17376	*Kabuli*	Ca_Kabuli_denovo	225042	(A/G)
17377	CakSNP17377	*Kabuli*	Ca_Kabuli_denovo	225043	(A/G)
17378	CakSNP17378	*Kabuli*	Ca_Kabuli_denovo	225408	(A/C)
17379	CakSNP17379	*Kabuli*	Ca_Kabuli_denovo	225409	(C/A)
17380	CakSNP17380	*Kabuli*	Ca_Kabuli_denovo	225414	(T/A)
17381	CakSNP17381	*Kabuli*	Ca_Kabuli_denovo	225417	(G/T)
17382	CakSNP17382	*Kabuli*	Ca_Kabuli_denovo	225422	(G/C)
17383	CakSNP17383	*Kabuli*	Ca_Kabuli_denovo	225425	(T/G)
17384	CakSNP17384	*Kabuli*	Ca_Kabuli_denovo	230711	(G/T)
17385	CakSNP17385	*Kabuli*	Ca_Kabuli_denovo	230712	(C/T)
17386	CakSNP17386	*Kabuli*	Ca_Kabuli_denovo	232397	(G/C)
17387	CakSNP17387	*Kabuli*	Ca_Kabuli_denovo	232398	(C/G)
17388	CakSNP17388	*Kabuli*	Ca_Kabuli_denovo	232399	(C/A)
17389	CakSNP17389	*Kabuli*	Ca_Kabuli_denovo	232400	(T/G)
17390	CakSNP17390	*Kabuli*	Ca_Kabuli_denovo	232401	(C/A)
17391	CakSNP17391	*Kabuli*	Ca_Kabuli_denovo	235464	(C/A)
17392	CakSNP17392	*Kabuli*	Ca_Kabuli_denovo	238447	(A/G)

S.N.	SNP IDs	Cultivars	Chromosomes/scaffolds	Physical positions (bp)	SNPs
17393	CakSNP17393	*Kabuli*	Ca_Kabuli_denovo	238449	(A/G)
17394	CakSNP17394	*Kabuli*	Ca_Kabuli_denovo	238451	(T/A)
17395	CakSNP17395	*Kabuli*	Ca_Kabuli_denovo	238452	(G/T)
17396	CakSNP17396	*Kabuli*	Ca_Kabuli_denovo	239728	(G/T)
17397	CakSNP17397	*Kabuli*	Ca_Kabuli_denovo	240943	(C/T)
17398	CakSNP17398	*Kabuli*	Ca_Kabuli_denovo	248697	(A/C)
17399	CakSNP17399	*Kabuli*	Ca_Kabuli_denovo	248719	(T/C)
17400	CakSNP17400	*Kabuli*	Ca_Kabuli_denovo	248775	(T/G)
17401	CakSNP17401	*Kabuli*	Ca_Kabuli_denovo	256228	(A/G)
17402	CakSNP17402	*Kabuli*	Ca_Kabuli_denovo	256262	(T/G)
17403	CakSNP17403	*Kabuli*	Ca_Kabuli_denovo	256272	(G/A)
17404	CakSNP17404	*Kabuli*	Ca_Kabuli_denovo	264802	(G/C)
17405	CakSNP17405	*Kabuli*	Ca_Kabuli_denovo	264812	(T/G)
17406	CakSNP17406	*Kabuli*	Ca_Kabuli_denovo	264815	(C/G)
17407	CakSNP17407	*Kabuli*	Ca_Kabuli_denovo	264817	(G/C)
17408	CakSNP17408	*Kabuli*	Ca_Kabuli_denovo	264819	(A/G)
17409	CakSNP17409	*Kabuli*	Ca_Kabuli_denovo	265972	(A/G)
17410	CakSNP17410	*Kabuli*	Ca_Kabuli_denovo	268126	(G/T)
17411	CakSNP17411	*Kabuli*	Ca_Kabuli_denovo	268171	(T/C)
17412	CakSNP17412	*Kabuli*	Ca_Kabuli_denovo	273531	(A/G)
17413	CakSNP17413	*Kabuli*	Ca_Kabuli_denovo	273541	(C/G)
17414	CakSNP17414	*Kabuli*	Ca_Kabuli_denovo	273543	(G/C)
17415	CakSNP17415	*Kabuli*	Ca_Kabuli_denovo	275658	(T/G)
17416	CakSNP17416	*Kabuli*	Ca_Kabuli_denovo	275671	(A/G)
17417	CakSNP17417	*Kabuli*	Ca_Kabuli_denovo	275698	(T/G)
17418	CakSNP17418	*Kabuli*	Ca_Kabuli_denovo	281765	(C/G)
17419	CakSNP17419	*Kabuli*	Ca_Kabuli_denovo	281766	(G/C)
17420	CakSNP17420	*Kabuli*	Ca_Kabuli_denovo	281767	(G/A)
17421	CakSNP17421	*Kabuli*	Ca_Kabuli_denovo	281768	(T/G)
17422	CakSNP17422	*Kabuli*	Ca_Kabuli_denovo	281769	(T/G)
17423	CakSNP17423	*Kabuli*	Ca_Kabuli_denovo	281770	(A/C)
17424	CakSNP17424	*Kabuli*	Ca_Kabuli_denovo	290341	(T/A)
17425	CakSNP17425	*Kabuli*	Ca_Kabuli_denovo	290342	(T/G)
17426	CakSNP17426	*Kabuli*	Ca_Kabuli_denovo	290344	(C/T)
17427	CakSNP17427	*Kabuli*	Ca_Kabuli_denovo	290345	(G/T)
17428	CakSNP17428	*Kabuli*	Ca_Kabuli_denovo	299355	(A/T)
17429	CakSNP17429	*Kabuli*	Ca_Kabuli_denovo	299356	(C/T)
17430	CakSNP17430	*Kabuli*	Ca_Kabuli_denovo	300954	(A/G)
17431	CakSNP17431	*Kabuli*	Ca_Kabuli_denovo	300963	(A/G)

S.N.	SNP IDs	Cultivars	Chromosomes/scaffolds	Physical positions (bp)	SNPs
17432	CakSNP17432	*Kabuli*	Ca_Kabuli_denovo	300979	(A/G)
17433	CakSNP17433	*Kabuli*	Ca_Kabuli_denovo	300980	(T/G)
17434	CakSNP17434	*Kabuli*	Ca_Kabuli_denovo	300982	(T/G)
17435	CakSNP17435	*Kabuli*	Ca_Kabuli_denovo	300994	(A/G)
17436	CakSNP17436	*Kabuli*	Ca_Kabuli_denovo	301008	(A/G)
17437	CakSNP17437	*Kabuli*	Ca_Kabuli_denovo	301011	(C/G)
17438	CakSNP17438	*Kabuli*	Ca_Kabuli_denovo	301012	(C/G)
17439	CakSNP17439	*Kabuli*	Ca_Kabuli_denovo	301021	(A/G)
17440	CakSNP17440	*Kabuli*	Ca_Kabuli_denovo	301027	(A/G)
17441	CakSNP17441	*Kabuli*	Ca_Kabuli_denovo	301037	(A/G)
17442	CakSNP17442	*Kabuli*	Ca_Kabuli_denovo	305833	(T/G)
17443	CakSNP17443	*Kabuli*	Ca_Kabuli_denovo	309250	(C/G)
17444	CakSNP17444	*Kabuli*	Ca_Kabuli_denovo	309283	(A/G)
17445	CakSNP17445	*Kabuli*	Ca_Kabuli_denovo	309293	(C/G)
17446	CakSNP17446	*Kabuli*	Ca_Kabuli_denovo	312821	(A/G)
17447	CakSNP17447	*Kabuli*	Ca_Kabuli_denovo	313026	(T/G)
17448	CakSNP17448	*Kabuli*	Ca_Kabuli_denovo	313030	(G/C)
17449	CakSNP17449	*Kabuli*	Ca_Kabuli_denovo	313032	(A/G)
17450	CakSNP17450	*Kabuli*	Ca_Kabuli_denovo	313033	(T/G)
17451	CakSNP17451	*Kabuli*	Ca_Kabuli_denovo	313034	(G/A)
17452	CakSNP17452	*Kabuli*	Ca_Kabuli_denovo	315534	(G/C)
17453	CakSNP17453	*Kabuli*	Ca_Kabuli_denovo	315535	(C/G)
17454	CakSNP17454	*Kabuli*	Ca_Kabuli_denovo	316184	(G/C)
17455	CakSNP17455	*Kabuli*	Ca_Kabuli_denovo	319444	(A/G)
17456	CakSNP17456	*Kabuli*	Ca_Kabuli_denovo	324057	(T/G)
17457	CakSNP17457	*Kabuli*	Ca_Kabuli_denovo	325498	(A/G)
17458	CakSNP17458	*Kabuli*	Ca_Kabuli_denovo	325500	(G/A)
17459	CakSNP17459	*Kabuli*	Ca_Kabuli_denovo	327160	(G/A)
17460	CakSNP17460	*Kabuli*	Ca_Kabuli_denovo	327161	(A/T)
17461	CakSNP17461	*Kabuli*	Ca_Kabuli_denovo	327162	(G/A)
17462	CakSNP17462	*Kabuli*	Ca_Kabuli_denovo	328553	(A/G)
17463	CakSNP17463	*Kabuli*	Ca_Kabuli_denovo	328554	(A/G)
17464	CakSNP17464	*Kabuli*	Ca_Kabuli_denovo	328555	(G/T)
17465	CakSNP17465	*Kabuli*	Ca_Kabuli_denovo	328556	(A/C)
17466	CakSNP17466	*Kabuli*	Ca_Kabuli_denovo	328867	(G/A)
17467	CakSNP17467	*Kabuli*	Ca_Kabuli_denovo	328887	(A/G)
17468	CakSNP17468	*Kabuli*	Ca_Kabuli_denovo	328899	(A/C)
17469	CakSNP17469	*Kabuli*	Ca_Kabuli_denovo	328908	(C/A)
17470	CakSNP17470	*Kabuli*	Ca_Kabuli_denovo	329973	(C/A)

S.N.	SNP IDs	Cultivars	Chromosomes/scaffolds	Physical positions (bp)	SNPs
17471	CakSNP17471	*Kabuli*	Ca_Kabuli_denovo	329977	(A/G)
17472	CakSNP17472	*Kabuli*	Ca_Kabuli_denovo	329986	(A/G)
17473	CakSNP17473	*Kabuli*	Ca_Kabuli_denovo	329993	(T/G)
17474	CakSNP17474	*Kabuli*	Ca_Kabuli_denovo	329999	(G/A)
17475	CakSNP17475	*Kabuli*	Ca_Kabuli_denovo	330000	(A/G)
17476	CakSNP17476	*Kabuli*	Ca_Kabuli_denovo	332747	(G/T)
17477	CakSNP17477	*Kabuli*	Ca_Kabuli_denovo	332748	(A/C)
17478	CakSNP17478	*Kabuli*	Ca_Kabuli_denovo	332751	(G/C)
17479	CakSNP17479	*Kabuli*	Ca_Kabuli_denovo	332755	(G/C)
17480	CakSNP17480	*Kabuli*	Ca_Kabuli_denovo	337250	(T/C)
17481	CakSNP17481	*Kabuli*	Ca_Kabuli_denovo	337257	(T/G)
17482	CakSNP17482	*Kabuli*	Ca_Kabuli_denovo	337269	(T/G)
17483	CakSNP17483	*Kabuli*	Ca_Kabuli_denovo	337289	(T/G)
17484	CakSNP17484	*Kabuli*	Ca_Kabuli_denovo	337405	(T/C)
17485	CakSNP17485	*Kabuli*	Ca_Kabuli_denovo	341733	(T/G)
17486	CakSNP17486	*Kabuli*	Ca_Kabuli_denovo	341774	(A/G)
17487	CakSNP17487	*Kabuli*	Ca_Kabuli_denovo	348683	(C/A)
17488	CakSNP17488	*Kabuli*	Ca_Kabuli_denovo	348717	(C/G)
17489	CakSNP17489	*Kabuli*	Ca_Kabuli_denovo	352430	(T/G)
17490	CakSNP17490	*Kabuli*	Ca_Kabuli_denovo	352452	(T/G)
17491	CakSNP17491	*Kabuli*	Ca_Kabuli_denovo	352471	(C/A)
17492	CakSNP17492	*Kabuli*	Ca_Kabuli_denovo	356259	(G/A)
17493	CakSNP17493	*Kabuli*	Ca_Kabuli_denovo	356260	(G/A)
17494	CakSNP17494	*Kabuli*	Ca_Kabuli_denovo	356262	(T/G)
17495	CakSNP17495	*Kabuli*	Ca_Kabuli_denovo	356264	(A/C)
17496	CakSNP17496	*Kabuli*	Ca_Kabuli_denovo	356266	(A/C)
17497	CakSNP17497	*Kabuli*	Ca_Kabuli_denovo	356835	(G/A)
17498	CakSNP17498	*Kabuli*	Ca_Kabuli_denovo	356836	(T/A)
17499	CakSNP17499	*Kabuli*	Ca_Kabuli_denovo	357513	(C/A)
17500	CakSNP17500	*Kabuli*	Ca_Kabuli_denovo	357533	(T/G)
17501	CakSNP17501	*Kabuli*	Ca_Kabuli_denovo	357578	(G/C)
17502	CakSNP17502	*Kabuli*	Ca_Kabuli_denovo	357580	(G/A)
17503	CakSNP17503	*Kabuli*	Ca_Kabuli_denovo	357581	(A/T)
17504	CakSNP17504	*Kabuli*	Ca_Kabuli_denovo	357582	(C/A)
17505	CakSNP17505	*Kabuli*	Ca_Kabuli_denovo	357583	(T/C)
17506	CakSNP17506	*Kabuli*	Ca_Kabuli_denovo	357584	(C/G)
17507	CakSNP17507	*Kabuli*	Ca_Kabuli_denovo	357585	(G/T)
17508	CakSNP17508	*Kabuli*	Ca_Kabuli_denovo	357586	(T/C)
17509	CakSNP17509	*Kabuli*	Ca_Kabuli_denovo	358284	(T/G)

S.N.	SNP IDs	Cultivars	Chromosomes/scaffolds	Physical positions (bp)	SNPs
17510	CakSNP17510	*Kabuli*	Ca_Kabuli_denovo	358878	(C/G)
17511	CakSNP17511	*Kabuli*	Ca_Kabuli_denovo	358879	(A/C)
17512	CakSNP17512	*Kabuli*	Ca_Kabuli_denovo	358882	(C/G)
17513	CakSNP17513	*Kabuli*	Ca_Kabuli_denovo	359691	(C/G)
17514	CakSNP17514	*Kabuli*	Ca_Kabuli_denovo	359693	(G/A)
17515	CakSNP17515	*Kabuli*	Ca_Kabuli_denovo	359695	(A/G)
17516	CakSNP17516	*Kabuli*	Ca_Kabuli_denovo	359696	(G/A)
17517	CakSNP17517	*Kabuli*	Ca_Kabuli_denovo	359697	(A/G)
17518	CakSNP17518	*Kabuli*	Ca_Kabuli_denovo	359698	(G/C)
17519	CakSNP17519	*Kabuli*	Ca_Kabuli_denovo	359699	(C/G)
17520	CakSNP17520	*Kabuli*	Ca_Kabuli_denovo	359701	(G/T)
17521	CakSNP17521	*Kabuli*	Ca_Kabuli_denovo	359703	(T/C)
17522	CakSNP17522	*Kabuli*	Ca_Kabuli_denovo	359704	(C/A)
17523	CakSNP17523	*Kabuli*	Ca_Kabuli_denovo	359705	(A/G)
17524	CakSNP17524	*Kabuli*	Ca_Kabuli_denovo	359706	(G/C)
17525	CakSNP17525	*Kabuli*	Ca_Kabuli_denovo	359707	(C/A)
17526	CakSNP17526	*Kabuli*	Ca_Kabuli_denovo	359708	(A/G)
17527	CakSNP17527	*Kabuli*	Ca_Kabuli_denovo	359710	(G/A)
17528	CakSNP17528	*Kabuli*	Ca_Kabuli_denovo	359712	(A/T)
17529	CakSNP17529	*Kabuli*	Ca_Kabuli_denovo	359713	(T/G)
17530	CakSNP17530	*Kabuli*	Ca_Kabuli_denovo	359714	(G/C)
17531	CakSNP17531	*Kabuli*	Ca_Kabuli_denovo	359716	(C/G)
17532	CakSNP17532	*Kabuli*	Ca_Kabuli_denovo	359717	(G/A)
17533	CakSNP17533	*Kabuli*	Ca_Kabuli_denovo	359718	(A/G)
17534	CakSNP17534	*Kabuli*	Ca_Kabuli_denovo	359719	(G/A)
17535	CakSNP17535	*Kabuli*	Ca_Kabuli_denovo	359720	(A/C)
17536	CakSNP17536	*Kabuli*	Ca_Kabuli_denovo	359722	(C/G)
17537	CakSNP17537	*Kabuli*	Ca_Kabuli_denovo	359724	(G/T)
17538	CakSNP17538	*Kabuli*	Ca_Kabuli_denovo	359725	(A/C)
17539	CakSNP17539	*Kabuli*	Ca_Kabuli_denovo	364778	(T/G)
17540	CakSNP17540	*Kabuli*	Ca_Kabuli_denovo	364839	(A/C)
17541	CakSNP17541	*Kabuli*	Ca_Kabuli_denovo	366496	(A/G)
17542	CakSNP17542	*Kabuli*	Ca_Kabuli_denovo	366498	(C/A)
17543	CakSNP17543	*Kabuli*	Ca_Kabuli_denovo	369650	(A/G)
17544	CakSNP17544	*Kabuli*	Ca_Kabuli_denovo	375270	(G/A)
17545	CakSNP17545	*Kabuli*	Ca_Kabuli_denovo	375282	(T/G)
17546	CakSNP17546	*Kabuli*	Ca_Kabuli_denovo	381255	(T/G)
17547	CakSNP17547	*Kabuli*	Ca_Kabuli_denovo	381258	(T/G)
17548	CakSNP17548	*Kabuli*	Ca_Kabuli_denovo	381275	(T/G)

S.N.	SNP IDs	Cultivars	Chromosomes/scaffolds	Physical positions (bp)	SNPs
17549	CakSNP17549	*Kabuli*	Ca_Kabuli_denovo	382497	(G/A)
17550	CakSNP17550	*Kabuli*	Ca_Kabuli_denovo	386867	(G/C)
17551	CakSNP17551	*Kabuli*	Ca_Kabuli_denovo	386970	(G/C)
17552	CakSNP17552	*Kabuli*	Ca_Kabuli_denovo	389031	(T/G)
17553	CakSNP17553	*Kabuli*	Ca_Kabuli_denovo	389037	(A/G)
17554	CakSNP17554	*Kabuli*	Ca_Kabuli_denovo	389049	(C/G)
17555	CakSNP17555	*Kabuli*	Ca_Kabuli_denovo	389058	(T/G)
17556	CakSNP17556	*Kabuli*	Ca_Kabuli_denovo	389063	(T/G)
17557	CakSNP17557	*Kabuli*	Ca_Kabuli_denovo	389071	(C/G)
17558	CakSNP17558	*Kabuli*	Ca_Kabuli_denovo	389076	(A/G)
17559	CakSNP17559	*Kabuli*	Ca_Kabuli_denovo	389084	(C/G)
17560	CakSNP17560	*Kabuli*	Ca_Kabuli_denovo	392493	(T/G)
17561	CakSNP17561	*Kabuli*	Ca_Kabuli_denovo	396462	(A/T)
17562	CakSNP17562	*Kabuli*	Ca_Kabuli_denovo	396485	(T/G)
17563	CakSNP17563	*Kabuli*	Ca_Kabuli_denovo	396487	(T/G)
17564	CakSNP17564	*Kabuli*	Ca_Kabuli_denovo	396520	(T/G)
17565	CakSNP17565	*Kabuli*	Ca_Kabuli_denovo	396522	(T/G)
17566	CakSNP17566	*Kabuli*	Ca_Kabuli_denovo	396537	(T/A)
17567	CakSNP17567	*Kabuli*	Ca_Kabuli_denovo	401937	(C/G)
17568	CakSNP17568	*Kabuli*	Ca_Kabuli_denovo	401939	(C/G)
17569	CakSNP17569	*Kabuli*	Ca_Kabuli_denovo	401940	(G/A)
17570	CakSNP17570	*Kabuli*	Ca_Kabuli_denovo	401941	(A/G)
17571	CakSNP17571	*Kabuli*	Ca_Kabuli_denovo	405694	(G/C)
17572	CakSNP17572	*Kabuli*	Ca_Kabuli_denovo	408057	(A/G)
17573	CakSNP17573	*Kabuli*	Ca_Kabuli_denovo	408058	(T/G)
17574	CakSNP17574	*Kabuli*	Ca_Kabuli_denovo	408059	(T/C)
17575	CakSNP17575	*Kabuli*	Ca_Kabuli_denovo	408060	(C/G)
17576	CakSNP17576	*Kabuli*	Ca_Kabuli_denovo	409398	(A/G)
17577	CakSNP17577	*Kabuli*	Ca_Kabuli_denovo	409408	(C/G)
17578	CakSNP17578	*Kabuli*	Ca_Kabuli_denovo	409485	(A/G)
17579	CakSNP17579	*Kabuli*	Ca_Kabuli_denovo	409491	(A/G)
17580	CakSNP17580	*Kabuli*	Ca_Kabuli_denovo	409503	(A/G)
17581	CakSNP17581	*Kabuli*	Ca_Kabuli_denovo	409516	(C/A)
17582	CakSNP17582	*Kabuli*	Ca_Kabuli_denovo	409517	(C/G)
17583	CakSNP17583	*Kabuli*	Ca_Kabuli_denovo	409538	(T/G)
17584	CakSNP17584	*Kabuli*	Ca_Kabuli_denovo	412542	(A/G)
17585	CakSNP17585	*Kabuli*	Ca_Kabuli_denovo	412543	(G/C)
17586	CakSNP17586	*Kabuli*	Ca_Kabuli_denovo	412544	(A/C)
17587	CakSNP17587	*Kabuli*	Ca_Kabuli_denovo	412545	(G/A)

S.N.	SNP IDs	Cultivars	Chromosomes/scaffolds	Physical positions (bp)	SNPs
17588	CakSNP17588	*Kabuli*	Ca_Kabuli_denovo	416852	(C/G)
17589	CakSNP17589	*Kabuli*	Ca_Kabuli_denovo	416855	(T/G)
17590	CakSNP17590	*Kabuli*	Ca_Kabuli_denovo	416876	(T/G)
17591	CakSNP17591	*Kabuli*	Ca_Kabuli_denovo	416887	(A/G)
17592	CakSNP17592	*Kabuli*	Ca_Kabuli_denovo	416915	(A/G)
17593	CakSNP17593	*Kabuli*	Ca_Kabuli_denovo	416999	(A/T)
17594	CakSNP17594	*Kabuli*	Ca_Kabuli_denovo	417000	(A/C)
17595	CakSNP17595	*Kabuli*	Ca_Kabuli_denovo	420045	(A/G)
17596	CakSNP17596	*Kabuli*	Ca_Kabuli_denovo	420049	(A/G)
17597	CakSNP17597	*Kabuli*	Ca_Kabuli_denovo	420278	(A/G)
17598	CakSNP17598	*Kabuli*	Ca_Kabuli_denovo	420290	(C/G)
17599	CakSNP17599	*Kabuli*	Ca_Kabuli_denovo	420297	(C/G)
17600	CakSNP17600	*Kabuli*	Ca_Kabuli_denovo	420313	(A/G)
17601	CakSNP17601	*Kabuli*	Ca_Kabuli_denovo	420318	(C/G)
17602	CakSNP17602	*Kabuli*	Ca_Kabuli_denovo	420329	(A/G)
17603	CakSNP17603	*Kabuli*	Ca_Kabuli_denovo	420360	(A/G)
17604	CakSNP17604	*Kabuli*	Ca_Kabuli_denovo	422246	(C/G)
17605	CakSNP17605	*Kabuli*	Ca_Kabuli_denovo	422283	(G/A)
17606	CakSNP17606	*Kabuli*	Ca_Kabuli_denovo	422291	(T/G)
17607	CakSNP17607	*Kabuli*	Ca_Kabuli_denovo	422304	(C/A)
17608	CakSNP17608	*Kabuli*	Ca_Kabuli_denovo	425114	(A/G)
17609	CakSNP17609	*Kabuli*	Ca_Kabuli_denovo	425192	(A/G)
17610	CakSNP17610	*Kabuli*	Ca_Kabuli_denovo	427123	(T/G)
17611	CakSNP17611	*Kabuli*	Ca_Kabuli_denovo	440472	(A/G)
17612	CakSNP17612	*Kabuli*	Ca_Kabuli_denovo	441668	(T/G)
17613	CakSNP17613	*Kabuli*	Ca_Kabuli_denovo	442080	(T/G)
17614	CakSNP17614	*Kabuli*	Ca_Kabuli_denovo	442120	(T/C)
17615	CakSNP17615	*Kabuli*	Ca_Kabuli_denovo	442121	(C/A)
17616	CakSNP17616	*Kabuli*	Ca_Kabuli_denovo	442122	(T/A)
17617	CakSNP17617	*Kabuli*	Ca_Kabuli_denovo	442213	(C/T)
17618	CakSNP17618	*Kabuli*	Ca_Kabuli_denovo	442216	(C/T)
17619	CakSNP17619	*Kabuli*	Ca_Kabuli_denovo	442391	(G/C)
17620	CakSNP17620	*Kabuli*	Ca_Kabuli_denovo	442393	(C/G)
17621	CakSNP17621	*Kabuli*	Ca_Kabuli_denovo	442394	(G/A)
17622	CakSNP17622	*Kabuli*	Ca_Kabuli_denovo	442395	(G/A)
17623	CakSNP17623	*Kabuli*	Ca_Kabuli_denovo	442398	(C/T)
17624	CakSNP17624	*Kabuli*	Ca_Kabuli_denovo	442399	(G/C)
17625	CakSNP17625	*Kabuli*	Ca_Kabuli_denovo	442400	(A/G)
17626	CakSNP17626	*Kabuli*	Ca_Kabuli_denovo	442402	(C/A)

S.N.	SNP IDs	Cultivars	Chromosomes/scaffolds	Physical positions (bp)	SNPs
17627	CakSNP17627	*Kabuli*	Ca_Kabuli_denovo	443991	(T/G)
17628	CakSNP17628	*Kabuli*	Ca_Kabuli_denovo	444015	(T/C)
17629	CakSNP17629	*Kabuli*	Ca_Kabuli_denovo	444023	(T/G)
17630	CakSNP17630	*Kabuli*	Ca_Kabuli_denovo	444031	(A/G)
17631	CakSNP17631	*Kabuli*	Ca_Kabuli_denovo	453291	(C/G)
17632	CakSNP17632	*Kabuli*	Ca_Kabuli_denovo	453300	(A/G)
17633	CakSNP17633	*Kabuli*	Ca_Kabuli_denovo	453318	(A/G)
17634	CakSNP17634	*Kabuli*	Ca_Kabuli_denovo	453861	(G/A)
17635	CakSNP17635	*Kabuli*	Ca_Kabuli_denovo	453862	(G/A)
17636	CakSNP17636	*Kabuli*	Ca_Kabuli_denovo	453900	(G/A)
17637	CakSNP17637	*Kabuli*	Ca_Kabuli_denovo	454466	(C/G)
17638	CakSNP17638	*Kabuli*	Ca_Kabuli_denovo	468460	(G/A)
17639	CakSNP17639	*Kabuli*	Ca_Kabuli_denovo	469180	(A/G)
17640	CakSNP17640	*Kabuli*	Ca_Kabuli_denovo	476805	(G/C)
17641	CakSNP17641	*Kabuli*	Ca_Kabuli_denovo	476807	(C/G)
17642	CakSNP17642	*Kabuli*	Ca_Kabuli_denovo	476808	(A/G)
17643	CakSNP17643	*Kabuli*	Ca_Kabuli_denovo	476809	(G/A)
17644	CakSNP17644	*Kabuli*	Ca_Kabuli_denovo	476811	(C/A)
17645	CakSNP17645	*Kabuli*	Ca_Kabuli_denovo	479038	(C/A)
17646	CakSNP17646	*Kabuli*	Ca_Kabuli_denovo	479043	(A/G)
17647	CakSNP17647	*Kabuli*	Ca_Kabuli_denovo	489994	(T/G)
17648	CakSNP17648	*Kabuli*	Ca_Kabuli_denovo	490709	(G/C)
17649	CakSNP17649	*Kabuli*	Ca_Kabuli_denovo	490829	(C/A)
17650	CakSNP17650	*Kabuli*	Ca_Kabuli_denovo	490851	(A/C)
17651	CakSNP17651	*Kabuli*	Ca_Kabuli_denovo	490863	(T/G)
17652	CakSNP17652	*Kabuli*	Ca_Kabuli_denovo	490944	(C/A)
17653	CakSNP17653	*Kabuli*	Ca_Kabuli_denovo	490945	(A/T)
17654	CakSNP17654	*Kabuli*	Ca_Kabuli_denovo	490946	(G/T)
17655	CakSNP17655	*Kabuli*	Ca_Kabuli_denovo	490947	(A/C)
17656	CakSNP17656	*Kabuli*	Ca_Kabuli_denovo	491027	(G/T)
17657	CakSNP17657	*Kabuli*	Ca_Kabuli_denovo	492735	(A/T)
17658	CakSNP17658	*Kabuli*	Ca_Kabuli_denovo	500805	(A/C)
17659	CakSNP17659	*Kabuli*	Ca_Kabuli_denovo	504532	(T/G)
17660	CakSNP17660	*Kabuli*	Ca_Kabuli_denovo	504535	(C/G)
17661	CakSNP17661	*Kabuli*	Ca_Kabuli_denovo	504543	(T/G)
17662	CakSNP17662	*Kabuli*	Ca_Kabuli_denovo	504570	(A/G)
17663	CakSNP17663	*Kabuli*	Ca_Kabuli_denovo	504576	(T/G)
17664	CakSNP17664	*Kabuli*	Ca_Kabuli_denovo	504608	(G/A)
17665	CakSNP17665	*Kabuli*	Ca_Kabuli_denovo	504615	(C/T)

S.N.	SNP IDs	Cultivars	Chromosomes/scaffolds	Physical positions (bp)	SNPs
17666	CakSNP17666	*Kabuli*	Ca_Kabuli_denovo	514036	(G/C)
17667	CakSNP17667	*Kabuli*	Ca_Kabuli_denovo	514044	(T/C)
17668	CakSNP17668	*Kabuli*	Ca_Kabuli_denovo	520412	(A/G)
17669	CakSNP17669	*Kabuli*	Ca_Kabuli_denovo	520413	(G/A)
17670	CakSNP17670	*Kabuli*	Ca_Kabuli_denovo	520414	(G/T)
17671	CakSNP17671	*Kabuli*	Ca_Kabuli_denovo	520415	(A/C)
17672	CakSNP17672	*Kabuli*	Ca_Kabuli_denovo	520883	(G/A)
17673	CakSNP17673	*Kabuli*	Ca_Kabuli_denovo	523230	(A/G)
17674	CakSNP17674	*Kabuli*	Ca_Kabuli_denovo	523242	(T/A)
17675	CakSNP17675	*Kabuli*	Ca_Kabuli_denovo	525830	(C/A)
17676	CakSNP17676	*Kabuli*	Ca_Kabuli_denovo	525854	(T/A)
17677	CakSNP17677	*Kabuli*	Ca_Kabuli_denovo	525877	(G/C)
17678	CakSNP17678	*Kabuli*	Ca_Kabuli_denovo	533049	(A/T)
17679	CakSNP17679	*Kabuli*	Ca_Kabuli_denovo	533050	(G/T)
17680	CakSNP17680	*Kabuli*	Ca_Kabuli_denovo	533054	(C/T)
17681	CakSNP17681	*Kabuli*	Ca_Kabuli_denovo	534272	(A/G)
17682	CakSNP17682	*Kabuli*	Ca_Kabuli_denovo	554834	(G/C)
17683	CakSNP17683	*Kabuli*	Ca_Kabuli_denovo	554836	(A/G)
17684	CakSNP17684	*Kabuli*	Ca_Kabuli_denovo	554837	(G/A)
17685	CakSNP17685	*Kabuli*	Ca_Kabuli_denovo	554838	(G/A)
17686	CakSNP17686	*Kabuli*	Ca_Kabuli_denovo	554839	(A/G)
17687	CakSNP17687	*Kabuli*	Ca_Kabuli_denovo	565001	(C/G)
17688	CakSNP17688	*Kabuli*	Ca_Kabuli_denovo	565008	(T/G)
17689	CakSNP17689	*Kabuli*	Ca_Kabuli_denovo	565056	(A/G)
17690	CakSNP17690	*Kabuli*	Ca_Kabuli_denovo	565065	(C/G)
17691	CakSNP17691	*Kabuli*	Ca_Kabuli_denovo	566223	(A/G)
17692	CakSNP17692	*Kabuli*	Ca_Kabuli_denovo	566225	(T/G)
17693	CakSNP17693	*Kabuli*	Ca_Kabuli_denovo	566238	(C/A)
17694	CakSNP17694	*Kabuli*	Ca_Kabuli_denovo	566255	(T/C)
17695	CakSNP17695	*Kabuli*	Ca_Kabuli_denovo	575792	(T/G)
17696	CakSNP17696	*Kabuli*	Ca_Kabuli_denovo	575809	(A/G)
17697	CakSNP17697	*Kabuli*	Ca_Kabuli_denovo	736077	(A/C)
17698	CakSNP17698	*Kabuli*	Ca_Kabuli_denovo	861305	(T/C)
17699	CakSNP17699	*Kabuli*	Ca_Kabuli_denovo	861308	(G/C)
17700	CakSNP17700	*Kabuli*	Ca_Kabuli_denovo	930198	(T/A)
17701	CakSNP17701	*Kabuli*	Ca_Kabuli_denovo	930227	(T/G)
17702	CakSNP17702	*Kabuli*	Ca_Kabuli_denovo	930235	(A/G)
17703	CakSNP17703	*Kabuli*	Ca_Kabuli_denovo	930257	(T/G)
17704	CakSNP17704	*Kabuli*	Ca_Kabuli_denovo	930278	(G/C)

S.N.	SNP IDs	Cultivars	Chromosomes/scaffolds	Physical positions (bp)	SNPs
17705	CakSNP17705	*Kabuli*	Ca_Kabuli_denovo	930279	(G/A)
17706	CakSNP17706	*Kabuli*	Ca_Kabuli_denovo	933718	(C/A)
17707	CakSNP17707	*Kabuli*	Ca_Kabuli_denovo	933719	(T/G)
17708	CakSNP17708	*Kabuli*	Ca_Kabuli_denovo	933720	(C/A)
17709	CakSNP17709	*Kabuli*	Ca_Kabuli_denovo	965223	(A/G)
17710	CakSNP17710	*Kabuli*	Ca_Kabuli_denovo	1158419	(G/C)
17711	CakSNP17711	*Kabuli*	Ca_Kabuli_denovo	1158466	(C/G)
17712	CakSNP17712	*Kabuli*	Ca_Kabuli_denovo	1188058	(C/A)
17713	CakSNP17713	*Kabuli*	Ca_Kabuli_denovo	1188059	(A/C)
17714	CakSNP17714	*Kabuli*	Ca_Kabuli_denovo	1188060	(G/C)
17715	CakSNP17715	*Kabuli*	Ca_Kabuli_denovo	1188063	(T/A)
17716	CakSNP17716	*Kabuli*	Ca_Kabuli_denovo	1188064	(C/T)
17717	CakSNP17717	*Kabuli*	Ca_Kabuli_denovo	1286765	(C/A)
17718	CakSNP17718	*Kabuli*	Ca_Kabuli_denovo	1286799	(T/G)
17719	CakSNP17719	*Kabuli*	Ca_Kabuli_denovo	1286839	(C/A)
17720	CakSNP17720	*Kabuli*	Ca_Kabuli_denovo	1286848	(T/G)
17721	CakSNP17721	*Kabuli*	Ca_Kabuli_denovo	1286873	(T/A)
17722	CakSNP17722	*Kabuli*	Ca_Kabuli_denovo	1286885	(A/G)
17723	CakSNP17723	*Kabuli*	Ca_Kabuli_denovo	1286892	(T/G)
17724	CakSNP17724	*Kabuli*	Ca_Kabuli_denovo	1286899	(A/G)
17725	CakSNP17725	*Kabuli*	Ca_Kabuli_denovo	1286901	(C/A)
17726	CakSNP17726	*Kabuli*	Ca_Kabuli_denovo	1541967	(C/G)
17727	CakSNP17727	*Kabuli*	Ca_Kabuli_denovo	1542153	(T/G)
17728	CakSNP17728	*Kabuli*	Ca_Kabuli_denovo	1602962	(T/A)
17729	CakSNP17729	*Kabuli*	Ca_Kabuli_denovo	1603019	(T/C)
17730	CakSNP17730	*Kabuli*	Ca_Kabuli_denovo	1603020	(T/A)
17731	CakSNP17731	*Kabuli*	Ca_Kabuli_denovo	1603038	(A/C)
17732	CakSNP17732	*Kabuli*	Ca_Kabuli_denovo	1603067	(C/G)
17733	CakSNP17733	*Kabuli*	Ca_Kabuli_denovo	1603073	(G/T)
17734	CakSNP17734	*Kabuli*	Ca_Kabuli_denovo	1603120	(G/C)
17735	CakSNP17735	*Kabuli*	Ca_Kabuli_denovo	1604436	(A/G)
17736	CakSNP17736	*Kabuli*	Ca_Kabuli_denovo	1604633	(G/A)
17737	CakSNP17737	*Kabuli*	Ca_Kabuli_denovo	1604645	(G/A)
17738	CakSNP17738	*Kabuli*	Ca_Kabuli_denovo	1604682	(T/G)
17739	CakSNP17739	*Kabuli*	Ca_Kabuli_denovo	1607526	(A/G)
17740	CakSNP17740	*Kabuli*	Ca_Kabuli_denovo	1621430	(C/G)
17741	CakSNP17741	*Kabuli*	Ca_Kabuli_denovo	1621431	(C/G)
17742	CakSNP17742	*Kabuli*	Ca_Kabuli_denovo	1622000	(T/A)
17743	CakSNP17743	*Kabuli*	Ca_Kabuli_denovo	1622001	(T/G)

S.N.	SNP IDs	Cultivars	Chromosomes/scaffolds	Physical positions (bp)	SNPs
17744	CakSNP17744	*Kabuli*	Ca_Kabuli_denovo	1622005	(G/C)
17745	CakSNP17745	*Kabuli*	Ca_Kabuli_denovo	1627804	(A/G)
17746	CakSNP17746	*Kabuli*	Ca_Kabuli_denovo	1627832	(C/G)
17747	CakSNP17747	*Kabuli*	Ca_Kabuli_denovo	1627845	(C/G)
17748	CakSNP17748	*Kabuli*	Ca_Kabuli_denovo	1629193	(C/G)
17749	CakSNP17749	*Kabuli*	Ca_Kabuli_denovo	1629194	(G/T)
17750	CakSNP17750	*Kabuli*	Ca_Kabuli_denovo	1629209	(C/G)
17751	CakSNP17751	*Kabuli*	Ca_Kabuli_denovo	1629225	(T/G)
17752	CakSNP17752	*Kabuli*	Ca_Kabuli_denovo	1645344	(A/C)
17753	CakSNP17753	*Kabuli*	Ca_Kabuli_denovo	1646822	(T/A)
17754	CakSNP17754	*Kabuli*	Ca_Kabuli_denovo	1646824	(C/G)
17755	CakSNP17755	*Kabuli*	Ca_Kabuli_denovo	1646825	(C/A)
17756	CakSNP17756	*Kabuli*	Ca_Kabuli_denovo	1646826	(C/G)
17757	CakSNP17757	*Kabuli*	Ca_Kabuli_denovo	1651702	(C/G)
17758	CakSNP17758	*Kabuli*	Ca_Kabuli_denovo	1651703	(G/A)
17759	CakSNP17759	*Kabuli*	Ca_Kabuli_denovo	1651738	(A/G)
17760	CakSNP17760	*Kabuli*	Ca_Kabuli_denovo	1657001	(C/G)
17761	CakSNP17761	*Kabuli*	Ca_Kabuli_denovo	1657040	(T/G)
17762	CakSNP17762	*Kabuli*	Ca_Kabuli_denovo	1662755	(C/G)
17763	CakSNP17763	*Kabuli*	Ca_Kabuli_denovo	1662758	(A/G)
17764	CakSNP17764	*Kabuli*	Ca_Kabuli_denovo	1662759	(T/G)
17765	CakSNP17765	*Kabuli*	Ca_Kabuli_denovo	1662766	(G/A)
17766	CakSNP17766	*Kabuli*	Ca_Kabuli_denovo	1662768	(A/G)
17767	CakSNP17767	*Kabuli*	Ca_Kabuli_denovo	1662796	(G/A)
17768	CakSNP17768	*Kabuli*	Ca_Kabuli_denovo	1664534	(T/G)
17769	CakSNP17769	*Kabuli*	Ca_Kabuli_denovo	1664562	(T/G)
17770	CakSNP17770	*Kabuli*	Ca_Kabuli_denovo	1664573	(A/G)
17771	CakSNP17771	*Kabuli*	Ca_Kabuli_denovo	1665734	(A/G)
17772	CakSNP17772	*Kabuli*	Ca_Kabuli_denovo	1665742	(G/A)
17773	CakSNP17773	*Kabuli*	Ca_Kabuli_denovo	1665774	(C/G)
17774	CakSNP17774	*Kabuli*	Ca_Kabuli_denovo	1665815	(T/G)
17775	CakSNP17775	*Kabuli*	Ca_Kabuli_denovo	1670929	(C/T)
17776	CakSNP17776	*Kabuli*	Ca_Kabuli_denovo	1670930	(C/G)
17777	CakSNP17777	*Kabuli*	Ca_Kabuli_denovo	1670931	(G/C)
17778	CakSNP17778	*Kabuli*	Ca_Kabuli_denovo	1670932	(A/C)
17779	CakSNP17779	*Kabuli*	Ca_Kabuli_denovo	1674472	(T/G)
17780	CakSNP17780	*Kabuli*	Ca_Kabuli_denovo	1674473	(T/G)
17781	CakSNP17781	*Kabuli*	Ca_Kabuli_denovo	1674498	(G/A)
17782	CakSNP17782	*Kabuli*	Ca_Kabuli_denovo	1674513	(A/G)

S.N.	SNP IDs	Cultivars	Chromosomes/scaffolds	Physical positions (bp)	SNPs
17783	CakSNP17783	*Kabuli*	Ca_Kabuli_denovo	1674786	(T/G)
17784	CakSNP17784	*Kabuli*	Ca_Kabuli_denovo	1674794	(G/T)
17785	CakSNP17785	*Kabuli*	Ca_Kabuli_denovo	1674824	(T/G)
17786	CakSNP17786	*Kabuli*	Ca_Kabuli_denovo	1676619	(A/C)
17787	CakSNP17787	*Kabuli*	Ca_Kabuli_denovo	1676622	(C/A)
17788	CakSNP17788	*Kabuli*	Ca_Kabuli_denovo	1676628	(C/G)
17789	CakSNP17789	*Kabuli*	Ca_Kabuli_denovo	1676641	(T/A)
17790	CakSNP17790	*Kabuli*	Ca_Kabuli_denovo	1676642	(T/G)
17791	CakSNP17791	*Kabuli*	Ca_Kabuli_denovo	1676643	(T/G)
17792	CakSNP17792	*Kabuli*	Ca_Kabuli_denovo	1676647	(A/G)
17793	CakSNP17793	*Kabuli*	Ca_Kabuli_denovo	1676665	(T/G)
17794	CakSNP17794	*Kabuli*	Ca_Kabuli_denovo	1676692	(T/G)
17795	CakSNP17795	*Kabuli*	Ca_Kabuli_denovo	1679945	(C/T)
17796	CakSNP17796	*Kabuli*	Ca_Kabuli_denovo	1684919	(A/T)
17797	CakSNP17797	*Kabuli*	Ca_Kabuli_denovo	1684920	(G/T)
17798	CakSNP17798	*Kabuli*	Ca_Kabuli_denovo	1684923	(T/G)
17799	CakSNP17799	*Kabuli*	Ca_Kabuli_denovo	1685743	(A/C)
17800	CakSNP17800	*Kabuli*	Ca_Kabuli_denovo	1685744	(C/T)
17801	CakSNP17801	*Kabuli*	Ca_Kabuli_denovo	1685745	(T/C)
17802	CakSNP17802	*Kabuli*	Ca_Kabuli_denovo	1687665	(A/G)
17803	CakSNP17803	*Kabuli*	Ca_Kabuli_denovo	1687667	(A/G)
17804	CakSNP17804	*Kabuli*	Ca_Kabuli_denovo	1687669	(A/T)
17805	CakSNP17805	*Kabuli*	Ca_Kabuli_denovo	1688595	(A/T)
17806	CakSNP17806	*Kabuli*	Ca_Kabuli_denovo	1688596	(T/C)
17807	CakSNP17807	*Kabuli*	Ca_Kabuli_denovo	1688598	(C/A)
17808	CakSNP17808	*Kabuli*	Ca_Kabuli_denovo	1688599	(A/T)
17809	CakSNP17809	*Kabuli*	Ca_Kabuli_denovo	1688600	(C/A)
17810	CakSNP17810	*Kabuli*	Ca_Kabuli_denovo	1690593	(G/A)
17811	CakSNP17811	*Kabuli*	Ca_Kabuli_denovo	1690595	(T/G)
17812	CakSNP17812	*Kabuli*	Ca_Kabuli_denovo	1690605	(T/A)
17813	CakSNP17813	*Kabuli*	Ca_Kabuli_denovo	1696698	(T/G)
17814	CakSNP17814	*Kabuli*	Ca_Kabuli_denovo	1696705	(C/G)
17815	CakSNP17815	*Kabuli*	Ca_Kabuli_denovo	1698932	(G/C)
17816	CakSNP17816	*Kabuli*	Ca_Kabuli_denovo	1698934	(A/G)
17817	CakSNP17817	*Kabuli*	Ca_Kabuli_denovo	1698960	(A/G)
17818	CakSNP17818	*Kabuli*	Ca_Kabuli_denovo	1699496	(C/G)
17819	CakSNP17819	*Kabuli*	Ca_Kabuli_denovo	1699498	(C/G)
17820	CakSNP17820	*Kabuli*	Ca_Kabuli_denovo	1699521	(T/G)
17821	CakSNP17821	*Kabuli*	Ca_Kabuli_denovo	1699556	(G/T)

S.N.	SNP IDs	Cultivars	Chromosomes/scaffolds	Physical positions (bp)	SNPs
17822	CakSNP17822	*Kabuli*	Ca_Kabuli_denovo	1699569	(A/T)
17823	CakSNP17823	*Kabuli*	Ca_Kabuli_denovo	1707083	(C/A)
17824	CakSNP17824	*Kabuli*	Ca_Kabuli_denovo	1707085	(C/G)
17825	CakSNP17825	*Kabuli*	Ca_Kabuli_denovo	1707086	(A/T)
17826	CakSNP17826	*Kabuli*	Ca_Kabuli_denovo	1707560	(C/A)
17827	CakSNP17827	*Kabuli*	Ca_Kabuli_denovo	1707561	(A/G)
17828	CakSNP17828	*Kabuli*	Ca_Kabuli_denovo	1707563	(A/G)
17829	CakSNP17829	*Kabuli*	Ca_Kabuli_denovo	1707565	(T/A)
17830	CakSNP17830	*Kabuli*	Ca_Kabuli_denovo	1732322	(A/G)
17831	CakSNP17831	*Kabuli*	Ca_Kabuli_denovo	1732338	(C/T)
17832	CakSNP17832	*Kabuli*	Ca_Kabuli_denovo	1734760	(G/C)
17833	CakSNP17833	*Kabuli*	Ca_Kabuli_denovo	1735490	(C/G)
17834	CakSNP17834	*Kabuli*	Ca_Kabuli_denovo	1735502	(C/T)
17835	CakSNP17835	*Kabuli*	Ca_Kabuli_denovo	1735523	(T/C)
17836	CakSNP17836	*Kabuli*	Ca_Kabuli_denovo	1735891	(T/G)
17837	CakSNP17837	*Kabuli*	Ca_Kabuli_denovo	1735929	(T/A)
17838	CakSNP17838	*Kabuli*	Ca_Kabuli_denovo	1735956	(G/C)
17839	CakSNP17839	*Kabuli*	Ca_Kabuli_denovo	1735988	(A/G)
17840	CakSNP17840	*Kabuli*	Ca_Kabuli_denovo	1737810	(A/G)
17841	CakSNP17841	*Kabuli*	Ca_Kabuli_denovo	1737898	(T/G)
17842	CakSNP17842	*Kabuli*	Ca_Kabuli_denovo	1737939	(G/A)
17843	CakSNP17843	*Kabuli*	Ca_Kabuli_denovo	1737964	(C/A)
17844	CakSNP17844	*Kabuli*	Ca_Kabuli_denovo	1739167	(G/C)
17845	CakSNP17845	*Kabuli*	Ca_Kabuli_denovo	1739168	(A/C)
17846	CakSNP17846	*Kabuli*	Ca_Kabuli_denovo	1739955	(G/A)
17847	CakSNP17847	*Kabuli*	Ca_Kabuli_denovo	1740013	(T/G)
17848	CakSNP17848	*Kabuli*	Ca_Kabuli_denovo	1741581	(G/A)
17849	CakSNP17849	*Kabuli*	Ca_Kabuli_denovo	1745745	(C/T)
17850	CakSNP17850	*Kabuli*	Ca_Kabuli_denovo	1745967	(G/T)
17851	CakSNP17851	*Kabuli*	Ca_Kabuli_denovo	1773627	(C/T)
17852	CakSNP17852	*Kabuli*	Ca_Kabuli_denovo	1773675	(T/G)
17853	CakSNP17853	*Kabuli*	Ca_Kabuli_denovo	1778702	(T/A)
17854	CakSNP17854	*Kabuli*	Ca_Kabuli_denovo	1778708	(G/A)
17855	CakSNP17855	*Kabuli*	Ca_Kabuli_denovo	1782502	(C/T)
17856	CakSNP17856	*Kabuli*	Ca_Kabuli_denovo	1782521	(T/C)
17857	CakSNP17857	*Kabuli*	Ca_Kabuli_denovo	1785710	(T/C)
17858	CakSNP17858	*Kabuli*	Ca_Kabuli_denovo	1799281	(G/T)
17859	CakSNP17859	*Kabuli*	Ca_Kabuli_denovo	1835504	(G/A)
17860	CakSNP17860	*Kabuli*	Ca_Kabuli_denovo	1848623	(T/C)

S.N.	SNP IDs	Cultivars	Chromosomes/scaffolds	Physical positions (bp)	SNPs
17861	CakSNP17861	*Kabuli*	Ca_Kabuli_denovo	1865044	(C/G)
17862	CakSNP17862	*Kabuli*	Ca_Kabuli_denovo	1865051	(T/C)
17863	CakSNP17863	*Kabuli*	Ca_Kabuli_denovo	1877655	(A/C)
17864	CakSNP17864	*Kabuli*	Ca_Kabuli_denovo	1885551	(C/T)
17865	CakSNP17865	*Kabuli*	Ca_Kabuli_denovo	1885575	(G/A)
17866	CakSNP17866	*Kabuli*	Ca_Kabuli_denovo	1892464	(C/T)
17867	CakSNP17867	*Kabuli*	Ca_Kabuli_denovo	1892470	(A/T)
17868	CakSNP17868	*Kabuli*	Ca_Kabuli_denovo	1898838	(A/G)
17869	CakSNP17869	*Kabuli*	Ca_Kabuli_denovo	1898842	(G/A)
17870	CakSNP17870	*Kabuli*	Ca_Kabuli_denovo	1907169	(C/T)
17871	CakSNP17871	*Kabuli*	Ca_Kabuli_denovo	1954938	(C/T)
17872	CakSNP17872	*Kabuli*	Ca_Kabuli_denovo	1973101	(A/C)
17873	CakSNP17873	*Kabuli*	Ca_Kabuli_denovo	1981659	(C/G)
17874	CakSNP17874	*Kabuli*	Ca_Kabuli_denovo	1981685	(C/T)
17875	CakSNP17875	*Kabuli*	Ca_Kabuli_denovo	2017269	(C/T)
17876	CakSNP17876	*Kabuli*	Ca_Kabuli_denovo	2017293	(G/A)
17877	CakSNP17877	*Kabuli*	Ca_Kabuli_denovo	2082507	(C/T)
17878	CakSNP17878	*Kabuli*	Ca_Kabuli_denovo	2082510	(T/C)
17879	CakSNP17879	*Kabuli*	Ca_Kabuli_denovo	2083031	(G/C)
17880	CakSNP17880	*Kabuli*	Ca_Kabuli_denovo	2083034	(G/A)
17881	CakSNP17881	*Kabuli*	Ca_Kabuli_denovo	2083043	(T/G)
17882	CakSNP17882	*Kabuli*	Ca_Kabuli_denovo	2086223	(A/C)
17883	CakSNP17883	*Kabuli*	Ca_Kabuli_denovo	2089458	(A/G)
17884	CakSNP17884	*Kabuli*	Ca_Kabuli_denovo	2089483	(G/C)
17885	CakSNP17885	*Kabuli*	Ca_Kabuli_denovo	2103496	(C/G)
17886	CakSNP17886	*Kabuli*	Ca_Kabuli_denovo	2117543	(A/G)
17887	CakSNP17887	*Kabuli*	Ca_Kabuli_denovo	2179176	(A/C)
17888	CakSNP17888	*Kabuli*	Ca_Kabuli_denovo	2196538	(T/A)
17889	CakSNP17889	*Kabuli*	Ca_Kabuli_denovo	2196959	(G/A)
17890	CakSNP17890	*Kabuli*	Ca_Kabuli_denovo	2196969	(G/A)
17891	CakSNP17891	*Kabuli*	Ca_Kabuli_denovo	2204023	(C/T)
17892	CakSNP17892	*Kabuli*	Ca_Kabuli_denovo	2212621	(C/T)
17893	CakSNP17893	*Kabuli*	Ca_Kabuli_denovo	2212895	(C/T)
17894	CakSNP17894	*Kabuli*	Ca_Kabuli_denovo	2238931	(C/T)
17895	CakSNP17895	*Kabuli*	Ca_Kabuli_denovo	2238976	(C/T)
17896	CakSNP17896	*Kabuli*	Ca_Kabuli_denovo	2272968	(G/A)
17897	CakSNP17897	*Kabuli*	Ca_Kabuli_denovo	2289863	(C/A)
17898	CakSNP17898	*Kabuli*	Ca_Kabuli_denovo	2329264	(G/A)
17899	CakSNP17899	*Kabuli*	Ca_Kabuli_denovo	2339668	(G/A)

S.N.	SNP IDs	Cultivars	Chromosomes/scaffolds	Physical positions (bp)	SNPs
17900	CakSNP17900	*Kabuli*	Ca_Kabuli_denovo	2345594	(A/T)
17901	CakSNP17901	*Kabuli*	Ca_Kabuli_denovo	2349919	(G/T)
17902	CakSNP17902	*Kabuli*	Ca_Kabuli_denovo	2349923	(T/G)
17903	CakSNP17903	*Kabuli*	Ca_Kabuli_denovo	2352980	(T/G)
17904	CakSNP17904	*Kabuli*	Ca_Kabuli_denovo	2368635	(A/T)
17905	CakSNP17905	*Kabuli*	Ca_Kabuli_denovo	2377565	(G/T)
17906	CakSNP17906	*Kabuli*	Ca_Kabuli_denovo	2378145	(G/A)
17907	CakSNP17907	*Kabuli*	Ca_Kabuli_denovo	2422055	(G/A)
17908	CakSNP17908	*Kabuli*	Ca_Kabuli_denovo	2424690	(C/T)
17909	CakSNP17909	*Kabuli*	Ca_Kabuli_denovo	2436733	(T/G)
17910	CakSNP17910	*Kabuli*	Ca_Kabuli_denovo	2442765	(G/C)
17911	CakSNP17911	*Kabuli*	Ca_Kabuli_denovo	2472151	(C/G)
17912	CakSNP17912	*Kabuli*	Ca_Kabuli_denovo	2472157	(T/C)
17913	CakSNP17913	*Kabuli*	Ca_Kabuli_denovo	2472160	(A/C)
17914	CakSNP17914	*Kabuli*	Ca_Kabuli_denovo	2481362	(C/G)
17915	CakSNP17915	*Kabuli*	Ca_Kabuli_denovo	2481371	(G/C)
17916	CakSNP17916	*Kabuli*	Ca_Kabuli_denovo	2484130	(G/C)
17917	CakSNP17917	*Kabuli*	Ca_Kabuli_denovo	2493981	(A/C)
17918	CakSNP17918	*Kabuli*	Ca_Kabuli_denovo	2521639	(T/C)
17919	CakSNP17919	*Kabuli*	Ca_Kabuli_denovo	2532212	(C/T)
17920	CakSNP17920	*Kabuli*	Ca_Kabuli_denovo	2534679	(T/C)
17921	CakSNP17921	*Kabuli*	Ca_Kabuli_denovo	2543790	(C/G)
17922	CakSNP17922	*Kabuli*	Ca_Kabuli_denovo	2543805	(G/A)
17923	CakSNP17923	*Kabuli*	Ca_Kabuli_denovo	2557532	(T/G)
17924	CakSNP17924	*Kabuli*	Ca_Kabuli_denovo	2566186	(C/T)
17925	CakSNP17925	*Kabuli*	Ca_Kabuli_denovo	2586184	(T/C)
17926	CakSNP17926	*Kabuli*	Ca_Kabuli_denovo	2596306	(G/A)
17927	CakSNP17927	*Kabuli*	Ca_Kabuli_denovo	2599278	(C/T)
17928	CakSNP17928	*Kabuli*	Ca_Kabuli_denovo	2599284	(T/C)
17929	CakSNP17929	*Kabuli*	Ca_Kabuli_denovo	2810251	(C/G)
17930	CakSNP17930	*Kabuli*	Ca_Kabuli_denovo	2822788	(C/T)
17931	CakSNP17931	*Kabuli*	Ca_Kabuli_denovo	2822794	(G/A)
17932	CakSNP17932	*Kabuli*	Ca_Kabuli_denovo	2841190	(C/G)
17933	CakSNP17933	*Kabuli*	Ca_Kabuli_denovo	2899276	(A/G)
17934	CakSNP17934	*Kabuli*	Ca_Kabuli_denovo	2905320	(A/C)
17935	CakSNP17935	*Kabuli*	Ca_Kabuli_denovo	2920107	(A/C)
17936	CakSNP17936	*Kabuli*	Ca_Kabuli_denovo	2943170	(A/C)
17937	CakSNP17937	*Kabuli*	Ca_Kabuli_denovo	2950044	(T/C)
17938	CakSNP17938	*Kabuli*	Ca_Kabuli_denovo	2967013	(A/G)

S.N.	SNP IDs	Cultivars	Chromosomes/scaffolds	Physical positions (bp)	SNPs
17939	CakSNP17939	*Kabuli*	Ca_Kabuli_denovo	2986733	(G/C)
17940	CakSNP17940	*Kabuli*	Ca_Kabuli_denovo	2988473	(G/A)
17941	CakSNP17941	*Kabuli*	Ca_Kabuli_denovo	3002912	(G/C)
17942	CakSNP17942	*Kabuli*	Ca_Kabuli_denovo	3004600	(C/G)
17943	CakSNP17943	*Kabuli*	Ca_Kabuli_denovo	3028757	(A/G)
17944	CakSNP17944	*Kabuli*	Ca_Kabuli_denovo	3058052	(G/A)
17945	CakSNP17945	*Kabuli*	Ca_Kabuli_denovo	3067960	(A/G)
17946	CakSNP17946	*Kabuli*	Ca_Kabuli_denovo	3075746	(C/G)
17947	CakSNP17947	*Kabuli*	Ca_Kabuli_denovo	3075749	(A/G)
17948	CakSNP17948	*Kabuli*	Ca_Kabuli_denovo	3082312	(T/A)
17949	CakSNP17949	*Kabuli*	Ca_Kabuli_denovo	3102755	(C/T)
17950	CakSNP17950	*Kabuli*	Ca_Kabuli_denovo	3112730	(C/T)
17951	CakSNP17951	*Kabuli*	Ca_Kabuli_denovo	3119860	(G/A)
17952	CakSNP17952	*Kabuli*	Ca_Kabuli_denovo	3119864	(A/G)
17953	CakSNP17953	*Kabuli*	Ca_Kabuli_denovo	3122405	(A/G)
17954	CakSNP17954	*Kabuli*	Ca_Kabuli_denovo	3158126	(G/T)
17955	CakSNP17955	*Kabuli*	Ca_Kabuli_denovo	3158176	(G/A)
17956	CakSNP17956	*Kabuli*	Ca_Kabuli_denovo	3195629	(G/A)
17957	CakSNP17957	*Kabuli*	Ca_Kabuli_denovo	3234354	(G/A)
17958	CakSNP17958	*Kabuli*	Ca_Kabuli_denovo	3236873	(A/G)
17959	CakSNP17959	*Kabuli*	Ca_Kabuli_denovo	3236875	(T/C)
17960	CakSNP17960	*Kabuli*	Ca_Kabuli_denovo	3236878	(T/G)
17961	CakSNP17961	*Kabuli*	Ca_Kabuli_denovo	3303558	(A/C)
17962	CakSNP17962	*Kabuli*	Ca_Kabuli_denovo	3303631	(G/C)
17963	CakSNP17963	*Kabuli*	Ca_Kabuli_denovo	3315370	(A/G)
17964	CakSNP17964	*Kabuli*	Ca_Kabuli_denovo	3315379	(A/G)
17965	CakSNP17965	*Kabuli*	Ca_Kabuli_denovo	3366344	(C/T)
17966	CakSNP17966	*Kabuli*	Ca_Kabuli_denovo	3366389	(G/T)
17967	CakSNP17967	*Kabuli*	Ca_Kabuli_denovo	3376003	(A/G)
17968	CakSNP17968	*Kabuli*	Ca_Kabuli_denovo	3376309	(T/C)
17969	CakSNP17969	*Kabuli*	Ca_Kabuli_denovo	3381616	(T/C)
17970	CakSNP17970	*Kabuli*	Ca_Kabuli_denovo	3383148	(T/C)
17971	CakSNP17971	*Kabuli*	Ca_Kabuli_denovo	3397038	(T/C)
17972	CakSNP17972	*Kabuli*	Ca_Kabuli_denovo	3401848	(A/C)
17973	CakSNP17973	*Kabuli*	Ca_Kabuli_denovo	3419633	(C/G)
17974	CakSNP17974	*Kabuli*	Ca_Kabuli_denovo	3427074	(A/G)
17975	CakSNP17975	*Kabuli*	Ca_Kabuli_denovo	3438889	(C/T)
17976	CakSNP17976	*Kabuli*	Ca_Kabuli_denovo	3520157	(T/C)
17977	CakSNP17977	*Kabuli*	Ca_Kabuli_denovo	3520187	(A/G)

S.N.	SNP IDs	Cultivars	Chromosomes/scaffolds	Physical positions (bp)	SNPs
17978	CakSNP17978	*Kabuli*	Ca_Kabuli_denovo	3563125	(C/T)
17979	CakSNP17979	*Kabuli*	Ca_Kabuli_denovo	3569168	(A/G)
17980	CakSNP17980	*Kabuli*	Ca_Kabuli_denovo	3569183	(A/G)
17981	CakSNP17981	*Kabuli*	Ca_Kabuli_denovo	3593979	(G/T)
17982	CakSNP17982	*Kabuli*	Ca_Kabuli_denovo	3608269	(C/T)
17983	CakSNP17983	*Kabuli*	Ca_Kabuli_denovo	3608277	(G/T)
17984	CakSNP17984	*Kabuli*	Ca_Kabuli_denovo	3619659	(C/T)
17985	CakSNP17985	*Kabuli*	Ca_Kabuli_denovo	3619740	(A/G)
17986	CakSNP17986	*Kabuli*	Ca_Kabuli_denovo	3621163	(G/A)
17987	CakSNP17987	*Kabuli*	Ca_Kabuli_denovo	3621166	(T/G)
17988	CakSNP17988	*Kabuli*	Ca_Kabuli_denovo	3627246	(T/A)
17989	CakSNP17989	*Kabuli*	Ca_Kabuli_denovo	3640534	(G/A)
17990	CakSNP17990	*Kabuli*	Ca_Kabuli_denovo	3640537	(A/G)
17991	CakSNP17991	*Kabuli*	Ca_Kabuli_denovo	3643962	(C/T)
17992	CakSNP17992	*Kabuli*	Ca_Kabuli_denovo	3649832	(T/G)
17993	CakSNP17993	*Kabuli*	Ca_Kabuli_denovo	3649844	(A/G)
17994	CakSNP17994	*Kabuli*	Ca_Kabuli_denovo	3656017	(A/C)
17995	CakSNP17995	*Kabuli*	Ca_Kabuli_denovo	3733774	(C/T)
17996	CakSNP17996	*Kabuli*	Ca_Kabuli_denovo	3733795	(C/G)
17997	CakSNP17997	*Kabuli*	Ca_Kabuli_denovo	3733959	(G/A)
17998	CakSNP17998	*Kabuli*	Ca_Kabuli_denovo	3865215	(A/G)
17999	CakSNP17999	*Kabuli*	Ca_Kabuli_denovo	3882576	(G/A)
18000	CakSNP18000	*Kabuli*	Ca_Kabuli_denovo	3882602	(G/C)
18001	CakSNP18001	*Kabuli*	Ca_Kabuli_denovo	3891592	(A/G)
18002	CakSNP18002	*Kabuli*	Ca_Kabuli_denovo	3926567	(T/C)
18003	CakSNP18003	*Kabuli*	Ca_Kabuli_denovo	3932481	(T/A)
18004	CakSNP18004	*Kabuli*	Ca_Kabuli_denovo	3962628	(C/A)
18005	CakSNP18005	*Kabuli*	Ca_Kabuli_denovo	3971486	(C/G)
18006	CakSNP18006	*Kabuli*	Ca_Kabuli_denovo	3971511	(T/C)
18007	CakSNP18007	*Kabuli*	Ca_Kabuli_denovo	3985191	(G/A)
18008	CakSNP18008	*Kabuli*	Ca_Kabuli_denovo	3985194	(C/T)
18009	CakSNP18009	*Kabuli*	Ca_Kabuli_denovo	3988996	(G/C)
18010	CakSNP18010	*Kabuli*	Ca_Kabuli_denovo	3989017	(G/A)
18011	CakSNP18011	*Kabuli*	Ca_Kabuli_denovo	4058620	(G/C)
18012	CakSNP18012	*Kabuli*	Ca_Kabuli_denovo	4058680	(A/C)
18013	CakSNP18013	*Kabuli*	Ca_Kabuli_denovo	4069641	(G/C)
18014	CakSNP18014	*Kabuli*	Ca_Kabuli_denovo	4069659	(C/T)
18015	CakSNP18015	*Kabuli*	Ca_Kabuli_denovo	4074763	(A/G)
18016	CakSNP18016	*Kabuli*	Ca_Kabuli_denovo	4074764	(C/G)

S.N.	SNP IDs	Cultivars	Chromosomes/scaffolds	Physical positions (bp)	SNPs
18017	CakSNP18017	*Kabuli*	Ca_Kabuli_denovo	4102951	(A/G)
18018	CakSNP18018	*Kabuli*	Ca_Kabuli_denovo	4131618	(A/C)
18019	CakSNP18019	*Kabuli*	Ca_Kabuli_denovo	4327961	(T/C)
18020	CakSNP18020	*Kabuli*	Ca_Kabuli_denovo	4354794	(C/T)
18021	CakSNP18021	*Kabuli*	Ca_Kabuli_denovo	4354804	(C/T)
18022	CakSNP18022	*Kabuli*	Ca_Kabuli_denovo	4384538	(T/C)
18023	CakSNP18023	*Kabuli*	Ca_Kabuli_denovo	4423469	(A/G)
18024	CakSNP18024	*Kabuli*	Ca_Kabuli_denovo	4423478	(C/G)
18025	CakSNP18025	*Kabuli*	Ca_Kabuli_denovo	4462086	(C/A)
18026	CakSNP18026	*Kabuli*	Ca_Kabuli_denovo	4531890	(A/C)
18027	CakSNP18027	*Kabuli*	Ca_Kabuli_denovo	4533618	(T/C)
18028	CakSNP18028	*Kabuli*	Ca_Kabuli_denovo	4565146	(G/A)
18029	CakSNP18029	*Kabuli*	Ca_Kabuli_denovo	4575382	(C/T)
18030	CakSNP18030	*Kabuli*	Ca_Kabuli_denovo	4581997	(C/G)
18031	CakSNP18031	*Kabuli*	Ca_Kabuli_denovo	4581998	(C/A)
18032	CakSNP18032	*Kabuli*	Ca_Kabuli_denovo	4633334	(G/A)
18033	CakSNP18033	*Kabuli*	Ca_Kabuli_denovo	4634189	(A/C)
18034	CakSNP18034	*Kabuli*	Ca_Kabuli_denovo	4645619	(G/A)
18035	CakSNP18035	*Kabuli*	Ca_Kabuli_denovo	4659307	(G/C)
18036	CakSNP18036	*Kabuli*	Ca_Kabuli_denovo	4659309	(C/A)
18037	CakSNP18037	*Kabuli*	Ca_Kabuli_denovo	4659328	(G/A)
18038	CakSNP18038	*Kabuli*	Ca_Kabuli_denovo	4704245	(G/A)
18039	CakSNP18039	*Kabuli*	Ca_Kabuli_denovo	4704305	(C/T)
18040	CakSNP18040	*Kabuli*	Ca_Kabuli_denovo	4738377	(T/A)
18041	CakSNP18041	*Kabuli*	Ca_Kabuli_denovo	4738379	(G/T)
18042	CakSNP18042	*Kabuli*	Ca_Kabuli_denovo	4738831	(G/A)
18043	CakSNP18043	*Kabuli*	Ca_Kabuli_denovo	4756479	(G/A)
18044	CakSNP18044	*Kabuli*	Ca_Kabuli_denovo	4763483	(C/A)
18045	CakSNP18045	*Kabuli*	Ca_Kabuli_denovo	4769505	(C/A)
18046	CakSNP18046	*Kabuli*	Ca_Kabuli_denovo	4769506	(A/G)
18047	CakSNP18047	*Kabuli*	Ca_Kabuli_denovo	4779386	(G/T)
18048	CakSNP18048	*Kabuli*	Ca_Kabuli_denovo	4779389	(G/A)
18049	CakSNP18049	*Kabuli*	Ca_Kabuli_denovo	4801328	(G/A)
18050	CakSNP18050	*Kabuli*	Ca_Kabuli_denovo	4801337	(G/A)
18051	CakSNP18051	*Kabuli*	Ca_Kabuli_denovo	4801346	(C/T)
18052	CakSNP18052	*Kabuli*	Ca_Kabuli_denovo	4804447	(A/G)
18053	CakSNP18053	*Kabuli*	Ca_Kabuli_denovo	4829417	(G/C)
18054	CakSNP18054	*Kabuli*	Ca_Kabuli_denovo	4829418	(T/C)
18055	CakSNP18055	*Kabuli*	Ca_Kabuli_denovo	4837576	(C/T)

**S.N.**	**SNP IDs**	**Cultivars**	**Chromosomes/scaffolds**	**Physical positions (bp)**	**SNPs**
18056	CakSNP18056	*Kabuli*	Ca_Kabuli_denovo	4848221	(C/T)
18057	CakSNP18057	*Kabuli*	Ca_Kabuli_denovo	4848227	(G/A)
18058	CakSNP18058	*Kabuli*	Ca_Kabuli_denovo	4849347	(C/G)
18059	CakSNP18059	*Kabuli*	Ca_Kabuli_denovo	4870801	(G/A)
18060	CakSNP18060	*Kabuli*	Ca_Kabuli_denovo	4910257	(G/A)
18061	CakSNP18061	*Kabuli*	Ca_Kabuli_denovo	4931630	(C/T)
18062	CakSNP18062	*Kabuli*	Ca_Kabuli_denovo	4956229	(A/G)
18063	CakSNP18063	*Kabuli*	Ca_Kabuli_denovo	4970754	(A/C)
18064	CakSNP18064	*Kabuli*	Ca_Kabuli_denovo	4984720	(G/A)
18065	CakSNP18065	*Kabuli*	Ca_Kabuli_denovo	4984729	(C/T)
18066	CakSNP18066	*Kabuli*	Ca_Kabuli_denovo	4986581	(T/C)
18067	CakSNP18067	*Kabuli*	Ca_Kabuli_denovo	4997664	(G/A)
18068	CakSNP18068	*Kabuli*	Ca_Kabuli_denovo	4999327	(A/G)
18069	CakSNP18069	*Kabuli*	Ca_Kabuli_denovo	4999329	(G/A)
18070	CakSNP18070	*Kabuli*	Ca_Kabuli_denovo	4999334	(G/C)
18071	CakSNP18071	*Kabuli*	Ca_Kabuli_denovo	4999336	(C/G)
18072	CakSNP18072	*Kabuli*	Ca_Kabuli_denovo	4999337	(A/C)
18073	CakSNP18073	*Kabuli*	Ca_Kabuli_denovo	4999367	(T/G)
18074	CakSNP18074	*Kabuli*	Ca_Kabuli_denovo	5021916	(A/T)
18075	CakSNP18075	*Kabuli*	Ca_Kabuli_denovo	5021954	(C/T)
18076	CakSNP18076	*Kabuli*	Ca_Kabuli_denovo	5036439	(C/A)
18077	CakSNP18077	*Kabuli*	Ca_Kabuli_denovo	5085488	(C/T)
18078	CakSNP18078	*Kabuli*	Ca_Kabuli_denovo	5104591	(A/G)
18079	CakSNP18079	*Kabuli*	Ca_Kabuli_denovo	5109203	(T/C)
18080	CakSNP18080	*Kabuli*	Ca_Kabuli_denovo	5128398	(C/G)
18081	CakSNP18081	*Kabuli*	Ca_Kabuli_denovo	5128413	(A/G)
18082	CakSNP18082	*Kabuli*	Ca_Kabuli_denovo	5154944	(C/G)
18083	CakSNP18083	*Kabuli*	Ca_Kabuli_denovo	5155600	(C/G)
18084	CakSNP18084	*Kabuli*	Ca_Kabuli_denovo	5191898	(C/T)
18085	CakSNP18085	*Kabuli*	Ca_Kabuli_denovo	5196708	(T/A)
18086	CakSNP18086	*Kabuli*	Ca_Kabuli_denovo	5229436	(G/C)
18087	CakSNP18087	*Kabuli*	Ca_Kabuli_denovo	5230153	(C/G)
18088	CakSNP18088	*Kabuli*	Ca_Kabuli_denovo	5230160	(T/C)
18089	CakSNP18089	*Kabuli*	Ca_Kabuli_denovo	5236751	(G/A)
18090	CakSNP18090	*Kabuli*	Ca_Kabuli_denovo	5236789	(T/A)
18091	CakSNP18091	*Kabuli*	Ca_Kabuli_denovo	5238535	(T/A)
18092	CakSNP18092	*Kabuli*	Ca_Kabuli_denovo	5238598	(A/G)
18093	CakSNP18093	*Kabuli*	Ca_Kabuli_denovo	5263444	(C/T)
18094	CakSNP18094	*Kabuli*	Ca_Kabuli_denovo	5273594	(G/A)

S.N.	SNP IDs	Cultivars	Chromosomes/scaffolds	Physical positions (bp)	SNPs
18095	CakSNP18095	*Kabuli*	Ca_Kabuli_denovo	5273656	(C/G)
18096	CakSNP18096	*Kabuli*	Ca_Kabuli_denovo	5275340	(G/A)
18097	CakSNP18097	*Kabuli*	Ca_Kabuli_denovo	5280137	(G/A)
18098	CakSNP18098	*Kabuli*	Ca_Kabuli_denovo	5305760	(G/C)
18099	CakSNP18099	*Kabuli*	Ca_Kabuli_denovo	5329084	(G/C)
18100	CakSNP18100	*Kabuli*	Ca_Kabuli_denovo	5329559	(T/C)
18101	CakSNP18101	*Kabuli*	Ca_Kabuli_denovo	5329562	(C/G)
18102	CakSNP18102	*Kabuli*	Ca_Kabuli_denovo	5332558	(C/T)
18103	CakSNP18103	*Kabuli*	Ca_Kabuli_denovo	5332561	(C/A)
18104	CakSNP18104	*Kabuli*	Ca_Kabuli_denovo	5337094	(G/C)
18105	CakSNP18105	*Kabuli*	Ca_Kabuli_denovo	5357564	(C/T)
18106	CakSNP18106	*Kabuli*	Ca_Kabuli_denovo	5358806	(A/G)
18107	CakSNP18107	*Kabuli*	Ca_Kabuli_denovo	5388664	(A/T)
18108	CakSNP18108	*Kabuli*	Ca_Kabuli_denovo	5402790	(G/A)
18109	CakSNP18109	*Kabuli*	Ca_Kabuli_denovo	5402793	(C/T)
18110	CakSNP18110	*Kabuli*	Ca_Kabuli_denovo	5470445	(A/C)
18111	CakSNP18111	*Kabuli*	Ca_Kabuli_denovo	5470446	(G/C)
18112	CakSNP18112	*Kabuli*	Ca_Kabuli_denovo	5470447	(A/G)
18113	CakSNP18113	*Kabuli*	Ca_Kabuli_denovo	5471395	(C/T)
18114	CakSNP18114	*Kabuli*	Ca_Kabuli_denovo	5477856	(C/A)
18115	CakSNP18115	*Kabuli*	Ca_Kabuli_denovo	5529333	(C/G)
18116	CakSNP18116	*Kabuli*	Ca_Kabuli_denovo	5529359	(C/T)
18117	CakSNP18117	*Kabuli*	Ca_Kabuli_denovo	5558990	(C/T)
18118	CakSNP18118	*Kabuli*	Ca_Kabuli_denovo	5559026	(C/A)
18119	CakSNP18119	*Kabuli*	Ca_Kabuli_denovo	5568535	(C/G)
18120	CakSNP18120	*Kabuli*	Ca_Kabuli_denovo	5582991	(A/T)
18121	CakSNP18121	*Kabuli*	Ca_Kabuli_denovo	5592088	(C/T)
18122	CakSNP18122	*Kabuli*	Ca_Kabuli_denovo	5592090	(G/T)
18123	CakSNP18123	*Kabuli*	Ca_Kabuli_denovo	5596926	(A/C)
18124	CakSNP18124	*Kabuli*	Ca_Kabuli_denovo	5681190	(A/T)
18125	CakSNP18125	*Kabuli*	Ca_Kabuli_denovo	5681191	(C/T)
18126	CakSNP18126	*Kabuli*	Ca_Kabuli_denovo	5725564	(C/T)
18127	CakSNP18127	*Kabuli*	Ca_Kabuli_denovo	5725609	(G/A)
18128	CakSNP18128	*Kabuli*	Ca_Kabuli_denovo	5766882	(T/G)
18129	CakSNP18129	*Kabuli*	Ca_Kabuli_denovo	5766883	(T/A)
18130	CakSNP18130	*Kabuli*	Ca_Kabuli_denovo	5773213	(C/T)
18131	CakSNP18131	*Kabuli*	Ca_Kabuli_denovo	5781658	(C/G)
18132	CakSNP18132	*Kabuli*	Ca_Kabuli_denovo	5806886	(T/C)
18133	CakSNP18133	*Kabuli*	Ca_Kabuli_denovo	5816220	(A/C)

S.N.	SNP IDs	Cultivars	Chromosomes/scaffolds	Physical positions (bp)	SNPs
18134	CakSNP18134	*Kabuli*	Ca_Kabuli_denovo	5819283	(G/C)
18135	CakSNP18135	*Kabuli*	Ca_Kabuli_denovo	5848818	(T/A)
18136	CakSNP18136	*Kabuli*	Ca_Kabuli_denovo	5856817	(G/A)
18137	CakSNP18137	*Kabuli*	Ca_Kabuli_denovo	5863013	(C/A)
18138	CakSNP18138	*Kabuli*	Ca_Kabuli_denovo	5863024	(G/A)
18139	CakSNP18139	*Kabuli*	Ca_Kabuli_denovo	5864127	(G/A)
18140	CakSNP18140	*Kabuli*	Ca_Kabuli_denovo	5864153	(G/C)
18141	CakSNP18141	*Kabuli*	Ca_Kabuli_denovo	5873238	(C/A)
18142	CakSNP18142	*Kabuli*	Ca_Kabuli_denovo	5873308	(A/G)
18143	CakSNP18143	*Kabuli*	Ca_Kabuli_denovo	5879375	(G/T)
18144	CakSNP18144	*Kabuli*	Ca_Kabuli_denovo	5879387	(C/G)
18145	CakSNP18145	*Kabuli*	Ca_Kabuli_denovo	5879389	(C/A)
18146	CakSNP18146	*Kabuli*	Ca_Kabuli_denovo	5879418	(T/G)
18147	CakSNP18147	*Kabuli*	Ca_Kabuli_denovo	5879435	(C/A)
18148	CakSNP18148	*Kabuli*	Ca_Kabuli_denovo	5879445	(G/T)
18149	CakSNP18149	*Kabuli*	Ca_Kabuli_denovo	5879451	(C/A)
18150	CakSNP18150	*Kabuli*	Ca_Kabuli_denovo	5890893	(T/A)
18151	CakSNP18151	*Kabuli*	Ca_Kabuli_denovo	5890894	(G/A)
18152	CakSNP18152	*Kabuli*	Ca_Kabuli_denovo	5890895	(G/T)
18153	CakSNP18153	*Kabuli*	Ca_Kabuli_denovo	5890898	(C/T)
18154	CakSNP18154	*Kabuli*	Ca_Kabuli_denovo	5890899	(A/T)
18155	CakSNP18155	*Kabuli*	Ca_Kabuli_denovo	5919819	(C/G)
18156	CakSNP18156	*Kabuli*	Ca_Kabuli_denovo	5952126	(A/G)
18157	CakSNP18157	*Kabuli*	Ca_Kabuli_denovo	5952154	(A/C)
18158	CakSNP18158	*Kabuli*	Ca_Kabuli_denovo	5952171	(A/G)
18159	CakSNP18159	*Kabuli*	Ca_Kabuli_denovo	5963529	(G/A)
18160	CakSNP18160	*Kabuli*	Ca_Kabuli_denovo	5963530	(T/A)
18161	CakSNP18161	*Kabuli*	Ca_Kabuli_denovo	5963531	(T/C)
18162	CakSNP18162	*Kabuli*	Ca_Kabuli_denovo	5963533	(G/A)
18163	CakSNP18163	*Kabuli*	Ca_Kabuli_denovo	5971199	(T/G)
18164	CakSNP18164	*Kabuli*	Ca_Kabuli_denovo	6010227	(T/A)
18165	CakSNP18165	*Kabuli*	Ca_Kabuli_denovo	6010251	(T/G)
18166	CakSNP18166	*Kabuli*	Ca_Kabuli_denovo	6010287	(G/T)
18167	CakSNP18167	*Kabuli*	Ca_Kabuli_denovo	6010311	(A/G)
18168	CakSNP18168	*Kabuli*	Ca_Kabuli_denovo	6010573	(G/A)
18169	CakSNP18169	*Kabuli*	Ca_Kabuli_denovo	6012251	(A/C)
18170	CakSNP18170	*Kabuli*	Ca_Kabuli_denovo	6014521	(C/T)
18171	CakSNP18171	*Kabuli*	Ca_Kabuli_denovo	6014733	(T/G)
18172	CakSNP18172	*Kabuli*	Ca_Kabuli_denovo	6014753	(T/G)

S.N.	SNP IDs	Cultivars	Chromosomes/scaffolds	Physical positions (bp)	SNPs
18173	CakSNP18173	*Kabuli*	Ca_Kabuli_denovo	6014754	(T/A)
18174	CakSNP18174	*Kabuli*	Ca_Kabuli_denovo	6014921	(A/C)
18175	CakSNP18175	*Kabuli*	Ca_Kabuli_denovo	6017294	(G/T)
18176	CakSNP18176	*Kabuli*	Ca_Kabuli_denovo	6017306	(T/G)
18177	CakSNP18177	*Kabuli*	Ca_Kabuli_denovo	6017326	(C/G)
18178	CakSNP18178	*Kabuli*	Ca_Kabuli_denovo	6017328	(T/G)
18179	CakSNP18179	*Kabuli*	Ca_Kabuli_denovo	6017335	(A/G)
18180	CakSNP18180	*Kabuli*	Ca_Kabuli_denovo	6017341	(A/G)
18181	CakSNP18181	*Kabuli*	Ca_Kabuli_denovo	6017344	(C/A)
18182	CakSNP18182	*Kabuli*	Ca_Kabuli_denovo	6017345	(C/G)
18183	CakSNP18183	*Kabuli*	Ca_Kabuli_denovo	6017348	(T/G)
18184	CakSNP18184	*Kabuli*	Ca_Kabuli_denovo	6034062	(T/C)
18185	CakSNP18185	*Kabuli*	Ca_Kabuli_denovo	6034071	(A/G)
18186	CakSNP18186	*Kabuli*	Ca_Kabuli_denovo	6043616	(A/G)
18187	CakSNP18187	*Kabuli*	Ca_Kabuli_denovo	6043627	(G/T)
18188	CakSNP18188	*Kabuli*	Ca_Kabuli_denovo	6047989	(T/G)
18189	CakSNP18189	*Kabuli*	Ca_Kabuli_denovo	6051272	(T/C)
18190	CakSNP18190	*Kabuli*	Ca_Kabuli_denovo	6057777	(G/A)
18191	CakSNP18191	*Kabuli*	Ca_Kabuli_denovo	6059536	(A/G)
18192	CakSNP18192	*Kabuli*	Ca_Kabuli_denovo	6059551	(A/G)
18193	CakSNP18193	*Kabuli*	Ca_Kabuli_denovo	6059588	(A/C)
18194	CakSNP18194	*Kabuli*	Ca_Kabuli_denovo	6098413	(G/A)
18195	CakSNP18195	*Kabuli*	Ca_Kabuli_denovo	6103875	(G/A)
18196	CakSNP18196	*Kabuli*	Ca_Kabuli_denovo	6117175	(C/A)
18197	CakSNP18197	*Kabuli*	Ca_Kabuli_denovo	6117188	(C/A)
18198	CakSNP18198	*Kabuli*	Ca_Kabuli_denovo	6117213	(C/G)
18199	CakSNP18199	*Kabuli*	Ca_Kabuli_denovo	6117215	(A/G)
18200	CakSNP18200	*Kabuli*	Ca_Kabuli_denovo	6165667	(T/C)
18201	CakSNP18201	*Kabuli*	Ca_Kabuli_denovo	6182275	(T/G)
18202	CakSNP18202	*Kabuli*	Ca_Kabuli_denovo	6182281	(G/C)
18203	CakSNP18203	*Kabuli*	Ca_Kabuli_denovo	6190775	(T/C)
18204	CakSNP18204	*Kabuli*	Ca_Kabuli_denovo	6191143	(G/C)
18205	CakSNP18205	*Kabuli*	Ca_Kabuli_denovo	6191170	(G/A)
18206	CakSNP18206	*Kabuli*	Ca_Kabuli_denovo	6237722	(G/A)
18207	CakSNP18207	*Kabuli*	Ca_Kabuli_denovo	6258541	(C/G)
18208	CakSNP18208	*Kabuli*	Ca_Kabuli_denovo	6264608	(C/T)
18209	CakSNP18209	*Kabuli*	Ca_Kabuli_denovo	6300610	(A/G)
18210	CakSNP18210	*Kabuli*	Ca_Kabuli_denovo	6300657	(T/C)
18211	CakSNP18211	*Kabuli*	Ca_Kabuli_denovo	6310736	(G/C)

S.N.	SNP IDs	Cultivars	Chromosomes/scaffolds	Physical positions (bp)	SNPs
18212	CakSNP18212	*Kabuli*	Ca_Kabuli_denovo	6310744	(T/C)
18213	CakSNP18213	*Kabuli*	Ca_Kabuli_denovo	6321154	(G/A)
18214	CakSNP18214	*Kabuli*	Ca_Kabuli_denovo	6355193	(T/G)
18215	CakSNP18215	*Kabuli*	Ca_Kabuli_denovo	6368181	(G/A)
18216	CakSNP18216	*Kabuli*	Ca_Kabuli_denovo	6377203	(C/G)
18217	CakSNP18217	*Kabuli*	Ca_Kabuli_denovo	6399060	(G/A)
18218	CakSNP18218	*Kabuli*	Ca_Kabuli_denovo	6402317	(G/C)
18219	CakSNP18219	*Kabuli*	Ca_Kabuli_denovo	6402384	(C/T)
18220	CakSNP18220	*Kabuli*	Ca_Kabuli_denovo	6402397	(A/C)
18221	CakSNP18221	*Kabuli*	Ca_Kabuli_denovo	6409872	(T/A)
18222	CakSNP18222	*Kabuli*	Ca_Kabuli_denovo	6411767	(C/G)
18223	CakSNP18223	*Kabuli*	Ca_Kabuli_denovo	6411770	(C/T)
18224	CakSNP18224	*Kabuli*	Ca_Kabuli_denovo	6419754	(G/A)
18225	CakSNP18225	*Kabuli*	Ca_Kabuli_denovo	6427894	(T/C)
18226	CakSNP18226	*Kabuli*	Ca_Kabuli_denovo	6462028	(C/T)
18227	CakSNP18227	*Kabuli*	Ca_Kabuli_denovo	6475346	(G/A)
18228	CakSNP18228	*Kabuli*	Ca_Kabuli_denovo	6475348	(T/C)
18229	CakSNP18229	*Kabuli*	Ca_Kabuli_denovo	6480559	(A/G)
18230	CakSNP18230	*Kabuli*	Ca_Kabuli_denovo	6480580	(C/G)
18231	CakSNP18231	*Kabuli*	Ca_Kabuli_denovo	6486758	(G/A)
18232	CakSNP18232	*Kabuli*	Ca_Kabuli_denovo	6486782	(C/T)
18233	CakSNP18233	*Kabuli*	Ca_Kabuli_denovo	6526925	(A/C)
18234	CakSNP18234	*Kabuli*	Ca_Kabuli_denovo	6531409	(T/C)
18235	CakSNP18235	*Kabuli*	Ca_Kabuli_denovo	6545167	(G/C)
18236	CakSNP18236	*Kabuli*	Ca_Kabuli_denovo	6545175	(A/G)
18237	CakSNP18237	*Kabuli*	Ca_Kabuli_denovo	6552139	(C/T)
18238	CakSNP18238	*Kabuli*	Ca_Kabuli_denovo	6552142	(G/T)
18239	CakSNP18239	*Kabuli*	Ca_Kabuli_denovo	6552157	(G/T)
18240	CakSNP18240	*Kabuli*	Ca_Kabuli_denovo	6552161	(T/A)
18241	CakSNP18241	*Kabuli*	Ca_Kabuli_denovo	6559984	(C/T)
18242	CakSNP18242	*Kabuli*	Ca_Kabuli_denovo	6559996	(G/A)
18243	CakSNP18243	*Kabuli*	Ca_Kabuli_denovo	6560006	(A/G)
18244	CakSNP18244	*Kabuli*	Ca_Kabuli_denovo	6565348	(T/C)
18245	CakSNP18245	*Kabuli*	Ca_Kabuli_denovo	6581608	(A/C)
18246	CakSNP18246	*Kabuli*	Ca_Kabuli_denovo	6583973	(T/G)
18247	CakSNP18247	*Kabuli*	Ca_Kabuli_denovo	6590097	(C/T)
18248	CakSNP18248	*Kabuli*	Ca_Kabuli_denovo	6590129	(G/A)
18249	CakSNP18249	*Kabuli*	Ca_Kabuli_denovo	6590156	(T/G)
18250	CakSNP18250	*Kabuli*	Ca_Kabuli_denovo	6599573	(A/G)

S.N.	SNP IDs	Cultivars	Chromosomes/scaffolds	Physical positions (bp)	SNPs
18251	CakSNP18251	*Kabuli*	Ca_Kabuli_denovo	6612721	(A/G)
18252	CakSNP18252	*Kabuli*	Ca_Kabuli_denovo	6663521	(C/G)
18253	CakSNP18253	*Kabuli*	Ca_Kabuli_denovo	6663527	(G/A)
18254	CakSNP18254	*Kabuli*	Ca_Kabuli_denovo	6688010	(C/G)
18255	CakSNP18255	*Kabuli*	Ca_Kabuli_denovo	6706345	(A/G)
18256	CakSNP18256	*Kabuli*	Ca_Kabuli_denovo	6707272	(T/C)
18257	CakSNP18257	*Kabuli*	Ca_Kabuli_denovo	6711061	(A/G)
18258	CakSNP18258	*Kabuli*	Ca_Kabuli_denovo	6712133	(C/G)
18259	CakSNP18259	*Kabuli*	Ca_Kabuli_denovo	6712135	(G/C)
18260	CakSNP18260	*Kabuli*	Ca_Kabuli_denovo	6712138	(C/A)
18261	CakSNP18261	*Kabuli*	Ca_Kabuli_denovo	6712141	(A/T)
18262	CakSNP18262	*Kabuli*	Ca_Kabuli_denovo	6712146	(G/T)
18263	CakSNP18263	*Kabuli*	Ca_Kabuli_denovo	6712147	(T/G)
18264	CakSNP18264	*Kabuli*	Ca_Kabuli_denovo	6720667	(A/G)
18265	CakSNP18265	*Kabuli*	Ca_Kabuli_denovo	6785596	(T/A)
18266	CakSNP18266	*Kabuli*	Ca_Kabuli_denovo	6791052	(C/A)
18267	CakSNP18267	*Kabuli*	Ca_Kabuli_denovo	6791071	(A/C)
18268	CakSNP18268	*Kabuli*	Ca_Kabuli_denovo	6800910	(A/T)
18269	CakSNP18269	*Kabuli*	Ca_Kabuli_denovo	6800913	(A/G)
18270	CakSNP18270	*Kabuli*	Ca_Kabuli_denovo	6800915	(G/A)
18271	CakSNP18271	*Kabuli*	Ca_Kabuli_denovo	6800927	(G/C)
18272	CakSNP18272	*Kabuli*	Ca_Kabuli_denovo	6800944	(G/C)
18273	CakSNP18273	*Kabuli*	Ca_Kabuli_denovo	6800949	(T/C)
18274	CakSNP18274	*Kabuli*	Ca_Kabuli_denovo	6811156	(G/T)
18275	CakSNP18275	*Kabuli*	Ca_Kabuli_denovo	6811456	(A/T)
18276	CakSNP18276	*Kabuli*	Ca_Kabuli_denovo	6811472	(A/T)
18277	CakSNP18277	*Kabuli*	Ca_Kabuli_denovo	6813703	(A/G)
18278	CakSNP18278	*Kabuli*	Ca_Kabuli_denovo	6861843	(T/C)
18279	CakSNP18279	*Kabuli*	Ca_Kabuli_denovo	6861855	(G/A)
18280	CakSNP18280	*Kabuli*	Ca_Kabuli_denovo	6869394	(T/C)
18281	CakSNP18281	*Kabuli*	Ca_Kabuli_denovo	6870117	(C/T)
18282	CakSNP18282	*Kabuli*	Ca_Kabuli_denovo	6870139	(G/A)
18283	CakSNP18283	*Kabuli*	Ca_Kabuli_denovo	6873819	(A/T)
18284	CakSNP18284	*Kabuli*	Ca_Kabuli_denovo	6873823	(T/G)
18285	CakSNP18285	*Kabuli*	Ca_Kabuli_denovo	6873825	(T/C)
18286	CakSNP18286	*Kabuli*	Ca_Kabuli_denovo	6873826	(C/T)
18287	CakSNP18287	*Kabuli*	Ca_Kabuli_denovo	6874179	(C/A)
18288	CakSNP18288	*Kabuli*	Ca_Kabuli_denovo	6874189	(G/T)
18289	CakSNP18289	*Kabuli*	Ca_Kabuli_denovo	6877071	(G/A)

S.N.	SNP IDs	Cultivars	Chromosomes/scaffolds	Physical positions (bp)	SNPs
18290	CakSNP18290	*Kabuli*	Ca_Kabuli_denovo	6882102	(C/G)
18291	CakSNP18291	*Kabuli*	Ca_Kabuli_denovo	6882109	(T/C)
18292	CakSNP18292	*Kabuli*	Ca_Kabuli_denovo	6882113	(T/A)
18293	CakSNP18293	*Kabuli*	Ca_Kabuli_denovo	6882804	(T/A)
18294	CakSNP18294	*Kabuli*	Ca_Kabuli_denovo	6894538	(C/G)
18295	CakSNP18295	*Kabuli*	Ca_Kabuli_denovo	6894541	(A/G)
18296	CakSNP18296	*Kabuli*	Ca_Kabuli_denovo	6894542	(C/A)
18297	CakSNP18297	*Kabuli*	Ca_Kabuli_denovo	6898197	(A/G)
18298	CakSNP18298	*Kabuli*	Ca_Kabuli_denovo	6907485	(T/A)
18299	CakSNP18299	*Kabuli*	Ca_Kabuli_denovo	6907494	(A/G)
18300	CakSNP18300	*Kabuli*	Ca_Kabuli_denovo	6916345	(C/G)
18301	CakSNP18301	*Kabuli*	Ca_Kabuli_denovo	6916346	(T/A)
18302	CakSNP18302	*Kabuli*	Ca_Kabuli_denovo	6916357	(C/T)
18303	CakSNP18303	*Kabuli*	Ca_Kabuli_denovo	6916359	(T/C)
18304	CakSNP18304	*Kabuli*	Ca_Kabuli_denovo	6918579	(A/G)
18305	CakSNP18305	*Kabuli*	Ca_Kabuli_denovo	6918593	(C/G)
18306	CakSNP18306	*Kabuli*	Ca_Kabuli_denovo	6923821	(A/T)
18307	CakSNP18307	*Kabuli*	Ca_Kabuli_denovo	6929548	(A/T)
18308	CakSNP18308	*Kabuli*	Ca_Kabuli_denovo	6939673	(G/A)
18309	CakSNP18309	*Kabuli*	Ca_Kabuli_denovo	6939691	(A/T)
18310	CakSNP18310	*Kabuli*	Ca_Kabuli_denovo	6944977	(A/C)
18311	CakSNP18311	*Kabuli*	Ca_Kabuli_denovo	6945035	(C/G)
18312	CakSNP18312	*Kabuli*	Ca_Kabuli_denovo	6945036	(A/G)
18313	CakSNP18313	*Kabuli*	Ca_Kabuli_denovo	6956695	(C/T)
18314	CakSNP18314	*Kabuli*	Ca_Kabuli_denovo	6956707	(A/G)
18315	CakSNP18315	*Kabuli*	Ca_Kabuli_denovo	6959682	(C/G)
18316	CakSNP18316	*Kabuli*	Ca_Kabuli_denovo	6959727	(T/C)
18317	CakSNP18317	*Kabuli*	Ca_Kabuli_denovo	6960786	(G/A)
18318	CakSNP18318	*Kabuli*	Ca_Kabuli_denovo	6960796	(C/A)
18319	CakSNP18319	*Kabuli*	Ca_Kabuli_denovo	6960820	(C/G)
18320	CakSNP18320	*Kabuli*	Ca_Kabuli_denovo	6975387	(A/G)
18321	CakSNP18321	*Kabuli*	Ca_Kabuli_denovo	6975388	(T/A)
18322	CakSNP18322	*Kabuli*	Ca_Kabuli_denovo	6975430	(T/C)
18323	CakSNP18323	*Kabuli*	Ca_Kabuli_denovo	6975851	(T/C)
18324	CakSNP18324	*Kabuli*	Ca_Kabuli_denovo	6975869	(C/G)
18325	CakSNP18325	*Kabuli*	Ca_Kabuli_denovo	6976418	(C/G)
18326	CakSNP18326	*Kabuli*	Ca_Kabuli_denovo	6976441	(T/C)
18327	CakSNP18327	*Kabuli*	Ca_Kabuli_denovo	6985651	(T/C)
18328	CakSNP18328	*Kabuli*	Ca_Kabuli_denovo	6998248	(A/G)

S.N.	SNP IDs	Cultivars	Chromosomes/scaffolds	Physical positions (bp)	SNPs
18329	CakSNP18329	*Kabuli*	Ca_Kabuli_denovo	6998250	(C/A)
18330	CakSNP18330	*Kabuli*	Ca_Kabuli_denovo	7000764	(A/T)
18331	CakSNP18331	*Kabuli*	Ca_Kabuli_denovo	7012691	(A/G)
18332	CakSNP18332	*Kabuli*	Ca_Kabuli_denovo	7017046	(T/C)
18333	CakSNP18333	*Kabuli*	Ca_Kabuli_denovo	7017051	(A/G)
18334	CakSNP18334	*Kabuli*	Ca_Kabuli_denovo	7017052	(T/C)
18335	CakSNP18335	*Kabuli*	Ca_Kabuli_denovo	7017053	(A/C)
18336	CakSNP18336	*Kabuli*	Ca_Kabuli_denovo	7017058	(G/C)
18337	CakSNP18337	*Kabuli*	Ca_Kabuli_denovo	7017061	(A/T)
18338	CakSNP18338	*Kabuli*	Ca_Kabuli_denovo	7017065	(T/C)
18339	CakSNP18339	*Kabuli*	Ca_Kabuli_denovo	7017071	(C/T)
18340	CakSNP18340	*Kabuli*	Ca_Kabuli_denovo	7017079	(C/G)
18341	CakSNP18341	*Kabuli*	Ca_Kabuli_denovo	7017080	(A/G)
18342	CakSNP18342	*Kabuli*	Ca_Kabuli_denovo	7017081	(G/T)
18343	CakSNP18343	*Kabuli*	Ca_Kabuli_denovo	7069247	(G/A)
18344	CakSNP18344	*Kabuli*	Ca_Kabuli_denovo	7069273	(C/T)
18345	CakSNP18345	*Kabuli*	Ca_Kabuli_denovo	7069930	(G/C)
18346	CakSNP18346	*Kabuli*	Ca_Kabuli_denovo	7070108	(A/G)
18347	CakSNP18347	*Kabuli*	Ca_Kabuli_denovo	7070110	(G/A)
18348	CakSNP18348	*Kabuli*	Ca_Kabuli_denovo	7070111	(A/G)
18349	CakSNP18349	*Kabuli*	Ca_Kabuli_denovo	7070113	(C/G)
18350	CakSNP18350	*Kabuli*	Ca_Kabuli_denovo	7070115	(T/G)
18351	CakSNP18351	*Kabuli*	Ca_Kabuli_denovo	7070117	(C/T)
18352	CakSNP18352	*Kabuli*	Ca_Kabuli_denovo	7086244	(A/G)
18353	CakSNP18353	*Kabuli*	Ca_Kabuli_denovo	7103380	(A/C)
18354	CakSNP18354	*Kabuli*	Ca_Kabuli_denovo	7127589	(G/A)
18355	CakSNP18355	*Kabuli*	Ca_Kabuli_denovo	7131343	(G/A)
18356	CakSNP18356	*Kabuli*	Ca_Kabuli_denovo	7141293	(A/G)
18357	CakSNP18357	*Kabuli*	Ca_Kabuli_denovo	7141324	(A/G)
18358	CakSNP18358	*Kabuli*	Ca_Kabuli_denovo	7143296	(G/A)
18359	CakSNP18359	*Kabuli*	Ca_Kabuli_denovo	7143640	(G/A)
18360	CakSNP18360	*Kabuli*	Ca_Kabuli_denovo	7143649	(G/A)
18361	CakSNP18361	*Kabuli*	Ca_Kabuli_denovo	7143693	(T/C)
18362	CakSNP18362	*Kabuli*	Ca_Kabuli_denovo	7146142	(C/T)
18363	CakSNP18363	*Kabuli*	Ca_Kabuli_denovo	7146173	(C/T)
18364	CakSNP18364	*Kabuli*	Ca_Kabuli_denovo	7149196	(C/T)
18365	CakSNP18365	*Kabuli*	Ca_Kabuli_denovo	7152489	(C/T)
18366	CakSNP18366	*Kabuli*	Ca_Kabuli_denovo	7183627	(A/G)
18367	CakSNP18367	*Kabuli*	Ca_Kabuli_denovo	7191526	(C/T)

S.N.	SNP IDs	Cultivars	Chromosomes/scaffolds	Physical positions (bp)	SNPs
18368	CakSNP18368	*Kabuli*	Ca_Kabuli_denovo	7246580	(C/T)
18369	CakSNP18369	*Kabuli*	Ca_Kabuli_denovo	7254020	(C/G)
18370	CakSNP18370	*Kabuli*	Ca_Kabuli_denovo	7254039	(T/C)
18371	CakSNP18371	*Kabuli*	Ca_Kabuli_denovo	7257747	(G/C)
18372	CakSNP18372	*Kabuli*	Ca_Kabuli_denovo	7267238	(C/T)
18373	CakSNP18373	*Kabuli*	Ca_Kabuli_denovo	7267256	(C/A)
18374	CakSNP18374	*Kabuli*	Ca_Kabuli_denovo	7267260	(A/T)
18375	CakSNP18375	*Kabuli*	Ca_Kabuli_denovo	7311958	(G/A)
18376	CakSNP18376	*Kabuli*	Ca_Kabuli_denovo	7328061	(A/G)
18377	CakSNP18377	*Kabuli*	Ca_Kabuli_denovo	7344433	(T/A)
18378	CakSNP18378	*Kabuli*	Ca_Kabuli_denovo	7369005	(G/A)
18379	CakSNP18379	*Kabuli*	Ca_Kabuli_denovo	7375018	(T/C)
18380	CakSNP18380	*Kabuli*	Ca_Kabuli_denovo	7382267	(T/C)
18381	CakSNP18381	*Kabuli*	Ca_Kabuli_denovo	7382310	(G/T)
18382	CakSNP18382	*Kabuli*	Ca_Kabuli_denovo	7391587	(C/G)
18383	CakSNP18383	*Kabuli*	Ca_Kabuli_denovo	7391613	(T/G)
18384	CakSNP18384	*Kabuli*	Ca_Kabuli_denovo	7391949	(C/T)
18385	CakSNP18385	*Kabuli*	Ca_Kabuli_denovo	7401051	(C/G)
18386	CakSNP18386	*Kabuli*	Ca_Kabuli_denovo	7401054	(G/T)
18387	CakSNP18387	*Kabuli*	Ca_Kabuli_denovo	7401077	(A/G)
18388	CakSNP18388	*Kabuli*	Ca_Kabuli_denovo	7401092	(G/A)
18389	CakSNP18389	*Kabuli*	Ca_Kabuli_denovo	7401100	(A/G)
18390	CakSNP18390	*Kabuli*	Ca_Kabuli_denovo	7401103	(C/T)
18391	CakSNP18391	*Kabuli*	Ca_Kabuli_denovo	7401244	(T/A)
18392	CakSNP18392	*Kabuli*	Ca_Kabuli_denovo	7416312	(C/T)
18393	CakSNP18393	*Kabuli*	Ca_Kabuli_denovo	7416345	(A/G)
18394	CakSNP18394	*Kabuli*	Ca_Kabuli_denovo	7427788	(G/A)
18395	CakSNP18395	*Kabuli*	Ca_Kabuli_denovo	7430435	(C/T)
18396	CakSNP18396	*Kabuli*	Ca_Kabuli_denovo	7430455	(A/T)
18397	CakSNP18397	*Kabuli*	Ca_Kabuli_denovo	7430464	(A/G)
18398	CakSNP18398	*Kabuli*	Ca_Kabuli_denovo	7452895	(C/G)
18399	CakSNP18399	*Kabuli*	Ca_Kabuli_denovo	7467529	(G/T)
18400	CakSNP18400	*Kabuli*	Ca_Kabuli_denovo	7470943	(T/G)
18401	CakSNP18401	*Kabuli*	Ca_Kabuli_denovo	7473234	(A/G)
18402	CakSNP18402	*Kabuli*	Ca_Kabuli_denovo	7477213	(G/A)
18403	CakSNP18403	*Kabuli*	Ca_Kabuli_denovo	7477273	(A/G)
18404	CakSNP18404	*Kabuli*	Ca_Kabuli_denovo	7477350	(C/T)
18405	CakSNP18405	*Kabuli*	Ca_Kabuli_denovo	7480213	(C/G)
18406	CakSNP18406	*Kabuli*	Ca_Kabuli_denovo	7480219	(C/T)

S.N.	SNP IDs	Cultivars	Chromosomes/scaffolds	Physical positions (bp)	SNPs
18407	CakSNP18407	*Kabuli*	Ca_Kabuli_denovo	7482161	(T/G)
18408	CakSNP18408	*Kabuli*	Ca_Kabuli_denovo	7494282	(T/A)
18409	CakSNP18409	*Kabuli*	Ca_Kabuli_denovo	7496672	(C/G)
18410	CakSNP18410	*Kabuli*	Ca_Kabuli_denovo	7496846	(A/G)
18411	CakSNP18411	*Kabuli*	Ca_Kabuli_denovo	7496849	(C/G)
18412	CakSNP18412	*Kabuli*	Ca_Kabuli_denovo	7500180	(G/A)
18413	CakSNP18413	*Kabuli*	Ca_Kabuli_denovo	7503626	(G/A)
18414	CakSNP18414	*Kabuli*	Ca_Kabuli_denovo	7504369	(C/G)
18415	CakSNP18415	*Kabuli*	Ca_Kabuli_denovo	7508116	(T/C)
18416	CakSNP18416	*Kabuli*	Ca_Kabuli_denovo	7511720	(C/G)
18417	CakSNP18417	*Kabuli*	Ca_Kabuli_denovo	7511741	(G/A)
18418	CakSNP18418	*Kabuli*	Ca_Kabuli_denovo	7511744	(G/C)
18419	CakSNP18419	*Kabuli*	Ca_Kabuli_denovo	7516002	(A/G)
18420	CakSNP18420	*Kabuli*	Ca_Kabuli_denovo	7516053	(G/A)
18421	CakSNP18421	*Kabuli*	Ca_Kabuli_denovo	7516067	(T/C)
18422	CakSNP18422	*Kabuli*	Ca_Kabuli_denovo	7518038	(A/G)
18423	CakSNP18423	*Kabuli*	Ca_Kabuli_denovo	7518231	(C/T)
18424	CakSNP18424	*Kabuli*	Ca_Kabuli_denovo	7519248	(G/A)
18425	CakSNP18425	*Kabuli*	Ca_Kabuli_denovo	7520456	(A/G)
18426	CakSNP18426	*Kabuli*	Ca_Kabuli_denovo	7520467	(T/C)
18427	CakSNP18427	*Kabuli*	Ca_Kabuli_denovo	7522835	(G/A)
18428	CakSNP18428	*Kabuli*	Ca_Kabuli_denovo	7532746	(T/C)
18429	CakSNP18429	*Kabuli*	Ca_Kabuli_denovo	7534604	(C/T)
18430	CakSNP18430	*Kabuli*	Ca_Kabuli_denovo	7537117	(G/A)
18431	CakSNP18431	*Kabuli*	Ca_Kabuli_denovo	7537138	(C/T)
18432	CakSNP18432	*Kabuli*	Ca_Kabuli_denovo	7539822	(C/T)
18433	CakSNP18433	*Kabuli*	Ca_Kabuli_denovo	7539825	(C/G)
18434	CakSNP18434	*Kabuli*	Ca_Kabuli_denovo	7546313	(A/G)
18435	CakSNP18435	*Kabuli*	Ca_Kabuli_denovo	7548311	(A/G)
18436	CakSNP18436	*Kabuli*	Ca_Kabuli_denovo	7549471	(T/C)
18437	CakSNP18437	*Kabuli*	Ca_Kabuli_denovo	7549507	(T/G)
18438	CakSNP18438	*Kabuli*	Ca_Kabuli_denovo	7553420	(A/G)
18439	CakSNP18439	*Kabuli*	Ca_Kabuli_denovo	7557108	(C/G)
18440	CakSNP18440	*Kabuli*	Ca_Kabuli_denovo	7557111	(G/C)
18441	CakSNP18441	*Kabuli*	Ca_Kabuli_denovo	7563251	(A/G)
18442	CakSNP18442	*Kabuli*	Ca_Kabuli_denovo	7563332	(C/G)
18443	CakSNP18443	*Kabuli*	Ca_Kabuli_denovo	7569104	(T/A)
18444	CakSNP18444	*Kabuli*	Ca_Kabuli_denovo	7569136	(A/C)
18445	CakSNP18445	*Kabuli*	Ca_Kabuli_denovo	7571356	(C/G)

S.N.	SNP IDs	Cultivars	Chromosomes/scaffolds	Physical positions (bp)	SNPs
18446	CakSNP18446	*Kabuli*	Ca_Kabuli_denovo	7581291	(T/C)
18447	CakSNP18447	*Kabuli*	Ca_Kabuli_denovo	7583443	(T/G)
18448	CakSNP18448	*Kabuli*	Ca_Kabuli_denovo	7586717	(T/C)
18449	CakSNP18449	*Kabuli*	Ca_Kabuli_denovo	7587722	(T/G)
18450	CakSNP18450	*Kabuli*	Ca_Kabuli_denovo	7590523	(A/G)
18451	CakSNP18451	*Kabuli*	Ca_Kabuli_denovo	7597523	(G/A)
18452	CakSNP18452	*Kabuli*	Ca_Kabuli_denovo	7597543	(G/T)
18453	CakSNP18453	*Kabuli*	Ca_Kabuli_denovo	7599351	(A/G)
18454	CakSNP18454	*Kabuli*	Ca_Kabuli_denovo	7599355	(G/A)
18455	CakSNP18455	*Kabuli*	Ca_Kabuli_denovo	7607382	(C/T)
18456	CakSNP18456	*Kabuli*	Ca_Kabuli_denovo	7619139	(G/A)
18457	CakSNP18457	*Kabuli*	Ca_Kabuli_denovo	7619163	(G/A)
18458	CakSNP18458	*Kabuli*	Ca_Kabuli_denovo	7621274	(A/G)
18459	CakSNP18459	*Kabuli*	Ca_Kabuli_denovo	7621346	(T/C)
18460	CakSNP18460	*Kabuli*	Ca_Kabuli_denovo	7632661	(A/G)
18461	CakSNP18461	*Kabuli*	Ca_Kabuli_denovo	7632682	(C/T)
18462	CakSNP18462	*Kabuli*	Ca_Kabuli_denovo	7634464	(G/A)
18463	CakSNP18463	*Kabuli*	Ca_Kabuli_denovo	7638848	(C/G)
18464	CakSNP18464	*Kabuli*	Ca_Kabuli_denovo	7638917	(A/C)
18465	CakSNP18465	*Kabuli*	Ca_Kabuli_denovo	7642288	(A/G)
18466	CakSNP18466	*Kabuli*	Ca_Kabuli_denovo	7644157	(G/A)
18467	CakSNP18467	*Kabuli*	Ca_Kabuli_denovo	7644247	(C/G)
18468	CakSNP18468	*Kabuli*	Ca_Kabuli_denovo	7644265	(C/T)
18469	CakSNP18469	*Kabuli*	Ca_Kabuli_denovo	7644275	(G/A)
18470	CakSNP18470	*Kabuli*	Ca_Kabuli_denovo	7644306	(G/A)
18471	CakSNP18471	*Kabuli*	Ca_Kabuli_denovo	7647077	(G/A)
18472	CakSNP18472	*Kabuli*	Ca_Kabuli_denovo	7659948	(T/A)
18473	CakSNP18473	*Kabuli*	Ca_Kabuli_denovo	7660004	(A/G)
18474	CakSNP18474	*Kabuli*	Ca_Kabuli_denovo	7661617	(G/T)
18475	CakSNP18475	*Kabuli*	Ca_Kabuli_denovo	7662572	(C/G)
18476	CakSNP18476	*Kabuli*	Ca_Kabuli_denovo	7662608	(A/G)
18477	CakSNP18477	*Kabuli*	Ca_Kabuli_denovo	7663940	(A/C)
18478	CakSNP18478	*Kabuli*	Ca_Kabuli_denovo	7663942	(A/G)
18479	CakSNP18479	*Kabuli*	Ca_Kabuli_denovo	7665276	(G/A)
18480	CakSNP18480	*Kabuli*	Ca_Kabuli_denovo	7668261	(C/T)
18481	CakSNP18481	*Kabuli*	Ca_Kabuli_denovo	7668301	(T/C)
18482	CakSNP18482	*Kabuli*	Ca_Kabuli_denovo	7671493	(C/T)
18483	CakSNP18483	*Kabuli*	Ca_Kabuli_denovo	7671508	(T/C)
18484	CakSNP18484	*Kabuli*	Ca_Kabuli_denovo	7674683	(A/C)

S.N.	SNP IDs	Cultivars	Chromosomes/scaffolds	Physical positions (bp)	SNPs
18485	CakSNP18485	*Kabuli*	Ca_Kabuli_denovo	7679874	(G/C)
18486	CakSNP18486	*Kabuli*	Ca_Kabuli_denovo	7681981	(G/T)
18487	CakSNP18487	*Kabuli*	Ca_Kabuli_denovo	7686913	(A/C)
18488	CakSNP18488	*Kabuli*	Ca_Kabuli_denovo	7686915	(G/A)
18489	CakSNP18489	*Kabuli*	Ca_Kabuli_denovo	7689434	(G/A)
18490	CakSNP18490	*Kabuli*	Ca_Kabuli_denovo	7689491	(G/A)
18491	CakSNP18491	*Kabuli*	Ca_Kabuli_denovo	7694767	(C/T)
18492	CakSNP18492	*Kabuli*	Ca_Kabuli_denovo	7694773	(T/C)
18493	CakSNP18493	*Kabuli*	Ca_Kabuli_denovo	7696282	(C/T)
18494	CakSNP18494	*Kabuli*	Ca_Kabuli_denovo	7698789	(G/C)
18495	CakSNP18495	*Kabuli*	Ca_Kabuli_denovo	7707323	(G/A)
18496	CakSNP18496	*Kabuli*	Ca_Kabuli_denovo	7713250	(C/T)
18497	CakSNP18497	*Kabuli*	Ca_Kabuli_denovo	7713283	(C/A)
18498	CakSNP18498	*Kabuli*	Ca_Kabuli_denovo	7714197	(A/G)
18499	CakSNP18499	*Kabuli*	Ca_Kabuli_denovo	7719731	(G/A)
18500	CakSNP18500	*Kabuli*	Ca_Kabuli_denovo	7722023	(T/G)
18501	CakSNP18501	*Kabuli*	Ca_Kabuli_denovo	7722035	(G/T)
18502	CakSNP18502	*Kabuli*	Ca_Kabuli_denovo	7736605	(G/A)
18503	CakSNP18503	*Kabuli*	Ca_Kabuli_denovo	7740036	(T/A)
18504	CakSNP18504	*Kabuli*	Ca_Kabuli_denovo	7742921	(A/G)
18505	CakSNP18505	*Kabuli*	Ca_Kabuli_denovo	7742924	(T/G)
18506	CakSNP18506	*Kabuli*	Ca_Kabuli_denovo	7744414	(C/T)
18507	CakSNP18507	*Kabuli*	Ca_Kabuli_denovo	7749013	(C/T)
18508	CakSNP18508	*Kabuli*	Ca_Kabuli_denovo	7755838	(A/G)
18509	CakSNP18509	*Kabuli*	Ca_Kabuli_denovo	7757665	(G/T)
18510	CakSNP18510	*Kabuli*	Ca_Kabuli_denovo	7763638	(A/C)
18511	CakSNP18511	*Kabuli*	Ca_Kabuli_denovo	7763641	(T/A)
18512	CakSNP18512	*Kabuli*	Ca_Kabuli_denovo	7773005	(C/G)
18513	CakSNP18513	*Kabuli*	Ca_Kabuli_denovo	7773497	(C/T)
18514	CakSNP18514	*Kabuli*	Ca_Kabuli_denovo	7775131	(C/A)
18515	CakSNP18515	*Kabuli*	Ca_Kabuli_denovo	7777349	(G/A)
18516	CakSNP18516	*Kabuli*	Ca_Kabuli_denovo	7777355	(C/G)
18517	CakSNP18517	*Kabuli*	Ca_Kabuli_denovo	7780110	(T/C)
18518	CakSNP18518	*Kabuli*	Ca_Kabuli_denovo	7780147	(T/C)
18519	CakSNP18519	*Kabuli*	Ca_Kabuli_denovo	7780424	(T/G)
18520	CakSNP18520	*Kabuli*	Ca_Kabuli_denovo	7783550	(T/C)
18521	CakSNP18521	*Kabuli*	Ca_Kabuli_denovo	7784298	(A/G)
18522	CakSNP18522	*Kabuli*	Ca_Kabuli_denovo	7784355	(G/A)
18523	CakSNP18523	*Kabuli*	Ca_Kabuli_denovo	7786066	(A/G)

S.N.	SNP IDs	Cultivars	Chromosomes/scaffolds	Physical positions (bp)	SNPs
18524	CakSNP18524	*Kabuli*	Ca_Kabuli_denovo	7786092	(T/C)
18525	CakSNP18525	*Kabuli*	Ca_Kabuli_denovo	7788204	(T/C)
18526	CakSNP18526	*Kabuli*	Ca_Kabuli_denovo	7789221	(T/C)
18527	CakSNP18527	*Kabuli*	Ca_Kabuli_denovo	7789301	(C/T)
18528	CakSNP18528	*Kabuli*	Ca_Kabuli_denovo	7793891	(G/C)
18529	CakSNP18529	*Kabuli*	Ca_Kabuli_denovo	7793893	(C/A)
18530	CakSNP18530	*Kabuli*	Ca_Kabuli_denovo	7797598	(A/G)
18531	CakSNP18531	*Kabuli*	Ca_Kabuli_denovo	7801499	(T/C)
18532	CakSNP18532	*Kabuli*	Ca_Kabuli_denovo	7803930	(G/A)
18533	CakSNP18533	*Kabuli*	Ca_Kabuli_denovo	7803960	(T/C)
18534	CakSNP18534	*Kabuli*	Ca_Kabuli_denovo	7808478	(T/C)
18535	CakSNP18535	*Kabuli*	Ca_Kabuli_denovo	7811193	(T/C)
18536	CakSNP18536	*Kabuli*	Ca_Kabuli_denovo	7811229	(T/C)
18537	CakSNP18537	*Kabuli*	Ca_Kabuli_denovo	7815667	(G/T)
18538	CakSNP18538	*Kabuli*	Ca_Kabuli_denovo	7817621	(G/C)
18539	CakSNP18539	*Kabuli*	Ca_Kabuli_denovo	7817663	(T/C)
18540	CakSNP18540	*Kabuli*	Ca_Kabuli_denovo	7819661	(A/G)
18541	CakSNP18541	*Kabuli*	Ca_Kabuli_denovo	7819688	(C/T)
18542	CakSNP18542	*Kabuli*	Ca_Kabuli_denovo	7821392	(A/T)
18543	CakSNP18543	*Kabuli*	Ca_Kabuli_denovo	7822168	(T/C)
18544	CakSNP18544	*Kabuli*	Ca_Kabuli_denovo	7823280	(G/A)
18545	CakSNP18545	*Kabuli*	Ca_Kabuli_denovo	7825450	(C/A)
18546	CakSNP18546	*Kabuli*	Ca_Kabuli_denovo	7832200	(T/G)
18547	CakSNP18547	*Kabuli*	Ca_Kabuli_denovo	7833031	(G/A)
18548	CakSNP18548	*Kabuli*	Ca_Kabuli_denovo	7842059	(A/G)
18549	CakSNP18549	*Kabuli*	Ca_Kabuli_denovo	7845807	(C/A)
18550	CakSNP18550	*Kabuli*	Ca_Kabuli_denovo	7845827	(C/A)
18551	CakSNP18551	*Kabuli*	Ca_Kabuli_denovo	7851012	(G/A)
18552	CakSNP18552	*Kabuli*	Ca_Kabuli_denovo	7851036	(T/C)
18553	CakSNP18553	*Kabuli*	Ca_Kabuli_denovo	7851360	(G/A)
18554	CakSNP18554	*Kabuli*	Ca_Kabuli_denovo	7851364	(C/G)
18555	CakSNP18555	*Kabuli*	Ca_Kabuli_denovo	7851413	(A/G)
18556	CakSNP18556	*Kabuli*	Ca_Kabuli_denovo	7855438	(T/C)
18557	CakSNP18557	*Kabuli*	Ca_Kabuli_denovo	7855500	(A/G)
18558	CakSNP18558	*Kabuli*	Ca_Kabuli_denovo	7858167	(T/C)
18559	CakSNP18559	*Kabuli*	Ca_Kabuli_denovo	7866944	(C/T)
18560	CakSNP18560	*Kabuli*	Ca_Kabuli_denovo	7867542	(A/G)
18561	CakSNP18561	*Kabuli*	Ca_Kabuli_denovo	7867799	(A/C)
18562	CakSNP18562	*Kabuli*	Ca_Kabuli_denovo	7876080	(A/T)

S.N.	SNP IDs	Cultivars	Chromosomes/scaffolds	Physical positions (bp)	SNPs
18563	CakSNP18563	*Kabuli*	Ca_Kabuli_denovo	7880716	(A/T)
18564	CakSNP18564	*Kabuli*	Ca_Kabuli_denovo	7880717	(C/T)
18565	CakSNP18565	*Kabuli*	Ca_Kabuli_denovo	7881296	(G/A)
18566	CakSNP18566	*Kabuli*	Ca_Kabuli_denovo	7881314	(G/A)
18567	CakSNP18567	*Kabuli*	Ca_Kabuli_denovo	7887564	(C/T)
18568	CakSNP18568	*Kabuli*	Ca_Kabuli_denovo	7888564	(A/G)
18569	CakSNP18569	*Kabuli*	Ca_Kabuli_denovo	7892719	(T/C)
18570	CakSNP18570	*Kabuli*	Ca_Kabuli_denovo	7894702	(C/A)
18571	CakSNP18571	*Kabuli*	Ca_Kabuli_denovo	7894708	(G/C)
18572	CakSNP18572	*Kabuli*	Ca_Kabuli_denovo	7897351	(G/A)
18573	CakSNP18573	*Kabuli*	Ca_Kabuli_denovo	7897373	(A/C)
18574	CakSNP18574	*Kabuli*	Ca_Kabuli_denovo	7900400	(T/C)
18575	CakSNP18575	*Kabuli*	Ca_Kabuli_denovo	7901584	(A/G)
18576	CakSNP18576	*Kabuli*	Ca_Kabuli_denovo	7901585	(T/C)
18577	CakSNP18577	*Kabuli*	Ca_Kabuli_denovo	7910500	(C/T)
18578	CakSNP18578	*Kabuli*	Ca_Kabuli_denovo	7910554	(G/A)
18579	CakSNP18579	*Kabuli*	Ca_Kabuli_denovo	7920197	(C/T)
18580	CakSNP18580	*Kabuli*	Ca_Kabuli_denovo	7920209	(G/A)
18581	CakSNP18581	*Kabuli*	Ca_Kabuli_denovo	7930088	(T/G)
18582	CakSNP18582	*Kabuli*	Ca_Kabuli_denovo	7936283	(C/T)
18583	CakSNP18583	*Kabuli*	Ca_Kabuli_denovo	7936329	(T/G)
18584	CakSNP18584	*Kabuli*	Ca_Kabuli_denovo	7941133	(C/T)
18585	CakSNP18585	*Kabuli*	Ca_Kabuli_denovo	7941147	(T/C)
18586	CakSNP18586	*Kabuli*	Ca_Kabuli_denovo	7945108	(G/A)
18587	CakSNP18587	*Kabuli*	Ca_Kabuli_denovo	7949240	(G/C)
18588	CakSNP18588	*Kabuli*	Ca_Kabuli_denovo	7949247	(G/C)
18589	CakSNP18589	*Kabuli*	Ca_Kabuli_denovo	7952539	(A/G)
18590	CakSNP18590	*Kabuli*	Ca_Kabuli_denovo	7952565	(T/C)
18591	CakSNP18591	*Kabuli*	Ca_Kabuli_denovo	7953341	(G/C)
18592	CakSNP18592	*Kabuli*	Ca_Kabuli_denovo	7955422	(T/C)
18593	CakSNP18593	*Kabuli*	Ca_Kabuli_denovo	7955973	(G/C)
18594	CakSNP18594	*Kabuli*	Ca_Kabuli_denovo	7955994	(G/T)
18595	CakSNP18595	*Kabuli*	Ca_Kabuli_denovo	7956631	(T/C)
18596	CakSNP18596	*Kabuli*	Ca_Kabuli_denovo	7960635	(C/T)
18597	CakSNP18597	*Kabuli*	Ca_Kabuli_denovo	7962856	(C/T)
18598	CakSNP18598	*Kabuli*	Ca_Kabuli_denovo	7962883	(G/C)
18599	CakSNP18599	*Kabuli*	Ca_Kabuli_denovo	7963369	(A/G)
18600	CakSNP18600	*Kabuli*	Ca_Kabuli_denovo	7963390	(G/C)
18601	CakSNP18601	*Kabuli*	Ca_Kabuli_denovo	7970950	(G/C)

S.N.	SNP IDs	Cultivars	Chromosomes/scaffolds	Physical positions (bp)	SNPs
18602	CakSNP18602	*Kabuli*	Ca_Kabuli_denovo	7974031	(G/C)
18603	CakSNP18603	*Kabuli*	Ca_Kabuli_denovo	7974052	(C/G)
18604	CakSNP18604	*Kabuli*	Ca_Kabuli_denovo	7982597	(A/G)
18605	CakSNP18605	*Kabuli*	Ca_Kabuli_denovo	7988467	(G/A)
18606	CakSNP18606	*Kabuli*	Ca_Kabuli_denovo	7995056	(A/G)
18607	CakSNP18607	*Kabuli*	Ca_Kabuli_denovo	7995068	(C/T)
18608	CakSNP18608	*Kabuli*	Ca_Kabuli_denovo	7997461	(T/C)
18609	CakSNP18609	*Kabuli*	Ca_Kabuli_denovo	7999842	(T/G)
18610	CakSNP18610	*Kabuli*	Ca_Kabuli_denovo	7999848	(T/A)
18611	CakSNP18611	*Kabuli*	Ca_Kabuli_denovo	8001503	(T/C)
18612	CakSNP18612	*Kabuli*	Ca_Kabuli_denovo	8003727	(A/G)
18613	CakSNP18613	*Kabuli*	Ca_Kabuli_denovo	8003736	(A/G)
18614	CakSNP18614	*Kabuli*	Ca_Kabuli_denovo	8011242	(G/C)
18615	CakSNP18615	*Kabuli*	Ca_Kabuli_denovo	8011262	(C/T)
18616	CakSNP18616	*Kabuli*	Ca_Kabuli_denovo	8013352	(C/T)
18617	CakSNP18617	*Kabuli*	Ca_Kabuli_denovo	8019793	(C/A)
18618	CakSNP18618	*Kabuli*	Ca_Kabuli_denovo	8022307	(G/A)
18619	CakSNP18619	*Kabuli*	Ca_Kabuli_denovo	8022322	(C/G)
18620	CakSNP18620	*Kabuli*	Ca_Kabuli_denovo	8022996	(A/G)
18621	CakSNP18621	*Kabuli*	Ca_Kabuli_denovo	8026787	(T/G)
18622	CakSNP18622	*Kabuli*	Ca_Kabuli_denovo	8026830	(G/C)
18623	CakSNP18623	*Kabuli*	Ca_Kabuli_denovo	8027198	(T/C)
18624	CakSNP18624	*Kabuli*	Ca_Kabuli_denovo	8027199	(T/C)
18625	CakSNP18625	*Kabuli*	Ca_Kabuli_denovo	8039817	(T/C)
18626	CakSNP18626	*Kabuli*	Ca_Kabuli_denovo	8041747	(T/C)
18627	CakSNP18627	*Kabuli*	Ca_Kabuli_denovo	8041759	(C/A)
18628	CakSNP18628	*Kabuli*	Ca_Kabuli_denovo	8043209	(A/G)
18629	CakSNP18629	*Kabuli*	Ca_Kabuli_denovo	8043253	(T/C)
18630	CakSNP18630	*Kabuli*	Ca_Kabuli_denovo	8044069	(A/G)
18631	CakSNP18631	*Kabuli*	Ca_Kabuli_denovo	8044129	(G/A)
18632	CakSNP18632	*Kabuli*	Ca_Kabuli_denovo	8045895	(A/G)
18633	CakSNP18633	*Kabuli*	Ca_Kabuli_denovo	8051714	(T/C)
18634	CakSNP18634	*Kabuli*	Ca_Kabuli_denovo	8057562	(G/A)
18635	CakSNP18635	*Kabuli*	Ca_Kabuli_denovo	8063940	(A/T)
18636	CakSNP18636	*Kabuli*	Ca_Kabuli_denovo	8069087	(C/A)
18637	CakSNP18637	*Kabuli*	Ca_Kabuli_denovo	8069369	(G/C)
18638	CakSNP18638	*Kabuli*	Ca_Kabuli_denovo	8070193	(G/C)
18639	CakSNP18639	*Kabuli*	Ca_Kabuli_denovo	8070240	(T/C)
18640	CakSNP18640	*Kabuli*	Ca_Kabuli_denovo	8072550	(C/T)

S.N.	SNP IDs	Cultivars	Chromosomes/scaffolds	Physical positions (bp)	SNPs
18641	CakSNP18641	*Kabuli*	Ca_Kabuli_denovo	8082932	(G/A)
18642	CakSNP18642	*Kabuli*	Ca_Kabuli_denovo	8083232	(A/G)
18643	CakSNP18643	*Kabuli*	Ca_Kabuli_denovo	8087512	(A/G)
18644	CakSNP18644	*Kabuli*	Ca_Kabuli_denovo	8087573	(A/G)
18645	CakSNP18645	*Kabuli*	Ca_Kabuli_denovo	8088037	(C/G)
18646	CakSNP18646	*Kabuli*	Ca_Kabuli_denovo	8088041	(T/C)
18647	CakSNP18647	*Kabuli*	Ca_Kabuli_denovo	8088319	(C/T)
18648	CakSNP18648	*Kabuli*	Ca_Kabuli_denovo	8088322	(G/C)
18649	CakSNP18649	*Kabuli*	Ca_Kabuli_denovo	8094162	(A/C)
18650	CakSNP18650	*Kabuli*	Ca_Kabuli_denovo	8098520	(A/G)
18651	CakSNP18651	*Kabuli*	Ca_Kabuli_denovo	8105899	(T/G)
18652	CakSNP18652	*Kabuli*	Ca_Kabuli_denovo	8105968	(T/C)
18653	CakSNP18653	*Kabuli*	Ca_Kabuli_denovo	8109763	(A/C)
18654	CakSNP18654	*Kabuli*	Ca_Kabuli_denovo	8112263	(A/G)
18655	CakSNP18655	*Kabuli*	Ca_Kabuli_denovo	8112296	(A/C)
18656	CakSNP18656	*Kabuli*	Ca_Kabuli_denovo	8112297	(G/C)
18657	CakSNP18657	*Kabuli*	Ca_Kabuli_denovo	8115320	(A/G)
18658	CakSNP18658	*Kabuli*	Ca_Kabuli_denovo	8122096	(T/C)
18659	CakSNP18659	*Kabuli*	Ca_Kabuli_denovo	8130858	(G/A)
18660	CakSNP18660	*Kabuli*	Ca_Kabuli_denovo	8130859	(C/G)
18661	CakSNP18661	*Kabuli*	Ca_Kabuli_denovo	8137324	(C/T)
18662	CakSNP18662	*Kabuli*	Ca_Kabuli_denovo	8137330	(T/G)
18663	CakSNP18663	*Kabuli*	Ca_Kabuli_denovo	8141933	(A/G)
18664	CakSNP18664	*Kabuli*	Ca_Kabuli_denovo	8144021	(A/C)
18665	CakSNP18665	*Kabuli*	Ca_Kabuli_denovo	8144078	(G/A)
18666	CakSNP18666	*Kabuli*	Ca_Kabuli_denovo	8148859	(G/A)
18667	CakSNP18667	*Kabuli*	Ca_Kabuli_denovo	8148868	(C/A)
18668	CakSNP18668	*Kabuli*	Ca_Kabuli_denovo	8149952	(C/G)
18669	CakSNP18669	*Kabuli*	Ca_Kabuli_denovo	8149954	(A/G)
18670	CakSNP18670	*Kabuli*	Ca_Kabuli_denovo	8154225	(C/A)
18671	CakSNP18671	*Kabuli*	Ca_Kabuli_denovo	8158725	(C/T)
18672	CakSNP18672	*Kabuli*	Ca_Kabuli_denovo	8158779	(T/C)
18673	CakSNP18673	*Kabuli*	Ca_Kabuli_denovo	8159110	(A/G)
18674	CakSNP18674	*Kabuli*	Ca_Kabuli_denovo	8159113	(A/G)
18675	CakSNP18675	*Kabuli*	Ca_Kabuli_denovo	8168485	(A/G)
18676	CakSNP18676	*Kabuli*	Ca_Kabuli_denovo	8169341	(C/G)
18677	CakSNP18677	*Kabuli*	Ca_Kabuli_denovo	8171387	(G/C)
18678	CakSNP18678	*Kabuli*	Ca_Kabuli_denovo	8178754	(T/C)
18679	CakSNP18679	*Kabuli*	Ca_Kabuli_denovo	8184848	(C/A)

S.N.	SNP IDs	Cultivars	Chromosomes/scaffolds	Physical positions (bp)	SNPs
18680	CakSNP18680	*Kabuli*	Ca_Kabuli_denovo	8189540	(A/C)
18681	CakSNP18681	*Kabuli*	Ca_Kabuli_denovo	8196300	(T/C)
18682	CakSNP18682	*Kabuli*	Ca_Kabuli_denovo	8196341	(C/G)
18683	CakSNP18683	*Kabuli*	Ca_Kabuli_denovo	8203367	(G/A)
18684	CakSNP18684	*Kabuli*	Ca_Kabuli_denovo	8207563	(C/T)
18685	CakSNP18685	*Kabuli*	Ca_Kabuli_denovo	8210958	(C/G)
18686	CakSNP18686	*Kabuli*	Ca_Kabuli_denovo	8211907	(A/G)
18687	CakSNP18687	*Kabuli*	Ca_Kabuli_denovo	8214133	(A/G)
18688	CakSNP18688	*Kabuli*	Ca_Kabuli_denovo	8214554	(T/C)
18689	CakSNP18689	*Kabuli*	Ca_Kabuli_denovo	8214590	(A/G)
18690	CakSNP18690	*Kabuli*	Ca_Kabuli_denovo	8217362	(A/G)
18691	CakSNP18691	*Kabuli*	Ca_Kabuli_denovo	8218000	(C/G)
18692	CakSNP18692	*Kabuli*	Ca_Kabuli_denovo	8218005	(A/C)
18693	CakSNP18693	*Kabuli*	Ca_Kabuli_denovo	8219090	(G/T)
18694	CakSNP18694	*Kabuli*	Ca_Kabuli_denovo	8223822	(C/G)
18695	CakSNP18695	*Kabuli*	Ca_Kabuli_denovo	8226812	(C/T)
18696	CakSNP18696	*Kabuli*	Ca_Kabuli_denovo	8230958	(C/G)
18697	CakSNP18697	*Kabuli*	Ca_Kabuli_denovo	8230966	(A/T)
18698	CakSNP18698	*Kabuli*	Ca_Kabuli_denovo	8232844	(G/A)
18699	CakSNP18699	*Kabuli*	Ca_Kabuli_denovo	8232880	(G/A)
18700	CakSNP18700	*Kabuli*	Ca_Kabuli_denovo	8234144	(A/G)
18701	CakSNP18701	*Kabuli*	Ca_Kabuli_denovo	8234153	(C/G)
18702	CakSNP18702	*Kabuli*	Ca_Kabuli_denovo	8241946	(C/T)
18703	CakSNP18703	*Kabuli*	Ca_Kabuli_denovo	8241952	(C/T)
18704	CakSNP18704	*Kabuli*	Ca_Kabuli_denovo	8242371	(G/A)
18705	CakSNP18705	*Kabuli*	Ca_Kabuli_denovo	8243836	(T/C)
18706	CakSNP18706	*Kabuli*	Ca_Kabuli_denovo	8244667	(C/T)
18707	CakSNP18707	*Kabuli*	Ca_Kabuli_denovo	8249352	(C/A)
18708	CakSNP18708	*Kabuli*	Ca_Kabuli_denovo	8256558	(C/T)
18709	CakSNP18709	*Kabuli*	Ca_Kabuli_denovo	8256561	(C/T)
18710	CakSNP18710	*Kabuli*	Ca_Kabuli_denovo	8257059	(C/T)
18711	CakSNP18711	*Kabuli*	Ca_Kabuli_denovo	8257088	(A/G)
18712	CakSNP18712	*Kabuli*	Ca_Kabuli_denovo	8261284	(C/G)
18713	CakSNP18713	*Kabuli*	Ca_Kabuli_denovo	8266889	(C/T)
18714	CakSNP18714	*Kabuli*	Ca_Kabuli_denovo	8269821	(A/G)
18715	CakSNP18715	*Kabuli*	Ca_Kabuli_denovo	8271190	(C/G)
18716	CakSNP18716	*Kabuli*	Ca_Kabuli_denovo	8271195	(G/C)
18717	CakSNP18717	*Kabuli*	Ca_Kabuli_denovo	8283720	(C/T)
18718	CakSNP18718	*Kabuli*	Ca_Kabuli_denovo	8283723	(T/C)

S.N.	SNP IDs	Cultivars	Chromosomes/scaffolds	Physical positions (bp)	SNPs
18719	CakSNP18719	*Kabuli*	Ca_Kabuli_denovo	8287800	(G/T)
18720	CakSNP18720	*Kabuli*	Ca_Kabuli_denovo	8289104	(C/T)
18721	CakSNP18721	*Kabuli*	Ca_Kabuli_denovo	8293206	(G/T)
18722	CakSNP18722	*Kabuli*	Ca_Kabuli_denovo	8297749	(A/C)
18723	CakSNP18723	*Kabuli*	Ca_Kabuli_denovo	8300470	(T/G)
18724	CakSNP18724	*Kabuli*	Ca_Kabuli_denovo	8300931	(A/G)
18725	CakSNP18725	*Kabuli*	Ca_Kabuli_denovo	8300939	(T/A)
18726	CakSNP18726	*Kabuli*	Ca_Kabuli_denovo	8303497	(T/C)
18727	CakSNP18727	*Kabuli*	Ca_Kabuli_denovo	8303505	(C/G)
18728	CakSNP18728	*Kabuli*	Ca_Kabuli_denovo	8305655	(A/G)
18729	CakSNP18729	*Kabuli*	Ca_Kabuli_denovo	8305661	(G/A)
18730	CakSNP18730	*Kabuli*	Ca_Kabuli_denovo	8306153	(G/A)
18731	CakSNP18731	*Kabuli*	Ca_Kabuli_denovo	8317400	(G/C)
18732	CakSNP18732	*Kabuli*	Ca_Kabuli_denovo	8317564	(G/C)
18733	CakSNP18733	*Kabuli*	Ca_Kabuli_denovo	8317582	(C/T)
18734	CakSNP18734	*Kabuli*	Ca_Kabuli_denovo	8321061	(C/T)
18735	CakSNP18735	*Kabuli*	Ca_Kabuli_denovo	8322931	(G/A)
18736	CakSNP18736	*Kabuli*	Ca_Kabuli_denovo	8325194	(C/T)
18737	CakSNP18737	*Kabuli*	Ca_Kabuli_denovo	8325213	(C/A)
18738	CakSNP18738	*Kabuli*	Ca_Kabuli_denovo	8327042	(C/T)
18739	CakSNP18739	*Kabuli*	Ca_Kabuli_denovo	8327887	(A/G)
18740	CakSNP18740	*Kabuli*	Ca_Kabuli_denovo	8327890	(A/G)
18741	CakSNP18741	*Kabuli*	Ca_Kabuli_denovo	8328850	(G/A)
18742	CakSNP18742	*Kabuli*	Ca_Kabuli_denovo	8329853	(G/T)
18743	CakSNP18743	*Kabuli*	Ca_Kabuli_denovo	8329859	(G/A)
18744	CakSNP18744	*Kabuli*	Ca_Kabuli_denovo	8331379	(C/T)
18745	CakSNP18745	*Kabuli*	Ca_Kabuli_denovo	8335396	(T/C)
18746	CakSNP18746	*Kabuli*	Ca_Kabuli_denovo	8335402	(T/G)
18747	CakSNP18747	*Kabuli*	Ca_Kabuli_denovo	8336646	(G/C)
18748	CakSNP18748	*Kabuli*	Ca_Kabuli_denovo	8336655	(T/C)
18749	CakSNP18749	*Kabuli*	Ca_Kabuli_denovo	8336819	(G/A)
18750	CakSNP18750	*Kabuli*	Ca_Kabuli_denovo	8336864	(A/G)
18751	CakSNP18751	*Kabuli*	Ca_Kabuli_denovo	8337449	(T/G)
18752	CakSNP18752	*Kabuli*	Ca_Kabuli_denovo	8343165	(A/G)
18753	CakSNP18753	*Kabuli*	Ca_Kabuli_denovo	8349391	(G/C)
18754	CakSNP18754	*Kabuli*	Ca_Kabuli_denovo	8349400	(C/T)
18755	CakSNP18755	*Kabuli*	Ca_Kabuli_denovo	8350117	(T/G)
18756	CakSNP18756	*Kabuli*	Ca_Kabuli_denovo	8350148	(C/G)
18757	CakSNP18757	*Kabuli*	Ca_Kabuli_denovo	8353015	(G/T)

S.N.	SNP IDs	Cultivars	Chromosomes/scaffolds	Physical positions (bp)	SNPs
18758	CakSNP18758	*Kabuli*	Ca_Kabuli_denovo	8354669	(A/T)
18759	CakSNP18759	*Kabuli*	Ca_Kabuli_denovo	8354690	(T/C)
18760	CakSNP18760	*Kabuli*	Ca_Kabuli_denovo	8355827	(T/G)
18761	CakSNP18761	*Kabuli*	Ca_Kabuli_denovo	8360991	(C/A)
18762	CakSNP18762	*Kabuli*	Ca_Kabuli_denovo	8361061	(G/A)
18763	CakSNP18763	*Kabuli*	Ca_Kabuli_denovo	8365868	(T/C)
18764	CakSNP18764	*Kabuli*	Ca_Kabuli_denovo	8365899	(G/A)
18765	CakSNP18765	*Kabuli*	Ca_Kabuli_denovo	8379869	(A/C)
18766	CakSNP18766	*Kabuli*	Ca_Kabuli_denovo	8380718	(C/G)
18767	CakSNP18767	*Kabuli*	Ca_Kabuli_denovo	8380722	(G/A)
18768	CakSNP18768	*Kabuli*	Ca_Kabuli_denovo	8391995	(A/G)
18769	CakSNP18769	*Kabuli*	Ca_Kabuli_denovo	8392015	(A/C)
18770	CakSNP18770	*Kabuli*	Ca_Kabuli_denovo	8400805	(C/T)
18771	CakSNP18771	*Kabuli*	Ca_Kabuli_denovo	8405558	(A/G)
18772	CakSNP18772	*Kabuli*	Ca_Kabuli_denovo	8405615	(T/C)
18773	CakSNP18773	*Kabuli*	Ca_Kabuli_denovo	8409196	(T/C)
18774	CakSNP18774	*Kabuli*	Ca_Kabuli_denovo	8409374	(T/C)
18775	CakSNP18775	*Kabuli*	Ca_Kabuli_denovo	8411035	(T/G)
18776	CakSNP18776	*Kabuli*	Ca_Kabuli_denovo	8411059	(T/G)
18777	CakSNP18777	*Kabuli*	Ca_Kabuli_denovo	8413267	(G/C)
18778	CakSNP18778	*Kabuli*	Ca_Kabuli_denovo	8413291	(A/C)
18779	CakSNP18779	*Kabuli*	Ca_Kabuli_denovo	8416177	(T/C)
18780	CakSNP18780	*Kabuli*	Ca_Kabuli_denovo	8416180	(T/C)
18781	CakSNP18781	*Kabuli*	Ca_Kabuli_denovo	8417405	(T/G)
18782	CakSNP18782	*Kabuli*	Ca_Kabuli_denovo	8417948	(G/T)
18783	CakSNP18783	*Kabuli*	Ca_Kabuli_denovo	8418116	(G/C)
18784	CakSNP18784	*Kabuli*	Ca_Kabuli_denovo	8418159	(T/C)
18785	CakSNP18785	*Kabuli*	Ca_Kabuli_denovo	8419456	(C/G)
18786	CakSNP18786	*Kabuli*	Ca_Kabuli_denovo	8420072	(A/C)
18787	CakSNP18787	*Kabuli*	Ca_Kabuli_denovo	8422556	(T/C)
18788	CakSNP18788	*Kabuli*	Ca_Kabuli_denovo	8423156	(T/C)
18789	CakSNP18789	*Kabuli*	Ca_Kabuli_denovo	8429766	(A/C)
18790	CakSNP18790	*Kabuli*	Ca_Kabuli_denovo	8436507	(C/T)
18791	CakSNP18791	*Kabuli*	Ca_Kabuli_denovo	8436561	(G/A)
18792	CakSNP18792	*Kabuli*	Ca_Kabuli_denovo	8438196	(T/G)
18793	CakSNP18793	*Kabuli*	Ca_Kabuli_denovo	8438207	(G/C)
18794	CakSNP18794	*Kabuli*	Ca_Kabuli_denovo	8448425	(G/C)
18795	CakSNP18795	*Kabuli*	Ca_Kabuli_denovo	8448815	(C/A)
18796	CakSNP18796	*Kabuli*	Ca_Kabuli_denovo	8452890	(T/G)

S.N.	SNP IDs	Cultivars	Chromosomes/scaffolds	Physical positions (bp)	SNPs
18797	CakSNP18797	*Kabuli*	Ca_Kabuli_denovo	8454266	(T/C)
18798	CakSNP18798	*Kabuli*	Ca_Kabuli_denovo	8460560	(C/A)
18799	CakSNP18799	*Kabuli*	Ca_Kabuli_denovo	8461914	(C/G)
18800	CakSNP18800	*Kabuli*	Ca_Kabuli_denovo	8466689	(A/G)
18801	CakSNP18801	*Kabuli*	Ca_Kabuli_denovo	8469963	(A/G)
18802	CakSNP18802	*Kabuli*	Ca_Kabuli_denovo	8469975	(C/A)
18803	CakSNP18803	*Kabuli*	Ca_Kabuli_denovo	8479007	(G/T)
18804	CakSNP18804	*Kabuli*	Ca_Kabuli_denovo	8479010	(C/G)
18805	CakSNP18805	*Kabuli*	Ca_Kabuli_denovo	8480432	(C/G)
18806	CakSNP18806	*Kabuli*	Ca_Kabuli_denovo	8480589	(C/T)
18807	CakSNP18807	*Kabuli*	Ca_Kabuli_denovo	8483863	(T/C)
18808	CakSNP18808	*Kabuli*	Ca_Kabuli_denovo	8486588	(G/T)
18809	CakSNP18809	*Kabuli*	Ca_Kabuli_denovo	8489504	(G/A)
18810	CakSNP18810	*Kabuli*	Ca_Kabuli_denovo	8492446	(C/G)
18811	CakSNP18811	*Kabuli*	Ca_Kabuli_denovo	8492462	(A/T)
18812	CakSNP18812	*Kabuli*	Ca_Kabuli_denovo	8496663	(T/G)
18813	CakSNP18813	*Kabuli*	Ca_Kabuli_denovo	8503771	(T/C)
18814	CakSNP18814	*Kabuli*	Ca_Kabuli_denovo	8503785	(T/G)
18815	CakSNP18815	*Kabuli*	Ca_Kabuli_denovo	8503798	(A/G)
18816	CakSNP18816	*Kabuli*	Ca_Kabuli_denovo	8505482	(T/C)
18817	CakSNP18817	*Kabuli*	Ca_Kabuli_denovo	8505765	(G/A)
18818	CakSNP18818	*Kabuli*	Ca_Kabuli_denovo	8509639	(A/G)
18819	CakSNP18819	*Kabuli*	Ca_Kabuli_denovo	8509669	(G/A)
18820	CakSNP18820	*Kabuli*	Ca_Kabuli_denovo	8513119	(A/C)
18821	CakSNP18821	*Kabuli*	Ca_Kabuli_denovo	8530754	(T/C)
18822	CakSNP18822	*Kabuli*	Ca_Kabuli_denovo	8530755	(T/G)
18823	CakSNP18823	*Kabuli*	Ca_Kabuli_denovo	8531674	(C/T)
18824	CakSNP18824	*Kabuli*	Ca_Kabuli_denovo	8531701	(A/T)
18825	CakSNP18825	*Kabuli*	Ca_Kabuli_denovo	8531753	(G/C)
18826	CakSNP18826	*Kabuli*	Ca_Kabuli_denovo	8531795	(G/A)
18827	CakSNP18827	*Kabuli*	Ca_Kabuli_denovo	8533432	(C/T)
18828	CakSNP18828	*Kabuli*	Ca_Kabuli_denovo	8533441	(A/C)
18829	CakSNP18829	*Kabuli*	Ca_Kabuli_denovo	8533970	(A/C)
18830	CakSNP18830	*Kabuli*	Ca_Kabuli_denovo	8533990	(T/C)
18831	CakSNP18831	*Kabuli*	Ca_Kabuli_denovo	8534449	(G/A)
18832	CakSNP18832	*Kabuli*	Ca_Kabuli_denovo	8550060	(A/G)
18833	CakSNP18833	*Kabuli*	Ca_Kabuli_denovo	8554253	(A/T)
18834	CakSNP18834	*Kabuli*	Ca_Kabuli_denovo	8554325	(G/A)
18835	CakSNP18835	*Kabuli*	Ca_Kabuli_denovo	8556197	(G/T)

S.N.	SNP IDs	Cultivars	Chromosomes/scaffolds	Physical positions (bp)	SNPs
18836	CakSNP18836	*Kabuli*	Ca_Kabuli_denovo	8556200	(A/G)
18837	CakSNP18837	*Kabuli*	Ca_Kabuli_denovo	8556235	(C/T)
18838	CakSNP18838	*Kabuli*	Ca_Kabuli_denovo	8560474	(C/G)
18839	CakSNP18839	*Kabuli*	Ca_Kabuli_denovo	8560544	(A/C)
18840	CakSNP18840	*Kabuli*	Ca_Kabuli_denovo	8562341	(G/C)
18841	CakSNP18841	*Kabuli*	Ca_Kabuli_denovo	8566053	(G/T)
18842	CakSNP18842	*Kabuli*	Ca_Kabuli_denovo	8566099	(C/T)
18843	CakSNP18843	*Kabuli*	Ca_Kabuli_denovo	8571259	(A/G)
18844	CakSNP18844	*Kabuli*	Ca_Kabuli_denovo	8573599	(A/T)
18845	CakSNP18845	*Kabuli*	Ca_Kabuli_denovo	8578805	(A/C)
18846	CakSNP18846	*Kabuli*	Ca_Kabuli_denovo	8580661	(G/C)
18847	CakSNP18847	*Kabuli*	Ca_Kabuli_denovo	8580697	(G/T)
18848	CakSNP18848	*Kabuli*	Ca_Kabuli_denovo	8581281	(G/A)
18849	CakSNP18849	*Kabuli*	Ca_Kabuli_denovo	8584477	(A/G)
18850	CakSNP18850	*Kabuli*	Ca_Kabuli_denovo	8584479	(C/A)
18851	CakSNP18851	*Kabuli*	Ca_Kabuli_denovo	8584858	(T/C)
18852	CakSNP18852	*Kabuli*	Ca_Kabuli_denovo	8584877	(G/A)
18853	CakSNP18853	*Kabuli*	Ca_Kabuli_denovo	8593352	(G/C)
18854	CakSNP18854	*Kabuli*	Ca_Kabuli_denovo	8593359	(A/C)
18855	CakSNP18855	*Kabuli*	Ca_Kabuli_denovo	8603638	(C/T)
18856	CakSNP18856	*Kabuli*	Ca_Kabuli_denovo	8603707	(C/A)
18857	CakSNP18857	*Kabuli*	Ca_Kabuli_denovo	8607036	(T/C)
18858	CakSNP18858	*Kabuli*	Ca_Kabuli_denovo	8623968	(C/A)
18859	CakSNP18859	*Kabuli*	Ca_Kabuli_denovo	8626120	(T/C)
18860	CakSNP18860	*Kabuli*	Ca_Kabuli_denovo	8626123	(C/T)
18861	CakSNP18861	*Kabuli*	Ca_Kabuli_denovo	8629836	(A/C)
18862	CakSNP18862	*Kabuli*	Ca_Kabuli_denovo	8629837	(G/C)
18863	CakSNP18863	*Kabuli*	Ca_Kabuli_denovo	8629839	(T/C)
18864	CakSNP18864	*Kabuli*	Ca_Kabuli_denovo	8630141	(T/G)
18865	CakSNP18865	*Kabuli*	Ca_Kabuli_denovo	8630199	(G/C)
18866	CakSNP18866	*Kabuli*	Ca_Kabuli_denovo	8633226	(G/A)
18867	CakSNP18867	*Kabuli*	Ca_Kabuli_denovo	8633271	(G/T)
18868	CakSNP18868	*Kabuli*	Ca_Kabuli_denovo	8634180	(T/C)
18869	CakSNP18869	*Kabuli*	Ca_Kabuli_denovo	8634188	(A/G)
18870	CakSNP18870	*Kabuli*	Ca_Kabuli_denovo	8642988	(A/C)
18871	CakSNP18871	*Kabuli*	Ca_Kabuli_denovo	8652511	(T/A)
18872	CakSNP18872	*Kabuli*	Ca_Kabuli_denovo	8656524	(G/C)
18873	CakSNP18873	*Kabuli*	Ca_Kabuli_denovo	8657680	(G/T)
18874	CakSNP18874	*Kabuli*	Ca_Kabuli_denovo	8661307	(A/G)

S.N.	SNP IDs	Cultivars	Chromosomes/scaffolds	Physical positions (bp)	SNPs
18875	CakSNP18875	*Kabuli*	Ca_Kabuli_denovo	8661312	(G/A)
18876	CakSNP18876	*Kabuli*	Ca_Kabuli_denovo	8665424	(G/A)
18877	CakSNP18877	*Kabuli*	Ca_Kabuli_denovo	8677202	(C/T)
18878	CakSNP18878	*Kabuli*	Ca_Kabuli_denovo	8677253	(T/C)
18879	CakSNP18879	*Kabuli*	Ca_Kabuli_denovo	8683447	(T/C)
18880	CakSNP18880	*Kabuli*	Ca_Kabuli_denovo	8683473	(T/C)
18881	CakSNP18881	*Kabuli*	Ca_Kabuli_denovo	8688554	(G/A)
18882	CakSNP18882	*Kabuli*	Ca_Kabuli_denovo	8692558	(A/G)
18883	CakSNP18883	*Kabuli*	Ca_Kabuli_denovo	8697009	(A/G)
18884	CakSNP18884	*Kabuli*	Ca_Kabuli_denovo	8701182	(C/T)
18885	CakSNP18885	*Kabuli*	Ca_Kabuli_denovo	8706399	(C/T)
18886	CakSNP18886	*Kabuli*	Ca_Kabuli_denovo	8712116	(C/T)
18887	CakSNP18887	*Kabuli*	Ca_Kabuli_denovo	8712137	(C/G)
18888	CakSNP18888	*Kabuli*	Ca_Kabuli_denovo	8713270	(C/A)
18889	CakSNP18889	*Kabuli*	Ca_Kabuli_denovo	8713287	(T/G)
18890	CakSNP18890	*Kabuli*	Ca_Kabuli_denovo	8714821	(G/A)
18891	CakSNP18891	*Kabuli*	Ca_Kabuli_denovo	8717740	(T/C)
18892	CakSNP18892	*Kabuli*	Ca_Kabuli_denovo	8717768	(G/C)
18893	CakSNP18893	*Kabuli*	Ca_Kabuli_denovo	8718318	(T/G)
18894	CakSNP18894	*Kabuli*	Ca_Kabuli_denovo	8723450	(C/T)
18895	CakSNP18895	*Kabuli*	Ca_Kabuli_denovo	8728039	(C/T)
18896	CakSNP18896	*Kabuli*	Ca_Kabuli_denovo	8728049	(T/G)
18897	CakSNP18897	*Kabuli*	Ca_Kabuli_denovo	8733667	(G/T)
18898	CakSNP18898	*Kabuli*	Ca_Kabuli_denovo	8737564	(A/G)
18899	CakSNP18899	*Kabuli*	Ca_Kabuli_denovo	8740252	(C/T)
18900	CakSNP18900	*Kabuli*	Ca_Kabuli_denovo	8741945	(G/A)
18901	CakSNP18901	*Kabuli*	Ca_Kabuli_denovo	8743682	(C/A)
18902	CakSNP18902	*Kabuli*	Ca_Kabuli_denovo	8745896	(T/G)
18903	CakSNP18903	*Kabuli*	Ca_Kabuli_denovo	8746884	(A/G)
18904	CakSNP18904	*Kabuli*	Ca_Kabuli_denovo	8749660	(G/A)
18905	CakSNP18905	*Kabuli*	Ca_Kabuli_denovo	8749690	(C/G)
18906	CakSNP18906	*Kabuli*	Ca_Kabuli_denovo	8751619	(C/A)
18907	CakSNP18907	*Kabuli*	Ca_Kabuli_denovo	8751625	(C/T)
18908	CakSNP18908	*Kabuli*	Ca_Kabuli_denovo	8754866	(C/G)
18909	CakSNP18909	*Kabuli*	Ca_Kabuli_denovo	8756405	(T/A)
18910	CakSNP18910	*Kabuli*	Ca_Kabuli_denovo	8762906	(T/C)
18911	CakSNP18911	*Kabuli*	Ca_Kabuli_denovo	8764006	(C/G)
18912	CakSNP18912	*Kabuli*	Ca_Kabuli_denovo	8773383	(C/A)
18913	CakSNP18913	*Kabuli*	Ca_Kabuli_denovo	8783399	(C/T)

S.N.	SNP IDs	Cultivars	Chromosomes/scaffolds	Physical positions (bp)	SNPs
18914	CakSNP18914	*Kabuli*	Ca_Kabuli_denovo	8785543	(T/A)
18915	CakSNP18915	*Kabuli*	Ca_Kabuli_denovo	8785573	(G/A)
18916	CakSNP18916	*Kabuli*	Ca_Kabuli_denovo	8785912	(A/G)
18917	CakSNP18917	*Kabuli*	Ca_Kabuli_denovo	8785983	(A/C)
18918	CakSNP18918	*Kabuli*	Ca_Kabuli_denovo	8786375	(C/T)
18919	CakSNP18919	*Kabuli*	Ca_Kabuli_denovo	8786405	(T/C)
18920	CakSNP18920	*Kabuli*	Ca_Kabuli_denovo	8790559	(G/A)
18921	CakSNP18921	*Kabuli*	Ca_Kabuli_denovo	8793365	(T/C)
18922	CakSNP18922	*Kabuli*	Ca_Kabuli_denovo	8797171	(C/A)
18923	CakSNP18923	*Kabuli*	Ca_Kabuli_denovo	8797219	(T/C)
18924	CakSNP18924	*Kabuli*	Ca_Kabuli_denovo	8802899	(C/T)
18925	CakSNP18925	*Kabuli*	Ca_Kabuli_denovo	8802968	(C/A)
18926	CakSNP18926	*Kabuli*	Ca_Kabuli_denovo	8802970	(C/G)
18927	CakSNP18927	*Kabuli*	Ca_Kabuli_denovo	8817440	(T/C)
18928	CakSNP18928	*Kabuli*	Ca_Kabuli_denovo	8817510	(C/A)
18929	CakSNP18929	*Kabuli*	Ca_Kabuli_denovo	8821101	(C/T)
18930	CakSNP18930	*Kabuli*	Ca_Kabuli_denovo	8835782	(A/C)
18931	CakSNP18931	*Kabuli*	Ca_Kabuli_denovo	8837267	(C/G)
18932	CakSNP18932	*Kabuli*	Ca_Kabuli_denovo	8837276	(C/G)
18933	CakSNP18933	*Kabuli*	Ca_Kabuli_denovo	8839300	(G/C)
18934	CakSNP18934	*Kabuli*	Ca_Kabuli_denovo	8839943	(C/T)
18935	CakSNP18935	*Kabuli*	Ca_Kabuli_denovo	8839950	(G/A)
18936	CakSNP18936	*Kabuli*	Ca_Kabuli_denovo	8841891	(C/G)
18937	CakSNP18937	*Kabuli*	Ca_Kabuli_denovo	8843216	(T/C)
18938	CakSNP18938	*Kabuli*	Ca_Kabuli_denovo	8846463	(T/C)
18939	CakSNP18939	*Kabuli*	Ca_Kabuli_denovo	8847644	(T/G)
18940	CakSNP18940	*Kabuli*	Ca_Kabuli_denovo	8847757	(A/T)
18941	CakSNP18941	*Kabuli*	Ca_Kabuli_denovo	8847796	(A/G)
18942	CakSNP18942	*Kabuli*	Ca_Kabuli_denovo	8852097	(T/G)
18943	CakSNP18943	*Kabuli*	Ca_Kabuli_denovo	8855774	(G/C)
18944	CakSNP18944	*Kabuli*	Ca_Kabuli_denovo	8856170	(A/C)
18945	CakSNP18945	*Kabuli*	Ca_Kabuli_denovo	8857655	(A/T)
18946	CakSNP18946	*Kabuli*	Ca_Kabuli_denovo	8858498	(A/G)
18947	CakSNP18947	*Kabuli*	Ca_Kabuli_denovo	8864720	(T/G)
18948	CakSNP18948	*Kabuli*	Ca_Kabuli_denovo	8869813	(G/A)
18949	CakSNP18949	*Kabuli*	Ca_Kabuli_denovo	8881500	(A/C)
18950	CakSNP18950	*Kabuli*	Ca_Kabuli_denovo	8881501	(G/A)
18951	CakSNP18951	*Kabuli*	Ca_Kabuli_denovo	8882179	(C/T)
18952	CakSNP18952	*Kabuli*	Ca_Kabuli_denovo	8882231	(G/A)

S.N.	SNP IDs	Cultivars	Chromosomes/scaffolds	Physical positions (bp)	SNPs
18953	CakSNP18953	*Kabuli*	Ca_Kabuli_denovo	8885140	(A/C)
18954	CakSNP18954	*Kabuli*	Ca_Kabuli_denovo	8885204	(C/T)
18955	CakSNP18955	*Kabuli*	Ca_Kabuli_denovo	8887438	(T/G)
18956	CakSNP18956	*Kabuli*	Ca_Kabuli_denovo	8891101	(C/T)
18957	CakSNP18957	*Kabuli*	Ca_Kabuli_denovo	8898477	(T/C)
18958	CakSNP18958	*Kabuli*	Ca_Kabuli_denovo	8903642	(A/G)
18959	CakSNP18959	*Kabuli*	Ca_Kabuli_denovo	8903702	(G/A)
18960	CakSNP18960	*Kabuli*	Ca_Kabuli_denovo	8904259	(T/G)
18961	CakSNP18961	*Kabuli*	Ca_Kabuli_denovo	8910990	(A/G)
18962	CakSNP18962	*Kabuli*	Ca_Kabuli_denovo	8911135	(G/A)
18963	CakSNP18963	*Kabuli*	Ca_Kabuli_denovo	8911140	(G/A)
18964	CakSNP18964	*Kabuli*	Ca_Kabuli_denovo	8916332	(C/A)
18965	CakSNP18965	*Kabuli*	Ca_Kabuli_denovo	8918015	(A/G)
18966	CakSNP18966	*Kabuli*	Ca_Kabuli_denovo	8918028	(C/T)
18967	CakSNP18967	*Kabuli*	Ca_Kabuli_denovo	8918993	(C/T)
18968	CakSNP18968	*Kabuli*	Ca_Kabuli_denovo	8922620	(C/G)
18969	CakSNP18969	*Kabuli*	Ca_Kabuli_denovo	8922642	(T/G)
18970	CakSNP18970	*Kabuli*	Ca_Kabuli_denovo	8923604	(A/G)
18971	CakSNP18971	*Kabuli*	Ca_Kabuli_denovo	8935945	(G/T)
18972	CakSNP18972	*Kabuli*	Ca_Kabuli_denovo	8941714	(G/A)
18973	CakSNP18973	*Kabuli*	Ca_Kabuli_denovo	8941759	(G/A)
18974	CakSNP18974	*Kabuli*	Ca_Kabuli_denovo	8946705	(C/T)
18975	CakSNP18975	*Kabuli*	Ca_Kabuli_denovo	8946759	(C/T)
18976	CakSNP18976	*Kabuli*	Ca_Kabuli_denovo	8955101	(C/T)
18977	CakSNP18977	*Kabuli*	Ca_Kabuli_denovo	8956392	(T/C)
18978	CakSNP18978	*Kabuli*	Ca_Kabuli_denovo	8956416	(A/G)
18979	CakSNP18979	*Kabuli*	Ca_Kabuli_denovo	8958868	(C/A)
18980	CakSNP18980	*Kabuli*	Ca_Kabuli_denovo	8962549	(T/C)
18981	CakSNP18981	*Kabuli*	Ca_Kabuli_denovo	8969882	(C/G)
18982	CakSNP18982	*Kabuli*	Ca_Kabuli_denovo	8969927	(G/A)
18983	CakSNP18983	*Kabuli*	Ca_Kabuli_denovo	8972239	(C/G)
18984	CakSNP18984	*Kabuli*	Ca_Kabuli_denovo	8979187	(A/G)
18985	CakSNP18985	*Kabuli*	Ca_Kabuli_denovo	8991933	(T/C)
18986	CakSNP18986	*Kabuli*	Ca_Kabuli_denovo	8998274	(C/T)
18987	CakSNP18987	*Kabuli*	Ca_Kabuli_denovo	8998283	(G/C)
18988	CakSNP18988	*Kabuli*	Ca_Kabuli_denovo	8998729	(C/G)
18989	CakSNP18989	*Kabuli*	Ca_Kabuli_denovo	8998743	(G/T)
18990	CakSNP18990	*Kabuli*	Ca_Kabuli_denovo	8999684	(C/T)
18991	CakSNP18991	*Kabuli*	Ca_Kabuli_denovo	8999695	(T/C)

S.N.	SNP IDs	Cultivars	Chromosomes/scaffolds	Physical positions (bp)	SNPs
18992	CakSNP18992	*Kabuli*	Ca_Kabuli_denovo	9007535	(C/A)
18993	CakSNP18993	*Kabuli*	Ca_Kabuli_denovo	9007546	(A/C)
18994	CakSNP18994	*Kabuli*	Ca_Kabuli_denovo	9008111	(G/A)
18995	CakSNP18995	*Kabuli*	Ca_Kabuli_denovo	9008153	(A/G)
18996	CakSNP18996	*Kabuli*	Ca_Kabuli_denovo	9009853	(T/G)
18997	CakSNP18997	*Kabuli*	Ca_Kabuli_denovo	9010359	(T/C)
18998	CakSNP18998	*Kabuli*	Ca_Kabuli_denovo	9016466	(A/C)
18999	CakSNP18999	*Kabuli*	Ca_Kabuli_denovo	9016479	(C/T)
19000	CakSNP19000	*Kabuli*	Ca_Kabuli_denovo	9020583	(C/G)
19001	CakSNP19001	*Kabuli*	Ca_Kabuli_denovo	9020621	(A/G)
19002	CakSNP19002	*Kabuli*	Ca_Kabuli_denovo	9020928	(A/G)
19003	CakSNP19003	*Kabuli*	Ca_Kabuli_denovo	9021373	(C/T)
19004	CakSNP19004	*Kabuli*	Ca_Kabuli_denovo	9025243	(C/A)
19005	CakSNP19005	*Kabuli*	Ca_Kabuli_denovo	9025246	(G/C)
19006	CakSNP19006	*Kabuli*	Ca_Kabuli_denovo	9026799	(A/C)
19007	CakSNP19007	*Kabuli*	Ca_Kabuli_denovo	9028405	(C/G)
19008	CakSNP19008	*Kabuli*	Ca_Kabuli_denovo	9029661	(C/T)
19009	CakSNP19009	*Kabuli*	Ca_Kabuli_denovo	9030509	(T/C)
19010	CakSNP19010	*Kabuli*	Ca_Kabuli_denovo	9034404	(A/G)
19011	CakSNP19011	*Kabuli*	Ca_Kabuli_denovo	9034429	(A/C)
19012	CakSNP19012	*Kabuli*	Ca_Kabuli_denovo	9043273	(C/G)
19013	CakSNP19013	*Kabuli*	Ca_Kabuli_denovo	9043321	(C/T)
19014	CakSNP19014	*Kabuli*	Ca_Kabuli_denovo	9050711	(C/G)
19015	CakSNP19015	*Kabuli*	Ca_Kabuli_denovo	9050747	(G/A)
19016	CakSNP19016	*Kabuli*	Ca_Kabuli_denovo	9056377	(A/G)
19017	CakSNP19017	*Kabuli*	Ca_Kabuli_denovo	9067796	(A/G)
19018	CakSNP19018	*Kabuli*	Ca_Kabuli_denovo	9067797	(A/G)
19019	CakSNP19019	*Kabuli*	Ca_Kabuli_denovo	9068479	(A/G)
19020	CakSNP19020	*Kabuli*	Ca_Kabuli_denovo	9071784	(G/A)
19021	CakSNP19021	*Kabuli*	Ca_Kabuli_denovo	9077263	(A/C)
19022	CakSNP19022	*Kabuli*	Ca_Kabuli_denovo	9077474	(T/G)
19023	CakSNP19023	*Kabuli*	Ca_Kabuli_denovo	9077488	(T/C)
19024	CakSNP19024	*Kabuli*	Ca_Kabuli_denovo	9077513	(T/G)
19025	CakSNP19025	*Kabuli*	Ca_Kabuli_denovo	9077529	(T/G)
19026	CakSNP19026	*Kabuli*	Ca_Kabuli_denovo	9077813	(C/T)
19027	CakSNP19027	*Kabuli*	Ca_Kabuli_denovo	9081380	(G/A)
19028	CakSNP19028	*Kabuli*	Ca_Kabuli_denovo	9081383	(C/T)
19029	CakSNP19029	*Kabuli*	Ca_Kabuli_denovo	9085000	(T/C)
19030	CakSNP19030	*Kabuli*	Ca_Kabuli_denovo	9085051	(T/C)

S.N.	SNP IDs	Cultivars	Chromosomes/scaffolds	Physical positions (bp)	SNPs
19031	CakSNP19031	*Kabuli*	Ca_Kabuli_denovo	9087707	(T/C)
19032	CakSNP19032	*Kabuli*	Ca_Kabuli_denovo	9089170	(A/C)
19033	CakSNP19033	*Kabuli*	Ca_Kabuli_denovo	9089960	(G/A)
19034	CakSNP19034	*Kabuli*	Ca_Kabuli_denovo	9093663	(G/A)
19035	CakSNP19035	*Kabuli*	Ca_Kabuli_denovo	9094477	(C/T)
19036	CakSNP19036	*Kabuli*	Ca_Kabuli_denovo	9096275	(G/A)
19037	CakSNP19037	*Kabuli*	Ca_Kabuli_denovo	9096314	(A/G)
19038	CakSNP19038	*Kabuli*	Ca_Kabuli_denovo	9099899	(G/C)
19039	CakSNP19039	*Kabuli*	Ca_Kabuli_denovo	9099908	(C/G)
19040	CakSNP19040	*Kabuli*	Ca_Kabuli_denovo	9101826	(A/G)
19041	CakSNP19041	*Kabuli*	Ca_Kabuli_denovo	9110057	(T/G)
19042	CakSNP19042	*Kabuli*	Ca_Kabuli_denovo	9116771	(G/A)
19043	CakSNP19043	*Kabuli*	Ca_Kabuli_denovo	9116864	(C/T)
19044	CakSNP19044	*Kabuli*	Ca_Kabuli_denovo	9116876	(C/G)
19045	CakSNP19045	*Kabuli*	Ca_Kabuli_denovo	9118126	(A/G)
19046	CakSNP19046	*Kabuli*	Ca_Kabuli_denovo	9119868	(A/C)
19047	CakSNP19047	*Kabuli*	Ca_Kabuli_denovo	9119949	(G/A)
19048	CakSNP19048	*Kabuli*	Ca_Kabuli_denovo	9120924	(C/T)
19049	CakSNP19049	*Kabuli*	Ca_Kabuli_denovo	9120942	(C/G)
19050	CakSNP19050	*Kabuli*	Ca_Kabuli_denovo	9124892	(A/G)
19051	CakSNP19051	*Kabuli*	Ca_Kabuli_denovo	9124895	(G/T)
19052	CakSNP19052	*Kabuli*	Ca_Kabuli_denovo	9125565	(C/T)
19053	CakSNP19053	*Kabuli*	Ca_Kabuli_denovo	9128353	(A/G)
19054	CakSNP19054	*Kabuli*	Ca_Kabuli_denovo	9128371	(C/T)
19055	CakSNP19055	*Kabuli*	Ca_Kabuli_denovo	9130620	(G/T)
19056	CakSNP19056	*Kabuli*	Ca_Kabuli_denovo	9140141	(G/A)
19057	CakSNP19057	*Kabuli*	Ca_Kabuli_denovo	9140451	(G/C)
19058	CakSNP19058	*Kabuli*	Ca_Kabuli_denovo	9140463	(A/G)
19059	CakSNP19059	*Kabuli*	Ca_Kabuli_denovo	9142027	(T/C)
19060	CakSNP19060	*Kabuli*	Ca_Kabuli_denovo	9147040	(G/C)
19061	CakSNP19061	*Kabuli*	Ca_Kabuli_denovo	9147082	(G/C)
19062	CakSNP19062	*Kabuli*	Ca_Kabuli_denovo	9148606	(G/C)
19063	CakSNP19063	*Kabuli*	Ca_Kabuli_denovo	9150620	(G/C)
19064	CakSNP19064	*Kabuli*	Ca_Kabuli_denovo	9156588	(G/T)
19065	CakSNP19065	*Kabuli*	Ca_Kabuli_denovo	9156594	(C/T)
19066	CakSNP19066	*Kabuli*	Ca_Kabuli_denovo	9158920	(T/G)
19067	CakSNP19067	*Kabuli*	Ca_Kabuli_denovo	9160264	(G/C)
19068	CakSNP19068	*Kabuli*	Ca_Kabuli_denovo	9160267	(T/G)
19069	CakSNP19069	*Kabuli*	Ca_Kabuli_denovo	9172662	(A/C)

S.N.	SNP IDs	Cultivars	Chromosomes/scaffolds	Physical positions (bp)	SNPs
19070	CakSNP19070	*Kabuli*	Ca_Kabuli_denovo	9173737	(C/T)
19071	CakSNP19071	*Kabuli*	Ca_Kabuli_denovo	9173759	(C/A)
19072	CakSNP19072	*Kabuli*	Ca_Kabuli_denovo	9176774	(A/C)
19073	CakSNP19073	*Kabuli*	Ca_Kabuli_denovo	9176776	(A/G)
19074	CakSNP19074	*Kabuli*	Ca_Kabuli_denovo	9177251	(T/G)
19075	CakSNP19075	*Kabuli*	Ca_Kabuli_denovo	9177255	(A/C)
19076	CakSNP19076	*Kabuli*	Ca_Kabuli_denovo	9185606	(A/C)
19077	CakSNP19077	*Kabuli*	Ca_Kabuli_denovo	9185607	(G/C)
19078	CakSNP19078	*Kabuli*	Ca_Kabuli_denovo	9185932	(C/T)
19079	CakSNP19079	*Kabuli*	Ca_Kabuli_denovo	9187737	(T/G)
19080	CakSNP19080	*Kabuli*	Ca_Kabuli_denovo	9192222	(G/A)
19081	CakSNP19081	*Kabuli*	Ca_Kabuli_denovo	9192798	(G/A)
19082	CakSNP19082	*Kabuli*	Ca_Kabuli_denovo	9192851	(T/C)
19083	CakSNP19083	*Kabuli*	Ca_Kabuli_denovo	9198550	(A/G)
19084	CakSNP19084	*Kabuli*	Ca_Kabuli_denovo	9198616	(C/G)
19085	CakSNP19085	*Kabuli*	Ca_Kabuli_denovo	9201182	(T/C)
19086	CakSNP19086	*Kabuli*	Ca_Kabuli_denovo	9203603	(T/C)
19087	CakSNP19087	*Kabuli*	Ca_Kabuli_denovo	9205951	(A/G)
19088	CakSNP19088	*Kabuli*	Ca_Kabuli_denovo	9208178	(C/A)
19089	CakSNP19089	*Kabuli*	Ca_Kabuli_denovo	9208179	(A/G)
19090	CakSNP19090	*Kabuli*	Ca_Kabuli_denovo	9209336	(T/G)
19091	CakSNP19091	*Kabuli*	Ca_Kabuli_denovo	9214280	(G/C)
19092	CakSNP19092	*Kabuli*	Ca_Kabuli_denovo	9224701	(A/G)
19093	CakSNP19093	*Kabuli*	Ca_Kabuli_denovo	9224756	(A/G)
19094	CakSNP19094	*Kabuli*	Ca_Kabuli_denovo	9226486	(T/C)
19095	CakSNP19095	*Kabuli*	Ca_Kabuli_denovo	9239246	(T/C)
19096	CakSNP19096	*Kabuli*	Ca_Kabuli_denovo	9239264	(A/G)
19097	CakSNP19097	*Kabuli*	Ca_Kabuli_denovo	9239699	(G/A)
19098	CakSNP19098	*Kabuli*	Ca_Kabuli_denovo	9239708	(G/A)
19099	CakSNP19099	*Kabuli*	Ca_Kabuli_denovo	9248504	(T/C)
19100	CakSNP19100	*Kabuli*	Ca_Kabuli_denovo	9248531	(C/G)
19101	CakSNP19101	*Kabuli*	Ca_Kabuli_denovo	9250624	(A/G)
19102	CakSNP19102	*Kabuli*	Ca_Kabuli_denovo	9251558	(C/G)
19103	CakSNP19103	*Kabuli*	Ca_Kabuli_denovo	9252627	(C/T)
19104	CakSNP19104	*Kabuli*	Ca_Kabuli_denovo	9260377	(A/G)
19105	CakSNP19105	*Kabuli*	Ca_Kabuli_denovo	9275027	(C/T)
19106	CakSNP19106	*Kabuli*	Ca_Kabuli_denovo	9279086	(G/A)
19107	CakSNP19107	*Kabuli*	Ca_Kabuli_denovo	9279103	(C/G)
19108	CakSNP19108	*Kabuli*	Ca_Kabuli_denovo	9280476	(G/T)

S.N.	SNP IDs	Cultivars	Chromosomes/scaffolds	Physical positions (bp)	SNPs
19109	CakSNP19109	*Kabuli*	Ca_Kabuli_denovo	9285521	(A/G)
19110	CakSNP19110	*Kabuli*	Ca_Kabuli_denovo	9285552	(A/C)
19111	CakSNP19111	*Kabuli*	Ca_Kabuli_denovo	9290097	(C/T)
19112	CakSNP19112	*Kabuli*	Ca_Kabuli_denovo	9290124	(T/C)
19113	CakSNP19113	*Kabuli*	Ca_Kabuli_denovo	9291022	(T/C)
19114	CakSNP19114	*Kabuli*	Ca_Kabuli_denovo	9291386	(G/T)
19115	CakSNP19115	*Kabuli*	Ca_Kabuli_denovo	9295107	(C/T)
19116	CakSNP19116	*Kabuli*	Ca_Kabuli_denovo	9302031	(A/G)
19117	CakSNP19117	*Kabuli*	Ca_Kabuli_denovo	9303206	(G/C)
19118	CakSNP19118	*Kabuli*	Ca_Kabuli_denovo	9303255	(G/A)
19119	CakSNP19119	*Kabuli*	Ca_Kabuli_denovo	9306274	(T/C)
19120	CakSNP19120	*Kabuli*	Ca_Kabuli_denovo	9311223	(T/C)
19121	CakSNP19121	*Kabuli*	Ca_Kabuli_denovo	9311232	(T/C)
19122	CakSNP19122	*Kabuli*	Ca_Kabuli_denovo	9313124	(G/A)
19123	CakSNP19123	*Kabuli*	Ca_Kabuli_denovo	9313760	(A/C)
19124	CakSNP19124	*Kabuli*	Ca_Kabuli_denovo	9313761	(G/C)
19125	CakSNP19125	*Kabuli*	Ca_Kabuli_denovo	9313763	(T/A)
19126	CakSNP19126	*Kabuli*	Ca_Kabuli_denovo	9313764	(C/T)
19127	CakSNP19127	*Kabuli*	Ca_Kabuli_denovo	9323010	(A/G)
19128	CakSNP19128	*Kabuli*	Ca_Kabuli_denovo	9325321	(G/A)
19129	CakSNP19129	*Kabuli*	Ca_Kabuli_denovo	9326340	(C/G)
19130	CakSNP19130	*Kabuli*	Ca_Kabuli_denovo	9326358	(C/T)
19131	CakSNP19131	*Kabuli*	Ca_Kabuli_denovo	9327287	(G/A)
19132	CakSNP19132	*Kabuli*	Ca_Kabuli_denovo	9329987	(A/G)
19133	CakSNP19133	*Kabuli*	Ca_Kabuli_denovo	9331268	(T/A)
19134	CakSNP19134	*Kabuli*	Ca_Kabuli_denovo	9331322	(G/A)
19135	CakSNP19135	*Kabuli*	Ca_Kabuli_denovo	9336282	(C/T)
19136	CakSNP19136	*Kabuli*	Ca_Kabuli_denovo	9336314	(A/G)
19137	CakSNP19137	*Kabuli*	Ca_Kabuli_denovo	9337079	(A/G)
19138	CakSNP19138	*Kabuli*	Ca_Kabuli_denovo	9337886	(G/A)
19139	CakSNP19139	*Kabuli*	Ca_Kabuli_denovo	9337907	(G/A)
19140	CakSNP19140	*Kabuli*	Ca_Kabuli_denovo	9341919	(G/A)
19141	CakSNP19141	*Kabuli*	Ca_Kabuli_denovo	9343441	(C/T)
19142	CakSNP19142	*Kabuli*	Ca_Kabuli_denovo	9343510	(A/C)
19143	CakSNP19143	*Kabuli*	Ca_Kabuli_denovo	9343631	(G/A)
19144	CakSNP19144	*Kabuli*	Ca_Kabuli_denovo	9349136	(G/A)
19145	CakSNP19145	*Kabuli*	Ca_Kabuli_denovo	9349164	(C/T)
19146	CakSNP19146	*Kabuli*	Ca_Kabuli_denovo	9360445	(T/C)
19147	CakSNP19147	*Kabuli*	Ca_Kabuli_denovo	9360845	(C/G)

S.N.	SNP IDs	Cultivars	Chromosomes/scaffolds	Physical positions (bp)	SNPs
19148	CakSNP19148	*Kabuli*	Ca_Kabuli_denovo	9361697	(A/G)
19149	CakSNP19149	*Kabuli*	Ca_Kabuli_denovo	9362542	(T/G)
19150	CakSNP19150	*Kabuli*	Ca_Kabuli_denovo	9364583	(A/G)
19151	CakSNP19151	*Kabuli*	Ca_Kabuli_denovo	9364589	(C/G)
19152	CakSNP19152	*Kabuli*	Ca_Kabuli_denovo	9370733	(A/C)
19153	CakSNP19153	*Kabuli*	Ca_Kabuli_denovo	9373782	(A/G)
19154	CakSNP19154	*Kabuli*	Ca_Kabuli_denovo	9378726	(T/C)
19155	CakSNP19155	*Kabuli*	Ca_Kabuli_denovo	9380666	(A/G)
19156	CakSNP19156	*Kabuli*	Ca_Kabuli_denovo	9380693	(T/C)
19157	CakSNP19157	*Kabuli*	Ca_Kabuli_denovo	9383066	(T/C)
19158	CakSNP19158	*Kabuli*	Ca_Kabuli_denovo	9386351	(T/C)
19159	CakSNP19159	*Kabuli*	Ca_Kabuli_denovo	9389346	(C/T)
19160	CakSNP19160	*Kabuli*	Ca_Kabuli_denovo	9393532	(C/T)
19161	CakSNP19161	*Kabuli*	Ca_Kabuli_denovo	9394058	(G/A)
19162	CakSNP19162	*Kabuli*	Ca_Kabuli_denovo	9395983	(G/T)
19163	CakSNP19163	*Kabuli*	Ca_Kabuli_denovo	9396052	(G/A)
19164	CakSNP19164	*Kabuli*	Ca_Kabuli_denovo	9396286	(T/C)
19165	CakSNP19165	*Kabuli*	Ca_Kabuli_denovo	9396295	(T/C)
19166	CakSNP19166	*Kabuli*	Ca_Kabuli_denovo	9397435	(C/T)
19167	CakSNP19167	*Kabuli*	Ca_Kabuli_denovo	9397440	(T/A)
19168	CakSNP19168	*Kabuli*	Ca_Kabuli_denovo	9404461	(G/A)
19169	CakSNP19169	*Kabuli*	Ca_Kabuli_denovo	9404509	(T/C)
19170	CakSNP19170	*Kabuli*	Ca_Kabuli_denovo	9410160	(A/G)
19171	CakSNP19171	*Kabuli*	Ca_Kabuli_denovo	9412751	(G/C)
19172	CakSNP19172	*Kabuli*	Ca_Kabuli_denovo	9412760	(A/G)
19173	CakSNP19173	*Kabuli*	Ca_Kabuli_denovo	9414379	(T/G)
19174	CakSNP19174	*Kabuli*	Ca_Kabuli_denovo	9415076	(A/G)
19175	CakSNP19175	*Kabuli*	Ca_Kabuli_denovo	9415133	(G/A)
19176	CakSNP19176	*Kabuli*	Ca_Kabuli_denovo	9418681	(T/A)
19177	CakSNP19177	*Kabuli*	Ca_Kabuli_denovo	9421464	(A/C)
19178	CakSNP19178	*Kabuli*	Ca_Kabuli_denovo	9421521	(T/C)
19179	CakSNP19179	*Kabuli*	Ca_Kabuli_denovo	9425870	(T/C)
19180	CakSNP19180	*Kabuli*	Ca_Kabuli_denovo	9429363	(A/C)
19181	CakSNP19181	*Kabuli*	Ca_Kabuli_denovo	9432400	(T/C)
19182	CakSNP19182	*Kabuli*	Ca_Kabuli_denovo	9434348	(T/C)
19183	CakSNP19183	*Kabuli*	Ca_Kabuli_denovo	9434372	(T/G)
19184	CakSNP19184	*Kabuli*	Ca_Kabuli_denovo	9435488	(T/G)
19185	CakSNP19185	*Kabuli*	Ca_Kabuli_denovo	9437375	(A/G)
19186	CakSNP19186	*Kabuli*	Ca_Kabuli_denovo	9437396	(C/G)

S.N.	SNP IDs	Cultivars	Chromosomes/scaffolds	Physical positions (bp)	SNPs
19187	CakSNP19187	*Kabuli*	Ca_Kabuli_denovo	9437960	(C/T)
19188	CakSNP19188	*Kabuli*	Ca_Kabuli_denovo	9437977	(A/G)
19189	CakSNP19189	*Kabuli*	Ca_Kabuli_denovo	9438865	(G/C)
19190	CakSNP19190	*Kabuli*	Ca_Kabuli_denovo	9442353	(G/C)
19191	CakSNP19191	*Kabuli*	Ca_Kabuli_denovo	9442362	(A/G)
19192	CakSNP19192	*Kabuli*	Ca_Kabuli_denovo	9444085	(T/C)
19193	CakSNP19193	*Kabuli*	Ca_Kabuli_denovo	9448354	(G/T)
19194	CakSNP19194	*Kabuli*	Ca_Kabuli_denovo	9452904	(G/A)
19195	CakSNP19195	*Kabuli*	Ca_Kabuli_denovo	9456475	(T/C)
19196	CakSNP19196	*Kabuli*	Ca_Kabuli_denovo	9456505	(T/G)
19197	CakSNP19197	*Kabuli*	Ca_Kabuli_denovo	9457401	(G/C)
19198	CakSNP19198	*Kabuli*	Ca_Kabuli_denovo	9462647	(A/G)
19199	CakSNP19199	*Kabuli*	Ca_Kabuli_denovo	9462650	(C/G)
19200	CakSNP19200	*Kabuli*	Ca_Kabuli_denovo	9469427	(G/C)
19201	CakSNP19201	*Kabuli*	Ca_Kabuli_denovo	9475012	(A/G)
19202	CakSNP19202	*Kabuli*	Ca_Kabuli_denovo	9479197	(G/C)
19203	CakSNP19203	*Kabuli*	Ca_Kabuli_denovo	9479611	(A/T)
19204	CakSNP19204	*Kabuli*	Ca_Kabuli_denovo	9482483	(T/C)
19205	CakSNP19205	*Kabuli*	Ca_Kabuli_denovo	9493569	(T/G)
19206	CakSNP19206	*Kabuli*	Ca_Kabuli_denovo	9493584	(T/G)
19207	CakSNP19207	*Kabuli*	Ca_Kabuli_denovo	9495891	(C/G)
19208	CakSNP19208	*Kabuli*	Ca_Kabuli_denovo	9495933	(T/G)
19209	CakSNP19209	*Kabuli*	Ca_Kabuli_denovo	9501269	(T/C)
19210	CakSNP19210	*Kabuli*	Ca_Kabuli_denovo	9503039	(G/T)
19211	CakSNP19211	*Kabuli*	Ca_Kabuli_denovo	9510912	(A/G)
19212	CakSNP19212	*Kabuli*	Ca_Kabuli_denovo	9511963	(C/T)
19213	CakSNP19213	*Kabuli*	Ca_Kabuli_denovo	9511976	(G/A)
19214	CakSNP19214	*Kabuli*	Ca_Kabuli_denovo	9513069	(C/T)
19215	CakSNP19215	*Kabuli*	Ca_Kabuli_denovo	9513373	(T/C)
19216	CakSNP19216	*Kabuli*	Ca_Kabuli_denovo	9513845	(G/A)
19217	CakSNP19217	*Kabuli*	Ca_Kabuli_denovo	9518015	(G/A)
19218	CakSNP19218	*Kabuli*	Ca_Kabuli_denovo	9519581	(C/T)
19219	CakSNP19219	*Kabuli*	Ca_Kabuli_denovo	9519617	(C/T)
19220	CakSNP19220	*Kabuli*	Ca_Kabuli_denovo	9519815	(A/G)
19221	CakSNP19221	*Kabuli*	Ca_Kabuli_denovo	9519824	(A/G)
19222	CakSNP19222	*Kabuli*	Ca_Kabuli_denovo	9522876	(C/G)
19223	CakSNP19223	*Kabuli*	Ca_Kabuli_denovo	9523870	(C/G)
19224	CakSNP19224	*Kabuli*	Ca_Kabuli_denovo	9523885	(C/T)
19225	CakSNP19225	*Kabuli*	Ca_Kabuli_denovo	9523894	(C/A)

S.N.	SNP IDs	Cultivars	Chromosomes/scaffolds	Physical positions (bp)	SNPs
19226	CakSNP19226	*Kabuli*	Ca_Kabuli_denovo	9524048	(A/G)
19227	CakSNP19227	*Kabuli*	Ca_Kabuli_denovo	9527559	(T/A)
19228	CakSNP19228	*Kabuli*	Ca_Kabuli_denovo	9533107	(C/T)
19229	CakSNP19229	*Kabuli*	Ca_Kabuli_denovo	9533143	(C/T)
19230	CakSNP19230	*Kabuli*	Ca_Kabuli_denovo	9533629	(G/A)
19231	CakSNP19231	*Kabuli*	Ca_Kabuli_denovo	9533635	(A/G)
19232	CakSNP19232	*Kabuli*	Ca_Kabuli_denovo	9533903	(G/C)
19233	CakSNP19233	*Kabuli*	Ca_Kabuli_denovo	9533953	(G/T)
19234	CakSNP19234	*Kabuli*	Ca_Kabuli_denovo	9535405	(A/C)
19235	CakSNP19235	*Kabuli*	Ca_Kabuli_denovo	9535430	(A/C)
19236	CakSNP19236	*Kabuli*	Ca_Kabuli_denovo	9545146	(T/G)
19237	CakSNP19237	*Kabuli*	Ca_Kabuli_denovo	9545486	(A/T)
19238	CakSNP19238	*Kabuli*	Ca_Kabuli_denovo	9551035	(G/C)
19239	CakSNP19239	*Kabuli*	Ca_Kabuli_denovo	9554120	(A/G)
19240	CakSNP19240	*Kabuli*	Ca_Kabuli_denovo	9555017	(C/T)
19241	CakSNP19241	*Kabuli*	Ca_Kabuli_denovo	9555080	(T/C)
19242	CakSNP19242	*Kabuli*	Ca_Kabuli_denovo	9565532	(C/T)
19243	CakSNP19243	*Kabuli*	Ca_Kabuli_denovo	9565561	(T/G)
19244	CakSNP19244	*Kabuli*	Ca_Kabuli_denovo	9573858	(T/C)
19245	CakSNP19245	*Kabuli*	Ca_Kabuli_denovo	9585179	(T/C)
19246	CakSNP19246	*Kabuli*	Ca_Kabuli_denovo	9586184	(G/C)
19247	CakSNP19247	*Kabuli*	Ca_Kabuli_denovo	9595407	(C/G)
19248	CakSNP19248	*Kabuli*	Ca_Kabuli_denovo	9595430	(C/T)
19249	CakSNP19249	*Kabuli*	Ca_Kabuli_denovo	9595612	(T/G)
19250	CakSNP19250	*Kabuli*	Ca_Kabuli_denovo	9595624	(C/G)
19251	CakSNP19251	*Kabuli*	Ca_Kabuli_denovo	9602675	(T/C)
19252	CakSNP19252	*Kabuli*	Ca_Kabuli_denovo	9607619	(G/A)
19253	CakSNP19253	*Kabuli*	Ca_Kabuli_denovo	9608629	(C/T)
19254	CakSNP19254	*Kabuli*	Ca_Kabuli_denovo	9611662	(C/T)
19255	CakSNP19255	*Kabuli*	Ca_Kabuli_denovo	9611695	(A/C)
19256	CakSNP19256	*Kabuli*	Ca_Kabuli_denovo	9612481	(C/T)
19257	CakSNP19257	*Kabuli*	Ca_Kabuli_denovo	9615028	(G/T)
19258	CakSNP19258	*Kabuli*	Ca_Kabuli_denovo	9619205	(G/A)
19259	CakSNP19259	*Kabuli*	Ca_Kabuli_denovo	9622636	(A/C)
19260	CakSNP19260	*Kabuli*	Ca_Kabuli_denovo	9624793	(T/C)
19261	CakSNP19261	*Kabuli*	Ca_Kabuli_denovo	9626464	(G/A)
19262	CakSNP19262	*Kabuli*	Ca_Kabuli_denovo	9626469	(G/A)
19263	CakSNP19263	*Kabuli*	Ca_Kabuli_denovo	9626640	(G/T)
19264	CakSNP19264	*Kabuli*	Ca_Kabuli_denovo	9626684	(C/G)

S.N.	SNP IDs	Cultivars	Chromosomes/scaffolds	Physical positions (bp)	SNPs
19265	CakSNP19265	*Kabuli*	Ca_Kabuli_denovo	9627699	(T/C)
19266	CakSNP19266	*Kabuli*	Ca_Kabuli_denovo	9628193	(A/G)
19267	CakSNP19267	*Kabuli*	Ca_Kabuli_denovo	9628195	(T/G)
19268	CakSNP19268	*Kabuli*	Ca_Kabuli_denovo	9633018	(A/C)
19269	CakSNP19269	*Kabuli*	Ca_Kabuli_denovo	9633019	(G/A)
19270	CakSNP19270	*Kabuli*	Ca_Kabuli_denovo	9634980	(C/G)
19271	CakSNP19271	*Kabuli*	Ca_Kabuli_denovo	9634986	(C/T)
19272	CakSNP19272	*Kabuli*	Ca_Kabuli_denovo	9635479	(G/A)
19273	CakSNP19273	*Kabuli*	Ca_Kabuli_denovo	9639661	(A/G)
19274	CakSNP19274	*Kabuli*	Ca_Kabuli_denovo	9639705	(C/A)
19275	CakSNP19275	*Kabuli*	Ca_Kabuli_denovo	9648137	(G/A)
19276	CakSNP19276	*Kabuli*	Ca_Kabuli_denovo	9650779	(A/G)
19277	CakSNP19277	*Kabuli*	Ca_Kabuli_denovo	9653500	(G/A)
19278	CakSNP19278	*Kabuli*	Ca_Kabuli_denovo	9653548	(G/T)
19279	CakSNP19279	*Kabuli*	Ca_Kabuli_denovo	9655416	(G/C)
19280	CakSNP19280	*Kabuli*	Ca_Kabuli_denovo	9655928	(C/G)
19281	CakSNP19281	*Kabuli*	Ca_Kabuli_denovo	9658278	(G/A)
19282	CakSNP19282	*Kabuli*	Ca_Kabuli_denovo	9660064	(C/G)
19283	CakSNP19283	*Kabuli*	Ca_Kabuli_denovo	9661613	(G/C)
19284	CakSNP19284	*Kabuli*	Ca_Kabuli_denovo	9664866	(C/T)
19285	CakSNP19285	*Kabuli*	Ca_Kabuli_denovo	9664901	(G/C)
19286	CakSNP19286	*Kabuli*	Ca_Kabuli_denovo	9668776	(G/T)
19287	CakSNP19287	*Kabuli*	Ca_Kabuli_denovo	9672444	(T/C)
19288	CakSNP19288	*Kabuli*	Ca_Kabuli_denovo	9672812	(G/A)
19289	CakSNP19289	*Kabuli*	Ca_Kabuli_denovo	9678908	(A/G)
19290	CakSNP19290	*Kabuli*	Ca_Kabuli_denovo	9678917	(C/T)
19291	CakSNP19291	*Kabuli*	Ca_Kabuli_denovo	9679714	(T/G)
19292	CakSNP19292	*Kabuli*	Ca_Kabuli_denovo	9681415	(G/C)
19293	CakSNP19293	*Kabuli*	Ca_Kabuli_denovo	9685223	(G/C)
19294	CakSNP19294	*Kabuli*	Ca_Kabuli_denovo	9689620	(A/G)
19295	CakSNP19295	*Kabuli*	Ca_Kabuli_denovo	9689670	(A/G)
19296	CakSNP19296	*Kabuli*	Ca_Kabuli_denovo	9699577	(C/T)
19297	CakSNP19297	*Kabuli*	Ca_Kabuli_denovo	9702980	(T/C)
19298	CakSNP19298	*Kabuli*	Ca_Kabuli_denovo	9702994	(T/C)
19299	CakSNP19299	*Kabuli*	Ca_Kabuli_denovo	9718247	(G/A)
19300	CakSNP19300	*Kabuli*	Ca_Kabuli_denovo	9718330	(C/A)
19301	CakSNP19301	*Kabuli*	Ca_Kabuli_denovo	9718375	(C/T)
19302	CakSNP19302	*Kabuli*	Ca_Kabuli_denovo	9719868	(T/C)
19303	CakSNP19303	*Kabuli*	Ca_Kabuli_denovo	9720016	(T/C)

S.N.	SNP IDs	Cultivars	Chromosomes/scaffolds	Physical positions (bp)	SNPs
19304	CakSNP19304	*Kabuli*	Ca_Kabuli_denovo	9720034	(A/G)
19305	CakSNP19305	*Kabuli*	Ca_Kabuli_denovo	9725329	(A/C)
19306	CakSNP19306	*Kabuli*	Ca_Kabuli_denovo	9726829	(G/T)
19307	CakSNP19307	*Kabuli*	Ca_Kabuli_denovo	9726834	(T/C)
19308	CakSNP19308	*Kabuli*	Ca_Kabuli_denovo	9730519	(A/G)
19309	CakSNP19309	*Kabuli*	Ca_Kabuli_denovo	9730534	(C/A)
19310	CakSNP19310	*Kabuli*	Ca_Kabuli_denovo	9737591	(A/G)
19311	CakSNP19311	*Kabuli*	Ca_Kabuli_denovo	9738624	(A/C)
19312	CakSNP19312	*Kabuli*	Ca_Kabuli_denovo	9738660	(G/A)
19313	CakSNP19313	*Kabuli*	Ca_Kabuli_denovo	9739260	(A/C)
19314	CakSNP19314	*Kabuli*	Ca_Kabuli_denovo	9739831	(G/A)
19315	CakSNP19315	*Kabuli*	Ca_Kabuli_denovo	9741292	(C/A)
19316	CakSNP19316	*Kabuli*	Ca_Kabuli_denovo	9751264	(G/C)
19317	CakSNP19317	*Kabuli*	Ca_Kabuli_denovo	9751267	(C/T)
19318	CakSNP19318	*Kabuli*	Ca_Kabuli_denovo	9751700	(A/C)
19319	CakSNP19319	*Kabuli*	Ca_Kabuli_denovo	9751701	(G/A)
19320	CakSNP19320	*Kabuli*	Ca_Kabuli_denovo	9758471	(C/T)
19321	CakSNP19321	*Kabuli*	Ca_Kabuli_denovo	9765484	(C/G)
19322	CakSNP19322	*Kabuli*	Ca_Kabuli_denovo	9765523	(T/C)
19323	CakSNP19323	*Kabuli*	Ca_Kabuli_denovo	9766654	(C/T)
19324	CakSNP19324	*Kabuli*	Ca_Kabuli_denovo	9780824	(T/C)
19325	CakSNP19325	*Kabuli*	Ca_Kabuli_denovo	9780872	(T/C)
19326	CakSNP19326	*Kabuli*	Ca_Kabuli_denovo	9781196	(C/T)
19327	CakSNP19327	*Kabuli*	Ca_Kabuli_denovo	9781236	(A/G)
19328	CakSNP19328	*Kabuli*	Ca_Kabuli_denovo	9783341	(A/G)
19329	CakSNP19329	*Kabuli*	Ca_Kabuli_denovo	9786959	(G/C)
19330	CakSNP19330	*Kabuli*	Ca_Kabuli_denovo	9788350	(G/A)
19331	CakSNP19331	*Kabuli*	Ca_Kabuli_denovo	9788437	(T/C)
19332	CakSNP19332	*Kabuli*	Ca_Kabuli_denovo	9793854	(C/G)
19333	CakSNP19333	*Kabuli*	Ca_Kabuli_denovo	9802771	(T/G)
19334	CakSNP19334	*Kabuli*	Ca_Kabuli_denovo	9804090	(G/A)
19335	CakSNP19335	*Kabuli*	Ca_Kabuli_denovo	9804109	(C/T)
19336	CakSNP19336	*Kabuli*	Ca_Kabuli_denovo	9804576	(G/A)
19337	CakSNP19337	*Kabuli*	Ca_Kabuli_denovo	9809489	(T/C)
19338	CakSNP19338	*Kabuli*	Ca_Kabuli_denovo	9809493	(T/C)
19339	CakSNP19339	*Kabuli*	Ca_Kabuli_denovo	9815146	(G/T)
19340	CakSNP19340	*Kabuli*	Ca_Kabuli_denovo	9815151	(A/T)
19341	CakSNP19341	*Kabuli*	Ca_Kabuli_denovo	9823378	(C/G)
19342	CakSNP19342	*Kabuli*	Ca_Kabuli_denovo	9830944	(C/T)

S.N.	SNP IDs	Cultivars	Chromosomes/scaffolds	Physical positions (bp)	SNPs
19343	CakSNP19343	*Kabuli*	Ca_Kabuli_denovo	9830977	(T/C)
19344	CakSNP19344	*Kabuli*	Ca_Kabuli_denovo	9832549	(G/A)
19345	CakSNP19345	*Kabuli*	Ca_Kabuli_denovo	9833972	(G/A)
19346	CakSNP19346	*Kabuli*	Ca_Kabuli_denovo	9836572	(G/T)
19347	CakSNP19347	*Kabuli*	Ca_Kabuli_denovo	9845280	(A/C)
19348	CakSNP19348	*Kabuli*	Ca_Kabuli_denovo	9845868	(T/C)
19349	CakSNP19349	*Kabuli*	Ca_Kabuli_denovo	9847577	(C/A)
19350	CakSNP19350	*Kabuli*	Ca_Kabuli_denovo	9847585	(G/A)
19351	CakSNP19351	*Kabuli*	Ca_Kabuli_denovo	9848680	(G/C)
19352	CakSNP19352	*Kabuli*	Ca_Kabuli_denovo	9850046	(A/G)
19353	CakSNP19353	*Kabuli*	Ca_Kabuli_denovo	9853101	(A/C)
19354	CakSNP19354	*Kabuli*	Ca_Kabuli_denovo	9863140	(A/G)
19355	CakSNP19355	*Kabuli*	Ca_Kabuli_denovo	9863513	(T/C)
19356	CakSNP19356	*Kabuli*	Ca_Kabuli_denovo	9863525	(G/C)
19357	CakSNP19357	*Kabuli*	Ca_Kabuli_denovo	9865957	(C/T)
19358	CakSNP19358	*Kabuli*	Ca_Kabuli_denovo	9870575	(T/C)
19359	CakSNP19359	*Kabuli*	Ca_Kabuli_denovo	9872528	(T/G)
19360	CakSNP19360	*Kabuli*	Ca_Kabuli_denovo	9872591	(C/G)
19361	CakSNP19361	*Kabuli*	Ca_Kabuli_denovo	9878441	(A/G)
19362	CakSNP19362	*Kabuli*	Ca_Kabuli_denovo	9880795	(C/G)
19363	CakSNP19363	*Kabuli*	Ca_Kabuli_denovo	9881788	(G/T)
19364	CakSNP19364	*Kabuli*	Ca_Kabuli_denovo	9881800	(A/G)
19365	CakSNP19365	*Kabuli*	Ca_Kabuli_denovo	9884524	(T/G)
19366	CakSNP19366	*Kabuli*	Ca_Kabuli_denovo	9885838	(G/C)
19367	CakSNP19367	*Kabuli*	Ca_Kabuli_denovo	9889813	(T/C)
19368	CakSNP19368	*Kabuli*	Ca_Kabuli_denovo	9891114	(G/T)
19369	CakSNP19369	*Kabuli*	Ca_Kabuli_denovo	9891158	(T/C)
19370	CakSNP19370	*Kabuli*	Ca_Kabuli_denovo	9891452	(A/G)
19371	CakSNP19371	*Kabuli*	Ca_Kabuli_denovo	9896914	(G/A)
19372	CakSNP19372	*Kabuli*	Ca_Kabuli_denovo	9896968	(G/A)
19373	CakSNP19373	*Kabuli*	Ca_Kabuli_denovo	9899452	(C/G)
19374	CakSNP19374	*Kabuli*	Ca_Kabuli_denovo	9901759	(C/T)
19375	CakSNP19375	*Kabuli*	Ca_Kabuli_denovo	9901779	(G/C)
19376	CakSNP19376	*Kabuli*	Ca_Kabuli_denovo	9908975	(T/C)
19377	CakSNP19377	*Kabuli*	Ca_Kabuli_denovo	9913858	(G/T)
19378	CakSNP19378	*Kabuli*	Ca_Kabuli_denovo	9913864	(G/A)
19379	CakSNP19379	*Kabuli*	Ca_Kabuli_denovo	9914830	(G/T)
19380	CakSNP19380	*Kabuli*	Ca_Kabuli_denovo	9915716	(C/G)
19381	CakSNP19381	*Kabuli*	Ca_Kabuli_denovo	9915762	(T/G)

S.N.	SNP IDs	Cultivars	Chromosomes/scaffolds	Physical positions (bp)	SNPs
19382	CakSNP19382	*Kabuli*	Ca_Kabuli_denovo	9915772	(C/G)
19383	CakSNP19383	*Kabuli*	Ca_Kabuli_denovo	9918090	(C/A)
19384	CakSNP19384	*Kabuli*	Ca_Kabuli_denovo	9918838	(C/G)
19385	CakSNP19385	*Kabuli*	Ca_Kabuli_denovo	9918841	(A/G)
19386	CakSNP19386	*Kabuli*	Ca_Kabuli_denovo	9918853	(C/T)
19387	CakSNP19387	*Kabuli*	Ca_Kabuli_denovo	9923995	(C/T)
19388	CakSNP19388	*Kabuli*	Ca_Kabuli_denovo	9924598	(C/T)
19389	CakSNP19389	*Kabuli*	Ca_Kabuli_denovo	9924640	(T/A)
19390	CakSNP19390	*Kabuli*	Ca_Kabuli_denovo	9924819	(G/T)
19391	CakSNP19391	*Kabuli*	Ca_Kabuli_denovo	9924862	(G/A)
19392	CakSNP19392	*Kabuli*	Ca_Kabuli_denovo	9929093	(C/T)
19393	CakSNP19393	*Kabuli*	Ca_Kabuli_denovo	9932690	(G/A)
19394	CakSNP19394	*Kabuli*	Ca_Kabuli_denovo	9932696	(G/C)
19395	CakSNP19395	*Kabuli*	Ca_Kabuli_denovo	9933865	(G/C)
19396	CakSNP19396	*Kabuli*	Ca_Kabuli_denovo	9933889	(A/G)
19397	CakSNP19397	*Kabuli*	Ca_Kabuli_denovo	9937838	(T/C)
19398	CakSNP19398	*Kabuli*	Ca_Kabuli_denovo	9938641	(A/G)
19399	CakSNP19399	*Kabuli*	Ca_Kabuli_denovo	9938665	(G/C)
19400	CakSNP19400	*Kabuli*	Ca_Kabuli_denovo	9940609	(C/T)
19401	CakSNP19401	*Kabuli*	Ca_Kabuli_denovo	9942826	(T/C)
19402	CakSNP19402	*Kabuli*	Ca_Kabuli_denovo	9942846	(A/G)
19403	CakSNP19403	*Kabuli*	Ca_Kabuli_denovo	9947560	(A/C)
19404	CakSNP19404	*Kabuli*	Ca_Kabuli_denovo	9951000	(A/G)
19405	CakSNP19405	*Kabuli*	Ca_Kabuli_denovo	9962011	(G/A)
19406	CakSNP19406	*Kabuli*	Ca_Kabuli_denovo	9962035	(C/T)
19407	CakSNP19407	*Kabuli*	Ca_Kabuli_denovo	9973611	(G/T)
19408	CakSNP19408	*Kabuli*	Ca_Kabuli_denovo	9973617	(T/C)
19409	CakSNP19409	*Kabuli*	Ca_Kabuli_denovo	9973620	(A/G)
19410	CakSNP19410	*Kabuli*	Ca_Kabuli_denovo	9974899	(G/A)
19411	CakSNP19411	*Kabuli*	Ca_Kabuli_denovo	9976962	(T/C)
19412	CakSNP19412	*Kabuli*	Ca_Kabuli_denovo	9976966	(A/G)
19413	CakSNP19413	*Kabuli*	Ca_Kabuli_denovo	9981376	(A/C)
19414	CakSNP19414	*Kabuli*	Ca_Kabuli_denovo	9981406	(G/T)
19415	CakSNP19415	*Kabuli*	Ca_Kabuli_denovo	9985529	(C/G)
19416	CakSNP19416	*Kabuli*	Ca_Kabuli_denovo	9985544	(C/G)
19417	CakSNP19417	*Kabuli*	Ca_Kabuli_denovo	9996966	(G/A)
19418	CakSNP19418	*Kabuli*	Ca_Kabuli_denovo	9996979	(G/T)
19419	CakSNP19419	*Kabuli*	Ca_Kabuli_denovo	10003680	(G/T)
19420	CakSNP19420	*Kabuli*	Ca_Kabuli_denovo	10008824	(T/C)

S.N.	SNP IDs	Cultivars	Chromosomes/scaffolds	Physical positions (bp)	SNPs
19421	CakSNP19421	*Kabuli*	Ca_Kabuli_denovo	10009526	(T/C)
19422	CakSNP19422	*Kabuli*	Ca_Kabuli_denovo	10012784	(A/G)
19423	CakSNP19423	*Kabuli*	Ca_Kabuli_denovo	10012811	(G/C)
19424	CakSNP19424	*Kabuli*	Ca_Kabuli_denovo	10013047	(C/T)
19425	CakSNP19425	*Kabuli*	Ca_Kabuli_denovo	10013311	(A/C)
19426	CakSNP19426	*Kabuli*	Ca_Kabuli_denovo	10013608	(T/C)
19427	CakSNP19427	*Kabuli*	Ca_Kabuli_denovo	10013617	(T/G)
19428	CakSNP19428	*Kabuli*	Ca_Kabuli_denovo	10015026	(C/G)
19429	CakSNP19429	*Kabuli*	Ca_Kabuli_denovo	10015729	(T/G)
19430	CakSNP19430	*Kabuli*	Ca_Kabuli_denovo	10016049	(C/A)
19431	CakSNP19431	*Kabuli*	Ca_Kabuli_denovo	10019860	(A/G)
19432	CakSNP19432	*Kabuli*	Ca_Kabuli_denovo	10019905	(A/G)
19433	CakSNP19433	*Kabuli*	Ca_Kabuli_denovo	10022721	(T/C)
19434	CakSNP19434	*Kabuli*	Ca_Kabuli_denovo	10022740	(C/A)
19435	CakSNP19435	*Kabuli*	Ca_Kabuli_denovo	10032574	(T/C)
19436	CakSNP19436	*Kabuli*	Ca_Kabuli_denovo	10032577	(T/G)
19437	CakSNP19437	*Kabuli*	Ca_Kabuli_denovo	10039375	(C/G)
19438	CakSNP19438	*Kabuli*	Ca_Kabuli_denovo	10039400	(C/T)
19439	CakSNP19439	*Kabuli*	Ca_Kabuli_denovo	10040960	(T/C)
19440	CakSNP19440	*Kabuli*	Ca_Kabuli_denovo	10041262	(T/G)
19441	CakSNP19441	*Kabuli*	Ca_Kabuli_denovo	10043768	(T/C)
19442	CakSNP19442	*Kabuli*	Ca_Kabuli_denovo	10045315	(A/G)
19443	CakSNP19443	*Kabuli*	Ca_Kabuli_denovo	10045339	(C/G)
19444	CakSNP19444	*Kabuli*	Ca_Kabuli_denovo	10061400	(T/C)
19445	CakSNP19445	*Kabuli*	Ca_Kabuli_denovo	10061401	(T/C)
19446	CakSNP19446	*Kabuli*	Ca_Kabuli_denovo	10061405	(C/G)
19447	CakSNP19447	*Kabuli*	Ca_Kabuli_denovo	10068192	(T/C)
19448	CakSNP19448	*Kabuli*	Ca_Kabuli_denovo	10070360	(G/C)
19449	CakSNP19449	*Kabuli*	Ca_Kabuli_denovo	10077413	(C/T)
19450	CakSNP19450	*Kabuli*	Ca_Kabuli_denovo	10077418	(C/T)
19451	CakSNP19451	*Kabuli*	Ca_Kabuli_denovo	10078621	(C/T)
19452	CakSNP19452	*Kabuli*	Ca_Kabuli_denovo	10084025	(T/C)
19453	CakSNP19453	*Kabuli*	Ca_Kabuli_denovo	10086513	(A/G)
19454	CakSNP19454	*Kabuli*	Ca_Kabuli_denovo	10086534	(C/T)
19455	CakSNP19455	*Kabuli*	Ca_Kabuli_denovo	10088290	(T/C)
19456	CakSNP19456	*Kabuli*	Ca_Kabuli_denovo	10090741	(G/C)
19457	CakSNP19457	*Kabuli*	Ca_Kabuli_denovo	10090793	(G/A)
19458	CakSNP19458	*Kabuli*	Ca_Kabuli_denovo	10091452	(G/C)
19459	CakSNP19459	*Kabuli*	Ca_Kabuli_denovo	10091458	(C/T)

S.N.	SNP IDs	Cultivars	Chromosomes/scaffolds	Physical positions (bp)	SNPs
19460	CakSNP19460	*Kabuli*	Ca_Kabuli_denovo	10093966	(A/G)
19461	CakSNP19461	*Kabuli*	Ca_Kabuli_denovo	10093969	(C/A)
19462	CakSNP19462	*Kabuli*	Ca_Kabuli_denovo	10096801	(A/G)
19463	CakSNP19463	*Kabuli*	Ca_Kabuli_denovo	10096810	(C/T)
19464	CakSNP19464	*Kabuli*	Ca_Kabuli_denovo	10097986	(T/C)
19465	CakSNP19465	*Kabuli*	Ca_Kabuli_denovo	10101482	(C/T)
19466	CakSNP19466	*Kabuli*	Ca_Kabuli_denovo	10101496	(C/T)
19467	CakSNP19467	*Kabuli*	Ca_Kabuli_denovo	10103354	(T/G)
19468	CakSNP19468	*Kabuli*	Ca_Kabuli_denovo	10103399	(A/C)
19469	CakSNP19469	*Kabuli*	Ca_Kabuli_denovo	10104830	(C/T)
19470	CakSNP19470	*Kabuli*	Ca_Kabuli_denovo	10104864	(G/A)
19471	CakSNP19471	*Kabuli*	Ca_Kabuli_denovo	10109044	(C/G)
19472	CakSNP19472	*Kabuli*	Ca_Kabuli_denovo	10112518	(A/C)
19473	CakSNP19473	*Kabuli*	Ca_Kabuli_denovo	10112928	(A/C)
19474	CakSNP19474	*Kabuli*	Ca_Kabuli_denovo	10112950	(G/C)
19475	CakSNP19475	*Kabuli*	Ca_Kabuli_denovo	10117322	(C/T)
19476	CakSNP19476	*Kabuli*	Ca_Kabuli_denovo	10117331	(T/G)
19477	CakSNP19477	*Kabuli*	Ca_Kabuli_denovo	10124748	(A/G)
19478	CakSNP19478	*Kabuli*	Ca_Kabuli_denovo	10124801	(G/A)
19479	CakSNP19479	*Kabuli*	Ca_Kabuli_denovo	10129566	(G/C)
19480	CakSNP19480	*Kabuli*	Ca_Kabuli_denovo	10129575	(T/C)
19481	CakSNP19481	*Kabuli*	Ca_Kabuli_denovo	10137026	(C/G)
19482	CakSNP19482	*Kabuli*	Ca_Kabuli_denovo	10137050	(T/C)
19483	CakSNP19483	*Kabuli*	Ca_Kabuli_denovo	10138779	(A/C)
19484	CakSNP19484	*Kabuli*	Ca_Kabuli_denovo	10141306	(G/T)
19485	CakSNP19485	*Kabuli*	Ca_Kabuli_denovo	10141307	(T/C)
19486	CakSNP19486	*Kabuli*	Ca_Kabuli_denovo	10141954	(A/G)
19487	CakSNP19487	*Kabuli*	Ca_Kabuli_denovo	10142443	(C/T)
19488	CakSNP19488	*Kabuli*	Ca_Kabuli_denovo	10145154	(A/G)
19489	CakSNP19489	*Kabuli*	Ca_Kabuli_denovo	10149666	(T/C)
19490	CakSNP19490	*Kabuli*	Ca_Kabuli_denovo	10149781	(G/A)
19491	CakSNP19491	*Kabuli*	Ca_Kabuli_denovo	10149790	(A/G)
19492	CakSNP19492	*Kabuli*	Ca_Kabuli_denovo	10149841	(T/C)
19493	CakSNP19493	*Kabuli*	Ca_Kabuli_denovo	10149869	(A/C)
19494	CakSNP19494	*Kabuli*	Ca_Kabuli_denovo	10156177	(T/C)
19495	CakSNP19495	*Kabuli*	Ca_Kabuli_denovo	10156216	(G/C)
19496	CakSNP19496	*Kabuli*	Ca_Kabuli_denovo	10168295	(G/A)
19497	CakSNP19497	*Kabuli*	Ca_Kabuli_denovo	10168335	(A/G)
19498	CakSNP19498	*Kabuli*	Ca_Kabuli_denovo	10169966	(G/A)

S.N.	SNP IDs	Cultivars	Chromosomes/scaffolds	Physical positions (bp)	SNPs
19499	CakSNP19499	*Kabuli*	Ca_Kabuli_denovo	10169996	(A/G)
19500	CakSNP19500	*Kabuli*	Ca_Kabuli_denovo	10171792	(A/C)
19501	CakSNP19501	*Kabuli*	Ca_Kabuli_denovo	10171816	(A/G)
19502	CakSNP19502	*Kabuli*	Ca_Kabuli_denovo	10175611	(A/C)
19503	CakSNP19503	*Kabuli*	Ca_Kabuli_denovo	10177607	(A/G)
19504	CakSNP19504	*Kabuli*	Ca_Kabuli_denovo	10177613	(T/C)
19505	CakSNP19505	*Kabuli*	Ca_Kabuli_denovo	10178789	(G/C)
19506	CakSNP19506	*Kabuli*	Ca_Kabuli_denovo	10178804	(C/T)
19507	CakSNP19507	*Kabuli*	Ca_Kabuli_denovo	10190673	(A/G)
19508	CakSNP19508	*Kabuli*	Ca_Kabuli_denovo	10194406	(C/T)
19509	CakSNP19509	*Kabuli*	Ca_Kabuli_denovo	10194475	(A/G)
19510	CakSNP19510	*Kabuli*	Ca_Kabuli_denovo	10205609	(G/A)
19511	CakSNP19511	*Kabuli*	Ca_Kabuli_denovo	10210572	(G/T)
19512	CakSNP19512	*Kabuli*	Ca_Kabuli_denovo	10213833	(A/C)
19513	CakSNP19513	*Kabuli*	Ca_Kabuli_denovo	10217093	(C/G)
19514	CakSNP19514	*Kabuli*	Ca_Kabuli_denovo	10217104	(A/C)
19515	CakSNP19515	*Kabuli*	Ca_Kabuli_denovo	10225142	(C/T)
19516	CakSNP19516	*Kabuli*	Ca_Kabuli_denovo	10225149	(G/A)
19517	CakSNP19517	*Kabuli*	Ca_Kabuli_denovo	10228761	(A/G)
19518	CakSNP19518	*Kabuli*	Ca_Kabuli_denovo	10228762	(G/A)
19519	CakSNP19519	*Kabuli*	Ca_Kabuli_denovo	10230849	(G/T)
19520	CakSNP19520	*Kabuli*	Ca_Kabuli_denovo	10230851	(T/C)
19521	CakSNP19521	*Kabuli*	Ca_Kabuli_denovo	10236737	(C/T)
19522	CakSNP19522	*Kabuli*	Ca_Kabuli_denovo	10254467	(C/T)
19523	CakSNP19523	*Kabuli*	Ca_Kabuli_denovo	10255898	(A/G)
19524	CakSNP19524	*Kabuli*	Ca_Kabuli_denovo	10256594	(C/G)
19525	CakSNP19525	*Kabuli*	Ca_Kabuli_denovo	10257196	(A/G)
19526	CakSNP19526	*Kabuli*	Ca_Kabuli_denovo	10261646	(T/C)
19527	CakSNP19527	*Kabuli*	Ca_Kabuli_denovo	10261700	(G/C)
19528	CakSNP19528	*Kabuli*	Ca_Kabuli_denovo	10262876	(C/T)
19529	CakSNP19529	*Kabuli*	Ca_Kabuli_denovo	10263678	(A/G)
19530	CakSNP19530	*Kabuli*	Ca_Kabuli_denovo	10265917	(C/T)
19531	CakSNP19531	*Kabuli*	Ca_Kabuli_denovo	10265974	(C/A)
19532	CakSNP19532	*Kabuli*	Ca_Kabuli_denovo	10269550	(C/T)
19533	CakSNP19533	*Kabuli*	Ca_Kabuli_denovo	10269607	(T/G)
19534	CakSNP19534	*Kabuli*	Ca_Kabuli_denovo	10274756	(G/T)
19535	CakSNP19535	*Kabuli*	Ca_Kabuli_denovo	10274772	(T/C)
19536	CakSNP19536	*Kabuli*	Ca_Kabuli_denovo	10279030	(C/T)
19537	CakSNP19537	*Kabuli*	Ca_Kabuli_denovo	10280444	(A/G)

S.N.	SNP IDs	Cultivars	Chromosomes/scaffolds	Physical positions (bp)	SNPs
19538	CakSNP19538	*Kabuli*	Ca_Kabuli_denovo	10286367	(T/C)
19539	CakSNP19539	*Kabuli*	Ca_Kabuli_denovo	10288899	(T/A)
19540	CakSNP19540	*Kabuli*	Ca_Kabuli_denovo	10288969	(C/T)
19541	CakSNP19541	*Kabuli*	Ca_Kabuli_denovo	10290935	(A/G)
19542	CakSNP19542	*Kabuli*	Ca_Kabuli_denovo	10291504	(G/A)
19543	CakSNP19543	*Kabuli*	Ca_Kabuli_denovo	10291534	(C/T)
19544	CakSNP19544	*Kabuli*	Ca_Kabuli_denovo	10292621	(T/C)
19545	CakSNP19545	*Kabuli*	Ca_Kabuli_denovo	10300039	(G/A)
19546	CakSNP19546	*Kabuli*	Ca_Kabuli_denovo	10309223	(C/G)
19547	CakSNP19547	*Kabuli*	Ca_Kabuli_denovo	10313066	(G/A)
19548	CakSNP19548	*Kabuli*	Ca_Kabuli_denovo	10313087	(A/G)
19549	CakSNP19549	*Kabuli*	Ca_Kabuli_denovo	10313600	(T/C)
19550	CakSNP19550	*Kabuli*	Ca_Kabuli_denovo	10320550	(G/C)
19551	CakSNP19551	*Kabuli*	Ca_Kabuli_denovo	10320553	(C/T)
19552	CakSNP19552	*Kabuli*	Ca_Kabuli_denovo	10321987	(T/A)
19553	CakSNP19553	*Kabuli*	Ca_Kabuli_denovo	10322002	(T/C)
19554	CakSNP19554	*Kabuli*	Ca_Kabuli_denovo	10324130	(C/G)
19555	CakSNP19555	*Kabuli*	Ca_Kabuli_denovo	10324205	(G/C)
19556	CakSNP19556	*Kabuli*	Ca_Kabuli_denovo	10328878	(T/C)
19557	CakSNP19557	*Kabuli*	Ca_Kabuli_denovo	10329076	(C/T)
19558	CakSNP19558	*Kabuli*	Ca_Kabuli_denovo	10332868	(G/A)
19559	CakSNP19559	*Kabuli*	Ca_Kabuli_denovo	10336212	(C/T)
19560	CakSNP19560	*Kabuli*	Ca_Kabuli_denovo	10336214	(C/T)
19561	CakSNP19561	*Kabuli*	Ca_Kabuli_denovo	10336794	(G/C)
19562	CakSNP19562	*Kabuli*	Ca_Kabuli_denovo	10339873	(C/T)
19563	CakSNP19563	*Kabuli*	Ca_Kabuli_denovo	10340283	(T/C)
19564	CakSNP19564	*Kabuli*	Ca_Kabuli_denovo	10349134	(G/T)
19565	CakSNP19565	*Kabuli*	Ca_Kabuli_denovo	10352881	(A/C)
19566	CakSNP19566	*Kabuli*	Ca_Kabuli_denovo	10352883	(A/G)
19567	CakSNP19567	*Kabuli*	Ca_Kabuli_denovo	10359340	(T/G)
19568	CakSNP19568	*Kabuli*	Ca_Kabuli_denovo	10362271	(C/T)
19569	CakSNP19569	*Kabuli*	Ca_Kabuli_denovo	10362307	(A/C)
19570	CakSNP19570	*Kabuli*	Ca_Kabuli_denovo	10371987	(T/G)
19571	CakSNP19571	*Kabuli*	Ca_Kabuli_denovo	10382340	(G/C)
19572	CakSNP19572	*Kabuli*	Ca_Kabuli_denovo	10382402	(T/C)
19573	CakSNP19573	*Kabuli*	Ca_Kabuli_denovo	10386409	(C/T)
19574	CakSNP19574	*Kabuli*	Ca_Kabuli_denovo	10387229	(G/A)
19575	CakSNP19575	*Kabuli*	Ca_Kabuli_denovo	10388512	(A/G)
19576	CakSNP19576	*Kabuli*	Ca_Kabuli_denovo	10389471	(G/A)

S.N.	SNP IDs	Cultivars	Chromosomes/scaffolds	Physical positions (bp)	SNPs
19577	CakSNP19577	*Kabuli*	Ca_Kabuli_denovo	10397284	(C/T)
19578	CakSNP19578	*Kabuli*	Ca_Kabuli_denovo	10397306	(C/G)
19579	CakSNP19579	*Kabuli*	Ca_Kabuli_denovo	10398136	(C/T)
19580	CakSNP19580	*Kabuli*	Ca_Kabuli_denovo	10400788	(G/C)
19581	CakSNP19581	*Kabuli*	Ca_Kabuli_denovo	10404768	(A/G)
19582	CakSNP19582	*Kabuli*	Ca_Kabuli_denovo	10404818	(G/A)
19583	CakSNP19583	*Kabuli*	Ca_Kabuli_denovo	10407328	(C/G)
19584	CakSNP19584	*Kabuli*	Ca_Kabuli_denovo	10413612	(A/G)
19585	CakSNP19585	*Kabuli*	Ca_Kabuli_denovo	10415506	(T/C)
19586	CakSNP19586	*Kabuli*	Ca_Kabuli_denovo	10417123	(G/C)
19587	CakSNP19587	*Kabuli*	Ca_Kabuli_denovo	10417126	(A/G)
19588	CakSNP19588	*Kabuli*	Ca_Kabuli_denovo	10420396	(C/T)
19589	CakSNP19589	*Kabuli*	Ca_Kabuli_denovo	10420453	(T/G)
19590	CakSNP19590	*Kabuli*	Ca_Kabuli_denovo	10421757	(G/A)
19591	CakSNP19591	*Kabuli*	Ca_Kabuli_denovo	10421896	(C/A)
19592	CakSNP19592	*Kabuli*	Ca_Kabuli_denovo	10423550	(C/T)
19593	CakSNP19593	*Kabuli*	Ca_Kabuli_denovo	10423580	(T/C)
19594	CakSNP19594	*Kabuli*	Ca_Kabuli_denovo	10426889	(T/C)
19595	CakSNP19595	*Kabuli*	Ca_Kabuli_denovo	10426931	(G/A)
19596	CakSNP19596	*Kabuli*	Ca_Kabuli_denovo	10427834	(G/C)
19597	CakSNP19597	*Kabuli*	Ca_Kabuli_denovo	10427835	(C/T)
19598	CakSNP19598	*Kabuli*	Ca_Kabuli_denovo	10427855	(T/C)
19599	CakSNP19599	*Kabuli*	Ca_Kabuli_denovo	10428671	(G/A)
19600	CakSNP19600	*Kabuli*	Ca_Kabuli_denovo	10428677	(G/T)
19601	CakSNP19601	*Kabuli*	Ca_Kabuli_denovo	10433563	(T/G)
19602	CakSNP19602	*Kabuli*	Ca_Kabuli_denovo	10434056	(G/A)
19603	CakSNP19603	*Kabuli*	Ca_Kabuli_denovo	10434071	(G/T)
19604	CakSNP19604	*Kabuli*	Ca_Kabuli_denovo	10434715	(T/C)
19605	CakSNP19605	*Kabuli*	Ca_Kabuli_denovo	10438263	(A/C)
19606	CakSNP19606	*Kabuli*	Ca_Kabuli_denovo	10438309	(A/C)
19607	CakSNP19607	*Kabuli*	Ca_Kabuli_denovo	10449585	(A/T)
19608	CakSNP19608	*Kabuli*	Ca_Kabuli_denovo	10453372	(G/A)
19609	CakSNP19609	*Kabuli*	Ca_Kabuli_denovo	10457782	(A/G)
19610	CakSNP19610	*Kabuli*	Ca_Kabuli_denovo	10462185	(T/G)
19611	CakSNP19611	*Kabuli*	Ca_Kabuli_denovo	10463932	(A/G)
19612	CakSNP19612	*Kabuli*	Ca_Kabuli_denovo	10466745	(C/T)
19613	CakSNP19613	*Kabuli*	Ca_Kabuli_denovo	10466768	(C/T)
19614	CakSNP19614	*Kabuli*	Ca_Kabuli_denovo	10471964	(A/G)
19615	CakSNP19615	*Kabuli*	Ca_Kabuli_denovo	10474267	(A/C)

S.N.	SNP IDs	Cultivars	Chromosomes/scaffolds	Physical positions (bp)	SNPs
19616	CakSNP19616	*Kabuli*	Ca_Kabuli_denovo	10474993	(G/A)
19617	CakSNP19617	*Kabuli*	Ca_Kabuli_denovo	10475020	(C/G)
19618	CakSNP19618	*Kabuli*	Ca_Kabuli_denovo	10476143	(A/C)
19619	CakSNP19619	*Kabuli*	Ca_Kabuli_denovo	10478166	(G/A)
19620	CakSNP19620	*Kabuli*	Ca_Kabuli_denovo	10478171	(C/T)
19621	CakSNP19621	*Kabuli*	Ca_Kabuli_denovo	10480186	(G/A)
19622	CakSNP19622	*Kabuli*	Ca_Kabuli_denovo	10480247	(G/A)
19623	CakSNP19623	*Kabuli*	Ca_Kabuli_denovo	10480562	(A/G)
19624	CakSNP19624	*Kabuli*	Ca_Kabuli_denovo	10487489	(C/T)
19625	CakSNP19625	*Kabuli*	Ca_Kabuli_denovo	10487501	(C/T)
19626	CakSNP19626	*Kabuli*	Ca_Kabuli_denovo	10487719	(C/A)
19627	CakSNP19627	*Kabuli*	Ca_Kabuli_denovo	10488191	(G/A)
19628	CakSNP19628	*Kabuli*	Ca_Kabuli_denovo	10488218	(T/C)
19629	CakSNP19629	*Kabuli*	Ca_Kabuli_denovo	10490833	(T/G)
19630	CakSNP19630	*Kabuli*	Ca_Kabuli_denovo	10490857	(C/T)
19631	CakSNP19631	*Kabuli*	Ca_Kabuli_denovo	10491343	(G/A)
19632	CakSNP19632	*Kabuli*	Ca_Kabuli_denovo	10491421	(A/C)
19633	CakSNP19633	*Kabuli*	Ca_Kabuli_denovo	10504586	(T/A)
19634	CakSNP19634	*Kabuli*	Ca_Kabuli_denovo	10509436	(C/T)
19635	CakSNP19635	*Kabuli*	Ca_Kabuli_denovo	10520172	(A/G)
19636	CakSNP19636	*Kabuli*	Ca_Kabuli_denovo	10521045	(C/T)
19637	CakSNP19637	*Kabuli*	Ca_Kabuli_denovo	10522423	(C/T)
19638	CakSNP19638	*Kabuli*	Ca_Kabuli_denovo	10526136	(T/C)
19639	CakSNP19639	*Kabuli*	Ca_Kabuli_denovo	10526145	(C/T)
19640	CakSNP19640	*Kabuli*	Ca_Kabuli_denovo	10527066	(C/A)
19641	CakSNP19641	*Kabuli*	Ca_Kabuli_denovo	10528013	(A/G)
19642	CakSNP19642	*Kabuli*	Ca_Kabuli_denovo	10538248	(G/A)
19643	CakSNP19643	*Kabuli*	Ca_Kabuli_denovo	10544632	(A/G)
19644	CakSNP19644	*Kabuli*	Ca_Kabuli_denovo	10546885	(A/G)
19645	CakSNP19645	*Kabuli*	Ca_Kabuli_denovo	10555780	(C/A)
19646	CakSNP19646	*Kabuli*	Ca_Kabuli_denovo	10558499	(C/T)
19647	CakSNP19647	*Kabuli*	Ca_Kabuli_denovo	10558812	(T/C)
19648	CakSNP19648	*Kabuli*	Ca_Kabuli_denovo	10559975	(A/G)
19649	CakSNP19649	*Kabuli*	Ca_Kabuli_denovo	10563440	(G/A)
19650	CakSNP19650	*Kabuli*	Ca_Kabuli_denovo	10566203	(C/G)
19651	CakSNP19651	*Kabuli*	Ca_Kabuli_denovo	10580621	(G/A)
19652	CakSNP19652	*Kabuli*	Ca_Kabuli_denovo	10583596	(G/A)
19653	CakSNP19653	*Kabuli*	Ca_Kabuli_denovo	10586396	(G/A)
19654	CakSNP19654	*Kabuli*	Ca_Kabuli_denovo	10586476	(A/G)

S.N.	SNP IDs	Cultivars	Chromosomes/scaffolds	Physical positions (bp)	SNPs
19655	CakSNP19655	*Kabuli*	Ca_Kabuli_denovo	10587347	(T/C)
19656	CakSNP19656	*Kabuli*	Ca_Kabuli_denovo	10587505	(C/G)
19657	CakSNP19657	*Kabuli*	Ca_Kabuli_denovo	10597388	(C/G)
19658	CakSNP19658	*Kabuli*	Ca_Kabuli_denovo	10597948	(T/C)
19659	CakSNP19659	*Kabuli*	Ca_Kabuli_denovo	10601860	(C/T)
19660	CakSNP19660	*Kabuli*	Ca_Kabuli_denovo	10602584	(A/G)
19661	CakSNP19661	*Kabuli*	Ca_Kabuli_denovo	10604019	(C/T)
19662	CakSNP19662	*Kabuli*	Ca_Kabuli_denovo	10608370	(C/T)
19663	CakSNP19663	*Kabuli*	Ca_Kabuli_denovo	10609543	(G/A)
19664	CakSNP19664	*Kabuli*	Ca_Kabuli_denovo	10612490	(G/A)
19665	CakSNP19665	*Kabuli*	Ca_Kabuli_denovo	10613546	(T/G)
19666	CakSNP19666	*Kabuli*	Ca_Kabuli_denovo	10614294	(C/T)
19667	CakSNP19667	*Kabuli*	Ca_Kabuli_denovo	10614905	(G/A)
19668	CakSNP19668	*Kabuli*	Ca_Kabuli_denovo	10619587	(G/A)
19669	CakSNP19669	*Kabuli*	Ca_Kabuli_denovo	10623248	(A/G)
19670	CakSNP19670	*Kabuli*	Ca_Kabuli_denovo	10625948	(C/A)
19671	CakSNP19671	*Kabuli*	Ca_Kabuli_denovo	10625976	(A/T)
19672	CakSNP19672	*Kabuli*	Ca_Kabuli_denovo	10633747	(C/T)
19673	CakSNP19673	*Kabuli*	Ca_Kabuli_denovo	10634473	(A/G)
19674	CakSNP19674	*Kabuli*	Ca_Kabuli_denovo	10634539	(C/G)
19675	CakSNP19675	*Kabuli*	Ca_Kabuli_denovo	10635906	(G/A)
19676	CakSNP19676	*Kabuli*	Ca_Kabuli_denovo	10636722	(T/G)
19677	CakSNP19677	*Kabuli*	Ca_Kabuli_denovo	10636753	(T/C)
19678	CakSNP19678	*Kabuli*	Ca_Kabuli_denovo	10638379	(C/T)
19679	CakSNP19679	*Kabuli*	Ca_Kabuli_denovo	10638416	(C/T)
19680	CakSNP19680	*Kabuli*	Ca_Kabuli_denovo	10639123	(T/C)
19681	CakSNP19681	*Kabuli*	Ca_Kabuli_denovo	10639199	(A/C)
19682	CakSNP19682	*Kabuli*	Ca_Kabuli_denovo	10639200	(G/T)
19683	CakSNP19683	*Kabuli*	Ca_Kabuli_denovo	10639201	(A/C)
19684	CakSNP19684	*Kabuli*	Ca_Kabuli_denovo	10645075	(G/A)
19685	CakSNP19685	*Kabuli*	Ca_Kabuli_denovo	10648894	(A/C)
19686	CakSNP19686	*Kabuli*	Ca_Kabuli_denovo	10648964	(A/G)
19687	CakSNP19687	*Kabuli*	Ca_Kabuli_denovo	10651838	(C/T)
19688	CakSNP19688	*Kabuli*	Ca_Kabuli_denovo	10652481	(A/G)
19689	CakSNP19689	*Kabuli*	Ca_Kabuli_denovo	10652486	(T/C)
19690	CakSNP19690	*Kabuli*	Ca_Kabuli_denovo	10660520	(G/A)
19691	CakSNP19691	*Kabuli*	Ca_Kabuli_denovo	10675025	(A/G)
19692	CakSNP19692	*Kabuli*	Ca_Kabuli_denovo	10675052	(G/A)
19693	CakSNP19693	*Kabuli*	Ca_Kabuli_denovo	10675879	(A/G)

S.N.	SNP IDs	Cultivars	Chromosomes/scaffolds	Physical positions (bp)	SNPs
19694	CakSNP19694	*Kabuli*	Ca_Kabuli_denovo	10682359	(A/G)
19695	CakSNP19695	*Kabuli*	Ca_Kabuli_denovo	10685319	(T/A)
19696	CakSNP19696	*Kabuli*	Ca_Kabuli_denovo	10690207	(C/A)
19697	CakSNP19697	*Kabuli*	Ca_Kabuli_denovo	10690236	(T/C)
19698	CakSNP19698	*Kabuli*	Ca_Kabuli_denovo	10704771	(C/T)
19699	CakSNP19699	*Kabuli*	Ca_Kabuli_denovo	10704772	(C/T)
19700	CakSNP19700	*Kabuli*	Ca_Kabuli_denovo	10709211	(G/A)
19701	CakSNP19701	*Kabuli*	Ca_Kabuli_denovo	10709212	(C/T)
19702	CakSNP19702	*Kabuli*	Ca_Kabuli_denovo	10711513	(C/T)
19703	CakSNP19703	*Kabuli*	Ca_Kabuli_denovo	10717240	(C/T)
19704	CakSNP19704	*Kabuli*	Ca_Kabuli_denovo	10725261	(T/C)
19705	CakSNP19705	*Kabuli*	Ca_Kabuli_denovo	10725280	(A/G)
19706	CakSNP19706	*Kabuli*	Ca_Kabuli_denovo	10726837	(T/G)
19707	CakSNP19707	*Kabuli*	Ca_Kabuli_denovo	10726857	(A/G)
19708	CakSNP19708	*Kabuli*	Ca_Kabuli_denovo	10727523	(C/T)
19709	CakSNP19709	*Kabuli*	Ca_Kabuli_denovo	10737510	(A/C)
19710	CakSNP19710	*Kabuli*	Ca_Kabuli_denovo	10737559	(A/G)
19711	CakSNP19711	*Kabuli*	Ca_Kabuli_denovo	10744775	(G/A)
19712	CakSNP19712	*Kabuli*	Ca_Kabuli_denovo	10748089	(T/C)
19713	CakSNP19713	*Kabuli*	Ca_Kabuli_denovo	10748469	(T/G)
19714	CakSNP19714	*Kabuli*	Ca_Kabuli_denovo	10750888	(A/G)
19715	CakSNP19715	*Kabuli*	Ca_Kabuli_denovo	10750911	(T/C)
19716	CakSNP19716	*Kabuli*	Ca_Kabuli_denovo	10750941	(G/A)
19717	CakSNP19717	*Kabuli*	Ca_Kabuli_denovo	10765332	(C/T)
19718	CakSNP19718	*Kabuli*	Ca_Kabuli_denovo	10774154	(C/A)
19719	CakSNP19719	*Kabuli*	Ca_Kabuli_denovo	10774193	(A/G)
19720	CakSNP19720	*Kabuli*	Ca_Kabuli_denovo	10779799	(C/G)
19721	CakSNP19721	*Kabuli*	Ca_Kabuli_denovo	10779817	(C/T)
19722	CakSNP19722	*Kabuli*	Ca_Kabuli_denovo	10784206	(C/G)
19723	CakSNP19723	*Kabuli*	Ca_Kabuli_denovo	10785716	(C/G)
19724	CakSNP19724	*Kabuli*	Ca_Kabuli_denovo	10793538	(C/T)
19725	CakSNP19725	*Kabuli*	Ca_Kabuli_denovo	10801968	(C/G)
19726	CakSNP19726	*Kabuli*	Ca_Kabuli_denovo	10802297	(T/C)
19727	CakSNP19727	*Kabuli*	Ca_Kabuli_denovo	10802312	(A/G)
19728	CakSNP19728	*Kabuli*	Ca_Kabuli_denovo	10804689	(A/C)
19729	CakSNP19729	*Kabuli*	Ca_Kabuli_denovo	10804725	(A/C)
19730	CakSNP19730	*Kabuli*	Ca_Kabuli_denovo	10815305	(C/T)
19731	CakSNP19731	*Kabuli*	Ca_Kabuli_denovo	10816024	(A/G)
19732	CakSNP19732	*Kabuli*	Ca_Kabuli_denovo	10820299	(T/C)

S.N.	SNP IDs	Cultivars	Chromosomes/scaffolds	Physical positions (bp)	SNPs
19733	CakSNP19733	*Kabuli*	Ca_Kabuli_denovo	10820305	(T/G)
19734	CakSNP19734	*Kabuli*	Ca_Kabuli_denovo	10827752	(T/C)
19735	CakSNP19735	*Kabuli*	Ca_Kabuli_denovo	10827766	(C/G)
19736	CakSNP19736	*Kabuli*	Ca_Kabuli_denovo	10829958	(G/C)
19737	CakSNP19737	*Kabuli*	Ca_Kabuli_denovo	10830004	(C/T)
19738	CakSNP19738	*Kabuli*	Ca_Kabuli_denovo	10836808	(T/G)
19739	CakSNP19739	*Kabuli*	Ca_Kabuli_denovo	10837882	(A/G)
19740	CakSNP19740	*Kabuli*	Ca_Kabuli_denovo	10837892	(A/C)
19741	CakSNP19741	*Kabuli*	Ca_Kabuli_denovo	10843751	(G/A)
19742	CakSNP19742	*Kabuli*	Ca_Kabuli_denovo	10852994	(G/T)
19743	CakSNP19743	*Kabuli*	Ca_Kabuli_denovo	10853440	(C/A)
19744	CakSNP19744	*Kabuli*	Ca_Kabuli_denovo	10855184	(A/C)
19745	CakSNP19745	*Kabuli*	Ca_Kabuli_denovo	10859383	(A/G)
19746	CakSNP19746	*Kabuli*	Ca_Kabuli_denovo	10859422	(C/T)
19747	CakSNP19747	*Kabuli*	Ca_Kabuli_denovo	10863716	(A/C)
19748	CakSNP19748	*Kabuli*	Ca_Kabuli_denovo	10863767	(C/T)
19749	CakSNP19749	*Kabuli*	Ca_Kabuli_denovo	10867451	(C/A)
19750	CakSNP19750	*Kabuli*	Ca_Kabuli_denovo	10868177	(C/T)
19751	CakSNP19751	*Kabuli*	Ca_Kabuli_denovo	10871629	(C/T)
19752	CakSNP19752	*Kabuli*	Ca_Kabuli_denovo	10871630	(G/A)
19753	CakSNP19753	*Kabuli*	Ca_Kabuli_denovo	10872857	(C/A)
19754	CakSNP19754	*Kabuli*	Ca_Kabuli_denovo	10872867	(T/G)
19755	CakSNP19755	*Kabuli*	Ca_Kabuli_denovo	10873672	(G/C)
19756	CakSNP19756	*Kabuli*	Ca_Kabuli_denovo	10879509	(A/G)
19757	CakSNP19757	*Kabuli*	Ca_Kabuli_denovo	10880917	(G/C)
19758	CakSNP19758	*Kabuli*	Ca_Kabuli_denovo	10882337	(C/G)
19759	CakSNP19759	*Kabuli*	Ca_Kabuli_denovo	10884357	(A/G)
19760	CakSNP19760	*Kabuli*	Ca_Kabuli_denovo	10884407	(A/T)
19761	CakSNP19761	*Kabuli*	Ca_Kabuli_denovo	10894351	(C/A)
19762	CakSNP19762	*Kabuli*	Ca_Kabuli_denovo	10894895	(A/G)
19763	CakSNP19763	*Kabuli*	Ca_Kabuli_denovo	10894913	(T/C)
19764	CakSNP19764	*Kabuli*	Ca_Kabuli_denovo	10897663	(C/T)
19765	CakSNP19765	*Kabuli*	Ca_Kabuli_denovo	10905659	(A/G)
19766	CakSNP19766	*Kabuli*	Ca_Kabuli_denovo	10929918	(A/T)
19767	CakSNP19767	*Kabuli*	Ca_Kabuli_denovo	10930163	(G/T)
19768	CakSNP19768	*Kabuli*	Ca_Kabuli_denovo	10935880	(C/T)
19769	CakSNP19769	*Kabuli*	Ca_Kabuli_denovo	10935937	(A/C)
19770	CakSNP19770	*Kabuli*	Ca_Kabuli_denovo	10936993	(A/G)
19771	CakSNP19771	*Kabuli*	Ca_Kabuli_denovo	10942394	(A/G)

S.N.	SNP IDs	Cultivars	Chromosomes/scaffolds	Physical positions (bp)	SNPs
19772	CakSNP19772	*Kabuli*	Ca_Kabuli_denovo	10942431	(A/G)
19773	CakSNP19773	*Kabuli*	Ca_Kabuli_denovo	10943375	(T/C)
19774	CakSNP19774	*Kabuli*	Ca_Kabuli_denovo	10943443	(C/T)
19775	CakSNP19775	*Kabuli*	Ca_Kabuli_denovo	10945287	(G/A)
19776	CakSNP19776	*Kabuli*	Ca_Kabuli_denovo	10945318	(G/C)
19777	CakSNP19777	*Kabuli*	Ca_Kabuli_denovo	10951393	(G/T)
19778	CakSNP19778	*Kabuli*	Ca_Kabuli_denovo	10951395	(A/G)
19779	CakSNP19779	*Kabuli*	Ca_Kabuli_denovo	10957310	(C/T)
19780	CakSNP19780	*Kabuli*	Ca_Kabuli_denovo	10958307	(C/T)
19781	CakSNP19781	*Kabuli*	Ca_Kabuli_denovo	10958312	(C/A)
19782	CakSNP19782	*Kabuli*	Ca_Kabuli_denovo	10959965	(C/G)
19783	CakSNP19783	*Kabuli*	Ca_Kabuli_denovo	10960754	(G/A)
19784	CakSNP19784	*Kabuli*	Ca_Kabuli_denovo	10964203	(C/T)
19785	CakSNP19785	*Kabuli*	Ca_Kabuli_denovo	10964227	(G/A)
19786	CakSNP19786	*Kabuli*	Ca_Kabuli_denovo	10971033	(G/A)
19787	CakSNP19787	*Kabuli*	Ca_Kabuli_denovo	10971053	(G/C)
19788	CakSNP19788	*Kabuli*	Ca_Kabuli_denovo	10977079	(A/C)
19789	CakSNP19789	*Kabuli*	Ca_Kabuli_denovo	10978239	(A/G)
19790	CakSNP19790	*Kabuli*	Ca_Kabuli_denovo	10978244	(G/A)
19791	CakSNP19791	*Kabuli*	Ca_Kabuli_denovo	10978256	(C/G)
19792	CakSNP19792	*Kabuli*	Ca_Kabuli_denovo	10985386	(T/C)
19793	CakSNP19793	*Kabuli*	Ca_Kabuli_denovo	10985566	(A/G)
19794	CakSNP19794	*Kabuli*	Ca_Kabuli_denovo	10985567	(T/C)
19795	CakSNP19795	*Kabuli*	Ca_Kabuli_denovo	10986704	(C/G)
19796	CakSNP19796	*Kabuli*	Ca_Kabuli_denovo	10986710	(C/G)
19797	CakSNP19797	*Kabuli*	Ca_Kabuli_denovo	10987146	(T/G)
19798	CakSNP19798	*Kabuli*	Ca_Kabuli_denovo	10987152	(T/A)
19799	CakSNP19799	*Kabuli*	Ca_Kabuli_denovo	10989989	(T/G)
19800	CakSNP19800	*Kabuli*	Ca_Kabuli_denovo	10994002	(C/T)
19801	CakSNP19801	*Kabuli*	Ca_Kabuli_denovo	10994032	(T/C)
19802	CakSNP19802	*Kabuli*	Ca_Kabuli_denovo	10996772	(C/T)
19803	CakSNP19803	*Kabuli*	Ca_Kabuli_denovo	11000849	(G/C)
19804	CakSNP19804	*Kabuli*	Ca_Kabuli_denovo	11000856	(C/G)
19805	CakSNP19805	*Kabuli*	Ca_Kabuli_denovo	11009676	(C/T)
19806	CakSNP19806	*Kabuli*	Ca_Kabuli_denovo	11015742	(G/A)
19807	CakSNP19807	*Kabuli*	Ca_Kabuli_denovo	11018664	(C/A)
19808	CakSNP19808	*Kabuli*	Ca_Kabuli_denovo	11018718	(C/G)
19809	CakSNP19809	*Kabuli*	Ca_Kabuli_denovo	11022673	(C/G)
19810	CakSNP19810	*Kabuli*	Ca_Kabuli_denovo	11022685	(T/C)

S.N.	SNP IDs	Cultivars	Chromosomes/scaffolds	Physical positions (bp)	SNPs
19811	CakSNP19811	*Kabuli*	Ca_Kabuli_denovo	11022766	(C/T)
19812	CakSNP19812	*Kabuli*	Ca_Kabuli_denovo	11025360	(A/C)
19813	CakSNP19813	*Kabuli*	Ca_Kabuli_denovo	11025765	(C/G)
19814	CakSNP19814	*Kabuli*	Ca_Kabuli_denovo	11035425	(C/T)
19815	CakSNP19815	*Kabuli*	Ca_Kabuli_denovo	11035455	(C/G)
19816	CakSNP19816	*Kabuli*	Ca_Kabuli_denovo	11038835	(C/G)
19817	CakSNP19817	*Kabuli*	Ca_Kabuli_denovo	11038859	(T/C)
19818	CakSNP19818	*Kabuli*	Ca_Kabuli_denovo	11041618	(G/A)
19819	CakSNP19819	*Kabuli*	Ca_Kabuli_denovo	11041631	(A/G)
19820	CakSNP19820	*Kabuli*	Ca_Kabuli_denovo	11049997	(A/G)
19821	CakSNP19821	*Kabuli*	Ca_Kabuli_denovo	11050024	(G/A)
19822	CakSNP19822	*Kabuli*	Ca_Kabuli_denovo	11052623	(T/G)
19823	CakSNP19823	*Kabuli*	Ca_Kabuli_denovo	11052626	(T/C)
19824	CakSNP19824	*Kabuli*	Ca_Kabuli_denovo	11059845	(G/A)
19825	CakSNP19825	*Kabuli*	Ca_Kabuli_denovo	11060901	(G/C)
19826	CakSNP19826	*Kabuli*	Ca_Kabuli_denovo	11063106	(T/A)
19827	CakSNP19827	*Kabuli*	Ca_Kabuli_denovo	11078845	(G/C)
19828	CakSNP19828	*Kabuli*	Ca_Kabuli_denovo	11081534	(T/C)
19829	CakSNP19829	*Kabuli*	Ca_Kabuli_denovo	11081662	(C/T)
19830	CakSNP19830	*Kabuli*	Ca_Kabuli_denovo	11083492	(G/A)
19831	CakSNP19831	*Kabuli*	Ca_Kabuli_denovo	11083548	(G/A)
19832	CakSNP19832	*Kabuli*	Ca_Kabuli_denovo	11086369	(G/A)
19833	CakSNP19833	*Kabuli*	Ca_Kabuli_denovo	11099095	(G/A)
19834	CakSNP19834	*Kabuli*	Ca_Kabuli_denovo	11101450	(A/G)
19835	CakSNP19835	*Kabuli*	Ca_Kabuli_denovo	11101462	(T/C)
19836	CakSNP19836	*Kabuli*	Ca_Kabuli_denovo	11114537	(G/C)
19837	CakSNP19837	*Kabuli*	Ca_Kabuli_denovo	11116590	(A/G)
19838	CakSNP19838	*Kabuli*	Ca_Kabuli_denovo	11116620	(T/C)
19839	CakSNP19839	*Kabuli*	Ca_Kabuli_denovo	11117655	(A/C)
19840	CakSNP19840	*Kabuli*	Ca_Kabuli_denovo	11127124	(T/C)
19841	CakSNP19841	*Kabuli*	Ca_Kabuli_denovo	11129839	(G/A)
19842	CakSNP19842	*Kabuli*	Ca_Kabuli_denovo	11132036	(G/C)
19843	CakSNP19843	*Kabuli*	Ca_Kabuli_denovo	11136790	(A/G)
19844	CakSNP19844	*Kabuli*	Ca_Kabuli_denovo	11136841	(C/T)
19845	CakSNP19845	*Kabuli*	Ca_Kabuli_denovo	11137317	(G/A)
19846	CakSNP19846	*Kabuli*	Ca_Kabuli_denovo	11137921	(G/T)
19847	CakSNP19847	*Kabuli*	Ca_Kabuli_denovo	11143568	(A/G)
19848	CakSNP19848	*Kabuli*	Ca_Kabuli_denovo	11144773	(A/G)
19849	CakSNP19849	*Kabuli*	Ca_Kabuli_denovo	11144842	(A/C)

S.N.	SNP IDs	Cultivars	Chromosomes/scaffolds	Physical positions (bp)	SNPs
19850	CakSNP19850	*Kabuli*	Ca_Kabuli_denovo	11146079	(C/A)
19851	CakSNP19851	*Kabuli*	Ca_Kabuli_denovo	11161889	(G/T)
19852	CakSNP19852	*Kabuli*	Ca_Kabuli_denovo	11162483	(G/T)
19853	CakSNP19853	*Kabuli*	Ca_Kabuli_denovo	11162500	(A/G)
19854	CakSNP19854	*Kabuli*	Ca_Kabuli_denovo	11165328	(A/G)
19855	CakSNP19855	*Kabuli*	Ca_Kabuli_denovo	11165347	(A/C)
19856	CakSNP19856	*Kabuli*	Ca_Kabuli_denovo	11171823	(G/T)
19857	CakSNP19857	*Kabuli*	Ca_Kabuli_denovo	11172625	(A/G)
19858	CakSNP19858	*Kabuli*	Ca_Kabuli_denovo	11172634	(A/G)
19859	CakSNP19859	*Kabuli*	Ca_Kabuli_denovo	11174544	(G/T)
19860	CakSNP19860	*Kabuli*	Ca_Kabuli_denovo	11174567	(A/G)
19861	CakSNP19861	*Kabuli*	Ca_Kabuli_denovo	11177330	(C/G)
19862	CakSNP19862	*Kabuli*	Ca_Kabuli_denovo	11185106	(C/G)
19863	CakSNP19863	*Kabuli*	Ca_Kabuli_denovo	11188583	(G/C)
19864	CakSNP19864	*Kabuli*	Ca_Kabuli_denovo	11193535	(C/G)
19865	CakSNP19865	*Kabuli*	Ca_Kabuli_denovo	11193573	(G/A)
19866	CakSNP19866	*Kabuli*	Ca_Kabuli_denovo	11195851	(G/T)
19867	CakSNP19867	*Kabuli*	Ca_Kabuli_denovo	11204126	(C/T)
19868	CakSNP19868	*Kabuli*	Ca_Kabuli_denovo	11204171	(A/C)
19869	CakSNP19869	*Kabuli*	Ca_Kabuli_denovo	11205211	(G/A)
19870	CakSNP19870	*Kabuli*	Ca_Kabuli_denovo	11205220	(C/A)
19871	CakSNP19871	*Kabuli*	Ca_Kabuli_denovo	11205976	(A/G)
19872	CakSNP19872	*Kabuli*	Ca_Kabuli_denovo	11205991	(C/T)
19873	CakSNP19873	*Kabuli*	Ca_Kabuli_denovo	11207799	(T/C)
19874	CakSNP19874	*Kabuli*	Ca_Kabuli_denovo	11210096	(T/C)
19875	CakSNP19875	*Kabuli*	Ca_Kabuli_denovo	11212029	(T/C)
19876	CakSNP19876	*Kabuli*	Ca_Kabuli_denovo	11212077	(A/G)
19877	CakSNP19877	*Kabuli*	Ca_Kabuli_denovo	11212273	(G/C)
19878	CakSNP19878	*Kabuli*	Ca_Kabuli_denovo	11212276	(A/G)
19879	CakSNP19879	*Kabuli*	Ca_Kabuli_denovo	11226427	(A/G)
19880	CakSNP19880	*Kabuli*	Ca_Kabuli_denovo	11226478	(C/G)
19881	CakSNP19881	*Kabuli*	Ca_Kabuli_denovo	11232178	(C/A)
19882	CakSNP19882	*Kabuli*	Ca_Kabuli_denovo	11232208	(T/C)
19883	CakSNP19883	*Kabuli*	Ca_Kabuli_denovo	11235535	(T/C)
19884	CakSNP19884	*Kabuli*	Ca_Kabuli_denovo	11238073	(G/A)
19885	CakSNP19885	*Kabuli*	Ca_Kabuli_denovo	11238076	(C/G)
19886	CakSNP19886	*Kabuli*	Ca_Kabuli_denovo	11243846	(C/T)
19887	CakSNP19887	*Kabuli*	Ca_Kabuli_denovo	11247995	(G/A)
19888	CakSNP19888	*Kabuli*	Ca_Kabuli_denovo	11251636	(A/G)

S.N.	SNP IDs	Cultivars	Chromosomes/scaffolds	Physical positions (bp)	SNPs
19889	CakSNP19889	*Kabuli*	Ca_Kabuli_denovo	11253863	(C/T)
19890	CakSNP19890	*Kabuli*	Ca_Kabuli_denovo	11253937	(C/T)
19891	CakSNP19891	*Kabuli*	Ca_Kabuli_denovo	11253941	(G/T)
19892	CakSNP19892	*Kabuli*	Ca_Kabuli_denovo	11254529	(C/A)
19893	CakSNP19893	*Kabuli*	Ca_Kabuli_denovo	11254535	(G/C)
19894	CakSNP19894	*Kabuli*	Ca_Kabuli_denovo	11257861	(A/G)
19895	CakSNP19895	*Kabuli*	Ca_Kabuli_denovo	11257890	(T/C)
19896	CakSNP19896	*Kabuli*	Ca_Kabuli_denovo	11260640	(C/T)
19897	CakSNP19897	*Kabuli*	Ca_Kabuli_denovo	11261285	(T/G)
19898	CakSNP19898	*Kabuli*	Ca_Kabuli_denovo	11263910	(C/T)
19899	CakSNP19899	*Kabuli*	Ca_Kabuli_denovo	11264488	(A/G)
19900	CakSNP19900	*Kabuli*	Ca_Kabuli_denovo	11266340	(C/G)
19901	CakSNP19901	*Kabuli*	Ca_Kabuli_denovo	11268597	(A/G)
19902	CakSNP19902	*Kabuli*	Ca_Kabuli_denovo	11269385	(C/A)
19903	CakSNP19903	*Kabuli*	Ca_Kabuli_denovo	11274782	(G/A)
19904	CakSNP19904	*Kabuli*	Ca_Kabuli_denovo	11274830	(A/G)
19905	CakSNP19905	*Kabuli*	Ca_Kabuli_denovo	11276474	(C/T)
19906	CakSNP19906	*Kabuli*	Ca_Kabuli_denovo	11281562	(A/G)
19907	CakSNP19907	*Kabuli*	Ca_Kabuli_denovo	11282591	(A/T)
19908	CakSNP19908	*Kabuli*	Ca_Kabuli_denovo	11284197	(C/T)
19909	CakSNP19909	*Kabuli*	Ca_Kabuli_denovo	11284218	(A/G)
19910	CakSNP19910	*Kabuli*	Ca_Kabuli_denovo	11294318	(T/C)
19911	CakSNP19911	*Kabuli*	Ca_Kabuli_denovo	11294360	(T/C)
19912	CakSNP19912	*Kabuli*	Ca_Kabuli_denovo	11298034	(A/G)
19913	CakSNP19913	*Kabuli*	Ca_Kabuli_denovo	11302009	(A/G)
19914	CakSNP19914	*Kabuli*	Ca_Kabuli_denovo	11303066	(G/A)
19915	CakSNP19915	*Kabuli*	Ca_Kabuli_denovo	11303067	(C/G)
19916	CakSNP19916	*Kabuli*	Ca_Kabuli_denovo	11303075	(A/G)
19917	CakSNP19917	*Kabuli*	Ca_Kabuli_denovo	11305242	(C/G)
19918	CakSNP19918	*Kabuli*	Ca_Kabuli_denovo	11311346	(G/A)
19919	CakSNP19919	*Kabuli*	Ca_Kabuli_denovo	11311355	(C/G)
19920	CakSNP19920	*Kabuli*	Ca_Kabuli_denovo	11313010	(G/A)
19921	CakSNP19921	*Kabuli*	Ca_Kabuli_denovo	11313019	(G/T)
19922	CakSNP19922	*Kabuli*	Ca_Kabuli_denovo	11320821	(C/T)
19923	CakSNP19923	*Kabuli*	Ca_Kabuli_denovo	11320852	(C/T)
19924	CakSNP19924	*Kabuli*	Ca_Kabuli_denovo	11320859	(C/G)
19925	CakSNP19925	*Kabuli*	Ca_Kabuli_denovo	11321893	(C/A)
19926	CakSNP19926	*Kabuli*	Ca_Kabuli_denovo	11322134	(A/G)
19927	CakSNP19927	*Kabuli*	Ca_Kabuli_denovo	11322171	(A/G)

S.N.	SNP IDs	Cultivars	Chromosomes/scaffolds	Physical positions (bp)	SNPs
19928	CakSNP19928	*Kabuli*	Ca_Kabuli_denovo	11322424	(G/A)
19929	CakSNP19929	*Kabuli*	Ca_Kabuli_denovo	11328453	(T/C)
19930	CakSNP19930	*Kabuli*	Ca_Kabuli_denovo	11328457	(A/G)
19931	CakSNP19931	*Kabuli*	Ca_Kabuli_denovo	11330932	(A/C)
19932	CakSNP19932	*Kabuli*	Ca_Kabuli_denovo	11335776	(T/C)
19933	CakSNP19933	*Kabuli*	Ca_Kabuli_denovo	11335791	(T/C)
19934	CakSNP19934	*Kabuli*	Ca_Kabuli_denovo	11336858	(T/C)
19935	CakSNP19935	*Kabuli*	Ca_Kabuli_denovo	11337191	(G/A)
19936	CakSNP19936	*Kabuli*	Ca_Kabuli_denovo	11338105	(G/C)
19937	CakSNP19937	*Kabuli*	Ca_Kabuli_denovo	11338164	(A/T)
19938	CakSNP19938	*Kabuli*	Ca_Kabuli_denovo	11338385	(G/A)
19939	CakSNP19939	*Kabuli*	Ca_Kabuli_denovo	11338391	(C/G)
19940	CakSNP19940	*Kabuli*	Ca_Kabuli_denovo	11346296	(G/T)
19941	CakSNP19941	*Kabuli*	Ca_Kabuli_denovo	11353664	(T/C)
19942	CakSNP19942	*Kabuli*	Ca_Kabuli_denovo	11361169	(G/C)
19943	CakSNP19943	*Kabuli*	Ca_Kabuli_denovo	11361193	(C/A)
19944	CakSNP19944	*Kabuli*	Ca_Kabuli_denovo	11361211	(A/G)
19945	CakSNP19945	*Kabuli*	Ca_Kabuli_denovo	11361500	(G/C)
19946	CakSNP19946	*Kabuli*	Ca_Kabuli_denovo	11361520	(C/T)
19947	CakSNP19947	*Kabuli*	Ca_Kabuli_denovo	11375757	(C/T)
19948	CakSNP19948	*Kabuli*	Ca_Kabuli_denovo	11378866	(C/T)
19949	CakSNP19949	*Kabuli*	Ca_Kabuli_denovo	11378886	(G/C)
19950	CakSNP19950	*Kabuli*	Ca_Kabuli_denovo	11386105	(C/T)
19951	CakSNP19951	*Kabuli*	Ca_Kabuli_denovo	11386154	(C/G)
19952	CakSNP19952	*Kabuli*	Ca_Kabuli_denovo	11389200	(G/C)
19953	CakSNP19953	*Kabuli*	Ca_Kabuli_denovo	11393292	(C/T)
19954	CakSNP19954	*Kabuli*	Ca_Kabuli_denovo	11400208	(C/G)
19955	CakSNP19955	*Kabuli*	Ca_Kabuli_denovo	11400582	(C/A)
19956	CakSNP19956	*Kabuli*	Ca_Kabuli_denovo	11402566	(C/T)
19957	CakSNP19957	*Kabuli*	Ca_Kabuli_denovo	11403286	(A/G)
19958	CakSNP19958	*Kabuli*	Ca_Kabuli_denovo	11407953	(A/G)
19959	CakSNP19959	*Kabuli*	Ca_Kabuli_denovo	11416225	(A/G)
19960	CakSNP19960	*Kabuli*	Ca_Kabuli_denovo	11430755	(A/G)
19961	CakSNP19961	*Kabuli*	Ca_Kabuli_denovo	11434250	(T/G)
19962	CakSNP19962	*Kabuli*	Ca_Kabuli_denovo	11441548	(G/C)
19963	CakSNP19963	*Kabuli*	Ca_Kabuli_denovo	11441557	(T/C)
19964	CakSNP19964	*Kabuli*	Ca_Kabuli_denovo	11452594	(T/C)
19965	CakSNP19965	*Kabuli*	Ca_Kabuli_denovo	11452603	(A/G)
19966	CakSNP19966	*Kabuli*	Ca_Kabuli_denovo	11455379	(G/T)

S.N.	SNP IDs	Cultivars	Chromosomes/scaffolds	Physical positions (bp)	SNPs
19967	CakSNP19967	*Kabuli*	Ca_Kabuli_denovo	11455475	(T/C)
19968	CakSNP19968	*Kabuli*	Ca_Kabuli_denovo	11455484	(C/G)
19969	CakSNP19969	*Kabuli*	Ca_Kabuli_denovo	11467373	(G/A)
19970	CakSNP19970	*Kabuli*	Ca_Kabuli_denovo	11483928	(G/A)
19971	CakSNP19971	*Kabuli*	Ca_Kabuli_denovo	11485151	(G/A)
19972	CakSNP19972	*Kabuli*	Ca_Kabuli_denovo	11485417	(C/A)
19973	CakSNP19973	*Kabuli*	Ca_Kabuli_denovo	11489691	(A/G)
19974	CakSNP19974	*Kabuli*	Ca_Kabuli_denovo	11489727	(A/G)
19975	CakSNP19975	*Kabuli*	Ca_Kabuli_denovo	11492137	(T/C)
19976	CakSNP19976	*Kabuli*	Ca_Kabuli_denovo	11500226	(C/T)
19977	CakSNP19977	*Kabuli*	Ca_Kabuli_denovo	11500286	(A/G)
19978	CakSNP19978	*Kabuli*	Ca_Kabuli_denovo	11500518	(C/T)
19979	CakSNP19979	*Kabuli*	Ca_Kabuli_denovo	11500549	(G/A)
19980	CakSNP19980	*Kabuli*	Ca_Kabuli_denovo	11509689	(A/G)
19981	CakSNP19981	*Kabuli*	Ca_Kabuli_denovo	11509710	(T/A)
19982	CakSNP19982	*Kabuli*	Ca_Kabuli_denovo	11520113	(T/C)
19983	CakSNP19983	*Kabuli*	Ca_Kabuli_denovo	11520121	(T/G)
19984	CakSNP19984	*Kabuli*	Ca_Kabuli_denovo	11522244	(T/C)
19985	CakSNP19985	*Kabuli*	Ca_Kabuli_denovo	11522247	(G/A)
19986	CakSNP19986	*Kabuli*	Ca_Kabuli_denovo	11524862	(A/G)
19987	CakSNP19987	*Kabuli*	Ca_Kabuli_denovo	11524904	(A/G)
19988	CakSNP19988	*Kabuli*	Ca_Kabuli_denovo	11526181	(G/C)
19989	CakSNP19989	*Kabuli*	Ca_Kabuli_denovo	11526429	(C/T)
19990	CakSNP19990	*Kabuli*	Ca_Kabuli_denovo	11526440	(C/T)
19991	CakSNP19991	*Kabuli*	Ca_Kabuli_denovo	11536624	(T/G)
19992	CakSNP19992	*Kabuli*	Ca_Kabuli_denovo	11537381	(G/A)
19993	CakSNP19993	*Kabuli*	Ca_Kabuli_denovo	11539912	(A/G)
19994	CakSNP19994	*Kabuli*	Ca_Kabuli_denovo	11539928	(C/A)
19995	CakSNP19995	*Kabuli*	Ca_Kabuli_denovo	11543736	(G/A)
19996	CakSNP19996	*Kabuli*	Ca_Kabuli_denovo	11546270	(G/A)
19997	CakSNP19997	*Kabuli*	Ca_Kabuli_denovo	11546282	(G/A)
19998	CakSNP19998	*Kabuli*	Ca_Kabuli_denovo	11560747	(A/T)
19999	CakSNP19999	*Kabuli*	Ca_Kabuli_denovo	11560777	(T/A)
20000	CakSNP20000	*Kabuli*	Ca_Kabuli_denovo	11562092	(A/C)
20001	CakSNP20001	*Kabuli*	Ca_Kabuli_denovo	11566483	(T/C)
20002	CakSNP20002	*Kabuli*	Ca_Kabuli_denovo	11570439	(G/C)
20003	CakSNP20003	*Kabuli*	Ca_Kabuli_denovo	11571669	(C/T)
20004	CakSNP20004	*Kabuli*	Ca_Kabuli_denovo	11571683	(C/T)
20005	CakSNP20005	*Kabuli*	Ca_Kabuli_denovo	11572107	(C/T)

S.N.	SNP IDs	Cultivars	Chromosomes/scaffolds	Physical positions (bp)	SNPs
20006	CakSNP20006	*Kabuli*	Ca_Kabuli_denovo	11576597	(G/A)
20007	CakSNP20007	*Kabuli*	Ca_Kabuli_denovo	11582680	(A/G)
20008	CakSNP20008	*Kabuli*	Ca_Kabuli_denovo	11584675	(A/G)
20009	CakSNP20009	*Kabuli*	Ca_Kabuli_denovo	11594624	(T/C)
20010	CakSNP20010	*Kabuli*	Ca_Kabuli_denovo	11596364	(G/A)
20011	CakSNP20011	*Kabuli*	Ca_Kabuli_denovo	11596442	(A/G)
20012	CakSNP20012	*Kabuli*	Ca_Kabuli_denovo	11597952	(C/T)
20013	CakSNP20013	*Kabuli*	Ca_Kabuli_denovo	11597994	(A/G)
20014	CakSNP20014	*Kabuli*	Ca_Kabuli_denovo	11601006	(G/T)
20015	CakSNP20015	*Kabuli*	Ca_Kabuli_denovo	11602335	(G/C)
20016	CakSNP20016	*Kabuli*	Ca_Kabuli_denovo	11602781	(C/G)
20017	CakSNP20017	*Kabuli*	Ca_Kabuli_denovo	11603266	(T/A)
20018	CakSNP20018	*Kabuli*	Ca_Kabuli_denovo	11621388	(A/G)
20019	CakSNP20019	*Kabuli*	Ca_Kabuli_denovo	11622352	(G/C)
20020	CakSNP20020	*Kabuli*	Ca_Kabuli_denovo	11622365	(A/T)
20021	CakSNP20021	*Kabuli*	Ca_Kabuli_denovo	11628118	(T/C)
20022	CakSNP20022	*Kabuli*	Ca_Kabuli_denovo	11628136	(A/C)
20023	CakSNP20023	*Kabuli*	Ca_Kabuli_denovo	11629142	(G/C)
20024	CakSNP20024	*Kabuli*	Ca_Kabuli_denovo	11629160	(T/G)
20025	CakSNP20025	*Kabuli*	Ca_Kabuli_denovo	11632641	(T/G)
20026	CakSNP20026	*Kabuli*	Ca_Kabuli_denovo	11632708	(T/C)
20027	CakSNP20027	*Kabuli*	Ca_Kabuli_denovo	11633909	(A/G)
20028	CakSNP20028	*Kabuli*	Ca_Kabuli_denovo	11635456	(T/C)
20029	CakSNP20029	*Kabuli*	Ca_Kabuli_denovo	11635493	(G/C)
20030	CakSNP20030	*Kabuli*	Ca_Kabuli_denovo	11637531	(C/T)
20031	CakSNP20031	*Kabuli*	Ca_Kabuli_denovo	11647295	(G/A)
20032	CakSNP20032	*Kabuli*	Ca_Kabuli_denovo	11647988	(A/G)
20033	CakSNP20033	*Kabuli*	Ca_Kabuli_denovo	11653902	(C/A)
20034	CakSNP20034	*Kabuli*	Ca_Kabuli_denovo	11654696	(G/T)
20035	CakSNP20035	*Kabuli*	Ca_Kabuli_denovo	11654699	(G/A)
20036	CakSNP20036	*Kabuli*	Ca_Kabuli_denovo	11657390	(G/A)
20037	CakSNP20037	*Kabuli*	Ca_Kabuli_denovo	11657404	(G/A)
20038	CakSNP20038	*Kabuli*	Ca_Kabuli_denovo	11657818	(C/T)
20039	CakSNP20039	*Kabuli*	Ca_Kabuli_denovo	11657827	(C/G)
20040	CakSNP20040	*Kabuli*	Ca_Kabuli_denovo	11658673	(G/C)
20041	CakSNP20041	*Kabuli*	Ca_Kabuli_denovo	11658712	(G/T)
20042	CakSNP20042	*Kabuli*	Ca_Kabuli_denovo	11658756	(C/G)
20043	CakSNP20043	*Kabuli*	Ca_Kabuli_denovo	11659710	(C/G)
20044	CakSNP20044	*Kabuli*	Ca_Kabuli_denovo	11663334	(T/C)

S.N.	SNP IDs	Cultivars	Chromosomes/scaffolds	Physical positions (bp)	SNPs
20045	CakSNP20045	*Kabuli*	Ca_Kabuli_denovo	11665747	(C/T)
20046	CakSNP20046	*Kabuli*	Ca_Kabuli_denovo	11672834	(T/C)
20047	CakSNP20047	*Kabuli*	Ca_Kabuli_denovo	11672842	(G/A)
20048	CakSNP20048	*Kabuli*	Ca_Kabuli_denovo	11676813	(G/C)
20049	CakSNP20049	*Kabuli*	Ca_Kabuli_denovo	11677203	(G/T)
20050	CakSNP20050	*Kabuli*	Ca_Kabuli_denovo	11677211	(C/T)
20051	CakSNP20051	*Kabuli*	Ca_Kabuli_denovo	11677396	(A/G)
20052	CakSNP20052	*Kabuli*	Ca_Kabuli_denovo	11678405	(A/G)
20053	CakSNP20053	*Kabuli*	Ca_Kabuli_denovo	11681261	(C/A)
20054	CakSNP20054	*Kabuli*	Ca_Kabuli_denovo	11681262	(T/G)
20055	CakSNP20055	*Kabuli*	Ca_Kabuli_denovo	11691933	(A/G)
20056	CakSNP20056	*Kabuli*	Ca_Kabuli_denovo	11691993	(C/A)
20057	CakSNP20057	*Kabuli*	Ca_Kabuli_denovo	11700734	(G/A)
20058	CakSNP20058	*Kabuli*	Ca_Kabuli_denovo	11705216	(A/C)
20059	CakSNP20059	*Kabuli*	Ca_Kabuli_denovo	11705234	(G/A)
20060	CakSNP20060	*Kabuli*	Ca_Kabuli_denovo	11718358	(T/G)
20061	CakSNP20061	*Kabuli*	Ca_Kabuli_denovo	11727596	(A/G)
20062	CakSNP20062	*Kabuli*	Ca_Kabuli_denovo	11729357	(A/T)
20063	CakSNP20063	*Kabuli*	Ca_Kabuli_denovo	11729711	(C/A)
20064	CakSNP20064	*Kabuli*	Ca_Kabuli_denovo	11731601	(C/T)
20065	CakSNP20065	*Kabuli*	Ca_Kabuli_denovo	11732939	(G/A)
20066	CakSNP20066	*Kabuli*	Ca_Kabuli_denovo	11751802	(T/C)
20067	CakSNP20067	*Kabuli*	Ca_Kabuli_denovo	11751841	(C/T)
20068	CakSNP20068	*Kabuli*	Ca_Kabuli_denovo	11756035	(G/T)
20069	CakSNP20069	*Kabuli*	Ca_Kabuli_denovo	11757946	(C/T)
20070	CakSNP20070	*Kabuli*	Ca_Kabuli_denovo	11758845	(C/T)
20071	CakSNP20071	*Kabuli*	Ca_Kabuli_denovo	11758846	(G/A)
20072	CakSNP20072	*Kabuli*	Ca_Kabuli_denovo	11765477	(T/A)
20073	CakSNP20073	*Kabuli*	Ca_Kabuli_denovo	11767720	(T/C)
20074	CakSNP20074	*Kabuli*	Ca_Kabuli_denovo	11767747	(C/G)
20075	CakSNP20075	*Kabuli*	Ca_Kabuli_denovo	11773745	(C/G)
20076	CakSNP20076	*Kabuli*	Ca_Kabuli_denovo	11777816	(G/C)
20077	CakSNP20077	*Kabuli*	Ca_Kabuli_denovo	11785619	(G/A)
20078	CakSNP20078	*Kabuli*	Ca_Kabuli_denovo	11785637	(C/G)
20079	CakSNP20079	*Kabuli*	Ca_Kabuli_denovo	11789993	(G/T)
20080	CakSNP20080	*Kabuli*	Ca_Kabuli_denovo	11789999	(A/C)
20081	CakSNP20081	*Kabuli*	Ca_Kabuli_denovo	11792609	(C/T)
20082	CakSNP20082	*Kabuli*	Ca_Kabuli_denovo	11792678	(A/C)
20083	CakSNP20083	*Kabuli*	Ca_Kabuli_denovo	11792680	(A/T)

S.N.	SNP IDs	Cultivars	Chromosomes/scaffolds	Physical positions (bp)	SNPs
20084	CakSNP20084	*Kabuli*	Ca_Kabuli_denovo	11797038	(T/C)
20085	CakSNP20085	*Kabuli*	Ca_Kabuli_denovo	11797045	(C/A)
20086	CakSNP20086	*Kabuli*	Ca_Kabuli_denovo	11797720	(C/T)
20087	CakSNP20087	*Kabuli*	Ca_Kabuli_denovo	11798474	(A/G)
20088	CakSNP20088	*Kabuli*	Ca_Kabuli_denovo	11800425	(C/T)
20089	CakSNP20089	*Kabuli*	Ca_Kabuli_denovo	11800433	(C/A)
20090	CakSNP20090	*Kabuli*	Ca_Kabuli_denovo	11801910	(G/C)
20091	CakSNP20091	*Kabuli*	Ca_Kabuli_denovo	11801949	(A/G)
20092	CakSNP20092	*Kabuli*	Ca_Kabuli_denovo	11801959	(C/G)
20093	CakSNP20093	*Kabuli*	Ca_Kabuli_denovo	11813358	(T/C)
20094	CakSNP20094	*Kabuli*	Ca_Kabuli_denovo	11813400	(T/A)
20095	CakSNP20095	*Kabuli*	Ca_Kabuli_denovo	11818388	(C/G)
20096	CakSNP20096	*Kabuli*	Ca_Kabuli_denovo	11818997	(A/C)
20097	CakSNP20097	*Kabuli*	Ca_Kabuli_denovo	11818999	(A/C)
20098	CakSNP20098	*Kabuli*	Ca_Kabuli_denovo	11821809	(C/T)
20099	CakSNP20099	*Kabuli*	Ca_Kabuli_denovo	11826617	(G/T)
20100	CakSNP20100	*Kabuli*	Ca_Kabuli_denovo	11826835	(C/T)
20101	CakSNP20101	*Kabuli*	Ca_Kabuli_denovo	11826865	(T/C)
20102	CakSNP20102	*Kabuli*	Ca_Kabuli_denovo	11828227	(C/A)
20103	CakSNP20103	*Kabuli*	Ca_Kabuli_denovo	11832922	(A/G)
20104	CakSNP20104	*Kabuli*	Ca_Kabuli_denovo	11836267	(T/C)
20105	CakSNP20105	*Kabuli*	Ca_Kabuli_denovo	11836286	(C/T)
20106	CakSNP20106	*Kabuli*	Ca_Kabuli_denovo	11839483	(T/C)
20107	CakSNP20107	*Kabuli*	Ca_Kabuli_denovo	11841284	(A/G)
20108	CakSNP20108	*Kabuli*	Ca_Kabuli_denovo	11842163	(T/C)
20109	CakSNP20109	*Kabuli*	Ca_Kabuli_denovo	11842187	(T/C)
20110	CakSNP20110	*Kabuli*	Ca_Kabuli_denovo	11844115	(G/C)
20111	CakSNP20111	*Kabuli*	Ca_Kabuli_denovo	11848053	(T/C)
20112	CakSNP20112	*Kabuli*	Ca_Kabuli_denovo	11850589	(C/G)
20113	CakSNP20113	*Kabuli*	Ca_Kabuli_denovo	11851324	(T/C)
20114	CakSNP20114	*Kabuli*	Ca_Kabuli_denovo	11851331	(G/A)
20115	CakSNP20115	*Kabuli*	Ca_Kabuli_denovo	11853095	(T/A)
20116	CakSNP20116	*Kabuli*	Ca_Kabuli_denovo	11855554	(A/G)
20117	CakSNP20117	*Kabuli*	Ca_Kabuli_denovo	11855592	(A/T)
20118	CakSNP20118	*Kabuli*	Ca_Kabuli_denovo	11857112	(A/G)
20119	CakSNP20119	*Kabuli*	Ca_Kabuli_denovo	11858823	(C/G)
20120	CakSNP20120	*Kabuli*	Ca_Kabuli_denovo	11858841	(C/T)
20121	CakSNP20121	*Kabuli*	Ca_Kabuli_denovo	11867607	(G/A)
20122	CakSNP20122	*Kabuli*	Ca_Kabuli_denovo	11868238	(A/G)

S.N.	SNP IDs	Cultivars	Chromosomes/scaffolds	Physical positions (bp)	SNPs
20123	CakSNP20123	*Kabuli*	Ca_Kabuli_denovo	11868277	(C/T)
20124	CakSNP20124	*Kabuli*	Ca_Kabuli_denovo	11870258	(T/C)
20125	CakSNP20125	*Kabuli*	Ca_Kabuli_denovo	11879386	(A/C)
20126	CakSNP20126	*Kabuli*	Ca_Kabuli_denovo	11879392	(T/C)
20127	CakSNP20127	*Kabuli*	Ca_Kabuli_denovo	11887761	(T/C)
20128	CakSNP20128	*Kabuli*	Ca_Kabuli_denovo	11887771	(T/C)
20129	CakSNP20129	*Kabuli*	Ca_Kabuli_denovo	11888785	(T/C)
20130	CakSNP20130	*Kabuli*	Ca_Kabuli_denovo	11888809	(T/C)
20131	CakSNP20131	*Kabuli*	Ca_Kabuli_denovo	11901103	(A/T)
20132	CakSNP20132	*Kabuli*	Ca_Kabuli_denovo	11904032	(G/T)
20133	CakSNP20133	*Kabuli*	Ca_Kabuli_denovo	11905598	(T/A)
20134	CakSNP20134	*Kabuli*	Ca_Kabuli_denovo	11905613	(C/G)
20135	CakSNP20135	*Kabuli*	Ca_Kabuli_denovo	11909810	(G/C)
20136	CakSNP20136	*Kabuli*	Ca_Kabuli_denovo	11913469	(A/G)
20137	CakSNP20137	*Kabuli*	Ca_Kabuli_denovo	11917788	(G/C)
20138	CakSNP20138	*Kabuli*	Ca_Kabuli_denovo	11918472	(T/C)
20139	CakSNP20139	*Kabuli*	Ca_Kabuli_denovo	11921650	(T/C)
20140	CakSNP20140	*Kabuli*	Ca_Kabuli_denovo	11921653	(C/T)
20141	CakSNP20141	*Kabuli*	Ca_Kabuli_denovo	11926343	(G/A)
20142	CakSNP20142	*Kabuli*	Ca_Kabuli_denovo	11926414	(C/G)
20143	CakSNP20143	*Kabuli*	Ca_Kabuli_denovo	11928286	(C/G)
20144	CakSNP20144	*Kabuli*	Ca_Kabuli_denovo	11928288	(A/G)
20145	CakSNP20145	*Kabuli*	Ca_Kabuli_denovo	11932767	(C/T)
20146	CakSNP20146	*Kabuli*	Ca_Kabuli_denovo	11933776	(T/C)
20147	CakSNP20147	*Kabuli*	Ca_Kabuli_denovo	11933779	(T/C)
20148	CakSNP20148	*Kabuli*	Ca_Kabuli_denovo	11934806	(A/G)
20149	CakSNP20149	*Kabuli*	Ca_Kabuli_denovo	11934835	(C/T)
20150	CakSNP20150	*Kabuli*	Ca_Kabuli_denovo	11935833	(C/T)
20151	CakSNP20151	*Kabuli*	Ca_Kabuli_denovo	11936229	(A/T)
20152	CakSNP20152	*Kabuli*	Ca_Kabuli_denovo	11937312	(A/G)
20153	CakSNP20153	*Kabuli*	Ca_Kabuli_denovo	11937322	(A/C)
20154	CakSNP20154	*Kabuli*	Ca_Kabuli_denovo	11937520	(G/T)
20155	CakSNP20155	*Kabuli*	Ca_Kabuli_denovo	11941139	(A/G)
20156	CakSNP20156	*Kabuli*	Ca_Kabuli_denovo	11942242	(T/A)
20157	CakSNP20157	*Kabuli*	Ca_Kabuli_denovo	11942299	(C/T)
20158	CakSNP20158	*Kabuli*	Ca_Kabuli_denovo	11947730	(T/A)
20159	CakSNP20159	*Kabuli*	Ca_Kabuli_denovo	11953049	(C/G)
20160	CakSNP20160	*Kabuli*	Ca_Kabuli_denovo	11962838	(A/G)
20161	CakSNP20161	*Kabuli*	Ca_Kabuli_denovo	11962840	(G/A)

S.N.	SNP IDs	Cultivars	Chromosomes/scaffolds	Physical positions (bp)	SNPs
20162	CakSNP20162	*Kabuli*	Ca_Kabuli_denovo	11965507	(A/G)
20163	CakSNP20163	*Kabuli*	Ca_Kabuli_denovo	11965510	(G/A)
20164	CakSNP20164	*Kabuli*	Ca_Kabuli_denovo	11970885	(C/G)
20165	CakSNP20165	*Kabuli*	Ca_Kabuli_denovo	11976757	(C/G)
20166	CakSNP20166	*Kabuli*	Ca_Kabuli_denovo	11983367	(T/C)
20167	CakSNP20167	*Kabuli*	Ca_Kabuli_denovo	11983391	(C/A)
20168	CakSNP20168	*Kabuli*	Ca_Kabuli_denovo	11986506	(G/A)
20169	CakSNP20169	*Kabuli*	Ca_Kabuli_denovo	11986536	(A/G)
20170	CakSNP20170	*Kabuli*	Ca_Kabuli_denovo	11986557	(A/G)
20171	CakSNP20171	*Kabuli*	Ca_Kabuli_denovo	11987561	(G/C)
20172	CakSNP20172	*Kabuli*	Ca_Kabuli_denovo	11993728	(G/A)
20173	CakSNP20173	*Kabuli*	Ca_Kabuli_denovo	12005056	(T/C)
20174	CakSNP20174	*Kabuli*	Ca_Kabuli_denovo	12008099	(T/C)
20175	CakSNP20175	*Kabuli*	Ca_Kabuli_denovo	12008111	(T/C)
20176	CakSNP20176	*Kabuli*	Ca_Kabuli_denovo	12010534	(A/T)
20177	CakSNP20177	*Kabuli*	Ca_Kabuli_denovo	12018239	(A/G)
20178	CakSNP20178	*Kabuli*	Ca_Kabuli_denovo	12019804	(C/T)
20179	CakSNP20179	*Kabuli*	Ca_Kabuli_denovo	12024854	(T/G)
20180	CakSNP20180	*Kabuli*	Ca_Kabuli_denovo	12027996	(C/G)
20181	CakSNP20181	*Kabuli*	Ca_Kabuli_denovo	12028061	(A/G)
20182	CakSNP20182	*Kabuli*	Ca_Kabuli_denovo	12033965	(G/A)
20183	CakSNP20183	*Kabuli*	Ca_Kabuli_denovo	12033971	(C/T)
20184	CakSNP20184	*Kabuli*	Ca_Kabuli_denovo	12036188	(A/G)
20185	CakSNP20185	*Kabuli*	Ca_Kabuli_denovo	12036224	(T/A)
20186	CakSNP20186	*Kabuli*	Ca_Kabuli_denovo	12037582	(A/G)
20187	CakSNP20187	*Kabuli*	Ca_Kabuli_denovo	12037583	(T/C)
20188	CakSNP20188	*Kabuli*	Ca_Kabuli_denovo	12041729	(T/G)
20189	CakSNP20189	*Kabuli*	Ca_Kabuli_denovo	12049740	(G/A)
20190	CakSNP20190	*Kabuli*	Ca_Kabuli_denovo	12054052	(T/G)
20191	CakSNP20191	*Kabuli*	Ca_Kabuli_denovo	12063452	(G/A)
20192	CakSNP20192	*Kabuli*	Ca_Kabuli_denovo	12063460	(C/T)
20193	CakSNP20193	*Kabuli*	Ca_Kabuli_denovo	12065643	(T/G)
20194	CakSNP20194	*Kabuli*	Ca_Kabuli_denovo	12066145	(T/C)
20195	CakSNP20195	*Kabuli*	Ca_Kabuli_denovo	12075499	(A/T)
20196	CakSNP20196	*Kabuli*	Ca_Kabuli_denovo	12075514	(A/T)
20197	CakSNP20197	*Kabuli*	Ca_Kabuli_denovo	12075516	(C/G)
20198	CakSNP20198	*Kabuli*	Ca_Kabuli_denovo	12075524	(C/G)
20199	CakSNP20199	*Kabuli*	Ca_Kabuli_denovo	12076633	(T/C)
20200	CakSNP20200	*Kabuli*	Ca_Kabuli_denovo	12076636	(A/G)

S.N.	SNP IDs	Cultivars	Chromosomes/scaffolds	Physical positions (bp)	SNPs
20201	CakSNP20201	*Kabuli*	Ca_Kabuli_denovo	12077380	(C/T)
20202	CakSNP20202	*Kabuli*	Ca_Kabuli_denovo	12079082	(G/A)
20203	CakSNP20203	*Kabuli*	Ca_Kabuli_denovo	12079352	(G/A)
20204	CakSNP20204	*Kabuli*	Ca_Kabuli_denovo	12079358	(G/C)
20205	CakSNP20205	*Kabuli*	Ca_Kabuli_denovo	12083899	(T/C)
20206	CakSNP20206	*Kabuli*	Ca_Kabuli_denovo	12083908	(G/T)
20207	CakSNP20207	*Kabuli*	Ca_Kabuli_denovo	12090488	(C/G)
20208	CakSNP20208	*Kabuli*	Ca_Kabuli_denovo	12100423	(C/T)
20209	CakSNP20209	*Kabuli*	Ca_Kabuli_denovo	12100504	(C/A)
20210	CakSNP20210	*Kabuli*	Ca_Kabuli_denovo	12105637	(T/C)
20211	CakSNP20211	*Kabuli*	Ca_Kabuli_denovo	12105669	(G/A)
20212	CakSNP20212	*Kabuli*	Ca_Kabuli_denovo	12108912	(G/A)
20213	CakSNP20213	*Kabuli*	Ca_Kabuli_denovo	12118782	(G/T)
20214	CakSNP20214	*Kabuli*	Ca_Kabuli_denovo	12118820	(T/A)
20215	CakSNP20215	*Kabuli*	Ca_Kabuli_denovo	12123015	(A/C)
20216	CakSNP20216	*Kabuli*	Ca_Kabuli_denovo	12123016	(G/C)
20217	CakSNP20217	*Kabuli*	Ca_Kabuli_denovo	12128221	(A/C)
20218	CakSNP20218	*Kabuli*	Ca_Kabuli_denovo	12129661	(T/C)
20219	CakSNP20219	*Kabuli*	Ca_Kabuli_denovo	12132152	(C/T)
20220	CakSNP20220	*Kabuli*	Ca_Kabuli_denovo	12140555	(C/T)
20221	CakSNP20221	*Kabuli*	Ca_Kabuli_denovo	12140573	(C/A)
20222	CakSNP20222	*Kabuli*	Ca_Kabuli_denovo	12141836	(G/A)
20223	CakSNP20223	*Kabuli*	Ca_Kabuli_denovo	12142574	(A/G)
20224	CakSNP20224	*Kabuli*	Ca_Kabuli_denovo	12151348	(A/G)
20225	CakSNP20225	*Kabuli*	Ca_Kabuli_denovo	12151375	(A/G)
20226	CakSNP20226	*Kabuli*	Ca_Kabuli_denovo	12157446	(T/G)
20227	CakSNP20227	*Kabuli*	Ca_Kabuli_denovo	12157447	(T/A)
20228	CakSNP20228	*Kabuli*	Ca_Kabuli_denovo	12157570	(G/A)
20229	CakSNP20229	*Kabuli*	Ca_Kabuli_denovo	12160442	(T/G)
20230	CakSNP20230	*Kabuli*	Ca_Kabuli_denovo	12160463	(C/T)
20231	CakSNP20231	*Kabuli*	Ca_Kabuli_denovo	12160547	(T/C)
20232	CakSNP20232	*Kabuli*	Ca_Kabuli_denovo	12160588	(A/G)
20233	CakSNP20233	*Kabuli*	Ca_Kabuli_denovo	12161049	(A/C)
20234	CakSNP20234	*Kabuli*	Ca_Kabuli_denovo	12163984	(G/C)
20235	CakSNP20235	*Kabuli*	Ca_Kabuli_denovo	12170666	(T/C)
20236	CakSNP20236	*Kabuli*	Ca_Kabuli_denovo	12174208	(C/G)
20237	CakSNP20237	*Kabuli*	Ca_Kabuli_denovo	12178363	(A/G)
20238	CakSNP20238	*Kabuli*	Ca_Kabuli_denovo	12181987	(G/A)
20239	CakSNP20239	*Kabuli*	Ca_Kabuli_denovo	12182026	(C/G)

S.N.	SNP IDs	Cultivars	Chromosomes/scaffolds	Physical positions (bp)	SNPs
20240	CakSNP20240	*Kabuli*	Ca_Kabuli_denovo	12186730	(T/C)
20241	CakSNP20241	*Kabuli*	Ca_Kabuli_denovo	12186760	(C/G)
20242	CakSNP20242	*Kabuli*	Ca_Kabuli_denovo	12187344	(A/G)
20243	CakSNP20243	*Kabuli*	Ca_Kabuli_denovo	12192872	(C/T)
20244	CakSNP20244	*Kabuli*	Ca_Kabuli_denovo	12201334	(A/G)
20245	CakSNP20245	*Kabuli*	Ca_Kabuli_denovo	12202915	(A/G)
20246	CakSNP20246	*Kabuli*	Ca_Kabuli_denovo	12209776	(T/C)
20247	CakSNP20247	*Kabuli*	Ca_Kabuli_denovo	12215372	(G/T)
20248	CakSNP20248	*Kabuli*	Ca_Kabuli_denovo	12216070	(C/T)
20249	CakSNP20249	*Kabuli*	Ca_Kabuli_denovo	12217982	(G/A)
20250	CakSNP20250	*Kabuli*	Ca_Kabuli_denovo	12234821	(C/T)
20251	CakSNP20251	*Kabuli*	Ca_Kabuli_denovo	12236422	(T/C)
20252	CakSNP20252	*Kabuli*	Ca_Kabuli_denovo	12239018	(C/G)
20253	CakSNP20253	*Kabuli*	Ca_Kabuli_denovo	12240512	(A/G)
20254	CakSNP20254	*Kabuli*	Ca_Kabuli_denovo	12240549	(A/G)
20255	CakSNP20255	*Kabuli*	Ca_Kabuli_denovo	12243380	(A/G)
20256	CakSNP20256	*Kabuli*	Ca_Kabuli_denovo	12248626	(A/G)
20257	CakSNP20257	*Kabuli*	Ca_Kabuli_denovo	12251127	(C/A)
20258	CakSNP20258	*Kabuli*	Ca_Kabuli_denovo	12252623	(A/G)
20259	CakSNP20259	*Kabuli*	Ca_Kabuli_denovo	12257991	(T/C)
20260	CakSNP20260	*Kabuli*	Ca_Kabuli_denovo	12257993	(T/G)
20261	CakSNP20261	*Kabuli*	Ca_Kabuli_denovo	12264308	(G/T)
20262	CakSNP20262	*Kabuli*	Ca_Kabuli_denovo	12266786	(A/G)
20263	CakSNP20263	*Kabuli*	Ca_Kabuli_denovo	12269505	(G/C)
20264	CakSNP20264	*Kabuli*	Ca_Kabuli_denovo	12292363	(G/C)
20265	CakSNP20265	*Kabuli*	Ca_Kabuli_denovo	12298060	(C/G)
20266	CakSNP20266	*Kabuli*	Ca_Kabuli_denovo	12298745	(C/T)
20267	CakSNP20267	*Kabuli*	Ca_Kabuli_denovo	12301582	(A/C)
20268	CakSNP20268	*Kabuli*	Ca_Kabuli_denovo	12309140	(G/A)
20269	CakSNP20269	*Kabuli*	Ca_Kabuli_denovo	12309148	(A/G)
20270	CakSNP20270	*Kabuli*	Ca_Kabuli_denovo	12318085	(T/C)
20271	CakSNP20271	*Kabuli*	Ca_Kabuli_denovo	12318112	(T/C)
20272	CakSNP20272	*Kabuli*	Ca_Kabuli_denovo	12319732	(G/C)
20273	CakSNP20273	*Kabuli*	Ca_Kabuli_denovo	12323652	(G/C)
20274	CakSNP20274	*Kabuli*	Ca_Kabuli_denovo	12329986	(G/A)
20275	CakSNP20275	*Kabuli*	Ca_Kabuli_denovo	12329992	(C/T)
20276	CakSNP20276	*Kabuli*	Ca_Kabuli_denovo	12331347	(C/G)
20277	CakSNP20277	*Kabuli*	Ca_Kabuli_denovo	12331761	(G/C)
20278	CakSNP20278	*Kabuli*	Ca_Kabuli_denovo	12331775	(C/G)

S.N.	SNP IDs	Cultivars	Chromosomes/scaffolds	Physical positions (bp)	SNPs
20279	CakSNP20279	*Kabuli*	Ca_Kabuli_denovo	12343528	(T/C)
20280	CakSNP20280	*Kabuli*	Ca_Kabuli_denovo	12343563	(T/C)
20281	CakSNP20281	*Kabuli*	Ca_Kabuli_denovo	12348038	(C/G)
20282	CakSNP20282	*Kabuli*	Ca_Kabuli_denovo	12348044	(C/G)
20283	CakSNP20283	*Kabuli*	Ca_Kabuli_denovo	12356015	(T/C)
20284	CakSNP20284	*Kabuli*	Ca_Kabuli_denovo	12360701	(T/G)
20285	CakSNP20285	*Kabuli*	Ca_Kabuli_denovo	12360722	(C/T)
20286	CakSNP20286	*Kabuli*	Ca_Kabuli_denovo	12364826	(G/T)
20287	CakSNP20287	*Kabuli*	Ca_Kabuli_denovo	12365459	(A/C)
20288	CakSNP20288	*Kabuli*	Ca_Kabuli_denovo	12368369	(C/T)
20289	CakSNP20289	*Kabuli*	Ca_Kabuli_denovo	12380551	(A/T)
20290	CakSNP20290	*Kabuli*	Ca_Kabuli_denovo	12380579	(G/C)
20291	CakSNP20291	*Kabuli*	Ca_Kabuli_denovo	12381201	(C/T)
20292	CakSNP20292	*Kabuli*	Ca_Kabuli_denovo	12381391	(C/T)
20293	CakSNP20293	*Kabuli*	Ca_Kabuli_denovo	12385312	(G/C)
20294	CakSNP20294	*Kabuli*	Ca_Kabuli_denovo	12385354	(A/C)
20295	CakSNP20295	*Kabuli*	Ca_Kabuli_denovo	12386113	(C/G)
20296	CakSNP20296	*Kabuli*	Ca_Kabuli_denovo	12387661	(C/A)
20297	CakSNP20297	*Kabuli*	Ca_Kabuli_denovo	12387670	(T/C)
20298	CakSNP20298	*Kabuli*	Ca_Kabuli_denovo	12391385	(G/A)
20299	CakSNP20299	*Kabuli*	Ca_Kabuli_denovo	12391414	(G/A)
20300	CakSNP20300	*Kabuli*	Ca_Kabuli_denovo	12394679	(T/C)
20301	CakSNP20301	*Kabuli*	Ca_Kabuli_denovo	12394728	(G/C)
20302	CakSNP20302	*Kabuli*	Ca_Kabuli_denovo	12394732	(C/T)
20303	CakSNP20303	*Kabuli*	Ca_Kabuli_denovo	12404927	(A/G)
20304	CakSNP20304	*Kabuli*	Ca_Kabuli_denovo	12411157	(C/G)
20305	CakSNP20305	*Kabuli*	Ca_Kabuli_denovo	12415971	(C/T)
20306	CakSNP20306	*Kabuli*	Ca_Kabuli_denovo	12417288	(C/A)
20307	CakSNP20307	*Kabuli*	Ca_Kabuli_denovo	12417294	(G/C)
20308	CakSNP20308	*Kabuli*	Ca_Kabuli_denovo	12420048	(G/C)
20309	CakSNP20309	*Kabuli*	Ca_Kabuli_denovo	12422409	(C/G)
20310	CakSNP20310	*Kabuli*	Ca_Kabuli_denovo	12422413	(A/C)
20311	CakSNP20311	*Kabuli*	Ca_Kabuli_denovo	12424936	(C/T)
20312	CakSNP20312	*Kabuli*	Ca_Kabuli_denovo	12424968	(A/T)
20313	CakSNP20313	*Kabuli*	Ca_Kabuli_denovo	12431462	(C/T)
20314	CakSNP20314	*Kabuli*	Ca_Kabuli_denovo	12440976	(C/T)
20315	CakSNP20315	*Kabuli*	Ca_Kabuli_denovo	12449927	(T/C)
20316	CakSNP20316	*Kabuli*	Ca_Kabuli_denovo	12449999	(C/T)
20317	CakSNP20317	*Kabuli*	Ca_Kabuli_denovo	12450760	(A/G)

S.N.	SNP IDs	Cultivars	Chromosomes/scaffolds	Physical positions (bp)	SNPs
20318	CakSNP20318	*Kabuli*	Ca_Kabuli_denovo	12450818	(G/C)
20319	CakSNP20319	*Kabuli*	Ca_Kabuli_denovo	12450967	(C/T)
20320	CakSNP20320	*Kabuli*	Ca_Kabuli_denovo	12450989	(A/G)
20321	CakSNP20321	*Kabuli*	Ca_Kabuli_denovo	12467411	(A/G)
20322	CakSNP20322	*Kabuli*	Ca_Kabuli_denovo	12468098	(C/G)
20323	CakSNP20323	*Kabuli*	Ca_Kabuli_denovo	12471420	(T/C)
20324	CakSNP20324	*Kabuli*	Ca_Kabuli_denovo	12472768	(G/A)
20325	CakSNP20325	*Kabuli*	Ca_Kabuli_denovo	12473457	(A/G)
20326	CakSNP20326	*Kabuli*	Ca_Kabuli_denovo	12484829	(T/G)
20327	CakSNP20327	*Kabuli*	Ca_Kabuli_denovo	12485751	(A/G)
20328	CakSNP20328	*Kabuli*	Ca_Kabuli_denovo	12485766	(C/T)
20329	CakSNP20329	*Kabuli*	Ca_Kabuli_denovo	12486851	(A/G)
20330	CakSNP20330	*Kabuli*	Ca_Kabuli_denovo	12486854	(G/A)
20331	CakSNP20331	*Kabuli*	Ca_Kabuli_denovo	12487619	(C/T)
20332	CakSNP20332	*Kabuli*	Ca_Kabuli_denovo	12487668	(A/G)
20333	CakSNP20333	*Kabuli*	Ca_Kabuli_denovo	12496808	(C/T)
20334	CakSNP20334	*Kabuli*	Ca_Kabuli_denovo	12500463	(G/A)
20335	CakSNP20335	*Kabuli*	Ca_Kabuli_denovo	12500466	(G/C)
20336	CakSNP20336	*Kabuli*	Ca_Kabuli_denovo	12502746	(A/T)
20337	CakSNP20337	*Kabuli*	Ca_Kabuli_denovo	12505149	(G/T)
20338	CakSNP20338	*Kabuli*	Ca_Kabuli_denovo	12506116	(A/C)
20339	CakSNP20339	*Kabuli*	Ca_Kabuli_denovo	12506158	(A/G)
20340	CakSNP20340	*Kabuli*	Ca_Kabuli_denovo	12507925	(C/T)
20341	CakSNP20341	*Kabuli*	Ca_Kabuli_denovo	12514887	(A/C)
20342	CakSNP20342	*Kabuli*	Ca_Kabuli_denovo	12527488	(G/C)
20343	CakSNP20343	*Kabuli*	Ca_Kabuli_denovo	12529187	(A/G)
20344	CakSNP20344	*Kabuli*	Ca_Kabuli_denovo	12530009	(C/T)
20345	CakSNP20345	*Kabuli*	Ca_Kabuli_denovo	12539987	(C/T)
20346	CakSNP20346	*Kabuli*	Ca_Kabuli_denovo	12541576	(C/G)
20347	CakSNP20347	*Kabuli*	Ca_Kabuli_denovo	12550942	(C/T)
20348	CakSNP20348	*Kabuli*	Ca_Kabuli_denovo	12562412	(C/T)
20349	CakSNP20349	*Kabuli*	Ca_Kabuli_denovo	12562414	(G/A)
20350	CakSNP20350	*Kabuli*	Ca_Kabuli_denovo	12567144	(T/C)
20351	CakSNP20351	*Kabuli*	Ca_Kabuli_denovo	12568213	(A/G)
20352	CakSNP20352	*Kabuli*	Ca_Kabuli_denovo	12568222	(G/A)
20353	CakSNP20353	*Kabuli*	Ca_Kabuli_denovo	12572669	(A/C)
20354	CakSNP20354	*Kabuli*	Ca_Kabuli_denovo	12572691	(C/T)
20355	CakSNP20355	*Kabuli*	Ca_Kabuli_denovo	12578294	(T/C)
20356	CakSNP20356	*Kabuli*	Ca_Kabuli_denovo	12578300	(A/C)

S.N.	SNP IDs	Cultivars	Chromosomes/scaffolds	Physical positions (bp)	SNPs
20357	CakSNP20357	*Kabuli*	Ca_Kabuli_denovo	12583593	(T/C)
20358	CakSNP20358	*Kabuli*	Ca_Kabuli_denovo	12588224	(G/A)
20359	CakSNP20359	*Kabuli*	Ca_Kabuli_denovo	12597602	(C/T)
20360	CakSNP20360	*Kabuli*	Ca_Kabuli_denovo	12597680	(C/T)
20361	CakSNP20361	*Kabuli*	Ca_Kabuli_denovo	12597698	(A/G)
20362	CakSNP20362	*Kabuli*	Ca_Kabuli_denovo	12598854	(G/A)
20363	CakSNP20363	*Kabuli*	Ca_Kabuli_denovo	12614448	(C/G)
20364	CakSNP20364	*Kabuli*	Ca_Kabuli_denovo	12614475	(T/G)
20365	CakSNP20365	*Kabuli*	Ca_Kabuli_denovo	12620203	(T/G)
20366	CakSNP20366	*Kabuli*	Ca_Kabuli_denovo	12625088	(G/A)
20367	CakSNP20367	*Kabuli*	Ca_Kabuli_denovo	12630071	(G/A)
20368	CakSNP20368	*Kabuli*	Ca_Kabuli_denovo	12630136	(C/G)
20369	CakSNP20369	*Kabuli*	Ca_Kabuli_denovo	12647378	(C/G)
20370	CakSNP20370	*Kabuli*	Ca_Kabuli_denovo	12647381	(C/T)
20371	CakSNP20371	*Kabuli*	Ca_Kabuli_denovo	12649202	(G/C)
20372	CakSNP20372	*Kabuli*	Ca_Kabuli_denovo	12649208	(G/A)
20373	CakSNP20373	*Kabuli*	Ca_Kabuli_denovo	12655462	(A/C)
20374	CakSNP20374	*Kabuli*	Ca_Kabuli_denovo	12655487	(A/C)
20375	CakSNP20375	*Kabuli*	Ca_Kabuli_denovo	12669133	(G/A)
20376	CakSNP20376	*Kabuli*	Ca_Kabuli_denovo	12672676	(T/C)
20377	CakSNP20377	*Kabuli*	Ca_Kabuli_denovo	12680590	(T/G)
20378	CakSNP20378	*Kabuli*	Ca_Kabuli_denovo	12680643	(T/C)
20379	CakSNP20379	*Kabuli*	Ca_Kabuli_denovo	12681610	(G/T)
20380	CakSNP20380	*Kabuli*	Ca_Kabuli_denovo	12683863	(C/A)
20381	CakSNP20381	*Kabuli*	Ca_Kabuli_denovo	12684098	(A/G)
20382	CakSNP20382	*Kabuli*	Ca_Kabuli_denovo	12684100	(C/T)
20383	CakSNP20383	*Kabuli*	Ca_Kabuli_denovo	12686753	(A/G)
20384	CakSNP20384	*Kabuli*	Ca_Kabuli_denovo	12686758	(C/A)
20385	CakSNP20385	*Kabuli*	Ca_Kabuli_denovo	12688024	(C/T)
20386	CakSNP20386	*Kabuli*	Ca_Kabuli_denovo	12688476	(G/C)
20387	CakSNP20387	*Kabuli*	Ca_Kabuli_denovo	12688489	(G/T)
20388	CakSNP20388	*Kabuli*	Ca_Kabuli_denovo	12689864	(G/A)
20389	CakSNP20389	*Kabuli*	Ca_Kabuli_denovo	12689914	(C/T)
20390	CakSNP20390	*Kabuli*	Ca_Kabuli_denovo	12691912	(C/A)
20391	CakSNP20391	*Kabuli*	Ca_Kabuli_denovo	12691926	(C/G)
20392	CakSNP20392	*Kabuli*	Ca_Kabuli_denovo	12692387	(A/G)
20393	CakSNP20393	*Kabuli*	Ca_Kabuli_denovo	12701219	(C/T)
20394	CakSNP20394	*Kabuli*	Ca_Kabuli_denovo	12701233	(A/G)
20395	CakSNP20395	*Kabuli*	Ca_Kabuli_denovo	12701423	(G/A)

**S.N.**	**SNP IDs**	**Cultivars**	**Chromosomes/scaffolds**	**Physical positions (bp)**	**SNPs**
20396	CakSNP20396	*Kabuli*	Ca_Kabuli_denovo	12711354	(C/G)
20397	CakSNP20397	*Kabuli*	Ca_Kabuli_denovo	12711370	(T/C)
20398	CakSNP20398	*Kabuli*	Ca_Kabuli_denovo	12711403	(G/C)
20399	CakSNP20399	*Kabuli*	Ca_Kabuli_denovo	12712367	(A/G)
20400	CakSNP20400	*Kabuli*	Ca_Kabuli_denovo	12712436	(A/G)
20401	CakSNP20401	*Kabuli*	Ca_Kabuli_denovo	12712492	(C/G)
20402	CakSNP20402	*Kabuli*	Ca_Kabuli_denovo	12719308	(C/T)
20403	CakSNP20403	*Kabuli*	Ca_Kabuli_denovo	12728194	(C/A)
20404	CakSNP20404	*Kabuli*	Ca_Kabuli_denovo	12728235	(C/G)
20405	CakSNP20405	*Kabuli*	Ca_Kabuli_denovo	12728761	(G/C)
20406	CakSNP20406	*Kabuli*	Ca_Kabuli_denovo	12728774	(G/C)
20407	CakSNP20407	*Kabuli*	Ca_Kabuli_denovo	12730181	(C/T)
20408	CakSNP20408	*Kabuli*	Ca_Kabuli_denovo	12735810	(A/G)
20409	CakSNP20409	*Kabuli*	Ca_Kabuli_denovo	12735840	(G/A)
20410	CakSNP20410	*Kabuli*	Ca_Kabuli_denovo	12738518	(C/T)
20411	CakSNP20411	*Kabuli*	Ca_Kabuli_denovo	12738520	(T/C)
20412	CakSNP20412	*Kabuli*	Ca_Kabuli_denovo	12739001	(C/G)
20413	CakSNP20413	*Kabuli*	Ca_Kabuli_denovo	12739011	(G/C)
20414	CakSNP20414	*Kabuli*	Ca_Kabuli_denovo	12739895	(T/G)
20415	CakSNP20415	*Kabuli*	Ca_Kabuli_denovo	12740080	(T/G)
20416	CakSNP20416	*Kabuli*	Ca_Kabuli_denovo	12740138	(G/C)
20417	CakSNP20417	*Kabuli*	Ca_Kabuli_denovo	12748520	(T/C)
20418	CakSNP20418	*Kabuli*	Ca_Kabuli_denovo	12754598	(G/A)
20419	CakSNP20419	*Kabuli*	Ca_Kabuli_denovo	12760788	(G/T)
20420	CakSNP20420	*Kabuli*	Ca_Kabuli_denovo	12763262	(C/T)
20421	CakSNP20421	*Kabuli*	Ca_Kabuli_denovo	12765002	(G/C)
20422	CakSNP20422	*Kabuli*	Ca_Kabuli_denovo	12765006	(G/A)
20423	CakSNP20423	*Kabuli*	Ca_Kabuli_denovo	12767039	(A/G)
20424	CakSNP20424	*Kabuli*	Ca_Kabuli_denovo	12769214	(A/T)
20425	CakSNP20425	*Kabuli*	Ca_Kabuli_denovo	12770684	(A/G)
20426	CakSNP20426	*Kabuli*	Ca_Kabuli_denovo	12770747	(C/G)
20427	CakSNP20427	*Kabuli*	Ca_Kabuli_denovo	12772557	(A/G)
20428	CakSNP20428	*Kabuli*	Ca_Kabuli_denovo	12772584	(T/C)
20429	CakSNP20429	*Kabuli*	Ca_Kabuli_denovo	12776581	(G/A)
20430	CakSNP20430	*Kabuli*	Ca_Kabuli_denovo	12780073	(C/A)
20431	CakSNP20431	*Kabuli*	Ca_Kabuli_denovo	12785221	(C/A)
20432	CakSNP20432	*Kabuli*	Ca_Kabuli_denovo	12785230	(G/C)
20433	CakSNP20433	*Kabuli*	Ca_Kabuli_denovo	12785482	(C/G)
20434	CakSNP20434	*Kabuli*	Ca_Kabuli_denovo	12785515	(A/G)

S.N.	SNP IDs	Cultivars	Chromosomes/scaffolds	Physical positions (bp)	SNPs
20435	CakSNP20435	*Kabuli*	Ca_Kabuli_denovo	12791066	(A/G)
20436	CakSNP20436	*Kabuli*	Ca_Kabuli_denovo	12791067	(A/G)
20437	CakSNP20437	*Kabuli*	Ca_Kabuli_denovo	12793473	(T/C)
20438	CakSNP20438	*Kabuli*	Ca_Kabuli_denovo	12793863	(G/A)
20439	CakSNP20439	*Kabuli*	Ca_Kabuli_denovo	12793884	(C/G)
20440	CakSNP20440	*Kabuli*	Ca_Kabuli_denovo	12795878	(C/T)
20441	CakSNP20441	*Kabuli*	Ca_Kabuli_denovo	12805396	(T/C)
20442	CakSNP20442	*Kabuli*	Ca_Kabuli_denovo	12812076	(C/G)
20443	CakSNP20443	*Kabuli*	Ca_Kabuli_denovo	12812111	(A/C)
20444	CakSNP20444	*Kabuli*	Ca_Kabuli_denovo	12817660	(C/T)
20445	CakSNP20445	*Kabuli*	Ca_Kabuli_denovo	12818175	(T/G)
20446	CakSNP20446	*Kabuli*	Ca_Kabuli_denovo	12829473	(G/A)
20447	CakSNP20447	*Kabuli*	Ca_Kabuli_denovo	12830550	(C/G)
20448	CakSNP20448	*Kabuli*	Ca_Kabuli_denovo	12830559	(G/A)
20449	CakSNP20449	*Kabuli*	Ca_Kabuli_denovo	12832898	(C/A)
20450	CakSNP20450	*Kabuli*	Ca_Kabuli_denovo	12832925	(C/G)
20451	CakSNP20451	*Kabuli*	Ca_Kabuli_denovo	12843699	(C/T)
20452	CakSNP20452	*Kabuli*	Ca_Kabuli_denovo	12843736	(T/A)
20453	CakSNP20453	*Kabuli*	Ca_Kabuli_denovo	12845113	(G/T)
20454	CakSNP20454	*Kabuli*	Ca_Kabuli_denovo	12848545	(T/C)
20455	CakSNP20455	*Kabuli*	Ca_Kabuli_denovo	12850100	(T/C)
20456	CakSNP20456	*Kabuli*	Ca_Kabuli_denovo	12850561	(A/G)
20457	CakSNP20457	*Kabuli*	Ca_Kabuli_denovo	12850591	(C/T)
20458	CakSNP20458	*Kabuli*	Ca_Kabuli_denovo	12854008	(A/G)
20459	CakSNP20459	*Kabuli*	Ca_Kabuli_denovo	12854011	(G/C)
20460	CakSNP20460	*Kabuli*	Ca_Kabuli_denovo	12856834	(T/G)
20461	CakSNP20461	*Kabuli*	Ca_Kabuli_denovo	12860525	(G/A)
20462	CakSNP20462	*Kabuli*	Ca_Kabuli_denovo	12862348	(T/G)
20463	CakSNP20463	*Kabuli*	Ca_Kabuli_denovo	12863560	(A/G)
20464	CakSNP20464	*Kabuli*	Ca_Kabuli_denovo	12865448	(C/T)
20465	CakSNP20465	*Kabuli*	Ca_Kabuli_denovo	12865468	(C/T)
20466	CakSNP20466	*Kabuli*	Ca_Kabuli_denovo	12869255	(A/G)
20467	CakSNP20467	*Kabuli*	Ca_Kabuli_denovo	12871791	(C/G)
20468	CakSNP20468	*Kabuli*	Ca_Kabuli_denovo	12872788	(G/A)
20469	CakSNP20469	*Kabuli*	Ca_Kabuli_denovo	12872842	(T/C)
20470	CakSNP20470	*Kabuli*	Ca_Kabuli_denovo	12876043	(A/G)
20471	CakSNP20471	*Kabuli*	Ca_Kabuli_denovo	12888592	(A/C)
20472	CakSNP20472	*Kabuli*	Ca_Kabuli_denovo	12897255	(A/G)
20473	CakSNP20473	*Kabuli*	Ca_Kabuli_denovo	12899024	(T/C)

S.N.	SNP IDs	Cultivars	Chromosomes/scaffolds	Physical positions (bp)	SNPs
20474	CakSNP20474	*Kabuli*	Ca_Kabuli_denovo	12903398	(C/A)
20475	CakSNP20475	*Kabuli*	Ca_Kabuli_denovo	12903437	(C/A)
20476	CakSNP20476	*Kabuli*	Ca_Kabuli_denovo	12904085	(T/C)
20477	CakSNP20477	*Kabuli*	Ca_Kabuli_denovo	12904118	(A/G)
20478	CakSNP20478	*Kabuli*	Ca_Kabuli_denovo	12910271	(C/T)
20479	CakSNP20479	*Kabuli*	Ca_Kabuli_denovo	12910286	(A/G)
20480	CakSNP20480	*Kabuli*	Ca_Kabuli_denovo	12911244	(T/G)
20481	CakSNP20481	*Kabuli*	Ca_Kabuli_denovo	12911275	(A/G)
20482	CakSNP20482	*Kabuli*	Ca_Kabuli_denovo	12914398	(C/T)
20483	CakSNP20483	*Kabuli*	Ca_Kabuli_denovo	12914424	(C/A)
20484	CakSNP20484	*Kabuli*	Ca_Kabuli_denovo	12927462	(A/G)
20485	CakSNP20485	*Kabuli*	Ca_Kabuli_denovo	12933253	(T/C)
20486	CakSNP20486	*Kabuli*	Ca_Kabuli_denovo	12935128	(T/C)
20487	CakSNP20487	*Kabuli*	Ca_Kabuli_denovo	12935673	(C/G)
20488	CakSNP20488	*Kabuli*	Ca_Kabuli_denovo	12936805	(T/C)
20489	CakSNP20489	*Kabuli*	Ca_Kabuli_denovo	12939572	(T/C)
20490	CakSNP20490	*Kabuli*	Ca_Kabuli_denovo	12939580	(G/A)
20491	CakSNP20491	*Kabuli*	Ca_Kabuli_denovo	12974203	(T/C)
20492	CakSNP20492	*Kabuli*	Ca_Kabuli_denovo	12975846	(T/C)
20493	CakSNP20493	*Kabuli*	Ca_Kabuli_denovo	12976965	(C/G)
20494	CakSNP20494	*Kabuli*	Ca_Kabuli_denovo	12977012	(C/G)
20495	CakSNP20495	*Kabuli*	Ca_Kabuli_denovo	12980401	(G/A)
20496	CakSNP20496	*Kabuli*	Ca_Kabuli_denovo	12980404	(G/C)
20497	CakSNP20497	*Kabuli*	Ca_Kabuli_denovo	12981713	(C/T)
20498	CakSNP20498	*Kabuli*	Ca_Kabuli_denovo	12983088	(A/G)
20499	CakSNP20499	*Kabuli*	Ca_Kabuli_denovo	12997717	(G/A)
20500	CakSNP20500	*Kabuli*	Ca_Kabuli_denovo	13000283	(A/C)
20501	CakSNP20501	*Kabuli*	Ca_Kabuli_denovo	13013951	(A/C)
20502	CakSNP20502	*Kabuli*	Ca_Kabuli_denovo	13013952	(G/A)
20503	CakSNP20503	*Kabuli*	Ca_Kabuli_denovo	13013955	(C/T)
20504	CakSNP20504	*Kabuli*	Ca_Kabuli_denovo	13014166	(T/C)
20505	CakSNP20505	*Kabuli*	Ca_Kabuli_denovo	13016388	(C/T)
20506	CakSNP20506	*Kabuli*	Ca_Kabuli_denovo	13016391	(G/C)
20507	CakSNP20507	*Kabuli*	Ca_Kabuli_denovo	13020665	(G/A)
20508	CakSNP20508	*Kabuli*	Ca_Kabuli_denovo	13021311	(G/A)
20509	CakSNP20509	*Kabuli*	Ca_Kabuli_denovo	13028389	(A/G)
20510	CakSNP20510	*Kabuli*	Ca_Kabuli_denovo	13028463	(G/A)
20511	CakSNP20511	*Kabuli*	Ca_Kabuli_denovo	13030260	(G/A)
20512	CakSNP20512	*Kabuli*	Ca_Kabuli_denovo	13031950	(A/C)

S.N.	SNP IDs	Cultivars	Chromosomes/scaffolds	Physical positions (bp)	SNPs
20513	CakSNP20513	*Kabuli*	Ca_Kabuli_denovo	13032980	(G/A)
20514	CakSNP20514	*Kabuli*	Ca_Kabuli_denovo	13033007	(C/T)
20515	CakSNP20515	*Kabuli*	Ca_Kabuli_denovo	13044230	(G/C)
20516	CakSNP20516	*Kabuli*	Ca_Kabuli_denovo	13047420	(G/A)
20517	CakSNP20517	*Kabuli*	Ca_Kabuli_denovo	13048862	(C/T)
20518	CakSNP20518	*Kabuli*	Ca_Kabuli_denovo	13049519	(T/C)
20519	CakSNP20519	*Kabuli*	Ca_Kabuli_denovo	13051397	(C/G)
20520	CakSNP20520	*Kabuli*	Ca_Kabuli_denovo	13055832	(A/G)
20521	CakSNP20521	*Kabuli*	Ca_Kabuli_denovo	13060385	(C/A)
20522	CakSNP20522	*Kabuli*	Ca_Kabuli_denovo	13060407	(G/C)
20523	CakSNP20523	*Kabuli*	Ca_Kabuli_denovo	13064433	(G/A)
20524	CakSNP20524	*Kabuli*	Ca_Kabuli_denovo	13065262	(G/C)
20525	CakSNP20525	*Kabuli*	Ca_Kabuli_denovo	13082032	(G/C)
20526	CakSNP20526	*Kabuli*	Ca_Kabuli_denovo	13088614	(G/C)
20527	CakSNP20527	*Kabuli*	Ca_Kabuli_denovo	13101486	(C/G)
20528	CakSNP20528	*Kabuli*	Ca_Kabuli_denovo	13101540	(G/C)
20529	CakSNP20529	*Kabuli*	Ca_Kabuli_denovo	13107551	(T/G)
20530	CakSNP20530	*Kabuli*	Ca_Kabuli_denovo	13108297	(C/T)
20531	CakSNP20531	*Kabuli*	Ca_Kabuli_denovo	13108376	(A/G)
20532	CakSNP20532	*Kabuli*	Ca_Kabuli_denovo	13109480	(G/C)
20533	CakSNP20533	*Kabuli*	Ca_Kabuli_denovo	13109533	(C/G)
20534	CakSNP20534	*Kabuli*	Ca_Kabuli_denovo	13113390	(A/T)
20535	CakSNP20535	*Kabuli*	Ca_Kabuli_denovo	13113393	(T/C)
20536	CakSNP20536	*Kabuli*	Ca_Kabuli_denovo	13113408	(G/A)
20537	CakSNP20537	*Kabuli*	Ca_Kabuli_denovo	13114873	(A/C)
20538	CakSNP20538	*Kabuli*	Ca_Kabuli_denovo	13115242	(T/C)
20539	CakSNP20539	*Kabuli*	Ca_Kabuli_denovo	13117588	(T/G)
20540	CakSNP20540	*Kabuli*	Ca_Kabuli_denovo	13117614	(A/G)
20541	CakSNP20541	*Kabuli*	Ca_Kabuli_denovo	13119370	(T/C)
20542	CakSNP20542	*Kabuli*	Ca_Kabuli_denovo	13121866	(T/C)
20543	CakSNP20543	*Kabuli*	Ca_Kabuli_denovo	13121875	(A/G)
20544	CakSNP20544	*Kabuli*	Ca_Kabuli_denovo	13123297	(A/G)
20545	CakSNP20545	*Kabuli*	Ca_Kabuli_denovo	13124552	(A/G)
20546	CakSNP20546	*Kabuli*	Ca_Kabuli_denovo	13124612	(G/A)
20547	CakSNP20547	*Kabuli*	Ca_Kabuli_denovo	13127534	(G/T)
20548	CakSNP20548	*Kabuli*	Ca_Kabuli_denovo	13127558	(C/G)
20549	CakSNP20549	*Kabuli*	Ca_Kabuli_denovo	13131624	(G/A)
20550	CakSNP20550	*Kabuli*	Ca_Kabuli_denovo	13131674	(G/A)
20551	CakSNP20551	*Kabuli*	Ca_Kabuli_denovo	13138680	(A/G)

S.N.	SNP IDs	Cultivars	Chromosomes/scaffolds	Physical positions (bp)	SNPs
20552	CakSNP20552	*Kabuli*	Ca_Kabuli_denovo	13138756	(G/A)
20553	CakSNP20553	*Kabuli*	Ca_Kabuli_denovo	13141952	(T/C)
20554	CakSNP20554	*Kabuli*	Ca_Kabuli_denovo	13143666	(C/G)
20555	CakSNP20555	*Kabuli*	Ca_Kabuli_denovo	13145752	(T/G)
20556	CakSNP20556	*Kabuli*	Ca_Kabuli_denovo	13145765	(C/T)
20557	CakSNP20557	*Kabuli*	Ca_Kabuli_denovo	13146212	(A/T)
20558	CakSNP20558	*Kabuli*	Ca_Kabuli_denovo	13146263	(T/A)
20559	CakSNP20559	*Kabuli*	Ca_Kabuli_denovo	13147196	(A/G)
20560	CakSNP20560	*Kabuli*	Ca_Kabuli_denovo	13147202	(G/A)
20561	CakSNP20561	*Kabuli*	Ca_Kabuli_denovo	13155547	(G/T)
20562	CakSNP20562	*Kabuli*	Ca_Kabuli_denovo	13162784	(G/C)
20563	CakSNP20563	*Kabuli*	Ca_Kabuli_denovo	13169513	(C/T)
20564	CakSNP20564	*Kabuli*	Ca_Kabuli_denovo	13169527	(C/T)
20565	CakSNP20565	*Kabuli*	Ca_Kabuli_denovo	13171259	(A/G)
20566	CakSNP20566	*Kabuli*	Ca_Kabuli_denovo	13171283	(A/G)
20567	CakSNP20567	*Kabuli*	Ca_Kabuli_denovo	13172040	(A/G)
20568	CakSNP20568	*Kabuli*	Ca_Kabuli_denovo	13173271	(C/T)
20569	CakSNP20569	*Kabuli*	Ca_Kabuli_denovo	13173276	(G/A)
20570	CakSNP20570	*Kabuli*	Ca_Kabuli_denovo	13178980	(C/G)
20571	CakSNP20571	*Kabuli*	Ca_Kabuli_denovo	13179014	(C/G)
20572	CakSNP20572	*Kabuli*	Ca_Kabuli_denovo	13182701	(C/G)
20573	CakSNP20573	*Kabuli*	Ca_Kabuli_denovo	13184993	(G/C)
20574	CakSNP20574	*Kabuli*	Ca_Kabuli_denovo	13187926	(C/T)
20575	CakSNP20575	*Kabuli*	Ca_Kabuli_denovo	13189933	(A/G)
20576	CakSNP20576	*Kabuli*	Ca_Kabuli_denovo	13194655	(A/C)
20577	CakSNP20577	*Kabuli*	Ca_Kabuli_denovo	13198712	(T/C)
20578	CakSNP20578	*Kabuli*	Ca_Kabuli_denovo	13198718	(C/T)
20579	CakSNP20579	*Kabuli*	Ca_Kabuli_denovo	13203576	(T/G)
20580	CakSNP20580	*Kabuli*	Ca_Kabuli_denovo	13205570	(T/C)
20581	CakSNP20581	*Kabuli*	Ca_Kabuli_denovo	13205676	(A/C)
20582	CakSNP20582	*Kabuli*	Ca_Kabuli_denovo	13206342	(G/C)
20583	CakSNP20583	*Kabuli*	Ca_Kabuli_denovo	13208511	(A/C)
20584	CakSNP20584	*Kabuli*	Ca_Kabuli_denovo	13208512	(G/A)
20585	CakSNP20585	*Kabuli*	Ca_Kabuli_denovo	13211264	(A/G)
20586	CakSNP20586	*Kabuli*	Ca_Kabuli_denovo	13219900	(G/T)
20587	CakSNP20587	*Kabuli*	Ca_Kabuli_denovo	13220434	(C/T)
20588	CakSNP20588	*Kabuli*	Ca_Kabuli_denovo	13220440	(A/G)
20589	CakSNP20589	*Kabuli*	Ca_Kabuli_denovo	13223422	(C/G)
20590	CakSNP20590	*Kabuli*	Ca_Kabuli_denovo	13223458	(C/T)

S.N.	SNP IDs	Cultivars	Chromosomes/scaffolds	Physical positions (bp)	SNPs
20591	CakSNP20591	*Kabuli*	Ca_Kabuli_denovo	13233187	(T/A)
20592	CakSNP20592	*Kabuli*	Ca_Kabuli_denovo	13233773	(C/T)
20593	CakSNP20593	*Kabuli*	Ca_Kabuli_denovo	13236038	(A/C)
20594	CakSNP20594	*Kabuli*	Ca_Kabuli_denovo	13236125	(T/C)
20595	CakSNP20595	*Kabuli*	Ca_Kabuli_denovo	13236608	(A/G)
20596	CakSNP20596	*Kabuli*	Ca_Kabuli_denovo	13243606	(G/T)
20597	CakSNP20597	*Kabuli*	Ca_Kabuli_denovo	13243624	(C/A)
20598	CakSNP20598	*Kabuli*	Ca_Kabuli_denovo	13255115	(G/A)
20599	CakSNP20599	*Kabuli*	Ca_Kabuli_denovo	13256716	(C/T)
20600	CakSNP20600	*Kabuli*	Ca_Kabuli_denovo	13257182	(A/G)
20601	CakSNP20601	*Kabuli*	Ca_Kabuli_denovo	13257218	(C/T)
20602	CakSNP20602	*Kabuli*	Ca_Kabuli_denovo	13257741	(G/C)
20603	CakSNP20603	*Kabuli*	Ca_Kabuli_denovo	13257752	(C/T)
20604	CakSNP20604	*Kabuli*	Ca_Kabuli_denovo	13262646	(C/T)
20605	CakSNP20605	*Kabuli*	Ca_Kabuli_denovo	13262682	(G/C)
20606	CakSNP20606	*Kabuli*	Ca_Kabuli_denovo	13263857	(C/G)
20607	CakSNP20607	*Kabuli*	Ca_Kabuli_denovo	13263881	(T/C)
20608	CakSNP20608	*Kabuli*	Ca_Kabuli_denovo	13265069	(T/C)
20609	CakSNP20609	*Kabuli*	Ca_Kabuli_denovo	13265087	(G/C)
20610	CakSNP20610	*Kabuli*	Ca_Kabuli_denovo	13265898	(A/G)
20611	CakSNP20611	*Kabuli*	Ca_Kabuli_denovo	13265931	(G/A)
20612	CakSNP20612	*Kabuli*	Ca_Kabuli_denovo	13266100	(T/C)
20613	CakSNP20613	*Kabuli*	Ca_Kabuli_denovo	13266130	(A/T)
20614	CakSNP20614	*Kabuli*	Ca_Kabuli_denovo	13268055	(T/C)
20615	CakSNP20615	*Kabuli*	Ca_Kabuli_denovo	13268076	(G/T)
20616	CakSNP20616	*Kabuli*	Ca_Kabuli_denovo	13271245	(A/G)
20617	CakSNP20617	*Kabuli*	Ca_Kabuli_denovo	13271253	(T/C)
20618	CakSNP20618	*Kabuli*	Ca_Kabuli_denovo	13274178	(C/A)
20619	CakSNP20619	*Kabuli*	Ca_Kabuli_denovo	13274216	(C/G)
20620	CakSNP20620	*Kabuli*	Ca_Kabuli_denovo	13281091	(T/A)
20621	CakSNP20621	*Kabuli*	Ca_Kabuli_denovo	13283664	(C/A)
20622	CakSNP20622	*Kabuli*	Ca_Kabuli_denovo	13291126	(C/A)
20623	CakSNP20623	*Kabuli*	Ca_Kabuli_denovo	13298005	(C/T)
20624	CakSNP20624	*Kabuli*	Ca_Kabuli_denovo	13299371	(A/T)
20625	CakSNP20625	*Kabuli*	Ca_Kabuli_denovo	13299943	(G/A)
20626	CakSNP20626	*Kabuli*	Ca_Kabuli_denovo	13300502	(C/T)
20627	CakSNP20627	*Kabuli*	Ca_Kabuli_denovo	13300525	(C/T)
20628	CakSNP20628	*Kabuli*	Ca_Kabuli_denovo	13302088	(G/T)
20629	CakSNP20629	*Kabuli*	Ca_Kabuli_denovo	13302097	(A/G)

S.N.	SNP IDs	Cultivars	Chromosomes/scaffolds	Physical positions (bp)	SNPs
20630	CakSNP20630	*Kabuli*	Ca_Kabuli_denovo	13305822	(C/T)
20631	CakSNP20631	*Kabuli*	Ca_Kabuli_denovo	13309255	(A/G)
20632	CakSNP20632	*Kabuli*	Ca_Kabuli_denovo	13309265	(T/C)
20633	CakSNP20633	*Kabuli*	Ca_Kabuli_denovo	13318048	(T/C)
20634	CakSNP20634	*Kabuli*	Ca_Kabuli_denovo	13321431	(G/C)
20635	CakSNP20635	*Kabuli*	Ca_Kabuli_denovo	13321444	(T/A)
20636	CakSNP20636	*Kabuli*	Ca_Kabuli_denovo	13326443	(T/A)
20637	CakSNP20637	*Kabuli*	Ca_Kabuli_denovo	13326470	(A/G)
20638	CakSNP20638	*Kabuli*	Ca_Kabuli_denovo	13331285	(A/G)
20639	CakSNP20639	*Kabuli*	Ca_Kabuli_denovo	13331312	(C/G)
20640	CakSNP20640	*Kabuli*	Ca_Kabuli_denovo	13338641	(A/C)
20641	CakSNP20641	*Kabuli*	Ca_Kabuli_denovo	13338649	(T/G)
20642	CakSNP20642	*Kabuli*	Ca_Kabuli_denovo	13342906	(A/G)
20643	CakSNP20643	*Kabuli*	Ca_Kabuli_denovo	13347586	(G/C)
20644	CakSNP20644	*Kabuli*	Ca_Kabuli_denovo	13347619	(C/T)
20645	CakSNP20645	*Kabuli*	Ca_Kabuli_denovo	13350368	(G/C)
20646	CakSNP20646	*Kabuli*	Ca_Kabuli_denovo	13350390	(A/G)
20647	CakSNP20647	*Kabuli*	Ca_Kabuli_denovo	13350733	(A/G)
20648	CakSNP20648	*Kabuli*	Ca_Kabuli_denovo	13350766	(T/C)
20649	CakSNP20649	*Kabuli*	Ca_Kabuli_denovo	13351946	(A/G)
20650	CakSNP20650	*Kabuli*	Ca_Kabuli_denovo	13354654	(C/G)
20651	CakSNP20651	*Kabuli*	Ca_Kabuli_denovo	13361155	(T/C)
20652	CakSNP20652	*Kabuli*	Ca_Kabuli_denovo	13368691	(G/T)
20653	CakSNP20653	*Kabuli*	Ca_Kabuli_denovo	13373609	(C/T)
20654	CakSNP20654	*Kabuli*	Ca_Kabuli_denovo	13373661	(G/A)
20655	CakSNP20655	*Kabuli*	Ca_Kabuli_denovo	13375276	(T/C)
20656	CakSNP20656	*Kabuli*	Ca_Kabuli_denovo	13376177	(C/T)
20657	CakSNP20657	*Kabuli*	Ca_Kabuli_denovo	13376187	(C/T)
20658	CakSNP20658	*Kabuli*	Ca_Kabuli_denovo	13390756	(T/C)
20659	CakSNP20659	*Kabuli*	Ca_Kabuli_denovo	13390759	(A/G)
20660	CakSNP20660	*Kabuli*	Ca_Kabuli_denovo	13400505	(T/C)
20661	CakSNP20661	*Kabuli*	Ca_Kabuli_denovo	13400514	(C/A)
20662	CakSNP20662	*Kabuli*	Ca_Kabuli_denovo	13402676	(C/T)
20663	CakSNP20663	*Kabuli*	Ca_Kabuli_denovo	13402685	(C/T)
20664	CakSNP20664	*Kabuli*	Ca_Kabuli_denovo	13402948	(T/C)
20665	CakSNP20665	*Kabuli*	Ca_Kabuli_denovo	13402956	(G/C)
20666	CakSNP20666	*Kabuli*	Ca_Kabuli_denovo	13402957	(C/T)
20667	CakSNP20667	*Kabuli*	Ca_Kabuli_denovo	13403374	(C/T)
20668	CakSNP20668	*Kabuli*	Ca_Kabuli_denovo	13404299	(C/T)

S.N.	SNP IDs	Cultivars	Chromosomes/scaffolds	Physical positions (bp)	SNPs
20669	CakSNP20669	*Kabuli*	Ca_Kabuli_denovo	13405999	(T/C)
20670	CakSNP20670	*Kabuli*	Ca_Kabuli_denovo	13408133	(G/A)
20671	CakSNP20671	*Kabuli*	Ca_Kabuli_denovo	13413523	(C/T)
20672	CakSNP20672	*Kabuli*	Ca_Kabuli_denovo	13421129	(A/G)
20673	CakSNP20673	*Kabuli*	Ca_Kabuli_denovo	13423674	(G/A)
20674	CakSNP20674	*Kabuli*	Ca_Kabuli_denovo	13424125	(A/T)
20675	CakSNP20675	*Kabuli*	Ca_Kabuli_denovo	13425959	(C/G)
20676	CakSNP20676	*Kabuli*	Ca_Kabuli_denovo	13431139	(T/C)
20677	CakSNP20677	*Kabuli*	Ca_Kabuli_denovo	13434517	(C/G)
20678	CakSNP20678	*Kabuli*	Ca_Kabuli_denovo	13437430	(T/C)
20679	CakSNP20679	*Kabuli*	Ca_Kabuli_denovo	13438296	(C/A)
20680	CakSNP20680	*Kabuli*	Ca_Kabuli_denovo	13444653	(A/G)
20681	CakSNP20681	*Kabuli*	Ca_Kabuli_denovo	13449681	(C/T)
20682	CakSNP20682	*Kabuli*	Ca_Kabuli_denovo	13450012	(G/A)
20683	CakSNP20683	*Kabuli*	Ca_Kabuli_denovo	13451539	(T/C)
20684	CakSNP20684	*Kabuli*	Ca_Kabuli_denovo	13451572	(G/T)
20685	CakSNP20685	*Kabuli*	Ca_Kabuli_denovo	13452839	(A/G)
20686	CakSNP20686	*Kabuli*	Ca_Kabuli_denovo	13462372	(A/C)
20687	CakSNP20687	*Kabuli*	Ca_Kabuli_denovo	13465634	(C/G)
20688	CakSNP20688	*Kabuli*	Ca_Kabuli_denovo	13467173	(G/A)
20689	CakSNP20689	*Kabuli*	Ca_Kabuli_denovo	13475729	(C/T)
20690	CakSNP20690	*Kabuli*	Ca_Kabuli_denovo	13475780	(T/C)
20691	CakSNP20691	*Kabuli*	Ca_Kabuli_denovo	13477849	(T/C)
20692	CakSNP20692	*Kabuli*	Ca_Kabuli_denovo	13477879	(C/T)
20693	CakSNP20693	*Kabuli*	Ca_Kabuli_denovo	13482416	(C/T)
20694	CakSNP20694	*Kabuli*	Ca_Kabuli_denovo	13483653	(C/G)
20695	CakSNP20695	*Kabuli*	Ca_Kabuli_denovo	13483915	(C/T)
20696	CakSNP20696	*Kabuli*	Ca_Kabuli_denovo	13483957	(C/T)
20697	CakSNP20697	*Kabuli*	Ca_Kabuli_denovo	13484597	(G/A)
20698	CakSNP20698	*Kabuli*	Ca_Kabuli_denovo	13486160	(G/A)
20699	CakSNP20699	*Kabuli*	Ca_Kabuli_denovo	13486322	(C/T)
20700	CakSNP20700	*Kabuli*	Ca_Kabuli_denovo	13487264	(C/T)
20701	CakSNP20701	*Kabuli*	Ca_Kabuli_denovo	13487276	(C/T)
20702	CakSNP20702	*Kabuli*	Ca_Kabuli_denovo	13489767	(C/G)
20703	CakSNP20703	*Kabuli*	Ca_Kabuli_denovo	13489770	(A/G)
20704	CakSNP20704	*Kabuli*	Ca_Kabuli_denovo	13489794	(C/T)
20705	CakSNP20705	*Kabuli*	Ca_Kabuli_denovo	13494557	(G/C)
20706	CakSNP20706	*Kabuli*	Ca_Kabuli_denovo	13494571	(A/G)
20707	CakSNP20707	*Kabuli*	Ca_Kabuli_denovo	13500382	(C/A)

S.N.	SNP IDs	Cultivars	Chromosomes/scaffolds	Physical positions (bp)	SNPs
20708	CakSNP20708	*Kabuli*	Ca_Kabuli_denovo	13511809	(A/G)
20709	CakSNP20709	*Kabuli*	Ca_Kabuli_denovo	13511857	(A/G)
20710	CakSNP20710	*Kabuli*	Ca_Kabuli_denovo	13515765	(T/C)
20711	CakSNP20711	*Kabuli*	Ca_Kabuli_denovo	13528252	(G/A)
20712	CakSNP20712	*Kabuli*	Ca_Kabuli_denovo	13528261	(G/A)
20713	CakSNP20713	*Kabuli*	Ca_Kabuli_denovo	13536814	(A/G)
20714	CakSNP20714	*Kabuli*	Ca_Kabuli_denovo	13536909	(C/T)
20715	CakSNP20715	*Kabuli*	Ca_Kabuli_denovo	13536915	(G/C)
20716	CakSNP20716	*Kabuli*	Ca_Kabuli_denovo	13539057	(G/C)
20717	CakSNP20717	*Kabuli*	Ca_Kabuli_denovo	13544201	(G/A)
20718	CakSNP20718	*Kabuli*	Ca_Kabuli_denovo	13550350	(T/C)
20719	CakSNP20719	*Kabuli*	Ca_Kabuli_denovo	13560452	(G/A)
20720	CakSNP20720	*Kabuli*	Ca_Kabuli_denovo	13565367	(C/G)
20721	CakSNP20721	*Kabuli*	Ca_Kabuli_denovo	13565390	(A/G)
20722	CakSNP20722	*Kabuli*	Ca_Kabuli_denovo	13575988	(T/C)
20723	CakSNP20723	*Kabuli*	Ca_Kabuli_denovo	13576027	(G/T)
20724	CakSNP20724	*Kabuli*	Ca_Kabuli_denovo	13602770	(G/T)
20725	CakSNP20725	*Kabuli*	Ca_Kabuli_denovo	13604914	(T/C)
20726	CakSNP20726	*Kabuli*	Ca_Kabuli_denovo	13604935	(C/T)
20727	CakSNP20727	*Kabuli*	Ca_Kabuli_denovo	13606541	(A/G)
20728	CakSNP20728	*Kabuli*	Ca_Kabuli_denovo	13613180	(T/C)
20729	CakSNP20729	*Kabuli*	Ca_Kabuli_denovo	13613224	(A/G)
20730	CakSNP20730	*Kabuli*	Ca_Kabuli_denovo	13613225	(A/G)
20731	CakSNP20731	*Kabuli*	Ca_Kabuli_denovo	13613231	(C/G)
20732	CakSNP20732	*Kabuli*	Ca_Kabuli_denovo	13617653	(C/T)
20733	CakSNP20733	*Kabuli*	Ca_Kabuli_denovo	13617683	(A/T)
20734	CakSNP20734	*Kabuli*	Ca_Kabuli_denovo	13623029	(G/A)
20735	CakSNP20735	*Kabuli*	Ca_Kabuli_denovo	13623080	(G/C)
20736	CakSNP20736	*Kabuli*	Ca_Kabuli_denovo	13625592	(C/G)
20737	CakSNP20737	*Kabuli*	Ca_Kabuli_denovo	13625871	(G/T)
20738	CakSNP20738	*Kabuli*	Ca_Kabuli_denovo	13626117	(C/T)
20739	CakSNP20739	*Kabuli*	Ca_Kabuli_denovo	13629263	(T/C)
20740	CakSNP20740	*Kabuli*	Ca_Kabuli_denovo	13629308	(C/T)
20741	CakSNP20741	*Kabuli*	Ca_Kabuli_denovo	13632321	(T/C)
20742	CakSNP20742	*Kabuli*	Ca_Kabuli_denovo	13632391	(A/G)
20743	CakSNP20743	*Kabuli*	Ca_Kabuli_denovo	13641188	(A/G)
20744	CakSNP20744	*Kabuli*	Ca_Kabuli_denovo	13641203	(G/A)
20745	CakSNP20745	*Kabuli*	Ca_Kabuli_denovo	13641400	(G/A)
20746	CakSNP20746	*Kabuli*	Ca_Kabuli_denovo	13643385	(G/C)

S.N.	SNP IDs	Cultivars	Chromosomes/scaffolds	Physical positions (bp)	SNPs
20747	CakSNP20747	*Kabuli*	Ca_Kabuli_denovo	13643444	(A/G)
20748	CakSNP20748	*Kabuli*	Ca_Kabuli_denovo	13651337	(T/G)
20749	CakSNP20749	*Kabuli*	Ca_Kabuli_denovo	13655503	(A/G)
20750	CakSNP20750	*Kabuli*	Ca_Kabuli_denovo	13655518	(A/G)
20751	CakSNP20751	*Kabuli*	Ca_Kabuli_denovo	13666470	(C/T)
20752	CakSNP20752	*Kabuli*	Ca_Kabuli_denovo	13666480	(A/G)
20753	CakSNP20753	*Kabuli*	Ca_Kabuli_denovo	13673377	(T/C)
20754	CakSNP20754	*Kabuli*	Ca_Kabuli_denovo	13675125	(C/T)
20755	CakSNP20755	*Kabuli*	Ca_Kabuli_denovo	13675292	(G/A)
20756	CakSNP20756	*Kabuli*	Ca_Kabuli_denovo	13677592	(A/C)
20757	CakSNP20757	*Kabuli*	Ca_Kabuli_denovo	13687869	(G/C)
20758	CakSNP20758	*Kabuli*	Ca_Kabuli_denovo	13691846	(G/A)
20759	CakSNP20759	*Kabuli*	Ca_Kabuli_denovo	13691919	(T/A)
20760	CakSNP20760	*Kabuli*	Ca_Kabuli_denovo	13698536	(C/T)
20761	CakSNP20761	*Kabuli*	Ca_Kabuli_denovo	13698551	(G/C)
20762	CakSNP20762	*Kabuli*	Ca_Kabuli_denovo	13701338	(G/C)
20763	CakSNP20763	*Kabuli*	Ca_Kabuli_denovo	13701377	(C/T)
20764	CakSNP20764	*Kabuli*	Ca_Kabuli_denovo	13704680	(T/C)
20765	CakSNP20765	*Kabuli*	Ca_Kabuli_denovo	13706750	(T/C)
20766	CakSNP20766	*Kabuli*	Ca_Kabuli_denovo	13706908	(T/C)
20767	CakSNP20767	*Kabuli*	Ca_Kabuli_denovo	13706944	(C/T)
20768	CakSNP20768	*Kabuli*	Ca_Kabuli_denovo	13709609	(A/G)
20769	CakSNP20769	*Kabuli*	Ca_Kabuli_denovo	13710121	(G/T)
20770	CakSNP20770	*Kabuli*	Ca_Kabuli_denovo	13711298	(T/C)
20771	CakSNP20771	*Kabuli*	Ca_Kabuli_denovo	13711325	(C/G)
20772	CakSNP20772	*Kabuli*	Ca_Kabuli_denovo	13718021	(G/A)
20773	CakSNP20773	*Kabuli*	Ca_Kabuli_denovo	13721788	(T/C)
20774	CakSNP20774	*Kabuli*	Ca_Kabuli_denovo	13721815	(C/T)
20775	CakSNP20775	*Kabuli*	Ca_Kabuli_denovo	13732598	(C/T)
20776	CakSNP20776	*Kabuli*	Ca_Kabuli_denovo	13732658	(C/T)
20777	CakSNP20777	*Kabuli*	Ca_Kabuli_denovo	13736037	(G/A)
20778	CakSNP20778	*Kabuli*	Ca_Kabuli_denovo	13736092	(C/G)
20779	CakSNP20779	*Kabuli*	Ca_Kabuli_denovo	13739207	(A/G)
20780	CakSNP20780	*Kabuli*	Ca_Kabuli_denovo	13739213	(C/T)
20781	CakSNP20781	*Kabuli*	Ca_Kabuli_denovo	13740208	(A/G)
20782	CakSNP20782	*Kabuli*	Ca_Kabuli_denovo	13745703	(T/C)
20783	CakSNP20783	*Kabuli*	Ca_Kabuli_denovo	13745704	(T/C)
20784	CakSNP20784	*Kabuli*	Ca_Kabuli_denovo	13757579	(G/T)
20785	CakSNP20785	*Kabuli*	Ca_Kabuli_denovo	13762433	(G/A)

S.N.	SNP IDs	Cultivars	Chromosomes/scaffolds	Physical positions (bp)	SNPs
20786	CakSNP20786	*Kabuli*	Ca_Kabuli_denovo	13762450	(C/T)
20787	CakSNP20787	*Kabuli*	Ca_Kabuli_denovo	13764490	(C/A)
20788	CakSNP20788	*Kabuli*	Ca_Kabuli_denovo	13770447	(C/T)
20789	CakSNP20789	*Kabuli*	Ca_Kabuli_denovo	13770484	(T/C)
20790	CakSNP20790	*Kabuli*	Ca_Kabuli_denovo	13788645	(T/G)
20791	CakSNP20791	*Kabuli*	Ca_Kabuli_denovo	13788697	(G/A)
20792	CakSNP20792	*Kabuli*	Ca_Kabuli_denovo	13788721	(T/C)
20793	CakSNP20793	*Kabuli*	Ca_Kabuli_denovo	13794543	(T/C)
20794	CakSNP20794	*Kabuli*	Ca_Kabuli_denovo	13799629	(G/C)
20795	CakSNP20795	*Kabuli*	Ca_Kabuli_denovo	13799704	(T/C)
20796	CakSNP20796	*Kabuli*	Ca_Kabuli_denovo	13817434	(G/A)
20797	CakSNP20797	*Kabuli*	Ca_Kabuli_denovo	13819277	(C/A)
20798	CakSNP20798	*Kabuli*	Ca_Kabuli_denovo	13819286	(G/C)
20799	CakSNP20799	*Kabuli*	Ca_Kabuli_denovo	13820035	(C/T)
20800	CakSNP20800	*Kabuli*	Ca_Kabuli_denovo	13826976	(G/T)
20801	CakSNP20801	*Kabuli*	Ca_Kabuli_denovo	13826995	(G/A)
20802	CakSNP20802	*Kabuli*	Ca_Kabuli_denovo	13831242	(A/T)
20803	CakSNP20803	*Kabuli*	Ca_Kabuli_denovo	13838447	(T/C)
20804	CakSNP20804	*Kabuli*	Ca_Kabuli_denovo	13841687	(C/T)
20805	CakSNP20805	*Kabuli*	Ca_Kabuli_denovo	13841700	(A/G)
20806	CakSNP20806	*Kabuli*	Ca_Kabuli_denovo	13841822	(T/G)
20807	CakSNP20807	*Kabuli*	Ca_Kabuli_denovo	13849092	(C/T)
20808	CakSNP20808	*Kabuli*	Ca_Kabuli_denovo	13851470	(A/G)
20809	CakSNP20809	*Kabuli*	Ca_Kabuli_denovo	13854412	(G/C)
20810	CakSNP20810	*Kabuli*	Ca_Kabuli_denovo	13868476	(A/C)
20811	CakSNP20811	*Kabuli*	Ca_Kabuli_denovo	13868494	(C/G)
20812	CakSNP20812	*Kabuli*	Ca_Kabuli_denovo	13868588	(G/A)
20813	CakSNP20813	*Kabuli*	Ca_Kabuli_denovo	13870026	(T/C)
20814	CakSNP20814	*Kabuli*	Ca_Kabuli_denovo	13870068	(A/C)
20815	CakSNP20815	*Kabuli*	Ca_Kabuli_denovo	13878412	(A/C)
20816	CakSNP20816	*Kabuli*	Ca_Kabuli_denovo	13879822	(G/T)
20817	CakSNP20817	*Kabuli*	Ca_Kabuli_denovo	13882414	(G/A)
20818	CakSNP20818	*Kabuli*	Ca_Kabuli_denovo	13882450	(T/C)
20819	CakSNP20819	*Kabuli*	Ca_Kabuli_denovo	13884785	(T/C)
20820	CakSNP20820	*Kabuli*	Ca_Kabuli_denovo	13885854	(T/C)
20821	CakSNP20821	*Kabuli*	Ca_Kabuli_denovo	13892083	(A/G)
20822	CakSNP20822	*Kabuli*	Ca_Kabuli_denovo	13892084	(C/A)
20823	CakSNP20823	*Kabuli*	Ca_Kabuli_denovo	13898027	(G/A)
20824	CakSNP20824	*Kabuli*	Ca_Kabuli_denovo	13901395	(T/C)

S.N.	SNP IDs	Cultivars	Chromosomes/scaffolds	Physical positions (bp)	SNPs
20825	CakSNP20825	*Kabuli*	Ca_Kabuli_denovo	13902570	(A/C)
20826	CakSNP20826	*Kabuli*	Ca_Kabuli_denovo	13902571	(A/G)
20827	CakSNP20827	*Kabuli*	Ca_Kabuli_denovo	13911052	(T/A)
20828	CakSNP20828	*Kabuli*	Ca_Kabuli_denovo	13911082	(G/A)
20829	CakSNP20829	*Kabuli*	Ca_Kabuli_denovo	13912343	(T/C)
20830	CakSNP20830	*Kabuli*	Ca_Kabuli_denovo	13912361	(A/G)
20831	CakSNP20831	*Kabuli*	Ca_Kabuli_denovo	13915445	(G/A)
20832	CakSNP20832	*Kabuli*	Ca_Kabuli_denovo	13928830	(C/T)
20833	CakSNP20833	*Kabuli*	Ca_Kabuli_denovo	13935332	(G/A)
20834	CakSNP20834	*Kabuli*	Ca_Kabuli_denovo	13938219	(G/A)
20835	CakSNP20835	*Kabuli*	Ca_Kabuli_denovo	13938220	(G/A)
20836	CakSNP20836	*Kabuli*	Ca_Kabuli_denovo	13942437	(G/A)
20837	CakSNP20837	*Kabuli*	Ca_Kabuli_denovo	13942467	(G/A)
20838	CakSNP20838	*Kabuli*	Ca_Kabuli_denovo	13945517	(C/T)
20839	CakSNP20839	*Kabuli*	Ca_Kabuli_denovo	13948830	(T/C)
20840	CakSNP20840	*Kabuli*	Ca_Kabuli_denovo	13955773	(T/C)
20841	CakSNP20841	*Kabuli*	Ca_Kabuli_denovo	13955776	(G/C)
20842	CakSNP20842	*Kabuli*	Ca_Kabuli_denovo	13958314	(A/T)
20843	CakSNP20843	*Kabuli*	Ca_Kabuli_denovo	13960720	(C/T)
20844	CakSNP20844	*Kabuli*	Ca_Kabuli_denovo	13963694	(C/A)
20845	CakSNP20845	*Kabuli*	Ca_Kabuli_denovo	13963760	(A/G)
20846	CakSNP20846	*Kabuli*	Ca_Kabuli_denovo	13965291	(C/G)
20847	CakSNP20847	*Kabuli*	Ca_Kabuli_denovo	13965316	(C/T)
20848	CakSNP20848	*Kabuli*	Ca_Kabuli_denovo	13974263	(G/A)
20849	CakSNP20849	*Kabuli*	Ca_Kabuli_denovo	13977184	(G/A)
20850	CakSNP20850	*Kabuli*	Ca_Kabuli_denovo	13978845	(A/G)
20851	CakSNP20851	*Kabuli*	Ca_Kabuli_denovo	13978865	(A/G)
20852	CakSNP20852	*Kabuli*	Ca_Kabuli_denovo	13978866	(C/G)
20853	CakSNP20853	*Kabuli*	Ca_Kabuli_denovo	13980992	(G/A)
20854	CakSNP20854	*Kabuli*	Ca_Kabuli_denovo	13981007	(T/C)
20855	CakSNP20855	*Kabuli*	Ca_Kabuli_denovo	13988791	(G/C)
20856	CakSNP20856	*Kabuli*	Ca_Kabuli_denovo	13988810	(C/G)
20857	CakSNP20857	*Kabuli*	Ca_Kabuli_denovo	13989848	(A/G)
20858	CakSNP20858	*Kabuli*	Ca_Kabuli_denovo	13989891	(G/A)
20859	CakSNP20859	*Kabuli*	Ca_Kabuli_denovo	13991399	(T/C)
20860	CakSNP20860	*Kabuli*	Ca_Kabuli_denovo	13991444	(A/G)
20861	CakSNP20861	*Kabuli*	Ca_Kabuli_denovo	13991699	(A/G)
20862	CakSNP20862	*Kabuli*	Ca_Kabuli_denovo	13993112	(G/A)
20863	CakSNP20863	*Kabuli*	Ca_Kabuli_denovo	13994280	(C/T)

S.N.	SNP IDs	Cultivars	Chromosomes/scaffolds	Physical positions (bp)	SNPs
20864	CakSNP20864	*Kabuli*	Ca_Kabuli_denovo	13994560	(C/G)
20865	CakSNP20865	*Kabuli*	Ca_Kabuli_denovo	13994609	(A/G)
20866	CakSNP20866	*Kabuli*	Ca_Kabuli_denovo	13997710	(C/A)
20867	CakSNP20867	*Kabuli*	Ca_Kabuli_denovo	14013165	(T/C)
20868	CakSNP20868	*Kabuli*	Ca_Kabuli_denovo	14024171	(A/G)
20869	CakSNP20869	*Kabuli*	Ca_Kabuli_denovo	14024173	(T/C)
20870	CakSNP20870	*Kabuli*	Ca_Kabuli_denovo	14031255	(G/C)
20871	CakSNP20871	*Kabuli*	Ca_Kabuli_denovo	14031282	(A/G)
20872	CakSNP20872	*Kabuli*	Ca_Kabuli_denovo	14038882	(A/G)
20873	CakSNP20873	*Kabuli*	Ca_Kabuli_denovo	14047682	(A/G)
20874	CakSNP20874	*Kabuli*	Ca_Kabuli_denovo	14047694	(G/A)
20875	CakSNP20875	*Kabuli*	Ca_Kabuli_denovo	14051292	(C/A)
20876	CakSNP20876	*Kabuli*	Ca_Kabuli_denovo	14054007	(C/T)
20877	CakSNP20877	*Kabuli*	Ca_Kabuli_denovo	14054913	(C/T)
20878	CakSNP20878	*Kabuli*	Ca_Kabuli_denovo	14063953	(G/C)
20879	CakSNP20879	*Kabuli*	Ca_Kabuli_denovo	14069422	(A/G)
20880	CakSNP20880	*Kabuli*	Ca_Kabuli_denovo	14069482	(C/T)
20881	CakSNP20881	*Kabuli*	Ca_Kabuli_denovo	14070071	(A/G)
20882	CakSNP20882	*Kabuli*	Ca_Kabuli_denovo	14070080	(T/C)
20883	CakSNP20883	*Kabuli*	Ca_Kabuli_denovo	14090644	(C/G)
20884	CakSNP20884	*Kabuli*	Ca_Kabuli_denovo	14094627	(C/T)
20885	CakSNP20885	*Kabuli*	Ca_Kabuli_denovo	14094666	(A/G)
20886	CakSNP20886	*Kabuli*	Ca_Kabuli_denovo	14095149	(T/G)
20887	CakSNP20887	*Kabuli*	Ca_Kabuli_denovo	14097307	(T/G)
20888	CakSNP20888	*Kabuli*	Ca_Kabuli_denovo	14097992	(G/A)
20889	CakSNP20889	*Kabuli*	Ca_Kabuli_denovo	14099100	(A/G)
20890	CakSNP20890	*Kabuli*	Ca_Kabuli_denovo	14101707	(G/C)
20891	CakSNP20891	*Kabuli*	Ca_Kabuli_denovo	14102440	(C/T)
20892	CakSNP20892	*Kabuli*	Ca_Kabuli_denovo	14102489	(G/T)
20893	CakSNP20893	*Kabuli*	Ca_Kabuli_denovo	14104688	(T/A)
20894	CakSNP20894	*Kabuli*	Ca_Kabuli_denovo	14109844	(G/A)
20895	CakSNP20895	*Kabuli*	Ca_Kabuli_denovo	14109866	(A/C)
20896	CakSNP20896	*Kabuli*	Ca_Kabuli_denovo	14109868	(A/C)
20897	CakSNP20897	*Kabuli*	Ca_Kabuli_denovo	14112239	(C/T)
20898	CakSNP20898	*Kabuli*	Ca_Kabuli_denovo	14112675	(G/T)
20899	CakSNP20899	*Kabuli*	Ca_Kabuli_denovo	14112693	(C/T)
20900	CakSNP20900	*Kabuli*	Ca_Kabuli_denovo	14115751	(C/T)
20901	CakSNP20901	*Kabuli*	Ca_Kabuli_denovo	14121780	(G/T)
20902	CakSNP20902	*Kabuli*	Ca_Kabuli_denovo	14121828	(C/T)

S.N.	SNP IDs	Cultivars	Chromosomes/scaffolds	Physical positions (bp)	SNPs
20903	CakSNP20903	*Kabuli*	Ca_Kabuli_denovo	14123266	(C/T)
20904	CakSNP20904	*Kabuli*	Ca_Kabuli_denovo	14132147	(C/G)
20905	CakSNP20905	*Kabuli*	Ca_Kabuli_denovo	14134459	(C/T)
20906	CakSNP20906	*Kabuli*	Ca_Kabuli_denovo	14136009	(A/G)
20907	CakSNP20907	*Kabuli*	Ca_Kabuli_denovo	14136078	(A/G)
20908	CakSNP20908	*Kabuli*	Ca_Kabuli_denovo	14141764	(T/C)
20909	CakSNP20909	*Kabuli*	Ca_Kabuli_denovo	14146205	(T/C)
20910	CakSNP20910	*Kabuli*	Ca_Kabuli_denovo	14146512	(A/T)
20911	CakSNP20911	*Kabuli*	Ca_Kabuli_denovo	14146533	(C/T)
20912	CakSNP20912	*Kabuli*	Ca_Kabuli_denovo	14151634	(A/G)
20913	CakSNP20913	*Kabuli*	Ca_Kabuli_denovo	14162150	(G/C)
20914	CakSNP20914	*Kabuli*	Ca_Kabuli_denovo	14162186	(G/A)
20915	CakSNP20915	*Kabuli*	Ca_Kabuli_denovo	14162459	(C/T)
20916	CakSNP20916	*Kabuli*	Ca_Kabuli_denovo	14165612	(A/G)
20917	CakSNP20917	*Kabuli*	Ca_Kabuli_denovo	14170796	(C/T)
20918	CakSNP20918	*Kabuli*	Ca_Kabuli_denovo	14170853	(G/A)
20919	CakSNP20919	*Kabuli*	Ca_Kabuli_denovo	14173237	(C/T)
20920	CakSNP20920	*Kabuli*	Ca_Kabuli_denovo	14175735	(C/G)
20921	CakSNP20921	*Kabuli*	Ca_Kabuli_denovo	14178519	(A/C)
20922	CakSNP20922	*Kabuli*	Ca_Kabuli_denovo	14178529	(C/T)
20923	CakSNP20923	*Kabuli*	Ca_Kabuli_denovo	14180447	(A/G)
20924	CakSNP20924	*Kabuli*	Ca_Kabuli_denovo	14186276	(T/C)
20925	CakSNP20925	*Kabuli*	Ca_Kabuli_denovo	14188666	(T/C)
20926	CakSNP20926	*Kabuli*	Ca_Kabuli_denovo	14204954	(T/C)
20927	CakSNP20927	*Kabuli*	Ca_Kabuli_denovo	14204970	(A/G)
20928	CakSNP20928	*Kabuli*	Ca_Kabuli_denovo	14210876	(A/G)
20929	CakSNP20929	*Kabuli*	Ca_Kabuli_denovo	14210938	(C/T)
20930	CakSNP20930	*Kabuli*	Ca_Kabuli_denovo	14219449	(A/G)
20931	CakSNP20931	*Kabuli*	Ca_Kabuli_denovo	14219489	(G/A)
20932	CakSNP20932	*Kabuli*	Ca_Kabuli_denovo	14219623	(T/C)
20933	CakSNP20933	*Kabuli*	Ca_Kabuli_denovo	14221319	(G/A)
20934	CakSNP20934	*Kabuli*	Ca_Kabuli_denovo	14221501	(T/C)
20935	CakSNP20935	*Kabuli*	Ca_Kabuli_denovo	14229007	(C/A)
20936	CakSNP20936	*Kabuli*	Ca_Kabuli_denovo	14231935	(T/C)
20937	CakSNP20937	*Kabuli*	Ca_Kabuli_denovo	14231950	(G/T)
20938	CakSNP20938	*Kabuli*	Ca_Kabuli_denovo	14235265	(T/G)
20939	CakSNP20939	*Kabuli*	Ca_Kabuli_denovo	14237166	(G/A)
20940	CakSNP20940	*Kabuli*	Ca_Kabuli_denovo	14237557	(A/C)
20941	CakSNP20941	*Kabuli*	Ca_Kabuli_denovo	14241384	(A/G)

S.N.	SNP IDs	Cultivars	Chromosomes/scaffolds	Physical positions (bp)	SNPs
20942	CakSNP20942	*Kabuli*	Ca_Kabuli_denovo	14244846	(A/G)
20943	CakSNP20943	*Kabuli*	Ca_Kabuli_denovo	14246524	(T/G)
20944	CakSNP20944	*Kabuli*	Ca_Kabuli_denovo	14251791	(G/C)
20945	CakSNP20945	*Kabuli*	Ca_Kabuli_denovo	14251806	(A/G)
20946	CakSNP20946	*Kabuli*	Ca_Kabuli_denovo	14255378	(G/A)
20947	CakSNP20947	*Kabuli*	Ca_Kabuli_denovo	14257965	(C/A)
20948	CakSNP20948	*Kabuli*	Ca_Kabuli_denovo	14264833	(G/C)
20949	CakSNP20949	*Kabuli*	Ca_Kabuli_denovo	14264890	(T/C)
20950	CakSNP20950	*Kabuli*	Ca_Kabuli_denovo	14265359	(A/G)
20951	CakSNP20951	*Kabuli*	Ca_Kabuli_denovo	14275421	(G/T)
20952	CakSNP20952	*Kabuli*	Ca_Kabuli_denovo	14276179	(C/T)
20953	CakSNP20953	*Kabuli*	Ca_Kabuli_denovo	14276201	(G/A)
20954	CakSNP20954	*Kabuli*	Ca_Kabuli_denovo	14282422	(T/G)
20955	CakSNP20955	*Kabuli*	Ca_Kabuli_denovo	14282784	(G/A)
20956	CakSNP20956	*Kabuli*	Ca_Kabuli_denovo	14282796	(G/A)
20957	CakSNP20957	*Kabuli*	Ca_Kabuli_denovo	14286253	(T/C)
20958	CakSNP20958	*Kabuli*	Ca_Kabuli_denovo	14286269	(T/C)
20959	CakSNP20959	*Kabuli*	Ca_Kabuli_denovo	14289279	(C/G)
20960	CakSNP20960	*Kabuli*	Ca_Kabuli_denovo	14289309	(A/G)
20961	CakSNP20961	*Kabuli*	Ca_Kabuli_denovo	14292246	(C/G)
20962	CakSNP20962	*Kabuli*	Ca_Kabuli_denovo	14296223	(C/T)
20963	CakSNP20963	*Kabuli*	Ca_Kabuli_denovo	14297461	(G/C)
20964	CakSNP20964	*Kabuli*	Ca_Kabuli_denovo	14297530	(G/C)
20965	CakSNP20965	*Kabuli*	Ca_Kabuli_denovo	14303045	(C/T)
20966	CakSNP20966	*Kabuli*	Ca_Kabuli_denovo	14307152	(A/G)
20967	CakSNP20967	*Kabuli*	Ca_Kabuli_denovo	14308158	(C/G)
20968	CakSNP20968	*Kabuli*	Ca_Kabuli_denovo	14308820	(C/T)
20969	CakSNP20969	*Kabuli*	Ca_Kabuli_denovo	14310581	(A/C)
20970	CakSNP20970	*Kabuli*	Ca_Kabuli_denovo	14310632	(T/C)
20971	CakSNP20971	*Kabuli*	Ca_Kabuli_denovo	14311760	(A/G)
20972	CakSNP20972	*Kabuli*	Ca_Kabuli_denovo	14313417	(C/G)
20973	CakSNP20973	*Kabuli*	Ca_Kabuli_denovo	14320707	(G/A)
20974	CakSNP20974	*Kabuli*	Ca_Kabuli_denovo	14320709	(T/A)
20975	CakSNP20975	*Kabuli*	Ca_Kabuli_denovo	14328623	(A/C)
20976	CakSNP20976	*Kabuli*	Ca_Kabuli_denovo	14328917	(G/T)
20977	CakSNP20977	*Kabuli*	Ca_Kabuli_denovo	14333931	(A/G)
20978	CakSNP20978	*Kabuli*	Ca_Kabuli_denovo	14337745	(A/G)
20979	CakSNP20979	*Kabuli*	Ca_Kabuli_denovo	14337746	(T/C)
20980	CakSNP20980	*Kabuli*	Ca_Kabuli_denovo	14341574	(A/G)

S.N.	SNP IDs	Cultivars	Chromosomes/scaffolds	Physical positions (bp)	SNPs
20981	CakSNP20981	*Kabuli*	Ca_Kabuli_denovo	14341617	(A/C)
20982	CakSNP20982	*Kabuli*	Ca_Kabuli_denovo	14346701	(A/G)
20983	CakSNP20983	*Kabuli*	Ca_Kabuli_denovo	14346747	(G/T)
20984	CakSNP20984	*Kabuli*	Ca_Kabuli_denovo	14347271	(T/C)
20985	CakSNP20985	*Kabuli*	Ca_Kabuli_denovo	14348435	(A/T)
20986	CakSNP20986	*Kabuli*	Ca_Kabuli_denovo	14352328	(T/C)
20987	CakSNP20987	*Kabuli*	Ca_Kabuli_denovo	14352669	(A/G)
20988	CakSNP20988	*Kabuli*	Ca_Kabuli_denovo	14353348	(G/T)
20989	CakSNP20989	*Kabuli*	Ca_Kabuli_denovo	14353366	(G/A)
20990	CakSNP20990	*Kabuli*	Ca_Kabuli_denovo	14364703	(C/G)
20991	CakSNP20991	*Kabuli*	Ca_Kabuli_denovo	14369102	(C/G)
20992	CakSNP20992	*Kabuli*	Ca_Kabuli_denovo	14369921	(T/C)
20993	CakSNP20993	*Kabuli*	Ca_Kabuli_denovo	14380810	(C/G)
20994	CakSNP20994	*Kabuli*	Ca_Kabuli_denovo	14380823	(T/C)
20995	CakSNP20995	*Kabuli*	Ca_Kabuli_denovo	14383046	(C/T)
20996	CakSNP20996	*Kabuli*	Ca_Kabuli_denovo	14388649	(G/A)
20997	CakSNP20997	*Kabuli*	Ca_Kabuli_denovo	14388663	(T/A)
20998	CakSNP20998	*Kabuli*	Ca_Kabuli_denovo	14394158	(C/T)
20999	CakSNP20999	*Kabuli*	Ca_Kabuli_denovo	14397942	(G/A)
21000	CakSNP21000	*Kabuli*	Ca_Kabuli_denovo	14401170	(T/C)
21001	CakSNP21001	*Kabuli*	Ca_Kabuli_denovo	14402239	(T/G)
21002	CakSNP21002	*Kabuli*	Ca_Kabuli_denovo	14403229	(T/G)
21003	CakSNP21003	*Kabuli*	Ca_Kabuli_denovo	14405364	(C/T)
21004	CakSNP21004	*Kabuli*	Ca_Kabuli_denovo	14405406	(T/C)
21005	CakSNP21005	*Kabuli*	Ca_Kabuli_denovo	14406925	(C/G)
21006	CakSNP21006	*Kabuli*	Ca_Kabuli_denovo	14406958	(A/G)
21007	CakSNP21007	*Kabuli*	Ca_Kabuli_denovo	14406974	(G/C)
21008	CakSNP21008	*Kabuli*	Ca_Kabuli_denovo	14407215	(C/A)
21009	CakSNP21009	*Kabuli*	Ca_Kabuli_denovo	14407218	(T/C)
21010	CakSNP21010	*Kabuli*	Ca_Kabuli_denovo	14414831	(A/C)
21011	CakSNP21011	*Kabuli*	Ca_Kabuli_denovo	14416795	(G/A)
21012	CakSNP21012	*Kabuli*	Ca_Kabuli_denovo	14429719	(T/C)
21013	CakSNP21013	*Kabuli*	Ca_Kabuli_denovo	14433591	(C/T)
21014	CakSNP21014	*Kabuli*	Ca_Kabuli_denovo	14437122	(T/G)
21015	CakSNP21015	*Kabuli*	Ca_Kabuli_denovo	14437433	(T/C)
21016	CakSNP21016	*Kabuli*	Ca_Kabuli_denovo	14442419	(T/C)
21017	CakSNP21017	*Kabuli*	Ca_Kabuli_denovo	14442430	(C/T)
21018	CakSNP21018	*Kabuli*	Ca_Kabuli_denovo	14443760	(T/G)
21019	CakSNP21019	*Kabuli*	Ca_Kabuli_denovo	14443778	(A/G)

S.N.	SNP IDs	Cultivars	Chromosomes/scaffolds	Physical positions (bp)	SNPs
21020	CakSNP21020	*Kabuli*	Ca_Kabuli_denovo	14444490	(T/C)
21021	CakSNP21021	*Kabuli*	Ca_Kabuli_denovo	14444536	(C/G)
21022	CakSNP21022	*Kabuli*	Ca_Kabuli_denovo	14445505	(A/C)
21023	CakSNP21023	*Kabuli*	Ca_Kabuli_denovo	14448509	(T/G)
21024	CakSNP21024	*Kabuli*	Ca_Kabuli_denovo	14449521	(A/T)
21025	CakSNP21025	*Kabuli*	Ca_Kabuli_denovo	14449530	(G/A)
21026	CakSNP21026	*Kabuli*	Ca_Kabuli_denovo	14452270	(T/C)
21027	CakSNP21027	*Kabuli*	Ca_Kabuli_denovo	14457529	(C/T)
21028	CakSNP21028	*Kabuli*	Ca_Kabuli_denovo	14467081	(T/G)
21029	CakSNP21029	*Kabuli*	Ca_Kabuli_denovo	14469056	(T/C)
21030	CakSNP21030	*Kabuli*	Ca_Kabuli_denovo	14469127	(C/G)
21031	CakSNP21031	*Kabuli*	Ca_Kabuli_denovo	14469420	(A/G)
21032	CakSNP21032	*Kabuli*	Ca_Kabuli_denovo	14483569	(C/T)
21033	CakSNP21033	*Kabuli*	Ca_Kabuli_denovo	14495553	(A/G)
21034	CakSNP21034	*Kabuli*	Ca_Kabuli_denovo	14495559	(T/G)
21035	CakSNP21035	*Kabuli*	Ca_Kabuli_denovo	14513052	(T/C)
21036	CakSNP21036	*Kabuli*	Ca_Kabuli_denovo	14513906	(G/T)
21037	CakSNP21037	*Kabuli*	Ca_Kabuli_denovo	14514776	(T/A)
21038	CakSNP21038	*Kabuli*	Ca_Kabuli_denovo	14514787	(T/C)
21039	CakSNP21039	*Kabuli*	Ca_Kabuli_denovo	14515738	(G/C)
21040	CakSNP21040	*Kabuli*	Ca_Kabuli_denovo	14530041	(A/C)
21041	CakSNP21041	*Kabuli*	Ca_Kabuli_denovo	14537319	(T/C)
21042	CakSNP21042	*Kabuli*	Ca_Kabuli_denovo	14543760	(C/T)
21043	CakSNP21043	*Kabuli*	Ca_Kabuli_denovo	14546700	(A/G)
21044	CakSNP21044	*Kabuli*	Ca_Kabuli_denovo	14547594	(C/T)
21045	CakSNP21045	*Kabuli*	Ca_Kabuli_denovo	14550152	(G/A)
21046	CakSNP21046	*Kabuli*	Ca_Kabuli_denovo	14551303	(G/T)
21047	CakSNP21047	*Kabuli*	Ca_Kabuli_denovo	14561183	(A/G)
21048	CakSNP21048	*Kabuli*	Ca_Kabuli_denovo	14562250	(C/T)
21049	CakSNP21049	*Kabuli*	Ca_Kabuli_denovo	14566607	(A/G)
21050	CakSNP21050	*Kabuli*	Ca_Kabuli_denovo	14570440	(C/G)
21051	CakSNP21051	*Kabuli*	Ca_Kabuli_denovo	14570663	(G/T)
21052	CakSNP21052	*Kabuli*	Ca_Kabuli_denovo	14571952	(T/A)
21053	CakSNP21053	*Kabuli*	Ca_Kabuli_denovo	14572744	(T/C)
21054	CakSNP21054	*Kabuli*	Ca_Kabuli_denovo	14573976	(C/T)
21055	CakSNP21055	*Kabuli*	Ca_Kabuli_denovo	14578027	(A/C)
21056	CakSNP21056	*Kabuli*	Ca_Kabuli_denovo	14578231	(A/G)
21057	CakSNP21057	*Kabuli*	Ca_Kabuli_denovo	14578249	(C/T)
21058	CakSNP21058	*Kabuli*	Ca_Kabuli_denovo	14578273	(G/A)

S.N.	SNP IDs	Cultivars	Chromosomes/scaffolds	Physical positions (bp)	SNPs
21059	CakSNP21059	*Kabuli*	Ca_Kabuli_denovo	14582337	(C/T)
21060	CakSNP21060	*Kabuli*	Ca_Kabuli_denovo	14587510	(T/C)
21061	CakSNP21061	*Kabuli*	Ca_Kabuli_denovo	14589670	(G/A)
21062	CakSNP21062	*Kabuli*	Ca_Kabuli_denovo	14589693	(G/C)
21063	CakSNP21063	*Kabuli*	Ca_Kabuli_denovo	14590243	(A/C)
21064	CakSNP21064	*Kabuli*	Ca_Kabuli_denovo	14596822	(T/G)
21065	CakSNP21065	*Kabuli*	Ca_Kabuli_denovo	14596886	(T/C)
21066	CakSNP21066	*Kabuli*	Ca_Kabuli_denovo	14597698	(T/C)
21067	CakSNP21067	*Kabuli*	Ca_Kabuli_denovo	14599704	(A/G)
21068	CakSNP21068	*Kabuli*	Ca_Kabuli_denovo	14609759	(C/G)
21069	CakSNP21069	*Kabuli*	Ca_Kabuli_denovo	14609792	(T/C)
21070	CakSNP21070	*Kabuli*	Ca_Kabuli_denovo	14614842	(G/T)
21071	CakSNP21071	*Kabuli*	Ca_Kabuli_denovo	14615031	(T/G)
21072	CakSNP21072	*Kabuli*	Ca_Kabuli_denovo	14617523	(T/C)
21073	CakSNP21073	*Kabuli*	Ca_Kabuli_denovo	14619462	(T/G)
21074	CakSNP21074	*Kabuli*	Ca_Kabuli_denovo	14619490	(T/C)
21075	CakSNP21075	*Kabuli*	Ca_Kabuli_denovo	14620013	(C/T)
21076	CakSNP21076	*Kabuli*	Ca_Kabuli_denovo	14631563	(T/G)
21077	CakSNP21077	*Kabuli*	Ca_Kabuli_denovo	14636201	(G/A)
21078	CakSNP21078	*Kabuli*	Ca_Kabuli_denovo	14636204	(A/G)
21079	CakSNP21079	*Kabuli*	Ca_Kabuli_denovo	14636266	(G/A)
21080	CakSNP21080	*Kabuli*	Ca_Kabuli_denovo	14636270	(A/C)
21081	CakSNP21081	*Kabuli*	Ca_Kabuli_denovo	14641177	(A/C)
21082	CakSNP21082	*Kabuli*	Ca_Kabuli_denovo	14648080	(C/G)
21083	CakSNP21083	*Kabuli*	Ca_Kabuli_denovo	14648723	(T/C)
21084	CakSNP21084	*Kabuli*	Ca_Kabuli_denovo	14648727	(C/T)
21085	CakSNP21085	*Kabuli*	Ca_Kabuli_denovo	14652198	(G/T)
21086	CakSNP21086	*Kabuli*	Ca_Kabuli_denovo	14660915	(A/G)
21087	CakSNP21087	*Kabuli*	Ca_Kabuli_denovo	14668713	(A/C)
21088	CakSNP21088	*Kabuli*	Ca_Kabuli_denovo	14670691	(C/T)
21089	CakSNP21089	*Kabuli*	Ca_Kabuli_denovo	14670709	(C/T)
21090	CakSNP21090	*Kabuli*	Ca_Kabuli_denovo	14673778	(T/C)
21091	CakSNP21091	*Kabuli*	Ca_Kabuli_denovo	14682478	(C/G)
21092	CakSNP21092	*Kabuli*	Ca_Kabuli_denovo	14682505	(T/C)
21093	CakSNP21093	*Kabuli*	Ca_Kabuli_denovo	14685915	(G/A)
21094	CakSNP21094	*Kabuli*	Ca_Kabuli_denovo	14689649	(G/C)
21095	CakSNP21095	*Kabuli*	Ca_Kabuli_denovo	14696938	(A/G)
21096	CakSNP21096	*Kabuli*	Ca_Kabuli_denovo	14698942	(T/C)
21097	CakSNP21097	*Kabuli*	Ca_Kabuli_denovo	14703530	(C/G)

S.N.	SNP IDs	Cultivars	Chromosomes/scaffolds	Physical positions (bp)	SNPs
21098	CakSNP21098	*Kabuli*	Ca_Kabuli_denovo	14703575	(A/G)
21099	CakSNP21099	*Kabuli*	Ca_Kabuli_denovo	14704072	(T/C)
21100	CakSNP21100	*Kabuli*	Ca_Kabuli_denovo	14704120	(T/C)
21101	CakSNP21101	*Kabuli*	Ca_Kabuli_denovo	14704731	(C/T)
21102	CakSNP21102	*Kabuli*	Ca_Kabuli_denovo	14704732	(T/C)
21103	CakSNP21103	*Kabuli*	Ca_Kabuli_denovo	14715112	(A/G)
21104	CakSNP21104	*Kabuli*	Ca_Kabuli_denovo	14715215	(G/A)
21105	CakSNP21105	*Kabuli*	Ca_Kabuli_denovo	14715220	(C/G)
21106	CakSNP21106	*Kabuli*	Ca_Kabuli_denovo	14726401	(A/G)
21107	CakSNP21107	*Kabuli*	Ca_Kabuli_denovo	14728979	(G/T)
21108	CakSNP21108	*Kabuli*	Ca_Kabuli_denovo	14729758	(C/G)
21109	CakSNP21109	*Kabuli*	Ca_Kabuli_denovo	14729891	(A/T)
21110	CakSNP21110	*Kabuli*	Ca_Kabuli_denovo	14735636	(T/G)
21111	CakSNP21111	*Kabuli*	Ca_Kabuli_denovo	14736247	(A/G)
21112	CakSNP21112	*Kabuli*	Ca_Kabuli_denovo	14736289	(C/T)
21113	CakSNP21113	*Kabuli*	Ca_Kabuli_denovo	14741708	(A/C)
21114	CakSNP21114	*Kabuli*	Ca_Kabuli_denovo	14743692	(C/G)
21115	CakSNP21115	*Kabuli*	Ca_Kabuli_denovo	14743756	(T/A)
21116	CakSNP21116	*Kabuli*	Ca_Kabuli_denovo	14745532	(A/C)
21117	CakSNP21117	*Kabuli*	Ca_Kabuli_denovo	14746118	(A/G)
21118	CakSNP21118	*Kabuli*	Ca_Kabuli_denovo	14746154	(A/G)
21119	CakSNP21119	*Kabuli*	Ca_Kabuli_denovo	14748412	(G/A)
21120	CakSNP21120	*Kabuli*	Ca_Kabuli_denovo	14748417	(G/C)
21121	CakSNP21121	*Kabuli*	Ca_Kabuli_denovo	14749636	(G/A)
21122	CakSNP21122	*Kabuli*	Ca_Kabuli_denovo	14749704	(T/G)
21123	CakSNP21123	*Kabuli*	Ca_Kabuli_denovo	14749705	(G/T)
21124	CakSNP21124	*Kabuli*	Ca_Kabuli_denovo	14752787	(T/C)
21125	CakSNP21125	*Kabuli*	Ca_Kabuli_denovo	14755314	(C/T)
21126	CakSNP21126	*Kabuli*	Ca_Kabuli_denovo	14757725	(C/G)
21127	CakSNP21127	*Kabuli*	Ca_Kabuli_denovo	14759576	(C/T)
21128	CakSNP21128	*Kabuli*	Ca_Kabuli_denovo	14782372	(C/T)
21129	CakSNP21129	*Kabuli*	Ca_Kabuli_denovo	14782399	(C/T)
21130	CakSNP21130	*Kabuli*	Ca_Kabuli_denovo	14787071	(G/T)
21131	CakSNP21131	*Kabuli*	Ca_Kabuli_denovo	14788336	(T/C)
21132	CakSNP21132	*Kabuli*	Ca_Kabuli_denovo	14792248	(G/C)
21133	CakSNP21133	*Kabuli*	Ca_Kabuli_denovo	14792775	(G/A)
21134	CakSNP21134	*Kabuli*	Ca_Kabuli_denovo	14801212	(C/G)
21135	CakSNP21135	*Kabuli*	Ca_Kabuli_denovo	14801218	(C/T)
21136	CakSNP21136	*Kabuli*	Ca_Kabuli_denovo	14805457	(T/C)

S.N.	SNP IDs	Cultivars	Chromosomes/scaffolds	Physical positions (bp)	SNPs
21137	CakSNP21137	*Kabuli*	Ca_Kabuli_denovo	14805477	(G/C)
21138	CakSNP21138	*Kabuli*	Ca_Kabuli_denovo	14805855	(T/C)
21139	CakSNP21139	*Kabuli*	Ca_Kabuli_denovo	14805865	(A/G)
21140	CakSNP21140	*Kabuli*	Ca_Kabuli_denovo	14811884	(G/C)
21141	CakSNP21141	*Kabuli*	Ca_Kabuli_denovo	14825428	(C/G)
21142	CakSNP21142	*Kabuli*	Ca_Kabuli_denovo	14825440	(G/C)
21143	CakSNP21143	*Kabuli*	Ca_Kabuli_denovo	14833055	(C/A)
21144	CakSNP21144	*Kabuli*	Ca_Kabuli_denovo	14833103	(A/G)
21145	CakSNP21145	*Kabuli*	Ca_Kabuli_denovo	14834045	(G/C)
21146	CakSNP21146	*Kabuli*	Ca_Kabuli_denovo	14838314	(G/C)
21147	CakSNP21147	*Kabuli*	Ca_Kabuli_denovo	14841355	(G/A)
21148	CakSNP21148	*Kabuli*	Ca_Kabuli_denovo	14841976	(C/T)
21149	CakSNP21149	*Kabuli*	Ca_Kabuli_denovo	14845633	(A/C)
21150	CakSNP21150	*Kabuli*	Ca_Kabuli_denovo	14861241	(T/C)
21151	CakSNP21151	*Kabuli*	Ca_Kabuli_denovo	14861255	(T/C)
21152	CakSNP21152	*Kabuli*	Ca_Kabuli_denovo	14864054	(G/T)
21153	CakSNP21153	*Kabuli*	Ca_Kabuli_denovo	14864425	(C/T)
21154	CakSNP21154	*Kabuli*	Ca_Kabuli_denovo	14872625	(C/T)
21155	CakSNP21155	*Kabuli*	Ca_Kabuli_denovo	14872631	(C/T)
21156	CakSNP21156	*Kabuli*	Ca_Kabuli_denovo	14877101	(G/A)
21157	CakSNP21157	*Kabuli*	Ca_Kabuli_denovo	14877962	(G/A)
21158	CakSNP21158	*Kabuli*	Ca_Kabuli_denovo	14878041	(C/G)
21159	CakSNP21159	*Kabuli*	Ca_Kabuli_denovo	14881946	(C/T)
21160	CakSNP21160	*Kabuli*	Ca_Kabuli_denovo	14883255	(A/C)
21161	CakSNP21161	*Kabuli*	Ca_Kabuli_denovo	14883746	(C/T)
21162	CakSNP21162	*Kabuli*	Ca_Kabuli_denovo	14883748	(G/A)
21163	CakSNP21163	*Kabuli*	Ca_Kabuli_denovo	14886823	(T/C)
21164	CakSNP21164	*Kabuli*	Ca_Kabuli_denovo	14888070	(A/G)
21165	CakSNP21165	*Kabuli*	Ca_Kabuli_denovo	14891224	(G/A)
21166	CakSNP21166	*Kabuli*	Ca_Kabuli_denovo	14893670	(A/C)
21167	CakSNP21167	*Kabuli*	Ca_Kabuli_denovo	14893855	(T/G)
21168	CakSNP21168	*Kabuli*	Ca_Kabuli_denovo	14895580	(A/C)
21169	CakSNP21169	*Kabuli*	Ca_Kabuli_denovo	14898622	(C/T)
21170	CakSNP21170	*Kabuli*	Ca_Kabuli_denovo	14898764	(C/A)
21171	CakSNP21171	*Kabuli*	Ca_Kabuli_denovo	14899131	(T/G)
21172	CakSNP21172	*Kabuli*	Ca_Kabuli_denovo	14899150	(T/C)
21173	CakSNP21173	*Kabuli*	Ca_Kabuli_denovo	14899484	(T/C)
21174	CakSNP21174	*Kabuli*	Ca_Kabuli_denovo	14905145	(C/G)
21175	CakSNP21175	*Kabuli*	Ca_Kabuli_denovo	14906147	(C/G)

S.N.	SNP IDs	Cultivars	Chromosomes/scaffolds	Physical positions (bp)	SNPs
21176	CakSNP21176	*Kabuli*	Ca_Kabuli_denovo	14907584	(C/T)
21177	CakSNP21177	*Kabuli*	Ca_Kabuli_denovo	14909654	(T/G)
21178	CakSNP21178	*Kabuli*	Ca_Kabuli_denovo	14909803	(T/G)
21179	CakSNP21179	*Kabuli*	Ca_Kabuli_denovo	14912143	(A/G)
21180	CakSNP21180	*Kabuli*	Ca_Kabuli_denovo	14912144	(A/G)
21181	CakSNP21181	*Kabuli*	Ca_Kabuli_denovo	14913605	(C/T)
21182	CakSNP21182	*Kabuli*	Ca_Kabuli_denovo	14914323	(G/A)
21183	CakSNP21183	*Kabuli*	Ca_Kabuli_denovo	14916673	(T/C)
21184	CakSNP21184	*Kabuli*	Ca_Kabuli_denovo	14916715	(A/G)
21185	CakSNP21185	*Kabuli*	Ca_Kabuli_denovo	14918881	(G/C)
21186	CakSNP21186	*Kabuli*	Ca_Kabuli_denovo	14926796	(A/G)
21187	CakSNP21187	*Kabuli*	Ca_Kabuli_denovo	14927585	(T/C)
21188	CakSNP21188	*Kabuli*	Ca_Kabuli_denovo	14929577	(A/C)
21189	CakSNP21189	*Kabuli*	Ca_Kabuli_denovo	14935159	(C/A)
21190	CakSNP21190	*Kabuli*	Ca_Kabuli_denovo	14942882	(A/G)
21191	CakSNP21191	*Kabuli*	Ca_Kabuli_denovo	14942888	(A/C)
21192	CakSNP21192	*Kabuli*	Ca_Kabuli_denovo	14943646	(G/A)
21193	CakSNP21193	*Kabuli*	Ca_Kabuli_denovo	14950163	(T/G)
21194	CakSNP21194	*Kabuli*	Ca_Kabuli_denovo	14962978	(T/C)
21195	CakSNP21195	*Kabuli*	Ca_Kabuli_denovo	14968986	(C/A)
21196	CakSNP21196	*Kabuli*	Ca_Kabuli_denovo	14971785	(C/T)
21197	CakSNP21197	*Kabuli*	Ca_Kabuli_denovo	14974801	(G/A)
21198	CakSNP21198	*Kabuli*	Ca_Kabuli_denovo	14974806	(G/A)
21199	CakSNP21199	*Kabuli*	Ca_Kabuli_denovo	14977193	(T/C)
21200	CakSNP21200	*Kabuli*	Ca_Kabuli_denovo	14978299	(A/C)
21201	CakSNP21201	*Kabuli*	Ca_Kabuli_denovo	14981709	(C/T)
21202	CakSNP21202	*Kabuli*	Ca_Kabuli_denovo	14981741	(A/G)
21203	CakSNP21203	*Kabuli*	Ca_Kabuli_denovo	14982459	(G/A)
21204	CakSNP21204	*Kabuli*	Ca_Kabuli_denovo	14982465	(A/C)
21205	CakSNP21205	*Kabuli*	Ca_Kabuli_denovo	14985193	(G/A)
21206	CakSNP21206	*Kabuli*	Ca_Kabuli_denovo	14985249	(G/A)
21207	CakSNP21207	*Kabuli*	Ca_Kabuli_denovo	14991461	(T/C)
21208	CakSNP21208	*Kabuli*	Ca_Kabuli_denovo	14993274	(T/G)
21209	CakSNP21209	*Kabuli*	Ca_Kabuli_denovo	14998061	(T/C)
21210	CakSNP21210	*Kabuli*	Ca_Kabuli_denovo	15002955	(T/C)
21211	CakSNP21211	*Kabuli*	Ca_Kabuli_denovo	15006029	(T/A)
21212	CakSNP21212	*Kabuli*	Ca_Kabuli_denovo	15007070	(G/T)
21213	CakSNP21213	*Kabuli*	Ca_Kabuli_denovo	15011020	(G/A)
21214	CakSNP21214	*Kabuli*	Ca_Kabuli_denovo	15012544	(C/A)

S.N.	SNP IDs	Cultivars	Chromosomes/scaffolds	Physical positions (bp)	SNPs
21215	CakSNP21215	*Kabuli*	Ca_Kabuli_denovo	15012589	(C/T)
21216	CakSNP21216	*Kabuli*	Ca_Kabuli_denovo	15019523	(T/C)
21217	CakSNP21217	*Kabuli*	Ca_Kabuli_denovo	15019572	(G/T)
21218	CakSNP21218	*Kabuli*	Ca_Kabuli_denovo	15029564	(G/A)
21219	CakSNP21219	*Kabuli*	Ca_Kabuli_denovo	15029594	(G/A)
21220	CakSNP21220	*Kabuli*	Ca_Kabuli_denovo	15029893	(T/G)
21221	CakSNP21221	*Kabuli*	Ca_Kabuli_denovo	15029901	(T/C)
21222	CakSNP21222	*Kabuli*	Ca_Kabuli_denovo	15033418	(G/A)
21223	CakSNP21223	*Kabuli*	Ca_Kabuli_denovo	15048640	(G/C)
21224	CakSNP21224	*Kabuli*	Ca_Kabuli_denovo	15053938	(G/C)
21225	CakSNP21225	*Kabuli*	Ca_Kabuli_denovo	15053965	(T/C)
21226	CakSNP21226	*Kabuli*	Ca_Kabuli_denovo	15055093	(G/C)
21227	CakSNP21227	*Kabuli*	Ca_Kabuli_denovo	15055828	(C/A)
21228	CakSNP21228	*Kabuli*	Ca_Kabuli_denovo	15056085	(T/C)
21229	CakSNP21229	*Kabuli*	Ca_Kabuli_denovo	15056124	(C/T)
21230	CakSNP21230	*Kabuli*	Ca_Kabuli_denovo	15057672	(A/G)
21231	CakSNP21231	*Kabuli*	Ca_Kabuli_denovo	15058226	(C/A)
21232	CakSNP21232	*Kabuli*	Ca_Kabuli_denovo	15061165	(A/C)
21233	CakSNP21233	*Kabuli*	Ca_Kabuli_denovo	15063461	(C/T)
21234	CakSNP21234	*Kabuli*	Ca_Kabuli_denovo	15064384	(C/T)
21235	CakSNP21235	*Kabuli*	Ca_Kabuli_denovo	15068536	(G/C)
21236	CakSNP21236	*Kabuli*	Ca_Kabuli_denovo	15072879	(C/T)
21237	CakSNP21237	*Kabuli*	Ca_Kabuli_denovo	15072882	(A/C)
21238	CakSNP21238	*Kabuli*	Ca_Kabuli_denovo	15076991	(G/C)
21239	CakSNP21239	*Kabuli*	Ca_Kabuli_denovo	15077061	(T/C)
21240	CakSNP21240	*Kabuli*	Ca_Kabuli_denovo	15078562	(T/C)
21241	CakSNP21241	*Kabuli*	Ca_Kabuli_denovo	15078586	(G/C)
21242	CakSNP21242	*Kabuli*	Ca_Kabuli_denovo	15078864	(A/G)
21243	CakSNP21243	*Kabuli*	Ca_Kabuli_denovo	15079985	(C/A)
21244	CakSNP21244	*Kabuli*	Ca_Kabuli_denovo	15079994	(A/G)
21245	CakSNP21245	*Kabuli*	Ca_Kabuli_denovo	15080664	(G/A)
21246	CakSNP21246	*Kabuli*	Ca_Kabuli_denovo	15081288	(C/T)
21247	CakSNP21247	*Kabuli*	Ca_Kabuli_denovo	15081298	(G/A)
21248	CakSNP21248	*Kabuli*	Ca_Kabuli_denovo	15084526	(C/T)
21249	CakSNP21249	*Kabuli*	Ca_Kabuli_denovo	15084576	(C/T)
21250	CakSNP21250	*Kabuli*	Ca_Kabuli_denovo	15097373	(A/C)
21251	CakSNP21251	*Kabuli*	Ca_Kabuli_denovo	15101968	(T/C)
21252	CakSNP21252	*Kabuli*	Ca_Kabuli_denovo	15109364	(G/A)
21253	CakSNP21253	*Kabuli*	Ca_Kabuli_denovo	15111061	(T/C)

**S.N.**	**SNP IDs**	**Cultivars**	**Chromosomes/scaffolds**	**Physical positions (bp)**	**SNPs**
21254	CakSNP21254	*Kabuli*	Ca_Kabuli_denovo	15121640	(T/C)
21255	CakSNP21255	*Kabuli*	Ca_Kabuli_denovo	15122674	(A/C)
21256	CakSNP21256	*Kabuli*	Ca_Kabuli_denovo	15125029	(A/C)
21257	CakSNP21257	*Kabuli*	Ca_Kabuli_denovo	15125256	(A/G)
21258	CakSNP21258	*Kabuli*	Ca_Kabuli_denovo	15128950	(C/G)
21259	CakSNP21259	*Kabuli*	Ca_Kabuli_denovo	15136143	(G/A)
21260	CakSNP21260	*Kabuli*	Ca_Kabuli_denovo	15146085	(T/C)
21261	CakSNP21261	*Kabuli*	Ca_Kabuli_denovo	15146116	(C/G)
21262	CakSNP21262	*Kabuli*	Ca_Kabuli_denovo	15147215	(G/C)
21263	CakSNP21263	*Kabuli*	Ca_Kabuli_denovo	15148861	(A/C)
21264	CakSNP21264	*Kabuli*	Ca_Kabuli_denovo	15148863	(A/C)
21265	CakSNP21265	*Kabuli*	Ca_Kabuli_denovo	15149093	(G/T)
21266	CakSNP21266	*Kabuli*	Ca_Kabuli_denovo	15149102	(C/T)
21267	CakSNP21267	*Kabuli*	Ca_Kabuli_denovo	15162466	(C/G)
21268	CakSNP21268	*Kabuli*	Ca_Kabuli_denovo	15162535	(C/G)
21269	CakSNP21269	*Kabuli*	Ca_Kabuli_denovo	15172412	(A/G)
21270	CakSNP21270	*Kabuli*	Ca_Kabuli_denovo	15173713	(G/T)
21271	CakSNP21271	*Kabuli*	Ca_Kabuli_denovo	15173771	(T/G)
21272	CakSNP21272	*Kabuli*	Ca_Kabuli_denovo	15175666	(T/C)
21273	CakSNP21273	*Kabuli*	Ca_Kabuli_denovo	15178364	(A/C)
21274	CakSNP21274	*Kabuli*	Ca_Kabuli_denovo	15178367	(C/T)
21275	CakSNP21275	*Kabuli*	Ca_Kabuli_denovo	15181189	(C/G)
21276	CakSNP21276	*Kabuli*	Ca_Kabuli_denovo	15193542	(A/G)
21277	CakSNP21277	*Kabuli*	Ca_Kabuli_denovo	15193595	(T/G)
21278	CakSNP21278	*Kabuli*	Ca_Kabuli_denovo	15194474	(A/C)
21279	CakSNP21279	*Kabuli*	Ca_Kabuli_denovo	15194482	(A/G)
21280	CakSNP21280	*Kabuli*	Ca_Kabuli_denovo	15198160	(T/C)
21281	CakSNP21281	*Kabuli*	Ca_Kabuli_denovo	15198174	(G/A)
21282	CakSNP21282	*Kabuli*	Ca_Kabuli_denovo	15198245	(A/G)
21283	CakSNP21283	*Kabuli*	Ca_Kabuli_denovo	15199468	(T/C)
21284	CakSNP21284	*Kabuli*	Ca_Kabuli_denovo	15199489	(T/C)
21285	CakSNP21285	*Kabuli*	Ca_Kabuli_denovo	15199495	(G/A)
21286	CakSNP21286	*Kabuli*	Ca_Kabuli_denovo	15206923	(G/A)
21287	CakSNP21287	*Kabuli*	Ca_Kabuli_denovo	15206935	(A/G)
21288	CakSNP21288	*Kabuli*	Ca_Kabuli_denovo	15209330	(A/G)
21289	CakSNP21289	*Kabuli*	Ca_Kabuli_denovo	15210193	(G/A)
21290	CakSNP21290	*Kabuli*	Ca_Kabuli_denovo	15210344	(C/T)
21291	CakSNP21291	*Kabuli*	Ca_Kabuli_denovo	15211610	(A/G)
21292	CakSNP21292	*Kabuli*	Ca_Kabuli_denovo	15212128	(T/C)

S.N.	SNP IDs	Cultivars	Chromosomes/scaffolds	Physical positions (bp)	SNPs
21293	CakSNP21293	*Kabuli*	Ca_Kabuli_denovo	15212149	(T/C)
21294	CakSNP21294	*Kabuli*	Ca_Kabuli_denovo	15212161	(G/C)
21295	CakSNP21295	*Kabuli*	Ca_Kabuli_denovo	15213006	(A/T)
21296	CakSNP21296	*Kabuli*	Ca_Kabuli_denovo	15226225	(G/A)
21297	CakSNP21297	*Kabuli*	Ca_Kabuli_denovo	15228338	(A/C)
21298	CakSNP21298	*Kabuli*	Ca_Kabuli_denovo	15233099	(A/G)
21299	CakSNP21299	*Kabuli*	Ca_Kabuli_denovo	15237165	(T/C)
21300	CakSNP21300	*Kabuli*	Ca_Kabuli_denovo	15238403	(C/G)
21301	CakSNP21301	*Kabuli*	Ca_Kabuli_denovo	15244019	(C/T)
21302	CakSNP21302	*Kabuli*	Ca_Kabuli_denovo	15245249	(G/C)
21303	CakSNP21303	*Kabuli*	Ca_Kabuli_denovo	15250791	(A/C)
21304	CakSNP21304	*Kabuli*	Ca_Kabuli_denovo	15261871	(A/G)
21305	CakSNP21305	*Kabuli*	Ca_Kabuli_denovo	15262432	(T/C)
21306	CakSNP21306	*Kabuli*	Ca_Kabuli_denovo	15262463	(T/C)
21307	CakSNP21307	*Kabuli*	Ca_Kabuli_denovo	15267483	(C/T)
21308	CakSNP21308	*Kabuli*	Ca_Kabuli_denovo	15267510	(T/C)
21309	CakSNP21309	*Kabuli*	Ca_Kabuli_denovo	15270517	(A/G)
21310	CakSNP21310	*Kabuli*	Ca_Kabuli_denovo	15270541	(G/C)
21311	CakSNP21311	*Kabuli*	Ca_Kabuli_denovo	15271966	(G/T)
21312	CakSNP21312	*Kabuli*	Ca_Kabuli_denovo	15277962	(C/A)
21313	CakSNP21313	*Kabuli*	Ca_Kabuli_denovo	15278013	(A/C)
21314	CakSNP21314	*Kabuli*	Ca_Kabuli_denovo	15278377	(C/G)
21315	CakSNP21315	*Kabuli*	Ca_Kabuli_denovo	15278389	(T/C)
21316	CakSNP21316	*Kabuli*	Ca_Kabuli_denovo	15281124	(A/C)
21317	CakSNP21317	*Kabuli*	Ca_Kabuli_denovo	15281657	(C/A)
21318	CakSNP21318	*Kabuli*	Ca_Kabuli_denovo	15281660	(C/G)
21319	CakSNP21319	*Kabuli*	Ca_Kabuli_denovo	15285712	(T/A)
21320	CakSNP21320	*Kabuli*	Ca_Kabuli_denovo	15286336	(C/T)
21321	CakSNP21321	*Kabuli*	Ca_Kabuli_denovo	15289465	(C/T)
21322	CakSNP21322	*Kabuli*	Ca_Kabuli_denovo	15289612	(A/G)
21323	CakSNP21323	*Kabuli*	Ca_Kabuli_denovo	15298450	(G/T)
21324	CakSNP21324	*Kabuli*	Ca_Kabuli_denovo	15298471	(T/A)
21325	CakSNP21325	*Kabuli*	Ca_Kabuli_denovo	15301323	(C/T)
21326	CakSNP21326	*Kabuli*	Ca_Kabuli_denovo	15301328	(C/T)
21327	CakSNP21327	*Kabuli*	Ca_Kabuli_denovo	15307943	(G/C)
21328	CakSNP21328	*Kabuli*	Ca_Kabuli_denovo	15308754	(C/G)
21329	CakSNP21329	*Kabuli*	Ca_Kabuli_denovo	15310682	(A/C)
21330	CakSNP21330	*Kabuli*	Ca_Kabuli_denovo	15310711	(T/C)
21331	CakSNP21331	*Kabuli*	Ca_Kabuli_denovo	15310717	(G/A)

S.N.	SNP IDs	Cultivars	Chromosomes/scaffolds	Physical positions (bp)	SNPs
21332	CakSNP21332	*Kabuli*	Ca_Kabuli_denovo	15311372	(A/T)
21333	CakSNP21333	*Kabuli*	Ca_Kabuli_denovo	15321205	(G/A)
21334	CakSNP21334	*Kabuli*	Ca_Kabuli_denovo	15325303	(C/G)
21335	CakSNP21335	*Kabuli*	Ca_Kabuli_denovo	15325309	(A/C)
21336	CakSNP21336	*Kabuli*	Ca_Kabuli_denovo	15328163	(G/C)
21337	CakSNP21337	*Kabuli*	Ca_Kabuli_denovo	15339934	(C/T)
21338	CakSNP21338	*Kabuli*	Ca_Kabuli_denovo	15341482	(A/G)
21339	CakSNP21339	*Kabuli*	Ca_Kabuli_denovo	15342107	(G/A)
21340	CakSNP21340	*Kabuli*	Ca_Kabuli_denovo	15343248	(C/G)
21341	CakSNP21341	*Kabuli*	Ca_Kabuli_denovo	15343266	(A/G)
21342	CakSNP21342	*Kabuli*	Ca_Kabuli_denovo	15345196	(T/C)
21343	CakSNP21343	*Kabuli*	Ca_Kabuli_denovo	15345239	(T/A)
21344	CakSNP21344	*Kabuli*	Ca_Kabuli_denovo	15349210	(C/T)
21345	CakSNP21345	*Kabuli*	Ca_Kabuli_denovo	15349219	(C/T)
21346	CakSNP21346	*Kabuli*	Ca_Kabuli_denovo	15350042	(C/T)
21347	CakSNP21347	*Kabuli*	Ca_Kabuli_denovo	15350057	(C/G)
21348	CakSNP21348	*Kabuli*	Ca_Kabuli_denovo	15352453	(G/A)
21349	CakSNP21349	*Kabuli*	Ca_Kabuli_denovo	15352531	(A/G)
21350	CakSNP21350	*Kabuli*	Ca_Kabuli_denovo	15357660	(G/C)
21351	CakSNP21351	*Kabuli*	Ca_Kabuli_denovo	15357668	(C/T)
21352	CakSNP21352	*Kabuli*	Ca_Kabuli_denovo	15358078	(C/T)
21353	CakSNP21353	*Kabuli*	Ca_Kabuli_denovo	15358348	(C/T)
21354	CakSNP21354	*Kabuli*	Ca_Kabuli_denovo	15361024	(C/T)
21355	CakSNP21355	*Kabuli*	Ca_Kabuli_denovo	15361027	(A/G)
21356	CakSNP21356	*Kabuli*	Ca_Kabuli_denovo	15361033	(A/C)
21357	CakSNP21357	*Kabuli*	Ca_Kabuli_denovo	15363103	(C/T)
21358	CakSNP21358	*Kabuli*	Ca_Kabuli_denovo	15363133	(A/G)
21359	CakSNP21359	*Kabuli*	Ca_Kabuli_denovo	15366397	(G/A)
21360	CakSNP21360	*Kabuli*	Ca_Kabuli_denovo	15368258	(T/C)
21361	CakSNP21361	*Kabuli*	Ca_Kabuli_denovo	15368306	(A/G)
21362	CakSNP21362	*Kabuli*	Ca_Kabuli_denovo	15379238	(T/C)
21363	CakSNP21363	*Kabuli*	Ca_Kabuli_denovo	15379241	(G/A)
21364	CakSNP21364	*Kabuli*	Ca_Kabuli_denovo	15383419	(T/C)
21365	CakSNP21365	*Kabuli*	Ca_Kabuli_denovo	15386024	(G/A)
21366	CakSNP21366	*Kabuli*	Ca_Kabuli_denovo	15386083	(C/A)
21367	CakSNP21367	*Kabuli*	Ca_Kabuli_denovo	15387519	(C/T)
21368	CakSNP21368	*Kabuli*	Ca_Kabuli_denovo	15390193	(T/G)
21369	CakSNP21369	*Kabuli*	Ca_Kabuli_denovo	15390614	(G/C)
21370	CakSNP21370	*Kabuli*	Ca_Kabuli_denovo	15390631	(G/C)

S.N.	SNP IDs	Cultivars	Chromosomes/scaffolds	Physical positions (bp)	SNPs
21371	CakSNP21371	*Kabuli*	Ca_Kabuli_denovo	15390776	(C/G)
21372	CakSNP21372	*Kabuli*	Ca_Kabuli_denovo	15390791	(G/A)
21373	CakSNP21373	*Kabuli*	Ca_Kabuli_denovo	15391286	(G/A)
21374	CakSNP21374	*Kabuli*	Ca_Kabuli_denovo	15391314	(A/C)
21375	CakSNP21375	*Kabuli*	Ca_Kabuli_denovo	15397292	(G/C)
21376	CakSNP21376	*Kabuli*	Ca_Kabuli_denovo	15397334	(C/G)
21377	CakSNP21377	*Kabuli*	Ca_Kabuli_denovo	15400127	(G/A)
21378	CakSNP21378	*Kabuli*	Ca_Kabuli_denovo	15400133	(A/C)
21379	CakSNP21379	*Kabuli*	Ca_Kabuli_denovo	15401000	(A/G)
21380	CakSNP21380	*Kabuli*	Ca_Kabuli_denovo	15401033	(G/T)
21381	CakSNP21381	*Kabuli*	Ca_Kabuli_denovo	15406282	(A/C)
21382	CakSNP21382	*Kabuli*	Ca_Kabuli_denovo	15406287	(G/C)
21383	CakSNP21383	*Kabuli*	Ca_Kabuli_denovo	15406288	(G/T)
21384	CakSNP21384	*Kabuli*	Ca_Kabuli_denovo	15407414	(T/G)
21385	CakSNP21385	*Kabuli*	Ca_Kabuli_denovo	15407444	(A/G)
21386	CakSNP21386	*Kabuli*	Ca_Kabuli_denovo	15414998	(A/G)
21387	CakSNP21387	*Kabuli*	Ca_Kabuli_denovo	15417467	(A/G)
21388	CakSNP21388	*Kabuli*	Ca_Kabuli_denovo	15424718	(C/G)
21389	CakSNP21389	*Kabuli*	Ca_Kabuli_denovo	15425714	(A/G)
21390	CakSNP21390	*Kabuli*	Ca_Kabuli_denovo	15426125	(A/G)
21391	CakSNP21391	*Kabuli*	Ca_Kabuli_denovo	15428336	(G/C)
21392	CakSNP21392	*Kabuli*	Ca_Kabuli_denovo	15430885	(T/G)
21393	CakSNP21393	*Kabuli*	Ca_Kabuli_denovo	15431873	(G/C)
21394	CakSNP21394	*Kabuli*	Ca_Kabuli_denovo	15431888	(C/T)
21395	CakSNP21395	*Kabuli*	Ca_Kabuli_denovo	15431906	(C/G)
21396	CakSNP21396	*Kabuli*	Ca_Kabuli_denovo	15431918	(C/T)
21397	CakSNP21397	*Kabuli*	Ca_Kabuli_denovo	15440132	(A/C)
21398	CakSNP21398	*Kabuli*	Ca_Kabuli_denovo	15441290	(A/C)
21399	CakSNP21399	*Kabuli*	Ca_Kabuli_denovo	15441305	(A/G)
21400	CakSNP21400	*Kabuli*	Ca_Kabuli_denovo	15455235	(G/T)
21401	CakSNP21401	*Kabuli*	Ca_Kabuli_denovo	15455296	(C/G)
21402	CakSNP21402	*Kabuli*	Ca_Kabuli_denovo	15456996	(G/A)
21403	CakSNP21403	*Kabuli*	Ca_Kabuli_denovo	15458395	(C/T)
21404	CakSNP21404	*Kabuli*	Ca_Kabuli_denovo	15458942	(G/A)
21405	CakSNP21405	*Kabuli*	Ca_Kabuli_denovo	15460422	(A/G)
21406	CakSNP21406	*Kabuli*	Ca_Kabuli_denovo	15460460	(G/A)
21407	CakSNP21407	*Kabuli*	Ca_Kabuli_denovo	15464054	(C/T)
21408	CakSNP21408	*Kabuli*	Ca_Kabuli_denovo	15464063	(A/G)
21409	CakSNP21409	*Kabuli*	Ca_Kabuli_denovo	15464257	(C/T)

S.N.	SNP IDs	Cultivars	Chromosomes/scaffolds	Physical positions (bp)	SNPs
21410	CakSNP21410	*Kabuli*	Ca_Kabuli_denovo	15464599	(A/G)
21411	CakSNP21411	*Kabuli*	Ca_Kabuli_denovo	15468646	(C/T)
21412	CakSNP21412	*Kabuli*	Ca_Kabuli_denovo	15469155	(C/T)
21413	CakSNP21413	*Kabuli*	Ca_Kabuli_denovo	15474029	(A/G)
21414	CakSNP21414	*Kabuli*	Ca_Kabuli_denovo	15474040	(T/G)
21415	CakSNP21415	*Kabuli*	Ca_Kabuli_denovo	15474052	(C/A)
21416	CakSNP21416	*Kabuli*	Ca_Kabuli_denovo	15474082	(G/T)
21417	CakSNP21417	*Kabuli*	Ca_Kabuli_denovo	15475209	(C/G)
21418	CakSNP21418	*Kabuli*	Ca_Kabuli_denovo	15475225	(T/C)
21419	CakSNP21419	*Kabuli*	Ca_Kabuli_denovo	15478410	(T/G)
21420	CakSNP21420	*Kabuli*	Ca_Kabuli_denovo	15480813	(C/T)
21421	CakSNP21421	*Kabuli*	Ca_Kabuli_denovo	15480822	(C/T)
21422	CakSNP21422	*Kabuli*	Ca_Kabuli_denovo	15491990	(T/C)
21423	CakSNP21423	*Kabuli*	Ca_Kabuli_denovo	15491996	(G/A)
21424	CakSNP21424	*Kabuli*	Ca_Kabuli_denovo	15499304	(G/T)
21425	CakSNP21425	*Kabuli*	Ca_Kabuli_denovo	15499316	(T/C)
21426	CakSNP21426	*Kabuli*	Ca_Kabuli_denovo	15499338	(T/G)
21427	CakSNP21427	*Kabuli*	Ca_Kabuli_denovo	15506868	(C/T)
21428	CakSNP21428	*Kabuli*	Ca_Kabuli_denovo	15507953	(G/T)
21429	CakSNP21429	*Kabuli*	Ca_Kabuli_denovo	15507965	(A/G)
21430	CakSNP21430	*Kabuli*	Ca_Kabuli_denovo	15521689	(T/C)
21431	CakSNP21431	*Kabuli*	Ca_Kabuli_denovo	15522761	(G/A)
21432	CakSNP21432	*Kabuli*	Ca_Kabuli_denovo	15522773	(G/A)
21433	CakSNP21433	*Kabuli*	Ca_Kabuli_denovo	15524716	(G/T)
21434	CakSNP21434	*Kabuli*	Ca_Kabuli_denovo	15524717	(T/C)
21435	CakSNP21435	*Kabuli*	Ca_Kabuli_denovo	15524990	(T/C)
21436	CakSNP21436	*Kabuli*	Ca_Kabuli_denovo	15526190	(A/G)
21437	CakSNP21437	*Kabuli*	Ca_Kabuli_denovo	15530285	(T/G)
21438	CakSNP21438	*Kabuli*	Ca_Kabuli_denovo	15531595	(T/C)
21439	CakSNP21439	*Kabuli*	Ca_Kabuli_denovo	15552032	(A/G)
21440	CakSNP21440	*Kabuli*	Ca_Kabuli_denovo	15564979	(C/T)
21441	CakSNP21441	*Kabuli*	Ca_Kabuli_denovo	15566724	(T/A)
21442	CakSNP21442	*Kabuli*	Ca_Kabuli_denovo	15568183	(T/C)
21443	CakSNP21443	*Kabuli*	Ca_Kabuli_denovo	15568192	(G/T)
21444	CakSNP21444	*Kabuli*	Ca_Kabuli_denovo	15575379	(A/G)
21445	CakSNP21445	*Kabuli*	Ca_Kabuli_denovo	15575382	(G/C)
21446	CakSNP21446	*Kabuli*	Ca_Kabuli_denovo	15576520	(G/C)
21447	CakSNP21447	*Kabuli*	Ca_Kabuli_denovo	15582787	(T/C)
21448	CakSNP21448	*Kabuli*	Ca_Kabuli_denovo	15582800	(C/G)

S.N.	SNP IDs	Cultivars	Chromosomes/scaffolds	Physical positions (bp)	SNPs
21449	CakSNP21449	*Kabuli*	Ca_Kabuli_denovo	15590817	(A/G)
21450	CakSNP21450	*Kabuli*	Ca_Kabuli_denovo	15591832	(T/C)
21451	CakSNP21451	*Kabuli*	Ca_Kabuli_denovo	15595473	(C/A)
21452	CakSNP21452	*Kabuli*	Ca_Kabuli_denovo	15597791	(G/A)
21453	CakSNP21453	*Kabuli*	Ca_Kabuli_denovo	15597799	(C/A)
21454	CakSNP21454	*Kabuli*	Ca_Kabuli_denovo	15603179	(A/G)
21455	CakSNP21455	*Kabuli*	Ca_Kabuli_denovo	15603193	(T/C)
21456	CakSNP21456	*Kabuli*	Ca_Kabuli_denovo	15606500	(G/A)
21457	CakSNP21457	*Kabuli*	Ca_Kabuli_denovo	15609823	(G/A)
21458	CakSNP21458	*Kabuli*	Ca_Kabuli_denovo	15611115	(G/C)
21459	CakSNP21459	*Kabuli*	Ca_Kabuli_denovo	15611121	(C/T)
21460	CakSNP21460	*Kabuli*	Ca_Kabuli_denovo	15620902	(C/T)
21461	CakSNP21461	*Kabuli*	Ca_Kabuli_denovo	15620920	(G/T)
21462	CakSNP21462	*Kabuli*	Ca_Kabuli_denovo	15620944	(C/G)
21463	CakSNP21463	*Kabuli*	Ca_Kabuli_denovo	15621127	(G/A)
21464	CakSNP21464	*Kabuli*	Ca_Kabuli_denovo	15628387	(T/C)
21465	CakSNP21465	*Kabuli*	Ca_Kabuli_denovo	15631264	(G/T)
21466	CakSNP21466	*Kabuli*	Ca_Kabuli_denovo	15631270	(A/G)
21467	CakSNP21467	*Kabuli*	Ca_Kabuli_denovo	15631562	(C/A)
21468	CakSNP21468	*Kabuli*	Ca_Kabuli_denovo	15633158	(C/A)
21469	CakSNP21469	*Kabuli*	Ca_Kabuli_denovo	15637429	(G/T)
21470	CakSNP21470	*Kabuli*	Ca_Kabuli_denovo	15638521	(C/G)
21471	CakSNP21471	*Kabuli*	Ca_Kabuli_denovo	15638548	(A/G)
21472	CakSNP21472	*Kabuli*	Ca_Kabuli_denovo	15640495	(C/T)
21473	CakSNP21473	*Kabuli*	Ca_Kabuli_denovo	15643259	(G/A)
21474	CakSNP21474	*Kabuli*	Ca_Kabuli_denovo	15643271	(T/C)
21475	CakSNP21475	*Kabuli*	Ca_Kabuli_denovo	15643735	(C/T)
21476	CakSNP21476	*Kabuli*	Ca_Kabuli_denovo	15643743	(A/G)
21477	CakSNP21477	*Kabuli*	Ca_Kabuli_denovo	15643912	(T/C)
21478	CakSNP21478	*Kabuli*	Ca_Kabuli_denovo	15643960	(C/T)
21479	CakSNP21479	*Kabuli*	Ca_Kabuli_denovo	15644967	(A/C)
21480	CakSNP21480	*Kabuli*	Ca_Kabuli_denovo	15647635	(A/G)
21481	CakSNP21481	*Kabuli*	Ca_Kabuli_denovo	15650816	(G/A)
21482	CakSNP21482	*Kabuli*	Ca_Kabuli_denovo	15656144	(T/C)
21483	CakSNP21483	*Kabuli*	Ca_Kabuli_denovo	15662922	(A/G)
21484	CakSNP21484	*Kabuli*	Ca_Kabuli_denovo	15662923	(A/G)
21485	CakSNP21485	*Kabuli*	Ca_Kabuli_denovo	15668904	(C/G)
21486	CakSNP21486	*Kabuli*	Ca_Kabuli_denovo	15672757	(G/A)
21487	CakSNP21487	*Kabuli*	Ca_Kabuli_denovo	15672819	(C/A)

S.N.	SNP IDs	Cultivars	Chromosomes/scaffolds	Physical positions (bp)	SNPs
21488	CakSNP21488	*Kabuli*	Ca_Kabuli_denovo	15676871	(A/G)
21489	CakSNP21489	*Kabuli*	Ca_Kabuli_denovo	15679048	(C/T)
21490	CakSNP21490	*Kabuli*	Ca_Kabuli_denovo	15682338	(A/G)
21491	CakSNP21491	*Kabuli*	Ca_Kabuli_denovo	15686389	(G/A)
21492	CakSNP21492	*Kabuli*	Ca_Kabuli_denovo	15689140	(T/G)
21493	CakSNP21493	*Kabuli*	Ca_Kabuli_denovo	15689179	(A/G)
21494	CakSNP21494	*Kabuli*	Ca_Kabuli_denovo	15693017	(A/G)
21495	CakSNP21495	*Kabuli*	Ca_Kabuli_denovo	15693033	(A/C)
21496	CakSNP21496	*Kabuli*	Ca_Kabuli_denovo	15696764	(T/G)
21497	CakSNP21497	*Kabuli*	Ca_Kabuli_denovo	15710031	(A/C)
21498	CakSNP21498	*Kabuli*	Ca_Kabuli_denovo	15724677	(T/C)
21499	CakSNP21499	*Kabuli*	Ca_Kabuli_denovo	15729201	(C/T)
21500	CakSNP21500	*Kabuli*	Ca_Kabuli_denovo	15730162	(T/C)
21501	CakSNP21501	*Kabuli*	Ca_Kabuli_denovo	15731996	(T/C)
21502	CakSNP21502	*Kabuli*	Ca_Kabuli_denovo	15732030	(C/T)
21503	CakSNP21503	*Kabuli*	Ca_Kabuli_denovo	15737951	(G/C)
21504	CakSNP21504	*Kabuli*	Ca_Kabuli_denovo	15738002	(A/G)
21505	CakSNP21505	*Kabuli*	Ca_Kabuli_denovo	15738908	(T/C)
21506	CakSNP21506	*Kabuli*	Ca_Kabuli_denovo	15738922	(T/C)
21507	CakSNP21507	*Kabuli*	Ca_Kabuli_denovo	15742863	(C/T)
21508	CakSNP21508	*Kabuli*	Ca_Kabuli_denovo	15746595	(T/G)
21509	CakSNP21509	*Kabuli*	Ca_Kabuli_denovo	15746721	(A/G)
21510	CakSNP21510	*Kabuli*	Ca_Kabuli_denovo	15748786	(T/C)
21511	CakSNP21511	*Kabuli*	Ca_Kabuli_denovo	15750852	(A/C)
21512	CakSNP21512	*Kabuli*	Ca_Kabuli_denovo	15750903	(T/A)
21513	CakSNP21513	*Kabuli*	Ca_Kabuli_denovo	15750933	(C/T)
21514	CakSNP21514	*Kabuli*	Ca_Kabuli_denovo	15756831	(C/A)
21515	CakSNP21515	*Kabuli*	Ca_Kabuli_denovo	15760646	(C/G)
21516	CakSNP21516	*Kabuli*	Ca_Kabuli_denovo	15760923	(A/C)
21517	CakSNP21517	*Kabuli*	Ca_Kabuli_denovo	15766715	(C/T)
21518	CakSNP21518	*Kabuli*	Ca_Kabuli_denovo	15766718	(G/T)
21519	CakSNP21519	*Kabuli*	Ca_Kabuli_denovo	15769597	(C/T)
21520	CakSNP21520	*Kabuli*	Ca_Kabuli_denovo	15775001	(G/A)
21521	CakSNP21521	*Kabuli*	Ca_Kabuli_denovo	15777288	(T/G)
21522	CakSNP21522	*Kabuli*	Ca_Kabuli_denovo	15779882	(G/C)
21523	CakSNP21523	*Kabuli*	Ca_Kabuli_denovo	15780781	(C/T)
21524	CakSNP21524	*Kabuli*	Ca_Kabuli_denovo	15781142	(G/A)
21525	CakSNP21525	*Kabuli*	Ca_Kabuli_denovo	15781160	(G/A)
21526	CakSNP21526	*Kabuli*	Ca_Kabuli_denovo	15791427	(C/A)

S.N.	SNP IDs	Cultivars	Chromosomes/scaffolds	Physical positions (bp)	SNPs
21527	CakSNP21527	*Kabuli*	Ca_Kabuli_denovo	15791472	(C/G)
21528	CakSNP21528	*Kabuli*	Ca_Kabuli_denovo	15792255	(C/T)
21529	CakSNP21529	*Kabuli*	Ca_Kabuli_denovo	15806405	(G/A)
21530	CakSNP21530	*Kabuli*	Ca_Kabuli_denovo	15816444	(G/C)
21531	CakSNP21531	*Kabuli*	Ca_Kabuli_denovo	15816474	(A/C)
21532	CakSNP21532	*Kabuli*	Ca_Kabuli_denovo	15817830	(A/G)
21533	CakSNP21533	*Kabuli*	Ca_Kabuli_denovo	15820384	(T/C)
21534	CakSNP21534	*Kabuli*	Ca_Kabuli_denovo	15827646	(G/C)
21535	CakSNP21535	*Kabuli*	Ca_Kabuli_denovo	15835243	(A/T)
21536	CakSNP21536	*Kabuli*	Ca_Kabuli_denovo	15840418	(C/T)
21537	CakSNP21537	*Kabuli*	Ca_Kabuli_denovo	15843233	(T/A)
21538	CakSNP21538	*Kabuli*	Ca_Kabuli_denovo	15846580	(C/T)
21539	CakSNP21539	*Kabuli*	Ca_Kabuli_denovo	15846593	(T/C)
21540	CakSNP21540	*Kabuli*	Ca_Kabuli_denovo	15848981	(A/G)
21541	CakSNP21541	*Kabuli*	Ca_Kabuli_denovo	15848984	(G/C)
21542	CakSNP21542	*Kabuli*	Ca_Kabuli_denovo	15853479	(G/C)
21543	CakSNP21543	*Kabuli*	Ca_Kabuli_denovo	15855332	(G/T)
21544	CakSNP21544	*Kabuli*	Ca_Kabuli_denovo	15857901	(A/G)
21545	CakSNP21545	*Kabuli*	Ca_Kabuli_denovo	15858482	(A/G)
21546	CakSNP21546	*Kabuli*	Ca_Kabuli_denovo	15858484	(C/G)
21547	CakSNP21547	*Kabuli*	Ca_Kabuli_denovo	15866668	(A/G)
21548	CakSNP21548	*Kabuli*	Ca_Kabuli_denovo	15867686	(T/G)
21549	CakSNP21549	*Kabuli*	Ca_Kabuli_denovo	15873998	(C/T)
21550	CakSNP21550	*Kabuli*	Ca_Kabuli_denovo	15874929	(G/A)
21551	CakSNP21551	*Kabuli*	Ca_Kabuli_denovo	15874948	(A/G)
21552	CakSNP21552	*Kabuli*	Ca_Kabuli_denovo	15875007	(G/A)
21553	CakSNP21553	*Kabuli*	Ca_Kabuli_denovo	15876783	(A/G)
21554	CakSNP21554	*Kabuli*	Ca_Kabuli_denovo	15884976	(C/G)
21555	CakSNP21555	*Kabuli*	Ca_Kabuli_denovo	15887807	(G/A)
21556	CakSNP21556	*Kabuli*	Ca_Kabuli_denovo	15892953	(T/C)
21557	CakSNP21557	*Kabuli*	Ca_Kabuli_denovo	15893254	(T/C)
21558	CakSNP21558	*Kabuli*	Ca_Kabuli_denovo	15893260	(A/G)
21559	CakSNP21559	*Kabuli*	Ca_Kabuli_denovo	15893327	(A/C)
21560	CakSNP21560	*Kabuli*	Ca_Kabuli_denovo	15893329	(A/C)
21561	CakSNP21561	*Kabuli*	Ca_Kabuli_denovo	15897534	(A/G)
21562	CakSNP21562	*Kabuli*	Ca_Kabuli_denovo	15897602	(C/T)
21563	CakSNP21563	*Kabuli*	Ca_Kabuli_denovo	15903773	(G/T)
21564	CakSNP21564	*Kabuli*	Ca_Kabuli_denovo	15903815	(A/G)
21565	CakSNP21565	*Kabuli*	Ca_Kabuli_denovo	15905649	(C/G)

S.N.	SNP IDs	Cultivars	Chromosomes/scaffolds	Physical positions (bp)	SNPs
21566	CakSNP21566	*Kabuli*	Ca_Kabuli_denovo	15907232	(T/C)
21567	CakSNP21567	*Kabuli*	Ca_Kabuli_denovo	15908094	(G/C)
21568	CakSNP21568	*Kabuli*	Ca_Kabuli_denovo	15911035	(T/C)
21569	CakSNP21569	*Kabuli*	Ca_Kabuli_denovo	15924073	(C/T)
21570	CakSNP21570	*Kabuli*	Ca_Kabuli_denovo	15929633	(T/A)
21571	CakSNP21571	*Kabuli*	Ca_Kabuli_denovo	15929674	(T/C)
21572	CakSNP21572	*Kabuli*	Ca_Kabuli_denovo	15930740	(G/A)
21573	CakSNP21573	*Kabuli*	Ca_Kabuli_denovo	15930808	(C/T)
21574	CakSNP21574	*Kabuli*	Ca_Kabuli_denovo	15931690	(C/G)
21575	CakSNP21575	*Kabuli*	Ca_Kabuli_denovo	15931714	(G/T)
21576	CakSNP21576	*Kabuli*	Ca_Kabuli_denovo	15932330	(A/G)
21577	CakSNP21577	*Kabuli*	Ca_Kabuli_denovo	15934836	(C/T)
21578	CakSNP21578	*Kabuli*	Ca_Kabuli_denovo	15937570	(A/G)
21579	CakSNP21579	*Kabuli*	Ca_Kabuli_denovo	15951873	(G/A)
21580	CakSNP21580	*Kabuli*	Ca_Kabuli_denovo	15952688	(A/G)
21581	CakSNP21581	*Kabuli*	Ca_Kabuli_denovo	15952703	(A/G)
21582	CakSNP21582	*Kabuli*	Ca_Kabuli_denovo	15970245	(G/A)
21583	CakSNP21583	*Kabuli*	Ca_Kabuli_denovo	15972570	(G/T)
21584	CakSNP21584	*Kabuli*	Ca_Kabuli_denovo	15972591	(C/G)
21585	CakSNP21585	*Kabuli*	Ca_Kabuli_denovo	15977624	(G/T)
21586	CakSNP21586	*Kabuli*	Ca_Kabuli_denovo	15980240	(A/T)
21587	CakSNP21587	*Kabuli*	Ca_Kabuli_denovo	15992568	(C/G)
21588	CakSNP21588	*Kabuli*	Ca_Kabuli_denovo	15996800	(A/C)
21589	CakSNP21589	*Kabuli*	Ca_Kabuli_denovo	15997796	(A/T)
21590	CakSNP21590	*Kabuli*	Ca_Kabuli_denovo	15997817	(G/A)
21591	CakSNP21591	*Kabuli*	Ca_Kabuli_denovo	16007356	(G/C)
21592	CakSNP21592	*Kabuli*	Ca_Kabuli_denovo	16016229	(T/C)
21593	CakSNP21593	*Kabuli*	Ca_Kabuli_denovo	16018177	(C/T)
21594	CakSNP21594	*Kabuli*	Ca_Kabuli_denovo	16019462	(G/C)
21595	CakSNP21595	*Kabuli*	Ca_Kabuli_denovo	16032952	(G/C)
21596	CakSNP21596	*Kabuli*	Ca_Kabuli_denovo	16035104	(T/A)
21597	CakSNP21597	*Kabuli*	Ca_Kabuli_denovo	16035142	(T/C)
21598	CakSNP21598	*Kabuli*	Ca_Kabuli_denovo	16042128	(A/C)
21599	CakSNP21599	*Kabuli*	Ca_Kabuli_denovo	16045168	(G/T)
21600	CakSNP21600	*Kabuli*	Ca_Kabuli_denovo	16046908	(A/G)
21601	CakSNP21601	*Kabuli*	Ca_Kabuli_denovo	16051304	(C/T)
21602	CakSNP21602	*Kabuli*	Ca_Kabuli_denovo	16053222	(G/A)
21603	CakSNP21603	*Kabuli*	Ca_Kabuli_denovo	16059216	(G/C)
21604	CakSNP21604	*Kabuli*	Ca_Kabuli_denovo	16063343	(G/A)

S.N.	SNP IDs	Cultivars	Chromosomes/scaffolds	Physical positions (bp)	SNPs
21605	CakSNP21605	*Kabuli*	Ca_Kabuli_denovo	16063447	(T/G)
21606	CakSNP21606	*Kabuli*	Ca_Kabuli_denovo	16063460	(A/C)
21607	CakSNP21607	*Kabuli*	Ca_Kabuli_denovo	16064860	(C/G)
21608	CakSNP21608	*Kabuli*	Ca_Kabuli_denovo	16072684	(A/G)
21609	CakSNP21609	*Kabuli*	Ca_Kabuli_denovo	16077406	(A/C)
21610	CakSNP21610	*Kabuli*	Ca_Kabuli_denovo	16083241	(T/G)
21611	CakSNP21611	*Kabuli*	Ca_Kabuli_denovo	16088745	(T/C)
21612	CakSNP21612	*Kabuli*	Ca_Kabuli_denovo	16088999	(A/C)
21613	CakSNP21613	*Kabuli*	Ca_Kabuli_denovo	16089001	(A/C)
21614	CakSNP21614	*Kabuli*	Ca_Kabuli_denovo	16091858	(T/G)
21615	CakSNP21615	*Kabuli*	Ca_Kabuli_denovo	16104177	(G/A)
21616	CakSNP21616	*Kabuli*	Ca_Kabuli_denovo	16107450	(C/T)
21617	CakSNP21617	*Kabuli*	Ca_Kabuli_denovo	16107506	(G/A)
21618	CakSNP21618	*Kabuli*	Ca_Kabuli_denovo	16114582	(A/G)
21619	CakSNP21619	*Kabuli*	Ca_Kabuli_denovo	16115155	(C/T)
21620	CakSNP21620	*Kabuli*	Ca_Kabuli_denovo	16115164	(C/A)
21621	CakSNP21621	*Kabuli*	Ca_Kabuli_denovo	16116836	(T/G)
21622	CakSNP21622	*Kabuli*	Ca_Kabuli_denovo	16116890	(T/G)
21623	CakSNP21623	*Kabuli*	Ca_Kabuli_denovo	16118811	(T/C)
21624	CakSNP21624	*Kabuli*	Ca_Kabuli_denovo	16118838	(G/A)
21625	CakSNP21625	*Kabuli*	Ca_Kabuli_denovo	16119632	(A/G)
21626	CakSNP21626	*Kabuli*	Ca_Kabuli_denovo	16134211	(G/A)
21627	CakSNP21627	*Kabuli*	Ca_Kabuli_denovo	16134249	(G/A)
21628	CakSNP21628	*Kabuli*	Ca_Kabuli_denovo	16134580	(G/A)
21629	CakSNP21629	*Kabuli*	Ca_Kabuli_denovo	16143666	(A/G)
21630	CakSNP21630	*Kabuli*	Ca_Kabuli_denovo	16144928	(A/G)
21631	CakSNP21631	*Kabuli*	Ca_Kabuli_denovo	16146300	(C/G)
21632	CakSNP21632	*Kabuli*	Ca_Kabuli_denovo	16152938	(G/A)
21633	CakSNP21633	*Kabuli*	Ca_Kabuli_denovo	16159144	(T/C)
21634	CakSNP21634	*Kabuli*	Ca_Kabuli_denovo	16165826	(C/G)
21635	CakSNP21635	*Kabuli*	Ca_Kabuli_denovo	16165837	(G/A)
21636	CakSNP21636	*Kabuli*	Ca_Kabuli_denovo	16167983	(C/T)
21637	CakSNP21637	*Kabuli*	Ca_Kabuli_denovo	16171075	(G/A)
21638	CakSNP21638	*Kabuli*	Ca_Kabuli_denovo	16173457	(T/G)
21639	CakSNP21639	*Kabuli*	Ca_Kabuli_denovo	16173469	(A/G)
21640	CakSNP21640	*Kabuli*	Ca_Kabuli_denovo	16180337	(A/T)
21641	CakSNP21641	*Kabuli*	Ca_Kabuli_denovo	16180369	(G/C)
21642	CakSNP21642	*Kabuli*	Ca_Kabuli_denovo	16180679	(T/C)
21643	CakSNP21643	*Kabuli*	Ca_Kabuli_denovo	16180694	(G/A)

S.N.	SNP IDs	Cultivars	Chromosomes/scaffolds	Physical positions (bp)	SNPs
21644	CakSNP21644	*Kabuli*	Ca_Kabuli_denovo	16180697	(G/A)
21645	CakSNP21645	*Kabuli*	Ca_Kabuli_denovo	16192364	(T/G)
21646	CakSNP21646	*Kabuli*	Ca_Kabuli_denovo	16195262	(A/G)
21647	CakSNP21647	*Kabuli*	Ca_Kabuli_denovo	16195265	(C/A)
21648	CakSNP21648	*Kabuli*	Ca_Kabuli_denovo	16202129	(C/G)
21649	CakSNP21649	*Kabuli*	Ca_Kabuli_denovo	16202257	(G/T)
21650	CakSNP21650	*Kabuli*	Ca_Kabuli_denovo	16203514	(A/G)
21651	CakSNP21651	*Kabuli*	Ca_Kabuli_denovo	16212822	(A/G)
21652	CakSNP21652	*Kabuli*	Ca_Kabuli_denovo	16212833	(G/T)
21653	CakSNP21653	*Kabuli*	Ca_Kabuli_denovo	16223803	(C/T)
21654	CakSNP21654	*Kabuli*	Ca_Kabuli_denovo	16223835	(A/T)
21655	CakSNP21655	*Kabuli*	Ca_Kabuli_denovo	16226517	(T/G)
21656	CakSNP21656	*Kabuli*	Ca_Kabuli_denovo	16226520	(A/C)
21657	CakSNP21657	*Kabuli*	Ca_Kabuli_denovo	16228102	(T/G)
21658	CakSNP21658	*Kabuli*	Ca_Kabuli_denovo	16231770	(C/T)
21659	CakSNP21659	*Kabuli*	Ca_Kabuli_denovo	16232648	(C/A)
21660	CakSNP21660	*Kabuli*	Ca_Kabuli_denovo	16234566	(G/T)
21661	CakSNP21661	*Kabuli*	Ca_Kabuli_denovo	16234575	(G/A)
21662	CakSNP21662	*Kabuli*	Ca_Kabuli_denovo	16240526	(G/A)
21663	CakSNP21663	*Kabuli*	Ca_Kabuli_denovo	16244719	(A/T)
21664	CakSNP21664	*Kabuli*	Ca_Kabuli_denovo	16247333	(G/A)
21665	CakSNP21665	*Kabuli*	Ca_Kabuli_denovo	16247374	(C/T)
21666	CakSNP21666	*Kabuli*	Ca_Kabuli_denovo	16260126	(G/C)
21667	CakSNP21667	*Kabuli*	Ca_Kabuli_denovo	16260137	(A/G)
21668	CakSNP21668	*Kabuli*	Ca_Kabuli_denovo	16260601	(C/T)
21669	CakSNP21669	*Kabuli*	Ca_Kabuli_denovo	16260619	(G/A)
21670	CakSNP21670	*Kabuli*	Ca_Kabuli_denovo	16262566	(G/C)
21671	CakSNP21671	*Kabuli*	Ca_Kabuli_denovo	16263211	(C/T)
21672	CakSNP21672	*Kabuli*	Ca_Kabuli_denovo	16263606	(A/T)
21673	CakSNP21673	*Kabuli*	Ca_Kabuli_denovo	16263609	(T/C)
21674	CakSNP21674	*Kabuli*	Ca_Kabuli_denovo	16265172	(C/G)
21675	CakSNP21675	*Kabuli*	Ca_Kabuli_denovo	16266042	(T/G)
21676	CakSNP21676	*Kabuli*	Ca_Kabuli_denovo	16280261	(C/A)
21677	CakSNP21677	*Kabuli*	Ca_Kabuli_denovo	16280269	(C/T)
21678	CakSNP21678	*Kabuli*	Ca_Kabuli_denovo	16285150	(C/T)
21679	CakSNP21679	*Kabuli*	Ca_Kabuli_denovo	16285196	(C/T)
21680	CakSNP21680	*Kabuli*	Ca_Kabuli_denovo	16285228	(G/C)
21681	CakSNP21681	*Kabuli*	Ca_Kabuli_denovo	16287350	(G/C)
21682	CakSNP21682	*Kabuli*	Ca_Kabuli_denovo	16287361	(C/T)

S.N.	SNP IDs	Cultivars	Chromosomes/scaffolds	Physical positions (bp)	SNPs
21683	CakSNP21683	*Kabuli*	Ca_Kabuli_denovo	16293927	(C/T)
21684	CakSNP21684	*Kabuli*	Ca_Kabuli_denovo	16304638	(C/G)
21685	CakSNP21685	*Kabuli*	Ca_Kabuli_denovo	16304644	(G/A)
21686	CakSNP21686	*Kabuli*	Ca_Kabuli_denovo	16309405	(C/T)
21687	CakSNP21687	*Kabuli*	Ca_Kabuli_denovo	16310098	(A/C)
21688	CakSNP21688	*Kabuli*	Ca_Kabuli_denovo	16311865	(A/G)
21689	CakSNP21689	*Kabuli*	Ca_Kabuli_denovo	16318334	(A/C)
21690	CakSNP21690	*Kabuli*	Ca_Kabuli_denovo	16321322	(G/A)
21691	CakSNP21691	*Kabuli*	Ca_Kabuli_denovo	16321357	(G/T)
21692	CakSNP21692	*Kabuli*	Ca_Kabuli_denovo	16323646	(C/T)
21693	CakSNP21693	*Kabuli*	Ca_Kabuli_denovo	16323653	(G/A)
21694	CakSNP21694	*Kabuli*	Ca_Kabuli_denovo	16324174	(T/C)
21695	CakSNP21695	*Kabuli*	Ca_Kabuli_denovo	16324198	(G/C)
21696	CakSNP21696	*Kabuli*	Ca_Kabuli_denovo	16326296	(A/G)
21697	CakSNP21697	*Kabuli*	Ca_Kabuli_denovo	16326326	(T/C)
21698	CakSNP21698	*Kabuli*	Ca_Kabuli_denovo	16327612	(G/T)
21699	CakSNP21699	*Kabuli*	Ca_Kabuli_denovo	16329733	(T/G)
21700	CakSNP21700	*Kabuli*	Ca_Kabuli_denovo	16329739	(T/C)
21701	CakSNP21701	*Kabuli*	Ca_Kabuli_denovo	16331645	(T/C)
21702	CakSNP21702	*Kabuli*	Ca_Kabuli_denovo	16332497	(A/G)
21703	CakSNP21703	*Kabuli*	Ca_Kabuli_denovo	16335146	(A/G)
21704	CakSNP21704	*Kabuli*	Ca_Kabuli_denovo	16337697	(A/C)
21705	CakSNP21705	*Kabuli*	Ca_Kabuli_denovo	16338451	(T/C)
21706	CakSNP21706	*Kabuli*	Ca_Kabuli_denovo	16338474	(T/G)
21707	CakSNP21707	*Kabuli*	Ca_Kabuli_denovo	16345856	(C/G)
21708	CakSNP21708	*Kabuli*	Ca_Kabuli_denovo	16360422	(C/T)
21709	CakSNP21709	*Kabuli*	Ca_Kabuli_denovo	16366147	(A/C)
21710	CakSNP21710	*Kabuli*	Ca_Kabuli_denovo	16366177	(T/C)
21711	CakSNP21711	*Kabuli*	Ca_Kabuli_denovo	16374055	(C/T)
21712	CakSNP21712	*Kabuli*	Ca_Kabuli_denovo	16374136	(C/A)
21713	CakSNP21713	*Kabuli*	Ca_Kabuli_denovo	16379800	(G/A)
21714	CakSNP21714	*Kabuli*	Ca_Kabuli_denovo	16380555	(A/G)
21715	CakSNP21715	*Kabuli*	Ca_Kabuli_denovo	16382611	(C/T)
21716	CakSNP21716	*Kabuli*	Ca_Kabuli_denovo	16386909	(T/G)
21717	CakSNP21717	*Kabuli*	Ca_Kabuli_denovo	16386939	(A/G)
21718	CakSNP21718	*Kabuli*	Ca_Kabuli_denovo	16395993	(A/C)
21719	CakSNP21719	*Kabuli*	Ca_Kabuli_denovo	16395994	(G/A)
21720	CakSNP21720	*Kabuli*	Ca_Kabuli_denovo	16396510	(A/G)
21721	CakSNP21721	*Kabuli*	Ca_Kabuli_denovo	16396541	(A/G)

S.N.	SNP IDs	Cultivars	Chromosomes/scaffolds	Physical positions (bp)	SNPs
21722	CakSNP21722	*Kabuli*	Ca_Kabuli_denovo	16399727	(C/G)
21723	CakSNP21723	*Kabuli*	Ca_Kabuli_denovo	16400456	(A/C)
21724	CakSNP21724	*Kabuli*	Ca_Kabuli_denovo	16400458	(A/G)
21725	CakSNP21725	*Kabuli*	Ca_Kabuli_denovo	16403710	(T/G)
21726	CakSNP21726	*Kabuli*	Ca_Kabuli_denovo	16407355	(T/G)
21727	CakSNP21727	*Kabuli*	Ca_Kabuli_denovo	16407358	(G/C)
21728	CakSNP21728	*Kabuli*	Ca_Kabuli_denovo	16408072	(T/C)
21729	CakSNP21729	*Kabuli*	Ca_Kabuli_denovo	16419017	(G/T)
21730	CakSNP21730	*Kabuli*	Ca_Kabuli_denovo	16419044	(T/C)
21731	CakSNP21731	*Kabuli*	Ca_Kabuli_denovo	16422703	(G/C)
21732	CakSNP21732	*Kabuli*	Ca_Kabuli_denovo	16423796	(C/G)
21733	CakSNP21733	*Kabuli*	Ca_Kabuli_denovo	16431153	(A/G)
21734	CakSNP21734	*Kabuli*	Ca_Kabuli_denovo	16431161	(G/A)
21735	CakSNP21735	*Kabuli*	Ca_Kabuli_denovo	16432329	(A/G)
21736	CakSNP21736	*Kabuli*	Ca_Kabuli_denovo	16433327	(G/A)
21737	CakSNP21737	*Kabuli*	Ca_Kabuli_denovo	16433379	(A/G)
21738	CakSNP21738	*Kabuli*	Ca_Kabuli_denovo	16433532	(T/C)
21739	CakSNP21739	*Kabuli*	Ca_Kabuli_denovo	16433951	(A/G)
21740	CakSNP21740	*Kabuli*	Ca_Kabuli_denovo	16433958	(T/C)
21741	CakSNP21741	*Kabuli*	Ca_Kabuli_denovo	16433964	(T/C)
21742	CakSNP21742	*Kabuli*	Ca_Kabuli_denovo	16438515	(G/C)
21743	CakSNP21743	*Kabuli*	Ca_Kabuli_denovo	16438572	(G/T)
21744	CakSNP21744	*Kabuli*	Ca_Kabuli_denovo	16439398	(G/C)
21745	CakSNP21745	*Kabuli*	Ca_Kabuli_denovo	16439416	(T/C)
21746	CakSNP21746	*Kabuli*	Ca_Kabuli_denovo	16442634	(G/A)
21747	CakSNP21747	*Kabuli*	Ca_Kabuli_denovo	16442868	(A/T)
21748	CakSNP21748	*Kabuli*	Ca_Kabuli_denovo	16443463	(G/A)
21749	CakSNP21749	*Kabuli*	Ca_Kabuli_denovo	16443487	(C/T)
21750	CakSNP21750	*Kabuli*	Ca_Kabuli_denovo	16446001	(T/C)
21751	CakSNP21751	*Kabuli*	Ca_Kabuli_denovo	16446028	(A/T)
21752	CakSNP21752	*Kabuli*	Ca_Kabuli_denovo	16446612	(C/G)
21753	CakSNP21753	*Kabuli*	Ca_Kabuli_denovo	16446660	(C/T)
21754	CakSNP21754	*Kabuli*	Ca_Kabuli_denovo	16448652	(C/T)
21755	CakSNP21755	*Kabuli*	Ca_Kabuli_denovo	16448673	(C/T)
21756	CakSNP21756	*Kabuli*	Ca_Kabuli_denovo	16450928	(C/T)
21757	CakSNP21757	*Kabuli*	Ca_Kabuli_denovo	16450936	(G/C)
21758	CakSNP21758	*Kabuli*	Ca_Kabuli_denovo	16454737	(A/G)
21759	CakSNP21759	*Kabuli*	Ca_Kabuli_denovo	16454742	(C/T)
21760	CakSNP21760	*Kabuli*	Ca_Kabuli_denovo	16463528	(C/G)

S.N.	SNP IDs	Cultivars	Chromosomes/scaffolds	Physical positions (bp)	SNPs
21761	CakSNP21761	*Kabuli*	Ca_Kabuli_denovo	16463533	(A/C)
21762	CakSNP21762	*Kabuli*	Ca_Kabuli_denovo	16466561	(G/C)
21763	CakSNP21763	*Kabuli*	Ca_Kabuli_denovo	16478129	(C/T)
21764	CakSNP21764	*Kabuli*	Ca_Kabuli_denovo	16480207	(G/A)
21765	CakSNP21765	*Kabuli*	Ca_Kabuli_denovo	16481213	(T/C)
21766	CakSNP21766	*Kabuli*	Ca_Kabuli_denovo	16481216	(C/G)
21767	CakSNP21767	*Kabuli*	Ca_Kabuli_denovo	16488089	(C/G)
21768	CakSNP21768	*Kabuli*	Ca_Kabuli_denovo	16491339	(G/A)
21769	CakSNP21769	*Kabuli*	Ca_Kabuli_denovo	16493110	(C/G)
21770	CakSNP21770	*Kabuli*	Ca_Kabuli_denovo	16493176	(T/C)
21771	CakSNP21771	*Kabuli*	Ca_Kabuli_denovo	16493859	(C/T)
21772	CakSNP21772	*Kabuli*	Ca_Kabuli_denovo	16493864	(G/A)
21773	CakSNP21773	*Kabuli*	Ca_Kabuli_denovo	16495795	(T/G)
21774	CakSNP21774	*Kabuli*	Ca_Kabuli_denovo	16495801	(A/G)
21775	CakSNP21775	*Kabuli*	Ca_Kabuli_denovo	16499520	(A/G)
21776	CakSNP21776	*Kabuli*	Ca_Kabuli_denovo	16499555	(C/A)
21777	CakSNP21777	*Kabuli*	Ca_Kabuli_denovo	16500677	(A/G)
21778	CakSNP21778	*Kabuli*	Ca_Kabuli_denovo	16500683	(A/G)
21779	CakSNP21779	*Kabuli*	Ca_Kabuli_denovo	16501449	(T/G)
21780	CakSNP21780	*Kabuli*	Ca_Kabuli_denovo	16503695	(G/C)
21781	CakSNP21781	*Kabuli*	Ca_Kabuli_denovo	16506410	(C/G)
21782	CakSNP21782	*Kabuli*	Ca_Kabuli_denovo	16506452	(C/T)
21783	CakSNP21783	*Kabuli*	Ca_Kabuli_denovo	16506584	(C/G)
21784	CakSNP21784	*Kabuli*	Ca_Kabuli_denovo	16506595	(A/C)
21785	CakSNP21785	*Kabuli*	Ca_Kabuli_denovo	16506801	(C/T)
21786	CakSNP21786	*Kabuli*	Ca_Kabuli_denovo	16506848	(T/G)
21787	CakSNP21787	*Kabuli*	Ca_Kabuli_denovo	16507564	(T/G)
21788	CakSNP21788	*Kabuli*	Ca_Kabuli_denovo	16507589	(T/G)
21789	CakSNP21789	*Kabuli*	Ca_Kabuli_denovo	16511992	(G/A)
21790	CakSNP21790	*Kabuli*	Ca_Kabuli_denovo	16516079	(T/C)
21791	CakSNP21791	*Kabuli*	Ca_Kabuli_denovo	16518646	(A/G)
21792	CakSNP21792	*Kabuli*	Ca_Kabuli_denovo	16521054	(G/C)
21793	CakSNP21793	*Kabuli*	Ca_Kabuli_denovo	16523681	(A/G)
21794	CakSNP21794	*Kabuli*	Ca_Kabuli_denovo	16527540	(A/G)
21795	CakSNP21795	*Kabuli*	Ca_Kabuli_denovo	16527703	(T/C)
21796	CakSNP21796	*Kabuli*	Ca_Kabuli_denovo	16527716	(G/A)
21797	CakSNP21797	*Kabuli*	Ca_Kabuli_denovo	16531688	(G/C)
21798	CakSNP21798	*Kabuli*	Ca_Kabuli_denovo	16531733	(G/C)
21799	CakSNP21799	*Kabuli*	Ca_Kabuli_denovo	16535101	(C/A)

S.N.	SNP IDs	Cultivars	Chromosomes/scaffolds	Physical positions (bp)	SNPs
21800	CakSNP21800	*Kabuli*	Ca_Kabuli_denovo	16537066	(C/A)
21801	CakSNP21801	*Kabuli*	Ca_Kabuli_denovo	16538701	(C/T)
21802	CakSNP21802	*Kabuli*	Ca_Kabuli_denovo	16538725	(G/T)
21803	CakSNP21803	*Kabuli*	Ca_Kabuli_denovo	16540897	(G/T)
21804	CakSNP21804	*Kabuli*	Ca_Kabuli_denovo	16540932	(T/C)
21805	CakSNP21805	*Kabuli*	Ca_Kabuli_denovo	16541788	(T/C)
21806	CakSNP21806	*Kabuli*	Ca_Kabuli_denovo	16541815	(C/G)
21807	CakSNP21807	*Kabuli*	Ca_Kabuli_denovo	16541818	(C/T)
21808	CakSNP21808	*Kabuli*	Ca_Kabuli_denovo	16542667	(T/C)
21809	CakSNP21809	*Kabuli*	Ca_Kabuli_denovo	16543444	(T/C)
21810	CakSNP21810	*Kabuli*	Ca_Kabuli_denovo	16548536	(G/A)
21811	CakSNP21811	*Kabuli*	Ca_Kabuli_denovo	16548706	(T/G)
21812	CakSNP21812	*Kabuli*	Ca_Kabuli_denovo	16548709	(C/T)
21813	CakSNP21813	*Kabuli*	Ca_Kabuli_denovo	16550594	(A/G)
21814	CakSNP21814	*Kabuli*	Ca_Kabuli_denovo	16550597	(G/C)
21815	CakSNP21815	*Kabuli*	Ca_Kabuli_denovo	16550753	(T/G)
21816	CakSNP21816	*Kabuli*	Ca_Kabuli_denovo	16551596	(T/C)
21817	CakSNP21817	*Kabuli*	Ca_Kabuli_denovo	16551608	(C/T)
21818	CakSNP21818	*Kabuli*	Ca_Kabuli_denovo	16551984	(C/G)
21819	CakSNP21819	*Kabuli*	Ca_Kabuli_denovo	16552001	(C/G)
21820	CakSNP21820	*Kabuli*	Ca_Kabuli_denovo	16555320	(A/G)
21821	CakSNP21821	*Kabuli*	Ca_Kabuli_denovo	16555331	(A/G)
21822	CakSNP21822	*Kabuli*	Ca_Kabuli_denovo	16555347	(C/T)
21823	CakSNP21823	*Kabuli*	Ca_Kabuli_denovo	16563232	(C/G)
21824	CakSNP21824	*Kabuli*	Ca_Kabuli_denovo	16568993	(G/T)
21825	CakSNP21825	*Kabuli*	Ca_Kabuli_denovo	16568994	(T/C)
21826	CakSNP21826	*Kabuli*	Ca_Kabuli_denovo	16573820	(G/C)
21827	CakSNP21827	*Kabuli*	Ca_Kabuli_denovo	16573891	(C/T)
21828	CakSNP21828	*Kabuli*	Ca_Kabuli_denovo	16573900	(C/G)
21829	CakSNP21829	*Kabuli*	Ca_Kabuli_denovo	16573903	(C/T)
21830	CakSNP21830	*Kabuli*	Ca_Kabuli_denovo	16575551	(A/G)
21831	CakSNP21831	*Kabuli*	Ca_Kabuli_denovo	16577951	(G/A)
21832	CakSNP21832	*Kabuli*	Ca_Kabuli_denovo	16577959	(G/T)
21833	CakSNP21833	*Kabuli*	Ca_Kabuli_denovo	16578568	(C/T)
21834	CakSNP21834	*Kabuli*	Ca_Kabuli_denovo	16585761	(C/T)
21835	CakSNP21835	*Kabuli*	Ca_Kabuli_denovo	16589452	(G/A)
21836	CakSNP21836	*Kabuli*	Ca_Kabuli_denovo	16590210	(G/C)
21837	CakSNP21837	*Kabuli*	Ca_Kabuli_denovo	16590229	(C/G)
21838	CakSNP21838	*Kabuli*	Ca_Kabuli_denovo	16611748	(C/T)

S.N.	SNP IDs	Cultivars	Chromosomes/scaffolds	Physical positions (bp)	SNPs
21839	CakSNP21839	*Kabuli*	Ca_Kabuli_denovo	16613548	(A/G)
21840	CakSNP21840	*Kabuli*	Ca_Kabuli_denovo	16616090	(G/A)
21841	CakSNP21841	*Kabuli*	Ca_Kabuli_denovo	16616105	(A/G)
21842	CakSNP21842	*Kabuli*	Ca_Kabuli_denovo	16616806	(C/A)
21843	CakSNP21843	*Kabuli*	Ca_Kabuli_denovo	16619801	(C/A)
21844	CakSNP21844	*Kabuli*	Ca_Kabuli_denovo	16623076	(A/G)
21845	CakSNP21845	*Kabuli*	Ca_Kabuli_denovo	16626028	(C/A)
21846	CakSNP21846	*Kabuli*	Ca_Kabuli_denovo	16626043	(A/G)
21847	CakSNP21847	*Kabuli*	Ca_Kabuli_denovo	16629782	(C/T)
21848	CakSNP21848	*Kabuli*	Ca_Kabuli_denovo	16643323	(A/G)
21849	CakSNP21849	*Kabuli*	Ca_Kabuli_denovo	16648155	(A/G)
21850	CakSNP21850	*Kabuli*	Ca_Kabuli_denovo	16653157	(T/C)
21851	CakSNP21851	*Kabuli*	Ca_Kabuli_denovo	16665169	(T/C)
21852	CakSNP21852	*Kabuli*	Ca_Kabuli_denovo	16669271	(T/G)
21853	CakSNP21853	*Kabuli*	Ca_Kabuli_denovo	16669353	(G/C)
21854	CakSNP21854	*Kabuli*	Ca_Kabuli_denovo	16669354	(G/C)
21855	CakSNP21855	*Kabuli*	Ca_Kabuli_denovo	16672658	(C/G)
21856	CakSNP21856	*Kabuli*	Ca_Kabuli_denovo	16673523	(C/T)
21857	CakSNP21857	*Kabuli*	Ca_Kabuli_denovo	16673532	(T/A)
21858	CakSNP21858	*Kabuli*	Ca_Kabuli_denovo	16673632	(C/A)
21859	CakSNP21859	*Kabuli*	Ca_Kabuli_denovo	16678096	(A/G)
21860	CakSNP21860	*Kabuli*	Ca_Kabuli_denovo	16678143	(G/T)
21861	CakSNP21861	*Kabuli*	Ca_Kabuli_denovo	16691722	(T/A)
21862	CakSNP21862	*Kabuli*	Ca_Kabuli_denovo	16700868	(G/C)
21863	CakSNP21863	*Kabuli*	Ca_Kabuli_denovo	16706187	(G/C)
21864	CakSNP21864	*Kabuli*	Ca_Kabuli_denovo	16706241	(G/A)
21865	CakSNP21865	*Kabuli*	Ca_Kabuli_denovo	16707329	(G/A)
21866	CakSNP21866	*Kabuli*	Ca_Kabuli_denovo	16707356	(A/C)
21867	CakSNP21867	*Kabuli*	Ca_Kabuli_denovo	16708337	(A/G)
21868	CakSNP21868	*Kabuli*	Ca_Kabuli_denovo	16715210	(A/G)
21869	CakSNP21869	*Kabuli*	Ca_Kabuli_denovo	16715216	(A/C)
21870	CakSNP21870	*Kabuli*	Ca_Kabuli_denovo	16720077	(T/G)
21871	CakSNP21871	*Kabuli*	Ca_Kabuli_denovo	16720096	(C/A)
21872	CakSNP21872	*Kabuli*	Ca_Kabuli_denovo	16723012	(G/A)
21873	CakSNP21873	*Kabuli*	Ca_Kabuli_denovo	16724754	(A/G)
21874	CakSNP21874	*Kabuli*	Ca_Kabuli_denovo	16724832	(T/C)
21875	CakSNP21875	*Kabuli*	Ca_Kabuli_denovo	16725436	(T/C)
21876	CakSNP21876	*Kabuli*	Ca_Kabuli_denovo	16728254	(A/C)
21877	CakSNP21877	*Kabuli*	Ca_Kabuli_denovo	16734735	(A/G)

S.N.	SNP IDs	Cultivars	Chromosomes/scaffolds	Physical positions (bp)	SNPs
21878	CakSNP21878	*Kabuli*	Ca_Kabuli_denovo	16738628	(C/T)
21879	CakSNP21879	*Kabuli*	Ca_Kabuli_denovo	16741741	(T/C)
21880	CakSNP21880	*Kabuli*	Ca_Kabuli_denovo	16745312	(T/G)
21881	CakSNP21881	*Kabuli*	Ca_Kabuli_denovo	16745357	(C/T)
21882	CakSNP21882	*Kabuli*	Ca_Kabuli_denovo	16749543	(C/T)
21883	CakSNP21883	*Kabuli*	Ca_Kabuli_denovo	16753524	(T/C)
21884	CakSNP21884	*Kabuli*	Ca_Kabuli_denovo	16756754	(T/G)
21885	CakSNP21885	*Kabuli*	Ca_Kabuli_denovo	16756778	(C/G)
21886	CakSNP21886	*Kabuli*	Ca_Kabuli_denovo	16759066	(A/C)
21887	CakSNP21887	*Kabuli*	Ca_Kabuli_denovo	16763119	(G/C)
21888	CakSNP21888	*Kabuli*	Ca_Kabuli_denovo	16768916	(C/T)
21889	CakSNP21889	*Kabuli*	Ca_Kabuli_denovo	16770864	(C/T)
21890	CakSNP21890	*Kabuli*	Ca_Kabuli_denovo	16773167	(G/A)
21891	CakSNP21891	*Kabuli*	Ca_Kabuli_denovo	16773169	(T/C)
21892	CakSNP21892	*Kabuli*	Ca_Kabuli_denovo	16775909	(G/C)
21893	CakSNP21893	*Kabuli*	Ca_Kabuli_denovo	16792384	(G/T)
21894	CakSNP21894	*Kabuli*	Ca_Kabuli_denovo	16792385	(T/C)
21895	CakSNP21895	*Kabuli*	Ca_Kabuli_denovo	16795334	(A/C)
21896	CakSNP21896	*Kabuli*	Ca_Kabuli_denovo	16796009	(G/C)
21897	CakSNP21897	*Kabuli*	Ca_Kabuli_denovo	16797381	(A/C)
21898	CakSNP21898	*Kabuli*	Ca_Kabuli_denovo	16798070	(T/G)
21899	CakSNP21899	*Kabuli*	Ca_Kabuli_denovo	16800967	(G/C)
21900	CakSNP21900	*Kabuli*	Ca_Kabuli_denovo	16800982	(G/A)
21901	CakSNP21901	*Kabuli*	Ca_Kabuli_denovo	16805285	(A/C)
21902	CakSNP21902	*Kabuli*	Ca_Kabuli_denovo	16806851	(A/G)
21903	CakSNP21903	*Kabuli*	Ca_Kabuli_denovo	16810319	(C/G)
21904	CakSNP21904	*Kabuli*	Ca_Kabuli_denovo	16810328	(G/T)
21905	CakSNP21905	*Kabuli*	Ca_Kabuli_denovo	16811019	(A/G)
21906	CakSNP21906	*Kabuli*	Ca_Kabuli_denovo	16817728	(C/T)
21907	CakSNP21907	*Kabuli*	Ca_Kabuli_denovo	16818477	(G/T)
21908	CakSNP21908	*Kabuli*	Ca_Kabuli_denovo	16822925	(G/A)
21909	CakSNP21909	*Kabuli*	Ca_Kabuli_denovo	16839161	(T/C)
21910	CakSNP21910	*Kabuli*	Ca_Kabuli_denovo	16847104	(G/A)
21911	CakSNP21911	*Kabuli*	Ca_Kabuli_denovo	16847905	(T/G)
21912	CakSNP21912	*Kabuli*	Ca_Kabuli_denovo	16847915	(T/C)
21913	CakSNP21913	*Kabuli*	Ca_Kabuli_denovo	16848678	(A/G)
21914	CakSNP21914	*Kabuli*	Ca_Kabuli_denovo	16848818	(C/G)
21915	CakSNP21915	*Kabuli*	Ca_Kabuli_denovo	16849886	(C/A)
21916	CakSNP21916	*Kabuli*	Ca_Kabuli_denovo	16849916	(G/C)

S.N.	SNP IDs	Cultivars	Chromosomes/scaffolds	Physical positions (bp)	SNPs
21917	CakSNP21917	*Kabuli*	Ca_Kabuli_denovo	16854862	(C/T)
21918	CakSNP21918	*Kabuli*	Ca_Kabuli_denovo	16854880	(A/G)
21919	CakSNP21919	*Kabuli*	Ca_Kabuli_denovo	16856071	(T/C)
21920	CakSNP21920	*Kabuli*	Ca_Kabuli_denovo	16857724	(G/T)
21921	CakSNP21921	*Kabuli*	Ca_Kabuli_denovo	16866134	(C/T)
21922	CakSNP21922	*Kabuli*	Ca_Kabuli_denovo	16866191	(T/G)
21923	CakSNP21923	*Kabuli*	Ca_Kabuli_denovo	16867690	(G/A)
21924	CakSNP21924	*Kabuli*	Ca_Kabuli_denovo	16884460	(G/C)
21925	CakSNP21925	*Kabuli*	Ca_Kabuli_denovo	16890964	(T/C)
21926	CakSNP21926	*Kabuli*	Ca_Kabuli_denovo	16890974	(A/G)
21927	CakSNP21927	*Kabuli*	Ca_Kabuli_denovo	16900104	(G/A)
21928	CakSNP21928	*Kabuli*	Ca_Kabuli_denovo	16900127	(G/A)
21929	CakSNP21929	*Kabuli*	Ca_Kabuli_denovo	16901597	(T/G)
21930	CakSNP21930	*Kabuli*	Ca_Kabuli_denovo	16901600	(T/A)
21931	CakSNP21931	*Kabuli*	Ca_Kabuli_denovo	16901701	(C/T)
21932	CakSNP21932	*Kabuli*	Ca_Kabuli_denovo	16904740	(A/C)
21933	CakSNP21933	*Kabuli*	Ca_Kabuli_denovo	16909393	(C/G)
21934	CakSNP21934	*Kabuli*	Ca_Kabuli_denovo	16909447	(C/A)
21935	CakSNP21935	*Kabuli*	Ca_Kabuli_denovo	16913920	(G/T)
21936	CakSNP21936	*Kabuli*	Ca_Kabuli_denovo	16917202	(G/C)
21937	CakSNP21937	*Kabuli*	Ca_Kabuli_denovo	16917212	(G/A)
21938	CakSNP21938	*Kabuli*	Ca_Kabuli_denovo	16921376	(A/G)
21939	CakSNP21939	*Kabuli*	Ca_Kabuli_denovo	16929094	(C/T)
21940	CakSNP21940	*Kabuli*	Ca_Kabuli_denovo	16930382	(T/C)
21941	CakSNP21941	*Kabuli*	Ca_Kabuli_denovo	16936177	(A/G)
21942	CakSNP21942	*Kabuli*	Ca_Kabuli_denovo	16938854	(T/C)
21943	CakSNP21943	*Kabuli*	Ca_Kabuli_denovo	16938863	(A/G)
21944	CakSNP21944	*Kabuli*	Ca_Kabuli_denovo	16947610	(G/A)
21945	CakSNP21945	*Kabuli*	Ca_Kabuli_denovo	16947664	(T/C)
21946	CakSNP21946	*Kabuli*	Ca_Kabuli_denovo	16947792	(G/A)
21947	CakSNP21947	*Kabuli*	Ca_Kabuli_denovo	16950010	(T/C)
21948	CakSNP21948	*Kabuli*	Ca_Kabuli_denovo	16959044	(C/G)
21949	CakSNP21949	*Kabuli*	Ca_Kabuli_denovo	16959070	(C/G)
21950	CakSNP21950	*Kabuli*	Ca_Kabuli_denovo	16959077	(G/A)
21951	CakSNP21951	*Kabuli*	Ca_Kabuli_denovo	16972762	(T/C)
21952	CakSNP21952	*Kabuli*	Ca_Kabuli_denovo	16973548	(C/T)
21953	CakSNP21953	*Kabuli*	Ca_Kabuli_denovo	16973590	(C/G)
21954	CakSNP21954	*Kabuli*	Ca_Kabuli_denovo	16984599	(C/G)
21955	CakSNP21955	*Kabuli*	Ca_Kabuli_denovo	16984785	(C/G)

S.N.	SNP IDs	Cultivars	Chromosomes/scaffolds	Physical positions (bp)	SNPs
21956	CakSNP21956	*Kabuli*	Ca_Kabuli_denovo	16984812	(T/C)
21957	CakSNP21957	*Kabuli*	Ca_Kabuli_denovo	17004468	(G/A)
21958	CakSNP21958	*Kabuli*	Ca_Kabuli_denovo	17004504	(C/T)
21959	CakSNP21959	*Kabuli*	Ca_Kabuli_denovo	17004540	(C/T)
21960	CakSNP21960	*Kabuli*	Ca_Kabuli_denovo	17005753	(G/A)
21961	CakSNP21961	*Kabuli*	Ca_Kabuli_denovo	17005756	(A/G)
21962	CakSNP21962	*Kabuli*	Ca_Kabuli_denovo	17016529	(C/T)
21963	CakSNP21963	*Kabuli*	Ca_Kabuli_denovo	17016536	(G/T)
21964	CakSNP21964	*Kabuli*	Ca_Kabuli_denovo	17016995	(A/T)
21965	CakSNP21965	*Kabuli*	Ca_Kabuli_denovo	17021436	(T/G)
21966	CakSNP21966	*Kabuli*	Ca_Kabuli_denovo	17021442	(G/T)
21967	CakSNP21967	*Kabuli*	Ca_Kabuli_denovo	17024552	(C/A)
21968	CakSNP21968	*Kabuli*	Ca_Kabuli_denovo	17034564	(T/C)
21969	CakSNP21969	*Kabuli*	Ca_Kabuli_denovo	17034585	(G/A)
21970	CakSNP21970	*Kabuli*	Ca_Kabuli_denovo	17035472	(G/A)
21971	CakSNP21971	*Kabuli*	Ca_Kabuli_denovo	17039549	(C/G)
21972	CakSNP21972	*Kabuli*	Ca_Kabuli_denovo	17039551	(T/G)
21973	CakSNP21973	*Kabuli*	Ca_Kabuli_denovo	17042770	(C/G)
21974	CakSNP21974	*Kabuli*	Ca_Kabuli_denovo	17043941	(G/A)
21975	CakSNP21975	*Kabuli*	Ca_Kabuli_denovo	17045500	(T/G)
21976	CakSNP21976	*Kabuli*	Ca_Kabuli_denovo	17050364	(G/A)
21977	CakSNP21977	*Kabuli*	Ca_Kabuli_denovo	17052384	(T/G)
21978	CakSNP21978	*Kabuli*	Ca_Kabuli_denovo	17056471	(C/T)
21979	CakSNP21979	*Kabuli*	Ca_Kabuli_denovo	17056477	(C/G)
21980	CakSNP21980	*Kabuli*	Ca_Kabuli_denovo	17057732	(C/T)
21981	CakSNP21981	*Kabuli*	Ca_Kabuli_denovo	17057823	(T/C)
21982	CakSNP21982	*Kabuli*	Ca_Kabuli_denovo	17057850	(G/A)
21983	CakSNP21983	*Kabuli*	Ca_Kabuli_denovo	17069033	(A/C)
21984	CakSNP21984	*Kabuli*	Ca_Kabuli_denovo	17069653	(C/T)
21985	CakSNP21985	*Kabuli*	Ca_Kabuli_denovo	17069674	(T/C)
21986	CakSNP21986	*Kabuli*	Ca_Kabuli_denovo	17072513	(T/C)
21987	CakSNP21987	*Kabuli*	Ca_Kabuli_denovo	17072515	(G/C)
21988	CakSNP21988	*Kabuli*	Ca_Kabuli_denovo	17072813	(T/C)
21989	CakSNP21989	*Kabuli*	Ca_Kabuli_denovo	17074228	(A/G)
21990	CakSNP21990	*Kabuli*	Ca_Kabuli_denovo	17074250	(T/A)
21991	CakSNP21991	*Kabuli*	Ca_Kabuli_denovo	17074307	(A/T)
21992	CakSNP21992	*Kabuli*	Ca_Kabuli_denovo	17077410	(A/G)
21993	CakSNP21993	*Kabuli*	Ca_Kabuli_denovo	17082462	(T/C)
21994	CakSNP21994	*Kabuli*	Ca_Kabuli_denovo	17082822	(A/G)

S.N.	SNP IDs	Cultivars	Chromosomes/scaffolds	Physical positions (bp)	SNPs
21995	CakSNP21995	*Kabuli*	Ca_Kabuli_denovo	17083441	(G/T)
21996	CakSNP21996	*Kabuli*	Ca_Kabuli_denovo	17085224	(T/C)
21997	CakSNP21997	*Kabuli*	Ca_Kabuli_denovo	17094726	(G/A)
21998	CakSNP21998	*Kabuli*	Ca_Kabuli_denovo	17095757	(A/G)
21999	CakSNP21999	*Kabuli*	Ca_Kabuli_denovo	17100012	(C/G)
22000	CakSNP22000	*Kabuli*	Ca_Kabuli_denovo	17100020	(A/C)
22001	CakSNP22001	*Kabuli*	Ca_Kabuli_denovo	17100026	(C/G)
22002	CakSNP22002	*Kabuli*	Ca_Kabuli_denovo	17100254	(A/T)
22003	CakSNP22003	*Kabuli*	Ca_Kabuli_denovo	17100270	(C/G)
22004	CakSNP22004	*Kabuli*	Ca_Kabuli_denovo	17100331	(T/C)
22005	CakSNP22005	*Kabuli*	Ca_Kabuli_denovo	17105131	(T/G)
22006	CakSNP22006	*Kabuli*	Ca_Kabuli_denovo	17124123	(G/C)
22007	CakSNP22007	*Kabuli*	Ca_Kabuli_denovo	17124179	(G/A)
22008	CakSNP22008	*Kabuli*	Ca_Kabuli_denovo	17135908	(C/G)
22009	CakSNP22009	*Kabuli*	Ca_Kabuli_denovo	17135911	(T/C)
22010	CakSNP22010	*Kabuli*	Ca_Kabuli_denovo	17137643	(A/C)
22011	CakSNP22011	*Kabuli*	Ca_Kabuli_denovo	17145502	(C/T)
22012	CakSNP22012	*Kabuli*	Ca_Kabuli_denovo	17149807	(C/G)
22013	CakSNP22013	*Kabuli*	Ca_Kabuli_denovo	17153306	(G/A)
22014	CakSNP22014	*Kabuli*	Ca_Kabuli_denovo	17153538	(A/G)
22015	CakSNP22015	*Kabuli*	Ca_Kabuli_denovo	17153539	(T/C)
22016	CakSNP22016	*Kabuli*	Ca_Kabuli_denovo	17158844	(T/G)
22017	CakSNP22017	*Kabuli*	Ca_Kabuli_denovo	17158845	(C/A)
22018	CakSNP22018	*Kabuli*	Ca_Kabuli_denovo	17164315	(G/T)
22019	CakSNP22019	*Kabuli*	Ca_Kabuli_denovo	17169559	(C/T)
22020	CakSNP22020	*Kabuli*	Ca_Kabuli_denovo	17173889	(C/T)
22021	CakSNP22021	*Kabuli*	Ca_Kabuli_denovo	17175017	(C/G)
22022	CakSNP22022	*Kabuli*	Ca_Kabuli_denovo	17175032	(C/T)
22023	CakSNP22023	*Kabuli*	Ca_Kabuli_denovo	17183208	(C/T)
22024	CakSNP22024	*Kabuli*	Ca_Kabuli_denovo	17183478	(C/G)
22025	CakSNP22025	*Kabuli*	Ca_Kabuli_denovo	17183481	(T/C)
22026	CakSNP22026	*Kabuli*	Ca_Kabuli_denovo	17191143	(A/C)
22027	CakSNP22027	*Kabuli*	Ca_Kabuli_denovo	17191144	(G/A)
22028	CakSNP22028	*Kabuli*	Ca_Kabuli_denovo	17193826	(G/A)
22029	CakSNP22029	*Kabuli*	Ca_Kabuli_denovo	17194069	(G/T)
22030	CakSNP22030	*Kabuli*	Ca_Kabuli_denovo	17197111	(T/G)
22031	CakSNP22031	*Kabuli*	Ca_Kabuli_denovo	17197112	(C/T)
22032	CakSNP22032	*Kabuli*	Ca_Kabuli_denovo	17211956	(G/A)
22033	CakSNP22033	*Kabuli*	Ca_Kabuli_denovo	17221166	(T/C)

S.N.	SNP IDs	Cultivars	Chromosomes/scaffolds	Physical positions (bp)	SNPs
22034	CakSNP22034	*Kabuli*	Ca_Kabuli_denovo	17222126	(G/T)
22035	CakSNP22035	*Kabuli*	Ca_Kabuli_denovo	17228590	(A/G)
22036	CakSNP22036	*Kabuli*	Ca_Kabuli_denovo	17228617	(C/G)
22037	CakSNP22037	*Kabuli*	Ca_Kabuli_denovo	17229757	(T/C)
22038	CakSNP22038	*Kabuli*	Ca_Kabuli_denovo	17229851	(A/G)
22039	CakSNP22039	*Kabuli*	Ca_Kabuli_denovo	17229856	(T/C)
22040	CakSNP22040	*Kabuli*	Ca_Kabuli_denovo	17230023	(C/T)
22041	CakSNP22041	*Kabuli*	Ca_Kabuli_denovo	17232772	(G/A)
22042	CakSNP22042	*Kabuli*	Ca_Kabuli_denovo	17234123	(C/T)
22043	CakSNP22043	*Kabuli*	Ca_Kabuli_denovo	17234152	(T/G)
22044	CakSNP22044	*Kabuli*	Ca_Kabuli_denovo	17234682	(G/A)
22045	CakSNP22045	*Kabuli*	Ca_Kabuli_denovo	17234713	(C/A)
22046	CakSNP22046	*Kabuli*	Ca_Kabuli_denovo	17236651	(C/T)
22047	CakSNP22047	*Kabuli*	Ca_Kabuli_denovo	17236681	(T/C)
22048	CakSNP22048	*Kabuli*	Ca_Kabuli_denovo	17237146	(A/G)
22049	CakSNP22049	*Kabuli*	Ca_Kabuli_denovo	17259898	(C/G)
22050	CakSNP22050	*Kabuli*	Ca_Kabuli_denovo	17263412	(T/G)
22051	CakSNP22051	*Kabuli*	Ca_Kabuli_denovo	17264715	(G/C)
22052	CakSNP22052	*Kabuli*	Ca_Kabuli_denovo	17264725	(T/C)
22053	CakSNP22053	*Kabuli*	Ca_Kabuli_denovo	17264921	(G/A)
22054	CakSNP22054	*Kabuli*	Ca_Kabuli_denovo	17265470	(A/G)
22055	CakSNP22055	*Kabuli*	Ca_Kabuli_denovo	17270108	(G/A)
22056	CakSNP22056	*Kabuli*	Ca_Kabuli_denovo	17275057	(G/A)
22057	CakSNP22057	*Kabuli*	Ca_Kabuli_denovo	17275063	(A/G)
22058	CakSNP22058	*Kabuli*	Ca_Kabuli_denovo	17284324	(A/G)
22059	CakSNP22059	*Kabuli*	Ca_Kabuli_denovo	17286294	(C/T)
22060	CakSNP22060	*Kabuli*	Ca_Kabuli_denovo	17286314	(A/G)
22061	CakSNP22061	*Kabuli*	Ca_Kabuli_denovo	17288522	(C/T)
22062	CakSNP22062	*Kabuli*	Ca_Kabuli_denovo	17288523	(T/C)
22063	CakSNP22063	*Kabuli*	Ca_Kabuli_denovo	17289626	(T/G)
22064	CakSNP22064	*Kabuli*	Ca_Kabuli_denovo	17290132	(C/T)
22065	CakSNP22065	*Kabuli*	Ca_Kabuli_denovo	17291686	(A/C)
22066	CakSNP22066	*Kabuli*	Ca_Kabuli_denovo	17297188	(C/T)
22067	CakSNP22067	*Kabuli*	Ca_Kabuli_denovo	17297200	(G/A)
22068	CakSNP22068	*Kabuli*	Ca_Kabuli_denovo	17309055	(A/G)
22069	CakSNP22069	*Kabuli*	Ca_Kabuli_denovo	17310208	(T/C)
22070	CakSNP22070	*Kabuli*	Ca_Kabuli_denovo	17313517	(C/A)
22071	CakSNP22071	*Kabuli*	Ca_Kabuli_denovo	17313528	(A/C)
22072	CakSNP22072	*Kabuli*	Ca_Kabuli_denovo	17313529	(G/C)

S.N.	SNP IDs	Cultivars	Chromosomes/scaffolds	Physical positions (bp)	SNPs
22073	CakSNP22073	*Kabuli*	Ca_Kabuli_denovo	17317567	(C/T)
22074	CakSNP22074	*Kabuli*	Ca_Kabuli_denovo	17318919	(T/G)
22075	CakSNP22075	*Kabuli*	Ca_Kabuli_denovo	17319872	(C/T)
22076	CakSNP22076	*Kabuli*	Ca_Kabuli_denovo	17321416	(C/A)
22077	CakSNP22077	*Kabuli*	Ca_Kabuli_denovo	17330189	(T/C)
22078	CakSNP22078	*Kabuli*	Ca_Kabuli_denovo	17330572	(T/G)
22079	CakSNP22079	*Kabuli*	Ca_Kabuli_denovo	17335969	(T/C)
22080	CakSNP22080	*Kabuli*	Ca_Kabuli_denovo	17335978	(C/T)
22081	CakSNP22081	*Kabuli*	Ca_Kabuli_denovo	17336333	(C/T)
22082	CakSNP22082	*Kabuli*	Ca_Kabuli_denovo	17336409	(T/C)
22083	CakSNP22083	*Kabuli*	Ca_Kabuli_denovo	17339624	(A/G)
22084	CakSNP22084	*Kabuli*	Ca_Kabuli_denovo	17347047	(G/T)
22085	CakSNP22085	*Kabuli*	Ca_Kabuli_denovo	17347065	(G/C)
22086	CakSNP22086	*Kabuli*	Ca_Kabuli_denovo	17349179	(C/A)
22087	CakSNP22087	*Kabuli*	Ca_Kabuli_denovo	17350379	(C/T)
22088	CakSNP22088	*Kabuli*	Ca_Kabuli_denovo	17350403	(G/C)
22089	CakSNP22089	*Kabuli*	Ca_Kabuli_denovo	17355331	(C/T)
22090	CakSNP22090	*Kabuli*	Ca_Kabuli_denovo	17355343	(T/G)
22091	CakSNP22091	*Kabuli*	Ca_Kabuli_denovo	17363326	(A/G)
22092	CakSNP22092	*Kabuli*	Ca_Kabuli_denovo	17363362	(C/G)
22093	CakSNP22093	*Kabuli*	Ca_Kabuli_denovo	17363588	(G/C)
22094	CakSNP22094	*Kabuli*	Ca_Kabuli_denovo	17363607	(G/A)
22095	CakSNP22095	*Kabuli*	Ca_Kabuli_denovo	17365595	(A/G)
22096	CakSNP22096	*Kabuli*	Ca_Kabuli_denovo	17367130	(T/G)
22097	CakSNP22097	*Kabuli*	Ca_Kabuli_denovo	17372448	(G/A)
22098	CakSNP22098	*Kabuli*	Ca_Kabuli_denovo	17372474	(G/T)
22099	CakSNP22099	*Kabuli*	Ca_Kabuli_denovo	17374148	(T/C)
22100	CakSNP22100	*Kabuli*	Ca_Kabuli_denovo	17374150	(G/A)
22101	CakSNP22101	*Kabuli*	Ca_Kabuli_denovo	17374982	(C/G)
22102	CakSNP22102	*Kabuli*	Ca_Kabuli_denovo	17383714	(G/C)
22103	CakSNP22103	*Kabuli*	Ca_Kabuli_denovo	17390158	(C/T)
22104	CakSNP22104	*Kabuli*	Ca_Kabuli_denovo	17391946	(G/A)
22105	CakSNP22105	*Kabuli*	Ca_Kabuli_denovo	17392021	(A/G)
22106	CakSNP22106	*Kabuli*	Ca_Kabuli_denovo	17396458	(G/A)
22107	CakSNP22107	*Kabuli*	Ca_Kabuli_denovo	17400281	(G/C)
22108	CakSNP22108	*Kabuli*	Ca_Kabuli_denovo	17418177	(G/A)
22109	CakSNP22109	*Kabuli*	Ca_Kabuli_denovo	17418195	(G/A)
22110	CakSNP22110	*Kabuli*	Ca_Kabuli_denovo	17423923	(A/C)
22111	CakSNP22111	*Kabuli*	Ca_Kabuli_denovo	17425180	(T/A)

**S.N.**	**SNP IDs**	**Cultivars**	**Chromosomes/scaffolds**	**Physical positions (bp)**	**SNPs**
22112	CakSNP22112	*Kabuli*	Ca_Kabuli_denovo	17425534	(G/C)
22113	CakSNP22113	*Kabuli*	Ca_Kabuli_denovo	17426379	(C/T)
22114	CakSNP22114	*Kabuli*	Ca_Kabuli_denovo	17427763	(A/G)
22115	CakSNP22115	*Kabuli*	Ca_Kabuli_denovo	17427807	(G/A)
22116	CakSNP22116	*Kabuli*	Ca_Kabuli_denovo	17429423	(A/G)
22117	CakSNP22117	*Kabuli*	Ca_Kabuli_denovo	17429444	(G/A)
22118	CakSNP22118	*Kabuli*	Ca_Kabuli_denovo	17432893	(G/A)
22119	CakSNP22119	*Kabuli*	Ca_Kabuli_denovo	17438905	(A/G)
22120	CakSNP22120	*Kabuli*	Ca_Kabuli_denovo	17454408	(G/A)
22121	CakSNP22121	*Kabuli*	Ca_Kabuli_denovo	17454527	(C/G)
22122	CakSNP22122	*Kabuli*	Ca_Kabuli_denovo	17454584	(G/A)
22123	CakSNP22123	*Kabuli*	Ca_Kabuli_denovo	17458338	(C/G)
22124	CakSNP22124	*Kabuli*	Ca_Kabuli_denovo	17458378	(A/C)
22125	CakSNP22125	*Kabuli*	Ca_Kabuli_denovo	17460854	(C/A)
22126	CakSNP22126	*Kabuli*	Ca_Kabuli_denovo	17462015	(T/C)
22127	CakSNP22127	*Kabuli*	Ca_Kabuli_denovo	17462108	(G/T)
22128	CakSNP22128	*Kabuli*	Ca_Kabuli_denovo	17463999	(T/G)
22129	CakSNP22129	*Kabuli*	Ca_Kabuli_denovo	17464342	(A/G)
22130	CakSNP22130	*Kabuli*	Ca_Kabuli_denovo	17465423	(C/T)
22131	CakSNP22131	*Kabuli*	Ca_Kabuli_denovo	17465429	(A/G)
22132	CakSNP22132	*Kabuli*	Ca_Kabuli_denovo	17467389	(A/G)
22133	CakSNP22133	*Kabuli*	Ca_Kabuli_denovo	17468575	(A/T)
22134	CakSNP22134	*Kabuli*	Ca_Kabuli_denovo	17472963	(C/A)
22135	CakSNP22135	*Kabuli*	Ca_Kabuli_denovo	17475553	(A/G)
22136	CakSNP22136	*Kabuli*	Ca_Kabuli_denovo	17475769	(A/C)
22137	CakSNP22137	*Kabuli*	Ca_Kabuli_denovo	17484863	(A/G)
22138	CakSNP22138	*Kabuli*	Ca_Kabuli_denovo	17484890	(C/A)
22139	CakSNP22139	*Kabuli*	Ca_Kabuli_denovo	17490392	(G/A)
22140	CakSNP22140	*Kabuli*	Ca_Kabuli_denovo	17491797	(C/T)
22141	CakSNP22141	*Kabuli*	Ca_Kabuli_denovo	17494871	(A/T)
22142	CakSNP22142	*Kabuli*	Ca_Kabuli_denovo	17495308	(T/C)
22143	CakSNP22143	*Kabuli*	Ca_Kabuli_denovo	17495506	(A/T)
22144	CakSNP22144	*Kabuli*	Ca_Kabuli_denovo	17495658	(A/G)
22145	CakSNP22145	*Kabuli*	Ca_Kabuli_denovo	17495911	(C/A)
22146	CakSNP22146	*Kabuli*	Ca_Kabuli_denovo	17495980	(C/G)
22147	CakSNP22147	*Kabuli*	Ca_Kabuli_denovo	17496047	(A/G)
22148	CakSNP22148	*Kabuli*	Ca_Kabuli_denovo	17496086	(C/T)
22149	CakSNP22149	*Kabuli*	Ca_Kabuli_denovo	17498540	(G/C)
22150	CakSNP22150	*Kabuli*	Ca_Kabuli_denovo	17502327	(C/G)

S.N.	SNP IDs	Cultivars	Chromosomes/scaffolds	Physical positions (bp)	SNPs
22151	CakSNP22151	*Kabuli*	Ca_Kabuli_denovo	17502354	(C/T)
22152	CakSNP22152	*Kabuli*	Ca_Kabuli_denovo	17509865	(C/G)
22153	CakSNP22153	*Kabuli*	Ca_Kabuli_denovo	17509874	(G/T)
22154	CakSNP22154	*Kabuli*	Ca_Kabuli_denovo	17512372	(G/C)
22155	CakSNP22155	*Kabuli*	Ca_Kabuli_denovo	17512443	(A/G)
22156	CakSNP22156	*Kabuli*	Ca_Kabuli_denovo	17514272	(T/G)
22157	CakSNP22157	*Kabuli*	Ca_Kabuli_denovo	17514319	(A/C)
22158	CakSNP22158	*Kabuli*	Ca_Kabuli_denovo	17515725	(T/C)
22159	CakSNP22159	*Kabuli*	Ca_Kabuli_denovo	17515728	(A/G)
22160	CakSNP22160	*Kabuli*	Ca_Kabuli_denovo	17516278	(A/G)
22161	CakSNP22161	*Kabuli*	Ca_Kabuli_denovo	17517744	(A/G)
22162	CakSNP22162	*Kabuli*	Ca_Kabuli_denovo	17519081	(A/G)
22163	CakSNP22163	*Kabuli*	Ca_Kabuli_denovo	17519153	(C/G)
22164	CakSNP22164	*Kabuli*	Ca_Kabuli_denovo	17522335	(A/G)
22165	CakSNP22165	*Kabuli*	Ca_Kabuli_denovo	17522341	(C/G)
22166	CakSNP22166	*Kabuli*	Ca_Kabuli_denovo	17543443	(A/G)
22167	CakSNP22167	*Kabuli*	Ca_Kabuli_denovo	17554118	(A/G)
22168	CakSNP22168	*Kabuli*	Ca_Kabuli_denovo	17554879	(G/A)
22169	CakSNP22169	*Kabuli*	Ca_Kabuli_denovo	17555105	(G/A)
22170	CakSNP22170	*Kabuli*	Ca_Kabuli_denovo	17555680	(C/T)
22171	CakSNP22171	*Kabuli*	Ca_Kabuli_denovo	17563276	(A/C)
22172	CakSNP22172	*Kabuli*	Ca_Kabuli_denovo	17563281	(G/A)
22173	CakSNP22173	*Kabuli*	Ca_Kabuli_denovo	17568185	(A/G)
22174	CakSNP22174	*Kabuli*	Ca_Kabuli_denovo	17568195	(A/G)
22175	CakSNP22175	*Kabuli*	Ca_Kabuli_denovo	17568481	(A/C)
22176	CakSNP22176	*Kabuli*	Ca_Kabuli_denovo	17568536	(A/G)
22177	CakSNP22177	*Kabuli*	Ca_Kabuli_denovo	17569512	(C/G)
22178	CakSNP22178	*Kabuli*	Ca_Kabuli_denovo	17571190	(T/C)
22179	CakSNP22179	*Kabuli*	Ca_Kabuli_denovo	17575857	(G/C)
22180	CakSNP22180	*Kabuli*	Ca_Kabuli_denovo	17575861	(A/G)
22181	CakSNP22181	*Kabuli*	Ca_Kabuli_denovo	17575862	(A/G)
22182	CakSNP22182	*Kabuli*	Ca_Kabuli_denovo	17575990	(A/G)
22183	CakSNP22183	*Kabuli*	Ca_Kabuli_denovo	17576348	(T/C)
22184	CakSNP22184	*Kabuli*	Ca_Kabuli_denovo	17577311	(G/T)
22185	CakSNP22185	*Kabuli*	Ca_Kabuli_denovo	17577949	(T/G)
22186	CakSNP22186	*Kabuli*	Ca_Kabuli_denovo	17578019	(C/G)
22187	CakSNP22187	*Kabuli*	Ca_Kabuli_denovo	17587745	(A/G)
22188	CakSNP22188	*Kabuli*	Ca_Kabuli_denovo	17587759	(A/G)
22189	CakSNP22189	*Kabuli*	Ca_Kabuli_denovo	17588013	(C/A)

S.N.	SNP IDs	Cultivars	Chromosomes/scaffolds	Physical positions (bp)	SNPs
22190	CakSNP22190	*Kabuli*	Ca_Kabuli_denovo	17588037	(A/G)
22191	CakSNP22191	*Kabuli*	Ca_Kabuli_denovo	17588065	(C/T)
22192	CakSNP22192	*Kabuli*	Ca_Kabuli_denovo	17589665	(C/T)
22193	CakSNP22193	*Kabuli*	Ca_Kabuli_denovo	17589862	(C/G)
22194	CakSNP22194	*Kabuli*	Ca_Kabuli_denovo	17593933	(G/A)
22195	CakSNP22195	*Kabuli*	Ca_Kabuli_denovo	17593960	(A/G)
22196	CakSNP22196	*Kabuli*	Ca_Kabuli_denovo	17605730	(G/A)
22197	CakSNP22197	*Kabuli*	Ca_Kabuli_denovo	17609678	(G/C)
22198	CakSNP22198	*Kabuli*	Ca_Kabuli_denovo	17609709	(A/G)
22199	CakSNP22199	*Kabuli*	Ca_Kabuli_denovo	17610652	(A/G)
22200	CakSNP22200	*Kabuli*	Ca_Kabuli_denovo	17610664	(G/C)
22201	CakSNP22201	*Kabuli*	Ca_Kabuli_denovo	17610812	(G/A)
22202	CakSNP22202	*Kabuli*	Ca_Kabuli_denovo	17610820	(C/T)
22203	CakSNP22203	*Kabuli*	Ca_Kabuli_denovo	17611216	(C/T)
22204	CakSNP22204	*Kabuli*	Ca_Kabuli_denovo	17611222	(A/G)
22205	CakSNP22205	*Kabuli*	Ca_Kabuli_denovo	17621283	(C/T)
22206	CakSNP22206	*Kabuli*	Ca_Kabuli_denovo	17621351	(T/C)
22207	CakSNP22207	*Kabuli*	Ca_Kabuli_denovo	17625679	(A/G)
22208	CakSNP22208	*Kabuli*	Ca_Kabuli_denovo	17626513	(T/G)
22209	CakSNP22209	*Kabuli*	Ca_Kabuli_denovo	17627257	(C/T)
22210	CakSNP22210	*Kabuli*	Ca_Kabuli_denovo	17634324	(G/T)
22211	CakSNP22211	*Kabuli*	Ca_Kabuli_denovo	17634364	(G/A)
22212	CakSNP22212	*Kabuli*	Ca_Kabuli_denovo	17643307	(G/A)
22213	CakSNP22213	*Kabuli*	Ca_Kabuli_denovo	17645604	(A/G)
22214	CakSNP22214	*Kabuli*	Ca_Kabuli_denovo	17648275	(C/T)
22215	CakSNP22215	*Kabuli*	Ca_Kabuli_denovo	17651790	(C/T)
22216	CakSNP22216	*Kabuli*	Ca_Kabuli_denovo	17651792	(G/C)
22217	CakSNP22217	*Kabuli*	Ca_Kabuli_denovo	17653212	(C/A)
22218	CakSNP22218	*Kabuli*	Ca_Kabuli_denovo	17653385	(A/G)
22219	CakSNP22219	*Kabuli*	Ca_Kabuli_denovo	17653412	(T/C)
22220	CakSNP22220	*Kabuli*	Ca_Kabuli_denovo	17655910	(G/A)
22221	CakSNP22221	*Kabuli*	Ca_Kabuli_denovo	17659610	(C/T)
22222	CakSNP22222	*Kabuli*	Ca_Kabuli_denovo	17660788	(G/T)
22223	CakSNP22223	*Kabuli*	Ca_Kabuli_denovo	17664464	(G/A)
22224	CakSNP22224	*Kabuli*	Ca_Kabuli_denovo	17664469	(C/T)
22225	CakSNP22225	*Kabuli*	Ca_Kabuli_denovo	17667464	(A/C)
22226	CakSNP22226	*Kabuli*	Ca_Kabuli_denovo	17667796	(C/G)
22227	CakSNP22227	*Kabuli*	Ca_Kabuli_denovo	17667823	(A/G)
22228	CakSNP22228	*Kabuli*	Ca_Kabuli_denovo	17668198	(T/C)

S.N.	SNP IDs	Cultivars	Chromosomes/scaffolds	Physical positions (bp)	SNPs
22229	CakSNP22229	*Kabuli*	Ca_Kabuli_denovo	17675941	(C/T)
22230	CakSNP22230	*Kabuli*	Ca_Kabuli_denovo	17675953	(C/G)
22231	CakSNP22231	*Kabuli*	Ca_Kabuli_denovo	17681320	(T/C)
22232	CakSNP22232	*Kabuli*	Ca_Kabuli_denovo	17686750	(C/T)
22233	CakSNP22233	*Kabuli*	Ca_Kabuli_denovo	17686802	(C/T)
22234	CakSNP22234	*Kabuli*	Ca_Kabuli_denovo	17690386	(A/G)
22235	CakSNP22235	*Kabuli*	Ca_Kabuli_denovo	17690457	(T/G)
22236	CakSNP22236	*Kabuli*	Ca_Kabuli_denovo	17695830	(T/C)
22237	CakSNP22237	*Kabuli*	Ca_Kabuli_denovo	17696236	(A/G)
22238	CakSNP22238	*Kabuli*	Ca_Kabuli_denovo	17703446	(C/T)
22239	CakSNP22239	*Kabuli*	Ca_Kabuli_denovo	17707089	(T/G)
22240	CakSNP22240	*Kabuli*	Ca_Kabuli_denovo	17714825	(A/C)
22241	CakSNP22241	*Kabuli*	Ca_Kabuli_denovo	17719103	(A/C)
22242	CakSNP22242	*Kabuli*	Ca_Kabuli_denovo	17729630	(G/A)
22243	CakSNP22243	*Kabuli*	Ca_Kabuli_denovo	17729707	(G/T)
22244	CakSNP22244	*Kabuli*	Ca_Kabuli_denovo	17730593	(G/C)
22245	CakSNP22245	*Kabuli*	Ca_Kabuli_denovo	17730605	(A/C)
22246	CakSNP22246	*Kabuli*	Ca_Kabuli_denovo	17730836	(A/C)
22247	CakSNP22247	*Kabuli*	Ca_Kabuli_denovo	17737454	(G/A)
22248	CakSNP22248	*Kabuli*	Ca_Kabuli_denovo	17737466	(A/G)
22249	CakSNP22249	*Kabuli*	Ca_Kabuli_denovo	17741720	(G/C)
22250	CakSNP22250	*Kabuli*	Ca_Kabuli_denovo	17743579	(C/T)
22251	CakSNP22251	*Kabuli*	Ca_Kabuli_denovo	17745440	(T/C)
22252	CakSNP22252	*Kabuli*	Ca_Kabuli_denovo	17745478	(C/T)
22253	CakSNP22253	*Kabuli*	Ca_Kabuli_denovo	17746537	(T/G)
22254	CakSNP22254	*Kabuli*	Ca_Kabuli_denovo	17749390	(C/G)
22255	CakSNP22255	*Kabuli*	Ca_Kabuli_denovo	17749399	(T/C)
22256	CakSNP22256	*Kabuli*	Ca_Kabuli_denovo	17753739	(T/A)
22257	CakSNP22257	*Kabuli*	Ca_Kabuli_denovo	17760588	(A/G)
22258	CakSNP22258	*Kabuli*	Ca_Kabuli_denovo	17760620	(C/T)
22259	CakSNP22259	*Kabuli*	Ca_Kabuli_denovo	17760650	(T/G)
22260	CakSNP22260	*Kabuli*	Ca_Kabuli_denovo	17761956	(G/A)
22261	CakSNP22261	*Kabuli*	Ca_Kabuli_denovo	17765524	(G/T)
22262	CakSNP22262	*Kabuli*	Ca_Kabuli_denovo	17765543	(G/C)
22263	CakSNP22263	*Kabuli*	Ca_Kabuli_denovo	17781649	(G/C)
22264	CakSNP22264	*Kabuli*	Ca_Kabuli_denovo	17785598	(G/C)
22265	CakSNP22265	*Kabuli*	Ca_Kabuli_denovo	17785614	(T/G)
22266	CakSNP22266	*Kabuli*	Ca_Kabuli_denovo	17785643	(T/C)
22267	CakSNP22267	*Kabuli*	Ca_Kabuli_denovo	17787071	(A/G)

S.N.	SNP IDs	Cultivars	Chromosomes/scaffolds	Physical positions (bp)	SNPs
22268	CakSNP22268	*Kabuli*	Ca_Kabuli_denovo	17790731	(G/A)
22269	CakSNP22269	*Kabuli*	Ca_Kabuli_denovo	17792770	(C/T)
22270	CakSNP22270	*Kabuli*	Ca_Kabuli_denovo	17792777	(G/A)
22271	CakSNP22271	*Kabuli*	Ca_Kabuli_denovo	17792957	(C/T)
22272	CakSNP22272	*Kabuli*	Ca_Kabuli_denovo	17792964	(G/A)
22273	CakSNP22273	*Kabuli*	Ca_Kabuli_denovo	17809880	(T/C)
22274	CakSNP22274	*Kabuli*	Ca_Kabuli_denovo	17809913	(G/A)
22275	CakSNP22275	*Kabuli*	Ca_Kabuli_denovo	17813020	(G/A)
22276	CakSNP22276	*Kabuli*	Ca_Kabuli_denovo	17813023	(C/G)
22277	CakSNP22277	*Kabuli*	Ca_Kabuli_denovo	17814716	(C/T)
22278	CakSNP22278	*Kabuli*	Ca_Kabuli_denovo	17814788	(C/G)
22279	CakSNP22279	*Kabuli*	Ca_Kabuli_denovo	17818318	(C/A)
22280	CakSNP22280	*Kabuli*	Ca_Kabuli_denovo	17830073	(C/T)
22281	CakSNP22281	*Kabuli*	Ca_Kabuli_denovo	17831657	(A/G)
22282	CakSNP22282	*Kabuli*	Ca_Kabuli_denovo	17831663	(G/T)
22283	CakSNP22283	*Kabuli*	Ca_Kabuli_denovo	17832301	(G/A)
22284	CakSNP22284	*Kabuli*	Ca_Kabuli_denovo	17832314	(G/A)
22285	CakSNP22285	*Kabuli*	Ca_Kabuli_denovo	17837354	(T/C)
22286	CakSNP22286	*Kabuli*	Ca_Kabuli_denovo	17838454	(G/A)
22287	CakSNP22287	*Kabuli*	Ca_Kabuli_denovo	17844685	(A/C)
22288	CakSNP22288	*Kabuli*	Ca_Kabuli_denovo	17844706	(T/C)
22289	CakSNP22289	*Kabuli*	Ca_Kabuli_denovo	17846171	(C/T)
22290	CakSNP22290	*Kabuli*	Ca_Kabuli_denovo	17846249	(A/G)
22291	CakSNP22291	*Kabuli*	Ca_Kabuli_denovo	17846303	(C/T)
22292	CakSNP22292	*Kabuli*	Ca_Kabuli_denovo	17848306	(T/C)
22293	CakSNP22293	*Kabuli*	Ca_Kabuli_denovo	17852659	(G/A)
22294	CakSNP22294	*Kabuli*	Ca_Kabuli_denovo	17863496	(T/C)
22295	CakSNP22295	*Kabuli*	Ca_Kabuli_denovo	17863520	(G/C)
22296	CakSNP22296	*Kabuli*	Ca_Kabuli_denovo	17864834	(C/G)
22297	CakSNP22297	*Kabuli*	Ca_Kabuli_denovo	17865968	(T/C)
22298	CakSNP22298	*Kabuli*	Ca_Kabuli_denovo	17866529	(C/G)
22299	CakSNP22299	*Kabuli*	Ca_Kabuli_denovo	17870735	(G/A)
22300	CakSNP22300	*Kabuli*	Ca_Kabuli_denovo	17870747	(A/G)
22301	CakSNP22301	*Kabuli*	Ca_Kabuli_denovo	17871285	(G/A)
22302	CakSNP22302	*Kabuli*	Ca_Kabuli_denovo	17871294	(C/T)
22303	CakSNP22303	*Kabuli*	Ca_Kabuli_denovo	17872306	(A/C)
22304	CakSNP22304	*Kabuli*	Ca_Kabuli_denovo	17872945	(A/T)
22305	CakSNP22305	*Kabuli*	Ca_Kabuli_denovo	17872976	(A/G)
22306	CakSNP22306	*Kabuli*	Ca_Kabuli_denovo	17884474	(T/G)

S.N.	SNP IDs	Cultivars	Chromosomes/scaffolds	Physical positions (bp)	SNPs
22307	CakSNP22307	*Kabuli*	Ca_Kabuli_denovo	17889232	(G/C)
22308	CakSNP22308	*Kabuli*	Ca_Kabuli_denovo	17889257	(A/G)
22309	CakSNP22309	*Kabuli*	Ca_Kabuli_denovo	17891779	(T/C)
22310	CakSNP22310	*Kabuli*	Ca_Kabuli_denovo	17892202	(T/C)
22311	CakSNP22311	*Kabuli*	Ca_Kabuli_denovo	17901128	(C/T)
22312	CakSNP22312	*Kabuli*	Ca_Kabuli_denovo	17901641	(A/G)
22313	CakSNP22313	*Kabuli*	Ca_Kabuli_denovo	17901648	(T/A)
22314	CakSNP22314	*Kabuli*	Ca_Kabuli_denovo	17910343	(A/G)
22315	CakSNP22315	*Kabuli*	Ca_Kabuli_denovo	17910397	(A/C)
22316	CakSNP22316	*Kabuli*	Ca_Kabuli_denovo	17913667	(A/G)
22317	CakSNP22317	*Kabuli*	Ca_Kabuli_denovo	17913745	(T/C)
22318	CakSNP22318	*Kabuli*	Ca_Kabuli_denovo	17919055	(A/C)
22319	CakSNP22319	*Kabuli*	Ca_Kabuli_denovo	17925237	(G/A)
22320	CakSNP22320	*Kabuli*	Ca_Kabuli_denovo	17925240	(T/G)
22321	CakSNP22321	*Kabuli*	Ca_Kabuli_denovo	17928555	(G/T)
22322	CakSNP22322	*Kabuli*	Ca_Kabuli_denovo	17928573	(C/G)
22323	CakSNP22323	*Kabuli*	Ca_Kabuli_denovo	17932364	(C/T)
22324	CakSNP22324	*Kabuli*	Ca_Kabuli_denovo	17935615	(C/A)
22325	CakSNP22325	*Kabuli*	Ca_Kabuli_denovo	17935649	(T/G)
22326	CakSNP22326	*Kabuli*	Ca_Kabuli_denovo	17935692	(C/T)
22327	CakSNP22327	*Kabuli*	Ca_Kabuli_denovo	17935701	(A/G)
22328	CakSNP22328	*Kabuli*	Ca_Kabuli_denovo	17942231	(A/G)
22329	CakSNP22329	*Kabuli*	Ca_Kabuli_denovo	17949666	(A/G)
22330	CakSNP22330	*Kabuli*	Ca_Kabuli_denovo	17950774	(A/G)
22331	CakSNP22331	*Kabuli*	Ca_Kabuli_denovo	17957321	(C/A)
22332	CakSNP22332	*Kabuli*	Ca_Kabuli_denovo	17957334	(C/G)
22333	CakSNP22333	*Kabuli*	Ca_Kabuli_denovo	17963997	(T/G)
22334	CakSNP22334	*Kabuli*	Ca_Kabuli_denovo	17968728	(G/A)
22335	CakSNP22335	*Kabuli*	Ca_Kabuli_denovo	17969758	(G/C)
22336	CakSNP22336	*Kabuli*	Ca_Kabuli_denovo	17971133	(G/C)
22337	CakSNP22337	*Kabuli*	Ca_Kabuli_denovo	17971802	(G/T)
22338	CakSNP22338	*Kabuli*	Ca_Kabuli_denovo	17971807	(G/C)
22339	CakSNP22339	*Kabuli*	Ca_Kabuli_denovo	17972205	(A/G)
22340	CakSNP22340	*Kabuli*	Ca_Kabuli_denovo	17974116	(G/A)
22341	CakSNP22341	*Kabuli*	Ca_Kabuli_denovo	17974129	(C/T)
22342	CakSNP22342	*Kabuli*	Ca_Kabuli_denovo	17985164	(C/T)
22343	CakSNP22343	*Kabuli*	Ca_Kabuli_denovo	17986886	(T/A)
22344	CakSNP22344	*Kabuli*	Ca_Kabuli_denovo	17991233	(C/G)
22345	CakSNP22345	*Kabuli*	Ca_Kabuli_denovo	17991279	(G/T)

S.N.	SNP IDs	Cultivars	Chromosomes/scaffolds	Physical positions (bp)	SNPs
22346	CakSNP22346	*Kabuli*	Ca_Kabuli_denovo	17999993	(A/G)
22347	CakSNP22347	*Kabuli*	Ca_Kabuli_denovo	18000376	(T/C)
22348	CakSNP22348	*Kabuli*	Ca_Kabuli_denovo	18009494	(G/A)
22349	CakSNP22349	*Kabuli*	Ca_Kabuli_denovo	18009530	(G/A)
22350	CakSNP22350	*Kabuli*	Ca_Kabuli_denovo	18015260	(C/T)
22351	CakSNP22351	*Kabuli*	Ca_Kabuli_denovo	18015266	(T/C)
22352	CakSNP22352	*Kabuli*	Ca_Kabuli_denovo	18019036	(G/T)
22353	CakSNP22353	*Kabuli*	Ca_Kabuli_denovo	18019054	(G/C)
22354	CakSNP22354	*Kabuli*	Ca_Kabuli_denovo	18021175	(A/C)
22355	CakSNP22355	*Kabuli*	Ca_Kabuli_denovo	18021208	(C/T)
22356	CakSNP22356	*Kabuli*	Ca_Kabuli_denovo	18022504	(A/G)
22357	CakSNP22357	*Kabuli*	Ca_Kabuli_denovo	18030218	(A/G)
22358	CakSNP22358	*Kabuli*	Ca_Kabuli_denovo	18030245	(G/C)
22359	CakSNP22359	*Kabuli*	Ca_Kabuli_denovo	18045236	(A/G)
22360	CakSNP22360	*Kabuli*	Ca_Kabuli_denovo	18045254	(G/C)
22361	CakSNP22361	*Kabuli*	Ca_Kabuli_denovo	18049288	(A/G)
22362	CakSNP22362	*Kabuli*	Ca_Kabuli_denovo	18057460	(A/G)
22363	CakSNP22363	*Kabuli*	Ca_Kabuli_denovo	18057493	(A/G)
22364	CakSNP22364	*Kabuli*	Ca_Kabuli_denovo	18062095	(T/C)
22365	CakSNP22365	*Kabuli*	Ca_Kabuli_denovo	18062116	(C/T)
22366	CakSNP22366	*Kabuli*	Ca_Kabuli_denovo	18063677	(T/G)
22367	CakSNP22367	*Kabuli*	Ca_Kabuli_denovo	18065873	(T/G)
22368	CakSNP22368	*Kabuli*	Ca_Kabuli_denovo	18073526	(A/C)
22369	CakSNP22369	*Kabuli*	Ca_Kabuli_denovo	18073553	(C/T)
22370	CakSNP22370	*Kabuli*	Ca_Kabuli_denovo	18075293	(G/C)
22371	CakSNP22371	*Kabuli*	Ca_Kabuli_denovo	18078197	(G/A)
22372	CakSNP22372	*Kabuli*	Ca_Kabuli_denovo	18078269	(C/T)
22373	CakSNP22373	*Kabuli*	Ca_Kabuli_denovo	18085071	(C/T)
22374	CakSNP22374	*Kabuli*	Ca_Kabuli_denovo	18086849	(A/C)
22375	CakSNP22375	*Kabuli*	Ca_Kabuli_denovo	18095969	(C/T)
22376	CakSNP22376	*Kabuli*	Ca_Kabuli_denovo	18096648	(T/C)
22377	CakSNP22377	*Kabuli*	Ca_Kabuli_denovo	18100045	(T/C)
22378	CakSNP22378	*Kabuli*	Ca_Kabuli_denovo	18100062	(G/C)
22379	CakSNP22379	*Kabuli*	Ca_Kabuli_denovo	18109396	(C/A)
22380	CakSNP22380	*Kabuli*	Ca_Kabuli_denovo	18109402	(T/C)
22381	CakSNP22381	*Kabuli*	Ca_Kabuli_denovo	18111873	(G/A)
22382	CakSNP22382	*Kabuli*	Ca_Kabuli_denovo	18111898	(C/T)
22383	CakSNP22383	*Kabuli*	Ca_Kabuli_denovo	18113153	(C/A)
22384	CakSNP22384	*Kabuli*	Ca_Kabuli_denovo	18113171	(G/A)

S.N.	SNP IDs	Cultivars	Chromosomes/scaffolds	Physical positions (bp)	SNPs
22385	CakSNP22385	*Kabuli*	Ca_Kabuli_denovo	18113367	(C/A)
22386	CakSNP22386	*Kabuli*	Ca_Kabuli_denovo	18113809	(G/A)
22387	CakSNP22387	*Kabuli*	Ca_Kabuli_denovo	18115789	(T/A)
22388	CakSNP22388	*Kabuli*	Ca_Kabuli_denovo	18115810	(G/C)
22389	CakSNP22389	*Kabuli*	Ca_Kabuli_denovo	18116597	(T/C)
22390	CakSNP22390	*Kabuli*	Ca_Kabuli_denovo	18116666	(A/T)
22391	CakSNP22391	*Kabuli*	Ca_Kabuli_denovo	18118195	(T/G)
22392	CakSNP22392	*Kabuli*	Ca_Kabuli_denovo	18120678	(A/C)
22393	CakSNP22393	*Kabuli*	Ca_Kabuli_denovo	18126053	(T/C)
22394	CakSNP22394	*Kabuli*	Ca_Kabuli_denovo	18127484	(G/C)
22395	CakSNP22395	*Kabuli*	Ca_Kabuli_denovo	18130665	(G/A)
22396	CakSNP22396	*Kabuli*	Ca_Kabuli_denovo	18131139	(C/A)
22397	CakSNP22397	*Kabuli*	Ca_Kabuli_denovo	18131168	(G/A)
22398	CakSNP22398	*Kabuli*	Ca_Kabuli_denovo	18132154	(A/G)
22399	CakSNP22399	*Kabuli*	Ca_Kabuli_denovo	18136870	(T/G)
22400	CakSNP22400	*Kabuli*	Ca_Kabuli_denovo	18136875	(G/C)
22401	CakSNP22401	*Kabuli*	Ca_Kabuli_denovo	18143261	(C/T)
22402	CakSNP22402	*Kabuli*	Ca_Kabuli_denovo	18145646	(T/C)
22403	CakSNP22403	*Kabuli*	Ca_Kabuli_denovo	18145676	(C/T)
22404	CakSNP22404	*Kabuli*	Ca_Kabuli_denovo	18150116	(A/G)
22405	CakSNP22405	*Kabuli*	Ca_Kabuli_denovo	18150140	(G/T)
22406	CakSNP22406	*Kabuli*	Ca_Kabuli_denovo	18152696	(G/C)
22407	CakSNP22407	*Kabuli*	Ca_Kabuli_denovo	18155798	(A/G)
22408	CakSNP22408	*Kabuli*	Ca_Kabuli_denovo	18155801	(A/G)
22409	CakSNP22409	*Kabuli*	Ca_Kabuli_denovo	18157850	(A/C)
22410	CakSNP22410	*Kabuli*	Ca_Kabuli_denovo	18159030	(C/T)
22411	CakSNP22411	*Kabuli*	Ca_Kabuli_denovo	18161129	(C/G)
22412	CakSNP22412	*Kabuli*	Ca_Kabuli_denovo	18164824	(T/A)
22413	CakSNP22413	*Kabuli*	Ca_Kabuli_denovo	18166118	(G/C)
22414	CakSNP22414	*Kabuli*	Ca_Kabuli_denovo	18166365	(T/C)
22415	CakSNP22415	*Kabuli*	Ca_Kabuli_denovo	18166405	(G/A)
22416	CakSNP22416	*Kabuli*	Ca_Kabuli_denovo	18167705	(A/T)
22417	CakSNP22417	*Kabuli*	Ca_Kabuli_denovo	18169954	(C/T)
22418	CakSNP22418	*Kabuli*	Ca_Kabuli_denovo	18170385	(A/G)
22419	CakSNP22419	*Kabuli*	Ca_Kabuli_denovo	18173531	(C/T)
22420	CakSNP22420	*Kabuli*	Ca_Kabuli_denovo	18178644	(G/T)
22421	CakSNP22421	*Kabuli*	Ca_Kabuli_denovo	18178846	(C/T)
22422	CakSNP22422	*Kabuli*	Ca_Kabuli_denovo	18183712	(T/G)
22423	CakSNP22423	*Kabuli*	Ca_Kabuli_denovo	18185898	(T/C)

S.N.	SNP IDs	Cultivars	Chromosomes/scaffolds	Physical positions (bp)	SNPs
22424	CakSNP22424	*Kabuli*	Ca_Kabuli_denovo	18185916	(A/G)
22425	CakSNP22425	*Kabuli*	Ca_Kabuli_denovo	18196616	(T/C)
22426	CakSNP22426	*Kabuli*	Ca_Kabuli_denovo	18196660	(G/C)
22427	CakSNP22427	*Kabuli*	Ca_Kabuli_denovo	18199026	(C/T)
22428	CakSNP22428	*Kabuli*	Ca_Kabuli_denovo	18209704	(G/A)
22429	CakSNP22429	*Kabuli*	Ca_Kabuli_denovo	18209734	(A/C)
22430	CakSNP22430	*Kabuli*	Ca_Kabuli_denovo	18214361	(T/C)
22431	CakSNP22431	*Kabuli*	Ca_Kabuli_denovo	18214422	(A/C)
22432	CakSNP22432	*Kabuli*	Ca_Kabuli_denovo	18214627	(C/G)
22433	CakSNP22433	*Kabuli*	Ca_Kabuli_denovo	18214630	(C/T)
22434	CakSNP22434	*Kabuli*	Ca_Kabuli_denovo	18215680	(A/T)
22435	CakSNP22435	*Kabuli*	Ca_Kabuli_denovo	18215725	(G/A)
22436	CakSNP22436	*Kabuli*	Ca_Kabuli_denovo	18225599	(A/G)
22437	CakSNP22437	*Kabuli*	Ca_Kabuli_denovo	18225626	(G/A)
22438	CakSNP22438	*Kabuli*	Ca_Kabuli_denovo	18226822	(C/T)
22439	CakSNP22439	*Kabuli*	Ca_Kabuli_denovo	18229442	(C/A)
22440	CakSNP22440	*Kabuli*	Ca_Kabuli_denovo	18232712	(C/T)
22441	CakSNP22441	*Kabuli*	Ca_Kabuli_denovo	18232715	(A/G)
22442	CakSNP22442	*Kabuli*	Ca_Kabuli_denovo	18240760	(G/C)
22443	CakSNP22443	*Kabuli*	Ca_Kabuli_denovo	18240772	(C/T)
22444	CakSNP22444	*Kabuli*	Ca_Kabuli_denovo	18244217	(G/C)
22445	CakSNP22445	*Kabuli*	Ca_Kabuli_denovo	18244244	(A/G)
22446	CakSNP22446	*Kabuli*	Ca_Kabuli_denovo	18249035	(G/T)
22447	CakSNP22447	*Kabuli*	Ca_Kabuli_denovo	18254434	(C/G)
22448	CakSNP22448	*Kabuli*	Ca_Kabuli_denovo	18254479	(A/G)
22449	CakSNP22449	*Kabuli*	Ca_Kabuli_denovo	18255571	(T/C)
22450	CakSNP22450	*Kabuli*	Ca_Kabuli_denovo	18255580	(G/T)
22451	CakSNP22451	*Kabuli*	Ca_Kabuli_denovo	18255938	(T/C)
22452	CakSNP22452	*Kabuli*	Ca_Kabuli_denovo	18259685	(T/C)
22453	CakSNP22453	*Kabuli*	Ca_Kabuli_denovo	18259712	(G/C)
22454	CakSNP22454	*Kabuli*	Ca_Kabuli_denovo	18260199	(G/A)
22455	CakSNP22455	*Kabuli*	Ca_Kabuli_denovo	18260208	(A/C)
22456	CakSNP22456	*Kabuli*	Ca_Kabuli_denovo	18268558	(T/G)
22457	CakSNP22457	*Kabuli*	Ca_Kabuli_denovo	18268564	(T/C)
22458	CakSNP22458	*Kabuli*	Ca_Kabuli_denovo	18269034	(A/G)
22459	CakSNP22459	*Kabuli*	Ca_Kabuli_denovo	18270676	(T/C)
22460	CakSNP22460	*Kabuli*	Ca_Kabuli_denovo	18270688	(T/G)
22461	CakSNP22461	*Kabuli*	Ca_Kabuli_denovo	18270828	(G/A)
22462	CakSNP22462	*Kabuli*	Ca_Kabuli_denovo	18270846	(C/G)

S.N.	SNP IDs	Cultivars	Chromosomes/scaffolds	Physical positions (bp)	SNPs
22463	CakSNP22463	*Kabuli*	Ca_Kabuli_denovo	18274147	(T/C)
22464	CakSNP22464	*Kabuli*	Ca_Kabuli_denovo	18275905	(T/C)
22465	CakSNP22465	*Kabuli*	Ca_Kabuli_denovo	18276932	(A/G)
22466	CakSNP22466	*Kabuli*	Ca_Kabuli_denovo	18279539	(C/T)
22467	CakSNP22467	*Kabuli*	Ca_Kabuli_denovo	18284434	(G/A)
22468	CakSNP22468	*Kabuli*	Ca_Kabuli_denovo	18284503	(G/C)
22469	CakSNP22469	*Kabuli*	Ca_Kabuli_denovo	18288645	(T/C)
22470	CakSNP22470	*Kabuli*	Ca_Kabuli_denovo	18292460	(G/A)
22471	CakSNP22471	*Kabuli*	Ca_Kabuli_denovo	18293034	(A/G)
22472	CakSNP22472	*Kabuli*	Ca_Kabuli_denovo	18303244	(T/C)
22473	CakSNP22473	*Kabuli*	Ca_Kabuli_denovo	18312877	(G/A)
22474	CakSNP22474	*Kabuli*	Ca_Kabuli_denovo	18315388	(T/C)
22475	CakSNP22475	*Kabuli*	Ca_Kabuli_denovo	18326563	(C/G)
22476	CakSNP22476	*Kabuli*	Ca_Kabuli_denovo	18326575	(C/T)
22477	CakSNP22477	*Kabuli*	Ca_Kabuli_denovo	18326977	(G/A)
22478	CakSNP22478	*Kabuli*	Ca_Kabuli_denovo	18330217	(T/C)
22479	CakSNP22479	*Kabuli*	Ca_Kabuli_denovo	18330247	(C/T)
22480	CakSNP22480	*Kabuli*	Ca_Kabuli_denovo	18349380	(C/G)
22481	CakSNP22481	*Kabuli*	Ca_Kabuli_denovo	18351620	(T/G)
22482	CakSNP22482	*Kabuli*	Ca_Kabuli_denovo	18352549	(T/C)
22483	CakSNP22483	*Kabuli*	Ca_Kabuli_denovo	18352558	(A/C)
22484	CakSNP22484	*Kabuli*	Ca_Kabuli_denovo	18354146	(A/G)
22485	CakSNP22485	*Kabuli*	Ca_Kabuli_denovo	18354163	(T/C)
22486	CakSNP22486	*Kabuli*	Ca_Kabuli_denovo	18357472	(A/G)
22487	CakSNP22487	*Kabuli*	Ca_Kabuli_denovo	18368995	(G/A)
22488	CakSNP22488	*Kabuli*	Ca_Kabuli_denovo	18369476	(G/T)
22489	CakSNP22489	*Kabuli*	Ca_Kabuli_denovo	18369482	(T/C)
22490	CakSNP22490	*Kabuli*	Ca_Kabuli_denovo	18371972	(A/C)
22491	CakSNP22491	*Kabuli*	Ca_Kabuli_denovo	18371974	(T/C)
22492	CakSNP22492	*Kabuli*	Ca_Kabuli_denovo	18378184	(A/G)
22493	CakSNP22493	*Kabuli*	Ca_Kabuli_denovo	18378189	(G/A)
22494	CakSNP22494	*Kabuli*	Ca_Kabuli_denovo	18379267	(G/A)
22495	CakSNP22495	*Kabuli*	Ca_Kabuli_denovo	18380355	(A/G)
22496	CakSNP22496	*Kabuli*	Ca_Kabuli_denovo	18382345	(G/C)
22497	CakSNP22497	*Kabuli*	Ca_Kabuli_denovo	18383189	(C/T)
22498	CakSNP22498	*Kabuli*	Ca_Kabuli_denovo	18387158	(T/C)
22499	CakSNP22499	*Kabuli*	Ca_Kabuli_denovo	18387172	(G/A)
22500	CakSNP22500	*Kabuli*	Ca_Kabuli_denovo	18395703	(G/A)
22501	CakSNP22501	*Kabuli*	Ca_Kabuli_denovo	18399965	(G/C)

S.N.	SNP IDs	Cultivars	Chromosomes/scaffolds	Physical positions (bp)	SNPs
22502	CakSNP22502	*Kabuli*	Ca_Kabuli_denovo	18399994	(T/C)
22503	CakSNP22503	*Kabuli*	Ca_Kabuli_denovo	18400927	(G/C)
22504	CakSNP22504	*Kabuli*	Ca_Kabuli_denovo	18400934	(G/A)
22505	CakSNP22505	*Kabuli*	Ca_Kabuli_denovo	18411242	(T/C)
22506	CakSNP22506	*Kabuli*	Ca_Kabuli_denovo	18411256	(G/A)
22507	CakSNP22507	*Kabuli*	Ca_Kabuli_denovo	18416998	(G/C)
22508	CakSNP22508	*Kabuli*	Ca_Kabuli_denovo	18416999	(C/G)
22509	CakSNP22509	*Kabuli*	Ca_Kabuli_denovo	18417740	(C/G)
22510	CakSNP22510	*Kabuli*	Ca_Kabuli_denovo	18417754	(T/C)
22511	CakSNP22511	*Kabuli*	Ca_Kabuli_denovo	18423214	(C/T)
22512	CakSNP22512	*Kabuli*	Ca_Kabuli_denovo	18423619	(C/T)
22513	CakSNP22513	*Kabuli*	Ca_Kabuli_denovo	18423628	(T/C)
22514	CakSNP22514	*Kabuli*	Ca_Kabuli_denovo	18423994	(G/A)
22515	CakSNP22515	*Kabuli*	Ca_Kabuli_denovo	18428416	(G/T)
22516	CakSNP22516	*Kabuli*	Ca_Kabuli_denovo	18429367	(T/G)
22517	CakSNP22517	*Kabuli*	Ca_Kabuli_denovo	18429383	(A/G)
22518	CakSNP22518	*Kabuli*	Ca_Kabuli_denovo	18432130	(T/C)
22519	CakSNP22519	*Kabuli*	Ca_Kabuli_denovo	18432188	(T/C)
22520	CakSNP22520	*Kabuli*	Ca_Kabuli_denovo	18437366	(C/A)
22521	CakSNP22521	*Kabuli*	Ca_Kabuli_denovo	18438204	(A/G)
22522	CakSNP22522	*Kabuli*	Ca_Kabuli_denovo	18440440	(G/A)
22523	CakSNP22523	*Kabuli*	Ca_Kabuli_denovo	18451604	(G/A)
22524	CakSNP22524	*Kabuli*	Ca_Kabuli_denovo	18451622	(C/A)
22525	CakSNP22525	*Kabuli*	Ca_Kabuli_denovo	18455332	(G/A)
22526	CakSNP22526	*Kabuli*	Ca_Kabuli_denovo	18455350	(C/A)
22527	CakSNP22527	*Kabuli*	Ca_Kabuli_denovo	18455356	(T/C)
22528	CakSNP22528	*Kabuli*	Ca_Kabuli_denovo	18459497	(C/G)
22529	CakSNP22529	*Kabuli*	Ca_Kabuli_denovo	18462101	(A/C)
22530	CakSNP22530	*Kabuli*	Ca_Kabuli_denovo	18464620	(A/G)
22531	CakSNP22531	*Kabuli*	Ca_Kabuli_denovo	18464648	(G/A)
22532	CakSNP22532	*Kabuli*	Ca_Kabuli_denovo	18466527	(G/C)
22533	CakSNP22533	*Kabuli*	Ca_Kabuli_denovo	18466539	(T/C)
22534	CakSNP22534	*Kabuli*	Ca_Kabuli_denovo	18469553	(G/T)
22535	CakSNP22535	*Kabuli*	Ca_Kabuli_denovo	18470860	(T/C)
22536	CakSNP22536	*Kabuli*	Ca_Kabuli_denovo	18474096	(A/G)
22537	CakSNP22537	*Kabuli*	Ca_Kabuli_denovo	18476944	(T/G)
22538	CakSNP22538	*Kabuli*	Ca_Kabuli_denovo	18477935	(A/G)
22539	CakSNP22539	*Kabuli*	Ca_Kabuli_denovo	18479017	(G/C)
22540	CakSNP22540	*Kabuli*	Ca_Kabuli_denovo	18484876	(C/T)

S.N.	SNP IDs	Cultivars	Chromosomes/scaffolds	Physical positions (bp)	SNPs
22541	CakSNP22541	*Kabuli*	Ca_Kabuli_denovo	18492425	(G/C)
22542	CakSNP22542	*Kabuli*	Ca_Kabuli_denovo	18492458	(A/G)
22543	CakSNP22543	*Kabuli*	Ca_Kabuli_denovo	18492911	(G/C)
22544	CakSNP22544	*Kabuli*	Ca_Kabuli_denovo	18497528	(G/A)
22545	CakSNP22545	*Kabuli*	Ca_Kabuli_denovo	18497537	(T/C)
22546	CakSNP22546	*Kabuli*	Ca_Kabuli_denovo	18501632	(G/A)
22547	CakSNP22547	*Kabuli*	Ca_Kabuli_denovo	18501638	(G/A)
22548	CakSNP22548	*Kabuli*	Ca_Kabuli_denovo	18504613	(C/A)
22549	CakSNP22549	*Kabuli*	Ca_Kabuli_denovo	18509084	(C/T)
22550	CakSNP22550	*Kabuli*	Ca_Kabuli_denovo	18512044	(A/C)
22551	CakSNP22551	*Kabuli*	Ca_Kabuli_denovo	18512047	(G/A)
22552	CakSNP22552	*Kabuli*	Ca_Kabuli_denovo	18512138	(A/G)
22553	CakSNP22553	*Kabuli*	Ca_Kabuli_denovo	18515042	(A/G)
22554	CakSNP22554	*Kabuli*	Ca_Kabuli_denovo	18518254	(A/C)
22555	CakSNP22555	*Kabuli*	Ca_Kabuli_denovo	18520034	(C/G)
22556	CakSNP22556	*Kabuli*	Ca_Kabuli_denovo	18520055	(T/C)
22557	CakSNP22557	*Kabuli*	Ca_Kabuli_denovo	18524435	(C/T)
22558	CakSNP22558	*Kabuli*	Ca_Kabuli_denovo	18524445	(A/C)
22559	CakSNP22559	*Kabuli*	Ca_Kabuli_denovo	18524597	(C/G)
22560	CakSNP22560	*Kabuli*	Ca_Kabuli_denovo	18529410	(T/C)
22561	CakSNP22561	*Kabuli*	Ca_Kabuli_denovo	18539598	(C/A)
22562	CakSNP22562	*Kabuli*	Ca_Kabuli_denovo	18542780	(T/A)
22563	CakSNP22563	*Kabuli*	Ca_Kabuli_denovo	18543466	(C/G)
22564	CakSNP22564	*Kabuli*	Ca_Kabuli_denovo	18543550	(G/T)
22565	CakSNP22565	*Kabuli*	Ca_Kabuli_denovo	18543835	(T/C)
22566	CakSNP22566	*Kabuli*	Ca_Kabuli_denovo	18545659	(A/G)
22567	CakSNP22567	*Kabuli*	Ca_Kabuli_denovo	18552228	(C/G)
22568	CakSNP22568	*Kabuli*	Ca_Kabuli_denovo	18558448	(C/A)
22569	CakSNP22569	*Kabuli*	Ca_Kabuli_denovo	18559193	(C/T)
22570	CakSNP22570	*Kabuli*	Ca_Kabuli_denovo	18559231	(G/A)
22571	CakSNP22571	*Kabuli*	Ca_Kabuli_denovo	18559927	(G/A)
22572	CakSNP22572	*Kabuli*	Ca_Kabuli_denovo	18563930	(G/A)
22573	CakSNP22573	*Kabuli*	Ca_Kabuli_denovo	18563978	(C/T)
22574	CakSNP22574	*Kabuli*	Ca_Kabuli_denovo	18566071	(C/T)
22575	CakSNP22575	*Kabuli*	Ca_Kabuli_denovo	18568152	(T/C)
22576	CakSNP22576	*Kabuli*	Ca_Kabuli_denovo	18568157	(T/C)
22577	CakSNP22577	*Kabuli*	Ca_Kabuli_denovo	18569485	(C/T)
22578	CakSNP22578	*Kabuli*	Ca_Kabuli_denovo	18569488	(T/C)
22579	CakSNP22579	*Kabuli*	Ca_Kabuli_denovo	18573632	(G/A)

S.N.	SNP IDs	Cultivars	Chromosomes/scaffolds	Physical positions (bp)	SNPs
22580	CakSNP22580	*Kabuli*	Ca_Kabuli_denovo	18579933	(C/T)
22581	CakSNP22581	*Kabuli*	Ca_Kabuli_denovo	18581122	(A/C)
22582	CakSNP22582	*Kabuli*	Ca_Kabuli_denovo	18586183	(G/T)
22583	CakSNP22583	*Kabuli*	Ca_Kabuli_denovo	18586211	(A/G)
22584	CakSNP22584	*Kabuli*	Ca_Kabuli_denovo	18587105	(C/T)
22585	CakSNP22585	*Kabuli*	Ca_Kabuli_denovo	18589602	(T/C)
22586	CakSNP22586	*Kabuli*	Ca_Kabuli_denovo	18591097	(A/G)
22587	CakSNP22587	*Kabuli*	Ca_Kabuli_denovo	18592866	(C/G)
22588	CakSNP22588	*Kabuli*	Ca_Kabuli_denovo	18592869	(C/T)
22589	CakSNP22589	*Kabuli*	Ca_Kabuli_denovo	18596290	(T/C)
22590	CakSNP22590	*Kabuli*	Ca_Kabuli_denovo	18596296	(T/G)
22591	CakSNP22591	*Kabuli*	Ca_Kabuli_denovo	18597427	(G/A)
22592	CakSNP22592	*Kabuli*	Ca_Kabuli_denovo	18597485	(C/T)
22593	CakSNP22593	*Kabuli*	Ca_Kabuli_denovo	18598249	(C/G)
22594	CakSNP22594	*Kabuli*	Ca_Kabuli_denovo	18606359	(C/A)
22595	CakSNP22595	*Kabuli*	Ca_Kabuli_denovo	18608180	(G/A)
22596	CakSNP22596	*Kabuli*	Ca_Kabuli_denovo	18608195	(T/C)
22597	CakSNP22597	*Kabuli*	Ca_Kabuli_denovo	18614037	(T/C)
22598	CakSNP22598	*Kabuli*	Ca_Kabuli_denovo	18618464	(A/G)
22599	CakSNP22599	*Kabuli*	Ca_Kabuli_denovo	18620233	(T/C)
22600	CakSNP22600	*Kabuli*	Ca_Kabuli_denovo	18623050	(G/A)
22601	CakSNP22601	*Kabuli*	Ca_Kabuli_denovo	18623064	(G/A)
22602	CakSNP22602	*Kabuli*	Ca_Kabuli_denovo	18623808	(T/C)
22603	CakSNP22603	*Kabuli*	Ca_Kabuli_denovo	18624662	(G/A)
22604	CakSNP22604	*Kabuli*	Ca_Kabuli_denovo	18624688	(T/C)
22605	CakSNP22605	*Kabuli*	Ca_Kabuli_denovo	18627935	(A/G)
22606	CakSNP22606	*Kabuli*	Ca_Kabuli_denovo	18632591	(A/C)
22607	CakSNP22607	*Kabuli*	Ca_Kabuli_denovo	18633248	(G/A)
22608	CakSNP22608	*Kabuli*	Ca_Kabuli_denovo	18639938	(A/G)
22609	CakSNP22609	*Kabuli*	Ca_Kabuli_denovo	18641122	(C/G)
22610	CakSNP22610	*Kabuli*	Ca_Kabuli_denovo	18641197	(C/G)
22611	CakSNP22611	*Kabuli*	Ca_Kabuli_denovo	18645319	(C/T)
22612	CakSNP22612	*Kabuli*	Ca_Kabuli_denovo	18646865	(T/C)
22613	CakSNP22613	*Kabuli*	Ca_Kabuli_denovo	18650506	(G/A)
22614	CakSNP22614	*Kabuli*	Ca_Kabuli_denovo	18651436	(C/A)
22615	CakSNP22615	*Kabuli*	Ca_Kabuli_denovo	18651511	(T/C)
22616	CakSNP22616	*Kabuli*	Ca_Kabuli_denovo	18663629	(G/A)
22617	CakSNP22617	*Kabuli*	Ca_Kabuli_denovo	18663648	(T/A)
22618	CakSNP22618	*Kabuli*	Ca_Kabuli_denovo	18670780	(T/G)

S.N.	SNP IDs	Cultivars	Chromosomes/scaffolds	Physical positions (bp)	SNPs
22619	CakSNP22619	*Kabuli*	Ca_Kabuli_denovo	18671340	(T/C)
22620	CakSNP22620	*Kabuli*	Ca_Kabuli_denovo	18671551	(A/G)
22621	CakSNP22621	*Kabuli*	Ca_Kabuli_denovo	18671557	(G/C)
22622	CakSNP22622	*Kabuli*	Ca_Kabuli_denovo	18675353	(A/G)
22623	CakSNP22623	*Kabuli*	Ca_Kabuli_denovo	18675369	(G/C)
22624	CakSNP22624	*Kabuli*	Ca_Kabuli_denovo	18676288	(G/A)
22625	CakSNP22625	*Kabuli*	Ca_Kabuli_denovo	18676421	(T/C)
22626	CakSNP22626	*Kabuli*	Ca_Kabuli_denovo	18676427	(T/C)
22627	CakSNP22627	*Kabuli*	Ca_Kabuli_denovo	18678872	(A/G)
22628	CakSNP22628	*Kabuli*	Ca_Kabuli_denovo	18680021	(C/T)
22629	CakSNP22629	*Kabuli*	Ca_Kabuli_denovo	18680071	(C/A)
22630	CakSNP22630	*Kabuli*	Ca_Kabuli_denovo	18680107	(A/C)
22631	CakSNP22631	*Kabuli*	Ca_Kabuli_denovo	18685944	(A/C)
22632	CakSNP22632	*Kabuli*	Ca_Kabuli_denovo	18690041	(C/A)
22633	CakSNP22633	*Kabuli*	Ca_Kabuli_denovo	18690059	(A/T)
22634	CakSNP22634	*Kabuli*	Ca_Kabuli_denovo	18696550	(C/T)
22635	CakSNP22635	*Kabuli*	Ca_Kabuli_denovo	18696580	(G/A)
22636	CakSNP22636	*Kabuli*	Ca_Kabuli_denovo	18700033	(G/C)
22637	CakSNP22637	*Kabuli*	Ca_Kabuli_denovo	18704924	(A/C)
22638	CakSNP22638	*Kabuli*	Ca_Kabuli_denovo	18705814	(A/C)
22639	CakSNP22639	*Kabuli*	Ca_Kabuli_denovo	18712035	(G/T)
22640	CakSNP22640	*Kabuli*	Ca_Kabuli_denovo	18713763	(A/T)
22641	CakSNP22641	*Kabuli*	Ca_Kabuli_denovo	18713777	(G/A)
22642	CakSNP22642	*Kabuli*	Ca_Kabuli_denovo	18713783	(T/C)
22643	CakSNP22643	*Kabuli*	Ca_Kabuli_denovo	18716914	(A/G)
22644	CakSNP22644	*Kabuli*	Ca_Kabuli_denovo	18722230	(G/T)
22645	CakSNP22645	*Kabuli*	Ca_Kabuli_denovo	18724934	(G/A)
22646	CakSNP22646	*Kabuli*	Ca_Kabuli_denovo	18726509	(A/G)
22647	CakSNP22647	*Kabuli*	Ca_Kabuli_denovo	18727704	(A/G)
22648	CakSNP22648	*Kabuli*	Ca_Kabuli_denovo	18727738	(G/C)
22649	CakSNP22649	*Kabuli*	Ca_Kabuli_denovo	18728749	(T/G)
22650	CakSNP22650	*Kabuli*	Ca_Kabuli_denovo	18728774	(T/G)
22651	CakSNP22651	*Kabuli*	Ca_Kabuli_denovo	18733070	(C/T)
22652	CakSNP22652	*Kabuli*	Ca_Kabuli_denovo	18733079	(C/G)
22653	CakSNP22653	*Kabuli*	Ca_Kabuli_denovo	18739407	(A/C)
22654	CakSNP22654	*Kabuli*	Ca_Kabuli_denovo	18739408	(A/G)
22655	CakSNP22655	*Kabuli*	Ca_Kabuli_denovo	18747163	(G/A)
22656	CakSNP22656	*Kabuli*	Ca_Kabuli_denovo	18748718	(C/A)
22657	CakSNP22657	*Kabuli*	Ca_Kabuli_denovo	18748786	(T/C)

S.N.	SNP IDs	Cultivars	Chromosomes/scaffolds	Physical positions (bp)	SNPs
22658	CakSNP22658	*Kabuli*	Ca_Kabuli_denovo	18753052	(A/G)
22659	CakSNP22659	*Kabuli*	Ca_Kabuli_denovo	18756362	(C/T)
22660	CakSNP22660	*Kabuli*	Ca_Kabuli_denovo	18762726	(G/A)
22661	CakSNP22661	*Kabuli*	Ca_Kabuli_denovo	18762744	(C/A)
22662	CakSNP22662	*Kabuli*	Ca_Kabuli_denovo	18763792	(G/T)
22663	CakSNP22663	*Kabuli*	Ca_Kabuli_denovo	18770479	(C/T)
22664	CakSNP22664	*Kabuli*	Ca_Kabuli_denovo	18770536	(T/C)
22665	CakSNP22665	*Kabuli*	Ca_Kabuli_denovo	18770586	(C/T)
22666	CakSNP22666	*Kabuli*	Ca_Kabuli_denovo	18773521	(G/A)
22667	CakSNP22667	*Kabuli*	Ca_Kabuli_denovo	18775835	(T/C)
22668	CakSNP22668	*Kabuli*	Ca_Kabuli_denovo	18775837	(A/G)
22669	CakSNP22669	*Kabuli*	Ca_Kabuli_denovo	18777081	(C/G)
22670	CakSNP22670	*Kabuli*	Ca_Kabuli_denovo	18787985	(C/G)
22671	CakSNP22671	*Kabuli*	Ca_Kabuli_denovo	18788003	(T/C)
22672	CakSNP22672	*Kabuli*	Ca_Kabuli_denovo	18790370	(A/C)
22673	CakSNP22673	*Kabuli*	Ca_Kabuli_denovo	18791098	(C/T)
22674	CakSNP22674	*Kabuli*	Ca_Kabuli_denovo	18791101	(C/A)
22675	CakSNP22675	*Kabuli*	Ca_Kabuli_denovo	18793121	(T/C)
22676	CakSNP22676	*Kabuli*	Ca_Kabuli_denovo	18803897	(A/G)
22677	CakSNP22677	*Kabuli*	Ca_Kabuli_denovo	18803935	(G/C)
22678	CakSNP22678	*Kabuli*	Ca_Kabuli_denovo	18806483	(C/T)
22679	CakSNP22679	*Kabuli*	Ca_Kabuli_denovo	18808206	(T/G)
22680	CakSNP22680	*Kabuli*	Ca_Kabuli_denovo	18808227	(T/G)
22681	CakSNP22681	*Kabuli*	Ca_Kabuli_denovo	18809542	(C/T)
22682	CakSNP22682	*Kabuli*	Ca_Kabuli_denovo	18812003	(G/T)
22683	CakSNP22683	*Kabuli*	Ca_Kabuli_denovo	18813549	(G/A)
22684	CakSNP22684	*Kabuli*	Ca_Kabuli_denovo	18814122	(G/A)
22685	CakSNP22685	*Kabuli*	Ca_Kabuli_denovo	18816705	(T/C)
22686	CakSNP22686	*Kabuli*	Ca_Kabuli_denovo	18816713	(G/A)
22687	CakSNP22687	*Kabuli*	Ca_Kabuli_denovo	18828492	(G/A)
22688	CakSNP22688	*Kabuli*	Ca_Kabuli_denovo	18829915	(C/A)
22689	CakSNP22689	*Kabuli*	Ca_Kabuli_denovo	18829924	(A/G)
22690	CakSNP22690	*Kabuli*	Ca_Kabuli_denovo	18832361	(G/T)
22691	CakSNP22691	*Kabuli*	Ca_Kabuli_denovo	18832368	(G/C)
22692	CakSNP22692	*Kabuli*	Ca_Kabuli_denovo	18834096	(C/G)
22693	CakSNP22693	*Kabuli*	Ca_Kabuli_denovo	18835613	(T/C)
22694	CakSNP22694	*Kabuli*	Ca_Kabuli_denovo	18836177	(C/T)
22695	CakSNP22695	*Kabuli*	Ca_Kabuli_denovo	18841637	(A/G)
22696	CakSNP22696	*Kabuli*	Ca_Kabuli_denovo	18841640	(T/G)

S.N.	SNP IDs	Cultivars	Chromosomes/scaffolds	Physical positions (bp)	SNPs
22697	CakSNP22697	*Kabuli*	Ca_Kabuli_denovo	18849251	(G/A)
22698	CakSNP22698	*Kabuli*	Ca_Kabuli_denovo	18850197	(A/G)
22699	CakSNP22699	*Kabuli*	Ca_Kabuli_denovo	18850203	(C/T)
22700	CakSNP22700	*Kabuli*	Ca_Kabuli_denovo	18850655	(G/A)
22701	CakSNP22701	*Kabuli*	Ca_Kabuli_denovo	18850691	(G/C)
22702	CakSNP22702	*Kabuli*	Ca_Kabuli_denovo	18852209	(A/G)
22703	CakSNP22703	*Kabuli*	Ca_Kabuli_denovo	18852950	(A/G)
22704	CakSNP22704	*Kabuli*	Ca_Kabuli_denovo	18856357	(T/C)
22705	CakSNP22705	*Kabuli*	Ca_Kabuli_denovo	18857764	(C/T)
22706	CakSNP22706	*Kabuli*	Ca_Kabuli_denovo	18857773	(T/C)
22707	CakSNP22707	*Kabuli*	Ca_Kabuli_denovo	18860305	(A/G)
22708	CakSNP22708	*Kabuli*	Ca_Kabuli_denovo	18867206	(A/G)
22709	CakSNP22709	*Kabuli*	Ca_Kabuli_denovo	18869392	(G/A)
22710	CakSNP22710	*Kabuli*	Ca_Kabuli_denovo	18869409	(A/C)
22711	CakSNP22711	*Kabuli*	Ca_Kabuli_denovo	18872425	(C/T)
22712	CakSNP22712	*Kabuli*	Ca_Kabuli_denovo	18872431	(T/C)
22713	CakSNP22713	*Kabuli*	Ca_Kabuli_denovo	18872443	(T/C)
22714	CakSNP22714	*Kabuli*	Ca_Kabuli_denovo	18873043	(G/A)
22715	CakSNP22715	*Kabuli*	Ca_Kabuli_denovo	18873726	(T/C)
22716	CakSNP22716	*Kabuli*	Ca_Kabuli_denovo	18873766	(G/A)
22717	CakSNP22717	*Kabuli*	Ca_Kabuli_denovo	18878910	(T/A)
22718	CakSNP22718	*Kabuli*	Ca_Kabuli_denovo	18879388	(G/T)
22719	CakSNP22719	*Kabuli*	Ca_Kabuli_denovo	18880593	(T/C)
22720	CakSNP22720	*Kabuli*	Ca_Kabuli_denovo	18881435	(T/G)
22721	CakSNP22721	*Kabuli*	Ca_Kabuli_denovo	18882519	(T/C)
22722	CakSNP22722	*Kabuli*	Ca_Kabuli_denovo	18891748	(T/G)
22723	CakSNP22723	*Kabuli*	Ca_Kabuli_denovo	18891754	(T/C)
22724	CakSNP22724	*Kabuli*	Ca_Kabuli_denovo	18892953	(A/T)
22725	CakSNP22725	*Kabuli*	Ca_Kabuli_denovo	18896152	(A/G)
22726	CakSNP22726	*Kabuli*	Ca_Kabuli_denovo	18898353	(C/A)
22727	CakSNP22727	*Kabuli*	Ca_Kabuli_denovo	18898800	(C/T)
22728	CakSNP22728	*Kabuli*	Ca_Kabuli_denovo	18899861	(A/G)
22729	CakSNP22729	*Kabuli*	Ca_Kabuli_denovo	18899909	(C/G)
22730	CakSNP22730	*Kabuli*	Ca_Kabuli_denovo	18900482	(A/C)
22731	CakSNP22731	*Kabuli*	Ca_Kabuli_denovo	18900483	(G/T)
22732	CakSNP22732	*Kabuli*	Ca_Kabuli_denovo	18900484	(A/G)
22733	CakSNP22733	*Kabuli*	Ca_Kabuli_denovo	18905479	(G/A)
22734	CakSNP22734	*Kabuli*	Ca_Kabuli_denovo	18911372	(C/G)
22735	CakSNP22735	*Kabuli*	Ca_Kabuli_denovo	18911381	(A/G)

S.N.	SNP IDs	Cultivars	Chromosomes/scaffolds	Physical positions (bp)	SNPs
22736	CakSNP22736	*Kabuli*	Ca_Kabuli_denovo	18924368	(C/A)
22737	CakSNP22737	*Kabuli*	Ca_Kabuli_denovo	18927874	(G/C)
22738	CakSNP22738	*Kabuli*	Ca_Kabuli_denovo	18930752	(A/G)
22739	CakSNP22739	*Kabuli*	Ca_Kabuli_denovo	18930761	(T/C)
22740	CakSNP22740	*Kabuli*	Ca_Kabuli_denovo	18930885	(A/G)
22741	CakSNP22741	*Kabuli*	Ca_Kabuli_denovo	18930894	(A/G)
22742	CakSNP22742	*Kabuli*	Ca_Kabuli_denovo	18934921	(C/T)
22743	CakSNP22743	*Kabuli*	Ca_Kabuli_denovo	18934964	(C/T)
22744	CakSNP22744	*Kabuli*	Ca_Kabuli_denovo	18935278	(C/T)
22745	CakSNP22745	*Kabuli*	Ca_Kabuli_denovo	18938911	(G/A)
22746	CakSNP22746	*Kabuli*	Ca_Kabuli_denovo	18938964	(G/C)
22747	CakSNP22747	*Kabuli*	Ca_Kabuli_denovo	18940227	(G/C)
22748	CakSNP22748	*Kabuli*	Ca_Kabuli_denovo	18949813	(C/T)
22749	CakSNP22749	*Kabuli*	Ca_Kabuli_denovo	18951660	(G/C)
22750	CakSNP22750	*Kabuli*	Ca_Kabuli_denovo	18951699	(G/C)
22751	CakSNP22751	*Kabuli*	Ca_Kabuli_denovo	18951736	(A/G)
22752	CakSNP22752	*Kabuli*	Ca_Kabuli_denovo	18951760	(A/G)
22753	CakSNP22753	*Kabuli*	Ca_Kabuli_denovo	18968953	(G/C)
22754	CakSNP22754	*Kabuli*	Ca_Kabuli_denovo	18969406	(A/G)
22755	CakSNP22755	*Kabuli*	Ca_Kabuli_denovo	18969451	(A/G)
22756	CakSNP22756	*Kabuli*	Ca_Kabuli_denovo	18975086	(G/A)
22757	CakSNP22757	*Kabuli*	Ca_Kabuli_denovo	18975094	(T/C)
22758	CakSNP22758	*Kabuli*	Ca_Kabuli_denovo	18975107	(T/C)
22759	CakSNP22759	*Kabuli*	Ca_Kabuli_denovo	18975761	(T/C)
22760	CakSNP22760	*Kabuli*	Ca_Kabuli_denovo	18975813	(A/G)
22761	CakSNP22761	*Kabuli*	Ca_Kabuli_denovo	18976289	(G/A)
22762	CakSNP22762	*Kabuli*	Ca_Kabuli_denovo	18976292	(G/A)
22763	CakSNP22763	*Kabuli*	Ca_Kabuli_denovo	18977055	(C/G)
22764	CakSNP22764	*Kabuli*	Ca_Kabuli_denovo	18978483	(T/C)
22765	CakSNP22765	*Kabuli*	Ca_Kabuli_denovo	18981320	(T/C)
22766	CakSNP22766	*Kabuli*	Ca_Kabuli_denovo	18987252	(G/T)
22767	CakSNP22767	*Kabuli*	Ca_Kabuli_denovo	18992585	(G/A)
22768	CakSNP22768	*Kabuli*	Ca_Kabuli_denovo	18997411	(T/C)
22769	CakSNP22769	*Kabuli*	Ca_Kabuli_denovo	19005025	(A/C)
22770	CakSNP22770	*Kabuli*	Ca_Kabuli_denovo	19005067	(G/T)
22771	CakSNP22771	*Kabuli*	Ca_Kabuli_denovo	19006159	(T/C)
22772	CakSNP22772	*Kabuli*	Ca_Kabuli_denovo	19008722	(A/T)
22773	CakSNP22773	*Kabuli*	Ca_Kabuli_denovo	19010698	(T/C)
22774	CakSNP22774	*Kabuli*	Ca_Kabuli_denovo	19010774	(G/A)

S.N.	SNP IDs	Cultivars	Chromosomes/scaffolds	Physical positions (bp)	SNPs
22775	CakSNP22775	*Kabuli*	Ca_Kabuli_denovo	19016555	(G/A)
22776	CakSNP22776	*Kabuli*	Ca_Kabuli_denovo	19016573	(A/G)
22777	CakSNP22777	*Kabuli*	Ca_Kabuli_denovo	19017747	(T/G)
22778	CakSNP22778	*Kabuli*	Ca_Kabuli_denovo	19019311	(C/G)
22779	CakSNP22779	*Kabuli*	Ca_Kabuli_denovo	19026033	(A/C)
22780	CakSNP22780	*Kabuli*	Ca_Kabuli_denovo	19026034	(G/C)
22781	CakSNP22781	*Kabuli*	Ca_Kabuli_denovo	19032739	(G/A)
22782	CakSNP22782	*Kabuli*	Ca_Kabuli_denovo	19034113	(A/T)
22783	CakSNP22783	*Kabuli*	Ca_Kabuli_denovo	19040086	(T/C)
22784	CakSNP22784	*Kabuli*	Ca_Kabuli_denovo	19040170	(G/T)
22785	CakSNP22785	*Kabuli*	Ca_Kabuli_denovo	19045310	(G/A)
22786	CakSNP22786	*Kabuli*	Ca_Kabuli_denovo	19049477	(T/C)
22787	CakSNP22787	*Kabuli*	Ca_Kabuli_denovo	19050458	(A/T)
22788	CakSNP22788	*Kabuli*	Ca_Kabuli_denovo	19056692	(A/G)
22789	CakSNP22789	*Kabuli*	Ca_Kabuli_denovo	19056836	(C/T)
22790	CakSNP22790	*Kabuli*	Ca_Kabuli_denovo	19056854	(G/C)
22791	CakSNP22791	*Kabuli*	Ca_Kabuli_denovo	19063347	(C/G)
22792	CakSNP22792	*Kabuli*	Ca_Kabuli_denovo	19066239	(C/A)
22793	CakSNP22793	*Kabuli*	Ca_Kabuli_denovo	19069211	(T/C)
22794	CakSNP22794	*Kabuli*	Ca_Kabuli_denovo	19069247	(A/G)
22795	CakSNP22795	*Kabuli*	Ca_Kabuli_denovo	19069451	(T/C)
22796	CakSNP22796	*Kabuli*	Ca_Kabuli_denovo	19079071	(C/T)
22797	CakSNP22797	*Kabuli*	Ca_Kabuli_denovo	19079086	(C/G)
22798	CakSNP22798	*Kabuli*	Ca_Kabuli_denovo	19087156	(T/G)
22799	CakSNP22799	*Kabuli*	Ca_Kabuli_denovo	19088934	(C/G)
22800	CakSNP22800	*Kabuli*	Ca_Kabuli_denovo	19088942	(C/T)
22801	CakSNP22801	*Kabuli*	Ca_Kabuli_denovo	19089410	(G/A)
22802	CakSNP22802	*Kabuli*	Ca_Kabuli_denovo	19089454	(G/T)
22803	CakSNP22803	*Kabuli*	Ca_Kabuli_denovo	19090604	(G/A)
22804	CakSNP22804	*Kabuli*	Ca_Kabuli_denovo	19091010	(T/C)
22805	CakSNP22805	*Kabuli*	Ca_Kabuli_denovo	19091040	(A/C)
22806	CakSNP22806	*Kabuli*	Ca_Kabuli_denovo	19091083	(T/C)
22807	CakSNP22807	*Kabuli*	Ca_Kabuli_denovo	19093687	(G/T)
22808	CakSNP22808	*Kabuli*	Ca_Kabuli_denovo	19096458	(A/G)
22809	CakSNP22809	*Kabuli*	Ca_Kabuli_denovo	19108084	(G/A)
22810	CakSNP22810	*Kabuli*	Ca_Kabuli_denovo	19108134	(G/A)
22811	CakSNP22811	*Kabuli*	Ca_Kabuli_denovo	19108151	(G/C)
22812	CakSNP22812	*Kabuli*	Ca_Kabuli_denovo	19110319	(C/T)
22813	CakSNP22813	*Kabuli*	Ca_Kabuli_denovo	19110332	(A/G)

S.N.	SNP IDs	Cultivars	Chromosomes/scaffolds	Physical positions (bp)	SNPs
22814	CakSNP22814	*Kabuli*	Ca_Kabuli_denovo	19110398	(C/A)
22815	CakSNP22815	*Kabuli*	Ca_Kabuli_denovo	19126783	(T/A)
22816	CakSNP22816	*Kabuli*	Ca_Kabuli_denovo	19132339	(A/C)
22817	CakSNP22817	*Kabuli*	Ca_Kabuli_denovo	19132396	(T/C)
22818	CakSNP22818	*Kabuli*	Ca_Kabuli_denovo	19135975	(G/A)
22819	CakSNP22819	*Kabuli*	Ca_Kabuli_denovo	19136023	(A/G)
22820	CakSNP22820	*Kabuli*	Ca_Kabuli_denovo	19138028	(T/C)
22821	CakSNP22821	*Kabuli*	Ca_Kabuli_denovo	19138056	(G/A)
22822	CakSNP22822	*Kabuli*	Ca_Kabuli_denovo	19147683	(A/G)
22823	CakSNP22823	*Kabuli*	Ca_Kabuli_denovo	19147701	(T/C)
22824	CakSNP22824	*Kabuli*	Ca_Kabuli_denovo	19149268	(C/T)
22825	CakSNP22825	*Kabuli*	Ca_Kabuli_denovo	19154228	(G/A)
22826	CakSNP22826	*Kabuli*	Ca_Kabuli_denovo	19154258	(A/G)
22827	CakSNP22827	*Kabuli*	Ca_Kabuli_denovo	19155218	(T/C)
22828	CakSNP22828	*Kabuli*	Ca_Kabuli_denovo	19155611	(A/G)
22829	CakSNP22829	*Kabuli*	Ca_Kabuli_denovo	19155614	(G/A)
22830	CakSNP22830	*Kabuli*	Ca_Kabuli_denovo	19156125	(T/C)
22831	CakSNP22831	*Kabuli*	Ca_Kabuli_denovo	19158322	(T/C)
22832	CakSNP22832	*Kabuli*	Ca_Kabuli_denovo	19160518	(C/T)
22833	CakSNP22833	*Kabuli*	Ca_Kabuli_denovo	19160524	(T/A)
22834	CakSNP22834	*Kabuli*	Ca_Kabuli_denovo	19161853	(C/A)
22835	CakSNP22835	*Kabuli*	Ca_Kabuli_denovo	19163941	(C/G)
22836	CakSNP22836	*Kabuli*	Ca_Kabuli_denovo	19169207	(G/T)
22837	CakSNP22837	*Kabuli*	Ca_Kabuli_denovo	19169216	(C/T)
22838	CakSNP22838	*Kabuli*	Ca_Kabuli_denovo	19171720	(G/A)
22839	CakSNP22839	*Kabuli*	Ca_Kabuli_denovo	19171726	(A/G)
22840	CakSNP22840	*Kabuli*	Ca_Kabuli_denovo	19177383	(C/A)
22841	CakSNP22841	*Kabuli*	Ca_Kabuli_denovo	19177386	(C/A)
22842	CakSNP22842	*Kabuli*	Ca_Kabuli_denovo	19184471	(C/T)
22843	CakSNP22843	*Kabuli*	Ca_Kabuli_denovo	19185765	(A/G)
22844	CakSNP22844	*Kabuli*	Ca_Kabuli_denovo	19185789	(A/G)
22845	CakSNP22845	*Kabuli*	Ca_Kabuli_denovo	19191108	(A/C)
22846	CakSNP22846	*Kabuli*	Ca_Kabuli_denovo	19191109	(A/G)
22847	CakSNP22847	*Kabuli*	Ca_Kabuli_denovo	19192438	(G/A)
22848	CakSNP22848	*Kabuli*	Ca_Kabuli_denovo	19192486	(C/T)
22849	CakSNP22849	*Kabuli*	Ca_Kabuli_denovo	19195741	(A/G)
22850	CakSNP22850	*Kabuli*	Ca_Kabuli_denovo	19195744	(G/A)
22851	CakSNP22851	*Kabuli*	Ca_Kabuli_denovo	19198315	(T/C)
22852	CakSNP22852	*Kabuli*	Ca_Kabuli_denovo	19198343	(T/G)

S.N.	SNP IDs	Cultivars	Chromosomes/scaffolds	Physical positions (bp)	SNPs
22853	CakSNP22853	*Kabuli*	Ca_Kabuli_denovo	19200266	(A/G)
22854	CakSNP22854	*Kabuli*	Ca_Kabuli_denovo	19201724	(T/G)
22855	CakSNP22855	*Kabuli*	Ca_Kabuli_denovo	19206320	(T/C)
22856	CakSNP22856	*Kabuli*	Ca_Kabuli_denovo	19223412	(C/T)
22857	CakSNP22857	*Kabuli*	Ca_Kabuli_denovo	19223413	(G/T)
22858	CakSNP22858	*Kabuli*	Ca_Kabuli_denovo	19229621	(A/G)
22859	CakSNP22859	*Kabuli*	Ca_Kabuli_denovo	19234774	(T/C)
22860	CakSNP22860	*Kabuli*	Ca_Kabuli_denovo	19238116	(A/C)
22861	CakSNP22861	*Kabuli*	Ca_Kabuli_denovo	19238124	(C/G)
22862	CakSNP22862	*Kabuli*	Ca_Kabuli_denovo	19238946	(C/T)
22863	CakSNP22863	*Kabuli*	Ca_Kabuli_denovo	19240781	(T/C)
22864	CakSNP22864	*Kabuli*	Ca_Kabuli_denovo	19240800	(G/A)
22865	CakSNP22865	*Kabuli*	Ca_Kabuli_denovo	19247383	(G/C)
22866	CakSNP22866	*Kabuli*	Ca_Kabuli_denovo	19248118	(A/C)
22867	CakSNP22867	*Kabuli*	Ca_Kabuli_denovo	19251244	(C/T)
22868	CakSNP22868	*Kabuli*	Ca_Kabuli_denovo	19254548	(C/A)
22869	CakSNP22869	*Kabuli*	Ca_Kabuli_denovo	19254587	(A/G)
22870	CakSNP22870	*Kabuli*	Ca_Kabuli_denovo	19256586	(C/T)
22871	CakSNP22871	*Kabuli*	Ca_Kabuli_denovo	19274063	(T/G)
22872	CakSNP22872	*Kabuli*	Ca_Kabuli_denovo	19275686	(C/T)
22873	CakSNP22873	*Kabuli*	Ca_Kabuli_denovo	19275704	(T/C)
22874	CakSNP22874	*Kabuli*	Ca_Kabuli_denovo	19279916	(T/G)
22875	CakSNP22875	*Kabuli*	Ca_Kabuli_denovo	19284224	(A/G)
22876	CakSNP22876	*Kabuli*	Ca_Kabuli_denovo	19284257	(G/C)
22877	CakSNP22877	*Kabuli*	Ca_Kabuli_denovo	19287074	(C/T)
22878	CakSNP22878	*Kabuli*	Ca_Kabuli_denovo	19294446	(G/A)
22879	CakSNP22879	*Kabuli*	Ca_Kabuli_denovo	19294650	(G/A)
22880	CakSNP22880	*Kabuli*	Ca_Kabuli_denovo	19295751	(T/C)
22881	CakSNP22881	*Kabuli*	Ca_Kabuli_denovo	19302256	(A/G)
22882	CakSNP22882	*Kabuli*	Ca_Kabuli_denovo	19303277	(T/C)
22883	CakSNP22883	*Kabuli*	Ca_Kabuli_denovo	19304858	(T/C)
22884	CakSNP22884	*Kabuli*	Ca_Kabuli_denovo	19304894	(T/C)
22885	CakSNP22885	*Kabuli*	Ca_Kabuli_denovo	19304897	(C/T)
22886	CakSNP22886	*Kabuli*	Ca_Kabuli_denovo	19315474	(G/A)
22887	CakSNP22887	*Kabuli*	Ca_Kabuli_denovo	19315480	(G/A)
22888	CakSNP22888	*Kabuli*	Ca_Kabuli_denovo	19323757	(A/G)
22889	CakSNP22889	*Kabuli*	Ca_Kabuli_denovo	19328354	(C/A)
22890	CakSNP22890	*Kabuli*	Ca_Kabuli_denovo	19331804	(G/A)
22891	CakSNP22891	*Kabuli*	Ca_Kabuli_denovo	19332683	(G/T)

S.N.	SNP IDs	Cultivars	Chromosomes/scaffolds	Physical positions (bp)	SNPs
22892	CakSNP22892	*Kabuli*	Ca_Kabuli_denovo	19335471	(C/G)
22893	CakSNP22893	*Kabuli*	Ca_Kabuli_denovo	19346404	(T/C)
22894	CakSNP22894	*Kabuli*	Ca_Kabuli_denovo	19348192	(A/C)
22895	CakSNP22895	*Kabuli*	Ca_Kabuli_denovo	19348233	(C/G)
22896	CakSNP22896	*Kabuli*	Ca_Kabuli_denovo	19349645	(T/G)
22897	CakSNP22897	*Kabuli*	Ca_Kabuli_denovo	19350085	(G/A)
22898	CakSNP22898	*Kabuli*	Ca_Kabuli_denovo	19352147	(G/A)
22899	CakSNP22899	*Kabuli*	Ca_Kabuli_denovo	19356539	(C/T)
22900	CakSNP22900	*Kabuli*	Ca_Kabuli_denovo	19361046	(A/G)
22901	CakSNP22901	*Kabuli*	Ca_Kabuli_denovo	19367179	(T/C)
22902	CakSNP22902	*Kabuli*	Ca_Kabuli_denovo	19370551	(T/C)
22903	CakSNP22903	*Kabuli*	Ca_Kabuli_denovo	19374728	(A/G)
22904	CakSNP22904	*Kabuli*	Ca_Kabuli_denovo	19376336	(G/A)
22905	CakSNP22905	*Kabuli*	Ca_Kabuli_denovo	19376363	(G/A)
22906	CakSNP22906	*Kabuli*	Ca_Kabuli_denovo	19379073	(C/T)
22907	CakSNP22907	*Kabuli*	Ca_Kabuli_denovo	19379306	(C/A)
22908	CakSNP22908	*Kabuli*	Ca_Kabuli_denovo	19379735	(T/C)
22909	CakSNP22909	*Kabuli*	Ca_Kabuli_denovo	19385230	(C/G)
22910	CakSNP22910	*Kabuli*	Ca_Kabuli_denovo	19385511	(G/C)
22911	CakSNP22911	*Kabuli*	Ca_Kabuli_denovo	19385523	(A/G)
22912	CakSNP22912	*Kabuli*	Ca_Kabuli_denovo	19390162	(C/T)
22913	CakSNP22913	*Kabuli*	Ca_Kabuli_denovo	19390333	(T/C)
22914	CakSNP22914	*Kabuli*	Ca_Kabuli_denovo	19391252	(C/T)
22915	CakSNP22915	*Kabuli*	Ca_Kabuli_denovo	19398054	(C/A)
22916	CakSNP22916	*Kabuli*	Ca_Kabuli_denovo	19398085	(T/C)
22917	CakSNP22917	*Kabuli*	Ca_Kabuli_denovo	19398318	(G/A)
22918	CakSNP22918	*Kabuli*	Ca_Kabuli_denovo	19400571	(C/T)
22919	CakSNP22919	*Kabuli*	Ca_Kabuli_denovo	19401445	(G/C)
22920	CakSNP22920	*Kabuli*	Ca_Kabuli_denovo	19401475	(A/G)
22921	CakSNP22921	*Kabuli*	Ca_Kabuli_denovo	19402744	(C/G)
22922	CakSNP22922	*Kabuli*	Ca_Kabuli_denovo	19407853	(C/G)
22923	CakSNP22923	*Kabuli*	Ca_Kabuli_denovo	19416866	(C/T)
22924	CakSNP22924	*Kabuli*	Ca_Kabuli_denovo	19421553	(C/T)
22925	CakSNP22925	*Kabuli*	Ca_Kabuli_denovo	19421556	(A/G)
22926	CakSNP22926	*Kabuli*	Ca_Kabuli_denovo	19422615	(G/A)
22927	CakSNP22927	*Kabuli*	Ca_Kabuli_denovo	19422629	(G/A)
22928	CakSNP22928	*Kabuli*	Ca_Kabuli_denovo	19429950	(T/G)
22929	CakSNP22929	*Kabuli*	Ca_Kabuli_denovo	19429960	(G/A)
22930	CakSNP22930	*Kabuli*	Ca_Kabuli_denovo	19430010	(C/G)

S.N.	SNP IDs	Cultivars	Chromosomes/scaffolds	Physical positions (bp)	SNPs
22931	CakSNP22931	*Kabuli*	Ca_Kabuli_denovo	19430159	(G/A)
22932	CakSNP22932	*Kabuli*	Ca_Kabuli_denovo	19430182	(C/A)
22933	CakSNP22933	*Kabuli*	Ca_Kabuli_denovo	19432428	(G/T)
22934	CakSNP22934	*Kabuli*	Ca_Kabuli_denovo	19434335	(A/G)
22935	CakSNP22935	*Kabuli*	Ca_Kabuli_denovo	19435393	(G/T)
22936	CakSNP22936	*Kabuli*	Ca_Kabuli_denovo	19435406	(T/C)
22937	CakSNP22937	*Kabuli*	Ca_Kabuli_denovo	19446380	(G/C)
22938	CakSNP22938	*Kabuli*	Ca_Kabuli_denovo	19449849	(A/G)
22939	CakSNP22939	*Kabuli*	Ca_Kabuli_denovo	19459653	(A/G)
22940	CakSNP22940	*Kabuli*	Ca_Kabuli_denovo	19459666	(G/C)
22941	CakSNP22941	*Kabuli*	Ca_Kabuli_denovo	19461469	(A/G)
22942	CakSNP22942	*Kabuli*	Ca_Kabuli_denovo	19467652	(C/G)
22943	CakSNP22943	*Kabuli*	Ca_Kabuli_denovo	19482253	(T/C)
22944	CakSNP22944	*Kabuli*	Ca_Kabuli_denovo	19482283	(G/C)
22945	CakSNP22945	*Kabuli*	Ca_Kabuli_denovo	19490764	(A/G)
22946	CakSNP22946	*Kabuli*	Ca_Kabuli_denovo	19490794	(G/A)
22947	CakSNP22947	*Kabuli*	Ca_Kabuli_denovo	19494880	(C/T)
22948	CakSNP22948	*Kabuli*	Ca_Kabuli_denovo	19496641	(C/G)
22949	CakSNP22949	*Kabuli*	Ca_Kabuli_denovo	19496704	(C/T)
22950	CakSNP22950	*Kabuli*	Ca_Kabuli_denovo	19497856	(G/A)
22951	CakSNP22951	*Kabuli*	Ca_Kabuli_denovo	19497910	(C/T)
22952	CakSNP22952	*Kabuli*	Ca_Kabuli_denovo	19499330	(G/T)
22953	CakSNP22953	*Kabuli*	Ca_Kabuli_denovo	19499333	(A/G)
22954	CakSNP22954	*Kabuli*	Ca_Kabuli_denovo	19500292	(C/T)
22955	CakSNP22955	*Kabuli*	Ca_Kabuli_denovo	19500454	(A/G)
22956	CakSNP22956	*Kabuli*	Ca_Kabuli_denovo	19500513	(G/A)
22957	CakSNP22957	*Kabuli*	Ca_Kabuli_denovo	19504817	(C/T)
22958	CakSNP22958	*Kabuli*	Ca_Kabuli_denovo	19504856	(T/G)
22959	CakSNP22959	*Kabuli*	Ca_Kabuli_denovo	19505980	(A/G)
22960	CakSNP22960	*Kabuli*	Ca_Kabuli_denovo	19508060	(C/T)
22961	CakSNP22961	*Kabuli*	Ca_Kabuli_denovo	19508077	(G/T)
22962	CakSNP22962	*Kabuli*	Ca_Kabuli_denovo	19508114	(A/G)
22963	CakSNP22963	*Kabuli*	Ca_Kabuli_denovo	19509112	(C/A)
22964	CakSNP22964	*Kabuli*	Ca_Kabuli_denovo	19515877	(T/C)
22965	CakSNP22965	*Kabuli*	Ca_Kabuli_denovo	19519198	(A/G)
22966	CakSNP22966	*Kabuli*	Ca_Kabuli_denovo	19519203	(G/A)
22967	CakSNP22967	*Kabuli*	Ca_Kabuli_denovo	19521213	(C/G)
22968	CakSNP22968	*Kabuli*	Ca_Kabuli_denovo	19521958	(T/A)
22969	CakSNP22969	*Kabuli*	Ca_Kabuli_denovo	19521965	(A/G)

S.N.	SNP IDs	Cultivars	Chromosomes/scaffolds	Physical positions (bp)	SNPs
22970	CakSNP22970	*Kabuli*	Ca_Kabuli_denovo	19525451	(G/A)
22971	CakSNP22971	*Kabuli*	Ca_Kabuli_denovo	19526859	(G/C)
22972	CakSNP22972	*Kabuli*	Ca_Kabuli_denovo	19526871	(T/C)
22973	CakSNP22973	*Kabuli*	Ca_Kabuli_denovo	19532983	(T/C)
22974	CakSNP22974	*Kabuli*	Ca_Kabuli_denovo	19537330	(A/G)
22975	CakSNP22975	*Kabuli*	Ca_Kabuli_denovo	19547897	(G/A)
22976	CakSNP22976	*Kabuli*	Ca_Kabuli_denovo	19549075	(C/T)
22977	CakSNP22977	*Kabuli*	Ca_Kabuli_denovo	19549145	(A/G)
22978	CakSNP22978	*Kabuli*	Ca_Kabuli_denovo	19554267	(A/G)
22979	CakSNP22979	*Kabuli*	Ca_Kabuli_denovo	19558394	(C/T)
22980	CakSNP22980	*Kabuli*	Ca_Kabuli_denovo	19558883	(A/G)
22981	CakSNP22981	*Kabuli*	Ca_Kabuli_denovo	19559530	(T/C)
22982	CakSNP22982	*Kabuli*	Ca_Kabuli_denovo	19569592	(C/G)
22983	CakSNP22983	*Kabuli*	Ca_Kabuli_denovo	19578021	(C/T)
22984	CakSNP22984	*Kabuli*	Ca_Kabuli_denovo	19580031	(C/G)
22985	CakSNP22985	*Kabuli*	Ca_Kabuli_denovo	19583318	(G/A)
22986	CakSNP22986	*Kabuli*	Ca_Kabuli_denovo	19584237	(C/G)
22987	CakSNP22987	*Kabuli*	Ca_Kabuli_denovo	19592563	(A/C)
22988	CakSNP22988	*Kabuli*	Ca_Kabuli_denovo	19594005	(A/T)
22989	CakSNP22989	*Kabuli*	Ca_Kabuli_denovo	19594048	(G/C)
22990	CakSNP22990	*Kabuli*	Ca_Kabuli_denovo	19596700	(C/G)
22991	CakSNP22991	*Kabuli*	Ca_Kabuli_denovo	19596703	(G/A)
22992	CakSNP22992	*Kabuli*	Ca_Kabuli_denovo	19596726	(G/C)
22993	CakSNP22993	*Kabuli*	Ca_Kabuli_denovo	19599063	(G/A)
22994	CakSNP22994	*Kabuli*	Ca_Kabuli_denovo	19607491	(A/C)
22995	CakSNP22995	*Kabuli*	Ca_Kabuli_denovo	19607505	(G/C)
22996	CakSNP22996	*Kabuli*	Ca_Kabuli_denovo	19607787	(T/C)
22997	CakSNP22997	*Kabuli*	Ca_Kabuli_denovo	19607832	(G/A)
22998	CakSNP22998	*Kabuli*	Ca_Kabuli_denovo	19609996	(C/T)
22999	CakSNP22999	*Kabuli*	Ca_Kabuli_denovo	19610895	(C/G)
23000	CakSNP23000	*Kabuli*	Ca_Kabuli_denovo	19613068	(C/T)
23001	CakSNP23001	*Kabuli*	Ca_Kabuli_denovo	19629262	(A/C)
23002	CakSNP23002	*Kabuli*	Ca_Kabuli_denovo	19630852	(G/A)
23003	CakSNP23003	*Kabuli*	Ca_Kabuli_denovo	19632166	(T/C)
23004	CakSNP23004	*Kabuli*	Ca_Kabuli_denovo	19632919	(C/T)
23005	CakSNP23005	*Kabuli*	Ca_Kabuli_denovo	19634087	(T/C)
23006	CakSNP23006	*Kabuli*	Ca_Kabuli_denovo	19640222	(A/G)
23007	CakSNP23007	*Kabuli*	Ca_Kabuli_denovo	19640228	(C/T)
23008	CakSNP23008	*Kabuli*	Ca_Kabuli_denovo	19645993	(C/T)

S.N.	SNP IDs	Cultivars	Chromosomes/scaffolds	Physical positions (bp)	SNPs
23009	CakSNP23009	*Kabuli*	Ca_Kabuli_denovo	19652525	(G/A)
23010	CakSNP23010	*Kabuli*	Ca_Kabuli_denovo	19657004	(G/A)
23011	CakSNP23011	*Kabuli*	Ca_Kabuli_denovo	19658542	(T/C)
23012	CakSNP23012	*Kabuli*	Ca_Kabuli_denovo	19659001	(T/C)
23013	CakSNP23013	*Kabuli*	Ca_Kabuli_denovo	19661154	(C/G)
23014	CakSNP23014	*Kabuli*	Ca_Kabuli_denovo	19664674	(T/A)
23015	CakSNP23015	*Kabuli*	Ca_Kabuli_denovo	19664737	(T/G)
23016	CakSNP23016	*Kabuli*	Ca_Kabuli_denovo	19666531	(A/C)
23017	CakSNP23017	*Kabuli*	Ca_Kabuli_denovo	19666855	(A/G)
23018	CakSNP23018	*Kabuli*	Ca_Kabuli_denovo	19666861	(C/A)
23019	CakSNP23019	*Kabuli*	Ca_Kabuli_denovo	19670099	(G/C)
23020	CakSNP23020	*Kabuli*	Ca_Kabuli_denovo	19687054	(T/C)
23021	CakSNP23021	*Kabuli*	Ca_Kabuli_denovo	19692882	(G/T)
23022	CakSNP23022	*Kabuli*	Ca_Kabuli_denovo	19692883	(A/C)
23023	CakSNP23023	*Kabuli*	Ca_Kabuli_denovo	19694833	(A/G)
23024	CakSNP23024	*Kabuli*	Ca_Kabuli_denovo	19694872	(G/T)
23025	CakSNP23025	*Kabuli*	Ca_Kabuli_denovo	19695841	(C/T)
23026	CakSNP23026	*Kabuli*	Ca_Kabuli_denovo	19695891	(C/T)
23027	CakSNP23027	*Kabuli*	Ca_Kabuli_denovo	19699736	(G/A)
23028	CakSNP23028	*Kabuli*	Ca_Kabuli_denovo	19713310	(A/G)
23029	CakSNP23029	*Kabuli*	Ca_Kabuli_denovo	19713350	(T/C)
23030	CakSNP23030	*Kabuli*	Ca_Kabuli_denovo	19715189	(C/G)
23031	CakSNP23031	*Kabuli*	Ca_Kabuli_denovo	19715195	(C/T)
23032	CakSNP23032	*Kabuli*	Ca_Kabuli_denovo	19720566	(C/G)
23033	CakSNP23033	*Kabuli*	Ca_Kabuli_denovo	19720598	(T/A)
23034	CakSNP23034	*Kabuli*	Ca_Kabuli_denovo	19721704	(G/A)
23035	CakSNP23035	*Kabuli*	Ca_Kabuli_denovo	19721717	(C/T)
23036	CakSNP23036	*Kabuli*	Ca_Kabuli_denovo	19728777	(G/A)
23037	CakSNP23037	*Kabuli*	Ca_Kabuli_denovo	19728792	(A/G)
23038	CakSNP23038	*Kabuli*	Ca_Kabuli_denovo	19731087	(C/G)
23039	CakSNP23039	*Kabuli*	Ca_Kabuli_denovo	19734795	(G/A)
23040	CakSNP23040	*Kabuli*	Ca_Kabuli_denovo	19734828	(C/G)
23041	CakSNP23041	*Kabuli*	Ca_Kabuli_denovo	19738169	(A/C)
23042	CakSNP23042	*Kabuli*	Ca_Kabuli_denovo	19741005	(T/G)
23043	CakSNP23043	*Kabuli*	Ca_Kabuli_denovo	19744691	(C/T)
23044	CakSNP23044	*Kabuli*	Ca_Kabuli_denovo	19750612	(G/A)
23045	CakSNP23045	*Kabuli*	Ca_Kabuli_denovo	19756173	(A/G)
23046	CakSNP23046	*Kabuli*	Ca_Kabuli_denovo	19761671	(G/A)
23047	CakSNP23047	*Kabuli*	Ca_Kabuli_denovo	19761695	(A/T)

S.N.	SNP IDs	Cultivars	Chromosomes/scaffolds	Physical positions (bp)	SNPs
23048	CakSNP23048	*Kabuli*	Ca_Kabuli_denovo	19762343	(C/T)
23049	CakSNP23049	*Kabuli*	Ca_Kabuli_denovo	19762346	(T/C)
23050	CakSNP23050	*Kabuli*	Ca_Kabuli_denovo	19764134	(G/C)
23051	CakSNP23051	*Kabuli*	Ca_Kabuli_denovo	19769234	(G/T)
23052	CakSNP23052	*Kabuli*	Ca_Kabuli_denovo	19774332	(C/T)
23053	CakSNP23053	*Kabuli*	Ca_Kabuli_denovo	19776405	(T/G)
23054	CakSNP23054	*Kabuli*	Ca_Kabuli_denovo	19776889	(T/G)
23055	CakSNP23055	*Kabuli*	Ca_Kabuli_denovo	19781120	(C/T)
23056	CakSNP23056	*Kabuli*	Ca_Kabuli_denovo	19788782	(A/G)
23057	CakSNP23057	*Kabuli*	Ca_Kabuli_denovo	19788806	(C/G)
23058	CakSNP23058	*Kabuli*	Ca_Kabuli_denovo	19793263	(G/A)
23059	CakSNP23059	*Kabuli*	Ca_Kabuli_denovo	19794417	(A/G)
23060	CakSNP23060	*Kabuli*	Ca_Kabuli_denovo	19797482	(C/T)
23061	CakSNP23061	*Kabuli*	Ca_Kabuli_denovo	19797497	(T/C)
23062	CakSNP23062	*Kabuli*	Ca_Kabuli_denovo	19806614	(T/G)
23063	CakSNP23063	*Kabuli*	Ca_Kabuli_denovo	19806651	(G/C)
23064	CakSNP23064	*Kabuli*	Ca_Kabuli_denovo	19808095	(A/C)
23065	CakSNP23065	*Kabuli*	Ca_Kabuli_denovo	19808101	(A/G)
23066	CakSNP23066	*Kabuli*	Ca_Kabuli_denovo	19814186	(C/A)
23067	CakSNP23067	*Kabuli*	Ca_Kabuli_denovo	19825655	(A/G)
23068	CakSNP23068	*Kabuli*	Ca_Kabuli_denovo	19825684	(C/G)
23069	CakSNP23069	*Kabuli*	Ca_Kabuli_denovo	19831346	(T/C)
23070	CakSNP23070	*Kabuli*	Ca_Kabuli_denovo	19831394	(A/G)
23071	CakSNP23071	*Kabuli*	Ca_Kabuli_denovo	19833191	(C/T)
23072	CakSNP23072	*Kabuli*	Ca_Kabuli_denovo	19835711	(C/T)
23073	CakSNP23073	*Kabuli*	Ca_Kabuli_denovo	19837847	(T/C)
23074	CakSNP23074	*Kabuli*	Ca_Kabuli_denovo	19837862	(C/A)
23075	CakSNP23075	*Kabuli*	Ca_Kabuli_denovo	19838759	(T/C)
23076	CakSNP23076	*Kabuli*	Ca_Kabuli_denovo	19842335	(T/G)
23077	CakSNP23077	*Kabuli*	Ca_Kabuli_denovo	19842348	(G/A)
23078	CakSNP23078	*Kabuli*	Ca_Kabuli_denovo	19848450	(G/C)
23079	CakSNP23079	*Kabuli*	Ca_Kabuli_denovo	19848471	(T/C)
23080	CakSNP23080	*Kabuli*	Ca_Kabuli_denovo	19852642	(C/T)
23081	CakSNP23081	*Kabuli*	Ca_Kabuli_denovo	19852670	(C/G)
23082	CakSNP23082	*Kabuli*	Ca_Kabuli_denovo	19857655	(C/T)
23083	CakSNP23083	*Kabuli*	Ca_Kabuli_denovo	19857667	(T/C)
23084	CakSNP23084	*Kabuli*	Ca_Kabuli_denovo	19858311	(C/G)
23085	CakSNP23085	*Kabuli*	Ca_Kabuli_denovo	19858321	(G/C)
23086	CakSNP23086	*Kabuli*	Ca_Kabuli_denovo	19866905	(G/C)

S.N.	SNP IDs	Cultivars	Chromosomes/scaffolds	Physical positions (bp)	SNPs
23087	CakSNP23087	*Kabuli*	Ca_Kabuli_denovo	19873840	(C/A)
23088	CakSNP23088	*Kabuli*	Ca_Kabuli_denovo	19873946	(T/C)
23089	CakSNP23089	*Kabuli*	Ca_Kabuli_denovo	19875607	(T/C)
23090	CakSNP23090	*Kabuli*	Ca_Kabuli_denovo	19878301	(T/A)
23091	CakSNP23091	*Kabuli*	Ca_Kabuli_denovo	19878370	(C/T)
23092	CakSNP23092	*Kabuli*	Ca_Kabuli_denovo	19879644	(A/G)
23093	CakSNP23093	*Kabuli*	Ca_Kabuli_denovo	19879658	(C/T)
23094	CakSNP23094	*Kabuli*	Ca_Kabuli_denovo	19880110	(G/A)
23095	CakSNP23095	*Kabuli*	Ca_Kabuli_denovo	19888886	(T/G)
23096	CakSNP23096	*Kabuli*	Ca_Kabuli_denovo	19895426	(C/T)
23097	CakSNP23097	*Kabuli*	Ca_Kabuli_denovo	19895429	(C/T)
23098	CakSNP23098	*Kabuli*	Ca_Kabuli_denovo	19896219	(A/G)
23099	CakSNP23099	*Kabuli*	Ca_Kabuli_denovo	19897592	(A/G)
23100	CakSNP23100	*Kabuli*	Ca_Kabuli_denovo	19898670	(C/T)
23101	CakSNP23101	*Kabuli*	Ca_Kabuli_denovo	19900442	(G/A)
23102	CakSNP23102	*Kabuli*	Ca_Kabuli_denovo	19901441	(A/G)
23103	CakSNP23103	*Kabuli*	Ca_Kabuli_denovo	19904162	(G/C)
23104	CakSNP23104	*Kabuli*	Ca_Kabuli_denovo	19906133	(C/T)
23105	CakSNP23105	*Kabuli*	Ca_Kabuli_denovo	19906160	(C/G)
23106	CakSNP23106	*Kabuli*	Ca_Kabuli_denovo	19913298	(A/G)
23107	CakSNP23107	*Kabuli*	Ca_Kabuli_denovo	19913325	(T/G)
23108	CakSNP23108	*Kabuli*	Ca_Kabuli_denovo	19917542	(T/C)
23109	CakSNP23109	*Kabuli*	Ca_Kabuli_denovo	19917611	(T/G)
23110	CakSNP23110	*Kabuli*	Ca_Kabuli_denovo	19917912	(C/T)
23111	CakSNP23111	*Kabuli*	Ca_Kabuli_denovo	19917922	(G/A)
23112	CakSNP23112	*Kabuli*	Ca_Kabuli_denovo	19919217	(T/C)
23113	CakSNP23113	*Kabuli*	Ca_Kabuli_denovo	19919234	(G/C)
23114	CakSNP23114	*Kabuli*	Ca_Kabuli_denovo	19921011	(C/A)
23115	CakSNP23115	*Kabuli*	Ca_Kabuli_denovo	19921041	(A/G)
23116	CakSNP23116	*Kabuli*	Ca_Kabuli_denovo	19926525	(G/C)
23117	CakSNP23117	*Kabuli*	Ca_Kabuli_denovo	19931580	(C/A)
23118	CakSNP23118	*Kabuli*	Ca_Kabuli_denovo	19931658	(C/G)
23119	CakSNP23119	*Kabuli*	Ca_Kabuli_denovo	19937324	(G/A)
23120	CakSNP23120	*Kabuli*	Ca_Kabuli_denovo	19937333	(G/C)
23121	CakSNP23121	*Kabuli*	Ca_Kabuli_denovo	19938269	(C/T)
23122	CakSNP23122	*Kabuli*	Ca_Kabuli_denovo	19942267	(A/C)
23123	CakSNP23123	*Kabuli*	Ca_Kabuli_denovo	19945803	(G/T)
23124	CakSNP23124	*Kabuli*	Ca_Kabuli_denovo	19948365	(C/G)
23125	CakSNP23125	*Kabuli*	Ca_Kabuli_denovo	19948387	(C/A)

S.N.	SNP IDs	Cultivars	Chromosomes/scaffolds	Physical positions (bp)	SNPs
23126	CakSNP23126	*Kabuli*	Ca_Kabuli_denovo	19948522	(C/T)
23127	CakSNP23127	*Kabuli*	Ca_Kabuli_denovo	19948538	(T/A)
23128	CakSNP23128	*Kabuli*	Ca_Kabuli_denovo	19952071	(T/A)
23129	CakSNP23129	*Kabuli*	Ca_Kabuli_denovo	19953836	(C/T)
23130	CakSNP23130	*Kabuli*	Ca_Kabuli_denovo	19953839	(T/G)
23131	CakSNP23131	*Kabuli*	Ca_Kabuli_denovo	19954188	(C/G)
23132	CakSNP23132	*Kabuli*	Ca_Kabuli_denovo	19955948	(G/C)
23133	CakSNP23133	*Kabuli*	Ca_Kabuli_denovo	19956797	(C/T)
23134	CakSNP23134	*Kabuli*	Ca_Kabuli_denovo	19958097	(C/G)
23135	CakSNP23135	*Kabuli*	Ca_Kabuli_denovo	19958902	(A/G)
23136	CakSNP23136	*Kabuli*	Ca_Kabuli_denovo	19963409	(G/A)
23137	CakSNP23137	*Kabuli*	Ca_Kabuli_denovo	19968457	(G/A)
23138	CakSNP23138	*Kabuli*	Ca_Kabuli_denovo	19975434	(T/A)
23139	CakSNP23139	*Kabuli*	Ca_Kabuli_denovo	19975893	(G/A)
23140	CakSNP23140	*Kabuli*	Ca_Kabuli_denovo	19975905	(G/C)
23141	CakSNP23141	*Kabuli*	Ca_Kabuli_denovo	19984691	(C/T)
23142	CakSNP23142	*Kabuli*	Ca_Kabuli_denovo	19989240	(G/A)
23143	CakSNP23143	*Kabuli*	Ca_Kabuli_denovo	19989273	(A/G)
23144	CakSNP23144	*Kabuli*	Ca_Kabuli_denovo	19997189	(A/C)
23145	CakSNP23145	*Kabuli*	Ca_Kabuli_denovo	19997192	(C/G)
23146	CakSNP23146	*Kabuli*	Ca_Kabuli_denovo	19998340	(T/C)
23147	CakSNP23147	*Kabuli*	Ca_Kabuli_denovo	20000681	(G/A)
23148	CakSNP23148	*Kabuli*	Ca_Kabuli_denovo	20000697	(T/C)
23149	CakSNP23149	*Kabuli*	Ca_Kabuli_denovo	20000708	(T/C)
23150	CakSNP23150	*Kabuli*	Ca_Kabuli_denovo	20005079	(G/A)
23151	CakSNP23151	*Kabuli*	Ca_Kabuli_denovo	20017910	(C/G)
23152	CakSNP23152	*Kabuli*	Ca_Kabuli_denovo	20017928	(T/C)
23153	CakSNP23153	*Kabuli*	Ca_Kabuli_denovo	20018908	(C/G)
23154	CakSNP23154	*Kabuli*	Ca_Kabuli_denovo	20018920	(C/T)
23155	CakSNP23155	*Kabuli*	Ca_Kabuli_denovo	20024896	(C/G)
23156	CakSNP23156	*Kabuli*	Ca_Kabuli_denovo	20024947	(C/A)
23157	CakSNP23157	*Kabuli*	Ca_Kabuli_denovo	20025608	(A/G)
23158	CakSNP23158	*Kabuli*	Ca_Kabuli_denovo	20025654	(G/A)
23159	CakSNP23159	*Kabuli*	Ca_Kabuli_denovo	20026305	(T/C)
23160	CakSNP23160	*Kabuli*	Ca_Kabuli_denovo	20027451	(G/C)
23161	CakSNP23161	*Kabuli*	Ca_Kabuli_denovo	20035598	(C/T)
23162	CakSNP23162	*Kabuli*	Ca_Kabuli_denovo	20035633	(G/C)
23163	CakSNP23163	*Kabuli*	Ca_Kabuli_denovo	20038648	(G/C)
23164	CakSNP23164	*Kabuli*	Ca_Kabuli_denovo	20038704	(A/G)

S.N.	SNP IDs	Cultivars	Chromosomes/scaffolds	Physical positions (bp)	SNPs
23165	CakSNP23165	*Kabuli*	Ca_Kabuli_denovo	20040215	(T/A)
23166	CakSNP23166	*Kabuli*	Ca_Kabuli_denovo	20042344	(T/C)
23167	CakSNP23167	*Kabuli*	Ca_Kabuli_denovo	20044804	(C/G)
23168	CakSNP23168	*Kabuli*	Ca_Kabuli_denovo	20044831	(C/T)
23169	CakSNP23169	*Kabuli*	Ca_Kabuli_denovo	20048966	(G/A)
23170	CakSNP23170	*Kabuli*	Ca_Kabuli_denovo	20056233	(G/C)
23171	CakSNP23171	*Kabuli*	Ca_Kabuli_denovo	20063573	(G/A)
23172	CakSNP23172	*Kabuli*	Ca_Kabuli_denovo	20068026	(G/C)
23173	CakSNP23173	*Kabuli*	Ca_Kabuli_denovo	20069077	(C/G)
23174	CakSNP23174	*Kabuli*	Ca_Kabuli_denovo	20074843	(C/A)
23175	CakSNP23175	*Kabuli*	Ca_Kabuli_denovo	20078987	(G/T)
23176	CakSNP23176	*Kabuli*	Ca_Kabuli_denovo	20081620	(C/A)
23177	CakSNP23177	*Kabuli*	Ca_Kabuli_denovo	20083394	(T/A)
23178	CakSNP23178	*Kabuli*	Ca_Kabuli_denovo	20083396	(T/C)
23179	CakSNP23179	*Kabuli*	Ca_Kabuli_denovo	20085531	(T/C)
23180	CakSNP23180	*Kabuli*	Ca_Kabuli_denovo	20085546	(G/T)
23181	CakSNP23181	*Kabuli*	Ca_Kabuli_denovo	20087213	(T/C)
23182	CakSNP23182	*Kabuli*	Ca_Kabuli_denovo	20087214	(T/G)
23183	CakSNP23183	*Kabuli*	Ca_Kabuli_denovo	20101340	(C/G)
23184	CakSNP23184	*Kabuli*	Ca_Kabuli_denovo	20104647	(A/C)
23185	CakSNP23185	*Kabuli*	Ca_Kabuli_denovo	20104648	(G/C)
23186	CakSNP23186	*Kabuli*	Ca_Kabuli_denovo	20110626	(G/T)
23187	CakSNP23187	*Kabuli*	Ca_Kabuli_denovo	20110644	(C/T)
23188	CakSNP23188	*Kabuli*	Ca_Kabuli_denovo	20116483	(C/T)
23189	CakSNP23189	*Kabuli*	Ca_Kabuli_denovo	20119877	(A/G)
23190	CakSNP23190	*Kabuli*	Ca_Kabuli_denovo	20123545	(T/C)
23191	CakSNP23191	*Kabuli*	Ca_Kabuli_denovo	20123553	(A/G)
23192	CakSNP23192	*Kabuli*	Ca_Kabuli_denovo	20126594	(T/G)
23193	CakSNP23193	*Kabuli*	Ca_Kabuli_denovo	20126610	(G/C)
23194	CakSNP23194	*Kabuli*	Ca_Kabuli_denovo	20129287	(A/G)
23195	CakSNP23195	*Kabuli*	Ca_Kabuli_denovo	20129288	(A/T)
23196	CakSNP23196	*Kabuli*	Ca_Kabuli_denovo	20129305	(C/G)
23197	CakSNP23197	*Kabuli*	Ca_Kabuli_denovo	20131006	(G/A)
23198	CakSNP23198	*Kabuli*	Ca_Kabuli_denovo	20131170	(A/T)
23199	CakSNP23199	*Kabuli*	Ca_Kabuli_denovo	20134798	(A/C)
23200	CakSNP23200	*Kabuli*	Ca_Kabuli_denovo	20136511	(A/G)
23201	CakSNP23201	*Kabuli*	Ca_Kabuli_denovo	20137771	(G/A)
23202	CakSNP23202	*Kabuli*	Ca_Kabuli_denovo	20141248	(C/G)
23203	CakSNP23203	*Kabuli*	Ca_Kabuli_denovo	20142644	(G/A)

S.N.	SNP IDs	Cultivars	Chromosomes/scaffolds	Physical positions (bp)	SNPs
23204	CakSNP23204	*Kabuli*	Ca_Kabuli_denovo	20145625	(A/G)
23205	CakSNP23205	*Kabuli*	Ca_Kabuli_denovo	20145669	(C/G)
23206	CakSNP23206	*Kabuli*	Ca_Kabuli_denovo	20145687	(G/T)
23207	CakSNP23207	*Kabuli*	Ca_Kabuli_denovo	20150919	(C/T)
23208	CakSNP23208	*Kabuli*	Ca_Kabuli_denovo	20152100	(A/T)
23209	CakSNP23209	*Kabuli*	Ca_Kabuli_denovo	20156472	(T/C)
23210	CakSNP23210	*Kabuli*	Ca_Kabuli_denovo	20157125	(G/A)
23211	CakSNP23211	*Kabuli*	Ca_Kabuli_denovo	20157152	(T/C)
23212	CakSNP23212	*Kabuli*	Ca_Kabuli_denovo	20158810	(T/C)
23213	CakSNP23213	*Kabuli*	Ca_Kabuli_denovo	20159644	(T/C)
23214	CakSNP23214	*Kabuli*	Ca_Kabuli_denovo	20159683	(G/T)
23215	CakSNP23215	*Kabuli*	Ca_Kabuli_denovo	20161993	(T/C)
23216	CakSNP23216	*Kabuli*	Ca_Kabuli_denovo	20162803	(C/T)
23217	CakSNP23217	*Kabuli*	Ca_Kabuli_denovo	20162851	(C/T)
23218	CakSNP23218	*Kabuli*	Ca_Kabuli_denovo	20164653	(T/C)
23219	CakSNP23219	*Kabuli*	Ca_Kabuli_denovo	20164656	(G/A)
23220	CakSNP23220	*Kabuli*	Ca_Kabuli_denovo	20170406	(A/G)
23221	CakSNP23221	*Kabuli*	Ca_Kabuli_denovo	20170473	(A/C)
23222	CakSNP23222	*Kabuli*	Ca_Kabuli_denovo	20180858	(T/G)
23223	CakSNP23223	*Kabuli*	Ca_Kabuli_denovo	20194916	(G/C)
23224	CakSNP23224	*Kabuli*	Ca_Kabuli_denovo	20194934	(A/C)
23225	CakSNP23225	*Kabuli*	Ca_Kabuli_denovo	20196346	(T/C)
23226	CakSNP23226	*Kabuli*	Ca_Kabuli_denovo	20200393	(T/C)
23227	CakSNP23227	*Kabuli*	Ca_Kabuli_denovo	20202734	(A/T)
23228	CakSNP23228	*Kabuli*	Ca_Kabuli_denovo	20202761	(A/G)
23229	CakSNP23229	*Kabuli*	Ca_Kabuli_denovo	20212724	(A/G)
23230	CakSNP23230	*Kabuli*	Ca_Kabuli_denovo	20212763	(G/A)
23231	CakSNP23231	*Kabuli*	Ca_Kabuli_denovo	20219887	(G/A)
23232	CakSNP23232	*Kabuli*	Ca_Kabuli_denovo	20220084	(G/A)
23233	CakSNP23233	*Kabuli*	Ca_Kabuli_denovo	20220098	(C/G)
23234	CakSNP23234	*Kabuli*	Ca_Kabuli_denovo	20230591	(C/T)
23235	CakSNP23235	*Kabuli*	Ca_Kabuli_denovo	20230657	(C/T)
23236	CakSNP23236	*Kabuli*	Ca_Kabuli_denovo	20236388	(A/G)
23237	CakSNP23237	*Kabuli*	Ca_Kabuli_denovo	20240633	(T/G)
23238	CakSNP23238	*Kabuli*	Ca_Kabuli_denovo	20240645	(C/G)
23239	CakSNP23239	*Kabuli*	Ca_Kabuli_denovo	20241466	(T/C)
23240	CakSNP23240	*Kabuli*	Ca_Kabuli_denovo	20241469	(G/A)
23241	CakSNP23241	*Kabuli*	Ca_Kabuli_denovo	20244579	(G/A)
23242	CakSNP23242	*Kabuli*	Ca_Kabuli_denovo	20249119	(C/A)

S.N.	SNP IDs	Cultivars	Chromosomes/scaffolds	Physical positions (bp)	SNPs
23243	CakSNP23243	*Kabuli*	Ca_Kabuli_denovo	20249316	(T/C)
23244	CakSNP23244	*Kabuli*	Ca_Kabuli_denovo	20249645	(T/C)
23245	CakSNP23245	*Kabuli*	Ca_Kabuli_denovo	20249668	(C/A)
23246	CakSNP23246	*Kabuli*	Ca_Kabuli_denovo	20257527	(C/G)
23247	CakSNP23247	*Kabuli*	Ca_Kabuli_denovo	20257528	(G/A)
23248	CakSNP23248	*Kabuli*	Ca_Kabuli_denovo	20260076	(C/G)
23249	CakSNP23249	*Kabuli*	Ca_Kabuli_denovo	20260108	(G/A)
23250	CakSNP23250	*Kabuli*	Ca_Kabuli_denovo	20264839	(T/C)
23251	CakSNP23251	*Kabuli*	Ca_Kabuli_denovo	20269376	(C/T)
23252	CakSNP23252	*Kabuli*	Ca_Kabuli_denovo	20269682	(T/G)
23253	CakSNP23253	*Kabuli*	Ca_Kabuli_denovo	20272800	(A/C)
23254	CakSNP23254	*Kabuli*	Ca_Kabuli_denovo	20272866	(C/G)
23255	CakSNP23255	*Kabuli*	Ca_Kabuli_denovo	20274219	(C/T)
23256	CakSNP23256	*Kabuli*	Ca_Kabuli_denovo	20281838	(T/G)
23257	CakSNP23257	*Kabuli*	Ca_Kabuli_denovo	20283791	(G/A)
23258	CakSNP23258	*Kabuli*	Ca_Kabuli_denovo	20286365	(C/T)
23259	CakSNP23259	*Kabuli*	Ca_Kabuli_denovo	20286656	(G/A)
23260	CakSNP23260	*Kabuli*	Ca_Kabuli_denovo	20287498	(C/T)
23261	CakSNP23261	*Kabuli*	Ca_Kabuli_denovo	20287522	(T/C)
23262	CakSNP23262	*Kabuli*	Ca_Kabuli_denovo	20295694	(T/C)
23263	CakSNP23263	*Kabuli*	Ca_Kabuli_denovo	20295877	(A/G)
23264	CakSNP23264	*Kabuli*	Ca_Kabuli_denovo	20297276	(C/G)
23265	CakSNP23265	*Kabuli*	Ca_Kabuli_denovo	20297287	(T/C)
23266	CakSNP23266	*Kabuli*	Ca_Kabuli_denovo	20304659	(G/C)
23267	CakSNP23267	*Kabuli*	Ca_Kabuli_denovo	20307614	(C/T)
23268	CakSNP23268	*Kabuli*	Ca_Kabuli_denovo	20308015	(C/T)
23269	CakSNP23269	*Kabuli*	Ca_Kabuli_denovo	20309478	(T/C)
23270	CakSNP23270	*Kabuli*	Ca_Kabuli_denovo	20309524	(C/T)
23271	CakSNP23271	*Kabuli*	Ca_Kabuli_denovo	20309582	(A/C)
23272	CakSNP23272	*Kabuli*	Ca_Kabuli_denovo	20313491	(T/C)
23273	CakSNP23273	*Kabuli*	Ca_Kabuli_denovo	20317455	(G/T)
23274	CakSNP23274	*Kabuli*	Ca_Kabuli_denovo	20318783	(C/T)
23275	CakSNP23275	*Kabuli*	Ca_Kabuli_denovo	20319883	(T/G)
23276	CakSNP23276	*Kabuli*	Ca_Kabuli_denovo	20321059	(T/G)
23277	CakSNP23277	*Kabuli*	Ca_Kabuli_denovo	20333539	(G/T)
23278	CakSNP23278	*Kabuli*	Ca_Kabuli_denovo	20333593	(G/C)
23279	CakSNP23279	*Kabuli*	Ca_Kabuli_denovo	20338885	(G/A)
23280	CakSNP23280	*Kabuli*	Ca_Kabuli_denovo	20339572	(A/T)
23281	CakSNP23281	*Kabuli*	Ca_Kabuli_denovo	20355762	(C/T)

S.N.	SNP IDs	Cultivars	Chromosomes/scaffolds	Physical positions (bp)	SNPs
23282	CakSNP23282	*Kabuli*	Ca_Kabuli_denovo	20359839	(A/G)
23283	CakSNP23283	*Kabuli*	Ca_Kabuli_denovo	20360563	(C/T)
23284	CakSNP23284	*Kabuli*	Ca_Kabuli_denovo	20362457	(C/T)
23285	CakSNP23285	*Kabuli*	Ca_Kabuli_denovo	20367494	(C/T)
23286	CakSNP23286	*Kabuli*	Ca_Kabuli_denovo	20368379	(A/T)
23287	CakSNP23287	*Kabuli*	Ca_Kabuli_denovo	20370821	(T/C)
23288	CakSNP23288	*Kabuli*	Ca_Kabuli_denovo	20375797	(A/G)
23289	CakSNP23289	*Kabuli*	Ca_Kabuli_denovo	20375808	(G/A)
23290	CakSNP23290	*Kabuli*	Ca_Kabuli_denovo	20380039	(G/C)
23291	CakSNP23291	*Kabuli*	Ca_Kabuli_denovo	20386266	(G/A)
23292	CakSNP23292	*Kabuli*	Ca_Kabuli_denovo	20386332	(A/G)
23293	CakSNP23293	*Kabuli*	Ca_Kabuli_denovo	20393328	(C/T)
23294	CakSNP23294	*Kabuli*	Ca_Kabuli_denovo	20396493	(A/G)
23295	CakSNP23295	*Kabuli*	Ca_Kabuli_denovo	20407673	(G/C)
23296	CakSNP23296	*Kabuli*	Ca_Kabuli_denovo	20407694	(C/G)
23297	CakSNP23297	*Kabuli*	Ca_Kabuli_denovo	20411690	(A/G)
23298	CakSNP23298	*Kabuli*	Ca_Kabuli_denovo	20416894	(G/A)
23299	CakSNP23299	*Kabuli*	Ca_Kabuli_denovo	20416922	(T/C)
23300	CakSNP23300	*Kabuli*	Ca_Kabuli_denovo	20418994	(G/A)
23301	CakSNP23301	*Kabuli*	Ca_Kabuli_denovo	20424469	(A/G)
23302	CakSNP23302	*Kabuli*	Ca_Kabuli_denovo	20424475	(T/G)
23303	CakSNP23303	*Kabuli*	Ca_Kabuli_denovo	20426830	(C/G)
23304	CakSNP23304	*Kabuli*	Ca_Kabuli_denovo	20426890	(G/C)
23305	CakSNP23305	*Kabuli*	Ca_Kabuli_denovo	20450527	(C/T)
23306	CakSNP23306	*Kabuli*	Ca_Kabuli_denovo	20450551	(T/C)
23307	CakSNP23307	*Kabuli*	Ca_Kabuli_denovo	20462740	(T/C)
23308	CakSNP23308	*Kabuli*	Ca_Kabuli_denovo	20463827	(T/C)
23309	CakSNP23309	*Kabuli*	Ca_Kabuli_denovo	20463839	(C/T)
23310	CakSNP23310	*Kabuli*	Ca_Kabuli_denovo	20464124	(C/T)
23311	CakSNP23311	*Kabuli*	Ca_Kabuli_denovo	20464330	(G/T)
23312	CakSNP23312	*Kabuli*	Ca_Kabuli_denovo	20464352	(G/A)
23313	CakSNP23313	*Kabuli*	Ca_Kabuli_denovo	20466839	(C/T)
23314	CakSNP23314	*Kabuli*	Ca_Kabuli_denovo	20471359	(G/C)
23315	CakSNP23315	*Kabuli*	Ca_Kabuli_denovo	20473596	(G/A)
23316	CakSNP23316	*Kabuli*	Ca_Kabuli_denovo	20473597	(T/C)
23317	CakSNP23317	*Kabuli*	Ca_Kabuli_denovo	20474659	(A/G)
23318	CakSNP23318	*Kabuli*	Ca_Kabuli_denovo	20474660	(G/A)
23319	CakSNP23319	*Kabuli*	Ca_Kabuli_denovo	20486435	(C/A)
23320	CakSNP23320	*Kabuli*	Ca_Kabuli_denovo	20486468	(A/G)

S.N.	SNP IDs	Cultivars	Chromosomes/scaffolds	Physical positions (bp)	SNPs
23321	CakSNP23321	*Kabuli*	Ca_Kabuli_denovo	20490102	(C/A)
23322	CakSNP23322	*Kabuli*	Ca_Kabuli_denovo	20501163	(C/T)
23323	CakSNP23323	*Kabuli*	Ca_Kabuli_denovo	20511427	(A/C)
23324	CakSNP23324	*Kabuli*	Ca_Kabuli_denovo	20511808	(G/A)
23325	CakSNP23325	*Kabuli*	Ca_Kabuli_denovo	20516470	(C/T)
23326	CakSNP23326	*Kabuli*	Ca_Kabuli_denovo	20517433	(C/T)
23327	CakSNP23327	*Kabuli*	Ca_Kabuli_denovo	20522644	(C/T)
23328	CakSNP23328	*Kabuli*	Ca_Kabuli_denovo	20522665	(T/A)
23329	CakSNP23329	*Kabuli*	Ca_Kabuli_denovo	20541587	(C/G)
23330	CakSNP23330	*Kabuli*	Ca_Kabuli_denovo	20542211	(A/G)
23331	CakSNP23331	*Kabuli*	Ca_Kabuli_denovo	20546414	(A/G)
23332	CakSNP23332	*Kabuli*	Ca_Kabuli_denovo	20546702	(G/A)
23333	CakSNP23333	*Kabuli*	Ca_Kabuli_denovo	20546714	(G/A)
23334	CakSNP23334	*Kabuli*	Ca_Kabuli_denovo	20549051	(C/G)
23335	CakSNP23335	*Kabuli*	Ca_Kabuli_denovo	20554469	(G/T)
23336	CakSNP23336	*Kabuli*	Ca_Kabuli_denovo	20555467	(T/G)
23337	CakSNP23337	*Kabuli*	Ca_Kabuli_denovo	20555488	(T/C)
23338	CakSNP23338	*Kabuli*	Ca_Kabuli_denovo	20567620	(G/T)
23339	CakSNP23339	*Kabuli*	Ca_Kabuli_denovo	20567674	(G/C)
23340	CakSNP23340	*Kabuli*	Ca_Kabuli_denovo	20568646	(A/G)
23341	CakSNP23341	*Kabuli*	Ca_Kabuli_denovo	20569289	(G/A)
23342	CakSNP23342	*Kabuli*	Ca_Kabuli_denovo	20571818	(T/C)
23343	CakSNP23343	*Kabuli*	Ca_Kabuli_denovo	20571867	(C/A)
23344	CakSNP23344	*Kabuli*	Ca_Kabuli_denovo	20577212	(G/A)
23345	CakSNP23345	*Kabuli*	Ca_Kabuli_denovo	20577218	(C/T)
23346	CakSNP23346	*Kabuli*	Ca_Kabuli_denovo	20581578	(G/A)
23347	CakSNP23347	*Kabuli*	Ca_Kabuli_denovo	20584509	(T/C)
23348	CakSNP23348	*Kabuli*	Ca_Kabuli_denovo	20588181	(G/C)
23349	CakSNP23349	*Kabuli*	Ca_Kabuli_denovo	20588184	(T/C)
23350	CakSNP23350	*Kabuli*	Ca_Kabuli_denovo	20588933	(A/G)
23351	CakSNP23351	*Kabuli*	Ca_Kabuli_denovo	20588955	(C/T)
23352	CakSNP23352	*Kabuli*	Ca_Kabuli_denovo	20591069	(C/T)
23353	CakSNP23353	*Kabuli*	Ca_Kabuli_denovo	20591637	(G/A)
23354	CakSNP23354	*Kabuli*	Ca_Kabuli_denovo	20591679	(C/G)
23355	CakSNP23355	*Kabuli*	Ca_Kabuli_denovo	20597291	(C/T)
23356	CakSNP23356	*Kabuli*	Ca_Kabuli_denovo	20603510	(C/G)
23357	CakSNP23357	*Kabuli*	Ca_Kabuli_denovo	20608014	(T/C)
23358	CakSNP23358	*Kabuli*	Ca_Kabuli_denovo	20608016	(G/C)
23359	CakSNP23359	*Kabuli*	Ca_Kabuli_denovo	20608286	(G/A)

S.N.	SNP IDs	Cultivars	Chromosomes/scaffolds	Physical positions (bp)	SNPs
23360	CakSNP23360	*Kabuli*	Ca_Kabuli_denovo	20608691	(A/G)
23361	CakSNP23361	*Kabuli*	Ca_Kabuli_denovo	20610844	(G/A)
23362	CakSNP23362	*Kabuli*	Ca_Kabuli_denovo	20618225	(C/A)
23363	CakSNP23363	*Kabuli*	Ca_Kabuli_denovo	20620363	(T/C)
23364	CakSNP23364	*Kabuli*	Ca_Kabuli_denovo	20621301	(A/G)
23365	CakSNP23365	*Kabuli*	Ca_Kabuli_denovo	20622012	(G/A)
23366	CakSNP23366	*Kabuli*	Ca_Kabuli_denovo	20624246	(T/C)
23367	CakSNP23367	*Kabuli*	Ca_Kabuli_denovo	20624249	(A/G)
23368	CakSNP23368	*Kabuli*	Ca_Kabuli_denovo	20626274	(T/C)
23369	CakSNP23369	*Kabuli*	Ca_Kabuli_denovo	20626284	(G/A)
23370	CakSNP23370	*Kabuli*	Ca_Kabuli_denovo	20629298	(G/A)
23371	CakSNP23371	*Kabuli*	Ca_Kabuli_denovo	20629762	(T/C)
23372	CakSNP23372	*Kabuli*	Ca_Kabuli_denovo	20629816	(C/T)
23373	CakSNP23373	*Kabuli*	Ca_Kabuli_denovo	20629867	(T/C)
23374	CakSNP23374	*Kabuli*	Ca_Kabuli_denovo	20631581	(A/C)
23375	CakSNP23375	*Kabuli*	Ca_Kabuli_denovo	20632830	(A/T)
23376	CakSNP23376	*Kabuli*	Ca_Kabuli_denovo	20632874	(G/A)
23377	CakSNP23377	*Kabuli*	Ca_Kabuli_denovo	20644947	(G/A)
23378	CakSNP23378	*Kabuli*	Ca_Kabuli_denovo	20647462	(G/A)
23379	CakSNP23379	*Kabuli*	Ca_Kabuli_denovo	20647463	(T/A)
23380	CakSNP23380	*Kabuli*	Ca_Kabuli_denovo	20649939	(C/T)
23381	CakSNP23381	*Kabuli*	Ca_Kabuli_denovo	20649985	(G/A)
23382	CakSNP23382	*Kabuli*	Ca_Kabuli_denovo	20651807	(T/C)
23383	CakSNP23383	*Kabuli*	Ca_Kabuli_denovo	20652253	(G/A)
23384	CakSNP23384	*Kabuli*	Ca_Kabuli_denovo	20652319	(C/G)
23385	CakSNP23385	*Kabuli*	Ca_Kabuli_denovo	20655181	(A/G)
23386	CakSNP23386	*Kabuli*	Ca_Kabuli_denovo	20655205	(C/T)
23387	CakSNP23387	*Kabuli*	Ca_Kabuli_denovo	20658706	(G/C)
23388	CakSNP23388	*Kabuli*	Ca_Kabuli_denovo	20659695	(A/G)
23389	CakSNP23389	*Kabuli*	Ca_Kabuli_denovo	20660543	(A/G)
23390	CakSNP23390	*Kabuli*	Ca_Kabuli_denovo	20661734	(G/A)
23391	CakSNP23391	*Kabuli*	Ca_Kabuli_denovo	20663129	(C/T)
23392	CakSNP23392	*Kabuli*	Ca_Kabuli_denovo	20663431	(A/G)
23393	CakSNP23393	*Kabuli*	Ca_Kabuli_denovo	20663481	(G/C)
23394	CakSNP23394	*Kabuli*	Ca_Kabuli_denovo	20664732	(A/G)
23395	CakSNP23395	*Kabuli*	Ca_Kabuli_denovo	20666474	(G/A)
23396	CakSNP23396	*Kabuli*	Ca_Kabuli_denovo	20666533	(T/C)
23397	CakSNP23397	*Kabuli*	Ca_Kabuli_denovo	20671435	(G/T)
23398	CakSNP23398	*Kabuli*	Ca_Kabuli_denovo	20671438	(T/C)

S.N.	SNP IDs	Cultivars	Chromosomes/scaffolds	Physical positions (bp)	SNPs
23399	CakSNP23399	*Kabuli*	Ca_Kabuli_denovo	20671699	(G/A)
23400	CakSNP23400	*Kabuli*	Ca_Kabuli_denovo	20674290	(T/C)
23401	CakSNP23401	*Kabuli*	Ca_Kabuli_denovo	20674470	(G/T)
23402	CakSNP23402	*Kabuli*	Ca_Kabuli_denovo	20674471	(G/T)
23403	CakSNP23403	*Kabuli*	Ca_Kabuli_denovo	20674987	(G/T)
23404	CakSNP23404	*Kabuli*	Ca_Kabuli_denovo	20675794	(T/G)
23405	CakSNP23405	*Kabuli*	Ca_Kabuli_denovo	20675819	(T/G)
23406	CakSNP23406	*Kabuli*	Ca_Kabuli_denovo	20676549	(T/C)
23407	CakSNP23407	*Kabuli*	Ca_Kabuli_denovo	20679128	(T/G)
23408	CakSNP23408	*Kabuli*	Ca_Kabuli_denovo	20681826	(C/T)
23409	CakSNP23409	*Kabuli*	Ca_Kabuli_denovo	20681874	(G/C)
23410	CakSNP23410	*Kabuli*	Ca_Kabuli_denovo	20682581	(T/C)
23411	CakSNP23411	*Kabuli*	Ca_Kabuli_denovo	20682638	(C/T)
23412	CakSNP23412	*Kabuli*	Ca_Kabuli_denovo	20692335	(G/C)
23413	CakSNP23413	*Kabuli*	Ca_Kabuli_denovo	20692407	(A/C)
23414	CakSNP23414	*Kabuli*	Ca_Kabuli_denovo	20693892	(G/T)
23415	CakSNP23415	*Kabuli*	Ca_Kabuli_denovo	20697315	(A/G)
23416	CakSNP23416	*Kabuli*	Ca_Kabuli_denovo	20698586	(G/T)
23417	CakSNP23417	*Kabuli*	Ca_Kabuli_denovo	20702486	(G/C)
23418	CakSNP23418	*Kabuli*	Ca_Kabuli_denovo	20702493	(C/G)
23419	CakSNP23419	*Kabuli*	Ca_Kabuli_denovo	20706779	(G/T)
23420	CakSNP23420	*Kabuli*	Ca_Kabuli_denovo	20706816	(A/T)
23421	CakSNP23421	*Kabuli*	Ca_Kabuli_denovo	20710940	(G/A)
23422	CakSNP23422	*Kabuli*	Ca_Kabuli_denovo	20712713	(G/C)
23423	CakSNP23423	*Kabuli*	Ca_Kabuli_denovo	20712728	(C/T)
23424	CakSNP23424	*Kabuli*	Ca_Kabuli_denovo	20713275	(G/A)
23425	CakSNP23425	*Kabuli*	Ca_Kabuli_denovo	20715271	(G/A)
23426	CakSNP23426	*Kabuli*	Ca_Kabuli_denovo	20720557	(A/G)
23427	CakSNP23427	*Kabuli*	Ca_Kabuli_denovo	20723873	(C/G)
23428	CakSNP23428	*Kabuli*	Ca_Kabuli_denovo	20723876	(A/G)
23429	CakSNP23429	*Kabuli*	Ca_Kabuli_denovo	20725341	(A/G)
23430	CakSNP23430	*Kabuli*	Ca_Kabuli_denovo	20725359	(C/G)
23431	CakSNP23431	*Kabuli*	Ca_Kabuli_denovo	20727759	(C/G)
23432	CakSNP23432	*Kabuli*	Ca_Kabuli_denovo	20727764	(G/C)
23433	CakSNP23433	*Kabuli*	Ca_Kabuli_denovo	20740597	(A/G)
23434	CakSNP23434	*Kabuli*	Ca_Kabuli_denovo	20740642	(C/G)
23435	CakSNP23435	*Kabuli*	Ca_Kabuli_denovo	20743843	(A/G)
23436	CakSNP23436	*Kabuli*	Ca_Kabuli_denovo	20760598	(T/G)
23437	CakSNP23437	*Kabuli*	Ca_Kabuli_denovo	20765039	(A/C)

S.N.	SNP IDs	Cultivars	Chromosomes/scaffolds	Physical positions (bp)	SNPs
23438	CakSNP23438	*Kabuli*	Ca_Kabuli_denovo	20765041	(A/G)
23439	CakSNP23439	*Kabuli*	Ca_Kabuli_denovo	20773882	(C/A)
23440	CakSNP23440	*Kabuli*	Ca_Kabuli_denovo	20777272	(G/T)
23441	CakSNP23441	*Kabuli*	Ca_Kabuli_denovo	20777274	(T/C)
23442	CakSNP23442	*Kabuli*	Ca_Kabuli_denovo	20782360	(T/C)
23443	CakSNP23443	*Kabuli*	Ca_Kabuli_denovo	20789033	(T/C)
23444	CakSNP23444	*Kabuli*	Ca_Kabuli_denovo	20790116	(A/C)
23445	CakSNP23445	*Kabuli*	Ca_Kabuli_denovo	20790127	(C/T)
23446	CakSNP23446	*Kabuli*	Ca_Kabuli_denovo	20800334	(G/C)
23447	CakSNP23447	*Kabuli*	Ca_Kabuli_denovo	20801433	(G/A)
23448	CakSNP23448	*Kabuli*	Ca_Kabuli_denovo	20801457	(A/C)
23449	CakSNP23449	*Kabuli*	Ca_Kabuli_denovo	20806916	(G/A)
23450	CakSNP23450	*Kabuli*	Ca_Kabuli_denovo	20820671	(G/A)
23451	CakSNP23451	*Kabuli*	Ca_Kabuli_denovo	20820733	(T/C)
23452	CakSNP23452	*Kabuli*	Ca_Kabuli_denovo	20822191	(C/G)
23453	CakSNP23453	*Kabuli*	Ca_Kabuli_denovo	20827366	(C/G)
23454	CakSNP23454	*Kabuli*	Ca_Kabuli_denovo	20827408	(C/G)
23455	CakSNP23455	*Kabuli*	Ca_Kabuli_denovo	20828077	(G/T)
23456	CakSNP23456	*Kabuli*	Ca_Kabuli_denovo	20840036	(A/T)
23457	CakSNP23457	*Kabuli*	Ca_Kabuli_denovo	20848569	(C/T)
23458	CakSNP23458	*Kabuli*	Ca_Kabuli_denovo	20850730	(C/G)
23459	CakSNP23459	*Kabuli*	Ca_Kabuli_denovo	20858746	(C/T)
23460	CakSNP23460	*Kabuli*	Ca_Kabuli_denovo	20858776	(G/T)
23461	CakSNP23461	*Kabuli*	Ca_Kabuli_denovo	20859779	(C/G)
23462	CakSNP23462	*Kabuli*	Ca_Kabuli_denovo	20859806	(A/C)
23463	CakSNP23463	*Kabuli*	Ca_Kabuli_denovo	20865497	(C/T)
23464	CakSNP23464	*Kabuli*	Ca_Kabuli_denovo	20869412	(A/G)
23465	CakSNP23465	*Kabuli*	Ca_Kabuli_denovo	20870518	(G/T)
23466	CakSNP23466	*Kabuli*	Ca_Kabuli_denovo	20870556	(T/C)
23467	CakSNP23467	*Kabuli*	Ca_Kabuli_denovo	20877900	(G/C)
23468	CakSNP23468	*Kabuli*	Ca_Kabuli_denovo	20881291	(A/C)
23469	CakSNP23469	*Kabuli*	Ca_Kabuli_denovo	20889434	(C/T)
23470	CakSNP23470	*Kabuli*	Ca_Kabuli_denovo	20889463	(A/G)
23471	CakSNP23471	*Kabuli*	Ca_Kabuli_denovo	20890276	(G/A)
23472	CakSNP23472	*Kabuli*	Ca_Kabuli_denovo	20890735	(C/G)
23473	CakSNP23473	*Kabuli*	Ca_Kabuli_denovo	20891812	(G/T)
23474	CakSNP23474	*Kabuli*	Ca_Kabuli_denovo	20896658	(T/C)
23475	CakSNP23475	*Kabuli*	Ca_Kabuli_denovo	20899817	(G/A)
23476	CakSNP23476	*Kabuli*	Ca_Kabuli_denovo	20899873	(C/T)

S.N.	SNP IDs	Cultivars	Chromosomes/scaffolds	Physical positions (bp)	SNPs
23477	CakSNP23477	*Kabuli*	Ca_Kabuli_denovo	20901410	(G/A)
23478	CakSNP23478	*Kabuli*	Ca_Kabuli_denovo	20902136	(G/T)
23479	CakSNP23479	*Kabuli*	Ca_Kabuli_denovo	20902138	(C/G)
23480	CakSNP23480	*Kabuli*	Ca_Kabuli_denovo	20905216	(C/G)
23481	CakSNP23481	*Kabuli*	Ca_Kabuli_denovo	20909680	(A/G)
23482	CakSNP23482	*Kabuli*	Ca_Kabuli_denovo	20918685	(A/G)
23483	CakSNP23483	*Kabuli*	Ca_Kabuli_denovo	20920301	(T/C)
23484	CakSNP23484	*Kabuli*	Ca_Kabuli_denovo	20927066	(C/T)
23485	CakSNP23485	*Kabuli*	Ca_Kabuli_denovo	20927075	(C/G)
23486	CakSNP23486	*Kabuli*	Ca_Kabuli_denovo	20930235	(A/G)
23487	CakSNP23487	*Kabuli*	Ca_Kabuli_denovo	20931160	(C/G)
23488	CakSNP23488	*Kabuli*	Ca_Kabuli_denovo	20932009	(C/G)
23489	CakSNP23489	*Kabuli*	Ca_Kabuli_denovo	20932069	(A/G)
23490	CakSNP23490	*Kabuli*	Ca_Kabuli_denovo	20935272	(C/T)
23491	CakSNP23491	*Kabuli*	Ca_Kabuli_denovo	20939058	(G/A)
23492	CakSNP23492	*Kabuli*	Ca_Kabuli_denovo	20942369	(T/G)
23493	CakSNP23493	*Kabuli*	Ca_Kabuli_denovo	20943817	(C/G)
23494	CakSNP23494	*Kabuli*	Ca_Kabuli_denovo	20943829	(C/T)
23495	CakSNP23495	*Kabuli*	Ca_Kabuli_denovo	20947352	(G/A)
23496	CakSNP23496	*Kabuli*	Ca_Kabuli_denovo	20952554	(G/C)
23497	CakSNP23497	*Kabuli*	Ca_Kabuli_denovo	20954312	(C/T)
23498	CakSNP23498	*Kabuli*	Ca_Kabuli_denovo	20954315	(T/C)
23499	CakSNP23499	*Kabuli*	Ca_Kabuli_denovo	20956282	(G/C)
23500	CakSNP23500	*Kabuli*	Ca_Kabuli_denovo	20956459	(C/T)
23501	CakSNP23501	*Kabuli*	Ca_Kabuli_denovo	20957959	(T/C)
23502	CakSNP23502	*Kabuli*	Ca_Kabuli_denovo	20959789	(T/A)
23503	CakSNP23503	*Kabuli*	Ca_Kabuli_denovo	20962222	(G/A)
23504	CakSNP23504	*Kabuli*	Ca_Kabuli_denovo	20962285	(A/G)
23505	CakSNP23505	*Kabuli*	Ca_Kabuli_denovo	20967037	(G/A)
23506	CakSNP23506	*Kabuli*	Ca_Kabuli_denovo	20975342	(A/G)
23507	CakSNP23507	*Kabuli*	Ca_Kabuli_denovo	20976175	(G/A)
23508	CakSNP23508	*Kabuli*	Ca_Kabuli_denovo	20976187	(A/G)
23509	CakSNP23509	*Kabuli*	Ca_Kabuli_denovo	20976285	(G/A)
23510	CakSNP23510	*Kabuli*	Ca_Kabuli_denovo	20978728	(G/A)
23511	CakSNP23511	*Kabuli*	Ca_Kabuli_denovo	20980830	(T/C)
23512	CakSNP23512	*Kabuli*	Ca_Kabuli_denovo	20980872	(T/C)
23513	CakSNP23513	*Kabuli*	Ca_Kabuli_denovo	20982688	(A/G)
23514	CakSNP23514	*Kabuli*	Ca_Kabuli_denovo	20982709	(G/T)
23515	CakSNP23515	*Kabuli*	Ca_Kabuli_denovo	20986531	(C/T)

S.N.	SNP IDs	Cultivars	Chromosomes/scaffolds	Physical positions (bp)	SNPs
23516	CakSNP23516	*Kabuli*	Ca_Kabuli_denovo	20987836	(A/G)
23517	CakSNP23517	*Kabuli*	Ca_Kabuli_denovo	20990243	(A/G)
23518	CakSNP23518	*Kabuli*	Ca_Kabuli_denovo	20990278	(C/A)
23519	CakSNP23519	*Kabuli*	Ca_Kabuli_denovo	20994487	(A/G)
23520	CakSNP23520	*Kabuli*	Ca_Kabuli_denovo	20994648	(A/C)
23521	CakSNP23521	*Kabuli*	Ca_Kabuli_denovo	20994656	(T/C)
23522	CakSNP23522	*Kabuli*	Ca_Kabuli_denovo	20996617	(A/G)
23523	CakSNP23523	*Kabuli*	Ca_Kabuli_denovo	21002777	(T/C)
23524	CakSNP23524	*Kabuli*	Ca_Kabuli_denovo	21005000	(G/A)
23525	CakSNP23525	*Kabuli*	Ca_Kabuli_denovo	21005039	(G/A)
23526	CakSNP23526	*Kabuli*	Ca_Kabuli_denovo	21005600	(G/C)
23527	CakSNP23527	*Kabuli*	Ca_Kabuli_denovo	21005601	(C/T)
23528	CakSNP23528	*Kabuli*	Ca_Kabuli_denovo	21008057	(T/G)
23529	CakSNP23529	*Kabuli*	Ca_Kabuli_denovo	21008099	(G/C)
23530	CakSNP23530	*Kabuli*	Ca_Kabuli_denovo	21008633	(A/C)
23531	CakSNP23531	*Kabuli*	Ca_Kabuli_denovo	21008674	(C/G)
23532	CakSNP23532	*Kabuli*	Ca_Kabuli_denovo	21012356	(C/T)
23533	CakSNP23533	*Kabuli*	Ca_Kabuli_denovo	21012365	(C/T)
23534	CakSNP23534	*Kabuli*	Ca_Kabuli_denovo	21017170	(C/G)
23535	CakSNP23535	*Kabuli*	Ca_Kabuli_denovo	21017204	(G/A)
23536	CakSNP23536	*Kabuli*	Ca_Kabuli_denovo	21017243	(A/G)
23537	CakSNP23537	*Kabuli*	Ca_Kabuli_denovo	21020518	(G/A)
23538	CakSNP23538	*Kabuli*	Ca_Kabuli_denovo	21020548	(A/T)
23539	CakSNP23539	*Kabuli*	Ca_Kabuli_denovo	21024546	(T/C)
23540	CakSNP23540	*Kabuli*	Ca_Kabuli_denovo	21030016	(C/T)
23541	CakSNP23541	*Kabuli*	Ca_Kabuli_denovo	21044341	(C/T)
23542	CakSNP23542	*Kabuli*	Ca_Kabuli_denovo	21047977	(T/C)
23543	CakSNP23543	*Kabuli*	Ca_Kabuli_denovo	21061094	(T/A)
23544	CakSNP23544	*Kabuli*	Ca_Kabuli_denovo	21068365	(G/C)
23545	CakSNP23545	*Kabuli*	Ca_Kabuli_denovo	21068404	(G/T)
23546	CakSNP23546	*Kabuli*	Ca_Kabuli_denovo	21071286	(C/T)
23547	CakSNP23547	*Kabuli*	Ca_Kabuli_denovo	21077257	(A/G)
23548	CakSNP23548	*Kabuli*	Ca_Kabuli_denovo	21077281	(T/C)
23549	CakSNP23549	*Kabuli*	Ca_Kabuli_denovo	21082230	(G/T)
23550	CakSNP23550	*Kabuli*	Ca_Kabuli_denovo	21082236	(G/T)
23551	CakSNP23551	*Kabuli*	Ca_Kabuli_denovo	21082241	(T/G)
23552	CakSNP23552	*Kabuli*	Ca_Kabuli_denovo	21082258	(T/G)
23553	CakSNP23553	*Kabuli*	Ca_Kabuli_denovo	21084346	(T/G)
23554	CakSNP23554	*Kabuli*	Ca_Kabuli_denovo	21084718	(T/G)

S.N.	SNP IDs	Cultivars	Chromosomes/scaffolds	Physical positions (bp)	SNPs
23555	CakSNP23555	*Kabuli*	Ca_Kabuli_denovo	21084761	(A/G)
23556	CakSNP23556	*Kabuli*	Ca_Kabuli_denovo	21090649	(T/C)
23557	CakSNP23557	*Kabuli*	Ca_Kabuli_denovo	21090888	(C/G)
23558	CakSNP23558	*Kabuli*	Ca_Kabuli_denovo	21090964	(T/G)
23559	CakSNP23559	*Kabuli*	Ca_Kabuli_denovo	21090985	(G/A)
23560	CakSNP23560	*Kabuli*	Ca_Kabuli_denovo	21100610	(C/T)
23561	CakSNP23561	*Kabuli*	Ca_Kabuli_denovo	21106642	(C/A)
23562	CakSNP23562	*Kabuli*	Ca_Kabuli_denovo	21106699	(A/C)
23563	CakSNP23563	*Kabuli*	Ca_Kabuli_denovo	21110848	(T/C)
23564	CakSNP23564	*Kabuli*	Ca_Kabuli_denovo	21113900	(T/C)
23565	CakSNP23565	*Kabuli*	Ca_Kabuli_denovo	21115327	(A/G)
23566	CakSNP23566	*Kabuli*	Ca_Kabuli_denovo	21115333	(A/G)
23567	CakSNP23567	*Kabuli*	Ca_Kabuli_denovo	21117191	(C/A)
23568	CakSNP23568	*Kabuli*	Ca_Kabuli_denovo	21117193	(A/G)
23569	CakSNP23569	*Kabuli*	Ca_Kabuli_denovo	21126652	(G/A)
23570	CakSNP23570	*Kabuli*	Ca_Kabuli_denovo	21126679	(A/G)
23571	CakSNP23571	*Kabuli*	Ca_Kabuli_denovo	21134870	(G/C)
23572	CakSNP23572	*Kabuli*	Ca_Kabuli_denovo	21144962	(T/A)
23573	CakSNP23573	*Kabuli*	Ca_Kabuli_denovo	21144985	(C/A)
23574	CakSNP23574	*Kabuli*	Ca_Kabuli_denovo	21147449	(T/C)
23575	CakSNP23575	*Kabuli*	Ca_Kabuli_denovo	21150332	(C/T)
23576	CakSNP23576	*Kabuli*	Ca_Kabuli_denovo	21154187	(C/T)
23577	CakSNP23577	*Kabuli*	Ca_Kabuli_denovo	21158231	(C/G)
23578	CakSNP23578	*Kabuli*	Ca_Kabuli_denovo	21160880	(G/A)
23579	CakSNP23579	*Kabuli*	Ca_Kabuli_denovo	21162260	(T/C)
23580	CakSNP23580	*Kabuli*	Ca_Kabuli_denovo	21162266	(A/G)
23581	CakSNP23581	*Kabuli*	Ca_Kabuli_denovo	21169177	(C/T)
23582	CakSNP23582	*Kabuli*	Ca_Kabuli_denovo	21173866	(C/A)
23583	CakSNP23583	*Kabuli*	Ca_Kabuli_denovo	21176854	(A/G)
23584	CakSNP23584	*Kabuli*	Ca_Kabuli_denovo	21176884	(G/A)
23585	CakSNP23585	*Kabuli*	Ca_Kabuli_denovo	21178432	(G/A)
23586	CakSNP23586	*Kabuli*	Ca_Kabuli_denovo	21179437	(A/G)
23587	CakSNP23587	*Kabuli*	Ca_Kabuli_denovo	21179446	(C/G)
23588	CakSNP23588	*Kabuli*	Ca_Kabuli_denovo	21181786	(T/C)
23589	CakSNP23589	*Kabuli*	Ca_Kabuli_denovo	21184619	(T/C)
23590	CakSNP23590	*Kabuli*	Ca_Kabuli_denovo	21184628	(T/C)
23591	CakSNP23591	*Kabuli*	Ca_Kabuli_denovo	21186065	(G/A)
23592	CakSNP23592	*Kabuli*	Ca_Kabuli_denovo	21186539	(G/T)
23593	CakSNP23593	*Kabuli*	Ca_Kabuli_denovo	21190874	(G/T)

S.N.	SNP IDs	Cultivars	Chromosomes/scaffolds	Physical positions (bp)	SNPs
23594	CakSNP23594	*Kabuli*	Ca_Kabuli_denovo	21194997	(C/T)
23595	CakSNP23595	*Kabuli*	Ca_Kabuli_denovo	21199835	(G/C)
23596	CakSNP23596	*Kabuli*	Ca_Kabuli_denovo	21200833	(T/G)
23597	CakSNP23597	*Kabuli*	Ca_Kabuli_denovo	21204235	(G/C)
23598	CakSNP23598	*Kabuli*	Ca_Kabuli_denovo	21208112	(C/A)
23599	CakSNP23599	*Kabuli*	Ca_Kabuli_denovo	21208135	(A/G)
23600	CakSNP23600	*Kabuli*	Ca_Kabuli_denovo	21213971	(C/T)
23601	CakSNP23601	*Kabuli*	Ca_Kabuli_denovo	21221451	(T/G)
23602	CakSNP23602	*Kabuli*	Ca_Kabuli_denovo	21225048	(C/G)
23603	CakSNP23603	*Kabuli*	Ca_Kabuli_denovo	21225421	(G/C)
23604	CakSNP23604	*Kabuli*	Ca_Kabuli_denovo	21231167	(T/G)
23605	CakSNP23605	*Kabuli*	Ca_Kabuli_denovo	21232376	(C/T)
23606	CakSNP23606	*Kabuli*	Ca_Kabuli_denovo	21232394	(C/T)
23607	CakSNP23607	*Kabuli*	Ca_Kabuli_denovo	21244441	(C/T)
23608	CakSNP23608	*Kabuli*	Ca_Kabuli_denovo	21250052	(G/A)
23609	CakSNP23609	*Kabuli*	Ca_Kabuli_denovo	21250842	(G/A)
23610	CakSNP23610	*Kabuli*	Ca_Kabuli_denovo	21251182	(T/G)
23611	CakSNP23611	*Kabuli*	Ca_Kabuli_denovo	21254233	(T/C)
23612	CakSNP23612	*Kabuli*	Ca_Kabuli_denovo	21255077	(G/A)
23613	CakSNP23613	*Kabuli*	Ca_Kabuli_denovo	21255104	(G/C)
23614	CakSNP23614	*Kabuli*	Ca_Kabuli_denovo	21256294	(T/C)
23615	CakSNP23615	*Kabuli*	Ca_Kabuli_denovo	21262614	(C/G)
23616	CakSNP23616	*Kabuli*	Ca_Kabuli_denovo	21262652	(T/C)
23617	CakSNP23617	*Kabuli*	Ca_Kabuli_denovo	21267786	(A/G)
23618	CakSNP23618	*Kabuli*	Ca_Kabuli_denovo	21270928	(G/A)
23619	CakSNP23619	*Kabuli*	Ca_Kabuli_denovo	21271173	(G/A)
23620	CakSNP23620	*Kabuli*	Ca_Kabuli_denovo	21271385	(C/A)
23621	CakSNP23621	*Kabuli*	Ca_Kabuli_denovo	21272775	(A/G)
23622	CakSNP23622	*Kabuli*	Ca_Kabuli_denovo	21277217	(C/T)
23623	CakSNP23623	*Kabuli*	Ca_Kabuli_denovo	21277218	(C/T)
23624	CakSNP23624	*Kabuli*	Ca_Kabuli_denovo	21279072	(C/T)
23625	CakSNP23625	*Kabuli*	Ca_Kabuli_denovo	21279083	(A/G)
23626	CakSNP23626	*Kabuli*	Ca_Kabuli_denovo	21282944	(G/A)
23627	CakSNP23627	*Kabuli*	Ca_Kabuli_denovo	21283436	(C/T)
23628	CakSNP23628	*Kabuli*	Ca_Kabuli_denovo	21284173	(C/G)
23629	CakSNP23629	*Kabuli*	Ca_Kabuli_denovo	21295065	(G/C)
23630	CakSNP23630	*Kabuli*	Ca_Kabuli_denovo	21295070	(T/C)
23631	CakSNP23631	*Kabuli*	Ca_Kabuli_denovo	21296946	(T/A)
23632	CakSNP23632	*Kabuli*	Ca_Kabuli_denovo	21296983	(G/A)

S.N.	SNP IDs	Cultivars	Chromosomes/scaffolds	Physical positions (bp)	SNPs
23633	CakSNP23633	*Kabuli*	Ca_Kabuli_denovo	21298616	(T/C)
23634	CakSNP23634	*Kabuli*	Ca_Kabuli_denovo	21307635	(G/A)
23635	CakSNP23635	*Kabuli*	Ca_Kabuli_denovo	21308677	(T/G)
23636	CakSNP23636	*Kabuli*	Ca_Kabuli_denovo	21318213	(A/C)
23637	CakSNP23637	*Kabuli*	Ca_Kabuli_denovo	21329472	(A/G)
23638	CakSNP23638	*Kabuli*	Ca_Kabuli_denovo	21331823	(C/A)
23639	CakSNP23639	*Kabuli*	Ca_Kabuli_denovo	21331826	(C/G)
23640	CakSNP23640	*Kabuli*	Ca_Kabuli_denovo	21332074	(C/T)
23641	CakSNP23641	*Kabuli*	Ca_Kabuli_denovo	21332076	(A/G)
23642	CakSNP23642	*Kabuli*	Ca_Kabuli_denovo	21341423	(C/T)
23643	CakSNP23643	*Kabuli*	Ca_Kabuli_denovo	21341964	(T/C)
23644	CakSNP23644	*Kabuli*	Ca_Kabuli_denovo	21343141	(C/T)
23645	CakSNP23645	*Kabuli*	Ca_Kabuli_denovo	21344835	(G/C)
23646	CakSNP23646	*Kabuli*	Ca_Kabuli_denovo	21348438	(G/A)
23647	CakSNP23647	*Kabuli*	Ca_Kabuli_denovo	21348450	(T/C)
23648	CakSNP23648	*Kabuli*	Ca_Kabuli_denovo	21349005	(C/A)
23649	CakSNP23649	*Kabuli*	Ca_Kabuli_denovo	21351422	(A/T)
23650	CakSNP23650	*Kabuli*	Ca_Kabuli_denovo	21356826	(T/C)
23651	CakSNP23651	*Kabuli*	Ca_Kabuli_denovo	21356851	(C/G)
23652	CakSNP23652	*Kabuli*	Ca_Kabuli_denovo	21358482	(G/A)
23653	CakSNP23653	*Kabuli*	Ca_Kabuli_denovo	21358524	(C/T)
23654	CakSNP23654	*Kabuli*	Ca_Kabuli_denovo	21361581	(C/T)
23655	CakSNP23655	*Kabuli*	Ca_Kabuli_denovo	21366756	(G/T)
23656	CakSNP23656	*Kabuli*	Ca_Kabuli_denovo	21367515	(G/C)
23657	CakSNP23657	*Kabuli*	Ca_Kabuli_denovo	21367533	(T/C)
23658	CakSNP23658	*Kabuli*	Ca_Kabuli_denovo	21367537	(C/A)
23659	CakSNP23659	*Kabuli*	Ca_Kabuli_denovo	21372130	(G/A)
23660	CakSNP23660	*Kabuli*	Ca_Kabuli_denovo	21377916	(G/A)
23661	CakSNP23661	*Kabuli*	Ca_Kabuli_denovo	21377970	(T/C)
23662	CakSNP23662	*Kabuli*	Ca_Kabuli_denovo	21380467	(C/T)
23663	CakSNP23663	*Kabuli*	Ca_Kabuli_denovo	21381098	(G/A)
23664	CakSNP23664	*Kabuli*	Ca_Kabuli_denovo	21381144	(C/A)
23665	CakSNP23665	*Kabuli*	Ca_Kabuli_denovo	21384365	(C/T)
23666	CakSNP23666	*Kabuli*	Ca_Kabuli_denovo	21384367	(G/A)
23667	CakSNP23667	*Kabuli*	Ca_Kabuli_denovo	21385344	(T/G)
23668	CakSNP23668	*Kabuli*	Ca_Kabuli_denovo	21393480	(A/G)
23669	CakSNP23669	*Kabuli*	Ca_Kabuli_denovo	21393528	(G/A)
23670	CakSNP23670	*Kabuli*	Ca_Kabuli_denovo	21399407	(A/T)
23671	CakSNP23671	*Kabuli*	Ca_Kabuli_denovo	21399412	(G/A)

**S.N.**	**SNP IDs**	**Cultivars**	**Chromosomes/scaffolds**	**Physical positions (bp)**	**SNPs**
23672	CakSNP23672	*Kabuli*	Ca_Kabuli_denovo	21400517	(C/T)
23673	CakSNP23673	*Kabuli*	Ca_Kabuli_denovo	21400573	(C/T)
23674	CakSNP23674	*Kabuli*	Ca_Kabuli_denovo	21407519	(A/G)
23675	CakSNP23675	*Kabuli*	Ca_Kabuli_denovo	21421791	(A/G)
23676	CakSNP23676	*Kabuli*	Ca_Kabuli_denovo	21424958	(T/G)
23677	CakSNP23677	*Kabuli*	Ca_Kabuli_denovo	21440438	(T/G)
23678	CakSNP23678	*Kabuli*	Ca_Kabuli_denovo	21445256	(G/A)
23679	CakSNP23679	*Kabuli*	Ca_Kabuli_denovo	21445286	(A/G)
23680	CakSNP23680	*Kabuli*	Ca_Kabuli_denovo	21451496	(T/C)
23681	CakSNP23681	*Kabuli*	Ca_Kabuli_denovo	21452183	(A/G)
23682	CakSNP23682	*Kabuli*	Ca_Kabuli_denovo	21455208	(T/C)
23683	CakSNP23683	*Kabuli*	Ca_Kabuli_denovo	21455244	(C/A)
23684	CakSNP23684	*Kabuli*	Ca_Kabuli_denovo	21459122	(C/A)
23685	CakSNP23685	*Kabuli*	Ca_Kabuli_denovo	21459187	(G/A)
23686	CakSNP23686	*Kabuli*	Ca_Kabuli_denovo	21467527	(T/G)
23687	CakSNP23687	*Kabuli*	Ca_Kabuli_denovo	21467545	(C/T)
23688	CakSNP23688	*Kabuli*	Ca_Kabuli_denovo	21475924	(A/G)
23689	CakSNP23689	*Kabuli*	Ca_Kabuli_denovo	21478754	(G/A)
23690	CakSNP23690	*Kabuli*	Ca_Kabuli_denovo	21482973	(G/C)
23691	CakSNP23691	*Kabuli*	Ca_Kabuli_denovo	21484952	(C/T)
23692	CakSNP23692	*Kabuli*	Ca_Kabuli_denovo	21490667	(C/T)
23693	CakSNP23693	*Kabuli*	Ca_Kabuli_denovo	21491669	(C/G)
23694	CakSNP23694	*Kabuli*	Ca_Kabuli_denovo	21493435	(C/A)
23695	CakSNP23695	*Kabuli*	Ca_Kabuli_denovo	21501063	(A/G)
23696	CakSNP23696	*Kabuli*	Ca_Kabuli_denovo	21503867	(A/G)
23697	CakSNP23697	*Kabuli*	Ca_Kabuli_denovo	21505441	(A/T)
23698	CakSNP23698	*Kabuli*	Ca_Kabuli_denovo	21506174	(G/A)
23699	CakSNP23699	*Kabuli*	Ca_Kabuli_denovo	21506198	(C/T)
23700	CakSNP23700	*Kabuli*	Ca_Kabuli_denovo	21510879	(G/C)
23701	CakSNP23701	*Kabuli*	Ca_Kabuli_denovo	21512410	(G/A)
23702	CakSNP23702	*Kabuli*	Ca_Kabuli_denovo	21512440	(T/C)
23703	CakSNP23703	*Kabuli*	Ca_Kabuli_denovo	21517339	(T/C)
23704	CakSNP23704	*Kabuli*	Ca_Kabuli_denovo	21517364	(G/A)
23705	CakSNP23705	*Kabuli*	Ca_Kabuli_denovo	21521543	(T/C)
23706	CakSNP23706	*Kabuli*	Ca_Kabuli_denovo	21529901	(C/T)
23707	CakSNP23707	*Kabuli*	Ca_Kabuli_denovo	21533729	(C/A)
23708	CakSNP23708	*Kabuli*	Ca_Kabuli_denovo	21536504	(G/A)
23709	CakSNP23709	*Kabuli*	Ca_Kabuli_denovo	21536561	(C/G)
23710	CakSNP23710	*Kabuli*	Ca_Kabuli_denovo	21536878	(T/C)

S.N.	SNP IDs	Cultivars	Chromosomes/scaffolds	Physical positions (bp)	SNPs
23711	CakSNP23711	*Kabuli*	Ca_Kabuli_denovo	21541280	(C/T)
23712	CakSNP23712	*Kabuli*	Ca_Kabuli_denovo	21541298	(C/T)
23713	CakSNP23713	*Kabuli*	Ca_Kabuli_denovo	21541870	(T/C)
23714	CakSNP23714	*Kabuli*	Ca_Kabuli_denovo	21549651	(C/G)
23715	CakSNP23715	*Kabuli*	Ca_Kabuli_denovo	21558166	(C/T)
23716	CakSNP23716	*Kabuli*	Ca_Kabuli_denovo	21559750	(A/T)
23717	CakSNP23717	*Kabuli*	Ca_Kabuli_denovo	21560733	(T/G)
23718	CakSNP23718	*Kabuli*	Ca_Kabuli_denovo	21561543	(G/T)
23719	CakSNP23719	*Kabuli*	Ca_Kabuli_denovo	21565201	(A/G)
23720	CakSNP23720	*Kabuli*	Ca_Kabuli_denovo	21565213	(A/T)
23721	CakSNP23721	*Kabuli*	Ca_Kabuli_denovo	21576483	(G/A)
23722	CakSNP23722	*Kabuli*	Ca_Kabuli_denovo	21577694	(T/G)
23723	CakSNP23723	*Kabuli*	Ca_Kabuli_denovo	21577730	(G/A)
23724	CakSNP23724	*Kabuli*	Ca_Kabuli_denovo	21577736	(A/G)
23725	CakSNP23725	*Kabuli*	Ca_Kabuli_denovo	21577930	(G/T)
23726	CakSNP23726	*Kabuli*	Ca_Kabuli_denovo	21587730	(C/A)
23727	CakSNP23727	*Kabuli*	Ca_Kabuli_denovo	21587742	(G/C)
23728	CakSNP23728	*Kabuli*	Ca_Kabuli_denovo	21588175	(C/G)
23729	CakSNP23729	*Kabuli*	Ca_Kabuli_denovo	21588402	(G/T)
23730	CakSNP23730	*Kabuli*	Ca_Kabuli_denovo	21588479	(A/G)
23731	CakSNP23731	*Kabuli*	Ca_Kabuli_denovo	21589510	(C/A)
23732	CakSNP23732	*Kabuli*	Ca_Kabuli_denovo	21591421	(C/T)
23733	CakSNP23733	*Kabuli*	Ca_Kabuli_denovo	21595077	(C/T)
23734	CakSNP23734	*Kabuli*	Ca_Kabuli_denovo	21595111	(C/T)
23735	CakSNP23735	*Kabuli*	Ca_Kabuli_denovo	21595640	(C/G)
23736	CakSNP23736	*Kabuli*	Ca_Kabuli_denovo	21611670	(T/A)
23737	CakSNP23737	*Kabuli*	Ca_Kabuli_denovo	21611698	(T/G)
23738	CakSNP23738	*Kabuli*	Ca_Kabuli_denovo	21633676	(C/T)
23739	CakSNP23739	*Kabuli*	Ca_Kabuli_denovo	21633691	(T/C)
23740	CakSNP23740	*Kabuli*	Ca_Kabuli_denovo	21651168	(C/T)
23741	CakSNP23741	*Kabuli*	Ca_Kabuli_denovo	21651210	(A/G)
23742	CakSNP23742	*Kabuli*	Ca_Kabuli_denovo	21652622	(C/G)
23743	CakSNP23743	*Kabuli*	Ca_Kabuli_denovo	21655049	(A/C)
23744	CakSNP23744	*Kabuli*	Ca_Kabuli_denovo	21655050	(G/C)
23745	CakSNP23745	*Kabuli*	Ca_Kabuli_denovo	21659196	(C/G)
23746	CakSNP23746	*Kabuli*	Ca_Kabuli_denovo	21664740	(T/C)
23747	CakSNP23747	*Kabuli*	Ca_Kabuli_denovo	21675772	(C/T)
23748	CakSNP23748	*Kabuli*	Ca_Kabuli_denovo	21676093	(C/G)
23749	CakSNP23749	*Kabuli*	Ca_Kabuli_denovo	21676147	(T/G)

S.N.	SNP IDs	Cultivars	Chromosomes/scaffolds	Physical positions (bp)	SNPs
23750	CakSNP23750	*Kabuli*	Ca_Kabuli_denovo	21676158	(T/G)
23751	CakSNP23751	*Kabuli*	Ca_Kabuli_denovo	21683180	(A/T)
23752	CakSNP23752	*Kabuli*	Ca_Kabuli_denovo	21683186	(G/A)
23753	CakSNP23753	*Kabuli*	Ca_Kabuli_denovo	21690203	(T/G)
23754	CakSNP23754	*Kabuli*	Ca_Kabuli_denovo	21691850	(T/C)
23755	CakSNP23755	*Kabuli*	Ca_Kabuli_denovo	21691851	(T/C)
23756	CakSNP23756	*Kabuli*	Ca_Kabuli_denovo	21701022	(C/G)
23757	CakSNP23757	*Kabuli*	Ca_Kabuli_denovo	21702075	(G/A)
23758	CakSNP23758	*Kabuli*	Ca_Kabuli_denovo	21702811	(T/G)
23759	CakSNP23759	*Kabuli*	Ca_Kabuli_denovo	21705038	(C/G)
23760	CakSNP23760	*Kabuli*	Ca_Kabuli_denovo	21707912	(C/A)
23761	CakSNP23761	*Kabuli*	Ca_Kabuli_denovo	21713585	(T/C)
23762	CakSNP23762	*Kabuli*	Ca_Kabuli_denovo	21713624	(A/G)
23763	CakSNP23763	*Kabuli*	Ca_Kabuli_denovo	21717589	(T/C)
23764	CakSNP23764	*Kabuli*	Ca_Kabuli_denovo	21717626	(A/G)
23765	CakSNP23765	*Kabuli*	Ca_Kabuli_denovo	21724227	(A/G)
23766	CakSNP23766	*Kabuli*	Ca_Kabuli_denovo	21724237	(T/C)
23767	CakSNP23767	*Kabuli*	Ca_Kabuli_denovo	21725945	(A/C)
23768	CakSNP23768	*Kabuli*	Ca_Kabuli_denovo	21725951	(A/G)
23769	CakSNP23769	*Kabuli*	Ca_Kabuli_denovo	21726047	(C/A)
23770	CakSNP23770	*Kabuli*	Ca_Kabuli_denovo	21726056	(C/T)
23771	CakSNP23771	*Kabuli*	Ca_Kabuli_denovo	21726967	(G/A)
23772	CakSNP23772	*Kabuli*	Ca_Kabuli_denovo	21729583	(A/G)
23773	CakSNP23773	*Kabuli*	Ca_Kabuli_denovo	21730951	(C/T)
23774	CakSNP23774	*Kabuli*	Ca_Kabuli_denovo	21730966	(C/T)
23775	CakSNP23775	*Kabuli*	Ca_Kabuli_denovo	21745463	(A/T)
23776	CakSNP23776	*Kabuli*	Ca_Kabuli_denovo	21745487	(T/C)
23777	CakSNP23777	*Kabuli*	Ca_Kabuli_denovo	21748246	(C/T)
23778	CakSNP23778	*Kabuli*	Ca_Kabuli_denovo	21748466	(G/C)
23779	CakSNP23779	*Kabuli*	Ca_Kabuli_denovo	21759102	(T/G)
23780	CakSNP23780	*Kabuli*	Ca_Kabuli_denovo	21759330	(C/T)
23781	CakSNP23781	*Kabuli*	Ca_Kabuli_denovo	21759386	(C/T)
23782	CakSNP23782	*Kabuli*	Ca_Kabuli_denovo	21761283	(T/C)
23783	CakSNP23783	*Kabuli*	Ca_Kabuli_denovo	21762479	(A/C)
23784	CakSNP23784	*Kabuli*	Ca_Kabuli_denovo	21764026	(C/G)
23785	CakSNP23785	*Kabuli*	Ca_Kabuli_denovo	21765047	(G/A)
23786	CakSNP23786	*Kabuli*	Ca_Kabuli_denovo	21768651	(C/A)
23787	CakSNP23787	*Kabuli*	Ca_Kabuli_denovo	21768663	(G/A)
23788	CakSNP23788	*Kabuli*	Ca_Kabuli_denovo	21778525	(G/C)

S.N.	SNP IDs	Cultivars	Chromosomes/scaffolds	Physical positions (bp)	SNPs
23789	CakSNP23789	*Kabuli*	Ca_Kabuli_denovo	21778543	(A/G)
23790	CakSNP23790	*Kabuli*	Ca_Kabuli_denovo	21778657	(T/C)
23791	CakSNP23791	*Kabuli*	Ca_Kabuli_denovo	21788450	(C/G)
23792	CakSNP23792	*Kabuli*	Ca_Kabuli_denovo	21788489	(A/G)
23793	CakSNP23793	*Kabuli*	Ca_Kabuli_denovo	21798025	(A/C)
23794	CakSNP23794	*Kabuli*	Ca_Kabuli_denovo	21798919	(T/G)
23795	CakSNP23795	*Kabuli*	Ca_Kabuli_denovo	21800681	(A/G)
23796	CakSNP23796	*Kabuli*	Ca_Kabuli_denovo	21801770	(G/T)
23797	CakSNP23797	*Kabuli*	Ca_Kabuli_denovo	21801797	(C/T)
23798	CakSNP23798	*Kabuli*	Ca_Kabuli_denovo	21804246	(G/A)
23799	CakSNP23799	*Kabuli*	Ca_Kabuli_denovo	21804254	(C/G)
23800	CakSNP23800	*Kabuli*	Ca_Kabuli_denovo	21805673	(C/T)
23801	CakSNP23801	*Kabuli*	Ca_Kabuli_denovo	21807860	(G/T)
23802	CakSNP23802	*Kabuli*	Ca_Kabuli_denovo	21807880	(T/G)
23803	CakSNP23803	*Kabuli*	Ca_Kabuli_denovo	21808391	(C/G)
23804	CakSNP23804	*Kabuli*	Ca_Kabuli_denovo	21811794	(A/C)
23805	CakSNP23805	*Kabuli*	Ca_Kabuli_denovo	21811800	(A/G)
23806	CakSNP23806	*Kabuli*	Ca_Kabuli_denovo	21815122	(G/C)
23807	CakSNP23807	*Kabuli*	Ca_Kabuli_denovo	21815146	(G/A)
23808	CakSNP23808	*Kabuli*	Ca_Kabuli_denovo	21817157	(C/T)
23809	CakSNP23809	*Kabuli*	Ca_Kabuli_denovo	21817167	(A/G)
23810	CakSNP23810	*Kabuli*	Ca_Kabuli_denovo	21818738	(T/C)
23811	CakSNP23811	*Kabuli*	Ca_Kabuli_denovo	21818765	(T/C)
23812	CakSNP23812	*Kabuli*	Ca_Kabuli_denovo	21822406	(T/C)
23813	CakSNP23813	*Kabuli*	Ca_Kabuli_denovo	21822476	(A/G)
23814	CakSNP23814	*Kabuli*	Ca_Kabuli_denovo	21822488	(G/C)
23815	CakSNP23815	*Kabuli*	Ca_Kabuli_denovo	21824609	(T/C)
23816	CakSNP23816	*Kabuli*	Ca_Kabuli_denovo	21824648	(C/G)
23817	CakSNP23817	*Kabuli*	Ca_Kabuli_denovo	21826085	(A/G)
23818	CakSNP23818	*Kabuli*	Ca_Kabuli_denovo	21826147	(C/T)
23819	CakSNP23819	*Kabuli*	Ca_Kabuli_denovo	21827387	(A/C)
23820	CakSNP23820	*Kabuli*	Ca_Kabuli_denovo	21830766	(T/C)
23821	CakSNP23821	*Kabuli*	Ca_Kabuli_denovo	21830792	(A/C)
23822	CakSNP23822	*Kabuli*	Ca_Kabuli_denovo	21832533	(G/A)
23823	CakSNP23823	*Kabuli*	Ca_Kabuli_denovo	21832563	(A/G)
23824	CakSNP23824	*Kabuli*	Ca_Kabuli_denovo	21833724	(A/G)
23825	CakSNP23825	*Kabuli*	Ca_Kabuli_denovo	21833757	(C/T)
23826	CakSNP23826	*Kabuli*	Ca_Kabuli_denovo	21833915	(T/C)
23827	CakSNP23827	*Kabuli*	Ca_Kabuli_denovo	21839422	(A/G)

S.N.	SNP IDs	Cultivars	Chromosomes/scaffolds	Physical positions (bp)	SNPs
23828	CakSNP23828	*Kabuli*	Ca_Kabuli_denovo	21842425	(C/T)
23829	CakSNP23829	*Kabuli*	Ca_Kabuli_denovo	21857124	(G/A)
23830	CakSNP23830	*Kabuli*	Ca_Kabuli_denovo	21860971	(C/G)
23831	CakSNP23831	*Kabuli*	Ca_Kabuli_denovo	21860983	(G/T)
23832	CakSNP23832	*Kabuli*	Ca_Kabuli_denovo	21862785	(A/G)
23833	CakSNP23833	*Kabuli*	Ca_Kabuli_denovo	21863197	(T/G)
23834	CakSNP23834	*Kabuli*	Ca_Kabuli_denovo	21863232	(A/G)
23835	CakSNP23835	*Kabuli*	Ca_Kabuli_denovo	21868496	(C/T)
23836	CakSNP23836	*Kabuli*	Ca_Kabuli_denovo	21876586	(T/C)
23837	CakSNP23837	*Kabuli*	Ca_Kabuli_denovo	21876590	(G/C)
23838	CakSNP23838	*Kabuli*	Ca_Kabuli_denovo	21876611	(G/A)
23839	CakSNP23839	*Kabuli*	Ca_Kabuli_denovo	21880120	(C/T)
23840	CakSNP23840	*Kabuli*	Ca_Kabuli_denovo	21880140	(G/C)
23841	CakSNP23841	*Kabuli*	Ca_Kabuli_denovo	21882551	(C/T)
23842	CakSNP23842	*Kabuli*	Ca_Kabuli_denovo	21883006	(C/G)
23843	CakSNP23843	*Kabuli*	Ca_Kabuli_denovo	21883012	(G/T)
23844	CakSNP23844	*Kabuli*	Ca_Kabuli_denovo	21885351	(G/C)
23845	CakSNP23845	*Kabuli*	Ca_Kabuli_denovo	21886844	(G/A)
23846	CakSNP23846	*Kabuli*	Ca_Kabuli_denovo	21886849	(C/T)
23847	CakSNP23847	*Kabuli*	Ca_Kabuli_denovo	21888586	(G/A)
23848	CakSNP23848	*Kabuli*	Ca_Kabuli_denovo	21893756	(C/T)
23849	CakSNP23849	*Kabuli*	Ca_Kabuli_denovo	21894934	(T/C)
23850	CakSNP23850	*Kabuli*	Ca_Kabuli_denovo	21894955	(A/G)
23851	CakSNP23851	*Kabuli*	Ca_Kabuli_denovo	21898260	(G/A)
23852	CakSNP23852	*Kabuli*	Ca_Kabuli_denovo	21898280	(A/G)
23853	CakSNP23853	*Kabuli*	Ca_Kabuli_denovo	21903472	(C/G)
23854	CakSNP23854	*Kabuli*	Ca_Kabuli_denovo	21903496	(T/C)
23855	CakSNP23855	*Kabuli*	Ca_Kabuli_denovo	21905287	(A/G)
23856	CakSNP23856	*Kabuli*	Ca_Kabuli_denovo	21905310	(G/A)
23857	CakSNP23857	*Kabuli*	Ca_Kabuli_denovo	21907451	(A/C)
23858	CakSNP23858	*Kabuli*	Ca_Kabuli_denovo	21907453	(A/C)
23859	CakSNP23859	*Kabuli*	Ca_Kabuli_denovo	21913758	(T/C)
23860	CakSNP23860	*Kabuli*	Ca_Kabuli_denovo	21915275	(G/A)
23861	CakSNP23861	*Kabuli*	Ca_Kabuli_denovo	21915344	(A/T)
23862	CakSNP23862	*Kabuli*	Ca_Kabuli_denovo	21916354	(G/C)
23863	CakSNP23863	*Kabuli*	Ca_Kabuli_denovo	21916363	(G/C)
23864	CakSNP23864	*Kabuli*	Ca_Kabuli_denovo	21917642	(C/T)
23865	CakSNP23865	*Kabuli*	Ca_Kabuli_denovo	21923496	(C/T)
23866	CakSNP23866	*Kabuli*	Ca_Kabuli_denovo	21926068	(G/A)

S.N.	SNP IDs	Cultivars	Chromosomes/scaffolds	Physical positions (bp)	SNPs
23867	CakSNP23867	*Kabuli*	Ca_Kabuli_denovo	21929425	(G/A)
23868	CakSNP23868	*Kabuli*	Ca_Kabuli_denovo	21931570	(C/T)
23869	CakSNP23869	*Kabuli*	Ca_Kabuli_denovo	21931574	(C/G)
23870	CakSNP23870	*Kabuli*	Ca_Kabuli_denovo	21933266	(G/C)
23871	CakSNP23871	*Kabuli*	Ca_Kabuli_denovo	21943045	(C/T)
23872	CakSNP23872	*Kabuli*	Ca_Kabuli_denovo	21943062	(C/T)
23873	CakSNP23873	*Kabuli*	Ca_Kabuli_denovo	21944105	(T/G)
23874	CakSNP23874	*Kabuli*	Ca_Kabuli_denovo	21947457	(T/C)
23875	CakSNP23875	*Kabuli*	Ca_Kabuli_denovo	21959356	(C/T)
23876	CakSNP23876	*Kabuli*	Ca_Kabuli_denovo	21959383	(T/C)
23877	CakSNP23877	*Kabuli*	Ca_Kabuli_denovo	21959872	(G/T)
23878	CakSNP23878	*Kabuli*	Ca_Kabuli_denovo	21968331	(A/G)
23879	CakSNP23879	*Kabuli*	Ca_Kabuli_denovo	21968856	(T/C)
23880	CakSNP23880	*Kabuli*	Ca_Kabuli_denovo	21968860	(A/G)
23881	CakSNP23881	*Kabuli*	Ca_Kabuli_denovo	21976577	(G/A)
23882	CakSNP23882	*Kabuli*	Ca_Kabuli_denovo	21976623	(C/A)
23883	CakSNP23883	*Kabuli*	Ca_Kabuli_denovo	21978703	(A/C)
23884	CakSNP23884	*Kabuli*	Ca_Kabuli_denovo	21978727	(A/C)
23885	CakSNP23885	*Kabuli*	Ca_Kabuli_denovo	21983999	(A/G)
23886	CakSNP23886	*Kabuli*	Ca_Kabuli_denovo	21984023	(A/G)
23887	CakSNP23887	*Kabuli*	Ca_Kabuli_denovo	21984026	(G/A)
23888	CakSNP23888	*Kabuli*	Ca_Kabuli_denovo	21984665	(A/T)
23889	CakSNP23889	*Kabuli*	Ca_Kabuli_denovo	21984673	(T/C)
23890	CakSNP23890	*Kabuli*	Ca_Kabuli_denovo	21984871	(A/G)
23891	CakSNP23891	*Kabuli*	Ca_Kabuli_denovo	21984916	(A/G)
23892	CakSNP23892	*Kabuli*	Ca_Kabuli_denovo	21993694	(A/G)
23893	CakSNP23893	*Kabuli*	Ca_Kabuli_denovo	21993700	(A/C)
23894	CakSNP23894	*Kabuli*	Ca_Kabuli_denovo	21995519	(T/G)
23895	CakSNP23895	*Kabuli*	Ca_Kabuli_denovo	21999081	(C/G)
23896	CakSNP23896	*Kabuli*	Ca_Kabuli_denovo	21999091	(G/T)
23897	CakSNP23897	*Kabuli*	Ca_Kabuli_denovo	22001325	(C/A)
23898	CakSNP23898	*Kabuli*	Ca_Kabuli_denovo	22001329	(G/A)
23899	CakSNP23899	*Kabuli*	Ca_Kabuli_denovo	22003126	(A/G)
23900	CakSNP23900	*Kabuli*	Ca_Kabuli_denovo	22005228	(A/G)
23901	CakSNP23901	*Kabuli*	Ca_Kabuli_denovo	22010799	(C/A)
23902	CakSNP23902	*Kabuli*	Ca_Kabuli_denovo	22010803	(A/C)
23903	CakSNP23903	*Kabuli*	Ca_Kabuli_denovo	22011716	(T/G)
23904	CakSNP23904	*Kabuli*	Ca_Kabuli_denovo	22011794	(C/T)
23905	CakSNP23905	*Kabuli*	Ca_Kabuli_denovo	22016922	(A/C)

S.N.	SNP IDs	Cultivars	Chromosomes/scaffolds	Physical positions (bp)	SNPs
23906	CakSNP23906	*Kabuli*	Ca_Kabuli_denovo	22020149	(C/T)
23907	CakSNP23907	*Kabuli*	Ca_Kabuli_denovo	22020151	(G/A)
23908	CakSNP23908	*Kabuli*	Ca_Kabuli_denovo	22026815	(G/A)
23909	CakSNP23909	*Kabuli*	Ca_Kabuli_denovo	22027645	(C/T)
23910	CakSNP23910	*Kabuli*	Ca_Kabuli_denovo	22028368	(C/T)
23911	CakSNP23911	*Kabuli*	Ca_Kabuli_denovo	22028371	(A/G)
23912	CakSNP23912	*Kabuli*	Ca_Kabuli_denovo	22028386	(T/C)
23913	CakSNP23913	*Kabuli*	Ca_Kabuli_denovo	22028745	(T/G)
23914	CakSNP23914	*Kabuli*	Ca_Kabuli_denovo	22031438	(T/G)
23915	CakSNP23915	*Kabuli*	Ca_Kabuli_denovo	22040585	(G/T)
23916	CakSNP23916	*Kabuli*	Ca_Kabuli_denovo	22044687	(C/T)
23917	CakSNP23917	*Kabuli*	Ca_Kabuli_denovo	22048634	(C/A)
23918	CakSNP23918	*Kabuli*	Ca_Kabuli_denovo	22048658	(G/A)
23919	CakSNP23919	*Kabuli*	Ca_Kabuli_denovo	22054161	(A/G)
23920	CakSNP23920	*Kabuli*	Ca_Kabuli_denovo	22057228	(T/C)
23921	CakSNP23921	*Kabuli*	Ca_Kabuli_denovo	22059344	(C/G)
23922	CakSNP23922	*Kabuli*	Ca_Kabuli_denovo	22059356	(A/G)
23923	CakSNP23923	*Kabuli*	Ca_Kabuli_denovo	22070684	(T/G)
23924	CakSNP23924	*Kabuli*	Ca_Kabuli_denovo	22071511	(G/A)
23925	CakSNP23925	*Kabuli*	Ca_Kabuli_denovo	22072977	(T/G)
23926	CakSNP23926	*Kabuli*	Ca_Kabuli_denovo	22074563	(A/T)
23927	CakSNP23927	*Kabuli*	Ca_Kabuli_denovo	22078330	(T/C)
23928	CakSNP23928	*Kabuli*	Ca_Kabuli_denovo	22078518	(T/C)
23929	CakSNP23929	*Kabuli*	Ca_Kabuli_denovo	22078563	(G/C)
23930	CakSNP23930	*Kabuli*	Ca_Kabuli_denovo	22082588	(T/C)
23931	CakSNP23931	*Kabuli*	Ca_Kabuli_denovo	22082621	(C/T)
23932	CakSNP23932	*Kabuli*	Ca_Kabuli_denovo	22083454	(C/T)
23933	CakSNP23933	*Kabuli*	Ca_Kabuli_denovo	22086659	(T/G)
23934	CakSNP23934	*Kabuli*	Ca_Kabuli_denovo	22093836	(G/A)
23935	CakSNP23935	*Kabuli*	Ca_Kabuli_denovo	22095169	(C/T)
23936	CakSNP23936	*Kabuli*	Ca_Kabuli_denovo	22099049	(A/T)
23937	CakSNP23937	*Kabuli*	Ca_Kabuli_denovo	22099091	(G/A)
23938	CakSNP23938	*Kabuli*	Ca_Kabuli_denovo	22105626	(T/C)
23939	CakSNP23939	*Kabuli*	Ca_Kabuli_denovo	22109925	(C/G)
23940	CakSNP23940	*Kabuli*	Ca_Kabuli_denovo	22109955	(T/C)
23941	CakSNP23941	*Kabuli*	Ca_Kabuli_denovo	22115972	(C/G)
23942	CakSNP23942	*Kabuli*	Ca_Kabuli_denovo	22127527	(C/G)
23943	CakSNP23943	*Kabuli*	Ca_Kabuli_denovo	22145936	(C/A)
23944	CakSNP23944	*Kabuli*	Ca_Kabuli_denovo	22145951	(A/G)

S.N.	SNP IDs	Cultivars	Chromosomes/scaffolds	Physical positions (bp)	SNPs
23945	CakSNP23945	*Kabuli*	Ca_Kabuli_denovo	22147873	(A/G)
23946	CakSNP23946	*Kabuli*	Ca_Kabuli_denovo	22150196	(A/G)
23947	CakSNP23947	*Kabuli*	Ca_Kabuli_denovo	22150199	(G/C)
23948	CakSNP23948	*Kabuli*	Ca_Kabuli_denovo	22154593	(C/T)
23949	CakSNP23949	*Kabuli*	Ca_Kabuli_denovo	22155073	(T/A)
23950	CakSNP23950	*Kabuli*	Ca_Kabuli_denovo	22155343	(G/C)
23951	CakSNP23951	*Kabuli*	Ca_Kabuli_denovo	22155358	(C/A)
23952	CakSNP23952	*Kabuli*	Ca_Kabuli_denovo	22156352	(G/A)
23953	CakSNP23953	*Kabuli*	Ca_Kabuli_denovo	22156384	(C/G)
23954	CakSNP23954	*Kabuli*	Ca_Kabuli_denovo	22162484	(A/G)
23955	CakSNP23955	*Kabuli*	Ca_Kabuli_denovo	22166305	(T/C)
23956	CakSNP23956	*Kabuli*	Ca_Kabuli_denovo	22175865	(C/T)
23957	CakSNP23957	*Kabuli*	Ca_Kabuli_denovo	22175884	(T/C)
23958	CakSNP23958	*Kabuli*	Ca_Kabuli_denovo	22177441	(A/C)
23959	CakSNP23959	*Kabuli*	Ca_Kabuli_denovo	22177732	(T/C)
23960	CakSNP23960	*Kabuli*	Ca_Kabuli_denovo	22177762	(T/C)
23961	CakSNP23961	*Kabuli*	Ca_Kabuli_denovo	22185743	(C/T)
23962	CakSNP23962	*Kabuli*	Ca_Kabuli_denovo	22185763	(T/G)
23963	CakSNP23963	*Kabuli*	Ca_Kabuli_denovo	22187682	(T/C)
23964	CakSNP23964	*Kabuli*	Ca_Kabuli_denovo	22187734	(G/T)
23965	CakSNP23965	*Kabuli*	Ca_Kabuli_denovo	22191206	(C/A)
23966	CakSNP23966	*Kabuli*	Ca_Kabuli_denovo	22191233	(G/C)
23967	CakSNP23967	*Kabuli*	Ca_Kabuli_denovo	22194019	(T/C)
23968	CakSNP23968	*Kabuli*	Ca_Kabuli_denovo	22194078	(G/C)
23969	CakSNP23969	*Kabuli*	Ca_Kabuli_denovo	22199275	(T/C)
23970	CakSNP23970	*Kabuli*	Ca_Kabuli_denovo	22204436	(G/A)
23971	CakSNP23971	*Kabuli*	Ca_Kabuli_denovo	22204475	(A/G)
23972	CakSNP23972	*Kabuli*	Ca_Kabuli_denovo	22206003	(G/A)
23973	CakSNP23973	*Kabuli*	Ca_Kabuli_denovo	22210115	(C/G)
23974	CakSNP23974	*Kabuli*	Ca_Kabuli_denovo	22213095	(C/A)
23975	CakSNP23975	*Kabuli*	Ca_Kabuli_denovo	22213116	(A/G)
23976	CakSNP23976	*Kabuli*	Ca_Kabuli_denovo	22213901	(T/C)
23977	CakSNP23977	*Kabuli*	Ca_Kabuli_denovo	22213930	(G/C)
23978	CakSNP23978	*Kabuli*	Ca_Kabuli_denovo	22216043	(A/G)
23979	CakSNP23979	*Kabuli*	Ca_Kabuli_denovo	22223582	(G/A)
23980	CakSNP23980	*Kabuli*	Ca_Kabuli_denovo	22223614	(T/C)
23981	CakSNP23981	*Kabuli*	Ca_Kabuli_denovo	22223700	(T/A)
23982	CakSNP23982	*Kabuli*	Ca_Kabuli_denovo	22223707	(A/C)
23983	CakSNP23983	*Kabuli*	Ca_Kabuli_denovo	22224352	(G/T)

S.N.	SNP IDs	Cultivars	Chromosomes/scaffolds	Physical positions (bp)	SNPs
23984	CakSNP23984	*Kabuli*	Ca_Kabuli_denovo	22224391	(G/T)
23985	CakSNP23985	*Kabuli*	Ca_Kabuli_denovo	22236255	(C/T)
23986	CakSNP23986	*Kabuli*	Ca_Kabuli_denovo	22236273	(T/G)
23987	CakSNP23987	*Kabuli*	Ca_Kabuli_denovo	22237102	(G/A)
23988	CakSNP23988	*Kabuli*	Ca_Kabuli_denovo	22242259	(C/T)
23989	CakSNP23989	*Kabuli*	Ca_Kabuli_denovo	22242265	(C/T)
23990	CakSNP23990	*Kabuli*	Ca_Kabuli_denovo	22258645	(A/G)
23991	CakSNP23991	*Kabuli*	Ca_Kabuli_denovo	22266067	(G/C)
23992	CakSNP23992	*Kabuli*	Ca_Kabuli_denovo	22274978	(T/C)
23993	CakSNP23993	*Kabuli*	Ca_Kabuli_denovo	22277631	(G/C)
23994	CakSNP23994	*Kabuli*	Ca_Kabuli_denovo	22277682	(C/T)
23995	CakSNP23995	*Kabuli*	Ca_Kabuli_denovo	22279871	(C/A)
23996	CakSNP23996	*Kabuli*	Ca_Kabuli_denovo	22279916	(C/T)
23997	CakSNP23997	*Kabuli*	Ca_Kabuli_denovo	22280798	(G/A)
23998	CakSNP23998	*Kabuli*	Ca_Kabuli_denovo	22288948	(T/C)
23999	CakSNP23999	*Kabuli*	Ca_Kabuli_denovo	22290167	(C/A)
24000	CakSNP24000	*Kabuli*	Ca_Kabuli_denovo	22290169	(A/G)
24001	CakSNP24001	*Kabuli*	Ca_Kabuli_denovo	22297049	(C/A)
24002	CakSNP24002	*Kabuli*	Ca_Kabuli_denovo	22297053	(G/C)
24003	CakSNP24003	*Kabuli*	Ca_Kabuli_denovo	22297315	(T/C)
24004	CakSNP24004	*Kabuli*	Ca_Kabuli_denovo	22297334	(A/G)
24005	CakSNP24005	*Kabuli*	Ca_Kabuli_denovo	22302898	(G/A)
24006	CakSNP24006	*Kabuli*	Ca_Kabuli_denovo	22309863	(A/C)
24007	CakSNP24007	*Kabuli*	Ca_Kabuli_denovo	22315446	(A/T)
24008	CakSNP24008	*Kabuli*	Ca_Kabuli_denovo	22315449	(A/C)
24009	CakSNP24009	*Kabuli*	Ca_Kabuli_denovo	22324880	(T/G)
24010	CakSNP24010	*Kabuli*	Ca_Kabuli_denovo	22324883	(G/A)
24011	CakSNP24011	*Kabuli*	Ca_Kabuli_denovo	22328246	(A/G)
24012	CakSNP24012	*Kabuli*	Ca_Kabuli_denovo	22328273	(C/T)
24013	CakSNP24013	*Kabuli*	Ca_Kabuli_denovo	22332214	(G/T)
24014	CakSNP24014	*Kabuli*	Ca_Kabuli_denovo	22337605	(C/A)
24015	CakSNP24015	*Kabuli*	Ca_Kabuli_denovo	22340311	(C/A)
24016	CakSNP24016	*Kabuli*	Ca_Kabuli_denovo	22340317	(T/G)
24017	CakSNP24017	*Kabuli*	Ca_Kabuli_denovo	22342839	(G/C)
24018	CakSNP24018	*Kabuli*	Ca_Kabuli_denovo	22343917	(T/C)
24019	CakSNP24019	*Kabuli*	Ca_Kabuli_denovo	22343947	(A/C)
24020	CakSNP24020	*Kabuli*	Ca_Kabuli_denovo	22344811	(T/C)
24021	CakSNP24021	*Kabuli*	Ca_Kabuli_denovo	22347675	(T/C)
24022	CakSNP24022	*Kabuli*	Ca_Kabuli_denovo	22355630	(A/G)

S.N.	SNP IDs	Cultivars	Chromosomes/scaffolds	Physical positions (bp)	SNPs
24023	CakSNP24023	*Kabuli*	Ca_Kabuli_denovo	22355651	(A/C)
24024	CakSNP24024	*Kabuli*	Ca_Kabuli_denovo	22356787	(T/C)
24025	CakSNP24025	*Kabuli*	Ca_Kabuli_denovo	22359477	(T/A)
24026	CakSNP24026	*Kabuli*	Ca_Kabuli_denovo	22364608	(C/T)
24027	CakSNP24027	*Kabuli*	Ca_Kabuli_denovo	22370629	(G/A)
24028	CakSNP24028	*Kabuli*	Ca_Kabuli_denovo	22370640	(C/T)
24029	CakSNP24029	*Kabuli*	Ca_Kabuli_denovo	22379899	(G/C)
24030	CakSNP24030	*Kabuli*	Ca_Kabuli_denovo	22383680	(A/G)
24031	CakSNP24031	*Kabuli*	Ca_Kabuli_denovo	22384415	(A/C)
24032	CakSNP24032	*Kabuli*	Ca_Kabuli_denovo	22388447	(T/G)
24033	CakSNP24033	*Kabuli*	Ca_Kabuli_denovo	22390212	(A/C)
24034	CakSNP24034	*Kabuli*	Ca_Kabuli_denovo	22390214	(A/C)
24035	CakSNP24035	*Kabuli*	Ca_Kabuli_denovo	22396027	(G/A)
24036	CakSNP24036	*Kabuli*	Ca_Kabuli_denovo	22399918	(G/C)
24037	CakSNP24037	*Kabuli*	Ca_Kabuli_denovo	22402729	(A/G)
24038	CakSNP24038	*Kabuli*	Ca_Kabuli_denovo	22409040	(G/A)
24039	CakSNP24039	*Kabuli*	Ca_Kabuli_denovo	22409090	(T/G)
24040	CakSNP24040	*Kabuli*	Ca_Kabuli_denovo	22415065	(A/G)
24041	CakSNP24041	*Kabuli*	Ca_Kabuli_denovo	22415068	(T/C)
24042	CakSNP24042	*Kabuli*	Ca_Kabuli_denovo	22421870	(G/A)
24043	CakSNP24043	*Kabuli*	Ca_Kabuli_denovo	22423241	(C/G)
24044	CakSNP24044	*Kabuli*	Ca_Kabuli_denovo	22423263	(G/T)
24045	CakSNP24045	*Kabuli*	Ca_Kabuli_denovo	22424926	(G/C)
24046	CakSNP24046	*Kabuli*	Ca_Kabuli_denovo	22426360	(C/G)
24047	CakSNP24047	*Kabuli*	Ca_Kabuli_denovo	22427046	(A/T)
24048	CakSNP24048	*Kabuli*	Ca_Kabuli_denovo	22431626	(C/A)
24049	CakSNP24049	*Kabuli*	Ca_Kabuli_denovo	22431652	(C/T)
24050	CakSNP24050	*Kabuli*	Ca_Kabuli_denovo	22432380	(C/T)
24051	CakSNP24051	*Kabuli*	Ca_Kabuli_denovo	22432382	(C/G)
24052	CakSNP24052	*Kabuli*	Ca_Kabuli_denovo	22433280	(G/A)
24053	CakSNP24053	*Kabuli*	Ca_Kabuli_denovo	22433295	(A/G)
24054	CakSNP24054	*Kabuli*	Ca_Kabuli_denovo	22436951	(C/G)
24055	CakSNP24055	*Kabuli*	Ca_Kabuli_denovo	22436975	(T/G)
24056	CakSNP24056	*Kabuli*	Ca_Kabuli_denovo	22439163	(C/T)
24057	CakSNP24057	*Kabuli*	Ca_Kabuli_denovo	22443182	(T/C)
24058	CakSNP24058	*Kabuli*	Ca_Kabuli_denovo	22444030	(C/G)
24059	CakSNP24059	*Kabuli*	Ca_Kabuli_denovo	22449467	(G/T)
24060	CakSNP24060	*Kabuli*	Ca_Kabuli_denovo	22449512	(C/G)
24061	CakSNP24061	*Kabuli*	Ca_Kabuli_denovo	22450397	(C/G)

S.N.	SNP IDs	Cultivars	Chromosomes/scaffolds	Physical positions (bp)	SNPs
24062	CakSNP24062	*Kabuli*	Ca_Kabuli_denovo	22451704	(C/T)
24063	CakSNP24063	*Kabuli*	Ca_Kabuli_denovo	22452438	(A/G)
24064	CakSNP24064	*Kabuli*	Ca_Kabuli_denovo	22452449	(C/G)
24065	CakSNP24065	*Kabuli*	Ca_Kabuli_denovo	22453019	(T/C)
24066	CakSNP24066	*Kabuli*	Ca_Kabuli_denovo	22453049	(A/G)
24067	CakSNP24067	*Kabuli*	Ca_Kabuli_denovo	22453067	(G/A)
24068	CakSNP24068	*Kabuli*	Ca_Kabuli_denovo	22454097	(A/G)
24069	CakSNP24069	*Kabuli*	Ca_Kabuli_denovo	22454100	(G/A)
24070	CakSNP24070	*Kabuli*	Ca_Kabuli_denovo	22462238	(C/G)
24071	CakSNP24071	*Kabuli*	Ca_Kabuli_denovo	22462279	(T/C)
24072	CakSNP24072	*Kabuli*	Ca_Kabuli_denovo	22463261	(G/C)
24073	CakSNP24073	*Kabuli*	Ca_Kabuli_denovo	22464550	(C/A)
24074	CakSNP24074	*Kabuli*	Ca_Kabuli_denovo	22464930	(T/C)
24075	CakSNP24075	*Kabuli*	Ca_Kabuli_denovo	22464938	(G/C)
24076	CakSNP24076	*Kabuli*	Ca_Kabuli_denovo	22465107	(T/C)
24077	CakSNP24077	*Kabuli*	Ca_Kabuli_denovo	22465162	(A/G)
24078	CakSNP24078	*Kabuli*	Ca_Kabuli_denovo	22466096	(G/T)
24079	CakSNP24079	*Kabuli*	Ca_Kabuli_denovo	22476717	(A/C)
24080	CakSNP24080	*Kabuli*	Ca_Kabuli_denovo	22476744	(T/C)
24081	CakSNP24081	*Kabuli*	Ca_Kabuli_denovo	22479050	(A/G)
24082	CakSNP24082	*Kabuli*	Ca_Kabuli_denovo	22479122	(T/C)
24083	CakSNP24083	*Kabuli*	Ca_Kabuli_denovo	22480360	(C/T)
24084	CakSNP24084	*Kabuli*	Ca_Kabuli_denovo	22480405	(C/T)
24085	CakSNP24085	*Kabuli*	Ca_Kabuli_denovo	22486514	(G/A)
24086	CakSNP24086	*Kabuli*	Ca_Kabuli_denovo	22486522	(C/T)
24087	CakSNP24087	*Kabuli*	Ca_Kabuli_denovo	22493767	(T/C)
24088	CakSNP24088	*Kabuli*	Ca_Kabuli_denovo	22504660	(C/T)
24089	CakSNP24089	*Kabuli*	Ca_Kabuli_denovo	22514807	(C/A)
24090	CakSNP24090	*Kabuli*	Ca_Kabuli_denovo	22516434	(A/G)
24091	CakSNP24091	*Kabuli*	Ca_Kabuli_denovo	22520823	(G/C)
24092	CakSNP24092	*Kabuli*	Ca_Kabuli_denovo	22520875	(T/C)
24093	CakSNP24093	*Kabuli*	Ca_Kabuli_denovo	22521766	(G/C)
24094	CakSNP24094	*Kabuli*	Ca_Kabuli_denovo	22521796	(C/T)
24095	CakSNP24095	*Kabuli*	Ca_Kabuli_denovo	22529928	(A/G)
24096	CakSNP24096	*Kabuli*	Ca_Kabuli_denovo	22531422	(A/C)
24097	CakSNP24097	*Kabuli*	Ca_Kabuli_denovo	22531463	(A/G)
24098	CakSNP24098	*Kabuli*	Ca_Kabuli_denovo	22534289	(A/G)
24099	CakSNP24099	*Kabuli*	Ca_Kabuli_denovo	22542381	(T/G)
24100	CakSNP24100	*Kabuli*	Ca_Kabuli_denovo	22542441	(G/A)

S.N.	SNP IDs	Cultivars	Chromosomes/scaffolds	Physical positions (bp)	SNPs
24101	CakSNP24101	*Kabuli*	Ca_Kabuli_denovo	22546566	(A/C)
24102	CakSNP24102	*Kabuli*	Ca_Kabuli_denovo	22556801	(T/C)
24103	CakSNP24103	*Kabuli*	Ca_Kabuli_denovo	22561762	(G/C)
24104	CakSNP24104	*Kabuli*	Ca_Kabuli_denovo	22561771	(T/G)
24105	CakSNP24105	*Kabuli*	Ca_Kabuli_denovo	22567017	(T/C)
24106	CakSNP24106	*Kabuli*	Ca_Kabuli_denovo	22567026	(G/A)
24107	CakSNP24107	*Kabuli*	Ca_Kabuli_denovo	22567059	(T/C)
24108	CakSNP24108	*Kabuli*	Ca_Kabuli_denovo	22567219	(T/C)
24109	CakSNP24109	*Kabuli*	Ca_Kabuli_denovo	22567221	(C/T)
24110	CakSNP24110	*Kabuli*	Ca_Kabuli_denovo	22568144	(C/G)
24111	CakSNP24111	*Kabuli*	Ca_Kabuli_denovo	22568186	(C/A)
24112	CakSNP24112	*Kabuli*	Ca_Kabuli_denovo	22568434	(C/G)
24113	CakSNP24113	*Kabuli*	Ca_Kabuli_denovo	22578643	(C/T)
24114	CakSNP24114	*Kabuli*	Ca_Kabuli_denovo	22579945	(G/C)
24115	CakSNP24115	*Kabuli*	Ca_Kabuli_denovo	22581626	(T/C)
24116	CakSNP24116	*Kabuli*	Ca_Kabuli_denovo	22586002	(C/T)
24117	CakSNP24117	*Kabuli*	Ca_Kabuli_denovo	22590746	(G/A)
24118	CakSNP24118	*Kabuli*	Ca_Kabuli_denovo	22590761	(T/C)
24119	CakSNP24119	*Kabuli*	Ca_Kabuli_denovo	22602100	(C/T)
24120	CakSNP24120	*Kabuli*	Ca_Kabuli_denovo	22614086	(C/T)
24121	CakSNP24121	*Kabuli*	Ca_Kabuli_denovo	22614088	(C/G)
24122	CakSNP24122	*Kabuli*	Ca_Kabuli_denovo	22615811	(G/T)
24123	CakSNP24123	*Kabuli*	Ca_Kabuli_denovo	22635214	(G/C)
24124	CakSNP24124	*Kabuli*	Ca_Kabuli_denovo	22635612	(T/C)
24125	CakSNP24125	*Kabuli*	Ca_Kabuli_denovo	22635633	(G/A)
24126	CakSNP24126	*Kabuli*	Ca_Kabuli_denovo	22639106	(A/G)
24127	CakSNP24127	*Kabuli*	Ca_Kabuli_denovo	22639177	(T/C)
24128	CakSNP24128	*Kabuli*	Ca_Kabuli_denovo	22642069	(G/C)
24129	CakSNP24129	*Kabuli*	Ca_Kabuli_denovo	22643291	(C/G)
24130	CakSNP24130	*Kabuli*	Ca_Kabuli_denovo	22643294	(C/G)
24131	CakSNP24131	*Kabuli*	Ca_Kabuli_denovo	22647446	(C/G)
24132	CakSNP24132	*Kabuli*	Ca_Kabuli_denovo	22647449	(T/C)
24133	CakSNP24133	*Kabuli*	Ca_Kabuli_denovo	22650472	(A/G)
24134	CakSNP24134	*Kabuli*	Ca_Kabuli_denovo	22651126	(A/G)
24135	CakSNP24135	*Kabuli*	Ca_Kabuli_denovo	22651157	(A/G)
24136	CakSNP24136	*Kabuli*	Ca_Kabuli_denovo	22658903	(C/T)
24137	CakSNP24137	*Kabuli*	Ca_Kabuli_denovo	22658906	(C/T)
24138	CakSNP24138	*Kabuli*	Ca_Kabuli_denovo	22663217	(G/C)
24139	CakSNP24139	*Kabuli*	Ca_Kabuli_denovo	22663232	(T/C)

S.N.	SNP IDs	Cultivars	Chromosomes/scaffolds	Physical positions (bp)	SNPs
24140	CakSNP24140	*Kabuli*	Ca_Kabuli_denovo	22665794	(C/G)
24141	CakSNP24141	*Kabuli*	Ca_Kabuli_denovo	22667929	(T/C)
24142	CakSNP24142	*Kabuli*	Ca_Kabuli_denovo	22698389	(C/G)
24143	CakSNP24143	*Kabuli*	Ca_Kabuli_denovo	22701613	(A/T)
24144	CakSNP24144	*Kabuli*	Ca_Kabuli_denovo	22701626	(C/T)
24145	CakSNP24145	*Kabuli*	Ca_Kabuli_denovo	22704938	(C/G)
24146	CakSNP24146	*Kabuli*	Ca_Kabuli_denovo	22706294	(A/G)
24147	CakSNP24147	*Kabuli*	Ca_Kabuli_denovo	22706309	(G/A)
24148	CakSNP24148	*Kabuli*	Ca_Kabuli_denovo	22711654	(T/C)
24149	CakSNP24149	*Kabuli*	Ca_Kabuli_denovo	22712338	(G/C)
24150	CakSNP24150	*Kabuli*	Ca_Kabuli_denovo	22722943	(T/C)
24151	CakSNP24151	*Kabuli*	Ca_Kabuli_denovo	22722952	(G/C)
24152	CakSNP24152	*Kabuli*	Ca_Kabuli_denovo	22725798	(G/C)
24153	CakSNP24153	*Kabuli*	Ca_Kabuli_denovo	22725833	(G/T)
24154	CakSNP24154	*Kabuli*	Ca_Kabuli_denovo	22733117	(A/G)
24155	CakSNP24155	*Kabuli*	Ca_Kabuli_denovo	22733125	(C/G)
24156	CakSNP24156	*Kabuli*	Ca_Kabuli_denovo	22735553	(A/G)
24157	CakSNP24157	*Kabuli*	Ca_Kabuli_denovo	22735578	(A/G)
24158	CakSNP24158	*Kabuli*	Ca_Kabuli_denovo	22749173	(G/T)
24159	CakSNP24159	*Kabuli*	Ca_Kabuli_denovo	22749177	(G/C)
24160	CakSNP24160	*Kabuli*	Ca_Kabuli_denovo	22749186	(T/G)
24161	CakSNP24161	*Kabuli*	Ca_Kabuli_denovo	22762717	(C/G)
24162	CakSNP24162	*Kabuli*	Ca_Kabuli_denovo	22762805	(T/G)
24163	CakSNP24163	*Kabuli*	Ca_Kabuli_denovo	22762824	(G/T)
24164	CakSNP24164	*Kabuli*	Ca_Kabuli_denovo	22762857	(C/G)
24165	CakSNP24165	*Kabuli*	Ca_Kabuli_denovo	22767279	(T/G)
24166	CakSNP24166	*Kabuli*	Ca_Kabuli_denovo	22767322	(C/A)
24167	CakSNP24167	*Kabuli*	Ca_Kabuli_denovo	22770414	(T/C)
24168	CakSNP24168	*Kabuli*	Ca_Kabuli_denovo	22770468	(A/G)
24169	CakSNP24169	*Kabuli*	Ca_Kabuli_denovo	22772193	(G/C)
24170	CakSNP24170	*Kabuli*	Ca_Kabuli_denovo	22772208	(T/G)
24171	CakSNP24171	*Kabuli*	Ca_Kabuli_denovo	22774872	(A/G)
24172	CakSNP24172	*Kabuli*	Ca_Kabuli_denovo	22774887	(C/A)
24173	CakSNP24173	*Kabuli*	Ca_Kabuli_denovo	22774929	(T/G)
24174	CakSNP24174	*Kabuli*	Ca_Kabuli_denovo	22774938	(C/G)
24175	CakSNP24175	*Kabuli*	Ca_Kabuli_denovo	22779099	(G/T)
24176	CakSNP24176	*Kabuli*	Ca_Kabuli_denovo	22786218	(T/G)
24177	CakSNP24177	*Kabuli*	Ca_Kabuli_denovo	22786232	(A/G)
24178	CakSNP24178	*Kabuli*	Ca_Kabuli_denovo	22786258	(G/A)

S.N.	SNP IDs	Cultivars	Chromosomes/scaffolds	Physical positions (bp)	SNPs
24179	CakSNP24179	*Kabuli*	Ca_Kabuli_denovo	22786269	(A/G)
24180	CakSNP24180	*Kabuli*	Ca_Kabuli_denovo	23389902	(A/G)
24181	CakSNP24181	*Kabuli*	Ca_Kabuli_denovo	26279213	(G/C)
24182	CakSNP24182	*Kabuli*	Ca_Kabuli_denovo	26279215	(G/T)
24183	CakSNP24183	*Kabuli*	Ca_Kabuli_denovo	26279249	(T/C)
24184	CakSNP24184	*Kabuli*	Ca_Kabuli_denovo	28463588	(A/G)
24185	CakSNP24185	*Kabuli*	Ca_Kabuli_denovo	28463605	(G/A)
24186	CakSNP24186	*Kabuli*	Ca_Kabuli_denovo	30521758	(C/T)
24187	CakSNP24187	*Kabuli*	Ca_Kabuli_denovo	30521760	(T/C)
24188	CakSNP24188	*Kabuli*	Ca_Kabuli_denovo	30521761	(G/A)
24189	CakSNP24189	*Kabuli*	Ca_Kabuli_denovo	30521763	(T/C)
24190	CakSNP24190	*Kabuli*	Ca_Kabuli_denovo	30542416	(G/A)
24191	CakSNP24191	*Kabuli*	Ca_Kabuli_denovo	30542450	(A/G)
24192	CakSNP24192	*Kabuli*	Ca_Kabuli_denovo	30542467	(C/G)
24193	CakSNP24193	*Kabuli*	Ca_Kabuli_denovo	30542477	(A/G)
24194	CakSNP24194	*Kabuli*	Ca_Kabuli_denovo	30544320	(T/G)
24195	CakSNP24195	*Kabuli*	Ca_Kabuli_denovo	30544340	(T/C)
24196	CakSNP24196	*Kabuli*	Ca_Kabuli_denovo	30544349	(A/G)
24197	CakSNP24197	*Kabuli*	Ca_Kabuli_denovo	30544978	(T/A)
24198	CakSNP24198	*Kabuli*	Ca_Kabuli_denovo	30544987	(T/A)
24199	CakSNP24199	*Kabuli*	Ca_Kabuli_denovo	30544997	(G/C)
24200	CakSNP24200	*Kabuli*	Ca_Kabuli_denovo	30556074	(T/C)
24201	CakSNP24201	*Kabuli*	Ca_Kabuli_denovo	30556076	(T/C)
24202	CakSNP24202	*Kabuli*	Ca_Kabuli_denovo	30556080	(T/A)
24203	CakSNP24203	*Kabuli*	Ca_Kabuli_denovo	30556081	(C/A)
24204	CakSNP24204	*Kabuli*	Ca_Kabuli_denovo	30562605	(A/G)
24205	CakSNP24205	*Kabuli*	Ca_Kabuli_denovo	30562663	(G/A)
24206	CakSNP24206	*Kabuli*	Ca_Kabuli_denovo	30564215	(C/G)
24207	CakSNP24207	*Kabuli*	Ca_Kabuli_denovo	30564217	(T/G)
24208	CakSNP24208	*Kabuli*	Ca_Kabuli_denovo	30565114	(T/G)
24209	CakSNP24209	*Kabuli*	Ca_Kabuli_denovo	30565484	(C/A)
24210	CakSNP24210	*Kabuli*	Ca_Kabuli_denovo	30565552	(G/T)
24211	CakSNP24211	*Kabuli*	Ca_Kabuli_denovo	30585112	(C/G)
24212	CakSNP24212	*Kabuli*	Ca_Kabuli_denovo	30595715	(C/G)
24213	CakSNP24213	*Kabuli*	Ca_Kabuli_denovo	30595726	(C/G)
24214	CakSNP24214	*Kabuli*	Ca_Kabuli_denovo	30595772	(A/G)
24215	CakSNP24215	*Kabuli*	Ca_Kabuli_denovo	30595815	(C/A)
24216	CakSNP24216	*Kabuli*	Ca_Kabuli_denovo	30595832	(T/A)
24217	CakSNP24217	*Kabuli*	Ca_Kabuli_denovo	30608911	(G/A)

S.N.	SNP IDs	Cultivars	Chromosomes/scaffolds	Physical positions (bp)	SNPs
24218	CakSNP24218	*Kabuli*	Ca_Kabuli_denovo	30608992	(G/T)
24219	CakSNP24219	*Kabuli*	Ca_Kabuli_denovo	30608994	(T/C)
24220	CakSNP24220	*Kabuli*	Ca_Kabuli_denovo	30624438	(A/T)
24221	CakSNP24221	*Kabuli*	Ca_Kabuli_denovo	30624449	(G/A)
24222	CakSNP24222	*Kabuli*	Ca_Kabuli_denovo	30654546	(A/G)
24223	CakSNP24223	*Kabuli*	Ca_Kabuli_denovo	30654549	(A/C)
24224	CakSNP24224	*Kabuli*	Ca_Kabuli_denovo	30654551	(C/G)
24225	CakSNP24225	*Kabuli*	Ca_Kabuli_denovo	30654552	(T/G)
24226	CakSNP24226	*Kabuli*	Ca_Kabuli_denovo	30689389	(C/T)
24227	CakSNP24227	*Kabuli*	Ca_Kabuli_denovo	30692542	(C/T)
24228	CakSNP24228	*Kabuli*	Ca_Kabuli_denovo	30701261	(G/A)
24229	CakSNP24229	*Kabuli*	Ca_Kabuli_denovo	30701345	(T/A)
24230	CakSNP24230	*Kabuli*	Ca_Kabuli_denovo	30713916	(A/G)
24231	CakSNP24231	*Kabuli*	Ca_Kabuli_denovo	30713943	(A/G)
24232	CakSNP24232	*Kabuli*	Ca_Kabuli_denovo	30717672	(T/G)
24233	CakSNP24233	*Kabuli*	Ca_Kabuli_denovo	30720426	(T/G)
24234	CakSNP24234	*Kabuli*	Ca_Kabuli_denovo	30720438	(G/T)
24235	CakSNP24235	*Kabuli*	Ca_Kabuli_denovo	30720473	(A/G)
24236	CakSNP24236	*Kabuli*	Ca_Kabuli_denovo	30722929	(G/T)
24237	CakSNP24237	*Kabuli*	Ca_Kabuli_denovo	30722957	(T/G)
24238	CakSNP24238	*Kabuli*	Ca_Kabuli_denovo	30722963	(A/G)
24239	CakSNP24239	*Kabuli*	Ca_Kabuli_denovo	30722980	(G/A)
24240	CakSNP24240	*Kabuli*	Ca_Kabuli_denovo	30726959	(G/A)
24241	CakSNP24241	*Kabuli*	Ca_Kabuli_denovo	30726961	(G/A)
24242	CakSNP24242	*Kabuli*	Ca_Kabuli_denovo	30726962	(A/G)
24243	CakSNP24243	*Kabuli*	Ca_Kabuli_denovo	30726963	(G/A)
24244	CakSNP24244	*Kabuli*	Ca_Kabuli_denovo	30728437	(T/G)
24245	CakSNP24245	*Kabuli*	Ca_Kabuli_denovo	30728439	(A/G)
24246	CakSNP24246	*Kabuli*	Ca_Kabuli_denovo	30728469	(A/G)
24247	CakSNP24247	*Kabuli*	Ca_Kabuli_denovo	30730294	(G/C)
24248	CakSNP24248	*Kabuli*	Ca_Kabuli_denovo	30730741	(C/A)
24249	CakSNP24249	*Kabuli*	Ca_Kabuli_denovo	30730742	(C/G)
24250	CakSNP24250	*Kabuli*	Ca_Kabuli_denovo	30730743	(A/C)
24251	CakSNP24251	*Kabuli*	Ca_Kabuli_denovo	30730744	(G/A)
24252	CakSNP24252	*Kabuli*	Ca_Kabuli_denovo	30730790	(A/C)
24253	CakSNP24253	*Kabuli*	Ca_Kabuli_denovo	30731368	(C/G)
24254	CakSNP24254	*Kabuli*	Ca_Kabuli_denovo	30731905	(T/G)
24255	CakSNP24255	*Kabuli*	Ca_Kabuli_denovo	30731938	(T/G)
24256	CakSNP24256	*Kabuli*	Ca_Kabuli_denovo	30733630	(T/G)

S.N.	SNP IDs	Cultivars	Chromosomes/scaffolds	Physical positions (bp)	SNPs
24257	CakSNP24257	*Kabuli*	Ca_Kabuli_denovo	30733690	(C/G)
24258	CakSNP24258	*Kabuli*	Ca_Kabuli_denovo	30733694	(A/T)
24259	CakSNP24259	*Kabuli*	Ca_Kabuli_denovo	30733703	(G/T)
24260	CakSNP24260	*Kabuli*	Ca_Kabuli_denovo	30742989	(T/G)
24261	CakSNP24261	*Kabuli*	Ca_Kabuli_denovo	30742991	(A/C)
24262	CakSNP24262	*Kabuli*	Ca_Kabuli_denovo	30742993	(A/C)
24263	CakSNP24263	*Kabuli*	Ca_Kabuli_denovo	30742994	(G/A)
24264	CakSNP24264	*Kabuli*	Ca_Kabuli_denovo	30742995	(A/G)
24265	CakSNP24265	*Kabuli*	Ca_Kabuli_denovo	30746022	(G/A)
24266	CakSNP24266	*Kabuli*	Ca_Kabuli_denovo	30746046	(A/G)
24267	CakSNP24267	*Kabuli*	Ca_Kabuli_denovo	30752607	(A/G)
24268	CakSNP24268	*Kabuli*	Ca_Kabuli_denovo	30756090	(A/G)
24269	CakSNP24269	*Kabuli*	Ca_Kabuli_denovo	30756108	(A/C)
24270	CakSNP24270	*Kabuli*	Ca_Kabuli_denovo	30756117	(A/G)
24271	CakSNP24271	*Kabuli*	Ca_Kabuli_denovo	30760173	(G/C)
24272	CakSNP24272	*Kabuli*	Ca_Kabuli_denovo	30760215	(C/G)
24273	CakSNP24273	*Kabuli*	Ca_Kabuli_denovo	30765681	(T/C)
24274	CakSNP24274	*Kabuli*	Ca_Kabuli_denovo	30765682	(C/G)
24275	CakSNP24275	*Kabuli*	Ca_Kabuli_denovo	30765685	(A/C)
24276	CakSNP24276	*Kabuli*	Ca_Kabuli_denovo	30765686	(A/C)
24277	CakSNP24277	*Kabuli*	Ca_Kabuli_denovo	30766057	(G/A)
24278	CakSNP24278	*Kabuli*	Ca_Kabuli_denovo	30768470	(A/T)
24279	CakSNP24279	*Kabuli*	Ca_Kabuli_denovo	30772256	(C/G)
24280	CakSNP24280	*Kabuli*	Ca_Kabuli_denovo	30772258	(G/T)
24281	CakSNP24281	*Kabuli*	Ca_Kabuli_denovo	30772260	(C/T)
24282	CakSNP24282	*Kabuli*	Ca_Kabuli_denovo	30772261	(A/C)
24283	CakSNP24283	*Kabuli*	Ca_Kabuli_denovo	30772262	(G/A)
24284	CakSNP24284	*Kabuli*	Ca_Kabuli_denovo	30774518	(A/G)
24285	CakSNP24285	*Kabuli*	Ca_Kabuli_denovo	30774519	(G/A)
24286	CakSNP24286	*Kabuli*	Ca_Kabuli_denovo	30774521	(A/C)
24287	CakSNP24287	*Kabuli*	Ca_Kabuli_denovo	30774523	(G/A)
24288	CakSNP24288	*Kabuli*	Ca_Kabuli_denovo	30774524	(T/A)
24289	CakSNP24289	*Kabuli*	Ca_Kabuli_denovo	30774525	(C/A)
24290	CakSNP24290	*Kabuli*	Ca_Kabuli_denovo	30775207	(A/G)
24291	CakSNP24291	*Kabuli*	Ca_Kabuli_denovo	30775239	(A/G)
24292	CakSNP24292	*Kabuli*	Ca_Kabuli_denovo	30775240	(T/G)
24293	CakSNP24293	*Kabuli*	Ca_Kabuli_denovo	30775682	(C/G)
24294	CakSNP24294	*Kabuli*	Ca_Kabuli_denovo	30775707	(C/G)
24295	CakSNP24295	*Kabuli*	Ca_Kabuli_denovo	30775728	(A/G)

S.N.	SNP IDs	Cultivars	Chromosomes/scaffolds	Physical positions (bp)	SNPs
24296	CakSNP24296	*Kabuli*	Ca_Kabuli_denovo	30776185	(C/G)
24297	CakSNP24297	*Kabuli*	Ca_Kabuli_denovo	30776186	(A/G)
24298	CakSNP24298	*Kabuli*	Ca_Kabuli_denovo	30776187	(A/T)
24299	CakSNP24299	*Kabuli*	Ca_Kabuli_denovo	30776188	(C/T)
24300	CakSNP24300	*Kabuli*	Ca_Kabuli_denovo	30780155	(C/A)
24301	CakSNP24301	*Kabuli*	Ca_Kabuli_denovo	30780159	(A/G)
24302	CakSNP24302	*Kabuli*	Ca_Kabuli_denovo	30780168	(T/G)
24303	CakSNP24303	*Kabuli*	Ca_Kabuli_denovo	30780869	(T/G)
24304	CakSNP24304	*Kabuli*	Ca_Kabuli_denovo	30780871	(C/G)
24305	CakSNP24305	*Kabuli*	Ca_Kabuli_denovo	30780877	(A/G)
24306	CakSNP24306	*Kabuli*	Ca_Kabuli_denovo	30780881	(C/G)
24307	CakSNP24307	*Kabuli*	Ca_Kabuli_denovo	30780893	(T/G)
24308	CakSNP24308	*Kabuli*	Ca_Kabuli_denovo	30786169	(A/G)
24309	CakSNP24309	*Kabuli*	Ca_Kabuli_denovo	30786176	(G/A)
24310	CakSNP24310	*Kabuli*	Ca_Kabuli_denovo	30786230	(T/G)
24311	CakSNP24311	*Kabuli*	Ca_Kabuli_denovo	30786234	(A/G)
24312	CakSNP24312	*Kabuli*	Ca_Kabuli_denovo	30792759	(T/C)
24313	CakSNP24313	*Kabuli*	Ca_Kabuli_denovo	30792760	(A/C)
24314	CakSNP24314	*Kabuli*	Ca_Kabuli_denovo	30792764	(A/C)
24315	CakSNP24315	*Kabuli*	Ca_Kabuli_denovo	30792765	(A/G)
24316	CakSNP24316	*Kabuli*	Ca_Kabuli_denovo	30797378	(T/G)
24317	CakSNP24317	*Kabuli*	Ca_Kabuli_denovo	30797410	(A/G)
24318	CakSNP24318	*Kabuli*	Ca_Kabuli_denovo	30798168	(C/G)
24319	CakSNP24319	*Kabuli*	Ca_Kabuli_denovo	30798174	(A/G)
24320	CakSNP24320	*Kabuli*	Ca_Kabuli_denovo	30798186	(G/A)
24321	CakSNP24321	*Kabuli*	Ca_Kabuli_denovo	30798221	(A/G)
24322	CakSNP24322	*Kabuli*	Ca_Kabuli_denovo	30806118	(A/G)
24323	CakSNP24323	*Kabuli*	Ca_Kabuli_denovo	30807009	(C/G)
24324	CakSNP24324	*Kabuli*	Ca_Kabuli_denovo	30814984	(C/A)
24325	CakSNP24325	*Kabuli*	Ca_Kabuli_denovo	30814986	(G/T)
24326	CakSNP24326	*Kabuli*	Ca_Kabuli_denovo	30814988	(C/A)
24327	CakSNP24327	*Kabuli*	Ca_Kabuli_denovo	30814989	(C/A)
24328	CakSNP24328	*Kabuli*	Ca_Kabuli_denovo	30814991	(A/G)
24329	CakSNP24329	*Kabuli*	Ca_Kabuli_denovo	30817556	(C/G)
24330	CakSNP24330	*Kabuli*	Ca_Kabuli_denovo	30820587	(G/A)
24331	CakSNP24331	*Kabuli*	Ca_Kabuli_denovo	30820674	(A/G)
24332	CakSNP24332	*Kabuli*	Ca_Kabuli_denovo	30820693	(T/G)
24333	CakSNP24333	*Kabuli*	Ca_Kabuli_denovo	30820725	(A/G)
24334	CakSNP24334	*Kabuli*	Ca_Kabuli_denovo	30820857	(G/A)

S.N.	SNP IDs	Cultivars	Chromosomes/scaffolds	Physical positions (bp)	SNPs
24335	CakSNP24335	*Kabuli*	Ca_Kabuli_denovo	30820885	(T/G)
24336	CakSNP24336	*Kabuli*	Ca_Kabuli_denovo	30820935	(G/C)
24337	CakSNP24337	*Kabuli*	Ca_Kabuli_denovo	30821907	(A/G)
24338	CakSNP24338	*Kabuli*	Ca_Kabuli_denovo	30821961	(T/G)
24339	CakSNP24339	*Kabuli*	Ca_Kabuli_denovo	30824952	(C/A)
24340	CakSNP24340	*Kabuli*	Ca_Kabuli_denovo	30825037	(G/T)
24341	CakSNP24341	*Kabuli*	Ca_Kabuli_denovo	30825073	(C/G)
24342	CakSNP24342	*Kabuli*	Ca_Kabuli_denovo	30825087	(T/G)
24343	CakSNP24343	*Kabuli*	Ca_Kabuli_denovo	30825459	(C/G)
24344	CakSNP24344	*Kabuli*	Ca_Kabuli_denovo	30826626	(A/G)
24345	CakSNP24345	*Kabuli*	Ca_Kabuli_denovo	30826708	(T/A)
24346	CakSNP24346	*Kabuli*	Ca_Kabuli_denovo	30826821	(C/A)
24347	CakSNP24347	*Kabuli*	Ca_Kabuli_denovo	30826826	(T/G)
24348	CakSNP24348	*Kabuli*	Ca_Kabuli_denovo	30826840	(G/T)
24349	CakSNP24349	*Kabuli*	Ca_Kabuli_denovo	30826859	(A/G)
24350	CakSNP24350	*Kabuli*	Ca_Kabuli_denovo	30826896	(T/G)
24351	CakSNP24351	*Kabuli*	Ca_Kabuli_denovo	30829200	(G/A)
24352	CakSNP24352	*Kabuli*	Ca_Kabuli_denovo	30829213	(C/G)
24353	CakSNP24353	*Kabuli*	Ca_Kabuli_denovo	30831665	(G/T)
24354	CakSNP24354	*Kabuli*	Ca_Kabuli_denovo	30832766	(C/G)
24355	CakSNP24355	*Kabuli*	Ca_Kabuli_denovo	30833069	(G/A)
24356	CakSNP24356	*Kabuli*	Ca_Kabuli_denovo	30835039	(G/A)
24357	CakSNP24357	*Kabuli*	Ca_Kabuli_denovo	30835060	(G/C)
24358	CakSNP24358	*Kabuli*	Ca_Kabuli_denovo	30836884	(A/G)
24359	CakSNP24359	*Kabuli*	Ca_Kabuli_denovo	30836903	(T/C)
24360	CakSNP24360	*Kabuli*	Ca_Kabuli_denovo	30836937	(T/A)
24361	CakSNP24361	*Kabuli*	Ca_Kabuli_denovo	30836941	(A/C)
24362	CakSNP24362	*Kabuli*	Ca_Kabuli_denovo	30846063	(T/G)
24363	CakSNP24363	*Kabuli*	Ca_Kabuli_denovo	30846909	(T/G)
24364	CakSNP24364	*Kabuli*	Ca_Kabuli_denovo	30886176	(A/G)
24365	CakSNP24365	*Kabuli*	Ca_Kabuli_denovo	30886221	(C/T)
24366	CakSNP24366	*Kabuli*	Ca_Kabuli_denovo	30914188	(A/T)
24367	CakSNP24367	*Kabuli*	Ca_Kabuli_denovo	30914189	(T/C)
24368	CakSNP24368	*Kabuli*	Ca_Kabuli_denovo	30914190	(C/G)
24369	CakSNP24369	*Kabuli*	Ca_Kabuli_denovo	30914192	(A/G)
24370	CakSNP24370	*Kabuli*	Ca_Kabuli_denovo	30914194	(G/A)
24371	CakSNP24371	*Kabuli*	Ca_Kabuli_denovo	30914211	(T/A)
24372	CakSNP24372	*Kabuli*	Ca_Kabuli_denovo	30914217	(G/A)
24373	CakSNP24373	*Kabuli*	Ca_Kabuli_denovo	30914223	(T/C)

S.N.	SNP IDs	Cultivars	Chromosomes/scaffolds	Physical positions (bp)	SNPs
24374	CakSNP24374	*Kabuli*	Ca_Kabuli_denovo	30914224	(C/T)
24375	CakSNP24375	*Kabuli*	Ca_Kabuli_denovo	30915856	(G/A)
24376	CakSNP24376	*Kabuli*	Ca_Kabuli_denovo	30920569	(C/A)
24377	CakSNP24377	*Kabuli*	Ca_Kabuli_denovo	30920603	(A/G)
24378	CakSNP24378	*Kabuli*	Ca_Kabuli_denovo	30920620	(C/G)
24379	CakSNP24379	*Kabuli*	Ca_Kabuli_denovo	30920641	(T/G)
24380	CakSNP24380	*Kabuli*	Ca_Kabuli_denovo	30948570	(A/G)
24381	CakSNP24381	*Kabuli*	Ca_Kabuli_denovo	30948583	(C/T)
24382	CakSNP24382	*Kabuli*	Ca_Kabuli_denovo	39369006	(C/G)
24383	CakSNP24383	*Kabuli*	Ca_Kabuli_denovo	43194868	(T/A)
24384	CakSNP24384	*Kabuli*	Ca_Kabuli_denovo	43209602	(C/T)
24385	CakSNP24385	*Kabuli*	Ca_Kabuli_denovo	43209612	(T/G)
24386	CakSNP24386	*Kabuli*	Ca_Kabuli_denovo	43706574	(C/T)
24387	CakSNP24387	*Kabuli*	Ca_Kabuli_denovo	43711784	(A/C)
24388	CakSNP24388	*Kabuli*	Ca_Kabuli_denovo	43711813	(A/G)
24389	CakSNP24389	*Kabuli*	Ca_Kabuli_denovo	43990689	(T/C)
24390	CakSNP24390	*Kabuli*	Ca_Kabuli_denovo	43990690	(C/G)
24391	CakSNP24391	*Kabuli*	Ca_Kabuli_denovo	43990720	(C/A)
24392	CakSNP24392	*Kabuli*	Ca_Kabuli_denovo	43990743	(G/A)
24393	CakSNP24393	*Kabuli*	Ca_Kabuli_denovo	43990748	(T/G)
24394	CakSNP24394	*Kabuli*	Ca_Kabuli_denovo	43990751	(C/T)
24395	CakSNP24395	*Kabuli*	Ca_Kabuli_denovo	44029557	(G/C)
24396	CakSNP24396	*Kabuli*	Ca_Kabuli_denovo	44029593	(G/T)
24397	CakSNP24397	*Kabuli*	Ca_Kabuli_denovo	44071541	(A/G)
24398	CakSNP24398	*Kabuli*	Ca_Kabuli_denovo	44449557	(A/G)
24399	CakSNP24399	*Kabuli*	Ca_Kabuli_denovo	44449575	(A/T)
24400	CakSNP24400	*Kabuli*	Ca_Kabuli_denovo	44449577	(G/A)
24401	CakSNP24401	*Kabuli*	Ca_Kabuli_denovo	44449597	(C/T)
24402	CakSNP24402	*Kabuli*	Ca_Kabuli_denovo	67025915	(G/T)
24403	CakSNP24403	*Kabuli*	Ca_Kabuli_denovo	67025928	(G/T)
24404	CakSNP24404	*Kabuli*	Ca_Kabuli_denovo	67030173	(T/G)
24405	CakSNP24405	*Kabuli*	Ca_Kabuli_denovo	67030216	(A/G)
24406	CadSNP1	*Desi*	Ca_Desi_Chr01	179189	(A/C)
24407	CadSNP2	*Desi*	Ca_Desi_Chr01	382862	(T/C)
24408	CadSNP3	*Desi*	Ca_Desi_Chr01	382872	(G/A)
24409	CadSNP4	*Desi*	Ca_Desi_Chr01	382898	(T/C)
24410	CadSNP5	*Desi*	Ca_Desi_Chr01	436895	(A/C)
24411	CadSNP6	*Desi*	Ca_Desi_Chr01	461870	(A/G)
24412	CadSNP7	*Desi*	Ca_Desi_Chr01	498862	(A/G)

S.N.	SNP IDs	Cultivars	Chromosomes/scaffolds	Physical positions (bp)	SNPs
24413	CadSNP8	*Desi*	Ca_Desi_Chr01	517512	(T/C)
24414	CadSNP9	*Desi*	Ca_Desi_Chr01	517439	(A/G)
24415	CadSNP10	*Desi*	Ca_Desi_Chr01	527099	(A/G)
24416	CadSNP11	*Desi*	Ca_Desi_Chr01	527113	(G/T)
24417	CadSNP12	*Desi*	Ca_Desi_Chr01	527118	(T/C)
24418	CadSNP13	*Desi*	Ca_Desi_Chr01	527135	(G/A)
24419	CadSNP14	*Desi*	Ca_Desi_Chr01	527142	(G/T)
24420	CadSNP15	*Desi*	Ca_Desi_Chr01	527181	(C/A)
24421	CadSNP16	*Desi*	Ca_Desi_Chr01	527176	(T/C)
24422	CadSNP17	*Desi*	Ca_Desi_Chr01	527104	(G/T)
24423	CadSNP18	*Desi*	Ca_Desi_Chr01	533122	(G/A)
24424	CadSNP19	*Desi*	Ca_Desi_Chr01	533125	(C/T)
24425	CadSNP20	*Desi*	Ca_Desi_Chr01	533127	(T/A)
24426	CadSNP21	*Desi*	Ca_Desi_Chr01	533182	(T/A)
24427	CadSNP22	*Desi*	Ca_Desi_Chr01	537937	(T/C)
24428	CadSNP23	*Desi*	Ca_Desi_Chr01	537971	(A/G)
24429	CadSNP24	*Desi*	Ca_Desi_Chr01	538012	(C/T)
24430	CadSNP25	*Desi*	Ca_Desi_Chr01	538086	(G/T)
24431	CadSNP26	*Desi*	Ca_Desi_Chr01	548203	(T/C)
24432	CadSNP27	*Desi*	Ca_Desi_Chr01	551496	(T/G)
24433	CadSNP28	*Desi*	Ca_Desi_Chr01	559934	(C/T)
24434	CadSNP29	*Desi*	Ca_Desi_Chr01	559964	(C/T)
24435	CadSNP30	*Desi*	Ca_Desi_Chr01	620260	(T/G)
24436	CadSNP31	*Desi*	Ca_Desi_Chr01	620310	(C/T)
24437	CadSNP32	*Desi*	Ca_Desi_Chr01	647919	(G/T)
24438	CadSNP33	*Desi*	Ca_Desi_Chr01	648015	(G/A)
24439	CadSNP34	*Desi*	Ca_Desi_Chr01	664787	(T/G)
24440	CadSNP35	*Desi*	Ca_Desi_Chr01	685373	(T/C)
24441	CadSNP36	*Desi*	Ca_Desi_Chr01	704852	(A/G)
24442	CadSNP37	*Desi*	Ca_Desi_Chr01	704814	(C/T)
24443	CadSNP38	*Desi*	Ca_Desi_Chr01	704812	(C/G)
24444	CadSNP39	*Desi*	Ca_Desi_Chr01	739330	(C/T)
24445	CadSNP40	*Desi*	Ca_Desi_Chr01	739365	(G/A)
24446	CadSNP41	*Desi*	Ca_Desi_Chr01	739371	(T/A)
24447	CadSNP42	*Desi*	Ca_Desi_Chr01	739452	(T/C)
24448	CadSNP43	*Desi*	Ca_Desi_Chr01	739437	(G/A)
24449	CadSNP44	*Desi*	Ca_Desi_Chr01	756894	(A/G)
24450	CadSNP45	*Desi*	Ca_Desi_Chr01	757353	(A/C)
24451	CadSNP46	*Desi*	Ca_Desi_Chr01	757501	(T/A)

S.N.	SNP IDs	Cultivars	Chromosomes/scaffolds	Physical positions (bp)	SNPs
24452	CadSNP47	*Desi*	Ca_Desi_Chr01	766231	(C/A)
24453	CadSNP48	*Desi*	Ca_Desi_Chr01	766221	(T/C)
24454	CadSNP49	*Desi*	Ca_Desi_Chr01	766202	(T/C)
24455	CadSNP50	*Desi*	Ca_Desi_Chr01	766200	(A/G)
24456	CadSNP51	*Desi*	Ca_Desi_Chr01	766199	(A/T)
24457	CadSNP52	*Desi*	Ca_Desi_Chr01	785375	(C/A)
24458	CadSNP53	*Desi*	Ca_Desi_Chr01	785650	(G/A)
24459	CadSNP54	*Desi*	Ca_Desi_Chr01	786321	(A/T)
24460	CadSNP55	*Desi*	Ca_Desi_Chr01	791180	(A/C)
24461	CadSNP56	*Desi*	Ca_Desi_Chr01	807232	(T/C)
24462	CadSNP57	*Desi*	Ca_Desi_Chr01	807195	(C/T)
24463	CadSNP58	*Desi*	Ca_Desi_Chr01	836330	(G/T)
24464	CadSNP59	*Desi*	Ca_Desi_Chr01	836351	(C/T)
24465	CadSNP60	*Desi*	Ca_Desi_Chr01	836381	(T/C)
24466	CadSNP61	*Desi*	Ca_Desi_Chr01	836365	(C/T)
24467	CadSNP62	*Desi*	Ca_Desi_Chr01	836796	(C/G)
24468	CadSNP63	*Desi*	Ca_Desi_Chr01	836859	(G/A)
24469	CadSNP64	*Desi*	Ca_Desi_Chr01	836842	(T/G)
24470	CadSNP65	*Desi*	Ca_Desi_Chr01	845508	(C/T)
24471	CadSNP66	*Desi*	Ca_Desi_Chr01	872280	(C/T)
24472	CadSNP67	*Desi*	Ca_Desi_Chr01	872247	(G/A)
24473	CadSNP68	*Desi*	Ca_Desi_Chr01	885647	(A/C)
24474	CadSNP69	*Desi*	Ca_Desi_Chr01	885643	(C/T)
24475	CadSNP70	*Desi*	Ca_Desi_Chr01	885745	(G/T)
24476	CadSNP71	*Desi*	Ca_Desi_Chr01	965708	(T/C)
24477	CadSNP72	*Desi*	Ca_Desi_Chr01	965885	(T/C)
24478	CadSNP73	*Desi*	Ca_Desi_Chr01	965884	(G/A)
24479	CadSNP74	*Desi*	Ca_Desi_Chr01	965854	(T/C)
24480	CadSNP75	*Desi*	Ca_Desi_Chr01	968375	(T/C)
24481	CadSNP76	*Desi*	Ca_Desi_Chr01	973945	(G/T)
24482	CadSNP77	*Desi*	Ca_Desi_Chr01	973954	(C/T)
24483	CadSNP78	*Desi*	Ca_Desi_Chr01	978116	(T/C)
24484	CadSNP79	*Desi*	Ca_Desi_Chr01	978133	(T/A)
24485	CadSNP80	*Desi*	Ca_Desi_Chr01	983043	(C/A)
24486	CadSNP81	*Desi*	Ca_Desi_Chr01	983173	(G/A)
24487	CadSNP82	*Desi*	Ca_Desi_Chr01	983203	(G/T)
24488	CadSNP83	*Desi*	Ca_Desi_Chr01	992175	(G/A)
24489	CadSNP84	*Desi*	Ca_Desi_Chr01	992174	(C/T)
24490	CadSNP85	*Desi*	Ca_Desi_Chr01	999613	(T/C)

S.N.	SNP IDs	Cultivars	Chromosomes/scaffolds	Physical positions (bp)	SNPs
24491	CadSNP86	*Desi*	Ca_Desi_Chr01	1025432	(T/C)
24492	CadSNP87	*Desi*	Ca_Desi_Chr01	1025458	(C/T)
24493	CadSNP88	*Desi*	Ca_Desi_Chr01	1025593	(C/A)
24494	CadSNP89	*Desi*	Ca_Desi_Chr01	1025590	(A/G)
24495	CadSNP90	*Desi*	Ca_Desi_Chr01	1069295	(A/C)
24496	CadSNP91	*Desi*	Ca_Desi_Chr01	1069436	(G/A)
24497	CadSNP92	*Desi*	Ca_Desi_Chr01	1069425	(A/C)
24498	CadSNP93	*Desi*	Ca_Desi_Chr01	1069410	(A/T)
24499	CadSNP94	*Desi*	Ca_Desi_Chr01	1069403	(A/G)
24500	CadSNP95	*Desi*	Ca_Desi_Chr01	1071218	(C/T)
24501	CadSNP96	*Desi*	Ca_Desi_Chr01	1071230	(C/T)
24502	CadSNP97	*Desi*	Ca_Desi_Chr01	1071343	(G/A)
24503	CadSNP98	*Desi*	Ca_Desi_Chr01	1071275	(T/C)
24504	CadSNP99	*Desi*	Ca_Desi_Chr01	1072524	(A/T)
24505	CadSNP100	*Desi*	Ca_Desi_Chr01	1072532	(C/T)
24506	CadSNP101	*Desi*	Ca_Desi_Chr01	1072623	(G/A)
24507	CadSNP102	*Desi*	Ca_Desi_Chr01	1072955	(A/G)
24508	CadSNP103	*Desi*	Ca_Desi_Chr01	1072973	(T/C)
24509	CadSNP104	*Desi*	Ca_Desi_Chr01	1072976	(A/G)
24510	CadSNP105	*Desi*	Ca_Desi_Chr01	1073000	(A/T)
24511	CadSNP106	*Desi*	Ca_Desi_Chr01	1073059	(G/A)
24512	CadSNP107	*Desi*	Ca_Desi_Chr01	1073054	(A/G)
24513	CadSNP108	*Desi*	Ca_Desi_Chr01	1073048	(G/A)
24514	CadSNP109	*Desi*	Ca_Desi_Chr01	1126922	(G/C)
24515	CadSNP110	*Desi*	Ca_Desi_Chr01	1140358	(A/G)
24516	CadSNP111	*Desi*	Ca_Desi_Chr01	1140485	(A/T)
24517	CadSNP112	*Desi*	Ca_Desi_Chr01	1145410	(A/G)
24518	CadSNP113	*Desi*	Ca_Desi_Chr01	1145493	(G/T)
24519	CadSNP114	*Desi*	Ca_Desi_Chr01	1146141	(C/T)
24520	CadSNP115	*Desi*	Ca_Desi_Chr01	1146138	(T/G)
24521	CadSNP116	*Desi*	Ca_Desi_Chr01	1146211	(C/T)
24522	CadSNP117	*Desi*	Ca_Desi_Chr01	1146466	(C/T)
24523	CadSNP118	*Desi*	Ca_Desi_Chr01	1146471	(A/G)
24524	CadSNP119	*Desi*	Ca_Desi_Chr01	1146489	(C/T)
24525	CadSNP120	*Desi*	Ca_Desi_Chr01	1146490	(G/A)
24526	CadSNP121	*Desi*	Ca_Desi_Chr01	1146659	(C/T)
24527	CadSNP122	*Desi*	Ca_Desi_Chr01	1146626	(C/A)
24528	CadSNP123	*Desi*	Ca_Desi_Chr01	1146611	(C/G)
24529	CadSNP124	*Desi*	Ca_Desi_Chr01	1156112	(A/G)

S.N.	SNP IDs	Cultivars	Chromosomes/scaffolds	Physical positions (bp)	SNPs
24530	CadSNP125	*Desi*	Ca_Desi_Chr01	1189681	(C/G)
24531	CadSNP126	*Desi*	Ca_Desi_Chr01	1189628	(C/A)
24532	CadSNP127	*Desi*	Ca_Desi_Chr01	1189627	(T/A)
24533	CadSNP128	*Desi*	Ca_Desi_Chr01	1189626	(G/C)
24534	CadSNP129	*Desi*	Ca_Desi_Chr01	1234998	(A/T)
24535	CadSNP130	*Desi*	Ca_Desi_Chr01	1234995	(G/A)
24536	CadSNP131	*Desi*	Ca_Desi_Chr01	1234986	(A/G)
24537	CadSNP132	*Desi*	Ca_Desi_Chr01	1234985	(A/G)
24538	CadSNP133	*Desi*	Ca_Desi_Chr01	1234977	(G/T)
24539	CadSNP134	*Desi*	Ca_Desi_Chr01	1271491	(C/T)
24540	CadSNP135	*Desi*	Ca_Desi_Chr01	1271498	(A/T)
24541	CadSNP136	*Desi*	Ca_Desi_Chr01	1271686	(G/A)
24542	CadSNP137	*Desi*	Ca_Desi_Chr01	1271654	(T/G)
24543	CadSNP138	*Desi*	Ca_Desi_Chr01	1375921	(C/G)
24544	CadSNP139	*Desi*	Ca_Desi_Chr01	1384553	(C/T)
24545	CadSNP140	*Desi*	Ca_Desi_Chr01	1389951	(C/T)
24546	CadSNP141	*Desi*	Ca_Desi_Chr01	1390763	(T/C)
24547	CadSNP142	*Desi*	Ca_Desi_Chr01	1445548	(T/C)
24548	CadSNP143	*Desi*	Ca_Desi_Chr01	1451002	(A/G)
24549	CadSNP144	*Desi*	Ca_Desi_Chr01	1451028	(A/T)
24550	CadSNP145	*Desi*	Ca_Desi_Chr01	1463203	(T/C)
24551	CadSNP146	*Desi*	Ca_Desi_Chr01	1463234	(C/T)
24552	CadSNP147	*Desi*	Ca_Desi_Chr01	1463270	(A/C)
24553	CadSNP148	*Desi*	Ca_Desi_Chr01	1463335	(C/T)
24554	CadSNP149	*Desi*	Ca_Desi_Chr01	1479425	(T/C)
24555	CadSNP150	*Desi*	Ca_Desi_Chr01	1479437	(T/C)
24556	CadSNP151	*Desi*	Ca_Desi_Chr01	1480187	(T/C)
24557	CadSNP152	*Desi*	Ca_Desi_Chr01	1486086	(G/C)
24558	CadSNP153	*Desi*	Ca_Desi_Chr01	1486057	(A/C)
24559	CadSNP154	*Desi*	Ca_Desi_Chr01	1522221	(T/C)
24560	CadSNP155	*Desi*	Ca_Desi_Chr01	1526742	(G/T)
24561	CadSNP156	*Desi*	Ca_Desi_Chr01	1526741	(C/A)
24562	CadSNP157	*Desi*	Ca_Desi_Chr01	1553951	(A/G)
24563	CadSNP158	*Desi*	Ca_Desi_Chr01	1553958	(A/G)
24564	CadSNP159	*Desi*	Ca_Desi_Chr01	1598211	(C/T)
24565	CadSNP160	*Desi*	Ca_Desi_Chr01	1627093	(G/T)
24566	CadSNP161	*Desi*	Ca_Desi_Chr01	1632231	(G/A)
24567	CadSNP162	*Desi*	Ca_Desi_Chr01	1638965	(T/A)
24568	CadSNP163	*Desi*	Ca_Desi_Chr01	1639227	(C/G)

S.N.	SNP IDs	Cultivars	Chromosomes/scaffolds	Physical positions (bp)	SNPs
24569	CadSNP164	*Desi*	Ca_Desi_Chr01	1727845	(T/A)
24570	CadSNP165	*Desi*	Ca_Desi_Chr01	1787370	(A/T)
24571	CadSNP166	*Desi*	Ca_Desi_Chr01	1815665	(T/C)
24572	CadSNP167	*Desi*	Ca_Desi_Chr01	1826921	(A/G)
24573	CadSNP168	*Desi*	Ca_Desi_Chr01	1828118	(A/G)
24574	CadSNP169	*Desi*	Ca_Desi_Chr01	1828194	(C/G)
24575	CadSNP170	*Desi*	Ca_Desi_Chr01	1828281	(A/C)
24576	CadSNP171	*Desi*	Ca_Desi_Chr01	1838149	(C/G)
24577	CadSNP172	*Desi*	Ca_Desi_Chr01	1839807	(A/G)
24578	CadSNP173	*Desi*	Ca_Desi_Chr01	1842557	(C/T)
24579	CadSNP174	*Desi*	Ca_Desi_Chr01	1842545	(T/C)
24580	CadSNP175	*Desi*	Ca_Desi_Chr01	1851780	(A/T)
24581	CadSNP176	*Desi*	Ca_Desi_Chr01	1881482	(A/G)
24582	CadSNP177	*Desi*	Ca_Desi_Chr01	1881584	(A/G)
24583	CadSNP178	*Desi*	Ca_Desi_Chr01	1904344	(T/C)
24584	CadSNP179	*Desi*	Ca_Desi_Chr01	1904458	(G/A)
24585	CadSNP180	*Desi*	Ca_Desi_Chr01	1904473	(C/T)
24586	CadSNP181	*Desi*	Ca_Desi_Chr01	1906298	(A/C)
24587	CadSNP182	*Desi*	Ca_Desi_Chr01	1906300	(T/G)
24588	CadSNP183	*Desi*	Ca_Desi_Chr01	1906308	(A/G)
24589	CadSNP184	*Desi*	Ca_Desi_Chr01	1906324	(A/C)
24590	CadSNP185	*Desi*	Ca_Desi_Chr01	1906333	(A/C)
24591	CadSNP186	*Desi*	Ca_Desi_Chr01	1906363	(G/A)
24592	CadSNP187	*Desi*	Ca_Desi_Chr01	1906417	(G/A)
24593	CadSNP188	*Desi*	Ca_Desi_Chr01	1912864	(T/A)
24594	CadSNP189	*Desi*	Ca_Desi_Chr01	1912850	(A/C)
24595	CadSNP190	*Desi*	Ca_Desi_Chr01	1912820	(C/T)
24596	CadSNP191	*Desi*	Ca_Desi_Chr01	1920014	(C/G)
24597	CadSNP192	*Desi*	Ca_Desi_Chr01	1920100	(A/G)
24598	CadSNP193	*Desi*	Ca_Desi_Chr01	1936976	(C/T)
24599	CadSNP194	*Desi*	Ca_Desi_Chr01	1937117	(A/G)
24600	CadSNP195	*Desi*	Ca_Desi_Chr01	1937351	(C/T)
24601	CadSNP196	*Desi*	Ca_Desi_Chr01	1960896	(A/C)
24602	CadSNP197	*Desi*	Ca_Desi_Chr01	1960988	(A/T)
24603	CadSNP198	*Desi*	Ca_Desi_Chr01	1971575	(C/T)
24604	CadSNP199	*Desi*	Ca_Desi_Chr01	1982239	(C/A)
24605	CadSNP200	*Desi*	Ca_Desi_Chr01	1982267	(T/G)
24606	CadSNP201	*Desi*	Ca_Desi_Chr01	2021813	(A/G)
24607	CadSNP202	*Desi*	Ca_Desi_Chr01	2021754	(T/A)

S.N.	SNP IDs	Cultivars	Chromosomes/scaffolds	Physical positions (bp)	SNPs
24608	CadSNP203	*Desi*	Ca_Desi_Chr01	2044121	(A/G)
24609	CadSNP204	*Desi*	Ca_Desi_Chr01	2067290	(C/A)
24610	CadSNP205	*Desi*	Ca_Desi_Chr01	2084464	(G/A)
24611	CadSNP206	*Desi*	Ca_Desi_Chr01	2084449	(A/G)
24612	CadSNP207	*Desi*	Ca_Desi_Chr01	2084396	(T/G)
24613	CadSNP208	*Desi*	Ca_Desi_Chr01	2084474	(C/A)
24614	CadSNP209	*Desi*	Ca_Desi_Chr01	2084506	(G/T)
24615	CadSNP210	*Desi*	Ca_Desi_Chr01	2084548	(C/G)
24616	CadSNP211	*Desi*	Ca_Desi_Chr01	2185003	(C/T)
24617	CadSNP212	*Desi*	Ca_Desi_Chr01	2213640	(A/G)
24618	CadSNP213	*Desi*	Ca_Desi_Chr01	2213694	(C/T)
24619	CadSNP214	*Desi*	Ca_Desi_Chr01	2213700	(C/T)
24620	CadSNP215	*Desi*	Ca_Desi_Chr01	2213762	(T/C)
24621	CadSNP216	*Desi*	Ca_Desi_Chr01	2213817	(A/G)
24622	CadSNP217	*Desi*	Ca_Desi_Chr01	2214078	(A/T)
24623	CadSNP218	*Desi*	Ca_Desi_Chr01	2214038	(A/T)
24624	CadSNP219	*Desi*	Ca_Desi_Chr01	2214019	(A/G)
24625	CadSNP220	*Desi*	Ca_Desi_Chr01	2249232	(A/G)
24626	CadSNP221	*Desi*	Ca_Desi_Chr01	2263604	(C/A)
24627	CadSNP222	*Desi*	Ca_Desi_Chr01	2281554	(T/C)
24628	CadSNP223	*Desi*	Ca_Desi_Chr01	2281552	(C/T)
24629	CadSNP224	*Desi*	Ca_Desi_Chr01	2292933	(T/C)
24630	CadSNP225	*Desi*	Ca_Desi_Chr01	2322362	(G/A)
24631	CadSNP226	*Desi*	Ca_Desi_Chr01	2322455	(A/C)
24632	CadSNP227	*Desi*	Ca_Desi_Chr01	2327411	(T/G)
24633	CadSNP228	*Desi*	Ca_Desi_Chr01	2349214	(C/A)
24634	CadSNP229	*Desi*	Ca_Desi_Chr01	2349258	(G/A)
24635	CadSNP230	*Desi*	Ca_Desi_Chr01	2349306	(C/T)
24636	CadSNP231	*Desi*	Ca_Desi_Chr01	2350002	(T/C)
24637	CadSNP232	*Desi*	Ca_Desi_Chr01	2356768	(A/G)
24638	CadSNP233	*Desi*	Ca_Desi_Chr01	2356772	(A/T)
24639	CadSNP234	*Desi*	Ca_Desi_Chr01	2438649	(T/A)
24640	CadSNP235	*Desi*	Ca_Desi_Chr01	2438719	(T/A)
24641	CadSNP236	*Desi*	Ca_Desi_Chr01	2440248	(C/T)
24642	CadSNP237	*Desi*	Ca_Desi_Chr01	2448997	(C/T)
24643	CadSNP238	*Desi*	Ca_Desi_Chr01	2456844	(T/G)
24644	CadSNP239	*Desi*	Ca_Desi_Chr01	2457014	(A/C)
24645	CadSNP240	*Desi*	Ca_Desi_Chr01	2471770	(G/T)
24646	CadSNP241	*Desi*	Ca_Desi_Chr01	2471753	(G/A)

S.N.	SNP IDs	Cultivars	Chromosomes/scaffolds	Physical positions (bp)	SNPs
24647	CadSNP242	*Desi*	Ca_Desi_Chr01	2471803	(A/G)
24648	CadSNP243	*Desi*	Ca_Desi_Chr01	2471865	(T/C)
24649	CadSNP244	*Desi*	Ca_Desi_Chr01	2471996	(G/A)
24650	CadSNP245	*Desi*	Ca_Desi_Chr01	2471946	(C/A)
24651	CadSNP246	*Desi*	Ca_Desi_Chr01	2471938	(G/T)
24652	CadSNP247	*Desi*	Ca_Desi_Chr01	2471924	(G/A)
24653	CadSNP248	*Desi*	Ca_Desi_Chr01	2471923	(C/A)
24654	CadSNP249	*Desi*	Ca_Desi_Chr01	2491534	(T/C)
24655	CadSNP250	*Desi*	Ca_Desi_Chr01	2491494	(G/A)
24656	CadSNP251	*Desi*	Ca_Desi_Chr01	2491479	(T/C)
24657	CadSNP252	*Desi*	Ca_Desi_Chr01	2536793	(A/G)
24658	CadSNP253	*Desi*	Ca_Desi_Chr01	2541722	(T/C)
24659	CadSNP254	*Desi*	Ca_Desi_Chr01	2553114	(C/T)
24660	CadSNP255	*Desi*	Ca_Desi_Chr01	2553120	(A/C)
24661	CadSNP256	*Desi*	Ca_Desi_Chr01	2553187	(A/G)
24662	CadSNP257	*Desi*	Ca_Desi_Chr01	2553150	(C/T)
24663	CadSNP258	*Desi*	Ca_Desi_Chr01	2554326	(T/G)
24664	CadSNP259	*Desi*	Ca_Desi_Chr01	2555171	(C/G)
24665	CadSNP260	*Desi*	Ca_Desi_Chr01	2573180	(A/C)
24666	CadSNP261	*Desi*	Ca_Desi_Chr01	2593870	(C/T)
24667	CadSNP262	*Desi*	Ca_Desi_Chr01	2637647	(C/G)
24668	CadSNP263	*Desi*	Ca_Desi_Chr01	2637591	(T/A)
24669	CadSNP264	*Desi*	Ca_Desi_Chr01	2637890	(G/C)
24670	CadSNP265	*Desi*	Ca_Desi_Chr01	2637849	(C/T)
24671	CadSNP266	*Desi*	Ca_Desi_Chr01	2775191	(G/A)
24672	CadSNP267	*Desi*	Ca_Desi_Chr01	2824614	(G/A)
24673	CadSNP268	*Desi*	Ca_Desi_Chr01	2856666	(T/C)
24674	CadSNP269	*Desi*	Ca_Desi_Chr01	2887261	(C/G)
24675	CadSNP270	*Desi*	Ca_Desi_Chr01	3010959	(T/C)
24676	CadSNP271	*Desi*	Ca_Desi_Chr01	3011053	(T/G)
24677	CadSNP272	*Desi*	Ca_Desi_Chr01	3073657	(C/T)
24678	CadSNP273	*Desi*	Ca_Desi_Chr01	3083080	(G/T)
24679	CadSNP274	*Desi*	Ca_Desi_Chr01	3088883	(G/T)
24680	CadSNP275	*Desi*	Ca_Desi_Chr01	3151378	(A/C)
24681	CadSNP276	*Desi*	Ca_Desi_Chr01	3151834	(G/C)
24682	CadSNP277	*Desi*	Ca_Desi_Chr01	3192625	(C/T)
24683	CadSNP278	*Desi*	Ca_Desi_Chr01	3192622	(C/G)
24684	CadSNP279	*Desi*	Ca_Desi_Chr01	3192562	(C/G)
24685	CadSNP280	*Desi*	Ca_Desi_Chr01	3328510	(C/A)

S.N.	SNP IDs	Cultivars	Chromosomes/scaffolds	Physical positions (bp)	SNPs
24686	CadSNP281	*Desi*	Ca_Desi_Chr01	3328604	(G/A)
24687	CadSNP282	*Desi*	Ca_Desi_Chr01	3366300	(T/C)
24688	CadSNP283	*Desi*	Ca_Desi_Chr01	3368165	(A/C)
24689	CadSNP284	*Desi*	Ca_Desi_Chr01	3370851	(A/T)
24690	CadSNP285	*Desi*	Ca_Desi_Chr01	3370914	(G/A)
24691	CadSNP286	*Desi*	Ca_Desi_Chr01	3390238	(G/T)
24692	CadSNP287	*Desi*	Ca_Desi_Chr01	3425891	(C/T)
24693	CadSNP288	*Desi*	Ca_Desi_Chr01	3427492	(G/A)
24694	CadSNP289	*Desi*	Ca_Desi_Chr01	3435226	(G/A)
24695	CadSNP290	*Desi*	Ca_Desi_Chr01	3435227	(C/T)
24696	CadSNP291	*Desi*	Ca_Desi_Chr01	3439224	(T/C)
24697	CadSNP292	*Desi*	Ca_Desi_Chr01	3440875	(C/A)
24698	CadSNP293	*Desi*	Ca_Desi_Chr01	3444610	(A/G)
24699	CadSNP294	*Desi*	Ca_Desi_Chr01	3444681	(G/T)
24700	CadSNP295	*Desi*	Ca_Desi_Chr01	3444708	(T/C)
24701	CadSNP296	*Desi*	Ca_Desi_Chr01	3444696	(C/A)
24702	CadSNP297	*Desi*	Ca_Desi_Chr01	3452397	(C/T)
24703	CadSNP298	*Desi*	Ca_Desi_Chr01	3545305	(T/G)
24704	CadSNP299	*Desi*	Ca_Desi_Chr01	3545332	(T/G)
24705	CadSNP300	*Desi*	Ca_Desi_Chr01	3585574	(G/T)
24706	CadSNP301	*Desi*	Ca_Desi_Chr01	3606976	(C/T)
24707	CadSNP302	*Desi*	Ca_Desi_Chr01	3633117	(T/C)
24708	CadSNP303	*Desi*	Ca_Desi_Chr01	3674413	(A/C)
24709	CadSNP304	*Desi*	Ca_Desi_Chr01	3674854	(A/C)
24710	CadSNP305	*Desi*	Ca_Desi_Chr01	3675786	(T/C)
24711	CadSNP306	*Desi*	Ca_Desi_Chr01	3677607	(G/A)
24712	CadSNP307	*Desi*	Ca_Desi_Chr01	3743152	(G/A)
24713	CadSNP308	*Desi*	Ca_Desi_Chr01	3771750	(C/T)
24714	CadSNP309	*Desi*	Ca_Desi_Chr01	3771899	(T/C)
24715	CadSNP310	*Desi*	Ca_Desi_Chr01	3783317	(G/A)
24716	CadSNP311	*Desi*	Ca_Desi_Chr01	3786526	(T/C)
24717	CadSNP312	*Desi*	Ca_Desi_Chr01	3786523	(C/T)
24718	CadSNP313	*Desi*	Ca_Desi_Chr01	3804312	(G/T)
24719	CadSNP314	*Desi*	Ca_Desi_Chr01	3877417	(G/A)
24720	CadSNP315	*Desi*	Ca_Desi_Chr01	3877438	(A/T)
24721	CadSNP316	*Desi*	Ca_Desi_Chr01	3911580	(A/G)
24722	CadSNP317	*Desi*	Ca_Desi_Chr01	3953451	(C/T)
24723	CadSNP318	*Desi*	Ca_Desi_Chr01	3963327	(T/C)
24724	CadSNP319	*Desi*	Ca_Desi_Chr01	3993197	(C/T)

S.N.	SNP IDs	Cultivars	Chromosomes/scaffolds	Physical positions (bp)	SNPs
24725	CadSNP320	*Desi*	Ca_Desi_Chr01	3993242	(T/G)
24726	CadSNP321	*Desi*	Ca_Desi_Chr01	3994131	(G/A)
24727	CadSNP322	*Desi*	Ca_Desi_Chr01	3995579	(C/T)
24728	CadSNP323	*Desi*	Ca_Desi_Chr01	3996356	(C/T)
24729	CadSNP324	*Desi*	Ca_Desi_Chr01	3996916	(C/T)
24730	CadSNP325	*Desi*	Ca_Desi_Chr01	5004852	(G/A)
24731	CadSNP326	*Desi*	Ca_Desi_Chr01	5004855	(A/C)
24732	CadSNP327	*Desi*	Ca_Desi_Chr01	5004857	(G/C)
24733	CadSNP328	*Desi*	Ca_Desi_Chr01	5004917	(C/A)
24734	CadSNP329	*Desi*	Ca_Desi_Chr01	5022262	(G/T)
24735	CadSNP330	*Desi*	Ca_Desi_Chr01	5022319	(G/A)
24736	CadSNP331	*Desi*	Ca_Desi_Chr01	5022390	(C/T)
24737	CadSNP332	*Desi*	Ca_Desi_Chr01	5039591	(A/G)
24738	CadSNP333	*Desi*	Ca_Desi_Chr01	5039614	(C/A)
24739	CadSNP334	*Desi*	Ca_Desi_Chr01	5040610	(A/G)
24740	CadSNP335	*Desi*	Ca_Desi_Chr01	5040663	(A/G)
24741	CadSNP336	*Desi*	Ca_Desi_Chr01	5056212	(T/C)
24742	CadSNP337	*Desi*	Ca_Desi_Chr01	5056260	(A/C)
24743	CadSNP338	*Desi*	Ca_Desi_Chr01	5056328	(T/C)
24744	CadSNP339	*Desi*	Ca_Desi_Chr01	5059326	(A/G)
24745	CadSNP340	*Desi*	Ca_Desi_Chr01	5061066	(T/A)
24746	CadSNP341	*Desi*	Ca_Desi_Chr01	5061125	(G/C)
24747	CadSNP342	*Desi*	Ca_Desi_Chr01	5069716	(A/G)
24748	CadSNP343	*Desi*	Ca_Desi_Chr01	5069743	(A/G)
24749	CadSNP344	*Desi*	Ca_Desi_Chr01	5094757	(G/C)
24750	CadSNP345	*Desi*	Ca_Desi_Chr01	5094752	(G/A)
24751	CadSNP346	*Desi*	Ca_Desi_Chr01	5161857	(C/T)
24752	CadSNP347	*Desi*	Ca_Desi_Chr01	5197254	(A/G)
24753	CadSNP348	*Desi*	Ca_Desi_Chr01	5197445	(C/A)
24754	CadSNP349	*Desi*	Ca_Desi_Chr01	5202104	(T/C)
24755	CadSNP350	*Desi*	Ca_Desi_Chr01	5202057	(A/T)
24756	CadSNP351	*Desi*	Ca_Desi_Chr01	5218664	(T/C)
24757	CadSNP352	*Desi*	Ca_Desi_Chr01	5227055	(C/A)
24758	CadSNP353	*Desi*	Ca_Desi_Chr01	5240171	(G/A)
24759	CadSNP354	*Desi*	Ca_Desi_Chr01	5292807	(A/G)
24760	CadSNP355	*Desi*	Ca_Desi_Chr01	5292810	(A/T)
24761	CadSNP356	*Desi*	Ca_Desi_Chr01	5348734	(A/G)
24762	CadSNP357	*Desi*	Ca_Desi_Chr01	5348941	(T/C)
24763	CadSNP358	*Desi*	Ca_Desi_Chr01	5428980	(C/A)

S.N.	SNP IDs	Cultivars	Chromosomes/scaffolds	Physical positions (bp)	SNPs
24764	CadSNP359	*Desi*	Ca_Desi_Chr01	5446596	(A/C)
24765	CadSNP360	*Desi*	Ca_Desi_Chr01	5446549	(A/C)
24766	CadSNP361	*Desi*	Ca_Desi_Chr01	5498868	(A/G)
24767	CadSNP362	*Desi*	Ca_Desi_Chr01	5498890	(A/G)
24768	CadSNP363	*Desi*	Ca_Desi_Chr01	5506578	(G/C)
24769	CadSNP364	*Desi*	Ca_Desi_Chr01	5511110	(A/T)
24770	CadSNP365	*Desi*	Ca_Desi_Chr01	5511140	(A/T)
24771	CadSNP366	*Desi*	Ca_Desi_Chr01	5511162	(T/G)
24772	CadSNP367	*Desi*	Ca_Desi_Chr01	5511149	(T/C)
24773	CadSNP368	*Desi*	Ca_Desi_Chr01	5537208	(C/T)
24774	CadSNP369	*Desi*	Ca_Desi_Chr01	5537211	(G/A)
24775	CadSNP370	*Desi*	Ca_Desi_Chr01	5537394	(A/G)
24776	CadSNP371	*Desi*	Ca_Desi_Chr01	5537397	(T/A)
24777	CadSNP372	*Desi*	Ca_Desi_Chr01	5537508	(C/T)
24778	CadSNP373	*Desi*	Ca_Desi_Chr01	5555967	(C/T)
24779	CadSNP374	*Desi*	Ca_Desi_Chr01	5556081	(G/A)
24780	CadSNP375	*Desi*	Ca_Desi_Chr01	5568121	(A/C)
24781	CadSNP376	*Desi*	Ca_Desi_Chr01	5580120	(C/T)
24782	CadSNP377	*Desi*	Ca_Desi_Chr01	5580193	(C/T)
24783	CadSNP378	*Desi*	Ca_Desi_Chr01	5605911	(G/C)
24784	CadSNP379	*Desi*	Ca_Desi_Chr01	5605923	(G/T)
24785	CadSNP380	*Desi*	Ca_Desi_Chr01	5613829	(C/A)
24786	CadSNP381	*Desi*	Ca_Desi_Chr01	5644131	(G/C)
24787	CadSNP382	*Desi*	Ca_Desi_Chr01	5645053	(C/T)
24788	CadSNP383	*Desi*	Ca_Desi_Chr01	5645050	(T/C)
24789	CadSNP384	*Desi*	Ca_Desi_Chr01	5645015	(T/C)
24790	CadSNP385	*Desi*	Ca_Desi_Chr01	5667771	(A/G)
24791	CadSNP386	*Desi*	Ca_Desi_Chr01	5676601	(T/C)
24792	CadSNP387	*Desi*	Ca_Desi_Chr01	5704265	(C/A)
24793	CadSNP388	*Desi*	Ca_Desi_Chr01	5753424	(G/A)
24794	CadSNP389	*Desi*	Ca_Desi_Chr01	5753449	(T/G)
24795	CadSNP390	*Desi*	Ca_Desi_Chr01	5753484	(T/G)
24796	CadSNP391	*Desi*	Ca_Desi_Chr01	5776338	(C/A)
24797	CadSNP392	*Desi*	Ca_Desi_Chr01	5776339	(A/G)
24798	CadSNP393	*Desi*	Ca_Desi_Chr01	5776369	(T/C)
24799	CadSNP394	*Desi*	Ca_Desi_Chr01	5776540	(T/C)
24800	CadSNP395	*Desi*	Ca_Desi_Chr01	5778096	(C/G)
24801	CadSNP396	*Desi*	Ca_Desi_Chr01	5807567	(C/G)
24802	CadSNP397	*Desi*	Ca_Desi_Chr01	5807642	(G/A)

S.N.	SNP IDs	Cultivars	Chromosomes/scaffolds	Physical positions (bp)	SNPs
24803	CadSNP398	*Desi*	Ca_Desi_Chr01	5823260	(C/T)
24804	CadSNP399	*Desi*	Ca_Desi_Chr01	5853627	(T/A)
24805	CadSNP400	*Desi*	Ca_Desi_Chr01	5853706	(G/A)
24806	CadSNP401	*Desi*	Ca_Desi_Chr01	5854373	(C/T)
24807	CadSNP402	*Desi*	Ca_Desi_Chr01	6037738	(T/G)
24808	CadSNP403	*Desi*	Ca_Desi_Chr01	6072236	(A/G)
24809	CadSNP404	*Desi*	Ca_Desi_Chr01	6072205	(A/G)
24810	CadSNP405	*Desi*	Ca_Desi_Chr01	6080835	(G/C)
24811	CadSNP406	*Desi*	Ca_Desi_Chr01	6080870	(T/C)
24812	CadSNP407	*Desi*	Ca_Desi_Chr01	6084027	(C/T)
24813	CadSNP408	*Desi*	Ca_Desi_Chr01	6083987	(T/G)
24814	CadSNP409	*Desi*	Ca_Desi_Chr01	6107667	(T/C)
24815	CadSNP410	*Desi*	Ca_Desi_Chr01	6107673	(A/C)
24816	CadSNP411	*Desi*	Ca_Desi_Chr01	6108004	(C/A)
24817	CadSNP412	*Desi*	Ca_Desi_Chr01	6108008	(T/G)
24818	CadSNP413	*Desi*	Ca_Desi_Chr01	6108016	(C/T)
24819	CadSNP414	*Desi*	Ca_Desi_Chr01	6108035	(T/A)
24820	CadSNP415	*Desi*	Ca_Desi_Chr01	6108091	(T/G)
24821	CadSNP416	*Desi*	Ca_Desi_Chr01	6108094	(C/T)
24822	CadSNP417	*Desi*	Ca_Desi_Chr01	6108141	(T/A)
24823	CadSNP418	*Desi*	Ca_Desi_Chr01	6108140	(A/G)
24824	CadSNP419	*Desi*	Ca_Desi_Chr01	6111677	(A/G)
24825	CadSNP420	*Desi*	Ca_Desi_Chr01	6111732	(T/G)
24826	CadSNP421	*Desi*	Ca_Desi_Chr01	6112469	(C/T)
24827	CadSNP422	*Desi*	Ca_Desi_Chr01	6112462	(G/A)
24828	CadSNP423	*Desi*	Ca_Desi_Chr01	6112461	(A/G)
24829	CadSNP424	*Desi*	Ca_Desi_Chr01	6112451	(A/G)
24830	CadSNP425	*Desi*	Ca_Desi_Chr01	6112443	(A/G)
24831	CadSNP426	*Desi*	Ca_Desi_Chr01	6112435	(C/A)
24832	CadSNP427	*Desi*	Ca_Desi_Chr01	6112434	(T/A)
24833	CadSNP428	*Desi*	Ca_Desi_Chr01	6112419	(T/G)
24834	CadSNP429	*Desi*	Ca_Desi_Chr01	6113487	(T/C)
24835	CadSNP430	*Desi*	Ca_Desi_Chr01	6113445	(T/C)
24836	CadSNP431	*Desi*	Ca_Desi_Chr01	6135902	(A/G)
24837	CadSNP432	*Desi*	Ca_Desi_Chr01	6141133	(A/C)
24838	CadSNP433	*Desi*	Ca_Desi_Chr01	6141126	(A/C)
24839	CadSNP434	*Desi*	Ca_Desi_Chr01	6149357	(C/A)
24840	CadSNP435	*Desi*	Ca_Desi_Chr01	6262953	(A/C)
24841	CadSNP436	*Desi*	Ca_Desi_Chr01	6262893	(T/A)

S.N.	SNP IDs	Cultivars	Chromosomes/scaffolds	Physical positions (bp)	SNPs
24842	CadSNP437	*Desi*	Ca_Desi_Chr01	6303938	(A/G)
24843	CadSNP438	*Desi*	Ca_Desi_Chr01	6303965	(T/G)
24844	CadSNP439	*Desi*	Ca_Desi_Chr01	6304541	(A/G)
24845	CadSNP440	*Desi*	Ca_Desi_Chr01	6313746	(T/A)
24846	CadSNP441	*Desi*	Ca_Desi_Chr01	6313759	(T/A)
24847	CadSNP442	*Desi*	Ca_Desi_Chr01	6318871	(G/T)
24848	CadSNP443	*Desi*	Ca_Desi_Chr01	6319012	(A/G)
24849	CadSNP444	*Desi*	Ca_Desi_Chr01	6319042	(A/C)
24850	CadSNP445	*Desi*	Ca_Desi_Chr01	6322174	(A/G)
24851	CadSNP446	*Desi*	Ca_Desi_Chr01	6322202	(G/T)
24852	CadSNP447	*Desi*	Ca_Desi_Chr01	6333247	(A/G)
24853	CadSNP448	*Desi*	Ca_Desi_Chr01	6333238	(C/T)
24854	CadSNP449	*Desi*	Ca_Desi_Chr01	6333418	(G/A)
24855	CadSNP450	*Desi*	Ca_Desi_Chr01	6341237	(C/T)
24856	CadSNP451	*Desi*	Ca_Desi_Chr01	6341213	(A/C)
24857	CadSNP452	*Desi*	Ca_Desi_Chr01	6351975	(T/C)
24858	CadSNP453	*Desi*	Ca_Desi_Chr01	6379951	(G/A)
24859	CadSNP454	*Desi*	Ca_Desi_Chr01	6399918	(C/T)
24860	CadSNP455	*Desi*	Ca_Desi_Chr01	6399989	(C/T)
24861	CadSNP456	*Desi*	Ca_Desi_Chr01	6400007	(C/T)
24862	CadSNP457	*Desi*	Ca_Desi_Chr01	6747779	(C/T)
24863	CadSNP458	*Desi*	Ca_Desi_Chr01	6750146	(A/G)
24864	CadSNP459	*Desi*	Ca_Desi_Chr01	6836227	(A/T)
24865	CadSNP460	*Desi*	Ca_Desi_Chr01	6858906	(T/A)
24866	CadSNP461	*Desi*	Ca_Desi_Chr01	6858922	(C/T)
24867	CadSNP462	*Desi*	Ca_Desi_Chr01	6858925	(C/T)
24868	CadSNP463	*Desi*	Ca_Desi_Chr01	6858952	(A/C)
24869	CadSNP464	*Desi*	Ca_Desi_Chr01	6858964	(A/C)
24870	CadSNP465	*Desi*	Ca_Desi_Chr01	6859408	(C/A)
24871	CadSNP466	*Desi*	Ca_Desi_Chr01	6859661	(C/A)
24872	CadSNP467	*Desi*	Ca_Desi_Chr01	6859634	(T/C)
24873	CadSNP468	*Desi*	Ca_Desi_Chr01	6859628	(G/A)
24874	CadSNP469	*Desi*	Ca_Desi_Chr01	6859622	(A/G)
24875	CadSNP470	*Desi*	Ca_Desi_Chr01	6861332	(T/A)
24876	CadSNP471	*Desi*	Ca_Desi_Chr01	6862834	(C/A)
24877	CadSNP472	*Desi*	Ca_Desi_Chr01	6862836	(T/G)
24878	CadSNP473	*Desi*	Ca_Desi_Chr01	6862866	(A/G)
24879	CadSNP474	*Desi*	Ca_Desi_Chr01	6862869	(C/T)
24880	CadSNP475	*Desi*	Ca_Desi_Chr01	6862872	(A/T)

S.N.	SNP IDs	Cultivars	Chromosomes/scaffolds	Physical positions (bp)	SNPs
24881	CadSNP476	*Desi*	Ca_Desi_Chr01	6862873	(A/C)
24882	CadSNP477	*Desi*	Ca_Desi_Chr01	6862881	(A/C)
24883	CadSNP478	*Desi*	Ca_Desi_Chr01	6862888	(T/A)
24884	CadSNP479	*Desi*	Ca_Desi_Chr01	6862889	(A/C)
24885	CadSNP480	*Desi*	Ca_Desi_Chr01	6862893	(T/C)
24886	CadSNP481	*Desi*	Ca_Desi_Chr01	7013189	(A/G)
24887	CadSNP482	*Desi*	Ca_Desi_Chr01	7142598	(C/T)
24888	CadSNP483	*Desi*	Ca_Desi_Chr01	7168613	(G/A)
24889	CadSNP484	*Desi*	Ca_Desi_Chr01	7274246	(C/T)
24890	CadSNP485	*Desi*	Ca_Desi_Chr01	7279148	(G/A)
24891	CadSNP486	*Desi*	Ca_Desi_Chr01	7279147	(G/A)
24892	CadSNP487	*Desi*	Ca_Desi_Chr01	7279116	(G/A)
24893	CadSNP488	*Desi*	Ca_Desi_Chr01	7279115	(T/C)
24894	CadSNP489	*Desi*	Ca_Desi_Chr01	7279106	(G/T)
24895	CadSNP490	*Desi*	Ca_Desi_Chr01	7279097	(G/A)
24896	CadSNP491	*Desi*	Ca_Desi_Chr01	7289926	(G/A)
24897	CadSNP492	*Desi*	Ca_Desi_Chr01	7289965	(G/A)
24898	CadSNP493	*Desi*	Ca_Desi_Chr01	7331882	(T/C)
24899	CadSNP494	*Desi*	Ca_Desi_Chr01	7331912	(C/G)
24900	CadSNP495	*Desi*	Ca_Desi_Chr01	7331936	(C/T)
24901	CadSNP496	*Desi*	Ca_Desi_Chr01	7331994	(G/A)
24902	CadSNP497	*Desi*	Ca_Desi_Chr01	7331987	(C/T)
24903	CadSNP498	*Desi*	Ca_Desi_Chr01	7383196	(A/C)
24904	CadSNP499	*Desi*	Ca_Desi_Chr01	7383206	(A/G)
24905	CadSNP500	*Desi*	Ca_Desi_Chr01	7383207	(G/A)
24906	CadSNP501	*Desi*	Ca_Desi_Chr01	7383208	(T/A)
24907	CadSNP502	*Desi*	Ca_Desi_Chr01	7383224	(A/G)
24908	CadSNP503	*Desi*	Ca_Desi_Chr01	7383225	(G/A)
24909	CadSNP504	*Desi*	Ca_Desi_Chr01	7383226	(C/A)
24910	CadSNP505	*Desi*	Ca_Desi_Chr01	7383229	(G/A)
24911	CadSNP506	*Desi*	Ca_Desi_Chr01	7383232	(G/A)
24912	CadSNP507	*Desi*	Ca_Desi_Chr01	7383255	(A/G)
24913	CadSNP508	*Desi*	Ca_Desi_Chr01	7383256	(G/A)
24914	CadSNP509	*Desi*	Ca_Desi_Chr01	7383259	(G/A)
24915	CadSNP510	*Desi*	Ca_Desi_Chr01	7383265	(T/G)
24916	CadSNP511	*Desi*	Ca_Desi_Chr01	7383271	(A/G)
24917	CadSNP512	*Desi*	Ca_Desi_Chr01	7383274	(C/T)
24918	CadSNP513	*Desi*	Ca_Desi_Chr01	7383281	(G/A)
24919	CadSNP514	*Desi*	Ca_Desi_Chr01	7383437	(T/C)

S.N.	SNP IDs	Cultivars	Chromosomes/scaffolds	Physical positions (bp)	SNPs
24920	CadSNP515	*Desi*	Ca_Desi_Chr01	7383436	(G/A)
24921	CadSNP516	*Desi*	Ca_Desi_Chr01	7383433	(T/A)
24922	CadSNP517	*Desi*	Ca_Desi_Chr01	7383406	(C/T)
24923	CadSNP518	*Desi*	Ca_Desi_Chr01	7383808	(A/T)
24924	CadSNP519	*Desi*	Ca_Desi_Chr01	7436170	(T/A)
24925	CadSNP520	*Desi*	Ca_Desi_Chr01	7436190	(T/G)
24926	CadSNP521	*Desi*	Ca_Desi_Chr01	7436196	(G/A)
24927	CadSNP522	*Desi*	Ca_Desi_Chr01	7436205	(T/G)
24928	CadSNP523	*Desi*	Ca_Desi_Chr01	7448000	(C/G)
24929	CadSNP524	*Desi*	Ca_Desi_Chr01	7489519	(T/A)
24930	CadSNP525	*Desi*	Ca_Desi_Chr01	7489492	(T/A)
24931	CadSNP526	*Desi*	Ca_Desi_Chr01	7489471	(T/A)
24932	CadSNP527	*Desi*	Ca_Desi_Chr01	7489452	(T/G)
24933	CadSNP528	*Desi*	Ca_Desi_Chr01	7489451	(A/T)
24934	CadSNP529	*Desi*	Ca_Desi_Chr01	7489447	(C/A)
24935	CadSNP530	*Desi*	Ca_Desi_Chr01	7606885	(C/G)
24936	CadSNP531	*Desi*	Ca_Desi_Chr01	7606933	(C/T)
24937	CadSNP532	*Desi*	Ca_Desi_Chr01	7632307	(A/G)
24938	CadSNP533	*Desi*	Ca_Desi_Chr01	7632356	(C/T)
24939	CadSNP534	*Desi*	Ca_Desi_Chr01	7668717	(G/A)
24940	CadSNP535	*Desi*	Ca_Desi_Chr01	7669506	(G/C)
24941	CadSNP536	*Desi*	Ca_Desi_Chr01	7669603	(G/C)
24942	CadSNP537	*Desi*	Ca_Desi_Chr01	7671799	(A/G)
24943	CadSNP538	*Desi*	Ca_Desi_Chr01	7671815	(G/A)
24944	CadSNP539	*Desi*	Ca_Desi_Chr01	7671819	(T/C)
24945	CadSNP540	*Desi*	Ca_Desi_Chr01	7671842	(A/T)
24946	CadSNP541	*Desi*	Ca_Desi_Chr01	7671878	(T/G)
24947	CadSNP542	*Desi*	Ca_Desi_Chr01	7671931	(C/A)
24948	CadSNP543	*Desi*	Ca_Desi_Chr01	7671947	(C/T)
24949	CadSNP544	*Desi*	Ca_Desi_Chr01	7671963	(C/T)
24950	CadSNP545	*Desi*	Ca_Desi_Chr01	7676039	(G/C)
24951	CadSNP546	*Desi*	Ca_Desi_Chr01	7680425	(A/G)
24952	CadSNP547	*Desi*	Ca_Desi_Chr01	7680457	(C/T)
24953	CadSNP548	*Desi*	Ca_Desi_Chr01	7680543	(C/T)
24954	CadSNP549	*Desi*	Ca_Desi_Chr01	7712801	(A/C)
24955	CadSNP550	*Desi*	Ca_Desi_Chr01	7712821	(C/T)
24956	CadSNP551	*Desi*	Ca_Desi_Chr01	7956977	(T/G)
24957	CadSNP552	*Desi*	Ca_Desi_Chr01	7957053	(G/A)
24958	CadSNP553	*Desi*	Ca_Desi_Chr01	8163297	(C/A)

S.N.	SNP IDs	Cultivars	Chromosomes/scaffolds	Physical positions (bp)	SNPs
24959	CadSNP554	*Desi*	Ca_Desi_Chr01	8173005	(T/C)
24960	CadSNP555	*Desi*	Ca_Desi_Chr01	8219717	(A/G)
24961	CadSNP556	*Desi*	Ca_Desi_Chr01	8220943	(G/A)
24962	CadSNP557	*Desi*	Ca_Desi_Chr01	8220952	(C/T)
24963	CadSNP558	*Desi*	Ca_Desi_Chr01	8221152	(G/A)
24964	CadSNP559	*Desi*	Ca_Desi_Chr01	8279866	(T/G)
24965	CadSNP560	*Desi*	Ca_Desi_Chr01	8280837	(C/A)
24966	CadSNP561	*Desi*	Ca_Desi_Chr01	8280838	(C/G)
24967	CadSNP562	*Desi*	Ca_Desi_Chr01	8297933	(A/G)
24968	CadSNP563	*Desi*	Ca_Desi_Chr01	8365636	(A/C)
24969	CadSNP564	*Desi*	Ca_Desi_Chr01	8390528	(C/T)
24970	CadSNP565	*Desi*	Ca_Desi_Chr01	8397872	(C/T)
24971	CadSNP566	*Desi*	Ca_Desi_Chr01	8398204	(G/T)
24972	CadSNP567	*Desi*	Ca_Desi_Chr01	8416775	(G/A)
24973	CadSNP568	*Desi*	Ca_Desi_Chr01	8422096	(G/T)
24974	CadSNP569	*Desi*	Ca_Desi_Chr01	8422113	(G/T)
24975	CadSNP570	*Desi*	Ca_Desi_Chr01	8426569	(G/A)
24976	CadSNP571	*Desi*	Ca_Desi_Chr01	8492439	(T/A)
24977	CadSNP572	*Desi*	Ca_Desi_Chr01	8492480	(A/T)
24978	CadSNP573	*Desi*	Ca_Desi_Chr01	8492496	(A/T)
24979	CadSNP574	*Desi*	Ca_Desi_Chr01	8492493	(A/C)
24980	CadSNP575	*Desi*	Ca_Desi_Chr01	8492492	(A/G)
24981	CadSNP576	*Desi*	Ca_Desi_Chr01	8506952	(G/A)
24982	CadSNP577	*Desi*	Ca_Desi_Chr01	8506982	(A/G)
24983	CadSNP578	*Desi*	Ca_Desi_Chr01	8561851	(G/A)
24984	CadSNP579	*Desi*	Ca_Desi_Chr01	8689520	(A/C)
24985	CadSNP580	*Desi*	Ca_Desi_Chr01	8689473	(C/T)
24986	CadSNP581	*Desi*	Ca_Desi_Chr01	8725597	(G/A)
24987	CadSNP582	*Desi*	Ca_Desi_Chr01	8725599	(C/A)
24988	CadSNP583	*Desi*	Ca_Desi_Chr01	8725654	(A/T)
24989	CadSNP584	*Desi*	Ca_Desi_Chr01	8772201	(A/G)
24990	CadSNP585	*Desi*	Ca_Desi_Chr01	8786857	(C/A)
24991	CadSNP586	*Desi*	Ca_Desi_Chr01	8871945	(C/T)
24992	CadSNP587	*Desi*	Ca_Desi_Chr01	8902125	(C/A)
24993	CadSNP588	*Desi*	Ca_Desi_Chr01	8952943	(A/G)
24994	CadSNP589	*Desi*	Ca_Desi_Chr01	8988064	(G/A)
24995	CadSNP590	*Desi*	Ca_Desi_Chr01	8988049	(G/A)
24996	CadSNP591	*Desi*	Ca_Desi_Chr01	9014610	(C/A)
24997	CadSNP592	*Desi*	Ca_Desi_Chr01	9022986	(G/A)

S.N.	SNP IDs	Cultivars	Chromosomes/scaffolds	Physical positions (bp)	SNPs
24998	CadSNP593	*Desi*	Ca_Desi_Chr01	9023240	(T/C)
24999	CadSNP594	*Desi*	Ca_Desi_Chr01	9023290	(G/A)
25000	CadSNP595	*Desi*	Ca_Desi_Chr01	9033783	(T/C)
25001	CadSNP596	*Desi*	Ca_Desi_Chr01	9089686	(T/C)
25002	CadSNP597	*Desi*	Ca_Desi_Chr01	9193835	(G/A)
25003	CadSNP598	*Desi*	Ca_Desi_Chr01	9217460	(C/T)
25004	CadSNP599	*Desi*	Ca_Desi_Chr01	9217685	(A/G)
25005	CadSNP600	*Desi*	Ca_Desi_Chr01	9217840	(G/T)
25006	CadSNP601	*Desi*	Ca_Desi_Chr01	9218827	(T/G)
25007	CadSNP602	*Desi*	Ca_Desi_Chr01	9218838	(A/C)
25008	CadSNP603	*Desi*	Ca_Desi_Chr01	9218853	(G/A)
25009	CadSNP604	*Desi*	Ca_Desi_Chr01	9218890	(C/A)
25010	CadSNP605	*Desi*	Ca_Desi_Chr01	9298126	(T/G)
25011	CadSNP606	*Desi*	Ca_Desi_Chr01	9298398	(A/G)
25012	CadSNP607	*Desi*	Ca_Desi_Chr01	9306517	(A/C)
25013	CadSNP608	*Desi*	Ca_Desi_Chr01	9308560	(T/C)
25014	CadSNP609	*Desi*	Ca_Desi_Chr01	9308653	(C/T)
25015	CadSNP610	*Desi*	Ca_Desi_Chr01	9354522	(G/A)
25016	CadSNP611	*Desi*	Ca_Desi_Chr01	9354518	(T/G)
25017	CadSNP612	*Desi*	Ca_Desi_Chr01	9361145	(G/T)
25018	CadSNP613	*Desi*	Ca_Desi_Chr01	9362408	(G/A)
25019	CadSNP614	*Desi*	Ca_Desi_Chr01	9761124	(A/G)
25020	CadSNP615	*Desi*	Ca_Desi_Chr01	9761183	(A/G)
25021	CadSNP616	*Desi*	Ca_Desi_Chr01	9761192	(T/G)
25022	CadSNP617	*Desi*	Ca_Desi_Chr01	9761256	(T/C)
25023	CadSNP618	*Desi*	Ca_Desi_Chr01	10000773	(T/C)
25024	CadSNP619	*Desi*	Ca_Desi_Chr01	10002060	(A/C)
25025	CadSNP620	*Desi*	Ca_Desi_Chr01	10012819	(T/C)
25026	CadSNP621	*Desi*	Ca_Desi_Chr01	10012821	(G/A)
25027	CadSNP622	*Desi*	Ca_Desi_Chr01	10039308	(T/A)
25028	CadSNP623	*Desi*	Ca_Desi_Chr01	10078804	(A/G)
25029	CadSNP624	*Desi*	Ca_Desi_Chr01	10106781	(G/C)
25030	CadSNP625	*Desi*	Ca_Desi_Chr01	10311695	(T/C)
25031	CadSNP626	*Desi*	Ca_Desi_Chr01	10322769	(C/T)
25032	CadSNP627	*Desi*	Ca_Desi_Chr01	10331572	(A/G)
25033	CadSNP628	*Desi*	Ca_Desi_Chr01	10331598	(G/A)
25034	CadSNP629	*Desi*	Ca_Desi_Chr01	10371032	(G/A)
25035	CadSNP630	*Desi*	Ca_Desi_Chr01	10441613	(G/A)
25036	CadSNP631	*Desi*	Ca_Desi_Chr01	10441719	(A/G)

S.N.	SNP IDs	Cultivars	Chromosomes/scaffolds	Physical positions (bp)	SNPs
25037	CadSNP632	*Desi*	Ca_Desi_Chr01	10441699	(G/A)
25038	CadSNP633	*Desi*	Ca_Desi_Chr01	10441697	(A/C)
25039	CadSNP634	*Desi*	Ca_Desi_Chr01	10441800	(C/A)
25040	CadSNP635	*Desi*	Ca_Desi_Chr01	10441806	(C/A)
25041	CadSNP636	*Desi*	Ca_Desi_Chr01	10441837	(G/A)
25042	CadSNP637	*Desi*	Ca_Desi_Chr01	10474199	(G/A)
25043	CadSNP638	*Desi*	Ca_Desi_Chr01	10530999	(T/C)
25044	CadSNP639	*Desi*	Ca_Desi_Chr01	10608390	(A/T)
25045	CadSNP640	*Desi*	Ca_Desi_Chr01	10707824	(G/T)
25046	CadSNP641	*Desi*	Ca_Desi_Chr01	10719893	(T/G)
25047	CadSNP642	*Desi*	Ca_Desi_Chr01	10719894	(T/A)
25048	CadSNP643	*Desi*	Ca_Desi_Chr01	10720003	(A/T)
25049	CadSNP644	*Desi*	Ca_Desi_Chr01	10723301	(C/T)
25050	CadSNP645	*Desi*	Ca_Desi_Chr01	10741069	(G/C)
25051	CadSNP646	*Desi*	Ca_Desi_Chr01	10741089	(A/G)
25052	CadSNP647	*Desi*	Ca_Desi_Chr01	10754555	(C/G)
25053	CadSNP648	*Desi*	Ca_Desi_Chr01	10763266	(T/C)
25054	CadSNP649	*Desi*	Ca_Desi_Chr01	10827427	(C/T)
25055	CadSNP650	*Desi*	Ca_Desi_Chr01	10876770	(A/G)
25056	CadSNP651	*Desi*	Ca_Desi_Chr01	10876828	(C/A)
25057	CadSNP652	*Desi*	Ca_Desi_Chr01	10943212	(T/C)
25058	CadSNP653	*Desi*	Ca_Desi_Chr01	10972557	(C/T)
25059	CadSNP654	*Desi*	Ca_Desi_Chr01	10972556	(G/A)
25060	CadSNP655	*Desi*	Ca_Desi_Chr01	11104634	(G/T)
25061	CadSNP656	*Desi*	Ca_Desi_Chr01	11104890	(C/T)
25062	CadSNP657	*Desi*	Ca_Desi_Chr01	11106063	(A/T)
25063	CadSNP658	*Desi*	Ca_Desi_Chr01	11106278	(C/T)
25064	CadSNP659	*Desi*	Ca_Desi_Chr01	11109156	(G/C)
25065	CadSNP660	*Desi*	Ca_Desi_Chr01	11109187	(A/T)
25066	CadSNP661	*Desi*	Ca_Desi_Chr01	11140259	(G/T)
25067	CadSNP662	*Desi*	Ca_Desi_Chr01	11140249	(A/C)
25068	CadSNP663	*Desi*	Ca_Desi_Chr01	11140319	(A/T)
25069	CadSNP664	*Desi*	Ca_Desi_Chr01	11140333	(G/A)
25070	CadSNP665	*Desi*	Ca_Desi_Chr01	11153637	(G/T)
25071	CadSNP666	*Desi*	Ca_Desi_Chr01	11188515	(T/C)
25072	CadSNP667	*Desi*	Ca_Desi_Chr01	11188519	(A/G)
25073	CadSNP668	*Desi*	Ca_Desi_Chr01	11203214	(A/G)
25074	CadSNP669	*Desi*	Ca_Desi_Chr01	11242051	(C/A)
25075	CadSNP670	*Desi*	Ca_Desi_Chr01	11242086	(A/T)

S.N.	SNP IDs	Cultivars	Chromosomes/scaffolds	Physical positions (bp)	SNPs
25076	CadSNP671	*Desi*	Ca_Desi_Chr01	11242117	(C/T)
25077	CadSNP672	*Desi*	Ca_Desi_Chr01	11255650	(A/C)
25078	CadSNP673	*Desi*	Ca_Desi_Chr01	11255645	(A/C)
25079	CadSNP674	*Desi*	Ca_Desi_Chr01	11258827	(A/T)
25080	CadSNP675	*Desi*	Ca_Desi_Chr01	11302818	(G/A)
25081	CadSNP676	*Desi*	Ca_Desi_Chr01	11302861	(G/A)
25082	CadSNP677	*Desi*	Ca_Desi_Chr01	11302870	(A/G)
25083	CadSNP678	*Desi*	Ca_Desi_Chr01	11305587	(T/G)
25084	CadSNP679	*Desi*	Ca_Desi_Chr01	11368294	(C/A)
25085	CadSNP680	*Desi*	Ca_Desi_Chr01	11411238	(A/G)
25086	CadSNP681	*Desi*	Ca_Desi_Chr01	11411433	(G/C)
25087	CadSNP682	*Desi*	Ca_Desi_Chr01	11447597	(C/T)
25088	CadSNP683	*Desi*	Ca_Desi_Chr01	11447611	(C/T)
25089	CadSNP684	*Desi*	Ca_Desi_Chr01	11447639	(T/C)
25090	CadSNP685	*Desi*	Ca_Desi_Chr01	11447647	(C/T)
25091	CadSNP686	*Desi*	Ca_Desi_Chr01	11447673	(T/C)
25092	CadSNP687	*Desi*	Ca_Desi_Chr01	11470235	(A/G)
25093	CadSNP688	*Desi*	Ca_Desi_Chr01	11479650	(A/T)
25094	CadSNP689	*Desi*	Ca_Desi_Chr01	11520563	(T/A)
25095	CadSNP690	*Desi*	Ca_Desi_Chr01	11520595	(T/C)
25096	CadSNP691	*Desi*	Ca_Desi_Chr01	11568882	(C/T)
25097	CadSNP692	*Desi*	Ca_Desi_Chr01	11569800	(A/G)
25098	CadSNP693	*Desi*	Ca_Desi_Chr01	11569811	(A/C)
25099	CadSNP694	*Desi*	Ca_Desi_Chr01	11586934	(G/T)
25100	CadSNP695	*Desi*	Ca_Desi_Chr01	11588193	(C/T)
25101	CadSNP696	*Desi*	Ca_Desi_Chr01	11588259	(G/A)
25102	CadSNP697	*Desi*	Ca_Desi_Chr01	11900706	(G/C)
25103	CadSNP698	*Desi*	Ca_Desi_Chr01	11906460	(A/C)
25104	CadSNP699	*Desi*	Ca_Desi_Chr01	11909644	(A/C)
25105	CadSNP700	*Desi*	Ca_Desi_Chr01	12045526	(G/A)
25106	CadSNP701	*Desi*	Ca_Desi_Chr01	12155983	(T/G)
25107	CadSNP702	*Desi*	Ca_Desi_Chr01	12159674	(A/C)
25108	CadSNP703	*Desi*	Ca_Desi_Chr01	12163294	(C/A)
25109	CadSNP704	*Desi*	Ca_Desi_Chr01	12171484	(A/G)
25110	CadSNP705	*Desi*	Ca_Desi_Chr01	12171505	(T/C)
25111	CadSNP706	*Desi*	Ca_Desi_Chr01	12191974	(C/T)
25112	CadSNP707	*Desi*	Ca_Desi_Chr01	12192017	(G/C)
25113	CadSNP708	*Desi*	Ca_Desi_Chr01	12192106	(A/C)
25114	CadSNP709	*Desi*	Ca_Desi_Chr01	12192068	(C/G)

S.N.	SNP IDs	Cultivars	Chromosomes/scaffolds	Physical positions (bp)	SNPs
25115	CadSNP710	*Desi*	Ca_Desi_Chr01	13563876	(T/G)
25116	CadSNP711	*Desi*	Ca_Desi_Chr01	13585102	(C/G)
25117	CadSNP712	*Desi*	Ca_Desi_Chr01	13585162	(T/A)
25118	CadSNP713	*Desi*	Ca_Desi_Chr01	13590335	(A/G)
25119	CadSNP714	*Desi*	Ca_Desi_Chr01	13602425	(C/A)
25120	CadSNP715	*Desi*	Ca_Desi_Chr01	13620048	(C/T)
25121	CadSNP716	*Desi*	Ca_Desi_Chr01	13641367	(C/T)
25122	CadSNP717	*Desi*	Ca_Desi_Chr01	13661302	(A/G)
25123	CadSNP718	*Desi*	Ca_Desi_Chr01	13661337	(G/A)
25124	CadSNP719	*Desi*	Ca_Desi_Chr01	13690923	(G/A)
25125	CadSNP720	*Desi*	Ca_Desi_Chr01	13737053	(G/A)
25126	CadSNP721	*Desi*	Ca_Desi_Chr01	13748421	(C/G)
25127	CadSNP722	*Desi*	Ca_Desi_Chr01	13766314	(G/A)
25128	CadSNP723	*Desi*	Ca_Desi_Chr01	13798808	(A/T)
25129	CadSNP724	*Desi*	Ca_Desi_Chr01	13834782	(G/C)
25130	CadSNP725	*Desi*	Ca_Desi_Chr01	13843833	(C/T)
25131	CadSNP726	*Desi*	Ca_Desi_Chr01	13843906	(G/A)
25132	CadSNP727	*Desi*	Ca_Desi_Chr01	13843980	(C/A)
25133	CadSNP728	*Desi*	Ca_Desi_Chr01	13843964	(C/G)
25134	CadSNP729	*Desi*	Ca_Desi_Chr01	13843940	(C/T)
25135	CadSNP730	*Desi*	Ca_Desi_Chr01	13844080	(C/T)
25136	CadSNP731	*Desi*	Ca_Desi_Chr01	13844081	(A/T)
25137	CadSNP732	*Desi*	Ca_Desi_Chr01	13844082	(A/C)
25138	CadSNP733	*Desi*	Ca_Desi_Chr01	13844150	(G/C)
25139	CadSNP734	*Desi*	Ca_Desi_Chr01	13852748	(A/G)
25140	CadSNP735	*Desi*	Ca_Desi_Chr01	13852733	(G/A)
25141	CadSNP736	*Desi*	Ca_Desi_Chr01	13852713	(C/T)
25142	CadSNP737	*Desi*	Ca_Desi_Chr01	13853257	(T/C)
25143	CadSNP738	*Desi*	Ca_Desi_Chr01	13853344	(T/C)
25144	CadSNP739	*Desi*	Ca_Desi_Chr01	13864949	(G/A)
25145	CadSNP740	*Desi*	Ca_Desi_Chr01	13927517	(T/C)
25146	CadSNP741	*Desi*	Ca_Desi_Chr01	13927520	(T/C)
25147	CadSNP742	*Desi*	Ca_Desi_Chr01	13927698	(T/G)
25148	CadSNP743	*Desi*	Ca_Desi_Chr01	13934729	(G/T)
25149	CadSNP744	*Desi*	Ca_Desi_Chr01	13935583	(G/A)
25150	CadSNP745	*Desi*	Ca_Desi_Chr01	13969163	(T/G)
25151	CadSNP746	*Desi*	Ca_Desi_Chr01	13969143	(A/C)
25152	CadSNP747	*Desi*	Ca_Desi_Chr01	14093206	(T/A)
25153	CadSNP748	*Desi*	Ca_Desi_Chr01	14113511	(G/C)

S.N.	SNP IDs	Cultivars	Chromosomes/scaffolds	Physical positions (bp)	SNPs
25154	CadSNP749	*Desi*	Ca_Desi_Chr01	14113485	(A/G)
25155	CadSNP750	*Desi*	Ca_Desi_Chr01	14113598	(C/T)
25156	CadSNP751	*Desi*	Ca_Desi_Chr01	14114903	(A/C)
25157	CadSNP752	*Desi*	Ca_Desi_Chr01	14128326	(T/G)
25158	CadSNP753	*Desi*	Ca_Desi_Chr01	14161553	(G/T)
25159	CadSNP754	*Desi*	Ca_Desi_Chr02	16503	(A/C)
25160	CadSNP755	*Desi*	Ca_Desi_Chr02	16504	(G/A)
25161	CadSNP756	*Desi*	Ca_Desi_Chr02	82708	(C/G)
25162	CadSNP757	*Desi*	Ca_Desi_Chr02	179194	(C/G)
25163	CadSNP758	*Desi*	Ca_Desi_Chr02	179173	(G/T)
25164	CadSNP759	*Desi*	Ca_Desi_Chr02	179260	(T/A)
25165	CadSNP760	*Desi*	Ca_Desi_Chr02	187800	(A/G)
25166	CadSNP761	*Desi*	Ca_Desi_Chr02	201869	(T/C)
25167	CadSNP762	*Desi*	Ca_Desi_Chr02	205617	(C/T)
25168	CadSNP763	*Desi*	Ca_Desi_Chr02	281905	(T/C)
25169	CadSNP764	*Desi*	Ca_Desi_Chr02	303118	(C/G)
25170	CadSNP765	*Desi*	Ca_Desi_Chr02	303138	(A/G)
25171	CadSNP766	*Desi*	Ca_Desi_Chr02	318397	(A/C)
25172	CadSNP767	*Desi*	Ca_Desi_Chr02	318414	(A/G)
25173	CadSNP768	*Desi*	Ca_Desi_Chr02	328850	(G/A)
25174	CadSNP769	*Desi*	Ca_Desi_Chr02	328828	(C/T)
25175	CadSNP770	*Desi*	Ca_Desi_Chr02	398016	(C/T)
25176	CadSNP771	*Desi*	Ca_Desi_Chr02	429417	(T/C)
25177	CadSNP772	*Desi*	Ca_Desi_Chr02	440499	(C/G)
25178	CadSNP773	*Desi*	Ca_Desi_Chr02	440520	(G/A)
25179	CadSNP774	*Desi*	Ca_Desi_Chr02	440592	(A/T)
25180	CadSNP775	*Desi*	Ca_Desi_Chr02	440595	(T/C)
25181	CadSNP776	*Desi*	Ca_Desi_Chr02	440600	(T/A)
25182	CadSNP777	*Desi*	Ca_Desi_Chr02	440607	(A/G)
25183	CadSNP778	*Desi*	Ca_Desi_Chr02	440754	(T/A)
25184	CadSNP779	*Desi*	Ca_Desi_Chr02	440724	(C/T)
25185	CadSNP780	*Desi*	Ca_Desi_Chr02	440718	(G/T)
25186	CadSNP781	*Desi*	Ca_Desi_Chr02	527661	(G/A)
25187	CadSNP782	*Desi*	Ca_Desi_Chr02	527635	(C/A)
25188	CadSNP783	*Desi*	Ca_Desi_Chr02	527634	(C/A)
25189	CadSNP784	*Desi*	Ca_Desi_Chr02	527633	(T/A)
25190	CadSNP785	*Desi*	Ca_Desi_Chr02	575365	(T/G)
25191	CadSNP786	*Desi*	Ca_Desi_Chr02	575518	(A/G)
25192	CadSNP787	*Desi*	Ca_Desi_Chr02	576524	(G/A)

S.N.	SNP IDs	Cultivars	Chromosomes/scaffolds	Physical positions (bp)	SNPs
25193	CadSNP788	*Desi*	Ca_Desi_Chr02	576624	(A/G)
25194	CadSNP789	*Desi*	Ca_Desi_Chr02	579705	(G/A)
25195	CadSNP790	*Desi*	Ca_Desi_Chr02	580263	(C/T)
25196	CadSNP791	*Desi*	Ca_Desi_Chr02	595836	(T/A)
25197	CadSNP792	*Desi*	Ca_Desi_Chr02	595837	(C/A)
25198	CadSNP793	*Desi*	Ca_Desi_Chr02	595842	(T/A)
25199	CadSNP794	*Desi*	Ca_Desi_Chr02	595845	(G/A)
25200	CadSNP795	*Desi*	Ca_Desi_Chr02	595850	(A/T)
25201	CadSNP796	*Desi*	Ca_Desi_Chr02	595851	(T/A)
25202	CadSNP797	*Desi*	Ca_Desi_Chr02	595853	(C/A)
25203	CadSNP798	*Desi*	Ca_Desi_Chr02	595863	(G/A)
25204	CadSNP799	*Desi*	Ca_Desi_Chr02	727262	(G/C)
25205	CadSNP800	*Desi*	Ca_Desi_Chr02	749831	(C/G)
25206	CadSNP801	*Desi*	Ca_Desi_Chr02	760148	(C/T)
25207	CadSNP802	*Desi*	Ca_Desi_Chr02	842935	(T/C)
25208	CadSNP803	*Desi*	Ca_Desi_Chr02	842908	(T/C)
25209	CadSNP804	*Desi*	Ca_Desi_Chr02	842943	(T/C)
25210	CadSNP805	*Desi*	Ca_Desi_Chr02	930040	(T/C)
25211	CadSNP806	*Desi*	Ca_Desi_Chr02	972909	(A/T)
25212	CadSNP807	*Desi*	Ca_Desi_Chr02	972918	(C/A)
25213	CadSNP808	*Desi*	Ca_Desi_Chr02	972921	(T/C)
25214	CadSNP809	*Desi*	Ca_Desi_Chr02	972930	(G/A)
25215	CadSNP810	*Desi*	Ca_Desi_Chr02	972933	(A/G)
25216	CadSNP811	*Desi*	Ca_Desi_Chr02	992292	(C/A)
25217	CadSNP812	*Desi*	Ca_Desi_Chr02	1019748	(G/A)
25218	CadSNP813	*Desi*	Ca_Desi_Chr02	1019708	(A/T)
25219	CadSNP814	*Desi*	Ca_Desi_Chr02	1019692	(G/T)
25220	CadSNP815	*Desi*	Ca_Desi_Chr02	1019793	(C/T)
25221	CadSNP816	*Desi*	Ca_Desi_Chr02	1048681	(G/T)
25222	CadSNP817	*Desi*	Ca_Desi_Chr02	1048657	(G/A)
25223	CadSNP818	*Desi*	Ca_Desi_Chr02	1066639	(A/C)
25224	CadSNP819	*Desi*	Ca_Desi_Chr02	1066693	(A/T)
25225	CadSNP820	*Desi*	Ca_Desi_Chr02	1066672	(T/A)
25226	CadSNP821	*Desi*	Ca_Desi_Chr02	1066647	(A/T)
25227	CadSNP822	*Desi*	Ca_Desi_Chr02	1081581	(T/C)
25228	CadSNP823	*Desi*	Ca_Desi_Chr02	1147386	(A/G)
25229	CadSNP824	*Desi*	Ca_Desi_Chr02	1147402	(T/C)
25230	CadSNP825	*Desi*	Ca_Desi_Chr02	1151458	(G/T)
25231	CadSNP826	*Desi*	Ca_Desi_Chr02	1205892	(A/C)

S.N.	SNP IDs	Cultivars	Chromosomes/scaffolds	Physical positions (bp)	SNPs
25232	CadSNP827	*Desi*	Ca_Desi_Chr02	1220563	(G/C)
25233	CadSNP828	*Desi*	Ca_Desi_Chr02	1367073	(C/A)
25234	CadSNP829	*Desi*	Ca_Desi_Chr02	1369594	(G/T)
25235	CadSNP830	*Desi*	Ca_Desi_Chr02	1369598	(G/T)
25236	CadSNP831	*Desi*	Ca_Desi_Chr02	1521985	(A/C)
25237	CadSNP832	*Desi*	Ca_Desi_Chr02	1521957	(A/C)
25238	CadSNP833	*Desi*	Ca_Desi_Chr02	1557630	(G/T)
25239	CadSNP834	*Desi*	Ca_Desi_Chr02	1565698	(G/A)
25240	CadSNP835	*Desi*	Ca_Desi_Chr02	1565686	(A/G)
25241	CadSNP836	*Desi*	Ca_Desi_Chr02	1565676	(C/T)
25242	CadSNP837	*Desi*	Ca_Desi_Chr02	1621762	(C/A)
25243	CadSNP838	*Desi*	Ca_Desi_Chr02	1846506	(G/T)
25244	CadSNP839	*Desi*	Ca_Desi_Chr02	1857220	(G/A)
25245	CadSNP840	*Desi*	Ca_Desi_Chr02	1857224	(A/G)
25246	CadSNP841	*Desi*	Ca_Desi_Chr02	1861385	(A/G)
25247	CadSNP842	*Desi*	Ca_Desi_Chr02	1861412	(G/A)
25248	CadSNP843	*Desi*	Ca_Desi_Chr02	1861525	(G/A)
25249	CadSNP844	*Desi*	Ca_Desi_Chr02	1861519	(C/T)
25250	CadSNP845	*Desi*	Ca_Desi_Chr02	1868000	(A/G)
25251	CadSNP846	*Desi*	Ca_Desi_Chr02	1883048	(A/G)
25252	CadSNP847	*Desi*	Ca_Desi_Chr02	1883019	(A/G)
25253	CadSNP848	*Desi*	Ca_Desi_Chr02	1894083	(A/T)
25254	CadSNP849	*Desi*	Ca_Desi_Chr02	1954750	(A/C)
25255	CadSNP850	*Desi*	Ca_Desi_Chr02	1958652	(A/C)
25256	CadSNP851	*Desi*	Ca_Desi_Chr02	1959776	(C/T)
25257	CadSNP852	*Desi*	Ca_Desi_Chr02	1996590	(T/C)
25258	CadSNP853	*Desi*	Ca_Desi_Chr02	1996699	(G/A)
25259	CadSNP854	*Desi*	Ca_Desi_Chr02	2100229	(C/T)
25260	CadSNP855	*Desi*	Ca_Desi_Chr02	2308597	(A/G)
25261	CadSNP856	*Desi*	Ca_Desi_Chr02	2308730	(G/A)
25262	CadSNP857	*Desi*	Ca_Desi_Chr02	2326287	(G/C)
25263	CadSNP858	*Desi*	Ca_Desi_Chr02	2344280	(G/A)
25264	CadSNP859	*Desi*	Ca_Desi_Chr02	2344333	(T/C)
25265	CadSNP860	*Desi*	Ca_Desi_Chr02	2361308	(G/A)
25266	CadSNP861	*Desi*	Ca_Desi_Chr02	2361249	(T/C)
25267	CadSNP862	*Desi*	Ca_Desi_Chr02	2363610	(C/G)
25268	CadSNP863	*Desi*	Ca_Desi_Chr02	2379453	(A/G)
25269	CadSNP864	*Desi*	Ca_Desi_Chr02	2406652	(A/G)
25270	CadSNP865	*Desi*	Ca_Desi_Chr02	2449541	(T/G)

S.N.	SNP IDs	Cultivars	Chromosomes/scaffolds	Physical positions (bp)	SNPs
25271	CadSNP866	*Desi*	Ca_Desi_Chr02	2449576	(T/A)
25272	CadSNP867	*Desi*	Ca_Desi_Chr02	2461994	(T/A)
25273	CadSNP868	*Desi*	Ca_Desi_Chr02	2488147	(A/G)
25274	CadSNP869	*Desi*	Ca_Desi_Chr02	2488151	(C/T)
25275	CadSNP870	*Desi*	Ca_Desi_Chr02	2488181	(T/G)
25276	CadSNP871	*Desi*	Ca_Desi_Chr02	2527417	(C/T)
25277	CadSNP872	*Desi*	Ca_Desi_Chr02	2527461	(C/T)
25278	CadSNP873	*Desi*	Ca_Desi_Chr02	2565668	(G/A)
25279	CadSNP874	*Desi*	Ca_Desi_Chr02	2574544	(T/C)
25280	CadSNP875	*Desi*	Ca_Desi_Chr02	2600740	(G/T)
25281	CadSNP876	*Desi*	Ca_Desi_Chr02	2600744	(G/T)
25282	CadSNP877	*Desi*	Ca_Desi_Chr02	2600751	(C/T)
25283	CadSNP878	*Desi*	Ca_Desi_Chr02	2607635	(G/C)
25284	CadSNP879	*Desi*	Ca_Desi_Chr02	2607620	(T/G)
25285	CadSNP880	*Desi*	Ca_Desi_Chr02	2607603	(T/G)
25286	CadSNP881	*Desi*	Ca_Desi_Chr02	2607737	(T/A)
25287	CadSNP882	*Desi*	Ca_Desi_Chr02	2607804	(A/C)
25288	CadSNP883	*Desi*	Ca_Desi_Chr02	2607820	(G/T)
25289	CadSNP884	*Desi*	Ca_Desi_Chr02	2607827	(T/G)
25290	CadSNP885	*Desi*	Ca_Desi_Chr02	2607828	(A/T)
25291	CadSNP886	*Desi*	Ca_Desi_Chr02	2607993	(A/T)
25292	CadSNP887	*Desi*	Ca_Desi_Chr02	2608058	(G/A)
25293	CadSNP888	*Desi*	Ca_Desi_Chr02	2608080	(C/T)
25294	CadSNP889	*Desi*	Ca_Desi_Chr02	2608756	(A/G)
25295	CadSNP890	*Desi*	Ca_Desi_Chr02	2704878	(C/T)
25296	CadSNP891	*Desi*	Ca_Desi_Chr02	2764423	(C/G)
25297	CadSNP892	*Desi*	Ca_Desi_Chr02	2764465	(A/G)
25298	CadSNP893	*Desi*	Ca_Desi_Chr02	2764476	(T/C)
25299	CadSNP894	*Desi*	Ca_Desi_Chr02	2764617	(C/T)
25300	CadSNP895	*Desi*	Ca_Desi_Chr02	2764833	(T/C)
25301	CadSNP896	*Desi*	Ca_Desi_Chr02	2849625	(G/A)
25302	CadSNP897	*Desi*	Ca_Desi_Chr02	2879030	(T/A)
25303	CadSNP898	*Desi*	Ca_Desi_Chr02	2890359	(G/A)
25304	CadSNP899	*Desi*	Ca_Desi_Chr02	2956401	(A/G)
25305	CadSNP900	*Desi*	Ca_Desi_Chr02	3068073	(T/A)
25306	CadSNP901	*Desi*	Ca_Desi_Chr02	3069887	(A/G)
25307	CadSNP902	*Desi*	Ca_Desi_Chr02	3069889	(T/A)
25308	CadSNP903	*Desi*	Ca_Desi_Chr02	3118713	(T/G)
25309	CadSNP904	*Desi*	Ca_Desi_Chr02	3331580	(G/T)

S.N.	SNP IDs	Cultivars	Chromosomes/scaffolds	Physical positions (bp)	SNPs
25310	CadSNP905	*Desi*	Ca_Desi_Chr02	3360033	(A/T)
25311	CadSNP906	*Desi*	Ca_Desi_Chr02	3589210	(C/T)
25312	CadSNP907	*Desi*	Ca_Desi_Chr02	3589227	(G/A)
25313	CadSNP908	*Desi*	Ca_Desi_Chr02	3589397	(G/A)
25314	CadSNP909	*Desi*	Ca_Desi_Chr02	3604220	(C/A)
25315	CadSNP910	*Desi*	Ca_Desi_Chr02	3604390	(G/A)
25316	CadSNP911	*Desi*	Ca_Desi_Chr02	3604373	(A/G)
25317	CadSNP912	*Desi*	Ca_Desi_Chr02	3604348	(G/A)
25318	CadSNP913	*Desi*	Ca_Desi_Chr02	3604347	(T/C)
25319	CadSNP914	*Desi*	Ca_Desi_Chr02	3604341	(G/A)
25320	CadSNP915	*Desi*	Ca_Desi_Chr02	3604339	(A/C)
25321	CadSNP916	*Desi*	Ca_Desi_Chr02	3604329	(G/A)
25322	CadSNP917	*Desi*	Ca_Desi_Chr02	3604402	(A/C)
25323	CadSNP918	*Desi*	Ca_Desi_Chr02	3604419	(T/C)
25324	CadSNP919	*Desi*	Ca_Desi_Chr02	3722112	(T/C)
25325	CadSNP920	*Desi*	Ca_Desi_Chr02	3722164	(T/A)
25326	CadSNP921	*Desi*	Ca_Desi_Chr02	3722146	(A/G)
25327	CadSNP922	*Desi*	Ca_Desi_Chr02	3750227	(C/A)
25328	CadSNP923	*Desi*	Ca_Desi_Chr02	3750343	(T/A)
25329	CadSNP924	*Desi*	Ca_Desi_Chr02	3750312	(A/G)
25330	CadSNP925	*Desi*	Ca_Desi_Chr02	3750295	(T/C)
25331	CadSNP926	*Desi*	Ca_Desi_Chr02	3756279	(C/A)
25332	CadSNP927	*Desi*	Ca_Desi_Chr02	3756269	(A/C)
25333	CadSNP928	*Desi*	Ca_Desi_Chr02	3756677	(T/C)
25334	CadSNP929	*Desi*	Ca_Desi_Chr02	3854174	(C/T)
25335	CadSNP930	*Desi*	Ca_Desi_Chr02	3854173	(A/T)
25336	CadSNP931	*Desi*	Ca_Desi_Chr02	3930203	(C/G)
25337	CadSNP932	*Desi*	Ca_Desi_Chr02	4041350	(C/T)
25338	CadSNP933	*Desi*	Ca_Desi_Chr02	4041319	(A/G)
25339	CadSNP934	*Desi*	Ca_Desi_Chr02	4058389	(G/A)
25340	CadSNP935	*Desi*	Ca_Desi_Chr02	4125467	(G/A)
25341	CadSNP936	*Desi*	Ca_Desi_Chr02	4185142	(A/G)
25342	CadSNP937	*Desi*	Ca_Desi_Chr02	4185428	(T/A)
25343	CadSNP938	*Desi*	Ca_Desi_Chr02	4185422	(T/C)
25344	CadSNP939	*Desi*	Ca_Desi_Chr02	4185408	(A/G)
25345	CadSNP940	*Desi*	Ca_Desi_Chr02	4185394	(A/T)
25346	CadSNP941	*Desi*	Ca_Desi_Chr02	4190614	(C/T)
25347	CadSNP942	*Desi*	Ca_Desi_Chr02	4190767	(T/A)
25348	CadSNP943	*Desi*	Ca_Desi_Chr02	4212810	(A/G)

S.N.	SNP IDs	Cultivars	Chromosomes/scaffolds	Physical positions (bp)	SNPs
25349	CadSNP944	*Desi*	Ca_Desi_Chr02	4238261	(G/A)
25350	CadSNP945	*Desi*	Ca_Desi_Chr02	4527225	(T/A)
25351	CadSNP946	*Desi*	Ca_Desi_Chr02	4527227	(A/G)
25352	CadSNP947	*Desi*	Ca_Desi_Chr02	4527234	(A/G)
25353	CadSNP948	*Desi*	Ca_Desi_Chr02	4528767	(A/T)
25354	CadSNP949	*Desi*	Ca_Desi_Chr02	4552112	(C/T)
25355	CadSNP950	*Desi*	Ca_Desi_Chr02	4727092	(G/C)
25356	CadSNP951	*Desi*	Ca_Desi_Chr02	5116790	(C/T)
25357	CadSNP952	*Desi*	Ca_Desi_Chr02	5116882	(G/A)
25358	CadSNP953	*Desi*	Ca_Desi_Chr02	5168628	(T/A)
25359	CadSNP954	*Desi*	Ca_Desi_Chr02	5168645	(C/A)
25360	CadSNP955	*Desi*	Ca_Desi_Chr02	5213222	(G/A)
25361	CadSNP956	*Desi*	Ca_Desi_Chr02	5223443	(G/A)
25362	CadSNP957	*Desi*	Ca_Desi_Chr02	5400370	(A/T)
25363	CadSNP958	*Desi*	Ca_Desi_Chr02	5400364	(C/T)
25364	CadSNP959	*Desi*	Ca_Desi_Chr02	5448589	(C/A)
25365	CadSNP960	*Desi*	Ca_Desi_Chr02	5462566	(C/G)
25366	CadSNP961	*Desi*	Ca_Desi_Chr02	5466959	(C/T)
25367	CadSNP962	*Desi*	Ca_Desi_Chr02	5466961	(A/G)
25368	CadSNP963	*Desi*	Ca_Desi_Chr02	5466969	(T/A)
25369	CadSNP964	*Desi*	Ca_Desi_Chr02	5466993	(C/T)
25370	CadSNP965	*Desi*	Ca_Desi_Chr02	5467020	(G/A)
25371	CadSNP966	*Desi*	Ca_Desi_Chr02	5472865	(A/G)
25372	CadSNP967	*Desi*	Ca_Desi_Chr02	5472802	(T/C)
25373	CadSNP968	*Desi*	Ca_Desi_Chr02	5503519	(G/A)
25374	CadSNP969	*Desi*	Ca_Desi_Chr02	5503511	(G/T)
25375	CadSNP970	*Desi*	Ca_Desi_Chr02	5503509	(A/G)
25376	CadSNP971	*Desi*	Ca_Desi_Chr02	5503507	(C/A)
25377	CadSNP972	*Desi*	Ca_Desi_Chr02	5503492	(G/A)
25378	CadSNP973	*Desi*	Ca_Desi_Chr02	5503490	(T/G)
25379	CadSNP974	*Desi*	Ca_Desi_Chr02	5503486	(G/A)
25380	CadSNP975	*Desi*	Ca_Desi_Chr02	5503637	(C/G)
25381	CadSNP976	*Desi*	Ca_Desi_Chr02	5503732	(G/C)
25382	CadSNP977	*Desi*	Ca_Desi_Chr02	5503727	(T/G)
25383	CadSNP978	*Desi*	Ca_Desi_Chr02	5513741	(T/C)
25384	CadSNP979	*Desi*	Ca_Desi_Chr02	5530121	(C/T)
25385	CadSNP980	*Desi*	Ca_Desi_Chr02	5548819	(G/A)
25386	CadSNP981	*Desi*	Ca_Desi_Chr02	5548807	(C/G)
25387	CadSNP982	*Desi*	Ca_Desi_Chr02	5549174	(G/A)

S.N.	SNP IDs	Cultivars	Chromosomes/scaffolds	Physical positions (bp)	SNPs
25388	CadSNP983	*Desi*	Ca_Desi_Chr02	5549164	(T/C)
25389	CadSNP984	*Desi*	Ca_Desi_Chr02	5549156	(T/A)
25390	CadSNP985	*Desi*	Ca_Desi_Chr02	5549150	(C/A)
25391	CadSNP986	*Desi*	Ca_Desi_Chr02	5549966	(A/T)
25392	CadSNP987	*Desi*	Ca_Desi_Chr02	5550007	(G/A)
25393	CadSNP988	*Desi*	Ca_Desi_Chr02	5550009	(A/C)
25394	CadSNP989	*Desi*	Ca_Desi_Chr02	5577178	(A/T)
25395	CadSNP990	*Desi*	Ca_Desi_Chr02	5632043	(A/G)
25396	CadSNP991	*Desi*	Ca_Desi_Chr02	5635080	(T/C)
25397	CadSNP992	*Desi*	Ca_Desi_Chr02	5635215	(A/C)
25398	CadSNP993	*Desi*	Ca_Desi_Chr02	5635178	(A/T)
25399	CadSNP994	*Desi*	Ca_Desi_Chr02	5648550	(A/G)
25400	CadSNP995	*Desi*	Ca_Desi_Chr02	5661404	(C/T)
25401	CadSNP996	*Desi*	Ca_Desi_Chr02	5661407	(A/G)
25402	CadSNP997	*Desi*	Ca_Desi_Chr02	5661416	(A/G)
25403	CadSNP998	*Desi*	Ca_Desi_Chr02	5661427	(A/G)
25404	CadSNP999	*Desi*	Ca_Desi_Chr02	5661431	(G/T)
25405	CadSNP1000	*Desi*	Ca_Desi_Chr02	5661464	(A/T)
25406	CadSNP1001	*Desi*	Ca_Desi_Chr02	5661470	(A/T)
25407	CadSNP1002	*Desi*	Ca_Desi_Chr02	5661482	(A/G)
25408	CadSNP1003	*Desi*	Ca_Desi_Chr02	5661485	(G/C)
25409	CadSNP1004	*Desi*	Ca_Desi_Chr02	5661500	(A/G)
25410	CadSNP1005	*Desi*	Ca_Desi_Chr02	5661498	(T/C)
25411	CadSNP1006	*Desi*	Ca_Desi_Chr02	5661494	(T/A)
25412	CadSNP1007	*Desi*	Ca_Desi_Chr02	5694128	(G/T)
25413	CadSNP1008	*Desi*	Ca_Desi_Chr02	5694127	(C/T)
25414	CadSNP1009	*Desi*	Ca_Desi_Chr02	5694118	(T/C)
25415	CadSNP1010	*Desi*	Ca_Desi_Chr02	5698170	(T/A)
25416	CadSNP1011	*Desi*	Ca_Desi_Chr02	5698197	(G/A)
25417	CadSNP1012	*Desi*	Ca_Desi_Chr02	5698994	(G/C)
25418	CadSNP1013	*Desi*	Ca_Desi_Chr02	5698976	(C/T)
25419	CadSNP1014	*Desi*	Ca_Desi_Chr02	5699101	(G/A)
25420	CadSNP1015	*Desi*	Ca_Desi_Chr02	5699314	(A/G)
25421	CadSNP1016	*Desi*	Ca_Desi_Chr02	5699361	(G/A)
25422	CadSNP1017	*Desi*	Ca_Desi_Chr02	5699391	(T/A)
25423	CadSNP1018	*Desi*	Ca_Desi_Chr02	5699393	(G/A)
25424	CadSNP1019	*Desi*	Ca_Desi_Chr02	5733756	(C/A)
25425	CadSNP1020	*Desi*	Ca_Desi_Chr02	5740309	(C/A)
25426	CadSNP1021	*Desi*	Ca_Desi_Chr02	5745142	(C/T)

S.N.	SNP IDs	Cultivars	Chromosomes/scaffolds	Physical positions (bp)	SNPs
25427	CadSNP1022	*Desi*	Ca_Desi_Chr02	5917902	(G/A)
25428	CadSNP1023	*Desi*	Ca_Desi_Chr02	5965393	(G/T)
25429	CadSNP1024	*Desi*	Ca_Desi_Chr02	5965391	(C/T)
25430	CadSNP1025	*Desi*	Ca_Desi_Chr02	5966192	(C/T)
25431	CadSNP1026	*Desi*	Ca_Desi_Chr02	5988875	(T/A)
25432	CadSNP1027	*Desi*	Ca_Desi_Chr02	6010840	(G/A)
25433	CadSNP1028	*Desi*	Ca_Desi_Chr02	6011026	(G/A)
25434	CadSNP1029	*Desi*	Ca_Desi_Chr02	6018033	(C/T)
25435	CadSNP1030	*Desi*	Ca_Desi_Chr02	6019934	(G/T)
25436	CadSNP1031	*Desi*	Ca_Desi_Chr02	6020224	(G/A)
25437	CadSNP1032	*Desi*	Ca_Desi_Chr02	6022635	(G/A)
25438	CadSNP1033	*Desi*	Ca_Desi_Chr02	6022627	(T/G)
25439	CadSNP1034	*Desi*	Ca_Desi_Chr02	6022619	(C/T)
25440	CadSNP1035	*Desi*	Ca_Desi_Chr02	6048386	(T/A)
25441	CadSNP1036	*Desi*	Ca_Desi_Chr02	6053043	(A/T)
25442	CadSNP1037	*Desi*	Ca_Desi_Chr02	6071359	(C/T)
25443	CadSNP1038	*Desi*	Ca_Desi_Chr02	6088304	(C/T)
25444	CadSNP1039	*Desi*	Ca_Desi_Chr02	6089048	(T/C)
25445	CadSNP1040	*Desi*	Ca_Desi_Chr02	6089257	(G/A)
25446	CadSNP1041	*Desi*	Ca_Desi_Chr02	6091962	(C/T)
25447	CadSNP1042	*Desi*	Ca_Desi_Chr02	6091970	(A/C)
25448	CadSNP1043	*Desi*	Ca_Desi_Chr02	6091973	(T/G)
25449	CadSNP1044	*Desi*	Ca_Desi_Chr02	6092094	(A/G)
25450	CadSNP1045	*Desi*	Ca_Desi_Chr02	6092092	(G/A)
25451	CadSNP1046	*Desi*	Ca_Desi_Chr02	6092158	(G/C)
25452	CadSNP1047	*Desi*	Ca_Desi_Chr02	6092265	(A/G)
25453	CadSNP1048	*Desi*	Ca_Desi_Chr02	6092376	(T/C)
25454	CadSNP1049	*Desi*	Ca_Desi_Chr02	6092347	(T/G)
25455	CadSNP1050	*Desi*	Ca_Desi_Chr02	6092444	(C/T)
25456	CadSNP1051	*Desi*	Ca_Desi_Chr02	6094379	(C/G)
25457	CadSNP1052	*Desi*	Ca_Desi_Chr02	6103902	(T/C)
25458	CadSNP1053	*Desi*	Ca_Desi_Chr02	6107446	(A/C)
25459	CadSNP1054	*Desi*	Ca_Desi_Chr02	6107432	(G/T)
25460	CadSNP1055	*Desi*	Ca_Desi_Chr02	6131587	(T/C)
25461	CadSNP1056	*Desi*	Ca_Desi_Chr02	6135210	(G/A)
25462	CadSNP1057	*Desi*	Ca_Desi_Chr02	6138794	(T/G)
25463	CadSNP1058	*Desi*	Ca_Desi_Chr02	6138861	(C/T)
25464	CadSNP1059	*Desi*	Ca_Desi_Chr02	6138912	(C/T)
25465	CadSNP1060	*Desi*	Ca_Desi_Chr02	6138975	(A/T)

S.N.	SNP IDs	Cultivars	Chromosomes/scaffolds	Physical positions (bp)	SNPs
25466	CadSNP1061	*Desi*	Ca_Desi_Chr02	6138976	(G/T)
25467	CadSNP1062	*Desi*	Ca_Desi_Chr02	6139045	(A/G)
25468	CadSNP1063	*Desi*	Ca_Desi_Chr02	6140423	(G/A)
25469	CadSNP1064	*Desi*	Ca_Desi_Chr02	6140387	(T/C)
25470	CadSNP1065	*Desi*	Ca_Desi_Chr02	6145109	(C/T)
25471	CadSNP1066	*Desi*	Ca_Desi_Chr02	6145149	(T/A)
25472	CadSNP1067	*Desi*	Ca_Desi_Chr02	6145148	(C/A)
25473	CadSNP1068	*Desi*	Ca_Desi_Chr02	6145235	(C/T)
25474	CadSNP1069	*Desi*	Ca_Desi_Chr02	6145253	(G/A)
25475	CadSNP1070	*Desi*	Ca_Desi_Chr02	6145263	(G/A)
25476	CadSNP1071	*Desi*	Ca_Desi_Chr02	6179369	(A/C)
25477	CadSNP1072	*Desi*	Ca_Desi_Chr02	6243184	(C/T)
25478	CadSNP1073	*Desi*	Ca_Desi_Chr02	6243219	(T/G)
25479	CadSNP1074	*Desi*	Ca_Desi_Chr02	6249860	(G/A)
25480	CadSNP1075	*Desi*	Ca_Desi_Chr02	6249876	(C/T)
25481	CadSNP1076	*Desi*	Ca_Desi_Chr02	6249888	(C/A)
25482	CadSNP1077	*Desi*	Ca_Desi_Chr02	6249889	(A/C)
25483	CadSNP1078	*Desi*	Ca_Desi_Chr02	6249975	(T/C)
25484	CadSNP1079	*Desi*	Ca_Desi_Chr02	6255732	(T/C)
25485	CadSNP1080	*Desi*	Ca_Desi_Chr02	6265666	(A/G)
25486	CadSNP1081	*Desi*	Ca_Desi_Chr02	6275717	(C/T)
25487	CadSNP1082	*Desi*	Ca_Desi_Chr02	6436332	(T/A)
25488	CadSNP1083	*Desi*	Ca_Desi_Chr02	6436365	(A/C)
25489	CadSNP1084	*Desi*	Ca_Desi_Chr02	6436390	(G/T)
25490	CadSNP1085	*Desi*	Ca_Desi_Chr02	6436396	(G/T)
25491	CadSNP1086	*Desi*	Ca_Desi_Chr02	6437062	(A/T)
25492	CadSNP1087	*Desi*	Ca_Desi_Chr02	6437145	(T/C)
25493	CadSNP1088	*Desi*	Ca_Desi_Chr02	6532962	(C/T)
25494	CadSNP1089	*Desi*	Ca_Desi_Chr02	6532976	(G/T)
25495	CadSNP1090	*Desi*	Ca_Desi_Chr02	6535217	(C/T)
25496	CadSNP1091	*Desi*	Ca_Desi_Chr02	6589542	(A/G)
25497	CadSNP1092	*Desi*	Ca_Desi_Chr02	6589551	(T/C)
25498	CadSNP1093	*Desi*	Ca_Desi_Chr02	6589573	(G/A)
25499	CadSNP1094	*Desi*	Ca_Desi_Chr02	6601251	(T/A)
25500	CadSNP1095	*Desi*	Ca_Desi_Chr02	6608684	(T/C)
25501	CadSNP1096	*Desi*	Ca_Desi_Chr02	6608737	(G/A)
25502	CadSNP1097	*Desi*	Ca_Desi_Chr02	6609027	(G/T)
25503	CadSNP1098	*Desi*	Ca_Desi_Chr02	6609053	(A/T)
25504	CadSNP1099	*Desi*	Ca_Desi_Chr02	6614650	(G/A)

S.N.	SNP IDs	Cultivars	Chromosomes/scaffolds	Physical positions (bp)	SNPs
25505	CadSNP1100	*Desi*	Ca_Desi_Chr02	6614735	(T/G)
25506	CadSNP1101	*Desi*	Ca_Desi_Chr02	6614685	(G/A)
25507	CadSNP1102	*Desi*	Ca_Desi_Chr02	6614760	(G/C)
25508	CadSNP1103	*Desi*	Ca_Desi_Chr02	6622281	(T/A)
25509	CadSNP1104	*Desi*	Ca_Desi_Chr02	6622600	(A/G)
25510	CadSNP1105	*Desi*	Ca_Desi_Chr02	6624856	(T/C)
25511	CadSNP1106	*Desi*	Ca_Desi_Chr02	6624907	(C/G)
25512	CadSNP1107	*Desi*	Ca_Desi_Chr02	6651178	(A/T)
25513	CadSNP1108	*Desi*	Ca_Desi_Chr02	6653902	(C/T)
25514	CadSNP1109	*Desi*	Ca_Desi_Chr02	6653887	(A/G)
25515	CadSNP1110	*Desi*	Ca_Desi_Chr02	6653879	(G/T)
25516	CadSNP1111	*Desi*	Ca_Desi_Chr02	6669823	(C/G)
25517	CadSNP1112	*Desi*	Ca_Desi_Chr02	6671859	(A/G)
25518	CadSNP1113	*Desi*	Ca_Desi_Chr02	6701392	(G/A)
25519	CadSNP1114	*Desi*	Ca_Desi_Chr02	6701394	(G/A)
25520	CadSNP1115	*Desi*	Ca_Desi_Chr02	6711702	(C/T)
25521	CadSNP1116	*Desi*	Ca_Desi_Chr02	6711747	(C/T)
25522	CadSNP1117	*Desi*	Ca_Desi_Chr02	6711829	(T/G)
25523	CadSNP1118	*Desi*	Ca_Desi_Chr02	6713000	(T/A)
25524	CadSNP1119	*Desi*	Ca_Desi_Chr02	6908990	(A/G)
25525	CadSNP1120	*Desi*	Ca_Desi_Chr02	6968294	(T/G)
25526	CadSNP1121	*Desi*	Ca_Desi_Chr02	6968382	(C/G)
25527	CadSNP1122	*Desi*	Ca_Desi_Chr02	6992535	(A/G)
25528	CadSNP1123	*Desi*	Ca_Desi_Chr02	7059796	(C/T)
25529	CadSNP1124	*Desi*	Ca_Desi_Chr02	7059783	(G/C)
25530	CadSNP1125	*Desi*	Ca_Desi_Chr02	7062006	(A/G)
25531	CadSNP1126	*Desi*	Ca_Desi_Chr02	7062059	(G/A)
25532	CadSNP1127	*Desi*	Ca_Desi_Chr02	7082429	(A/G)
25533	CadSNP1128	*Desi*	Ca_Desi_Chr02	7082437	(T/C)
25534	CadSNP1129	*Desi*	Ca_Desi_Chr02	7082452	(A/C)
25535	CadSNP1130	*Desi*	Ca_Desi_Chr02	7082486	(A/C)
25536	CadSNP1131	*Desi*	Ca_Desi_Chr02	7082872	(A/C)
25537	CadSNP1132	*Desi*	Ca_Desi_Chr02	7082948	(C/G)
25538	CadSNP1133	*Desi*	Ca_Desi_Chr02	7085006	(A/G)
25539	CadSNP1134	*Desi*	Ca_Desi_Chr02	7084987	(T/C)
25540	CadSNP1135	*Desi*	Ca_Desi_Chr02	7084976	(A/C)
25541	CadSNP1136	*Desi*	Ca_Desi_Chr02	7113287	(G/T)
25542	CadSNP1137	*Desi*	Ca_Desi_Chr02	7113358	(G/T)
25543	CadSNP1138	*Desi*	Ca_Desi_Chr02	7222282	(T/A)

S.N.	SNP IDs	Cultivars	Chromosomes/scaffolds	Physical positions (bp)	SNPs
25544	CadSNP1139	*Desi*	Ca_Desi_Chr02	7222474	(T/G)
25545	CadSNP1140	*Desi*	Ca_Desi_Chr02	7222511	(T/A)
25546	CadSNP1141	*Desi*	Ca_Desi_Chr02	7241676	(G/A)
25547	CadSNP1142	*Desi*	Ca_Desi_Chr02	7241824	(G/A)
25548	CadSNP1143	*Desi*	Ca_Desi_Chr02	7242108	(A/G)
25549	CadSNP1144	*Desi*	Ca_Desi_Chr02	7242124	(G/A)
25550	CadSNP1145	*Desi*	Ca_Desi_Chr02	7316884	(A/T)
25551	CadSNP1146	*Desi*	Ca_Desi_Chr02	7317863	(G/A)
25552	CadSNP1147	*Desi*	Ca_Desi_Chr02	7317845	(A/G)
25553	CadSNP1148	*Desi*	Ca_Desi_Chr02	7327612	(C/T)
25554	CadSNP1149	*Desi*	Ca_Desi_Chr02	7327751	(T/C)
25555	CadSNP1150	*Desi*	Ca_Desi_Chr02	7353195	(G/A)
25556	CadSNP1151	*Desi*	Ca_Desi_Chr02	7358767	(C/A)
25557	CadSNP1152	*Desi*	Ca_Desi_Chr02	7389166	(G/A)
25558	CadSNP1153	*Desi*	Ca_Desi_Chr02	7397445	(T/A)
25559	CadSNP1154	*Desi*	Ca_Desi_Chr02	7409824	(C/T)
25560	CadSNP1155	*Desi*	Ca_Desi_Chr02	7419315	(A/G)
25561	CadSNP1156	*Desi*	Ca_Desi_Chr02	7438626	(G/A)
25562	CadSNP1157	*Desi*	Ca_Desi_Chr02	7457010	(C/T)
25563	CadSNP1158	*Desi*	Ca_Desi_Chr02	7458342	(G/A)
25564	CadSNP1159	*Desi*	Ca_Desi_Chr02	7459382	(G/A)
25565	CadSNP1160	*Desi*	Ca_Desi_Chr02	7462051	(A/C)
25566	CadSNP1161	*Desi*	Ca_Desi_Chr02	7471326	(T/C)
25567	CadSNP1162	*Desi*	Ca_Desi_Chr02	7471357	(T/C)
25568	CadSNP1163	*Desi*	Ca_Desi_Chr02	7585941	(G/A)
25569	CadSNP1164	*Desi*	Ca_Desi_Chr02	7610221	(T/C)
25570	CadSNP1165	*Desi*	Ca_Desi_Chr02	7610503	(T/C)
25571	CadSNP1166	*Desi*	Ca_Desi_Chr02	7630044	(G/A)
25572	CadSNP1167	*Desi*	Ca_Desi_Chr02	7659346	(C/T)
25573	CadSNP1168	*Desi*	Ca_Desi_Chr02	7659312	(G/T)
25574	CadSNP1169	*Desi*	Ca_Desi_Chr02	7659401	(C/T)
25575	CadSNP1170	*Desi*	Ca_Desi_Chr02	7659625	(T/C)
25576	CadSNP1171	*Desi*	Ca_Desi_Chr02	7675697	(A/G)
25577	CadSNP1172	*Desi*	Ca_Desi_Chr02	7697803	(G/C)
25578	CadSNP1173	*Desi*	Ca_Desi_Chr02	7708782	(G/A)
25579	CadSNP1174	*Desi*	Ca_Desi_Chr02	7708761	(C/T)
25580	CadSNP1175	*Desi*	Ca_Desi_Chr02	7708760	(A/C)
25581	CadSNP1176	*Desi*	Ca_Desi_Chr02	7708749	(G/A)
25582	CadSNP1177	*Desi*	Ca_Desi_Chr02	7708765	(A/C)

S.N.	SNP IDs	Cultivars	Chromosomes/scaffolds	Physical positions (bp)	SNPs
25583	CadSNP1178	*Desi*	Ca_Desi_Chr02	7709532	(C/T)
25584	CadSNP1179	*Desi*	Ca_Desi_Chr02	7709745	(A/G)
25585	CadSNP1180	*Desi*	Ca_Desi_Chr02	7709743	(A/G)
25586	CadSNP1181	*Desi*	Ca_Desi_Chr02	7728470	(C/G)
25587	CadSNP1182	*Desi*	Ca_Desi_Chr02	7728474	(A/G)
25588	CadSNP1183	*Desi*	Ca_Desi_Chr02	7813999	(C/A)
25589	CadSNP1184	*Desi*	Ca_Desi_Chr02	7813998	(T/A)
25590	CadSNP1185	*Desi*	Ca_Desi_Chr02	7869084	(A/C)
25591	CadSNP1186	*Desi*	Ca_Desi_Chr02	7875419	(A/G)
25592	CadSNP1187	*Desi*	Ca_Desi_Chr02	7894116	(C/T)
25593	CadSNP1188	*Desi*	Ca_Desi_Chr02	7897881	(C/A)
25594	CadSNP1189	*Desi*	Ca_Desi_Chr02	7944911	(T/C)
25595	CadSNP1190	*Desi*	Ca_Desi_Chr02	7956738	(T/C)
25596	CadSNP1191	*Desi*	Ca_Desi_Chr02	7958321	(A/C)
25597	CadSNP1192	*Desi*	Ca_Desi_Chr02	7958494	(C/T)
25598	CadSNP1193	*Desi*	Ca_Desi_Chr02	7958522	(A/G)
25599	CadSNP1194	*Desi*	Ca_Desi_Chr02	7958529	(T/A)
25600	CadSNP1195	*Desi*	Ca_Desi_Chr02	7960966	(A/T)
25601	CadSNP1196	*Desi*	Ca_Desi_Chr02	8013247	(C/T)
25602	CadSNP1197	*Desi*	Ca_Desi_Chr02	8037211	(C/A)
25603	CadSNP1198	*Desi*	Ca_Desi_Chr02	8065988	(C/A)
25604	CadSNP1199	*Desi*	Ca_Desi_Chr02	8073314	(G/A)
25605	CadSNP1200	*Desi*	Ca_Desi_Chr02	8113745	(G/T)
25606	CadSNP1201	*Desi*	Ca_Desi_Chr02	8113738	(C/T)
25607	CadSNP1202	*Desi*	Ca_Desi_Chr02	8113723	(C/T)
25608	CadSNP1203	*Desi*	Ca_Desi_Chr02	8113708	(T/G)
25609	CadSNP1204	*Desi*	Ca_Desi_Chr02	8113696	(C/G)
25610	CadSNP1205	*Desi*	Ca_Desi_Chr02	8149881	(T/G)
25611	CadSNP1206	*Desi*	Ca_Desi_Chr02	8160645	(C/A)
25612	CadSNP1207	*Desi*	Ca_Desi_Chr02	8160750	(G/A)
25613	CadSNP1208	*Desi*	Ca_Desi_Chr02	8160737	(A/C)
25614	CadSNP1209	*Desi*	Ca_Desi_Chr02	8160733	(C/T)
25615	CadSNP1210	*Desi*	Ca_Desi_Chr02	8167177	(A/C)
25616	CadSNP1211	*Desi*	Ca_Desi_Chr02	8167702	(T/C)
25617	CadSNP1212	*Desi*	Ca_Desi_Chr02	8167708	(C/T)
25618	CadSNP1213	*Desi*	Ca_Desi_Chr02	8167709	(C/T)
25619	CadSNP1214	*Desi*	Ca_Desi_Chr02	8167714	(A/G)
25620	CadSNP1215	*Desi*	Ca_Desi_Chr02	8167719	(T/C)
25621	CadSNP1216	*Desi*	Ca_Desi_Chr02	8167720	(G/A)

S.N.	SNP IDs	Cultivars	Chromosomes/scaffolds	Physical positions (bp)	SNPs
25622	CadSNP1217	*Desi*	Ca_Desi_Chr02	8167721	(A/C)
25623	CadSNP1218	*Desi*	Ca_Desi_Chr02	8167724	(C/T)
25624	CadSNP1219	*Desi*	Ca_Desi_Chr02	8167729	(A/G)
25625	CadSNP1220	*Desi*	Ca_Desi_Chr02	8167735	(G/A)
25626	CadSNP1221	*Desi*	Ca_Desi_Chr02	8167740	(C/A)
25627	CadSNP1222	*Desi*	Ca_Desi_Chr02	8167744	(G/A)
25628	CadSNP1223	*Desi*	Ca_Desi_Chr02	8167753	(G/A)
25629	CadSNP1224	*Desi*	Ca_Desi_Chr02	8167754	(A/T)
25630	CadSNP1225	*Desi*	Ca_Desi_Chr02	8167759	(C/G)
25631	CadSNP1226	*Desi*	Ca_Desi_Chr02	8167760	(C/G)
25632	CadSNP1227	*Desi*	Ca_Desi_Chr02	8167764	(G/A)
25633	CadSNP1228	*Desi*	Ca_Desi_Chr02	8174448	(G/A)
25634	CadSNP1229	*Desi*	Ca_Desi_Chr02	8174458	(C/T)
25635	CadSNP1230	*Desi*	Ca_Desi_Chr02	8234099	(A/C)
25636	CadSNP1231	*Desi*	Ca_Desi_Chr02	8294583	(T/C)
25637	CadSNP1232	*Desi*	Ca_Desi_Chr02	8294721	(C/A)
25638	CadSNP1233	*Desi*	Ca_Desi_Chr02	8294715	(C/T)
25639	CadSNP1234	*Desi*	Ca_Desi_Chr02	8294709	(C/T)
25640	CadSNP1235	*Desi*	Ca_Desi_Chr02	8294706	(C/T)
25641	CadSNP1236	*Desi*	Ca_Desi_Chr02	8303613	(G/A)
25642	CadSNP1237	*Desi*	Ca_Desi_Chr02	8322723	(A/G)
25643	CadSNP1238	*Desi*	Ca_Desi_Chr02	8322740	(G/A)
25644	CadSNP1239	*Desi*	Ca_Desi_Chr02	8322763	(A/G)
25645	CadSNP1240	*Desi*	Ca_Desi_Chr02	8397884	(A/G)
25646	CadSNP1241	*Desi*	Ca_Desi_Chr02	8397955	(G/C)
25647	CadSNP1242	*Desi*	Ca_Desi_Chr02	8528763	(G/A)
25648	CadSNP1243	*Desi*	Ca_Desi_Chr02	8530649	(C/T)
25649	CadSNP1244	*Desi*	Ca_Desi_Chr02	9840097	(G/A)
25650	CadSNP1245	*Desi*	Ca_Desi_Chr02	9840141	(C/T)
25651	CadSNP1246	*Desi*	Ca_Desi_Chr02	9845051	(A/C)
25652	CadSNP1247	*Desi*	Ca_Desi_Chr02	9861790	(A/T)
25653	CadSNP1248	*Desi*	Ca_Desi_Chr02	9861771	(A/C)
25654	CadSNP1249	*Desi*	Ca_Desi_Chr02	9864788	(G/T)
25655	CadSNP1250	*Desi*	Ca_Desi_Chr02	9873103	(A/C)
25656	CadSNP1251	*Desi*	Ca_Desi_Chr02	9873154	(C/T)
25657	CadSNP1252	*Desi*	Ca_Desi_Chr02	9886040	(C/T)
25658	CadSNP1253	*Desi*	Ca_Desi_Chr02	10091173	(T/C)
25659	CadSNP1254	*Desi*	Ca_Desi_Chr02	10091208	(C/T)
25660	CadSNP1255	*Desi*	Ca_Desi_Chr02	10091494	(T/C)

S.N.	SNP IDs	Cultivars	Chromosomes/scaffolds	Physical positions (bp)	SNPs
25661	CadSNP1256	*Desi*	Ca_Desi_Chr02	10091528	(A/G)
25662	CadSNP1257	*Desi*	Ca_Desi_Chr02	10091558	(A/G)
25663	CadSNP1258	*Desi*	Ca_Desi_Chr02	10091566	(C/A)
25664	CadSNP1259	*Desi*	Ca_Desi_Chr02	10208337	(T/G)
25665	CadSNP1260	*Desi*	Ca_Desi_Chr02	10286615	(C/G)
25666	CadSNP1261	*Desi*	Ca_Desi_Chr02	10436482	(C/T)
25667	CadSNP1262	*Desi*	Ca_Desi_Chr02	10436528	(T/C)
25668	CadSNP1263	*Desi*	Ca_Desi_Chr02	10436644	(G/T)
25669	CadSNP1264	*Desi*	Ca_Desi_Chr02	10439843	(G/A)
25670	CadSNP1265	*Desi*	Ca_Desi_Chr02	10864591	(G/A)
25671	CadSNP1266	*Desi*	Ca_Desi_Chr02	10864593	(A/T)
25672	CadSNP1267	*Desi*	Ca_Desi_Chr02	10864609	(G/A)
25673	CadSNP1268	*Desi*	Ca_Desi_Chr02	10864621	(T/A)
25674	CadSNP1269	*Desi*	Ca_Desi_Chr02	10864624	(C/T)
25675	CadSNP1270	*Desi*	Ca_Desi_Chr02	10864631	(C/T)
25676	CadSNP1271	*Desi*	Ca_Desi_Chr02	10864664	(A/G)
25677	CadSNP1272	*Desi*	Ca_Desi_Chr02	10864728	(C/A)
25678	CadSNP1273	*Desi*	Ca_Desi_Chr02	10864722	(T/C)
25679	CadSNP1274	*Desi*	Ca_Desi_Chr02	10864699	(G/A)
25680	CadSNP1275	*Desi*	Ca_Desi_Chr02	10864698	(G/A)
25681	CadSNP1276	*Desi*	Ca_Desi_Chr02	11110650	(A/T)
25682	CadSNP1277	*Desi*	Ca_Desi_Chr02	11110651	(T/C)
25683	CadSNP1278	*Desi*	Ca_Desi_Chr02	11170269	(C/T)
25684	CadSNP1279	*Desi*	Ca_Desi_Chr02	11268822	(C/T)
25685	CadSNP1280	*Desi*	Ca_Desi_Chr02	11276069	(G/A)
25686	CadSNP1281	*Desi*	Ca_Desi_Chr02	11393742	(A/G)
25687	CadSNP1282	*Desi*	Ca_Desi_Chr02	11393800	(C/T)
25688	CadSNP1283	*Desi*	Ca_Desi_Chr02	11393824	(C/T)
25689	CadSNP1284	*Desi*	Ca_Desi_Chr02	11393816	(G/A)
25690	CadSNP1285	*Desi*	Ca_Desi_Chr02	11393862	(C/G)
25691	CadSNP1286	*Desi*	Ca_Desi_Chr02	11394075	(A/G)
25692	CadSNP1287	*Desi*	Ca_Desi_Chr02	11394066	(G/A)
25693	CadSNP1288	*Desi*	Ca_Desi_Chr02	11394036	(T/C)
25694	CadSNP1289	*Desi*	Ca_Desi_Chr02	11394024	(A/G)
25695	CadSNP1290	*Desi*	Ca_Desi_Chr02	11394153	(T/C)
25696	CadSNP1291	*Desi*	Ca_Desi_Chr02	11394247	(G/A)
25697	CadSNP1292	*Desi*	Ca_Desi_Chr02	11403900	(C/A)
25698	CadSNP1293	*Desi*	Ca_Desi_Chr02	11404024	(A/G)
25699	CadSNP1294	*Desi*	Ca_Desi_Chr02	11403986	(C/T)

S.N.	SNP IDs	Cultivars	Chromosomes/scaffolds	Physical positions (bp)	SNPs
25700	CadSNP1295	*Desi*	Ca_Desi_Chr02	11405477	(C/T)
25701	CadSNP1296	*Desi*	Ca_Desi_Chr02	11422984	(G/A)
25702	CadSNP1297	*Desi*	Ca_Desi_Chr02	11423000	(C/A)
25703	CadSNP1298	*Desi*	Ca_Desi_Chr02	11423014	(G/A)
25704	CadSNP1299	*Desi*	Ca_Desi_Chr02	11423022	(T/C)
25705	CadSNP1300	*Desi*	Ca_Desi_Chr02	11423023	(C/T)
25706	CadSNP1301	*Desi*	Ca_Desi_Chr02	11423041	(T/G)
25707	CadSNP1302	*Desi*	Ca_Desi_Chr02	11423053	(T/C)
25708	CadSNP1303	*Desi*	Ca_Desi_Chr02	11423165	(A/T)
25709	CadSNP1304	*Desi*	Ca_Desi_Chr02	11423145	(A/G)
25710	CadSNP1305	*Desi*	Ca_Desi_Chr02	11636237	(T/A)
25711	CadSNP1306	*Desi*	Ca_Desi_Chr02	11636243	(C/G)
25712	CadSNP1307	*Desi*	Ca_Desi_Chr02	11636275	(C/T)
25713	CadSNP1308	*Desi*	Ca_Desi_Chr02	11636378	(A/C)
25714	CadSNP1309	*Desi*	Ca_Desi_Chr02	11636495	(T/C)
25715	CadSNP1310	*Desi*	Ca_Desi_Chr02	11957822	(A/G)
25716	CadSNP1311	*Desi*	Ca_Desi_Chr02	11957812	(C/T)
25717	CadSNP1312	*Desi*	Ca_Desi_Chr02	11957805	(C/A)
25718	CadSNP1313	*Desi*	Ca_Desi_Chr02	11957800	(T/C)
25719	CadSNP1314	*Desi*	Ca_Desi_Chr02	11957789	(G/T)
25720	CadSNP1315	*Desi*	Ca_Desi_Chr02	11957784	(T/C)
25721	CadSNP1316	*Desi*	Ca_Desi_Chr02	11984016	(C/T)
25722	CadSNP1317	*Desi*	Ca_Desi_Chr02	11985665	(A/C)
25723	CadSNP1318	*Desi*	Ca_Desi_Chr02	11992316	(A/G)
25724	CadSNP1319	*Desi*	Ca_Desi_Chr02	12071159	(T/C)
25725	CadSNP1320	*Desi*	Ca_Desi_Chr02	12071213	(G/A)
25726	CadSNP1321	*Desi*	Ca_Desi_Chr02	12072110	(A/C)
25727	CadSNP1322	*Desi*	Ca_Desi_Chr02	12357679	(A/G)
25728	CadSNP1323	*Desi*	Ca_Desi_Chr02	12462629	(C/T)
25729	CadSNP1324	*Desi*	Ca_Desi_Chr02	12462654	(G/T)
25730	CadSNP1325	*Desi*	Ca_Desi_Chr02	12490110	(G/C)
25731	CadSNP1326	*Desi*	Ca_Desi_Chr02	12490080	(A/G)
25732	CadSNP1327	*Desi*	Ca_Desi_Chr02	12490043	(G/A)
25733	CadSNP1328	*Desi*	Ca_Desi_Chr02	12518521	(G/T)
25734	CadSNP1329	*Desi*	Ca_Desi_Chr02	12560789	(T/G)
25735	CadSNP1330	*Desi*	Ca_Desi_Chr02	12726887	(A/G)
25736	CadSNP1331	*Desi*	Ca_Desi_Chr02	12729502	(C/T)
25737	CadSNP1332	*Desi*	Ca_Desi_Chr02	12737222	(G/T)
25738	CadSNP1333	*Desi*	Ca_Desi_Chr02	12760068	(C/T)

S.N.	SNP IDs	Cultivars	Chromosomes/scaffolds	Physical positions (bp)	SNPs
25739	CadSNP1334	*Desi*	Ca_Desi_Chr02	12797455	(A/T)
25740	CadSNP1335	*Desi*	Ca_Desi_Chr02	12802226	(C/A)
25741	CadSNP1336	*Desi*	Ca_Desi_Chr02	12802223	(T/C)
25742	CadSNP1337	*Desi*	Ca_Desi_Chr02	12802202	(G/T)
25743	CadSNP1338	*Desi*	Ca_Desi_Chr02	12827722	(C/T)
25744	CadSNP1339	*Desi*	Ca_Desi_Chr02	12827705	(A/G)
25745	CadSNP1340	*Desi*	Ca_Desi_Chr02	12830428	(A/C)
25746	CadSNP1341	*Desi*	Ca_Desi_Chr02	12985136	(G/A)
25747	CadSNP1342	*Desi*	Ca_Desi_Chr02	13036994	(G/C)
25748	CadSNP1343	*Desi*	Ca_Desi_Chr02	13036951	(A/C)
25749	CadSNP1344	*Desi*	Ca_Desi_Chr02	13039200	(T/C)
25750	CadSNP1345	*Desi*	Ca_Desi_Chr02	13039869	(C/G)
25751	CadSNP1346	*Desi*	Ca_Desi_Chr02	13040155	(C/T)
25752	CadSNP1347	*Desi*	Ca_Desi_Chr02	13100484	(A/C)
25753	CadSNP1348	*Desi*	Ca_Desi_Chr02	13122853	(T/G)
25754	CadSNP1349	*Desi*	Ca_Desi_Chr02	13127113	(T/G)
25755	CadSNP1350	*Desi*	Ca_Desi_Chr02	13147204	(A/T)
25756	CadSNP1351	*Desi*	Ca_Desi_Chr02	13162836	(T/C)
25757	CadSNP1352	*Desi*	Ca_Desi_Chr02	13162848	(G/C)
25758	CadSNP1353	*Desi*	Ca_Desi_Chr02	13178463	(T/C)
25759	CadSNP1354	*Desi*	Ca_Desi_Chr02	13260498	(G/A)
25760	CadSNP1355	*Desi*	Ca_Desi_Chr02	13331702	(A/G)
25761	CadSNP1356	*Desi*	Ca_Desi_Chr02	13379964	(A/T)
25762	CadSNP1357	*Desi*	Ca_Desi_Chr02	13421370	(C/T)
25763	CadSNP1358	*Desi*	Ca_Desi_Chr02	13572362	(G/A)
25764	CadSNP1359	*Desi*	Ca_Desi_Chr02	13840468	(T/G)
25765	CadSNP1360	*Desi*	Ca_Desi_Chr02	13840749	(T/A)
25766	CadSNP1361	*Desi*	Ca_Desi_Chr02	13900392	(A/G)
25767	CadSNP1362	*Desi*	Ca_Desi_Chr02	13924598	(A/C)
25768	CadSNP1363	*Desi*	Ca_Desi_Chr02	13924579	(A/T)
25769	CadSNP1364	*Desi*	Ca_Desi_Chr02	14061466	(T/C)
25770	CadSNP1365	*Desi*	Ca_Desi_Chr02	14064995	(G/T)
25771	CadSNP1366	*Desi*	Ca_Desi_Chr02	14163542	(A/C)
25772	CadSNP1367	*Desi*	Ca_Desi_Chr02	14163647	(C/T)
25773	CadSNP1368	*Desi*	Ca_Desi_Chr02	14209016	(A/G)
25774	CadSNP1369	*Desi*	Ca_Desi_Chr02	14215276	(G/A)
25775	CadSNP1370	*Desi*	Ca_Desi_Chr02	14215293	(G/C)
25776	CadSNP1371	*Desi*	Ca_Desi_Chr02	14215344	(A/T)
25777	CadSNP1372	*Desi*	Ca_Desi_Chr02	14215466	(T/C)

S.N.	SNP IDs	Cultivars	Chromosomes/scaffolds	Physical positions (bp)	SNPs
25778	CadSNP1373	*Desi*	Ca_Desi_Chr02	14215453	(A/T)
25779	CadSNP1374	*Desi*	Ca_Desi_Chr02	14453533	(T/C)
25780	CadSNP1375	*Desi*	Ca_Desi_Chr02	14453917	(T/C)
25781	CadSNP1376	*Desi*	Ca_Desi_Chr02	14463390	(T/A)
25782	CadSNP1377	*Desi*	Ca_Desi_Chr02	14474102	(C/T)
25783	CadSNP1378	*Desi*	Ca_Desi_Chr02	14474157	(T/A)
25784	CadSNP1379	*Desi*	Ca_Desi_Chr02	14474167	(A/T)
25785	CadSNP1380	*Desi*	Ca_Desi_Chr02	14492206	(T/C)
25786	CadSNP1381	*Desi*	Ca_Desi_Chr02	14492207	(A/G)
25787	CadSNP1382	*Desi*	Ca_Desi_Chr02	14492297	(T/C)
25788	CadSNP1383	*Desi*	Ca_Desi_Chr02	14493784	(T/A)
25789	CadSNP1384	*Desi*	Ca_Desi_Chr02	14510462	(G/T)
25790	CadSNP1385	*Desi*	Ca_Desi_Chr02	14510458	(C/A)
25791	CadSNP1386	*Desi*	Ca_Desi_Chr02	14577347	(G/A)
25792	CadSNP1387	*Desi*	Ca_Desi_Chr02	14577488	(C/T)
25793	CadSNP1388	*Desi*	Ca_Desi_Chr02	14729007	(T/A)
25794	CadSNP1389	*Desi*	Ca_Desi_Chr02	14765296	(T/C)
25795	CadSNP1390	*Desi*	Ca_Desi_Chr02	14766612	(T/G)
25796	CadSNP1391	*Desi*	Ca_Desi_Chr02	14766747	(G/A)
25797	CadSNP1392	*Desi*	Ca_Desi_Chr02	14766780	(C/T)
25798	CadSNP1393	*Desi*	Ca_Desi_Chr02	14784192	(C/T)
25799	CadSNP1394	*Desi*	Ca_Desi_Chr02	14784195	(A/G)
25800	CadSNP1395	*Desi*	Ca_Desi_Chr02	14784208	(G/C)
25801	CadSNP1396	*Desi*	Ca_Desi_Chr02	14784243	(A/G)
25802	CadSNP1397	*Desi*	Ca_Desi_Chr02	14784247	(G/T)
25803	CadSNP1398	*Desi*	Ca_Desi_Chr02	14811203	(A/G)
25804	CadSNP1399	*Desi*	Ca_Desi_Chr02	14861116	(A/C)
25805	CadSNP1400	*Desi*	Ca_Desi_Chr02	14861159	(T/G)
25806	CadSNP1401	*Desi*	Ca_Desi_Chr02	14978631	(T/C)
25807	CadSNP1402	*Desi*	Ca_Desi_Chr02	14978653	(C/T)
25808	CadSNP1403	*Desi*	Ca_Desi_Chr02	15006255	(C/A)
25809	CadSNP1404	*Desi*	Ca_Desi_Chr02	15006256	(G/T)
25810	CadSNP1405	*Desi*	Ca_Desi_Chr02	15014922	(C/T)
25811	CadSNP1406	*Desi*	Ca_Desi_Chr02	15222856	(C/T)
25812	CadSNP1407	*Desi*	Ca_Desi_Chr02	15279307	(A/G)
25813	CadSNP1408	*Desi*	Ca_Desi_Chr02	15284031	(T/A)
25814	CadSNP1409	*Desi*	Ca_Desi_Chr02	15313773	(A/G)
25815	CadSNP1410	*Desi*	Ca_Desi_Chr02	15318170	(C/T)
25816	CadSNP1411	*Desi*	Ca_Desi_Chr02	15318202	(A/G)

S.N.	SNP IDs	Cultivars	Chromosomes/scaffolds	Physical positions (bp)	SNPs
25817	CadSNP1412	*Desi*	Ca_Desi_Chr02	15391794	(T/C)
25818	CadSNP1413	*Desi*	Ca_Desi_Chr02	15392614	(C/T)
25819	CadSNP1414	*Desi*	Ca_Desi_Chr02	15392855	(T/G)
25820	CadSNP1415	*Desi*	Ca_Desi_Chr02	15427877	(G/A)
25821	CadSNP1416	*Desi*	Ca_Desi_Chr02	15438733	(A/C)
25822	CadSNP1417	*Desi*	Ca_Desi_Chr02	15470642	(C/T)
25823	CadSNP1418	*Desi*	Ca_Desi_Chr02	15488586	(G/A)
25824	CadSNP1419	*Desi*	Ca_Desi_Chr02	15488553	(C/T)
25825	CadSNP1420	*Desi*	Ca_Desi_Chr02	15502293	(T/C)
25826	CadSNP1421	*Desi*	Ca_Desi_Chr02	15510668	(C/A)
25827	CadSNP1422	*Desi*	Ca_Desi_Chr02	15510675	(G/A)
25828	CadSNP1423	*Desi*	Ca_Desi_Chr02	15526715	(G/T)
25829	CadSNP1424	*Desi*	Ca_Desi_Chr02	15526673	(G/A)
25830	CadSNP1425	*Desi*	Ca_Desi_Chr02	15526670	(C/T)
25831	CadSNP1426	*Desi*	Ca_Desi_Chr02	15551741	(T/C)
25832	CadSNP1427	*Desi*	Ca_Desi_Chr02	15551733	(A/G)
25833	CadSNP1428	*Desi*	Ca_Desi_Chr02	15575093	(A/G)
25834	CadSNP1429	*Desi*	Ca_Desi_Chr02	15575141	(A/G)
25835	CadSNP1430	*Desi*	Ca_Desi_Chr02	15627570	(T/G)
25836	CadSNP1431	*Desi*	Ca_Desi_Chr02	15653215	(A/T)
25837	CadSNP1432	*Desi*	Ca_Desi_Chr02	15656951	(C/T)
25838	CadSNP1433	*Desi*	Ca_Desi_Chr02	15671766	(C/A)
25839	CadSNP1434	*Desi*	Ca_Desi_Chr02	15677639	(C/T)
25840	CadSNP1435	*Desi*	Ca_Desi_Chr02	15757574	(T/A)
25841	CadSNP1436	*Desi*	Ca_Desi_Chr02	15769880	(A/T)
25842	CadSNP1437	*Desi*	Ca_Desi_Chr02	15769883	(A/C)
25843	CadSNP1438	*Desi*	Ca_Desi_Chr02	15772497	(G/A)
25844	CadSNP1439	*Desi*	Ca_Desi_Chr02	15786439	(G/A)
25845	CadSNP1440	*Desi*	Ca_Desi_Chr02	15874395	(A/G)
25846	CadSNP1441	*Desi*	Ca_Desi_Chr02	15878454	(C/A)
25847	CadSNP1442	*Desi*	Ca_Desi_Chr02	15878469	(C/A)
25848	CadSNP1443	*Desi*	Ca_Desi_Chr02	15878472	(A/T)
25849	CadSNP1444	*Desi*	Ca_Desi_Chr02	15878509	(A/T)
25850	CadSNP1445	*Desi*	Ca_Desi_Chr02	15905306	(T/C)
25851	CadSNP1446	*Desi*	Ca_Desi_Chr02	15905388	(T/A)
25852	CadSNP1447	*Desi*	Ca_Desi_Chr02	15959857	(A/G)
25853	CadSNP1448	*Desi*	Ca_Desi_Chr02	15970026	(C/G)
25854	CadSNP1449	*Desi*	Ca_Desi_Chr02	15970055	(T/C)
25855	CadSNP1450	*Desi*	Ca_Desi_Chr02	15970076	(A/T)

S.N.	SNP IDs	Cultivars	Chromosomes/scaffolds	Physical positions (bp)	SNPs
25856	CadSNP1451	*Desi*	Ca_Desi_Chr02	15970077	(A/G)
25857	CadSNP1452	*Desi*	Ca_Desi_Chr02	15970117	(A/G)
25858	CadSNP1453	*Desi*	Ca_Desi_Chr02	15970114	(A/G)
25859	CadSNP1454	*Desi*	Ca_Desi_Chr02	16066784	(G/A)
25860	CadSNP1455	*Desi*	Ca_Desi_Chr02	16066788	(C/T)
25861	CadSNP1456	*Desi*	Ca_Desi_Chr02	16113340	(C/A)
25862	CadSNP1457	*Desi*	Ca_Desi_Chr02	16114802	(T/C)
25863	CadSNP1458	*Desi*	Ca_Desi_Chr02	16114821	(A/T)
25864	CadSNP1459	*Desi*	Ca_Desi_Chr02	16116122	(C/G)
25865	CadSNP1460	*Desi*	Ca_Desi_Chr02	16130330	(A/G)
25866	CadSNP1461	*Desi*	Ca_Desi_Chr02	16136692	(A/T)
25867	CadSNP1462	*Desi*	Ca_Desi_Chr02	16136703	(T/C)
25868	CadSNP1463	*Desi*	Ca_Desi_Chr02	16136908	(C/T)
25869	CadSNP1464	*Desi*	Ca_Desi_Chr02	16137005	(C/T)
25870	CadSNP1465	*Desi*	Ca_Desi_Chr02	16138793	(T/C)
25871	CadSNP1466	*Desi*	Ca_Desi_Chr02	16138943	(A/C)
25872	CadSNP1467	*Desi*	Ca_Desi_Chr02	16140958	(A/C)
25873	CadSNP1468	*Desi*	Ca_Desi_Chr02	16157597	(A/C)
25874	CadSNP1469	*Desi*	Ca_Desi_Chr02	16172626	(G/A)
25875	CadSNP1470	*Desi*	Ca_Desi_Chr02	16172603	(A/G)
25876	CadSNP1471	*Desi*	Ca_Desi_Chr02	16310713	(C/G)
25877	CadSNP1472	*Desi*	Ca_Desi_Chr02	16637277	(T/G)
25878	CadSNP1473	*Desi*	Ca_Desi_Chr02	16689981	(C/T)
25879	CadSNP1474	*Desi*	Ca_Desi_Chr02	16690001	(T/G)
25880	CadSNP1475	*Desi*	Ca_Desi_Chr02	16690008	(C/T)
25881	CadSNP1476	*Desi*	Ca_Desi_Chr02	16690011	(A/T)
25882	CadSNP1477	*Desi*	Ca_Desi_Chr02	16690097	(G/A)
25883	CadSNP1478	*Desi*	Ca_Desi_Chr02	16712209	(A/G)
25884	CadSNP1479	*Desi*	Ca_Desi_Chr02	16741302	(C/T)
25885	CadSNP1480	*Desi*	Ca_Desi_Chr02	16741251	(C/T)
25886	CadSNP1481	*Desi*	Ca_Desi_Chr02	16741238	(G/T)
25887	CadSNP1482	*Desi*	Ca_Desi_Chr02	16748554	(G/T)
25888	CadSNP1483	*Desi*	Ca_Desi_Chr02	16748556	(T/G)
25889	CadSNP1484	*Desi*	Ca_Desi_Chr02	16748700	(G/A)
25890	CadSNP1485	*Desi*	Ca_Desi_Chr02	16748679	(T/A)
25891	CadSNP1486	*Desi*	Ca_Desi_Chr02	16748726	(T/A)
25892	CadSNP1487	*Desi*	Ca_Desi_Chr02	16748758	(C/A)
25893	CadSNP1488	*Desi*	Ca_Desi_Chr02	16748779	(T/A)
25894	CadSNP1489	*Desi*	Ca_Desi_Chr02	16748803	(T/G)

S.N.	SNP IDs	Cultivars	Chromosomes/scaffolds	Physical positions (bp)	SNPs
25895	CadSNP1490	*Desi*	Ca_Desi_Chr02	16838445	(C/G)
25896	CadSNP1491	*Desi*	Ca_Desi_Chr02	16856357	(A/C)
25897	CadSNP1492	*Desi*	Ca_Desi_Chr02	16872692	(T/G)
25898	CadSNP1493	*Desi*	Ca_Desi_Chr02	16872691	(A/T)
25899	CadSNP1494	*Desi*	Ca_Desi_Chr02	16872685	(A/G)
25900	CadSNP1495	*Desi*	Ca_Desi_Chr02	16872684	(A/T)
25901	CadSNP1496	*Desi*	Ca_Desi_Chr02	16872680	(T/A)
25902	CadSNP1497	*Desi*	Ca_Desi_Chr02	16872674	(T/C)
25903	CadSNP1498	*Desi*	Ca_Desi_Chr02	16872671	(A/G)
25904	CadSNP1499	*Desi*	Ca_Desi_Chr02	16872665	(T/A)
25905	CadSNP1500	*Desi*	Ca_Desi_Chr02	16872659	(T/C)
25906	CadSNP1501	*Desi*	Ca_Desi_Chr02	16872657	(C/T)
25907	CadSNP1502	*Desi*	Ca_Desi_Chr02	16872653	(C/T)
25908	CadSNP1503	*Desi*	Ca_Desi_Chr02	16954914	(G/A)
25909	CadSNP1504	*Desi*	Ca_Desi_Chr02	17060800	(T/C)
25910	CadSNP1505	*Desi*	Ca_Desi_Chr02	17063464	(G/C)
25911	CadSNP1506	*Desi*	Ca_Desi_Chr02	17063468	(T/C)
25912	CadSNP1507	*Desi*	Ca_Desi_Chr02	17167481	(A/G)
25913	CadSNP1508	*Desi*	Ca_Desi_Chr02	17184026	(C/T)
25914	CadSNP1509	*Desi*	Ca_Desi_Chr02	17187208	(T/C)
25915	CadSNP1510	*Desi*	Ca_Desi_Chr02	17187245	(G/C)
25916	CadSNP1511	*Desi*	Ca_Desi_Chr02	17187980	(C/A)
25917	CadSNP1512	*Desi*	Ca_Desi_Chr03	44873	(T/G)
25918	CadSNP1513	*Desi*	Ca_Desi_Chr03	44877	(T/C)
25919	CadSNP1514	*Desi*	Ca_Desi_Chr03	45008	(C/T)
25920	CadSNP1515	*Desi*	Ca_Desi_Chr03	45009	(C/A)
25921	CadSNP1516	*Desi*	Ca_Desi_Chr03	45013	(G/A)
25922	CadSNP1517	*Desi*	Ca_Desi_Chr03	76445	(G/A)
25923	CadSNP1518	*Desi*	Ca_Desi_Chr03	80216	(A/C)
25924	CadSNP1519	*Desi*	Ca_Desi_Chr03	126485	(T/G)
25925	CadSNP1520	*Desi*	Ca_Desi_Chr03	126527	(C/T)
25926	CadSNP1521	*Desi*	Ca_Desi_Chr03	126619	(G/A)
25927	CadSNP1522	*Desi*	Ca_Desi_Chr03	335668	(T/C)
25928	CadSNP1523	*Desi*	Ca_Desi_Chr03	347892	(C/T)
25929	CadSNP1524	*Desi*	Ca_Desi_Chr03	347865	(C/A)
25930	CadSNP1525	*Desi*	Ca_Desi_Chr03	645024	(A/C)
25931	CadSNP1526	*Desi*	Ca_Desi_Chr03	669270	(C/T)
25932	CadSNP1527	*Desi*	Ca_Desi_Chr03	684601	(T/C)
25933	CadSNP1528	*Desi*	Ca_Desi_Chr03	684572	(G/A)

S.N.	SNP IDs	Cultivars	Chromosomes/scaffolds	Physical positions (bp)	SNPs
25934	CadSNP1529	*Desi*	Ca_Desi_Chr03	690339	(G/A)
25935	CadSNP1530	*Desi*	Ca_Desi_Chr03	690372	(G/C)
25936	CadSNP1531	*Desi*	Ca_Desi_Chr03	691472	(C/T)
25937	CadSNP1532	*Desi*	Ca_Desi_Chr03	693294	(G/A)
25938	CadSNP1533	*Desi*	Ca_Desi_Chr03	695497	(C/T)
25939	CadSNP1534	*Desi*	Ca_Desi_Chr03	695494	(T/A)
25940	CadSNP1535	*Desi*	Ca_Desi_Chr03	697967	(T/G)
25941	CadSNP1536	*Desi*	Ca_Desi_Chr03	698068	(G/A)
25942	CadSNP1537	*Desi*	Ca_Desi_Chr03	698170	(T/G)
25943	CadSNP1538	*Desi*	Ca_Desi_Chr03	753812	(T/G)
25944	CadSNP1539	*Desi*	Ca_Desi_Chr03	762935	(G/A)
25945	CadSNP1540	*Desi*	Ca_Desi_Chr03	768344	(G/A)
25946	CadSNP1541	*Desi*	Ca_Desi_Chr03	768325	(C/G)
25947	CadSNP1542	*Desi*	Ca_Desi_Chr03	768323	(A/T)
25948	CadSNP1543	*Desi*	Ca_Desi_Chr03	782934	(C/T)
25949	CadSNP1544	*Desi*	Ca_Desi_Chr03	786406	(A/G)
25950	CadSNP1545	*Desi*	Ca_Desi_Chr03	854698	(C/T)
25951	CadSNP1546	*Desi*	Ca_Desi_Chr03	954899	(T/C)
25952	CadSNP1547	*Desi*	Ca_Desi_Chr03	993205	(A/G)
25953	CadSNP1548	*Desi*	Ca_Desi_Chr03	993204	(G/A)
25954	CadSNP1549	*Desi*	Ca_Desi_Chr03	993159	(C/T)
25955	CadSNP1550	*Desi*	Ca_Desi_Chr03	1000520	(T/G)
25956	CadSNP1551	*Desi*	Ca_Desi_Chr03	1001326	(A/G)
25957	CadSNP1552	*Desi*	Ca_Desi_Chr03	1006408	(C/T)
25958	CadSNP1553	*Desi*	Ca_Desi_Chr03	1009680	(A/C)
25959	CadSNP1554	*Desi*	Ca_Desi_Chr03	1037187	(A/G)
25960	CadSNP1555	*Desi*	Ca_Desi_Chr03	1132867	(G/A)
25961	CadSNP1556	*Desi*	Ca_Desi_Chr03	1138482	(A/G)
25962	CadSNP1557	*Desi*	Ca_Desi_Chr03	1142176	(T/C)
25963	CadSNP1558	*Desi*	Ca_Desi_Chr03	1142293	(C/A)
25964	CadSNP1559	*Desi*	Ca_Desi_Chr03	1146163	(T/C)
25965	CadSNP1560	*Desi*	Ca_Desi_Chr03	1147189	(C/T)
25966	CadSNP1561	*Desi*	Ca_Desi_Chr03	1148157	(G/T)
25967	CadSNP1562	*Desi*	Ca_Desi_Chr03	1148176	(T/G)
25968	CadSNP1563	*Desi*	Ca_Desi_Chr03	1148257	(A/G)
25969	CadSNP1564	*Desi*	Ca_Desi_Chr03	1148353	(A/C)
25970	CadSNP1565	*Desi*	Ca_Desi_Chr03	1156273	(T/C)
25971	CadSNP1566	*Desi*	Ca_Desi_Chr03	1156347	(G/C)
25972	CadSNP1567	*Desi*	Ca_Desi_Chr03	1198705	(A/C)

S.N.	SNP IDs	Cultivars	Chromosomes/scaffolds	Physical positions (bp)	SNPs
25973	CadSNP1568	*Desi*	Ca_Desi_Chr03	1198729	(T/C)
25974	CadSNP1569	*Desi*	Ca_Desi_Chr03	1198799	(T/A)
25975	CadSNP1570	*Desi*	Ca_Desi_Chr03	1199241	(C/A)
25976	CadSNP1571	*Desi*	Ca_Desi_Chr03	1205701	(A/G)
25977	CadSNP1572	*Desi*	Ca_Desi_Chr03	1205850	(A/T)
25978	CadSNP1573	*Desi*	Ca_Desi_Chr03	1205847	(G/C)
25979	CadSNP1574	*Desi*	Ca_Desi_Chr03	1205826	(T/G)
25980	CadSNP1575	*Desi*	Ca_Desi_Chr03	1205824	(T/A)
25981	CadSNP1576	*Desi*	Ca_Desi_Chr03	1205811	(T/A)
25982	CadSNP1577	*Desi*	Ca_Desi_Chr03	1205789	(A/C)
25983	CadSNP1578	*Desi*	Ca_Desi_Chr03	1208905	(A/G)
25984	CadSNP1579	*Desi*	Ca_Desi_Chr03	1208869	(T/C)
25985	CadSNP1580	*Desi*	Ca_Desi_Chr03	1223420	(C/A)
25986	CadSNP1581	*Desi*	Ca_Desi_Chr03	1228195	(A/G)
25987	CadSNP1582	*Desi*	Ca_Desi_Chr03	1228218	(T/C)
25988	CadSNP1583	*Desi*	Ca_Desi_Chr03	1228231	(A/C)
25989	CadSNP1584	*Desi*	Ca_Desi_Chr03	1233409	(A/C)
25990	CadSNP1585	*Desi*	Ca_Desi_Chr03	1255515	(C/T)
25991	CadSNP1586	*Desi*	Ca_Desi_Chr03	1267770	(T/C)
25992	CadSNP1587	*Desi*	Ca_Desi_Chr03	1286463	(T/C)
25993	CadSNP1588	*Desi*	Ca_Desi_Chr03	1286513	(G/A)
25994	CadSNP1589	*Desi*	Ca_Desi_Chr03	1298429	(G/T)
25995	CadSNP1590	*Desi*	Ca_Desi_Chr03	1298524	(G/A)
25996	CadSNP1591	*Desi*	Ca_Desi_Chr03	1298497	(C/T)
25997	CadSNP1592	*Desi*	Ca_Desi_Chr03	1298488	(A/G)
25998	CadSNP1593	*Desi*	Ca_Desi_Chr03	1348788	(C/G)
25999	CadSNP1594	*Desi*	Ca_Desi_Chr03	1348911	(T/C)
26000	CadSNP1595	*Desi*	Ca_Desi_Chr03	1392395	(A/G)
26001	CadSNP1596	*Desi*	Ca_Desi_Chr03	1404434	(T/A)
26002	CadSNP1597	*Desi*	Ca_Desi_Chr03	1404499	(A/G)
26003	CadSNP1598	*Desi*	Ca_Desi_Chr03	1414321	(A/G)
26004	CadSNP1599	*Desi*	Ca_Desi_Chr03	1416925	(C/T)
26005	CadSNP1600	*Desi*	Ca_Desi_Chr03	1416904	(T/A)
26006	CadSNP1601	*Desi*	Ca_Desi_Chr03	1416895	(A/T)
26007	CadSNP1602	*Desi*	Ca_Desi_Chr03	1427742	(C/T)
26008	CadSNP1603	*Desi*	Ca_Desi_Chr03	1427752	(C/T)
26009	CadSNP1604	*Desi*	Ca_Desi_Chr03	1427760	(A/G)
26010	CadSNP1605	*Desi*	Ca_Desi_Chr03	1427788	(C/T)
26011	CadSNP1606	*Desi*	Ca_Desi_Chr03	1427793	(G/A)

S.N.	SNP IDs	Cultivars	Chromosomes/scaffolds	Physical positions (bp)	SNPs
26012	CadSNP1607	*Desi*	Ca_Desi_Chr03	1427796	(A/G)
26013	CadSNP1608	*Desi*	Ca_Desi_Chr03	1427799	(T/C)
26014	CadSNP1609	*Desi*	Ca_Desi_Chr03	1427811	(A/T)
26015	CadSNP1610	*Desi*	Ca_Desi_Chr03	1427822	(A/T)
26016	CadSNP1611	*Desi*	Ca_Desi_Chr03	1427904	(A/G)
26017	CadSNP1612	*Desi*	Ca_Desi_Chr03	1427901	(A/T)
26018	CadSNP1613	*Desi*	Ca_Desi_Chr03	1427892	(A/T)
26019	CadSNP1614	*Desi*	Ca_Desi_Chr03	1427874	(T/C)
26020	CadSNP1615	*Desi*	Ca_Desi_Chr03	1427865	(T/C)
26021	CadSNP1616	*Desi*	Ca_Desi_Chr03	1427862	(T/A)
26022	CadSNP1617	*Desi*	Ca_Desi_Chr03	1427850	(A/G)
26023	CadSNP1618	*Desi*	Ca_Desi_Chr03	1427826	(T/C)
26024	CadSNP1619	*Desi*	Ca_Desi_Chr03	1427905	(T/C)
26025	CadSNP1620	*Desi*	Ca_Desi_Chr03	1463047	(A/G)
26026	CadSNP1621	*Desi*	Ca_Desi_Chr03	1503173	(A/T)
26027	CadSNP1622	*Desi*	Ca_Desi_Chr03	1516868	(C/A)
26028	CadSNP1623	*Desi*	Ca_Desi_Chr03	1533891	(A/C)
26029	CadSNP1624	*Desi*	Ca_Desi_Chr03	1536663	(G/T)
26030	CadSNP1625	*Desi*	Ca_Desi_Chr03	1536675	(G/A)
26031	CadSNP1626	*Desi*	Ca_Desi_Chr03	1536708	(A/G)
26032	CadSNP1627	*Desi*	Ca_Desi_Chr03	1536748	(A/G)
26033	CadSNP1628	*Desi*	Ca_Desi_Chr03	1536750	(A/G)
26034	CadSNP1629	*Desi*	Ca_Desi_Chr03	1536753	(C/T)
26035	CadSNP1630	*Desi*	Ca_Desi_Chr03	1536804	(A/T)
26036	CadSNP1631	*Desi*	Ca_Desi_Chr03	1554464	(T/C)
26037	CadSNP1632	*Desi*	Ca_Desi_Chr03	1554518	(T/C)
26038	CadSNP1633	*Desi*	Ca_Desi_Chr03	1554578	(C/T)
26039	CadSNP1634	*Desi*	Ca_Desi_Chr03	1633031	(T/A)
26040	CadSNP1635	*Desi*	Ca_Desi_Chr03	1659975	(A/G)
26041	CadSNP1636	*Desi*	Ca_Desi_Chr03	1667821	(C/T)
26042	CadSNP1637	*Desi*	Ca_Desi_Chr03	1670337	(C/T)
26043	CadSNP1638	*Desi*	Ca_Desi_Chr03	1686934	(G/C)
26044	CadSNP1639	*Desi*	Ca_Desi_Chr03	1686974	(G/A)
26045	CadSNP1640	*Desi*	Ca_Desi_Chr03	2660813	(A/G)
26046	CadSNP1641	*Desi*	Ca_Desi_Chr03	2660782	(C/T)
26047	CadSNP1642	*Desi*	Ca_Desi_Chr03	2672499	(G/A)
26048	CadSNP1643	*Desi*	Ca_Desi_Chr03	2674284	(T/A)
26049	CadSNP1644	*Desi*	Ca_Desi_Chr03	2674302	(A/G)
26050	CadSNP1645	*Desi*	Ca_Desi_Chr03	2674439	(A/C)

S.N.	SNP IDs	Cultivars	Chromosomes/scaffolds	Physical positions (bp)	SNPs
26051	CadSNP1646	*Desi*	Ca_Desi_Chr03	2679323	(A/G)
26052	CadSNP1647	*Desi*	Ca_Desi_Chr03	2683637	(T/G)
26053	CadSNP1648	*Desi*	Ca_Desi_Chr03	2693535	(C/G)
26054	CadSNP1649	*Desi*	Ca_Desi_Chr03	2693537	(C/T)
26055	CadSNP1650	*Desi*	Ca_Desi_Chr03	2700887	(T/C)
26056	CadSNP1651	*Desi*	Ca_Desi_Chr03	2713239	(T/A)
26057	CadSNP1652	*Desi*	Ca_Desi_Chr03	2713248	(A/C)
26058	CadSNP1653	*Desi*	Ca_Desi_Chr03	2716549	(G/A)
26059	CadSNP1654	*Desi*	Ca_Desi_Chr03	2718128	(G/C)
26060	CadSNP1655	*Desi*	Ca_Desi_Chr03	2718129	(G/A)
26061	CadSNP1656	*Desi*	Ca_Desi_Chr03	2718182	(A/T)
26062	CadSNP1657	*Desi*	Ca_Desi_Chr03	2725750	(A/G)
26063	CadSNP1658	*Desi*	Ca_Desi_Chr03	2729400	(A/G)
26064	CadSNP1659	*Desi*	Ca_Desi_Chr03	2737288	(T/C)
26065	CadSNP1660	*Desi*	Ca_Desi_Chr03	2754108	(C/T)
26066	CadSNP1661	*Desi*	Ca_Desi_Chr03	2754162	(C/G)
26067	CadSNP1662	*Desi*	Ca_Desi_Chr03	2758011	(G/T)
26068	CadSNP1663	*Desi*	Ca_Desi_Chr03	2758003	(A/C)
26069	CadSNP1664	*Desi*	Ca_Desi_Chr03	2787196	(C/G)
26070	CadSNP1665	*Desi*	Ca_Desi_Chr03	2787283	(G/C)
26071	CadSNP1666	*Desi*	Ca_Desi_Chr03	2787268	(T/C)
26072	CadSNP1667	*Desi*	Ca_Desi_Chr03	2787355	(G/C)
26073	CadSNP1668	*Desi*	Ca_Desi_Chr03	2793058	(G/A)
26074	CadSNP1669	*Desi*	Ca_Desi_Chr03	2795523	(C/T)
26075	CadSNP1670	*Desi*	Ca_Desi_Chr03	2799304	(T/C)
26076	CadSNP1671	*Desi*	Ca_Desi_Chr03	2803750	(A/G)
26077	CadSNP1672	*Desi*	Ca_Desi_Chr03	2812122	(G/A)
26078	CadSNP1673	*Desi*	Ca_Desi_Chr03	2814757	(A/G)
26079	CadSNP1674	*Desi*	Ca_Desi_Chr03	2814908	(G/A)
26080	CadSNP1675	*Desi*	Ca_Desi_Chr03	2840621	(G/A)
26081	CadSNP1676	*Desi*	Ca_Desi_Chr03	2873563	(G/T)
26082	CadSNP1677	*Desi*	Ca_Desi_Chr03	2920219	(A/G)
26083	CadSNP1678	*Desi*	Ca_Desi_Chr03	2920379	(G/A)
26084	CadSNP1679	*Desi*	Ca_Desi_Chr03	2953036	(C/T)
26085	CadSNP1680	*Desi*	Ca_Desi_Chr03	2986236	(C/T)
26086	CadSNP1681	*Desi*	Ca_Desi_Chr03	2986233	(T/C)
26087	CadSNP1682	*Desi*	Ca_Desi_Chr03	2991036	(A/C)
26088	CadSNP1683	*Desi*	Ca_Desi_Chr03	3002455	(A/C)
26089	CadSNP1684	*Desi*	Ca_Desi_Chr03	3015825	(A/G)

S.N.	SNP IDs	Cultivars	Chromosomes/scaffolds	Physical positions (bp)	SNPs
26090	CadSNP1685	*Desi*	Ca_Desi_Chr03	3015816	(A/G)
26091	CadSNP1686	*Desi*	Ca_Desi_Chr03	3048193	(C/T)
26092	CadSNP1687	*Desi*	Ca_Desi_Chr03	3048229	(T/C)
26093	CadSNP1688	*Desi*	Ca_Desi_Chr03	3130784	(G/T)
26094	CadSNP1689	*Desi*	Ca_Desi_Chr03	3179253	(C/T)
26095	CadSNP1690	*Desi*	Ca_Desi_Chr03	3187918	(A/T)
26096	CadSNP1691	*Desi*	Ca_Desi_Chr03	3218964	(A/G)
26097	CadSNP1692	*Desi*	Ca_Desi_Chr03	3224433	(T/C)
26098	CadSNP1693	*Desi*	Ca_Desi_Chr03	3254955	(C/T)
26099	CadSNP1694	*Desi*	Ca_Desi_Chr03	3255060	(C/T)
26100	CadSNP1695	*Desi*	Ca_Desi_Chr03	3255029	(C/T)
26101	CadSNP1696	*Desi*	Ca_Desi_Chr03	3267987	(T/G)
26102	CadSNP1697	*Desi*	Ca_Desi_Chr03	3295800	(T/A)
26103	CadSNP1698	*Desi*	Ca_Desi_Chr03	3333129	(A/G)
26104	CadSNP1699	*Desi*	Ca_Desi_Chr03	3398702	(A/G)
26105	CadSNP1700	*Desi*	Ca_Desi_Chr03	3398708	(C/A)
26106	CadSNP1701	*Desi*	Ca_Desi_Chr03	3404200	(C/A)
26107	CadSNP1702	*Desi*	Ca_Desi_Chr03	3424969	(T/C)
26108	CadSNP1703	*Desi*	Ca_Desi_Chr03	3427256	(G/T)
26109	CadSNP1704	*Desi*	Ca_Desi_Chr03	3455564	(G/A)
26110	CadSNP1705	*Desi*	Ca_Desi_Chr03	3479333	(A/C)
26111	CadSNP1706	*Desi*	Ca_Desi_Chr03	3488455	(C/A)
26112	CadSNP1707	*Desi*	Ca_Desi_Chr03	3493823	(T/G)
26113	CadSNP1708	*Desi*	Ca_Desi_Chr03	3493826	(C/T)
26114	CadSNP1709	*Desi*	Ca_Desi_Chr03	3493840	(G/C)
26115	CadSNP1710	*Desi*	Ca_Desi_Chr03	3495518	(T/A)
26116	CadSNP1711	*Desi*	Ca_Desi_Chr03	3495509	(T/C)
26117	CadSNP1712	*Desi*	Ca_Desi_Chr03	3495557	(A/G)
26118	CadSNP1713	*Desi*	Ca_Desi_Chr03	3512869	(A/G)
26119	CadSNP1714	*Desi*	Ca_Desi_Chr03	3523403	(T/C)
26120	CadSNP1715	*Desi*	Ca_Desi_Chr03	3523411	(T/A)
26121	CadSNP1716	*Desi*	Ca_Desi_Chr03	3524914	(A/G)
26122	CadSNP1717	*Desi*	Ca_Desi_Chr03	3524855	(T/C)
26123	CadSNP1718	*Desi*	Ca_Desi_Chr03	3524842	(C/T)
26124	CadSNP1719	*Desi*	Ca_Desi_Chr03	3538547	(C/A)
26125	CadSNP1720	*Desi*	Ca_Desi_Chr03	3567295	(T/C)
26126	CadSNP1721	*Desi*	Ca_Desi_Chr03	3567298	(C/T)
26127	CadSNP1722	*Desi*	Ca_Desi_Chr03	3567301	(C/T)
26128	CadSNP1723	*Desi*	Ca_Desi_Chr03	3567305	(C/T)

S.N.	SNP IDs	Cultivars	Chromosomes/scaffolds	Physical positions (bp)	SNPs
26129	CadSNP1724	*Desi*	Ca_Desi_Chr03	3567444	(C/T)
26130	CadSNP1725	*Desi*	Ca_Desi_Chr03	3576516	(C/T)
26131	CadSNP1726	*Desi*	Ca_Desi_Chr03	3576517	(T/C)
26132	CadSNP1727	*Desi*	Ca_Desi_Chr03	3576686	(A/G)
26133	CadSNP1728	*Desi*	Ca_Desi_Chr03	3580883	(T/C)
26134	CadSNP1729	*Desi*	Ca_Desi_Chr03	3593781	(T/C)
26135	CadSNP1730	*Desi*	Ca_Desi_Chr03	3593776	(A/G)
26136	CadSNP1731	*Desi*	Ca_Desi_Chr03	3609578	(C/T)
26137	CadSNP1732	*Desi*	Ca_Desi_Chr03	3621996	(T/C)
26138	CadSNP1733	*Desi*	Ca_Desi_Chr03	3630551	(A/G)
26139	CadSNP1734	*Desi*	Ca_Desi_Chr03	3631362	(C/T)
26140	CadSNP1735	*Desi*	Ca_Desi_Chr03	3713712	(C/G)
26141	CadSNP1736	*Desi*	Ca_Desi_Chr03	3773822	(A/G)
26142	CadSNP1737	*Desi*	Ca_Desi_Chr03	3773865	(T/C)
26143	CadSNP1738	*Desi*	Ca_Desi_Chr03	3774007	(A/T)
26144	CadSNP1739	*Desi*	Ca_Desi_Chr03	3794458	(A/G)
26145	CadSNP1740	*Desi*	Ca_Desi_Chr03	3860790	(G/C)
26146	CadSNP1741	*Desi*	Ca_Desi_Chr03	3860844	(G/A)
26147	CadSNP1742	*Desi*	Ca_Desi_Chr03	3861051	(C/T)
26148	CadSNP1743	*Desi*	Ca_Desi_Chr03	3884489	(A/C)
26149	CadSNP1744	*Desi*	Ca_Desi_Chr03	3884665	(C/A)
26150	CadSNP1745	*Desi*	Ca_Desi_Chr03	3913855	(G/A)
26151	CadSNP1746	*Desi*	Ca_Desi_Chr03	3946342	(G/A)
26152	CadSNP1747	*Desi*	Ca_Desi_Chr03	3946457	(G/T)
26153	CadSNP1748	*Desi*	Ca_Desi_Chr03	3967388	(G/A)
26154	CadSNP1749	*Desi*	Ca_Desi_Chr03	3967371	(G/T)
26155	CadSNP1750	*Desi*	Ca_Desi_Chr03	3973351	(C/T)
26156	CadSNP1751	*Desi*	Ca_Desi_Chr03	3973376	(T/C)
26157	CadSNP1752	*Desi*	Ca_Desi_Chr03	3973381	(A/G)
26158	CadSNP1753	*Desi*	Ca_Desi_Chr03	4015979	(G/A)
26159	CadSNP1754	*Desi*	Ca_Desi_Chr03	4016158	(C/T)
26160	CadSNP1755	*Desi*	Ca_Desi_Chr03	4016176	(A/C)
26161	CadSNP1756	*Desi*	Ca_Desi_Chr03	4019445	(T/G)
26162	CadSNP1757	*Desi*	Ca_Desi_Chr03	4019505	(T/C)
26163	CadSNP1758	*Desi*	Ca_Desi_Chr03	4039042	(A/G)
26164	CadSNP1759	*Desi*	Ca_Desi_Chr03	4053024	(G/A)
26165	CadSNP1760	*Desi*	Ca_Desi_Chr03	4085844	(A/G)
26166	CadSNP1761	*Desi*	Ca_Desi_Chr03	4086443	(T/C)
26167	CadSNP1762	*Desi*	Ca_Desi_Chr03	4086479	(C/T)

S.N.	SNP IDs	Cultivars	Chromosomes/scaffolds	Physical positions (bp)	SNPs
26168	CadSNP1763	*Desi*	Ca_Desi_Chr03	4087924	(T/C)
26169	CadSNP1764	*Desi*	Ca_Desi_Chr03	4087934	(C/A)
26170	CadSNP1765	*Desi*	Ca_Desi_Chr03	4098735	(T/A)
26171	CadSNP1766	*Desi*	Ca_Desi_Chr03	4098784	(C/A)
26172	CadSNP1767	*Desi*	Ca_Desi_Chr03	4100611	(T/C)
26173	CadSNP1768	*Desi*	Ca_Desi_Chr03	4100608	(C/T)
26174	CadSNP1769	*Desi*	Ca_Desi_Chr03	4100572	(C/T)
26175	CadSNP1770	*Desi*	Ca_Desi_Chr03	4100651	(C/T)
26176	CadSNP1771	*Desi*	Ca_Desi_Chr03	4100710	(C/T)
26177	CadSNP1772	*Desi*	Ca_Desi_Chr03	4100757	(T/G)
26178	CadSNP1773	*Desi*	Ca_Desi_Chr03	4100741	(G/A)
26179	CadSNP1774	*Desi*	Ca_Desi_Chr03	4100725	(A/T)
26180	CadSNP1775	*Desi*	Ca_Desi_Chr03	4118888	(G/A)
26181	CadSNP1776	*Desi*	Ca_Desi_Chr03	4118883	(T/C)
26182	CadSNP1777	*Desi*	Ca_Desi_Chr03	4133177	(T/G)
26183	CadSNP1778	*Desi*	Ca_Desi_Chr03	4133180	(T/C)
26184	CadSNP1779	*Desi*	Ca_Desi_Chr03	4134312	(C/G)
26185	CadSNP1780	*Desi*	Ca_Desi_Chr03	4135452	(G/T)
26186	CadSNP1781	*Desi*	Ca_Desi_Chr03	4143474	(G/A)
26187	CadSNP1782	*Desi*	Ca_Desi_Chr03	4145509	(T/C)
26188	CadSNP1783	*Desi*	Ca_Desi_Chr03	4174824	(T/G)
26189	CadSNP1784	*Desi*	Ca_Desi_Chr03	4174837	(A/G)
26190	CadSNP1785	*Desi*	Ca_Desi_Chr03	4174863	(A/G)
26191	CadSNP1786	*Desi*	Ca_Desi_Chr03	4175622	(A/C)
26192	CadSNP1787	*Desi*	Ca_Desi_Chr03	4182312	(A/T)
26193	CadSNP1788	*Desi*	Ca_Desi_Chr03	4182322	(G/A)
26194	CadSNP1789	*Desi*	Ca_Desi_Chr03	4182356	(T/A)
26195	CadSNP1790	*Desi*	Ca_Desi_Chr03	4187139	(G/A)
26196	CadSNP1791	*Desi*	Ca_Desi_Chr03	4197023	(A/T)
26197	CadSNP1792	*Desi*	Ca_Desi_Chr03	4197024	(A/T)
26198	CadSNP1793	*Desi*	Ca_Desi_Chr03	4197306	(A/G)
26199	CadSNP1794	*Desi*	Ca_Desi_Chr03	4197307	(C/T)
26200	CadSNP1795	*Desi*	Ca_Desi_Chr03	4197439	(G/A)
26201	CadSNP1796	*Desi*	Ca_Desi_Chr03	4197390	(G/T)
26202	CadSNP1797	*Desi*	Ca_Desi_Chr03	4197482	(T/A)
26203	CadSNP1798	*Desi*	Ca_Desi_Chr03	4197487	(T/A)
26204	CadSNP1799	*Desi*	Ca_Desi_Chr03	4211457	(C/T)
26205	CadSNP1800	*Desi*	Ca_Desi_Chr03	4211455	(C/T)
26206	CadSNP1801	*Desi*	Ca_Desi_Chr03	4211441	(G/T)

S.N.	SNP IDs	Cultivars	Chromosomes/scaffolds	Physical positions (bp)	SNPs
26207	CadSNP1802	*Desi*	Ca_Desi_Chr03	4211431	(G/C)
26208	CadSNP1803	*Desi*	Ca_Desi_Chr03	4216738	(T/C)
26209	CadSNP1804	*Desi*	Ca_Desi_Chr03	4229802	(C/A)
26210	CadSNP1805	*Desi*	Ca_Desi_Chr03	4250045	(T/G)
26211	CadSNP1806	*Desi*	Ca_Desi_Chr03	4261374	(A/G)
26212	CadSNP1807	*Desi*	Ca_Desi_Chr03	4261410	(C/T)
26213	CadSNP1808	*Desi*	Ca_Desi_Chr03	4261422	(C/T)
26214	CadSNP1809	*Desi*	Ca_Desi_Chr03	4300046	(A/G)
26215	CadSNP1810	*Desi*	Ca_Desi_Chr03	4300111	(T/C)
26216	CadSNP1811	*Desi*	Ca_Desi_Chr03	4314473	(T/C)
26217	CadSNP1812	*Desi*	Ca_Desi_Chr03	4316822	(T/A)
26218	CadSNP1813	*Desi*	Ca_Desi_Chr03	4330756	(C/T)
26219	CadSNP1814	*Desi*	Ca_Desi_Chr03	4347515	(G/T)
26220	CadSNP1815	*Desi*	Ca_Desi_Chr03	4347520	(C/G)
26221	CadSNP1816	*Desi*	Ca_Desi_Chr03	4347579	(G/A)
26222	CadSNP1817	*Desi*	Ca_Desi_Chr03	4356878	(A/T)
26223	CadSNP1818	*Desi*	Ca_Desi_Chr03	4356995	(G/A)
26224	CadSNP1819	*Desi*	Ca_Desi_Chr03	4356981	(C/T)
26225	CadSNP1820	*Desi*	Ca_Desi_Chr03	4365790	(C/A)
26226	CadSNP1821	*Desi*	Ca_Desi_Chr03	4389997	(T/C)
26227	CadSNP1822	*Desi*	Ca_Desi_Chr03	4401074	(A/G)
26228	CadSNP1823	*Desi*	Ca_Desi_Chr03	4415458	(G/A)
26229	CadSNP1824	*Desi*	Ca_Desi_Chr03	4415548	(T/A)
26230	CadSNP1825	*Desi*	Ca_Desi_Chr03	4415704	(T/A)
26231	CadSNP1826	*Desi*	Ca_Desi_Chr03	4415709	(C/T)
26232	CadSNP1827	*Desi*	Ca_Desi_Chr03	4451536	(A/C)
26233	CadSNP1828	*Desi*	Ca_Desi_Chr03	4572951	(T/C)
26234	CadSNP1829	*Desi*	Ca_Desi_Chr03	4573221	(A/T)
26235	CadSNP1830	*Desi*	Ca_Desi_Chr03	4580231	(A/G)
26236	CadSNP1831	*Desi*	Ca_Desi_Chr03	4581060	(G/A)
26237	CadSNP1832	*Desi*	Ca_Desi_Chr03	4581079	(T/G)
26238	CadSNP1833	*Desi*	Ca_Desi_Chr03	4664491	(G/A)
26239	CadSNP1834	*Desi*	Ca_Desi_Chr03	4664461	(G/C)
26240	CadSNP1835	*Desi*	Ca_Desi_Chr03	4698954	(A/G)
26241	CadSNP1836	*Desi*	Ca_Desi_Chr03	4721302	(C/T)
26242	CadSNP1837	*Desi*	Ca_Desi_Chr03	4736130	(A/T)
26243	CadSNP1838	*Desi*	Ca_Desi_Chr03	4741278	(C/T)
26244	CadSNP1839	*Desi*	Ca_Desi_Chr03	4741284	(C/T)
26245	CadSNP1840	*Desi*	Ca_Desi_Chr03	4741395	(G/C)

S.N.	SNP IDs	Cultivars	Chromosomes/scaffolds	Physical positions (bp)	SNPs
26246	CadSNP1841	*Desi*	Ca_Desi_Chr03	4787269	(G/T)
26247	CadSNP1842	*Desi*	Ca_Desi_Chr03	4787368	(A/T)
26248	CadSNP1843	*Desi*	Ca_Desi_Chr03	4801104	(T/C)
26249	CadSNP1844	*Desi*	Ca_Desi_Chr03	4801073	(A/C)
26250	CadSNP1845	*Desi*	Ca_Desi_Chr03	5804745	(G/C)
26251	CadSNP1846	*Desi*	Ca_Desi_Chr03	5850077	(T/G)
26252	CadSNP1847	*Desi*	Ca_Desi_Chr03	5851070	(C/T)
26253	CadSNP1848	*Desi*	Ca_Desi_Chr03	5851142	(A/G)
26254	CadSNP1849	*Desi*	Ca_Desi_Chr03	5944499	(G/T)
26255	CadSNP1850	*Desi*	Ca_Desi_Chr03	5976239	(C/T)
26256	CadSNP1851	*Desi*	Ca_Desi_Chr03	5990527	(G/A)
26257	CadSNP1852	*Desi*	Ca_Desi_Chr03	5990581	(A/G)
26258	CadSNP1853	*Desi*	Ca_Desi_Chr03	5990565	(T/C)
26259	CadSNP1854	*Desi*	Ca_Desi_Chr03	6018129	(A/G)
26260	CadSNP1855	*Desi*	Ca_Desi_Chr03	6019858	(T/C)
26261	CadSNP1856	*Desi*	Ca_Desi_Chr03	6027089	(A/G)
26262	CadSNP1857	*Desi*	Ca_Desi_Chr03	6078359	(A/G)
26263	CadSNP1858	*Desi*	Ca_Desi_Chr03	6078356	(A/G)
26264	CadSNP1859	*Desi*	Ca_Desi_Chr03	6078513	(C/T)
26265	CadSNP1860	*Desi*	Ca_Desi_Chr03	6078557	(A/C)
26266	CadSNP1861	*Desi*	Ca_Desi_Chr03	6109024	(T/C)
26267	CadSNP1862	*Desi*	Ca_Desi_Chr03	6108969	(T/C)
26268	CadSNP1863	*Desi*	Ca_Desi_Chr03	6119240	(G/A)
26269	CadSNP1864	*Desi*	Ca_Desi_Chr03	6119374	(G/T)
26270	CadSNP1865	*Desi*	Ca_Desi_Chr03	6119459	(G/A)
26271	CadSNP1866	*Desi*	Ca_Desi_Chr03	6120706	(C/T)
26272	CadSNP1867	*Desi*	Ca_Desi_Chr03	6139595	(C/G)
26273	CadSNP1868	*Desi*	Ca_Desi_Chr03	6139652	(A/T)
26274	CadSNP1869	*Desi*	Ca_Desi_Chr03	6172165	(G/T)
26275	CadSNP1870	*Desi*	Ca_Desi_Chr03	6172170	(A/G)
26276	CadSNP1871	*Desi*	Ca_Desi_Chr03	6172185	(G/A)
26277	CadSNP1872	*Desi*	Ca_Desi_Chr03	6172225	(A/T)
26278	CadSNP1873	*Desi*	Ca_Desi_Chr03	6172249	(G/T)
26279	CadSNP1874	*Desi*	Ca_Desi_Chr03	6172800	(C/A)
26280	CadSNP1875	*Desi*	Ca_Desi_Chr03	6172834	(T/C)
26281	CadSNP1876	*Desi*	Ca_Desi_Chr03	6195683	(A/G)
26282	CadSNP1877	*Desi*	Ca_Desi_Chr03	6260072	(T/C)
26283	CadSNP1878	*Desi*	Ca_Desi_Chr03	6260171	(C/A)
26284	CadSNP1879	*Desi*	Ca_Desi_Chr03	6262005	(C/T)

S.N.	SNP IDs	Cultivars	Chromosomes/scaffolds	Physical positions (bp)	SNPs
26285	CadSNP1880	*Desi*	Ca_Desi_Chr03	6295042	(T/A)
26286	CadSNP1881	*Desi*	Ca_Desi_Chr03	6330548	(C/A)
26287	CadSNP1882	*Desi*	Ca_Desi_Chr03	6330729	(C/T)
26288	CadSNP1883	*Desi*	Ca_Desi_Chr03	6330673	(A/G)
26289	CadSNP1884	*Desi*	Ca_Desi_Chr03	6390861	(A/G)
26290	CadSNP1885	*Desi*	Ca_Desi_Chr03	6390842	(C/T)
26291	CadSNP1886	*Desi*	Ca_Desi_Chr03	6390835	(C/T)
26292	CadSNP1887	*Desi*	Ca_Desi_Chr03	6392638	(G/A)
26293	CadSNP1888	*Desi*	Ca_Desi_Chr03	6408930	(C/T)
26294	CadSNP1889	*Desi*	Ca_Desi_Chr03	6434624	(T/A)
26295	CadSNP1890	*Desi*	Ca_Desi_Chr03	6434627	(G/T)
26296	CadSNP1891	*Desi*	Ca_Desi_Chr03	6434644	(T/C)
26297	CadSNP1892	*Desi*	Ca_Desi_Chr03	6435914	(A/C)
26298	CadSNP1893	*Desi*	Ca_Desi_Chr03	6435907	(T/G)
26299	CadSNP1894	*Desi*	Ca_Desi_Chr03	6436024	(G/A)
26300	CadSNP1895	*Desi*	Ca_Desi_Chr03	6435997	(A/G)
26301	CadSNP1896	*Desi*	Ca_Desi_Chr03	6436085	(G/A)
26302	CadSNP1897	*Desi*	Ca_Desi_Chr03	6436108	(A/C)
26303	CadSNP1898	*Desi*	Ca_Desi_Chr03	6449814	(C/T)
26304	CadSNP1899	*Desi*	Ca_Desi_Chr03	6449896	(C/T)
26305	CadSNP1900	*Desi*	Ca_Desi_Chr03	6449881	(G/A)
26306	CadSNP1901	*Desi*	Ca_Desi_Chr03	6485692	(A/G)
26307	CadSNP1902	*Desi*	Ca_Desi_Chr03	6490909	(C/T)
26308	CadSNP1903	*Desi*	Ca_Desi_Chr03	6499799	(A/C)
26309	CadSNP1904	*Desi*	Ca_Desi_Chr03	6499971	(A/T)
26310	CadSNP1905	*Desi*	Ca_Desi_Chr03	6499977	(A/G)
26311	CadSNP1906	*Desi*	Ca_Desi_Chr03	6499989	(A/T)
26312	CadSNP1907	*Desi*	Ca_Desi_Chr03	6499995	(A/G)
26313	CadSNP1908	*Desi*	Ca_Desi_Chr03	6500010	(A/G)
26314	CadSNP1909	*Desi*	Ca_Desi_Chr03	6500013	(A/C)
26315	CadSNP1910	*Desi*	Ca_Desi_Chr03	6500019	(A/G)
26316	CadSNP1911	*Desi*	Ca_Desi_Chr03	6500028	(A/G)
26317	CadSNP1912	*Desi*	Ca_Desi_Chr03	6501197	(A/T)
26318	CadSNP1913	*Desi*	Ca_Desi_Chr03	6501233	(T/A)
26319	CadSNP1914	*Desi*	Ca_Desi_Chr03	6501239	(G/A)
26320	CadSNP1915	*Desi*	Ca_Desi_Chr03	6501241	(T/C)
26321	CadSNP1916	*Desi*	Ca_Desi_Chr03	6501247	(G/A)
26322	CadSNP1917	*Desi*	Ca_Desi_Chr03	6501251	(C/T)
26323	CadSNP1918	*Desi*	Ca_Desi_Chr03	6501259	(T/C)

S.N.	SNP IDs	Cultivars	Chromosomes/scaffolds	Physical positions (bp)	SNPs
26324	CadSNP1919	*Desi*	Ca_Desi_Chr03	6501274	(C/A)
26325	CadSNP1920	*Desi*	Ca_Desi_Chr03	6501442	(C/T)
26326	CadSNP1921	*Desi*	Ca_Desi_Chr03	6501440	(T/A)
26327	CadSNP1922	*Desi*	Ca_Desi_Chr03	6501431	(G/T)
26328	CadSNP1923	*Desi*	Ca_Desi_Chr03	6501428	(C/T)
26329	CadSNP1924	*Desi*	Ca_Desi_Chr03	6501410	(A/G)
26330	CadSNP1925	*Desi*	Ca_Desi_Chr03	6501383	(G/A)
26331	CadSNP1926	*Desi*	Ca_Desi_Chr03	6501376	(C/G)
26332	CadSNP1927	*Desi*	Ca_Desi_Chr03	6501371	(G/A)
26333	CadSNP1928	*Desi*	Ca_Desi_Chr03	6501368	(T/G)
26334	CadSNP1929	*Desi*	Ca_Desi_Chr03	6543102	(A/G)
26335	CadSNP1930	*Desi*	Ca_Desi_Chr03	6543089	(A/T)
26336	CadSNP1931	*Desi*	Ca_Desi_Chr03	6543414	(C/T)
26337	CadSNP1932	*Desi*	Ca_Desi_Chr03	6544021	(T/A)
26338	CadSNP1933	*Desi*	Ca_Desi_Chr03	6544048	(C/T)
26339	CadSNP1934	*Desi*	Ca_Desi_Chr03	6544056	(A/G)
26340	CadSNP1935	*Desi*	Ca_Desi_Chr03	6544127	(G/A)
26341	CadSNP1936	*Desi*	Ca_Desi_Chr03	6547513	(A/G)
26342	CadSNP1937	*Desi*	Ca_Desi_Chr03	6547617	(C/A)
26343	CadSNP1938	*Desi*	Ca_Desi_Chr03	6592195	(A/G)
26344	CadSNP1939	*Desi*	Ca_Desi_Chr03	6593914	(G/A)
26345	CadSNP1940	*Desi*	Ca_Desi_Chr03	6593992	(A/G)
26346	CadSNP1941	*Desi*	Ca_Desi_Chr03	6636057	(C/T)
26347	CadSNP1942	*Desi*	Ca_Desi_Chr03	6641323	(G/A)
26348	CadSNP1943	*Desi*	Ca_Desi_Chr03	6721053	(T/A)
26349	CadSNP1944	*Desi*	Ca_Desi_Chr03	6723774	(T/A)
26350	CadSNP1945	*Desi*	Ca_Desi_Chr03	6723852	(C/G)
26351	CadSNP1946	*Desi*	Ca_Desi_Chr03	6723839	(G/A)
26352	CadSNP1947	*Desi*	Ca_Desi_Chr03	6723814	(A/G)
26353	CadSNP1948	*Desi*	Ca_Desi_Chr03	6738215	(A/T)
26354	CadSNP1949	*Desi*	Ca_Desi_Chr03	6739004	(A/G)
26355	CadSNP1950	*Desi*	Ca_Desi_Chr03	6738992	(G/A)
26356	CadSNP1951	*Desi*	Ca_Desi_Chr03	6758389	(T/C)
26357	CadSNP1952	*Desi*	Ca_Desi_Chr03	6758514	(T/C)
26358	CadSNP1953	*Desi*	Ca_Desi_Chr03	6758461	(C/T)
26359	CadSNP1954	*Desi*	Ca_Desi_Chr03	6776135	(G/A)
26360	CadSNP1955	*Desi*	Ca_Desi_Chr03	6776192	(A/G)
26361	CadSNP1956	*Desi*	Ca_Desi_Chr03	6776469	(T/C)
26362	CadSNP1957	*Desi*	Ca_Desi_Chr03	6776529	(C/T)

S.N.	SNP IDs	Cultivars	Chromosomes/scaffolds	Physical positions (bp)	SNPs
26363	CadSNP1958	*Desi*	Ca_Desi_Chr03	6790404	(G/A)
26364	CadSNP1959	*Desi*	Ca_Desi_Chr03	6790441	(T/G)
26365	CadSNP1960	*Desi*	Ca_Desi_Chr03	6790700	(G/A)
26366	CadSNP1961	*Desi*	Ca_Desi_Chr03	6790766	(G/T)
26367	CadSNP1962	*Desi*	Ca_Desi_Chr03	6799069	(G/A)
26368	CadSNP1963	*Desi*	Ca_Desi_Chr03	6799079	(G/C)
26369	CadSNP1964	*Desi*	Ca_Desi_Chr03	6799080	(A/G)
26370	CadSNP1965	*Desi*	Ca_Desi_Chr03	6799092	(A/T)
26371	CadSNP1966	*Desi*	Ca_Desi_Chr03	6799102	(C/T)
26372	CadSNP1967	*Desi*	Ca_Desi_Chr03	6799103	(C/T)
26373	CadSNP1968	*Desi*	Ca_Desi_Chr03	6799104	(G/C)
26374	CadSNP1969	*Desi*	Ca_Desi_Chr03	6799108	(A/G)
26375	CadSNP1970	*Desi*	Ca_Desi_Chr03	6799122	(T/C)
26376	CadSNP1971	*Desi*	Ca_Desi_Chr03	6799206	(G/A)
26377	CadSNP1972	*Desi*	Ca_Desi_Chr03	6799167	(T/C)
26378	CadSNP1973	*Desi*	Ca_Desi_Chr03	6812141	(C/T)
26379	CadSNP1974	*Desi*	Ca_Desi_Chr03	6812138	(C/A)
26380	CadSNP1975	*Desi*	Ca_Desi_Chr03	6812125	(C/T)
26381	CadSNP1976	*Desi*	Ca_Desi_Chr03	6812111	(A/G)
26382	CadSNP1977	*Desi*	Ca_Desi_Chr03	6812104	(C/G)
26383	CadSNP1978	*Desi*	Ca_Desi_Chr03	6812091	(G/A)
26384	CadSNP1979	*Desi*	Ca_Desi_Chr03	6812090	(C/T)
26385	CadSNP1980	*Desi*	Ca_Desi_Chr03	6812089	(G/T)
26386	CadSNP1981	*Desi*	Ca_Desi_Chr03	6812085	(G/A)
26387	CadSNP1982	*Desi*	Ca_Desi_Chr03	6812195	(C/T)
26388	CadSNP1983	*Desi*	Ca_Desi_Chr03	6812249	(G/A)
26389	CadSNP1984	*Desi*	Ca_Desi_Chr03	6812306	(A/C)
26390	CadSNP1985	*Desi*	Ca_Desi_Chr03	6812334	(G/A)
26391	CadSNP1986	*Desi*	Ca_Desi_Chr03	6833787	(G/A)
26392	CadSNP1987	*Desi*	Ca_Desi_Chr03	6833812	(C/A)
26393	CadSNP1988	*Desi*	Ca_Desi_Chr03	6833831	(A/G)
26394	CadSNP1989	*Desi*	Ca_Desi_Chr03	6833840	(G/T)
26395	CadSNP1990	*Desi*	Ca_Desi_Chr03	7012818	(T/C)
26396	CadSNP1991	*Desi*	Ca_Desi_Chr03	7012976	(A/G)
26397	CadSNP1992	*Desi*	Ca_Desi_Chr03	7059071	(G/A)
26398	CadSNP1993	*Desi*	Ca_Desi_Chr03	7059090	(A/G)
26399	CadSNP1994	*Desi*	Ca_Desi_Chr03	7059208	(T/C)
26400	CadSNP1995	*Desi*	Ca_Desi_Chr03	7101708	(G/T)
26401	CadSNP1996	*Desi*	Ca_Desi_Chr03	7101702	(T/G)

S.N.	SNP IDs	Cultivars	Chromosomes/scaffolds	Physical positions (bp)	SNPs
26402	CadSNP1997	*Desi*	Ca_Desi_Chr03	7104055	(G/A)
26403	CadSNP1998	*Desi*	Ca_Desi_Chr03	7104519	(C/T)
26404	CadSNP1999	*Desi*	Ca_Desi_Chr03	7104579	(T/A)
26405	CadSNP2000	*Desi*	Ca_Desi_Chr03	7144144	(T/A)
26406	CadSNP2001	*Desi*	Ca_Desi_Chr03	7144200	(T/G)
26407	CadSNP2002	*Desi*	Ca_Desi_Chr03	7177530	(C/A)
26408	CadSNP2003	*Desi*	Ca_Desi_Chr03	7207213	(C/G)
26409	CadSNP2004	*Desi*	Ca_Desi_Chr03	7207239	(G/T)
26410	CadSNP2005	*Desi*	Ca_Desi_Chr03	7207240	(T/G)
26411	CadSNP2006	*Desi*	Ca_Desi_Chr03	7207270	(T/C)
26412	CadSNP2007	*Desi*	Ca_Desi_Chr03	7207273	(A/G)
26413	CadSNP2008	*Desi*	Ca_Desi_Chr03	7207388	(A/C)
26414	CadSNP2009	*Desi*	Ca_Desi_Chr03	7207370	(C/T)
26415	CadSNP2010	*Desi*	Ca_Desi_Chr03	7207355	(A/G)
26416	CadSNP2011	*Desi*	Ca_Desi_Chr03	7207306	(T/G)
26417	CadSNP2012	*Desi*	Ca_Desi_Chr03	7212795	(T/C)
26418	CadSNP2013	*Desi*	Ca_Desi_Chr03	7212796	(T/C)
26419	CadSNP2014	*Desi*	Ca_Desi_Chr03	7218359	(A/C)
26420	CadSNP2015	*Desi*	Ca_Desi_Chr03	7234381	(A/T)
26421	CadSNP2016	*Desi*	Ca_Desi_Chr03	7241614	(G/A)
26422	CadSNP2017	*Desi*	Ca_Desi_Chr03	7241639	(A/G)
26423	CadSNP2018	*Desi*	Ca_Desi_Chr03	7241675	(G/T)
26424	CadSNP2019	*Desi*	Ca_Desi_Chr03	8455273	(A/T)
26425	CadSNP2020	*Desi*	Ca_Desi_Chr03	8456386	(C/A)
26426	CadSNP2021	*Desi*	Ca_Desi_Chr03	8499689	(C/T)
26427	CadSNP2022	*Desi*	Ca_Desi_Chr03	8540339	(T/A)
26428	CadSNP2023	*Desi*	Ca_Desi_Chr03	8540535	(T/C)
26429	CadSNP2024	*Desi*	Ca_Desi_Chr03	8548425	(C/T)
26430	CadSNP2025	*Desi*	Ca_Desi_Chr03	8556886	(G/T)
26431	CadSNP2026	*Desi*	Ca_Desi_Chr03	8556931	(G/T)
26432	CadSNP2027	*Desi*	Ca_Desi_Chr03	8814590	(A/G)
26433	CadSNP2028	*Desi*	Ca_Desi_Chr03	8815626	(A/T)
26434	CadSNP2029	*Desi*	Ca_Desi_Chr03	8815663	(C/T)
26435	CadSNP2030	*Desi*	Ca_Desi_Chr03	8815664	(G/A)
26436	CadSNP2031	*Desi*	Ca_Desi_Chr03	8815694	(G/A)
26437	CadSNP2032	*Desi*	Ca_Desi_Chr03	8826351	(G/C)
26438	CadSNP2033	*Desi*	Ca_Desi_Chr03	8826641	(A/G)
26439	CadSNP2034	*Desi*	Ca_Desi_Chr03	8828383	(C/T)
26440	CadSNP2035	*Desi*	Ca_Desi_Chr03	8844120	(T/C)

S.N.	SNP IDs	Cultivars	Chromosomes/scaffolds	Physical positions (bp)	SNPs
26441	CadSNP2036	*Desi*	Ca_Desi_Chr03	8844165	(G/A)
26442	CadSNP2037	*Desi*	Ca_Desi_Chr03	8844171	(C/T)
26443	CadSNP2038	*Desi*	Ca_Desi_Chr03	8844245	(C/A)
26444	CadSNP2039	*Desi*	Ca_Desi_Chr03	8852584	(C/T)
26445	CadSNP2040	*Desi*	Ca_Desi_Chr03	8907433	(T/G)
26446	CadSNP2041	*Desi*	Ca_Desi_Chr03	8907468	(T/C)
26447	CadSNP2042	*Desi*	Ca_Desi_Chr03	8907459	(A/G)
26448	CadSNP2043	*Desi*	Ca_Desi_Chr03	8907661	(C/T)
26449	CadSNP2044	*Desi*	Ca_Desi_Chr03	8989825	(A/T)
26450	CadSNP2045	*Desi*	Ca_Desi_Chr03	9852607	(T/A)
26451	CadSNP2046	*Desi*	Ca_Desi_Chr03	9934450	(C/A)
26452	CadSNP2047	*Desi*	Ca_Desi_Chr03	9934613	(A/G)
26453	CadSNP2048	*Desi*	Ca_Desi_Chr03	9938011	(A/T)
26454	CadSNP2049	*Desi*	Ca_Desi_Chr03	10075249	(G/A)
26455	CadSNP2050	*Desi*	Ca_Desi_Chr03	10078325	(A/C)
26456	CadSNP2051	*Desi*	Ca_Desi_Chr03	10247487	(C/G)
26457	CadSNP2052	*Desi*	Ca_Desi_Chr03	10304204	(T/A)
26458	CadSNP2053	*Desi*	Ca_Desi_Chr03	10304224	(A/T)
26459	CadSNP2054	*Desi*	Ca_Desi_Chr03	10304244	(C/A)
26460	CadSNP2055	*Desi*	Ca_Desi_Chr03	10308956	(A/C)
26461	CadSNP2056	*Desi*	Ca_Desi_Chr03	10349421	(A/G)
26462	CadSNP2057	*Desi*	Ca_Desi_Chr03	10350151	(C/T)
26463	CadSNP2058	*Desi*	Ca_Desi_Chr03	10350133	(G/T)
26464	CadSNP2059	*Desi*	Ca_Desi_Chr03	10387956	(A/G)
26465	CadSNP2060	*Desi*	Ca_Desi_Chr03	10388061	(T/C)
26466	CadSNP2061	*Desi*	Ca_Desi_Chr03	10389534	(T/G)
26467	CadSNP2062	*Desi*	Ca_Desi_Chr03	10413004	(A/C)
26468	CadSNP2063	*Desi*	Ca_Desi_Chr03	10468011	(C/T)
26469	CadSNP2064	*Desi*	Ca_Desi_Chr03	10509285	(A/T)
26470	CadSNP2065	*Desi*	Ca_Desi_Chr03	10509304	(T/C)
26471	CadSNP2066	*Desi*	Ca_Desi_Chr03	10509352	(C/A)
26472	CadSNP2067	*Desi*	Ca_Desi_Chr03	10509908	(T/G)
26473	CadSNP2068	*Desi*	Ca_Desi_Chr03	10509938	(A/C)
26474	CadSNP2069	*Desi*	Ca_Desi_Chr03	10597811	(T/G)
26475	CadSNP2070	*Desi*	Ca_Desi_Chr03	10597847	(C/T)
26476	CadSNP2071	*Desi*	Ca_Desi_Chr03	10641927	(G/A)
26477	CadSNP2072	*Desi*	Ca_Desi_Chr03	10656917	(G/A)
26478	CadSNP2073	*Desi*	Ca_Desi_Chr03	10676655	(G/A)
26479	CadSNP2074	*Desi*	Ca_Desi_Chr03	10676659	(G/A)

S.N.	SNP IDs	Cultivars	Chromosomes/scaffolds	Physical positions (bp)	SNPs
26480	CadSNP2075	*Desi*	Ca_Desi_Chr03	10811691	(G/T)
26481	CadSNP2076	*Desi*	Ca_Desi_Chr03	10811721	(G/C)
26482	CadSNP2077	*Desi*	Ca_Desi_Chr03	10812216	(G/A)
26483	CadSNP2078	*Desi*	Ca_Desi_Chr03	10832501	(T/G)
26484	CadSNP2079	*Desi*	Ca_Desi_Chr03	10869140	(G/A)
26485	CadSNP2080	*Desi*	Ca_Desi_Chr03	10881083	(C/T)
26486	CadSNP2081	*Desi*	Ca_Desi_Chr03	10915073	(G/A)
26487	CadSNP2082	*Desi*	Ca_Desi_Chr03	11064391	(C/G)
26488	CadSNP2083	*Desi*	Ca_Desi_Chr03	11115658	(G/A)
26489	CadSNP2084	*Desi*	Ca_Desi_Chr03	11121805	(G/T)
26490	CadSNP2085	*Desi*	Ca_Desi_Chr03	11121776	(T/C)
26491	CadSNP2086	*Desi*	Ca_Desi_Chr03	11146489	(T/C)
26492	CadSNP2087	*Desi*	Ca_Desi_Chr03	11146442	(A/C)
26493	CadSNP2088	*Desi*	Ca_Desi_Chr03	11147829	(G/C)
26494	CadSNP2089	*Desi*	Ca_Desi_Chr03	11147853	(T/G)
26495	CadSNP2090	*Desi*	Ca_Desi_Chr03	11148357	(C/T)
26496	CadSNP2091	*Desi*	Ca_Desi_Chr03	11163988	(C/T)
26497	CadSNP2092	*Desi*	Ca_Desi_Chr03	11220397	(T/C)
26498	CadSNP2093	*Desi*	Ca_Desi_Chr03	11223968	(C/G)
26499	CadSNP2094	*Desi*	Ca_Desi_Chr03	11251354	(C/T)
26500	CadSNP2095	*Desi*	Ca_Desi_Chr03	11251333	(A/C)
26501	CadSNP2096	*Desi*	Ca_Desi_Chr03	11295835	(C/T)
26502	CadSNP2097	*Desi*	Ca_Desi_Chr03	11330443	(T/G)
26503	CadSNP2098	*Desi*	Ca_Desi_Chr03	11370420	(G/A)
26504	CadSNP2099	*Desi*	Ca_Desi_Chr03	11390003	(A/G)
26505	CadSNP2100	*Desi*	Ca_Desi_Chr03	11390004	(T/C)
26506	CadSNP2101	*Desi*	Ca_Desi_Chr03	11390007	(C/T)
26507	CadSNP2102	*Desi*	Ca_Desi_Chr03	11390016	(C/A)
26508	CadSNP2103	*Desi*	Ca_Desi_Chr03	11390049	(A/G)
26509	CadSNP2104	*Desi*	Ca_Desi_Chr03	11390057	(A/G)
26510	CadSNP2105	*Desi*	Ca_Desi_Chr03	11390970	(A/C)
26511	CadSNP2106	*Desi*	Ca_Desi_Chr03	11392062	(A/G)
26512	CadSNP2107	*Desi*	Ca_Desi_Chr03	11392061	(C/T)
26513	CadSNP2108	*Desi*	Ca_Desi_Chr03	11392059	(G/A)
26514	CadSNP2109	*Desi*	Ca_Desi_Chr03	11392056	(G/A)
26515	CadSNP2110	*Desi*	Ca_Desi_Chr03	11392049	(A/G)
26516	CadSNP2111	*Desi*	Ca_Desi_Chr03	11392045	(T/A)
26517	CadSNP2112	*Desi*	Ca_Desi_Chr03	11392037	(T/C)
26518	CadSNP2113	*Desi*	Ca_Desi_Chr03	11392024	(T/C)

S.N.	SNP IDs	Cultivars	Chromosomes/scaffolds	Physical positions (bp)	SNPs
26519	CadSNP2114	*Desi*	Ca_Desi_Chr03	11396406	(A/C)
26520	CadSNP2115	*Desi*	Ca_Desi_Chr03	11396407	(T/A)
26521	CadSNP2116	*Desi*	Ca_Desi_Chr03	11400666	(A/C)
26522	CadSNP2117	*Desi*	Ca_Desi_Chr03	11404484	(C/G)
26523	CadSNP2118	*Desi*	Ca_Desi_Chr03	11404536	(G/A)
26524	CadSNP2119	*Desi*	Ca_Desi_Chr03	11418718	(G/A)
26525	CadSNP2120	*Desi*	Ca_Desi_Chr03	11418716	(T/C)
26526	CadSNP2121	*Desi*	Ca_Desi_Chr03	11418710	(C/A)
26527	CadSNP2122	*Desi*	Ca_Desi_Chr03	11418707	(T/A)
26528	CadSNP2123	*Desi*	Ca_Desi_Chr03	11418699	(T/C)
26529	CadSNP2124	*Desi*	Ca_Desi_Chr03	11432450	(G/A)
26530	CadSNP2125	*Desi*	Ca_Desi_Chr03	11437660	(A/C)
26531	CadSNP2126	*Desi*	Ca_Desi_Chr03	11444564	(A/C)
26532	CadSNP2127	*Desi*	Ca_Desi_Chr03	11457594	(C/G)
26533	CadSNP2128	*Desi*	Ca_Desi_Chr03	11457591	(G/C)
26534	CadSNP2129	*Desi*	Ca_Desi_Chr03	11457652	(A/C)
26535	CadSNP2130	*Desi*	Ca_Desi_Chr03	11497356	(C/T)
26536	CadSNP2131	*Desi*	Ca_Desi_Chr03	11547118	(A/G)
26537	CadSNP2132	*Desi*	Ca_Desi_Chr03	11548485	(G/A)
26538	CadSNP2133	*Desi*	Ca_Desi_Chr03	11567306	(G/A)
26539	CadSNP2134	*Desi*	Ca_Desi_Chr03	11567364	(A/C)
26540	CadSNP2135	*Desi*	Ca_Desi_Chr03	11569456	(C/T)
26541	CadSNP2136	*Desi*	Ca_Desi_Chr03	11599239	(G/A)
26542	CadSNP2137	*Desi*	Ca_Desi_Chr03	11599240	(A/T)
26543	CadSNP2138	*Desi*	Ca_Desi_Chr03	11607367	(T/C)
26544	CadSNP2139	*Desi*	Ca_Desi_Chr03	11624139	(T/C)
26545	CadSNP2140	*Desi*	Ca_Desi_Chr03	11625131	(C/T)
26546	CadSNP2141	*Desi*	Ca_Desi_Chr03	11625124	(G/A)
26547	CadSNP2142	*Desi*	Ca_Desi_Chr03	11656953	(A/G)
26548	CadSNP2143	*Desi*	Ca_Desi_Chr03	11656922	(T/G)
26549	CadSNP2144	*Desi*	Ca_Desi_Chr03	11676785	(A/G)
26550	CadSNP2145	*Desi*	Ca_Desi_Chr03	11676764	(A/G)
26551	CadSNP2146	*Desi*	Ca_Desi_Chr03	11722037	(T/C)
26552	CadSNP2147	*Desi*	Ca_Desi_Chr03	11728702	(A/C)
26553	CadSNP2148	*Desi*	Ca_Desi_Chr03	11728698	(A/G)
26554	CadSNP2149	*Desi*	Ca_Desi_Chr03	11740570	(C/T)
26555	CadSNP2150	*Desi*	Ca_Desi_Chr03	11740579	(T/C)
26556	CadSNP2151	*Desi*	Ca_Desi_Chr03	11740600	(C/A)
26557	CadSNP2152	*Desi*	Ca_Desi_Chr03	11740672	(C/T)

S.N.	SNP IDs	Cultivars	Chromosomes/scaffolds	Physical positions (bp)	SNPs
26558	CadSNP2153	*Desi*	Ca_Desi_Chr03	11740669	(A/G)
26559	CadSNP2154	*Desi*	Ca_Desi_Chr03	11748297	(T/A)
26560	CadSNP2155	*Desi*	Ca_Desi_Chr03	11781845	(C/T)
26561	CadSNP2156	*Desi*	Ca_Desi_Chr03	11782814	(G/T)
26562	CadSNP2157	*Desi*	Ca_Desi_Chr03	11783341	(C/A)
26563	CadSNP2158	*Desi*	Ca_Desi_Chr03	11783336	(C/A)
26564	CadSNP2159	*Desi*	Ca_Desi_Chr03	11783335	(G/A)
26565	CadSNP2160	*Desi*	Ca_Desi_Chr03	11783334	(T/C)
26566	CadSNP2161	*Desi*	Ca_Desi_Chr03	11783331	(C/T)
26567	CadSNP2162	*Desi*	Ca_Desi_Chr03	11783328	(C/T)
26568	CadSNP2163	*Desi*	Ca_Desi_Chr03	11828957	(C/A)
26569	CadSNP2164	*Desi*	Ca_Desi_Chr03	11900779	(G/A)
26570	CadSNP2165	*Desi*	Ca_Desi_Chr03	11900840	(T/C)
26571	CadSNP2166	*Desi*	Ca_Desi_Chr03	11900841	(G/A)
26572	CadSNP2167	*Desi*	Ca_Desi_Chr03	11900930	(T/A)
26573	CadSNP2168	*Desi*	Ca_Desi_Chr03	11900889	(A/G)
26574	CadSNP2169	*Desi*	Ca_Desi_Chr03	11900887	(T/C)
26575	CadSNP2170	*Desi*	Ca_Desi_Chr03	11974197	(T/C)
26576	CadSNP2171	*Desi*	Ca_Desi_Chr03	11974244	(A/T)
26577	CadSNP2172	*Desi*	Ca_Desi_Chr03	11974241	(G/T)
26578	CadSNP2173	*Desi*	Ca_Desi_Chr03	11974238	(A/G)
26579	CadSNP2174	*Desi*	Ca_Desi_Chr03	12051872	(G/C)
26580	CadSNP2175	*Desi*	Ca_Desi_Chr03	12051878	(A/C)
26581	CadSNP2176	*Desi*	Ca_Desi_Chr03	12095970	(G/T)
26582	CadSNP2177	*Desi*	Ca_Desi_Chr03	12096007	(C/T)
26583	CadSNP2178	*Desi*	Ca_Desi_Chr03	12096018	(G/T)
26584	CadSNP2179	*Desi*	Ca_Desi_Chr03	12163332	(T/C)
26585	CadSNP2180	*Desi*	Ca_Desi_Chr03	12163323	(G/A)
26586	CadSNP2181	*Desi*	Ca_Desi_Chr03	12277447	(C/G)
26587	CadSNP2182	*Desi*	Ca_Desi_Chr03	12277580	(A/G)
26588	CadSNP2183	*Desi*	Ca_Desi_Chr03	12277558	(A/G)
26589	CadSNP2184	*Desi*	Ca_Desi_Chr03	12353499	(A/G)
26590	CadSNP2185	*Desi*	Ca_Desi_Chr03	12354035	(A/G)
26591	CadSNP2186	*Desi*	Ca_Desi_Chr03	12385670	(G/T)
26592	CadSNP2187	*Desi*	Ca_Desi_Chr03	12386707	(A/G)
26593	CadSNP2188	*Desi*	Ca_Desi_Chr03	12387498	(G/T)
26594	CadSNP2189	*Desi*	Ca_Desi_Chr03	12388427	(T/C)
26595	CadSNP2190	*Desi*	Ca_Desi_Chr03	12388434	(C/T)
26596	CadSNP2191	*Desi*	Ca_Desi_Chr03	12389294	(G/A)

S.N.	SNP IDs	Cultivars	Chromosomes/scaffolds	Physical positions (bp)	SNPs
26597	CadSNP2192	*Desi*	Ca_Desi_Chr03	12389297	(G/A)
26598	CadSNP2193	*Desi*	Ca_Desi_Chr03	12389303	(C/T)
26599	CadSNP2194	*Desi*	Ca_Desi_Chr03	12399041	(C/T)
26600	CadSNP2195	*Desi*	Ca_Desi_Chr03	12399107	(T/A)
26601	CadSNP2196	*Desi*	Ca_Desi_Chr03	12401439	(C/G)
26602	CadSNP2197	*Desi*	Ca_Desi_Chr03	12401650	(T/C)
26603	CadSNP2198	*Desi*	Ca_Desi_Chr03	12460531	(T/C)
26604	CadSNP2199	*Desi*	Ca_Desi_Chr03	12472072	(G/A)
26605	CadSNP2200	*Desi*	Ca_Desi_Chr03	12498074	(G/A)
26606	CadSNP2201	*Desi*	Ca_Desi_Chr03	12535870	(T/C)
26607	CadSNP2202	*Desi*	Ca_Desi_Chr03	12535887	(T/C)
26608	CadSNP2203	*Desi*	Ca_Desi_Chr03	12535894	(C/T)
26609	CadSNP2204	*Desi*	Ca_Desi_Chr03	12535919	(G/A)
26610	CadSNP2205	*Desi*	Ca_Desi_Chr03	12543791	(G/A)
26611	CadSNP2206	*Desi*	Ca_Desi_Chr03	12543837	(A/C)
26612	CadSNP2207	*Desi*	Ca_Desi_Chr03	12863764	(C/T)
26613	CadSNP2208	*Desi*	Ca_Desi_Chr03	12863779	(T/C)
26614	CadSNP2209	*Desi*	Ca_Desi_Chr03	12863789	(C/A)
26615	CadSNP2210	*Desi*	Ca_Desi_Chr03	12863810	(C/T)
26616	CadSNP2211	*Desi*	Ca_Desi_Chr03	12863812	(C/T)
26617	CadSNP2212	*Desi*	Ca_Desi_Chr03	12897333	(T/A)
26618	CadSNP2213	*Desi*	Ca_Desi_Chr03	12897345	(C/G)
26619	CadSNP2214	*Desi*	Ca_Desi_Chr03	12930500	(C/A)
26620	CadSNP2215	*Desi*	Ca_Desi_Chr03	12930626	(C/T)
26621	CadSNP2216	*Desi*	Ca_Desi_Chr03	12979506	(T/C)
26622	CadSNP2217	*Desi*	Ca_Desi_Chr03	12979551	(C/A)
26623	CadSNP2218	*Desi*	Ca_Desi_Chr03	13008395	(A/C)
26624	CadSNP2219	*Desi*	Ca_Desi_Chr03	13191097	(A/T)
26625	CadSNP2220	*Desi*	Ca_Desi_Chr03	13191072	(C/G)
26626	CadSNP2221	*Desi*	Ca_Desi_Chr03	13191049	(A/G)
26627	CadSNP2222	*Desi*	Ca_Desi_Chr03	13207036	(C/A)
26628	CadSNP2223	*Desi*	Ca_Desi_Chr03	13208922	(T/G)
26629	CadSNP2224	*Desi*	Ca_Desi_Chr03	13407432	(T/A)
26630	CadSNP2225	*Desi*	Ca_Desi_Chr03	13407459	(T/G)
26631	CadSNP2226	*Desi*	Ca_Desi_Chr03	13423127	(T/G)
26632	CadSNP2227	*Desi*	Ca_Desi_Chr03	13517775	(T/C)
26633	CadSNP2228	*Desi*	Ca_Desi_Chr03	13517888	(C/T)
26634	CadSNP2229	*Desi*	Ca_Desi_Chr03	13518296	(A/G)
26635	CadSNP2230	*Desi*	Ca_Desi_Chr03	13538576	(A/C)

S.N.	SNP IDs	Cultivars	Chromosomes/scaffolds	Physical positions (bp)	SNPs
26636	CadSNP2231	*Desi*	Ca_Desi_Chr03	13664273	(G/A)
26637	CadSNP2232	*Desi*	Ca_Desi_Chr03	13664267	(A/G)
26638	CadSNP2233	*Desi*	Ca_Desi_Chr03	13664213	(A/C)
26639	CadSNP2234	*Desi*	Ca_Desi_Chr03	13825261	(G/A)
26640	CadSNP2235	*Desi*	Ca_Desi_Chr03	13839597	(C/T)
26641	CadSNP2236	*Desi*	Ca_Desi_Chr03	13841817	(T/C)
26642	CadSNP2237	*Desi*	Ca_Desi_Chr03	13841895	(A/C)
26643	CadSNP2238	*Desi*	Ca_Desi_Chr03	13866595	(G/T)
26644	CadSNP2239	*Desi*	Ca_Desi_Chr03	13892242	(T/C)
26645	CadSNP2240	*Desi*	Ca_Desi_Chr03	13894638	(T/A)
26646	CadSNP2241	*Desi*	Ca_Desi_Chr03	13909204	(C/T)
26647	CadSNP2242	*Desi*	Ca_Desi_Chr03	13909208	(A/C)
26648	CadSNP2243	*Desi*	Ca_Desi_Chr03	13909226	(T/C)
26649	CadSNP2244	*Desi*	Ca_Desi_Chr03	13909264	(A/T)
26650	CadSNP2245	*Desi*	Ca_Desi_Chr03	13994137	(A/C)
26651	CadSNP2246	*Desi*	Ca_Desi_Chr03	14005265	(C/T)
26652	CadSNP2247	*Desi*	Ca_Desi_Chr03	14005543	(C/G)
26653	CadSNP2248	*Desi*	Ca_Desi_Chr03	14005497	(T/C)
26654	CadSNP2249	*Desi*	Ca_Desi_Chr03	14006868	(G/A)
26655	CadSNP2250	*Desi*	Ca_Desi_Chr03	14006814	(T/G)
26656	CadSNP2251	*Desi*	Ca_Desi_Chr03	14172661	(C/T)
26657	CadSNP2252	*Desi*	Ca_Desi_Chr03	14172702	(G/A)
26658	CadSNP2253	*Desi*	Ca_Desi_Chr03	14469822	(G/A)
26659	CadSNP2254	*Desi*	Ca_Desi_Chr03	14469811	(T/C)
26660	CadSNP2255	*Desi*	Ca_Desi_Chr03	14469806	(A/G)
26661	CadSNP2256	*Desi*	Ca_Desi_Chr03	14469801	(G/A)
26662	CadSNP2257	*Desi*	Ca_Desi_Chr03	14469790	(C/A)
26663	CadSNP2258	*Desi*	Ca_Desi_Chr03	14469745	(A/T)
26664	CadSNP2259	*Desi*	Ca_Desi_Chr03	14469761	(G/A)
26665	CadSNP2260	*Desi*	Ca_Desi_Chr03	14490484	(C/T)
26666	CadSNP2261	*Desi*	Ca_Desi_Chr03	14619725	(G/A)
26667	CadSNP2262	*Desi*	Ca_Desi_Chr03	14672190	(A/G)
26668	CadSNP2263	*Desi*	Ca_Desi_Chr03	14694727	(A/G)
26669	CadSNP2264	*Desi*	Ca_Desi_Chr03	14702839	(C/T)
26670	CadSNP2265	*Desi*	Ca_Desi_Chr03	14732794	(T/C)
26671	CadSNP2266	*Desi*	Ca_Desi_Chr03	14733079	(G/C)
26672	CadSNP2267	*Desi*	Ca_Desi_Chr03	14734975	(A/C)
26673	CadSNP2268	*Desi*	Ca_Desi_Chr03	14790196	(C/T)
26674	CadSNP2269	*Desi*	Ca_Desi_Chr03	14817852	(G/A)

S.N.	SNP IDs	Cultivars	Chromosomes/scaffolds	Physical positions (bp)	SNPs
26675	CadSNP2270	*Desi*	Ca_Desi_Chr03	14828680	(T/A)
26676	CadSNP2271	*Desi*	Ca_Desi_Chr03	14828628	(T/C)
26677	CadSNP2272	*Desi*	Ca_Desi_Chr03	14860205	(G/T)
26678	CadSNP2273	*Desi*	Ca_Desi_Chr03	14860342	(T/C)
26679	CadSNP2274	*Desi*	Ca_Desi_Chr03	14890519	(G/A)
26680	CadSNP2275	*Desi*	Ca_Desi_Chr03	14890526	(A/G)
26681	CadSNP2276	*Desi*	Ca_Desi_Chr03	14890671	(G/T)
26682	CadSNP2277	*Desi*	Ca_Desi_Chr03	14890670	(T/A)
26683	CadSNP2278	*Desi*	Ca_Desi_Chr03	14890662	(T/G)
26684	CadSNP2279	*Desi*	Ca_Desi_Chr03	14890652	(G/A)
26685	CadSNP2280	*Desi*	Ca_Desi_Chr03	14890643	(C/A)
26686	CadSNP2281	*Desi*	Ca_Desi_Chr03	14890606	(G/T)
26687	CadSNP2282	*Desi*	Ca_Desi_Chr03	14890599	(G/T)
26688	CadSNP2283	*Desi*	Ca_Desi_Chr03	14927251	(G/A)
26689	CadSNP2284	*Desi*	Ca_Desi_Chr03	15066955	(A/G)
26690	CadSNP2285	*Desi*	Ca_Desi_Chr03	15101360	(G/A)
26691	CadSNP2286	*Desi*	Ca_Desi_Chr03	15101362	(G/T)
26692	CadSNP2287	*Desi*	Ca_Desi_Chr03	15101368	(A/G)
26693	CadSNP2288	*Desi*	Ca_Desi_Chr03	15101369	(G/C)
26694	CadSNP2289	*Desi*	Ca_Desi_Chr03	15101397	(G/A)
26695	CadSNP2290	*Desi*	Ca_Desi_Chr03	15107917	(T/C)
26696	CadSNP2291	*Desi*	Ca_Desi_Chr03	15115080	(G/A)
26697	CadSNP2292	*Desi*	Ca_Desi_Chr03	15115083	(T/C)
26698	CadSNP2293	*Desi*	Ca_Desi_Chr03	15115094	(C/T)
26699	CadSNP2294	*Desi*	Ca_Desi_Chr03	15115099	(A/T)
26700	CadSNP2295	*Desi*	Ca_Desi_Chr03	15115111	(C/T)
26701	CadSNP2296	*Desi*	Ca_Desi_Chr03	15115114	(A/T)
26702	CadSNP2297	*Desi*	Ca_Desi_Chr03	15115115	(C/T)
26703	CadSNP2298	*Desi*	Ca_Desi_Chr03	15115122	(A/T)
26704	CadSNP2299	*Desi*	Ca_Desi_Chr03	15115126	(T/C)
26705	CadSNP2300	*Desi*	Ca_Desi_Chr03	15115128	(G/T)
26706	CadSNP2301	*Desi*	Ca_Desi_Chr03	15115141	(T/A)
26707	CadSNP2302	*Desi*	Ca_Desi_Chr03	15134512	(A/G)
26708	CadSNP2303	*Desi*	Ca_Desi_Chr03	15134527	(T/C)
26709	CadSNP2304	*Desi*	Ca_Desi_Chr03	15134699	(G/A)
26710	CadSNP2305	*Desi*	Ca_Desi_Chr03	15140391	(A/G)
26711	CadSNP2306	*Desi*	Ca_Desi_Chr03	15140451	(C/G)
26712	CadSNP2307	*Desi*	Ca_Desi_Chr03	15140454	(A/G)
26713	CadSNP2308	*Desi*	Ca_Desi_Chr03	15206796	(A/T)

S.N.	SNP IDs	Cultivars	Chromosomes/scaffolds	Physical positions (bp)	SNPs
26714	CadSNP2309	*Desi*	Ca_Desi_Chr03	15281781	(C/T)
26715	CadSNP2310	*Desi*	Ca_Desi_Chr03	15281745	(T/C)
26716	CadSNP2311	*Desi*	Ca_Desi_Chr03	15332475	(C/G)
26717	CadSNP2312	*Desi*	Ca_Desi_Chr03	15332490	(C/G)
26718	CadSNP2313	*Desi*	Ca_Desi_Chr03	15332493	(C/A)
26719	CadSNP2314	*Desi*	Ca_Desi_Chr03	15332539	(G/A)
26720	CadSNP2315	*Desi*	Ca_Desi_Chr03	15376304	(G/C)
26721	CadSNP2316	*Desi*	Ca_Desi_Chr03	15385992	(G/C)
26722	CadSNP2317	*Desi*	Ca_Desi_Chr03	15386093	(A/C)
26723	CadSNP2318	*Desi*	Ca_Desi_Chr03	15386074	(T/C)
26724	CadSNP2319	*Desi*	Ca_Desi_Chr03	15411640	(A/T)
26725	CadSNP2320	*Desi*	Ca_Desi_Chr03	15411677	(A/G)
26726	CadSNP2321	*Desi*	Ca_Desi_Chr03	15412922	(A/C)
26727	CadSNP2322	*Desi*	Ca_Desi_Chr03	15445856	(A/G)
26728	CadSNP2323	*Desi*	Ca_Desi_Chr03	15449309	(T/C)
26729	CadSNP2324	*Desi*	Ca_Desi_Chr03	15458600	(C/T)
26730	CadSNP2325	*Desi*	Ca_Desi_Chr03	15459712	(T/G)
26731	CadSNP2326	*Desi*	Ca_Desi_Chr03	15459709	(T/C)
26732	CadSNP2327	*Desi*	Ca_Desi_Chr03	15466277	(A/G)
26733	CadSNP2328	*Desi*	Ca_Desi_Chr03	15487859	(A/T)
26734	CadSNP2329	*Desi*	Ca_Desi_Chr03	15492126	(A/G)
26735	CadSNP2330	*Desi*	Ca_Desi_Chr03	15497431	(A/G)
26736	CadSNP2331	*Desi*	Ca_Desi_Chr03	15502488	(C/T)
26737	CadSNP2332	*Desi*	Ca_Desi_Chr03	15506011	(A/G)
26738	CadSNP2333	*Desi*	Ca_Desi_Chr03	15505991	(C/T)
26739	CadSNP2334	*Desi*	Ca_Desi_Chr03	15505987	(C/A)
26740	CadSNP2335	*Desi*	Ca_Desi_Chr03	15505983	(C/A)
26741	CadSNP2336	*Desi*	Ca_Desi_Chr03	15813048	(C/T)
26742	CadSNP2337	*Desi*	Ca_Desi_Chr03	15825478	(G/A)
26743	CadSNP2338	*Desi*	Ca_Desi_Chr03	15828104	(C/A)
26744	CadSNP2339	*Desi*	Ca_Desi_Chr03	15835391	(T/A)
26745	CadSNP2340	*Desi*	Ca_Desi_Chr03	15875198	(A/G)
26746	CadSNP2341	*Desi*	Ca_Desi_Chr03	15877087	(A/G)
26747	CadSNP2342	*Desi*	Ca_Desi_Chr03	15877222	(T/G)
26748	CadSNP2343	*Desi*	Ca_Desi_Chr03	15877185	(A/G)
26749	CadSNP2344	*Desi*	Ca_Desi_Chr03	15879088	(T/G)
26750	CadSNP2345	*Desi*	Ca_Desi_Chr03	15885833	(G/T)
26751	CadSNP2346	*Desi*	Ca_Desi_Chr03	15885849	(G/T)
26752	CadSNP2347	*Desi*	Ca_Desi_Chr03	15946686	(T/G)

S.N.	SNP IDs	Cultivars	Chromosomes/scaffolds	Physical positions (bp)	SNPs
26753	CadSNP2348	*Desi*	Ca_Desi_Chr03	15946765	(G/C)
26754	CadSNP2349	*Desi*	Ca_Desi_Chr03	15991427	(C/T)
26755	CadSNP2350	*Desi*	Ca_Desi_Chr03	15991589	(T/C)
26756	CadSNP2351	*Desi*	Ca_Desi_Chr03	16001126	(T/A)
26757	CadSNP2352	*Desi*	Ca_Desi_Chr03	16050465	(G/T)
26758	CadSNP2353	*Desi*	Ca_Desi_Chr03	16050429	(G/A)
26759	CadSNP2354	*Desi*	Ca_Desi_Chr03	16104825	(G/T)
26760	CadSNP2355	*Desi*	Ca_Desi_Chr03	16108691	(A/G)
26761	CadSNP2356	*Desi*	Ca_Desi_Chr03	16137168	(G/C)
26762	CadSNP2357	*Desi*	Ca_Desi_Chr03	16139116	(C/T)
26763	CadSNP2358	*Desi*	Ca_Desi_Chr03	16145409	(T/C)
26764	CadSNP2359	*Desi*	Ca_Desi_Chr03	16145494	(C/T)
26765	CadSNP2360	*Desi*	Ca_Desi_Chr03	16145469	(T/A)
26766	CadSNP2361	*Desi*	Ca_Desi_Chr03	16181589	(G/C)
26767	CadSNP2362	*Desi*	Ca_Desi_Chr03	16181578	(G/A)
26768	CadSNP2363	*Desi*	Ca_Desi_Chr03	16209416	(G/A)
26769	CadSNP2364	*Desi*	Ca_Desi_Chr03	16209498	(A/G)
26770	CadSNP2365	*Desi*	Ca_Desi_Chr03	16218534	(T/C)
26771	CadSNP2366	*Desi*	Ca_Desi_Chr03	16232554	(G/A)
26772	CadSNP2367	*Desi*	Ca_Desi_Chr03	16307552	(C/T)
26773	CadSNP2368	*Desi*	Ca_Desi_Chr03	16337251	(C/T)
26774	CadSNP2369	*Desi*	Ca_Desi_Chr03	16373373	(C/A)
26775	CadSNP2370	*Desi*	Ca_Desi_Chr03	16408351	(A/G)
26776	CadSNP2371	*Desi*	Ca_Desi_Chr03	16408411	(A/G)
26777	CadSNP2372	*Desi*	Ca_Desi_Chr03	16410820	(C/T)
26778	CadSNP2373	*Desi*	Ca_Desi_Chr03	16423933	(T/C)
26779	CadSNP2374	*Desi*	Ca_Desi_Chr03	16429296	(G/A)
26780	CadSNP2375	*Desi*	Ca_Desi_Chr03	16429290	(A/G)
26781	CadSNP2376	*Desi*	Ca_Desi_Chr03	16429260	(A/C)
26782	CadSNP2377	*Desi*	Ca_Desi_Chr03	16429257	(A/C)
26783	CadSNP2378	*Desi*	Ca_Desi_Chr03	16457210	(T/C)
26784	CadSNP2379	*Desi*	Ca_Desi_Chr03	16546902	(G/A)
26785	CadSNP2380	*Desi*	Ca_Desi_Chr03	16588500	(A/G)
26786	CadSNP2381	*Desi*	Ca_Desi_Chr03	16588533	(G/C)
26787	CadSNP2382	*Desi*	Ca_Desi_Chr03	16588555	(T/C)
26788	CadSNP2383	*Desi*	Ca_Desi_Chr03	16588661	(T/C)
26789	CadSNP2384	*Desi*	Ca_Desi_Chr03	16588648	(T/G)
26790	CadSNP2385	*Desi*	Ca_Desi_Chr03	16588642	(T/C)
26791	CadSNP2386	*Desi*	Ca_Desi_Chr03	16608969	(G/A)

S.N.	SNP IDs	Cultivars	Chromosomes/scaffolds	Physical positions (bp)	SNPs
26792	CadSNP2387	*Desi*	Ca_Desi_Chr03	16612390	(A/C)
26793	CadSNP2388	*Desi*	Ca_Desi_Chr03	16612523	(G/T)
26794	CadSNP2389	*Desi*	Ca_Desi_Chr03	16612522	(T/A)
26795	CadSNP2390	*Desi*	Ca_Desi_Chr03	16647962	(G/C)
26796	CadSNP2391	*Desi*	Ca_Desi_Chr03	16650388	(T/C)
26797	CadSNP2392	*Desi*	Ca_Desi_Chr03	16654383	(T/C)
26798	CadSNP2393	*Desi*	Ca_Desi_Chr03	16665981	(T/C)
26799	CadSNP2394	*Desi*	Ca_Desi_Chr03	16665928	(G/C)
26800	CadSNP2395	*Desi*	Ca_Desi_Chr03	16779590	(C/T)
26801	CadSNP2396	*Desi*	Ca_Desi_Chr03	16786527	(A/C)
26802	CadSNP2397	*Desi*	Ca_Desi_Chr03	16829269	(G/A)
26803	CadSNP2398	*Desi*	Ca_Desi_Chr03	16829284	(T/C)
26804	CadSNP2399	*Desi*	Ca_Desi_Chr03	16869021	(C/T)
26805	CadSNP2400	*Desi*	Ca_Desi_Chr03	16885534	(C/G)
26806	CadSNP2401	*Desi*	Ca_Desi_Chr03	16885685	(A/G)
26807	CadSNP2402	*Desi*	Ca_Desi_Chr03	16894386	(A/G)
26808	CadSNP2403	*Desi*	Ca_Desi_Chr03	16894455	(G/C)
26809	CadSNP2404	*Desi*	Ca_Desi_Chr03	16925661	(A/C)
26810	CadSNP2405	*Desi*	Ca_Desi_Chr03	16934270	(G/T)
26811	CadSNP2406	*Desi*	Ca_Desi_Chr03	16934316	(T/G)
26812	CadSNP2407	*Desi*	Ca_Desi_Chr03	16968147	(A/C)
26813	CadSNP2408	*Desi*	Ca_Desi_Chr03	16968093	(G/C)
26814	CadSNP2409	*Desi*	Ca_Desi_Chr03	17011107	(C/A)
26815	CadSNP2410	*Desi*	Ca_Desi_Chr03	17011108	(A/G)
26816	CadSNP2411	*Desi*	Ca_Desi_Chr03	17014193	(T/A)
26817	CadSNP2412	*Desi*	Ca_Desi_Chr03	17088351	(A/C)
26818	CadSNP2413	*Desi*	Ca_Desi_Chr03	17096881	(T/G)
26819	CadSNP2414	*Desi*	Ca_Desi_Chr03	17117877	(T/G)
26820	CadSNP2415	*Desi*	Ca_Desi_Chr03	17135055	(A/C)
26821	CadSNP2416	*Desi*	Ca_Desi_Chr03	17151326	(A/T)
26822	CadSNP2417	*Desi*	Ca_Desi_Chr03	17151439	(C/A)
26823	CadSNP2418	*Desi*	Ca_Desi_Chr03	17160362	(G/C)
26824	CadSNP2419	*Desi*	Ca_Desi_Chr03	17160394	(G/T)
26825	CadSNP2420	*Desi*	Ca_Desi_Chr03	17202494	(A/G)
26826	CadSNP2421	*Desi*	Ca_Desi_Chr03	17296670	(A/T)
26827	CadSNP2422	*Desi*	Ca_Desi_Chr03	17619289	(G/C)
26828	CadSNP2423	*Desi*	Ca_Desi_Chr03	17619292	(T/C)
26829	CadSNP2424	*Desi*	Ca_Desi_Chr03	17648928	(C/T)
26830	CadSNP2425	*Desi*	Ca_Desi_Chr03	17664224	(C/T)

S.N.	SNP IDs	Cultivars	Chromosomes/scaffolds	Physical positions (bp)	SNPs
26831	CadSNP2426	*Desi*	Ca_Desi_Chr03	17681284	(G/C)
26832	CadSNP2427	*Desi*	Ca_Desi_Chr03	17681331	(A/G)
26833	CadSNP2428	*Desi*	Ca_Desi_Chr03	17732900	(T/A)
26834	CadSNP2429	*Desi*	Ca_Desi_Chr03	17732871	(C/T)
26835	CadSNP2430	*Desi*	Ca_Desi_Chr03	17800447	(T/C)
26836	CadSNP2431	*Desi*	Ca_Desi_Chr03	17815074	(A/C)
26837	CadSNP2432	*Desi*	Ca_Desi_Chr03	17815081	(A/G)
26838	CadSNP2433	*Desi*	Ca_Desi_Chr03	17821525	(G/A)
26839	CadSNP2434	*Desi*	Ca_Desi_Chr03	17833336	(T/C)
26840	CadSNP2435	*Desi*	Ca_Desi_Chr03	17838091	(T/C)
26841	CadSNP2436	*Desi*	Ca_Desi_Chr03	17840051	(A/C)
26842	CadSNP2437	*Desi*	Ca_Desi_Chr03	17840040	(A/G)
26843	CadSNP2438	*Desi*	Ca_Desi_Chr03	17847712	(G/A)
26844	CadSNP2439	*Desi*	Ca_Desi_Chr03	17848961	(T/C)
26845	CadSNP2440	*Desi*	Ca_Desi_Chr03	17853430	(A/C)
26846	CadSNP2441	*Desi*	Ca_Desi_Chr03	17853418	(C/A)
26847	CadSNP2442	*Desi*	Ca_Desi_Chr03	17853412	(A/G)
26848	CadSNP2443	*Desi*	Ca_Desi_Chr03	17854089	(A/C)
26849	CadSNP2444	*Desi*	Ca_Desi_Chr03	17855179	(G/C)
26850	CadSNP2445	*Desi*	Ca_Desi_Chr03	17858263	(C/A)
26851	CadSNP2446	*Desi*	Ca_Desi_Chr03	17872943	(A/G)
26852	CadSNP2447	*Desi*	Ca_Desi_Chr03	17872966	(A/C)
26853	CadSNP2448	*Desi*	Ca_Desi_Chr03	17895451	(A/G)
26854	CadSNP2449	*Desi*	Ca_Desi_Chr03	17895430	(G/T)
26855	CadSNP2450	*Desi*	Ca_Desi_Chr03	17920666	(C/G)
26856	CadSNP2451	*Desi*	Ca_Desi_Chr03	17920734	(T/C)
26857	CadSNP2452	*Desi*	Ca_Desi_Chr03	17944044	(T/G)
26858	CadSNP2453	*Desi*	Ca_Desi_Chr03	17944090	(A/G)
26859	CadSNP2454	*Desi*	Ca_Desi_Chr03	17952424	(G/T)
26860	CadSNP2455	*Desi*	Ca_Desi_Chr03	17974469	(G/A)
26861	CadSNP2456	*Desi*	Ca_Desi_Chr03	17974517	(T/C)
26862	CadSNP2457	*Desi*	Ca_Desi_Chr03	17974556	(T/G)
26863	CadSNP2458	*Desi*	Ca_Desi_Chr03	17974550	(T/G)
26864	CadSNP2459	*Desi*	Ca_Desi_Chr03	17974629	(A/G)
26865	CadSNP2460	*Desi*	Ca_Desi_Chr03	17974623	(T/C)
26866	CadSNP2461	*Desi*	Ca_Desi_Chr03	18004723	(G/A)
26867	CadSNP2462	*Desi*	Ca_Desi_Chr03	18017145	(A/C)
26868	CadSNP2463	*Desi*	Ca_Desi_Chr03	18017201	(C/T)
26869	CadSNP2464	*Desi*	Ca_Desi_Chr03	18017326	(A/G)

S.N.	SNP IDs	Cultivars	Chromosomes/scaffolds	Physical positions (bp)	SNPs
26870	CadSNP2465	*Desi*	Ca_Desi_Chr03	18018792	(G/A)
26871	CadSNP2466	*Desi*	Ca_Desi_Chr03	18018827	(T/C)
26872	CadSNP2467	*Desi*	Ca_Desi_Chr03	18021905	(C/A)
26873	CadSNP2468	*Desi*	Ca_Desi_Chr03	18045434	(A/T)
26874	CadSNP2469	*Desi*	Ca_Desi_Chr03	18046766	(A/G)
26875	CadSNP2470	*Desi*	Ca_Desi_Chr03	18046910	(A/G)
26876	CadSNP2471	*Desi*	Ca_Desi_Chr03	18057610	(T/A)
26877	CadSNP2472	*Desi*	Ca_Desi_Chr03	18101880	(A/T)
26878	CadSNP2473	*Desi*	Ca_Desi_Chr03	18101900	(T/C)
26879	CadSNP2474	*Desi*	Ca_Desi_Chr03	18140360	(A/C)
26880	CadSNP2475	*Desi*	Ca_Desi_Chr03	18140427	(G/A)
26881	CadSNP2476	*Desi*	Ca_Desi_Chr03	18140618	(G/A)
26882	CadSNP2477	*Desi*	Ca_Desi_Chr03	18150924	(C/T)
26883	CadSNP2478	*Desi*	Ca_Desi_Chr03	18150894	(C/T)
26884	CadSNP2479	*Desi*	Ca_Desi_Chr03	18171597	(C/T)
26885	CadSNP2480	*Desi*	Ca_Desi_Chr03	18171672	(A/G)
26886	CadSNP2481	*Desi*	Ca_Desi_Chr03	18172204	(T/C)
26887	CadSNP2482	*Desi*	Ca_Desi_Chr03	18192715	(T/C)
26888	CadSNP2483	*Desi*	Ca_Desi_Chr03	18193117	(T/A)
26889	CadSNP2484	*Desi*	Ca_Desi_Chr03	18193119	(G/C)
26890	CadSNP2485	*Desi*	Ca_Desi_Chr03	18195900	(A/C)
26891	CadSNP2486	*Desi*	Ca_Desi_Chr03	18195888	(G/A)
26892	CadSNP2487	*Desi*	Ca_Desi_Chr03	18207344	(T/C)
26893	CadSNP2488	*Desi*	Ca_Desi_Chr03	18207347	(T/C)
26894	CadSNP2489	*Desi*	Ca_Desi_Chr03	18207408	(C/T)
26895	CadSNP2490	*Desi*	Ca_Desi_Chr03	18273088	(A/G)
26896	CadSNP2491	*Desi*	Ca_Desi_Chr03	18273114	(T/C)
26897	CadSNP2492	*Desi*	Ca_Desi_Chr03	18285050	(A/G)
26898	CadSNP2493	*Desi*	Ca_Desi_Chr03	18285039	(G/A)
26899	CadSNP2494	*Desi*	Ca_Desi_Chr03	18299725	(A/C)
26900	CadSNP2495	*Desi*	Ca_Desi_Chr03	18302796	(A/G)
26901	CadSNP2496	*Desi*	Ca_Desi_Chr03	18340225	(T/A)
26902	CadSNP2497	*Desi*	Ca_Desi_Chr03	18370632	(T/A)
26903	CadSNP2498	*Desi*	Ca_Desi_Chr03	18416125	(A/G)
26904	CadSNP2499	*Desi*	Ca_Desi_Chr03	18416269	(C/T)
26905	CadSNP2500	*Desi*	Ca_Desi_Chr03	18437879	(C/A)
26906	CadSNP2501	*Desi*	Ca_Desi_Chr03	18439972	(A/G)
26907	CadSNP2502	*Desi*	Ca_Desi_Chr03	18440169	(A/G)
26908	CadSNP2503	*Desi*	Ca_Desi_Chr03	18440231	(C/T)

S.N.	SNP IDs	Cultivars	Chromosomes/scaffolds	Physical positions (bp)	SNPs
26909	CadSNP2504	*Desi*	Ca_Desi_Chr03	18440247	(A/C)
26910	CadSNP2505	*Desi*	Ca_Desi_Chr03	18440955	(A/C)
26911	CadSNP2506	*Desi*	Ca_Desi_Chr03	18444954	(G/A)
26912	CadSNP2507	*Desi*	Ca_Desi_Chr03	18444987	(C/T)
26913	CadSNP2508	*Desi*	Ca_Desi_Chr03	18445034	(G/A)
26914	CadSNP2509	*Desi*	Ca_Desi_Chr03	18445098	(G/A)
26915	CadSNP2510	*Desi*	Ca_Desi_Chr03	18445067	(G/A)
26916	CadSNP2511	*Desi*	Ca_Desi_Chr03	18446744	(A/C)
26917	CadSNP2512	*Desi*	Ca_Desi_Chr03	18448735	(G/T)
26918	CadSNP2513	*Desi*	Ca_Desi_Chr03	18448878	(A/C)
26919	CadSNP2514	*Desi*	Ca_Desi_Chr03	18461505	(T/C)
26920	CadSNP2515	*Desi*	Ca_Desi_Chr03	18461526	(T/C)
26921	CadSNP2516	*Desi*	Ca_Desi_Chr03	18461555	(T/C)
26922	CadSNP2517	*Desi*	Ca_Desi_Chr03	18461745	(G/A)
26923	CadSNP2518	*Desi*	Ca_Desi_Chr03	18461805	(A/G)
26924	CadSNP2519	*Desi*	Ca_Desi_Chr03	18486133	(T/C)
26925	CadSNP2520	*Desi*	Ca_Desi_Chr03	18486193	(T/G)
26926	CadSNP2521	*Desi*	Ca_Desi_Chr03	18486244	(C/A)
26927	CadSNP2522	*Desi*	Ca_Desi_Chr03	18500277	(C/G)
26928	CadSNP2523	*Desi*	Ca_Desi_Chr03	18530390	(A/G)
26929	CadSNP2524	*Desi*	Ca_Desi_Chr03	18530461	(A/G)
26930	CadSNP2525	*Desi*	Ca_Desi_Chr03	18573749	(A/C)
26931	CadSNP2526	*Desi*	Ca_Desi_Chr03	18652129	(T/C)
26932	CadSNP2527	*Desi*	Ca_Desi_Chr03	18667652	(A/G)
26933	CadSNP2528	*Desi*	Ca_Desi_Chr03	18667770	(T/A)
26934	CadSNP2529	*Desi*	Ca_Desi_Chr03	18682250	(A/G)
26935	CadSNP2530	*Desi*	Ca_Desi_Chr03	18702954	(C/T)
26936	CadSNP2531	*Desi*	Ca_Desi_Chr03	18703009	(G/T)
26937	CadSNP2532	*Desi*	Ca_Desi_Chr03	18703008	(C/A)
26938	CadSNP2533	*Desi*	Ca_Desi_Chr03	18703005	(G/A)
26939	CadSNP2534	*Desi*	Ca_Desi_Chr03	18715729	(G/C)
26940	CadSNP2535	*Desi*	Ca_Desi_Chr03	18818986	(A/C)
26941	CadSNP2536	*Desi*	Ca_Desi_Chr03	18818967	(T/C)
26942	CadSNP2537	*Desi*	Ca_Desi_Chr03	18968448	(T/C)
26943	CadSNP2538	*Desi*	Ca_Desi_Chr03	18976059	(C/T)
26944	CadSNP2539	*Desi*	Ca_Desi_Chr03	18976309	(C/A)
26945	CadSNP2540	*Desi*	Ca_Desi_Chr03	18976346	(A/C)
26946	CadSNP2541	*Desi*	Ca_Desi_Chr03	18989078	(G/C)
26947	CadSNP2542	*Desi*	Ca_Desi_Chr03	18989029	(C/T)

S.N.	SNP IDs	Cultivars	Chromosomes/scaffolds	Physical positions (bp)	SNPs
26948	CadSNP2543	*Desi*	Ca_Desi_Chr03	19054144	(G/C)
26949	CadSNP2544	*Desi*	Ca_Desi_Chr03	19061973	(G/A)
26950	CadSNP2545	*Desi*	Ca_Desi_Chr03	19083604	(G/A)
26951	CadSNP2546	*Desi*	Ca_Desi_Chr03	19113194	(A/G)
26952	CadSNP2547	*Desi*	Ca_Desi_Chr03	19113403	(T/C)
26953	CadSNP2548	*Desi*	Ca_Desi_Chr03	19113488	(C/T)
26954	CadSNP2549	*Desi*	Ca_Desi_Chr03	19114097	(G/C)
26955	CadSNP2550	*Desi*	Ca_Desi_Chr03	19114100	(T/G)
26956	CadSNP2551	*Desi*	Ca_Desi_Chr03	19114103	(T/C)
26957	CadSNP2552	*Desi*	Ca_Desi_Chr03	19114106	(T/A)
26958	CadSNP2553	*Desi*	Ca_Desi_Chr03	19114160	(A/G)
26959	CadSNP2554	*Desi*	Ca_Desi_Chr03	19148843	(C/G)
26960	CadSNP2555	*Desi*	Ca_Desi_Chr03	19166405	(G/T)
26961	CadSNP2556	*Desi*	Ca_Desi_Chr03	19172991	(A/C)
26962	CadSNP2557	*Desi*	Ca_Desi_Chr03	19201730	(T/A)
26963	CadSNP2558	*Desi*	Ca_Desi_Chr03	19562105	(C/T)
26964	CadSNP2559	*Desi*	Ca_Desi_Chr03	19562079	(C/T)
26965	CadSNP2560	*Desi*	Ca_Desi_Chr03	19562232	(T/G)
26966	CadSNP2561	*Desi*	Ca_Desi_Chr03	19562374	(A/G)
26967	CadSNP2562	*Desi*	Ca_Desi_Chr03	19567077	(A/G)
26968	CadSNP2563	*Desi*	Ca_Desi_Chr03	19567072	(C/T)
26969	CadSNP2564	*Desi*	Ca_Desi_Chr03	19567059	(A/G)
26970	CadSNP2565	*Desi*	Ca_Desi_Chr03	19593350	(C/T)
26971	CadSNP2566	*Desi*	Ca_Desi_Chr03	19612103	(G/A)
26972	CadSNP2567	*Desi*	Ca_Desi_Chr03	19617421	(T/C)
26973	CadSNP2568	*Desi*	Ca_Desi_Chr03	19634559	(C/T)
26974	CadSNP2569	*Desi*	Ca_Desi_Chr03	19634540	(G/A)
26975	CadSNP2570	*Desi*	Ca_Desi_Chr03	19634515	(G/T)
26976	CadSNP2571	*Desi*	Ca_Desi_Chr03	19634618	(C/G)
26977	CadSNP2572	*Desi*	Ca_Desi_Chr03	19649177	(A/C)
26978	CadSNP2573	*Desi*	Ca_Desi_Chr03	19660296	(T/G)
26979	CadSNP2574	*Desi*	Ca_Desi_Chr03	19666249	(G/A)
26980	CadSNP2575	*Desi*	Ca_Desi_Chr03	19666243	(A/T)
26981	CadSNP2576	*Desi*	Ca_Desi_Chr03	19666239	(T/A)
26982	CadSNP2577	*Desi*	Ca_Desi_Chr03	19666229	(T/G)
26983	CadSNP2578	*Desi*	Ca_Desi_Chr03	19672916	(C/T)
26984	CadSNP2579	*Desi*	Ca_Desi_Chr03	19673774	(A/G)
26985	CadSNP2580	*Desi*	Ca_Desi_Chr03	19674856	(T/G)
26986	CadSNP2581	*Desi*	Ca_Desi_Chr03	19702745	(C/T)

S.N.	SNP IDs	Cultivars	Chromosomes/scaffolds	Physical positions (bp)	SNPs
26987	CadSNP2582	*Desi*	Ca_Desi_Chr03	19702753	(T/G)
26988	CadSNP2583	*Desi*	Ca_Desi_Chr03	19702779	(G/A)
26989	CadSNP2584	*Desi*	Ca_Desi_Chr03	19707456	(T/C)
26990	CadSNP2585	*Desi*	Ca_Desi_Chr03	19707424	(G/C)
26991	CadSNP2586	*Desi*	Ca_Desi_Chr03	19707556	(C/T)
26992	CadSNP2587	*Desi*	Ca_Desi_Chr03	19707735	(A/G)
26993	CadSNP2588	*Desi*	Ca_Desi_Chr03	19707742	(G/A)
26994	CadSNP2589	*Desi*	Ca_Desi_Chr03	19710005	(T/C)
26995	CadSNP2590	*Desi*	Ca_Desi_Chr03	19710036	(T/C)
26996	CadSNP2591	*Desi*	Ca_Desi_Chr03	19710066	(A/T)
26997	CadSNP2592	*Desi*	Ca_Desi_Chr03	19715908	(A/G)
26998	CadSNP2593	*Desi*	Ca_Desi_Chr03	19715915	(G/A)
26999	CadSNP2594	*Desi*	Ca_Desi_Chr03	19779276	(A/T)
27000	CadSNP2595	*Desi*	Ca_Desi_Chr03	19779288	(G/A)
27001	CadSNP2596	*Desi*	Ca_Desi_Chr03	19779292	(C/G)
27002	CadSNP2597	*Desi*	Ca_Desi_Chr03	19781105	(C/G)
27003	CadSNP2598	*Desi*	Ca_Desi_Chr03	19781127	(T/C)
27004	CadSNP2599	*Desi*	Ca_Desi_Chr03	19781156	(T/C)
27005	CadSNP2600	*Desi*	Ca_Desi_Chr03	19821918	(T/G)
27006	CadSNP2601	*Desi*	Ca_Desi_Chr03	19826899	(A/G)
27007	CadSNP2602	*Desi*	Ca_Desi_Chr03	19827539	(T/G)
27008	CadSNP2603	*Desi*	Ca_Desi_Chr03	19827573	(C/T)
27009	CadSNP2604	*Desi*	Ca_Desi_Chr03	19844515	(C/G)
27010	CadSNP2605	*Desi*	Ca_Desi_Chr03	19857684	(G/A)
27011	CadSNP2606	*Desi*	Ca_Desi_Chr03	19868544	(A/C)
27012	CadSNP2607	*Desi*	Ca_Desi_Chr03	19873907	(C/G)
27013	CadSNP2608	*Desi*	Ca_Desi_Chr03	19877477	(T/G)
27014	CadSNP2609	*Desi*	Ca_Desi_Chr03	19895631	(C/A)
27015	CadSNP2610	*Desi*	Ca_Desi_Chr03	19897330	(G/A)
27016	CadSNP2611	*Desi*	Ca_Desi_Chr03	19897394	(C/T)
27017	CadSNP2612	*Desi*	Ca_Desi_Chr03	19898864	(T/C)
27018	CadSNP2613	*Desi*	Ca_Desi_Chr03	19899536	(C/T)
27019	CadSNP2614	*Desi*	Ca_Desi_Chr03	19899597	(C/A)
27020	CadSNP2615	*Desi*	Ca_Desi_Chr03	19899599	(A/G)
27021	CadSNP2616	*Desi*	Ca_Desi_Chr03	19899620	(G/T)
27022	CadSNP2617	*Desi*	Ca_Desi_Chr03	19899611	(G/T)
27023	CadSNP2618	*Desi*	Ca_Desi_Chr03	19899673	(C/G)
27024	CadSNP2619	*Desi*	Ca_Desi_Chr03	19899698	(G/T)
27025	CadSNP2620	*Desi*	Ca_Desi_Chr03	19921122	(T/C)

S.N.	SNP IDs	Cultivars	Chromosomes/scaffolds	Physical positions (bp)	SNPs
27026	CadSNP2621	*Desi*	Ca_Desi_Chr03	19925033	(T/C)
27027	CadSNP2622	*Desi*	Ca_Desi_Chr03	19925933	(G/T)
27028	CadSNP2623	*Desi*	Ca_Desi_Chr03	19925894	(C/T)
27029	CadSNP2624	*Desi*	Ca_Desi_Chr03	19925928	(A/G)
27030	CadSNP2625	*Desi*	Ca_Desi_Chr03	19925945	(G/T)
27031	CadSNP2626	*Desi*	Ca_Desi_Chr03	19965806	(T/C)
27032	CadSNP2627	*Desi*	Ca_Desi_Chr03	19965795	(T/G)
27033	CadSNP2628	*Desi*	Ca_Desi_Chr03	20002679	(C/T)
27034	CadSNP2629	*Desi*	Ca_Desi_Chr03	20002666	(G/T)
27035	CadSNP2630	*Desi*	Ca_Desi_Chr03	20002673	(G/A)
27036	CadSNP2631	*Desi*	Ca_Desi_Chr03	20023325	(T/C)
27037	CadSNP2632	*Desi*	Ca_Desi_Chr03	20023632	(T/A)
27038	CadSNP2633	*Desi*	Ca_Desi_Chr03	20023610	(T/C)
27039	CadSNP2634	*Desi*	Ca_Desi_Chr03	20023606	(G/T)
27040	CadSNP2635	*Desi*	Ca_Desi_Chr03	20023598	(A/G)
27041	CadSNP2636	*Desi*	Ca_Desi_Chr03	20023597	(C/T)
27042	CadSNP2637	*Desi*	Ca_Desi_Chr03	20023592	(A/G)
27043	CadSNP2638	*Desi*	Ca_Desi_Chr03	20023586	(G/T)
27044	CadSNP2639	*Desi*	Ca_Desi_Chr03	20023572	(T/C)
27045	CadSNP2640	*Desi*	Ca_Desi_Chr03	20040675	(A/G)
27046	CadSNP2641	*Desi*	Ca_Desi_Chr03	20040669	(C/T)
27047	CadSNP2642	*Desi*	Ca_Desi_Chr03	20040729	(T/A)
27048	CadSNP2643	*Desi*	Ca_Desi_Chr03	20085780	(T/C)
27049	CadSNP2644	*Desi*	Ca_Desi_Chr03	20085993	(A/G)
27050	CadSNP2645	*Desi*	Ca_Desi_Chr03	20085985	(T/G)
27051	CadSNP2646	*Desi*	Ca_Desi_Chr03	20085973	(G/T)
27052	CadSNP2647	*Desi*	Ca_Desi_Chr03	20103164	(G/A)
27053	CadSNP2648	*Desi*	Ca_Desi_Chr03	20103283	(A/G)
27054	CadSNP2649	*Desi*	Ca_Desi_Chr03	20120485	(C/T)
27055	CadSNP2650	*Desi*	Ca_Desi_Chr03	20120524	(T/C)
27056	CadSNP2651	*Desi*	Ca_Desi_Chr03	20120527	(T/A)
27057	CadSNP2652	*Desi*	Ca_Desi_Chr03	20120529	(C/G)
27058	CadSNP2653	*Desi*	Ca_Desi_Chr03	20139253	(A/G)
27059	CadSNP2654	*Desi*	Ca_Desi_Chr03	20159856	(C/T)
27060	CadSNP2655	*Desi*	Ca_Desi_Chr03	20161445	(G/A)
27061	CadSNP2656	*Desi*	Ca_Desi_Chr03	20161447	(A/G)
27062	CadSNP2657	*Desi*	Ca_Desi_Chr03	20205815	(T/C)
27063	CadSNP2658	*Desi*	Ca_Desi_Chr03	20241816	(T/C)
27064	CadSNP2659	*Desi*	Ca_Desi_Chr03	20251494	(G/C)

S.N.	SNP IDs	Cultivars	Chromosomes/scaffolds	Physical positions (bp)	SNPs
27065	CadSNP2660	*Desi*	Ca_Desi_Chr03	20263158	(C/T)
27066	CadSNP2661	*Desi*	Ca_Desi_Chr03	20263132	(C/T)
27067	CadSNP2662	*Desi*	Ca_Desi_Chr03	20263125	(T/C)
27068	CadSNP2663	*Desi*	Ca_Desi_Chr03	20264396	(A/G)
27069	CadSNP2664	*Desi*	Ca_Desi_Chr03	20267244	(T/G)
27070	CadSNP2665	*Desi*	Ca_Desi_Chr03	20267317	(G/C)
27071	CadSNP2666	*Desi*	Ca_Desi_Chr03	20276932	(A/C)
27072	CadSNP2667	*Desi*	Ca_Desi_Chr03	20276944	(T/G)
27073	CadSNP2668	*Desi*	Ca_Desi_Chr03	20277002	(G/A)
27074	CadSNP2669	*Desi*	Ca_Desi_Chr03	20293230	(A/C)
27075	CadSNP2670	*Desi*	Ca_Desi_Chr03	20303278	(C/T)
27076	CadSNP2671	*Desi*	Ca_Desi_Chr03	20313862	(T/A)
27077	CadSNP2672	*Desi*	Ca_Desi_Chr03	20313923	(A/G)
27078	CadSNP2673	*Desi*	Ca_Desi_Chr03	20316744	(T/C)
27079	CadSNP2674	*Desi*	Ca_Desi_Chr03	20316797	(C/A)
27080	CadSNP2675	*Desi*	Ca_Desi_Chr03	20316902	(A/G)
27081	CadSNP2676	*Desi*	Ca_Desi_Chr03	20319272	(C/T)
27082	CadSNP2677	*Desi*	Ca_Desi_Chr03	20335521	(G/T)
27083	CadSNP2678	*Desi*	Ca_Desi_Chr03	20372617	(G/A)
27084	CadSNP2679	*Desi*	Ca_Desi_Chr03	20372609	(A/G)
27085	CadSNP2680	*Desi*	Ca_Desi_Chr03	20372731	(C/G)
27086	CadSNP2681	*Desi*	Ca_Desi_Chr03	20418933	(T/C)
27087	CadSNP2682	*Desi*	Ca_Desi_Chr03	20439532	(A/T)
27088	CadSNP2683	*Desi*	Ca_Desi_Chr03	20440511	(T/C)
27089	CadSNP2684	*Desi*	Ca_Desi_Chr03	20603607	(C/T)
27090	CadSNP2685	*Desi*	Ca_Desi_Chr03	20614613	(T/G)
27091	CadSNP2686	*Desi*	Ca_Desi_Chr03	20614542	(A/G)
27092	CadSNP2687	*Desi*	Ca_Desi_Chr03	21029404	(G/A)
27093	CadSNP2688	*Desi*	Ca_Desi_Chr03	21054058	(G/A)
27094	CadSNP2689	*Desi*	Ca_Desi_Chr03	21058243	(A/C)
27095	CadSNP2690	*Desi*	Ca_Desi_Chr03	21063851	(T/G)
27096	CadSNP2691	*Desi*	Ca_Desi_Chr03	21063811	(T/A)
27097	CadSNP2692	*Desi*	Ca_Desi_Chr03	21114837	(C/T)
27098	CadSNP2693	*Desi*	Ca_Desi_Chr03	21114951	(T/C)
27099	CadSNP2694	*Desi*	Ca_Desi_Chr03	21114912	(C/A)
27100	CadSNP2695	*Desi*	Ca_Desi_Chr03	21115138	(A/G)
27101	CadSNP2696	*Desi*	Ca_Desi_Chr03	21180741	(T/G)
27102	CadSNP2697	*Desi*	Ca_Desi_Chr03	21180891	(A/G)
[truncated: 581,560 more chars]
