# Supplementary material for: Employing genome-wide SNP discovery and genotyping strategy to extrapolate the natural allelic diversity and domestication patterns in chickpea
Source: Front Plant Sci. 2015 Mar 31;6:162. doi: 10.3389/fpls.2015.00162 (PMC4379880; doi:10.3389/fpls.2015.00162)
Supplement: Supplementary file 14 [file Table4.PDF]

Table S4: Structural and functional annotation of 22542 GBS-based SNPs in *desi* and *kabuli* chickpea genomes

S.N.	SNP IDs	Chickpea cultivars	Chromosomes	Physical positions (bp)	SNPs	Gene accession IDs	Putative functions	Sequence components of genome
1	CakSNP1	*Kabuli*	Ca_Kabuli_Chr01	523	[G/C]		--	INTERGENIC
2	CakSNP2	*Kabuli*	Ca_Kabuli_Chr01	462	[G/C]		--	INTERGENIC
3	CakSNP3	*Kabuli*	Ca_Kabuli_Chr01	548	[G/C]		--	INTERGENIC
4	CakSNP4	*Kabuli*	Ca_Kabuli_Chr01	578	[G/A]		--	INTERGENIC
5	CakSNP5	*Kabuli*	Ca_Kabuli_Chr01	579	[T/C]		--	INTERGENIC
6	CakSNP6	*Kabuli*	Ca_Kabuli_Chr01	589	[A/C]		--	INTERGENIC
7	CakSNP7	*Kabuli*	Ca_Kabuli_Chr01	627	[C/A]		--	INTERGENIC
8	CakSNP8	*Kabuli*	Ca_Kabuli_Chr01	839	[A/T]		--	INTERGENIC
9	CakSNP9	*Kabuli*	Ca_Kabuli_Chr01	903	[G/T]		--	INTERGENIC
10	CakSNP10	*Kabuli*	Ca_Kabuli_Chr01	927	[C/A]		--	INTERGENIC
11	CakSNP11	*Kabuli*	Ca_Kabuli_Chr01	98930	[T/C]	Ca_00007	RNA recognition motif domain	NON_SYNONYMOUS_CODING

S.N.	SNP IDs	Chickpea cultivars	Chromosomes	Physical positions (bp)	SNPs	Gene accession IDs	Putative functions	Sequence components of genome
12	CakSNP12	*Kabuli*	Ca_Kabuli_Chr01	98992	[G/T]	Ca_00007	RNA recognition motif domain	INTRON
13	CakSNP13	*Kabuli*	Ca_Kabuli_Chr01	99242	[G/A]	Ca_00007	RNA recognition motif domain	INTRON
14	CakSNP14	*Kabuli*	Ca_Kabuli_Chr01	108882	[C/A]	Ca_00008	--	INTRON
15	CakSNP15	*Kabuli*	Ca_Kabuli_Chr01	108984	[G/A]	Ca_00008	--	INTRON
16	CakSNP16	*Kabuli*	Ca_Kabuli_Chr01	108979	[T/G]	Ca_00008	--	INTRON
17	CakSNP17	*Kabuli*	Ca_Kabuli_Chr01	129394	[A/G]	Ca_00009	Oligopeptide transporter	INTRON
18	CakSNP18	*Kabuli*	Ca_Kabuli_Chr01	129401	[A/G]	Ca_00009	Oligopeptide transporter	INTRON
19	CakSNP19	*Kabuli*	Ca_Kabuli_Chr01	173669	[C/T]	Ca_00013	Protein kinase, catalytic domain	SYNONYMOUS_CODING
20	CakSNP20	*Kabuli*	Ca_Kabuli_Chr01	202954	[G/T]	Ca_00016	RNA recognition motif domain	SYNONYMOUS_CODING
21	CakSNP21	*Kabuli*	Ca_Kabuli_Chr01	208244	[G/A]	Ca_00016	RNA recognition motif domain	INTRON

S.N.	SNP IDs	Chickpea cultivars	Chromosomes	Physical positions (bp)	SNPs	Gene accession IDs	Putative functions	Sequence components of genome
22	CakSNP22	*Kabuli*	Ca_Kabuli_Chr01	214979	[T/A]	Ca_00017	Protein kinase, catalytic domain	INTRON
23	CakSNP23	*Kabuli*	Ca_Kabuli_Chr01	215066	[G/T]	Ca_00017	Protein kinase, catalytic domain	INTRON
24	CakSNP24	*Kabuli*	Ca_Kabuli_Chr01	215058	[T/C]	Ca_00017	Protein kinase, catalytic domain	INTRON
25	CakSNP25	*Kabuli*	Ca_Kabuli_Chr01	215053	[G/T]	Ca_00017	Protein kinase, catalytic domain	INTRON
26	CakSNP26	*Kabuli*	Ca_Kabuli_Chr01	215048	[C/T]	Ca_00017	Protein kinase, catalytic domain	INTRON
27	CakSNP27	*Kabuli*	Ca_Kabuli_Chr01	215029	[C/A]	Ca_00017	Protein kinase, catalytic domain	INTRON
28	CakSNP28	*Kabuli*	Ca_Kabuli_Chr01	215027	[T/C]	Ca_00017	Protein kinase, catalytic domain	INTRON
29	CakSNP29	*Kabuli*	Ca_Kabuli_Chr01	215006	[C/T]	Ca_00017	Protein kinase, catalytic domain	INTRON
30	CakSNP30	*Kabuli*	Ca_Kabuli_Chr01	215239	[C/G]	Ca_00017	Protein kinase, catalytic domain	SYNONYMOUS_CODING
31	CakSNP31	*Kabuli*	Ca_Kabuli_Chr01	292962	[T/C]	Ca_00028	Proteasome component (PCI) domain	SYNONYMOUS_CODING

S.N.	SNP IDs	Chickpea cultivars	Chromosomes	Physical positions (bp)	SNPs	Gene accession IDs	Putative functions	Sequence components of genome
32	CakSNP32	*Kabuli*	Ca_Kabuli_Chr01	292972	[G/A]	Ca_00028	Proteasome component (PCI) domain	NON_SYNONYMOUS_CODING
33	CakSNP33	*Kabuli*	Ca_Kabuli_Chr01	292998	[T/C]	Ca_00028	Proteasome component (PCI) domain	SYNONYMOUS_CODING
34	CakSNP34	*Kabuli*	Ca_Kabuli_Chr01	347620	[A/C]	Ca_00035	NIF system FeS cluster assembly, NifU, C-terminal	SYNONYMOUS_CODING
35	CakSNP35	*Kabuli*	Ca_Kabuli_Chr01	372530	[A/G]	Ca_00038	Protein of unknown function DUF803	INTRON
36	CakSNP36	*Kabuli*	Ca_Kabuli_Chr01	406570	[A/G]	Ca_00043	Glycosyl-phosphatidyl inositol-anchored, plant	SYNONYMOUS_CODING
37	CakSNP37	*Kabuli*	Ca_Kabuli_Chr01	425149	[T/C]		--	INTERGENIC
38	CakSNP38	*Kabuli*	Ca_Kabuli_Chr01	425076	[A/G]		--	INTERGENIC
39	CakSNP39	*Kabuli*	Ca_Kabuli_Chr01	435259	[A/G]	Ca_00049	Protein kinase, catalytic domain	INTRON
40	CakSNP40	*Kabuli*	Ca_Kabuli_Chr01	435274	[G/T]	Ca_00049	Protein kinase, catalytic domain	INTRON
41	CakSNP41	*Kabuli*	Ca_Kabuli_Chr01	435280	[T/C]	Ca_00049	Protein kinase, catalytic domain	INTRON

S.N.	SNP IDs	Chickpea cultivars	Chromosomes	Physical positions (bp)	SNPs	Gene accession IDs	Putative functions	Sequence components of genome
42	CakSNP42	*Kabuli*	Ca_Kabuli_Chr01	435297	[G/A]	Ca_00049	Protein kinase, catalytic domain	INTRON
43	CakSNP43	*Kabuli*	Ca_Kabuli_Chr01	435304	[G/T]	Ca_00049	Protein kinase, catalytic domain	INTRON
44	CakSNP44	*Kabuli*	Ca_Kabuli_Chr01	435343	[C/A]	Ca_00049	Protein kinase, catalytic domain	INTRON
45	CakSNP45	*Kabuli*	Ca_Kabuli_Chr01	435338	[T/C]	Ca_00049	Protein kinase, catalytic domain	INTRON
46	CakSNP46	*Kabuli*	Ca_Kabuli_Chr01	435264	[G/T]	Ca_00049	Protein kinase, catalytic domain	INTRON
47	CakSNP47	*Kabuli*	Ca_Kabuli_Chr01	441283	[G/A]	Ca_00050	Thiamine monophosphate synthase	INTRON
48	CakSNP48	*Kabuli*	Ca_Kabuli_Chr01	441286	[C/T]	Ca_00050	Thiamine monophosphate synthase	INTRON
49	CakSNP49	*Kabuli*	Ca_Kabuli_Chr01	441288	[T/A]	Ca_00050	Thiamine monophosphate synthase	INTRON
50	CakSNP50	*Kabuli*	Ca_Kabuli_Chr01	441343	[T/A]	Ca_00050	Thiamine monophosphate synthase	SYNONYMOUS_CODING
51	CakSNP51	*Kabuli*	Ca_Kabuli_Chr01	446409	[T/C]	Ca_00051	Helicase, C-terminal	SYNONYMOUS_CODING

S.N.	SNP IDs	Chickpea cultivars	Chromosomes	Physical positions (bp)	SNPs	Gene accession IDs	Putative functions	Sequence components of genome
52	CakSNP52	*Kabuli*	Ca_Kabuli_Chr01	446443	[A/G]	Ca_00051	Helicase, C-terminal	NON_SYNONYMOUS_CODING
53	CakSNP53	*Kabuli*	Ca_Kabuli_Chr01	446484	[C/T]	Ca_00051	Helicase, C-terminal	SYNONYMOUS_CODING
54	CakSNP54	*Kabuli*	Ca_Kabuli_Chr01	446558	[G/T]	Ca_00051	Helicase, C-terminal	INTRON
55	CakSNP55	*Kabuli*	Ca_Kabuli_Chr01	459968	[T/G]	Ca_00053	CMP/dCMP deaminase, zinc-binding	INTRON
56	CakSNP56	*Kabuli*	Ca_Kabuli_Chr01	464039	[A/C]	Ca_00053	CMP/dCMP deaminase, zinc-binding	SYNONYMOUS_CODING
57	CakSNP57	*Kabuli*	Ca_Kabuli_Chr01	464040	[G/C]	Ca_00053	CMP/dCMP deaminase, zinc-binding	SYNONYMOUS_CODING
58	CakSNP58	*Kabuli*	Ca_Kabuli_Chr01	464043	[C/G]	Ca_00053	CMP/dCMP deaminase, zinc-binding	SYNONYMOUS_CODING
59	CakSNP59	*Kabuli*	Ca_Kabuli_Chr01	471007	[C/T]	Ca_00053	CMP/dCMP deaminase, zinc-binding	SYNONYMOUS_CODING
60	CakSNP60	*Kabuli*	Ca_Kabuli_Chr01	471037	[C/T]	Ca_00053	CMP/dCMP deaminase, zinc-binding	SYNONYMOUS_CODING
61	CakSNP61	*Kabuli*	Ca_Kabuli_Chr01	527271	[T/G]	Ca_00060	Membralin	INTRON

S.N.	SNP IDs	Chickpea cultivars	Chromosomes	Physical positions (bp)	SNPs	Gene accession IDs	Putative functions	Sequence components of genome
62	CakSNP62	*Kabuli*	Ca_Kabuli_Chr01	527321	[C/T]	Ca_00060	Membralin	INTRON
63	CakSNP63	*Kabuli*	Ca_Kabuli_Chr01	554581	[G/T]	Ca_00064	Glycosyl transferase, family 8	INTRON
64	CakSNP64	*Kabuli*	Ca_Kabuli_Chr01	554677	[G/A]	Ca_00064	Glycosyl transferase, family 8	INTRON
65	CakSNP65	*Kabuli*	Ca_Kabuli_Chr01	571020	[T/G]	Ca_00064	Glycosyl transferase, family 8	INTRON
66	CakSNP66	*Kabuli*	Ca_Kabuli_Chr01	591609	[T/C]	Ca_00066	Peptidase S16, Lon protease, C-terminal	INTRON
67	CakSNP67	*Kabuli*	Ca_Kabuli_Chr01	609709	[A/G]		--	INTERGENIC
68	CakSNP68	*Kabuli*	Ca_Kabuli_Chr01	609671	[C/T]		--	INTERGENIC
69	CakSNP69	*Kabuli*	Ca_Kabuli_Chr01	609669	[C/G]		--	INTERGENIC
70	CakSNP70	*Kabuli*	Ca_Kabuli_Chr01	644318	[C/T]	Ca_00075	Protein of unknown function DUF639	NON_SYNONYMOUS_CODING
71	CakSNP71	*Kabuli*	Ca_Kabuli_Chr01	644353	[G/A]	Ca_00075	Protein of unknown function DUF639	INTRON

S.N.	SNP IDs	Chickpea cultivars	Chromosomes	Physical positions (bp)	SNPs	Gene accession IDs	Putative functions	Sequence components of genome
72	CakSNP72	*Kabuli*	Ca_Kabuli_Chr01	644359	[T/A]	Ca_00075	Protein of unknown function DUF639	INTRON
73	CakSNP73	*Kabuli*	Ca_Kabuli_Chr01	644440	[T/C]	Ca_00075	Protein of unknown function DUF639	INTRON
74	CakSNP74	*Kabuli*	Ca_Kabuli_Chr01	644425	[G/A]	Ca_00075	Protein of unknown function DUF639	INTRON
75	CakSNP75	*Kabuli*	Ca_Kabuli_Chr01	661882	[A/G]		--	INTERGENIC
76	CakSNP76	*Kabuli*	Ca_Kabuli_Chr01	662341	[A/C]		--	INTERGENIC
77	CakSNP77	*Kabuli*	Ca_Kabuli_Chr01	662489	[T/A]	Ca_00079	--	NON_SYNONYMOUS_CODING
78	CakSNP78	*Kabuli*	Ca_Kabuli_Chr01	671420	[C/A]	Ca_00080	Plastid lipid-associated protein/fibrillin conserved domain	INTRON
79	CakSNP79	*Kabuli*	Ca_Kabuli_Chr01	671410	[T/C]	Ca_00080	Plastid lipid-associated protein/fibrillin conserved domain	INTRON
80	CakSNP80	*Kabuli*	Ca_Kabuli_Chr01	671391	[T/C]	Ca_00080	Plastid lipid-associated protein/fibrillin conserved domain	INTRON
81	CakSNP81	*Kabuli*	Ca_Kabuli_Chr01	671389	[A/G]	Ca_00080	Plastid lipid-associated protein/fibrillin conserved domain	INTRON

S.N.	SNP IDs	Chickpea cultivars	Chromosomes	Physical positions (bp)	SNPs	Gene accession IDs	Putative functions	Sequence components of genome
82	CakSNP82	*Kabuli*	Ca_Kabuli_Chr01	671388	[A/T]	Ca_00080	Plastid lipid-associated protein/fibrillin conserved domain	INTRON
83	CakSNP83	*Kabuli*	Ca_Kabuli_Chr01	690156	[C/A]		--	INTERGENIC
84	CakSNP84	*Kabuli*	Ca_Kabuli_Chr01	690431	[G/A]		--	INTERGENIC
85	CakSNP85	*Kabuli*	Ca_Kabuli_Chr01	691102	[A/T]	Ca_00083	Transmembrane receptor, eukaryota	SYNONYMOUS_CODING
86	CakSNP86	*Kabuli*	Ca_Kabuli_Chr01	695962	[A/C]	Ca_00084	Exon junction complex, Pym	INTRON
87	CakSNP87	*Kabuli*	Ca_Kabuli_Chr01	712110	[T/C]	Ca_00086	Peptidase C50, separase	NON_SYNONYMOUS_CODING
88	CakSNP88	*Kabuli*	Ca_Kabuli_Chr01	712073	[C/T]	Ca_00086	Peptidase C50, separase	SYNONYMOUS_CODING
89	CakSNP89	*Kabuli*	Ca_Kabuli_Chr01	741514	[G/T]	Ca_00090	COP9 signalosome, subunit CSN8	NON_SYNONYMOUS_CODING
90	CakSNP90	*Kabuli*	Ca_Kabuli_Chr01	741535	[C/T]	Ca_00090	COP9 signalosome, subunit CSN8	INTRON
91	CakSNP91	*Kabuli*	Ca_Kabuli_Chr01	741565	[T/C]	Ca_00090	COP9 signalosome, subunit CSN8	INTRON

S.N.	SNP IDs	Chickpea cultivars	Chromosomes	Physical positions (bp)	SNPs	Gene accession IDs	Putative functions	Sequence components of genome
92	CakSNP92	*Kabuli*	Ca_Kabuli_Chr01	741549	[C/T]	Ca_00090	COP9 signalosome, subunit CSN8	INTRON
93	CakSNP93	*Kabuli*	Ca_Kabuli_Chr01	741980	[C/G]	Ca_00090	COP9 signalosome, subunit CSN8	SYNONYMOUS_CODING
94	CakSNP94	*Kabuli*	Ca_Kabuli_Chr01	742043	[G/A]	Ca_00090	COP9 signalosome, subunit CSN8	SYNONYMOUS_CODING
95	CakSNP95	*Kabuli*	Ca_Kabuli_Chr01	742026	[T/G]	Ca_00090	COP9 signalosome, subunit CSN8	NON_SYNONYMOUS_CODING
96	CakSNP96	*Kabuli*	Ca_Kabuli_Chr01	750693	[C/T]	Ca_00092	FAS1 domain	NON_SYNONYMOUS_CODING
97	CakSNP97	*Kabuli*	Ca_Kabuli_Chr01	777273	[C/T]	Ca_00096	Tubby, C-terminal	SYNONYMOUS_CODING
98	CakSNP98	*Kabuli*	Ca_Kabuli_Chr01	777240	[G/A]	Ca_00096	Tubby, C-terminal	SYNONYMOUS_CODING
99	CakSNP99	*Kabuli*	Ca_Kabuli_Chr01	790004	[A/C]	Ca_00098	Drug/metabolite transporter	NON_SYNONYMOUS_CODING
100	CakSNP100	*Kabuli*	Ca_Kabuli_Chr01	790000	[C/T]	Ca_00098	Drug/metabolite transporter	NON_SYNONYMOUS_CODING
101	CakSNP101	*Kabuli*	Ca_Kabuli_Chr01	790102	[G/T]	Ca_00098	Drug/metabolite transporter	NON_SYNONYMOUS_CODING

S.N.	SNP IDs	Chickpea cultivars	Chromosomes	Physical positions (bp)	SNPs	Gene accession IDs	Putative functions	Sequence components of genome
102	CakSNP102	*Kabuli*	Ca_Kabuli_Chr01	864205	[T/C]		--	INTERGENIC
103	CakSNP103	*Kabuli*	Ca_Kabuli_Chr01	864382	[T/C]		--	INTERGENIC
104	CakSNP104	*Kabuli*	Ca_Kabuli_Chr01	864381	[G/A]		--	INTERGENIC
105	CakSNP105	*Kabuli*	Ca_Kabuli_Chr01	864351	[T/C]		--	INTERGENIC
106	CakSNP106	*Kabuli*	Ca_Kabuli_Chr01	866872	[T/C]	Ca_00105	Pyridoxal phosphate-dependent decarboxylase	SYNONYMOUS_CODING
107	CakSNP107	*Kabuli*	Ca_Kabuli_Chr01	872443	[G/T]	Ca_00106	--	SYNONYMOUS_CODING
108	CakSNP108	*Kabuli*	Ca_Kabuli_Chr01	872452	[C/T]	Ca_00106	--	SYNONYMOUS_CODING
109	CakSNP109	*Kabuli*	Ca_Kabuli_Chr01	876616	[T/C]	Ca_00107	Plant peroxidase	INTRON
110	CakSNP110	*Kabuli*	Ca_Kabuli_Chr01	876633	[T/A]	Ca_00107	Plant peroxidase	INTRON
111	CakSNP111	*Kabuli*	Ca_Kabuli_Chr01	881543	[C/A]	Ca_00108	ANTH	NON_SYNONYMOUS_CODING

S.N.	SNP IDs	Chickpea cultivars	Chromosomes	Physical positions (bp)	SNPs	Gene accession IDs	Putative functions	Sequence components of genome
112	CakSNP112	*Kabuli*	Ca_Kabuli_Chr01	881673	[G/A]	Ca_00108	ANTH	SYNONYMOUS_CODING
113	CakSNP113	*Kabuli*	Ca_Kabuli_Chr01	881703	[G/T]	Ca_00108	ANTH	SYNONYMOUS_CODING
114	CakSNP114	*Kabuli*	Ca_Kabuli_Chr01	890949	[G/A]	Ca_00110	Calcium-binding EF-hand	INTRON
115	CakSNP115	*Kabuli*	Ca_Kabuli_Chr01	890948	[C/T]	Ca_00110	Calcium-binding EF-hand	INTRON
116	CakSNP116	*Kabuli*	Ca_Kabuli_Chr01	910486	[T/A]	Ca_00115	Glycoside hydrolase, family 3, N-terminal	NON_SYNONYMOUS_CODING
117	CakSNP117	*Kabuli*	Ca_Kabuli_Chr01	970315	[A/T]	Ca_00120	Sulphate transporter/antisigma-factor antagonist STAS	SYNONYMOUS_CODING
118	CakSNP118	*Kabuli*	Ca_Kabuli_Chr01	996439	[T/C]	Ca_00123	Chlorophyll A-B binding protein, plant	SYNONYMOUS_CODING
119	CakSNP119	*Kabuli*	Ca_Kabuli_Chr01	1007698	[A/G]	Ca_00127	--	INTRON
120	CakSNP120	*Kabuli*	Ca_Kabuli_Chr01	1008895	[A/G]	Ca_00127	--	SYNONYMOUS_CODING
121	CakSNP121	*Kabuli*	Ca_Kabuli_Chr01	1008971	[C/G]	Ca_00127	--	NON_SYNONYMOUS_CODING

S.N.	SNP IDs	Chickpea cultivars	Chromosomes	Physical positions (bp)	SNPs	Gene accession IDs	Putative functions	Sequence components of genome
122	CakSNP122	*Kabuli*	Ca_Kabuli_Chr01	1009058	[A/C]	Ca_00127	--	NON_SYNONYMOUS_CODING
123	CakSNP123	*Kabuli*	Ca_Kabuli_Chr01	1018966	[C/G]		--	INTERGENIC
124	CakSNP124	*Kabuli*	Ca_Kabuli_Chr01	1020800	[A/G]		--	INTERGENIC
125	CakSNP125	*Kabuli*	Ca_Kabuli_Chr01	1023550	[C/T]		--	INTERGENIC
126	CakSNP126	*Kabuli*	Ca_Kabuli_Chr01	1023538	[T/C]		--	INTERGENIC
127	CakSNP127	*Kabuli*	Ca_Kabuli_Chr01	1032734	[A/T]	Ca_00128	K+ potassium transporter	NON_SYNONYMOUS_CODING
128	CakSNP128	*Kabuli*	Ca_Kabuli_Chr01	1059209	[A/G]	Ca_00129	IQ motif, EF-hand binding site	NON_SYNONYMOUS_CODING
129	CakSNP129	*Kabuli*	Ca_Kabuli_Chr01	1059311	[A/G]	Ca_00129	IQ motif, EF-hand binding site	NON_SYNONYMOUS_CODING
130	CakSNP130	*Kabuli*	Ca_Kabuli_Chr01	1083274	[T/C]	Ca_00134	Peptidase C14, caspase catalytic	NON_SYNONYMOUS_CODING
131	CakSNP131	*Kabuli*	Ca_Kabuli_Chr01	1083388	[G/A]	Ca_00134	Peptidase C14, caspase catalytic	INTRON

S.N.	SNP IDs	Chickpea cultivars	Chromosomes	Physical positions (bp)	SNPs	Gene accession IDs	Putative functions	Sequence components of genome
132	CakSNP132	*Kabuli*	Ca_Kabuli_Chr01	1083403	[C/T]	Ca_00134	Peptidase C14, caspase catalytic	INTRON
133	CakSNP133	*Kabuli*	Ca_Kabuli_Chr01	1085228	[A/C]	Ca_00134	Peptidase C14, caspase catalytic	INTRON
134	CakSNP134	*Kabuli*	Ca_Kabuli_Chr01	1085230	[T/G]	Ca_00134	Peptidase C14, caspase catalytic	INTRON
135	CakSNP135	*Kabuli*	Ca_Kabuli_Chr01	1085238	[A/G]	Ca_00134	Peptidase C14, caspase catalytic	INTRON
136	CakSNP136	*Kabuli*	Ca_Kabuli_Chr01	1085254	[A/C]	Ca_00134	Peptidase C14, caspase catalytic	INTRON
137	CakSNP137	*Kabuli*	Ca_Kabuli_Chr01	1085263	[A/C]	Ca_00134	Peptidase C14, caspase catalytic	INTRON
138	CakSNP138	*Kabuli*	Ca_Kabuli_Chr01	1085293	[G/A]	Ca_00134	Peptidase C14, caspase catalytic	INTRON
139	CakSNP139	*Kabuli*	Ca_Kabuli_Chr01	1085348	[G/A]	Ca_00134	Peptidase C14, caspase catalytic	INTRON
140	CakSNP140	*Kabuli*	Ca_Kabuli_Chr01	1091548	[T/A]	Ca_00135	Methyl-CpG DNA binding	INTRON
141	CakSNP141	*Kabuli*	Ca_Kabuli_Chr01	1091534	[A/C]	Ca_00135	Methyl-CpG DNA binding	INTRON

S.N.	SNP IDs	Chickpea cultivars	Chromosomes	Physical positions (bp)	SNPs	Gene accession IDs	Putative functions	Sequence components of genome
142	CakSNP142	*Kabuli*	Ca_Kabuli_Chr01	1091504	[C/T]	Ca_00135	Methyl-CpG DNA binding	NON_SYNONYMOUS_CODING
143	CakSNP143	*Kabuli*	Ca_Kabuli_Chr01	1098703	[C/G]		--	INTERGENIC
144	CakSNP144	*Kabuli*	Ca_Kabuli_Chr01	1098789	[A/G]		--	INTERGENIC
145	CakSNP145	*Kabuli*	Ca_Kabuli_Chr01	1115668	[C/T]	Ca_00138	HEAT	SYNONYMOUS_CODING
146	CakSNP146	*Kabuli*	Ca_Kabuli_Chr01	1115809	[A/G]	Ca_00138	HEAT	SYNONYMOUS_CODING
147	CakSNP147	*Kabuli*	Ca_Kabuli_Chr01	1116043	[C/T]	Ca_00138	HEAT	SYNONYMOUS_CODING
148	CakSNP148	*Kabuli*	Ca_Kabuli_Chr01	1140313	[A/C]	Ca_00141	Sodium/sulphate symporter	INTRON
149	CakSNP149	*Kabuli*	Ca_Kabuli_Chr01	1140405	[A/T]	Ca_00141	Sodium/sulphate symporter	NON_SYNONYMOUS_CODING
150	CakSNP150	*Kabuli*	Ca_Kabuli_Chr01	1150973	[C/T]	Ca_00143	--	NON_SYNONYMOUS_CODING
151	CakSNP151	*Kabuli*	Ca_Kabuli_Chr01	1159383	[C/A]	Ca_00144	--	NON_SYNONYMOUS_CODING

S.N.	SNP IDs	Chickpea cultivars	Chromosomes	Physical positions (bp)	SNPs	Gene accession IDs	Putative functions	Sequence components of genome
152	CakSNP152	*Kabuli*	Ca_Kabuli_Chr01	1159411	[T/G]	Ca_00144	--	SYNONYMOUS_CODING
153	CakSNP153	*Kabuli*	Ca_Kabuli_Chr01	1215024	[T/C]	Ca_00149	BTB/POZ-like	SYNONYMOUS_CODING
154	CakSNP154	*Kabuli*	Ca_Kabuli_Chr01	1269212	[C/G]	Ca_00155	Peptidase C48, SUMO/Sentrin/Ubl3	NON_SYNONYMOUS_CODING
155	CakSNP155	*Kabuli*	Ca_Kabuli_Chr01	1269180	[C/A]	Ca_00155	Peptidase C48, SUMO/Sentrin/Ubl2	NON_SYNONYMOUS_CODING
156	CakSNP156	*Kabuli*	Ca_Kabuli_Chr01	1278884	[G/T]		--	INTERGENIC
157	CakSNP157	*Kabuli*	Ca_Kabuli_Chr01	1278997	[T/A]		--	INTERGENIC
158	CakSNP158	*Kabuli*	Ca_Kabuli_Chr01	1295527	[T/G]	Ca_00160	--	NON_SYNONYMOUS_CODING
159	CakSNP159	*Kabuli*	Ca_Kabuli_Chr01	1335518	[A/C]		--	INTERGENIC
160	CakSNP160	*Kabuli*	Ca_Kabuli_Chr01	1385553	[C/A]		--	INTERGENIC
161	CakSNP161	*Kabuli*	Ca_Kabuli_Chr01	1385554	[G/T]		--	INTERGENIC

S.N.	SNP IDs	Chickpea cultivars	Chromosomes	Physical positions (bp)	SNPs	Gene accession IDs	Putative functions	Sequence components of genome
162	CakSNP162	*Kabuli*	Ca_Kabuli_Chr01	1390073	[A/G]		--	INTERGENIC
163	CakSNP163	*Kabuli*	Ca_Kabuli_Chr01	1411348	[C/T]		--	INTERGENIC
164	CakSNP164	*Kabuli*	Ca_Kabuli_Chr01	1411351	[A/C]		--	INTERGENIC
165	CakSNP165	*Kabuli*	Ca_Kabuli_Chr01	1411501	[C/T]		--	INTERGENIC
166	CakSNP166	*Kabuli*	Ca_Kabuli_Chr01	1411498	[T/A]		--	INTERGENIC
167	CakSNP167	*Kabuli*	Ca_Kabuli_Chr01	1411495	[A/G]		--	INTERGENIC
168	CakSNP168	*Kabuli*	Ca_Kabuli_Chr01	1411490	[T/C]		--	INTERGENIC
169	CakSNP169	*Kabuli*	Ca_Kabuli_Chr01	1428222	[C/G]	Ca_00174	DNA polymerase V	NON_SYNONYMOUS_CODING
170	CakSNP170	*Kabuli*	Ca_Kabuli_Chr01	1428251	[T/G]	Ca_00174	DNA polymerase V	SYNONYMOUS_CODING
171	CakSNP171	*Kabuli*	Ca_Kabuli_Chr01	1428264	[T/G]	Ca_00174	DNA polymerase V	NON_SYNONYMOUS_CODING

S.N.	SNP IDs	Chickpea cultivars	Chromosomes	Physical positions (bp)	SNPs	Gene accession IDs	Putative functions	Sequence components of genome
172	CakSNP172	*Kabuli*	Ca_Kabuli_Chr01	1434121	[A/G]	Ca_00174	DNA polymerase V	NON_SYNONYMOUS_CODING
173	CakSNP173	*Kabuli*	Ca_Kabuli_Chr01	1434883	[A/G]		--	INTERGENIC
174	CakSNP174	*Kabuli*	Ca_Kabuli_Chr01	1434875	[A/G]		--	INTERGENIC
175	CakSNP175	*Kabuli*	Ca_Kabuli_Chr01	1449848	[G/A]	Ca_00177	Phox/Bem1p	SYNONYMOUS_CODING
176	CakSNP176	*Kabuli*	Ca_Kabuli_Chr01	1449913	[T/G]	Ca_00177	Phox/Bem1p	NON_SYNONYMOUS_CODING
177	CakSNP177	*Kabuli*	Ca_Kabuli_Chr01	1449949	[G/A]	Ca_00177	Phox/Bem1p	NON_SYNONYMOUS_CODING
178	CakSNP178	*Kabuli*	Ca_Kabuli_Chr01	1449980	[A/G]	Ca_00177	Phox/Bem1p	SYNONYMOUS_CODING
179	CakSNP179	*Kabuli*	Ca_Kabuli_Chr01	1461574	[T/A]	Ca_00178	Amidase	INTRON
180	CakSNP180	*Kabuli*	Ca_Kabuli_Chr01	1461600	[T/C]	Ca_00178	Amidase	INTRON
181	CakSNP181	*Kabuli*	Ca_Kabuli_Chr01	1467056	[A/G]	Ca_00180	Zinc finger, RING-type	SYNONYMOUS_CODING

S.N.	SNP IDs	Chickpea cultivars	Chromosomes	Physical positions (bp)	SNPs	Gene accession IDs	Putative functions	Sequence components of genome
182	CakSNP182	*Kabuli*	Ca_Kabuli_Chr01	1527513	[A/G]	Ca_00185	Inorganic pyrophosphatase	INTRON
183	CakSNP183	*Kabuli*	Ca_Kabuli_Chr01	1528325	[G/A]	Ca_00185	Inorganic pyrophosphatase	INTRON
184	CakSNP184	*Kabuli*	Ca_Kabuli_Chr01	1533723	[G/A]	Ca_00186	Helix-loop-helix DNA-binding domain	NON_SYNONYMOUS_CODING
185	CakSNP185	*Kabuli*	Ca_Kabuli_Chr01	1546679	[G/C]	Ca_00187	--	SYNONYMOUS_CODING
186	CakSNP186	*Kabuli*	Ca_Kabuli_Chr01	1599821	[A/G]		--	INTERGENIC
187	CakSNP187	*Kabuli*	Ca_Kabuli_Chr01	1654823	[C/T]	Ca_00203	Kinesin, motor domain	SYNONYMOUS_CODING
188	CakSNP188	*Kabuli*	Ca_Kabuli_Chr01	1654855	[A/C]	Ca_00203	Kinesin, motor domain	INTRON
189	CakSNP189	*Kabuli*	Ca_Kabuli_Chr01	1655018	[G/A]	Ca_00203	Kinesin, motor domain	NON_SYNONYMOUS_CODING
190	CakSNP190	*Kabuli*	Ca_Kabuli_Chr01	1655012	[G/T]	Ca_00203	Kinesin, motor domain	NON_SYNONYMOUS_CODING
191	CakSNP191	*Kabuli*	Ca_Kabuli_Chr01	1693010	[T/A]		--	INTERGENIC

S.N.	SNP IDs	Chickpea cultivars	Chromosomes	Physical positions (bp)	SNPs	Gene accession IDs	Putative functions	Sequence components of genome
192	CakSNP192	*Kabuli*	Ca_Kabuli_Chr01	1693013	[C/T]		--	INTERGENIC
193	CakSNP193	*Kabuli*	Ca_Kabuli_Chr01	1693022	[T/C]		--	INTERGENIC
194	CakSNP194	*Kabuli*	Ca_Kabuli_Chr01	1693023	[T/C]		--	INTERGENIC
195	CakSNP195	*Kabuli*	Ca_Kabuli_Chr01	1693031	[C/A]		--	INTERGENIC
196	CakSNP196	*Kabuli*	Ca_Kabuli_Chr01	1738179	[G/C]		--	INTERGENIC
197	CakSNP197	*Kabuli*	Ca_Kabuli_Chr01	1772813	[T/C]	Ca_00223	Ovarian tumour, otubain	INTRON
198	CakSNP198	*Kabuli*	Ca_Kabuli_Chr01	1782760	[G/A]	Ca_00224	Zinc finger, RING-type	INTRON
199	CakSNP199	*Kabuli*	Ca_Kabuli_Chr01	1782793	[G/T]	Ca_00224	Zinc finger, RING-type	INTRON
200	CakSNP200	*Kabuli*	Ca_Kabuli_Chr01	1782808	[G/C]	Ca_00224	Zinc finger, RING-type	INTRON
201	CakSNP201	*Kabuli*	Ca_Kabuli_Chr01	1782953	[G/A]	Ca_00224	Zinc finger, RING-type	INTRON

S.N.	SNP IDs	Chickpea cultivars	Chromosomes	Physical positions (bp)	SNPs	Gene accession IDs	Putative functions	Sequence components of genome
202	CakSNP202	*Kabuli*	Ca_Kabuli_Chr01	1782948	[T/C]	Ca_00224	Zinc finger, RING-type	INTRON
203	CakSNP203	*Kabuli*	Ca_Kabuli_Chr01	1782930	[G/A]	Ca_00224	Zinc finger, RING-type	INTRON
204	CakSNP204	*Kabuli*	Ca_Kabuli_Chr01	1782929	[C/T]	Ca_00224	Zinc finger, RING-type	INTRON
205	CakSNP205	*Kabuli*	Ca_Kabuli_Chr01	1783207	[G/A]	Ca_00224	Zinc finger, RING-type	INTRON
206	CakSNP206	*Kabuli*	Ca_Kabuli_Chr01	1783277	[G/A]	Ca_00224	Zinc finger, RING-type	INTRON
207	CakSNP207	*Kabuli*	Ca_Kabuli_Chr01	1783280	[A/C]	Ca_00224	Zinc finger, RING-type	INTRON
208	CakSNP208	*Kabuli*	Ca_Kabuli_Chr01	1783342	[A/T]	Ca_00224	Zinc finger, RING-type	INTRON
209	CakSNP209	*Kabuli*	Ca_Kabuli_Chr01	1783923	[C/A]	Ca_00224	Zinc finger, RING-type	NON_SYNONYMOUS_CODING
210	CakSNP210	*Kabuli*	Ca_Kabuli_Chr01	1784006	[T/C]	Ca_00224	Zinc finger, RING-type	SYNONYMOUS_CODING
211	CakSNP211	*Kabuli*	Ca_Kabuli_Chr01	1788958	[T/A]	Ca_00225	Uncharacterised protein family Cys-rich	INTRON

S.N.	SNP IDs	Chickpea cultivars	Chromosomes	Physical positions (bp)	SNPs	Gene accession IDs	Putative functions	Sequence components of genome
212	CakSNP212	*Kabuli*	Ca_Kabuli_Chr01	1789087	[T/C]	Ca_00225	Uncharacterised protein family Cys-rich	INTRON
213	CakSNP213	*Kabuli*	Ca_Kabuli_Chr01	1802992	[C/G]	Ca_00228	Delayed-early response protein/equilibrative nucleoside transporter	SYNONYMOUS_CODING
214	CakSNP214	*Kabuli*	Ca_Kabuli_Chr01	1858073	[C/T]	Ca_00233	Inositol monophosphatase	INTRON
215	CakSNP215	*Kabuli*	Ca_Kabuli_Chr01	1858078	[T/C]	Ca_00233	Inositol monophosphatase	INTRON
216	CakSNP216	*Kabuli*	Ca_Kabuli_Chr01	1858084	[C/T]	Ca_00233	Inositol monophosphatase	INTRON
217	CakSNP217	*Kabuli*	Ca_Kabuli_Chr01	1858132	[T/A]	Ca_00233	Inositol monophosphatase	INTRON
218	CakSNP218	*Kabuli*	Ca_Kabuli_Chr01	1858156	[C/T]	Ca_00233	Inositol monophosphatase	INTRON
219	CakSNP219	*Kabuli*	Ca_Kabuli_Chr01	1858177	[T/C]	Ca_00233	Inositol monophosphatase	SYNONYMOUS_CODING
220	CakSNP220	*Kabuli*	Ca_Kabuli_Chr01	1858159	[A/G]	Ca_00233	Inositol monophosphatase	INTRON
221	CakSNP221	*Kabuli*	Ca_Kabuli_Chr01	1858509	[C/T]	Ca_00233	Inositol monophosphatase	SYNONYMOUS_CODING

S.N.	SNP IDs	Chickpea cultivars	Chromosomes	Physical positions (bp)	SNPs	Gene accession IDs	Putative functions	Sequence components of genome
222	CakSNP222	*Kabuli*	Ca_Kabuli_Chr01	1858608	[T/A]	Ca_00233	Inositol monophosphatase	INTRON
223	CakSNP223	*Kabuli*	Ca_Kabuli_Chr01	1858600	[G/A]	Ca_00233	Inositol monophosphatase	INTRON
224	CakSNP224	*Kabuli*	Ca_Kabuli_Chr01	1859789	[C/T]	Ca_00233	Inositol monophosphatase	INTRON
225	CakSNP225	*Kabuli*	Ca_Kabuli_Chr01	1859857	[A/G]	Ca_00233	Inositol monophosphatase	INTRON
226	CakSNP226	*Kabuli*	Ca_Kabuli_Chr01	1859914	[G/A]	Ca_00233	Inositol monophosphatase	INTRON
227	CakSNP227	*Kabuli*	Ca_Kabuli_Chr01	1859902	[G/A]	Ca_00233	Inositol monophosphatase	INTRON
228	CakSNP228	*Kabuli*	Ca_Kabuli_Chr01	1861696	[C/T]	Ca_00233	Inositol monophosphatase	SYNONYMOUS_CODING
229	CakSNP229	*Kabuli*	Ca_Kabuli_Chr01	1861707	[T/G]	Ca_00233	Inositol monophosphatase	INTRON
230	CakSNP230	*Kabuli*	Ca_Kabuli_Chr01	1861722	[T/A]	Ca_00233	Inositol monophosphatase	INTRON
231	CakSNP231	*Kabuli*	Ca_Kabuli_Chr01	1861729	[T/C]	Ca_00233	Inositol monophosphatase	INTRON

S.N.	SNP IDs	Chickpea cultivars	Chromosomes	Physical positions (bp)	SNPs	Gene accession IDs	Putative functions	Sequence components of genome
232	CakSNP232	*Kabuli*	Ca_Kabuli_Chr01	1861837	[T/G]	Ca_00233	Inositol monophosphatase	SYNONYMOUS_CODING
233	CakSNP233	*Kabuli*	Ca_Kabuli_Chr01	1904233	[T/C]	Ca_00238	Reverse transcriptase	INTRON
234	CakSNP234	*Kabuli*	Ca_Kabuli_Chr01	1904230	[G/T]	Ca_00238	Reverse transcriptase	INTRON
235	CakSNP235	*Kabuli*	Ca_Kabuli_Chr01	1904365	[G/A]	Ca_00238	Reverse transcriptase	INTRON
236	CakSNP236	*Kabuli*	Ca_Kabuli_Chr01	1904391	[A/G]	Ca_00238	Reverse transcriptase	INTRON
237	CakSNP237	*Kabuli*	Ca_Kabuli_Chr01	1935729	[A/G]		--	INTERGENIC
238	CakSNP238	*Kabuli*	Ca_Kabuli_Chr01	1962876	[A/G]	Ca_00244	Transcriptional factor B3	NON_SYNONYMOUS_CODING
239	CakSNP239	*Kabuli*	Ca_Kabuli_Chr01	1968828	[G/A]		--	INTERGENIC
240	CakSNP240	*Kabuli*	Ca_Kabuli_Chr01	2002844	[G/A]	Ca_00251	Ribosomal protein L26/L24P, eukaryotic/archaeal	SYNONYMOUS_CODING
241	CakSNP241	*Kabuli*	Ca_Kabuli_Chr01	2002922	[G/T]	Ca_00251	Ribosomal protein L26/L24P, eukaryotic/archaeal	NON_SYNONYMOUS_CODING

S.N.	SNP IDs	Chickpea cultivars	Chromosomes	Physical positions (bp)	SNPs	Gene accession IDs	Putative functions	Sequence components of genome
242	CakSNP242	*Kabuli*	Ca_Kabuli_Chr01	2040943	[C/T]	Ca_00258	Disease resistance protein	SYNONYMOUS_CODING
243	CakSNP243	*Kabuli*	Ca_Kabuli_Chr01	2040946	[G/T]	Ca_00258	Disease resistance protein	SYNONYMOUS_CODING
244	CakSNP244	*Kabuli*	Ca_Kabuli_Chr01	2041027	[C/G]	Ca_00258	Disease resistance protein	NON_SYNONYMOUS_CODING
245	CakSNP245	*Kabuli*	Ca_Kabuli_Chr01	2041190	[A/C]	Ca_00258	Disease resistance protein	NON_SYNONYMOUS_CODING
246	CakSNP246	*Kabuli*	Ca_Kabuli_Chr01	2041180	[T/C]	Ca_00258	Disease resistance protein	SYNONYMOUS_CODING
247	CakSNP247	*Kabuli*	Ca_Kabuli_Chr01	2041178	[A/C]	Ca_00258	Disease resistance protein	NON_SYNONYMOUS_CODING
248	CakSNP248	*Kabuli*	Ca_Kabuli_Chr01	2041174	[C/T]	Ca_00258	Disease resistance protein	SYNONYMOUS_CODING
249	CakSNP249	*Kabuli*	Ca_Kabuli_Chr01	2041154	[G/C]	Ca_00258	Disease resistance protein	NON_SYNONYMOUS_CODING
250	CakSNP250	*Kabuli*	Ca_Kabuli_Chr01	2041132	[C/T]	Ca_00258	Disease resistance protein	SYNONYMOUS_CODING
251	CakSNP251	*Kabuli*	Ca_Kabuli_Chr01	2042071	[C/G]	Ca_00258	Disease resistance protein	SYNONYMOUS_CODING

S.N.	SNP IDs	Chickpea cultivars	Chromosomes	Physical positions (bp)	SNPs	Gene accession IDs	Putative functions	Sequence components of genome
252	CakSNP252	*Kabuli*	Ca_Kabuli_Chr01	2042185	[T/C]	Ca_00258	Disease resistance protein	SYNONYMOUS_CODING
253	CakSNP253	*Kabuli*	Ca_Kabuli_Chr01	2042152	[G/C]	Ca_00258	Disease resistance protein	NON_SYNONYMOUS_CODING
254	CakSNP254	*Kabuli*	Ca_Kabuli_Chr01	2042149	[G/T]	Ca_00258	Disease resistance protein	NON_SYNONYMOUS_CODING
255	CakSNP255	*Kabuli*	Ca_Kabuli_Chr01	2044338	[G/A]	Ca_00258	Disease resistance protein	NON_SYNONYMOUS_CODING
256	CakSNP256	*Kabuli*	Ca_Kabuli_Chr01	2044304	[A/C]	Ca_00258	Disease resistance protein	NON_SYNONYMOUS_CODING
257	CakSNP257	*Kabuli*	Ca_Kabuli_Chr01	2044289	[A/G]	Ca_00258	Disease resistance protein	NON_SYNONYMOUS_CODING
258	CakSNP258	*Kabuli*	Ca_Kabuli_Chr01	2044263	[A/C]	Ca_00258	Disease resistance protein	SYNONYMOUS_CODING
259	CakSNP259	*Kabuli*	Ca_Kabuli_Chr01	2044359	[C/T]	Ca_00258	Disease resistance protein	SYNONYMOUS_CODING
260	CakSNP260	*Kabuli*	Ca_Kabuli_Chr01	2044374	[C/G]	Ca_00258	Disease resistance protein	NON_SYNONYMOUS_CODING
261	CakSNP261	*Kabuli*	Ca_Kabuli_Chr01	2044380	[G/C]	Ca_00258	Disease resistance protein	NON_SYNONYMOUS_CODING

S.N.	SNP IDs	Chickpea cultivars	Chromosomes	Physical positions (bp)	SNPs	Gene accession IDs	Putative functions	Sequence components of genome
262	CakSNP262	*Kabuli*	Ca_Kabuli_Chr01	2044408	[A/C]	Ca_00258	Disease resistance protein	NON_SYNONYMOUS_CODING
263	CakSNP263	*Kabuli*	Ca_Kabuli_Chr01	2044414	[G/A]	Ca_00258	Disease resistance protein	SYNONYMOUS_CODING
264	CakSNP264	*Kabuli*	Ca_Kabuli_Chr01	2044474	[A/G]	Ca_00258	Disease resistance protein	SYNONYMOUS_CODING
265	CakSNP265	*Kabuli*	Ca_Kabuli_Chr01	2050232	[A/T]	Ca_00260	Disease resistance protein	SYNONYMOUS_CODING
266	CakSNP266	*Kabuli*	Ca_Kabuli_Chr01	2050187	[C/G]		--	INTERGENIC
267	CakSNP267	*Kabuli*	Ca_Kabuli_Chr01	2050479	[A/C]	Ca_00260	Disease resistance protein	NON_SYNONYMOUS_CODING
268	CakSNP268	*Kabuli*	Ca_Kabuli_Chr01	2050469	[T/C]	Ca_00260	Disease resistance protein	SYNONYMOUS_CODING
269	CakSNP269	*Kabuli*	Ca_Kabuli_Chr01	2050467	[A/C]	Ca_00260	Disease resistance protein	NON_SYNONYMOUS_CODING
270	CakSNP270	*Kabuli*	Ca_Kabuli_Chr01	2050465	[A/G]	Ca_00260	Disease resistance protein	NON_SYNONYMOUS_CODING
271	CakSNP271	*Kabuli*	Ca_Kabuli_Chr01	2050463	[C/T]	Ca_00260	Disease resistance protein	SYNONYMOUS_CODING

S.N.	SNP IDs	Chickpea cultivars	Chromosomes	Physical positions (bp)	SNPs	Gene accession IDs	Putative functions	Sequence components of genome
272	CakSNP272	*Kabuli*	Ca_Kabuli_Chr01	2050443	[G/C]	Ca_00260	Disease resistance protein	NON_SYNONYMOUS_CODING
273	CakSNP273	*Kabuli*	Ca_Kabuli_Chr01	2050421	[C/T]	Ca_00260	Disease resistance protein	SYNONYMOUS_CODING
274	CakSNP274	*Kabuli*	Ca_Kabuli_Chr01	2051360	[A/G]	Ca_00260	Disease resistance protein	SYNONYMOUS_CODING
275	CakSNP275	*Kabuli*	Ca_Kabuli_Chr01	2051375	[G/T]	Ca_00260	Disease resistance protein	NON_SYNONYMOUS_CODING
276	CakSNP276	*Kabuli*	Ca_Kabuli_Chr01	2051379	[C/T]	Ca_00260	Disease resistance protein	NON_SYNONYMOUS_CODING
277	CakSNP277	*Kabuli*	Ca_Kabuli_Chr01	2053732	[C/T]	Ca_00260	Disease resistance protein	NON_SYNONYMOUS_CODING
278	CakSNP278	*Kabuli*	Ca_Kabuli_Chr01	2053744	[C/A]	Ca_00260	Disease resistance protein	NON_SYNONYMOUS_CODING
279	CakSNP279	*Kabuli*	Ca_Kabuli_Chr01	2053749	[T/A]	Ca_00260	Disease resistance protein	NON_SYNONYMOUS_CODING
280	CakSNP280	*Kabuli*	Ca_Kabuli_Chr01	2053756	[G/T]	Ca_00260	Disease resistance protein	NON_SYNONYMOUS_CODING
281	CakSNP281	*Kabuli*	Ca_Kabuli_Chr01	2053757	[C/T]	Ca_00260	Disease resistance protein	SYNONYMOUS_CODING

S.N.	SNP IDs	Chickpea cultivars	Chromosomes	Physical positions (bp)	SNPs	Gene accession IDs	Putative functions	Sequence components of genome
282	CakSNP282	*Kabuli*	Ca_Kabuli_Chr01	2053758	[A/G]	Ca_00260	Disease resistance protein	NON_SYNONYMOUS_CODING
283	CakSNP283	*Kabuli*	Ca_Kabuli_Chr01	2053784	[C/A]	Ca_00260	Disease resistance protein	NON_SYNONYMOUS_CODING
284	CakSNP284	*Kabuli*	Ca_Kabuli_Chr01	2053788	[A/G]	Ca_00260	Disease resistance protein	NON_SYNONYMOUS_CODING
285	CakSNP285	*Kabuli*	Ca_Kabuli_Chr01	2053790	[G/A]	Ca_00260	Disease resistance protein	SYNONYMOUS_CODING
286	CakSNP286	*Kabuli*	Ca_Kabuli_Chr01	2053808	[G/T]	Ca_00260	Disease resistance protein	SYNONYMOUS_CODING
287	CakSNP287	*Kabuli*	Ca_Kabuli_Chr01	2053856	[T/G]	Ca_00260	Disease resistance protein	SYNONYMOUS_CODING
288	CakSNP288	*Kabuli*	Ca_Kabuli_Chr01	2053843	[T/A]	Ca_00260	Disease resistance protein	NON_SYNONYMOUS_CODING
289	CakSNP289	*Kabuli*	Ca_Kabuli_Chr01	2053841	[C/A]	Ca_00260	Disease resistance protein	STOP_GAINED
290	CakSNP290	*Kabuli*	Ca_Kabuli_Chr01	2053840	[A/C]	Ca_00260	Disease resistance protein	NON_SYNONYMOUS_CODING
291	CakSNP291	*Kabuli*	Ca_Kabuli_Chr01	2053833	[T/G]	Ca_00260	Disease resistance protein	SYNONYMOUS_CODING

S.N.	SNP IDs	Chickpea cultivars	Chromosomes	Physical positions (bp)	SNPs	Gene accession IDs	Putative functions	Sequence components of genome
292	CakSNP292	*Kabuli*	Ca_Kabuli_Chr01	2053830	[A/G]	Ca_00260	Disease resistance protein	SYNONYMOUS_CODING
293	CakSNP293	*Kabuli*	Ca_Kabuli_Chr01	2053819	[C/A]	Ca_00260	Disease resistance protein	SYNONYMOUS_CODING
294	CakSNP294	*Kabuli*	Ca_Kabuli_Chr01	2053818	[G/C]	Ca_00260	Disease resistance protein	NON_SYNONYMOUS_CODING
295	CakSNP295	*Kabuli*	Ca_Kabuli_Chr01	2071456	[T/A]		--	INTERGENIC
296	CakSNP296	*Kabuli*	Ca_Kabuli_Chr01	2071448	[A/T]		--	INTERGENIC
297	CakSNP297	*Kabuli*	Ca_Kabuli_Chr01	2071424	[T/A]		--	INTERGENIC
298	CakSNP298	*Kabuli*	Ca_Kabuli_Chr01	2071708	[C/G]		--	INTERGENIC
299	CakSNP299	*Kabuli*	Ca_Kabuli_Chr01	2071703	[A/C]		--	INTERGENIC
300	CakSNP300	*Kabuli*	Ca_Kabuli_Chr01	2071699	[T/C]		--	INTERGENIC
301	CakSNP301	*Kabuli*	Ca_Kabuli_Chr01	2071693	[T/C]		--	INTERGENIC

S.N.	SNP IDs	Chickpea cultivars	Chromosomes	Physical positions (bp)	SNPs	Gene accession IDs	Putative functions	Sequence components of genome
302	CakSNP302	*Kabuli*	Ca_Kabuli_Chr01	2071691	[A/C]		--	INTERGENIC
303	CakSNP303	*Kabuli*	Ca_Kabuli_Chr01	2071689	[A/G]		--	INTERGENIC
304	CakSNP304	*Kabuli*	Ca_Kabuli_Chr01	2071687	[C/T]		--	INTERGENIC
305	CakSNP305	*Kabuli*	Ca_Kabuli_Chr01	2071667	[G/C]		--	INTERGENIC
306	CakSNP306	*Kabuli*	Ca_Kabuli_Chr01	2071645	[C/T]		--	INTERGENIC
307	CakSNP307	*Kabuli*	Ca_Kabuli_Chr01	2072613	[G/A]	Ca_00263	Disease resistance protein	NON_SYNONYMOUS_CODING
308	CakSNP308	*Kabuli*	Ca_Kabuli_Chr01	2072627	[A/T]	Ca_00263	Disease resistance protein	NON_SYNONYMOUS_CODING
309	CakSNP309	*Kabuli*	Ca_Kabuli_Chr01	2074998	[A/C]	Ca_00264	--	NON_SYNONYMOUS_CODING
310	CakSNP310	*Kabuli*	Ca_Kabuli_Chr01	2075087	[A/T]	Ca_00264	--	SYNONYMOUS_CODING
311	CakSNP311	*Kabuli*	Ca_Kabuli_Chr01	2075057	[G/T]	Ca_00264	--	NON_SYNONYMOUS_CODING

S.N.	SNP IDs	Chickpea cultivars	Chromosomes	Physical positions (bp)	SNPs	Gene accession IDs	Putative functions	Sequence components of genome
312	CakSNP312	*Kabuli*	Ca_Kabuli_Chr01	2075045	[C/A]	Ca_00264	--	NON_SYNONYMOUS_CODING
313	CakSNP313	*Kabuli*	Ca_Kabuli_Chr01	2075044	[A/G]	Ca_00264	--	NON_SYNONYMOUS_CODING
314	CakSNP314	*Kabuli*	Ca_Kabuli_Chr01	2075043	[C/A]	Ca_00264	--	NON_SYNONYMOUS_CODING
315	CakSNP315	*Kabuli*	Ca_Kabuli_Chr01	2075015	[G/A]	Ca_00264	--	SYNONYMOUS_CODING
316	CakSNP316	*Kabuli*	Ca_Kabuli_Chr01	2075013	[A/G]	Ca_00264	--	NON_SYNONYMOUS_CODING
317	CakSNP317	*Kabuli*	Ca_Kabuli_Chr01	2075009	[C/A]	Ca_00264	--	NON_SYNONYMOUS_CODING
318	CakSNP318	*Kabuli*	Ca_Kabuli_Chr01	2082760	[C/G]	Ca_00265	Protein of unknown function DUF707	INTRON
319	CakSNP319	*Kabuli*	Ca_Kabuli_Chr01	2082801	[G/A]	Ca_00265	Protein of unknown function DUF707	INTRON
320	CakSNP320	*Kabuli*	Ca_Kabuli_Chr01	2083003	[G/C]	Ca_00265	Protein of unknown function DUF707	INTRON
321	CakSNP321	*Kabuli*	Ca_Kabuli_Chr01	2083059	[A/T]	Ca_00265	Protein of unknown function DUF707	INTRON

S.N.	SNP IDs	Chickpea cultivars	Chromosomes	Physical positions (bp)	SNPs	Gene accession IDs	Putative functions	Sequence components of genome
322	CakSNP322	*Kabuli*	Ca_Kabuli_Chr01	2126267	[G/A]	Ca_00268	Protein kinase, catalytic domain	INTRON
323	CakSNP323	*Kabuli*	Ca_Kabuli_Chr01	2148583	[T/G]		--	INTERGENIC
324	CakSNP324	*Kabuli*	Ca_Kabuli_Chr01	2170090	[G/C]	Ca_00272	--	NON_SYNONYMOUS_CODING
325	CakSNP325	*Kabuli*	Ca_Kabuli_Chr01	2170935	[A/C]		--	INTERGENIC
326	CakSNP326	*Kabuli*	Ca_Kabuli_Chr01	2172076	[T/C]	Ca_00273	LisH dimerisation motif	INTRON
327	CakSNP327	*Kabuli*	Ca_Kabuli_Chr01	2172113	[G/A]	Ca_00273	LisH dimerisation motif	INTRON
328	CakSNP328	*Kabuli*	Ca_Kabuli_Chr01	2172143	[T/G]	Ca_00273	LisH dimerisation motif	INTRON
329	CakSNP329	*Kabuli*	Ca_Kabuli_Chr01	2172149	[G/A]	Ca_00273	LisH dimerisation motif	INTRON
330	CakSNP330	*Kabuli*	Ca_Kabuli_Chr01	2183544	[A/G]	Ca_00274	SNF2-related	INTRON
331	CakSNP331	*Kabuli*	Ca_Kabuli_Chr01	2188472	[T/C]	Ca_00274	SNF2-related	NON_SYNONYMOUS_CODING

S.N.	SNP IDs	Chickpea cultivars	Chromosomes	Physical positions (bp)	SNPs	Gene accession IDs	Putative functions	Sequence components of genome
332	CakSNP332	*Kabuli*	Ca_Kabuli_Chr01	2232724	[A/G]	Ca_00279	Amidohydrolase 1	INTRON
333	CakSNP333	*Kabuli*	Ca_Kabuli_Chr01	2232764	[C/T]	Ca_00279	Amidohydrolase 1	INTRON
334	CakSNP334	*Kabuli*	Ca_Kabuli_Chr01	2232779	[A/G]	Ca_00279	Amidohydrolase 1	INTRON
335	CakSNP335	*Kabuli*	Ca_Kabuli_Chr01	2252270	[C/T]	Ca_00281	Zinc finger, RING-type	INTRON
336	CakSNP336	*Kabuli*	Ca_Kabuli_Chr01	2252320	[G/T]	Ca_00281	Zinc finger, RING-type	INTRON
337	CakSNP337	*Kabuli*	Ca_Kabuli_Chr01	2252328	[C/A]	Ca_00281	Zinc finger, RING-type	INTRON
338	CakSNP338	*Kabuli*	Ca_Kabuli_Chr01	2252342	[C/T]	Ca_00281	Zinc finger, RING-type	INTRON
339	CakSNP339	*Kabuli*	Ca_Kabuli_Chr01	2252343	[G/T]	Ca_00281	Zinc finger, RING-type	INTRON
340	CakSNP340	*Kabuli*	Ca_Kabuli_Chr01	2252463	[T/C]	Ca_00281	Zinc finger, RING-type	INTRON
341	CakSNP341	*Kabuli*	Ca_Kabuli_Chr01	2252401	[A/G]	Ca_00281	Zinc finger, RING-type	INTRON

S.N.	SNP IDs	Chickpea cultivars	Chromosomes	Physical positions (bp)	SNPs	Gene accession IDs	Putative functions	Sequence components of genome
342	CakSNP342	*Kabuli*	Ca_Kabuli_Chr01	2252496	[C/A]	Ca_00281	Zinc finger, RING-type	INTRON
343	CakSNP343	*Kabuli*	Ca_Kabuli_Chr01	2252513	[C/T]	Ca_00281	Zinc finger, RING-type	INTRON
344	CakSNP344	*Kabuli*	Ca_Kabuli_Chr01	2269319	[T/G]	Ca_00284	TPP-binding enzyme, conserved site	SYNONYMOUS_CODING
345	CakSNP345	*Kabuli*	Ca_Kabuli_Chr01	2269489	[A/C]	Ca_00284	TPP-binding enzyme, conserved site	NON_SYNONYMOUS_CODING
346	CakSNP346	*Kabuli*	Ca_Kabuli_Chr01	2277459	[G/A]	Ca_00285	RNA recognition motif domain	INTRON
347	CakSNP347	*Kabuli*	Ca_Kabuli_Chr01	2285879	[G/A]		--	INTERGENIC
348	CakSNP348	*Kabuli*	Ca_Kabuli_Chr01	2287408	[A/T]	Ca_00287	--	NON_SYNONYMOUS_CODING
349	CakSNP349	*Kabuli*	Ca_Kabuli_Chr01	2287478	[A/T]	Ca_00287	--	SYNONYMOUS_CODING
350	CakSNP350	*Kabuli*	Ca_Kabuli_Chr01	2366413	[T/C]	Ca_00300	Crotonase, core	SYNONYMOUS_CODING
351	CakSNP351	*Kabuli*	Ca_Kabuli_Chr01	2366409	[T/A]	Ca_00300	Crotonase, core	NON_SYNONYMOUS_CODING

S.N.	SNP IDs	Chickpea cultivars	Chromosomes	Physical positions (bp)	SNPs	Gene accession IDs	Putative functions	Sequence components of genome
352	CakSNP352	*Kabuli*	Ca_Kabuli_Chr01	2373166	[A/G]	Ca_00301	Ribosomal RNA methyltransferase RrmJ/FtsJ	INTRON
353	CakSNP353	*Kabuli*	Ca_Kabuli_Chr01	2373862	[G/A]	Ca_00301	Ribosomal RNA methyltransferase RrmJ/FtsJ	INTRON
354	CakSNP354	*Kabuli*	Ca_Kabuli_Chr01	2373910	[C/T]	Ca_00301	Ribosomal RNA methyltransferase RrmJ/FtsJ	INTRON
355	CakSNP355	*Kabuli*	Ca_Kabuli_Chr01	2373954	[G/T]	Ca_00301	Ribosomal RNA methyltransferase RrmJ/FtsJ	INTRON
356	CakSNP356	*Kabuli*	Ca_Kabuli_Chr01	2395842	[A/C]		--	INTERGENIC
357	CakSNP357	*Kabuli*	Ca_Kabuli_Chr01	2400799	[T/G]	Ca_00306	Ubiquitin-conjugating enzyme, E2	NON_SYNONYMOUS_CODING
358	CakSNP358	*Kabuli*	Ca_Kabuli_Chr01	2400892	[C/T]	Ca_00306	Ubiquitin-conjugating enzyme, E2	SYNONYMOUS_CODING
359	CakSNP359	*Kabuli*	Ca_Kabuli_Chr01	2428531	[A/G]		--	INTERGENIC
360	CakSNP360	*Kabuli*	Ca_Kabuli_Chr01	2439915	[A/G]		--	INTERGENIC
361	CakSNP361	*Kabuli*	Ca_Kabuli_Chr01	2439917	[G/A]		--	INTERGENIC

S.N.	SNP IDs	Chickpea cultivars	Chromosomes	Physical positions (bp)	SNPs	Gene accession IDs	Putative functions	Sequence components of genome
362	CakSNP362	*Kabuli*	Ca_Kabuli_Chr01	2473309	[T/C]		--	INTERGENIC
363	CakSNP363	*Kabuli*	Ca_Kabuli_Chr01	2513476	[T/A]	Ca_00314	Telomerase activating protein Est1	SYNONYMOUS_CODING
364	CakSNP364	*Kabuli*	Ca_Kabuli_Chr01	2513516	[T/A]	Ca_00314	Telomerase activating protein Est1	NON_SYNONYMOUS_CODING
365	CakSNP365	*Kabuli*	Ca_Kabuli_Chr01	2513535	[T/C]	Ca_00314	Telomerase activating protein Est1	NON_SYNONYMOUS_CODING
366	CakSNP366	*Kabuli*	Ca_Kabuli_Chr01	2513737	[T/C]	Ca_00314	Telomerase activating protein Est1	SYNONYMOUS_CODING
367	CakSNP367	*Kabuli*	Ca_Kabuli_Chr01	2513860	[G/A]	Ca_00314	Telomerase activating protein Est1	SYNONYMOUS_CODING
368	CakSNP368	*Kabuli*	Ca_Kabuli_Chr01	2513854	[G/A]	Ca_00314	Telomerase activating protein Est1	SYNONYMOUS_CODING
369	CakSNP369	*Kabuli*	Ca_Kabuli_Chr01	2513792	[A/G]	Ca_00314	Telomerase activating protein Est1	NON_SYNONYMOUS_CODING
370	CakSNP370	*Kabuli*	Ca_Kabuli_Chr01	2513914	[T/C]	Ca_00314	Telomerase activating protein Est1	SYNONYMOUS_CODING
371	CakSNP371	*Kabuli*	Ca_Kabuli_Chr01	2542652	[G/A]	Ca_00317	--	NON_SYNONYMOUS_CODING

S.N.	SNP IDs	Chickpea cultivars	Chromosomes	Physical positions (bp)	SNPs	Gene accession IDs	Putative functions	Sequence components of genome
372	CakSNP372	*Kabuli*	Ca_Kabuli_Chr01	2649822	[G/C]	Ca_00326	Pathogenesis-related transcriptional factor/ERF, DNA-binding	SYNONYMOUS_CODING
373	CakSNP373	*Kabuli*	Ca_Kabuli_Chr01	2649864	[C/A]	Ca_00326	Pathogenesis-related transcriptional factor/ERF, DNA-binding	SYNONYMOUS_CODING
374	CakSNP374	*Kabuli*	Ca_Kabuli_Chr01	2649921	[T/C]	Ca_00326	Pathogenesis-related transcriptional factor/ERF, DNA-binding	SYNONYMOUS_CODING
375	CakSNP375	*Kabuli*	Ca_Kabuli_Chr01	2649906	[C/T]	Ca_00326	Pathogenesis-related transcriptional factor/ERF, DNA-binding	SYNONYMOUS_CODING
376	CakSNP376	*Kabuli*	Ca_Kabuli_Chr01	2649974	[A/C]	Ca_00326	Pathogenesis-related transcriptional factor/ERF, DNA-binding	SYNONYMOUS_CODING
377	CakSNP377	*Kabuli*	Ca_Kabuli_Chr01	2671403	[G/T]	Ca_00327	Pathogenesis-related transcriptional factor/ERF, DNA-binding	SYNONYMOUS_CODING
378	CakSNP378	*Kabuli*	Ca_Kabuli_Chr01	2694584	[T/C]	Ca_00330	--	SYNONYMOUS_CODING
379	CakSNP379	*Kabuli*	Ca_Kabuli_Chr01	2745499	[C/A]		--	INTERGENIC
380	CakSNP380	*Kabuli*	Ca_Kabuli_Chr01	2745514	[A/C]		--	INTERGENIC
381	CakSNP381	*Kabuli*	Ca_Kabuli_Chr01	2745519	[G/C]		--	INTERGENIC

S.N.	SNP IDs	Chickpea cultivars	Chromosomes	Physical positions (bp)	SNPs	Gene accession IDs	Putative functions	Sequence components of genome
382	CakSNP382	*Kabuli*	Ca_Kabuli_Chr01	2745486	[C/T]		--	INTERGENIC
383	CakSNP383	*Kabuli*	Ca_Kabuli_Chr01	2754998	[C/T]	Ca_00340	Disease resistance protein	SYNONYMOUS_CODING
384	CakSNP384	*Kabuli*	Ca_Kabuli_Chr01	2755012	[C/T]	Ca_00340	Disease resistance protein	SYNONYMOUS_CODING
385	CakSNP385	*Kabuli*	Ca_Kabuli_Chr01	2755013	[T/A]	Ca_00340	Disease resistance protein	SYNONYMOUS_CODING
386	CakSNP386	*Kabuli*	Ca_Kabuli_Chr01	2755015	[T/C]	Ca_00340	Disease resistance protein	SYNONYMOUS_CODING
387	CakSNP387	*Kabuli*	Ca_Kabuli_Chr01	2755443	[T/A]	Ca_00340	Disease resistance protein	NON_SYNONYMOUS_CODING
388	CakSNP388	*Kabuli*	Ca_Kabuli_Chr01	2755455	[G/A]	Ca_00340	Disease resistance protein	SYNONYMOUS_CODING
389	CakSNP389	*Kabuli*	Ca_Kabuli_Chr01	2755457	[C/T]	Ca_00340	Disease resistance protein	NON_SYNONYMOUS_CODING
390	CakSNP390	*Kabuli*	Ca_Kabuli_Chr01	2755459	[T/G]	Ca_00340	Disease resistance protein	SYNONYMOUS_CODING
391	CakSNP391	*Kabuli*	Ca_Kabuli_Chr01	2755465	[T/A]	Ca_00340	Disease resistance protein	SYNONYMOUS_CODING

S.N.	SNP IDs	Chickpea cultivars	Chromosomes	Physical positions (bp)	SNPs	Gene accession IDs	Putative functions	Sequence components of genome
392	CakSNP392	*Kabuli*	Ca_Kabuli_Chr01	2755466	[C/G]	Ca_00340	Disease resistance protein	SYNONYMOUS_CODING
393	CakSNP393	*Kabuli*	Ca_Kabuli_Chr01	2755470	[A/T]	Ca_00340	Disease resistance protein	SYNONYMOUS_CODING
394	CakSNP394	*Kabuli*	Ca_Kabuli_Chr01	2755471	[C/A]	Ca_00340	Disease resistance protein	SYNONYMOUS_CODING
395	CakSNP395	*Kabuli*	Ca_Kabuli_Chr01	2755489	[G/A]	Ca_00340	Disease resistance protein	NON_SYNONYMOUS_CODING
396	CakSNP396	*Kabuli*	Ca_Kabuli_Chr01	2755494	[T/C]	Ca_00340	Disease resistance protein	SYNONYMOUS_CODING
397	CakSNP397	*Kabuli*	Ca_Kabuli_Chr01	2755496	[A/G]	Ca_00340	Disease resistance protein	NON_SYNONYMOUS_CODING
398	CakSNP398	*Kabuli*	Ca_Kabuli_Chr01	2755613	[G/T]	Ca_00340	Disease resistance protein	NON_SYNONYMOUS_CODING
399	CakSNP399	*Kabuli*	Ca_Kabuli_Chr01	2755604	[A/C]	Ca_00340	Disease resistance protein	NON_SYNONYMOUS_CODING
400	CakSNP400	*Kabuli*	Ca_Kabuli_Chr01	2755596	[C/A]	Ca_00340	Disease resistance protein	NON_SYNONYMOUS_CODING
401	CakSNP401	*Kabuli*	Ca_Kabuli_Chr01	2757711	[T/G]	Ca_00340	Disease resistance protein	SYNONYMOUS_CODING

S.N.	SNP IDs	Chickpea cultivars	Chromosomes	Physical positions (bp)	SNPs	Gene accession IDs	Putative functions	Sequence components of genome
402	CakSNP402	*Kabuli*	Ca_Kabuli_Chr01	2757696	[A/G]	Ca_00340	Disease resistance protein	SYNONYMOUS_CODING
403	CakSNP403	*Kabuli*	Ca_Kabuli_Chr01	2757693	[C/T]	Ca_00340	Disease resistance protein	SYNONYMOUS_CODING
404	CakSNP404	*Kabuli*	Ca_Kabuli_Chr01	2757678	[A/G]	Ca_00340	Disease resistance protein	SYNONYMOUS_CODING
405	CakSNP405	*Kabuli*	Ca_Kabuli_Chr01	2758781	[G/C]	Ca_00340	Disease resistance protein	NON_SYNONYMOUS_CODING
406	CakSNP406	*Kabuli*	Ca_Kabuli_Chr01	2758759	[G/C]	Ca_00340	Disease resistance protein	NON_SYNONYMOUS_CODING
407	CakSNP407	*Kabuli*	Ca_Kabuli_Chr01	2758750	[A/G]	Ca_00340	Disease resistance protein	NON_SYNONYMOUS_CODING
408	CakSNP408	*Kabuli*	Ca_Kabuli_Chr01	2758746	[G/A]	Ca_00340	Disease resistance protein	SYNONYMOUS_CODING
409	CakSNP409	*Kabuli*	Ca_Kabuli_Chr01	2758739	[A/T]	Ca_00340	Disease resistance protein	NON_SYNONYMOUS_CODING
410	CakSNP410	*Kabuli*	Ca_Kabuli_Chr01	2758738	[T/G]	Ca_00340	Disease resistance protein	NON_SYNONYMOUS_CODING
411	CakSNP411	*Kabuli*	Ca_Kabuli_Chr01	2758859	[A/T]		--	INTERGENIC

S.N.	SNP IDs	Chickpea cultivars	Chromosomes	Physical positions (bp)	SNPs	Gene accession IDs	Putative functions	Sequence components of genome
412	CakSNP412	*Kabuli*	Ca_Kabuli_Chr01	2758835	[T/A]		--	INTERGENIC
413	CakSNP413	*Kabuli*	Ca_Kabuli_Chr01	2758827	[A/T]	Ca_00340	Disease resistance protein	NON_SYNONYMOUS_CODING
414	CakSNP414	*Kabuli*	Ca_Kabuli_Chr01	2787006	[C/T]	Ca_00342	tRNA methyltransferase complex GCD14 subunit	SYNONYMOUS_CODING
415	CakSNP415	*Kabuli*	Ca_Kabuli_Chr01	2786994	[A/C]	Ca_00342	tRNA methyltransferase complex GCD14 subunit	SYNONYMOUS_CODING
416	CakSNP416	*Kabuli*	Ca_Kabuli_Chr01	2786989	[T/C]	Ca_00342	tRNA methyltransferase complex GCD14 subunit	NON_SYNONYMOUS_CODING
417	CakSNP417	*Kabuli*	Ca_Kabuli_Chr01	2786976	[A/C]	Ca_00342	tRNA methyltransferase complex GCD14 subunit	SYNONYMOUS_CODING
418	CakSNP418	*Kabuli*	Ca_Kabuli_Chr01	2786974	[T/C]	Ca_00342	tRNA methyltransferase complex GCD14 subunit	NON_SYNONYMOUS_CODING
419	CakSNP419	*Kabuli*	Ca_Kabuli_Chr01	2786970	[T/A]	Ca_00342	tRNA methyltransferase complex GCD14 subunit	NON_SYNONYMOUS_CODING
420	CakSNP420	*Kabuli*	Ca_Kabuli_Chr01	2807605	[G/C]	Ca_00345	--	NON_SYNONYMOUS_CODING
421	CakSNP421	*Kabuli*	Ca_Kabuli_Chr01	2807616	[G/A]	Ca_00345	--	NON_SYNONYMOUS_CODING

S.N.	SNP IDs	Chickpea cultivars	Chromosomes	Physical positions (bp)	SNPs	Gene accession IDs	Putative functions	Sequence components of genome
422	CakSNP422	*Kabuli*	Ca_Kabuli_Chr01	2808197	[T/C]	Ca_00345	--	SYNONYMOUS_CODING
423	CakSNP423	*Kabuli*	Ca_Kabuli_Chr01	2808170	[T/G]	Ca_00345	--	SYNONYMOUS_CODING
424	CakSNP424	*Kabuli*	Ca_Kabuli_Chr01	2832792	[G/A]		--	INTERGENIC
425	CakSNP425	*Kabuli*	Ca_Kabuli_Chr01	2832969	[G/A]	Ca_00347	Transcriptional factor B3	SYNONYMOUS_CODING
426	CakSNP426	*Kabuli*	Ca_Kabuli_Chr01	2832939	[G/T]	Ca_00347	Transcriptional factor B3	NON_SYNONYMOUS_CODING
427	CakSNP427	*Kabuli*	Ca_Kabuli_Chr01	2833448	[C/A]	Ca_00347	Transcriptional factor B3	NON_SYNONYMOUS_CODING
428	CakSNP428	*Kabuli*	Ca_Kabuli_Chr01	2855919	[T/G]		--	INTERGENIC
429	CakSNP429	*Kabuli*	Ca_Kabuli_Chr01	2855926	[T/A]		--	INTERGENIC
430	CakSNP430	*Kabuli*	Ca_Kabuli_Chr01	2855927	[T/A]		--	INTERGENIC
431	CakSNP431	*Kabuli*	Ca_Kabuli_Chr01	2855941	[T/G]		--	INTERGENIC

S.N.	SNP IDs	Chickpea cultivars	Chromosomes	Physical positions (bp)	SNPs	Gene accession IDs	Putative functions	Sequence components of genome
432	CakSNP432	*Kabuli*	Ca_Kabuli_Chr01	2855962	[A/G]		--	INTERGENIC
433	CakSNP433	*Kabuli*	Ca_Kabuli_Chr01	2855969	[C/T]		--	INTERGENIC
434	CakSNP434	*Kabuli*	Ca_Kabuli_Chr01	2856003	[T/C]		--	INTERGENIC
435	CakSNP435	*Kabuli*	Ca_Kabuli_Chr01	2856045	[C/T]		--	INTERGENIC
436	CakSNP436	*Kabuli*	Ca_Kabuli_Chr01	2856079	[T/G]		--	INTERGENIC
437	CakSNP437	*Kabuli*	Ca_Kabuli_Chr01	2856094	[T/G]		--	INTERGENIC
438	CakSNP438	*Kabuli*	Ca_Kabuli_Chr01	2856120	[T/G]		--	INTERGENIC
439	CakSNP439	*Kabuli*	Ca_Kabuli_Chr01	2856534	[T/A]		--	INTERGENIC
440	CakSNP440	*Kabuli*	Ca_Kabuli_Chr01	2858263	[A/G]		--	INTERGENIC
441	CakSNP441	*Kabuli*	Ca_Kabuli_Chr01	2858278	[T/A]		--	INTERGENIC

S.N.	SNP IDs	Chickpea cultivars	Chromosomes	Physical positions (bp)	SNPs	Gene accession IDs	Putative functions	Sequence components of genome
442	CakSNP442	*Kabuli*	Ca_Kabuli_Chr01	2858287	[G/T]		--	INTERGENIC
443	CakSNP443	*Kabuli*	Ca_Kabuli_Chr01	2858299	[C/A]		--	INTERGENIC
444	CakSNP444	*Kabuli*	Ca_Kabuli_Chr01	2868942	[G/A]	Ca_00349	--	INTRON
445	CakSNP445	*Kabuli*	Ca_Kabuli_Chr01	2881315	[T/C]	Ca_00351	Disease resistance protein	SYNONYMOUS_CODING
446	CakSNP446	*Kabuli*	Ca_Kabuli_Chr01	2881321	[C/A]	Ca_00351	Disease resistance protein	SYNONYMOUS_CODING
447	CakSNP447	*Kabuli*	Ca_Kabuli_Chr01	2881323	[A/T]	Ca_00351	Disease resistance protein	SYNONYMOUS_CODING
448	CakSNP448	*Kabuli*	Ca_Kabuli_Chr01	2881327	[C/T]	Ca_00351	Disease resistance protein	NON_SYNONYMOUS_CODING
449	CakSNP449	*Kabuli*	Ca_Kabuli_Chr01	2881356	[C/A]	Ca_00351	Disease resistance protein	SYNONYMOUS_CODING
450	CakSNP450	*Kabuli*	Ca_Kabuli_Chr01	2881357	[A/T]	Ca_00351	Disease resistance protein	NON_SYNONYMOUS_CODING
451	CakSNP451	*Kabuli*	Ca_Kabuli_Chr01	2881422	[T/C]	Ca_00351	Disease resistance protein	SYNONYMOUS_CODING

S.N.	SNP IDs	Chickpea cultivars	Chromosomes	Physical positions (bp)	SNPs	Gene accession IDs	Putative functions	Sequence components of genome
452	CakSNP452	*Kabuli*	Ca_Kabuli_Chr01	2881389	[G/C]	Ca_00351	Disease resistance protein	NON_SYNONYMOUS_CODING
453	CakSNP453	*Kabuli*	Ca_Kabuli_Chr01	2881386	[G/T]	Ca_00351	Disease resistance protein	NON_SYNONYMOUS_CODING
454	CakSNP454	*Kabuli*	Ca_Kabuli_Chr01	2883625	[T/A]	Ca_00351	Disease resistance protein	STOP_GAINED
455	CakSNP455	*Kabuli*	Ca_Kabuli_Chr01	2883634	[T/A]		--	INTERGENIC
456	CakSNP456	*Kabuli*	Ca_Kabuli_Chr01	2883635	[A/G]		--	INTERGENIC
457	CakSNP457	*Kabuli*	Ca_Kabuli_Chr01	2883660	[C/G]		--	INTERGENIC
458	CakSNP458	*Kabuli*	Ca_Kabuli_Chr01	2883666	[G/A]		--	INTERGENIC
459	CakSNP459	*Kabuli*	Ca_Kabuli_Chr01	2883684	[G/T]		--	INTERGENIC
460	CakSNP460	*Kabuli*	Ca_Kabuli_Chr01	2883717	[C/A]		--	INTERGENIC
461	CakSNP461	*Kabuli*	Ca_Kabuli_Chr01	2883716	[A/C]		--	INTERGENIC

S.N.	SNP IDs	Chickpea cultivars	Chromosomes	Physical positions (bp)	SNPs	Gene accession IDs	Putative functions	Sequence components of genome
462	CakSNP462	*Kabuli*	Ca_Kabuli_Chr01	2883709	[A/G]		--	INTERGENIC
463	CakSNP463	*Kabuli*	Ca_Kabuli_Chr01	2885508	[T/C]		--	INTERGENIC
464	CakSNP464	*Kabuli*	Ca_Kabuli_Chr01	2885525	[T/C]		--	INTERGENIC
465	CakSNP465	*Kabuli*	Ca_Kabuli_Chr01	2885531	[C/T]		--	INTERGENIC
466	CakSNP466	*Kabuli*	Ca_Kabuli_Chr01	2885626	[A/G]		--	INTERGENIC
467	CakSNP467	*Kabuli*	Ca_Kabuli_Chr01	2885673	[G/C]		--	INTERGENIC
468	CakSNP468	*Kabuli*	Ca_Kabuli_Chr01	2885892	[T/A]		--	INTERGENIC
469	CakSNP469	*Kabuli*	Ca_Kabuli_Chr01	2885906	[C/T]		--	INTERGENIC
470	CakSNP470	*Kabuli*	Ca_Kabuli_Chr01	2885923	[C/G]		--	INTERGENIC
471	CakSNP471	*Kabuli*	Ca_Kabuli_Chr01	2886025	[A/G]		--	INTERGENIC

S.N.	SNP IDs	Chickpea cultivars	Chromosomes	Physical positions (bp)	SNPs	Gene accession IDs	Putative functions	Sequence components of genome
472	CakSNP472	*Kabuli*	Ca_Kabuli_Chr01	2886020	[G/T]		--	INTERGENIC
473	CakSNP473	*Kabuli*	Ca_Kabuli_Chr01	2886015	[G/A]		--	INTERGENIC
474	CakSNP474	*Kabuli*	Ca_Kabuli_Chr01	2886005	[G/A]		--	INTERGENIC
475	CakSNP475	*Kabuli*	Ca_Kabuli_Chr01	2885997	[G/A]		--	INTERGENIC
476	CakSNP476	*Kabuli*	Ca_Kabuli_Chr01	2885988	[C/G]		--	INTERGENIC
477	CakSNP477	*Kabuli*	Ca_Kabuli_Chr01	2885987	[T/A]		--	INTERGENIC
478	CakSNP478	*Kabuli*	Ca_Kabuli_Chr01	2948211	[C/T]	Ca_00359	Pathogenesis-related transcriptional factor/ERF, DNA-binding	SYNONYMOUS_CODING
479	CakSNP479	*Kabuli*	Ca_Kabuli_Chr01	3009417	[C/T]		--	INTERGENIC
480	CakSNP480	*Kabuli*	Ca_Kabuli_Chr01	3032061	[C/A]	Ca_00368	NUDIX hydrolase domain	NON_SYNONYMOUS_CODING
481	CakSNP481	*Kabuli*	Ca_Kabuli_Chr01	3081723	[G/T]	Ca_00376	Lipase, GDSL	SYNONYMOUS_CODING

S.N.	SNP IDs	Chickpea cultivars	Chromosomes	Physical positions (bp)	SNPs	Gene accession IDs	Putative functions	Sequence components of genome
482	CakSNP482	*Kabuli*	Ca_Kabuli_Chr01	3081851	[T/G]	Ca_00376	Lipase, GDSL	INTRON
483	CakSNP483	*Kabuli*	Ca_Kabuli_Chr01	3081850	[C/A]	Ca_00376	Lipase, GDSL	INTRON
484	CakSNP484	*Kabuli*	Ca_Kabuli_Chr01	3082280	[G/A]	Ca_00376	Lipase, GDSL	SYNONYMOUS_CODING
485	CakSNP485	*Kabuli*	Ca_Kabuli_Chr01	3151007	[G/A]		--	INTERGENIC
486	CakSNP486	*Kabuli*	Ca_Kabuli_Chr01	3150986	[T/C]		--	INTERGENIC
487	CakSNP487	*Kabuli*	Ca_Kabuli_Chr01	3150964	[C/G]		--	INTERGENIC
488	CakSNP488	*Kabuli*	Ca_Kabuli_Chr01	3259114	[G/A]	Ca_00397	Granulin	INTRON
489	CakSNP489	*Kabuli*	Ca_Kabuli_Chr01	3259117	[G/C]	Ca_00397	Granulin	INTRON
490	CakSNP490	*Kabuli*	Ca_Kabuli_Chr01	3259177	[G/C]	Ca_00397	Granulin	INTRON
491	CakSNP491	*Kabuli*	Ca_Kabuli_Chr01	3298327	[C/G]	Ca_00399	--	NON_SYNONYMOUS_CODING

S.N.	SNP IDs	Chickpea cultivars	Chromosomes	Physical positions (bp)	SNPs	Gene accession IDs	Putative functions	Sequence components of genome
492	CakSNP492	*Kabuli*	Ca_Kabuli_Chr01	3298783	[T/G]		--	INTERGENIC
493	CakSNP493	*Kabuli*	Ca_Kabuli_Chr01	3359148	[C/A]	Ca_00406	Protein kinase, catalytic domain	INTRON
494	CakSNP494	*Kabuli*	Ca_Kabuli_Chr01	3364952	[A/C]	Ca_00407	Protein kinase, catalytic domain	SYNONYMOUS_CODING
495	CakSNP495	*Kabuli*	Ca_Kabuli_Chr01	3374375	[G/A]	Ca_00409	Protein kinase, catalytic domain	NON_SYNONYMOUS_CODING
496	CakSNP496	*Kabuli*	Ca_Kabuli_Chr01	3427555	[A/C]	Ca_00416	Drug/metabolite transporter	INTRON
497	CakSNP497	*Kabuli*	Ca_Kabuli_Chr01	3427649	[A/G]	Ca_00416	Drug/metabolite transporter	SYNONYMOUS_CODING
498	CakSNP498	*Kabuli*	Ca_Kabuli_Chr01	3503059	[C/A]	Ca_00425	Armadillo	NON_SYNONYMOUS_CODING
499	CakSNP499	*Kabuli*	Ca_Kabuli_Chr01	3503153	[G/A]	Ca_00425	Armadillo	SYNONYMOUS_CODING
500	CakSNP500	*Kabuli*	Ca_Kabuli_Chr01	3542263	[A/C]	Ca_00430	Ankyrin repeat	INTRON
501	CakSNP501	*Kabuli*	Ca_Kabuli_Chr01	3544948	[A/T]	Ca_00431	Zinc finger, FYVE-type	SYNONYMOUS_CODING

S.N.	SNP IDs	Chickpea cultivars	Chromosomes	Physical positions (bp)	SNPs	Gene accession IDs	Putative functions	Sequence components of genome
502	CakSNP502	*Kabuli*	Ca_Kabuli_Chr01	3545011	[G/A]	Ca_00431	Zinc finger, FYVE-type	SYNONYMOUS_CODING
503	CakSNP503	*Kabuli*	Ca_Kabuli_Chr01	3564342	[G/T]	Ca_00433	Vitamin B6 biosynthesis protein	SYNONYMOUS_CODING
504	CakSNP504	*Kabuli*	Ca_Kabuli_Chr01	3596241	[C/T]		--	INTERGENIC
505	CakSNP505	*Kabuli*	Ca_Kabuli_Chr01	3597842	[G/A]	Ca_00440	Mov34/MPN/PAD-1	INTRON
506	CakSNP506	*Kabuli*	Ca_Kabuli_Chr01	3605582	[G/A]	Ca_00441	Membrane attack complex component/perforin (MACPF) domain	INTRON
507	CakSNP507	*Kabuli*	Ca_Kabuli_Chr01	3605583	[C/T]	Ca_00441	Membrane attack complex component/perforin (MACPF) domain	INTRON
508	CakSNP508	*Kabuli*	Ca_Kabuli_Chr01	3609580	[T/C]	Ca_00442	Oligopeptide transporter	SYNONYMOUS_CODING
509	CakSNP509	*Kabuli*	Ca_Kabuli_Chr01	3611231	[C/A]	Ca_00442	Oligopeptide transporter	NON_SYNONYMOUS_CODING
510	CakSNP510	*Kabuli*	Ca_Kabuli_Chr01	3614966	[A/G]		--	INTERGENIC
511	CakSNP511	*Kabuli*	Ca_Kabuli_Chr01	3615037	[G/T]		--	INTERGENIC

S.N.	SNP IDs	Chickpea cultivars	Chromosomes	Physical positions (bp)	SNPs	Gene accession IDs	Putative functions	Sequence components of genome
512	CakSNP512	*Kabuli*	Ca_Kabuli_Chr01	3615064	[T/C]		--	INTERGENIC
513	CakSNP513	*Kabuli*	Ca_Kabuli_Chr01	3615052	[C/A]		--	INTERGENIC
514	CakSNP514	*Kabuli*	Ca_Kabuli_Chr01	3622757	[C/T]	Ca_00444	Protein kinase, catalytic domain	SYNONYMOUS_CODING
515	CakSNP515	*Kabuli*	Ca_Kabuli_Chr01	3711498	[T/G]		--	INTERGENIC
516	CakSNP516	*Kabuli*	Ca_Kabuli_Chr01	3711525	[T/G]		--	INTERGENIC
517	CakSNP517	*Kabuli*	Ca_Kabuli_Chr01	3751865	[G/T]	Ca_00456	Proteasome-interacting thioredoxin-like domain, C-terminal	INTRON
518	CakSNP518	*Kabuli*	Ca_Kabuli_Chr01	3777223	[C/T]	Ca_00458	--	INTRON
519	CakSNP519	*Kabuli*	Ca_Kabuli_Chr01	3810931	[T/C]	Ca_00461	ABC transporter, transmembrane domain	SYNONYMOUS_CODING
520	CakSNP520	*Kabuli*	Ca_Kabuli_Chr01	3852035	[A/C]	Ca_00467	AUX/IAA protein	INTRON
521	CakSNP521	*Kabuli*	Ca_Kabuli_Chr01	3852476	[A/C]	Ca_00467	AUX/IAA protein	INTRON

S.N.	SNP IDs	Chickpea cultivars	Chromosomes	Physical positions (bp)	SNPs	Gene accession IDs	Putative functions	Sequence components of genome
522	CakSNP522	*Kabuli*	Ca_Kabuli_Chr01	3853408	[T/C]	Ca_00467	AUX/IAA protein	SYNONYMOUS_CODING
523	CakSNP523	*Kabuli*	Ca_Kabuli_Chr01	3855229	[G/A]	Ca_00467	AUX/IAA protein	INTRON
524	CakSNP524	*Kabuli*	Ca_Kabuli_Chr01	3891319	[T/C]	Ca_00470	Galectin, carbohydrate recognition domain	NON_SYNONYMOUS_CODING
525	CakSNP525	*Kabuli*	Ca_Kabuli_Chr01	3911547	[G/A]	Ca_00473	Nucleoporin protein Ndc1-Nup	NON_SYNONYMOUS_CODING
526	CakSNP526	*Kabuli*	Ca_Kabuli_Chr01	3941250	[C/T]	Ca_00478	Cytochrome b561, eukaryote	NON_SYNONYMOUS_CODING
527	CakSNP527	*Kabuli*	Ca_Kabuli_Chr01	3941399	[T/C]	Ca_00478	Cytochrome b561, eukaryote	SYNONYMOUS_CODING
528	CakSNP528	*Kabuli*	Ca_Kabuli_Chr01	3952819	[G/A]	Ca_00480	Protein kinase, catalytic domain	SYNONYMOUS_CODING
529	CakSNP529	*Kabuli*	Ca_Kabuli_Chr01	3956031	[T/C]		--	INTERGENIC
530	CakSNP530	*Kabuli*	Ca_Kabuli_Chr01	3956028	[C/T]		--	INTERGENIC
531	CakSNP531	*Kabuli*	Ca_Kabuli_Chr01	3972671	[G/T]	Ca_00481	ABC transporter, transmembrane domain	SYNONYMOUS_CODING

S.N.	SNP IDs	Chickpea cultivars	Chromosomes	Physical positions (bp)	SNPs	Gene accession IDs	Putative functions	Sequence components of genome
532	CakSNP532	*Kabuli*	Ca_Kabuli_Chr01	4048854	[G/A]	Ca_00487	Peptidase S1/S6, chymotrypsin/Hap	INTRON
533	CakSNP533	*Kabuli*	Ca_Kabuli_Chr01	4048875	[A/T]	Ca_00487	Peptidase S1/S6, chymotrypsin/Hap	INTRON
534	CakSNP534	*Kabuli*	Ca_Kabuli_Chr01	4073941	[A/C]		--	INTERGENIC
535	CakSNP535	*Kabuli*	Ca_Kabuli_Chr01	4112929	[A/T]	Ca_00493	Cullin, N-terminal	INTRON
536	CakSNP536	*Kabuli*	Ca_Kabuli_Chr01	4189333	[C/T]	Ca_00500	Reverse transcriptase	SYNONYMOUS_CODING
537	CakSNP537	*Kabuli*	Ca_Kabuli_Chr01	4189297	[T/C]	Ca_00500	Reverse transcriptase	SYNONYMOUS_CODING
538	CakSNP538	*Kabuli*	Ca_Kabuli_Chr01	4245058	[C/G]	Ca_00505	--	NON_SYNONYMOUS_CODING
539	CakSNP539	*Kabuli*	Ca_Kabuli_Chr01	4245073	[C/G]	Ca_00505	--	NON_SYNONYMOUS_CODING
540	CakSNP540	*Kabuli*	Ca_Kabuli_Chr01	4245176	[G/T]	Ca_00505	--	SYNONYMOUS_CODING
541	CakSNP541	*Kabuli*	Ca_Kabuli_Chr01	4292981	[G/C]		--	INTERGENIC

S.N.	SNP IDs	Chickpea cultivars	Chromosomes	Physical positions (bp)	SNPs	Gene accession IDs	Putative functions	Sequence components of genome
542	CakSNP542	*Kabuli*	Ca_Kabuli_Chr01	4302669	[G/C]	Ca_00512	Zinc finger, CCCH-type	NON_SYNONYMOUS_CODING
543	CakSNP543	*Kabuli*	Ca_Kabuli_Chr01	4302770	[A/C]	Ca_00512	Zinc finger, CCCH-type	NON_SYNONYMOUS_CODING
544	CakSNP544	*Kabuli*	Ca_Kabuli_Chr01	4302751	[T/C]	Ca_00512	Zinc finger, CCCH-type	SYNONYMOUS_CODING
545	CakSNP545	*Kabuli*	Ca_Kabuli_Chr01	4334299	[A/T]	Ca_00517	Tetratricopeptide repeat-containing	INTRON
546	CakSNP546	*Kabuli*	Ca_Kabuli_Chr01	4334336	[A/G]	Ca_00517	Tetratricopeptide repeat-containing	INTRON
547	CakSNP547	*Kabuli*	Ca_Kabuli_Chr01	4372257	[A/G]	Ca_00522	Protein kinase, catalytic domain	INTRON
548	CakSNP548	*Kabuli*	Ca_Kabuli_Chr01	4375710	[T/C]	Ca_00522	Protein kinase, catalytic domain	SYNONYMOUS_CODING
549	CakSNP549	*Kabuli*	Ca_Kabuli_Chr01	4385002	[C/T]	Ca_00523	SNF2-related	INTRON
550	CakSNP550	*Kabuli*	Ca_Kabuli_Chr01	4386114	[T/G]	Ca_00523	SNF2-related	INTRON
551	CakSNP551	*Kabuli*	Ca_Kabuli_Chr01	4386111	[T/C]	Ca_00523	SNF2-related	INTRON

S.N.	SNP IDs	Chickpea cultivars	Chromosomes	Physical positions (bp)	SNPs	Gene accession IDs	Putative functions	Sequence components of genome
552	CakSNP552	*Kabuli*	Ca_Kabuli_Chr01	4392680	[A/G]	Ca_00524	Lipase, class 3	INTRON
553	CakSNP553	*Kabuli*	Ca_Kabuli_Chr01	4414410	[A/T]	Ca_00528	Ribosomal protein S7	INTRON
554	CakSNP554	*Kabuli*	Ca_Kabuli_Chr01	4423984	[A/G]		--	INTERGENIC
555	CakSNP555	*Kabuli*	Ca_Kabuli_Chr01	4429044	[C/T]	Ca_00530	DNA-directed RNA polymerase, subunit 2, domain 6	NON_SYNONYMOUS_CODING
556	CakSNP556	*Kabuli*	Ca_Kabuli_Chr01	4432569	[A/G]	Ca_00530	DNA-directed RNA polymerase, subunit 2, domain 6	INTRON
557	CakSNP557	*Kabuli*	Ca_Kabuli_Chr01	4432549	[C/T]	Ca_00530	DNA-directed RNA polymerase, subunit 2, domain 6	INTRON
558	CakSNP558	*Kabuli*	Ca_Kabuli_Chr01	4432545	[C/A]	Ca_00530	DNA-directed RNA polymerase, subunit 2, domain 6	INTRON
559	CakSNP559	*Kabuli*	Ca_Kabuli_Chr01	4432541	[C/A]	Ca_00530	DNA-directed RNA polymerase, subunit 2, domain 6	INTRON
560	CakSNP560	*Kabuli*	Ca_Kabuli_Chr01	4456662	[G/A]	Ca_00531	DNA-directed RNA polymerase, subunit 2, domain 6	NON_SYNONYMOUS_CODING
561	CakSNP561	*Kabuli*	Ca_Kabuli_Chr01	4456654	[A/C]	Ca_00531	DNA-directed RNA polymerase, subunit 2, domain 6	SYNONYMOUS_CODING

S.N.	SNP IDs	Chickpea cultivars	Chromosomes	Physical positions (bp)	SNPs	Gene accession IDs	Putative functions	Sequence components of genome
562	CakSNP562	*Kabuli*	Ca_Kabuli_Chr01	4456648	[T/C]	Ca_00531	DNA-directed RNA polymerase, subunit 2, domain 6	NON_SYNONYMOUS_CODING
563	CakSNP563	*Kabuli*	Ca_Kabuli_Chr01	4457588	[A/G]	Ca_00531	DNA-directed RNA polymerase, subunit 2, domain 6	INTRON
564	CakSNP564	*Kabuli*	Ca_Kabuli_Chr01	4460112	[A/T]	Ca_00531	DNA-directed RNA polymerase, subunit 2, domain 6	INTRON
565	CakSNP565	*Kabuli*	Ca_Kabuli_Chr01	4494270	[C/T]	Ca_00534	Protein kinase, catalytic domain	SYNONYMOUS_CODING
566	CakSNP566	*Kabuli*	Ca_Kabuli_Chr01	4494467	[A/G]	Ca_00534	Protein kinase, catalytic domain	INTRON
567	CakSNP567	*Kabuli*	Ca_Kabuli_Chr01	4512566	[T/C]	Ca_00536	Tubby, C-terminal	SYNONYMOUS_CODING
568	CakSNP568	*Kabuli*	Ca_Kabuli_Chr01	4528117	[A/G]	Ca_00537	--	INTRON
569	CakSNP569	*Kabuli*	Ca_Kabuli_Chr01	4528184	[C/T]	Ca_00537	--	INTRON
570	CakSNP570	*Kabuli*	Ca_Kabuli_Chr01	4530304	[T/C]	Ca_00537	--	SYNONYMOUS_CODING
571	CakSNP571	*Kabuli*	Ca_Kabuli_Chr01	4560792	[C/T]	Ca_00541	Protein kinase, catalytic domain	SYNONYMOUS_CODING

S.N.	SNP IDs	Chickpea cultivars	Chromosomes	Physical positions (bp)	SNPs	Gene accession IDs	Putative functions	Sequence components of genome
572	CakSNP572	*Kabuli*	Ca_Kabuli_Chr01	4560920	[G/A]	Ca_00541	Protein kinase, catalytic domain	NON_SYNONYMOUS_CODING
573	CakSNP573	*Kabuli*	Ca_Kabuli_Chr01	4569669	[T/A]	Ca_00542	--	NON_SYNONYMOUS_CODING
574	CakSNP574	*Kabuli*	Ca_Kabuli_Chr01	4569670	[T/A]	Ca_00542	--	NON_SYNONYMOUS_CODING
575	CakSNP575	*Kabuli*	Ca_Kabuli_Chr01	4569690	[A/G]	Ca_00542	--	SYNONYMOUS_CODING
576	CakSNP576	*Kabuli*	Ca_Kabuli_Chr01	4571615	[T/C]		--	INTERGENIC
577	CakSNP577	*Kabuli*	Ca_Kabuli_Chr01	4571540	[C/G]		--	INTERGENIC
578	CakSNP578	*Kabuli*	Ca_Kabuli_Chr01	4571539	[G/A]		--	INTERGENIC
579	CakSNP579	*Kabuli*	Ca_Kabuli_Chr01	4576888	[A/G]	Ca_00543	BRCT	NON_SYNONYMOUS_CODING
580	CakSNP580	*Kabuli*	Ca_Kabuli_Chr01	4578967	[T/G]	Ca_00543	BRCT	INTRON
581	CakSNP581	*Kabuli*	Ca_Kabuli_Chr01	4578999	[T/A]	Ca_00543	BRCT	INTRON

S.N.	SNP IDs	Chickpea cultivars	Chromosomes	Physical positions (bp)	SNPs	Gene accession IDs	Putative functions	Sequence components of genome
582	CakSNP582	*Kabuli*	Ca_Kabuli_Chr01	4582189	[C/T]	Ca_00544	Ankyrin repeat	SYNONYMOUS_CODING
583	CakSNP583	*Kabuli*	Ca_Kabuli_Chr01	4626099	[T/G]	Ca_00547	Transketolase-like, pyrimidine-binding domain	SYNONYMOUS_CODING
584	CakSNP584	*Kabuli*	Ca_Kabuli_Chr01	4626098	[C/G]	Ca_00547	Transketolase-like, pyrimidine-binding domain	SYNONYMOUS_CODING
585	CakSNP585	*Kabuli*	Ca_Kabuli_Chr01	4631467	[G/A]	Ca_00548	Dehydrogenase, E1 component	SYNONYMOUS_CODING
586	CakSNP586	*Kabuli*	Ca_Kabuli_Chr01	4632188	[G/A]	Ca_00548	Dehydrogenase, E1 component	SYNONYMOUS_CODING
587	CakSNP587	*Kabuli*	Ca_Kabuli_Chr01	4632965	[G/A]	Ca_00548	Dehydrogenase, E1 component	SYNONYMOUS_CODING
588	CakSNP588	*Kabuli*	Ca_Kabuli_Chr01	4634413	[C/T]	Ca_00548	Dehydrogenase, E1 component	SYNONYMOUS_CODING
589	CakSNP589	*Kabuli*	Ca_Kabuli_Chr01	4635347	[G/A]	Ca_00548	Dehydrogenase, E1 component	INTRON
590	CakSNP590	*Kabuli*	Ca_Kabuli_Chr01	4635302	[A/C]	Ca_00548	Dehydrogenase, E1 component	INTRON
591	CakSNP591	*Kabuli*	Ca_Kabuli_Chr01	4662564	[A/G]	Ca_00550	Transcription factor, SBP-box	INTRON

S.N.	SNP IDs	Chickpea cultivars	Chromosomes	Physical positions (bp)	SNPs	Gene accession IDs	Putative functions	Sequence components of genome
592	CakSNP592	*Kabuli*	Ca_Kabuli_Chr01	4672439	[G/A]	Ca_00551	Sugar/inositol transporter	NON_SYNONYMOUS_CODING
593	CakSNP593	*Kabuli*	Ca_Kabuli_Chr01	4715563	[T/C]	Ca_00555	Protein kinase, catalytic domain	NON_SYNONYMOUS_CODING
594	CakSNP594	*Kabuli*	Ca_Kabuli_Chr01	4780330	[C/T]	Ca_00562	Zinc finger, RING-type	NON_SYNONYMOUS_CODING
595	CakSNP595	*Kabuli*	Ca_Kabuli_Chr01	4780345	[G/T]	Ca_00562	Zinc finger, RING-type	NON_SYNONYMOUS_CODING
596	CakSNP596	*Kabuli*	Ca_Kabuli_Chr01	4781362	[C/T]	Ca_00562	Zinc finger, RING-type	SYNONYMOUS_CODING
597	CakSNP597	*Kabuli*	Ca_Kabuli_Chr01	4781416	[G/A]	Ca_00562	Zinc finger, RING-type	SYNONYMOUS_CODING
598	CakSNP598	*Kabuli*	Ca_Kabuli_Chr01	4781704	[G/A]	Ca_00562	Zinc finger, RING-type	SYNONYMOUS_CODING
599	CakSNP599	*Kabuli*	Ca_Kabuli_Chr01	4808910	[A/T]	Ca_00566	SAC3/GANP/Nin1/mts3/eIF-3 p25	INTRON
600	CakSNP600	*Kabuli*	Ca_Kabuli_Chr01	4878683	[T/C]		--	INTERGENIC
601	CakSNP601	*Kabuli*	Ca_Kabuli_Chr01	4878691	[A/C]		--	INTERGENIC

S.N.	SNP IDs	Chickpea cultivars	Chromosomes	Physical positions (bp)	SNPs	Gene accession IDs	Putative functions	Sequence components of genome
602	CakSNP602	*Kabuli*	Ca_Kabuli_Chr01	4878695	[T/C]		--	INTERGENIC
603	CakSNP603	*Kabuli*	Ca_Kabuli_Chr01	4878823	[A/G]		--	INTERGENIC
604	CakSNP604	*Kabuli*	Ca_Kabuli_Chr01	4903185	[T/A]	Ca_00576	Leucine-rich repeat	NON_SYNONYMOUS_CODING
605	CakSNP605	*Kabuli*	Ca_Kabuli_Chr01	4903203	[A/G]	Ca_00576	Leucine-rich repeat	SYNONYMOUS_CODING
606	CakSNP606	*Kabuli*	Ca_Kabuli_Chr01	4903239	[G/A]	Ca_00576	Leucine-rich repeat	SYNONYMOUS_CODING
607	CakSNP607	*Kabuli*	Ca_Kabuli_Chr01	4906759	[G/C]		--	INTERGENIC
608	CakSNP608	*Kabuli*	Ca_Kabuli_Chr01	4906775	[A/T]		--	INTERGENIC
609	CakSNP609	*Kabuli*	Ca_Kabuli_Chr01	4906777	[G/T]		--	INTERGENIC
610	CakSNP610	*Kabuli*	Ca_Kabuli_Chr01	4906778	[T/G]		--	INTERGENIC
611	CakSNP611	*Kabuli*	Ca_Kabuli_Chr01	4906800	[T/G]		--	INTERGENIC

S.N.	SNP IDs	Chickpea cultivars	Chromosomes	Physical positions (bp)	SNPs	Gene accession IDs	Putative functions	Sequence components of genome
612	CakSNP612	*Kabuli*	Ca_Kabuli_Chr01	4906816	[A/G]		--	INTERGENIC
613	CakSNP613	*Kabuli*	Ca_Kabuli_Chr01	4906868	[G/C]		--	INTERGENIC
614	CakSNP614	*Kabuli*	Ca_Kabuli_Chr01	4906859	[T/C]		--	INTERGENIC
615	CakSNP615	*Kabuli*	Ca_Kabuli_Chr01	4906854	[A/G]		--	INTERGENIC
616	CakSNP616	*Kabuli*	Ca_Kabuli_Chr01	4909155	[T/C]	Ca_00577	Disease resistance protein	NON_SYNONYMOUS_CODING
617	CakSNP617	*Kabuli*	Ca_Kabuli_Chr01	4909286	[T/G]	Ca_00577	Disease resistance protein	SYNONYMOUS_CODING
618	CakSNP618	*Kabuli*	Ca_Kabuli_Chr01	4909280	[T/G]	Ca_00577	Disease resistance protein	SYNONYMOUS_CODING
619	CakSNP619	*Kabuli*	Ca_Kabuli_Chr01	4909271	[A/G]	Ca_00577	Disease resistance protein	SYNONYMOUS_CODING
620	CakSNP620	*Kabuli*	Ca_Kabuli_Chr01	4909268	[C/T]	Ca_00577	Disease resistance protein	SYNONYMOUS_CODING
621	CakSNP621	*Kabuli*	Ca_Kabuli_Chr01	4909261	[G/T]	Ca_00577	Disease resistance protein	SYNONYMOUS_CODING

S.N.	SNP IDs	Chickpea cultivars	Chromosomes	Physical positions (bp)	SNPs	Gene accession IDs	Putative functions	Sequence components of genome
622	CakSNP622	*Kabuli*	Ca_Kabuli_Chr01	4909255	[G/A]	Ca_00577	Disease resistance protein	NON_SYNONYMOUS_CODING
623	CakSNP623	*Kabuli*	Ca_Kabuli_Chr01	4909253	[A/G]	Ca_00577	Disease resistance protein	SYNONYMOUS_CODING
624	CakSNP624	*Kabuli*	Ca_Kabuli_Chr01	4910497	[A/G]	Ca_00577	Disease resistance protein	SYNONYMOUS_CODING
625	CakSNP625	*Kabuli*	Ca_Kabuli_Chr01	4910494	[C/A]	Ca_00577	Disease resistance protein	SYNONYMOUS_CODING
626	CakSNP626	*Kabuli*	Ca_Kabuli_Chr01	4948740	[A/T]	Ca_00584	Armadillo	INTRON
627	CakSNP627	*Kabuli*	Ca_Kabuli_Chr01	4949578	[A/T]	Ca_00584	Armadillo	SYNONYMOUS_CODING
628	CakSNP628	*Kabuli*	Ca_Kabuli_Chr01	4950457	[G/A]	Ca_00584	Armadillo	SYNONYMOUS_CODING
629	CakSNP629	*Kabuli*	Ca_Kabuli_Chr01	4950726	[G/T]	Ca_00584	Armadillo	SYNONYMOUS_CODING
630	CakSNP630	*Kabuli*	Ca_Kabuli_Chr01	4959562	[A/C]	Ca_00586	Protein kinase, catalytic domain	NON_SYNONYMOUS_CODING
631	CakSNP631	*Kabuli*	Ca_Kabuli_Chr01	4995240	[T/A]	Ca_00587	--	SYNONYMOUS_CODING

S.N.	SNP IDs	Chickpea cultivars	Chromosomes	Physical positions (bp)	SNPs	Gene accession IDs	Putative functions	Sequence components of genome
632	CakSNP632	*Kabuli*	Ca_Kabuli_Chr01	4996404	[A/C]	Ca_00587	--	SYNONYMOUS_CODING
633	CakSNP633	*Kabuli*	Ca_Kabuli_Chr01	4996459	[T/C]	Ca_00587	--	SYNONYMOUS_CODING
634	CakSNP634	*Kabuli*	Ca_Kabuli_Chr01	4996476	[G/A]	Ca_00587	--	SYNONYMOUS_CODING
635	CakSNP635	*Kabuli*	Ca_Kabuli_Chr01	4996536	[C/T]	Ca_00587	--	SYNONYMOUS_CODING
636	CakSNP636	*Kabuli*	Ca_Kabuli_Chr01	5071210	[A/G]	Ca_00593	RNA recognition motif domain	INTRON
637	CakSNP637	*Kabuli*	Ca_Kabuli_Chr01	5071195	[T/A]	Ca_00593	RNA recognition motif domain	INTRON
638	CakSNP638	*Kabuli*	Ca_Kabuli_Chr01	5071177	[A/G]	Ca_00593	RNA recognition motif domain	INTRON
639	CakSNP639	*Kabuli*	Ca_Kabuli_Chr01	5071158	[G/A]	Ca_00593	RNA recognition motif domain	INTRON
640	CakSNP640	*Kabuli*	Ca_Kabuli_Chr01	5072022	[C/A]	Ca_00593	RNA recognition motif domain	SYNONYMOUS_CODING
641	CakSNP641	*Kabuli*	Ca_Kabuli_Chr01	5080342	[A/T]	Ca_00594	Derlin	INTRON

S.N.	SNP IDs	Chickpea cultivars	Chromosomes	Physical positions (bp)	SNPs	Gene accession IDs	Putative functions	Sequence components of genome
642	CakSNP642	*Kabuli*	Ca_Kabuli_Chr01	5080358	[G/A]	Ca_00594	Derlin	INTRON
643	CakSNP643	*Kabuli*	Ca_Kabuli_Chr01	5087428	[T/C]	Ca_00596	CDP-alcohol phosphatidyltransferase	INTRON
644	CakSNP644	*Kabuli*	Ca_Kabuli_Chr01	5096676	[A/T]	Ca_00598	Protein kinase, catalytic domain	NON_SYNONYMOUS_CODING
645	CakSNP645	*Kabuli*	Ca_Kabuli_Chr01	5096650	[C/T]	Ca_00598	Protein kinase, catalytic domain	SYNONYMOUS_CODING
646	CakSNP646	*Kabuli*	Ca_Kabuli_Chr01	5122663	[A/G]		--	INTERGENIC
647	CakSNP647	*Kabuli*	Ca_Kabuli_Chr01	5129940	[G/A]	Ca_00603	Drug/metabolite transporter	SYNONYMOUS_CODING
648	CakSNP648	*Kabuli*	Ca_Kabuli_Chr01	5138013	[A/C]		--	INTERGENIC
649	CakSNP649	*Kabuli*	Ca_Kabuli_Chr01	5140801	[C/A]		--	INTERGENIC
650	CakSNP650	*Kabuli*	Ca_Kabuli_Chr01	5140837	[A/T]		--	INTERGENIC
651	CakSNP651	*Kabuli*	Ca_Kabuli_Chr01	5141009	[G/C]		--	INTERGENIC

S.N.	SNP IDs	Chickpea cultivars	Chromosomes	Physical positions (bp)	SNPs	Gene accession IDs	Putative functions	Sequence components of genome
652	CakSNP652	*Kabuli*	Ca_Kabuli_Chr01	5140944	[C/G]		--	INTERGENIC
653	CakSNP653	*Kabuli*	Ca_Kabuli_Chr01	5146658	[C/A]		--	INTERGENIC
654	CakSNP654	*Kabuli*	Ca_Kabuli_Chr01	5146667	[T/G]		--	INTERGENIC
655	CakSNP655	*Kabuli*	Ca_Kabuli_Chr01	5146696	[T/A]		--	INTERGENIC
656	CakSNP656	*Kabuli*	Ca_Kabuli_Chr01	5164127	[A/G]	Ca_00606	Short-chain dehydrogenase/reductase SDR	SYNONYMOUS_CODING
657	CakSNP657	*Kabuli*	Ca_Kabuli_Chr01	5164136	[C/T]	Ca_00606	Short-chain dehydrogenase/reductase SDR	SYNONYMOUS_CODING
658	CakSNP658	*Kabuli*	Ca_Kabuli_Chr01	5164198	[C/A]	Ca_00606	Short-chain dehydrogenase/reductase SDR	NON_SYNONYMOUS_CODING
659	CakSNP659	*Kabuli*	Ca_Kabuli_Chr01	5197461	[C/T]	Ca_00608	Uncharacterised protein family, carbohydrate kinase-related	INTRON
660	CakSNP660	*Kabuli*	Ca_Kabuli_Chr01	5197475	[T/A]	Ca_00608	Uncharacterised protein family, carbohydrate kinase-related	INTRON
661	CakSNP661	*Kabuli*	Ca_Kabuli_Chr01	5200250	[T/G]	Ca_00608	Uncharacterised protein family, carbohydrate kinase-related	INTRON

S.N.	SNP IDs	Chickpea cultivars	Chromosomes	Physical positions (bp)	SNPs	Gene accession IDs	Putative functions	Sequence components of genome
662	CakSNP662	*Kabuli*	Ca_Kabuli_Chr01	5200289	[G/T]	Ca_00608	Uncharacterised protein family, carbohydrate kinase-related	INTRON
663	CakSNP663	*Kabuli*	Ca_Kabuli_Chr01	5249839	[G/A]	Ca_00613	Protein of unknown function DUF584	NON_SYNONYMOUS_CODING
664	CakSNP664	*Kabuli*	Ca_Kabuli_Chr01	5249846	[A/C]	Ca_00613	Protein of unknown function DUF584	SYNONYMOUS_CODING
665	CakSNP665	*Kabuli*	Ca_Kabuli_Chr01	5249868	[G/A]	Ca_00613	Protein of unknown function DUF584	SYNONYMOUS_CODING
666	CakSNP666	*Kabuli*	Ca_Kabuli_Chr01	5288427	[T/C]	Ca_00616	Heavy metal transport/detoxification protein	SYNONYMOUS_CODING
667	CakSNP667	*Kabuli*	Ca_Kabuli_Chr01	5288421	[A/C]	Ca_00616	Heavy metal transport/detoxification protein	SYNONYMOUS_CODING
668	CakSNP668	*Kabuli*	Ca_Kabuli_Chr01	5304576	[A/C]	Ca_00617	Zinc finger, RING-type	INTRON
669	CakSNP669	*Kabuli*	Ca_Kabuli_Chr01	5321398	[A/G]		--	INTERGENIC
670	CakSNP670	*Kabuli*	Ca_Kabuli_Chr01	5430700	[A/C]	Ca_00628	Protein of unknown function DUF584	SYNONYMOUS_CODING
671	CakSNP671	*Kabuli*	Ca_Kabuli_Chr01	5430703	[T/G]	Ca_00628	Protein of unknown function DUF584	SYNONYMOUS_CODING

S.N.	SNP IDs	Chickpea cultivars	Chromosomes	Physical positions (bp)	SNPs	Gene accession IDs	Putative functions	Sequence components of genome
672	CakSNP672	*Kabuli*	Ca_Kabuli_Chr01	5430809	[G/A]	Ca_00628	Protein of unknown function DUF584	NON_SYNONYMOUS_CODING
673	CakSNP673	*Kabuli*	Ca_Kabuli_Chr01	5444130	[A/C]	Ca_00629	Uncharacterised protein family, carbohydrate kinase-related	INTRON
674	CakSNP674	*Kabuli*	Ca_Kabuli_Chr01	5444142	[A/T]	Ca_00629	Uncharacterised protein family, carbohydrate kinase-related	INTRON
675	CakSNP675	*Kabuli*	Ca_Kabuli_Chr01	5474604	[G/A]	Ca_00632	Peptidyl-prolyl cis-trans isomerase, cyclophilin-type	INTRON
676	CakSNP676	*Kabuli*	Ca_Kabuli_Chr01	5477196	[T/C]	Ca_00632	Peptidyl-prolyl cis-trans isomerase, cyclophilin-type	INTRON
677	CakSNP677	*Kabuli*	Ca_Kabuli_Chr01	5487535	[A/G]	Ca_00634	--	NON_SYNONYMOUS_CODING
678	CakSNP678	*Kabuli*	Ca_Kabuli_Chr01	5489806	[C/G]	Ca_00634	--	INTRON
679	CakSNP679	*Kabuli*	Ca_Kabuli_Chr01	5577080	[T/C]	Ca_00642	Glyoxalase/bleomycin resistance protein/dioxygenase	INTRON
680	CakSNP680	*Kabuli*	Ca_Kabuli_Chr01	5629027	[G/A]	Ca_00649	--	SYNONYMOUS_CODING
681	CakSNP681	*Kabuli*	Ca_Kabuli_Chr01	5637388	[T/A]	Ca_00651	--	SYNONYMOUS_CODING

S.N.	SNP IDs	Chickpea cultivars	Chromosomes	Physical positions (bp)	SNPs	Gene accession IDs	Putative functions	Sequence components of genome
682	CakSNP682	*Kabuli*	Ca_Kabuli_Chr01	5637442	[C/T]	Ca_00651	--	SYNONYMOUS_CODING
683	CakSNP683	*Kabuli*	Ca_Kabuli_Chr01	5643313	[G/C]		--	INTERGENIC
684	CakSNP684	*Kabuli*	Ca_Kabuli_Chr01	5666178	[A/C]	Ca_00656	DNA-binding WRKY	SYNONYMOUS_CODING
685	CakSNP685	*Kabuli*	Ca_Kabuli_Chr01	5666217	[C/A]	Ca_00656	DNA-binding WRKY	NON_SYNONYMOUS_CODING
686	CakSNP686	*Kabuli*	Ca_Kabuli_Chr01	5666260	[T/G]	Ca_00656	DNA-binding WRKY	SYNONYMOUS_CODING
687	CakSNP687	*Kabuli*	Ca_Kabuli_Chr01	5666266	[T/G]	Ca_00656	DNA-binding WRKY	NON_SYNONYMOUS_CODING
688	CakSNP688	*Kabuli*	Ca_Kabuli_Chr01	5666263	[T/G]	Ca_00656	DNA-binding WRKY	SYNONYMOUS_CODING
689	CakSNP689	*Kabuli*	Ca_Kabuli_Chr01	5666355	[T/G]	Ca_00656	DNA-binding WRKY	NON_SYNONYMOUS_CODING
690	CakSNP690	*Kabuli*	Ca_Kabuli_Chr01	5667605	[G/A]	Ca_00656	DNA-binding WRKY	SYNONYMOUS_CODING
691	CakSNP691	*Kabuli*	Ca_Kabuli_Chr01	5702638	[C/T]	Ca_00662	Glycosyl transferase, family 14	NON_SYNONYMOUS_CODING

S.N.	SNP IDs	Chickpea cultivars	Chromosomes	Physical positions (bp)	SNPs	Gene accession IDs	Putative functions	Sequence components of genome
692	CakSNP692	*Kabuli*	Ca_Kabuli_Chr01	5706858	[A/T]		--	INTERGENIC
693	CakSNP693	*Kabuli*	Ca_Kabuli_Chr01	5706961	[G/T]		--	INTERGENIC
694	CakSNP694	*Kabuli*	Ca_Kabuli_Chr01	5706948	[A/T]		--	INTERGENIC
695	CakSNP695	*Kabuli*	Ca_Kabuli_Chr01	5706944	[T/G]		--	INTERGENIC
696	CakSNP696	*Kabuli*	Ca_Kabuli_Chr01	5706920	[G/A]		--	INTERGENIC
697	CakSNP697	*Kabuli*	Ca_Kabuli_Chr01	5721616	[A/T]	Ca_00664	Protein kinase, catalytic domain	INTRON
698	CakSNP698	*Kabuli*	Ca_Kabuli_Chr01	5749456	[C/T]		--	INTERGENIC
699	CakSNP699	*Kabuli*	Ca_Kabuli_Chr01	5791118	[T/G]	Ca_00669	UBX	NON_SYNONYMOUS_CODING
700	CakSNP700	*Kabuli*	Ca_Kabuli_Chr01	5796146	[C/T]	Ca_00670	UBX	INTRON
701	CakSNP701	*Kabuli*	Ca_Kabuli_Chr01	5796886	[A/G]	Ca_00670	UBX	INTRON

S.N.	SNP IDs	Chickpea cultivars	Chromosomes	Physical positions (bp)	SNPs	Gene accession IDs	Putative functions	Sequence components of genome
702	CakSNP702	*Kabuli*	Ca_Kabuli_Chr01	5807492	[T/C]		--	INTERGENIC
703	CakSNP703	*Kabuli*	Ca_Kabuli_Chr01	5812283	[G/T]	Ca_00672	--	SYNONYMOUS_CODING
704	CakSNP704	*Kabuli*	Ca_Kabuli_Chr01	5812298	[G/A]	Ca_00672	--	SYNONYMOUS_CODING
705	CakSNP705	*Kabuli*	Ca_Kabuli_Chr01	5862565	[C/T]		--	INTERGENIC
706	CakSNP706	*Kabuli*	Ca_Kabuli_Chr01	5960264	[T/C]	Ca_00683	--	SYNONYMOUS_CODING
707	CakSNP707	*Kabuli*	Ca_Kabuli_Chr01	5978146	[G/A]	Ca_00685	--	INTRON
708	CakSNP708	*Kabuli*	Ca_Kabuli_Chr01	5978289	[C/T]	Ca_00685	--	SYNONYMOUS_CODING
709	CakSNP709	*Kabuli*	Ca_Kabuli_Chr01	5981127	[C/T]	Ca_00685	--	SYNONYMOUS_CODING
710	CakSNP710	*Kabuli*	Ca_Kabuli_Chr01	5981178	[A/G]	Ca_00685	--	SYNONYMOUS_CODING
711	CakSNP711	*Kabuli*	Ca_Kabuli_Chr01	6011727	[G/T]		--	INTERGENIC

S.N.	SNP IDs	Chickpea cultivars	Chromosomes	Physical positions (bp)	SNPs	Gene accession IDs	Putative functions	Sequence components of genome
712	CakSNP712	*Kabuli*	Ca_Kabuli_Chr01	6011697	[T/G]		--	INTERGENIC
713	CakSNP713	*Kabuli*	Ca_Kabuli_Chr01	6011689	[G/A]		--	INTERGENIC
714	CakSNP714	*Kabuli*	Ca_Kabuli_Chr01	6011657	[T/C]		--	INTERGENIC
715	CakSNP715	*Kabuli*	Ca_Kabuli_Chr01	6011761	[C/T]		--	INTERGENIC
716	CakSNP716	*Kabuli*	Ca_Kabuli_Chr01	6017854	[A/C]	Ca_00689	Phosphatidate cytidylyltransferase	INTRON
717	CakSNP717	*Kabuli*	Ca_Kabuli_Chr01	6066972	[C/T]	Ca_00693	RHO protein GDP dissociation inhibitor	INTRON
718	CakSNP718	*Kabuli*	Ca_Kabuli_Chr01	6066936	[A/G]	Ca_00693	RHO protein GDP dissociation inhibitor	INTRON
719	CakSNP719	*Kabuli*	Ca_Kabuli_Chr01	6066933	[C/T]	Ca_00693	RHO protein GDP dissociation inhibitor	INTRON
720	CakSNP720	*Kabuli*	Ca_Kabuli_Chr01	6067846	[T/C]	Ca_00693	RHO protein GDP dissociation inhibitor	INTRON
721	CakSNP721	*Kabuli*	Ca_Kabuli_Chr01	6088283	[C/T]		--	INTERGENIC

S.N.	SNP IDs	Chickpea cultivars	Chromosomes	Physical positions (bp)	SNPs	Gene accession IDs	Putative functions	Sequence components of genome
722	CakSNP722	*Kabuli*	Ca_Kabuli_Chr01	6101205	[C/T]	Ca_00695	HECT	NON_SYNONYMOUS_CODING
723	CakSNP723	*Kabuli*	Ca_Kabuli_Chr01	6126761	[T/A]	Ca_00697	Ribosomal protein L14b/L23e	INTRON
724	CakSNP724	*Kabuli*	Ca_Kabuli_Chr01	6126772	[G/A]	Ca_00697	Ribosomal protein L14b/L23e	INTRON
725	CakSNP725	*Kabuli*	Ca_Kabuli_Chr01	6155341	[A/G]	Ca_00700	Inositol monophosphatase	INTRON
726	CakSNP726	*Kabuli*	Ca_Kabuli_Chr01	6224010	[C/G]		--	INTERGENIC
727	CakSNP727	*Kabuli*	Ca_Kabuli_Chr01	6224015	[C/T]		--	INTERGENIC
728	CakSNP728	*Kabuli*	Ca_Kabuli_Chr01	6249062	[T/C]	Ca_07884	RNA recognition motif domain	SYNONYMOUS_CODING
729	CakSNP729	*Kabuli*	Ca_Kabuli_Chr01	6249035	[T/C]	Ca_07884	RNA recognition motif domain	SYNONYMOUS_CODING
730	CakSNP730	*Kabuli*	Ca_Kabuli_Chr01	6257653	[C/G]	Ca_07885	Thymidine kinase	INTRON
731	CakSNP731	*Kabuli*	Ca_Kabuli_Chr01	6257712	[A/T]	Ca_07885	Thymidine kinase	INTRON

S.N.	SNP IDs	Chickpea cultivars	Chromosomes	Physical positions (bp)	SNPs	Gene accession IDs	Putative functions	Sequence components of genome
732	CakSNP732	*Kabuli*	Ca_Kabuli_Chr01	6259458	[T/C]	Ca_07885	Thymidine kinase	SYNONYMOUS_CODING
733	CakSNP733	*Kabuli*	Ca_Kabuli_Chr01	6262461	[A/G]	Ca_07886	--	INTRON
734	CakSNP734	*Kabuli*	Ca_Kabuli_Chr01	6262529	[T/G]	Ca_07886	--	NON_SYNONYMOUS_CODING
735	CakSNP735	*Kabuli*	Ca_Kabuli_Chr01	6262577	[A/G]	Ca_07886	--	NON_SYNONYMOUS_CODING
736	CakSNP736	*Kabuli*	Ca_Kabuli_Chr01	6278572	[T/C]		--	INTERGENIC
737	CakSNP737	*Kabuli*	Ca_Kabuli_Chr01	6278625	[T/C]		--	INTERGENIC
738	CakSNP738	*Kabuli*	Ca_Kabuli_Chr01	6279644	[G/T]		--	INTERGENIC
739	CakSNP739	*Kabuli*	Ca_Kabuli_Chr01	6296858	[G/A]	Ca_07890	Uncharacterised protein family UPF0497, trans-membrane plant	INTRON
740	CakSNP740	*Kabuli*	Ca_Kabuli_Chr01	6296986	[C/A]	Ca_07890	Uncharacterised protein family UPF0497, trans-membrane plant	INTRON
741	CakSNP741	*Kabuli*	Ca_Kabuli_Chr01	6296929	[C/T]	Ca_07890	Uncharacterised protein family UPF0497, trans-membrane plant	INTRON

S.N.	SNP IDs	Chickpea cultivars	Chromosomes	Physical positions (bp)	SNPs	Gene accession IDs	Putative functions	Sequence components of genome
742	CakSNP742	*Kabuli*	Ca_Kabuli_Chr01	6315337	[G/T]	Ca_07892	Zinc finger, ZZ-type	INTRON
743	CakSNP743	*Kabuli*	Ca_Kabuli_Chr01	6315402	[C/T]	Ca_07892	Zinc finger, ZZ-type	INTRON
744	CakSNP744	*Kabuli*	Ca_Kabuli_Chr01	6315399	[T/G]	Ca_07892	Zinc finger, ZZ-type	INTRON
745	CakSNP745	*Kabuli*	Ca_Kabuli_Chr01	6315397	[C/G]	Ca_07892	Zinc finger, ZZ-type	INTRON
746	CakSNP746	*Kabuli*	Ca_Kabuli_Chr01	6328801	[T/A]		--	INTERGENIC
747	CakSNP747	*Kabuli*	Ca_Kabuli_Chr01	6328957	[C/T]		--	INTERGENIC
748	CakSNP748	*Kabuli*	Ca_Kabuli_Chr01	6328963	[A/G]		--	INTERGENIC
749	CakSNP749	*Kabuli*	Ca_Kabuli_Chr01	6329805	[A/G]	Ca_07893	Bromo adjacent homology (BAH) domain	SYNONYMOUS_CODING
750	CakSNP750	*Kabuli*	Ca_Kabuli_Chr01	6329814	[T/A]	Ca_07893	Bromo adjacent homology (BAH) domain	SYNONYMOUS_CODING
751	CakSNP751	*Kabuli*	Ca_Kabuli_Chr01	6329844	[C/T]	Ca_07893	Bromo adjacent homology (BAH) domain	NON_SYNONYMOUS_CODING

S.N.	SNP IDs	Chickpea cultivars	Chromosomes	Physical positions (bp)	SNPs	Gene accession IDs	Putative functions	Sequence components of genome
752	CakSNP752	*Kabuli*	Ca_Kabuli_Chr01	6329847	[A/G]	Ca_07893	Bromo adjacent homology (BAH) domain	SYNONYMOUS_CODING
753	CakSNP753	*Kabuli*	Ca_Kabuli_Chr01	6332229	[T/C]	Ca_07893	Bromo adjacent homology (BAH) domain	INTRON
754	CakSNP754	*Kabuli*	Ca_Kabuli_Chr01	6347626	[C/T]		--	INTERGENIC
755	CakSNP755	*Kabuli*	Ca_Kabuli_Chr01	6347720	[G/A]		--	INTERGENIC
756	CakSNP756	*Kabuli*	Ca_Kabuli_Chr01	6381114	[T/G]	Ca_07899	Myosin II heavy chain-like	INTRON
757	CakSNP757	*Kabuli*	Ca_Kabuli_Chr01	6381191	[T/C]	Ca_07899	Myosin II heavy chain-like	SYNONYMOUS_CODING
758	CakSNP758	*Kabuli*	Ca_Kabuli_Chr01	6390541	[T/A]		--	INTERGENIC
759	CakSNP759	*Kabuli*	Ca_Kabuli_Chr01	6390849	[G/A]	Ca_07902	Smr protein/MutS2 C-terminal	NON_SYNONYMOUS_CODING
760	CakSNP760	*Kabuli*	Ca_Kabuli_Chr01	6391017	[T/C]	Ca_07902	Smr protein/MutS2 C-terminal	NON_SYNONYMOUS_CODING
761	CakSNP761	*Kabuli*	Ca_Kabuli_Chr01	6433592	[T/G]		--	INTERGENIC

S.N.	SNP IDs	Chickpea cultivars	Chromosomes	Physical positions (bp)	SNPs	Gene accession IDs	Putative functions	Sequence components of genome
762	CakSNP762	*Kabuli*	Ca_Kabuli_Chr01	6433826	[G/A]	Ca_07907	NUDIX hydrolase domain	SYNONYMOUS_CODING
763	CakSNP763	*Kabuli*	Ca_Kabuli_Chr01	6433838	[C/T]	Ca_07907	NUDIX hydrolase domain	SYNONYMOUS_CODING
764	CakSNP764	*Kabuli*	Ca_Kabuli_Chr01	6433841	[C/T]	Ca_07907	NUDIX hydrolase domain	SYNONYMOUS_CODING
765	CakSNP765	*Kabuli*	Ca_Kabuli_Chr01	6433883	[A/G]	Ca_07907	NUDIX hydrolase domain	SYNONYMOUS_CODING
766	CakSNP766	*Kabuli*	Ca_Kabuli_Chr01	6433934	[T/A]	Ca_07907	NUDIX hydrolase domain	SYNONYMOUS_CODING
767	CakSNP767	*Kabuli*	Ca_Kabuli_Chr01	6433925	[C/T]	Ca_07907	NUDIX hydrolase domain	SYNONYMOUS_CODING
768	CakSNP768	*Kabuli*	Ca_Kabuli_Chr01	6478736	[A/G]		--	INTERGENIC
769	CakSNP769	*Kabuli*	Ca_Kabuli_Chr01	6478758	[A/G]		--	INTERGENIC
770	CakSNP770	*Kabuli*	Ca_Kabuli_Chr01	6486446	[G/C]	Ca_07911	--	INTRON
771	CakSNP771	*Kabuli*	Ca_Kabuli_Chr01	6490978	[A/T]	Ca_07911	--	INTRON

S.N.	SNP IDs	Chickpea cultivars	Chromosomes	Physical positions (bp)	SNPs	Gene accession IDs	Putative functions	Sequence components of genome
772	CakSNP772	*Kabuli*	Ca_Kabuli_Chr01	6491008	[A/T]	Ca_07911	--	INTRON
773	CakSNP773	*Kabuli*	Ca_Kabuli_Chr01	6491030	[T/G]	Ca_07911	--	INTRON
774	CakSNP774	*Kabuli*	Ca_Kabuli_Chr01	6491017	[T/C]	Ca_07911	--	INTRON
775	CakSNP775	*Kabuli*	Ca_Kabuli_Chr01	6521670	[C/T]	Ca_07916	Protein of unknown function DUF827, plant	SYNONYMOUS_CODING
776	CakSNP776	*Kabuli*	Ca_Kabuli_Chr01	6521673	[G/A]	Ca_07916	Protein of unknown function DUF827, plant	SYNONYMOUS_CODING
777	CakSNP777	*Kabuli*	Ca_Kabuli_Chr01	6521856	[A/G]	Ca_07916	Protein of unknown function DUF827, plant	SYNONYMOUS_CODING
778	CakSNP778	*Kabuli*	Ca_Kabuli_Chr01	6521859	[T/A]	Ca_07916	Protein of unknown function DUF827, plant	SYNONYMOUS_CODING
779	CakSNP779	*Kabuli*	Ca_Kabuli_Chr01	6521970	[C/T]	Ca_07916	Protein of unknown function DUF827, plant	SYNONYMOUS_CODING
780	CakSNP780	*Kabuli*	Ca_Kabuli_Chr01	6540056	[C/T]	Ca_07919	Protein kinase, catalytic domain	SYNONYMOUS_CODING
781	CakSNP781	*Kabuli*	Ca_Kabuli_Chr01	6540170	[G/A]	Ca_07919	Protein kinase, catalytic domain	SYNONYMOUS_CODING

S.N.	SNP IDs	Chickpea cultivars	Chromosomes	Physical positions (bp)	SNPs	Gene accession IDs	Putative functions	Sequence components of genome
782	CakSNP782	*Kabuli*	Ca_Kabuli_Chr01	6553107	[A/C]	Ca_07920	--	INTRON
783	CakSNP783	*Kabuli*	Ca_Kabuli_Chr01	6564716	[C/T]	Ca_07921	Polyprenyl synthetase	SYNONYMOUS_CODING
784	CakSNP784	*Kabuli*	Ca_Kabuli_Chr01	6564789	[C/T]	Ca_07921	Polyprenyl synthetase	INTRON
785	CakSNP785	*Kabuli*	Ca_Kabuli_Chr01	6591506	[G/C]	Ca_07923	Forkhead-associated (FHA) domain	NON_SYNONYMOUS_CODING
786	CakSNP786	*Kabuli*	Ca_Kabuli_Chr01	6591518	[G/T]	Ca_07923	Forkhead-associated (FHA) domain	SYNONYMOUS_CODING
787	CakSNP787	*Kabuli*	Ca_Kabuli_Chr01	6599917	[C/A]		--	INTERGENIC
788	CakSNP788	*Kabuli*	Ca_Kabuli_Chr01	6634482	[G/C]		--	INTERGENIC
789	CakSNP789	*Kabuli*	Ca_Kabuli_Chr01	6635404	[C/T]	Ca_07930	Glycoside hydrolase, family 17	SYNONYMOUS_CODING
790	CakSNP790	*Kabuli*	Ca_Kabuli_Chr01	6635401	[T/C]	Ca_07930	Glycoside hydrolase, family 17	SYNONYMOUS_CODING
791	CakSNP791	*Kabuli*	Ca_Kabuli_Chr01	6635366	[T/C]	Ca_07930	Glycoside hydrolase, family 17	NON_SYNONYMOUS_CODING

S.N.	SNP IDs	Chickpea cultivars	Chromosomes	Physical positions (bp)	SNPs	Gene accession IDs	Putative functions	Sequence components of genome
792	CakSNP792	*Kabuli*	Ca_Kabuli_Chr01	6679253	[A/G]	Ca_07932	WD40 repeat	SYNONYMOUS_CODING
793	CakSNP793	*Kabuli*	Ca_Kabuli_Chr01	6679329	[A/G]	Ca_07932	WD40 repeat	NON_SYNONYMOUS_CODING
794	CakSNP794	*Kabuli*	Ca_Kabuli_Chr01	6803364	[G/A]		--	INTERGENIC
795	CakSNP795	*Kabuli*	Ca_Kabuli_Chr01	6804031	[C/T]	Ca_07946	Peptidase A1	SYNONYMOUS_CODING
796	CakSNP796	*Kabuli*	Ca_Kabuli_Chr01	6804110	[A/T]	Ca_07946	Peptidase A1	NON_SYNONYMOUS_CODING
797	CakSNP797	*Kabuli*	Ca_Kabuli_Chr01	6849437	[C/T]	Ca_07951	Vps53-like, N-terminal	INTRON
798	CakSNP798	*Kabuli*	Ca_Kabuli_Chr01	6849512	[G/C]	Ca_07951	Vps53-like, N-terminal	INTRON
799	CakSNP799	*Kabuli*	Ca_Kabuli_Chr01	6879110	[G/C]	Ca_07954	Glycoside hydrolase, family 9	SYNONYMOUS_CODING
800	CakSNP800	*Kabuli*	Ca_Kabuli_Chr01	6880666	[A/G]	Ca_07954	Glycoside hydrolase, family 9	SYNONYMOUS_CODING
801	CakSNP801	*Kabuli*	Ca_Kabuli_Chr01	6880837	[A/G]	Ca_07954	Glycoside hydrolase, family 9	SYNONYMOUS_CODING

S.N.	SNP IDs	Chickpea cultivars	Chromosomes	Physical positions (bp)	SNPs	Gene accession IDs	Putative functions	Sequence components of genome
802	CakSNP802	*Kabuli*	Ca_Kabuli_Chr01	6904278	[A/C]	Ca_07956	Protein kinase, catalytic domain	NON_SYNONYMOUS_CODING
803	CakSNP803	*Kabuli*	Ca_Kabuli_Chr01	6904313	[A/C]	Ca_07956	Protein kinase, catalytic domain	INTRON
804	CakSNP804	*Kabuli*	Ca_Kabuli_Chr01	6904338	[C/T]	Ca_07956	Protein kinase, catalytic domain	INTRON
805	CakSNP805	*Kabuli*	Ca_Kabuli_Chr01	6952382	[G/T]	Ca_07963	Protein of unknown function DUF1296	NON_SYNONYMOUS_CODING
806	CakSNP806	*Kabuli*	Ca_Kabuli_Chr01	7050825	[G/A]		--	INTERGENIC
807	CakSNP807	*Kabuli*	Ca_Kabuli_Chr01	7050958	[C/T]		--	INTERGENIC
808	CakSNP808	*Kabuli*	Ca_Kabuli_Chr01	7050953	[C/T]		--	INTERGENIC
809	CakSNP809	*Kabuli*	Ca_Kabuli_Chr01	7104985	[G/T]		--	INTERGENIC
810	CakSNP810	*Kabuli*	Ca_Kabuli_Chr01	7127843	[A/G]	Ca_07973	Sec8 exocyst complex component specific domain	SYNONYMOUS_CODING
811	CakSNP811	*Kabuli*	Ca_Kabuli_Chr01	7136677	[T/C]		--	INTERGENIC

S.N.	SNP IDs	Chickpea cultivars	Chromosomes	Physical positions (bp)	SNPs	Gene accession IDs	Putative functions	Sequence components of genome
812	CakSNP812	*Kabuli*	Ca_Kabuli_Chr01	7147651	[T/A]	Ca_07974	--	INTRON
813	CakSNP813	*Kabuli*	Ca_Kabuli_Chr01	7147643	[A/G]	Ca_07974	--	INTRON
814	CakSNP814	*Kabuli*	Ca_Kabuli_Chr01	7155344	[A/G]	Ca_07974	--	INTRON
815	CakSNP815	*Kabuli*	Ca_Kabuli_Chr01	7155302	[A/G]	Ca_07974	--	INTRON
816	CakSNP816	*Kabuli*	Ca_Kabuli_Chr01	7156354	[G/T]	Ca_07974	--	INTRON
817	CakSNP817	*Kabuli*	Ca_Kabuli_Chr01	7156355	[A/T]	Ca_07974	--	INTRON
818	CakSNP818	*Kabuli*	Ca_Kabuli_Chr01	7157057	[A/C]	Ca_07974	--	INTRON
819	CakSNP819	*Kabuli*	Ca_Kabuli_Chr01	7157112	[T/C]	Ca_07974	--	INTRON
820	CakSNP820	*Kabuli*	Ca_Kabuli_Chr01	7160649	[T/C]	Ca_07974	--	INTRON
821	CakSNP821	*Kabuli*	Ca_Kabuli_Chr01	7160648	[A/T]	Ca_07974	--	INTRON

S.N.	SNP IDs	Chickpea cultivars	Chromosomes	Physical positions (bp)	SNPs	Gene accession IDs	Putative functions	Sequence components of genome
822	CakSNP822	*Kabuli*	Ca_Kabuli_Chr01	7160695	[G/A]	Ca_07974	--	INTRON
823	CakSNP823	*Kabuli*	Ca_Kabuli_Chr01	7160698	[A/C]	Ca_07974	--	INTRON
824	CakSNP824	*Kabuli*	Ca_Kabuli_Chr01	7160754	[A/T]	Ca_07974	--	INTRON
825	CakSNP825	*Kabuli*	Ca_Kabuli_Chr01	7160781	[A/C]	Ca_07974	--	INTRON
826	CakSNP826	*Kabuli*	Ca_Kabuli_Chr01	7160773	[G/A]	Ca_07974	--	INTRON
827	CakSNP827	*Kabuli*	Ca_Kabuli_Chr01	7161122	[A/G]	Ca_07974	--	INTRON
828	CakSNP828	*Kabuli*	Ca_Kabuli_Chr01	7184813	[A/C]	Ca_07975	Root hair defective 3 GTP-binding	SYNONYMOUS_CODING
829	CakSNP829	*Kabuli*	Ca_Kabuli_Chr01	7184773	[G/A]	Ca_07975	Root hair defective 3 GTP-binding	NON_SYNONYMOUS_CODING
830	CakSNP830	*Kabuli*	Ca_Kabuli_Chr01	7187934	[A/G]		--	INTERGENIC
831	CakSNP831	*Kabuli*	Ca_Kabuli_Chr01	7187969	[C/G]		--	INTERGENIC

S.N.	SNP IDs	Chickpea cultivars	Chromosomes	Physical positions (bp)	SNPs	Gene accession IDs	Putative functions	Sequence components of genome
832	CakSNP832	*Kabuli*	Ca_Kabuli_Chr01	7196570	[T/C]	Ca_07977	Pectinesterase inhibitor	NON_SYNONYMOUS_CODING
833	CakSNP833	*Kabuli*	Ca_Kabuli_Chr01	7196601	[T/C]	Ca_07977	Pectinesterase inhibitor	NON_SYNONYMOUS_CODING
834	CakSNP834	*Kabuli*	Ca_Kabuli_Chr01	7231750	[A/C]		--	INTERGENIC
835	CakSNP835	*Kabuli*	Ca_Kabuli_Chr01	7325164	[T/A]	Ca_07989	WD40 repeat	NON_SYNONYMOUS_CODING
836	CakSNP836	*Kabuli*	Ca_Kabuli_Chr01	7417717	[G/A]	Ca_07994	Homeobox	SYNONYMOUS_CODING
837	CakSNP837	*Kabuli*	Ca_Kabuli_Chr01	7417699	[G/A]	Ca_07994	Homeobox	SYNONYMOUS_CODING
838	CakSNP838	*Kabuli*	Ca_Kabuli_Chr01	7417788	[G/A]	Ca_07994	Homeobox	INTRON
839	CakSNP839	*Kabuli*	Ca_Kabuli_Chr01	7439421	[C/T]	Ca_07995	CCAAT-binding transcription factor, subunit B	SYNONYMOUS_CODING
840	CakSNP840	*Kabuli*	Ca_Kabuli_Chr01	7467280	[A/G]	Ca_07999	AMP-dependent synthetase/ligase	INTRON
841	CakSNP841	*Kabuli*	Ca_Kabuli_Chr01	7478022	[G/A]	Ca_08000	--	SYNONYMOUS_CODING

S.N.	SNP IDs	Chickpea cultivars	Chromosomes	Physical positions (bp)	SNPs	Gene accession IDs	Putative functions	Sequence components of genome
842	CakSNP842	*Kabuli*	Ca_Kabuli_Chr01	7478046	[T/G]	Ca_08000	--	NON_SYNONYMOUS_CODING
843	CakSNP843	*Kabuli*	Ca_Kabuli_Chr01	7486102	[C/T]	Ca_08001	Protein kinase, catalytic domain	SYNONYMOUS_CODING
844	CakSNP844	*Kabuli*	Ca_Kabuli_Chr01	7486273	[T/C]	Ca_08001	Protein kinase, catalytic domain	SYNONYMOUS_CODING
845	CakSNP845	*Kabuli*	Ca_Kabuli_Chr01	7486282	[G/A]	Ca_08001	Protein kinase, catalytic domain	SYNONYMOUS_CODING
846	CakSNP846	*Kabuli*	Ca_Kabuli_Chr01	7497334	[C/A]		--	INTERGENIC
847	CakSNP847	*Kabuli*	Ca_Kabuli_Chr01	7497362	[T/C]		--	INTERGENIC
848	CakSNP848	*Kabuli*	Ca_Kabuli_Chr01	7500494	[T/G]	Ca_08003	Protein kinase, catalytic domain	SYNONYMOUS_CODING
849	CakSNP849	*Kabuli*	Ca_Kabuli_Chr01	7500524	[T/C]	Ca_08003	Protein kinase, catalytic domain	SYNONYMOUS_CODING
850	CakSNP850	*Kabuli*	Ca_Kabuli_Chr01	7500665	[C/A]	Ca_08003	Protein kinase, catalytic domain	SYNONYMOUS_CODING
851	CakSNP851	*Kabuli*	Ca_Kabuli_Chr01	7505779	[A/T]		--	INTERGENIC

S.N.	SNP IDs	Chickpea cultivars	Chromosomes	Physical positions (bp)	SNPs	Gene accession IDs	Putative functions	Sequence components of genome
852	CakSNP852	*Kabuli*	Ca_Kabuli_Chr01	7505792	[A/T]		--	INTERGENIC
853	CakSNP853	*Kabuli*	Ca_Kabuli_Chr01	7515002	[T/C]	Ca_08006	SKI-interacting protein SKIP, SNW domain	SYNONYMOUS_CODING
854	CakSNP854	*Kabuli*	Ca_Kabuli_Chr01	7515605	[T/C]	Ca_08006	SKI-interacting protein SKIP, SNW domain	SYNONYMOUS_CODING
855	CakSNP855	*Kabuli*	Ca_Kabuli_Chr01	7515578	[A/C]	Ca_08006	SKI-interacting protein SKIP, SNW domain	SYNONYMOUS_CODING
856	CakSNP856	*Kabuli*	Ca_Kabuli_Chr01	7557751	[T/G]	Ca_08009	Histidinol dehydrogenase, conserved site	INTRON
857	CakSNP857	*Kabuli*	Ca_Kabuli_Chr01	7557811	[A/T]	Ca_08009	Histidinol dehydrogenase, conserved site	INTRON
858	CakSNP858	*Kabuli*	Ca_Kabuli_Chr01	7690613	[T/G]	Ca_08021	--	NON_SYNONYMOUS_CODING
859	CakSNP859	*Kabuli*	Ca_Kabuli_Chr01	7690620	[T/G]	Ca_08021	--	NON_SYNONYMOUS_CODING
860	CakSNP860	*Kabuli*	Ca_Kabuli_Chr01	7690625	[A/C]	Ca_08021	--	SYNONYMOUS_CODING
861	CakSNP861	*Kabuli*	Ca_Kabuli_Chr01	7695844	[T/C]	Ca_08021	--	INTRON

S.N.	SNP IDs	Chickpea cultivars	Chromosomes	Physical positions (bp)	SNPs	Gene accession IDs	Putative functions	Sequence components of genome
862	CakSNP862	*Kabuli*	Ca_Kabuli_Chr01	7811388	[C/T]	Ca_08032	Protein of unknown function DUF185	INTRON
863	CakSNP863	*Kabuli*	Ca_Kabuli_Chr01	7925666	[A/C]	Ca_08042	Biopterin transport-related protein BT1	SYNONYMOUS_CODING
864	CakSNP864	*Kabuli*	Ca_Kabuli_Chr01	7925801	[C/T]	Ca_08042	Biopterin transport-related protein BT1	SYNONYMOUS_CODING
865	CakSNP865	*Kabuli*	Ca_Kabuli_Chr01	7925774	[C/T]	Ca_08042	Biopterin transport-related protein BT1	SYNONYMOUS_CODING
866	CakSNP866	*Kabuli*	Ca_Kabuli_Chr01	7925738	[C/T]	Ca_08042	Biopterin transport-related protein BT1	SYNONYMOUS_CODING
867	CakSNP867	*Kabuli*	Ca_Kabuli_Chr01	7969202	[A/C]	Ca_08046	Ribosomal protein L7/L12, C-terminal	SYNONYMOUS_CODING
868	CakSNP868	*Kabuli*	Ca_Kabuli_Chr01	7969694	[A/G]		--	INTERGENIC
869	CakSNP869	*Kabuli*	Ca_Kabuli_Chr01	7969647	[G/A]		--	INTERGENIC
870	CakSNP870	*Kabuli*	Ca_Kabuli_Chr01	8036666	[C/T]	Ca_08051	NAD-dependent epimerase/dehydratase	INTRON
871	CakSNP871	*Kabuli*	Ca_Kabuli_Chr01	8036706	[A/G]	Ca_08051	NAD-dependent epimerase/dehydratase	INTRON

S.N.	SNP IDs	Chickpea cultivars	Chromosomes	Physical positions (bp)	SNPs	Gene accession IDs	Putative functions	Sequence components of genome
872	CakSNP872	*Kabuli*	Ca_Kabuli_Chr01	8036917	[T/G]	Ca_08051	NAD-dependent epimerase/dehydratase	INTRON
873	CakSNP873	*Kabuli*	Ca_Kabuli_Chr01	8036903	[A/T]	Ca_08051	NAD-dependent epimerase/dehydratase	INTRON
874	CakSNP874	*Kabuli*	Ca_Kabuli_Chr01	8075631	[A/T]	Ca_08055	Ribose 5-phosphate isomerase, type A	SYNONYMOUS_CODING
875	CakSNP875	*Kabuli*	Ca_Kabuli_Chr01	8103090	[G/T]	Ca_08056	Mitochondrial carrier protein	SYNONYMOUS_CODING
876	CakSNP876	*Kabuli*	Ca_Kabuli_Chr01	8103339	[T/C]	Ca_08056	Mitochondrial carrier protein	INTRON
877	CakSNP877	*Kabuli*	Ca_Kabuli_Chr01	8103401	[T/A]	Ca_08056	Mitochondrial carrier protein	INTRON
878	CakSNP878	*Kabuli*	Ca_Kabuli_Chr01	8107967	[A/C]	Ca_08057	RNA helicase, ATP-dependent, DEAD-box, conserved site	NON_SYNONYMOUS_CODING
879	CakSNP879	*Kabuli*	Ca_Kabuli_Chr01	8108014	[C/T]	Ca_08057	RNA helicase, ATP-dependent, DEAD-box, conserved site	NON_SYNONYMOUS_CODING
880	CakSNP880	*Kabuli*	Ca_Kabuli_Chr01	8111588	[G/A]	Ca_08057	RNA helicase, ATP-dependent, DEAD-box, conserved site	INTRON
881	CakSNP881	*Kabuli*	Ca_Kabuli_Chr01	8112343	[C/T]	Ca_08057	RNA helicase, ATP-dependent, DEAD-box, conserved site	SYNONYMOUS_CODING

S.N.	SNP IDs	Chickpea cultivars	Chromosomes	Physical positions (bp)	SNPs	Gene accession IDs	Putative functions	Sequence components of genome
882	CakSNP882	*Kabuli*	Ca_Kabuli_Chr01	8113235	[G/T]	Ca_08057	RNA helicase, ATP-dependent, DEAD-box, conserved site	NON_SYNONYMOUS_CODING
883	CakSNP883	*Kabuli*	Ca_Kabuli_Chr01	8113383	[T/G]	Ca_08057	RNA helicase, ATP-dependent, DEAD-box, conserved site	SYNONYMOUS_CODING
884	CakSNP884	*Kabuli*	Ca_Kabuli_Chr01	8113449	[T/A]	Ca_08057	RNA helicase, ATP-dependent, DEAD-box, conserved site	SYNONYMOUS_CODING
885	CakSNP885	*Kabuli*	Ca_Kabuli_Chr01	8113403	[A/T]	Ca_08057	RNA helicase, ATP-dependent, DEAD-box, conserved site	NON_SYNONYMOUS_CODING
886	CakSNP886	*Kabuli*	Ca_Kabuli_Chr01	8113398	[C/G]	Ca_08057	RNA helicase, ATP-dependent, DEAD-box, conserved site	NON_SYNONYMOUS_CODING
887	CakSNP887	*Kabuli*	Ca_Kabuli_Chr01	8123033	[A/G]		--	INTERGENIC
888	CakSNP888	*Kabuli*	Ca_Kabuli_Chr01	8123257	[A/G]		--	INTERGENIC
889	CakSNP889	*Kabuli*	Ca_Kabuli_Chr01	8123481	[A/G]		--	INTERGENIC
890	CakSNP890	*Kabuli*	Ca_Kabuli_Chr01	8134368	[T/C]	Ca_08060	--	INTRON
891	CakSNP891	*Kabuli*	Ca_Kabuli_Chr01	8178109	[T/C]		--	INTERGENIC

S.N.	SNP IDs	Chickpea cultivars	Chromosomes	Physical positions (bp)	SNPs	Gene accession IDs	Putative functions	Sequence components of genome
892	CakSNP892	*Kabuli*	Ca_Kabuli_Chr01	8178146	[G/A]		--	INTERGENIC
893	CakSNP893	*Kabuli*	Ca_Kabuli_Chr01	8192580	[T/C]	Ca_08067	Zinc finger, CCHC-type	NON_SYNONYMOUS_CODING
894	CakSNP894	*Kabuli*	Ca_Kabuli_Chr01	8220996	[T/A]	Ca_08068	HECT	INTRON
895	CakSNP895	*Kabuli*	Ca_Kabuli_Chr01	8220968	[A/G]	Ca_08068	HECT	INTRON
896	CakSNP896	*Kabuli*	Ca_Kabuli_Chr01	8343356	[G/A]		--	INTERGENIC
897	CakSNP897	*Kabuli*	Ca_Kabuli_Chr01	8389242	[G/A]	Ca_08075	Ribosomal protein S6	INTRON
898	CakSNP898	*Kabuli*	Ca_Kabuli_Chr01	8464621	[C/G]	Ca_08082	--	INTRON
899	CakSNP899	*Kabuli*	Ca_Kabuli_Chr01	8582341	[T/C]		--	INTERGENIC
900	CakSNP900	*Kabuli*	Ca_Kabuli_Chr01	8607820	[T/C]	Ca_08096	PAS	SYNONYMOUS_CODING
901	CakSNP901	*Kabuli*	Ca_Kabuli_Chr01	8607919	[A/C]	Ca_08096	PAS	SYNONYMOUS_CODING

S.N.	SNP IDs	Chickpea cultivars	Chromosomes	Physical positions (bp)	SNPs	Gene accession IDs	Putative functions	Sequence components of genome
902	CakSNP902	*Kabuli*	Ca_Kabuli_Chr01	8633598	[G/A]		--	INTERGENIC
903	CakSNP903	*Kabuli*	Ca_Kabuli_Chr01	8665705	[T/G]	Ca_02944	ATPase, P-type, K/Mg/Cd/Cu/Zn/Na/Ca/Na/H-transporter	INTRON
904	CakSNP904	*Kabuli*	Ca_Kabuli_Chr01	8697177	[G/T]	Ca_02942	Sodium/calcium exchanger membrane region	INTRON
905	CakSNP905	*Kabuli*	Ca_Kabuli_Chr01	8698059	[T/C]	Ca_02942	Sodium/calcium exchanger membrane region	SYNONYMOUS_CODING
906	CakSNP906	*Kabuli*	Ca_Kabuli_Chr01	8735740	[G/T]	Ca_02937	Ubiquitin-associated/translation elongation factor EF1B, N-terminal	INTRON
907	CakSNP907	*Kabuli*	Ca_Kabuli_Chr01	8735727	[G/C]	Ca_02937	Ubiquitin-associated/translation elongation factor EF1B, N-terminal	INTRON
908	CakSNP908	*Kabuli*	Ca_Kabuli_Chr01	8736733	[G/A]	Ca_02937	Ubiquitin-associated/translation elongation factor EF1B, N-terminal	INTRON
909	CakSNP909	*Kabuli*	Ca_Kabuli_Chr01	8736711	[G/T]	Ca_02937	Ubiquitin-associated/translation elongation factor EF1B, N-terminal	INTRON
910	CakSNP910	*Kabuli*	Ca_Kabuli_Chr01	8737380	[A/G]	Ca_02937	Ubiquitin-associated/translation elongation factor EF1B, N-terminal	SYNONYMOUS_CODING
911	CakSNP911	*Kabuli*	Ca_Kabuli_Chr01	8738691	[T/G]		--	INTERGENIC

S.N.	SNP IDs	Chickpea cultivars	Chromosomes	Physical positions (bp)	SNPs	Gene accession IDs	Putative functions	Sequence components of genome
912	CakSNP912	*Kabuli*	Ca_Kabuli_Chr01	8738695	[G/C]		--	INTERGENIC
913	CakSNP913	*Kabuli*	Ca_Kabuli_Chr01	8767122	[T/A]	Ca_02933	Pentatricopeptide repeat	INTRON
914	CakSNP914	*Kabuli*	Ca_Kabuli_Chr01	8767121	[A/T]	Ca_02933	Pentatricopeptide repeat	INTRON
915	CakSNP915	*Kabuli*	Ca_Kabuli_Chr01	8799685	[T/G]		--	INTERGENIC
916	CakSNP916	*Kabuli*	Ca_Kabuli_Chr01	8799701	[T/C]		--	INTERGENIC
917	CakSNP917	*Kabuli*	Ca_Kabuli_Chr01	8799708	[T/C]		--	INTERGENIC
918	CakSNP918	*Kabuli*	Ca_Kabuli_Chr01	8799758	[C/G]		--	INTERGENIC
919	CakSNP919	*Kabuli*	Ca_Kabuli_Chr01	8809188	[T/A]	Ca_02928	ABC transporter, transmembrane domain	INTRON
920	CakSNP920	*Kabuli*	Ca_Kabuli_Chr01	8809191	[C/T]	Ca_02928	ABC transporter, transmembrane domain	INTRON
921	CakSNP921	*Kabuli*	Ca_Kabuli_Chr01	8809295	[A/T]	Ca_02928	ABC transporter, transmembrane domain	INTRON

S.N.	SNP IDs	Chickpea cultivars	Chromosomes	Physical positions (bp)	SNPs	Gene accession IDs	Putative functions	Sequence components of genome
922	CakSNP922	*Kabuli*	Ca_Kabuli_Chr01	8809517	[C/G]	Ca_02928	ABC transporter, transmembrane domain	INTRON
923	CakSNP923	*Kabuli*	Ca_Kabuli_Chr01	8822121	[G/T]	Ca_02927	3-Oxoacyl-[acyl-carrier-protein (ACP)] synthase III C-terminal	INTRON
924	CakSNP924	*Kabuli*	Ca_Kabuli_Chr01	8822375	[G/A]	Ca_02927	3-Oxoacyl-[acyl-carrier-protein (ACP)] synthase III C-terminal	SYNONYMOUS_CODING
925	CakSNP925	*Kabuli*	Ca_Kabuli_Chr01	8822366	[T/A]	Ca_02927	3-Oxoacyl-[acyl-carrier-protein (ACP)] synthase III C-terminal	SYNONYMOUS_CODING
926	CakSNP926	*Kabuli*	Ca_Kabuli_Chr01	8824856	[T/C]	Ca_02926	--	INTRON
927	CakSNP927	*Kabuli*	Ca_Kabuli_Chr01	8852863	[G/T]		--	INTERGENIC
928	CakSNP928	*Kabuli*	Ca_Kabuli_Chr01	8852928	[T/A]		--	INTERGENIC
929	CakSNP929	*Kabuli*	Ca_Kabuli_Chr01	8852981	[C/A]		--	INTERGENIC
930	CakSNP930	*Kabuli*	Ca_Kabuli_Chr01	8852957	[C/A]		--	INTERGENIC
931	CakSNP931	*Kabuli*	Ca_Kabuli_Chr01	8852943	[C/A]		--	INTERGENIC

S.N.	SNP IDs	Chickpea cultivars	Chromosomes	Physical positions (bp)	SNPs	Gene accession IDs	Putative functions	Sequence components of genome
932	CakSNP932	*Kabuli*	Ca_Kabuli_Chr01	8853010	[A/G]		--	INTERGENIC
933	CakSNP933	*Kabuli*	Ca_Kabuli_Chr01	8854211	[G/T]	Ca_02923	Natural resistance-associated macrophage protein	INTRON
934	CakSNP934	*Kabuli*	Ca_Kabuli_Chr01	8891438	[T/G]	Ca_02918	ATPase, P-type, H+ transporting proton pump	SYNONYMOUS_CODING
935	CakSNP935	*Kabuli*	Ca_Kabuli_Chr01	8891485	[T/G]	Ca_02918	ATPase, P-type, H+ transporting proton pump	INTRON
936	CakSNP936	*Kabuli*	Ca_Kabuli_Chr01	8891562	[C/G]	Ca_02918	ATPase, P-type, H+ transporting proton pump	NON_SYNONYMOUS_CODING
937	CakSNP937	*Kabuli*	Ca_Kabuli_Chr01	8891561	[G/A]	Ca_02918	ATPase, P-type, H+ transporting proton pump	NON_SYNONYMOUS_CODING
938	CakSNP938	*Kabuli*	Ca_Kabuli_Chr01	8893985	[G/A]	Ca_02918	ATPase, P-type, H+ transporting proton pump	INTRON
939	CakSNP939	*Kabuli*	Ca_Kabuli_Chr01	8901769	[C/A]	Ca_02915	Protein kinase, catalytic domain	NON_SYNONYMOUS_CODING
940	CakSNP940	*Kabuli*	Ca_Kabuli_Chr01	8901842	[T/A]	Ca_02915	Protein kinase, catalytic domain	SYNONYMOUS_CODING
941	CakSNP941	*Kabuli*	Ca_Kabuli_Chr01	8941004	[A/C]		--	INTERGENIC

S.N.	SNP IDs	Chickpea cultivars	Chromosomes	Physical positions (bp)	SNPs	Gene accession IDs	Putative functions	Sequence components of genome
942	CakSNP942	*Kabuli*	Ca_Kabuli_Chr01	8941029	[G/A]		--	INTERGENIC
943	CakSNP943	*Kabuli*	Ca_Kabuli_Chr01	8941031	[G/C]		--	INTERGENIC
944	CakSNP944	*Kabuli*	Ca_Kabuli_Chr01	8942137	[T/C]	Ca_02909	Zinc finger, UBP-type	NON_SYNONYMOUS_CODING
945	CakSNP945	*Kabuli*	Ca_Kabuli_Chr01	8942131	[T/C]	Ca_02909	Zinc finger, UBP-type	NON_SYNONYMOUS_CODING
946	CakSNP946	*Kabuli*	Ca_Kabuli_Chr01	8942198	[A/G]	Ca_02909	Zinc finger, UBP-type	SYNONYMOUS_CODING
947	CakSNP947	*Kabuli*	Ca_Kabuli_Chr01	8942179	[C/G]	Ca_02909	Zinc finger, UBP-type	NON_SYNONYMOUS_CODING
948	CakSNP948	*Kabuli*	Ca_Kabuli_Chr01	8949723	[G/T]	Ca_02908	Isocitrate/isopropylmalate dehydrogenase	NON_SYNONYMOUS_CODING
949	CakSNP949	*Kabuli*	Ca_Kabuli_Chr01	8960630	[T/C]	Ca_02905	--	INTRON
950	CakSNP950	*Kabuli*	Ca_Kabuli_Chr01	8969321	[C/T]	Ca_02904	--	INTRON
951	CakSNP951	*Kabuli*	Ca_Kabuli_Chr01	8971718	[C/G]	Ca_02904	--	NON_SYNONYMOUS_CODING

S.N.	SNP IDs	Chickpea cultivars	Chromosomes	Physical positions (bp)	SNPs	Gene accession IDs	Putative functions	Sequence components of genome
952	CakSNP952	*Kabuli*	Ca_Kabuli_Chr01	8971776	[A/G]	Ca_02904	--	SYNONYMOUS_CODING
953	CakSNP953	*Kabuli*	Ca_Kabuli_Chr01	8976169	[A/T]	Ca_02904	--	NON_SYNONYMOUS_CODING
954	CakSNP954	*Kabuli*	Ca_Kabuli_Chr01	8980112	[C/A]		--	INTERGENIC
955	CakSNP955	*Kabuli*	Ca_Kabuli_Chr01	8993300	[T/C]		--	INTERGENIC
956	CakSNP956	*Kabuli*	Ca_Kabuli_Chr01	8993329	[A/C]		--	INTERGENIC
957	CakSNP957	*Kabuli*	Ca_Kabuli_Chr01	9018113	[A/C]	Ca_02899	WD40 repeat	INTRON
958	CakSNP958	*Kabuli*	Ca_Kabuli_Chr01	9031417	[G/T]	Ca_02897	NADH:ubiquinone oxidoreductase, 24kDa subunit	INTRON
959	CakSNP959	*Kabuli*	Ca_Kabuli_Chr01	9031825	[G/T]		--	INTERGENIC
960	CakSNP960	*Kabuli*	Ca_Kabuli_Chr01	9032126	[G/A]	Ca_02896	DNA glycosylase/AP lyase, catalytic domain	SYNONYMOUS_CODING
961	CakSNP961	*Kabuli*	Ca_Kabuli_Chr01	9035744	[G/A]	Ca_02896	DNA glycosylase/AP lyase, catalytic domain	SYNONYMOUS_CODING

S.N.	SNP IDs	Chickpea cultivars	Chromosomes	Physical positions (bp)	SNPs	Gene accession IDs	Putative functions	Sequence components of genome
962	CakSNP962	*Kabuli*	Ca_Kabuli_Chr01	9040419	[T/A]	Ca_02895	HAT dimerisation	SYNONYMOUS_CODING
963	CakSNP963	*Kabuli*	Ca_Kabuli_Chr01	9048675	[A/T]	Ca_02894	Phox homologous domain	INTRON
964	CakSNP964	*Kabuli*	Ca_Kabuli_Chr01	9048682	[T/G]	Ca_02894	Phox homologous domain	INTRON
965	CakSNP965	*Kabuli*	Ca_Kabuli_Chr01	9054490	[G/A]	Ca_02893	Signal recognition particle, SRP54 subunit, GTPase	INTRON
966	CakSNP966	*Kabuli*	Ca_Kabuli_Chr01	9065933	[T/C]	Ca_02890	Ubiquitin system component Cue	INTRON
967	CakSNP967	*Kabuli*	Ca_Kabuli_Chr01	9093812	[C/G]		--	INTERGENIC
968	CakSNP968	*Kabuli*	Ca_Kabuli_Chr01	9093964	[T/A]	Ca_02887	Cystathionine beta-synthase, core	SYNONYMOUS_CODING
969	CakSNP969	*Kabuli*	Ca_Kabuli_Chr01	9097885	[A/G]		--	INTERGENIC
970	CakSNP970	*Kabuli*	Ca_Kabuli_Chr01	9097881	[C/G]		--	INTERGENIC
971	CakSNP971	*Kabuli*	Ca_Kabuli_Chr01	9097879	[C/A]		--	INTERGENIC

S.N.	SNP IDs	Chickpea cultivars	Chromosomes	Physical positions (bp)	SNPs	Gene accession IDs	Putative functions	Sequence components of genome
972	CakSNP972	*Kabuli*	Ca_Kabuli_Chr01	9097878	[A/G]		--	INTERGENIC
973	CakSNP973	*Kabuli*	Ca_Kabuli_Chr01	9097877	[C/A]		--	INTERGENIC
974	CakSNP974	*Kabuli*	Ca_Kabuli_Chr01	9097869	[G/T]		--	INTERGENIC
975	CakSNP975	*Kabuli*	Ca_Kabuli_Chr01	9097947	[C/A]		--	INTERGENIC
976	CakSNP976	*Kabuli*	Ca_Kabuli_Chr01	9097949	[A/C]		--	INTERGENIC
977	CakSNP977	*Kabuli*	Ca_Kabuli_Chr01	9098003	[C/T]		--	INTERGENIC
978	CakSNP978	*Kabuli*	Ca_Kabuli_Chr01	9098051	[A/G]		--	INTERGENIC
979	CakSNP979	*Kabuli*	Ca_Kabuli_Chr01	9105432	[C/T]	Ca_02885	Initiation factor 2B-related	SYNONYMOUS_CODING
980	CakSNP980	*Kabuli*	Ca_Kabuli_Chr01	9195312	[G/A]	Ca_02876	Pentatricopeptide repeat	NON_SYNONYMOUS_CODING
981	CakSNP981	*Kabuli*	Ca_Kabuli_Chr01	9237766	[T/G]		--	INTERGENIC

S.N.	SNP IDs	Chickpea cultivars	Chromosomes	Physical positions (bp)	SNPs	Gene accession IDs	Putative functions	Sequence components of genome
982	CakSNP982	*Kabuli*	Ca_Kabuli_Chr01	9237813	[G/T]		--	INTERGENIC
983	CakSNP983	*Kabuli*	Ca_Kabuli_Chr01	9269888	[T/A]	Ca_02867	Nucleoporin interacting component Nup93/Nic96	NON_SYNONYMOUS_CODING
984	CakSNP984	*Kabuli*	Ca_Kabuli_Chr01	9468041	[C/T]	Ca_02850	NPH3	SYNONYMOUS_CODING
985	CakSNP985	*Kabuli*	Ca_Kabuli_Chr01	9468895	[C/A]	Ca_02850	NPH3	SYNONYMOUS_CODING
986	CakSNP986	*Kabuli*	Ca_Kabuli_Chr01	9476207	[A/C]	Ca_02849	--	INTRON
987	CakSNP987	*Kabuli*	Ca_Kabuli_Chr01	9476388	[A/G]	Ca_02849	--	SYNONYMOUS_CODING
988	CakSNP988	*Kabuli*	Ca_Kabuli_Chr01	9476385	[A/G]	Ca_02849	--	SYNONYMOUS_CODING
989	CakSNP989	*Kabuli*	Ca_Kabuli_Chr01	9535616	[C/T]	Ca_02844	Cyclin, C-terminal	NON_SYNONYMOUS_CODING
990	CakSNP990	*Kabuli*	Ca_Kabuli_Chr01	9547223	[A/G]	Ca_02843	Phenylalanyl-tRNA synthetase	INTRON
991	CakSNP991	*Kabuli*	Ca_Kabuli_Chr01	9547310	[A/G]	Ca_02843	Phenylalanyl-tRNA synthetase	SYNONYMOUS_CODING

S.N.	SNP IDs	Chickpea cultivars	Chromosomes	Physical positions (bp)	SNPs	Gene accession IDs	Putative functions	Sequence components of genome
992	CakSNP992	*Kabuli*	Ca_Kabuli_Chr01	9547854	[G/A]	Ca_02843	Phenylalanyl-tRNA synthetase	INTRON
993	CakSNP993	*Kabuli*	Ca_Kabuli_Chr01	9547834	[C/T]	Ca_02843	Phenylalanyl-tRNA synthetase	INTRON
994	CakSNP994	*Kabuli*	Ca_Kabuli_Chr01	9547819	[T/C]	Ca_02843	Phenylalanyl-tRNA synthetase	INTRON
995	CakSNP995	*Kabuli*	Ca_Kabuli_Chr01	9556418	[C/G]		--	INTERGENIC
996	CakSNP996	*Kabuli*	Ca_Kabuli_Chr01	9556486	[T/G]		--	INTERGENIC
997	CakSNP997	*Kabuli*	Ca_Kabuli_Chr01	9556487	[T/A]		--	INTERGENIC
998	CakSNP998	*Kabuli*	Ca_Kabuli_Chr01	9556488	[G/A]		--	INTERGENIC
999	CakSNP999	*Kabuli*	Ca_Kabuli_Chr01	9556588	[G/T]		--	INTERGENIC
1000	CakSNP1000	*Kabuli*	Ca_Kabuli_Chr01	9556604	[G/C]		--	INTERGENIC
1001	CakSNP1001	*Kabuli*	Ca_Kabuli_Chr01	9556628	[G/A]		--	INTERGENIC

S.N.	SNP IDs	Chickpea cultivars	Chromosomes	Physical positions (bp)	SNPs	Gene accession IDs	Putative functions	Sequence components of genome
1002	CakSNP1002	*Kabuli*	Ca_Kabuli_Chr01	9556735	[G/A]		--	INTERGENIC
1003	CakSNP1003	*Kabuli*	Ca_Kabuli_Chr01	9556662	[C/T]		--	INTERGENIC
1004	CakSNP1004	*Kabuli*	Ca_Kabuli_Chr01	9567049	[C/G]	Ca_02841	Tetratricopeptide repeat-containing	INTRON
1005	CakSNP1005	*Kabuli*	Ca_Kabuli_Chr01	9603896	[T/A]	Ca_02837	WD40 repeat	SYNONYMOUS_CODING
1006	CakSNP1006	*Kabuli*	Ca_Kabuli_Chr01	9634823	[C/T]	Ca_02835	Peptidase C14, caspase non-catalytic subunit p10	SYNONYMOUS_CODING
1007	CakSNP1007	*Kabuli*	Ca_Kabuli_Chr01	9647861	[G/C]		--	INTERGENIC
1008	CakSNP1008	*Kabuli*	Ca_Kabuli_Chr01	9659316	[C/T]		--	INTERGENIC
1009	CakSNP1009	*Kabuli*	Ca_Kabuli_Chr01	9705162	[A/C]	Ca_02827	Domain of unknown function DUF632	SYNONYMOUS_CODING
1010	CakSNP1010	*Kabuli*	Ca_Kabuli_Chr01	9706910	[C/T]	Ca_02826	Nicotinamide N-methyltransferase, putative	INTRON
1011	CakSNP1011	*Kabuli*	Ca_Kabuli_Chr01	9737032	[C/T]		--	INTERGENIC

S.N.	SNP IDs	Chickpea cultivars	Chromosomes	Physical positions (bp)	SNPs	Gene accession IDs	Putative functions	Sequence components of genome
1012	CakSNP1012	*Kabuli*	Ca_Kabuli_Chr01	9737067	[T/C]		--	INTERGENIC
1013	CakSNP1013	*Kabuli*	Ca_Kabuli_Chr01	9757006	[G/A]	Ca_02817	D-isomer specific 2-hydroxyacid dehydrogenase, catalytic domain	SYNONYMOUS_CODING
1014	CakSNP1014	*Kabuli*	Ca_Kabuli_Chr01	9779398	[G/A]	Ca_02813	--	NON_SYNONYMOUS_CODING
1015	CakSNP1015	*Kabuli*	Ca_Kabuli_Chr01	9797342	[G/T]	Ca_02811	Pentatricopeptide repeat	SYNONYMOUS_CODING
1016	CakSNP1016	*Kabuli*	Ca_Kabuli_Chr01	9810275	[T/C]	Ca_02811	Pentatricopeptide repeat	SYNONYMOUS_CODING
1017	CakSNP1017	*Kabuli*	Ca_Kabuli_Chr01	9815448	[A/T]	Ca_02809	Oligopeptide transporter	NON_SYNONYMOUS_CODING
1018	CakSNP1018	*Kabuli*	Ca_Kabuli_Chr01	9815508	[G/C]	Ca_02809	Oligopeptide transporter	NON_SYNONYMOUS_CODING
1019	CakSNP1019	*Kabuli*	Ca_Kabuli_Chr01	9836198	[A/C]		--	INTERGENIC
1020	CakSNP1020	*Kabuli*	Ca_Kabuli_Chr01	9896511	[C/T]	Ca_02797	Major intrinsic protein	SYNONYMOUS_CODING
1021	CakSNP1021	*Kabuli*	Ca_Kabuli_Chr01	9920951	[G/A]		--	INTERGENIC

S.N.	SNP IDs	Chickpea cultivars	Chromosomes	Physical positions (bp)	SNPs	Gene accession IDs	Putative functions	Sequence components of genome
1022	CakSNP1022	*Kabuli*	Ca_Kabuli_Chr01	9920986	[A/C]	Ca_02795	K Homology	STOP_LOST
1023	CakSNP1023	*Kabuli*	Ca_Kabuli_Chr01	9932690	[C/G]	Ca_02792	Peptidase M41	NON_SYNONYMOUS_CODING
1024	CakSNP1024	*Kabuli*	Ca_Kabuli_Chr01	10038486	[T/G]	Ca_02784	--	NON_SYNONYMOUS_CODING
1025	CakSNP1025	*Kabuli*	Ca_Kabuli_Chr01	10049364	[A/G]	Ca_02783	Protein kinase, catalytic domain	SYNONYMOUS_CODING
1026	CakSNP1026	*Kabuli*	Ca_Kabuli_Chr01	10049469	[A/G]	Ca_02783	Protein kinase, catalytic domain	INTRON
1027	CakSNP1027	*Kabuli*	Ca_Kabuli_Chr01	10142688	[A/G]	Ca_02774	--	NON_SYNONYMOUS_CODING
1028	CakSNP1028	*Kabuli*	Ca_Kabuli_Chr01	10155496	[T/G]	Ca_02771	--	SYNONYMOUS_CODING
1029	CakSNP1029	*Kabuli*	Ca_Kabuli_Chr01	10155584	[T/C]	Ca_02771	--	NON_SYNONYMOUS_CODING
1030	CakSNP1030	*Kabuli*	Ca_Kabuli_Chr01	10191578	[A/C]		--	INTERGENIC
1031	CakSNP1031	*Kabuli*	Ca_Kabuli_Chr01	10201074	[C/G]	Ca_02765	--	INTRON

S.N.	SNP IDs	Chickpea cultivars	Chromosomes	Physical positions (bp)	SNPs	Gene accession IDs	Putative functions	Sequence components of genome
1032	CakSNP1032	*Kabuli*	Ca_Kabuli_Chr01	10212925	[G/C]	Ca_02763	Ribosomal protein L28e	INTRON
1033	CakSNP1033	*Kabuli*	Ca_Kabuli_Chr01	10212926	[A/C]	Ca_02763	Ribosomal protein L28e	INTRON
1034	CakSNP1034	*Kabuli*	Ca_Kabuli_Chr01	10212948	[T/C]	Ca_02763	Ribosomal protein L28e	INTRON
1035	CakSNP1035	*Kabuli*	Ca_Kabuli_Chr01	10212976	[T/A]	Ca_02763	Ribosomal protein L28e	INTRON
1036	CakSNP1036	*Kabuli*	Ca_Kabuli_Chr01	10213111	[T/C]	Ca_02763	Ribosomal protein L28e	NON_SYNONYMOUS_CODING
1037	CakSNP1037	*Kabuli*	Ca_Kabuli_Chr01	10266774	[C/T]	Ca_02757	Protein kinase, catalytic domain	INTRON
1038	CakSNP1038	*Kabuli*	Ca_Kabuli_Chr01	10289781	[G/A]	Ca_02752	--	NON_SYNONYMOUS_CODING
1039	CakSNP1039	*Kabuli*	Ca_Kabuli_Chr01	10340870	[G/C]	Ca_02744	Protein kinase, catalytic domain	NON_SYNONYMOUS_CODING
1040	CakSNP1040	*Kabuli*	Ca_Kabuli_Chr01	10364986	[C/G]		--	INTERGENIC
1041	CakSNP1041	*Kabuli*	Ca_Kabuli_Chr01	10365024	[G/A]		--	INTERGENIC

S.N.	SNP IDs	Chickpea cultivars	Chromosomes	Physical positions (bp)	SNPs	Gene accession IDs	Putative functions	Sequence components of genome
1042	CakSNP1042	*Kabuli*	Ca_Kabuli_Chr01	10365029	[T/A]		--	INTERGENIC
1043	CakSNP1043	*Kabuli*	Ca_Kabuli_Chr01	10366383	[G/A]	Ca_02741	Lateral organ boundaries, LOB	SYNONYMOUS_CODING
1044	CakSNP1044	*Kabuli*	Ca_Kabuli_Chr01	10377700	[G/C]	Ca_02740	Glycoside hydrolase, family 35	INTRON
1045	CakSNP1045	*Kabuli*	Ca_Kabuli_Chr01	10377698	[T/C]	Ca_02740	Glycoside hydrolase, family 35	INTRON
1046	CakSNP1046	*Kabuli*	Ca_Kabuli_Chr01	10377691	[T/C]	Ca_02740	Glycoside hydrolase, family 35	INTRON
1047	CakSNP1047	*Kabuli*	Ca_Kabuli_Chr01	10377860	[T/A]	Ca_02740	Glycoside hydrolase, family 35	INTRON
1048	CakSNP1048	*Kabuli*	Ca_Kabuli_Chr01	10408322	[A/G]	Ca_02737	Glycoside hydrolase, family 32	NON_SYNONYMOUS_CODING
1049	CakSNP1049	*Kabuli*	Ca_Kabuli_Chr01	10408349	[G/T]	Ca_02737	Glycoside hydrolase, family 32	NON_SYNONYMOUS_CODING
1050	CakSNP1050	*Kabuli*	Ca_Kabuli_Chr01	10410479	[T/C]	Ca_02736	Nucleosome assembly protein (NAP)	NON_SYNONYMOUS_CODING
1051	CakSNP1051	*Kabuli*	Ca_Kabuli_Chr01	10439153	[C/G]	Ca_02731	IQ motif, EF-hand binding site	INTRON

S.N.	SNP IDs	Chickpea cultivars	Chromosomes	Physical positions (bp)	SNPs	Gene accession IDs	Putative functions	Sequence components of genome
1052	CakSNP1052	*Kabuli*	Ca_Kabuli_Chr01	10439177	[T/C]	Ca_02731	IQ motif, EF-hand binding site	INTRON
1053	CakSNP1053	*Kabuli*	Ca_Kabuli_Chr01	10439190	[C/A]	Ca_02731	IQ motif, EF-hand binding site	INTRON
1054	CakSNP1054	*Kabuli*	Ca_Kabuli_Chr01	10440393	[C/T]	Ca_02731	IQ motif, EF-hand binding site	INTRON
1055	CakSNP1055	*Kabuli*	Ca_Kabuli_Chr01	10440430	[G/T]	Ca_02731	IQ motif, EF-hand binding site	INTRON
1056	CakSNP1056	*Kabuli*	Ca_Kabuli_Chr01	10440533	[C/A]	Ca_02731	IQ motif, EF-hand binding site	SYNONYMOUS_CODING
1057	CakSNP1057	*Kabuli*	Ca_Kabuli_Chr01	10440479	[C/T]	Ca_02731	IQ motif, EF-hand binding site	INTRON
1058	CakSNP1058	*Kabuli*	Ca_Kabuli_Chr01	10489197	[A/G]		--	INTERGENIC
1059	CakSNP1059	*Kabuli*	Ca_Kabuli_Chr01	10527733	[A/G]		--	INTERGENIC
1060	CakSNP1060	*Kabuli*	Ca_Kabuli_Chr01	10551440	[T/C]	Ca_02715	Serine/threonine dehydratase, pyridoxal-phosphate-binding site	SYNONYMOUS_CODING
1061	CakSNP1061	*Kabuli*	Ca_Kabuli_Chr01	10556610	[T/G]	Ca_02714	Zinc finger, PHD-type	INTRON

S.N.	SNP IDs	Chickpea cultivars	Chromosomes	Physical positions (bp)	SNPs	Gene accession IDs	Putative functions	Sequence components of genome
1062	CakSNP1062	*Kabuli*	Ca_Kabuli_Chr01	10560693	[A/G]	Ca_02713	CCAAT-binding transcription factor, subunit B	SYNONYMOUS_CODING
1063	CakSNP1063	*Kabuli*	Ca_Kabuli_Chr01	10560690	[T/G]	Ca_02713	CCAAT-binding transcription factor, subunit B	SYNONYMOUS_CODING
1064	CakSNP1064	*Kabuli*	Ca_Kabuli_Chr01	10567303	[A/C]	Ca_02712	Pentatricopeptide repeat	NON_SYNONYMOUS_CODING
1065	CakSNP1065	*Kabuli*	Ca_Kabuli_Chr01	10571949	[G/A]	Ca_02712	Pentatricopeptide repeat	SYNONYMOUS_CODING
1066	CakSNP1066	*Kabuli*	Ca_Kabuli_Chr01	10601318	[T/G]	Ca_02710	Pentatricopeptide repeat	NON_SYNONYMOUS_CODING
1067	CakSNP1067	*Kabuli*	Ca_Kabuli_Chr01	10605977	[G/A]	Ca_02710	Pentatricopeptide repeat	SYNONYMOUS_CODING
1068	CakSNP1068	*Kabuli*	Ca_Kabuli_Chr01	10623359	[T/G]	Ca_02704	--	NON_SYNONYMOUS_CODING
1069	CakSNP1069	*Kabuli*	Ca_Kabuli_Chr01	10630658	[A/T]	Ca_02703	Phox/Bem1p	INTRON
1070	CakSNP1070	*Kabuli*	Ca_Kabuli_Chr01	10649758	[C/T]	Ca_02701	Plant peroxidase	NON_SYNONYMOUS_CODING
1071	CakSNP1071	*Kabuli*	Ca_Kabuli_Chr01	10655633	[G/T]	Ca_02700	Protein of unknown function DUF3755	INTRON

S.N.	SNP IDs	Chickpea cultivars	Chromosomes	Physical positions (bp)	SNPs	Gene accession IDs	Putative functions	Sequence components of genome
1072	CakSNP1072	*Kabuli*	Ca_Kabuli_Chr01	10657464	[T/A]	Ca_02700	Protein of unknown function DUF3755	INTRON
1073	CakSNP1073	*Kabuli*	Ca_Kabuli_Chr01	10697972	[C/G]	Ca_02694	Protein of unknown function DUF936, plant	INTRON
1074	CakSNP1074	*Kabuli*	Ca_Kabuli_Chr01	10710672	[T/G]	Ca_02692	Basic-leucine zipper (bZIP) transcription factor	SYNONYMOUS_CODING
1075	CakSNP1075	*Kabuli*	Ca_Kabuli_Chr01	10712235	[G/A]		--	INTERGENIC
1076	CakSNP1076	*Kabuli*	Ca_Kabuli_Chr01	10713001	[T/A]		--	INTERGENIC
1077	CakSNP1077	*Kabuli*	Ca_Kabuli_Chr01	10734511	[G/A]		--	INTERGENIC
1078	CakSNP1078	*Kabuli*	Ca_Kabuli_Chr01	10734570	[G/A]		--	INTERGENIC
1079	CakSNP1079	*Kabuli*	Ca_Kabuli_Chr01	10739374	[T/A]	Ca_02687	Ribokinase	INTRON
1080	CakSNP1080	*Kabuli*	Ca_Kabuli_Chr01	10761609	[G/T]		--	INTERGENIC
1081	CakSNP1081	*Kabuli*	Ca_Kabuli_Chr01	10761648	[C/T]		--	INTERGENIC

S.N.	SNP IDs	Chickpea cultivars	Chromosomes	Physical positions (bp)	SNPs	Gene accession IDs	Putative functions	Sequence components of genome
1082	CakSNP1082	*Kabuli*	Ca_Kabuli_Chr01	10814166	[T/C]	Ca_02677	Zinc finger, RING-type	INTRON
1083	CakSNP1083	*Kabuli*	Ca_Kabuli_Chr01	10837203	[C/T]	Ca_02675	--	NON_SYNONYMOUS_CODING
1084	CakSNP1084	*Kabuli*	Ca_Kabuli_Chr01	10861334	[A/G]	Ca_02673	Glycoside hydrolase, family 1	INTRON
1085	CakSNP1085	*Kabuli*	Ca_Kabuli_Chr01	10861332	[C/G]	Ca_02673	Glycoside hydrolase, family 1	INTRON
1086	CakSNP1086	*Kabuli*	Ca_Kabuli_Chr01	10861326	[T/G]	Ca_02673	Glycoside hydrolase, family 1	INTRON
1087	CakSNP1087	*Kabuli*	Ca_Kabuli_Chr01	10869338	[A/T]	Ca_02671	Oxidoreductase, N-terminal	INTRON
1088	CakSNP1088	*Kabuli*	Ca_Kabuli_Chr01	10869336	[G/A]	Ca_02671	Oxidoreductase, N-terminal	INTRON
1089	CakSNP1089	*Kabuli*	Ca_Kabuli_Chr01	10887927	[C/A]	Ca_02670	AMP-dependent synthetase/ligase	NON_SYNONYMOUS_CODING
1090	CakSNP1090	*Kabuli*	Ca_Kabuli_Chr01	10939428	[T/C]	Ca_02666	--	NON_SYNONYMOUS_CODING
1091	CakSNP1091	*Kabuli*	Ca_Kabuli_Chr01	10973356	[C/T]		--	INTERGENIC

S.N.	SNP IDs	Chickpea cultivars	Chromosomes	Physical positions (bp)	SNPs	Gene accession IDs	Putative functions	Sequence components of genome
1092	CakSNP1092	*Kabuli*	Ca_Kabuli_Chr01	11065411	[T/A]	Ca_02653	Zinc/iron permease	INTRON
1093	CakSNP1093	*Kabuli*	Ca_Kabuli_Chr01	11103176	[T/C]		--	INTERGENIC
1094	CakSNP1094	*Kabuli*	Ca_Kabuli_Chr01	11121162	[G/A]	Ca_02646	Phospholipid/glycerol acyltransferase	INTRON
1095	CakSNP1095	*Kabuli*	Ca_Kabuli_Chr01	11122665	[G/T]	Ca_02646	Phospholipid/glycerol acyltransferase	INTRON
1096	CakSNP1096	*Kabuli*	Ca_Kabuli_Chr01	11191023	[G/A]	Ca_02639	--	INTRON
1097	CakSNP1097	*Kabuli*	Ca_Kabuli_Chr01	11193334	[G/C]	Ca_02638	--	NON_SYNONYMOUS_CODING
1098	CakSNP1098	*Kabuli*	Ca_Kabuli_Chr01	11225828	[T/C]	Ca_02632	Pumilio RNA-binding repeat	INTRON
1099	CakSNP1099	*Kabuli*	Ca_Kabuli_Chr01	11227614	[T/C]	Ca_02632	Pumilio RNA-binding repeat	INTRON
1100	CakSNP1100	*Kabuli*	Ca_Kabuli_Chr01	11230086	[A/G]	Ca_02632	Pumilio RNA-binding repeat	SYNONYMOUS_CODING
1101	CakSNP1101	*Kabuli*	Ca_Kabuli_Chr01	11230109	[T/C]	Ca_02632	Pumilio RNA-binding repeat	NON_SYNONYMOUS_CODING

S.N.	SNP IDs	Chickpea cultivars	Chromosomes	Physical positions (bp)	SNPs	Gene accession IDs	Putative functions	Sequence components of genome
1102	CakSNP1102	*Kabuli*	Ca_Kabuli_Chr01	11230115	[G/C]	Ca_02632	Pumilio RNA-binding repeat	NON_SYNONYMOUS_CODING
1103	CakSNP1103	*Kabuli*	Ca_Kabuli_Chr01	11230116	[T/C]	Ca_02632	Pumilio RNA-binding repeat	SYNONYMOUS_CODING
1104	CakSNP1104	*Kabuli*	Ca_Kabuli_Chr01	11230118	[A/T]	Ca_02632	Pumilio RNA-binding repeat	NON_SYNONYMOUS_CODING
1105	CakSNP1105	*Kabuli*	Ca_Kabuli_Chr01	11230125	[A/C]	Ca_02632	Pumilio RNA-binding repeat	SYNONYMOUS_CODING
1106	CakSNP1106	*Kabuli*	Ca_Kabuli_Chr01	11230126	[G/T]	Ca_02632	Pumilio RNA-binding repeat	NON_SYNONYMOUS_CODING
1107	CakSNP1107	*Kabuli*	Ca_Kabuli_Chr01	11230127	[G/T]	Ca_02632	Pumilio RNA-binding repeat	NON_SYNONYMOUS_CODING
1108	CakSNP1108	*Kabuli*	Ca_Kabuli_Chr01	11230128	[T/G]	Ca_02632	Pumilio RNA-binding repeat	SYNONYMOUS_CODING
1109	CakSNP1109	*Kabuli*	Ca_Kabuli_Chr01	11230133	[G/A]	Ca_02632	Pumilio RNA-binding repeat	NON_SYNONYMOUS_CODING
1110	CakSNP1110	*Kabuli*	Ca_Kabuli_Chr01	11230972	[C/T]	Ca_02632	Pumilio RNA-binding repeat	NON_SYNONYMOUS_CODING
1111	CakSNP1111	*Kabuli*	Ca_Kabuli_Chr01	11230998	[A/T]	Ca_02632	Pumilio RNA-binding repeat	SYNONYMOUS_CODING

S.N.	SNP IDs	Chickpea cultivars	Chromosomes	Physical positions (bp)	SNPs	Gene accession IDs	Putative functions	Sequence components of genome
1112	CakSNP1112	*Kabuli*	Ca_Kabuli_Chr01	11231052	[A/G]	Ca_02632	Pumilio RNA-binding repeat	SYNONYMOUS_CODING
1113	CakSNP1113	*Kabuli*	Ca_Kabuli_Chr01	11231064	[A/G]	Ca_02632	Pumilio RNA-binding repeat	SYNONYMOUS_CODING
1114	CakSNP1114	*Kabuli*	Ca_Kabuli_Chr01	11231077	[T/C]	Ca_02632	Pumilio RNA-binding repeat	NON_SYNONYMOUS_CODING
1115	CakSNP1115	*Kabuli*	Ca_Kabuli_Chr01	11235211	[C/T]		--	INTERGENIC
1116	CakSNP1116	*Kabuli*	Ca_Kabuli_Chr01	11265367	[T/C]		--	INTERGENIC
1117	CakSNP1117	*Kabuli*	Ca_Kabuli_Chr01	11277703	[T/A]	Ca_02626	Transcription factor GRAS	SYNONYMOUS_CODING
1118	CakSNP1118	*Kabuli*	Ca_Kabuli_Chr01	11277964	[C/T]	Ca_02626	Transcription factor GRAS	SYNONYMOUS_CODING
1119	CakSNP1119	*Kabuli*	Ca_Kabuli_Chr01	11284183	[T/C]		--	INTERGENIC
1120	CakSNP1120	*Kabuli*	Ca_Kabuli_Chr01	11285192	[G/A]	Ca_02625	Transcription factor GRAS	SYNONYMOUS_CODING
1121	CakSNP1121	*Kabuli*	Ca_Kabuli_Chr01	11285247	[T/C]	Ca_02625	Transcription factor GRAS	SYNONYMOUS_CODING

S.N.	SNP IDs	Chickpea cultivars	Chromosomes	Physical positions (bp)	SNPs	Gene accession IDs	Putative functions	Sequence components of genome
1122	CakSNP1122	*Kabuli*	Ca_Kabuli_Chr01	11285291	[C/A]	Ca_02625	Transcription factor GRAS	NON_SYNONYMOUS_CODING
1123	CakSNP1123	*Kabuli*	Ca_Kabuli_Chr01	11291516	[G/A]	Ca_02624	SANT domain, DNA binding	INTRON
1124	CakSNP1124	*Kabuli*	Ca_Kabuli_Chr01	11291741	[A/C]	Ca_02624	SANT domain, DNA binding	INTRON
1125	CakSNP1125	*Kabuli*	Ca_Kabuli_Chr01	11291740	[T/A]	Ca_02624	SANT domain, DNA binding	INTRON
1126	CakSNP1126	*Kabuli*	Ca_Kabuli_Chr01	11291737	[C/A]	Ca_02624	SANT domain, DNA binding	INTRON
1127	CakSNP1127	*Kabuli*	Ca_Kabuli_Chr01	11319264	[A/C]	Ca_02623	Rab5-interacting	INTRON
1128	CakSNP1128	*Kabuli*	Ca_Kabuli_Chr01	11321670	[G/C]		--	INTERGENIC
1129	CakSNP1129	*Kabuli*	Ca_Kabuli_Chr01	11324018	[C/A]	Ca_02622	--	INTRON
1130	CakSNP1130	*Kabuli*	Ca_Kabuli_Chr01	11324023	[C/T]	Ca_02622	--	INTRON
1131	CakSNP1131	*Kabuli*	Ca_Kabuli_Chr01	11324035	[C/T]	Ca_02622	--	INTRON

S.N.	SNP IDs	Chickpea cultivars	Chromosomes	Physical positions (bp)	SNPs	Gene accession IDs	Putative functions	Sequence components of genome
1132	CakSNP1132	*Kabuli*	Ca_Kabuli_Chr01	11324050	[G/T]	Ca_02622	--	INTRON
1133	CakSNP1133	*Kabuli*	Ca_Kabuli_Chr01	11342388	[T/C]	Ca_02619	Uncharacterised protein domain XH	NON_SYNONYMOUS_CODING
1134	CakSNP1134	*Kabuli*	Ca_Kabuli_Chr01	11361646	[G/A]	Ca_02617	Protein kinase, catalytic domain	SYNONYMOUS_CODING
1135	CakSNP1135	*Kabuli*	Ca_Kabuli_Chr01	11361588	[T/G]	Ca_02617	Protein kinase, catalytic domain	NON_SYNONYMOUS_CODING
1136	CakSNP1136	*Kabuli*	Ca_Kabuli_Chr01	11361583	[T/C]	Ca_02617	Protein kinase, catalytic domain	SYNONYMOUS_CODING
1137	CakSNP1137	*Kabuli*	Ca_Kabuli_Chr01	11374707	[G/A]	Ca_02616	Triosephosphate isomerase	INTRON
1138	CakSNP1138	*Kabuli*	Ca_Kabuli_Chr01	11405015	[A/G]	Ca_02611	Protein kinase, catalytic domain	NON_SYNONYMOUS_CODING
1139	CakSNP1139	*Kabuli*	Ca_Kabuli_Chr01	11405118	[G/A]	Ca_02611	Protein kinase, catalytic domain	SYNONYMOUS_CODING
1140	CakSNP1140	*Kabuli*	Ca_Kabuli_Chr01	11423677	[C/A]	Ca_02609	Zinc finger, RING-type	INTRON
1141	CakSNP1141	*Kabuli*	Ca_Kabuli_Chr01	11487689	[A/G]	Ca_02602	NAD-dependent epimerase/dehydratase	INTRON

S.N.	SNP IDs	Chickpea cultivars	Chromosomes	Physical positions (bp)	SNPs	Gene accession IDs	Putative functions	Sequence components of genome
1142	CakSNP1142	*Kabuli*	Ca_Kabuli_Chr01	11487630	[C/A]	Ca_02602	NAD-dependent epimerase/dehydratase	SYNONYMOUS_CODING
1143	CakSNP1143	*Kabuli*	Ca_Kabuli_Chr01	11487752	[G/T]	Ca_02602	NAD-dependent epimerase/dehydratase	INTRON
1144	CakSNP1144	*Kabuli*	Ca_Kabuli_Chr01	11502148	[C/A]	Ca_02600	Histone H2A	INTRON
1145	CakSNP1145	*Kabuli*	Ca_Kabuli_Chr01	11514888	[T/A]	Ca_02599	Histone H2A	INTRON
1146	CakSNP1146	*Kabuli*	Ca_Kabuli_Chr01	11514898	[T/C]	Ca_02599	Histone H2A	INTRON
1147	CakSNP1147	*Kabuli*	Ca_Kabuli_Chr01	11514903	[A/G]	Ca_02599	Histone H2A	INTRON
1148	CakSNP1148	*Kabuli*	Ca_Kabuli_Chr01	11515055	[G/A]	Ca_02599	Histone H2A	SYNONYMOUS_CODING
1149	CakSNP1149	*Kabuli*	Ca_Kabuli_Chr01	11515130	[T/G]	Ca_02599	Histone H2A	SYNONYMOUS_CODING
1150	CakSNP1150	*Kabuli*	Ca_Kabuli_Chr01	11522656	[G/T]	Ca_02598	Histone H2A	INTRON
1151	CakSNP1151	*Kabuli*	Ca_Kabuli_Chr01	11522743	[T/C]	Ca_02598	Histone H2A	SYNONYMOUS_CODING

S.N.	SNP IDs	Chickpea cultivars	Chromosomes	Physical positions (bp)	SNPs	Gene accession IDs	Putative functions	Sequence components of genome
1152	CakSNP1152	*Kabuli*	Ca_Kabuli_Chr01	11522875	[C/T]	Ca_02598	Histone H2A	SYNONYMOUS_CODING
1153	CakSNP1153	*Kabuli*	Ca_Kabuli_Chr01	11601347	[T/C]	Ca_02586	RNA recognition motif domain	INTRON
1154	CakSNP1154	*Kabuli*	Ca_Kabuli_Chr01	11624419	[C/G]		--	INTERGENIC
1155	CakSNP1155	*Kabuli*	Ca_Kabuli_Chr01	11624464	[C/T]		--	INTERGENIC
1156	CakSNP1156	*Kabuli*	Ca_Kabuli_Chr01	11669794	[T/G]	Ca_02583	Ankyrin repeat	SYNONYMOUS_CODING
1157	CakSNP1157	*Kabuli*	Ca_Kabuli_Chr01	11673478	[A/C]		--	INTERGENIC
1158	CakSNP1158	*Kabuli*	Ca_Kabuli_Chr01	11677097	[C/A]		--	INTERGENIC
1159	CakSNP1159	*Kabuli*	Ca_Kabuli_Chr01	11685769	[A/G]	Ca_02582	Protein kinase, catalytic domain	SYNONYMOUS_CODING
1160	CakSNP1160	*Kabuli*	Ca_Kabuli_Chr01	11685790	[T/C]	Ca_02582	Protein kinase, catalytic domain	SYNONYMOUS_CODING
1161	CakSNP1161	*Kabuli*	Ca_Kabuli_Chr01	11706451	[C/T]		--	INTERGENIC

S.N.	SNP IDs	Chickpea cultivars	Chromosomes	Physical positions (bp)	SNPs	Gene accession IDs	Putative functions	Sequence components of genome
1162	CakSNP1162	*Kabuli*	Ca_Kabuli_Chr01	11706494	[G/C]		--	INTERGENIC
1163	CakSNP1163	*Kabuli*	Ca_Kabuli_Chr01	11706583	[A/C]		--	INTERGENIC
1164	CakSNP1164	*Kabuli*	Ca_Kabuli_Chr01	11706545	[C/G]		--	INTERGENIC
1165	CakSNP1165	*Kabuli*	Ca_Kabuli_Chr01	11741448	[T/A]	Ca_02578	Kinesin, motor domain	INTRON
1166	CakSNP1166	*Kabuli*	Ca_Kabuli_Chr01	11793443	[C/A]	Ca_02573	Alcohol dehydrogenase superfamily, zinc-containing	NON_SYNONYMOUS_CODING
1167	CakSNP1167	*Kabuli*	Ca_Kabuli_Chr01	11822248	[A/T]	Ca_02568	Peptidase S10, serine carboxypeptidase	INTRON
1168	CakSNP1168	*Kabuli*	Ca_Kabuli_Chr01	11822305	[G/T]	Ca_02568	Peptidase S10, serine carboxypeptidase	INTRON
1169	CakSNP1169	*Kabuli*	Ca_Kabuli_Chr01	11822309	[C/A]	Ca_02568	Peptidase S10, serine carboxypeptidase	INTRON
1170	CakSNP1170	*Kabuli*	Ca_Kabuli_Chr01	11870401	[A/G]	Ca_02565	Heavy metal transport/detoxification protein	NON_SYNONYMOUS_CODING
1171	CakSNP1171	*Kabuli*	Ca_Kabuli_Chr01	11935333	[A/T]		--	INTERGENIC

S.N.	SNP IDs	Chickpea cultivars	Chromosomes	Physical positions (bp)	SNPs	Gene accession IDs	Putative functions	Sequence components of genome
1172	CakSNP1172	*Kabuli*	Ca_Kabuli_Chr01	11955896	[G/T]		--	INTERGENIC
1173	CakSNP1173	*Kabuli*	Ca_Kabuli_Chr01	11955866	[G/A]		--	INTERGENIC
1174	CakSNP1174	*Kabuli*	Ca_Kabuli_Chr01	11955853	[A/T]		--	INTERGENIC
1175	CakSNP1175	*Kabuli*	Ca_Kabuli_Chr01	12054809	[C/T]		--	INTERGENIC
1176	CakSNP1176	*Kabuli*	Ca_Kabuli_Chr01	12189822	[T/G]	Ca_02538	Double-stranded RNA-binding	SYNONYMOUS_CODING
1177	CakSNP1177	*Kabuli*	Ca_Kabuli_Chr01	12189825	[T/G]	Ca_02538	Double-stranded RNA-binding	SYNONYMOUS_CODING
1178	CakSNP1178	*Kabuli*	Ca_Kabuli_Chr01	12193933	[T/G]	Ca_02538	Double-stranded RNA-binding	SYNONYMOUS_CODING
1179	CakSNP1179	*Kabuli*	Ca_Kabuli_Chr01	12199691	[C/G]	Ca_02537	Vacuolar fusion protein MON1	INTRON
1180	CakSNP1180	*Kabuli*	Ca_Kabuli_Chr01	12236387	[T/A]	Ca_02531	Protein kinase, catalytic domain	INTRON
1181	CakSNP1181	*Kabuli*	Ca_Kabuli_Chr01	12264697	[T/G]	Ca_02527	Zinc finger, RING-CH-type	INTRON

S.N.	SNP IDs	Chickpea cultivars	Chromosomes	Physical positions (bp)	SNPs	Gene accession IDs	Putative functions	Sequence components of genome
1182	CakSNP1182	*Kabuli*	Ca_Kabuli_Chr01	12326230	[A/G]	Ca_02517	Mlo-related protein	NON_SYNONYMOUS_CODING
1183	CakSNP1183	*Kabuli*	Ca_Kabuli_Chr01	12326234	[T/A]	Ca_02517	Mlo-related protein	NON_SYNONYMOUS_CODING
1184	CakSNP1184	*Kabuli*	Ca_Kabuli_Chr01	12326318	[C/G]	Ca_02517	Mlo-related protein	NON_SYNONYMOUS_CODING
1185	CakSNP1185	*Kabuli*	Ca_Kabuli_Chr01	12334259	[C/T]	Ca_02516	AUX/IAA protein	INTRON
1186	CakSNP1186	*Kabuli*	Ca_Kabuli_Chr01	12340884	[C/T]		--	INTERGENIC
1187	CakSNP1187	*Kabuli*	Ca_Kabuli_Chr01	12390756	[G/T]		--	INTERGENIC
1188	CakSNP1188	*Kabuli*	Ca_Kabuli_Chr01	12390750	[T/C]		--	INTERGENIC
1189	CakSNP1189	*Kabuli*	Ca_Kabuli_Chr01	12391116	[G/C]		--	INTERGENIC
1190	CakSNP1190	*Kabuli*	Ca_Kabuli_Chr01	12391074	[G/A]		--	INTERGENIC
1191	CakSNP1191	*Kabuli*	Ca_Kabuli_Chr01	12391067	[C/A]		--	INTERGENIC

S.N.	SNP IDs	Chickpea cultivars	Chromosomes	Physical positions (bp)	SNPs	Gene accession IDs	Putative functions	Sequence components of genome
1192	CakSNP1192	*Kabuli*	Ca_Kabuli_Chr01	12413012	[G/T]	Ca_02509	--	NON_SYNONYMOUS_CODING
1193	CakSNP1193	*Kabuli*	Ca_Kabuli_Chr01	12413016	[T/A]	Ca_02509	--	NON_SYNONYMOUS_CODING
1194	CakSNP1194	*Kabuli*	Ca_Kabuli_Chr01	12413025	[G/C]	Ca_02509	--	NON_SYNONYMOUS_CODING
1195	CakSNP1195	*Kabuli*	Ca_Kabuli_Chr01	12413116	[G/A]	Ca_02509	--	SYNONYMOUS_CODING
1196	CakSNP1196	*Kabuli*	Ca_Kabuli_Chr01	12414040	[G/A]		--	INTERGENIC
1197	CakSNP1197	*Kabuli*	Ca_Kabuli_Chr01	12481879	[C/A]	Ca_02504	ATPase, V0/A0 complex, 116kDa subunit	INTRON
1198	CakSNP1198	*Kabuli*	Ca_Kabuli_Chr01	12525445	[A/G]		--	INTERGENIC
1199	CakSNP1199	*Kabuli*	Ca_Kabuli_Chr01	12525640	[G/C]		--	INTERGENIC
1200	CakSNP1200	*Kabuli*	Ca_Kabuli_Chr01	12566565	[C/T]		--	INTERGENIC
1201	CakSNP1201	*Kabuli*	Ca_Kabuli_Chr01	12566579	[C/T]		--	INTERGENIC

S.N.	SNP IDs	Chickpea cultivars	Chromosomes	Physical positions (bp)	SNPs	Gene accession IDs	Putative functions	Sequence components of genome
1202	CakSNP1202	*Kabuli*	Ca_Kabuli_Chr01	12566607	[T/C]		--	INTERGENIC
1203	CakSNP1203	*Kabuli*	Ca_Kabuli_Chr01	12566615	[C/T]		--	INTERGENIC
1204	CakSNP1204	*Kabuli*	Ca_Kabuli_Chr01	12566641	[T/C]		--	INTERGENIC
1205	CakSNP1205	*Kabuli*	Ca_Kabuli_Chr01	12588525	[A/G]	Ca_02496	--	INTRON
1206	CakSNP1206	*Kabuli*	Ca_Kabuli_Chr01	12597939	[A/T]	Ca_02495	Protein kinase, catalytic domain	INTRON
1207	CakSNP1207	*Kabuli*	Ca_Kabuli_Chr01	12639552	[T/A]	Ca_02491	Phosphoenolpyruvate carboxylase, active site	INTRON
1208	CakSNP1208	*Kabuli*	Ca_Kabuli_Chr01	12639584	[T/C]	Ca_02491	Phosphoenolpyruvate carboxylase, active site	INTRON
1209	CakSNP1209	*Kabuli*	Ca_Kabuli_Chr01	12687506	[C/T]	Ca_02484	Tetratricopeptide TPR-1	NON_SYNONYMOUS_CODING
1210	CakSNP1210	*Kabuli*	Ca_Kabuli_Chr01	12688424	[A/G]	Ca_02484	Tetratricopeptide TPR-1	INTRON
1211	CakSNP1211	*Kabuli*	Ca_Kabuli_Chr01	12688435	[A/C]	Ca_02484	Tetratricopeptide TPR-1	INTRON

S.N.	SNP IDs	Chickpea cultivars	Chromosomes	Physical positions (bp)	SNPs	Gene accession IDs	Putative functions	Sequence components of genome
1212	CakSNP1212	*Kabuli*	Ca_Kabuli_Chr01	12704406	[G/T]	Ca_02483	Acetohydroxy acid isomeroreductase C-terminal	INTRON
1213	CakSNP1213	*Kabuli*	Ca_Kabuli_Chr01	12705665	[C/T]	Ca_02483	Acetohydroxy acid isomeroreductase C-terminal	SYNONYMOUS_CODING
1214	CakSNP1214	*Kabuli*	Ca_Kabuli_Chr01	12705731	[G/A]	Ca_02483	Acetohydroxy acid isomeroreductase C-terminal	SYNONYMOUS_CODING
1215	CakSNP1215	*Kabuli*	Ca_Kabuli_Chr01	12715427	[C/T]	Ca_02481	Adaptin ear-binding coat-associated protein 1 NECAP-1	INTRON
1216	CakSNP1216	*Kabuli*	Ca_Kabuli_Chr01	12719734	[T/A]	Ca_02480	--	INTRON
1217	CakSNP1217	*Kabuli*	Ca_Kabuli_Chr01	12719735	[G/T]	Ca_02480	--	INTRON
1218	CakSNP1218	*Kabuli*	Ca_Kabuli_Chr01	12719752	[G/C]	Ca_02480	--	INTRON
1219	CakSNP1219	*Kabuli*	Ca_Kabuli_Chr01	12736298	[A/G]	Ca_02478	--	NON_SYNONYMOUS_CODING
1220	CakSNP1220	*Kabuli*	Ca_Kabuli_Chr01	12736353	[A/C]	Ca_02478	--	SYNONYMOUS_CODING
1221	CakSNP1221	*Kabuli*	Ca_Kabuli_Chr01	12736622	[T/G]	Ca_02478	--	NON_SYNONYMOUS_CODING

S.N.	SNP IDs	Chickpea cultivars	Chromosomes	Physical positions (bp)	SNPs	Gene accession IDs	Putative functions	Sequence components of genome
1222	CakSNP1222	*Kabuli*	Ca_Kabuli_Chr01	12738564	[C/T]	Ca_02477	--	SYNONYMOUS_CODING
1223	CakSNP1223	*Kabuli*	Ca_Kabuli_Chr01	12754422	[G/T]		--	INTERGENIC
1224	CakSNP1224	*Kabuli*	Ca_Kabuli_Chr01	12794075	[C/A]		--	INTERGENIC
1225	CakSNP1225	*Kabuli*	Ca_Kabuli_Chr01	12794147	[G/T]	Ca_02472	Basic-leucine zipper (bZIP) transcription factor	NON_SYNONYMOUS_CODING
1226	CakSNP1226	*Kabuli*	Ca_Kabuli_Chr01	12794251	[A/C]	Ca_02472	Basic-leucine zipper (bZIP) transcription factor	NON_SYNONYMOUS_CODING
1227	CakSNP1227	*Kabuli*	Ca_Kabuli_Chr01	12800638	[T/C]	Ca_02471	Peptidase, cysteine peptidase active site	SYNONYMOUS_CODING
1228	CakSNP1228	*Kabuli*	Ca_Kabuli_Chr01	12800610	[A/C]	Ca_02471	Peptidase, cysteine peptidase active site	NON_SYNONYMOUS_CODING
1229	CakSNP1229	*Kabuli*	Ca_Kabuli_Chr01	12800607	[T/A]	Ca_02471	Peptidase, cysteine peptidase active site	NON_SYNONYMOUS_CODING
1230	CakSNP1230	*Kabuli*	Ca_Kabuli_Chr01	12800601	[T/A]	Ca_02471	Peptidase, cysteine peptidase active site	NON_SYNONYMOUS_CODING
1231	CakSNP1231	*Kabuli*	Ca_Kabuli_Chr01	12800597	[G/T]	Ca_02471	Peptidase, cysteine peptidase active site	NON_SYNONYMOUS_CODING

S.N.	SNP IDs	Chickpea cultivars	Chromosomes	Physical positions (bp)	SNPs	Gene accession IDs	Putative functions	Sequence components of genome
1232	CakSNP1232	*Kabuli*	Ca_Kabuli_Chr01	12831717	[G/T]		--	INTERGENIC
1233	CakSNP1233	*Kabuli*	Ca_Kabuli_Chr01	12845445	[A/C]		--	INTERGENIC
1234	CakSNP1234	*Kabuli*	Ca_Kabuli_Chr01	12875506	[G/A]	Ca_02459	Zinc finger, C2H2-type	NON_SYNONYMOUS_CODING
1235	CakSNP1235	*Kabuli*	Ca_Kabuli_Chr01	12880408	[G/A]		--	INTERGENIC
1236	CakSNP1236	*Kabuli*	Ca_Kabuli_Chr01	12912621	[C/T]		--	INTERGENIC
1237	CakSNP1237	*Kabuli*	Ca_Kabuli_Chr01	12912625	[T/C]		--	INTERGENIC
1238	CakSNP1238	*Kabuli*	Ca_Kabuli_Chr01	12912670	[C/T]		--	INTERGENIC
1239	CakSNP1239	*Kabuli*	Ca_Kabuli_Chr01	12947377	[C/T]		--	INTERGENIC
1240	CakSNP1240	*Kabuli*	Ca_Kabuli_Chr01	12947376	[C/T]		--	INTERGENIC
1241	CakSNP1241	*Kabuli*	Ca_Kabuli_Chr01	12947358	[A/T]		--	INTERGENIC

S.N.	SNP IDs	Chickpea cultivars	Chromosomes	Physical positions (bp)	SNPs	Gene accession IDs	Putative functions	Sequence components of genome
1242	CakSNP1242	*Kabuli*	Ca_Kabuli_Chr01	12947347	[A/G]		--	INTERGENIC
1243	CakSNP1243	*Kabuli*	Ca_Kabuli_Chr01	12992445	[A/C]		--	INTERGENIC
1244	CakSNP1244	*Kabuli*	Ca_Kabuli_Chr01	12992423	[A/C]		--	INTERGENIC
1245	CakSNP1245	*Kabuli*	Ca_Kabuli_Chr01	12992569	[A/G]		--	INTERGENIC
1246	CakSNP1246	*Kabuli*	Ca_Kabuli_Chr01	13028543	[T/C]	Ca_14066	1-aminocyclopropane-1-carboxylate synthase	NON_SYNONYMOUS_CODING
1247	CakSNP1247	*Kabuli*	Ca_Kabuli_Chr01	13028517	[G/A]	Ca_14066	1-aminocyclopropane-1-carboxylate synthase	NON_SYNONYMOUS_CODING
1248	CakSNP1248	*Kabuli*	Ca_Kabuli_Chr01	13028505	[A/G]	Ca_14066	1-aminocyclopropane-1-carboxylate synthase	NON_SYNONYMOUS_CODING
1249	CakSNP1249	*Kabuli*	Ca_Kabuli_Chr01	13034708	[T/G]	Ca_14067	--	SYNONYMOUS_CODING
1250	CakSNP1250	*Kabuli*	Ca_Kabuli_Chr01	13034810	[G/A]		--	INTERGENIC
1251	CakSNP1251	*Kabuli*	Ca_Kabuli_Chr01	13040664	[T/C]	Ca_14068	ATPase, P-type, K/Mg/Cd/Cu/Zn/Na/Ca/Na/H-transporter	INTRON

S.N.	SNP IDs	Chickpea cultivars	Chromosomes	Physical positions (bp)	SNPs	Gene accession IDs	Putative functions	Sequence components of genome
1252	CakSNP1252	*Kabuli*	Ca_Kabuli_Chr01	13066954	[C/T]		--	INTERGENIC
1253	CakSNP1253	*Kabuli*	Ca_Kabuli_Chr01	13066968	[C/G]		--	INTERGENIC
1254	CakSNP1254	*Kabuli*	Ca_Kabuli_Chr01	13076647	[T/A]	Ca_14071	Zinc finger, RING-type	INTRON
1255	CakSNP1255	*Kabuli*	Ca_Kabuli_Chr01	13076695	[G/A]	Ca_14071	Zinc finger, RING-type	NON_SYNONYMOUS_CODING
1256	CakSNP1256	*Kabuli*	Ca_Kabuli_Chr01	13156473	[A/C]		--	INTERGENIC
1257	CakSNP1257	*Kabuli*	Ca_Kabuli_Chr01	13158244	[C/T]		--	INTERGENIC
1258	CakSNP1258	*Kabuli*	Ca_Kabuli_Chr01	13158201	[C/T]		--	INTERGENIC
1259	CakSNP1259	*Kabuli*	Ca_Kabuli_Chr01	13158192	[T/C]		--	INTERGENIC
1260	CakSNP1260	*Kabuli*	Ca_Kabuli_Chr01	13199761	[T/A]	Ca_14081	DTW	INTRON
1261	CakSNP1261	*Kabuli*	Ca_Kabuli_Chr01	13202986	[T/G]		--	INTERGENIC

S.N.	SNP IDs	Chickpea cultivars	Chromosomes	Physical positions (bp)	SNPs	Gene accession IDs	Putative functions	Sequence components of genome
1262	CakSNP1262	*Kabuli*	Ca_Kabuli_Chr01	13202991	[T/G]		--	INTERGENIC
1263	CakSNP1263	*Kabuli*	Ca_Kabuli_Chr01	13216776	[G/T]		--	INTERGENIC
1264	CakSNP1264	*Kabuli*	Ca_Kabuli_Chr01	13216741	[T/A]		--	INTERGENIC
1265	CakSNP1265	*Kabuli*	Ca_Kabuli_Chr01	13216710	[G/A]		--	INTERGENIC
1266	CakSNP1266	*Kabuli*	Ca_Kabuli_Chr01	13267896	[T/C]	Ca_14089	Pathogenesis-related transcriptional factor/ERF, DNA-binding	SYNONYMOUS_CODING
1267	CakSNP1267	*Kabuli*	Ca_Kabuli_Chr01	13282556	[A/G]	Ca_14090	Domain of unknown function DUF292, eukaryotic	INTRON
1268	CakSNP1268	*Kabuli*	Ca_Kabuli_Chr01	13282552	[T/C]	Ca_14090	Domain of unknown function DUF292, eukaryotic	INTRON
1269	CakSNP1269	*Kabuli*	Ca_Kabuli_Chr01	13316992	[C/A]		--	INTERGENIC
1270	CakSNP1270	*Kabuli*	Ca_Kabuli_Chr01	13331955	[T/A]		--	INTERGENIC
1271	CakSNP1271	*Kabuli*	Ca_Kabuli_Chr01	13331941	[C/T]		--	INTERGENIC

S.N.	SNP IDs	Chickpea cultivars	Chromosomes	Physical positions (bp)	SNPs	Gene accession IDs	Putative functions	Sequence components of genome
1272	CakSNP1272	*Kabuli*	Ca_Kabuli_Chr01	13332015	[C/A]		--	INTERGENIC
1273	CakSNP1273	*Kabuli*	Ca_Kabuli_Chr01	13332025	[T/G]		--	INTERGENIC
1274	CakSNP1274	*Kabuli*	Ca_Kabuli_Chr01	13365594	[T/A]	Ca_14100	Tetratricopeptide TPR-1	NON_SYNONYMOUS_CODING
1275	CakSNP1275	*Kabuli*	Ca_Kabuli_Chr01	13365625	[C/G]	Ca_14100	Tetratricopeptide TPR-1	NON_SYNONYMOUS_CODING
1276	CakSNP1276	*Kabuli*	Ca_Kabuli_Chr01	13368341	[G/A]		--	INTERGENIC
1277	CakSNP1277	*Kabuli*	Ca_Kabuli_Chr01	13368556	[T/A]		--	INTERGENIC
1278	CakSNP1278	*Kabuli*	Ca_Kabuli_Chr01	13369730	[G/A]	Ca_14101	Protein kinase, catalytic domain	INTRON
1279	CakSNP1279	*Kabuli*	Ca_Kabuli_Chr01	13369986	[C/A]	Ca_14101	Protein kinase, catalytic domain	INTRON
1280	CakSNP1280	*Kabuli*	Ca_Kabuli_Chr01	13497572	[G/T]	Ca_14116	Photosystem II Psp29, biogenesis	INTRON
1281	CakSNP1281	*Kabuli*	Ca_Kabuli_Chr01	13549635	[T/C]	Ca_14121	Kinesin, motor domain	SYNONYMOUS_CODING

S.N.	SNP IDs	Chickpea cultivars	Chromosomes	Physical positions (bp)	SNPs	Gene accession IDs	Putative functions	Sequence components of genome
1282	CakSNP1282	*Kabuli*	Ca_Kabuli_Chr01	13550600	[G/A]	Ca_14121	Kinesin, motor domain	INTRON
1283	CakSNP1283	*Kabuli*	Ca_Kabuli_Chr01	13560364	[T/G]		--	INTERGENIC
1284	CakSNP1284	*Kabuli*	Ca_Kabuli_Chr01	13590903	[G/T]	Ca_14125	Protein kinase, catalytic domain	SYNONYMOUS_CODING
1285	CakSNP1285	*Kabuli*	Ca_Kabuli_Chr01	13591177	[G/A]	Ca_14125	Protein kinase, catalytic domain	NON_SYNONYMOUS_CODING
1286	CakSNP1286	*Kabuli*	Ca_Kabuli_Chr01	13591203	[C/G]	Ca_14125	Protein kinase, catalytic domain	NON_SYNONYMOUS_CODING
1287	CakSNP1287	*Kabuli*	Ca_Kabuli_Chr01	13591314	[C/T]	Ca_14125	Protein kinase, catalytic domain	SYNONYMOUS_CODING
1288	CakSNP1288	*Kabuli*	Ca_Kabuli_Chr01	13593931	[T/A]		--	INTERGENIC
1289	CakSNP1289	*Kabuli*	Ca_Kabuli_Chr01	13596411	[G/T]	Ca_14126	Protein kinase, catalytic domain	SYNONYMOUS_CODING
1290	CakSNP1290	*Kabuli*	Ca_Kabuli_Chr01	13596528	[T/A]	Ca_14126	Protein kinase, catalytic domain	NON_SYNONYMOUS_CODING
1291	CakSNP1291	*Kabuli*	Ca_Kabuli_Chr01	13596539	[A/G]	Ca_14126	Protein kinase, catalytic domain	NON_SYNONYMOUS_CODING

S.N.	SNP IDs	Chickpea cultivars	Chromosomes	Physical positions (bp)	SNPs	Gene accession IDs	Putative functions	Sequence components of genome
1292	CakSNP1292	*Kabuli*	Ca_Kabuli_Chr01	13596666	[T/A]	Ca_14126	Protein kinase, catalytic domain	SYNONYMOUS_CODING
1293	CakSNP1293	*Kabuli*	Ca_Kabuli_Chr01	13596659	[T/A]	Ca_14126	Protein kinase, catalytic domain	NON_SYNONYMOUS_CODING
1294	CakSNP1294	*Kabuli*	Ca_Kabuli_Chr01	13596631	[C/T]	Ca_14126	Protein kinase, catalytic domain	NON_SYNONYMOUS_CODING
1295	CakSNP1295	*Kabuli*	Ca_Kabuli_Chr01	13596621	[C/T]	Ca_14126	Protein kinase, catalytic domain	SYNONYMOUS_CODING
1296	CakSNP1296	*Kabuli*	Ca_Kabuli_Chr01	13596741	[C/T]	Ca_14126	Protein kinase, catalytic domain	SYNONYMOUS_CODING
1297	CakSNP1297	*Kabuli*	Ca_Kabuli_Chr01	13596771	[C/A]	Ca_14126	Protein kinase, catalytic domain	SYNONYMOUS_CODING
1298	CakSNP1298	*Kabuli*	Ca_Kabuli_Chr01	13607486	[A/C]	Ca_14127	Protein kinase, catalytic domain	SYNONYMOUS_CODING
1299	CakSNP1299	*Kabuli*	Ca_Kabuli_Chr01	13608414	[T/C]		--	INTERGENIC
1300	CakSNP1300	*Kabuli*	Ca_Kabuli_Chr01	13637705	[G/T]	Ca_14129	CCAAT-binding factor	INTRON
1301	CakSNP1301	*Kabuli*	Ca_Kabuli_Chr01	13637688	[A/G]	Ca_14129	CCAAT-binding factor	INTRON

S.N.	SNP IDs	Chickpea cultivars	Chromosomes	Physical positions (bp)	SNPs	Gene accession IDs	Putative functions	Sequence components of genome
1302	CakSNP1302	*Kabuli*	Ca_Kabuli_Chr01	13637687	[C/T]	Ca_14129	CCAAT-binding factor	INTRON
1303	CakSNP1303	*Kabuli*	Ca_Kabuli_Chr01	13637684	[C/T]	Ca_14129	CCAAT-binding factor	INTRON
1304	CakSNP1304	*Kabuli*	Ca_Kabuli_Chr01	13639157	[T/C]	Ca_14129	CCAAT-binding factor	INTRON
1305	CakSNP1305	*Kabuli*	Ca_Kabuli_Chr01	13641517	[A/G]	Ca_14129	CCAAT-binding factor	INTRON
1306	CakSNP1306	*Kabuli*	Ca_Kabuli_Chr01	13641644	[G/A]	Ca_14129	CCAAT-binding factor	INTRON
1307	CakSNP1307	*Kabuli*	Ca_Kabuli_Chr01	13641615	[A/G]	Ca_14129	CCAAT-binding factor	INTRON
1308	CakSNP1308	*Kabuli*	Ca_Kabuli_Chr01	13641608	[G/T]	Ca_14129	CCAAT-binding factor	INTRON
1309	CakSNP1309	*Kabuli*	Ca_Kabuli_Chr01	13641574	[A/C]	Ca_14129	CCAAT-binding factor	INTRON
1310	CakSNP1310	*Kabuli*	Ca_Kabuli_Chr01	13641564	[G/A]	Ca_14129	CCAAT-binding factor	INTRON
1311	CakSNP1311	*Kabuli*	Ca_Kabuli_Chr01	13668759	[T/A]		--	INTERGENIC

S.N.	SNP IDs	Chickpea cultivars	Chromosomes	Physical positions (bp)	SNPs	Gene accession IDs	Putative functions	Sequence components of genome
1312	CakSNP1312	*Kabuli*	Ca_Kabuli_Chr01	13680002	[G/T]	Ca_14133	Pathogenesis-related transcriptional factor/ERF, DNA-binding	SYNONYMOUS_CODING
1313	CakSNP1313	*Kabuli*	Ca_Kabuli_Chr01	13680008	[G/C]	Ca_14133	Pathogenesis-related transcriptional factor/ERF, DNA-binding	SYNONYMOUS_CODING
1314	CakSNP1314	*Kabuli*	Ca_Kabuli_Chr01	13762410	[C/A]	Ca_14138	SET domain	NON_SYNONYMOUS_CODING
1315	CakSNP1315	*Kabuli*	Ca_Kabuli_Chr01	13769805	[C/T]		--	INTERGENIC
1316	CakSNP1316	*Kabuli*	Ca_Kabuli_Chr01	13769771	[T/C]		--	INTERGENIC
1317	CakSNP1317	*Kabuli*	Ca_Kabuli_Chr01	13782049	[C/T]	Ca_14141	Glycoside hydrolase, family 14B, plant	SYNONYMOUS_CODING
1318	CakSNP1318	*Kabuli*	Ca_Kabuli_Chr01	13814790	[T/C]	Ca_14146	Protein of unknown function DUF604	INTRON
1319	CakSNP1319	*Kabuli*	Ca_Kabuli_Chr01	13828703	[C/T]	Ca_14148	CCT domain	INTRON
1320	CakSNP1320	*Kabuli*	Ca_Kabuli_Chr01	13828659	[G/A]	Ca_14148	CCT domain	INTRON
1321	CakSNP1321	*Kabuli*	Ca_Kabuli_Chr01	13828657	[G/A]	Ca_14148	CCT domain	INTRON

S.N.	SNP IDs	Chickpea cultivars	Chromosomes	Physical positions (bp)	SNPs	Gene accession IDs	Putative functions	Sequence components of genome
1322	CakSNP1322	*Kabuli*	Ca_Kabuli_Chr01	13828744	[A/T]	Ca_14148	CCT domain	INTRON
1323	CakSNP1323	*Kabuli*	Ca_Kabuli_Chr01	13991356	[T/G]	Ca_07131	Homeobox	SYNONYMOUS_CODING
1324	CakSNP1324	*Kabuli*	Ca_Kabuli_Chr01	14014566	[C/A]	Ca_07128	Ionotropic glutamate receptor	NON_SYNONYMOUS_CODING
1325	CakSNP1325	*Kabuli*	Ca_Kabuli_Chr01	14015757	[T/C]	Ca_07128	Ionotropic glutamate receptor	NON_SYNONYMOUS_CODING
1326	CakSNP1326	*Kabuli*	Ca_Kabuli_Chr01	14015781	[G/C]	Ca_07128	Ionotropic glutamate receptor	NON_SYNONYMOUS_CODING
1327	CakSNP1327	*Kabuli*	Ca_Kabuli_Chr01	14015818	[C/T]	Ca_07128	Ionotropic glutamate receptor	SYNONYMOUS_CODING
1328	CakSNP1328	*Kabuli*	Ca_Kabuli_Chr01	14022892	[C/A]	Ca_07127	Multi antimicrobial extrusion protein	NON_SYNONYMOUS_CODING
1329	CakSNP1329	*Kabuli*	Ca_Kabuli_Chr01	14022890	[A/T]	Ca_07127	Multi antimicrobial extrusion protein	SYNONYMOUS_CODING
1330	CakSNP1330	*Kabuli*	Ca_Kabuli_Chr01	14023097	[A/C]	Ca_07127	Multi antimicrobial extrusion protein	INTRON
1331	CakSNP1331	*Kabuli*	Ca_Kabuli_Chr01	14023095	[T/A]	Ca_07127	Multi antimicrobial extrusion protein	INTRON

S.N.	SNP IDs	Chickpea cultivars	Chromosomes	Physical positions (bp)	SNPs	Gene accession IDs	Putative functions	Sequence components of genome
1332	CakSNP1332	*Kabuli*	Ca_Kabuli_Chr01	14023078	[T/A]	Ca_07127	Multi antimicrobial extrusion protein	INTRON
1333	CakSNP1333	*Kabuli*	Ca_Kabuli_Chr01	14025015	[G/A]	Ca_07127	Multi antimicrobial extrusion protein	SYNONYMOUS_CODING
1334	CakSNP1334	*Kabuli*	Ca_Kabuli_Chr01	14025075	[A/G]	Ca_07127	Multi antimicrobial extrusion protein	SYNONYMOUS_CODING
1335	CakSNP1335	*Kabuli*	Ca_Kabuli_Chr01	14026347	[C/G]		--	INTERGENIC
1336	CakSNP1336	*Kabuli*	Ca_Kabuli_Chr01	14026391	[T/C]		--	INTERGENIC
1337	CakSNP1337	*Kabuli*	Ca_Kabuli_Chr01	14026467	[C/T]		--	INTERGENIC
1338	CakSNP1338	*Kabuli*	Ca_Kabuli_Chr01	14026444	[G/T]		--	INTERGENIC
1339	CakSNP1339	*Kabuli*	Ca_Kabuli_Chr01	14026437	[C/A]		--	INTERGENIC
1340	CakSNP1340	*Kabuli*	Ca_Kabuli_Chr01	14033226	[C/T]	Ca_07126	Multi antimicrobial extrusion protein	SYNONYMOUS_CODING
1341	CakSNP1341	*Kabuli*	Ca_Kabuli_Chr01	14033285	[C/T]	Ca_07126	Multi antimicrobial extrusion protein	NON_SYNONYMOUS_CODING

S.N.	SNP IDs	Chickpea cultivars	Chromosomes	Physical positions (bp)	SNPs	Gene accession IDs	Putative functions	Sequence components of genome
1342	CakSNP1342	*Kabuli*	Ca_Kabuli_Chr01	14033277	[G/C]	Ca_07126	Multi antimicrobial extrusion protein	SYNONYMOUS_CODING
1343	CakSNP1343	*Kabuli*	Ca_Kabuli_Chr01	14033951	[G/A]	Ca_07126	Multi antimicrobial extrusion protein	SYNONYMOUS_CODING
1344	CakSNP1344	*Kabuli*	Ca_Kabuli_Chr01	14034011	[A/G]	Ca_07126	Multi antimicrobial extrusion protein	SYNONYMOUS_CODING
1345	CakSNP1345	*Kabuli*	Ca_Kabuli_Chr01	14045110	[T/C]	Ca_07124	Aconitase A/isopropylmalate dehydratase small subunit, swivel	SYNONYMOUS_CODING
1346	CakSNP1346	*Kabuli*	Ca_Kabuli_Chr01	14045239	[A/G]	Ca_07124	Aconitase A/isopropylmalate dehydratase small subunit, swivel	SYNONYMOUS_CODING
1347	CakSNP1347	*Kabuli*	Ca_Kabuli_Chr01	14076924	[T/C]		--	INTERGENIC
1348	CakSNP1348	*Kabuli*	Ca_Kabuli_Chr01	14101608	[C/T]	Ca_07122	Glycosyl transferase, family 31	INTRON
1349	CakSNP1349	*Kabuli*	Ca_Kabuli_Chr01	14101621	[T/C]	Ca_07122	Glycosyl transferase, family 31	INTRON
1350	CakSNP1350	*Kabuli*	Ca_Kabuli_Chr01	14101624	[C/T]	Ca_07122	Glycosyl transferase, family 31	INTRON
1351	CakSNP1351	*Kabuli*	Ca_Kabuli_Chr01	14101644	[A/G]	Ca_07122	Glycosyl transferase, family 31	INTRON

S.N.	SNP IDs	Chickpea cultivars	Chromosomes	Physical positions (bp)	SNPs	Gene accession IDs	Putative functions	Sequence components of genome
1352	CakSNP1352	*Kabuli*	Ca_Kabuli_Chr01	14163638	[C/T]	Ca_07118	Prephenate dehydratase	INTRON
1353	CakSNP1353	*Kabuli*	Ca_Kabuli_Chr01	14163721	[T/C]	Ca_07118	Prephenate dehydratase	SYNONYMOUS_CODING
1354	CakSNP1354	*Kabuli*	Ca_Kabuli_Chr01	14163836	[A/C]	Ca_07118	Prephenate dehydratase	INTRON
1355	CakSNP1355	*Kabuli*	Ca_Kabuli_Chr01	14225210	[G/T]	Ca_07113	DNA-binding WRKY	SYNONYMOUS_CODING
1356	CakSNP1356	*Kabuli*	Ca_Kabuli_Chr01	14237163	[T/G]	Ca_07112	Cellulose synthase	NON_SYNONYMOUS_CODING
1357	CakSNP1357	*Kabuli*	Ca_Kabuli_Chr01	14237164	[T/A]	Ca_07112	Cellulose synthase	SYNONYMOUS_CODING
1358	CakSNP1358	*Kabuli*	Ca_Kabuli_Chr01	14237273	[A/T]	Ca_07112	Cellulose synthase	NON_SYNONYMOUS_CODING
1359	CakSNP1359	*Kabuli*	Ca_Kabuli_Chr01	14240571	[C/T]	Ca_07111	RNA polymerase, Rpb8	INTRON
1360	CakSNP1360	*Kabuli*	Ca_Kabuli_Chr01	14259435	[G/C]	Ca_07108	Condensin complex subunit 2/barren	NON_SYNONYMOUS_CODING
1361	CakSNP1361	*Kabuli*	Ca_Kabuli_Chr01	14259455	[A/G]	Ca_07108	Condensin complex subunit 2/barren	INTRON

S.N.	SNP IDs	Chickpea cultivars	Chromosomes	Physical positions (bp)	SNPs	Gene accession IDs	Putative functions	Sequence components of genome
1362	CakSNP1362	*Kabuli*	Ca_Kabuli_Chr01	14281643	[T/C]	Ca_07106	Kinesin, motor domain	SYNONYMOUS_CODING
1363	CakSNP1363	*Kabuli*	Ca_Kabuli_Chr01	14348207	[C/T]	Ca_07100	Peptidase C19, ubiquitin carboxyl-terminal hydrolase 2	INTRON
1364	CakSNP1364	*Kabuli*	Ca_Kabuli_Chr01	14393255	[A/G]	Ca_07095	Bacterial surface antigen (D15)	SYNONYMOUS_CODING
1365	CakSNP1365	*Kabuli*	Ca_Kabuli_Chr01	14393313	[C/A]	Ca_07095	Bacterial surface antigen (D15)	NON_SYNONYMOUS_CODING
1366	CakSNP1366	*Kabuli*	Ca_Kabuli_Chr01	14459908	[T/C]	Ca_07091	Helix-loop-helix DNA-binding domain	SYNONYMOUS_CODING
1367	CakSNP1367	*Kabuli*	Ca_Kabuli_Chr01	14576525	[C/T]		--	INTERGENIC
1368	CakSNP1368	*Kabuli*	Ca_Kabuli_Chr01	14623271	[T/C]	Ca_07071	Ovarian tumour, otubain	NON_SYNONYMOUS_CODING
1369	CakSNP1369	*Kabuli*	Ca_Kabuli_Chr01	14698461	[A/T]	Ca_07061	Glycoside hydrolase, family 17	INTRON
1370	CakSNP1370	*Kabuli*	Ca_Kabuli_Chr01	14801406	[A/G]	Ca_07050	Zinc finger, AN1-type	SYNONYMOUS_CODING
1371	CakSNP1371	*Kabuli*	Ca_Kabuli_Chr01	14884940	[G/T]	Ca_07044	Prohibitin	SYNONYMOUS_CODING

S.N.	SNP IDs	Chickpea cultivars	Chromosomes	Physical positions (bp)	SNPs	Gene accession IDs	Putative functions	Sequence components of genome
1372	CakSNP1372	*Kabuli*	Ca_Kabuli_Chr01	14916633	[G/C]		--	INTERGENIC
1373	CakSNP1373	*Kabuli*	Ca_Kabuli_Chr01	15126244	[T/C]	Ca_07021	SNF2-related	INTRON
1374	CakSNP1374	*Kabuli*	Ca_Kabuli_Chr01	15137319	[C/T]	Ca_07020	Zinc finger, PHD-type	INTRON
1375	CakSNP1375	*Kabuli*	Ca_Kabuli_Chr01	15146123	[A/G]	Ca_07019	Clathrin/coatomer adaptor, adaptin-like, N-terminal	NON_SYNONYMOUS_CODING
1376	CakSNP1376	*Kabuli*	Ca_Kabuli_Chr01	15146149	[G/A]	Ca_07019	Clathrin/coatomer adaptor, adaptin-like, N-terminal	SYNONYMOUS_CODING
1377	CakSNP1377	*Kabuli*	Ca_Kabuli_Chr01	15167641	[T/C]	Ca_07018	Protein of unknown function DUF3411	SYNONYMOUS_CODING
1378	CakSNP1378	*Kabuli*	Ca_Kabuli_Chr01	15167643	[G/A]	Ca_07018	Protein of unknown function DUF3411	SYNONYMOUS_CODING
1379	CakSNP1379	*Kabuli*	Ca_Kabuli_Chr01	15196154	[T/A]	Ca_07016	Lipase, GDSL	NON_SYNONYMOUS_CODING
1380	CakSNP1380	*Kabuli*	Ca_Kabuli_Chr01	15236202	[A/G]	Ca_07011	Peptidase A1	SYNONYMOUS_CODING
1381	CakSNP1381	*Kabuli*	Ca_Kabuli_Chr01	15253283	[G/A]		--	INTERGENIC

S.N.	SNP IDs	Chickpea cultivars	Chromosomes	Physical positions (bp)	SNPs	Gene accession IDs	Putative functions	Sequence components of genome
1382	CakSNP1382	*Kabuli*	Ca_Kabuli_Chr01	15253285	[A/G]		--	INTERGENIC
1383	CakSNP1383	*Kabuli*	Ca_Kabuli_Chr01	15291748	[C/T]	Ca_07004	--	SYNONYMOUS_CODING
1384	CakSNP1384	*Kabuli*	Ca_Kabuli_Chr01	15319181	[T/C]	Ca_07002	Zinc finger, PHD-type	SYNONYMOUS_CODING
1385	CakSNP1385	*Kabuli*	Ca_Kabuli_Chr01	15611784	[T/C]	Ca_06983	Ras GTPase	INTRON
1386	CakSNP1386	*Kabuli*	Ca_Kabuli_Chr01	15613071	[A/C]	Ca_06983	Ras GTPase	INTRON
1387	CakSNP1387	*Kabuli*	Ca_Kabuli_Chr01	15683091	[G/A]		--	INTERGENIC
1388	CakSNP1388	*Kabuli*	Ca_Kabuli_Chr01	15683101	[A/C]		--	INTERGENIC
1389	CakSNP1389	*Kabuli*	Ca_Kabuli_Chr01	15683106	[C/A]		--	INTERGENIC
1390	CakSNP1390	*Kabuli*	Ca_Kabuli_Chr01	15683117	[G/T]		--	INTERGENIC
1391	CakSNP1391	*Kabuli*	Ca_Kabuli_Chr01	15683138	[A/G]		--	INTERGENIC

S.N.	SNP IDs	Chickpea cultivars	Chromosomes	Physical positions (bp)	SNPs	Gene accession IDs	Putative functions	Sequence components of genome
1392	CakSNP1392	*Kabuli*	Ca_Kabuli_Chr01	15760638	[C/A]	Ca_06971	Domain of unknown function DUF1692	INTRON
1393	CakSNP1393	*Kabuli*	Ca_Kabuli_Chr01	15784021	[C/T]	Ca_06969	Rab-GAP/TBC domain	NON_SYNONYMOUS_CODING
1394	CakSNP1394	*Kabuli*	Ca_Kabuli_Chr01	15802393	[G/A]	Ca_06967	DDRGK domain containing protein	SYNONYMOUS_CODING
1395	CakSNP1395	*Kabuli*	Ca_Kabuli_Chr01	15862493	[C/T]	Ca_06964	Oxoglutarate/iron-dependent oxygenase	SYNONYMOUS_CODING
1396	CakSNP1396	*Kabuli*	Ca_Kabuli_Chr01	15960425	[A/C]	Ca_06958	Glycosyl transferase, family 8	INTRON
1397	CakSNP1397	*Kabuli*	Ca_Kabuli_Chr01	15965278	[G/A]	Ca_06956	Helix-loop-helix DNA-binding domain	INTRON
1398	CakSNP1398	*Kabuli*	Ca_Kabuli_Chr01	16061242	[A/G]	Ca_06945	BTB/Kelch-associated	INTRON
1399	CakSNP1399	*Kabuli*	Ca_Kabuli_Chr01	16061293	[C/G]	Ca_06945	BTB/Kelch-associated	INTRON
1400	CakSNP1400	*Kabuli*	Ca_Kabuli_Chr01	16061294	[T/A]	Ca_06945	BTB/Kelch-associated	INTRON
1401	CakSNP1401	*Kabuli*	Ca_Kabuli_Chr01	16061295	[A/G]	Ca_06945	BTB/Kelch-associated	INTRON

S.N.	SNP IDs	Chickpea cultivars	Chromosomes	Physical positions (bp)	SNPs	Gene accession IDs	Putative functions	Sequence components of genome
1402	CakSNP1402	*Kabuli*	Ca_Kabuli_Chr01	16061300	[A/G]	Ca_06945	BTB/Kelch-associated	INTRON
1403	CakSNP1403	*Kabuli*	Ca_Kabuli_Chr01	16061385	[A/G]	Ca_06945	BTB/Kelch-associated	INTRON
1404	CakSNP1404	*Kabuli*	Ca_Kabuli_Chr01	16061486	[G/A]	Ca_06945	BTB/Kelch-associated	INTRON
1405	CakSNP1405	*Kabuli*	Ca_Kabuli_Chr01	16061541	[G/A]	Ca_06945	BTB/Kelch-associated	SYNONYMOUS_CODING
1406	CakSNP1406	*Kabuli*	Ca_Kabuli_Chr01	16061536	[A/C]	Ca_06945	BTB/Kelch-associated	NON_SYNONYMOUS_CODING
1407	CakSNP1407	*Kabuli*	Ca_Kabuli_Chr01	16081438	[T/C]	Ca_06943	Transcription factor GRAS	SYNONYMOUS_CODING
1408	CakSNP1408	*Kabuli*	Ca_Kabuli_Chr01	16099437	[T/G]		--	INTERGENIC
1409	CakSNP1409	*Kabuli*	Ca_Kabuli_Chr01	16099438	[G/A]		--	INTERGENIC
1410	CakSNP1410	*Kabuli*	Ca_Kabuli_Chr01	16099468	[G/A]		--	INTERGENIC
1411	CakSNP1411	*Kabuli*	Ca_Kabuli_Chr01	16099444	[T/C]		--	INTERGENIC

S.N.	SNP IDs	Chickpea cultivars	Chromosomes	Physical positions (bp)	SNPs	Gene accession IDs	Putative functions	Sequence components of genome
1412	CakSNP1412	*Kabuli*	Ca_Kabuli_Chr01	16099459	[T/C]		--	INTERGENIC
1413	CakSNP1413	*Kabuli*	Ca_Kabuli_Chr01	16099457	[T/C]		--	INTERGENIC
1414	CakSNP1414	*Kabuli*	Ca_Kabuli_Chr01	16104255	[G/T]		--	INTERGENIC
1415	CakSNP1415	*Kabuli*	Ca_Kabuli_Chr01	16104351	[G/A]		--	INTERGENIC
1416	CakSNP1416	*Kabuli*	Ca_Kabuli_Chr01	16104334	[T/G]		--	INTERGENIC
1417	CakSNP1417	*Kabuli*	Ca_Kabuli_Chr01	16104298	[C/T]		--	INTERGENIC
1418	CakSNP1418	*Kabuli*	Ca_Kabuli_Chr01	16114607	[C/A]	Ca_06940	General substrate transporter	NON_SYNONYMOUS_CODING
1419	CakSNP1419	*Kabuli*	Ca_Kabuli_Chr01	16196930	[A/G]		--	INTERGENIC
1420	CakSNP1420	*Kabuli*	Ca_Kabuli_Chr01	16228049	[C/A]	Ca_06935	NADPH-dependent FMN reductase	INTRON
1421	CakSNP1421	*Kabuli*	Ca_Kabuli_Chr01	16228039	[C/A]	Ca_06935	NADPH-dependent FMN reductase	INTRON

S.N.	SNP IDs	Chickpea cultivars	Chromosomes	Physical positions (bp)	SNPs	Gene accession IDs	Putative functions	Sequence components of genome
1422	CakSNP1422	*Kabuli*	Ca_Kabuli_Chr01	16289397	[G/A]		--	INTERGENIC
1423	CakSNP1423	*Kabuli*	Ca_Kabuli_Chr01	16329161	[G/A]		--	INTERGENIC
1424	CakSNP1424	*Kabuli*	Ca_Kabuli_Chr01	16376254	[G/T]	Ca_06927	Ubiquitin-associated/translation elongation factor EF1B, N-terminal	NON_SYNONYMOUS_CODING
1425	CakSNP1425	*Kabuli*	Ca_Kabuli_Chr01	16379239	[A/G]	Ca_06927	Ubiquitin-associated/translation elongation factor EF1B, N-terminal	INTRON
1426	CakSNP1426	*Kabuli*	Ca_Kabuli_Chr01	16379258	[C/T]	Ca_06927	Ubiquitin-associated/translation elongation factor EF1B, N-terminal	INTRON
1427	CakSNP1427	*Kabuli*	Ca_Kabuli_Chr01	16410186	[C/T]	Ca_06922	Major sperm protein	INTRON
1428	CakSNP1428	*Kabuli*	Ca_Kabuli_Chr01	16419469	[C/T]	Ca_06921	Cyclic nucleotide-binding domain	SYNONYMOUS_CODING
1429	CakSNP1429	*Kabuli*	Ca_Kabuli_Chr01	16454432	[C/T]	Ca_06919	Integrase, catalytic core	SYNONYMOUS_CODING
1430	CakSNP1430	*Kabuli*	Ca_Kabuli_Chr01	16454487	[G/C]	Ca_06919	Integrase, catalytic core	NON_SYNONYMOUS_CODING
1431	CakSNP1431	*Kabuli*	Ca_Kabuli_Chr01	16493894	[A/G]	Ca_06914	Peptidase S54, rhomboid	SYNONYMOUS_CODING

S.N.	SNP IDs	Chickpea cultivars	Chromosomes	Physical positions (bp)	SNPs	Gene accession IDs	Putative functions	Sequence components of genome
1432	CakSNP1432	*Kabuli*	Ca_Kabuli_Chr01	16512011	[G/T]	Ca_06913	Protein of unknown function DUF3411	INTRON
1433	CakSNP1433	*Kabuli*	Ca_Kabuli_Chr01	16512026	[C/T]	Ca_06913	Protein of unknown function DUF3411	INTRON
1434	CakSNP1434	*Kabuli*	Ca_Kabuli_Chr01	16512171	[G/A]	Ca_06913	Protein of unknown function DUF3411	SYNONYMOUS_CODING
1435	CakSNP1435	*Kabuli*	Ca_Kabuli_Chr01	16512195	[G/A]	Ca_06913	Protein of unknown function DUF3411	SYNONYMOUS_CODING
1436	CakSNP1436	*Kabuli*	Ca_Kabuli_Chr01	16515112	[T/C]		--	INTERGENIC
1437	CakSNP1437	*Kabuli*	Ca_Kabuli_Chr01	16527347	[T/A]	Ca_06911	Arf GTPase activating protein	INTRON
1438	CakSNP1438	*Kabuli*	Ca_Kabuli_Chr01	16527366	[G/A]	Ca_06911	Arf GTPase activating protein	INTRON
1439	CakSNP1439	*Kabuli*	Ca_Kabuli_Chr01	16575167	[A/G]	Ca_06906	Protein phosphatase 2C-like	INTRON
1440	CakSNP1440	*Kabuli*	Ca_Kabuli_Chr01	16636587	[G/A]	Ca_06901	No apical meristem (NAM) protein	SYNONYMOUS_CODING
1441	CakSNP1441	*Kabuli*	Ca_Kabuli_Chr01	16743569	[C/T]	Ca_06894	Protein of unknown function DUF1218	SYNONYMOUS_CODING

S.N.	SNP IDs	Chickpea cultivars	Chromosomes	Physical positions (bp)	SNPs	Gene accession IDs	Putative functions	Sequence components of genome
1442	CakSNP1442	*Kabuli*	Ca_Kabuli_Chr01	16799112	[T/C]	Ca_06892	Short-chain dehydrogenase/reductase SDR	SYNONYMOUS_CODING
1443	CakSNP1443	*Kabuli*	Ca_Kabuli_Chr01	16799142	[C/T]	Ca_06892	Short-chain dehydrogenase/reductase SDR	SYNONYMOUS_CODING
1444	CakSNP1444	*Kabuli*	Ca_Kabuli_Chr01	16813600	[T/A]	Ca_06890	--	INTRON
1445	CakSNP1445	*Kabuli*	Ca_Kabuli_Chr01	16813603	[T/G]	Ca_06890	--	INTRON
1446	CakSNP1446	*Kabuli*	Ca_Kabuli_Chr01	16813604	[T/C]	Ca_06890	--	INTRON
1447	CakSNP1447	*Kabuli*	Ca_Kabuli_Chr01	16813657	[A/T]	Ca_06890	--	INTRON
1448	CakSNP1448	*Kabuli*	Ca_Kabuli_Chr01	16813616	[T/A]	Ca_06890	--	INTRON
1449	CakSNP1449	*Kabuli*	Ca_Kabuli_Chr01	16873356	[C/T]	Ca_06886	Regulator of chromosome condensation, RCC1	INTRON
1450	CakSNP1450	*Kabuli*	Ca_Kabuli_Chr01	16877812	[C/A]	Ca_06885	Transcription factor TFIIIC, tau55-related	INTRON
1451	CakSNP1451	*Kabuli*	Ca_Kabuli_Chr01	16877829	[C/A]	Ca_06885	Transcription factor TFIIIC, tau55-related	INTRON

S.N.	SNP IDs	Chickpea cultivars	Chromosomes	Physical positions (bp)	SNPs	Gene accession IDs	Putative functions	Sequence components of genome
1452	CakSNP1452	*Kabuli*	Ca_Kabuli_Chr01	16883153	[C/T]	Ca_06884	Leucine-rich repeat	SYNONYMOUS_CODING
1453	CakSNP1453	*Kabuli*	Ca_Kabuli_Chr01	16901798	[C/A]	Ca_06882	GTP-binding protein, HSR1-related	NON_SYNONYMOUS_CODING
1454	CakSNP1454	*Kabuli*	Ca_Kabuli_Chr01	16902129	[G/A]		--	INTERGENIC
1455	CakSNP1455	*Kabuli*	Ca_Kabuli_Chr01	16908636	[G/A]	Ca_06881	GTP-binding protein, HSR1-related	NON_SYNONYMOUS_CODING
1456	CakSNP1456	*Kabuli*	Ca_Kabuli_Chr01	16933423	[T/G]	Ca_06878	--	INTRON
1457	CakSNP1457	*Kabuli*	Ca_Kabuli_Chr01	16933431	[T/G]	Ca_06878	--	INTRON
1458	CakSNP1458	*Kabuli*	Ca_Kabuli_Chr01	17006391	[T/C]	Ca_06867	Protein of unknown function DUF616	SYNONYMOUS_CODING
1459	CakSNP1459	*Kabuli*	Ca_Kabuli_Chr01	17023111	[G/C]	Ca_06866	Protein kinase, catalytic domain	NON_SYNONYMOUS_CODING
1460	CakSNP1460	*Kabuli*	Ca_Kabuli_Chr01	17024083	[A/C]	Ca_06866	Protein kinase, catalytic domain	SYNONYMOUS_CODING
1461	CakSNP1461	*Kabuli*	Ca_Kabuli_Chr01	17068798	[A/C]		--	INTERGENIC

S.N.	SNP IDs	Chickpea cultivars	Chromosomes	Physical positions (bp)	SNPs	Gene accession IDs	Putative functions	Sequence components of genome
1462	CakSNP1462	*Kabuli*	Ca_Kabuli_Chr01	17092204	[C/T]	Ca_06863	--	NON_SYNONYMOUS_CODING
1463	CakSNP1463	*Kabuli*	Ca_Kabuli_Chr01	17092404	[G/A]	Ca_06863	--	SYNONYMOUS_CODING
1464	CakSNP1464	*Kabuli*	Ca_Kabuli_Chr01	17092413	[C/T]	Ca_06863	--	SYNONYMOUS_CODING
1465	CakSNP1465	*Kabuli*	Ca_Kabuli_Chr01	17093640	[T/C]	Ca_06863	--	NON_SYNONYMOUS_CODING
1466	CakSNP1466	*Kabuli*	Ca_Kabuli_Chr01	17167019	[G/A]		--	INTERGENIC
1467	CakSNP1467	*Kabuli*	Ca_Kabuli_Chr01	17170443	[A/T]		--	INTERGENIC
1468	CakSNP1468	*Kabuli*	Ca_Kabuli_Chr01	17170727	[T/C]		--	INTERGENIC
1469	CakSNP1469	*Kabuli*	Ca_Kabuli_Chr01	17182711	[C/T]	Ca_22768	Universal stress protein A	INTRON
1470	CakSNP1470	*Kabuli*	Ca_Kabuli_Chr01	17182697	[A/T]	Ca_22768	Universal stress protein A	INTRON
1471	CakSNP1471	*Kabuli*	Ca_Kabuli_Chr01	17182867	[G/A]	Ca_22768	Universal stress protein A	INTRON

S.N.	SNP IDs	Chickpea cultivars	Chromosomes	Physical positions (bp)	SNPs	Gene accession IDs	Putative functions	Sequence components of genome
1472	CakSNP1472	*Kabuli*	Ca_Kabuli_Chr01	17194835	[C/T]		--	INTERGENIC
1473	CakSNP1473	*Kabuli*	Ca_Kabuli_Chr01	17272338	[A/C]	Ca_22778	AIG1	NON_SYNONYMOUS_CODING
1474	CakSNP1474	*Kabuli*	Ca_Kabuli_Chr01	17272401	[C/T]	Ca_22778	AIG1	SYNONYMOUS_CODING
1475	CakSNP1475	*Kabuli*	Ca_Kabuli_Chr01	17314880	[A/C]	Ca_22780	WD40 repeat	INTRON
1476	CakSNP1476	*Kabuli*	Ca_Kabuli_Chr01	17314911	[A/G]	Ca_22780	WD40 repeat	NON_SYNONYMOUS_CODING
1477	CakSNP1477	*Kabuli*	Ca_Kabuli_Chr01	17314945	[T/C]	Ca_22780	WD40 repeat	SYNONYMOUS_CODING
1478	CakSNP1478	*Kabuli*	Ca_Kabuli_Chr01	17315046	[G/A]	Ca_22780	WD40 repeat	INTRON
1479	CakSNP1479	*Kabuli*	Ca_Kabuli_Chr01	17328721	[G/A]	Ca_22781	Bromodomain	SYNONYMOUS_CODING
1480	CakSNP1480	*Kabuli*	Ca_Kabuli_Chr01	17328808	[G/A]	Ca_22781	Bromodomain	SYNONYMOUS_CODING
1481	CakSNP1481	*Kabuli*	Ca_Kabuli_Chr01	17332981	[C/T]	Ca_22782	Zinc finger, TFIIS-type	INTRON

S.N.	SNP IDs	Chickpea cultivars	Chromosomes	Physical positions (bp)	SNPs	Gene accession IDs	Putative functions	Sequence components of genome
1482	CakSNP1482	*Kabuli*	Ca_Kabuli_Chr01	17333056	[C/G]	Ca_22782	Zinc finger, TFIIS-type	INTRON
1483	CakSNP1483	*Kabuli*	Ca_Kabuli_Chr01	17333049	[C/T]	Ca_22782	Zinc finger, TFIIS-type	INTRON
1484	CakSNP1484	*Kabuli*	Ca_Kabuli_Chr01	17340321	[T/C]		--	INTERGENIC
1485	CakSNP1485	*Kabuli*	Ca_Kabuli_Chr01	17340350	[G/T]		--	INTERGENIC
1486	CakSNP1486	*Kabuli*	Ca_Kabuli_Chr01	17340398	[G/T]		--	INTERGENIC
1487	CakSNP1487	*Kabuli*	Ca_Kabuli_Chr01	17359712	[A/G]	Ca_22785	Domain of unknown function DUF625	INTRON
1488	CakSNP1488	*Kabuli*	Ca_Kabuli_Chr01	17359688	[A/C]	Ca_22785	Domain of unknown function DUF625	INTRON
1489	CakSNP1489	*Kabuli*	Ca_Kabuli_Chr01	17359686	[C/T]	Ca_22785	Domain of unknown function DUF625	INTRON
1490	CakSNP1490	*Kabuli*	Ca_Kabuli_Chr01	17359682	[A/C]	Ca_22785	Domain of unknown function DUF625	INTRON
1491	CakSNP1491	*Kabuli*	Ca_Kabuli_Chr01	17386549	[C/T]	Ca_22785	Domain of unknown function DUF625	INTRON

S.N.	SNP IDs	Chickpea cultivars	Chromosomes	Physical positions (bp)	SNPs	Gene accession IDs	Putative functions	Sequence components of genome
1492	CakSNP1492	*Kabuli*	Ca_Kabuli_Chr01	17386603	[A/T]	Ca_22785	Domain of unknown function DUF625	INTRON
1493	CakSNP1493	*Kabuli*	Ca_Kabuli_Chr01	17395543	[C/A]	Ca_22785	Domain of unknown function DUF625	NON_SYNONYMOUS_CODING
1494	CakSNP1494	*Kabuli*	Ca_Kabuli_Chr01	17437336	[G/C]	Ca_14789	Glycoside hydrolase, family 19, catalytic	SYNONYMOUS_CODING
1495	CakSNP1495	*Kabuli*	Ca_Kabuli_Chr01	17558885	[T/C]	Ca_14780	Signal transduction response regulator, receiver domain	SYNONYMOUS_CODING
1496	CakSNP1496	*Kabuli*	Ca_Kabuli_Chr01	17558940	[A/G]	Ca_14780	Signal transduction response regulator, receiver domain	NON_SYNONYMOUS_CODING
1497	CakSNP1497	*Kabuli*	Ca_Kabuli_Chr01	17558975	[A/G]	Ca_14780	Signal transduction response regulator, receiver domain	SYNONYMOUS_CODING
1498	CakSNP1498	*Kabuli*	Ca_Kabuli_Chr01	17576793	[T/G]	Ca_14778	Protein of unknown function DUF789	SYNONYMOUS_CODING
1499	CakSNP1499	*Kabuli*	Ca_Kabuli_Chr01	17761244	[T/C]	Ca_14761	Lipase, class 3	SYNONYMOUS_CODING
1500	CakSNP1500	*Kabuli*	Ca_Kabuli_Chr01	17763949	[G/A]	Ca_14761	Lipase, class 3	SYNONYMOUS_CODING
1501	CakSNP1501	*Kabuli*	Ca_Kabuli_Chr01	17819374	[A/C]		--	INTERGENIC

S.N.	SNP IDs	Chickpea cultivars	Chromosomes	Physical positions (bp)	SNPs	Gene accession IDs	Putative functions	Sequence components of genome
1502	CakSNP1502	*Kabuli*	Ca_Kabuli_Chr01	17856892	[A/G]		--	INTERGENIC
1503	CakSNP1503	*Kabuli*	Ca_Kabuli_Chr01	17857089	[T/A]	Ca_14756	Transcription initiation factor IID, 18kDa subunit	INTRON
1504	CakSNP1504	*Kabuli*	Ca_Kabuli_Chr01	17880143	[T/C]	Ca_14753	Non-SMC condensin II complex, subunit H2-like	INTRON
1505	CakSNP1505	*Kabuli*	Ca_Kabuli_Chr01	17880185	[T/C]	Ca_14753	Non-SMC condensin II complex, subunit H2-like	INTRON
1506	CakSNP1506	*Kabuli*	Ca_Kabuli_Chr01	17961779	[T/C]	Ca_14746	Crotonase, core	INTRON
1507	CakSNP1507	*Kabuli*	Ca_Kabuli_Chr01	17983301	[T/G]	Ca_14744	RAG1-activating protein-1-related	SYNONYMOUS_CODING
1508	CakSNP1508	*Kabuli*	Ca_Kabuli_Chr01	17983310	[T/G]	Ca_14744	RAG1-activating protein-1-related	SYNONYMOUS_CODING
1509	CakSNP1509	*Kabuli*	Ca_Kabuli_Chr01	18009665	[C/G]		--	INTERGENIC
1510	CakSNP1510	*Kabuli*	Ca_Kabuli_Chr01	18050590	[G/T]		--	INTERGENIC
1511	CakSNP1511	*Kabuli*	Ca_Kabuli_Chr01	18064930	[C/T]	Ca_14736	--	NON_SYNONYMOUS_CODING

S.N.	SNP IDs	Chickpea cultivars	Chromosomes	Physical positions (bp)	SNPs	Gene accession IDs	Putative functions	Sequence components of genome
1512	CakSNP1512	*Kabuli*	Ca_Kabuli_Chr01	18065239	[T/C]	Ca_14736	--	NON_SYNONYMOUS_CODING
1513	CakSNP1513	*Kabuli*	Ca_Kabuli_Chr01	18092704	[T/G]		--	INTERGENIC
1514	CakSNP1514	*Kabuli*	Ca_Kabuli_Chr01	18092761	[C/T]		--	INTERGENIC
1515	CakSNP1515	*Kabuli*	Ca_Kabuli_Chr01	18098516	[C/T]	Ca_14734	Kinesin, motor domain	SYNONYMOUS_CODING
1516	CakSNP1516	*Kabuli*	Ca_Kabuli_Chr01	18099331	[C/T]	Ca_14734	Kinesin, motor domain	INTRON
1517	CakSNP1517	*Kabuli*	Ca_Kabuli_Chr01	18099334	[T/A]	Ca_14734	Kinesin, motor domain	INTRON
1518	CakSNP1518	*Kabuli*	Ca_Kabuli_Chr01	18112318	[A/G]	Ca_14732	ABC transporter, transmembrane domain	NON_SYNONYMOUS_CODING
1519	CakSNP1519	*Kabuli*	Ca_Kabuli_Chr01	18112353	[T/C]	Ca_14732	ABC transporter, transmembrane domain	SYNONYMOUS_CODING
1520	CakSNP1520	*Kabuli*	Ca_Kabuli_Chr01	18131516	[T/G]	Ca_14730	--	NON_SYNONYMOUS_CODING
1521	CakSNP1521	*Kabuli*	Ca_Kabuli_Chr01	18131545	[A/T]	Ca_14730	--	NON_SYNONYMOUS_CODING

S.N.	SNP IDs	Chickpea cultivars	Chromosomes	Physical positions (bp)	SNPs	Gene accession IDs	Putative functions	Sequence components of genome
1522	CakSNP1522	*Kabuli*	Ca_Kabuli_Chr01	18131898	[G/A]	Ca_14730	--	SYNONYMOUS_CODING
1523	CakSNP1523	*Kabuli*	Ca_Kabuli_Chr01	18141990	[C/A]	Ca_14729	Aminoacyl-tRNA synthetase, class I, conserved site	INTRON
1524	CakSNP1524	*Kabuli*	Ca_Kabuli_Chr01	18144107	[C/G]	Ca_14729	Aminoacyl-tRNA synthetase, class I, conserved site	INTRON
1525	CakSNP1525	*Kabuli*	Ca_Kabuli_Chr01	18144137	[T/A]	Ca_14729	Aminoacyl-tRNA synthetase, class I, conserved site	INTRON
1526	CakSNP1526	*Kabuli*	Ca_Kabuli_Chr01	18147011	[C/G]	Ca_14729	Aminoacyl-tRNA synthetase, class I, conserved site	NON_SYNONYMOUS_CODING
1527	CakSNP1527	*Kabuli*	Ca_Kabuli_Chr01	18150151	[G/A]	Ca_14728	Skb1 methyltransferase	INTRON
1528	CakSNP1528	*Kabuli*	Ca_Kabuli_Chr01	18153870	[T/G]	Ca_14728	Skb1 methyltransferase	INTRON
1529	CakSNP1529	*Kabuli*	Ca_Kabuli_Chr01	18154083	[A/G]	Ca_14728	Skb1 methyltransferase	INTRON
1530	CakSNP1530	*Kabuli*	Ca_Kabuli_Chr01	18156634	[A/G]		--	INTERGENIC
1531	CakSNP1531	*Kabuli*	Ca_Kabuli_Chr01	18172762	[G/T]	Ca_14725	Protein phosphatase 2C-like	NON_SYNONYMOUS_CODING

S.N.	SNP IDs	Chickpea cultivars	Chromosomes	Physical positions (bp)	SNPs	Gene accession IDs	Putative functions	Sequence components of genome
1532	CakSNP1532	*Kabuli*	Ca_Kabuli_Chr01	18198444	[C/T]	Ca_14723	--	INTRON
1533	CakSNP1533	*Kabuli*	Ca_Kabuli_Chr01	18488172	[G/A]		--	INTERGENIC
1534	CakSNP1534	*Kabuli*	Ca_Kabuli_Chr01	18746045	[T/G]		--	INTERGENIC
1535	CakSNP1535	*Kabuli*	Ca_Kabuli_Chr01	18763916	[G/A]	Ca_17380	SET domain	INTRON
1536	CakSNP1536	*Kabuli*	Ca_Kabuli_Chr01	18763911	[C/A]	Ca_17380	SET domain	INTRON
1537	CakSNP1537	*Kabuli*	Ca_Kabuli_Chr01	18764225	[C/T]	Ca_17380	SET domain	INTRON
1538	CakSNP1538	*Kabuli*	Ca_Kabuli_Chr01	18768563	[C/A]		--	INTERGENIC
1539	CakSNP1539	*Kabuli*	Ca_Kabuli_Chr01	18797330	[T/C]	Ca_17376	Amino acid/polyamine transporter I	SYNONYMOUS_CODING
1540	CakSNP1540	*Kabuli*	Ca_Kabuli_Chr01	18890559	[A/G]	Ca_17371	Parallel beta-helix repeat	SYNONYMOUS_CODING
1541	CakSNP1541	*Kabuli*	Ca_Kabuli_Chr01	18890616	[G/A]	Ca_17371	Parallel beta-helix repeat	SYNONYMOUS_CODING

S.N.	SNP IDs	Chickpea cultivars	Chromosomes	Physical positions (bp)	SNPs	Gene accession IDs	Putative functions	Sequence components of genome
1542	CakSNP1542	*Kabuli*	Ca_Kabuli_Chr01	18890833	[G/A]	Ca_17371	Parallel beta-helix repeat	INTRON
1543	CakSNP1543	*Kabuli*	Ca_Kabuli_Chr01	18910213	[C/T]	Ca_17368	Methyltransferase type 11	INTRON
1544	CakSNP1544	*Kabuli*	Ca_Kabuli_Chr01	18910202	[G/A]	Ca_17368	Methyltransferase type 11	INTRON
1545	CakSNP1545	*Kabuli*	Ca_Kabuli_Chr01	18910173	[T/C]	Ca_17368	Methyltransferase type 11	INTRON
1546	CakSNP1546	*Kabuli*	Ca_Kabuli_Chr01	19000445	[T/C]		--	INTERGENIC
1547	CakSNP1547	*Kabuli*	Ca_Kabuli_Chr01	19055085	[C/T]	Ca_17357	Peptidase M41	SYNONYMOUS_CODING
1548	CakSNP1548	*Kabuli*	Ca_Kabuli_Chr01	19130894	[T/C]		--	INTERGENIC
1549	CakSNP1549	*Kabuli*	Ca_Kabuli_Chr01	19130884	[C/A]		--	INTERGENIC
1550	CakSNP1550	*Kabuli*	Ca_Kabuli_Chr01	19130883	[C/A]		--	INTERGENIC
1551	CakSNP1551	*Kabuli*	Ca_Kabuli_Chr01	19130872	[G/A]		--	INTERGENIC

S.N.	SNP IDs	Chickpea cultivars	Chromosomes	Physical positions (bp)	SNPs	Gene accession IDs	Putative functions	Sequence components of genome
1552	CakSNP1552	*Kabuli*	Ca_Kabuli_Chr01	19130833	[G/T]		--	INTERGENIC
1553	CakSNP1553	*Kabuli*	Ca_Kabuli_Chr01	19130831	[A/G]		--	INTERGENIC
1554	CakSNP1554	*Kabuli*	Ca_Kabuli_Chr01	19130848	[C/T]		--	INTERGENIC
1555	CakSNP1555	*Kabuli*	Ca_Kabuli_Chr01	19130853	[G/A]		--	INTERGENIC
1556	CakSNP1556	*Kabuli*	Ca_Kabuli_Chr01	19164976	[T/C]	Ca_17353	Phospholipid/glycerol acyltransferase	INTRON
1557	CakSNP1557	*Kabuli*	Ca_Kabuli_Chr01	19166597	[A/G]		--	INTERGENIC
1558	CakSNP1558	*Kabuli*	Ca_Kabuli_Chr01	19218067	[G/A]		--	INTERGENIC
1559	CakSNP1559	*Kabuli*	Ca_Kabuli_Chr01	19218093	[T/G]		--	INTERGENIC
1560	CakSNP1560	*Kabuli*	Ca_Kabuli_Chr01	19252952	[G/T]	Ca_17349	--	SYNONYMOUS_CODING
1561	CakSNP1561	*Kabuli*	Ca_Kabuli_Chr01	19252938	[G/A]	Ca_17349	--	SYNONYMOUS_CODING

S.N.	SNP IDs	Chickpea cultivars	Chromosomes	Physical positions (bp)	SNPs	Gene accession IDs	Putative functions	Sequence components of genome
1562	CakSNP1562	*Kabuli*	Ca_Kabuli_Chr01	19253655	[C/T]	Ca_17349	--	INTRON
1563	CakSNP1563	*Kabuli*	Ca_Kabuli_Chr01	19254161	[G/A]	Ca_17349	--	INTRON
1564	CakSNP1564	*Kabuli*	Ca_Kabuli_Chr01	19276489	[A/G]	Ca_17347	Cof protein	INTRON
1565	CakSNP1565	*Kabuli*	Ca_Kabuli_Chr01	19276697	[C/G]	Ca_17347	Cof protein	SYNONYMOUS_CODING
1566	CakSNP1566	*Kabuli*	Ca_Kabuli_Chr01	19359812	[A/C]	Ca_17340	Pentatricopeptide repeat	SYNONYMOUS_CODING
1567	CakSNP1567	*Kabuli*	Ca_Kabuli_Chr01	19359844	[C/A]	Ca_17340	Pentatricopeptide repeat	NON_SYNONYMOUS_CODING
1568	CakSNP1568	*Kabuli*	Ca_Kabuli_Chr01	19399112	[C/T]	Ca_17339	ATPase, P-type, H+ transporting proton pump	SYNONYMOUS_CODING
1569	CakSNP1569	*Kabuli*	Ca_Kabuli_Chr01	19526320	[T/C]	Ca_08868	Aldose 1-epimerase	NON_SYNONYMOUS_CODING
1570	CakSNP1570	*Kabuli*	Ca_Kabuli_Chr01	19526531	[C/G]	Ca_08868	Aldose 1-epimerase	INTRON
1571	CakSNP1571	*Kabuli*	Ca_Kabuli_Chr01	19526548	[G/A]	Ca_08868	Aldose 1-epimerase	INTRON

S.N.	SNP IDs	Chickpea cultivars	Chromosomes	Physical positions (bp)	SNPs	Gene accession IDs	Putative functions	Sequence components of genome
1572	CakSNP1572	*Kabuli*	Ca_Kabuli_Chr01	19526561	[T/C]	Ca_08868	Aldose 1-epimerase	INTRON
1573	CakSNP1573	*Kabuli*	Ca_Kabuli_Chr01	19526592	[T/A]	Ca_08868	Aldose 1-epimerase	INTRON
1574	CakSNP1574	*Kabuli*	Ca_Kabuli_Chr01	19526652	[G/T]	Ca_08868	Aldose 1-epimerase	INTRON
1575	CakSNP1575	*Kabuli*	Ca_Kabuli_Chr01	19526632	[C/T]	Ca_08868	Aldose 1-epimerase	INTRON
1576	CakSNP1576	*Kabuli*	Ca_Kabuli_Chr01	19572408	[C/T]	Ca_08866	Pumilio RNA-binding repeat	INTRON
1577	CakSNP1577	*Kabuli*	Ca_Kabuli_Chr01	19572494	[G/C]	Ca_08866	Pumilio RNA-binding repeat	INTRON
1578	CakSNP1578	*Kabuli*	Ca_Kabuli_Chr01	19591671	[A/G]		--	INTERGENIC
1579	CakSNP1579	*Kabuli*	Ca_Kabuli_Chr01	19877654	[A/T]	Ca_08848	Peptidase S8/S53, subtilisin/kexin/sedolisin	INTRON
1580	CakSNP1580	*Kabuli*	Ca_Kabuli_Chr01	19902735	[T/A]	Ca_08847	RNA polymerase I associated factor, A49-like	INTRON
1581	CakSNP1581	*Kabuli*	Ca_Kabuli_Chr01	19903665	[A/G]	Ca_08847	RNA polymerase I associated factor, A49-like	INTRON

S.N.	SNP IDs	Chickpea cultivars	Chromosomes	Physical positions (bp)	SNPs	Gene accession IDs	Putative functions	Sequence components of genome
1582	CakSNP1582	*Kabuli*	Ca_Kabuli_Chr01	19903683	[G/A]	Ca_08847	RNA polymerase I associated factor, A49-like	INTRON
1583	CakSNP1583	*Kabuli*	Ca_Kabuli_Chr01	19903686	[T/A]	Ca_08847	RNA polymerase I associated factor, A49-like	INTRON
1584	CakSNP1584	*Kabuli*	Ca_Kabuli_Chr01	19903690	[A/G]	Ca_08847	RNA polymerase I associated factor, A49-like	INTRON
1585	CakSNP1585	*Kabuli*	Ca_Kabuli_Chr01	19903693	[G/T]	Ca_08847	RNA polymerase I associated factor, A49-like	INTRON
1586	CakSNP1586	*Kabuli*	Ca_Kabuli_Chr01	19903701	[G/A]	Ca_08847	RNA polymerase I associated factor, A49-like	INTRON
1587	CakSNP1587	*Kabuli*	Ca_Kabuli_Chr01	19903704	[G/A]	Ca_08847	RNA polymerase I associated factor, A49-like	INTRON
1588	CakSNP1588	*Kabuli*	Ca_Kabuli_Chr01	20004655	[A/G]	Ca_08843	--	INTRON
1589	CakSNP1589	*Kabuli*	Ca_Kabuli_Chr01	20005747	[C/T]	Ca_08843	--	INTRON
1590	CakSNP1590	*Kabuli*	Ca_Kabuli_Chr01	20016525	[C/T]		--	INTERGENIC
1591	CakSNP1591	*Kabuli*	Ca_Kabuli_Chr01	20070045	[A/G]		--	INTERGENIC

S.N.	SNP IDs	Chickpea cultivars	Chromosomes	Physical positions (bp)	SNPs	Gene accession IDs	Putative functions	Sequence components of genome
1592	CakSNP1592	*Kabuli*	Ca_Kabuli_Chr01	20070197	[T/C]	Ca_08835	--	START_LOST
1593	CakSNP1593	*Kabuli*	Ca_Kabuli_Chr01	20091291	[T/G]	Ca_08834	BRCT	NON_SYNONYMOUS_CODING
1594	CakSNP1594	*Kabuli*	Ca_Kabuli_Chr01	20092571	[C/G]	Ca_08834	BRCT	INTRON
1595	CakSNP1595	*Kabuli*	Ca_Kabuli_Chr01	20092677	[C/A]	Ca_08834	BRCT	NON_SYNONYMOUS_CODING
1596	CakSNP1596	*Kabuli*	Ca_Kabuli_Chr01	21822975	[A/G]	Ca_22111	Preprotein translocase Sec, Sec61-beta subunit	SYNONYMOUS_CODING
1597	CakSNP1597	*Kabuli*	Ca_Kabuli_Chr01	21881133	[G/A]	Ca_22109	--	NON_SYNONYMOUS_CODING
1598	CakSNP1598	*Kabuli*	Ca_Kabuli_Chr01	21881112	[C/T]	Ca_22109	--	NON_SYNONYMOUS_CODING
1599	CakSNP1599	*Kabuli*	Ca_Kabuli_Chr01	21881103	[G/A]	Ca_22109	--	NON_SYNONYMOUS_CODING
1600	CakSNP1600	*Kabuli*	Ca_Kabuli_Chr01	21881094	[G/A]	Ca_22109	--	NON_SYNONYMOUS_CODING
1601	CakSNP1601	*Kabuli*	Ca_Kabuli_Chr01	21918747	[G/A]	Ca_22107	ATPase, P-type, K/Mg/Cd/Cu/Zn/Na/Ca/Na/H-transporter	INTRON

S.N.	SNP IDs	Chickpea cultivars	Chromosomes	Physical positions (bp)	SNPs	Gene accession IDs	Putative functions	Sequence components of genome
1602	CakSNP1602	*Kabuli*	Ca_Kabuli_Chr01	22344003	[G/A]	Ca_20629	WD40 repeat	INTRON
1603	CakSNP1603	*Kabuli*	Ca_Kabuli_Chr01	22437033	[C/T]		--	INTERGENIC
1604	CakSNP1604	*Kabuli*	Ca_Kabuli_Chr01	22465555	[C/T]		--	INTERGENIC
1605	CakSNP1605	*Kabuli*	Ca_Kabuli_Chr01	22465692	[A/G]		--	INTERGENIC
1606	CakSNP1606	*Kabuli*	Ca_Kabuli_Chr01	22719581	[T/G]		--	INTERGENIC
1607	CakSNP1607	*Kabuli*	Ca_Kabuli_Chr01	22743361	[T/G]	Ca_20613	Nucleosome assembly protein (NAP)	NON_SYNONYMOUS_CODING
1608	CakSNP1608	*Kabuli*	Ca_Kabuli_Chr01	23122951	[A/G]	Ca_22358	Helicase, C-terminal	INTRON
1609	CakSNP1609	*Kabuli*	Ca_Kabuli_Chr01	23128921	[T/A]	Ca_22358	Helicase, C-terminal	INTRON
1610	CakSNP1610	*Kabuli*	Ca_Kabuli_Chr01	23128979	[G/T]	Ca_22358	Helicase, C-terminal	INTRON
1611	CakSNP1611	*Kabuli*	Ca_Kabuli_Chr01	23129094	[C/T]	Ca_22358	Helicase, C-terminal	INTRON

S.N.	SNP IDs	Chickpea cultivars	Chromosomes	Physical positions (bp)	SNPs	Gene accession IDs	Putative functions	Sequence components of genome
1612	CakSNP1612	*Kabuli*	Ca_Kabuli_Chr01	23131178	[A/C]	Ca_22358	Helicase, C-terminal	INTRON
1613	CakSNP1613	*Kabuli*	Ca_Kabuli_Chr01	23131914	[T/C]	Ca_22358	Helicase, C-terminal	NON_SYNONYMOUS_CODING
1614	CakSNP1614	*Kabuli*	Ca_Kabuli_Chr01	23148736	[A/C]	Ca_22359	Initiation factor 2B-related	SYNONYMOUS_CODING
1615	CakSNP1615	*Kabuli*	Ca_Kabuli_Chr01	23149053	[A/G]	Ca_22359	Initiation factor 2B-related	SYNONYMOUS_CODING
1616	CakSNP1616	*Kabuli*	Ca_Kabuli_Chr01	23149096	[A/C]	Ca_22359	Initiation factor 2B-related	NON_SYNONYMOUS_CODING
1617	CakSNP1617	*Kabuli*	Ca_Kabuli_Chr01	23341499	[C/G]		--	INTERGENIC
1618	CakSNP1618	*Kabuli*	Ca_Kabuli_Chr01	23382483	[T/A]		--	INTERGENIC
1619	CakSNP1619	*Kabuli*	Ca_Kabuli_Chr01	23382454	[G/T]		--	INTERGENIC
1620	CakSNP1620	*Kabuli*	Ca_Kabuli_Chr01	23382664	[C/T]		--	INTERGENIC
1621	CakSNP1621	*Kabuli*	Ca_Kabuli_Chr01	23382645	[C/G]		--	INTERGENIC

S.N.	SNP IDs	Chickpea cultivars	Chromosomes	Physical positions (bp)	SNPs	Gene accession IDs	Putative functions	Sequence components of genome
1622	CakSNP1622	*Kabuli*	Ca_Kabuli_Chr01	23915127	[C/G]	Ca_19294	ATPase, P-type, K/Mg/Cd/Cu/Zn/Na/Ca/Na/H-transporter	SYNONYMOUS_CODING
1623	CakSNP1623	*Kabuli*	Ca_Kabuli_Chr01	23941438	[G/A]		--	INTERGENIC
1624	CakSNP1624	*Kabuli*	Ca_Kabuli_Chr01	23941518	[C/T]		--	INTERGENIC
1625	CakSNP1625	*Kabuli*	Ca_Kabuli_Chr01	24070908	[C/T]	Ca_19289	AUX/IAA protein	INTRON
1626	CakSNP1626	*Kabuli*	Ca_Kabuli_Chr01	24070968	[C/T]	Ca_19289	AUX/IAA protein	INTRON
1627	CakSNP1627	*Kabuli*	Ca_Kabuli_Chr01	24071007	[C/A]	Ca_19289	AUX/IAA protein	INTRON
1628	CakSNP1628	*Kabuli*	Ca_Kabuli_Chr01	24071014	[T/G]	Ca_19289	AUX/IAA protein	INTRON
1629	CakSNP1629	*Kabuli*	Ca_Kabuli_Chr01	24173273	[A/G]		--	INTERGENIC
1630	CakSNP1630	*Kabuli*	Ca_Kabuli_Chr01	24450415	[T/C]	Ca_18343	Ribulose-phosphate 3-epimerase	INTRON
1631	CakSNP1631	*Kabuli*	Ca_Kabuli_Chr01	24450511	[A/G]	Ca_18343	Ribulose-phosphate 3-epimerase	INTRON

S.N.	SNP IDs	Chickpea cultivars	Chromosomes	Physical positions (bp)	SNPs	Gene accession IDs	Putative functions	Sequence components of genome
1632	CakSNP1632	*Kabuli*	Ca_Kabuli_Chr01	24450483	[T/G]	Ca_18343	Ribulose-phosphate 3-epimerase	INTRON
1633	CakSNP1633	*Kabuli*	Ca_Kabuli_Chr01	24693506	[C/T]	Ca_18336	--	NON_SYNONYMOUS_CODING
1634	CakSNP1634	*Kabuli*	Ca_Kabuli_Chr01	24693602	[A/G]	Ca_18336	--	NON_SYNONYMOUS_CODING
1635	CakSNP1635	*Kabuli*	Ca_Kabuli_Chr01	24693601	[C/T]	Ca_18336	--	SYNONYMOUS_CODING
1636	CakSNP1636	*Kabuli*	Ca_Kabuli_Chr01	24693586	[C/T]	Ca_18336	--	SYNONYMOUS_CODING
1637	CakSNP1637	*Kabuli*	Ca_Kabuli_Chr01	24693571	[G/A]	Ca_18336	--	SYNONYMOUS_CODING
1638	CakSNP1638	*Kabuli*	Ca_Kabuli_Chr01	24693550	[G/A]	Ca_18336	--	SYNONYMOUS_CODING
1639	CakSNP1639	*Kabuli*	Ca_Kabuli_Chr01	24693533	[G/A]	Ca_18336	--	NON_SYNONYMOUS_CODING
1640	CakSNP1640	*Kabuli*	Ca_Kabuli_Chr01	24693521	[G/A]	Ca_18336	--	NON_SYNONYMOUS_CODING
1641	CakSNP1641	*Kabuli*	Ca_Kabuli_Chr01	24828169	[C/T]	Ca_18326	ARID/BRIGHT DNA-binding domain	INTRON

S.N.	SNP IDs	Chickpea cultivars	Chromosomes	Physical positions (bp)	SNPs	Gene accession IDs	Putative functions	Sequence components of genome
1642	CakSNP1642	*Kabuli*	Ca_Kabuli_Chr01	24828245	[A/C]	Ca_18326	ARID/BRIGHT DNA-binding domain	SYNONYMOUS_CODING
1643	CakSNP1643	*Kabuli*	Ca_Kabuli_Chr01	24845148	[A/C]	Ca_18325	Zinc finger, RING-type	SYNONYMOUS_CODING
1644	CakSNP1644	*Kabuli*	Ca_Kabuli_Chr01	24983908	[A/G]	Ca_18319	Pectinesterase, catalytic	NON_SYNONYMOUS_CODING
1645	CakSNP1645	*Kabuli*	Ca_Kabuli_Chr01	25039836	[T/C]		--	INTERGENIC
1646	CakSNP1646	*Kabuli*	Ca_Kabuli_Chr01	25083482	[A/C]	Ca_18313	Pectinesterase, catalytic	INTRON
1647	CakSNP1647	*Kabuli*	Ca_Kabuli_Chr01	25083534	[A/C]	Ca_18313	Pectinesterase, catalytic	INTRON
1648	CakSNP1648	*Kabuli*	Ca_Kabuli_Chr01	25175124	[C/T]		--	INTERGENIC
1649	CakSNP1649	*Kabuli*	Ca_Kabuli_Chr01	25176162	[C/A]	Ca_18308	5'(3')-deoxyribonucleotidase	NON_SYNONYMOUS_CODING
1650	CakSNP1650	*Kabuli*	Ca_Kabuli_Chr01	25192363	[T/C]		--	INTERGENIC
1651	CakSNP1651	*Kabuli*	Ca_Kabuli_Chr01	25524382	[C/T]		--	INTERGENIC

S.N.	SNP IDs	Chickpea cultivars	Chromosomes	Physical positions (bp)	SNPs	Gene accession IDs	Putative functions	Sequence components of genome
1652	CakSNP1652	*Kabuli*	Ca_Kabuli_Chr01	25524425	[C/T]		--	INTERGENIC
1653	CakSNP1653	*Kabuli*	Ca_Kabuli_Chr01	25524441	[T/C]		--	INTERGENIC
1654	CakSNP1654	*Kabuli*	Ca_Kabuli_Chr01	25524459	[C/G]		--	INTERGENIC
1655	CakSNP1655	*Kabuli*	Ca_Kabuli_Chr01	25524532	[T/C]		--	INTERGENIC
1656	CakSNP1656	*Kabuli*	Ca_Kabuli_Chr01	25524519	[C/T]		--	INTERGENIC
1657	CakSNP1657	*Kabuli*	Ca_Kabuli_Chr01	25524481	[T/A]		--	INTERGENIC
1658	CakSNP1658	*Kabuli*	Ca_Kabuli_Chr01	25524480	[G/A]		--	INTERGENIC
1659	CakSNP1659	*Kabuli*	Ca_Kabuli_Chr01	25524473	[C/T]		--	INTERGENIC
1660	CakSNP1660	*Kabuli*	Ca_Kabuli_Chr01	25524465	[A/G]		--	INTERGENIC
1661	CakSNP1661	*Kabuli*	Ca_Kabuli_Chr01	25524460	[G/A]		--	INTERGENIC

S.N.	SNP IDs	Chickpea cultivars	Chromosomes	Physical positions (bp)	SNPs	Gene accession IDs	Putative functions	Sequence components of genome
1662	CakSNP1662	*Kabuli*	Ca_Kabuli_Chr01	25524458	[G/A]		--	INTERGENIC
1663	CakSNP1663	*Kabuli*	Ca_Kabuli_Chr01	25524456	[C/T]		--	INTERGENIC
1664	CakSNP1664	*Kabuli*	Ca_Kabuli_Chr01	25524455	[G/A]		--	INTERGENIC
1665	CakSNP1665	*Kabuli*	Ca_Kabuli_Chr01	25524453	[C/T]		--	INTERGENIC
1666	CakSNP1666	*Kabuli*	Ca_Kabuli_Chr01	25524451	[A/T]		--	INTERGENIC
1667	CakSNP1667	*Kabuli*	Ca_Kabuli_Chr01	25524450	[C/T]		--	INTERGENIC
1668	CakSNP1668	*Kabuli*	Ca_Kabuli_Chr01	25524464	[G/A]		--	INTERGENIC
1669	CakSNP1669	*Kabuli*	Ca_Kabuli_Chr01	25524478	[C/A]		--	INTERGENIC
1670	CakSNP1670	*Kabuli*	Ca_Kabuli_Chr01	25524488	[G/T]		--	INTERGENIC
1671	CakSNP1671	*Kabuli*	Ca_Kabuli_Chr01	25524498	[G/C]		--	INTERGENIC

S.N.	SNP IDs	Chickpea cultivars	Chromosomes	Physical positions (bp)	SNPs	Gene accession IDs	Putative functions	Sequence components of genome
1672	CakSNP1672	*Kabuli*	Ca_Kabuli_Chr01	25524505	[A/G]		--	INTERGENIC
1673	CakSNP1673	*Kabuli*	Ca_Kabuli_Chr01	25524520	[C/T]		--	INTERGENIC
1674	CakSNP1674	*Kabuli*	Ca_Kabuli_Chr01	25524522	[G/A]		--	INTERGENIC
1675	CakSNP1675	*Kabuli*	Ca_Kabuli_Chr01	25524524	[C/T]		--	INTERGENIC
1676	CakSNP1676	*Kabuli*	Ca_Kabuli_Chr01	25732004	[T/C]		--	INTERGENIC
1677	CakSNP1677	*Kabuli*	Ca_Kabuli_Chr01	25887539	[G/A]		--	INTERGENIC
1678	CakSNP1678	*Kabuli*	Ca_Kabuli_Chr01	25987201	[A/C]		--	INTERGENIC
1679	CakSNP1679	*Kabuli*	Ca_Kabuli_Chr01	26026817	[C/T]	Ca_18591	Transcription factor, SBP-box	SYNONYMOUS_CODING
1680	CakSNP1680	*Kabuli*	Ca_Kabuli_Chr01	26190096	[G/A]		--	INTERGENIC
1681	CakSNP1681	*Kabuli*	Ca_Kabuli_Chr01	26190050	[G/A]		--	INTERGENIC

S.N.	SNP IDs	Chickpea cultivars	Chromosomes	Physical positions (bp)	SNPs	Gene accession IDs	Putative functions	Sequence components of genome
1682	CakSNP1682	*Kabuli*	Ca_Kabuli_Chr01	26481730	[C/T]	Ca_18610	NSF attachment protein	INTRON
1683	CakSNP1683	*Kabuli*	Ca_Kabuli_Chr01	26481747	[T/G]	Ca_18610	NSF attachment protein	INTRON
1684	CakSNP1684	*Kabuli*	Ca_Kabuli_Chr01	26484868	[G/C]	Ca_18610	NSF attachment protein	NON_SYNONYMOUS_CODING
1685	CakSNP1685	*Kabuli*	Ca_Kabuli_Chr01	26574098	[C/T]	Ca_18614	Peptidase C48, SUMO/Sentrin/Ubl1	NON_SYNONYMOUS_CODING
1686	CakSNP1686	*Kabuli*	Ca_Kabuli_Chr01	26574146	[C/T]	Ca_18614	Peptidase C48, SUMO/Sentrin/Ubl1	NON_SYNONYMOUS_CODING
1687	CakSNP1687	*Kabuli*	Ca_Kabuli_Chr01	26579621	[A/C]		--	INTERGENIC
1688	CakSNP1688	*Kabuli*	Ca_Kabuli_Chr01	26746094	[A/T]		--	INTERGENIC
1689	CakSNP1689	*Kabuli*	Ca_Kabuli_Chr01	26767522	[A/G]	Ca_20282	Protein kinase, catalytic domain	NON_SYNONYMOUS_CODING
1690	CakSNP1690	*Kabuli*	Ca_Kabuli_Chr01	26767514	[A/G]	Ca_20282	Protein kinase, catalytic domain	SYNONYMOUS_CODING
1691	CakSNP1691	*Kabuli*	Ca_Kabuli_Chr01	26784404	[G/A]		--	INTERGENIC

S.N.	SNP IDs	Chickpea cultivars	Chromosomes	Physical positions (bp)	SNPs	Gene accession IDs	Putative functions	Sequence components of genome
1692	CakSNP1692	*Kabuli*	Ca_Kabuli_Chr01	26784421	[G/T]		--	INTERGENIC
1693	CakSNP1693	*Kabuli*	Ca_Kabuli_Chr01	26784422	[C/T]		--	INTERGENIC
1694	CakSNP1694	*Kabuli*	Ca_Kabuli_Chr01	26784464	[C/T]		--	INTERGENIC
1695	CakSNP1695	*Kabuli*	Ca_Kabuli_Chr01	26784548	[C/A]		--	INTERGENIC
1696	CakSNP1696	*Kabuli*	Ca_Kabuli_Chr01	26784570	[A/G]		--	INTERGENIC
1697	CakSNP1697	*Kabuli*	Ca_Kabuli_Chr01	26784592	[A/G]		--	INTERGENIC
1698	CakSNP1698	*Kabuli*	Ca_Kabuli_Chr01	26784618	[G/A]		--	INTERGENIC
1699	CakSNP1699	*Kabuli*	Ca_Kabuli_Chr01	26784732	[C/G]		--	INTERGENIC
1700	CakSNP1700	*Kabuli*	Ca_Kabuli_Chr01	26785566	[T/G]	Ca_20284	FAS1 domain	NON_SYNONYMOUS_CODING
1701	CakSNP1701	*Kabuli*	Ca_Kabuli_Chr01	26807401	[A/G]		--	INTERGENIC

S.N.	SNP IDs	Chickpea cultivars	Chromosomes	Physical positions (bp)	SNPs	Gene accession IDs	Putative functions	Sequence components of genome
1702	CakSNP1702	*Kabuli*	Ca_Kabuli_Chr01	26807395	[T/G]		--	INTERGENIC
1703	CakSNP1703	*Kabuli*	Ca_Kabuli_Chr01	26856541	[A/T]	Ca_20289	--	NON_SYNONYMOUS_CODING
1704	CakSNP1704	*Kabuli*	Ca_Kabuli_Chr01	26856691	[G/A]	Ca_20289	--	SYNONYMOUS_CODING
1705	CakSNP1705	*Kabuli*	Ca_Kabuli_Chr01	26921471	[C/T]		--	INTERGENIC
1706	CakSNP1706	*Kabuli*	Ca_Kabuli_Chr01	26921487	[C/A]		--	INTERGENIC
1707	CakSNP1707	*Kabuli*	Ca_Kabuli_Chr01	26922011	[T/G]		--	INTERGENIC
1708	CakSNP1708	*Kabuli*	Ca_Kabuli_Chr01	26922631	[C/T]	Ca_20293	Pentatricopeptide repeat	SYNONYMOUS_CODING
1709	CakSNP1709	*Kabuli*	Ca_Kabuli_Chr01	26953535	[C/T]		--	INTERGENIC
1710	CakSNP1710	*Kabuli*	Ca_Kabuli_Chr01	27012495	[C/T]		--	INTERGENIC
1711	CakSNP1711	*Kabuli*	Ca_Kabuli_Chr01	27012660	[G/C]		--	INTERGENIC

S.N.	SNP IDs	Chickpea cultivars	Chromosomes	Physical positions (bp)	SNPs	Gene accession IDs	Putative functions	Sequence components of genome
1712	CakSNP1712	*Kabuli*	Ca_Kabuli_Chr01	27014895	[T/G]	Ca_20299	Cystathionine beta-synthase, core	INTRON
1713	CakSNP1713	*Kabuli*	Ca_Kabuli_Chr01	27014981	[A/G]	Ca_20299	Cystathionine beta-synthase, core	INTRON
1714	CakSNP1714	*Kabuli*	Ca_Kabuli_Chr01	27014951	[A/C]	Ca_20299	Cystathionine beta-synthase, core	INTRON
1715	CakSNP1715	*Kabuli*	Ca_Kabuli_Chr01	27016101	[G/A]	Ca_20299	Cystathionine beta-synthase, core	INTRON
1716	CakSNP1716	*Kabuli*	Ca_Kabuli_Chr01	27016206	[T/A]	Ca_20299	Cystathionine beta-synthase, core	INTRON
1717	CakSNP1717	*Kabuli*	Ca_Kabuli_Chr01	27017559	[G/A]	Ca_20299	Cystathionine beta-synthase, core	INTRON
1718	CakSNP1718	*Kabuli*	Ca_Kabuli_Chr01	27017560	[G/A]	Ca_20299	Cystathionine beta-synthase, core	INTRON
1719	CakSNP1719	*Kabuli*	Ca_Kabuli_Chr01	27017570	[A/G]	Ca_20299	Cystathionine beta-synthase, core	INTRON
1720	CakSNP1720	*Kabuli*	Ca_Kabuli_Chr01	27017604	[T/G]	Ca_20299	Cystathionine beta-synthase, core	INTRON
1721	CakSNP1721	*Kabuli*	Ca_Kabuli_Chr01	27017606	[C/T]	Ca_20299	Cystathionine beta-synthase, core	INTRON

S.N.	SNP IDs	Chickpea cultivars	Chromosomes	Physical positions (bp)	SNPs	Gene accession IDs	Putative functions	Sequence components of genome
1722	CakSNP1722	*Kabuli*	Ca_Kabuli_Chr01	27017617	[G/A]	Ca_20299	Cystathionine beta-synthase, core	INTRON
1723	CakSNP1723	*Kabuli*	Ca_Kabuli_Chr01	27017626	[A/C]	Ca_20299	Cystathionine beta-synthase, core	INTRON
1724	CakSNP1724	*Kabuli*	Ca_Kabuli_Chr01	27017628	[C/A]	Ca_20299	Cystathionine beta-synthase, core	INTRON
1725	CakSNP1725	*Kabuli*	Ca_Kabuli_Chr01	27017734	[T/C]	Ca_20299	Cystathionine beta-synthase, core	INTRON
1726	CakSNP1726	*Kabuli*	Ca_Kabuli_Chr01	27017765	[A/T]	Ca_20299	Cystathionine beta-synthase, core	INTRON
1727	CakSNP1727	*Kabuli*	Ca_Kabuli_Chr01	27017771	[G/A]	Ca_20299	Cystathionine beta-synthase, core	INTRON
1728	CakSNP1728	*Kabuli*	Ca_Kabuli_Chr01	27020193	[T/C]	Ca_20299	Cystathionine beta-synthase, core	INTRON
1729	CakSNP1729	*Kabuli*	Ca_Kabuli_Chr01	27020354	[C/A]	Ca_20299	Cystathionine beta-synthase, core	INTRON
1730	CakSNP1730	*Kabuli*	Ca_Kabuli_Chr01	28088501	[T/C]	Ca_18523	Vps54-like	SYNONYMOUS_CODING
1731	CakSNP1731	*Kabuli*	Ca_Kabuli_Chr01	28106756	[G/A]		--	INTERGENIC

S.N.	SNP IDs	Chickpea cultivars	Chromosomes	Physical positions (bp)	SNPs	Gene accession IDs	Putative functions	Sequence components of genome
1732	CakSNP1732	*Kabuli*	Ca_Kabuli_Chr01	28133847	[A/G]	Ca_18521	Leucine-rich repeat	INTRON
1733	CakSNP1733	*Kabuli*	Ca_Kabuli_Chr01	28133830	[A/T]	Ca_18521	Leucine-rich repeat	INTRON
1734	CakSNP1734	*Kabuli*	Ca_Kabuli_Chr01	28133814	[C/T]	Ca_18521	Leucine-rich repeat	INTRON
1735	CakSNP1735	*Kabuli*	Ca_Kabuli_Chr01	28143955	[G/A]	Ca_18521	Leucine-rich repeat	INTRON
1736	CakSNP1736	*Kabuli*	Ca_Kabuli_Chr01	28152260	[A/G]	Ca_18521	Leucine-rich repeat	INTRON
1737	CakSNP1737	*Kabuli*	Ca_Kabuli_Chr01	28656197	[C/A]		--	INTERGENIC
1738	CakSNP1738	*Kabuli*	Ca_Kabuli_Chr01	28832810	[C/A]		--	INTERGENIC
1739	CakSNP1739	*Kabuli*	Ca_Kabuli_Chr01	28907617	[G/A]	Ca_18486	Sugar/inositol transporter	NON_SYNONYMOUS_CODING
1740	CakSNP1740	*Kabuli*	Ca_Kabuli_Chr01	28909059	[T/A]	Ca_18486	Sugar/inositol transporter	NON_SYNONYMOUS_CODING
1741	CakSNP1741	*Kabuli*	Ca_Kabuli_Chr01	29020220	[T/G]		--	INTERGENIC

S.N.	SNP IDs	Chickpea cultivars	Chromosomes	Physical positions (bp)	SNPs	Gene accession IDs	Putative functions	Sequence components of genome
1742	CakSNP1742	*Kabuli*	Ca_Kabuli_Chr01	29116430	[T/C]		--	INTERGENIC
1743	CakSNP1743	*Kabuli*	Ca_Kabuli_Chr01	29460440	[C/A]	Ca_22559	--	NON_SYNONYMOUS_CODING
1744	CakSNP1744	*Kabuli*	Ca_Kabuli_Chr01	29634320	[G/T]	Ca_24382	K Homology, type 1	SYNONYMOUS_CODING
1745	CakSNP1745	*Kabuli*	Ca_Kabuli_Chr01	29637509	[A/C]	Ca_24382	K Homology, type 1	INTRON
1746	CakSNP1746	*Kabuli*	Ca_Kabuli_Chr01	29637526	[C/T]	Ca_24382	K Homology, type 1	INTRON
1747	CakSNP1747	*Kabuli*	Ca_Kabuli_Chr01	29637572	[T/G]	Ca_24382	K Homology, type 1	INTRON
1748	CakSNP1748	*Kabuli*	Ca_Kabuli_Chr01	29637571	[T/G]	Ca_24382	K Homology, type 1	INTRON
1749	CakSNP1749	*Kabuli*	Ca_Kabuli_Chr01	30262124	[A/G]	Ca_22465	Kinesin, motor domain	NON_SYNONYMOUS_CODING
1750	CakSNP1750	*Kabuli*	Ca_Kabuli_Chr01	31080673	[T/G]	Ca_25827	Enolase	INTRON
1751	CakSNP1751	*Kabuli*	Ca_Kabuli_Chr01	31080792	[T/C]	Ca_25827	Enolase	INTRON

S.N.	SNP IDs	Chickpea cultivars	Chromosomes	Physical positions (bp)	SNPs	Gene accession IDs	Putative functions	Sequence components of genome
1752	CakSNP1752	*Kabuli*	Ca_Kabuli_Chr01	31240370	[A/G]		--	INTERGENIC
1753	CakSNP1753	*Kabuli*	Ca_Kabuli_Chr01	31359861	[C/A]		--	INTERGENIC
1754	CakSNP1754	*Kabuli*	Ca_Kabuli_Chr01	31369599	[T/C]	Ca_21855	Peptidase C13, legumain	INTRON
1755	CakSNP1755	*Kabuli*	Ca_Kabuli_Chr01	31590263	[C/G]	Ca_21868	Orotidine 5'-phosphate decarboxylase domain	INTRON
1756	CakSNP1756	*Kabuli*	Ca_Kabuli_Chr01	31590510	[G/A]	Ca_21868	Orotidine 5'-phosphate decarboxylase domain	SYNONYMOUS_CODING
1757	CakSNP1757	*Kabuli*	Ca_Kabuli_Chr01	31590548	[C/T]	Ca_21868	Orotidine 5'-phosphate decarboxylase domain	NON_SYNONYMOUS_CODING
1758	CakSNP1758	*Kabuli*	Ca_Kabuli_Chr01	31838239	[G/A]	Ca_26401	Peptidase C48, SUMO/Sentrin/Ubl1	NON_SYNONYMOUS_CODING
1759	CakSNP1759	*Kabuli*	Ca_Kabuli_Chr01	32286538	[C/T]		--	INTERGENIC
1760	CakSNP1760	*Kabuli*	Ca_Kabuli_Chr01	32286534	[G/C]		--	INTERGENIC
1761	CakSNP1761	*Kabuli*	Ca_Kabuli_Chr01	32286518	[C/A]		--	INTERGENIC

S.N.	SNP IDs	Chickpea cultivars	Chromosomes	Physical positions (bp)	SNPs	Gene accession IDs	Putative functions	Sequence components of genome
1762	CakSNP1762	*Kabuli*	Ca_Kabuli_Chr01	32286517	[G/A]		--	INTERGENIC
1763	CakSNP1763	*Kabuli*	Ca_Kabuli_Chr01	32286469	[G/A]		--	INTERGENIC
1764	CakSNP1764	*Kabuli*	Ca_Kabuli_Chr01	32286466	[A/G]		--	INTERGENIC
1765	CakSNP1765	*Kabuli*	Ca_Kabuli_Chr01	32286568	[A/C]		--	INTERGENIC
1766	CakSNP1766	*Kabuli*	Ca_Kabuli_Chr01	32286558	[A/G]		--	INTERGENIC
1767	CakSNP1767	*Kabuli*	Ca_Kabuli_Chr01	32286550	[G/A]		--	INTERGENIC
1768	CakSNP1768	*Kabuli*	Ca_Kabuli_Chr01	32286536	[G/A]		--	INTERGENIC
1769	CakSNP1769	*Kabuli*	Ca_Kabuli_Chr01	32286551	[C/T]		--	INTERGENIC
1770	CakSNP1770	*Kabuli*	Ca_Kabuli_Chr01	32286567	[C/T]		--	INTERGENIC
1771	CakSNP1771	*Kabuli*	Ca_Kabuli_Chr01	32467929	[G/A]		--	INTERGENIC

S.N.	SNP IDs	Chickpea cultivars	Chromosomes	Physical positions (bp)	SNPs	Gene accession IDs	Putative functions	Sequence components of genome
1772	CakSNP1772	*Kabuli*	Ca_Kabuli_Chr01	32467954	[A/C]		--	INTERGENIC
1773	CakSNP1773	*Kabuli*	Ca_Kabuli_Chr01	32468053	[A/C]		--	INTERGENIC
1774	CakSNP1774	*Kabuli*	Ca_Kabuli_Chr01	32468080	[A/T]		--	INTERGENIC
1775	CakSNP1775	*Kabuli*	Ca_Kabuli_Chr01	32468105	[C/G]		--	INTERGENIC
1776	CakSNP1776	*Kabuli*	Ca_Kabuli_Chr01	32468116	[A/G]		--	INTERGENIC
1777	CakSNP1777	*Kabuli*	Ca_Kabuli_Chr01	32468119	[T/C]		--	INTERGENIC
1778	CakSNP1778	*Kabuli*	Ca_Kabuli_Chr01	33058743	[T/C]	Ca_19437	Argonaute/Dicer protein, PAZ	INTRON
1779	CakSNP1779	*Kabuli*	Ca_Kabuli_Chr01	33063207	[C/T]	Ca_19437	Argonaute/Dicer protein, PAZ	INTRON
1780	CakSNP1780	*Kabuli*	Ca_Kabuli_Chr01	33194600	[T/C]	Ca_19440	Helicase, C-terminal	INTRON
1781	CakSNP1781	*Kabuli*	Ca_Kabuli_Chr01	33804152	[C/T]	Ca_19463	--	NON_SYNONYMOUS_CODING

S.N.	SNP IDs	Chickpea cultivars	Chromosomes	Physical positions (bp)	SNPs	Gene accession IDs	Putative functions	Sequence components of genome
1782	CakSNP1782	*Kabuli*	Ca_Kabuli_Chr01	33804153	[C/T]	Ca_19463	--	SYNONYMOUS_CODING
1783	CakSNP1783	*Kabuli*	Ca_Kabuli_Chr01	33804189	[C/T]	Ca_19463	--	NON_SYNONYMOUS_CODING
1784	CakSNP1784	*Kabuli*	Ca_Kabuli_Chr01	33804201	[C/T]	Ca_19463	--	NON_SYNONYMOUS_CODING
1785	CakSNP1785	*Kabuli*	Ca_Kabuli_Chr01	33804232	[T/G]	Ca_19463	--	SYNONYMOUS_CODING
1786	CakSNP1786	*Kabuli*	Ca_Kabuli_Chr01	33804247	[A/G]	Ca_19463	--	SYNONYMOUS_CODING
1787	CakSNP1787	*Kabuli*	Ca_Kabuli_Chr01	33804262	[C/G]	Ca_19463	--	SYNONYMOUS_CODING
1788	CakSNP1788	*Kabuli*	Ca_Kabuli_Chr01	34161921	[G/A]		--	INTERGENIC
1789	CakSNP1789	*Kabuli*	Ca_Kabuli_Chr01	34261166	[C/A]	Ca_25475	Glycosyl transferase, family 48	INTRON
1790	CakSNP1790	*Kabuli*	Ca_Kabuli_Chr01	34261158	[C/A]	Ca_25475	Glycosyl transferase, family 48	INTRON
1791	CakSNP1791	*Kabuli*	Ca_Kabuli_Chr01	34311145	[T/G]	Ca_25478	GTP-binding protein, HSR1-related	SYNONYMOUS_CODING

S.N.	SNP IDs	Chickpea cultivars	Chromosomes	Physical positions (bp)	SNPs	Gene accession IDs	Putative functions	Sequence components of genome
1792	CakSNP1792	*Kabuli*	Ca_Kabuli_Chr01	34437114	[C/T]		--	INTERGENIC
1793	CakSNP1793	*Kabuli*	Ca_Kabuli_Chr01	34727251	[T/G]	Ca_21419	Zinc finger, CCCH-type	SYNONYMOUS_CODING
1794	CakSNP1794	*Kabuli*	Ca_Kabuli_Chr01	34772813	[T/A]	Ca_21416	--	NON_SYNONYMOUS_CODING
1795	CakSNP1795	*Kabuli*	Ca_Kabuli_Chr01	34866709	[C/A]	Ca_21411	SANT domain, DNA binding	NON_SYNONYMOUS_CODING
1796	CakSNP1796	*Kabuli*	Ca_Kabuli_Chr01	34905580	[A/G]	Ca_21409	--	INTRON
1797	CakSNP1797	*Kabuli*	Ca_Kabuli_Chr01	34911065	[T/A]		--	INTERGENIC
1798	CakSNP1798	*Kabuli*	Ca_Kabuli_Chr01	34948934	[A/G]	Ca_21408	Proteasome, alpha-subunit, conserved site	INTRON
1799	CakSNP1799	*Kabuli*	Ca_Kabuli_Chr01	35041241	[T/G]		--	INTERGENIC
1800	CakSNP1800	*Kabuli*	Ca_Kabuli_Chr01	35041324	[G/T]		--	INTERGENIC
1801	CakSNP1801	*Kabuli*	Ca_Kabuli_Chr01	35313277	[A/G]		--	INTERGENIC

S.N.	SNP IDs	Chickpea cultivars	Chromosomes	Physical positions (bp)	SNPs	Gene accession IDs	Putative functions	Sequence components of genome
1802	CakSNP1802	*Kabuli*	Ca_Kabuli_Chr01	35315381	[T/A]		--	INTERGENIC
1803	CakSNP1803	*Kabuli*	Ca_Kabuli_Chr01	35315379	[A/T]		--	INTERGENIC
1804	CakSNP1804	*Kabuli*	Ca_Kabuli_Chr01	35315347	[C/T]		--	INTERGENIC
1805	CakSNP1805	*Kabuli*	Ca_Kabuli_Chr01	35315327	[T/A]		--	INTERGENIC
1806	CakSNP1806	*Kabuli*	Ca_Kabuli_Chr01	35315436	[G/A]		--	INTERGENIC
1807	CakSNP1807	*Kabuli*	Ca_Kabuli_Chr01	35708841	[C/T]	Ca_24110	Mediator complex, subunit Med10	SYNONYMOUS_CODING
1808	CakSNP1808	*Kabuli*	Ca_Kabuli_Chr01	36126090	[C/T]		--	INTERGENIC
1809	CakSNP1809	*Kabuli*	Ca_Kabuli_Chr01	36794731	[A/G]	Ca_21700	Peptidase C48, SUMO/Sentrin/Ubl1	NON_SYNONYMOUS_CODING
1810	CakSNP1810	*Kabuli*	Ca_Kabuli_Chr01	36794797	[G/A]	Ca_21700	Peptidase C48, SUMO/Sentrin/Ubl1	NON_SYNONYMOUS_CODING
1811	CakSNP1811	*Kabuli*	Ca_Kabuli_Chr01	36794800	[G/T]	Ca_21700	Peptidase C48, SUMO/Sentrin/Ubl1	NON_SYNONYMOUS_CODING

S.N.	SNP IDs	Chickpea cultivars	Chromosomes	Physical positions (bp)	SNPs	Gene accession IDs	Putative functions	Sequence components of genome
1812	CakSNP1812	*Kabuli*	Ca_Kabuli_Chr01	36794806	[G/T]	Ca_21700	Peptidase C48, SUMO/Sentrin/Ubl1	NON_SYNONYMOUS_CODING
1813	CakSNP1813	*Kabuli*	Ca_Kabuli_Chr01	36794813	[C/T]	Ca_21700	Peptidase C48, SUMO/Sentrin/Ubl1	SYNONYMOUS_CODING
1814	CakSNP1814	*Kabuli*	Ca_Kabuli_Chr01	36794887	[G/A]	Ca_21700	Peptidase C48, SUMO/Sentrin/Ubl1	NON_SYNONYMOUS_CODING
1815	CakSNP1815	*Kabuli*	Ca_Kabuli_Chr01	36794870	[C/T]	Ca_21700	Peptidase C48, SUMO/Sentrin/Ubl1	NON_SYNONYMOUS_CODING
1816	CakSNP1816	*Kabuli*	Ca_Kabuli_Chr01	36794900	[C/T]	Ca_21700	Peptidase C48, SUMO/Sentrin/Ubl1	NON_SYNONYMOUS_CODING
1817	CakSNP1817	*Kabuli*	Ca_Kabuli_Chr01	36794906	[A/C]	Ca_21700	Peptidase C48, SUMO/Sentrin/Ubl1	NON_SYNONYMOUS_CODING
1818	CakSNP1818	*Kabuli*	Ca_Kabuli_Chr01	36794912	[G/T]	Ca_21700	Peptidase C48, SUMO/Sentrin/Ubl1	NON_SYNONYMOUS_CODING
1819	CakSNP1819	*Kabuli*	Ca_Kabuli_Chr01	36794913	[A/T]	Ca_21700	Peptidase C48, SUMO/Sentrin/Ubl1	SYNONYMOUS_CODING
1820	CakSNP1820	*Kabuli*	Ca_Kabuli_Chr01	36794915	[G/A]	Ca_21700	Peptidase C48, SUMO/Sentrin/Ubl1	NON_SYNONYMOUS_CODING
1821	CakSNP1821	*Kabuli*	Ca_Kabuli_Chr01	36892533	[C/T]	Ca_27231	Peptidase C48, SUMO/Sentrin/Ubl1	NON_SYNONYMOUS_CODING

S.N.	SNP IDs	Chickpea cultivars	Chromosomes	Physical positions (bp)	SNPs	Gene accession IDs	Putative functions	Sequence components of genome
1822	CakSNP1822	*Kabuli*	Ca_Kabuli_Chr01	37026302	[C/T]		--	INTERGENIC
1823	CakSNP1823	*Kabuli*	Ca_Kabuli_Chr01	37608030	[G/A]	Ca_23528	Ribosomal protein L7/L12, C-terminal	SYNONYMOUS_CODING
1824	CakSNP1824	*Kabuli*	Ca_Kabuli_Chr01	37608050	[T/G]	Ca_23528	Ribosomal protein L7/L12, C-terminal	NON_SYNONYMOUS_CODING
1825	CakSNP1825	*Kabuli*	Ca_Kabuli_Chr01	37642546	[G/A]		--	INTERGENIC
1826	CakSNP1826	*Kabuli*	Ca_Kabuli_Chr01	37643213	[T/C]		--	INTERGENIC
1827	CakSNP1827	*Kabuli*	Ca_Kabuli_Chr01	37647602	[C/G]	Ca_23531	Aldehyde dehydrogenase domain	INTRON
1828	CakSNP1828	*Kabuli*	Ca_Kabuli_Chr01	37651679	[G/A]	Ca_23531	Aldehyde dehydrogenase domain	INTRON
1829	CakSNP1829	*Kabuli*	Ca_Kabuli_Chr01	37651695	[G/A]	Ca_23531	Aldehyde dehydrogenase domain	INTRON
1830	CakSNP1830	*Kabuli*	Ca_Kabuli_Chr01	37651764	[A/C]	Ca_23531	Aldehyde dehydrogenase domain	INTRON
1831	CakSNP1831	*Kabuli*	Ca_Kabuli_Chr01	37651711	[G/T]	Ca_23531	Aldehyde dehydrogenase domain	INTRON

S.N.	SNP IDs	Chickpea cultivars	Chromosomes	Physical positions (bp)	SNPs	Gene accession IDs	Putative functions	Sequence components of genome
1832	CakSNP1832	*Kabuli*	Ca_Kabuli_Chr01	37651800	[T/A]	Ca_23531	Aldehyde dehydrogenase domain	INTRON
1833	CakSNP1833	*Kabuli*	Ca_Kabuli_Chr01	37651823	[A/G]	Ca_23531	Aldehyde dehydrogenase domain	INTRON
1834	CakSNP1834	*Kabuli*	Ca_Kabuli_Chr01	37651827	[C/T]	Ca_23531	Aldehyde dehydrogenase domain	INTRON
1835	CakSNP1835	*Kabuli*	Ca_Kabuli_Chr01	37651843	[T/C]	Ca_23531	Aldehyde dehydrogenase domain	INTRON
1836	CakSNP1836	*Kabuli*	Ca_Kabuli_Chr01	37654039	[C/G]	Ca_23531	Aldehyde dehydrogenase domain	INTRON
1837	CakSNP1837	*Kabuli*	Ca_Kabuli_Chr01	37654136	[C/G]	Ca_23531	Aldehyde dehydrogenase domain	INTRON
1838	CakSNP1838	*Kabuli*	Ca_Kabuli_Chr01	37654926	[C/T]	Ca_23531	Aldehyde dehydrogenase domain	INTRON
1839	CakSNP1839	*Kabuli*	Ca_Kabuli_Chr01	37690416	[G/A]	Ca_23533	Myb, DNA-binding	INTRON
1840	CakSNP1840	*Kabuli*	Ca_Kabuli_Chr01	37690465	[T/C]	Ca_23533	Myb, DNA-binding	INTRON
1841	CakSNP1841	*Kabuli*	Ca_Kabuli_Chr01	37690498	[C/A]	Ca_23533	Myb, DNA-binding	INTRON

S.N.	SNP IDs	Chickpea cultivars	Chromosomes	Physical positions (bp)	SNPs	Gene accession IDs	Putative functions	Sequence components of genome
1842	CakSNP1842	*Kabuli*	Ca_Kabuli_Chr01	37690497	[A/T]	Ca_23533	Myb, DNA-binding	INTRON
1843	CakSNP1843	*Kabuli*	Ca_Kabuli_Chr01	37699039	[T/G]		--	INTERGENIC
1844	CakSNP1844	*Kabuli*	Ca_Kabuli_Chr01	37699036	[T/C]		--	INTERGENIC
1845	CakSNP1845	*Kabuli*	Ca_Kabuli_Chr01	37722095	[G/A]	Ca_23534	Protein of unknown function DUF620	SYNONYMOUS_CODING
1846	CakSNP1846	*Kabuli*	Ca_Kabuli_Chr01	37722143	[G/C]	Ca_23534	Protein of unknown function DUF620	SYNONYMOUS_CODING
1847	CakSNP1847	*Kabuli*	Ca_Kabuli_Chr01	37768179	[T/A]		--	INTERGENIC
1848	CakSNP1848	*Kabuli*	Ca_Kabuli_Chr01	37768206	[A/T]		--	INTERGENIC
1849	CakSNP1849	*Kabuli*	Ca_Kabuli_Chr01	37768238	[A/G]		--	INTERGENIC
1850	CakSNP1850	*Kabuli*	Ca_Kabuli_Chr01	37768333	[C/T]		--	INTERGENIC
1851	CakSNP1851	*Kabuli*	Ca_Kabuli_Chr01	37768299	[A/G]		--	INTERGENIC

S.N.	SNP IDs	Chickpea cultivars	Chromosomes	Physical positions (bp)	SNPs	Gene accession IDs	Putative functions	Sequence components of genome
1852	CakSNP1852	*Kabuli*	Ca_Kabuli_Chr01	37768276	[G/C]		--	INTERGENIC
1853	CakSNP1853	*Kabuli*	Ca_Kabuli_Chr01	37768264	[A/G]		--	INTERGENIC
1854	CakSNP1854	*Kabuli*	Ca_Kabuli_Chr01	37862342	[T/G]	Ca_23539	Zinc finger, PHD-type	SYNONYMOUS_CODING
1855	CakSNP1855	*Kabuli*	Ca_Kabuli_Chr01	37880995	[T/C]	Ca_23539	Zinc finger, PHD-type	INTRON
1856	CakSNP1856	*Kabuli*	Ca_Kabuli_Chr01	37892088	[A/C]		--	INTERGENIC
1857	CakSNP1857	*Kabuli*	Ca_Kabuli_Chr01	37892089	[C/T]		--	INTERGENIC
1858	CakSNP1858	*Kabuli*	Ca_Kabuli_Chr01	37892095	[A/G]		--	INTERGENIC
1859	CakSNP1859	*Kabuli*	Ca_Kabuli_Chr01	37892139	[A/T]		--	INTERGENIC
1860	CakSNP1860	*Kabuli*	Ca_Kabuli_Chr01	38049721	[C/T]	Ca_27444	Cation efflux protein	NON_SYNONYMOUS_CODING
1861	CakSNP1861	*Kabuli*	Ca_Kabuli_Chr01	38678712	[T/C]		--	INTERGENIC

S.N.	SNP IDs	Chickpea cultivars	Chromosomes	Physical positions (bp)	SNPs	Gene accession IDs	Putative functions	Sequence components of genome
1862	CakSNP1862	*Kabuli*	Ca_Kabuli_Chr01	38877670	[T/C]		--	INTERGENIC
1863	CakSNP1863	*Kabuli*	Ca_Kabuli_Chr01	38877692	[G/T]		--	INTERGENIC
1864	CakSNP1864	*Kabuli*	Ca_Kabuli_Chr01	39436291	[T/C]	Ca_13931	IQ motif, EF-hand binding site	INTRON
1865	CakSNP1865	*Kabuli*	Ca_Kabuli_Chr01	39601982	[T/G]		--	INTERGENIC
1866	CakSNP1866	*Kabuli*	Ca_Kabuli_Chr01	39602029	[G/T]		--	INTERGENIC
1867	CakSNP1867	*Kabuli*	Ca_Kabuli_Chr01	39616139	[A/C]	Ca_13924	CTP synthase, N-terminal	SYNONYMOUS_CODING
1868	CakSNP1868	*Kabuli*	Ca_Kabuli_Chr01	39663432	[C/G]	Ca_13922	ABC transporter-like	INTRON
1869	CakSNP1869	*Kabuli*	Ca_Kabuli_Chr01	40224835	[T/A]		--	INTERGENIC
1870	CakSNP1870	*Kabuli*	Ca_Kabuli_Chr01	40224834	[C/G]		--	INTERGENIC
1871	CakSNP1871	*Kabuli*	Ca_Kabuli_Chr01	40224804	[A/G]		--	INTERGENIC

S.N.	SNP IDs	Chickpea cultivars	Chromosomes	Physical positions (bp)	SNPs	Gene accession IDs	Putative functions	Sequence components of genome
1872	CakSNP1872	*Kabuli*	Ca_Kabuli_Chr01	40343216	[G/T]	Ca_13893	Tetratricopeptide, MLP1/MLP2-like	INTRON
1873	CakSNP1873	*Kabuli*	Ca_Kabuli_Chr01	40346280	[A/C]	Ca_13893	Tetratricopeptide, MLP1/MLP2-like	INTRON
1874	CakSNP1874	*Kabuli*	Ca_Kabuli_Chr01	40358693	[C/T]	Ca_13893	Tetratricopeptide, MLP1/MLP2-like	INTRON
1875	CakSNP1875	*Kabuli*	Ca_Kabuli_Chr01	40358747	[T/G]	Ca_13893	Tetratricopeptide, MLP1/MLP2-like	INTRON
1876	CakSNP1876	*Kabuli*	Ca_Kabuli_Chr01	40367940	[G/A]	Ca_13893	Tetratricopeptide, MLP1/MLP2-like	INTRON
1877	CakSNP1877	*Kabuli*	Ca_Kabuli_Chr01	40368845	[C/T]	Ca_13893	Tetratricopeptide, MLP1/MLP2-like	NON_SYNONYMOUS_CODING
1878	CakSNP1878	*Kabuli*	Ca_Kabuli_Chr01	40502077	[T/C]	Ca_18443	CWC16 protein	INTRON
1879	CakSNP1879	*Kabuli*	Ca_Kabuli_Chr01	40570811	[G/T]	Ca_18445	Peptidase C19, ubiquitin carboxyl-terminal hydrolase 2	NON_SYNONYMOUS_CODING
1880	CakSNP1880	*Kabuli*	Ca_Kabuli_Chr01	40585026	[T/C]	Ca_18446	--	INTRON
1881	CakSNP1881	*Kabuli*	Ca_Kabuli_Chr01	40630215	[C/T]		--	INTERGENIC

S.N.	SNP IDs	Chickpea cultivars	Chromosomes	Physical positions (bp)	SNPs	Gene accession IDs	Putative functions	Sequence components of genome
1882	CakSNP1882	*Kabuli*	Ca_Kabuli_Chr01	40630213	[G/T]		--	INTERGENIC
1883	CakSNP1883	*Kabuli*	Ca_Kabuli_Chr01	40630158	[T/A]		--	INTERGENIC
1884	CakSNP1884	*Kabuli*	Ca_Kabuli_Chr01	40660069	[T/G]	Ca_18454	C2 calcium-dependent membrane targeting	INTRON
1885	CakSNP1885	*Kabuli*	Ca_Kabuli_Chr01	40660116	[G/A]	Ca_18454	C2 calcium-dependent membrane targeting	INTRON
1886	CakSNP1886	*Kabuli*	Ca_Kabuli_Chr01	40745421	[G/T]		--	INTERGENIC
1887	CakSNP1887	*Kabuli*	Ca_Kabuli_Chr01	40745452	[T/C]		--	INTERGENIC
1888	CakSNP1888	*Kabuli*	Ca_Kabuli_Chr01	40745492	[A/G]		--	INTERGENIC
1889	CakSNP1889	*Kabuli*	Ca_Kabuli_Chr01	40745846	[T/C]		--	INTERGENIC
1890	CakSNP1890	*Kabuli*	Ca_Kabuli_Chr01	41029732	[C/T]		--	INTERGENIC
1891	CakSNP1891	*Kabuli*	Ca_Kabuli_Chr01	41065797	[G/C]		--	INTERGENIC

S.N.	SNP IDs	Chickpea cultivars	Chromosomes	Physical positions (bp)	SNPs	Gene accession IDs	Putative functions	Sequence components of genome
1892	CakSNP1892	*Kabuli*	Ca_Kabuli_Chr01	41125493	[T/C]		--	INTERGENIC
1893	CakSNP1893	*Kabuli*	Ca_Kabuli_Chr01	41169218	[G/T]		--	INTERGENIC
1894	CakSNP1894	*Kabuli*	Ca_Kabuli_Chr01	41169430	[T/G]	Ca_18480	--	NON_SYNONYMOUS_CODING
1895	CakSNP1895	*Kabuli*	Ca_Kabuli_Chr01	41169372	[T/A]	Ca_18480	--	NON_SYNONYMOUS_CODING
1896	CakSNP1896	*Kabuli*	Ca_Kabuli_Chr01	41178168	[G/A]	Ca_18481	Ribokinase	SYNONYMOUS_CODING
1897	CakSNP1897	*Kabuli*	Ca_Kabuli_Chr01	41178183	[C/A]	Ca_18481	Ribokinase	SYNONYMOUS_CODING
1898	CakSNP1898	*Kabuli*	Ca_Kabuli_Chr01	41179660	[G/T]	Ca_18481	Ribokinase	INTRON
1899	CakSNP1899	*Kabuli*	Ca_Kabuli_Chr01	41179650	[A/T]	Ca_18481	Ribokinase	INTRON
1900	CakSNP1900	*Kabuli*	Ca_Kabuli_Chr01	41185512	[T/G]		--	INTERGENIC
1901	CakSNP1901	*Kabuli*	Ca_Kabuli_Chr01	41202173	[T/G]	Ca_18482	Protein kinase, catalytic domain	INTRON

S.N.	SNP IDs	Chickpea cultivars	Chromosomes	Physical positions (bp)	SNPs	Gene accession IDs	Putative functions	Sequence components of genome
1902	CakSNP1902	*Kabuli*	Ca_Kabuli_Chr01	41202206	[A/G]	Ca_18482	Protein kinase, catalytic domain	INTRON
1903	CakSNP1903	*Kabuli*	Ca_Kabuli_Chr01	41310401	[T/G]	Ca_23106	FMN-dependent dehydrogenase	INTRON
1904	CakSNP1904	*Kabuli*	Ca_Kabuli_Chr01	41339408	[T/A]	Ca_23108	Domain of unknown function DUF250	INTRON
1905	CakSNP1905	*Kabuli*	Ca_Kabuli_Chr01	41339445	[C/A]	Ca_23108	Domain of unknown function DUF250	INTRON
1906	CakSNP1906	*Kabuli*	Ca_Kabuli_Chr01	41380061	[G/T]	Ca_23111	Glutathione S-transferase, N-terminal	SYNONYMOUS_CODING
1907	CakSNP1907	*Kabuli*	Ca_Kabuli_Chr01	41437222	[A/G]	Ca_23118	Protein kinase, catalytic domain	INTRON
1908	CakSNP1908	*Kabuli*	Ca_Kabuli_Chr01	41437366	[T/C]	Ca_23118	Protein kinase, catalytic domain	INTRON
1909	CakSNP1909	*Kabuli*	Ca_Kabuli_Chr01	41437453	[T/G]	Ca_23118	Protein kinase, catalytic domain	INTRON
1910	CakSNP1910	*Kabuli*	Ca_Kabuli_Chr01	41437483	[G/A]	Ca_23118	Protein kinase, catalytic domain	INTRON
1911	CakSNP1911	*Kabuli*	Ca_Kabuli_Chr01	41439539	[A/G]	Ca_23118	Protein kinase, catalytic domain	SYNONYMOUS_CODING

S.N.	SNP IDs	Chickpea cultivars	Chromosomes	Physical positions (bp)	SNPs	Gene accession IDs	Putative functions	Sequence components of genome
1912	CakSNP1912	*Kabuli*	Ca_Kabuli_Chr01	41439593	[A/G]	Ca_23118	Protein kinase, catalytic domain	SYNONYMOUS_CODING
1913	CakSNP1913	*Kabuli*	Ca_Kabuli_Chr01	41451910	[C/T]	Ca_23119	Peptidase C48, SUMO/Sentrin/Ubl1	SYNONYMOUS_CODING
1914	CakSNP1914	*Kabuli*	Ca_Kabuli_Chr01	41451977	[C/T]	Ca_23119	Peptidase C48, SUMO/Sentrin/Ubl1	NON_SYNONYMOUS_CODING
1915	CakSNP1915	*Kabuli*	Ca_Kabuli_Chr01	41475442	[T/C]	Ca_23121	Kinesin, motor domain	SYNONYMOUS_CODING
1916	CakSNP1916	*Kabuli*	Ca_Kabuli_Chr01	41484033	[T/C]		--	INTERGENIC
1917	CakSNP1917	*Kabuli*	Ca_Kabuli_Chr01	41484085	[C/T]		--	INTERGENIC
1918	CakSNP1918	*Kabuli*	Ca_Kabuli_Chr01	41564809	[T/C]	Ca_25794	--	INTRON
1919	CakSNP1919	*Kabuli*	Ca_Kabuli_Chr01	41795343	[G/A]	Ca_22519	Plastocyanin-like	NON_SYNONYMOUS_CODING
1920	CakSNP1920	*Kabuli*	Ca_Kabuli_Chr01	41795367	[C/T]	Ca_22519	Plastocyanin-like	NON_SYNONYMOUS_CODING
1921	CakSNP1921	*Kabuli*	Ca_Kabuli_Chr01	41822419	[C/T]	Ca_22520	Surfeit locus 6	SYNONYMOUS_CODING

S.N.	SNP IDs	Chickpea cultivars	Chromosomes	Physical positions (bp)	SNPs	Gene accession IDs	Putative functions	Sequence components of genome
1922	CakSNP1922	*Kabuli*	Ca_Kabuli_Chr01	41827388	[T/C]	Ca_22521	Multi antimicrobial extrusion protein	NON_SYNONYMOUS_CODING
1923	CakSNP1923	*Kabuli*	Ca_Kabuli_Chr01	41867035	[C/T]	Ca_22524	--	NON_SYNONYMOUS_CODING
1924	CakSNP1924	*Kabuli*	Ca_Kabuli_Chr01	41897216	[C/A]	Ca_22528	--	INTRON
1925	CakSNP1925	*Kabuli*	Ca_Kabuli_Chr01	41946005	[A/G]		--	INTERGENIC
1926	CakSNP1926	*Kabuli*	Ca_Kabuli_Chr01	41981244	[G/A]	Ca_22534	Pathogenesis-related transcriptional factor/ERF, DNA-binding	SYNONYMOUS_CODING
1927	CakSNP1927	*Kabuli*	Ca_Kabuli_Chr01	41981259	[G/A]	Ca_22534	Pathogenesis-related transcriptional factor/ERF, DNA-binding	SYNONYMOUS_CODING
1928	CakSNP1928	*Kabuli*	Ca_Kabuli_Chr01	42189380	[A/G]		--	INTERGENIC
1929	CakSNP1929	*Kabuli*	Ca_Kabuli_Chr01	42452801	[G/A]	Ca_13620	Transcription factor CBF/NF-Y/archaeal histone	NON_SYNONYMOUS_CODING
1930	CakSNP1930	*Kabuli*	Ca_Kabuli_Chr01	42452939	[G/T]	Ca_13620	Transcription factor CBF/NF-Y/archaeal histone	NON_SYNONYMOUS_CODING
1931	CakSNP1931	*Kabuli*	Ca_Kabuli_Chr01	42515406	[C/T]	Ca_13617	--	NON_SYNONYMOUS_CODING

S.N.	SNP IDs	Chickpea cultivars	Chromosomes	Physical positions (bp)	SNPs	Gene accession IDs	Putative functions	Sequence components of genome
1932	CakSNP1932	*Kabuli*	Ca_Kabuli_Chr01	42551574	[T/G]	Ca_13614	--	NON_SYNONYMOUS_CODING
1933	CakSNP1933	*Kabuli*	Ca_Kabuli_Chr01	42551585	[C/A]	Ca_13614	--	SYNONYMOUS_CODING
1934	CakSNP1934	*Kabuli*	Ca_Kabuli_Chr01	42552658	[A/C]		--	INTERGENIC
1935	CakSNP1935	*Kabuli*	Ca_Kabuli_Chr01	42605239	[T/G]	Ca_13610	Helix-loop-helix DNA-binding domain	NON_SYNONYMOUS_CODING
1936	CakSNP1936	*Kabuli*	Ca_Kabuli_Chr01	42610115	[A/C]	Ca_13609	Putative rRNA methylase	SYNONYMOUS_CODING
1937	CakSNP1937	*Kabuli*	Ca_Kabuli_Chr01	42613060	[T/G]	Ca_13608	Helix-loop-helix DNA-binding domain	NON_SYNONYMOUS_CODING
1938	CakSNP1938	*Kabuli*	Ca_Kabuli_Chr01	42894518	[T/C]	Ca_13593	Plant peroxidase	INTRON
1939	CakSNP1939	*Kabuli*	Ca_Kabuli_Chr01	42894525	[T/C]	Ca_13593	Plant peroxidase	INTRON
1940	CakSNP1940	*Kabuli*	Ca_Kabuli_Chr01	42894536	[G/T]	Ca_13593	Plant peroxidase	INTRON
1941	CakSNP1941	*Kabuli*	Ca_Kabuli_Chr01	43053424	[G/C]		--	INTERGENIC

S.N.	SNP IDs	Chickpea cultivars	Chromosomes	Physical positions (bp)	SNPs	Gene accession IDs	Putative functions	Sequence components of genome
1942	CakSNP1942	*Kabuli*	Ca_Kabuli_Chr01	43273910	[G/T]		--	INTERGENIC
1943	CakSNP1943	*Kabuli*	Ca_Kabuli_Chr01	43273922	[T/C]		--	INTERGENIC
1944	CakSNP1944	*Kabuli*	Ca_Kabuli_Chr01	43273925	[A/G]		--	INTERGENIC
1945	CakSNP1945	*Kabuli*	Ca_Kabuli_Chr01	43273931	[G/A]		--	INTERGENIC
1946	CakSNP1946	*Kabuli*	Ca_Kabuli_Chr01	43273941	[C/G]		--	INTERGENIC
1947	CakSNP1947	*Kabuli*	Ca_Kabuli_Chr01	43273943	[A/G]		--	INTERGENIC
1948	CakSNP1948	*Kabuli*	Ca_Kabuli_Chr01	43273961	[C/T]		--	INTERGENIC
1949	CakSNP1949	*Kabuli*	Ca_Kabuli_Chr01	43273962	[A/G]		--	INTERGENIC
1950	CakSNP1950	*Kabuli*	Ca_Kabuli_Chr01	43273968	[T/G]		--	INTERGENIC
1951	CakSNP1951	*Kabuli*	Ca_Kabuli_Chr01	43273972	[C/T]		--	INTERGENIC

S.N.	SNP IDs	Chickpea cultivars	Chromosomes	Physical positions (bp)	SNPs	Gene accession IDs	Putative functions	Sequence components of genome
1952	CakSNP1952	*Kabuli*	Ca_Kabuli_Chr01	43273981	[G/T]		--	INTERGENIC
1953	CakSNP1953	*Kabuli*	Ca_Kabuli_Chr01	43273985	[G/A]		--	INTERGENIC
1954	CakSNP1954	*Kabuli*	Ca_Kabuli_Chr01	43304584	[A/C]	Ca_13580	Tetratricopeptide repeat-containing	NON_SYNONYMOUS_CODING
1955	CakSNP1955	*Kabuli*	Ca_Kabuli_Chr01	43304573	[C/T]	Ca_13580	Tetratricopeptide repeat-containing	SYNONYMOUS_CODING
1956	CakSNP1956	*Kabuli*	Ca_Kabuli_Chr01	43305858	[T/C]		--	INTERGENIC
1957	CakSNP1957	*Kabuli*	Ca_Kabuli_Chr01	43305870	[G/A]		--	INTERGENIC
1958	CakSNP1958	*Kabuli*	Ca_Kabuli_Chr01	43305886	[T/C]		--	INTERGENIC
1959	CakSNP1959	*Kabuli*	Ca_Kabuli_Chr01	43305904	[C/A]		--	INTERGENIC
1960	CakSNP1960	*Kabuli*	Ca_Kabuli_Chr01	43306048	[G/A]		--	INTERGENIC
1961	CakSNP1961	*Kabuli*	Ca_Kabuli_Chr01	43306045	[C/A]		--	INTERGENIC

S.N.	SNP IDs	Chickpea cultivars	Chromosomes	Physical positions (bp)	SNPs	Gene accession IDs	Putative functions	Sequence components of genome
1962	CakSNP1962	*Kabuli*	Ca_Kabuli_Chr01	43306036	[G/A]		--	INTERGENIC
1963	CakSNP1963	*Kabuli*	Ca_Kabuli_Chr01	43306012	[G/A]		--	INTERGENIC
1964	CakSNP1964	*Kabuli*	Ca_Kabuli_Chr01	43306221	[A/G]		--	INTERGENIC
1965	CakSNP1965	*Kabuli*	Ca_Kabuli_Chr01	43509159	[A/G]	Ca_13571	No apical meristem (NAM) protein	INTRON
1966	CakSNP1966	*Kabuli*	Ca_Kabuli_Chr01	43509409	[T/C]		--	INTERGENIC
1967	CakSNP1967	*Kabuli*	Ca_Kabuli_Chr01	43576059	[A/G]		--	INTERGENIC
1968	CakSNP1968	*Kabuli*	Ca_Kabuli_Chr01	43585657	[T/G]	Ca_13566	Translation elongation factor EFG/EF2, C-terminal	SYNONYMOUS_CODING
1969	CakSNP1969	*Kabuli*	Ca_Kabuli_Chr01	43590106	[C/T]		--	INTERGENIC
1970	CakSNP1970	*Kabuli*	Ca_Kabuli_Chr01	43590258	[T/C]		--	INTERGENIC
1971	CakSNP1971	*Kabuli*	Ca_Kabuli_Chr01	43724824	[C/T]		--	INTERGENIC

S.N.	SNP IDs	Chickpea cultivars	Chromosomes	Physical positions (bp)	SNPs	Gene accession IDs	Putative functions	Sequence components of genome
1972	CakSNP1972	*Kabuli*	Ca_Kabuli_Chr01	43724839	[C/T]		--	INTERGENIC
1973	CakSNP1973	*Kabuli*	Ca_Kabuli_Chr01	43724963	[C/T]	Ca_13554	--	SYNONYMOUS_CODING
1974	CakSNP1974	*Kabuli*	Ca_Kabuli_Chr01	43725059	[C/T]	Ca_13554	--	SYNONYMOUS_CODING
1975	CakSNP1975	*Kabuli*	Ca_Kabuli_Chr01	43725104	[T/C]	Ca_13554	--	SYNONYMOUS_CODING
1976	CakSNP1976	*Kabuli*	Ca_Kabuli_Chr01	43727804	[C/T]	Ca_13554	--	SYNONYMOUS_CODING
1977	CakSNP1977	*Kabuli*	Ca_Kabuli_Chr01	43792565	[G/A]		--	INTERGENIC
1978	CakSNP1978	*Kabuli*	Ca_Kabuli_Chr01	43800367	[C/T]	Ca_13548	BTB/POZ-like	SYNONYMOUS_CODING
1979	CakSNP1979	*Kabuli*	Ca_Kabuli_Chr01	43930143	[A/T]		--	INTERGENIC
1980	CakSNP1980	*Kabuli*	Ca_Kabuli_Chr01	43930138	[T/C]		--	INTERGENIC
1981	CakSNP1981	*Kabuli*	Ca_Kabuli_Chr01	43933881	[G/C]		--	INTERGENIC

S.N.	SNP IDs	Chickpea cultivars	Chromosomes	Physical positions (bp)	SNPs	Gene accession IDs	Putative functions	Sequence components of genome
1982	CakSNP1982	*Kabuli*	Ca_Kabuli_Chr01	43933904	[A/G]		--	INTERGENIC
1983	CakSNP1983	*Kabuli*	Ca_Kabuli_Chr01	43934024	[G/T]		--	INTERGENIC
1984	CakSNP1984	*Kabuli*	Ca_Kabuli_Chr01	43934120	[A/G]		--	INTERGENIC
1985	CakSNP1985	*Kabuli*	Ca_Kabuli_Chr01	43934117	[G/T]		--	INTERGENIC
1986	CakSNP1986	*Kabuli*	Ca_Kabuli_Chr01	43934081	[C/T]		--	INTERGENIC
1987	CakSNP1987	*Kabuli*	Ca_Kabuli_Chr01	43943035	[G/T]		--	INTERGENIC
1988	CakSNP1988	*Kabuli*	Ca_Kabuli_Chr01	43954743	[C/G]	Ca_13537	No apical meristem (NAM) protein	INTRON
1989	CakSNP1989	*Kabuli*	Ca_Kabuli_Chr01	44043281	[G/A]		--	INTERGENIC
1990	CakSNP1990	*Kabuli*	Ca_Kabuli_Chr01	44085069	[C/T]	Ca_13530	Endosulphine	INTRON
1991	CakSNP1991	*Kabuli*	Ca_Kabuli_Chr01	44348947	[C/T]	Ca_23498	Ribonuclease II/R	NON_SYNONYMOUS_CODING

S.N.	SNP IDs	Chickpea cultivars	Chromosomes	Physical positions (bp)	SNPs	Gene accession IDs	Putative functions	Sequence components of genome
1992	CakSNP1992	*Kabuli*	Ca_Kabuli_Chr01	44349004	[T/C]	Ca_23498	Ribonuclease II/R	NON_SYNONYMOUS_CODING
1993	CakSNP1993	*Kabuli*	Ca_Kabuli_Chr01	44350953	[G/T]	Ca_23498	Ribonuclease II/R	INTRON
1994	CakSNP1994	*Kabuli*	Ca_Kabuli_Chr01	44635939	[T/C]		--	INTERGENIC
1995	CakSNP1995	*Kabuli*	Ca_Kabuli_Chr01	44635926	[A/G]		--	INTERGENIC
1996	CakSNP1996	*Kabuli*	Ca_Kabuli_Chr01	44635893	[A/G]		--	INTERGENIC
1997	CakSNP1997	*Kabuli*	Ca_Kabuli_Chr01	44976738	[T/A]	Ca_25713	Protein kinase, catalytic domain	INTRON
1998	CakSNP1998	*Kabuli*	Ca_Kabuli_Chr01	44976735	[C/T]	Ca_25713	Protein kinase, catalytic domain	INTRON
1999	CakSNP1999	*Kabuli*	Ca_Kabuli_Chr01	44988344	[A/G]		--	INTERGENIC
2000	CakSNP2000	*Kabuli*	Ca_Kabuli_Chr01	44988387	[C/A]		--	INTERGENIC
2001	CakSNP2001	*Kabuli*	Ca_Kabuli_Chr01	45003190	[G/A]	Ca_25714	--	NON_SYNONYMOUS_CODING

S.N.	SNP IDs	Chickpea cultivars	Chromosomes	Physical positions (bp)	SNPs	Gene accession IDs	Putative functions	Sequence components of genome
2002	CakSNP2002	*Kabuli*	Ca_Kabuli_Chr01	45003514	[G/A]	Ca_25714	--	NON_SYNONYMOUS_CODING
2003	CakSNP2003	*Kabuli*	Ca_Kabuli_Chr01	45003509	[C/A]	Ca_25714	--	INTRON
2004	CakSNP2004	*Kabuli*	Ca_Kabuli_Chr01	45003505	[A/C]	Ca_25714	--	INTRON
2005	CakSNP2005	*Kabuli*	Ca_Kabuli_Chr01	45003938	[A/G]	Ca_25714	--	INTRON
2006	CakSNP2006	*Kabuli*	Ca_Kabuli_Chr01	45003939	[T/A]	Ca_25714	--	INTRON
2007	CakSNP2007	*Kabuli*	Ca_Kabuli_Chr01	45003940	[T/G]	Ca_25714	--	INTRON
2008	CakSNP2008	*Kabuli*	Ca_Kabuli_Chr01	45003944	[C/T]	Ca_25714	--	INTRON
2009	CakSNP2009	*Kabuli*	Ca_Kabuli_Chr01	45003945	[T/A]	Ca_25714	--	INTRON
2010	CakSNP2010	*Kabuli*	Ca_Kabuli_Chr01	45003946	[T/G]	Ca_25714	--	INTRON
2011	CakSNP2011	*Kabuli*	Ca_Kabuli_Chr01	45003947	[C/A]	Ca_25714	--	INTRON

S.N.	SNP IDs	Chickpea cultivars	Chromosomes	Physical positions (bp)	SNPs	Gene accession IDs	Putative functions	Sequence components of genome
2012	CakSNP2012	*Kabuli*	Ca_Kabuli_Chr01	45003973	[T/C]	Ca_25714	--	INTRON
2013	CakSNP2013	*Kabuli*	Ca_Kabuli_Chr01	45003985	[G/T]	Ca_25714	--	INTRON
2014	CakSNP2014	*Kabuli*	Ca_Kabuli_Chr01	45003998	[G/A]	Ca_25714	--	INTRON
2015	CakSNP2015	*Kabuli*	Ca_Kabuli_Chr01	45004001	[C/T]	Ca_25714	--	INTRON
2016	CakSNP2016	*Kabuli*	Ca_Kabuli_Chr01	45007032	[A/C]	Ca_25714	--	INTRON
2017	CakSNP2017	*Kabuli*	Ca_Kabuli_Chr01	45007227	[T/C]	Ca_25714	--	INTRON
2018	CakSNP2018	*Kabuli*	Ca_Kabuli_Chr01	45007259	[A/G]	Ca_25714	--	INTRON
2019	CakSNP2019	*Kabuli*	Ca_Kabuli_Chr01	45007331	[C/T]	Ca_25714	--	INTRON
2020	CakSNP2020	*Kabuli*	Ca_Kabuli_Chr01	45008076	[G/A]	Ca_25714	--	INTRON
2021	CakSNP2021	*Kabuli*	Ca_Kabuli_Chr01	45008075	[T/C]	Ca_25714	--	INTRON

S.N.	SNP IDs	Chickpea cultivars	Chromosomes	Physical positions (bp)	SNPs	Gene accession IDs	Putative functions	Sequence components of genome
2022	CakSNP2022	*Kabuli*	Ca_Kabuli_Chr01	45008073	[T/G]	Ca_25714	--	INTRON
2023	CakSNP2023	*Kabuli*	Ca_Kabuli_Chr01	45008071	[G/C]	Ca_25714	--	INTRON
2024	CakSNP2024	*Kabuli*	Ca_Kabuli_Chr01	45008050	[C/G]	Ca_25714	--	INTRON
2025	CakSNP2025	*Kabuli*	Ca_Kabuli_Chr01	45008044	[T/C]	Ca_25714	--	INTRON
2026	CakSNP2026	*Kabuli*	Ca_Kabuli_Chr01	45008041	[C/G]	Ca_25714	--	INTRON
2027	CakSNP2027	*Kabuli*	Ca_Kabuli_Chr01	45008040	[G/C]	Ca_25714	--	INTRON
2028	CakSNP2028	*Kabuli*	Ca_Kabuli_Chr01	45008039	[C/T]	Ca_25714	--	INTRON
2029	CakSNP2029	*Kabuli*	Ca_Kabuli_Chr01	45008016	[T/A]	Ca_25714	--	INTRON
2030	CakSNP2030	*Kabuli*	Ca_Kabuli_Chr01	45008015	[G/A]	Ca_25714	--	INTRON
2031	CakSNP2031	*Kabuli*	Ca_Kabuli_Chr01	45008009	[C/T]	Ca_25714	--	INTRON

S.N.	SNP IDs	Chickpea cultivars	Chromosomes	Physical positions (bp)	SNPs	Gene accession IDs	Putative functions	Sequence components of genome
2032	CakSNP2032	*Kabuli*	Ca_Kabuli_Chr01	45008006	[C/A]	Ca_25714	--	INTRON
2033	CakSNP2033	*Kabuli*	Ca_Kabuli_Chr01	45008004	[C/T]	Ca_25714	--	INTRON
2034	CakSNP2034	*Kabuli*	Ca_Kabuli_Chr01	45008074	[C/T]	Ca_25714	--	INTRON
2035	CakSNP2035	*Kabuli*	Ca_Kabuli_Chr01	45250874	[G/A]		--	INTERGENIC
2036	CakSNP2036	*Kabuli*	Ca_Kabuli_Chr01	45250872	[G/T]		--	INTERGENIC
2037	CakSNP2037	*Kabuli*	Ca_Kabuli_Chr01	45250869	[G/A]		--	INTERGENIC
2038	CakSNP2038	*Kabuli*	Ca_Kabuli_Chr01	45250863	[C/T]		--	INTERGENIC
2039	CakSNP2039	*Kabuli*	Ca_Kabuli_Chr01	45250862	[A/T]		--	INTERGENIC
2040	CakSNP2040	*Kabuli*	Ca_Kabuli_Chr01	45250834	[A/G]		--	INTERGENIC
2041	CakSNP2041	*Kabuli*	Ca_Kabuli_Chr01	45250828	[G/C]		--	INTERGENIC

S.N.	SNP IDs	Chickpea cultivars	Chromosomes	Physical positions (bp)	SNPs	Gene accession IDs	Putative functions	Sequence components of genome
2042	CakSNP2042	*Kabuli*	Ca_Kabuli_Chr01	45250807	[C/G]		--	INTERGENIC
2043	CakSNP2043	*Kabuli*	Ca_Kabuli_Chr01	45250805	[A/C]		--	INTERGENIC
2044	CakSNP2044	*Kabuli*	Ca_Kabuli_Chr01	45250803	[A/G]		--	INTERGENIC
2045	CakSNP2045	*Kabuli*	Ca_Kabuli_Chr01	45250802	[C/T]		--	INTERGENIC
2046	CakSNP2046	*Kabuli*	Ca_Kabuli_Chr01	46437023	[T/G]	Ca_21547	Adenylate kinase	SYNONYMOUS_CODING
2047	CakSNP2047	*Kabuli*	Ca_Kabuli_Chr01	46523221	[C/T]	Ca_21550	--	SYNONYMOUS_CODING
2048	CakSNP2048	*Kabuli*	Ca_Kabuli_Chr01	46597131	[T/G]		--	INTERGENIC
2049	CakSNP2049	*Kabuli*	Ca_Kabuli_Chr01	46745628	[C/A]	Ca_25111	Protein of unknown function DUF3754	INTRON
2050	CakSNP2050	*Kabuli*	Ca_Kabuli_Chr01	46778814	[A/C]	Ca_25114	Protein kinase, catalytic domain	NON_SYNONYMOUS_CODING
2051	CakSNP2051	*Kabuli*	Ca_Kabuli_Chr01	46791939	[T/G]		--	INTERGENIC

S.N.	SNP IDs	Chickpea cultivars	Chromosomes	Physical positions (bp)	SNPs	Gene accession IDs	Putative functions	Sequence components of genome
2052	CakSNP2052	*Kabuli*	Ca_Kabuli_Chr01	46793244	[G/A]		--	INTERGENIC
2053	CakSNP2053	*Kabuli*	Ca_Kabuli_Chr01	46793357	[T/C]		--	INTERGENIC
2054	CakSNP2054	*Kabuli*	Ca_Kabuli_Chr01	46793331	[C/G]		--	INTERGENIC
2055	CakSNP2055	*Kabuli*	Ca_Kabuli_Chr01	46816041	[A/T]		--	INTERGENIC
2056	CakSNP2056	*Kabuli*	Ca_Kabuli_Chr01	46825211	[G/A]	Ca_25116	--	INTRON
2057	CakSNP2057	*Kabuli*	Ca_Kabuli_Chr01	46829662	[C/T]	Ca_25116	--	INTRON
2058	CakSNP2058	*Kabuli*	Ca_Kabuli_Chr01	46832306	[T/G]	Ca_25116	--	INTRON
2059	CakSNP2059	*Kabuli*	Ca_Kabuli_Chr01	46832395	[A/G]	Ca_25116	--	INTRON
2060	CakSNP2060	*Kabuli*	Ca_Kabuli_Chr01	46832383	[T/C]	Ca_25116	--	INTRON
2061	CakSNP2061	*Kabuli*	Ca_Kabuli_Chr01	46920524	[C/T]	Ca_12949	--	INTRON

S.N.	SNP IDs	Chickpea cultivars	Chromosomes	Physical positions (bp)	SNPs	Gene accession IDs	Putative functions	Sequence components of genome
2062	CakSNP2062	*Kabuli*	Ca_Kabuli_Chr01	46920554	[T/C]	Ca_12949	--	INTRON
2063	CakSNP2063	*Kabuli*	Ca_Kabuli_Chr01	46936196	[C/A]	Ca_12947	Bile acid:sodium symporter	INTRON
2064	CakSNP2064	*Kabuli*	Ca_Kabuli_Chr01	46936201	[C/T]	Ca_12947	Bile acid:sodium symporter	INTRON
2065	CakSNP2065	*Kabuli*	Ca_Kabuli_Chr01	46936207	[C/A]	Ca_12947	Bile acid:sodium symporter	INTRON
2066	CakSNP2066	*Kabuli*	Ca_Kabuli_Chr01	46936209	[C/T]	Ca_12947	Bile acid:sodium symporter	INTRON
2067	CakSNP2067	*Kabuli*	Ca_Kabuli_Chr01	46936329	[C/A]	Ca_12947	Bile acid:sodium symporter	INTRON
2068	CakSNP2068	*Kabuli*	Ca_Kabuli_Chr01	46936297	[A/G]	Ca_12947	Bile acid:sodium symporter	INTRON
2069	CakSNP2069	*Kabuli*	Ca_Kabuli_Chr01	46936428	[T/C]	Ca_12947	Bile acid:sodium symporter	INTRON
2070	CakSNP2070	*Kabuli*	Ca_Kabuli_Chr01	46936460	[A/C]	Ca_12947	Bile acid:sodium symporter	INTRON
2071	CakSNP2071	*Kabuli*	Ca_Kabuli_Chr01	46936456	[T/C]	Ca_12947	Bile acid:sodium symporter	INTRON

S.N.	SNP IDs	Chickpea cultivars	Chromosomes	Physical positions (bp)	SNPs	Gene accession IDs	Putative functions	Sequence components of genome
2072	CakSNP2072	*Kabuli*	Ca_Kabuli_Chr01	46936448	[A/C]	Ca_12947	Bile acid:sodium symporter	INTRON
2073	CakSNP2073	*Kabuli*	Ca_Kabuli_Chr01	46936447	[G/A]	Ca_12947	Bile acid:sodium symporter	INTRON
2074	CakSNP2074	*Kabuli*	Ca_Kabuli_Chr01	46941875	[T/C]		--	INTERGENIC
2075	CakSNP2075	*Kabuli*	Ca_Kabuli_Chr01	46968740	[T/C]	Ca_12942	Glycosyl transferase, family 20	SYNONYMOUS_CODING
2076	CakSNP2076	*Kabuli*	Ca_Kabuli_Chr01	46969221	[T/A]	Ca_12942	Glycosyl transferase, family 20	INTRON
2077	CakSNP2077	*Kabuli*	Ca_Kabuli_Chr01	46976508	[A/G]	Ca_12942	Glycosyl transferase, family 20	SYNONYMOUS_CODING
2078	CakSNP2078	*Kabuli*	Ca_Kabuli_Chr01	47042798	[T/A]	Ca_12938	Protein of unknown function DUF92, TMEM19	NON_SYNONYMOUS_CODING
2079	CakSNP2079	*Kabuli*	Ca_Kabuli_Chr01	47203123	[A/C]		--	INTERGENIC
2080	CakSNP2080	*Kabuli*	Ca_Kabuli_Chr01	47263589	[C/T]		--	INTERGENIC
2081	CakSNP2081	*Kabuli*	Ca_Kabuli_Chr01	47278505	[T/C]	Ca_12926	--	INTRON

S.N.	SNP IDs	Chickpea cultivars	Chromosomes	Physical positions (bp)	SNPs	Gene accession IDs	Putative functions	Sequence components of genome
2082	CakSNP2082	*Kabuli*	Ca_Kabuli_Chr01	47331082	[T/C]		--	INTERGENIC
2083	CakSNP2083	*Kabuli*	Ca_Kabuli_Chr01	47331080	[T/C]		--	INTERGENIC
2084	CakSNP2084	*Kabuli*	Ca_Kabuli_Chr01	47423715	[A/T]		--	INTERGENIC
2085	CakSNP2085	*Kabuli*	Ca_Kabuli_Chr01	47449818	[A/G]	Ca_12910	--	INTRON
2086	CakSNP2086	*Kabuli*	Ca_Kabuli_Chr01	47449950	[T/C]	Ca_12910	--	INTRON
2087	CakSNP2087	*Kabuli*	Ca_Kabuli_Chr01	47449891	[T/C]	Ca_12910	--	INTRON
2088	CakSNP2088	*Kabuli*	Ca_Kabuli_Chr01	47449882	[A/C]	Ca_12910	--	INTRON
2089	CakSNP2089	*Kabuli*	Ca_Kabuli_Chr01	47801491	[A/G]	Ca_12892	Uroporphyrinogen decarboxylase (URO-D)	SYNONYMOUS_CODING
2090	CakSNP2090	*Kabuli*	Ca_Kabuli_Chr01	47890026	[C/T]	Ca_12888	Phosphoenolpyruvate carboxylase, active site	SYNONYMOUS_CODING
2091	CakSNP2091	*Kabuli*	Ca_Kabuli_Chr01	47897914	[C/T]	Ca_12887	GPI mannosyltransferase	INTRON

S.N.	SNP IDs	Chickpea cultivars	Chromosomes	Physical positions (bp)	SNPs	Gene accession IDs	Putative functions	Sequence components of genome
2092	CakSNP2092	*Kabuli*	Ca_Kabuli_Chr01	47936748	[G/A]	Ca_12883	Ubiquitin-conjugating enzyme, E2	INTRON
2093	CakSNP2093	*Kabuli*	Ca_Kabuli_Chr01	47936841	[A/G]	Ca_12883	Ubiquitin-conjugating enzyme, E2	INTRON
2094	CakSNP2094	*Kabuli*	Ca_Kabuli_Chr01	47938884	[T/G]	Ca_12883	Ubiquitin-conjugating enzyme, E2	INTRON
2095	CakSNP2095	*Kabuli*	Ca_Kabuli_Chr01	47947009	[T/C]	Ca_12882	Tetratricopeptide TPR-1	SYNONYMOUS_CODING
2096	CakSNP2096	*Kabuli*	Ca_Kabuli_Chr01	47947281	[A/C]	Ca_12882	Tetratricopeptide TPR-1	SYNONYMOUS_CODING
2097	CakSNP2097	*Kabuli*	Ca_Kabuli_Chr01	48026664	[G/T]		--	INTERGENIC
2098	CakSNP2098	*Kabuli*	Ca_Kabuli_Chr01	48026701	[C/T]		--	INTERGENIC
2099	CakSNP2099	*Kabuli*	Ca_Kabuli_Chr01	48026716	[T/G]		--	INTERGENIC
2100	CakSNP2100	*Kabuli*	Ca_Kabuli_Chr01	48026727	[A/C]		--	INTERGENIC
2101	CakSNP2101	*Kabuli*	Ca_Kabuli_Chr01	48027714	[C/A]	Ca_12875	GTP1/OBG	SYNONYMOUS_CODING

S.N.	SNP IDs	Chickpea cultivars	Chromosomes	Physical positions (bp)	SNPs	Gene accession IDs	Putative functions	Sequence components of genome
2102	CakSNP2102	*Kabuli*	Ca_Kabuli_Chr01	48027869	[T/C]	Ca_12875	GTP1/OBG	SYNONYMOUS_CODING
2103	CakSNP2103	*Kabuli*	Ca_Kabuli_Chr01	48028094	[G/A]	Ca_12875	GTP1/OBG	SYNONYMOUS_CODING
2104	CakSNP2104	*Kabuli*	Ca_Kabuli_Chr01	48049293	[C/T]	Ca_12870	Peptidase S8, subtilisin-related	SYNONYMOUS_CODING
2105	CakSNP2105	*Kabuli*	Ca_Kabuli_Chr01	48132217	[G/A]	Ca_12865	IQ motif, EF-hand binding site	SYNONYMOUS_CODING
2106	CakSNP2106	*Kabuli*	Ca_Kabuli_Chr01	48132521	[A/C]	Ca_12865	IQ motif, EF-hand binding site	NON_SYNONYMOUS_CODING
2107	CakSNP2107	*Kabuli*	Ca_Kabuli_Chr01	48154321	[A/C]	Ca_12863	Calcium-binding EF-hand	INTRON
2108	CakSNP2108	*Kabuli*	Ca_Kabuli_Chr01	48191460	[T/A]		--	INTERGENIC
2109	CakSNP2109	*Kabuli*	Ca_Kabuli_Chr01	48195174	[T/A]	Ca_12860	Peptidase M24, structural domain	NON_SYNONYMOUS_CODING
2110	CakSNP2110	*Kabuli*	Ca_Kabuli_Chr01	48197895	[A/T]	Ca_12860	Peptidase M24, structural domain	INTRON
2111	CakSNP2111	*Kabuli*	Ca_Kabuli_Chr01	48197911	[T/G]	Ca_12860	Peptidase M24, structural domain	INTRON

S.N.	SNP IDs	Chickpea cultivars	Chromosomes	Physical positions (bp)	SNPs	Gene accession IDs	Putative functions	Sequence components of genome
2112	CakSNP2112	*Kabuli*	Ca_Kabuli_Chr01	48197979	[A/G]	Ca_12860	Peptidase M24, structural domain	INTRON
2113	CakSNP2113	*Kabuli*	Ca_Kabuli_Chr01	48197969	[G/A]	Ca_12860	Peptidase M24, structural domain	INTRON
2114	CakSNP2114	*Kabuli*	Ca_Kabuli_Chr01	48198932	[A/T]	Ca_12860	Peptidase M24, structural domain	SYNONYMOUS_CODING
2115	CakSNP2115	*Kabuli*	Ca_Kabuli_Chr01	48265590	[T/G]	Ca_12856	--	SYNONYMOUS_CODING
2116	CakSNP2116	*Kabuli*	Ca_Kabuli_Chr01	48273097	[G/C]	Ca_12856	--	INTRON
2117	CakSNP2117	*Kabuli*	Ca_Kabuli_Chr01	48273307	[C/T]	Ca_12856	--	INTRON
2118	CakSNP2118	*Kabuli*	Ca_Kabuli_Chr02	113330	[C/T]	Ca_16962	Armadillo	INTRON
2119	CakSNP2119	*Kabuli*	Ca_Kabuli_Chr02	149250	[C/A]	Ca_16968	Uncharacterised protein family UPF0565	INTRON
2120	CakSNP2120	*Kabuli*	Ca_Kabuli_Chr02	149251	[A/T]	Ca_16968	Uncharacterised protein family UPF0565	INTRON
2121	CakSNP2121	*Kabuli*	Ca_Kabuli_Chr02	149259	[A/C]	Ca_16968	Uncharacterised protein family UPF0565	INTRON

S.N.	SNP IDs	Chickpea cultivars	Chromosomes	Physical positions (bp)	SNPs	Gene accession IDs	Putative functions	Sequence components of genome
2122	CakSNP2122	*Kabuli*	Ca_Kabuli_Chr02	149269	[C/T]	Ca_16968	Uncharacterised protein family UPF0565	INTRON
2123	CakSNP2123	*Kabuli*	Ca_Kabuli_Chr02	149278	[G/T]	Ca_16968	Uncharacterised protein family UPF0565	INTRON
2124	CakSNP2124	*Kabuli*	Ca_Kabuli_Chr02	149315	[C/A]	Ca_16968	Uncharacterised protein family UPF0565	INTRON
2125	CakSNP2125	*Kabuli*	Ca_Kabuli_Chr02	149322	[C/A]	Ca_16968	Uncharacterised protein family UPF0565	INTRON
2126	CakSNP2126	*Kabuli*	Ca_Kabuli_Chr02	149402	[C/T]	Ca_16968	Uncharacterised protein family UPF0565	INTRON
2127	CakSNP2127	*Kabuli*	Ca_Kabuli_Chr02	149395	[T/C]	Ca_16968	Uncharacterised protein family UPF0565	INTRON
2128	CakSNP2128	*Kabuli*	Ca_Kabuli_Chr02	189287	[A/G]		--	INTERGENIC
2129	CakSNP2129	*Kabuli*	Ca_Kabuli_Chr02	189784	[C/A]		--	INTERGENIC
2130	CakSNP2130	*Kabuli*	Ca_Kabuli_Chr02	221903	[A/T]	Ca_16974	Alpha/beta hydrolase fold-1	SYNONYMOUS_CODING
2131	CakSNP2131	*Kabuli*	Ca_Kabuli_Chr02	221955	[A/G]	Ca_16974	Alpha/beta hydrolase fold-1	INTRON

S.N.	SNP IDs	Chickpea cultivars	Chromosomes	Physical positions (bp)	SNPs	Gene accession IDs	Putative functions	Sequence components of genome
2132	CakSNP2132	*Kabuli*	Ca_Kabuli_Chr02	233589	[C/T]	Ca_16976	--	NON_SYNONYMOUS_CODING
2133	CakSNP2133	*Kabuli*	Ca_Kabuli_Chr02	270139	[G/A]		--	INTERGENIC
2134	CakSNP2134	*Kabuli*	Ca_Kabuli_Chr02	354795	[T/C]	Ca_16982	Lateral organ boundaries, LOB	SYNONYMOUS_CODING
2135	CakSNP2135	*Kabuli*	Ca_Kabuli_Chr02	355905	[T/C]	Ca_16982	Lateral organ boundaries, LOB	SYNONYMOUS_CODING
2136	CakSNP2136	*Kabuli*	Ca_Kabuli_Chr02	507857	[G/T]	Ca_16990	--	SYNONYMOUS_CODING
2137	CakSNP2137	*Kabuli*	Ca_Kabuli_Chr02	522943	[A/G]		--	INTERGENIC
2138	CakSNP2138	*Kabuli*	Ca_Kabuli_Chr02	543360	[G/T]	Ca_16992	--	INTRON
2139	CakSNP2139	*Kabuli*	Ca_Kabuli_Chr02	616251	[C/T]	Ca_16995	Alcohol dehydrogenase superfamily, zinc-containing	SYNONYMOUS_CODING
2140	CakSNP2140	*Kabuli*	Ca_Kabuli_Chr02	616410	[C/G]	Ca_16995	Alcohol dehydrogenase superfamily, zinc-containing	INTRON
2141	CakSNP2141	*Kabuli*	Ca_Kabuli_Chr02	669304	[A/T]	Ca_16997	14-3-3 protein	INTRON

S.N.	SNP IDs	Chickpea cultivars	Chromosomes	Physical positions (bp)	SNPs	Gene accession IDs	Putative functions	Sequence components of genome
2142	CakSNP2142	*Kabuli*	Ca_Kabuli_Chr02	728957	[T/C]	Ca_16999	SNARE associated Golgi protein	SYNONYMOUS_CODING
2143	CakSNP2143	*Kabuli*	Ca_Kabuli_Chr02	737183	[T/G]	Ca_17000	--	SYNONYMOUS_CODING
2144	CakSNP2144	*Kabuli*	Ca_Kabuli_Chr02	770164	[A/C]	Ca_17001	Peptidase C48, SUMO/Sentrin/Ubl1	SYNONYMOUS_CODING
2145	CakSNP2145	*Kabuli*	Ca_Kabuli_Chr02	770183	[C/T]	Ca_17001	Peptidase C48, SUMO/Sentrin/Ubl1	NON_SYNONYMOUS_CODING
2146	CakSNP2146	*Kabuli*	Ca_Kabuli_Chr02	770234	[C/T]	Ca_17001	Peptidase C48, SUMO/Sentrin/Ubl1	NON_SYNONYMOUS_CODING
2147	CakSNP2147	*Kabuli*	Ca_Kabuli_Chr02	770237	[C/T]	Ca_17001	Peptidase C48, SUMO/Sentrin/Ubl1	NON_SYNONYMOUS_CODING
2148	CakSNP2148	*Kabuli*	Ca_Kabuli_Chr02	770225	[C/T]	Ca_17001	Peptidase C48, SUMO/Sentrin/Ubl1	NON_SYNONYMOUS_CODING
2149	CakSNP2149	*Kabuli*	Ca_Kabuli_Chr02	770260	[C/T]	Ca_17001	Peptidase C48, SUMO/Sentrin/Ubl1	NON_SYNONYMOUS_CODING
2150	CakSNP2150	*Kabuli*	Ca_Kabuli_Chr02	770266	[C/A]	Ca_17001	Peptidase C48, SUMO/Sentrin/Ubl1	NON_SYNONYMOUS_CODING
2151	CakSNP2151	*Kabuli*	Ca_Kabuli_Chr02	1231486	[A/G]		--	INTERGENIC

S.N.	SNP IDs	Chickpea cultivars	Chromosomes	Physical positions (bp)	SNPs	Gene accession IDs	Putative functions	Sequence components of genome
2152	CakSNP2152	*Kabuli*	Ca_Kabuli_Chr02	1231500	[A/G]		--	INTERGENIC
2153	CakSNP2153	*Kabuli*	Ca_Kabuli_Chr02	1231519	[T/C]		--	INTERGENIC
2154	CakSNP2154	*Kabuli*	Ca_Kabuli_Chr02	1231618	[G/A]		--	INTERGENIC
2155	CakSNP2155	*Kabuli*	Ca_Kabuli_Chr02	1234932	[C/T]		--	INTERGENIC
2156	CakSNP2156	*Kabuli*	Ca_Kabuli_Chr02	1235018	[G/A]		--	INTERGENIC
2157	CakSNP2157	*Kabuli*	Ca_Kabuli_Chr02	1234983	[T/C]		--	INTERGENIC
2158	CakSNP2158	*Kabuli*	Ca_Kabuli_Chr02	1314367	[G/A]		--	INTERGENIC
2159	CakSNP2159	*Kabuli*	Ca_Kabuli_Chr02	1368911	[A/G]	Ca_20691	Leucine-rich repeat	NON_SYNONYMOUS_CODING
2160	CakSNP2160	*Kabuli*	Ca_Kabuli_Chr02	1389661	[A/G]	Ca_20695	KDPG/KHG aldolase	INTRON
2161	CakSNP2161	*Kabuli*	Ca_Kabuli_Chr02	1397779	[C/T]	Ca_20696	Lipase, GDSL	SYNONYMOUS_CODING

S.N.	SNP IDs	Chickpea cultivars	Chromosomes	Physical positions (bp)	SNPs	Gene accession IDs	Putative functions	Sequence components of genome
2162	CakSNP2162	*Kabuli*	Ca_Kabuli_Chr02	1429186	[T/C]	Ca_20698	TRAUB	INTRON
2163	CakSNP2163	*Kabuli*	Ca_Kabuli_Chr02	1429471	[G/C]	Ca_20698	TRAUB	INTRON
2164	CakSNP2164	*Kabuli*	Ca_Kabuli_Chr02	1431368	[A/C]	Ca_20698	TRAUB	INTRON
2165	CakSNP2165	*Kabuli*	Ca_Kabuli_Chr02	1493971	[A/G]		--	INTERGENIC
2166	CakSNP2166	*Kabuli*	Ca_Kabuli_Chr02	1610477	[G/T]	Ca_12629	Diacylglycerol kinase, catalytic domain	NON_SYNONYMOUS_CODING
2167	CakSNP2167	*Kabuli*	Ca_Kabuli_Chr02	1668116	[A/G]	Ca_12621	HEAT	SYNONYMOUS_CODING
2168	CakSNP2168	*Kabuli*	Ca_Kabuli_Chr02	1713343	[T/C]	Ca_12615	Protein kinase, catalytic domain	SYNONYMOUS_CODING
2169	CakSNP2169	*Kabuli*	Ca_Kabuli_Chr02	1797399	[A/G]	Ca_12605	Domain of unknown function DUF3730	SYNONYMOUS_CODING
2170	CakSNP2170	*Kabuli*	Ca_Kabuli_Chr02	1801341	[C/T]	Ca_12605	Domain of unknown function DUF3730	SYNONYMOUS_CODING
2171	CakSNP2171	*Kabuli*	Ca_Kabuli_Chr02	1828318	[T/C]	Ca_12602	Protein of unknown function DUF1421	SYNONYMOUS_CODING

S.N.	SNP IDs	Chickpea cultivars	Chromosomes	Physical positions (bp)	SNPs	Gene accession IDs	Putative functions	Sequence components of genome
2172	CakSNP2172	*Kabuli*	Ca_Kabuli_Chr02	1828406	[G/A]	Ca_12602	Protein of unknown function DUF1421	NON_SYNONYMOUS_CODING
2173	CakSNP2173	*Kabuli*	Ca_Kabuli_Chr02	1829772	[C/T]	Ca_12602	Protein of unknown function DUF1421	INTRON
2174	CakSNP2174	*Kabuli*	Ca_Kabuli_Chr02	1835055	[G/A]	Ca_12601	Raffinose synthase	INTRON
2175	CakSNP2175	*Kabuli*	Ca_Kabuli_Chr02	1841290	[A/T]	Ca_12600	Glycosyl transferase, family 14	NON_SYNONYMOUS_CODING
2176	CakSNP2176	*Kabuli*	Ca_Kabuli_Chr02	1841267	[C/T]	Ca_12600	Glycosyl transferase, family 14	SYNONYMOUS_CODING
2177	CakSNP2177	*Kabuli*	Ca_Kabuli_Chr02	1867041	[C/A]	Ca_12598	Bacterial Ig-like, group 2	INTRON
2178	CakSNP2178	*Kabuli*	Ca_Kabuli_Chr02	1882691	[C/T]	Ca_12597	RNA recognition motif domain	SYNONYMOUS_CODING
2179	CakSNP2179	*Kabuli*	Ca_Kabuli_Chr02	1884583	[C/T]	Ca_12597	RNA recognition motif domain	SYNONYMOUS_CODING
2180	CakSNP2180	*Kabuli*	Ca_Kabuli_Chr02	1884704	[A/G]	Ca_12597	RNA recognition motif domain	NON_SYNONYMOUS_CODING
2181	CakSNP2181	*Kabuli*	Ca_Kabuli_Chr02	1888657	[C/G]	Ca_12596	Ribosomal protein S11	NON_SYNONYMOUS_CODING

S.N.	SNP IDs	Chickpea cultivars	Chromosomes	Physical positions (bp)	SNPs	Gene accession IDs	Putative functions	Sequence components of genome
2182	CakSNP2182	*Kabuli*	Ca_Kabuli_Chr02	1953614	[T/C]	Ca_12586	Cobalamin (vitamin B12) biosynthesis CbiX	INTRON
2183	CakSNP2183	*Kabuli*	Ca_Kabuli_Chr02	1997914	[C/T]	Ca_12579	--	INTRON
2184	CakSNP2184	*Kabuli*	Ca_Kabuli_Chr02	1998003	[C/T]	Ca_12579	--	NON_SYNONYMOUS_CODING
2185	CakSNP2185	*Kabuli*	Ca_Kabuli_Chr02	2086763	[T/G]	Ca_12571	ATPase, AAA+ type, core	NON_SYNONYMOUS_CODING
2186	CakSNP2186	*Kabuli*	Ca_Kabuli_Chr02	2220871	[C/T]	Ca_12559	Lipase, GDSL	SYNONYMOUS_CODING
2187	CakSNP2187	*Kabuli*	Ca_Kabuli_Chr02	2227118	[A/G]	Ca_12557	Tetratricopeptide repeat-containing	SYNONYMOUS_CODING
2188	CakSNP2188	*Kabuli*	Ca_Kabuli_Chr02	2227074	[T/C]	Ca_12557	Tetratricopeptide repeat-containing	NON_SYNONYMOUS_CODING
2189	CakSNP2189	*Kabuli*	Ca_Kabuli_Chr02	2227465	[G/A]	Ca_12557	Tetratricopeptide repeat-containing	SYNONYMOUS_CODING
2190	CakSNP2190	*Kabuli*	Ca_Kabuli_Chr02	2230089	[G/A]		--	INTERGENIC
2191	CakSNP2191	*Kabuli*	Ca_Kabuli_Chr02	2230056	[T/C]		--	INTERGENIC

S.N.	SNP IDs	Chickpea cultivars	Chromosomes	Physical positions (bp)	SNPs	Gene accession IDs	Putative functions	Sequence components of genome
2192	CakSNP2192	*Kabuli*	Ca_Kabuli_Chr02	2230984	[T/G]		--	INTERGENIC
2193	CakSNP2193	*Kabuli*	Ca_Kabuli_Chr02	2230980	[C/A]		--	INTERGENIC
2194	CakSNP2194	*Kabuli*	Ca_Kabuli_Chr02	2230976	[T/G]		--	INTERGENIC
2195	CakSNP2195	*Kabuli*	Ca_Kabuli_Chr02	2246678	[T/C]	Ca_12554	Prenyltransferase/squalene oxidase	INTRON
2196	CakSNP2196	*Kabuli*	Ca_Kabuli_Chr02	2311915	[C/A]		--	INTERGENIC
2197	CakSNP2197	*Kabuli*	Ca_Kabuli_Chr02	2311917	[A/C]		--	INTERGENIC
2198	CakSNP2198	*Kabuli*	Ca_Kabuli_Chr02	2312104	[C/T]		--	INTERGENIC
2199	CakSNP2199	*Kabuli*	Ca_Kabuli_Chr02	2312037	[T/G]		--	INTERGENIC
2200	CakSNP2200	*Kabuli*	Ca_Kabuli_Chr02	2344777	[G/A]	Ca_12544	Oligopeptide transporter	SYNONYMOUS_CODING
2201	CakSNP2201	*Kabuli*	Ca_Kabuli_Chr02	2344778	[A/G]	Ca_12544	Oligopeptide transporter	NON_SYNONYMOUS_CODING

S.N.	SNP IDs	Chickpea cultivars	Chromosomes	Physical positions (bp)	SNPs	Gene accession IDs	Putative functions	Sequence components of genome
2202	CakSNP2202	*Kabuli*	Ca_Kabuli_Chr02	2344786	[G/A]	Ca_12544	Oligopeptide transporter	SYNONYMOUS_CODING
2203	CakSNP2203	*Kabuli*	Ca_Kabuli_Chr02	2344835	[G/T]	Ca_12544	Oligopeptide transporter	STOP_GAINED
2204	CakSNP2204	*Kabuli*	Ca_Kabuli_Chr02	2414265	[A/G]		--	INTERGENIC
2205	CakSNP2205	*Kabuli*	Ca_Kabuli_Chr02	2416422	[C/T]	Ca_12538	Methyl-CpG DNA binding	NON_SYNONYMOUS_CODING
2206	CakSNP2206	*Kabuli*	Ca_Kabuli_Chr02	2441571	[G/A]		--	INTERGENIC
2207	CakSNP2207	*Kabuli*	Ca_Kabuli_Chr02	2602831	[A/C]		--	INTERGENIC
2208	CakSNP2208	*Kabuli*	Ca_Kabuli_Chr02	2650375	[T/C]	Ca_10580	Lipase, class 3	INTRON
2209	CakSNP2209	*Kabuli*	Ca_Kabuli_Chr02	2650373	[A/T]	Ca_10580	Lipase, class 3	INTRON
2210	CakSNP2210	*Kabuli*	Ca_Kabuli_Chr02	2652189	[A/T]	Ca_10580	Lipase, class 3	INTRON
2211	CakSNP2211	*Kabuli*	Ca_Kabuli_Chr02	2682235	[T/C]	Ca_10575	Ferredoxin thioredoxin reductase, beta subunit	INTRON

S.N.	SNP IDs	Chickpea cultivars	Chromosomes	Physical positions (bp)	SNPs	Gene accession IDs	Putative functions	Sequence components of genome
2212	CakSNP2212	*Kabuli*	Ca_Kabuli_Chr02	2682293	[T/C]	Ca_10575	Ferredoxin thioredoxin reductase, beta subunit	INTRON
2213	CakSNP2213	*Kabuli*	Ca_Kabuli_Chr02	2682321	[G/A]	Ca_10575	Ferredoxin thioredoxin reductase, beta subunit	INTRON
2214	CakSNP2214	*Kabuli*	Ca_Kabuli_Chr02	2682375	[A/G]	Ca_10575	Ferredoxin thioredoxin reductase, beta subunit	SYNONYMOUS_CODING
2215	CakSNP2215	*Kabuli*	Ca_Kabuli_Chr02	2682408	[G/A]	Ca_10575	Ferredoxin thioredoxin reductase, beta subunit	SYNONYMOUS_CODING
2216	CakSNP2216	*Kabuli*	Ca_Kabuli_Chr02	2682471	[A/G]	Ca_10575	Ferredoxin thioredoxin reductase, beta subunit	INTRON
2217	CakSNP2217	*Kabuli*	Ca_Kabuli_Chr02	2685299	[T/C]	Ca_10575	Ferredoxin thioredoxin reductase, beta subunit	INTRON
2218	CakSNP2218	*Kabuli*	Ca_Kabuli_Chr02	2697688	[G/C]	Ca_10574	RNA-binding, CRM domain	NON_SYNONYMOUS_CODING
2219	CakSNP2219	*Kabuli*	Ca_Kabuli_Chr02	2708805	[G/C]		--	INTERGENIC
2220	CakSNP2220	*Kabuli*	Ca_Kabuli_Chr02	2780283	[C/T]	Ca_10564	--	SYNONYMOUS_CODING
2221	CakSNP2221	*Kabuli*	Ca_Kabuli_Chr02	2783727	[G/A]	Ca_10563	Zinc finger, RING-type	NON_SYNONYMOUS_CODING

S.N.	SNP IDs	Chickpea cultivars	Chromosomes	Physical positions (bp)	SNPs	Gene accession IDs	Putative functions	Sequence components of genome
2222	CakSNP2222	*Kabuli*	Ca_Kabuli_Chr02	2846667	[T/C]	Ca_10559	Phosphoglucose isomerase (PGI)	INTRON
2223	CakSNP2223	*Kabuli*	Ca_Kabuli_Chr02	2846774	[C/A]	Ca_10559	Phosphoglucose isomerase (PGI)	NON_SYNONYMOUS_CODING
2224	CakSNP2224	*Kabuli*	Ca_Kabuli_Chr02	2965867	[G/A]		--	INTERGENIC
2225	CakSNP2225	*Kabuli*	Ca_Kabuli_Chr02	3099959	[C/A]	Ca_10540	Band 7 protein	INTRON
2226	CakSNP2226	*Kabuli*	Ca_Kabuli_Chr02	3099997	[A/T]	Ca_10540	Band 7 protein	INTRON
2227	CakSNP2227	*Kabuli*	Ca_Kabuli_Chr02	3100116	[A/G]	Ca_10540	Band 7 protein	INTRON
2228	CakSNP2228	*Kabuli*	Ca_Kabuli_Chr02	3113560	[G/A]	Ca_10538	Auxin efflux carrier	INTRON
2229	CakSNP2229	*Kabuli*	Ca_Kabuli_Chr02	3141128	[A/C]		--	INTERGENIC
2230	CakSNP2230	*Kabuli*	Ca_Kabuli_Chr02	3164404	[A/G]		--	INTERGENIC
2231	CakSNP2231	*Kabuli*	Ca_Kabuli_Chr02	3164355	[G/A]		--	INTERGENIC

S.N.	SNP IDs	Chickpea cultivars	Chromosomes	Physical positions (bp)	SNPs	Gene accession IDs	Putative functions	Sequence components of genome
2232	CakSNP2232	*Kabuli*	Ca_Kabuli_Chr02	3366023	[C/T]	Ca_10517	ZF-HD homeobox protein, Cys/His-rich dimerisation domain	NON_SYNONYMOUS_CODING
2233	CakSNP2233	*Kabuli*	Ca_Kabuli_Chr02	3410375	[C/T]	Ca_10515	ZF-HD homeobox protein, Cys/His-rich dimerisation domain	NON_SYNONYMOUS_CODING
2234	CakSNP2234	*Kabuli*	Ca_Kabuli_Chr02	3450904	[C/A]	Ca_10514	ZF-HD homeobox protein, Cys/His-rich dimerisation domain	SYNONYMOUS_CODING
2235	CakSNP2235	*Kabuli*	Ca_Kabuli_Chr02	3503763	[T/G]	Ca_10509	Reticulon	INTRON
2236	CakSNP2236	*Kabuli*	Ca_Kabuli_Chr02	3503759	[T/A]	Ca_10509	Reticulon	INTRON
2237	CakSNP2237	*Kabuli*	Ca_Kabuli_Chr02	3503753	[C/G]	Ca_10509	Reticulon	INTRON
2238	CakSNP2238	*Kabuli*	Ca_Kabuli_Chr02	3503750	[C/G]	Ca_10509	Reticulon	INTRON
2239	CakSNP2239	*Kabuli*	Ca_Kabuli_Chr02	3789808	[G/T]	Ca_10481	U box domain	SYNONYMOUS_CODING
2240	CakSNP2240	*Kabuli*	Ca_Kabuli_Chr02	3843814	[C/T]	Ca_10475	Chaperonin ClpA/B	INTRON
2241	CakSNP2241	*Kabuli*	Ca_Kabuli_Chr02	3843826	[T/C]	Ca_10475	Chaperonin ClpA/B	INTRON

S.N.	SNP IDs	Chickpea cultivars	Chromosomes	Physical positions (bp)	SNPs	Gene accession IDs	Putative functions	Sequence components of genome
2242	CakSNP2242	*Kabuli*	Ca_Kabuli_Chr02	3843836	[G/A]	Ca_10475	Chaperonin ClpA/B	INTRON
2243	CakSNP2243	*Kabuli*	Ca_Kabuli_Chr02	3852203	[C/A]		--	INTERGENIC
2244	CakSNP2244	*Kabuli*	Ca_Kabuli_Chr02	3894041	[G/T]	Ca_10467	Glycosyl transferase, family 35	INTRON
2245	CakSNP2245	*Kabuli*	Ca_Kabuli_Chr02	3904757	[G/A]	Ca_10466	UDP-glucose/GDP-mannose dehydrogenase, N-terminal	SYNONYMOUS_CODING
2246	CakSNP2246	*Kabuli*	Ca_Kabuli_Chr02	3904761	[A/G]	Ca_10466	UDP-glucose/GDP-mannose dehydrogenase, N-terminal	NON_SYNONYMOUS_CODING
2247	CakSNP2247	*Kabuli*	Ca_Kabuli_Chr02	3908928	[A/G]	Ca_10465	Xklp2 targeting protein	INTRON
2248	CakSNP2248	*Kabuli*	Ca_Kabuli_Chr02	3908955	[G/A]	Ca_10465	Xklp2 targeting protein	INTRON
2249	CakSNP2249	*Kabuli*	Ca_Kabuli_Chr02	3909068	[G/A]	Ca_10465	Xklp2 targeting protein	INTRON
2250	CakSNP2250	*Kabuli*	Ca_Kabuli_Chr02	3909062	[C/T]	Ca_10465	Xklp2 targeting protein	INTRON
2251	CakSNP2251	*Kabuli*	Ca_Kabuli_Chr02	3915549	[A/G]	Ca_10464	Biotin/lipoyl attachment	NON_SYNONYMOUS_CODING

S.N.	SNP IDs	Chickpea cultivars	Chromosomes	Physical positions (bp)	SNPs	Gene accession IDs	Putative functions	Sequence components of genome
2252	CakSNP2252	*Kabuli*	Ca_Kabuli_Chr02	3930211	[A/G]	Ca_10463	Zinc finger, PHD-type	NON_SYNONYMOUS_CODING
2253	CakSNP2253	*Kabuli*	Ca_Kabuli_Chr02	3930182	[A/G]	Ca_10463	Zinc finger, PHD-type	SYNONYMOUS_CODING
2254	CakSNP2254	*Kabuli*	Ca_Kabuli_Chr02	3941397	[A/T]		--	INTERGENIC
2255	CakSNP2255	*Kabuli*	Ca_Kabuli_Chr02	3994150	[A/C]		--	INTERGENIC
2256	CakSNP2256	*Kabuli*	Ca_Kabuli_Chr02	3998491	[A/C]	Ca_10456	--	SYNONYMOUS_CODING
2257	CakSNP2257	*Kabuli*	Ca_Kabuli_Chr02	3999615	[C/T]	Ca_10456	--	NON_SYNONYMOUS_CODING
2258	CakSNP2258	*Kabuli*	Ca_Kabuli_Chr02	4039745	[T/C]		--	INTERGENIC
2259	CakSNP2259	*Kabuli*	Ca_Kabuli_Chr02	4039854	[G/A]		--	INTERGENIC
2260	CakSNP2260	*Kabuli*	Ca_Kabuli_Chr02	4325658	[A/C]		--	INTERGENIC
2261	CakSNP2261	*Kabuli*	Ca_Kabuli_Chr02	4325684	[C/A]		--	INTERGENIC

S.N.	SNP IDs	Chickpea cultivars	Chromosomes	Physical positions (bp)	SNPs	Gene accession IDs	Putative functions	Sequence components of genome
2262	CakSNP2262	*Kabuli*	Ca_Kabuli_Chr02	4404813	[C/T]	Ca_21106	Glycosyl hydrolase, family 13, catalytic domain	INTRON
2263	CakSNP2263	*Kabuli*	Ca_Kabuli_Chr02	4404797	[G/A]	Ca_21106	Glycosyl hydrolase, family 13, catalytic domain	INTRON
2264	CakSNP2264	*Kabuli*	Ca_Kabuli_Chr02	4966216	[C/T]	Ca_14714	DNA ligase, ATP-dependent, N-terminal	NON_SYNONYMOUS_CODING
2265	CakSNP2265	*Kabuli*	Ca_Kabuli_Chr02	4966168	[G/C]		--	INTERGENIC
2266	CakSNP2266	*Kabuli*	Ca_Kabuli_Chr02	4981626	[A/G]	Ca_14713	--	SYNONYMOUS_CODING
2267	CakSNP2267	*Kabuli*	Ca_Kabuli_Chr02	4981698	[C/T]	Ca_14713	--	SYNONYMOUS_CODING
2268	CakSNP2268	*Kabuli*	Ca_Kabuli_Chr02	4981686	[T/C]	Ca_14713	--	SYNONYMOUS_CODING
2269	CakSNP2269	*Kabuli*	Ca_Kabuli_Chr02	4981666	[G/C]	Ca_14713	--	NON_SYNONYMOUS_CODING
2270	CakSNP2270	*Kabuli*	Ca_Kabuli_Chr02	5017291	[T/A]	Ca_14707	--	INTRON
2271	CakSNP2271	*Kabuli*	Ca_Kabuli_Chr02	5017330	[G/C]	Ca_14707	--	INTRON

S.N.	SNP IDs	Chickpea cultivars	Chromosomes	Physical positions (bp)	SNPs	Gene accession IDs	Putative functions	Sequence components of genome
2272	CakSNP2272	*Kabuli*	Ca_Kabuli_Chr02	5078323	[T/C]	Ca_14702	Zinc finger, RING-type	INTRON
2273	CakSNP2273	*Kabuli*	Ca_Kabuli_Chr02	5079639	[C/T]	Ca_14702	Zinc finger, RING-type	NON_SYNONYMOUS_CODING
2274	CakSNP2274	*Kabuli*	Ca_Kabuli_Chr02	5085761	[A/C]	Ca_14701	Pentatricopeptide repeat	INTRON
2275	CakSNP2275	*Kabuli*	Ca_Kabuli_Chr02	5085836	[G/C]	Ca_14701	Pentatricopeptide repeat	INTRON
2276	CakSNP2276	*Kabuli*	Ca_Kabuli_Chr02	5100017	[T/A]	Ca_14699	Ras GTPase	INTRON
2277	CakSNP2277	*Kabuli*	Ca_Kabuli_Chr02	5099985	[A/G]	Ca_14699	Ras GTPase	INTRON
2278	CakSNP2278	*Kabuli*	Ca_Kabuli_Chr02	5118370	[A/T]	Ca_14698	Protein synthesis factor, GTP-binding	SYNONYMOUS_CODING
2279	CakSNP2279	*Kabuli*	Ca_Kabuli_Chr02	5118364	[C/T]	Ca_14698	Protein synthesis factor, GTP-binding	SYNONYMOUS_CODING
2280	CakSNP2280	*Kabuli*	Ca_Kabuli_Chr02	5141075	[C/T]	Ca_14697	Protein synthesis factor, GTP-binding	SYNONYMOUS_CODING
2281	CakSNP2281	*Kabuli*	Ca_Kabuli_Chr02	5141081	[A/T]	Ca_14697	Protein synthesis factor, GTP-binding	SYNONYMOUS_CODING

S.N.	SNP IDs	Chickpea cultivars	Chromosomes	Physical positions (bp)	SNPs	Gene accession IDs	Putative functions	Sequence components of genome
2282	CakSNP2282	*Kabuli*	Ca_Kabuli_Chr02	5247305	[T/C]	Ca_14684	Xklp2 targeting protein	INTRON
2283	CakSNP2283	*Kabuli*	Ca_Kabuli_Chr02	5247548	[G/T]	Ca_14684	Xklp2 targeting protein	INTRON
2284	CakSNP2284	*Kabuli*	Ca_Kabuli_Chr02	5247547	[A/G]	Ca_14684	Xklp2 targeting protein	INTRON
2285	CakSNP2285	*Kabuli*	Ca_Kabuli_Chr02	5247540	[T/C]	Ca_14684	Xklp2 targeting protein	INTRON
2286	CakSNP2286	*Kabuli*	Ca_Kabuli_Chr02	5247481	[T/A]	Ca_14684	Xklp2 targeting protein	INTRON
2287	CakSNP2287	*Kabuli*	Ca_Kabuli_Chr02	5247557	[A/C]	Ca_14684	Xklp2 targeting protein	INTRON
2288	CakSNP2288	*Kabuli*	Ca_Kabuli_Chr02	5247558	[G/T]	Ca_14684	Xklp2 targeting protein	INTRON
2289	CakSNP2289	*Kabuli*	Ca_Kabuli_Chr02	5259254	[T/C]	Ca_14682	--	NON_SYNONYMOUS_CODING
2290	CakSNP2290	*Kabuli*	Ca_Kabuli_Chr02	5276208	[T/C]		--	INTERGENIC
2291	CakSNP2291	*Kabuli*	Ca_Kabuli_Chr02	5342864	[C/A]		--	INTERGENIC

S.N.	SNP IDs	Chickpea cultivars	Chromosomes	Physical positions (bp)	SNPs	Gene accession IDs	Putative functions	Sequence components of genome
2292	CakSNP2292	*Kabuli*	Ca_Kabuli_Chr02	5342923	[A/G]		--	INTERGENIC
2293	CakSNP2293	*Kabuli*	Ca_Kabuli_Chr02	5359177	[C/A]	Ca_14671	Ankyrin repeat	NON_SYNONYMOUS_CODING
2294	CakSNP2294	*Kabuli*	Ca_Kabuli_Chr02	5449886	[C/T]	Ca_14662	Cytochrome c1	INTRON
2295	CakSNP2295	*Kabuli*	Ca_Kabuli_Chr02	5449978	[T/C]	Ca_14662	Cytochrome c1	SYNONYMOUS_CODING
2296	CakSNP2296	*Kabuli*	Ca_Kabuli_Chr02	5449907	[G/A]	Ca_14662	Cytochrome c1	INTRON
2297	CakSNP2297	*Kabuli*	Ca_Kabuli_Chr02	5462902	[A/T]	Ca_14661	Rubisco LS methyltransferase, substrate-binding domain	INTRON
2298	CakSNP2298	*Kabuli*	Ca_Kabuli_Chr02	5468575	[A/T]		--	INTERGENIC
2299	CakSNP2299	*Kabuli*	Ca_Kabuli_Chr02	5475329	[C/A]	Ca_14660	Proteasome component (PCI) domain	INTRON
2300	CakSNP2300	*Kabuli*	Ca_Kabuli_Chr02	5475344	[A/G]	Ca_14660	Proteasome component (PCI) domain	INTRON
2301	CakSNP2301	*Kabuli*	Ca_Kabuli_Chr02	5475365	[T/C]	Ca_14660	Proteasome component (PCI) domain	INTRON

S.N.	SNP IDs	Chickpea cultivars	Chromosomes	Physical positions (bp)	SNPs	Gene accession IDs	Putative functions	Sequence components of genome
2302	CakSNP2302	*Kabuli*	Ca_Kabuli_Chr02	5475407	[C/T]	Ca_14660	Proteasome component (PCI) domain	INTRON
2303	CakSNP2303	*Kabuli*	Ca_Kabuli_Chr02	5504300	[T/G]	Ca_14658	Protein kinase, catalytic domain	INTRON
2304	CakSNP2304	*Kabuli*	Ca_Kabuli_Chr02	5601092	[G/C]	Ca_14654	--	NON_SYNONYMOUS_CODING
2305	CakSNP2305	*Kabuli*	Ca_Kabuli_Chr02	5787862	[G/A]	Ca_19683	Plant specific mitochondrial import receptor subunit TOM21	SYNONYMOUS_CODING
2306	CakSNP2306	*Kabuli*	Ca_Kabuli_Chr02	5787887	[A/C]	Ca_19683	Plant specific mitochondrial import receptor subunit TOM22	INTRON
2307	CakSNP2307	*Kabuli*	Ca_Kabuli_Chr02	5877688	[A/G]	Ca_19692	Heat shock protein DnaJ, N-terminal	INTRON
2308	CakSNP2308	*Kabuli*	Ca_Kabuli_Chr02	5881503	[A/G]	Ca_19692	Heat shock protein DnaJ, N-terminal	INTRON
2309	CakSNP2309	*Kabuli*	Ca_Kabuli_Chr02	5897314	[T/C]	Ca_19695	--	INTRON
2310	CakSNP2310	*Kabuli*	Ca_Kabuli_Chr02	5904368	[T/C]	Ca_19695	--	SYNONYMOUS_CODING
2311	CakSNP2311	*Kabuli*	Ca_Kabuli_Chr02	5936796	[C/A]	Ca_19698	Arf GTPase activating protein	NON_SYNONYMOUS_CODING

S.N.	SNP IDs	Chickpea cultivars	Chromosomes	Physical positions (bp)	SNPs	Gene accession IDs	Putative functions	Sequence components of genome
2312	CakSNP2312	*Kabuli*	Ca_Kabuli_Chr02	5936902	[A/T]	Ca_19698	Arf GTPase activating protein	INTRON
2313	CakSNP2313	*Kabuli*	Ca_Kabuli_Chr02	6042131	[G/A]		--	INTERGENIC
2314	CakSNP2314	*Kabuli*	Ca_Kabuli_Chr02	6042139	[C/A]		--	INTERGENIC
2315	CakSNP2315	*Kabuli*	Ca_Kabuli_Chr02	6042210	[C/A]		--	INTERGENIC
2316	CakSNP2316	*Kabuli*	Ca_Kabuli_Chr02	6060259	[G/C]	Ca_19707	Transcription factor TFIIB-related	INTRON
2317	CakSNP2317	*Kabuli*	Ca_Kabuli_Chr02	6130301	[G/C]	Ca_19712	--	NON_SYNONYMOUS_CODING
2318	CakSNP2318	*Kabuli*	Ca_Kabuli_Chr02	6130257	[C/A]	Ca_19712	--	NON_SYNONYMOUS_CODING
2319	CakSNP2319	*Kabuli*	Ca_Kabuli_Chr02	6130247	[G/C]	Ca_19712	--	NON_SYNONYMOUS_CODING
2320	CakSNP2320	*Kabuli*	Ca_Kabuli_Chr02	6162378	[T/G]	Ca_19714	Amino acid/polyamine transporter I	INTRON
2321	CakSNP2321	*Kabuli*	Ca_Kabuli_Chr02	6162406	[G/C]	Ca_19714	Amino acid/polyamine transporter I	INTRON

S.N.	SNP IDs	Chickpea cultivars	Chromosomes	Physical positions (bp)	SNPs	Gene accession IDs	Putative functions	Sequence components of genome
2322	CakSNP2322	*Kabuli*	Ca_Kabuli_Chr02	6213582	[T/A]	Ca_20925	--	NON_SYNONYMOUS_CODING
2323	CakSNP2323	*Kabuli*	Ca_Kabuli_Chr02	6213580	[C/A]	Ca_20925	--	NON_SYNONYMOUS_CODING
2324	CakSNP2324	*Kabuli*	Ca_Kabuli_Chr02	6213566	[C/T]	Ca_20925	--	NON_SYNONYMOUS_CODING
2325	CakSNP2325	*Kabuli*	Ca_Kabuli_Chr02	6213530	[C/A]	Ca_20925	--	NON_SYNONYMOUS_CODING
2326	CakSNP2326	*Kabuli*	Ca_Kabuli_Chr02	6213520	[G/T]	Ca_20925	--	NON_SYNONYMOUS_CODING
2327	CakSNP2327	*Kabuli*	Ca_Kabuli_Chr02	6349208	[T/G]	Ca_20931	Helicase/RNase D C-terminal, HRDC domain	INTRON
2328	CakSNP2328	*Kabuli*	Ca_Kabuli_Chr02	6367649	[A/G]	Ca_20933	BTB/POZ-like	INTRON
2329	CakSNP2329	*Kabuli*	Ca_Kabuli_Chr02	6367704	[A/G]	Ca_20933	BTB/POZ-like	INTRON
2330	CakSNP2330	*Kabuli*	Ca_Kabuli_Chr02	6391819	[T/C]	Ca_20935	Ankyrin repeat	SYNONYMOUS_CODING
2331	CakSNP2331	*Kabuli*	Ca_Kabuli_Chr02	6392158	[A/T]	Ca_20935	Ankyrin repeat	SYNONYMOUS_CODING

S.N.	SNP IDs	Chickpea cultivars	Chromosomes	Physical positions (bp)	SNPs	Gene accession IDs	Putative functions	Sequence components of genome
2332	CakSNP2332	*Kabuli*	Ca_Kabuli_Chr02	6392209	[G/A]	Ca_20935	Ankyrin repeat	SYNONYMOUS_CODING
2333	CakSNP2333	*Kabuli*	Ca_Kabuli_Chr02	6392272	[T/C]	Ca_20935	Ankyrin repeat	SYNONYMOUS_CODING
2334	CakSNP2334	*Kabuli*	Ca_Kabuli_Chr02	6392254	[T/C]	Ca_20935	Ankyrin repeat	SYNONYMOUS_CODING
2335	CakSNP2335	*Kabuli*	Ca_Kabuli_Chr02	6392449	[A/G]	Ca_20935	Ankyrin repeat	SYNONYMOUS_CODING
2336	CakSNP2336	*Kabuli*	Ca_Kabuli_Chr02	6397551	[A/G]	Ca_20936	Methionine synthase, vitamin-B12 independent	NON_SYNONYMOUS_CODING
2337	CakSNP2337	*Kabuli*	Ca_Kabuli_Chr02	6397597	[A/C]	Ca_20936	Methionine synthase, vitamin-B12 independent	INTRON
2338	CakSNP2338	*Kabuli*	Ca_Kabuli_Chr02	6408406	[C/A]	Ca_20937	Double-stranded RNA-binding	INTRON
2339	CakSNP2339	*Kabuli*	Ca_Kabuli_Chr02	6534776	[C/A]	Ca_20948	Target SNARE coiled-coil domain	INTRON
2340	CakSNP2340	*Kabuli*	Ca_Kabuli_Chr02	6537296	[G/T]	Ca_20948	Target SNARE coiled-coil domain	INTRON
2341	CakSNP2341	*Kabuli*	Ca_Kabuli_Chr02	6537300	[G/T]	Ca_20948	Target SNARE coiled-coil domain	INTRON

S.N.	SNP IDs	Chickpea cultivars	Chromosomes	Physical positions (bp)	SNPs	Gene accession IDs	Putative functions	Sequence components of genome
2342	CakSNP2342	*Kabuli*	Ca_Kabuli_Chr02	6727932	[G/C]	Ca_19158	Glutamate/phenylalanine/leucine/valine dehydrogenase	INTRON
2343	CakSNP2343	*Kabuli*	Ca_Kabuli_Chr02	6727905	[G/C]	Ca_19158	Glutamate/phenylalanine/leucine/valine dehydrogenase	INTRON
2344	CakSNP2344	*Kabuli*	Ca_Kabuli_Chr02	6727900	[G/C]	Ca_19158	Glutamate/phenylalanine/leucine/valine dehydrogenase	INTRON
2345	CakSNP2345	*Kabuli*	Ca_Kabuli_Chr02	6728716	[A/T]	Ca_19158	Glutamate/phenylalanine/leucine/valine dehydrogenase	INTRON
2346	CakSNP2346	*Kabuli*	Ca_Kabuli_Chr02	6728719	[C/T]	Ca_19158	Glutamate/phenylalanine/leucine/valine dehydrogenase	INTRON
2347	CakSNP2347	*Kabuli*	Ca_Kabuli_Chr02	6728763	[T/C]	Ca_19158	Glutamate/phenylalanine/leucine/valine dehydrogenase	INTRON
2348	CakSNP2348	*Kabuli*	Ca_Kabuli_Chr02	6989950	[A/G]	Ca_19138	Ovarian tumour, otubain	SYNONYMOUS_CODING
2349	CakSNP2349	*Kabuli*	Ca_Kabuli_Chr02	6990083	[G/A]	Ca_19138	Ovarian tumour, otubain	INTRON
2350	CakSNP2350	*Kabuli*	Ca_Kabuli_Chr02	7007701	[G/C]		--	INTERGENIC
2351	CakSNP2351	*Kabuli*	Ca_Kabuli_Chr02	7027093	[G/A]	Ca_19136	RNA recognition motif domain	INTRON

S.N.	SNP IDs	Chickpea cultivars	Chromosomes	Physical positions (bp)	SNPs	Gene accession IDs	Putative functions	Sequence components of genome
2352	CakSNP2352	*Kabuli*	Ca_Kabuli_Chr02	7027146	[T/C]	Ca_19136	RNA recognition motif domain	INTRON
2353	CakSNP2353	*Kabuli*	Ca_Kabuli_Chr02	7047195	[C/A]	Ca_19135	Pathogenesis-related transcriptional factor/ERF, DNA-binding	NON_SYNONYMOUS_CODING
2354	CakSNP2354	*Kabuli*	Ca_Kabuli_Chr02	7047196	[T/C]	Ca_19135	Pathogenesis-related transcriptional factor/ERF, DNA-binding	NON_SYNONYMOUS_CODING
2355	CakSNP2355	*Kabuli*	Ca_Kabuli_Chr02	7047197	[G/C]	Ca_19135	Pathogenesis-related transcriptional factor/ERF, DNA-binding	SYNONYMOUS_CODING
2356	CakSNP2356	*Kabuli*	Ca_Kabuli_Chr02	7051451	[G/A]	Ca_19134	Protein Transporter, Pam16	INTRON
2357	CakSNP2357	*Kabuli*	Ca_Kabuli_Chr02	7125992	[T/G]	Ca_19128	Oxidoreductase, N-terminal	NON_SYNONYMOUS_CODING
2358	CakSNP2358	*Kabuli*	Ca_Kabuli_Chr02	7126018	[T/G]	Ca_19128	Oxidoreductase, N-terminal	NON_SYNONYMOUS_CODING
2359	CakSNP2359	*Kabuli*	Ca_Kabuli_Chr02	7507259	[C/A]	Ca_18111	Peptidase C19, ubiquitin carboxyl-terminal hydrolase 2	NON_SYNONYMOUS_CODING
2360	CakSNP2360	*Kabuli*	Ca_Kabuli_Chr02	7633520	[G/A]		--	INTERGENIC
2361	CakSNP2361	*Kabuli*	Ca_Kabuli_Chr02	7633547	[C/T]		--	INTERGENIC

S.N.	SNP IDs	Chickpea cultivars	Chromosomes	Physical positions (bp)	SNPs	Gene accession IDs	Putative functions	Sequence components of genome
2362	CakSNP2362	*Kabuli*	Ca_Kabuli_Chr02	7633564	[G/T]		--	INTERGENIC
2363	CakSNP2363	*Kabuli*	Ca_Kabuli_Chr02	7633572	[T/C]		--	INTERGENIC
2364	CakSNP2364	*Kabuli*	Ca_Kabuli_Chr02	7633605	[T/A]		--	INTERGENIC
2365	CakSNP2365	*Kabuli*	Ca_Kabuli_Chr02	7633592	[G/A]		--	INTERGENIC
2366	CakSNP2366	*Kabuli*	Ca_Kabuli_Chr02	7633585	[G/A]		--	INTERGENIC
2367	CakSNP2367	*Kabuli*	Ca_Kabuli_Chr02	7633869	[G/A]		--	INTERGENIC
2368	CakSNP2368	*Kabuli*	Ca_Kabuli_Chr02	7633826	[G/A]		--	INTERGENIC
2369	CakSNP2369	*Kabuli*	Ca_Kabuli_Chr02	7633818	[G/A]		--	INTERGENIC
2370	CakSNP2370	*Kabuli*	Ca_Kabuli_Chr02	7633816	[G/A]		--	INTERGENIC
2371	CakSNP2371	*Kabuli*	Ca_Kabuli_Chr02	7633813	[G/T]		--	INTERGENIC

S.N.	SNP IDs	Chickpea cultivars	Chromosomes	Physical positions (bp)	SNPs	Gene accession IDs	Putative functions	Sequence components of genome
2372	CakSNP2372	*Kabuli*	Ca_Kabuli_Chr02	7633793	[C/T]		--	INTERGENIC
2373	CakSNP2373	*Kabuli*	Ca_Kabuli_Chr02	7634041	[G/A]		--	INTERGENIC
2374	CakSNP2374	*Kabuli*	Ca_Kabuli_Chr02	7679627	[A/T]	Ca_18121	Multi antimicrobial extrusion protein	NON_SYNONYMOUS_CODING
2375	CakSNP2375	*Kabuli*	Ca_Kabuli_Chr02	7679693	[C/G]	Ca_18121	Multi antimicrobial extrusion protein	NON_SYNONYMOUS_CODING
2376	CakSNP2376	*Kabuli*	Ca_Kabuli_Chr02	7701189	[T/G]	Ca_18123	Multi antimicrobial extrusion protein	NON_SYNONYMOUS_CODING
2377	CakSNP2377	*Kabuli*	Ca_Kabuli_Chr02	7701199	[T/G]	Ca_18123	Multi antimicrobial extrusion protein	NON_SYNONYMOUS_CODING
2378	CakSNP2378	*Kabuli*	Ca_Kabuli_Chr02	7701216	[T/G]	Ca_18123	Multi antimicrobial extrusion protein	NON_SYNONYMOUS_CODING
2379	CakSNP2379	*Kabuli*	Ca_Kabuli_Chr02	7701230	[T/G]	Ca_18123	Multi antimicrobial extrusion protein	SYNONYMOUS_CODING
2380	CakSNP2380	*Kabuli*	Ca_Kabuli_Chr02	7702468	[A/T]	Ca_18123	Multi antimicrobial extrusion protein	INTRON
2381	CakSNP2381	*Kabuli*	Ca_Kabuli_Chr02	7708668	[C/T]		--	INTERGENIC

S.N.	SNP IDs	Chickpea cultivars	Chromosomes	Physical positions (bp)	SNPs	Gene accession IDs	Putative functions	Sequence components of genome
2382	CakSNP2382	*Kabuli*	Ca_Kabuli_Chr02	7708663	[T/C]		--	INTERGENIC
2383	CakSNP2383	*Kabuli*	Ca_Kabuli_Chr02	7708660	[G/A]		--	INTERGENIC
2384	CakSNP2384	*Kabuli*	Ca_Kabuli_Chr02	7708630	[C/A]		--	INTERGENIC
2385	CakSNP2385	*Kabuli*	Ca_Kabuli_Chr02	7708617	[G/T]		--	INTERGENIC
2386	CakSNP2386	*Kabuli*	Ca_Kabuli_Chr02	7708603	[C/T]		--	INTERGENIC
2387	CakSNP2387	*Kabuli*	Ca_Kabuli_Chr02	7708598	[C/T]		--	INTERGENIC
2388	CakSNP2388	*Kabuli*	Ca_Kabuli_Chr02	7708596	[T/C]		--	INTERGENIC
2389	CakSNP2389	*Kabuli*	Ca_Kabuli_Chr02	7708590	[A/T]		--	INTERGENIC
2390	CakSNP2390	*Kabuli*	Ca_Kabuli_Chr02	7708678	[C/T]		--	INTERGENIC
2391	CakSNP2391	*Kabuli*	Ca_Kabuli_Chr02	7712181	[C/T]		--	INTERGENIC

S.N.	SNP IDs	Chickpea cultivars	Chromosomes	Physical positions (bp)	SNPs	Gene accession IDs	Putative functions	Sequence components of genome
2392	CakSNP2392	*Kabuli*	Ca_Kabuli_Chr02	7712155	[G/T]		--	INTERGENIC
2393	CakSNP2393	*Kabuli*	Ca_Kabuli_Chr02	7714392	[G/A]	Ca_18124	--	NON_SYNONYMOUS_CODING
2394	CakSNP2394	*Kabuli*	Ca_Kabuli_Chr02	7714454	[A/G]	Ca_18124	--	SYNONYMOUS_CODING
2395	CakSNP2395	*Kabuli*	Ca_Kabuli_Chr02	7714463	[T/C]	Ca_18124	--	SYNONYMOUS_CODING
2396	CakSNP2396	*Kabuli*	Ca_Kabuli_Chr02	7753419	[G/C]	Ca_18125	NPH3	INTRON
2397	CakSNP2397	*Kabuli*	Ca_Kabuli_Chr02	7753425	[T/C]	Ca_18125	NPH3	INTRON
2398	CakSNP2398	*Kabuli*	Ca_Kabuli_Chr02	7753440	[C/T]	Ca_18125	NPH3	INTRON
2399	CakSNP2399	*Kabuli*	Ca_Kabuli_Chr02	7819281	[T/C]		--	INTERGENIC
2400	CakSNP2400	*Kabuli*	Ca_Kabuli_Chr02	7870919	[G/T]	Ca_18134	F-box domain, cyclin-like	INTRON
2401	CakSNP2401	*Kabuli*	Ca_Kabuli_Chr02	7870943	[T/G]	Ca_18134	F-box domain, cyclin-like	INTRON

S.N.	SNP IDs	Chickpea cultivars	Chromosomes	Physical positions (bp)	SNPs	Gene accession IDs	Putative functions	Sequence components of genome
2402	CakSNP2402	*Kabuli*	Ca_Kabuli_Chr02	7938890	[T/C]	Ca_18144	Allinase, C-terminal	SYNONYMOUS_CODING
2403	CakSNP2403	*Kabuli*	Ca_Kabuli_Chr02	8337517	[C/T]	Ca_21468	Phosphatidylinositol 3-/4-kinase, catalytic	INTRON
2404	CakSNP2404	*Kabuli*	Ca_Kabuli_Chr02	8484840	[A/T]	Ca_21454	--	SYNONYMOUS_CODING
2405	CakSNP2405	*Kabuli*	Ca_Kabuli_Chr02	8487331	[T/A]		--	INTERGENIC
2406	CakSNP2406	*Kabuli*	Ca_Kabuli_Chr02	8487343	[C/T]		--	INTERGENIC
2407	CakSNP2407	*Kabuli*	Ca_Kabuli_Chr02	8489241	[C/A]		--	INTERGENIC
2408	CakSNP2408	*Kabuli*	Ca_Kabuli_Chr02	8489237	[G/A]		--	INTERGENIC
2409	CakSNP2409	*Kabuli*	Ca_Kabuli_Chr02	8489172	[C/A]		--	INTERGENIC
2410	CakSNP2410	*Kabuli*	Ca_Kabuli_Chr02	8600256	[C/G]	Ca_22315	Armadillo	NON_SYNONYMOUS_CODING
2411	CakSNP2411	*Kabuli*	Ca_Kabuli_Chr02	8600298	[A/G]	Ca_22315	Armadillo	SYNONYMOUS_CODING

S.N.	SNP IDs	Chickpea cultivars	Chromosomes	Physical positions (bp)	SNPs	Gene accession IDs	Putative functions	Sequence components of genome
2412	CakSNP2412	*Kabuli*	Ca_Kabuli_Chr02	8600309	[T/C]	Ca_22315	Armadillo	NON_SYNONYMOUS_CODING
2413	CakSNP2413	*Kabuli*	Ca_Kabuli_Chr02	8600450	[C/T]	Ca_22315	Armadillo	NON_SYNONYMOUS_CODING
2414	CakSNP2414	*Kabuli*	Ca_Kabuli_Chr02	8600666	[T/C]	Ca_22315	Armadillo	NON_SYNONYMOUS_CODING
2415	CakSNP2415	*Kabuli*	Ca_Kabuli_Chr02	8685674	[G/A]	Ca_22311	K+ potassium transporter	INTRON
2416	CakSNP2416	*Kabuli*	Ca_Kabuli_Chr02	8719725	[T/A]	Ca_22308	Clathrin/coatomer adaptor, adaptin-like, N-terminal	INTRON
2417	CakSNP2417	*Kabuli*	Ca_Kabuli_Chr02	8734136	[G/A]	Ca_22308	Clathrin/coatomer adaptor, adaptin-like, N-terminal	INTRON
2418	CakSNP2418	*Kabuli*	Ca_Kabuli_Chr02	8802884	[A/G]		--	INTERGENIC
2419	CakSNP2419	*Kabuli*	Ca_Kabuli_Chr02	9458933	[C/T]	Ca_20026	Putative thiol-disulphide oxidoreductase DCC	NON_SYNONYMOUS_CODING
2420	CakSNP2420	*Kabuli*	Ca_Kabuli_Chr02	9458985	[A/G]	Ca_20026	Putative thiol-disulphide oxidoreductase DCC	SYNONYMOUS_CODING
2421	CakSNP2421	*Kabuli*	Ca_Kabuli_Chr02	9473339	[T/C]	Ca_20024	NB-ARC	INTRON

S.N.	SNP IDs	Chickpea cultivars	Chromosomes	Physical positions (bp)	SNPs	Gene accession IDs	Putative functions	Sequence components of genome
2422	CakSNP2422	*Kabuli*	Ca_Kabuli_Chr02	9473353	[C/A]	Ca_20024	NB-ARC	INTRON
2423	CakSNP2423	*Kabuli*	Ca_Kabuli_Chr02	9473362	[G/A]	Ca_20024	NB-ARC	INTRON
2424	CakSNP2424	*Kabuli*	Ca_Kabuli_Chr02	9473380	[A/G]	Ca_20024	NB-ARC	INTRON
2425	CakSNP2425	*Kabuli*	Ca_Kabuli_Chr02	9473393	[G/A]	Ca_20024	NB-ARC	INTRON
2426	CakSNP2426	*Kabuli*	Ca_Kabuli_Chr02	9473474	[T/A]	Ca_20024	NB-ARC	INTRON
2427	CakSNP2427	*Kabuli*	Ca_Kabuli_Chr02	9473452	[A/G]	Ca_20024	NB-ARC	INTRON
2428	CakSNP2428	*Kabuli*	Ca_Kabuli_Chr02	9473430	[A/G]	Ca_20024	NB-ARC	INTRON
2429	CakSNP2429	*Kabuli*	Ca_Kabuli_Chr02	9621953	[A/C]	Ca_25089	Actin-binding FH2/DRF autoregulatory	SYNONYMOUS_CODING
2430	CakSNP2430	*Kabuli*	Ca_Kabuli_Chr02	9740018	[G/A]		--	INTERGENIC
2431	CakSNP2431	*Kabuli*	Ca_Kabuli_Chr02	9740017	[C/T]		--	INTERGENIC

S.N.	SNP IDs	Chickpea cultivars	Chromosomes	Physical positions (bp)	SNPs	Gene accession IDs	Putative functions	Sequence components of genome
2432	CakSNP2432	*Kabuli*	Ca_Kabuli_Chr02	9740010	[A/G]		--	INTERGENIC
2433	CakSNP2433	*Kabuli*	Ca_Kabuli_Chr02	9740007	[A/G]		--	INTERGENIC
2434	CakSNP2434	*Kabuli*	Ca_Kabuli_Chr02	9739980	[G/C]		--	INTERGENIC
2435	CakSNP2435	*Kabuli*	Ca_Kabuli_Chr02	9739974	[T/C]		--	INTERGENIC
2436	CakSNP2436	*Kabuli*	Ca_Kabuli_Chr02	9739959	[T/C]		--	INTERGENIC
2437	CakSNP2437	*Kabuli*	Ca_Kabuli_Chr02	9739965	[A/G]		--	INTERGENIC
2438	CakSNP2438	*Kabuli*	Ca_Kabuli_Chr02	9756955	[G/A]		--	INTERGENIC
2439	CakSNP2439	*Kabuli*	Ca_Kabuli_Chr02	9756946	[C/T]		--	INTERGENIC
2440	CakSNP2440	*Kabuli*	Ca_Kabuli_Chr02	9756910	[G/A]		--	INTERGENIC
2441	CakSNP2441	*Kabuli*	Ca_Kabuli_Chr02	9756887	[G/T]		--	INTERGENIC

S.N.	SNP IDs	Chickpea cultivars	Chromosomes	Physical positions (bp)	SNPs	Gene accession IDs	Putative functions	Sequence components of genome
2442	CakSNP2442	*Kabuli*	Ca_Kabuli_Chr02	9823224	[G/A]	Ca_20402	Mpv17/PMP22	INTRON
2443	CakSNP2443	*Kabuli*	Ca_Kabuli_Chr02	9823165	[T/C]	Ca_20402	Mpv17/PMP22	INTRON
2444	CakSNP2444	*Kabuli*	Ca_Kabuli_Chr02	9825526	[C/G]	Ca_20402	Mpv17/PMP22	INTRON
2445	CakSNP2445	*Kabuli*	Ca_Kabuli_Chr02	9841625	[A/G]		--	INTERGENIC
2446	CakSNP2446	*Kabuli*	Ca_Kabuli_Chr02	9862794	[A/G]	Ca_20401	Protein kinase, catalytic domain	INTRON
2447	CakSNP2447	*Kabuli*	Ca_Kabuli_Chr02	9906042	[T/G]		--	INTERGENIC
2448	CakSNP2448	*Kabuli*	Ca_Kabuli_Chr02	9906077	[T/A]		--	INTERGENIC
2449	CakSNP2449	*Kabuli*	Ca_Kabuli_Chr02	9918536	[T/A]	Ca_20395	Helicase, C-terminal	INTRON
2450	CakSNP2450	*Kabuli*	Ca_Kabuli_Chr02	9943370	[A/G]	Ca_20392	Choline/ethanolamine kinase	INTRON
2451	CakSNP2451	*Kabuli*	Ca_Kabuli_Chr02	9943374	[C/T]	Ca_20392	Choline/ethanolamine kinase	INTRON

S.N.	SNP IDs	Chickpea cultivars	Chromosomes	Physical positions (bp)	SNPs	Gene accession IDs	Putative functions	Sequence components of genome
2452	CakSNP2452	*Kabuli*	Ca_Kabuli_Chr02	9943404	[T/G]	Ca_20392	Choline/ethanolamine kinase	NON_SYNONYMOUS_CODING
2453	CakSNP2453	*Kabuli*	Ca_Kabuli_Chr02	9986639	[C/T]	Ca_20388	Ribosomal protein S1, RNA-binding domain	INTRON
2454	CakSNP2454	*Kabuli*	Ca_Kabuli_Chr02	9986683	[C/T]	Ca_20388	Ribosomal protein S1, RNA-binding domain	INTRON
2455	CakSNP2455	*Kabuli*	Ca_Kabuli_Chr02	10004694	[G/A]		--	INTERGENIC
2456	CakSNP2456	*Kabuli*	Ca_Kabuli_Chr02	10025451	[G/A]	Ca_20383	Protein of unknown function DUF810	SYNONYMOUS_CODING
2457	CakSNP2457	*Kabuli*	Ca_Kabuli_Chr02	10033991	[T/C]	Ca_20382	Ribosomal protein S13	INTRON
2458	CakSNP2458	*Kabuli*	Ca_Kabuli_Chr02	10060196	[G/T]		--	INTERGENIC
2459	CakSNP2459	*Kabuli*	Ca_Kabuli_Chr02	10060200	[G/T]		--	INTERGENIC
2460	CakSNP2460	*Kabuli*	Ca_Kabuli_Chr02	10060207	[C/T]		--	INTERGENIC
2461	CakSNP2461	*Kabuli*	Ca_Kabuli_Chr02	10067093	[G/C]	Ca_20380	SANT domain, DNA binding	INTRON

S.N.	SNP IDs	Chickpea cultivars	Chromosomes	Physical positions (bp)	SNPs	Gene accession IDs	Putative functions	Sequence components of genome
2462	CakSNP2462	*Kabuli*	Ca_Kabuli_Chr02	10067078	[T/G]	Ca_20380	SANT domain, DNA binding	INTRON
2463	CakSNP2463	*Kabuli*	Ca_Kabuli_Chr02	10067061	[T/G]	Ca_20380	SANT domain, DNA binding	INTRON
2464	CakSNP2464	*Kabuli*	Ca_Kabuli_Chr02	10067195	[T/A]	Ca_20380	SANT domain, DNA binding	INTRON
2465	CakSNP2465	*Kabuli*	Ca_Kabuli_Chr02	10067262	[A/C]	Ca_20380	SANT domain, DNA binding	INTRON
2466	CakSNP2466	*Kabuli*	Ca_Kabuli_Chr02	10067278	[G/T]	Ca_20380	SANT domain, DNA binding	INTRON
2467	CakSNP2467	*Kabuli*	Ca_Kabuli_Chr02	10067285	[T/G]	Ca_20380	SANT domain, DNA binding	INTRON
2468	CakSNP2468	*Kabuli*	Ca_Kabuli_Chr02	10067286	[A/T]	Ca_20380	SANT domain, DNA binding	INTRON
2469	CakSNP2469	*Kabuli*	Ca_Kabuli_Chr02	10067451	[A/T]	Ca_20380	SANT domain, DNA binding	INTRON
2470	CakSNP2470	*Kabuli*	Ca_Kabuli_Chr02	10067516	[G/A]	Ca_20380	SANT domain, DNA binding	INTRON
2471	CakSNP2471	*Kabuli*	Ca_Kabuli_Chr02	10067538	[C/T]	Ca_20380	SANT domain, DNA binding	INTRON

S.N.	SNP IDs	Chickpea cultivars	Chromosomes	Physical positions (bp)	SNPs	Gene accession IDs	Putative functions	Sequence components of genome
2472	CakSNP2472	*Kabuli*	Ca_Kabuli_Chr02	10068214	[A/G]	Ca_20380	SANT domain, DNA binding	NON_SYNONYMOUS_CODING
2473	CakSNP2473	*Kabuli*	Ca_Kabuli_Chr02	10220396	[T/C]		--	INTERGENIC
2474	CakSNP2474	*Kabuli*	Ca_Kabuli_Chr02	10270474	[C/A]		--	INTERGENIC
2475	CakSNP2475	*Kabuli*	Ca_Kabuli_Chr02	10284426	[C/G]		--	INTERGENIC
2476	CakSNP2476	*Kabuli*	Ca_Kabuli_Chr02	10294698	[A/G]	Ca_17594	ATPase, P-type, K/Mg/Cd/Cu/Zn/Na/Ca/Na/H-transporter	SYNONYMOUS_CODING
2477	CakSNP2477	*Kabuli*	Ca_Kabuli_Chr02	10294635	[T/C]	Ca_17594	ATPase, P-type, K/Mg/Cd/Cu/Zn/Na/Ca/Na/H-transporter	INTRON
2478	CakSNP2478	*Kabuli*	Ca_Kabuli_Chr02	10322943	[T/C]	Ca_17592	--	INTRON
2479	CakSNP2479	*Kabuli*	Ca_Kabuli_Chr02	10352705	[T/A]		--	INTERGENIC
2480	CakSNP2480	*Kabuli*	Ca_Kabuli_Chr02	10358484	[C/T]		--	INTERGENIC
2481	CakSNP2481	*Kabuli*	Ca_Kabuli_Chr02	10358496	[G/T]		--	INTERGENIC

S.N.	SNP IDs	Chickpea cultivars	Chromosomes	Physical positions (bp)	SNPs	Gene accession IDs	Putative functions	Sequence components of genome
2482	CakSNP2482	*Kabuli*	Ca_Kabuli_Chr02	10377695	[G/A]	Ca_17589	Domain of unknown function DUF1337	INTRON
2483	CakSNP2483	*Kabuli*	Ca_Kabuli_Chr02	10434727	[T/C]	Ca_17586	Glutathione S-transferase, N-terminal	NON_SYNONYMOUS_CODING
2484	CakSNP2484	*Kabuli*	Ca_Kabuli_Chr02	10434726	[G/C]	Ca_17586	Glutathione S-transferase, N-terminal	NON_SYNONYMOUS_CODING
2485	CakSNP2485	*Kabuli*	Ca_Kabuli_Chr02	10434714	[C/G]	Ca_17586	Glutathione S-transferase, N-terminal	NON_SYNONYMOUS_CODING
2486	CakSNP2486	*Kabuli*	Ca_Kabuli_Chr02	10540735	[C/T]	Ca_17577	--	NON_SYNONYMOUS_CODING
2487	CakSNP2487	*Kabuli*	Ca_Kabuli_Chr02	10637631	[C/A]	Ca_17571	Oligopeptide transporter	NON_SYNONYMOUS_CODING
2488	CakSNP2488	*Kabuli*	Ca_Kabuli_Chr02	10656518	[A/T]		--	INTERGENIC
2489	CakSNP2489	*Kabuli*	Ca_Kabuli_Chr02	10705902	[C/A]	Ca_17569	--	SYNONYMOUS_CODING
2490	CakSNP2490	*Kabuli*	Ca_Kabuli_Chr02	10705923	[T/A]	Ca_17569	--	SYNONYMOUS_CODING
2491	CakSNP2491	*Kabuli*	Ca_Kabuli_Chr02	10705979	[C/A]	Ca_17569	--	NON_SYNONYMOUS_CODING

S.N.	SNP IDs	Chickpea cultivars	Chromosomes	Physical positions (bp)	SNPs	Gene accession IDs	Putative functions	Sequence components of genome
2492	CakSNP2492	*Kabuli*	Ca_Kabuli_Chr02	10705982	[C/T]	Ca_17569	--	NON_SYNONYMOUS_CODING
2493	CakSNP2493	*Kabuli*	Ca_Kabuli_Chr02	10760939	[G/A]	Ca_17564	Zinc finger, C2H2-type	NON_SYNONYMOUS_CODING
2494	CakSNP2494	*Kabuli*	Ca_Kabuli_Chr02	10761036	[C/G]	Ca_17564	Zinc finger, C2H2-type	NON_SYNONYMOUS_CODING
2495	CakSNP2495	*Kabuli*	Ca_Kabuli_Chr02	11799472	[G/A]		--	INTERGENIC
2496	CakSNP2496	*Kabuli*	Ca_Kabuli_Chr02	11799462	[G/A]		--	INTERGENIC
2497	CakSNP2497	*Kabuli*	Ca_Kabuli_Chr02	12252285	[T/G]		--	INTERGENIC
2498	CakSNP2498	*Kabuli*	Ca_Kabuli_Chr02	12252370	[A/G]		--	INTERGENIC
2499	CakSNP2499	*Kabuli*	Ca_Kabuli_Chr02	12278583	[G/A]	Ca_18061	Zinc finger, RING-CH-type	INTRON
2500	CakSNP2500	*Kabuli*	Ca_Kabuli_Chr02	12278584	[T/C]	Ca_18061	Zinc finger, RING-CH-type	INTRON
2501	CakSNP2501	*Kabuli*	Ca_Kabuli_Chr02	12278606	[T/C]	Ca_18061	Zinc finger, RING-CH-type	INTRON

S.N.	SNP IDs	Chickpea cultivars	Chromosomes	Physical positions (bp)	SNPs	Gene accession IDs	Putative functions	Sequence components of genome
2502	CakSNP2502	*Kabuli*	Ca_Kabuli_Chr02	12281599	[A/C]	Ca_18061	Zinc finger, RING-CH-type	NON_SYNONYMOUS_CODING
2503	CakSNP2503	*Kabuli*	Ca_Kabuli_Chr02	12357732	[A/G]		--	INTERGENIC
2504	CakSNP2504	*Kabuli*	Ca_Kabuli_Chr02	12389166	[G/T]		--	INTERGENIC
2505	CakSNP2505	*Kabuli*	Ca_Kabuli_Chr02	12389112	[G/A]		--	INTERGENIC
2506	CakSNP2506	*Kabuli*	Ca_Kabuli_Chr02	12515595	[A/G]		--	INTERGENIC
2507	CakSNP2507	*Kabuli*	Ca_Kabuli_Chr02	12528825	[C/G]	Ca_18079	Zinc finger, Dof-type	NON_SYNONYMOUS_CODING
2508	CakSNP2508	*Kabuli*	Ca_Kabuli_Chr02	12710137	[G/A]	Ca_18087	Endosulphine	SYNONYMOUS_CODING
2509	CakSNP2509	*Kabuli*	Ca_Kabuli_Chr02	12737814	[T/C]		--	INTERGENIC
2510	CakSNP2510	*Kabuli*	Ca_Kabuli_Chr02	12794438	[G/A]	Ca_18093	EF-HAND 2	NON_SYNONYMOUS_CODING
2511	CakSNP2511	*Kabuli*	Ca_Kabuli_Chr02	12854610	[A/G]		--	INTERGENIC

S.N.	SNP IDs	Chickpea cultivars	Chromosomes	Physical positions (bp)	SNPs	Gene accession IDs	Putative functions	Sequence components of genome
2512	CakSNP2512	*Kabuli*	Ca_Kabuli_Chr02	12854591	[T/A]		--	INTERGENIC
2513	CakSNP2513	*Kabuli*	Ca_Kabuli_Chr02	12854586	[G/A]		--	INTERGENIC
2514	CakSNP2514	*Kabuli*	Ca_Kabuli_Chr02	12862414	[G/C]	Ca_18095	SPX, N-terminal	INTRON
2515	CakSNP2515	*Kabuli*	Ca_Kabuli_Chr02	13079417	[T/G]	Ca_11686	Protein of unknown function DUF3110	NON_SYNONYMOUS_CODING
2516	CakSNP2516	*Kabuli*	Ca_Kabuli_Chr02	13079445	[T/G]	Ca_11686	Protein of unknown function DUF3110	SYNONYMOUS_CODING
2517	CakSNP2517	*Kabuli*	Ca_Kabuli_Chr02	13217781	[T/C]	Ca_11681	Zinc finger, DHHC-type, palmitoyltransferase	SYNONYMOUS_CODING
2518	CakSNP2518	*Kabuli*	Ca_Kabuli_Chr02	13355018	[T/G]	Ca_11675	ATP-grasp fold	SYNONYMOUS_CODING
2519	CakSNP2519	*Kabuli*	Ca_Kabuli_Chr02	13355022	[G/T]	Ca_11675	ATP-grasp fold	NON_SYNONYMOUS_CODING
2520	CakSNP2520	*Kabuli*	Ca_Kabuli_Chr02	13355033	[A/T]	Ca_11675	ATP-grasp fold	NON_SYNONYMOUS_CODING
2521	CakSNP2521	*Kabuli*	Ca_Kabuli_Chr02	13355615	[T/G]	Ca_11675	ATP-grasp fold	SYNONYMOUS_CODING

S.N.	SNP IDs	Chickpea cultivars	Chromosomes	Physical positions (bp)	SNPs	Gene accession IDs	Putative functions	Sequence components of genome
2522	CakSNP2522	*Kabuli*	Ca_Kabuli_Chr02	13425568	[T/A]		--	INTERGENIC
2523	CakSNP2523	*Kabuli*	Ca_Kabuli_Chr02	13434904	[G/A]	Ca_11665	Glutamyl-tRNA(Gln) amidotransferase, subunit B/E, N-terminal	INTRON
2524	CakSNP2524	*Kabuli*	Ca_Kabuli_Chr02	13434884	[T/A]	Ca_11665	Glutamyl-tRNA(Gln) amidotransferase, subunit B/E, N-terminal	INTRON
2525	CakSNP2525	*Kabuli*	Ca_Kabuli_Chr02	13435043	[C/T]	Ca_11665	Glutamyl-tRNA(Gln) amidotransferase, subunit B/E, N-terminal	INTRON
2526	CakSNP2526	*Kabuli*	Ca_Kabuli_Chr02	13435030	[G/C]	Ca_11665	Glutamyl-tRNA(Gln) amidotransferase, subunit B/E, N-terminal	INTRON
2527	CakSNP2527	*Kabuli*	Ca_Kabuli_Chr02	13434995	[A/C]	Ca_11665	Glutamyl-tRNA(Gln) amidotransferase, subunit B/E, N-terminal	INTRON
2528	CakSNP2528	*Kabuli*	Ca_Kabuli_Chr02	13437317	[A/G]	Ca_11665	Glutamyl-tRNA(Gln) amidotransferase, subunit B/E, N-terminal	INTRON
2529	CakSNP2529	*Kabuli*	Ca_Kabuli_Chr02	13529057	[G/A]	Ca_11656	UvrB/UvrC protein	INTRON
2530	CakSNP2530	*Kabuli*	Ca_Kabuli_Chr02	13552505	[T/A]	Ca_11652	Ankyrin repeat	INTRON
2531	CakSNP2531	*Kabuli*	Ca_Kabuli_Chr02	13554038	[T/C]	Ca_11652	Ankyrin repeat	INTRON

S.N.	SNP IDs	Chickpea cultivars	Chromosomes	Physical positions (bp)	SNPs	Gene accession IDs	Putative functions	Sequence components of genome
2532	CakSNP2532	*Kabuli*	Ca_Kabuli_Chr02	13554045	[T/C]	Ca_11652	Ankyrin repeat	INTRON
2533	CakSNP2533	*Kabuli*	Ca_Kabuli_Chr02	13554047	[A/T]	Ca_11652	Ankyrin repeat	INTRON
2534	CakSNP2534	*Kabuli*	Ca_Kabuli_Chr02	13703083	[G/A]	Ca_11644	DNA-binding WRKY	INTRON
2535	CakSNP2535	*Kabuli*	Ca_Kabuli_Chr02	13782906	[C/A]	Ca_11639	Remorin, C-terminal	INTRON
2536	CakSNP2536	*Kabuli*	Ca_Kabuli_Chr02	14101913	[T/A]	Ca_11627	Alpha/beta hydrolase fold-1	SYNONYMOUS_CODING
2537	CakSNP2537	*Kabuli*	Ca_Kabuli_Chr02	14134413	[A/C]	Ca_11622	--	SYNONYMOUS_CODING
2538	CakSNP2538	*Kabuli*	Ca_Kabuli_Chr02	14134676	[T/C]	Ca_11622	--	NON_SYNONYMOUS_CODING
2539	CakSNP2539	*Kabuli*	Ca_Kabuli_Chr02	14136062	[C/A]	Ca_11621	--	NON_SYNONYMOUS_CODING
2540	CakSNP2540	*Kabuli*	Ca_Kabuli_Chr02	14136019	[A/G]	Ca_11621	--	SYNONYMOUS_CODING
2541	CakSNP2541	*Kabuli*	Ca_Kabuli_Chr02	14136013	[T/G]	Ca_11621	--	SYNONYMOUS_CODING

S.N.	SNP IDs	Chickpea cultivars	Chromosomes	Physical positions (bp)	SNPs	Gene accession IDs	Putative functions	Sequence components of genome
2542	CakSNP2542	*Kabuli*	Ca_Kabuli_Chr02	14136223	[A/C]	Ca_11621	--	SYNONYMOUS_CODING
2543	CakSNP2543	*Kabuli*	Ca_Kabuli_Chr02	15160301	[T/C]		--	INTERGENIC
2544	CakSNP2544	*Kabuli*	Ca_Kabuli_Chr02	15178565	[G/A]	Ca_18569	Metallo-dependent phosphatase	INTRON
2545	CakSNP2545	*Kabuli*	Ca_Kabuli_Chr02	15178564	[A/G]	Ca_18569	Metallo-dependent phosphatase	INTRON
2546	CakSNP2546	*Kabuli*	Ca_Kabuli_Chr02	15178540	[A/G]	Ca_18569	Metallo-dependent phosphatase	INTRON
2547	CakSNP2547	*Kabuli*	Ca_Kabuli_Chr02	15181635	[G/T]	Ca_18568	Mg2+ transporter protein, CorA-like	SYNONYMOUS_CODING
2548	CakSNP2548	*Kabuli*	Ca_Kabuli_Chr02	15234951	[C/A]	Ca_18564	Protein of unknown function DUF1675	SYNONYMOUS_CODING
2549	CakSNP2549	*Kabuli*	Ca_Kabuli_Chr02	15389412	[G/A]	Ca_18558	Protein kinase, catalytic domain	SYNONYMOUS_CODING
2550	CakSNP2550	*Kabuli*	Ca_Kabuli_Chr02	15390673	[A/G]	Ca_18558	Protein kinase, catalytic domain	INTRON
2551	CakSNP2551	*Kabuli*	Ca_Kabuli_Chr02	15390731	[G/A]	Ca_18558	Protein kinase, catalytic domain	INTRON

S.N.	SNP IDs	Chickpea cultivars	Chromosomes	Physical positions (bp)	SNPs	Gene accession IDs	Putative functions	Sequence components of genome
2552	CakSNP2552	*Kabuli*	Ca_Kabuli_Chr02	15390684	[A/G]	Ca_18558	Protein kinase, catalytic domain	INTRON
2553	CakSNP2553	*Kabuli*	Ca_Kabuli_Chr02	15688657	[G/T]	Ca_18540	Alpha-1,4-glucan-protein synthase, UDP-forming	INTRON
2554	CakSNP2554	*Kabuli*	Ca_Kabuli_Chr02	15817688	[T/C]	Ca_18533	YABBY protein	INTRON
2555	CakSNP2555	*Kabuli*	Ca_Kabuli_Chr02	16460174	[G/A]		--	INTERGENIC
2556	CakSNP2556	*Kabuli*	Ca_Kabuli_Chr02	16460142	[A/G]		--	INTERGENIC
2557	CakSNP2557	*Kabuli*	Ca_Kabuli_Chr02	16460097	[C/A]		--	INTERGENIC
2558	CakSNP2558	*Kabuli*	Ca_Kabuli_Chr02	16460106	[C/T]		--	INTERGENIC
2559	CakSNP2559	*Kabuli*	Ca_Kabuli_Chr02	16460175	[C/A]		--	INTERGENIC
2560	CakSNP2560	*Kabuli*	Ca_Kabuli_Chr02	16462095	[A/G]		--	INTERGENIC
2561	CakSNP2561	*Kabuli*	Ca_Kabuli_Chr02	16462089	[C/T]		--	INTERGENIC

S.N.	SNP IDs	Chickpea cultivars	Chromosomes	Physical positions (bp)	SNPs	Gene accession IDs	Putative functions	Sequence components of genome
2562	CakSNP2562	*Kabuli*	Ca_Kabuli_Chr02	16462056	[A/C]		--	INTERGENIC
2563	CakSNP2563	*Kabuli*	Ca_Kabuli_Chr02	16462038	[G/T]		--	INTERGENIC
2564	CakSNP2564	*Kabuli*	Ca_Kabuli_Chr02	16555733	[C/T]	Ca_22041	BSD	INTRON
2565	CakSNP2565	*Kabuli*	Ca_Kabuli_Chr02	16555790	[C/G]	Ca_22041	BSD	INTRON
2566	CakSNP2566	*Kabuli*	Ca_Kabuli_Chr02	16563117	[A/T]	Ca_22041	BSD	INTRON
2567	CakSNP2567	*Kabuli*	Ca_Kabuli_Chr02	16563121	[A/T]	Ca_22041	BSD	INTRON
2568	CakSNP2568	*Kabuli*	Ca_Kabuli_Chr02	16563123	[C/T]	Ca_22041	BSD	INTRON
2569	CakSNP2569	*Kabuli*	Ca_Kabuli_Chr02	16563125	[A/T]	Ca_22041	BSD	INTRON
2570	CakSNP2570	*Kabuli*	Ca_Kabuli_Chr02	16563133	[A/T]	Ca_22041	BSD	INTRON
2571	CakSNP2571	*Kabuli*	Ca_Kabuli_Chr02	16705119	[C/T]		--	INTERGENIC

S.N.	SNP IDs	Chickpea cultivars	Chromosomes	Physical positions (bp)	SNPs	Gene accession IDs	Putative functions	Sequence components of genome
2572	CakSNP2572	*Kabuli*	Ca_Kabuli_Chr02	17450780	[C/T]	Ca_16006	Importin-beta, N-terminal	INTRON
2573	CakSNP2573	*Kabuli*	Ca_Kabuli_Chr02	17578623	[G/A]		--	INTERGENIC
2574	CakSNP2574	*Kabuli*	Ca_Kabuli_Chr02	17578633	[C/G]		--	INTERGENIC
2575	CakSNP2575	*Kabuli*	Ca_Kabuli_Chr02	17578659	[A/G]		--	INTERGENIC
2576	CakSNP2576	*Kabuli*	Ca_Kabuli_Chr02	17578689	[G/A]		--	INTERGENIC
2577	CakSNP2577	*Kabuli*	Ca_Kabuli_Chr02	17578663	[C/A]		--	INTERGENIC
2578	CakSNP2578	*Kabuli*	Ca_Kabuli_Chr02	17578676	[T/C]		--	INTERGENIC
2579	CakSNP2579	*Kabuli*	Ca_Kabuli_Chr02	17578701	[A/C]		--	INTERGENIC
2580	CakSNP2580	*Kabuli*	Ca_Kabuli_Chr02	17578706	[A/T]		--	INTERGENIC
2581	CakSNP2581	*Kabuli*	Ca_Kabuli_Chr02	17578726	[A/T]		--	INTERGENIC

S.N.	SNP IDs	Chickpea cultivars	Chromosomes	Physical positions (bp)	SNPs	Gene accession IDs	Putative functions	Sequence components of genome
2582	CakSNP2582	*Kabuli*	Ca_Kabuli_Chr02	17578740	[A/G]		--	INTERGENIC
2583	CakSNP2583	*Kabuli*	Ca_Kabuli_Chr02	17681915	[C/T]		--	INTERGENIC
2584	CakSNP2584	*Kabuli*	Ca_Kabuli_Chr02	17716022	[A/T]	Ca_15995	S-adenosylmethionine decarboxylase	NON_SYNONYMOUS_CODING
2585	CakSNP2585	*Kabuli*	Ca_Kabuli_Chr02	17727340	[G/A]		--	INTERGENIC
2586	CakSNP2586	*Kabuli*	Ca_Kabuli_Chr02	17727362	[G/C]		--	INTERGENIC
2587	CakSNP2587	*Kabuli*	Ca_Kabuli_Chr02	17727403	[C/T]		--	INTERGENIC
2588	CakSNP2588	*Kabuli*	Ca_Kabuli_Chr02	17839209	[T/A]		--	INTERGENIC
2589	CakSNP2589	*Kabuli*	Ca_Kabuli_Chr02	17839297	[T/C]		--	INTERGENIC
2590	CakSNP2590	*Kabuli*	Ca_Kabuli_Chr02	17910111	[C/G]		--	INTERGENIC
2591	CakSNP2591	*Kabuli*	Ca_Kabuli_Chr02	17910145	[C/T]		--	INTERGENIC

S.N.	SNP IDs	Chickpea cultivars	Chromosomes	Physical positions (bp)	SNPs	Gene accession IDs	Putative functions	Sequence components of genome
2592	CakSNP2592	*Kabuli*	Ca_Kabuli_Chr02	17910783	[A/T]		--	INTERGENIC
2593	CakSNP2593	*Kabuli*	Ca_Kabuli_Chr02	18312200	[T/C]		--	INTERGENIC
2594	CakSNP2594	*Kabuli*	Ca_Kabuli_Chr02	18395345	[A/C]		--	INTERGENIC
2595	CakSNP2595	*Kabuli*	Ca_Kabuli_Chr02	18395410	[T/C]		--	INTERGENIC
2596	CakSNP2596	*Kabuli*	Ca_Kabuli_Chr02	18395378	[A/G]		--	INTERGENIC
2597	CakSNP2597	*Kabuli*	Ca_Kabuli_Chr02	18546345	[T/C]	Ca_15969	RNA recognition motif domain	INTRON
2598	CakSNP2598	*Kabuli*	Ca_Kabuli_Chr02	18546366	[A/T]	Ca_15969	RNA recognition motif domain	INTRON
2599	CakSNP2599	*Kabuli*	Ca_Kabuli_Chr02	18631367	[T/A]		--	INTERGENIC
2600	CakSNP2600	*Kabuli*	Ca_Kabuli_Chr02	18631383	[A/G]		--	INTERGENIC
2601	CakSNP2601	*Kabuli*	Ca_Kabuli_Chr02	18631404	[T/G]		--	INTERGENIC

S.N.	SNP IDs	Chickpea cultivars	Chromosomes	Physical positions (bp)	SNPs	Gene accession IDs	Putative functions	Sequence components of genome
2602	CakSNP2602	*Kabuli*	Ca_Kabuli_Chr02	18631412	[T/G]		--	INTERGENIC
2603	CakSNP2603	*Kabuli*	Ca_Kabuli_Chr02	18631522	[A/C]		--	INTERGENIC
2604	CakSNP2604	*Kabuli*	Ca_Kabuli_Chr02	18671692	[C/A]		--	INTERGENIC
2605	CakSNP2605	*Kabuli*	Ca_Kabuli_Chr02	18671672	[T/A]		--	INTERGENIC
2606	CakSNP2606	*Kabuli*	Ca_Kabuli_Chr02	18671666	[C/T]		--	INTERGENIC
2607	CakSNP2607	*Kabuli*	Ca_Kabuli_Chr02	18671637	[G/A]		--	INTERGENIC
2608	CakSNP2608	*Kabuli*	Ca_Kabuli_Chr02	18671618	[T/G]		--	INTERGENIC
2609	CakSNP2609	*Kabuli*	Ca_Kabuli_Chr02	18671723	[C/G]		--	INTERGENIC
2610	CakSNP2610	*Kabuli*	Ca_Kabuli_Chr02	18671746	[C/T]		--	INTERGENIC
2611	CakSNP2611	*Kabuli*	Ca_Kabuli_Chr02	18671756	[T/A]		--	INTERGENIC

S.N.	SNP IDs	Chickpea cultivars	Chromosomes	Physical positions (bp)	SNPs	Gene accession IDs	Putative functions	Sequence components of genome
2612	CakSNP2612	*Kabuli*	Ca_Kabuli_Chr02	18671765	[C/A]		--	INTERGENIC
2613	CakSNP2613	*Kabuli*	Ca_Kabuli_Chr02	18671767	[G/C]		--	INTERGENIC
2614	CakSNP2614	*Kabuli*	Ca_Kabuli_Chr02	18671785	[G/A]		--	INTERGENIC
2615	CakSNP2615	*Kabuli*	Ca_Kabuli_Chr02	18671824	[C/T]		--	INTERGENIC
2616	CakSNP2616	*Kabuli*	Ca_Kabuli_Chr02	20392224	[A/G]	Ca_25924	--	SYNONYMOUS_CODING
2617	CakSNP2617	*Kabuli*	Ca_Kabuli_Chr02	20762187	[A/G]	Ca_23762	--	SYNONYMOUS_CODING
2618	CakSNP2618	*Kabuli*	Ca_Kabuli_Chr02	20762205	[A/T]	Ca_23762	--	SYNONYMOUS_CODING
2619	CakSNP2619	*Kabuli*	Ca_Kabuli_Chr02	21000509	[C/T]		--	INTERGENIC
2620	CakSNP2620	*Kabuli*	Ca_Kabuli_Chr02	21000466	[C/T]		--	INTERGENIC
2621	CakSNP2621	*Kabuli*	Ca_Kabuli_Chr02	21000463	[C/T]		--	INTERGENIC

S.N.	SNP IDs	Chickpea cultivars	Chromosomes	Physical positions (bp)	SNPs	Gene accession IDs	Putative functions	Sequence components of genome
2622	CakSNP2622	*Kabuli*	Ca_Kabuli_Chr02	21849874	[A/T]	Ca_23059	--	INTRON
2623	CakSNP2623	*Kabuli*	Ca_Kabuli_Chr02	21906952	[A/G]	Ca_23061	Mitochondrial substrate/solute carrier	NON_SYNONYMOUS_CODING
2624	CakSNP2624	*Kabuli*	Ca_Kabuli_Chr02	21965365	[T/C]		--	INTERGENIC
2625	CakSNP2625	*Kabuli*	Ca_Kabuli_Chr02	22158593	[C/T]	Ca_15007	Peptidase C48, SUMO/Sentrin/Ubl1	NON_SYNONYMOUS_CODING
2626	CakSNP2626	*Kabuli*	Ca_Kabuli_Chr02	22158602	[C/T]	Ca_15007	Peptidase C48, SUMO/Sentrin/Ubl1	NON_SYNONYMOUS_CODING
2627	CakSNP2627	*Kabuli*	Ca_Kabuli_Chr02	22190700	[C/T]		--	INTERGENIC
2628	CakSNP2628	*Kabuli*	Ca_Kabuli_Chr02	22190743	[C/T]		--	INTERGENIC
2629	CakSNP2629	*Kabuli*	Ca_Kabuli_Chr02	22190748	[G/T]		--	INTERGENIC
2630	CakSNP2630	*Kabuli*	Ca_Kabuli_Chr02	22190751	[C/T]		--	INTERGENIC
2631	CakSNP2631	*Kabuli*	Ca_Kabuli_Chr02	22190756	[C/A]		--	INTERGENIC

S.N.	SNP IDs	Chickpea cultivars	Chromosomes	Physical positions (bp)	SNPs	Gene accession IDs	Putative functions	Sequence components of genome
2632	CakSNP2632	*Kabuli*	Ca_Kabuli_Chr02	22190753	[C/T]		--	INTERGENIC
2633	CakSNP2633	*Kabuli*	Ca_Kabuli_Chr02	22190790	[G/A]		--	INTERGENIC
2634	CakSNP2634	*Kabuli*	Ca_Kabuli_Chr02	22190815	[C/T]		--	INTERGENIC
2635	CakSNP2635	*Kabuli*	Ca_Kabuli_Chr02	22205024	[G/C]		--	INTERGENIC
2636	CakSNP2636	*Kabuli*	Ca_Kabuli_Chr02	22347405	[C/T]	Ca_15004	Cyclin, C-terminal	INTRON
2637	CakSNP2637	*Kabuli*	Ca_Kabuli_Chr02	22415930	[C/T]	Ca_15002	Ankyrin repeat	NON_SYNONYMOUS_CODING
2638	CakSNP2638	*Kabuli*	Ca_Kabuli_Chr02	22416899	[A/C]	Ca_15002	Ankyrin repeat	NON_SYNONYMOUS_CODING
2639	CakSNP2639	*Kabuli*	Ca_Kabuli_Chr02	22419501	[C/G]	Ca_15002	Ankyrin repeat	NON_SYNONYMOUS_CODING
2640	CakSNP2640	*Kabuli*	Ca_Kabuli_Chr02	22419513	[T/C]	Ca_15002	Ankyrin repeat	NON_SYNONYMOUS_CODING
2641	CakSNP2641	*Kabuli*	Ca_Kabuli_Chr02	22419514	[C/T]	Ca_15002	Ankyrin repeat	NON_SYNONYMOUS_CODING

S.N.	SNP IDs	Chickpea cultivars	Chromosomes	Physical positions (bp)	SNPs	Gene accession IDs	Putative functions	Sequence components of genome
2642	CakSNP2642	*Kabuli*	Ca_Kabuli_Chr02	22419529	[T/C]	Ca_15002	Ankyrin repeat	NON_SYNONYMOUS_CODING
2643	CakSNP2643	*Kabuli*	Ca_Kabuli_Chr02	22419625	[C/T]	Ca_15002	Ankyrin repeat	NON_SYNONYMOUS_CODING
2644	CakSNP2644	*Kabuli*	Ca_Kabuli_Chr02	22419633	[C/T]	Ca_15002	Ankyrin repeat	NON_SYNONYMOUS_CODING
2645	CakSNP2645	*Kabuli*	Ca_Kabuli_Chr02	22419634	[G/C]	Ca_15002	Ankyrin repeat	NON_SYNONYMOUS_CODING
2646	CakSNP2646	*Kabuli*	Ca_Kabuli_Chr02	22419679	[G/A]	Ca_15002	Ankyrin repeat	STOP_GAINED
2647	CakSNP2647	*Kabuli*	Ca_Kabuli_Chr02	22457234	[G/A]	Ca_14999	Peptidase C48, SUMO/Sentrin/Ubl1	NON_SYNONYMOUS_CODING
2648	CakSNP2648	*Kabuli*	Ca_Kabuli_Chr02	22457221	[T/A]	Ca_14999	Peptidase C48, SUMO/Sentrin/Ubl1	NON_SYNONYMOUS_CODING
2649	CakSNP2649	*Kabuli*	Ca_Kabuli_Chr02	22457215	[G/A]	Ca_14999	Peptidase C48, SUMO/Sentrin/Ubl1	NON_SYNONYMOUS_CODING
2650	CakSNP2650	*Kabuli*	Ca_Kabuli_Chr02	22457181	[G/T]	Ca_14999	Peptidase C48, SUMO/Sentrin/Ubl1	SYNONYMOUS_CODING
2651	CakSNP2651	*Kabuli*	Ca_Kabuli_Chr02	22457233	[C/T]	Ca_14999	Peptidase C48, SUMO/Sentrin/Ubl1	NON_SYNONYMOUS_CODING

S.N.	SNP IDs	Chickpea cultivars	Chromosomes	Physical positions (bp)	SNPs	Gene accession IDs	Putative functions	Sequence components of genome
2652	CakSNP2652	*Kabuli*	Ca_Kabuli_Chr02	22457255	[C/T]	Ca_14999	Peptidase C48, SUMO/Sentrin/Ubl1	NON_SYNONYMOUS_CODING
2653	CakSNP2653	*Kabuli*	Ca_Kabuli_Chr02	22457263	[G/A]	Ca_14999	Peptidase C48, SUMO/Sentrin/Ubl1	NON_SYNONYMOUS_CODING
2654	CakSNP2654	*Kabuli*	Ca_Kabuli_Chr02	22457257	[G/A]	Ca_14999	Peptidase C48, SUMO/Sentrin/Ubl1	NON_SYNONYMOUS_CODING
2655	CakSNP2655	*Kabuli*	Ca_Kabuli_Chr02	22483072	[C/T]		--	INTERGENIC
2656	CakSNP2656	*Kabuli*	Ca_Kabuli_Chr02	22483067	[G/A]		--	INTERGENIC
2657	CakSNP2657	*Kabuli*	Ca_Kabuli_Chr02	22483052	[C/T]		--	INTERGENIC
2658	CakSNP2658	*Kabuli*	Ca_Kabuli_Chr02	22483019	[A/T]		--	INTERGENIC
2659	CakSNP2659	*Kabuli*	Ca_Kabuli_Chr02	22703091	[A/C]	Ca_14990	Rho GTPase-activating protein domain	INTRON
2660	CakSNP2660	*Kabuli*	Ca_Kabuli_Chr02	22820616	[C/G]	Ca_14985	Peptidase C19, ubiquitin carboxyl-terminal hydrolase 2	INTRON
2661	CakSNP2661	*Kabuli*	Ca_Kabuli_Chr02	22820588	[A/C]	Ca_14985	Peptidase C19, ubiquitin carboxyl-terminal hydrolase 2	INTRON

S.N.	SNP IDs	Chickpea cultivars	Chromosomes	Physical positions (bp)	SNPs	Gene accession IDs	Putative functions	Sequence components of genome
2662	CakSNP2662	*Kabuli*	Ca_Kabuli_Chr02	22897694	[G/A]		--	INTERGENIC
2663	CakSNP2663	*Kabuli*	Ca_Kabuli_Chr02	22897647	[T/C]		--	INTERGENIC
2664	CakSNP2664	*Kabuli*	Ca_Kabuli_Chr02	23123734	[C/T]	Ca_14976	--	INTRON
2665	CakSNP2665	*Kabuli*	Ca_Kabuli_Chr02	23141564	[C/T]	Ca_14974	--	INTRON
2666	CakSNP2666	*Kabuli*	Ca_Kabuli_Chr02	23181353	[C/T]	Ca_14972	Protein of unknown function DUF1423, plant	SYNONYMOUS_CODING
2667	CakSNP2667	*Kabuli*	Ca_Kabuli_Chr02	23247801	[C/T]		--	INTERGENIC
2668	CakSNP2668	*Kabuli*	Ca_Kabuli_Chr02	23282374	[T/C]		--	INTERGENIC
2669	CakSNP2669	*Kabuli*	Ca_Kabuli_Chr02	23297412	[G/A]	Ca_14967	Domain of unknown function FMP27, domain-6, C-terminal	NON_SYNONYMOUS_CODING
2670	CakSNP2670	*Kabuli*	Ca_Kabuli_Chr02	23329325	[G/T]		--	INTERGENIC
2671	CakSNP2671	*Kabuli*	Ca_Kabuli_Chr02	23329424	[C/T]		--	INTERGENIC

S.N.	SNP IDs	Chickpea cultivars	Chromosomes	Physical positions (bp)	SNPs	Gene accession IDs	Putative functions	Sequence components of genome
2672	CakSNP2672	*Kabuli*	Ca_Kabuli_Chr02	23329440	[G/A]		--	INTERGENIC
2673	CakSNP2673	*Kabuli*	Ca_Kabuli_Chr02	23417518	[T/G]		--	INTERGENIC
2674	CakSNP2674	*Kabuli*	Ca_Kabuli_Chr02	23435939	[A/G]	Ca_14960	Ubiquitin-associated/translation elongation factor EF1B, N-terminal	SYNONYMOUS_CODING
2675	CakSNP2675	*Kabuli*	Ca_Kabuli_Chr02	23439966	[T/C]	Ca_14960	Ubiquitin-associated/translation elongation factor EF1B, N-terminal	NON_SYNONYMOUS_CODING
2676	CakSNP2676	*Kabuli*	Ca_Kabuli_Chr02	23441740	[A/G]	Ca_14960	Ubiquitin-associated/translation elongation factor EF1B, N-terminal	SYNONYMOUS_CODING
2677	CakSNP2677	*Kabuli*	Ca_Kabuli_Chr02	23910254	[T/C]	Ca_14938	Peptidase S8/S53, subtilisin/kexin/sedolisin	NON_SYNONYMOUS_CODING
2678	CakSNP2678	*Kabuli*	Ca_Kabuli_Chr02	23961003	[G/A]	Ca_21675	Quinate/shikimate 5-dehydrogenase/glutamyl-tRNA reductase	SYNONYMOUS_CODING
2679	CakSNP2679	*Kabuli*	Ca_Kabuli_Chr02	24048087	[A/C]	Ca_21673	PUCC protein	INTRON
2680	CakSNP2680	*Kabuli*	Ca_Kabuli_Chr02	24098243	[T/C]	Ca_21670	Transmembrane receptor, eukaryota	INTRON
2681	CakSNP2681	*Kabuli*	Ca_Kabuli_Chr02	24101371	[C/T]	Ca_21670	Transmembrane receptor, eukaryota	SYNONYMOUS_CODING

S.N.	SNP IDs	Chickpea cultivars	Chromosomes	Physical positions (bp)	SNPs	Gene accession IDs	Putative functions	Sequence components of genome
2682	CakSNP2682	*Kabuli*	Ca_Kabuli_Chr02	24164335	[A/T]	Ca_21668	Rhodanese-like	INTRON
2683	CakSNP2683	*Kabuli*	Ca_Kabuli_Chr02	24164840	[T/A]	Ca_21668	Rhodanese-like	SYNONYMOUS_CODING
2684	CakSNP2684	*Kabuli*	Ca_Kabuli_Chr02	24164903	[T/C]	Ca_21668	Rhodanese-like	SYNONYMOUS_CODING
2685	CakSNP2685	*Kabuli*	Ca_Kabuli_Chr02	24164900	[A/T]	Ca_21668	Rhodanese-like	SYNONYMOUS_CODING
2686	CakSNP2686	*Kabuli*	Ca_Kabuli_Chr02	24221498	[G/T]	Ca_21666	SNF2-related	INTRON
2687	CakSNP2687	*Kabuli*	Ca_Kabuli_Chr02	24291665	[T/C]	Ca_21663	Protein of unknown function DUF3133	NON_SYNONYMOUS_CODING
2688	CakSNP2688	*Kabuli*	Ca_Kabuli_Chr02	24353303	[A/G]		--	INTERGENIC
2689	CakSNP2689	*Kabuli*	Ca_Kabuli_Chr02	24353243	[A/T]		--	INTERGENIC
2690	CakSNP2690	*Kabuli*	Ca_Kabuli_Chr02	24511053	[G/A]		--	INTERGENIC
2691	CakSNP2691	*Kabuli*	Ca_Kabuli_Chr02	24511114	[G/A]		--	INTERGENIC

S.N.	SNP IDs	Chickpea cultivars	Chromosomes	Physical positions (bp)	SNPs	Gene accession IDs	Putative functions	Sequence components of genome
2692	CakSNP2692	*Kabuli*	Ca_Kabuli_Chr02	24511121	[G/A]		--	INTERGENIC
2693	CakSNP2693	*Kabuli*	Ca_Kabuli_Chr02	24511119	[T/G]		--	INTERGENIC
2694	CakSNP2694	*Kabuli*	Ca_Kabuli_Chr02	24511116	[C/A]		--	INTERGENIC
2695	CakSNP2695	*Kabuli*	Ca_Kabuli_Chr02	24586360	[T/C]	Ca_22901	Peptidase C78, ubiquitin fold modifier-specific peptidase 1/ 3	SYNONYMOUS_CODING
2696	CakSNP2696	*Kabuli*	Ca_Kabuli_Chr02	24709014	[C/G]	Ca_22892	DNA mismatch repair protein MutS, C-terminal domain	INTRON
2697	CakSNP2697	*Kabuli*	Ca_Kabuli_Chr02	24709103	[G/A]	Ca_22892	DNA mismatch repair protein MutS, C-terminal domain	INTRON
2698	CakSNP2698	*Kabuli*	Ca_Kabuli_Chr02	24709139	[A/G]	Ca_22892	DNA mismatch repair protein MutS, C-terminal domain	INTRON
2699	CakSNP2699	*Kabuli*	Ca_Kabuli_Chr02	24709159	[T/A]	Ca_22892	DNA mismatch repair protein MutS, C-terminal domain	INTRON
2700	CakSNP2700	*Kabuli*	Ca_Kabuli_Chr02	24709295	[G/A]	Ca_22892	DNA mismatch repair protein MutS, C-terminal domain	INTRON
2701	CakSNP2701	*Kabuli*	Ca_Kabuli_Chr02	24741195	[C/T]	Ca_22889	--	SYNONYMOUS_CODING

S.N.	SNP IDs	Chickpea cultivars	Chromosomes	Physical positions (bp)	SNPs	Gene accession IDs	Putative functions	Sequence components of genome
2702	CakSNP2702	*Kabuli*	Ca_Kabuli_Chr02	24744726	[A/T]	Ca_22888	Thaumatin, pathogenesis-related	SYNONYMOUS_CODING
2703	CakSNP2703	*Kabuli*	Ca_Kabuli_Chr02	24817957	[G/A]		--	INTERGENIC
2704	CakSNP2704	*Kabuli*	Ca_Kabuli_Chr02	24817945	[C/T]		--	INTERGENIC
2705	CakSNP2705	*Kabuli*	Ca_Kabuli_Chr02	24817940	[G/A]		--	INTERGENIC
2706	CakSNP2706	*Kabuli*	Ca_Kabuli_Chr02	24817934	[A/G]		--	INTERGENIC
2707	CakSNP2707	*Kabuli*	Ca_Kabuli_Chr02	24817921	[G/A]		--	INTERGENIC
2708	CakSNP2708	*Kabuli*	Ca_Kabuli_Chr02	24876355	[T/G]	Ca_24906	Glycoside hydrolase, carbohydrate-binding	NON_SYNONYMOUS_CODING
2709	CakSNP2709	*Kabuli*	Ca_Kabuli_Chr02	24911080	[T/C]	Ca_24902	Armadillo	NON_SYNONYMOUS_CODING
2710	CakSNP2710	*Kabuli*	Ca_Kabuli_Chr02	24911154	[G/C]	Ca_24902	Armadillo	NON_SYNONYMOUS_CODING
2711	CakSNP2711	*Kabuli*	Ca_Kabuli_Chr02	25022971	[A/G]	Ca_14236	Zinc finger, B-box	NON_SYNONYMOUS_CODING

S.N.	SNP IDs	Chickpea cultivars	Chromosomes	Physical positions (bp)	SNPs	Gene accession IDs	Putative functions	Sequence components of genome
2712	CakSNP2712	*Kabuli*	Ca_Kabuli_Chr02	25104319	[G/T]		--	INTERGENIC
2713	CakSNP2713	*Kabuli*	Ca_Kabuli_Chr02	25104291	[A/T]		--	INTERGENIC
2714	CakSNP2714	*Kabuli*	Ca_Kabuli_Chr02	25108249	[T/C]	Ca_14243	--	SYNONYMOUS_CODING
2715	CakSNP2715	*Kabuli*	Ca_Kabuli_Chr02	25126798	[C/T]	Ca_14246	Multicopper oxidase, type 1	SYNONYMOUS_CODING
2716	CakSNP2716	*Kabuli*	Ca_Kabuli_Chr02	25126804	[A/G]	Ca_14246	Multicopper oxidase, type 1	SYNONYMOUS_CODING
2717	CakSNP2717	*Kabuli*	Ca_Kabuli_Chr02	25126837	[G/T]	Ca_14246	Multicopper oxidase, type 1	SYNONYMOUS_CODING
2718	CakSNP2718	*Kabuli*	Ca_Kabuli_Chr02	25126910	[G/C]	Ca_14246	Multicopper oxidase, type 1	NON_SYNONYMOUS_CODING
2719	CakSNP2719	*Kabuli*	Ca_Kabuli_Chr02	25163062	[C/G]	Ca_14248	Glycoside hydrolase, family 3, N-terminal	INTRON
2720	CakSNP2720	*Kabuli*	Ca_Kabuli_Chr02	25194583	[G/A]		--	INTERGENIC
2721	CakSNP2721	*Kabuli*	Ca_Kabuli_Chr02	25317391	[A/G]	Ca_14265	ABC transporter-like	SYNONYMOUS_CODING

S.N.	SNP IDs	Chickpea cultivars	Chromosomes	Physical positions (bp)	SNPs	Gene accession IDs	Putative functions	Sequence components of genome
2722	CakSNP2722	*Kabuli*	Ca_Kabuli_Chr02	25321993	[C/A]	Ca_14266	Sodium/calcium exchanger membrane region	INTRON
2723	CakSNP2723	*Kabuli*	Ca_Kabuli_Chr02	25493973	[T/G]	Ca_14279	Chaperonin ClpA/B	SYNONYMOUS_CODING
2724	CakSNP2724	*Kabuli*	Ca_Kabuli_Chr02	25507637	[C/G]	Ca_14280	Zinc finger, CCCH-type	NON_SYNONYMOUS_CODING
2725	CakSNP2725	*Kabuli*	Ca_Kabuli_Chr02	25507681	[A/G]	Ca_14280	Zinc finger, CCCH-type	NON_SYNONYMOUS_CODING
2726	CakSNP2726	*Kabuli*	Ca_Kabuli_Chr02	25508579	[C/A]	Ca_14280	Zinc finger, CCCH-type	SYNONYMOUS_CODING
2727	CakSNP2727	*Kabuli*	Ca_Kabuli_Chr02	25508679	[A/G]	Ca_14280	Zinc finger, CCCH-type	NON_SYNONYMOUS_CODING
2728	CakSNP2728	*Kabuli*	Ca_Kabuli_Chr02	25508660	[G/C]	Ca_14280	Zinc finger, CCCH-type	NON_SYNONYMOUS_CODING
2729	CakSNP2729	*Kabuli*	Ca_Kabuli_Chr02	25508920	[G/A]	Ca_14280	Zinc finger, CCCH-type	NON_SYNONYMOUS_CODING
2730	CakSNP2730	*Kabuli*	Ca_Kabuli_Chr02	25508969	[T/C]	Ca_14280	Zinc finger, CCCH-type	SYNONYMOUS_CODING
2731	CakSNP2731	*Kabuli*	Ca_Kabuli_Chr02	25508964	[A/C]	Ca_14280	Zinc finger, CCCH-type	NON_SYNONYMOUS_CODING

S.N.	SNP IDs	Chickpea cultivars	Chromosomes	Physical positions (bp)	SNPs	Gene accession IDs	Putative functions	Sequence components of genome
2732	CakSNP2732	*Kabuli*	Ca_Kabuli_Chr02	25508963	[A/G]	Ca_14280	Zinc finger, CCCH-type	SYNONYMOUS_CODING
2733	CakSNP2733	*Kabuli*	Ca_Kabuli_Chr02	25508935	[A/T]	Ca_14280	Zinc finger, CCCH-type	NON_SYNONYMOUS_CODING
2734	CakSNP2734	*Kabuli*	Ca_Kabuli_Chr02	25509087	[C/A]	Ca_14280	Zinc finger, CCCH-type	NON_SYNONYMOUS_CODING
2735	CakSNP2735	*Kabuli*	Ca_Kabuli_Chr02	25509093	[C/G]	Ca_14280	Zinc finger, CCCH-type	NON_SYNONYMOUS_CODING
2736	CakSNP2736	*Kabuli*	Ca_Kabuli_Chr02	25509095	[G/A]	Ca_14280	Zinc finger, CCCH-type	SYNONYMOUS_CODING
2737	CakSNP2737	*Kabuli*	Ca_Kabuli_Chr02	25509203	[T/C]	Ca_14280	Zinc finger, CCCH-type	SYNONYMOUS_CODING
2738	CakSNP2738	*Kabuli*	Ca_Kabuli_Chr02	25509202	[T/A]	Ca_14280	Zinc finger, CCCH-type	NON_SYNONYMOUS_CODING
2739	CakSNP2739	*Kabuli*	Ca_Kabuli_Chr02	25667417	[G/C]	Ca_14291	Protein of unknown function DUF652	INTRON
2740	CakSNP2740	*Kabuli*	Ca_Kabuli_Chr02	25667427	[T/C]	Ca_14291	Protein of unknown function DUF652	INTRON
2741	CakSNP2741	*Kabuli*	Ca_Kabuli_Chr02	25788124	[T/A]	Ca_14295	RNA recognition motif domain	INTRON

S.N.	SNP IDs	Chickpea cultivars	Chromosomes	Physical positions (bp)	SNPs	Gene accession IDs	Putative functions	Sequence components of genome
2742	CakSNP2742	*Kabuli*	Ca_Kabuli_Chr02	25788139	[T/C]	Ca_14295	RNA recognition motif domain	INTRON
2743	CakSNP2743	*Kabuli*	Ca_Kabuli_Chr02	25880318	[T/C]		--	INTERGENIC
2744	CakSNP2744	*Kabuli*	Ca_Kabuli_Chr02	25898931	[T/C]	Ca_14302	K+ potassium transporter	INTRON
2745	CakSNP2745	*Kabuli*	Ca_Kabuli_Chr02	25930490	[G/A]	Ca_14304	Phosphoglycerate/bisphosphoglycerate mutase, active site	NON_SYNONYMOUS_CODING
2746	CakSNP2746	*Kabuli*	Ca_Kabuli_Chr02	26051667	[T/G]		--	INTERGENIC
2747	CakSNP2747	*Kabuli*	Ca_Kabuli_Chr02	26092881	[C/G]		--	INTERGENIC
2748	CakSNP2748	*Kabuli*	Ca_Kabuli_Chr02	26092913	[C/A]		--	INTERGENIC
2749	CakSNP2749	*Kabuli*	Ca_Kabuli_Chr02	26092928	[T/G]		--	INTERGENIC
2750	CakSNP2750	*Kabuli*	Ca_Kabuli_Chr02	26092991	[T/C]		--	INTERGENIC
2751	CakSNP2751	*Kabuli*	Ca_Kabuli_Chr02	26121495	[A/C]	Ca_14316	--	SYNONYMOUS_CODING

S.N.	SNP IDs	Chickpea cultivars	Chromosomes	Physical positions (bp)	SNPs	Gene accession IDs	Putative functions	Sequence components of genome
2752	CakSNP2752	*Kabuli*	Ca_Kabuli_Chr02	26121491	[T/G]	Ca_14316	--	SYNONYMOUS_CODING
2753	CakSNP2753	*Kabuli*	Ca_Kabuli_Chr02	26417311	[T/A]	Ca_17244	UDP-glucuronosyl/UDP-glucosyltransferase	NON_SYNONYMOUS_CODING
2754	CakSNP2754	*Kabuli*	Ca_Kabuli_Chr02	26417281	[A/G]	Ca_17244	UDP-glucuronosyl/UDP-glucosyltransferase	SYNONYMOUS_CODING
2755	CakSNP2755	*Kabuli*	Ca_Kabuli_Chr02	26417256	[T/C]	Ca_17244	UDP-glucuronosyl/UDP-glucosyltransferase	NON_SYNONYMOUS_CODING
2756	CakSNP2756	*Kabuli*	Ca_Kabuli_Chr02	26431061	[T/C]		--	INTERGENIC
2757	CakSNP2757	*Kabuli*	Ca_Kabuli_Chr02	26431058	[G/C]		--	INTERGENIC
2758	CakSNP2758	*Kabuli*	Ca_Kabuli_Chr02	26431052	[T/C]		--	INTERGENIC
2759	CakSNP2759	*Kabuli*	Ca_Kabuli_Chr02	26461867	[C/G]	Ca_17247	Metallo-dependent phosphatase	NON_SYNONYMOUS_CODING
2760	CakSNP2760	*Kabuli*	Ca_Kabuli_Chr02	26461986	[A/C]		--	INTERGENIC
2761	CakSNP2761	*Kabuli*	Ca_Kabuli_Chr02	26461960	[T/A]		--	INTERGENIC

S.N.	SNP IDs	Chickpea cultivars	Chromosomes	Physical positions (bp)	SNPs	Gene accession IDs	Putative functions	Sequence components of genome
2762	CakSNP2762	*Kabuli*	Ca_Kabuli_Chr02	26486049	[G/A]		--	INTERGENIC
2763	CakSNP2763	*Kabuli*	Ca_Kabuli_Chr02	26486065	[C/T]		--	INTERGENIC
2764	CakSNP2764	*Kabuli*	Ca_Kabuli_Chr02	26486109	[T/C]		--	INTERGENIC
2765	CakSNP2765	*Kabuli*	Ca_Kabuli_Chr02	26502153	[T/C]		--	INTERGENIC
2766	CakSNP2766	*Kabuli*	Ca_Kabuli_Chr02	26503262	[C/A]		--	INTERGENIC
2767	CakSNP2767	*Kabuli*	Ca_Kabuli_Chr02	26567088	[T/C]	Ca_17252	Multicopper oxidase, type 1	SYNONYMOUS_CODING
2768	CakSNP2768	*Kabuli*	Ca_Kabuli_Chr02	26585842	[T/A]	Ca_17253	Phox homologous domain	INTRON
2769	CakSNP2769	*Kabuli*	Ca_Kabuli_Chr02	26601337	[C/A]	Ca_17255	IQ motif, EF-hand binding site	SYNONYMOUS_CODING
2770	CakSNP2770	*Kabuli*	Ca_Kabuli_Chr02	26601331	[G/C]	Ca_17255	IQ motif, EF-hand binding site	SYNONYMOUS_CODING
2771	CakSNP2771	*Kabuli*	Ca_Kabuli_Chr02	26706849	[A/G]		--	INTERGENIC

S.N.	SNP IDs	Chickpea cultivars	Chromosomes	Physical positions (bp)	SNPs	Gene accession IDs	Putative functions	Sequence components of genome
2772	CakSNP2772	*Kabuli*	Ca_Kabuli_Chr02	27057582	[C/T]	Ca_15636	--	INTRON
2773	CakSNP2773	*Kabuli*	Ca_Kabuli_Chr02	27063749	[C/A]	Ca_15636	--	NON_SYNONYMOUS_CODING
2774	CakSNP2774	*Kabuli*	Ca_Kabuli_Chr02	27134574	[A/G]		--	INTERGENIC
2775	CakSNP2775	*Kabuli*	Ca_Kabuli_Chr02	27134560	[G/T]		--	INTERGENIC
2776	CakSNP2776	*Kabuli*	Ca_Kabuli_Chr02	27182855	[T/C]		--	INTERGENIC
2777	CakSNP2777	*Kabuli*	Ca_Kabuli_Chr02	27317272	[A/T]	Ca_15653	Nucleotidyl transferase domain	INTRON
2778	CakSNP2778	*Kabuli*	Ca_Kabuli_Chr02	27374064	[A/T]	Ca_15659	Transcriptional factor B3	INTRON
2779	CakSNP2779	*Kabuli*	Ca_Kabuli_Chr02	27374094	[C/A]	Ca_15659	Transcriptional factor B3	INTRON
2780	CakSNP2780	*Kabuli*	Ca_Kabuli_Chr02	27377809	[T/G]	Ca_15659	Transcriptional factor B3	INTRON
2781	CakSNP2781	*Kabuli*	Ca_Kabuli_Chr02	27623258	[A/G]	Ca_15667	KIP1-like	SYNONYMOUS_CODING

S.N.	SNP IDs	Chickpea cultivars	Chromosomes	Physical positions (bp)	SNPs	Gene accession IDs	Putative functions	Sequence components of genome
2782	CakSNP2782	*Kabuli*	Ca_Kabuli_Chr02	27703356	[G/A]		--	INTERGENIC
2783	CakSNP2783	*Kabuli*	Ca_Kabuli_Chr02	27724425	[G/T]	Ca_15674	BTB/POZ-like	SYNONYMOUS_CODING
2784	CakSNP2784	*Kabuli*	Ca_Kabuli_Chr02	27757490	[T/C]	Ca_15677	Cation efflux protein	SYNONYMOUS_CODING
2785	CakSNP2785	*Kabuli*	Ca_Kabuli_Chr02	27757824	[A/G]	Ca_15677	Cation efflux protein	NON_SYNONYMOUS_CODING
2786	CakSNP2786	*Kabuli*	Ca_Kabuli_Chr02	27757804	[A/G]	Ca_15677	Cation efflux protein	NON_SYNONYMOUS_CODING
2787	CakSNP2787	*Kabuli*	Ca_Kabuli_Chr02	27757795	[T/G]	Ca_15677	Cation efflux protein	NON_SYNONYMOUS_CODING
2788	CakSNP2788	*Kabuli*	Ca_Kabuli_Chr02	27758057	[C/T]	Ca_15677	Cation efflux protein	NON_SYNONYMOUS_CODING
2789	CakSNP2789	*Kabuli*	Ca_Kabuli_Chr02	27758058	[A/G]	Ca_15677	Cation efflux protein	NON_SYNONYMOUS_CODING
2790	CakSNP2790	*Kabuli*	Ca_Kabuli_Chr02	27758065	[C/G]	Ca_15677	Cation efflux protein	NON_SYNONYMOUS_CODING
2791	CakSNP2791	*Kabuli*	Ca_Kabuli_Chr02	27758093	[C/T]	Ca_15677	Cation efflux protein	SYNONYMOUS_CODING

S.N.	SNP IDs	Chickpea cultivars	Chromosomes	Physical positions (bp)	SNPs	Gene accession IDs	Putative functions	Sequence components of genome
2792	CakSNP2792	*Kabuli*	Ca_Kabuli_Chr02	27766561	[T/A]	Ca_15677	Cation efflux protein	NON_SYNONYMOUS_CODING
2793	CakSNP2793	*Kabuli*	Ca_Kabuli_Chr02	27766623	[T/C]	Ca_15677	Cation efflux protein	SYNONYMOUS_CODING
2794	CakSNP2794	*Kabuli*	Ca_Kabuli_Chr02	27766605	[A/C]	Ca_15677	Cation efflux protein	SYNONYMOUS_CODING
2795	CakSNP2795	*Kabuli*	Ca_Kabuli_Chr02	27766594	[C/A]	Ca_15677	Cation efflux protein	NON_SYNONYMOUS_CODING
2796	CakSNP2796	*Kabuli*	Ca_Kabuli_Chr02	27807994	[A/G]		--	INTERGENIC
2797	CakSNP2797	*Kabuli*	Ca_Kabuli_Chr02	27808070	[G/A]		--	INTERGENIC
2798	CakSNP2798	*Kabuli*	Ca_Kabuli_Chr02	27824962	[G/A]	Ca_15681	ABC transporter, transmembrane domain	INTRON
2799	CakSNP2799	*Kabuli*	Ca_Kabuli_Chr02	27831340	[A/C]	Ca_15681	ABC transporter, transmembrane domain	INTRON
2800	CakSNP2800	*Kabuli*	Ca_Kabuli_Chr02	27831398	[A/G]	Ca_15681	ABC transporter, transmembrane domain	SYNONYMOUS_CODING
2801	CakSNP2801	*Kabuli*	Ca_Kabuli_Chr02	27835871	[G/A]		--	INTERGENIC

S.N.	SNP IDs	Chickpea cultivars	Chromosomes	Physical positions (bp)	SNPs	Gene accession IDs	Putative functions	Sequence components of genome
2802	CakSNP2802	*Kabuli*	Ca_Kabuli_Chr02	27835850	[G/A]		--	INTERGENIC
2803	CakSNP2803	*Kabuli*	Ca_Kabuli_Chr02	27835843	[C/A]		--	INTERGENIC
2804	CakSNP2804	*Kabuli*	Ca_Kabuli_Chr02	27835837	[C/T]		--	INTERGENIC
2805	CakSNP2805	*Kabuli*	Ca_Kabuli_Chr02	27835832	[C/T]		--	INTERGENIC
2806	CakSNP2806	*Kabuli*	Ca_Kabuli_Chr02	27847670	[A/C]	Ca_15682	Cohesin loading factor	INTRON
2807	CakSNP2807	*Kabuli*	Ca_Kabuli_Chr02	28017163	[A/G]	Ca_15694	AUX/IAA protein	SYNONYMOUS_CODING
2808	CakSNP2808	*Kabuli*	Ca_Kabuli_Chr02	28018158	[T/C]	Ca_15694	AUX/IAA protein	NON_SYNONYMOUS_CODING
2809	CakSNP2809	*Kabuli*	Ca_Kabuli_Chr02	28060867	[T/G]	Ca_14397	Uncharacterised protein family UPF0497, trans-membrane plant	NON_SYNONYMOUS_CODING
2810	CakSNP2810	*Kabuli*	Ca_Kabuli_Chr02	28060850	[A/G]		--	INTERGENIC
2811	CakSNP2811	*Kabuli*	Ca_Kabuli_Chr02	28060879	[C/T]	Ca_14397	Uncharacterised protein family UPF0497, trans-membrane plant	NON_SYNONYMOUS_CODING

S.N.	SNP IDs	Chickpea cultivars	Chromosomes	Physical positions (bp)	SNPs	Gene accession IDs	Putative functions	Sequence components of genome
2812	CakSNP2812	*Kabuli*	Ca_Kabuli_Chr02	28060896	[T/C]	Ca_14397	Uncharacterised protein family UPF0497, trans-membrane plant	NON_SYNONYMOUS_CODING
2813	CakSNP2813	*Kabuli*	Ca_Kabuli_Chr02	28060921	[C/T]	Ca_14397	Uncharacterised protein family UPF0497, trans-membrane plant	NON_SYNONYMOUS_CODING
2814	CakSNP2814	*Kabuli*	Ca_Kabuli_Chr02	28060922	[A/G]	Ca_14397	Uncharacterised protein family UPF0497, trans-membrane plant	SYNONYMOUS_CODING
2815	CakSNP2815	*Kabuli*	Ca_Kabuli_Chr02	28060928	[C/T]	Ca_14397	Uncharacterised protein family UPF0497, trans-membrane plant	SYNONYMOUS_CODING
2816	CakSNP2816	*Kabuli*	Ca_Kabuli_Chr02	28060930	[T/G]	Ca_14397	Uncharacterised protein family UPF0497, trans-membrane plant	NON_SYNONYMOUS_CODING
2817	CakSNP2817	*Kabuli*	Ca_Kabuli_Chr02	28060940	[C/T]	Ca_14397	Uncharacterised protein family UPF0497, trans-membrane plant	SYNONYMOUS_CODING
2818	CakSNP2818	*Kabuli*	Ca_Kabuli_Chr02	28061049	[G/T]	Ca_14397	Uncharacterised protein family UPF0497, trans-membrane plant	STOP_GAINED
2819	CakSNP2819	*Kabuli*	Ca_Kabuli_Chr02	28076664	[C/T]	Ca_14396	Ras GTPase	SYNONYMOUS_CODING
2820	CakSNP2820	*Kabuli*	Ca_Kabuli_Chr02	28076834	[C/T]	Ca_14396	Ras GTPase	INTRON
2821	CakSNP2821	*Kabuli*	Ca_Kabuli_Chr02	28076851	[G/A]	Ca_14396	Ras GTPase	INTRON

S.N.	SNP IDs	Chickpea cultivars	Chromosomes	Physical positions (bp)	SNPs	Gene accession IDs	Putative functions	Sequence components of genome
2822	CakSNP2822	*Kabuli*	Ca_Kabuli_Chr02	28084910	[C/T]	Ca_14394	Squalene/phytoene synthase	SYNONYMOUS_CODING
2823	CakSNP2823	*Kabuli*	Ca_Kabuli_Chr02	28084913	[C/A]	Ca_14394	Squalene/phytoene synthase	SYNONYMOUS_CODING
2824	CakSNP2824	*Kabuli*	Ca_Kabuli_Chr02	28084953	[C/T]	Ca_14394	Squalene/phytoene synthase	NON_SYNONYMOUS_CODING
2825	CakSNP2825	*Kabuli*	Ca_Kabuli_Chr02	28229031	[G/A]	Ca_14380	Protein kinase, catalytic domain	SYNONYMOUS_CODING
2826	CakSNP2826	*Kabuli*	Ca_Kabuli_Chr02	28229046	[G/A]	Ca_14380	Protein kinase, catalytic domain	SYNONYMOUS_CODING
2827	CakSNP2827	*Kabuli*	Ca_Kabuli_Chr02	28324577	[T/A]	Ca_14377	PRP38	INTRON
2828	CakSNP2828	*Kabuli*	Ca_Kabuli_Chr02	28352533	[C/A]	Ca_14374	--	INTRON
2829	CakSNP2829	*Kabuli*	Ca_Kabuli_Chr02	28407316	[T/C]		--	INTERGENIC
2830	CakSNP2830	*Kabuli*	Ca_Kabuli_Chr02	28407324	[C/T]		--	INTERGENIC
2831	CakSNP2831	*Kabuli*	Ca_Kabuli_Chr02	28534235	[T/C]		--	INTERGENIC

S.N.	SNP IDs	Chickpea cultivars	Chromosomes	Physical positions (bp)	SNPs	Gene accession IDs	Putative functions	Sequence components of genome
2832	CakSNP2832	*Kabuli*	Ca_Kabuli_Chr02	28699149	[C/T]	Ca_14360	CCAAT-binding factor	INTRON
2833	CakSNP2833	*Kabuli*	Ca_Kabuli_Chr02	28699178	[T/C]	Ca_14360	CCAAT-binding factor	INTRON
2834	CakSNP2834	*Kabuli*	Ca_Kabuli_Chr02	28699185	[C/A]	Ca_14360	CCAAT-binding factor	INTRON
2835	CakSNP2835	*Kabuli*	Ca_Kabuli_Chr02	28699219	[T/G]	Ca_14360	CCAAT-binding factor	INTRON
2836	CakSNP2836	*Kabuli*	Ca_Kabuli_Chr02	28699229	[C/T]	Ca_14360	CCAAT-binding factor	INTRON
2837	CakSNP2837	*Kabuli*	Ca_Kabuli_Chr02	28699276	[T/C]	Ca_14360	CCAAT-binding factor	INTRON
2838	CakSNP2838	*Kabuli*	Ca_Kabuli_Chr02	28699383	[A/T]	Ca_14360	CCAAT-binding factor	INTRON
2839	CakSNP2839	*Kabuli*	Ca_Kabuli_Chr02	28699403	[C/T]	Ca_14360	CCAAT-binding factor	INTRON
2840	CakSNP2840	*Kabuli*	Ca_Kabuli_Chr02	28699414	[C/T]	Ca_14360	CCAAT-binding factor	INTRON
2841	CakSNP2841	*Kabuli*	Ca_Kabuli_Chr02	28699579	[A/C]	Ca_14360	CCAAT-binding factor	INTRON

S.N.	SNP IDs	Chickpea cultivars	Chromosomes	Physical positions (bp)	SNPs	Gene accession IDs	Putative functions	Sequence components of genome
2842	CakSNP2842	*Kabuli*	Ca_Kabuli_Chr02	28701724	[A/G]	Ca_14360	CCAAT-binding factor	INTRON
2843	CakSNP2843	*Kabuli*	Ca_Kabuli_Chr02	28741764	[T/C]		--	INTERGENIC
2844	CakSNP2844	*Kabuli*	Ca_Kabuli_Chr02	28931402	[T/C]	Ca_14345	--	SYNONYMOUS_CODING
2845	CakSNP2845	*Kabuli*	Ca_Kabuli_Chr02	28931340	[T/C]	Ca_14345	--	INTRON
2846	CakSNP2846	*Kabuli*	Ca_Kabuli_Chr02	28944712	[T/C]	Ca_14343	Lipase, GDSL	NON_SYNONYMOUS_CODING
2847	CakSNP2847	*Kabuli*	Ca_Kabuli_Chr02	28944707	[A/C]	Ca_14343	Lipase, GDSL	NON_SYNONYMOUS_CODING
2848	CakSNP2848	*Kabuli*	Ca_Kabuli_Chr02	28949900	[C/T]	Ca_14342	Glycoside hydrolase, family 17	SYNONYMOUS_CODING
2849	CakSNP2849	*Kabuli*	Ca_Kabuli_Chr02	28949909	[G/A]	Ca_14342	Glycoside hydrolase, family 17	SYNONYMOUS_CODING
2850	CakSNP2850	*Kabuli*	Ca_Kabuli_Chr02	28949911	[A/C]	Ca_14342	Glycoside hydrolase, family 17	NON_SYNONYMOUS_CODING
2851	CakSNP2851	*Kabuli*	Ca_Kabuli_Chr02	28949919	[A/T]	Ca_14342	Glycoside hydrolase, family 17	NON_SYNONYMOUS_CODING

S.N.	SNP IDs	Chickpea cultivars	Chromosomes	Physical positions (bp)	SNPs	Gene accession IDs	Putative functions	Sequence components of genome
2852	CakSNP2852	*Kabuli*	Ca_Kabuli_Chr02	28949954	[T/A]	Ca_14342	Glycoside hydrolase, family 17	SYNONYMOUS_CODING
2853	CakSNP2853	*Kabuli*	Ca_Kabuli_Chr02	28949948	[A/G]	Ca_14342	Glycoside hydrolase, family 17	SYNONYMOUS_CODING
2854	CakSNP2854	*Kabuli*	Ca_Kabuli_Chr02	28970621	[A/G]	Ca_14340	--	INTRON
2855	CakSNP2855	*Kabuli*	Ca_Kabuli_Chr02	28970618	[A/G]	Ca_14340	--	INTRON
2856	CakSNP2856	*Kabuli*	Ca_Kabuli_Chr02	29103993	[T/C]		--	INTERGENIC
2857	CakSNP2857	*Kabuli*	Ca_Kabuli_Chr02	29103970	[C/T]		--	INTERGENIC
2858	CakSNP2858	*Kabuli*	Ca_Kabuli_Chr02	29122581	[G/A]	Ca_14327	Peptidase A1	SYNONYMOUS_CODING
2859	CakSNP2859	*Kabuli*	Ca_Kabuli_Chr02	29122589	[T/G]	Ca_14327	Peptidase A1	NON_SYNONYMOUS_CODING
2860	CakSNP2860	*Kabuli*	Ca_Kabuli_Chr02	29592666	[G/A]	Ca_16128	Double-stranded RNA-binding	NON_SYNONYMOUS_CODING
2861	CakSNP2861	*Kabuli*	Ca_Kabuli_Chr02	29592694	[T/C]	Ca_16128	Double-stranded RNA-binding	NON_SYNONYMOUS_CODING

S.N.	SNP IDs	Chickpea cultivars	Chromosomes	Physical positions (bp)	SNPs	Gene accession IDs	Putative functions	Sequence components of genome
2862	CakSNP2862	*Kabuli*	Ca_Kabuli_Chr02	29601458	[C/T]	Ca_16127	A1 cistron-splicing factor, AAR2	INTRON
2863	CakSNP2863	*Kabuli*	Ca_Kabuli_Chr02	29601463	[C/T]	Ca_16127	A1 cistron-splicing factor, AAR2	INTRON
2864	CakSNP2864	*Kabuli*	Ca_Kabuli_Chr02	29614370	[C/T]	Ca_16126	A1 cistron-splicing factor, AAR2	INTRON
2865	CakSNP2865	*Kabuli*	Ca_Kabuli_Chr02	29614375	[C/T]	Ca_16126	A1 cistron-splicing factor, AAR2	INTRON
2866	CakSNP2866	*Kabuli*	Ca_Kabuli_Chr02	29693107	[A/G]	Ca_16122	Lateral organ boundaries, LOB	SYNONYMOUS_CODING
2867	CakSNP2867	*Kabuli*	Ca_Kabuli_Chr02	29948356	[G/A]	Ca_16096	Radical SAM	NON_SYNONYMOUS_CODING
2868	CakSNP2868	*Kabuli*	Ca_Kabuli_Chr02	29960049	[G/A]	Ca_16094	A1 cistron-splicing factor, AAR2	INTRON
2869	CakSNP2869	*Kabuli*	Ca_Kabuli_Chr02	29960054	[G/A]	Ca_16094	A1 cistron-splicing factor, AAR2	INTRON
2870	CakSNP2870	*Kabuli*	Ca_Kabuli_Chr02	29960130	[C/T]	Ca_16094	A1 cistron-splicing factor, AAR2	INTRON
2871	CakSNP2871	*Kabuli*	Ca_Kabuli_Chr02	30042255	[A/C]	Ca_16084	Globin, subset	INTRON

S.N.	SNP IDs	Chickpea cultivars	Chromosomes	Physical positions (bp)	SNPs	Gene accession IDs	Putative functions	Sequence components of genome
2872	CakSNP2872	*Kabuli*	Ca_Kabuli_Chr02	30042309	[T/G]	Ca_16084	Globin, subset	INTRON
2873	CakSNP2873	*Kabuli*	Ca_Kabuli_Chr02	30042302	[A/T]	Ca_16084	Globin, subset	INTRON
2874	CakSNP2874	*Kabuli*	Ca_Kabuli_Chr02	30042297	[C/T]	Ca_16084	Globin, subset	INTRON
2875	CakSNP2875	*Kabuli*	Ca_Kabuli_Chr02	30042267	[C/T]	Ca_16084	Globin, subset	INTRON
2876	CakSNP2876	*Kabuli*	Ca_Kabuli_Chr02	30045338	[C/G]		--	INTERGENIC
2877	CakSNP2877	*Kabuli*	Ca_Kabuli_Chr02	30091313	[G/A]	Ca_16081	Peptidase S8/S53, subtilisin/kexin/sedolisin	NON_SYNONYMOUS_CODING
2878	CakSNP2878	*Kabuli*	Ca_Kabuli_Chr02	30091311	[C/T]	Ca_16081	Peptidase S8/S53, subtilisin/kexin/sedolisin	SYNONYMOUS_CODING
2879	CakSNP2879	*Kabuli*	Ca_Kabuli_Chr02	30091308	[A/T]	Ca_16081	Peptidase S8/S53, subtilisin/kexin/sedolisin	SYNONYMOUS_CODING
2880	CakSNP2880	*Kabuli*	Ca_Kabuli_Chr02	30091307	[C/T]	Ca_16081	Peptidase S8/S53, subtilisin/kexin/sedolisin	NON_SYNONYMOUS_CODING
2881	CakSNP2881	*Kabuli*	Ca_Kabuli_Chr02	30091295	[T/A]	Ca_16081	Peptidase S8/S53, subtilisin/kexin/sedolisin	NON_SYNONYMOUS_CODING

S.N.	SNP IDs	Chickpea cultivars	Chromosomes	Physical positions (bp)	SNPs	Gene accession IDs	Putative functions	Sequence components of genome
2882	CakSNP2882	*Kabuli*	Ca_Kabuli_Chr02	30091290	[T/C]	Ca_16081	Peptidase S8/S53, subtilisin/kexin/sedolisin	SYNONYMOUS_CODING
2883	CakSNP2883	*Kabuli*	Ca_Kabuli_Chr02	30091286	[G/A]	Ca_16081	Peptidase S8/S53, subtilisin/kexin/sedolisin	NON_SYNONYMOUS_CODING
2884	CakSNP2884	*Kabuli*	Ca_Kabuli_Chr02	30091284	[A/C]	Ca_16081	Peptidase S8/S53, subtilisin/kexin/sedolisin	SYNONYMOUS_CODING
2885	CakSNP2885	*Kabuli*	Ca_Kabuli_Chr02	30091280	[C/G]	Ca_16081	Peptidase S8/S53, subtilisin/kexin/sedolisin	NON_SYNONYMOUS_CODING
2886	CakSNP2886	*Kabuli*	Ca_Kabuli_Chr02	30133162	[G/A]	Ca_16079	Peptidase S8/S53, subtilisin/kexin/sedolisin	NON_SYNONYMOUS_CODING
2887	CakSNP2887	*Kabuli*	Ca_Kabuli_Chr02	30133189	[G/A]	Ca_16079	Peptidase S8/S53, subtilisin/kexin/sedolisin	NON_SYNONYMOUS_CODING
2888	CakSNP2888	*Kabuli*	Ca_Kabuli_Chr02	30133215	[G/T]	Ca_16079	Peptidase S8/S53, subtilisin/kexin/sedolisin	SYNONYMOUS_CODING
2889	CakSNP2889	*Kabuli*	Ca_Kabuli_Chr02	30133221	[A/T]	Ca_16079	Peptidase S8/S53, subtilisin/kexin/sedolisin	SYNONYMOUS_CODING
2890	CakSNP2890	*Kabuli*	Ca_Kabuli_Chr02	30335160	[T/C]	Ca_12519	Ribosomal protein L35A	INTRON
2891	CakSNP2891	*Kabuli*	Ca_Kabuli_Chr02	30335212	[T/A]	Ca_12519	Ribosomal protein L35A	INTRON

S.N.	SNP IDs	Chickpea cultivars	Chromosomes	Physical positions (bp)	SNPs	Gene accession IDs	Putative functions	Sequence components of genome
2892	CakSNP2892	*Kabuli*	Ca_Kabuli_Chr02	30335194	[A/G]	Ca_12519	Ribosomal protein L35A	INTRON
2893	CakSNP2893	*Kabuli*	Ca_Kabuli_Chr02	30363957	[C/A]		--	INTERGENIC
2894	CakSNP2894	*Kabuli*	Ca_Kabuli_Chr02	30364073	[T/A]		--	INTERGENIC
2895	CakSNP2895	*Kabuli*	Ca_Kabuli_Chr02	30364042	[A/G]		--	INTERGENIC
2896	CakSNP2896	*Kabuli*	Ca_Kabuli_Chr02	30364025	[T/C]		--	INTERGENIC
2897	CakSNP2897	*Kabuli*	Ca_Kabuli_Chr02	30370013	[C/A]		--	INTERGENIC
2898	CakSNP2898	*Kabuli*	Ca_Kabuli_Chr02	30370003	[A/C]		--	INTERGENIC
2899	CakSNP2899	*Kabuli*	Ca_Kabuli_Chr02	30370411	[T/C]		--	INTERGENIC
2900	CakSNP2900	*Kabuli*	Ca_Kabuli_Chr02	30467552	[C/T]	Ca_12503	Mov34/MPN/PAD-1	INTRON
2901	CakSNP2901	*Kabuli*	Ca_Kabuli_Chr02	30467551	[A/T]	Ca_12503	Mov34/MPN/PAD-1	INTRON

S.N.	SNP IDs	Chickpea cultivars	Chromosomes	Physical positions (bp)	SNPs	Gene accession IDs	Putative functions	Sequence components of genome
2902	CakSNP2902	*Kabuli*	Ca_Kabuli_Chr02	30508454	[T/C]	Ca_12501	EXTL2, alpha-1,4-N-acetylhexosaminyltransferase	INTRON
2903	CakSNP2903	*Kabuli*	Ca_Kabuli_Chr02	30530642	[A/T]	Ca_12498	Ribosomal protein L10/acidic P0	SYNONYMOUS_CODING
2904	CakSNP2904	*Kabuli*	Ca_Kabuli_Chr02	30537667	[A/T]	Ca_12497	Ribosomal protein L10/acidic P0	INTRON
2905	CakSNP2905	*Kabuli*	Ca_Kabuli_Chr02	30537673	[A/G]	Ca_12497	Ribosomal protein L10/acidic P0	INTRON
2906	CakSNP2906	*Kabuli*	Ca_Kabuli_Chr02	30537687	[T/C]	Ca_12497	Ribosomal protein L10/acidic P0	INTRON
2907	CakSNP2907	*Kabuli*	Ca_Kabuli_Chr02	30537701	[T/A]	Ca_12497	Ribosomal protein L10/acidic P0	INTRON
2908	CakSNP2908	*Kabuli*	Ca_Kabuli_Chr02	30537953	[T/C]	Ca_12497	Ribosomal protein L10/acidic P0	SYNONYMOUS_CODING
2909	CakSNP2909	*Kabuli*	Ca_Kabuli_Chr02	30614086	[C/T]	Ca_12491	--	NON_SYNONYMOUS_CODING
2910	CakSNP2910	*Kabuli*	Ca_Kabuli_Chr02	30686920	[C/T]	Ca_12488	HAD-superfamily hydrolase, subfamily IG, 5'-nucleotidase	INTRON
2911	CakSNP2911	*Kabuli*	Ca_Kabuli_Chr02	30702121	[T/C]	Ca_12487	High mobility group, HMG1/HMG2	NON_SYNONYMOUS_CODING

S.N.	SNP IDs	Chickpea cultivars	Chromosomes	Physical positions (bp)	SNPs	Gene accession IDs	Putative functions	Sequence components of genome
2912	CakSNP2912	*Kabuli*	Ca_Kabuli_Chr02	30702090	[G/A]	Ca_12487	High mobility group, HMG1/HMG2	NON_SYNONYMOUS_CODING
2913	CakSNP2913	*Kabuli*	Ca_Kabuli_Chr02	30867547	[G/A]	Ca_12474	Zinc finger, CCCH-type	SYNONYMOUS_CODING
2914	CakSNP2914	*Kabuli*	Ca_Kabuli_Chr02	30880148	[A/G]	Ca_12473	Synaptobrevin	INTRON
2915	CakSNP2915	*Kabuli*	Ca_Kabuli_Chr02	30880243	[G/C]	Ca_12473	Synaptobrevin	INTRON
2916	CakSNP2916	*Kabuli*	Ca_Kabuli_Chr02	30880246	[G/A]	Ca_12473	Synaptobrevin	INTRON
2917	CakSNP2917	*Kabuli*	Ca_Kabuli_Chr02	30880303	[T/A]	Ca_12473	Synaptobrevin	INTRON
2918	CakSNP2918	*Kabuli*	Ca_Kabuli_Chr02	30880302	[T/C]	Ca_12473	Synaptobrevin	INTRON
2919	CakSNP2919	*Kabuli*	Ca_Kabuli_Chr02	30880282	[A/G]	Ca_12473	Synaptobrevin	INTRON
2920	CakSNP2920	*Kabuli*	Ca_Kabuli_Chr02	30921092	[C/A]	Ca_12470	--	SYNONYMOUS_CODING
2921	CakSNP2921	*Kabuli*	Ca_Kabuli_Chr02	30921758	[C/T]	Ca_12470	--	INTRON

S.N.	SNP IDs	Chickpea cultivars	Chromosomes	Physical positions (bp)	SNPs	Gene accession IDs	Putative functions	Sequence components of genome
2922	CakSNP2922	*Kabuli*	Ca_Kabuli_Chr02	30921888	[T/C]	Ca_12470	--	INTRON
2923	CakSNP2923	*Kabuli*	Ca_Kabuli_Chr02	30921882	[C/G]	Ca_12470	--	INTRON
2924	CakSNP2924	*Kabuli*	Ca_Kabuli_Chr02	30921917	[C/A]	Ca_12470	--	INTRON
2925	CakSNP2925	*Kabuli*	Ca_Kabuli_Chr02	30921923	[A/G]	Ca_12470	--	INTRON
2926	CakSNP2926	*Kabuli*	Ca_Kabuli_Chr02	30954317	[G/A]	Ca_12469	Clathrin, heavy chain/VPS, 7-fold repeat	INTRON
2927	CakSNP2927	*Kabuli*	Ca_Kabuli_Chr02	30954305	[T/G]	Ca_12469	Clathrin, heavy chain/VPS, 7-fold repeat	INTRON
2928	CakSNP2928	*Kabuli*	Ca_Kabuli_Chr02	30977572	[A/G]		--	INTERGENIC
2929	CakSNP2929	*Kabuli*	Ca_Kabuli_Chr02	30977528	[C/T]		--	INTERGENIC
2930	CakSNP2930	*Kabuli*	Ca_Kabuli_Chr02	30977522	[A/T]		--	INTERGENIC
2931	CakSNP2931	*Kabuli*	Ca_Kabuli_Chr02	30977508	[A/T]		--	INTERGENIC

S.N.	SNP IDs	Chickpea cultivars	Chromosomes	Physical positions (bp)	SNPs	Gene accession IDs	Putative functions	Sequence components of genome
2932	CakSNP2932	*Kabuli*	Ca_Kabuli_Chr02	30997769	[T/A]	Ca_12465	Pathogenesis-related transcriptional factor/ERF, DNA-binding	SYNONYMOUS_CODING
2933	CakSNP2933	*Kabuli*	Ca_Kabuli_Chr02	30997780	[A/C]	Ca_12465	Pathogenesis-related transcriptional factor/ERF, DNA-binding	SYNONYMOUS_CODING
2934	CakSNP2934	*Kabuli*	Ca_Kabuli_Chr02	30997784	[T/A]	Ca_12465	Pathogenesis-related transcriptional factor/ERF, DNA-binding	SYNONYMOUS_CODING
2935	CakSNP2935	*Kabuli*	Ca_Kabuli_Chr02	31005847	[T/G]	Ca_12464	Lateral organ boundaries, LOB	SYNONYMOUS_CODING
2936	CakSNP2936	*Kabuli*	Ca_Kabuli_Chr02	31005845	[C/T]	Ca_12464	Lateral organ boundaries, LOB	SYNONYMOUS_CODING
2937	CakSNP2937	*Kabuli*	Ca_Kabuli_Chr02	31014295	[A/T]		--	INTERGENIC
2938	CakSNP2938	*Kabuli*	Ca_Kabuli_Chr02	31055083	[C/T]	Ca_12459	Rad21/Rec8-like protein, C-terminal, eukaryotic	INTRON
2939	CakSNP2939	*Kabuli*	Ca_Kabuli_Chr02	31055007	[G/A]	Ca_12459	Rad21/Rec8-like protein, C-terminal, eukaryotic	INTRON
2940	CakSNP2940	*Kabuli*	Ca_Kabuli_Chr02	31088681	[G/T]	Ca_12454	Ribosomal protein S4e	INTRON
2941	CakSNP2941	*Kabuli*	Ca_Kabuli_Chr02	31088676	[G/C]	Ca_12454	Ribosomal protein S4e	INTRON

S.N.	SNP IDs	Chickpea cultivars	Chromosomes	Physical positions (bp)	SNPs	Gene accession IDs	Putative functions	Sequence components of genome
2942	CakSNP2942	*Kabuli*	Ca_Kabuli_Chr02	31088670	[C/T]	Ca_12454	Ribosomal protein S4e	INTRON
2943	CakSNP2943	*Kabuli*	Ca_Kabuli_Chr02	31088648	[A/G]	Ca_12454	Ribosomal protein S4e	INTRON
2944	CakSNP2944	*Kabuli*	Ca_Kabuli_Chr02	31088645	[A/C]	Ca_12454	Ribosomal protein S4e	INTRON
2945	CakSNP2945	*Kabuli*	Ca_Kabuli_Chr02	31088778	[A/T]	Ca_12454	Ribosomal protein S4e	INTRON
2946	CakSNP2946	*Kabuli*	Ca_Kabuli_Chr02	31088800	[T/C]	Ca_12454	Ribosomal protein S4e	INTRON
2947	CakSNP2947	*Kabuli*	Ca_Kabuli_Chr02	31088882	[A/G]	Ca_12454	Ribosomal protein S4e	INTRON
2948	CakSNP2948	*Kabuli*	Ca_Kabuli_Chr02	31109311	[G/T]		--	INTERGENIC
2949	CakSNP2949	*Kabuli*	Ca_Kabuli_Chr02	31109358	[C/T]		--	INTERGENIC
2950	CakSNP2950	*Kabuli*	Ca_Kabuli_Chr02	31109481	[G/A]		--	INTERGENIC
2951	CakSNP2951	*Kabuli*	Ca_Kabuli_Chr02	31109441	[C/G]		--	INTERGENIC

S.N.	SNP IDs	Chickpea cultivars	Chromosomes	Physical positions (bp)	SNPs	Gene accession IDs	Putative functions	Sequence components of genome
2952	CakSNP2952	*Kabuli*	Ca_Kabuli_Chr02	31109435	[G/A]		--	INTERGENIC
2953	CakSNP2953	*Kabuli*	Ca_Kabuli_Chr02	31109427	[C/T]		--	INTERGENIC
2954	CakSNP2954	*Kabuli*	Ca_Kabuli_Chr02	31141019	[G/A]		--	INTERGENIC
2955	CakSNP2955	*Kabuli*	Ca_Kabuli_Chr02	31167603	[G/A]	Ca_12446	Pathogenesis-related transcriptional factor/ERF, DNA-binding	SYNONYMOUS_CODING
2956	CakSNP2956	*Kabuli*	Ca_Kabuli_Chr02	31167594	[A/G]	Ca_12446	Pathogenesis-related transcriptional factor/ERF, DNA-binding	SYNONYMOUS_CODING
2957	CakSNP2957	*Kabuli*	Ca_Kabuli_Chr02	31188355	[A/G]	Ca_12445	Multicopper oxidase, type 1	SYNONYMOUS_CODING
2958	CakSNP2958	*Kabuli*	Ca_Kabuli_Chr02	31201872	[T/C]	Ca_12444	Phox homologous domain	INTRON
2959	CakSNP2959	*Kabuli*	Ca_Kabuli_Chr02	31201859	[T/A]	Ca_12444	Phox homologous domain	INTRON
2960	CakSNP2960	*Kabuli*	Ca_Kabuli_Chr02	31201795	[A/G]	Ca_12444	Phox homologous domain	INTRON
2961	CakSNP2961	*Kabuli*	Ca_Kabuli_Chr02	31203154	[G/A]	Ca_12444	Phox homologous domain	SYNONYMOUS_CODING

S.N.	SNP IDs	Chickpea cultivars	Chromosomes	Physical positions (bp)	SNPs	Gene accession IDs	Putative functions	Sequence components of genome
2962	CakSNP2962	*Kabuli*	Ca_Kabuli_Chr02	31205531	[C/T]	Ca_12443	Protein kinase, catalytic domain	NON_SYNONYMOUS_CODING
2963	CakSNP2963	*Kabuli*	Ca_Kabuli_Chr02	31427861	[A/G]	Ca_12419	Protein of unknown function DUF827, plant	NON_SYNONYMOUS_CODING
2964	CakSNP2964	*Kabuli*	Ca_Kabuli_Chr02	31427902	[G/C]	Ca_12419	Protein of unknown function DUF827, plant	NON_SYNONYMOUS_CODING
2965	CakSNP2965	*Kabuli*	Ca_Kabuli_Chr02	31427915	[A/G]	Ca_12419	Protein of unknown function DUF827, plant	NON_SYNONYMOUS_CODING
2966	CakSNP2966	*Kabuli*	Ca_Kabuli_Chr02	31428024	[T/G]	Ca_12419	Protein of unknown function DUF827, plant	NON_SYNONYMOUS_CODING
2967	CakSNP2967	*Kabuli*	Ca_Kabuli_Chr02	31428132	[G/A]	Ca_12419	Protein of unknown function DUF827, plant	SYNONYMOUS_CODING
2968	CakSNP2968	*Kabuli*	Ca_Kabuli_Chr02	31428405	[T/C]	Ca_12419	Protein of unknown function DUF827, plant	SYNONYMOUS_CODING
2969	CakSNP2969	*Kabuli*	Ca_Kabuli_Chr02	31428429	[T/G]	Ca_12419	Protein of unknown function DUF827, plant	NON_SYNONYMOUS_CODING
2970	CakSNP2970	*Kabuli*	Ca_Kabuli_Chr02	31534265	[A/C]		--	INTERGENIC
2971	CakSNP2971	*Kabuli*	Ca_Kabuli_Chr02	31534283	[A/G]		--	INTERGENIC

S.N.	SNP IDs	Chickpea cultivars	Chromosomes	Physical positions (bp)	SNPs	Gene accession IDs	Putative functions	Sequence components of genome
2972	CakSNP2972	*Kabuli*	Ca_Kabuli_Chr02	31534455	[T/C]		--	INTERGENIC
2973	CakSNP2973	*Kabuli*	Ca_Kabuli_Chr02	31686714	[C/T]		--	INTERGENIC
2974	CakSNP2974	*Kabuli*	Ca_Kabuli_Chr02	31686716	[C/T]		--	INTERGENIC
2975	CakSNP2975	*Kabuli*	Ca_Kabuli_Chr02	31686742	[A/C]		--	INTERGENIC
2976	CakSNP2976	*Kabuli*	Ca_Kabuli_Chr02	31686751	[A/T]		--	INTERGENIC
2977	CakSNP2977	*Kabuli*	Ca_Kabuli_Chr02	31686758	[C/A]		--	INTERGENIC
2978	CakSNP2978	*Kabuli*	Ca_Kabuli_Chr02	31686761	[A/C]		--	INTERGENIC
2979	CakSNP2979	*Kabuli*	Ca_Kabuli_Chr02	31686767	[C/T]		--	INTERGENIC
2980	CakSNP2980	*Kabuli*	Ca_Kabuli_Chr02	31686779	[G/A]		--	INTERGENIC
2981	CakSNP2981	*Kabuli*	Ca_Kabuli_Chr02	31686820	[T/C]		--	INTERGENIC

S.N.	SNP IDs	Chickpea cultivars	Chromosomes	Physical positions (bp)	SNPs	Gene accession IDs	Putative functions	Sequence components of genome
2982	CakSNP2982	*Kabuli*	Ca_Kabuli_Chr02	31756777	[G/T]		--	INTERGENIC
2983	CakSNP2983	*Kabuli*	Ca_Kabuli_Chr02	31847033	[A/C]		--	INTERGENIC
2984	CakSNP2984	*Kabuli*	Ca_Kabuli_Chr02	31881918	[G/T]	Ca_17830	RZZ complex, subunit Zw10	NON_SYNONYMOUS_CODING
2985	CakSNP2985	*Kabuli*	Ca_Kabuli_Chr02	31885934	[A/G]		--	INTERGENIC
2986	CakSNP2986	*Kabuli*	Ca_Kabuli_Chr02	31908694	[G/A]	Ca_17826	Zinc finger, RING-type	SYNONYMOUS_CODING
2987	CakSNP2987	*Kabuli*	Ca_Kabuli_Chr02	31908742	[G/A]	Ca_17826	Zinc finger, RING-type	SYNONYMOUS_CODING
2988	CakSNP2988	*Kabuli*	Ca_Kabuli_Chr02	31908803	[C/T]	Ca_17826	Zinc finger, RING-type	INTRON
2989	CakSNP2989	*Kabuli*	Ca_Kabuli_Chr02	31908787	[G/A]	Ca_17826	Zinc finger, RING-type	INTRON
2990	CakSNP2990	*Kabuli*	Ca_Kabuli_Chr02	31908845	[T/A]	Ca_17826	Zinc finger, RING-type	INTRON
2991	CakSNP2991	*Kabuli*	Ca_Kabuli_Chr02	31908857	[C/T]	Ca_17826	Zinc finger, RING-type	INTRON

S.N.	SNP IDs	Chickpea cultivars	Chromosomes	Physical positions (bp)	SNPs	Gene accession IDs	Putative functions	Sequence components of genome
2992	CakSNP2992	*Kabuli*	Ca_Kabuli_Chr02	31941463	[T/C]	Ca_17823	Zinc finger, CCCH-type	SYNONYMOUS_CODING
2993	CakSNP2993	*Kabuli*	Ca_Kabuli_Chr02	31941554	[A/T]	Ca_17823	Zinc finger, CCCH-type	INTRON
2994	CakSNP2994	*Kabuli*	Ca_Kabuli_Chr02	31975269	[C/T]		--	INTERGENIC
2995	CakSNP2995	*Kabuli*	Ca_Kabuli_Chr02	32003536	[G/T]		--	INTERGENIC
2996	CakSNP2996	*Kabuli*	Ca_Kabuli_Chr02	32017361	[G/A]		--	INTERGENIC
2997	CakSNP2997	*Kabuli*	Ca_Kabuli_Chr02	32049523	[C/A]	Ca_17817	--	NON_SYNONYMOUS_CODING
2998	CakSNP2998	*Kabuli*	Ca_Kabuli_Chr02	32049525	[A/C]	Ca_17817	--	SYNONYMOUS_CODING
2999	CakSNP2999	*Kabuli*	Ca_Kabuli_Chr02	32058591	[T/C]	Ca_17816	Ankyrin repeat	SYNONYMOUS_CODING
3000	CakSNP3000	*Kabuli*	Ca_Kabuli_Chr02	32061053	[C/T]	Ca_17816	Ankyrin repeat	INTRON
3001	CakSNP3001	*Kabuli*	Ca_Kabuli_Chr02	32061054	[T/C]	Ca_17816	Ankyrin repeat	INTRON

S.N.	SNP IDs	Chickpea cultivars	Chromosomes	Physical positions (bp)	SNPs	Gene accession IDs	Putative functions	Sequence components of genome
3002	CakSNP3002	*Kabuli*	Ca_Kabuli_Chr02	32062010	[A/C]	Ca_17816	Ankyrin repeat	SYNONYMOUS_CODING
3003	CakSNP3003	*Kabuli*	Ca_Kabuli_Chr02	32071107	[C/G]	Ca_17814	Protein of unknown function DUF423	INTRON
3004	CakSNP3004	*Kabuli*	Ca_Kabuli_Chr02	32080002	[T/C]		--	INTERGENIC
3005	CakSNP3005	*Kabuli*	Ca_Kabuli_Chr02	32080007	[T/C]		--	INTERGENIC
3006	CakSNP3006	*Kabuli*	Ca_Kabuli_Chr02	32080010	[G/C]		--	INTERGENIC
3007	CakSNP3007	*Kabuli*	Ca_Kabuli_Chr02	32080016	[A/T]		--	INTERGENIC
3008	CakSNP3008	*Kabuli*	Ca_Kabuli_Chr02	32080022	[C/A]		--	INTERGENIC
3009	CakSNP3009	*Kabuli*	Ca_Kabuli_Chr02	32080037	[A/G]		--	INTERGENIC
3010	CakSNP3010	*Kabuli*	Ca_Kabuli_Chr02	32096192	[A/C]		--	INTERGENIC
3011	CakSNP3011	*Kabuli*	Ca_Kabuli_Chr02	32096186	[T/A]		--	INTERGENIC

S.N.	SNP IDs	Chickpea cultivars	Chromosomes	Physical positions (bp)	SNPs	Gene accession IDs	Putative functions	Sequence components of genome
3012	CakSNP3012	*Kabuli*	Ca_Kabuli_Chr02	32096180	[C/G]		--	INTERGENIC
3013	CakSNP3013	*Kabuli*	Ca_Kabuli_Chr02	32096177	[C/T]		--	INTERGENIC
3014	CakSNP3014	*Kabuli*	Ca_Kabuli_Chr02	32096172	[C/T]		--	INTERGENIC
3015	CakSNP3015	*Kabuli*	Ca_Kabuli_Chr02	32099757	[C/T]		--	INTERGENIC
3016	CakSNP3016	*Kabuli*	Ca_Kabuli_Chr02	32099907	[T/C]		--	INTERGENIC
3017	CakSNP3017	*Kabuli*	Ca_Kabuli_Chr02	32109805	[G/A]		--	INTERGENIC
3018	CakSNP3018	*Kabuli*	Ca_Kabuli_Chr02	32109811	[G/A]		--	INTERGENIC
3019	CakSNP3019	*Kabuli*	Ca_Kabuli_Chr02	32109813	[C/T]		--	INTERGENIC
3020	CakSNP3020	*Kabuli*	Ca_Kabuli_Chr02	32109821	[G/A]		--	INTERGENIC
3021	CakSNP3021	*Kabuli*	Ca_Kabuli_Chr02	32109827	[T/C]		--	INTERGENIC

S.N.	SNP IDs	Chickpea cultivars	Chromosomes	Physical positions (bp)	SNPs	Gene accession IDs	Putative functions	Sequence components of genome
3022	CakSNP3022	*Kabuli*	Ca_Kabuli_Chr02	32109859	[A/C]		--	INTERGENIC
3023	CakSNP3023	*Kabuli*	Ca_Kabuli_Chr02	32109904	[T/A]		--	INTERGENIC
3024	CakSNP3024	*Kabuli*	Ca_Kabuli_Chr02	32109895	[A/G]		--	INTERGENIC
3025	CakSNP3025	*Kabuli*	Ca_Kabuli_Chr02	32109892	[T/A]		--	INTERGENIC
3026	CakSNP3026	*Kabuli*	Ca_Kabuli_Chr02	32119094	[A/T]		--	INTERGENIC
3027	CakSNP3027	*Kabuli*	Ca_Kabuli_Chr02	32119268	[T/A]		--	INTERGENIC
3028	CakSNP3028	*Kabuli*	Ca_Kabuli_Chr02	32119274	[T/C]		--	INTERGENIC
3029	CakSNP3029	*Kabuli*	Ca_Kabuli_Chr02	32119331	[A/T]		--	INTERGENIC
3030	CakSNP3030	*Kabuli*	Ca_Kabuli_Chr02	32129272	[T/C]	Ca_17811	Phenylalanyl-tRNA synthetase	INTRON
3031	CakSNP3031	*Kabuli*	Ca_Kabuli_Chr02	32129395	[C/G]	Ca_17811	Phenylalanyl-tRNA synthetase	INTRON

S.N.	SNP IDs	Chickpea cultivars	Chromosomes	Physical positions (bp)	SNPs	Gene accession IDs	Putative functions	Sequence components of genome
3032	CakSNP3032	*Kabuli*	Ca_Kabuli_Chr02	32140939	[A/T]	Ca_17808	Peptidase S8/S53, subtilisin/kexin/sedolisin	SYNONYMOUS_CODING
3033	CakSNP3033	*Kabuli*	Ca_Kabuli_Chr02	32140948	[A/C]	Ca_17808	Peptidase S8/S53, subtilisin/kexin/sedolisin	SYNONYMOUS_CODING
3034	CakSNP3034	*Kabuli*	Ca_Kabuli_Chr02	32140987	[A/G]	Ca_17808	Peptidase S8/S53, subtilisin/kexin/sedolisin	SYNONYMOUS_CODING
3035	CakSNP3035	*Kabuli*	Ca_Kabuli_Chr02	32141047	[A/G]	Ca_17808	Peptidase S8/S53, subtilisin/kexin/sedolisin	SYNONYMOUS_CODING
3036	CakSNP3036	*Kabuli*	Ca_Kabuli_Chr02	32141038	[T/C]	Ca_17808	Peptidase S8/S53, subtilisin/kexin/sedolisin	SYNONYMOUS_CODING
3037	CakSNP3037	*Kabuli*	Ca_Kabuli_Chr02	32141019	[G/A]	Ca_17808	Peptidase S8/S53, subtilisin/kexin/sedolisin	SYNONYMOUS_CODING
3038	CakSNP3038	*Kabuli*	Ca_Kabuli_Chr02	32141013	[G/C]	Ca_17808	Peptidase S8/S53, subtilisin/kexin/sedolisin	NON_SYNONYMOUS_CODING
3039	CakSNP3039	*Kabuli*	Ca_Kabuli_Chr02	32141002	[A/T]	Ca_17808	Peptidase S8/S53, subtilisin/kexin/sedolisin	SYNONYMOUS_CODING
3040	CakSNP3040	*Kabuli*	Ca_Kabuli_Chr02	32140998	[G/T]	Ca_17808	Peptidase S8/S53, subtilisin/kexin/sedolisin	NON_SYNONYMOUS_CODING
3041	CakSNP3041	*Kabuli*	Ca_Kabuli_Chr02	32140990	[G/A]	Ca_17808	Peptidase S8/S53, subtilisin/kexin/sedolisin	SYNONYMOUS_CODING

S.N.	SNP IDs	Chickpea cultivars	Chromosomes	Physical positions (bp)	SNPs	Gene accession IDs	Putative functions	Sequence components of genome
3042	CakSNP3042	*Kabuli*	Ca_Kabuli_Chr02	32203032	[A/G]	Ca_10295	Peptidase S8/S53, subtilisin/kexin/sedolisin	SYNONYMOUS_CODING
3043	CakSNP3043	*Kabuli*	Ca_Kabuli_Chr02	32203036	[A/T]	Ca_10295	Peptidase S8/S53, subtilisin/kexin/sedolisin	NON_SYNONYMOUS_CODING
3044	CakSNP3044	*Kabuli*	Ca_Kabuli_Chr02	32242818	[T/G]	Ca_10292	Protein kinase, catalytic domain	NON_SYNONYMOUS_CODING
3045	CakSNP3045	*Kabuli*	Ca_Kabuli_Chr02	32348054	[T/A]	Ca_10283	--	INTRON
3046	CakSNP3046	*Kabuli*	Ca_Kabuli_Chr02	32348087	[A/C]	Ca_10283	--	INTRON
3047	CakSNP3047	*Kabuli*	Ca_Kabuli_Chr02	32348119	[G/T]	Ca_10283	--	INTRON
3048	CakSNP3048	*Kabuli*	Ca_Kabuli_Chr02	32348793	[G/A]	Ca_10283	--	INTRON
3049	CakSNP3049	*Kabuli*	Ca_Kabuli_Chr02	32348870	[T/C]	Ca_10283	--	INTRON
3050	CakSNP3050	*Kabuli*	Ca_Kabuli_Chr02	32411994	[G/T]		--	INTERGENIC
3051	CakSNP3051	*Kabuli*	Ca_Kabuli_Chr02	32412000	[G/T]		--	INTERGENIC

S.N.	SNP IDs	Chickpea cultivars	Chromosomes	Physical positions (bp)	SNPs	Gene accession IDs	Putative functions	Sequence components of genome
3052	CakSNP3052	*Kabuli*	Ca_Kabuli_Chr02	32481969	[C/T]		--	INTERGENIC
3053	CakSNP3053	*Kabuli*	Ca_Kabuli_Chr02	32481983	[G/T]		--	INTERGENIC
3054	CakSNP3054	*Kabuli*	Ca_Kabuli_Chr02	32484223	[C/T]	Ca_10272	Domain of unknown function DUF221	INTRON
3055	CakSNP3055	*Kabuli*	Ca_Kabuli_Chr02	32545320	[A/G]	Ca_10268	Zinc finger, CCHC-type	SYNONYMOUS_CODING
3056	CakSNP3056	*Kabuli*	Ca_Kabuli_Chr02	32545329	[T/C]	Ca_10268	Zinc finger, CCHC-type	SYNONYMOUS_CODING
3057	CakSNP3057	*Kabuli*	Ca_Kabuli_Chr02	32545351	[G/A]	Ca_10268	Zinc finger, CCHC-type	NON_SYNONYMOUS_CODING
3058	CakSNP3058	*Kabuli*	Ca_Kabuli_Chr02	32556992	[T/A]	Ca_10267	Protein kinase, catalytic domain	INTRON
3059	CakSNP3059	*Kabuli*	Ca_Kabuli_Chr02	32564427	[T/C]	Ca_10266	RecF/RecN/SMC	INTRON
3060	CakSNP3060	*Kabuli*	Ca_Kabuli_Chr02	32564480	[G/A]	Ca_10266	RecF/RecN/SMC	INTRON
3061	CakSNP3061	*Kabuli*	Ca_Kabuli_Chr02	32564770	[G/T]	Ca_10266	RecF/RecN/SMC	INTRON

S.N.	SNP IDs	Chickpea cultivars	Chromosomes	Physical positions (bp)	SNPs	Gene accession IDs	Putative functions	Sequence components of genome
3062	CakSNP3062	*Kabuli*	Ca_Kabuli_Chr02	32564796	[A/T]	Ca_10266	RecF/RecN/SMC	INTRON
3063	CakSNP3063	*Kabuli*	Ca_Kabuli_Chr02	32570394	[G/A]	Ca_10266	RecF/RecN/SMC	INTRON
3064	CakSNP3064	*Kabuli*	Ca_Kabuli_Chr02	32570479	[T/G]	Ca_10266	RecF/RecN/SMC	INTRON
3065	CakSNP3065	*Kabuli*	Ca_Kabuli_Chr02	32570429	[G/A]	Ca_10266	RecF/RecN/SMC	INTRON
3066	CakSNP3066	*Kabuli*	Ca_Kabuli_Chr02	32570504	[G/C]	Ca_10266	RecF/RecN/SMC	INTRON
3067	CakSNP3067	*Kabuli*	Ca_Kabuli_Chr02	32577274	[T/A]	Ca_10266	RecF/RecN/SMC	INTRON
3068	CakSNP3068	*Kabuli*	Ca_Kabuli_Chr02	32577593	[A/G]	Ca_10266	RecF/RecN/SMC	SYNONYMOUS_CODING
3069	CakSNP3069	*Kabuli*	Ca_Kabuli_Chr02	32579849	[T/C]	Ca_10266	RecF/RecN/SMC	SYNONYMOUS_CODING
3070	CakSNP3070	*Kabuli*	Ca_Kabuli_Chr02	32579900	[C/G]	Ca_10266	RecF/RecN/SMC	SYNONYMOUS_CODING
3071	CakSNP3071	*Kabuli*	Ca_Kabuli_Chr02	32608900	[A/T]	Ca_10261	Aldehyde oxidase/xanthine dehydrogenase, a/b hammerhead	NON_SYNONYMOUS_CODING

S.N.	SNP IDs	Chickpea cultivars	Chromosomes	Physical positions (bp)	SNPs	Gene accession IDs	Putative functions	Sequence components of genome
3072	CakSNP3072	*Kabuli*	Ca_Kabuli_Chr02	32611624	[C/T]	Ca_10261	Aldehyde oxidase/xanthine dehydrogenase, a/b hammerhead	SYNONYMOUS_CODING
3073	CakSNP3073	*Kabuli*	Ca_Kabuli_Chr02	32611609	[A/G]	Ca_10261	Aldehyde oxidase/xanthine dehydrogenase, a/b hammerhead	SYNONYMOUS_CODING
3074	CakSNP3074	*Kabuli*	Ca_Kabuli_Chr02	32611601	[G/T]	Ca_10261	Aldehyde oxidase/xanthine dehydrogenase, a/b hammerhead	NON_SYNONYMOUS_CODING
3075	CakSNP3075	*Kabuli*	Ca_Kabuli_Chr02	32627727	[C/G]	Ca_10259	Aldehyde oxidase/xanthine dehydrogenase, a/b hammerhead	INTRON
3076	CakSNP3076	*Kabuli*	Ca_Kabuli_Chr02	32629763	[A/G]	Ca_10259	Aldehyde oxidase/xanthine dehydrogenase, a/b hammerhead	NON_SYNONYMOUS_CODING
3077	CakSNP3077	*Kabuli*	Ca_Kabuli_Chr02	32670054	[C/T]	Ca_10254	BTB/POZ-like	SYNONYMOUS_CODING
3078	CakSNP3078	*Kabuli*	Ca_Kabuli_Chr02	32670099	[C/T]	Ca_10254	BTB/POZ-like	SYNONYMOUS_CODING
3079	CakSNP3079	*Kabuli*	Ca_Kabuli_Chr02	32670181	[T/G]	Ca_10254	BTB/POZ-like	NON_SYNONYMOUS_CODING
3080	CakSNP3080	*Kabuli*	Ca_Kabuli_Chr02	32671354	[T/A]	Ca_10254	BTB/POZ-like	INTRON
3081	CakSNP3081	*Kabuli*	Ca_Kabuli_Chr02	32781880	[A/G]	Ca_10243	Monooxygenase, FAD-binding	SYNONYMOUS_CODING

S.N.	SNP IDs	Chickpea cultivars	Chromosomes	Physical positions (bp)	SNPs	Gene accession IDs	Putative functions	Sequence components of genome
3082	CakSNP3082	*Kabuli*	Ca_Kabuli_Chr02	32844754	[T/G]	Ca_10238	Late embryogenesis abundant protein, group 2	NON_SYNONYMOUS_CODING
3083	CakSNP3083	*Kabuli*	Ca_Kabuli_Chr02	32844842	[C/G]	Ca_10238	Late embryogenesis abundant protein, group 2	NON_SYNONYMOUS_CODING
3084	CakSNP3084	*Kabuli*	Ca_Kabuli_Chr02	32867125	[A/G]	Ca_10236	Ethylene insensitive 3	SYNONYMOUS_CODING
3085	CakSNP3085	*Kabuli*	Ca_Kabuli_Chr02	32934646	[C/T]	Ca_10230	Protein of unknown function DM15	INTRON
3086	CakSNP3086	*Kabuli*	Ca_Kabuli_Chr02	32934633	[G/C]	Ca_10230	Protein of unknown function DM15	INTRON
3087	CakSNP3087	*Kabuli*	Ca_Kabuli_Chr02	32936855	[A/G]	Ca_10230	Protein of unknown function DM15	NON_SYNONYMOUS_CODING
3088	CakSNP3088	*Kabuli*	Ca_Kabuli_Chr02	32936908	[G/A]	Ca_10230	Protein of unknown function DM15	NON_SYNONYMOUS_CODING
3089	CakSNP3089	*Kabuli*	Ca_Kabuli_Chr02	32957515	[A/G]	Ca_10228	Basic-leucine zipper (bZIP) transcription factor	INTRON
3090	CakSNP3090	*Kabuli*	Ca_Kabuli_Chr02	32957523	[T/C]	Ca_10228	Basic-leucine zipper (bZIP) transcription factor	INTRON
3091	CakSNP3091	*Kabuli*	Ca_Kabuli_Chr02	32957538	[A/C]	Ca_10228	Basic-leucine zipper (bZIP) transcription factor	INTRON

S.N.	SNP IDs	Chickpea cultivars	Chromosomes	Physical positions (bp)	SNPs	Gene accession IDs	Putative functions	Sequence components of genome
3092	CakSNP3092	*Kabuli*	Ca_Kabuli_Chr02	32957572	[A/C]	Ca_10228	Basic-leucine zipper (bZIP) transcription factor	INTRON
3093	CakSNP3093	*Kabuli*	Ca_Kabuli_Chr02	32957958	[A/C]	Ca_10228	Basic-leucine zipper (bZIP) transcription factor	NON_SYNONYMOUS_CODING
3094	CakSNP3094	*Kabuli*	Ca_Kabuli_Chr02	32958034	[C/G]		--	INTERGENIC
3095	CakSNP3095	*Kabuli*	Ca_Kabuli_Chr02	32989134	[G/T]	Ca_10225	UBX	NON_SYNONYMOUS_CODING
3096	CakSNP3096	*Kabuli*	Ca_Kabuli_Chr02	32989205	[G/T]	Ca_10225	UBX	SYNONYMOUS_CODING
3097	CakSNP3097	*Kabuli*	Ca_Kabuli_Chr02	33083903	[T/A]	Ca_10214	Translation elongation factor EF1B, beta/delta chains, conserved site	INTRON
3098	CakSNP3098	*Kabuli*	Ca_Kabuli_Chr02	33084095	[T/G]	Ca_10214	Translation elongation factor EF1B, beta/delta chains, conserved site	INTRON
3099	CakSNP3099	*Kabuli*	Ca_Kabuli_Chr02	33084132	[T/A]	Ca_10214	Translation elongation factor EF1B, beta/delta chains, conserved site	INTRON
3100	CakSNP3100	*Kabuli*	Ca_Kabuli_Chr02	33103318	[G/A]	Ca_10211	Ubiquitin-associated/translation elongation factor EF1B, N-terminal, eukaryote	SYNONYMOUS_CODING
3101	CakSNP3101	*Kabuli*	Ca_Kabuli_Chr02	33103466	[G/A]	Ca_10211	Ubiquitin-associated/translation elongation factor EF1B, N-terminal, eukaryote	NON_SYNONYMOUS_CODING

S.N.	SNP IDs	Chickpea cultivars	Chromosomes	Physical positions (bp)	SNPs	Gene accession IDs	Putative functions	Sequence components of genome
3102	CakSNP3102	*Kabuli*	Ca_Kabuli_Chr02	33103750	[A/G]	Ca_10211	Ubiquitin-associated/translation elongation factor EF1B, N-terminal, eukaryote	SYNONYMOUS_CODING
3103	CakSNP3103	*Kabuli*	Ca_Kabuli_Chr02	33103766	[G/A]	Ca_10211	Ubiquitin-associated/translation elongation factor EF1B, N-terminal, eukaryote	SYNONYMOUS_CODING
3104	CakSNP3104	*Kabuli*	Ca_Kabuli_Chr02	33172936	[C/T]	Ca_10204	Plastocyanin-like	SYNONYMOUS_CODING
3105	CakSNP3105	*Kabuli*	Ca_Kabuli_Chr02	33172971	[T/G]	Ca_10204	Plastocyanin-like	NON_SYNONYMOUS_CODING
3106	CakSNP3106	*Kabuli*	Ca_Kabuli_Chr02	33178391	[G/A]	Ca_10203	--	INTRON
3107	CakSNP3107	*Kabuli*	Ca_Kabuli_Chr02	33178407	[C/T]	Ca_10203	--	INTRON
3108	CakSNP3108	*Kabuli*	Ca_Kabuli_Chr02	33178419	[C/A]	Ca_10203	--	INTRON
3109	CakSNP3109	*Kabuli*	Ca_Kabuli_Chr02	33178420	[A/C]	Ca_10203	--	INTRON
3110	CakSNP3110	*Kabuli*	Ca_Kabuli_Chr02	33178506	[T/C]	Ca_10203	--	SYNONYMOUS_CODING
3111	CakSNP3111	*Kabuli*	Ca_Kabuli_Chr02	33184262	[T/C]	Ca_10203	--	NON_SYNONYMOUS_CODING

S.N.	SNP IDs	Chickpea cultivars	Chromosomes	Physical positions (bp)	SNPs	Gene accession IDs	Putative functions	Sequence components of genome
3112	CakSNP3112	*Kabuli*	Ca_Kabuli_Chr02	33194198	[A/G]	Ca_10203	--	SYNONYMOUS_CODING
3113	CakSNP3113	*Kabuli*	Ca_Kabuli_Chr02	33204253	[C/T]	Ca_10202	--	INTRON
3114	CakSNP3114	*Kabuli*	Ca_Kabuli_Chr02	33326392	[T/G]	Ca_10195	--	NON_SYNONYMOUS_CODING
3115	CakSNP3115	*Kabuli*	Ca_Kabuli_Chr02	33326398	[C/T]	Ca_10195	--	SYNONYMOUS_CODING
3116	CakSNP3116	*Kabuli*	Ca_Kabuli_Chr02	33326444	[G/A]	Ca_10195	--	NON_SYNONYMOUS_CODING
3117	CakSNP3117	*Kabuli*	Ca_Kabuli_Chr02	33326522	[G/A]	Ca_10195	--	NON_SYNONYMOUS_CODING
3118	CakSNP3118	*Kabuli*	Ca_Kabuli_Chr02	33326520	[T/C]	Ca_10195	--	NON_SYNONYMOUS_CODING
3119	CakSNP3119	*Kabuli*	Ca_Kabuli_Chr02	33326516	[G/A]	Ca_10195	--	NON_SYNONYMOUS_CODING
3120	CakSNP3120	*Kabuli*	Ca_Kabuli_Chr02	33326510	[C/G]	Ca_10195	--	NON_SYNONYMOUS_CODING
3121	CakSNP3121	*Kabuli*	Ca_Kabuli_Chr02	33393209	[T/C]		--	INTERGENIC

S.N.	SNP IDs	Chickpea cultivars	Chromosomes	Physical positions (bp)	SNPs	Gene accession IDs	Putative functions	Sequence components of genome
3122	CakSNP3122	*Kabuli*	Ca_Kabuli_Chr02	33398561	[C/T]		--	INTERGENIC
3123	CakSNP3123	*Kabuli*	Ca_Kabuli_Chr02	33398550	[G/A]		--	INTERGENIC
3124	CakSNP3124	*Kabuli*	Ca_Kabuli_Chr02	33398549	[T/C]		--	INTERGENIC
3125	CakSNP3125	*Kabuli*	Ca_Kabuli_Chr02	33398527	[T/G]		--	INTERGENIC
3126	CakSNP3126	*Kabuli*	Ca_Kabuli_Chr02	33399010	[C/T]	Ca_10186	Disease resistance protein	NON_SYNONYMOUS_CODING
3127	CakSNP3127	*Kabuli*	Ca_Kabuli_Chr02	33399019	[T/C]	Ca_10186	Disease resistance protein	NON_SYNONYMOUS_CODING
3128	CakSNP3128	*Kabuli*	Ca_Kabuli_Chr02	33399045	[A/C]	Ca_10186	Disease resistance protein	SYNONYMOUS_CODING
3129	CakSNP3129	*Kabuli*	Ca_Kabuli_Chr02	33399098	[G/A]	Ca_10186	Disease resistance protein	NON_SYNONYMOUS_CODING
3130	CakSNP3130	*Kabuli*	Ca_Kabuli_Chr02	33399063	[C/T]	Ca_10186	Disease resistance protein	SYNONYMOUS_CODING
3131	CakSNP3131	*Kabuli*	Ca_Kabuli_Chr02	33399405	[C/T]	Ca_10186	Disease resistance protein	SYNONYMOUS_CODING

S.N.	SNP IDs	Chickpea cultivars	Chromosomes	Physical positions (bp)	SNPs	Gene accession IDs	Putative functions	Sequence components of genome
3132	CakSNP3132	*Kabuli*	Ca_Kabuli_Chr02	33399404	[C/A]	Ca_10186	Disease resistance protein	NON_SYNONYMOUS_CODING
3133	CakSNP3133	*Kabuli*	Ca_Kabuli_Chr02	33399397	[C/T]	Ca_10186	Disease resistance protein	NON_SYNONYMOUS_CODING
3134	CakSNP3134	*Kabuli*	Ca_Kabuli_Chr02	33399393	[C/A]	Ca_10186	Disease resistance protein	NON_SYNONYMOUS_CODING
3135	CakSNP3135	*Kabuli*	Ca_Kabuli_Chr02	33399381	[C/T]	Ca_10186	Disease resistance protein	SYNONYMOUS_CODING
3136	CakSNP3136	*Kabuli*	Ca_Kabuli_Chr02	33399342	[A/C]	Ca_10186	Disease resistance protein	NON_SYNONYMOUS_CODING
3137	CakSNP3137	*Kabuli*	Ca_Kabuli_Chr02	33399465	[C/G]	Ca_10186	Disease resistance protein	NON_SYNONYMOUS_CODING
3138	CakSNP3138	*Kabuli*	Ca_Kabuli_Chr02	33399608	[C/A]	Ca_10186	Disease resistance protein	NON_SYNONYMOUS_CODING
3139	CakSNP3139	*Kabuli*	Ca_Kabuli_Chr02	33401608	[A/G]	Ca_10186	Disease resistance protein	SYNONYMOUS_CODING
3140	CakSNP3140	*Kabuli*	Ca_Kabuli_Chr02	33490058	[C/A]	Ca_10183	Telomerase activating protein Est1	NON_SYNONYMOUS_CODING
3141	CakSNP3141	*Kabuli*	Ca_Kabuli_Chr02	33489989	[T/C]	Ca_10183	Telomerase activating protein Est1	SYNONYMOUS_CODING

S.N.	SNP IDs	Chickpea cultivars	Chromosomes	Physical positions (bp)	SNPs	Gene accession IDs	Putative functions	Sequence components of genome
3142	CakSNP3142	*Kabuli*	Ca_Kabuli_Chr02	33575286	[A/C]		--	INTERGENIC
3143	CakSNP3143	*Kabuli*	Ca_Kabuli_Chr02	33582948	[G/A]	Ca_10177	Peptidase C48, SUMO/Sentrin/Ubl1	NON_SYNONYMOUS_CODING
3144	CakSNP3144	*Kabuli*	Ca_Kabuli_Chr02	33582900	[G/A]	Ca_10177	Peptidase C48, SUMO/Sentrin/Ubl1	NON_SYNONYMOUS_CODING
3145	CakSNP3145	*Kabuli*	Ca_Kabuli_Chr02	33650447	[A/T]		--	INTERGENIC
3146	CakSNP3146	*Kabuli*	Ca_Kabuli_Chr02	33651426	[G/A]	Ca_10173	Pentatricopeptide repeat	SYNONYMOUS_CODING
3147	CakSNP3147	*Kabuli*	Ca_Kabuli_Chr02	33651408	[A/G]		--	INTERGENIC
3148	CakSNP3148	*Kabuli*	Ca_Kabuli_Chr02	33661177	[C/T]	Ca_10172	--	SYNONYMOUS_CODING
3149	CakSNP3149	*Kabuli*	Ca_Kabuli_Chr02	33661316	[T/C]		--	INTERGENIC
3150	CakSNP3150	*Kabuli*	Ca_Kabuli_Chr02	33686794	[G/A]	Ca_10170	GTP-binding protein, HSR1-related	INTRON
3151	CakSNP3151	*Kabuli*	Ca_Kabuli_Chr02	33692368	[C/A]		--	INTERGENIC

S.N.	SNP IDs	Chickpea cultivars	Chromosomes	Physical positions (bp)	SNPs	Gene accession IDs	Putative functions	Sequence components of genome
3152	CakSNP3152	*Kabuli*	Ca_Kabuli_Chr02	33713018	[G/A]		--	INTERGENIC
3153	CakSNP3153	*Kabuli*	Ca_Kabuli_Chr02	33721515	[T/A]	Ca_10165	SNF2-related	INTRON
3154	CakSNP3154	*Kabuli*	Ca_Kabuli_Chr02	33733893	[C/T]		--	INTERGENIC
3155	CakSNP3155	*Kabuli*	Ca_Kabuli_Chr02	33745281	[A/G]	Ca_10163	Protein kinase, catalytic domain	NON_SYNONYMOUS_CODING
3156	CakSNP3156	*Kabuli*	Ca_Kabuli_Chr02	33764634	[G/A]	Ca_10161	ATPase, BadF/BadG/BcrA/BcrD type	SYNONYMOUS_CODING
3157	CakSNP3157	*Kabuli*	Ca_Kabuli_Chr02	33783871	[C/T]	Ca_10158	Multi antimicrobial extrusion protein	INTRON
3158	CakSNP3158	*Kabuli*	Ca_Kabuli_Chr02	33785203	[G/A]		--	INTERGENIC
3159	CakSNP3159	*Kabuli*	Ca_Kabuli_Chr02	33786243	[G/A]	Ca_10157	Amine oxidase	NON_SYNONYMOUS_CODING
3160	CakSNP3160	*Kabuli*	Ca_Kabuli_Chr02	33788913	[A/C]	Ca_10157	Amine oxidase	SYNONYMOUS_CODING
3161	CakSNP3161	*Kabuli*	Ca_Kabuli_Chr02	33798717	[T/C]		--	INTERGENIC

S.N.	SNP IDs	Chickpea cultivars	Chromosomes	Physical positions (bp)	SNPs	Gene accession IDs	Putative functions	Sequence components of genome
3162	CakSNP3162	*Kabuli*	Ca_Kabuli_Chr02	33798748	[T/C]		--	INTERGENIC
3163	CakSNP3163	*Kabuli*	Ca_Kabuli_Chr02	34152352	[T/C]	Ca_15238	--	INTRON
3164	CakSNP3164	*Kabuli*	Ca_Kabuli_Chr02	34152384	[T/C]	Ca_15238	--	SYNONYMOUS_CODING
3165	CakSNP3165	*Kabuli*	Ca_Kabuli_Chr02	34152476	[C/T]	Ca_15238	--	SYNONYMOUS_CODING
3166	CakSNP3166	*Kabuli*	Ca_Kabuli_Chr02	34155090	[A/G]	Ca_15238	--	INTRON
3167	CakSNP3167	*Kabuli*	Ca_Kabuli_Chr02	34155179	[A/G]	Ca_15238	--	INTRON
3168	CakSNP3168	*Kabuli*	Ca_Kabuli_Chr02	34178534	[G/A]	Ca_15241	Bacterial Fmu (Sun)/eukaryotic nucleolar NOL1/Nop2p	INTRON
3169	CakSNP3169	*Kabuli*	Ca_Kabuli_Chr02	34203162	[T/C]	Ca_15245	--	NON_SYNONYMOUS_CODING
3170	CakSNP3170	*Kabuli*	Ca_Kabuli_Chr02	34203444	[T/C]		--	INTERGENIC
3171	CakSNP3171	*Kabuli*	Ca_Kabuli_Chr02	34222585	[G/A]	Ca_15247	Conserved oligomeric Golgi complex, subunit 5	SYNONYMOUS_CODING

S.N.	SNP IDs	Chickpea cultivars	Chromosomes	Physical positions (bp)	SNPs	Gene accession IDs	Putative functions	Sequence components of genome
3172	CakSNP3172	*Kabuli*	Ca_Kabuli_Chr02	34249987	[C/T]	Ca_15249	Domain of unknown function DUF1084	INTRON
3173	CakSNP3173	*Kabuli*	Ca_Kabuli_Chr02	34249953	[G/T]	Ca_15249	Domain of unknown function DUF1084	INTRON
3174	CakSNP3174	*Kabuli*	Ca_Kabuli_Chr02	34250042	[C/T]	Ca_15249	Domain of unknown function DUF1084	SYNONYMOUS_CODING
3175	CakSNP3175	*Kabuli*	Ca_Kabuli_Chr02	34250266	[T/C]	Ca_15249	Domain of unknown function DUF1084	NON_SYNONYMOUS_CODING
3176	CakSNP3176	*Kabuli*	Ca_Kabuli_Chr02	34266109	[A/G]	Ca_15250	Protein kinase, catalytic domain	NON_SYNONYMOUS_CODING
3177	CakSNP3177	*Kabuli*	Ca_Kabuli_Chr02	34288116	[G/C]	Ca_15253	Potassium channel, two pore-domain	INTRON
3178	CakSNP3178	*Kabuli*	Ca_Kabuli_Chr02	34299096	[G/A]	Ca_15255	Domain of unknown function DUF296	SYNONYMOUS_CODING
3179	CakSNP3179	*Kabuli*	Ca_Kabuli_Chr02	34299075	[C/T]	Ca_15255	Domain of unknown function DUF296	SYNONYMOUS_CODING
3180	CakSNP3180	*Kabuli*	Ca_Kabuli_Chr02	34299074	[A/C]	Ca_15255	Domain of unknown function DUF296	NON_SYNONYMOUS_CODING
3181	CakSNP3181	*Kabuli*	Ca_Kabuli_Chr02	34299063	[G/A]	Ca_15255	Domain of unknown function DUF296	SYNONYMOUS_CODING

S.N.	SNP IDs	Chickpea cultivars	Chromosomes	Physical positions (bp)	SNPs	Gene accession IDs	Putative functions	Sequence components of genome
3182	CakSNP3182	*Kabuli*	Ca_Kabuli_Chr02	34299079	[A/C]	Ca_15255	Domain of unknown function DUF296	NON_SYNONYMOUS_CODING
3183	CakSNP3183	*Kabuli*	Ca_Kabuli_Chr02	34299846	[C/T]	Ca_15256	Pentatricopeptide repeat	NON_SYNONYMOUS_CODING
3184	CakSNP3184	*Kabuli*	Ca_Kabuli_Chr02	34300059	[A/G]	Ca_15256	Pentatricopeptide repeat	NON_SYNONYMOUS_CODING
3185	CakSNP3185	*Kabuli*	Ca_Kabuli_Chr02	34300057	[A/G]	Ca_15256	Pentatricopeptide repeat	SYNONYMOUS_CODING
3186	CakSNP3186	*Kabuli*	Ca_Kabuli_Chr02	34343478	[C/G]	Ca_15262	--	NON_SYNONYMOUS_CODING
3187	CakSNP3187	*Kabuli*	Ca_Kabuli_Chr02	34343705	[G/A]	Ca_15262	--	STOP_GAINED
3188	CakSNP3188	*Kabuli*	Ca_Kabuli_Chr02	34346849	[C/T]		--	INTERGENIC
3189	CakSNP3189	*Kabuli*	Ca_Kabuli_Chr02	34346842	[T/C]		--	INTERGENIC
3190	CakSNP3190	*Kabuli*	Ca_Kabuli_Chr02	34372503	[T/G]	Ca_15264	--	NON_SYNONYMOUS_CODING
3191	CakSNP3191	*Kabuli*	Ca_Kabuli_Chr02	34428978	[C/A]		--	INTERGENIC

S.N.	SNP IDs	Chickpea cultivars	Chromosomes	Physical positions (bp)	SNPs	Gene accession IDs	Putative functions	Sequence components of genome
3192	CakSNP3192	*Kabuli*	Ca_Kabuli_Chr02	34428979	[G/C]		--	INTERGENIC
3193	CakSNP3193	*Kabuli*	Ca_Kabuli_Chr02	34429029	[G/T]		--	INTERGENIC
3194	CakSNP3194	*Kabuli*	Ca_Kabuli_Chr02	34429030	[A/T]		--	INTERGENIC
3195	CakSNP3195	*Kabuli*	Ca_Kabuli_Chr02	34551849	[A/C]	Ca_15281	Like-Sm ribonucleoprotein (LSM) domain	SYNONYMOUS_CODING
3196	CakSNP3196	*Kabuli*	Ca_Kabuli_Chr02	34554164	[A/C]	Ca_15282	Drug/metabolite transporter	NON_SYNONYMOUS_CODING
3197	CakSNP3197	*Kabuli*	Ca_Kabuli_Chr02	34601444	[C/A]	Ca_15284	Lipocalin	SYNONYMOUS_CODING
3198	CakSNP3198	*Kabuli*	Ca_Kabuli_Chr02	34634128	[C/T]	Ca_15290	Cytochrome P450	NON_SYNONYMOUS_CODING
3199	CakSNP3199	*Kabuli*	Ca_Kabuli_Chr02	34638960	[A/C]	Ca_15291	Uncharacterised protein family UPF0136, Transmembrane	INTRON
3200	CakSNP3200	*Kabuli*	Ca_Kabuli_Chr02	34638955	[T/A]	Ca_15291	Uncharacterised protein family UPF0136, Transmembrane	INTRON
3201	CakSNP3201	*Kabuli*	Ca_Kabuli_Chr02	34734329	[G/A]	Ca_16897	Heat shock protein Hsp70	NON_SYNONYMOUS_CODING

S.N.	SNP IDs	Chickpea cultivars	Chromosomes	Physical positions (bp)	SNPs	Gene accession IDs	Putative functions	Sequence components of genome
3202	CakSNP3202	*Kabuli*	Ca_Kabuli_Chr02	34734379	[G/A]	Ca_16897	Heat shock protein Hsp70	SYNONYMOUS_CODING
3203	CakSNP3203	*Kabuli*	Ca_Kabuli_Chr02	34734439	[G/A]	Ca_16897	Heat shock protein Hsp70	SYNONYMOUS_CODING
3204	CakSNP3204	*Kabuli*	Ca_Kabuli_Chr02	34734472	[A/G]	Ca_16897	Heat shock protein Hsp70	SYNONYMOUS_CODING
3205	CakSNP3205	*Kabuli*	Ca_Kabuli_Chr02	34736649	[T/C]	Ca_16897	Heat shock protein Hsp70	INTRON
3206	CakSNP3206	*Kabuli*	Ca_Kabuli_Chr02	34736658	[A/C]	Ca_16897	Heat shock protein Hsp70	INTRON
3207	CakSNP3207	*Kabuli*	Ca_Kabuli_Chr02	34736753	[C/T]	Ca_16897	Heat shock protein Hsp70	NON_SYNONYMOUS_CODING
3208	CakSNP3208	*Kabuli*	Ca_Kabuli_Chr02	34754363	[T/C]		--	INTERGENIC
3209	CakSNP3209	*Kabuli*	Ca_Kabuli_Chr02	34754507	[C/T]		--	INTERGENIC
3210	CakSNP3210	*Kabuli*	Ca_Kabuli_Chr02	34820075	[C/T]	Ca_16887	Leucine-rich repeat, cysteine-containing subtype	SYNONYMOUS_CODING
3211	CakSNP3211	*Kabuli*	Ca_Kabuli_Chr02	34830995	[A/G]		--	INTERGENIC

S.N.	SNP IDs	Chickpea cultivars	Chromosomes	Physical positions (bp)	SNPs	Gene accession IDs	Putative functions	Sequence components of genome
3212	CakSNP3212	*Kabuli*	Ca_Kabuli_Chr02	34847681	[C/A]	Ca_16883	Bromodomain	SYNONYMOUS_CODING
3213	CakSNP3213	*Kabuli*	Ca_Kabuli_Chr02	34848053	[G/A]	Ca_16883	Bromodomain	SYNONYMOUS_CODING
3214	CakSNP3214	*Kabuli*	Ca_Kabuli_Chr02	34848480	[T/C]	Ca_16883	Bromodomain	NON_SYNONYMOUS_CODING
3215	CakSNP3215	*Kabuli*	Ca_Kabuli_Chr02	34862129	[G/T]	Ca_16882	Lipase, GDSL	SYNONYMOUS_CODING
3216	CakSNP3216	*Kabuli*	Ca_Kabuli_Chr02	34862135	[G/A]	Ca_16882	Lipase, GDSL	SYNONYMOUS_CODING
3217	CakSNP3217	*Kabuli*	Ca_Kabuli_Chr02	34862139	[A/G]	Ca_16882	Lipase, GDSL	NON_SYNONYMOUS_CODING
3218	CakSNP3218	*Kabuli*	Ca_Kabuli_Chr02	34862150	[C/T]	Ca_16882	Lipase, GDSL	SYNONYMOUS_CODING
3219	CakSNP3219	*Kabuli*	Ca_Kabuli_Chr02	34862276	[C/G]	Ca_16882	Lipase, GDSL	NON_SYNONYMOUS_CODING
3220	CakSNP3220	*Kabuli*	Ca_Kabuli_Chr02	34888414	[T/C]	Ca_16879	--	SYNONYMOUS_CODING
3221	CakSNP3221	*Kabuli*	Ca_Kabuli_Chr02	35013088	[G/C]		--	INTERGENIC

S.N.	SNP IDs	Chickpea cultivars	Chromosomes	Physical positions (bp)	SNPs	Gene accession IDs	Putative functions	Sequence components of genome
3222	CakSNP3222	*Kabuli*	Ca_Kabuli_Chr02	35013084	[T/C]		--	INTERGENIC
3223	CakSNP3223	*Kabuli*	Ca_Kabuli_Chr02	35052930	[C/A]	Ca_16868	Aspartate carbamoyltransferase, eukaryotic	INTRON
3224	CakSNP3224	*Kabuli*	Ca_Kabuli_Chr02	35054244	[C/A]	Ca_16868	Aspartate carbamoyltransferase, eukaryotic	INTRON
3225	CakSNP3225	*Kabuli*	Ca_Kabuli_Chr02	35059446	[A/T]	Ca_16867	UBA/THIF-type NAD/FAD binding fold	SYNONYMOUS_CODING
3226	CakSNP3226	*Kabuli*	Ca_Kabuli_Chr02	35059434	[T/C]	Ca_16867	UBA/THIF-type NAD/FAD binding fold	SYNONYMOUS_CODING
3227	CakSNP3227	*Kabuli*	Ca_Kabuli_Chr02	35059440	[A/C]	Ca_16867	UBA/THIF-type NAD/FAD binding fold	SYNONYMOUS_CODING
3228	CakSNP3228	*Kabuli*	Ca_Kabuli_Chr02	35059451	[C/A]	Ca_16867	UBA/THIF-type NAD/FAD binding fold	NON_SYNONYMOUS_CODING
3229	CakSNP3229	*Kabuli*	Ca_Kabuli_Chr02	35060171	[A/G]	Ca_16867	UBA/THIF-type NAD/FAD binding fold	INTRON
3230	CakSNP3230	*Kabuli*	Ca_Kabuli_Chr02	35060178	[A/T]	Ca_16867	UBA/THIF-type NAD/FAD binding fold	INTRON
3231	CakSNP3231	*Kabuli*	Ca_Kabuli_Chr02	35060228	[T/C]	Ca_16867	UBA/THIF-type NAD/FAD binding fold	INTRON

S.N.	SNP IDs	Chickpea cultivars	Chromosomes	Physical positions (bp)	SNPs	Gene accession IDs	Putative functions	Sequence components of genome
3232	CakSNP3232	*Kabuli*	Ca_Kabuli_Chr02	35060273	[G/A]	Ca_16867	UBA/THIF-type NAD/FAD binding fold	INTRON
3233	CakSNP3233	*Kabuli*	Ca_Kabuli_Chr02	35060264	[A/T]	Ca_16867	UBA/THIF-type NAD/FAD binding fold	INTRON
3234	CakSNP3234	*Kabuli*	Ca_Kabuli_Chr02	35066138	[G/A]	Ca_16866	Glycoside hydrolase, family 9	SYNONYMOUS_CODING
3235	CakSNP3235	*Kabuli*	Ca_Kabuli_Chr02	35095010	[A/G]	Ca_16862	Microtubule-associated protein, MAP65/ASE1-type	INTRON
3236	CakSNP3236	*Kabuli*	Ca_Kabuli_Chr02	35096648	[A/T]	Ca_16862	Microtubule-associated protein, MAP65/ASE1-type	INTRON
3237	CakSNP3237	*Kabuli*	Ca_Kabuli_Chr02	35096649	[A/T]	Ca_16862	Microtubule-associated protein, MAP65/ASE1-type	INTRON
3238	CakSNP3238	*Kabuli*	Ca_Kabuli_Chr02	35120761	[G/A]	Ca_16857	Metallo-dependent phosphatase	SYNONYMOUS_CODING
3239	CakSNP3239	*Kabuli*	Ca_Kabuli_Chr02	35122109	[C/G]	Ca_16857	Metallo-dependent phosphatase	INTRON
3240	CakSNP3240	*Kabuli*	Ca_Kabuli_Chr02	35154629	[C/A]	Ca_16852	Plant lipid transfer protein/seed storage/trypsin-alpha amylase inhibitor	NON_SYNONYMOUS_CODING
3241	CakSNP3241	*Kabuli*	Ca_Kabuli_Chr02	35154603	[G/A]	Ca_16852	Plant lipid transfer protein/seed storage/trypsin-alpha amylase inhibitor	NON_SYNONYMOUS_CODING

S.N.	SNP IDs	Chickpea cultivars	Chromosomes	Physical positions (bp)	SNPs	Gene accession IDs	Putative functions	Sequence components of genome
3242	CakSNP3242	*Kabuli*	Ca_Kabuli_Chr02	35171882	[T/G]	Ca_16849	Glycerophosphoryl diester phosphodiesterase	SYNONYMOUS_CODING
3243	CakSNP3243	*Kabuli*	Ca_Kabuli_Chr02	35189471	[C/T]		--	INTERGENIC
3244	CakSNP3244	*Kabuli*	Ca_Kabuli_Chr02	35195303	[T/C]	Ca_16845	Kinesin, motor domain	NON_SYNONYMOUS_CODING
3245	CakSNP3245	*Kabuli*	Ca_Kabuli_Chr02	35198352	[T/C]	Ca_16845	Kinesin, motor domain	INTRON
3246	CakSNP3246	*Kabuli*	Ca_Kabuli_Chr02	35209940	[C/T]	Ca_16844	C2 calcium-dependent membrane targeting	NON_SYNONYMOUS_CODING
3247	CakSNP3247	*Kabuli*	Ca_Kabuli_Chr02	35362587	[A/G]		--	INTERGENIC
3248	CakSNP3248	*Kabuli*	Ca_Kabuli_Chr02	35362642	[A/G]		--	INTERGENIC
3249	CakSNP3249	*Kabuli*	Ca_Kabuli_Chr02	35362657	[C/A]		--	INTERGENIC
3250	CakSNP3250	*Kabuli*	Ca_Kabuli_Chr02	35362729	[A/G]	Ca_09693	Frigida-like	SYNONYMOUS_CODING
3251	CakSNP3251	*Kabuli*	Ca_Kabuli_Chr02	35363488	[T/C]	Ca_09693	Frigida-like	SYNONYMOUS_CODING

S.N.	SNP IDs	Chickpea cultivars	Chromosomes	Physical positions (bp)	SNPs	Gene accession IDs	Putative functions	Sequence components of genome
3252	CakSNP3252	*Kabuli*	Ca_Kabuli_Chr02	35363501	[T/C]	Ca_09693	Frigida-like	NON_SYNONYMOUS_CODING
3253	CakSNP3253	*Kabuli*	Ca_Kabuli_Chr02	35365029	[C/T]	Ca_09693	Frigida-like	NON_SYNONYMOUS_CODING
3254	CakSNP3254	*Kabuli*	Ca_Kabuli_Chr02	35367161	[G/A]		--	INTERGENIC
3255	CakSNP3255	*Kabuli*	Ca_Kabuli_Chr02	35413400	[T/A]		--	INTERGENIC
3256	CakSNP3256	*Kabuli*	Ca_Kabuli_Chr02	35441184	[C/T]		--	INTERGENIC
3257	CakSNP3257	*Kabuli*	Ca_Kabuli_Chr02	35441180	[A/G]		--	INTERGENIC
3258	CakSNP3258	*Kabuli*	Ca_Kabuli_Chr02	35441174	[T/G]		--	INTERGENIC
3259	CakSNP3259	*Kabuli*	Ca_Kabuli_Chr02	35447976	[A/G]	Ca_09705	ABC transporter, transmembrane domain	SYNONYMOUS_CODING
3260	CakSNP3260	*Kabuli*	Ca_Kabuli_Chr02	35448018	[C/G]	Ca_09705	ABC transporter, transmembrane domain	SYNONYMOUS_CODING
3261	CakSNP3261	*Kabuli*	Ca_Kabuli_Chr02	35448126	[T/A]	Ca_09705	ABC transporter, transmembrane domain	SYNONYMOUS_CODING

S.N.	SNP IDs	Chickpea cultivars	Chromosomes	Physical positions (bp)	SNPs	Gene accession IDs	Putative functions	Sequence components of genome
3262	CakSNP3262	*Kabuli*	Ca_Kabuli_Chr02	35449653	[G/A]	Ca_09705	ABC transporter, transmembrane domain	INTRON
3263	CakSNP3263	*Kabuli*	Ca_Kabuli_Chr02	35473250	[C/T]		--	INTERGENIC
3264	CakSNP3264	*Kabuli*	Ca_Kabuli_Chr02	35505791	[C/G]	Ca_09713	NLI interacting factor	INTRON
3265	CakSNP3265	*Kabuli*	Ca_Kabuli_Chr02	35505874	[T/G]	Ca_09713	NLI interacting factor	INTRON
3266	CakSNP3266	*Kabuli*	Ca_Kabuli_Chr02	35533246	[G/A]	Ca_09716	Cysteine synthase/cystathionine beta-synthase P-phosphate-binding site	NON_SYNONYMOUS_CODING
3267	CakSNP3267	*Kabuli*	Ca_Kabuli_Chr02	35593439	[T/C]	Ca_09728	Bacterial Fmu (Sun)/eukaryotic nucleolar NOL1/Nop2p	SYNONYMOUS_STOP
3268	CakSNP3268	*Kabuli*	Ca_Kabuli_Chr02	35593797	[G/T]	Ca_09728	Bacterial Fmu (Sun)/eukaryotic nucleolar NOL1/Nop2p	SYNONYMOUS_CODING
3269	CakSNP3269	*Kabuli*	Ca_Kabuli_Chr02	35593824	[A/C]	Ca_09728	Bacterial Fmu (Sun)/eukaryotic nucleolar NOL1/Nop2p	SYNONYMOUS_CODING
3270	CakSNP3270	*Kabuli*	Ca_Kabuli_Chr02	35593827	[A/G]	Ca_09728	Bacterial Fmu (Sun)/eukaryotic nucleolar NOL1/Nop2p	SYNONYMOUS_CODING
3271	CakSNP3271	*Kabuli*	Ca_Kabuli_Chr02	35594018	[A/T]	Ca_09728	Bacterial Fmu (Sun)/eukaryotic nucleolar NOL1/Nop2p	INTRON

S.N.	SNP IDs	Chickpea cultivars	Chromosomes	Physical positions (bp)	SNPs	Gene accession IDs	Putative functions	Sequence components of genome
3272	CakSNP3272	*Kabuli*	Ca_Kabuli_Chr02	35594013	[C/T]	Ca_09728	Bacterial Fmu (Sun)/eukaryotic nucleolar NOL1/Nop2p	INTRON
3273	CakSNP3273	*Kabuli*	Ca_Kabuli_Chr02	35593985	[A/G]	Ca_09728	Bacterial Fmu (Sun)/eukaryotic nucleolar NOL1/Nop2p	INTRON
3274	CakSNP3274	*Kabuli*	Ca_Kabuli_Chr02	35593965	[C/T]	Ca_09728	Bacterial Fmu (Sun)/eukaryotic nucleolar NOL1/Nop2p	INTRON
3275	CakSNP3275	*Kabuli*	Ca_Kabuli_Chr02	35593956	[A/G]	Ca_09728	Bacterial Fmu (Sun)/eukaryotic nucleolar NOL1/Nop2p	INTRON
3276	CakSNP3276	*Kabuli*	Ca_Kabuli_Chr02	35601566	[T/C]		--	INTERGENIC
3277	CakSNP3277	*Kabuli*	Ca_Kabuli_Chr02	35601571	[A/C]		--	INTERGENIC
3278	CakSNP3278	*Kabuli*	Ca_Kabuli_Chr02	35601578	[C/T]		--	INTERGENIC
3279	CakSNP3279	*Kabuli*	Ca_Kabuli_Chr02	35601605	[G/A]		--	INTERGENIC
3280	CakSNP3280	*Kabuli*	Ca_Kabuli_Chr02	35601691	[A/G]		--	INTERGENIC
3281	CakSNP3281	*Kabuli*	Ca_Kabuli_Chr02	35601657	[T/C]		--	INTERGENIC

S.N.	SNP IDs	Chickpea cultivars	Chromosomes	Physical positions (bp)	SNPs	Gene accession IDs	Putative functions	Sequence components of genome
3282	CakSNP3282	*Kabuli*	Ca_Kabuli_Chr02	35601632	[G/T]		--	INTERGENIC
3283	CakSNP3283	*Kabuli*	Ca_Kabuli_Chr02	35612587	[C/A]	Ca_09731	Protein of unknown function DUF827, plant	SYNONYMOUS_CODING
3284	CakSNP3284	*Kabuli*	Ca_Kabuli_Chr02	35612590	[G/A]	Ca_09731	Protein of unknown function DUF827, plant	SYNONYMOUS_CODING
3285	CakSNP3285	*Kabuli*	Ca_Kabuli_Chr02	35612644	[G/A]	Ca_09731	Protein of unknown function DUF827, plant	SYNONYMOUS_CODING
3286	CakSNP3286	*Kabuli*	Ca_Kabuli_Chr02	35612788	[G/A]	Ca_09731	Protein of unknown function DUF827, plant	SYNONYMOUS_CODING
3287	CakSNP3287	*Kabuli*	Ca_Kabuli_Chr02	35612895	[T/C]	Ca_09731	Protein of unknown function DUF827, plant	NON_SYNONYMOUS_CODING
3288	CakSNP3288	*Kabuli*	Ca_Kabuli_Chr02	35612900	[C/T]	Ca_09731	Protein of unknown function DUF827, plant	NON_SYNONYMOUS_CODING
3289	CakSNP3289	*Kabuli*	Ca_Kabuli_Chr02	35613016	[G/A]	Ca_09731	Protein of unknown function DUF827, plant	SYNONYMOUS_CODING
3290	CakSNP3290	*Kabuli*	Ca_Kabuli_Chr02	35613135	[T/C]		--	INTERGENIC
3291	CakSNP3291	*Kabuli*	Ca_Kabuli_Chr02	35658426	[C/T]	Ca_09735	Oligopeptide transporter OPT superfamily	SYNONYMOUS_CODING

S.N.	SNP IDs	Chickpea cultivars	Chromosomes	Physical positions (bp)	SNPs	Gene accession IDs	Putative functions	Sequence components of genome
3292	CakSNP3292	*Kabuli*	Ca_Kabuli_Chr02	35658537	[T/C]	Ca_09735	Oligopeptide transporter OPT superfamily	SYNONYMOUS_CODING
3293	CakSNP3293	*Kabuli*	Ca_Kabuli_Chr02	35661053	[C/T]	Ca_09735	Oligopeptide transporter OPT superfamily	NON_SYNONYMOUS_CODING
3294	CakSNP3294	*Kabuli*	Ca_Kabuli_Chr02	35670478	[A/G]	Ca_09737	Protein of unknown function DUF1296	INTRON
3295	CakSNP3295	*Kabuli*	Ca_Kabuli_Chr02	35704018	[A/G]	Ca_09740	ABC transporter-like	SYNONYMOUS_CODING
3296	CakSNP3296	*Kabuli*	Ca_Kabuli_Chr02	35704069	[G/A]	Ca_09740	ABC transporter-like	SYNONYMOUS_CODING
3297	CakSNP3297	*Kabuli*	Ca_Kabuli_Chr02	35704228	[C/A]	Ca_09740	ABC transporter-like	INTRON
3298	CakSNP3298	*Kabuli*	Ca_Kabuli_Chr02	35704213	[A/G]	Ca_09740	ABC transporter-like	INTRON
3299	CakSNP3299	*Kabuli*	Ca_Kabuli_Chr02	35704204	[T/C]	Ca_09740	ABC transporter-like	INTRON
3300	CakSNP3300	*Kabuli*	Ca_Kabuli_Chr02	35704200	[T/C]	Ca_09740	ABC transporter-like	INTRON
3301	CakSNP3301	*Kabuli*	Ca_Kabuli_Chr02	35704180	[A/G]	Ca_09740	ABC transporter-like	INTRON

S.N.	SNP IDs	Chickpea cultivars	Chromosomes	Physical positions (bp)	SNPs	Gene accession IDs	Putative functions	Sequence components of genome
3302	CakSNP3302	*Kabuli*	Ca_Kabuli_Chr02	35704178	[G/A]	Ca_09740	ABC transporter-like	INTRON
3303	CakSNP3303	*Kabuli*	Ca_Kabuli_Chr02	35728285	[T/A]		--	INTERGENIC
3304	CakSNP3304	*Kabuli*	Ca_Kabuli_Chr02	35754617	[G/T]	Ca_09748	Ribosomal protein L26/L24P, eukaryotic/archaeal	SYNONYMOUS_CODING
3305	CakSNP3305	*Kabuli*	Ca_Kabuli_Chr02	35754557	[C/T]	Ca_09748	Ribosomal protein L26/L24P, eukaryotic/archaeal	SYNONYMOUS_CODING
3306	CakSNP3306	*Kabuli*	Ca_Kabuli_Chr02	35754680	[C/A]		--	INTERGENIC
3307	CakSNP3307	*Kabuli*	Ca_Kabuli_Chr02	35754682	[A/G]		--	INTERGENIC
3308	CakSNP3308	*Kabuli*	Ca_Kabuli_Chr02	35754806	[G/C]		--	INTERGENIC
3309	CakSNP3309	*Kabuli*	Ca_Kabuli_Chr02	35754757	[G/C]		--	INTERGENIC
3310	CakSNP3310	*Kabuli*	Ca_Kabuli_Chr02	35759932	[A/T]	Ca_09749	Regulatory protein RecX	INTRON
3311	CakSNP3311	*Kabuli*	Ca_Kabuli_Chr02	35759940	[T/C]	Ca_09749	Regulatory protein RecX	INTRON

S.N.	SNP IDs	Chickpea cultivars	Chromosomes	Physical positions (bp)	SNPs	Gene accession IDs	Putative functions	Sequence components of genome
3312	CakSNP3312	*Kabuli*	Ca_Kabuli_Chr02	35759981	[G/A]	Ca_09749	Regulatory protein RecX	INTRON
3313	CakSNP3313	*Kabuli*	Ca_Kabuli_Chr02	35760062	[G/C]	Ca_09749	Regulatory protein RecX	INTRON
3314	CakSNP3314	*Kabuli*	Ca_Kabuli_Chr02	35760073	[A/T]	Ca_09749	Regulatory protein RecX	INTRON
3315	CakSNP3315	*Kabuli*	Ca_Kabuli_Chr02	35760170	[A/G]	Ca_09749	Regulatory protein RecX	INTRON
3316	CakSNP3316	*Kabuli*	Ca_Kabuli_Chr02	35760155	[G/A]	Ca_09749	Regulatory protein RecX	INTRON
3317	CakSNP3317	*Kabuli*	Ca_Kabuli_Chr02	35770569	[A/G]	Ca_09751	Calponin homology domain	INTRON
3318	CakSNP3318	*Kabuli*	Ca_Kabuli_Chr02	35795446	[G/A]	Ca_09754	Peptidase S8/S53, subtilisin/kexin/sedolisin	SYNONYMOUS_CODING
3319	CakSNP3319	*Kabuli*	Ca_Kabuli_Chr02	35795395	[A/G]	Ca_09754	Peptidase S8/S53, subtilisin/kexin/sedolisin	SYNONYMOUS_CODING
3320	CakSNP3320	*Kabuli*	Ca_Kabuli_Chr02	35795494	[G/A]	Ca_09754	Peptidase S8/S53, subtilisin/kexin/sedolisin	SYNONYMOUS_CODING
3321	CakSNP3321	*Kabuli*	Ca_Kabuli_Chr02	35795496	[G/T]	Ca_09754	Peptidase S8/S53, subtilisin/kexin/sedolisin	NON_SYNONYMOUS_CODING

S.N.	SNP IDs	Chickpea cultivars	Chromosomes	Physical positions (bp)	SNPs	Gene accession IDs	Putative functions	Sequence components of genome
3322	CakSNP3322	*Kabuli*	Ca_Kabuli_Chr02	35795497	[G/T]	Ca_09754	Peptidase S8/S53, subtilisin/kexin/sedolisin	NON_SYNONYMOUS_CODING
3323	CakSNP3323	*Kabuli*	Ca_Kabuli_Chr02	35795521	[T/C]	Ca_09754	Peptidase S8/S53, subtilisin/kexin/sedolisin	SYNONYMOUS_CODING
3324	CakSNP3324	*Kabuli*	Ca_Kabuli_Chr02	35828553	[G/A]	Ca_09757	Raffinose synthase	INTRON
3325	CakSNP3325	*Kabuli*	Ca_Kabuli_Chr02	35829782	[A/G]	Ca_09757	Raffinose synthase	INTRON
3326	CakSNP3326	*Kabuli*	Ca_Kabuli_Chr02	35844790	[A/G]		--	INTERGENIC
3327	CakSNP3327	*Kabuli*	Ca_Kabuli_Chr02	35844762	[A/G]		--	INTERGENIC
3328	CakSNP3328	*Kabuli*	Ca_Kabuli_Chr02	35862852	[C/T]	Ca_09762	Armadillo	INTRON
3329	CakSNP3329	*Kabuli*	Ca_Kabuli_Chr02	35862878	[T/G]	Ca_09762	Armadillo	INTRON
3330	CakSNP3330	*Kabuli*	Ca_Kabuli_Chr02	35875666	[A/G]	Ca_09763	Peptidase A1	SYNONYMOUS_CODING
3331	CakSNP3331	*Kabuli*	Ca_Kabuli_Chr02	35875653	[C/T]	Ca_09763	Peptidase A1	NON_SYNONYMOUS_CODING

S.N.	SNP IDs	Chickpea cultivars	Chromosomes	Physical positions (bp)	SNPs	Gene accession IDs	Putative functions	Sequence components of genome
3332	CakSNP3332	*Kabuli*	Ca_Kabuli_Chr02	35930699	[T/G]		--	INTERGENIC
3333	CakSNP3333	*Kabuli*	Ca_Kabuli_Chr02	35993062	[C/T]	Ca_09773	Disease resistance protein	NON_SYNONYMOUS_CODING
3334	CakSNP3334	*Kabuli*	Ca_Kabuli_Chr02	35993082	[T/G]	Ca_09773	Disease resistance protein	NON_SYNONYMOUS_CODING
3335	CakSNP3335	*Kabuli*	Ca_Kabuli_Chr02	35993089	[C/T]	Ca_09773	Disease resistance protein	NON_SYNONYMOUS_CODING
3336	CakSNP3336	*Kabuli*	Ca_Kabuli_Chr02	35993092	[A/T]	Ca_09773	Disease resistance protein	NON_SYNONYMOUS_CODING
3337	CakSNP3337	*Kabuli*	Ca_Kabuli_Chr02	35993178	[G/A]	Ca_09773	Disease resistance protein	SYNONYMOUS_CODING
3338	CakSNP3338	*Kabuli*	Ca_Kabuli_Chr02	36013809	[A/G]	Ca_09776	Nucleoporin, Nup133/Nup155-like, C-terminal	INTRON
3339	CakSNP3339	*Kabuli*	Ca_Kabuli_Chr02	36039428	[C/T]	Ca_09780	--	SYNONYMOUS_CODING
3340	CakSNP3340	*Kabuli*	Ca_Kabuli_Chr02	36039377	[C/T]	Ca_09780	--	NON_SYNONYMOUS_CODING
3341	CakSNP3341	*Kabuli*	Ca_Kabuli_Chr02	36039364	[G/T]	Ca_09780	--	NON_SYNONYMOUS_CODING

S.N.	SNP IDs	Chickpea cultivars	Chromosomes	Physical positions (bp)	SNPs	Gene accession IDs	Putative functions	Sequence components of genome
3342	CakSNP3342	*Kabuli*	Ca_Kabuli_Chr02	36046593	[G/T]		--	INTERGENIC
3343	CakSNP3343	*Kabuli*	Ca_Kabuli_Chr02	36046595	[T/G]		--	INTERGENIC
3344	CakSNP3344	*Kabuli*	Ca_Kabuli_Chr02	36046741	[G/A]		--	INTERGENIC
3345	CakSNP3345	*Kabuli*	Ca_Kabuli_Chr02	36046720	[T/A]		--	INTERGENIC
3346	CakSNP3346	*Kabuli*	Ca_Kabuli_Chr02	36046767	[T/A]		--	INTERGENIC
3347	CakSNP3347	*Kabuli*	Ca_Kabuli_Chr02	36046799	[C/A]		--	INTERGENIC
3348	CakSNP3348	*Kabuli*	Ca_Kabuli_Chr02	36046820	[T/A]		--	INTERGENIC
3349	CakSNP3349	*Kabuli*	Ca_Kabuli_Chr02	36046844	[T/G]		--	INTERGENIC
3350	CakSNP3350	*Kabuli*	Ca_Kabuli_Chr02	36069340	[T/C]	Ca_09784	Signal transduction response regulator, receiver domain	SYNONYMOUS_CODING
3351	CakSNP3351	*Kabuli*	Ca_Kabuli_Chr02	36072247	[C/A]		--	INTERGENIC

S.N.	SNP IDs	Chickpea cultivars	Chromosomes	Physical positions (bp)	SNPs	Gene accession IDs	Putative functions	Sequence components of genome
3352	CakSNP3352	*Kabuli*	Ca_Kabuli_Chr02	36074147	[T/C]	Ca_09785	X8	SYNONYMOUS_CODING
3353	CakSNP3353	*Kabuli*	Ca_Kabuli_Chr02	36088577	[A/T]	Ca_09786	Transcription factor GRAS	SYNONYMOUS_CODING
3354	CakSNP3354	*Kabuli*	Ca_Kabuli_Chr02	36088623	[C/T]	Ca_09786	Transcription factor GRAS	NON_SYNONYMOUS_CODING
3355	CakSNP3355	*Kabuli*	Ca_Kabuli_Chr02	36088676	[T/C]	Ca_09786	Transcription factor GRAS	SYNONYMOUS_CODING
3356	CakSNP3356	*Kabuli*	Ca_Kabuli_Chr02	36088853	[G/A]	Ca_09786	Transcription factor GRAS	SYNONYMOUS_CODING
3357	CakSNP3357	*Kabuli*	Ca_Kabuli_Chr02	36101358	[A/G]	Ca_09788	Nuclear protein DGCR14	SYNONYMOUS_CODING
3358	CakSNP3358	*Kabuli*	Ca_Kabuli_Chr02	36101379	[A/G]	Ca_09788	Nuclear protein DGCR14	SYNONYMOUS_CODING
3359	CakSNP3359	*Kabuli*	Ca_Kabuli_Chr02	36101421	[C/T]	Ca_09788	Nuclear protein DGCR14	SYNONYMOUS_CODING
3360	CakSNP3360	*Kabuli*	Ca_Kabuli_Chr02	36117695	[T/A]		--	INTERGENIC
3361	CakSNP3361	*Kabuli*	Ca_Kabuli_Chr02	36117647	[G/A]		--	INTERGENIC

S.N.	SNP IDs	Chickpea cultivars	Chromosomes	Physical positions (bp)	SNPs	Gene accession IDs	Putative functions	Sequence components of genome
3362	CakSNP3362	*Kabuli*	Ca_Kabuli_Chr02	36253581	[C/T]	Ca_09809	FAS1 domain	SYNONYMOUS_CODING
3363	CakSNP3363	*Kabuli*	Ca_Kabuli_Chr02	36276791	[G/A]	Ca_09813	Pentatricopeptide repeat	NON_SYNONYMOUS_CODING
3364	CakSNP3364	*Kabuli*	Ca_Kabuli_Chr02	36323575	[G/T]		--	INTERGENIC
3365	CakSNP3365	*Kabuli*	Ca_Kabuli_Chr02	36326361	[A/C]	Ca_09817	Phenylalanine/histidine ammonia-lyase	SYNONYMOUS_CODING
3366	CakSNP3366	*Kabuli*	Ca_Kabuli_Chr02	36335252	[A/C]	Ca_09819	Chlorophyll A-B binding protein, plant	SYNONYMOUS_CODING
3367	CakSNP3367	*Kabuli*	Ca_Kabuli_Chr02	36343900	[C/T]	Ca_09821	Coproporphyrinogen III oxidase	INTRON
3368	CakSNP3368	*Kabuli*	Ca_Kabuli_Chr02	36343933	[T/C]	Ca_09821	Coproporphyrinogen III oxidase	INTRON
3369	CakSNP3369	*Kabuli*	Ca_Kabuli_Chr02	36346336	[A/G]	Ca_09822	CbxX/CfqX	SYNONYMOUS_CODING
3370	CakSNP3370	*Kabuli*	Ca_Kabuli_Chr02	36375224	[A/G]	Ca_09827	Domain of unknown function DUF632	SYNONYMOUS_CODING
3371	CakSNP3371	*Kabuli*	Ca_Kabuli_Chr02	36375638	[A/G]	Ca_09827	Domain of unknown function DUF632	SYNONYMOUS_CODING

S.N.	SNP IDs	Chickpea cultivars	Chromosomes	Physical positions (bp)	SNPs	Gene accession IDs	Putative functions	Sequence components of genome
3372	CakSNP3372	*Kabuli*	Ca_Kabuli_Chr02	36387189	[G/C]	Ca_09828	GDP-fucose protein O-fucosyltransferase	INTRON
3373	CakSNP3373	*Kabuli*	Ca_Kabuli_Chr02	36387245	[T/C]	Ca_09828	GDP-fucose protein O-fucosyltransferase	NON_SYNONYMOUS_CODING
3374	CakSNP3374	*Kabuli*	Ca_Kabuli_Chr02	36389041	[G/A]	Ca_09828	GDP-fucose protein O-fucosyltransferase	NON_SYNONYMOUS_CODING
3375	CakSNP3375	*Kabuli*	Ca_Kabuli_Chr02	36389096	[C/T]	Ca_09828	GDP-fucose protein O-fucosyltransferase	SYNONYMOUS_CODING
3376	CakSNP3376	*Kabuli*	Ca_Kabuli_Chr02	36403788	[A/G]		--	INTERGENIC
3377	CakSNP3377	*Kabuli*	Ca_Kabuli_Chr02	36427841	[C/T]	Ca_09832	Phox/Bem1p	SYNONYMOUS_CODING
3378	CakSNP3378	*Kabuli*	Ca_Kabuli_Chr02	36429512	[T/C]	Ca_09832	Phox/Bem1p	NON_SYNONYMOUS_CODING
3379	CakSNP3379	*Kabuli*	Ca_Kabuli_Chr02	36438939	[T/G]	Ca_09833	Sterol-sensing domain	INTRON
3380	CakSNP3380	*Kabuli*	Ca_Kabuli_Chr02	36453691	[A/G]	Ca_09833	Sterol-sensing domain	SYNONYMOUS_CODING
3381	CakSNP3381	*Kabuli*	Ca_Kabuli_Chr02	36453685	[G/A]	Ca_09833	Sterol-sensing domain	SYNONYMOUS_CODING

S.N.	SNP IDs	Chickpea cultivars	Chromosomes	Physical positions (bp)	SNPs	Gene accession IDs	Putative functions	Sequence components of genome
3382	CakSNP3382	*Kabuli*	Ca_Kabuli_Chr02	36466837	[C/T]	Ca_09835	EPS15 homology (EH)	NON_SYNONYMOUS_CODING
3383	CakSNP3383	*Kabuli*	Ca_Kabuli_Chr02	36466998	[A/G]	Ca_09835	EPS15 homology (EH)	SYNONYMOUS_CODING
3384	CakSNP3384	*Kabuli*	Ca_Kabuli_Chr02	36467100	[A/C]	Ca_09835	EPS15 homology (EH)	SYNONYMOUS_CODING
3385	CakSNP3385	*Kabuli*	Ca_Kabuli_Chr02	36468390	[A/G]	Ca_09835	EPS15 homology (EH)	NON_SYNONYMOUS_CODING
3386	CakSNP3386	*Kabuli*	Ca_Kabuli_Chr02	36468505	[C/A]	Ca_09835	EPS15 homology (EH)	NON_SYNONYMOUS_CODING
3387	CakSNP3387	*Kabuli*	Ca_Kabuli_Chr02	36469921	[A/G]	Ca_09835	EPS15 homology (EH)	INTRON
3388	CakSNP3388	*Kabuli*	Ca_Kabuli_Chr02	36505316	[C/A]	Ca_09839	Cystathionine beta-synthase, core	NON_SYNONYMOUS_CODING
3389	CakSNP3389	*Kabuli*	Ca_Kabuli_Chr02	36521200	[T/C]		--	INTERGENIC
3390	CakSNP3390	*Kabuli*	Ca_Kabuli_Chr02	36533821	[A/C]	Ca_09841	SEC7-like	INTRON
3391	CakSNP3391	*Kabuli*	Ca_Kabuli_Chr02	36533910	[G/A]	Ca_09841	SEC7-like	INTRON

S.N.	SNP IDs	Chickpea cultivars	Chromosomes	Physical positions (bp)	SNPs	Gene accession IDs	Putative functions	Sequence components of genome
3392	CakSNP3392	*Kabuli*	Ca_Kabuli_Chr02	36554430	[G/A]		--	INTERGENIC
3393	CakSNP3393	*Kabuli*	Ca_Kabuli_Chr02	36609730	[T/A]		--	INTERGENIC
3394	CakSNP3394	*Kabuli*	Ca_Kabuli_Chr02	36609743	[C/T]		--	INTERGENIC
3395	CakSNP3395	*Kabuli*	Ca_Kabuli_Chr02	36609744	[G/C]		--	INTERGENIC
3396	CakSNP3396	*Kabuli*	Ca_Kabuli_Chr02	36609858	[C/G]		--	INTERGENIC
3397	CakSNP3397	*Kabuli*	Ca_Kabuli_Chr02	36609950	[C/T]		--	INTERGENIC
3398	CakSNP3398	*Kabuli*	Ca_Kabuli_Chr02	36609968	[C/T]		--	INTERGENIC
3399	CakSNP3399	*Kabuli*	Ca_Kabuli_Chr02	36634234	[T/C]		--	INTERGENIC
3400	CakSNP3400	*Kabuli*	Ca_Kabuli_Chr02	36634297	[C/G]		--	INTERGENIC
3401	CakSNP3401	*Kabuli*	Ca_Kabuli_Chr03	171409	[A/T]	Ca_22938	NAD-dependent epimerase/dehydratase	NON_SYNONYMOUS_CODING

S.N.	SNP IDs	Chickpea cultivars	Chromosomes	Physical positions (bp)	SNPs	Gene accession IDs	Putative functions	Sequence components of genome
3402	CakSNP3402	*Kabuli*	Ca_Kabuli_Chr03	171468	[A/G]	Ca_22938	NAD-dependent epimerase/dehydratase	SYNONYMOUS_CODING
3403	CakSNP3403	*Kabuli*	Ca_Kabuli_Chr03	171574	[C/A]	Ca_22938	NAD-dependent epimerase/dehydratase	INTRON
3404	CakSNP3404	*Kabuli*	Ca_Kabuli_Chr03	171576	[G/T]	Ca_22938	NAD-dependent epimerase/dehydratase	INTRON
3405	CakSNP3405	*Kabuli*	Ca_Kabuli_Chr03	171579	[C/T]	Ca_22938	NAD-dependent epimerase/dehydratase	INTRON
3406	CakSNP3406	*Kabuli*	Ca_Kabuli_Chr03	171675	[T/A]	Ca_22938	NAD-dependent epimerase/dehydratase	SYNONYMOUS_CODING
3407	CakSNP3407	*Kabuli*	Ca_Kabuli_Chr03	208233	[C/A]	Ca_22936	U box domain	NON_SYNONYMOUS_CODING
3408	CakSNP3408	*Kabuli*	Ca_Kabuli_Chr03	220564	[A/G]	Ca_22935	Glycosyl transferase, family 48	INTRON
3409	CakSNP3409	*Kabuli*	Ca_Kabuli_Chr03	220568	[A/T]	Ca_22935	Glycosyl transferase, family 48	INTRON
3410	CakSNP3410	*Kabuli*	Ca_Kabuli_Chr03	220572	[T/C]	Ca_22935	Glycosyl transferase, family 48	INTRON
3411	CakSNP3411	*Kabuli*	Ca_Kabuli_Chr03	220573	[G/T]	Ca_22935	Glycosyl transferase, family 48	INTRON

S.N.	SNP IDs	Chickpea cultivars	Chromosomes	Physical positions (bp)	SNPs	Gene accession IDs	Putative functions	Sequence components of genome
3412	CakSNP3412	*Kabuli*	Ca_Kabuli_Chr03	223943	[G/A]	Ca_22935	Glycosyl transferase, family 48	INTRON
3413	CakSNP3413	*Kabuli*	Ca_Kabuli_Chr03	223976	[G/C]	Ca_22935	Glycosyl transferase, family 48	INTRON
3414	CakSNP3414	*Kabuli*	Ca_Kabuli_Chr03	286425	[G/T]	Ca_22933	Protein kinase, catalytic domain	INTRON
3415	CakSNP3415	*Kabuli*	Ca_Kabuli_Chr03	419715	[T/G]	Ca_22927	Peptidase C48, SUMO/Sentrin/Ubl1	SYNONYMOUS_CODING
3416	CakSNP3416	*Kabuli*	Ca_Kabuli_Chr03	988477	[G/A]		--	INTERGENIC
3417	CakSNP3417	*Kabuli*	Ca_Kabuli_Chr03	988471	[T/C]		--	INTERGENIC
3418	CakSNP3418	*Kabuli*	Ca_Kabuli_Chr03	1499038	[G/A]		--	INTERGENIC
3419	CakSNP3419	*Kabuli*	Ca_Kabuli_Chr03	1499006	[A/G]		--	INTERGENIC
3420	CakSNP3420	*Kabuli*	Ca_Kabuli_Chr03	1746388	[C/A]	Ca_19405	Helicase, C-terminal	INTRON
3421	CakSNP3421	*Kabuli*	Ca_Kabuli_Chr03	1746389	[A/G]	Ca_19405	Helicase, C-terminal	INTRON

S.N.	SNP IDs	Chickpea cultivars	Chromosomes	Physical positions (bp)	SNPs	Gene accession IDs	Putative functions	Sequence components of genome
3422	CakSNP3422	*Kabuli*	Ca_Kabuli_Chr03	1829412	[T/G]		--	INTERGENIC
3423	CakSNP3423	*Kabuli*	Ca_Kabuli_Chr03	1829358	[T/C]		--	INTERGENIC
3424	CakSNP3424	*Kabuli*	Ca_Kabuli_Chr03	1829352	[C/T]		--	INTERGENIC
3425	CakSNP3425	*Kabuli*	Ca_Kabuli_Chr03	1831920	[T/G]		--	INTERGENIC
3426	CakSNP3426	*Kabuli*	Ca_Kabuli_Chr03	1831866	[T/C]		--	INTERGENIC
3427	CakSNP3427	*Kabuli*	Ca_Kabuli_Chr03	1831860	[C/T]		--	INTERGENIC
3428	CakSNP3428	*Kabuli*	Ca_Kabuli_Chr03	1831931	[A/C]		--	INTERGENIC
3429	CakSNP3429	*Kabuli*	Ca_Kabuli_Chr03	2097376	[T/G]		--	INTERGENIC
3430	CakSNP3430	*Kabuli*	Ca_Kabuli_Chr03	2097413	[A/G]		--	INTERGENIC
3431	CakSNP3431	*Kabuli*	Ca_Kabuli_Chr03	2391515	[T/G]	Ca_26372	NHL repeat	SYNONYMOUS_CODING

S.N.	SNP IDs	Chickpea cultivars	Chromosomes	Physical positions (bp)	SNPs	Gene accession IDs	Putative functions	Sequence components of genome
3432	CakSNP3432	*Kabuli*	Ca_Kabuli_Chr03	2423760	[T/C]		--	INTERGENIC
3433	CakSNP3433	*Kabuli*	Ca_Kabuli_Chr03	2708703	[C/T]	Ca_22148	Zinc finger, B-box	NON_SYNONYMOUS_CODING
3434	CakSNP3434	*Kabuli*	Ca_Kabuli_Chr03	2825546	[G/T]		--	INTERGENIC
3435	CakSNP3435	*Kabuli*	Ca_Kabuli_Chr03	2927148	[T/A]	Ca_22158	Tetratricopeptide repeat-containing	INTRON
3436	CakSNP3436	*Kabuli*	Ca_Kabuli_Chr03	2927150	[A/T]	Ca_22158	Tetratricopeptide repeat-containing	INTRON
3437	CakSNP3437	*Kabuli*	Ca_Kabuli_Chr03	2931027	[G/C]	Ca_22158	Tetratricopeptide repeat-containing	INTRON
3438	CakSNP3438	*Kabuli*	Ca_Kabuli_Chr03	2931046	[C/T]	Ca_22158	Tetratricopeptide repeat-containing	INTRON
3439	CakSNP3439	*Kabuli*	Ca_Kabuli_Chr03	2975179	[T/C]	Ca_22161	--	SYNONYMOUS_CODING
3440	CakSNP3440	*Kabuli*	Ca_Kabuli_Chr03	3280478	[C/T]	Ca_24555	RNA recognition motif domain	NON_SYNONYMOUS_CODING
3441	CakSNP3441	*Kabuli*	Ca_Kabuli_Chr03	3280500	[C/T]	Ca_24555	RNA recognition motif domain	SYNONYMOUS_CODING

S.N.	SNP IDs	Chickpea cultivars	Chromosomes	Physical positions (bp)	SNPs	Gene accession IDs	Putative functions	Sequence components of genome
3442	CakSNP3442	*Kabuli*	Ca_Kabuli_Chr03	3280530	[G/A]	Ca_24555	RNA recognition motif domain	SYNONYMOUS_CODING
3443	CakSNP3443	*Kabuli*	Ca_Kabuli_Chr03	3280595	[G/A]	Ca_24555	RNA recognition motif domain	SYNONYMOUS_CODING
3444	CakSNP3444	*Kabuli*	Ca_Kabuli_Chr03	3617555	[G/A]	Ca_21346	ZF-HD homeobox protein, Cys/His-rich dimerisation domain	NON_SYNONYMOUS_CODING
3445	CakSNP3445	*Kabuli*	Ca_Kabuli_Chr03	3701394	[A/T]	Ca_21344	Gibberellin regulated protein	INTRON
3446	CakSNP3446	*Kabuli*	Ca_Kabuli_Chr03	3701396	[C/T]	Ca_21344	Gibberellin regulated protein	INTRON
3447	CakSNP3447	*Kabuli*	Ca_Kabuli_Chr03	3701398	[A/T]	Ca_21344	Gibberellin regulated protein	INTRON
3448	CakSNP3448	*Kabuli*	Ca_Kabuli_Chr03	3701401	[A/T]	Ca_21344	Gibberellin regulated protein	INTRON
3449	CakSNP3449	*Kabuli*	Ca_Kabuli_Chr03	3701403	[A/T]	Ca_21344	Gibberellin regulated protein	INTRON
3450	CakSNP3450	*Kabuli*	Ca_Kabuli_Chr03	3701405	[G/T]	Ca_21344	Gibberellin regulated protein	INTRON
3451	CakSNP3451	*Kabuli*	Ca_Kabuli_Chr03	3701415	[C/T]	Ca_21344	Gibberellin regulated protein	INTRON

S.N.	SNP IDs	Chickpea cultivars	Chromosomes	Physical positions (bp)	SNPs	Gene accession IDs	Putative functions	Sequence components of genome
3452	CakSNP3452	*Kabuli*	Ca_Kabuli_Chr03	3701422	[C/T]	Ca_21344	Gibberellin regulated protein	INTRON
3453	CakSNP3453	*Kabuli*	Ca_Kabuli_Chr03	3701426	[T/C]	Ca_21344	Gibberellin regulated protein	INTRON
3454	CakSNP3454	*Kabuli*	Ca_Kabuli_Chr03	3701435	[A/T]	Ca_21344	Gibberellin regulated protein	INTRON
3455	CakSNP3455	*Kabuli*	Ca_Kabuli_Chr03	3701436	[C/T]	Ca_21344	Gibberellin regulated protein	INTRON
3456	CakSNP3456	*Kabuli*	Ca_Kabuli_Chr03	3701437	[A/T]	Ca_21344	Gibberellin regulated protein	INTRON
3457	CakSNP3457	*Kabuli*	Ca_Kabuli_Chr03	3701440	[G/T]	Ca_21344	Gibberellin regulated protein	INTRON
3458	CakSNP3458	*Kabuli*	Ca_Kabuli_Chr03	3701450	[C/T]	Ca_21344	Gibberellin regulated protein	INTRON
3459	CakSNP3459	*Kabuli*	Ca_Kabuli_Chr03	3701375	[G/A]	Ca_21344	Gibberellin regulated protein	INTRON
3460	CakSNP3460	*Kabuli*	Ca_Kabuli_Chr03	3701370	[G/A]	Ca_21344	Gibberellin regulated protein	INTRON
3461	CakSNP3461	*Kabuli*	Ca_Kabuli_Chr03	3744908	[G/A]	Ca_21337	--	NON_SYNONYMOUS_CODING

S.N.	SNP IDs	Chickpea cultivars	Chromosomes	Physical positions (bp)	SNPs	Gene accession IDs	Putative functions	Sequence components of genome
3462	CakSNP3462	*Kabuli*	Ca_Kabuli_Chr03	3764420	[T/A]		--	INTERGENIC
3463	CakSNP3463	*Kabuli*	Ca_Kabuli_Chr03	3764508	[T/C]		--	INTERGENIC
3464	CakSNP3464	*Kabuli*	Ca_Kabuli_Chr03	3764545	[C/T]		--	INTERGENIC
3465	CakSNP3465	*Kabuli*	Ca_Kabuli_Chr03	4145743	[G/A]	Ca_22385	Domain of unknown function DUF298	INTRON
3466	CakSNP3466	*Kabuli*	Ca_Kabuli_Chr03	4178154	[C/T]		--	INTERGENIC
3467	CakSNP3467	*Kabuli*	Ca_Kabuli_Chr03	4266739	[G/A]		--	INTERGENIC
3468	CakSNP3468	*Kabuli*	Ca_Kabuli_Chr03	4266700	[T/G]		--	INTERGENIC
3469	CakSNP3469	*Kabuli*	Ca_Kabuli_Chr03	4266691	[C/T]		--	INTERGENIC
3470	CakSNP3470	*Kabuli*	Ca_Kabuli_Chr03	4266686	[C/T]		--	INTERGENIC
3471	CakSNP3471	*Kabuli*	Ca_Kabuli_Chr03	4266668	[G/A]		--	INTERGENIC

S.N.	SNP IDs	Chickpea cultivars	Chromosomes	Physical positions (bp)	SNPs	Gene accession IDs	Putative functions	Sequence components of genome
3472	CakSNP3472	*Kabuli*	Ca_Kabuli_Chr03	4555124	[G/A]	Ca_22079	SPX, N-terminal	SYNONYMOUS_CODING
3473	CakSNP3473	*Kabuli*	Ca_Kabuli_Chr03	4555142	[T/A]	Ca_22079	SPX, N-terminal	INTRON
3474	CakSNP3474	*Kabuli*	Ca_Kabuli_Chr03	4555167	[C/G]	Ca_22079	SPX, N-terminal	INTRON
3475	CakSNP3475	*Kabuli*	Ca_Kabuli_Chr03	4555177	[C/T]	Ca_22079	SPX, N-terminal	INTRON
3476	CakSNP3476	*Kabuli*	Ca_Kabuli_Chr03	4596815	[C/T]		--	INTERGENIC
3477	CakSNP3477	*Kabuli*	Ca_Kabuli_Chr03	4596782	[A/G]		--	INTERGENIC
3478	CakSNP3478	*Kabuli*	Ca_Kabuli_Chr03	4604649	[A/G]		--	INTERGENIC
3479	CakSNP3479	*Kabuli*	Ca_Kabuli_Chr03	4606129	[T/A]	Ca_22083	Vesicle transport v-SNARE, N-terminal	INTRON
3480	CakSNP3480	*Kabuli*	Ca_Kabuli_Chr03	4606128	[A/G]	Ca_22083	Vesicle transport v-SNARE, N-terminal	INTRON
3481	CakSNP3481	*Kabuli*	Ca_Kabuli_Chr03	4629742	[A/T]	Ca_22084	--	INTRON

S.N.	SNP IDs	Chickpea cultivars	Chromosomes	Physical positions (bp)	SNPs	Gene accession IDs	Putative functions	Sequence components of genome
3482	CakSNP3482	*Kabuli*	Ca_Kabuli_Chr03	4629747	[A/C]	Ca_22084	--	INTRON
3483	CakSNP3483	*Kabuli*	Ca_Kabuli_Chr03	4654906	[C/T]		--	INTERGENIC
3484	CakSNP3484	*Kabuli*	Ca_Kabuli_Chr03	4654898	[G/T]		--	INTERGENIC
3485	CakSNP3485	*Kabuli*	Ca_Kabuli_Chr03	4654896	[T/C]		--	INTERGENIC
3486	CakSNP3486	*Kabuli*	Ca_Kabuli_Chr03	4823828	[T/C]	Ca_22090	DNA-binding WRKY	SYNONYMOUS_CODING
3487	CakSNP3487	*Kabuli*	Ca_Kabuli_Chr03	5224781	[A/G]	Ca_23868	--	NON_SYNONYMOUS_CODING
3488	CakSNP3488	*Kabuli*	Ca_Kabuli_Chr03	5225052	[A/G]	Ca_23868	--	NON_SYNONYMOUS_CODING
3489	CakSNP3489	*Kabuli*	Ca_Kabuli_Chr03	5225063	[G/T]	Ca_23868	--	NON_SYNONYMOUS_CODING
3490	CakSNP3490	*Kabuli*	Ca_Kabuli_Chr03	5225108	[T/G]	Ca_23868	--	NON_SYNONYMOUS_CODING
3491	CakSNP3491	*Kabuli*	Ca_Kabuli_Chr03	5225079	[G/A]	Ca_23868	--	NON_SYNONYMOUS_CODING

S.N.	SNP IDs	Chickpea cultivars	Chromosomes	Physical positions (bp)	SNPs	Gene accession IDs	Putative functions	Sequence components of genome
3492	CakSNP3492	*Kabuli*	Ca_Kabuli_Chr03	5415782	[G/A]		--	INTERGENIC
3493	CakSNP3493	*Kabuli*	Ca_Kabuli_Chr03	5436267	[A/G]		--	INTERGENIC
3494	CakSNP3494	*Kabuli*	Ca_Kabuli_Chr03	5436272	[T/C]		--	INTERGENIC
3495	CakSNP3495	*Kabuli*	Ca_Kabuli_Chr03	5436277	[C/T]		--	INTERGENIC
3496	CakSNP3496	*Kabuli*	Ca_Kabuli_Chr03	5436288	[G/T]		--	INTERGENIC
3497	CakSNP3497	*Kabuli*	Ca_Kabuli_Chr03	5436295	[G/A]		--	INTERGENIC
3498	CakSNP3498	*Kabuli*	Ca_Kabuli_Chr03	5436296	[C/T]		--	INTERGENIC
3499	CakSNP3499	*Kabuli*	Ca_Kabuli_Chr03	5436299	[A/C]		--	INTERGENIC
3500	CakSNP3500	*Kabuli*	Ca_Kabuli_Chr03	5436331	[C/A]		--	INTERGENIC
3501	CakSNP3501	*Kabuli*	Ca_Kabuli_Chr03	5514176	[A/G]		--	INTERGENIC

S.N.	SNP IDs	Chickpea cultivars	Chromosomes	Physical positions (bp)	SNPs	Gene accession IDs	Putative functions	Sequence components of genome
3502	CakSNP3502	*Kabuli*	Ca_Kabuli_Chr03	5514287	[C/A]		--	INTERGENIC
3503	CakSNP3503	*Kabuli*	Ca_Kabuli_Chr03	6169456	[G/T]		--	INTERGENIC
3504	CakSNP3504	*Kabuli*	Ca_Kabuli_Chr03	6169539	[T/C]		--	INTERGENIC
3505	CakSNP3505	*Kabuli*	Ca_Kabuli_Chr03	6169510	[A/C]		--	INTERGENIC
3506	CakSNP3506	*Kabuli*	Ca_Kabuli_Chr03	6278104	[G/A]		--	INTERGENIC
3507	CakSNP3507	*Kabuli*	Ca_Kabuli_Chr03	6467014	[G/A]		--	INTERGENIC
3508	CakSNP3508	*Kabuli*	Ca_Kabuli_Chr03	6683646	[A/G]	Ca_25772	--	INTRON
3509	CakSNP3509	*Kabuli*	Ca_Kabuli_Chr03	6683587	[T/A]	Ca_25772	--	INTRON
3510	CakSNP3510	*Kabuli*	Ca_Kabuli_Chr03	6828181	[A/T]		--	INTERGENIC
3511	CakSNP3511	*Kabuli*	Ca_Kabuli_Chr03	6840644	[C/T]	Ca_16550	WD40 repeat	SYNONYMOUS_CODING

S.N.	SNP IDs	Chickpea cultivars	Chromosomes	Physical positions (bp)	SNPs	Gene accession IDs	Putative functions	Sequence components of genome
3512	CakSNP3512	*Kabuli*	Ca_Kabuli_Chr03	6894987	[C/A]		--	INTERGENIC
3513	CakSNP3513	*Kabuli*	Ca_Kabuli_Chr03	6895085	[T/C]		--	INTERGENIC
3514	CakSNP3514	*Kabuli*	Ca_Kabuli_Chr03	6899667	[T/C]		--	INTERGENIC
3515	CakSNP3515	*Kabuli*	Ca_Kabuli_Chr03	7013492	[G/A]	Ca_16540	Zinc finger, ZZ-type	INTRON
3516	CakSNP3516	*Kabuli*	Ca_Kabuli_Chr03	7015213	[T/G]	Ca_16540	Zinc finger, ZZ-type	NON_SYNONYMOUS_CODING
3517	CakSNP3517	*Kabuli*	Ca_Kabuli_Chr03	7024620	[G/A]	Ca_16540	Zinc finger, ZZ-type	SYNONYMOUS_CODING
3518	CakSNP3518	*Kabuli*	Ca_Kabuli_Chr03	7204053	[G/A]		--	INTERGENIC
3519	CakSNP3519	*Kabuli*	Ca_Kabuli_Chr03	7204071	[T/C]		--	INTERGENIC
3520	CakSNP3520	*Kabuli*	Ca_Kabuli_Chr03	7204087	[C/T]		--	INTERGENIC
3521	CakSNP3521	*Kabuli*	Ca_Kabuli_Chr03	7204088	[G/C]		--	INTERGENIC

S.N.	SNP IDs	Chickpea cultivars	Chromosomes	Physical positions (bp)	SNPs	Gene accession IDs	Putative functions	Sequence components of genome
3522	CakSNP3522	*Kabuli*	Ca_Kabuli_Chr03	7204098	[G/A]		--	INTERGENIC
3523	CakSNP3523	*Kabuli*	Ca_Kabuli_Chr03	7204099	[C/T]		--	INTERGENIC
3524	CakSNP3524	*Kabuli*	Ca_Kabuli_Chr03	7204111	[G/A]		--	INTERGENIC
3525	CakSNP3525	*Kabuli*	Ca_Kabuli_Chr03	7204112	[G/A]		--	INTERGENIC
3526	CakSNP3526	*Kabuli*	Ca_Kabuli_Chr03	7204133	[G/T]		--	INTERGENIC
3527	CakSNP3527	*Kabuli*	Ca_Kabuli_Chr03	7226689	[T/C]	Ca_16534	Peptidase C48, SUMO/Sentrin/Ubl1	NON_SYNONYMOUS_CODING
3528	CakSNP3528	*Kabuli*	Ca_Kabuli_Chr03	7226661	[G/T]	Ca_16534	Peptidase C48, SUMO/Sentrin/Ubl1	NON_SYNONYMOUS_CODING
3529	CakSNP3529	*Kabuli*	Ca_Kabuli_Chr03	7226632	[G/T]	Ca_16534	Peptidase C48, SUMO/Sentrin/Ubl1	NON_SYNONYMOUS_CODING
3530	CakSNP3530	*Kabuli*	Ca_Kabuli_Chr03	7246049	[T/G]	Ca_16533	Phosphatidylinositol 3-/4-kinase, catalytic	SYNONYMOUS_CODING
3531	CakSNP3531	*Kabuli*	Ca_Kabuli_Chr03	7420705	[G/A]		--	INTERGENIC

S.N.	SNP IDs	Chickpea cultivars	Chromosomes	Physical positions (bp)	SNPs	Gene accession IDs	Putative functions	Sequence components of genome
3532	CakSNP3532	*Kabuli*	Ca_Kabuli_Chr03	7433747	[C/A]	Ca_16525	Forkhead-associated (FHA) domain	SYNONYMOUS_CODING
3533	CakSNP3533	*Kabuli*	Ca_Kabuli_Chr03	7461833	[A/G]	Ca_16524	Smr protein/MutS2 C-terminal	NON_SYNONYMOUS_CODING
3534	CakSNP3534	*Kabuli*	Ca_Kabuli_Chr03	7482871	[C/T]		--	INTERGENIC
3535	CakSNP3535	*Kabuli*	Ca_Kabuli_Chr03	7606843	[A/G]	Ca_16518	--	INTRON
3536	CakSNP3536	*Kabuli*	Ca_Kabuli_Chr03	7606901	[T/C]	Ca_16518	--	INTRON
3537	CakSNP3537	*Kabuli*	Ca_Kabuli_Chr03	7606905	[C/G]	Ca_16518	--	INTRON
3538	CakSNP3538	*Kabuli*	Ca_Kabuli_Chr03	7608100	[A/G]	Ca_16518	--	INTRON
3539	CakSNP3539	*Kabuli*	Ca_Kabuli_Chr03	7665123	[G/A]	Ca_16510	Peptidase C48, SUMO/Sentrin/Ubl1	NON_SYNONYMOUS_CODING
3540	CakSNP3540	*Kabuli*	Ca_Kabuli_Chr03	7665112	[C/T]	Ca_16510	Peptidase C48, SUMO/Sentrin/Ubl1	NON_SYNONYMOUS_CODING
3541	CakSNP3541	*Kabuli*	Ca_Kabuli_Chr03	7665100	[G/T]	Ca_16510	Peptidase C48, SUMO/Sentrin/Ubl1	NON_SYNONYMOUS_CODING

S.N.	SNP IDs	Chickpea cultivars	Chromosomes	Physical positions (bp)	SNPs	Gene accession IDs	Putative functions	Sequence components of genome
3542	CakSNP3542	*Kabuli*	Ca_Kabuli_Chr03	7665098	[C/T]	Ca_16510	Peptidase C48, SUMO/Sentrin/Ubl1	SYNONYMOUS_CODING
3543	CakSNP3543	*Kabuli*	Ca_Kabuli_Chr03	7665090	[C/T]	Ca_16510	Peptidase C48, SUMO/Sentrin/Ubl1	NON_SYNONYMOUS_CODING
3544	CakSNP3544	*Kabuli*	Ca_Kabuli_Chr03	7665078	[G/A]	Ca_16510	Peptidase C48, SUMO/Sentrin/Ubl1	NON_SYNONYMOUS_CODING
3545	CakSNP3545	*Kabuli*	Ca_Kabuli_Chr03	7665075	[G/A]	Ca_16510	Peptidase C48, SUMO/Sentrin/Ubl1	NON_SYNONYMOUS_CODING
3546	CakSNP3546	*Kabuli*	Ca_Kabuli_Chr03	7665156	[G/A]	Ca_16510	Peptidase C48, SUMO/Sentrin/Ubl1	NON_SYNONYMOUS_CODING
3547	CakSNP3547	*Kabuli*	Ca_Kabuli_Chr03	7665149	[T/G]	Ca_16510	Peptidase C48, SUMO/Sentrin/Ubl1	SYNONYMOUS_CODING
3548	CakSNP3548	*Kabuli*	Ca_Kabuli_Chr03	7665142	[G/A]	Ca_16510	Peptidase C48, SUMO/Sentrin/Ubl1	NON_SYNONYMOUS_CODING
3549	CakSNP3549	*Kabuli*	Ca_Kabuli_Chr03	7665140	[G/T]	Ca_16510	Peptidase C48, SUMO/Sentrin/Ubl1	SYNONYMOUS_CODING
3550	CakSNP3550	*Kabuli*	Ca_Kabuli_Chr03	7665157	[C/T]	Ca_16510	Peptidase C48, SUMO/Sentrin/Ubl1	NON_SYNONYMOUS_CODING
3551	CakSNP3551	*Kabuli*	Ca_Kabuli_Chr03	7694198	[C/T]	Ca_16508	6-phosphogluconate dehydrogenase, C-terminal	SYNONYMOUS_CODING

S.N.	SNP IDs	Chickpea cultivars	Chromosomes	Physical positions (bp)	SNPs	Gene accession IDs	Putative functions	Sequence components of genome
3552	CakSNP3552	*Kabuli*	Ca_Kabuli_Chr03	7694297	[T/C]	Ca_16508	6-phosphogluconate dehydrogenase, C-terminal	SYNONYMOUS_CODING
3553	CakSNP3553	*Kabuli*	Ca_Kabuli_Chr03	8536213	[T/C]		--	INTERGENIC
3554	CakSNP3554	*Kabuli*	Ca_Kabuli_Chr03	8536275	[C/T]		--	INTERGENIC
3555	CakSNP3555	*Kabuli*	Ca_Kabuli_Chr03	8536295	[A/T]		--	INTERGENIC
3556	CakSNP3556	*Kabuli*	Ca_Kabuli_Chr03	8536301	[G/T]		--	INTERGENIC
3557	CakSNP3557	*Kabuli*	Ca_Kabuli_Chr03	8536425	[C/A]		--	INTERGENIC
3558	CakSNP3558	*Kabuli*	Ca_Kabuli_Chr03	8536498	[T/A]		--	INTERGENIC
3559	CakSNP3559	*Kabuli*	Ca_Kabuli_Chr03	8536559	[T/C]		--	INTERGENIC
3560	CakSNP3560	*Kabuli*	Ca_Kabuli_Chr03	8668628	[A/C]		--	INTERGENIC
3561	CakSNP3561	*Kabuli*	Ca_Kabuli_Chr03	8669004	[A/T]	Ca_23713	Protein kinase, catalytic domain	NON_SYNONYMOUS_CODING

S.N.	SNP IDs	Chickpea cultivars	Chromosomes	Physical positions (bp)	SNPs	Gene accession IDs	Putative functions	Sequence components of genome
3562	CakSNP3562	*Kabuli*	Ca_Kabuli_Chr03	8669132	[T/A]	Ca_23713	Protein kinase, catalytic domain	SYNONYMOUS_CODING
3563	CakSNP3563	*Kabuli*	Ca_Kabuli_Chr03	8697186	[A/G]	Ca_23715	Aldo/keto reductase	SYNONYMOUS_CODING
3564	CakSNP3564	*Kabuli*	Ca_Kabuli_Chr03	8712381	[A/C]	Ca_23716	Aspartate/glutamate/uridylate kinase	INTRON
3565	CakSNP3565	*Kabuli*	Ca_Kabuli_Chr03	8933037	[T/C]	Ca_25280	ABC transporter-like	INTRON
3566	CakSNP3566	*Kabuli*	Ca_Kabuli_Chr03	8933084	[G/A]	Ca_25280	ABC transporter-like	INTRON
3567	CakSNP3567	*Kabuli*	Ca_Kabuli_Chr03	8933086	[C/A]	Ca_25280	ABC transporter-like	INTRON
3568	CakSNP3568	*Kabuli*	Ca_Kabuli_Chr03	8933221	[T/A]	Ca_25280	ABC transporter-like	INTRON
3569	CakSNP3569	*Kabuli*	Ca_Kabuli_Chr03	8933183	[A/G]	Ca_25280	ABC transporter-like	INTRON
3570	CakSNP3570	*Kabuli*	Ca_Kabuli_Chr03	8933255	[A/T]	Ca_25280	ABC transporter-like	INTRON
3571	CakSNP3571	*Kabuli*	Ca_Kabuli_Chr03	8933266	[G/T]	Ca_25280	ABC transporter-like	INTRON

S.N.	SNP IDs	Chickpea cultivars	Chromosomes	Physical positions (bp)	SNPs	Gene accession IDs	Putative functions	Sequence components of genome
3572	CakSNP3572	*Kabuli*	Ca_Kabuli_Chr03	8933409	[A/C]	Ca_25280	ABC transporter-like	INTRON
3573	CakSNP3573	*Kabuli*	Ca_Kabuli_Chr03	8933370	[A/G]	Ca_25280	ABC transporter-like	INTRON
3574	CakSNP3574	*Kabuli*	Ca_Kabuli_Chr03	8933355	[A/T]	Ca_25280	ABC transporter-like	INTRON
3575	CakSNP3575	*Kabuli*	Ca_Kabuli_Chr03	9221403	[G/A]	Ca_20954	ABC transporter-like	NON_SYNONYMOUS_CODING
3576	CakSNP3576	*Kabuli*	Ca_Kabuli_Chr03	9221528	[C/T]	Ca_20954	ABC transporter-like	SYNONYMOUS_CODING
3577	CakSNP3577	*Kabuli*	Ca_Kabuli_Chr03	9221579	[G/C]	Ca_20954	ABC transporter-like	SYNONYMOUS_CODING
3578	CakSNP3578	*Kabuli*	Ca_Kabuli_Chr03	9221580	[T/G]	Ca_20954	ABC transporter-like	NON_SYNONYMOUS_CODING
3579	CakSNP3579	*Kabuli*	Ca_Kabuli_Chr03	9223444	[G/C]		--	INTERGENIC
3580	CakSNP3580	*Kabuli*	Ca_Kabuli_Chr03	9223875	[G/A]		--	INTERGENIC
3581	CakSNP3581	*Kabuli*	Ca_Kabuli_Chr03	9224033	[C/A]		--	INTERGENIC

S.N.	SNP IDs	Chickpea cultivars	Chromosomes	Physical positions (bp)	SNPs	Gene accession IDs	Putative functions	Sequence components of genome
3582	CakSNP3582	*Kabuli*	Ca_Kabuli_Chr03	9224011	[G/A]		--	INTERGENIC
3583	CakSNP3583	*Kabuli*	Ca_Kabuli_Chr03	9296469	[T/G]	Ca_20960	Arf GTPase activating protein	SYNONYMOUS_CODING
3584	CakSNP3584	*Kabuli*	Ca_Kabuli_Chr03	9448525	[T/C]	Ca_20971	Peptidase S10, serine carboxypeptidase	INTRON
3585	CakSNP3585	*Kabuli*	Ca_Kabuli_Chr03	9577402	[G/A]	Ca_20977	--	NON_SYNONYMOUS_CODING
3586	CakSNP3586	*Kabuli*	Ca_Kabuli_Chr03	9579805	[A/T]		--	INTERGENIC
3587	CakSNP3587	*Kabuli*	Ca_Kabuli_Chr03	9579808	[C/T]		--	INTERGENIC
3588	CakSNP3588	*Kabuli*	Ca_Kabuli_Chr03	9579811	[G/C]		--	INTERGENIC
3589	CakSNP3589	*Kabuli*	Ca_Kabuli_Chr03	9579817	[G/A]		--	INTERGENIC
3590	CakSNP3590	*Kabuli*	Ca_Kabuli_Chr03	9579818	[G/T]		--	INTERGENIC
3591	CakSNP3591	*Kabuli*	Ca_Kabuli_Chr03	9579824	[A/G]		--	INTERGENIC

S.N.	SNP IDs	Chickpea cultivars	Chromosomes	Physical positions (bp)	SNPs	Gene accession IDs	Putative functions	Sequence components of genome
3592	CakSNP3592	*Kabuli*	Ca_Kabuli_Chr03	9579830	[T/A]		--	INTERGENIC
3593	CakSNP3593	*Kabuli*	Ca_Kabuli_Chr03	9579831	[C/G]		--	INTERGENIC
3594	CakSNP3594	*Kabuli*	Ca_Kabuli_Chr03	9579839	[G/A]		--	INTERGENIC
3595	CakSNP3595	*Kabuli*	Ca_Kabuli_Chr03	9894316	[C/T]		--	INTERGENIC
3596	CakSNP3596	*Kabuli*	Ca_Kabuli_Chr03	9894310	[C/A]		--	INTERGENIC
3597	CakSNP3597	*Kabuli*	Ca_Kabuli_Chr03	10039539	[T/A]	Ca_18807	Aldo/keto reductase	NON_SYNONYMOUS_CODING
3598	CakSNP3598	*Kabuli*	Ca_Kabuli_Chr03	10105733	[A/G]		--	INTERGENIC
3599	CakSNP3599	*Kabuli*	Ca_Kabuli_Chr03	10142152	[C/A]	Ca_18811	WD40 repeat	INTRON
3600	CakSNP3600	*Kabuli*	Ca_Kabuli_Chr03	10159836	[A/C]		--	INTERGENIC
3601	CakSNP3601	*Kabuli*	Ca_Kabuli_Chr03	10159859	[G/A]		--	INTERGENIC

S.N.	SNP IDs	Chickpea cultivars	Chromosomes	Physical positions (bp)	SNPs	Gene accession IDs	Putative functions	Sequence components of genome
3602	CakSNP3602	*Kabuli*	Ca_Kabuli_Chr03	10159941	[C/A]		--	INTERGENIC
3603	CakSNP3603	*Kabuli*	Ca_Kabuli_Chr03	10159943	[G/A]		--	INTERGENIC
3604	CakSNP3604	*Kabuli*	Ca_Kabuli_Chr03	10159944	[T/C]		--	INTERGENIC
3605	CakSNP3605	*Kabuli*	Ca_Kabuli_Chr03	10186893	[G/C]	Ca_18812	--	INTRON
3606	CakSNP3606	*Kabuli*	Ca_Kabuli_Chr03	10186861	[G/T]	Ca_18812	--	INTRON
3607	CakSNP3607	*Kabuli*	Ca_Kabuli_Chr03	10186857	[A/G]	Ca_18812	--	INTRON
3608	CakSNP3608	*Kabuli*	Ca_Kabuli_Chr03	10186880	[A/G]	Ca_18812	--	INTRON
3609	CakSNP3609	*Kabuli*	Ca_Kabuli_Chr03	10186889	[G/A]	Ca_18812	--	INTRON
3610	CakSNP3610	*Kabuli*	Ca_Kabuli_Chr03	10186891	[C/G]	Ca_18812	--	INTRON
3611	CakSNP3611	*Kabuli*	Ca_Kabuli_Chr03	10201361	[G/T]	Ca_18813	VRR-NUC	SYNONYMOUS_CODING

S.N.	SNP IDs	Chickpea cultivars	Chromosomes	Physical positions (bp)	SNPs	Gene accession IDs	Putative functions	Sequence components of genome
3612	CakSNP3612	*Kabuli*	Ca_Kabuli_Chr03	10722780	[G/T]	Ca_18831	--	INTRON
3613	CakSNP3613	*Kabuli*	Ca_Kabuli_Chr03	10722849	[G/C]	Ca_18831	--	INTRON
3614	CakSNP3614	*Kabuli*	Ca_Kabuli_Chr03	10722859	[G/A]	Ca_18831	--	INTRON
3615	CakSNP3615	*Kabuli*	Ca_Kabuli_Chr03	11143751	[G/A]	Ca_19368	--	NON_SYNONYMOUS_CODING
3616	CakSNP3616	*Kabuli*	Ca_Kabuli_Chr03	11143818	[G/A]	Ca_19368	--	SYNONYMOUS_CODING
3617	CakSNP3617	*Kabuli*	Ca_Kabuli_Chr03	11143816	[C/T]	Ca_19368	--	SYNONYMOUS_CODING
3618	CakSNP3618	*Kabuli*	Ca_Kabuli_Chr03	11376752	[C/A]	Ca_19376	ABC transporter, transmembrane domain	NON_SYNONYMOUS_CODING
3619	CakSNP3619	*Kabuli*	Ca_Kabuli_Chr03	11378141	[C/T]	Ca_19376	ABC transporter, transmembrane domain	NON_SYNONYMOUS_CODING
3620	CakSNP3620	*Kabuli*	Ca_Kabuli_Chr03	11378425	[G/T]	Ca_19376	ABC transporter, transmembrane domain	NON_SYNONYMOUS_CODING
3621	CakSNP3621	*Kabuli*	Ca_Kabuli_Chr03	11590767	[C/T]	Ca_19380	--	NON_SYNONYMOUS_CODING

S.N.	SNP IDs	Chickpea cultivars	Chromosomes	Physical positions (bp)	SNPs	Gene accession IDs	Putative functions	Sequence components of genome
3622	CakSNP3622	*Kabuli*	Ca_Kabuli_Chr03	11590793	[C/T]	Ca_19380	--	NON_SYNONYMOUS_CODING
3623	CakSNP3623	*Kabuli*	Ca_Kabuli_Chr03	11590817	[C/G]	Ca_19380	--	NON_SYNONYMOUS_CODING
3624	CakSNP3624	*Kabuli*	Ca_Kabuli_Chr03	11590874	[A/G]	Ca_19380	--	NON_SYNONYMOUS_CODING
3625	CakSNP3625	*Kabuli*	Ca_Kabuli_Chr03	11590860	[G/A]	Ca_19380	--	SYNONYMOUS_CODING
3626	CakSNP3626	*Kabuli*	Ca_Kabuli_Chr03	11590832	[G/A]	Ca_19380	--	NON_SYNONYMOUS_CODING
3627	CakSNP3627	*Kabuli*	Ca_Kabuli_Chr03	11590814	[T/C]	Ca_19380	--	NON_SYNONYMOUS_CODING
3628	CakSNP3628	*Kabuli*	Ca_Kabuli_Chr03	11610500	[A/G]		--	INTERGENIC
3629	CakSNP3629	*Kabuli*	Ca_Kabuli_Chr03	11610494	[C/T]		--	INTERGENIC
3630	CakSNP3630	*Kabuli*	Ca_Kabuli_Chr03	11610460	[A/G]		--	INTERGENIC
3631	CakSNP3631	*Kabuli*	Ca_Kabuli_Chr03	11610441	[G/T]		--	INTERGENIC

S.N.	SNP IDs	Chickpea cultivars	Chromosomes	Physical positions (bp)	SNPs	Gene accession IDs	Putative functions	Sequence components of genome
3632	CakSNP3632	*Kabuli*	Ca_Kabuli_Chr03	11610481	[C/T]		--	INTERGENIC
3633	CakSNP3633	*Kabuli*	Ca_Kabuli_Chr03	11610451	[C/T]		--	INTERGENIC
3634	CakSNP3634	*Kabuli*	Ca_Kabuli_Chr03	11678625	[G/A]	Ca_19383	Homeobox	SYNONYMOUS_CODING
3635	CakSNP3635	*Kabuli*	Ca_Kabuli_Chr03	11684552	[G/A]	Ca_19385	Pentatricopeptide repeat	SYNONYMOUS_CODING
3636	CakSNP3636	*Kabuli*	Ca_Kabuli_Chr03	11709777	[G/T]		--	INTERGENIC
3637	CakSNP3637	*Kabuli*	Ca_Kabuli_Chr03	11811685	[A/G]	Ca_19392	Putative zinc-finger domain	INTRON
3638	CakSNP3638	*Kabuli*	Ca_Kabuli_Chr03	12051826	[A/C]		--	INTERGENIC
3639	CakSNP3639	*Kabuli*	Ca_Kabuli_Chr03	12058576	[T/C]	Ca_22246	--	INTRON
3640	CakSNP3640	*Kabuli*	Ca_Kabuli_Chr03	12058756	[A/G]	Ca_22246	--	INTRON
3641	CakSNP3641	*Kabuli*	Ca_Kabuli_Chr03	12058748	[A/T]	Ca_22246	--	INTRON

S.N.	SNP IDs	Chickpea cultivars	Chromosomes	Physical positions (bp)	SNPs	Gene accession IDs	Putative functions	Sequence components of genome
3642	CakSNP3642	*Kabuli*	Ca_Kabuli_Chr03	12067458	[A/C]	Ca_22246	--	INTRON
3643	CakSNP3643	*Kabuli*	Ca_Kabuli_Chr03	12069092	[T/G]	Ca_22246	--	SYNONYMOUS_CODING
3644	CakSNP3644	*Kabuli*	Ca_Kabuli_Chr03	12095571	[T/G]	Ca_22245	Crotonase, core	SYNONYMOUS_CODING
3645	CakSNP3645	*Kabuli*	Ca_Kabuli_Chr03	12124099	[G/A]		--	INTERGENIC
3646	CakSNP3646	*Kabuli*	Ca_Kabuli_Chr03	12394742	[G/A]		--	INTERGENIC
3647	CakSNP3647	*Kabuli*	Ca_Kabuli_Chr03	12599496	[A/C]	Ca_21021	Zinc finger, Dof-type	SYNONYMOUS_CODING
3648	CakSNP3648	*Kabuli*	Ca_Kabuli_Chr03	12622503	[A/G]		--	INTERGENIC
3649	CakSNP3649	*Kabuli*	Ca_Kabuli_Chr03	12622504	[G/T]		--	INTERGENIC
3650	CakSNP3650	*Kabuli*	Ca_Kabuli_Chr03	12622518	[T/G]		--	INTERGENIC
3651	CakSNP3651	*Kabuli*	Ca_Kabuli_Chr03	12622534	[C/T]		--	INTERGENIC

S.N.	SNP IDs	Chickpea cultivars	Chromosomes	Physical positions (bp)	SNPs	Gene accession IDs	Putative functions	Sequence components of genome
3652	CakSNP3652	*Kabuli*	Ca_Kabuli_Chr03	12622550	[A/T]		--	INTERGENIC
3653	CakSNP3653	*Kabuli*	Ca_Kabuli_Chr03	12622578	[G/A]		--	INTERGENIC
3654	CakSNP3654	*Kabuli*	Ca_Kabuli_Chr03	12622567	[A/C]		--	INTERGENIC
3655	CakSNP3655	*Kabuli*	Ca_Kabuli_Chr03	12622543	[G/A]		--	INTERGENIC
3656	CakSNP3656	*Kabuli*	Ca_Kabuli_Chr03	12627120	[C/T]		--	INTERGENIC
3657	CakSNP3657	*Kabuli*	Ca_Kabuli_Chr03	12627125	[T/C]		--	INTERGENIC
3658	CakSNP3658	*Kabuli*	Ca_Kabuli_Chr03	12627131	[G/A]		--	INTERGENIC
3659	CakSNP3659	*Kabuli*	Ca_Kabuli_Chr03	12627144	[G/A]		--	INTERGENIC
3660	CakSNP3660	*Kabuli*	Ca_Kabuli_Chr03	12627165	[T/C]		--	INTERGENIC
3661	CakSNP3661	*Kabuli*	Ca_Kabuli_Chr03	12627143	[T/C]		--	INTERGENIC

S.N.	SNP IDs	Chickpea cultivars	Chromosomes	Physical positions (bp)	SNPs	Gene accession IDs	Putative functions	Sequence components of genome
3662	CakSNP3662	*Kabuli*	Ca_Kabuli_Chr03	12627164	[G/A]		--	INTERGENIC
3663	CakSNP3663	*Kabuli*	Ca_Kabuli_Chr03	12627170	[C/G]		--	INTERGENIC
3664	CakSNP3664	*Kabuli*	Ca_Kabuli_Chr03	12809390	[A/G]	Ca_21015	Kinesin, motor domain	INTRON
3665	CakSNP3665	*Kabuli*	Ca_Kabuli_Chr03	12809794	[T/C]	Ca_21015	Kinesin, motor domain	NON_SYNONYMOUS_CODING
3666	CakSNP3666	*Kabuli*	Ca_Kabuli_Chr03	12814631	[C/T]		--	INTERGENIC
3667	CakSNP3667	*Kabuli*	Ca_Kabuli_Chr03	12814603	[T/C]		--	INTERGENIC
3668	CakSNP3668	*Kabuli*	Ca_Kabuli_Chr03	12814601	[A/C]		--	INTERGENIC
3669	CakSNP3669	*Kabuli*	Ca_Kabuli_Chr03	12814600	[A/G]		--	INTERGENIC
3670	CakSNP3670	*Kabuli*	Ca_Kabuli_Chr03	12814599	[A/T]		--	INTERGENIC
3671	CakSNP3671	*Kabuli*	Ca_Kabuli_Chr03	12814597	[T/A]		--	INTERGENIC

S.N.	SNP IDs	Chickpea cultivars	Chromosomes	Physical positions (bp)	SNPs	Gene accession IDs	Putative functions	Sequence components of genome
3672	CakSNP3672	*Kabuli*	Ca_Kabuli_Chr03	12814571	[C/A]		--	INTERGENIC
3673	CakSNP3673	*Kabuli*	Ca_Kabuli_Chr03	12814558	[A/G]		--	INTERGENIC
3674	CakSNP3674	*Kabuli*	Ca_Kabuli_Chr03	12897820	[C/T]		--	INTERGENIC
3675	CakSNP3675	*Kabuli*	Ca_Kabuli_Chr03	12897831	[C/A]		--	INTERGENIC
3676	CakSNP3676	*Kabuli*	Ca_Kabuli_Chr03	12897839	[G/A]		--	INTERGENIC
3677	CakSNP3677	*Kabuli*	Ca_Kabuli_Chr03	12897841	[C/A]		--	INTERGENIC
3678	CakSNP3678	*Kabuli*	Ca_Kabuli_Chr03	12897876	[C/T]		--	INTERGENIC
3679	CakSNP3679	*Kabuli*	Ca_Kabuli_Chr03	12897881	[C/T]		--	INTERGENIC
3680	CakSNP3680	*Kabuli*	Ca_Kabuli_Chr03	12897882	[T/G]		--	INTERGENIC
3681	CakSNP3681	*Kabuli*	Ca_Kabuli_Chr03	12897926	[A/C]		--	INTERGENIC

S.N.	SNP IDs	Chickpea cultivars	Chromosomes	Physical positions (bp)	SNPs	Gene accession IDs	Putative functions	Sequence components of genome
3682	CakSNP3682	*Kabuli*	Ca_Kabuli_Chr03	12897906	[C/A]		--	INTERGENIC
3683	CakSNP3683	*Kabuli*	Ca_Kabuli_Chr03	12897887	[T/C]		--	INTERGENIC
3684	CakSNP3684	*Kabuli*	Ca_Kabuli_Chr03	12897884	[C/T]		--	INTERGENIC
3685	CakSNP3685	*Kabuli*	Ca_Kabuli_Chr03	12897883	[G/T]		--	INTERGENIC
3686	CakSNP3686	*Kabuli*	Ca_Kabuli_Chr03	12897886	[G/A]		--	INTERGENIC
3687	CakSNP3687	*Kabuli*	Ca_Kabuli_Chr03	13185541	[G/A]	Ca_22687	--	NON_SYNONYMOUS_CODING
3688	CakSNP3688	*Kabuli*	Ca_Kabuli_Chr03	13185530	[C/T]	Ca_22687	--	NON_SYNONYMOUS_CODING
3689	CakSNP3689	*Kabuli*	Ca_Kabuli_Chr03	13185496	[G/A]	Ca_22687	--	NON_SYNONYMOUS_CODING
3690	CakSNP3690	*Kabuli*	Ca_Kabuli_Chr03	13185490	[G/A]	Ca_22687	--	NON_SYNONYMOUS_CODING
3691	CakSNP3691	*Kabuli*	Ca_Kabuli_Chr03	13185493	[G/A]	Ca_22687	--	NON_SYNONYMOUS_CODING

S.N.	SNP IDs	Chickpea cultivars	Chromosomes	Physical positions (bp)	SNPs	Gene accession IDs	Putative functions	Sequence components of genome
3692	CakSNP3692	*Kabuli*	Ca_Kabuli_Chr03	13185508	[C/T]	Ca_22687	--	NON_SYNONYMOUS_CODING
3693	CakSNP3693	*Kabuli*	Ca_Kabuli_Chr03	13185516	[C/T]	Ca_22687	--	SYNONYMOUS_CODING
3694	CakSNP3694	*Kabuli*	Ca_Kabuli_Chr03	13185583	[T/C]	Ca_22687	--	NON_SYNONYMOUS_CODING
3695	CakSNP3695	*Kabuli*	Ca_Kabuli_Chr03	13185581	[T/A]	Ca_22687	--	STOP_GAINED
3696	CakSNP3696	*Kabuli*	Ca_Kabuli_Chr03	13185574	[G/A]	Ca_22687	--	NON_SYNONYMOUS_CODING
3697	CakSNP3697	*Kabuli*	Ca_Kabuli_Chr03	13185560	[G/A]	Ca_22687	--	NON_SYNONYMOUS_CODING
3698	CakSNP3698	*Kabuli*	Ca_Kabuli_Chr03	13185575	[C/T]	Ca_22687	--	NON_SYNONYMOUS_CODING
3699	CakSNP3699	*Kabuli*	Ca_Kabuli_Chr03	13185582	[A/G]	Ca_22687	--	SYNONYMOUS_CODING
3700	CakSNP3700	*Kabuli*	Ca_Kabuli_Chr03	13185590	[C/A]	Ca_22687	--	STOP_GAINED
3701	CakSNP3701	*Kabuli*	Ca_Kabuli_Chr03	13185591	[C/T]	Ca_22687	--	SYNONYMOUS_CODING

S.N.	SNP IDs	Chickpea cultivars	Chromosomes	Physical positions (bp)	SNPs	Gene accession IDs	Putative functions	Sequence components of genome
3702	CakSNP3702	*Kabuli*	Ca_Kabuli_Chr03	13185592	[A/C]	Ca_22687	--	NON_SYNONYMOUS_CODING
3703	CakSNP3703	*Kabuli*	Ca_Kabuli_Chr03	13202989	[C/T]	Ca_22688	--	NON_SYNONYMOUS_CODING
3704	CakSNP3704	*Kabuli*	Ca_Kabuli_Chr03	13396925	[A/T]	Ca_22695	Glycosyl transferase, family 2	INTRON
3705	CakSNP3705	*Kabuli*	Ca_Kabuli_Chr03	13397207	[A/C]	Ca_22695	Glycosyl transferase, family 2	INTRON
3706	CakSNP3706	*Kabuli*	Ca_Kabuli_Chr03	13420776	[T/C]	Ca_22696	Zinc finger, C2H2-type	SYNONYMOUS_CODING
3707	CakSNP3707	*Kabuli*	Ca_Kabuli_Chr03	13420758	[A/G]	Ca_22696	Zinc finger, C2H2-type	NON_SYNONYMOUS_CODING
3708	CakSNP3708	*Kabuli*	Ca_Kabuli_Chr03	13420756	[A/G]	Ca_22696	Zinc finger, C2H2-type	NON_SYNONYMOUS_CODING
3709	CakSNP3709	*Kabuli*	Ca_Kabuli_Chr03	13617948	[A/C]	Ca_25319	--	SYNONYMOUS_CODING
3710	CakSNP3710	*Kabuli*	Ca_Kabuli_Chr03	14200381	[A/C]		--	INTERGENIC
3711	CakSNP3711	*Kabuli*	Ca_Kabuli_Chr03	14465236	[T/G]	Ca_22513	SNF2-related	INTRON

S.N.	SNP IDs	Chickpea cultivars	Chromosomes	Physical positions (bp)	SNPs	Gene accession IDs	Putative functions	Sequence components of genome
3712	CakSNP3712	*Kabuli*	Ca_Kabuli_Chr03	14497374	[C/A]		--	INTERGENIC
3713	CakSNP3713	*Kabuli*	Ca_Kabuli_Chr03	14653195	[G/T]	Ca_22505	Zinc finger, RING-type	INTRON
3714	CakSNP3714	*Kabuli*	Ca_Kabuli_Chr03	14653194	[C/T]	Ca_22505	Zinc finger, RING-type	INTRON
3715	CakSNP3715	*Kabuli*	Ca_Kabuli_Chr03	14653193	[T/G]	Ca_22505	Zinc finger, RING-type	INTRON
3716	CakSNP3716	*Kabuli*	Ca_Kabuli_Chr03	14653270	[C/A]	Ca_22505	Zinc finger, RING-type	INTRON
3717	CakSNP3717	*Kabuli*	Ca_Kabuli_Chr03	14653271	[G/A]	Ca_22505	Zinc finger, RING-type	INTRON
3718	CakSNP3718	*Kabuli*	Ca_Kabuli_Chr03	14658175	[A/C]	Ca_22505	Zinc finger, RING-type	INTRON
3719	CakSNP3719	*Kabuli*	Ca_Kabuli_Chr03	14812960	[A/C]	Ca_22500	UDP-glucuronosyl/UDP-glucosyltransferase	NON_SYNONYMOUS_CODING
3720	CakSNP3720	*Kabuli*	Ca_Kabuli_Chr03	14982667	[C/T]	Ca_22498	DNA mismatch repair protein MutS, C-terminal domain	INTRON
3721	CakSNP3721	*Kabuli*	Ca_Kabuli_Chr03	14982739	[A/T]	Ca_22498	DNA mismatch repair protein MutS, C-terminal domain	INTRON

S.N.	SNP IDs	Chickpea cultivars	Chromosomes	Physical positions (bp)	SNPs	Gene accession IDs	Putative functions	Sequence components of genome
3722	CakSNP3722	*Kabuli*	Ca_Kabuli_Chr03	15744906	[T/A]	Ca_17048	Forkhead-associated (FHA) domain	INTRON
3723	CakSNP3723	*Kabuli*	Ca_Kabuli_Chr03	15745010	[C/T]	Ca_17048	Forkhead-associated (FHA) domain	INTRON
3724	CakSNP3724	*Kabuli*	Ca_Kabuli_Chr03	15745017	[A/G]	Ca_17048	Forkhead-associated (FHA) domain	INTRON
3725	CakSNP3725	*Kabuli*	Ca_Kabuli_Chr03	15745264	[G/C]	Ca_17048	Forkhead-associated (FHA) domain	INTRON
3726	CakSNP3726	*Kabuli*	Ca_Kabuli_Chr03	15745311	[G/A]	Ca_17048	Forkhead-associated (FHA) domain	INTRON
3727	CakSNP3727	*Kabuli*	Ca_Kabuli_Chr03	15757804	[T/C]	Ca_17049	Pectinesterase, catalytic	INTRON
3728	CakSNP3728	*Kabuli*	Ca_Kabuli_Chr03	15757829	[C/T]	Ca_17049	Pectinesterase, catalytic	INTRON
3729	CakSNP3729	*Kabuli*	Ca_Kabuli_Chr03	15757852	[A/C]	Ca_17049	Pectinesterase, catalytic	INTRON
3730	CakSNP3730	*Kabuli*	Ca_Kabuli_Chr03	16071411	[G/C]	Ca_17064	MIF4G-like, type 3	NON_SYNONYMOUS_CODING
3731	CakSNP3731	*Kabuli*	Ca_Kabuli_Chr03	16396662	[C/G]	Ca_18261	Glyoxalase/bleomycin resistance protein/dioxygenase	SYNONYMOUS_CODING

S.N.	SNP IDs	Chickpea cultivars	Chromosomes	Physical positions (bp)	SNPs	Gene accession IDs	Putative functions	Sequence components of genome
3732	CakSNP3732	*Kabuli*	Ca_Kabuli_Chr03	16396673	[A/C]	Ca_18261	Glyoxalase/bleomycin resistance protein/dioxygenase	SYNONYMOUS_CODING
3733	CakSNP3733	*Kabuli*	Ca_Kabuli_Chr03	16436286	[T/A]	Ca_18265	--	NON_SYNONYMOUS_CODING
3734	CakSNP3734	*Kabuli*	Ca_Kabuli_Chr03	16458123	[G/T]	Ca_18266	Bile acid:sodium symporter	NON_SYNONYMOUS_CODING
3735	CakSNP3735	*Kabuli*	Ca_Kabuli_Chr03	16458413	[T/A]	Ca_18266	Bile acid:sodium symporter	INTRON
3736	CakSNP3736	*Kabuli*	Ca_Kabuli_Chr03	16540328	[T/G]	Ca_18272	Glycoside hydrolase, family 1	INTRON
3737	CakSNP3737	*Kabuli*	Ca_Kabuli_Chr03	16568567	[A/G]	Ca_18273	Glycoside hydrolase, family 1	NON_SYNONYMOUS_CODING
3738	CakSNP3738	*Kabuli*	Ca_Kabuli_Chr03	16621498	[G/A]		--	INTERGENIC
3739	CakSNP3739	*Kabuli*	Ca_Kabuli_Chr03	16621624	[G/T]		--	INTERGENIC
3740	CakSNP3740	*Kabuli*	Ca_Kabuli_Chr03	16768867	[A/G]		--	INTERGENIC
3741	CakSNP3741	*Kabuli*	Ca_Kabuli_Chr03	16768936	[A/G]		--	INTERGENIC

S.N.	SNP IDs	Chickpea cultivars	Chromosomes	Physical positions (bp)	SNPs	Gene accession IDs	Putative functions	Sequence components of genome
3742	CakSNP3742	*Kabuli*	Ca_Kabuli_Chr03	16768917	[C/T]		--	INTERGENIC
3743	CakSNP3743	*Kabuli*	Ca_Kabuli_Chr03	16948510	[G/T]		--	INTERGENIC
3744	CakSNP3744	*Kabuli*	Ca_Kabuli_Chr03	16948854	[A/T]	Ca_18293	Ribosomal protein L7/L12, C-terminal	NON_SYNONYMOUS_CODING
3745	CakSNP3745	*Kabuli*	Ca_Kabuli_Chr03	16995243	[C/T]		--	INTERGENIC
3746	CakSNP3746	*Kabuli*	Ca_Kabuli_Chr03	16997328	[G/A]	Ca_18297	Alcohol dehydrogenase superfamily, zinc-containing	SYNONYMOUS_CODING
3747	CakSNP3747	*Kabuli*	Ca_Kabuli_Chr03	16997331	[C/T]	Ca_18297	Alcohol dehydrogenase superfamily, zinc-containing	SYNONYMOUS_CODING
3748	CakSNP3748	*Kabuli*	Ca_Kabuli_Chr03	16997400	[T/C]	Ca_18297	Alcohol dehydrogenase superfamily, zinc-containing	SYNONYMOUS_CODING
3749	CakSNP3749	*Kabuli*	Ca_Kabuli_Chr03	17136071	[G/C]		--	INTERGENIC
3750	CakSNP3750	*Kabuli*	Ca_Kabuli_Chr03	17136097	[T/C]		--	INTERGENIC
3751	CakSNP3751	*Kabuli*	Ca_Kabuli_Chr03	17145639	[A/T]		--	INTERGENIC

S.N.	SNP IDs	Chickpea cultivars	Chromosomes	Physical positions (bp)	SNPs	Gene accession IDs	Putative functions	Sequence components of genome
3752	CakSNP3752	*Kabuli*	Ca_Kabuli_Chr03	17145643	[G/T]		--	INTERGENIC
3753	CakSNP3753	*Kabuli*	Ca_Kabuli_Chr03	17181213	[A/G]	Ca_24617	WD40 repeat	NON_SYNONYMOUS_CODING
3754	CakSNP3754	*Kabuli*	Ca_Kabuli_Chr03	17185682	[A/G]		--	INTERGENIC
3755	CakSNP3755	*Kabuli*	Ca_Kabuli_Chr03	17334924	[G/T]		--	INTERGENIC
3756	CakSNP3756	*Kabuli*	Ca_Kabuli_Chr03	17335851	[A/C]		--	INTERGENIC
3757	CakSNP3757	*Kabuli*	Ca_Kabuli_Chr03	17351553	[A/T]	Ca_23341	Multicopper oxidase, type 1	SYNONYMOUS_CODING
3758	CakSNP3758	*Kabuli*	Ca_Kabuli_Chr03	17351526	[A/C]	Ca_23341	Multicopper oxidase, type 1	SYNONYMOUS_CODING
3759	CakSNP3759	*Kabuli*	Ca_Kabuli_Chr03	17459986	[T/A]		--	INTERGENIC
3760	CakSNP3760	*Kabuli*	Ca_Kabuli_Chr03	17460002	[G/A]		--	INTERGENIC
3761	CakSNP3761	*Kabuli*	Ca_Kabuli_Chr03	17460012	[C/A]		--	INTERGENIC

S.N.	SNP IDs	Chickpea cultivars	Chromosomes	Physical positions (bp)	SNPs	Gene accession IDs	Putative functions	Sequence components of genome
3762	CakSNP3762	*Kabuli*	Ca_Kabuli_Chr03	17574247	[C/T]		--	INTERGENIC
3763	CakSNP3763	*Kabuli*	Ca_Kabuli_Chr03	17635591	[T/A]		--	INTERGENIC
3764	CakSNP3764	*Kabuli*	Ca_Kabuli_Chr03	17635531	[T/C]	Ca_23331	--	NON_SYNONYMOUS_CODING
3765	CakSNP3765	*Kabuli*	Ca_Kabuli_Chr03	17679409	[C/T]	Ca_21115	--	SYNONYMOUS_CODING
3766	CakSNP3766	*Kabuli*	Ca_Kabuli_Chr03	17679404	[C/T]	Ca_21115	--	SYNONYMOUS_CODING
3767	CakSNP3767	*Kabuli*	Ca_Kabuli_Chr03	17914677	[T/G]	Ca_21128	--	SYNONYMOUS_CODING
3768	CakSNP3768	*Kabuli*	Ca_Kabuli_Chr03	17936321	[T/C]	Ca_21130	SANT domain, DNA binding	SYNONYMOUS_CODING
3769	CakSNP3769	*Kabuli*	Ca_Kabuli_Chr03	17936729	[G/A]	Ca_21130	SANT domain, DNA binding	SYNONYMOUS_CODING
3770	CakSNP3770	*Kabuli*	Ca_Kabuli_Chr03	17936842	[A/G]	Ca_21130	SANT domain, DNA binding	SYNONYMOUS_CODING
3771	CakSNP3771	*Kabuli*	Ca_Kabuli_Chr03	17984978	[T/G]	Ca_21132	Protein kinase, catalytic domain	SYNONYMOUS_CODING

S.N.	SNP IDs	Chickpea cultivars	Chromosomes	Physical positions (bp)	SNPs	Gene accession IDs	Putative functions	Sequence components of genome
3772	CakSNP3772	*Kabuli*	Ca_Kabuli_Chr03	17986521	[G/C]	Ca_21132	Protein kinase, catalytic domain	INTRON
3773	CakSNP3773	*Kabuli*	Ca_Kabuli_Chr03	17986525	[G/T]	Ca_21132	Protein kinase, catalytic domain	INTRON
3774	CakSNP3774	*Kabuli*	Ca_Kabuli_Chr03	17986740	[T/C]	Ca_21132	Protein kinase, catalytic domain	SYNONYMOUS_CODING
3775	CakSNP3775	*Kabuli*	Ca_Kabuli_Chr03	18215321	[G/C]	Ca_19612	Oligopeptide transporter	SYNONYMOUS_CODING
3776	CakSNP3776	*Kabuli*	Ca_Kabuli_Chr03	18215332	[T/C]	Ca_19612	Oligopeptide transporter	NON_SYNONYMOUS_CODING
3777	CakSNP3777	*Kabuli*	Ca_Kabuli_Chr03	18355427	[T/C]	Ca_19620	Protein of unknown function DUF3411	SYNONYMOUS_CODING
3778	CakSNP3778	*Kabuli*	Ca_Kabuli_Chr03	18355445	[T/C]	Ca_19620	Protein of unknown function DUF3411	SYNONYMOUS_CODING
3779	CakSNP3779	*Kabuli*	Ca_Kabuli_Chr03	18375547	[T/C]	Ca_19622	Glutathione S-transferase, N-terminal	NON_SYNONYMOUS_CODING
3780	CakSNP3780	*Kabuli*	Ca_Kabuli_Chr03	18375581	[G/C]	Ca_19622	Glutathione S-transferase, N-terminal	NON_SYNONYMOUS_CODING
3781	CakSNP3781	*Kabuli*	Ca_Kabuli_Chr03	18382404	[T/C]	Ca_19624	Glutathione S-transferase, N-terminal	NON_SYNONYMOUS_CODING

S.N.	SNP IDs	Chickpea cultivars	Chromosomes	Physical positions (bp)	SNPs	Gene accession IDs	Putative functions	Sequence components of genome
3782	CakSNP3782	*Kabuli*	Ca_Kabuli_Chr03	18382438	[G/C]	Ca_19624	Glutathione S-transferase, N-terminal	NON_SYNONYMOUS_CODING
3783	CakSNP3783	*Kabuli*	Ca_Kabuli_Chr03	18383884	[T/C]	Ca_19625	Glutathione S-transferase, N-terminal	NON_SYNONYMOUS_CODING
3784	CakSNP3784	*Kabuli*	Ca_Kabuli_Chr03	18476006	[A/G]		--	INTERGENIC
3785	CakSNP3785	*Kabuli*	Ca_Kabuli_Chr03	18476065	[C/A]		--	INTERGENIC
3786	CakSNP3786	*Kabuli*	Ca_Kabuli_Chr03	18497633	[T/C]	Ca_19631	Ctr copper transporter	NON_SYNONYMOUS_CODING
3787	CakSNP3787	*Kabuli*	Ca_Kabuli_Chr03	18573701	[T/A]	Ca_19637	Zinc finger, RING-CH-type	INTRON
3788	CakSNP3788	*Kabuli*	Ca_Kabuli_Chr03	18645114	[T/C]	Ca_19638	Multicopper oxidase, type 1	SYNONYMOUS_CODING
3789	CakSNP3789	*Kabuli*	Ca_Kabuli_Chr03	18687994	[T/A]	Ca_19641	Fructose-1,6-bisphosphatase class 1/Sedoheputulose-1,7-bisphosphatase	NON_SYNONYMOUS_CODING
3790	CakSNP3790	*Kabuli*	Ca_Kabuli_Chr03	18709398	[G/A]		--	INTERGENIC
3791	CakSNP3791	*Kabuli*	Ca_Kabuli_Chr03	18731098	[T/C]	Ca_19642	Legume lectin, beta chain	SYNONYMOUS_CODING

S.N.	SNP IDs	Chickpea cultivars	Chromosomes	Physical positions (bp)	SNPs	Gene accession IDs	Putative functions	Sequence components of genome
3792	CakSNP3792	*Kabuli*	Ca_Kabuli_Chr03	18825708	[T/C]		--	INTERGENIC
3793	CakSNP3793	*Kabuli*	Ca_Kabuli_Chr03	18825687	[T/C]		--	INTERGENIC
3794	CakSNP3794	*Kabuli*	Ca_Kabuli_Chr03	18939425	[C/G]	Ca_19321	--	NON_SYNONYMOUS_CODING
3795	CakSNP3795	*Kabuli*	Ca_Kabuli_Chr03	19372722	[G/T]		--	INTERGENIC
3796	CakSNP3796	*Kabuli*	Ca_Kabuli_Chr03	19372739	[C/T]		--	INTERGENIC
3797	CakSNP3797	*Kabuli*	Ca_Kabuli_Chr03	19400104	[T/C]		--	INTERGENIC
3798	CakSNP3798	*Kabuli*	Ca_Kabuli_Chr03	19400121	[G/T]		--	INTERGENIC
3799	CakSNP3799	*Kabuli*	Ca_Kabuli_Chr03	19400276	[C/T]		--	INTERGENIC
3800	CakSNP3800	*Kabuli*	Ca_Kabuli_Chr03	19442839	[G/A]		--	INTERGENIC
3801	CakSNP3801	*Kabuli*	Ca_Kabuli_Chr03	19442884	[G/C]		--	INTERGENIC

S.N.	SNP IDs	Chickpea cultivars	Chromosomes	Physical positions (bp)	SNPs	Gene accession IDs	Putative functions	Sequence components of genome
3802	CakSNP3802	*Kabuli*	Ca_Kabuli_Chr03	19442941	[G/A]		--	INTERGENIC
3803	CakSNP3803	*Kabuli*	Ca_Kabuli_Chr03	19445925	[A/G]		--	INTERGENIC
3804	CakSNP3804	*Kabuli*	Ca_Kabuli_Chr03	19445920	[T/A]		--	INTERGENIC
3805	CakSNP3805	*Kabuli*	Ca_Kabuli_Chr03	19448140	[A/G]	Ca_19342	Protein of unknown function DUF647	INTRON
3806	CakSNP3806	*Kabuli*	Ca_Kabuli_Chr03	19475095	[G/T]	Ca_19345	Mitochondrial carrier protein	INTRON
3807	CakSNP3807	*Kabuli*	Ca_Kabuli_Chr03	19571396	[C/A]		--	INTERGENIC
3808	CakSNP3808	*Kabuli*	Ca_Kabuli_Chr03	19571382	[G/T]		--	INTERGENIC
3809	CakSNP3809	*Kabuli*	Ca_Kabuli_Chr03	19571371	[A/G]		--	INTERGENIC
3810	CakSNP3810	*Kabuli*	Ca_Kabuli_Chr03	19742600	[G/A]		--	INTERGENIC
3811	CakSNP3811	*Kabuli*	Ca_Kabuli_Chr03	19742530	[C/T]		--	INTERGENIC

S.N.	SNP IDs	Chickpea cultivars	Chromosomes	Physical positions (bp)	SNPs	Gene accession IDs	Putative functions	Sequence components of genome
3812	CakSNP3812	*Kabuli*	Ca_Kabuli_Chr03	19788512	[G/A]		--	INTERGENIC
3813	CakSNP3813	*Kabuli*	Ca_Kabuli_Chr03	19802763	[C/T]	Ca_09383	Adenosine/AMP deaminase	INTRON
3814	CakSNP3814	*Kabuli*	Ca_Kabuli_Chr03	19804985	[T/C]	Ca_09383	Adenosine/AMP deaminase	SYNONYMOUS_CODING
3815	CakSNP3815	*Kabuli*	Ca_Kabuli_Chr03	19805063	[A/C]	Ca_09383	Adenosine/AMP deaminase	SYNONYMOUS_CODING
3816	CakSNP3816	*Kabuli*	Ca_Kabuli_Chr03	19829510	[G/T]		--	INTERGENIC
3817	CakSNP3817	*Kabuli*	Ca_Kabuli_Chr03	19850532	[T/C]	Ca_09387	Stress induced protein	SYNONYMOUS_CODING
3818	CakSNP3818	*Kabuli*	Ca_Kabuli_Chr03	19850589	[C/T]	Ca_09387	Stress induced protein	SYNONYMOUS_CODING
3819	CakSNP3819	*Kabuli*	Ca_Kabuli_Chr03	19855217	[T/C]	Ca_09388	Peptidase M24, structural domain	NON_SYNONYMOUS_CODING
3820	CakSNP3820	*Kabuli*	Ca_Kabuli_Chr03	19857613	[T/A]	Ca_09388	Peptidase M24, structural domain	INTRON
3821	CakSNP3821	*Kabuli*	Ca_Kabuli_Chr03	19883898	[T/C]	Ca_09392	Importin-beta, N-terminal	SYNONYMOUS_CODING

S.N.	SNP IDs	Chickpea cultivars	Chromosomes	Physical positions (bp)	SNPs	Gene accession IDs	Putative functions	Sequence components of genome
3822	CakSNP3822	*Kabuli*	Ca_Kabuli_Chr03	19883927	[A/G]	Ca_09392	Importin-beta, N-terminal	NON_SYNONYMOUS_CODING
3823	CakSNP3823	*Kabuli*	Ca_Kabuli_Chr03	19883936	[T/G]	Ca_09392	Importin-beta, N-terminal	NON_SYNONYMOUS_CODING
3824	CakSNP3824	*Kabuli*	Ca_Kabuli_Chr03	19896800	[C/T]	Ca_09393	Peptidase C48, SUMO/Sentrin/Ubl1	SYNONYMOUS_CODING
3825	CakSNP3825	*Kabuli*	Ca_Kabuli_Chr03	19896849	[G/A]	Ca_09393	Peptidase C48, SUMO/Sentrin/Ubl1	NON_SYNONYMOUS_CODING
3826	CakSNP3826	*Kabuli*	Ca_Kabuli_Chr03	19907774	[T/C]		--	INTERGENIC
3827	CakSNP3827	*Kabuli*	Ca_Kabuli_Chr03	19956343	[C/T]	Ca_09398	Ubiquitin	INTRON
3828	CakSNP3828	*Kabuli*	Ca_Kabuli_Chr03	19956391	[C/T]	Ca_09398	Ubiquitin	INTRON
3829	CakSNP3829	*Kabuli*	Ca_Kabuli_Chr03	19978343	[A/C]	Ca_09399	Ammonium transporter	NON_SYNONYMOUS_CODING
3830	CakSNP3830	*Kabuli*	Ca_Kabuli_Chr03	19978334	[A/C]	Ca_09399	Ammonium transporter	NON_SYNONYMOUS_CODING
3831	CakSNP3831	*Kabuli*	Ca_Kabuli_Chr03	19978407	[C/T]	Ca_09399	Ammonium transporter	SYNONYMOUS_CODING

S.N.	SNP IDs	Chickpea cultivars	Chromosomes	Physical positions (bp)	SNPs	Gene accession IDs	Putative functions	Sequence components of genome
3832	CakSNP3832	*Kabuli*	Ca_Kabuli_Chr03	19979288	[A/T]	Ca_09399	Ammonium transporter	SYNONYMOUS_CODING
3833	CakSNP3833	*Kabuli*	Ca_Kabuli_Chr03	19979318	[C/A]	Ca_09399	Ammonium transporter	SYNONYMOUS_CODING
3834	CakSNP3834	*Kabuli*	Ca_Kabuli_Chr03	19979390	[A/T]	Ca_09399	Ammonium transporter	SYNONYMOUS_CODING
3835	CakSNP3835	*Kabuli*	Ca_Kabuli_Chr03	20000967	[C/G]	Ca_09402	F-box domain, cyclin-like	NON_SYNONYMOUS_CODING
3836	CakSNP3836	*Kabuli*	Ca_Kabuli_Chr03	20030262	[T/C]	Ca_09406	Protein of unknown function DUF688	SYNONYMOUS_CODING
3837	CakSNP3837	*Kabuli*	Ca_Kabuli_Chr03	20030298	[G/A]	Ca_09406	Protein of unknown function DUF688	SYNONYMOUS_CODING
3838	CakSNP3838	*Kabuli*	Ca_Kabuli_Chr03	20030484	[A/G]	Ca_09406	Protein of unknown function DUF688	SYNONYMOUS_CODING
3839	CakSNP3839	*Kabuli*	Ca_Kabuli_Chr03	20103039	[G/A]		--	INTERGENIC
3840	CakSNP3840	*Kabuli*	Ca_Kabuli_Chr03	20103060	[C/T]		--	INTERGENIC
3841	CakSNP3841	*Kabuli*	Ca_Kabuli_Chr03	20103126	[A/C]		--	INTERGENIC

S.N.	SNP IDs	Chickpea cultivars	Chromosomes	Physical positions (bp)	SNPs	Gene accession IDs	Putative functions	Sequence components of genome
3842	CakSNP3842	*Kabuli*	Ca_Kabuli_Chr03	20172492	[C/T]	Ca_09421	Protein kinase, catalytic domain	INTRON
3843	CakSNP3843	*Kabuli*	Ca_Kabuli_Chr03	20240118	[T/G]	Ca_09424	CCAAT-binding factor	INTRON
3844	CakSNP3844	*Kabuli*	Ca_Kabuli_Chr03	20242663	[C/T]	Ca_09424	CCAAT-binding factor	INTRON
3845	CakSNP3845	*Kabuli*	Ca_Kabuli_Chr03	20331659	[G/A]	Ca_09431	Pentatricopeptide repeat	SYNONYMOUS_CODING
3846	CakSNP3846	*Kabuli*	Ca_Kabuli_Chr03	20365442	[A/C]		--	INTERGENIC
3847	CakSNP3847	*Kabuli*	Ca_Kabuli_Chr03	20387399	[T/A]	Ca_09434	Protein phosphatase 2C-like	SYNONYMOUS_CODING
3848	CakSNP3848	*Kabuli*	Ca_Kabuli_Chr03	20387471	[A/G]	Ca_09434	Protein phosphatase 2C-like	SYNONYMOUS_CODING
3849	CakSNP3849	*Kabuli*	Ca_Kabuli_Chr03	20415660	[C/G]		--	INTERGENIC
3850	CakSNP3850	*Kabuli*	Ca_Kabuli_Chr03	20415615	[A/G]		--	INTERGENIC
3851	CakSNP3851	*Kabuli*	Ca_Kabuli_Chr03	20462715	[T/A]	Ca_09436	UDP-glucuronosyl/UDP-glucosyltransferase	SYNONYMOUS_CODING

S.N.	SNP IDs	Chickpea cultivars	Chromosomes	Physical positions (bp)	SNPs	Gene accession IDs	Putative functions	Sequence components of genome
3852	CakSNP3852	*Kabuli*	Ca_Kabuli_Chr03	20490488	[T/C]	Ca_09439	UDP-glucuronosyl/UDP-glucosyltransferase	SYNONYMOUS_CODING
3853	CakSNP3853	*Kabuli*	Ca_Kabuli_Chr03	20495810	[G/A]	Ca_09440	UDP-glucuronosyl/UDP-glucosyltransferase	SYNONYMOUS_CODING
3854	CakSNP3854	*Kabuli*	Ca_Kabuli_Chr03	20503939	[G/T]	Ca_09441	Uncharacterised protein family UPF0497, trans-membrane plant	INTRON
3855	CakSNP3855	*Kabuli*	Ca_Kabuli_Chr03	20506663	[C/G]	Ca_09442	DNA-binding WRKY	INTRON
3856	CakSNP3856	*Kabuli*	Ca_Kabuli_Chr03	20552836	[T/G]	Ca_09446	--	NON_SYNONYMOUS_CODING
3857	CakSNP3857	*Kabuli*	Ca_Kabuli_Chr03	20555942	[G/A]	Ca_09446	--	NON_SYNONYMOUS_CODING
3858	CakSNP3858	*Kabuli*	Ca_Kabuli_Chr03	20690207	[T/G]	Ca_09451	NSF attachment protein	INTRON
3859	CakSNP3859	*Kabuli*	Ca_Kabuli_Chr03	20690185	[A/T]	Ca_09451	NSF attachment protein	INTRON
3860	CakSNP3860	*Kabuli*	Ca_Kabuli_Chr03	20794236	[A/T]	Ca_09455	Integrase, catalytic core	INTRON
3861	CakSNP3861	*Kabuli*	Ca_Kabuli_Chr03	20794206	[A/T]	Ca_09455	Integrase, catalytic core	INTRON

S.N.	SNP IDs	Chickpea cultivars	Chromosomes	Physical positions (bp)	SNPs	Gene accession IDs	Putative functions	Sequence components of genome
3862	CakSNP3862	*Kabuli*	Ca_Kabuli_Chr03	20794984	[C/T]	Ca_09455	Integrase, catalytic core	INTRON
3863	CakSNP3863	*Kabuli*	Ca_Kabuli_Chr03	20795019	[C/T]	Ca_09455	Integrase, catalytic core	INTRON
3864	CakSNP3864	*Kabuli*	Ca_Kabuli_Chr03	20795014	[C/T]	Ca_09455	Integrase, catalytic core	INTRON
3865	CakSNP3865	*Kabuli*	Ca_Kabuli_Chr03	20794976	[A/C]	Ca_09455	Integrase, catalytic core	INTRON
3866	CakSNP3866	*Kabuli*	Ca_Kabuli_Chr03	20794993	[A/G]	Ca_09455	Integrase, catalytic core	INTRON
3867	CakSNP3867	*Kabuli*	Ca_Kabuli_Chr03	20794990	[G/A]	Ca_09455	Integrase, catalytic core	INTRON
3868	CakSNP3868	*Kabuli*	Ca_Kabuli_Chr03	20799767	[A/G]		--	INTERGENIC
3869	CakSNP3869	*Kabuli*	Ca_Kabuli_Chr03	20799778	[C/T]		--	INTERGENIC
3870	CakSNP3870	*Kabuli*	Ca_Kabuli_Chr03	20799811	[C/T]		--	INTERGENIC
3871	CakSNP3871	*Kabuli*	Ca_Kabuli_Chr03	20799860	[A/G]		--	INTERGENIC

S.N.	SNP IDs	Chickpea cultivars	Chromosomes	Physical positions (bp)	SNPs	Gene accession IDs	Putative functions	Sequence components of genome
3872	CakSNP3872	*Kabuli*	Ca_Kabuli_Chr03	20799841	[C/T]		--	INTERGENIC
3873	CakSNP3873	*Kabuli*	Ca_Kabuli_Chr03	20799819	[C/T]		--	INTERGENIC
3874	CakSNP3874	*Kabuli*	Ca_Kabuli_Chr03	20799820	[A/G]		--	INTERGENIC
3875	CakSNP3875	*Kabuli*	Ca_Kabuli_Chr03	20822676	[T/C]	Ca_09458	VHS	INTRON
3876	CakSNP3876	*Kabuli*	Ca_Kabuli_Chr03	20823146	[C/T]	Ca_09458	VHS	SYNONYMOUS_CODING
3877	CakSNP3877	*Kabuli*	Ca_Kabuli_Chr03	20828272	[G/T]	Ca_09459	HEAT	SYNONYMOUS_CODING
3878	CakSNP3878	*Kabuli*	Ca_Kabuli_Chr03	20829399	[A/G]	Ca_09459	HEAT	INTRON
3879	CakSNP3879	*Kabuli*	Ca_Kabuli_Chr03	20829407	[T/G]	Ca_09459	HEAT	INTRON
3880	CakSNP3880	*Kabuli*	Ca_Kabuli_Chr03	20841298	[T/G]	Ca_09459	HEAT	INTRON
3881	CakSNP3881	*Kabuli*	Ca_Kabuli_Chr03	20841352	[C/T]	Ca_09459	HEAT	INTRON

S.N.	SNP IDs	Chickpea cultivars	Chromosomes	Physical positions (bp)	SNPs	Gene accession IDs	Putative functions	Sequence components of genome
3882	CakSNP3882	*Kabuli*	Ca_Kabuli_Chr03	20880294	[T/A]	Ca_09462	Aminotransferase class-III	INTRON
3883	CakSNP3883	*Kabuli*	Ca_Kabuli_Chr03	21020182	[A/G]	Ca_09470	Protein of unknown function DUF827, plant	NON_SYNONYMOUS_CODING
3884	CakSNP3884	*Kabuli*	Ca_Kabuli_Chr03	21020191	[C/T]	Ca_09470	Protein of unknown function DUF827, plant	NON_SYNONYMOUS_CODING
3885	CakSNP3885	*Kabuli*	Ca_Kabuli_Chr03	21069672	[A/G]	Ca_09474	Aminoacyl-tRNA synthetase, class II (D/K/N)	SYNONYMOUS_CODING
3886	CakSNP3886	*Kabuli*	Ca_Kabuli_Chr03	21069714	[G/A]	Ca_09474	Aminoacyl-tRNA synthetase, class II (D/K/N)	INTRON
3887	CakSNP3887	*Kabuli*	Ca_Kabuli_Chr03	21069916	[C/T]	Ca_09474	Aminoacyl-tRNA synthetase, class II (D/K/N)	SYNONYMOUS_CODING
3888	CakSNP3888	*Kabuli*	Ca_Kabuli_Chr03	21069917	[G/A]	Ca_09474	Aminoacyl-tRNA synthetase, class II (D/K/N)	NON_SYNONYMOUS_CODING
3889	CakSNP3889	*Kabuli*	Ca_Kabuli_Chr03	21069938	[C/A]	Ca_09474	Aminoacyl-tRNA synthetase, class II (D/K/N)	INTRON
3890	CakSNP3890	*Kabuli*	Ca_Kabuli_Chr03	21069959	[G/A]	Ca_09474	Aminoacyl-tRNA synthetase, class II (D/K/N)	INTRON
3891	CakSNP3891	*Kabuli*	Ca_Kabuli_Chr03	21069986	[A/C]	Ca_09474	Aminoacyl-tRNA synthetase, class II (D/K/N)	INTRON

S.N.	SNP IDs	Chickpea cultivars	Chromosomes	Physical positions (bp)	SNPs	Gene accession IDs	Putative functions	Sequence components of genome
3892	CakSNP3892	*Kabuli*	Ca_Kabuli_Chr03	21127162	[C/G]		--	INTERGENIC
3893	CakSNP3893	*Kabuli*	Ca_Kabuli_Chr03	21127156	[T/G]		--	INTERGENIC
3894	CakSNP3894	*Kabuli*	Ca_Kabuli_Chr03	21198607	[A/T]	Ca_09482	Plant lipid transfer protein/Par allergen	SYNONYMOUS_CODING
3895	CakSNP3895	*Kabuli*	Ca_Kabuli_Chr03	21210002	[T/A]	Ca_09483	Plant lipid transfer protein/Par allergen	SYNONYMOUS_CODING
3896	CakSNP3896	*Kabuli*	Ca_Kabuli_Chr03	21210005	[C/A]	Ca_09483	Plant lipid transfer protein/Par allergen	SYNONYMOUS_CODING
3897	CakSNP3897	*Kabuli*	Ca_Kabuli_Chr03	21210008	[T/C]	Ca_09483	Plant lipid transfer protein/Par allergen	SYNONYMOUS_CODING
3898	CakSNP3898	*Kabuli*	Ca_Kabuli_Chr03	21210049	[A/G]	Ca_09483	Plant lipid transfer protein/Par allergen	NON_SYNONYMOUS_CODING
3899	CakSNP3899	*Kabuli*	Ca_Kabuli_Chr03	21273368	[A/G]	Ca_09489	--	INTRON
3900	CakSNP3900	*Kabuli*	Ca_Kabuli_Chr03	21288626	[C/A]		--	INTERGENIC
3901	CakSNP3901	*Kabuli*	Ca_Kabuli_Chr03	21288652	[G/C]		--	INTERGENIC

S.N.	SNP IDs	Chickpea cultivars	Chromosomes	Physical positions (bp)	SNPs	Gene accession IDs	Putative functions	Sequence components of genome
3902	CakSNP3902	*Kabuli*	Ca_Kabuli_Chr03	21288695	[G/A]		--	INTERGENIC
3903	CakSNP3903	*Kabuli*	Ca_Kabuli_Chr03	21335892	[C/T]	Ca_09494	Protein of unknown function DUF599	SYNONYMOUS_CODING
3904	CakSNP3904	*Kabuli*	Ca_Kabuli_Chr03	21382790	[G/A]	Ca_09496	ATPase, P-type, K/Mg/Cd/Cu/Zn/Na/Ca/Na/H-transporter	SYNONYMOUS_CODING
3905	CakSNP3905	*Kabuli*	Ca_Kabuli_Chr03	21421082	[A/G]		--	INTERGENIC
3906	CakSNP3906	*Kabuli*	Ca_Kabuli_Chr03	21421085	[G/A]		--	INTERGENIC
3907	CakSNP3907	*Kabuli*	Ca_Kabuli_Chr03	21421146	[C/A]		--	INTERGENIC
3908	CakSNP3908	*Kabuli*	Ca_Kabuli_Chr03	21421162	[G/A]		--	INTERGENIC
3909	CakSNP3909	*Kabuli*	Ca_Kabuli_Chr03	21421358	[G/A]		--	INTERGENIC
3910	CakSNP3910	*Kabuli*	Ca_Kabuli_Chr03	21421362	[T/A]		--	INTERGENIC
3911	CakSNP3911	*Kabuli*	Ca_Kabuli_Chr03	21421369	[G/A]		--	INTERGENIC

S.N.	SNP IDs	Chickpea cultivars	Chromosomes	Physical positions (bp)	SNPs	Gene accession IDs	Putative functions	Sequence components of genome
3912	CakSNP3912	*Kabuli*	Ca_Kabuli_Chr03	21421424	[G/A]		--	INTERGENIC
3913	CakSNP3913	*Kabuli*	Ca_Kabuli_Chr03	21421440	[C/T]		--	INTERGENIC
3914	CakSNP3914	*Kabuli*	Ca_Kabuli_Chr03	21450650	[A/G]	Ca_09500	Protein kinase, catalytic domain	NON_SYNONYMOUS_CODING
3915	CakSNP3915	*Kabuli*	Ca_Kabuli_Chr03	21452017	[G/A]	Ca_09500	Protein kinase, catalytic domain	SYNONYMOUS_CODING
3916	CakSNP3916	*Kabuli*	Ca_Kabuli_Chr03	21468634	[G/A]	Ca_09503	Protein kinase, catalytic domain	NON_SYNONYMOUS_CODING
3917	CakSNP3917	*Kabuli*	Ca_Kabuli_Chr03	21468692	[A/C]	Ca_09503	Protein kinase, catalytic domain	NON_SYNONYMOUS_CODING
3918	CakSNP3918	*Kabuli*	Ca_Kabuli_Chr03	21470784	[C/T]	Ca_09503	Protein kinase, catalytic domain	SYNONYMOUS_CODING
3919	CakSNP3919	*Kabuli*	Ca_Kabuli_Chr03	21500178	[G/A]	Ca_09507	Protein kinase, catalytic domain	NON_SYNONYMOUS_CODING
3920	CakSNP3920	*Kabuli*	Ca_Kabuli_Chr03	21500179	[A/T]	Ca_09507	Protein kinase, catalytic domain	NON_SYNONYMOUS_CODING
3921	CakSNP3921	*Kabuli*	Ca_Kabuli_Chr03	21507422	[T/C]	Ca_09508	--	INTRON

S.N.	SNP IDs	Chickpea cultivars	Chromosomes	Physical positions (bp)	SNPs	Gene accession IDs	Putative functions	Sequence components of genome
3922	CakSNP3922	*Kabuli*	Ca_Kabuli_Chr03	21524084	[T/C]		--	INTERGENIC
3923	CakSNP3923	*Kabuli*	Ca_Kabuli_Chr03	21524192	[T/C]		--	INTERGENIC
3924	CakSNP3924	*Kabuli*	Ca_Kabuli_Chr03	21524296	[G/T]		--	INTERGENIC
3925	CakSNP3925	*Kabuli*	Ca_Kabuli_Chr03	21524379	[A/G]		--	INTERGENIC
3926	CakSNP3926	*Kabuli*	Ca_Kabuli_Chr03	21525184	[C/T]		--	INTERGENIC
3927	CakSNP3927	*Kabuli*	Ca_Kabuli_Chr03	21525177	[G/A]		--	INTERGENIC
3928	CakSNP3928	*Kabuli*	Ca_Kabuli_Chr03	21558388	[A/G]	Ca_09512	Pentatricopeptide repeat	SYNONYMOUS_CODING
3929	CakSNP3929	*Kabuli*	Ca_Kabuli_Chr03	21558357	[T/G]	Ca_09512	Pentatricopeptide repeat	SYNONYMOUS_CODING
3930	CakSNP3930	*Kabuli*	Ca_Kabuli_Chr03	21627610	[T/C]	Ca_09515	--	NON_SYNONYMOUS_CODING
3931	CakSNP3931	*Kabuli*	Ca_Kabuli_Chr03	21634275	[A/C]	Ca_09516	--	INTRON

S.N.	SNP IDs	Chickpea cultivars	Chromosomes	Physical positions (bp)	SNPs	Gene accession IDs	Putative functions	Sequence components of genome
3932	CakSNP3932	*Kabuli*	Ca_Kabuli_Chr03	21634271	[A/G]	Ca_09516	--	INTRON
3933	CakSNP3933	*Kabuli*	Ca_Kabuli_Chr03	21646863	[C/T]	Ca_09518	Isy1-like splicing	SYNONYMOUS_CODING
3934	CakSNP3934	*Kabuli*	Ca_Kabuli_Chr03	21646872	[T/C]	Ca_09518	Isy1-like splicing	SYNONYMOUS_CODING
3935	CakSNP3935	*Kabuli*	Ca_Kabuli_Chr03	21646893	[C/A]	Ca_09518	Isy1-like splicing	SYNONYMOUS_CODING
3936	CakSNP3936	*Kabuli*	Ca_Kabuli_Chr03	21646965	[C/T]	Ca_09518	Isy1-like splicing	SYNONYMOUS_CODING
3937	CakSNP3937	*Kabuli*	Ca_Kabuli_Chr03	21646962	[A/G]	Ca_09518	Isy1-like splicing	SYNONYMOUS_CODING
3938	CakSNP3938	*Kabuli*	Ca_Kabuli_Chr03	21654592	[T/A]	Ca_09519	Auxin efflux carrier	INTRON
3939	CakSNP3939	*Kabuli*	Ca_Kabuli_Chr03	21686470	[C/T]	Ca_09523	--	SYNONYMOUS_CODING
3940	CakSNP3940	*Kabuli*	Ca_Kabuli_Chr03	21687439	[G/T]	Ca_09523	--	SYNONYMOUS_CODING
3941	CakSNP3941	*Kabuli*	Ca_Kabuli_Chr03	21687966	[C/A]	Ca_09523	--	NON_SYNONYMOUS_CODING

S.N.	SNP IDs	Chickpea cultivars	Chromosomes	Physical positions (bp)	SNPs	Gene accession IDs	Putative functions	Sequence components of genome
3942	CakSNP3942	*Kabuli*	Ca_Kabuli_Chr03	21687961	[C/A]	Ca_09523	--	STOP_GAINED
3943	CakSNP3943	*Kabuli*	Ca_Kabuli_Chr03	21687960	[G/A]	Ca_09523	--	NON_SYNONYMOUS_CODING
3944	CakSNP3944	*Kabuli*	Ca_Kabuli_Chr03	21687959	[T/C]	Ca_09523	--	NON_SYNONYMOUS_CODING
3945	CakSNP3945	*Kabuli*	Ca_Kabuli_Chr03	21687956	[C/T]	Ca_09523	--	SYNONYMOUS_CODING
3946	CakSNP3946	*Kabuli*	Ca_Kabuli_Chr03	21687953	[C/T]	Ca_09523	--	NON_SYNONYMOUS_CODING
3947	CakSNP3947	*Kabuli*	Ca_Kabuli_Chr03	21741914	[C/A]		--	INTERGENIC
3948	CakSNP3948	*Kabuli*	Ca_Kabuli_Chr03	21809395	[G/A]		--	INTERGENIC
3949	CakSNP3949	*Kabuli*	Ca_Kabuli_Chr03	21809456	[T/C]		--	INTERGENIC
3950	CakSNP3950	*Kabuli*	Ca_Kabuli_Chr03	21809457	[G/A]		--	INTERGENIC
3951	CakSNP3951	*Kabuli*	Ca_Kabuli_Chr03	21809546	[T/A]		--	INTERGENIC

S.N.	SNP IDs	Chickpea cultivars	Chromosomes	Physical positions (bp)	SNPs	Gene accession IDs	Putative functions	Sequence components of genome
3952	CakSNP3952	*Kabuli*	Ca_Kabuli_Chr03	21809505	[A/G]		--	INTERGENIC
3953	CakSNP3953	*Kabuli*	Ca_Kabuli_Chr03	21809503	[T/C]		--	INTERGENIC
3954	CakSNP3954	*Kabuli*	Ca_Kabuli_Chr03	21950053	[C/G]	Ca_20431	Domain of unknown function DUF296	INTRON
3955	CakSNP3955	*Kabuli*	Ca_Kabuli_Chr03	21950186	[A/G]	Ca_20431	Domain of unknown function DUF296	SYNONYMOUS_CODING
3956	CakSNP3956	*Kabuli*	Ca_Kabuli_Chr03	21950164	[A/G]	Ca_20431	Domain of unknown function DUF296	INTRON
3957	CakSNP3957	*Kabuli*	Ca_Kabuli_Chr03	22008087	[G/A]	Ca_20426	SEC7-like	NON_SYNONYMOUS_CODING
3958	CakSNP3958	*Kabuli*	Ca_Kabuli_Chr03	22026111	[A/G]	Ca_20423	Armadillo	INTRON
3959	CakSNP3959	*Kabuli*	Ca_Kabuli_Chr03	22026647	[A/G]	Ca_20423	Armadillo	INTRON
3960	CakSNP3960	*Kabuli*	Ca_Kabuli_Chr03	22060199	[G/T]	Ca_20420	Forkhead-associated (FHA) domain	NON_SYNONYMOUS_CODING
3961	CakSNP3961	*Kabuli*	Ca_Kabuli_Chr03	22061236	[A/G]	Ca_20420	Forkhead-associated (FHA) domain	INTRON

S.N.	SNP IDs	Chickpea cultivars	Chromosomes	Physical positions (bp)	SNPs	Gene accession IDs	Putative functions	Sequence components of genome
3962	CakSNP3962	*Kabuli*	Ca_Kabuli_Chr03	22062029	[G/T]	Ca_20420	Forkhead-associated (FHA) domain	INTRON
3963	CakSNP3963	*Kabuli*	Ca_Kabuli_Chr03	22062958	[T/C]	Ca_20420	Forkhead-associated (FHA) domain	SYNONYMOUS_CODING
3964	CakSNP3964	*Kabuli*	Ca_Kabuli_Chr03	22062965	[C/T]	Ca_20420	Forkhead-associated (FHA) domain	INTRON
3965	CakSNP3965	*Kabuli*	Ca_Kabuli_Chr03	22063826	[G/A]	Ca_20420	Forkhead-associated (FHA) domain	INTRON
3966	CakSNP3966	*Kabuli*	Ca_Kabuli_Chr03	22063829	[G/A]	Ca_20420	Forkhead-associated (FHA) domain	INTRON
3967	CakSNP3967	*Kabuli*	Ca_Kabuli_Chr03	22063835	[C/T]	Ca_20420	Forkhead-associated (FHA) domain	INTRON
3968	CakSNP3968	*Kabuli*	Ca_Kabuli_Chr03	22073381	[C/T]	Ca_20419	--	SYNONYMOUS_CODING
3969	CakSNP3969	*Kabuli*	Ca_Kabuli_Chr03	22073447	[T/A]	Ca_20419	--	NON_SYNONYMOUS_CODING
3970	CakSNP3970	*Kabuli*	Ca_Kabuli_Chr03	22075779	[C/G]	Ca_20418	Ankyrin repeat	INTRON
3971	CakSNP3971	*Kabuli*	Ca_Kabuli_Chr03	22075990	[T/C]	Ca_20418	Ankyrin repeat	SYNONYMOUS_CODING

S.N.	SNP IDs	Chickpea cultivars	Chromosomes	Physical positions (bp)	SNPs	Gene accession IDs	Putative functions	Sequence components of genome
3972	CakSNP3972	*Kabuli*	Ca_Kabuli_Chr03	22169816	[T/C]	Ca_20412	BEACH domain	SYNONYMOUS_CODING
3973	CakSNP3973	*Kabuli*	Ca_Kabuli_Chr03	22188124	[T/C]	Ca_20411	Domain of unknown function DUF1088	SYNONYMOUS_CODING
3974	CakSNP3974	*Kabuli*	Ca_Kabuli_Chr03	22188169	[T/A]	Ca_20411	Domain of unknown function DUF1088	SYNONYMOUS_CODING
3975	CakSNP3975	*Kabuli*	Ca_Kabuli_Chr03	22200927	[T/C]	Ca_20410	--	SYNONYMOUS_CODING
3976	CakSNP3976	*Kabuli*	Ca_Kabuli_Chr03	22201045	[T/A]	Ca_20410	--	NON_SYNONYMOUS_CODING
3977	CakSNP3977	*Kabuli*	Ca_Kabuli_Chr03	22202317	[C/T]	Ca_20410	--	NON_SYNONYMOUS_CODING
3978	CakSNP3978	*Kabuli*	Ca_Kabuli_Chr03	22202819	[T/C]	Ca_20410	--	NON_SYNONYMOUS_CODING
3979	CakSNP3979	*Kabuli*	Ca_Kabuli_Chr03	22203460	[A/G]	Ca_20410	--	SYNONYMOUS_CODING
3980	CakSNP3980	*Kabuli*	Ca_Kabuli_Chr03	22225186	[C/A]		--	INTERGENIC
3981	CakSNP3981	*Kabuli*	Ca_Kabuli_Chr03	22225154	[C/G]	Ca_20408	Plastocyanin-like	NON_SYNONYMOUS_CODING

S.N.	SNP IDs	Chickpea cultivars	Chromosomes	Physical positions (bp)	SNPs	Gene accession IDs	Putative functions	Sequence components of genome
3982	CakSNP3982	*Kabuli*	Ca_Kabuli_Chr03	22225129	[C/G]	Ca_20408	Plastocyanin-like	SYNONYMOUS_CODING
3983	CakSNP3983	*Kabuli*	Ca_Kabuli_Chr03	22233428	[G/A]		--	INTERGENIC
3984	CakSNP3984	*Kabuli*	Ca_Kabuli_Chr03	22233409	[T/C]		--	INTERGENIC
3985	CakSNP3985	*Kabuli*	Ca_Kabuli_Chr03	22235801	[G/A]	Ca_20407	C2 calcium-dependent membrane targeting	NON_SYNONYMOUS_CODING
3986	CakSNP3986	*Kabuli*	Ca_Kabuli_Chr03	22253842	[T/C]	Ca_20405	Glycosyl transferase, family 8	INTRON
3987	CakSNP3987	*Kabuli*	Ca_Kabuli_Chr03	22331057	[C/T]	Ca_06277	Peptidase S8/S53, subtilisin/kexin/sedolisin	INTRON
3988	CakSNP3988	*Kabuli*	Ca_Kabuli_Chr03	22346294	[T/C]	Ca_06276	Peptidase S8/S53, subtilisin/kexin/sedolisin	NON_SYNONYMOUS_CODING
3989	CakSNP3989	*Kabuli*	Ca_Kabuli_Chr03	22404660	[G/T]	Ca_06269	Pentatricopeptide repeat	NON_SYNONYMOUS_CODING
3990	CakSNP3990	*Kabuli*	Ca_Kabuli_Chr03	22527529	[A/C]		--	INTERGENIC
3991	CakSNP3991	*Kabuli*	Ca_Kabuli_Chr03	22539384	[G/A]	Ca_06260	Armadillo	SYNONYMOUS_CODING

S.N.	SNP IDs	Chickpea cultivars	Chromosomes	Physical positions (bp)	SNPs	Gene accession IDs	Putative functions	Sequence components of genome
3992	CakSNP3992	*Kabuli*	Ca_Kabuli_Chr03	22539702	[C/T]	Ca_06260	Armadillo	SYNONYMOUS_CODING
3993	CakSNP3993	*Kabuli*	Ca_Kabuli_Chr03	22539885	[C/T]	Ca_06260	Armadillo	SYNONYMOUS_CODING
3994	CakSNP3994	*Kabuli*	Ca_Kabuli_Chr03	22539952	[G/A]	Ca_06260	Armadillo	NON_SYNONYMOUS_CODING
3995	CakSNP3995	*Kabuli*	Ca_Kabuli_Chr03	22636201	[T/C]	Ca_06249	--	SYNONYMOUS_CODING
3996	CakSNP3996	*Kabuli*	Ca_Kabuli_Chr03	22646897	[T/C]		--	INTERGENIC
3997	CakSNP3997	*Kabuli*	Ca_Kabuli_Chr03	22659924	[G/A]	Ca_06245	SPX, N-terminal	SYNONYMOUS_CODING
3998	CakSNP3998	*Kabuli*	Ca_Kabuli_Chr03	22681658	[G/A]	Ca_06243	Glycosyl transferase, group 1	SYNONYMOUS_CODING
3999	CakSNP3999	*Kabuli*	Ca_Kabuli_Chr03	22719470	[T/C]		--	INTERGENIC
4000	CakSNP4000	*Kabuli*	Ca_Kabuli_Chr03	22719487	[T/C]		--	INTERGENIC
4001	CakSNP4001	*Kabuli*	Ca_Kabuli_Chr03	22719494	[C/T]		--	INTERGENIC

S.N.	SNP IDs	Chickpea cultivars	Chromosomes	Physical positions (bp)	SNPs	Gene accession IDs	Putative functions	Sequence components of genome
4002	CakSNP4002	*Kabuli*	Ca_Kabuli_Chr03	22719519	[G/A]		--	INTERGENIC
4003	CakSNP4003	*Kabuli*	Ca_Kabuli_Chr03	22727393	[G/A]	Ca_06238	Malic oxidoreductase	NON_SYNONYMOUS_CODING
4004	CakSNP4004	*Kabuli*	Ca_Kabuli_Chr03	22727439	[A/C]	Ca_06238	Malic oxidoreductase	SYNONYMOUS_CODING
4005	CakSNP4005	*Kabuli*	Ca_Kabuli_Chr03	22765806	[G/A]	Ca_06235	Eukaryotic phosphomannomutase	INTRON
4006	CakSNP4006	*Kabuli*	Ca_Kabuli_Chr03	22765862	[T/C]	Ca_06235	Eukaryotic phosphomannomutase	INTRON
4007	CakSNP4007	*Kabuli*	Ca_Kabuli_Chr03	22765987	[G/T]	Ca_06235	Eukaryotic phosphomannomutase	NON_SYNONYMOUS_CODING
4008	CakSNP4008	*Kabuli*	Ca_Kabuli_Chr03	22798281	[T/G]		--	INTERGENIC
4009	CakSNP4009	*Kabuli*	Ca_Kabuli_Chr03	22812717	[A/T]		--	INTERGENIC
4010	CakSNP4010	*Kabuli*	Ca_Kabuli_Chr03	22846051	[G/A]	Ca_06225	RNA helicase, ATP-dependent, DEAD-box, conserved site	INTRON
4011	CakSNP4011	*Kabuli*	Ca_Kabuli_Chr03	22847885	[G/T]	Ca_06225	RNA helicase, ATP-dependent, DEAD-box, conserved site	SYNONYMOUS_CODING

S.N.	SNP IDs	Chickpea cultivars	Chromosomes	Physical positions (bp)	SNPs	Gene accession IDs	Putative functions	Sequence components of genome
4012	CakSNP4012	*Kabuli*	Ca_Kabuli_Chr03	22847984	[A/G]	Ca_06225	RNA helicase, ATP-dependent, DEAD-box, conserved site	SYNONYMOUS_CODING
4013	CakSNP4013	*Kabuli*	Ca_Kabuli_Chr03	22990523	[C/T]	Ca_06210	Glycoside hydrolase, family 14	NON_SYNONYMOUS_CODING
4014	CakSNP4014	*Kabuli*	Ca_Kabuli_Chr03	23028855	[A/G]	Ca_06206	Mediator complex, subunit Med12	NON_SYNONYMOUS_CODING
4015	CakSNP4015	*Kabuli*	Ca_Kabuli_Chr03	23033373	[A/C]	Ca_06206	Mediator complex, subunit Med12	NON_SYNONYMOUS_CODING
4016	CakSNP4016	*Kabuli*	Ca_Kabuli_Chr03	23042249	[A/C]	Ca_06206	Mediator complex, subunit Med12	NON_SYNONYMOUS_CODING
4017	CakSNP4017	*Kabuli*	Ca_Kabuli_Chr03	23053008	[C/G]		--	INTERGENIC
4018	CakSNP4018	*Kabuli*	Ca_Kabuli_Chr03	23053005	[G/C]		--	INTERGENIC
4019	CakSNP4019	*Kabuli*	Ca_Kabuli_Chr03	23053066	[A/C]		--	INTERGENIC
4020	CakSNP4020	*Kabuli*	Ca_Kabuli_Chr03	23095498	[C/T]	Ca_06203	Down-regulated-in-metastasis protein	INTRON
4021	CakSNP4021	*Kabuli*	Ca_Kabuli_Chr03	23273057	[T/A]	Ca_06189	SNF2-related	SYNONYMOUS_CODING

S.N.	SNP IDs	Chickpea cultivars	Chromosomes	Physical positions (bp)	SNPs	Gene accession IDs	Putative functions	Sequence components of genome
4022	CakSNP4022	*Kabuli*	Ca_Kabuli_Chr03	23273069	[C/G]	Ca_06189	SNF2-related	NON_SYNONYMOUS_CODING
4023	CakSNP4023	*Kabuli*	Ca_Kabuli_Chr03	23327648	[C/T]	Ca_06187	Zinc finger, RING-type	INTRON
4024	CakSNP4024	*Kabuli*	Ca_Kabuli_Chr03	23327700	[G/C]	Ca_06187	Zinc finger, RING-type	INTRON
4025	CakSNP4025	*Kabuli*	Ca_Kabuli_Chr03	23331530	[T/G]	Ca_06187	Zinc finger, RING-type	INTRON
4026	CakSNP4026	*Kabuli*	Ca_Kabuli_Chr03	23335788	[A/T]		--	INTERGENIC
4027	CakSNP4027	*Kabuli*	Ca_Kabuli_Chr03	23335789	[T/G]		--	INTERGENIC
4028	CakSNP4028	*Kabuli*	Ca_Kabuli_Chr03	23340171	[A/G]	Ca_06186	Protein of unknown function DUF3522	INTRON
4029	CakSNP4029	*Kabuli*	Ca_Kabuli_Chr03	23340134	[G/A]	Ca_06186	Protein of unknown function DUF3522	INTRON
4030	CakSNP4030	*Kabuli*	Ca_Kabuli_Chr03	23340133	[T/C]	Ca_06186	Protein of unknown function DUF3522	INTRON
4031	CakSNP4031	*Kabuli*	Ca_Kabuli_Chr03	23341384	[T/G]	Ca_06186	Protein of unknown function DUF3522	INTRON

S.N.	SNP IDs	Chickpea cultivars	Chromosomes	Physical positions (bp)	SNPs	Gene accession IDs	Putative functions	Sequence components of genome
4032	CakSNP4032	*Kabuli*	Ca_Kabuli_Chr03	23342297	[T/C]	Ca_06186	Protein of unknown function DUF3522	INTRON
4033	CakSNP4033	*Kabuli*	Ca_Kabuli_Chr03	23342305	[T/C]	Ca_06186	Protein of unknown function DUF3522	INTRON
4034	CakSNP4034	*Kabuli*	Ca_Kabuli_Chr03	23342338	[G/T]	Ca_06186	Protein of unknown function DUF3522	INTRON
4035	CakSNP4035	*Kabuli*	Ca_Kabuli_Chr03	23342347	[G/A]	Ca_06186	Protein of unknown function DUF3522	INTRON
4036	CakSNP4036	*Kabuli*	Ca_Kabuli_Chr03	23342350	[A/G]	Ca_06186	Protein of unknown function DUF3522	INTRON
4037	CakSNP4037	*Kabuli*	Ca_Kabuli_Chr03	23342351	[T/C]	Ca_06186	Protein of unknown function DUF3522	INTRON
4038	CakSNP4038	*Kabuli*	Ca_Kabuli_Chr03	23360857	[C/T]		--	INTERGENIC
4039	CakSNP4039	*Kabuli*	Ca_Kabuli_Chr03	23400864	[A/C]		--	INTERGENIC
4040	CakSNP4040	*Kabuli*	Ca_Kabuli_Chr03	23455008	[A/G]	Ca_06179	Protein kinase, catalytic domain	SYNONYMOUS_CODING
4041	CakSNP4041	*Kabuli*	Ca_Kabuli_Chr03	23455128	[A/G]	Ca_06179	Protein kinase, catalytic domain	SYNONYMOUS_CODING

S.N.	SNP IDs	Chickpea cultivars	Chromosomes	Physical positions (bp)	SNPs	Gene accession IDs	Putative functions	Sequence components of genome
4042	CakSNP4042	*Kabuli*	Ca_Kabuli_Chr03	23461370	[C/A]		--	INTERGENIC
4043	CakSNP4043	*Kabuli*	Ca_Kabuli_Chr03	23555586	[C/A]		--	INTERGENIC
4044	CakSNP4044	*Kabuli*	Ca_Kabuli_Chr03	23593288	[G/A]		--	INTERGENIC
4045	CakSNP4045	*Kabuli*	Ca_Kabuli_Chr03	23593294	[T/C]		--	INTERGENIC
4046	CakSNP4046	*Kabuli*	Ca_Kabuli_Chr03	23593309	[T/C]		--	INTERGENIC
4047	CakSNP4047	*Kabuli*	Ca_Kabuli_Chr03	23593269	[T/C]		--	INTERGENIC
4048	CakSNP4048	*Kabuli*	Ca_Kabuli_Chr03	23714158	[T/A]		--	INTERGENIC
4049	CakSNP4049	*Kabuli*	Ca_Kabuli_Chr03	23714129	[C/A]		--	INTERGENIC
4050	CakSNP4050	*Kabuli*	Ca_Kabuli_Chr03	23745324	[C/T]	Ca_06154	UDP-glucuronosyl/UDP-glucosyltransferase	INTRON
4051	CakSNP4051	*Kabuli*	Ca_Kabuli_Chr03	23792975	[A/T]		--	INTERGENIC

S.N.	SNP IDs	Chickpea cultivars	Chromosomes	Physical positions (bp)	SNPs	Gene accession IDs	Putative functions	Sequence components of genome
4052	CakSNP4052	*Kabuli*	Ca_Kabuli_Chr03	23833683	[G/A]	Ca_06141	Cystathionine beta-synthase, core	SYNONYMOUS_CODING
4053	CakSNP4053	*Kabuli*	Ca_Kabuli_Chr03	23833680	[G/C]	Ca_06141	Cystathionine beta-synthase, core	SYNONYMOUS_CODING
4054	CakSNP4054	*Kabuli*	Ca_Kabuli_Chr03	23833668	[G/A]	Ca_06141	Cystathionine beta-synthase, core	SYNONYMOUS_CODING
4055	CakSNP4055	*Kabuli*	Ca_Kabuli_Chr03	23886582	[A/G]	Ca_06135	Cupin 1	NON_SYNONYMOUS_CODING
4056	CakSNP4056	*Kabuli*	Ca_Kabuli_Chr03	23918105	[T/C]	Ca_06131	Uncharacterised protein family UPF0016	INTRON
4057	CakSNP4057	*Kabuli*	Ca_Kabuli_Chr03	23966110	[G/A]	Ca_06124	DNA-binding WRKY	SYNONYMOUS_CODING
4058	CakSNP4058	*Kabuli*	Ca_Kabuli_Chr03	23997287	[A/T]	Ca_06121	NUDIX hydrolase domain	INTRON
4059	CakSNP4059	*Kabuli*	Ca_Kabuli_Chr03	24000847	[G/T]	Ca_06121	NUDIX hydrolase domain	INTRON
4060	CakSNP4060	*Kabuli*	Ca_Kabuli_Chr03	24000818	[T/C]	Ca_06121	NUDIX hydrolase domain	INTRON
4061	CakSNP4061	*Kabuli*	Ca_Kabuli_Chr03	24024560	[T/C]	Ca_06119	CMP/dCMP deaminase, zinc-binding	SYNONYMOUS_CODING

S.N.	SNP IDs	Chickpea cultivars	Chromosomes	Physical positions (bp)	SNPs	Gene accession IDs	Putative functions	Sequence components of genome
4062	CakSNP4062	*Kabuli*	Ca_Kabuli_Chr03	24024513	[A/C]	Ca_06119	CMP/dCMP deaminase, zinc-binding	NON_SYNONYMOUS_CODING
4063	CakSNP4063	*Kabuli*	Ca_Kabuli_Chr03	24025900	[G/C]	Ca_06119	CMP/dCMP deaminase, zinc-binding	INTRON
4064	CakSNP4064	*Kabuli*	Ca_Kabuli_Chr03	24025924	[T/G]	Ca_06119	CMP/dCMP deaminase, zinc-binding	INTRON
4065	CakSNP4065	*Kabuli*	Ca_Kabuli_Chr03	24026428	[C/T]	Ca_06119	CMP/dCMP deaminase, zinc-binding	SYNONYMOUS_CODING
4066	CakSNP4066	*Kabuli*	Ca_Kabuli_Chr03	24041806	[C/T]	Ca_06118	Protein phosphatase 2C-like	SYNONYMOUS_CODING
4067	CakSNP4067	*Kabuli*	Ca_Kabuli_Chr03	24095268	[T/C]	Ca_06111	Biotin/lipoyl attachment	INTRON
4068	CakSNP4068	*Kabuli*	Ca_Kabuli_Chr03	24099191	[C/G]	Ca_06110	--	NON_SYNONYMOUS_CODING
4069	CakSNP4069	*Kabuli*	Ca_Kabuli_Chr03	24127222	[C/T]		--	INTERGENIC
4070	CakSNP4070	*Kabuli*	Ca_Kabuli_Chr03	24127201	[A/C]		--	INTERGENIC
4071	CakSNP4071	*Kabuli*	Ca_Kabuli_Chr03	24162153	[C/T]	Ca_06103	Lecithin:cholesterol acyltransferase	INTRON

S.N.	SNP IDs	Chickpea cultivars	Chromosomes	Physical positions (bp)	SNPs	Gene accession IDs	Putative functions	Sequence components of genome
4072	CakSNP4072	*Kabuli*	Ca_Kabuli_Chr03	24194574	[C/T]	Ca_06100	Aminoacyl-tRNA synthetase, class II (D/K/N)	INTRON
4073	CakSNP4073	*Kabuli*	Ca_Kabuli_Chr03	24247161	[G/C]	Ca_06095	Rad21/Rec8-like protein, C-terminal, eukaryotic	NON_SYNONYMOUS_CODING
4074	CakSNP4074	*Kabuli*	Ca_Kabuli_Chr03	24297842	[T/G]	Ca_06091	--	SYNONYMOUS_CODING
4075	CakSNP4075	*Kabuli*	Ca_Kabuli_Chr03	24297894	[C/A]	Ca_06091	--	NON_SYNONYMOUS_CODING
4076	CakSNP4076	*Kabuli*	Ca_Kabuli_Chr03	24417477	[T/G]	Ca_06076	Actin-binding FH2/DRF autoregulatory	NON_SYNONYMOUS_CODING
4077	CakSNP4077	*Kabuli*	Ca_Kabuli_Chr03	24417507	[A/T]	Ca_06076	Actin-binding FH2/DRF autoregulatory	SYNONYMOUS_CODING
4078	CakSNP4078	*Kabuli*	Ca_Kabuli_Chr03	24418063	[G/T]	Ca_06076	Actin-binding FH2/DRF autoregulatory	INTRON
4079	CakSNP4079	*Kabuli*	Ca_Kabuli_Chr03	24418111	[A/G]	Ca_06076	Actin-binding FH2/DRF autoregulatory	NON_SYNONYMOUS_CODING
4080	CakSNP4080	*Kabuli*	Ca_Kabuli_Chr03	24418130	[T/A]	Ca_06076	Actin-binding FH2/DRF autoregulatory	NON_SYNONYMOUS_CODING
4081	CakSNP4081	*Kabuli*	Ca_Kabuli_Chr03	24459669	[G/A]	Ca_06071	ATPase, alpha/beta subunit, nucleotide-binding domain	SYNONYMOUS_CODING

S.N.	SNP IDs	Chickpea cultivars	Chromosomes	Physical positions (bp)	SNPs	Gene accession IDs	Putative functions	Sequence components of genome
4082	CakSNP4082	*Kabuli*	Ca_Kabuli_Chr03	24515489	[T/G]		--	INTERGENIC
4083	CakSNP4083	*Kabuli*	Ca_Kabuli_Chr03	24538726	[A/C]	Ca_06062	Smr protein/MutS2 C-terminal	NON_SYNONYMOUS_CODING
4084	CakSNP4084	*Kabuli*	Ca_Kabuli_Chr03	24540199	[A/G]	Ca_06062	Smr protein/MutS2 C-terminal	SYNONYMOUS_CODING
4085	CakSNP4085	*Kabuli*	Ca_Kabuli_Chr03	24540304	[T/C]	Ca_06062	Smr protein/MutS2 C-terminal	SYNONYMOUS_CODING
4086	CakSNP4086	*Kabuli*	Ca_Kabuli_Chr03	24637834	[G/T]	Ca_06053	Peptidase S8/S53, subtilisin/kexin/sedolisin	SYNONYMOUS_CODING
4087	CakSNP4087	*Kabuli*	Ca_Kabuli_Chr03	24637837	[A/C]	Ca_06053	Peptidase S8/S53, subtilisin/kexin/sedolisin	SYNONYMOUS_CODING
4088	CakSNP4088	*Kabuli*	Ca_Kabuli_Chr03	24664582	[G/T]		--	INTERGENIC
4089	CakSNP4089	*Kabuli*	Ca_Kabuli_Chr03	24664550	[T/G]		--	INTERGENIC
4090	CakSNP4090	*Kabuli*	Ca_Kabuli_Chr03	24664651	[G/T]		--	INTERGENIC
4091	CakSNP4091	*Kabuli*	Ca_Kabuli_Chr03	24666594	[T/C]	Ca_06051	Peptidase S8/S53, subtilisin/kexin/sedolisin	NON_SYNONYMOUS_CODING

S.N.	SNP IDs	Chickpea cultivars	Chromosomes	Physical positions (bp)	SNPs	Gene accession IDs	Putative functions	Sequence components of genome
4092	CakSNP4092	*Kabuli*	Ca_Kabuli_Chr03	24763730	[A/G]	Ca_06046	Peptidase S8/S53, subtilisin/kexin/sedolisin	SYNONYMOUS_CODING
4093	CakSNP4093	*Kabuli*	Ca_Kabuli_Chr03	24933940	[T/C]	Ca_06035	Proteasome component (PCI) domain	INTRON
4094	CakSNP4094	*Kabuli*	Ca_Kabuli_Chr03	24933901	[C/A]	Ca_06035	Proteasome component (PCI) domain	INTRON
4095	CakSNP4095	*Kabuli*	Ca_Kabuli_Chr03	24933899	[G/T]	Ca_06035	Proteasome component (PCI) domain	INTRON
4096	CakSNP4096	*Kabuli*	Ca_Kabuli_Chr03	24998316	[C/G]	Ca_06031	--	INTRON
4097	CakSNP4097	*Kabuli*	Ca_Kabuli_Chr03	24998300	[C/T]	Ca_06031	--	INTRON
4098	CakSNP4098	*Kabuli*	Ca_Kabuli_Chr03	25037746	[G/A]	Ca_06025	Pentatricopeptide repeat	SYNONYMOUS_CODING
4099	CakSNP4099	*Kabuli*	Ca_Kabuli_Chr03	25046423	[C/G]	Ca_06024	Peptidyl-tRNA hydrolase	NON_SYNONYMOUS_CODING
4100	CakSNP4100	*Kabuli*	Ca_Kabuli_Chr03	25046421	[T/C]	Ca_06024	Peptidyl-tRNA hydrolase	NON_SYNONYMOUS_CODING
4101	CakSNP4101	*Kabuli*	Ca_Kabuli_Chr03	25055049	[C/T]	Ca_06023	Sugar/inositol transporter	INTRON

S.N.	SNP IDs	Chickpea cultivars	Chromosomes	Physical positions (bp)	SNPs	Gene accession IDs	Putative functions	Sequence components of genome
4102	CakSNP4102	*Kabuli*	Ca_Kabuli_Chr03	25062250	[C/A]	Ca_06022	Protein kinase, catalytic domain	INTRON
4103	CakSNP4103	*Kabuli*	Ca_Kabuli_Chr03	25110037	[T/G]	Ca_06018	Pentatricopeptide repeat	NON_SYNONYMOUS_CODING
4104	CakSNP4104	*Kabuli*	Ca_Kabuli_Chr03	25137192	[T/A]	Ca_06015	LisH dimerisation motif	INTRON
4105	CakSNP4105	*Kabuli*	Ca_Kabuli_Chr03	25147096	[G/A]	Ca_06015	LisH dimerisation motif	SYNONYMOUS_CODING
4106	CakSNP4106	*Kabuli*	Ca_Kabuli_Chr03	25205548	[C/A]		--	INTERGENIC
4107	CakSNP4107	*Kabuli*	Ca_Kabuli_Chr03	25241115	[G/A]	Ca_06005	DNA repair metallo-beta-lactamase	NON_SYNONYMOUS_CODING
4108	CakSNP4108	*Kabuli*	Ca_Kabuli_Chr03	25244194	[A/C]	Ca_06004	Methyltransferase small	SYNONYMOUS_CODING
4109	CakSNP4109	*Kabuli*	Ca_Kabuli_Chr03	25270592	[C/G]		--	INTERGENIC
4110	CakSNP4110	*Kabuli*	Ca_Kabuli_Chr03	25399911	[C/G]	Ca_05991	BAR	INTRON
4111	CakSNP4111	*Kabuli*	Ca_Kabuli_Chr03	25457474	[T/A]		--	INTERGENIC

S.N.	SNP IDs	Chickpea cultivars	Chromosomes	Physical positions (bp)	SNPs	Gene accession IDs	Putative functions	Sequence components of genome
4112	CakSNP4112	*Kabuli*	Ca_Kabuli_Chr03	25457494	[A/T]		--	INTERGENIC
4113	CakSNP4113	*Kabuli*	Ca_Kabuli_Chr03	25457514	[C/A]		--	INTERGENIC
4114	CakSNP4114	*Kabuli*	Ca_Kabuli_Chr03	25462227	[A/C]	Ca_05988	Glycerophosphoryl diester phosphodiesterase	NON_SYNONYMOUS_CODING
4115	CakSNP4115	*Kabuli*	Ca_Kabuli_Chr03	25504708	[A/G]		--	INTERGENIC
4116	CakSNP4116	*Kabuli*	Ca_Kabuli_Chr03	25505438	[C/T]		--	INTERGENIC
4117	CakSNP4117	*Kabuli*	Ca_Kabuli_Chr03	25505420	[G/T]		--	INTERGENIC
4118	CakSNP4118	*Kabuli*	Ca_Kabuli_Chr03	25534044	[C/T]		--	INTERGENIC
4119	CakSNP4119	*Kabuli*	Ca_Kabuli_Chr03	25538867	[T/C]	Ca_05981	F-box domain, cyclin-like	INTRON
4120	CakSNP4120	*Kabuli*	Ca_Kabuli_Chr03	25580072	[A/T]	Ca_05977	Nodulin-like	NON_SYNONYMOUS_CODING
4121	CakSNP4121	*Kabuli*	Ca_Kabuli_Chr03	25614515	[T/G]		--	INTERGENIC

S.N.	SNP IDs	Chickpea cultivars	Chromosomes	Physical positions (bp)	SNPs	Gene accession IDs	Putative functions	Sequence components of genome
4122	CakSNP4122	*Kabuli*	Ca_Kabuli_Chr03	25641434	[C/A]	Ca_23894	MIF4G-like, type 3	INTRON
4123	CakSNP4123	*Kabuli*	Ca_Kabuli_Chr03	25641433	[G/A]	Ca_23894	MIF4G-like, type 3	INTRON
4124	CakSNP4124	*Kabuli*	Ca_Kabuli_Chr03	25688341	[G/A]	Ca_23889	Pentatricopeptide repeat	SYNONYMOUS_CODING
4125	CakSNP4125	*Kabuli*	Ca_Kabuli_Chr03	25702238	[G/A]	Ca_23886	Zinc finger, RING-type	INTRON
4126	CakSNP4126	*Kabuli*	Ca_Kabuli_Chr03	25718829	[C/A]		--	INTERGENIC
4127	CakSNP4127	*Kabuli*	Ca_Kabuli_Chr03	25718827	[T/A]		--	INTERGENIC
4128	CakSNP4128	*Kabuli*	Ca_Kabuli_Chr03	25757693	[C/T]	Ca_23882	SET domain	SYNONYMOUS_CODING
4129	CakSNP4129	*Kabuli*	Ca_Kabuli_Chr03	25987920	[T/G]	Ca_08296	Protein of unknown function DUF677	SYNONYMOUS_CODING
4130	CakSNP4130	*Kabuli*	Ca_Kabuli_Chr03	26119425	[T/G]		--	INTERGENIC
4131	CakSNP4131	*Kabuli*	Ca_Kabuli_Chr03	26119461	[C/T]		--	INTERGENIC

S.N.	SNP IDs	Chickpea cultivars	Chromosomes	Physical positions (bp)	SNPs	Gene accession IDs	Putative functions	Sequence components of genome
4132	CakSNP4132	*Kabuli*	Ca_Kabuli_Chr03	26163449	[G/A]	Ca_08289	Calcium-binding EF-hand	NON_SYNONYMOUS_CODING
4133	CakSNP4133	*Kabuli*	Ca_Kabuli_Chr03	26178592	[G/A]		--	INTERGENIC
4134	CakSNP4134	*Kabuli*	Ca_Kabuli_Chr03	26198368	[G/A]		--	INTERGENIC
4135	CakSNP4135	*Kabuli*	Ca_Kabuli_Chr03	26198372	[G/A]		--	INTERGENIC
4136	CakSNP4136	*Kabuli*	Ca_Kabuli_Chr03	26293846	[A/G]	Ca_08273	Glyceraldehyde 3-phosphate dehydrogenase, NAD(P) binding domain	SYNONYMOUS_CODING
4137	CakSNP4137	*Kabuli*	Ca_Kabuli_Chr03	26335889	[T/A]	Ca_08268	Exportin-1/Importin-beta-like	SYNONYMOUS_CODING
4138	CakSNP4138	*Kabuli*	Ca_Kabuli_Chr03	26339286	[T/C]	Ca_08268	Exportin-1/Importin-beta-like	NON_SYNONYMOUS_CODING
4139	CakSNP4139	*Kabuli*	Ca_Kabuli_Chr03	26339449	[G/T]	Ca_08268	Exportin-1/Importin-beta-like	INTRON
4140	CakSNP4140	*Kabuli*	Ca_Kabuli_Chr03	26562004	[A/G]	Ca_08253	Thioredoxin	INTRON
4141	CakSNP4141	*Kabuli*	Ca_Kabuli_Chr03	26573902	[A/G]	Ca_08252	Not CCR4-Not complex component, N-terminal	NON_SYNONYMOUS_CODING

S.N.	SNP IDs	Chickpea cultivars	Chromosomes	Physical positions (bp)	SNPs	Gene accession IDs	Putative functions	Sequence components of genome
4142	CakSNP4142	*Kabuli*	Ca_Kabuli_Chr03	26574056	[C/T]	Ca_08252	Not CCR4-Not complex component, N-terminal	SYNONYMOUS_CODING
4143	CakSNP4143	*Kabuli*	Ca_Kabuli_Chr03	26574068	[T/G]	Ca_08252	Not CCR4-Not complex component, N-terminal	SYNONYMOUS_CODING
4144	CakSNP4144	*Kabuli*	Ca_Kabuli_Chr03	26574831	[A/G]	Ca_08252	Not CCR4-Not complex component, N-terminal	SYNONYMOUS_CODING
4145	CakSNP4145	*Kabuli*	Ca_Kabuli_Chr03	26589678	[T/A]		--	INTERGENIC
4146	CakSNP4146	*Kabuli*	Ca_Kabuli_Chr03	26627150	[T/G]	Ca_08246	YTH domain	INTRON
4147	CakSNP4147	*Kabuli*	Ca_Kabuli_Chr03	26636553	[A/G]	Ca_08245	Protein kinase, catalytic domain	INTRON
4148	CakSNP4148	*Kabuli*	Ca_Kabuli_Chr03	26646152	[C/T]	Ca_08244	IMP dehydrogenase/GMP reductase	NON_SYNONYMOUS_CODING
4149	CakSNP4149	*Kabuli*	Ca_Kabuli_Chr03	26678343	[T/C]	Ca_08242	Glutamate/phenylalanine/leucine/valine dehydrogenase	INTRON
4150	CakSNP4150	*Kabuli*	Ca_Kabuli_Chr03	26678433	[T/G]	Ca_08242	Glutamate/phenylalanine/leucine/valine dehydrogenase	SYNONYMOUS_CODING
4151	CakSNP4151	*Kabuli*	Ca_Kabuli_Chr03	26726897	[C/A]		--	INTERGENIC

S.N.	SNP IDs	Chickpea cultivars	Chromosomes	Physical positions (bp)	SNPs	Gene accession IDs	Putative functions	Sequence components of genome
4152	CakSNP4152	*Kabuli*	Ca_Kabuli_Chr03	26758452	[C/A]	Ca_08231	HEAT	NON_SYNONYMOUS_CODING
4153	CakSNP4153	*Kabuli*	Ca_Kabuli_Chr03	26848870	[G/C]	Ca_08222	Regulator of chromosome condensation, RCC1	NON_SYNONYMOUS_CODING
4154	CakSNP4154	*Kabuli*	Ca_Kabuli_Chr03	26849288	[T/A]	Ca_08222	Regulator of chromosome condensation, RCC1	NON_SYNONYMOUS_CODING
4155	CakSNP4155	*Kabuli*	Ca_Kabuli_Chr03	26878028	[A/T]	Ca_08221	Plastocyanin-like	SYNONYMOUS_CODING
4156	CakSNP4156	*Kabuli*	Ca_Kabuli_Chr03	26900271	[T/A]	Ca_08215	MIF4G-like, type 3	NON_SYNONYMOUS_CODING
4157	CakSNP4157	*Kabuli*	Ca_Kabuli_Chr03	26911443	[A/T]	Ca_08214	Methionine synthase, vitamin-B12 independent	INTRON
4158	CakSNP4158	*Kabuli*	Ca_Kabuli_Chr03	26911514	[A/G]	Ca_08214	Methionine synthase, vitamin-B12 independent	INTRON
4159	CakSNP4159	*Kabuli*	Ca_Kabuli_Chr03	26911723	[T/A]	Ca_08214	Methionine synthase, vitamin-B12 independent	INTRON
4160	CakSNP4160	*Kabuli*	Ca_Kabuli_Chr03	26911792	[A/G]	Ca_08214	Methionine synthase, vitamin-B12 independent	SYNONYMOUS_CODING
4161	CakSNP4161	*Kabuli*	Ca_Kabuli_Chr03	26976249	[A/G]		--	INTERGENIC

S.N.	SNP IDs	Chickpea cultivars	Chromosomes	Physical positions (bp)	SNPs	Gene accession IDs	Putative functions	Sequence components of genome
4162	CakSNP4162	*Kabuli*	Ca_Kabuli_Chr03	26976323	[G/A]		--	INTERGENIC
4163	CakSNP4163	*Kabuli*	Ca_Kabuli_Chr03	27024215	[T/C]	Ca_08201	Transmembrane protein 194	SYNONYMOUS_CODING
4164	CakSNP4164	*Kabuli*	Ca_Kabuli_Chr03	27047840	[A/G]	Ca_08199	BTB/POZ-like	SYNONYMOUS_CODING
4165	CakSNP4165	*Kabuli*	Ca_Kabuli_Chr03	27047847	[A/C]	Ca_08199	BTB/POZ-like	SYNONYMOUS_CODING
4166	CakSNP4166	*Kabuli*	Ca_Kabuli_Chr03	27063150	[A/C]	Ca_08198	Amino acid/polyamine transporter I	INTRON
4167	CakSNP4167	*Kabuli*	Ca_Kabuli_Chr03	27074842	[C/A]	Ca_08196	Disease resistance protein	NON_SYNONYMOUS_CODING
4168	CakSNP4168	*Kabuli*	Ca_Kabuli_Chr03	27074896	[A/T]	Ca_08196	Disease resistance protein	NON_SYNONYMOUS_CODING
4169	CakSNP4169	*Kabuli*	Ca_Kabuli_Chr03	27074931	[A/T]	Ca_08196	Disease resistance protein	NON_SYNONYMOUS_CODING
4170	CakSNP4170	*Kabuli*	Ca_Kabuli_Chr03	27074930	[A/C]	Ca_08196	Disease resistance protein	NON_SYNONYMOUS_CODING
4171	CakSNP4171	*Kabuli*	Ca_Kabuli_Chr03	27076716	[T/C]	Ca_08196	Disease resistance protein	SYNONYMOUS_CODING

S.N.	SNP IDs	Chickpea cultivars	Chromosomes	Physical positions (bp)	SNPs	Gene accession IDs	Putative functions	Sequence components of genome
4172	CakSNP4172	*Kabuli*	Ca_Kabuli_Chr03	27076735	[T/G]	Ca_08196	Disease resistance protein	NON_SYNONYMOUS_CODING
4173	CakSNP4173	*Kabuli*	Ca_Kabuli_Chr03	27076744	[C/A]	Ca_08196	Disease resistance protein	NON_SYNONYMOUS_CODING
4174	CakSNP4174	*Kabuli*	Ca_Kabuli_Chr03	27105637	[C/T]	Ca_08194	EMSY N-terminal	INTRON
4175	CakSNP4175	*Kabuli*	Ca_Kabuli_Chr03	27157840	[T/A]	Ca_08188	Transcription factor TFIIB-related	INTRON
4176	CakSNP4176	*Kabuli*	Ca_Kabuli_Chr03	27157848	[C/T]	Ca_08188	Transcription factor TFIIB-related	INTRON
4177	CakSNP4177	*Kabuli*	Ca_Kabuli_Chr03	27161813	[T/G]	Ca_08188	Transcription factor TFIIB-related	INTRON
4178	CakSNP4178	*Kabuli*	Ca_Kabuli_Chr03	27161801	[A/G]	Ca_08188	Transcription factor TFIIB-related	INTRON
4179	CakSNP4179	*Kabuli*	Ca_Kabuli_Chr03	27194033	[C/T]	Ca_08186	Zinc finger, Dof-type	SYNONYMOUS_CODING
4180	CakSNP4180	*Kabuli*	Ca_Kabuli_Chr03	27194069	[T/C]	Ca_08186	Zinc finger, Dof-type	SYNONYMOUS_CODING
4181	CakSNP4181	*Kabuli*	Ca_Kabuli_Chr03	27338521	[T/C]		--	INTERGENIC

S.N.	SNP IDs	Chickpea cultivars	Chromosomes	Physical positions (bp)	SNPs	Gene accession IDs	Putative functions	Sequence components of genome
4182	CakSNP4182	*Kabuli*	Ca_Kabuli_Chr03	27342919	[G/A]	Ca_08176	WD40 repeat	INTRON
4183	CakSNP4183	*Kabuli*	Ca_Kabuli_Chr03	27391199	[G/A]		--	INTERGENIC
4184	CakSNP4184	*Kabuli*	Ca_Kabuli_Chr03	27391354	[G/A]		--	INTERGENIC
4185	CakSNP4185	*Kabuli*	Ca_Kabuli_Chr03	27391321	[A/G]		--	INTERGENIC
4186	CakSNP4186	*Kabuli*	Ca_Kabuli_Chr03	27417561	[A/T]	Ca_08165	Multi antimicrobial extrusion protein	NON_SYNONYMOUS_CODING
4187	CakSNP4187	*Kabuli*	Ca_Kabuli_Chr03	27420276	[T/C]	Ca_08165	Multi antimicrobial extrusion protein	SYNONYMOUS_CODING
4188	CakSNP4188	*Kabuli*	Ca_Kabuli_Chr03	27420330	[C/T]	Ca_08165	Multi antimicrobial extrusion protein	SYNONYMOUS_CODING
4189	CakSNP4189	*Kabuli*	Ca_Kabuli_Chr03	27435210	[T/C]	Ca_08164	Multi antimicrobial extrusion protein	INTRON
4190	CakSNP4190	*Kabuli*	Ca_Kabuli_Chr03	27435209	[G/C]	Ca_08164	Multi antimicrobial extrusion protein	INTRON
4191	CakSNP4191	*Kabuli*	Ca_Kabuli_Chr03	27435198	[T/A]	Ca_08164	Multi antimicrobial extrusion protein	INTRON

S.N.	SNP IDs	Chickpea cultivars	Chromosomes	Physical positions (bp)	SNPs	Gene accession IDs	Putative functions	Sequence components of genome
4192	CakSNP4192	*Kabuli*	Ca_Kabuli_Chr03	27447685	[C/A]	Ca_08162	RNA polymerase II, large subunit, CTD	NON_SYNONYMOUS_CODING
4193	CakSNP4193	*Kabuli*	Ca_Kabuli_Chr03	27470443	[A/C]	Ca_08160	--	NON_SYNONYMOUS_CODING
4194	CakSNP4194	*Kabuli*	Ca_Kabuli_Chr03	27470439	[G/T]	Ca_08160	--	NON_SYNONYMOUS_CODING
4195	CakSNP4195	*Kabuli*	Ca_Kabuli_Chr03	27597331	[A/C]	Ca_08149	--	SYNONYMOUS_CODING
4196	CakSNP4196	*Kabuli*	Ca_Kabuli_Chr03	27597409	[A/G]	Ca_08149	--	SYNONYMOUS_CODING
4197	CakSNP4197	*Kabuli*	Ca_Kabuli_Chr03	27632100	[G/A]	Ca_08144	Myb, DNA-binding	NON_SYNONYMOUS_CODING
4198	CakSNP4198	*Kabuli*	Ca_Kabuli_Chr03	27632169	[T/C]	Ca_08144	Myb, DNA-binding	INTRON
4199	CakSNP4199	*Kabuli*	Ca_Kabuli_Chr03	27654798	[A/C]	Ca_08142	RNA recognition motif domain	SYNONYMOUS_CODING
4200	CakSNP4200	*Kabuli*	Ca_Kabuli_Chr03	27673727	[C/A]	Ca_08139	--	NON_SYNONYMOUS_CODING
4201	CakSNP4201	*Kabuli*	Ca_Kabuli_Chr03	27681551	[T/C]	Ca_08138	Protein of unknown function DUF309	INTRON

S.N.	SNP IDs	Chickpea cultivars	Chromosomes	Physical positions (bp)	SNPs	Gene accession IDs	Putative functions	Sequence components of genome
4202	CakSNP4202	*Kabuli*	Ca_Kabuli_Chr03	27716396	[C/T]	Ca_08135	Rubrerythrin	SYNONYMOUS_CODING
4203	CakSNP4203	*Kabuli*	Ca_Kabuli_Chr03	27763236	[C/T]	Ca_08130	Alpha-L-arabinofuranosidase, C-terminal	SYNONYMOUS_CODING
4204	CakSNP4204	*Kabuli*	Ca_Kabuli_Chr03	27812571	[A/T]		--	INTERGENIC
4205	CakSNP4205	*Kabuli*	Ca_Kabuli_Chr03	27876405	[A/G]	Ca_08121	Ubiquitin-associated/translation elongation factor EF1B, N-terminal	SYNONYMOUS_CODING
4206	CakSNP4206	*Kabuli*	Ca_Kabuli_Chr03	27877442	[A/T]	Ca_08121	Ubiquitin-associated/translation elongation factor EF1B, N-terminal	INTRON
4207	CakSNP4207	*Kabuli*	Ca_Kabuli_Chr03	27877479	[C/T]	Ca_08121	Ubiquitin-associated/translation elongation factor EF1B, N-terminal	SYNONYMOUS_CODING
4208	CakSNP4208	*Kabuli*	Ca_Kabuli_Chr03	27877480	[G/A]	Ca_08121	Ubiquitin-associated/translation elongation factor EF1B, N-terminal	SYNONYMOUS_CODING
4209	CakSNP4209	*Kabuli*	Ca_Kabuli_Chr03	27877510	[G/A]	Ca_08121	Ubiquitin-associated/translation elongation factor EF1B, N-terminal	SYNONYMOUS_CODING
4210	CakSNP4210	*Kabuli*	Ca_Kabuli_Chr03	27888817	[G/C]	Ca_08120	Ribosomal protein S1, RNA-binding domain	SYNONYMOUS_CODING
4211	CakSNP4211	*Kabuli*	Ca_Kabuli_Chr03	27889107	[A/G]	Ca_08120	Ribosomal protein S1, RNA-binding domain	NON_SYNONYMOUS_CODING

S.N.	SNP IDs	Chickpea cultivars	Chromosomes	Physical positions (bp)	SNPs	Gene accession IDs	Putative functions	Sequence components of genome
4212	CakSNP4212	*Kabuli*	Ca_Kabuli_Chr03	27890849	[C/T]	Ca_08120	Ribosomal protein S1, RNA-binding domain	SYNONYMOUS_CODING
4213	CakSNP4213	*Kabuli*	Ca_Kabuli_Chr03	27905944	[T/C]	Ca_08119	--	SYNONYMOUS_CODING
4214	CakSNP4214	*Kabuli*	Ca_Kabuli_Chr03	27905989	[G/A]	Ca_08119	--	SYNONYMOUS_CODING
4215	CakSNP4215	*Kabuli*	Ca_Kabuli_Chr03	27905995	[C/T]	Ca_08119	--	SYNONYMOUS_CODING
4216	CakSNP4216	*Kabuli*	Ca_Kabuli_Chr03	27906069	[C/A]	Ca_08119	--	NON_SYNONYMOUS_CODING
4217	CakSNP4217	*Kabuli*	Ca_Kabuli_Chr03	27914409	[C/T]	Ca_08118	Glycoside hydrolase, carbohydrate-binding	INTRON
4218	CakSNP4218	*Kabuli*	Ca_Kabuli_Chr03	27968979	[T/G]	Ca_08113	Zinc finger, C2H2-type	NON_SYNONYMOUS_CODING
4219	CakSNP4219	*Kabuli*	Ca_Kabuli_Chr03	27969014	[T/C]	Ca_08113	Zinc finger, C2H2-type	NON_SYNONYMOUS_CODING
4220	CakSNP4220	*Kabuli*	Ca_Kabuli_Chr03	27969005	[A/G]	Ca_08113	Zinc finger, C2H2-type	NON_SYNONYMOUS_CODING
4221	CakSNP4221	*Kabuli*	Ca_Kabuli_Chr03	27969207	[C/T]	Ca_08113	Zinc finger, C2H2-type	NON_SYNONYMOUS_CODING

S.N.	SNP IDs	Chickpea cultivars	Chromosomes	Physical positions (bp)	SNPs	Gene accession IDs	Putative functions	Sequence components of genome
4222	CakSNP4222	*Kabuli*	Ca_Kabuli_Chr03	28044035	[A/T]	Ca_08106	Oligopeptide transporter	NON_SYNONYMOUS_CODING
4223	CakSNP4223	*Kabuli*	Ca_Kabuli_Chr03	28176380	[A/C]	Ca_26950	Oleosin	SYNONYMOUS_CODING
4224	CakSNP4224	*Kabuli*	Ca_Kabuli_Chr03	28176333	[C/A]	Ca_26950	Oleosin	SYNONYMOUS_CODING
4225	CakSNP4225	*Kabuli*	Ca_Kabuli_Chr03	28277991	[C/G]	Ca_07143	Single-stranded nucleic acid binding R3H	INTRON
4226	CakSNP4226	*Kabuli*	Ca_Kabuli_Chr03	28278018	[C/G]	Ca_07143	Single-stranded nucleic acid binding R3H	INTRON
4227	CakSNP4227	*Kabuli*	Ca_Kabuli_Chr03	28374024	[C/T]	Ca_07151	Transcription elongation factor, TFIIS/CRSP70, N-terminal, sub-type	SYNONYMOUS_CODING
4228	CakSNP4228	*Kabuli*	Ca_Kabuli_Chr03	28375082	[G/A]	Ca_07151	Transcription elongation factor, TFIIS/CRSP70, N-terminal, sub-type	INTRON
4229	CakSNP4229	*Kabuli*	Ca_Kabuli_Chr03	28409001	[G/A]		--	INTERGENIC
4230	CakSNP4230	*Kabuli*	Ca_Kabuli_Chr03	28420948	[C/T]		--	INTERGENIC
4231	CakSNP4231	*Kabuli*	Ca_Kabuli_Chr03	28478044	[C/T]	Ca_07159	Helix-loop-helix DNA-binding domain	SYNONYMOUS_CODING

S.N.	SNP IDs	Chickpea cultivars	Chromosomes	Physical positions (bp)	SNPs	Gene accession IDs	Putative functions	Sequence components of genome
4232	CakSNP4232	*Kabuli*	Ca_Kabuli_Chr03	28478539	[C/G]	Ca_07159	Helix-loop-helix DNA-binding domain	SYNONYMOUS_CODING
4233	CakSNP4233	*Kabuli*	Ca_Kabuli_Chr03	28478569	[C/A]	Ca_07159	Helix-loop-helix DNA-binding domain	SYNONYMOUS_CODING
4234	CakSNP4234	*Kabuli*	Ca_Kabuli_Chr03	28602055	[C/A]	Ca_07169	DNA topoisomerase, type IIA, subunit B	INTRON
4235	CakSNP4235	*Kabuli*	Ca_Kabuli_Chr03	28603892	[T/C]	Ca_07169	DNA topoisomerase, type IIA, subunit B	INTRON
4236	CakSNP4236	*Kabuli*	Ca_Kabuli_Chr03	28613067	[A/G]	Ca_07170	Basic-leucine zipper (bZIP) transcription factor	INTRON
4237	CakSNP4237	*Kabuli*	Ca_Kabuli_Chr03	28614784	[A/G]	Ca_07170	Basic-leucine zipper (bZIP) transcription factor	SYNONYMOUS_CODING
4238	CakSNP4238	*Kabuli*	Ca_Kabuli_Chr03	28614919	[A/T]	Ca_07170	Basic-leucine zipper (bZIP) transcription factor	NON_SYNONYMOUS_CODING
4239	CakSNP4239	*Kabuli*	Ca_Kabuli_Chr03	28680858	[C/T]	Ca_07175	Glutamine amidotransferase, class-II	INTRON
4240	CakSNP4240	*Kabuli*	Ca_Kabuli_Chr03	28706106	[T/A]	Ca_07176	BTB/POZ-like	SYNONYMOUS_CODING
4241	CakSNP4241	*Kabuli*	Ca_Kabuli_Chr03	28706292	[G/C]	Ca_07176	BTB/POZ-like	SYNONYMOUS_CODING

S.N.	SNP IDs	Chickpea cultivars	Chromosomes	Physical positions (bp)	SNPs	Gene accession IDs	Putative functions	Sequence components of genome
4242	CakSNP4242	*Kabuli*	Ca_Kabuli_Chr03	28712993	[T/C]		--	INTERGENIC
4243	CakSNP4243	*Kabuli*	Ca_Kabuli_Chr03	28716003	[C/T]	Ca_07177	Cellular retinaldehyde-binding/triple function, C-terminal	INTRON
4244	CakSNP4244	*Kabuli*	Ca_Kabuli_Chr03	28716027	[A/T]	Ca_07177	Cellular retinaldehyde-binding/triple function, C-terminal	INTRON
4245	CakSNP4245	*Kabuli*	Ca_Kabuli_Chr03	28716028	[A/T]	Ca_07177	Cellular retinaldehyde-binding/triple function, C-terminal	INTRON
4246	CakSNP4246	*Kabuli*	Ca_Kabuli_Chr03	28716029	[A/G]	Ca_07177	Cellular retinaldehyde-binding/triple function, C-terminal	INTRON
4247	CakSNP4247	*Kabuli*	Ca_Kabuli_Chr03	28716038	[T/G]	Ca_07177	Cellular retinaldehyde-binding/triple function, C-terminal	INTRON
4248	CakSNP4248	*Kabuli*	Ca_Kabuli_Chr03	28716047	[G/A]	Ca_07177	Cellular retinaldehyde-binding/triple function, C-terminal	INTRON
4249	CakSNP4249	*Kabuli*	Ca_Kabuli_Chr03	28755111	[A/G]	Ca_07181	Pyrroline-5-carboxylate reductase	INTRON
4250	CakSNP4250	*Kabuli*	Ca_Kabuli_Chr03	28755149	[G/A]	Ca_07181	Pyrroline-5-carboxylate reductase	INTRON
4251	CakSNP4251	*Kabuli*	Ca_Kabuli_Chr03	28845958	[A/C]	Ca_07193	Strictosidine synthase, conserved region	NON_SYNONYMOUS_CODING

S.N.	SNP IDs	Chickpea cultivars	Chromosomes	Physical positions (bp)	SNPs	Gene accession IDs	Putative functions	Sequence components of genome
4252	CakSNP4252	*Kabuli*	Ca_Kabuli_Chr03	28926094	[T/A]	Ca_07198	Patatin/Phospholipase A2-related	INTRON
4253	CakSNP4253	*Kabuli*	Ca_Kabuli_Chr03	28926290	[T/C]	Ca_07198	Patatin/Phospholipase A2-related	SYNONYMOUS_CODING
4254	CakSNP4254	*Kabuli*	Ca_Kabuli_Chr03	28934196	[C/T]	Ca_07199	Calmodulin-binding protein, plant	SYNONYMOUS_CODING
4255	CakSNP4255	*Kabuli*	Ca_Kabuli_Chr03	28943956	[C/T]	Ca_07201	Protein phosphatase 2C, manganese/magnesium aspartate binding site	NON_SYNONYMOUS_CODING
4256	CakSNP4256	*Kabuli*	Ca_Kabuli_Chr03	28943989	[G/T]	Ca_07201	Protein phosphatase 2C, manganese/magnesium aspartate binding site	NON_SYNONYMOUS_CODING
4257	CakSNP4257	*Kabuli*	Ca_Kabuli_Chr03	29056451	[T/C]		--	INTERGENIC
4258	CakSNP4258	*Kabuli*	Ca_Kabuli_Chr03	29056528	[G/T]		--	INTERGENIC
4259	CakSNP4259	*Kabuli*	Ca_Kabuli_Chr03	29056768	[T/C]	Ca_07211	ATPase, AAA+ type, core	SYNONYMOUS_CODING
4260	CakSNP4260	*Kabuli*	Ca_Kabuli_Chr03	29107776	[A/G]	Ca_07215	Heat shock protein DnaJ, N-terminal	INTRON
4261	CakSNP4261	*Kabuli*	Ca_Kabuli_Chr03	29107947	[C/A]	Ca_07215	Heat shock protein DnaJ, N-terminal	NON_SYNONYMOUS_CODING

S.N.	SNP IDs	Chickpea cultivars	Chromosomes	Physical positions (bp)	SNPs	Gene accession IDs	Putative functions	Sequence components of genome
4262	CakSNP4262	*Kabuli*	Ca_Kabuli_Chr03	29155741	[C/G]	Ca_07220	Rho termination factor, N-terminal	INTRON
4263	CakSNP4263	*Kabuli*	Ca_Kabuli_Chr03	29177885	[A/C]		--	INTERGENIC
4264	CakSNP4264	*Kabuli*	Ca_Kabuli_Chr03	29188300	[T/G]		--	INTERGENIC
4265	CakSNP4265	*Kabuli*	Ca_Kabuli_Chr03	29188308	[G/A]		--	INTERGENIC
4266	CakSNP4266	*Kabuli*	Ca_Kabuli_Chr03	29207338	[G/A]		--	INTERGENIC
4267	CakSNP4267	*Kabuli*	Ca_Kabuli_Chr03	29293770	[G/T]	Ca_07236	--	INTRON
4268	CakSNP4268	*Kabuli*	Ca_Kabuli_Chr03	29293783	[G/A]	Ca_07236	--	INTRON
4269	CakSNP4269	*Kabuli*	Ca_Kabuli_Chr03	29293840	[C/A]	Ca_07236	--	INTRON
4270	CakSNP4270	*Kabuli*	Ca_Kabuli_Chr03	29302251	[C/T]	Ca_07237	Pathogenesis-related transcriptional factor/ERF, DNA-binding	INTRON
4271	CakSNP4271	*Kabuli*	Ca_Kabuli_Chr03	29302448	[A/C]	Ca_07237	Pathogenesis-related transcriptional factor/ERF, DNA-binding	INTRON

S.N.	SNP IDs	Chickpea cultivars	Chromosomes	Physical positions (bp)	SNPs	Gene accession IDs	Putative functions	Sequence components of genome
4272	CakSNP4272	*Kabuli*	Ca_Kabuli_Chr03	29303144	[A/C]	Ca_07237	Pathogenesis-related transcriptional factor/ERF, DNA-binding	INTRON
4273	CakSNP4273	*Kabuli*	Ca_Kabuli_Chr03	29304116	[A/G]		--	INTERGENIC
4274	CakSNP4274	*Kabuli*	Ca_Kabuli_Chr03	29304161	[C/T]		--	INTERGENIC
4275	CakSNP4275	*Kabuli*	Ca_Kabuli_Chr03	29311672	[C/A]	Ca_07238	Pentatricopeptide repeat	NON_SYNONYMOUS_CODING
4276	CakSNP4276	*Kabuli*	Ca_Kabuli_Chr03	29348267	[C/T]	Ca_07239	Villin headpiece	SYNONYMOUS_CODING
4277	CakSNP4277	*Kabuli*	Ca_Kabuli_Chr03	29355268	[T/A]		--	INTERGENIC
4278	CakSNP4278	*Kabuli*	Ca_Kabuli_Chr03	29376025	[A/G]		--	INTERGENIC
4279	CakSNP4279	*Kabuli*	Ca_Kabuli_Chr03	29376008	[G/A]		--	INTERGENIC
4280	CakSNP4280	*Kabuli*	Ca_Kabuli_Chr03	29439505	[A/G]	Ca_07248	Protein kinase, catalytic domain	SYNONYMOUS_CODING
4281	CakSNP4281	*Kabuli*	Ca_Kabuli_Chr03	29485590	[T/C]	Ca_07254	Glycoside hydrolase, family 28	SYNONYMOUS_CODING

S.N.	SNP IDs	Chickpea cultivars	Chromosomes	Physical positions (bp)	SNPs	Gene accession IDs	Putative functions	Sequence components of genome
4282	CakSNP4282	*Kabuli*	Ca_Kabuli_Chr03	29485566	[T/A]	Ca_07254	Glycoside hydrolase, family 28	NON_SYNONYMOUS_CODING
4283	CakSNP4283	*Kabuli*	Ca_Kabuli_Chr03	29508831	[C/T]	Ca_07256	Protein kinase, catalytic domain	NON_SYNONYMOUS_CODING
4284	CakSNP4284	*Kabuli*	Ca_Kabuli_Chr03	29509323	[G/A]	Ca_07256	Protein kinase, catalytic domain	SYNONYMOUS_CODING
4285	CakSNP4285	*Kabuli*	Ca_Kabuli_Chr03	29509310	[G/T]	Ca_07256	Protein kinase, catalytic domain	SYNONYMOUS_CODING
4286	CakSNP4286	*Kabuli*	Ca_Kabuli_Chr03	29547528	[G/A]	Ca_07260	Protein kinase, catalytic domain	SYNONYMOUS_CODING
4287	CakSNP4287	*Kabuli*	Ca_Kabuli_Chr03	29588940	[C/T]	Ca_07263	--	INTRON
4288	CakSNP4288	*Kabuli*	Ca_Kabuli_Chr03	29588934	[T/C]	Ca_07263	--	INTRON
4289	CakSNP4289	*Kabuli*	Ca_Kabuli_Chr03	29588930	[T/C]	Ca_07263	--	INTRON
4290	CakSNP4290	*Kabuli*	Ca_Kabuli_Chr03	29591575	[T/G]	Ca_07263	--	NON_SYNONYMOUS_CODING
4291	CakSNP4291	*Kabuli*	Ca_Kabuli_Chr03	29591613	[G/T]	Ca_07263	--	SYNONYMOUS_CODING

S.N.	SNP IDs	Chickpea cultivars	Chromosomes	Physical positions (bp)	SNPs	Gene accession IDs	Putative functions	Sequence components of genome
4292	CakSNP4292	*Kabuli*	Ca_Kabuli_Chr03	29603720	[T/C]	Ca_07265	ATPase, AAA+ type, core	NON_SYNONYMOUS_CODING
4293	CakSNP4293	*Kabuli*	Ca_Kabuli_Chr03	29640075	[A/C]		--	INTERGENIC
4294	CakSNP4294	*Kabuli*	Ca_Kabuli_Chr03	29640071	[T/C]		--	INTERGENIC
4295	CakSNP4295	*Kabuli*	Ca_Kabuli_Chr03	29666633	[C/T]		--	INTERGENIC
4296	CakSNP4296	*Kabuli*	Ca_Kabuli_Chr03	29690732	[C/A]	Ca_07274	Zinc finger, AN1-type	SYNONYMOUS_CODING
4297	CakSNP4297	*Kabuli*	Ca_Kabuli_Chr03	29698166	[G/T]		--	INTERGENIC
4298	CakSNP4298	*Kabuli*	Ca_Kabuli_Chr03	29698245	[T/A]		--	INTERGENIC
4299	CakSNP4299	*Kabuli*	Ca_Kabuli_Chr03	29698289	[G/A]		--	INTERGENIC
4300	CakSNP4300	*Kabuli*	Ca_Kabuli_Chr03	29698273	[A/C]		--	INTERGENIC
4301	CakSNP4301	*Kabuli*	Ca_Kabuli_Chr03	29730019	[G/T]		--	INTERGENIC

S.N.	SNP IDs	Chickpea cultivars	Chromosomes	Physical positions (bp)	SNPs	Gene accession IDs	Putative functions	Sequence components of genome
4302	CakSNP4302	*Kabuli*	Ca_Kabuli_Chr03	29744878	[T/G]	Ca_07282	--	SYNONYMOUS_CODING
4303	CakSNP4303	*Kabuli*	Ca_Kabuli_Chr03	29744890	[A/C]	Ca_07282	--	SYNONYMOUS_CODING
4304	CakSNP4304	*Kabuli*	Ca_Kabuli_Chr03	29803866	[C/T]	Ca_07288	Protein of unknown function DUF2048	SYNONYMOUS_CODING
4305	CakSNP4305	*Kabuli*	Ca_Kabuli_Chr03	29803962	[A/G]	Ca_07288	Protein of unknown function DUF2048	SYNONYMOUS_CODING
4306	CakSNP4306	*Kabuli*	Ca_Kabuli_Chr03	29807091	[C/G]		--	INTERGENIC
4307	CakSNP4307	*Kabuli*	Ca_Kabuli_Chr03	29807248	[A/G]		--	INTERGENIC
4308	CakSNP4308	*Kabuli*	Ca_Kabuli_Chr03	29836337	[C/A]		--	INTERGENIC
4309	CakSNP4309	*Kabuli*	Ca_Kabuli_Chr03	29840029	[C/G]		--	INTERGENIC
4310	CakSNP4310	*Kabuli*	Ca_Kabuli_Chr03	29840028	[T/C]		--	INTERGENIC
4311	CakSNP4311	*Kabuli*	Ca_Kabuli_Chr03	29840022	[C/A]		--	INTERGENIC

S.N.	SNP IDs	Chickpea cultivars	Chromosomes	Physical positions (bp)	SNPs	Gene accession IDs	Putative functions	Sequence components of genome
4312	CakSNP4312	*Kabuli*	Ca_Kabuli_Chr03	29840012	[A/T]		--	INTERGENIC
4313	CakSNP4313	*Kabuli*	Ca_Kabuli_Chr03	29844509	[T/A]	Ca_07292	Basic-leucine zipper (bZIP) transcription factor	INTRON
4314	CakSNP4314	*Kabuli*	Ca_Kabuli_Chr03	29869190	[G/T]	Ca_07295	CARP motif	INTRON
4315	CakSNP4315	*Kabuli*	Ca_Kabuli_Chr03	29869199	[T/C]	Ca_07295	CARP motif	INTRON
4316	CakSNP4316	*Kabuli*	Ca_Kabuli_Chr03	29869428	[G/T]	Ca_07295	CARP motif	INTRON
4317	CakSNP4317	*Kabuli*	Ca_Kabuli_Chr03	29869381	[G/A]	Ca_07295	CARP motif	NON_SYNONYMOUS_CODING
4318	CakSNP4318	*Kabuli*	Ca_Kabuli_Chr03	29869380	[G/A]	Ca_07295	CARP motif	NON_SYNONYMOUS_CODING
4319	CakSNP4319	*Kabuli*	Ca_Kabuli_Chr03	29872643	[A/G]	Ca_07295	CARP motif	INTRON
4320	CakSNP4320	*Kabuli*	Ca_Kabuli_Chr03	29872682	[G/A]	Ca_07295	CARP motif	INTRON
4321	CakSNP4321	*Kabuli*	Ca_Kabuli_Chr03	29898396	[A/G]	Ca_07299	Transcription factor, SBP-box	SYNONYMOUS_CODING

S.N.	SNP IDs	Chickpea cultivars	Chromosomes	Physical positions (bp)	SNPs	Gene accession IDs	Putative functions	Sequence components of genome
4322	CakSNP4322	*Kabuli*	Ca_Kabuli_Chr03	29912488	[A/G]	Ca_07302	RNA recognition motif domain	SYNONYMOUS_CODING
4323	CakSNP4323	*Kabuli*	Ca_Kabuli_Chr03	29915510	[G/A]	Ca_07302	RNA recognition motif domain	INTRON
4324	CakSNP4324	*Kabuli*	Ca_Kabuli_Chr03	29932378	[C/G]	Ca_07304	Myb, DNA-binding	INTRON
4325	CakSNP4325	*Kabuli*	Ca_Kabuli_Chr03	29932504	[T/A]	Ca_07304	Myb, DNA-binding	INTRON
4326	CakSNP4326	*Kabuli*	Ca_Kabuli_Chr03	29932502	[G/C]	Ca_07304	Myb, DNA-binding	INTRON
4327	CakSNP4327	*Kabuli*	Ca_Kabuli_Chr03	29954498	[G/A]		--	INTERGENIC
4328	CakSNP4328	*Kabuli*	Ca_Kabuli_Chr03	29954533	[A/G]		--	INTERGENIC
4329	CakSNP4329	*Kabuli*	Ca_Kabuli_Chr03	29954648	[A/T]		--	INTERGENIC
4330	CakSNP4330	*Kabuli*	Ca_Kabuli_Chr03	29954612	[T/G]		--	INTERGENIC
4331	CakSNP4331	*Kabuli*	Ca_Kabuli_Chr03	29974532	[A/G]	Ca_07307	Zinc finger, CCCH-type	SYNONYMOUS_CODING

S.N.	SNP IDs	Chickpea cultivars	Chromosomes	Physical positions (bp)	SNPs	Gene accession IDs	Putative functions	Sequence components of genome
4332	CakSNP4332	*Kabuli*	Ca_Kabuli_Chr03	29974535	[A/G]	Ca_07307	Zinc finger, CCCH-type	SYNONYMOUS_CODING
4333	CakSNP4333	*Kabuli*	Ca_Kabuli_Chr03	29974595	[C/T]	Ca_07307	Zinc finger, CCCH-type	SYNONYMOUS_CODING
4334	CakSNP4334	*Kabuli*	Ca_Kabuli_Chr03	29982638	[A/G]		--	INTERGENIC
4335	CakSNP4335	*Kabuli*	Ca_Kabuli_Chr03	29982644	[T/C]		--	INTERGENIC
4336	CakSNP4336	*Kabuli*	Ca_Kabuli_Chr03	29982718	[C/A]		--	INTERGENIC
4337	CakSNP4337	*Kabuli*	Ca_Kabuli_Chr03	30004352	[T/A]	Ca_07313	Protein phosphatase 2C-like	SYNONYMOUS_CODING
4338	CakSNP4338	*Kabuli*	Ca_Kabuli_Chr03	30004311	[T/G]	Ca_07313	Protein phosphatase 2C-like	INTRON
4339	CakSNP4339	*Kabuli*	Ca_Kabuli_Chr03	30004295	[A/G]	Ca_07313	Protein phosphatase 2C-like	INTRON
4340	CakSNP4340	*Kabuli*	Ca_Kabuli_Chr03	30004279	[G/T]	Ca_07313	Protein phosphatase 2C-like	INTRON
4341	CakSNP4341	*Kabuli*	Ca_Kabuli_Chr03	30009698	[C/T]		--	INTERGENIC

S.N.	SNP IDs	Chickpea cultivars	Chromosomes	Physical positions (bp)	SNPs	Gene accession IDs	Putative functions	Sequence components of genome
4342	CakSNP4342	*Kabuli*	Ca_Kabuli_Chr03	30009751	[A/C]		--	INTERGENIC
4343	CakSNP4343	*Kabuli*	Ca_Kabuli_Chr03	30011584	[T/C]	Ca_07314	Protein phosphatase 2C-like	INTRON
4344	CakSNP4344	*Kabuli*	Ca_Kabuli_Chr03	30011669	[A/G]	Ca_07314	Protein phosphatase 2C-like	NON_SYNONYMOUS_CODING
4345	CakSNP4345	*Kabuli*	Ca_Kabuli_Chr03	30011642	[T/A]	Ca_07314	Protein phosphatase 2C-like	INTRON
4346	CakSNP4346	*Kabuli*	Ca_Kabuli_Chr03	30011621	[C/T]	Ca_07314	Protein phosphatase 2C-like	INTRON
4347	CakSNP4347	*Kabuli*	Ca_Kabuli_Chr03	30029874	[C/T]	Ca_07318	NUDIX hydrolase domain	NON_SYNONYMOUS_CODING
4348	CakSNP4348	*Kabuli*	Ca_Kabuli_Chr03	30029858	[T/A]	Ca_07318	NUDIX hydrolase domain	NON_SYNONYMOUS_CODING
4349	CakSNP4349	*Kabuli*	Ca_Kabuli_Chr03	30029842	[G/C]	Ca_07318	NUDIX hydrolase domain	INTRON
4350	CakSNP4350	*Kabuli*	Ca_Kabuli_Chr03	30029826	[T/C]	Ca_07318	NUDIX hydrolase domain	INTRON
4351	CakSNP4351	*Kabuli*	Ca_Kabuli_Chr03	30029817	[T/G]	Ca_07318	NUDIX hydrolase domain	INTRON

S.N.	SNP IDs	Chickpea cultivars	Chromosomes	Physical positions (bp)	SNPs	Gene accession IDs	Putative functions	Sequence components of genome
4352	CakSNP4352	*Kabuli*	Ca_Kabuli_Chr03	30084154	[T/C]		--	INTERGENIC
4353	CakSNP4353	*Kabuli*	Ca_Kabuli_Chr03	30101412	[T/C]	Ca_07324	Methyltransferase-related	INTRON
4354	CakSNP4354	*Kabuli*	Ca_Kabuli_Chr03	30101476	[A/C]	Ca_07324	Methyltransferase-related	INTRON
4355	CakSNP4355	*Kabuli*	Ca_Kabuli_Chr03	30111154	[A/C]		--	INTERGENIC
4356	CakSNP4356	*Kabuli*	Ca_Kabuli_Chr03	30111146	[G/C]		--	INTERGENIC
4357	CakSNP4357	*Kabuli*	Ca_Kabuli_Chr03	30148907	[T/C]	Ca_07332	--	SYNONYMOUS_CODING
4358	CakSNP4358	*Kabuli*	Ca_Kabuli_Chr03	30194810	[C/T]	Ca_07336	Kinesin, motor domain	SYNONYMOUS_CODING
4359	CakSNP4359	*Kabuli*	Ca_Kabuli_Chr03	30206121	[T/A]	Ca_07336	Kinesin, motor domain	INTRON
4360	CakSNP4360	*Kabuli*	Ca_Kabuli_Chr03	30206266	[G/A]	Ca_07336	Kinesin, motor domain	INTRON
4361	CakSNP4361	*Kabuli*	Ca_Kabuli_Chr03	30206281	[A/T]	Ca_07336	Kinesin, motor domain	INTRON

S.N.	SNP IDs	Chickpea cultivars	Chromosomes	Physical positions (bp)	SNPs	Gene accession IDs	Putative functions	Sequence components of genome
4362	CakSNP4362	*Kabuli*	Ca_Kabuli_Chr03	30206436	[G/A]	Ca_07336	Kinesin, motor domain	INTRON
4363	CakSNP4363	*Kabuli*	Ca_Kabuli_Chr03	30267761	[T/G]		--	INTERGENIC
4364	CakSNP4364	*Kabuli*	Ca_Kabuli_Chr03	30271022	[G/T]	Ca_07340	Histone H2A	INTRON
4365	CakSNP4365	*Kabuli*	Ca_Kabuli_Chr03	30271028	[G/A]	Ca_07340	Histone H2A	INTRON
4366	CakSNP4366	*Kabuli*	Ca_Kabuli_Chr03	30271034	[G/A]	Ca_07340	Histone H2A	INTRON
4367	CakSNP4367	*Kabuli*	Ca_Kabuli_Chr03	30309625	[G/C]	Ca_07345	Sugar/inositol transporter	NON_SYNONYMOUS_CODING
4368	CakSNP4368	*Kabuli*	Ca_Kabuli_Chr03	30309624	[A/T]	Ca_07345	Sugar/inositol transporter	SYNONYMOUS_CODING
4369	CakSNP4369	*Kabuli*	Ca_Kabuli_Chr03	30321177	[C/G]		--	INTERGENIC
4370	CakSNP4370	*Kabuli*	Ca_Kabuli_Chr03	30321245	[C/T]		--	INTERGENIC
4371	CakSNP4371	*Kabuli*	Ca_Kabuli_Chr03	30321272	[C/A]		--	INTERGENIC

S.N.	SNP IDs	Chickpea cultivars	Chromosomes	Physical positions (bp)	SNPs	Gene accession IDs	Putative functions	Sequence components of genome
4372	CakSNP4372	*Kabuli*	Ca_Kabuli_Chr03	30323585	[G/A]	Ca_07350	Oxoglutarate/iron-dependent oxygenase	INTRON
4373	CakSNP4373	*Kabuli*	Ca_Kabuli_Chr03	30376319	[G/A]	Ca_07358	Short-chain dehydrogenase/reductase SDR	SYNONYMOUS_CODING
4374	CakSNP4374	*Kabuli*	Ca_Kabuli_Chr03	30385144	[G/A]	Ca_07359	--	INTRON
4375	CakSNP4375	*Kabuli*	Ca_Kabuli_Chr03	30385153	[T/A]	Ca_07359	--	INTRON
4376	CakSNP4376	*Kabuli*	Ca_Kabuli_Chr03	30390663	[C/T]	Ca_07359	--	INTRON
4377	CakSNP4377	*Kabuli*	Ca_Kabuli_Chr03	30397262	[A/T]	Ca_07359	--	INTRON
4378	CakSNP4378	*Kabuli*	Ca_Kabuli_Chr03	30425496	[G/T]	Ca_07362	PWWP	INTRON
4379	CakSNP4379	*Kabuli*	Ca_Kabuli_Chr03	30510392	[T/A]		--	INTERGENIC
4380	CakSNP4380	*Kabuli*	Ca_Kabuli_Chr03	30510333	[C/T]		--	INTERGENIC
4381	CakSNP4381	*Kabuli*	Ca_Kabuli_Chr03	30522442	[A/C]		--	INTERGENIC

S.N.	SNP IDs	Chickpea cultivars	Chromosomes	Physical positions (bp)	SNPs	Gene accession IDs	Putative functions	Sequence components of genome
4382	CakSNP4382	*Kabuli*	Ca_Kabuli_Chr03	30522485	[C/T]		--	INTERGENIC
4383	CakSNP4383	*Kabuli*	Ca_Kabuli_Chr03	30522498	[T/C]		--	INTERGENIC
4384	CakSNP4384	*Kabuli*	Ca_Kabuli_Chr03	30650598	[C/A]		--	INTERGENIC
4385	CakSNP4385	*Kabuli*	Ca_Kabuli_Chr03	30650634	[T/C]		--	INTERGENIC
4386	CakSNP4386	*Kabuli*	Ca_Kabuli_Chr03	30650659	[C/T]		--	INTERGENIC
4387	CakSNP4387	*Kabuli*	Ca_Kabuli_Chr03	30659438	[T/A]	Ca_07384	Splicing factor motif	INTRON
4388	CakSNP4388	*Kabuli*	Ca_Kabuli_Chr03	30675468	[T/G]		--	INTERGENIC
4389	CakSNP4389	*Kabuli*	Ca_Kabuli_Chr03	30681031	[A/G]	Ca_07388	Helicase, C-terminal	INTRON
4390	CakSNP4390	*Kabuli*	Ca_Kabuli_Chr03	30681032	[A/G]	Ca_07388	Helicase, C-terminal	INTRON
4391	CakSNP4391	*Kabuli*	Ca_Kabuli_Chr03	30688336	[T/G]	Ca_07389	--	INTRON

S.N.	SNP IDs	Chickpea cultivars	Chromosomes	Physical positions (bp)	SNPs	Gene accession IDs	Putative functions	Sequence components of genome
4392	CakSNP4392	*Kabuli*	Ca_Kabuli_Chr03	30688354	[G/A]	Ca_07389	--	INTRON
4393	CakSNP4393	*Kabuli*	Ca_Kabuli_Chr03	30688369	[T/C]	Ca_07389	--	INTRON
4394	CakSNP4394	*Kabuli*	Ca_Kabuli_Chr03	30688418	[A/C]	Ca_07389	--	INTRON
4395	CakSNP4395	*Kabuli*	Ca_Kabuli_Chr03	30688511	[G/C]	Ca_07389	--	INTRON
4396	CakSNP4396	*Kabuli*	Ca_Kabuli_Chr03	30688485	[C/A]	Ca_07389	--	INTRON
4397	CakSNP4397	*Kabuli*	Ca_Kabuli_Chr03	30688484	[A/C]	Ca_07389	--	INTRON
4398	CakSNP4398	*Kabuli*	Ca_Kabuli_Chr03	30688454	[A/G]	Ca_07389	--	INTRON
4399	CakSNP4399	*Kabuli*	Ca_Kabuli_Chr03	30688451	[T/C]	Ca_07389	--	INTRON
4400	CakSNP4400	*Kabuli*	Ca_Kabuli_Chr03	30718334	[G/T]		--	INTERGENIC
4401	CakSNP4401	*Kabuli*	Ca_Kabuli_Chr03	30751893	[A/C]		--	INTERGENIC

S.N.	SNP IDs	Chickpea cultivars	Chromosomes	Physical positions (bp)	SNPs	Gene accession IDs	Putative functions	Sequence components of genome
4402	CakSNP4402	*Kabuli*	Ca_Kabuli_Chr03	30751949	[A/T]		--	INTERGENIC
4403	CakSNP4403	*Kabuli*	Ca_Kabuli_Chr03	30791169	[A/T]	Ca_07400	KIP1-like	NON_SYNONYMOUS_CODING
4404	CakSNP4404	*Kabuli*	Ca_Kabuli_Chr03	30791229	[G/A]	Ca_07400	KIP1-like	NON_SYNONYMOUS_CODING
4405	CakSNP4405	*Kabuli*	Ca_Kabuli_Chr03	30791693	[C/T]	Ca_07400	KIP1-like	SYNONYMOUS_CODING
4406	CakSNP4406	*Kabuli*	Ca_Kabuli_Chr03	30794040	[C/A]	Ca_07400	KIP1-like	NON_SYNONYMOUS_CODING
4407	CakSNP4407	*Kabuli*	Ca_Kabuli_Chr03	30794046	[A/C]	Ca_07400	KIP1-like	NON_SYNONYMOUS_CODING
4408	CakSNP4408	*Kabuli*	Ca_Kabuli_Chr03	30837147	[A/G]	Ca_12181	Domain of unknown function DUF828	SYNONYMOUS_CODING
4409	CakSNP4409	*Kabuli*	Ca_Kabuli_Chr03	30837284	[C/T]	Ca_12181	Domain of unknown function DUF828	NON_SYNONYMOUS_CODING
4410	CakSNP4410	*Kabuli*	Ca_Kabuli_Chr03	30837265	[T/C]	Ca_12181	Domain of unknown function DUF828	NON_SYNONYMOUS_CODING
4411	CakSNP4411	*Kabuli*	Ca_Kabuli_Chr03	30880452	[T/C]	Ca_12185	ATPase, P-type, H+ transporting proton pump	NON_SYNONYMOUS_CODING

S.N.	SNP IDs	Chickpea cultivars	Chromosomes	Physical positions (bp)	SNPs	Gene accession IDs	Putative functions	Sequence components of genome
4412	CakSNP4412	*Kabuli*	Ca_Kabuli_Chr03	30880610	[A/G]	Ca_12185	ATPase, P-type, H+ transporting proton pump	SYNONYMOUS_CODING
4413	CakSNP4413	*Kabuli*	Ca_Kabuli_Chr03	31020357	[C/A]	Ca_12200	Glycosyl transferase, family 8	NON_SYNONYMOUS_CODING
4414	CakSNP4414	*Kabuli*	Ca_Kabuli_Chr03	31020358	[C/G]	Ca_12200	Glycosyl transferase, family 8	NON_SYNONYMOUS_CODING
4415	CakSNP4415	*Kabuli*	Ca_Kabuli_Chr03	31073396	[T/C]		--	INTERGENIC
4416	CakSNP4416	*Kabuli*	Ca_Kabuli_Chr03	31073425	[G/C]		--	INTERGENIC
4417	CakSNP4417	*Kabuli*	Ca_Kabuli_Chr03	31073451	[A/G]		--	INTERGENIC
4418	CakSNP4418	*Kabuli*	Ca_Kabuli_Chr03	31091875	[T/G]	Ca_12206	Histone H1/H5	NON_SYNONYMOUS_CODING
4419	CakSNP4419	*Kabuli*	Ca_Kabuli_Chr03	31267639	[T/A]	Ca_12222	General substrate transporter	SYNONYMOUS_CODING
4420	CakSNP4420	*Kabuli*	Ca_Kabuli_Chr03	31267675	[A/G]	Ca_12222	General substrate transporter	SYNONYMOUS_CODING
4421	CakSNP4421	*Kabuli*	Ca_Kabuli_Chr03	31267681	[G/A]	Ca_12222	General substrate transporter	SYNONYMOUS_CODING

S.N.	SNP IDs	Chickpea cultivars	Chromosomes	Physical positions (bp)	SNPs	Gene accession IDs	Putative functions	Sequence components of genome
4422	CakSNP4422	*Kabuli*	Ca_Kabuli_Chr03	31267828	[A/G]	Ca_12222	General substrate transporter	SYNONYMOUS_CODING
4423	CakSNP4423	*Kabuli*	Ca_Kabuli_Chr03	31267927	[T/G]	Ca_12222	General substrate transporter	SYNONYMOUS_CODING
4424	CakSNP4424	*Kabuli*	Ca_Kabuli_Chr03	31268143	[A/G]	Ca_12222	General substrate transporter	SYNONYMOUS_CODING
4425	CakSNP4425	*Kabuli*	Ca_Kabuli_Chr03	31279502	[C/T]	Ca_12224	Protein of unknown function DUF647	SYNONYMOUS_CODING
4426	CakSNP4426	*Kabuli*	Ca_Kabuli_Chr03	31279541	[A/G]	Ca_12224	Protein of unknown function DUF647	SYNONYMOUS_CODING
4427	CakSNP4427	*Kabuli*	Ca_Kabuli_Chr03	31279639	[C/T]	Ca_12224	Protein of unknown function DUF647	INTRON
4428	CakSNP4428	*Kabuli*	Ca_Kabuli_Chr03	31279629	[C/G]	Ca_12224	Protein of unknown function DUF647	INTRON
4429	CakSNP4429	*Kabuli*	Ca_Kabuli_Chr03	31279628	[T/C]	Ca_12224	Protein of unknown function DUF647	INTRON
4430	CakSNP4430	*Kabuli*	Ca_Kabuli_Chr03	31279616	[T/A]	Ca_12224	Protein of unknown function DUF647	INTRON
4431	CakSNP4431	*Kabuli*	Ca_Kabuli_Chr03	31279606	[G/A]	Ca_12224	Protein of unknown function DUF647	INTRON

S.N.	SNP IDs	Chickpea cultivars	Chromosomes	Physical positions (bp)	SNPs	Gene accession IDs	Putative functions	Sequence components of genome
4432	CakSNP4432	*Kabuli*	Ca_Kabuli_Chr03	31279605	[G/A]	Ca_12224	Protein of unknown function DUF647	INTRON
4433	CakSNP4433	*Kabuli*	Ca_Kabuli_Chr03	31279604	[C/G]	Ca_12224	Protein of unknown function DUF647	INTRON
4434	CakSNP4434	*Kabuli*	Ca_Kabuli_Chr03	31279600	[T/C]	Ca_12224	Protein of unknown function DUF647	INTRON
4435	CakSNP4435	*Kabuli*	Ca_Kabuli_Chr03	31279586	[A/G]	Ca_12224	Protein of unknown function DUF647	SYNONYMOUS_CODING
4436	CakSNP4436	*Kabuli*	Ca_Kabuli_Chr03	31287618	[C/A]	Ca_12225	General substrate transporter	SYNONYMOUS_CODING
4437	CakSNP4437	*Kabuli*	Ca_Kabuli_Chr03	31287909	[A/G]	Ca_12225	General substrate transporter	SYNONYMOUS_CODING
4438	CakSNP4438	*Kabuli*	Ca_Kabuli_Chr03	31288143	[T/C]	Ca_12225	General substrate transporter	SYNONYMOUS_CODING
4439	CakSNP4439	*Kabuli*	Ca_Kabuli_Chr03	31301723	[G/A]	Ca_12227	Pre-mRNA splicing Prp18-interacting factor	SYNONYMOUS_CODING
4440	CakSNP4440	*Kabuli*	Ca_Kabuli_Chr03	31301783	[A/G]	Ca_12227	Pre-mRNA splicing Prp18-interacting factor	SYNONYMOUS_CODING
4441	CakSNP4441	*Kabuli*	Ca_Kabuli_Chr03	31302060	[T/C]	Ca_12227	Pre-mRNA splicing Prp18-interacting factor	INTRON

S.N.	SNP IDs	Chickpea cultivars	Chromosomes	Physical positions (bp)	SNPs	Gene accession IDs	Putative functions	Sequence components of genome
4442	CakSNP4442	*Kabuli*	Ca_Kabuli_Chr03	31302117	[C/T]	Ca_12227	Pre-mRNA splicing Prp18-interacting factor	SYNONYMOUS_CODING
4443	CakSNP4443	*Kabuli*	Ca_Kabuli_Chr03	31313021	[G/T]	Ca_12230	11-S seed storage protein, plant	NON_SYNONYMOUS_CODING
4444	CakSNP4444	*Kabuli*	Ca_Kabuli_Chr03	31317521	[T/G]	Ca_12231	11-S seed storage protein, plant	SYNONYMOUS_CODING
4445	CakSNP4445	*Kabuli*	Ca_Kabuli_Chr03	31321725	[A/G]	Ca_12232	VHS	NON_SYNONYMOUS_CODING
4446	CakSNP4446	*Kabuli*	Ca_Kabuli_Chr03	31321778	[G/A]	Ca_12232	VHS	SYNONYMOUS_CODING
4447	CakSNP4447	*Kabuli*	Ca_Kabuli_Chr03	31321850	[A/G]	Ca_12232	VHS	SYNONYMOUS_CODING
4448	CakSNP4448	*Kabuli*	Ca_Kabuli_Chr03	31341335	[C/T]	Ca_12234	Domain of unknown function DUF640	SYNONYMOUS_CODING
4449	CakSNP4449	*Kabuli*	Ca_Kabuli_Chr03	31341323	[T/C]	Ca_12234	Domain of unknown function DUF640	SYNONYMOUS_CODING
4450	CakSNP4450	*Kabuli*	Ca_Kabuli_Chr03	31342119	[A/T]		--	INTERGENIC
4451	CakSNP4451	*Kabuli*	Ca_Kabuli_Chr03	31354988	[G/C]	Ca_12235	Cystathionine beta-synthase, core	INTRON

S.N.	SNP IDs	Chickpea cultivars	Chromosomes	Physical positions (bp)	SNPs	Gene accession IDs	Putative functions	Sequence components of genome
4452	CakSNP4452	*Kabuli*	Ca_Kabuli_Chr03	31355001	[C/T]	Ca_12235	Cystathionine beta-synthase, core	INTRON
4453	CakSNP4453	*Kabuli*	Ca_Kabuli_Chr03	31355026	[T/C]	Ca_12235	Cystathionine beta-synthase, core	INTRON
4454	CakSNP4454	*Kabuli*	Ca_Kabuli_Chr03	31355066	[A/T]	Ca_12235	Cystathionine beta-synthase, core	INTRON
4455	CakSNP4455	*Kabuli*	Ca_Kabuli_Chr03	31357787	[A/T]	Ca_12236	Transcription factor jumonji/aspartyl beta-hydroxylase	SYNONYMOUS_CODING
4456	CakSNP4456	*Kabuli*	Ca_Kabuli_Chr03	31431216	[C/T]	Ca_12240	Protein kinase, catalytic domain	INTRON
4457	CakSNP4457	*Kabuli*	Ca_Kabuli_Chr03	31478804	[T/C]	Ca_12246	Glucose-methanol-choline oxidoreductase, N-terminal	SYNONYMOUS_CODING
4458	CakSNP4458	*Kabuli*	Ca_Kabuli_Chr03	31478882	[C/T]	Ca_12246	Glucose-methanol-choline oxidoreductase, N-terminal	SYNONYMOUS_CODING
4459	CakSNP4459	*Kabuli*	Ca_Kabuli_Chr03	31480603	[T/C]		--	INTERGENIC
4460	CakSNP4460	*Kabuli*	Ca_Kabuli_Chr03	31516662	[G/T]	Ca_12250	Oligopeptide transporter	SYNONYMOUS_CODING
4461	CakSNP4461	*Kabuli*	Ca_Kabuli_Chr03	31516766	[T/C]	Ca_12250	Oligopeptide transporter	INTRON

S.N.	SNP IDs	Chickpea cultivars	Chromosomes	Physical positions (bp)	SNPs	Gene accession IDs	Putative functions	Sequence components of genome
4462	CakSNP4462	*Kabuli*	Ca_Kabuli_Chr03	31520119	[C/T]	Ca_12251	--	INTRON
4463	CakSNP4463	*Kabuli*	Ca_Kabuli_Chr03	31520225	[A/T]	Ca_12251	--	INTRON
4464	CakSNP4464	*Kabuli*	Ca_Kabuli_Chr03	31520198	[G/A]	Ca_12251	--	INTRON
4465	CakSNP4465	*Kabuli*	Ca_Kabuli_Chr03	31520190	[T/C]	Ca_12251	--	INTRON
4466	CakSNP4466	*Kabuli*	Ca_Kabuli_Chr03	31520832	[G/A]	Ca_12251	--	INTRON
4467	CakSNP4467	*Kabuli*	Ca_Kabuli_Chr03	31521142	[T/C]		--	INTERGENIC
4468	CakSNP4468	*Kabuli*	Ca_Kabuli_Chr03	31521155	[T/A]		--	INTERGENIC
4469	CakSNP4469	*Kabuli*	Ca_Kabuli_Chr03	31589355	[A/G]	Ca_12258	SET domain	INTRON
4470	CakSNP4470	*Kabuli*	Ca_Kabuli_Chr03	31589329	[C/T]	Ca_12258	SET domain	INTRON
4471	CakSNP4471	*Kabuli*	Ca_Kabuli_Chr03	31591065	[G/A]	Ca_12258	SET domain	SYNONYMOUS_CODING

S.N.	SNP IDs	Chickpea cultivars	Chromosomes	Physical positions (bp)	SNPs	Gene accession IDs	Putative functions	Sequence components of genome
4472	CakSNP4472	*Kabuli*	Ca_Kabuli_Chr03	31606359	[C/T]	Ca_12260	Gnk2-homologous domain	NON_SYNONYMOUS_CODING
4473	CakSNP4473	*Kabuli*	Ca_Kabuli_Chr03	31630175	[T/A]	Ca_12262	RNA recognition motif domain	INTRON
4474	CakSNP4474	*Kabuli*	Ca_Kabuli_Chr03	31630178	[G/T]	Ca_12262	RNA recognition motif domain	INTRON
4475	CakSNP4475	*Kabuli*	Ca_Kabuli_Chr03	31630195	[T/C]	Ca_12262	RNA recognition motif domain	INTRON
4476	CakSNP4476	*Kabuli*	Ca_Kabuli_Chr03	31631451	[A/C]	Ca_12262	RNA recognition motif domain	NON_SYNONYMOUS_CODING
4477	CakSNP4477	*Kabuli*	Ca_Kabuli_Chr03	31631444	[T/G]	Ca_12262	RNA recognition motif domain	NON_SYNONYMOUS_CODING
4478	CakSNP4478	*Kabuli*	Ca_Kabuli_Chr03	31631561	[G/A]	Ca_12262	RNA recognition motif domain	SYNONYMOUS_CODING
4479	CakSNP4479	*Kabuli*	Ca_Kabuli_Chr03	31631534	[A/G]	Ca_12262	RNA recognition motif domain	SYNONYMOUS_CODING
4480	CakSNP4480	*Kabuli*	Ca_Kabuli_Chr03	31631622	[G/A]	Ca_12262	RNA recognition motif domain	INTRON
4481	CakSNP4481	*Kabuli*	Ca_Kabuli_Chr03	31631644	[A/C]	Ca_12262	RNA recognition motif domain	INTRON

S.N.	SNP IDs	Chickpea cultivars	Chromosomes	Physical positions (bp)	SNPs	Gene accession IDs	Putative functions	Sequence components of genome
4482	CakSNP4482	*Kabuli*	Ca_Kabuli_Chr03	31646735	[C/T]		--	INTERGENIC
4483	CakSNP4483	*Kabuli*	Ca_Kabuli_Chr03	31646817	[C/T]		--	INTERGENIC
4484	CakSNP4484	*Kabuli*	Ca_Kabuli_Chr03	31646802	[G/A]		--	INTERGENIC
4485	CakSNP4485	*Kabuli*	Ca_Kabuli_Chr03	31683906	[A/G]	Ca_12270	Peptidase S8/S53, subtilisin/kexin/sedolisin	NON_SYNONYMOUS_CODING
4486	CakSNP4486	*Kabuli*	Ca_Kabuli_Chr03	31689128	[C/T]	Ca_12271	Exostosin-like	NON_SYNONYMOUS_CODING
4487	CakSNP4487	*Kabuli*	Ca_Kabuli_Chr03	31698493	[A/C]	Ca_12272	ABC transporter, transmembrane domain	INTRON
4488	CakSNP4488	*Kabuli*	Ca_Kabuli_Chr03	31766363	[G/A]	Ca_12278	Ammonium transporter	SYNONYMOUS_CODING
4489	CakSNP4489	*Kabuli*	Ca_Kabuli_Chr03	31770365	[A/C]	Ca_12279	--	SYNONYMOUS_CODING
4490	CakSNP4490	*Kabuli*	Ca_Kabuli_Chr03	31771314	[C/A]	Ca_12279	--	INTRON
4491	CakSNP4491	*Kabuli*	Ca_Kabuli_Chr03	31790189	[C/T]	Ca_12281	Alcohol dehydrogenase superfamily, zinc-containing	INTRON

S.N.	SNP IDs	Chickpea cultivars	Chromosomes	Physical positions (bp)	SNPs	Gene accession IDs	Putative functions	Sequence components of genome
4492	CakSNP4492	*Kabuli*	Ca_Kabuli_Chr03	31790216	[G/A]	Ca_12281	Alcohol dehydrogenase superfamily, zinc-containing	INTRON
4493	CakSNP4493	*Kabuli*	Ca_Kabuli_Chr03	31790239	[G/T]	Ca_12281	Alcohol dehydrogenase superfamily, zinc-containing	INTRON
4494	CakSNP4494	*Kabuli*	Ca_Kabuli_Chr03	31790352	[G/A]	Ca_12281	Alcohol dehydrogenase superfamily, zinc-containing	SYNONYMOUS_CODING
4495	CakSNP4495	*Kabuli*	Ca_Kabuli_Chr03	31814300	[A/G]	Ca_12284	WD40 repeat	INTRON
4496	CakSNP4496	*Kabuli*	Ca_Kabuli_Chr03	31852191	[T/C]	Ca_12289	Ribokinase	INTRON
4497	CakSNP4497	*Kabuli*	Ca_Kabuli_Chr03	31857877	[C/T]		--	INTERGENIC
4498	CakSNP4498	*Kabuli*	Ca_Kabuli_Chr03	31859152	[C/G]		--	INTERGENIC
4499	CakSNP4499	*Kabuli*	Ca_Kabuli_Chr03	31859251	[T/C]		--	INTERGENIC
4500	CakSNP4500	*Kabuli*	Ca_Kabuli_Chr03	31926694	[C/G]	Ca_12294	--	INTRON
4501	CakSNP4501	*Kabuli*	Ca_Kabuli_Chr03	31931014	[A/C]	Ca_12294	--	SYNONYMOUS_CODING

S.N.	SNP IDs	Chickpea cultivars	Chromosomes	Physical positions (bp)	SNPs	Gene accession IDs	Putative functions	Sequence components of genome
4502	CakSNP4502	*Kabuli*	Ca_Kabuli_Chr03	31945504	[C/T]	Ca_12295	HEAT	INTRON
4503	CakSNP4503	*Kabuli*	Ca_Kabuli_Chr03	31945634	[A/G]	Ca_12295	HEAT	INTRON
4504	CakSNP4504	*Kabuli*	Ca_Kabuli_Chr03	31951994	[G/A]	Ca_12295	HEAT	SYNONYMOUS_CODING
4505	CakSNP4505	*Kabuli*	Ca_Kabuli_Chr03	31953466	[G/A]	Ca_12295	HEAT	INTRON
4506	CakSNP4506	*Kabuli*	Ca_Kabuli_Chr03	31953549	[C/T]	Ca_12295	HEAT	INTRON
4507	CakSNP4507	*Kabuli*	Ca_Kabuli_Chr03	32351052	[C/T]	Ca_11958	Heat shock protein Hsp70	SYNONYMOUS_CODING
4508	CakSNP4508	*Kabuli*	Ca_Kabuli_Chr03	32351031	[G/C]	Ca_11958	Heat shock protein Hsp70	SYNONYMOUS_CODING
4509	CakSNP4509	*Kabuli*	Ca_Kabuli_Chr03	32357599	[C/T]	Ca_11959	Heat shock protein Hsp70	SYNONYMOUS_CODING
4510	CakSNP4510	*Kabuli*	Ca_Kabuli_Chr03	32357578	[G/C]	Ca_11959	Heat shock protein Hsp70	SYNONYMOUS_CODING
4511	CakSNP4511	*Kabuli*	Ca_Kabuli_Chr03	32448959	[T/C]	Ca_11968	Ion channel regulatory protein, UNC-93	NON_SYNONYMOUS_CODING

S.N.	SNP IDs	Chickpea cultivars	Chromosomes	Physical positions (bp)	SNPs	Gene accession IDs	Putative functions	Sequence components of genome
4512	CakSNP4512	*Kabuli*	Ca_Kabuli_Chr03	32449654	[T/G]	Ca_11968	Ion channel regulatory protein, UNC-93	NON_SYNONYMOUS_CODING
4513	CakSNP4513	*Kabuli*	Ca_Kabuli_Chr03	32449700	[C/T]	Ca_11968	Ion channel regulatory protein, UNC-93	INTRON
4514	CakSNP4514	*Kabuli*	Ca_Kabuli_Chr03	32510033	[T/G]		--	INTERGENIC
4515	CakSNP4515	*Kabuli*	Ca_Kabuli_Chr03	32510090	[A/G]		--	INTERGENIC
4516	CakSNP4516	*Kabuli*	Ca_Kabuli_Chr03	32510130	[A/G]		--	INTERGENIC
4517	CakSNP4517	*Kabuli*	Ca_Kabuli_Chr03	32510124	[C/T]		--	INTERGENIC
4518	CakSNP4518	*Kabuli*	Ca_Kabuli_Chr03	32510111	[C/T]		--	INTERGENIC
4519	CakSNP4519	*Kabuli*	Ca_Kabuli_Chr03	32510071	[G/T]		--	INTERGENIC
4520	CakSNP4520	*Kabuli*	Ca_Kabuli_Chr03	32510081	[C/T]		--	INTERGENIC
4521	CakSNP4521	*Kabuli*	Ca_Kabuli_Chr03	32514908	[A/G]		--	INTERGENIC

S.N.	SNP IDs	Chickpea cultivars	Chromosomes	Physical positions (bp)	SNPs	Gene accession IDs	Putative functions	Sequence components of genome
4522	CakSNP4522	*Kabuli*	Ca_Kabuli_Chr03	32514890	[A/G]		--	INTERGENIC
4523	CakSNP4523	*Kabuli*	Ca_Kabuli_Chr03	32514889	[C/T]		--	INTERGENIC
4524	CakSNP4524	*Kabuli*	Ca_Kabuli_Chr03	32514869	[C/T]		--	INTERGENIC
4525	CakSNP4525	*Kabuli*	Ca_Kabuli_Chr03	32514868	[A/G]		--	INTERGENIC
4526	CakSNP4526	*Kabuli*	Ca_Kabuli_Chr03	32514867	[C/T]		--	INTERGENIC
4527	CakSNP4527	*Kabuli*	Ca_Kabuli_Chr03	32514863	[G/C]		--	INTERGENIC
4528	CakSNP4528	*Kabuli*	Ca_Kabuli_Chr03	32514859	[C/T]		--	INTERGENIC
4529	CakSNP4529	*Kabuli*	Ca_Kabuli_Chr03	32514916	[G/C]		--	INTERGENIC
4530	CakSNP4530	*Kabuli*	Ca_Kabuli_Chr03	32525128	[G/T]	Ca_11978	Ras GTPase	NON_SYNONYMOUS_CODING
4531	CakSNP4531	*Kabuli*	Ca_Kabuli_Chr03	32557850	[C/T]	Ca_11982	Diaminopimelate epimerase	INTRON

S.N.	SNP IDs	Chickpea cultivars	Chromosomes	Physical positions (bp)	SNPs	Gene accession IDs	Putative functions	Sequence components of genome
4532	CakSNP4532	*Kabuli*	Ca_Kabuli_Chr03	32571237	[G/A]		--	INTERGENIC
4533	CakSNP4533	*Kabuli*	Ca_Kabuli_Chr03	32571291	[A/G]		--	INTERGENIC
4534	CakSNP4534	*Kabuli*	Ca_Kabuli_Chr03	32571275	[T/C]		--	INTERGENIC
4535	CakSNP4535	*Kabuli*	Ca_Kabuli_Chr03	32600003	[A/G]	Ca_11986	Ankyrin repeat	SYNONYMOUS_CODING
4536	CakSNP4536	*Kabuli*	Ca_Kabuli_Chr03	32601732	[T/C]		--	INTERGENIC
4537	CakSNP4537	*Kabuli*	Ca_Kabuli_Chr03	32608947	[A/G]	Ca_11988	Lipin, N-terminal	SYNONYMOUS_CODING
4538	CakSNP4538	*Kabuli*	Ca_Kabuli_Chr03	32662861	[A/G]	Ca_11994	RNA recognition motif domain	INTRON
4539	CakSNP4539	*Kabuli*	Ca_Kabuli_Chr03	32662858	[A/G]	Ca_11994	RNA recognition motif domain	INTRON
4540	CakSNP4540	*Kabuli*	Ca_Kabuli_Chr03	32663015	[C/T]	Ca_11994	RNA recognition motif domain	SYNONYMOUS_CODING
4541	CakSNP4541	*Kabuli*	Ca_Kabuli_Chr03	32663059	[A/C]	Ca_11994	RNA recognition motif domain	NON_SYNONYMOUS_CODING

S.N.	SNP IDs	Chickpea cultivars	Chromosomes	Physical positions (bp)	SNPs	Gene accession IDs	Putative functions	Sequence components of genome
4542	CakSNP4542	*Kabuli*	Ca_Kabuli_Chr03	32694509	[T/C]	Ca_11998	Protein phosphatase 2C, manganese/magnesium aspartate binding site	INTRON
4543	CakSNP4543	*Kabuli*	Ca_Kabuli_Chr03	32694454	[T/C]	Ca_11998	Protein phosphatase 2C, manganese/magnesium aspartate binding site	INTRON
4544	CakSNP4544	*Kabuli*	Ca_Kabuli_Chr03	32704734	[G/A]	Ca_12000	--	INTRON
4545	CakSNP4545	*Kabuli*	Ca_Kabuli_Chr03	32704868	[G/T]	Ca_12000	--	INTRON
4546	CakSNP4546	*Kabuli*	Ca_Kabuli_Chr03	32704953	[G/A]	Ca_12000	--	INTRON
4547	CakSNP4547	*Kabuli*	Ca_Kabuli_Chr03	32706200	[C/T]	Ca_12000	--	INTRON
4548	CakSNP4548	*Kabuli*	Ca_Kabuli_Chr03	32725039	[C/G]	Ca_12004	Protein kinase, catalytic domain	SYNONYMOUS_CODING
4549	CakSNP4549	*Kabuli*	Ca_Kabuli_Chr03	32725096	[A/T]	Ca_12004	Protein kinase, catalytic domain	INTRON
4550	CakSNP4550	*Kabuli*	Ca_Kabuli_Chr03	32758259	[C/A]	Ca_12010	Pathogenesis-related transcriptional factor/ERF, DNA-binding	INTRON
4551	CakSNP4551	*Kabuli*	Ca_Kabuli_Chr03	32758293	[T/C]	Ca_12010	Pathogenesis-related transcriptional factor/ERF, DNA-binding	INTRON

S.N.	SNP IDs	Chickpea cultivars	Chromosomes	Physical positions (bp)	SNPs	Gene accession IDs	Putative functions	Sequence components of genome
4552	CakSNP4552	*Kabuli*	Ca_Kabuli_Chr03	32785228	[A/G]	Ca_12012	Protein kinase, catalytic domain	SYNONYMOUS_CODING
4553	CakSNP4553	*Kabuli*	Ca_Kabuli_Chr03	32827067	[C/G]	Ca_12018	--	NON_SYNONYMOUS_CODING
4554	CakSNP4554	*Kabuli*	Ca_Kabuli_Chr03	32827065	[T/G]	Ca_12018	--	SYNONYMOUS_CODING
4555	CakSNP4555	*Kabuli*	Ca_Kabuli_Chr03	32845361	[A/G]	Ca_12020	IQ motif, EF-hand binding site	SYNONYMOUS_CODING
4556	CakSNP4556	*Kabuli*	Ca_Kabuli_Chr03	32845395	[C/A]	Ca_12020	IQ motif, EF-hand binding site	NON_SYNONYMOUS_CODING
4557	CakSNP4557	*Kabuli*	Ca_Kabuli_Chr03	32847120	[T/A]	Ca_12020	IQ motif, EF-hand binding site	INTRON
4558	CakSNP4558	*Kabuli*	Ca_Kabuli_Chr03	32847216	[A/G]	Ca_12020	IQ motif, EF-hand binding site	INTRON
4559	CakSNP4559	*Kabuli*	Ca_Kabuli_Chr03	32865549	[C/T]	Ca_12023	Helicase, C-terminal	INTRON
4560	CakSNP4560	*Kabuli*	Ca_Kabuli_Chr03	32865530	[C/G]	Ca_12023	Helicase, C-terminal	INTRON
4561	CakSNP4561	*Kabuli*	Ca_Kabuli_Chr03	32954428	[T/C]		--	INTERGENIC

S.N.	SNP IDs	Chickpea cultivars	Chromosomes	Physical positions (bp)	SNPs	Gene accession IDs	Putative functions	Sequence components of genome
4562	CakSNP4562	*Kabuli*	Ca_Kabuli_Chr03	33033245	[T/C]		--	INTERGENIC
4563	CakSNP4563	*Kabuli*	Ca_Kabuli_Chr03	33091737	[A/C]	Ca_12046	SNF2-related	INTRON
4564	CakSNP4564	*Kabuli*	Ca_Kabuli_Chr03	33120619	[G/A]	Ca_12048	Translin	INTRON
4565	CakSNP4565	*Kabuli*	Ca_Kabuli_Chr03	33120825	[G/A]	Ca_12048	Translin	NON_SYNONYMOUS_CODING
4566	CakSNP4566	*Kabuli*	Ca_Kabuli_Chr03	33520615	[A/C]	Ca_23669	--	NON_SYNONYMOUS_CODING
4567	CakSNP4567	*Kabuli*	Ca_Kabuli_Chr03	33520887	[G/A]	Ca_23669	--	INTRON
4568	CakSNP4568	*Kabuli*	Ca_Kabuli_Chr03	33522974	[G/A]	Ca_23669	--	INTRON
4569	CakSNP4569	*Kabuli*	Ca_Kabuli_Chr03	33533273	[G/A]		--	INTERGENIC
4570	CakSNP4570	*Kabuli*	Ca_Kabuli_Chr03	33627826	[A/C]		--	INTERGENIC
4571	CakSNP4571	*Kabuli*	Ca_Kabuli_Chr03	33715215	[G/T]	Ca_20869	Alpha/beta hydrolase fold-1	INTRON

S.N.	SNP IDs	Chickpea cultivars	Chromosomes	Physical positions (bp)	SNPs	Gene accession IDs	Putative functions	Sequence components of genome
4572	CakSNP4572	*Kabuli*	Ca_Kabuli_Chr03	33898250	[C/T]	Ca_20884	ABC transporter, transmembrane domain	SYNONYMOUS_CODING
4573	CakSNP4573	*Kabuli*	Ca_Kabuli_Chr03	33900522	[A/G]	Ca_20884	ABC transporter, transmembrane domain	INTRON
4574	CakSNP4574	*Kabuli*	Ca_Kabuli_Chr03	33900533	[A/G]	Ca_20884	ABC transporter, transmembrane domain	INTRON
4575	CakSNP4575	*Kabuli*	Ca_Kabuli_Chr03	33904394	[T/A]	Ca_20885	--	INTRON
4576	CakSNP4576	*Kabuli*	Ca_Kabuli_Chr03	34247736	[T/G]	Ca_00702	ABC transporter, transmembrane domain	NON_SYNONYMOUS_CODING
4577	CakSNP4577	*Kabuli*	Ca_Kabuli_Chr03	34247831	[G/A]	Ca_00702	ABC transporter, transmembrane domain	SYNONYMOUS_CODING
4578	CakSNP4578	*Kabuli*	Ca_Kabuli_Chr03	34247828	[T/C]	Ca_00702	ABC transporter, transmembrane domain	SYNONYMOUS_CODING
4579	CakSNP4579	*Kabuli*	Ca_Kabuli_Chr03	34247893	[A/G]	Ca_00702	ABC transporter, transmembrane domain	INTRON
4580	CakSNP4580	*Kabuli*	Ca_Kabuli_Chr03	34268356	[A/T]		--	INTERGENIC
4581	CakSNP4581	*Kabuli*	Ca_Kabuli_Chr03	34282419	[C/T]	Ca_00705	Thioredoxin	NON_SYNONYMOUS_CODING

S.N.	SNP IDs	Chickpea cultivars	Chromosomes	Physical positions (bp)	SNPs	Gene accession IDs	Putative functions	Sequence components of genome
4582	CakSNP4582	*Kabuli*	Ca_Kabuli_Chr03	34290129	[T/C]	Ca_00706	Molybdopterin binding	INTRON
4583	CakSNP4583	*Kabuli*	Ca_Kabuli_Chr03	34290135	[G/A]	Ca_00706	Molybdopterin binding	INTRON
4584	CakSNP4584	*Kabuli*	Ca_Kabuli_Chr03	34290173	[C/T]	Ca_00706	Molybdopterin binding	INTRON
4585	CakSNP4585	*Kabuli*	Ca_Kabuli_Chr03	34352385	[A/C]		--	INTERGENIC
4586	CakSNP4586	*Kabuli*	Ca_Kabuli_Chr03	34356844	[A/C]		--	INTERGENIC
4587	CakSNP4587	*Kabuli*	Ca_Kabuli_Chr03	34357171	[T/G]		--	INTERGENIC
4588	CakSNP4588	*Kabuli*	Ca_Kabuli_Chr03	34357296	[C/G]		--	INTERGENIC
4589	CakSNP4589	*Kabuli*	Ca_Kabuli_Chr03	34496982	[G/C]	Ca_00725	WD40 repeat	SYNONYMOUS_CODING
4590	CakSNP4590	*Kabuli*	Ca_Kabuli_Chr03	34524656	[C/A]	Ca_00726	--	NON_SYNONYMOUS_CODING
4591	CakSNP4591	*Kabuli*	Ca_Kabuli_Chr03	34542046	[T/G]	Ca_00726	--	INTRON

S.N.	SNP IDs	Chickpea cultivars	Chromosomes	Physical positions (bp)	SNPs	Gene accession IDs	Putative functions	Sequence components of genome
4592	CakSNP4592	*Kabuli*	Ca_Kabuli_Chr03	34543039	[C/T]	Ca_00726	--	NON_SYNONYMOUS_CODING
4593	CakSNP4593	*Kabuli*	Ca_Kabuli_Chr03	34543111	[A/G]	Ca_00726	--	NON_SYNONYMOUS_CODING
4594	CakSNP4594	*Kabuli*	Ca_Kabuli_Chr03	34547052	[A/T]	Ca_00726	--	NON_SYNONYMOUS_CODING
4595	CakSNP4595	*Kabuli*	Ca_Kabuli_Chr03	34547069	[G/T]	Ca_00726	--	NON_SYNONYMOUS_CODING
4596	CakSNP4596	*Kabuli*	Ca_Kabuli_Chr03	34547141	[A/G]	Ca_00726	--	NON_SYNONYMOUS_CODING
4597	CakSNP4597	*Kabuli*	Ca_Kabuli_Chr03	34576769	[G/A]	Ca_00729	Transcription factor, MADS-box	NON_SYNONYMOUS_CODING
4598	CakSNP4598	*Kabuli*	Ca_Kabuli_Chr03	34595487	[A/G]	Ca_00732	Zinc finger, PHD-type	INTRON
4599	CakSNP4599	*Kabuli*	Ca_Kabuli_Chr03	34601038	[T/C]	Ca_00732	Zinc finger, PHD-type	INTRON
4600	CakSNP4600	*Kabuli*	Ca_Kabuli_Chr03	34603669	[T/A]	Ca_00732	Zinc finger, PHD-type	NON_SYNONYMOUS_CODING
4601	CakSNP4601	*Kabuli*	Ca_Kabuli_Chr03	34650308	[G/A]	Ca_00739	Peptidase A1	SYNONYMOUS_CODING

S.N.	SNP IDs	Chickpea cultivars	Chromosomes	Physical positions (bp)	SNPs	Gene accession IDs	Putative functions	Sequence components of genome
4602	CakSNP4602	*Kabuli*	Ca_Kabuli_Chr03	34650245	[G/A]	Ca_00739	Peptidase A1	SYNONYMOUS_CODING
4603	CakSNP4603	*Kabuli*	Ca_Kabuli_Chr03	34662736	[T/G]	Ca_00740	RNA recognition motif domain	INTRON
4604	CakSNP4604	*Kabuli*	Ca_Kabuli_Chr03	34699114	[C/A]	Ca_00745	HECT	NON_SYNONYMOUS_CODING
4605	CakSNP4605	*Kabuli*	Ca_Kabuli_Chr03	34699076	[T/C]	Ca_00745	HECT	NON_SYNONYMOUS_CODING
4606	CakSNP4606	*Kabuli*	Ca_Kabuli_Chr03	34722508	[A/T]	Ca_00747	Late embryogenesis abundant protein, group 4	SYNONYMOUS_CODING
4607	CakSNP4607	*Kabuli*	Ca_Kabuli_Chr03	34722931	[A/G]	Ca_00747	Late embryogenesis abundant protein, group 4	SYNONYMOUS_CODING
4608	CakSNP4608	*Kabuli*	Ca_Kabuli_Chr03	34722979	[T/C]	Ca_00747	Late embryogenesis abundant protein, group 4	SYNONYMOUS_CODING
4609	CakSNP4609	*Kabuli*	Ca_Kabuli_Chr03	34722992	[T/G]	Ca_00747	Late embryogenesis abundant protein, group 4	NON_SYNONYMOUS_CODING
4610	CakSNP4610	*Kabuli*	Ca_Kabuli_Chr03	34730463	[T/C]	Ca_00748	Protein of unknown function DUF81	INTRON
4611	CakSNP4611	*Kabuli*	Ca_Kabuli_Chr03	34730954	[G/A]		--	INTERGENIC

S.N.	SNP IDs	Chickpea cultivars	Chromosomes	Physical positions (bp)	SNPs	Gene accession IDs	Putative functions	Sequence components of genome
4612	CakSNP4612	*Kabuli*	Ca_Kabuli_Chr03	34800277	[G/A]	Ca_00756	Protein of unknown function DUF668	NON_SYNONYMOUS_CODING
4613	CakSNP4613	*Kabuli*	Ca_Kabuli_Chr03	34820472	[T/A]	Ca_00758	Glycosyl transferase, family 2	INTRON
4614	CakSNP4614	*Kabuli*	Ca_Kabuli_Chr03	34820532	[T/A]	Ca_00758	Glycosyl transferase, family 2	INTRON
4615	CakSNP4615	*Kabuli*	Ca_Kabuli_Chr03	34852030	[G/A]	Ca_00763	Zinc finger, C2H2-type	INTRON
4616	CakSNP4616	*Kabuli*	Ca_Kabuli_Chr03	34852070	[T/G]	Ca_00763	Zinc finger, C2H2-type	INTRON
4617	CakSNP4617	*Kabuli*	Ca_Kabuli_Chr03	34852047	[T/A]	Ca_00763	Zinc finger, C2H2-type	INTRON
4618	CakSNP4618	*Kabuli*	Ca_Kabuli_Chr03	34889316	[C/G]	Ca_00767	Fructose-bisphosphate aldolase, class-I	SYNONYMOUS_CODING
4619	CakSNP4619	*Kabuli*	Ca_Kabuli_Chr03	34889361	[T/C]	Ca_00767	Fructose-bisphosphate aldolase, class-I	SYNONYMOUS_CODING
4620	CakSNP4620	*Kabuli*	Ca_Kabuli_Chr03	34911621	[G/A]	Ca_00768	Pathogenesis-related transcriptional factor/ERF, DNA-binding	SYNONYMOUS_CODING
4621	CakSNP4621	*Kabuli*	Ca_Kabuli_Chr03	35071821	[A/G]	Ca_00779	Protein kinase, catalytic domain	INTRON

S.N.	SNP IDs	Chickpea cultivars	Chromosomes	Physical positions (bp)	SNPs	Gene accession IDs	Putative functions	Sequence components of genome
4622	CakSNP4622	*Kabuli*	Ca_Kabuli_Chr03	35071852	[T/G]	Ca_00779	Protein kinase, catalytic domain	INTRON
4623	CakSNP4623	*Kabuli*	Ca_Kabuli_Chr03	35085559	[T/A]	Ca_00781	Peptidase S8/S53, subtilisin/kexin/sedolisin	SYNONYMOUS_CODING
4624	CakSNP4624	*Kabuli*	Ca_Kabuli_Chr03	35085658	[C/A]	Ca_00781	Peptidase S8/S53, subtilisin/kexin/sedolisin	SYNONYMOUS_CODING
4625	CakSNP4625	*Kabuli*	Ca_Kabuli_Chr03	35126649	[C/G]	Ca_00786	--	NON_SYNONYMOUS_CODING
4626	CakSNP4626	*Kabuli*	Ca_Kabuli_Chr03	35126766	[G/A]	Ca_00786	--	NON_SYNONYMOUS_CODING
4627	CakSNP4627	*Kabuli*	Ca_Kabuli_Chr03	35126760	[G/A]	Ca_00786	--	NON_SYNONYMOUS_CODING
4628	CakSNP4628	*Kabuli*	Ca_Kabuli_Chr03	35167312	[T/G]		--	INTERGENIC
4629	CakSNP4629	*Kabuli*	Ca_Kabuli_Chr03	35167359	[T/G]		--	INTERGENIC
4630	CakSNP4630	*Kabuli*	Ca_Kabuli_Chr03	35185001	[G/T]	Ca_00791	Basic-leucine zipper (bZIP) transcription factor	SYNONYMOUS_CODING
4631	CakSNP4631	*Kabuli*	Ca_Kabuli_Chr03	35274992	[A/G]	Ca_00805	F-box domain, cyclin-like	NON_SYNONYMOUS_CODING

S.N.	SNP IDs	Chickpea cultivars	Chromosomes	Physical positions (bp)	SNPs	Gene accession IDs	Putative functions	Sequence components of genome
4632	CakSNP4632	*Kabuli*	Ca_Kabuli_Chr03	35275199	[T/C]	Ca_00805	F-box domain, cyclin-like	SYNONYMOUS_CODING
4633	CakSNP4633	*Kabuli*	Ca_Kabuli_Chr03	35332088	[T/A]	Ca_00812	Kinesin, motor domain	NON_SYNONYMOUS_CODING
4634	CakSNP4634	*Kabuli*	Ca_Kabuli_Chr03	35332091	[T/C]	Ca_00812	Kinesin, motor domain	SYNONYMOUS_CODING
4635	CakSNP4635	*Kabuli*	Ca_Kabuli_Chr03	35383318	[C/T]	Ca_00819	Peptidyl-prolyl cis-trans isomerase, cyclophilin-type	INTRON
4636	CakSNP4636	*Kabuli*	Ca_Kabuli_Chr03	35394684	[G/T]		--	INTERGENIC
4637	CakSNP4637	*Kabuli*	Ca_Kabuli_Chr03	35403074	[A/G]		--	INTERGENIC
4638	CakSNP4638	*Kabuli*	Ca_Kabuli_Chr03	35420061	[A/G]	Ca_00824	LETM1-like	INTRON
4639	CakSNP4639	*Kabuli*	Ca_Kabuli_Chr03	35420108	[T/A]	Ca_00824	LETM1-like	INTRON
4640	CakSNP4640	*Kabuli*	Ca_Kabuli_Chr03	35424994	[T/C]		--	INTERGENIC
4641	CakSNP4641	*Kabuli*	Ca_Kabuli_Chr03	35483932	[T/C]	Ca_00831	Zinc finger, MYND-type	INTRON

S.N.	SNP IDs	Chickpea cultivars	Chromosomes	Physical positions (bp)	SNPs	Gene accession IDs	Putative functions	Sequence components of genome
4642	CakSNP4642	*Kabuli*	Ca_Kabuli_Chr03	35483960	[T/C]	Ca_00831	Zinc finger, MYND-type	INTRON
4643	CakSNP4643	*Kabuli*	Ca_Kabuli_Chr03	35483995	[T/C]	Ca_00831	Zinc finger, MYND-type	INTRON
4644	CakSNP4644	*Kabuli*	Ca_Kabuli_Chr03	35483999	[C/T]	Ca_00831	Zinc finger, MYND-type	INTRON
4645	CakSNP4645	*Kabuli*	Ca_Kabuli_Chr03	35484034	[G/C]	Ca_00831	Zinc finger, MYND-type	INTRON
4646	CakSNP4646	*Kabuli*	Ca_Kabuli_Chr03	35487736	[T/C]		--	INTERGENIC
4647	CakSNP4647	*Kabuli*	Ca_Kabuli_Chr03	35488503	[A/C]	Ca_00832	TLDc	INTRON
4648	CakSNP4648	*Kabuli*	Ca_Kabuli_Chr03	35488597	[T/C]	Ca_00832	TLDc	INTRON
4649	CakSNP4649	*Kabuli*	Ca_Kabuli_Chr03	35488570	[G/T]	Ca_00832	TLDc	INTRON
4650	CakSNP4650	*Kabuli*	Ca_Kabuli_Chr03	35490991	[A/G]	Ca_00832	TLDc	SYNONYMOUS_CODING
4651	CakSNP4651	*Kabuli*	Ca_Kabuli_Chr03	35490951	[C/T]	Ca_00832	TLDc	NON_SYNONYMOUS_CODING

S.N.	SNP IDs	Chickpea cultivars	Chromosomes	Physical positions (bp)	SNPs	Gene accession IDs	Putative functions	Sequence components of genome
4652	CakSNP4652	*Kabuli*	Ca_Kabuli_Chr03	35543614	[C/T]		--	INTERGENIC
4653	CakSNP4653	*Kabuli*	Ca_Kabuli_Chr03	35631679	[C/G]		--	INTERGENIC
4654	CakSNP4654	*Kabuli*	Ca_Kabuli_Chr03	35631689	[C/A]		--	INTERGENIC
4655	CakSNP4655	*Kabuli*	Ca_Kabuli_Chr03	35631723	[A/G]		--	INTERGENIC
4656	CakSNP4656	*Kabuli*	Ca_Kabuli_Chr03	35647986	[C/G]	Ca_00852	--	INTRON
4657	CakSNP4657	*Kabuli*	Ca_Kabuli_Chr03	35747394	[C/G]		--	INTERGENIC
4658	CakSNP4658	*Kabuli*	Ca_Kabuli_Chr03	35807222	[A/G]	Ca_00868	--	NON_SYNONYMOUS_CODING
4659	CakSNP4659	*Kabuli*	Ca_Kabuli_Chr03	35807265	[T/C]	Ca_00868	--	NON_SYNONYMOUS_CODING
4660	CakSNP4660	*Kabuli*	Ca_Kabuli_Chr03	35807407	[A/T]	Ca_00868	--	SYNONYMOUS_CODING
4661	CakSNP4661	*Kabuli*	Ca_Kabuli_Chr03	35829834	[A/G]	Ca_00870	--	NON_SYNONYMOUS_CODING

S.N.	SNP IDs	Chickpea cultivars	Chromosomes	Physical positions (bp)	SNPs	Gene accession IDs	Putative functions	Sequence components of genome
4662	CakSNP4662	*Kabuli*	Ca_Kabuli_Chr03	35898320	[G/C]	Ca_00881	WD40 repeat	SYNONYMOUS_CODING
4663	CakSNP4663	*Kabuli*	Ca_Kabuli_Chr03	35898374	[G/A]	Ca_00881	WD40 repeat	SYNONYMOUS_CODING
4664	CakSNP4664	*Kabuli*	Ca_Kabuli_Chr03	35898581	[C/T]	Ca_00881	WD40 repeat	SYNONYMOUS_CODING
4665	CakSNP4665	*Kabuli*	Ca_Kabuli_Chr03	35920684	[A/C]	Ca_00884	--	INTRON
4666	CakSNP4666	*Kabuli*	Ca_Kabuli_Chr03	35920860	[C/A]	Ca_00884	--	NON_SYNONYMOUS_CODING
4667	CakSNP4667	*Kabuli*	Ca_Kabuli_Chr03	35948487	[G/A]		--	INTERGENIC
4668	CakSNP4668	*Kabuli*	Ca_Kabuli_Chr03	35979412	[G/T]	Ca_00892	FAD-linked oxidase, C-terminal	SYNONYMOUS_CODING
4669	CakSNP4669	*Kabuli*	Ca_Kabuli_Chr03	36003699	[G/A]	Ca_00896	Protease-associated domain, PA	INTRON
4670	CakSNP4670	*Kabuli*	Ca_Kabuli_Chr03	36003682	[G/T]	Ca_00896	Protease-associated domain, PA	INTRON
4671	CakSNP4671	*Kabuli*	Ca_Kabuli_Chr03	36004638	[C/T]	Ca_00896	Protease-associated domain, PA	INTRON

S.N.	SNP IDs	Chickpea cultivars	Chromosomes	Physical positions (bp)	SNPs	Gene accession IDs	Putative functions	Sequence components of genome
4672	CakSNP4672	*Kabuli*	Ca_Kabuli_Chr03	36004663	[T/C]	Ca_00896	Protease-associated domain, PA	INTRON
4673	CakSNP4673	*Kabuli*	Ca_Kabuli_Chr03	36004668	[A/G]	Ca_00896	Protease-associated domain, PA	INTRON
4674	CakSNP4674	*Kabuli*	Ca_Kabuli_Chr03	36046759	[G/A]	Ca_00903	SWAP/Surp	NON_SYNONYMOUS_CODING
4675	CakSNP4675	*Kabuli*	Ca_Kabuli_Chr03	36046938	[C/T]	Ca_00903	SWAP/Surp	SYNONYMOUS_CODING
4676	CakSNP4676	*Kabuli*	Ca_Kabuli_Chr03	36046956	[A/C]	Ca_00903	SWAP/Surp	SYNONYMOUS_CODING
4677	CakSNP4677	*Kabuli*	Ca_Kabuli_Chr03	36050259	[T/G]	Ca_00903	SWAP/Surp	SYNONYMOUS_CODING
4678	CakSNP4678	*Kabuli*	Ca_Kabuli_Chr03	36050319	[T/C]	Ca_00903	SWAP/Surp	SYNONYMOUS_CODING
4679	CakSNP4679	*Kabuli*	Ca_Kabuli_Chr03	36071033	[A/G]		--	INTERGENIC
4680	CakSNP4680	*Kabuli*	Ca_Kabuli_Chr03	36085966	[G/A]	Ca_00907	Phosphatidic acid phosphatase type 2/haloperoxidase	INTRON
4681	CakSNP4681	*Kabuli*	Ca_Kabuli_Chr03	36117521	[A/G]	Ca_00911	--	SYNONYMOUS_CODING

S.N.	SNP IDs	Chickpea cultivars	Chromosomes	Physical positions (bp)	SNPs	Gene accession IDs	Putative functions	Sequence components of genome
4682	CakSNP4682	*Kabuli*	Ca_Kabuli_Chr03	36118120	[T/C]	Ca_00911	--	INTRON
4683	CakSNP4683	*Kabuli*	Ca_Kabuli_Chr03	36118156	[C/T]	Ca_00911	--	INTRON
4684	CakSNP4684	*Kabuli*	Ca_Kabuli_Chr03	36119592	[T/C]	Ca_00911	--	NON_SYNONYMOUS_CODING
4685	CakSNP4685	*Kabuli*	Ca_Kabuli_Chr03	36119602	[C/A]	Ca_00911	--	NON_SYNONYMOUS_CODING
4686	CakSNP4686	*Kabuli*	Ca_Kabuli_Chr03	36130561	[T/A]		--	INTERGENIC
4687	CakSNP4687	*Kabuli*	Ca_Kabuli_Chr03	36130610	[C/A]		--	INTERGENIC
4688	CakSNP4688	*Kabuli*	Ca_Kabuli_Chr03	36132440	[T/C]	Ca_00912	NPL4	INTRON
4689	CakSNP4689	*Kabuli*	Ca_Kabuli_Chr03	36132437	[C/T]	Ca_00912	NPL4	INTRON
4690	CakSNP4690	*Kabuli*	Ca_Kabuli_Chr03	36132401	[C/T]	Ca_00912	NPL4	INTRON
4691	CakSNP4691	*Kabuli*	Ca_Kabuli_Chr03	36132480	[C/T]	Ca_00912	NPL4	INTRON

S.N.	SNP IDs	Chickpea cultivars	Chromosomes	Physical positions (bp)	SNPs	Gene accession IDs	Putative functions	Sequence components of genome
4692	CakSNP4692	*Kabuli*	Ca_Kabuli_Chr03	36132539	[C/T]	Ca_00912	NPL4	INTRON
4693	CakSNP4693	*Kabuli*	Ca_Kabuli_Chr03	36132586	[T/G]	Ca_00912	NPL4	INTRON
4694	CakSNP4694	*Kabuli*	Ca_Kabuli_Chr03	36132570	[G/A]	Ca_00912	NPL4	INTRON
4695	CakSNP4695	*Kabuli*	Ca_Kabuli_Chr03	36132554	[A/T]	Ca_00912	NPL4	INTRON
4696	CakSNP4696	*Kabuli*	Ca_Kabuli_Chr03	36150722	[G/A]		--	INTERGENIC
4697	CakSNP4697	*Kabuli*	Ca_Kabuli_Chr03	36150717	[T/C]		--	INTERGENIC
4698	CakSNP4698	*Kabuli*	Ca_Kabuli_Chr03	36164863	[T/G]	Ca_00917	Zinc finger, C2H2-type matrin	INTRON
4699	CakSNP4699	*Kabuli*	Ca_Kabuli_Chr03	36164866	[T/C]	Ca_00917	Zinc finger, C2H2-type matrin	INTRON
4700	CakSNP4700	*Kabuli*	Ca_Kabuli_Chr03	36165997	[C/G]	Ca_00917	Zinc finger, C2H2-type matrin	NON_SYNONYMOUS_CODING
4701	CakSNP4701	*Kabuli*	Ca_Kabuli_Chr03	36167136	[G/T]		--	INTERGENIC

S.N.	SNP IDs	Chickpea cultivars	Chromosomes	Physical positions (bp)	SNPs	Gene accession IDs	Putative functions	Sequence components of genome
4702	CakSNP4702	*Kabuli*	Ca_Kabuli_Chr03	36175164	[G/A]	Ca_00919	Short-chain dehydrogenase/reductase SDR	NON_SYNONYMOUS_CODING
4703	CakSNP4703	*Kabuli*	Ca_Kabuli_Chr03	36177201	[T/C]	Ca_00920	--	NON_SYNONYMOUS_CODING
4704	CakSNP4704	*Kabuli*	Ca_Kabuli_Chr03	36205806	[T/G]		--	INTERGENIC
4705	CakSNP4705	*Kabuli*	Ca_Kabuli_Chr03	36205819	[A/G]		--	INTERGENIC
4706	CakSNP4706	*Kabuli*	Ca_Kabuli_Chr03	36205845	[A/G]		--	INTERGENIC
4707	CakSNP4707	*Kabuli*	Ca_Kabuli_Chr03	36206615	[A/C]	Ca_00925	rRNA processing	INTRON
4708	CakSNP4708	*Kabuli*	Ca_Kabuli_Chr03	36212799	[A/T]	Ca_00926	Uncharacterised protein family UPF0497, trans-membrane plant	SYNONYMOUS_CODING
4709	CakSNP4709	*Kabuli*	Ca_Kabuli_Chr03	36212809	[G/A]	Ca_00926	Uncharacterised protein family UPF0497, trans-membrane plant	NON_SYNONYMOUS_CODING
4710	CakSNP4710	*Kabuli*	Ca_Kabuli_Chr03	36212843	[T/A]	Ca_00926	Uncharacterised protein family UPF0497, trans-membrane plant	NON_SYNONYMOUS_CODING
4711	CakSNP4711	*Kabuli*	Ca_Kabuli_Chr03	36217686	[G/A]	Ca_00927	Zinc finger, B-box	INTRON

S.N.	SNP IDs	Chickpea cultivars	Chromosomes	Physical positions (bp)	SNPs	Gene accession IDs	Putative functions	Sequence components of genome
4712	CakSNP4712	*Kabuli*	Ca_Kabuli_Chr03	36227565	[A/T]		--	INTERGENIC
4713	CakSNP4713	*Kabuli*	Ca_Kabuli_Chr03	36227566	[A/T]		--	INTERGENIC
4714	CakSNP4714	*Kabuli*	Ca_Kabuli_Chr03	36227948	[A/G]		--	INTERGENIC
4715	CakSNP4715	*Kabuli*	Ca_Kabuli_Chr03	36227949	[C/T]		--	INTERGENIC
4716	CakSNP4716	*Kabuli*	Ca_Kabuli_Chr03	36228078	[G/A]		--	INTERGENIC
4717	CakSNP4717	*Kabuli*	Ca_Kabuli_Chr03	36228032	[G/T]		--	INTERGENIC
4718	CakSNP4718	*Kabuli*	Ca_Kabuli_Chr03	36228121	[T/A]	Ca_00929	Ribosomal protein S19/S15	INTRON
4719	CakSNP4719	*Kabuli*	Ca_Kabuli_Chr03	36228126	[T/A]	Ca_00929	Ribosomal protein S19/S15	INTRON
4720	CakSNP4720	*Kabuli*	Ca_Kabuli_Chr03	36242506	[C/T]		--	INTERGENIC
4721	CakSNP4721	*Kabuli*	Ca_Kabuli_Chr03	36242504	[C/T]		--	INTERGENIC

S.N.	SNP IDs	Chickpea cultivars	Chromosomes	Physical positions (bp)	SNPs	Gene accession IDs	Putative functions	Sequence components of genome
4722	CakSNP4722	*Kabuli*	Ca_Kabuli_Chr03	36242490	[G/T]		--	INTERGENIC
4723	CakSNP4723	*Kabuli*	Ca_Kabuli_Chr03	36242480	[G/C]		--	INTERGENIC
4724	CakSNP4724	*Kabuli*	Ca_Kabuli_Chr03	36247792	[T/C]		--	INTERGENIC
4725	CakSNP4725	*Kabuli*	Ca_Kabuli_Chr03	36260865	[C/A]	Ca_00936	BTB/POZ-like	SYNONYMOUS_CODING
4726	CakSNP4726	*Kabuli*	Ca_Kabuli_Chr03	36282091	[T/G]	Ca_00938	Domain of unknown function DUF255	NON_SYNONYMOUS_CODING
4727	CakSNP4727	*Kabuli*	Ca_Kabuli_Chr03	36293589	[A/G]	Ca_00939	Zinc finger, CW-type	SYNONYMOUS_CODING
4728	CakSNP4728	*Kabuli*	Ca_Kabuli_Chr03	36293625	[C/T]	Ca_00939	Zinc finger, CW-type	SYNONYMOUS_CODING
4729	CakSNP4729	*Kabuli*	Ca_Kabuli_Chr03	36293637	[C/T]	Ca_00939	Zinc finger, CW-type	SYNONYMOUS_CODING
4730	CakSNP4730	*Kabuli*	Ca_Kabuli_Chr03	36330750	[A/G]	Ca_00942	Homeobox	NON_SYNONYMOUS_CODING
4731	CakSNP4731	*Kabuli*	Ca_Kabuli_Chr03	36330815	[T/C]	Ca_00942	Homeobox	SYNONYMOUS_CODING

S.N.	SNP IDs	Chickpea cultivars	Chromosomes	Physical positions (bp)	SNPs	Gene accession IDs	Putative functions	Sequence components of genome
4732	CakSNP4732	*Kabuli*	Ca_Kabuli_Chr03	36345158	[T/C]	Ca_00943	Zinc finger, C2H2-type	NON_SYNONYMOUS_CODING
4733	CakSNP4733	*Kabuli*	Ca_Kabuli_Chr03	36347203	[T/A]	Ca_00943	Zinc finger, C2H2-type	NON_SYNONYMOUS_CODING
4734	CakSNP4734	*Kabuli*	Ca_Kabuli_Chr03	36361339	[C/T]		--	INTERGENIC
4735	CakSNP4735	*Kabuli*	Ca_Kabuli_Chr03	36378294	[G/T]	Ca_00946	Protein kinase, catalytic domain	SYNONYMOUS_CODING
4736	CakSNP4736	*Kabuli*	Ca_Kabuli_Chr03	36378299	[C/G]	Ca_00946	Protein kinase, catalytic domain	INTRON
4737	CakSNP4737	*Kabuli*	Ca_Kabuli_Chr03	36378358	[G/A]	Ca_00946	Protein kinase, catalytic domain	INTRON
4738	CakSNP4738	*Kabuli*	Ca_Kabuli_Chr03	36387615	[A/T]	Ca_00947	WD40 repeat	SYNONYMOUS_CODING
4739	CakSNP4739	*Kabuli*	Ca_Kabuli_Chr03	36387732	[G/A]	Ca_00947	WD40 repeat	SYNONYMOUS_CODING
4740	CakSNP4740	*Kabuli*	Ca_Kabuli_Chr03	36387718	[C/T]	Ca_00947	WD40 repeat	SYNONYMOUS_CODING
4741	CakSNP4741	*Kabuli*	Ca_Kabuli_Chr03	36396559	[C/A]	Ca_00950	Heavy metal transport/detoxification protein	NON_SYNONYMOUS_CODING

S.N.	SNP IDs	Chickpea cultivars	Chromosomes	Physical positions (bp)	SNPs	Gene accession IDs	Putative functions	Sequence components of genome
4742	CakSNP4742	*Kabuli*	Ca_Kabuli_Chr03	36420902	[T/C]		--	INTERGENIC
4743	CakSNP4743	*Kabuli*	Ca_Kabuli_Chr03	36432389	[A/G]	Ca_00957	Thiamin pyrophosphokinase, catalytic domain	SYNONYMOUS_CODING
4744	CakSNP4744	*Kabuli*	Ca_Kabuli_Chr03	36447274	[G/A]		--	INTERGENIC
4745	CakSNP4745	*Kabuli*	Ca_Kabuli_Chr03	36447364	[T/A]		--	INTERGENIC
4746	CakSNP4746	*Kabuli*	Ca_Kabuli_Chr03	36447520	[T/A]	Ca_00960	CS-like domain	INTRON
4747	CakSNP4747	*Kabuli*	Ca_Kabuli_Chr03	36447525	[C/T]	Ca_00960	CS-like domain	INTRON
4748	CakSNP4748	*Kabuli*	Ca_Kabuli_Chr03	36488722	[T/A]	Ca_00965	Pectinesterase inhibitor	SYNONYMOUS_CODING
4749	CakSNP4749	*Kabuli*	Ca_Kabuli_Chr03	36488736	[T/C]	Ca_00965	Pectinesterase inhibitor	NON_SYNONYMOUS_CODING
4750	CakSNP4750	*Kabuli*	Ca_Kabuli_Chr03	36488853	[A/G]	Ca_00965	Pectinesterase inhibitor	NON_SYNONYMOUS_CODING
4751	CakSNP4751	*Kabuli*	Ca_Kabuli_Chr03	36488890	[A/G]	Ca_00965	Pectinesterase inhibitor	SYNONYMOUS_CODING

S.N.	SNP IDs	Chickpea cultivars	Chromosomes	Physical positions (bp)	SNPs	Gene accession IDs	Putative functions	Sequence components of genome
4752	CakSNP4752	*Kabuli*	Ca_Kabuli_Chr03	36503906	[C/T]	Ca_00967	Small-subunit processome, Utp14	NON_SYNONYMOUS_CODING
4753	CakSNP4753	*Kabuli*	Ca_Kabuli_Chr03	36503912	[T/C]	Ca_00967	Small-subunit processome, Utp14	NON_SYNONYMOUS_CODING
4754	CakSNP4754	*Kabuli*	Ca_Kabuli_Chr03	36524598	[G/C]	Ca_00969	Late embryogenesis abundant protein, group 3	NON_SYNONYMOUS_CODING
4755	CakSNP4755	*Kabuli*	Ca_Kabuli_Chr03	36524635	[A/G]	Ca_00969	Late embryogenesis abundant protein, group 3	SYNONYMOUS_CODING
4756	CakSNP4756	*Kabuli*	Ca_Kabuli_Chr03	36524659	[T/C]	Ca_00969	Late embryogenesis abundant protein, group 3	SYNONYMOUS_CODING
4757	CakSNP4757	*Kabuli*	Ca_Kabuli_Chr03	36531151	[C/A]	Ca_00971	Exocyst complex subunit Sec15-like	NON_SYNONYMOUS_CODING
4758	CakSNP4758	*Kabuli*	Ca_Kabuli_Chr03	36588856	[C/T]	Ca_00978	Pectinesterase, catalytic	SYNONYMOUS_CODING
4759	CakSNP4759	*Kabuli*	Ca_Kabuli_Chr03	36588896	[C/G]	Ca_00978	Pectinesterase, catalytic	NON_SYNONYMOUS_CODING
4760	CakSNP4760	*Kabuli*	Ca_Kabuli_Chr03	36606527	[G/A]	Ca_00980	Metal-dependent phosphohydrolase, HD domain	INTRON
4761	CakSNP4761	*Kabuli*	Ca_Kabuli_Chr03	36609043	[G/A]	Ca_00980	Metal-dependent phosphohydrolase, HD domain	NON_SYNONYMOUS_CODING

S.N.	SNP IDs	Chickpea cultivars	Chromosomes	Physical positions (bp)	SNPs	Gene accession IDs	Putative functions	Sequence components of genome
4762	CakSNP4762	*Kabuli*	Ca_Kabuli_Chr03	36616855	[T/C]	Ca_00982	Ribosomal protein L19/L19e	INTRON
4763	CakSNP4763	*Kabuli*	Ca_Kabuli_Chr03	36644117	[A/T]	Ca_00985	Target SNARE coiled-coil domain	SYNONYMOUS_CODING
4764	CakSNP4764	*Kabuli*	Ca_Kabuli_Chr03	36733097	[G/A]	Ca_00997	Heat shock protein DnaJ, N-terminal	SYNONYMOUS_CODING
4765	CakSNP4765	*Kabuli*	Ca_Kabuli_Chr03	36733157	[A/G]	Ca_00997	Heat shock protein DnaJ, N-terminal	SYNONYMOUS_CODING
4766	CakSNP4766	*Kabuli*	Ca_Kabuli_Chr03	36733211	[A/G]	Ca_00997	Heat shock protein DnaJ, N-terminal	SYNONYMOUS_CODING
4767	CakSNP4767	*Kabuli*	Ca_Kabuli_Chr03	36749775	[T/A]	Ca_00998	Glycoside hydrolase, family 3, N-terminal	SYNONYMOUS_CODING
4768	CakSNP4768	*Kabuli*	Ca_Kabuli_Chr03	36749826	[G/A]	Ca_00998	Glycoside hydrolase, family 3, N-terminal	SYNONYMOUS_CODING
4769	CakSNP4769	*Kabuli*	Ca_Kabuli_Chr03	36749829	[T/C]	Ca_00998	Glycoside hydrolase, family 3, N-terminal	NON_SYNONYMOUS_CODING
4770	CakSNP4770	*Kabuli*	Ca_Kabuli_Chr03	36749831	[T/C]	Ca_00998	Glycoside hydrolase, family 3, N-terminal	NON_SYNONYMOUS_CODING
4771	CakSNP4771	*Kabuli*	Ca_Kabuli_Chr03	36749871	[T/C]	Ca_00998	Glycoside hydrolase, family 3, N-terminal	SYNONYMOUS_CODING

S.N.	SNP IDs	Chickpea cultivars	Chromosomes	Physical positions (bp)	SNPs	Gene accession IDs	Putative functions	Sequence components of genome
4772	CakSNP4772	*Kabuli*	Ca_Kabuli_Chr03	36749904	[C/T]	Ca_00998	Glycoside hydrolase, family 3, N-terminal	SYNONYMOUS_CODING
4773	CakSNP4773	*Kabuli*	Ca_Kabuli_Chr03	36749916	[C/A]	Ca_00998	Glycoside hydrolase, family 3, N-terminal	NON_SYNONYMOUS_CODING
4774	CakSNP4774	*Kabuli*	Ca_Kabuli_Chr03	36752689	[T/G]	Ca_00998	Glycoside hydrolase, family 3, N-terminal	NON_SYNONYMOUS_CODING
4775	CakSNP4775	*Kabuli*	Ca_Kabuli_Chr03	36769775	[G/T]		--	INTERGENIC
4776	CakSNP4776	*Kabuli*	Ca_Kabuli_Chr03	36783494	[T/A]	Ca_01001	DNA mismatch repair protein MutS, C-terminal domain	INTRON
4777	CakSNP4777	*Kabuli*	Ca_Kabuli_Chr03	36825463	[T/C]	Ca_01006	Peptidase S9, prolyl oligopeptidase, catalytic domain	SYNONYMOUS_CODING
4778	CakSNP4778	*Kabuli*	Ca_Kabuli_Chr03	36860134	[A/G]	Ca_01011	Peptidase S14, ClpP	NON_SYNONYMOUS_CODING
4779	CakSNP4779	*Kabuli*	Ca_Kabuli_Chr03	36860246	[T/C]	Ca_01011	Peptidase S14, ClpP	SYNONYMOUS_CODING
4780	CakSNP4780	*Kabuli*	Ca_Kabuli_Chr03	36873720	[T/C]	Ca_01013	Protein kinase, catalytic domain	SYNONYMOUS_CODING
4781	CakSNP4781	*Kabuli*	Ca_Kabuli_Chr03	36883268	[T/C]	Ca_01014	RNA helicase, ATP-dependent, DEAD-box, conserved site	SYNONYMOUS_CODING

S.N.	SNP IDs	Chickpea cultivars	Chromosomes	Physical positions (bp)	SNPs	Gene accession IDs	Putative functions	Sequence components of genome
4782	CakSNP4782	*Kabuli*	Ca_Kabuli_Chr03	36883333	[A/T]	Ca_01014	RNA helicase, ATP-dependent, DEAD-box, conserved site	NON_SYNONYMOUS_CODING
4783	CakSNP4783	*Kabuli*	Ca_Kabuli_Chr03	36895362	[T/C]	Ca_01015	--	INTRON
4784	CakSNP4784	*Kabuli*	Ca_Kabuli_Chr03	36938148	[A/G]	Ca_01019	RNA recognition motif domain	INTRON
4785	CakSNP4785	*Kabuli*	Ca_Kabuli_Chr03	36938271	[G/C]	Ca_01019	RNA recognition motif domain	INTRON
4786	CakSNP4786	*Kabuli*	Ca_Kabuli_Chr03	36943522	[A/G]		--	INTERGENIC
4787	CakSNP4787	*Kabuli*	Ca_Kabuli_Chr03	36991718	[C/A]	Ca_01027	Ras small GTPase, Rab type	INTRON
4788	CakSNP4788	*Kabuli*	Ca_Kabuli_Chr03	37003976	[C/T]		--	INTERGENIC
4789	CakSNP4789	*Kabuli*	Ca_Kabuli_Chr03	37004026	[A/G]		--	INTERGENIC
4790	CakSNP4790	*Kabuli*	Ca_Kabuli_Chr03	37022354	[A/G]	Ca_01030	Bicarbonate transporter, eukaryotic	NON_SYNONYMOUS_CODING
4791	CakSNP4791	*Kabuli*	Ca_Kabuli_Chr03	37034618	[G/A]	Ca_01031	Protein kinase, catalytic domain	INTRON

S.N.	SNP IDs	Chickpea cultivars	Chromosomes	Physical positions (bp)	SNPs	Gene accession IDs	Putative functions	Sequence components of genome
4792	CakSNP4792	*Kabuli*	Ca_Kabuli_Chr03	37067095	[T/G]	Ca_01033	Zinc finger, RING-type	SYNONYMOUS_CODING
4793	CakSNP4793	*Kabuli*	Ca_Kabuli_Chr03	37072289	[A/G]	Ca_01034	WD40 repeat	INTRON
4794	CakSNP4794	*Kabuli*	Ca_Kabuli_Chr03	37072276	[T/G]	Ca_01034	WD40 repeat	INTRON
4795	CakSNP4795	*Kabuli*	Ca_Kabuli_Chr03	37072312	[T/C]	Ca_01034	WD40 repeat	INTRON
4796	CakSNP4796	*Kabuli*	Ca_Kabuli_Chr03	37077087	[G/T]	Ca_01034	WD40 repeat	INTRON
4797	CakSNP4797	*Kabuli*	Ca_Kabuli_Chr03	37090968	[T/C]	Ca_01035	S-adenosylmethionine synthetase	SYNONYMOUS_CODING
4798	CakSNP4798	*Kabuli*	Ca_Kabuli_Chr03	37091004	[A/G]	Ca_01035	S-adenosylmethionine synthetase	SYNONYMOUS_CODING
4799	CakSNP4799	*Kabuli*	Ca_Kabuli_Chr03	37094437	[T/C]	Ca_01036	Transcription factor, SBP-box	NON_SYNONYMOUS_CODING
4800	CakSNP4800	*Kabuli*	Ca_Kabuli_Chr03	37100897	[G/T]	Ca_01036	Transcription factor, SBP-box	INTRON
4801	CakSNP4801	*Kabuli*	Ca_Kabuli_Chr03	37101339	[A/T]	Ca_01036	Transcription factor, SBP-box	NON_SYNONYMOUS_CODING

S.N.	SNP IDs	Chickpea cultivars	Chromosomes	Physical positions (bp)	SNPs	Gene accession IDs	Putative functions	Sequence components of genome
4802	CakSNP4802	*Kabuli*	Ca_Kabuli_Chr03	37101433	[T/G]	Ca_01036	Transcription factor, SBP-box	NON_SYNONYMOUS_CODING
4803	CakSNP4803	*Kabuli*	Ca_Kabuli_Chr03	37101409	[A/G]	Ca_01036	Transcription factor, SBP-box	SYNONYMOUS_CODING
4804	CakSNP4804	*Kabuli*	Ca_Kabuli_Chr03	37108762	[A/G]	Ca_01037	Protein kinase, catalytic domain	INTRON
4805	CakSNP4805	*Kabuli*	Ca_Kabuli_Chr03	37108908	[A/G]	Ca_01037	Protein kinase, catalytic domain	INTRON
4806	CakSNP4806	*Kabuli*	Ca_Kabuli_Chr03	37108904	[A/T]	Ca_01037	Protein kinase, catalytic domain	INTRON
4807	CakSNP4807	*Kabuli*	Ca_Kabuli_Chr03	37108876	[C/A]	Ca_01037	Protein kinase, catalytic domain	INTRON
4808	CakSNP4808	*Kabuli*	Ca_Kabuli_Chr03	37110070	[A/G]	Ca_01037	Protein kinase, catalytic domain	NON_SYNONYMOUS_CODING
4809	CakSNP4809	*Kabuli*	Ca_Kabuli_Chr03	37110124	[A/T]	Ca_01037	Protein kinase, catalytic domain	INTRON
4810	CakSNP4810	*Kabuli*	Ca_Kabuli_Chr03	37146766	[C/G]	Ca_01044	Potassium channel, two pore-domain	NON_SYNONYMOUS_CODING
4811	CakSNP4811	*Kabuli*	Ca_Kabuli_Chr03	37146840	[A/G]	Ca_01044	Potassium channel, two pore-domain	SYNONYMOUS_CODING

S.N.	SNP IDs	Chickpea cultivars	Chromosomes	Physical positions (bp)	SNPs	Gene accession IDs	Putative functions	Sequence components of genome
4812	CakSNP4812	*Kabuli*	Ca_Kabuli_Chr03	37154761	[T/G]	Ca_01046	NEFA-interacting nuclear protein NIP30, N-terminal	INTRON
4813	CakSNP4813	*Kabuli*	Ca_Kabuli_Chr03	37154857	[T/C]	Ca_01046	NEFA-interacting nuclear protein NIP30, N-terminal	INTRON
4814	CakSNP4814	*Kabuli*	Ca_Kabuli_Chr03	37154938	[A/C]	Ca_01046	NEFA-interacting nuclear protein NIP30, N-terminal	INTRON
4815	CakSNP4815	*Kabuli*	Ca_Kabuli_Chr03	37154957	[C/A]	Ca_01046	NEFA-interacting nuclear protein NIP30, N-terminal	INTRON
4816	CakSNP4816	*Kabuli*	Ca_Kabuli_Chr03	37155925	[G/A]	Ca_01046	NEFA-interacting nuclear protein NIP30, N-terminal	SYNONYMOUS_CODING
4817	CakSNP4817	*Kabuli*	Ca_Kabuli_Chr03	37156951	[A/G]	Ca_01046	NEFA-interacting nuclear protein NIP30, N-terminal	INTRON
4818	CakSNP4818	*Kabuli*	Ca_Kabuli_Chr03	37160822	[G/T]	Ca_01047	Zinc finger, Sec23/Sec24-type	SYNONYMOUS_CODING
4819	CakSNP4819	*Kabuli*	Ca_Kabuli_Chr03	37160939	[A/G]	Ca_01047	Zinc finger, Sec23/Sec24-type	SYNONYMOUS_CODING
4820	CakSNP4820	*Kabuli*	Ca_Kabuli_Chr03	37164633	[T/C]	Ca_01047	Zinc finger, Sec23/Sec24-type	INTRON
4821	CakSNP4821	*Kabuli*	Ca_Kabuli_Chr03	37170249	[C/T]	Ca_01048	Aminoacyl-tRNA synthetase, class II (G/ H/ P/ S), conserved domain	INTRON

S.N.	SNP IDs	Chickpea cultivars	Chromosomes	Physical positions (bp)	SNPs	Gene accession IDs	Putative functions	Sequence components of genome
4822	CakSNP4822	*Kabuli*	Ca_Kabuli_Chr03	37271869	[T/C]	Ca_01061	--	INTRON
4823	CakSNP4823	*Kabuli*	Ca_Kabuli_Chr03	37300206	[T/G]		--	INTERGENIC
4824	CakSNP4824	*Kabuli*	Ca_Kabuli_Chr03	37303478	[G/A]	Ca_01064	WD40 repeat	SYNONYMOUS_CODING
4825	CakSNP4825	*Kabuli*	Ca_Kabuli_Chr03	37308614	[T/C]	Ca_01064	WD40 repeat	SYNONYMOUS_CODING
4826	CakSNP4826	*Kabuli*	Ca_Kabuli_Chr03	37309420	[A/C]	Ca_01064	WD40 repeat	INTRON
4827	CakSNP4827	*Kabuli*	Ca_Kabuli_Chr03	37316736	[T/C]		--	INTERGENIC
4828	CakSNP4828	*Kabuli*	Ca_Kabuli_Chr03	37316737	[C/T]		--	INTERGENIC
4829	CakSNP4829	*Kabuli*	Ca_Kabuli_Chr03	37316782	[G/A]		--	INTERGENIC
4830	CakSNP4830	*Kabuli*	Ca_Kabuli_Chr03	37355013	[A/G]	Ca_01069	PAS	NON_SYNONYMOUS_CODING
4831	CakSNP4831	*Kabuli*	Ca_Kabuli_Chr03	37444451	[C/A]	Ca_01076	Peptidase A1	SYNONYMOUS_CODING

S.N.	SNP IDs	Chickpea cultivars	Chromosomes	Physical positions (bp)	SNPs	Gene accession IDs	Putative functions	Sequence components of genome
4832	CakSNP4832	*Kabuli*	Ca_Kabuli_Chr03	37566557	[C/A]	Ca_01092	NAD-dependent epimerase/dehydratase	NON_SYNONYMOUS_CODING
4833	CakSNP4833	*Kabuli*	Ca_Kabuli_Chr03	37599142	[T/C]	Ca_01097	Armadillo	NON_SYNONYMOUS_CODING
4834	CakSNP4834	*Kabuli*	Ca_Kabuli_Chr03	37604672	[T/A]	Ca_01098	Armadillo	INTRON
4835	CakSNP4835	*Kabuli*	Ca_Kabuli_Chr03	37630502	[T/C]	Ca_01101	Thioredoxin	SYNONYMOUS_CODING
4836	CakSNP4836	*Kabuli*	Ca_Kabuli_Chr03	37635909	[G/A]	Ca_01102	Zinc finger, U1-type	NON_SYNONYMOUS_CODING
4837	CakSNP4837	*Kabuli*	Ca_Kabuli_Chr03	37635961	[C/T]		--	INTERGENIC
4838	CakSNP4838	*Kabuli*	Ca_Kabuli_Chr03	37639300	[A/G]	Ca_01103	Small GTPase SAR1-type	INTRON
4839	CakSNP4839	*Kabuli*	Ca_Kabuli_Chr03	37682816	[T/C]	Ca_01107	NHL repeat	INTRON
4840	CakSNP4840	*Kabuli*	Ca_Kabuli_Chr03	37682824	[G/A]	Ca_01107	NHL repeat	INTRON
4841	CakSNP4841	*Kabuli*	Ca_Kabuli_Chr03	37723959	[C/T]		--	INTERGENIC

S.N.	SNP IDs	Chickpea cultivars	Chromosomes	Physical positions (bp)	SNPs	Gene accession IDs	Putative functions	Sequence components of genome
4842	CakSNP4842	*Kabuli*	Ca_Kabuli_Chr03	37759329	[G/A]	Ca_01114	Armadillo	INTRON
4843	CakSNP4843	*Kabuli*	Ca_Kabuli_Chr03	37765767	[A/C]		--	INTERGENIC
4844	CakSNP4844	*Kabuli*	Ca_Kabuli_Chr03	37806690	[G/A]		--	INTERGENIC
4845	CakSNP4845	*Kabuli*	Ca_Kabuli_Chr03	37814955	[A/T]	Ca_01119	Calponin homology domain	INTRON
4846	CakSNP4846	*Kabuli*	Ca_Kabuli_Chr03	37814954	[T/A]	Ca_01119	Calponin homology domain	INTRON
4847	CakSNP4847	*Kabuli*	Ca_Kabuli_Chr03	37816895	[T/A]	Ca_01119	Calponin homology domain	INTRON
4848	CakSNP4848	*Kabuli*	Ca_Kabuli_Chr03	37859943	[T/C]	Ca_01125	WD40 repeat	INTRON
4849	CakSNP4849	*Kabuli*	Ca_Kabuli_Chr03	37864390	[C/T]	Ca_01125	WD40 repeat	INTRON
4850	CakSNP4850	*Kabuli*	Ca_Kabuli_Chr03	37866258	[A/G]	Ca_01125	WD40 repeat	INTRON
4851	CakSNP4851	*Kabuli*	Ca_Kabuli_Chr03	37886983	[G/T]		--	INTERGENIC

S.N.	SNP IDs	Chickpea cultivars	Chromosomes	Physical positions (bp)	SNPs	Gene accession IDs	Putative functions	Sequence components of genome
4852	CakSNP4852	*Kabuli*	Ca_Kabuli_Chr03	37886971	[A/G]		--	INTERGENIC
4853	CakSNP4853	*Kabuli*	Ca_Kabuli_Chr03	37886942	[A/C]		--	INTERGENIC
4854	CakSNP4854	*Kabuli*	Ca_Kabuli_Chr03	37908317	[T/C]	Ca_01128	Leucine-rich repeat, cysteine-containing subtype	NON_SYNONYMOUS_CODING
4855	CakSNP4855	*Kabuli*	Ca_Kabuli_Chr03	37908492	[A/C]	Ca_01128	Leucine-rich repeat, cysteine-containing subtype	INTRON
4856	CakSNP4856	*Kabuli*	Ca_Kabuli_Chr03	37910881	[A/G]	Ca_01129	Pectinesterase, catalytic	SYNONYMOUS_CODING
4857	CakSNP4857	*Kabuli*	Ca_Kabuli_Chr03	37910899	[T/C]	Ca_01129	Pectinesterase, catalytic	SYNONYMOUS_CODING
4858	CakSNP4858	*Kabuli*	Ca_Kabuli_Chr03	37910911	[C/T]	Ca_01129	Pectinesterase, catalytic	SYNONYMOUS_CODING
4859	CakSNP4859	*Kabuli*	Ca_Kabuli_Chr03	37913266	[C/T]	Ca_01130	Pectinesterase, catalytic	SYNONYMOUS_CODING
4860	CakSNP4860	*Kabuli*	Ca_Kabuli_Chr03	37919880	[C/T]		--	INTERGENIC
4861	CakSNP4861	*Kabuli*	Ca_Kabuli_Chr03	37942807	[A/G]		--	INTERGENIC

S.N.	SNP IDs	Chickpea cultivars	Chromosomes	Physical positions (bp)	SNPs	Gene accession IDs	Putative functions	Sequence components of genome
4862	CakSNP4862	*Kabuli*	Ca_Kabuli_Chr03	37942962	[T/C]		--	INTERGENIC
4863	CakSNP4863	*Kabuli*	Ca_Kabuli_Chr03	37985612	[A/C]	Ca_01139	Metallo-dependent phosphatase	INTRON
4864	CakSNP4864	*Kabuli*	Ca_Kabuli_Chr03	37985652	[C/T]	Ca_01139	Metallo-dependent phosphatase	INTRON
4865	CakSNP4865	*Kabuli*	Ca_Kabuli_Chr03	37985776	[T/A]	Ca_01139	Metallo-dependent phosphatase	SYNONYMOUS_CODING
4866	CakSNP4866	*Kabuli*	Ca_Kabuli_Chr03	37985977	[A/G]	Ca_01139	Metallo-dependent phosphatase	SYNONYMOUS_CODING
4867	CakSNP4867	*Kabuli*	Ca_Kabuli_Chr03	37985947	[A/T]	Ca_01139	Metallo-dependent phosphatase	SYNONYMOUS_CODING
4868	CakSNP4868	*Kabuli*	Ca_Kabuli_Chr03	38026470	[G/C]	Ca_01143	--	INTRON
4869	CakSNP4869	*Kabuli*	Ca_Kabuli_Chr03	38026539	[A/G]	Ca_01143	--	NON_SYNONYMOUS_CODING
4870	CakSNP4870	*Kabuli*	Ca_Kabuli_Chr03	38026734	[G/C]	Ca_01143	--	SYNONYMOUS_CODING
4871	CakSNP4871	*Kabuli*	Ca_Kabuli_Chr03	38026725	[G/A]	Ca_01143	--	SYNONYMOUS_CODING

S.N.	SNP IDs	Chickpea cultivars	Chromosomes	Physical positions (bp)	SNPs	Gene accession IDs	Putative functions	Sequence components of genome
4872	CakSNP4872	*Kabuli*	Ca_Kabuli_Chr03	38029095	[G/C]	Ca_01143	--	NON_SYNONYMOUS_CODING
4873	CakSNP4873	*Kabuli*	Ca_Kabuli_Chr03	38033965	[G/A]		--	INTERGENIC
4874	CakSNP4874	*Kabuli*	Ca_Kabuli_Chr03	38033969	[A/G]		--	INTERGENIC
4875	CakSNP4875	*Kabuli*	Ca_Kabuli_Chr03	38046510	[T/C]	Ca_01147	Tafazzin	NON_SYNONYMOUS_CODING
4876	CakSNP4876	*Kabuli*	Ca_Kabuli_Chr03	38046575	[A/C]	Ca_01147	Tafazzin	SYNONYMOUS_CODING
4877	CakSNP4877	*Kabuli*	Ca_Kabuli_Chr03	38049996	[A/G]	Ca_01147	Tafazzin	INTRON
4878	CakSNP4878	*Kabuli*	Ca_Kabuli_Chr03	38059303	[T/C]		--	INTERGENIC
4879	CakSNP4879	*Kabuli*	Ca_Kabuli_Chr03	38059311	[T/A]		--	INTERGENIC
4880	CakSNP4880	*Kabuli*	Ca_Kabuli_Chr03	38060814	[A/G]	Ca_01149	Peptidase S14, ClpP	SYNONYMOUS_CODING
4881	CakSNP4881	*Kabuli*	Ca_Kabuli_Chr03	38060755	[T/C]	Ca_01149	Peptidase S14, ClpP	NON_SYNONYMOUS_CODING

S.N.	SNP IDs	Chickpea cultivars	Chromosomes	Physical positions (bp)	SNPs	Gene accession IDs	Putative functions	Sequence components of genome
4882	CakSNP4882	*Kabuli*	Ca_Kabuli_Chr03	38060742	[C/T]	Ca_01149	Peptidase S14, ClpP	INTRON
4883	CakSNP4883	*Kabuli*	Ca_Kabuli_Chr03	38074259	[C/A]		--	INTERGENIC
4884	CakSNP4884	*Kabuli*	Ca_Kabuli_Chr03	38103375	[T/C]	Ca_01154	Peptidase S1/S6, chymotrypsin/Hap	INTRON
4885	CakSNP4885	*Kabuli*	Ca_Kabuli_Chr03	38103378	[C/T]	Ca_01154	Peptidase S1/S6, chymotrypsin/Hap	INTRON
4886	CakSNP4886	*Kabuli*	Ca_Kabuli_Chr03	38103381	[C/T]	Ca_01154	Peptidase S1/S6, chymotrypsin/Hap	INTRON
4887	CakSNP4887	*Kabuli*	Ca_Kabuli_Chr03	38103385	[C/T]	Ca_01154	Peptidase S1/S6, chymotrypsin/Hap	INTRON
4888	CakSNP4888	*Kabuli*	Ca_Kabuli_Chr03	38103524	[C/T]	Ca_01154	Peptidase S1/S6, chymotrypsin/Hap	SYNONYMOUS_CODING
4889	CakSNP4889	*Kabuli*	Ca_Kabuli_Chr03	38112801	[C/T]	Ca_01155	PWWP	NON_SYNONYMOUS_CODING
4890	CakSNP4890	*Kabuli*	Ca_Kabuli_Chr03	38112802	[T/C]	Ca_01155	PWWP	SYNONYMOUS_CODING
4891	CakSNP4891	*Kabuli*	Ca_Kabuli_Chr03	38112971	[A/G]	Ca_01155	PWWP	INTRON

S.N.	SNP IDs	Chickpea cultivars	Chromosomes	Physical positions (bp)	SNPs	Gene accession IDs	Putative functions	Sequence components of genome
4892	CakSNP4892	*Kabuli*	Ca_Kabuli_Chr03	38117169	[T/C]	Ca_01156	Acyl-CoA-binding protein, ACBP	INTRON
4893	CakSNP4893	*Kabuli*	Ca_Kabuli_Chr03	38130415	[T/C]		--	INTERGENIC
4894	CakSNP4894	*Kabuli*	Ca_Kabuli_Chr03	38130410	[A/G]		--	INTERGENIC
4895	CakSNP4895	*Kabuli*	Ca_Kabuli_Chr03	38145115	[C/T]	Ca_01158	Cystathionine beta-synthase, core	SYNONYMOUS_CODING
4896	CakSNP4896	*Kabuli*	Ca_Kabuli_Chr03	38157519	[T/C]	Ca_01160	DNA-directed DNA polymerase, family B, exonuclease domain	INTRON
4897	CakSNP4897	*Kabuli*	Ca_Kabuli_Chr03	38166074	[A/G]	Ca_01160	DNA-directed DNA polymerase, family B, exonuclease domain	INTRON
4898	CakSNP4898	*Kabuli*	Ca_Kabuli_Chr03	38166881	[C/T]	Ca_01160	DNA-directed DNA polymerase, family B, exonuclease domain	INTRON
4899	CakSNP4899	*Kabuli*	Ca_Kabuli_Chr03	38202223	[C/T]	Ca_01163	Creatinase	INTRON
4900	CakSNP4900	*Kabuli*	Ca_Kabuli_Chr03	38220910	[T/C]	Ca_01166	Kinesin, motor domain	SYNONYMOUS_CODING
4901	CakSNP4901	*Kabuli*	Ca_Kabuli_Chr03	38220949	[A/T]	Ca_01166	Kinesin, motor domain	SYNONYMOUS_CODING

S.N.	SNP IDs	Chickpea cultivars	Chromosomes	Physical positions (bp)	SNPs	Gene accession IDs	Putative functions	Sequence components of genome
4902	CakSNP4902	*Kabuli*	Ca_Kabuli_Chr03	38220958	[A/G]	Ca_01166	Kinesin, motor domain	SYNONYMOUS_CODING
4903	CakSNP4903	*Kabuli*	Ca_Kabuli_Chr03	38222750	[A/C]	Ca_01166	Kinesin, motor domain	INTRON
4904	CakSNP4904	*Kabuli*	Ca_Kabuli_Chr03	38222747	[G/A]	Ca_01166	Kinesin, motor domain	INTRON
4905	CakSNP4905	*Kabuli*	Ca_Kabuli_Chr03	38222733	[C/G]	Ca_01166	Kinesin, motor domain	INTRON
4906	CakSNP4906	*Kabuli*	Ca_Kabuli_Chr03	38228118	[G/T]	Ca_01167	Heat shock protein DnaJ, N-terminal	INTRON
4907	CakSNP4907	*Kabuli*	Ca_Kabuli_Chr03	38237246	[T/G]	Ca_01169	Glycoside hydrolase, family 2, immunoglobulin-like beta-sandwich	INTRON
4908	CakSNP4908	*Kabuli*	Ca_Kabuli_Chr03	38251660	[C/A]	Ca_01171	Yip1 domain	INTRON
4909	CakSNP4909	*Kabuli*	Ca_Kabuli_Chr03	38253654	[T/C]	Ca_01171	Yip1 domain	INTRON
4910	CakSNP4910	*Kabuli*	Ca_Kabuli_Chr03	38258645	[C/A]		--	INTERGENIC
4911	CakSNP4911	*Kabuli*	Ca_Kabuli_Chr03	38264250	[C/T]		--	INTERGENIC

S.N.	SNP IDs	Chickpea cultivars	Chromosomes	Physical positions (bp)	SNPs	Gene accession IDs	Putative functions	Sequence components of genome
4912	CakSNP4912	*Kabuli*	Ca_Kabuli_Chr03	38294286	[C/A]	Ca_01176	Sodium/calcium exchanger membrane region	SYNONYMOUS_CODING
4913	CakSNP4913	*Kabuli*	Ca_Kabuli_Chr03	38296573	[A/G]	Ca_01177	Signal peptidase 22kDa subunit	INTRON
4914	CakSNP4914	*Kabuli*	Ca_Kabuli_Chr03	38315560	[G/T]	Ca_01180	Zinc finger, RING-type	SYNONYMOUS_CODING
4915	CakSNP4915	*Kabuli*	Ca_Kabuli_Chr03	38321159	[T/C]		--	INTERGENIC
4916	CakSNP4916	*Kabuli*	Ca_Kabuli_Chr03	38321153	[G/T]		--	INTERGENIC
4917	CakSNP4917	*Kabuli*	Ca_Kabuli_Chr03	38386392	[T/C]	Ca_01189	Protein of unknown function DUF3595	NON_SYNONYMOUS_CODING
4918	CakSNP4918	*Kabuli*	Ca_Kabuli_Chr03	38424238	[A/T]	Ca_01195	--	SYNONYMOUS_CODING
4919	CakSNP4919	*Kabuli*	Ca_Kabuli_Chr03	38452060	[A/C]	Ca_01198	Heat shock protein DnaJ, N-terminal	NON_SYNONYMOUS_CODING
4920	CakSNP4920	*Kabuli*	Ca_Kabuli_Chr03	38465366	[G/A]	Ca_01200	--	INTRON
4921	CakSNP4921	*Kabuli*	Ca_Kabuli_Chr03	38465335	[G/A]	Ca_01200	--	INTRON

S.N.	SNP IDs	Chickpea cultivars	Chromosomes	Physical positions (bp)	SNPs	Gene accession IDs	Putative functions	Sequence components of genome
4922	CakSNP4922	*Kabuli*	Ca_Kabuli_Chr03	38465440	[G/A]	Ca_01200	--	INTRON
4923	CakSNP4923	*Kabuli*	Ca_Kabuli_Chr03	38496236	[A/G]	Ca_01203	Exportin-1/Importin-beta-like	INTRON
4924	CakSNP4924	*Kabuli*	Ca_Kabuli_Chr03	38501705	[T/C]	Ca_01204	Protein kinase, catalytic domain	SYNONYMOUS_CODING
4925	CakSNP4925	*Kabuli*	Ca_Kabuli_Chr03	38539721	[T/G]	Ca_01207	Alpha/beta hydrolase fold-1	NON_SYNONYMOUS_CODING
4926	CakSNP4926	*Kabuli*	Ca_Kabuli_Chr03	38551142	[C/T]	Ca_01208	WD40 repeat	INTRON
4927	CakSNP4927	*Kabuli*	Ca_Kabuli_Chr03	38555941	[G/A]	Ca_01208	WD40 repeat	SYNONYMOUS_CODING
4928	CakSNP4928	*Kabuli*	Ca_Kabuli_Chr03	38555944	[A/G]	Ca_01208	WD40 repeat	SYNONYMOUS_CODING
4929	CakSNP4929	*Kabuli*	Ca_Kabuli_Chr03	38592077	[G/A]	Ca_01213	Proteasome, subunit alpha/beta	INTRON
4930	CakSNP4930	*Kabuli*	Ca_Kabuli_Chr03	38624786	[C/T]	Ca_01218	--	NON_SYNONYMOUS_CODING
4931	CakSNP4931	*Kabuli*	Ca_Kabuli_Chr03	38624934	[T/C]	Ca_01218	--	SYNONYMOUS_CODING

S.N.	SNP IDs	Chickpea cultivars	Chromosomes	Physical positions (bp)	SNPs	Gene accession IDs	Putative functions	Sequence components of genome
4932	CakSNP4932	*Kabuli*	Ca_Kabuli_Chr03	38672863	[C/A]	Ca_01222	Tetratricopeptide repeat-containing	INTRON
4933	CakSNP4933	*Kabuli*	Ca_Kabuli_Chr03	38704356	[C/T]	Ca_01227	Transglutaminase-like	NON_SYNONYMOUS_CODING
4934	CakSNP4934	*Kabuli*	Ca_Kabuli_Chr03	38728528	[C/T]		--	INTERGENIC
4935	CakSNP4935	*Kabuli*	Ca_Kabuli_Chr03	38728679	[T/C]		--	INTERGENIC
4936	CakSNP4936	*Kabuli*	Ca_Kabuli_Chr03	38731315	[C/T]	Ca_01231	Ammonium transporter	INTRON
4937	CakSNP4937	*Kabuli*	Ca_Kabuli_Chr03	38731466	[T/G]	Ca_01231	Ammonium transporter	NON_SYNONYMOUS_CODING
4938	CakSNP4938	*Kabuli*	Ca_Kabuli_Chr03	38764372	[G/A]	Ca_01235	Bacterial sec-independent translocation protein MttA/Hcf106	SYNONYMOUS_CODING
4939	CakSNP4939	*Kabuli*	Ca_Kabuli_Chr03	38764390	[A/C]	Ca_01235	Bacterial sec-independent translocation protein MttA/Hcf106	SYNONYMOUS_CODING
4940	CakSNP4940	*Kabuli*	Ca_Kabuli_Chr03	38766156	[T/A]	Ca_01235	Bacterial sec-independent translocation protein MttA/Hcf106	INTRON
4941	CakSNP4941	*Kabuli*	Ca_Kabuli_Chr03	38766174	[A/G]	Ca_01235	Bacterial sec-independent translocation protein MttA/Hcf106	INTRON

S.N.	SNP IDs	Chickpea cultivars	Chromosomes	Physical positions (bp)	SNPs	Gene accession IDs	Putative functions	Sequence components of genome
4942	CakSNP4942	*Kabuli*	Ca_Kabuli_Chr03	38766311	[A/C]	Ca_01235	Bacterial sec-independent translocation protein MttA/Hcf106	SYNONYMOUS_CODING
4943	CakSNP4943	*Kabuli*	Ca_Kabuli_Chr03	38771817	[A/G]	Ca_01236	Mediator complex, subunit Med15	SYNONYMOUS_CODING
4944	CakSNP4944	*Kabuli*	Ca_Kabuli_Chr03	38776366	[T/G]	Ca_01236	Mediator complex, subunit Med16	SYNONYMOUS_CODING
4945	CakSNP4945	*Kabuli*	Ca_Kabuli_Chr03	38785843	[C/G]	Ca_01237	Chitin-binding, type 1	NON_SYNONYMOUS_CODING
4946	CakSNP4946	*Kabuli*	Ca_Kabuli_Chr03	38785845	[C/T]	Ca_01237	Chitin-binding, type 1	SYNONYMOUS_CODING
4947	CakSNP4947	*Kabuli*	Ca_Kabuli_Chr03	38793194	[T/C]	Ca_01238	Zinc finger, RING-type	INTRON
4948	CakSNP4948	*Kabuli*	Ca_Kabuli_Chr03	38805822	[T/A]	Ca_01240	Ankyrin repeat	INTRON
4949	CakSNP4949	*Kabuli*	Ca_Kabuli_Chr03	38805831	[A/C]	Ca_01240	Ankyrin repeat	INTRON
4950	CakSNP4950	*Kabuli*	Ca_Kabuli_Chr03	38809132	[G/A]	Ca_01240	Ankyrin repeat	SYNONYMOUS_CODING
4951	CakSNP4951	*Kabuli*	Ca_Kabuli_Chr03	38810765	[A/T]	Ca_01240	Ankyrin repeat	SYNONYMOUS_CODING

S.N.	SNP IDs	Chickpea cultivars	Chromosomes	Physical positions (bp)	SNPs	Gene accession IDs	Putative functions	Sequence components of genome
4952	CakSNP4952	*Kabuli*	Ca_Kabuli_Chr03	38818340	[A/G]	Ca_01242	Protein kinase, catalytic domain	NON_SYNONYMOUS_CODING
4953	CakSNP4953	*Kabuli*	Ca_Kabuli_Chr03	38821990	[A/G]	Ca_01243	--	NON_SYNONYMOUS_CODING
4954	CakSNP4954	*Kabuli*	Ca_Kabuli_Chr03	38831533	[T/C]		--	INTERGENIC
4955	CakSNP4955	*Kabuli*	Ca_Kabuli_Chr03	38848358	[C/T]	Ca_01245	K Homology	SYNONYMOUS_CODING
4956	CakSNP4956	*Kabuli*	Ca_Kabuli_Chr03	38848412	[C/G]	Ca_01245	K Homology	SYNONYMOUS_CODING
4957	CakSNP4957	*Kabuli*	Ca_Kabuli_Chr03	38878577	[C/G]	Ca_01247	SSXT	INTRON
4958	CakSNP4958	*Kabuli*	Ca_Kabuli_Chr03	38878664	[G/C]	Ca_01247	SSXT	INTRON
4959	CakSNP4959	*Kabuli*	Ca_Kabuli_Chr03	38878649	[T/C]	Ca_01247	SSXT	INTRON
4960	CakSNP4960	*Kabuli*	Ca_Kabuli_Chr03	38878736	[G/C]	Ca_01247	SSXT	NON_SYNONYMOUS_CODING
4961	CakSNP4961	*Kabuli*	Ca_Kabuli_Chr03	38887640	[C/T]	Ca_01248	IQ motif, EF-hand binding site	NON_SYNONYMOUS_CODING

S.N.	SNP IDs	Chickpea cultivars	Chromosomes	Physical positions (bp)	SNPs	Gene accession IDs	Putative functions	Sequence components of genome
4962	CakSNP4962	*Kabuli*	Ca_Kabuli_Chr03	38891567	[T/C]	Ca_01248	IQ motif, EF-hand binding site	INTRON
4963	CakSNP4963	*Kabuli*	Ca_Kabuli_Chr03	38896013	[A/G]		--	INTERGENIC
4964	CakSNP4964	*Kabuli*	Ca_Kabuli_Chr03	38913449	[T/C]		--	INTERGENIC
4965	CakSNP4965	*Kabuli*	Ca_Kabuli_Chr03	38914528	[A/G]	Ca_01250	--	NON_SYNONYMOUS_CODING
4966	CakSNP4966	*Kabuli*	Ca_Kabuli_Chr03	38923321	[C/T]	Ca_01252	Glycosyl transferase, family 8	SYNONYMOUS_CODING
4967	CakSNP4967	*Kabuli*	Ca_Kabuli_Chr03	38951428	[G/A]	Ca_01256	Myb, DNA-binding	INTRON
4968	CakSNP4968	*Kabuli*	Ca_Kabuli_Chr03	38951698	[A/C]	Ca_01256	Myb, DNA-binding	INTRON
4969	CakSNP4969	*Kabuli*	Ca_Kabuli_Chr03	38965610	[T/A]	Ca_01258	SANT domain, DNA binding	NON_SYNONYMOUS_CODING
4970	CakSNP4970	*Kabuli*	Ca_Kabuli_Chr03	38982481	[A/G]	Ca_01260	--	INTRON
4971	CakSNP4971	*Kabuli*	Ca_Kabuli_Chr03	38987810	[G/A]	Ca_01261	Ankyrin repeat	SYNONYMOUS_CODING

S.N.	SNP IDs	Chickpea cultivars	Chromosomes	Physical positions (bp)	SNPs	Gene accession IDs	Putative functions	Sequence components of genome
4972	CakSNP4972	*Kabuli*	Ca_Kabuli_Chr03	38987916	[A/C]	Ca_01261	Ankyrin repeat	NON_SYNONYMOUS_CODING
4973	CakSNP4973	*Kabuli*	Ca_Kabuli_Chr03	39003771	[G/A]	Ca_01263	SANT domain, DNA binding	INTRON
4974	CakSNP4974	*Kabuli*	Ca_Kabuli_Chr03	39013988	[T/G]	Ca_01264	Aromatic amino acid beta-eliminating lyase/threonine aldolase	INTRON
4975	CakSNP4975	*Kabuli*	Ca_Kabuli_Chr03	39029251	[A/C]	Ca_01265	--	INTRON
4976	CakSNP4976	*Kabuli*	Ca_Kabuli_Chr03	39034246	[C/T]	Ca_01265	--	NON_SYNONYMOUS_CODING
4977	CakSNP4977	*Kabuli*	Ca_Kabuli_Chr03	39034433	[A/G]	Ca_01265	--	SYNONYMOUS_CODING
4978	CakSNP4978	*Kabuli*	Ca_Kabuli_Chr03	39036130	[T/C]	Ca_01265	--	INTRON
4979	CakSNP4979	*Kabuli*	Ca_Kabuli_Chr03	39052024	[T/C]		--	INTERGENIC
4980	CakSNP4980	*Kabuli*	Ca_Kabuli_Chr03	39055529	[C/T]	Ca_01268	EF-Hand 1, calcium-binding site	INTRON
4981	CakSNP4981	*Kabuli*	Ca_Kabuli_Chr03	39055455	[T/C]	Ca_01268	EF-Hand 1, calcium-binding site	SYNONYMOUS_CODING

S.N.	SNP IDs	Chickpea cultivars	Chromosomes	Physical positions (bp)	SNPs	Gene accession IDs	Putative functions	Sequence components of genome
4982	CakSNP4982	*Kabuli*	Ca_Kabuli_Chr03	39081040	[A/G]		--	INTERGENIC
4983	CakSNP4983	*Kabuli*	Ca_Kabuli_Chr03	39084916	[T/A]	Ca_01271	Domain of unknown function DUF296	STOP_GAINED
4984	CakSNP4984	*Kabuli*	Ca_Kabuli_Chr03	39084979	[A/C]	Ca_01271	Domain of unknown function DUF296	SYNONYMOUS_CODING
4985	CakSNP4985	*Kabuli*	Ca_Kabuli_Chr03	39093242	[G/A]		--	INTERGENIC
4986	CakSNP4986	*Kabuli*	Ca_Kabuli_Chr03	39093243	[C/A]		--	INTERGENIC
4987	CakSNP4987	*Kabuli*	Ca_Kabuli_Chr03	39093245	[T/A]		--	INTERGENIC
4988	CakSNP4988	*Kabuli*	Ca_Kabuli_Chr03	39093215	[G/A]		--	INTERGENIC
4989	CakSNP4989	*Kabuli*	Ca_Kabuli_Chr03	39115274	[A/C]	Ca_01274	HEAT	INTRON
4990	CakSNP4990	*Kabuli*	Ca_Kabuli_Chr03	39115986	[C/G]	Ca_01274	HEAT	INTRON
4991	CakSNP4991	*Kabuli*	Ca_Kabuli_Chr03	39115979	[A/G]	Ca_01274	HEAT	INTRON

S.N.	SNP IDs	Chickpea cultivars	Chromosomes	Physical positions (bp)	SNPs	Gene accession IDs	Putative functions	Sequence components of genome
4992	CakSNP4992	*Kabuli*	Ca_Kabuli_Chr03	39115972	[T/A]	Ca_01274	HEAT	INTRON
4993	CakSNP4993	*Kabuli*	Ca_Kabuli_Chr03	39115970	[T/G]	Ca_01274	HEAT	INTRON
4994	CakSNP4994	*Kabuli*	Ca_Kabuli_Chr03	39118510	[C/T]	Ca_01274	HEAT	INTRON
4995	CakSNP4995	*Kabuli*	Ca_Kabuli_Chr03	39200823	[C/A]	Ca_01277	Cytoplasmic FMR1-interacting	INTRON
4996	CakSNP4996	*Kabuli*	Ca_Kabuli_Chr03	39200847	[A/C]	Ca_01277	Cytoplasmic FMR1-interacting	INTRON
4997	CakSNP4997	*Kabuli*	Ca_Kabuli_Chr03	39202479	[G/C]	Ca_01277	Cytoplasmic FMR1-interacting	INTRON
4998	CakSNP4998	*Kabuli*	Ca_Kabuli_Chr03	39202481	[A/T]	Ca_01277	Cytoplasmic FMR1-interacting	INTRON
4999	CakSNP4999	*Kabuli*	Ca_Kabuli_Chr03	39207112	[A/C]	Ca_01277	Cytoplasmic FMR1-interacting	SYNONYMOUS_CODING
5000	CakSNP5000	*Kabuli*	Ca_Kabuli_Chr03	39215327	[C/T]	Ca_01278	Armadillo	NON_SYNONYMOUS_CODING
5001	CakSNP5001	*Kabuli*	Ca_Kabuli_Chr03	39215476	[G/A]	Ca_01278	Armadillo	SYNONYMOUS_CODING

S.N.	SNP IDs	Chickpea cultivars	Chromosomes	Physical positions (bp)	SNPs	Gene accession IDs	Putative functions	Sequence components of genome
5002	CakSNP5002	*Kabuli*	Ca_Kabuli_Chr03	39215558	[C/T]	Ca_01278	Armadillo	SYNONYMOUS_CODING
5003	CakSNP5003	*Kabuli*	Ca_Kabuli_Chr03	39233774	[A/T]		--	INTERGENIC
5004	CakSNP5004	*Kabuli*	Ca_Kabuli_Chr03	39233852	[A/C]	Ca_01281	Protein kinase, catalytic domain	SYNONYMOUS_CODING
5005	CakSNP5005	*Kabuli*	Ca_Kabuli_Chr03	39233885	[A/T]	Ca_01281	Protein kinase, catalytic domain	NON_SYNONYMOUS_CODING
5006	CakSNP5006	*Kabuli*	Ca_Kabuli_Chr03	39234161	[A/G]	Ca_01281	Protein kinase, catalytic domain	SYNONYMOUS_CODING
5007	CakSNP5007	*Kabuli*	Ca_Kabuli_Chr03	39254527	[T/G]		--	INTERGENIC
5008	CakSNP5008	*Kabuli*	Ca_Kabuli_Chr03	39254532	[C/G]		--	INTERGENIC
5009	CakSNP5009	*Kabuli*	Ca_Kabuli_Chr03	39255721	[A/C]		--	INTERGENIC
5010	CakSNP5010	*Kabuli*	Ca_Kabuli_Chr03	39255715	[G/A]		--	INTERGENIC
5011	CakSNP5011	*Kabuli*	Ca_Kabuli_Chr03	39255713	[C/A]		--	INTERGENIC

S.N.	SNP IDs	Chickpea cultivars	Chromosomes	Physical positions (bp)	SNPs	Gene accession IDs	Putative functions	Sequence components of genome
5012	CakSNP5012	*Kabuli*	Ca_Kabuli_Chr03	39255709	[A/T]		--	INTERGENIC
5013	CakSNP5013	*Kabuli*	Ca_Kabuli_Chr03	39255729	[C/T]		--	INTERGENIC
5014	CakSNP5014	*Kabuli*	Ca_Kabuli_Chr03	39255792	[C/T]		--	INTERGENIC
5015	CakSNP5015	*Kabuli*	Ca_Kabuli_Chr03	39257226	[A/G]	Ca_01285	--	INTRON
5016	CakSNP5016	*Kabuli*	Ca_Kabuli_Chr03	39258671	[A/C]	Ca_01285	--	INTRON
5017	CakSNP5017	*Kabuli*	Ca_Kabuli_Chr03	39265412	[A/C]	Ca_01286	Nucleotide binding protein, PINc	INTRON
5018	CakSNP5018	*Kabuli*	Ca_Kabuli_Chr03	39289840	[T/A]	Ca_01290	BTB/POZ-like	INTRON
5019	CakSNP5019	*Kabuli*	Ca_Kabuli_Chr03	39303386	[C/G]		--	INTERGENIC
5020	CakSNP5020	*Kabuli*	Ca_Kabuli_Chr03	39303416	[G/T]		--	INTERGENIC
5021	CakSNP5021	*Kabuli*	Ca_Kabuli_Chr03	39303407	[C/A]		--	INTERGENIC

S.N.	SNP IDs	Chickpea cultivars	Chromosomes	Physical positions (bp)	SNPs	Gene accession IDs	Putative functions	Sequence components of genome
5022	CakSNP5022	*Kabuli*	Ca_Kabuli_Chr03	39323779	[A/G]	Ca_01294	Aldose 1-epimerase	SYNONYMOUS_CODING
5023	CakSNP5023	*Kabuli*	Ca_Kabuli_Chr03	39326387	[C/T]	Ca_01295	Exoribonuclease, phosphorolytic domain 1	NON_SYNONYMOUS_CODING
5024	CakSNP5024	*Kabuli*	Ca_Kabuli_Chr03	39405252	[C/A]	Ca_01306	Lipase, class 3	NON_SYNONYMOUS_CODING
5025	CakSNP5025	*Kabuli*	Ca_Kabuli_Chr03	39405873	[C/G]	Ca_01306	Lipase, class 3	INTRON
5026	CakSNP5026	*Kabuli*	Ca_Kabuli_Chr03	39472291	[G/C]	Ca_01315	Ribosomal protein S18	INTRON
5027	CakSNP5027	*Kabuli*	Ca_Kabuli_Chr03	39481371	[A/C]	Ca_01317	Protein of unknown function DUF2043	SYNONYMOUS_CODING
5028	CakSNP5028	*Kabuli*	Ca_Kabuli_Chr03	39485182	[G/A]	Ca_01318	Peptidase A1	SYNONYMOUS_CODING
5029	CakSNP5029	*Kabuli*	Ca_Kabuli_Chr03	39504242	[A/G]		--	INTERGENIC
5030	CakSNP5030	*Kabuli*	Ca_Kabuli_Chr03	39515531	[A/G]		--	INTERGENIC
5031	CakSNP5031	*Kabuli*	Ca_Kabuli_Chr03	39515710	[T/A]		--	INTERGENIC

S.N.	SNP IDs	Chickpea cultivars	Chromosomes	Physical positions (bp)	SNPs	Gene accession IDs	Putative functions	Sequence components of genome
5032	CakSNP5032	*Kabuli*	Ca_Kabuli_Chr03	39519640	[A/G]	Ca_01323	--	SYNONYMOUS_CODING
5033	CakSNP5033	*Kabuli*	Ca_Kabuli_Chr03	39556535	[T/A]	Ca_01327	--	INTRON
5034	CakSNP5034	*Kabuli*	Ca_Kabuli_Chr03	39587620	[T/G]	Ca_01333	GHMP kinase	NON_SYNONYMOUS_CODING
5035	CakSNP5035	*Kabuli*	Ca_Kabuli_Chr03	39592340	[C/T]	Ca_01334	Armadillo	SYNONYMOUS_CODING
5036	CakSNP5036	*Kabuli*	Ca_Kabuli_Chr03	39612466	[T/C]	Ca_01338	Profilin/allergen	INTRON
5037	CakSNP5037	*Kabuli*	Ca_Kabuli_Chr03	39612458	[C/A]	Ca_01338	Profilin/allergen	INTRON
5038	CakSNP5038	*Kabuli*	Ca_Kabuli_Chr03	39612447	[C/A]	Ca_01338	Profilin/allergen	INTRON
5039	CakSNP5039	*Kabuli*	Ca_Kabuli_Chr03	39612568	[A/T]	Ca_01338	Profilin/allergen	NON_SYNONYMOUS_CODING
5040	CakSNP5040	*Kabuli*	Ca_Kabuli_Chr03	39628916	[G/A]		--	INTERGENIC
5041	CakSNP5041	*Kabuli*	Ca_Kabuli_Chr03	39698060	[T/C]	Ca_01350	FAD linked oxidase, N-terminal	SYNONYMOUS_CODING

S.N.	SNP IDs	Chickpea cultivars	Chromosomes	Physical positions (bp)	SNPs	Gene accession IDs	Putative functions	Sequence components of genome
5042	CakSNP5042	*Kabuli*	Ca_Kabuli_Chr03	39701532	[G/A]	Ca_01351	Peptidase S8/S53, subtilisin/kexin/sedolisin	SYNONYMOUS_CODING
5043	CakSNP5043	*Kabuli*	Ca_Kabuli_Chr03	39722314	[C/T]	Ca_01355	Dehydrogenase, E1 component	NON_SYNONYMOUS_CODING
5044	CakSNP5044	*Kabuli*	Ca_Kabuli_Chr03	39731437	[A/C]	Ca_01357	RNA recognition motif domain	NON_SYNONYMOUS_CODING
5045	CakSNP5045	*Kabuli*	Ca_Kabuli_Chr03	39787513	[A/C]	Ca_01365	--	SYNONYMOUS_CODING
5046	CakSNP5046	*Kabuli*	Ca_Kabuli_Chr03	39787615	[C/T]	Ca_01365	--	SYNONYMOUS_CODING
5047	CakSNP5047	*Kabuli*	Ca_Kabuli_Chr03	39787716	[A/C]	Ca_01365	--	NON_SYNONYMOUS_CODING
5048	CakSNP5048	*Kabuli*	Ca_Kabuli_Chr03	39791717	[C/T]	Ca_01366	Inorganic pyrophosphatase	INTRON
5049	CakSNP5049	*Kabuli*	Ca_Kabuli_Chr03	39793539	[G/A]	Ca_01367	Ribonuclease III	SYNONYMOUS_CODING
5050	CakSNP5050	*Kabuli*	Ca_Kabuli_Chr03	39794672	[C/T]	Ca_01367	Ribonuclease III	SYNONYMOUS_CODING
5051	CakSNP5051	*Kabuli*	Ca_Kabuli_Chr03	39794639	[C/G]	Ca_01367	Ribonuclease III	NON_SYNONYMOUS_CODING

S.N.	SNP IDs	Chickpea cultivars	Chromosomes	Physical positions (bp)	SNPs	Gene accession IDs	Putative functions	Sequence components of genome
5052	CakSNP5052	*Kabuli*	Ca_Kabuli_Chr03	39801213	[A/G]	Ca_01367	Ribonuclease III	INTRON
5053	CakSNP5053	*Kabuli*	Ca_Kabuli_Chr03	39801242	[C/T]	Ca_01367	Ribonuclease III	INTRON
5054	CakSNP5054	*Kabuli*	Ca_Kabuli_Chr03	39817122	[G/A]	Ca_01368	Transcriptional factor B3	SYNONYMOUS_CODING
5055	CakSNP5055	*Kabuli*	Ca_Kabuli_Chr03	39839763	[T/G]	Ca_01370	ATPase, P-type, H+ transporting proton pump	INTRON
5056	CakSNP5056	*Kabuli*	Ca_Kabuli_Chr03	39891075	[G/A]	Ca_01375	Exportin-1/Importin-beta-like	INTRON
5057	CakSNP5057	*Kabuli*	Ca_Kabuli_Chr03	39890991	[A/T]	Ca_01375	Exportin-1/Importin-beta-like	INTRON
5058	CakSNP5058	*Kabuli*	Ca_Kabuli_Chr03	39896395	[A/G]	Ca_01375	Exportin-1/Importin-beta-like	INTRON
5059	CakSNP5059	*Kabuli*	Ca_Kabuli_Chr03	39908983	[C/G]		--	INTERGENIC
5060	CakSNP5060	*Kabuli*	Ca_Kabuli_Chr03	39909824	[C/G]		--	INTERGENIC
5061	CakSNP5061	*Kabuli*	Ca_Kabuli_Chr03	39909839	[T/C]		--	INTERGENIC

S.N.	SNP IDs	Chickpea cultivars	Chromosomes	Physical positions (bp)	SNPs	Gene accession IDs	Putative functions	Sequence components of genome
5062	CakSNP5062	*Kabuli*	Ca_Kabuli_Chr03	39914344	[T/G]	Ca_01376	RNA recognition motif domain	INTRON
5063	CakSNP5063	*Kabuli*	Ca_Kabuli_Chr03	39941101	[T/G]	Ca_01378	Ankyrin repeat-containing domain	INTRON
5064	CakSNP5064	*Kabuli*	Ca_Kabuli_Chr03	39941090	[G/A]	Ca_01378	Ankyrin repeat-containing domain	INTRON
5065	CakSNP5065	*Kabuli*	Ca_Kabuli_Chr04	20297	[A/G]	Ca_27693	Basic-leucine zipper (bZIP) transcription factor	SYNONYMOUS_CODING
5066	CakSNP5066	*Kabuli*	Ca_Kabuli_Chr04	20495	[G/C]	Ca_27693	Basic-leucine zipper (bZIP) transcription factor	INTRON
5067	CakSNP5067	*Kabuli*	Ca_Kabuli_Chr04	167229	[T/G]	Ca_07652	LNS2, Lipin/Ned1/Smp2	SYNONYMOUS_CODING
5068	CakSNP5068	*Kabuli*	Ca_Kabuli_Chr04	228864	[G/A]	Ca_07660	Protein of unknown function DUF3741	INTRON
5069	CakSNP5069	*Kabuli*	Ca_Kabuli_Chr04	305558	[C/T]	Ca_07664	Drug/metabolite transporter	SYNONYMOUS_CODING
5070	CakSNP5070	*Kabuli*	Ca_Kabuli_Chr04	335941	[C/T]		--	INTERGENIC
5071	CakSNP5071	*Kabuli*	Ca_Kabuli_Chr04	336055	[G/A]		--	INTERGENIC

S.N.	SNP IDs	Chickpea cultivars	Chromosomes	Physical positions (bp)	SNPs	Gene accession IDs	Putative functions	Sequence components of genome
5072	CakSNP5072	*Kabuli*	Ca_Kabuli_Chr04	336050	[T/C]		--	INTERGENIC
5073	CakSNP5073	*Kabuli*	Ca_Kabuli_Chr04	336029	[G/A]		--	INTERGENIC
5074	CakSNP5074	*Kabuli*	Ca_Kabuli_Chr04	338641	[A/G]		--	INTERGENIC
5075	CakSNP5075	*Kabuli*	Ca_Kabuli_Chr04	338721	[C/T]		--	INTERGENIC
5076	CakSNP5076	*Kabuli*	Ca_Kabuli_Chr04	338707	[G/T]		--	INTERGENIC
5077	CakSNP5077	*Kabuli*	Ca_Kabuli_Chr04	349309	[C/T]	Ca_07667	Multi antimicrobial extrusion protein	NON_SYNONYMOUS_CODING
5078	CakSNP5078	*Kabuli*	Ca_Kabuli_Chr04	353069	[C/A]		--	INTERGENIC
5079	CakSNP5079	*Kabuli*	Ca_Kabuli_Chr04	353079	[C/T]		--	INTERGENIC
5080	CakSNP5080	*Kabuli*	Ca_Kabuli_Chr04	353119	[G/A]		--	INTERGENIC
5081	CakSNP5081	*Kabuli*	Ca_Kabuli_Chr04	353120	[C/G]		--	INTERGENIC

S.N.	SNP IDs	Chickpea cultivars	Chromosomes	Physical positions (bp)	SNPs	Gene accession IDs	Putative functions	Sequence components of genome
5082	CakSNP5082	*Kabuli*	Ca_Kabuli_Chr04	353124	[A/G]		--	INTERGENIC
5083	CakSNP5083	*Kabuli*	Ca_Kabuli_Chr04	353126	[T/G]		--	INTERGENIC
5084	CakSNP5084	*Kabuli*	Ca_Kabuli_Chr04	369258	[C/T]	Ca_07669	Multi antimicrobial extrusion protein	NON_SYNONYMOUS_CODING
5085	CakSNP5085	*Kabuli*	Ca_Kabuli_Chr04	394661	[C/G]	Ca_07671	Inorganic pyrophosphatase	NON_SYNONYMOUS_CODING
5086	CakSNP5086	*Kabuli*	Ca_Kabuli_Chr04	411208	[A/C]	Ca_07673	Arf GTPase activating protein	INTRON
5087	CakSNP5087	*Kabuli*	Ca_Kabuli_Chr04	412017	[C/A]	Ca_07673	Arf GTPase activating protein	NON_SYNONYMOUS_CODING
5088	CakSNP5088	*Kabuli*	Ca_Kabuli_Chr04	455361	[A/G]	Ca_07679	Arginine biosynthesis protein ArgJ	INTRON
5089	CakSNP5089	*Kabuli*	Ca_Kabuli_Chr04	459360	[T/C]	Ca_07679	Arginine biosynthesis protein ArgJ	INTRON
5090	CakSNP5090	*Kabuli*	Ca_Kabuli_Chr04	480138	[C/A]	Ca_07682	--	NON_SYNONYMOUS_CODING
5091	CakSNP5091	*Kabuli*	Ca_Kabuli_Chr04	481556	[A/G]	Ca_07682	--	NON_SYNONYMOUS_CODING

S.N.	SNP IDs	Chickpea cultivars	Chromosomes	Physical positions (bp)	SNPs	Gene accession IDs	Putative functions	Sequence components of genome
5092	CakSNP5092	*Kabuli*	Ca_Kabuli_Chr04	481687	[T/G]	Ca_07682	--	NON_SYNONYMOUS_CODING
5093	CakSNP5093	*Kabuli*	Ca_Kabuli_Chr04	504928	[C/A]	Ca_07683	SANT associated	NON_SYNONYMOUS_CODING
5094	CakSNP5094	*Kabuli*	Ca_Kabuli_Chr04	586273	[A/G]	Ca_07690	--	INTRON
5095	CakSNP5095	*Kabuli*	Ca_Kabuli_Chr04	586703	[T/C]	Ca_07690	--	INTRON
5096	CakSNP5096	*Kabuli*	Ca_Kabuli_Chr04	589140	[T/C]		--	INTERGENIC
5097	CakSNP5097	*Kabuli*	Ca_Kabuli_Chr04	589191	[A/G]		--	INTERGENIC
5098	CakSNP5098	*Kabuli*	Ca_Kabuli_Chr04	589204	[G/A]		--	INTERGENIC
5099	CakSNP5099	*Kabuli*	Ca_Kabuli_Chr04	589220	[A/C]		--	INTERGENIC
5100	CakSNP5100	*Kabuli*	Ca_Kabuli_Chr04	589224	[T/C]		--	INTERGENIC
5101	CakSNP5101	*Kabuli*	Ca_Kabuli_Chr04	595982	[C/G]	Ca_07691	Helix-loop-helix DNA-binding domain	SYNONYMOUS_CODING

S.N.	SNP IDs	Chickpea cultivars	Chromosomes	Physical positions (bp)	SNPs	Gene accession IDs	Putative functions	Sequence components of genome
5102	CakSNP5102	*Kabuli*	Ca_Kabuli_Chr04	613150	[G/A]	Ca_07692	HEAT, type 2	INTRON
5103	CakSNP5103	*Kabuli*	Ca_Kabuli_Chr04	613166	[A/G]	Ca_07692	HEAT, type 2	INTRON
5104	CakSNP5104	*Kabuli*	Ca_Kabuli_Chr04	645103	[T/A]		--	INTERGENIC
5105	CakSNP5105	*Kabuli*	Ca_Kabuli_Chr04	645075	[T/A]		--	INTERGENIC
5106	CakSNP5106	*Kabuli*	Ca_Kabuli_Chr04	646567	[C/T]		--	INTERGENIC
5107	CakSNP5107	*Kabuli*	Ca_Kabuli_Chr04	646576	[A/G]		--	INTERGENIC
5108	CakSNP5108	*Kabuli*	Ca_Kabuli_Chr04	651040	[C/T]		--	INTERGENIC
5109	CakSNP5109	*Kabuli*	Ca_Kabuli_Chr04	660034	[A/C]	Ca_07697	Photosystem I PsaE, reaction centre subunit IV	SYNONYMOUS_CODING
5110	CakSNP5110	*Kabuli*	Ca_Kabuli_Chr04	660029	[T/G]	Ca_07697	Photosystem I PsaE, reaction centre subunit IV	SYNONYMOUS_CODING
5111	CakSNP5111	*Kabuli*	Ca_Kabuli_Chr04	703978	[A/T]	Ca_07701	IQ motif, EF-hand binding site	INTRON

S.N.	SNP IDs	Chickpea cultivars	Chromosomes	Physical positions (bp)	SNPs	Gene accession IDs	Putative functions	Sequence components of genome
5112	CakSNP5112	*Kabuli*	Ca_Kabuli_Chr04	703975	[G/T]	Ca_07701	IQ motif, EF-hand binding site	INTRON
5113	CakSNP5113	*Kabuli*	Ca_Kabuli_Chr04	704009	[G/A]	Ca_07701	IQ motif, EF-hand binding site	INTRON
5114	CakSNP5114	*Kabuli*	Ca_Kabuli_Chr04	705336	[T/G]	Ca_07701	IQ motif, EF-hand binding site	SYNONYMOUS_CODING
5115	CakSNP5115	*Kabuli*	Ca_Kabuli_Chr04	783302	[C/T]	Ca_07707	Beta-ketoacyl synthase	INTRON
5116	CakSNP5116	*Kabuli*	Ca_Kabuli_Chr04	783382	[G/T]	Ca_07707	Beta-ketoacyl synthase	INTRON
5117	CakSNP5117	*Kabuli*	Ca_Kabuli_Chr04	827630	[T/C]		--	INTERGENIC
5118	CakSNP5118	*Kabuli*	Ca_Kabuli_Chr04	848192	[G/A]	Ca_07716	Protein kinase, catalytic domain	SYNONYMOUS_CODING
5119	CakSNP5119	*Kabuli*	Ca_Kabuli_Chr04	913689	[A/G]		--	INTERGENIC
5120	CakSNP5120	*Kabuli*	Ca_Kabuli_Chr04	951389	[T/G]	Ca_07728	--	NON_SYNONYMOUS_CODING
5121	CakSNP5121	*Kabuli*	Ca_Kabuli_Chr04	951390	[G/A]	Ca_07728	--	NON_SYNONYMOUS_CODING

S.N.	SNP IDs	Chickpea cultivars	Chromosomes	Physical positions (bp)	SNPs	Gene accession IDs	Putative functions	Sequence components of genome
5122	CakSNP5122	*Kabuli*	Ca_Kabuli_Chr04	1035328	[G/C]	Ca_07736	Protein kinase, catalytic domain	INTRON
5123	CakSNP5123	*Kabuli*	Ca_Kabuli_Chr04	1047639	[G/T]	Ca_07738	SNF2-related	SYNONYMOUS_CODING
5124	CakSNP5124	*Kabuli*	Ca_Kabuli_Chr04	1065107	[C/T]	Ca_07740	Ribophorin II	INTRON
5125	CakSNP5125	*Kabuli*	Ca_Kabuli_Chr04	1065245	[A/G]	Ca_07740	Ribophorin II	SYNONYMOUS_CODING
5126	CakSNP5126	*Kabuli*	Ca_Kabuli_Chr04	1189997	[C/T]	Ca_07749	Ribosomal protein L21e	INTRON
5127	CakSNP5127	*Kabuli*	Ca_Kabuli_Chr04	1190007	[C/G]	Ca_07749	Ribosomal protein L21e	INTRON
5128	CakSNP5128	*Kabuli*	Ca_Kabuli_Chr04	1190038	[G/T]	Ca_07749	Ribosomal protein L21e	INTRON
5129	CakSNP5129	*Kabuli*	Ca_Kabuli_Chr04	1190208	[T/C]	Ca_07749	Ribosomal protein L21e	INTRON
5130	CakSNP5130	*Kabuli*	Ca_Kabuli_Chr04	1190184	[A/C]	Ca_07749	Ribosomal protein L21e	INTRON
5131	CakSNP5131	*Kabuli*	Ca_Kabuli_Chr04	1190158	[T/G]	Ca_07749	Ribosomal protein L21e	INTRON

S.N.	SNP IDs	Chickpea cultivars	Chromosomes	Physical positions (bp)	SNPs	Gene accession IDs	Putative functions	Sequence components of genome
5132	CakSNP5132	*Kabuli*	Ca_Kabuli_Chr04	1259917	[G/T]	Ca_07757	Tetratricopeptide repeat-containing	NON_SYNONYMOUS_CODING
5133	CakSNP5133	*Kabuli*	Ca_Kabuli_Chr04	1259915	[T/G]	Ca_07757	Tetratricopeptide repeat-containing	SYNONYMOUS_CODING
5134	CakSNP5134	*Kabuli*	Ca_Kabuli_Chr04	1264361	[T/C]		--	INTERGENIC
5135	CakSNP5135	*Kabuli*	Ca_Kabuli_Chr04	1266175	[A/G]	Ca_07758	Autophagy-related protein 9	INTRON
5136	CakSNP5136	*Kabuli*	Ca_Kabuli_Chr04	1266174	[C/T]	Ca_07758	Autophagy-related protein 9	INTRON
5137	CakSNP5137	*Kabuli*	Ca_Kabuli_Chr04	1283369	[A/G]	Ca_07759	DNA replication factor CDT1-like	NON_SYNONYMOUS_CODING
5138	CakSNP5138	*Kabuli*	Ca_Kabuli_Chr04	1344151	[T/C]		--	INTERGENIC
5139	CakSNP5139	*Kabuli*	Ca_Kabuli_Chr04	1396281	[G/C]		--	INTERGENIC
5140	CakSNP5140	*Kabuli*	Ca_Kabuli_Chr04	1396425	[C/A]		--	INTERGENIC
5141	CakSNP5141	*Kabuli*	Ca_Kabuli_Chr04	1474771	[T/C]	Ca_07777	Histone H1/H5	SYNONYMOUS_CODING

S.N.	SNP IDs	Chickpea cultivars	Chromosomes	Physical positions (bp)	SNPs	Gene accession IDs	Putative functions	Sequence components of genome
5142	CakSNP5142	*Kabuli*	Ca_Kabuli_Chr04	1474898	[A/G]	Ca_07777	Histone H1/H5	NON_SYNONYMOUS_CODING
5143	CakSNP5143	*Kabuli*	Ca_Kabuli_Chr04	1474922	[G/T]	Ca_07777	Histone H1/H5	NON_SYNONYMOUS_CODING
5144	CakSNP5144	*Kabuli*	Ca_Kabuli_Chr04	1493927	[T/C]	Ca_07779	DNA-binding WRKY	SYNONYMOUS_CODING
5145	CakSNP5145	*Kabuli*	Ca_Kabuli_Chr04	1513664	[T/A]		--	INTERGENIC
5146	CakSNP5146	*Kabuli*	Ca_Kabuli_Chr04	1526270	[T/C]	Ca_07780	Amino acid transporter, transmembrane	SYNONYMOUS_CODING
5147	CakSNP5147	*Kabuli*	Ca_Kabuli_Chr04	1526731	[T/G]	Ca_07780	Amino acid transporter, transmembrane	INTRON
5148	CakSNP5148	*Kabuli*	Ca_Kabuli_Chr04	1613153	[G/A]	Ca_07789	Primosome PriB/single-strand DNA-binding	NON_SYNONYMOUS_CODING
5149	CakSNP5149	*Kabuli*	Ca_Kabuli_Chr04	1613193	[G/A]	Ca_07789	Primosome PriB/single-strand DNA-binding	INTRON
5150	CakSNP5150	*Kabuli*	Ca_Kabuli_Chr04	1613303	[G/A]	Ca_07789	Primosome PriB/single-strand DNA-binding	NON_SYNONYMOUS_CODING
5151	CakSNP5151	*Kabuli*	Ca_Kabuli_Chr04	1687346	[A/G]		--	INTERGENIC

S.N.	SNP IDs	Chickpea cultivars	Chromosomes	Physical positions (bp)	SNPs	Gene accession IDs	Putative functions	Sequence components of genome
5152	CakSNP5152	*Kabuli*	Ca_Kabuli_Chr04	1691555	[T/G]		--	INTERGENIC
5153	CakSNP5153	*Kabuli*	Ca_Kabuli_Chr04	1708370	[C/T]	Ca_07797	BEACH domain	SYNONYMOUS_CODING
5154	CakSNP5154	*Kabuli*	Ca_Kabuli_Chr04	1711864	[A/C]	Ca_07797	BEACH domain	SYNONYMOUS_CODING
5155	CakSNP5155	*Kabuli*	Ca_Kabuli_Chr04	1711865	[G/A]	Ca_07797	BEACH domain	SYNONYMOUS_CODING
5156	CakSNP5156	*Kabuli*	Ca_Kabuli_Chr04	1720544	[A/C]	Ca_07797	BEACH domain	INTRON
5157	CakSNP5157	*Kabuli*	Ca_Kabuli_Chr04	1720658	[A/G]	Ca_07797	BEACH domain	INTRON
5158	CakSNP5158	*Kabuli*	Ca_Kabuli_Chr04	1720642	[A/G]	Ca_07797	BEACH domain	INTRON
5159	CakSNP5159	*Kabuli*	Ca_Kabuli_Chr04	1720619	[A/C]	Ca_07797	BEACH domain	INTRON
5160	CakSNP5160	*Kabuli*	Ca_Kabuli_Chr04	1742468	[C/T]	Ca_07798	Transcription factor jumonji/aspartyl beta-hydroxylase	INTRON
5161	CakSNP5161	*Kabuli*	Ca_Kabuli_Chr04	1747181	[A/T]	Ca_07798	Transcription factor jumonji/aspartyl beta-hydroxylase	NON_SYNONYMOUS_CODING

S.N.	SNP IDs	Chickpea cultivars	Chromosomes	Physical positions (bp)	SNPs	Gene accession IDs	Putative functions	Sequence components of genome
5162	CakSNP5162	*Kabuli*	Ca_Kabuli_Chr04	1754555	[C/T]		--	INTERGENIC
5163	CakSNP5163	*Kabuli*	Ca_Kabuli_Chr04	1755066	[G/C]		--	INTERGENIC
5164	CakSNP5164	*Kabuli*	Ca_Kabuli_Chr04	1773886	[C/G]		--	INTERGENIC
5165	CakSNP5165	*Kabuli*	Ca_Kabuli_Chr04	1773939	[C/T]		--	INTERGENIC
5166	CakSNP5166	*Kabuli*	Ca_Kabuli_Chr04	1773951	[C/G]		--	INTERGENIC
5167	CakSNP5167	*Kabuli*	Ca_Kabuli_Chr04	1785705	[G/C]	Ca_07801	Protein kinase, catalytic domain	INTRON
5168	CakSNP5168	*Kabuli*	Ca_Kabuli_Chr04	1785680	[C/T]	Ca_07801	Protein kinase, catalytic domain	INTRON
5169	CakSNP5169	*Kabuli*	Ca_Kabuli_Chr04	1807484	[G/A]	Ca_07801	Protein kinase, catalytic domain	INTRON
5170	CakSNP5170	*Kabuli*	Ca_Kabuli_Chr04	1817779	[G/C]	Ca_07802	Tetratricopeptide TPR-1	SYNONYMOUS_CODING
5171	CakSNP5171	*Kabuli*	Ca_Kabuli_Chr04	1833570	[T/C]	Ca_07804	Domain of unknown function DUF828	SYNONYMOUS_CODING

S.N.	SNP IDs	Chickpea cultivars	Chromosomes	Physical positions (bp)	SNPs	Gene accession IDs	Putative functions	Sequence components of genome
5172	CakSNP5172	*Kabuli*	Ca_Kabuli_Chr04	1833678	[A/G]	Ca_07804	Domain of unknown function DUF828	SYNONYMOUS_CODING
5173	CakSNP5173	*Kabuli*	Ca_Kabuli_Chr04	1833725	[C/T]	Ca_07804	Domain of unknown function DUF828	NON_SYNONYMOUS_CODING
5174	CakSNP5174	*Kabuli*	Ca_Kabuli_Chr04	1845301	[G/A]	Ca_07806	Protein kinase, catalytic domain	INTRON
5175	CakSNP5175	*Kabuli*	Ca_Kabuli_Chr04	1867921	[G/A]	Ca_07807	Sulphate transporter/antisigma-factor antagonist STAS	INTRON
5176	CakSNP5176	*Kabuli*	Ca_Kabuli_Chr04	1869577	[C/T]	Ca_07808	KIP1-like	SYNONYMOUS_CODING
5177	CakSNP5177	*Kabuli*	Ca_Kabuli_Chr04	1870333	[A/G]	Ca_07808	KIP1-like	INTRON
5178	CakSNP5178	*Kabuli*	Ca_Kabuli_Chr04	1870317	[T/C]	Ca_07808	KIP1-like	INTRON
5179	CakSNP5179	*Kabuli*	Ca_Kabuli_Chr04	1870666	[G/C]	Ca_07808	KIP1-like	SYNONYMOUS_CODING
5180	CakSNP5180	*Kabuli*	Ca_Kabuli_Chr04	1870806	[C/A]	Ca_07808	KIP1-like	SYNONYMOUS_CODING
5181	CakSNP5181	*Kabuli*	Ca_Kabuli_Chr04	1870802	[C/T]	Ca_07808	KIP1-like	SYNONYMOUS_CODING

S.N.	SNP IDs	Chickpea cultivars	Chromosomes	Physical positions (bp)	SNPs	Gene accession IDs	Putative functions	Sequence components of genome
5182	CakSNP5182	*Kabuli*	Ca_Kabuli_Chr04	1873733	[T/C]	Ca_07808	KIP1-like	NON_SYNONYMOUS_CODING
5183	CakSNP5183	*Kabuli*	Ca_Kabuli_Chr04	1890220	[G/C]	Ca_07809	Biotin/lipoyl attachment	SYNONYMOUS_CODING
5184	CakSNP5184	*Kabuli*	Ca_Kabuli_Chr04	1941001	[T/G]	Ca_07811	Cupin 1	SYNONYMOUS_CODING
5185	CakSNP5185	*Kabuli*	Ca_Kabuli_Chr04	1941089	[C/T]	Ca_07811	Cupin 1	NON_SYNONYMOUS_CODING
5186	CakSNP5186	*Kabuli*	Ca_Kabuli_Chr04	1970471	[A/C]		--	INTERGENIC
5187	CakSNP5187	*Kabuli*	Ca_Kabuli_Chr04	1973375	[A/C]		--	INTERGENIC
5188	CakSNP5188	*Kabuli*	Ca_Kabuli_Chr04	2009707	[A/G]	Ca_07819	--	NON_SYNONYMOUS_CODING
5189	CakSNP5189	*Kabuli*	Ca_Kabuli_Chr04	2025214	[A/C]		--	INTERGENIC
5190	CakSNP5190	*Kabuli*	Ca_Kabuli_Chr04	2027063	[G/A]		--	INTERGENIC
5191	CakSNP5191	*Kabuli*	Ca_Kabuli_Chr04	2027101	[T/A]		--	INTERGENIC

S.N.	SNP IDs	Chickpea cultivars	Chromosomes	Physical positions (bp)	SNPs	Gene accession IDs	Putative functions	Sequence components of genome
5192	CakSNP5192	*Kabuli*	Ca_Kabuli_Chr04	2034429	[C/A]	Ca_07822	RNA-processing protein, HAT helix	INTRON
5193	CakSNP5193	*Kabuli*	Ca_Kabuli_Chr04	2034398	[C/T]	Ca_07822	RNA-processing protein, HAT helix	INTRON
5194	CakSNP5194	*Kabuli*	Ca_Kabuli_Chr04	2034396	[A/G]	Ca_07822	RNA-processing protein, HAT helix	INTRON
5195	CakSNP5195	*Kabuli*	Ca_Kabuli_Chr04	2034739	[A/C]	Ca_07822	RNA-processing protein, HAT helix	INTRON
5196	CakSNP5196	*Kabuli*	Ca_Kabuli_Chr04	2035797	[G/A]	Ca_07822	RNA-processing protein, HAT helix	SYNONYMOUS_CODING
5197	CakSNP5197	*Kabuli*	Ca_Kabuli_Chr04	2035801	[G/A]	Ca_07822	RNA-processing protein, HAT helix	SYNONYMOUS_CODING
5198	CakSNP5198	*Kabuli*	Ca_Kabuli_Chr04	2035841	[C/T]		--	INTERGENIC
5199	CakSNP5199	*Kabuli*	Ca_Kabuli_Chr04	2035894	[C/A]		--	INTERGENIC
5200	CakSNP5200	*Kabuli*	Ca_Kabuli_Chr04	2051684	[T/C]		--	INTERGENIC
5201	CakSNP5201	*Kabuli*	Ca_Kabuli_Chr04	2051642	[G/C]		--	INTERGENIC

S.N.	SNP IDs	Chickpea cultivars	Chromosomes	Physical positions (bp)	SNPs	Gene accession IDs	Putative functions	Sequence components of genome
5202	CakSNP5202	*Kabuli*	Ca_Kabuli_Chr04	2060244	[G/A]	Ca_07827	--	SYNONYMOUS_CODING
5203	CakSNP5203	*Kabuli*	Ca_Kabuli_Chr04	2060262	[C/T]	Ca_07827	--	SYNONYMOUS_CODING
5204	CakSNP5204	*Kabuli*	Ca_Kabuli_Chr04	2113564	[A/G]	Ca_07831	NAD-dependent epimerase/dehydratase	SYNONYMOUS_CODING
5205	CakSNP5205	*Kabuli*	Ca_Kabuli_Chr04	2125774	[G/A]	Ca_07833	Ras GTPase	NON_SYNONYMOUS_CODING
5206	CakSNP5206	*Kabuli*	Ca_Kabuli_Chr04	2143155	[A/G]	Ca_07834	Pentatricopeptide repeat	NON_SYNONYMOUS_CODING
5207	CakSNP5207	*Kabuli*	Ca_Kabuli_Chr04	2213710	[G/A]	Ca_07844	Helix-loop-helix DNA-binding domain	NON_SYNONYMOUS_CODING
5208	CakSNP5208	*Kabuli*	Ca_Kabuli_Chr04	2238480	[A/T]	Ca_07848	--	SYNONYMOUS_CODING
5209	CakSNP5209	*Kabuli*	Ca_Kabuli_Chr04	2270360	[T/C]		--	INTERGENIC
5210	CakSNP5210	*Kabuli*	Ca_Kabuli_Chr04	2277421	[A/T]		--	INTERGENIC
5211	CakSNP5211	*Kabuli*	Ca_Kabuli_Chr04	2311977	[A/G]	Ca_07853	Glucosylceramidase	INTRON

S.N.	SNP IDs	Chickpea cultivars	Chromosomes	Physical positions (bp)	SNPs	Gene accession IDs	Putative functions	Sequence components of genome
5212	CakSNP5212	*Kabuli*	Ca_Kabuli_Chr04	2313969	[G/A]	Ca_07853	Glucosylceramidase	SYNONYMOUS_CODING
5213	CakSNP5213	*Kabuli*	Ca_Kabuli_Chr04	2361088	[T/A]	Ca_07858	ABC transporter-like	INTRON
5214	CakSNP5214	*Kabuli*	Ca_Kabuli_Chr04	2380206	[A/G]		--	INTERGENIC
5215	CakSNP5215	*Kabuli*	Ca_Kabuli_Chr04	2393033	[T/G]	Ca_07861	DNA helicase, UvrD/REP type	INTRON
5216	CakSNP5216	*Kabuli*	Ca_Kabuli_Chr04	2396883	[A/G]	Ca_07861	DNA helicase, UvrD/REP type	INTRON
5217	CakSNP5217	*Kabuli*	Ca_Kabuli_Chr04	2411925	[G/T]	Ca_07861	DNA helicase, UvrD/REP type	INTRON
5218	CakSNP5218	*Kabuli*	Ca_Kabuli_Chr04	2422087	[A/G]		--	INTERGENIC
5219	CakSNP5219	*Kabuli*	Ca_Kabuli_Chr04	2422092	[G/A]		--	INTERGENIC
5220	CakSNP5220	*Kabuli*	Ca_Kabuli_Chr04	2422949	[C/T]		--	INTERGENIC
5221	CakSNP5221	*Kabuli*	Ca_Kabuli_Chr04	2463064	[A/G]		--	INTERGENIC

S.N.	SNP IDs	Chickpea cultivars	Chromosomes	Physical positions (bp)	SNPs	Gene accession IDs	Putative functions	Sequence components of genome
5222	CakSNP5222	*Kabuli*	Ca_Kabuli_Chr04	2463038	[T/C]		--	INTERGENIC
5223	CakSNP5223	*Kabuli*	Ca_Kabuli_Chr04	2492726	[A/G]	Ca_07866	Uncharacterised protein domain NUC173	SYNONYMOUS_CODING
5224	CakSNP5224	*Kabuli*	Ca_Kabuli_Chr04	2493195	[T/C]	Ca_07866	Uncharacterised protein domain NUC173	INTRON
5225	CakSNP5225	*Kabuli*	Ca_Kabuli_Chr04	2494101	[C/A]	Ca_07866	Uncharacterised protein domain NUC173	NON_SYNONYMOUS_CODING
5226	CakSNP5226	*Kabuli*	Ca_Kabuli_Chr04	2494140	[C/A]	Ca_07866	Uncharacterised protein domain NUC173	NON_SYNONYMOUS_CODING
5227	CakSNP5227	*Kabuli*	Ca_Kabuli_Chr04	2499257	[A/C]	Ca_07866	Uncharacterised protein domain NUC173	INTRON
5228	CakSNP5228	*Kabuli*	Ca_Kabuli_Chr04	2562541	[G/C]	Ca_07875	RNA-processing protein, HAT helix	INTRON
5229	CakSNP5229	*Kabuli*	Ca_Kabuli_Chr04	2564500	[T/A]		--	INTERGENIC
5230	CakSNP5230	*Kabuli*	Ca_Kabuli_Chr04	2609945	[C/T]		--	INTERGENIC
5231	CakSNP5231	*Kabuli*	Ca_Kabuli_Chr04	2676174	[C/T]	Ca_12071	Signal transduction response regulator, receiver domain	SYNONYMOUS_CODING

S.N.	SNP IDs	Chickpea cultivars	Chromosomes	Physical positions (bp)	SNPs	Gene accession IDs	Putative functions	Sequence components of genome
5232	CakSNP5232	*Kabuli*	Ca_Kabuli_Chr04	2740041	[T/G]	Ca_12077	HR-like lesion-inducer	INTRON
5233	CakSNP5233	*Kabuli*	Ca_Kabuli_Chr04	2740068	[A/T]	Ca_12077	HR-like lesion-inducer	INTRON
5234	CakSNP5234	*Kabuli*	Ca_Kabuli_Chr04	2740065	[A/G]	Ca_12077	HR-like lesion-inducer	INTRON
5235	CakSNP5235	*Kabuli*	Ca_Kabuli_Chr04	2752538	[G/A]		--	INTERGENIC
5236	CakSNP5236	*Kabuli*	Ca_Kabuli_Chr04	2752568	[C/T]		--	INTERGENIC
5237	CakSNP5237	*Kabuli*	Ca_Kabuli_Chr04	2886775	[T/G]	Ca_12085	General substrate transporter	SYNONYMOUS_CODING
5238	CakSNP5238	*Kabuli*	Ca_Kabuli_Chr04	2886863	[C/T]	Ca_12085	General substrate transporter	SYNONYMOUS_CODING
5239	CakSNP5239	*Kabuli*	Ca_Kabuli_Chr04	2978875	[T/C]		--	INTERGENIC
5240	CakSNP5240	*Kabuli*	Ca_Kabuli_Chr04	2978876	[T/G]		--	INTERGENIC
5241	CakSNP5241	*Kabuli*	Ca_Kabuli_Chr04	2978882	[G/T]		--	INTERGENIC

S.N.	SNP IDs	Chickpea cultivars	Chromosomes	Physical positions (bp)	SNPs	Gene accession IDs	Putative functions	Sequence components of genome
5242	CakSNP5242	*Kabuli*	Ca_Kabuli_Chr04	2978932	[A/C]		--	INTERGENIC
5243	CakSNP5243	*Kabuli*	Ca_Kabuli_Chr04	2978931	[A/T]		--	INTERGENIC
5244	CakSNP5244	*Kabuli*	Ca_Kabuli_Chr04	3002755	[A/G]	Ca_12098	Peptidase M20	NON_SYNONYMOUS_CODING
5245	CakSNP5245	*Kabuli*	Ca_Kabuli_Chr04	3002810	[T/C]	Ca_12098	Peptidase M20	NON_SYNONYMOUS_CODING
5246	CakSNP5246	*Kabuli*	Ca_Kabuli_Chr04	3037899	[A/C]	Ca_12102	--	NON_SYNONYMOUS_CODING
5247	CakSNP5247	*Kabuli*	Ca_Kabuli_Chr04	3037986	[T/A]	Ca_12102	--	NON_SYNONYMOUS_CODING
5248	CakSNP5248	*Kabuli*	Ca_Kabuli_Chr04	3037929	[T/G]	Ca_12102	--	SYNONYMOUS_CODING
5249	CakSNP5249	*Kabuli*	Ca_Kabuli_Chr04	3038022	[C/T]	Ca_12102	--	NON_SYNONYMOUS_CODING
5250	CakSNP5250	*Kabuli*	Ca_Kabuli_Chr04	3046920	[A/G]	Ca_12103	Tetratricopeptide TPR-1	INTRON
5251	CakSNP5251	*Kabuli*	Ca_Kabuli_Chr04	3144454	[T/C]	Ca_12112	Kinesin, motor domain	SYNONYMOUS_CODING

S.N.	SNP IDs	Chickpea cultivars	Chromosomes	Physical positions (bp)	SNPs	Gene accession IDs	Putative functions	Sequence components of genome
5252	CakSNP5252	*Kabuli*	Ca_Kabuli_Chr04	3158321	[A/G]	Ca_12115	Ribosomal protein L14	INTRON
5253	CakSNP5253	*Kabuli*	Ca_Kabuli_Chr04	3158323	[T/G]	Ca_12115	Ribosomal protein L14	INTRON
5254	CakSNP5254	*Kabuli*	Ca_Kabuli_Chr04	3158328	[G/T]	Ca_12115	Ribosomal protein L14	INTRON
5255	CakSNP5255	*Kabuli*	Ca_Kabuli_Chr04	3158331	[A/T]	Ca_12115	Ribosomal protein L14	INTRON
5256	CakSNP5256	*Kabuli*	Ca_Kabuli_Chr04	3158426	[G/A]	Ca_12115	Ribosomal protein L14	SYNONYMOUS_CODING
5257	CakSNP5257	*Kabuli*	Ca_Kabuli_Chr04	3158504	[A/T]	Ca_12115	Ribosomal protein L14	INTRON
5258	CakSNP5258	*Kabuli*	Ca_Kabuli_Chr04	3158506	[T/C]	Ca_12115	Ribosomal protein L14	INTRON
5259	CakSNP5259	*Kabuli*	Ca_Kabuli_Chr04	3158549	[A/G]	Ca_12115	Ribosomal protein L14	INTRON
5260	CakSNP5260	*Kabuli*	Ca_Kabuli_Chr04	3164115	[G/A]		--	INTERGENIC
5261	CakSNP5261	*Kabuli*	Ca_Kabuli_Chr04	3164083	[G/A]		--	INTERGENIC

S.N.	SNP IDs	Chickpea cultivars	Chromosomes	Physical positions (bp)	SNPs	Gene accession IDs	Putative functions	Sequence components of genome
5262	CakSNP5262	*Kabuli*	Ca_Kabuli_Chr04	3166657	[C/T]	Ca_12117	--	INTRON
5263	CakSNP5263	*Kabuli*	Ca_Kabuli_Chr04	3166663	[C/T]	Ca_12117	--	INTRON
5264	CakSNP5264	*Kabuli*	Ca_Kabuli_Chr04	3166674	[G/T]	Ca_12117	--	INTRON
5265	CakSNP5265	*Kabuli*	Ca_Kabuli_Chr04	3171162	[G/A]	Ca_12117	--	SYNONYMOUS_CODING
5266	CakSNP5266	*Kabuli*	Ca_Kabuli_Chr04	3171384	[T/G]	Ca_12117	--	INTRON
5267	CakSNP5267	*Kabuli*	Ca_Kabuli_Chr04	3171385	[A/G]	Ca_12117	--	INTRON
5268	CakSNP5268	*Kabuli*	Ca_Kabuli_Chr04	3171428	[T/C]	Ca_12117	--	INTRON
5269	CakSNP5269	*Kabuli*	Ca_Kabuli_Chr04	3171551	[C/T]	Ca_12117	--	INTRON
5270	CakSNP5270	*Kabuli*	Ca_Kabuli_Chr04	3264351	[T/C]	Ca_12126	Domain of unknown function DUF640	SYNONYMOUS_CODING
5271	CakSNP5271	*Kabuli*	Ca_Kabuli_Chr04	3280147	[G/A]		--	INTERGENIC

S.N.	SNP IDs	Chickpea cultivars	Chromosomes	Physical positions (bp)	SNPs	Gene accession IDs	Putative functions	Sequence components of genome
5272	CakSNP5272	*Kabuli*	Ca_Kabuli_Chr04	3280217	[A/G]		--	INTERGENIC
5273	CakSNP5273	*Kabuli*	Ca_Kabuli_Chr04	3281050	[T/G]		--	INTERGENIC
5274	CakSNP5274	*Kabuli*	Ca_Kabuli_Chr04	3290298	[T/G]		--	INTERGENIC
5275	CakSNP5275	*Kabuli*	Ca_Kabuli_Chr04	3353106	[T/C]	Ca_12138	Mg2+ transporter protein, CorA-like	SYNONYMOUS_CODING
5276	CakSNP5276	*Kabuli*	Ca_Kabuli_Chr04	3356252	[C/G]		--	INTERGENIC
5277	CakSNP5277	*Kabuli*	Ca_Kabuli_Chr04	3356242	[C/G]		--	INTERGENIC
5278	CakSNP5278	*Kabuli*	Ca_Kabuli_Chr04	3400828	[G/C]	Ca_12141	Glycoside hydrolase, family 47	INTRON
5279	CakSNP5279	*Kabuli*	Ca_Kabuli_Chr04	3427396	[A/G]	Ca_12144	Castor/Pollux ion channel	INTRON
5280	CakSNP5280	*Kabuli*	Ca_Kabuli_Chr04	3429035	[G/A]	Ca_12144	Castor/Pollux ion channel	INTRON
5281	CakSNP5281	*Kabuli*	Ca_Kabuli_Chr04	3429059	[C/A]	Ca_12144	Castor/Pollux ion channel	INTRON

S.N.	SNP IDs	Chickpea cultivars	Chromosomes	Physical positions (bp)	SNPs	Gene accession IDs	Putative functions	Sequence components of genome
5282	CakSNP5282	*Kabuli*	Ca_Kabuli_Chr04	3455029	[T/C]	Ca_12146	Armadillo	SYNONYMOUS_CODING
5283	CakSNP5283	*Kabuli*	Ca_Kabuli_Chr04	3455047	[G/A]	Ca_12146	Armadillo	NON_SYNONYMOUS_CODING
5284	CakSNP5284	*Kabuli*	Ca_Kabuli_Chr04	3456450	[G/T]	Ca_12146	Armadillo	NON_SYNONYMOUS_CODING
5285	CakSNP5285	*Kabuli*	Ca_Kabuli_Chr04	3456501	[C/T]	Ca_12146	Armadillo	SYNONYMOUS_CODING
5286	CakSNP5286	*Kabuli*	Ca_Kabuli_Chr04	3483687	[A/C]		--	INTERGENIC
5287	CakSNP5287	*Kabuli*	Ca_Kabuli_Chr04	3627810	[G/T]	Ca_12162	Domain of unknown function DUF89	INTRON
5288	CakSNP5288	*Kabuli*	Ca_Kabuli_Chr04	3642051	[A/T]		--	INTERGENIC
5289	CakSNP5289	*Kabuli*	Ca_Kabuli_Chr04	3642070	[T/C]		--	INTERGENIC
5290	CakSNP5290	*Kabuli*	Ca_Kabuli_Chr04	3642116	[A/T]		--	INTERGENIC
5291	CakSNP5291	*Kabuli*	Ca_Kabuli_Chr04	3689840	[T/C]	Ca_12165	ATPase, AAA+ type, core	INTRON

S.N.	SNP IDs	Chickpea cultivars	Chromosomes	Physical positions (bp)	SNPs	Gene accession IDs	Putative functions	Sequence components of genome
5292	CakSNP5292	*Kabuli*	Ca_Kabuli_Chr04	3689871	[G/A]	Ca_12165	ATPase, AAA+ type, core	INTRON
5293	CakSNP5293	*Kabuli*	Ca_Kabuli_Chr04	3739386	[C/A]		--	INTERGENIC
5294	CakSNP5294	*Kabuli*	Ca_Kabuli_Chr04	3741522	[T/G]	Ca_12175	Histone deacetylation protein Rxt3	SYNONYMOUS_CODING
5295	CakSNP5295	*Kabuli*	Ca_Kabuli_Chr04	3741539	[T/G]	Ca_12175	Histone deacetylation protein Rxt3	NON_SYNONYMOUS_CODING
5296	CakSNP5296	*Kabuli*	Ca_Kabuli_Chr04	3752141	[C/G]	Ca_12177	--	NON_SYNONYMOUS_CODING
5297	CakSNP5297	*Kabuli*	Ca_Kabuli_Chr04	3762559	[T/C]		--	INTERGENIC
5298	CakSNP5298	*Kabuli*	Ca_Kabuli_Chr04	3762561	[C/A]		--	INTERGENIC
5299	CakSNP5299	*Kabuli*	Ca_Kabuli_Chr04	3762585	[G/T]		--	INTERGENIC
5300	CakSNP5300	*Kabuli*	Ca_Kabuli_Chr04	3794764	[G/A]		--	INTERGENIC
5301	CakSNP5301	*Kabuli*	Ca_Kabuli_Chr04	3794885	[C/A]		--	INTERGENIC

S.N.	SNP IDs	Chickpea cultivars	Chromosomes	Physical positions (bp)	SNPs	Gene accession IDs	Putative functions	Sequence components of genome
5302	CakSNP5302	*Kabuli*	Ca_Kabuli_Chr04	3885444	[G/C]		--	INTERGENIC
5303	CakSNP5303	*Kabuli*	Ca_Kabuli_Chr04	3888010	[G/C]		--	INTERGENIC
5304	CakSNP5304	*Kabuli*	Ca_Kabuli_Chr04	3888053	[C/A]		--	INTERGENIC
5305	CakSNP5305	*Kabuli*	Ca_Kabuli_Chr04	3888164	[T/A]		--	INTERGENIC
5306	CakSNP5306	*Kabuli*	Ca_Kabuli_Chr04	3888185	[A/G]		--	INTERGENIC
5307	CakSNP5307	*Kabuli*	Ca_Kabuli_Chr04	3888236	[A/G]		--	INTERGENIC
5308	CakSNP5308	*Kabuli*	Ca_Kabuli_Chr04	3888328	[C/T]		--	INTERGENIC
5309	CakSNP5309	*Kabuli*	Ca_Kabuli_Chr04	3888462	[T/C]		--	INTERGENIC
5310	CakSNP5310	*Kabuli*	Ca_Kabuli_Chr04	3888510	[G/A]		--	INTERGENIC
5311	CakSNP5311	*Kabuli*	Ca_Kabuli_Chr04	3888514	[G/A]		--	INTERGENIC

S.N.	SNP IDs	Chickpea cultivars	Chromosomes	Physical positions (bp)	SNPs	Gene accession IDs	Putative functions	Sequence components of genome
5312	CakSNP5312	*Kabuli*	Ca_Kabuli_Chr04	3888529	[T/G]		--	INTERGENIC
5313	CakSNP5313	*Kabuli*	Ca_Kabuli_Chr04	3890556	[A/G]		--	INTERGENIC
5314	CakSNP5314	*Kabuli*	Ca_Kabuli_Chr04	3890709	[A/G]		--	INTERGENIC
5315	CakSNP5315	*Kabuli*	Ca_Kabuli_Chr04	3990810	[C/G]	Ca_03854	Major intrinsic protein	NON_SYNONYMOUS_CODING
5316	CakSNP5316	*Kabuli*	Ca_Kabuli_Chr04	3990809	[G/A]	Ca_03854	Major intrinsic protein	NON_SYNONYMOUS_CODING
5317	CakSNP5317	*Kabuli*	Ca_Kabuli_Chr04	4002917	[T/C]	Ca_03850	BRO1 domain	SYNONYMOUS_CODING
5318	CakSNP5318	*Kabuli*	Ca_Kabuli_Chr04	4006213	[A/C]		--	INTERGENIC
5319	CakSNP5319	*Kabuli*	Ca_Kabuli_Chr04	4027829	[A/T]		--	INTERGENIC
5320	CakSNP5320	*Kabuli*	Ca_Kabuli_Chr04	4027872	[A/C]		--	INTERGENIC
5321	CakSNP5321	*Kabuli*	Ca_Kabuli_Chr04	4097989	[G/A]	Ca_03846	UBX	SYNONYMOUS_CODING

S.N.	SNP IDs	Chickpea cultivars	Chromosomes	Physical positions (bp)	SNPs	Gene accession IDs	Putative functions	Sequence components of genome
5322	CakSNP5322	*Kabuli*	Ca_Kabuli_Chr04	4097935	[T/C]	Ca_03846	UBX	INTRON
5323	CakSNP5323	*Kabuli*	Ca_Kabuli_Chr04	4116386	[G/C]		--	INTERGENIC
5324	CakSNP5324	*Kabuli*	Ca_Kabuli_Chr04	4148061	[T/C]	Ca_03841	Plant specific mitochondrial import receptor subunit TOM21	SYNONYMOUS_CODING
5325	CakSNP5325	*Kabuli*	Ca_Kabuli_Chr04	4148250	[A/G]	Ca_03841	Plant specific mitochondrial import receptor subunit TOM22	INTRON
5326	CakSNP5326	*Kabuli*	Ca_Kabuli_Chr04	4154417	[A/C]	Ca_03840	--	SYNONYMOUS_CODING
5327	CakSNP5327	*Kabuli*	Ca_Kabuli_Chr04	4154507	[G/T]	Ca_03840	--	INTRON
5328	CakSNP5328	*Kabuli*	Ca_Kabuli_Chr04	4229295	[T/A]		--	INTERGENIC
5329	CakSNP5329	*Kabuli*	Ca_Kabuli_Chr04	4229345	[C/T]		--	INTERGENIC
5330	CakSNP5330	*Kabuli*	Ca_Kabuli_Chr04	4229381	[T/C]		--	INTERGENIC
5331	CakSNP5331	*Kabuli*	Ca_Kabuli_Chr04	4229371	[C/T]		--	INTERGENIC

S.N.	SNP IDs	Chickpea cultivars	Chromosomes	Physical positions (bp)	SNPs	Gene accession IDs	Putative functions	Sequence components of genome
5332	CakSNP5332	*Kabuli*	Ca_Kabuli_Chr04	4255863	[T/G]	Ca_03832	Peptidase S59, nucleoporin	NON_SYNONYMOUS_CODING
5333	CakSNP5333	*Kabuli*	Ca_Kabuli_Chr04	4256023	[C/T]	Ca_03832	Peptidase S59, nucleoporin	SYNONYMOUS_CODING
5334	CakSNP5334	*Kabuli*	Ca_Kabuli_Chr04	4258002	[A/G]	Ca_03832	Peptidase S59, nucleoporin	SYNONYMOUS_CODING
5335	CakSNP5335	*Kabuli*	Ca_Kabuli_Chr04	4258149	[A/G]	Ca_03832	Peptidase S59, nucleoporin	SYNONYMOUS_CODING
5336	CakSNP5336	*Kabuli*	Ca_Kabuli_Chr04	4305891	[A/C]	Ca_03829	Inositol polyphosphate-related phosphatase	SYNONYMOUS_CODING
5337	CakSNP5337	*Kabuli*	Ca_Kabuli_Chr04	4320615	[C/T]		--	INTERGENIC
5338	CakSNP5338	*Kabuli*	Ca_Kabuli_Chr04	4320743	[A/G]		--	INTERGENIC
5339	CakSNP5339	*Kabuli*	Ca_Kabuli_Chr04	4327268	[A/G]	Ca_03827	RNA recognition motif domain	INTRON
5340	CakSNP5340	*Kabuli*	Ca_Kabuli_Chr04	4334307	[T/G]	Ca_03827	RNA recognition motif domain	NON_SYNONYMOUS_CODING
5341	CakSNP5341	*Kabuli*	Ca_Kabuli_Chr04	4343586	[C/T]	Ca_03826	Peptidase A1	INTRON

S.N.	SNP IDs	Chickpea cultivars	Chromosomes	Physical positions (bp)	SNPs	Gene accession IDs	Putative functions	Sequence components of genome
5342	CakSNP5342	*Kabuli*	Ca_Kabuli_Chr04	4343540	[C/A]	Ca_03826	Peptidase A1	INTRON
5343	CakSNP5343	*Kabuli*	Ca_Kabuli_Chr04	4410351	[T/C]	Ca_03820	Single-stranded nucleic acid binding R3H	SYNONYMOUS_CODING
5344	CakSNP5344	*Kabuli*	Ca_Kabuli_Chr04	4426659	[C/T]	Ca_03817	Peptidase S8/S53, subtilisin/kexin/sedolisin	INTRON
5345	CakSNP5345	*Kabuli*	Ca_Kabuli_Chr04	4426760	[T/C]	Ca_03817	Peptidase S8/S53, subtilisin/kexin/sedolisin	SYNONYMOUS_CODING
5346	CakSNP5346	*Kabuli*	Ca_Kabuli_Chr04	4435621	[T/C]	Ca_03815	F-box domain, cyclin-like	INTRON
5347	CakSNP5347	*Kabuli*	Ca_Kabuli_Chr04	4451928	[T/C]	Ca_03813	Protein kinase, catalytic domain	SYNONYMOUS_CODING
5348	CakSNP5348	*Kabuli*	Ca_Kabuli_Chr04	4452018	[T/A]	Ca_03813	Protein kinase, catalytic domain	NON_SYNONYMOUS_CODING
5349	CakSNP5349	*Kabuli*	Ca_Kabuli_Chr04	4452380	[C/G]		--	INTERGENIC
5350	CakSNP5350	*Kabuli*	Ca_Kabuli_Chr04	4454305	[A/C]	Ca_03812	Armadillo	SYNONYMOUS_CODING
5351	CakSNP5351	*Kabuli*	Ca_Kabuli_Chr04	4454464	[A/G]	Ca_03812	Armadillo	SYNONYMOUS_CODING

S.N.	SNP IDs	Chickpea cultivars	Chromosomes	Physical positions (bp)	SNPs	Gene accession IDs	Putative functions	Sequence components of genome
5352	CakSNP5352	*Kabuli*	Ca_Kabuli_Chr04	4461562	[G/C]	Ca_03811	TRM13/UPF0224 family, U11-48K-like CHHC zinc finger domain	INTRON
5353	CakSNP5353	*Kabuli*	Ca_Kabuli_Chr04	4468394	[C/T]	Ca_03810	Tetratricopeptide TPR-1	INTRON
5354	CakSNP5354	*Kabuli*	Ca_Kabuli_Chr04	4536310	[A/G]	Ca_03805	Ammonium transporter	SYNONYMOUS_CODING
5355	CakSNP5355	*Kabuli*	Ca_Kabuli_Chr04	4536286	[G/C]	Ca_03805	Ammonium transporter	SYNONYMOUS_CODING
5356	CakSNP5356	*Kabuli*	Ca_Kabuli_Chr04	4578836	[C/T]	Ca_03800	Signal transduction response regulator, receiver domain	SYNONYMOUS_CODING
5357	CakSNP5357	*Kabuli*	Ca_Kabuli_Chr04	4579274	[C/T]	Ca_03800	Signal transduction response regulator, receiver domain	SYNONYMOUS_CODING
5358	CakSNP5358	*Kabuli*	Ca_Kabuli_Chr04	4605340	[A/C]	Ca_03797	--	SYNONYMOUS_CODING
5359	CakSNP5359	*Kabuli*	Ca_Kabuli_Chr04	4622932	[T/G]	Ca_03794	Pentatricopeptide repeat	INTRON
5360	CakSNP5360	*Kabuli*	Ca_Kabuli_Chr04	4622913	[C/T]	Ca_03794	Pentatricopeptide repeat	INTRON
5361	CakSNP5361	*Kabuli*	Ca_Kabuli_Chr04	4628221	[T/G]		--	INTERGENIC

S.N.	SNP IDs	Chickpea cultivars	Chromosomes	Physical positions (bp)	SNPs	Gene accession IDs	Putative functions	Sequence components of genome
5362	CakSNP5362	*Kabuli*	Ca_Kabuli_Chr04	4628232	[G/A]		--	INTERGENIC
5363	CakSNP5363	*Kabuli*	Ca_Kabuli_Chr04	4628240	[C/T]		--	INTERGENIC
5364	CakSNP5364	*Kabuli*	Ca_Kabuli_Chr04	4628243	[G/A]		--	INTERGENIC
5365	CakSNP5365	*Kabuli*	Ca_Kabuli_Chr04	4628309	[C/T]		--	INTERGENIC
5366	CakSNP5366	*Kabuli*	Ca_Kabuli_Chr04	4630407	[G/A]	Ca_03793	ASCH domain	NON_SYNONYMOUS_CODING
5367	CakSNP5367	*Kabuli*	Ca_Kabuli_Chr04	4630419	[A/C]	Ca_03793	ASCH domain	NON_SYNONYMOUS_CODING
5368	CakSNP5368	*Kabuli*	Ca_Kabuli_Chr04	4630514	[T/C]	Ca_03793	ASCH domain	SYNONYMOUS_CODING
5369	CakSNP5369	*Kabuli*	Ca_Kabuli_Chr04	4633913	[T/G]	Ca_03792	--	SYNONYMOUS_CODING
5370	CakSNP5370	*Kabuli*	Ca_Kabuli_Chr04	4634542	[A/T]	Ca_03792	--	INTRON
5371	CakSNP5371	*Kabuli*	Ca_Kabuli_Chr04	4660220	[A/G]		--	INTERGENIC

S.N.	SNP IDs	Chickpea cultivars	Chromosomes	Physical positions (bp)	SNPs	Gene accession IDs	Putative functions	Sequence components of genome
5372	CakSNP5372	*Kabuli*	Ca_Kabuli_Chr04	4670913	[A/G]	Ca_03789	EF-Hand 1, calcium-binding site	INTRON
5373	CakSNP5373	*Kabuli*	Ca_Kabuli_Chr04	4670975	[C/A]	Ca_03789	EF-Hand 1, calcium-binding site	INTRON
5374	CakSNP5374	*Kabuli*	Ca_Kabuli_Chr04	4675182	[T/G]		--	INTERGENIC
5375	CakSNP5375	*Kabuli*	Ca_Kabuli_Chr04	4675177	[T/A]		--	INTERGENIC
5376	CakSNP5376	*Kabuli*	Ca_Kabuli_Chr04	4675176	[T/G]		--	INTERGENIC
5377	CakSNP5377	*Kabuli*	Ca_Kabuli_Chr04	4675172	[T/A]		--	INTERGENIC
5378	CakSNP5378	*Kabuli*	Ca_Kabuli_Chr04	4675171	[T/A]		--	INTERGENIC
5379	CakSNP5379	*Kabuli*	Ca_Kabuli_Chr04	4675166	[A/T]		--	INTERGENIC
5380	CakSNP5380	*Kabuli*	Ca_Kabuli_Chr04	4679236	[A/G]	Ca_03788	Pentatricopeptide repeat	SYNONYMOUS_CODING
5381	CakSNP5381	*Kabuli*	Ca_Kabuli_Chr04	4707682	[A/G]	Ca_03785	Aromatic amino acid beta-eliminating lyase/threonine aldolase	INTRON

S.N.	SNP IDs	Chickpea cultivars	Chromosomes	Physical positions (bp)	SNPs	Gene accession IDs	Putative functions	Sequence components of genome
5382	CakSNP5382	*Kabuli*	Ca_Kabuli_Chr04	4722348	[G/A]	Ca_03784	RNA polymerase III Rpc4	INTRON
5383	CakSNP5383	*Kabuli*	Ca_Kabuli_Chr04	4757357	[T/C]	Ca_03781	SET domain	SYNONYMOUS_CODING
5384	CakSNP5384	*Kabuli*	Ca_Kabuli_Chr04	4757416	[G/A]	Ca_03781	SET domain	NON_SYNONYMOUS_CODING
5385	CakSNP5385	*Kabuli*	Ca_Kabuli_Chr04	4757498	[C/G]	Ca_03781	SET domain	INTRON
5386	CakSNP5386	*Kabuli*	Ca_Kabuli_Chr04	4794122	[G/A]	Ca_03778	SNF2-related	INTRON
5387	CakSNP5387	*Kabuli*	Ca_Kabuli_Chr04	4794192	[G/C]	Ca_03778	SNF2-related	INTRON
5388	CakSNP5388	*Kabuli*	Ca_Kabuli_Chr04	4799122	[C/T]	Ca_03778	SNF2-related	NON_SYNONYMOUS_CODING
5389	CakSNP5389	*Kabuli*	Ca_Kabuli_Chr04	4799116	[C/T]	Ca_03778	SNF2-related	NON_SYNONYMOUS_CODING
5390	CakSNP5390	*Kabuli*	Ca_Kabuli_Chr04	4803168	[T/A]	Ca_03778	SNF2-related	INTRON
5391	CakSNP5391	*Kabuli*	Ca_Kabuli_Chr04	4803213	[C/T]	Ca_03778	SNF2-related	INTRON

S.N.	SNP IDs	Chickpea cultivars	Chromosomes	Physical positions (bp)	SNPs	Gene accession IDs	Putative functions	Sequence components of genome
5392	CakSNP5392	*Kabuli*	Ca_Kabuli_Chr04	4874780	[A/T]		--	INTERGENIC
5393	CakSNP5393	*Kabuli*	Ca_Kabuli_Chr04	4874782	[C/A]		--	INTERGENIC
5394	CakSNP5394	*Kabuli*	Ca_Kabuli_Chr04	4874836	[C/T]		--	INTERGENIC
5395	CakSNP5395	*Kabuli*	Ca_Kabuli_Chr04	4882813	[C/G]	Ca_03769	IQ motif, EF-hand binding site	INTRON
5396	CakSNP5396	*Kabuli*	Ca_Kabuli_Chr04	4897934	[C/G]		--	INTERGENIC
5397	CakSNP5397	*Kabuli*	Ca_Kabuli_Chr04	4907987	[G/A]		--	INTERGENIC
5398	CakSNP5398	*Kabuli*	Ca_Kabuli_Chr04	4911083	[T/C]	Ca_03767	Phosphoribosylaminoimidazole carboxylase, core	INTRON
5399	CakSNP5399	*Kabuli*	Ca_Kabuli_Chr04	4911130	[T/C]	Ca_03767	Phosphoribosylaminoimidazole carboxylase, core	INTRON
5400	CakSNP5400	*Kabuli*	Ca_Kabuli_Chr04	4911209	[A/G]	Ca_03767	Phosphoribosylaminoimidazole carboxylase, core	SYNONYMOUS_CODING
5401	CakSNP5401	*Kabuli*	Ca_Kabuli_Chr04	4911217	[A/C]	Ca_03767	Phosphoribosylaminoimidazole carboxylase, core	SYNONYMOUS_CODING

S.N.	SNP IDs	Chickpea cultivars	Chromosomes	Physical positions (bp)	SNPs	Gene accession IDs	Putative functions	Sequence components of genome
5402	CakSNP5402	*Kabuli*	Ca_Kabuli_Chr04	4920706	[T/C]	Ca_03766	Zinc finger, PMZ-type	NON_SYNONYMOUS_CODING
5403	CakSNP5403	*Kabuli*	Ca_Kabuli_Chr04	4920712	[A/T]	Ca_03766	Zinc finger, PMZ-type	NON_SYNONYMOUS_CODING
5404	CakSNP5404	*Kabuli*	Ca_Kabuli_Chr04	4920719	[A/G]	Ca_03766	Zinc finger, PMZ-type	NON_SYNONYMOUS_CODING
5405	CakSNP5405	*Kabuli*	Ca_Kabuli_Chr04	4922290	[A/C]		--	INTERGENIC
5406	CakSNP5406	*Kabuli*	Ca_Kabuli_Chr04	4928764	[T/C]		--	INTERGENIC
5407	CakSNP5407	*Kabuli*	Ca_Kabuli_Chr04	4928890	[A/G]		--	INTERGENIC
5408	CakSNP5408	*Kabuli*	Ca_Kabuli_Chr04	4929387	[T/C]		--	INTERGENIC
5409	CakSNP5409	*Kabuli*	Ca_Kabuli_Chr04	4953629	[C/A]	Ca_03763	Histone H1/H6	NON_SYNONYMOUS_CODING
5410	CakSNP5410	*Kabuli*	Ca_Kabuli_Chr04	4967336	[T/C]		--	INTERGENIC
5411	CakSNP5411	*Kabuli*	Ca_Kabuli_Chr04	4967342	[G/A]		--	INTERGENIC

S.N.	SNP IDs	Chickpea cultivars	Chromosomes	Physical positions (bp)	SNPs	Gene accession IDs	Putative functions	Sequence components of genome
5412	CakSNP5412	*Kabuli*	Ca_Kabuli_Chr04	4967347	[A/G]		--	INTERGENIC
5413	CakSNP5413	*Kabuli*	Ca_Kabuli_Chr04	5018591	[G/A]	Ca_03753	Basic-leucine zipper (bZIP) transcription factor	SYNONYMOUS_CODING
5414	CakSNP5414	*Kabuli*	Ca_Kabuli_Chr04	5038300	[G/C]		--	INTERGENIC
5415	CakSNP5415	*Kabuli*	Ca_Kabuli_Chr04	5051617	[A/G]	Ca_03750	--	SYNONYMOUS_CODING
5416	CakSNP5416	*Kabuli*	Ca_Kabuli_Chr04	5051574	[T/G]	Ca_03750	--	INTRON
5417	CakSNP5417	*Kabuli*	Ca_Kabuli_Chr04	5051573	[A/C]	Ca_03750	--	INTRON
5418	CakSNP5418	*Kabuli*	Ca_Kabuli_Chr04	5086705	[T/C]		--	INTERGENIC
5419	CakSNP5419	*Kabuli*	Ca_Kabuli_Chr04	5120207	[C/T]	Ca_03742	Amine oxidase	SYNONYMOUS_CODING
5420	CakSNP5420	*Kabuli*	Ca_Kabuli_Chr04	5186401	[A/G]	Ca_03734	Pyridoxal phosphate-dependent enzyme, beta subunit	NON_SYNONYMOUS_CODING
5421	CakSNP5421	*Kabuli*	Ca_Kabuli_Chr04	5187402	[G/C]	Ca_03734	Pyridoxal phosphate-dependent enzyme, beta subunit	INTRON

S.N.	SNP IDs	Chickpea cultivars	Chromosomes	Physical positions (bp)	SNPs	Gene accession IDs	Putative functions	Sequence components of genome
5422	CakSNP5422	*Kabuli*	Ca_Kabuli_Chr04	5187344	[C/G]	Ca_03734	Pyridoxal phosphate-dependent enzyme, beta subunit	INTRON
5423	CakSNP5423	*Kabuli*	Ca_Kabuli_Chr04	5188084	[G/A]	Ca_03734	Pyridoxal phosphate-dependent enzyme, beta subunit	SYNONYMOUS_CODING
5424	CakSNP5424	*Kabuli*	Ca_Kabuli_Chr04	5188040	[A/G]	Ca_03734	Pyridoxal phosphate-dependent enzyme, beta subunit	INTRON
5425	CakSNP5425	*Kabuli*	Ca_Kabuli_Chr04	5197824	[T/C]	Ca_03733	Domain of unknown function DUF1981, SEC7 associated	INTRON
5426	CakSNP5426	*Kabuli*	Ca_Kabuli_Chr04	5199907	[C/T]	Ca_03733	Domain of unknown function DUF1981, SEC7 associated	INTRON
5427	CakSNP5427	*Kabuli*	Ca_Kabuli_Chr04	5222669	[A/C]	Ca_03733	Domain of unknown function DUF1981, SEC7 associated	SYNONYMOUS_CODING
5428	CakSNP5428	*Kabuli*	Ca_Kabuli_Chr04	5222805	[G/C]	Ca_03733	Domain of unknown function DUF1981, SEC7 associated	INTRON
5429	CakSNP5429	*Kabuli*	Ca_Kabuli_Chr04	5222851	[C/T]	Ca_03733	Domain of unknown function DUF1981, SEC7 associated	INTRON
5430	CakSNP5430	*Kabuli*	Ca_Kabuli_Chr04	5223100	[G/A]	Ca_03733	Domain of unknown function DUF1981, SEC7 associated	INTRON
5431	CakSNP5431	*Kabuli*	Ca_Kabuli_Chr04	5223206	[A/T]	Ca_03733	Domain of unknown function DUF1981, SEC7 associated	INTRON

S.N.	SNP IDs	Chickpea cultivars	Chromosomes	Physical positions (bp)	SNPs	Gene accession IDs	Putative functions	Sequence components of genome
5432	CakSNP5432	*Kabuli*	Ca_Kabuli_Chr04	5228950	[G/T]	Ca_03733	Domain of unknown function DUF1981, SEC7 associated	INTRON
5433	CakSNP5433	*Kabuli*	Ca_Kabuli_Chr04	5232364	[T/C]	Ca_03732	Seed maturation protein	NON_SYNONYMOUS_CODING
5434	CakSNP5434	*Kabuli*	Ca_Kabuli_Chr04	5263199	[A/G]	Ca_03729	Kinesin, motor domain	SYNONYMOUS_CODING
5435	CakSNP5435	*Kabuli*	Ca_Kabuli_Chr04	5268995	[A/C]		--	INTERGENIC
5436	CakSNP5436	*Kabuli*	Ca_Kabuli_Chr04	5282131	[T/C]		--	INTERGENIC
5437	CakSNP5437	*Kabuli*	Ca_Kabuli_Chr04	5310873	[A/G]	Ca_03726	--	SYNONYMOUS_CODING
5438	CakSNP5438	*Kabuli*	Ca_Kabuli_Chr04	5345520	[T/G]	Ca_03723	Uncharacterised protein family, transmembrane-40	NON_SYNONYMOUS_CODING
5439	CakSNP5439	*Kabuli*	Ca_Kabuli_Chr04	5377067	[A/C]	Ca_03720	Heat shock protein DnaJ, N-terminal	SYNONYMOUS_CODING
5440	CakSNP5440	*Kabuli*	Ca_Kabuli_Chr04	5377400	[C/T]	Ca_03720	Heat shock protein DnaJ, N-terminal	SYNONYMOUS_CODING
5441	CakSNP5441	*Kabuli*	Ca_Kabuli_Chr04	5390236	[C/A]	Ca_03718	--	SYNONYMOUS_CODING

S.N.	SNP IDs	Chickpea cultivars	Chromosomes	Physical positions (bp)	SNPs	Gene accession IDs	Putative functions	Sequence components of genome
5442	CakSNP5442	*Kabuli*	Ca_Kabuli_Chr04	5390317	[G/A]	Ca_03718	--	SYNONYMOUS_CODING
5443	CakSNP5443	*Kabuli*	Ca_Kabuli_Chr04	5453008	[A/C]	Ca_03707	--	INTRON
5444	CakSNP5444	*Kabuli*	Ca_Kabuli_Chr04	5473174	[C/T]		--	INTERGENIC
5445	CakSNP5445	*Kabuli*	Ca_Kabuli_Chr04	5484259	[G/T]		--	INTERGENIC
5446	CakSNP5446	*Kabuli*	Ca_Kabuli_Chr04	5484257	[G/C]		--	INTERGENIC
5447	CakSNP5447	*Kabuli*	Ca_Kabuli_Chr04	5484238	[G/A]		--	INTERGENIC
5448	CakSNP5448	*Kabuli*	Ca_Kabuli_Chr04	5484236	[C/T]		--	INTERGENIC
5449	CakSNP5449	*Kabuli*	Ca_Kabuli_Chr04	5484220	[A/G]		--	INTERGENIC
5450	CakSNP5450	*Kabuli*	Ca_Kabuli_Chr04	5485052	[G/T]		--	INTERGENIC
5451	CakSNP5451	*Kabuli*	Ca_Kabuli_Chr04	5485050	[G/C]		--	INTERGENIC

S.N.	SNP IDs	Chickpea cultivars	Chromosomes	Physical positions (bp)	SNPs	Gene accession IDs	Putative functions	Sequence components of genome
5452	CakSNP5452	*Kabuli*	Ca_Kabuli_Chr04	5485031	[G/A]		--	INTERGENIC
5453	CakSNP5453	*Kabuli*	Ca_Kabuli_Chr04	5485029	[C/T]		--	INTERGENIC
5454	CakSNP5454	*Kabuli*	Ca_Kabuli_Chr04	5485013	[A/G]		--	INTERGENIC
5455	CakSNP5455	*Kabuli*	Ca_Kabuli_Chr04	5528421	[T/G]	Ca_03702	Zinc finger, RING-type	SYNONYMOUS_CODING
5456	CakSNP5456	*Kabuli*	Ca_Kabuli_Chr04	5530612	[A/G]		--	INTERGENIC
5457	CakSNP5457	*Kabuli*	Ca_Kabuli_Chr04	5535661	[A/G]	Ca_03701	DNA repair protein rad10	INTRON
5458	CakSNP5458	*Kabuli*	Ca_Kabuli_Chr04	5575846	[G/C]	Ca_03697	Heat shock protein Hsp20	SYNONYMOUS_CODING
5459	CakSNP5459	*Kabuli*	Ca_Kabuli_Chr04	5595079	[T/C]	Ca_03692	Short-chain dehydrogenase/reductase SDR	SYNONYMOUS_CODING
5460	CakSNP5460	*Kabuli*	Ca_Kabuli_Chr04	5595077	[T/A]	Ca_03692	Short-chain dehydrogenase/reductase SDR	NON_SYNONYMOUS_CODING
5461	CakSNP5461	*Kabuli*	Ca_Kabuli_Chr04	5623571	[A/G]	Ca_03688	Ras GTPase	INTRON

S.N.	SNP IDs	Chickpea cultivars	Chromosomes	Physical positions (bp)	SNPs	Gene accession IDs	Putative functions	Sequence components of genome
5462	CakSNP5462	*Kabuli*	Ca_Kabuli_Chr04	5627096	[C/T]		--	INTERGENIC
5463	CakSNP5463	*Kabuli*	Ca_Kabuli_Chr04	5701873	[A/C]		--	INTERGENIC
5464	CakSNP5464	*Kabuli*	Ca_Kabuli_Chr04	5701900	[G/T]		--	INTERGENIC
5465	CakSNP5465	*Kabuli*	Ca_Kabuli_Chr04	5701902	[C/T]		--	INTERGENIC
5466	CakSNP5466	*Kabuli*	Ca_Kabuli_Chr04	5701907	[G/T]		--	INTERGENIC
5467	CakSNP5467	*Kabuli*	Ca_Kabuli_Chr04	5779698	[A/G]		--	INTERGENIC
5468	CakSNP5468	*Kabuli*	Ca_Kabuli_Chr04	5779765	[A/T]		--	INTERGENIC
5469	CakSNP5469	*Kabuli*	Ca_Kabuli_Chr04	5787727	[G/A]		--	INTERGENIC
5470	CakSNP5470	*Kabuli*	Ca_Kabuli_Chr04	5787722	[G/A]		--	INTERGENIC
5471	CakSNP5471	*Kabuli*	Ca_Kabuli_Chr04	5841444	[C/G]	Ca_03665	Pathogenesis-related transcriptional factor/ERF, DNA-binding	NON_SYNONYMOUS_CODING

S.N.	SNP IDs	Chickpea cultivars	Chromosomes	Physical positions (bp)	SNPs	Gene accession IDs	Putative functions	Sequence components of genome
5472	CakSNP5472	*Kabuli*	Ca_Kabuli_Chr04	5841462	[A/C]	Ca_03665	Pathogenesis-related transcriptional factor/ERF, DNA-binding	NON_SYNONYMOUS_CODING
5473	CakSNP5473	*Kabuli*	Ca_Kabuli_Chr04	5853789	[C/T]	Ca_03664	Zinc finger, RING-type	INTRON
5474	CakSNP5474	*Kabuli*	Ca_Kabuli_Chr04	5853798	[T/G]	Ca_03664	Zinc finger, RING-type	INTRON
5475	CakSNP5475	*Kabuli*	Ca_Kabuli_Chr04	5900194	[C/A]		--	INTERGENIC
5476	CakSNP5476	*Kabuli*	Ca_Kabuli_Chr04	5900290	[T/C]		--	INTERGENIC
5477	CakSNP5477	*Kabuli*	Ca_Kabuli_Chr04	5905252	[C/T]		--	INTERGENIC
5478	CakSNP5478	*Kabuli*	Ca_Kabuli_Chr04	5905276	[C/T]		--	INTERGENIC
5479	CakSNP5479	*Kabuli*	Ca_Kabuli_Chr04	5905303	[A/C]		--	INTERGENIC
5480	CakSNP5480	*Kabuli*	Ca_Kabuli_Chr04	5905385	[G/A]	Ca_03659	Ribosomal protein L22e	NON_SYNONYMOUS_CODING
5481	CakSNP5481	*Kabuli*	Ca_Kabuli_Chr04	5907409	[T/G]		--	INTERGENIC

S.N.	SNP IDs	Chickpea cultivars	Chromosomes	Physical positions (bp)	SNPs	Gene accession IDs	Putative functions	Sequence components of genome
5482	CakSNP5482	*Kabuli*	Ca_Kabuli_Chr04	5907421	[T/G]		--	INTERGENIC
5483	CakSNP5483	*Kabuli*	Ca_Kabuli_Chr04	5907437	[C/A]		--	INTERGENIC
5484	CakSNP5484	*Kabuli*	Ca_Kabuli_Chr04	5907465	[C/A]		--	INTERGENIC
5485	CakSNP5485	*Kabuli*	Ca_Kabuli_Chr04	5940176	[C/T]	Ca_03654	Pectinesterase, catalytic	SYNONYMOUS_CODING
5486	CakSNP5486	*Kabuli*	Ca_Kabuli_Chr04	5999964	[T/C]	Ca_03648	WD40 repeat	INTRON
5487	CakSNP5487	*Kabuli*	Ca_Kabuli_Chr04	6000010	[T/A]	Ca_03648	WD40 repeat	INTRON
5488	CakSNP5488	*Kabuli*	Ca_Kabuli_Chr04	6000013	[A/C]	Ca_03648	WD40 repeat	INTRON
5489	CakSNP5489	*Kabuli*	Ca_Kabuli_Chr04	6000037	[T/G]	Ca_03648	WD40 repeat	INTRON
5490	CakSNP5490	*Kabuli*	Ca_Kabuli_Chr04	6006849	[T/G]		--	INTERGENIC
5491	CakSNP5491	*Kabuli*	Ca_Kabuli_Chr04	6030948	[T/G]		--	INTERGENIC

S.N.	SNP IDs	Chickpea cultivars	Chromosomes	Physical positions (bp)	SNPs	Gene accession IDs	Putative functions	Sequence components of genome
5492	CakSNP5492	*Kabuli*	Ca_Kabuli_Chr04	6030966	[A/G]		--	INTERGENIC
5493	CakSNP5493	*Kabuli*	Ca_Kabuli_Chr04	6041134	[A/C]	Ca_03643	Drought induced 19/ RING finger protein 114	INTRON
5494	CakSNP5494	*Kabuli*	Ca_Kabuli_Chr04	6041130	[C/A]	Ca_03643	Drought induced 19/ RING finger protein 114	INTRON
5495	CakSNP5495	*Kabuli*	Ca_Kabuli_Chr04	6041126	[A/G]	Ca_03643	Drought induced 19/ RING finger protein 114	INTRON
5496	CakSNP5496	*Kabuli*	Ca_Kabuli_Chr04	6041120	[G/T]	Ca_03643	Drought induced 19/ RING finger protein 114	INTRON
5497	CakSNP5497	*Kabuli*	Ca_Kabuli_Chr04	6041117	[C/T]	Ca_03643	Drought induced 19/ RING finger protein 114	INTRON
5498	CakSNP5498	*Kabuli*	Ca_Kabuli_Chr04	6076530	[T/C]		--	INTERGENIC
5499	CakSNP5499	*Kabuli*	Ca_Kabuli_Chr04	6076594	[G/C]		--	INTERGENIC
5500	CakSNP5500	*Kabuli*	Ca_Kabuli_Chr04	6076614	[A/G]		--	INTERGENIC
5501	CakSNP5501	*Kabuli*	Ca_Kabuli_Chr04	6077103	[G/A]		--	INTERGENIC

S.N.	SNP IDs	Chickpea cultivars	Chromosomes	Physical positions (bp)	SNPs	Gene accession IDs	Putative functions	Sequence components of genome
5502	CakSNP5502	*Kabuli*	Ca_Kabuli_Chr04	6077113	[G/T]		--	INTERGENIC
5503	CakSNP5503	*Kabuli*	Ca_Kabuli_Chr04	6077156	[G/T]		--	INTERGENIC
5504	CakSNP5504	*Kabuli*	Ca_Kabuli_Chr04	6077168	[T/C]		--	INTERGENIC
5505	CakSNP5505	*Kabuli*	Ca_Kabuli_Chr04	6157625	[T/C]	Ca_03628	Peptidase S16, Lon protease, C-terminal	NON_SYNONYMOUS_CODING
5506	CakSNP5506	*Kabuli*	Ca_Kabuli_Chr04	6201137	[T/C]		--	INTERGENIC
5507	CakSNP5507	*Kabuli*	Ca_Kabuli_Chr04	6238354	[G/A]	Ca_03621	High mobility group, HMG1/HMG2	SYNONYMOUS_CODING
5508	CakSNP5508	*Kabuli*	Ca_Kabuli_Chr04	6238367	[T/A]	Ca_03621	High mobility group, HMG1/HMG2	NON_SYNONYMOUS_CODING
5509	CakSNP5509	*Kabuli*	Ca_Kabuli_Chr04	6238376	[A/G]	Ca_03621	High mobility group, HMG1/HMG2	NON_SYNONYMOUS_CODING
5510	CakSNP5510	*Kabuli*	Ca_Kabuli_Chr04	6238414	[T/A]	Ca_03621	High mobility group, HMG1/HMG2	SYNONYMOUS_CODING
5511	CakSNP5511	*Kabuli*	Ca_Kabuli_Chr04	6277398	[C/T]	Ca_03616	Rhodanese-like	NON_SYNONYMOUS_CODING

S.N.	SNP IDs	Chickpea cultivars	Chromosomes	Physical positions (bp)	SNPs	Gene accession IDs	Putative functions	Sequence components of genome
5512	CakSNP5512	*Kabuli*	Ca_Kabuli_Chr04	6281228	[A/C]	Ca_03615	Peptidase M24, structural domain	INTRON
5513	CakSNP5513	*Kabuli*	Ca_Kabuli_Chr04	6296089	[G/T]		--	INTERGENIC
5514	CakSNP5514	*Kabuli*	Ca_Kabuli_Chr04	6387938	[T/G]	Ca_03602	X8	NON_SYNONYMOUS_CODING
5515	CakSNP5515	*Kabuli*	Ca_Kabuli_Chr04	6403910	[A/C]		--	INTERGENIC
5516	CakSNP5516	*Kabuli*	Ca_Kabuli_Chr04	6408543	[T/G]		--	INTERGENIC
5517	CakSNP5517	*Kabuli*	Ca_Kabuli_Chr04	6415058	[A/T]		--	INTERGENIC
5518	CakSNP5518	*Kabuli*	Ca_Kabuli_Chr04	6462228	[C/T]	Ca_03597	Peptidase A1	INTRON
5519	CakSNP5519	*Kabuli*	Ca_Kabuli_Chr04	6507540	[G/A]	Ca_03591	Helix-loop-helix DNA-binding domain	SYNONYMOUS_CODING
5520	CakSNP5520	*Kabuli*	Ca_Kabuli_Chr04	6509701	[T/A]	Ca_03591	Helix-loop-helix DNA-binding domain	INTRON
5521	CakSNP5521	*Kabuli*	Ca_Kabuli_Chr04	6509761	[C/A]	Ca_03591	Helix-loop-helix DNA-binding domain	INTRON

S.N.	SNP IDs	Chickpea cultivars	Chromosomes	Physical positions (bp)	SNPs	Gene accession IDs	Putative functions	Sequence components of genome
5522	CakSNP5522	*Kabuli*	Ca_Kabuli_Chr04	6511977	[C/T]	Ca_03590	--	SYNONYMOUS_CODING
5523	CakSNP5523	*Kabuli*	Ca_Kabuli_Chr04	6512060	[A/T]	Ca_03590	--	NON_SYNONYMOUS_CODING
5524	CakSNP5524	*Kabuli*	Ca_Kabuli_Chr04	6513321	[A/T]	Ca_03590	--	NON_SYNONYMOUS_CODING
5525	CakSNP5525	*Kabuli*	Ca_Kabuli_Chr04	6513493	[C/T]	Ca_03590	--	SYNONYMOUS_CODING
5526	CakSNP5526	*Kabuli*	Ca_Kabuli_Chr04	6513576	[A/T]	Ca_03590	--	NON_SYNONYMOUS_CODING
5527	CakSNP5527	*Kabuli*	Ca_Kabuli_Chr04	6544385	[G/A]		--	INTERGENIC
5528	CakSNP5528	*Kabuli*	Ca_Kabuli_Chr04	6544342	[A/C]		--	INTERGENIC
5529	CakSNP5529	*Kabuli*	Ca_Kabuli_Chr04	6544337	[A/C]		--	INTERGENIC
5530	CakSNP5530	*Kabuli*	Ca_Kabuli_Chr04	6552776	[A/G]	Ca_03588	NIF system FeS cluster assembly, NifU, C-terminal	INTRON
5531	CakSNP5531	*Kabuli*	Ca_Kabuli_Chr04	6562373	[T/A]	Ca_03588	NIF system FeS cluster assembly, NifU, C-terminal	INTRON

S.N.	SNP IDs	Chickpea cultivars	Chromosomes	Physical positions (bp)	SNPs	Gene accession IDs	Putative functions	Sequence components of genome
5532	CakSNP5532	*Kabuli*	Ca_Kabuli_Chr04	6562391	[A/C]	Ca_03588	NIF system FeS cluster assembly, NifU, C-terminal	INTRON
5533	CakSNP5533	*Kabuli*	Ca_Kabuli_Chr04	6562402	[G/C]	Ca_03588	NIF system FeS cluster assembly, NifU, C-terminal	INTRON
5534	CakSNP5534	*Kabuli*	Ca_Kabuli_Chr04	6611820	[G/A]	Ca_03585	5'-3' exonuclease, N-terminal	INTRON
5535	CakSNP5535	*Kabuli*	Ca_Kabuli_Chr04	6616016	[C/T]		--	INTERGENIC
5536	CakSNP5536	*Kabuli*	Ca_Kabuli_Chr04	6616071	[C/T]		--	INTERGENIC
5537	CakSNP5537	*Kabuli*	Ca_Kabuli_Chr04	6688218	[G/A]		--	INTERGENIC
5538	CakSNP5538	*Kabuli*	Ca_Kabuli_Chr04	6715326	[C/T]		--	INTERGENIC
5539	CakSNP5539	*Kabuli*	Ca_Kabuli_Chr04	6723103	[G/T]	Ca_03571	Protein phosphatase 2C-like	INTRON
5540	CakSNP5540	*Kabuli*	Ca_Kabuli_Chr04	6749334	[A/G]	Ca_03567	ABC-1	INTRON
5541	CakSNP5541	*Kabuli*	Ca_Kabuli_Chr04	6749481	[C/T]	Ca_03567	ABC-1	INTRON

S.N.	SNP IDs	Chickpea cultivars	Chromosomes	Physical positions (bp)	SNPs	Gene accession IDs	Putative functions	Sequence components of genome
5542	CakSNP5542	*Kabuli*	Ca_Kabuli_Chr04	6749550	[G/A]	Ca_03567	ABC-1	INTRON
5543	CakSNP5543	*Kabuli*	Ca_Kabuli_Chr04	6749522	[T/C]	Ca_03567	ABC-1	INTRON
5544	CakSNP5544	*Kabuli*	Ca_Kabuli_Chr04	6751549	[A/T]		--	INTERGENIC
5545	CakSNP5545	*Kabuli*	Ca_Kabuli_Chr04	6761778	[C/A]	Ca_03565	Pectinacetylesterase	STOP_GAINED
5546	CakSNP5546	*Kabuli*	Ca_Kabuli_Chr04	6765922	[T/G]	Ca_03564	SANT domain, DNA binding	NON_SYNONYMOUS_CODING
5547	CakSNP5547	*Kabuli*	Ca_Kabuli_Chr04	6765884	[T/C]	Ca_03564	SANT domain, DNA binding	NON_SYNONYMOUS_CODING
5548	CakSNP5548	*Kabuli*	Ca_Kabuli_Chr04	6792858	[T/G]	Ca_03562	Cytochrome P450	NON_SYNONYMOUS_CODING
5549	CakSNP5549	*Kabuli*	Ca_Kabuli_Chr04	6801632	[C/A]	Ca_03560	--	INTRON
5550	CakSNP5550	*Kabuli*	Ca_Kabuli_Chr04	6841854	[G/A]		--	INTERGENIC
5551	CakSNP5551	*Kabuli*	Ca_Kabuli_Chr04	6841856	[G/A]		--	INTERGENIC

S.N.	SNP IDs	Chickpea cultivars	Chromosomes	Physical positions (bp)	SNPs	Gene accession IDs	Putative functions	Sequence components of genome
5552	CakSNP5552	*Kabuli*	Ca_Kabuli_Chr04	6841858	[G/A]		--	INTERGENIC
5553	CakSNP5553	*Kabuli*	Ca_Kabuli_Chr04	6841859	[A/C]		--	INTERGENIC
5554	CakSNP5554	*Kabuli*	Ca_Kabuli_Chr04	6841860	[G/A]		--	INTERGENIC
5555	CakSNP5555	*Kabuli*	Ca_Kabuli_Chr04	6841861	[A/C]		--	INTERGENIC
5556	CakSNP5556	*Kabuli*	Ca_Kabuli_Chr04	6843659	[T/C]		--	INTERGENIC
5557	CakSNP5557	*Kabuli*	Ca_Kabuli_Chr04	6852908	[T/C]	Ca_03554	ABC transporter, transmembrane domain	SYNONYMOUS_CODING
5558	CakSNP5558	*Kabuli*	Ca_Kabuli_Chr04	6889964	[T/G]	Ca_03550	Pectinesterase, catalytic	NON_SYNONYMOUS_CODING
5559	CakSNP5559	*Kabuli*	Ca_Kabuli_Chr04	6889974	[T/G]	Ca_03550	Pectinesterase, catalytic	NON_SYNONYMOUS_CODING
5560	CakSNP5560	*Kabuli*	Ca_Kabuli_Chr04	6890000	[A/G]	Ca_03550	Pectinesterase, catalytic	SYNONYMOUS_CODING
5561	CakSNP5561	*Kabuli*	Ca_Kabuli_Chr04	6890005	[C/T]	Ca_03550	Pectinesterase, catalytic	NON_SYNONYMOUS_CODING

S.N.	SNP IDs	Chickpea cultivars	Chromosomes	Physical positions (bp)	SNPs	Gene accession IDs	Putative functions	Sequence components of genome
5562	CakSNP5562	*Kabuli*	Ca_Kabuli_Chr04	6890014	[G/T]	Ca_03550	Pectinesterase, catalytic	NON_SYNONYMOUS_CODING
5563	CakSNP5563	*Kabuli*	Ca_Kabuli_Chr04	6889994	[T/G]	Ca_03550	Pectinesterase, catalytic	NON_SYNONYMOUS_CODING
5564	CakSNP5564	*Kabuli*	Ca_Kabuli_Chr04	6895804	[G/A]	Ca_03549	Protein kinase, catalytic domain	SYNONYMOUS_CODING
5565	CakSNP5565	*Kabuli*	Ca_Kabuli_Chr04	6919896	[C/T]	Ca_03548	Transcription factor, SBP-box	NON_SYNONYMOUS_CODING
5566	CakSNP5566	*Kabuli*	Ca_Kabuli_Chr04	6920068	[G/A]	Ca_03548	Transcription factor, SBP-box	INTRON
5567	CakSNP5567	*Kabuli*	Ca_Kabuli_Chr04	6931668	[T/A]		--	INTERGENIC
5568	CakSNP5568	*Kabuli*	Ca_Kabuli_Chr04	6960336	[G/A]	Ca_03541	Glycoside hydrolase, family 28	INTRON
5569	CakSNP5569	*Kabuli*	Ca_Kabuli_Chr04	6969771	[G/T]		--	INTERGENIC
5570	CakSNP5570	*Kabuli*	Ca_Kabuli_Chr04	6969799	[T/C]		--	INTERGENIC
5571	CakSNP5571	*Kabuli*	Ca_Kabuli_Chr04	6970808	[A/G]		--	INTERGENIC

S.N.	SNP IDs	Chickpea cultivars	Chromosomes	Physical positions (bp)	SNPs	Gene accession IDs	Putative functions	Sequence components of genome
5572	CakSNP5572	*Kabuli*	Ca_Kabuli_Chr04	6970800	[A/G]		--	INTERGENIC
5573	CakSNP5573	*Kabuli*	Ca_Kabuli_Chr04	6970792	[T/A]		--	INTERGENIC
5574	CakSNP5574	*Kabuli*	Ca_Kabuli_Chr04	6970781	[G/A]		--	INTERGENIC
5575	CakSNP5575	*Kabuli*	Ca_Kabuli_Chr04	6970856	[T/C]		--	INTERGENIC
5576	CakSNP5576	*Kabuli*	Ca_Kabuli_Chr04	7013498	[A/G]	Ca_03533	Isopenicillin N synthase	SYNONYMOUS_CODING
5577	CakSNP5577	*Kabuli*	Ca_Kabuli_Chr04	7040229	[A/T]	Ca_03532	DNA methyltransferase 1-associated 1	INTRON
5578	CakSNP5578	*Kabuli*	Ca_Kabuli_Chr04	7142498	[G/A]	Ca_03522	Pentatricopeptide repeat	SYNONYMOUS_CODING
5579	CakSNP5579	*Kabuli*	Ca_Kabuli_Chr04	7143207	[G/T]	Ca_03522	Pentatricopeptide repeat	NON_SYNONYMOUS_CODING
5580	CakSNP5580	*Kabuli*	Ca_Kabuli_Chr04	7143214	[T/C]	Ca_03522	Pentatricopeptide repeat	NON_SYNONYMOUS_CODING
5581	CakSNP5581	*Kabuli*	Ca_Kabuli_Chr04	7143238	[C/T]	Ca_03522	Pentatricopeptide repeat	NON_SYNONYMOUS_CODING

S.N.	SNP IDs	Chickpea cultivars	Chromosomes	Physical positions (bp)	SNPs	Gene accession IDs	Putative functions	Sequence components of genome
5582	CakSNP5582	*Kabuli*	Ca_Kabuli_Chr04	7155826	[G/A]	Ca_03520	Protein kinase, catalytic domain	NON_SYNONYMOUS_CODING
5583	CakSNP5583	*Kabuli*	Ca_Kabuli_Chr04	7185974	[G/A]	Ca_03516	SANT domain, DNA binding	NON_SYNONYMOUS_CODING
5584	CakSNP5584	*Kabuli*	Ca_Kabuli_Chr04	7231299	[C/T]	Ca_03511	Vps51/Vps67	INTRON
5585	CakSNP5585	*Kabuli*	Ca_Kabuli_Chr04	7239917	[T/G]	Ca_03510	WD40 repeat	SYNONYMOUS_CODING
5586	CakSNP5586	*Kabuli*	Ca_Kabuli_Chr04	7244301	[G/A]	Ca_03510	WD40 repeat	INTRON
5587	CakSNP5587	*Kabuli*	Ca_Kabuli_Chr04	7335587	[C/T]	Ca_03503	BRCT	NON_SYNONYMOUS_CODING
5588	CakSNP5588	*Kabuli*	Ca_Kabuli_Chr04	7349958	[T/C]	Ca_03501	Lipase, class 3	NON_SYNONYMOUS_CODING
5589	CakSNP5589	*Kabuli*	Ca_Kabuli_Chr04	7349948	[T/C]	Ca_03501	Lipase, class 3	SYNONYMOUS_CODING
5590	CakSNP5590	*Kabuli*	Ca_Kabuli_Chr04	7349900	[G/A]	Ca_03501	Lipase, class 3	SYNONYMOUS_CODING
5591	CakSNP5591	*Kabuli*	Ca_Kabuli_Chr04	7349885	[G/A]	Ca_03501	Lipase, class 3	SYNONYMOUS_CODING

S.N.	SNP IDs	Chickpea cultivars	Chromosomes	Physical positions (bp)	SNPs	Gene accession IDs	Putative functions	Sequence components of genome
5592	CakSNP5592	*Kabuli*	Ca_Kabuli_Chr04	7350192	[G/T]	Ca_03501	Lipase, class 3	NON_SYNONYMOUS_CODING
5593	CakSNP5593	*Kabuli*	Ca_Kabuli_Chr04	7371667	[G/A]	Ca_03499	Exocyst complex subunit Sec15-like	SYNONYMOUS_CODING
5594	CakSNP5594	*Kabuli*	Ca_Kabuli_Chr04	7371708	[T/C]	Ca_03499	Exocyst complex subunit Sec15-like	NON_SYNONYMOUS_CODING
5595	CakSNP5595	*Kabuli*	Ca_Kabuli_Chr04	7416949	[C/T]		--	INTERGENIC
5596	CakSNP5596	*Kabuli*	Ca_Kabuli_Chr04	7496298	[A/G]	Ca_03486	Mitochondrial carrier protein	NON_SYNONYMOUS_CODING
5597	CakSNP5597	*Kabuli*	Ca_Kabuli_Chr04	7520182	[A/T]	Ca_03485	Calponin homology domain	INTRON
5598	CakSNP5598	*Kabuli*	Ca_Kabuli_Chr04	7520184	[A/C]	Ca_03485	Calponin homology domain	INTRON
5599	CakSNP5599	*Kabuli*	Ca_Kabuli_Chr04	7522047	[C/T]	Ca_03485	Calponin homology domain	SYNONYMOUS_CODING
5600	CakSNP5600	*Kabuli*	Ca_Kabuli_Chr04	7522033	[A/G]	Ca_03485	Calponin homology domain	INTRON
5601	CakSNP5601	*Kabuli*	Ca_Kabuli_Chr04	7587317	[A/G]	Ca_03477	--	NON_SYNONYMOUS_CODING

S.N.	SNP IDs	Chickpea cultivars	Chromosomes	Physical positions (bp)	SNPs	Gene accession IDs	Putative functions	Sequence components of genome
5602	CakSNP5602	*Kabuli*	Ca_Kabuli_Chr04	7587516	[T/C]	Ca_03477	--	NON_SYNONYMOUS_CODING
5603	CakSNP5603	*Kabuli*	Ca_Kabuli_Chr04	7598474	[G/C]	Ca_03475	Sucrose synthase	INTRON
5604	CakSNP5604	*Kabuli*	Ca_Kabuli_Chr04	7699429	[C/T]		--	INTERGENIC
5605	CakSNP5605	*Kabuli*	Ca_Kabuli_Chr04	7717770	[A/G]	Ca_03469	DNA-binding SAP	INTRON
5606	CakSNP5606	*Kabuli*	Ca_Kabuli_Chr04	7717935	[T/G]	Ca_03469	DNA-binding SAP	INTRON
5607	CakSNP5607	*Kabuli*	Ca_Kabuli_Chr04	7717978	[G/A]	Ca_03469	DNA-binding SAP	INTRON
5608	CakSNP5608	*Kabuli*	Ca_Kabuli_Chr04	7718270	[T/C]	Ca_03469	DNA-binding SAP	INTRON
5609	CakSNP5609	*Kabuli*	Ca_Kabuli_Chr04	7718277	[G/A]	Ca_03469	DNA-binding SAP	INTRON
5610	CakSNP5610	*Kabuli*	Ca_Kabuli_Chr04	7739202	[C/T]	Ca_03468	Spc97/Spc98	INTRON
5611	CakSNP5611	*Kabuli*	Ca_Kabuli_Chr04	7746230	[A/C]	Ca_03467	DNA photolyase, FAD-binding/Cryptochrome, C-terminal	INTRON

S.N.	SNP IDs	Chickpea cultivars	Chromosomes	Physical positions (bp)	SNPs	Gene accession IDs	Putative functions	Sequence components of genome
5612	CakSNP5612	*Kabuli*	Ca_Kabuli_Chr04	7746242	[C/G]	Ca_03467	DNA photolyase, FAD-binding/Cryptochrome, C-terminal	INTRON
5613	CakSNP5613	*Kabuli*	Ca_Kabuli_Chr04	7812468	[C/T]		--	INTERGENIC
5614	CakSNP5614	*Kabuli*	Ca_Kabuli_Chr04	7812491	[T/A]		--	INTERGENIC
5615	CakSNP5615	*Kabuli*	Ca_Kabuli_Chr04	7812521	[A/G]		--	INTERGENIC
5616	CakSNP5616	*Kabuli*	Ca_Kabuli_Chr04	7819865	[T/C]	Ca_03457	Glycoside hydrolase, family 1	INTRON
5617	CakSNP5617	*Kabuli*	Ca_Kabuli_Chr04	7821269	[G/A]	Ca_03457	Glycoside hydrolase, family 1	INTRON
5618	CakSNP5618	*Kabuli*	Ca_Kabuli_Chr04	7868480	[A/T]	Ca_03450	CS-like domain	INTRON
5619	CakSNP5619	*Kabuli*	Ca_Kabuli_Chr04	7883707	[T/A]	Ca_03448	DNA mismatch repair protein	NON_SYNONYMOUS_CODING
5620	CakSNP5620	*Kabuli*	Ca_Kabuli_Chr04	7892596	[A/C]	Ca_03447	ATPase, AAA+ type, core	INTRON
5621	CakSNP5621	*Kabuli*	Ca_Kabuli_Chr04	7975577	[C/G]		--	INTERGENIC

S.N.	SNP IDs	Chickpea cultivars	Chromosomes	Physical positions (bp)	SNPs	Gene accession IDs	Putative functions	Sequence components of genome
5622	CakSNP5622	*Kabuli*	Ca_Kabuli_Chr04	7975586	[T/G]		--	INTERGENIC
5623	CakSNP5623	*Kabuli*	Ca_Kabuli_Chr04	7988367	[A/G]		--	INTERGENIC
5624	CakSNP5624	*Kabuli*	Ca_Kabuli_Chr04	7988396	[C/T]		--	INTERGENIC
5625	CakSNP5625	*Kabuli*	Ca_Kabuli_Chr04	7988399	[G/A]		--	INTERGENIC
5626	CakSNP5626	*Kabuli*	Ca_Kabuli_Chr04	7988422	[T/G]		--	INTERGENIC
5627	CakSNP5627	*Kabuli*	Ca_Kabuli_Chr04	7988444	[T/G]		--	INTERGENIC
5628	CakSNP5628	*Kabuli*	Ca_Kabuli_Chr04	8025363	[T/C]	Ca_03430	F-box domain, cyclin-like	SYNONYMOUS_CODING
5629	CakSNP5629	*Kabuli*	Ca_Kabuli_Chr04	8025348	[A/T]	Ca_03430	F-box domain, cyclin-like	SYNONYMOUS_CODING
5630	CakSNP5630	*Kabuli*	Ca_Kabuli_Chr04	8025530	[A/G]	Ca_03430	F-box domain, cyclin-like	INTRON
5631	CakSNP5631	*Kabuli*	Ca_Kabuli_Chr04	8050770	[T/C]	Ca_03429	Tubby, C-terminal	NON_SYNONYMOUS_CODING

S.N.	SNP IDs	Chickpea cultivars	Chromosomes	Physical positions (bp)	SNPs	Gene accession IDs	Putative functions	Sequence components of genome
5632	CakSNP5632	*Kabuli*	Ca_Kabuli_Chr04	8051306	[T/C]	Ca_03429	Tubby, C-terminal	INTRON
5633	CakSNP5633	*Kabuli*	Ca_Kabuli_Chr04	8117401	[G/A]		--	INTERGENIC
5634	CakSNP5634	*Kabuli*	Ca_Kabuli_Chr04	8178043	[G/A]	Ca_08306	WD40 repeat	INTRON
5635	CakSNP5635	*Kabuli*	Ca_Kabuli_Chr04	8198702	[T/G]	Ca_08309	WD40 repeat	NON_SYNONYMOUS_CODING
5636	CakSNP5636	*Kabuli*	Ca_Kabuli_Chr04	8203586	[C/T]		--	INTERGENIC
5637	CakSNP5637	*Kabuli*	Ca_Kabuli_Chr04	8236449	[C/A]	Ca_08313	Zinc finger, RING-type	SYNONYMOUS_CODING
5638	CakSNP5638	*Kabuli*	Ca_Kabuli_Chr04	8237145	[C/T]	Ca_08313	Zinc finger, RING-type	SYNONYMOUS_CODING
5639	CakSNP5639	*Kabuli*	Ca_Kabuli_Chr04	8269099	[T/G]	Ca_08315	Protein of unknown function DUF3595	INTRON
5640	CakSNP5640	*Kabuli*	Ca_Kabuli_Chr04	8269152	[C/T]	Ca_08315	Protein of unknown function DUF3595	INTRON
5641	CakSNP5641	*Kabuli*	Ca_Kabuli_Chr04	8269347	[C/G]	Ca_08315	Protein of unknown function DUF3595	INTRON

S.N.	SNP IDs	Chickpea cultivars	Chromosomes	Physical positions (bp)	SNPs	Gene accession IDs	Putative functions	Sequence components of genome
5642	CakSNP5642	*Kabuli*	Ca_Kabuli_Chr04	8302198	[A/G]		--	INTERGENIC
5643	CakSNP5643	*Kabuli*	Ca_Kabuli_Chr04	8383890	[G/A]	Ca_08327	BSD	INTRON
5644	CakSNP5644	*Kabuli*	Ca_Kabuli_Chr04	8396861	[T/A]	Ca_08329	Chaperonin Cpn60	INTRON
5645	CakSNP5645	*Kabuli*	Ca_Kabuli_Chr04	8396837	[T/G]	Ca_08329	Chaperonin Cpn60	INTRON
5646	CakSNP5646	*Kabuli*	Ca_Kabuli_Chr04	8401109	[A/G]	Ca_08329	Chaperonin Cpn60	INTRON
5647	CakSNP5647	*Kabuli*	Ca_Kabuli_Chr04	8401126	[G/T]	Ca_08329	Chaperonin Cpn60	INTRON
5648	CakSNP5648	*Kabuli*	Ca_Kabuli_Chr04	8401143	[A/T]	Ca_08329	Chaperonin Cpn60	SYNONYMOUS_CODING
5649	CakSNP5649	*Kabuli*	Ca_Kabuli_Chr04	8401248	[T/A]		--	INTERGENIC
5650	CakSNP5650	*Kabuli*	Ca_Kabuli_Chr04	8401230	[A/G]		--	INTERGENIC
5651	CakSNP5651	*Kabuli*	Ca_Kabuli_Chr04	8404108	[T/C]	Ca_08330	Chaperonin Cpn60	INTRON

S.N.	SNP IDs	Chickpea cultivars	Chromosomes	Physical positions (bp)	SNPs	Gene accession IDs	Putative functions	Sequence components of genome
5652	CakSNP5652	*Kabuli*	Ca_Kabuli_Chr04	8404165	[C/A]	Ca_08330	Chaperonin Cpn60	NON_SYNONYMOUS_CODING
5653	CakSNP5653	*Kabuli*	Ca_Kabuli_Chr04	8404244	[C/G]	Ca_08330	Chaperonin Cpn60	INTRON
5654	CakSNP5654	*Kabuli*	Ca_Kabuli_Chr04	8471029	[G/A]		--	INTERGENIC
5655	CakSNP5655	*Kabuli*	Ca_Kabuli_Chr04	8473120	[G/A]	Ca_08339	Phosphofructokinase domain	INTRON
5656	CakSNP5656	*Kabuli*	Ca_Kabuli_Chr04	8473127	[G/A]	Ca_08339	Phosphofructokinase domain	INTRON
5657	CakSNP5657	*Kabuli*	Ca_Kabuli_Chr04	8473294	[G/A]	Ca_08339	Phosphofructokinase domain	INTRON
5658	CakSNP5658	*Kabuli*	Ca_Kabuli_Chr04	8473288	[A/G]	Ca_08339	Phosphofructokinase domain	INTRON
5659	CakSNP5659	*Kabuli*	Ca_Kabuli_Chr04	8473246	[T/C]	Ca_08339	Phosphofructokinase domain	INTRON
5660	CakSNP5660	*Kabuli*	Ca_Kabuli_Chr04	8474442	[C/T]	Ca_08339	Phosphofructokinase domain	INTRON
5661	CakSNP5661	*Kabuli*	Ca_Kabuli_Chr04	8474441	[G/A]	Ca_08339	Phosphofructokinase domain	INTRON

S.N.	SNP IDs	Chickpea cultivars	Chromosomes	Physical positions (bp)	SNPs	Gene accession IDs	Putative functions	Sequence components of genome
5662	CakSNP5662	*Kabuli*	Ca_Kabuli_Chr04	8474401	[T/A]	Ca_08339	Phosphofructokinase domain	INTRON
5663	CakSNP5663	*Kabuli*	Ca_Kabuli_Chr04	8475634	[A/C]	Ca_08339	Phosphofructokinase domain	SYNONYMOUS_CODING
5664	CakSNP5664	*Kabuli*	Ca_Kabuli_Chr04	8483482	[T/C]	Ca_08340	WD40 repeat	SYNONYMOUS_CODING
5665	CakSNP5665	*Kabuli*	Ca_Kabuli_Chr04	8483494	[T/C]	Ca_08340	WD40 repeat	SYNONYMOUS_CODING
5666	CakSNP5666	*Kabuli*	Ca_Kabuli_Chr04	8484976	[G/A]	Ca_08340	WD40 repeat	NON_SYNONYMOUS_CODING
5667	CakSNP5667	*Kabuli*	Ca_Kabuli_Chr04	8484978	[A/G]	Ca_08340	WD40 repeat	NON_SYNONYMOUS_CODING
5668	CakSNP5668	*Kabuli*	Ca_Kabuli_Chr04	8496641	[C/A]	Ca_08341	WD40 repeat	SYNONYMOUS_CODING
5669	CakSNP5669	*Kabuli*	Ca_Kabuli_Chr04	8510011	[A/C]	Ca_08343	--	SYNONYMOUS_CODING
5670	CakSNP5670	*Kabuli*	Ca_Kabuli_Chr04	8512095	[G/A]	Ca_08343	--	SYNONYMOUS_CODING
5671	CakSNP5671	*Kabuli*	Ca_Kabuli_Chr04	8512280	[C/T]	Ca_08343	--	NON_SYNONYMOUS_CODING

S.N.	SNP IDs	Chickpea cultivars	Chromosomes	Physical positions (bp)	SNPs	Gene accession IDs	Putative functions	Sequence components of genome
5672	CakSNP5672	*Kabuli*	Ca_Kabuli_Chr04	8518979	[G/A]		--	INTERGENIC
5673	CakSNP5673	*Kabuli*	Ca_Kabuli_Chr04	8534120	[C/T]	Ca_08347	HAD-superfamily hydrolase, subfamily IE	INTRON
5674	CakSNP5674	*Kabuli*	Ca_Kabuli_Chr04	8567380	[A/T]		--	INTERGENIC
5675	CakSNP5675	*Kabuli*	Ca_Kabuli_Chr04	8567431	[C/T]		--	INTERGENIC
5676	CakSNP5676	*Kabuli*	Ca_Kabuli_Chr04	8567490	[C/A]		--	INTERGENIC
5677	CakSNP5677	*Kabuli*	Ca_Kabuli_Chr04	8567478	[A/C]		--	INTERGENIC
5678	CakSNP5678	*Kabuli*	Ca_Kabuli_Chr04	8567477	[T/C]		--	INTERGENIC
5679	CakSNP5679	*Kabuli*	Ca_Kabuli_Chr04	8567465	[C/T]		--	INTERGENIC
5680	CakSNP5680	*Kabuli*	Ca_Kabuli_Chr04	8570458	[T/G]	Ca_08352	NUDIX hydrolase domain	INTRON
5681	CakSNP5681	*Kabuli*	Ca_Kabuli_Chr04	8570501	[G/C]	Ca_08352	NUDIX hydrolase domain	INTRON

S.N.	SNP IDs	Chickpea cultivars	Chromosomes	Physical positions (bp)	SNPs	Gene accession IDs	Putative functions	Sequence components of genome
5682	CakSNP5682	*Kabuli*	Ca_Kabuli_Chr04	8595427	[C/T]		--	INTERGENIC
5683	CakSNP5683	*Kabuli*	Ca_Kabuli_Chr04	8601364	[T/G]	Ca_08354	Exo70 exocyst complex subunit	INTRON
5684	CakSNP5684	*Kabuli*	Ca_Kabuli_Chr04	8659774	[G/A]	Ca_08360	Disease resistance protein	SYNONYMOUS_CODING
5685	CakSNP5685	*Kabuli*	Ca_Kabuli_Chr04	8659935	[T/A]	Ca_08360	Disease resistance protein	NON_SYNONYMOUS_CODING
5686	CakSNP5686	*Kabuli*	Ca_Kabuli_Chr04	8659946	[C/T]	Ca_08360	Disease resistance protein	NON_SYNONYMOUS_CODING
5687	CakSNP5687	*Kabuli*	Ca_Kabuli_Chr04	8659985	[T/C]	Ca_08360	Disease resistance protein	NON_SYNONYMOUS_CODING
5688	CakSNP5688	*Kabuli*	Ca_Kabuli_Chr04	8660115	[A/T]	Ca_08360	Disease resistance protein	SYNONYMOUS_CODING
5689	CakSNP5689	*Kabuli*	Ca_Kabuli_Chr04	8660136	[G/A]	Ca_08360	Disease resistance protein	SYNONYMOUS_CODING
5690	CakSNP5690	*Kabuli*	Ca_Kabuli_Chr04	8660608	[G/T]	Ca_08360	Disease resistance protein	NON_SYNONYMOUS_CODING
5691	CakSNP5691	*Kabuli*	Ca_Kabuli_Chr04	8660599	[A/G]	Ca_08360	Disease resistance protein	NON_SYNONYMOUS_CODING

S.N.	SNP IDs	Chickpea cultivars	Chromosomes	Physical positions (bp)	SNPs	Gene accession IDs	Putative functions	Sequence components of genome
5692	CakSNP5692	*Kabuli*	Ca_Kabuli_Chr04	8668648	[G/A]	Ca_08361	Disease resistance protein	SYNONYMOUS_CODING
5693	CakSNP5693	*Kabuli*	Ca_Kabuli_Chr04	8668753	[A/G]	Ca_08361	Disease resistance protein	SYNONYMOUS_CODING
5694	CakSNP5694	*Kabuli*	Ca_Kabuli_Chr04	8668945	[T/C]	Ca_08361	Disease resistance protein	SYNONYMOUS_CODING
5695	CakSNP5695	*Kabuli*	Ca_Kabuli_Chr04	8669014	[C/G]	Ca_08361	Disease resistance protein	NON_SYNONYMOUS_CODING
5696	CakSNP5696	*Kabuli*	Ca_Kabuli_Chr04	8669104	[T/C]	Ca_08361	Disease resistance protein	SYNONYMOUS_CODING
5697	CakSNP5697	*Kabuli*	Ca_Kabuli_Chr04	8669515	[C/T]	Ca_08361	Disease resistance protein	SYNONYMOUS_CODING
5698	CakSNP5698	*Kabuli*	Ca_Kabuli_Chr04	8669513	[G/A]	Ca_08361	Disease resistance protein	SYNONYMOUS_CODING
5699	CakSNP5699	*Kabuli*	Ca_Kabuli_Chr04	8669505	[G/T]	Ca_08361	Disease resistance protein	SYNONYMOUS_CODING
5700	CakSNP5700	*Kabuli*	Ca_Kabuli_Chr04	8681949	[G/A]	Ca_08364	Disease resistance protein	SYNONYMOUS_CODING
5701	CakSNP5701	*Kabuli*	Ca_Kabuli_Chr04	8695562	[C/T]	Ca_08365	Glycoside hydrolase, family 13, N-terminal	INTRON

S.N.	SNP IDs	Chickpea cultivars	Chromosomes	Physical positions (bp)	SNPs	Gene accession IDs	Putative functions	Sequence components of genome
5702	CakSNP5702	*Kabuli*	Ca_Kabuli_Chr04	8751638	[A/C]	Ca_08371	No apical meristem (NAM) protein	SYNONYMOUS_CODING
5703	CakSNP5703	*Kabuli*	Ca_Kabuli_Chr04	8751629	[T/C]	Ca_08371	No apical meristem (NAM) protein	SYNONYMOUS_CODING
5704	CakSNP5704	*Kabuli*	Ca_Kabuli_Chr04	8751618	[A/C]	Ca_08371	No apical meristem (NAM) protein	NON_SYNONYMOUS_CODING
5705	CakSNP5705	*Kabuli*	Ca_Kabuli_Chr04	8751591	[A/C]		--	INTERGENIC
5706	CakSNP5706	*Kabuli*	Ca_Kabuli_Chr04	8772215	[A/C]	Ca_08372	No apical meristem (NAM) protein	SYNONYMOUS_CODING
5707	CakSNP5707	*Kabuli*	Ca_Kabuli_Chr04	8782879	[G/C]	Ca_08374	AMP-dependent synthetase/ligase	SYNONYMOUS_CODING
5708	CakSNP5708	*Kabuli*	Ca_Kabuli_Chr04	8791654	[A/G]	Ca_08375	Kelch repeat type 2	SYNONYMOUS_CODING
5709	CakSNP5709	*Kabuli*	Ca_Kabuli_Chr04	8805504	[A/G]	Ca_08378	K Homology	INTRON
5710	CakSNP5710	*Kabuli*	Ca_Kabuli_Chr04	8805578	[A/C]	Ca_08378	K Homology	INTRON
5711	CakSNP5711	*Kabuli*	Ca_Kabuli_Chr04	8805667	[C/T]	Ca_08378	K Homology	INTRON

S.N.	SNP IDs	Chickpea cultivars	Chromosomes	Physical positions (bp)	SNPs	Gene accession IDs	Putative functions	Sequence components of genome
5712	CakSNP5712	*Kabuli*	Ca_Kabuli_Chr04	8805867	[A/C]	Ca_08378	K Homology	INTRON
5713	CakSNP5713	*Kabuli*	Ca_Kabuli_Chr04	8805839	[G/A]	Ca_08378	K Homology	INTRON
5714	CakSNP5714	*Kabuli*	Ca_Kabuli_Chr04	8809032	[C/T]	Ca_08378	K Homology	INTRON
5715	CakSNP5715	*Kabuli*	Ca_Kabuli_Chr04	8809064	[T/C]	Ca_08378	K Homology	SYNONYMOUS_CODING
5716	CakSNP5716	*Kabuli*	Ca_Kabuli_Chr04	8809010	[A/G]	Ca_08378	K Homology	INTRON
5717	CakSNP5717	*Kabuli*	Ca_Kabuli_Chr04	8809456	[A/C]		--	INTERGENIC
5718	CakSNP5718	*Kabuli*	Ca_Kabuli_Chr04	8844846	[A/G]	Ca_08381	Zinc finger, RING-type	SYNONYMOUS_CODING
5719	CakSNP5719	*Kabuli*	Ca_Kabuli_Chr04	8848654	[C/T]	Ca_08381	Zinc finger, RING-type	SYNONYMOUS_CODING
5720	CakSNP5720	*Kabuli*	Ca_Kabuli_Chr04	8848653	[T/C]	Ca_08381	Zinc finger, RING-type	NON_SYNONYMOUS_CODING
5721	CakSNP5721	*Kabuli*	Ca_Kabuli_Chr04	8852334	[T/A]	Ca_08382	--	SYNONYMOUS_CODING

S.N.	SNP IDs	Chickpea cultivars	Chromosomes	Physical positions (bp)	SNPs	Gene accession IDs	Putative functions	Sequence components of genome
5722	CakSNP5722	*Kabuli*	Ca_Kabuli_Chr04	8852420	[T/C]	Ca_08382	--	SYNONYMOUS_CODING
5723	CakSNP5723	*Kabuli*	Ca_Kabuli_Chr04	8873494	[G/T]	Ca_08385	WD40 repeat	SYNONYMOUS_CODING
5724	CakSNP5724	*Kabuli*	Ca_Kabuli_Chr04	8873509	[G/C]	Ca_08385	WD40 repeat	SYNONYMOUS_CODING
5725	CakSNP5725	*Kabuli*	Ca_Kabuli_Chr04	8919264	[C/T]	Ca_08388	--	NON_SYNONYMOUS_CODING
5726	CakSNP5726	*Kabuli*	Ca_Kabuli_Chr04	8919300	[C/T]	Ca_08388	--	NON_SYNONYMOUS_CODING
5727	CakSNP5727	*Kabuli*	Ca_Kabuli_Chr04	8919395	[A/G]	Ca_08388	--	NON_SYNONYMOUS_CODING
5728	CakSNP5728	*Kabuli*	Ca_Kabuli_Chr04	8952236	[A/G]	Ca_08391	WD40 repeat	INTRON
5729	CakSNP5729	*Kabuli*	Ca_Kabuli_Chr04	8985949	[C/T]	Ca_08393	Protein kinase, catalytic domain	SYNONYMOUS_CODING
5730	CakSNP5730	*Kabuli*	Ca_Kabuli_Chr04	8985961	[C/T]	Ca_08393	Protein kinase, catalytic domain	SYNONYMOUS_CODING
5731	CakSNP5731	*Kabuli*	Ca_Kabuli_Chr04	8986031	[T/A]	Ca_08393	Protein kinase, catalytic domain	INTRON

S.N.	SNP IDs	Chickpea cultivars	Chromosomes	Physical positions (bp)	SNPs	Gene accession IDs	Putative functions	Sequence components of genome
5732	CakSNP5732	*Kabuli*	Ca_Kabuli_Chr04	8986026	[T/A]	Ca_08393	Protein kinase, catalytic domain	INTRON
5733	CakSNP5733	*Kabuli*	Ca_Kabuli_Chr04	8986024	[T/A]	Ca_08393	Protein kinase, catalytic domain	INTRON
5734	CakSNP5734	*Kabuli*	Ca_Kabuli_Chr04	8986018	[G/A]	Ca_08393	Protein kinase, catalytic domain	INTRON
5735	CakSNP5735	*Kabuli*	Ca_Kabuli_Chr04	8986008	[T/A]	Ca_08393	Protein kinase, catalytic domain	INTRON
5736	CakSNP5736	*Kabuli*	Ca_Kabuli_Chr04	8986002	[T/A]	Ca_08393	Protein kinase, catalytic domain	INTRON
5737	CakSNP5737	*Kabuli*	Ca_Kabuli_Chr04	8985998	[T/A]	Ca_08393	Protein kinase, catalytic domain	INTRON
5738	CakSNP5738	*Kabuli*	Ca_Kabuli_Chr04	8985994	[C/A]	Ca_08393	Protein kinase, catalytic domain	INTRON
5739	CakSNP5739	*Kabuli*	Ca_Kabuli_Chr04	8986207	[G/C]	Ca_08393	Protein kinase, catalytic domain	INTRON
5740	CakSNP5740	*Kabuli*	Ca_Kabuli_Chr04	8986217	[T/A]	Ca_08393	Protein kinase, catalytic domain	INTRON
5741	CakSNP5741	*Kabuli*	Ca_Kabuli_Chr04	9075398	[G/A]		--	INTERGENIC

S.N.	SNP IDs	Chickpea cultivars	Chromosomes	Physical positions (bp)	SNPs	Gene accession IDs	Putative functions	Sequence components of genome
5742	CakSNP5742	*Kabuli*	Ca_Kabuli_Chr04	9075395	[A/G]		--	INTERGENIC
5743	CakSNP5743	*Kabuli*	Ca_Kabuli_Chr04	9075459	[C/T]		--	INTERGENIC
5744	CakSNP5744	*Kabuli*	Ca_Kabuli_Chr04	9075520	[C/T]		--	INTERGENIC
5745	CakSNP5745	*Kabuli*	Ca_Kabuli_Chr04	9075565	[A/G]		--	INTERGENIC
5746	CakSNP5746	*Kabuli*	Ca_Kabuli_Chr04	9075543	[T/C]		--	INTERGENIC
5747	CakSNP5747	*Kabuli*	Ca_Kabuli_Chr04	9075671	[A/G]		--	INTERGENIC
5748	CakSNP5748	*Kabuli*	Ca_Kabuli_Chr04	9075677	[T/C]		--	INTERGENIC
5749	CakSNP5749	*Kabuli*	Ca_Kabuli_Chr04	9075682	[G/A]		--	INTERGENIC
5750	CakSNP5750	*Kabuli*	Ca_Kabuli_Chr04	9075737	[G/A]		--	INTERGENIC
5751	CakSNP5751	*Kabuli*	Ca_Kabuli_Chr04	9075744	[T/G]		--	INTERGENIC

S.N.	SNP IDs	Chickpea cultivars	Chromosomes	Physical positions (bp)	SNPs	Gene accession IDs	Putative functions	Sequence components of genome
5752	CakSNP5752	*Kabuli*	Ca_Kabuli_Chr04	9187887	[G/C]	Ca_08410	Vps51/Vps67	NON_SYNONYMOUS_CODING
5753	CakSNP5753	*Kabuli*	Ca_Kabuli_Chr04	9187973	[T/G]	Ca_08410	Vps51/Vps67	SYNONYMOUS_CODING
5754	CakSNP5754	*Kabuli*	Ca_Kabuli_Chr04	9189976	[T/A]	Ca_08410	Vps51/Vps67	NON_SYNONYMOUS_CODING
5755	CakSNP5755	*Kabuli*	Ca_Kabuli_Chr04	9211570	[A/G]	Ca_08411	Serine/threonine dehydratase, pyridoxal-phosphate-binding site	INTRON
5756	CakSNP5756	*Kabuli*	Ca_Kabuli_Chr04	9257131	[G/A]		--	INTERGENIC
5757	CakSNP5757	*Kabuli*	Ca_Kabuli_Chr04	9331949	[C/T]	Ca_08423	ABC transporter-like	INTRON
5758	CakSNP5758	*Kabuli*	Ca_Kabuli_Chr04	9392373	[A/T]	Ca_08428	--	NON_SYNONYMOUS_CODING
5759	CakSNP5759	*Kabuli*	Ca_Kabuli_Chr04	9392455	[A/G]	Ca_08428	--	SYNONYMOUS_CODING
5760	CakSNP5760	*Kabuli*	Ca_Kabuli_Chr04	9392427	[G/A]	Ca_08428	--	NON_SYNONYMOUS_CODING
5761	CakSNP5761	*Kabuli*	Ca_Kabuli_Chr04	9427890	[T/G]		--	INTERGENIC

S.N.	SNP IDs	Chickpea cultivars	Chromosomes	Physical positions (bp)	SNPs	Gene accession IDs	Putative functions	Sequence components of genome
5762	CakSNP5762	*Kabuli*	Ca_Kabuli_Chr04	9427892	[G/T]		--	INTERGENIC
5763	CakSNP5763	*Kabuli*	Ca_Kabuli_Chr04	9427894	[A/C]		--	INTERGENIC
5764	CakSNP5764	*Kabuli*	Ca_Kabuli_Chr04	9427964	[C/A]		--	INTERGENIC
5765	CakSNP5765	*Kabuli*	Ca_Kabuli_Chr04	9451895	[T/A]		--	INTERGENIC
5766	CakSNP5766	*Kabuli*	Ca_Kabuli_Chr04	9451850	[T/G]		--	INTERGENIC
5767	CakSNP5767	*Kabuli*	Ca_Kabuli_Chr04	9491741	[A/T]	Ca_08437	Malectin	SYNONYMOUS_CODING
5768	CakSNP5768	*Kabuli*	Ca_Kabuli_Chr04	9533658	[A/G]	Ca_08438	NmrA-like	SYNONYMOUS_CODING
5769	CakSNP5769	*Kabuli*	Ca_Kabuli_Chr04	9533688	[T/G]	Ca_08438	NmrA-like	NON_SYNONYMOUS_CODING
5770	CakSNP5770	*Kabuli*	Ca_Kabuli_Chr04	9579383	[C/A]		--	INTERGENIC
5771	CakSNP5771	*Kabuli*	Ca_Kabuli_Chr04	9579393	[G/A]		--	INTERGENIC

S.N.	SNP IDs	Chickpea cultivars	Chromosomes	Physical positions (bp)	SNPs	Gene accession IDs	Putative functions	Sequence components of genome
5772	CakSNP5772	*Kabuli*	Ca_Kabuli_Chr04	9579401	[G/T]		--	INTERGENIC
5773	CakSNP5773	*Kabuli*	Ca_Kabuli_Chr04	9579519	[A/T]		--	INTERGENIC
5774	CakSNP5774	*Kabuli*	Ca_Kabuli_Chr04	9579517	[T/C]		--	INTERGENIC
5775	CakSNP5775	*Kabuli*	Ca_Kabuli_Chr04	9580341	[T/A]	Ca_08441	CWC16 protein	SYNONYMOUS_CODING
5776	CakSNP5776	*Kabuli*	Ca_Kabuli_Chr04	9580339	[T/G]	Ca_08441	CWC16 protein	NON_SYNONYMOUS_CODING
5777	CakSNP5777	*Kabuli*	Ca_Kabuli_Chr04	9614897	[G/A]	Ca_08443	--	SYNONYMOUS_CODING
5778	CakSNP5778	*Kabuli*	Ca_Kabuli_Chr04	9614905	[C/A]	Ca_08443	--	NON_SYNONYMOUS_CODING
5779	CakSNP5779	*Kabuli*	Ca_Kabuli_Chr04	9661685	[G/A]		--	INTERGENIC
5780	CakSNP5780	*Kabuli*	Ca_Kabuli_Chr04	9670905	[C/T]	Ca_08446	Phosphoenolpyruvate carboxylase, active site	INTRON
5781	CakSNP5781	*Kabuli*	Ca_Kabuli_Chr04	9670996	[G/T]	Ca_08446	Phosphoenolpyruvate carboxylase, active site	INTRON

S.N.	SNP IDs	Chickpea cultivars	Chromosomes	Physical positions (bp)	SNPs	Gene accession IDs	Putative functions	Sequence components of genome
5782	CakSNP5782	*Kabuli*	Ca_Kabuli_Chr04	9671007	[G/T]	Ca_08446	Phosphoenolpyruvate carboxylase, active site	INTRON
5783	CakSNP5783	*Kabuli*	Ca_Kabuli_Chr04	9671089	[T/G]	Ca_08446	Phosphoenolpyruvate carboxylase, active site	INTRON
5784	CakSNP5784	*Kabuli*	Ca_Kabuli_Chr04	9706453	[T/G]	Ca_08449	--	NON_SYNONYMOUS_CODING
5785	CakSNP5785	*Kabuli*	Ca_Kabuli_Chr04	9706480	[T/A]		--	INTERGENIC
5786	CakSNP5786	*Kabuli*	Ca_Kabuli_Chr04	9706481	[A/C]		--	INTERGENIC
5787	CakSNP5787	*Kabuli*	Ca_Kabuli_Chr04	9740099	[A/G]	Ca_08453	Peptidyl-prolyl cis-trans isomerase, cyclophilin-type	INTRON
5788	CakSNP5788	*Kabuli*	Ca_Kabuli_Chr04	9800865	[C/T]		--	INTERGENIC
5789	CakSNP5789	*Kabuli*	Ca_Kabuli_Chr04	9802603	[T/C]	Ca_08457	Peptidase, cysteine peptidase active site	INTRON
5790	CakSNP5790	*Kabuli*	Ca_Kabuli_Chr04	9836559	[G/T]	Ca_08462	VHS	INTRON
5791	CakSNP5791	*Kabuli*	Ca_Kabuli_Chr04	9840763	[C/A]	Ca_08462	VHS	INTRON

S.N.	SNP IDs	Chickpea cultivars	Chromosomes	Physical positions (bp)	SNPs	Gene accession IDs	Putative functions	Sequence components of genome
5792	CakSNP5792	*Kabuli*	Ca_Kabuli_Chr04	9840906	[A/C]	Ca_08462	VHS	NON_SYNONYMOUS_CODING
5793	CakSNP5793	*Kabuli*	Ca_Kabuli_Chr04	9846321	[T/A]	Ca_08462	VHS	SYNONYMOUS_CODING
5794	CakSNP5794	*Kabuli*	Ca_Kabuli_Chr04	9947077	[A/G]		--	INTERGENIC
5795	CakSNP5795	*Kabuli*	Ca_Kabuli_Chr04	9973728	[C/G]		--	INTERGENIC
5796	CakSNP5796	*Kabuli*	Ca_Kabuli_Chr04	9976039	[C/T]	Ca_08471	Amino acid transporter, transmembrane	SYNONYMOUS_CODING
5797	CakSNP5797	*Kabuli*	Ca_Kabuli_Chr04	9999907	[A/T]		--	INTERGENIC
5798	CakSNP5798	*Kabuli*	Ca_Kabuli_Chr04	10007069	[A/G]		--	INTERGENIC
5799	CakSNP5799	*Kabuli*	Ca_Kabuli_Chr04	10007151	[C/T]		--	INTERGENIC
5800	CakSNP5800	*Kabuli*	Ca_Kabuli_Chr04	10007193	[T/A]		--	INTERGENIC
5801	CakSNP5801	*Kabuli*	Ca_Kabuli_Chr04	10007332	[T/C]		--	INTERGENIC

S.N.	SNP IDs	Chickpea cultivars	Chromosomes	Physical positions (bp)	SNPs	Gene accession IDs	Putative functions	Sequence components of genome
5802	CakSNP5802	*Kabuli*	Ca_Kabuli_Chr04	10007286	[C/T]		--	INTERGENIC
5803	CakSNP5803	*Kabuli*	Ca_Kabuli_Chr04	10007372	[C/A]		--	INTERGENIC
5804	CakSNP5804	*Kabuli*	Ca_Kabuli_Chr04	10009839	[G/A]		--	INTERGENIC
5805	CakSNP5805	*Kabuli*	Ca_Kabuli_Chr04	10013120	[G/A]	Ca_08474	--	INTRON
5806	CakSNP5806	*Kabuli*	Ca_Kabuli_Chr04	10013148	[G/T]	Ca_08474	--	NON_SYNONYMOUS_CODING
5807	CakSNP5807	*Kabuli*	Ca_Kabuli_Chr04	10017467	[A/C]		--	INTERGENIC
5808	CakSNP5808	*Kabuli*	Ca_Kabuli_Chr04	10024226	[G/A]	Ca_08477	Transcription factor jumonji/aspartyl beta-hydroxylase	SYNONYMOUS_CODING
5809	CakSNP5809	*Kabuli*	Ca_Kabuli_Chr04	10030134	[A/G]		--	INTERGENIC
5810	CakSNP5810	*Kabuli*	Ca_Kabuli_Chr04	10116516	[T/G]	Ca_08486	Pleckstrin homology domain	SYNONYMOUS_CODING
5811	CakSNP5811	*Kabuli*	Ca_Kabuli_Chr04	10151711	[A/C]	Ca_08487	Zinc finger, GATA-type	SYNONYMOUS_CODING

S.N.	SNP IDs	Chickpea cultivars	Chromosomes	Physical positions (bp)	SNPs	Gene accession IDs	Putative functions	Sequence components of genome
5812	CakSNP5812	*Kabuli*	Ca_Kabuli_Chr04	10151943	[C/T]	Ca_08487	Zinc finger, GATA-type	NON_SYNONYMOUS_CODING
5813	CakSNP5813	*Kabuli*	Ca_Kabuli_Chr04	10184428	[G/T]	Ca_08489	GCN5-related N-acetyltransferase (GNAT) domain	NON_SYNONYMOUS_CODING
5814	CakSNP5814	*Kabuli*	Ca_Kabuli_Chr04	10184427	[C/A]	Ca_08489	GCN5-related N-acetyltransferase (GNAT) domain	SYNONYMOUS_CODING
5815	CakSNP5815	*Kabuli*	Ca_Kabuli_Chr04	10185602	[T/G]	Ca_08489	GCN5-related N-acetyltransferase (GNAT) domain	NON_SYNONYMOUS_CODING
5816	CakSNP5816	*Kabuli*	Ca_Kabuli_Chr04	10185625	[A/G]	Ca_08489	GCN5-related N-acetyltransferase (GNAT) domain	SYNONYMOUS_CODING
5817	CakSNP5817	*Kabuli*	Ca_Kabuli_Chr04	10205246	[C/T]		--	INTERGENIC
5818	CakSNP5818	*Kabuli*	Ca_Kabuli_Chr04	10290706	[G/T]	Ca_08495	Chorismate synthase	INTRON
5819	CakSNP5819	*Kabuli*	Ca_Kabuli_Chr04	10293203	[T/C]		--	INTERGENIC
5820	CakSNP5820	*Kabuli*	Ca_Kabuli_Chr04	10293241	[C/G]	Ca_08496	Carbamoyl-phosphate synthase, GATase domain	NON_SYNONYMOUS_CODING
5821	CakSNP5821	*Kabuli*	Ca_Kabuli_Chr04	10294380	[A/G]	Ca_08496	Carbamoyl-phosphate synthase, GATase domain	INTRON

S.N.	SNP IDs	Chickpea cultivars	Chromosomes	Physical positions (bp)	SNPs	Gene accession IDs	Putative functions	Sequence components of genome
5822	CakSNP5822	*Kabuli*	Ca_Kabuli_Chr04	10305276	[G/A]	Ca_08496	Carbamoyl-phosphate synthase, GATase domain	INTRON
5823	CakSNP5823	*Kabuli*	Ca_Kabuli_Chr04	10305235	[A/G]	Ca_08496	Carbamoyl-phosphate synthase, GATase domain	INTRON
5824	CakSNP5824	*Kabuli*	Ca_Kabuli_Chr04	10305285	[C/A]	Ca_08496	Carbamoyl-phosphate synthase, GATase domain	INTRON
5825	CakSNP5825	*Kabuli*	Ca_Kabuli_Chr04	10411809	[G/A]		--	INTERGENIC
5826	CakSNP5826	*Kabuli*	Ca_Kabuli_Chr04	10422142	[A/T]	Ca_04278	Pseudouridine synthase, RsuA and RluB/C/D/E/F	INTRON
5827	CakSNP5827	*Kabuli*	Ca_Kabuli_Chr04	10422469	[C/A]	Ca_04278	Pseudouridine synthase, RsuA and RluB/C/D/E/F	INTRON
5828	CakSNP5828	*Kabuli*	Ca_Kabuli_Chr04	10430622	[A/C]	Ca_04279	--	NON_SYNONYMOUS_CODING
5829	CakSNP5829	*Kabuli*	Ca_Kabuli_Chr04	10430617	[A/C]	Ca_04279	--	SYNONYMOUS_CODING
5830	CakSNP5830	*Kabuli*	Ca_Kabuli_Chr04	10430613	[C/A]	Ca_04279	--	NON_SYNONYMOUS_CODING
5831	CakSNP5831	*Kabuli*	Ca_Kabuli_Chr04	10430603	[G/A]	Ca_04279	--	NON_SYNONYMOUS_CODING

S.N.	SNP IDs	Chickpea cultivars	Chromosomes	Physical positions (bp)	SNPs	Gene accession IDs	Putative functions	Sequence components of genome
5832	CakSNP5832	*Kabuli*	Ca_Kabuli_Chr04	10430595	[A/C]	Ca_04279	--	NON_SYNONYMOUS_CODING
5833	CakSNP5833	*Kabuli*	Ca_Kabuli_Chr04	10430588	[T/C]	Ca_04279	--	NON_SYNONYMOUS_CODING
5834	CakSNP5834	*Kabuli*	Ca_Kabuli_Chr04	10430574	[A/T]	Ca_04279	--	NON_SYNONYMOUS_CODING
5835	CakSNP5835	*Kabuli*	Ca_Kabuli_Chr04	10464927	[T/G]	Ca_04283	IQ motif, EF-hand binding site	SYNONYMOUS_CODING
5836	CakSNP5836	*Kabuli*	Ca_Kabuli_Chr04	10465328	[T/C]	Ca_04283	IQ motif, EF-hand binding site	SYNONYMOUS_CODING
5837	CakSNP5837	*Kabuli*	Ca_Kabuli_Chr04	10547575	[G/A]	Ca_04291	Uncharacterised protein family UPF0363	INTRON
5838	CakSNP5838	*Kabuli*	Ca_Kabuli_Chr04	10547561	[T/C]	Ca_04291	Uncharacterised protein family UPF0363	INTRON
5839	CakSNP5839	*Kabuli*	Ca_Kabuli_Chr04	10642002	[A/G]	Ca_04299	Forkhead-associated (FHA) domain	SYNONYMOUS_CODING
5840	CakSNP5840	*Kabuli*	Ca_Kabuli_Chr04	10642043	[T/G]	Ca_04299	Forkhead-associated (FHA) domain	NON_SYNONYMOUS_CODING
5841	CakSNP5841	*Kabuli*	Ca_Kabuli_Chr04	10642044	[A/T]	Ca_04299	Forkhead-associated (FHA) domain	SYNONYMOUS_CODING

S.N.	SNP IDs	Chickpea cultivars	Chromosomes	Physical positions (bp)	SNPs	Gene accession IDs	Putative functions	Sequence components of genome
5842	CakSNP5842	*Kabuli*	Ca_Kabuli_Chr04	10643339	[T/A]	Ca_04299	Forkhead-associated (FHA) domain	INTRON
5843	CakSNP5843	*Kabuli*	Ca_Kabuli_Chr04	10643331	[T/A]	Ca_04299	Forkhead-associated (FHA) domain	INTRON
5844	CakSNP5844	*Kabuli*	Ca_Kabuli_Chr04	10644245	[G/A]	Ca_04299	Forkhead-associated (FHA) domain	SYNONYMOUS_CODING
5845	CakSNP5845	*Kabuli*	Ca_Kabuli_Chr04	10670664	[C/A]	Ca_04303	Protein kinase, catalytic domain	SYNONYMOUS_CODING
5846	CakSNP5846	*Kabuli*	Ca_Kabuli_Chr04	10699624	[A/T]	Ca_04305	Protein of unknown function DUF616	INTRON
5847	CakSNP5847	*Kabuli*	Ca_Kabuli_Chr04	10780990	[A/G]	Ca_04314	Vps51/Vps67	INTRON
5848	CakSNP5848	*Kabuli*	Ca_Kabuli_Chr04	10807993	[T/C]	Ca_04315	ABC transporter-like	SYNONYMOUS_CODING
5849	CakSNP5849	*Kabuli*	Ca_Kabuli_Chr04	10827485	[G/A]	Ca_04317	Zinc finger, RING-type	INTRON
5850	CakSNP5850	*Kabuli*	Ca_Kabuli_Chr04	10827515	[C/G]	Ca_04317	Zinc finger, RING-type	INTRON
5851	CakSNP5851	*Kabuli*	Ca_Kabuli_Chr04	10901282	[T/C]		--	INTERGENIC

S.N.	SNP IDs	Chickpea cultivars	Chromosomes	Physical positions (bp)	SNPs	Gene accession IDs	Putative functions	Sequence components of genome
5852	CakSNP5852	*Kabuli*	Ca_Kabuli_Chr04	10917723	[T/C]		--	INTERGENIC
5853	CakSNP5853	*Kabuli*	Ca_Kabuli_Chr04	10917717	[C/G]		--	INTERGENIC
5854	CakSNP5854	*Kabuli*	Ca_Kabuli_Chr04	10925490	[G/A]	Ca_04326	Filamin/ABP280 repeat-like	SYNONYMOUS_CODING
5855	CakSNP5855	*Kabuli*	Ca_Kabuli_Chr04	10928923	[T/C]	Ca_04326	Filamin/ABP280 repeat-like	INTRON
5856	CakSNP5856	*Kabuli*	Ca_Kabuli_Chr04	10950823	[C/T]	Ca_04329	Chaperonin Cpn60/TCP-1	SYNONYMOUS_CODING
5857	CakSNP5857	*Kabuli*	Ca_Kabuli_Chr04	10971039	[C/A]	Ca_04330	WD40 repeat	INTRON
5858	CakSNP5858	*Kabuli*	Ca_Kabuli_Chr04	10990000	[T/C]	Ca_04331	Protein kinase, catalytic domain	INTRON
5859	CakSNP5859	*Kabuli*	Ca_Kabuli_Chr04	10990008	[A/T]	Ca_04331	Protein kinase, catalytic domain	INTRON
5860	CakSNP5860	*Kabuli*	Ca_Kabuli_Chr04	10990049	[G/T]	Ca_04331	Protein kinase, catalytic domain	INTRON
5861	CakSNP5861	*Kabuli*	Ca_Kabuli_Chr04	10990058	[T/C]	Ca_04331	Protein kinase, catalytic domain	INTRON

S.N.	SNP IDs	Chickpea cultivars	Chromosomes	Physical positions (bp)	SNPs	Gene accession IDs	Putative functions	Sequence components of genome
5862	CakSNP5862	*Kabuli*	Ca_Kabuli_Chr04	10990265	[T/A]	Ca_04331	Protein kinase, catalytic domain	INTRON
5863	CakSNP5863	*Kabuli*	Ca_Kabuli_Chr04	10990261	[A/G]	Ca_04331	Protein kinase, catalytic domain	INTRON
5864	CakSNP5864	*Kabuli*	Ca_Kabuli_Chr04	10991842	[G/A]	Ca_04331	Protein kinase, catalytic domain	INTRON
5865	CakSNP5865	*Kabuli*	Ca_Kabuli_Chr04	10991910	[C/T]	Ca_04331	Protein kinase, catalytic domain	INTRON
5866	CakSNP5866	*Kabuli*	Ca_Kabuli_Chr04	11040108	[C/T]	Ca_04336	RNA recognition motif domain	SYNONYMOUS_CODING
5867	CakSNP5867	*Kabuli*	Ca_Kabuli_Chr04	11051335	[C/T]	Ca_04337	No apical meristem (NAM) protein	NON_SYNONYMOUS_CODING
5868	CakSNP5868	*Kabuli*	Ca_Kabuli_Chr04	11059403	[T/G]		--	INTERGENIC
5869	CakSNP5869	*Kabuli*	Ca_Kabuli_Chr04	11059404	[T/G]		--	INTERGENIC
5870	CakSNP5870	*Kabuli*	Ca_Kabuli_Chr04	11059414	[T/G]		--	INTERGENIC
5871	CakSNP5871	*Kabuli*	Ca_Kabuli_Chr04	11059572	[G/A]	Ca_04338	Fatty acid hydroxylase	NON_SYNONYMOUS_CODING

S.N.	SNP IDs	Chickpea cultivars	Chromosomes	Physical positions (bp)	SNPs	Gene accession IDs	Putative functions	Sequence components of genome
5872	CakSNP5872	*Kabuli*	Ca_Kabuli_Chr04	11059540	[T/C]	Ca_04338	Fatty acid hydroxylase	SYNONYMOUS_CODING
5873	CakSNP5873	*Kabuli*	Ca_Kabuli_Chr04	11096124	[A/G]		--	INTERGENIC
5874	CakSNP5874	*Kabuli*	Ca_Kabuli_Chr04	11096225	[A/G]		--	INTERGENIC
5875	CakSNP5875	*Kabuli*	Ca_Kabuli_Chr04	11096290	[G/A]		--	INTERGENIC
5876	CakSNP5876	*Kabuli*	Ca_Kabuli_Chr04	11113277	[A/C]	Ca_04344	RNA recognition motif domain	SYNONYMOUS_CODING
5877	CakSNP5877	*Kabuli*	Ca_Kabuli_Chr04	11114238	[T/C]	Ca_04344	RNA recognition motif domain	SYNONYMOUS_CODING
5878	CakSNP5878	*Kabuli*	Ca_Kabuli_Chr04	11142202	[A/G]		--	INTERGENIC
5879	CakSNP5879	*Kabuli*	Ca_Kabuli_Chr04	11142227	[C/T]		--	INTERGENIC
5880	CakSNP5880	*Kabuli*	Ca_Kabuli_Chr04	11196803	[T/G]		--	INTERGENIC
5881	CakSNP5881	*Kabuli*	Ca_Kabuli_Chr04	11230403	[G/T]	Ca_04355	Major intrinsic protein	NON_SYNONYMOUS_CODING

S.N.	SNP IDs	Chickpea cultivars	Chromosomes	Physical positions (bp)	SNPs	Gene accession IDs	Putative functions	Sequence components of genome
5882	CakSNP5882	*Kabuli*	Ca_Kabuli_Chr04	11230400	[T/A]	Ca_04355	Major intrinsic protein	NON_SYNONYMOUS_CODING
5883	CakSNP5883	*Kabuli*	Ca_Kabuli_Chr04	11230397	[G/C]	Ca_04355	Major intrinsic protein	NON_SYNONYMOUS_CODING
5884	CakSNP5884	*Kabuli*	Ca_Kabuli_Chr04	11231142	[A/G]	Ca_04355	Major intrinsic protein	NON_SYNONYMOUS_CODING
5885	CakSNP5885	*Kabuli*	Ca_Kabuli_Chr04	11231137	[A/G]	Ca_04355	Major intrinsic protein	NON_SYNONYMOUS_CODING
5886	CakSNP5886	*Kabuli*	Ca_Kabuli_Chr04	11231135	[C/T]	Ca_04355	Major intrinsic protein	SYNONYMOUS_CODING
5887	CakSNP5887	*Kabuli*	Ca_Kabuli_Chr04	11244334	[T/G]		--	INTERGENIC
5888	CakSNP5888	*Kabuli*	Ca_Kabuli_Chr04	11244395	[G/T]		--	INTERGENIC
5889	CakSNP5889	*Kabuli*	Ca_Kabuli_Chr04	11246093	[G/A]		--	INTERGENIC
5890	CakSNP5890	*Kabuli*	Ca_Kabuli_Chr04	11246173	[C/T]		--	INTERGENIC
5891	CakSNP5891	*Kabuli*	Ca_Kabuli_Chr04	11246164	[G/A]		--	INTERGENIC

S.N.	SNP IDs	Chickpea cultivars	Chromosomes	Physical positions (bp)	SNPs	Gene accession IDs	Putative functions	Sequence components of genome
5892	CakSNP5892	*Kabuli*	Ca_Kabuli_Chr04	11273328	[T/C]	Ca_04359	--	INTRON
5893	CakSNP5893	*Kabuli*	Ca_Kabuli_Chr04	11273405	[A/T]	Ca_04359	--	INTRON
5894	CakSNP5894	*Kabuli*	Ca_Kabuli_Chr04	11274281	[T/C]	Ca_04359	--	SYNONYMOUS_CODING
5895	CakSNP5895	*Kabuli*	Ca_Kabuli_Chr04	11275171	[T/C]	Ca_04359	--	SYNONYMOUS_CODING
5896	CakSNP5896	*Kabuli*	Ca_Kabuli_Chr04	11276413	[C/G]	Ca_04359	--	INTRON
5897	CakSNP5897	*Kabuli*	Ca_Kabuli_Chr04	11276484	[T/C]	Ca_04359	--	SYNONYMOUS_CODING
5898	CakSNP5898	*Kabuli*	Ca_Kabuli_Chr04	11277138	[A/G]	Ca_04359	--	SYNONYMOUS_CODING
5899	CakSNP5899	*Kabuli*	Ca_Kabuli_Chr04	11277297	[G/A]	Ca_04359	--	SYNONYMOUS_CODING
5900	CakSNP5900	*Kabuli*	Ca_Kabuli_Chr04	11277574	[G/C]	Ca_04359	--	NON_SYNONYMOUS_CODING
5901	CakSNP5901	*Kabuli*	Ca_Kabuli_Chr04	11334350	[A/G]	Ca_04367	SET domain	INTRON

S.N.	SNP IDs	Chickpea cultivars	Chromosomes	Physical positions (bp)	SNPs	Gene accession IDs	Putative functions	Sequence components of genome
5902	CakSNP5902	*Kabuli*	Ca_Kabuli_Chr04	11334343	[A/T]	Ca_04367	SET domain	INTRON
5903	CakSNP5903	*Kabuli*	Ca_Kabuli_Chr04	11343257	[T/C]	Ca_04368	Zinc finger, DHHC-type, palmitoyltransferase	INTRON
5904	CakSNP5904	*Kabuli*	Ca_Kabuli_Chr04	11343230	[G/C]	Ca_04368	Zinc finger, DHHC-type, palmitoyltransferase	INTRON
5905	CakSNP5905	*Kabuli*	Ca_Kabuli_Chr04	11379077	[T/C]		--	INTERGENIC
5906	CakSNP5906	*Kabuli*	Ca_Kabuli_Chr04	11398699	[A/G]		--	INTERGENIC
5907	CakSNP5907	*Kabuli*	Ca_Kabuli_Chr04	11398682	[G/A]		--	INTERGENIC
5908	CakSNP5908	*Kabuli*	Ca_Kabuli_Chr04	11398889	[A/G]		--	INTERGENIC
5909	CakSNP5909	*Kabuli*	Ca_Kabuli_Chr04	11414506	[C/T]		--	INTERGENIC
5910	CakSNP5910	*Kabuli*	Ca_Kabuli_Chr04	11435651	[A/G]	Ca_04378	--	SYNONYMOUS_CODING
5911	CakSNP5911	*Kabuli*	Ca_Kabuli_Chr04	11441604	[T/A]	Ca_04380	Ribosomal protein S13	INTRON

S.N.	SNP IDs	Chickpea cultivars	Chromosomes	Physical positions (bp)	SNPs	Gene accession IDs	Putative functions	Sequence components of genome
5912	CakSNP5912	*Kabuli*	Ca_Kabuli_Chr04	11441615	[T/C]	Ca_04380	Ribosomal protein S13	INTRON
5913	CakSNP5913	*Kabuli*	Ca_Kabuli_Chr04	11441619	[G/A]	Ca_04380	Ribosomal protein S13	INTRON
5914	CakSNP5914	*Kabuli*	Ca_Kabuli_Chr04	11441655	[T/A]	Ca_04380	Ribosomal protein S13	INTRON
5915	CakSNP5915	*Kabuli*	Ca_Kabuli_Chr04	11441707	[A/C]	Ca_04380	Ribosomal protein S13	INTRON
5916	CakSNP5916	*Kabuli*	Ca_Kabuli_Chr04	11465057	[C/G]	Ca_04383	Protein BYPASS-related	SYNONYMOUS_CODING
5917	CakSNP5917	*Kabuli*	Ca_Kabuli_Chr04	11465075	[T/C]	Ca_04383	Protein BYPASS-related	SYNONYMOUS_CODING
5918	CakSNP5918	*Kabuli*	Ca_Kabuli_Chr04	11465113	[C/T]	Ca_04383	Protein BYPASS-related	NON_SYNONYMOUS_CODING
5919	CakSNP5919	*Kabuli*	Ca_Kabuli_Chr04	11490100	[C/T]	Ca_04384	Tubby, C-terminal	SYNONYMOUS_CODING
5920	CakSNP5920	*Kabuli*	Ca_Kabuli_Chr04	11490154	[A/T]	Ca_04384	Tubby, C-terminal	SYNONYMOUS_CODING
5921	CakSNP5921	*Kabuli*	Ca_Kabuli_Chr04	11490125	[G/T]	Ca_04384	Tubby, C-terminal	SYNONYMOUS_CODING

S.N.	SNP IDs	Chickpea cultivars	Chromosomes	Physical positions (bp)	SNPs	Gene accession IDs	Putative functions	Sequence components of genome
5922	CakSNP5922	*Kabuli*	Ca_Kabuli_Chr04	11490496	[G/A]	Ca_04384	Tubby, C-terminal	SYNONYMOUS_CODING
5923	CakSNP5923	*Kabuli*	Ca_Kabuli_Chr04	11517582	[C/T]		--	INTERGENIC
5924	CakSNP5924	*Kabuli*	Ca_Kabuli_Chr04	11517517	[C/A]		--	INTERGENIC
5925	CakSNP5925	*Kabuli*	Ca_Kabuli_Chr04	11572051	[C/T]		--	INTERGENIC
5926	CakSNP5926	*Kabuli*	Ca_Kabuli_Chr04	11645738	[A/G]	Ca_04392	Protein synthesis factor, GTP-binding	SYNONYMOUS_CODING
5927	CakSNP5927	*Kabuli*	Ca_Kabuli_Chr04	11646347	[C/G]	Ca_04392	Protein synthesis factor, GTP-binding	SYNONYMOUS_CODING
5928	CakSNP5928	*Kabuli*	Ca_Kabuli_Chr04	11646453	[G/A]	Ca_04392	Protein synthesis factor, GTP-binding	NON_SYNONYMOUS_CODING
5929	CakSNP5929	*Kabuli*	Ca_Kabuli_Chr04	11658323	[A/G]	Ca_04393	Domain of unknown function DUF125, transmembrane	SYNONYMOUS_CODING
5930	CakSNP5930	*Kabuli*	Ca_Kabuli_Chr04	11658314	[A/G]	Ca_04393	Domain of unknown function DUF125, transmembrane	SYNONYMOUS_CODING
5931	CakSNP5931	*Kabuli*	Ca_Kabuli_Chr04	11689611	[C/T]	Ca_04395	Basic-leucine zipper (bZIP) transcription factor	NON_SYNONYMOUS_CODING

S.N.	SNP IDs	Chickpea cultivars	Chromosomes	Physical positions (bp)	SNPs	Gene accession IDs	Putative functions	Sequence components of genome
5932	CakSNP5932	*Kabuli*	Ca_Kabuli_Chr04	11689647	[T/C]	Ca_04395	Basic-leucine zipper (bZIP) transcription factor	NON_SYNONYMOUS_CODING
5933	CakSNP5933	*Kabuli*	Ca_Kabuli_Chr04	11752862	[A/G]	Ca_04400	--	SYNONYMOUS_CODING
5934	CakSNP5934	*Kabuli*	Ca_Kabuli_Chr04	11752855	[A/G]	Ca_04400	--	NON_SYNONYMOUS_CODING
5935	CakSNP5935	*Kabuli*	Ca_Kabuli_Chr04	11772023	[G/T]	Ca_04401	Zinc finger, FYVE-type	NON_SYNONYMOUS_CODING
5936	CakSNP5936	*Kabuli*	Ca_Kabuli_Chr04	11838005	[C/T]	Ca_04404	ABC transporter, transmembrane domain	SYNONYMOUS_CODING
5937	CakSNP5937	*Kabuli*	Ca_Kabuli_Chr04	11852291	[A/G]	Ca_04406	Glycoside hydrolase, family 18, catalytic domain	SYNONYMOUS_CODING
5938	CakSNP5938	*Kabuli*	Ca_Kabuli_Chr04	11926352	[G/C]	Ca_04414	ATPase, AAA+ type, core	INTRON
5939	CakSNP5939	*Kabuli*	Ca_Kabuli_Chr04	11985628	[G/A]	Ca_04421	SPX, N-terminal	SYNONYMOUS_CODING
5940	CakSNP5940	*Kabuli*	Ca_Kabuli_Chr04	11985604	[C/T]	Ca_04421	SPX, N-terminal	SYNONYMOUS_CODING
5941	CakSNP5941	*Kabuli*	Ca_Kabuli_Chr04	12004082	[A/T]	Ca_04424	Ornithine cyclodeaminase/mu-crystallin	NON_SYNONYMOUS_CODING

S.N.	SNP IDs	Chickpea cultivars	Chromosomes	Physical positions (bp)	SNPs	Gene accession IDs	Putative functions	Sequence components of genome
5942	CakSNP5942	*Kabuli*	Ca_Kabuli_Chr04	12004209	[C/T]	Ca_04424	Ornithine cyclodeaminase/mu-crystallin	SYNONYMOUS_CODING
5943	CakSNP5943	*Kabuli*	Ca_Kabuli_Chr04	12004205	[A/C]	Ca_04424	Ornithine cyclodeaminase/mu-crystallin	NON_SYNONYMOUS_CODING
5944	CakSNP5944	*Kabuli*	Ca_Kabuli_Chr04	12023137	[A/T]	Ca_04428	Protein kinase, catalytic domain	NON_SYNONYMOUS_CODING
5945	CakSNP5945	*Kabuli*	Ca_Kabuli_Chr04	12023215	[G/A]	Ca_04428	Protein kinase, catalytic domain	SYNONYMOUS_CODING
5946	CakSNP5946	*Kabuli*	Ca_Kabuli_Chr04	12045899	[T/A]		--	INTERGENIC
5947	CakSNP5947	*Kabuli*	Ca_Kabuli_Chr04	12045903	[C/A]		--	INTERGENIC
5948	CakSNP5948	*Kabuli*	Ca_Kabuli_Chr04	12045906	[C/A]		--	INTERGENIC
5949	CakSNP5949	*Kabuli*	Ca_Kabuli_Chr04	12045910	[G/A]		--	INTERGENIC
5950	CakSNP5950	*Kabuli*	Ca_Kabuli_Chr04	12070990	[T/A]		--	INTERGENIC
5951	CakSNP5951	*Kabuli*	Ca_Kabuli_Chr04	12070994	[C/A]		--	INTERGENIC

S.N.	SNP IDs	Chickpea cultivars	Chromosomes	Physical positions (bp)	SNPs	Gene accession IDs	Putative functions	Sequence components of genome
5952	CakSNP5952	*Kabuli*	Ca_Kabuli_Chr04	12070997	[C/A]		--	INTERGENIC
5953	CakSNP5953	*Kabuli*	Ca_Kabuli_Chr04	12071001	[G/A]		--	INTERGENIC
5954	CakSNP5954	*Kabuli*	Ca_Kabuli_Chr04	12167328	[A/G]	Ca_04440	Ferric reductase, NAD binding	INTRON
5955	CakSNP5955	*Kabuli*	Ca_Kabuli_Chr04	12167568	[G/A]	Ca_04440	Ferric reductase, NAD binding	NON_SYNONYMOUS_CODING
5956	CakSNP5956	*Kabuli*	Ca_Kabuli_Chr04	12212917	[G/A]	Ca_04443	Multi antimicrobial extrusion protein	INTRON
5957	CakSNP5957	*Kabuli*	Ca_Kabuli_Chr04	12213071	[C/A]	Ca_04443	Multi antimicrobial extrusion protein	SYNONYMOUS_CODING
5958	CakSNP5958	*Kabuli*	Ca_Kabuli_Chr04	12213046	[T/C]	Ca_04443	Multi antimicrobial extrusion protein	INTRON
5959	CakSNP5959	*Kabuli*	Ca_Kabuli_Chr04	12234358	[C/A]	Ca_04446	Bacterial sec-independent translocation protein MttA/Hcf107	INTRON
5960	CakSNP5960	*Kabuli*	Ca_Kabuli_Chr04	12249494	[T/C]	Ca_04448	Protein kinase, catalytic domain	INTRON
5961	CakSNP5961	*Kabuli*	Ca_Kabuli_Chr04	12251184	[G/A]	Ca_04448	Protein kinase, catalytic domain	INTRON

S.N.	SNP IDs	Chickpea cultivars	Chromosomes	Physical positions (bp)	SNPs	Gene accession IDs	Putative functions	Sequence components of genome
5962	CakSNP5962	*Kabuli*	Ca_Kabuli_Chr04	12274290	[A/T]	Ca_04451	Zinc finger, C2H2-type	INTRON
5963	CakSNP5963	*Kabuli*	Ca_Kabuli_Chr04	12316847	[T/C]	Ca_04456	Phox/Bem1p	INTRON
5964	CakSNP5964	*Kabuli*	Ca_Kabuli_Chr04	12431895	[C/T]	Ca_04466	PAR1	SYNONYMOUS_CODING
5965	CakSNP5965	*Kabuli*	Ca_Kabuli_Chr04	12431915	[A/G]	Ca_04466	PAR1	SYNONYMOUS_CODING
5966	CakSNP5966	*Kabuli*	Ca_Kabuli_Chr04	12484911	[A/T]		--	INTERGENIC
5967	CakSNP5967	*Kabuli*	Ca_Kabuli_Chr04	12506440	[A/G]		--	INTERGENIC
5968	CakSNP5968	*Kabuli*	Ca_Kabuli_Chr04	12506450	[T/A]		--	INTERGENIC
5969	CakSNP5969	*Kabuli*	Ca_Kabuli_Chr04	12514728	[G/A]		--	INTERGENIC
5970	CakSNP5970	*Kabuli*	Ca_Kabuli_Chr04	12514726	[C/T]		--	INTERGENIC
5971	CakSNP5971	*Kabuli*	Ca_Kabuli_Chr04	12514724	[T/C]		--	INTERGENIC

S.N.	SNP IDs	Chickpea cultivars	Chromosomes	Physical positions (bp)	SNPs	Gene accession IDs	Putative functions	Sequence components of genome
5972	CakSNP5972	*Kabuli*	Ca_Kabuli_Chr04	12514706	[G/A]		--	INTERGENIC
5973	CakSNP5973	*Kabuli*	Ca_Kabuli_Chr04	12525328	[A/G]	Ca_04475	Cytochrome b561, eukaryote	SYNONYMOUS_CODING
5974	CakSNP5974	*Kabuli*	Ca_Kabuli_Chr04	12525358	[C/G]	Ca_04475	Cytochrome b561, eukaryote	SYNONYMOUS_CODING
5975	CakSNP5975	*Kabuli*	Ca_Kabuli_Chr04	12525639	[T/A]	Ca_04475	Cytochrome b561, eukaryote	INTRON
5976	CakSNP5976	*Kabuli*	Ca_Kabuli_Chr04	12534847	[T/C]	Ca_04477	SANT domain, DNA binding	INTRON
5977	CakSNP5977	*Kabuli*	Ca_Kabuli_Chr04	12558541	[T/C]	Ca_04478	NUDIX hydrolase domain	INTRON
5978	CakSNP5978	*Kabuli*	Ca_Kabuli_Chr04	12558680	[C/T]	Ca_04478	NUDIX hydrolase domain	INTRON
5979	CakSNP5979	*Kabuli*	Ca_Kabuli_Chr04	12568707	[G/A]	Ca_04479	Homoserine kinase	SYNONYMOUS_CODING
5980	CakSNP5980	*Kabuli*	Ca_Kabuli_Chr04	12568845	[G/T]	Ca_04479	Homoserine kinase	SYNONYMOUS_CODING
5981	CakSNP5981	*Kabuli*	Ca_Kabuli_Chr04	12568905	[G/A]	Ca_04479	Homoserine kinase	SYNONYMOUS_CODING

S.N.	SNP IDs	Chickpea cultivars	Chromosomes	Physical positions (bp)	SNPs	Gene accession IDs	Putative functions	Sequence components of genome
5982	CakSNP5982	*Kabuli*	Ca_Kabuli_Chr04	12568944	[A/G]	Ca_04479	Homoserine kinase	SYNONYMOUS_CODING
5983	CakSNP5983	*Kabuli*	Ca_Kabuli_Chr04	12568995	[G/A]	Ca_04479	Homoserine kinase	SYNONYMOUS_CODING
5984	CakSNP5984	*Kabuli*	Ca_Kabuli_Chr04	12569133	[G/A]	Ca_04479	Homoserine kinase	SYNONYMOUS_CODING
5985	CakSNP5985	*Kabuli*	Ca_Kabuli_Chr04	12574650	[G/A]	Ca_04480	DNA mismatch repair protein MutS, C-terminal domain	NON_SYNONYMOUS_CODING
5986	CakSNP5986	*Kabuli*	Ca_Kabuli_Chr04	12578259	[A/G]	Ca_04480	DNA mismatch repair protein MutS, C-terminal domain	SYNONYMOUS_CODING
5987	CakSNP5987	*Kabuli*	Ca_Kabuli_Chr04	12578350	[A/G]	Ca_04480	DNA mismatch repair protein MutS, C-terminal domain	INTRON
5988	CakSNP5988	*Kabuli*	Ca_Kabuli_Chr04	12581371	[G/T]	Ca_04480	DNA mismatch repair protein MutS, C-terminal domain	SYNONYMOUS_CODING
5989	CakSNP5989	*Kabuli*	Ca_Kabuli_Chr04	12692925	[C/T]		--	INTERGENIC
5990	CakSNP5990	*Kabuli*	Ca_Kabuli_Chr04	12692958	[G/C]		--	INTERGENIC
5991	CakSNP5991	*Kabuli*	Ca_Kabuli_Chr04	12693000	[T/C]		--	INTERGENIC

S.N.	SNP IDs	Chickpea cultivars	Chromosomes	Physical positions (bp)	SNPs	Gene accession IDs	Putative functions	Sequence components of genome
5992	CakSNP5992	*Kabuli*	Ca_Kabuli_Chr04	12740330	[A/G]	Ca_04493	IQ motif, EF-hand binding site	SYNONYMOUS_CODING
5993	CakSNP5993	*Kabuli*	Ca_Kabuli_Chr04	12740351	[T/G]	Ca_04493	IQ motif, EF-hand binding site	SYNONYMOUS_CODING
5994	CakSNP5994	*Kabuli*	Ca_Kabuli_Chr04	12779146	[A/C]	Ca_04497	Dihydrodipicolinate reductase, N-terminal	INTRON
5995	CakSNP5995	*Kabuli*	Ca_Kabuli_Chr04	12780274	[C/T]	Ca_04497	Dihydrodipicolinate reductase, N-terminal	INTRON
5996	CakSNP5996	*Kabuli*	Ca_Kabuli_Chr04	12795036	[G/A]		--	INTERGENIC
5997	CakSNP5997	*Kabuli*	Ca_Kabuli_Chr04	12795037	[G/A]		--	INTERGENIC
5998	CakSNP5998	*Kabuli*	Ca_Kabuli_Chr04	12869023	[C/T]	Ca_04506	Protein kinase, catalytic domain	NON_SYNONYMOUS_CODING
5999	CakSNP5999	*Kabuli*	Ca_Kabuli_Chr04	12869049	[C/T]	Ca_04506	Protein kinase, catalytic domain	SYNONYMOUS_CODING
6000	CakSNP6000	*Kabuli*	Ca_Kabuli_Chr04	12907910	[C/A]		--	INTERGENIC
6001	CakSNP6001	*Kabuli*	Ca_Kabuli_Chr04	12908080	[C/T]		--	INTERGENIC

S.N.	SNP IDs	Chickpea cultivars	Chromosomes	Physical positions (bp)	SNPs	Gene accession IDs	Putative functions	Sequence components of genome
6002	CakSNP6002	*Kabuli*	Ca_Kabuli_Chr04	12911860	[A/G]		--	INTERGENIC
6003	CakSNP6003	*Kabuli*	Ca_Kabuli_Chr04	12911874	[T/C]		--	INTERGENIC
6004	CakSNP6004	*Kabuli*	Ca_Kabuli_Chr04	12911888	[G/C]		--	INTERGENIC
6005	CakSNP6005	*Kabuli*	Ca_Kabuli_Chr04	12912015	[G/A]		--	INTERGENIC
6006	CakSNP6006	*Kabuli*	Ca_Kabuli_Chr04	12955130	[G/A]	Ca_04517	Ribokinase	INTRON
6007	CakSNP6007	*Kabuli*	Ca_Kabuli_Chr04	12973657	[C/A]	Ca_04519	GDP-fucose protein O-fucosyltransferase	NON_SYNONYMOUS_CODING
6008	CakSNP6008	*Kabuli*	Ca_Kabuli_Chr04	12982420	[G/A]	Ca_04520	Alpha-D-phosphohexomutase	INTRON
6009	CakSNP6009	*Kabuli*	Ca_Kabuli_Chr04	13018788	[C/G]	Ca_04523	Alpha/beta hydrolase fold-3	SYNONYMOUS_CODING
6010	CakSNP6010	*Kabuli*	Ca_Kabuli_Chr04	13020891	[A/G]	Ca_04524	Alpha/beta hydrolase fold-3	SYNONYMOUS_CODING
6011	CakSNP6011	*Kabuli*	Ca_Kabuli_Chr04	13050470	[T/C]		--	INTERGENIC

S.N.	SNP IDs	Chickpea cultivars	Chromosomes	Physical positions (bp)	SNPs	Gene accession IDs	Putative functions	Sequence components of genome
6012	CakSNP6012	*Kabuli*	Ca_Kabuli_Chr04	13050516	[A/G]		--	INTERGENIC
6013	CakSNP6013	*Kabuli*	Ca_Kabuli_Chr04	13050527	[C/T]		--	INTERGENIC
6014	CakSNP6014	*Kabuli*	Ca_Kabuli_Chr04	13050585	[C/T]		--	INTERGENIC
6015	CakSNP6015	*Kabuli*	Ca_Kabuli_Chr04	13062744	[T/A]	Ca_04529	Peptidase S8/S53, subtilisin/kexin/sedolisin	SYNONYMOUS_CODING
6016	CakSNP6016	*Kabuli*	Ca_Kabuli_Chr04	13062761	[A/T]	Ca_04529	Peptidase S8/S53, subtilisin/kexin/sedolisin	NON_SYNONYMOUS_CODING
6017	CakSNP6017	*Kabuli*	Ca_Kabuli_Chr04	13062805	[C/G]	Ca_04529	Peptidase S8/S53, subtilisin/kexin/sedolisin	NON_SYNONYMOUS_CODING
6018	CakSNP6018	*Kabuli*	Ca_Kabuli_Chr04	13062972	[A/T]	Ca_04529	Peptidase S8/S53, subtilisin/kexin/sedolisin	SYNONYMOUS_CODING
6019	CakSNP6019	*Kabuli*	Ca_Kabuli_Chr04	13063689	[T/A]	Ca_04529	Peptidase S8/S53, subtilisin/kexin/sedolisin	SYNONYMOUS_CODING
6020	CakSNP6020	*Kabuli*	Ca_Kabuli_Chr04	13063699	[G/T]	Ca_04529	Peptidase S8/S53, subtilisin/kexin/sedolisin	NON_SYNONYMOUS_CODING
6021	CakSNP6021	*Kabuli*	Ca_Kabuli_Chr04	13069574	[T/C]		--	INTERGENIC

S.N.	SNP IDs	Chickpea cultivars	Chromosomes	Physical positions (bp)	SNPs	Gene accession IDs	Putative functions	Sequence components of genome
6022	CakSNP6022	*Kabuli*	Ca_Kabuli_Chr04	13071169	[T/A]	Ca_04530	Peptidase S8/S53, subtilisin/kexin/sedolisin	SYNONYMOUS_CODING
6023	CakSNP6023	*Kabuli*	Ca_Kabuli_Chr04	13071199	[A/T]	Ca_04530	Peptidase S8/S53, subtilisin/kexin/sedolisin	SYNONYMOUS_CODING
6024	CakSNP6024	*Kabuli*	Ca_Kabuli_Chr04	13071230	[T/G]	Ca_04530	Peptidase S8/S53, subtilisin/kexin/sedolisin	NON_SYNONYMOUS_CODING
6025	CakSNP6025	*Kabuli*	Ca_Kabuli_Chr04	13072114	[T/A]	Ca_04530	Peptidase S8/S53, subtilisin/kexin/sedolisin	SYNONYMOUS_CODING
6026	CakSNP6026	*Kabuli*	Ca_Kabuli_Chr04	13072118	[T/G]	Ca_04530	Peptidase S8/S53, subtilisin/kexin/sedolisin	NON_SYNONYMOUS_CODING
6027	CakSNP6027	*Kabuli*	Ca_Kabuli_Chr04	13072208	[C/A]	Ca_04530	Peptidase S8/S53, subtilisin/kexin/sedolisin	NON_SYNONYMOUS_CODING
6028	CakSNP6028	*Kabuli*	Ca_Kabuli_Chr04	13082999	[A/G]	Ca_04531	FAE1/Type III polyketide synthase-like protein	SYNONYMOUS_CODING
6029	CakSNP6029	*Kabuli*	Ca_Kabuli_Chr04	13102381	[G/A]	Ca_04534	Cytochrome P450	INTRON
6030	CakSNP6030	*Kabuli*	Ca_Kabuli_Chr04	13102348	[G/T]	Ca_04534	Cytochrome P450	NON_SYNONYMOUS_CODING
6031	CakSNP6031	*Kabuli*	Ca_Kabuli_Chr04	13147948	[A/G]		--	INTERGENIC

S.N.	SNP IDs	Chickpea cultivars	Chromosomes	Physical positions (bp)	SNPs	Gene accession IDs	Putative functions	Sequence components of genome
6032	CakSNP6032	*Kabuli*	Ca_Kabuli_Chr04	13147951	[C/T]		--	INTERGENIC
6033	CakSNP6033	*Kabuli*	Ca_Kabuli_Chr04	13147960	[T/C]		--	INTERGENIC
6034	CakSNP6034	*Kabuli*	Ca_Kabuli_Chr04	13147965	[A/G]		--	INTERGENIC
6035	CakSNP6035	*Kabuli*	Ca_Kabuli_Chr04	13148016	[G/A]		--	INTERGENIC
6036	CakSNP6036	*Kabuli*	Ca_Kabuli_Chr04	13179829	[T/C]	Ca_04542	Sulphate adenylyltransferase	NON_SYNONYMOUS_CODING
6037	CakSNP6037	*Kabuli*	Ca_Kabuli_Chr04	13203008	[A/T]		--	INTERGENIC
6038	CakSNP6038	*Kabuli*	Ca_Kabuli_Chr04	13229035	[C/T]		--	INTERGENIC
6039	CakSNP6039	*Kabuli*	Ca_Kabuli_Chr04	13252015	[A/T]		--	INTERGENIC
6040	CakSNP6040	*Kabuli*	Ca_Kabuli_Chr04	13252077	[C/G]		--	INTERGENIC
6041	CakSNP6041	*Kabuli*	Ca_Kabuli_Chr04	13252126	[T/C]		--	INTERGENIC

S.N.	SNP IDs	Chickpea cultivars	Chromosomes	Physical positions (bp)	SNPs	Gene accession IDs	Putative functions	Sequence components of genome
6042	CakSNP6042	*Kabuli*	Ca_Kabuli_Chr04	13252212	[G/A]		--	INTERGENIC
6043	CakSNP6043	*Kabuli*	Ca_Kabuli_Chr04	13272282	[T/C]		--	INTERGENIC
6044	CakSNP6044	*Kabuli*	Ca_Kabuli_Chr04	13282984	[A/C]		--	INTERGENIC
6045	CakSNP6045	*Kabuli*	Ca_Kabuli_Chr04	13296221	[A/T]	Ca_04553	Dymeclin	INTRON
6046	CakSNP6046	*Kabuli*	Ca_Kabuli_Chr04	13296217	[G/T]	Ca_04553	Dymeclin	INTRON
6047	CakSNP6047	*Kabuli*	Ca_Kabuli_Chr04	13310700	[T/C]		--	INTERGENIC
6048	CakSNP6048	*Kabuli*	Ca_Kabuli_Chr04	13318811	[C/T]	Ca_04556	Domain of unknown function DUF3598	SYNONYMOUS_CODING
6049	CakSNP6049	*Kabuli*	Ca_Kabuli_Chr04	13331280	[A/G]	Ca_04558	Tify	NON_SYNONYMOUS_CODING
6050	CakSNP6050	*Kabuli*	Ca_Kabuli_Chr04	13332384	[G/A]	Ca_04558	Tify	INTRON
6051	CakSNP6051	*Kabuli*	Ca_Kabuli_Chr04	13332452	[T/C]	Ca_04558	Tify	INTRON

S.N.	SNP IDs	Chickpea cultivars	Chromosomes	Physical positions (bp)	SNPs	Gene accession IDs	Putative functions	Sequence components of genome
6052	CakSNP6052	*Kabuli*	Ca_Kabuli_Chr04	13376456	[A/T]	Ca_04560	Domain of unknown function DUF828	NON_SYNONYMOUS_CODING
6053	CakSNP6053	*Kabuli*	Ca_Kabuli_Chr04	13463481	[C/T]	Ca_04567	Protein kinase, catalytic domain	INTRON
6054	CakSNP6054	*Kabuli*	Ca_Kabuli_Chr04	13500679	[A/C]		--	INTERGENIC
6055	CakSNP6055	*Kabuli*	Ca_Kabuli_Chr04	13500693	[G/A]		--	INTERGENIC
6056	CakSNP6056	*Kabuli*	Ca_Kabuli_Chr04	13588956	[C/T]	Ca_04577	Biopterin transport-related protein BT1	SYNONYMOUS_CODING
6057	CakSNP6057	*Kabuli*	Ca_Kabuli_Chr04	13588928	[T/C]	Ca_04577	Biopterin transport-related protein BT1	INTRON
6058	CakSNP6058	*Kabuli*	Ca_Kabuli_Chr04	13646571	[T/C]	Ca_04583	Seed maturation protein	SYNONYMOUS_CODING
6059	CakSNP6059	*Kabuli*	Ca_Kabuli_Chr04	13646628	[C/T]	Ca_04583	Seed maturation protein	SYNONYMOUS_CODING
6060	CakSNP6060	*Kabuli*	Ca_Kabuli_Chr04	13687456	[G/A]	Ca_04589	Frigida-like	INTRON
6061	CakSNP6061	*Kabuli*	Ca_Kabuli_Chr04	13713380	[G/T]		--	INTERGENIC

S.N.	SNP IDs	Chickpea cultivars	Chromosomes	Physical positions (bp)	SNPs	Gene accession IDs	Putative functions	Sequence components of genome
6062	CakSNP6062	*Kabuli*	Ca_Kabuli_Chr04	13713367	[G/C]		--	INTERGENIC
6063	CakSNP6063	*Kabuli*	Ca_Kabuli_Chr04	13724666	[G/C]	Ca_04596	Zinc finger, PHD-type	NON_SYNONYMOUS_CODING
6064	CakSNP6064	*Kabuli*	Ca_Kabuli_Chr04	13787448	[A/C]	Ca_04603	Copper amine oxidase	INTRON
6065	CakSNP6065	*Kabuli*	Ca_Kabuli_Chr04	13787649	[T/C]	Ca_04603	Copper amine oxidase	SYNONYMOUS_CODING
6066	CakSNP6066	*Kabuli*	Ca_Kabuli_Chr04	13787720	[G/C]	Ca_04603	Copper amine oxidase	NON_SYNONYMOUS_CODING
6067	CakSNP6067	*Kabuli*	Ca_Kabuli_Chr04	13823667	[T/A]		--	INTERGENIC
6068	CakSNP6068	*Kabuli*	Ca_Kabuli_Chr04	13823672	[G/T]		--	INTERGENIC
6069	CakSNP6069	*Kabuli*	Ca_Kabuli_Chr04	13823695	[T/C]		--	INTERGENIC
6070	CakSNP6070	*Kabuli*	Ca_Kabuli_Chr04	13838241	[G/T]	Ca_04608	--	SYNONYMOUS_CODING
6071	CakSNP6071	*Kabuli*	Ca_Kabuli_Chr04	13838796	[A/G]	Ca_04608	--	SYNONYMOUS_CODING

S.N.	SNP IDs	Chickpea cultivars	Chromosomes	Physical positions (bp)	SNPs	Gene accession IDs	Putative functions	Sequence components of genome
6072	CakSNP6072	*Kabuli*	Ca_Kabuli_Chr04	13839294	[A/G]	Ca_04608	--	SYNONYMOUS_CODING
6073	CakSNP6073	*Kabuli*	Ca_Kabuli_Chr04	13839288	[G/A]	Ca_04608	--	SYNONYMOUS_CODING
6074	CakSNP6074	*Kabuli*	Ca_Kabuli_Chr04	13840191	[T/A]	Ca_04608	--	SYNONYMOUS_CODING
6075	CakSNP6075	*Kabuli*	Ca_Kabuli_Chr04	13840227	[C/A]	Ca_04608	--	SYNONYMOUS_CODING
6076	CakSNP6076	*Kabuli*	Ca_Kabuli_Chr04	13840251	[A/G]	Ca_04608	--	SYNONYMOUS_CODING
6077	CakSNP6077	*Kabuli*	Ca_Kabuli_Chr04	13840484	[T/C]	Ca_04608	--	SYNONYMOUS_CODING
6078	CakSNP6078	*Kabuli*	Ca_Kabuli_Chr04	13841340	[G/T]	Ca_04608	--	NON_SYNONYMOUS_CODING
6079	CakSNP6079	*Kabuli*	Ca_Kabuli_Chr04	13841401	[A/G]	Ca_04608	--	SYNONYMOUS_CODING
6080	CakSNP6080	*Kabuli*	Ca_Kabuli_Chr04	13845581	[A/T]		--	INTERGENIC
6081	CakSNP6081	*Kabuli*	Ca_Kabuli_Chr04	13845639	[A/C]		--	INTERGENIC

S.N.	SNP IDs	Chickpea cultivars	Chromosomes	Physical positions (bp)	SNPs	Gene accession IDs	Putative functions	Sequence components of genome
6082	CakSNP6082	*Kabuli*	Ca_Kabuli_Chr04	13845675	[T/A]		--	INTERGENIC
6083	CakSNP6083	*Kabuli*	Ca_Kabuli_Chr04	13900013	[A/G]		--	INTERGENIC
6084	CakSNP6084	*Kabuli*	Ca_Kabuli_Chr04	13923366	[T/C]	Ca_04614	--	SYNONYMOUS_CODING
6085	CakSNP6085	*Kabuli*	Ca_Kabuli_Chr04	13949513	[A/C]		--	INTERGENIC
6086	CakSNP6086	*Kabuli*	Ca_Kabuli_Chr04	13949580	[C/G]		--	INTERGENIC
6087	CakSNP6087	*Kabuli*	Ca_Kabuli_Chr04	13954832	[G/A]	Ca_04618	Protein kinase, catalytic domain	NON_SYNONYMOUS_CODING
6088	CakSNP6088	*Kabuli*	Ca_Kabuli_Chr04	13968291	[C/T]		--	INTERGENIC
6089	CakSNP6089	*Kabuli*	Ca_Kabuli_Chr04	13968397	[A/G]		--	INTERGENIC
6090	CakSNP6090	*Kabuli*	Ca_Kabuli_Chr04	13968354	[A/G]		--	INTERGENIC
6091	CakSNP6091	*Kabuli*	Ca_Kabuli_Chr04	13982970	[G/A]	Ca_04623	Protein of unknown function DUF171	SYNONYMOUS_CODING

S.N.	SNP IDs	Chickpea cultivars	Chromosomes	Physical positions (bp)	SNPs	Gene accession IDs	Putative functions	Sequence components of genome
6092	CakSNP6092	*Kabuli*	Ca_Kabuli_Chr04	14031548	[A/G]	Ca_04627	Mitochondrial carrier protein	INTRON
6093	CakSNP6093	*Kabuli*	Ca_Kabuli_Chr04	14056908	[A/G]		--	INTERGENIC
6094	CakSNP6094	*Kabuli*	Ca_Kabuli_Chr04	14073059	[C/T]	Ca_04634	Glycoside hydrolase, family 16	INTRON
6095	CakSNP6095	*Kabuli*	Ca_Kabuli_Chr04	14073056	[C/T]	Ca_04634	Glycoside hydrolase, family 16	INTRON
6096	CakSNP6096	*Kabuli*	Ca_Kabuli_Chr04	14073045	[G/A]	Ca_04634	Glycoside hydrolase, family 16	INTRON
6097	CakSNP6097	*Kabuli*	Ca_Kabuli_Chr04	14073044	[C/T]	Ca_04634	Glycoside hydrolase, family 16	INTRON
6098	CakSNP6098	*Kabuli*	Ca_Kabuli_Chr04	14073004	[C/A]	Ca_04634	Glycoside hydrolase, family 16	INTRON
6099	CakSNP6099	*Kabuli*	Ca_Kabuli_Chr04	14082157	[C/A]	Ca_04635	PDZ-binding protein, CRIPT	INTRON
6100	CakSNP6100	*Kabuli*	Ca_Kabuli_Chr04	14082158	[T/G]	Ca_04635	PDZ-binding protein, CRIPT	INTRON
6101	CakSNP6101	*Kabuli*	Ca_Kabuli_Chr04	14082186	[G/A]	Ca_04635	PDZ-binding protein, CRIPT	INTRON

S.N.	SNP IDs	Chickpea cultivars	Chromosomes	Physical positions (bp)	SNPs	Gene accession IDs	Putative functions	Sequence components of genome
6102	CakSNP6102	*Kabuli*	Ca_Kabuli_Chr04	14082210	[T/C]	Ca_04635	PDZ-binding protein, CRIPT	INTRON
6103	CakSNP6103	*Kabuli*	Ca_Kabuli_Chr04	14082285	[C/G]		--	INTERGENIC
6104	CakSNP6104	*Kabuli*	Ca_Kabuli_Chr04	14093027	[A/G]	Ca_04636	Ubiquitin	SYNONYMOUS_CODING
6105	CakSNP6105	*Kabuli*	Ca_Kabuli_Chr04	14104096	[A/C]		--	INTERGENIC
6106	CakSNP6106	*Kabuli*	Ca_Kabuli_Chr04	14112911	[C/A]		--	INTERGENIC
6107	CakSNP6107	*Kabuli*	Ca_Kabuli_Chr04	14132659	[A/C]		--	INTERGENIC
6108	CakSNP6108	*Kabuli*	Ca_Kabuli_Chr04	14149589	[G/A]	Ca_04645	Protein kinase, catalytic domain	NON_SYNONYMOUS_CODING
6109	CakSNP6109	*Kabuli*	Ca_Kabuli_Chr04	14149588	[A/G]	Ca_04645	Protein kinase, catalytic domain	NON_SYNONYMOUS_CODING
6110	CakSNP6110	*Kabuli*	Ca_Kabuli_Chr04	14155978	[A/G]	Ca_04646	Protein kinase, catalytic domain	SYNONYMOUS_CODING
6111	CakSNP6111	*Kabuli*	Ca_Kabuli_Chr04	14156014	[C/T]	Ca_04646	Protein kinase, catalytic domain	SYNONYMOUS_CODING

S.N.	SNP IDs	Chickpea cultivars	Chromosomes	Physical positions (bp)	SNPs	Gene accession IDs	Putative functions	Sequence components of genome
6112	CakSNP6112	*Kabuli*	Ca_Kabuli_Chr04	14163060	[C/T]	Ca_04647	Protein kinase, catalytic domain	SYNONYMOUS_CODING
6113	CakSNP6113	*Kabuli*	Ca_Kabuli_Chr04	14181527	[A/G]	Ca_04651	Protein kinase, catalytic domain	NON_SYNONYMOUS_CODING
6114	CakSNP6114	*Kabuli*	Ca_Kabuli_Chr04	14189356	[A/C]	Ca_04652	Protein kinase, catalytic domain	NON_SYNONYMOUS_CODING
6115	CakSNP6115	*Kabuli*	Ca_Kabuli_Chr04	14199191	[G/C]	Ca_04653	Protein kinase, catalytic domain	NON_SYNONYMOUS_CODING
6116	CakSNP6116	*Kabuli*	Ca_Kabuli_Chr04	14225284	[A/G]		--	INTERGENIC
6117	CakSNP6117	*Kabuli*	Ca_Kabuli_Chr04	14270811	[G/A]	Ca_04663	--	NON_SYNONYMOUS_CODING
6118	CakSNP6118	*Kabuli*	Ca_Kabuli_Chr04	14270763	[G/A]	Ca_04663	--	NON_SYNONYMOUS_CODING
6119	CakSNP6119	*Kabuli*	Ca_Kabuli_Chr04	14453942	[T/C]	Ca_05659	--	SYNONYMOUS_CODING
6120	CakSNP6120	*Kabuli*	Ca_Kabuli_Chr04	14515543	[T/A]		--	INTERGENIC
6121	CakSNP6121	*Kabuli*	Ca_Kabuli_Chr04	14515531	[T/A]		--	INTERGENIC

S.N.	SNP IDs	Chickpea cultivars	Chromosomes	Physical positions (bp)	SNPs	Gene accession IDs	Putative functions	Sequence components of genome
6122	CakSNP6122	*Kabuli*	Ca_Kabuli_Chr04	14517424	[C/T]		--	INTERGENIC
6123	CakSNP6123	*Kabuli*	Ca_Kabuli_Chr04	14529007	[A/G]		--	INTERGENIC
6124	CakSNP6124	*Kabuli*	Ca_Kabuli_Chr04	14565948	[A/C]		--	INTERGENIC
6125	CakSNP6125	*Kabuli*	Ca_Kabuli_Chr04	14566064	[G/A]		--	INTERGENIC
6126	CakSNP6126	*Kabuli*	Ca_Kabuli_Chr04	14566074	[T/A]		--	INTERGENIC
6127	CakSNP6127	*Kabuli*	Ca_Kabuli_Chr04	14566081	[A/G]		--	INTERGENIC
6128	CakSNP6128	*Kabuli*	Ca_Kabuli_Chr04	14611177	[A/G]		--	INTERGENIC
6129	CakSNP6129	*Kabuli*	Ca_Kabuli_Chr04	14657743	[C/T]	Ca_05639	Protein of unknown function DUF567	INTRON
6130	CakSNP6130	*Kabuli*	Ca_Kabuli_Chr04	14696775	[T/C]		--	INTERGENIC
6131	CakSNP6131	*Kabuli*	Ca_Kabuli_Chr04	14698825	[A/T]	Ca_05634	--	SYNONYMOUS_CODING

S.N.	SNP IDs	Chickpea cultivars	Chromosomes	Physical positions (bp)	SNPs	Gene accession IDs	Putative functions	Sequence components of genome
6132	CakSNP6132	*Kabuli*	Ca_Kabuli_Chr04	14699270	[T/C]	Ca_05634	--	NON_SYNONYMOUS_CODING
6133	CakSNP6133	*Kabuli*	Ca_Kabuli_Chr04	14748051	[A/C]	Ca_05628	Protein of unknown function DUF620	SYNONYMOUS_CODING
6134	CakSNP6134	*Kabuli*	Ca_Kabuli_Chr04	14770484	[C/T]	Ca_05627	Peptidase C48, SUMO/Sentrin/Ubl1	NON_SYNONYMOUS_CODING
6135	CakSNP6135	*Kabuli*	Ca_Kabuli_Chr04	14770524	[G/C]	Ca_05627	Peptidase C48, SUMO/Sentrin/Ubl1	SYNONYMOUS_CODING
6136	CakSNP6136	*Kabuli*	Ca_Kabuli_Chr04	14770525	[G/A]	Ca_05627	Peptidase C48, SUMO/Sentrin/Ubl1	NON_SYNONYMOUS_CODING
6137	CakSNP6137	*Kabuli*	Ca_Kabuli_Chr04	14770532	[C/T]	Ca_05627	Peptidase C48, SUMO/Sentrin/Ubl1	NON_SYNONYMOUS_CODING
6138	CakSNP6138	*Kabuli*	Ca_Kabuli_Chr04	14785530	[G/A]		--	INTERGENIC
6139	CakSNP6139	*Kabuli*	Ca_Kabuli_Chr04	14785557	[A/C]		--	INTERGENIC
6140	CakSNP6140	*Kabuli*	Ca_Kabuli_Chr04	14786093	[C/G]	Ca_05625	Gibberellin regulated protein	NON_SYNONYMOUS_CODING
6141	CakSNP6141	*Kabuli*	Ca_Kabuli_Chr04	14831204	[A/T]		--	INTERGENIC

S.N.	SNP IDs	Chickpea cultivars	Chromosomes	Physical positions (bp)	SNPs	Gene accession IDs	Putative functions	Sequence components of genome
6142	CakSNP6142	*Kabuli*	Ca_Kabuli_Chr04	14831298	[A/T]		--	INTERGENIC
6143	CakSNP6143	*Kabuli*	Ca_Kabuli_Chr04	14855406	[T/C]	Ca_05615	--	INTRON
6144	CakSNP6144	*Kabuli*	Ca_Kabuli_Chr04	14855417	[T/A]	Ca_05615	--	INTRON
6145	CakSNP6145	*Kabuli*	Ca_Kabuli_Chr04	14870658	[T/G]	Ca_05614	Aminoglycoside phosphotransferase	NON_SYNONYMOUS_CODING
6146	CakSNP6146	*Kabuli*	Ca_Kabuli_Chr04	14870641	[G/A]	Ca_05614	Aminoglycoside phosphotransferase	SYNONYMOUS_CODING
6147	CakSNP6147	*Kabuli*	Ca_Kabuli_Chr04	14989557	[C/T]	Ca_05600	CWC16 protein	SYNONYMOUS_CODING
6148	CakSNP6148	*Kabuli*	Ca_Kabuli_Chr04	14989544	[C/T]	Ca_05600	CWC16 protein	NON_SYNONYMOUS_CODING
6149	CakSNP6149	*Kabuli*	Ca_Kabuli_Chr04	14989526	[G/A]	Ca_05600	CWC16 protein	NON_SYNONYMOUS_CODING
6150	CakSNP6150	*Kabuli*	Ca_Kabuli_Chr04	15025765	[T/A]	Ca_05597	Na+/H+ antiporter	INTRON
6151	CakSNP6151	*Kabuli*	Ca_Kabuli_Chr04	15025785	[T/G]	Ca_05597	Na+/H+ antiporter	INTRON

S.N.	SNP IDs	Chickpea cultivars	Chromosomes	Physical positions (bp)	SNPs	Gene accession IDs	Putative functions	Sequence components of genome
6152	CakSNP6152	*Kabuli*	Ca_Kabuli_Chr04	15026058	[C/T]	Ca_05597	Na+/H+ antiporter	SYNONYMOUS_CODING
6153	CakSNP6153	*Kabuli*	Ca_Kabuli_Chr04	15036362	[A/G]	Ca_05596	--	INTRON
6154	CakSNP6154	*Kabuli*	Ca_Kabuli_Chr04	15039897	[C/G]		--	INTERGENIC
6155	CakSNP6155	*Kabuli*	Ca_Kabuli_Chr04	15039905	[T/C]		--	INTERGENIC
6156	CakSNP6156	*Kabuli*	Ca_Kabuli_Chr04	15039948	[G/C]		--	INTERGENIC
6157	CakSNP6157	*Kabuli*	Ca_Kabuli_Chr04	15039996	[C/T]		--	INTERGENIC
6158	CakSNP6158	*Kabuli*	Ca_Kabuli_Chr04	15040072	[A/C]		--	INTERGENIC
6159	CakSNP6159	*Kabuli*	Ca_Kabuli_Chr04	15053643	[T/C]	Ca_05594	--	SYNONYMOUS_CODING
6160	CakSNP6160	*Kabuli*	Ca_Kabuli_Chr04	15075489	[T/G]	Ca_05592	Putative cyclase	INTRON
6161	CakSNP6161	*Kabuli*	Ca_Kabuli_Chr04	15075502	[G/T]	Ca_05592	Putative cyclase	INTRON

S.N.	SNP IDs	Chickpea cultivars	Chromosomes	Physical positions (bp)	SNPs	Gene accession IDs	Putative functions	Sequence components of genome
6162	CakSNP6162	*Kabuli*	Ca_Kabuli_Chr04	15159143	[A/G]	Ca_05584	Cwf19-like protein, C-terminal domain-2	SYNONYMOUS_CODING
6163	CakSNP6163	*Kabuli*	Ca_Kabuli_Chr04	15190387	[T/G]	Ca_05579	ABC transporter-like	SYNONYMOUS_CODING
6164	CakSNP6164	*Kabuli*	Ca_Kabuli_Chr04	15190423	[T/A]	Ca_05579	ABC transporter-like	SYNONYMOUS_CODING
6165	CakSNP6165	*Kabuli*	Ca_Kabuli_Chr04	15212787	[G/A]		--	INTERGENIC
6166	CakSNP6166	*Kabuli*	Ca_Kabuli_Chr04	15240675	[G/A]	Ca_05575	Defects-in-morphology protein 1-like, mitochondrial	NON_SYNONYMOUS_CODING
6167	CakSNP6167	*Kabuli*	Ca_Kabuli_Chr04	15240678	[T/C]	Ca_05575	Defects-in-morphology protein 1-like, mitochondrial	NON_SYNONYMOUS_CODING
6168	CakSNP6168	*Kabuli*	Ca_Kabuli_Chr04	15299232	[G/A]	Ca_05569	Maf1 regulator	INTRON
6169	CakSNP6169	*Kabuli*	Ca_Kabuli_Chr04	15320777	[G/A]	Ca_05565	--	INTRON
6170	CakSNP6170	*Kabuli*	Ca_Kabuli_Chr04	15357651	[C/T]	Ca_05562	--	NON_SYNONYMOUS_CODING
6171	CakSNP6171	*Kabuli*	Ca_Kabuli_Chr04	15357695	[A/G]	Ca_05562	--	NON_SYNONYMOUS_CODING

S.N.	SNP IDs	Chickpea cultivars	Chromosomes	Physical positions (bp)	SNPs	Gene accession IDs	Putative functions	Sequence components of genome
6172	CakSNP6172	*Kabuli*	Ca_Kabuli_Chr04	15394470	[T/C]	Ca_05556	Multi antimicrobial extrusion protein	NON_SYNONYMOUS_CODING
6173	CakSNP6173	*Kabuli*	Ca_Kabuli_Chr04	15448826	[G/T]	Ca_05551	Double-stranded RNA-binding	INTRON
6174	CakSNP6174	*Kabuli*	Ca_Kabuli_Chr04	15449843	[A/G]	Ca_05551	Double-stranded RNA-binding	INTRON
6175	CakSNP6175	*Kabuli*	Ca_Kabuli_Chr04	15542543	[T/C]	Ca_05547	Zinc finger, CCCH-type	SYNONYMOUS_CODING
6176	CakSNP6176	*Kabuli*	Ca_Kabuli_Chr04	15542597	[A/G]	Ca_05547	Zinc finger, CCCH-type	SYNONYMOUS_CODING
6177	CakSNP6177	*Kabuli*	Ca_Kabuli_Chr04	15552690	[C/T]		--	INTERGENIC
6178	CakSNP6178	*Kabuli*	Ca_Kabuli_Chr04	15552683	[C/T]		--	INTERGENIC
6179	CakSNP6179	*Kabuli*	Ca_Kabuli_Chr04	15697128	[A/G]	Ca_05536	Phox/Bem1p	NON_SYNONYMOUS_CODING
6180	CakSNP6180	*Kabuli*	Ca_Kabuli_Chr04	15697303	[G/T]	Ca_05536	Phox/Bem1p	SYNONYMOUS_CODING
6181	CakSNP6181	*Kabuli*	Ca_Kabuli_Chr04	15698212	[G/T]		--	INTERGENIC

S.N.	SNP IDs	Chickpea cultivars	Chromosomes	Physical positions (bp)	SNPs	Gene accession IDs	Putative functions	Sequence components of genome
6182	CakSNP6182	*Kabuli*	Ca_Kabuli_Chr04	15698179	[G/A]		--	INTERGENIC
6183	CakSNP6183	*Kabuli*	Ca_Kabuli_Chr04	15698233	[A/C]		--	INTERGENIC
6184	CakSNP6184	*Kabuli*	Ca_Kabuli_Chr04	15698305	[T/C]		--	INTERGENIC
6185	CakSNP6185	*Kabuli*	Ca_Kabuli_Chr04	15698405	[G/A]		--	INTERGENIC
6186	CakSNP6186	*Kabuli*	Ca_Kabuli_Chr04	15698486	[T/C]		--	INTERGENIC
6187	CakSNP6187	*Kabuli*	Ca_Kabuli_Chr04	15698456	[T/C]		--	INTERGENIC
6188	CakSNP6188	*Kabuli*	Ca_Kabuli_Chr04	15698444	[C/T]		--	INTERGENIC
6189	CakSNP6189	*Kabuli*	Ca_Kabuli_Chr04	15698420	[G/C]		--	INTERGENIC
6190	CakSNP6190	*Kabuli*	Ca_Kabuli_Chr04	15700603	[A/T]		--	INTERGENIC
6191	CakSNP6191	*Kabuli*	Ca_Kabuli_Chr04	15700630	[G/A]		--	INTERGENIC

S.N.	SNP IDs	Chickpea cultivars	Chromosomes	Physical positions (bp)	SNPs	Gene accession IDs	Putative functions	Sequence components of genome
6192	CakSNP6192	*Kabuli*	Ca_Kabuli_Chr04	15713955	[G/A]	Ca_05534	Alpha/beta hydrolase fold-1	INTRON
6193	CakSNP6193	*Kabuli*	Ca_Kabuli_Chr04	15717281	[T/C]		--	INTERGENIC
6194	CakSNP6194	*Kabuli*	Ca_Kabuli_Chr04	15735993	[T/C]	Ca_05530	Cytidylyltransferase	INTRON
6195	CakSNP6195	*Kabuli*	Ca_Kabuli_Chr04	15736015	[C/T]	Ca_05530	Cytidylyltransferase	INTRON
6196	CakSNP6196	*Kabuli*	Ca_Kabuli_Chr04	15736562	[A/T]	Ca_05530	Cytidylyltransferase	INTRON
6197	CakSNP6197	*Kabuli*	Ca_Kabuli_Chr04	15736619	[T/G]	Ca_05530	Cytidylyltransferase	INTRON
6198	CakSNP6198	*Kabuli*	Ca_Kabuli_Chr04	15736627	[T/C]	Ca_05530	Cytidylyltransferase	INTRON
6199	CakSNP6199	*Kabuli*	Ca_Kabuli_Chr04	15744703	[T/G]		--	INTERGENIC
6200	CakSNP6200	*Kabuli*	Ca_Kabuli_Chr04	15752567	[G/C]	Ca_05529	Glycosyl transferase, family 20	NON_SYNONYMOUS_CODING
6201	CakSNP6201	*Kabuli*	Ca_Kabuli_Chr04	15766341	[A/G]		--	INTERGENIC

S.N.	SNP IDs	Chickpea cultivars	Chromosomes	Physical positions (bp)	SNPs	Gene accession IDs	Putative functions	Sequence components of genome
6202	CakSNP6202	*Kabuli*	Ca_Kabuli_Chr04	15766360	[G/A]		--	INTERGENIC
6203	CakSNP6203	*Kabuli*	Ca_Kabuli_Chr04	15772235	[C/A]	Ca_05528	Reticulon	NON_SYNONYMOUS_CODING
6204	CakSNP6204	*Kabuli*	Ca_Kabuli_Chr04	15772275	[A/G]	Ca_05528	Reticulon	SYNONYMOUS_CODING
6205	CakSNP6205	*Kabuli*	Ca_Kabuli_Chr04	15821282	[G/A]		--	INTERGENIC
6206	CakSNP6206	*Kabuli*	Ca_Kabuli_Chr04	15821311	[T/A]		--	INTERGENIC
6207	CakSNP6207	*Kabuli*	Ca_Kabuli_Chr04	15825621	[A/T]	Ca_05524	Protein of unknown function DUF789	INTRON
6208	CakSNP6208	*Kabuli*	Ca_Kabuli_Chr04	15860388	[A/G]	Ca_05522	Protein kinase, catalytic domain	INTRON
6209	CakSNP6209	*Kabuli*	Ca_Kabuli_Chr04	15860359	[C/A]	Ca_05522	Protein kinase, catalytic domain	INTRON
6210	CakSNP6210	*Kabuli*	Ca_Kabuli_Chr04	15900602	[G/A]	Ca_05518	Protein phosphatase 2C-like	SYNONYMOUS_CODING
6211	CakSNP6211	*Kabuli*	Ca_Kabuli_Chr04	15925936	[T/G]		--	INTERGENIC

S.N.	SNP IDs	Chickpea cultivars	Chromosomes	Physical positions (bp)	SNPs	Gene accession IDs	Putative functions	Sequence components of genome
6212	CakSNP6212	*Kabuli*	Ca_Kabuli_Chr04	15926160	[A/G]		--	INTERGENIC
6213	CakSNP6213	*Kabuli*	Ca_Kabuli_Chr04	15934607	[A/T]	Ca_05513	Beta-lactamase-like	INTRON
6214	CakSNP6214	*Kabuli*	Ca_Kabuli_Chr04	15934901	[T/G]	Ca_05513	Beta-lactamase-like	SYNONYMOUS_CODING
6215	CakSNP6215	*Kabuli*	Ca_Kabuli_Chr04	15957070	[T/A]		--	INTERGENIC
6216	CakSNP6216	*Kabuli*	Ca_Kabuli_Chr04	16027272	[G/A]	Ca_05507	Inhibitor of apoptosis-promoting Bax1-related	SYNONYMOUS_CODING
6217	CakSNP6217	*Kabuli*	Ca_Kabuli_Chr04	16027292	[A/C]		--	INTERGENIC
6218	CakSNP6218	*Kabuli*	Ca_Kabuli_Chr04	16027368	[G/T]		--	INTERGENIC
6219	CakSNP6219	*Kabuli*	Ca_Kabuli_Chr04	16027299	[T/A]		--	INTERGENIC
6220	CakSNP6220	*Kabuli*	Ca_Kabuli_Chr04	16045694	[T/G]	Ca_05504	Glutamyl/glutaminyl-tRNA synthetase, class Ic	SYNONYMOUS_CODING
6221	CakSNP6221	*Kabuli*	Ca_Kabuli_Chr04	16045870	[G/A]	Ca_05504	Glutamyl/glutaminyl-tRNA synthetase, class Ic	INTRON

S.N.	SNP IDs	Chickpea cultivars	Chromosomes	Physical positions (bp)	SNPs	Gene accession IDs	Putative functions	Sequence components of genome
6222	CakSNP6222	*Kabuli*	Ca_Kabuli_Chr04	16046928	[T/C]	Ca_05504	Glutamyl/glutaminyl-tRNA synthetase, class Ic	SYNONYMOUS_CODING
6223	CakSNP6223	*Kabuli*	Ca_Kabuli_Chr04	16278600	[C/T]	Ca_05487	Zinc finger, B-box	NON_SYNONYMOUS_CODING
6224	CakSNP6224	*Kabuli*	Ca_Kabuli_Chr04	16278671	[T/A]	Ca_05487	Zinc finger, B-box	SYNONYMOUS_CODING
6225	CakSNP6225	*Kabuli*	Ca_Kabuli_Chr04	16446305	[T/C]	Ca_05482	Ubiquitin-associated/translation elongation factor EF1B, N-terminal	INTRON
6226	CakSNP6226	*Kabuli*	Ca_Kabuli_Chr04	16446341	[T/G]	Ca_05482	Ubiquitin-associated/translation elongation factor EF1B, N-terminal	SYNONYMOUS_CODING
6227	CakSNP6227	*Kabuli*	Ca_Kabuli_Chr04	16446336	[C/G]	Ca_05482	Ubiquitin-associated/translation elongation factor EF1B, N-terminal	NON_SYNONYMOUS_CODING
6228	CakSNP6228	*Kabuli*	Ca_Kabuli_Chr04	16446956	[T/C]	Ca_05482	Ubiquitin-associated/translation elongation factor EF1B, N-terminal	SYNONYMOUS_CODING
6229	CakSNP6229	*Kabuli*	Ca_Kabuli_Chr04	16457940	[G/A]	Ca_05480	Biotin/lipoyl attachment	SYNONYMOUS_CODING
6230	CakSNP6230	*Kabuli*	Ca_Kabuli_Chr04	16501291	[T/A]	Ca_05477	Domain of unknown function DUF292, eukaryotic	NON_SYNONYMOUS_CODING
6231	CakSNP6231	*Kabuli*	Ca_Kabuli_Chr04	16501306	[T/A]	Ca_05477	Domain of unknown function DUF292, eukaryotic	INTRON

S.N.	SNP IDs	Chickpea cultivars	Chromosomes	Physical positions (bp)	SNPs	Gene accession IDs	Putative functions	Sequence components of genome
6232	CakSNP6232	*Kabuli*	Ca_Kabuli_Chr04	16501354	[C/T]	Ca_05477	Domain of unknown function DUF292, eukaryotic	INTRON
6233	CakSNP6233	*Kabuli*	Ca_Kabuli_Chr04	16501782	[G/A]	Ca_05477	Domain of unknown function DUF292, eukaryotic	NON_SYNONYMOUS_CODING
6234	CakSNP6234	*Kabuli*	Ca_Kabuli_Chr04	16501781	[C/A]	Ca_05477	Domain of unknown function DUF292, eukaryotic	SYNONYMOUS_CODING
6235	CakSNP6235	*Kabuli*	Ca_Kabuli_Chr04	16501769	[C/T]	Ca_05477	Domain of unknown function DUF292, eukaryotic	SYNONYMOUS_CODING
6236	CakSNP6236	*Kabuli*	Ca_Kabuli_Chr04	16501738	[G/A]	Ca_05477	Domain of unknown function DUF292, eukaryotic	NON_SYNONYMOUS_CODING
6237	CakSNP6237	*Kabuli*	Ca_Kabuli_Chr04	16501716	[T/C]	Ca_05477	Domain of unknown function DUF292, eukaryotic	SYNONYMOUS_CODING
6238	CakSNP6238	*Kabuli*	Ca_Kabuli_Chr04	16505159	[A/G]	Ca_05476	Cytochrome P450	SYNONYMOUS_CODING
6239	CakSNP6239	*Kabuli*	Ca_Kabuli_Chr04	16536754	[C/T]	Ca_05470	Choline transporter-like	SYNONYMOUS_CODING
6240	CakSNP6240	*Kabuli*	Ca_Kabuli_Chr04	16571288	[T/C]	Ca_05467	ATPase, AAA+ type, core	INTRON
6241	CakSNP6241	*Kabuli*	Ca_Kabuli_Chr04	16586863	[G/C]		--	INTERGENIC

S.N.	SNP IDs	Chickpea cultivars	Chromosomes	Physical positions (bp)	SNPs	Gene accession IDs	Putative functions	Sequence components of genome
6242	CakSNP6242	*Kabuli*	Ca_Kabuli_Chr04	16586913	[G/C]		--	INTERGENIC
6243	CakSNP6243	*Kabuli*	Ca_Kabuli_Chr04	16586929	[G/C]		--	INTERGENIC
6244	CakSNP6244	*Kabuli*	Ca_Kabuli_Chr04	16619312	[T/C]	Ca_05461	KOW	SYNONYMOUS_CODING
6245	CakSNP6245	*Kabuli*	Ca_Kabuli_Chr04	16622514	[C/A]	Ca_05461	KOW	NON_SYNONYMOUS_CODING
6246	CakSNP6246	*Kabuli*	Ca_Kabuli_Chr04	16625148	[T/G]		--	INTERGENIC
6247	CakSNP6247	*Kabuli*	Ca_Kabuli_Chr04	16625270	[T/G]		--	INTERGENIC
6248	CakSNP6248	*Kabuli*	Ca_Kabuli_Chr04	16625221	[G/A]		--	INTERGENIC
6249	CakSNP6249	*Kabuli*	Ca_Kabuli_Chr04	16628559	[T/G]	Ca_05460	Protein phosphatase 2A, regulatory B subunit, B56	INTRON
6250	CakSNP6250	*Kabuli*	Ca_Kabuli_Chr04	16628591	[T/G]	Ca_05460	Protein phosphatase 2A, regulatory B subunit, B56	INTRON
6251	CakSNP6251	*Kabuli*	Ca_Kabuli_Chr04	16628594	[T/G]	Ca_05460	Protein phosphatase 2A, regulatory B subunit, B56	INTRON

S.N.	SNP IDs	Chickpea cultivars	Chromosomes	Physical positions (bp)	SNPs	Gene accession IDs	Putative functions	Sequence components of genome
6252	CakSNP6252	*Kabuli*	Ca_Kabuli_Chr04	16628542	[G/A]	Ca_05460	Protein phosphatase 2A, regulatory B subunit, B56	INTRON
6253	CakSNP6253	*Kabuli*	Ca_Kabuli_Chr04	16628540	[G/A]	Ca_05460	Protein phosphatase 2A, regulatory B subunit, B56	INTRON
6254	CakSNP6254	*Kabuli*	Ca_Kabuli_Chr04	16628536	[T/A]	Ca_05460	Protein phosphatase 2A, regulatory B subunit, B56	INTRON
6255	CakSNP6255	*Kabuli*	Ca_Kabuli_Chr04	16628529	[C/A]	Ca_05460	Protein phosphatase 2A, regulatory B subunit, B56	INTRON
6256	CakSNP6256	*Kabuli*	Ca_Kabuli_Chr04	16628527	[G/A]	Ca_05460	Protein phosphatase 2A, regulatory B subunit, B56	INTRON
6257	CakSNP6257	*Kabuli*	Ca_Kabuli_Chr04	16628523	[T/C]	Ca_05460	Protein phosphatase 2A, regulatory B subunit, B56	INTRON
6258	CakSNP6258	*Kabuli*	Ca_Kabuli_Chr04	16629560	[G/T]	Ca_05460	Protein phosphatase 2A, regulatory B subunit, B56	SYNONYMOUS_CODING
6259	CakSNP6259	*Kabuli*	Ca_Kabuli_Chr04	16629559	[T/G]	Ca_05460	Protein phosphatase 2A, regulatory B subunit, B56	NON_SYNONYMOUS_CODING
6260	CakSNP6260	*Kabuli*	Ca_Kabuli_Chr04	16629638	[C/A]		--	INTERGENIC
6261	CakSNP6261	*Kabuli*	Ca_Kabuli_Chr04	16630385	[G/T]		--	INTERGENIC

S.N.	SNP IDs	Chickpea cultivars	Chromosomes	Physical positions (bp)	SNPs	Gene accession IDs	Putative functions	Sequence components of genome
6262	CakSNP6262	*Kabuli*	Ca_Kabuli_Chr04	16669916	[T/C]	Ca_05457	Inner centromere protein, ARK-binding domain	INTRON
6263	CakSNP6263	*Kabuli*	Ca_Kabuli_Chr04	16749740	[A/G]	Ca_05451	AMMECR1 domain	INTRON
6264	CakSNP6264	*Kabuli*	Ca_Kabuli_Chr04	16750416	[A/G]	Ca_05451	AMMECR1 domain	SYNONYMOUS_CODING
6265	CakSNP6265	*Kabuli*	Ca_Kabuli_Chr04	16751205	[T/C]	Ca_05450	RNA recognition motif domain	NON_SYNONYMOUS_CODING
6266	CakSNP6266	*Kabuli*	Ca_Kabuli_Chr04	16789476	[G/A]	Ca_05446	Haemerythrin/HHE cation-binding motif	SYNONYMOUS_CODING
6267	CakSNP6267	*Kabuli*	Ca_Kabuli_Chr04	16789532	[A/C]	Ca_05446	Haemerythrin/HHE cation-binding motif	NON_SYNONYMOUS_CODING
6268	CakSNP6268	*Kabuli*	Ca_Kabuli_Chr04	16820726	[C/A]		--	INTERGENIC
6269	CakSNP6269	*Kabuli*	Ca_Kabuli_Chr04	16837336	[T/A]	Ca_05438	HEAT, type 2	INTRON
6270	CakSNP6270	*Kabuli*	Ca_Kabuli_Chr04	16839081	[T/A]	Ca_05438	HEAT, type 2	NON_SYNONYMOUS_CODING
6271	CakSNP6271	*Kabuli*	Ca_Kabuli_Chr04	16845299	[G/A]		--	INTERGENIC

S.N.	SNP IDs	Chickpea cultivars	Chromosomes	Physical positions (bp)	SNPs	Gene accession IDs	Putative functions	Sequence components of genome
6272	CakSNP6272	*Kabuli*	Ca_Kabuli_Chr04	16889650	[A/T]		--	INTERGENIC
6273	CakSNP6273	*Kabuli*	Ca_Kabuli_Chr04	16926545	[T/C]	Ca_05430	Zinc finger, C2H2-type	SYNONYMOUS_CODING
6274	CakSNP6274	*Kabuli*	Ca_Kabuli_Chr04	16926527	[T/A]	Ca_05430	Zinc finger, C2H2-type	SYNONYMOUS_CODING
6275	CakSNP6275	*Kabuli*	Ca_Kabuli_Chr04	16926614	[A/T]	Ca_05430	Zinc finger, C2H2-type	NON_SYNONYMOUS_CODING
6276	CakSNP6276	*Kabuli*	Ca_Kabuli_Chr04	17028303	[A/G]		--	INTERGENIC
6277	CakSNP6277	*Kabuli*	Ca_Kabuli_Chr04	17028368	[G/A]		--	INTERGENIC
6278	CakSNP6278	*Kabuli*	Ca_Kabuli_Chr04	17028523	[C/T]		--	INTERGENIC
6279	CakSNP6279	*Kabuli*	Ca_Kabuli_Chr04	17028495	[T/A]		--	INTERGENIC
6280	CakSNP6280	*Kabuli*	Ca_Kabuli_Chr04	17032676	[T/C]	Ca_05420	Pentatricopeptide repeat	INTRON
6281	CakSNP6281	*Kabuli*	Ca_Kabuli_Chr04	17032673	[C/T]	Ca_05420	Pentatricopeptide repeat	INTRON

S.N.	SNP IDs	Chickpea cultivars	Chromosomes	Physical positions (bp)	SNPs	Gene accession IDs	Putative functions	Sequence components of genome
6282	CakSNP6282	*Kabuli*	Ca_Kabuli_Chr04	17059830	[G/A]	Ca_05417	Phospholipase-like, arabidopsis	INTRON
6283	CakSNP6283	*Kabuli*	Ca_Kabuli_Chr04	17059833	[A/T]	Ca_05417	Phospholipase-like, arabidopsis	INTRON
6284	CakSNP6284	*Kabuli*	Ca_Kabuli_Chr04	17059852	[T/C]	Ca_05417	Phospholipase-like, arabidopsis	INTRON
6285	CakSNP6285	*Kabuli*	Ca_Kabuli_Chr04	17059866	[G/A]	Ca_05417	Phospholipase-like, arabidopsis	INTRON
6286	CakSNP6286	*Kabuli*	Ca_Kabuli_Chr04	17059872	[A/G]	Ca_05417	Phospholipase-like, arabidopsis	INTRON
6287	CakSNP6287	*Kabuli*	Ca_Kabuli_Chr04	17059887	[G/A]	Ca_05417	Phospholipase-like, arabidopsis	INTRON
6288	CakSNP6288	*Kabuli*	Ca_Kabuli_Chr04	17093090	[C/G]		--	INTERGENIC
6289	CakSNP6289	*Kabuli*	Ca_Kabuli_Chr04	17147875	[C/T]		--	INTERGENIC
6290	CakSNP6290	*Kabuli*	Ca_Kabuli_Chr04	17147926	[T/C]		--	INTERGENIC
6291	CakSNP6291	*Kabuli*	Ca_Kabuli_Chr04	17187560	[C/G]		--	INTERGENIC

S.N.	SNP IDs	Chickpea cultivars	Chromosomes	Physical positions (bp)	SNPs	Gene accession IDs	Putative functions	Sequence components of genome
6292	CakSNP6292	*Kabuli*	Ca_Kabuli_Chr04	17191562	[T/A]	Ca_05405	Protein phosphatase 2C, manganese/magnesium aspartate binding site	SYNONYMOUS_CODING
6293	CakSNP6293	*Kabuli*	Ca_Kabuli_Chr04	17199316	[T/C]	Ca_05404	Domain of unknown function DUF632	SYNONYMOUS_CODING
6294	CakSNP6294	*Kabuli*	Ca_Kabuli_Chr04	17199477	[G/A]	Ca_05404	Domain of unknown function DUF632	SYNONYMOUS_CODING
6295	CakSNP6295	*Kabuli*	Ca_Kabuli_Chr04	17199432	[G/A]	Ca_05404	Domain of unknown function DUF632	SYNONYMOUS_CODING
6296	CakSNP6296	*Kabuli*	Ca_Kabuli_Chr04	17202770	[A/G]	Ca_05403	Protein of unknown function DUF3353	SYNONYMOUS_CODING
6297	CakSNP6297	*Kabuli*	Ca_Kabuli_Chr04	17225334	[G/A]	Ca_05400	HEAT	SYNONYMOUS_CODING
6298	CakSNP6298	*Kabuli*	Ca_Kabuli_Chr04	17264248	[C/A]	Ca_05398	Major facilitator superfamily MFS-1	SYNONYMOUS_CODING
6299	CakSNP6299	*Kabuli*	Ca_Kabuli_Chr04	17264592	[G/A]	Ca_05398	Major facilitator superfamily MFS-1	INTRON
6300	CakSNP6300	*Kabuli*	Ca_Kabuli_Chr04	17264621	[C/G]	Ca_05398	Major facilitator superfamily MFS-1	INTRON
6301	CakSNP6301	*Kabuli*	Ca_Kabuli_Chr04	17264637	[C/T]	Ca_05398	Major facilitator superfamily MFS-1	INTRON

S.N.	SNP IDs	Chickpea cultivars	Chromosomes	Physical positions (bp)	SNPs	Gene accession IDs	Putative functions	Sequence components of genome
6302	CakSNP6302	*Kabuli*	Ca_Kabuli_Chr04	17282119	[T/C]	Ca_05394	Ankyrin repeat	SYNONYMOUS_CODING
6303	CakSNP6303	*Kabuli*	Ca_Kabuli_Chr04	17282139	[G/C]	Ca_05394	Ankyrin repeat	NON_SYNONYMOUS_CODING
6304	CakSNP6304	*Kabuli*	Ca_Kabuli_Chr04	17282148	[G/C]	Ca_05394	Ankyrin repeat	SYNONYMOUS_CODING
6305	CakSNP6305	*Kabuli*	Ca_Kabuli_Chr04	17282336	[G/T]	Ca_05394	Ankyrin repeat	SYNONYMOUS_CODING
6306	CakSNP6306	*Kabuli*	Ca_Kabuli_Chr04	17282345	[G/C]	Ca_05394	Ankyrin repeat	SYNONYMOUS_CODING
6307	CakSNP6307	*Kabuli*	Ca_Kabuli_Chr04	17289594	[G/C]		--	INTERGENIC
6308	CakSNP6308	*Kabuli*	Ca_Kabuli_Chr04	17297498	[G/T]	Ca_05392	Ankyrin repeat-containing domain	INTRON
6309	CakSNP6309	*Kabuli*	Ca_Kabuli_Chr04	17297567	[G/A]	Ca_05392	Ankyrin repeat-containing domain	INTRON
6310	CakSNP6310	*Kabuli*	Ca_Kabuli_Chr04	17297563	[A/G]	Ca_05392	Ankyrin repeat-containing domain	INTRON
6311	CakSNP6311	*Kabuli*	Ca_Kabuli_Chr04	17309656	[A/C]	Ca_05391	Phosphatidylinositol 3-/4-kinase, catalytic	SYNONYMOUS_CODING

S.N.	SNP IDs	Chickpea cultivars	Chromosomes	Physical positions (bp)	SNPs	Gene accession IDs	Putative functions	Sequence components of genome
6312	CakSNP6312	*Kabuli*	Ca_Kabuli_Chr04	17345431	[T/C]	Ca_05389	Fibrillarin	INTRON
6313	CakSNP6313	*Kabuli*	Ca_Kabuli_Chr04	17345436	[G/T]	Ca_05389	Fibrillarin	INTRON
6314	CakSNP6314	*Kabuli*	Ca_Kabuli_Chr04	17345439	[G/A]	Ca_05389	Fibrillarin	INTRON
6315	CakSNP6315	*Kabuli*	Ca_Kabuli_Chr04	17345448	[T/G]	Ca_05389	Fibrillarin	INTRON
6316	CakSNP6316	*Kabuli*	Ca_Kabuli_Chr04	17345500	[G/A]	Ca_05389	Fibrillarin	SYNONYMOUS_CODING
6317	CakSNP6317	*Kabuli*	Ca_Kabuli_Chr04	17345483	[C/A]	Ca_05389	Fibrillarin	INTRON
6318	CakSNP6318	*Kabuli*	Ca_Kabuli_Chr04	17345454	[A/G]	Ca_05389	Fibrillarin	INTRON
6319	CakSNP6319	*Kabuli*	Ca_Kabuli_Chr04	17358736	[C/T]	Ca_05388	Protein kinase, catalytic domain	INTRON
6320	CakSNP6320	*Kabuli*	Ca_Kabuli_Chr04	17361143	[T/A]	Ca_05388	Protein kinase, catalytic domain	INTRON
6321	CakSNP6321	*Kabuli*	Ca_Kabuli_Chr04	17396325	[A/G]	Ca_05386	Zinc finger, RING-type	INTRON

S.N.	SNP IDs	Chickpea cultivars	Chromosomes	Physical positions (bp)	SNPs	Gene accession IDs	Putative functions	Sequence components of genome
6322	CakSNP6322	*Kabuli*	Ca_Kabuli_Chr04	17396349	[T/G]	Ca_05386	Zinc finger, RING-type	INTRON
6323	CakSNP6323	*Kabuli*	Ca_Kabuli_Chr04	17396352	[T/G]	Ca_05386	Zinc finger, RING-type	INTRON
6324	CakSNP6324	*Kabuli*	Ca_Kabuli_Chr04	17429456	[A/T]	Ca_05384	Transcription factor jumonji/aspartyl beta-hydroxylase	SYNONYMOUS_CODING
6325	CakSNP6325	*Kabuli*	Ca_Kabuli_Chr04	17429457	[A/T]	Ca_05384	Transcription factor jumonji/aspartyl beta-hydroxylase	SYNONYMOUS_CODING
6326	CakSNP6326	*Kabuli*	Ca_Kabuli_Chr04	17429468	[T/C]	Ca_05384	Transcription factor jumonji/aspartyl beta-hydroxylase	SYNONYMOUS_CODING
6327	CakSNP6327	*Kabuli*	Ca_Kabuli_Chr04	17429747	[G/A]	Ca_05384	Transcription factor jumonji/aspartyl beta-hydroxylase	SYNONYMOUS_CODING
6328	CakSNP6328	*Kabuli*	Ca_Kabuli_Chr04	17429742	[A/G]	Ca_05384	Transcription factor jumonji/aspartyl beta-hydroxylase	SYNONYMOUS_CODING
6329	CakSNP6329	*Kabuli*	Ca_Kabuli_Chr04	17429735	[A/G]	Ca_05384	Transcription factor jumonji/aspartyl beta-hydroxylase	SYNONYMOUS_CODING
6330	CakSNP6330	*Kabuli*	Ca_Kabuli_Chr04	17429699	[G/C]	Ca_05384	Transcription factor jumonji/aspartyl beta-hydroxylase	SYNONYMOUS_CODING
6331	CakSNP6331	*Kabuli*	Ca_Kabuli_Chr04	17433782	[G/A]	Ca_05384	Transcription factor jumonji/aspartyl beta-hydroxylase	INTRON

S.N.	SNP IDs	Chickpea cultivars	Chromosomes	Physical positions (bp)	SNPs	Gene accession IDs	Putative functions	Sequence components of genome
6332	CakSNP6332	*Kabuli*	Ca_Kabuli_Chr04	17433914	[G/A]	Ca_05384	Transcription factor jumonji/aspartyl beta-hydroxylase	INTRON
6333	CakSNP6333	*Kabuli*	Ca_Kabuli_Chr04	17444578	[A/C]		--	INTERGENIC
6334	CakSNP6334	*Kabuli*	Ca_Kabuli_Chr04	17472129	[T/A]		--	INTERGENIC
6335	CakSNP6335	*Kabuli*	Ca_Kabuli_Chr04	17472992	[T/G]	Ca_05380	SKI-interacting protein SKIP, SNW domain	SYNONYMOUS_CODING
6336	CakSNP6336	*Kabuli*	Ca_Kabuli_Chr04	17473174	[C/T]	Ca_05380	SKI-interacting protein SKIP, SNW domain	SYNONYMOUS_CODING
6337	CakSNP6337	*Kabuli*	Ca_Kabuli_Chr04	17473237	[T/C]	Ca_05380	SKI-interacting protein SKIP, SNW domain	SYNONYMOUS_CODING
6338	CakSNP6338	*Kabuli*	Ca_Kabuli_Chr04	17473234	[C/A]	Ca_05380	SKI-interacting protein SKIP, SNW domain	SYNONYMOUS_CODING
6339	CakSNP6339	*Kabuli*	Ca_Kabuli_Chr04	17603613	[T/C]	Ca_05371	E2F-associated phosphoprotein	INTRON
6340	CakSNP6340	*Kabuli*	Ca_Kabuli_Chr04	17603677	[G/C]	Ca_05371	E2F-associated phosphoprotein	INTRON
6341	CakSNP6341	*Kabuli*	Ca_Kabuli_Chr04	17603689	[T/C]	Ca_05371	E2F-associated phosphoprotein	INTRON

S.N.	SNP IDs	Chickpea cultivars	Chromosomes	Physical positions (bp)	SNPs	Gene accession IDs	Putative functions	Sequence components of genome
6342	CakSNP6342	*Kabuli*	Ca_Kabuli_Chr04	17603725	[T/C]	Ca_05371	E2F-associated phosphoprotein	SYNONYMOUS_CODING
6343	CakSNP6343	*Kabuli*	Ca_Kabuli_Chr04	17609554	[A/T]		--	INTERGENIC
6344	CakSNP6344	*Kabuli*	Ca_Kabuli_Chr04	17621035	[G/T]	Ca_05368	Dihydroorotate dehydrogenase, conserved site	INTRON
6345	CakSNP6345	*Kabuli*	Ca_Kabuli_Chr04	17621609	[G/A]	Ca_05368	Dihydroorotate dehydrogenase, conserved site	NON_SYNONYMOUS_CODING
6346	CakSNP6346	*Kabuli*	Ca_Kabuli_Chr04	17647558	[G/A]	Ca_05365	Molybdopterin cofactor biosynthesis C (MoaC) domain	SYNONYMOUS_CODING
6347	CakSNP6347	*Kabuli*	Ca_Kabuli_Chr04	17652735	[T/C]	Ca_05364	Protein of unknown function DUF829, TMEM53	SYNONYMOUS_CODING
6348	CakSNP6348	*Kabuli*	Ca_Kabuli_Chr04	17685058	[T/G]	Ca_05357	Ribosomal protein L7/L12, C-terminal	SYNONYMOUS_CODING
6349	CakSNP6349	*Kabuli*	Ca_Kabuli_Chr04	17685113	[C/A]	Ca_05357	Ribosomal protein L7/L12, C-terminal	NON_SYNONYMOUS_CODING
6350	CakSNP6350	*Kabuli*	Ca_Kabuli_Chr04	17718540	[C/T]	Ca_05353	Peptidase A1	SYNONYMOUS_CODING
6351	CakSNP6351	*Kabuli*	Ca_Kabuli_Chr04	17718536	[C/A]	Ca_05353	Peptidase A1	SYNONYMOUS_CODING

S.N.	SNP IDs	Chickpea cultivars	Chromosomes	Physical positions (bp)	SNPs	Gene accession IDs	Putative functions	Sequence components of genome
6352	CakSNP6352	*Kabuli*	Ca_Kabuli_Chr04	17718894	[G/A]	Ca_05353	Peptidase A1	SYNONYMOUS_CODING
6353	CakSNP6353	*Kabuli*	Ca_Kabuli_Chr04	17718922	[T/G]	Ca_05353	Peptidase A1	NON_SYNONYMOUS_CODING
6354	CakSNP6354	*Kabuli*	Ca_Kabuli_Chr04	17718997	[C/A]	Ca_05353	Peptidase A1	SYNONYMOUS_CODING
6355	CakSNP6355	*Kabuli*	Ca_Kabuli_Chr04	17729988	[A/C]	Ca_05352	SNF2-related	INTRON
6356	CakSNP6356	*Kabuli*	Ca_Kabuli_Chr04	17748171	[A/T]		--	INTERGENIC
6357	CakSNP6357	*Kabuli*	Ca_Kabuli_Chr04	17752013	[C/T]	Ca_05350	Leucine-rich repeat	SYNONYMOUS_CODING
6358	CakSNP6358	*Kabuli*	Ca_Kabuli_Chr04	17760841	[A/G]	Ca_05349	Peptidase S9, prolyl oligopeptidase, catalytic domain	INTRON
6359	CakSNP6359	*Kabuli*	Ca_Kabuli_Chr04	17760853	[T/G]	Ca_05349	Peptidase S9, prolyl oligopeptidase, catalytic domain	INTRON
6360	CakSNP6360	*Kabuli*	Ca_Kabuli_Chr04	17768507	[T/C]	Ca_05348	--	INTRON
6361	CakSNP6361	*Kabuli*	Ca_Kabuli_Chr04	17771708	[C/G]	Ca_05347	D111/G-patch	NON_SYNONYMOUS_CODING

S.N.	SNP IDs	Chickpea cultivars	Chromosomes	Physical positions (bp)	SNPs	Gene accession IDs	Putative functions	Sequence components of genome
6362	CakSNP6362	*Kabuli*	Ca_Kabuli_Chr04	17784792	[T/A]	Ca_05346	Protein of unknown function DUF639	INTRON
6363	CakSNP6363	*Kabuli*	Ca_Kabuli_Chr04	17826472	[G/A]		--	INTERGENIC
6364	CakSNP6364	*Kabuli*	Ca_Kabuli_Chr04	17826533	[A/C]		--	INTERGENIC
6365	CakSNP6365	*Kabuli*	Ca_Kabuli_Chr04	17826601	[T/C]		--	INTERGENIC
6366	CakSNP6366	*Kabuli*	Ca_Kabuli_Chr04	17826580	[C/G]		--	INTERGENIC
6367	CakSNP6367	*Kabuli*	Ca_Kabuli_Chr04	17826543	[T/C]		--	INTERGENIC
6368	CakSNP6368	*Kabuli*	Ca_Kabuli_Chr04	17843480	[G/T]		--	INTERGENIC
6369	CakSNP6369	*Kabuli*	Ca_Kabuli_Chr04	17847737	[G/A]	Ca_18621	Methionyl-tRNA synthetase	INTRON
6370	CakSNP6370	*Kabuli*	Ca_Kabuli_Chr04	17847854	[T/C]	Ca_18621	Methionyl-tRNA synthetase	INTRON
6371	CakSNP6371	*Kabuli*	Ca_Kabuli_Chr04	17856307	[T/C]	Ca_18622	IKI3	SYNONYMOUS_CODING

S.N.	SNP IDs	Chickpea cultivars	Chromosomes	Physical positions (bp)	SNPs	Gene accession IDs	Putative functions	Sequence components of genome
6372	CakSNP6372	*Kabuli*	Ca_Kabuli_Chr04	17856311	[T/G]	Ca_18622	IKI3	SYNONYMOUS_CODING
6373	CakSNP6373	*Kabuli*	Ca_Kabuli_Chr04	17856334	[A/G]	Ca_18622	IKI3	SYNONYMOUS_CODING
6374	CakSNP6374	*Kabuli*	Ca_Kabuli_Chr04	17857753	[C/T]	Ca_18622	IKI3	SYNONYMOUS_CODING
6375	CakSNP6375	*Kabuli*	Ca_Kabuli_Chr04	17858122	[T/G]	Ca_18622	IKI3	INTRON
6376	CakSNP6376	*Kabuli*	Ca_Kabuli_Chr04	17858133	[C/T]	Ca_18622	IKI3	INTRON
6377	CakSNP6377	*Kabuli*	Ca_Kabuli_Chr04	17858135	[G/A]	Ca_18622	IKI3	INTRON
6378	CakSNP6378	*Kabuli*	Ca_Kabuli_Chr04	17858140	[A/C]	Ca_18622	IKI3	INTRON
6379	CakSNP6379	*Kabuli*	Ca_Kabuli_Chr04	17858143	[A/T]	Ca_18622	IKI3	INTRON
6380	CakSNP6380	*Kabuli*	Ca_Kabuli_Chr04	17858144	[G/C]	Ca_18622	IKI3	INTRON
6381	CakSNP6381	*Kabuli*	Ca_Kabuli_Chr04	17858152	[A/T]	Ca_18622	IKI3	INTRON

S.N.	SNP IDs	Chickpea cultivars	Chromosomes	Physical positions (bp)	SNPs	Gene accession IDs	Putative functions	Sequence components of genome
6382	CakSNP6382	*Kabuli*	Ca_Kabuli_Chr04	17858159	[C/A]	Ca_18622	IKI3	INTRON
6383	CakSNP6383	*Kabuli*	Ca_Kabuli_Chr04	17858226	[C/T]	Ca_18622	IKI3	INTRON
6384	CakSNP6384	*Kabuli*	Ca_Kabuli_Chr04	17871769	[C/A]		--	INTERGENIC
6385	CakSNP6385	*Kabuli*	Ca_Kabuli_Chr04	17871763	[T/C]		--	INTERGENIC
6386	CakSNP6386	*Kabuli*	Ca_Kabuli_Chr04	17871804	[T/C]		--	INTERGENIC
6387	CakSNP6387	*Kabuli*	Ca_Kabuli_Chr04	17871828	[A/C]		--	INTERGENIC
6388	CakSNP6388	*Kabuli*	Ca_Kabuli_Chr04	17871919	[A/G]		--	INTERGENIC
6389	CakSNP6389	*Kabuli*	Ca_Kabuli_Chr04	17871915	[T/A]		--	INTERGENIC
6390	CakSNP6390	*Kabuli*	Ca_Kabuli_Chr04	17913731	[A/C]	Ca_18632	ABC transporter, transmembrane domain	SYNONYMOUS_CODING
6391	CakSNP6391	*Kabuli*	Ca_Kabuli_Chr04	17913726	[G/A]	Ca_18632	ABC transporter, transmembrane domain	NON_SYNONYMOUS_CODING

S.N.	SNP IDs	Chickpea cultivars	Chromosomes	Physical positions (bp)	SNPs	Gene accession IDs	Putative functions	Sequence components of genome
6392	CakSNP6392	*Kabuli*	Ca_Kabuli_Chr04	17913719	[G/C]	Ca_18632	ABC transporter, transmembrane domain	SYNONYMOUS_CODING
6393	CakSNP6393	*Kabuli*	Ca_Kabuli_Chr04	17913698	[C/T]	Ca_18632	ABC transporter, transmembrane domain	SYNONYMOUS_CODING
6394	CakSNP6394	*Kabuli*	Ca_Kabuli_Chr04	17913682	[C/A]	Ca_18632	ABC transporter, transmembrane domain	STOP_GAINED
6395	CakSNP6395	*Kabuli*	Ca_Kabuli_Chr04	17920616	[C/G]	Ca_18632	ABC transporter, transmembrane domain	SYNONYMOUS_CODING
6396	CakSNP6396	*Kabuli*	Ca_Kabuli_Chr04	17931955	[G/A]	Ca_18633	Protein of unknown function DUF674	SYNONYMOUS_CODING
6397	CakSNP6397	*Kabuli*	Ca_Kabuli_Chr04	17970218	[T/G]		--	INTERGENIC
6398	CakSNP6398	*Kabuli*	Ca_Kabuli_Chr04	17970221	[C/T]		--	INTERGENIC
6399	CakSNP6399	*Kabuli*	Ca_Kabuli_Chr04	17970225	[T/C]		--	INTERGENIC
6400	CakSNP6400	*Kabuli*	Ca_Kabuli_Chr04	17970232	[A/G]		--	INTERGENIC
6401	CakSNP6401	*Kabuli*	Ca_Kabuli_Chr04	17970280	[T/A]		--	INTERGENIC

S.N.	SNP IDs	Chickpea cultivars	Chromosomes	Physical positions (bp)	SNPs	Gene accession IDs	Putative functions	Sequence components of genome
6402	CakSNP6402	*Kabuli*	Ca_Kabuli_Chr04	18053756	[A/G]	Ca_18647	Protein phosphatase 2C, manganese/magnesium aspartate binding site	INTRON
6403	CakSNP6403	*Kabuli*	Ca_Kabuli_Chr04	18070293	[G/T]	Ca_18648	Ankyrin repeat	NON_SYNONYMOUS_CODING
6404	CakSNP6404	*Kabuli*	Ca_Kabuli_Chr04	18071114	[C/A]	Ca_18648	Ankyrin repeat	NON_SYNONYMOUS_CODING
6405	CakSNP6405	*Kabuli*	Ca_Kabuli_Chr04	18071324	[G/A]	Ca_18648	Ankyrin repeat	NON_SYNONYMOUS_CODING
6406	CakSNP6406	*Kabuli*	Ca_Kabuli_Chr04	18089423	[G/C]	Ca_18650	--	SYNONYMOUS_CODING
6407	CakSNP6407	*Kabuli*	Ca_Kabuli_Chr04	18089422	[T/C]	Ca_18650	--	NON_SYNONYMOUS_CODING
6408	CakSNP6408	*Kabuli*	Ca_Kabuli_Chr04	18117833	[T/C]	Ca_18651	Chalcone isomerase, subgroup	NON_SYNONYMOUS_CODING
6409	CakSNP6409	*Kabuli*	Ca_Kabuli_Chr04	18222762	[T/C]	Ca_18659	SET domain	NON_SYNONYMOUS_CODING
6410	CakSNP6410	*Kabuli*	Ca_Kabuli_Chr04	18231443	[T/C]	Ca_18659	SET domain	INTRON
6411	CakSNP6411	*Kabuli*	Ca_Kabuli_Chr04	18231463	[T/C]	Ca_18659	SET domain	INTRON

S.N.	SNP IDs	Chickpea cultivars	Chromosomes	Physical positions (bp)	SNPs	Gene accession IDs	Putative functions	Sequence components of genome
6412	CakSNP6412	*Kabuli*	Ca_Kabuli_Chr04	18231466	[T/C]	Ca_18659	SET domain	INTRON
6413	CakSNP6413	*Kabuli*	Ca_Kabuli_Chr04	18242020	[C/G]	Ca_18659	SET domain	INTRON
6414	CakSNP6414	*Kabuli*	Ca_Kabuli_Chr04	18241995	[C/G]	Ca_18659	SET domain	INTRON
6415	CakSNP6415	*Kabuli*	Ca_Kabuli_Chr04	18427385	[T/G]	Ca_21626	LisH dimerisation motif	INTRON
6416	CakSNP6416	*Kabuli*	Ca_Kabuli_Chr04	18427384	[C/T]	Ca_21626	LisH dimerisation motif	INTRON
6417	CakSNP6417	*Kabuli*	Ca_Kabuli_Chr04	18429046	[G/T]		--	INTERGENIC
6418	CakSNP6418	*Kabuli*	Ca_Kabuli_Chr04	18429139	[C/T]		--	INTERGENIC
6419	CakSNP6419	*Kabuli*	Ca_Kabuli_Chr04	18485782	[T/C]	Ca_21622	UbiA prenyltransferase family	INTRON
6420	CakSNP6420	*Kabuli*	Ca_Kabuli_Chr04	18489456	[G/C]		--	INTERGENIC
6421	CakSNP6421	*Kabuli*	Ca_Kabuli_Chr04	18495186	[A/G]		--	INTERGENIC

S.N.	SNP IDs	Chickpea cultivars	Chromosomes	Physical positions (bp)	SNPs	Gene accession IDs	Putative functions	Sequence components of genome
6422	CakSNP6422	*Kabuli*	Ca_Kabuli_Chr04	18545489	[A/T]	Ca_21616	Protein kinase, catalytic domain	INTRON
6423	CakSNP6423	*Kabuli*	Ca_Kabuli_Chr04	18546209	[T/G]	Ca_21616	Protein kinase, catalytic domain	SYNONYMOUS_CODING
6424	CakSNP6424	*Kabuli*	Ca_Kabuli_Chr04	18625387	[G/A]	Ca_21604	Glycosyl transferase, family 48	INTRON
6425	CakSNP6425	*Kabuli*	Ca_Kabuli_Chr04	18628582	[A/G]	Ca_21604	Glycosyl transferase, family 48	SYNONYMOUS_CODING
6426	CakSNP6426	*Kabuli*	Ca_Kabuli_Chr04	18710941	[T/G]	Ca_13978	--	INTRON
6427	CakSNP6427	*Kabuli*	Ca_Kabuli_Chr04	18719417	[A/G]	Ca_13978	--	INTRON
6428	CakSNP6428	*Kabuli*	Ca_Kabuli_Chr04	18721801	[G/A]	Ca_13978	--	INTRON
6429	CakSNP6429	*Kabuli*	Ca_Kabuli_Chr04	18779396	[A/C]		--	INTERGENIC
6430	CakSNP6430	*Kabuli*	Ca_Kabuli_Chr04	18812610	[T/C]	Ca_13982	--	SYNONYMOUS_CODING
6431	CakSNP6431	*Kabuli*	Ca_Kabuli_Chr04	18880757	[A/G]	Ca_13985	Pyruvate carboxyltransferase	INTRON

S.N.	SNP IDs	Chickpea cultivars	Chromosomes	Physical positions (bp)	SNPs	Gene accession IDs	Putative functions	Sequence components of genome
6432	CakSNP6432	*Kabuli*	Ca_Kabuli_Chr04	18904060	[G/A]	Ca_13988	Protein kinase, catalytic domain	SYNONYMOUS_CODING
6433	CakSNP6433	*Kabuli*	Ca_Kabuli_Chr04	18912038	[G/C]	Ca_13989	Survival motor neuron interacting protein 1	NON_SYNONYMOUS_CODING
6434	CakSNP6434	*Kabuli*	Ca_Kabuli_Chr04	18912137	[T/C]	Ca_13989	Survival motor neuron interacting protein 1	NON_SYNONYMOUS_CODING
6435	CakSNP6435	*Kabuli*	Ca_Kabuli_Chr04	18912124	[C/A]	Ca_13989	Survival motor neuron interacting protein 1	SYNONYMOUS_CODING
6436	CakSNP6436	*Kabuli*	Ca_Kabuli_Chr04	18912142	[A/G]	Ca_13989	Survival motor neuron interacting protein 1	SYNONYMOUS_CODING
6437	CakSNP6437	*Kabuli*	Ca_Kabuli_Chr04	18923641	[G/T]	Ca_13991	Nucleotide-sugar transporter	INTRON
6438	CakSNP6438	*Kabuli*	Ca_Kabuli_Chr04	18953354	[C/A]		--	INTERGENIC
6439	CakSNP6439	*Kabuli*	Ca_Kabuli_Chr04	18953423	[A/C]		--	INTERGENIC
6440	CakSNP6440	*Kabuli*	Ca_Kabuli_Chr04	18953511	[C/G]		--	INTERGENIC
6441	CakSNP6441	*Kabuli*	Ca_Kabuli_Chr04	18998727	[C/T]	Ca_14001	Nse5	INTRON

S.N.	SNP IDs	Chickpea cultivars	Chromosomes	Physical positions (bp)	SNPs	Gene accession IDs	Putative functions	Sequence components of genome
6442	CakSNP6442	*Kabuli*	Ca_Kabuli_Chr04	19005285	[T/G]	Ca_14002	Oligopeptide transporter	SYNONYMOUS_CODING
6443	CakSNP6443	*Kabuli*	Ca_Kabuli_Chr04	19005416	[T/G]	Ca_14002	Oligopeptide transporter	NON_SYNONYMOUS_CODING
6444	CakSNP6444	*Kabuli*	Ca_Kabuli_Chr04	19006493	[C/T]	Ca_14002	Oligopeptide transporter	INTRON
6445	CakSNP6445	*Kabuli*	Ca_Kabuli_Chr04	19024709	[C/T]		--	INTERGENIC
6446	CakSNP6446	*Kabuli*	Ca_Kabuli_Chr04	19024699	[T/C]		--	INTERGENIC
6447	CakSNP6447	*Kabuli*	Ca_Kabuli_Chr04	19206563	[A/G]	Ca_14019	Peptidyl-prolyl cis-trans isomerase, cyclophilin-type	INTRON
6448	CakSNP6448	*Kabuli*	Ca_Kabuli_Chr04	19206581	[C/T]	Ca_14019	Peptidyl-prolyl cis-trans isomerase, cyclophilin-type	INTRON
6449	CakSNP6449	*Kabuli*	Ca_Kabuli_Chr04	19206611	[T/C]	Ca_14019	Peptidyl-prolyl cis-trans isomerase, cyclophilin-type	INTRON
6450	CakSNP6450	*Kabuli*	Ca_Kabuli_Chr04	19206613	[T/G]	Ca_14019	Peptidyl-prolyl cis-trans isomerase, cyclophilin-type	INTRON
6451	CakSNP6451	*Kabuli*	Ca_Kabuli_Chr04	19206678	[A/T]	Ca_14019	Peptidyl-prolyl cis-trans isomerase, cyclophilin-type	INTRON

S.N.	SNP IDs	Chickpea cultivars	Chromosomes	Physical positions (bp)	SNPs	Gene accession IDs	Putative functions	Sequence components of genome
6452	CakSNP6452	*Kabuli*	Ca_Kabuli_Chr04	19207121	[A/T]	Ca_14019	Peptidyl-prolyl cis-trans isomerase, cyclophilin-type	INTRON
6453	CakSNP6453	*Kabuli*	Ca_Kabuli_Chr04	19227691	[G/C]	Ca_14021	Protein kinase, catalytic domain	NON_SYNONYMOUS_CODING
6454	CakSNP6454	*Kabuli*	Ca_Kabuli_Chr04	19263964	[G/C]	Ca_14024	--	INTRON
6455	CakSNP6455	*Kabuli*	Ca_Kabuli_Chr04	19263967	[T/G]	Ca_14024	--	INTRON
6456	CakSNP6456	*Kabuli*	Ca_Kabuli_Chr04	19267581	[A/G]	Ca_14024	--	INTRON
6457	CakSNP6457	*Kabuli*	Ca_Kabuli_Chr04	19481258	[A/G]	Ca_14038	Rab-GAP/TBC domain	SYNONYMOUS_CODING
6458	CakSNP6458	*Kabuli*	Ca_Kabuli_Chr04	19540632	[A/C]	Ca_14045	Spo11/DNA topoisomerase VI, subunit A	NON_SYNONYMOUS_CODING
6459	CakSNP6459	*Kabuli*	Ca_Kabuli_Chr04	19540646	[T/G]	Ca_14045	Spo11/DNA topoisomerase VI, subunit A	SYNONYMOUS_CODING
6460	CakSNP6460	*Kabuli*	Ca_Kabuli_Chr04	19579390	[G/T]		--	INTERGENIC
6461	CakSNP6461	*Kabuli*	Ca_Kabuli_Chr04	19579401	[C/A]		--	INTERGENIC

S.N.	SNP IDs	Chickpea cultivars	Chromosomes	Physical positions (bp)	SNPs	Gene accession IDs	Putative functions	Sequence components of genome
6462	CakSNP6462	*Kabuli*	Ca_Kabuli_Chr04	19579423	[A/G]		--	INTERGENIC
6463	CakSNP6463	*Kabuli*	Ca_Kabuli_Chr04	19579433	[G/A]		--	INTERGENIC
6464	CakSNP6464	*Kabuli*	Ca_Kabuli_Chr04	19579435	[A/G]		--	INTERGENIC
6465	CakSNP6465	*Kabuli*	Ca_Kabuli_Chr04	19579436	[A/T]		--	INTERGENIC
6466	CakSNP6466	*Kabuli*	Ca_Kabuli_Chr04	19579442	[C/A]		--	INTERGENIC
6467	CakSNP6467	*Kabuli*	Ca_Kabuli_Chr04	19649499	[A/G]		--	INTERGENIC
6468	CakSNP6468	*Kabuli*	Ca_Kabuli_Chr04	19668499	[T/C]	Ca_14053	Pentatricopeptide repeat	SYNONYMOUS_CODING
6469	CakSNP6469	*Kabuli*	Ca_Kabuli_Chr04	19668502	[T/G]	Ca_14053	Pentatricopeptide repeat	SYNONYMOUS_CODING
6470	CakSNP6470	*Kabuli*	Ca_Kabuli_Chr04	19668595	[T/C]	Ca_14053	Pentatricopeptide repeat	SYNONYMOUS_CODING
6471	CakSNP6471	*Kabuli*	Ca_Kabuli_Chr04	19688016	[A/G]	Ca_14055	PWWP	SYNONYMOUS_CODING

S.N.	SNP IDs	Chickpea cultivars	Chromosomes	Physical positions (bp)	SNPs	Gene accession IDs	Putative functions	Sequence components of genome
6472	CakSNP6472	*Kabuli*	Ca_Kabuli_Chr04	19712406	[C/T]	Ca_14059	--	SYNONYMOUS_CODING
6473	CakSNP6473	*Kabuli*	Ca_Kabuli_Chr04	19713311	[T/G]	Ca_14059	--	SYNONYMOUS_CODING
6474	CakSNP6474	*Kabuli*	Ca_Kabuli_Chr04	19734940	[G/A]	Ca_14061	Heat shock protein DnaJ, N-terminal	SYNONYMOUS_CODING
6475	CakSNP6475	*Kabuli*	Ca_Kabuli_Chr04	19844376	[A/G]		--	INTERGENIC
6476	CakSNP6476	*Kabuli*	Ca_Kabuli_Chr04	19869729	[G/T]	Ca_17123	Target SNARE coiled-coil domain	NON_SYNONYMOUS_CODING
6477	CakSNP6477	*Kabuli*	Ca_Kabuli_Chr04	19869722	[T/G]	Ca_17123	Target SNARE coiled-coil domain	NON_SYNONYMOUS_CODING
6478	CakSNP6478	*Kabuli*	Ca_Kabuli_Chr04	20218430	[T/G]	Ca_17141	Cyclic nucleotide-binding domain	INTRON
6479	CakSNP6479	*Kabuli*	Ca_Kabuli_Chr04	20308992	[C/A]	Ca_17144	Phenylalanine/histidine ammonia-lyase	SYNONYMOUS_CODING
6480	CakSNP6480	*Kabuli*	Ca_Kabuli_Chr04	20309151	[C/T]	Ca_17144	Phenylalanine/histidine ammonia-lyase	SYNONYMOUS_CODING
6481	CakSNP6481	*Kabuli*	Ca_Kabuli_Chr04	20398548	[T/G]	Ca_17149	--	SYNONYMOUS_CODING

S.N.	SNP IDs	Chickpea cultivars	Chromosomes	Physical positions (bp)	SNPs	Gene accession IDs	Putative functions	Sequence components of genome
6482	CakSNP6482	*Kabuli*	Ca_Kabuli_Chr04	20398685	[A/C]	Ca_17149	--	NON_SYNONYMOUS_CODING
6483	CakSNP6483	*Kabuli*	Ca_Kabuli_Chr04	20402361	[A/G]		--	INTERGENIC
6484	CakSNP6484	*Kabuli*	Ca_Kabuli_Chr04	20402391	[A/G]		--	INTERGENIC
6485	CakSNP6485	*Kabuli*	Ca_Kabuli_Chr04	20412329	[A/T]	Ca_17150	Protein kinase, catalytic domain	INTRON
6486	CakSNP6486	*Kabuli*	Ca_Kabuli_Chr04	20436136	[G/A]	Ca_17152	Transcription factor, TCP	SYNONYMOUS_CODING
6487	CakSNP6487	*Kabuli*	Ca_Kabuli_Chr04	20444953	[T/C]	Ca_17153	RNA polymerase sigma-70 factor	SYNONYMOUS_CODING
6488	CakSNP6488	*Kabuli*	Ca_Kabuli_Chr04	20599050	[A/G]		--	INTERGENIC
6489	CakSNP6489	*Kabuli*	Ca_Kabuli_Chr04	20599058	[T/C]		--	INTERGENIC
6490	CakSNP6490	*Kabuli*	Ca_Kabuli_Chr04	20599081	[C/T]		--	INTERGENIC
6491	CakSNP6491	*Kabuli*	Ca_Kabuli_Chr04	20599091	[T/G]		--	INTERGENIC

S.N.	SNP IDs	Chickpea cultivars	Chromosomes	Physical positions (bp)	SNPs	Gene accession IDs	Putative functions	Sequence components of genome
6492	CakSNP6492	*Kabuli*	Ca_Kabuli_Chr04	20656170	[T/G]	Ca_17165	Nucleoporin Nup120/160	SYNONYMOUS_CODING
6493	CakSNP6493	*Kabuli*	Ca_Kabuli_Chr04	20669174	[T/C]	Ca_17165	Nucleoporin Nup120/160	INTRON
6494	CakSNP6494	*Kabuli*	Ca_Kabuli_Chr04	20669442	[G/A]	Ca_17165	Nucleoporin Nup120/160	INTRON
6495	CakSNP6495	*Kabuli*	Ca_Kabuli_Chr04	20696913	[A/G]	Ca_17166	ABC transporter, transmembrane domain	SYNONYMOUS_CODING
6496	CakSNP6496	*Kabuli*	Ca_Kabuli_Chr04	20696952	[G/T]	Ca_17166	ABC transporter, transmembrane domain	SYNONYMOUS_CODING
6497	CakSNP6497	*Kabuli*	Ca_Kabuli_Chr04	20696979	[C/T]	Ca_17166	ABC transporter, transmembrane domain	SYNONYMOUS_CODING
6498	CakSNP6498	*Kabuli*	Ca_Kabuli_Chr04	20697338	[T/A]	Ca_17166	ABC transporter, transmembrane domain	INTRON
6499	CakSNP6499	*Kabuli*	Ca_Kabuli_Chr04	20697408	[A/G]	Ca_17166	ABC transporter, transmembrane domain	INTRON
6500	CakSNP6500	*Kabuli*	Ca_Kabuli_Chr04	20774180	[C/T]	Ca_17170	--	INTRON
6501	CakSNP6501	*Kabuli*	Ca_Kabuli_Chr04	20905105	[G/A]	Ca_19045	Serine/threonine dehydratase, pyridoxal-phosphate-binding site	INTRON

S.N.	SNP IDs	Chickpea cultivars	Chromosomes	Physical positions (bp)	SNPs	Gene accession IDs	Putative functions	Sequence components of genome
6502	CakSNP6502	*Kabuli*	Ca_Kabuli_Chr04	21096178	[G/A]		--	INTERGENIC
6503	CakSNP6503	*Kabuli*	Ca_Kabuli_Chr04	21385308	[A/T]	Ca_19066	Peptidase A1	SYNONYMOUS_CODING
6504	CakSNP6504	*Kabuli*	Ca_Kabuli_Chr04	21830534	[C/T]	Ca_20151	Helix-turn-helix motif, lambda-like repressor	NON_SYNONYMOUS_CODING
6505	CakSNP6505	*Kabuli*	Ca_Kabuli_Chr04	21835459	[T/A]		--	INTERGENIC
6506	CakSNP6506	*Kabuli*	Ca_Kabuli_Chr04	21835457	[T/A]		--	INTERGENIC
6507	CakSNP6507	*Kabuli*	Ca_Kabuli_Chr04	21835454	[C/A]		--	INTERGENIC
6508	CakSNP6508	*Kabuli*	Ca_Kabuli_Chr04	21835446	[T/A]		--	INTERGENIC
6509	CakSNP6509	*Kabuli*	Ca_Kabuli_Chr04	21931559	[A/G]		--	INTERGENIC
6510	CakSNP6510	*Kabuli*	Ca_Kabuli_Chr04	21931558	[G/A]		--	INTERGENIC
6511	CakSNP6511	*Kabuli*	Ca_Kabuli_Chr04	21931541	[C/T]		--	INTERGENIC

S.N.	SNP IDs	Chickpea cultivars	Chromosomes	Physical positions (bp)	SNPs	Gene accession IDs	Putative functions	Sequence components of genome
6512	CakSNP6512	*Kabuli*	Ca_Kabuli_Chr04	22079566	[G/A]	Ca_20137	HhH-GPD domain	SYNONYMOUS_CODING
6513	CakSNP6513	*Kabuli*	Ca_Kabuli_Chr04	22237989	[A/T]	Ca_20127	Pathogenesis-related transcriptional factor/ERF, DNA-binding	SYNONYMOUS_CODING
6514	CakSNP6514	*Kabuli*	Ca_Kabuli_Chr04	22237971	[C/T]	Ca_20127	Pathogenesis-related transcriptional factor/ERF, DNA-binding	SYNONYMOUS_CODING
6515	CakSNP6515	*Kabuli*	Ca_Kabuli_Chr04	22288433	[T/C]		--	INTERGENIC
6516	CakSNP6516	*Kabuli*	Ca_Kabuli_Chr04	22293495	[T/C]	Ca_20124	Protein kinase, catalytic domain	NON_SYNONYMOUS_CODING
6517	CakSNP6517	*Kabuli*	Ca_Kabuli_Chr04	22850596	[C/G]	Ca_14467	--	NON_SYNONYMOUS_CODING
6518	CakSNP6518	*Kabuli*	Ca_Kabuli_Chr04	22859422	[G/A]	Ca_14466	Protein of unknown function DUF408	SYNONYMOUS_CODING
6519	CakSNP6519	*Kabuli*	Ca_Kabuli_Chr04	22860281	[A/C]	Ca_14466	Protein of unknown function DUF408	SYNONYMOUS_CODING
6520	CakSNP6520	*Kabuli*	Ca_Kabuli_Chr04	22865436	[A/T]	Ca_14465	Xklp2 targeting protein	INTRON
6521	CakSNP6521	*Kabuli*	Ca_Kabuli_Chr04	22865390	[G/A]	Ca_14465	Xklp2 targeting protein	INTRON

S.N.	SNP IDs	Chickpea cultivars	Chromosomes	Physical positions (bp)	SNPs	Gene accession IDs	Putative functions	Sequence components of genome
6522	CakSNP6522	*Kabuli*	Ca_Kabuli_Chr04	22865389	[C/T]	Ca_14465	Xklp2 targeting protein	INTRON
6523	CakSNP6523	*Kabuli*	Ca_Kabuli_Chr04	22908360	[A/G]	Ca_14463	Pentatricopeptide repeat	SYNONYMOUS_CODING
6524	CakSNP6524	*Kabuli*	Ca_Kabuli_Chr04	22971660	[G/A]	Ca_14460	--	NON_SYNONYMOUS_CODING
6525	CakSNP6525	*Kabuli*	Ca_Kabuli_Chr04	22971766	[A/G]	Ca_14460	--	NON_SYNONYMOUS_CODING
6526	CakSNP6526	*Kabuli*	Ca_Kabuli_Chr04	22971746	[G/A]	Ca_14460	--	SYNONYMOUS_CODING
6527	CakSNP6527	*Kabuli*	Ca_Kabuli_Chr04	22971744	[A/C]	Ca_14460	--	NON_SYNONYMOUS_CODING
6528	CakSNP6528	*Kabuli*	Ca_Kabuli_Chr04	22971847	[C/A]	Ca_14460	--	NON_SYNONYMOUS_CODING
6529	CakSNP6529	*Kabuli*	Ca_Kabuli_Chr04	22971853	[C/A]	Ca_14460	--	NON_SYNONYMOUS_CODING
6530	CakSNP6530	*Kabuli*	Ca_Kabuli_Chr04	22971884	[G/A]	Ca_14460	--	SYNONYMOUS_CODING
6531	CakSNP6531	*Kabuli*	Ca_Kabuli_Chr04	23157656	[C/G]	Ca_14454	RNA recognition motif domain	NON_SYNONYMOUS_CODING

S.N.	SNP IDs	Chickpea cultivars	Chromosomes	Physical positions (bp)	SNPs	Gene accession IDs	Putative functions	Sequence components of genome
6532	CakSNP6532	*Kabuli*	Ca_Kabuli_Chr04	23267842	[T/C]		--	INTERGENIC
6533	CakSNP6533	*Kabuli*	Ca_Kabuli_Chr04	23267856	[C/T]		--	INTERGENIC
6534	CakSNP6534	*Kabuli*	Ca_Kabuli_Chr04	23267858	[C/T]		--	INTERGENIC
6535	CakSNP6535	*Kabuli*	Ca_Kabuli_Chr04	23267859	[T/C]		--	INTERGENIC
6536	CakSNP6536	*Kabuli*	Ca_Kabuli_Chr04	23314624	[C/T]		--	INTERGENIC
6537	CakSNP6537	*Kabuli*	Ca_Kabuli_Chr04	23314665	[A/C]		--	INTERGENIC
6538	CakSNP6538	*Kabuli*	Ca_Kabuli_Chr04	23318875	[T/C]		--	INTERGENIC
6539	CakSNP6539	*Kabuli*	Ca_Kabuli_Chr04	23319309	[G/C]		--	INTERGENIC
6540	CakSNP6540	*Kabuli*	Ca_Kabuli_Chr04	23333610	[C/T]		--	INTERGENIC
6541	CakSNP6541	*Kabuli*	Ca_Kabuli_Chr04	23333554	[T/C]		--	INTERGENIC

S.N.	SNP IDs	Chickpea cultivars	Chromosomes	Physical positions (bp)	SNPs	Gene accession IDs	Putative functions	Sequence components of genome
6542	CakSNP6542	*Kabuli*	Ca_Kabuli_Chr04	23337341	[T/C]		--	INTERGENIC
6543	CakSNP6543	*Kabuli*	Ca_Kabuli_Chr04	23337312	[T/G]		--	INTERGENIC
6544	CakSNP6544	*Kabuli*	Ca_Kabuli_Chr04	23481919	[G/A]		--	INTERGENIC
6545	CakSNP6545	*Kabuli*	Ca_Kabuli_Chr04	23481927	[G/T]		--	INTERGENIC
6546	CakSNP6546	*Kabuli*	Ca_Kabuli_Chr04	23481962	[G/A]		--	INTERGENIC
6547	CakSNP6547	*Kabuli*	Ca_Kabuli_Chr04	23482033	[A/G]		--	INTERGENIC
6548	CakSNP6548	*Kabuli*	Ca_Kabuli_Chr04	23481998	[T/C]		--	INTERGENIC
6549	CakSNP6549	*Kabuli*	Ca_Kabuli_Chr04	23486812	[A/G]		--	INTERGENIC
6550	CakSNP6550	*Kabuli*	Ca_Kabuli_Chr04	23486859	[A/G]		--	INTERGENIC
6551	CakSNP6551	*Kabuli*	Ca_Kabuli_Chr04	23486824	[T/C]		--	INTERGENIC

S.N.	SNP IDs	Chickpea cultivars	Chromosomes	Physical positions (bp)	SNPs	Gene accession IDs	Putative functions	Sequence components of genome
6552	CakSNP6552	*Kabuli*	Ca_Kabuli_Chr04	23671441	[T/G]	Ca_14430	NC	NON_SYNONYMOUS_CODING
6553	CakSNP6553	*Kabuli*	Ca_Kabuli_Chr04	23671443	[A/T]	Ca_14430	NC	NON_SYNONYMOUS_CODING
6554	CakSNP6554	*Kabuli*	Ca_Kabuli_Chr04	23731572	[C/T]	Ca_14425	Exo70 exocyst complex subunit	SYNONYMOUS_CODING
6555	CakSNP6555	*Kabuli*	Ca_Kabuli_Chr04	23732099	[A/T]	Ca_14425	Exo70 exocyst complex subunit	INTRON
6556	CakSNP6556	*Kabuli*	Ca_Kabuli_Chr04	24050459	[G/A]	Ca_14409	Phenylalanine/histidine ammonia-lyase	INTRON
6557	CakSNP6557	*Kabuli*	Ca_Kabuli_Chr04	24050464	[G/A]	Ca_14409	Phenylalanine/histidine ammonia-lyase	INTRON
6558	CakSNP6558	*Kabuli*	Ca_Kabuli_Chr04	24120866	[A/G]		--	INTERGENIC
6559	CakSNP6559	*Kabuli*	Ca_Kabuli_Chr04	24544345	[G/A]		--	INTERGENIC
6560	CakSNP6560	*Kabuli*	Ca_Kabuli_Chr04	24614582	[G/T]	Ca_20855	Cytoskeleton-associated protein, Gly-rich domain	NON_SYNONYMOUS_CODING
6561	CakSNP6561	*Kabuli*	Ca_Kabuli_Chr04	24729302	[T/C]	Ca_20853	Protein kinase, catalytic domain	INTRON

S.N.	SNP IDs	Chickpea cultivars	Chromosomes	Physical positions (bp)	SNPs	Gene accession IDs	Putative functions	Sequence components of genome
6562	CakSNP6562	*Kabuli*	Ca_Kabuli_Chr04	24732991	[G/T]	Ca_20853	Protein kinase, catalytic domain	INTRON
6563	CakSNP6563	*Kabuli*	Ca_Kabuli_Chr04	24732990	[T/C]	Ca_20853	Protein kinase, catalytic domain	INTRON
6564	CakSNP6564	*Kabuli*	Ca_Kabuli_Chr04	24771737	[T/A]	Ca_20851	--	NON_SYNONYMOUS_CODING
6565	CakSNP6565	*Kabuli*	Ca_Kabuli_Chr04	24782714	[C/A]	Ca_20850	--	NON_SYNONYMOUS_CODING
6566	CakSNP6566	*Kabuli*	Ca_Kabuli_Chr04	24782715	[A/G]	Ca_20850	--	NON_SYNONYMOUS_CODING
6567	CakSNP6567	*Kabuli*	Ca_Kabuli_Chr04	24856115	[A/C]	Ca_20847	Protein kinase, catalytic domain	NON_SYNONYMOUS_CODING
6568	CakSNP6568	*Kabuli*	Ca_Kabuli_Chr04	24857501	[A/G]	Ca_20847	Protein kinase, catalytic domain	INTRON
6569	CakSNP6569	*Kabuli*	Ca_Kabuli_Chr04	25401714	[T/C]		--	INTERGENIC
6570	CakSNP6570	*Kabuli*	Ca_Kabuli_Chr04	25446677	[G/C]		--	INTERGENIC
6571	CakSNP6571	*Kabuli*	Ca_Kabuli_Chr04	25446684	[T/C]		--	INTERGENIC

S.N.	SNP IDs	Chickpea cultivars	Chromosomes	Physical positions (bp)	SNPs	Gene accession IDs	Putative functions	Sequence components of genome
6572	CakSNP6572	*Kabuli*	Ca_Kabuli_Chr04	25449897	[T/G]		--	INTERGENIC
6573	CakSNP6573	*Kabuli*	Ca_Kabuli_Chr04	25455156	[G/A]		--	INTERGENIC
6574	CakSNP6574	*Kabuli*	Ca_Kabuli_Chr04	25455204	[A/G]		--	INTERGENIC
6575	CakSNP6575	*Kabuli*	Ca_Kabuli_Chr04	25455225	[G/A]		--	INTERGENIC
6576	CakSNP6576	*Kabuli*	Ca_Kabuli_Chr04	25592561	[T/C]		--	INTERGENIC
6577	CakSNP6577	*Kabuli*	Ca_Kabuli_Chr04	25592548	[A/G]		--	INTERGENIC
6578	CakSNP6578	*Kabuli*	Ca_Kabuli_Chr04	25592528	[A/C]		--	INTERGENIC
6579	CakSNP6579	*Kabuli*	Ca_Kabuli_Chr04	25592525	[C/A]		--	INTERGENIC
6580	CakSNP6580	*Kabuli*	Ca_Kabuli_Chr04	25592492	[T/C]		--	INTERGENIC
6581	CakSNP6581	*Kabuli*	Ca_Kabuli_Chr04	25592487	[A/G]		--	INTERGENIC

S.N.	SNP IDs	Chickpea cultivars	Chromosomes	Physical positions (bp)	SNPs	Gene accession IDs	Putative functions	Sequence components of genome
6582	CakSNP6582	*Kabuli*	Ca_Kabuli_Chr04	25592484	[A/C]		--	INTERGENIC
6583	CakSNP6583	*Kabuli*	Ca_Kabuli_Chr04	25592481	[T/C]		--	INTERGENIC
6584	CakSNP6584	*Kabuli*	Ca_Kabuli_Chr04	25729905	[C/A]		--	INTERGENIC
6585	CakSNP6585	*Kabuli*	Ca_Kabuli_Chr04	25729914	[C/A]		--	INTERGENIC
6586	CakSNP6586	*Kabuli*	Ca_Kabuli_Chr04	25729974	[G/C]		--	INTERGENIC
6587	CakSNP6587	*Kabuli*	Ca_Kabuli_Chr04	25729977	[C/T]		--	INTERGENIC
6588	CakSNP6588	*Kabuli*	Ca_Kabuli_Chr04	25747256	[T/C]		--	INTERGENIC
6589	CakSNP6589	*Kabuli*	Ca_Kabuli_Chr04	25747262	[G/A]		--	INTERGENIC
6590	CakSNP6590	*Kabuli*	Ca_Kabuli_Chr04	25747274	[T/G]		--	INTERGENIC
6591	CakSNP6591	*Kabuli*	Ca_Kabuli_Chr04	25747275	[G/A]		--	INTERGENIC

S.N.	SNP IDs	Chickpea cultivars	Chromosomes	Physical positions (bp)	SNPs	Gene accession IDs	Putative functions	Sequence components of genome
6592	CakSNP6592	*Kabuli*	Ca_Kabuli_Chr04	25747281	[T/C]		--	INTERGENIC
6593	CakSNP6593	*Kabuli*	Ca_Kabuli_Chr04	25747295	[G/A]		--	INTERGENIC
6594	CakSNP6594	*Kabuli*	Ca_Kabuli_Chr04	25747296	[T/C]		--	INTERGENIC
6595	CakSNP6595	*Kabuli*	Ca_Kabuli_Chr04	25747364	[T/A]		--	INTERGENIC
6596	CakSNP6596	*Kabuli*	Ca_Kabuli_Chr04	25747297	[G/A]		--	INTERGENIC
6597	CakSNP6597	*Kabuli*	Ca_Kabuli_Chr04	25809665	[T/C]	Ca_16599	Aminoglycoside phosphotransferase	INTRON
6598	CakSNP6598	*Kabuli*	Ca_Kabuli_Chr04	25921165	[T/C]	Ca_16606	Thiolase	INTRON
6599	CakSNP6599	*Kabuli*	Ca_Kabuli_Chr04	26000532	[C/T]	Ca_16610	Aldo/keto reductase	INTRON
6600	CakSNP6600	*Kabuli*	Ca_Kabuli_Chr04	26000483	[T/A]	Ca_16610	Aldo/keto reductase	INTRON
6601	CakSNP6601	*Kabuli*	Ca_Kabuli_Chr04	26002991	[C/A]	Ca_16610	Aldo/keto reductase	INTRON

S.N.	SNP IDs	Chickpea cultivars	Chromosomes	Physical positions (bp)	SNPs	Gene accession IDs	Putative functions	Sequence components of genome
6602	CakSNP6602	*Kabuli*	Ca_Kabuli_Chr04	26008744	[G/A]		--	INTERGENIC
6603	CakSNP6603	*Kabuli*	Ca_Kabuli_Chr04	26008745	[G/A]		--	INTERGENIC
6604	CakSNP6604	*Kabuli*	Ca_Kabuli_Chr04	26008748	[C/T]		--	INTERGENIC
6605	CakSNP6605	*Kabuli*	Ca_Kabuli_Chr04	26008768	[A/G]		--	INTERGENIC
6606	CakSNP6606	*Kabuli*	Ca_Kabuli_Chr04	26008816	[C/A]		--	INTERGENIC
6607	CakSNP6607	*Kabuli*	Ca_Kabuli_Chr04	26009438	[G/T]		--	INTERGENIC
6608	CakSNP6608	*Kabuli*	Ca_Kabuli_Chr04	26009439	[G/T]		--	INTERGENIC
6609	CakSNP6609	*Kabuli*	Ca_Kabuli_Chr04	26222180	[A/G]		--	INTERGENIC
6610	CakSNP6610	*Kabuli*	Ca_Kabuli_Chr04	26282236	[A/C]		--	INTERGENIC
6611	CakSNP6611	*Kabuli*	Ca_Kabuli_Chr04	26324460	[T/C]	Ca_25306	Alcohol dehydrogenase superfamily, zinc-containing	SYNONYMOUS_CODING

S.N.	SNP IDs	Chickpea cultivars	Chromosomes	Physical positions (bp)	SNPs	Gene accession IDs	Putative functions	Sequence components of genome
6612	CakSNP6612	*Kabuli*	Ca_Kabuli_Chr04	26529606	[C/T]	Ca_25003	RNA recognition motif domain	INTRON
6613	CakSNP6613	*Kabuli*	Ca_Kabuli_Chr04	26532763	[T/G]	Ca_25003	RNA recognition motif domain	INTRON
6614	CakSNP6614	*Kabuli*	Ca_Kabuli_Chr04	26536919	[C/G]		--	INTERGENIC
6615	CakSNP6615	*Kabuli*	Ca_Kabuli_Chr04	26537053	[C/T]		--	INTERGENIC
6616	CakSNP6616	*Kabuli*	Ca_Kabuli_Chr04	26537258	[A/C]		--	INTERGENIC
6617	CakSNP6617	*Kabuli*	Ca_Kabuli_Chr04	26869756	[A/G]	Ca_20441	--	NON_SYNONYMOUS_CODING
6618	CakSNP6618	*Kabuli*	Ca_Kabuli_Chr04	26915602	[G/A]	Ca_20444	Aminotransferase-like, plant mobile domain	INTRON
6619	CakSNP6619	*Kabuli*	Ca_Kabuli_Chr04	26915615	[A/G]	Ca_20444	Aminotransferase-like, plant mobile domain	INTRON
6620	CakSNP6620	*Kabuli*	Ca_Kabuli_Chr04	26915621	[G/A]	Ca_20444	Aminotransferase-like, plant mobile domain	INTRON
6621	CakSNP6621	*Kabuli*	Ca_Kabuli_Chr04	26915626	[C/T]	Ca_20444	Aminotransferase-like, plant mobile domain	INTRON

S.N.	SNP IDs	Chickpea cultivars	Chromosomes	Physical positions (bp)	SNPs	Gene accession IDs	Putative functions	Sequence components of genome
6622	CakSNP6622	*Kabuli*	Ca_Kabuli_Chr04	26989085	[G/A]	Ca_20453	Ribosomal protein S3, C-terminal	INTRON
6623	CakSNP6623	*Kabuli*	Ca_Kabuli_Chr04	26989090	[G/T]	Ca_20453	Ribosomal protein S3, C-terminal	INTRON
6624	CakSNP6624	*Kabuli*	Ca_Kabuli_Chr04	26989204	[A/C]	Ca_20453	Ribosomal protein S3, C-terminal	NON_SYNONYMOUS_CODING
6625	CakSNP6625	*Kabuli*	Ca_Kabuli_Chr04	27102807	[G/A]		--	INTERGENIC
6626	CakSNP6626	*Kabuli*	Ca_Kabuli_Chr04	27102817	[A/C]		--	INTERGENIC
6627	CakSNP6627	*Kabuli*	Ca_Kabuli_Chr04	27102833	[G/T]		--	INTERGENIC
6628	CakSNP6628	*Kabuli*	Ca_Kabuli_Chr04	27262241	[C/T]		--	INTERGENIC
6629	CakSNP6629	*Kabuli*	Ca_Kabuli_Chr04	27262893	[G/A]		--	INTERGENIC
6630	CakSNP6630	*Kabuli*	Ca_Kabuli_Chr04	27262870	[C/T]		--	INTERGENIC
6631	CakSNP6631	*Kabuli*	Ca_Kabuli_Chr04	27262830	[T/A]		--	INTERGENIC

S.N.	SNP IDs	Chickpea cultivars	Chromosomes	Physical positions (bp)	SNPs	Gene accession IDs	Putative functions	Sequence components of genome
6632	CakSNP6632	*Kabuli*	Ca_Kabuli_Chr04	27268301	[G/A]		--	INTERGENIC
6633	CakSNP6633	*Kabuli*	Ca_Kabuli_Chr04	27268278	[C/T]		--	INTERGENIC
6634	CakSNP6634	*Kabuli*	Ca_Kabuli_Chr04	27493010	[G/T]	Ca_21317	Protein of unknown function DUF716, TMEM45	SYNONYMOUS_CODING
6635	CakSNP6635	*Kabuli*	Ca_Kabuli_Chr04	27613450	[C/T]	Ca_21314	Peptidase C48, SUMO/Sentrin/Ubl1	INTRON
6636	CakSNP6636	*Kabuli*	Ca_Kabuli_Chr04	27617803	[A/G]	Ca_21313	--	INTRON
6637	CakSNP6637	*Kabuli*	Ca_Kabuli_Chr04	27626559	[G/A]	Ca_21312	Protein of unknown function DUF810	SYNONYMOUS_CODING
6638	CakSNP6638	*Kabuli*	Ca_Kabuli_Chr04	27626999	[C/T]	Ca_21312	Protein of unknown function DUF810	SYNONYMOUS_CODING
6639	CakSNP6639	*Kabuli*	Ca_Kabuli_Chr04	27661241	[T/G]	Ca_21309	--	SYNONYMOUS_CODING
6640	CakSNP6640	*Kabuli*	Ca_Kabuli_Chr04	27669829	[C/G]		--	INTERGENIC
6641	CakSNP6641	*Kabuli*	Ca_Kabuli_Chr04	27777968	[A/C]	Ca_21303	MATH	INTRON

S.N.	SNP IDs	Chickpea cultivars	Chromosomes	Physical positions (bp)	SNPs	Gene accession IDs	Putative functions	Sequence components of genome
6642	CakSNP6642	*Kabuli*	Ca_Kabuli_Chr04	27786162	[G/C]	Ca_21303	MATH	INTRON
6643	CakSNP6643	*Kabuli*	Ca_Kabuli_Chr04	27970989	[T/G]	Ca_21299	GAF	SYNONYMOUS_CODING
6644	CakSNP6644	*Kabuli*	Ca_Kabuli_Chr04	28061002	[A/C]	Ca_23178	--	SYNONYMOUS_CODING
6645	CakSNP6645	*Kabuli*	Ca_Kabuli_Chr04	28092609	[G/A]	Ca_23175	Crotonase, core	NON_SYNONYMOUS_CODING
6646	CakSNP6646	*Kabuli*	Ca_Kabuli_Chr04	28092727	[T/A]	Ca_23175	Crotonase, core	NON_SYNONYMOUS_CODING
6647	CakSNP6647	*Kabuli*	Ca_Kabuli_Chr04	28092725	[T/G]	Ca_23175	Crotonase, core	NON_SYNONYMOUS_CODING
6648	CakSNP6648	*Kabuli*	Ca_Kabuli_Chr04	28092724	[T/A]	Ca_23175	Crotonase, core	NON_SYNONYMOUS_CODING
6649	CakSNP6649	*Kabuli*	Ca_Kabuli_Chr04	28268788	[G/A]	Ca_23167	--	NON_SYNONYMOUS_CODING
6650	CakSNP6650	*Kabuli*	Ca_Kabuli_Chr04	28289900	[C/A]		--	INTERGENIC
6651	CakSNP6651	*Kabuli*	Ca_Kabuli_Chr04	28299238	[G/A]	Ca_23165	General substrate transporter	SYNONYMOUS_CODING

S.N.	SNP IDs	Chickpea cultivars	Chromosomes	Physical positions (bp)	SNPs	Gene accession IDs	Putative functions	Sequence components of genome
6652	CakSNP6652	*Kabuli*	Ca_Kabuli_Chr04	28301853	[T/C]	Ca_23164	Cryptochrome/DNA photolyase, class 1	NON_SYNONYMOUS_CODING
6653	CakSNP6653	*Kabuli*	Ca_Kabuli_Chr04	28423079	[T/C]		--	INTERGENIC
6654	CakSNP6654	*Kabuli*	Ca_Kabuli_Chr04	28423140	[A/G]		--	INTERGENIC
6655	CakSNP6655	*Kabuli*	Ca_Kabuli_Chr04	28431706	[G/A]		--	INTERGENIC
6656	CakSNP6656	*Kabuli*	Ca_Kabuli_Chr04	28790085	[A/G]	Ca_23698	NADH:ubiquinone oxidoreductase, 51kDa subunit, conserved site	NON_SYNONYMOUS_CODING
6657	CakSNP6657	*Kabuli*	Ca_Kabuli_Chr04	29582296	[C/T]		--	INTERGENIC
6658	CakSNP6658	*Kabuli*	Ca_Kabuli_Chr04	29582289	[G/A]		--	INTERGENIC
6659	CakSNP6659	*Kabuli*	Ca_Kabuli_Chr04	29582285	[C/A]		--	INTERGENIC
6660	CakSNP6660	*Kabuli*	Ca_Kabuli_Chr04	29582282	[C/A]		--	INTERGENIC
6661	CakSNP6661	*Kabuli*	Ca_Kabuli_Chr04	29582273	[G/A]		--	INTERGENIC

S.N.	SNP IDs	Chickpea cultivars	Chromosomes	Physical positions (bp)	SNPs	Gene accession IDs	Putative functions	Sequence components of genome
6662	CakSNP6662	*Kabuli*	Ca_Kabuli_Chr04	29582268	[C/G]		--	INTERGENIC
6663	CakSNP6663	*Kabuli*	Ca_Kabuli_Chr04	29582257	[G/A]		--	INTERGENIC
6664	CakSNP6664	*Kabuli*	Ca_Kabuli_Chr04	29582255	[C/T]		--	INTERGENIC
6665	CakSNP6665	*Kabuli*	Ca_Kabuli_Chr04	29582252	[G/A]		--	INTERGENIC
6666	CakSNP6666	*Kabuli*	Ca_Kabuli_Chr04	29582249	[C/T]		--	INTERGENIC
6667	CakSNP6667	*Kabuli*	Ca_Kabuli_Chr04	29582235	[T/A]		--	INTERGENIC
6668	CakSNP6668	*Kabuli*	Ca_Kabuli_Chr04	29582232	[C/T]		--	INTERGENIC
6669	CakSNP6669	*Kabuli*	Ca_Kabuli_Chr04	29582227	[G/A]		--	INTERGENIC
6670	CakSNP6670	*Kabuli*	Ca_Kabuli_Chr04	29582219	[A/C]		--	INTERGENIC
6671	CakSNP6671	*Kabuli*	Ca_Kabuli_Chr04	29582271	[C/T]		--	INTERGENIC

S.N.	SNP IDs	Chickpea cultivars	Chromosomes	Physical positions (bp)	SNPs	Gene accession IDs	Putative functions	Sequence components of genome
6672	CakSNP6672	*Kabuli*	Ca_Kabuli_Chr04	29582286	[T/A]		--	INTERGENIC
6673	CakSNP6673	*Kabuli*	Ca_Kabuli_Chr04	29582294	[G/A]		--	INTERGENIC
6674	CakSNP6674	*Kabuli*	Ca_Kabuli_Chr04	29582301	[G/T]		--	INTERGENIC
6675	CakSNP6675	*Kabuli*	Ca_Kabuli_Chr04	29671563	[G/A]		--	INTERGENIC
6676	CakSNP6676	*Kabuli*	Ca_Kabuli_Chr04	29671606	[A/G]		--	INTERGENIC
6677	CakSNP6677	*Kabuli*	Ca_Kabuli_Chr04	29683718	[A/T]	Ca_14166	Kinesin, motor domain	NON_SYNONYMOUS_CODING
6678	CakSNP6678	*Kabuli*	Ca_Kabuli_Chr04	29923380	[G/A]	Ca_14177	Plant peroxidase	SYNONYMOUS_CODING
6679	CakSNP6679	*Kabuli*	Ca_Kabuli_Chr04	29958122	[T/G]		--	INTERGENIC
6680	CakSNP6680	*Kabuli*	Ca_Kabuli_Chr04	29958113	[G/A]		--	INTERGENIC
6681	CakSNP6681	*Kabuli*	Ca_Kabuli_Chr04	29972268	[T/G]		--	INTERGENIC

S.N.	SNP IDs	Chickpea cultivars	Chromosomes	Physical positions (bp)	SNPs	Gene accession IDs	Putative functions	Sequence components of genome
6682	CakSNP6682	*Kabuli*	Ca_Kabuli_Chr04	30066903	[G/A]	Ca_14182	Heat shock protein DnaJ, N-terminal	NON_SYNONYMOUS_CODING
6683	CakSNP6683	*Kabuli*	Ca_Kabuli_Chr04	30153390	[G/A]		--	INTERGENIC
6684	CakSNP6684	*Kabuli*	Ca_Kabuli_Chr04	30153448	[T/C]		--	INTERGENIC
6685	CakSNP6685	*Kabuli*	Ca_Kabuli_Chr04	30153505	[C/G]		--	INTERGENIC
6686	CakSNP6686	*Kabuli*	Ca_Kabuli_Chr04	30258044	[A/T]		--	INTERGENIC
6687	CakSNP6687	*Kabuli*	Ca_Kabuli_Chr04	30258045	[A/T]		--	INTERGENIC
6688	CakSNP6688	*Kabuli*	Ca_Kabuli_Chr04	30258056	[A/G]		--	INTERGENIC
6689	CakSNP6689	*Kabuli*	Ca_Kabuli_Chr04	30258484	[G/C]	Ca_14189	Late embryogenesis abundant protein, group 1	SYNONYMOUS_CODING
6690	CakSNP6690	*Kabuli*	Ca_Kabuli_Chr04	30258613	[C/A]	Ca_14189	Late embryogenesis abundant protein, group 1	SYNONYMOUS_CODING
6691	CakSNP6691	*Kabuli*	Ca_Kabuli_Chr04	30258607	[C/A]	Ca_14189	Late embryogenesis abundant protein, group 1	NON_SYNONYMOUS_CODING

S.N.	SNP IDs	Chickpea cultivars	Chromosomes	Physical positions (bp)	SNPs	Gene accession IDs	Putative functions	Sequence components of genome
6692	CakSNP6692	*Kabuli*	Ca_Kabuli_Chr04	30258604	[C/A]	Ca_14189	Late embryogenesis abundant protein, group 1	NON_SYNONYMOUS_CODING
6693	CakSNP6693	*Kabuli*	Ca_Kabuli_Chr04	30258596	[G/C]	Ca_14189	Late embryogenesis abundant protein, group 1	SYNONYMOUS_CODING
6694	CakSNP6694	*Kabuli*	Ca_Kabuli_Chr04	30258567	[C/A]	Ca_14189	Late embryogenesis abundant protein, group 1	SYNONYMOUS_CODING
6695	CakSNP6695	*Kabuli*	Ca_Kabuli_Chr04	30260485	[C/A]	Ca_14190	Zinc finger, CCCH-type	SYNONYMOUS_CODING
6696	CakSNP6696	*Kabuli*	Ca_Kabuli_Chr04	30281991	[A/G]		--	INTERGENIC
6697	CakSNP6697	*Kabuli*	Ca_Kabuli_Chr04	30301002	[T/G]	Ca_14192	SNF2-related	SYNONYMOUS_CODING
6698	CakSNP6698	*Kabuli*	Ca_Kabuli_Chr04	30308719	[A/C]	Ca_14192	SNF2-related	INTRON
6699	CakSNP6699	*Kabuli*	Ca_Kabuli_Chr04	30308812	[G/A]	Ca_14192	SNF2-related	INTRON
6700	CakSNP6700	*Kabuli*	Ca_Kabuli_Chr04	30308788	[T/C]	Ca_14192	SNF2-related	INTRON
6701	CakSNP6701	*Kabuli*	Ca_Kabuli_Chr04	30463063	[T/C]		--	INTERGENIC

S.N.	SNP IDs	Chickpea cultivars	Chromosomes	Physical positions (bp)	SNPs	Gene accession IDs	Putative functions	Sequence components of genome
6702	CakSNP6702	*Kabuli*	Ca_Kabuli_Chr04	30463117	[C/A]		--	INTERGENIC
6703	CakSNP6703	*Kabuli*	Ca_Kabuli_Chr04	30599096	[C/A]	Ca_14207	Protein kinase, catalytic domain	SYNONYMOUS_CODING
6704	CakSNP6704	*Kabuli*	Ca_Kabuli_Chr04	30599188	[C/T]	Ca_14207	Protein kinase, catalytic domain	INTRON
6705	CakSNP6705	*Kabuli*	Ca_Kabuli_Chr04	30667916	[G/A]		--	INTERGENIC
6706	CakSNP6706	*Kabuli*	Ca_Kabuli_Chr04	30668893	[A/C]		--	INTERGENIC
6707	CakSNP6707	*Kabuli*	Ca_Kabuli_Chr04	30851027	[A/T]	Ca_14212	Basic-leucine zipper (bZIP) transcription factor	INTRON
6708	CakSNP6708	*Kabuli*	Ca_Kabuli_Chr04	30851411	[T/G]	Ca_14212	Basic-leucine zipper (bZIP) transcription factor	INTRON
6709	CakSNP6709	*Kabuli*	Ca_Kabuli_Chr04	30851840	[A/T]	Ca_14212	Basic-leucine zipper (bZIP) transcription factor	INTRON
6710	CakSNP6710	*Kabuli*	Ca_Kabuli_Chr04	30931586	[G/T]		--	INTERGENIC
6711	CakSNP6711	*Kabuli*	Ca_Kabuli_Chr04	31218647	[T/C]	Ca_14226	Ovarian tumour, otubain	INTRON

S.N.	SNP IDs	Chickpea cultivars	Chromosomes	Physical positions (bp)	SNPs	Gene accession IDs	Putative functions	Sequence components of genome
6712	CakSNP6712	*Kabuli*	Ca_Kabuli_Chr04	31221264	[T/G]		--	INTERGENIC
6713	CakSNP6713	*Kabuli*	Ca_Kabuli_Chr04	31221253	[G/A]		--	INTERGENIC
6714	CakSNP6714	*Kabuli*	Ca_Kabuli_Chr04	31221226	[T/A]		--	INTERGENIC
6715	CakSNP6715	*Kabuli*	Ca_Kabuli_Chr04	31224001	[G/A]		--	INTERGENIC
6716	CakSNP6716	*Kabuli*	Ca_Kabuli_Chr04	31262596	[T/A]		--	INTERGENIC
6717	CakSNP6717	*Kabuli*	Ca_Kabuli_Chr04	31263205	[T/A]	Ca_14230	Pentatricopeptide repeat	NON_SYNONYMOUS_CODING
6718	CakSNP6718	*Kabuli*	Ca_Kabuli_Chr04	31594126	[A/C]		--	INTERGENIC
6719	CakSNP6719	*Kabuli*	Ca_Kabuli_Chr04	31618949	[T/A]		--	INTERGENIC
6720	CakSNP6720	*Kabuli*	Ca_Kabuli_Chr04	31875713	[A/G]	Ca_15451	Tetratricopeptide TPR-1	INTRON
6721	CakSNP6721	*Kabuli*	Ca_Kabuli_Chr04	31987890	[A/C]		--	INTERGENIC

S.N.	SNP IDs	Chickpea cultivars	Chromosomes	Physical positions (bp)	SNPs	Gene accession IDs	Putative functions	Sequence components of genome
6722	CakSNP6722	*Kabuli*	Ca_Kabuli_Chr04	32042488	[T/G]	Ca_15459	Domain of unknown function DUF828	SYNONYMOUS_CODING
6723	CakSNP6723	*Kabuli*	Ca_Kabuli_Chr04	32042527	[C/T]	Ca_15459	Domain of unknown function DUF828	SYNONYMOUS_CODING
6724	CakSNP6724	*Kabuli*	Ca_Kabuli_Chr04	32068961	[A/G]	Ca_15461	Ankyrin repeat	SYNONYMOUS_CODING
6725	CakSNP6725	*Kabuli*	Ca_Kabuli_Chr04	32073552	[T/C]		--	INTERGENIC
6726	CakSNP6726	*Kabuli*	Ca_Kabuli_Chr04	32073550	[C/G]		--	INTERGENIC
6727	CakSNP6727	*Kabuli*	Ca_Kabuli_Chr04	32073549	[G/C]		--	INTERGENIC
6728	CakSNP6728	*Kabuli*	Ca_Kabuli_Chr04	32166559	[C/G]		--	INTERGENIC
6729	CakSNP6729	*Kabuli*	Ca_Kabuli_Chr04	32166557	[T/A]		--	INTERGENIC
6730	CakSNP6730	*Kabuli*	Ca_Kabuli_Chr04	32183307	[C/G]	Ca_15464	Pentatricopeptide repeat	SYNONYMOUS_CODING
6731	CakSNP6731	*Kabuli*	Ca_Kabuli_Chr04	32184628	[A/G]	Ca_15464	Pentatricopeptide repeat	SYNONYMOUS_CODING

S.N.	SNP IDs	Chickpea cultivars	Chromosomes	Physical positions (bp)	SNPs	Gene accession IDs	Putative functions	Sequence components of genome
6732	CakSNP6732	*Kabuli*	Ca_Kabuli_Chr04	32212899	[G/A]		--	INTERGENIC
6733	CakSNP6733	*Kabuli*	Ca_Kabuli_Chr04	32297780	[C/A]		--	INTERGENIC
6734	CakSNP6734	*Kabuli*	Ca_Kabuli_Chr04	32297758	[G/A]		--	INTERGENIC
6735	CakSNP6735	*Kabuli*	Ca_Kabuli_Chr04	32297734	[A/G]		--	INTERGENIC
6736	CakSNP6736	*Kabuli*	Ca_Kabuli_Chr04	32303394	[C/T]	Ca_15473	Manganese/iron superoxide dismutase	INTRON
6737	CakSNP6737	*Kabuli*	Ca_Kabuli_Chr04	32429228	[A/T]	Ca_15476	Armadillo	NON_SYNONYMOUS_CODING
6738	CakSNP6738	*Kabuli*	Ca_Kabuli_Chr04	32429222	[A/C]	Ca_15476	Armadillo	SYNONYMOUS_CODING
6739	CakSNP6739	*Kabuli*	Ca_Kabuli_Chr04	32521346	[T/C]	Ca_15480	--	INTRON
6740	CakSNP6740	*Kabuli*	Ca_Kabuli_Chr04	32602658	[C/T]		--	INTERGENIC
6741	CakSNP6741	*Kabuli*	Ca_Kabuli_Chr04	32604261	[G/A]	Ca_15484	Pentatricopeptide repeat	NON_SYNONYMOUS_CODING

S.N.	SNP IDs	Chickpea cultivars	Chromosomes	Physical positions (bp)	SNPs	Gene accession IDs	Putative functions	Sequence components of genome
6742	CakSNP6742	*Kabuli*	Ca_Kabuli_Chr04	32604538	[T/C]	Ca_15484	Pentatricopeptide repeat	SYNONYMOUS_CODING
6743	CakSNP6743	*Kabuli*	Ca_Kabuli_Chr04	32604510	[A/C]	Ca_15484	Pentatricopeptide repeat	NON_SYNONYMOUS_CODING
6744	CakSNP6744	*Kabuli*	Ca_Kabuli_Chr04	32636315	[T/C]		--	INTERGENIC
6745	CakSNP6745	*Kabuli*	Ca_Kabuli_Chr04	32636276	[T/C]		--	INTERGENIC
6746	CakSNP6746	*Kabuli*	Ca_Kabuli_Chr04	32636428	[C/A]		--	INTERGENIC
6747	CakSNP6747	*Kabuli*	Ca_Kabuli_Chr04	32636459	[A/G]		--	INTERGENIC
6748	CakSNP6748	*Kabuli*	Ca_Kabuli_Chr04	32636690	[C/T]		--	INTERGENIC
6749	CakSNP6749	*Kabuli*	Ca_Kabuli_Chr04	32636953	[A/G]		--	INTERGENIC
6750	CakSNP6750	*Kabuli*	Ca_Kabuli_Chr04	32639121	[C/T]		--	INTERGENIC
6751	CakSNP6751	*Kabuli*	Ca_Kabuli_Chr04	32639188	[C/G]		--	INTERGENIC

S.N.	SNP IDs	Chickpea cultivars	Chromosomes	Physical positions (bp)	SNPs	Gene accession IDs	Putative functions	Sequence components of genome
6752	CakSNP6752	*Kabuli*	Ca_Kabuli_Chr04	32639118	[A/C]		--	INTERGENIC
6753	CakSNP6753	*Kabuli*	Ca_Kabuli_Chr04	32639262	[A/G]		--	INTERGENIC
6754	CakSNP6754	*Kabuli*	Ca_Kabuli_Chr04	32639315	[A/G]		--	INTERGENIC
6755	CakSNP6755	*Kabuli*	Ca_Kabuli_Chr04	32639654	[T/C]		--	INTERGENIC
6756	CakSNP6756	*Kabuli*	Ca_Kabuli_Chr04	32639681	[C/T]		--	INTERGENIC
6757	CakSNP6757	*Kabuli*	Ca_Kabuli_Chr04	32639770	[C/G]		--	INTERGENIC
6758	CakSNP6758	*Kabuli*	Ca_Kabuli_Chr04	32640911	[A/G]		--	INTERGENIC
6759	CakSNP6759	*Kabuli*	Ca_Kabuli_Chr04	32853834	[T/C]	Ca_15495	DNA topoisomerase I, C-terminal	INTRON
6760	CakSNP6760	*Kabuli*	Ca_Kabuli_Chr04	32853838	[G/T]	Ca_15495	DNA topoisomerase I, C-terminal	INTRON
6761	CakSNP6761	*Kabuli*	Ca_Kabuli_Chr04	33068276	[G/A]		--	INTERGENIC

S.N.	SNP IDs	Chickpea cultivars	Chromosomes	Physical positions (bp)	SNPs	Gene accession IDs	Putative functions	Sequence components of genome
6762	CakSNP6762	*Kabuli*	Ca_Kabuli_Chr04	33221361	[T/C]	Ca_24018	ATPase, P-type, H+ transporting proton pump	INTRON
6763	CakSNP6763	*Kabuli*	Ca_Kabuli_Chr04	33221371	[T/C]	Ca_24018	ATPase, P-type, H+ transporting proton pump	INTRON
6764	CakSNP6764	*Kabuli*	Ca_Kabuli_Chr04	33221397	[T/G]	Ca_24018	ATPase, P-type, H+ transporting proton pump	INTRON
6765	CakSNP6765	*Kabuli*	Ca_Kabuli_Chr04	33509759	[A/C]		--	INTERGENIC
6766	CakSNP6766	*Kabuli*	Ca_Kabuli_Chr04	33509762	[G/A]		--	INTERGENIC
6767	CakSNP6767	*Kabuli*	Ca_Kabuli_Chr04	33509804	[A/G]		--	INTERGENIC
6768	CakSNP6768	*Kabuli*	Ca_Kabuli_Chr04	33641757	[C/T]	Ca_24030	SNF2-related	INTRON
6769	CakSNP6769	*Kabuli*	Ca_Kabuli_Chr04	33820657	[A/C]	Ca_20371	Clp, N-terminal	SYNONYMOUS_CODING
6770	CakSNP6770	*Kabuli*	Ca_Kabuli_Chr04	33874346	[T/C]		--	INTERGENIC
6771	CakSNP6771	*Kabuli*	Ca_Kabuli_Chr04	33874327	[T/C]		--	INTERGENIC

S.N.	SNP IDs	Chickpea cultivars	Chromosomes	Physical positions (bp)	SNPs	Gene accession IDs	Putative functions	Sequence components of genome
6772	CakSNP6772	*Kabuli*	Ca_Kabuli_Chr04	33874313	[G/A]		--	INTERGENIC
6773	CakSNP6773	*Kabuli*	Ca_Kabuli_Chr04	33929598	[A/C]	Ca_20365	Peptidase C19, ubiquitin carboxyl-terminal hydrolase 3	INTRON
6774	CakSNP6774	*Kabuli*	Ca_Kabuli_Chr04	33929777	[C/T]	Ca_20365	Peptidase C19, ubiquitin carboxyl-terminal hydrolase 4	INTRON
6775	CakSNP6775	*Kabuli*	Ca_Kabuli_Chr04	34032176	[C/T]		--	INTERGENIC
6776	CakSNP6776	*Kabuli*	Ca_Kabuli_Chr04	34155135	[A/C]	Ca_20360	Peptidase C48, SUMO/Sentrin/Ubl1	SYNONYMOUS_CODING
6777	CakSNP6777	*Kabuli*	Ca_Kabuli_Chr04	34155131	[T/A]	Ca_20360	Peptidase C48, SUMO/Sentrin/Ubl1	STOP_GAINED
6778	CakSNP6778	*Kabuli*	Ca_Kabuli_Chr04	34155128	[C/T]	Ca_20360	Peptidase C48, SUMO/Sentrin/Ubl1	NON_SYNONYMOUS_CODING
6779	CakSNP6779	*Kabuli*	Ca_Kabuli_Chr04	34155127	[G/A]	Ca_20360	Peptidase C48, SUMO/Sentrin/Ubl1	NON_SYNONYMOUS_CODING
6780	CakSNP6780	*Kabuli*	Ca_Kabuli_Chr04	34155124	[G/A]	Ca_20360	Peptidase C48, SUMO/Sentrin/Ubl1	NON_SYNONYMOUS_CODING
6781	CakSNP6781	*Kabuli*	Ca_Kabuli_Chr04	34155084	[C/G]	Ca_20360	Peptidase C48, SUMO/Sentrin/Ubl1	SYNONYMOUS_CODING

S.N.	SNP IDs	Chickpea cultivars	Chromosomes	Physical positions (bp)	SNPs	Gene accession IDs	Putative functions	Sequence components of genome
6782	CakSNP6782	*Kabuli*	Ca_Kabuli_Chr04	34155083	[C/T]	Ca_20360	Peptidase C48, SUMO/Sentrin/Ubl1	NON_SYNONYMOUS_CODING
6783	CakSNP6783	*Kabuli*	Ca_Kabuli_Chr04	34222792	[G/C]		--	INTERGENIC
6784	CakSNP6784	*Kabuli*	Ca_Kabuli_Chr04	34354519	[G/T]		--	INTERGENIC
6785	CakSNP6785	*Kabuli*	Ca_Kabuli_Chr04	34487064	[T/G]	Ca_20350	Domain of unknown function DUF676, hydrolase-like	INTRON
6786	CakSNP6786	*Kabuli*	Ca_Kabuli_Chr04	34744344	[T/G]	Ca_27238	Ribosomal protein L24e	SYNONYMOUS_CODING
6787	CakSNP6787	*Kabuli*	Ca_Kabuli_Chr04	34744398	[T/A]	Ca_27238	Ribosomal protein L24e	SYNONYMOUS_CODING
6788	CakSNP6788	*Kabuli*	Ca_Kabuli_Chr04	34784285	[G/A]	Ca_19990	Ribosomal protein L22/L17	NON_SYNONYMOUS_CODING
6789	CakSNP6789	*Kabuli*	Ca_Kabuli_Chr04	34798597	[C/A]	Ca_19992	Pentatricopeptide repeat	INTRON
6790	CakSNP6790	*Kabuli*	Ca_Kabuli_Chr04	34916032	[C/T]	Ca_19997	Inosine/uridine-preferring nucleoside hydrolase domain	INTRON
6791	CakSNP6791	*Kabuli*	Ca_Kabuli_Chr04	34924575	[C/T]	Ca_19998	Metallo-dependent phosphatase	NON_SYNONYMOUS_CODING

S.N.	SNP IDs	Chickpea cultivars	Chromosomes	Physical positions (bp)	SNPs	Gene accession IDs	Putative functions	Sequence components of genome
6792	CakSNP6792	*Kabuli*	Ca_Kabuli_Chr04	34955953	[A/G]	Ca_19999	Peptidase C48, SUMO/Sentrin/Ubl1	NON_SYNONYMOUS_CODING
6793	CakSNP6793	*Kabuli*	Ca_Kabuli_Chr04	34955971	[A/T]	Ca_19999	Peptidase C48, SUMO/Sentrin/Ubl1	NON_SYNONYMOUS_CODING
6794	CakSNP6794	*Kabuli*	Ca_Kabuli_Chr04	34956006	[C/T]	Ca_19999	Peptidase C48, SUMO/Sentrin/Ubl1	SYNONYMOUS_CODING
6795	CakSNP6795	*Kabuli*	Ca_Kabuli_Chr04	34956019	[G/A]	Ca_19999	Peptidase C48, SUMO/Sentrin/Ubl1	NON_SYNONYMOUS_CODING
6796	CakSNP6796	*Kabuli*	Ca_Kabuli_Chr04	34956035	[C/T]	Ca_19999	Peptidase C48, SUMO/Sentrin/Ubl1	NON_SYNONYMOUS_CODING
6797	CakSNP6797	*Kabuli*	Ca_Kabuli_Chr04	34956111	[G/T]	Ca_19999	Peptidase C48, SUMO/Sentrin/Ubl1	SYNONYMOUS_CODING
6798	CakSNP6798	*Kabuli*	Ca_Kabuli_Chr04	34956031	[G/A]	Ca_19999	Peptidase C48, SUMO/Sentrin/Ubl1	NON_SYNONYMOUS_CODING
6799	CakSNP6799	*Kabuli*	Ca_Kabuli_Chr04	35141281	[A/T]	Ca_20010	Glycoside hydrolase, family 9	NON_SYNONYMOUS_CODING
6800	CakSNP6800	*Kabuli*	Ca_Kabuli_Chr04	35141309	[A/C]	Ca_20010	Glycoside hydrolase, family 9	SYNONYMOUS_CODING
6801	CakSNP6801	*Kabuli*	Ca_Kabuli_Chr04	35141340	[G/A]	Ca_20010	Glycoside hydrolase, family 9	INTRON

S.N.	SNP IDs	Chickpea cultivars	Chromosomes	Physical positions (bp)	SNPs	Gene accession IDs	Putative functions	Sequence components of genome
6802	CakSNP6802	*Kabuli*	Ca_Kabuli_Chr04	35141353	[C/G]	Ca_20010	Glycoside hydrolase, family 9	INTRON
6803	CakSNP6803	*Kabuli*	Ca_Kabuli_Chr04	35141359	[C/G]	Ca_20010	Glycoside hydrolase, family 9	INTRON
6804	CakSNP6804	*Kabuli*	Ca_Kabuli_Chr04	35141375	[A/G]	Ca_20010	Glycoside hydrolase, family 9	INTRON
6805	CakSNP6805	*Kabuli*	Ca_Kabuli_Chr04	35141403	[C/T]	Ca_20010	Glycoside hydrolase, family 9	INTRON
6806	CakSNP6806	*Kabuli*	Ca_Kabuli_Chr04	35141425	[A/T]	Ca_20010	Glycoside hydrolase, family 9	INTRON
6807	CakSNP6807	*Kabuli*	Ca_Kabuli_Chr04	35141421	[T/A]	Ca_20010	Glycoside hydrolase, family 9	INTRON
6808	CakSNP6808	*Kabuli*	Ca_Kabuli_Chr04	35141408	[A/G]	Ca_20010	Glycoside hydrolase, family 9	INTRON
6809	CakSNP6809	*Kabuli*	Ca_Kabuli_Chr04	35168915	[C/T]	Ca_20011	--	INTRON
6810	CakSNP6810	*Kabuli*	Ca_Kabuli_Chr04	35168939	[C/T]	Ca_20011	--	INTRON
6811	CakSNP6811	*Kabuli*	Ca_Kabuli_Chr04	35229966	[C/T]	Ca_20015	--	NON_SYNONYMOUS_CODING

S.N.	SNP IDs	Chickpea cultivars	Chromosomes	Physical positions (bp)	SNPs	Gene accession IDs	Putative functions	Sequence components of genome
6812	CakSNP6812	*Kabuli*	Ca_Kabuli_Chr04	35230144	[A/G]		--	INTERGENIC
6813	CakSNP6813	*Kabuli*	Ca_Kabuli_Chr04	35231891	[C/T]		--	INTERGENIC
6814	CakSNP6814	*Kabuli*	Ca_Kabuli_Chr04	35304102	[C/T]	Ca_20017	--	SYNONYMOUS_CODING
6815	CakSNP6815	*Kabuli*	Ca_Kabuli_Chr04	35304108	[C/A]	Ca_20017	--	NON_SYNONYMOUS_CODING
6816	CakSNP6816	*Kabuli*	Ca_Kabuli_Chr04	35309709	[A/G]	Ca_20017	--	INTRON
6817	CakSNP6817	*Kabuli*	Ca_Kabuli_Chr04	35311815	[A/C]	Ca_20017	--	SYNONYMOUS_CODING
6818	CakSNP6818	*Kabuli*	Ca_Kabuli_Chr04	35337282	[A/G]	Ca_20018	IQ motif, EF-hand binding site	INTRON
6819	CakSNP6819	*Kabuli*	Ca_Kabuli_Chr04	35344356	[A/G]	Ca_20018	IQ motif, EF-hand binding site	INTRON
6820	CakSNP6820	*Kabuli*	Ca_Kabuli_Chr04	35344353	[T/C]	Ca_20018	IQ motif, EF-hand binding site	INTRON
6821	CakSNP6821	*Kabuli*	Ca_Kabuli_Chr04	35344323	[C/T]	Ca_20018	IQ motif, EF-hand binding site	INTRON

S.N.	SNP IDs	Chickpea cultivars	Chromosomes	Physical positions (bp)	SNPs	Gene accession IDs	Putative functions	Sequence components of genome
6822	CakSNP6822	*Kabuli*	Ca_Kabuli_Chr04	35346415	[G/A]	Ca_20018	IQ motif, EF-hand binding site	INTRON
6823	CakSNP6823	*Kabuli*	Ca_Kabuli_Chr04	35346417	[C/T]	Ca_20018	IQ motif, EF-hand binding site	INTRON
6824	CakSNP6824	*Kabuli*	Ca_Kabuli_Chr04	35346495	[C/T]	Ca_20018	IQ motif, EF-hand binding site	INTRON
6825	CakSNP6825	*Kabuli*	Ca_Kabuli_Chr04	35346465	[G/T]	Ca_20018	IQ motif, EF-hand binding site	INTRON
6826	CakSNP6826	*Kabuli*	Ca_Kabuli_Chr04	35346558	[G/T]	Ca_20018	IQ motif, EF-hand binding site	INTRON
6827	CakSNP6827	*Kabuli*	Ca_Kabuli_Chr04	35346563	[G/T]	Ca_20018	IQ motif, EF-hand binding site	INTRON
6828	CakSNP6828	*Kabuli*	Ca_Kabuli_Chr04	35346573	[A/G]	Ca_20018	IQ motif, EF-hand binding site	INTRON
6829	CakSNP6829	*Kabuli*	Ca_Kabuli_Chr04	35346576	[C/G]	Ca_20018	IQ motif, EF-hand binding site	INTRON
6830	CakSNP6830	*Kabuli*	Ca_Kabuli_Chr04	35346624	[G/A]	Ca_20018	IQ motif, EF-hand binding site	INTRON
6831	CakSNP6831	*Kabuli*	Ca_Kabuli_Chr04	35393505	[G/T]	Ca_20021	Transcription factor, TCP	SYNONYMOUS_CODING

S.N.	SNP IDs	Chickpea cultivars	Chromosomes	Physical positions (bp)	SNPs	Gene accession IDs	Putative functions	Sequence components of genome
6832	CakSNP6832	*Kabuli*	Ca_Kabuli_Chr04	35393643	[G/A]	Ca_20021	Transcription factor, TCP	SYNONYMOUS_CODING
6833	CakSNP6833	*Kabuli*	Ca_Kabuli_Chr04	35455095	[A/C]		--	INTERGENIC
6834	CakSNP6834	*Kabuli*	Ca_Kabuli_Chr04	35455062	[A/T]		--	INTERGENIC
6835	CakSNP6835	*Kabuli*	Ca_Kabuli_Chr04	35455708	[G/A]		--	INTERGENIC
6836	CakSNP6836	*Kabuli*	Ca_Kabuli_Chr04	35469308	[T/G]	Ca_22431	Sugar/inositol transporter	NON_SYNONYMOUS_CODING
6837	CakSNP6837	*Kabuli*	Ca_Kabuli_Chr04	35469304	[G/A]	Ca_22431	Sugar/inositol transporter	NON_SYNONYMOUS_CODING
6838	CakSNP6838	*Kabuli*	Ca_Kabuli_Chr04	35487504	[G/C]	Ca_22433	Sugar/inositol transporter	NON_SYNONYMOUS_CODING
6839	CakSNP6839	*Kabuli*	Ca_Kabuli_Chr04	35487496	[C/A]	Ca_22433	Sugar/inositol transporter	STOP_GAINED
6840	CakSNP6840	*Kabuli*	Ca_Kabuli_Chr04	35487471	[A/C]	Ca_22433	Sugar/inositol transporter	NON_SYNONYMOUS_CODING
6841	CakSNP6841	*Kabuli*	Ca_Kabuli_Chr04	35528758	[C/A]	Ca_22434	--	INTRON

S.N.	SNP IDs	Chickpea cultivars	Chromosomes	Physical positions (bp)	SNPs	Gene accession IDs	Putative functions	Sequence components of genome
6842	CakSNP6842	*Kabuli*	Ca_Kabuli_Chr04	35534777	[G/C]	Ca_22434	--	INTRON
6843	CakSNP6843	*Kabuli*	Ca_Kabuli_Chr04	35534877	[T/C]	Ca_22434	--	INTRON
6844	CakSNP6844	*Kabuli*	Ca_Kabuli_Chr04	35534891	[C/T]	Ca_22434	--	INTRON
6845	CakSNP6845	*Kabuli*	Ca_Kabuli_Chr04	35574379	[C/A]	Ca_22438	Transcription factor, MADS-box	NON_SYNONYMOUS_CODING
6846	CakSNP6846	*Kabuli*	Ca_Kabuli_Chr04	35634712	[A/G]		--	INTERGENIC
6847	CakSNP6847	*Kabuli*	Ca_Kabuli_Chr04	35634751	[A/G]		--	INTERGENIC
6848	CakSNP6848	*Kabuli*	Ca_Kabuli_Chr04	35634873	[G/T]		--	INTERGENIC
6849	CakSNP6849	*Kabuli*	Ca_Kabuli_Chr04	35961593	[A/C]	Ca_14859	Zinc/iron permease	SYNONYMOUS_CODING
6850	CakSNP6850	*Kabuli*	Ca_Kabuli_Chr04	35977422	[G/A]	Ca_14857	Ribosomal protein L19	INTRON
6851	CakSNP6851	*Kabuli*	Ca_Kabuli_Chr04	36005847	[T/G]		--	INTERGENIC

S.N.	SNP IDs	Chickpea cultivars	Chromosomes	Physical positions (bp)	SNPs	Gene accession IDs	Putative functions	Sequence components of genome
6852	CakSNP6852	*Kabuli*	Ca_Kabuli_Chr04	36010756	[T/G]		--	INTERGENIC
6853	CakSNP6853	*Kabuli*	Ca_Kabuli_Chr04	36153818	[A/G]	Ca_14853	Protein of unknown function DUF827, plant	NON_SYNONYMOUS_CODING
6854	CakSNP6854	*Kabuli*	Ca_Kabuli_Chr04	36154771	[T/C]	Ca_14853	Protein of unknown function DUF827, plant	SYNONYMOUS_CODING
6855	CakSNP6855	*Kabuli*	Ca_Kabuli_Chr04	36154820	[G/A]	Ca_14853	Protein of unknown function DUF827, plant	NON_SYNONYMOUS_CODING
6856	CakSNP6856	*Kabuli*	Ca_Kabuli_Chr04	36154822	[T/G]	Ca_14853	Protein of unknown function DUF827, plant	SYNONYMOUS_CODING
6857	CakSNP6857	*Kabuli*	Ca_Kabuli_Chr04	36184969	[A/G]	Ca_14850	Bacterial Fmu (Sun)/eukaryotic nucleolar NOL1/Nop2p	INTRON
6858	CakSNP6858	*Kabuli*	Ca_Kabuli_Chr04	36277383	[C/T]	Ca_14843	Helicase, C-terminal	NON_SYNONYMOUS_CODING
6859	CakSNP6859	*Kabuli*	Ca_Kabuli_Chr04	36368713	[T/C]	Ca_14840	Domain of unknown function DUF1084	SYNONYMOUS_CODING
6860	CakSNP6860	*Kabuli*	Ca_Kabuli_Chr04	36445345	[G/A]	Ca_14833	Armadillo	SYNONYMOUS_CODING
6861	CakSNP6861	*Kabuli*	Ca_Kabuli_Chr04	36445367	[A/G]	Ca_14833	Armadillo	NON_SYNONYMOUS_CODING

S.N.	SNP IDs	Chickpea cultivars	Chromosomes	Physical positions (bp)	SNPs	Gene accession IDs	Putative functions	Sequence components of genome
6862	CakSNP6862	*Kabuli*	Ca_Kabuli_Chr04	36449101	[C/T]	Ca_14833	Armadillo	INTRON
6863	CakSNP6863	*Kabuli*	Ca_Kabuli_Chr04	36461836	[A/G]	Ca_14831	--	SYNONYMOUS_CODING
6864	CakSNP6864	*Kabuli*	Ca_Kabuli_Chr04	36467928	[C/T]	Ca_14830	Semialdehyde dehydrogenase, NAD-binding	INTRON
6865	CakSNP6865	*Kabuli*	Ca_Kabuli_Chr04	36467943	[A/G]	Ca_14830	Semialdehyde dehydrogenase, NAD-binding	INTRON
6866	CakSNP6866	*Kabuli*	Ca_Kabuli_Chr04	36467964	[G/A]	Ca_14830	Semialdehyde dehydrogenase, NAD-binding	INTRON
6867	CakSNP6867	*Kabuli*	Ca_Kabuli_Chr04	36467967	[T/C]	Ca_14830	Semialdehyde dehydrogenase, NAD-binding	INTRON
6868	CakSNP6868	*Kabuli*	Ca_Kabuli_Chr04	36467980	[C/A]	Ca_14830	Semialdehyde dehydrogenase, NAD-binding	INTRON
6869	CakSNP6869	*Kabuli*	Ca_Kabuli_Chr04	36467988	[C/T]	Ca_14830	Semialdehyde dehydrogenase, NAD-binding	INTRON
6870	CakSNP6870	*Kabuli*	Ca_Kabuli_Chr04	36467990	[T/G]	Ca_14830	Semialdehyde dehydrogenase, NAD-binding	INTRON
6871	CakSNP6871	*Kabuli*	Ca_Kabuli_Chr04	36468003	[T/A]	Ca_14830	Semialdehyde dehydrogenase, NAD-binding	INTRON

S.N.	SNP IDs	Chickpea cultivars	Chromosomes	Physical positions (bp)	SNPs	Gene accession IDs	Putative functions	Sequence components of genome
6872	CakSNP6872	*Kabuli*	Ca_Kabuli_Chr04	36468053	[A/G]	Ca_14830	Semialdehyde dehydrogenase, NAD-binding	INTRON
6873	CakSNP6873	*Kabuli*	Ca_Kabuli_Chr04	36488966	[A/G]	Ca_14828	--	SYNONYMOUS_CODING
6874	CakSNP6874	*Kabuli*	Ca_Kabuli_Chr04	36537118	[C/T]	Ca_14825	Transcriptional factor B3	SYNONYMOUS_CODING
6875	CakSNP6875	*Kabuli*	Ca_Kabuli_Chr04	36537124	[C/T]	Ca_14825	Transcriptional factor B3	SYNONYMOUS_CODING
6876	CakSNP6876	*Kabuli*	Ca_Kabuli_Chr04	36537170	[C/T]	Ca_14825	Transcriptional factor B3	NON_SYNONYMOUS_CODING
6877	CakSNP6877	*Kabuli*	Ca_Kabuli_Chr04	36541088	[G/T]	Ca_14824	PetM of cytochrome b6/f complex subunit 7	NON_SYNONYMOUS_CODING
6878	CakSNP6878	*Kabuli*	Ca_Kabuli_Chr04	36541087	[T/C]	Ca_14824	PetM of cytochrome b6/f complex subunit 7	SYNONYMOUS_CODING
6879	CakSNP6879	*Kabuli*	Ca_Kabuli_Chr04	36541083	[A/C]	Ca_14824	PetM of cytochrome b6/f complex subunit 7	NON_SYNONYMOUS_CODING
6880	CakSNP6880	*Kabuli*	Ca_Kabuli_Chr04	36639333	[A/T]		--	INTERGENIC
6881	CakSNP6881	*Kabuli*	Ca_Kabuli_Chr04	36639382	[T/C]		--	INTERGENIC

S.N.	SNP IDs	Chickpea cultivars	Chromosomes	Physical positions (bp)	SNPs	Gene accession IDs	Putative functions	Sequence components of genome
6882	CakSNP6882	*Kabuli*	Ca_Kabuli_Chr04	36639474	[G/C]		--	INTERGENIC
6883	CakSNP6883	*Kabuli*	Ca_Kabuli_Chr04	36673677	[C/A]		--	INTERGENIC
6884	CakSNP6884	*Kabuli*	Ca_Kabuli_Chr04	36682215	[G/A]	Ca_14812	--	NON_SYNONYMOUS_CODING
6885	CakSNP6885	*Kabuli*	Ca_Kabuli_Chr04	36682213	[C/T]	Ca_14812	--	NON_SYNONYMOUS_CODING
6886	CakSNP6886	*Kabuli*	Ca_Kabuli_Chr04	36682211	[G/A]	Ca_14812	--	SYNONYMOUS_CODING
6887	CakSNP6887	*Kabuli*	Ca_Kabuli_Chr04	36682253	[T/G]	Ca_14812	--	SYNONYMOUS_CODING
6888	CakSNP6888	*Kabuli*	Ca_Kabuli_Chr04	36682231	[G/T]	Ca_14812	--	NON_SYNONYMOUS_CODING
6889	CakSNP6889	*Kabuli*	Ca_Kabuli_Chr04	36709731	[G/A]		--	INTERGENIC
6890	CakSNP6890	*Kabuli*	Ca_Kabuli_Chr04	36754121	[T/C]	Ca_14808	Phosphofructokinase domain	INTRON
6891	CakSNP6891	*Kabuli*	Ca_Kabuli_Chr04	36754149	[G/A]	Ca_14808	Phosphofructokinase domain	INTRON

S.N.	SNP IDs	Chickpea cultivars	Chromosomes	Physical positions (bp)	SNPs	Gene accession IDs	Putative functions	Sequence components of genome
6892	CakSNP6892	*Kabuli*	Ca_Kabuli_Chr04	36754290	[T/A]	Ca_14808	Phosphofructokinase domain	INTRON
6893	CakSNP6893	*Kabuli*	Ca_Kabuli_Chr04	36754378	[C/T]	Ca_14808	Phosphofructokinase domain	INTRON
6894	CakSNP6894	*Kabuli*	Ca_Kabuli_Chr04	36754377	[T/A]	Ca_14808	Phosphofructokinase domain	INTRON
6895	CakSNP6895	*Kabuli*	Ca_Kabuli_Chr04	36754348	[C/T]	Ca_14808	Phosphofructokinase domain	INTRON
6896	CakSNP6896	*Kabuli*	Ca_Kabuli_Chr04	36754343	[A/C]	Ca_14808	Phosphofructokinase domain	INTRON
6897	CakSNP6897	*Kabuli*	Ca_Kabuli_Chr04	36754327	[G/A]	Ca_14808	Phosphofructokinase domain	INTRON
6898	CakSNP6898	*Kabuli*	Ca_Kabuli_Chr04	36754468	[G/A]	Ca_14808	Phosphofructokinase domain	SYNONYMOUS_CODING
6899	CakSNP6899	*Kabuli*	Ca_Kabuli_Chr04	36754453	[A/G]	Ca_14808	Phosphofructokinase domain	SYNONYMOUS_CODING
6900	CakSNP6900	*Kabuli*	Ca_Kabuli_Chr04	36799551	[C/T]	Ca_14807	RNA recognition motif domain	SYNONYMOUS_CODING
6901	CakSNP6901	*Kabuli*	Ca_Kabuli_Chr04	36831035	[A/G]	Ca_14805	--	NON_SYNONYMOUS_CODING

S.N.	SNP IDs	Chickpea cultivars	Chromosomes	Physical positions (bp)	SNPs	Gene accession IDs	Putative functions	Sequence components of genome
6902	CakSNP6902	*Kabuli*	Ca_Kabuli_Chr04	36831199	[G/A]	Ca_14805	--	NON_SYNONYMOUS_CODING
6903	CakSNP6903	*Kabuli*	Ca_Kabuli_Chr04	36831267	[A/C]	Ca_14805	--	SYNONYMOUS_CODING
6904	CakSNP6904	*Kabuli*	Ca_Kabuli_Chr04	36955706	[G/T]	Ca_14797	Zinc finger, C2H2-type	NON_SYNONYMOUS_CODING
6905	CakSNP6905	*Kabuli*	Ca_Kabuli_Chr04	36977192	[C/T]		--	INTERGENIC
6906	CakSNP6906	*Kabuli*	Ca_Kabuli_Chr04	36977194	[G/A]		--	INTERGENIC
6907	CakSNP6907	*Kabuli*	Ca_Kabuli_Chr04	36979866	[T/G]		--	INTERGENIC
6908	CakSNP6908	*Kabuli*	Ca_Kabuli_Chr04	36979926	[G/T]		--	INTERGENIC
6909	CakSNP6909	*Kabuli*	Ca_Kabuli_Chr04	36979879	[C/T]		--	INTERGENIC
6910	CakSNP6910	*Kabuli*	Ca_Kabuli_Chr04	36982955	[A/G]	Ca_14795	SNF2-related	INTRON
6911	CakSNP6911	*Kabuli*	Ca_Kabuli_Chr04	36984727	[C/G]	Ca_14795	SNF2-related	INTRON

S.N.	SNP IDs	Chickpea cultivars	Chromosomes	Physical positions (bp)	SNPs	Gene accession IDs	Putative functions	Sequence components of genome
6912	CakSNP6912	*Kabuli*	Ca_Kabuli_Chr04	36984829	[C/T]	Ca_14795	SNF2-related	INTRON
6913	CakSNP6913	*Kabuli*	Ca_Kabuli_Chr04	36984795	[T/A]	Ca_14795	SNF2-related	INTRON
6914	CakSNP6914	*Kabuli*	Ca_Kabuli_Chr04	36998201	[A/C]		--	INTERGENIC
6915	CakSNP6915	*Kabuli*	Ca_Kabuli_Chr04	36998209	[C/T]		--	INTERGENIC
6916	CakSNP6916	*Kabuli*	Ca_Kabuli_Chr04	37064588	[A/G]		--	INTERGENIC
6917	CakSNP6917	*Kabuli*	Ca_Kabuli_Chr04	37064630	[G/A]		--	INTERGENIC
6918	CakSNP6918	*Kabuli*	Ca_Kabuli_Chr04	37185576	[T/A]	Ca_15153	RNA-dependent RNA polymerase, eukaryotic-type	INTRON
6919	CakSNP6919	*Kabuli*	Ca_Kabuli_Chr04	37185577	[T/A]	Ca_15153	RNA-dependent RNA polymerase, eukaryotic-type	INTRON
6920	CakSNP6920	*Kabuli*	Ca_Kabuli_Chr04	37185578	[T/A]	Ca_15153	RNA-dependent RNA polymerase, eukaryotic-type	INTRON
6921	CakSNP6921	*Kabuli*	Ca_Kabuli_Chr04	37237211	[G/A]		--	INTERGENIC

S.N.	SNP IDs	Chickpea cultivars	Chromosomes	Physical positions (bp)	SNPs	Gene accession IDs	Putative functions	Sequence components of genome
6922	CakSNP6922	*Kabuli*	Ca_Kabuli_Chr04	37237276	[T/C]		--	INTERGENIC
6923	CakSNP6923	*Kabuli*	Ca_Kabuli_Chr04	37241235	[A/G]	Ca_15151	Signal transduction response regulator, receiver domain	SYNONYMOUS_CODING
6924	CakSNP6924	*Kabuli*	Ca_Kabuli_Chr04	37241262	[G/A]	Ca_15151	Signal transduction response regulator, receiver domain	SYNONYMOUS_CODING
6925	CakSNP6925	*Kabuli*	Ca_Kabuli_Chr04	37260697	[T/C]	Ca_15149	Dynamin central domain	INTRON
6926	CakSNP6926	*Kabuli*	Ca_Kabuli_Chr04	37260873	[A/G]	Ca_15149	Dynamin central domain	SYNONYMOUS_CODING
6927	CakSNP6927	*Kabuli*	Ca_Kabuli_Chr04	37260841	[T/A]	Ca_15149	Dynamin central domain	INTRON
6928	CakSNP6928	*Kabuli*	Ca_Kabuli_Chr04	37264628	[G/A]	Ca_15149	Dynamin central domain	INTRON
6929	CakSNP6929	*Kabuli*	Ca_Kabuli_Chr04	37286591	[T/A]	Ca_15147	--	INTRON
6930	CakSNP6930	*Kabuli*	Ca_Kabuli_Chr04	37286625	[C/A]	Ca_15147	--	INTRON
6931	CakSNP6931	*Kabuli*	Ca_Kabuli_Chr04	37286632	[G/A]	Ca_15147	--	INTRON

S.N.	SNP IDs	Chickpea cultivars	Chromosomes	Physical positions (bp)	SNPs	Gene accession IDs	Putative functions	Sequence components of genome
6932	CakSNP6932	*Kabuli*	Ca_Kabuli_Chr04	37286663	[A/G]	Ca_15147	--	INTRON
6933	CakSNP6933	*Kabuli*	Ca_Kabuli_Chr04	37293752	[G/A]		--	INTERGENIC
6934	CakSNP6934	*Kabuli*	Ca_Kabuli_Chr04	37293780	[T/A]		--	INTERGENIC
6935	CakSNP6935	*Kabuli*	Ca_Kabuli_Chr04	37293773	[A/T]		--	INTERGENIC
6936	CakSNP6936	*Kabuli*	Ca_Kabuli_Chr04	37293769	[C/A]		--	INTERGENIC
6937	CakSNP6937	*Kabuli*	Ca_Kabuli_Chr04	37294720	[A/G]		--	INTERGENIC
6938	CakSNP6938	*Kabuli*	Ca_Kabuli_Chr04	37316574	[T/A]	Ca_15144	Zinc finger, RING-type	SYNONYMOUS_CODING
6939	CakSNP6939	*Kabuli*	Ca_Kabuli_Chr04	37316748	[T/A]	Ca_15144	Zinc finger, RING-type	SYNONYMOUS_CODING
6940	CakSNP6940	*Kabuli*	Ca_Kabuli_Chr04	37316738	[C/T]	Ca_15144	Zinc finger, RING-type	NON_SYNONYMOUS_CODING
6941	CakSNP6941	*Kabuli*	Ca_Kabuli_Chr04	37316713	[A/T]	Ca_15144	Zinc finger, RING-type	NON_SYNONYMOUS_CODING

S.N.	SNP IDs	Chickpea cultivars	Chromosomes	Physical positions (bp)	SNPs	Gene accession IDs	Putative functions	Sequence components of genome
6942	CakSNP6942	*Kabuli*	Ca_Kabuli_Chr04	37316700	[C/A]	Ca_15144	Zinc finger, RING-type	NON_SYNONYMOUS_CODING
6943	CakSNP6943	*Kabuli*	Ca_Kabuli_Chr04	37349071	[T/C]	Ca_15141	Methyltransferase TRM13	INTRON
6944	CakSNP6944	*Kabuli*	Ca_Kabuli_Chr04	37349212	[A/C]	Ca_15141	Methyltransferase TRM13	SYNONYMOUS_CODING
6945	CakSNP6945	*Kabuli*	Ca_Kabuli_Chr04	37349239	[C/G]	Ca_15141	Methyltransferase TRM13	INTRON
6946	CakSNP6946	*Kabuli*	Ca_Kabuli_Chr04	37349304	[T/C]	Ca_15141	Methyltransferase TRM13	INTRON
6947	CakSNP6947	*Kabuli*	Ca_Kabuli_Chr04	37349331	[C/T]	Ca_15141	Methyltransferase TRM13	INTRON
6948	CakSNP6948	*Kabuli*	Ca_Kabuli_Chr04	37349321	[T/C]	Ca_15141	Methyltransferase TRM13	INTRON
6949	CakSNP6949	*Kabuli*	Ca_Kabuli_Chr04	37356865	[C/A]	Ca_15140	HEAT	SYNONYMOUS_CODING
6950	CakSNP6950	*Kabuli*	Ca_Kabuli_Chr04	37356889	[C/T]	Ca_15140	HEAT	INTRON
6951	CakSNP6951	*Kabuli*	Ca_Kabuli_Chr04	37356892	[C/T]	Ca_15140	HEAT	INTRON

S.N.	SNP IDs	Chickpea cultivars	Chromosomes	Physical positions (bp)	SNPs	Gene accession IDs	Putative functions	Sequence components of genome
6952	CakSNP6952	*Kabuli*	Ca_Kabuli_Chr04	37356919	[A/T]	Ca_15140	HEAT	INTRON
6953	CakSNP6953	*Kabuli*	Ca_Kabuli_Chr04	37356951	[C/T]	Ca_15140	HEAT	INTRON
6954	CakSNP6954	*Kabuli*	Ca_Kabuli_Chr04	37364980	[G/A]	Ca_15140	HEAT	SYNONYMOUS_CODING
6955	CakSNP6955	*Kabuli*	Ca_Kabuli_Chr04	37365020	[C/T]	Ca_15140	HEAT	NON_SYNONYMOUS_CODING
6956	CakSNP6956	*Kabuli*	Ca_Kabuli_Chr04	37371262	[G/A]	Ca_15139	Ankyrin repeat	SYNONYMOUS_CODING
6957	CakSNP6957	*Kabuli*	Ca_Kabuli_Chr04	37371263	[G/A]	Ca_15139	Ankyrin repeat	NON_SYNONYMOUS_CODING
6958	CakSNP6958	*Kabuli*	Ca_Kabuli_Chr04	37433459	[G/T]		--	INTERGENIC
6959	CakSNP6959	*Kabuli*	Ca_Kabuli_Chr04	37450206	[A/C]	Ca_15131	Oxoglutarate/iron-dependent oxygenase	SYNONYMOUS_CODING
6960	CakSNP6960	*Kabuli*	Ca_Kabuli_Chr04	37450326	[A/T]	Ca_15131	Oxoglutarate/iron-dependent oxygenase	SYNONYMOUS_CODING
6961	CakSNP6961	*Kabuli*	Ca_Kabuli_Chr04	37450308	[C/T]	Ca_15131	Oxoglutarate/iron-dependent oxygenase	SYNONYMOUS_CODING

S.N.	SNP IDs	Chickpea cultivars	Chromosomes	Physical positions (bp)	SNPs	Gene accession IDs	Putative functions	Sequence components of genome
6962	CakSNP6962	*Kabuli*	Ca_Kabuli_Chr04	37450294	[T/C]	Ca_15131	Oxoglutarate/iron-dependent oxygenase	SYNONYMOUS_CODING
6963	CakSNP6963	*Kabuli*	Ca_Kabuli_Chr04	37453497	[C/G]	Ca_15130	--	SYNONYMOUS_CODING
6964	CakSNP6964	*Kabuli*	Ca_Kabuli_Chr04	37453449	[A/G]	Ca_15130	--	SYNONYMOUS_CODING
6965	CakSNP6965	*Kabuli*	Ca_Kabuli_Chr04	37453590	[C/A]	Ca_15130	--	SYNONYMOUS_CODING
6966	CakSNP6966	*Kabuli*	Ca_Kabuli_Chr04	37453586	[C/A]	Ca_15130	--	SYNONYMOUS_CODING
6967	CakSNP6967	*Kabuli*	Ca_Kabuli_Chr04	37454296	[A/G]	Ca_15130	--	INTRON
6968	CakSNP6968	*Kabuli*	Ca_Kabuli_Chr04	37492422	[C/A]		--	INTERGENIC
6969	CakSNP6969	*Kabuli*	Ca_Kabuli_Chr04	37549883	[T/C]	Ca_15125	Mediator complex subunit Med23	INTRON
6970	CakSNP6970	*Kabuli*	Ca_Kabuli_Chr04	37549871	[A/T]	Ca_15125	Mediator complex subunit Med23	INTRON
6971	CakSNP6971	*Kabuli*	Ca_Kabuli_Chr04	37550122	[G/A]	Ca_15125	Mediator complex subunit Med23	INTRON

S.N.	SNP IDs	Chickpea cultivars	Chromosomes	Physical positions (bp)	SNPs	Gene accession IDs	Putative functions	Sequence components of genome
6972	CakSNP6972	*Kabuli*	Ca_Kabuli_Chr04	37552973	[C/T]	Ca_15125	Mediator complex subunit Med23	INTRON
6973	CakSNP6973	*Kabuli*	Ca_Kabuli_Chr04	37558880	[C/T]	Ca_15125	Mediator complex subunit Med23	INTRON
6974	CakSNP6974	*Kabuli*	Ca_Kabuli_Chr04	37558881	[G/T]	Ca_15125	Mediator complex subunit Med23	INTRON
6975	CakSNP6975	*Kabuli*	Ca_Kabuli_Chr04	37575636	[T/C]	Ca_15125	Mediator complex subunit Med23	INTRON
6976	CakSNP6976	*Kabuli*	Ca_Kabuli_Chr04	37575670	[C/T]	Ca_15125	Mediator complex subunit Med23	INTRON
6977	CakSNP6977	*Kabuli*	Ca_Kabuli_Chr04	37575709	[C/T]	Ca_15125	Mediator complex subunit Med23	INTRON
6978	CakSNP6978	*Kabuli*	Ca_Kabuli_Chr04	37612154	[A/G]	Ca_15121	Glycoside hydrolase, family 18	SYNONYMOUS_CODING
6979	CakSNP6979	*Kabuli*	Ca_Kabuli_Chr04	37630404	[A/G]	Ca_15119	Exoribonuclease, phosphorolytic domain 1	INTRON
6980	CakSNP6980	*Kabuli*	Ca_Kabuli_Chr04	37630445	[A/G]	Ca_15119	Exoribonuclease, phosphorolytic domain 1	INTRON
6981	CakSNP6981	*Kabuli*	Ca_Kabuli_Chr04	37630527	[G/A]	Ca_15119	Exoribonuclease, phosphorolytic domain 1	INTRON

S.N.	SNP IDs	Chickpea cultivars	Chromosomes	Physical positions (bp)	SNPs	Gene accession IDs	Putative functions	Sequence components of genome
6982	CakSNP6982	*Kabuli*	Ca_Kabuli_Chr04	37630520	[G/C]	Ca_15119	Exoribonuclease, phosphorolytic domain 1	INTRON
6983	CakSNP6983	*Kabuli*	Ca_Kabuli_Chr04	37634624	[C/T]	Ca_15118	--	NON_SYNONYMOUS_CODING
6984	CakSNP6984	*Kabuli*	Ca_Kabuli_Chr04	37635300	[T/A]		--	INTERGENIC
6985	CakSNP6985	*Kabuli*	Ca_Kabuli_Chr04	37685824	[T/C]	Ca_15113	Armadillo	SYNONYMOUS_CODING
6986	CakSNP6986	*Kabuli*	Ca_Kabuli_Chr04	37685842	[T/G]	Ca_15113	Armadillo	SYNONYMOUS_CODING
6987	CakSNP6987	*Kabuli*	Ca_Kabuli_Chr04	37699176	[G/A]	Ca_15111	ATP-grasp fold	SYNONYMOUS_CODING
6988	CakSNP6988	*Kabuli*	Ca_Kabuli_Chr04	37699141	[T/G]	Ca_15111	ATP-grasp fold	SYNONYMOUS_CODING
6989	CakSNP6989	*Kabuli*	Ca_Kabuli_Chr04	37699140	[C/T]	Ca_15111	ATP-grasp fold	SYNONYMOUS_CODING
6990	CakSNP6990	*Kabuli*	Ca_Kabuli_Chr04	37703656	[G/T]		--	INTERGENIC
6991	CakSNP6991	*Kabuli*	Ca_Kabuli_Chr04	37703653	[A/C]		--	INTERGENIC

S.N.	SNP IDs	Chickpea cultivars	Chromosomes	Physical positions (bp)	SNPs	Gene accession IDs	Putative functions	Sequence components of genome
6992	CakSNP6992	*Kabuli*	Ca_Kabuli_Chr04	37703651	[T/C]		--	INTERGENIC
6993	CakSNP6993	*Kabuli*	Ca_Kabuli_Chr04	37703649	[A/C]		--	INTERGENIC
6994	CakSNP6994	*Kabuli*	Ca_Kabuli_Chr04	37703648	[C/T]		--	INTERGENIC
6995	CakSNP6995	*Kabuli*	Ca_Kabuli_Chr04	37707115	[G/A]		--	INTERGENIC
6996	CakSNP6996	*Kabuli*	Ca_Kabuli_Chr04	37708260	[A/G]		--	INTERGENIC
6997	CakSNP6997	*Kabuli*	Ca_Kabuli_Chr04	37708348	[T/G]		--	INTERGENIC
6998	CakSNP6998	*Kabuli*	Ca_Kabuli_Chr04	37708334	[T/A]		--	INTERGENIC
6999	CakSNP6999	*Kabuli*	Ca_Kabuli_Chr04	37753479	[C/T]	Ca_15104	--	INTRON
7000	CakSNP7000	*Kabuli*	Ca_Kabuli_Chr04	37806689	[T/C]		--	INTERGENIC
7001	CakSNP7001	*Kabuli*	Ca_Kabuli_Chr04	37812917	[G/C]		--	INTERGENIC

S.N.	SNP IDs	Chickpea cultivars	Chromosomes	Physical positions (bp)	SNPs	Gene accession IDs	Putative functions	Sequence components of genome
7002	CakSNP7002	*Kabuli*	Ca_Kabuli_Chr04	37859069	[T/G]	Ca_15089	Auxin efflux carrier	SYNONYMOUS_CODING
7003	CakSNP7003	*Kabuli*	Ca_Kabuli_Chr04	37897261	[G/T]		--	INTERGENIC
7004	CakSNP7004	*Kabuli*	Ca_Kabuli_Chr04	37897266	[A/T]		--	INTERGENIC
7005	CakSNP7005	*Kabuli*	Ca_Kabuli_Chr04	37897351	[C/A]		--	INTERGENIC
7006	CakSNP7006	*Kabuli*	Ca_Kabuli_Chr04	37898404	[A/G]		--	INTERGENIC
7007	CakSNP7007	*Kabuli*	Ca_Kabuli_Chr04	37899597	[C/T]		--	INTERGENIC
7008	CakSNP7008	*Kabuli*	Ca_Kabuli_Chr04	37901584	[C/T]		--	INTERGENIC
7009	CakSNP7009	*Kabuli*	Ca_Kabuli_Chr04	37901575	[G/A]		--	INTERGENIC
7010	CakSNP7010	*Kabuli*	Ca_Kabuli_Chr04	38008062	[T/A]	Ca_13145	Quinolinate phosphoribosyl transferase, C-terminal domain	INTRON
7011	CakSNP7011	*Kabuli*	Ca_Kabuli_Chr04	38008082	[T/C]	Ca_13145	Quinolinate phosphoribosyl transferase, C-terminal domain	INTRON

S.N.	SNP IDs	Chickpea cultivars	Chromosomes	Physical positions (bp)	SNPs	Gene accession IDs	Putative functions	Sequence components of genome
7012	CakSNP7012	*Kabuli*	Ca_Kabuli_Chr04	38008243	[C/T]	Ca_13145	Quinolinate phosphoribosyl transferase, C-terminal domain	INTRON
7013	CakSNP7013	*Kabuli*	Ca_Kabuli_Chr04	38008227	[G/T]	Ca_13145	Quinolinate phosphoribosyl transferase, C-terminal domain	INTRON
7014	CakSNP7014	*Kabuli*	Ca_Kabuli_Chr04	38008213	[C/T]	Ca_13145	Quinolinate phosphoribosyl transferase, C-terminal domain	INTRON
7015	CakSNP7015	*Kabuli*	Ca_Kabuli_Chr04	38008205	[A/G]	Ca_13145	Quinolinate phosphoribosyl transferase, C-terminal domain	INTRON
7016	CakSNP7016	*Kabuli*	Ca_Kabuli_Chr04	38008204	[G/A]	Ca_13145	Quinolinate phosphoribosyl transferase, C-terminal domain	INTRON
7017	CakSNP7017	*Kabuli*	Ca_Kabuli_Chr04	38008186	[A/C]	Ca_13145	Quinolinate phosphoribosyl transferase, C-terminal domain	INTRON
7018	CakSNP7018	*Kabuli*	Ca_Kabuli_Chr04	38008174	[A/G]	Ca_13145	Quinolinate phosphoribosyl transferase, C-terminal domain	INTRON
7019	CakSNP7019	*Kabuli*	Ca_Kabuli_Chr04	38027560	[G/A]	Ca_13142	Chaperonin TCP-1, conserved site	INTRON
7020	CakSNP7020	*Kabuli*	Ca_Kabuli_Chr04	38029012	[T/C]	Ca_13142	Chaperonin TCP-1, conserved site	INTRON
7021	CakSNP7021	*Kabuli*	Ca_Kabuli_Chr04	38029050	[G/A]	Ca_13142	Chaperonin TCP-1, conserved site	INTRON

S.N.	SNP IDs	Chickpea cultivars	Chromosomes	Physical positions (bp)	SNPs	Gene accession IDs	Putative functions	Sequence components of genome
7022	CakSNP7022	*Kabuli*	Ca_Kabuli_Chr04	38029136	[G/T]	Ca_13142	Chaperonin TCP-1, conserved site	NON_SYNONYMOUS_CODING
7023	CakSNP7023	*Kabuli*	Ca_Kabuli_Chr04	38038865	[C/T]	Ca_13141	Protein of unknown function DUF3326	NON_SYNONYMOUS_CODING
7024	CakSNP7024	*Kabuli*	Ca_Kabuli_Chr04	38038959	[A/G]	Ca_13141	Protein of unknown function DUF3326	INTRON
7025	CakSNP7025	*Kabuli*	Ca_Kabuli_Chr04	38039037	[T/C]	Ca_13141	Protein of unknown function DUF3326	INTRON
7026	CakSNP7026	*Kabuli*	Ca_Kabuli_Chr04	38039046	[C/T]	Ca_13141	Protein of unknown function DUF3326	INTRON
7027	CakSNP7027	*Kabuli*	Ca_Kabuli_Chr04	38039076	[A/G]	Ca_13141	Protein of unknown function DUF3326	INTRON
7028	CakSNP7028	*Kabuli*	Ca_Kabuli_Chr04	38039088	[T/C]	Ca_13141	Protein of unknown function DUF3326	INTRON
7029	CakSNP7029	*Kabuli*	Ca_Kabuli_Chr04	38039251	[G/C]	Ca_13141	Protein of unknown function DUF3326	INTRON
7030	CakSNP7030	*Kabuli*	Ca_Kabuli_Chr04	38039289	[G/A]	Ca_13141	Protein of unknown function DUF3326	INTRON
7031	CakSNP7031	*Kabuli*	Ca_Kabuli_Chr04	38039297	[C/T]	Ca_13141	Protein of unknown function DUF3326	NON_SYNONYMOUS_CODING

S.N.	SNP IDs	Chickpea cultivars	Chromosomes	Physical positions (bp)	SNPs	Gene accession IDs	Putative functions	Sequence components of genome
7032	CakSNP7032	*Kabuli*	Ca_Kabuli_Chr04	38039313	[G/A]	Ca_13141	Protein of unknown function DUF3326	NON_SYNONYMOUS_CODING
7033	CakSNP7033	*Kabuli*	Ca_Kabuli_Chr04	38039371	[T/C]	Ca_13141	Protein of unknown function DUF3326	SYNONYMOUS_CODING
7034	CakSNP7034	*Kabuli*	Ca_Kabuli_Chr04	38153183	[C/A]	Ca_13138	Tetrahydrofolate dehydrogenase/cyclohydrolase	SYNONYMOUS_CODING
7035	CakSNP7035	*Kabuli*	Ca_Kabuli_Chr04	38153195	[T/C]	Ca_13138	Tetrahydrofolate dehydrogenase/cyclohydrolase	SYNONYMOUS_CODING
7036	CakSNP7036	*Kabuli*	Ca_Kabuli_Chr04	38153216	[T/C]	Ca_13138	Tetrahydrofolate dehydrogenase/cyclohydrolase	SYNONYMOUS_CODING
7037	CakSNP7037	*Kabuli*	Ca_Kabuli_Chr04	38153277	[A/G]	Ca_13138	Tetrahydrofolate dehydrogenase/cyclohydrolase	INTRON
7038	CakSNP7038	*Kabuli*	Ca_Kabuli_Chr04	38153275	[C/A]	Ca_13138	Tetrahydrofolate dehydrogenase/cyclohydrolase	INTRON
7039	CakSNP7039	*Kabuli*	Ca_Kabuli_Chr04	38153269	[A/C]	Ca_13138	Tetrahydrofolate dehydrogenase/cyclohydrolase	INTRON
7040	CakSNP7040	*Kabuli*	Ca_Kabuli_Chr04	38179583	[A/C]		--	INTERGENIC
7041	CakSNP7041	*Kabuli*	Ca_Kabuli_Chr04	38179614	[G/C]		--	INTERGENIC

S.N.	SNP IDs	Chickpea cultivars	Chromosomes	Physical positions (bp)	SNPs	Gene accession IDs	Putative functions	Sequence components of genome
7042	CakSNP7042	*Kabuli*	Ca_Kabuli_Chr04	38181292	[G/A]	Ca_13135	--	NON_SYNONYMOUS_CODING
7043	CakSNP7043	*Kabuli*	Ca_Kabuli_Chr04	38195411	[T/A]	Ca_13134	Domain of unknown function DUF250	NON_SYNONYMOUS_CODING
7044	CakSNP7044	*Kabuli*	Ca_Kabuli_Chr04	38195438	[C/T]	Ca_13134	Domain of unknown function DUF250	NON_SYNONYMOUS_CODING
7045	CakSNP7045	*Kabuli*	Ca_Kabuli_Chr04	38215267	[G/A]	Ca_13132	AMP-dependent synthetase/ligase	INTRON
7046	CakSNP7046	*Kabuli*	Ca_Kabuli_Chr04	38221009	[A/G]	Ca_13131	Lipase, GDSL	SYNONYMOUS_CODING
7047	CakSNP7047	*Kabuli*	Ca_Kabuli_Chr04	38288823	[G/T]	Ca_13128	Protein of unknown function DUF869, plant	SYNONYMOUS_CODING
7048	CakSNP7048	*Kabuli*	Ca_Kabuli_Chr04	38288877	[C/G]	Ca_13128	Protein of unknown function DUF869, plant	NON_SYNONYMOUS_CODING
7049	CakSNP7049	*Kabuli*	Ca_Kabuli_Chr04	38289066	[G/A]	Ca_13128	Protein of unknown function DUF869, plant	SYNONYMOUS_CODING
7050	CakSNP7050	*Kabuli*	Ca_Kabuli_Chr04	38289052	[G/A]	Ca_13128	Protein of unknown function DUF869, plant	SYNONYMOUS_CODING
7051	CakSNP7051	*Kabuli*	Ca_Kabuli_Chr04	38288997	[T/A]	Ca_13128	Protein of unknown function DUF869, plant	SYNONYMOUS_CODING

S.N.	SNP IDs	Chickpea cultivars	Chromosomes	Physical positions (bp)	SNPs	Gene accession IDs	Putative functions	Sequence components of genome
7052	CakSNP7052	*Kabuli*	Ca_Kabuli_Chr04	38343633	[A/T]		--	INTERGENIC
7053	CakSNP7053	*Kabuli*	Ca_Kabuli_Chr04	38343750	[C/T]		--	INTERGENIC
7054	CakSNP7054	*Kabuli*	Ca_Kabuli_Chr04	38343687	[C/T]		--	INTERGENIC
7055	CakSNP7055	*Kabuli*	Ca_Kabuli_Chr04	38358067	[A/G]	Ca_13120	C2 calcium-dependent membrane targeting	NON_SYNONYMOUS_CODING
7056	CakSNP7056	*Kabuli*	Ca_Kabuli_Chr04	38358398	[A/G]	Ca_13120	C2 calcium-dependent membrane targeting	SYNONYMOUS_CODING
7057	CakSNP7057	*Kabuli*	Ca_Kabuli_Chr04	38358677	[G/A]	Ca_13120	C2 calcium-dependent membrane targeting	SYNONYMOUS_CODING
7058	CakSNP7058	*Kabuli*	Ca_Kabuli_Chr04	38358887	[G/A]	Ca_13120	C2 calcium-dependent membrane targeting	SYNONYMOUS_CODING
7059	CakSNP7059	*Kabuli*	Ca_Kabuli_Chr04	38358869	[G/A]	Ca_13120	C2 calcium-dependent membrane targeting	SYNONYMOUS_CODING
7060	CakSNP7060	*Kabuli*	Ca_Kabuli_Chr04	38362132	[T/A]	Ca_13120	C2 calcium-dependent membrane targeting	SYNONYMOUS_CODING
7061	CakSNP7061	*Kabuli*	Ca_Kabuli_Chr04	38362139	[T/A]	Ca_13120	C2 calcium-dependent membrane targeting	NON_SYNONYMOUS_CODING

S.N.	SNP IDs	Chickpea cultivars	Chromosomes	Physical positions (bp)	SNPs	Gene accession IDs	Putative functions	Sequence components of genome
7062	CakSNP7062	*Kabuli*	Ca_Kabuli_Chr04	38364369	[C/T]	Ca_13120	C2 calcium-dependent membrane targeting	INTRON
7063	CakSNP7063	*Kabuli*	Ca_Kabuli_Chr04	38370848	[A/G]		--	INTERGENIC
7064	CakSNP7064	*Kabuli*	Ca_Kabuli_Chr04	38370864	[G/A]		--	INTERGENIC
7065	CakSNP7065	*Kabuli*	Ca_Kabuli_Chr04	38370941	[A/G]		--	INTERGENIC
7066	CakSNP7066	*Kabuli*	Ca_Kabuli_Chr04	38370930	[C/T]		--	INTERGENIC
7067	CakSNP7067	*Kabuli*	Ca_Kabuli_Chr04	38370926	[T/C]		--	INTERGENIC
7068	CakSNP7068	*Kabuli*	Ca_Kabuli_Chr04	38370925	[G/A]		--	INTERGENIC
7069	CakSNP7069	*Kabuli*	Ca_Kabuli_Chr04	38370922	[G/A]		--	INTERGENIC
7070	CakSNP7070	*Kabuli*	Ca_Kabuli_Chr04	38370921	[T/G]		--	INTERGENIC
7071	CakSNP7071	*Kabuli*	Ca_Kabuli_Chr04	38382809	[T/C]	Ca_13119	Amino acid transporter, transmembrane	SYNONYMOUS_CODING

S.N.	SNP IDs	Chickpea cultivars	Chromosomes	Physical positions (bp)	SNPs	Gene accession IDs	Putative functions	Sequence components of genome
7072	CakSNP7072	*Kabuli*	Ca_Kabuli_Chr04	38382890	[C/G]	Ca_13119	Amino acid transporter, transmembrane	SYNONYMOUS_CODING
7073	CakSNP7073	*Kabuli*	Ca_Kabuli_Chr04	38421798	[A/C]	Ca_13115	Zinc finger, CCCH-type	INTRON
7074	CakSNP7074	*Kabuli*	Ca_Kabuli_Chr04	38422037	[C/T]	Ca_13115	Zinc finger, CCCH-type	INTRON
7075	CakSNP7075	*Kabuli*	Ca_Kabuli_Chr04	38421984	[C/T]	Ca_13115	Zinc finger, CCCH-type	INTRON
7076	CakSNP7076	*Kabuli*	Ca_Kabuli_Chr04	38425675	[A/G]	Ca_13115	Zinc finger, CCCH-type	SYNONYMOUS_CODING
7077	CakSNP7077	*Kabuli*	Ca_Kabuli_Chr04	38425720	[T/A]	Ca_13115	Zinc finger, CCCH-type	NON_SYNONYMOUS_CODING
7078	CakSNP7078	*Kabuli*	Ca_Kabuli_Chr04	38473540	[A/G]	Ca_13112	--	INTRON
7079	CakSNP7079	*Kabuli*	Ca_Kabuli_Chr04	38473653	[T/C]	Ca_13112	--	INTRON
7080	CakSNP7080	*Kabuli*	Ca_Kabuli_Chr04	38474123	[A/T]	Ca_13112	--	INTRON
7081	CakSNP7081	*Kabuli*	Ca_Kabuli_Chr04	38474108	[T/C]	Ca_13112	--	INTRON

S.N.	SNP IDs	Chickpea cultivars	Chromosomes	Physical positions (bp)	SNPs	Gene accession IDs	Putative functions	Sequence components of genome
7082	CakSNP7082	*Kabuli*	Ca_Kabuli_Chr04	38474192	[T/C]	Ca_13112	--	INTRON
7083	CakSNP7083	*Kabuli*	Ca_Kabuli_Chr04	38475310	[A/C]	Ca_13112	--	INTRON
7084	CakSNP7084	*Kabuli*	Ca_Kabuli_Chr04	38475425	[A/G]	Ca_13112	--	INTRON
7085	CakSNP7085	*Kabuli*	Ca_Kabuli_Chr04	38475449	[T/A]	Ca_13112	--	INTRON
7086	CakSNP7086	*Kabuli*	Ca_Kabuli_Chr04	38475442	[A/G]	Ca_13112	--	INTRON
7087	CakSNP7087	*Kabuli*	Ca_Kabuli_Chr04	38475424	[C/G]	Ca_13112	--	INTRON
7088	CakSNP7088	*Kabuli*	Ca_Kabuli_Chr04	38475547	[C/T]	Ca_13112	--	INTRON
7089	CakSNP7089	*Kabuli*	Ca_Kabuli_Chr04	38540201	[C/T]	Ca_13109	Pentatricopeptide repeat	INTRON
7090	CakSNP7090	*Kabuli*	Ca_Kabuli_Chr04	38540236	[C/T]	Ca_13109	Pentatricopeptide repeat	INTRON
7091	CakSNP7091	*Kabuli*	Ca_Kabuli_Chr04	38540247	[G/A]	Ca_13109	Pentatricopeptide repeat	INTRON

S.N.	SNP IDs	Chickpea cultivars	Chromosomes	Physical positions (bp)	SNPs	Gene accession IDs	Putative functions	Sequence components of genome
7092	CakSNP7092	*Kabuli*	Ca_Kabuli_Chr04	38620579	[C/T]		--	INTERGENIC
7093	CakSNP7093	*Kabuli*	Ca_Kabuli_Chr04	38620620	[A/T]		--	INTERGENIC
7094	CakSNP7094	*Kabuli*	Ca_Kabuli_Chr04	38659377	[T/C]	Ca_13102	Transcription factor GRAS	SYNONYMOUS_CODING
7095	CakSNP7095	*Kabuli*	Ca_Kabuli_Chr04	38659398	[G/A]	Ca_13102	Transcription factor GRAS	SYNONYMOUS_CODING
7096	CakSNP7096	*Kabuli*	Ca_Kabuli_Chr04	38659534	[T/A]	Ca_13102	Transcription factor GRAS	NON_SYNONYMOUS_CODING
7097	CakSNP7097	*Kabuli*	Ca_Kabuli_Chr04	38679987	[A/G]	Ca_13099	LIM binding protein	INTRON
7098	CakSNP7098	*Kabuli*	Ca_Kabuli_Chr04	38721154	[A/G]	Ca_13098	Exostosin-like	INTRON
7099	CakSNP7099	*Kabuli*	Ca_Kabuli_Chr04	38721160	[A/G]	Ca_13098	Exostosin-like	INTRON
7100	CakSNP7100	*Kabuli*	Ca_Kabuli_Chr04	38721207	[A/C]	Ca_13098	Exostosin-like	INTRON
7101	CakSNP7101	*Kabuli*	Ca_Kabuli_Chr04	38756507	[T/C]	Ca_13097	Auxin efflux carrier	NON_SYNONYMOUS_CODING

S.N.	SNP IDs	Chickpea cultivars	Chromosomes	Physical positions (bp)	SNPs	Gene accession IDs	Putative functions	Sequence components of genome
7102	CakSNP7102	*Kabuli*	Ca_Kabuli_Chr04	38756510	[C/G]	Ca_13097	Auxin efflux carrier	NON_SYNONYMOUS_CODING
7103	CakSNP7103	*Kabuli*	Ca_Kabuli_Chr04	38759640	[A/C]	Ca_13097	Auxin efflux carrier	INTRON
7104	CakSNP7104	*Kabuli*	Ca_Kabuli_Chr04	38770348	[T/A]	Ca_13096	Fatty acid desaturase, type 2	NON_SYNONYMOUS_CODING
7105	CakSNP7105	*Kabuli*	Ca_Kabuli_Chr04	38770370	[G/C]	Ca_13096	Fatty acid desaturase, type 2	NON_SYNONYMOUS_CODING
7106	CakSNP7106	*Kabuli*	Ca_Kabuli_Chr04	38770450	[C/G]	Ca_13096	Fatty acid desaturase, type 2	SYNONYMOUS_CODING
7107	CakSNP7107	*Kabuli*	Ca_Kabuli_Chr04	38770510	[G/A]	Ca_13096	Fatty acid desaturase, type 2	SYNONYMOUS_CODING
7108	CakSNP7108	*Kabuli*	Ca_Kabuli_Chr04	38770606	[G/A]	Ca_13096	Fatty acid desaturase, type 2	SYNONYMOUS_CODING
7109	CakSNP7109	*Kabuli*	Ca_Kabuli_Chr04	38770582	[G/A]	Ca_13096	Fatty acid desaturase, type 2	SYNONYMOUS_CODING
7110	CakSNP7110	*Kabuli*	Ca_Kabuli_Chr04	38785535	[G/A]	Ca_13094	Glycosyl transferase, family 8	NON_SYNONYMOUS_CODING
7111	CakSNP7111	*Kabuli*	Ca_Kabuli_Chr04	38829959	[T/G]		--	INTERGENIC

S.N.	SNP IDs	Chickpea cultivars	Chromosomes	Physical positions (bp)	SNPs	Gene accession IDs	Putative functions	Sequence components of genome
7112	CakSNP7112	*Kabuli*	Ca_Kabuli_Chr04	38833304	[C/A]	Ca_13091	--	INTRON
7113	CakSNP7113	*Kabuli*	Ca_Kabuli_Chr04	38833280	[C/T]	Ca_13091	--	INTRON
7114	CakSNP7114	*Kabuli*	Ca_Kabuli_Chr04	38833333	[A/C]	Ca_13091	--	INTRON
7115	CakSNP7115	*Kabuli*	Ca_Kabuli_Chr04	38833414	[A/C]	Ca_13091	--	INTRON
7116	CakSNP7116	*Kabuli*	Ca_Kabuli_Chr04	38834383	[G/A]	Ca_13091	--	INTRON
7117	CakSNP7117	*Kabuli*	Ca_Kabuli_Chr04	38834405	[G/A]	Ca_13091	--	INTRON
7118	CakSNP7118	*Kabuli*	Ca_Kabuli_Chr04	38834423	[G/C]	Ca_13091	--	INTRON
7119	CakSNP7119	*Kabuli*	Ca_Kabuli_Chr04	38834431	[G/A]	Ca_13091	--	INTRON
7120	CakSNP7120	*Kabuli*	Ca_Kabuli_Chr04	38834448	[A/T]	Ca_13091	--	INTRON
7121	CakSNP7121	*Kabuli*	Ca_Kabuli_Chr04	38834477	[A/G]	Ca_13091	--	INTRON

S.N.	SNP IDs	Chickpea cultivars	Chromosomes	Physical positions (bp)	SNPs	Gene accession IDs	Putative functions	Sequence components of genome
7122	CakSNP7122	*Kabuli*	Ca_Kabuli_Chr04	38834474	[G/A]	Ca_13091	--	INTRON
7123	CakSNP7123	*Kabuli*	Ca_Kabuli_Chr04	38834453	[C/T]	Ca_13091	--	INTRON
7124	CakSNP7124	*Kabuli*	Ca_Kabuli_Chr04	38888779	[C/T]	Ca_13087	Protein kinase, catalytic domain	SYNONYMOUS_CODING
7125	CakSNP7125	*Kabuli*	Ca_Kabuli_Chr04	38888791	[G/C]	Ca_13087	Protein kinase, catalytic domain	SYNONYMOUS_CODING
7126	CakSNP7126	*Kabuli*	Ca_Kabuli_Chr04	38889023	[C/T]	Ca_13087	Protein kinase, catalytic domain	NON_SYNONYMOUS_CODING
7127	CakSNP7127	*Kabuli*	Ca_Kabuli_Chr04	38918394	[A/T]	Ca_13086	General substrate transporter	SYNONYMOUS_CODING
7128	CakSNP7128	*Kabuli*	Ca_Kabuli_Chr04	38918424	[G/A]	Ca_13086	General substrate transporter	SYNONYMOUS_CODING
7129	CakSNP7129	*Kabuli*	Ca_Kabuli_Chr04	38918430	[C/A]	Ca_13086	General substrate transporter	SYNONYMOUS_CODING
7130	CakSNP7130	*Kabuli*	Ca_Kabuli_Chr04	38918628	[C/T]	Ca_13086	General substrate transporter	SYNONYMOUS_CODING
7131	CakSNP7131	*Kabuli*	Ca_Kabuli_Chr04	38918547	[T/C]	Ca_13086	General substrate transporter	SYNONYMOUS_CODING

S.N.	SNP IDs	Chickpea cultivars	Chromosomes	Physical positions (bp)	SNPs	Gene accession IDs	Putative functions	Sequence components of genome
7132	CakSNP7132	*Kabuli*	Ca_Kabuli_Chr04	38918649	[G/C]	Ca_13086	General substrate transporter	SYNONYMOUS_CODING
7133	CakSNP7133	*Kabuli*	Ca_Kabuli_Chr04	38929681	[A/G]	Ca_13085	--	SYNONYMOUS_CODING
7134	CakSNP7134	*Kabuli*	Ca_Kabuli_Chr04	38948414	[C/A]	Ca_13084	Pathogenesis-related transcriptional factor/ERF, DNA-binding	NON_SYNONYMOUS_CODING
7135	CakSNP7135	*Kabuli*	Ca_Kabuli_Chr04	38961521	[G/A]	Ca_13083	Ubiquitin-conjugating enzyme, E2	INTRON
7136	CakSNP7136	*Kabuli*	Ca_Kabuli_Chr04	38978462	[A/G]	Ca_13081	Folylpolyglutamate synthetase	SYNONYMOUS_CODING
7137	CakSNP7137	*Kabuli*	Ca_Kabuli_Chr04	39031801	[C/T]	Ca_13078	RNA recognition motif domain	NON_SYNONYMOUS_CODING
7138	CakSNP7138	*Kabuli*	Ca_Kabuli_Chr04	39031823	[G/A]	Ca_13078	RNA recognition motif domain	SYNONYMOUS_CODING
7139	CakSNP7139	*Kabuli*	Ca_Kabuli_Chr04	39042938	[T/G]	Ca_13077	Peptidase M41	INTRON
7140	CakSNP7140	*Kabuli*	Ca_Kabuli_Chr04	39042921	[T/C]	Ca_13077	Peptidase M41	INTRON
7141	CakSNP7141	*Kabuli*	Ca_Kabuli_Chr04	39057991	[T/C]	Ca_13075	Heat shock protein DnaJ, N-terminal	NON_SYNONYMOUS_CODING

S.N.	SNP IDs	Chickpea cultivars	Chromosomes	Physical positions (bp)	SNPs	Gene accession IDs	Putative functions	Sequence components of genome
7142	CakSNP7142	*Kabuli*	Ca_Kabuli_Chr04	39058011	[G/C]	Ca_13075	Heat shock protein DnaJ, N-terminal	NON_SYNONYMOUS_CODING
7143	CakSNP7143	*Kabuli*	Ca_Kabuli_Chr04	39081951	[A/G]	Ca_13074	Cyclic nucleotide-binding domain	INTRON
7144	CakSNP7144	*Kabuli*	Ca_Kabuli_Chr04	39110408	[T/C]	Ca_13072	Glucosamine/galactosamine-6-phosphate isomerase	SYNONYMOUS_CODING
7145	CakSNP7145	*Kabuli*	Ca_Kabuli_Chr04	39110448	[A/G]	Ca_13072	Glucosamine/galactosamine-6-phosphate isomerase	NON_SYNONYMOUS_CODING
7146	CakSNP7146	*Kabuli*	Ca_Kabuli_Chr04	39110735	[A/G]	Ca_13072	Glucosamine/galactosamine-6-phosphate isomerase	SYNONYMOUS_CODING
7147	CakSNP7147	*Kabuli*	Ca_Kabuli_Chr04	39113309	[T/C]	Ca_13071	WD40 repeat	INTRON
7148	CakSNP7148	*Kabuli*	Ca_Kabuli_Chr04	39113327	[G/A]	Ca_13071	WD40 repeat	INTRON
7149	CakSNP7149	*Kabuli*	Ca_Kabuli_Chr04	39113330	[G/A]	Ca_13071	WD40 repeat	INTRON
7150	CakSNP7150	*Kabuli*	Ca_Kabuli_Chr04	39113401	[A/T]	Ca_13071	WD40 repeat	INTRON
7151	CakSNP7151	*Kabuli*	Ca_Kabuli_Chr04	39114000	[A/C]	Ca_13071	WD40 repeat	INTRON

S.N.	SNP IDs	Chickpea cultivars	Chromosomes	Physical positions (bp)	SNPs	Gene accession IDs	Putative functions	Sequence components of genome
7152	CakSNP7152	*Kabuli*	Ca_Kabuli_Chr04	39136826	[C/T]		--	INTERGENIC
7153	CakSNP7153	*Kabuli*	Ca_Kabuli_Chr04	39189012	[A/G]		--	INTERGENIC
7154	CakSNP7154	*Kabuli*	Ca_Kabuli_Chr04	39189016	[G/A]		--	INTERGENIC
7155	CakSNP7155	*Kabuli*	Ca_Kabuli_Chr04	39268505	[T/C]	Ca_13059	Protein kinase, catalytic domain	SYNONYMOUS_CODING
7156	CakSNP7156	*Kabuli*	Ca_Kabuli_Chr04	39306900	[G/A]		--	INTERGENIC
7157	CakSNP7157	*Kabuli*	Ca_Kabuli_Chr04	39635746	[T/A]		--	INTERGENIC
7158	CakSNP7158	*Kabuli*	Ca_Kabuli_Chr04	39635721	[A/G]		--	INTERGENIC
7159	CakSNP7159	*Kabuli*	Ca_Kabuli_Chr04	39635720	[G/A]		--	INTERGENIC
7160	CakSNP7160	*Kabuli*	Ca_Kabuli_Chr04	39635718	[C/T]		--	INTERGENIC
7161	CakSNP7161	*Kabuli*	Ca_Kabuli_Chr04	39650872	[T/C]		--	INTERGENIC

S.N.	SNP IDs	Chickpea cultivars	Chromosomes	Physical positions (bp)	SNPs	Gene accession IDs	Putative functions	Sequence components of genome
7162	CakSNP7162	*Kabuli*	Ca_Kabuli_Chr04	39659757	[C/T]	Ca_14936	--	NON_SYNONYMOUS_CODING
7163	CakSNP7163	*Kabuli*	Ca_Kabuli_Chr04	39659750	[G/A]	Ca_14936	--	NON_SYNONYMOUS_CODING
7164	CakSNP7164	*Kabuli*	Ca_Kabuli_Chr04	39659832	[C/T]	Ca_14936	--	NON_SYNONYMOUS_CODING
7165	CakSNP7165	*Kabuli*	Ca_Kabuli_Chr04	39715087	[A/G]	Ca_14929	Glutamine amidotransferase, class-II	SYNONYMOUS_CODING
7166	CakSNP7166	*Kabuli*	Ca_Kabuli_Chr04	39719606	[C/G]	Ca_14929	Glutamine amidotransferase, class-II	SYNONYMOUS_CODING
7167	CakSNP7167	*Kabuli*	Ca_Kabuli_Chr04	39719747	[G/T]	Ca_14929	Glutamine amidotransferase, class-II	SYNONYMOUS_CODING
7168	CakSNP7168	*Kabuli*	Ca_Kabuli_Chr04	39779796	[G/A]	Ca_14927	Protein of unknown function DUF81	INTRON
7169	CakSNP7169	*Kabuli*	Ca_Kabuli_Chr04	39803305	[T/C]	Ca_14925	SANT domain, DNA binding	SYNONYMOUS_CODING
7170	CakSNP7170	*Kabuli*	Ca_Kabuli_Chr04	39849612	[G/T]	Ca_14922	VHS	SYNONYMOUS_CODING
7171	CakSNP7171	*Kabuli*	Ca_Kabuli_Chr04	39849657	[G/A]	Ca_14922	VHS	SYNONYMOUS_CODING

S.N.	SNP IDs	Chickpea cultivars	Chromosomes	Physical positions (bp)	SNPs	Gene accession IDs	Putative functions	Sequence components of genome
7172	CakSNP7172	*Kabuli*	Ca_Kabuli_Chr04	39964930	[T/C]	Ca_14914	Exostosin-like	SYNONYMOUS_CODING
7173	CakSNP7173	*Kabuli*	Ca_Kabuli_Chr04	40010119	[A/C]	Ca_14911	Pathogenesis-related transcriptional factor/ERF, DNA-binding	NON_SYNONYMOUS_CODING
7174	CakSNP7174	*Kabuli*	Ca_Kabuli_Chr04	40010201	[A/G]	Ca_14911	Pathogenesis-related transcriptional factor/ERF, DNA-binding	SYNONYMOUS_CODING
7175	CakSNP7175	*Kabuli*	Ca_Kabuli_Chr04	40010177	[G/A]	Ca_14911	Pathogenesis-related transcriptional factor/ERF, DNA-binding	SYNONYMOUS_CODING
7176	CakSNP7176	*Kabuli*	Ca_Kabuli_Chr04	40010165	[A/C]	Ca_14911	Pathogenesis-related transcriptional factor/ERF, DNA-binding	NON_SYNONYMOUS_CODING
7177	CakSNP7177	*Kabuli*	Ca_Kabuli_Chr04	40059880	[C/T]		--	INTERGENIC
7178	CakSNP7178	*Kabuli*	Ca_Kabuli_Chr04	40060022	[C/T]	Ca_14910	Nuclear pore complex protein, Nucleoporin Nup85-like	SYNONYMOUS_CODING
7179	CakSNP7179	*Kabuli*	Ca_Kabuli_Chr04	40060063	[T/G]	Ca_14910	Nuclear pore complex protein, Nucleoporin Nup85-like	SYNONYMOUS_CODING
7180	CakSNP7180	*Kabuli*	Ca_Kabuli_Chr04	40061195	[T/C]	Ca_14910	Nuclear pore complex protein, Nucleoporin Nup85-like	INTRON
7181	CakSNP7181	*Kabuli*	Ca_Kabuli_Chr04	40061262	[A/C]	Ca_14910	Nuclear pore complex protein, Nucleoporin Nup85-like	INTRON

S.N.	SNP IDs	Chickpea cultivars	Chromosomes	Physical positions (bp)	SNPs	Gene accession IDs	Putative functions	Sequence components of genome
7182	CakSNP7182	*Kabuli*	Ca_Kabuli_Chr04	40065505	[T/A]	Ca_14910	Nuclear pore complex protein, Nucleoporin Nup85-like	INTRON
7183	CakSNP7183	*Kabuli*	Ca_Kabuli_Chr04	40134460	[A/G]	Ca_14906	Exoribonuclease, phosphorolytic domain 1	INTRON
7184	CakSNP7184	*Kabuli*	Ca_Kabuli_Chr04	40134518	[T/C]	Ca_14906	Exoribonuclease, phosphorolytic domain 1	INTRON
7185	CakSNP7185	*Kabuli*	Ca_Kabuli_Chr04	40149064	[C/T]	Ca_14905	--	INTRON
7186	CakSNP7186	*Kabuli*	Ca_Kabuli_Chr04	40149132	[G/C]	Ca_14905	--	INTRON
7187	CakSNP7187	*Kabuli*	Ca_Kabuli_Chr04	40149324	[G/C]	Ca_14905	--	INTRON
7188	CakSNP7188	*Kabuli*	Ca_Kabuli_Chr04	40169176	[T/G]		--	INTERGENIC
7189	CakSNP7189	*Kabuli*	Ca_Kabuli_Chr04	40185606	[A/G]	Ca_14901	Amino acid transporter, transmembrane	NON_SYNONYMOUS_CODING
7190	CakSNP7190	*Kabuli*	Ca_Kabuli_Chr04	40199692	[A/C]	Ca_14900	Cof protein	INTRON
7191	CakSNP7191	*Kabuli*	Ca_Kabuli_Chr04	40218375	[G/T]	Ca_14897	Early nodulin 93 ENOD93 protein	INTRON

S.N.	SNP IDs	Chickpea cultivars	Chromosomes	Physical positions (bp)	SNPs	Gene accession IDs	Putative functions	Sequence components of genome
7192	CakSNP7192	*Kabuli*	Ca_Kabuli_Chr04	40218410	[G/A]	Ca_14897	Early nodulin 93 ENOD93 protein	INTRON
7193	CakSNP7193	*Kabuli*	Ca_Kabuli_Chr04	40267713	[G/T]		--	INTERGENIC
7194	CakSNP7194	*Kabuli*	Ca_Kabuli_Chr04	40267719	[G/A]		--	INTERGENIC
7195	CakSNP7195	*Kabuli*	Ca_Kabuli_Chr04	40279513	[G/T]		--	INTERGENIC
7196	CakSNP7196	*Kabuli*	Ca_Kabuli_Chr04	40313721	[A/G]		--	INTERGENIC
7197	CakSNP7197	*Kabuli*	Ca_Kabuli_Chr04	40349170	[C/T]		--	INTERGENIC
7198	CakSNP7198	*Kabuli*	Ca_Kabuli_Chr04	40368028	[C/T]		--	INTERGENIC
7199	CakSNP7199	*Kabuli*	Ca_Kabuli_Chr04	40368278	[T/C]	Ca_14885	Rab-GAP/TBC domain	SYNONYMOUS_CODING
7200	CakSNP7200	*Kabuli*	Ca_Kabuli_Chr04	40368765	[G/A]	Ca_14885	Rab-GAP/TBC domain	NON_SYNONYMOUS_CODING
7201	CakSNP7201	*Kabuli*	Ca_Kabuli_Chr04	40375386	[C/T]		--	INTERGENIC

S.N.	SNP IDs	Chickpea cultivars	Chromosomes	Physical positions (bp)	SNPs	Gene accession IDs	Putative functions	Sequence components of genome
7202	CakSNP7202	*Kabuli*	Ca_Kabuli_Chr04	40375361	[A/G]		--	INTERGENIC
7203	CakSNP7203	*Kabuli*	Ca_Kabuli_Chr04	40429387	[G/A]		--	INTERGENIC
7204	CakSNP7204	*Kabuli*	Ca_Kabuli_Chr04	40471412	[G/C]		--	INTERGENIC
7205	CakSNP7205	*Kabuli*	Ca_Kabuli_Chr04	40472097	[A/G]		--	INTERGENIC
7206	CakSNP7206	*Kabuli*	Ca_Kabuli_Chr04	40513733	[C/T]	Ca_14874	IQ motif, EF-hand binding site	SYNONYMOUS_CODING
7207	CakSNP7207	*Kabuli*	Ca_Kabuli_Chr04	40583205	[G/T]	Ca_14868	Zinc finger, RING-type	STOP_GAINED
7208	CakSNP7208	*Kabuli*	Ca_Kabuli_Chr04	40583206	[A/T]	Ca_14868	Zinc finger, RING-type	NON_SYNONYMOUS_CODING
7209	CakSNP7209	*Kabuli*	Ca_Kabuli_Chr04	40583211	[G/T]	Ca_14868	Zinc finger, RING-type	STOP_GAINED
7210	CakSNP7210	*Kabuli*	Ca_Kabuli_Chr04	40583214	[G/A]	Ca_14868	Zinc finger, RING-type	NON_SYNONYMOUS_CODING
7211	CakSNP7211	*Kabuli*	Ca_Kabuli_Chr04	40583216	[C/A]	Ca_14868	Zinc finger, RING-type	NON_SYNONYMOUS_CODING

S.N.	SNP IDs	Chickpea cultivars	Chromosomes	Physical positions (bp)	SNPs	Gene accession IDs	Putative functions	Sequence components of genome
7212	CakSNP7212	*Kabuli*	Ca_Kabuli_Chr04	40583217	[G/A]	Ca_14868	Zinc finger, RING-type	NON_SYNONYMOUS_CODING
7213	CakSNP7213	*Kabuli*	Ca_Kabuli_Chr04	40583219	[G/A]	Ca_14868	Zinc finger, RING-type	SYNONYMOUS_CODING
7214	CakSNP7214	*Kabuli*	Ca_Kabuli_Chr04	40597192	[G/A]	Ca_14866	Pectinesterase, catalytic	SYNONYMOUS_CODING
7215	CakSNP7215	*Kabuli*	Ca_Kabuli_Chr04	40597534	[A/G]	Ca_14866	Pectinesterase, catalytic	SYNONYMOUS_CODING
7216	CakSNP7216	*Kabuli*	Ca_Kabuli_Chr04	40597464	[G/A]	Ca_14866	Pectinesterase, catalytic	NON_SYNONYMOUS_CODING
7217	CakSNP7217	*Kabuli*	Ca_Kabuli_Chr04	40723208	[C/G]	Ca_23910	Peptidase A22, presenilin signal peptide	INTRON
7218	CakSNP7218	*Kabuli*	Ca_Kabuli_Chr04	40723212	[G/A]	Ca_23910	Peptidase A22, presenilin signal peptide	INTRON
7219	CakSNP7219	*Kabuli*	Ca_Kabuli_Chr04	40734572	[T/G]	Ca_23909	Peptidase S54, rhomboid	INTRON
7220	CakSNP7220	*Kabuli*	Ca_Kabuli_Chr04	40734765	[G/A]	Ca_23909	Peptidase S54, rhomboid	SYNONYMOUS_CODING
7221	CakSNP7221	*Kabuli*	Ca_Kabuli_Chr04	40738927	[C/T]	Ca_23908	Pentatricopeptide repeat	SYNONYMOUS_CODING

S.N.	SNP IDs	Chickpea cultivars	Chromosomes	Physical positions (bp)	SNPs	Gene accession IDs	Putative functions	Sequence components of genome
7222	CakSNP7222	*Kabuli*	Ca_Kabuli_Chr04	40795060	[G/A]		--	INTERGENIC
7223	CakSNP7223	*Kabuli*	Ca_Kabuli_Chr04	40795085	[C/G]		--	INTERGENIC
7224	CakSNP7224	*Kabuli*	Ca_Kabuli_Chr04	40795137	[T/G]		--	INTERGENIC
7225	CakSNP7225	*Kabuli*	Ca_Kabuli_Chr04	40801783	[T/C]	Ca_23902	DNA-directed DNA polymerase, family A, palm domain	SYNONYMOUS_CODING
7226	CakSNP7226	*Kabuli*	Ca_Kabuli_Chr04	40801791	[C/G]	Ca_23902	DNA-directed DNA polymerase, family A, palm domain	NON_SYNONYMOUS_CODING
7227	CakSNP7227	*Kabuli*	Ca_Kabuli_Chr04	40805740	[C/A]	Ca_23902	DNA-directed DNA polymerase, family A, palm domain	SYNONYMOUS_CODING
7228	CakSNP7228	*Kabuli*	Ca_Kabuli_Chr04	40806993	[A/G]	Ca_23902	DNA-directed DNA polymerase, family A, palm domain	SYNONYMOUS_CODING
7229	CakSNP7229	*Kabuli*	Ca_Kabuli_Chr04	40839967	[G/A]		--	INTERGENIC
7230	CakSNP7230	*Kabuli*	Ca_Kabuli_Chr04	40839949	[G/A]		--	INTERGENIC
7231	CakSNP7231	*Kabuli*	Ca_Kabuli_Chr04	40852381	[A/C]	Ca_23897	Globin, truncated bacterial-like	SYNONYMOUS_CODING

S.N.	SNP IDs	Chickpea cultivars	Chromosomes	Physical positions (bp)	SNPs	Gene accession IDs	Putative functions	Sequence components of genome
7232	CakSNP7232	*Kabuli*	Ca_Kabuli_Chr04	40852475	[T/C]	Ca_23897	Globin, truncated bacterial-like	INTRON
7233	CakSNP7233	*Kabuli*	Ca_Kabuli_Chr04	40912677	[A/G]	Ca_11018	--	SYNONYMOUS_CODING
7234	CakSNP7234	*Kabuli*	Ca_Kabuli_Chr04	40912681	[A/G]	Ca_11018	--	NON_SYNONYMOUS_CODING
7235	CakSNP7235	*Kabuli*	Ca_Kabuli_Chr04	40912714	[C/G]	Ca_11018	--	SYNONYMOUS_CODING
7236	CakSNP7236	*Kabuli*	Ca_Kabuli_Chr04	40912731	[C/T]	Ca_11018	--	SYNONYMOUS_CODING
7237	CakSNP7237	*Kabuli*	Ca_Kabuli_Chr04	40917776	[C/A]		--	INTERGENIC
7238	CakSNP7238	*Kabuli*	Ca_Kabuli_Chr04	40917777	[T/A]		--	INTERGENIC
7239	CakSNP7239	*Kabuli*	Ca_Kabuli_Chr04	40917807	[C/T]		--	INTERGENIC
7240	CakSNP7240	*Kabuli*	Ca_Kabuli_Chr04	40941283	[A/G]	Ca_11014	Proteasome component (PCI) domain	SYNONYMOUS_CODING
7241	CakSNP7241	*Kabuli*	Ca_Kabuli_Chr04	41032564	[A/G]	Ca_11007	GHMP kinase	INTRON

S.N.	SNP IDs	Chickpea cultivars	Chromosomes	Physical positions (bp)	SNPs	Gene accession IDs	Putative functions	Sequence components of genome
7242	CakSNP7242	*Kabuli*	Ca_Kabuli_Chr04	41032590	[C/A]	Ca_11007	GHMP kinase	INTRON
7243	CakSNP7243	*Kabuli*	Ca_Kabuli_Chr04	41032599	[T/C]	Ca_11007	GHMP kinase	INTRON
7244	CakSNP7244	*Kabuli*	Ca_Kabuli_Chr04	41032608	[T/C]	Ca_11007	GHMP kinase	INTRON
7245	CakSNP7245	*Kabuli*	Ca_Kabuli_Chr04	41070048	[C/G]	Ca_11002	Tetratricopeptide TPR-1	NON_SYNONYMOUS_CODING
7246	CakSNP7246	*Kabuli*	Ca_Kabuli_Chr04	41070017	[T/A]	Ca_11002	Tetratricopeptide TPR-1	NON_SYNONYMOUS_CODING
7247	CakSNP7247	*Kabuli*	Ca_Kabuli_Chr04	41094082	[T/C]	Ca_10999	Protein kinase, catalytic domain	NON_SYNONYMOUS_CODING
7248	CakSNP7248	*Kabuli*	Ca_Kabuli_Chr04	41094554	[A/C]	Ca_10999	Protein kinase, catalytic domain	INTRON
7249	CakSNP7249	*Kabuli*	Ca_Kabuli_Chr04	41094685	[G/C]	Ca_10999	Protein kinase, catalytic domain	SYNONYMOUS_CODING
7250	CakSNP7250	*Kabuli*	Ca_Kabuli_Chr04	41117822	[C/A]	Ca_10997	DNA topoisomerase, type IA	INTRON
7251	CakSNP7251	*Kabuli*	Ca_Kabuli_Chr04	41141086	[G/T]	Ca_10994	--	NON_SYNONYMOUS_CODING

S.N.	SNP IDs	Chickpea cultivars	Chromosomes	Physical positions (bp)	SNPs	Gene accession IDs	Putative functions	Sequence components of genome
7252	CakSNP7252	*Kabuli*	Ca_Kabuli_Chr04	41142299	[T/C]	Ca_10994	--	SYNONYMOUS_CODING
7253	CakSNP7253	*Kabuli*	Ca_Kabuli_Chr04	41142290	[A/G]	Ca_10994	--	SYNONYMOUS_CODING
7254	CakSNP7254	*Kabuli*	Ca_Kabuli_Chr04	41142288	[T/C]	Ca_10994	--	NON_SYNONYMOUS_CODING
7255	CakSNP7255	*Kabuli*	Ca_Kabuli_Chr04	41144277	[T/A]	Ca_10994	--	NON_SYNONYMOUS_CODING
7256	CakSNP7256	*Kabuli*	Ca_Kabuli_Chr04	41145767	[C/T]	Ca_10994	--	SYNONYMOUS_CODING
7257	CakSNP7257	*Kabuli*	Ca_Kabuli_Chr04	41145875	[G/A]	Ca_10994	--	SYNONYMOUS_CODING
7258	CakSNP7258	*Kabuli*	Ca_Kabuli_Chr04	41207328	[C/T]	Ca_10990	Cystathionine beta-synthase, core	INTRON
7259	CakSNP7259	*Kabuli*	Ca_Kabuli_Chr04	41207356	[C/T]	Ca_10990	Cystathionine beta-synthase, core	INTRON
7260	CakSNP7260	*Kabuli*	Ca_Kabuli_Chr04	41207392	[T/G]	Ca_10990	Cystathionine beta-synthase, core	INTRON
7261	CakSNP7261	*Kabuli*	Ca_Kabuli_Chr04	41207354	[A/C]	Ca_10990	Cystathionine beta-synthase, core	INTRON

S.N.	SNP IDs	Chickpea cultivars	Chromosomes	Physical positions (bp)	SNPs	Gene accession IDs	Putative functions	Sequence components of genome
7262	CakSNP7262	*Kabuli*	Ca_Kabuli_Chr04	41209521	[T/G]	Ca_10990	Cystathionine beta-synthase, core	INTRON
7263	CakSNP7263	*Kabuli*	Ca_Kabuli_Chr04	41209711	[C/A]	Ca_10990	Cystathionine beta-synthase, core	INTRON
7264	CakSNP7264	*Kabuli*	Ca_Kabuli_Chr04	41215850	[A/C]		--	INTERGENIC
7265	CakSNP7265	*Kabuli*	Ca_Kabuli_Chr04	41228918	[T/C]	Ca_10988	Zinc finger, RING-type	NON_SYNONYMOUS_CODING
7266	CakSNP7266	*Kabuli*	Ca_Kabuli_Chr04	41229605	[C/T]		--	INTERGENIC
7267	CakSNP7267	*Kabuli*	Ca_Kabuli_Chr04	41277786	[G/A]	Ca_10982	ATPase, P-type, H+ transporting proton pump	INTRON
7268	CakSNP7268	*Kabuli*	Ca_Kabuli_Chr04	41277799	[T/G]	Ca_10982	ATPase, P-type, H+ transporting proton pump	INTRON
7269	CakSNP7269	*Kabuli*	Ca_Kabuli_Chr04	41292152	[T/C]	Ca_10980	ATPase, P-type, H+ transporting proton pump	NON_SYNONYMOUS_CODING
7270	CakSNP7270	*Kabuli*	Ca_Kabuli_Chr04	41319598	[T/C]	Ca_10978	Zinc finger, U1-type	SYNONYMOUS_CODING
7271	CakSNP7271	*Kabuli*	Ca_Kabuli_Chr04	41319610	[G/A]	Ca_10978	Zinc finger, U1-type	SYNONYMOUS_CODING

S.N.	SNP IDs	Chickpea cultivars	Chromosomes	Physical positions (bp)	SNPs	Gene accession IDs	Putative functions	Sequence components of genome
7272	CakSNP7272	*Kabuli*	Ca_Kabuli_Chr04	41344739	[A/T]	Ca_10974	Protein kinase, catalytic domain	INTRON
7273	CakSNP7273	*Kabuli*	Ca_Kabuli_Chr04	41427081	[A/T]		--	INTERGENIC
7274	CakSNP7274	*Kabuli*	Ca_Kabuli_Chr04	41427086	[C/A]		--	INTERGENIC
7275	CakSNP7275	*Kabuli*	Ca_Kabuli_Chr04	41427093	[T/G]		--	INTERGENIC
7276	CakSNP7276	*Kabuli*	Ca_Kabuli_Chr04	41445171	[T/C]	Ca_10963	Peptidase C19, ubiquitin carboxyl-terminal hydrolase 2	SYNONYMOUS_CODING
7277	CakSNP7277	*Kabuli*	Ca_Kabuli_Chr04	41445212	[A/G]	Ca_10963	Peptidase C19, ubiquitin carboxyl-terminal hydrolase 2	NON_SYNONYMOUS_CODING
7278	CakSNP7278	*Kabuli*	Ca_Kabuli_Chr04	41445225	[A/T]	Ca_10963	Peptidase C19, ubiquitin carboxyl-terminal hydrolase 2	INTRON
7279	CakSNP7279	*Kabuli*	Ca_Kabuli_Chr04	41445242	[C/T]	Ca_10963	Peptidase C19, ubiquitin carboxyl-terminal hydrolase 2	INTRON
7280	CakSNP7280	*Kabuli*	Ca_Kabuli_Chr04	41458426	[A/G]		--	INTERGENIC
7281	CakSNP7281	*Kabuli*	Ca_Kabuli_Chr04	41500284	[A/C]		--	INTERGENIC

S.N.	SNP IDs	Chickpea cultivars	Chromosomes	Physical positions (bp)	SNPs	Gene accession IDs	Putative functions	Sequence components of genome
7282	CakSNP7282	*Kabuli*	Ca_Kabuli_Chr04	41500286	[A/G]		--	INTERGENIC
7283	CakSNP7283	*Kabuli*	Ca_Kabuli_Chr04	41512840	[G/A]	Ca_10956	Skb1 methyltransferase	INTRON
7284	CakSNP7284	*Kabuli*	Ca_Kabuli_Chr04	41568682	[A/C]		--	INTERGENIC
7285	CakSNP7285	*Kabuli*	Ca_Kabuli_Chr04	41629852	[T/A]		--	INTERGENIC
7286	CakSNP7286	*Kabuli*	Ca_Kabuli_Chr04	41648927	[C/T]	Ca_10946	--	SYNONYMOUS_CODING
7287	CakSNP7287	*Kabuli*	Ca_Kabuli_Chr04	41648998	[A/G]	Ca_10946	--	SYNONYMOUS_CODING
7288	CakSNP7288	*Kabuli*	Ca_Kabuli_Chr04	41675299	[A/T]	Ca_10945	EGF-type aspartate/asparagine hydroxylation site	NON_SYNONYMOUS_CODING
7289	CakSNP7289	*Kabuli*	Ca_Kabuli_Chr04	41698291	[A/G]		--	INTERGENIC
7290	CakSNP7290	*Kabuli*	Ca_Kabuli_Chr04	41698386	[A/G]		--	INTERGENIC
7291	CakSNP7291	*Kabuli*	Ca_Kabuli_Chr04	41700712	[C/A]	Ca_10942	Enhancer of polycomb-like, N-terminal	SYNONYMOUS_CODING

S.N.	SNP IDs	Chickpea cultivars	Chromosomes	Physical positions (bp)	SNPs	Gene accession IDs	Putative functions	Sequence components of genome
7292	CakSNP7292	*Kabuli*	Ca_Kabuli_Chr04	41721289	[G/A]	Ca_10941	--	SYNONYMOUS_CODING
7293	CakSNP7293	*Kabuli*	Ca_Kabuli_Chr04	41772690	[C/T]	Ca_10938	Protein phosphatase 2A, regulatory B subunit, B56	SYNONYMOUS_CODING
7294	CakSNP7294	*Kabuli*	Ca_Kabuli_Chr04	41772823	[G/A]		--	INTERGENIC
7295	CakSNP7295	*Kabuli*	Ca_Kabuli_Chr04	41772796	[T/A]		--	INTERGENIC
7296	CakSNP7296	*Kabuli*	Ca_Kabuli_Chr04	41772794	[T/C]		--	INTERGENIC
7297	CakSNP7297	*Kabuli*	Ca_Kabuli_Chr04	41864826	[G/A]	Ca_10932	WD40 repeat	SYNONYMOUS_CODING
7298	CakSNP7298	*Kabuli*	Ca_Kabuli_Chr04	41864846	[C/G]	Ca_10932	WD40 repeat	NON_SYNONYMOUS_CODING
7299	CakSNP7299	*Kabuli*	Ca_Kabuli_Chr04	41938784	[A/G]	Ca_10929	ABC transporter-like	INTRON
7300	CakSNP7300	*Kabuli*	Ca_Kabuli_Chr04	41938812	[T/G]	Ca_10929	ABC transporter-like	INTRON
7301	CakSNP7301	*Kabuli*	Ca_Kabuli_Chr04	41956050	[C/G]	Ca_10926	ATPase, V0 complex, proteolipid subunit C	INTRON

S.N.	SNP IDs	Chickpea cultivars	Chromosomes	Physical positions (bp)	SNPs	Gene accession IDs	Putative functions	Sequence components of genome
7302	CakSNP7302	*Kabuli*	Ca_Kabuli_Chr04	41956085	[T/C]	Ca_10926	ATPase, V0 complex, proteolipid subunit C	INTRON
7303	CakSNP7303	*Kabuli*	Ca_Kabuli_Chr04	41987193	[A/G]	Ca_10923	Histone H2A	INTRON
7304	CakSNP7304	*Kabuli*	Ca_Kabuli_Chr04	42056479	[G/A]	Ca_10917	Peptidyl-prolyl cis-trans isomerase, cyclophilin-type	INTRON
7305	CakSNP7305	*Kabuli*	Ca_Kabuli_Chr04	42092379	[T/G]	Ca_10914	Tesmin/TSO1-like, CXC	INTRON
7306	CakSNP7306	*Kabuli*	Ca_Kabuli_Chr04	42092381	[A/G]	Ca_10914	Tesmin/TSO1-like, CXC	INTRON
7307	CakSNP7307	*Kabuli*	Ca_Kabuli_Chr04	42092385	[G/A]	Ca_10914	Tesmin/TSO1-like, CXC	INTRON
7308	CakSNP7308	*Kabuli*	Ca_Kabuli_Chr04	42105737	[C/T]	Ca_10912	ATPase, AAA+ type, core	INTRON
7309	CakSNP7309	*Kabuli*	Ca_Kabuli_Chr04	42105701	[A/T]	Ca_10912	ATPase, AAA+ type, core	INTRON
7310	CakSNP7310	*Kabuli*	Ca_Kabuli_Chr04	42108980	[C/A]	Ca_10912	ATPase, AAA+ type, core	INTRON
7311	CakSNP7311	*Kabuli*	Ca_Kabuli_Chr04	42246499	[C/T]		--	INTERGENIC

S.N.	SNP IDs	Chickpea cultivars	Chromosomes	Physical positions (bp)	SNPs	Gene accession IDs	Putative functions	Sequence components of genome
7312	CakSNP7312	*Kabuli*	Ca_Kabuli_Chr04	42246527	[C/A]		--	INTERGENIC
7313	CakSNP7313	*Kabuli*	Ca_Kabuli_Chr04	42246565	[T/G]		--	INTERGENIC
7314	CakSNP7314	*Kabuli*	Ca_Kabuli_Chr04	42246566	[T/G]		--	INTERGENIC
7315	CakSNP7315	*Kabuli*	Ca_Kabuli_Chr04	42246572	[T/G]		--	INTERGENIC
7316	CakSNP7316	*Kabuli*	Ca_Kabuli_Chr04	42246653	[C/G]		--	INTERGENIC
7317	CakSNP7317	*Kabuli*	Ca_Kabuli_Chr04	42273252	[G/A]	Ca_10903	Methyltransferase type 11	INTRON
7318	CakSNP7318	*Kabuli*	Ca_Kabuli_Chr04	42353949	[C/T]		--	INTERGENIC
7319	CakSNP7319	*Kabuli*	Ca_Kabuli_Chr04	42499699	[A/T]	Ca_10898	Protein kinase, catalytic domain	NON_SYNONYMOUS_CODING
7320	CakSNP7320	*Kabuli*	Ca_Kabuli_Chr04	42603642	[T/C]	Ca_10894	Thioredoxin	INTRON
7321	CakSNP7321	*Kabuli*	Ca_Kabuli_Chr04	42768142	[T/G]		--	INTERGENIC

S.N.	SNP IDs	Chickpea cultivars	Chromosomes	Physical positions (bp)	SNPs	Gene accession IDs	Putative functions	Sequence components of genome
7322	CakSNP7322	*Kabuli*	Ca_Kabuli_Chr04	42807483	[G/A]		--	INTERGENIC
7323	CakSNP7323	*Kabuli*	Ca_Kabuli_Chr04	42807518	[A/G]		--	INTERGENIC
7324	CakSNP7324	*Kabuli*	Ca_Kabuli_Chr04	42807509	[C/T]		--	INTERGENIC
7325	CakSNP7325	*Kabuli*	Ca_Kabuli_Chr04	43319846	[A/G]	Ca_23683	Cyclin, C-terminal	SYNONYMOUS_CODING
7326	CakSNP7326	*Kabuli*	Ca_Kabuli_Chr04	43324547	[G/C]		--	INTERGENIC
7327	CakSNP7327	*Kabuli*	Ca_Kabuli_Chr04	43431770	[C/T]		--	INTERGENIC
7328	CakSNP7328	*Kabuli*	Ca_Kabuli_Chr04	43431772	[T/A]		--	INTERGENIC
7329	CakSNP7329	*Kabuli*	Ca_Kabuli_Chr04	43431815	[G/T]		--	INTERGENIC
7330	CakSNP7330	*Kabuli*	Ca_Kabuli_Chr04	43446753	[T/A]		--	INTERGENIC
7331	CakSNP7331	*Kabuli*	Ca_Kabuli_Chr04	43447262	[A/T]	Ca_23690	--	SYNONYMOUS_CODING

S.N.	SNP IDs	Chickpea cultivars	Chromosomes	Physical positions (bp)	SNPs	Gene accession IDs	Putative functions	Sequence components of genome
7332	CakSNP7332	*Kabuli*	Ca_Kabuli_Chr04	43476193	[T/C]	Ca_23692	Pollen Ole e 1 allergen/extensin	SYNONYMOUS_CODING
7333	CakSNP7333	*Kabuli*	Ca_Kabuli_Chr04	43653225	[A/G]		--	INTERGENIC
7334	CakSNP7334	*Kabuli*	Ca_Kabuli_Chr04	43653226	[C/T]		--	INTERGENIC
7335	CakSNP7335	*Kabuli*	Ca_Kabuli_Chr04	43662224	[A/C]		--	INTERGENIC
7336	CakSNP7336	*Kabuli*	Ca_Kabuli_Chr04	43677006	[T/G]	Ca_09052	Leucine-rich repeat	NON_SYNONYMOUS_CODING
7337	CakSNP7337	*Kabuli*	Ca_Kabuli_Chr04	43677222	[C/T]		--	INTERGENIC
7338	CakSNP7338	*Kabuli*	Ca_Kabuli_Chr04	43722728	[T/C]		--	INTERGENIC
7339	CakSNP7339	*Kabuli*	Ca_Kabuli_Chr04	43722727	[A/G]		--	INTERGENIC
7340	CakSNP7340	*Kabuli*	Ca_Kabuli_Chr04	43722704	[C/G]		--	INTERGENIC
7341	CakSNP7341	*Kabuli*	Ca_Kabuli_Chr04	43723219	[T/G]		--	INTERGENIC

S.N.	SNP IDs	Chickpea cultivars	Chromosomes	Physical positions (bp)	SNPs	Gene accession IDs	Putative functions	Sequence components of genome
7342	CakSNP7342	*Kabuli*	Ca_Kabuli_Chr04	43723224	[C/A]		--	INTERGENIC
7343	CakSNP7343	*Kabuli*	Ca_Kabuli_Chr04	43723248	[T/C]		--	INTERGENIC
7344	CakSNP7344	*Kabuli*	Ca_Kabuli_Chr04	43726406	[C/T]	Ca_09057	Protein kinase, catalytic domain	SYNONYMOUS_CODING
7345	CakSNP7345	*Kabuli*	Ca_Kabuli_Chr04	43729063	[G/T]	Ca_09058	--	SYNONYMOUS_CODING
7346	CakSNP7346	*Kabuli*	Ca_Kabuli_Chr04	43729261	[C/G]	Ca_09058	--	NON_SYNONYMOUS_CODING
7347	CakSNP7347	*Kabuli*	Ca_Kabuli_Chr04	43729266	[A/G]	Ca_09058	--	NON_SYNONYMOUS_CODING
7348	CakSNP7348	*Kabuli*	Ca_Kabuli_Chr04	43807399	[C/T]	Ca_09060	WD40 repeat	SYNONYMOUS_CODING
7349	CakSNP7349	*Kabuli*	Ca_Kabuli_Chr04	43807460	[C/T]	Ca_09060	WD40 repeat	INTRON
7350	CakSNP7350	*Kabuli*	Ca_Kabuli_Chr04	43840666	[C/G]		--	INTERGENIC
7351	CakSNP7351	*Kabuli*	Ca_Kabuli_Chr04	43840650	[C/T]		--	INTERGENIC

S.N.	SNP IDs	Chickpea cultivars	Chromosomes	Physical positions (bp)	SNPs	Gene accession IDs	Putative functions	Sequence components of genome
7352	CakSNP7352	*Kabuli*	Ca_Kabuli_Chr04	43979065	[A/G]	Ca_09074	Aspartate/other aminotransferase	INTRON
7353	CakSNP7353	*Kabuli*	Ca_Kabuli_Chr04	44008835	[T/C]		--	INTERGENIC
7354	CakSNP7354	*Kabuli*	Ca_Kabuli_Chr04	44059060	[C/T]	Ca_09080	Signal transduction response regulator, receiver domain	NON_SYNONYMOUS_CODING
7355	CakSNP7355	*Kabuli*	Ca_Kabuli_Chr04	44059061	[G/A]	Ca_09080	Signal transduction response regulator, receiver domain	SYNONYMOUS_CODING
7356	CakSNP7356	*Kabuli*	Ca_Kabuli_Chr04	44067780	[G/A]	Ca_09081	SNF2-related	INTRON
7357	CakSNP7357	*Kabuli*	Ca_Kabuli_Chr04	44090929	[T/G]	Ca_09082	Armadillo	SYNONYMOUS_CODING
7358	CakSNP7358	*Kabuli*	Ca_Kabuli_Chr04	44186434	[C/G]	Ca_09093	Plastocyanin-like	NON_SYNONYMOUS_CODING
7359	CakSNP7359	*Kabuli*	Ca_Kabuli_Chr04	44239783	[A/C]	Ca_09097	BTB/POZ-like	SYNONYMOUS_CODING
7360	CakSNP7360	*Kabuli*	Ca_Kabuli_Chr04	44239883	[A/T]	Ca_09097	BTB/POZ-like	NON_SYNONYMOUS_CODING
7361	CakSNP7361	*Kabuli*	Ca_Kabuli_Chr04	44250159	[C/T]	Ca_09099	Chorismate mutase, AroQ class, eukaryotic type	SYNONYMOUS_CODING

S.N.	SNP IDs	Chickpea cultivars	Chromosomes	Physical positions (bp)	SNPs	Gene accession IDs	Putative functions	Sequence components of genome
7362	CakSNP7362	*Kabuli*	Ca_Kabuli_Chr04	44261184	[A/C]	Ca_09100	Actin-binding FH2/DRF autoregulatory	SYNONYMOUS_CODING
7363	CakSNP7363	*Kabuli*	Ca_Kabuli_Chr04	44261909	[T/A]	Ca_09100	Actin-binding FH2/DRF autoregulatory	NON_SYNONYMOUS_CODING
7364	CakSNP7364	*Kabuli*	Ca_Kabuli_Chr04	44266692	[T/A]		--	INTERGENIC
7365	CakSNP7365	*Kabuli*	Ca_Kabuli_Chr04	44266695	[G/A]		--	INTERGENIC
7366	CakSNP7366	*Kabuli*	Ca_Kabuli_Chr04	44266758	[C/T]	Ca_09101	DNA-binding WRKY	SYNONYMOUS_CODING
7367	CakSNP7367	*Kabuli*	Ca_Kabuli_Chr04	44266819	[G/A]	Ca_09101	DNA-binding WRKY	NON_SYNONYMOUS_CODING
7368	CakSNP7368	*Kabuli*	Ca_Kabuli_Chr04	44353354	[C/T]	Ca_09105	Protein of unknown function DUF1077, TMEM85	INTRON
7369	CakSNP7369	*Kabuli*	Ca_Kabuli_Chr04	44353343	[G/A]	Ca_09105	Protein of unknown function DUF1077, TMEM85	INTRON
7370	CakSNP7370	*Kabuli*	Ca_Kabuli_Chr04	44375761	[T/C]	Ca_09108	--	SYNONYMOUS_CODING
7371	CakSNP7371	*Kabuli*	Ca_Kabuli_Chr04	44375831	[T/C]	Ca_09108	--	INTRON

S.N.	SNP IDs	Chickpea cultivars	Chromosomes	Physical positions (bp)	SNPs	Gene accession IDs	Putative functions	Sequence components of genome
7372	CakSNP7372	*Kabuli*	Ca_Kabuli_Chr04	44382515	[A/G]		--	INTERGENIC
7373	CakSNP7373	*Kabuli*	Ca_Kabuli_Chr04	44382677	[C/T]		--	INTERGENIC
7374	CakSNP7374	*Kabuli*	Ca_Kabuli_Chr04	44382671	[C/G]		--	INTERGENIC
7375	CakSNP7375	*Kabuli*	Ca_Kabuli_Chr04	44382614	[C/A]		--	INTERGENIC
7376	CakSNP7376	*Kabuli*	Ca_Kabuli_Chr04	44382602	[A/G]		--	INTERGENIC
7377	CakSNP7377	*Kabuli*	Ca_Kabuli_Chr04	44386579	[A/G]	Ca_09109	Protein phosphatase 2C, manganese/magnesium aspartate binding site	SYNONYMOUS_CODING
7378	CakSNP7378	*Kabuli*	Ca_Kabuli_Chr04	44431319	[A/C]	Ca_09114	Peptidase C19, ubiquitin carboxyl-terminal hydrolase 2	INTRON
7379	CakSNP7379	*Kabuli*	Ca_Kabuli_Chr04	44431353	[G/A]	Ca_09114	Peptidase C19, ubiquitin carboxyl-terminal hydrolase 2	INTRON
7380	CakSNP7380	*Kabuli*	Ca_Kabuli_Chr04	44439372	[C/T]	Ca_09115	Methyltransferase type 11	INTRON
7381	CakSNP7381	*Kabuli*	Ca_Kabuli_Chr04	44439503	[C/A]	Ca_09115	Methyltransferase type 11	INTRON

S.N.	SNP IDs	Chickpea cultivars	Chromosomes	Physical positions (bp)	SNPs	Gene accession IDs	Putative functions	Sequence components of genome
7382	CakSNP7382	*Kabuli*	Ca_Kabuli_Chr04	44439495	[G/A]	Ca_09115	Methyltransferase type 11	INTRON
7383	CakSNP7383	*Kabuli*	Ca_Kabuli_Chr04	44439493	[A/G]	Ca_09115	Methyltransferase type 11	INTRON
7384	CakSNP7384	*Kabuli*	Ca_Kabuli_Chr04	44439492	[G/T]	Ca_09115	Methyltransferase type 11	INTRON
7385	CakSNP7385	*Kabuli*	Ca_Kabuli_Chr04	44439448	[T/C]	Ca_09115	Methyltransferase type 11	INTRON
7386	CakSNP7386	*Kabuli*	Ca_Kabuli_Chr04	44446468	[G/A]	Ca_09116	F-box domain, cyclin-like	NON_SYNONYMOUS_CODING
7387	CakSNP7387	*Kabuli*	Ca_Kabuli_Chr04	44446575	[G/A]	Ca_09116	F-box domain, cyclin-like	SYNONYMOUS_CODING
7388	CakSNP7388	*Kabuli*	Ca_Kabuli_Chr04	44446506	[C/T]	Ca_09116	F-box domain, cyclin-like	SYNONYMOUS_CODING
7389	CakSNP7389	*Kabuli*	Ca_Kabuli_Chr04	44446503	[T/G]	Ca_09116	F-box domain, cyclin-like	SYNONYMOUS_CODING
7390	CakSNP7390	*Kabuli*	Ca_Kabuli_Chr04	44446497	[C/T]	Ca_09116	F-box domain, cyclin-like	SYNONYMOUS_CODING
7391	CakSNP7391	*Kabuli*	Ca_Kabuli_Chr04	44472282	[G/A]	Ca_09120	Protein of unknown function DUF1350	SYNONYMOUS_CODING

S.N.	SNP IDs	Chickpea cultivars	Chromosomes	Physical positions (bp)	SNPs	Gene accession IDs	Putative functions	Sequence components of genome
7392	CakSNP7392	*Kabuli*	Ca_Kabuli_Chr04	44472278	[A/G]	Ca_09120	Protein of unknown function DUF1350	NON_SYNONYMOUS_CODING
7393	CakSNP7393	*Kabuli*	Ca_Kabuli_Chr04	44472237	[T/A]	Ca_09120	Protein of unknown function DUF1350	NON_SYNONYMOUS_CODING
7394	CakSNP7394	*Kabuli*	Ca_Kabuli_Chr04	44590652	[T/C]	Ca_09131	Protein phosphatase 2C, manganese/magnesium aspartate binding site	SYNONYMOUS_CODING
7395	CakSNP7395	*Kabuli*	Ca_Kabuli_Chr04	44590678	[G/A]	Ca_09131	Protein phosphatase 2C, manganese/magnesium aspartate binding site	NON_SYNONYMOUS_CODING
7396	CakSNP7396	*Kabuli*	Ca_Kabuli_Chr04	44590697	[A/C]	Ca_09131	Protein phosphatase 2C, manganese/magnesium aspartate binding site	SYNONYMOUS_CODING
7397	CakSNP7397	*Kabuli*	Ca_Kabuli_Chr04	44591405	[G/T]	Ca_09131	Protein phosphatase 2C, manganese/magnesium aspartate binding site	INTRON
7398	CakSNP7398	*Kabuli*	Ca_Kabuli_Chr04	44754355	[C/T]	Ca_09143	F-box domain, cyclin-like	INTRON
7399	CakSNP7399	*Kabuli*	Ca_Kabuli_Chr04	44802374	[G/A]	Ca_09145	Phosphoribosyltransferase C-terminal	INTRON
7400	CakSNP7400	*Kabuli*	Ca_Kabuli_Chr04	44802364	[A/C]	Ca_09145	Phosphoribosyltransferase C-terminal	INTRON
7401	CakSNP7401	*Kabuli*	Ca_Kabuli_Chr04	44803694	[T/A]	Ca_09145	Phosphoribosyltransferase C-terminal	INTRON

S.N.	SNP IDs	Chickpea cultivars	Chromosomes	Physical positions (bp)	SNPs	Gene accession IDs	Putative functions	Sequence components of genome
7402	CakSNP7402	*Kabuli*	Ca_Kabuli_Chr04	44811519	[G/A]		--	INTERGENIC
7403	CakSNP7403	*Kabuli*	Ca_Kabuli_Chr04	44814857	[A/G]	Ca_09146	IQ motif, EF-hand binding site	SYNONYMOUS_CODING
7404	CakSNP7404	*Kabuli*	Ca_Kabuli_Chr04	44814981	[T/C]	Ca_09146	IQ motif, EF-hand binding site	INTRON
7405	CakSNP7405	*Kabuli*	Ca_Kabuli_Chr04	44814982	[A/G]	Ca_09146	IQ motif, EF-hand binding site	INTRON
7406	CakSNP7406	*Kabuli*	Ca_Kabuli_Chr04	44824103	[G/T]		--	INTERGENIC
7407	CakSNP7407	*Kabuli*	Ca_Kabuli_Chr04	44824105	[T/G]		--	INTERGENIC
7408	CakSNP7408	*Kabuli*	Ca_Kabuli_Chr04	44824178	[C/G]	Ca_09147	Dilute	NON_SYNONYMOUS_CODING
7409	CakSNP7409	*Kabuli*	Ca_Kabuli_Chr04	44824258	[A/G]	Ca_09147	Dilute	NON_SYNONYMOUS_CODING
7410	CakSNP7410	*Kabuli*	Ca_Kabuli_Chr04	44832121	[G/C]	Ca_09148	--	NON_SYNONYMOUS_CODING
7411	CakSNP7411	*Kabuli*	Ca_Kabuli_Chr04	44832144	[T/C]	Ca_09148	--	SYNONYMOUS_CODING

S.N.	SNP IDs	Chickpea cultivars	Chromosomes	Physical positions (bp)	SNPs	Gene accession IDs	Putative functions	Sequence components of genome
7412	CakSNP7412	*Kabuli*	Ca_Kabuli_Chr04	44906128	[C/A]	Ca_09153	Protein of unknown function DUF803	INTRON
7413	CakSNP7413	*Kabuli*	Ca_Kabuli_Chr04	44906255	[A/G]	Ca_09153	Protein of unknown function DUF803	SYNONYMOUS_CODING
7414	CakSNP7414	*Kabuli*	Ca_Kabuli_Chr04	44979108	[G/A]		--	INTERGENIC
7415	CakSNP7415	*Kabuli*	Ca_Kabuli_Chr04	45019764	[T/C]		--	INTERGENIC
7416	CakSNP7416	*Kabuli*	Ca_Kabuli_Chr04	45019778	[T/C]		--	INTERGENIC
7417	CakSNP7417	*Kabuli*	Ca_Kabuli_Chr04	45019787	[G/C]		--	INTERGENIC
7418	CakSNP7418	*Kabuli*	Ca_Kabuli_Chr04	45019805	[C/A]		--	INTERGENIC
7419	CakSNP7419	*Kabuli*	Ca_Kabuli_Chr04	45019807	[C/T]		--	INTERGENIC
7420	CakSNP7420	*Kabuli*	Ca_Kabuli_Chr04	45019814	[C/A]		--	INTERGENIC
7421	CakSNP7421	*Kabuli*	Ca_Kabuli_Chr04	45019820	[C/T]		--	INTERGENIC

S.N.	SNP IDs	Chickpea cultivars	Chromosomes	Physical positions (bp)	SNPs	Gene accession IDs	Putative functions	Sequence components of genome
7422	CakSNP7422	*Kabuli*	Ca_Kabuli_Chr04	45019824	[A/C]		--	INTERGENIC
7423	CakSNP7423	*Kabuli*	Ca_Kabuli_Chr04	45019867	[C/G]		--	INTERGENIC
7424	CakSNP7424	*Kabuli*	Ca_Kabuli_Chr04	45019858	[G/T]		--	INTERGENIC
7425	CakSNP7425	*Kabuli*	Ca_Kabuli_Chr04	45019848	[C/T]		--	INTERGENIC
7426	CakSNP7426	*Kabuli*	Ca_Kabuli_Chr04	45035589	[G/T]		--	INTERGENIC
7427	CakSNP7427	*Kabuli*	Ca_Kabuli_Chr04	45035629	[A/C]		--	INTERGENIC
7428	CakSNP7428	*Kabuli*	Ca_Kabuli_Chr04	45035745	[A/G]		--	INTERGENIC
7429	CakSNP7429	*Kabuli*	Ca_Kabuli_Chr04	45067534	[C/T]		--	INTERGENIC
7430	CakSNP7430	*Kabuli*	Ca_Kabuli_Chr04	45104767	[G/A]		--	INTERGENIC
7431	CakSNP7431	*Kabuli*	Ca_Kabuli_Chr04	45104779	[C/A]		--	INTERGENIC

S.N.	SNP IDs	Chickpea cultivars	Chromosomes	Physical positions (bp)	SNPs	Gene accession IDs	Putative functions	Sequence components of genome
7432	CakSNP7432	*Kabuli*	Ca_Kabuli_Chr04	45104805	[A/G]		--	INTERGENIC
7433	CakSNP7433	*Kabuli*	Ca_Kabuli_Chr04	45141976	[C/A]	Ca_09177	Recoverin	INTRON
7434	CakSNP7434	*Kabuli*	Ca_Kabuli_Chr04	45141990	[A/G]	Ca_09177	Recoverin	INTRON
7435	CakSNP7435	*Kabuli*	Ca_Kabuli_Chr04	45142020	[T/C]	Ca_09177	Recoverin	INTRON
7436	CakSNP7436	*Kabuli*	Ca_Kabuli_Chr04	45142009	[C/T]	Ca_09177	Recoverin	INTRON
7437	CakSNP7437	*Kabuli*	Ca_Kabuli_Chr04	45142003	[A/G]	Ca_09177	Recoverin	INTRON
7438	CakSNP7438	*Kabuli*	Ca_Kabuli_Chr04	45142077	[C/A]	Ca_09177	Recoverin	INTRON
7439	CakSNP7439	*Kabuli*	Ca_Kabuli_Chr04	45142087	[T/A]	Ca_09177	Recoverin	INTRON
7440	CakSNP7440	*Kabuli*	Ca_Kabuli_Chr04	45142104	[T/C]	Ca_09177	Recoverin	INTRON
7441	CakSNP7441	*Kabuli*	Ca_Kabuli_Chr04	45165191	[T/C]		--	INTERGENIC

S.N.	SNP IDs	Chickpea cultivars	Chromosomes	Physical positions (bp)	SNPs	Gene accession IDs	Putative functions	Sequence components of genome
7442	CakSNP7442	*Kabuli*	Ca_Kabuli_Chr04	45165246	[C/G]		--	INTERGENIC
7443	CakSNP7443	*Kabuli*	Ca_Kabuli_Chr04	45165240	[T/C]		--	INTERGENIC
7444	CakSNP7444	*Kabuli*	Ca_Kabuli_Chr04	45165231	[A/C]		--	INTERGENIC
7445	CakSNP7445	*Kabuli*	Ca_Kabuli_Chr04	45206203	[G/T]		--	INTERGENIC
7446	CakSNP7446	*Kabuli*	Ca_Kabuli_Chr04	45206209	[G/C]		--	INTERGENIC
7447	CakSNP7447	*Kabuli*	Ca_Kabuli_Chr04	45220084	[G/T]	Ca_09184	K Homology	SYNONYMOUS_CODING
7448	CakSNP7448	*Kabuli*	Ca_Kabuli_Chr04	45220642	[C/T]	Ca_09184	K Homology	SYNONYMOUS_CODING
7449	CakSNP7449	*Kabuli*	Ca_Kabuli_Chr04	45220916	[T/C]	Ca_09184	K Homology	NON_SYNONYMOUS_CODING
7450	CakSNP7450	*Kabuli*	Ca_Kabuli_Chr04	45225063	[T/C]	Ca_09185	Protein of unknown function DUF647	INTRON
7451	CakSNP7451	*Kabuli*	Ca_Kabuli_Chr04	45225059	[G/A]	Ca_09185	Protein of unknown function DUF647	INTRON

S.N.	SNP IDs	Chickpea cultivars	Chromosomes	Physical positions (bp)	SNPs	Gene accession IDs	Putative functions	Sequence components of genome
7452	CakSNP7452	*Kabuli*	Ca_Kabuli_Chr04	45224997	[G/C]	Ca_09185	Protein of unknown function DUF647	NON_SYNONYMOUS_CODING
7453	CakSNP7453	*Kabuli*	Ca_Kabuli_Chr04	45225077	[A/T]	Ca_09185	Protein of unknown function DUF647	INTRON
7454	CakSNP7454	*Kabuli*	Ca_Kabuli_Chr04	45225093	[G/A]	Ca_09185	Protein of unknown function DUF647	INTRON
7455	CakSNP7455	*Kabuli*	Ca_Kabuli_Chr04	45225145	[T/A]	Ca_09185	Protein of unknown function DUF647	INTRON
7456	CakSNP7456	*Kabuli*	Ca_Kabuli_Chr04	45225154	[T/A]	Ca_09185	Protein of unknown function DUF647	INTRON
7457	CakSNP7457	*Kabuli*	Ca_Kabuli_Chr04	45225155	[C/A]	Ca_09185	Protein of unknown function DUF647	INTRON
7458	CakSNP7458	*Kabuli*	Ca_Kabuli_Chr04	45225210	[G/T]	Ca_09185	Protein of unknown function DUF647	NON_SYNONYMOUS_CODING
7459	CakSNP7459	*Kabuli*	Ca_Kabuli_Chr04	45225193	[A/G]	Ca_09185	Protein of unknown function DUF647	INTRON
7460	CakSNP7460	*Kabuli*	Ca_Kabuli_Chr04	45225165	[T/C]	Ca_09185	Protein of unknown function DUF647	INTRON
7461	CakSNP7461	*Kabuli*	Ca_Kabuli_Chr04	45231839	[T/C]	Ca_09186	Protein phosphatase 2C-like	NON_SYNONYMOUS_CODING

S.N.	SNP IDs	Chickpea cultivars	Chromosomes	Physical positions (bp)	SNPs	Gene accession IDs	Putative functions	Sequence components of genome
7462	CakSNP7462	*Kabuli*	Ca_Kabuli_Chr04	45231867	[A/T]	Ca_09186	Protein phosphatase 2C-like	SYNONYMOUS_CODING
7463	CakSNP7463	*Kabuli*	Ca_Kabuli_Chr04	45231868	[C/A]	Ca_09186	Protein phosphatase 2C-like	NON_SYNONYMOUS_CODING
7464	CakSNP7464	*Kabuli*	Ca_Kabuli_Chr04	45231893	[G/T]	Ca_09186	Protein phosphatase 2C-like	NON_SYNONYMOUS_CODING
7465	CakSNP7465	*Kabuli*	Ca_Kabuli_Chr04	45292852	[G/T]	Ca_09192	--	NON_SYNONYMOUS_CODING
7466	CakSNP7466	*Kabuli*	Ca_Kabuli_Chr04	45292918	[T/G]		--	INTERGENIC
7467	CakSNP7467	*Kabuli*	Ca_Kabuli_Chr04	45292981	[G/T]		--	INTERGENIC
7468	CakSNP7468	*Kabuli*	Ca_Kabuli_Chr04	45292969	[T/A]		--	INTERGENIC
7469	CakSNP7469	*Kabuli*	Ca_Kabuli_Chr04	45292960	[A/T]		--	INTERGENIC
7470	CakSNP7470	*Kabuli*	Ca_Kabuli_Chr04	45552675	[C/A]	Ca_09214	Pathogenesis-related transcriptional factor/ERF, DNA-binding	INTRON
7471	CakSNP7471	*Kabuli*	Ca_Kabuli_Chr04	45595233	[G/A]	Ca_09216	WRC	INTRON

S.N.	SNP IDs	Chickpea cultivars	Chromosomes	Physical positions (bp)	SNPs	Gene accession IDs	Putative functions	Sequence components of genome
7472	CakSNP7472	*Kabuli*	Ca_Kabuli_Chr04	45612178	[T/G]	Ca_09217	WW/Rsp5/WWP	NON_SYNONYMOUS_CODING
7473	CakSNP7473	*Kabuli*	Ca_Kabuli_Chr04	45615465	[C/T]	Ca_09217	WW/Rsp5/WWP	INTRON
7474	CakSNP7474	*Kabuli*	Ca_Kabuli_Chr04	45615457	[C/T]	Ca_09217	WW/Rsp5/WWP	INTRON
7475	CakSNP7475	*Kabuli*	Ca_Kabuli_Chr04	45615452	[A/C]	Ca_09217	WW/Rsp5/WWP	INTRON
7476	CakSNP7476	*Kabuli*	Ca_Kabuli_Chr04	45615401	[T/G]	Ca_09217	WW/Rsp5/WWP	INTRON
7477	CakSNP7477	*Kabuli*	Ca_Kabuli_Chr04	45646003	[A/G]		--	INTERGENIC
7478	CakSNP7478	*Kabuli*	Ca_Kabuli_Chr04	45645996	[A/G]		--	INTERGENIC
7479	CakSNP7479	*Kabuli*	Ca_Kabuli_Chr04	45645957	[A/C]		--	INTERGENIC
7480	CakSNP7480	*Kabuli*	Ca_Kabuli_Chr04	45708895	[C/A]		--	INTERGENIC
7481	CakSNP7481	*Kabuli*	Ca_Kabuli_Chr04	45735754	[G/C]		--	INTERGENIC

S.N.	SNP IDs	Chickpea cultivars	Chromosomes	Physical positions (bp)	SNPs	Gene accession IDs	Putative functions	Sequence components of genome
7482	CakSNP7482	*Kabuli*	Ca_Kabuli_Chr04	45735819	[T/C]		--	INTERGENIC
7483	CakSNP7483	*Kabuli*	Ca_Kabuli_Chr04	45751623	[G/A]	Ca_18386	--	SYNONYMOUS_CODING
7484	CakSNP7484	*Kabuli*	Ca_Kabuli_Chr04	45751618	[C/T]	Ca_18386	--	NON_SYNONYMOUS_CODING
7485	CakSNP7485	*Kabuli*	Ca_Kabuli_Chr04	45752844	[G/A]		--	INTERGENIC
7486	CakSNP7486	*Kabuli*	Ca_Kabuli_Chr04	45752869	[G/T]		--	INTERGENIC
7487	CakSNP7487	*Kabuli*	Ca_Kabuli_Chr04	45752874	[A/T]		--	INTERGENIC
7488	CakSNP7488	*Kabuli*	Ca_Kabuli_Chr04	45782453	[A/T]	Ca_18384	WD40 repeat	SYNONYMOUS_CODING
7489	CakSNP7489	*Kabuli*	Ca_Kabuli_Chr04	45870087	[T/C]	Ca_18373	Tetratricopeptide repeat-containing	INTRON
7490	CakSNP7490	*Kabuli*	Ca_Kabuli_Chr04	45870042	[C/T]	Ca_18373	Tetratricopeptide repeat-containing	INTRON
7491	CakSNP7491	*Kabuli*	Ca_Kabuli_Chr04	45918351	[A/C]	Ca_18368	Porin, eukaryotic type	INTRON

S.N.	SNP IDs	Chickpea cultivars	Chromosomes	Physical positions (bp)	SNPs	Gene accession IDs	Putative functions	Sequence components of genome
7492	CakSNP7492	*Kabuli*	Ca_Kabuli_Chr04	45935594	[A/C]	Ca_18367	RNA recognition motif domain	NON_SYNONYMOUS_CODING
7493	CakSNP7493	*Kabuli*	Ca_Kabuli_Chr04	45942252	[G/A]	Ca_18367	RNA recognition motif domain	SYNONYMOUS_CODING
7494	CakSNP7494	*Kabuli*	Ca_Kabuli_Chr04	45942341	[T/C]	Ca_18367	RNA recognition motif domain	NON_SYNONYMOUS_CODING
7495	CakSNP7495	*Kabuli*	Ca_Kabuli_Chr04	45942729	[G/A]	Ca_18367	RNA recognition motif domain	SYNONYMOUS_CODING
7496	CakSNP7496	*Kabuli*	Ca_Kabuli_Chr04	46134302	[C/G]	Ca_18358	Peptidase S8/S53, subtilisin/kexin/sedolisin	NON_SYNONYMOUS_CODING
7497	CakSNP7497	*Kabuli*	Ca_Kabuli_Chr04	46140729	[A/T]	Ca_18357	Zinc finger, RING-type	INTRON
7498	CakSNP7498	*Kabuli*	Ca_Kabuli_Chr04	46148985	[A/T]	Ca_18357	Zinc finger, RING-type	INTRON
7499	CakSNP7499	*Kabuli*	Ca_Kabuli_Chr04	46149013	[A/G]	Ca_18357	Zinc finger, RING-type	INTRON
7500	CakSNP7500	*Kabuli*	Ca_Kabuli_Chr04	46150670	[T/C]	Ca_18357	Zinc finger, RING-type	INTRON
7501	CakSNP7501	*Kabuli*	Ca_Kabuli_Chr04	46202862	[A/G]		--	INTERGENIC

S.N.	SNP IDs	Chickpea cultivars	Chromosomes	Physical positions (bp)	SNPs	Gene accession IDs	Putative functions	Sequence components of genome
7502	CakSNP7502	*Kabuli*	Ca_Kabuli_Chr04	46344790	[G/A]	Ca_19277	Kinesin, motor domain	INTRON
7503	CakSNP7503	*Kabuli*	Ca_Kabuli_Chr04	46344826	[A/G]	Ca_19277	Kinesin, motor domain	INTRON
7504	CakSNP7504	*Kabuli*	Ca_Kabuli_Chr04	46345042	[A/G]	Ca_19277	Kinesin, motor domain	INTRON
7505	CakSNP7505	*Kabuli*	Ca_Kabuli_Chr04	46402035	[G/A]		--	INTERGENIC
7506	CakSNP7506	*Kabuli*	Ca_Kabuli_Chr04	46430326	[C/T]	Ca_19271	RNA recognition motif domain	INTRON
7507	CakSNP7507	*Kabuli*	Ca_Kabuli_Chr04	46430311	[C/A]	Ca_19271	RNA recognition motif domain	INTRON
7508	CakSNP7508	*Kabuli*	Ca_Kabuli_Chr04	46430288	[G/C]	Ca_19271	RNA recognition motif domain	INTRON
7509	CakSNP7509	*Kabuli*	Ca_Kabuli_Chr04	46605167	[A/G]	Ca_19258	Protein of unknown function DUF827, plant	SYNONYMOUS_CODING
7510	CakSNP7510	*Kabuli*	Ca_Kabuli_Chr04	46605131	[T/A]	Ca_19258	Protein of unknown function DUF827, plant	SYNONYMOUS_CODING
7511	CakSNP7511	*Kabuli*	Ca_Kabuli_Chr04	46612175	[G/A]	Ca_19256	Protein of unknown function DUF827, plant	SYNONYMOUS_CODING

S.N.	SNP IDs	Chickpea cultivars	Chromosomes	Physical positions (bp)	SNPs	Gene accession IDs	Putative functions	Sequence components of genome
7512	CakSNP7512	*Kabuli*	Ca_Kabuli_Chr04	46612197	[G/A]	Ca_19256	Protein of unknown function DUF827, plant	SYNONYMOUS_CODING
7513	CakSNP7513	*Kabuli*	Ca_Kabuli_Chr04	46612244	[C/T]	Ca_19256	Protein of unknown function DUF827, plant	NON_SYNONYMOUS_CODING
7514	CakSNP7514	*Kabuli*	Ca_Kabuli_Chr04	46613068	[T/G]	Ca_19256	Protein of unknown function DUF827, plant	NON_SYNONYMOUS_CODING
7515	CakSNP7515	*Kabuli*	Ca_Kabuli_Chr04	46694919	[T/A]		--	INTERGENIC
7516	CakSNP7516	*Kabuli*	Ca_Kabuli_Chr04	46695043	[A/C]	Ca_19249	--	NON_SYNONYMOUS_CODING
7517	CakSNP7517	*Kabuli*	Ca_Kabuli_Chr04	46695008	[T/C]		--	INTERGENIC
7518	CakSNP7518	*Kabuli*	Ca_Kabuli_Chr04	46705181	[G/T]	Ca_19247	Patatin/Phospholipase A2-related	SYNONYMOUS_CODING
7519	CakSNP7519	*Kabuli*	Ca_Kabuli_Chr04	46705178	[A/T]	Ca_19247	Patatin/Phospholipase A2-related	SYNONYMOUS_CODING
7520	CakSNP7520	*Kabuli*	Ca_Kabuli_Chr04	46705225	[A/C]	Ca_19247	Patatin/Phospholipase A2-related	INTRON
7521	CakSNP7521	*Kabuli*	Ca_Kabuli_Chr04	46713972	[C/T]	Ca_19246	PGAP1-like	NON_SYNONYMOUS_CODING

S.N.	SNP IDs	Chickpea cultivars	Chromosomes	Physical positions (bp)	SNPs	Gene accession IDs	Putative functions	Sequence components of genome
7522	CakSNP7522	*Kabuli*	Ca_Kabuli_Chr04	46715350	[T/C]	Ca_19246	PGAP1-like	SYNONYMOUS_CODING
7523	CakSNP7523	*Kabuli*	Ca_Kabuli_Chr04	46715518	[G/C]	Ca_19246	PGAP1-like	NON_SYNONYMOUS_CODING
7524	CakSNP7524	*Kabuli*	Ca_Kabuli_Chr04	46717155	[T/C]	Ca_19246	PGAP1-like	INTRON
7525	CakSNP7525	*Kabuli*	Ca_Kabuli_Chr04	46717153	[C/T]	Ca_19246	PGAP1-like	INTRON
7526	CakSNP7526	*Kabuli*	Ca_Kabuli_Chr04	46717200	[C/T]	Ca_19246	PGAP1-like	INTRON
7527	CakSNP7527	*Kabuli*	Ca_Kabuli_Chr04	46717508	[G/A]	Ca_19246	PGAP1-like	INTRON
7528	CakSNP7528	*Kabuli*	Ca_Kabuli_Chr04	46763540	[C/T]	Ca_19242	RNA recognition motif domain	SYNONYMOUS_CODING
7529	CakSNP7529	*Kabuli*	Ca_Kabuli_Chr04	46763555	[C/T]	Ca_19242	RNA recognition motif domain	SYNONYMOUS_CODING
7530	CakSNP7530	*Kabuli*	Ca_Kabuli_Chr04	46763868	[T/C]	Ca_19242	RNA recognition motif domain	INTRON
7531	CakSNP7531	*Kabuli*	Ca_Kabuli_Chr04	46763841	[A/C]	Ca_19242	RNA recognition motif domain	INTRON

S.N.	SNP IDs	Chickpea cultivars	Chromosomes	Physical positions (bp)	SNPs	Gene accession IDs	Putative functions	Sequence components of genome
7532	CakSNP7532	*Kabuli*	Ca_Kabuli_Chr04	47223016	[T/C]	Ca_10859	Xanthine/uracil/vitamin C permease	INTRON
7533	CakSNP7533	*Kabuli*	Ca_Kabuli_Chr04	47223047	[A/G]	Ca_10859	Xanthine/uracil/vitamin C permease	INTRON
7534	CakSNP7534	*Kabuli*	Ca_Kabuli_Chr04	47223635	[G/A]	Ca_10859	Xanthine/uracil/vitamin C permease	SYNONYMOUS_CODING
7535	CakSNP7535	*Kabuli*	Ca_Kabuli_Chr04	47223668	[T/G]	Ca_10859	Xanthine/uracil/vitamin C permease	SYNONYMOUS_CODING
7536	CakSNP7536	*Kabuli*	Ca_Kabuli_Chr04	47296400	[C/T]		--	INTERGENIC
7537	CakSNP7537	*Kabuli*	Ca_Kabuli_Chr04	47379992	[C/T]		--	INTERGENIC
7538	CakSNP7538	*Kabuli*	Ca_Kabuli_Chr04	47405685	[T/A]		--	INTERGENIC
7539	CakSNP7539	*Kabuli*	Ca_Kabuli_Chr04	47419514	[A/G]	Ca_10847	Protein of unknown function DUF616	SYNONYMOUS_CODING
7540	CakSNP7540	*Kabuli*	Ca_Kabuli_Chr04	47419517	[C/T]	Ca_10847	Protein of unknown function DUF616	SYNONYMOUS_CODING
7541	CakSNP7541	*Kabuli*	Ca_Kabuli_Chr04	47428580	[C/T]	Ca_10845	Multi antimicrobial extrusion protein	INTRON

S.N.	SNP IDs	Chickpea cultivars	Chromosomes	Physical positions (bp)	SNPs	Gene accession IDs	Putative functions	Sequence components of genome
7542	CakSNP7542	*Kabuli*	Ca_Kabuli_Chr04	47428599	[C/T]	Ca_10845	Multi antimicrobial extrusion protein	INTRON
7543	CakSNP7543	*Kabuli*	Ca_Kabuli_Chr04	47428631	[A/G]	Ca_10845	Multi antimicrobial extrusion protein	INTRON
7544	CakSNP7544	*Kabuli*	Ca_Kabuli_Chr04	47428733	[A/G]	Ca_10845	Multi antimicrobial extrusion protein	SYNONYMOUS_CODING
7545	CakSNP7545	*Kabuli*	Ca_Kabuli_Chr04	47440207	[G/A]	Ca_10843	Inositol polyphosphate-related phosphatase	INTRON
7546	CakSNP7546	*Kabuli*	Ca_Kabuli_Chr04	47450409	[C/T]	Ca_10841	Protein kinase, catalytic domain	NON_SYNONYMOUS_CODING
7547	CakSNP7547	*Kabuli*	Ca_Kabuli_Chr04	47450479	[C/G]	Ca_10841	Protein kinase, catalytic domain	NON_SYNONYMOUS_CODING
7548	CakSNP7548	*Kabuli*	Ca_Kabuli_Chr04	47562991	[A/T]	Ca_10833	--	INTRON
7549	CakSNP7549	*Kabuli*	Ca_Kabuli_Chr04	47572228	[G/A]		--	INTERGENIC
7550	CakSNP7550	*Kabuli*	Ca_Kabuli_Chr04	47572276	[C/T]		--	INTERGENIC
7551	CakSNP7551	*Kabuli*	Ca_Kabuli_Chr04	47578508	[C/T]	Ca_10832	MT-A70	SYNONYMOUS_CODING

S.N.	SNP IDs	Chickpea cultivars	Chromosomes	Physical positions (bp)	SNPs	Gene accession IDs	Putative functions	Sequence components of genome
7552	CakSNP7552	*Kabuli*	Ca_Kabuli_Chr04	47578556	[T/G]	Ca_10832	MT-A70	INTRON
7553	CakSNP7553	*Kabuli*	Ca_Kabuli_Chr04	47578655	[C/T]	Ca_10832	MT-A70	INTRON
7554	CakSNP7554	*Kabuli*	Ca_Kabuli_Chr04	47578596	[C/T]	Ca_10832	MT-A70	INTRON
7555	CakSNP7555	*Kabuli*	Ca_Kabuli_Chr04	47588760	[G/A]	Ca_10830	Phospholipid/glycerol acyltransferase	INTRON
7556	CakSNP7556	*Kabuli*	Ca_Kabuli_Chr04	47612395	[C/A]	Ca_10827	Protein phosphatase 2C-like	NON_SYNONYMOUS_CODING
7557	CakSNP7557	*Kabuli*	Ca_Kabuli_Chr04	47612396	[G/T]	Ca_10827	Protein phosphatase 2C-like	SYNONYMOUS_CODING
7558	CakSNP7558	*Kabuli*	Ca_Kabuli_Chr04	47615800	[G/C]	Ca_10827	Protein phosphatase 2C-like	INTRON
7559	CakSNP7559	*Kabuli*	Ca_Kabuli_Chr04	47616120	[C/T]	Ca_10827	Protein phosphatase 2C-like	SYNONYMOUS_CODING
7560	CakSNP7560	*Kabuli*	Ca_Kabuli_Chr04	47616216	[A/G]	Ca_10827	Protein phosphatase 2C-like	SYNONYMOUS_CODING
7561	CakSNP7561	*Kabuli*	Ca_Kabuli_Chr04	47622112	[G/T]	Ca_10826	--	NON_SYNONYMOUS_CODING

S.N.	SNP IDs	Chickpea cultivars	Chromosomes	Physical positions (bp)	SNPs	Gene accession IDs	Putative functions	Sequence components of genome
7562	CakSNP7562	*Kabuli*	Ca_Kabuli_Chr04	47675446	[A/G]	Ca_10821	GYF	SYNONYMOUS_CODING
7563	CakSNP7563	*Kabuli*	Ca_Kabuli_Chr04	47675758	[A/T]	Ca_10821	GYF	SYNONYMOUS_CODING
7564	CakSNP7564	*Kabuli*	Ca_Kabuli_Chr04	47675860	[G/A]	Ca_10821	GYF	SYNONYMOUS_CODING
7565	CakSNP7565	*Kabuli*	Ca_Kabuli_Chr04	47697239	[C/T]	Ca_10819	Glycoside hydrolase, family 17	SYNONYMOUS_CODING
7566	CakSNP7566	*Kabuli*	Ca_Kabuli_Chr04	47798580	[A/G]	Ca_10815	Flavodoxin	SYNONYMOUS_CODING
7567	CakSNP7567	*Kabuli*	Ca_Kabuli_Chr04	47798622	[G/A]	Ca_10815	Flavodoxin	SYNONYMOUS_CODING
7568	CakSNP7568	*Kabuli*	Ca_Kabuli_Chr04	47819943	[T/C]	Ca_10812	Protein of unknown function DUF248, methyltransferase putative	SYNONYMOUS_CODING
7569	CakSNP7569	*Kabuli*	Ca_Kabuli_Chr04	48008987	[T/C]	Ca_10788	Heat shock protein DnaJ, N-terminal	SYNONYMOUS_CODING
7570	CakSNP7570	*Kabuli*	Ca_Kabuli_Chr04	48009201	[C/T]	Ca_10788	Heat shock protein DnaJ, N-terminal	NON_SYNONYMOUS_CODING
7571	CakSNP7571	*Kabuli*	Ca_Kabuli_Chr04	48011446	[A/G]	Ca_10788	Heat shock protein DnaJ, N-terminal	SYNONYMOUS_CODING

S.N.	SNP IDs	Chickpea cultivars	Chromosomes	Physical positions (bp)	SNPs	Gene accession IDs	Putative functions	Sequence components of genome
7572	CakSNP7572	*Kabuli*	Ca_Kabuli_Chr04	48098570	[G/A]	Ca_10778	WD40 repeat	INTRON
7573	CakSNP7573	*Kabuli*	Ca_Kabuli_Chr04	48098562	[G/A]	Ca_10778	WD40 repeat	INTRON
7574	CakSNP7574	*Kabuli*	Ca_Kabuli_Chr04	48103202	[G/T]	Ca_10777	Plant peroxidase	NON_SYNONYMOUS_CODING
7575	CakSNP7575	*Kabuli*	Ca_Kabuli_Chr04	48103196	[G/A]	Ca_10777	Plant peroxidase	NON_SYNONYMOUS_CODING
7576	CakSNP7576	*Kabuli*	Ca_Kabuli_Chr04	48103159	[G/A]	Ca_10777	Plant peroxidase	SYNONYMOUS_CODING
7577	CakSNP7577	*Kabuli*	Ca_Kabuli_Chr04	48103153	[A/C]	Ca_10777	Plant peroxidase	SYNONYMOUS_CODING
7578	CakSNP7578	*Kabuli*	Ca_Kabuli_Chr04	48150277	[C/T]	Ca_10772	LisH dimerisation motif	INTRON
7579	CakSNP7579	*Kabuli*	Ca_Kabuli_Chr04	48150481	[T/C]	Ca_10772	LisH dimerisation motif	INTRON
7580	CakSNP7580	*Kabuli*	Ca_Kabuli_Chr04	48150480	[C/A]	Ca_10772	LisH dimerisation motif	INTRON
7581	CakSNP7581	*Kabuli*	Ca_Kabuli_Chr04	48150553	[A/G]	Ca_10772	LisH dimerisation motif	SYNONYMOUS_CODING

S.N.	SNP IDs	Chickpea cultivars	Chromosomes	Physical positions (bp)	SNPs	Gene accession IDs	Putative functions	Sequence components of genome
7582	CakSNP7582	*Kabuli*	Ca_Kabuli_Chr04	48150641	[G/T]	Ca_10772	LisH dimerisation motif	INTRON
7583	CakSNP7583	*Kabuli*	Ca_Kabuli_Chr04	48238919	[C/T]	Ca_10764	--	NON_SYNONYMOUS_CODING
7584	CakSNP7584	*Kabuli*	Ca_Kabuli_Chr04	48239048	[A/G]	Ca_10764	--	INTRON
7585	CakSNP7585	*Kabuli*	Ca_Kabuli_Chr04	48263603	[G/A]	Ca_10761	DNA-directed RNA polymerase, subunit 2, domain 6	SYNONYMOUS_CODING
7586	CakSNP7586	*Kabuli*	Ca_Kabuli_Chr04	48269138	[G/C]	Ca_10760	Amine oxidase	INTRON
7587	CakSNP7587	*Kabuli*	Ca_Kabuli_Chr04	48275016	[C/T]	Ca_10760	Amine oxidase	SYNONYMOUS_CODING
7588	CakSNP7588	*Kabuli*	Ca_Kabuli_Chr04	48322502	[C/T]	Ca_10755	Basic-leucine zipper (bZIP) transcription factor	SYNONYMOUS_CODING
7589	CakSNP7589	*Kabuli*	Ca_Kabuli_Chr04	48322507	[C/T]	Ca_10755	Basic-leucine zipper (bZIP) transcription factor	SYNONYMOUS_CODING
7590	CakSNP7590	*Kabuli*	Ca_Kabuli_Chr04	48322633	[T/C]	Ca_10755	Basic-leucine zipper (bZIP) transcription factor	SYNONYMOUS_CODING
7591	CakSNP7591	*Kabuli*	Ca_Kabuli_Chr04	48331069	[C/A]	Ca_10753	Ribosomal protein L13	NON_SYNONYMOUS_CODING

S.N.	SNP IDs	Chickpea cultivars	Chromosomes	Physical positions (bp)	SNPs	Gene accession IDs	Putative functions	Sequence components of genome
7592	CakSNP7592	*Kabuli*	Ca_Kabuli_Chr04	48345604	[A/G]	Ca_10752	IQ motif, EF-hand binding site	INTRON
7593	CakSNP7593	*Kabuli*	Ca_Kabuli_Chr04	48345608	[G/T]	Ca_10752	IQ motif, EF-hand binding site	INTRON
7594	CakSNP7594	*Kabuli*	Ca_Kabuli_Chr04	48345655	[A/C]	Ca_10752	IQ motif, EF-hand binding site	INTRON
7595	CakSNP7595	*Kabuli*	Ca_Kabuli_Chr04	48345697	[C/G]	Ca_10752	IQ motif, EF-hand binding site	NON_SYNONYMOUS_CODING
7596	CakSNP7596	*Kabuli*	Ca_Kabuli_Chr04	48355358	[A/T]	Ca_10751	Exonuclease	INTRON
7597	CakSNP7597	*Kabuli*	Ca_Kabuli_Chr04	48355468	[G/T]	Ca_10751	Exonuclease	INTRON
7598	CakSNP7598	*Kabuli*	Ca_Kabuli_Chr04	48364170	[G/A]	Ca_10750	Helicase, C-terminal	INTRON
7599	CakSNP7599	*Kabuli*	Ca_Kabuli_Chr04	48365208	[C/A]		--	INTERGENIC
7600	CakSNP7600	*Kabuli*	Ca_Kabuli_Chr04	48365388	[G/A]		--	INTERGENIC
7601	CakSNP7601	*Kabuli*	Ca_Kabuli_Chr04	48382666	[G/C]	Ca_10748	AUX/IAA protein	SYNONYMOUS_CODING

S.N.	SNP IDs	Chickpea cultivars	Chromosomes	Physical positions (bp)	SNPs	Gene accession IDs	Putative functions	Sequence components of genome
7602	CakSNP7602	*Kabuli*	Ca_Kabuli_Chr04	48464493	[T/C]	Ca_10746	Zinc finger, CCHC-type	NON_SYNONYMOUS_CODING
7603	CakSNP7603	*Kabuli*	Ca_Kabuli_Chr04	48464434	[G/C]	Ca_10746	Zinc finger, CCHC-type	SYNONYMOUS_CODING
7604	CakSNP7604	*Kabuli*	Ca_Kabuli_Chr04	48470477	[A/G]	Ca_10746	Zinc finger, CCHC-type	INTRON
7605	CakSNP7605	*Kabuli*	Ca_Kabuli_Chr04	48475447	[T/C]	Ca_10745	Appr-1-p processing	SYNONYMOUS_CODING
7606	CakSNP7606	*Kabuli*	Ca_Kabuli_Chr04	48475461	[G/A]	Ca_10745	Appr-1-p processing	SYNONYMOUS_CODING
7607	CakSNP7607	*Kabuli*	Ca_Kabuli_Chr04	48478018	[A/G]	Ca_10745	Appr-1-p processing	SYNONYMOUS_CODING
7608	CakSNP7608	*Kabuli*	Ca_Kabuli_Chr04	48478058	[T/C]	Ca_10745	Appr-1-p processing	NON_SYNONYMOUS_CODING
7609	CakSNP7609	*Kabuli*	Ca_Kabuli_Chr04	48478303	[G/A]	Ca_10745	Appr-1-p processing	SYNONYMOUS_CODING
7610	CakSNP7610	*Kabuli*	Ca_Kabuli_Chr04	48478524	[C/T]	Ca_10745	Appr-1-p processing	NON_SYNONYMOUS_CODING
7611	CakSNP7611	*Kabuli*	Ca_Kabuli_Chr04	48484453	[A/T]		--	INTERGENIC

S.N.	SNP IDs	Chickpea cultivars	Chromosomes	Physical positions (bp)	SNPs	Gene accession IDs	Putative functions	Sequence components of genome
7612	CakSNP7612	*Kabuli*	Ca_Kabuli_Chr04	48497731	[A/C]	Ca_10742	TB2/DP1/HVA22-related protein	SYNONYMOUS_CODING
7613	CakSNP7613	*Kabuli*	Ca_Kabuli_Chr04	48497725	[A/C]	Ca_10742	TB2/DP1/HVA22-related protein	NON_SYNONYMOUS_CODING
7614	CakSNP7614	*Kabuli*	Ca_Kabuli_Chr04	48497756	[A/G]	Ca_10742	TB2/DP1/HVA22-related protein	SYNONYMOUS_CODING
7615	CakSNP7615	*Kabuli*	Ca_Kabuli_Chr04	48497765	[G/A]	Ca_10742	TB2/DP1/HVA22-related protein	SYNONYMOUS_CODING
7616	CakSNP7616	*Kabuli*	Ca_Kabuli_Chr04	48497839	[G/A]	Ca_10742	TB2/DP1/HVA22-related protein	SYNONYMOUS_CODING
7617	CakSNP7617	*Kabuli*	Ca_Kabuli_Chr04	48498133	[C/T]	Ca_10742	TB2/DP1/HVA22-related protein	SYNONYMOUS_CODING
7618	CakSNP7618	*Kabuli*	Ca_Kabuli_Chr04	48498181	[G/C]		--	INTERGENIC
7619	CakSNP7619	*Kabuli*	Ca_Kabuli_Chr04	48498166	[A/G]	Ca_10742	TB2/DP1/HVA22-related protein	SYNONYMOUS_CODING
7620	CakSNP7620	*Kabuli*	Ca_Kabuli_Chr04	48498368	[C/T]		--	INTERGENIC
7621	CakSNP7621	*Kabuli*	Ca_Kabuli_Chr04	48498334	[T/G]		--	INTERGENIC

S.N.	SNP IDs	Chickpea cultivars	Chromosomes	Physical positions (bp)	SNPs	Gene accession IDs	Putative functions	Sequence components of genome
7622	CakSNP7622	*Kabuli*	Ca_Kabuli_Chr04	48498331	[T/A]		--	INTERGENIC
7623	CakSNP7623	*Kabuli*	Ca_Kabuli_Chr04	48498312	[T/C]		--	INTERGENIC
7624	CakSNP7624	*Kabuli*	Ca_Kabuli_Chr04	48501567	[G/T]	Ca_10741	Eukaryotic initiation factor 3, gamma subunit	INTRON
7625	CakSNP7625	*Kabuli*	Ca_Kabuli_Chr04	48501618	[C/A]	Ca_10741	Eukaryotic initiation factor 3, gamma subunit	INTRON
7626	CakSNP7626	*Kabuli*	Ca_Kabuli_Chr04	48501662	[A/T]	Ca_10741	Eukaryotic initiation factor 3, gamma subunit	SYNONYMOUS_CODING
7627	CakSNP7627	*Kabuli*	Ca_Kabuli_Chr04	48502554	[G/T]	Ca_10741	Eukaryotic initiation factor 3, gamma subunit	INTRON
7628	CakSNP7628	*Kabuli*	Ca_Kabuli_Chr04	48502718	[G/C]	Ca_10741	Eukaryotic initiation factor 3, gamma subunit	INTRON
7629	CakSNP7629	*Kabuli*	Ca_Kabuli_Chr04	48502714	[T/C]	Ca_10741	Eukaryotic initiation factor 3, gamma subunit	INTRON
7630	CakSNP7630	*Kabuli*	Ca_Kabuli_Chr04	48502687	[G/A]	Ca_10741	Eukaryotic initiation factor 3, gamma subunit	INTRON
7631	CakSNP7631	*Kabuli*	Ca_Kabuli_Chr04	48591482	[T/G]	Ca_23011	--	SYNONYMOUS_CODING

S.N.	SNP IDs	Chickpea cultivars	Chromosomes	Physical positions (bp)	SNPs	Gene accession IDs	Putative functions	Sequence components of genome
7632	CakSNP7632	*Kabuli*	Ca_Kabuli_Chr04	48591539	[T/C]	Ca_23011	--	SYNONYMOUS_CODING
7633	CakSNP7633	*Kabuli*	Ca_Kabuli_Chr04	48591578	[T/C]	Ca_23011	--	SYNONYMOUS_CODING
7634	CakSNP7634	*Kabuli*	Ca_Kabuli_Chr04	48591625	[G/T]	Ca_23011	--	SYNONYMOUS_CODING
7635	CakSNP7635	*Kabuli*	Ca_Kabuli_Chr04	48678278	[T/C]	Ca_23018	Transcription factor IIS, N-terminal	SYNONYMOUS_CODING
7636	CakSNP7636	*Kabuli*	Ca_Kabuli_Chr04	48678692	[T/C]	Ca_23018	Transcription factor IIS, N-terminal	SYNONYMOUS_CODING
7637	CakSNP7637	*Kabuli*	Ca_Kabuli_Chr04	48688766	[A/G]	Ca_23019	C2 calcium-dependent membrane targeting	SYNONYMOUS_CODING
7638	CakSNP7638	*Kabuli*	Ca_Kabuli_Chr04	48705815	[C/T]	Ca_23020	ABC transporter, transmembrane domain	INTRON
7639	CakSNP7639	*Kabuli*	Ca_Kabuli_Chr04	48705856	[A/T]	Ca_23020	ABC transporter, transmembrane domain	INTRON
7640	CakSNP7640	*Kabuli*	Ca_Kabuli_Chr04	48705957	[C/T]	Ca_23020	ABC transporter, transmembrane domain	SYNONYMOUS_CODING
7641	CakSNP7641	*Kabuli*	Ca_Kabuli_Chr04	48714912	[G/A]	Ca_23021	Vacuolar protein sorting-associated protein 35	INTRON

S.N.	SNP IDs	Chickpea cultivars	Chromosomes	Physical positions (bp)	SNPs	Gene accession IDs	Putative functions	Sequence components of genome
7642	CakSNP7642	*Kabuli*	Ca_Kabuli_Chr04	48715028	[A/G]	Ca_23021	Vacuolar protein sorting-associated protein 35	INTRON
7643	CakSNP7643	*Kabuli*	Ca_Kabuli_Chr04	48720330	[C/T]	Ca_23022	--	SYNONYMOUS_CODING
7644	CakSNP7644	*Kabuli*	Ca_Kabuli_Chr04	48720266	[C/T]	Ca_23022	--	INTRON
7645	CakSNP7645	*Kabuli*	Ca_Kabuli_Chr04	48784151	[T/G]		--	INTERGENIC
7646	CakSNP7646	*Kabuli*	Ca_Kabuli_Chr04	48784176	[T/C]		--	INTERGENIC
7647	CakSNP7647	*Kabuli*	Ca_Kabuli_Chr04	48784178	[C/G]		--	INTERGENIC
7648	CakSNP7648	*Kabuli*	Ca_Kabuli_Chr04	48784179	[G/A]		--	INTERGENIC
7649	CakSNP7649	*Kabuli*	Ca_Kabuli_Chr04	48784189	[A/T]		--	INTERGENIC
7650	CakSNP7650	*Kabuli*	Ca_Kabuli_Chr04	48784202	[A/C]		--	INTERGENIC
7651	CakSNP7651	*Kabuli*	Ca_Kabuli_Chr04	48784204	[G/C]		--	INTERGENIC

S.N.	SNP IDs	Chickpea cultivars	Chromosomes	Physical positions (bp)	SNPs	Gene accession IDs	Putative functions	Sequence components of genome
7652	CakSNP7652	*Kabuli*	Ca_Kabuli_Chr04	48931112	[T/G]	Ca_20761	Proteasome component (PCI) domain	INTRON
7653	CakSNP7653	*Kabuli*	Ca_Kabuli_Chr04	48931109	[A/G]	Ca_20761	Proteasome component (PCI) domain	INTRON
7654	CakSNP7654	*Kabuli*	Ca_Kabuli_Chr04	48936643	[G/A]	Ca_20762	Pentatricopeptide repeat	SYNONYMOUS_CODING
7655	CakSNP7655	*Kabuli*	Ca_Kabuli_Chr04	48936669	[G/A]	Ca_20762	Pentatricopeptide repeat	NON_SYNONYMOUS_CODING
7656	CakSNP7656	*Kabuli*	Ca_Kabuli_Chr04	48936710	[A/G]	Ca_20762	Pentatricopeptide repeat	NON_SYNONYMOUS_CODING
7657	CakSNP7657	*Kabuli*	Ca_Kabuli_Chr04	48936819	[C/T]	Ca_20762	Pentatricopeptide repeat	NON_SYNONYMOUS_CODING
7658	CakSNP7658	*Kabuli*	Ca_Kabuli_Chr04	48964200	[T/C]		--	INTERGENIC
7659	CakSNP7659	*Kabuli*	Ca_Kabuli_Chr04	48964260	[G/C]		--	INTERGENIC
7660	CakSNP7660	*Kabuli*	Ca_Kabuli_Chr04	48964248	[C/T]		--	INTERGENIC
7661	CakSNP7661	*Kabuli*	Ca_Kabuli_Chr04	49012733	[T/A]		--	INTERGENIC

S.N.	SNP IDs	Chickpea cultivars	Chromosomes	Physical positions (bp)	SNPs	Gene accession IDs	Putative functions	Sequence components of genome
7662	CakSNP7662	*Kabuli*	Ca_Kabuli_Chr04	49012756	[C/T]		--	INTERGENIC
7663	CakSNP7663	*Kabuli*	Ca_Kabuli_Chr05	95087	[G/A]		--	INTERGENIC
7664	CakSNP7664	*Kabuli*	Ca_Kabuli_Chr05	95253	[A/G]		--	INTERGENIC
7665	CakSNP7665	*Kabuli*	Ca_Kabuli_Chr05	131558	[A/C]	Ca_18169	Lipid-binding START	SYNONYMOUS_CODING
7666	CakSNP7666	*Kabuli*	Ca_Kabuli_Chr05	154084	[T/G]		--	INTERGENIC
7667	CakSNP7667	*Kabuli*	Ca_Kabuli_Chr05	154095	[C/G]		--	INTERGENIC
7668	CakSNP7668	*Kabuli*	Ca_Kabuli_Chr05	154097	[A/T]		--	INTERGENIC
7669	CakSNP7669	*Kabuli*	Ca_Kabuli_Chr05	154162	[C/T]		--	INTERGENIC
7670	CakSNP7670	*Kabuli*	Ca_Kabuli_Chr05	154128	[G/T]		--	INTERGENIC
7671	CakSNP7671	*Kabuli*	Ca_Kabuli_Chr05	166222	[T/G]	Ca_18171	No apical meristem (NAM) protein	SYNONYMOUS_CODING

S.N.	SNP IDs	Chickpea cultivars	Chromosomes	Physical positions (bp)	SNPs	Gene accession IDs	Putative functions	Sequence components of genome
7672	CakSNP7672	*Kabuli*	Ca_Kabuli_Chr05	166217	[G/C]	Ca_18171	No apical meristem (NAM) protein	NON_SYNONYMOUS_CODING
7673	CakSNP7673	*Kabuli*	Ca_Kabuli_Chr05	166216	[T/G]	Ca_18171	No apical meristem (NAM) protein	SYNONYMOUS_CODING
7674	CakSNP7674	*Kabuli*	Ca_Kabuli_Chr05	166341	[C/A]	Ca_18171	No apical meristem (NAM) protein	NON_SYNONYMOUS_CODING
7675	CakSNP7675	*Kabuli*	Ca_Kabuli_Chr05	166381	[A/G]	Ca_18171	No apical meristem (NAM) protein	SYNONYMOUS_CODING
7676	CakSNP7676	*Kabuli*	Ca_Kabuli_Chr05	210625	[T/C]		--	INTERGENIC
7677	CakSNP7677	*Kabuli*	Ca_Kabuli_Chr05	210649	[C/A]		--	INTERGENIC
7678	CakSNP7678	*Kabuli*	Ca_Kabuli_Chr05	222651	[A/C]		--	INTERGENIC
7679	CakSNP7679	*Kabuli*	Ca_Kabuli_Chr05	222630	[A/G]		--	INTERGENIC
7680	CakSNP7680	*Kabuli*	Ca_Kabuli_Chr05	222641	[C/T]		--	INTERGENIC
7681	CakSNP7681	*Kabuli*	Ca_Kabuli_Chr05	222653	[A/C]		--	INTERGENIC

S.N.	SNP IDs	Chickpea cultivars	Chromosomes	Physical positions (bp)	SNPs	Gene accession IDs	Putative functions	Sequence components of genome
7682	CakSNP7682	*Kabuli*	Ca_Kabuli_Chr05	222657	[G/A]		--	INTERGENIC
7683	CakSNP7683	*Kabuli*	Ca_Kabuli_Chr05	223384	[C/G]	Ca_18177	ZF-HD homeobox protein, Cys/His-rich dimerisation domain	SYNONYMOUS_CODING
7684	CakSNP7684	*Kabuli*	Ca_Kabuli_Chr05	234247	[T/C]	Ca_18178	Protein of unknown function DUF647	INTRON
7685	CakSNP7685	*Kabuli*	Ca_Kabuli_Chr05	234319	[T/A]	Ca_18178	Protein of unknown function DUF647	INTRON
7686	CakSNP7686	*Kabuli*	Ca_Kabuli_Chr05	234311	[T/A]	Ca_18178	Protein of unknown function DUF647	INTRON
7687	CakSNP7687	*Kabuli*	Ca_Kabuli_Chr05	234310	[G/A]	Ca_18178	Protein of unknown function DUF647	INTRON
7688	CakSNP7688	*Kabuli*	Ca_Kabuli_Chr05	234305	[T/A]	Ca_18178	Protein of unknown function DUF647	INTRON
7689	CakSNP7689	*Kabuli*	Ca_Kabuli_Chr05	234303	[C/A]	Ca_18178	Protein of unknown function DUF647	INTRON
7690	CakSNP7690	*Kabuli*	Ca_Kabuli_Chr05	234410	[A/C]	Ca_18178	Protein of unknown function DUF647	NON_SYNONYMOUS_CODING
7691	CakSNP7691	*Kabuli*	Ca_Kabuli_Chr05	245850	[T/G]	Ca_18179	HEAT	INTRON

S.N.	SNP IDs	Chickpea cultivars	Chromosomes	Physical positions (bp)	SNPs	Gene accession IDs	Putative functions	Sequence components of genome
7692	CakSNP7692	*Kabuli*	Ca_Kabuli_Chr05	245863	[A/T]	Ca_18179	HEAT	INTRON
7693	CakSNP7693	*Kabuli*	Ca_Kabuli_Chr05	245896	[A/T]	Ca_18179	HEAT	INTRON
7694	CakSNP7694	*Kabuli*	Ca_Kabuli_Chr05	246466	[A/T]	Ca_18179	HEAT	INTRON
7695	CakSNP7695	*Kabuli*	Ca_Kabuli_Chr05	246654	[A/G]	Ca_18179	HEAT	INTRON
7696	CakSNP7696	*Kabuli*	Ca_Kabuli_Chr05	256745	[G/A]	Ca_18179	HEAT	SYNONYMOUS_CODING
7697	CakSNP7697	*Kabuli*	Ca_Kabuli_Chr05	272972	[T/A]	Ca_18181	Mitotic checkpoint	INTRON
7698	CakSNP7698	*Kabuli*	Ca_Kabuli_Chr05	277040	[G/A]	Ca_18181	Mitotic checkpoint	SYNONYMOUS_CODING
7699	CakSNP7699	*Kabuli*	Ca_Kabuli_Chr05	277316	[T/A]	Ca_18181	Mitotic checkpoint	INTRON
7700	CakSNP7700	*Kabuli*	Ca_Kabuli_Chr05	343517	[C/T]		--	INTERGENIC
7701	CakSNP7701	*Kabuli*	Ca_Kabuli_Chr05	353792	[T/A]		--	INTERGENIC

S.N.	SNP IDs	Chickpea cultivars	Chromosomes	Physical positions (bp)	SNPs	Gene accession IDs	Putative functions	Sequence components of genome
7702	CakSNP7702	*Kabuli*	Ca_Kabuli_Chr05	438448	[C/A]	Ca_18196	--	INTRON
7703	CakSNP7703	*Kabuli*	Ca_Kabuli_Chr05	438445	[C/A]	Ca_18196	--	INTRON
7704	CakSNP7704	*Kabuli*	Ca_Kabuli_Chr05	438442	[T/C]	Ca_18196	--	INTRON
7705	CakSNP7705	*Kabuli*	Ca_Kabuli_Chr05	443309	[A/T]	Ca_18197	--	SYNONYMOUS_CODING
7706	CakSNP7706	*Kabuli*	Ca_Kabuli_Chr05	469397	[G/A]	Ca_18200	Inositol polyphosphate-related phosphatase	SYNONYMOUS_CODING
7707	CakSNP7707	*Kabuli*	Ca_Kabuli_Chr05	805859	[C/T]		--	INTERGENIC
7708	CakSNP7708	*Kabuli*	Ca_Kabuli_Chr05	805882	[A/T]		--	INTERGENIC
7709	CakSNP7709	*Kabuli*	Ca_Kabuli_Chr05	805883	[G/T]		--	INTERGENIC
7710	CakSNP7710	*Kabuli*	Ca_Kabuli_Chr05	805910	[G/A]		--	INTERGENIC
7711	CakSNP7711	*Kabuli*	Ca_Kabuli_Chr05	870867	[A/G]		--	INTERGENIC

S.N.	SNP IDs	Chickpea cultivars	Chromosomes	Physical positions (bp)	SNPs	Gene accession IDs	Putative functions	Sequence components of genome
7712	CakSNP7712	*Kabuli*	Ca_Kabuli_Chr05	870909	[G/A]		--	INTERGENIC
7713	CakSNP7713	*Kabuli*	Ca_Kabuli_Chr05	925554	[T/C]		--	INTERGENIC
7714	CakSNP7714	*Kabuli*	Ca_Kabuli_Chr05	925493	[T/C]		--	INTERGENIC
7715	CakSNP7715	*Kabuli*	Ca_Kabuli_Chr05	1141755	[C/T]	Ca_23234	--	NON_SYNONYMOUS_CODING
7716	CakSNP7716	*Kabuli*	Ca_Kabuli_Chr05	1220867	[A/G]	Ca_26284	YbaK/aminoacyl-tRNA synthetase-associated domain	INTRON
7717	CakSNP7717	*Kabuli*	Ca_Kabuli_Chr05	1252394	[G/A]	Ca_26282	MORN motif	INTRON
7718	CakSNP7718	*Kabuli*	Ca_Kabuli_Chr05	1252388	[A/T]	Ca_26282	MORN motif	INTRON
7719	CakSNP7719	*Kabuli*	Ca_Kabuli_Chr05	1270830	[T/C]	Ca_26280	1-aminocyclopropane-1-carboxylate synthase	SYNONYMOUS_CODING
7720	CakSNP7720	*Kabuli*	Ca_Kabuli_Chr05	1272581	[T/C]		--	INTERGENIC
7721	CakSNP7721	*Kabuli*	Ca_Kabuli_Chr05	1280977	[T/G]	Ca_26279	Transcription factor GRAS	NON_SYNONYMOUS_CODING

S.N.	SNP IDs	Chickpea cultivars	Chromosomes	Physical positions (bp)	SNPs	Gene accession IDs	Putative functions	Sequence components of genome
7722	CakSNP7722	*Kabuli*	Ca_Kabuli_Chr05	1281006	[A/C]	Ca_26279	Transcription factor GRAS	SYNONYMOUS_CODING
7723	CakSNP7723	*Kabuli*	Ca_Kabuli_Chr05	1281017	[G/A]	Ca_26279	Transcription factor GRAS	SYNONYMOUS_CODING
7724	CakSNP7724	*Kabuli*	Ca_Kabuli_Chr05	1281021	[C/T]	Ca_26279	Transcription factor GRAS	SYNONYMOUS_CODING
7725	CakSNP7725	*Kabuli*	Ca_Kabuli_Chr05	1534904	[C/T]		--	INTERGENIC
7726	CakSNP7726	*Kabuli*	Ca_Kabuli_Chr05	1534901	[C/T]		--	INTERGENIC
7727	CakSNP7727	*Kabuli*	Ca_Kabuli_Chr05	1534888	[T/C]		--	INTERGENIC
7728	CakSNP7728	*Kabuli*	Ca_Kabuli_Chr05	2166586	[A/G]	Ca_26506	SNF2-related	INTRON
7729	CakSNP7729	*Kabuli*	Ca_Kabuli_Chr05	2519977	[C/T]	Ca_18666	Peptidase C48, SUMO/Sentrin/Ubl1	NON_SYNONYMOUS_CODING
7730	CakSNP7730	*Kabuli*	Ca_Kabuli_Chr05	2519949	[C/T]	Ca_18666	Peptidase C48, SUMO/Sentrin/Ubl1	NON_SYNONYMOUS_CODING
7731	CakSNP7731	*Kabuli*	Ca_Kabuli_Chr05	2519961	[G/A]	Ca_18666	Peptidase C48, SUMO/Sentrin/Ubl1	NON_SYNONYMOUS_CODING

S.N.	SNP IDs	Chickpea cultivars	Chromosomes	Physical positions (bp)	SNPs	Gene accession IDs	Putative functions	Sequence components of genome
7732	CakSNP7732	*Kabuli*	Ca_Kabuli_Chr05	2519976	[G/A]	Ca_18666	Peptidase C48, SUMO/Sentrin/Ubl1	NON_SYNONYMOUS_CODING
7733	CakSNP7733	*Kabuli*	Ca_Kabuli_Chr05	2520035	[G/A]	Ca_18666	Peptidase C48, SUMO/Sentrin/Ubl1	SYNONYMOUS_CODING
7734	CakSNP7734	*Kabuli*	Ca_Kabuli_Chr05	2520058	[C/T]	Ca_18666	Peptidase C48, SUMO/Sentrin/Ubl1	NON_SYNONYMOUS_CODING
7735	CakSNP7735	*Kabuli*	Ca_Kabuli_Chr05	2520015	[C/G]	Ca_18666	Peptidase C48, SUMO/Sentrin/Ubl1	NON_SYNONYMOUS_CODING
7736	CakSNP7736	*Kabuli*	Ca_Kabuli_Chr05	2520049	[C/T]	Ca_18666	Peptidase C48, SUMO/Sentrin/Ubl1	NON_SYNONYMOUS_CODING
7737	CakSNP7737	*Kabuli*	Ca_Kabuli_Chr05	2520050	[G/C]	Ca_18666	Peptidase C48, SUMO/Sentrin/Ubl1	SYNONYMOUS_CODING
7738	CakSNP7738	*Kabuli*	Ca_Kabuli_Chr05	2520051	[G/T]	Ca_18666	Peptidase C48, SUMO/Sentrin/Ubl1	NON_SYNONYMOUS_CODING
7739	CakSNP7739	*Kabuli*	Ca_Kabuli_Chr05	2520055	[C/T]	Ca_18666	Peptidase C48, SUMO/Sentrin/Ubl1	NON_SYNONYMOUS_CODING
7740	CakSNP7740	*Kabuli*	Ca_Kabuli_Chr05	2520068	[T/A]	Ca_18666	Peptidase C48, SUMO/Sentrin/Ubl1	SYNONYMOUS_CODING
7741	CakSNP7741	*Kabuli*	Ca_Kabuli_Chr05	2520069	[C/T]	Ca_18666	Peptidase C48, SUMO/Sentrin/Ubl1	NON_SYNONYMOUS_CODING

S.N.	SNP IDs	Chickpea cultivars	Chromosomes	Physical positions (bp)	SNPs	Gene accession IDs	Putative functions	Sequence components of genome
7742	CakSNP7742	*Kabuli*	Ca_Kabuli_Chr05	2520094	[G/A]	Ca_18666	Peptidase C48, SUMO/Sentrin/Ubl1	NON_SYNONYMOUS_CODING
7743	CakSNP7743	*Kabuli*	Ca_Kabuli_Chr05	2520061	[C/T]	Ca_18666	Peptidase C48, SUMO/Sentrin/Ubl1	NON_SYNONYMOUS_CODING
7744	CakSNP7744	*Kabuli*	Ca_Kabuli_Chr05	2520093	[G/A]	Ca_18666	Peptidase C48, SUMO/Sentrin/Ubl1	NON_SYNONYMOUS_CODING
7745	CakSNP7745	*Kabuli*	Ca_Kabuli_Chr05	2520123	[A/G]	Ca_18666	Peptidase C48, SUMO/Sentrin/Ubl1	NON_SYNONYMOUS_CODING
7746	CakSNP7746	*Kabuli*	Ca_Kabuli_Chr05	2520114	[G/A]	Ca_18666	Peptidase C48, SUMO/Sentrin/Ubl1	NON_SYNONYMOUS_CODING
7747	CakSNP7747	*Kabuli*	Ca_Kabuli_Chr05	2520109	[G/A]	Ca_18666	Peptidase C48, SUMO/Sentrin/Ubl1	NON_SYNONYMOUS_CODING
7748	CakSNP7748	*Kabuli*	Ca_Kabuli_Chr05	2520101	[C/T]	Ca_18666	Peptidase C48, SUMO/Sentrin/Ubl1	SYNONYMOUS_CODING
7749	CakSNP7749	*Kabuli*	Ca_Kabuli_Chr05	2613423	[T/G]		--	INTERGENIC
7750	CakSNP7750	*Kabuli*	Ca_Kabuli_Chr05	2948530	[G/T]	Ca_18681	--	INTRON
7751	CakSNP7751	*Kabuli*	Ca_Kabuli_Chr05	2948531	[T/C]	Ca_18681	--	INTRON

S.N.	SNP IDs	Chickpea cultivars	Chromosomes	Physical positions (bp)	SNPs	Gene accession IDs	Putative functions	Sequence components of genome
7752	CakSNP7752	*Kabuli*	Ca_Kabuli_Chr05	2948549	[C/A]	Ca_18681	--	INTRON
7753	CakSNP7753	*Kabuli*	Ca_Kabuli_Chr05	2948592	[T/C]	Ca_18681	--	INTRON
7754	CakSNP7754	*Kabuli*	Ca_Kabuli_Chr05	2948596	[C/T]	Ca_18681	--	INTRON
7755	CakSNP7755	*Kabuli*	Ca_Kabuli_Chr05	2948616	[G/A]	Ca_18681	--	INTRON
7756	CakSNP7756	*Kabuli*	Ca_Kabuli_Chr05	2948698	[G/A]	Ca_18681	--	INTRON
7757	CakSNP7757	*Kabuli*	Ca_Kabuli_Chr05	2948977	[G/A]	Ca_18681	--	INTRON
7758	CakSNP7758	*Kabuli*	Ca_Kabuli_Chr05	3007213	[A/G]	Ca_18684	ABC transporter-like	INTRON
7759	CakSNP7759	*Kabuli*	Ca_Kabuli_Chr05	3007316	[T/C]	Ca_18684	ABC transporter-like	INTRON
7760	CakSNP7760	*Kabuli*	Ca_Kabuli_Chr05	3040264	[G/T]	Ca_18685	ABC transporter-like	SYNONYMOUS_CODING
7761	CakSNP7761	*Kabuli*	Ca_Kabuli_Chr05	3040948	[G/C]	Ca_18685	ABC transporter-like	INTRON

S.N.	SNP IDs	Chickpea cultivars	Chromosomes	Physical positions (bp)	SNPs	Gene accession IDs	Putative functions	Sequence components of genome
7762	CakSNP7762	*Kabuli*	Ca_Kabuli_Chr05	3159061	[G/A]	Ca_18692	Calponin homology domain	INTRON
7763	CakSNP7763	*Kabuli*	Ca_Kabuli_Chr05	3348291	[G/A]		--	INTERGENIC
7764	CakSNP7764	*Kabuli*	Ca_Kabuli_Chr05	3348310	[A/G]		--	INTERGENIC
7765	CakSNP7765	*Kabuli*	Ca_Kabuli_Chr05	3348366	[G/C]		--	INTERGENIC
7766	CakSNP7766	*Kabuli*	Ca_Kabuli_Chr05	3353052	[A/G]		--	INTERGENIC
7767	CakSNP7767	*Kabuli*	Ca_Kabuli_Chr05	4122395	[T/C]		--	INTERGENIC
7768	CakSNP7768	*Kabuli*	Ca_Kabuli_Chr05	4122342	[T/C]		--	INTERGENIC
7769	CakSNP7769	*Kabuli*	Ca_Kabuli_Chr05	5133644	[A/G]	Ca_25551	Zinc finger, U1-type	INTRON
7770	CakSNP7770	*Kabuli*	Ca_Kabuli_Chr05	5205261	[T/G]	Ca_22620	Domain of unknown function DUF641, plant	NON_SYNONYMOUS_CODING
7771	CakSNP7771	*Kabuli*	Ca_Kabuli_Chr05	5404261	[G/A]		--	INTERGENIC

S.N.	SNP IDs	Chickpea cultivars	Chromosomes	Physical positions (bp)	SNPs	Gene accession IDs	Putative functions	Sequence components of genome
7772	CakSNP7772	*Kabuli*	Ca_Kabuli_Chr05	6190494	[T/C]		--	INTERGENIC
7773	CakSNP7773	*Kabuli*	Ca_Kabuli_Chr05	6225426	[T/A]		--	INTERGENIC
7774	CakSNP7774	*Kabuli*	Ca_Kabuli_Chr05	6225415	[G/C]		--	INTERGENIC
7775	CakSNP7775	*Kabuli*	Ca_Kabuli_Chr05	6225410	[T/A]		--	INTERGENIC
7776	CakSNP7776	*Kabuli*	Ca_Kabuli_Chr05	6290453	[G/A]	Ca_20739	RNA recognition motif domain	INTRON
7777	CakSNP7777	*Kabuli*	Ca_Kabuli_Chr05	6300337	[C/A]	Ca_20739	RNA recognition motif domain	INTRON
7778	CakSNP7778	*Kabuli*	Ca_Kabuli_Chr05	6300360	[C/A]	Ca_20739	RNA recognition motif domain	INTRON
7779	CakSNP7779	*Kabuli*	Ca_Kabuli_Chr05	6300502	[C/A]	Ca_20739	RNA recognition motif domain	INTRON
7780	CakSNP7780	*Kabuli*	Ca_Kabuli_Chr05	6316787	[C/T]	Ca_20740	--	SYNONYMOUS_CODING
7781	CakSNP7781	*Kabuli*	Ca_Kabuli_Chr05	6317014	[C/T]	Ca_20740	--	NON_SYNONYMOUS_CODING

S.N.	SNP IDs	Chickpea cultivars	Chromosomes	Physical positions (bp)	SNPs	Gene accession IDs	Putative functions	Sequence components of genome
7782	CakSNP7782	*Kabuli*	Ca_Kabuli_Chr05	6931865	[A/G]		--	INTERGENIC
7783	CakSNP7783	*Kabuli*	Ca_Kabuli_Chr05	7082295	[G/A]		--	INTERGENIC
7784	CakSNP7784	*Kabuli*	Ca_Kabuli_Chr05	7082296	[T/G]		--	INTERGENIC
7785	CakSNP7785	*Kabuli*	Ca_Kabuli_Chr05	7082301	[G/T]		--	INTERGENIC
7786	CakSNP7786	*Kabuli*	Ca_Kabuli_Chr05	7278953	[C/T]		--	INTERGENIC
7787	CakSNP7787	*Kabuli*	Ca_Kabuli_Chr05	7398246	[A/C]		--	INTERGENIC
7788	CakSNP7788	*Kabuli*	Ca_Kabuli_Chr05	7425116	[C/T]		--	INTERGENIC
7789	CakSNP7789	*Kabuli*	Ca_Kabuli_Chr05	7617914	[T/A]		--	INTERGENIC
7790	CakSNP7790	*Kabuli*	Ca_Kabuli_Chr05	7628097	[C/A]	Ca_18740	Alpha/beta hydrolase fold-1	INTRON
7791	CakSNP7791	*Kabuli*	Ca_Kabuli_Chr05	7641993	[T/C]		--	INTERGENIC

S.N.	SNP IDs	Chickpea cultivars	Chromosomes	Physical positions (bp)	SNPs	Gene accession IDs	Putative functions	Sequence components of genome
7792	CakSNP7792	*Kabuli*	Ca_Kabuli_Chr05	7641925	[G/T]	Ca_18738	Transcriptional factor B3	INTRON
7793	CakSNP7793	*Kabuli*	Ca_Kabuli_Chr05	7641923	[A/G]	Ca_18738	Transcriptional factor B3	INTRON
7794	CakSNP7794	*Kabuli*	Ca_Kabuli_Chr05	7664421	[T/C]	Ca_18734	Regulator of nonsense-mediated decay, UPF3	NON_SYNONYMOUS_CODING
7795	CakSNP7795	*Kabuli*	Ca_Kabuli_Chr05	7775286	[C/A]		--	INTERGENIC
7796	CakSNP7796	*Kabuli*	Ca_Kabuli_Chr05	7775290	[A/T]		--	INTERGENIC
7797	CakSNP7797	*Kabuli*	Ca_Kabuli_Chr05	7775298	[A/T]		--	INTERGENIC
7798	CakSNP7798	*Kabuli*	Ca_Kabuli_Chr05	7775329	[A/G]		--	INTERGENIC
7799	CakSNP7799	*Kabuli*	Ca_Kabuli_Chr05	7775516	[A/G]		--	INTERGENIC
7800	CakSNP7800	*Kabuli*	Ca_Kabuli_Chr05	7775497	[G/C]		--	INTERGENIC
7801	CakSNP7801	*Kabuli*	Ca_Kabuli_Chr05	7775472	[G/A]		--	INTERGENIC

S.N.	SNP IDs	Chickpea cultivars	Chromosomes	Physical positions (bp)	SNPs	Gene accession IDs	Putative functions	Sequence components of genome
7802	CakSNP7802	*Kabuli*	Ca_Kabuli_Chr05	7775456	[T/C]		--	INTERGENIC
7803	CakSNP7803	*Kabuli*	Ca_Kabuli_Chr05	7775918	[C/T]		--	INTERGENIC
7804	CakSNP7804	*Kabuli*	Ca_Kabuli_Chr05	7789021	[G/T]		--	INTERGENIC
7805	CakSNP7805	*Kabuli*	Ca_Kabuli_Chr05	7789065	[A/G]		--	INTERGENIC
7806	CakSNP7806	*Kabuli*	Ca_Kabuli_Chr05	7826336	[G/T]		--	INTERGENIC
7807	CakSNP7807	*Kabuli*	Ca_Kabuli_Chr05	7826338	[T/G]		--	INTERGENIC
7808	CakSNP7808	*Kabuli*	Ca_Kabuli_Chr05	7826362	[C/T]		--	INTERGENIC
7809	CakSNP7809	*Kabuli*	Ca_Kabuli_Chr05	7826366	[A/G]		--	INTERGENIC
7810	CakSNP7810	*Kabuli*	Ca_Kabuli_Chr05	7925356	[A/C]		--	INTERGENIC
7811	CakSNP7811	*Kabuli*	Ca_Kabuli_Chr05	8263483	[A/G]		--	INTERGENIC

S.N.	SNP IDs	Chickpea cultivars	Chromosomes	Physical positions (bp)	SNPs	Gene accession IDs	Putative functions	Sequence components of genome
7812	CakSNP7812	*Kabuli*	Ca_Kabuli_Chr05	8653283	[G/A]	Ca_20324	--	INTRON
7813	CakSNP7813	*Kabuli*	Ca_Kabuli_Chr05	8676750	[T/G]	Ca_20327	Heat shock protein DnaJ, N-terminal	NON_SYNONYMOUS_CODING
7814	CakSNP7814	*Kabuli*	Ca_Kabuli_Chr05	8676807	[G/A]	Ca_20327	Heat shock protein DnaJ, N-terminal	SYNONYMOUS_CODING
7815	CakSNP7815	*Kabuli*	Ca_Kabuli_Chr05	8676789	[T/C]	Ca_20327	Heat shock protein DnaJ, N-terminal	SYNONYMOUS_CODING
7816	CakSNP7816	*Kabuli*	Ca_Kabuli_Chr05	8676771	[G/A]	Ca_20327	Heat shock protein DnaJ, N-terminal	SYNONYMOUS_CODING
7817	CakSNP7817	*Kabuli*	Ca_Kabuli_Chr05	8946218	[G/A]		--	INTERGENIC
7818	CakSNP7818	*Kabuli*	Ca_Kabuli_Chr05	9034100	[C/G]	Ca_19959	Peptidase T2, asparaginase 2	NON_SYNONYMOUS_CODING
7819	CakSNP7819	*Kabuli*	Ca_Kabuli_Chr05	9076709	[C/T]		--	INTERGENIC
7820	CakSNP7820	*Kabuli*	Ca_Kabuli_Chr05	9076712	[G/A]		--	INTERGENIC
7821	CakSNP7821	*Kabuli*	Ca_Kabuli_Chr05	9076737	[C/A]		--	INTERGENIC

S.N.	SNP IDs	Chickpea cultivars	Chromosomes	Physical positions (bp)	SNPs	Gene accession IDs	Putative functions	Sequence components of genome
7822	CakSNP7822	*Kabuli*	Ca_Kabuli_Chr05	9076738	[G/C]		--	INTERGENIC
7823	CakSNP7823	*Kabuli*	Ca_Kabuli_Chr05	9076767	[C/A]		--	INTERGENIC
7824	CakSNP7824	*Kabuli*	Ca_Kabuli_Chr05	9076824	[G/A]		--	INTERGENIC
7825	CakSNP7825	*Kabuli*	Ca_Kabuli_Chr05	9076821	[G/T]		--	INTERGENIC
7826	CakSNP7826	*Kabuli*	Ca_Kabuli_Chr05	9076809	[T/A]		--	INTERGENIC
7827	CakSNP7827	*Kabuli*	Ca_Kabuli_Chr05	9077489	[T/C]	Ca_19962	Prohibitin	SYNONYMOUS_CODING
7828	CakSNP7828	*Kabuli*	Ca_Kabuli_Chr05	9519382	[C/A]		--	INTERGENIC
7829	CakSNP7829	*Kabuli*	Ca_Kabuli_Chr05	9519390	[G/A]		--	INTERGENIC
7830	CakSNP7830	*Kabuli*	Ca_Kabuli_Chr05	9609055	[T/C]	Ca_23788	Pyridine nucleotide-disulphide oxidoreductase, NAD-binding region	SYNONYMOUS_CODING
7831	CakSNP7831	*Kabuli*	Ca_Kabuli_Chr05	9620998	[A/C]		--	INTERGENIC

S.N.	SNP IDs	Chickpea cultivars	Chromosomes	Physical positions (bp)	SNPs	Gene accession IDs	Putative functions	Sequence components of genome
7832	CakSNP7832	*Kabuli*	Ca_Kabuli_Chr05	9728498	[A/T]		--	INTERGENIC
7833	CakSNP7833	*Kabuli*	Ca_Kabuli_Chr05	9728609	[T/A]		--	INTERGENIC
7834	CakSNP7834	*Kabuli*	Ca_Kabuli_Chr05	9728591	[A/G]		--	INTERGENIC
7835	CakSNP7835	*Kabuli*	Ca_Kabuli_Chr05	9786213	[C/A]	Ca_20498	Protein of unknown function DUF842, eukaryotic	INTRON
7836	CakSNP7836	*Kabuli*	Ca_Kabuli_Chr05	9868796	[T/C]	Ca_20504	Ankyrin repeat	INTRON
7837	CakSNP7837	*Kabuli*	Ca_Kabuli_Chr05	9869005	[T/G]	Ca_20504	Ankyrin repeat	SYNONYMOUS_CODING
7838	CakSNP7838	*Kabuli*	Ca_Kabuli_Chr05	9961273	[T/C]	Ca_20508	DNA-binding WRKY	SYNONYMOUS_CODING
7839	CakSNP7839	*Kabuli*	Ca_Kabuli_Chr05	9961396	[T/C]	Ca_20508	DNA-binding WRKY	SYNONYMOUS_CODING
7840	CakSNP7840	*Kabuli*	Ca_Kabuli_Chr05	9961380	[C/T]	Ca_20508	DNA-binding WRKY	NON_SYNONYMOUS_CODING
7841	CakSNP7841	*Kabuli*	Ca_Kabuli_Chr05	9965880	[G/C]	Ca_20509	UDP-glucuronosyl/UDP-glucosyltransferase	NON_SYNONYMOUS_CODING

S.N.	SNP IDs	Chickpea cultivars	Chromosomes	Physical positions (bp)	SNPs	Gene accession IDs	Putative functions	Sequence components of genome
7842	CakSNP7842	*Kabuli*	Ca_Kabuli_Chr05	9965890	[T/C]	Ca_20509	UDP-glucuronosyl/UDP-glucosyltransferase	SYNONYMOUS_CODING
7843	CakSNP7843	*Kabuli*	Ca_Kabuli_Chr05	9965913	[A/C]	Ca_20509	UDP-glucuronosyl/UDP-glucosyltransferase	NON_SYNONYMOUS_CODING
7844	CakSNP7844	*Kabuli*	Ca_Kabuli_Chr05	9965848	[T/C]	Ca_20509	UDP-glucuronosyl/UDP-glucosyltransferase	SYNONYMOUS_CODING
7845	CakSNP7845	*Kabuli*	Ca_Kabuli_Chr05	9965846	[A/C]	Ca_20509	UDP-glucuronosyl/UDP-glucosyltransferase	NON_SYNONYMOUS_CODING
7846	CakSNP7846	*Kabuli*	Ca_Kabuli_Chr05	10004386	[G/T]	Ca_20512	--	NON_SYNONYMOUS_CODING
7847	CakSNP7847	*Kabuli*	Ca_Kabuli_Chr05	10004427	[T/G]	Ca_20512	--	SYNONYMOUS_CODING
7848	CakSNP7848	*Kabuli*	Ca_Kabuli_Chr05	10004430	[T/G]	Ca_20512	--	SYNONYMOUS_CODING
7849	CakSNP7849	*Kabuli*	Ca_Kabuli_Chr05	10004451	[T/G]	Ca_20512	--	SYNONYMOUS_CODING
7850	CakSNP7850	*Kabuli*	Ca_Kabuli_Chr05	10054140	[G/C]	Ca_20515	--	NON_SYNONYMOUS_CODING
7851	CakSNP7851	*Kabuli*	Ca_Kabuli_Chr05	10054146	[A/G]	Ca_20515	--	NON_SYNONYMOUS_CODING

S.N.	SNP IDs	Chickpea cultivars	Chromosomes	Physical positions (bp)	SNPs	Gene accession IDs	Putative functions	Sequence components of genome
7852	CakSNP7852	*Kabuli*	Ca_Kabuli_Chr05	10054266	[T/C]		--	INTERGENIC
7853	CakSNP7853	*Kabuli*	Ca_Kabuli_Chr05	10054213	[A/C]		--	INTERGENIC
7854	CakSNP7854	*Kabuli*	Ca_Kabuli_Chr05	10072358	[G/A]		--	INTERGENIC
7855	CakSNP7855	*Kabuli*	Ca_Kabuli_Chr05	10072403	[G/C]		--	INTERGENIC
7856	CakSNP7856	*Kabuli*	Ca_Kabuli_Chr05	10154016	[A/G]		--	INTERGENIC
7857	CakSNP7857	*Kabuli*	Ca_Kabuli_Chr05	10247223	[T/C]	Ca_21244	--	NON_SYNONYMOUS_CODING
7858	CakSNP7858	*Kabuli*	Ca_Kabuli_Chr05	10247224	[T/A]	Ca_21244	--	SYNONYMOUS_CODING
7859	CakSNP7859	*Kabuli*	Ca_Kabuli_Chr05	10247233	[C/T]	Ca_21244	--	SYNONYMOUS_CODING
7860	CakSNP7860	*Kabuli*	Ca_Kabuli_Chr05	10247244	[C/T]	Ca_21244	--	NON_SYNONYMOUS_CODING
7861	CakSNP7861	*Kabuli*	Ca_Kabuli_Chr05	10390882	[C/T]		--	INTERGENIC

S.N.	SNP IDs	Chickpea cultivars	Chromosomes	Physical positions (bp)	SNPs	Gene accession IDs	Putative functions	Sequence components of genome
7862	CakSNP7862	*Kabuli*	Ca_Kabuli_Chr05	10406562	[C/A]		--	INTERGENIC
7863	CakSNP7863	*Kabuli*	Ca_Kabuli_Chr05	10406602	[C/T]		--	INTERGENIC
7864	CakSNP7864	*Kabuli*	Ca_Kabuli_Chr05	10406613	[C/T]		--	INTERGENIC
7865	CakSNP7865	*Kabuli*	Ca_Kabuli_Chr05	10406636	[A/G]		--	INTERGENIC
7866	CakSNP7866	*Kabuli*	Ca_Kabuli_Chr05	10406649	[A/C]		--	INTERGENIC
7867	CakSNP7867	*Kabuli*	Ca_Kabuli_Chr05	10406650	[A/G]		--	INTERGENIC
7868	CakSNP7868	*Kabuli*	Ca_Kabuli_Chr05	10406754	[G/T]		--	INTERGENIC
7869	CakSNP7869	*Kabuli*	Ca_Kabuli_Chr05	10510166	[G/A]	Ca_21230	--	INTRON
7870	CakSNP7870	*Kabuli*	Ca_Kabuli_Chr05	10544823	[A/G]	Ca_21227	D-isomer specific 2-hydroxyacid dehydrogenase, catalytic domain	NON_SYNONYMOUS_CODING
7871	CakSNP7871	*Kabuli*	Ca_Kabuli_Chr05	10546944	[A/T]	Ca_21227	D-isomer specific 2-hydroxyacid dehydrogenase, catalytic domain	INTRON

S.N.	SNP IDs	Chickpea cultivars	Chromosomes	Physical positions (bp)	SNPs	Gene accession IDs	Putative functions	Sequence components of genome
7872	CakSNP7872	*Kabuli*	Ca_Kabuli_Chr05	10675252	[A/G]		--	INTERGENIC
7873	CakSNP7873	*Kabuli*	Ca_Kabuli_Chr05	10675279	[C/G]		--	INTERGENIC
7874	CakSNP7874	*Kabuli*	Ca_Kabuli_Chr05	10675294	[A/G]		--	INTERGENIC
7875	CakSNP7875	*Kabuli*	Ca_Kabuli_Chr05	10769765	[C/T]		--	INTERGENIC
7876	CakSNP7876	*Kabuli*	Ca_Kabuli_Chr05	10832275	[T/C]	Ca_23753	--	SYNONYMOUS_CODING
7877	CakSNP7877	*Kabuli*	Ca_Kabuli_Chr05	10858490	[C/T]		--	INTERGENIC
7878	CakSNP7878	*Kabuli*	Ca_Kabuli_Chr05	10918368	[A/G]	Ca_23750	MoeA, C-terminal, domain IV	NON_SYNONYMOUS_CODING
7879	CakSNP7879	*Kabuli*	Ca_Kabuli_Chr05	10919246	[C/G]	Ca_23750	MoeA, C-terminal, domain IV	INTRON
7880	CakSNP7880	*Kabuli*	Ca_Kabuli_Chr05	11230617	[C/G]		--	INTERGENIC
7881	CakSNP7881	*Kabuli*	Ca_Kabuli_Chr05	11230627	[A/G]		--	INTERGENIC

S.N.	SNP IDs	Chickpea cultivars	Chromosomes	Physical positions (bp)	SNPs	Gene accession IDs	Putative functions	Sequence components of genome
7882	CakSNP7882	*Kabuli*	Ca_Kabuli_Chr05	11230648	[G/A]		--	INTERGENIC
7883	CakSNP7883	*Kabuli*	Ca_Kabuli_Chr05	11230671	[T/C]		--	INTERGENIC
7884	CakSNP7884	*Kabuli*	Ca_Kabuli_Chr05	11263571	[C/G]		--	INTERGENIC
7885	CakSNP7885	*Kabuli*	Ca_Kabuli_Chr05	11263574	[C/A]		--	INTERGENIC
7886	CakSNP7886	*Kabuli*	Ca_Kabuli_Chr05	11317487	[C/T]		--	INTERGENIC
7887	CakSNP7887	*Kabuli*	Ca_Kabuli_Chr05	11317502	[C/A]		--	INTERGENIC
7888	CakSNP7888	*Kabuli*	Ca_Kabuli_Chr05	11317534	[C/A]		--	INTERGENIC
7889	CakSNP7889	*Kabuli*	Ca_Kabuli_Chr05	11385780	[A/C]	Ca_23356	RNA recognition motif domain	NON_SYNONYMOUS_CODING
7890	CakSNP7890	*Kabuli*	Ca_Kabuli_Chr05	11385874	[A/G]	Ca_23356	RNA recognition motif domain	SYNONYMOUS_CODING
7891	CakSNP7891	*Kabuli*	Ca_Kabuli_Chr05	11765841	[C/T]	Ca_17117	ABC transporter-like	SYNONYMOUS_CODING

S.N.	SNP IDs	Chickpea cultivars	Chromosomes	Physical positions (bp)	SNPs	Gene accession IDs	Putative functions	Sequence components of genome
7892	CakSNP7892	*Kabuli*	Ca_Kabuli_Chr05	11777112	[C/T]	Ca_17116	Integrase, catalytic core	SYNONYMOUS_CODING
7893	CakSNP7893	*Kabuli*	Ca_Kabuli_Chr05	11807822	[G/T]		--	INTERGENIC
7894	CakSNP7894	*Kabuli*	Ca_Kabuli_Chr05	11807842	[T/C]		--	INTERGENIC
7895	CakSNP7895	*Kabuli*	Ca_Kabuli_Chr05	11845515	[C/T]	Ca_17111	ABC transporter, transmembrane domain	NON_SYNONYMOUS_CODING
7896	CakSNP7896	*Kabuli*	Ca_Kabuli_Chr05	11853090	[C/T]	Ca_17111	ABC transporter, transmembrane domain	INTRON
7897	CakSNP7897	*Kabuli*	Ca_Kabuli_Chr05	11864959	[T/C]	Ca_17110	Zinc finger, CCHC-type	NON_SYNONYMOUS_CODING
7898	CakSNP7898	*Kabuli*	Ca_Kabuli_Chr05	12044714	[A/G]	Ca_17103	Cytochrome P450	NON_SYNONYMOUS_CODING
7899	CakSNP7899	*Kabuli*	Ca_Kabuli_Chr05	12157678	[A/G]		--	INTERGENIC
7900	CakSNP7900	*Kabuli*	Ca_Kabuli_Chr05	12157683	[T/C]		--	INTERGENIC
7901	CakSNP7901	*Kabuli*	Ca_Kabuli_Chr05	12157688	[C/T]		--	INTERGENIC

S.N.	SNP IDs	Chickpea cultivars	Chromosomes	Physical positions (bp)	SNPs	Gene accession IDs	Putative functions	Sequence components of genome
7902	CakSNP7902	*Kabuli*	Ca_Kabuli_Chr05	12157706	[G/A]		--	INTERGENIC
7903	CakSNP7903	*Kabuli*	Ca_Kabuli_Chr05	12157707	[C/T]		--	INTERGENIC
7904	CakSNP7904	*Kabuli*	Ca_Kabuli_Chr05	12157710	[A/C]		--	INTERGENIC
7905	CakSNP7905	*Kabuli*	Ca_Kabuli_Chr05	12157742	[C/A]		--	INTERGENIC
7906	CakSNP7906	*Kabuli*	Ca_Kabuli_Chr05	12157699	[G/T]		--	INTERGENIC
7907	CakSNP7907	*Kabuli*	Ca_Kabuli_Chr05	12162108	[T/G]		--	INTERGENIC
7908	CakSNP7908	*Kabuli*	Ca_Kabuli_Chr05	12162156	[T/C]		--	INTERGENIC
7909	CakSNP7909	*Kabuli*	Ca_Kabuli_Chr05	12162160	[G/T]		--	INTERGENIC
7910	CakSNP7910	*Kabuli*	Ca_Kabuli_Chr05	12162164	[C/G]		--	INTERGENIC
7911	CakSNP7911	*Kabuli*	Ca_Kabuli_Chr05	12198014	[G/A]		--	INTERGENIC

S.N.	SNP IDs	Chickpea cultivars	Chromosomes	Physical positions (bp)	SNPs	Gene accession IDs	Putative functions	Sequence components of genome
7912	CakSNP7912	*Kabuli*	Ca_Kabuli_Chr05	12204521	[T/A]	Ca_17094	Protein kinase, catalytic domain	INTRON
7913	CakSNP7913	*Kabuli*	Ca_Kabuli_Chr05	12204588	[A/G]	Ca_17094	Protein kinase, catalytic domain	INTRON
7914	CakSNP7914	*Kabuli*	Ca_Kabuli_Chr05	12427931	[C/T]		--	INTERGENIC
7915	CakSNP7915	*Kabuli*	Ca_Kabuli_Chr05	12427940	[T/A]		--	INTERGENIC
7916	CakSNP7916	*Kabuli*	Ca_Kabuli_Chr05	12427970	[T/C]		--	INTERGENIC
7917	CakSNP7917	*Kabuli*	Ca_Kabuli_Chr05	12427969	[T/C]		--	INTERGENIC
7918	CakSNP7918	*Kabuli*	Ca_Kabuli_Chr05	12427966	[T/C]		--	INTERGENIC
7919	CakSNP7919	*Kabuli*	Ca_Kabuli_Chr05	12428027	[G/A]		--	INTERGENIC
7920	CakSNP7920	*Kabuli*	Ca_Kabuli_Chr05	12428121	[A/T]		--	INTERGENIC
7921	CakSNP7921	*Kabuli*	Ca_Kabuli_Chr05	12428120	[G/A]		--	INTERGENIC

S.N.	SNP IDs	Chickpea cultivars	Chromosomes	Physical positions (bp)	SNPs	Gene accession IDs	Putative functions	Sequence components of genome
7922	CakSNP7922	*Kabuli*	Ca_Kabuli_Chr05	12428102	[A/G]		--	INTERGENIC
7923	CakSNP7923	*Kabuli*	Ca_Kabuli_Chr05	12480948	[T/G]	Ca_17080	Choline/ethanolamine kinase	INTRON
7924	CakSNP7924	*Kabuli*	Ca_Kabuli_Chr05	12553469	[A/C]		--	INTERGENIC
7925	CakSNP7925	*Kabuli*	Ca_Kabuli_Chr05	12553470	[G/C]		--	INTERGENIC
7926	CakSNP7926	*Kabuli*	Ca_Kabuli_Chr05	12590330	[A/G]	Ca_17072	Glycerophosphoryl diester phosphodiesterase	NON_SYNONYMOUS_CODING
7927	CakSNP7927	*Kabuli*	Ca_Kabuli_Chr05	12590361	[T/C]	Ca_17072	Glycerophosphoryl diester phosphodiesterase	SYNONYMOUS_CODING
7928	CakSNP7928	*Kabuli*	Ca_Kabuli_Chr05	12590478	[T/C]	Ca_17072	Glycerophosphoryl diester phosphodiesterase	SYNONYMOUS_CODING
7929	CakSNP7929	*Kabuli*	Ca_Kabuli_Chr05	12662138	[G/T]	Ca_17066	Protein kinase, catalytic domain	INTRON
7930	CakSNP7930	*Kabuli*	Ca_Kabuli_Chr05	13982000	[T/G]	Ca_20821	Protein kinase, catalytic domain	NON_SYNONYMOUS_CODING
7931	CakSNP7931	*Kabuli*	Ca_Kabuli_Chr05	13982041	[C/T]	Ca_20821	Protein kinase, catalytic domain	SYNONYMOUS_CODING

S.N.	SNP IDs	Chickpea cultivars	Chromosomes	Physical positions (bp)	SNPs	Gene accession IDs	Putative functions	Sequence components of genome
7932	CakSNP7932	*Kabuli*	Ca_Kabuli_Chr05	15401230	[C/T]	Ca_21639	Haloacid dehalogenase-like hydrolase	INTRON
7933	CakSNP7933	*Kabuli*	Ca_Kabuli_Chr05	15500782	[G/C]		--	INTERGENIC
7934	CakSNP7934	*Kabuli*	Ca_Kabuli_Chr05	15548719	[G/A]		--	INTERGENIC
7935	CakSNP7935	*Kabuli*	Ca_Kabuli_Chr05	15952395	[C/T]		--	INTERGENIC
7936	CakSNP7936	*Kabuli*	Ca_Kabuli_Chr05	16227319	[G/T]	Ca_17915	Peptidase C48, SUMO/Sentrin/Ubl1	SYNONYMOUS_CODING
7937	CakSNP7937	*Kabuli*	Ca_Kabuli_Chr05	16227321	[C/T]	Ca_17915	Peptidase C48, SUMO/Sentrin/Ubl1	NON_SYNONYMOUS_CODING
7938	CakSNP7938	*Kabuli*	Ca_Kabuli_Chr05	16227353	[G/A]	Ca_17915	Peptidase C48, SUMO/Sentrin/Ubl1	NON_SYNONYMOUS_CODING
7939	CakSNP7939	*Kabuli*	Ca_Kabuli_Chr05	16227395	[G/A]	Ca_17915	Peptidase C48, SUMO/Sentrin/Ubl1	NON_SYNONYMOUS_CODING
7940	CakSNP7940	*Kabuli*	Ca_Kabuli_Chr05	16227401	[G/A]	Ca_17915	Peptidase C48, SUMO/Sentrin/Ubl1	NON_SYNONYMOUS_CODING
7941	CakSNP7941	*Kabuli*	Ca_Kabuli_Chr05	16227408	[C/T]	Ca_17915	Peptidase C48, SUMO/Sentrin/Ubl1	NON_SYNONYMOUS_CODING

S.N.	SNP IDs	Chickpea cultivars	Chromosomes	Physical positions (bp)	SNPs	Gene accession IDs	Putative functions	Sequence components of genome
7942	CakSNP7942	*Kabuli*	Ca_Kabuli_Chr05	16426578	[G/A]	Ca_17927	Peptidase C48, SUMO/Sentrin/Ubl1	NON_SYNONYMOUS_CODING
7943	CakSNP7943	*Kabuli*	Ca_Kabuli_Chr05	16426575	[G/A]	Ca_17927	Peptidase C48, SUMO/Sentrin/Ubl1	NON_SYNONYMOUS_CODING
7944	CakSNP7944	*Kabuli*	Ca_Kabuli_Chr05	16426549	[G/A]	Ca_17927	Peptidase C48, SUMO/Sentrin/Ubl1	NON_SYNONYMOUS_CODING
7945	CakSNP7945	*Kabuli*	Ca_Kabuli_Chr05	16426662	[C/T]	Ca_17927	Peptidase C48, SUMO/Sentrin/Ubl1	NON_SYNONYMOUS_CODING
7946	CakSNP7946	*Kabuli*	Ca_Kabuli_Chr05	16426657	[C/T]	Ca_17927	Peptidase C48, SUMO/Sentrin/Ubl1	NON_SYNONYMOUS_CODING
7947	CakSNP7947	*Kabuli*	Ca_Kabuli_Chr05	16426656	[A/G]	Ca_17927	Peptidase C48, SUMO/Sentrin/Ubl1	SYNONYMOUS_CODING
7948	CakSNP7948	*Kabuli*	Ca_Kabuli_Chr05	16426642	[A/G]	Ca_17927	Peptidase C48, SUMO/Sentrin/Ubl1	SYNONYMOUS_CODING
7949	CakSNP7949	*Kabuli*	Ca_Kabuli_Chr05	16593880	[A/G]	Ca_17931	Pentatricopeptide repeat	NON_SYNONYMOUS_CODING
7950	CakSNP7950	*Kabuli*	Ca_Kabuli_Chr05	16751536	[C/T]	Ca_17939	Pentatricopeptide repeat	SYNONYMOUS_CODING
7951	CakSNP7951	*Kabuli*	Ca_Kabuli_Chr05	16767134	[C/T]	Ca_17942	Cellulose synthase	INTRON

S.N.	SNP IDs	Chickpea cultivars	Chromosomes	Physical positions (bp)	SNPs	Gene accession IDs	Putative functions	Sequence components of genome
7952	CakSNP7952	*Kabuli*	Ca_Kabuli_Chr05	16821406	[A/G]		--	INTERGENIC
7953	CakSNP7953	*Kabuli*	Ca_Kabuli_Chr05	16929903	[C/G]	Ca_17949	tRNA-dihydrouridine synthase	INTRON
7954	CakSNP7954	*Kabuli*	Ca_Kabuli_Chr05	16929897	[A/G]	Ca_17949	tRNA-dihydrouridine synthase	INTRON
7955	CakSNP7955	*Kabuli*	Ca_Kabuli_Chr05	17037918	[G/T]		--	INTERGENIC
7956	CakSNP7956	*Kabuli*	Ca_Kabuli_Chr05	17044340	[G/A]	Ca_17955	Disease resistance protein	INTRON
7957	CakSNP7957	*Kabuli*	Ca_Kabuli_Chr05	17044338	[G/A]	Ca_17955	Disease resistance protein	INTRON
7958	CakSNP7958	*Kabuli*	Ca_Kabuli_Chr05	17044332	[A/G]	Ca_17955	Disease resistance protein	INTRON
7959	CakSNP7959	*Kabuli*	Ca_Kabuli_Chr05	17045235	[T/C]	Ca_17955	Disease resistance protein	SYNONYMOUS_CODING
7960	CakSNP7960	*Kabuli*	Ca_Kabuli_Chr05	17045244	[A/G]	Ca_17955	Disease resistance protein	SYNONYMOUS_CODING
7961	CakSNP7961	*Kabuli*	Ca_Kabuli_Chr05	17045485	[T/G]	Ca_17955	Disease resistance protein	NON_SYNONYMOUS_CODING

S.N.	SNP IDs	Chickpea cultivars	Chromosomes	Physical positions (bp)	SNPs	Gene accession IDs	Putative functions	Sequence components of genome
7962	CakSNP7962	*Kabuli*	Ca_Kabuli_Chr05	17239612	[T/C]		--	INTERGENIC
7963	CakSNP7963	*Kabuli*	Ca_Kabuli_Chr05	17494714	[A/G]		--	INTERGENIC
7964	CakSNP7964	*Kabuli*	Ca_Kabuli_Chr05	17494702	[G/A]		--	INTERGENIC
7965	CakSNP7965	*Kabuli*	Ca_Kabuli_Chr05	17494700	[T/C]		--	INTERGENIC
7966	CakSNP7966	*Kabuli*	Ca_Kabuli_Chr05	17494697	[C/T]		--	INTERGENIC
7967	CakSNP7967	*Kabuli*	Ca_Kabuli_Chr05	17494672	[C/T]		--	INTERGENIC
7968	CakSNP7968	*Kabuli*	Ca_Kabuli_Chr05	17494699	[C/A]		--	INTERGENIC
7969	CakSNP7969	*Kabuli*	Ca_Kabuli_Chr05	17494705	[A/T]		--	INTERGENIC
7970	CakSNP7970	*Kabuli*	Ca_Kabuli_Chr05	17494710	[C/T]		--	INTERGENIC
7971	CakSNP7971	*Kabuli*	Ca_Kabuli_Chr05	17494731	[A/T]		--	INTERGENIC

S.N.	SNP IDs	Chickpea cultivars	Chromosomes	Physical positions (bp)	SNPs	Gene accession IDs	Putative functions	Sequence components of genome
7972	CakSNP7972	*Kabuli*	Ca_Kabuli_Chr05	18044808	[C/G]		--	INTERGENIC
7973	CakSNP7973	*Kabuli*	Ca_Kabuli_Chr05	18664706	[C/T]	Ca_24483	--	NON_SYNONYMOUS_CODING
7974	CakSNP7974	*Kabuli*	Ca_Kabuli_Chr05	18667782	[T/C]		--	INTERGENIC
7975	CakSNP7975	*Kabuli*	Ca_Kabuli_Chr05	18667775	[T/C]		--	INTERGENIC
7976	CakSNP7976	*Kabuli*	Ca_Kabuli_Chr05	18682788	[A/C]		--	INTERGENIC
7977	CakSNP7977	*Kabuli*	Ca_Kabuli_Chr05	18682792	[T/C]		--	INTERGENIC
7978	CakSNP7978	*Kabuli*	Ca_Kabuli_Chr05	18682793	[A/G]		--	INTERGENIC
7979	CakSNP7979	*Kabuli*	Ca_Kabuli_Chr05	18682794	[A/G]		--	INTERGENIC
7980	CakSNP7980	*Kabuli*	Ca_Kabuli_Chr05	18682812	[A/G]		--	INTERGENIC
7981	CakSNP7981	*Kabuli*	Ca_Kabuli_Chr05	18682814	[T/A]		--	INTERGENIC

S.N.	SNP IDs	Chickpea cultivars	Chromosomes	Physical positions (bp)	SNPs	Gene accession IDs	Putative functions	Sequence components of genome
7982	CakSNP7982	*Kabuli*	Ca_Kabuli_Chr05	18682822	[A/G]		--	INTERGENIC
7983	CakSNP7983	*Kabuli*	Ca_Kabuli_Chr05	18682827	[C/G]		--	INTERGENIC
7984	CakSNP7984	*Kabuli*	Ca_Kabuli_Chr05	18682830	[G/A]		--	INTERGENIC
7985	CakSNP7985	*Kabuli*	Ca_Kabuli_Chr05	18684334	[C/T]		--	INTERGENIC
7986	CakSNP7986	*Kabuli*	Ca_Kabuli_Chr05	18971085	[G/A]	Ca_24492	Peptidase S8, subtilisin-related	INTRON
7987	CakSNP7987	*Kabuli*	Ca_Kabuli_Chr05	18971088	[C/A]	Ca_24492	Peptidase S8, subtilisin-related	INTRON
7988	CakSNP7988	*Kabuli*	Ca_Kabuli_Chr05	19421820	[C/T]	Ca_23514	Peptidase C48, SUMO/Sentrin/Ubl1	NON_SYNONYMOUS_CODING
7989	CakSNP7989	*Kabuli*	Ca_Kabuli_Chr05	19421868	[C/T]	Ca_23514	Peptidase C48, SUMO/Sentrin/Ubl1	NON_SYNONYMOUS_CODING
7990	CakSNP7990	*Kabuli*	Ca_Kabuli_Chr05	19423387	[G/A]		--	INTERGENIC
7991	CakSNP7991	*Kabuli*	Ca_Kabuli_Chr05	19423357	[C/T]		--	INTERGENIC

S.N.	SNP IDs	Chickpea cultivars	Chromosomes	Physical positions (bp)	SNPs	Gene accession IDs	Putative functions	Sequence components of genome
7992	CakSNP7992	*Kabuli*	Ca_Kabuli_Chr05	19828124	[G/A]		--	INTERGENIC
7993	CakSNP7993	*Kabuli*	Ca_Kabuli_Chr05	19828151	[C/A]		--	INTERGENIC
7994	CakSNP7994	*Kabuli*	Ca_Kabuli_Chr05	19828163	[C/T]		--	INTERGENIC
7995	CakSNP7995	*Kabuli*	Ca_Kabuli_Chr05	19828166	[C/T]		--	INTERGENIC
7996	CakSNP7996	*Kabuli*	Ca_Kabuli_Chr05	19828167	[A/C]		--	INTERGENIC
7997	CakSNP7997	*Kabuli*	Ca_Kabuli_Chr05	19828226	[G/A]		--	INTERGENIC
7998	CakSNP7998	*Kabuli*	Ca_Kabuli_Chr05	19828190	[T/C]		--	INTERGENIC
7999	CakSNP7999	*Kabuli*	Ca_Kabuli_Chr05	19828184	[C/A]		--	INTERGENIC
8000	CakSNP8000	*Kabuli*	Ca_Kabuli_Chr05	19828172	[T/G]		--	INTERGENIC
8001	CakSNP8001	*Kabuli*	Ca_Kabuli_Chr05	19828144	[A/G]		--	INTERGENIC

S.N.	SNP IDs	Chickpea cultivars	Chromosomes	Physical positions (bp)	SNPs	Gene accession IDs	Putative functions	Sequence components of genome
8002	CakSNP8002	*Kabuli*	Ca_Kabuli_Chr05	19884132	[C/T]	Ca_22815	Vps16, C-terminal	INTRON
8003	CakSNP8003	*Kabuli*	Ca_Kabuli_Chr05	19884124	[A/G]	Ca_22815	Vps16, C-terminal	INTRON
8004	CakSNP8004	*Kabuli*	Ca_Kabuli_Chr05	20008545	[C/T]	Ca_22810	Sucrose synthase	SYNONYMOUS_CODING
8005	CakSNP8005	*Kabuli*	Ca_Kabuli_Chr05	20031711	[A/G]	Ca_22809	Glycosyl transferase, family 48	SYNONYMOUS_CODING
8006	CakSNP8006	*Kabuli*	Ca_Kabuli_Chr05	20354352	[T/C]		--	INTERGENIC
8007	CakSNP8007	*Kabuli*	Ca_Kabuli_Chr05	20359674	[G/A]	Ca_18204	Ribosomal protein L14b/L23e	SYNONYMOUS_CODING
8008	CakSNP8008	*Kabuli*	Ca_Kabuli_Chr05	20460857	[G/A]	Ca_18208	Tetratricopeptide TPR-1	SYNONYMOUS_CODING
8009	CakSNP8009	*Kabuli*	Ca_Kabuli_Chr05	20460859	[T/G]	Ca_18208	Tetratricopeptide TPR-1	NON_SYNONYMOUS_CODING
8010	CakSNP8010	*Kabuli*	Ca_Kabuli_Chr05	20546402	[T/C]		--	INTERGENIC
8011	CakSNP8011	*Kabuli*	Ca_Kabuli_Chr05	20546385	[T/G]		--	INTERGENIC

S.N.	SNP IDs	Chickpea cultivars	Chromosomes	Physical positions (bp)	SNPs	Gene accession IDs	Putative functions	Sequence components of genome
8012	CakSNP8012	*Kabuli*	Ca_Kabuli_Chr05	20644984	[C/A]	Ca_18219	Amidase	NON_SYNONYMOUS_CODING
8013	CakSNP8013	*Kabuli*	Ca_Kabuli_Chr05	20834519	[A/G]	Ca_18226	Domain of unknown function DUF231, plant	INTRON
8014	CakSNP8014	*Kabuli*	Ca_Kabuli_Chr05	21163371	[G/A]	Ca_18235	Pentatricopeptide repeat	INTRON
8015	CakSNP8015	*Kabuli*	Ca_Kabuli_Chr05	21385948	[T/C]	Ca_18243	Signal transduction response regulator, receiver domain	SYNONYMOUS_CODING
8016	CakSNP8016	*Kabuli*	Ca_Kabuli_Chr05	21495583	[C/A]		--	INTERGENIC
8017	CakSNP8017	*Kabuli*	Ca_Kabuli_Chr05	21495631	[C/T]		--	INTERGENIC
8018	CakSNP8018	*Kabuli*	Ca_Kabuli_Chr05	21495672	[G/A]		--	INTERGENIC
8019	CakSNP8019	*Kabuli*	Ca_Kabuli_Chr05	21495707	[A/G]		--	INTERGENIC
8020	CakSNP8020	*Kabuli*	Ca_Kabuli_Chr05	21495679	[G/A]		--	INTERGENIC
8021	CakSNP8021	*Kabuli*	Ca_Kabuli_Chr05	21495674	[G/A]		--	INTERGENIC

S.N.	SNP IDs	Chickpea cultivars	Chromosomes	Physical positions (bp)	SNPs	Gene accession IDs	Putative functions	Sequence components of genome
8022	CakSNP8022	*Kabuli*	Ca_Kabuli_Chr05	21769327	[G/A]		--	INTERGENIC
8023	CakSNP8023	*Kabuli*	Ca_Kabuli_Chr05	21769419	[C/G]		--	INTERGENIC
8024	CakSNP8024	*Kabuli*	Ca_Kabuli_Chr05	21769794	[A/C]	Ca_24459	Pyridoxal phosphate-dependent enzyme, beta subunit	INTRON
8025	CakSNP8025	*Kabuli*	Ca_Kabuli_Chr05	22027215	[T/C]	Ca_21166	Pyridoxal phosphate-dependent enzyme, beta subunit	INTRON
8026	CakSNP8026	*Kabuli*	Ca_Kabuli_Chr05	22027257	[G/T]	Ca_21166	Pyridoxal phosphate-dependent enzyme, beta subunit	INTRON
8027	CakSNP8027	*Kabuli*	Ca_Kabuli_Chr05	22027265	[A/T]	Ca_21166	Pyridoxal phosphate-dependent enzyme, beta subunit	INTRON
8028	CakSNP8028	*Kabuli*	Ca_Kabuli_Chr05	22029684	[C/A]	Ca_21166	Pyridoxal phosphate-dependent enzyme, beta subunit	INTRON
8029	CakSNP8029	*Kabuli*	Ca_Kabuli_Chr05	22029686	[G/A]	Ca_21166	Pyridoxal phosphate-dependent enzyme, beta subunit	INTRON
8030	CakSNP8030	*Kabuli*	Ca_Kabuli_Chr05	22029761	[C/T]	Ca_21166	Pyridoxal phosphate-dependent enzyme, beta subunit	INTRON
8031	CakSNP8031	*Kabuli*	Ca_Kabuli_Chr05	22065518	[C/T]	Ca_21161	--	INTRON

S.N.	SNP IDs	Chickpea cultivars	Chromosomes	Physical positions (bp)	SNPs	Gene accession IDs	Putative functions	Sequence components of genome
8032	CakSNP8032	*Kabuli*	Ca_Kabuli_Chr05	22065859	[G/A]	Ca_21161	--	SYNONYMOUS_CODING
8033	CakSNP8033	*Kabuli*	Ca_Kabuli_Chr05	22066034	[A/T]	Ca_21161	--	INTRON
8034	CakSNP8034	*Kabuli*	Ca_Kabuli_Chr05	22116961	[T/G]		--	INTERGENIC
8035	CakSNP8035	*Kabuli*	Ca_Kabuli_Chr05	22188341	[C/T]		--	INTERGENIC
8036	CakSNP8036	*Kabuli*	Ca_Kabuli_Chr05	22188318	[C/T]		--	INTERGENIC
8037	CakSNP8037	*Kabuli*	Ca_Kabuli_Chr05	22296731	[G/A]	Ca_21151	Ribosome 60S biogenesis N-terminal	SYNONYMOUS_CODING
8038	CakSNP8038	*Kabuli*	Ca_Kabuli_Chr05	22296728	[G/T]	Ca_21151	Ribosome 60S biogenesis N-terminal	SYNONYMOUS_CODING
8039	CakSNP8039	*Kabuli*	Ca_Kabuli_Chr05	22302350	[A/C]	Ca_21151	Ribosome 60S biogenesis N-terminal	SYNONYMOUS_CODING
8040	CakSNP8040	*Kabuli*	Ca_Kabuli_Chr05	22344868	[A/G]	Ca_21149	--	SYNONYMOUS_CODING
8041	CakSNP8041	*Kabuli*	Ca_Kabuli_Chr05	22445954	[T/C]	Ca_21142	--	INTRON

S.N.	SNP IDs	Chickpea cultivars	Chromosomes	Physical positions (bp)	SNPs	Gene accession IDs	Putative functions	Sequence components of genome
8042	CakSNP8042	*Kabuli*	Ca_Kabuli_Chr05	22679380	[C/A]		--	INTERGENIC
8043	CakSNP8043	*Kabuli*	Ca_Kabuli_Chr05	23039312	[C/T]	Ca_21971	Lipid-binding serum glycoprotein, C-terminal	SYNONYMOUS_CODING
8044	CakSNP8044	*Kabuli*	Ca_Kabuli_Chr05	23049683	[C/A]		--	INTERGENIC
8045	CakSNP8045	*Kabuli*	Ca_Kabuli_Chr05	23157899	[A/G]	Ca_21977	Zinc finger, PHD-type	INTRON
8046	CakSNP8046	*Kabuli*	Ca_Kabuli_Chr05	23161492	[A/T]	Ca_21977	Zinc finger, PHD-type	SYNONYMOUS_CODING
8047	CakSNP8047	*Kabuli*	Ca_Kabuli_Chr05	23261964	[A/G]		--	INTERGENIC
8048	CakSNP8048	*Kabuli*	Ca_Kabuli_Chr05	23262114	[C/A]		--	INTERGENIC
8049	CakSNP8049	*Kabuli*	Ca_Kabuli_Chr05	23262264	[C/A]		--	INTERGENIC
8050	CakSNP8050	*Kabuli*	Ca_Kabuli_Chr05	23323538	[G/A]		--	INTERGENIC
8051	CakSNP8051	*Kabuli*	Ca_Kabuli_Chr05	23323520	[C/A]		--	INTERGENIC

S.N.	SNP IDs	Chickpea cultivars	Chromosomes	Physical positions (bp)	SNPs	Gene accession IDs	Putative functions	Sequence components of genome
8052	CakSNP8052	*Kabuli*	Ca_Kabuli_Chr05	23323514	[C/A]		--	INTERGENIC
8053	CakSNP8053	*Kabuli*	Ca_Kabuli_Chr05	23323479	[A/T]		--	INTERGENIC
8054	CakSNP8054	*Kabuli*	Ca_Kabuli_Chr05	23323478	[A/T]		--	INTERGENIC
8055	CakSNP8055	*Kabuli*	Ca_Kabuli_Chr05	23440293	[C/T]	Ca_17699	HEAT	SYNONYMOUS_CODING
8056	CakSNP8056	*Kabuli*	Ca_Kabuli_Chr05	23449048	[T/C]		--	INTERGENIC
8057	CakSNP8057	*Kabuli*	Ca_Kabuli_Chr05	23449134	[A/C]		--	INTERGENIC
8058	CakSNP8058	*Kabuli*	Ca_Kabuli_Chr05	23449127	[C/A]		--	INTERGENIC
8059	CakSNP8059	*Kabuli*	Ca_Kabuli_Chr05	23562595	[G/A]	Ca_17690	--	NON_SYNONYMOUS_CODING
8060	CakSNP8060	*Kabuli*	Ca_Kabuli_Chr05	23562827	[G/A]	Ca_17690	--	INTRON
8061	CakSNP8061	*Kabuli*	Ca_Kabuli_Chr05	23674814	[C/G]		--	INTERGENIC

S.N.	SNP IDs	Chickpea cultivars	Chromosomes	Physical positions (bp)	SNPs	Gene accession IDs	Putative functions	Sequence components of genome
8062	CakSNP8062	*Kabuli*	Ca_Kabuli_Chr05	23674815	[C/T]		--	INTERGENIC
8063	CakSNP8063	*Kabuli*	Ca_Kabuli_Chr05	23674845	[G/C]		--	INTERGENIC
8064	CakSNP8064	*Kabuli*	Ca_Kabuli_Chr05	23783140	[T/C]	Ca_17673	Uridine kinase	INTRON
8065	CakSNP8065	*Kabuli*	Ca_Kabuli_Chr05	23783156	[C/A]	Ca_17673	Uridine kinase	INTRON
8066	CakSNP8066	*Kabuli*	Ca_Kabuli_Chr05	23783157	[G/C]	Ca_17673	Uridine kinase	INTRON
8067	CakSNP8067	*Kabuli*	Ca_Kabuli_Chr05	23783188	[G/A]	Ca_17673	Uridine kinase	INTRON
8068	CakSNP8068	*Kabuli*	Ca_Kabuli_Chr05	23783195	[C/G]	Ca_17673	Uridine kinase	INTRON
8069	CakSNP8069	*Kabuli*	Ca_Kabuli_Chr05	23783254	[A/G]	Ca_17673	Uridine kinase	INTRON
8070	CakSNP8070	*Kabuli*	Ca_Kabuli_Chr05	23783252	[T/A]	Ca_17673	Uridine kinase	INTRON
8071	CakSNP8071	*Kabuli*	Ca_Kabuli_Chr05	23884909	[C/T]	Ca_17668	Peptidase S8/S53, subtilisin/kexin/sedolisin	INTRON

S.N.	SNP IDs	Chickpea cultivars	Chromosomes	Physical positions (bp)	SNPs	Gene accession IDs	Putative functions	Sequence components of genome
8072	CakSNP8072	*Kabuli*	Ca_Kabuli_Chr05	23884936	[G/A]	Ca_17668	Peptidase S8/S53, subtilisin/kexin/sedolisin	INTRON
8073	CakSNP8073	*Kabuli*	Ca_Kabuli_Chr05	23930933	[C/T]		--	INTERGENIC
8074	CakSNP8074	*Kabuli*	Ca_Kabuli_Chr05	23930988	[G/A]		--	INTERGENIC
8075	CakSNP8075	*Kabuli*	Ca_Kabuli_Chr05	23932354	[C/T]	Ca_17665	WD40 repeat	INTRON
8076	CakSNP8076	*Kabuli*	Ca_Kabuli_Chr05	23934198	[G/T]	Ca_17665	WD40 repeat	SYNONYMOUS_CODING
8077	CakSNP8077	*Kabuli*	Ca_Kabuli_Chr05	24059182	[C/G]		--	INTERGENIC
8078	CakSNP8078	*Kabuli*	Ca_Kabuli_Chr05	24090533	[C/T]	Ca_17657	SANT domain, DNA binding	NON_SYNONYMOUS_CODING
8079	CakSNP8079	*Kabuli*	Ca_Kabuli_Chr05	24090523	[A/C]	Ca_17657	SANT domain, DNA binding	NON_SYNONYMOUS_CODING
8080	CakSNP8080	*Kabuli*	Ca_Kabuli_Chr05	24090515	[A/C]	Ca_17657	SANT domain, DNA binding	NON_SYNONYMOUS_CODING
8081	CakSNP8081	*Kabuli*	Ca_Kabuli_Chr05	24090749	[A/C]	Ca_17657	SANT domain, DNA binding	NON_SYNONYMOUS_CODING

S.N.	SNP IDs	Chickpea cultivars	Chromosomes	Physical positions (bp)	SNPs	Gene accession IDs	Putative functions	Sequence components of genome
8082	CakSNP8082	*Kabuli*	Ca_Kabuli_Chr05	24097823	[T/C]	Ca_17656	Protein of unknown function DUF3437	NON_SYNONYMOUS_CODING
8083	CakSNP8083	*Kabuli*	Ca_Kabuli_Chr05	24153061	[G/C]	Ca_17653	Protein of unknown function DUF3437	NON_SYNONYMOUS_CODING
8084	CakSNP8084	*Kabuli*	Ca_Kabuli_Chr05	24153205	[T/C]	Ca_17653	Protein of unknown function DUF3437	NON_SYNONYMOUS_CODING
8085	CakSNP8085	*Kabuli*	Ca_Kabuli_Chr05	24153273	[T/C]	Ca_17653	Protein of unknown function DUF3437	NON_SYNONYMOUS_CODING
8086	CakSNP8086	*Kabuli*	Ca_Kabuli_Chr05	24153202	[A/C]	Ca_17653	Protein of unknown function DUF3437	NON_SYNONYMOUS_CODING
8087	CakSNP8087	*Kabuli*	Ca_Kabuli_Chr05	24153199	[C/T]	Ca_17653	Protein of unknown function DUF3437	NON_SYNONYMOUS_CODING
8088	CakSNP8088	*Kabuli*	Ca_Kabuli_Chr05	24166103	[C/T]	Ca_17652	Protein of unknown function DUF3437	INTRON
8089	CakSNP8089	*Kabuli*	Ca_Kabuli_Chr05	24166095	[G/A]	Ca_17652	Protein of unknown function DUF3437	INTRON
8090	CakSNP8090	*Kabuli*	Ca_Kabuli_Chr05	24166093	[A/G]	Ca_17652	Protein of unknown function DUF3437	INTRON
8091	CakSNP8091	*Kabuli*	Ca_Kabuli_Chr05	24166046	[G/A]	Ca_17652	Protein of unknown function DUF3437	INTRON

S.N.	SNP IDs	Chickpea cultivars	Chromosomes	Physical positions (bp)	SNPs	Gene accession IDs	Putative functions	Sequence components of genome
8092	CakSNP8092	*Kabuli*	Ca_Kabuli_Chr05	24166047	[C/T]	Ca_17652	Protein of unknown function DUF3437	INTRON
8093	CakSNP8093	*Kabuli*	Ca_Kabuli_Chr05	24166082	[G/A]	Ca_17652	Protein of unknown function DUF3437	INTRON
8094	CakSNP8094	*Kabuli*	Ca_Kabuli_Chr05	24181232	[C/A]	Ca_17652	Protein of unknown function DUF3437	INTRON
8095	CakSNP8095	*Kabuli*	Ca_Kabuli_Chr05	24181228	[C/A]	Ca_17652	Protein of unknown function DUF3437	INTRON
8096	CakSNP8096	*Kabuli*	Ca_Kabuli_Chr05	24245312	[C/A]	Ca_15567	Protein of unknown function DUF3437	INTRON
8097	CakSNP8097	*Kabuli*	Ca_Kabuli_Chr05	24245308	[C/A]	Ca_15567	Protein of unknown function DUF3437	INTRON
8098	CakSNP8098	*Kabuli*	Ca_Kabuli_Chr05	24346350	[A/T]	Ca_15574	CCAAT-binding transcription factor, subunit B	INTRON
8099	CakSNP8099	*Kabuli*	Ca_Kabuli_Chr05	24576960	[C/T]		--	INTERGENIC
8100	CakSNP8100	*Kabuli*	Ca_Kabuli_Chr05	24576970	[A/G]		--	INTERGENIC
8101	CakSNP8101	*Kabuli*	Ca_Kabuli_Chr05	24576993	[T/G]		--	INTERGENIC

S.N.	SNP IDs	Chickpea cultivars	Chromosomes	Physical positions (bp)	SNPs	Gene accession IDs	Putative functions	Sequence components of genome
8102	CakSNP8102	*Kabuli*	Ca_Kabuli_Chr05	24596227	[T/C]	Ca_15595	Protein kinase, catalytic domain	SYNONYMOUS_CODING
8103	CakSNP8103	*Kabuli*	Ca_Kabuli_Chr05	24596257	[C/T]	Ca_15595	Protein kinase, catalytic domain	SYNONYMOUS_CODING
8104	CakSNP8104	*Kabuli*	Ca_Kabuli_Chr05	24596269	[G/A]	Ca_15595	Protein kinase, catalytic domain	SYNONYMOUS_CODING
8105	CakSNP8105	*Kabuli*	Ca_Kabuli_Chr05	24605438	[A/C]	Ca_15595	Protein kinase, catalytic domain	INTRON
8106	CakSNP8106	*Kabuli*	Ca_Kabuli_Chr05	24843015	[G/T]		--	INTERGENIC
8107	CakSNP8107	*Kabuli*	Ca_Kabuli_Chr05	25003171	[C/T]	Ca_15616	RNA-processing protein, HAT helix	SYNONYMOUS_CODING
8108	CakSNP8108	*Kabuli*	Ca_Kabuli_Chr05	25006110	[A/C]		--	INTERGENIC
8109	CakSNP8109	*Kabuli*	Ca_Kabuli_Chr05	25006123	[C/T]		--	INTERGENIC
8110	CakSNP8110	*Kabuli*	Ca_Kabuli_Chr05	25184089	[T/C]	Ca_15627	Smr protein/MutS2 C-terminal	INTRON
8111	CakSNP8111	*Kabuli*	Ca_Kabuli_Chr05	25184133	[G/A]	Ca_15627	Smr protein/MutS2 C-terminal	INTRON

S.N.	SNP IDs	Chickpea cultivars	Chromosomes	Physical positions (bp)	SNPs	Gene accession IDs	Putative functions	Sequence components of genome
8112	CakSNP8112	*Kabuli*	Ca_Kabuli_Chr05	25399115	[T/G]	Ca_09042	Zinc finger, CCCH-type	SYNONYMOUS_CODING
8113	CakSNP8113	*Kabuli*	Ca_Kabuli_Chr05	25400604	[A/G]	Ca_09042	Zinc finger, CCCH-type	NON_SYNONYMOUS_CODING
8114	CakSNP8114	*Kabuli*	Ca_Kabuli_Chr05	25474110	[A/C]	Ca_09038	Pyridine nucleotide-disulphide oxidoreductase, NAD-binding region	INTRON
8115	CakSNP8115	*Kabuli*	Ca_Kabuli_Chr05	25474105	[C/T]	Ca_09038	Pyridine nucleotide-disulphide oxidoreductase, NAD-binding region	INTRON
8116	CakSNP8116	*Kabuli*	Ca_Kabuli_Chr05	25474093	[G/C]	Ca_09038	Pyridine nucleotide-disulphide oxidoreductase, NAD-binding region	INTRON
8117	CakSNP8117	*Kabuli*	Ca_Kabuli_Chr05	25474089	[C/A]	Ca_09038	Pyridine nucleotide-disulphide oxidoreductase, NAD-binding region	INTRON
8118	CakSNP8118	*Kabuli*	Ca_Kabuli_Chr05	25597077	[A/G]	Ca_09030	Asparagine synthase	SYNONYMOUS_CODING
8119	CakSNP8119	*Kabuli*	Ca_Kabuli_Chr05	25678975	[G/T]		--	INTERGENIC
8120	CakSNP8120	*Kabuli*	Ca_Kabuli_Chr05	25697487	[C/G]	Ca_09026	Ribosomal protein S21	NON_SYNONYMOUS_CODING
8121	CakSNP8121	*Kabuli*	Ca_Kabuli_Chr05	25805020	[A/G]	Ca_09020	Peptidase C48, SUMO/Sentrin/Ubl1	INTRON

S.N.	SNP IDs	Chickpea cultivars	Chromosomes	Physical positions (bp)	SNPs	Gene accession IDs	Putative functions	Sequence components of genome
8122	CakSNP8122	*Kabuli*	Ca_Kabuli_Chr05	26025243	[C/T]	Ca_09011	Tetratricopeptide TPR-1	SYNONYMOUS_CODING
8123	CakSNP8123	*Kabuli*	Ca_Kabuli_Chr05	26025282	[A/G]	Ca_09011	Tetratricopeptide TPR-1	SYNONYMOUS_CODING
8124	CakSNP8124	*Kabuli*	Ca_Kabuli_Chr05	26025356	[A/T]	Ca_09011	Tetratricopeptide TPR-1	NON_SYNONYMOUS_CODING
8125	CakSNP8125	*Kabuli*	Ca_Kabuli_Chr05	26027313	[C/A]	Ca_09010	Protein kinase, catalytic domain	NON_SYNONYMOUS_CODING
8126	CakSNP8126	*Kabuli*	Ca_Kabuli_Chr05	26095084	[T/G]	Ca_09007	Ubiquitin/SUMO-activating enzyme E1	INTRON
8127	CakSNP8127	*Kabuli*	Ca_Kabuli_Chr05	26124240	[T/C]	Ca_09005	--	INTRON
8128	CakSNP8128	*Kabuli*	Ca_Kabuli_Chr05	26191896	[T/C]	Ca_09001	Glycoside hydrolase, family 17	SYNONYMOUS_CODING
8129	CakSNP8129	*Kabuli*	Ca_Kabuli_Chr05	26194579	[A/G]	Ca_09001	Glycoside hydrolase, family 17	NON_SYNONYMOUS_CODING
8130	CakSNP8130	*Kabuli*	Ca_Kabuli_Chr05	26238764	[T/G]		--	INTERGENIC
8131	CakSNP8131	*Kabuli*	Ca_Kabuli_Chr05	26238752	[A/T]		--	INTERGENIC

S.N.	SNP IDs	Chickpea cultivars	Chromosomes	Physical positions (bp)	SNPs	Gene accession IDs	Putative functions	Sequence components of genome
8132	CakSNP8132	*Kabuli*	Ca_Kabuli_Chr05	26238748	[A/T]		--	INTERGENIC
8133	CakSNP8133	*Kabuli*	Ca_Kabuli_Chr05	26238741	[G/T]		--	INTERGENIC
8134	CakSNP8134	*Kabuli*	Ca_Kabuli_Chr05	26238739	[C/T]		--	INTERGENIC
8135	CakSNP8135	*Kabuli*	Ca_Kabuli_Chr05	26384636	[C/G]		--	INTERGENIC
8136	CakSNP8136	*Kabuli*	Ca_Kabuli_Chr05	26467869	[A/C]		--	INTERGENIC
8137	CakSNP8137	*Kabuli*	Ca_Kabuli_Chr05	26474910	[A/C]	Ca_08972	--	INTRON
8138	CakSNP8138	*Kabuli*	Ca_Kabuli_Chr05	26475067	[T/C]	Ca_08972	--	INTRON
8139	CakSNP8139	*Kabuli*	Ca_Kabuli_Chr05	26477751	[A/T]		--	INTERGENIC
8140	CakSNP8140	*Kabuli*	Ca_Kabuli_Chr05	26494631	[A/G]	Ca_08970	Aminotransferase class-III	SYNONYMOUS_CODING
8141	CakSNP8141	*Kabuli*	Ca_Kabuli_Chr05	26494778	[T/C]	Ca_08970	Aminotransferase class-III	INTRON

S.N.	SNP IDs	Chickpea cultivars	Chromosomes	Physical positions (bp)	SNPs	Gene accession IDs	Putative functions	Sequence components of genome
8142	CakSNP8142	*Kabuli*	Ca_Kabuli_Chr05	26494769	[T/C]	Ca_08970	Aminotransferase class-III	INTRON
8143	CakSNP8143	*Kabuli*	Ca_Kabuli_Chr05	26496197	[A/G]	Ca_08970	Aminotransferase class-III	NON_SYNONYMOUS_CODING
8144	CakSNP8144	*Kabuli*	Ca_Kabuli_Chr05	26558406	[A/G]	Ca_08966	--	INTRON
8145	CakSNP8145	*Kabuli*	Ca_Kabuli_Chr05	26558366	[A/G]	Ca_08966	--	INTRON
8146	CakSNP8146	*Kabuli*	Ca_Kabuli_Chr05	26558363	[C/G]	Ca_08966	--	INTRON
8147	CakSNP8147	*Kabuli*	Ca_Kabuli_Chr05	26558357	[G/A]	Ca_08966	--	INTRON
8148	CakSNP8148	*Kabuli*	Ca_Kabuli_Chr05	26558356	[G/T]	Ca_08966	--	INTRON
8149	CakSNP8149	*Kabuli*	Ca_Kabuli_Chr05	26569400	[G/C]	Ca_08965	--	NON_SYNONYMOUS_CODING
8150	CakSNP8150	*Kabuli*	Ca_Kabuli_Chr05	26609014	[T/C]	Ca_08962	Helicase, C-terminal	INTRON
8151	CakSNP8151	*Kabuli*	Ca_Kabuli_Chr05	26608993	[A/G]	Ca_08962	Helicase, C-terminal	INTRON

S.N.	SNP IDs	Chickpea cultivars	Chromosomes	Physical positions (bp)	SNPs	Gene accession IDs	Putative functions	Sequence components of genome
8152	CakSNP8152	*Kabuli*	Ca_Kabuli_Chr05	26621093	[C/G]		--	INTERGENIC
8153	CakSNP8153	*Kabuli*	Ca_Kabuli_Chr05	26626861	[A/G]	Ca_08960	Aminoacyl-tRNA synthetase, class I, conserved site	NON_SYNONYMOUS_CODING
8154	CakSNP8154	*Kabuli*	Ca_Kabuli_Chr05	26626855	[T/G]	Ca_08960	Aminoacyl-tRNA synthetase, class I, conserved site	NON_SYNONYMOUS_CODING
8155	CakSNP8155	*Kabuli*	Ca_Kabuli_Chr05	26638041	[G/A]	Ca_08959	Phosphatidate cytidylyltransferase	INTRON
8156	CakSNP8156	*Kabuli*	Ca_Kabuli_Chr05	26638042	[C/T]	Ca_08959	Phosphatidate cytidylyltransferase	INTRON
8157	CakSNP8157	*Kabuli*	Ca_Kabuli_Chr05	26638080	[A/G]	Ca_08959	Phosphatidate cytidylyltransferase	INTRON
8158	CakSNP8158	*Kabuli*	Ca_Kabuli_Chr05	26638126	[C/T]	Ca_08959	Phosphatidate cytidylyltransferase	INTRON
8159	CakSNP8159	*Kabuli*	Ca_Kabuli_Chr05	26638118	[C/T]	Ca_08959	Phosphatidate cytidylyltransferase	INTRON
8160	CakSNP8160	*Kabuli*	Ca_Kabuli_Chr05	26679836	[C/T]	Ca_08952	Protein kinase, catalytic domain	NON_SYNONYMOUS_CODING
8161	CakSNP8161	*Kabuli*	Ca_Kabuli_Chr05	26696390	[G/A]		--	INTERGENIC

S.N.	SNP IDs	Chickpea cultivars	Chromosomes	Physical positions (bp)	SNPs	Gene accession IDs	Putative functions	Sequence components of genome
8162	CakSNP8162	*Kabuli*	Ca_Kabuli_Chr05	26696412	[C/A]		--	INTERGENIC
8163	CakSNP8163	*Kabuli*	Ca_Kabuli_Chr05	26696455	[G/A]		--	INTERGENIC
8164	CakSNP8164	*Kabuli*	Ca_Kabuli_Chr05	26696547	[C/T]		--	INTERGENIC
8165	CakSNP8165	*Kabuli*	Ca_Kabuli_Chr05	26696533	[C/T]		--	INTERGENIC
8166	CakSNP8166	*Kabuli*	Ca_Kabuli_Chr05	26696494	[G/A]		--	INTERGENIC
8167	CakSNP8167	*Kabuli*	Ca_Kabuli_Chr05	26696578	[G/A]		--	INTERGENIC
8168	CakSNP8168	*Kabuli*	Ca_Kabuli_Chr05	26696602	[C/T]		--	INTERGENIC
8169	CakSNP8169	*Kabuli*	Ca_Kabuli_Chr05	26696620	[C/T]		--	INTERGENIC
8170	CakSNP8170	*Kabuli*	Ca_Kabuli_Chr05	26696624	[G/A]		--	INTERGENIC
8171	CakSNP8171	*Kabuli*	Ca_Kabuli_Chr05	26785140	[G/A]		--	INTERGENIC

S.N.	SNP IDs	Chickpea cultivars	Chromosomes	Physical positions (bp)	SNPs	Gene accession IDs	Putative functions	Sequence components of genome
8172	CakSNP8172	*Kabuli*	Ca_Kabuli_Chr05	26785226	[C/A]		--	INTERGENIC
8173	CakSNP8173	*Kabuli*	Ca_Kabuli_Chr05	26997335	[T/G]		--	INTERGENIC
8174	CakSNP8174	*Kabuli*	Ca_Kabuli_Chr05	26999793	[C/T]		--	INTERGENIC
8175	CakSNP8175	*Kabuli*	Ca_Kabuli_Chr05	27000127	[C/G]		--	INTERGENIC
8176	CakSNP8176	*Kabuli*	Ca_Kabuli_Chr05	27000104	[C/A]		--	INTERGENIC
8177	CakSNP8177	*Kabuli*	Ca_Kabuli_Chr05	27000278	[T/A]		--	INTERGENIC
8178	CakSNP8178	*Kabuli*	Ca_Kabuli_Chr05	27000280	[T/G]		--	INTERGENIC
8179	CakSNP8179	*Kabuli*	Ca_Kabuli_Chr05	27025766	[A/G]		--	INTERGENIC
8180	CakSNP8180	*Kabuli*	Ca_Kabuli_Chr05	27025752	[A/C]		--	INTERGENIC
8181	CakSNP8181	*Kabuli*	Ca_Kabuli_Chr05	27042679	[G/A]	Ca_08930	MYB-like	INTRON

S.N.	SNP IDs	Chickpea cultivars	Chromosomes	Physical positions (bp)	SNPs	Gene accession IDs	Putative functions	Sequence components of genome
8182	CakSNP8182	*Kabuli*	Ca_Kabuli_Chr05	27117953	[A/G]	Ca_08929	Basic-leucine zipper (bZIP) transcription factor	INTRON
8183	CakSNP8183	*Kabuli*	Ca_Kabuli_Chr05	27150550	[A/G]	Ca_08924	Transcriptional factor B3	INTRON
8184	CakSNP8184	*Kabuli*	Ca_Kabuli_Chr05	27230515	[T/A]	Ca_08918	RNA helicase, ATP-dependent, DEAD-box, conserved site	INTRON
8185	CakSNP8185	*Kabuli*	Ca_Kabuli_Chr05	27230514	[A/G]	Ca_08918	RNA helicase, ATP-dependent, DEAD-box, conserved site	INTRON
8186	CakSNP8186	*Kabuli*	Ca_Kabuli_Chr05	27231394	[T/C]	Ca_08918	RNA helicase, ATP-dependent, DEAD-box, conserved site	INTRON
8187	CakSNP8187	*Kabuli*	Ca_Kabuli_Chr05	27231641	[T/A]	Ca_08918	RNA helicase, ATP-dependent, DEAD-box, conserved site	SYNONYMOUS_CODING
8188	CakSNP8188	*Kabuli*	Ca_Kabuli_Chr05	27232304	[T/A]	Ca_08918	RNA helicase, ATP-dependent, DEAD-box, conserved site	SYNONYMOUS_CODING
8189	CakSNP8189	*Kabuli*	Ca_Kabuli_Chr05	27234806	[G/A]	Ca_08918	RNA helicase, ATP-dependent, DEAD-box, conserved site	SYNONYMOUS_CODING
8190	CakSNP8190	*Kabuli*	Ca_Kabuli_Chr05	27235002	[T/G]		--	INTERGENIC
8191	CakSNP8191	*Kabuli*	Ca_Kabuli_Chr05	27328552	[G/A]	Ca_08909	DNA primase, small subunit	INTRON

S.N.	SNP IDs	Chickpea cultivars	Chromosomes	Physical positions (bp)	SNPs	Gene accession IDs	Putative functions	Sequence components of genome
8192	CakSNP8192	*Kabuli*	Ca_Kabuli_Chr05	27328737	[G/T]	Ca_08909	DNA primase, small subunit	INTRON
8193	CakSNP8193	*Kabuli*	Ca_Kabuli_Chr05	27334056	[A/C]		--	INTERGENIC
8194	CakSNP8194	*Kabuli*	Ca_Kabuli_Chr05	27334057	[G/C]		--	INTERGENIC
8195	CakSNP8195	*Kabuli*	Ca_Kabuli_Chr05	27334061	[G/C]		--	INTERGENIC
8196	CakSNP8196	*Kabuli*	Ca_Kabuli_Chr05	27349210	[T/A]	Ca_08908	ATPase, AAA+ type, core	SYNONYMOUS_CODING
8197	CakSNP8197	*Kabuli*	Ca_Kabuli_Chr05	27349237	[C/T]	Ca_08908	ATPase, AAA+ type, core	INTRON
8198	CakSNP8198	*Kabuli*	Ca_Kabuli_Chr05	27361515	[C/T]	Ca_08907	BTB/POZ-like	SYNONYMOUS_CODING
8199	CakSNP8199	*Kabuli*	Ca_Kabuli_Chr05	27361579	[T/G]	Ca_08907	BTB/POZ-like	SYNONYMOUS_CODING
8200	CakSNP8200	*Kabuli*	Ca_Kabuli_Chr05	27362517	[C/G]	Ca_08907	BTB/POZ-like	NON_SYNONYMOUS_CODING
8201	CakSNP8201	*Kabuli*	Ca_Kabuli_Chr05	27362570	[G/T]	Ca_08907	BTB/POZ-like	NON_SYNONYMOUS_CODING

S.N.	SNP IDs	Chickpea cultivars	Chromosomes	Physical positions (bp)	SNPs	Gene accession IDs	Putative functions	Sequence components of genome
8202	CakSNP8202	*Kabuli*	Ca_Kabuli_Chr05	27362573	[A/G]	Ca_08907	BTB/POZ-like	NON_SYNONYMOUS_CODING
8203	CakSNP8203	*Kabuli*	Ca_Kabuli_Chr05	27362584	[T/G]	Ca_08907	BTB/POZ-like	SYNONYMOUS_CODING
8204	CakSNP8204	*Kabuli*	Ca_Kabuli_Chr05	27452484	[C/G]	Ca_08906	Protein kinase, catalytic domain	SYNONYMOUS_CODING
8205	CakSNP8205	*Kabuli*	Ca_Kabuli_Chr05	27516054	[T/A]		--	INTERGENIC
8206	CakSNP8206	*Kabuli*	Ca_Kabuli_Chr05	27552106	[A/C]	Ca_08899	--	INTRON
8207	CakSNP8207	*Kabuli*	Ca_Kabuli_Chr05	27555637	[T/G]	Ca_08899	--	SYNONYMOUS_CODING
8208	CakSNP8208	*Kabuli*	Ca_Kabuli_Chr05	27568101	[T/G]	Ca_08898	--	NON_SYNONYMOUS_CODING
8209	CakSNP8209	*Kabuli*	Ca_Kabuli_Chr05	27568095	[C/A]	Ca_08898	--	NON_SYNONYMOUS_CODING
8210	CakSNP8210	*Kabuli*	Ca_Kabuli_Chr05	27568064	[A/C]	Ca_08898	--	NON_SYNONYMOUS_CODING
8211	CakSNP8211	*Kabuli*	Ca_Kabuli_Chr05	27568059	[C/T]	Ca_08898	--	NON_SYNONYMOUS_CODING

S.N.	SNP IDs	Chickpea cultivars	Chromosomes	Physical positions (bp)	SNPs	Gene accession IDs	Putative functions	Sequence components of genome
8212	CakSNP8212	*Kabuli*	Ca_Kabuli_Chr05	27568028	[A/T]	Ca_08898	--	NON_SYNONYMOUS_CODING
8213	CakSNP8213	*Kabuli*	Ca_Kabuli_Chr05	27568033	[G/A]	Ca_08898	--	SYNONYMOUS_CODING
8214	CakSNP8214	*Kabuli*	Ca_Kabuli_Chr05	27568082	[G/A]	Ca_08898	--	NON_SYNONYMOUS_CODING
8215	CakSNP8215	*Kabuli*	Ca_Kabuli_Chr05	27568097	[G/A]	Ca_08898	--	NON_SYNONYMOUS_CODING
8216	CakSNP8216	*Kabuli*	Ca_Kabuli_Chr05	27568098	[C/T]	Ca_08898	--	NON_SYNONYMOUS_CODING
8217	CakSNP8217	*Kabuli*	Ca_Kabuli_Chr05	27568118	[A/G]	Ca_08898	--	NON_SYNONYMOUS_CODING
8218	CakSNP8218	*Kabuli*	Ca_Kabuli_Chr05	27568133	[G/T]	Ca_08898	--	NON_SYNONYMOUS_CODING
8219	CakSNP8219	*Kabuli*	Ca_Kabuli_Chr05	27568081	[G/C]	Ca_08898	--	SYNONYMOUS_CODING
8220	CakSNP8220	*Kabuli*	Ca_Kabuli_Chr05	27568107	[C/G]	Ca_08898	--	NON_SYNONYMOUS_CODING
8221	CakSNP8221	*Kabuli*	Ca_Kabuli_Chr05	27568156	[C/G]	Ca_08898	--	NON_SYNONYMOUS_CODING

S.N.	SNP IDs	Chickpea cultivars	Chromosomes	Physical positions (bp)	SNPs	Gene accession IDs	Putative functions	Sequence components of genome
8222	CakSNP8222	*Kabuli*	Ca_Kabuli_Chr05	27568141	[C/T]	Ca_08898	--	SYNONYMOUS_CODING
8223	CakSNP8223	*Kabuli*	Ca_Kabuli_Chr05	27568180	[C/T]	Ca_08898	--	SYNONYMOUS_CODING
8224	CakSNP8224	*Kabuli*	Ca_Kabuli_Chr05	27568176	[T/C]	Ca_08898	--	NON_SYNONYMOUS_CODING
8225	CakSNP8225	*Kabuli*	Ca_Kabuli_Chr05	27579347	[C/T]		--	INTERGENIC
8226	CakSNP8226	*Kabuli*	Ca_Kabuli_Chr05	27605037	[A/T]	Ca_08895	Reticulon	NON_SYNONYMOUS_CODING
8227	CakSNP8227	*Kabuli*	Ca_Kabuli_Chr05	27605063	[T/A]	Ca_08895	Reticulon	INTRON
8228	CakSNP8228	*Kabuli*	Ca_Kabuli_Chr05	27605081	[C/T]	Ca_08895	Reticulon	INTRON
8229	CakSNP8229	*Kabuli*	Ca_Kabuli_Chr05	27605095	[G/C]	Ca_08895	Reticulon	INTRON
8230	CakSNP8230	*Kabuli*	Ca_Kabuli_Chr05	27692816	[C/T]		--	INTERGENIC
8231	CakSNP8231	*Kabuli*	Ca_Kabuli_Chr05	27692818	[C/T]		--	INTERGENIC

S.N.	SNP IDs	Chickpea cultivars	Chromosomes	Physical positions (bp)	SNPs	Gene accession IDs	Putative functions	Sequence components of genome
8232	CakSNP8232	*Kabuli*	Ca_Kabuli_Chr05	27692822	[C/T]		--	INTERGENIC
8233	CakSNP8233	*Kabuli*	Ca_Kabuli_Chr05	27692823	[A/T]		--	INTERGENIC
8234	CakSNP8234	*Kabuli*	Ca_Kabuli_Chr05	27692831	[A/T]		--	INTERGENIC
8235	CakSNP8235	*Kabuli*	Ca_Kabuli_Chr05	27694205	[C/A]	Ca_08882	--	NON_SYNONYMOUS_CODING
8236	CakSNP8236	*Kabuli*	Ca_Kabuli_Chr05	27750155	[C/T]	Ca_08872	AUX/IAA protein	SYNONYMOUS_CODING
8237	CakSNP8237	*Kabuli*	Ca_Kabuli_Chr05	27786025	[T/C]	Ca_08871	--	INTRON
8238	CakSNP8238	*Kabuli*	Ca_Kabuli_Chr05	27838003	[T/C]		--	INTERGENIC
8239	CakSNP8239	*Kabuli*	Ca_Kabuli_Chr05	27850760	[T/C]	Ca_13432	Protein kinase, catalytic domain	SYNONYMOUS_CODING
8240	CakSNP8240	*Kabuli*	Ca_Kabuli_Chr05	27850735	[C/A]	Ca_13432	Protein kinase, catalytic domain	NON_SYNONYMOUS_CODING
8241	CakSNP8241	*Kabuli*	Ca_Kabuli_Chr05	27853444	[C/T]	Ca_13432	Protein kinase, catalytic domain	SYNONYMOUS_CODING

S.N.	SNP IDs	Chickpea cultivars	Chromosomes	Physical positions (bp)	SNPs	Gene accession IDs	Putative functions	Sequence components of genome
8242	CakSNP8242	*Kabuli*	Ca_Kabuli_Chr05	27872968	[C/T]	Ca_13430	ThiJ/PfpI	SYNONYMOUS_CODING
8243	CakSNP8243	*Kabuli*	Ca_Kabuli_Chr05	27873001	[T/C]	Ca_13430	ThiJ/PfpI	SYNONYMOUS_CODING
8244	CakSNP8244	*Kabuli*	Ca_Kabuli_Chr05	27874414	[C/A]	Ca_13430	ThiJ/PfpI	SYNONYMOUS_CODING
8245	CakSNP8245	*Kabuli*	Ca_Kabuli_Chr05	27936521	[A/G]		--	INTERGENIC
8246	CakSNP8246	*Kabuli*	Ca_Kabuli_Chr05	27936524	[C/T]		--	INTERGENIC
8247	CakSNP8247	*Kabuli*	Ca_Kabuli_Chr05	27939162	[G/A]		--	INTERGENIC
8248	CakSNP8248	*Kabuli*	Ca_Kabuli_Chr05	27939135	[G/C]		--	INTERGENIC
8249	CakSNP8249	*Kabuli*	Ca_Kabuli_Chr05	27939134	[A/T]		--	INTERGENIC
8250	CakSNP8250	*Kabuli*	Ca_Kabuli_Chr05	27939128	[A/G]		--	INTERGENIC
8251	CakSNP8251	*Kabuli*	Ca_Kabuli_Chr05	27974691	[G/T]	Ca_13421	ABC-1	INTRON

S.N.	SNP IDs	Chickpea cultivars	Chromosomes	Physical positions (bp)	SNPs	Gene accession IDs	Putative functions	Sequence components of genome
8252	CakSNP8252	*Kabuli*	Ca_Kabuli_Chr05	28016628	[C/T]	Ca_13415	Peptidyl-prolyl cis-trans isomerase, cyclophilin-type	INTRON
8253	CakSNP8253	*Kabuli*	Ca_Kabuli_Chr05	28016669	[A/G]	Ca_13415	Peptidyl-prolyl cis-trans isomerase, cyclophilin-type	INTRON
8254	CakSNP8254	*Kabuli*	Ca_Kabuli_Chr05	28071467	[A/G]		--	INTERGENIC
8255	CakSNP8255	*Kabuli*	Ca_Kabuli_Chr05	28116818	[A/G]	Ca_13409	--	NON_SYNONYMOUS_CODING
8256	CakSNP8256	*Kabuli*	Ca_Kabuli_Chr05	28116988	[A/T]		--	INTERGENIC
8257	CakSNP8257	*Kabuli*	Ca_Kabuli_Chr05	28117011	[C/T]		--	INTERGENIC
8258	CakSNP8258	*Kabuli*	Ca_Kabuli_Chr05	28164985	[G/C]	Ca_13404	Clathrin, heavy chain/VPS, 7-fold repeat	INTRON
8259	CakSNP8259	*Kabuli*	Ca_Kabuli_Chr05	28168910	[A/G]		--	INTERGENIC
8260	CakSNP8260	*Kabuli*	Ca_Kabuli_Chr05	28169475	[T/C]		--	INTERGENIC
8261	CakSNP8261	*Kabuli*	Ca_Kabuli_Chr05	28175039	[A/G]	Ca_13403	--	NON_SYNONYMOUS_CODING

S.N.	SNP IDs	Chickpea cultivars	Chromosomes	Physical positions (bp)	SNPs	Gene accession IDs	Putative functions	Sequence components of genome
8262	CakSNP8262	*Kabuli*	Ca_Kabuli_Chr05	28266046	[T/G]	Ca_13399	--	SYNONYMOUS_CODING
8263	CakSNP8263	*Kabuli*	Ca_Kabuli_Chr05	28271638	[G/C]	Ca_13398	Bacterial surface antigen (D15)	INTRON
8264	CakSNP8264	*Kabuli*	Ca_Kabuli_Chr05	28271732	[C/T]	Ca_13398	Bacterial surface antigen (D15)	INTRON
8265	CakSNP8265	*Kabuli*	Ca_Kabuli_Chr05	28271733	[G/A]	Ca_13398	Bacterial surface antigen (D15)	INTRON
8266	CakSNP8266	*Kabuli*	Ca_Kabuli_Chr05	28271742	[G/A]	Ca_13398	Bacterial surface antigen (D15)	INTRON
8267	CakSNP8267	*Kabuli*	Ca_Kabuli_Chr05	28271767	[G/C]	Ca_13398	Bacterial surface antigen (D15)	INTRON
8268	CakSNP8268	*Kabuli*	Ca_Kabuli_Chr05	28284640	[T/C]	Ca_13395	Protein kinase, catalytic domain	SYNONYMOUS_CODING
8269	CakSNP8269	*Kabuli*	Ca_Kabuli_Chr05	28327832	[T/G]	Ca_13390	Ribosomal protein S1, RNA-binding domain	INTRON
8270	CakSNP8270	*Kabuli*	Ca_Kabuli_Chr05	28327883	[G/A]	Ca_13390	Ribosomal protein S1, RNA-binding domain	INTRON
8271	CakSNP8271	*Kabuli*	Ca_Kabuli_Chr05	28327870	[T/C]	Ca_13390	Ribosomal protein S1, RNA-binding domain	INTRON

S.N.	SNP IDs	Chickpea cultivars	Chromosomes	Physical positions (bp)	SNPs	Gene accession IDs	Putative functions	Sequence components of genome
8272	CakSNP8272	*Kabuli*	Ca_Kabuli_Chr05	28327868	[G/A]	Ca_13390	Ribosomal protein S1, RNA-binding domain	INTRON
8273	CakSNP8273	*Kabuli*	Ca_Kabuli_Chr05	28327856	[T/C]	Ca_13390	Ribosomal protein S1, RNA-binding domain	INTRON
8274	CakSNP8274	*Kabuli*	Ca_Kabuli_Chr05	28335455	[A/C]	Ca_13389	Histidine phosphatase superfamily, clade-2	SYNONYMOUS_CODING
8275	CakSNP8275	*Kabuli*	Ca_Kabuli_Chr05	28346497	[C/T]	Ca_13388	Lateral organ boundaries, LOB	SYNONYMOUS_CODING
8276	CakSNP8276	*Kabuli*	Ca_Kabuli_Chr05	28360495	[C/T]	Ca_13386	RNA helicase, ATP-dependent, DEAD-box, conserved site	SYNONYMOUS_CODING
8277	CakSNP8277	*Kabuli*	Ca_Kabuli_Chr05	28360492	[C/T]	Ca_13386	RNA helicase, ATP-dependent, DEAD-box, conserved site	SYNONYMOUS_CODING
8278	CakSNP8278	*Kabuli*	Ca_Kabuli_Chr05	28360585	[A/G]	Ca_13386	RNA helicase, ATP-dependent, DEAD-box, conserved site	INTRON
8279	CakSNP8279	*Kabuli*	Ca_Kabuli_Chr05	28383374	[G/T]	Ca_13385	Autophagy-related protein 27	INTRON
8280	CakSNP8280	*Kabuli*	Ca_Kabuli_Chr05	28491113	[G/A]	Ca_13376	Multicopper oxidase, type 1	SYNONYMOUS_CODING
8281	CakSNP8281	*Kabuli*	Ca_Kabuli_Chr05	28491131	[G/A]	Ca_13376	Multicopper oxidase, type 1	SYNONYMOUS_CODING

S.N.	SNP IDs	Chickpea cultivars	Chromosomes	Physical positions (bp)	SNPs	Gene accession IDs	Putative functions	Sequence components of genome
8282	CakSNP8282	*Kabuli*	Ca_Kabuli_Chr05	28509048	[G/A]	Ca_13375	Multicopper oxidase, type 1	SYNONYMOUS_CODING
8283	CakSNP8283	*Kabuli*	Ca_Kabuli_Chr05	28509066	[G/A]	Ca_13375	Multicopper oxidase, type 1	SYNONYMOUS_CODING
8284	CakSNP8284	*Kabuli*	Ca_Kabuli_Chr05	28562734	[G/A]	Ca_13370	Domain of unknown function DUF399	NON_SYNONYMOUS_CODING
8285	CakSNP8285	*Kabuli*	Ca_Kabuli_Chr05	28581814	[A/G]		--	INTERGENIC
8286	CakSNP8286	*Kabuli*	Ca_Kabuli_Chr05	28581815	[C/G]		--	INTERGENIC
8287	CakSNP8287	*Kabuli*	Ca_Kabuli_Chr05	28581827	[T/C]		--	INTERGENIC
8288	CakSNP8288	*Kabuli*	Ca_Kabuli_Chr05	28581866	[T/C]		--	INTERGENIC
8289	CakSNP8289	*Kabuli*	Ca_Kabuli_Chr05	28581926	[C/A]		--	INTERGENIC
8290	CakSNP8290	*Kabuli*	Ca_Kabuli_Chr05	28582525	[C/T]		--	INTERGENIC
8291	CakSNP8291	*Kabuli*	Ca_Kabuli_Chr05	28582806	[T/C]		--	INTERGENIC

S.N.	SNP IDs	Chickpea cultivars	Chromosomes	Physical positions (bp)	SNPs	Gene accession IDs	Putative functions	Sequence components of genome
8292	CakSNP8292	*Kabuli*	Ca_Kabuli_Chr05	28582763	[A/G]		--	INTERGENIC
8293	CakSNP8293	*Kabuli*	Ca_Kabuli_Chr05	28583402	[G/A]	Ca_13367	Sel1-like	INTRON
8294	CakSNP8294	*Kabuli*	Ca_Kabuli_Chr05	28583375	[C/G]	Ca_13367	Sel1-like	INTRON
8295	CakSNP8295	*Kabuli*	Ca_Kabuli_Chr05	28682973	[A/C]	Ca_13361	BTB/POZ-like	SYNONYMOUS_CODING
8296	CakSNP8296	*Kabuli*	Ca_Kabuli_Chr05	28682995	[G/A]	Ca_13361	BTB/POZ-like	NON_SYNONYMOUS_CODING
8297	CakSNP8297	*Kabuli*	Ca_Kabuli_Chr05	28683084	[A/C]	Ca_13361	BTB/POZ-like	SYNONYMOUS_CODING
8298	CakSNP8298	*Kabuli*	Ca_Kabuli_Chr05	28683070	[T/A]	Ca_13361	BTB/POZ-like	NON_SYNONYMOUS_CODING
8299	CakSNP8299	*Kabuli*	Ca_Kabuli_Chr05	28683069	[A/T]	Ca_13361	BTB/POZ-like	NON_SYNONYMOUS_CODING
8300	CakSNP8300	*Kabuli*	Ca_Kabuli_Chr05	28714658	[C/T]	Ca_13358	Oligopeptide transporter	INTRON
8301	CakSNP8301	*Kabuli*	Ca_Kabuli_Chr05	28717362	[C/T]	Ca_13358	Oligopeptide transporter	NON_SYNONYMOUS_CODING

S.N.	SNP IDs	Chickpea cultivars	Chromosomes	Physical positions (bp)	SNPs	Gene accession IDs	Putative functions	Sequence components of genome
8302	CakSNP8302	*Kabuli*	Ca_Kabuli_Chr05	28717384	[C/T]	Ca_13358	Oligopeptide transporter	SYNONYMOUS_CODING
8303	CakSNP8303	*Kabuli*	Ca_Kabuli_Chr05	28721236	[C/T]	Ca_13357	Protein kinase, catalytic domain	NON_SYNONYMOUS_CODING
8304	CakSNP8304	*Kabuli*	Ca_Kabuli_Chr05	28721237	[A/G]	Ca_13357	Protein kinase, catalytic domain	NON_SYNONYMOUS_CODING
8305	CakSNP8305	*Kabuli*	Ca_Kabuli_Chr05	28721386	[T/C]	Ca_13357	Protein kinase, catalytic domain	SYNONYMOUS_CODING
8306	CakSNP8306	*Kabuli*	Ca_Kabuli_Chr05	28721383	[A/C]	Ca_13357	Protein kinase, catalytic domain	NON_SYNONYMOUS_CODING
8307	CakSNP8307	*Kabuli*	Ca_Kabuli_Chr05	28746139	[A/G]	Ca_13355	Haloacid dehalogenase-like hydrolase	NON_SYNONYMOUS_CODING
8308	CakSNP8308	*Kabuli*	Ca_Kabuli_Chr05	28757467	[A/T]	Ca_13354	Protein of unknown function DUF827, plant	NON_SYNONYMOUS_CODING
8309	CakSNP8309	*Kabuli*	Ca_Kabuli_Chr05	28757474	[T/C]	Ca_13354	Protein of unknown function DUF827, plant	NON_SYNONYMOUS_CODING
8310	CakSNP8310	*Kabuli*	Ca_Kabuli_Chr05	28757479	[T/C]	Ca_13354	Protein of unknown function DUF827, plant	NON_SYNONYMOUS_CODING
8311	CakSNP8311	*Kabuli*	Ca_Kabuli_Chr05	28757485	[A/C]	Ca_13354	Protein of unknown function DUF827, plant	NON_SYNONYMOUS_CODING

S.N.	SNP IDs	Chickpea cultivars	Chromosomes	Physical positions (bp)	SNPs	Gene accession IDs	Putative functions	Sequence components of genome
8312	CakSNP8312	*Kabuli*	Ca_Kabuli_Chr05	28757507	[A/T]	Ca_13354	Protein of unknown function DUF827, plant	NON_SYNONYMOUS_CODING
8313	CakSNP8313	*Kabuli*	Ca_Kabuli_Chr05	28758723	[A/G]	Ca_13354	Protein of unknown function DUF827, plant	INTRON
8314	CakSNP8314	*Kabuli*	Ca_Kabuli_Chr05	28817832	[A/G]	Ca_13350	UspA	INTRON
8315	CakSNP8315	*Kabuli*	Ca_Kabuli_Chr05	28827467	[A/G]		--	INTERGENIC
8316	CakSNP8316	*Kabuli*	Ca_Kabuli_Chr05	28843854	[G/A]	Ca_13348	Peptidoglycan-binding Lysin subgroup	SYNONYMOUS_CODING
8317	CakSNP8317	*Kabuli*	Ca_Kabuli_Chr05	28843801	[A/G]	Ca_13348	Peptidoglycan-binding Lysin subgroup	NON_SYNONYMOUS_CODING
8318	CakSNP8318	*Kabuli*	Ca_Kabuli_Chr05	28843795	[A/T]	Ca_13348	Peptidoglycan-binding Lysin subgroup	NON_SYNONYMOUS_CODING
8319	CakSNP8319	*Kabuli*	Ca_Kabuli_Chr05	28843782	[C/G]	Ca_13348	Peptidoglycan-binding Lysin subgroup	SYNONYMOUS_CODING
8320	CakSNP8320	*Kabuli*	Ca_Kabuli_Chr05	28843775	[C/T]	Ca_13348	Peptidoglycan-binding Lysin subgroup	NON_SYNONYMOUS_CODING
8321	CakSNP8321	*Kabuli*	Ca_Kabuli_Chr05	28854486	[C/T]		--	INTERGENIC

S.N.	SNP IDs	Chickpea cultivars	Chromosomes	Physical positions (bp)	SNPs	Gene accession IDs	Putative functions	Sequence components of genome
8322	CakSNP8322	*Kabuli*	Ca_Kabuli_Chr05	28905203	[A/C]	Ca_13344	Drug/metabolite transporter	NON_SYNONYMOUS_CODING
8323	CakSNP8323	*Kabuli*	Ca_Kabuli_Chr05	29007895	[T/C]	Ca_23834	Camphor resistance CrcB protein	SYNONYMOUS_CODING
8324	CakSNP8324	*Kabuli*	Ca_Kabuli_Chr05	29007886	[C/A]	Ca_23834	Camphor resistance CrcB protein	SYNONYMOUS_CODING
8325	CakSNP8325	*Kabuli*	Ca_Kabuli_Chr05	29007884	[A/C]	Ca_23834	Camphor resistance CrcB protein	NON_SYNONYMOUS_CODING
8326	CakSNP8326	*Kabuli*	Ca_Kabuli_Chr05	29007882	[A/C]	Ca_23834	Camphor resistance CrcB protein	NON_SYNONYMOUS_CODING
8327	CakSNP8327	*Kabuli*	Ca_Kabuli_Chr05	29007879	[T/C]	Ca_23834	Camphor resistance CrcB protein	NON_SYNONYMOUS_CODING
8328	CakSNP8328	*Kabuli*	Ca_Kabuli_Chr05	29007847	[A/C]	Ca_23834	Camphor resistance CrcB protein	SYNONYMOUS_CODING
8329	CakSNP8329	*Kabuli*	Ca_Kabuli_Chr05	29007844	[T/C]	Ca_23834	Camphor resistance CrcB protein	SYNONYMOUS_CODING
8330	CakSNP8330	*Kabuli*	Ca_Kabuli_Chr05	29061056	[T/A]	Ca_23831	Pentatricopeptide repeat	INTRON
8331	CakSNP8331	*Kabuli*	Ca_Kabuli_Chr05	29061025	[A/C]	Ca_23831	Pentatricopeptide repeat	INTRON

S.N.	SNP IDs	Chickpea cultivars	Chromosomes	Physical positions (bp)	SNPs	Gene accession IDs	Putative functions	Sequence components of genome
8332	CakSNP8332	*Kabuli*	Ca_Kabuli_Chr05	29060992	[C/T]	Ca_23831	Pentatricopeptide repeat	INTRON
8333	CakSNP8333	*Kabuli*	Ca_Kabuli_Chr05	29062696	[G/A]		--	INTERGENIC
8334	CakSNP8334	*Kabuli*	Ca_Kabuli_Chr05	29062738	[A/G]		--	INTERGENIC
8335	CakSNP8335	*Kabuli*	Ca_Kabuli_Chr05	29079931	[G/A]	Ca_23829	Protein kinase, catalytic domain	SYNONYMOUS_CODING
8336	CakSNP8336	*Kabuli*	Ca_Kabuli_Chr05	29079962	[A/G]	Ca_23829	Protein kinase, catalytic domain	NON_SYNONYMOUS_CODING
8337	CakSNP8337	*Kabuli*	Ca_Kabuli_Chr05	29080066	[G/C]	Ca_23829	Protein kinase, catalytic domain	NON_SYNONYMOUS_CODING
8338	CakSNP8338	*Kabuli*	Ca_Kabuli_Chr05	29080060	[C/T]	Ca_23829	Protein kinase, catalytic domain	SYNONYMOUS_CODING
8339	CakSNP8339	*Kabuli*	Ca_Kabuli_Chr05	29111292	[T/G]	Ca_23826	Oxysterol-binding protein	INTRON
8340	CakSNP8340	*Kabuli*	Ca_Kabuli_Chr05	29111293	[T/C]	Ca_23826	Oxysterol-binding protein	INTRON
8341	CakSNP8341	*Kabuli*	Ca_Kabuli_Chr05	29186179	[T/G]	Ca_23822	Glycoside hydrolase, family 28	NON_SYNONYMOUS_CODING

S.N.	SNP IDs	Chickpea cultivars	Chromosomes	Physical positions (bp)	SNPs	Gene accession IDs	Putative functions	Sequence components of genome
8342	CakSNP8342	*Kabuli*	Ca_Kabuli_Chr05	29220980	[T/A]	Ca_16618	--	INTRON
8343	CakSNP8343	*Kabuli*	Ca_Kabuli_Chr05	29221003	[T/C]	Ca_16618	--	INTRON
8344	CakSNP8344	*Kabuli*	Ca_Kabuli_Chr05	29221919	[T/A]	Ca_16618	--	INTRON
8345	CakSNP8345	*Kabuli*	Ca_Kabuli_Chr05	29272117	[C/G]	Ca_16622	GTP-binding protein, HSR1-related	INTRON
8346	CakSNP8346	*Kabuli*	Ca_Kabuli_Chr05	29272125	[C/T]	Ca_16622	GTP-binding protein, HSR1-related	INTRON
8347	CakSNP8347	*Kabuli*	Ca_Kabuli_Chr05	29354269	[A/T]		--	INTERGENIC
8348	CakSNP8348	*Kabuli*	Ca_Kabuli_Chr05	29422029	[T/A]	Ca_16633	Trehalose-phosphatase	INTRON
8349	CakSNP8349	*Kabuli*	Ca_Kabuli_Chr05	29422066	[A/T]	Ca_16633	Trehalose-phosphatase	INTRON
8350	CakSNP8350	*Kabuli*	Ca_Kabuli_Chr05	29422091	[C/T]	Ca_16633	Trehalose-phosphatase	INTRON
8351	CakSNP8351	*Kabuli*	Ca_Kabuli_Chr05	29437139	[C/T]		--	INTERGENIC

S.N.	SNP IDs	Chickpea cultivars	Chromosomes	Physical positions (bp)	SNPs	Gene accession IDs	Putative functions	Sequence components of genome
8352	CakSNP8352	*Kabuli*	Ca_Kabuli_Chr05	29437146	[C/A]		--	INTERGENIC
8353	CakSNP8353	*Kabuli*	Ca_Kabuli_Chr05	29437159	[C/T]		--	INTERGENIC
8354	CakSNP8354	*Kabuli*	Ca_Kabuli_Chr05	29437162	[A/C]		--	INTERGENIC
8355	CakSNP8355	*Kabuli*	Ca_Kabuli_Chr05	29437171	[A/T]		--	INTERGENIC
8356	CakSNP8356	*Kabuli*	Ca_Kabuli_Chr05	29437181	[A/C]		--	INTERGENIC
8357	CakSNP8357	*Kabuli*	Ca_Kabuli_Chr05	29437189	[C/T]		--	INTERGENIC
8358	CakSNP8358	*Kabuli*	Ca_Kabuli_Chr05	29437193	[A/C]		--	INTERGENIC
8359	CakSNP8359	*Kabuli*	Ca_Kabuli_Chr05	29437182	[C/A]		--	INTERGENIC
8360	CakSNP8360	*Kabuli*	Ca_Kabuli_Chr05	29528859	[A/T]		--	INTERGENIC
8361	CakSNP8361	*Kabuli*	Ca_Kabuli_Chr05	29528890	[T/A]		--	INTERGENIC

S.N.	SNP IDs	Chickpea cultivars	Chromosomes	Physical positions (bp)	SNPs	Gene accession IDs	Putative functions	Sequence components of genome
8362	CakSNP8362	*Kabuli*	Ca_Kabuli_Chr05	29528904	[T/G]	Ca_16640	Sugar/inositol transporter	NON_SYNONYMOUS_CODING
8363	CakSNP8363	*Kabuli*	Ca_Kabuli_Chr05	29530996	[G/A]	Ca_16640	Sugar/inositol transporter	SYNONYMOUS_CODING
8364	CakSNP8364	*Kabuli*	Ca_Kabuli_Chr05	29531122	[A/T]	Ca_16640	Sugar/inositol transporter	SYNONYMOUS_CODING
8365	CakSNP8365	*Kabuli*	Ca_Kabuli_Chr05	29531113	[G/A]	Ca_16640	Sugar/inositol transporter	SYNONYMOUS_CODING
8366	CakSNP8366	*Kabuli*	Ca_Kabuli_Chr05	29531218	[A/T]	Ca_16640	Sugar/inositol transporter	SYNONYMOUS_CODING
8367	CakSNP8367	*Kabuli*	Ca_Kabuli_Chr05	29531416	[A/G]	Ca_16640	Sugar/inositol transporter	SYNONYMOUS_CODING
8368	CakSNP8368	*Kabuli*	Ca_Kabuli_Chr05	29567676	[T/C]	Ca_16643	--	SYNONYMOUS_CODING
8369	CakSNP8369	*Kabuli*	Ca_Kabuli_Chr05	29567820	[T/C]		--	INTERGENIC
8370	CakSNP8370	*Kabuli*	Ca_Kabuli_Chr05	29576690	[A/T]		--	INTERGENIC
8371	CakSNP8371	*Kabuli*	Ca_Kabuli_Chr05	29576689	[G/A]		--	INTERGENIC

S.N.	SNP IDs	Chickpea cultivars	Chromosomes	Physical positions (bp)	SNPs	Gene accession IDs	Putative functions	Sequence components of genome
8372	CakSNP8372	*Kabuli*	Ca_Kabuli_Chr05	29576716	[A/C]		--	INTERGENIC
8373	CakSNP8373	*Kabuli*	Ca_Kabuli_Chr05	29608657	[T/C]		--	INTERGENIC
8374	CakSNP8374	*Kabuli*	Ca_Kabuli_Chr05	29641659	[T/C]	Ca_16648	Leucine-rich repeat	SYNONYMOUS_CODING
8375	CakSNP8375	*Kabuli*	Ca_Kabuli_Chr05	29658258	[G/C]	Ca_16650	Protein of unknown function DUF248, methyltransferase putative	INTRON
8376	CakSNP8376	*Kabuli*	Ca_Kabuli_Chr05	29658280	[A/C]	Ca_16650	Protein of unknown function DUF248, methyltransferase putative	INTRON
8377	CakSNP8377	*Kabuli*	Ca_Kabuli_Chr05	29667787	[C/G]		--	INTERGENIC
8378	CakSNP8378	*Kabuli*	Ca_Kabuli_Chr05	29667783	[C/T]		--	INTERGENIC
8379	CakSNP8379	*Kabuli*	Ca_Kabuli_Chr05	29667774	[G/C]		--	INTERGENIC
8380	CakSNP8380	*Kabuli*	Ca_Kabuli_Chr05	29809862	[A/C]	Ca_16663	Peptidase T1A, proteasome beta-subunit	SYNONYMOUS_CODING
8381	CakSNP8381	*Kabuli*	Ca_Kabuli_Chr05	29816799	[T/C]	Ca_16664	Beta-lactamase-like	INTRON

S.N.	SNP IDs	Chickpea cultivars	Chromosomes	Physical positions (bp)	SNPs	Gene accession IDs	Putative functions	Sequence components of genome
8382	CakSNP8382	*Kabuli*	Ca_Kabuli_Chr05	29816800	[T/A]	Ca_16664	Beta-lactamase-like	INTRON
8383	CakSNP8383	*Kabuli*	Ca_Kabuli_Chr05	29824218	[T/G]	Ca_16665	Cycloeucalenol cycloisomerase	INTRON
8384	CakSNP8384	*Kabuli*	Ca_Kabuli_Chr05	29852130	[T/C]	Ca_16668	Peptidase M18	INTRON
8385	CakSNP8385	*Kabuli*	Ca_Kabuli_Chr05	29852105	[T/G]	Ca_16668	Peptidase M18	INTRON
8386	CakSNP8386	*Kabuli*	Ca_Kabuli_Chr05	29886086	[T/A]	Ca_16670	Bromodomain	SYNONYMOUS_CODING
8387	CakSNP8387	*Kabuli*	Ca_Kabuli_Chr05	29981826	[A/G]	Ca_04676	Arginine-tRNA-protein transferase, N-terminal	NON_SYNONYMOUS_CODING
8388	CakSNP8388	*Kabuli*	Ca_Kabuli_Chr05	29999414	[C/T]		--	INTERGENIC
8389	CakSNP8389	*Kabuli*	Ca_Kabuli_Chr05	29999427	[G/A]		--	INTERGENIC
8390	CakSNP8390	*Kabuli*	Ca_Kabuli_Chr05	29999433	[C/A]		--	INTERGENIC
8391	CakSNP8391	*Kabuli*	Ca_Kabuli_Chr05	29999465	[T/G]		--	INTERGENIC

S.N.	SNP IDs	Chickpea cultivars	Chromosomes	Physical positions (bp)	SNPs	Gene accession IDs	Putative functions	Sequence components of genome
8392	CakSNP8392	*Kabuli*	Ca_Kabuli_Chr05	29999476	[G/A]		--	INTERGENIC
8393	CakSNP8393	*Kabuli*	Ca_Kabuli_Chr05	29999523	[A/T]		--	INTERGENIC
8394	CakSNP8394	*Kabuli*	Ca_Kabuli_Chr05	30035763	[A/T]	Ca_04680	Sugar/inositol transporter	SYNONYMOUS_CODING
8395	CakSNP8395	*Kabuli*	Ca_Kabuli_Chr05	30049265	[G/A]		--	INTERGENIC
8396	CakSNP8396	*Kabuli*	Ca_Kabuli_Chr05	30053540	[C/G]	Ca_04682	--	NON_SYNONYMOUS_CODING
8397	CakSNP8397	*Kabuli*	Ca_Kabuli_Chr05	30060160	[C/T]		--	INTERGENIC
8398	CakSNP8398	*Kabuli*	Ca_Kabuli_Chr05	30086073	[A/G]		--	INTERGENIC
8399	CakSNP8399	*Kabuli*	Ca_Kabuli_Chr05	30086232	[A/C]	Ca_04684	Protein of unknown function DUF300	NON_SYNONYMOUS_CODING
8400	CakSNP8400	*Kabuli*	Ca_Kabuli_Chr05	30090906	[A/G]	Ca_04685	Protein of unknown function DUF300	INTRON
8401	CakSNP8401	*Kabuli*	Ca_Kabuli_Chr05	30091063	[G/A]	Ca_04685	Protein of unknown function DUF300	INTRON

S.N.	SNP IDs	Chickpea cultivars	Chromosomes	Physical positions (bp)	SNPs	Gene accession IDs	Putative functions	Sequence components of genome
8402	CakSNP8402	*Kabuli*	Ca_Kabuli_Chr05	30116978	[C/T]	Ca_04687	Glycoside hydrolase, family 19, catalytic	SYNONYMOUS_CODING
8403	CakSNP8403	*Kabuli*	Ca_Kabuli_Chr05	30116992	[T/G]	Ca_04687	Glycoside hydrolase, family 19, catalytic	NON_SYNONYMOUS_CODING
8404	CakSNP8404	*Kabuli*	Ca_Kabuli_Chr05	30159226	[C/T]		--	INTERGENIC
8405	CakSNP8405	*Kabuli*	Ca_Kabuli_Chr05	30159173	[G/A]		--	INTERGENIC
8406	CakSNP8406	*Kabuli*	Ca_Kabuli_Chr05	30159161	[G/A]		--	INTERGENIC
8407	CakSNP8407	*Kabuli*	Ca_Kabuli_Chr05	30191389	[A/C]		--	INTERGENIC
8408	CakSNP8408	*Kabuli*	Ca_Kabuli_Chr05	30248447	[C/A]	Ca_04701	--	NON_SYNONYMOUS_CODING
8409	CakSNP8409	*Kabuli*	Ca_Kabuli_Chr05	30258178	[T/G]		--	INTERGENIC
8410	CakSNP8410	*Kabuli*	Ca_Kabuli_Chr05	30287853	[C/T]	Ca_04705	Ribosomal protein L18/L5	INTRON
8411	CakSNP8411	*Kabuli*	Ca_Kabuli_Chr05	30304076	[A/T]	Ca_04707	Major intrinsic protein	INTRON

S.N.	SNP IDs	Chickpea cultivars	Chromosomes	Physical positions (bp)	SNPs	Gene accession IDs	Putative functions	Sequence components of genome
8412	CakSNP8412	*Kabuli*	Ca_Kabuli_Chr05	30319316	[A/G]	Ca_04709	Nickel/cobalt transporter, high-affinity	SYNONYMOUS_CODING
8413	CakSNP8413	*Kabuli*	Ca_Kabuli_Chr05	30359047	[T/C]	Ca_04713	D-galactoside/L-rhamnose binding SUEL lectin domain	SYNONYMOUS_CODING
8414	CakSNP8414	*Kabuli*	Ca_Kabuli_Chr05	30359022	[G/A]	Ca_04713	D-galactoside/L-rhamnose binding SUEL lectin domain	NON_SYNONYMOUS_CODING
8415	CakSNP8415	*Kabuli*	Ca_Kabuli_Chr05	30360185	[T/C]	Ca_04713	D-galactoside/L-rhamnose binding SUEL lectin domain	SYNONYMOUS_CODING
8416	CakSNP8416	*Kabuli*	Ca_Kabuli_Chr05	30360348	[T/C]	Ca_04713	D-galactoside/L-rhamnose binding SUEL lectin domain	NON_SYNONYMOUS_CODING
8417	CakSNP8417	*Kabuli*	Ca_Kabuli_Chr05	30367349	[G/T]	Ca_04714	HECT	NON_SYNONYMOUS_CODING
8418	CakSNP8418	*Kabuli*	Ca_Kabuli_Chr05	30373542	[A/G]	Ca_04715	Armadillo	NON_SYNONYMOUS_CODING
8419	CakSNP8419	*Kabuli*	Ca_Kabuli_Chr05	30373783	[T/A]	Ca_04715	Armadillo	SYNONYMOUS_CODING
8420	CakSNP8420	*Kabuli*	Ca_Kabuli_Chr05	30376661	[C/G]	Ca_04715	Armadillo	INTRON
8421	CakSNP8421	*Kabuli*	Ca_Kabuli_Chr05	30376722	[T/C]	Ca_04715	Armadillo	INTRON

S.N.	SNP IDs	Chickpea cultivars	Chromosomes	Physical positions (bp)	SNPs	Gene accession IDs	Putative functions	Sequence components of genome
8422	CakSNP8422	*Kabuli*	Ca_Kabuli_Chr05	30376723	[T/C]	Ca_04715	Armadillo	INTRON
8423	CakSNP8423	*Kabuli*	Ca_Kabuli_Chr05	30378114	[A/G]	Ca_04715	Armadillo	INTRON
8424	CakSNP8424	*Kabuli*	Ca_Kabuli_Chr05	30378184	[A/G]	Ca_04715	Armadillo	INTRON
8425	CakSNP8425	*Kabuli*	Ca_Kabuli_Chr05	30407725	[C/G]	Ca_04716	C2 calcium-dependent membrane targeting	INTRON
8426	CakSNP8426	*Kabuli*	Ca_Kabuli_Chr05	30408006	[C/T]	Ca_04716	C2 calcium-dependent membrane targeting	INTRON
8427	CakSNP8427	*Kabuli*	Ca_Kabuli_Chr05	30438913	[G/A]	Ca_04722	SANT domain, DNA binding	NON_SYNONYMOUS_CODING
8428	CakSNP8428	*Kabuli*	Ca_Kabuli_Chr05	30443858	[A/T]	Ca_04723	Pollen Ole e 1 allergen/extensin	INTRON
8429	CakSNP8429	*Kabuli*	Ca_Kabuli_Chr05	30562430	[C/G]		--	INTERGENIC
8430	CakSNP8430	*Kabuli*	Ca_Kabuli_Chr05	30562524	[G/T]		--	INTERGENIC
8431	CakSNP8431	*Kabuli*	Ca_Kabuli_Chr05	30601932	[T/C]	Ca_04753	--	NON_SYNONYMOUS_CODING

S.N.	SNP IDs	Chickpea cultivars	Chromosomes	Physical positions (bp)	SNPs	Gene accession IDs	Putative functions	Sequence components of genome
8432	CakSNP8432	*Kabuli*	Ca_Kabuli_Chr05	30601926	[T/C]	Ca_04753	--	NON_SYNONYMOUS_CODING
8433	CakSNP8433	*Kabuli*	Ca_Kabuli_Chr05	30601920	[T/C]	Ca_04753	--	NON_SYNONYMOUS_CODING
8434	CakSNP8434	*Kabuli*	Ca_Kabuli_Chr05	30627937	[G/T]	Ca_04755	Iron hydrogenase, small subunit-like	INTRON
8435	CakSNP8435	*Kabuli*	Ca_Kabuli_Chr05	30632697	[A/G]	Ca_04756	--	INTRON
8436	CakSNP8436	*Kabuli*	Ca_Kabuli_Chr05	30655941	[T/C]	Ca_04757	Kinesin, motor domain	SYNONYMOUS_CODING
8437	CakSNP8437	*Kabuli*	Ca_Kabuli_Chr05	30656058	[A/T]	Ca_04757	Kinesin, motor domain	INTRON
8438	CakSNP8438	*Kabuli*	Ca_Kabuli_Chr05	30690891	[T/G]		--	INTERGENIC
8439	CakSNP8439	*Kabuli*	Ca_Kabuli_Chr05	30690852	[G/A]		--	INTERGENIC
8440	CakSNP8440	*Kabuli*	Ca_Kabuli_Chr05	30690885	[A/G]		--	INTERGENIC
8441	CakSNP8441	*Kabuli*	Ca_Kabuli_Chr05	30691510	[A/G]	Ca_04762	ATPase, AAA+ type, core	INTRON

S.N.	SNP IDs	Chickpea cultivars	Chromosomes	Physical positions (bp)	SNPs	Gene accession IDs	Putative functions	Sequence components of genome
8442	CakSNP8442	*Kabuli*	Ca_Kabuli_Chr05	30691515	[G/A]	Ca_04762	ATPase, AAA+ type, core	INTRON
8443	CakSNP8443	*Kabuli*	Ca_Kabuli_Chr05	30691543	[A/G]	Ca_04762	ATPase, AAA+ type, core	INTRON
8444	CakSNP8444	*Kabuli*	Ca_Kabuli_Chr05	30697511	[G/A]	Ca_04764	Zinc finger, RING-type	SYNONYMOUS_CODING
8445	CakSNP8445	*Kabuli*	Ca_Kabuli_Chr05	30745571	[G/T]		--	INTERGENIC
8446	CakSNP8446	*Kabuli*	Ca_Kabuli_Chr05	30749553	[A/T]	Ca_04769	Cellular retinaldehyde binding/alpha-tocopherol transport	INTRON
8447	CakSNP8447	*Kabuli*	Ca_Kabuli_Chr05	30749696	[A/T]	Ca_04769	Cellular retinaldehyde binding/alpha-tocopherol transport	INTRON
8448	CakSNP8448	*Kabuli*	Ca_Kabuli_Chr05	30755644	[A/G]	Ca_04770	Thymidylate synthase	SYNONYMOUS_CODING
8449	CakSNP8449	*Kabuli*	Ca_Kabuli_Chr05	30755584	[G/A]	Ca_04770	Thymidylate synthase	SYNONYMOUS_CODING
8450	CakSNP8450	*Kabuli*	Ca_Kabuli_Chr05	30811250	[A/T]	Ca_04777	UspA	INTRON
8451	CakSNP8451	*Kabuli*	Ca_Kabuli_Chr05	30864010	[T/C]	Ca_04782	Protein kinase, catalytic domain	SYNONYMOUS_CODING

S.N.	SNP IDs	Chickpea cultivars	Chromosomes	Physical positions (bp)	SNPs	Gene accession IDs	Putative functions	Sequence components of genome
8452	CakSNP8452	*Kabuli*	Ca_Kabuli_Chr05	30868770	[G/C]		--	INTERGENIC
8453	CakSNP8453	*Kabuli*	Ca_Kabuli_Chr05	30868695	[G/C]		--	INTERGENIC
8454	CakSNP8454	*Kabuli*	Ca_Kabuli_Chr05	30878047	[C/T]	Ca_04784	G-protein, beta subunit	INTRON
8455	CakSNP8455	*Kabuli*	Ca_Kabuli_Chr05	30878092	[G/T]	Ca_04784	G-protein, beta subunit	INTRON
8456	CakSNP8456	*Kabuli*	Ca_Kabuli_Chr05	30878100	[T/A]	Ca_04784	G-protein, beta subunit	INTRON
8457	CakSNP8457	*Kabuli*	Ca_Kabuli_Chr05	30890480	[G/C]	Ca_04786	Clathrin/coatomer adaptor, adaptin-like, N-terminal	INTRON
8458	CakSNP8458	*Kabuli*	Ca_Kabuli_Chr05	30962640	[A/T]	Ca_04795	Sugar/inositol transporter	NON_SYNONYMOUS_CODING
8459	CakSNP8459	*Kabuli*	Ca_Kabuli_Chr05	30962641	[C/A]	Ca_04795	Sugar/inositol transporter	NON_SYNONYMOUS_CODING
8460	CakSNP8460	*Kabuli*	Ca_Kabuli_Chr05	30962650	[G/A]	Ca_04795	Sugar/inositol transporter	NON_SYNONYMOUS_CODING
8461	CakSNP8461	*Kabuli*	Ca_Kabuli_Chr05	30964628	[A/G]	Ca_04796	Phosphatidylinositol 3-/4-kinase, catalytic	SYNONYMOUS_CODING

S.N.	SNP IDs	Chickpea cultivars	Chromosomes	Physical positions (bp)	SNPs	Gene accession IDs	Putative functions	Sequence components of genome
8462	CakSNP8462	*Kabuli*	Ca_Kabuli_Chr05	31024408	[T/C]	Ca_04800	RNA recognition motif domain	SYNONYMOUS_CODING
8463	CakSNP8463	*Kabuli*	Ca_Kabuli_Chr05	31025764	[G/T]		--	INTERGENIC
8464	CakSNP8464	*Kabuli*	Ca_Kabuli_Chr05	31034500	[T/C]		--	INTERGENIC
8465	CakSNP8465	*Kabuli*	Ca_Kabuli_Chr05	31034898	[A/G]	Ca_04802	Photosystem II PsbP, oxygen evolving complex	INTRON
8466	CakSNP8466	*Kabuli*	Ca_Kabuli_Chr05	31064173	[C/G]	Ca_04805	Sugar/inositol transporter	SYNONYMOUS_CODING
8467	CakSNP8467	*Kabuli*	Ca_Kabuli_Chr05	31072213	[T/C]	Ca_04806	--	SYNONYMOUS_CODING
8468	CakSNP8468	*Kabuli*	Ca_Kabuli_Chr05	31072276	[G/A]	Ca_04806	--	INTRON
8469	CakSNP8469	*Kabuli*	Ca_Kabuli_Chr05	31084839	[A/C]	Ca_04808	--	INTRON
8470	CakSNP8470	*Kabuli*	Ca_Kabuli_Chr05	31088698	[T/G]	Ca_04809	Ribophorin I	NON_SYNONYMOUS_CODING
8471	CakSNP8471	*Kabuli*	Ca_Kabuli_Chr05	31126125	[C/T]		--	INTERGENIC

S.N.	SNP IDs	Chickpea cultivars	Chromosomes	Physical positions (bp)	SNPs	Gene accession IDs	Putative functions	Sequence components of genome
8472	CakSNP8472	*Kabuli*	Ca_Kabuli_Chr05	31131830	[T/C]	Ca_04815	--	INTRON
8473	CakSNP8473	*Kabuli*	Ca_Kabuli_Chr05	31132568	[A/T]	Ca_04815	--	SYNONYMOUS_CODING
8474	CakSNP8474	*Kabuli*	Ca_Kabuli_Chr05	31138650	[G/A]	Ca_04816	Domain of unknown function CP12	SYNONYMOUS_CODING
8475	CakSNP8475	*Kabuli*	Ca_Kabuli_Chr05	31140531	[T/C]	Ca_04817	GPCR, family 3	SYNONYMOUS_CODING
8476	CakSNP8476	*Kabuli*	Ca_Kabuli_Chr05	31162533	[G/A]	Ca_04820	Armadillo	SYNONYMOUS_CODING
8477	CakSNP8477	*Kabuli*	Ca_Kabuli_Chr05	31164387	[G/C]	Ca_04820	Armadillo	INTRON
8478	CakSNP8478	*Kabuli*	Ca_Kabuli_Chr05	31164539	[T/C]	Ca_04820	Armadillo	SYNONYMOUS_CODING
8479	CakSNP8479	*Kabuli*	Ca_Kabuli_Chr05	31165048	[T/C]	Ca_04820	Armadillo	SYNONYMOUS_CODING
8480	CakSNP8480	*Kabuli*	Ca_Kabuli_Chr05	31165039	[T/C]	Ca_04820	Armadillo	SYNONYMOUS_CODING
8481	CakSNP8481	*Kabuli*	Ca_Kabuli_Chr05	31165035	[T/C]	Ca_04820	Armadillo	NON_SYNONYMOUS_CODING

S.N.	SNP IDs	Chickpea cultivars	Chromosomes	Physical positions (bp)	SNPs	Gene accession IDs	Putative functions	Sequence components of genome
8482	CakSNP8482	*Kabuli*	Ca_Kabuli_Chr05	31165002	[A/C]	Ca_04820	Armadillo	NON_SYNONYMOUS_CODING
8483	CakSNP8483	*Kabuli*	Ca_Kabuli_Chr05	31171555	[G/A]	Ca_04822	EPS15 homology (EH)	SYNONYMOUS_CODING
8484	CakSNP8484	*Kabuli*	Ca_Kabuli_Chr05	31176722	[T/G]	Ca_04822	EPS15 homology (EH)	NON_SYNONYMOUS_CODING
8485	CakSNP8485	*Kabuli*	Ca_Kabuli_Chr05	31176810	[C/T]	Ca_04822	EPS15 homology (EH)	INTRON
8486	CakSNP8486	*Kabuli*	Ca_Kabuli_Chr05	31176844	[G/A]	Ca_04822	EPS15 homology (EH)	INTRON
8487	CakSNP8487	*Kabuli*	Ca_Kabuli_Chr05	31192415	[C/T]	Ca_04824	NADH:flavin oxidoreductase/NADH oxidase, N-terminal	SYNONYMOUS_CODING
8488	CakSNP8488	*Kabuli*	Ca_Kabuli_Chr05	31192413	[G/A]	Ca_04824	NADH:flavin oxidoreductase/NADH oxidase, N-terminal	NON_SYNONYMOUS_CODING
8489	CakSNP8489	*Kabuli*	Ca_Kabuli_Chr05	31194845	[G/T]	Ca_04825	High mobility group, HMG1/HMG2	INTRON
8490	CakSNP8490	*Kabuli*	Ca_Kabuli_Chr05	31194974	[T/C]	Ca_04825	High mobility group, HMG1/HMG2	NON_SYNONYMOUS_CODING
8491	CakSNP8491	*Kabuli*	Ca_Kabuli_Chr05	31194988	[G/T]	Ca_04825	High mobility group, HMG1/HMG2	INTRON

S.N.	SNP IDs	Chickpea cultivars	Chromosomes	Physical positions (bp)	SNPs	Gene accession IDs	Putative functions	Sequence components of genome
8492	CakSNP8492	*Kabuli*	Ca_Kabuli_Chr05	31194989	[A/C]	Ca_04825	High mobility group, HMG1/HMG2	INTRON
8493	CakSNP8493	*Kabuli*	Ca_Kabuli_Chr05	31215109	[C/T]	Ca_04827	Armadillo	NON_SYNONYMOUS_CODING
8494	CakSNP8494	*Kabuli*	Ca_Kabuli_Chr05	31310698	[C/T]		--	INTERGENIC
8495	CakSNP8495	*Kabuli*	Ca_Kabuli_Chr05	31310702	[C/G]		--	INTERGENIC
8496	CakSNP8496	*Kabuli*	Ca_Kabuli_Chr05	31310703	[A/G]		--	INTERGENIC
8497	CakSNP8497	*Kabuli*	Ca_Kabuli_Chr05	31310741	[A/G]		--	INTERGENIC
8498	CakSNP8498	*Kabuli*	Ca_Kabuli_Chr05	31314012	[A/C]	Ca_04834	Phox/Bem1p	SYNONYMOUS_CODING
8499	CakSNP8499	*Kabuli*	Ca_Kabuli_Chr05	31314056	[A/C]	Ca_04834	Phox/Bem1p	SYNONYMOUS_CODING
8500	CakSNP8500	*Kabuli*	Ca_Kabuli_Chr05	31344725	[G/A]	Ca_04836	Glycoside hydrolase, family 13, N-terminal	NON_SYNONYMOUS_CODING
8501	CakSNP8501	*Kabuli*	Ca_Kabuli_Chr05	31377103	[C/T]	Ca_04840	Ribosome biogenesis protein BMS1/TSR1, C-terminal	INTRON

S.N.	SNP IDs	Chickpea cultivars	Chromosomes	Physical positions (bp)	SNPs	Gene accession IDs	Putative functions	Sequence components of genome
8502	CakSNP8502	*Kabuli*	Ca_Kabuli_Chr05	31377122	[C/T]	Ca_04840	Ribosome biogenesis protein BMS1/TSR1, C-terminal	INTRON
8503	CakSNP8503	*Kabuli*	Ca_Kabuli_Chr05	31377134	[A/T]	Ca_04840	Ribosome biogenesis protein BMS1/TSR1, C-terminal	INTRON
8504	CakSNP8504	*Kabuli*	Ca_Kabuli_Chr05	31390221	[A/G]	Ca_04841	--	INTRON
8505	CakSNP8505	*Kabuli*	Ca_Kabuli_Chr05	31390319	[C/T]	Ca_04841	--	INTRON
8506	CakSNP8506	*Kabuli*	Ca_Kabuli_Chr05	31522389	[T/G]	Ca_04855	Peptidase S9, prolyl oligopeptidase, catalytic domain	INTRON
8507	CakSNP8507	*Kabuli*	Ca_Kabuli_Chr05	31548555	[G/A]		--	INTERGENIC
8508	CakSNP8508	*Kabuli*	Ca_Kabuli_Chr05	31651335	[G/T]	Ca_04866	RNA-binding S4	NON_SYNONYMOUS_CODING
8509	CakSNP8509	*Kabuli*	Ca_Kabuli_Chr05	31651336	[T/G]	Ca_04866	RNA-binding S4	NON_SYNONYMOUS_CODING
8510	CakSNP8510	*Kabuli*	Ca_Kabuli_Chr05	31653418	[T/C]	Ca_04866	RNA-binding S4	INTRON
8511	CakSNP8511	*Kabuli*	Ca_Kabuli_Chr05	31653423	[T/C]	Ca_04866	RNA-binding S4	INTRON

S.N.	SNP IDs	Chickpea cultivars	Chromosomes	Physical positions (bp)	SNPs	Gene accession IDs	Putative functions	Sequence components of genome
8512	CakSNP8512	*Kabuli*	Ca_Kabuli_Chr05	31653432	[A/G]	Ca_04866	RNA-binding S4	INTRON
8513	CakSNP8513	*Kabuli*	Ca_Kabuli_Chr05	31653437	[C/T]	Ca_04866	RNA-binding S4	INTRON
8514	CakSNP8514	*Kabuli*	Ca_Kabuli_Chr05	31653452	[C/A]	Ca_04866	RNA-binding S4	INTRON
8515	CakSNP8515	*Kabuli*	Ca_Kabuli_Chr05	31687881	[C/T]	Ca_04871	RNA-binding protein Lupus La	NON_SYNONYMOUS_CODING
8516	CakSNP8516	*Kabuli*	Ca_Kabuli_Chr05	31687891	[A/C]	Ca_04871	RNA-binding protein Lupus La	NON_SYNONYMOUS_CODING
8517	CakSNP8517	*Kabuli*	Ca_Kabuli_Chr05	31696236	[T/C]	Ca_04872	Peptidase S8/S53, subtilisin/kexin/sedolisin	NON_SYNONYMOUS_CODING
8518	CakSNP8518	*Kabuli*	Ca_Kabuli_Chr05	31710670	[G/A]	Ca_04874	--	SYNONYMOUS_CODING
8519	CakSNP8519	*Kabuli*	Ca_Kabuli_Chr05	31724217	[G/A]	Ca_04876	Pentatricopeptide repeat	INTRON
8520	CakSNP8520	*Kabuli*	Ca_Kabuli_Chr05	31724272	[C/T]	Ca_04876	Pentatricopeptide repeat	INTRON
8521	CakSNP8521	*Kabuli*	Ca_Kabuli_Chr05	31724332	[G/A]	Ca_04876	Pentatricopeptide repeat	INTRON

S.N.	SNP IDs	Chickpea cultivars	Chromosomes	Physical positions (bp)	SNPs	Gene accession IDs	Putative functions	Sequence components of genome
8522	CakSNP8522	*Kabuli*	Ca_Kabuli_Chr05	31750150	[T/G]	Ca_04878	Glutaredoxin	SYNONYMOUS_CODING
8523	CakSNP8523	*Kabuli*	Ca_Kabuli_Chr05	31750242	[A/G]	Ca_04878	Glutaredoxin	INTRON
8524	CakSNP8524	*Kabuli*	Ca_Kabuli_Chr05	31758166	[T/C]	Ca_04880	SANT domain, DNA binding	NON_SYNONYMOUS_CODING
8525	CakSNP8525	*Kabuli*	Ca_Kabuli_Chr05	31802684	[G/T]	Ca_04885	Protein of unknown function DUF668	INTRON
8526	CakSNP8526	*Kabuli*	Ca_Kabuli_Chr05	31802727	[G/A]	Ca_04885	Protein of unknown function DUF668	INTRON
8527	CakSNP8527	*Kabuli*	Ca_Kabuli_Chr05	31816202	[A/C]		--	INTERGENIC
8528	CakSNP8528	*Kabuli*	Ca_Kabuli_Chr05	31816228	[A/G]		--	INTERGENIC
8529	CakSNP8529	*Kabuli*	Ca_Kabuli_Chr05	31935674	[T/C]	Ca_04898	Protein of unknown function DUF869, plant	NON_SYNONYMOUS_CODING
8530	CakSNP8530	*Kabuli*	Ca_Kabuli_Chr05	31951925	[G/A]	Ca_04899	Regulator of chromosome condensation, RCC1	INTRON
8531	CakSNP8531	*Kabuli*	Ca_Kabuli_Chr05	31951944	[C/T]	Ca_04899	Regulator of chromosome condensation, RCC1	INTRON

S.N.	SNP IDs	Chickpea cultivars	Chromosomes	Physical positions (bp)	SNPs	Gene accession IDs	Putative functions	Sequence components of genome
8532	CakSNP8532	*Kabuli*	Ca_Kabuli_Chr05	31985390	[G/C]	Ca_04901	WD40 repeat	INTRON
8533	CakSNP8533	*Kabuli*	Ca_Kabuli_Chr05	32011781	[G/T]	Ca_04904	Kinesin, motor domain	INTRON
8534	CakSNP8534	*Kabuli*	Ca_Kabuli_Chr05	32011792	[T/G]	Ca_04904	Kinesin, motor domain	INTRON
8535	CakSNP8535	*Kabuli*	Ca_Kabuli_Chr05	32015967	[T/C]	Ca_04905	--	NON_SYNONYMOUS_CODING
8536	CakSNP8536	*Kabuli*	Ca_Kabuli_Chr05	32019864	[G/A]	Ca_04906	Protein of unknown function DUF726	SYNONYMOUS_CODING
8537	CakSNP8537	*Kabuli*	Ca_Kabuli_Chr05	32023925	[T/C]		--	INTERGENIC
8538	CakSNP8538	*Kabuli*	Ca_Kabuli_Chr05	32090295	[C/A]		--	INTERGENIC
8539	CakSNP8539	*Kabuli*	Ca_Kabuli_Chr05	32144081	[G/C]		--	INTERGENIC
8540	CakSNP8540	*Kabuli*	Ca_Kabuli_Chr05	32144108	[A/C]		--	INTERGENIC
8541	CakSNP8541	*Kabuli*	Ca_Kabuli_Chr05	32144120	[C/T]		--	INTERGENIC

S.N.	SNP IDs	Chickpea cultivars	Chromosomes	Physical positions (bp)	SNPs	Gene accession IDs	Putative functions	Sequence components of genome
8542	CakSNP8542	*Kabuli*	Ca_Kabuli_Chr05	32147522	[C/T]	Ca_04916	Thioredoxin	INTRON
8543	CakSNP8543	*Kabuli*	Ca_Kabuli_Chr05	32171632	[G/A]	Ca_04917	Tetrapyrrole methylase	INTRON
8544	CakSNP8544	*Kabuli*	Ca_Kabuli_Chr05	32171652	[T/A]	Ca_04917	Tetrapyrrole methylase	INTRON
8545	CakSNP8545	*Kabuli*	Ca_Kabuli_Chr05	32209547	[C/G]	Ca_04921	ATPase, AAA+ type, core	SYNONYMOUS_CODING
8546	CakSNP8546	*Kabuli*	Ca_Kabuli_Chr05	32209750	[T/C]	Ca_04921	ATPase, AAA+ type, core	INTRON
8547	CakSNP8547	*Kabuli*	Ca_Kabuli_Chr05	32229485	[A/T]	Ca_04923	Protein kinase, catalytic domain	INTRON
8548	CakSNP8548	*Kabuli*	Ca_Kabuli_Chr05	32291158	[C/T]	Ca_04927	Aminotransferase-like, plant mobile domain	STOP_GAINED
8549	CakSNP8549	*Kabuli*	Ca_Kabuli_Chr05	32291118	[G/A]		--	INTERGENIC
8550	CakSNP8550	*Kabuli*	Ca_Kabuli_Chr05	32328110	[T/C]	Ca_04931	Inositol-pentakisphosphate 2-kinase	NON_SYNONYMOUS_CODING
8551	CakSNP8551	*Kabuli*	Ca_Kabuli_Chr05	32346655	[A/C]	Ca_04932	Cytochrome P450	INTRON

S.N.	SNP IDs	Chickpea cultivars	Chromosomes	Physical positions (bp)	SNPs	Gene accession IDs	Putative functions	Sequence components of genome
8552	CakSNP8552	*Kabuli*	Ca_Kabuli_Chr05	32357511	[G/A]	Ca_04934	Pseudouridine synthase/archaeosine transglycosylase	NON_SYNONYMOUS_CODING
8553	CakSNP8553	*Kabuli*	Ca_Kabuli_Chr05	32368485	[G/C]	Ca_04935	Nucleotidyl transferase	INTRON
8554	CakSNP8554	*Kabuli*	Ca_Kabuli_Chr05	32369197	[C/A]	Ca_04935	Nucleotidyl transferase	INTRON
8555	CakSNP8555	*Kabuli*	Ca_Kabuli_Chr05	32399391	[T/A]	Ca_04939	Ornithine carbamoyltransferase	NON_SYNONYMOUS_CODING
8556	CakSNP8556	*Kabuli*	Ca_Kabuli_Chr05	32476432	[A/G]	Ca_04951	--	NON_SYNONYMOUS_CODING
8557	CakSNP8557	*Kabuli*	Ca_Kabuli_Chr05	32476423	[A/G]	Ca_04951	--	INTRON
8558	CakSNP8558	*Kabuli*	Ca_Kabuli_Chr05	32516773	[T/A]	Ca_04958	--	INTRON
8559	CakSNP8559	*Kabuli*	Ca_Kabuli_Chr05	32524646	[T/C]	Ca_04959	--	INTRON
8560	CakSNP8560	*Kabuli*	Ca_Kabuli_Chr05	32566131	[C/G]	Ca_04964	--	INTRON
8561	CakSNP8561	*Kabuli*	Ca_Kabuli_Chr05	32598221	[C/A]	Ca_04966	Thaumatin, pathogenesis-related	NON_SYNONYMOUS_CODING

S.N.	SNP IDs	Chickpea cultivars	Chromosomes	Physical positions (bp)	SNPs	Gene accession IDs	Putative functions	Sequence components of genome
8562	CakSNP8562	*Kabuli*	Ca_Kabuli_Chr05	32623507	[T/G]	Ca_04968	Exocyst complex, component Exoc1	INTRON
8563	CakSNP8563	*Kabuli*	Ca_Kabuli_Chr05	32631930	[C/A]	Ca_04968	Exocyst complex, component Exoc1	INTRON
8564	CakSNP8564	*Kabuli*	Ca_Kabuli_Chr05	32643759	[A/G]	Ca_04970	--	INTRON
8565	CakSNP8565	*Kabuli*	Ca_Kabuli_Chr05	32732862	[A/T]	Ca_04978	Oligopeptide transporter OPT superfamily	SYNONYMOUS_CODING
8566	CakSNP8566	*Kabuli*	Ca_Kabuli_Chr05	32732913	[A/T]	Ca_04978	Oligopeptide transporter OPT superfamily	SYNONYMOUS_CODING
8567	CakSNP8567	*Kabuli*	Ca_Kabuli_Chr05	32759420	[T/A]	Ca_04979	Alcohol dehydrogenase superfamily, zinc-containing	INTRON
8568	CakSNP8568	*Kabuli*	Ca_Kabuli_Chr05	32761420	[G/A]	Ca_04979	Alcohol dehydrogenase superfamily, zinc-containing	INTRON
8569	CakSNP8569	*Kabuli*	Ca_Kabuli_Chr05	32794288	[G/A]		--	INTERGENIC
8570	CakSNP8570	*Kabuli*	Ca_Kabuli_Chr05	32816626	[G/T]		--	INTERGENIC
8571	CakSNP8571	*Kabuli*	Ca_Kabuli_Chr05	32839347	[C/A]	Ca_04983	Quinate/shikimate 5-dehydrogenase/glutamyl-tRNA reductase	SYNONYMOUS_CODING

S.N.	SNP IDs	Chickpea cultivars	Chromosomes	Physical positions (bp)	SNPs	Gene accession IDs	Putative functions	Sequence components of genome
8572	CakSNP8572	*Kabuli*	Ca_Kabuli_Chr05	32840512	[A/T]	Ca_04983	Quinate/shikimate 5-dehydrogenase/glutamyl-tRNA reductase	SYNONYMOUS_CODING
8573	CakSNP8573	*Kabuli*	Ca_Kabuli_Chr05	32840522	[T/C]	Ca_04983	Quinate/shikimate 5-dehydrogenase/glutamyl-tRNA reductase	NON_SYNONYMOUS_CODING
8574	CakSNP8574	*Kabuli*	Ca_Kabuli_Chr05	32840541	[T/G]	Ca_04983	Quinate/shikimate 5-dehydrogenase/glutamyl-tRNA reductase	NON_SYNONYMOUS_CODING
8575	CakSNP8575	*Kabuli*	Ca_Kabuli_Chr05	32859483	[T/A]	Ca_04985	Protein of unknown function DUF3453	INTRON
8576	CakSNP8576	*Kabuli*	Ca_Kabuli_Chr05	32859504	[T/G]	Ca_04985	Protein of unknown function DUF3453	INTRON
8577	CakSNP8577	*Kabuli*	Ca_Kabuli_Chr05	32859515	[T/G]	Ca_04985	Protein of unknown function DUF3453	INTRON
8578	CakSNP8578	*Kabuli*	Ca_Kabuli_Chr05	32860087	[C/T]	Ca_04985	Protein of unknown function DUF3453	INTRON
8579	CakSNP8579	*Kabuli*	Ca_Kabuli_Chr05	32978975	[A/T]	Ca_04996	Domain of unknown function DUF676, hydrolase-like	SYNONYMOUS_CODING
8580	CakSNP8580	*Kabuli*	Ca_Kabuli_Chr05	33052329	[C/G]		--	INTERGENIC
8581	CakSNP8581	*Kabuli*	Ca_Kabuli_Chr05	33143038	[A/T]		--	INTERGENIC

S.N.	SNP IDs	Chickpea cultivars	Chromosomes	Physical positions (bp)	SNPs	Gene accession IDs	Putative functions	Sequence components of genome
8582	CakSNP8582	*Kabuli*	Ca_Kabuli_Chr05	33189733	[A/G]	Ca_05010	Nucleotidyl transferase domain	NON_SYNONYMOUS_CODING
8583	CakSNP8583	*Kabuli*	Ca_Kabuli_Chr05	33189791	[G/A]	Ca_05010	Nucleotidyl transferase domain	NON_SYNONYMOUS_CODING
8584	CakSNP8584	*Kabuli*	Ca_Kabuli_Chr05	33189808	[A/G]	Ca_05010	Nucleotidyl transferase domain	NON_SYNONYMOUS_CODING
8585	CakSNP8585	*Kabuli*	Ca_Kabuli_Chr05	33255823	[T/G]		--	INTERGENIC
8586	CakSNP8586	*Kabuli*	Ca_Kabuli_Chr05	33255971	[G/T]	Ca_05018	Glycoside hydrolase, family 18, catalytic domain	NON_SYNONYMOUS_CODING
8587	CakSNP8587	*Kabuli*	Ca_Kabuli_Chr05	33256053	[C/G]	Ca_05018	Glycoside hydrolase, family 18, catalytic domain	SYNONYMOUS_CODING
8588	CakSNP8588	*Kabuli*	Ca_Kabuli_Chr05	33256089	[T/C]	Ca_05018	Glycoside hydrolase, family 18, catalytic domain	SYNONYMOUS_CODING
8589	CakSNP8589	*Kabuli*	Ca_Kabuli_Chr05	33256245	[T/C]	Ca_05018	Glycoside hydrolase, family 18, catalytic domain	SYNONYMOUS_CODING
8590	CakSNP8590	*Kabuli*	Ca_Kabuli_Chr05	33256412	[A/T]	Ca_05018	Glycoside hydrolase, family 18, catalytic domain	NON_SYNONYMOUS_CODING
8591	CakSNP8591	*Kabuli*	Ca_Kabuli_Chr05	33256388	[T/A]	Ca_05018	Glycoside hydrolase, family 18, catalytic domain	NON_SYNONYMOUS_CODING

S.N.	SNP IDs	Chickpea cultivars	Chromosomes	Physical positions (bp)	SNPs	Gene accession IDs	Putative functions	Sequence components of genome
8592	CakSNP8592	*Kabuli*	Ca_Kabuli_Chr05	33336127	[C/G]		--	INTERGENIC
8593	CakSNP8593	*Kabuli*	Ca_Kabuli_Chr05	33336135	[G/T]		--	INTERGENIC
8594	CakSNP8594	*Kabuli*	Ca_Kabuli_Chr05	33349598	[C/T]	Ca_01921	Alpha/beta hydrolase, N-terminal	SYNONYMOUS_CODING
8595	CakSNP8595	*Kabuli*	Ca_Kabuli_Chr05	33349700	[A/G]	Ca_01921	Alpha/beta hydrolase, N-terminal	INTRON
8596	CakSNP8596	*Kabuli*	Ca_Kabuli_Chr05	33379904	[A/G]	Ca_01919	Homeobox	SYNONYMOUS_CODING
8597	CakSNP8597	*Kabuli*	Ca_Kabuli_Chr05	33379873	[C/T]	Ca_01919	Homeobox	NON_SYNONYMOUS_CODING
8598	CakSNP8598	*Kabuli*	Ca_Kabuli_Chr05	33381774	[C/T]	Ca_01919	Homeobox	SYNONYMOUS_CODING
8599	CakSNP8599	*Kabuli*	Ca_Kabuli_Chr05	33422813	[C/T]	Ca_01914	Regulator of chromosome condensation, RCC1	SYNONYMOUS_CODING
8600	CakSNP8600	*Kabuli*	Ca_Kabuli_Chr05	33422780	[A/G]	Ca_01914	Regulator of chromosome condensation, RCC1	SYNONYMOUS_CODING
8601	CakSNP8601	*Kabuli*	Ca_Kabuli_Chr05	33422779	[C/T]	Ca_01914	Regulator of chromosome condensation, RCC1	NON_SYNONYMOUS_CODING

S.N.	SNP IDs	Chickpea cultivars	Chromosomes	Physical positions (bp)	SNPs	Gene accession IDs	Putative functions	Sequence components of genome
8602	CakSNP8602	*Kabuli*	Ca_Kabuli_Chr05	33422767	[T/C]	Ca_01914	Regulator of chromosome condensation, RCC1	NON_SYNONYMOUS_CODING
8603	CakSNP8603	*Kabuli*	Ca_Kabuli_Chr05	33431897	[T/C]	Ca_01913	Protein of unknown function DUF810	SYNONYMOUS_CODING
8604	CakSNP8604	*Kabuli*	Ca_Kabuli_Chr05	33431996	[T/C]	Ca_01913	Protein of unknown function DUF810	SYNONYMOUS_CODING
8605	CakSNP8605	*Kabuli*	Ca_Kabuli_Chr05	33523676	[T/C]		--	INTERGENIC
8606	CakSNP8606	*Kabuli*	Ca_Kabuli_Chr05	33523686	[G/A]		--	INTERGENIC
8607	CakSNP8607	*Kabuli*	Ca_Kabuli_Chr05	33523691	[T/C]		--	INTERGENIC
8608	CakSNP8608	*Kabuli*	Ca_Kabuli_Chr05	33532807	[A/G]	Ca_01905	Exostosin-like	INTRON
8609	CakSNP8609	*Kabuli*	Ca_Kabuli_Chr05	33532804	[A/T]	Ca_01905	Exostosin-like	INTRON
8610	CakSNP8610	*Kabuli*	Ca_Kabuli_Chr05	33532798	[A/T]	Ca_01905	Exostosin-like	INTRON
8611	CakSNP8611	*Kabuli*	Ca_Kabuli_Chr05	33574726	[T/C]	Ca_01901	Glyoxalase/bleomycin resistance protein/dioxygenase	SYNONYMOUS_CODING

S.N.	SNP IDs	Chickpea cultivars	Chromosomes	Physical positions (bp)	SNPs	Gene accession IDs	Putative functions	Sequence components of genome
8612	CakSNP8612	*Kabuli*	Ca_Kabuli_Chr05	33578061	[T/A]	Ca_01900	Pleckstrin homology domain	INTRON
8613	CakSNP8613	*Kabuli*	Ca_Kabuli_Chr05	33578167	[A/T]	Ca_01900	Pleckstrin homology domain	INTRON
8614	CakSNP8614	*Kabuli*	Ca_Kabuli_Chr05	33578438	[T/C]	Ca_01900	Pleckstrin homology domain	SYNONYMOUS_CODING
8615	CakSNP8615	*Kabuli*	Ca_Kabuli_Chr05	33578477	[A/G]	Ca_01900	Pleckstrin homology domain	SYNONYMOUS_CODING
8616	CakSNP8616	*Kabuli*	Ca_Kabuli_Chr05	33581040	[A/G]		--	INTERGENIC
8617	CakSNP8617	*Kabuli*	Ca_Kabuli_Chr05	33626841	[C/T]	Ca_01894	--	NON_SYNONYMOUS_CODING
8618	CakSNP8618	*Kabuli*	Ca_Kabuli_Chr05	33627132	[T/C]		--	INTERGENIC
8619	CakSNP8619	*Kabuli*	Ca_Kabuli_Chr05	33631108	[A/G]		--	INTERGENIC
8620	CakSNP8620	*Kabuli*	Ca_Kabuli_Chr05	33631266	[T/A]		--	INTERGENIC
8621	CakSNP8621	*Kabuli*	Ca_Kabuli_Chr05	33656560	[C/G]	Ca_01892	Tesmin/TSO1-like, CXC	NON_SYNONYMOUS_CODING

S.N.	SNP IDs	Chickpea cultivars	Chromosomes	Physical positions (bp)	SNPs	Gene accession IDs	Putative functions	Sequence components of genome
8622	CakSNP8622	*Kabuli*	Ca_Kabuli_Chr05	33673894	[T/C]	Ca_01888	VHS	NON_SYNONYMOUS_CODING
8623	CakSNP8623	*Kabuli*	Ca_Kabuli_Chr05	33700181	[G/C]	Ca_01885	Aspartate/other aminotransferase	INTRON
8624	CakSNP8624	*Kabuli*	Ca_Kabuli_Chr05	33700196	[A/T]	Ca_01885	Aspartate/other aminotransferase	INTRON
8625	CakSNP8625	*Kabuli*	Ca_Kabuli_Chr05	33721487	[C/T]	Ca_01882	Protein of unknown function DUF81	INTRON
8626	CakSNP8626	*Kabuli*	Ca_Kabuli_Chr05	33721544	[A/G]	Ca_01882	Protein of unknown function DUF81	SYNONYMOUS_CODING
8627	CakSNP8627	*Kabuli*	Ca_Kabuli_Chr05	33771359	[T/C]		--	INTERGENIC
8628	CakSNP8628	*Kabuli*	Ca_Kabuli_Chr05	33812108	[T/C]	Ca_01876	--	INTRON
8629	CakSNP8629	*Kabuli*	Ca_Kabuli_Chr05	33812213	[A/C]	Ca_01876	--	INTRON
8630	CakSNP8630	*Kabuli*	Ca_Kabuli_Chr05	33812363	[T/A]	Ca_01876	--	INTRON
8631	CakSNP8631	*Kabuli*	Ca_Kabuli_Chr05	33818742	[T/A]	Ca_01875	RNA recognition motif domain	NON_SYNONYMOUS_CODING

S.N.	SNP IDs	Chickpea cultivars	Chromosomes	Physical positions (bp)	SNPs	Gene accession IDs	Putative functions	Sequence components of genome
8632	CakSNP8632	*Kabuli*	Ca_Kabuli_Chr05	33818789	[C/G]	Ca_01875	RNA recognition motif domain	NON_SYNONYMOUS_CODING
8633	CakSNP8633	*Kabuli*	Ca_Kabuli_Chr05	33818823	[A/G]	Ca_01875	RNA recognition motif domain	NON_SYNONYMOUS_CODING
8634	CakSNP8634	*Kabuli*	Ca_Kabuli_Chr05	33824516	[G/T]		--	INTERGENIC
8635	CakSNP8635	*Kabuli*	Ca_Kabuli_Chr05	33824513	[G/A]		--	INTERGENIC
8636	CakSNP8636	*Kabuli*	Ca_Kabuli_Chr05	33824503	[G/A]		--	INTERGENIC
8637	CakSNP8637	*Kabuli*	Ca_Kabuli_Chr05	33835816	[T/C]		--	INTERGENIC
8638	CakSNP8638	*Kabuli*	Ca_Kabuli_Chr05	33841544	[T/G]	Ca_01870	Protein kinase, catalytic domain	INTRON
8639	CakSNP8639	*Kabuli*	Ca_Kabuli_Chr05	33844056	[A/T]		--	INTERGENIC
8640	CakSNP8640	*Kabuli*	Ca_Kabuli_Chr05	33844059	[G/C]		--	INTERGENIC
8641	CakSNP8641	*Kabuli*	Ca_Kabuli_Chr05	33844222	[T/C]		--	INTERGENIC

S.N.	SNP IDs	Chickpea cultivars	Chromosomes	Physical positions (bp)	SNPs	Gene accession IDs	Putative functions	Sequence components of genome
8642	CakSNP8642	*Kabuli*	Ca_Kabuli_Chr05	33844436	[T/A]		--	INTERGENIC
8643	CakSNP8643	*Kabuli*	Ca_Kabuli_Chr05	33892596	[A/C]	Ca_01864	Proteasome component (PCI) domain	NON_SYNONYMOUS_CODING
8644	CakSNP8644	*Kabuli*	Ca_Kabuli_Chr05	33937307	[G/A]		--	INTERGENIC
8645	CakSNP8645	*Kabuli*	Ca_Kabuli_Chr05	33937316	[G/A]		--	INTERGENIC
8646	CakSNP8646	*Kabuli*	Ca_Kabuli_Chr05	33963525	[G/C]		--	INTERGENIC
8647	CakSNP8647	*Kabuli*	Ca_Kabuli_Chr05	33985819	[A/G]	Ca_01852	Protein kinase, catalytic domain	SYNONYMOUS_CODING
8648	CakSNP8648	*Kabuli*	Ca_Kabuli_Chr05	33986234	[C/T]	Ca_01852	Protein kinase, catalytic domain	SYNONYMOUS_CODING
8649	CakSNP8649	*Kabuli*	Ca_Kabuli_Chr05	33986253	[A/C]	Ca_01852	Protein kinase, catalytic domain	SYNONYMOUS_CODING
8650	CakSNP8650	*Kabuli*	Ca_Kabuli_Chr05	34022362	[G/T]	Ca_01849	Glycosyl transferase, family 2	SYNONYMOUS_CODING
8651	CakSNP8651	*Kabuli*	Ca_Kabuli_Chr05	34040479	[A/G]		--	INTERGENIC

S.N.	SNP IDs	Chickpea cultivars	Chromosomes	Physical positions (bp)	SNPs	Gene accession IDs	Putative functions	Sequence components of genome
8652	CakSNP8652	*Kabuli*	Ca_Kabuli_Chr05	34040508	[A/C]		--	INTERGENIC
8653	CakSNP8653	*Kabuli*	Ca_Kabuli_Chr05	34040535	[G/A]		--	INTERGENIC
8654	CakSNP8654	*Kabuli*	Ca_Kabuli_Chr05	34041705	[G/T]		--	INTERGENIC
8655	CakSNP8655	*Kabuli*	Ca_Kabuli_Chr05	34073378	[A/T]	Ca_01841	Peptidyl-tRNA hydrolase, PTH2	INTRON
8656	CakSNP8656	*Kabuli*	Ca_Kabuli_Chr05	34073395	[G/A]	Ca_01841	Peptidyl-tRNA hydrolase, PTH2	INTRON
8657	CakSNP8657	*Kabuli*	Ca_Kabuli_Chr05	34073445	[A/T]	Ca_01841	Peptidyl-tRNA hydrolase, PTH2	INTRON
8658	CakSNP8658	*Kabuli*	Ca_Kabuli_Chr05	34201989	[C/G]	Ca_01827	Peptidase C19, ubiquitin carboxyl-terminal hydrolase 2	NON_SYNONYMOUS_CODING
8659	CakSNP8659	*Kabuli*	Ca_Kabuli_Chr05	34202000	[T/C]	Ca_01827	Peptidase C19, ubiquitin carboxyl-terminal hydrolase 2	SYNONYMOUS_CODING
8660	CakSNP8660	*Kabuli*	Ca_Kabuli_Chr05	34202075	[T/G]	Ca_01827	Peptidase C19, ubiquitin carboxyl-terminal hydrolase 2	NON_SYNONYMOUS_CODING
8661	CakSNP8661	*Kabuli*	Ca_Kabuli_Chr05	34202222	[G/A]	Ca_01827	Peptidase C19, ubiquitin carboxyl-terminal hydrolase 2	INTRON

S.N.	SNP IDs	Chickpea cultivars	Chromosomes	Physical positions (bp)	SNPs	Gene accession IDs	Putative functions	Sequence components of genome
8662	CakSNP8662	*Kabuli*	Ca_Kabuli_Chr05	34241269	[C/G]	Ca_01824	WD40 repeat	SYNONYMOUS_CODING
8663	CakSNP8663	*Kabuli*	Ca_Kabuli_Chr05	34244499	[C/T]	Ca_01824	WD40 repeat	INTRON
8664	CakSNP8664	*Kabuli*	Ca_Kabuli_Chr05	34244681	[G/A]	Ca_01824	WD40 repeat	INTRON
8665	CakSNP8665	*Kabuli*	Ca_Kabuli_Chr05	34244670	[A/G]	Ca_01824	WD40 repeat	INTRON
8666	CakSNP8666	*Kabuli*	Ca_Kabuli_Chr05	34244662	[C/G]	Ca_01824	WD40 repeat	INTRON
8667	CakSNP8667	*Kabuli*	Ca_Kabuli_Chr05	34244754	[T/A]	Ca_01824	WD40 repeat	INTRON
8668	CakSNP8668	*Kabuli*	Ca_Kabuli_Chr05	34313779	[A/G]		--	INTERGENIC
8669	CakSNP8669	*Kabuli*	Ca_Kabuli_Chr05	34313758	[T/G]		--	INTERGENIC
8670	CakSNP8670	*Kabuli*	Ca_Kabuli_Chr05	34334980	[A/G]	Ca_01813	Ribosomal protein L10/acidic P0	INTRON
8671	CakSNP8671	*Kabuli*	Ca_Kabuli_Chr05	34334993	[A/C]	Ca_01813	Ribosomal protein L10/acidic P0	INTRON

S.N.	SNP IDs	Chickpea cultivars	Chromosomes	Physical positions (bp)	SNPs	Gene accession IDs	Putative functions	Sequence components of genome
8672	CakSNP8672	*Kabuli*	Ca_Kabuli_Chr05	34335252	[T/A]	Ca_01813	Ribosomal protein L10/acidic P0	SYNONYMOUS_CODING
8673	CakSNP8673	*Kabuli*	Ca_Kabuli_Chr05	34418798	[G/C]	Ca_01803	Protein of unknown function DUF490	INTRON
8674	CakSNP8674	*Kabuli*	Ca_Kabuli_Chr05	34441581	[A/C]	Ca_01800	Transcription elongation factor S-II, central domain	NON_SYNONYMOUS_CODING
8675	CakSNP8675	*Kabuli*	Ca_Kabuli_Chr05	34461159	[T/C]	Ca_01797	Cyclin, C-terminal	NON_SYNONYMOUS_CODING
8676	CakSNP8676	*Kabuli*	Ca_Kabuli_Chr05	34482245	[G/T]	Ca_01795	Glycosyl transferase, family 8	NON_SYNONYMOUS_CODING
8677	CakSNP8677	*Kabuli*	Ca_Kabuli_Chr05	34482217	[G/T]	Ca_01795	Glycosyl transferase, family 8	SYNONYMOUS_CODING
8678	CakSNP8678	*Kabuli*	Ca_Kabuli_Chr05	34511669	[G/T]	Ca_01790	WD40 repeat	NON_SYNONYMOUS_CODING
8679	CakSNP8679	*Kabuli*	Ca_Kabuli_Chr05	34511678	[T/C]	Ca_01790	WD40 repeat	SYNONYMOUS_CODING
8680	CakSNP8680	*Kabuli*	Ca_Kabuli_Chr05	34511693	[C/T]	Ca_01790	WD40 repeat	SYNONYMOUS_CODING
8681	CakSNP8681	*Kabuli*	Ca_Kabuli_Chr05	34511705	[G/T]	Ca_01790	WD40 repeat	SYNONYMOUS_CODING

S.N.	SNP IDs	Chickpea cultivars	Chromosomes	Physical positions (bp)	SNPs	Gene accession IDs	Putative functions	Sequence components of genome
8682	CakSNP8682	*Kabuli*	Ca_Kabuli_Chr05	34521655	[T/C]		--	INTERGENIC
8683	CakSNP8683	*Kabuli*	Ca_Kabuli_Chr05	34531222	[A/G]	Ca_01787	ABC transporter-like	INTRON
8684	CakSNP8684	*Kabuli*	Ca_Kabuli_Chr05	34535758	[T/C]	Ca_01787	ABC transporter-like	NON_SYNONYMOUS_CODING
8685	CakSNP8685	*Kabuli*	Ca_Kabuli_Chr05	34535893	[G/T]		--	INTERGENIC
8686	CakSNP8686	*Kabuli*	Ca_Kabuli_Chr05	34535878	[G/A]		--	INTERGENIC
8687	CakSNP8687	*Kabuli*	Ca_Kabuli_Chr05	34629028	[A/G]		--	INTERGENIC
8688	CakSNP8688	*Kabuli*	Ca_Kabuli_Chr05	34673784	[T/A]	Ca_01773	--	INTRON
8689	CakSNP8689	*Kabuli*	Ca_Kabuli_Chr05	34691234	[A/C]		--	INTERGENIC
8690	CakSNP8690	*Kabuli*	Ca_Kabuli_Chr05	34691302	[T/C]		--	INTERGENIC
8691	CakSNP8691	*Kabuli*	Ca_Kabuli_Chr05	34691453	[G/A]	Ca_01770	Mitochondrial carrier protein	SYNONYMOUS_CODING

S.N.	SNP IDs	Chickpea cultivars	Chromosomes	Physical positions (bp)	SNPs	Gene accession IDs	Putative functions	Sequence components of genome
8692	CakSNP8692	*Kabuli*	Ca_Kabuli_Chr05	34807071	[C/T]	Ca_01760	F-box domain, cyclin-like	SYNONYMOUS_CODING
8693	CakSNP8693	*Kabuli*	Ca_Kabuli_Chr05	34814216	[T/C]	Ca_01759	Xklp2 targeting protein	SYNONYMOUS_CODING
8694	CakSNP8694	*Kabuli*	Ca_Kabuli_Chr05	34824647	[C/G]	Ca_01758	Haem peroxidase, plant/fungal/bacterial	SYNONYMOUS_CODING
8695	CakSNP8695	*Kabuli*	Ca_Kabuli_Chr05	34824749	[A/T]	Ca_01758	Haem peroxidase, plant/fungal/bacterial	INTRON
8696	CakSNP8696	*Kabuli*	Ca_Kabuli_Chr05	34845398	[T/G]	Ca_01755	ANTH	SYNONYMOUS_CODING
8697	CakSNP8697	*Kabuli*	Ca_Kabuli_Chr05	34879731	[A/G]	Ca_01752	Ubiquitin supergroup	INTRON
8698	CakSNP8698	*Kabuli*	Ca_Kabuli_Chr05	34974615	[T/A]	Ca_01738	Cysteinyl-tRNA synthetase, class Ia	INTRON
8699	CakSNP8699	*Kabuli*	Ca_Kabuli_Chr05	34974703	[C/T]	Ca_01738	Cysteinyl-tRNA synthetase, class Ia	STOP_GAINED
8700	CakSNP8700	*Kabuli*	Ca_Kabuli_Chr05	34974795	[A/G]	Ca_01738	Cysteinyl-tRNA synthetase, class Ia	SYNONYMOUS_CODING
8701	CakSNP8701	*Kabuli*	Ca_Kabuli_Chr05	34975712	[T/A]		--	INTERGENIC

S.N.	SNP IDs	Chickpea cultivars	Chromosomes	Physical positions (bp)	SNPs	Gene accession IDs	Putative functions	Sequence components of genome
8702	CakSNP8702	*Kabuli*	Ca_Kabuli_Chr05	34998725	[G/A]	Ca_01735	--	INTRON
8703	CakSNP8703	*Kabuli*	Ca_Kabuli_Chr05	35126544	[A/C]	Ca_01725	Domain of unknown function DUF1086	INTRON
8704	CakSNP8704	*Kabuli*	Ca_Kabuli_Chr05	35164295	[C/T]	Ca_01720	Chaperonin ClpA/B	SYNONYMOUS_CODING
8705	CakSNP8705	*Kabuli*	Ca_Kabuli_Chr05	35191371	[C/G]	Ca_01716	Proteasome component (PCI) domain	NON_SYNONYMOUS_CODING
8706	CakSNP8706	*Kabuli*	Ca_Kabuli_Chr05	35209511	[T/C]	Ca_01715	--	SYNONYMOUS_CODING
8707	CakSNP8707	*Kabuli*	Ca_Kabuli_Chr05	35213601	[C/T]	Ca_01715	--	NON_SYNONYMOUS_CODING
8708	CakSNP8708	*Kabuli*	Ca_Kabuli_Chr05	35226049	[G/C]		--	INTERGENIC
8709	CakSNP8709	*Kabuli*	Ca_Kabuli_Chr05	35290452	[A/G]	Ca_01709	Phospholipase D/Transphosphatidylase	SYNONYMOUS_CODING
8710	CakSNP8710	*Kabuli*	Ca_Kabuli_Chr05	35296135	[A/C]	Ca_01708	Nonaspanin (TM9SF)	INTRON
8711	CakSNP8711	*Kabuli*	Ca_Kabuli_Chr05	35330946	[G/A]	Ca_01701	--	SYNONYMOUS_CODING

S.N.	SNP IDs	Chickpea cultivars	Chromosomes	Physical positions (bp)	SNPs	Gene accession IDs	Putative functions	Sequence components of genome
8712	CakSNP8712	*Kabuli*	Ca_Kabuli_Chr05	35330995	[A/C]		--	INTERGENIC
8713	CakSNP8713	*Kabuli*	Ca_Kabuli_Chr05	35340068	[A/G]	Ca_01699	Metallo-dependent phosphatase	SYNONYMOUS_CODING
8714	CakSNP8714	*Kabuli*	Ca_Kabuli_Chr05	35340065	[G/A]	Ca_01699	Metallo-dependent phosphatase	SYNONYMOUS_CODING
8715	CakSNP8715	*Kabuli*	Ca_Kabuli_Chr05	35374184	[G/A]	Ca_01696	ABC-1	NON_SYNONYMOUS_CODING
8716	CakSNP8716	*Kabuli*	Ca_Kabuli_Chr05	35438327	[G/T]	Ca_01691	Kinase binding protein CGI-121	SYNONYMOUS_CODING
8717	CakSNP8717	*Kabuli*	Ca_Kabuli_Chr05	35495900	[T/A]	Ca_01683	Pathogenesis-related transcriptional factor/ERF, DNA-binding	SYNONYMOUS_CODING
8718	CakSNP8718	*Kabuli*	Ca_Kabuli_Chr05	35500751	[T/C]	Ca_01682	Sterile alpha motif domain	SYNONYMOUS_CODING
8719	CakSNP8719	*Kabuli*	Ca_Kabuli_Chr05	35534923	[T/A]	Ca_01677	Ribonuclease III	NON_SYNONYMOUS_CODING
8720	CakSNP8720	*Kabuli*	Ca_Kabuli_Chr05	35534897	[G/A]		--	INTERGENIC
8721	CakSNP8721	*Kabuli*	Ca_Kabuli_Chr05	35540372	[T/C]	Ca_01677	Ribonuclease III	NON_SYNONYMOUS_CODING

S.N.	SNP IDs	Chickpea cultivars	Chromosomes	Physical positions (bp)	SNPs	Gene accession IDs	Putative functions	Sequence components of genome
8722	CakSNP8722	*Kabuli*	Ca_Kabuli_Chr05	35543648	[T/C]	Ca_01677	Ribonuclease III	INTRON
8723	CakSNP8723	*Kabuli*	Ca_Kabuli_Chr05	35554534	[C/A]		--	INTERGENIC
8724	CakSNP8724	*Kabuli*	Ca_Kabuli_Chr05	35554593	[C/T]		--	INTERGENIC
8725	CakSNP8725	*Kabuli*	Ca_Kabuli_Chr05	35555184	[A/G]		--	INTERGENIC
8726	CakSNP8726	*Kabuli*	Ca_Kabuli_Chr05	35576495	[A/G]		--	INTERGENIC
8727	CakSNP8727	*Kabuli*	Ca_Kabuli_Chr05	35617798	[A/C]	Ca_01670	Glycoside hydrolase, family 9	NON_SYNONYMOUS_CODING
8728	CakSNP8728	*Kabuli*	Ca_Kabuli_Chr05	35688558	[T/C]		--	INTERGENIC
8729	CakSNP8729	*Kabuli*	Ca_Kabuli_Chr05	35690996	[G/A]	Ca_01660	Mechanosensitive ion channel MscS	NON_SYNONYMOUS_CODING
8730	CakSNP8730	*Kabuli*	Ca_Kabuli_Chr05	35691581	[T/C]	Ca_01660	Mechanosensitive ion channel MscS	INTRON
8731	CakSNP8731	*Kabuli*	Ca_Kabuli_Chr05	35715495	[C/T]		--	INTERGENIC

S.N.	SNP IDs	Chickpea cultivars	Chromosomes	Physical positions (bp)	SNPs	Gene accession IDs	Putative functions	Sequence components of genome
8732	CakSNP8732	*Kabuli*	Ca_Kabuli_Chr05	35715550	[G/C]		--	INTERGENIC
8733	CakSNP8733	*Kabuli*	Ca_Kabuli_Chr05	35715571	[A/C]		--	INTERGENIC
8734	CakSNP8734	*Kabuli*	Ca_Kabuli_Chr05	35715563	[G/A]		--	INTERGENIC
8735	CakSNP8735	*Kabuli*	Ca_Kabuli_Chr05	35715548	[T/C]		--	INTERGENIC
8736	CakSNP8736	*Kabuli*	Ca_Kabuli_Chr05	35773040	[C/A]	Ca_01648	Peptidase M1, alanine aminopeptidase/leukotriene A4 hydrolase	SYNONYMOUS_CODING
8737	CakSNP8737	*Kabuli*	Ca_Kabuli_Chr05	35773044	[A/G]	Ca_01648	Peptidase M1, alanine aminopeptidase/leukotriene A4 hydrolase	NON_SYNONYMOUS_CODING
8738	CakSNP8738	*Kabuli*	Ca_Kabuli_Chr05	35775200	[T/C]	Ca_01648	Peptidase M1, alanine aminopeptidase/leukotriene A4 hydrolase	INTRON
8739	CakSNP8739	*Kabuli*	Ca_Kabuli_Chr05	35775191	[T/G]	Ca_01648	Peptidase M1, alanine aminopeptidase/leukotriene A4 hydrolase	INTRON
8740	CakSNP8740	*Kabuli*	Ca_Kabuli_Chr05	35775266	[G/A]	Ca_01648	Peptidase M1, alanine aminopeptidase/leukotriene A4 hydrolase	NON_SYNONYMOUS_CODING
8741	CakSNP8741	*Kabuli*	Ca_Kabuli_Chr05	35789373	[T/A]	Ca_01646	Protein kinase, catalytic domain	INTRON

S.N.	SNP IDs	Chickpea cultivars	Chromosomes	Physical positions (bp)	SNPs	Gene accession IDs	Putative functions	Sequence components of genome
8742	CakSNP8742	*Kabuli*	Ca_Kabuli_Chr05	35789775	[A/G]	Ca_01646	Protein kinase, catalytic domain	INTRON
8743	CakSNP8743	*Kabuli*	Ca_Kabuli_Chr05	35839230	[T/C]	Ca_01642	Mitochondrial carrier protein	SYNONYMOUS_CODING
8744	CakSNP8744	*Kabuli*	Ca_Kabuli_Chr05	35839233	[G/A]	Ca_01642	Mitochondrial carrier protein	SYNONYMOUS_CODING
8745	CakSNP8745	*Kabuli*	Ca_Kabuli_Chr05	35844295	[G/T]	Ca_01641	IQ motif, EF-hand binding site	NON_SYNONYMOUS_CODING
8746	CakSNP8746	*Kabuli*	Ca_Kabuli_Chr05	35845094	[G/A]	Ca_01641	IQ motif, EF-hand binding site	SYNONYMOUS_CODING
8747	CakSNP8747	*Kabuli*	Ca_Kabuli_Chr05	35845780	[T/C]	Ca_01641	IQ motif, EF-hand binding site	SYNONYMOUS_CODING
8748	CakSNP8748	*Kabuli*	Ca_Kabuli_Chr05	35845801	[T/C]	Ca_01641	IQ motif, EF-hand binding site	SYNONYMOUS_CODING
8749	CakSNP8749	*Kabuli*	Ca_Kabuli_Chr05	35846404	[A/G]		--	INTERGENIC
8750	CakSNP8750	*Kabuli*	Ca_Kabuli_Chr05	35868865	[G/A]	Ca_01639	Glutaredoxin	NON_SYNONYMOUS_CODING
8751	CakSNP8751	*Kabuli*	Ca_Kabuli_Chr05	35877505	[T/C]	Ca_01638	Mitochondrial carrier protein	SYNONYMOUS_CODING

S.N.	SNP IDs	Chickpea cultivars	Chromosomes	Physical positions (bp)	SNPs	Gene accession IDs	Putative functions	Sequence components of genome
8752	CakSNP8752	*Kabuli*	Ca_Kabuli_Chr05	35926105	[T/C]		--	INTERGENIC
8753	CakSNP8753	*Kabuli*	Ca_Kabuli_Chr05	35937904	[C/T]	Ca_01631	NADH:ubiquinone oxidoreductase-like, 20kDa subunit	SYNONYMOUS_CODING
8754	CakSNP8754	*Kabuli*	Ca_Kabuli_Chr05	35937842	[G/T]	Ca_01631	NADH:ubiquinone oxidoreductase-like, 20kDa subunit	NON_SYNONYMOUS_CODING
8755	CakSNP8755	*Kabuli*	Ca_Kabuli_Chr05	35947574	[G/A]	Ca_01630	K Homology	NON_SYNONYMOUS_CODING
8756	CakSNP8756	*Kabuli*	Ca_Kabuli_Chr05	35954656	[T/C]	Ca_01628	Phosphorylated adapter RNA export protein, RNA-binding domain	INTRON
8757	CakSNP8757	*Kabuli*	Ca_Kabuli_Chr05	35956534	[C/T]	Ca_01628	Phosphorylated adapter RNA export protein, RNA-binding domain	SYNONYMOUS_CODING
8758	CakSNP8758	*Kabuli*	Ca_Kabuli_Chr05	36012698	[C/G]	Ca_01620	--	NON_SYNONYMOUS_CODING
8759	CakSNP8759	*Kabuli*	Ca_Kabuli_Chr05	36039365	[A/C]	Ca_01618	Glycoside hydrolase, family 10	NON_SYNONYMOUS_CODING
8760	CakSNP8760	*Kabuli*	Ca_Kabuli_Chr05	36128655	[G/T]	Ca_01611	--	INTRON
8761	CakSNP8761	*Kabuli*	Ca_Kabuli_Chr05	36131156	[A/C]	Ca_01611	--	INTRON

S.N.	SNP IDs	Chickpea cultivars	Chromosomes	Physical positions (bp)	SNPs	Gene accession IDs	Putative functions	Sequence components of genome
8762	CakSNP8762	*Kabuli*	Ca_Kabuli_Chr05	36217803	[C/T]		--	INTERGENIC
8763	CakSNP8763	*Kabuli*	Ca_Kabuli_Chr05	36238656	[G/A]	Ca_01601	ATPase, P-type, K/Mg/Cd/Cu/Zn/Na/Ca/Na/H-transporter	INTRON
8764	CakSNP8764	*Kabuli*	Ca_Kabuli_Chr05	36238655	[G/C]	Ca_01601	ATPase, P-type, K/Mg/Cd/Cu/Zn/Na/Ca/Na/H-transporter	INTRON
8765	CakSNP8765	*Kabuli*	Ca_Kabuli_Chr05	36248568	[C/T]	Ca_01601	ATPase, P-type, K/Mg/Cd/Cu/Zn/Na/Ca/Na/H-transporter	INTRON
8766	CakSNP8766	*Kabuli*	Ca_Kabuli_Chr05	36292611	[C/G]	Ca_01595	WD40 repeat	SYNONYMOUS_CODING
8767	CakSNP8767	*Kabuli*	Ca_Kabuli_Chr05	36334569	[C/A]		--	INTERGENIC
8768	CakSNP8768	*Kabuli*	Ca_Kabuli_Chr05	36334592	[C/T]		--	INTERGENIC
8769	CakSNP8769	*Kabuli*	Ca_Kabuli_Chr05	36334625	[A/G]		--	INTERGENIC
8770	CakSNP8770	*Kabuli*	Ca_Kabuli_Chr05	36338598	[A/C]	Ca_01591	ATPase, P-type, H+ transporting proton pump	INTRON
8771	CakSNP8771	*Kabuli*	Ca_Kabuli_Chr05	36338658	[A/G]	Ca_01591	ATPase, P-type, H+ transporting proton pump	INTRON

S.N.	SNP IDs	Chickpea cultivars	Chromosomes	Physical positions (bp)	SNPs	Gene accession IDs	Putative functions	Sequence components of genome
8772	CakSNP8772	*Kabuli*	Ca_Kabuli_Chr05	36397489	[A/C]	Ca_01585	Transcription initiation factor TFIID component TAF4	SYNONYMOUS_CODING
8773	CakSNP8773	*Kabuli*	Ca_Kabuli_Chr05	36400943	[C/T]	Ca_01585	Transcription initiation factor TFIID component TAF4	INTRON
8774	CakSNP8774	*Kabuli*	Ca_Kabuli_Chr05	36408701	[G/A]	Ca_01584	Peptidase S8/S53, subtilisin/kexin/sedolisin	NON_SYNONYMOUS_CODING
8775	CakSNP8775	*Kabuli*	Ca_Kabuli_Chr05	36408719	[G/A]	Ca_01584	Peptidase S8/S53, subtilisin/kexin/sedolisin	NON_SYNONYMOUS_CODING
8776	CakSNP8776	*Kabuli*	Ca_Kabuli_Chr05	36408724	[T/C]	Ca_01584	Peptidase S8/S53, subtilisin/kexin/sedolisin	SYNONYMOUS_CODING
8777	CakSNP8777	*Kabuli*	Ca_Kabuli_Chr05	36408736	[C/A]	Ca_01584	Peptidase S8/S53, subtilisin/kexin/sedolisin	SYNONYMOUS_CODING
8778	CakSNP8778	*Kabuli*	Ca_Kabuli_Chr05	36430677	[A/C]	Ca_01578	NAD-dependent epimerase/dehydratase	SYNONYMOUS_CODING
8779	CakSNP8779	*Kabuli*	Ca_Kabuli_Chr05	36441063	[C/T]	Ca_01576	Protein of unknown function DUF620	SYNONYMOUS_CODING
8780	CakSNP8780	*Kabuli*	Ca_Kabuli_Chr05	36533996	[T/A]	Ca_01566	MRG	SYNONYMOUS_CODING
8781	CakSNP8781	*Kabuli*	Ca_Kabuli_Chr05	36534056	[G/C]	Ca_01566	MRG	INTRON

S.N.	SNP IDs	Chickpea cultivars	Chromosomes	Physical positions (bp)	SNPs	Gene accession IDs	Putative functions	Sequence components of genome
8782	CakSNP8782	*Kabuli*	Ca_Kabuli_Chr05	36534057	[T/G]	Ca_01566	MRG	INTRON
8783	CakSNP8783	*Kabuli*	Ca_Kabuli_Chr05	36534060	[G/A]	Ca_01566	MRG	INTRON
8784	CakSNP8784	*Kabuli*	Ca_Kabuli_Chr05	36534063	[C/T]	Ca_01566	MRG	INTRON
8785	CakSNP8785	*Kabuli*	Ca_Kabuli_Chr05	36534126	[A/G]	Ca_01566	MRG	INTRON
8786	CakSNP8786	*Kabuli*	Ca_Kabuli_Chr05	36534110	[T/C]	Ca_01566	MRG	INTRON
8787	CakSNP8787	*Kabuli*	Ca_Kabuli_Chr05	36566175	[G/A]	Ca_01562	Peptidase T2, asparaginase 2	SYNONYMOUS_CODING
8788	CakSNP8788	*Kabuli*	Ca_Kabuli_Chr05	36613610	[T/C]	Ca_01559	Protein kinase, catalytic domain	SYNONYMOUS_CODING
8789	CakSNP8789	*Kabuli*	Ca_Kabuli_Chr05	36619837	[A/G]	Ca_01558	Kinesin, motor domain	INTRON
8790	CakSNP8790	*Kabuli*	Ca_Kabuli_Chr05	36621872	[A/T]	Ca_01558	Kinesin, motor domain	NON_SYNONYMOUS_CODING
8791	CakSNP8791	*Kabuli*	Ca_Kabuli_Chr05	36672147	[C/T]	Ca_01555	RNA recognition motif domain	NON_SYNONYMOUS_CODING

S.N.	SNP IDs	Chickpea cultivars	Chromosomes	Physical positions (bp)	SNPs	Gene accession IDs	Putative functions	Sequence components of genome
8792	CakSNP8792	*Kabuli*	Ca_Kabuli_Chr05	36672164	[A/G]	Ca_01555	RNA recognition motif domain	SYNONYMOUS_CODING
8793	CakSNP8793	*Kabuli*	Ca_Kabuli_Chr05	36673950	[C/A]	Ca_01554	Transferase	NON_SYNONYMOUS_CODING
8794	CakSNP8794	*Kabuli*	Ca_Kabuli_Chr05	36731961	[T/C]		--	INTERGENIC
8795	CakSNP8795	*Kabuli*	Ca_Kabuli_Chr05	36735459	[A/T]	Ca_01550	Helix-loop-helix DNA-binding domain	NON_SYNONYMOUS_CODING
8796	CakSNP8796	*Kabuli*	Ca_Kabuli_Chr05	36751625	[C/T]		--	INTERGENIC
8797	CakSNP8797	*Kabuli*	Ca_Kabuli_Chr05	36751851	[T/A]		--	INTERGENIC
8798	CakSNP8798	*Kabuli*	Ca_Kabuli_Chr05	36757408	[G/A]		--	INTERGENIC
8799	CakSNP8799	*Kabuli*	Ca_Kabuli_Chr05	36797173	[A/G]	Ca_01541	Chlorophyll A-B binding protein, plant	NON_SYNONYMOUS_CODING
8800	CakSNP8800	*Kabuli*	Ca_Kabuli_Chr05	36797187	[G/T]	Ca_01541	Chlorophyll A-B binding protein, plant	NON_SYNONYMOUS_CODING
8801	CakSNP8801	*Kabuli*	Ca_Kabuli_Chr05	36800497	[G/T]	Ca_01540	Protein of unknown function DUF827, plant	SYNONYMOUS_CODING

S.N.	SNP IDs	Chickpea cultivars	Chromosomes	Physical positions (bp)	SNPs	Gene accession IDs	Putative functions	Sequence components of genome
8802	CakSNP8802	*Kabuli*	Ca_Kabuli_Chr05	36842784	[G/T]	Ca_01537	Protein phosphatase 2C-like	INTRON
8803	CakSNP8803	*Kabuli*	Ca_Kabuli_Chr05	36855028	[G/A]	Ca_01536	Transcriptional factor B3	INTRON
8804	CakSNP8804	*Kabuli*	Ca_Kabuli_Chr05	36916560	[T/C]	Ca_01528	Tetrapyrrole biosynthesis, uroporphyrinogen III synthase	INTRON
8805	CakSNP8805	*Kabuli*	Ca_Kabuli_Chr05	36999074	[G/C]	Ca_01518	Protein kinase, catalytic domain	NON_SYNONYMOUS_CODING
8806	CakSNP8806	*Kabuli*	Ca_Kabuli_Chr05	37016704	[C/G]	Ca_01515	Oligopeptide transporter	SYNONYMOUS_CODING
8807	CakSNP8807	*Kabuli*	Ca_Kabuli_Chr05	37039246	[A/T]		--	INTERGENIC
8808	CakSNP8808	*Kabuli*	Ca_Kabuli_Chr05	37039300	[A/G]		--	INTERGENIC
8809	CakSNP8809	*Kabuli*	Ca_Kabuli_Chr05	37040045	[G/A]	Ca_01510	Protein of unknown function DUF2053, membrane	SYNONYMOUS_CODING
8810	CakSNP8810	*Kabuli*	Ca_Kabuli_Chr05	37067293	[A/G]	Ca_01509	Aminoacyl-tRNA synthetase, class II (G/ H/ P/ S), conserved domain	INTRON
8811	CakSNP8811	*Kabuli*	Ca_Kabuli_Chr05	37067371	[T/C]	Ca_01509	Aminoacyl-tRNA synthetase, class II (G/ H/ P/ S), conserved domain	INTRON

S.N.	SNP IDs	Chickpea cultivars	Chromosomes	Physical positions (bp)	SNPs	Gene accession IDs	Putative functions	Sequence components of genome
8812	CakSNP8812	*Kabuli*	Ca_Kabuli_Chr05	37067384	[G/C]	Ca_01509	Aminoacyl-tRNA synthetase, class II (G/ H/ P/ S), conserved domain	INTRON
8813	CakSNP8813	*Kabuli*	Ca_Kabuli_Chr05	37083276	[T/G]	Ca_01507	GRAM	INTRON
8814	CakSNP8814	*Kabuli*	Ca_Kabuli_Chr05	37083275	[G/T]	Ca_01507	GRAM	INTRON
8815	CakSNP8815	*Kabuli*	Ca_Kabuli_Chr05	37114574	[A/G]	Ca_01505	WD40 repeat	INTRON
8816	CakSNP8816	*Kabuli*	Ca_Kabuli_Chr05	37126371	[A/G]		--	INTERGENIC
8817	CakSNP8817	*Kabuli*	Ca_Kabuli_Chr05	37126354	[T/C]		--	INTERGENIC
8818	CakSNP8818	*Kabuli*	Ca_Kabuli_Chr05	37147572	[C/T]	Ca_01503	ZF-HD homeobox protein, Cys/His-rich dimerisation domain	SYNONYMOUS_CODING
8819	CakSNP8819	*Kabuli*	Ca_Kabuli_Chr05	37167061	[A/G]	Ca_01500	Rho GTPase-activating protein domain	INTRON
8820	CakSNP8820	*Kabuli*	Ca_Kabuli_Chr05	37167114	[A/T]	Ca_01500	Rho GTPase-activating protein domain	INTRON
8821	CakSNP8821	*Kabuli*	Ca_Kabuli_Chr05	37167108	[A/T]	Ca_01500	Rho GTPase-activating protein domain	INTRON

S.N.	SNP IDs	Chickpea cultivars	Chromosomes	Physical positions (bp)	SNPs	Gene accession IDs	Putative functions	Sequence components of genome
8822	CakSNP8822	*Kabuli*	Ca_Kabuli_Chr05	37194939	[G/A]	Ca_01498	--	NON_SYNONYMOUS_CODING
8823	CakSNP8823	*Kabuli*	Ca_Kabuli_Chr05	37194917	[G/A]	Ca_01498	--	SYNONYMOUS_CODING
8824	CakSNP8824	*Kabuli*	Ca_Kabuli_Chr05	37197321	[C/A]	Ca_01497	Peptidase A1	SYNONYMOUS_CODING
8825	CakSNP8825	*Kabuli*	Ca_Kabuli_Chr05	37207821	[A/T]	Ca_01496	Peptidase C19, ubiquitin carboxyl-terminal hydrolase 2	SYNONYMOUS_CODING
8826	CakSNP8826	*Kabuli*	Ca_Kabuli_Chr05	37207837	[G/T]	Ca_01496	Peptidase C19, ubiquitin carboxyl-terminal hydrolase 2	NON_SYNONYMOUS_CODING
8827	CakSNP8827	*Kabuli*	Ca_Kabuli_Chr05	37211645	[T/G]	Ca_01496	Peptidase C19, ubiquitin carboxyl-terminal hydrolase 2	INTRON
8828	CakSNP8828	*Kabuli*	Ca_Kabuli_Chr05	37213853	[C/T]	Ca_01496	Peptidase C19, ubiquitin carboxyl-terminal hydrolase 2	SYNONYMOUS_CODING
8829	CakSNP8829	*Kabuli*	Ca_Kabuli_Chr05	37232307	[T/C]		--	INTERGENIC
8830	CakSNP8830	*Kabuli*	Ca_Kabuli_Chr05	37254596	[A/G]	Ca_01492	Peptidase C48, SUMO/Sentrin/Ubl1	SYNONYMOUS_CODING
8831	CakSNP8831	*Kabuli*	Ca_Kabuli_Chr05	37260601	[T/C]		--	INTERGENIC

S.N.	SNP IDs	Chickpea cultivars	Chromosomes	Physical positions (bp)	SNPs	Gene accession IDs	Putative functions	Sequence components of genome
8832	CakSNP8832	*Kabuli*	Ca_Kabuli_Chr05	37274572	[C/T]		--	INTERGENIC
8833	CakSNP8833	*Kabuli*	Ca_Kabuli_Chr05	37274583	[C/T]		--	INTERGENIC
8834	CakSNP8834	*Kabuli*	Ca_Kabuli_Chr05	37274616	[C/G]		--	INTERGENIC
8835	CakSNP8835	*Kabuli*	Ca_Kabuli_Chr05	37309917	[T/G]	Ca_01488	GDP-fucose protein O-fucosyltransferase	NON_SYNONYMOUS_CODING
8836	CakSNP8836	*Kabuli*	Ca_Kabuli_Chr05	37309935	[T/G]	Ca_01488	GDP-fucose protein O-fucosyltransferase	NON_SYNONYMOUS_CODING
8837	CakSNP8837	*Kabuli*	Ca_Kabuli_Chr05	37332101	[G/T]	Ca_01486	Kinesin, motor domain	NON_SYNONYMOUS_CODING
8838	CakSNP8838	*Kabuli*	Ca_Kabuli_Chr05	37332198	[G/A]	Ca_01486	Kinesin, motor domain	SYNONYMOUS_CODING
8839	CakSNP8839	*Kabuli*	Ca_Kabuli_Chr05	37332304	[G/A]		--	INTERGENIC
8840	CakSNP8840	*Kabuli*	Ca_Kabuli_Chr05	37332303	[A/C]		--	INTERGENIC
8841	CakSNP8841	*Kabuli*	Ca_Kabuli_Chr05	37332370	[T/C]		--	INTERGENIC

S.N.	SNP IDs	Chickpea cultivars	Chromosomes	Physical positions (bp)	SNPs	Gene accession IDs	Putative functions	Sequence components of genome
8842	CakSNP8842	*Kabuli*	Ca_Kabuli_Chr05	37332441	[T/C]		--	INTERGENIC
8843	CakSNP8843	*Kabuli*	Ca_Kabuli_Chr05	37332501	[C/T]		--	INTERGENIC
8844	CakSNP8844	*Kabuli*	Ca_Kabuli_Chr05	37339022	[T/G]	Ca_01485	--	NON_SYNONYMOUS_CODING
8845	CakSNP8845	*Kabuli*	Ca_Kabuli_Chr05	37339358	[T/G]	Ca_01485	--	INTRON
8846	CakSNP8846	*Kabuli*	Ca_Kabuli_Chr05	37339327	[A/G]	Ca_01485	--	INTRON
8847	CakSNP8847	*Kabuli*	Ca_Kabuli_Chr05	37339300	[A/G]	Ca_01485	--	INTRON
8848	CakSNP8848	*Kabuli*	Ca_Kabuli_Chr05	37342358	[G/T]	Ca_01484	Fumarate lyase	INTRON
8849	CakSNP8849	*Kabuli*	Ca_Kabuli_Chr05	37363626	[C/T]	Ca_01481	SANT domain, DNA binding	INTRON
8850	CakSNP8850	*Kabuli*	Ca_Kabuli_Chr05	37375575	[C/G]	Ca_01480	Flavodoxin	INTRON
8851	CakSNP8851	*Kabuli*	Ca_Kabuli_Chr05	37375620	[C/T]	Ca_01480	Flavodoxin	INTRON

S.N.	SNP IDs	Chickpea cultivars	Chromosomes	Physical positions (bp)	SNPs	Gene accession IDs	Putative functions	Sequence components of genome
8852	CakSNP8852	*Kabuli*	Ca_Kabuli_Chr05	37445226	[A/G]	Ca_01474	--	SYNONYMOUS_CODING
8853	CakSNP8853	*Kabuli*	Ca_Kabuli_Chr05	37460980	[A/G]	Ca_01473	BEACH domain	NON_SYNONYMOUS_CODING
8854	CakSNP8854	*Kabuli*	Ca_Kabuli_Chr05	37468727	[T/C]	Ca_01473	BEACH domain	INTRON
8855	CakSNP8855	*Kabuli*	Ca_Kabuli_Chr05	37468755	[G/A]	Ca_01473	BEACH domain	INTRON
8856	CakSNP8856	*Kabuli*	Ca_Kabuli_Chr05	37555816	[T/A]	Ca_01465	Domain of unknown function FMP27, domain-4	INTRON
8857	CakSNP8857	*Kabuli*	Ca_Kabuli_Chr05	37555846	[T/A]	Ca_01465	Domain of unknown function FMP27, domain-4	INTRON
8858	CakSNP8858	*Kabuli*	Ca_Kabuli_Chr05	37578999	[T/G]	Ca_01463	Protein of unknown function DUF914, eukaryotic	INTRON
8859	CakSNP8859	*Kabuli*	Ca_Kabuli_Chr05	37591782	[A/T]	Ca_01462	Ubiquitin-associated/translation elongation factor EF1B, N-terminal	NON_SYNONYMOUS_CODING
8860	CakSNP8860	*Kabuli*	Ca_Kabuli_Chr05	37593444	[A/T]	Ca_01462	Ubiquitin-associated/translation elongation factor EF1B, N-terminal	SYNONYMOUS_CODING
8861	CakSNP8861	*Kabuli*	Ca_Kabuli_Chr05	37594347	[T/C]	Ca_01462	Ubiquitin-associated/translation elongation factor EF1B, N-terminal	SYNONYMOUS_CODING

S.N.	SNP IDs	Chickpea cultivars	Chromosomes	Physical positions (bp)	SNPs	Gene accession IDs	Putative functions	Sequence components of genome
8862	CakSNP8862	*Kabuli*	Ca_Kabuli_Chr05	37604030	[T/C]		--	INTERGENIC
8863	CakSNP8863	*Kabuli*	Ca_Kabuli_Chr05	37604090	[G/T]		--	INTERGENIC
8864	CakSNP8864	*Kabuli*	Ca_Kabuli_Chr05	37604780	[C/T]		--	INTERGENIC
8865	CakSNP8865	*Kabuli*	Ca_Kabuli_Chr05	37796337	[T/G]	Ca_01444	VQ	SYNONYMOUS_CODING
8866	CakSNP8866	*Kabuli*	Ca_Kabuli_Chr05	37798313	[T/G]	Ca_01443	VQ	SYNONYMOUS_CODING
8867	CakSNP8867	*Kabuli*	Ca_Kabuli_Chr05	37868250	[C/T]	Ca_01437	UBA/THIF-type NAD/FAD binding fold	INTRON
8868	CakSNP8868	*Kabuli*	Ca_Kabuli_Chr05	37868256	[T/A]	Ca_01437	UBA/THIF-type NAD/FAD binding fold	INTRON
8869	CakSNP8869	*Kabuli*	Ca_Kabuli_Chr05	37869744	[T/C]	Ca_01437	UBA/THIF-type NAD/FAD binding fold	INTRON
8870	CakSNP8870	*Kabuli*	Ca_Kabuli_Chr05	37872223	[C/G]		--	INTERGENIC
8871	CakSNP8871	*Kabuli*	Ca_Kabuli_Chr05	37874564	[T/A]		--	INTERGENIC

S.N.	SNP IDs	Chickpea cultivars	Chromosomes	Physical positions (bp)	SNPs	Gene accession IDs	Putative functions	Sequence components of genome
8872	CakSNP8872	*Kabuli*	Ca_Kabuli_Chr05	37874725	[A/C]		--	INTERGENIC
8873	CakSNP8873	*Kabuli*	Ca_Kabuli_Chr05	37874694	[C/T]		--	INTERGENIC
8874	CakSNP8874	*Kabuli*	Ca_Kabuli_Chr05	37886417	[T/G]	Ca_01434	TPP-binding enzyme, conserved site	SYNONYMOUS_CODING
8875	CakSNP8875	*Kabuli*	Ca_Kabuli_Chr05	37899098	[T/C]	Ca_01432	Heat shock protein DnaJ, N-terminal	NON_SYNONYMOUS_CODING
8876	CakSNP8876	*Kabuli*	Ca_Kabuli_Chr05	37899238	[T/C]	Ca_01432	Heat shock protein DnaJ, N-terminal	SYNONYMOUS_CODING
8877	CakSNP8877	*Kabuli*	Ca_Kabuli_Chr05	37908818	[A/C]		--	INTERGENIC
8878	CakSNP8878	*Kabuli*	Ca_Kabuli_Chr05	37908807	[A/T]		--	INTERGENIC
8879	CakSNP8879	*Kabuli*	Ca_Kabuli_Chr05	37911813	[A/C]	Ca_01430	--	SYNONYMOUS_CODING
8880	CakSNP8880	*Kabuli*	Ca_Kabuli_Chr05	38018552	[A/T]		--	INTERGENIC
8881	CakSNP8881	*Kabuli*	Ca_Kabuli_Chr05	38018558	[G/C]		--	INTERGENIC

S.N.	SNP IDs	Chickpea cultivars	Chromosomes	Physical positions (bp)	SNPs	Gene accession IDs	Putative functions	Sequence components of genome
8882	CakSNP8882	*Kabuli*	Ca_Kabuli_Chr05	38057582	[C/T]	Ca_01417	Zinc finger, DHHC-type, palmitoyltransferase	NON_SYNONYMOUS_CODING
8883	CakSNP8883	*Kabuli*	Ca_Kabuli_Chr05	38080226	[A/G]	Ca_01411	Protein kinase, catalytic domain	SYNONYMOUS_CODING
8884	CakSNP8884	*Kabuli*	Ca_Kabuli_Chr05	38080349	[C/T]	Ca_01411	Protein kinase, catalytic domain	SYNONYMOUS_CODING
8885	CakSNP8885	*Kabuli*	Ca_Kabuli_Chr05	38092606	[G/A]		--	INTERGENIC
8886	CakSNP8886	*Kabuli*	Ca_Kabuli_Chr05	38092619	[T/C]		--	INTERGENIC
8887	CakSNP8887	*Kabuli*	Ca_Kabuli_Chr05	38092732	[C/T]		--	INTERGENIC
8888	CakSNP8888	*Kabuli*	Ca_Kabuli_Chr05	38092735	[C/T]		--	INTERGENIC
8889	CakSNP8889	*Kabuli*	Ca_Kabuli_Chr05	38092793	[C/T]		--	INTERGENIC
8890	CakSNP8890	*Kabuli*	Ca_Kabuli_Chr05	38102941	[T/G]	Ca_01408	--	SYNONYMOUS_CODING
8891	CakSNP8891	*Kabuli*	Ca_Kabuli_Chr05	38108396	[G/A]	Ca_01407	--	NON_SYNONYMOUS_CODING

S.N.	SNP IDs	Chickpea cultivars	Chromosomes	Physical positions (bp)	SNPs	Gene accession IDs	Putative functions	Sequence components of genome
8892	CakSNP8892	*Kabuli*	Ca_Kabuli_Chr05	38110219	[T/G]		--	INTERGENIC
8893	CakSNP8893	*Kabuli*	Ca_Kabuli_Chr05	38220843	[G/A]		--	INTERGENIC
8894	CakSNP8894	*Kabuli*	Ca_Kabuli_Chr05	38220908	[G/T]		--	INTERGENIC
8895	CakSNP8895	*Kabuli*	Ca_Kabuli_Chr05	38248895	[A/C]	Ca_01398	Dual specificity phosphatase, catalytic domain	NON_SYNONYMOUS_CODING
8896	CakSNP8896	*Kabuli*	Ca_Kabuli_Chr05	38249134	[T/C]		--	INTERGENIC
8897	CakSNP8897	*Kabuli*	Ca_Kabuli_Chr05	38249132	[A/C]		--	INTERGENIC
8898	CakSNP8898	*Kabuli*	Ca_Kabuli_Chr05	38266153	[G/A]	Ca_01397	Dual specificity phosphatase, catalytic domain	NON_SYNONYMOUS_CODING
8899	CakSNP8899	*Kabuli*	Ca_Kabuli_Chr05	38307036	[G/A]	Ca_01392	Glycoside hydrolase, family 38, core	SYNONYMOUS_CODING
8900	CakSNP8900	*Kabuli*	Ca_Kabuli_Chr05	38333718	[C/T]	Ca_01390	RNA recognition motif domain	INTRON
8901	CakSNP8901	*Kabuli*	Ca_Kabuli_Chr05	38333746	[G/A]	Ca_01390	RNA recognition motif domain	INTRON

S.N.	SNP IDs	Chickpea cultivars	Chromosomes	Physical positions (bp)	SNPs	Gene accession IDs	Putative functions	Sequence components of genome
8902	CakSNP8902	*Kabuli*	Ca_Kabuli_Chr05	38333774	[A/G]	Ca_01390	RNA recognition motif domain	INTRON
8903	CakSNP8903	*Kabuli*	Ca_Kabuli_Chr05	38347201	[A/C]	Ca_01389	WD40 repeat	SYNONYMOUS_CODING
8904	CakSNP8904	*Kabuli*	Ca_Kabuli_Chr05	38455563	[A/C]	Ca_07405	Telomere length regulation protein, conserved domain	SYNONYMOUS_CODING
8905	CakSNP8905	*Kabuli*	Ca_Kabuli_Chr05	38509657	[A/C]	Ca_07410	Transcriptional factor B3	INTRON
8906	CakSNP8906	*Kabuli*	Ca_Kabuli_Chr05	38538904	[A/G]	Ca_07411	Glycosyl transferase, family 48	INTRON
8907	CakSNP8907	*Kabuli*	Ca_Kabuli_Chr05	38551438	[C/T]		--	INTERGENIC
8908	CakSNP8908	*Kabuli*	Ca_Kabuli_Chr05	38551381	[G/A]		--	INTERGENIC
8909	CakSNP8909	*Kabuli*	Ca_Kabuli_Chr05	38647040	[A/C]	Ca_07417	Tetratricopeptide TPR-1	INTRON
8910	CakSNP8910	*Kabuli*	Ca_Kabuli_Chr05	38656599	[C/T]	Ca_07418	Peptidase C78, ubiquitin fold modifier-specific peptidase 1/ 3	INTRON
8911	CakSNP8911	*Kabuli*	Ca_Kabuli_Chr05	38783604	[C/A]	Ca_07428	Mediator complex subunit Med28	NON_SYNONYMOUS_CODING

S.N.	SNP IDs	Chickpea cultivars	Chromosomes	Physical positions (bp)	SNPs	Gene accession IDs	Putative functions	Sequence components of genome
8912	CakSNP8912	*Kabuli*	Ca_Kabuli_Chr05	38784078	[G/A]	Ca_07428	Mediator complex subunit Med28	INTRON
8913	CakSNP8913	*Kabuli*	Ca_Kabuli_Chr05	38784074	[A/G]	Ca_07428	Mediator complex subunit Med28	INTRON
8914	CakSNP8914	*Kabuli*	Ca_Kabuli_Chr05	38784150	[T/A]	Ca_07428	Mediator complex subunit Med28	INTRON
8915	CakSNP8915	*Kabuli*	Ca_Kabuli_Chr05	38784129	[G/C]	Ca_07428	Mediator complex subunit Med28	INTRON
8916	CakSNP8916	*Kabuli*	Ca_Kabuli_Chr05	38857904	[T/C]	Ca_07438	Membrane protein,Tapt1/CMV receptor	NON_SYNONYMOUS_CODING
8917	CakSNP8917	*Kabuli*	Ca_Kabuli_Chr05	38872143	[A/G]	Ca_07440	Metallo-dependent phosphatase	SYNONYMOUS_CODING
8918	CakSNP8918	*Kabuli*	Ca_Kabuli_Chr05	38892604	[A/C]		--	INTERGENIC
8919	CakSNP8919	*Kabuli*	Ca_Kabuli_Chr05	38898971	[G/A]		--	INTERGENIC
8920	CakSNP8920	*Kabuli*	Ca_Kabuli_Chr05	38909701	[C/T]	Ca_07443	Drought induced 19/ RING finger protein 114	NON_SYNONYMOUS_CODING
8921	CakSNP8921	*Kabuli*	Ca_Kabuli_Chr05	38937304	[C/G]	Ca_07446	Chaperonin ClpA/B	INTRON

S.N.	SNP IDs	Chickpea cultivars	Chromosomes	Physical positions (bp)	SNPs	Gene accession IDs	Putative functions	Sequence components of genome
8922	CakSNP8922	*Kabuli*	Ca_Kabuli_Chr05	38937398	[C/A]	Ca_07446	Chaperonin ClpA/B	INTRON
8923	CakSNP8923	*Kabuli*	Ca_Kabuli_Chr05	38939578	[C/A]	Ca_07446	Chaperonin ClpA/B	INTRON
8924	CakSNP8924	*Kabuli*	Ca_Kabuli_Chr05	38984762	[A/G]	Ca_07451	Phox/Bem1p	SYNONYMOUS_CODING
8925	CakSNP8925	*Kabuli*	Ca_Kabuli_Chr05	38984769	[A/C]	Ca_07451	Phox/Bem1p	NON_SYNONYMOUS_CODING
8926	CakSNP8926	*Kabuli*	Ca_Kabuli_Chr05	38984776	[A/G]	Ca_07451	Phox/Bem1p	SYNONYMOUS_CODING
8927	CakSNP8927	*Kabuli*	Ca_Kabuli_Chr05	39008980	[G/A]		--	INTERGENIC
8928	CakSNP8928	*Kabuli*	Ca_Kabuli_Chr05	39137170	[A/G]		--	INTERGENIC
8929	CakSNP8929	*Kabuli*	Ca_Kabuli_Chr05	39175007	[A/T]	Ca_07464	Allinase, C-terminal	SYNONYMOUS_CODING
8930	CakSNP8930	*Kabuli*	Ca_Kabuli_Chr05	39176391	[T/A]		--	INTERGENIC
8931	CakSNP8931	*Kabuli*	Ca_Kabuli_Chr05	39266333	[T/C]	Ca_07473	--	INTRON

S.N.	SNP IDs	Chickpea cultivars	Chromosomes	Physical positions (bp)	SNPs	Gene accession IDs	Putative functions	Sequence components of genome
8932	CakSNP8932	*Kabuli*	Ca_Kabuli_Chr05	39277581	[T/C]		--	INTERGENIC
8933	CakSNP8933	*Kabuli*	Ca_Kabuli_Chr05	39283856	[G/A]	Ca_07474	--	INTRON
8934	CakSNP8934	*Kabuli*	Ca_Kabuli_Chr05	39326933	[G/T]		--	INTERGENIC
8935	CakSNP8935	*Kabuli*	Ca_Kabuli_Chr05	39353755	[T/G]	Ca_07477	Glutaredoxin	INTRON
8936	CakSNP8936	*Kabuli*	Ca_Kabuli_Chr05	39364522	[T/C]		--	INTERGENIC
8937	CakSNP8937	*Kabuli*	Ca_Kabuli_Chr05	39364569	[G/A]		--	INTERGENIC
8938	CakSNP8938	*Kabuli*	Ca_Kabuli_Chr05	39364571	[C/A]		--	INTERGENIC
8939	CakSNP8939	*Kabuli*	Ca_Kabuli_Chr05	39364720	[A/T]		--	INTERGENIC
8940	CakSNP8940	*Kabuli*	Ca_Kabuli_Chr05	39364700	[C/A]		--	INTERGENIC
8941	CakSNP8941	*Kabuli*	Ca_Kabuli_Chr05	39364695	[A/C]		--	INTERGENIC

S.N.	SNP IDs	Chickpea cultivars	Chromosomes	Physical positions (bp)	SNPs	Gene accession IDs	Putative functions	Sequence components of genome
8942	CakSNP8942	*Kabuli*	Ca_Kabuli_Chr05	39364667	[T/A]		--	INTERGENIC
8943	CakSNP8943	*Kabuli*	Ca_Kabuli_Chr05	39364741	[A/T]		--	INTERGENIC
8944	CakSNP8944	*Kabuli*	Ca_Kabuli_Chr05	39364752	[G/T]		--	INTERGENIC
8945	CakSNP8945	*Kabuli*	Ca_Kabuli_Chr05	39364895	[A/C]		--	INTERGENIC
8946	CakSNP8946	*Kabuli*	Ca_Kabuli_Chr05	39364856	[A/G]		--	INTERGENIC
8947	CakSNP8947	*Kabuli*	Ca_Kabuli_Chr05	39364848	[C/T]		--	INTERGENIC
8948	CakSNP8948	*Kabuli*	Ca_Kabuli_Chr05	39364841	[A/T]		--	INTERGENIC
8949	CakSNP8949	*Kabuli*	Ca_Kabuli_Chr05	39366353	[C/G]		--	INTERGENIC
8950	CakSNP8950	*Kabuli*	Ca_Kabuli_Chr05	39385603	[T/G]		--	INTERGENIC
8951	CakSNP8951	*Kabuli*	Ca_Kabuli_Chr05	39385630	[C/T]		--	INTERGENIC

S.N.	SNP IDs	Chickpea cultivars	Chromosomes	Physical positions (bp)	SNPs	Gene accession IDs	Putative functions	Sequence components of genome
8952	CakSNP8952	*Kabuli*	Ca_Kabuli_Chr05	39410377	[T/G]	Ca_07478	Glycosyl transferase, family 3	INTRON
8953	CakSNP8953	*Kabuli*	Ca_Kabuli_Chr05	39410329	[T/G]	Ca_07478	Glycosyl transferase, family 3	INTRON
8954	CakSNP8954	*Kabuli*	Ca_Kabuli_Chr05	39460567	[A/C]	Ca_07481	Drug/metabolite transporter	INTRON
8955	CakSNP8955	*Kabuli*	Ca_Kabuli_Chr05	39475960	[T/C]	Ca_07483	Bicarbonate transporter, eukaryotic	SYNONYMOUS_CODING
8956	CakSNP8956	*Kabuli*	Ca_Kabuli_Chr05	39476040	[T/G]	Ca_07483	Bicarbonate transporter, eukaryotic	SYNONYMOUS_CODING
8957	CakSNP8957	*Kabuli*	Ca_Kabuli_Chr05	39498665	[G/A]	Ca_07486	NIF system FeS cluster assembly, NifU, N-terminal	SYNONYMOUS_CODING
8958	CakSNP8958	*Kabuli*	Ca_Kabuli_Chr05	39513563	[C/A]	Ca_07487	Domain of unknown function DUF89	INTRON
8959	CakSNP8959	*Kabuli*	Ca_Kabuli_Chr05	39543929	[C/T]	Ca_07489	Phosphatidylinositol 3-/4-kinase, catalytic	NON_SYNONYMOUS_CODING
8960	CakSNP8960	*Kabuli*	Ca_Kabuli_Chr05	39576823	[C/T]	Ca_07489	Phosphatidylinositol 3-/4-kinase, catalytic	INTRON
8961	CakSNP8961	*Kabuli*	Ca_Kabuli_Chr05	39576836	[T/A]	Ca_07489	Phosphatidylinositol 3-/4-kinase, catalytic	INTRON

S.N.	SNP IDs	Chickpea cultivars	Chromosomes	Physical positions (bp)	SNPs	Gene accession IDs	Putative functions	Sequence components of genome
8962	CakSNP8962	*Kabuli*	Ca_Kabuli_Chr05	39576839	[C/T]	Ca_07489	Phosphatidylinositol 3-/4-kinase, catalytic	INTRON
8963	CakSNP8963	*Kabuli*	Ca_Kabuli_Chr05	39576864	[C/A]	Ca_07489	Phosphatidylinositol 3-/4-kinase, catalytic	INTRON
8964	CakSNP8964	*Kabuli*	Ca_Kabuli_Chr05	39576870	[C/T]	Ca_07489	Phosphatidylinositol 3-/4-kinase, catalytic	INTRON
8965	CakSNP8965	*Kabuli*	Ca_Kabuli_Chr05	39576874	[G/T]	Ca_07489	Phosphatidylinositol 3-/4-kinase, catalytic	INTRON
8966	CakSNP8966	*Kabuli*	Ca_Kabuli_Chr05	39576841	[G/A]	Ca_07489	Phosphatidylinositol 3-/4-kinase, catalytic	INTRON
8967	CakSNP8967	*Kabuli*	Ca_Kabuli_Chr05	39592439	[A/G]	Ca_07489	Phosphatidylinositol 3-/4-kinase, catalytic	INTRON
8968	CakSNP8968	*Kabuli*	Ca_Kabuli_Chr05	39597485	[C/T]	Ca_07489	Phosphatidylinositol 3-/4-kinase, catalytic	INTRON
8969	CakSNP8969	*Kabuli*	Ca_Kabuli_Chr05	39597459	[T/C]	Ca_07489	Phosphatidylinositol 3-/4-kinase, catalytic	INTRON
8970	CakSNP8970	*Kabuli*	Ca_Kabuli_Chr05	39608204	[A/G]	Ca_07489	Phosphatidylinositol 3-/4-kinase, catalytic	SYNONYMOUS_CODING
8971	CakSNP8971	*Kabuli*	Ca_Kabuli_Chr05	39608209	[G/T]	Ca_07489	Phosphatidylinositol 3-/4-kinase, catalytic	INTRON

S.N.	SNP IDs	Chickpea cultivars	Chromosomes	Physical positions (bp)	SNPs	Gene accession IDs	Putative functions	Sequence components of genome
8972	CakSNP8972	*Kabuli*	Ca_Kabuli_Chr05	39684480	[A/G]		--	INTERGENIC
8973	CakSNP8973	*Kabuli*	Ca_Kabuli_Chr05	39684592	[A/T]		--	INTERGENIC
8974	CakSNP8974	*Kabuli*	Ca_Kabuli_Chr05	39713021	[G/A]	Ca_07497	Protein of unknown function DUF3506	SYNONYMOUS_CODING
8975	CakSNP8975	*Kabuli*	Ca_Kabuli_Chr05	39776794	[G/A]		--	INTERGENIC
8976	CakSNP8976	*Kabuli*	Ca_Kabuli_Chr05	39776795	[G/A]		--	INTERGENIC
8977	CakSNP8977	*Kabuli*	Ca_Kabuli_Chr05	39776818	[C/T]		--	INTERGENIC
8978	CakSNP8978	*Kabuli*	Ca_Kabuli_Chr05	39776850	[G/A]		--	INTERGENIC
8979	CakSNP8979	*Kabuli*	Ca_Kabuli_Chr05	39776869	[C/T]		--	INTERGENIC
8980	CakSNP8980	*Kabuli*	Ca_Kabuli_Chr05	39794718	[C/T]	Ca_07506	Armadillo	SYNONYMOUS_CODING
8981	CakSNP8981	*Kabuli*	Ca_Kabuli_Chr05	39851570	[T/G]		--	INTERGENIC

S.N.	SNP IDs	Chickpea cultivars	Chromosomes	Physical positions (bp)	SNPs	Gene accession IDs	Putative functions	Sequence components of genome
8982	CakSNP8982	*Kabuli*	Ca_Kabuli_Chr05	39869398	[C/T]	Ca_07512	Xanthine/uracil/vitamin C permease	SYNONYMOUS_CODING
8983	CakSNP8983	*Kabuli*	Ca_Kabuli_Chr05	39943298	[C/T]		--	INTERGENIC
8984	CakSNP8984	*Kabuli*	Ca_Kabuli_Chr05	39985042	[G/A]		--	INTERGENIC
8985	CakSNP8985	*Kabuli*	Ca_Kabuli_Chr05	39985332	[A/G]	Ca_07525	B12D	INTRON
8986	CakSNP8986	*Kabuli*	Ca_Kabuli_Chr05	40018264	[A/C]	Ca_07530	Glycine cleavage T-protein, N-terminal	SYNONYMOUS_CODING
8987	CakSNP8987	*Kabuli*	Ca_Kabuli_Chr05	40020062	[C/T]		--	INTERGENIC
8988	CakSNP8988	*Kabuli*	Ca_Kabuli_Chr05	40020073	[C/G]		--	INTERGENIC
8989	CakSNP8989	*Kabuli*	Ca_Kabuli_Chr05	40025250	[G/A]	Ca_07531	Argonaute/Dicer protein, PAZ	SYNONYMOUS_CODING
8990	CakSNP8990	*Kabuli*	Ca_Kabuli_Chr05	40025319	[T/C]	Ca_07531	Argonaute/Dicer protein, PAZ	SYNONYMOUS_CODING
8991	CakSNP8991	*Kabuli*	Ca_Kabuli_Chr05	40025326	[C/T]	Ca_07531	Argonaute/Dicer protein, PAZ	SYNONYMOUS_CODING

S.N.	SNP IDs	Chickpea cultivars	Chromosomes	Physical positions (bp)	SNPs	Gene accession IDs	Putative functions	Sequence components of genome
8992	CakSNP8992	*Kabuli*	Ca_Kabuli_Chr05	40025376	[A/G]	Ca_07531	Argonaute/Dicer protein, PAZ	INTRON
8993	CakSNP8993	*Kabuli*	Ca_Kabuli_Chr05	40025377	[T/A]	Ca_07531	Argonaute/Dicer protein, PAZ	INTRON
8994	CakSNP8994	*Kabuli*	Ca_Kabuli_Chr05	40027707	[A/G]	Ca_07531	Argonaute/Dicer protein, PAZ	INTRON
8995	CakSNP8995	*Kabuli*	Ca_Kabuli_Chr05	40078085	[C/G]	Ca_07534	Ethylene insensitive 3	NON_SYNONYMOUS_CODING
8996	CakSNP8996	*Kabuli*	Ca_Kabuli_Chr05	40078077	[A/G]	Ca_07534	Ethylene insensitive 3	NON_SYNONYMOUS_CODING
8997	CakSNP8997	*Kabuli*	Ca_Kabuli_Chr05	40159260	[C/A]	Ca_07540	Nse1 non-SMC component of SMC5-6 complex	NON_SYNONYMOUS_CODING
8998	CakSNP8998	*Kabuli*	Ca_Kabuli_Chr05	40159348	[G/A]	Ca_07540	Nse1 non-SMC component of SMC5-6 complex	NON_SYNONYMOUS_CODING
8999	CakSNP8999	*Kabuli*	Ca_Kabuli_Chr05	40162755	[T/C]		--	INTERGENIC
9000	CakSNP9000	*Kabuli*	Ca_Kabuli_Chr05	40162995	[C/T]		--	INTERGENIC
9001	CakSNP9001	*Kabuli*	Ca_Kabuli_Chr05	40164176	[C/G]		--	INTERGENIC

S.N.	SNP IDs	Chickpea cultivars	Chromosomes	Physical positions (bp)	SNPs	Gene accession IDs	Putative functions	Sequence components of genome
9002	CakSNP9002	*Kabuli*	Ca_Kabuli_Chr05	40239397	[G/C]	Ca_07550	Protein of unknown function DUF247, plant	NON_SYNONYMOUS_CODING
9003	CakSNP9003	*Kabuli*	Ca_Kabuli_Chr05	40290082	[G/A]		--	INTERGENIC
9004	CakSNP9004	*Kabuli*	Ca_Kabuli_Chr05	40290066	[A/G]		--	INTERGENIC
9005	CakSNP9005	*Kabuli*	Ca_Kabuli_Chr05	40404136	[C/T]	Ca_07559	Oxidoreductase FAD/NAD(P)-binding	INTRON
9006	CakSNP9006	*Kabuli*	Ca_Kabuli_Chr05	40404105	[G/C]	Ca_07559	Oxidoreductase FAD/NAD(P)-binding	INTRON
9007	CakSNP9007	*Kabuli*	Ca_Kabuli_Chr05	40410891	[C/A]	Ca_07561	HAD-superfamily phosphatase, subfamily IIIA	NON_SYNONYMOUS_CODING
9008	CakSNP9008	*Kabuli*	Ca_Kabuli_Chr05	40410895	[G/T]	Ca_07561	HAD-superfamily phosphatase, subfamily IIIA	SYNONYMOUS_CODING
9009	CakSNP9009	*Kabuli*	Ca_Kabuli_Chr05	40411325	[G/C]	Ca_07561	HAD-superfamily phosphatase, subfamily IIIA	NON_SYNONYMOUS_CODING
9010	CakSNP9010	*Kabuli*	Ca_Kabuli_Chr05	40464608	[A/G]	Ca_07566	Methionine synthase, vitamin-B12 independent	NON_SYNONYMOUS_CODING
9011	CakSNP9011	*Kabuli*	Ca_Kabuli_Chr05	40505498	[T/G]	Ca_07571	Zinc finger, CCHC-type	INTRON

S.N.	SNP IDs	Chickpea cultivars	Chromosomes	Physical positions (bp)	SNPs	Gene accession IDs	Putative functions	Sequence components of genome
9012	CakSNP9012	*Kabuli*	Ca_Kabuli_Chr05	40507018	[T/A]	Ca_07571	Zinc finger, CCHC-type	INTRON
9013	CakSNP9013	*Kabuli*	Ca_Kabuli_Chr05	40507107	[A/G]	Ca_07571	Zinc finger, CCHC-type	SYNONYMOUS_CODING
9014	CakSNP9014	*Kabuli*	Ca_Kabuli_Chr05	40507116	[A/G]	Ca_07571	Zinc finger, CCHC-type	SYNONYMOUS_CODING
9015	CakSNP9015	*Kabuli*	Ca_Kabuli_Chr05	40507158	[A/G]	Ca_07571	Zinc finger, CCHC-type	SYNONYMOUS_CODING
9016	CakSNP9016	*Kabuli*	Ca_Kabuli_Chr05	40507653	[G/C]	Ca_07571	Zinc finger, CCHC-type	SYNONYMOUS_CODING
9017	CakSNP9017	*Kabuli*	Ca_Kabuli_Chr05	40507662	[C/T]	Ca_07571	Zinc finger, CCHC-type	SYNONYMOUS_CODING
9018	CakSNP9018	*Kabuli*	Ca_Kabuli_Chr05	40507683	[G/T]	Ca_07571	Zinc finger, CCHC-type	SYNONYMOUS_CODING
9019	CakSNP9019	*Kabuli*	Ca_Kabuli_Chr05	40532435	[A/G]		--	INTERGENIC
9020	CakSNP9020	*Kabuli*	Ca_Kabuli_Chr05	40543989	[G/T]	Ca_07573	Ribonuclease III	NON_SYNONYMOUS_CODING
9021	CakSNP9021	*Kabuli*	Ca_Kabuli_Chr05	40553279	[C/G]		--	INTERGENIC

S.N.	SNP IDs	Chickpea cultivars	Chromosomes	Physical positions (bp)	SNPs	Gene accession IDs	Putative functions	Sequence components of genome
9022	CakSNP9022	*Kabuli*	Ca_Kabuli_Chr05	40631069	[G/C]		--	INTERGENIC
9023	CakSNP9023	*Kabuli*	Ca_Kabuli_Chr05	40666160	[T/A]	Ca_07586	Sugar/inositol transporter	INTRON
9024	CakSNP9024	*Kabuli*	Ca_Kabuli_Chr05	40666171	[T/C]	Ca_07586	Sugar/inositol transporter	INTRON
9025	CakSNP9025	*Kabuli*	Ca_Kabuli_Chr05	40666174	[C/T]	Ca_07586	Sugar/inositol transporter	INTRON
9026	CakSNP9026	*Kabuli*	Ca_Kabuli_Chr05	40673241	[A/G]		--	INTERGENIC
9027	CakSNP9027	*Kabuli*	Ca_Kabuli_Chr05	40700278	[C/G]	Ca_07589	Glycoside hydrolase, family 17	NON_SYNONYMOUS_CODING
9028	CakSNP9028	*Kabuli*	Ca_Kabuli_Chr05	40700260	[T/C]	Ca_07589	Glycoside hydrolase, family 17	NON_SYNONYMOUS_CODING
9029	CakSNP9029	*Kabuli*	Ca_Kabuli_Chr05	40700318	[C/A]	Ca_07589	Glycoside hydrolase, family 17	NON_SYNONYMOUS_CODING
9030	CakSNP9030	*Kabuli*	Ca_Kabuli_Chr05	40700772	[C/T]	Ca_07589	Glycoside hydrolase, family 17	SYNONYMOUS_CODING
9031	CakSNP9031	*Kabuli*	Ca_Kabuli_Chr05	40736291	[C/G]	Ca_07594	--	INTRON

S.N.	SNP IDs	Chickpea cultivars	Chromosomes	Physical positions (bp)	SNPs	Gene accession IDs	Putative functions	Sequence components of genome
9032	CakSNP9032	*Kabuli*	Ca_Kabuli_Chr05	40828566	[A/C]	Ca_07603	Cys/Met metabolism, pyridoxal phosphate-dependent enzyme	INTRON
9033	CakSNP9033	*Kabuli*	Ca_Kabuli_Chr05	40828567	[G/C]	Ca_07603	Cys/Met metabolism, pyridoxal phosphate-dependent enzyme	INTRON
9034	CakSNP9034	*Kabuli*	Ca_Kabuli_Chr05	40841690	[C/T]	Ca_07604	Oligopeptide transporter OPT superfamily	NON_SYNONYMOUS_CODING
9035	CakSNP9035	*Kabuli*	Ca_Kabuli_Chr05	40845320	[C/T]		--	INTERGENIC
9036	CakSNP9036	*Kabuli*	Ca_Kabuli_Chr05	40883438	[C/A]		--	INTERGENIC
9037	CakSNP9037	*Kabuli*	Ca_Kabuli_Chr05	40955055	[A/T]		--	INTERGENIC
9038	CakSNP9038	*Kabuli*	Ca_Kabuli_Chr05	40966058	[G/A]	Ca_07617	Heat shock protein Hsp70	SYNONYMOUS_CODING
9039	CakSNP9039	*Kabuli*	Ca_Kabuli_Chr05	41024079	[A/G]	Ca_07621	Domain of unknown function DUF1692	INTRON
9040	CakSNP9040	*Kabuli*	Ca_Kabuli_Chr05	41024135	[C/G]	Ca_07621	Domain of unknown function DUF1692	INTRON
9041	CakSNP9041	*Kabuli*	Ca_Kabuli_Chr05	41024201	[G/A]	Ca_07621	Domain of unknown function DUF1692	INTRON

S.N.	SNP IDs	Chickpea cultivars	Chromosomes	Physical positions (bp)	SNPs	Gene accession IDs	Putative functions	Sequence components of genome
9042	CakSNP9042	*Kabuli*	Ca_Kabuli_Chr05	41054578	[A/G]	Ca_07624	Cytidylyltransferase	INTRON
9043	CakSNP9043	*Kabuli*	Ca_Kabuli_Chr05	41055111	[T/A]	Ca_07624	Cytidylyltransferase	INTRON
9044	CakSNP9044	*Kabuli*	Ca_Kabuli_Chr05	41063657	[C/T]		--	INTERGENIC
9045	CakSNP9045	*Kabuli*	Ca_Kabuli_Chr05	41066644	[C/G]		--	INTERGENIC
9046	CakSNP9046	*Kabuli*	Ca_Kabuli_Chr05	41066664	[T/C]		--	INTERGENIC
9047	CakSNP9047	*Kabuli*	Ca_Kabuli_Chr05	41066667	[C/A]		--	INTERGENIC
9048	CakSNP9048	*Kabuli*	Ca_Kabuli_Chr05	41080035	[C/T]	Ca_07626	Zinc/iron permease	SYNONYMOUS_CODING
9049	CakSNP9049	*Kabuli*	Ca_Kabuli_Chr05	41191566	[A/T]		--	INTERGENIC
9050	CakSNP9050	*Kabuli*	Ca_Kabuli_Chr05	41191676	[G/C]		--	INTERGENIC
9051	CakSNP9051	*Kabuli*	Ca_Kabuli_Chr05	41197909	[A/T]		--	INTERGENIC

S.N.	SNP IDs	Chickpea cultivars	Chromosomes	Physical positions (bp)	SNPs	Gene accession IDs	Putative functions	Sequence components of genome
9052	CakSNP9052	*Kabuli*	Ca_Kabuli_Chr05	41203594	[C/T]		--	INTERGENIC
9053	CakSNP9053	*Kabuli*	Ca_Kabuli_Chr05	41253742	[A/G]		--	INTERGENIC
9054	CakSNP9054	*Kabuli*	Ca_Kabuli_Chr05	41283253	[T/C]	Ca_07642	Protein of unknown function DUF1296	NON_SYNONYMOUS_CODING
9055	CakSNP9055	*Kabuli*	Ca_Kabuli_Chr05	41283453	[A/C]	Ca_07642	Protein of unknown function DUF1296	NON_SYNONYMOUS_CODING
9056	CakSNP9056	*Kabuli*	Ca_Kabuli_Chr05	41285223	[A/C]	Ca_07642	Protein of unknown function DUF1296	NON_SYNONYMOUS_CODING
9057	CakSNP9057	*Kabuli*	Ca_Kabuli_Chr05	41302544	[C/G]	Ca_07644	AMP-dependent synthetase/ligase	INTRON
9058	CakSNP9058	*Kabuli*	Ca_Kabuli_Chr05	41302601	[G/A]	Ca_07644	AMP-dependent synthetase/ligase	INTRON
9059	CakSNP9059	*Kabuli*	Ca_Kabuli_Chr05	41302733	[T/C]	Ca_07644	AMP-dependent synthetase/ligase	INTRON
9060	CakSNP9060	*Kabuli*	Ca_Kabuli_Chr05	41302804	[A/G]	Ca_07644	AMP-dependent synthetase/ligase	INTRON
9061	CakSNP9061	*Kabuli*	Ca_Kabuli_Chr05	41302859	[C/A]	Ca_07644	AMP-dependent synthetase/ligase	INTRON

S.N.	SNP IDs	Chickpea cultivars	Chromosomes	Physical positions (bp)	SNPs	Gene accession IDs	Putative functions	Sequence components of genome
9062	CakSNP9062	*Kabuli*	Ca_Kabuli_Chr05	41302901	[C/A]	Ca_07644	AMP-dependent synthetase/ligase	INTRON
9063	CakSNP9063	*Kabuli*	Ca_Kabuli_Chr05	41302900	[T/A]	Ca_07644	AMP-dependent synthetase/ligase	INTRON
9064	CakSNP9064	*Kabuli*	Ca_Kabuli_Chr05	41302895	[G/A]	Ca_07644	AMP-dependent synthetase/ligase	INTRON
9065	CakSNP9065	*Kabuli*	Ca_Kabuli_Chr05	41304103	[G/A]	Ca_07644	AMP-dependent synthetase/ligase	INTRON
9066	CakSNP9066	*Kabuli*	Ca_Kabuli_Chr05	41399241	[C/T]	Ca_19236	ABC-1	SYNONYMOUS_CODING
9067	CakSNP9067	*Kabuli*	Ca_Kabuli_Chr05	41407077	[A/C]	Ca_19235	Ribosomal protein L13	INTRON
9068	CakSNP9068	*Kabuli*	Ca_Kabuli_Chr05	41407253	[T/C]	Ca_19235	Ribosomal protein L13	INTRON
9069	CakSNP9069	*Kabuli*	Ca_Kabuli_Chr05	41519141	[C/T]	Ca_19228	PUG domain	INTRON
9070	CakSNP9070	*Kabuli*	Ca_Kabuli_Chr05	41521854	[T/G]	Ca_19228	PUG domain	INTRON
9071	CakSNP9071	*Kabuli*	Ca_Kabuli_Chr05	41526026	[T/G]	Ca_19227	Protein of unknown function DUF1664	INTRON

S.N.	SNP IDs	Chickpea cultivars	Chromosomes	Physical positions (bp)	SNPs	Gene accession IDs	Putative functions	Sequence components of genome
9072	CakSNP9072	*Kabuli*	Ca_Kabuli_Chr05	41635262	[C/T]	Ca_19218	Zinc finger, RING-type	NON_SYNONYMOUS_CODING
9073	CakSNP9073	*Kabuli*	Ca_Kabuli_Chr05	41678144	[C/T]	Ca_19215	Argonaute/Dicer protein, PAZ	SYNONYMOUS_CODING
9074	CakSNP9074	*Kabuli*	Ca_Kabuli_Chr05	41696426	[T/C]	Ca_19213	--	INTRON
9075	CakSNP9075	*Kabuli*	Ca_Kabuli_Chr05	41696496	[G/C]	Ca_19213	--	INTRON
9076	CakSNP9076	*Kabuli*	Ca_Kabuli_Chr05	41701479	[T/C]	Ca_19212	Argonaute/Dicer protein, PAZ	SYNONYMOUS_CODING
9077	CakSNP9077	*Kabuli*	Ca_Kabuli_Chr05	41772759	[G/A]	Ca_19203	S-locus glycoprotein	SYNONYMOUS_CODING
9078	CakSNP9078	*Kabuli*	Ca_Kabuli_Chr05	41887214	[A/T]	Ca_11426	RNA recognition motif domain	INTRON
9079	CakSNP9079	*Kabuli*	Ca_Kabuli_Chr05	41902419	[A/C]	Ca_11423	Peptidase C19, ubiquitin carboxyl-terminal hydrolase 2	SYNONYMOUS_CODING
9080	CakSNP9080	*Kabuli*	Ca_Kabuli_Chr05	41945316	[A/G]	Ca_11418	Helicase, C-terminal	SYNONYMOUS_CODING
9081	CakSNP9081	*Kabuli*	Ca_Kabuli_Chr05	41945373	[C/T]	Ca_11418	Helicase, C-terminal	SYNONYMOUS_CODING

S.N.	SNP IDs	Chickpea cultivars	Chromosomes	Physical positions (bp)	SNPs	Gene accession IDs	Putative functions	Sequence components of genome
9082	CakSNP9082	*Kabuli*	Ca_Kabuli_Chr05	41945308	[G/A]	Ca_11418	Helicase, C-terminal	NON_SYNONYMOUS_CODING
9083	CakSNP9083	*Kabuli*	Ca_Kabuli_Chr05	41974690	[T/C]	Ca_11415	K Homology	NON_SYNONYMOUS_CODING
9084	CakSNP9084	*Kabuli*	Ca_Kabuli_Chr05	42004600	[T/G]	Ca_11412	Rab-GAP/TBC domain	INTRON
9085	CakSNP9085	*Kabuli*	Ca_Kabuli_Chr05	42004602	[A/G]	Ca_11412	Rab-GAP/TBC domain	INTRON
9086	CakSNP9086	*Kabuli*	Ca_Kabuli_Chr05	42004734	[G/A]	Ca_11412	Rab-GAP/TBC domain	INTRON
9087	CakSNP9087	*Kabuli*	Ca_Kabuli_Chr05	42004742	[G/A]	Ca_11412	Rab-GAP/TBC domain	INTRON
9088	CakSNP9088	*Kabuli*	Ca_Kabuli_Chr05	42004751	[G/A]	Ca_11412	Rab-GAP/TBC domain	INTRON
9089	CakSNP9089	*Kabuli*	Ca_Kabuli_Chr05	42004753	[C/A]	Ca_11412	Rab-GAP/TBC domain	INTRON
9090	CakSNP9090	*Kabuli*	Ca_Kabuli_Chr05	42008626	[A/C]	Ca_11412	Rab-GAP/TBC domain	SYNONYMOUS_CODING
9091	CakSNP9091	*Kabuli*	Ca_Kabuli_Chr05	42012972	[C/A]	Ca_11411	TAFII55 protein, conserved region	NON_SYNONYMOUS_CODING

S.N.	SNP IDs	Chickpea cultivars	Chromosomes	Physical positions (bp)	SNPs	Gene accession IDs	Putative functions	Sequence components of genome
9092	CakSNP9092	*Kabuli*	Ca_Kabuli_Chr05	42012983	[G/A]	Ca_11411	TAFII55 protein, conserved region	SYNONYMOUS_CODING
9093	CakSNP9093	*Kabuli*	Ca_Kabuli_Chr05	42012998	[C/T]	Ca_11411	TAFII55 protein, conserved region	SYNONYMOUS_CODING
9094	CakSNP9094	*Kabuli*	Ca_Kabuli_Chr05	42020435	[A/G]	Ca_11409	--	NON_SYNONYMOUS_CODING
9095	CakSNP9095	*Kabuli*	Ca_Kabuli_Chr05	42100070	[A/G]	Ca_11403	NAD-dependent epimerase/dehydratase	SYNONYMOUS_CODING
9096	CakSNP9096	*Kabuli*	Ca_Kabuli_Chr05	42117516	[C/T]	Ca_11402	Sugar/inositol transporter	SYNONYMOUS_CODING
9097	CakSNP9097	*Kabuli*	Ca_Kabuli_Chr05	42120698	[T/C]	Ca_11402	Sugar/inositol transporter	NON_SYNONYMOUS_CODING
9098	CakSNP9098	*Kabuli*	Ca_Kabuli_Chr05	42120735	[G/C]	Ca_11402	Sugar/inositol transporter	SYNONYMOUS_CODING
9099	CakSNP9099	*Kabuli*	Ca_Kabuli_Chr05	42121472	[C/A]	Ca_11402	Sugar/inositol transporter	INTRON
9100	CakSNP9100	*Kabuli*	Ca_Kabuli_Chr05	42225634	[G/A]		--	INTERGENIC
9101	CakSNP9101	*Kabuli*	Ca_Kabuli_Chr05	42275523	[G/A]	Ca_11388	Pentatricopeptide repeat	SYNONYMOUS_CODING

S.N.	SNP IDs	Chickpea cultivars	Chromosomes	Physical positions (bp)	SNPs	Gene accession IDs	Putative functions	Sequence components of genome
9102	CakSNP9102	*Kabuli*	Ca_Kabuli_Chr05	42282297	[G/A]	Ca_11387	AMP-dependent synthetase/ligase	INTRON
9103	CakSNP9103	*Kabuli*	Ca_Kabuli_Chr05	42413430	[A/C]	Ca_11377	Zinc finger, Sec23/Sec24-type	INTRON
9104	CakSNP9104	*Kabuli*	Ca_Kabuli_Chr05	42422867	[C/A]	Ca_11376	Zinc finger, C2H2-type	INTRON
9105	CakSNP9105	*Kabuli*	Ca_Kabuli_Chr05	42422845	[T/C]	Ca_11376	Zinc finger, C2H2-type	INTRON
9106	CakSNP9106	*Kabuli*	Ca_Kabuli_Chr05	42422837	[G/A]	Ca_11376	Zinc finger, C2H2-type	INTRON
9107	CakSNP9107	*Kabuli*	Ca_Kabuli_Chr05	42422808	[A/G]	Ca_11376	Zinc finger, C2H2-type	INTRON
9108	CakSNP9108	*Kabuli*	Ca_Kabuli_Chr05	42422932	[C/A]	Ca_11376	Zinc finger, C2H2-type	NON_SYNONYMOUS_CODING
9109	CakSNP9109	*Kabuli*	Ca_Kabuli_Chr05	42435263	[C/T]	Ca_11374	Kinesin, motor domain	SYNONYMOUS_CODING
9110	CakSNP9110	*Kabuli*	Ca_Kabuli_Chr05	42436972	[A/G]		--	INTERGENIC
9111	CakSNP9111	*Kabuli*	Ca_Kabuli_Chr05	42436976	[A/T]		--	INTERGENIC

S.N.	SNP IDs	Chickpea cultivars	Chromosomes	Physical positions (bp)	SNPs	Gene accession IDs	Putative functions	Sequence components of genome
9112	CakSNP9112	*Kabuli*	Ca_Kabuli_Chr05	42443786	[A/G]	Ca_11372	DNA topoisomerase, type IA	INTRON
9113	CakSNP9113	*Kabuli*	Ca_Kabuli_Chr05	42477712	[G/A]	Ca_11367	Membrane attack complex component/perforin (MACPF) domain	SYNONYMOUS_CODING
9114	CakSNP9114	*Kabuli*	Ca_Kabuli_Chr05	42485130	[T/C]	Ca_11365	NnrU	SYNONYMOUS_CODING
9115	CakSNP9115	*Kabuli*	Ca_Kabuli_Chr05	42528744	[T/G]	Ca_11361	IQ motif, EF-hand binding site	NON_SYNONYMOUS_CODING
9116	CakSNP9116	*Kabuli*	Ca_Kabuli_Chr05	42528934	[A/G]	Ca_11361	IQ motif, EF-hand binding site	NON_SYNONYMOUS_CODING
9117	CakSNP9117	*Kabuli*	Ca_Kabuli_Chr05	42540675	[A/T]	Ca_11358	Signal transduction response regulator, receiver domain	SYNONYMOUS_CODING
9118	CakSNP9118	*Kabuli*	Ca_Kabuli_Chr05	42582262	[T/A]	Ca_11356	Transcription factor, SBP-box	NON_SYNONYMOUS_CODING
9119	CakSNP9119	*Kabuli*	Ca_Kabuli_Chr05	42655824	[A/G]	Ca_11348	Rubber elongation factor	INTRON
9120	CakSNP9120	*Kabuli*	Ca_Kabuli_Chr05	42749499	[C/T]	Ca_11334	--	INTRON
9121	CakSNP9121	*Kabuli*	Ca_Kabuli_Chr05	42792635	[G/A]	Ca_11331	Ankyrin repeat	NON_SYNONYMOUS_CODING

S.N.	SNP IDs	Chickpea cultivars	Chromosomes	Physical positions (bp)	SNPs	Gene accession IDs	Putative functions	Sequence components of genome
9122	CakSNP9122	*Kabuli*	Ca_Kabuli_Chr05	42814621	[C/T]		--	INTERGENIC
9123	CakSNP9123	*Kabuli*	Ca_Kabuli_Chr05	42814623	[A/G]		--	INTERGENIC
9124	CakSNP9124	*Kabuli*	Ca_Kabuli_Chr05	42857977	[G/C]		--	INTERGENIC
9125	CakSNP9125	*Kabuli*	Ca_Kabuli_Chr05	42863366	[C/T]	Ca_11322	Short-chain dehydrogenase/reductase SDR	INTRON
9126	CakSNP9126	*Kabuli*	Ca_Kabuli_Chr05	42872074	[A/C]	Ca_11321	DNA polymerase alpha/epsilon, subunit B	INTRON
9127	CakSNP9127	*Kabuli*	Ca_Kabuli_Chr05	42872091	[A/G]	Ca_11321	DNA polymerase alpha/epsilon, subunit B	INTRON
9128	CakSNP9128	*Kabuli*	Ca_Kabuli_Chr05	42881185	[C/T]	Ca_11319	Ankyrin repeat	NON_SYNONYMOUS_CODING
9129	CakSNP9129	*Kabuli*	Ca_Kabuli_Chr05	42882945	[C/T]	Ca_11319	Ankyrin repeat	SYNONYMOUS_CODING
9130	CakSNP9130	*Kabuli*	Ca_Kabuli_Chr05	42882965	[C/T]	Ca_11319	Ankyrin repeat	NON_SYNONYMOUS_CODING
9131	CakSNP9131	*Kabuli*	Ca_Kabuli_Chr05	42883163	[T/C]	Ca_11319	Ankyrin repeat	NON_SYNONYMOUS_CODING

S.N.	SNP IDs	Chickpea cultivars	Chromosomes	Physical positions (bp)	SNPs	Gene accession IDs	Putative functions	Sequence components of genome
9132	CakSNP9132	*Kabuli*	Ca_Kabuli_Chr05	42910653	[A/G]	Ca_11316	DNA-binding HORMA	INTRON
9133	CakSNP9133	*Kabuli*	Ca_Kabuli_Chr05	42910661	[A/G]	Ca_11316	DNA-binding HORMA	INTRON
9134	CakSNP9134	*Kabuli*	Ca_Kabuli_Chr05	42910732	[A/T]	Ca_11316	DNA-binding HORMA	INTRON
9135	CakSNP9135	*Kabuli*	Ca_Kabuli_Chr05	42924113	[C/T]		--	INTERGENIC
9136	CakSNP9136	*Kabuli*	Ca_Kabuli_Chr05	42928986	[T/G]	Ca_11315	SANT domain, DNA binding	SYNONYMOUS_CODING
9137	CakSNP9137	*Kabuli*	Ca_Kabuli_Chr05	42929087	[A/C]		--	INTERGENIC
9138	CakSNP9138	*Kabuli*	Ca_Kabuli_Chr05	42946580	[T/C]	Ca_11314	Peptidase M3A/M3B, thimet/oligopeptidase F	INTRON
9139	CakSNP9139	*Kabuli*	Ca_Kabuli_Chr05	42946716	[A/T]	Ca_11314	Peptidase M3A/M3B, thimet/oligopeptidase F	INTRON
9140	CakSNP9140	*Kabuli*	Ca_Kabuli_Chr05	42999704	[C/T]		--	INTERGENIC
9141	CakSNP9141	*Kabuli*	Ca_Kabuli_Chr05	43000210	[A/G]	Ca_11308	Protein kinase, catalytic domain	SYNONYMOUS_CODING

S.N.	SNP IDs	Chickpea cultivars	Chromosomes	Physical positions (bp)	SNPs	Gene accession IDs	Putative functions	Sequence components of genome
9142	CakSNP9142	*Kabuli*	Ca_Kabuli_Chr05	43034618	[T/A]	Ca_11305	Regulator of G protein signalling	INTRON
9143	CakSNP9143	*Kabuli*	Ca_Kabuli_Chr05	43037863	[G/A]	Ca_11305	Regulator of G protein signalling	NON_SYNONYMOUS_CODING
9144	CakSNP9144	*Kabuli*	Ca_Kabuli_Chr05	43060091	[C/T]	Ca_11301	Photosystem II PsbY	SYNONYMOUS_CODING
9145	CakSNP9145	*Kabuli*	Ca_Kabuli_Chr05	43067894	[T/C]	Ca_11300	Membrane insertion protein, OxaA/YidC	SYNONYMOUS_CODING
9146	CakSNP9146	*Kabuli*	Ca_Kabuli_Chr05	43067891	[G/C]	Ca_11300	Membrane insertion protein, OxaA/YidC	SYNONYMOUS_CODING
9147	CakSNP9147	*Kabuli*	Ca_Kabuli_Chr05	43067905	[A/C]	Ca_11300	Membrane insertion protein, OxaA/YidC	SYNONYMOUS_CODING
9148	CakSNP9148	*Kabuli*	Ca_Kabuli_Chr05	43080493	[G/A]	Ca_11299	Regulator of chromosome condensation, RCC1	NON_SYNONYMOUS_CODING
9149	CakSNP9149	*Kabuli*	Ca_Kabuli_Chr05	43081783	[G/A]	Ca_11299	Regulator of chromosome condensation, RCC1	SYNONYMOUS_CODING
9150	CakSNP9150	*Kabuli*	Ca_Kabuli_Chr05	43110660	[G/A]	Ca_11297	Pectate lyase/Amb allergen	INTRON
9151	CakSNP9151	*Kabuli*	Ca_Kabuli_Chr05	43110669	[A/T]	Ca_11297	Pectate lyase/Amb allergen	INTRON

S.N.	SNP IDs	Chickpea cultivars	Chromosomes	Physical positions (bp)	SNPs	Gene accession IDs	Putative functions	Sequence components of genome
9152	CakSNP9152	*Kabuli*	Ca_Kabuli_Chr05	43110726	[T/G]	Ca_11297	Pectate lyase/Amb allergen	INTRON
9153	CakSNP9153	*Kabuli*	Ca_Kabuli_Chr05	43110737	[A/G]	Ca_11297	Pectate lyase/Amb allergen	SYNONYMOUS_CODING
9154	CakSNP9154	*Kabuli*	Ca_Kabuli_Chr05	43175781	[G/A]	Ca_12741	Pyridoxal phosphate-dependent enzyme, beta subunit	NON_SYNONYMOUS_CODING
9155	CakSNP9155	*Kabuli*	Ca_Kabuli_Chr05	43188824	[T/G]		--	INTERGENIC
9156	CakSNP9156	*Kabuli*	Ca_Kabuli_Chr05	43188853	[C/A]		--	INTERGENIC
9157	CakSNP9157	*Kabuli*	Ca_Kabuli_Chr05	43210793	[T/C]		--	INTERGENIC
9158	CakSNP9158	*Kabuli*	Ca_Kabuli_Chr05	43212083	[G/A]	Ca_12737	--	NON_SYNONYMOUS_CODING
9159	CakSNP9159	*Kabuli*	Ca_Kabuli_Chr05	43212066	[C/T]	Ca_12737	--	NON_SYNONYMOUS_CODING
9160	CakSNP9160	*Kabuli*	Ca_Kabuli_Chr05	43221125	[T/C]		--	INTERGENIC
9161	CakSNP9161	*Kabuli*	Ca_Kabuli_Chr05	43231220	[T/C]	Ca_12734	Protein kinase, catalytic domain	SYNONYMOUS_CODING

S.N.	SNP IDs	Chickpea cultivars	Chromosomes	Physical positions (bp)	SNPs	Gene accession IDs	Putative functions	Sequence components of genome
9162	CakSNP9162	*Kabuli*	Ca_Kabuli_Chr05	43231265	[C/T]	Ca_12734	Protein kinase, catalytic domain	INTRON
9163	CakSNP9163	*Kabuli*	Ca_Kabuli_Chr05	43253586	[C/A]	Ca_12732	--	INTRON
9164	CakSNP9164	*Kabuli*	Ca_Kabuli_Chr05	43275472	[T/C]		--	INTERGENIC
9165	CakSNP9165	*Kabuli*	Ca_Kabuli_Chr05	43334903	[T/C]		--	INTERGENIC
9166	CakSNP9166	*Kabuli*	Ca_Kabuli_Chr05	43341646	[A/C]	Ca_12721	Helicase, C-terminal	SYNONYMOUS_CODING
9167	CakSNP9167	*Kabuli*	Ca_Kabuli_Chr05	43402230	[C/T]	Ca_12716	--	SYNONYMOUS_CODING
9168	CakSNP9168	*Kabuli*	Ca_Kabuli_Chr05	43410326	[T/G]		--	INTERGENIC
9169	CakSNP9169	*Kabuli*	Ca_Kabuli_Chr05	43423593	[G/C]	Ca_12713	Bacterial Fmu (Sun)/eukaryotic nucleolar NOL1/Nop2p	INTRON
9170	CakSNP9170	*Kabuli*	Ca_Kabuli_Chr05	43521774	[A/T]	Ca_12700	Asparagine synthase	NON_SYNONYMOUS_CODING
9171	CakSNP9171	*Kabuli*	Ca_Kabuli_Chr05	43521789	[G/A]	Ca_12700	Asparagine synthase	SYNONYMOUS_CODING

S.N.	SNP IDs	Chickpea cultivars	Chromosomes	Physical positions (bp)	SNPs	Gene accession IDs	Putative functions	Sequence components of genome
9172	CakSNP9172	*Kabuli*	Ca_Kabuli_Chr05	43521830	[T/C]	Ca_12700	Asparagine synthase	INTRON
9173	CakSNP9173	*Kabuli*	Ca_Kabuli_Chr05	43521851	[G/A]	Ca_12700	Asparagine synthase	INTRON
9174	CakSNP9174	*Kabuli*	Ca_Kabuli_Chr05	43523560	[A/G]	Ca_12700	Asparagine synthase	NON_SYNONYMOUS_CODING
9175	CakSNP9175	*Kabuli*	Ca_Kabuli_Chr05	43530567	[C/T]	Ca_12698	--	NON_SYNONYMOUS_CODING
9176	CakSNP9176	*Kabuli*	Ca_Kabuli_Chr05	43565712	[T/C]	Ca_12691	ABC transporter-like	INTRON
9177	CakSNP9177	*Kabuli*	Ca_Kabuli_Chr05	43632249	[C/A]	Ca_12688	Tetratricopeptide TPR-1	SYNONYMOUS_CODING
9178	CakSNP9178	*Kabuli*	Ca_Kabuli_Chr05	43632281	[C/G]	Ca_12688	Tetratricopeptide TPR-1	INTRON
9179	CakSNP9179	*Kabuli*	Ca_Kabuli_Chr05	43632349	[T/A]	Ca_12688	Tetratricopeptide TPR-1	SYNONYMOUS_CODING
9180	CakSNP9180	*Kabuli*	Ca_Kabuli_Chr05	43632400	[A/G]	Ca_12688	Tetratricopeptide TPR-1	SYNONYMOUS_CODING
9181	CakSNP9181	*Kabuli*	Ca_Kabuli_Chr05	43632377	[C/T]	Ca_12688	Tetratricopeptide TPR-1	SYNONYMOUS_CODING

S.N.	SNP IDs	Chickpea cultivars	Chromosomes	Physical positions (bp)	SNPs	Gene accession IDs	Putative functions	Sequence components of genome
9182	CakSNP9182	*Kabuli*	Ca_Kabuli_Chr05	43632481	[G/T]	Ca_12688	Tetratricopeptide TPR-1	SYNONYMOUS_CODING
9183	CakSNP9183	*Kabuli*	Ca_Kabuli_Chr05	43632508	[A/G]	Ca_12688	Tetratricopeptide TPR-1	SYNONYMOUS_CODING
9184	CakSNP9184	*Kabuli*	Ca_Kabuli_Chr05	43710359	[T/G]		--	INTERGENIC
9185	CakSNP9185	*Kabuli*	Ca_Kabuli_Chr05	43710362	[A/T]		--	INTERGENIC
9186	CakSNP9186	*Kabuli*	Ca_Kabuli_Chr05	43710366	[G/T]		--	INTERGENIC
9187	CakSNP9187	*Kabuli*	Ca_Kabuli_Chr05	43710391	[A/G]		--	INTERGENIC
9188	CakSNP9188	*Kabuli*	Ca_Kabuli_Chr05	43742448	[C/T]		--	INTERGENIC
9189	CakSNP9189	*Kabuli*	Ca_Kabuli_Chr05	43742456	[G/A]		--	INTERGENIC
9190	CakSNP9190	*Kabuli*	Ca_Kabuli_Chr05	43755074	[A/G]	Ca_12676	C2 calcium-dependent membrane targeting	NON_SYNONYMOUS_CODING
9191	CakSNP9191	*Kabuli*	Ca_Kabuli_Chr05	43755756	[A/G]	Ca_12676	C2 calcium-dependent membrane targeting	SYNONYMOUS_CODING

S.N.	SNP IDs	Chickpea cultivars	Chromosomes	Physical positions (bp)	SNPs	Gene accession IDs	Putative functions	Sequence components of genome
9192	CakSNP9192	*Kabuli*	Ca_Kabuli_Chr05	43759059	[A/C]	Ca_12676	C2 calcium-dependent membrane targeting	NON_SYNONYMOUS_CODING
9193	CakSNP9193	*Kabuli*	Ca_Kabuli_Chr05	43786883	[A/G]	Ca_12674	RNA polymerase II, large subunit, CTD	SYNONYMOUS_CODING
9194	CakSNP9194	*Kabuli*	Ca_Kabuli_Chr05	43789875	[C/G]	Ca_12673	Short-chain dehydrogenase/reductase SDR	NON_SYNONYMOUS_CODING
9195	CakSNP9195	*Kabuli*	Ca_Kabuli_Chr05	43790108	[G/A]	Ca_12673	Short-chain dehydrogenase/reductase SDR	INTRON
9196	CakSNP9196	*Kabuli*	Ca_Kabuli_Chr05	43812119	[G/A]		--	INTERGENIC
9197	CakSNP9197	*Kabuli*	Ca_Kabuli_Chr05	43812213	[C/T]		--	INTERGENIC
9198	CakSNP9198	*Kabuli*	Ca_Kabuli_Chr05	43859301	[G/A]	Ca_12668	AMP-dependent synthetase/ligase	INTRON
9199	CakSNP9199	*Kabuli*	Ca_Kabuli_Chr05	43904489	[T/A]		--	INTERGENIC
9200	CakSNP9200	*Kabuli*	Ca_Kabuli_Chr05	43919009	[C/T]		--	INTERGENIC
9201	CakSNP9201	*Kabuli*	Ca_Kabuli_Chr05	43919017	[T/G]		--	INTERGENIC

S.N.	SNP IDs	Chickpea cultivars	Chromosomes	Physical positions (bp)	SNPs	Gene accession IDs	Putative functions	Sequence components of genome
9202	CakSNP9202	*Kabuli*	Ca_Kabuli_Chr05	43919022	[G/C]		--	INTERGENIC
9203	CakSNP9203	*Kabuli*	Ca_Kabuli_Chr05	43919028	[A/G]		--	INTERGENIC
9204	CakSNP9204	*Kabuli*	Ca_Kabuli_Chr05	43982994	[C/T]	Ca_12658	Peptidase A1	NON_SYNONYMOUS_CODING
9205	CakSNP9205	*Kabuli*	Ca_Kabuli_Chr05	43995843	[T/C]	Ca_12657	Peptidase A1	NON_SYNONYMOUS_CODING
9206	CakSNP9206	*Kabuli*	Ca_Kabuli_Chr05	44002824	[T/G]	Ca_12655	Aldehyde dehydrogenase NAD(P)-dependent	SYNONYMOUS_CODING
9207	CakSNP9207	*Kabuli*	Ca_Kabuli_Chr05	44002862	[C/A]	Ca_12655	Aldehyde dehydrogenase NAD(P)-dependent	INTRON
9208	CakSNP9208	*Kabuli*	Ca_Kabuli_Chr05	44038229	[T/C]	Ca_12653	Haem peroxidase, plant/fungal/bacterial	INTRON
9209	CakSNP9209	*Kabuli*	Ca_Kabuli_Chr05	44064495	[G/A]	Ca_12651	Domain of unknown function DUF632	SYNONYMOUS_CODING
9210	CakSNP9210	*Kabuli*	Ca_Kabuli_Chr05	44080450	[G/C]	Ca_12649	Signal transduction response regulator, receiver domain	INTRON
9211	CakSNP9211	*Kabuli*	Ca_Kabuli_Chr05	44109260	[G/T]	Ca_12646	--	STOP_GAINED

S.N.	SNP IDs	Chickpea cultivars	Chromosomes	Physical positions (bp)	SNPs	Gene accession IDs	Putative functions	Sequence components of genome
9212	CakSNP9212	*Kabuli*	Ca_Kabuli_Chr05	44154671	[A/G]	Ca_12641	SANT domain, DNA binding	INTRON
9213	CakSNP9213	*Kabuli*	Ca_Kabuli_Chr05	44159237	[C/G]	Ca_12641	SANT domain, DNA binding	NON_SYNONYMOUS_CODING
9214	CakSNP9214	*Kabuli*	Ca_Kabuli_Chr05	44289612	[T/C]		--	INTERGENIC
9215	CakSNP9215	*Kabuli*	Ca_Kabuli_Chr05	44334286	[C/G]	Ca_03882	SecA protein	NON_SYNONYMOUS_CODING
9216	CakSNP9216	*Kabuli*	Ca_Kabuli_Chr05	44334318	[C/T]	Ca_03882	SecA protein	INTRON
9217	CakSNP9217	*Kabuli*	Ca_Kabuli_Chr05	44364134	[C/T]	Ca_03884	Inorganic polyphosphate/ATP-NAD kinase, predicted	INTRON
9218	CakSNP9218	*Kabuli*	Ca_Kabuli_Chr05	44373968	[G/T]	Ca_03885	--	NON_SYNONYMOUS_CODING
9219	CakSNP9219	*Kabuli*	Ca_Kabuli_Chr05	44378178	[C/A]	Ca_03885	--	INTRON
9220	CakSNP9220	*Kabuli*	Ca_Kabuli_Chr05	44428965	[G/A]	Ca_03889	SET domain	NON_SYNONYMOUS_CODING
9221	CakSNP9221	*Kabuli*	Ca_Kabuli_Chr05	44461250	[A/G]	Ca_03893	UTP--glucose-1-phosphate uridylyltransferase	INTRON

S.N.	SNP IDs	Chickpea cultivars	Chromosomes	Physical positions (bp)	SNPs	Gene accession IDs	Putative functions	Sequence components of genome
9222	CakSNP9222	*Kabuli*	Ca_Kabuli_Chr05	44491489	[G/T]	Ca_03896	--	INTRON
9223	CakSNP9223	*Kabuli*	Ca_Kabuli_Chr05	44555718	[A/C]		--	INTERGENIC
9224	CakSNP9224	*Kabuli*	Ca_Kabuli_Chr05	44561095	[T/C]	Ca_03899	Protein of unknown function DUF248, methyltransferase putative	SYNONYMOUS_CODING
9225	CakSNP9225	*Kabuli*	Ca_Kabuli_Chr05	44656921	[G/A]	Ca_03910	Pentatricopeptide repeat	NON_SYNONYMOUS_CODING
9226	CakSNP9226	*Kabuli*	Ca_Kabuli_Chr05	44667285	[C/T]	Ca_03911	Dehydrogenase, E1 component	SYNONYMOUS_CODING
9227	CakSNP9227	*Kabuli*	Ca_Kabuli_Chr05	44667297	[C/T]	Ca_03911	Dehydrogenase, E1 component	SYNONYMOUS_CODING
9228	CakSNP9228	*Kabuli*	Ca_Kabuli_Chr05	44667359	[C/A]	Ca_03911	Dehydrogenase, E1 component	INTRON
9229	CakSNP9229	*Kabuli*	Ca_Kabuli_Chr05	44760403	[A/G]	Ca_03920	Transcription factor GRAS	SYNONYMOUS_CODING
9230	CakSNP9230	*Kabuli*	Ca_Kabuli_Chr05	44760347	[G/T]	Ca_03920	Transcription factor GRAS	NON_SYNONYMOUS_CODING
9231	CakSNP9231	*Kabuli*	Ca_Kabuli_Chr05	44760469	[C/T]	Ca_03920	Transcription factor GRAS	SYNONYMOUS_CODING

S.N.	SNP IDs	Chickpea cultivars	Chromosomes	Physical positions (bp)	SNPs	Gene accession IDs	Putative functions	Sequence components of genome
9232	CakSNP9232	*Kabuli*	Ca_Kabuli_Chr05	44760490	[C/A]	Ca_03920	Transcription factor GRAS	NON_SYNONYMOUS_CODING
9233	CakSNP9233	*Kabuli*	Ca_Kabuli_Chr05	44772326	[G/A]	Ca_03922	LIM binding protein	SYNONYMOUS_CODING
9234	CakSNP9234	*Kabuli*	Ca_Kabuli_Chr05	44884779	[G/C]	Ca_03932	Sec1-like protein	NON_SYNONYMOUS_CODING
9235	CakSNP9235	*Kabuli*	Ca_Kabuli_Chr05	44887105	[T/G]	Ca_03932	Sec1-like protein	INTRON
9236	CakSNP9236	*Kabuli*	Ca_Kabuli_Chr05	44887241	[T/C]	Ca_03932	Sec1-like protein	INTRON
9237	CakSNP9237	*Kabuli*	Ca_Kabuli_Chr05	44887234	[A/C]	Ca_03932	Sec1-like protein	INTRON
9238	CakSNP9238	*Kabuli*	Ca_Kabuli_Chr05	44891426	[G/C]		--	INTERGENIC
9239	CakSNP9239	*Kabuli*	Ca_Kabuli_Chr05	44911842	[C/T]		--	INTERGENIC
9240	CakSNP9240	*Kabuli*	Ca_Kabuli_Chr05	44911868	[C/G]		--	INTERGENIC
9241	CakSNP9241	*Kabuli*	Ca_Kabuli_Chr05	44911881	[G/A]		--	INTERGENIC

S.N.	SNP IDs	Chickpea cultivars	Chromosomes	Physical positions (bp)	SNPs	Gene accession IDs	Putative functions	Sequence components of genome
9242	CakSNP9242	*Kabuli*	Ca_Kabuli_Chr05	44911906	[C/T]		--	INTERGENIC
9243	CakSNP9243	*Kabuli*	Ca_Kabuli_Chr05	44918947	[C/A]	Ca_03936	Glycoside hydrolase, family 9	STOP_GAINED
9244	CakSNP9244	*Kabuli*	Ca_Kabuli_Chr05	44938814	[A/T]	Ca_03940	Protein kinase, catalytic domain	NON_SYNONYMOUS_CODING
9245	CakSNP9245	*Kabuli*	Ca_Kabuli_Chr05	44938832	[A/G]	Ca_03940	Protein kinase, catalytic domain	SYNONYMOUS_CODING
9246	CakSNP9246	*Kabuli*	Ca_Kabuli_Chr05	44939058	[C/G]	Ca_03940	Protein kinase, catalytic domain	SYNONYMOUS_CODING
9247	CakSNP9247	*Kabuli*	Ca_Kabuli_Chr05	44939033	[G/A]	Ca_03940	Protein kinase, catalytic domain	NON_SYNONYMOUS_CODING
9248	CakSNP9248	*Kabuli*	Ca_Kabuli_Chr05	45087170	[G/A]		--	INTERGENIC
9249	CakSNP9249	*Kabuli*	Ca_Kabuli_Chr05	45087156	[A/G]		--	INTERGENIC
9250	CakSNP9250	*Kabuli*	Ca_Kabuli_Chr05	45087146	[C/G]		--	INTERGENIC
9251	CakSNP9251	*Kabuli*	Ca_Kabuli_Chr05	45087140	[C/A]		--	INTERGENIC

S.N.	SNP IDs	Chickpea cultivars	Chromosomes	Physical positions (bp)	SNPs	Gene accession IDs	Putative functions	Sequence components of genome
9252	CakSNP9252	*Kabuli*	Ca_Kabuli_Chr05	45087202	[G/C]		--	INTERGENIC
9253	CakSNP9253	*Kabuli*	Ca_Kabuli_Chr05	45087267	[A/G]		--	INTERGENIC
9254	CakSNP9254	*Kabuli*	Ca_Kabuli_Chr05	45087228	[A/C]		--	INTERGENIC
9255	CakSNP9255	*Kabuli*	Ca_Kabuli_Chr05	45087215	[G/A]		--	INTERGENIC
9256	CakSNP9256	*Kabuli*	Ca_Kabuli_Chr05	45215617	[G/A]	Ca_03964	Protein of unknown function DUF455	INTRON
9257	CakSNP9257	*Kabuli*	Ca_Kabuli_Chr05	45219125	[C/T]	Ca_03964	Protein of unknown function DUF455	INTRON
9258	CakSNP9258	*Kabuli*	Ca_Kabuli_Chr05	45219258	[G/T]	Ca_03964	Protein of unknown function DUF455	NON_SYNONYMOUS_CODING
9259	CakSNP9259	*Kabuli*	Ca_Kabuli_Chr05	45219355	[T/G]		--	INTERGENIC
9260	CakSNP9260	*Kabuli*	Ca_Kabuli_Chr05	45254102	[C/T]	Ca_03969	Zinc finger, C2H2-type matrin	SYNONYMOUS_CODING
9261	CakSNP9261	*Kabuli*	Ca_Kabuli_Chr05	45254112	[T/G]	Ca_03969	Zinc finger, C2H2-type matrin	NON_SYNONYMOUS_CODING

S.N.	SNP IDs	Chickpea cultivars	Chromosomes	Physical positions (bp)	SNPs	Gene accession IDs	Putative functions	Sequence components of genome
9262	CakSNP9262	*Kabuli*	Ca_Kabuli_Chr05	45301772	[T/G]	Ca_03973	PWWP	INTRON
9263	CakSNP9263	*Kabuli*	Ca_Kabuli_Chr05	45337274	[C/G]	Ca_03976	Pectinesterase, catalytic	SYNONYMOUS_CODING
9264	CakSNP9264	*Kabuli*	Ca_Kabuli_Chr05	45337307	[C/T]	Ca_03976	Pectinesterase, catalytic	SYNONYMOUS_CODING
9265	CakSNP9265	*Kabuli*	Ca_Kabuli_Chr05	45406853	[C/T]	Ca_03983	--	INTRON
9266	CakSNP9266	*Kabuli*	Ca_Kabuli_Chr05	45407063	[G/A]	Ca_03983	--	INTRON
9267	CakSNP9267	*Kabuli*	Ca_Kabuli_Chr05	45407044	[A/C]	Ca_03983	--	INTRON
9268	CakSNP9268	*Kabuli*	Ca_Kabuli_Chr05	45414089	[C/G]	Ca_03984	IQ motif, EF-hand binding site	SYNONYMOUS_CODING
9269	CakSNP9269	*Kabuli*	Ca_Kabuli_Chr05	45428229	[A/G]	Ca_03986	Longin	SYNONYMOUS_CODING
9270	CakSNP9270	*Kabuli*	Ca_Kabuli_Chr05	45429513	[A/G]	Ca_03987	--	SYNONYMOUS_CODING
9271	CakSNP9271	*Kabuli*	Ca_Kabuli_Chr05	45429518	[T/G]	Ca_03987	--	NON_SYNONYMOUS_CODING

S.N.	SNP IDs	Chickpea cultivars	Chromosomes	Physical positions (bp)	SNPs	Gene accession IDs	Putative functions	Sequence components of genome
9272	CakSNP9272	*Kabuli*	Ca_Kabuli_Chr05	45441223	[C/T]	Ca_03989	TAP42-like protein	INTRON
9273	CakSNP9273	*Kabuli*	Ca_Kabuli_Chr05	45444675	[G/T]	Ca_03989	TAP42-like protein	SYNONYMOUS_CODING
9274	CakSNP9274	*Kabuli*	Ca_Kabuli_Chr05	45444924	[G/T]	Ca_03989	TAP42-like protein	INTRON
9275	CakSNP9275	*Kabuli*	Ca_Kabuli_Chr05	45480175	[T/G]	Ca_03994	--	NON_SYNONYMOUS_CODING
9276	CakSNP9276	*Kabuli*	Ca_Kabuli_Chr05	45501379	[C/T]		--	INTERGENIC
9277	CakSNP9277	*Kabuli*	Ca_Kabuli_Chr05	45540473	[G/A]	Ca_04002	Pentapeptide repeat	INTRON
9278	CakSNP9278	*Kabuli*	Ca_Kabuli_Chr05	45559874	[C/A]	Ca_04004	WD40 repeat	NON_SYNONYMOUS_CODING
9279	CakSNP9279	*Kabuli*	Ca_Kabuli_Chr05	45601196	[A/G]		--	INTERGENIC
9280	CakSNP9280	*Kabuli*	Ca_Kabuli_Chr05	45601313	[G/A]		--	INTERGENIC
9281	CakSNP9281	*Kabuli*	Ca_Kabuli_Chr05	45642344	[C/A]	Ca_04010	Secretory pathway Sec39	NON_SYNONYMOUS_CODING

S.N.	SNP IDs	Chickpea cultivars	Chromosomes	Physical positions (bp)	SNPs	Gene accession IDs	Putative functions	Sequence components of genome
9282	CakSNP9282	*Kabuli*	Ca_Kabuli_Chr05	45646235	[A/G]	Ca_04010	Secretory pathway Sec39	INTRON
9283	CakSNP9283	*Kabuli*	Ca_Kabuli_Chr05	45649180	[G/A]	Ca_04010	Secretory pathway Sec39	NON_SYNONYMOUS_CODING
9284	CakSNP9284	*Kabuli*	Ca_Kabuli_Chr05	45649189	[G/A]	Ca_04010	Secretory pathway Sec39	NON_SYNONYMOUS_CODING
9285	CakSNP9285	*Kabuli*	Ca_Kabuli_Chr05	45661109	[T/C]	Ca_04012	Protein of unknown function DUF791	SYNONYMOUS_CODING
9286	CakSNP9286	*Kabuli*	Ca_Kabuli_Chr05	45694453	[T/G]	Ca_04017	ATPase, AAA+ type, core	SYNONYMOUS_CODING
9287	CakSNP9287	*Kabuli*	Ca_Kabuli_Chr05	45710509	[G/A]	Ca_04019	Aminotransferase-like, plant mobile domain	INTRON
9288	CakSNP9288	*Kabuli*	Ca_Kabuli_Chr05	45710476	[A/G]	Ca_04019	Aminotransferase-like, plant mobile domain	INTRON
9289	CakSNP9289	*Kabuli*	Ca_Kabuli_Chr05	45726539	[T/A]	Ca_04021	Cytochrome b5	INTRON
9290	CakSNP9290	*Kabuli*	Ca_Kabuli_Chr05	45726995	[C/T]	Ca_04021	Cytochrome b5	INTRON
9291	CakSNP9291	*Kabuli*	Ca_Kabuli_Chr05	45727022	[T/C]	Ca_04021	Cytochrome b5	INTRON

S.N.	SNP IDs	Chickpea cultivars	Chromosomes	Physical positions (bp)	SNPs	Gene accession IDs	Putative functions	Sequence components of genome
9292	CakSNP9292	*Kabuli*	Ca_Kabuli_Chr05	45750121	[A/T]	Ca_04024	Glucosamine/galactosamine-6-phosphate isomerase	INTRON
9293	CakSNP9293	*Kabuli*	Ca_Kabuli_Chr05	45798850	[C/A]		--	INTERGENIC
9294	CakSNP9294	*Kabuli*	Ca_Kabuli_Chr05	45798966	[T/G]		--	INTERGENIC
9295	CakSNP9295	*Kabuli*	Ca_Kabuli_Chr05	45798950	[C/T]		--	INTERGENIC
9296	CakSNP9296	*Kabuli*	Ca_Kabuli_Chr05	45798919	[C/A]		--	INTERGENIC
9297	CakSNP9297	*Kabuli*	Ca_Kabuli_Chr05	45808905	[G/A]	Ca_04030	Protein kinase, catalytic domain	INTRON
9298	CakSNP9298	*Kabuli*	Ca_Kabuli_Chr05	45832009	[T/G]	Ca_04031	--	INTRON
9299	CakSNP9299	*Kabuli*	Ca_Kabuli_Chr05	45832048	[C/G]	Ca_04031	--	INTRON
9300	CakSNP9300	*Kabuli*	Ca_Kabuli_Chr05	45832070	[A/T]	Ca_04031	--	NON_SYNONYMOUS_CODING
9301	CakSNP9301	*Kabuli*	Ca_Kabuli_Chr05	45842438	[A/C]	Ca_04032	SSXT	INTRON

S.N.	SNP IDs	Chickpea cultivars	Chromosomes	Physical positions (bp)	SNPs	Gene accession IDs	Putative functions	Sequence components of genome
9302	CakSNP9302	*Kabuli*	Ca_Kabuli_Chr05	45845081	[A/C]	Ca_04033	--	INTRON
9303	CakSNP9303	*Kabuli*	Ca_Kabuli_Chr05	45848030	[A/G]	Ca_04033	--	INTRON
9304	CakSNP9304	*Kabuli*	Ca_Kabuli_Chr05	45848087	[T/G]	Ca_04033	--	INTRON
9305	CakSNP9305	*Kabuli*	Ca_Kabuli_Chr05	45952107	[A/G]	Ca_04042	RNA recognition motif domain	SYNONYMOUS_CODING
9306	CakSNP9306	*Kabuli*	Ca_Kabuli_Chr05	45952842	[A/C]	Ca_04042	RNA recognition motif domain	INTRON
9307	CakSNP9307	*Kabuli*	Ca_Kabuli_Chr05	45971560	[G/A]	Ca_04043	RNA recognition motif domain	SYNONYMOUS_CODING
9308	CakSNP9308	*Kabuli*	Ca_Kabuli_Chr05	45978758	[A/G]	Ca_04044	RNA recognition motif domain	NON_SYNONYMOUS_CODING
9309	CakSNP9309	*Kabuli*	Ca_Kabuli_Chr05	46002514	[C/T]		--	INTERGENIC
9310	CakSNP9310	*Kabuli*	Ca_Kabuli_Chr05	46002497	[G/A]		--	INTERGENIC
9311	CakSNP9311	*Kabuli*	Ca_Kabuli_Chr05	46018939	[C/T]	Ca_04049	Ribosomal protein 60S	NON_SYNONYMOUS_CODING

S.N.	SNP IDs	Chickpea cultivars	Chromosomes	Physical positions (bp)	SNPs	Gene accession IDs	Putative functions	Sequence components of genome
9312	CakSNP9312	*Kabuli*	Ca_Kabuli_Chr05	46019052	[T/A]		--	INTERGENIC
9313	CakSNP9313	*Kabuli*	Ca_Kabuli_Chr05	46022566	[C/T]	Ca_04050	K Homology	INTRON
9314	CakSNP9314	*Kabuli*	Ca_Kabuli_Chr05	46022598	[T/C]	Ca_04050	K Homology	SYNONYMOUS_CODING
9315	CakSNP9315	*Kabuli*	Ca_Kabuli_Chr05	46022551	[G/A]	Ca_04050	K Homology	INTRON
9316	CakSNP9316	*Kabuli*	Ca_Kabuli_Chr05	46022549	[G/A]	Ca_04050	K Homology	INTRON
9317	CakSNP9317	*Kabuli*	Ca_Kabuli_Chr05	46022547	[G/A]	Ca_04050	K Homology	INTRON
9318	CakSNP9318	*Kabuli*	Ca_Kabuli_Chr05	46022544	[A/G]	Ca_04050	K Homology	INTRON
9319	CakSNP9319	*Kabuli*	Ca_Kabuli_Chr05	46022531	[T/A]	Ca_04050	K Homology	INTRON
9320	CakSNP9320	*Kabuli*	Ca_Kabuli_Chr05	46049587	[A/G]		--	INTERGENIC
9321	CakSNP9321	*Kabuli*	Ca_Kabuli_Chr05	46050168	[A/T]		--	INTERGENIC

S.N.	SNP IDs	Chickpea cultivars	Chromosomes	Physical positions (bp)	SNPs	Gene accession IDs	Putative functions	Sequence components of genome
9322	CakSNP9322	*Kabuli*	Ca_Kabuli_Chr05	46077190	[T/C]	Ca_04056	Signal transduction response regulator, receiver domain	SYNONYMOUS_CODING
9323	CakSNP9323	*Kabuli*	Ca_Kabuli_Chr05	46077302	[G/A]	Ca_04056	Signal transduction response regulator, receiver domain	NON_SYNONYMOUS_CODING
9324	CakSNP9324	*Kabuli*	Ca_Kabuli_Chr05	46083283	[G/A]		--	INTERGENIC
9325	CakSNP9325	*Kabuli*	Ca_Kabuli_Chr05	46083343	[T/G]		--	INTERGENIC
9326	CakSNP9326	*Kabuli*	Ca_Kabuli_Chr05	46083414	[T/G]		--	INTERGENIC
9327	CakSNP9327	*Kabuli*	Ca_Kabuli_Chr05	46119122	[G/A]	Ca_04060	O-methyltransferase, family 2	SYNONYMOUS_CODING
9328	CakSNP9328	*Kabuli*	Ca_Kabuli_Chr05	46119182	[A/G]	Ca_04060	O-methyltransferase, family 2	SYNONYMOUS_CODING
9329	CakSNP9329	*Kabuli*	Ca_Kabuli_Chr05	46130289	[A/C]	Ca_04062	--	SYNONYMOUS_CODING
9330	CakSNP9330	*Kabuli*	Ca_Kabuli_Chr05	46155262	[C/T]		--	INTERGENIC
9331	CakSNP9331	*Kabuli*	Ca_Kabuli_Chr05	46176366	[T/C]	Ca_04069	No apical meristem (NAM) protein	NON_SYNONYMOUS_CODING

S.N.	SNP IDs	Chickpea cultivars	Chromosomes	Physical positions (bp)	SNPs	Gene accession IDs	Putative functions	Sequence components of genome
9332	CakSNP9332	*Kabuli*	Ca_Kabuli_Chr05	46176443	[G/A]	Ca_04069	No apical meristem (NAM) protein	NON_SYNONYMOUS_CODING
9333	CakSNP9333	*Kabuli*	Ca_Kabuli_Chr05	46214229	[G/T]		--	INTERGENIC
9334	CakSNP9334	*Kabuli*	Ca_Kabuli_Chr05	46214233	[C/T]		--	INTERGENIC
9335	CakSNP9335	*Kabuli*	Ca_Kabuli_Chr05	46230010	[G/C]	Ca_04075	Ion channel regulatory protein, UNC-93	NON_SYNONYMOUS_CODING
9336	CakSNP9336	*Kabuli*	Ca_Kabuli_Chr05	46230011	[G/A]	Ca_04075	Ion channel regulatory protein, UNC-93	SYNONYMOUS_CODING
9337	CakSNP9337	*Kabuli*	Ca_Kabuli_Chr05	46230109	[T/G]	Ca_04075	Ion channel regulatory protein, UNC-93	SYNONYMOUS_CODING
9338	CakSNP9338	*Kabuli*	Ca_Kabuli_Chr05	46246419	[G/C]		--	INTERGENIC
9339	CakSNP9339	*Kabuli*	Ca_Kabuli_Chr05	46252617	[C/T]		--	INTERGENIC
9340	CakSNP9340	*Kabuli*	Ca_Kabuli_Chr05	46252643	[T/A]		--	INTERGENIC
9341	CakSNP9341	*Kabuli*	Ca_Kabuli_Chr05	46252605	[C/A]		--	INTERGENIC

S.N.	SNP IDs	Chickpea cultivars	Chromosomes	Physical positions (bp)	SNPs	Gene accession IDs	Putative functions	Sequence components of genome
9342	CakSNP9342	*Kabuli*	Ca_Kabuli_Chr05	46260822	[C/T]	Ca_04079	Mitochondrial inner membrane translocase complex, subunit Tim17/23	SYNONYMOUS_CODING
9343	CakSNP9343	*Kabuli*	Ca_Kabuli_Chr05	46294000	[G/A]		--	INTERGENIC
9344	CakSNP9344	*Kabuli*	Ca_Kabuli_Chr05	46300789	[G/A]	Ca_04083	--	SYNONYMOUS_CODING
9345	CakSNP9345	*Kabuli*	Ca_Kabuli_Chr05	46301639	[A/G]	Ca_04083	--	SYNONYMOUS_CODING
9346	CakSNP9346	*Kabuli*	Ca_Kabuli_Chr05	46321992	[C/T]	Ca_04085	Cytochrome P450	NON_SYNONYMOUS_CODING
9347	CakSNP9347	*Kabuli*	Ca_Kabuli_Chr05	46328589	[C/A]	Ca_04087	C2 calcium-dependent membrane targeting	NON_SYNONYMOUS_CODING
9348	CakSNP9348	*Kabuli*	Ca_Kabuli_Chr05	46328627	[A/G]	Ca_04087	C2 calcium-dependent membrane targeting	NON_SYNONYMOUS_CODING
9349	CakSNP9349	*Kabuli*	Ca_Kabuli_Chr05	46328895	[T/C]	Ca_04087	C2 calcium-dependent membrane targeting	NON_SYNONYMOUS_CODING
9350	CakSNP9350	*Kabuli*	Ca_Kabuli_Chr05	46328902	[C/T]	Ca_04087	C2 calcium-dependent membrane targeting	SYNONYMOUS_CODING
9351	CakSNP9351	*Kabuli*	Ca_Kabuli_Chr05	46344109	[G/T]	Ca_04090	Alcohol dehydrogenase superfamily, zinc-containing	INTRON

S.N.	SNP IDs	Chickpea cultivars	Chromosomes	Physical positions (bp)	SNPs	Gene accession IDs	Putative functions	Sequence components of genome
9352	CakSNP9352	*Kabuli*	Ca_Kabuli_Chr05	46344121	[G/A]	Ca_04090	Alcohol dehydrogenase superfamily, zinc-containing	INTRON
9353	CakSNP9353	*Kabuli*	Ca_Kabuli_Chr05	46356764	[G/A]	Ca_04091	Zinc finger, RING-type	INTRON
9354	CakSNP9354	*Kabuli*	Ca_Kabuli_Chr05	46366598	[G/T]		--	INTERGENIC
9355	CakSNP9355	*Kabuli*	Ca_Kabuli_Chr05	46368606	[G/T]		--	INTERGENIC
9356	CakSNP9356	*Kabuli*	Ca_Kabuli_Chr05	46376496	[G/T]	Ca_04094	Protein kinase, catalytic domain	NON_SYNONYMOUS_CODING
9357	CakSNP9357	*Kabuli*	Ca_Kabuli_Chr05	46376532	[A/C]	Ca_04094	Protein kinase, catalytic domain	NON_SYNONYMOUS_CODING
9358	CakSNP9358	*Kabuli*	Ca_Kabuli_Chr05	46403922	[G/T]	Ca_04097	Protein of unknown function DUF3133	SYNONYMOUS_CODING
9359	CakSNP9359	*Kabuli*	Ca_Kabuli_Chr05	46422788	[C/G]		--	INTERGENIC
9360	CakSNP9360	*Kabuli*	Ca_Kabuli_Chr05	46422869	[A/T]		--	INTERGENIC
9361	CakSNP9361	*Kabuli*	Ca_Kabuli_Chr05	46445611	[A/T]	Ca_04103	tRNA-binding domain	INTRON

S.N.	SNP IDs	Chickpea cultivars	Chromosomes	Physical positions (bp)	SNPs	Gene accession IDs	Putative functions	Sequence components of genome
9362	CakSNP9362	*Kabuli*	Ca_Kabuli_Chr05	46445651	[G/T]	Ca_04103	tRNA-binding domain	INTRON
9363	CakSNP9363	*Kabuli*	Ca_Kabuli_Chr05	46491761	[C/G]		--	INTERGENIC
9364	CakSNP9364	*Kabuli*	Ca_Kabuli_Chr05	46526963	[A/C]	Ca_04110	Protein of unknown function DUF2044, membrane	SYNONYMOUS_CODING
9365	CakSNP9365	*Kabuli*	Ca_Kabuli_Chr05	46621351	[G/A]	Ca_04121	Domain of unknown function DUF250	INTRON
9366	CakSNP9366	*Kabuli*	Ca_Kabuli_Chr05	46621455	[C/T]	Ca_04121	Domain of unknown function DUF250	INTRON
9367	CakSNP9367	*Kabuli*	Ca_Kabuli_Chr05	46668462	[T/C]	Ca_04125	Plant peroxidase	INTRON
9368	CakSNP9368	*Kabuli*	Ca_Kabuli_Chr05	46792131	[C/T]	Ca_04139	ABC transporter-like	INTRON
9369	CakSNP9369	*Kabuli*	Ca_Kabuli_Chr05	46945916	[G/A]	Ca_04149	RNA recognition motif domain	SYNONYMOUS_CODING
9370	CakSNP9370	*Kabuli*	Ca_Kabuli_Chr05	46956474	[T/C]	Ca_04151	DNA/RNA-binding protein Alba-like	INTRON
9371	CakSNP9371	*Kabuli*	Ca_Kabuli_Chr05	47061531	[C/T]		--	INTERGENIC

S.N.	SNP IDs	Chickpea cultivars	Chromosomes	Physical positions (bp)	SNPs	Gene accession IDs	Putative functions	Sequence components of genome
9372	CakSNP9372	*Kabuli*	Ca_Kabuli_Chr05	47073011	[A/T]	Ca_04164	Photosystem I PsaN, reaction centre subunit N	NON_SYNONYMOUS_CODING
9373	CakSNP9373	*Kabuli*	Ca_Kabuli_Chr05	47073016	[G/C]	Ca_04164	Photosystem I PsaN, reaction centre subunit N	SYNONYMOUS_CODING
9374	CakSNP9374	*Kabuli*	Ca_Kabuli_Chr05	47091499	[C/T]		--	INTERGENIC
9375	CakSNP9375	*Kabuli*	Ca_Kabuli_Chr05	47151660	[C/G]	Ca_04172	WD40 repeat	SYNONYMOUS_CODING
9376	CakSNP9376	*Kabuli*	Ca_Kabuli_Chr05	47151840	[T/G]	Ca_04172	WD40 repeat	SYNONYMOUS_CODING
9377	CakSNP9377	*Kabuli*	Ca_Kabuli_Chr05	47165299	[A/C]	Ca_04173	Ribosomal protein S2	SYNONYMOUS_CODING
9378	CakSNP9378	*Kabuli*	Ca_Kabuli_Chr05	47256002	[G/T]	Ca_04182	GDP-fucose protein O-fucosyltransferase	INTRON
9379	CakSNP9379	*Kabuli*	Ca_Kabuli_Chr05	47256038	[A/T]	Ca_04182	GDP-fucose protein O-fucosyltransferase	INTRON
9380	CakSNP9380	*Kabuli*	Ca_Kabuli_Chr05	47289004	[A/C]	Ca_04185	Ribosomal protein S2	NON_SYNONYMOUS_CODING
9381	CakSNP9381	*Kabuli*	Ca_Kabuli_Chr05	47298345	[A/G]	Ca_04186	Phosphatidylinositol 3-/4-kinase, catalytic	INTRON

S.N.	SNP IDs	Chickpea cultivars	Chromosomes	Physical positions (bp)	SNPs	Gene accession IDs	Putative functions	Sequence components of genome
9382	CakSNP9382	*Kabuli*	Ca_Kabuli_Chr05	47329979	[T/G]	Ca_04190	Glycosyl transferase, family 48	NON_SYNONYMOUS_CODING
9383	CakSNP9383	*Kabuli*	Ca_Kabuli_Chr05	47362666	[T/G]		--	INTERGENIC
9384	CakSNP9384	*Kabuli*	Ca_Kabuli_Chr05	47362814	[C/T]		--	INTERGENIC
9385	CakSNP9385	*Kabuli*	Ca_Kabuli_Chr05	47375789	[C/T]		--	INTERGENIC
9386	CakSNP9386	*Kabuli*	Ca_Kabuli_Chr05	47422536	[G/A]	Ca_04203	Dehydrogenase, E1 component	SYNONYMOUS_CODING
9387	CakSNP9387	*Kabuli*	Ca_Kabuli_Chr05	47518533	[G/A]	Ca_04211	Zinc finger, RING-type	NON_SYNONYMOUS_CODING
9388	CakSNP9388	*Kabuli*	Ca_Kabuli_Chr05	47539345	[T/C]	Ca_04213	Protein kinase, catalytic domain	NON_SYNONYMOUS_CODING
9389	CakSNP9389	*Kabuli*	Ca_Kabuli_Chr05	47539505	[G/T]	Ca_04213	Protein kinase, catalytic domain	NON_SYNONYMOUS_CODING
9390	CakSNP9390	*Kabuli*	Ca_Kabuli_Chr05	47570939	[T/C]	Ca_04215	DNA topoisomerase, type IA	INTRON
9391	CakSNP9391	*Kabuli*	Ca_Kabuli_Chr05	47591327	[G/T]	Ca_04217	Prenylcysteine lyase	SYNONYMOUS_CODING

S.N.	SNP IDs	Chickpea cultivars	Chromosomes	Physical positions (bp)	SNPs	Gene accession IDs	Putative functions	Sequence components of genome
9392	CakSNP9392	*Kabuli*	Ca_Kabuli_Chr05	47594471	[T/C]	Ca_04218	Snf7	INTRON
9393	CakSNP9393	*Kabuli*	Ca_Kabuli_Chr05	47641224	[A/C]	Ca_04224	Heat shock protein DnaJ, N-terminal	INTRON
9394	CakSNP9394	*Kabuli*	Ca_Kabuli_Chr05	47641472	[T/C]	Ca_04224	Heat shock protein DnaJ, N-terminal	SYNONYMOUS_CODING
9395	CakSNP9395	*Kabuli*	Ca_Kabuli_Chr05	47641628	[T/C]	Ca_04224	Heat shock protein DnaJ, N-terminal	SYNONYMOUS_CODING
9396	CakSNP9396	*Kabuli*	Ca_Kabuli_Chr05	47641567	[A/G]	Ca_04224	Heat shock protein DnaJ, N-terminal	NON_SYNONYMOUS_CODING
9397	CakSNP9397	*Kabuli*	Ca_Kabuli_Chr05	47643992	[G/T]	Ca_04224	Heat shock protein DnaJ, N-terminal	INTRON
9398	CakSNP9398	*Kabuli*	Ca_Kabuli_Chr05	47669124	[A/G]	Ca_04227	Amino acid transporter, transmembrane	SYNONYMOUS_CODING
9399	CakSNP9399	*Kabuli*	Ca_Kabuli_Chr05	47670649	[G/A]	Ca_04227	Amino acid transporter, transmembrane	NON_SYNONYMOUS_CODING
9400	CakSNP9400	*Kabuli*	Ca_Kabuli_Chr05	47706780	[G/A]	Ca_04231	D-galactoside/L-rhamnose binding SUEL lectin domain	INTRON
9401	CakSNP9401	*Kabuli*	Ca_Kabuli_Chr05	47718830	[A/G]	Ca_04232	Glycoside hydrolase, family 35	INTRON

S.N.	SNP IDs	Chickpea cultivars	Chromosomes	Physical positions (bp)	SNPs	Gene accession IDs	Putative functions	Sequence components of genome
9402	CakSNP9402	*Kabuli*	Ca_Kabuli_Chr05	47718839	[A/G]	Ca_04232	Glycoside hydrolase, family 35	INTRON
9403	CakSNP9403	*Kabuli*	Ca_Kabuli_Chr05	47733645	[A/G]	Ca_04233	No apical meristem (NAM) protein	NON_SYNONYMOUS_CODING
9404	CakSNP9404	*Kabuli*	Ca_Kabuli_Chr05	47759169	[T/G]	Ca_04237	Protein kinase, catalytic domain	INTRON
9405	CakSNP9405	*Kabuli*	Ca_Kabuli_Chr05	47759745	[G/A]	Ca_04237	Protein kinase, catalytic domain	INTRON
9406	CakSNP9406	*Kabuli*	Ca_Kabuli_Chr05	47761348	[A/G]	Ca_04237	Protein kinase, catalytic domain	INTRON
9407	CakSNP9407	*Kabuli*	Ca_Kabuli_Chr05	47761322	[C/A]	Ca_04237	Protein kinase, catalytic domain	INTRON
9408	CakSNP9408	*Kabuli*	Ca_Kabuli_Chr05	47770909	[T/G]	Ca_04238	Amidase	INTRON
9409	CakSNP9409	*Kabuli*	Ca_Kabuli_Chr05	47774516	[A/G]	Ca_04239	Protein of unknown function DUF869, plant	NON_SYNONYMOUS_CODING
9410	CakSNP9410	*Kabuli*	Ca_Kabuli_Chr05	47774559	[C/T]	Ca_04239	Protein of unknown function DUF869, plant	NON_SYNONYMOUS_CODING
9411	CakSNP9411	*Kabuli*	Ca_Kabuli_Chr05	47774718	[C/G]	Ca_04239	Protein of unknown function DUF869, plant	NON_SYNONYMOUS_CODING

S.N.	SNP IDs	Chickpea cultivars	Chromosomes	Physical positions (bp)	SNPs	Gene accession IDs	Putative functions	Sequence components of genome
9412	CakSNP9412	*Kabuli*	Ca_Kabuli_Chr05	47775175	[C/T]	Ca_04239	Protein of unknown function DUF869, plant	SYNONYMOUS_CODING
9413	CakSNP9413	*Kabuli*	Ca_Kabuli_Chr05	47782605	[G/A]	Ca_04240	Phox/Bem1p	INTRON
9414	CakSNP9414	*Kabuli*	Ca_Kabuli_Chr05	47782606	[C/T]	Ca_04240	Phox/Bem1p	INTRON
9415	CakSNP9415	*Kabuli*	Ca_Kabuli_Chr05	47782697	[T/C]	Ca_04240	Phox/Bem1p	SYNONYMOUS_CODING
9416	CakSNP9416	*Kabuli*	Ca_Kabuli_Chr05	47782843	[T/C]	Ca_04240	Phox/Bem1p	INTRON
9417	CakSNP9417	*Kabuli*	Ca_Kabuli_Chr05	47803636	[C/A]		--	INTERGENIC
9418	CakSNP9418	*Kabuli*	Ca_Kabuli_Chr05	47812041	[C/T]		--	INTERGENIC
9419	CakSNP9419	*Kabuli*	Ca_Kabuli_Chr05	47813712	[T/C]	Ca_04243	Regulator of chromosome condensation, RCC1	NON_SYNONYMOUS_CODING
9420	CakSNP9420	*Kabuli*	Ca_Kabuli_Chr05	47813767	[A/G]	Ca_04243	Regulator of chromosome condensation, RCC1	INTRON
9421	CakSNP9421	*Kabuli*	Ca_Kabuli_Chr05	47829261	[T/C]		--	INTERGENIC

S.N.	SNP IDs	Chickpea cultivars	Chromosomes	Physical positions (bp)	SNPs	Gene accession IDs	Putative functions	Sequence components of genome
9422	CakSNP9422	*Kabuli*	Ca_Kabuli_Chr05	47829331	[T/A]		--	INTERGENIC
9423	CakSNP9423	*Kabuli*	Ca_Kabuli_Chr05	47871094	[C/T]		--	INTERGENIC
9424	CakSNP9424	*Kabuli*	Ca_Kabuli_Chr05	47871137	[G/A]		--	INTERGENIC
9425	CakSNP9425	*Kabuli*	Ca_Kabuli_Chr05	47871248	[G/T]		--	INTERGENIC
9426	CakSNP9426	*Kabuli*	Ca_Kabuli_Chr05	47871234	[A/G]		--	INTERGENIC
9427	CakSNP9427	*Kabuli*	Ca_Kabuli_Chr05	47871194	[T/C]		--	INTERGENIC
9428	CakSNP9428	*Kabuli*	Ca_Kabuli_Chr05	47878997	[C/T]	Ca_04250	Protein of unknown function DUF668	INTRON
9429	CakSNP9429	*Kabuli*	Ca_Kabuli_Chr05	47901017	[G/T]	Ca_04251	Translation elongation factor EFG/EF2, C-terminal	INTRON
9430	CakSNP9430	*Kabuli*	Ca_Kabuli_Chr05	47931566	[T/C]	Ca_04255	Ferrochelatase	SYNONYMOUS_CODING
9431	CakSNP9431	*Kabuli*	Ca_Kabuli_Chr05	47941783	[C/T]	Ca_04256	Protein kinase, catalytic domain	SYNONYMOUS_CODING

S.N.	SNP IDs	Chickpea cultivars	Chromosomes	Physical positions (bp)	SNPs	Gene accession IDs	Putative functions	Sequence components of genome
9432	CakSNP9432	*Kabuli*	Ca_Kabuli_Chr05	47943743	[G/A]	Ca_04256	Protein kinase, catalytic domain	INTRON
9433	CakSNP9433	*Kabuli*	Ca_Kabuli_Chr05	47965279	[G/A]	Ca_04258	--	NON_SYNONYMOUS_CODING
9434	CakSNP9434	*Kabuli*	Ca_Kabuli_Chr05	47994780	[A/C]	Ca_04259	Cof protein	INTRON
9435	CakSNP9435	*Kabuli*	Ca_Kabuli_Chr05	47997751	[A/T]	Ca_04259	Cof protein	INTRON
9436	CakSNP9436	*Kabuli*	Ca_Kabuli_Chr05	48005123	[A/C]		--	INTERGENIC
9437	CakSNP9437	*Kabuli*	Ca_Kabuli_Chr05	48005073	[G/A]		--	INTERGENIC
9438	CakSNP9438	*Kabuli*	Ca_Kabuli_Chr05	48042132	[A/C]	Ca_04264	Thioesterase superfamily	SYNONYMOUS_CODING
9439	CakSNP9439	*Kabuli*	Ca_Kabuli_Chr05	48063028	[A/T]	Ca_04267	XS domain	SYNONYMOUS_CODING
9440	CakSNP9440	*Kabuli*	Ca_Kabuli_Chr05	48069744	[G/A]		--	INTERGENIC
9441	CakSNP9441	*Kabuli*	Ca_Kabuli_Chr05	48069811	[G/C]		--	INTERGENIC

S.N.	SNP IDs	Chickpea cultivars	Chromosomes	Physical positions (bp)	SNPs	Gene accession IDs	Putative functions	Sequence components of genome
9442	CakSNP9442	*Kabuli*	Ca_Kabuli_Chr05	48069859	[A/T]		--	INTERGENIC
9443	CakSNP9443	*Kabuli*	Ca_Kabuli_Chr05	48069844	[T/C]		--	INTERGENIC
9444	CakSNP9444	*Kabuli*	Ca_Kabuli_Chr05	48069833	[G/A]		--	INTERGENIC
9445	CakSNP9445	*Kabuli*	Ca_Kabuli_Chr06	1916	[T/C]		--	INTERGENIC
9446	CakSNP9446	*Kabuli*	Ca_Kabuli_Chr06	1847	[G/T]		--	INTERGENIC
9447	CakSNP9447	*Kabuli*	Ca_Kabuli_Chr06	140189	[A/G]		--	INTERGENIC
9448	CakSNP9448	*Kabuli*	Ca_Kabuli_Chr06	140279	[T/C]		--	INTERGENIC
9449	CakSNP9449	*Kabuli*	Ca_Kabuli_Chr06	140320	[G/A]		--	INTERGENIC
9450	CakSNP9450	*Kabuli*	Ca_Kabuli_Chr06	152513	[C/T]		--	INTERGENIC
9451	CakSNP9451	*Kabuli*	Ca_Kabuli_Chr06	152556	[T/C]		--	INTERGENIC

S.N.	SNP IDs	Chickpea cultivars	Chromosomes	Physical positions (bp)	SNPs	Gene accession IDs	Putative functions	Sequence components of genome
9452	CakSNP9452	*Kabuli*	Ca_Kabuli_Chr06	166823	[G/A]	Ca_13247	Peptidase C48, SUMO/Sentrin/Ubl1	NON_SYNONYMOUS_CODING
9453	CakSNP9453	*Kabuli*	Ca_Kabuli_Chr06	166806	[C/T]	Ca_13247	Peptidase C48, SUMO/Sentrin/Ubl1	NON_SYNONYMOUS_CODING
9454	CakSNP9454	*Kabuli*	Ca_Kabuli_Chr06	166959	[G/A]	Ca_13247	Peptidase C48, SUMO/Sentrin/Ubl1	NON_SYNONYMOUS_CODING
9455	CakSNP9455	*Kabuli*	Ca_Kabuli_Chr06	166943	[G/A]	Ca_13247	Peptidase C48, SUMO/Sentrin/Ubl1	NON_SYNONYMOUS_CODING
9456	CakSNP9456	*Kabuli*	Ca_Kabuli_Chr06	166938	[G/T]	Ca_13247	Peptidase C48, SUMO/Sentrin/Ubl1	NON_SYNONYMOUS_CODING
9457	CakSNP9457	*Kabuli*	Ca_Kabuli_Chr06	166934	[G/A]	Ca_13247	Peptidase C48, SUMO/Sentrin/Ubl1	NON_SYNONYMOUS_CODING
9458	CakSNP9458	*Kabuli*	Ca_Kabuli_Chr06	166932	[G/A]	Ca_13247	Peptidase C48, SUMO/Sentrin/Ubl1	NON_SYNONYMOUS_CODING
9459	CakSNP9459	*Kabuli*	Ca_Kabuli_Chr06	166911	[A/G]	Ca_13247	Peptidase C48, SUMO/Sentrin/Ubl1	NON_SYNONYMOUS_CODING
9460	CakSNP9460	*Kabuli*	Ca_Kabuli_Chr06	204368	[G/A]	Ca_13250	Flavin-containing amine oxidase	INTRON
9461	CakSNP9461	*Kabuli*	Ca_Kabuli_Chr06	204412	[G/C]	Ca_13250	Flavin-containing amine oxidase	INTRON

S.N.	SNP IDs	Chickpea cultivars	Chromosomes	Physical positions (bp)	SNPs	Gene accession IDs	Putative functions	Sequence components of genome
9462	CakSNP9462	*Kabuli*	Ca_Kabuli_Chr06	204438	[T/C]	Ca_13250	Flavin-containing amine oxidase	INTRON
9463	CakSNP9463	*Kabuli*	Ca_Kabuli_Chr06	267151	[A/G]	Ca_13253	Peptidase M24, structural domain	SYNONYMOUS_CODING
9464	CakSNP9464	*Kabuli*	Ca_Kabuli_Chr06	269269	[T/G]		--	INTERGENIC
9465	CakSNP9465	*Kabuli*	Ca_Kabuli_Chr06	324301	[C/T]	Ca_13257	Zinc finger, FYVE-type	NON_SYNONYMOUS_CODING
9466	CakSNP9466	*Kabuli*	Ca_Kabuli_Chr06	348283	[C/T]		--	INTERGENIC
9467	CakSNP9467	*Kabuli*	Ca_Kabuli_Chr06	348295	[G/C]		--	INTERGENIC
9468	CakSNP9468	*Kabuli*	Ca_Kabuli_Chr06	375684	[T/G]	Ca_13262	Pyridoxal phosphate-dependent decarboxylase	SYNONYMOUS_CODING
9469	CakSNP9469	*Kabuli*	Ca_Kabuli_Chr06	386250	[A/G]		--	INTERGENIC
9470	CakSNP9470	*Kabuli*	Ca_Kabuli_Chr06	474489	[C/A]	Ca_13268	NMD3	NON_SYNONYMOUS_CODING
9471	CakSNP9471	*Kabuli*	Ca_Kabuli_Chr06	505875	[T/C]	Ca_13271	--	NON_SYNONYMOUS_CODING

S.N.	SNP IDs	Chickpea cultivars	Chromosomes	Physical positions (bp)	SNPs	Gene accession IDs	Putative functions	Sequence components of genome
9472	CakSNP9472	*Kabuli*	Ca_Kabuli_Chr06	515221	[T/G]	Ca_13272	--	INTRON
9473	CakSNP9473	*Kabuli*	Ca_Kabuli_Chr06	548416	[A/T]	Ca_13276	Nicastrin	INTRON
9474	CakSNP9474	*Kabuli*	Ca_Kabuli_Chr06	551130	[T/G]	Ca_13276	Nicastrin	NON_SYNONYMOUS_CODING
9475	CakSNP9475	*Kabuli*	Ca_Kabuli_Chr06	561562	[A/G]	Ca_13277	NADH:ubiquinone oxidoreductase, 75kDa subunit, conserved site	NON_SYNONYMOUS_CODING
9476	CakSNP9476	*Kabuli*	Ca_Kabuli_Chr06	580616	[G/C]		--	INTERGENIC
9477	CakSNP9477	*Kabuli*	Ca_Kabuli_Chr06	588592	[C/A]	Ca_13280	RNA recognition motif domain	INTRON
9478	CakSNP9478	*Kabuli*	Ca_Kabuli_Chr06	638983	[T/A]	Ca_13283	--	INTRON
9479	CakSNP9479	*Kabuli*	Ca_Kabuli_Chr06	645755	[G/T]	Ca_13283	--	INTRON
9480	CakSNP9480	*Kabuli*	Ca_Kabuli_Chr06	645746	[G/T]	Ca_13283	--	INTRON
9481	CakSNP9481	*Kabuli*	Ca_Kabuli_Chr06	647322	[A/C]	Ca_13283	--	SYNONYMOUS_CODING

S.N.	SNP IDs	Chickpea cultivars	Chromosomes	Physical positions (bp)	SNPs	Gene accession IDs	Putative functions	Sequence components of genome
9482	CakSNP9482	*Kabuli*	Ca_Kabuli_Chr06	697187	[A/G]		--	INTERGENIC
9483	CakSNP9483	*Kabuli*	Ca_Kabuli_Chr06	697169	[A/G]		--	INTERGENIC
9484	CakSNP9484	*Kabuli*	Ca_Kabuli_Chr06	697168	[C/T]		--	INTERGENIC
9485	CakSNP9485	*Kabuli*	Ca_Kabuli_Chr06	697162	[A/G]		--	INTERGENIC
9486	CakSNP9486	*Kabuli*	Ca_Kabuli_Chr06	697158	[G/A]		--	INTERGENIC
9487	CakSNP9487	*Kabuli*	Ca_Kabuli_Chr06	697148	[C/T]		--	INTERGENIC
9488	CakSNP9488	*Kabuli*	Ca_Kabuli_Chr06	697147	[A/G]		--	INTERGENIC
9489	CakSNP9489	*Kabuli*	Ca_Kabuli_Chr06	697142	[G/C]		--	INTERGENIC
9490	CakSNP9490	*Kabuli*	Ca_Kabuli_Chr06	894921	[A/G]	Ca_13301	PDZ/DHR/GLGF	INTRON
9491	CakSNP9491	*Kabuli*	Ca_Kabuli_Chr06	902403	[G/A]		--	INTERGENIC

S.N.	SNP IDs	Chickpea cultivars	Chromosomes	Physical positions (bp)	SNPs	Gene accession IDs	Putative functions	Sequence components of genome
9492	CakSNP9492	*Kabuli*	Ca_Kabuli_Chr06	902402	[T/C]		--	INTERGENIC
9493	CakSNP9493	*Kabuli*	Ca_Kabuli_Chr06	949263	[C/A]	Ca_13307	Protein kinase, catalytic domain	INTRON
9494	CakSNP9494	*Kabuli*	Ca_Kabuli_Chr06	961652	[C/T]		--	INTERGENIC
9495	CakSNP9495	*Kabuli*	Ca_Kabuli_Chr06	961805	[C/T]		--	INTERGENIC
9496	CakSNP9496	*Kabuli*	Ca_Kabuli_Chr06	961733	[T/A]		--	INTERGENIC
9497	CakSNP9497	*Kabuli*	Ca_Kabuli_Chr06	997997	[T/A]	Ca_13310	WD40 repeat	INTRON
9498	CakSNP9498	*Kabuli*	Ca_Kabuli_Chr06	1130293	[C/T]	Ca_13320	Domain of unknown function DUF296	SYNONYMOUS_CODING
9499	CakSNP9499	*Kabuli*	Ca_Kabuli_Chr06	1212889	[G/A]	Ca_13328	Short-chain dehydrogenase/reductase SDR	INTRON
9500	CakSNP9500	*Kabuli*	Ca_Kabuli_Chr06	1220425	[C/T]		--	INTERGENIC
9501	CakSNP9501	*Kabuli*	Ca_Kabuli_Chr06	1220460	[G/C]		--	INTERGENIC

S.N.	SNP IDs	Chickpea cultivars	Chromosomes	Physical positions (bp)	SNPs	Gene accession IDs	Putative functions	Sequence components of genome
9502	CakSNP9502	*Kabuli*	Ca_Kabuli_Chr06	1220465	[G/A]		--	INTERGENIC
9503	CakSNP9503	*Kabuli*	Ca_Kabuli_Chr06	1220510	[A/T]		--	INTERGENIC
9504	CakSNP9504	*Kabuli*	Ca_Kabuli_Chr06	1220499	[C/G]		--	INTERGENIC
9505	CakSNP9505	*Kabuli*	Ca_Kabuli_Chr06	1287168	[C/T]	Ca_13334	Regulator of chromosome condensation, RCC1	INTRON
9506	CakSNP9506	*Kabuli*	Ca_Kabuli_Chr06	1290413	[C/G]	Ca_13334	Regulator of chromosome condensation, RCC1	INTRON
9507	CakSNP9507	*Kabuli*	Ca_Kabuli_Chr06	1290538	[T/G]	Ca_13334	Regulator of chromosome condensation, RCC1	INTRON
9508	CakSNP9508	*Kabuli*	Ca_Kabuli_Chr06	1295748	[T/C]	Ca_13335	Protein kinase, catalytic domain	SYNONYMOUS_CODING
9509	CakSNP9509	*Kabuli*	Ca_Kabuli_Chr06	1312881	[T/C]	Ca_13338	Aspartate/glutamate/uridylate kinase	INTRON
9510	CakSNP9510	*Kabuli*	Ca_Kabuli_Chr06	1312932	[G/A]	Ca_13338	Aspartate/glutamate/uridylate kinase	INTRON
9511	CakSNP9511	*Kabuli*	Ca_Kabuli_Chr06	1312982	[C/A]	Ca_13338	Aspartate/glutamate/uridylate kinase	INTRON

S.N.	SNP IDs	Chickpea cultivars	Chromosomes	Physical positions (bp)	SNPs	Gene accession IDs	Putative functions	Sequence components of genome
9512	CakSNP9512	*Kabuli*	Ca_Kabuli_Chr06	1315583	[G/C]	Ca_13338	Aspartate/glutamate/uridylate kinase	INTRON
9513	CakSNP9513	*Kabuli*	Ca_Kabuli_Chr06	1626723	[T/C]		--	INTERGENIC
9514	CakSNP9514	*Kabuli*	Ca_Kabuli_Chr06	1626712	[C/T]		--	INTERGENIC
9515	CakSNP9515	*Kabuli*	Ca_Kabuli_Chr06	1817599	[C/T]	Ca_10308	Zinc finger, LIM-type	INTRON
9516	CakSNP9516	*Kabuli*	Ca_Kabuli_Chr06	1817602	[A/T]	Ca_10308	Zinc finger, LIM-type	INTRON
9517	CakSNP9517	*Kabuli*	Ca_Kabuli_Chr06	1817607	[T/C]	Ca_10308	Zinc finger, LIM-type	INTRON
9518	CakSNP9518	*Kabuli*	Ca_Kabuli_Chr06	1817628	[A/T]	Ca_10308	Zinc finger, LIM-type	INTRON
9519	CakSNP9519	*Kabuli*	Ca_Kabuli_Chr06	1932114	[C/G]	Ca_10319	Major intrinsic protein	SYNONYMOUS_CODING
9520	CakSNP9520	*Kabuli*	Ca_Kabuli_Chr06	1932175	[G/A]	Ca_10319	Major intrinsic protein	INTRON
9521	CakSNP9521	*Kabuli*	Ca_Kabuli_Chr06	1966137	[T/C]		--	INTERGENIC

S.N.	SNP IDs	Chickpea cultivars	Chromosomes	Physical positions (bp)	SNPs	Gene accession IDs	Putative functions	Sequence components of genome
9522	CakSNP9522	*Kabuli*	Ca_Kabuli_Chr06	1978781	[C/A]	Ca_10322	--	INTRON
9523	CakSNP9523	*Kabuli*	Ca_Kabuli_Chr06	1978864	[A/C]	Ca_10322	--	INTRON
9524	CakSNP9524	*Kabuli*	Ca_Kabuli_Chr06	1978867	[T/A]	Ca_10322	--	INTRON
9525	CakSNP9525	*Kabuli*	Ca_Kabuli_Chr06	2037575	[G/T]	Ca_10324	--	SYNONYMOUS_CODING
9526	CakSNP9526	*Kabuli*	Ca_Kabuli_Chr06	2037599	[A/G]	Ca_10324	--	SYNONYMOUS_CODING
9527	CakSNP9527	*Kabuli*	Ca_Kabuli_Chr06	2037620	[T/G]	Ca_10324	--	NON_SYNONYMOUS_CODING
9528	CakSNP9528	*Kabuli*	Ca_Kabuli_Chr06	2037623	[T/G]	Ca_10324	--	NON_SYNONYMOUS_CODING
9529	CakSNP9529	*Kabuli*	Ca_Kabuli_Chr06	2037626	[C/T]	Ca_10324	--	SYNONYMOUS_CODING
9530	CakSNP9530	*Kabuli*	Ca_Kabuli_Chr06	2050907	[A/G]		--	INTERGENIC
9531	CakSNP9531	*Kabuli*	Ca_Kabuli_Chr06	2061459	[A/G]	Ca_10326	Poly(A) polymerase, RNA-binding domain	SYNONYMOUS_CODING

S.N.	SNP IDs	Chickpea cultivars	Chromosomes	Physical positions (bp)	SNPs	Gene accession IDs	Putative functions	Sequence components of genome
9532	CakSNP9532	*Kabuli*	Ca_Kabuli_Chr06	2078416	[T/A]	Ca_10329	Protein of unknown function DUF760	SYNONYMOUS_CODING
9533	CakSNP9533	*Kabuli*	Ca_Kabuli_Chr06	2079137	[T/G]	Ca_10329	Protein of unknown function DUF760	NON_SYNONYMOUS_CODING
9534	CakSNP9534	*Kabuli*	Ca_Kabuli_Chr06	2079186	[G/T]	Ca_10329	Protein of unknown function DUF760	NON_SYNONYMOUS_CODING
9535	CakSNP9535	*Kabuli*	Ca_Kabuli_Chr06	2083784	[A/T]	Ca_10330	K+ potassium transporter	INTRON
9536	CakSNP9536	*Kabuli*	Ca_Kabuli_Chr06	2083805	[G/A]	Ca_10330	K+ potassium transporter	SYNONYMOUS_CODING
9537	CakSNP9537	*Kabuli*	Ca_Kabuli_Chr06	2083813	[T/C]	Ca_10330	K+ potassium transporter	NON_SYNONYMOUS_CODING
9538	CakSNP9538	*Kabuli*	Ca_Kabuli_Chr06	2126667	[C/T]	Ca_10335	ABC transporter-like	INTRON
9539	CakSNP9539	*Kabuli*	Ca_Kabuli_Chr06	2126655	[G/A]	Ca_10335	ABC transporter-like	INTRON
9540	CakSNP9540	*Kabuli*	Ca_Kabuli_Chr06	2140189	[G/T]	Ca_10336	Leucine-rich repeat	NON_SYNONYMOUS_CODING
9541	CakSNP9541	*Kabuli*	Ca_Kabuli_Chr06	2140406	[G/A]	Ca_10336	Leucine-rich repeat	SYNONYMOUS_CODING

S.N.	SNP IDs	Chickpea cultivars	Chromosomes	Physical positions (bp)	SNPs	Gene accession IDs	Putative functions	Sequence components of genome
9542	CakSNP9542	*Kabuli*	Ca_Kabuli_Chr06	2193748	[T/A]	Ca_10344	Auxin efflux carrier	INTRON
9543	CakSNP9543	*Kabuli*	Ca_Kabuli_Chr06	2193763	[A/C]	Ca_10344	Auxin efflux carrier	INTRON
9544	CakSNP9544	*Kabuli*	Ca_Kabuli_Chr06	2193803	[G/A]	Ca_10344	Auxin efflux carrier	SYNONYMOUS_CODING
9545	CakSNP9545	*Kabuli*	Ca_Kabuli_Chr06	2214131	[T/A]	Ca_10348	Amidase	INTRON
9546	CakSNP9546	*Kabuli*	Ca_Kabuli_Chr06	2214178	[T/C]	Ca_10348	Amidase	INTRON
9547	CakSNP9547	*Kabuli*	Ca_Kabuli_Chr06	2214247	[A/G]	Ca_10348	Amidase	INTRON
9548	CakSNP9548	*Kabuli*	Ca_Kabuli_Chr06	2223544	[A/T]		--	INTERGENIC
9549	CakSNP9549	*Kabuli*	Ca_Kabuli_Chr06	2223578	[T/G]		--	INTERGENIC
9550	CakSNP9550	*Kabuli*	Ca_Kabuli_Chr06	2223664	[A/C]		--	INTERGENIC
9551	CakSNP9551	*Kabuli*	Ca_Kabuli_Chr06	2244901	[A/C]		--	INTERGENIC

S.N.	SNP IDs	Chickpea cultivars	Chromosomes	Physical positions (bp)	SNPs	Gene accession IDs	Putative functions	Sequence components of genome
9552	CakSNP9552	*Kabuli*	Ca_Kabuli_Chr06	2275505	[A/G]	Ca_10356	--	NON_SYNONYMOUS_CODING
9553	CakSNP9553	*Kabuli*	Ca_Kabuli_Chr06	2275524	[G/A]	Ca_10356	--	SYNONYMOUS_CODING
9554	CakSNP9554	*Kabuli*	Ca_Kabuli_Chr06	2275553	[A/G]	Ca_10356	--	NON_SYNONYMOUS_CODING
9555	CakSNP9555	*Kabuli*	Ca_Kabuli_Chr06	2280734	[T/A]	Ca_10356	--	INTRON
9556	CakSNP9556	*Kabuli*	Ca_Kabuli_Chr06	2280752	[A/G]	Ca_10356	--	INTRON
9557	CakSNP9557	*Kabuli*	Ca_Kabuli_Chr06	2286205	[A/G]	Ca_10357	Pentatricopeptide repeat	INTRON
9558	CakSNP9558	*Kabuli*	Ca_Kabuli_Chr06	2286237	[C/T]	Ca_10357	Pentatricopeptide repeat	INTRON
9559	CakSNP9559	*Kabuli*	Ca_Kabuli_Chr06	2294911	[T/G]	Ca_10359	Peptidyl-prolyl cis-trans isomerase, FKBP-type, domain	NON_SYNONYMOUS_CODING
9560	CakSNP9560	*Kabuli*	Ca_Kabuli_Chr06	2303123	[T/G]	Ca_10360	Protein kinase, catalytic domain	INTRON
9561	CakSNP9561	*Kabuli*	Ca_Kabuli_Chr06	2313683	[A/C]	Ca_10361	Histone H1/H5	NON_SYNONYMOUS_CODING

S.N.	SNP IDs	Chickpea cultivars	Chromosomes	Physical positions (bp)	SNPs	Gene accession IDs	Putative functions	Sequence components of genome
9562	CakSNP9562	*Kabuli*	Ca_Kabuli_Chr06	2313828	[G/A]	Ca_10361	Histone H1/H5	NON_SYNONYMOUS_CODING
9563	CakSNP9563	*Kabuli*	Ca_Kabuli_Chr06	2314029	[T/C]	Ca_10361	Histone H1/H5	NON_SYNONYMOUS_CODING
9564	CakSNP9564	*Kabuli*	Ca_Kabuli_Chr06	2327842	[A/G]	Ca_10363	Pentatricopeptide repeat	SYNONYMOUS_CODING
9565	CakSNP9565	*Kabuli*	Ca_Kabuli_Chr06	2462481	[C/T]	Ca_10377	Chaperonin TCP-1, conserved site	NON_SYNONYMOUS_CODING
9566	CakSNP9566	*Kabuli*	Ca_Kabuli_Chr06	2502485	[A/G]		--	INTERGENIC
9567	CakSNP9567	*Kabuli*	Ca_Kabuli_Chr06	2527768	[A/G]		--	INTERGENIC
9568	CakSNP9568	*Kabuli*	Ca_Kabuli_Chr06	2542942	[A/T]		--	INTERGENIC
9569	CakSNP9569	*Kabuli*	Ca_Kabuli_Chr06	2542981	[T/C]		--	INTERGENIC
9570	CakSNP9570	*Kabuli*	Ca_Kabuli_Chr06	2543096	[C/T]		--	INTERGENIC
9571	CakSNP9571	*Kabuli*	Ca_Kabuli_Chr06	2543062	[A/G]		--	INTERGENIC

S.N.	SNP IDs	Chickpea cultivars	Chromosomes	Physical positions (bp)	SNPs	Gene accession IDs	Putative functions	Sequence components of genome
9572	CakSNP9572	*Kabuli*	Ca_Kabuli_Chr06	2548412	[A/G]	Ca_10387	Zinc finger, RING-type	SYNONYMOUS_CODING
9573	CakSNP9573	*Kabuli*	Ca_Kabuli_Chr06	2549997	[C/T]	Ca_10387	Zinc finger, RING-type	SYNONYMOUS_CODING
9574	CakSNP9574	*Kabuli*	Ca_Kabuli_Chr06	2549991	[T/C]	Ca_10387	Zinc finger, RING-type	SYNONYMOUS_CODING
9575	CakSNP9575	*Kabuli*	Ca_Kabuli_Chr06	2550154	[A/C]	Ca_10387	Zinc finger, RING-type	SYNONYMOUS_CODING
9576	CakSNP9576	*Kabuli*	Ca_Kabuli_Chr06	2623370	[T/C]	Ca_10393	THO complex, subunitTHOC2, N-terminal	SYNONYMOUS_CODING
9577	CakSNP9577	*Kabuli*	Ca_Kabuli_Chr06	2625793	[C/G]	Ca_10393	THO complex, subunitTHOC2, N-terminal	INTRON
9578	CakSNP9578	*Kabuli*	Ca_Kabuli_Chr06	2633941	[A/T]	Ca_10394	THO complex, subunitTHOC2, C-terminal	INTRON
9579	CakSNP9579	*Kabuli*	Ca_Kabuli_Chr06	2639558	[T/A]	Ca_10395	ANTH	SYNONYMOUS_CODING
9580	CakSNP9580	*Kabuli*	Ca_Kabuli_Chr06	2639606	[A/G]	Ca_10395	ANTH	SYNONYMOUS_CODING
9581	CakSNP9581	*Kabuli*	Ca_Kabuli_Chr06	2639607	[T/C]	Ca_10395	ANTH	NON_SYNONYMOUS_CODING

S.N.	SNP IDs	Chickpea cultivars	Chromosomes	Physical positions (bp)	SNPs	Gene accession IDs	Putative functions	Sequence components of genome
9582	CakSNP9582	*Kabuli*	Ca_Kabuli_Chr06	2676594	[C/T]		--	INTERGENIC
9583	CakSNP9583	*Kabuli*	Ca_Kabuli_Chr06	2714894	[T/C]	Ca_10405	Domain of unknown function DUF125, transmembrane	SYNONYMOUS_CODING
9584	CakSNP9584	*Kabuli*	Ca_Kabuli_Chr06	2714897	[C/A]	Ca_10405	Domain of unknown function DUF125, transmembrane	SYNONYMOUS_CODING
9585	CakSNP9585	*Kabuli*	Ca_Kabuli_Chr06	2714918	[T/G]	Ca_10405	Domain of unknown function DUF125, transmembrane	SYNONYMOUS_CODING
9586	CakSNP9586	*Kabuli*	Ca_Kabuli_Chr06	2717150	[G/A]		--	INTERGENIC
9587	CakSNP9587	*Kabuli*	Ca_Kabuli_Chr06	2740630	[A/G]		--	INTERGENIC
9588	CakSNP9588	*Kabuli*	Ca_Kabuli_Chr06	2740765	[A/G]		--	INTERGENIC
9589	CakSNP9589	*Kabuli*	Ca_Kabuli_Chr06	2740784	[T/C]		--	INTERGENIC
9590	CakSNP9590	*Kabuli*	Ca_Kabuli_Chr06	2741185	[G/A]		--	INTERGENIC
9591	CakSNP9591	*Kabuli*	Ca_Kabuli_Chr06	2777609	[A/G]	Ca_10411	Dynamin central domain	SYNONYMOUS_CODING

S.N.	SNP IDs	Chickpea cultivars	Chromosomes	Physical positions (bp)	SNPs	Gene accession IDs	Putative functions	Sequence components of genome
9592	CakSNP9592	*Kabuli*	Ca_Kabuli_Chr06	2802942	[C/A]	Ca_10415	--	SYNONYMOUS_CODING
9593	CakSNP9593	*Kabuli*	Ca_Kabuli_Chr06	2811345	[A/G]	Ca_10416	Nucleoporin, Nup155-like	NON_SYNONYMOUS_CODING
9594	CakSNP9594	*Kabuli*	Ca_Kabuli_Chr06	2812794	[A/C]	Ca_10416	Nucleoporin, Nup155-like	SYNONYMOUS_CODING
9595	CakSNP9595	*Kabuli*	Ca_Kabuli_Chr06	2812719	[G/A]	Ca_10416	Nucleoporin, Nup155-like	SYNONYMOUS_CODING
9596	CakSNP9596	*Kabuli*	Ca_Kabuli_Chr06	2813244	[G/A]	Ca_10416	Nucleoporin, Nup155-like	SYNONYMOUS_CODING
9597	CakSNP9597	*Kabuli*	Ca_Kabuli_Chr06	2821836	[G/T]	Ca_10417	HEAT	INTRON
9598	CakSNP9598	*Kabuli*	Ca_Kabuli_Chr06	2846051	[C/T]		--	INTERGENIC
9599	CakSNP9599	*Kabuli*	Ca_Kabuli_Chr06	2846018	[T/C]		--	INTERGENIC
9600	CakSNP9600	*Kabuli*	Ca_Kabuli_Chr06	2855691	[C/G]	Ca_10421	4Fe-4S binding domain	INTRON
9601	CakSNP9601	*Kabuli*	Ca_Kabuli_Chr06	2864713	[G/A]	Ca_10423	SET domain	SYNONYMOUS_CODING

S.N.	SNP IDs	Chickpea cultivars	Chromosomes	Physical positions (bp)	SNPs	Gene accession IDs	Putative functions	Sequence components of genome
9602	CakSNP9602	*Kabuli*	Ca_Kabuli_Chr06	2919355	[A/C]	Ca_10430	UBX	INTRON
9603	CakSNP9603	*Kabuli*	Ca_Kabuli_Chr06	2925908	[A/G]	Ca_10431	Tify	NON_SYNONYMOUS_CODING
9604	CakSNP9604	*Kabuli*	Ca_Kabuli_Chr06	2925962	[G/C]	Ca_10431	Tify	NON_SYNONYMOUS_CODING
9605	CakSNP9605	*Kabuli*	Ca_Kabuli_Chr06	2926006	[T/C]	Ca_10431	Tify	SYNONYMOUS_CODING
9606	CakSNP9606	*Kabuli*	Ca_Kabuli_Chr06	2927224	[G/A]	Ca_10431	Tify	NON_SYNONYMOUS_CODING
9607	CakSNP9607	*Kabuli*	Ca_Kabuli_Chr06	2927255	[C/A]	Ca_10431	Tify	NON_SYNONYMOUS_CODING
9608	CakSNP9608	*Kabuli*	Ca_Kabuli_Chr06	2927271	[G/A]	Ca_10431	Tify	SYNONYMOUS_CODING
9609	CakSNP9609	*Kabuli*	Ca_Kabuli_Chr06	2927386	[C/G]	Ca_10431	Tify	NON_SYNONYMOUS_CODING
9610	CakSNP9610	*Kabuli*	Ca_Kabuli_Chr06	2927387	[T/C]	Ca_10431	Tify	NON_SYNONYMOUS_CODING
9611	CakSNP9611	*Kabuli*	Ca_Kabuli_Chr06	2929355	[C/T]		--	INTERGENIC

S.N.	SNP IDs	Chickpea cultivars	Chromosomes	Physical positions (bp)	SNPs	Gene accession IDs	Putative functions	Sequence components of genome
9612	CakSNP9612	*Kabuli*	Ca_Kabuli_Chr06	2930453	[T/C]	Ca_10432	Glycoside hydrolase, family 17	SYNONYMOUS_CODING
9613	CakSNP9613	*Kabuli*	Ca_Kabuli_Chr06	2930459	[C/T]	Ca_10432	Glycoside hydrolase, family 17	SYNONYMOUS_CODING
9614	CakSNP9614	*Kabuli*	Ca_Kabuli_Chr06	2930504	[T/C]	Ca_10432	Glycoside hydrolase, family 17	SYNONYMOUS_CODING
9615	CakSNP9615	*Kabuli*	Ca_Kabuli_Chr06	2932029	[T/C]		--	INTERGENIC
9616	CakSNP9616	*Kabuli*	Ca_Kabuli_Chr06	2932179	[T/G]		--	INTERGENIC
9617	CakSNP9617	*Kabuli*	Ca_Kabuli_Chr06	2932168	[A/C]		--	INTERGENIC
9618	CakSNP9618	*Kabuli*	Ca_Kabuli_Chr06	2932143	[C/A]		--	INTERGENIC
9619	CakSNP9619	*Kabuli*	Ca_Kabuli_Chr06	2932130	[T/G]		--	INTERGENIC
9620	CakSNP9620	*Kabuli*	Ca_Kabuli_Chr06	2932777	[A/G]	Ca_10433	F-box domain, cyclin-like	NON_SYNONYMOUS_CODING
9621	CakSNP9621	*Kabuli*	Ca_Kabuli_Chr06	2972319	[C/T]	Ca_10439	Alpha-D-phosphohexomutase	INTRON

S.N.	SNP IDs	Chickpea cultivars	Chromosomes	Physical positions (bp)	SNPs	Gene accession IDs	Putative functions	Sequence components of genome
9622	CakSNP9622	*Kabuli*	Ca_Kabuli_Chr06	2972301	[T/C]	Ca_10439	Alpha-D-phosphohexomutase	INTRON
9623	CakSNP9623	*Kabuli*	Ca_Kabuli_Chr06	2973741	[A/G]	Ca_10439	Alpha-D-phosphohexomutase	INTRON
9624	CakSNP9624	*Kabuli*	Ca_Kabuli_Chr06	2973756	[A/C]	Ca_10439	Alpha-D-phosphohexomutase	INTRON
9625	CakSNP9625	*Kabuli*	Ca_Kabuli_Chr06	3060508	[G/A]		--	INTERGENIC
9626	CakSNP9626	*Kabuli*	Ca_Kabuli_Chr06	3060502	[G/A]		--	INTERGENIC
9627	CakSNP9627	*Kabuli*	Ca_Kabuli_Chr06	3288481	[C/A]		--	INTERGENIC
9628	CakSNP9628	*Kabuli*	Ca_Kabuli_Chr06	3288444	[G/A]		--	INTERGENIC
9629	CakSNP9629	*Kabuli*	Ca_Kabuli_Chr06	3288441	[A/G]		--	INTERGENIC
9630	CakSNP9630	*Kabuli*	Ca_Kabuli_Chr06	3288505	[G/T]		--	INTERGENIC
9631	CakSNP9631	*Kabuli*	Ca_Kabuli_Chr06	3297086	[G/A]	Ca_25243	Peptidyl-tRNA hydrolase	INTRON

S.N.	SNP IDs	Chickpea cultivars	Chromosomes	Physical positions (bp)	SNPs	Gene accession IDs	Putative functions	Sequence components of genome
9632	CakSNP9632	*Kabuli*	Ca_Kabuli_Chr06	3298952	[A/T]	Ca_25243	Peptidyl-tRNA hydrolase	INTRON
9633	CakSNP9633	*Kabuli*	Ca_Kabuli_Chr06	3395024	[A/G]	Ca_05973	Ras GTPase	INTRON
9634	CakSNP9634	*Kabuli*	Ca_Kabuli_Chr06	3395253	[G/A]	Ca_05973	Ras GTPase	SYNONYMOUS_CODING
9635	CakSNP9635	*Kabuli*	Ca_Kabuli_Chr06	3395370	[C/T]	Ca_05973	Ras GTPase	SYNONYMOUS_CODING
9636	CakSNP9636	*Kabuli*	Ca_Kabuli_Chr06	3397650	[A/G]		--	INTERGENIC
9637	CakSNP9637	*Kabuli*	Ca_Kabuli_Chr06	3401260	[A/G]	Ca_05972	Domain of unknown function DUF292, eukaryotic	INTRON
9638	CakSNP9638	*Kabuli*	Ca_Kabuli_Chr06	3401272	[G/A]	Ca_05972	Domain of unknown function DUF292, eukaryotic	INTRON
9639	CakSNP9639	*Kabuli*	Ca_Kabuli_Chr06	3401277	[C/T]	Ca_05972	Domain of unknown function DUF292, eukaryotic	INTRON
9640	CakSNP9640	*Kabuli*	Ca_Kabuli_Chr06	3482411	[A/G]	Ca_05962	Ribosomal protein L7Ae/L30e/S12e/Gadd45	SYNONYMOUS_CODING
9641	CakSNP9641	*Kabuli*	Ca_Kabuli_Chr06	3505662	[C/A]	Ca_05959	Oligopeptide transporter	NON_SYNONYMOUS_CODING

S.N.	SNP IDs	Chickpea cultivars	Chromosomes	Physical positions (bp)	SNPs	Gene accession IDs	Putative functions	Sequence components of genome
9642	CakSNP9642	*Kabuli*	Ca_Kabuli_Chr06	3530094	[T/C]	Ca_05957	FAD-dependent pyridine nucleotide-disulphide oxidoreductase	INTRON
9643	CakSNP9643	*Kabuli*	Ca_Kabuli_Chr06	3530137	[A/G]	Ca_05957	FAD-dependent pyridine nucleotide-disulphide oxidoreductase	INTRON
9644	CakSNP9644	*Kabuli*	Ca_Kabuli_Chr06	3548121	[C/A]	Ca_05955	CMP/dCMP deaminase, zinc-binding	NON_SYNONYMOUS_CODING
9645	CakSNP9645	*Kabuli*	Ca_Kabuli_Chr06	3548114	[A/C]	Ca_05955	CMP/dCMP deaminase, zinc-binding	NON_SYNONYMOUS_CODING
9646	CakSNP9646	*Kabuli*	Ca_Kabuli_Chr06	3786843	[A/G]		--	INTERGENIC
9647	CakSNP9647	*Kabuli*	Ca_Kabuli_Chr06	3787239	[C/A]	Ca_05924	Hexokinase	NON_SYNONYMOUS_CODING
9648	CakSNP9648	*Kabuli*	Ca_Kabuli_Chr06	3829070	[C/A]	Ca_05920	Glycoside hydrolase, family 17	NON_SYNONYMOUS_CODING
9649	CakSNP9649	*Kabuli*	Ca_Kabuli_Chr06	3962516	[A/C]		--	INTERGENIC
9650	CakSNP9650	*Kabuli*	Ca_Kabuli_Chr06	3985519	[G/A]		--	INTERGENIC
9651	CakSNP9651	*Kabuli*	Ca_Kabuli_Chr06	3992419	[C/T]	Ca_05901	Peptidase M16, zinc-binding site	NON_SYNONYMOUS_CODING

S.N.	SNP IDs	Chickpea cultivars	Chromosomes	Physical positions (bp)	SNPs	Gene accession IDs	Putative functions	Sequence components of genome
9652	CakSNP9652	*Kabuli*	Ca_Kabuli_Chr06	4112085	[T/C]		--	INTERGENIC
9653	CakSNP9653	*Kabuli*	Ca_Kabuli_Chr06	4226957	[G/A]		--	INTERGENIC
9654	CakSNP9654	*Kabuli*	Ca_Kabuli_Chr06	4226947	[A/T]		--	INTERGENIC
9655	CakSNP9655	*Kabuli*	Ca_Kabuli_Chr06	4226924	[G/T]		--	INTERGENIC
9656	CakSNP9656	*Kabuli*	Ca_Kabuli_Chr06	4226951	[C/A]		--	INTERGENIC
9657	CakSNP9657	*Kabuli*	Ca_Kabuli_Chr06	4228728	[T/C]		--	INTERGENIC
9658	CakSNP9658	*Kabuli*	Ca_Kabuli_Chr06	4228759	[C/G]		--	INTERGENIC
9659	CakSNP9659	*Kabuli*	Ca_Kabuli_Chr06	4228884	[T/C]		--	INTERGENIC
9660	CakSNP9660	*Kabuli*	Ca_Kabuli_Chr06	4344604	[T/C]	Ca_05866	Src homology-3 domain	SYNONYMOUS_CODING
9661	CakSNP9661	*Kabuli*	Ca_Kabuli_Chr06	4350822	[A/T]	Ca_05866	Src homology-3 domain	NON_SYNONYMOUS_CODING

S.N.	SNP IDs	Chickpea cultivars	Chromosomes	Physical positions (bp)	SNPs	Gene accession IDs	Putative functions	Sequence components of genome
9662	CakSNP9662	*Kabuli*	Ca_Kabuli_Chr06	4350867	[A/C]	Ca_05866	Src homology-3 domain	NON_SYNONYMOUS_CODING
9663	CakSNP9663	*Kabuli*	Ca_Kabuli_Chr06	4350889	[A/C]	Ca_05866	Src homology-3 domain	SYNONYMOUS_CODING
9664	CakSNP9664	*Kabuli*	Ca_Kabuli_Chr06	4440034	[T/G]	Ca_05857	Heat shock protein Hsp70	SYNONYMOUS_CODING
9665	CakSNP9665	*Kabuli*	Ca_Kabuli_Chr06	4562263	[C/A]	Ca_05843	--	NON_SYNONYMOUS_CODING
9666	CakSNP9666	*Kabuli*	Ca_Kabuli_Chr06	4562264	[C/G]	Ca_05843	--	NON_SYNONYMOUS_CODING
9667	CakSNP9667	*Kabuli*	Ca_Kabuli_Chr06	4601303	[A/C]	Ca_05837	ATPase, AAA+ type, core	SYNONYMOUS_CODING
9668	CakSNP9668	*Kabuli*	Ca_Kabuli_Chr06	4678523	[A/C]		--	INTERGENIC
9669	CakSNP9669	*Kabuli*	Ca_Kabuli_Chr06	4681686	[C/A]	Ca_05828	Pentatricopeptide repeat	NON_SYNONYMOUS_CODING
9670	CakSNP9670	*Kabuli*	Ca_Kabuli_Chr06	4743563	[C/G]		--	INTERGENIC
9671	CakSNP9671	*Kabuli*	Ca_Kabuli_Chr06	4743608	[C/T]		--	INTERGENIC

S.N.	SNP IDs	Chickpea cultivars	Chromosomes	Physical positions (bp)	SNPs	Gene accession IDs	Putative functions	Sequence components of genome
9672	CakSNP9672	*Kabuli*	Ca_Kabuli_Chr06	4807908	[G/C]	Ca_05817	--	SYNONYMOUS_CODING
9673	CakSNP9673	*Kabuli*	Ca_Kabuli_Chr06	4858109	[T/C]	Ca_05808	--	NON_SYNONYMOUS_CODING
9674	CakSNP9674	*Kabuli*	Ca_Kabuli_Chr06	4861184	[T/G]	Ca_05808	--	SYNONYMOUS_CODING
9675	CakSNP9675	*Kabuli*	Ca_Kabuli_Chr06	4981078	[G/T]	Ca_05796	--	NON_SYNONYMOUS_CODING
9676	CakSNP9676	*Kabuli*	Ca_Kabuli_Chr06	5185380	[A/G]	Ca_05778	Protein BYPASS-related	NON_SYNONYMOUS_CODING
9677	CakSNP9677	*Kabuli*	Ca_Kabuli_Chr06	5309445	[G/A]	Ca_05768	Xeroderma pigmentosum group B protein (XP-B)	INTRON
9678	CakSNP9678	*Kabuli*	Ca_Kabuli_Chr06	5338761	[T/G]	Ca_05766	ARID/BRIGHT DNA-binding domain	INTRON
9679	CakSNP9679	*Kabuli*	Ca_Kabuli_Chr06	5341510	[C/A]	Ca_05766	ARID/BRIGHT DNA-binding domain	NON_SYNONYMOUS_CODING
9680	CakSNP9680	*Kabuli*	Ca_Kabuli_Chr06	5366094	[A/G]	Ca_05763	--	SYNONYMOUS_CODING
9681	CakSNP9681	*Kabuli*	Ca_Kabuli_Chr06	5368455	[T/G]	Ca_05763	--	SYNONYMOUS_CODING

S.N.	SNP IDs	Chickpea cultivars	Chromosomes	Physical positions (bp)	SNPs	Gene accession IDs	Putative functions	Sequence components of genome
9682	CakSNP9682	*Kabuli*	Ca_Kabuli_Chr06	5392161	[G/A]	Ca_05761	Peptidase M1, alanine aminopeptidase/leukotriene A4 hydrolase	SYNONYMOUS_CODING
9683	CakSNP9683	*Kabuli*	Ca_Kabuli_Chr06	5392185	[T/C]	Ca_05761	Peptidase M1, alanine aminopeptidase/leukotriene A4 hydrolase	SYNONYMOUS_CODING
9684	CakSNP9684	*Kabuli*	Ca_Kabuli_Chr06	5396888	[T/G]	Ca_05761	Peptidase M1, alanine aminopeptidase/leukotriene A4 hydrolase	INTRON
9685	CakSNP9685	*Kabuli*	Ca_Kabuli_Chr06	5396890	[T/G]	Ca_05761	Peptidase M1, alanine aminopeptidase/leukotriene A4 hydrolase	INTRON
9686	CakSNP9686	*Kabuli*	Ca_Kabuli_Chr06	5396897	[T/G]	Ca_05761	Peptidase M1, alanine aminopeptidase/leukotriene A4 hydrolase	INTRON
9687	CakSNP9687	*Kabuli*	Ca_Kabuli_Chr06	5396913	[C/T]	Ca_05761	Peptidase M1, alanine aminopeptidase/leukotriene A4 hydrolase	SYNONYMOUS_CODING
9688	CakSNP9688	*Kabuli*	Ca_Kabuli_Chr06	5396916	[C/T]	Ca_05761	Peptidase M1, alanine aminopeptidase/leukotriene A4 hydrolase	SYNONYMOUS_CODING
9689	CakSNP9689	*Kabuli*	Ca_Kabuli_Chr06	5401307	[T/C]		--	INTERGENIC
9690	CakSNP9690	*Kabuli*	Ca_Kabuli_Chr06	5453512	[A/G]	Ca_05756	Protein kinase, catalytic domain	NON_SYNONYMOUS_CODING
9691	CakSNP9691	*Kabuli*	Ca_Kabuli_Chr06	5498898	[C/T]	Ca_05751	GMP synthase, C-terminal	SYNONYMOUS_CODING

S.N.	SNP IDs	Chickpea cultivars	Chromosomes	Physical positions (bp)	SNPs	Gene accession IDs	Putative functions	Sequence components of genome
9692	CakSNP9692	*Kabuli*	Ca_Kabuli_Chr06	5500791	[C/T]		--	INTERGENIC
9693	CakSNP9693	*Kabuli*	Ca_Kabuli_Chr06	5501363	[T/C]		--	INTERGENIC
9694	CakSNP9694	*Kabuli*	Ca_Kabuli_Chr06	5541264	[A/G]	Ca_05745	Bromo adjacent homology (BAH) domain	INTRON
9695	CakSNP9695	*Kabuli*	Ca_Kabuli_Chr06	5567967	[T/C]		--	INTERGENIC
9696	CakSNP9696	*Kabuli*	Ca_Kabuli_Chr06	5589373	[T/C]		--	INTERGENIC
9697	CakSNP9697	*Kabuli*	Ca_Kabuli_Chr06	5616372	[C/T]	Ca_05734	Glutathione S-transferase, N-terminal	INTRON
9698	CakSNP9698	*Kabuli*	Ca_Kabuli_Chr06	5616482	[A/G]	Ca_05734	Glutathione S-transferase, N-terminal	NON_SYNONYMOUS_CODING
9699	CakSNP9699	*Kabuli*	Ca_Kabuli_Chr06	5616454	[T/A]	Ca_05734	Glutathione S-transferase, N-terminal	SYNONYMOUS_CODING
9700	CakSNP9700	*Kabuli*	Ca_Kabuli_Chr06	5628948	[C/A]	Ca_05731	Nin one binding (NOB1) Zn-ribbon-like	SYNONYMOUS_CODING
9701	CakSNP9701	*Kabuli*	Ca_Kabuli_Chr06	5628956	[T/G]	Ca_05731	Nin one binding (NOB1) Zn-ribbon-like	INTRON

S.N.	SNP IDs	Chickpea cultivars	Chromosomes	Physical positions (bp)	SNPs	Gene accession IDs	Putative functions	Sequence components of genome
9702	CakSNP9702	*Kabuli*	Ca_Kabuli_Chr06	5692120	[C/A]	Ca_05725	Ribosomal protein S1, RNA-binding domain	INTRON
9703	CakSNP9703	*Kabuli*	Ca_Kabuli_Chr06	5694397	[G/T]	Ca_05725	Ribosomal protein S1, RNA-binding domain	INTRON
9704	CakSNP9704	*Kabuli*	Ca_Kabuli_Chr06	5701004	[A/G]	Ca_05724	Tetraspanin	INTRON
9705	CakSNP9705	*Kabuli*	Ca_Kabuli_Chr06	5704845	[T/C]	Ca_05723	Helicase, C-terminal	SYNONYMOUS_CODING
9706	CakSNP9706	*Kabuli*	Ca_Kabuli_Chr06	5704809	[T/A]	Ca_05723	Helicase, C-terminal	NON_SYNONYMOUS_CODING
9707	CakSNP9707	*Kabuli*	Ca_Kabuli_Chr06	5706649	[C/T]	Ca_05723	Helicase, C-terminal	INTRON
9708	CakSNP9708	*Kabuli*	Ca_Kabuli_Chr06	5841443	[G/C]		--	INTERGENIC
9709	CakSNP9709	*Kabuli*	Ca_Kabuli_Chr06	5871537	[G/A]	Ca_05707	Short-chain dehydrogenase/reductase SDR	SYNONYMOUS_CODING
9710	CakSNP9710	*Kabuli*	Ca_Kabuli_Chr06	5886393	[A/G]		--	INTERGENIC
9711	CakSNP9711	*Kabuli*	Ca_Kabuli_Chr06	5888423	[A/G]	Ca_05703	Domain of unknown function DUF1767	SYNONYMOUS_CODING

S.N.	SNP IDs	Chickpea cultivars	Chromosomes	Physical positions (bp)	SNPs	Gene accession IDs	Putative functions	Sequence components of genome
9712	CakSNP9712	*Kabuli*	Ca_Kabuli_Chr06	5924967	[G/T]	Ca_05701	ATPase-like, ATP-binding domain	INTRON
9713	CakSNP9713	*Kabuli*	Ca_Kabuli_Chr06	5925028	[G/T]	Ca_05701	ATPase-like, ATP-binding domain	INTRON
9714	CakSNP9714	*Kabuli*	Ca_Kabuli_Chr06	6110805	[T/C]	Ca_05684	Peptidase S10, serine carboxypeptidase	NON_SYNONYMOUS_CODING
9715	CakSNP9715	*Kabuli*	Ca_Kabuli_Chr06	6119644	[C/A]	Ca_05683	Dual specificity phosphatase, catalytic domain	SYNONYMOUS_CODING
9716	CakSNP9716	*Kabuli*	Ca_Kabuli_Chr06	6131564	[G/A]		--	INTERGENIC
9717	CakSNP9717	*Kabuli*	Ca_Kabuli_Chr06	6131518	[G/T]		--	INTERGENIC
9718	CakSNP9718	*Kabuli*	Ca_Kabuli_Chr06	6131514	[G/T]		--	INTERGENIC
9719	CakSNP9719	*Kabuli*	Ca_Kabuli_Chr06	6169291	[A/G]	Ca_05680	Arf GTPase activating protein	INTRON
9720	CakSNP9720	*Kabuli*	Ca_Kabuli_Chr06	6192895	[A/T]	Ca_05679	--	INTRON
9721	CakSNP9721	*Kabuli*	Ca_Kabuli_Chr06	6193105	[G/C]	Ca_05679	--	STOP_GAINED

S.N.	SNP IDs	Chickpea cultivars	Chromosomes	Physical positions (bp)	SNPs	Gene accession IDs	Putative functions	Sequence components of genome
9722	CakSNP9722	*Kabuli*	Ca_Kabuli_Chr06	6216766	[T/G]	Ca_05675	Amidase	INTRON
9723	CakSNP9723	*Kabuli*	Ca_Kabuli_Chr06	6216746	[G/C]	Ca_05675	Amidase	INTRON
9724	CakSNP9724	*Kabuli*	Ca_Kabuli_Chr06	6227887	[A/G]		--	INTERGENIC
9725	CakSNP9725	*Kabuli*	Ca_Kabuli_Chr06	6248260	[T/C]	Ca_05669	Protein of unknown function DUF339	SYNONYMOUS_CODING
9726	CakSNP9726	*Kabuli*	Ca_Kabuli_Chr06	6248203	[A/G]	Ca_05669	Protein of unknown function DUF339	SYNONYMOUS_CODING
9727	CakSNP9727	*Kabuli*	Ca_Kabuli_Chr06	6415818	[A/G]		--	INTERGENIC
9728	CakSNP9728	*Kabuli*	Ca_Kabuli_Chr06	6443828	[C/G]	Ca_16913	SANT domain, DNA binding	NON_SYNONYMOUS_CODING
9729	CakSNP9729	*Kabuli*	Ca_Kabuli_Chr06	6444169	[A/G]	Ca_16913	SANT domain, DNA binding	SYNONYMOUS_CODING
9730	CakSNP9730	*Kabuli*	Ca_Kabuli_Chr06	6444226	[T/G]	Ca_16913	SANT domain, DNA binding	INTRON
9731	CakSNP9731	*Kabuli*	Ca_Kabuli_Chr06	6444243	[G/A]	Ca_16913	SANT domain, DNA binding	INTRON

S.N.	SNP IDs	Chickpea cultivars	Chromosomes	Physical positions (bp)	SNPs	Gene accession IDs	Putative functions	Sequence components of genome
9732	CakSNP9732	*Kabuli*	Ca_Kabuli_Chr06	6444220	[G/A]	Ca_16913	SANT domain, DNA binding	INTRON
9733	CakSNP9733	*Kabuli*	Ca_Kabuli_Chr06	6517785	[T/C]		--	INTERGENIC
9734	CakSNP9734	*Kabuli*	Ca_Kabuli_Chr06	6517849	[C/T]		--	INTERGENIC
9735	CakSNP9735	*Kabuli*	Ca_Kabuli_Chr06	6538236	[C/T]	Ca_16915	Protein kinase, catalytic domain	INTRON
9736	CakSNP9736	*Kabuli*	Ca_Kabuli_Chr06	6604713	[G/A]	Ca_16921	Membrane attack complex component/perforin (MACPF) domain	NON_SYNONYMOUS_CODING
9737	CakSNP9737	*Kabuli*	Ca_Kabuli_Chr06	6604766	[C/A]	Ca_16921	Membrane attack complex component/perforin (MACPF) domain	NON_SYNONYMOUS_CODING
9738	CakSNP9738	*Kabuli*	Ca_Kabuli_Chr06	6607773	[A/G]	Ca_16921	Membrane attack complex component/perforin (MACPF) domain	SYNONYMOUS_CODING
9739	CakSNP9739	*Kabuli*	Ca_Kabuli_Chr06	6631216	[C/T]	Ca_16923	Protein of unknown function DUF668	NON_SYNONYMOUS_CODING
9740	CakSNP9740	*Kabuli*	Ca_Kabuli_Chr06	6654917	[T/C]	Ca_16925	Ammonium transporter	SYNONYMOUS_CODING
9741	CakSNP9741	*Kabuli*	Ca_Kabuli_Chr06	6764292	[G/A]	Ca_16936	Streptomyces cyclase/dehydrase	SYNONYMOUS_CODING

S.N.	SNP IDs	Chickpea cultivars	Chromosomes	Physical positions (bp)	SNPs	Gene accession IDs	Putative functions	Sequence components of genome
9742	CakSNP9742	*Kabuli*	Ca_Kabuli_Chr06	6779147	[G/C]	Ca_16938	Protein of unknown function DUF869, plant	NON_SYNONYMOUS_CODING
9743	CakSNP9743	*Kabuli*	Ca_Kabuli_Chr06	6779982	[C/T]	Ca_16938	Protein of unknown function DUF869, plant	NON_SYNONYMOUS_CODING
9744	CakSNP9744	*Kabuli*	Ca_Kabuli_Chr06	6792012	[C/T]	Ca_16939	Amidase	SYNONYMOUS_CODING
9745	CakSNP9745	*Kabuli*	Ca_Kabuli_Chr06	6800935	[A/T]	Ca_16940	Pyridine nucleotide-disulphide oxidoreductase, NAD-binding region	INTRON
9746	CakSNP9746	*Kabuli*	Ca_Kabuli_Chr06	6800927	[G/T]	Ca_16940	Pyridine nucleotide-disulphide oxidoreductase, NAD-binding region	INTRON
9747	CakSNP9747	*Kabuli*	Ca_Kabuli_Chr06	6800877	[T/A]	Ca_16940	Pyridine nucleotide-disulphide oxidoreductase, NAD-binding region	INTRON
9748	CakSNP9748	*Kabuli*	Ca_Kabuli_Chr06	6802259	[C/T]	Ca_16940	Pyridine nucleotide-disulphide oxidoreductase, NAD-binding region	INTRON
9749	CakSNP9749	*Kabuli*	Ca_Kabuli_Chr06	6802651	[A/G]	Ca_16940	Pyridine nucleotide-disulphide oxidoreductase, NAD-binding region	INTRON
9750	CakSNP9750	*Kabuli*	Ca_Kabuli_Chr06	6803433	[A/G]	Ca_16940	Pyridine nucleotide-disulphide oxidoreductase, NAD-binding region	SYNONYMOUS_CODING
9751	CakSNP9751	*Kabuli*	Ca_Kabuli_Chr06	6815594	[G/A]		--	INTERGENIC

S.N.	SNP IDs	Chickpea cultivars	Chromosomes	Physical positions (bp)	SNPs	Gene accession IDs	Putative functions	Sequence components of genome
9752	CakSNP9752	*Kabuli*	Ca_Kabuli_Chr06	6840803	[T/C]		--	INTERGENIC
9753	CakSNP9753	*Kabuli*	Ca_Kabuli_Chr06	6853983	[G/A]		--	INTERGENIC
9754	CakSNP9754	*Kabuli*	Ca_Kabuli_Chr06	6871801	[A/G]		--	INTERGENIC
9755	CakSNP9755	*Kabuli*	Ca_Kabuli_Chr06	6877020	[C/G]	Ca_16950	Heat shock protein DnaJ, N-terminal	SYNONYMOUS_CODING
9756	CakSNP9756	*Kabuli*	Ca_Kabuli_Chr06	6877088	[C/A]	Ca_16950	Heat shock protein DnaJ, N-terminal	NON_SYNONYMOUS_CODING
9757	CakSNP9757	*Kabuli*	Ca_Kabuli_Chr06	6877163	[T/C]	Ca_16950	Heat shock protein DnaJ, N-terminal	NON_SYNONYMOUS_CODING
9758	CakSNP9758	*Kabuli*	Ca_Kabuli_Chr06	6877246	[C/A]	Ca_16950	Heat shock protein DnaJ, N-terminal	NON_SYNONYMOUS_CODING
9759	CakSNP9759	*Kabuli*	Ca_Kabuli_Chr06	6877284	[C/T]	Ca_16950	Heat shock protein DnaJ, N-terminal	SYNONYMOUS_CODING
9760	CakSNP9760	*Kabuli*	Ca_Kabuli_Chr06	6899977	[G/A]		--	INTERGENIC
9761	CakSNP9761	*Kabuli*	Ca_Kabuli_Chr06	6899949	[T/G]		--	INTERGENIC

S.N.	SNP IDs	Chickpea cultivars	Chromosomes	Physical positions (bp)	SNPs	Gene accession IDs	Putative functions	Sequence components of genome
9762	CakSNP9762	*Kabuli*	Ca_Kabuli_Chr06	6913639	[T/C]	Ca_16954	SNF2-related	NON_SYNONYMOUS_CODING
9763	CakSNP9763	*Kabuli*	Ca_Kabuli_Chr06	6913622	[A/C]	Ca_16954	SNF2-related	NON_SYNONYMOUS_CODING
9764	CakSNP9764	*Kabuli*	Ca_Kabuli_Chr06	6913695	[G/A]	Ca_16954	SNF2-related	NON_SYNONYMOUS_CODING
9765	CakSNP9765	*Kabuli*	Ca_Kabuli_Chr06	6913825	[G/A]	Ca_16954	SNF2-related	INTRON
9766	CakSNP9766	*Kabuli*	Ca_Kabuli_Chr06	6913821	[A/T]	Ca_16954	SNF2-related	INTRON
9767	CakSNP9767	*Kabuli*	Ca_Kabuli_Chr06	6954468	[A/G]	Ca_09688	Protein of unknown function DUF248, methyltransferase putative	INTRON
9768	CakSNP9768	*Kabuli*	Ca_Kabuli_Chr06	6954480	[A/G]	Ca_09688	Protein of unknown function DUF248, methyltransferase putative	INTRON
9769	CakSNP9769	*Kabuli*	Ca_Kabuli_Chr06	7026495	[G/A]	Ca_09681	Ribosomal protein S12e	INTRON
9770	CakSNP9770	*Kabuli*	Ca_Kabuli_Chr06	7026481	[G/C]	Ca_09681	Ribosomal protein S12e	INTRON
9771	CakSNP9771	*Kabuli*	Ca_Kabuli_Chr06	7039578	[C/T]	Ca_09678	Protein kinase, catalytic domain	NON_SYNONYMOUS_CODING

S.N.	SNP IDs	Chickpea cultivars	Chromosomes	Physical positions (bp)	SNPs	Gene accession IDs	Putative functions	Sequence components of genome
9772	CakSNP9772	*Kabuli*	Ca_Kabuli_Chr06	7040231	[A/C]	Ca_09678	Protein kinase, catalytic domain	INTRON
9773	CakSNP9773	*Kabuli*	Ca_Kabuli_Chr06	7138381	[G/A]	Ca_09669	G-protein, beta subunit	SYNONYMOUS_CODING
9774	CakSNP9774	*Kabuli*	Ca_Kabuli_Chr06	7259533	[T/A]	Ca_09658	Leucine-rich repeat	SYNONYMOUS_CODING
9775	CakSNP9775	*Kabuli*	Ca_Kabuli_Chr06	7271634	[C/T]	Ca_09657	Protein kinase, catalytic domain	NON_SYNONYMOUS_CODING
9776	CakSNP9776	*Kabuli*	Ca_Kabuli_Chr06	7332579	[T/C]	Ca_09647	BolA protein	INTRON
9777	CakSNP9777	*Kabuli*	Ca_Kabuli_Chr06	7332585	[T/C]	Ca_09647	BolA protein	INTRON
9778	CakSNP9778	*Kabuli*	Ca_Kabuli_Chr06	7332762	[C/T]	Ca_09647	BolA protein	SYNONYMOUS_CODING
9779	CakSNP9779	*Kabuli*	Ca_Kabuli_Chr06	7349517	[A/C]	Ca_09644	Drug/metabolite transporter	SYNONYMOUS_CODING
9780	CakSNP9780	*Kabuli*	Ca_Kabuli_Chr06	7377229	[G/A]	Ca_09642	WD40 repeat	INTRON
9781	CakSNP9781	*Kabuli*	Ca_Kabuli_Chr06	7377276	[G/A]	Ca_09642	WD40 repeat	INTRON

S.N.	SNP IDs	Chickpea cultivars	Chromosomes	Physical positions (bp)	SNPs	Gene accession IDs	Putative functions	Sequence components of genome
9782	CakSNP9782	*Kabuli*	Ca_Kabuli_Chr06	7406219	[A/G]		--	INTERGENIC
9783	CakSNP9783	*Kabuli*	Ca_Kabuli_Chr06	7406233	[G/A]		--	INTERGENIC
9784	CakSNP9784	*Kabuli*	Ca_Kabuli_Chr06	7406301	[T/C]		--	INTERGENIC
9785	CakSNP9785	*Kabuli*	Ca_Kabuli_Chr06	7531353	[G/A]	Ca_09629	Plant neutral invertase	INTRON
9786	CakSNP9786	*Kabuli*	Ca_Kabuli_Chr06	7531445	[G/A]	Ca_09629	Plant neutral invertase	INTRON
9787	CakSNP9787	*Kabuli*	Ca_Kabuli_Chr06	7531456	[T/A]	Ca_09629	Plant neutral invertase	INTRON
9788	CakSNP9788	*Kabuli*	Ca_Kabuli_Chr06	7534360	[G/T]		--	INTERGENIC
9789	CakSNP9789	*Kabuli*	Ca_Kabuli_Chr06	7543580	[C/T]		--	INTERGENIC
9790	CakSNP9790	*Kabuli*	Ca_Kabuli_Chr06	7554094	[T/A]	Ca_09627	--	INTRON
9791	CakSNP9791	*Kabuli*	Ca_Kabuli_Chr06	7571950	[T/C]	Ca_09627	--	NON_SYNONYMOUS_CODING

S.N.	SNP IDs	Chickpea cultivars	Chromosomes	Physical positions (bp)	SNPs	Gene accession IDs	Putative functions	Sequence components of genome
9792	CakSNP9792	*Kabuli*	Ca_Kabuli_Chr06	7582726	[G/T]	Ca_09626	Mitochondrial carrier protein	INTRON
9793	CakSNP9793	*Kabuli*	Ca_Kabuli_Chr06	7600817	[G/A]	Ca_09624	Domain of unknown function DUF699, exodeoxyribonuclease V alpha chain	INTRON
9794	CakSNP9794	*Kabuli*	Ca_Kabuli_Chr06	7603195	[G/A]	Ca_09624	Domain of unknown function DUF699, exodeoxyribonuclease V alpha chain	NON_SYNONYMOUS_CODING
9795	CakSNP9795	*Kabuli*	Ca_Kabuli_Chr06	7603178	[A/C]	Ca_09624	Domain of unknown function DUF699, exodeoxyribonuclease V alpha chain	INTRON
9796	CakSNP9796	*Kabuli*	Ca_Kabuli_Chr06	7609814	[C/T]	Ca_09623	--	SYNONYMOUS_CODING
9797	CakSNP9797	*Kabuli*	Ca_Kabuli_Chr06	7647907	[T/G]	Ca_09618	Ras GTPase	SYNONYMOUS_CODING
9798	CakSNP9798	*Kabuli*	Ca_Kabuli_Chr06	7647931	[G/A]	Ca_09618	Ras GTPase	SYNONYMOUS_CODING
9799	CakSNP9799	*Kabuli*	Ca_Kabuli_Chr06	7661930	[T/G]		--	INTERGENIC
9800	CakSNP9800	*Kabuli*	Ca_Kabuli_Chr06	7661924	[T/C]		--	INTERGENIC
9801	CakSNP9801	*Kabuli*	Ca_Kabuli_Chr06	7667301	[T/C]	Ca_09615	Ribosomal protein L6E	INTRON

S.N.	SNP IDs	Chickpea cultivars	Chromosomes	Physical positions (bp)	SNPs	Gene accession IDs	Putative functions	Sequence components of genome
9802	CakSNP9802	*Kabuli*	Ca_Kabuli_Chr06	7667328	[A/T]	Ca_09615	Ribosomal protein L6E	INTRON
9803	CakSNP9803	*Kabuli*	Ca_Kabuli_Chr06	7673403	[C/T]	Ca_09614	SANT domain, DNA binding	INTRON
9804	CakSNP9804	*Kabuli*	Ca_Kabuli_Chr06	7694609	[A/T]		--	INTERGENIC
9805	CakSNP9805	*Kabuli*	Ca_Kabuli_Chr06	7694625	[G/T]		--	INTERGENIC
9806	CakSNP9806	*Kabuli*	Ca_Kabuli_Chr06	7694696	[T/C]		--	INTERGENIC
9807	CakSNP9807	*Kabuli*	Ca_Kabuli_Chr06	7744730	[A/G]	Ca_09604	ABC-1	INTRON
9808	CakSNP9808	*Kabuli*	Ca_Kabuli_Chr06	7744752	[T/C]	Ca_09604	ABC-1	INTRON
9809	CakSNP9809	*Kabuli*	Ca_Kabuli_Chr06	7745641	[A/G]	Ca_09604	ABC-1	INTRON
9810	CakSNP9810	*Kabuli*	Ca_Kabuli_Chr06	7747254	[G/A]	Ca_09604	ABC-1	SYNONYMOUS_CODING
9811	CakSNP9811	*Kabuli*	Ca_Kabuli_Chr06	7764968	[A/G]	Ca_09601	Aminotransferase class-III	SYNONYMOUS_CODING

S.N.	SNP IDs	Chickpea cultivars	Chromosomes	Physical positions (bp)	SNPs	Gene accession IDs	Putative functions	Sequence components of genome
9812	CakSNP9812	*Kabuli*	Ca_Kabuli_Chr06	7770357	[C/T]	Ca_09600	Basic-leucine zipper (bZIP) transcription factor	SYNONYMOUS_CODING
9813	CakSNP9813	*Kabuli*	Ca_Kabuli_Chr06	7805886	[G/T]	Ca_09597	--	INTRON
9814	CakSNP9814	*Kabuli*	Ca_Kabuli_Chr06	7821850	[A/G]	Ca_09596	Mitochondrial inner membrane protein Mitofilin	INTRON
9815	CakSNP9815	*Kabuli*	Ca_Kabuli_Chr06	7822666	[C/A]	Ca_09596	Mitochondrial inner membrane protein Mitofilin	NON_SYNONYMOUS_CODING
9816	CakSNP9816	*Kabuli*	Ca_Kabuli_Chr06	7829485	[G/A]	Ca_09595	C2 calcium-dependent membrane targeting	SYNONYMOUS_CODING
9817	CakSNP9817	*Kabuli*	Ca_Kabuli_Chr06	7829627	[T/G]	Ca_09595	C2 calcium-dependent membrane targeting	NON_SYNONYMOUS_CODING
9818	CakSNP9818	*Kabuli*	Ca_Kabuli_Chr06	7831214	[C/T]	Ca_09595	C2 calcium-dependent membrane targeting	SYNONYMOUS_CODING
9819	CakSNP9819	*Kabuli*	Ca_Kabuli_Chr06	7831409	[C/T]	Ca_09595	C2 calcium-dependent membrane targeting	SYNONYMOUS_CODING
9820	CakSNP9820	*Kabuli*	Ca_Kabuli_Chr06	7831585	[G/A]	Ca_09595	C2 calcium-dependent membrane targeting	NON_SYNONYMOUS_CODING
9821	CakSNP9821	*Kabuli*	Ca_Kabuli_Chr06	7831874	[A/G]	Ca_09595	C2 calcium-dependent membrane targeting	SYNONYMOUS_CODING

S.N.	SNP IDs	Chickpea cultivars	Chromosomes	Physical positions (bp)	SNPs	Gene accession IDs	Putative functions	Sequence components of genome
9822	CakSNP9822	*Kabuli*	Ca_Kabuli_Chr06	7831898	[G/A]	Ca_09595	C2 calcium-dependent membrane targeting	SYNONYMOUS_CODING
9823	CakSNP9823	*Kabuli*	Ca_Kabuli_Chr06	7831899	[G/A]	Ca_09595	C2 calcium-dependent membrane targeting	NON_SYNONYMOUS_CODING
9824	CakSNP9824	*Kabuli*	Ca_Kabuli_Chr06	7832029	[C/T]	Ca_09595	C2 calcium-dependent membrane targeting	NON_SYNONYMOUS_CODING
9825	CakSNP9825	*Kabuli*	Ca_Kabuli_Chr06	7832023	[C/T]	Ca_09595	C2 calcium-dependent membrane targeting	NON_SYNONYMOUS_CODING
9826	CakSNP9826	*Kabuli*	Ca_Kabuli_Chr06	7834595	[C/T]	Ca_09595	C2 calcium-dependent membrane targeting	SYNONYMOUS_CODING
9827	CakSNP9827	*Kabuli*	Ca_Kabuli_Chr06	7834922	[G/A]	Ca_09595	C2 calcium-dependent membrane targeting	SYNONYMOUS_CODING
9828	CakSNP9828	*Kabuli*	Ca_Kabuli_Chr06	7834895	[G/T]	Ca_09595	C2 calcium-dependent membrane targeting	SYNONYMOUS_CODING
9829	CakSNP9829	*Kabuli*	Ca_Kabuli_Chr06	7834976	[G/A]	Ca_09595	C2 calcium-dependent membrane targeting	SYNONYMOUS_CODING
9830	CakSNP9830	*Kabuli*	Ca_Kabuli_Chr06	7835427	[G/A]	Ca_09595	C2 calcium-dependent membrane targeting	NON_SYNONYMOUS_CODING
9831	CakSNP9831	*Kabuli*	Ca_Kabuli_Chr06	7835738	[C/T]	Ca_09595	C2 calcium-dependent membrane targeting	SYNONYMOUS_CODING

S.N.	SNP IDs	Chickpea cultivars	Chromosomes	Physical positions (bp)	SNPs	Gene accession IDs	Putative functions	Sequence components of genome
9832	CakSNP9832	*Kabuli*	Ca_Kabuli_Chr06	7835984	[T/A]	Ca_09595	C2 calcium-dependent membrane targeting	NON_SYNONYMOUS_CODING
9833	CakSNP9833	*Kabuli*	Ca_Kabuli_Chr06	7851000	[G/C]	Ca_09592	FAS1 domain	NON_SYNONYMOUS_CODING
9834	CakSNP9834	*Kabuli*	Ca_Kabuli_Chr06	7929263	[C/T]	Ca_09581	Acyl-CoA oxidase, C-terminal	NON_SYNONYMOUS_CODING
9835	CakSNP9835	*Kabuli*	Ca_Kabuli_Chr06	7929338	[T/C]	Ca_09581	Acyl-CoA oxidase, C-terminal	INTRON
9836	CakSNP9836	*Kabuli*	Ca_Kabuli_Chr06	7929339	[G/A]	Ca_09581	Acyl-CoA oxidase, C-terminal	INTRON
9837	CakSNP9837	*Kabuli*	Ca_Kabuli_Chr06	7929348	[C/T]	Ca_09581	Acyl-CoA oxidase, C-terminal	INTRON
9838	CakSNP9838	*Kabuli*	Ca_Kabuli_Chr06	7929384	[T/C]	Ca_09581	Acyl-CoA oxidase, C-terminal	INTRON
9839	CakSNP9839	*Kabuli*	Ca_Kabuli_Chr06	7929493	[A/G]	Ca_09581	Acyl-CoA oxidase, C-terminal	INTRON
9840	CakSNP9840	*Kabuli*	Ca_Kabuli_Chr06	7929629	[G/A]	Ca_09581	Acyl-CoA oxidase, C-terminal	INTRON
9841	CakSNP9841	*Kabuli*	Ca_Kabuli_Chr06	7929628	[C/T]	Ca_09581	Acyl-CoA oxidase, C-terminal	INTRON

S.N.	SNP IDs	Chickpea cultivars	Chromosomes	Physical positions (bp)	SNPs	Gene accession IDs	Putative functions	Sequence components of genome
9842	CakSNP9842	*Kabuli*	Ca_Kabuli_Chr06	7929607	[A/C]	Ca_09581	Acyl-CoA oxidase, C-terminal	INTRON
9843	CakSNP9843	*Kabuli*	Ca_Kabuli_Chr06	7939277	[T/A]	Ca_09580	--	SYNONYMOUS_CODING
9844	CakSNP9844	*Kabuli*	Ca_Kabuli_Chr06	7939281	[T/G]	Ca_09580	--	NON_SYNONYMOUS_CODING
9845	CakSNP9845	*Kabuli*	Ca_Kabuli_Chr06	7939465	[A/G]	Ca_09580	--	INTRON
9846	CakSNP9846	*Kabuli*	Ca_Kabuli_Chr06	7943546	[C/G]		--	INTERGENIC
9847	CakSNP9847	*Kabuli*	Ca_Kabuli_Chr06	7994732	[G/T]	Ca_09576	Late embryogenesis abundant protein, group 2	SYNONYMOUS_CODING
9848	CakSNP9848	*Kabuli*	Ca_Kabuli_Chr06	8010016	[T/C]	Ca_09573	Major facilitator superfamily MFS-1	SYNONYMOUS_CODING
9849	CakSNP9849	*Kabuli*	Ca_Kabuli_Chr06	8011685	[G/T]		--	INTERGENIC
9850	CakSNP9850	*Kabuli*	Ca_Kabuli_Chr06	8029226	[C/T]	Ca_09571	--	SYNONYMOUS_CODING
9851	CakSNP9851	*Kabuli*	Ca_Kabuli_Chr06	8041582	[A/G]	Ca_09569	Annexin	SYNONYMOUS_CODING

S.N.	SNP IDs	Chickpea cultivars	Chromosomes	Physical positions (bp)	SNPs	Gene accession IDs	Putative functions	Sequence components of genome
9852	CakSNP9852	*Kabuli*	Ca_Kabuli_Chr06	8052987	[G/C]	Ca_09568	TLDc	INTRON
9853	CakSNP9853	*Kabuli*	Ca_Kabuli_Chr06	8076394	[T/C]	Ca_09564	Ras GTPase	INTRON
9854	CakSNP9854	*Kabuli*	Ca_Kabuli_Chr06	8076446	[G/A]	Ca_09564	Ras GTPase	NON_SYNONYMOUS_CODING
9855	CakSNP9855	*Kabuli*	Ca_Kabuli_Chr06	8081800	[C/G]	Ca_09563	Mitochondrial carrier protein	SYNONYMOUS_CODING
9856	CakSNP9856	*Kabuli*	Ca_Kabuli_Chr06	8084950	[T/A]		--	INTERGENIC
9857	CakSNP9857	*Kabuli*	Ca_Kabuli_Chr06	8090739	[T/A]	Ca_09562	IQ motif, EF-hand binding site	NON_SYNONYMOUS_CODING
9858	CakSNP9858	*Kabuli*	Ca_Kabuli_Chr06	8090980	[A/G]	Ca_09562	IQ motif, EF-hand binding site	SYNONYMOUS_CODING
9859	CakSNP9859	*Kabuli*	Ca_Kabuli_Chr06	8091050	[A/G]	Ca_09562	IQ motif, EF-hand binding site	NON_SYNONYMOUS_CODING
9860	CakSNP9860	*Kabuli*	Ca_Kabuli_Chr06	8139434	[G/A]	Ca_09555	ATPase, P-type, H+ transporting proton pump	INTRON
9861	CakSNP9861	*Kabuli*	Ca_Kabuli_Chr06	8139446	[C/A]	Ca_09555	ATPase, P-type, H+ transporting proton pump	INTRON

S.N.	SNP IDs	Chickpea cultivars	Chromosomes	Physical positions (bp)	SNPs	Gene accession IDs	Putative functions	Sequence components of genome
9862	CakSNP9862	*Kabuli*	Ca_Kabuli_Chr06	8166317	[T/C]	Ca_09552	Enhancer of polycomb-like, N-terminal	INTRON
9863	CakSNP9863	*Kabuli*	Ca_Kabuli_Chr06	8166339	[T/C]	Ca_09552	Enhancer of polycomb-like, N-terminal	INTRON
9864	CakSNP9864	*Kabuli*	Ca_Kabuli_Chr06	8166435	[A/G]	Ca_09552	Enhancer of polycomb-like, N-terminal	INTRON
9865	CakSNP9865	*Kabuli*	Ca_Kabuli_Chr06	8166462	[A/T]	Ca_09552	Enhancer of polycomb-like, N-terminal	INTRON
9866	CakSNP9866	*Kabuli*	Ca_Kabuli_Chr06	8170583	[C/T]	Ca_09552	Enhancer of polycomb-like, N-terminal	INTRON
9867	CakSNP9867	*Kabuli*	Ca_Kabuli_Chr06	8170633	[A/G]	Ca_09552	Enhancer of polycomb-like, N-terminal	INTRON
9868	CakSNP9868	*Kabuli*	Ca_Kabuli_Chr06	8179815	[T/C]		--	INTERGENIC
9869	CakSNP9869	*Kabuli*	Ca_Kabuli_Chr06	8179797	[C/T]		--	INTERGENIC
9870	CakSNP9870	*Kabuli*	Ca_Kabuli_Chr06	8221204	[C/A]	Ca_09549	DNA-dependent ATPase MCM	NON_SYNONYMOUS_CODING
9871	CakSNP9871	*Kabuli*	Ca_Kabuli_Chr06	8222666	[T/C]	Ca_09549	DNA-dependent ATPase MCM	INTRON

S.N.	SNP IDs	Chickpea cultivars	Chromosomes	Physical positions (bp)	SNPs	Gene accession IDs	Putative functions	Sequence components of genome
9872	CakSNP9872	*Kabuli*	Ca_Kabuli_Chr06	8222685	[A/T]	Ca_09549	DNA-dependent ATPase MCM	INTRON
9873	CakSNP9873	*Kabuli*	Ca_Kabuli_Chr06	8223986	[C/G]	Ca_09549	DNA-dependent ATPase MCM	NON_SYNONYMOUS_CODING
9874	CakSNP9874	*Kabuli*	Ca_Kabuli_Chr06	8237286	[A/G]	Ca_09547	RNA recognition motif domain	INTRON
9875	CakSNP9875	*Kabuli*	Ca_Kabuli_Chr06	8243936	[A/T]	Ca_09546	Ras GTPase	INTRON
9876	CakSNP9876	*Kabuli*	Ca_Kabuli_Chr06	8243947	[T/C]	Ca_09546	Ras GTPase	INTRON
9877	CakSNP9877	*Kabuli*	Ca_Kabuli_Chr06	8244152	[C/T]	Ca_09546	Ras GTPase	INTRON
9878	CakSNP9878	*Kabuli*	Ca_Kabuli_Chr06	8244249	[C/T]	Ca_09546	Ras GTPase	INTRON
9879	CakSNP9879	*Kabuli*	Ca_Kabuli_Chr06	8246037	[T/C]	Ca_09545	Bacterial Fmu (Sun)/eukaryotic nucleolar NOL1/Nop2p	NON_SYNONYMOUS_CODING
9880	CakSNP9880	*Kabuli*	Ca_Kabuli_Chr06	8246187	[A/C]	Ca_09545	Bacterial Fmu (Sun)/eukaryotic nucleolar NOL1/Nop2p	NON_SYNONYMOUS_CODING
9881	CakSNP9881	*Kabuli*	Ca_Kabuli_Chr06	8248202	[A/C]	Ca_09545	Bacterial Fmu (Sun)/eukaryotic nucleolar NOL1/Nop2p	INTRON

S.N.	SNP IDs	Chickpea cultivars	Chromosomes	Physical positions (bp)	SNPs	Gene accession IDs	Putative functions	Sequence components of genome
9882	CakSNP9882	*Kabuli*	Ca_Kabuli_Chr06	8251365	[T/C]	Ca_09545	Bacterial Fmu (Sun)/eukaryotic nucleolar NOL1/Nop2p	INTRON
9883	CakSNP9883	*Kabuli*	Ca_Kabuli_Chr06	8269326	[A/C]	Ca_09543	Pentatricopeptide repeat	NON_SYNONYMOUS_CODING
9884	CakSNP9884	*Kabuli*	Ca_Kabuli_Chr06	8285170	[G/A]	Ca_09541	Protein of unknown function DUF3550/UPF0682	INTRON
9885	CakSNP9885	*Kabuli*	Ca_Kabuli_Chr06	8285147	[A/G]	Ca_09541	Protein of unknown function DUF3550/UPF0682	INTRON
9886	CakSNP9886	*Kabuli*	Ca_Kabuli_Chr06	8633231	[C/A]		--	INTERGENIC
9887	CakSNP9887	*Kabuli*	Ca_Kabuli_Chr06	8633215	[C/A]		--	INTERGENIC
9888	CakSNP9888	*Kabuli*	Ca_Kabuli_Chr06	8633187	[A/C]		--	INTERGENIC
9889	CakSNP9889	*Kabuli*	Ca_Kabuli_Chr06	8634513	[G/A]	Ca_16775	Ceramidase	SYNONYMOUS_CODING
9890	CakSNP9890	*Kabuli*	Ca_Kabuli_Chr06	8634494	[T/C]	Ca_16775	Ceramidase	NON_SYNONYMOUS_CODING
9891	CakSNP9891	*Kabuli*	Ca_Kabuli_Chr06	8643286	[T/C]	Ca_16774	Dual specificity phosphatase, catalytic domain	NON_SYNONYMOUS_CODING

S.N.	SNP IDs	Chickpea cultivars	Chromosomes	Physical positions (bp)	SNPs	Gene accession IDs	Putative functions	Sequence components of genome
9892	CakSNP9892	*Kabuli*	Ca_Kabuli_Chr06	8645079	[A/G]	Ca_16774	Dual specificity phosphatase, catalytic domain	SYNONYMOUS_CODING
9893	CakSNP9893	*Kabuli*	Ca_Kabuli_Chr06	8710064	[A/T]		--	INTERGENIC
9894	CakSNP9894	*Kabuli*	Ca_Kabuli_Chr06	8787556	[C/T]	Ca_16756	Protein kinase, catalytic domain	SYNONYMOUS_CODING
9895	CakSNP9895	*Kabuli*	Ca_Kabuli_Chr06	8787557	[T/C]	Ca_16756	Protein kinase, catalytic domain	NON_SYNONYMOUS_CODING
9896	CakSNP9896	*Kabuli*	Ca_Kabuli_Chr06	8787562	[C/T]	Ca_16756	Protein kinase, catalytic domain	NON_SYNONYMOUS_CODING
9897	CakSNP9897	*Kabuli*	Ca_Kabuli_Chr06	8787564	[G/T]	Ca_16756	Protein kinase, catalytic domain	SYNONYMOUS_CODING
9898	CakSNP9898	*Kabuli*	Ca_Kabuli_Chr06	8787568	[G/T]	Ca_16756	Protein kinase, catalytic domain	STOP_GAINED
9899	CakSNP9899	*Kabuli*	Ca_Kabuli_Chr06	8787572	[T/A]	Ca_16756	Protein kinase, catalytic domain	NON_SYNONYMOUS_CODING
9900	CakSNP9900	*Kabuli*	Ca_Kabuli_Chr06	8787573	[G/A]	Ca_16756	Protein kinase, catalytic domain	SYNONYMOUS_CODING
9901	CakSNP9901	*Kabuli*	Ca_Kabuli_Chr06	8794430	[A/G]		--	INTERGENIC

S.N.	SNP IDs	Chickpea cultivars	Chromosomes	Physical positions (bp)	SNPs	Gene accession IDs	Putative functions	Sequence components of genome
9902	CakSNP9902	*Kabuli*	Ca_Kabuli_Chr06	8794552	[C/G]		--	INTERGENIC
9903	CakSNP9903	*Kabuli*	Ca_Kabuli_Chr06	8890548	[A/G]	Ca_16747	NSF attachment protein	INTRON
9904	CakSNP9904	*Kabuli*	Ca_Kabuli_Chr06	8890552	[C/G]	Ca_16747	NSF attachment protein	INTRON
9905	CakSNP9905	*Kabuli*	Ca_Kabuli_Chr06	8893216	[A/G]	Ca_16746	Peptidase S8/S53, subtilisin/kexin/sedolisin	NON_SYNONYMOUS_CODING
9906	CakSNP9906	*Kabuli*	Ca_Kabuli_Chr06	8968068	[G/A]	Ca_16741	Phosphatidylinositol-4-phosphate 5-kinase, core	INTRON
9907	CakSNP9907	*Kabuli*	Ca_Kabuli_Chr06	8969711	[C/T]		--	INTERGENIC
9908	CakSNP9908	*Kabuli*	Ca_Kabuli_Chr06	8969725	[C/A]		--	INTERGENIC
9909	CakSNP9909	*Kabuli*	Ca_Kabuli_Chr06	9053279	[A/G]		--	INTERGENIC
9910	CakSNP9910	*Kabuli*	Ca_Kabuli_Chr06	9054596	[T/C]		--	INTERGENIC
9911	CakSNP9911	*Kabuli*	Ca_Kabuli_Chr06	9088121	[T/C]		--	INTERGENIC

S.N.	SNP IDs	Chickpea cultivars	Chromosomes	Physical positions (bp)	SNPs	Gene accession IDs	Putative functions	Sequence components of genome
9912	CakSNP9912	*Kabuli*	Ca_Kabuli_Chr06	9088151	[G/A]		--	INTERGENIC
9913	CakSNP9913	*Kabuli*	Ca_Kabuli_Chr06	9088163	[A/G]		--	INTERGENIC
9914	CakSNP9914	*Kabuli*	Ca_Kabuli_Chr06	9088184	[G/A]		--	INTERGENIC
9915	CakSNP9915	*Kabuli*	Ca_Kabuli_Chr06	9088234	[A/C]		--	INTERGENIC
9916	CakSNP9916	*Kabuli*	Ca_Kabuli_Chr06	9190620	[G/T]	Ca_08678	LETM1-like	SYNONYMOUS_CODING
9917	CakSNP9917	*Kabuli*	Ca_Kabuli_Chr06	9221201	[A/G]		--	INTERGENIC
9918	CakSNP9918	*Kabuli*	Ca_Kabuli_Chr06	9227767	[C/A]		--	INTERGENIC
9919	CakSNP9919	*Kabuli*	Ca_Kabuli_Chr06	9228004	[C/A]		--	INTERGENIC
9920	CakSNP9920	*Kabuli*	Ca_Kabuli_Chr06	9323775	[A/G]	Ca_08667	Homeobox	SYNONYMOUS_CODING
9921	CakSNP9921	*Kabuli*	Ca_Kabuli_Chr06	9324122	[A/C]	Ca_08667	Homeobox	NON_SYNONYMOUS_CODING

S.N.	SNP IDs	Chickpea cultivars	Chromosomes	Physical positions (bp)	SNPs	Gene accession IDs	Putative functions	Sequence components of genome
9922	CakSNP9922	*Kabuli*	Ca_Kabuli_Chr06	9373240	[C/A]		--	INTERGENIC
9923	CakSNP9923	*Kabuli*	Ca_Kabuli_Chr06	9373383	[G/C]		--	INTERGENIC
9924	CakSNP9924	*Kabuli*	Ca_Kabuli_Chr06	9378508	[A/T]	Ca_08661	Transposase, Ptta/En/Spm, plant	INTRON
9925	CakSNP9925	*Kabuli*	Ca_Kabuli_Chr06	9378557	[G/C]	Ca_08661	Transposase, Ptta/En/Spm, plant	INTRON
9926	CakSNP9926	*Kabuli*	Ca_Kabuli_Chr06	9382091	[C/T]	Ca_08660	Chaperonin TCP-1, conserved site	INTRON
9927	CakSNP9927	*Kabuli*	Ca_Kabuli_Chr06	9423222	[T/C]	Ca_08655	Aldo/keto reductase	NON_SYNONYMOUS_CODING
9928	CakSNP9928	*Kabuli*	Ca_Kabuli_Chr06	9424317	[T/C]	Ca_08655	Aldo/keto reductase	INTRON
9929	CakSNP9929	*Kabuli*	Ca_Kabuli_Chr06	9428522	[C/T]	Ca_08654	PDZ/DHR/GLGF	INTRON
9930	CakSNP9930	*Kabuli*	Ca_Kabuli_Chr06	9428517	[G/A]	Ca_08654	PDZ/DHR/GLGF	INTRON
9931	CakSNP9931	*Kabuli*	Ca_Kabuli_Chr06	9523228	[T/C]	Ca_08648	RNA-processing protein, HAT helix	INTRON

S.N.	SNP IDs	Chickpea cultivars	Chromosomes	Physical positions (bp)	SNPs	Gene accession IDs	Putative functions	Sequence components of genome
9932	CakSNP9932	*Kabuli*	Ca_Kabuli_Chr06	9544233	[T/C]	Ca_08646	Neutrophil cytosol factor 2 p67phox	INTRON
9933	CakSNP9933	*Kabuli*	Ca_Kabuli_Chr06	9565932	[A/G]	Ca_08643	DNA repair protein (XPGC)/yeast Rad	INTRON
9934	CakSNP9934	*Kabuli*	Ca_Kabuli_Chr06	9568243	[A/C]	Ca_08643	DNA repair protein (XPGC)/yeast Rad	NON_SYNONYMOUS_CODING
9935	CakSNP9935	*Kabuli*	Ca_Kabuli_Chr06	9568247	[G/A]	Ca_08643	DNA repair protein (XPGC)/yeast Rad	SYNONYMOUS_CODING
9936	CakSNP9936	*Kabuli*	Ca_Kabuli_Chr06	9568289	[A/C]	Ca_08643	DNA repair protein (XPGC)/yeast Rad	INTRON
9937	CakSNP9937	*Kabuli*	Ca_Kabuli_Chr06	9569170	[A/G]	Ca_08643	DNA repair protein (XPGC)/yeast Rad	SYNONYMOUS_CODING
9938	CakSNP9938	*Kabuli*	Ca_Kabuli_Chr06	9570753	[C/T]		--	INTERGENIC
9939	CakSNP9939	*Kabuli*	Ca_Kabuli_Chr06	9617218	[A/G]	Ca_08636	Oligopeptide transporter	SYNONYMOUS_CODING
9940	CakSNP9940	*Kabuli*	Ca_Kabuli_Chr06	9617233	[A/G]	Ca_08636	Oligopeptide transporter	SYNONYMOUS_CODING
9941	CakSNP9941	*Kabuli*	Ca_Kabuli_Chr06	9714240	[T/C]	Ca_08622	ER lumen protein retaining receptor	INTRON

S.N.	SNP IDs	Chickpea cultivars	Chromosomes	Physical positions (bp)	SNPs	Gene accession IDs	Putative functions	Sequence components of genome
9942	CakSNP9942	*Kabuli*	Ca_Kabuli_Chr06	9728195	[G/T]		--	INTERGENIC
9943	CakSNP9943	*Kabuli*	Ca_Kabuli_Chr06	9728181	[A/G]		--	INTERGENIC
9944	CakSNP9944	*Kabuli*	Ca_Kabuli_Chr06	9814378	[G/T]	Ca_08611	Protein Transporter, Pam16	INTRON
9945	CakSNP9945	*Kabuli*	Ca_Kabuli_Chr06	9834199	[T/C]	Ca_08608	WD40 repeat	SYNONYMOUS_CODING
9946	CakSNP9946	*Kabuli*	Ca_Kabuli_Chr06	9834198	[C/A]	Ca_08608	WD40 repeat	NON_SYNONYMOUS_CODING
9947	CakSNP9947	*Kabuli*	Ca_Kabuli_Chr06	9834187	[A/T]	Ca_08608	WD40 repeat	NON_SYNONYMOUS_CODING
9948	CakSNP9948	*Kabuli*	Ca_Kabuli_Chr06	9834184	[A/T]	Ca_08608	WD40 repeat	SYNONYMOUS_CODING
9949	CakSNP9949	*Kabuli*	Ca_Kabuli_Chr06	9834183	[C/T]	Ca_08608	WD40 repeat	NON_SYNONYMOUS_CODING
9950	CakSNP9950	*Kabuli*	Ca_Kabuli_Chr06	9841836	[G/A]	Ca_08607	Cellulose synthase	SYNONYMOUS_CODING
9951	CakSNP9951	*Kabuli*	Ca_Kabuli_Chr06	9841890	[G/A]	Ca_08607	Cellulose synthase	SYNONYMOUS_CODING

S.N.	SNP IDs	Chickpea cultivars	Chromosomes	Physical positions (bp)	SNPs	Gene accession IDs	Putative functions	Sequence components of genome
9952	CakSNP9952	*Kabuli*	Ca_Kabuli_Chr06	9845070	[C/T]	Ca_08607	Cellulose synthase	SYNONYMOUS_CODING
9953	CakSNP9953	*Kabuli*	Ca_Kabuli_Chr06	9845076	[T/C]	Ca_08607	Cellulose synthase	SYNONYMOUS_CODING
9954	CakSNP9954	*Kabuli*	Ca_Kabuli_Chr06	9872557	[G/A]	Ca_08602	Xanthine/uracil/vitamin C permease	INTRON
9955	CakSNP9955	*Kabuli*	Ca_Kabuli_Chr06	9872657	[C/T]	Ca_08602	Xanthine/uracil/vitamin C permease	NON_SYNONYMOUS_CODING
9956	CakSNP9956	*Kabuli*	Ca_Kabuli_Chr06	9872988	[A/T]	Ca_08602	Xanthine/uracil/vitamin C permease	INTRON
9957	CakSNP9957	*Kabuli*	Ca_Kabuli_Chr06	9888237	[G/A]		--	INTERGENIC
9958	CakSNP9958	*Kabuli*	Ca_Kabuli_Chr06	9938833	[T/C]	Ca_08597	Protein kinase, catalytic domain	SYNONYMOUS_CODING
9959	CakSNP9959	*Kabuli*	Ca_Kabuli_Chr06	9939017	[G/A]	Ca_08597	Protein kinase, catalytic domain	NON_SYNONYMOUS_CODING
9960	CakSNP9960	*Kabuli*	Ca_Kabuli_Chr06	9959686	[T/C]	Ca_08594	Preprotein translocase Sec, Sec61-beta subunit	SYNONYMOUS_CODING
9961	CakSNP9961	*Kabuli*	Ca_Kabuli_Chr06	10020177	[C/G]		--	INTERGENIC

S.N.	SNP IDs	Chickpea cultivars	Chromosomes	Physical positions (bp)	SNPs	Gene accession IDs	Putative functions	Sequence components of genome
9962	CakSNP9962	*Kabuli*	Ca_Kabuli_Chr06	10020187	[A/G]		--	INTERGENIC
9963	CakSNP9963	*Kabuli*	Ca_Kabuli_Chr06	10025115	[A/C]	Ca_08590	Protein kinase, catalytic domain	SYNONYMOUS_CODING
9964	CakSNP9964	*Kabuli*	Ca_Kabuli_Chr06	10025113	[C/G]	Ca_08590	Protein kinase, catalytic domain	SYNONYMOUS_CODING
9965	CakSNP9965	*Kabuli*	Ca_Kabuli_Chr06	10027019	[G/A]		--	INTERGENIC
9966	CakSNP9966	*Kabuli*	Ca_Kabuli_Chr06	10027015	[G/C]		--	INTERGENIC
9967	CakSNP9967	*Kabuli*	Ca_Kabuli_Chr06	10028504	[A/G]		--	INTERGENIC
9968	CakSNP9968	*Kabuli*	Ca_Kabuli_Chr06	10028508	[G/C]		--	INTERGENIC
9969	CakSNP9969	*Kabuli*	Ca_Kabuli_Chr06	10028515	[A/G]		--	INTERGENIC
9970	CakSNP9970	*Kabuli*	Ca_Kabuli_Chr06	10045644	[A/C]	Ca_08587	AMP-dependent synthetase/ligase	SYNONYMOUS_CODING
9971	CakSNP9971	*Kabuli*	Ca_Kabuli_Chr06	10045844	[A/G]		--	INTERGENIC

S.N.	SNP IDs	Chickpea cultivars	Chromosomes	Physical positions (bp)	SNPs	Gene accession IDs	Putative functions	Sequence components of genome
9972	CakSNP9972	*Kabuli*	Ca_Kabuli_Chr06	10088963	[G/T]	Ca_08583	Tubulin	SYNONYMOUS_CODING
9973	CakSNP9973	*Kabuli*	Ca_Kabuli_Chr06	10088984	[A/G]	Ca_08583	Tubulin	SYNONYMOUS_CODING
9974	CakSNP9974	*Kabuli*	Ca_Kabuli_Chr06	10100863	[C/G]	Ca_08581	HEAT	SYNONYMOUS_CODING
9975	CakSNP9975	*Kabuli*	Ca_Kabuli_Chr06	10111488	[C/G]	Ca_08580	Peptidyl-tRNA hydrolase	INTRON
9976	CakSNP9976	*Kabuli*	Ca_Kabuli_Chr06	10111520	[G/C]	Ca_08580	Peptidyl-tRNA hydrolase	INTRON
9977	CakSNP9977	*Kabuli*	Ca_Kabuli_Chr06	10111870	[A/G]	Ca_08580	Peptidyl-tRNA hydrolase	INTRON
9978	CakSNP9978	*Kabuli*	Ca_Kabuli_Chr06	10111938	[G/T]	Ca_08580	Peptidyl-tRNA hydrolase	INTRON
9979	CakSNP9979	*Kabuli*	Ca_Kabuli_Chr06	10116403	[A/T]	Ca_08580	Peptidyl-tRNA hydrolase	INTRON
9980	CakSNP9980	*Kabuli*	Ca_Kabuli_Chr06	10116475	[C/A]	Ca_08580	Peptidyl-tRNA hydrolase	INTRON
9981	CakSNP9981	*Kabuli*	Ca_Kabuli_Chr06	10138984	[C/T]	Ca_08579	--	NON_SYNONYMOUS_CODING

S.N.	SNP IDs	Chickpea cultivars	Chromosomes	Physical positions (bp)	SNPs	Gene accession IDs	Putative functions	Sequence components of genome
9982	CakSNP9982	*Kabuli*	Ca_Kabuli_Chr06	10151973	[A/G]	Ca_08577	Protein of unknown function DUF616	INTRON
9983	CakSNP9983	*Kabuli*	Ca_Kabuli_Chr06	10151974	[A/G]	Ca_08577	Protein of unknown function DUF616	INTRON
9984	CakSNP9984	*Kabuli*	Ca_Kabuli_Chr06	10158850	[T/C]	Ca_08576	Pheophorbide a oxygenase	INTRON
9985	CakSNP9985	*Kabuli*	Ca_Kabuli_Chr06	10188400	[G/T]		--	INTERGENIC
9986	CakSNP9986	*Kabuli*	Ca_Kabuli_Chr06	10191941	[A/G]	Ca_08570	AMP-dependent synthetase/ligase	NON_SYNONYMOUS_CODING
9987	CakSNP9987	*Kabuli*	Ca_Kabuli_Chr06	10215262	[T/G]		--	INTERGENIC
9988	CakSNP9988	*Kabuli*	Ca_Kabuli_Chr06	10230617	[A/G]	Ca_08566	Metallo-dependent phosphatase	INTRON
9989	CakSNP9989	*Kabuli*	Ca_Kabuli_Chr06	10230657	[A/G]	Ca_08566	Metallo-dependent phosphatase	INTRON
9990	CakSNP9990	*Kabuli*	Ca_Kabuli_Chr06	10231092	[T/G]	Ca_08566	Metallo-dependent phosphatase	SYNONYMOUS_CODING
9991	CakSNP9991	*Kabuli*	Ca_Kabuli_Chr06	10231095	[G/T]	Ca_08566	Metallo-dependent phosphatase	SYNONYMOUS_CODING

S.N.	SNP IDs	Chickpea cultivars	Chromosomes	Physical positions (bp)	SNPs	Gene accession IDs	Putative functions	Sequence components of genome
9992	CakSNP9992	*Kabuli*	Ca_Kabuli_Chr06	10234241	[A/G]	Ca_08566	Metallo-dependent phosphatase	INTRON
9993	CakSNP9993	*Kabuli*	Ca_Kabuli_Chr06	10259614	[C/T]	Ca_08563	Mediator complex, subunit Med10	SYNONYMOUS_CODING
9994	CakSNP9994	*Kabuli*	Ca_Kabuli_Chr06	10260274	[C/T]	Ca_08563	Mediator complex, subunit Med10	SYNONYMOUS_CODING
9995	CakSNP9995	*Kabuli*	Ca_Kabuli_Chr06	10314993	[C/G]		--	INTERGENIC
9996	CakSNP9996	*Kabuli*	Ca_Kabuli_Chr06	10395411	[G/A]	Ca_08553	Small ubiquitin-related modifier, SUMO	INTRON
9997	CakSNP9997	*Kabuli*	Ca_Kabuli_Chr06	10395379	[T/C]	Ca_08553	Small ubiquitin-related modifier, SUMO	INTRON
9998	CakSNP9998	*Kabuli*	Ca_Kabuli_Chr06	10395356	[T/A]	Ca_08553	Small ubiquitin-related modifier, SUMO	INTRON
9999	CakSNP9999	*Kabuli*	Ca_Kabuli_Chr06	10441736	[A/C]	Ca_08551	--	NON_SYNONYMOUS_CODING
10000	CakSNP10000	*Kabuli*	Ca_Kabuli_Chr06	10441705	[T/C]		--	INTERGENIC
10001	CakSNP10001	*Kabuli*	Ca_Kabuli_Chr06	10442148	[A/C]	Ca_08551	--	SYNONYMOUS_CODING

S.N.	SNP IDs	Chickpea cultivars	Chromosomes	Physical positions (bp)	SNPs	Gene accession IDs	Putative functions	Sequence components of genome
10002	CakSNP10002	*Kabuli*	Ca_Kabuli_Chr06	10445185	[C/T]	Ca_08551	--	INTRON
10003	CakSNP10003	*Kabuli*	Ca_Kabuli_Chr06	10445274	[G/A]	Ca_08551	--	INTRON
10004	CakSNP10004	*Kabuli*	Ca_Kabuli_Chr06	10456774	[A/G]	Ca_08551	--	SYNONYMOUS_CODING
10005	CakSNP10005	*Kabuli*	Ca_Kabuli_Chr06	10456854	[G/T]	Ca_08551	--	SYNONYMOUS_CODING
10006	CakSNP10006	*Kabuli*	Ca_Kabuli_Chr06	10494134	[C/T]	Ca_08547	D-galactoside/L-rhamnose binding SUEL lectin domain	INTRON
10007	CakSNP10007	*Kabuli*	Ca_Kabuli_Chr06	10494057	[C/A]	Ca_08547	D-galactoside/L-rhamnose binding SUEL lectin domain	INTRON
10008	CakSNP10008	*Kabuli*	Ca_Kabuli_Chr06	10494055	[T/A]	Ca_08547	D-galactoside/L-rhamnose binding SUEL lectin domain	INTRON
10009	CakSNP10009	*Kabuli*	Ca_Kabuli_Chr06	10495747	[G/T]	Ca_08547	D-galactoside/L-rhamnose binding SUEL lectin domain	INTRON
10010	CakSNP10010	*Kabuli*	Ca_Kabuli_Chr06	10495804	[C/T]	Ca_08547	D-galactoside/L-rhamnose binding SUEL lectin domain	INTRON
10011	CakSNP10011	*Kabuli*	Ca_Kabuli_Chr06	10502381	[A/G]		--	INTERGENIC

S.N.	SNP IDs	Chickpea cultivars	Chromosomes	Physical positions (bp)	SNPs	Gene accession IDs	Putative functions	Sequence components of genome
10012	CakSNP10012	*Kabuli*	Ca_Kabuli_Chr06	10502387	[C/A]		--	INTERGENIC
10013	CakSNP10013	*Kabuli*	Ca_Kabuli_Chr06	10510996	[T/C]	Ca_08544	Protein of unknown function DUF803	NON_SYNONYMOUS_CODING
10014	CakSNP10014	*Kabuli*	Ca_Kabuli_Chr06	10520958	[C/T]	Ca_08543	RNA recognition motif domain	INTRON
10015	CakSNP10015	*Kabuli*	Ca_Kabuli_Chr06	10521741	[A/T]	Ca_08543	RNA recognition motif domain	NON_SYNONYMOUS_CODING
10016	CakSNP10016	*Kabuli*	Ca_Kabuli_Chr06	10531311	[A/G]	Ca_08542	RNA recognition motif domain	INTRON
10017	CakSNP10017	*Kabuli*	Ca_Kabuli_Chr06	10531399	[C/T]	Ca_08542	RNA recognition motif domain	INTRON
10018	CakSNP10018	*Kabuli*	Ca_Kabuli_Chr06	10532182	[A/T]	Ca_08542	RNA recognition motif domain	NON_SYNONYMOUS_CODING
10019	CakSNP10019	*Kabuli*	Ca_Kabuli_Chr06	10532739	[A/G]	Ca_08542	RNA recognition motif domain	SYNONYMOUS_CODING
10020	CakSNP10020	*Kabuli*	Ca_Kabuli_Chr06	10539918	[A/C]		--	INTERGENIC
10021	CakSNP10021	*Kabuli*	Ca_Kabuli_Chr06	10539977	[C/T]		--	INTERGENIC

S.N.	SNP IDs	Chickpea cultivars	Chromosomes	Physical positions (bp)	SNPs	Gene accession IDs	Putative functions	Sequence components of genome
10022	CakSNP10022	*Kabuli*	Ca_Kabuli_Chr06	10539994	[A/C]		--	INTERGENIC
10023	CakSNP10023	*Kabuli*	Ca_Kabuli_Chr06	10567518	[A/G]		--	INTERGENIC
10024	CakSNP10024	*Kabuli*	Ca_Kabuli_Chr06	10588030	[C/T]		--	INTERGENIC
10025	CakSNP10025	*Kabuli*	Ca_Kabuli_Chr06	10613546	[A/G]	Ca_08536	Heat shock factor (HSF)-type, DNA-binding	SYNONYMOUS_CODING
10026	CakSNP10026	*Kabuli*	Ca_Kabuli_Chr06	10613612	[A/G]	Ca_08536	Heat shock factor (HSF)-type, DNA-binding	SYNONYMOUS_CODING
10027	CakSNP10027	*Kabuli*	Ca_Kabuli_Chr06	10652739	[C/T]	Ca_08531	AMP-dependent synthetase/ligase	INTRON
10028	CakSNP10028	*Kabuli*	Ca_Kabuli_Chr06	10667504	[A/T]		--	INTERGENIC
10029	CakSNP10029	*Kabuli*	Ca_Kabuli_Chr06	10667559	[T/C]		--	INTERGENIC
10030	CakSNP10030	*Kabuli*	Ca_Kabuli_Chr06	10667640	[T/G]	Ca_08530	Aspartate/glutamate/uridylate kinase	SYNONYMOUS_CODING
10031	CakSNP10031	*Kabuli*	Ca_Kabuli_Chr06	10670343	[G/A]	Ca_08530	Aspartate/glutamate/uridylate kinase	INTRON

S.N.	SNP IDs	Chickpea cultivars	Chromosomes	Physical positions (bp)	SNPs	Gene accession IDs	Putative functions	Sequence components of genome
10032	CakSNP10032	*Kabuli*	Ca_Kabuli_Chr06	10670368	[G/A]	Ca_08530	Aspartate/glutamate/uridylate kinase	INTRON
10033	CakSNP10033	*Kabuli*	Ca_Kabuli_Chr06	10670409	[G/A]	Ca_08530	Aspartate/glutamate/uridylate kinase	INTRON
10034	CakSNP10034	*Kabuli*	Ca_Kabuli_Chr06	10670412	[A/C]	Ca_08530	Aspartate/glutamate/uridylate kinase	INTRON
10035	CakSNP10035	*Kabuli*	Ca_Kabuli_Chr06	10670482	[C/T]	Ca_08530	Aspartate/glutamate/uridylate kinase	INTRON
10036	CakSNP10036	*Kabuli*	Ca_Kabuli_Chr06	10670582	[G/A]	Ca_08530	Aspartate/glutamate/uridylate kinase	INTRON
10037	CakSNP10037	*Kabuli*	Ca_Kabuli_Chr06	10670675	[C/T]	Ca_08530	Aspartate/glutamate/uridylate kinase	INTRON
10038	CakSNP10038	*Kabuli*	Ca_Kabuli_Chr06	10670694	[G/A]	Ca_08530	Aspartate/glutamate/uridylate kinase	INTRON
10039	CakSNP10039	*Kabuli*	Ca_Kabuli_Chr06	10670773	[A/C]	Ca_08530	Aspartate/glutamate/uridylate kinase	INTRON
10040	CakSNP10040	*Kabuli*	Ca_Kabuli_Chr06	10670959	[T/C]	Ca_08530	Aspartate/glutamate/uridylate kinase	INTRON
10041	CakSNP10041	*Kabuli*	Ca_Kabuli_Chr06	10671458	[T/C]	Ca_08530	Aspartate/glutamate/uridylate kinase	INTRON

S.N.	SNP IDs	Chickpea cultivars	Chromosomes	Physical positions (bp)	SNPs	Gene accession IDs	Putative functions	Sequence components of genome
10042	CakSNP10042	*Kabuli*	Ca_Kabuli_Chr06	10671444	[A/C]	Ca_08530	Aspartate/glutamate/uridylate kinase	INTRON
10043	CakSNP10043	*Kabuli*	Ca_Kabuli_Chr06	10672468	[C/T]	Ca_08530	Aspartate/glutamate/uridylate kinase	INTRON
10044	CakSNP10044	*Kabuli*	Ca_Kabuli_Chr06	10677469	[A/G]	Ca_08530	Aspartate/glutamate/uridylate kinase	INTRON
10045	CakSNP10045	*Kabuli*	Ca_Kabuli_Chr06	10677501	[C/T]	Ca_08530	Aspartate/glutamate/uridylate kinase	INTRON
10046	CakSNP10046	*Kabuli*	Ca_Kabuli_Chr06	10682221	[G/T]	Ca_08529	Peptidase S8/S53, subtilisin/kexin/sedolisin	SYNONYMOUS_CODING
10047	CakSNP10047	*Kabuli*	Ca_Kabuli_Chr06	10682201	[C/G]	Ca_08529	Peptidase S8/S53, subtilisin/kexin/sedolisin	SYNONYMOUS_CODING
10048	CakSNP10048	*Kabuli*	Ca_Kabuli_Chr06	10683193	[C/T]	Ca_08529	Peptidase S8/S53, subtilisin/kexin/sedolisin	SYNONYMOUS_CODING
10049	CakSNP10049	*Kabuli*	Ca_Kabuli_Chr06	10696986	[A/T]	Ca_08528	Protein kinase, catalytic domain	INTRON
10050	CakSNP10050	*Kabuli*	Ca_Kabuli_Chr06	10696990	[T/C]	Ca_08528	Protein kinase, catalytic domain	INTRON
10051	CakSNP10051	*Kabuli*	Ca_Kabuli_Chr06	10697064	[T/A]	Ca_08528	Protein kinase, catalytic domain	INTRON

S.N.	SNP IDs	Chickpea cultivars	Chromosomes	Physical positions (bp)	SNPs	Gene accession IDs	Putative functions	Sequence components of genome
10052	CakSNP10052	*Kabuli*	Ca_Kabuli_Chr06	10716986	[G/A]	Ca_08526	--	SYNONYMOUS_CODING
10053	CakSNP10053	*Kabuli*	Ca_Kabuli_Chr06	10716968	[G/A]	Ca_08526	--	SYNONYMOUS_CODING
10054	CakSNP10054	*Kabuli*	Ca_Kabuli_Chr06	10744029	[C/A]		--	INTERGENIC
10055	CakSNP10055	*Kabuli*	Ca_Kabuli_Chr06	10743996	[A/T]		--	INTERGENIC
10056	CakSNP10056	*Kabuli*	Ca_Kabuli_Chr06	10748965	[G/T]	Ca_08523	--	NON_SYNONYMOUS_CODING
10057	CakSNP10057	*Kabuli*	Ca_Kabuli_Chr06	10779368	[G/C]	Ca_08519	Kinesin, motor domain	NON_SYNONYMOUS_CODING
10058	CakSNP10058	*Kabuli*	Ca_Kabuli_Chr06	10779465	[A/T]	Ca_08519	Kinesin, motor domain	NON_SYNONYMOUS_CODING
10059	CakSNP10059	*Kabuli*	Ca_Kabuli_Chr06	10779547	[T/G]	Ca_08519	Kinesin, motor domain	NON_SYNONYMOUS_CODING
10060	CakSNP10060	*Kabuli*	Ca_Kabuli_Chr06	10779636	[C/A]	Ca_08519	Kinesin, motor domain	NON_SYNONYMOUS_CODING
10061	CakSNP10061	*Kabuli*	Ca_Kabuli_Chr06	10785480	[T/G]		--	INTERGENIC

S.N.	SNP IDs	Chickpea cultivars	Chromosomes	Physical positions (bp)	SNPs	Gene accession IDs	Putative functions	Sequence components of genome
10062	CakSNP10062	*Kabuli*	Ca_Kabuli_Chr06	10870187	[T/G]	Ca_08511	IQ motif, EF-hand binding site	NON_SYNONYMOUS_CODING
10063	CakSNP10063	*Kabuli*	Ca_Kabuli_Chr06	10870534	[A/G]	Ca_08511	IQ motif, EF-hand binding site	SYNONYMOUS_CODING
10064	CakSNP10064	*Kabuli*	Ca_Kabuli_Chr06	10880791	[C/T]	Ca_08509	Signal transduction response regulator, receiver domain	INTRON
10065	CakSNP10065	*Kabuli*	Ca_Kabuli_Chr06	10880819	[T/G]	Ca_08509	Signal transduction response regulator, receiver domain	INTRON
10066	CakSNP10066	*Kabuli*	Ca_Kabuli_Chr06	10881002	[A/G]	Ca_08509	Signal transduction response regulator, receiver domain	INTRON
10067	CakSNP10067	*Kabuli*	Ca_Kabuli_Chr06	10880985	[C/T]	Ca_08509	Signal transduction response regulator, receiver domain	INTRON
10068	CakSNP10068	*Kabuli*	Ca_Kabuli_Chr06	10880962	[G/T]	Ca_08509	Signal transduction response regulator, receiver domain	INTRON
10069	CakSNP10069	*Kabuli*	Ca_Kabuli_Chr06	10991652	[C/A]	Ca_20467	Zinc finger, MYND-type	NON_SYNONYMOUS_CODING
10070	CakSNP10070	*Kabuli*	Ca_Kabuli_Chr06	10991717	[T/A]	Ca_20467	Zinc finger, MYND-type	NON_SYNONYMOUS_CODING
10071	CakSNP10071	*Kabuli*	Ca_Kabuli_Chr06	10991737	[A/C]	Ca_20467	Zinc finger, MYND-type	SYNONYMOUS_CODING

S.N.	SNP IDs	Chickpea cultivars	Chromosomes	Physical positions (bp)	SNPs	Gene accession IDs	Putative functions	Sequence components of genome
10072	CakSNP10072	*Kabuli*	Ca_Kabuli_Chr06	11011847	[C/G]	Ca_20469	Rubber elongation factor	NON_SYNONYMOUS_CODING
10073	CakSNP10073	*Kabuli*	Ca_Kabuli_Chr06	11128158	[A/G]	Ca_20479	Glycoside hydrolase, family 63	INTRON
10074	CakSNP10074	*Kabuli*	Ca_Kabuli_Chr06	11128170	[T/C]	Ca_20479	Glycoside hydrolase, family 63	INTRON
10075	CakSNP10075	*Kabuli*	Ca_Kabuli_Chr06	11128204	[G/A]	Ca_20479	Glycoside hydrolase, family 63	INTRON
10076	CakSNP10076	*Kabuli*	Ca_Kabuli_Chr06	11131010	[T/A]	Ca_20479	Glycoside hydrolase, family 63	INTRON
10077	CakSNP10077	*Kabuli*	Ca_Kabuli_Chr06	11131464	[G/A]	Ca_20479	Glycoside hydrolase, family 63	INTRON
10078	CakSNP10078	*Kabuli*	Ca_Kabuli_Chr06	11131462	[G/A]	Ca_20479	Glycoside hydrolase, family 63	INTRON
10079	CakSNP10079	*Kabuli*	Ca_Kabuli_Chr06	11131436	[T/G]	Ca_20479	Glycoside hydrolase, family 63	INTRON
10080	CakSNP10080	*Kabuli*	Ca_Kabuli_Chr06	11163819	[T/G]	Ca_20482	Helicase, C-terminal	INTRON
10081	CakSNP10081	*Kabuli*	Ca_Kabuli_Chr06	11163822	[C/T]	Ca_20482	Helicase, C-terminal	INTRON

S.N.	SNP IDs	Chickpea cultivars	Chromosomes	Physical positions (bp)	SNPs	Gene accession IDs	Putative functions	Sequence components of genome
10082	CakSNP10082	*Kabuli*	Ca_Kabuli_Chr06	11163836	[C/T]	Ca_20482	Helicase, C-terminal	INTRON
10083	CakSNP10083	*Kabuli*	Ca_Kabuli_Chr06	11188803	[G/A]	Ca_20485	Protein kinase, catalytic domain	NON_SYNONYMOUS_CODING
10084	CakSNP10084	*Kabuli*	Ca_Kabuli_Chr06	11189051	[A/G]	Ca_20485	Protein kinase, catalytic domain	SYNONYMOUS_CODING
10085	CakSNP10085	*Kabuli*	Ca_Kabuli_Chr06	11229102	[C/T]	Ca_20489	Ankyrin repeat	SYNONYMOUS_CODING
10086	CakSNP10086	*Kabuli*	Ca_Kabuli_Chr06	11229143	[A/C]	Ca_20489	Ankyrin repeat	NON_SYNONYMOUS_CODING
10087	CakSNP10087	*Kabuli*	Ca_Kabuli_Chr06	11229302	[A/G]	Ca_20489	Ankyrin repeat	NON_SYNONYMOUS_CODING
10088	CakSNP10088	*Kabuli*	Ca_Kabuli_Chr06	11229388	[C/T]	Ca_20489	Ankyrin repeat	NON_SYNONYMOUS_CODING
10089	CakSNP10089	*Kabuli*	Ca_Kabuli_Chr06	11285884	[G/A]		--	INTERGENIC
10090	CakSNP10090	*Kabuli*	Ca_Kabuli_Chr06	11300597	[C/T]	Ca_20493	Signal recognition particle, SRP72 subunit, RNA-binding	SYNONYMOUS_CODING
10091	CakSNP10091	*Kabuli*	Ca_Kabuli_Chr06	11300549	[A/C]	Ca_20493	Signal recognition particle, SRP72 subunit, RNA-binding	SYNONYMOUS_CODING

S.N.	SNP IDs	Chickpea cultivars	Chromosomes	Physical positions (bp)	SNPs	Gene accession IDs	Putative functions	Sequence components of genome
10092	CakSNP10092	*Kabuli*	Ca_Kabuli_Chr06	11302037	[A/C]	Ca_20493	Signal recognition particle, SRP72 subunit, RNA-binding	NON_SYNONYMOUS_CODING
10093	CakSNP10093	*Kabuli*	Ca_Kabuli_Chr06	11302033	[T/C]	Ca_20493	Signal recognition particle, SRP72 subunit, RNA-binding	NON_SYNONYMOUS_CODING
10094	CakSNP10094	*Kabuli*	Ca_Kabuli_Chr06	11314581	[T/C]	Ca_20494	Pentatricopeptide repeat	SYNONYMOUS_CODING
10095	CakSNP10095	*Kabuli*	Ca_Kabuli_Chr06	11394963	[A/G]	Ca_23423	Zinc finger, MYND-type	NON_SYNONYMOUS_CODING
10096	CakSNP10096	*Kabuli*	Ca_Kabuli_Chr06	11395330	[C/T]	Ca_23423	Zinc finger, MYND-type	INTRON
10097	CakSNP10097	*Kabuli*	Ca_Kabuli_Chr06	11508828	[C/T]	Ca_23431	Heat shock protein DnaJ, N-terminal	NON_SYNONYMOUS_CODING
10098	CakSNP10098	*Kabuli*	Ca_Kabuli_Chr06	11508824	[T/G]	Ca_23431	Heat shock protein DnaJ, N-terminal	STOP_GAINED
10099	CakSNP10099	*Kabuli*	Ca_Kabuli_Chr06	11508817	[G/A]	Ca_23431	Heat shock protein DnaJ, N-terminal	NON_SYNONYMOUS_CODING
10100	CakSNP10100	*Kabuli*	Ca_Kabuli_Chr06	11508816	[A/C]	Ca_23431	Heat shock protein DnaJ, N-terminal	NON_SYNONYMOUS_CODING
10101	CakSNP10101	*Kabuli*	Ca_Kabuli_Chr06	11514926	[T/C]	Ca_23432	--	NON_SYNONYMOUS_CODING

S.N.	SNP IDs	Chickpea cultivars	Chromosomes	Physical positions (bp)	SNPs	Gene accession IDs	Putative functions	Sequence components of genome
10102	CakSNP10102	*Kabuli*	Ca_Kabuli_Chr06	11514883	[C/T]	Ca_23432	--	NON_SYNONYMOUS_CODING
10103	CakSNP10103	*Kabuli*	Ca_Kabuli_Chr06	11514935	[G/A]	Ca_23432	--	SYNONYMOUS_CODING
10104	CakSNP10104	*Kabuli*	Ca_Kabuli_Chr06	11543615	[G/C]		--	INTERGENIC
10105	CakSNP10105	*Kabuli*	Ca_Kabuli_Chr06	11543618	[T/G]		--	INTERGENIC
10106	CakSNP10106	*Kabuli*	Ca_Kabuli_Chr06	11665233	[G/A]	Ca_25061	Amine oxidase	SYNONYMOUS_CODING
10107	CakSNP10107	*Kabuli*	Ca_Kabuli_Chr06	11665338	[T/G]	Ca_25061	Amine oxidase	SYNONYMOUS_CODING
10108	CakSNP10108	*Kabuli*	Ca_Kabuli_Chr06	12408955	[A/G]		--	INTERGENIC
10109	CakSNP10109	*Kabuli*	Ca_Kabuli_Chr06	12408964	[G/C]		--	INTERGENIC
10110	CakSNP10110	*Kabuli*	Ca_Kabuli_Chr06	12434183	[G/T]	Ca_05025	AUX/IAA protein	INTRON
10111	CakSNP10111	*Kabuli*	Ca_Kabuli_Chr06	12437275	[A/T]	Ca_05025	AUX/IAA protein	NON_SYNONYMOUS_CODING

S.N.	SNP IDs	Chickpea cultivars	Chromosomes	Physical positions (bp)	SNPs	Gene accession IDs	Putative functions	Sequence components of genome
10112	CakSNP10112	*Kabuli*	Ca_Kabuli_Chr06	12437285	[A/G]	Ca_05025	AUX/IAA protein	SYNONYMOUS_CODING
10113	CakSNP10113	*Kabuli*	Ca_Kabuli_Chr06	12437288	[G/A]	Ca_05025	AUX/IAA protein	SYNONYMOUS_CODING
10114	CakSNP10114	*Kabuli*	Ca_Kabuli_Chr06	12437319	[C/T]	Ca_05025	AUX/IAA protein	STOP_GAINED
10115	CakSNP10115	*Kabuli*	Ca_Kabuli_Chr06	12494824	[G/T]	Ca_05028	Zinc finger, C3HC-like	INTRON
10116	CakSNP10116	*Kabuli*	Ca_Kabuli_Chr06	12494972	[A/G]	Ca_05028	Zinc finger, C3HC-like	INTRON
10117	CakSNP10117	*Kabuli*	Ca_Kabuli_Chr06	12532947	[T/A]		--	INTERGENIC
10118	CakSNP10118	*Kabuli*	Ca_Kabuli_Chr06	12535733	[C/G]	Ca_05031	Sugar/inositol transporter	NON_SYNONYMOUS_CODING
10119	CakSNP10119	*Kabuli*	Ca_Kabuli_Chr06	12619757	[C/T]	Ca_05038	FMN-dependent dehydrogenase	INTRON
10120	CakSNP10120	*Kabuli*	Ca_Kabuli_Chr06	12619786	[C/A]	Ca_05038	FMN-dependent dehydrogenase	INTRON
10121	CakSNP10121	*Kabuli*	Ca_Kabuli_Chr06	12619829	[A/T]	Ca_05038	FMN-dependent dehydrogenase	INTRON

S.N.	SNP IDs	Chickpea cultivars	Chromosomes	Physical positions (bp)	SNPs	Gene accession IDs	Putative functions	Sequence components of genome
10122	CakSNP10122	*Kabuli*	Ca_Kabuli_Chr06	12647539	[G/A]	Ca_05041	Transketolase-like, pyrimidine-binding domain	SYNONYMOUS_CODING
10123	CakSNP10123	*Kabuli*	Ca_Kabuli_Chr06	12799890	[T/G]	Ca_05052	Protein of unknown function DUF2359, TMEM214	NON_SYNONYMOUS_CODING
10124	CakSNP10124	*Kabuli*	Ca_Kabuli_Chr06	12844524	[G/A]	Ca_05057	Protein of unknown function DUF593	SYNONYMOUS_CODING
10125	CakSNP10125	*Kabuli*	Ca_Kabuli_Chr06	12893335	[C/A]		--	INTERGENIC
10126	CakSNP10126	*Kabuli*	Ca_Kabuli_Chr06	12893424	[C/T]		--	INTERGENIC
10127	CakSNP10127	*Kabuli*	Ca_Kabuli_Chr06	12906599	[G/A]	Ca_05064	Protein kinase, catalytic domain	INTRON
10128	CakSNP10128	*Kabuli*	Ca_Kabuli_Chr06	12906680	[T/C]	Ca_05064	Protein kinase, catalytic domain	INTRON
10129	CakSNP10129	*Kabuli*	Ca_Kabuli_Chr06	12906669	[G/A]	Ca_05064	Protein kinase, catalytic domain	INTRON
10130	CakSNP10130	*Kabuli*	Ca_Kabuli_Chr06	12973765	[A/G]		--	INTERGENIC
10131	CakSNP10131	*Kabuli*	Ca_Kabuli_Chr06	12976279	[T/A]		--	INTERGENIC

S.N.	SNP IDs	Chickpea cultivars	Chromosomes	Physical positions (bp)	SNPs	Gene accession IDs	Putative functions	Sequence components of genome
10132	CakSNP10132	*Kabuli*	Ca_Kabuli_Chr06	12977129	[A/G]		--	INTERGENIC
10133	CakSNP10133	*Kabuli*	Ca_Kabuli_Chr06	12977196	[A/G]		--	INTERGENIC
10134	CakSNP10134	*Kabuli*	Ca_Kabuli_Chr06	12977204	[T/G]		--	INTERGENIC
10135	CakSNP10135	*Kabuli*	Ca_Kabuli_Chr06	12985896	[A/C]	Ca_05072	Polyprenyl synthetase	NON_SYNONYMOUS_CODING
10136	CakSNP10136	*Kabuli*	Ca_Kabuli_Chr06	12985908	[C/G]	Ca_05072	Polyprenyl synthetase	NON_SYNONYMOUS_CODING
10137	CakSNP10137	*Kabuli*	Ca_Kabuli_Chr06	12985910	[C/T]	Ca_05072	Polyprenyl synthetase	SYNONYMOUS_CODING
10138	CakSNP10138	*Kabuli*	Ca_Kabuli_Chr06	12986776	[A/G]	Ca_05072	Polyprenyl synthetase	INTRON
10139	CakSNP10139	*Kabuli*	Ca_Kabuli_Chr06	12986833	[A/C]	Ca_05072	Polyprenyl synthetase	INTRON
10140	CakSNP10140	*Kabuli*	Ca_Kabuli_Chr06	13008333	[A/T]		--	INTERGENIC
10141	CakSNP10141	*Kabuli*	Ca_Kabuli_Chr06	13008360	[C/T]		--	INTERGENIC

S.N.	SNP IDs	Chickpea cultivars	Chromosomes	Physical positions (bp)	SNPs	Gene accession IDs	Putative functions	Sequence components of genome
10142	CakSNP10142	*Kabuli*	Ca_Kabuli_Chr06	13008465	[C/T]		--	INTERGENIC
10143	CakSNP10143	*Kabuli*	Ca_Kabuli_Chr06	13099658	[G/T]		--	INTERGENIC
10144	CakSNP10144	*Kabuli*	Ca_Kabuli_Chr06	13099656	[G/T]		--	INTERGENIC
10145	CakSNP10145	*Kabuli*	Ca_Kabuli_Chr06	13107887	[C/T]	Ca_05084	Protein kinase, catalytic domain	SYNONYMOUS_CODING
10146	CakSNP10146	*Kabuli*	Ca_Kabuli_Chr06	13117820	[A/T]		--	INTERGENIC
10147	CakSNP10147	*Kabuli*	Ca_Kabuli_Chr06	13131682	[C/G]	Ca_05086	Cyclic nucleotide-binding domain	NON_SYNONYMOUS_CODING
10148	CakSNP10148	*Kabuli*	Ca_Kabuli_Chr06	13136852	[C/T]	Ca_05086	Cyclic nucleotide-binding domain	INTRON
10149	CakSNP10149	*Kabuli*	Ca_Kabuli_Chr06	13136835	[C/T]	Ca_05086	Cyclic nucleotide-binding domain	INTRON
10150	CakSNP10150	*Kabuli*	Ca_Kabuli_Chr06	13136976	[A/G]	Ca_05086	Cyclic nucleotide-binding domain	INTRON
10151	CakSNP10151	*Kabuli*	Ca_Kabuli_Chr06	13136955	[G/A]	Ca_05086	Cyclic nucleotide-binding domain	INTRON

S.N.	SNP IDs	Chickpea cultivars	Chromosomes	Physical positions (bp)	SNPs	Gene accession IDs	Putative functions	Sequence components of genome
10152	CakSNP10152	*Kabuli*	Ca_Kabuli_Chr06	13187706	[A/G]	Ca_05090	Oligopeptide transporter	NON_SYNONYMOUS_CODING
10153	CakSNP10153	*Kabuli*	Ca_Kabuli_Chr06	13187709	[T/C]	Ca_05090	Oligopeptide transporter	NON_SYNONYMOUS_CODING
10154	CakSNP10154	*Kabuli*	Ca_Kabuli_Chr06	13188124	[G/C]	Ca_05090	Oligopeptide transporter	SYNONYMOUS_CODING
10155	CakSNP10155	*Kabuli*	Ca_Kabuli_Chr06	13188119	[A/C]	Ca_05090	Oligopeptide transporter	NON_SYNONYMOUS_CODING
10156	CakSNP10156	*Kabuli*	Ca_Kabuli_Chr06	13188480	[T/C]	Ca_05090	Oligopeptide transporter	INTRON
10157	CakSNP10157	*Kabuli*	Ca_Kabuli_Chr06	13192177	[G/A]	Ca_05091	Oligopeptide transporter	NON_SYNONYMOUS_CODING
10158	CakSNP10158	*Kabuli*	Ca_Kabuli_Chr06	13243282	[A/G]	Ca_05097	Zinc finger, CCCH-type	SYNONYMOUS_CODING
10159	CakSNP10159	*Kabuli*	Ca_Kabuli_Chr06	13252152	[C/T]	Ca_05098	Remorin, C-terminal	NON_SYNONYMOUS_CODING
10160	CakSNP10160	*Kabuli*	Ca_Kabuli_Chr06	13296128	[G/A]	Ca_05103	DNA helicase, UvrD/REP type	NON_SYNONYMOUS_CODING
10161	CakSNP10161	*Kabuli*	Ca_Kabuli_Chr06	13326969	[C/G]	Ca_05104	Zinc finger, RING-type	NON_SYNONYMOUS_CODING

S.N.	SNP IDs	Chickpea cultivars	Chromosomes	Physical positions (bp)	SNPs	Gene accession IDs	Putative functions	Sequence components of genome
10162	CakSNP10162	*Kabuli*	Ca_Kabuli_Chr06	13340012	[A/G]	Ca_05106	Protein kinase, catalytic domain	SYNONYMOUS_CODING
10163	CakSNP10163	*Kabuli*	Ca_Kabuli_Chr06	13340757	[T/C]	Ca_05106	Protein kinase, catalytic domain	SYNONYMOUS_CODING
10164	CakSNP10164	*Kabuli*	Ca_Kabuli_Chr06	13340762	[G/A]	Ca_05106	Protein kinase, catalytic domain	SYNONYMOUS_CODING
10165	CakSNP10165	*Kabuli*	Ca_Kabuli_Chr06	13340782	[G/A]	Ca_05106	Protein kinase, catalytic domain	NON_SYNONYMOUS_CODING
10166	CakSNP10166	*Kabuli*	Ca_Kabuli_Chr06	13413848	[C/G]		--	INTERGENIC
10167	CakSNP10167	*Kabuli*	Ca_Kabuli_Chr06	13483841	[T/G]		--	INTERGENIC
10168	CakSNP10168	*Kabuli*	Ca_Kabuli_Chr06	13565854	[T/C]	Ca_05127	HECT	SYNONYMOUS_CODING
10169	CakSNP10169	*Kabuli*	Ca_Kabuli_Chr06	13565990	[C/T]	Ca_05127	HECT	NON_SYNONYMOUS_CODING
10170	CakSNP10170	*Kabuli*	Ca_Kabuli_Chr06	13566007	[C/T]	Ca_05127	HECT	SYNONYMOUS_CODING
10171	CakSNP10171	*Kabuli*	Ca_Kabuli_Chr06	13566012	[T/G]	Ca_05127	HECT	NON_SYNONYMOUS_CODING

S.N.	SNP IDs	Chickpea cultivars	Chromosomes	Physical positions (bp)	SNPs	Gene accession IDs	Putative functions	Sequence components of genome
10172	CakSNP10172	*Kabuli*	Ca_Kabuli_Chr06	13566041	[C/T]	Ca_05127	HECT	NON_SYNONYMOUS_CODING
10173	CakSNP10173	*Kabuli*	Ca_Kabuli_Chr06	13566110	[A/G]	Ca_05127	HECT	NON_SYNONYMOUS_CODING
10174	CakSNP10174	*Kabuli*	Ca_Kabuli_Chr06	13566136	[A/C]	Ca_05127	HECT	SYNONYMOUS_CODING
10175	CakSNP10175	*Kabuli*	Ca_Kabuli_Chr06	13566130	[G/T]	Ca_05127	HECT	SYNONYMOUS_CODING
10176	CakSNP10176	*Kabuli*	Ca_Kabuli_Chr06	13572236	[A/T]	Ca_05127	HECT	INTRON
10177	CakSNP10177	*Kabuli*	Ca_Kabuli_Chr06	13572221	[A/C]	Ca_05127	HECT	INTRON
10178	CakSNP10178	*Kabuli*	Ca_Kabuli_Chr06	13576963	[T/A]	Ca_05127	HECT	INTRON
10179	CakSNP10179	*Kabuli*	Ca_Kabuli_Chr06	13591590	[T/G]		--	INTERGENIC
10180	CakSNP10180	*Kabuli*	Ca_Kabuli_Chr06	13593544	[A/G]		--	INTERGENIC
10181	CakSNP10181	*Kabuli*	Ca_Kabuli_Chr06	13593564	[C/A]		--	INTERGENIC

S.N.	SNP IDs	Chickpea cultivars	Chromosomes	Physical positions (bp)	SNPs	Gene accession IDs	Putative functions	Sequence components of genome
10182	CakSNP10182	*Kabuli*	Ca_Kabuli_Chr06	13593600	[T/C]		--	INTERGENIC
10183	CakSNP10183	*Kabuli*	Ca_Kabuli_Chr06	13593685	[G/A]		--	INTERGENIC
10184	CakSNP10184	*Kabuli*	Ca_Kabuli_Chr06	13593668	[A/G]		--	INTERGENIC
10185	CakSNP10185	*Kabuli*	Ca_Kabuli_Chr06	13593661	[T/C]		--	INTERGENIC
10186	CakSNP10186	*Kabuli*	Ca_Kabuli_Chr06	13593654	[T/C]		--	INTERGENIC
10187	CakSNP10187	*Kabuli*	Ca_Kabuli_Chr06	13619538	[C/T]	Ca_05131	HECT	NON_SYNONYMOUS_CODING
10188	CakSNP10188	*Kabuli*	Ca_Kabuli_Chr06	13619555	[C/T]	Ca_05131	HECT	SYNONYMOUS_CODING
10189	CakSNP10189	*Kabuli*	Ca_Kabuli_Chr06	13619560	[T/G]	Ca_05131	HECT	NON_SYNONYMOUS_CODING
10190	CakSNP10190	*Kabuli*	Ca_Kabuli_Chr06	13619589	[C/T]	Ca_05131	HECT	NON_SYNONYMOUS_CODING
10191	CakSNP10191	*Kabuli*	Ca_Kabuli_Chr06	13619658	[A/G]	Ca_05131	HECT	NON_SYNONYMOUS_CODING

S.N.	SNP IDs	Chickpea cultivars	Chromosomes	Physical positions (bp)	SNPs	Gene accession IDs	Putative functions	Sequence components of genome
10192	CakSNP10192	*Kabuli*	Ca_Kabuli_Chr06	13619684	[A/C]	Ca_05131	HECT	SYNONYMOUS_CODING
10193	CakSNP10193	*Kabuli*	Ca_Kabuli_Chr06	13622292	[C/T]	Ca_05131	HECT	NON_SYNONYMOUS_CODING
10194	CakSNP10194	*Kabuli*	Ca_Kabuli_Chr06	13625388	[A/G]		--	INTERGENIC
10195	CakSNP10195	*Kabuli*	Ca_Kabuli_Chr06	13637387	[T/G]		--	INTERGENIC
10196	CakSNP10196	*Kabuli*	Ca_Kabuli_Chr06	13637347	[C/T]		--	INTERGENIC
10197	CakSNP10197	*Kabuli*	Ca_Kabuli_Chr06	13668916	[T/A]		--	INTERGENIC
10198	CakSNP10198	*Kabuli*	Ca_Kabuli_Chr06	13668995	[A/G]		--	INTERGENIC
10199	CakSNP10199	*Kabuli*	Ca_Kabuli_Chr06	13669052	[G/C]		--	INTERGENIC
10200	CakSNP10200	*Kabuli*	Ca_Kabuli_Chr06	13669049	[C/T]		--	INTERGENIC
10201	CakSNP10201	*Kabuli*	Ca_Kabuli_Chr06	13669048	[T/A]		--	INTERGENIC

S.N.	SNP IDs	Chickpea cultivars	Chromosomes	Physical positions (bp)	SNPs	Gene accession IDs	Putative functions	Sequence components of genome
10202	CakSNP10202	*Kabuli*	Ca_Kabuli_Chr06	13669042	[A/G]		--	INTERGENIC
10203	CakSNP10203	*Kabuli*	Ca_Kabuli_Chr06	13669018	[G/A]		--	INTERGENIC
10204	CakSNP10204	*Kabuli*	Ca_Kabuli_Chr06	13669006	[G/C]		--	INTERGENIC
10205	CakSNP10205	*Kabuli*	Ca_Kabuli_Chr06	13714897	[A/T]	Ca_05136	Peptidase M41	SYNONYMOUS_CODING
10206	CakSNP10206	*Kabuli*	Ca_Kabuli_Chr06	13764980	[T/A]	Ca_05139	SNF2-related	INTRON
10207	CakSNP10207	*Kabuli*	Ca_Kabuli_Chr06	13827484	[C/T]	Ca_05146	Aspartate carbamoyltransferase, eukaryotic	SYNONYMOUS_CODING
10208	CakSNP10208	*Kabuli*	Ca_Kabuli_Chr06	13827452	[C/G]	Ca_05146	Aspartate carbamoyltransferase, eukaryotic	INTRON
10209	CakSNP10209	*Kabuli*	Ca_Kabuli_Chr06	13827444	[G/A]	Ca_05146	Aspartate carbamoyltransferase, eukaryotic	INTRON
10210	CakSNP10210	*Kabuli*	Ca_Kabuli_Chr06	13913539	[A/G]	Ca_05156	DNA-dependent ATPase MCM	INTRON
10211	CakSNP10211	*Kabuli*	Ca_Kabuli_Chr06	13917076	[T/C]	Ca_05156	DNA-dependent ATPase MCM	SYNONYMOUS_CODING

S.N.	SNP IDs	Chickpea cultivars	Chromosomes	Physical positions (bp)	SNPs	Gene accession IDs	Putative functions	Sequence components of genome
10212	CakSNP10212	*Kabuli*	Ca_Kabuli_Chr06	13917135	[T/A]	Ca_05156	DNA-dependent ATPase MCM	NON_SYNONYMOUS_CODING
10213	CakSNP10213	*Kabuli*	Ca_Kabuli_Chr06	13921954	[A/T]	Ca_05157	Beta-ketoacyl synthase	SYNONYMOUS_CODING
10214	CakSNP10214	*Kabuli*	Ca_Kabuli_Chr06	13941701	[A/T]	Ca_05159	Domain of unknown function DUF1084	INTRON
10215	CakSNP10215	*Kabuli*	Ca_Kabuli_Chr06	13942961	[A/G]		--	INTERGENIC
10216	CakSNP10216	*Kabuli*	Ca_Kabuli_Chr06	13948732	[G/T]		--	INTERGENIC
10217	CakSNP10217	*Kabuli*	Ca_Kabuli_Chr06	13948751	[T/G]		--	INTERGENIC
10218	CakSNP10218	*Kabuli*	Ca_Kabuli_Chr06	13948778	[A/C]		--	INTERGENIC
10219	CakSNP10219	*Kabuli*	Ca_Kabuli_Chr06	13986559	[A/G]	Ca_05163	Uncharacterised protein family Ycf23	SYNONYMOUS_CODING
10220	CakSNP10220	*Kabuli*	Ca_Kabuli_Chr06	14042859	[A/T]		--	INTERGENIC
10221	CakSNP10221	*Kabuli*	Ca_Kabuli_Chr06	14067312	[A/C]		--	INTERGENIC

S.N.	SNP IDs	Chickpea cultivars	Chromosomes	Physical positions (bp)	SNPs	Gene accession IDs	Putative functions	Sequence components of genome
10222	CakSNP10222	*Kabuli*	Ca_Kabuli_Chr06	14067326	[C/T]		--	INTERGENIC
10223	CakSNP10223	*Kabuli*	Ca_Kabuli_Chr06	14112542	[A/G]	Ca_05177	Stress up-regulated Nod 19	INTRON
10224	CakSNP10224	*Kabuli*	Ca_Kabuli_Chr06	14148771	[A/G]		--	INTERGENIC
10225	CakSNP10225	*Kabuli*	Ca_Kabuli_Chr06	14155543	[C/T]	Ca_05182	N-acetylglucosaminyl phosphatidylinositol deacetylase	INTRON
10226	CakSNP10226	*Kabuli*	Ca_Kabuli_Chr06	14155662	[T/C]	Ca_05182	N-acetylglucosaminyl phosphatidylinositol deacetylase	INTRON
10227	CakSNP10227	*Kabuli*	Ca_Kabuli_Chr06	14155643	[A/C]	Ca_05182	N-acetylglucosaminyl phosphatidylinositol deacetylase	INTRON
10228	CakSNP10228	*Kabuli*	Ca_Kabuli_Chr06	14161084	[G/C]	Ca_05182	N-acetylglucosaminyl phosphatidylinositol deacetylase	INTRON
10229	CakSNP10229	*Kabuli*	Ca_Kabuli_Chr06	14201001	[T/C]	Ca_05188	FAD linked oxidase, N-terminal	NON_SYNONYMOUS_CODING
10230	CakSNP10230	*Kabuli*	Ca_Kabuli_Chr06	14200997	[G/A]	Ca_05188	FAD linked oxidase, N-terminal	SYNONYMOUS_CODING
10231	CakSNP10231	*Kabuli*	Ca_Kabuli_Chr06	14204400	[T/C]		--	INTERGENIC

S.N.	SNP IDs	Chickpea cultivars	Chromosomes	Physical positions (bp)	SNPs	Gene accession IDs	Putative functions	Sequence components of genome
10232	CakSNP10232	*Kabuli*	Ca_Kabuli_Chr06	14204469	[G/A]		--	INTERGENIC
10233	CakSNP10233	*Kabuli*	Ca_Kabuli_Chr06	14254394	[A/G]	Ca_05194	Protein of unknown function DUF858, methyltransferase-like	INTRON
10234	CakSNP10234	*Kabuli*	Ca_Kabuli_Chr06	14309042	[C/T]	Ca_05200	Domain of unknown function DUF640	NON_SYNONYMOUS_CODING
10235	CakSNP10235	*Kabuli*	Ca_Kabuli_Chr06	14330533	[T/C]	Ca_05203	Protein kinase, catalytic domain	SYNONYMOUS_CODING
10236	CakSNP10236	*Kabuli*	Ca_Kabuli_Chr06	14332018	[A/C]	Ca_05203	Protein kinase, catalytic domain	NON_SYNONYMOUS_CODING
10237	CakSNP10237	*Kabuli*	Ca_Kabuli_Chr06	14332404	[A/G]	Ca_05203	Protein kinase, catalytic domain	INTRON
10238	CakSNP10238	*Kabuli*	Ca_Kabuli_Chr06	14353598	[C/T]		--	INTERGENIC
10239	CakSNP10239	*Kabuli*	Ca_Kabuli_Chr06	14353624	[G/A]		--	INTERGENIC
10240	CakSNP10240	*Kabuli*	Ca_Kabuli_Chr06	14371493	[C/T]	Ca_05207	Sec1-like protein	SYNONYMOUS_CODING
10241	CakSNP10241	*Kabuli*	Ca_Kabuli_Chr06	14371530	[C/T]	Ca_05207	Sec1-like protein	SYNONYMOUS_CODING

S.N.	SNP IDs	Chickpea cultivars	Chromosomes	Physical positions (bp)	SNPs	Gene accession IDs	Putative functions	Sequence components of genome
10242	CakSNP10242	*Kabuli*	Ca_Kabuli_Chr06	14375135	[G/A]		--	INTERGENIC
10243	CakSNP10243	*Kabuli*	Ca_Kabuli_Chr06	14375141	[G/C]		--	INTERGENIC
10244	CakSNP10244	*Kabuli*	Ca_Kabuli_Chr06	14375172	[A/G]		--	INTERGENIC
10245	CakSNP10245	*Kabuli*	Ca_Kabuli_Chr06	14375196	[G/A]		--	INTERGENIC
10246	CakSNP10246	*Kabuli*	Ca_Kabuli_Chr06	14375207	[C/T]		--	INTERGENIC
10247	CakSNP10247	*Kabuli*	Ca_Kabuli_Chr06	14401990	[G/A]	Ca_05211	cAMP response element binding (CREB) protein	SYNONYMOUS_CODING
10248	CakSNP10248	*Kabuli*	Ca_Kabuli_Chr06	14416997	[A/G]	Ca_05213	Domain of unknown function DUF676, hydrolase-like	NON_SYNONYMOUS_CODING
10249	CakSNP10249	*Kabuli*	Ca_Kabuli_Chr06	14416981	[A/G]	Ca_05213	Domain of unknown function DUF676, hydrolase-like	SYNONYMOUS_CODING
10250	CakSNP10250	*Kabuli*	Ca_Kabuli_Chr06	14419832	[C/T]	Ca_05213	Domain of unknown function DUF676, hydrolase-like	SYNONYMOUS_CODING
10251	CakSNP10251	*Kabuli*	Ca_Kabuli_Chr06	14526540	[A/T]		--	INTERGENIC

S.N.	SNP IDs	Chickpea cultivars	Chromosomes	Physical positions (bp)	SNPs	Gene accession IDs	Putative functions	Sequence components of genome
10252	CakSNP10252	*Kabuli*	Ca_Kabuli_Chr06	14589478	[T/G]		--	INTERGENIC
10253	CakSNP10253	*Kabuli*	Ca_Kabuli_Chr06	14589480	[T/C]		--	INTERGENIC
10254	CakSNP10254	*Kabuli*	Ca_Kabuli_Chr06	14589520	[G/C]		--	INTERGENIC
10255	CakSNP10255	*Kabuli*	Ca_Kabuli_Chr06	14589744	[C/T]	Ca_05228	Dynamin, GTPase domain	INTRON
10256	CakSNP10256	*Kabuli*	Ca_Kabuli_Chr06	14589807	[T/G]	Ca_05228	Dynamin, GTPase domain	SYNONYMOUS_CODING
10257	CakSNP10257	*Kabuli*	Ca_Kabuli_Chr06	14589873	[T/C]	Ca_05228	Dynamin, GTPase domain	SYNONYMOUS_CODING
10258	CakSNP10258	*Kabuli*	Ca_Kabuli_Chr06	14606892	[A/C]	Ca_05232	--	NON_SYNONYMOUS_CODING
10259	CakSNP10259	*Kabuli*	Ca_Kabuli_Chr06	14628213	[A/T]	Ca_05233	Glycoside hydrolase, family 3, N-terminal	SYNONYMOUS_CODING
10260	CakSNP10260	*Kabuli*	Ca_Kabuli_Chr06	14628215	[T/C]	Ca_05233	Glycoside hydrolase, family 3, N-terminal	NON_SYNONYMOUS_CODING
10261	CakSNP10261	*Kabuli*	Ca_Kabuli_Chr06	14628216	[A/C]	Ca_05233	Glycoside hydrolase, family 3, N-terminal	SYNONYMOUS_CODING

S.N.	SNP IDs	Chickpea cultivars	Chromosomes	Physical positions (bp)	SNPs	Gene accession IDs	Putative functions	Sequence components of genome
10262	CakSNP10262	*Kabuli*	Ca_Kabuli_Chr06	14628284	[G/A]	Ca_05233	Glycoside hydrolase, family 3, N-terminal	SYNONYMOUS_CODING
10263	CakSNP10263	*Kabuli*	Ca_Kabuli_Chr06	14719793	[T/C]	Ca_05242	--	INTRON
10264	CakSNP10264	*Kabuli*	Ca_Kabuli_Chr06	14719840	[G/T]	Ca_05242	--	INTRON
10265	CakSNP10265	*Kabuli*	Ca_Kabuli_Chr06	14747608	[T/C]	Ca_05248	RNA recognition motif domain	INTRON
10266	CakSNP10266	*Kabuli*	Ca_Kabuli_Chr06	14753191	[C/T]		--	INTERGENIC
10267	CakSNP10267	*Kabuli*	Ca_Kabuli_Chr06	14753190	[G/A]		--	INTERGENIC
10268	CakSNP10268	*Kabuli*	Ca_Kabuli_Chr06	14753158	[A/G]		--	INTERGENIC
10269	CakSNP10269	*Kabuli*	Ca_Kabuli_Chr06	14753113	[C/A]		--	INTERGENIC
10270	CakSNP10270	*Kabuli*	Ca_Kabuli_Chr06	14753145	[C/A]		--	INTERGENIC
10271	CakSNP10271	*Kabuli*	Ca_Kabuli_Chr06	14753150	[G/A]		--	INTERGENIC

S.N.	SNP IDs	Chickpea cultivars	Chromosomes	Physical positions (bp)	SNPs	Gene accession IDs	Putative functions	Sequence components of genome
10272	CakSNP10272	*Kabuli*	Ca_Kabuli_Chr06	14762493	[A/G]	Ca_05249	K+ potassium transporter	NON_SYNONYMOUS_CODING
10273	CakSNP10273	*Kabuli*	Ca_Kabuli_Chr06	14771564	[G/T]	Ca_05250	ATPase, P-type, K/Mg/Cd/Cu/Zn/Na/Ca/Na/H-transporter	INTRON
10274	CakSNP10274	*Kabuli*	Ca_Kabuli_Chr06	14771709	[G/A]	Ca_05250	ATPase, P-type, K/Mg/Cd/Cu/Zn/Na/Ca/Na/H-transporter	INTRON
10275	CakSNP10275	*Kabuli*	Ca_Kabuli_Chr06	14786015	[T/G]		--	INTERGENIC
10276	CakSNP10276	*Kabuli*	Ca_Kabuli_Chr06	14789964	[A/C]	Ca_05252	Alternative oxidase	INTRON
10277	CakSNP10277	*Kabuli*	Ca_Kabuli_Chr06	14846794	[C/T]	Ca_05257	Amidase	INTRON
10278	CakSNP10278	*Kabuli*	Ca_Kabuli_Chr06	14857722	[C/A]	Ca_05260	Integrase, catalytic core	STOP_GAINED
10279	CakSNP10279	*Kabuli*	Ca_Kabuli_Chr06	14857709	[A/G]	Ca_05260	Integrase, catalytic core	NON_SYNONYMOUS_CODING
10280	CakSNP10280	*Kabuli*	Ca_Kabuli_Chr06	14857680	[G/A]	Ca_05260	Integrase, catalytic core	SYNONYMOUS_CODING
10281	CakSNP10281	*Kabuli*	Ca_Kabuli_Chr06	14861487	[T/A]		--	INTERGENIC

S.N.	SNP IDs	Chickpea cultivars	Chromosomes	Physical positions (bp)	SNPs	Gene accession IDs	Putative functions	Sequence components of genome
10282	CakSNP10282	*Kabuli*	Ca_Kabuli_Chr06	14884604	[T/G]		--	INTERGENIC
10283	CakSNP10283	*Kabuli*	Ca_Kabuli_Chr06	14884606	[T/C]		--	INTERGENIC
10284	CakSNP10284	*Kabuli*	Ca_Kabuli_Chr06	14891588	[T/C]	Ca_05263	RNA recognition motif domain	INTRON
10285	CakSNP10285	*Kabuli*	Ca_Kabuli_Chr06	14891533	[T/C]	Ca_05263	RNA recognition motif domain	INTRON
10286	CakSNP10286	*Kabuli*	Ca_Kabuli_Chr06	14891597	[C/T]	Ca_05263	RNA recognition motif domain	INTRON
10287	CakSNP10287	*Kabuli*	Ca_Kabuli_Chr06	14891626	[C/A]	Ca_05263	RNA recognition motif domain	INTRON
10288	CakSNP10288	*Kabuli*	Ca_Kabuli_Chr06	14941348	[C/T]	Ca_05267	UbiA prenyltransferase family	INTRON
10289	CakSNP10289	*Kabuli*	Ca_Kabuli_Chr06	14951877	[C/T]	Ca_05269	3,4-dihydroxy-2-butanone 4-phosphate synthase, RibB	INTRON
10290	CakSNP10290	*Kabuli*	Ca_Kabuli_Chr06	14951860	[G/A]	Ca_05269	3,4-dihydroxy-2-butanone 4-phosphate synthase, RibB	INTRON
10291	CakSNP10291	*Kabuli*	Ca_Kabuli_Chr06	14982998	[T/C]	Ca_05273	BTB/POZ-like	SYNONYMOUS_CODING

S.N.	SNP IDs	Chickpea cultivars	Chromosomes	Physical positions (bp)	SNPs	Gene accession IDs	Putative functions	Sequence components of genome
10292	CakSNP10292	*Kabuli*	Ca_Kabuli_Chr06	14983391	[C/T]	Ca_05273	BTB/POZ-like	SYNONYMOUS_CODING
10293	CakSNP10293	*Kabuli*	Ca_Kabuli_Chr06	15024904	[C/A]	Ca_05281	Heat shock protein DnaJ, N-terminal	SYNONYMOUS_CODING
10294	CakSNP10294	*Kabuli*	Ca_Kabuli_Chr06	15024881	[C/A]	Ca_05281	Heat shock protein DnaJ, N-terminal	NON_SYNONYMOUS_CODING
10295	CakSNP10295	*Kabuli*	Ca_Kabuli_Chr06	15024979	[T/G]	Ca_05281	Heat shock protein DnaJ, N-terminal	SYNONYMOUS_CODING
10296	CakSNP10296	*Kabuli*	Ca_Kabuli_Chr06	15024999	[T/A]	Ca_05281	Heat shock protein DnaJ, N-terminal	NON_SYNONYMOUS_CODING
10297	CakSNP10297	*Kabuli*	Ca_Kabuli_Chr06	15025010	[T/C]	Ca_05281	Heat shock protein DnaJ, N-terminal	NON_SYNONYMOUS_CODING
10298	CakSNP10298	*Kabuli*	Ca_Kabuli_Chr06	15025012	[A/C]	Ca_05281	Heat shock protein DnaJ, N-terminal	SYNONYMOUS_CODING
10299	CakSNP10299	*Kabuli*	Ca_Kabuli_Chr06	15051094	[T/C]	Ca_05286	Protein of unknown function DUF3506	SYNONYMOUS_CODING
10300	CakSNP10300	*Kabuli*	Ca_Kabuli_Chr06	15061717	[G/A]	Ca_05286	Protein of unknown function DUF3506	INTRON
10301	CakSNP10301	*Kabuli*	Ca_Kabuli_Chr06	15123739	[C/T]	Ca_05293	Dynamin central domain	INTRON

S.N.	SNP IDs	Chickpea cultivars	Chromosomes	Physical positions (bp)	SNPs	Gene accession IDs	Putative functions	Sequence components of genome
10302	CakSNP10302	*Kabuli*	Ca_Kabuli_Chr06	15123771	[T/C]	Ca_05293	Dynamin central domain	INTRON
10303	CakSNP10303	*Kabuli*	Ca_Kabuli_Chr06	15123827	[T/G]	Ca_05293	Dynamin central domain	INTRON
10304	CakSNP10304	*Kabuli*	Ca_Kabuli_Chr06	15123947	[G/A]	Ca_05293	Dynamin central domain	INTRON
10305	CakSNP10305	*Kabuli*	Ca_Kabuli_Chr06	15173607	[G/A]	Ca_05299	Methylmalonate-semialdehyde dehydrogenase	INTRON
10306	CakSNP10306	*Kabuli*	Ca_Kabuli_Chr06	15185723	[T/C]	Ca_05300	Protein of unknown function DUF593	SYNONYMOUS_CODING
10307	CakSNP10307	*Kabuli*	Ca_Kabuli_Chr06	15204957	[G/C]	Ca_05303	Protein kinase, catalytic domain	INTRON
10308	CakSNP10308	*Kabuli*	Ca_Kabuli_Chr06	15235575	[A/G]		--	INTERGENIC
10309	CakSNP10309	*Kabuli*	Ca_Kabuli_Chr06	15235576	[C/T]		--	INTERGENIC
10310	CakSNP10310	*Kabuli*	Ca_Kabuli_Chr06	15235615	[C/T]		--	INTERGENIC
10311	CakSNP10311	*Kabuli*	Ca_Kabuli_Chr06	15243538	[A/T]		--	INTERGENIC

S.N.	SNP IDs	Chickpea cultivars	Chromosomes	Physical positions (bp)	SNPs	Gene accession IDs	Putative functions	Sequence components of genome
10312	CakSNP10312	*Kabuli*	Ca_Kabuli_Chr06	15243590	[A/G]		--	INTERGENIC
10313	CakSNP10313	*Kabuli*	Ca_Kabuli_Chr06	15243630	[C/T]		--	INTERGENIC
10314	CakSNP10314	*Kabuli*	Ca_Kabuli_Chr06	15243674	[T/A]		--	INTERGENIC
10315	CakSNP10315	*Kabuli*	Ca_Kabuli_Chr06	15268491	[T/G]		--	INTERGENIC
10316	CakSNP10316	*Kabuli*	Ca_Kabuli_Chr06	15294332	[T/G]		--	INTERGENIC
10317	CakSNP10317	*Kabuli*	Ca_Kabuli_Chr06	15294331	[C/T]		--	INTERGENIC
10318	CakSNP10318	*Kabuli*	Ca_Kabuli_Chr06	15294329	[A/C]		--	INTERGENIC
10319	CakSNP10319	*Kabuli*	Ca_Kabuli_Chr06	15301137	[C/T]	Ca_05314	Quinate/shikimate 5-dehydrogenase/glutamyl-tRNA reductase	SYNONYMOUS_CODING
10320	CakSNP10320	*Kabuli*	Ca_Kabuli_Chr06	15313655	[G/T]	Ca_05316	Regulator of K+ conductance, N-terminal	NON_SYNONYMOUS_CODING
10321	CakSNP10321	*Kabuli*	Ca_Kabuli_Chr06	15328759	[C/A]		--	INTERGENIC

S.N.	SNP IDs	Chickpea cultivars	Chromosomes	Physical positions (bp)	SNPs	Gene accession IDs	Putative functions	Sequence components of genome
10322	CakSNP10322	*Kabuli*	Ca_Kabuli_Chr06	15328805	[A/T]		--	INTERGENIC
10323	CakSNP10323	*Kabuli*	Ca_Kabuli_Chr06	15328948	[A/C]	Ca_05318	Zinc finger, DHHC-type, palmitoyltransferase	SYNONYMOUS_CODING
10324	CakSNP10324	*Kabuli*	Ca_Kabuli_Chr06	15347707	[C/T]	Ca_05321	Lactate/malate dehydrogenase, N-terminal	SYNONYMOUS_CODING
10325	CakSNP10325	*Kabuli*	Ca_Kabuli_Chr06	15393321	[A/T]	Ca_05325	Aminotransferase, class IV	SYNONYMOUS_CODING
10326	CakSNP10326	*Kabuli*	Ca_Kabuli_Chr06	15393349	[T/C]	Ca_05325	Aminotransferase, class IV	INTRON
10327	CakSNP10327	*Kabuli*	Ca_Kabuli_Chr06	15463326	[C/G]	Ca_05330	F-box domain, cyclin-like	NON_SYNONYMOUS_CODING
10328	CakSNP10328	*Kabuli*	Ca_Kabuli_Chr06	15475948	[A/G]		--	INTERGENIC
10329	CakSNP10329	*Kabuli*	Ca_Kabuli_Chr06	15488265	[C/T]	Ca_05332	Kinesin, motor domain	INTRON
10330	CakSNP10330	*Kabuli*	Ca_Kabuli_Chr06	15488314	[A/T]	Ca_05332	Kinesin, motor domain	INTRON
10331	CakSNP10331	*Kabuli*	Ca_Kabuli_Chr06	15488309	[G/A]	Ca_05332	Kinesin, motor domain	INTRON

S.N.	SNP IDs	Chickpea cultivars	Chromosomes	Physical positions (bp)	SNPs	Gene accession IDs	Putative functions	Sequence components of genome
10332	CakSNP10332	*Kabuli*	Ca_Kabuli_Chr06	15510433	[A/T]		--	INTERGENIC
10333	CakSNP10333	*Kabuli*	Ca_Kabuli_Chr06	15510397	[G/A]		--	INTERGENIC
10334	CakSNP10334	*Kabuli*	Ca_Kabuli_Chr06	15537908	[A/C]	Ca_05338	--	SYNONYMOUS_CODING
10335	CakSNP10335	*Kabuli*	Ca_Kabuli_Chr06	15543295	[C/G]	Ca_05339	Tetratricopeptide repeat	NON_SYNONYMOUS_CODING
10336	CakSNP10336	*Kabuli*	Ca_Kabuli_Chr06	15543293	[T/G]	Ca_05339	Tetratricopeptide repeat	NON_SYNONYMOUS_CODING
10337	CakSNP10337	*Kabuli*	Ca_Kabuli_Chr06	15543286	[G/A]	Ca_05339	Tetratricopeptide repeat	NON_SYNONYMOUS_CODING
10338	CakSNP10338	*Kabuli*	Ca_Kabuli_Chr06	15550044	[C/T]	Ca_05340	Terpene synthase-like	SYNONYMOUS_CODING
10339	CakSNP10339	*Kabuli*	Ca_Kabuli_Chr06	15589539	[A/T]	Ca_05341	Terpene synthase-like	INTRON
10340	CakSNP10340	*Kabuli*	Ca_Kabuli_Chr06	15589885	[G/T]	Ca_05341	Terpene synthase-like	INTRON
10341	CakSNP10341	*Kabuli*	Ca_Kabuli_Chr06	15635126	[A/T]		--	INTERGENIC

S.N.	SNP IDs	Chickpea cultivars	Chromosomes	Physical positions (bp)	SNPs	Gene accession IDs	Putative functions	Sequence components of genome
10342	CakSNP10342	*Kabuli*	Ca_Kabuli_Chr06	15644412	[A/T]	Ca_05343	Terpene synthase-like	INTRON
10343	CakSNP10343	*Kabuli*	Ca_Kabuli_Chr06	15644796	[A/T]	Ca_05343	Terpene synthase-like	INTRON
10344	CakSNP10344	*Kabuli*	Ca_Kabuli_Chr06	15857728	[T/C]	Ca_22020	ABC-1	SYNONYMOUS_CODING
10345	CakSNP10345	*Kabuli*	Ca_Kabuli_Chr06	15861278	[G/A]		--	INTERGENIC
10346	CakSNP10346	*Kabuli*	Ca_Kabuli_Chr06	16113523	[T/C]	Ca_19678	Haloacid dehydrogenase/epoxide hydrolase	NON_SYNONYMOUS_CODING
10347	CakSNP10347	*Kabuli*	Ca_Kabuli_Chr06	16113522	[A/T]	Ca_19678	Haloacid dehydrogenase/epoxide hydrolase	NON_SYNONYMOUS_CODING
10348	CakSNP10348	*Kabuli*	Ca_Kabuli_Chr06	16113521	[G/T]	Ca_19678	Haloacid dehydrogenase/epoxide hydrolase	SYNONYMOUS_CODING
10349	CakSNP10349	*Kabuli*	Ca_Kabuli_Chr06	16115933	[T/C]	Ca_19678	Haloacid dehydrogenase/epoxide hydrolase	INTRON
10350	CakSNP10350	*Kabuli*	Ca_Kabuli_Chr06	16147469	[A/G]	Ca_19676	Calmodulin binding protein-like	SYNONYMOUS_CODING
10351	CakSNP10351	*Kabuli*	Ca_Kabuli_Chr06	16148277	[A/C]	Ca_19676	Calmodulin binding protein-like	INTRON

S.N.	SNP IDs	Chickpea cultivars	Chromosomes	Physical positions (bp)	SNPs	Gene accession IDs	Putative functions	Sequence components of genome
10352	CakSNP10352	*Kabuli*	Ca_Kabuli_Chr06	16189692	[C/A]	Ca_19671	S1/P1 nuclease	NON_SYNONYMOUS_CODING
10353	CakSNP10353	*Kabuli*	Ca_Kabuli_Chr06	16189693	[G/T]	Ca_19671	S1/P1 nuclease	SYNONYMOUS_CODING
10354	CakSNP10354	*Kabuli*	Ca_Kabuli_Chr06	16195748	[T/C]	Ca_19670	Sugar/inositol transporter	SYNONYMOUS_CODING
10355	CakSNP10355	*Kabuli*	Ca_Kabuli_Chr06	16196582	[G/C]	Ca_19670	Sugar/inositol transporter	SYNONYMOUS_CODING
10356	CakSNP10356	*Kabuli*	Ca_Kabuli_Chr06	16315552	[T/A]		--	INTERGENIC
10357	CakSNP10357	*Kabuli*	Ca_Kabuli_Chr06	16332377	[G/A]	Ca_19665	Protein of unknown function DUF707	INTRON
10358	CakSNP10358	*Kabuli*	Ca_Kabuli_Chr06	16353698	[G/A]	Ca_19662	Armadillo	INTRON
10359	CakSNP10359	*Kabuli*	Ca_Kabuli_Chr06	16450107	[C/T]		--	INTERGENIC
10360	CakSNP10360	*Kabuli*	Ca_Kabuli_Chr06	16460495	[A/C]	Ca_19651	--	SYNONYMOUS_CODING
10361	CakSNP10361	*Kabuli*	Ca_Kabuli_Chr06	16561334	[C/T]	Ca_06286	Plus-3	NON_SYNONYMOUS_CODING

S.N.	SNP IDs	Chickpea cultivars	Chromosomes	Physical positions (bp)	SNPs	Gene accession IDs	Putative functions	Sequence components of genome
10362	CakSNP10362	*Kabuli*	Ca_Kabuli_Chr06	16635061	[C/T]	Ca_06293	Double-stranded RNA-binding	INTRON
10363	CakSNP10363	*Kabuli*	Ca_Kabuli_Chr06	16646769	[A/T]	Ca_06295	Clathrin light chain	NON_SYNONYMOUS_CODING
10364	CakSNP10364	*Kabuli*	Ca_Kabuli_Chr06	16646713	[T/G]	Ca_06295	Clathrin light chain	SYNONYMOUS_CODING
10365	CakSNP10365	*Kabuli*	Ca_Kabuli_Chr06	16665385	[G/C]	Ca_06297	--	INTRON
10366	CakSNP10366	*Kabuli*	Ca_Kabuli_Chr06	16665477	[T/C]	Ca_06297	--	INTRON
10367	CakSNP10367	*Kabuli*	Ca_Kabuli_Chr06	16665829	[G/C]	Ca_06297	--	INTRON
10368	CakSNP10368	*Kabuli*	Ca_Kabuli_Chr06	16677320	[C/A]	Ca_06297	--	INTRON
10369	CakSNP10369	*Kabuli*	Ca_Kabuli_Chr06	16717984	[G/A]	Ca_06303	DNA mismatch repair protein MutS, C-terminal domain	SYNONYMOUS_CODING
10370	CakSNP10370	*Kabuli*	Ca_Kabuli_Chr06	16718201	[G/A]	Ca_06303	DNA mismatch repair protein MutS, C-terminal domain	NON_SYNONYMOUS_CODING
10371	CakSNP10371	*Kabuli*	Ca_Kabuli_Chr06	16719918	[C/A]	Ca_06303	DNA mismatch repair protein MutS, C-terminal domain	NON_SYNONYMOUS_CODING

S.N.	SNP IDs	Chickpea cultivars	Chromosomes	Physical positions (bp)	SNPs	Gene accession IDs	Putative functions	Sequence components of genome
10372	CakSNP10372	*Kabuli*	Ca_Kabuli_Chr06	16719919	[T/G]	Ca_06303	DNA mismatch repair protein MutS, C-terminal domain	SYNONYMOUS_CODING
10373	CakSNP10373	*Kabuli*	Ca_Kabuli_Chr06	16741672	[C/T]	Ca_06306	Zinc finger, RING-type	INTRON
10374	CakSNP10374	*Kabuli*	Ca_Kabuli_Chr06	16748675	[A/C]	Ca_06306	Zinc finger, RING-type	INTRON
10375	CakSNP10375	*Kabuli*	Ca_Kabuli_Chr06	16768782	[G/A]	Ca_06307	--	INTRON
10376	CakSNP10376	*Kabuli*	Ca_Kabuli_Chr06	16823954	[A/G]		--	INTERGENIC
10377	CakSNP10377	*Kabuli*	Ca_Kabuli_Chr06	16932014	[C/G]	Ca_06319	WD40 repeat	NON_SYNONYMOUS_CODING
10378	CakSNP10378	*Kabuli*	Ca_Kabuli_Chr06	17048493	[A/C]	Ca_06326	Zinc finger, Dof-type	NON_SYNONYMOUS_CODING
10379	CakSNP10379	*Kabuli*	Ca_Kabuli_Chr06	17096214	[C/T]	Ca_06327	Mitochondrial substrate/solute carrier	INTRON
10380	CakSNP10380	*Kabuli*	Ca_Kabuli_Chr06	17096215	[G/A]	Ca_06327	Mitochondrial substrate/solute carrier	INTRON
10381	CakSNP10381	*Kabuli*	Ca_Kabuli_Chr06	17096224	[C/T]	Ca_06327	Mitochondrial substrate/solute carrier	INTRON

S.N.	SNP IDs	Chickpea cultivars	Chromosomes	Physical positions (bp)	SNPs	Gene accession IDs	Putative functions	Sequence components of genome
10382	CakSNP10382	*Kabuli*	Ca_Kabuli_Chr06	17129574	[A/G]	Ca_06330	--	NON_SYNONYMOUS_CODING
10383	CakSNP10383	*Kabuli*	Ca_Kabuli_Chr06	17148605	[G/A]	Ca_06333	Ubiquitin	INTRON
10384	CakSNP10384	*Kabuli*	Ca_Kabuli_Chr06	17148773	[A/G]	Ca_06333	Ubiquitin	INTRON
10385	CakSNP10385	*Kabuli*	Ca_Kabuli_Chr06	17175334	[C/A]		--	INTERGENIC
10386	CakSNP10386	*Kabuli*	Ca_Kabuli_Chr06	17175446	[G/A]	Ca_06337	Drought induced 19/ RING finger protein 114	SYNONYMOUS_CODING
10387	CakSNP10387	*Kabuli*	Ca_Kabuli_Chr06	17258827	[C/A]	Ca_06343	Homeobox	NON_SYNONYMOUS_CODING
10388	CakSNP10388	*Kabuli*	Ca_Kabuli_Chr06	17258886	[T/G]	Ca_06343	Homeobox	NON_SYNONYMOUS_CODING
10389	CakSNP10389	*Kabuli*	Ca_Kabuli_Chr06	17262283	[C/T]	Ca_06343	Homeobox	SYNONYMOUS_CODING
10390	CakSNP10390	*Kabuli*	Ca_Kabuli_Chr06	17263126	[G/A]	Ca_06343	Homeobox	SYNONYMOUS_CODING
10391	CakSNP10391	*Kabuli*	Ca_Kabuli_Chr06	17369613	[A/G]	Ca_06352	Transcriptional factor B3	SYNONYMOUS_CODING

S.N.	SNP IDs	Chickpea cultivars	Chromosomes	Physical positions (bp)	SNPs	Gene accession IDs	Putative functions	Sequence components of genome
10392	CakSNP10392	*Kabuli*	Ca_Kabuli_Chr06	17445945	[T/G]		--	INTERGENIC
10393	CakSNP10393	*Kabuli*	Ca_Kabuli_Chr06	17477741	[A/G]	Ca_06361	--	SYNONYMOUS_CODING
10394	CakSNP10394	*Kabuli*	Ca_Kabuli_Chr06	17478220	[G/A]	Ca_06361	--	SYNONYMOUS_CODING
10395	CakSNP10395	*Kabuli*	Ca_Kabuli_Chr06	17480486	[G/C]	Ca_06361	--	SYNONYMOUS_CODING
10396	CakSNP10396	*Kabuli*	Ca_Kabuli_Chr06	17481288	[T/C]		--	INTERGENIC
10397	CakSNP10397	*Kabuli*	Ca_Kabuli_Chr06	17481307	[C/A]		--	INTERGENIC
10398	CakSNP10398	*Kabuli*	Ca_Kabuli_Chr06	17481403	[T/G]		--	INTERGENIC
10399	CakSNP10399	*Kabuli*	Ca_Kabuli_Chr06	17481392	[A/G]		--	INTERGENIC
10400	CakSNP10400	*Kabuli*	Ca_Kabuli_Chr06	17482626	[A/G]		--	INTERGENIC
10401	CakSNP10401	*Kabuli*	Ca_Kabuli_Chr06	17482640	[C/T]		--	INTERGENIC

S.N.	SNP IDs	Chickpea cultivars	Chromosomes	Physical positions (bp)	SNPs	Gene accession IDs	Putative functions	Sequence components of genome
10402	CakSNP10402	*Kabuli*	Ca_Kabuli_Chr06	17482649	[A/G]		--	INTERGENIC
10403	CakSNP10403	*Kabuli*	Ca_Kabuli_Chr06	17482718	[G/A]		--	INTERGENIC
10404	CakSNP10404	*Kabuli*	Ca_Kabuli_Chr06	17482697	[A/G]		--	INTERGENIC
10405	CakSNP10405	*Kabuli*	Ca_Kabuli_Chr06	17573309	[A/G]	Ca_06374	Apoptosis inhibitory 5	INTRON
10406	CakSNP10406	*Kabuli*	Ca_Kabuli_Chr06	17659067	[A/C]	Ca_06383	Conserved hypothetical protein CHP02058	NON_SYNONYMOUS_CODING
10407	CakSNP10407	*Kabuli*	Ca_Kabuli_Chr06	17741078	[G/A]	Ca_06393	WW/Rsp5/WWP	SYNONYMOUS_CODING
10408	CakSNP10408	*Kabuli*	Ca_Kabuli_Chr06	18027391	[G/A]	Ca_06422	Myb, DNA-binding	INTRON
10409	CakSNP10409	*Kabuli*	Ca_Kabuli_Chr06	18094691	[C/A]		--	INTERGENIC
10410	CakSNP10410	*Kabuli*	Ca_Kabuli_Chr06	18125613	[C/A]		--	INTERGENIC
10411	CakSNP10411	*Kabuli*	Ca_Kabuli_Chr06	18232696	[G/A]	Ca_06440	Autophagy-related, C-terminal	SYNONYMOUS_CODING

S.N.	SNP IDs	Chickpea cultivars	Chromosomes	Physical positions (bp)	SNPs	Gene accession IDs	Putative functions	Sequence components of genome
10412	CakSNP10412	*Kabuli*	Ca_Kabuli_Chr06	18268960	[G/A]	Ca_06444	Leucine-rich repeat	INTRON
10413	CakSNP10413	*Kabuli*	Ca_Kabuli_Chr06	18268982	[A/G]	Ca_06444	Leucine-rich repeat	INTRON
10414	CakSNP10414	*Kabuli*	Ca_Kabuli_Chr06	18389704	[T/G]	Ca_06455	Glycosyl transferase, family 10	INTRON
10415	CakSNP10415	*Kabuli*	Ca_Kabuli_Chr06	18389661	[A/C]	Ca_06455	Glycosyl transferase, family 10	INTRON
10416	CakSNP10416	*Kabuli*	Ca_Kabuli_Chr06	18444725	[T/C]	Ca_06458	Basic-leucine zipper (bZIP) transcription factor	NON_SYNONYMOUS_CODING
10417	CakSNP10417	*Kabuli*	Ca_Kabuli_Chr06	18549073	[T/C]	Ca_06466	Helix-loop-helix DNA-binding domain	INTRON
10418	CakSNP10418	*Kabuli*	Ca_Kabuli_Chr06	18562366	[C/A]	Ca_06467	Zinc finger, RING-type	NON_SYNONYMOUS_CODING
10419	CakSNP10419	*Kabuli*	Ca_Kabuli_Chr06	18664114	[A/T]	Ca_06478	Cystathionine beta-synthase, core	INTRON
10420	CakSNP10420	*Kabuli*	Ca_Kabuli_Chr06	18747207	[G/A]	Ca_06486	Armadillo	SYNONYMOUS_CODING
10421	CakSNP10421	*Kabuli*	Ca_Kabuli_Chr06	18752554	[G/T]		--	INTERGENIC

S.N.	SNP IDs	Chickpea cultivars	Chromosomes	Physical positions (bp)	SNPs	Gene accession IDs	Putative functions	Sequence components of genome
10422	CakSNP10422	*Kabuli*	Ca_Kabuli_Chr06	18767357	[G/A]	Ca_06489	Chaperonin Cpn60/TCP-1	INTRON
10423	CakSNP10423	*Kabuli*	Ca_Kabuli_Chr06	18771093	[T/A]	Ca_06489	Chaperonin Cpn60/TCP-1	SYNONYMOUS_CODING
10424	CakSNP10424	*Kabuli*	Ca_Kabuli_Chr06	18798747	[A/C]	Ca_06490	Heat shock protein DnaJ, N-terminal	INTRON
10425	CakSNP10425	*Kabuli*	Ca_Kabuli_Chr06	18852806	[T/C]	Ca_06494	Auxin efflux carrier	SYNONYMOUS_CODING
10426	CakSNP10426	*Kabuli*	Ca_Kabuli_Chr06	18852854	[T/C]	Ca_06494	Auxin efflux carrier	SYNONYMOUS_CODING
10427	CakSNP10427	*Kabuli*	Ca_Kabuli_Chr06	18876221	[T/C]		--	INTERGENIC
10428	CakSNP10428	*Kabuli*	Ca_Kabuli_Chr06	18876213	[A/G]		--	INTERGENIC
10429	CakSNP10429	*Kabuli*	Ca_Kabuli_Chr06	18899989	[C/A]	Ca_06497	Peptidase S10, serine carboxypeptidase	SYNONYMOUS_CODING
10430	CakSNP10430	*Kabuli*	Ca_Kabuli_Chr06	18900031	[C/T]	Ca_06497	Peptidase S10, serine carboxypeptidase	SYNONYMOUS_CODING
10431	CakSNP10431	*Kabuli*	Ca_Kabuli_Chr06	18900034	[G/A]	Ca_06497	Peptidase S10, serine carboxypeptidase	SYNONYMOUS_CODING

S.N.	SNP IDs	Chickpea cultivars	Chromosomes	Physical positions (bp)	SNPs	Gene accession IDs	Putative functions	Sequence components of genome
10432	CakSNP10432	*Kabuli*	Ca_Kabuli_Chr06	18916626	[G/T]	Ca_06499	Uridine kinase	INTRON
10433	CakSNP10433	*Kabuli*	Ca_Kabuli_Chr06	18916619	[C/T]	Ca_06499	Uridine kinase	INTRON
10434	CakSNP10434	*Kabuli*	Ca_Kabuli_Chr06	18925002	[A/G]	Ca_06499	Uridine kinase	INTRON
10435	CakSNP10435	*Kabuli*	Ca_Kabuli_Chr06	18938479	[C/T]	Ca_06500	WD40 repeat	INTRON
10436	CakSNP10436	*Kabuli*	Ca_Kabuli_Chr06	18938512	[G/A]	Ca_06500	WD40 repeat	INTRON
10437	CakSNP10437	*Kabuli*	Ca_Kabuli_Chr06	18956194	[G/A]	Ca_06503	Sulphate transporter/antisigma-factor antagonist STAS	SYNONYMOUS_CODING
10438	CakSNP10438	*Kabuli*	Ca_Kabuli_Chr06	18987220	[T/G]	Ca_06505	Mitochondrial inner membrane translocase complex, subunit Tim17/23	NON_SYNONYMOUS_CODING
10439	CakSNP10439	*Kabuli*	Ca_Kabuli_Chr06	18999175	[C/T]	Ca_06506	GDP-fucose protein O-fucosyltransferase	SYNONYMOUS_CODING
10440	CakSNP10440	*Kabuli*	Ca_Kabuli_Chr06	19033068	[A/C]	Ca_06509	Alpha-D-phosphohexomutase	SYNONYMOUS_CODING
10441	CakSNP10441	*Kabuli*	Ca_Kabuli_Chr06	19033309	[G/A]	Ca_06509	Alpha-D-phosphohexomutase	INTRON

S.N.	SNP IDs	Chickpea cultivars	Chromosomes	Physical positions (bp)	SNPs	Gene accession IDs	Putative functions	Sequence components of genome
10442	CakSNP10442	*Kabuli*	Ca_Kabuli_Chr06	19034129	[A/G]	Ca_06509	Alpha-D-phosphohexomutase	NON_SYNONYMOUS_CODING
10443	CakSNP10443	*Kabuli*	Ca_Kabuli_Chr06	19125254	[G/A]	Ca_06518	DNA-directed RNA polymerase, 30-40kDa subunit, conserved site	INTRON
10444	CakSNP10444	*Kabuli*	Ca_Kabuli_Chr06	19125222	[T/C]	Ca_06518	DNA-directed RNA polymerase, 30-40kDa subunit, conserved site	INTRON
10445	CakSNP10445	*Kabuli*	Ca_Kabuli_Chr06	19129651	[T/C]	Ca_06519	Nck-associated protein 1	SYNONYMOUS_CODING
10446	CakSNP10446	*Kabuli*	Ca_Kabuli_Chr06	19169687	[T/C]	Ca_06524	Ribokinase	SYNONYMOUS_CODING
10447	CakSNP10447	*Kabuli*	Ca_Kabuli_Chr06	19195656	[A/T]		--	INTERGENIC
10448	CakSNP10448	*Kabuli*	Ca_Kabuli_Chr06	19195659	[A/C]		--	INTERGENIC
10449	CakSNP10449	*Kabuli*	Ca_Kabuli_Chr06	19198386	[G/A]	Ca_06528	Glutathione S-transferase, C-terminal	INTRON
10450	CakSNP10450	*Kabuli*	Ca_Kabuli_Chr06	19217820	[G/A]		--	INTERGENIC
10451	CakSNP10451	*Kabuli*	Ca_Kabuli_Chr06	19304834	[A/G]	Ca_06536	Zinc finger, ZPR1-type	SYNONYMOUS_CODING

S.N.	SNP IDs	Chickpea cultivars	Chromosomes	Physical positions (bp)	SNPs	Gene accession IDs	Putative functions	Sequence components of genome
10452	CakSNP10452	*Kabuli*	Ca_Kabuli_Chr06	19440988	[T/C]		--	INTERGENIC
10453	CakSNP10453	*Kabuli*	Ca_Kabuli_Chr06	19441372	[T/C]		--	INTERGENIC
10454	CakSNP10454	*Kabuli*	Ca_Kabuli_Chr06	19450845	[T/A]	Ca_06550	ATPase, AAA+ type, core	INTRON
10455	CakSNP10455	*Kabuli*	Ca_Kabuli_Chr06	19458843	[C/T]	Ca_06551	Protein of unknown function DUF833	INTRON
10456	CakSNP10456	*Kabuli*	Ca_Kabuli_Chr06	19458898	[T/A]	Ca_06551	Protein of unknown function DUF833	INTRON
10457	CakSNP10457	*Kabuli*	Ca_Kabuli_Chr06	19458908	[A/T]	Ca_06551	Protein of unknown function DUF833	INTRON
10458	CakSNP10458	*Kabuli*	Ca_Kabuli_Chr06	19476915	[T/C]	Ca_06554	NusB/RsmB/TIM44	INTRON
10459	CakSNP10459	*Kabuli*	Ca_Kabuli_Chr06	19476916	[A/G]	Ca_06554	NusB/RsmB/TIM44	INTRON
10460	CakSNP10460	*Kabuli*	Ca_Kabuli_Chr06	19477006	[T/C]	Ca_06554	NusB/RsmB/TIM44	INTRON
10461	CakSNP10461	*Kabuli*	Ca_Kabuli_Chr06	19478493	[T/A]	Ca_06554	NusB/RsmB/TIM44	SYNONYMOUS_CODING

S.N.	SNP IDs	Chickpea cultivars	Chromosomes	Physical positions (bp)	SNPs	Gene accession IDs	Putative functions	Sequence components of genome
10462	CakSNP10462	*Kabuli*	Ca_Kabuli_Chr06	19494588	[G/T]		--	INTERGENIC
10463	CakSNP10463	*Kabuli*	Ca_Kabuli_Chr06	19494584	[C/A]		--	INTERGENIC
10464	CakSNP10464	*Kabuli*	Ca_Kabuli_Chr06	19551459	[G/A]	Ca_06563	Asparagine synthase	INTRON
10465	CakSNP10465	*Kabuli*	Ca_Kabuli_Chr06	19551504	[G/C]	Ca_06563	Asparagine synthase	INTRON
10466	CakSNP10466	*Kabuli*	Ca_Kabuli_Chr06	19556904	[G/A]	Ca_06563	Asparagine synthase	INTRON
10467	CakSNP10467	*Kabuli*	Ca_Kabuli_Chr06	19557045	[C/T]	Ca_06563	Asparagine synthase	INTRON
10468	CakSNP10468	*Kabuli*	Ca_Kabuli_Chr06	19578972	[T/C]	Ca_06564	RNA recognition motif domain	SYNONYMOUS_CODING
10469	CakSNP10469	*Kabuli*	Ca_Kabuli_Chr06	19725007	[T/A]		--	INTERGENIC
10470	CakSNP10470	*Kabuli*	Ca_Kabuli_Chr06	20505641	[A/T]	Ca_16381	Cullin, N-terminal	NON_SYNONYMOUS_CODING
10471	CakSNP10471	*Kabuli*	Ca_Kabuli_Chr06	20558142	[C/T]		--	INTERGENIC

S.N.	SNP IDs	Chickpea cultivars	Chromosomes	Physical positions (bp)	SNPs	Gene accession IDs	Putative functions	Sequence components of genome
10472	CakSNP10472	*Kabuli*	Ca_Kabuli_Chr06	20558138	[G/A]		--	INTERGENIC
10473	CakSNP10473	*Kabuli*	Ca_Kabuli_Chr06	20660779	[T/C]	Ca_16368	--	SYNONYMOUS_CODING
10474	CakSNP10474	*Kabuli*	Ca_Kabuli_Chr06	20660782	[T/C]	Ca_16368	--	SYNONYMOUS_CODING
10475	CakSNP10475	*Kabuli*	Ca_Kabuli_Chr06	20660819	[T/C]	Ca_16368	--	NON_SYNONYMOUS_CODING
10476	CakSNP10476	*Kabuli*	Ca_Kabuli_Chr06	20660820	[T/A]	Ca_16368	--	NON_SYNONYMOUS_CODING
10477	CakSNP10477	*Kabuli*	Ca_Kabuli_Chr06	20660841	[A/G]	Ca_16368	--	NON_SYNONYMOUS_CODING
10478	CakSNP10478	*Kabuli*	Ca_Kabuli_Chr06	20660870	[G/C]	Ca_16368	--	NON_SYNONYMOUS_CODING
10479	CakSNP10479	*Kabuli*	Ca_Kabuli_Chr06	20671043	[T/C]	Ca_16366	Glycoside hydrolase, family 1	INTRON
10480	CakSNP10480	*Kabuli*	Ca_Kabuli_Chr06	20739258	[A/T]	Ca_16358	Actin-binding FH2/DRF autoregulatory	NON_SYNONYMOUS_CODING
10481	CakSNP10481	*Kabuli*	Ca_Kabuli_Chr06	20739340	[A/G]	Ca_16358	Actin-binding FH2/DRF autoregulatory	SYNONYMOUS_CODING

S.N.	SNP IDs	Chickpea cultivars	Chromosomes	Physical positions (bp)	SNPs	Gene accession IDs	Putative functions	Sequence components of genome
10482	CakSNP10482	*Kabuli*	Ca_Kabuli_Chr06	20766342	[T/A]	Ca_16355	Helix-loop-helix DNA-binding domain	SYNONYMOUS_CODING
10483	CakSNP10483	*Kabuli*	Ca_Kabuli_Chr06	20766377	[C/A]	Ca_16355	Helix-loop-helix DNA-binding domain	NON_SYNONYMOUS_CODING
10484	CakSNP10484	*Kabuli*	Ca_Kabuli_Chr06	20766397	[G/T]	Ca_16355	Helix-loop-helix DNA-binding domain	NON_SYNONYMOUS_CODING
10485	CakSNP10485	*Kabuli*	Ca_Kabuli_Chr06	20770728	[C/T]	Ca_16354	Glycosyl transferase, family 8	SYNONYMOUS_CODING
10486	CakSNP10486	*Kabuli*	Ca_Kabuli_Chr06	20776473	[A/G]	Ca_16353	SAC3/GANP/Nin1/mts3/eIF-3 p25	INTRON
10487	CakSNP10487	*Kabuli*	Ca_Kabuli_Chr06	20786079	[T/A]	Ca_16351	Toll-Interleukin receptor	NON_SYNONYMOUS_CODING
10488	CakSNP10488	*Kabuli*	Ca_Kabuli_Chr06	20802747	[G/C]	Ca_16348	Pentatricopeptide repeat	INTRON
10489	CakSNP10489	*Kabuli*	Ca_Kabuli_Chr06	20830353	[T/G]		--	INTERGENIC
10490	CakSNP10490	*Kabuli*	Ca_Kabuli_Chr06	20830634	[T/A]		--	INTERGENIC
10491	CakSNP10491	*Kabuli*	Ca_Kabuli_Chr06	20890714	[A/G]	Ca_16342	--	NON_SYNONYMOUS_CODING

S.N.	SNP IDs	Chickpea cultivars	Chromosomes	Physical positions (bp)	SNPs	Gene accession IDs	Putative functions	Sequence components of genome
10492	CakSNP10492	*Kabuli*	Ca_Kabuli_Chr06	20917713	[A/C]	Ca_16339	Plastocyanin-like	NON_SYNONYMOUS_CODING
10493	CakSNP10493	*Kabuli*	Ca_Kabuli_Chr06	20917694	[A/T]	Ca_16339	Plastocyanin-like	NON_SYNONYMOUS_CODING
10494	CakSNP10494	*Kabuli*	Ca_Kabuli_Chr06	21025495	[T/A]		--	INTERGENIC
10495	CakSNP10495	*Kabuli*	Ca_Kabuli_Chr06	21025494	[C/T]		--	INTERGENIC
10496	CakSNP10496	*Kabuli*	Ca_Kabuli_Chr06	21025540	[C/T]		--	INTERGENIC
10497	CakSNP10497	*Kabuli*	Ca_Kabuli_Chr06	21025677	[T/A]		--	INTERGENIC
10498	CakSNP10498	*Kabuli*	Ca_Kabuli_Chr06	21025742	[T/G]		--	INTERGENIC
10499	CakSNP10499	*Kabuli*	Ca_Kabuli_Chr06	21025706	[C/T]		--	INTERGENIC
10500	CakSNP10500	*Kabuli*	Ca_Kabuli_Chr06	21025673	[C/T]		--	INTERGENIC
10501	CakSNP10501	*Kabuli*	Ca_Kabuli_Chr06	21025680	[C/T]		--	INTERGENIC

S.N.	SNP IDs	Chickpea cultivars	Chromosomes	Physical positions (bp)	SNPs	Gene accession IDs	Putative functions	Sequence components of genome
10502	CakSNP10502	*Kabuli*	Ca_Kabuli_Chr06	21025682	[C/T]		--	INTERGENIC
10503	CakSNP10503	*Kabuli*	Ca_Kabuli_Chr06	21025712	[C/T]		--	INTERGENIC
10504	CakSNP10504	*Kabuli*	Ca_Kabuli_Chr06	21025718	[C/A]		--	INTERGENIC
10505	CakSNP10505	*Kabuli*	Ca_Kabuli_Chr06	21025894	[G/T]		--	INTERGENIC
10506	CakSNP10506	*Kabuli*	Ca_Kabuli_Chr06	21054758	[T/C]	Ca_16328	Peptidase, cysteine peptidase active site	SYNONYMOUS_CODING
10507	CakSNP10507	*Kabuli*	Ca_Kabuli_Chr06	21058286	[G/T]	Ca_16327	Heat shock protein DnaJ, N-terminal	NON_SYNONYMOUS_CODING
10508	CakSNP10508	*Kabuli*	Ca_Kabuli_Chr06	21135764	[C/T]	Ca_19889	--	NON_SYNONYMOUS_CODING
10509	CakSNP10509	*Kabuli*	Ca_Kabuli_Chr06	21135996	[C/T]	Ca_19889	--	NON_SYNONYMOUS_CODING
10510	CakSNP10510	*Kabuli*	Ca_Kabuli_Chr06	21139038	[C/G]		--	INTERGENIC
10511	CakSNP10511	*Kabuli*	Ca_Kabuli_Chr06	21165963	[A/C]		--	INTERGENIC

S.N.	SNP IDs	Chickpea cultivars	Chromosomes	Physical positions (bp)	SNPs	Gene accession IDs	Putative functions	Sequence components of genome
10512	CakSNP10512	*Kabuli*	Ca_Kabuli_Chr06	21165999	[G/T]		--	INTERGENIC
10513	CakSNP10513	*Kabuli*	Ca_Kabuli_Chr06	21170382	[G/A]	Ca_19887	Ribosomal protein L1	INTRON
10514	CakSNP10514	*Kabuli*	Ca_Kabuli_Chr06	21170401	[C/A]	Ca_19887	Ribosomal protein L1	INTRON
10515	CakSNP10515	*Kabuli*	Ca_Kabuli_Chr06	21177330	[C/A]	Ca_19886	Sec1-like protein	INTRON
10516	CakSNP10516	*Kabuli*	Ca_Kabuli_Chr06	21238178	[A/G]	Ca_19880	F-box domain, cyclin-like	SYNONYMOUS_CODING
10517	CakSNP10517	*Kabuli*	Ca_Kabuli_Chr06	21238711	[C/T]	Ca_19880	F-box domain, cyclin-like	NON_SYNONYMOUS_CODING
10518	CakSNP10518	*Kabuli*	Ca_Kabuli_Chr06	21238707	[G/T]	Ca_19880	F-box domain, cyclin-like	NON_SYNONYMOUS_CODING
10519	CakSNP10519	*Kabuli*	Ca_Kabuli_Chr06	21276479	[G/T]		--	INTERGENIC
10520	CakSNP10520	*Kabuli*	Ca_Kabuli_Chr06	21276551	[C/G]		--	INTERGENIC
10521	CakSNP10521	*Kabuli*	Ca_Kabuli_Chr06	21323438	[T/C]	Ca_19873	Phosphoenolpyruvate carboxylase, active site	SYNONYMOUS_CODING

S.N.	SNP IDs	Chickpea cultivars	Chromosomes	Physical positions (bp)	SNPs	Gene accession IDs	Putative functions	Sequence components of genome
10522	CakSNP10522	*Kabuli*	Ca_Kabuli_Chr06	21323390	[C/G]	Ca_19873	Phosphoenolpyruvate carboxylase, active site	INTRON
10523	CakSNP10523	*Kabuli*	Ca_Kabuli_Chr06	21367869	[A/G]	Ca_19867	Peptidase, cysteine peptidase active site	SYNONYMOUS_CODING
10524	CakSNP10524	*Kabuli*	Ca_Kabuli_Chr06	21367830	[T/A]	Ca_19867	Peptidase, cysteine peptidase active site	SYNONYMOUS_CODING
10525	CakSNP10525	*Kabuli*	Ca_Kabuli_Chr06	21368163	[C/T]	Ca_19867	Peptidase, cysteine peptidase active site	SYNONYMOUS_CODING
10526	CakSNP10526	*Kabuli*	Ca_Kabuli_Chr06	21385915	[C/A]	Ca_19864	Peptidase, cysteine peptidase active site	NON_SYNONYMOUS_CODING
10527	CakSNP10527	*Kabuli*	Ca_Kabuli_Chr06	21478820	[G/T]		--	INTERGENIC
10528	CakSNP10528	*Kabuli*	Ca_Kabuli_Chr06	21478816	[C/T]		--	INTERGENIC
10529	CakSNP10529	*Kabuli*	Ca_Kabuli_Chr06	21483425	[T/G]		--	INTERGENIC
10530	CakSNP10530	*Kabuli*	Ca_Kabuli_Chr06	21483429	[C/T]		--	INTERGENIC
10531	CakSNP10531	*Kabuli*	Ca_Kabuli_Chr06	21483494	[T/G]		--	INTERGENIC

S.N.	SNP IDs	Chickpea cultivars	Chromosomes	Physical positions (bp)	SNPs	Gene accession IDs	Putative functions	Sequence components of genome
10532	CakSNP10532	*Kabuli*	Ca_Kabuli_Chr06	21489498	[A/G]	Ca_11293	--	SYNONYMOUS_CODING
10533	CakSNP10533	*Kabuli*	Ca_Kabuli_Chr06	21506224	[A/C]	Ca_11291	Peptidase, cysteine peptidase active site	NON_SYNONYMOUS_CODING
10534	CakSNP10534	*Kabuli*	Ca_Kabuli_Chr06	21540338	[A/G]	Ca_11288	Translation elongation factor EFG/EF2, C-terminal	INTRON
10535	CakSNP10535	*Kabuli*	Ca_Kabuli_Chr06	21540382	[T/G]	Ca_11288	Translation elongation factor EFG/EF2, C-terminal	INTRON
10536	CakSNP10536	*Kabuli*	Ca_Kabuli_Chr06	21576745	[G/A]		--	INTERGENIC
10537	CakSNP10537	*Kabuli*	Ca_Kabuli_Chr06	21602874	[C/T]	Ca_11280	--	INTRON
10538	CakSNP10538	*Kabuli*	Ca_Kabuli_Chr06	21603102	[G/C]	Ca_11280	--	INTRON
10539	CakSNP10539	*Kabuli*	Ca_Kabuli_Chr06	21675734	[T/G]	Ca_11272	Homeobox	NON_SYNONYMOUS_CODING
10540	CakSNP10540	*Kabuli*	Ca_Kabuli_Chr06	21675757	[A/G]	Ca_11272	Homeobox	NON_SYNONYMOUS_CODING
10541	CakSNP10541	*Kabuli*	Ca_Kabuli_Chr06	21675762	[G/T]	Ca_11272	Homeobox	SYNONYMOUS_CODING

S.N.	SNP IDs	Chickpea cultivars	Chromosomes	Physical positions (bp)	SNPs	Gene accession IDs	Putative functions	Sequence components of genome
10542	CakSNP10542	*Kabuli*	Ca_Kabuli_Chr06	21675816	[C/G]	Ca_11272	Homeobox	NON_SYNONYMOUS_CODING
10543	CakSNP10543	*Kabuli*	Ca_Kabuli_Chr06	21718674	[G/T]	Ca_11268	Myb, DNA-binding	NON_SYNONYMOUS_CODING
10544	CakSNP10544	*Kabuli*	Ca_Kabuli_Chr06	21756666	[A/T]		--	INTERGENIC
10545	CakSNP10545	*Kabuli*	Ca_Kabuli_Chr06	21756706	[C/T]		--	INTERGENIC
10546	CakSNP10546	*Kabuli*	Ca_Kabuli_Chr06	21763468	[G/A]	Ca_11264	Mpv17/PMP22	NON_SYNONYMOUS_CODING
10547	CakSNP10547	*Kabuli*	Ca_Kabuli_Chr06	21763611	[C/T]		--	INTERGENIC
10548	CakSNP10548	*Kabuli*	Ca_Kabuli_Chr06	21763582	[C/T]		--	INTERGENIC
10549	CakSNP10549	*Kabuli*	Ca_Kabuli_Chr06	21804333	[T/A]		--	INTERGENIC
10550	CakSNP10550	*Kabuli*	Ca_Kabuli_Chr06	21804337	[G/C]		--	INTERGENIC
10551	CakSNP10551	*Kabuli*	Ca_Kabuli_Chr06	21834111	[C/G]	Ca_11258	Xklp2 targeting protein	INTRON

S.N.	SNP IDs	Chickpea cultivars	Chromosomes	Physical positions (bp)	SNPs	Gene accession IDs	Putative functions	Sequence components of genome
10552	CakSNP10552	*Kabuli*	Ca_Kabuli_Chr06	21861700	[T/A]	Ca_11255	Protein kinase, catalytic domain	SYNONYMOUS_CODING
10553	CakSNP10553	*Kabuli*	Ca_Kabuli_Chr06	21914387	[T/C]		--	INTERGENIC
10554	CakSNP10554	*Kabuli*	Ca_Kabuli_Chr06	21914380	[C/T]		--	INTERGENIC
10555	CakSNP10555	*Kabuli*	Ca_Kabuli_Chr06	21916465	[G/A]	Ca_11246	--	INTRON
10556	CakSNP10556	*Kabuli*	Ca_Kabuli_Chr06	21916476	[T/C]	Ca_11246	--	INTRON
10557	CakSNP10557	*Kabuli*	Ca_Kabuli_Chr06	21940793	[T/C]		--	INTERGENIC
10558	CakSNP10558	*Kabuli*	Ca_Kabuli_Chr06	21979846	[G/A]		--	INTERGENIC
10559	CakSNP10559	*Kabuli*	Ca_Kabuli_Chr06	21979860	[C/T]		--	INTERGENIC
10560	CakSNP10560	*Kabuli*	Ca_Kabuli_Chr06	21979873	[A/G]		--	INTERGENIC
10561	CakSNP10561	*Kabuli*	Ca_Kabuli_Chr06	21979887	[G/A]		--	INTERGENIC

S.N.	SNP IDs	Chickpea cultivars	Chromosomes	Physical positions (bp)	SNPs	Gene accession IDs	Putative functions	Sequence components of genome
10562	CakSNP10562	*Kabuli*	Ca_Kabuli_Chr06	21979889	[A/G]		--	INTERGENIC
10563	CakSNP10563	*Kabuli*	Ca_Kabuli_Chr06	21979893	[C/T]		--	INTERGENIC
10564	CakSNP10564	*Kabuli*	Ca_Kabuli_Chr06	21979903	[T/C]		--	INTERGENIC
10565	CakSNP10565	*Kabuli*	Ca_Kabuli_Chr06	22015915	[G/T]	Ca_11238	Glycosyl transferase, family 48	SYNONYMOUS_CODING
10566	CakSNP10566	*Kabuli*	Ca_Kabuli_Chr06	22016086	[A/G]	Ca_11238	Glycosyl transferase, family 48	SYNONYMOUS_CODING
10567	CakSNP10567	*Kabuli*	Ca_Kabuli_Chr06	22016767	[C/T]	Ca_11238	Glycosyl transferase, family 48	SYNONYMOUS_CODING
10568	CakSNP10568	*Kabuli*	Ca_Kabuli_Chr06	22016839	[A/G]	Ca_11238	Glycosyl transferase, family 48	SYNONYMOUS_CODING
10569	CakSNP10569	*Kabuli*	Ca_Kabuli_Chr06	22038857	[A/G]	Ca_11237	Prohibitin	INTRON
10570	CakSNP10570	*Kabuli*	Ca_Kabuli_Chr06	22038906	[A/T]	Ca_11237	Prohibitin	SYNONYMOUS_CODING
10571	CakSNP10571	*Kabuli*	Ca_Kabuli_Chr06	22074809	[T/C]	Ca_11231	--	SYNONYMOUS_CODING

S.N.	SNP IDs	Chickpea cultivars	Chromosomes	Physical positions (bp)	SNPs	Gene accession IDs	Putative functions	Sequence components of genome
10572	CakSNP10572	*Kabuli*	Ca_Kabuli_Chr06	22075447	[C/G]	Ca_11231	--	INTRON
10573	CakSNP10573	*Kabuli*	Ca_Kabuli_Chr06	22079148	[A/G]	Ca_11230	--	NON_SYNONYMOUS_CODING
10574	CakSNP10574	*Kabuli*	Ca_Kabuli_Chr06	22137477	[A/T]		--	INTERGENIC
10575	CakSNP10575	*Kabuli*	Ca_Kabuli_Chr06	22137464	[G/A]		--	INTERGENIC
10576	CakSNP10576	*Kabuli*	Ca_Kabuli_Chr06	22137569	[G/T]		--	INTERGENIC
10577	CakSNP10577	*Kabuli*	Ca_Kabuli_Chr06	22175547	[A/G]	Ca_11222	Heat shock factor (HSF)-type, DNA-binding	NON_SYNONYMOUS_CODING
10578	CakSNP10578	*Kabuli*	Ca_Kabuli_Chr06	22192503	[G/A]	Ca_11219	Exostosin-like	SYNONYMOUS_CODING
10579	CakSNP10579	*Kabuli*	Ca_Kabuli_Chr06	22192498	[A/C]	Ca_11219	Exostosin-like	NON_SYNONYMOUS_CODING
10580	CakSNP10580	*Kabuli*	Ca_Kabuli_Chr06	22194387	[C/A]	Ca_11219	Exostosin-like	STOP_GAINED
10581	CakSNP10581	*Kabuli*	Ca_Kabuli_Chr06	22201688	[C/T]	Ca_11219	Exostosin-like	INTRON

S.N.	SNP IDs	Chickpea cultivars	Chromosomes	Physical positions (bp)	SNPs	Gene accession IDs	Putative functions	Sequence components of genome
10582	CakSNP10582	*Kabuli*	Ca_Kabuli_Chr06	22206341	[G/T]		--	INTERGENIC
10583	CakSNP10583	*Kabuli*	Ca_Kabuli_Chr06	22208130	[G/C]	Ca_11218	Cellulose synthase	NON_SYNONYMOUS_CODING
10584	CakSNP10584	*Kabuli*	Ca_Kabuli_Chr06	22208125	[G/A]	Ca_11218	Cellulose synthase	NON_SYNONYMOUS_CODING
10585	CakSNP10585	*Kabuli*	Ca_Kabuli_Chr06	22208093	[G/A]	Ca_11218	Cellulose synthase	SYNONYMOUS_CODING
10586	CakSNP10586	*Kabuli*	Ca_Kabuli_Chr06	22211721	[C/T]	Ca_11217	ATPase, AAA+ type, core	INTRON
10587	CakSNP10587	*Kabuli*	Ca_Kabuli_Chr06	22211823	[T/C]	Ca_11217	ATPase, AAA+ type, core	SYNONYMOUS_CODING
10588	CakSNP10588	*Kabuli*	Ca_Kabuli_Chr06	22217158	[A/G]	Ca_11216	Zinc finger, CHCC-type	SYNONYMOUS_CODING
10589	CakSNP10589	*Kabuli*	Ca_Kabuli_Chr06	22217157	[T/G]	Ca_11216	Zinc finger, CHCC-type	NON_SYNONYMOUS_CODING
10590	CakSNP10590	*Kabuli*	Ca_Kabuli_Chr06	22217107	[G/A]	Ca_11216	Zinc finger, CHCC-type	SYNONYMOUS_CODING
10591	CakSNP10591	*Kabuli*	Ca_Kabuli_Chr06	22225288	[T/G]		--	INTERGENIC

S.N.	SNP IDs	Chickpea cultivars	Chromosomes	Physical positions (bp)	SNPs	Gene accession IDs	Putative functions	Sequence components of genome
10592	CakSNP10592	*Kabuli*	Ca_Kabuli_Chr06	22235449	[G/A]		--	INTERGENIC
10593	CakSNP10593	*Kabuli*	Ca_Kabuli_Chr06	22235421	[T/A]		--	INTERGENIC
10594	CakSNP10594	*Kabuli*	Ca_Kabuli_Chr06	22236475	[T/A]		--	INTERGENIC
10595	CakSNP10595	*Kabuli*	Ca_Kabuli_Chr06	22252149	[G/A]		--	INTERGENIC
10596	CakSNP10596	*Kabuli*	Ca_Kabuli_Chr06	22262999	[A/C]	Ca_11211	Sugar/inositol transporter	INTRON
10597	CakSNP10597	*Kabuli*	Ca_Kabuli_Chr06	22263011	[T/C]	Ca_11211	Sugar/inositol transporter	INTRON
10598	CakSNP10598	*Kabuli*	Ca_Kabuli_Chr06	22263388	[A/G]		--	INTERGENIC
10599	CakSNP10599	*Kabuli*	Ca_Kabuli_Chr06	22263452	[A/T]		--	INTERGENIC
10600	CakSNP10600	*Kabuli*	Ca_Kabuli_Chr06	22279906	[T/G]	Ca_11209	Aminoacyl-tRNA synthetase, class I, conserved site	INTRON
10601	CakSNP10601	*Kabuli*	Ca_Kabuli_Chr06	22287922	[C/T]	Ca_11208	Importin-beta, N-terminal	INTRON

S.N.	SNP IDs	Chickpea cultivars	Chromosomes	Physical positions (bp)	SNPs	Gene accession IDs	Putative functions	Sequence components of genome
10602	CakSNP10602	*Kabuli*	Ca_Kabuli_Chr06	22334733	[C/T]	Ca_11204	--	INTRON
10603	CakSNP10603	*Kabuli*	Ca_Kabuli_Chr06	22352976	[T/C]		--	INTERGENIC
10604	CakSNP10604	*Kabuli*	Ca_Kabuli_Chr06	22370242	[C/T]	Ca_11200	Protein of unknown function DUF1218	SYNONYMOUS_CODING
10605	CakSNP10605	*Kabuli*	Ca_Kabuli_Chr06	22371908	[A/C]		--	INTERGENIC
10606	CakSNP10606	*Kabuli*	Ca_Kabuli_Chr06	22446629	[G/A]	Ca_11195	Peptidase C48, SUMO/Sentrin/Ubl1	SYNONYMOUS_CODING
10607	CakSNP10607	*Kabuli*	Ca_Kabuli_Chr06	22446621	[A/C]	Ca_11195	Peptidase C48, SUMO/Sentrin/Ubl1	SYNONYMOUS_CODING
10608	CakSNP10608	*Kabuli*	Ca_Kabuli_Chr06	22446610	[G/A]	Ca_11195	Peptidase C48, SUMO/Sentrin/Ubl1	NON_SYNONYMOUS_CODING
10609	CakSNP10609	*Kabuli*	Ca_Kabuli_Chr06	22446570	[C/T]	Ca_11195	Peptidase C48, SUMO/Sentrin/Ubl1	SYNONYMOUS_CODING
10610	CakSNP10610	*Kabuli*	Ca_Kabuli_Chr06	22446649	[C/T]	Ca_11195	Peptidase C48, SUMO/Sentrin/Ubl1	NON_SYNONYMOUS_CODING
10611	CakSNP10611	*Kabuli*	Ca_Kabuli_Chr06	22446644	[C/T]	Ca_11195	Peptidase C48, SUMO/Sentrin/Ubl1	NON_SYNONYMOUS_CODING

S.N.	SNP IDs	Chickpea cultivars	Chromosomes	Physical positions (bp)	SNPs	Gene accession IDs	Putative functions	Sequence components of genome
10612	CakSNP10612	*Kabuli*	Ca_Kabuli_Chr06	22482366	[A/C]	Ca_11192	Nitrite/sulphite reductase, hemoprotein beta-component, ferrodoxin-like	INTRON
10613	CakSNP10613	*Kabuli*	Ca_Kabuli_Chr06	22498553	[T/C]	Ca_11190	NAD-dependent epimerase/dehydratase	NON_SYNONYMOUS_CODING
10614	CakSNP10614	*Kabuli*	Ca_Kabuli_Chr06	22498867	[T/C]	Ca_11190	NAD-dependent epimerase/dehydratase	INTRON
10615	CakSNP10615	*Kabuli*	Ca_Kabuli_Chr06	22518747	[G/T]	Ca_11188	Lipase, class 3	SYNONYMOUS_CODING
10616	CakSNP10616	*Kabuli*	Ca_Kabuli_Chr06	22879982	[C/T]	Ca_11157	Inorganic polyphosphate/ATP-NAD kinase, predicted	SYNONYMOUS_CODING
10617	CakSNP10617	*Kabuli*	Ca_Kabuli_Chr06	22881176	[A/T]	Ca_11157	Inorganic polyphosphate/ATP-NAD kinase, predicted	INTRON
10618	CakSNP10618	*Kabuli*	Ca_Kabuli_Chr06	22933938	[T/A]	Ca_11154	ABC transporter-like	SYNONYMOUS_CODING
10619	CakSNP10619	*Kabuli*	Ca_Kabuli_Chr06	22952185	[G/A]		--	INTERGENIC
10620	CakSNP10620	*Kabuli*	Ca_Kabuli_Chr06	22952253	[C/T]		--	INTERGENIC
10621	CakSNP10621	*Kabuli*	Ca_Kabuli_Chr06	22966001	[C/A]	Ca_11151	Elongator protein 3/MiaB/NifB	INTRON

S.N.	SNP IDs	Chickpea cultivars	Chromosomes	Physical positions (bp)	SNPs	Gene accession IDs	Putative functions	Sequence components of genome
10622	CakSNP10622	*Kabuli*	Ca_Kabuli_Chr06	22986891	[T/C]	Ca_11150	Aminoacyl-tRNA synthetase, class I, conserved site	INTRON
10623	CakSNP10623	*Kabuli*	Ca_Kabuli_Chr06	22986869	[G/C]	Ca_11150	Aminoacyl-tRNA synthetase, class I, conserved site	INTRON
10624	CakSNP10624	*Kabuli*	Ca_Kabuli_Chr06	23014724	[T/A]		--	INTERGENIC
10625	CakSNP10625	*Kabuli*	Ca_Kabuli_Chr06	23039165	[T/C]	Ca_11143	Glycoside hydrolase, family 17	SYNONYMOUS_CODING
10626	CakSNP10626	*Kabuli*	Ca_Kabuli_Chr06	23039147	[G/A]	Ca_11143	Glycoside hydrolase, family 17	SYNONYMOUS_CODING
10627	CakSNP10627	*Kabuli*	Ca_Kabuli_Chr06	23039145	[A/G]	Ca_11143	Glycoside hydrolase, family 17	SYNONYMOUS_CODING
10628	CakSNP10628	*Kabuli*	Ca_Kabuli_Chr06	23039125	[G/A]	Ca_11143	Glycoside hydrolase, family 17	NON_SYNONYMOUS_CODING
10629	CakSNP10629	*Kabuli*	Ca_Kabuli_Chr06	23039097	[C/T]	Ca_11143	Glycoside hydrolase, family 17	SYNONYMOUS_CODING
10630	CakSNP10630	*Kabuli*	Ca_Kabuli_Chr06	23046076	[C/T]		--	INTERGENIC
10631	CakSNP10631	*Kabuli*	Ca_Kabuli_Chr06	23113706	[A/G]	Ca_11140	Glycoside hydrolase, family 17	SYNONYMOUS_CODING

S.N.	SNP IDs	Chickpea cultivars	Chromosomes	Physical positions (bp)	SNPs	Gene accession IDs	Putative functions	Sequence components of genome
10632	CakSNP10632	*Kabuli*	Ca_Kabuli_Chr06	23129516	[A/G]	Ca_11139	Tetratricopeptide TPR-1	INTRON
10633	CakSNP10633	*Kabuli*	Ca_Kabuli_Chr06	23129504	[C/G]	Ca_11139	Tetratricopeptide TPR-1	INTRON
10634	CakSNP10634	*Kabuli*	Ca_Kabuli_Chr06	23145532	[T/A]	Ca_11137	Clathrin/coatomer adaptor, adaptin-like, N-terminal	SYNONYMOUS_CODING
10635	CakSNP10635	*Kabuli*	Ca_Kabuli_Chr06	23166355	[A/C]	Ca_11135	Zinc finger, PHD-type	NON_SYNONYMOUS_CODING
10636	CakSNP10636	*Kabuli*	Ca_Kabuli_Chr06	23253687	[G/A]	Ca_11131	--	NON_SYNONYMOUS_CODING
10637	CakSNP10637	*Kabuli*	Ca_Kabuli_Chr06	23253973	[G/C]	Ca_11131	--	SYNONYMOUS_CODING
10638	CakSNP10638	*Kabuli*	Ca_Kabuli_Chr06	23254642	[A/G]	Ca_11131	--	SYNONYMOUS_CODING
10639	CakSNP10639	*Kabuli*	Ca_Kabuli_Chr06	23256848	[C/G]	Ca_11131	--	NON_SYNONYMOUS_CODING
10640	CakSNP10640	*Kabuli*	Ca_Kabuli_Chr06	23256891	[T/G]	Ca_11131	--	INTRON
10641	CakSNP10641	*Kabuli*	Ca_Kabuli_Chr06	23380746	[G/A]	Ca_11121	Rop nucleotide exchanger, PRONE	SYNONYMOUS_CODING

S.N.	SNP IDs	Chickpea cultivars	Chromosomes	Physical positions (bp)	SNPs	Gene accession IDs	Putative functions	Sequence components of genome
10642	CakSNP10642	*Kabuli*	Ca_Kabuli_Chr06	23393426	[T/C]		--	INTERGENIC
10643	CakSNP10643	*Kabuli*	Ca_Kabuli_Chr06	23393446	[A/G]		--	INTERGENIC
10644	CakSNP10644	*Kabuli*	Ca_Kabuli_Chr06	23393469	[T/C]		--	INTERGENIC
10645	CakSNP10645	*Kabuli*	Ca_Kabuli_Chr06	23393491	[T/G]		--	INTERGENIC
10646	CakSNP10646	*Kabuli*	Ca_Kabuli_Chr06	23399103	[A/G]		--	INTERGENIC
10647	CakSNP10647	*Kabuli*	Ca_Kabuli_Chr06	23424061	[T/G]	Ca_11116	Nucleolar 27S pre-rRNA processing, Urb2/Npa2, C-terminal	INTRON
10648	CakSNP10648	*Kabuli*	Ca_Kabuli_Chr06	23549220	[A/T]	Ca_11100	Zinc finger, CCHC-type	INTRON
10649	CakSNP10649	*Kabuli*	Ca_Kabuli_Chr06	23638882	[A/G]	Ca_11091	--	SYNONYMOUS_CODING
10650	CakSNP10650	*Kabuli*	Ca_Kabuli_Chr06	23663204	[C/A]	Ca_11087	Glycoside hydrolase, family 9	INTRON
10651	CakSNP10651	*Kabuli*	Ca_Kabuli_Chr06	23663201	[T/C]	Ca_11087	Glycoside hydrolase, family 9	INTRON

S.N.	SNP IDs	Chickpea cultivars	Chromosomes	Physical positions (bp)	SNPs	Gene accession IDs	Putative functions	Sequence components of genome
10652	CakSNP10652	*Kabuli*	Ca_Kabuli_Chr06	23663180	[G/T]	Ca_11087	Glycoside hydrolase, family 9	INTRON
10653	CakSNP10653	*Kabuli*	Ca_Kabuli_Chr06	23688740	[C/T]	Ca_11084	RNA recognition motif domain	INTRON
10654	CakSNP10654	*Kabuli*	Ca_Kabuli_Chr06	23688723	[A/G]	Ca_11084	RNA recognition motif domain	INTRON
10655	CakSNP10655	*Kabuli*	Ca_Kabuli_Chr06	23691440	[A/C]	Ca_11084	RNA recognition motif domain	INTRON
10656	CakSNP10656	*Kabuli*	Ca_Kabuli_Chr06	23766943	[C/T]	Ca_11079	Tetratricopeptide repeat-containing	INTRON
10657	CakSNP10657	*Kabuli*	Ca_Kabuli_Chr06	23768142	[A/T]	Ca_11079	Tetratricopeptide repeat-containing	INTRON
10658	CakSNP10658	*Kabuli*	Ca_Kabuli_Chr06	23809120	[G/A]	Ca_11078	Alanyl-tRNA synthetase, class IIc	SYNONYMOUS_CODING
10659	CakSNP10659	*Kabuli*	Ca_Kabuli_Chr06	23890836	[A/G]	Ca_11072	WW/Rsp5/WWP	SYNONYMOUS_CODING
10660	CakSNP10660	*Kabuli*	Ca_Kabuli_Chr06	23891065	[G/A]	Ca_11072	WW/Rsp5/WWP	NON_SYNONYMOUS_CODING
10661	CakSNP10661	*Kabuli*	Ca_Kabuli_Chr06	23916260	[A/G]	Ca_11069	C2 calcium-dependent membrane targeting	INTRON

S.N.	SNP IDs	Chickpea cultivars	Chromosomes	Physical positions (bp)	SNPs	Gene accession IDs	Putative functions	Sequence components of genome
10662	CakSNP10662	*Kabuli*	Ca_Kabuli_Chr06	23963812	[T/C]		--	INTERGENIC
10663	CakSNP10663	*Kabuli*	Ca_Kabuli_Chr06	23973410	[G/A]	Ca_11064	RNA recognition motif domain	INTRON
10664	CakSNP10664	*Kabuli*	Ca_Kabuli_Chr06	24007185	[C/T]	Ca_11060	RNA recognition motif domain	NON_SYNONYMOUS_CODING
10665	CakSNP10665	*Kabuli*	Ca_Kabuli_Chr06	24007863	[T/C]	Ca_11060	RNA recognition motif domain	SYNONYMOUS_CODING
10666	CakSNP10666	*Kabuli*	Ca_Kabuli_Chr06	24061578	[G/T]	Ca_11054	Oxoglutarate/iron-dependent oxygenase	INTRON
10667	CakSNP10667	*Kabuli*	Ca_Kabuli_Chr06	24085747	[G/T]		--	INTERGENIC
10668	CakSNP10668	*Kabuli*	Ca_Kabuli_Chr06	24085795	[C/T]		--	INTERGENIC
10669	CakSNP10669	*Kabuli*	Ca_Kabuli_Chr06	24085807	[A/T]		--	INTERGENIC
10670	CakSNP10670	*Kabuli*	Ca_Kabuli_Chr06	24138357	[A/G]		--	INTERGENIC
10671	CakSNP10671	*Kabuli*	Ca_Kabuli_Chr06	24138388	[T/C]		--	INTERGENIC

**S.N.**	**SNP IDs**	**Chickpea cultivars**	**Chromosomes**	**Physical positions (bp)**	**SNPs**	**Gene accession IDs**	**Putative functions**	**Sequence components of genome**
10672	CakSNP10672	*Kabuli*	Ca_Kabuli_Chr06	24174940	[C/T]	Ca_11040	Armadillo	SYNONYMOUS_CODING
10673	CakSNP10673	*Kabuli*	Ca_Kabuli_Chr06	24260527	[T/C]		--	INTERGENIC
10674	CakSNP10674	*Kabuli*	Ca_Kabuli_Chr06	24301508	[T/A]	Ca_11035	GTP-binding protein, HSR1-related	SYNONYMOUS_CODING
10675	CakSNP10675	*Kabuli*	Ca_Kabuli_Chr06	24302205	[A/G]	Ca_11035	GTP-binding protein, HSR1-related	INTRON
10676	CakSNP10676	*Kabuli*	Ca_Kabuli_Chr06	24381949	[A/G]		--	INTERGENIC
10677	CakSNP10677	*Kabuli*	Ca_Kabuli_Chr06	24381937	[G/A]		--	INTERGENIC
10678	CakSNP10678	*Kabuli*	Ca_Kabuli_Chr06	24403316	[G/T]	Ca_11029	Peptidase S8/S53, subtilisin/kexin/sedolisin	NON_SYNONYMOUS_CODING
10679	CakSNP10679	*Kabuli*	Ca_Kabuli_Chr06	24413675	[T/C]	Ca_11028	Senescence/spartin-associated	SYNONYMOUS_CODING
10680	CakSNP10680	*Kabuli*	Ca_Kabuli_Chr06	24413714	[G/A]	Ca_11028	Senescence/spartin-associated	SYNONYMOUS_CODING
10681	CakSNP10681	*Kabuli*	Ca_Kabuli_Chr06	24414512	[G/C]	Ca_11028	Senescence/spartin-associated	SYNONYMOUS_CODING

S.N.	SNP IDs	Chickpea cultivars	Chromosomes	Physical positions (bp)	SNPs	Gene accession IDs	Putative functions	Sequence components of genome
10682	CakSNP10682	*Kabuli*	Ca_Kabuli_Chr06	24454583	[C/T]	Ca_11025	Haem peroxidase, plant/fungal/bacterial	INTRON
10683	CakSNP10683	*Kabuli*	Ca_Kabuli_Chr06	24454557	[T/C]	Ca_11025	Haem peroxidase, plant/fungal/bacterial	INTRON
10684	CakSNP10684	*Kabuli*	Ca_Kabuli_Chr06	24516523	[A/T]	Ca_27822	--	NON_SYNONYMOUS_CODING
10685	CakSNP10685	*Kabuli*	Ca_Kabuli_Chr06	24516528	[A/T]	Ca_27822	--	NON_SYNONYMOUS_CODING
10686	CakSNP10686	*Kabuli*	Ca_Kabuli_Chr06	24516532	[C/T]	Ca_27822	--	SYNONYMOUS_CODING
10687	CakSNP10687	*Kabuli*	Ca_Kabuli_Chr06	24547086	[T/G]	Ca_24172	D-galactoside/L-rhamnose binding SUEL lectin domain	NON_SYNONYMOUS_CODING
10688	CakSNP10688	*Kabuli*	Ca_Kabuli_Chr06	24644924	[T/G]	Ca_24163	Nickel/cobalt transporter, high-affinity	SYNONYMOUS_CODING
10689	CakSNP10689	*Kabuli*	Ca_Kabuli_Chr06	24661895	[G/A]		--	INTERGENIC
10690	CakSNP10690	*Kabuli*	Ca_Kabuli_Chr06	24661916	[C/T]		--	INTERGENIC
10691	CakSNP10691	*Kabuli*	Ca_Kabuli_Chr06	24661937	[C/T]		--	INTERGENIC

S.N.	SNP IDs	Chickpea cultivars	Chromosomes	Physical positions (bp)	SNPs	Gene accession IDs	Putative functions	Sequence components of genome
10692	CakSNP10692	*Kabuli*	Ca_Kabuli_Chr06	24661945	[T/A]		--	INTERGENIC
10693	CakSNP10693	*Kabuli*	Ca_Kabuli_Chr06	24661986	[G/A]		--	INTERGENIC
10694	CakSNP10694	*Kabuli*	Ca_Kabuli_Chr06	25575097	[T/C]	Ca_24008	--	SYNONYMOUS_CODING
10695	CakSNP10695	*Kabuli*	Ca_Kabuli_Chr06	25575078	[C/T]	Ca_24008	--	NON_SYNONYMOUS_CODING
10696	CakSNP10696	*Kabuli*	Ca_Kabuli_Chr06	25575062	[G/A]	Ca_24008	--	NON_SYNONYMOUS_CODING
10697	CakSNP10697	*Kabuli*	Ca_Kabuli_Chr06	25575045	[C/A]	Ca_24008	--	NON_SYNONYMOUS_CODING
10698	CakSNP10698	*Kabuli*	Ca_Kabuli_Chr06	25604942	[C/A]		--	INTERGENIC
10699	CakSNP10699	*Kabuli*	Ca_Kabuli_Chr06	25604936	[C/T]		--	INTERGENIC
10700	CakSNP10700	*Kabuli*	Ca_Kabuli_Chr06	25604931	[C/A]		--	INTERGENIC
10701	CakSNP10701	*Kabuli*	Ca_Kabuli_Chr06	25604922	[A/G]		--	INTERGENIC

S.N.	SNP IDs	Chickpea cultivars	Chromosomes	Physical positions (bp)	SNPs	Gene accession IDs	Putative functions	Sequence components of genome
10702	CakSNP10702	*Kabuli*	Ca_Kabuli_Chr06	25604954	[C/T]		--	INTERGENIC
10703	CakSNP10703	*Kabuli*	Ca_Kabuli_Chr06	25604962	[G/A]		--	INTERGENIC
10704	CakSNP10704	*Kabuli*	Ca_Kabuli_Chr06	25604994	[T/C]		--	INTERGENIC
10705	CakSNP10705	*Kabuli*	Ca_Kabuli_Chr06	25605006	[C/A]		--	INTERGENIC
10706	CakSNP10706	*Kabuli*	Ca_Kabuli_Chr06	25605023	[C/A]		--	INTERGENIC
10707	CakSNP10707	*Kabuli*	Ca_Kabuli_Chr06	25605022	[T/C]		--	INTERGENIC
10708	CakSNP10708	*Kabuli*	Ca_Kabuli_Chr06	25850717	[C/T]	Ca_24011	--	SYNONYMOUS_CODING
10709	CakSNP10709	*Kabuli*	Ca_Kabuli_Chr06	25850725	[T/G]	Ca_24011	--	SYNONYMOUS_CODING
10710	CakSNP10710	*Kabuli*	Ca_Kabuli_Chr06	25850736	[C/T]	Ca_24011	--	NON_SYNONYMOUS_CODING
10711	CakSNP10711	*Kabuli*	Ca_Kabuli_Chr06	25850760	[T/A]	Ca_24011	--	NON_SYNONYMOUS_CODING

S.N.	SNP IDs	Chickpea cultivars	Chromosomes	Physical positions (bp)	SNPs	Gene accession IDs	Putative functions	Sequence components of genome
10712	CakSNP10712	*Kabuli*	Ca_Kabuli_Chr06	25850759	[C/A]	Ca_24011	--	NON_SYNONYMOUS_CODING
10713	CakSNP10713	*Kabuli*	Ca_Kabuli_Chr06	25850783	[G/A]	Ca_24011	--	NON_SYNONYMOUS_CODING
10714	CakSNP10714	*Kabuli*	Ca_Kabuli_Chr06	25850816	[C/A]	Ca_24011	--	NON_SYNONYMOUS_CODING
10715	CakSNP10715	*Kabuli*	Ca_Kabuli_Chr06	25850813	[C/T]	Ca_24011	--	NON_SYNONYMOUS_CODING
10716	CakSNP10716	*Kabuli*	Ca_Kabuli_Chr06	25850866	[C/G]	Ca_24011	--	SYNONYMOUS_CODING
10717	CakSNP10717	*Kabuli*	Ca_Kabuli_Chr06	25850827	[C/T]	Ca_24011	--	SYNONYMOUS_CODING
10718	CakSNP10718	*Kabuli*	Ca_Kabuli_Chr06	26349363	[C/A]	Ca_16676	Transcription factor GRAS	NON_SYNONYMOUS_CODING
10719	CakSNP10719	*Kabuli*	Ca_Kabuli_Chr06	26349321	[C/T]	Ca_16676	Transcription factor GRAS	SYNONYMOUS_CODING
10720	CakSNP10720	*Kabuli*	Ca_Kabuli_Chr06	26349434	[G/A]	Ca_16676	Transcription factor GRAS	NON_SYNONYMOUS_CODING
10721	CakSNP10721	*Kabuli*	Ca_Kabuli_Chr06	26349451	[T/C]	Ca_16676	Transcription factor GRAS	SYNONYMOUS_CODING

S.N.	SNP IDs	Chickpea cultivars	Chromosomes	Physical positions (bp)	SNPs	Gene accession IDs	Putative functions	Sequence components of genome
10722	CakSNP10722	*Kabuli*	Ca_Kabuli_Chr06	26353512	[C/T]	Ca_16677	Transcription factor GRAS	SYNONYMOUS_CODING
10723	CakSNP10723	*Kabuli*	Ca_Kabuli_Chr06	26353740	[G/C]	Ca_16677	Transcription factor GRAS	SYNONYMOUS_CODING
10724	CakSNP10724	*Kabuli*	Ca_Kabuli_Chr06	26357149	[C/G]	Ca_16678	Transcription factor GRAS	SYNONYMOUS_CODING
10725	CakSNP10725	*Kabuli*	Ca_Kabuli_Chr06	26360367	[C/A]	Ca_16679	Oxysterol-binding protein	SYNONYMOUS_CODING
10726	CakSNP10726	*Kabuli*	Ca_Kabuli_Chr06	26385448	[A/G]		--	INTERGENIC
10727	CakSNP10727	*Kabuli*	Ca_Kabuli_Chr06	26385444	[C/T]		--	INTERGENIC
10728	CakSNP10728	*Kabuli*	Ca_Kabuli_Chr06	26385432	[T/C]		--	INTERGENIC
10729	CakSNP10729	*Kabuli*	Ca_Kabuli_Chr06	26426239	[C/A]	Ca_16684	Protein kinase, catalytic domain	INTRON
10730	CakSNP10730	*Kabuli*	Ca_Kabuli_Chr06	26426252	[A/G]	Ca_16684	Protein kinase, catalytic domain	INTRON
10731	CakSNP10731	*Kabuli*	Ca_Kabuli_Chr06	26426283	[G/A]	Ca_16684	Protein kinase, catalytic domain	INTRON

S.N.	SNP IDs	Chickpea cultivars	Chromosomes	Physical positions (bp)	SNPs	Gene accession IDs	Putative functions	Sequence components of genome
10732	CakSNP10732	*Kabuli*	Ca_Kabuli_Chr06	26527086	[T/C]	Ca_16688	Zinc finger, RING-type	INTRON
10733	CakSNP10733	*Kabuli*	Ca_Kabuli_Chr06	26527111	[G/T]	Ca_16688	Zinc finger, RING-type	INTRON
10734	CakSNP10734	*Kabuli*	Ca_Kabuli_Chr06	26527161	[C/G]	Ca_16688	Zinc finger, RING-type	INTRON
10735	CakSNP10735	*Kabuli*	Ca_Kabuli_Chr06	26527416	[C/T]	Ca_16688	Zinc finger, RING-type	INTRON
10736	CakSNP10736	*Kabuli*	Ca_Kabuli_Chr06	26549168	[T/G]	Ca_16689	Armadillo	INTRON
10737	CakSNP10737	*Kabuli*	Ca_Kabuli_Chr06	26549181	[C/T]	Ca_16689	Armadillo	INTRON
10738	CakSNP10738	*Kabuli*	Ca_Kabuli_Chr06	26551390	[C/T]	Ca_16689	Armadillo	NON_SYNONYMOUS_CODING
10739	CakSNP10739	*Kabuli*	Ca_Kabuli_Chr06	26551703	[T/G]	Ca_16689	Armadillo	SYNONYMOUS_CODING
10740	CakSNP10740	*Kabuli*	Ca_Kabuli_Chr06	26567360	[A/G]	Ca_16690	Zinc finger, Dof-type	SYNONYMOUS_CODING
10741	CakSNP10741	*Kabuli*	Ca_Kabuli_Chr06	26656016	[T/C]		--	INTERGENIC

S.N.	SNP IDs	Chickpea cultivars	Chromosomes	Physical positions (bp)	SNPs	Gene accession IDs	Putative functions	Sequence components of genome
10742	CakSNP10742	*Kabuli*	Ca_Kabuli_Chr06	26698265	[A/C]	Ca_16698	Cellular retinaldehyde-binding/triple function, C-terminal	SYNONYMOUS_CODING
10743	CakSNP10743	*Kabuli*	Ca_Kabuli_Chr06	26703130	[A/G]	Ca_16698	Cellular retinaldehyde-binding/triple function, C-terminal	NON_SYNONYMOUS_CODING
10744	CakSNP10744	*Kabuli*	Ca_Kabuli_Chr06	26703132	[T/C]	Ca_16698	Cellular retinaldehyde-binding/triple function, C-terminal	SYNONYMOUS_CODING
10745	CakSNP10745	*Kabuli*	Ca_Kabuli_Chr06	26767389	[T/C]	Ca_16707	Ras GTPase	INTRON
10746	CakSNP10746	*Kabuli*	Ca_Kabuli_Chr06	26769087	[C/A]	Ca_16707	Ras GTPase	NON_SYNONYMOUS_CODING
10747	CakSNP10747	*Kabuli*	Ca_Kabuli_Chr06	26928255	[T/C]		--	INTERGENIC
10748	CakSNP10748	*Kabuli*	Ca_Kabuli_Chr06	27053217	[A/G]	Ca_14636	Ankyrin repeat	SYNONYMOUS_CODING
10749	CakSNP10749	*Kabuli*	Ca_Kabuli_Chr06	27053288	[G/C]	Ca_14636	Ankyrin repeat	NON_SYNONYMOUS_CODING
10750	CakSNP10750	*Kabuli*	Ca_Kabuli_Chr06	27139791	[T/C]	Ca_14630	CCT domain	INTRON
10751	CakSNP10751	*Kabuli*	Ca_Kabuli_Chr06	27139816	[T/C]	Ca_14630	CCT domain	INTRON

S.N.	SNP IDs	Chickpea cultivars	Chromosomes	Physical positions (bp)	SNPs	Gene accession IDs	Putative functions	Sequence components of genome
10752	CakSNP10752	*Kabuli*	Ca_Kabuli_Chr06	27190671	[G/A]		--	INTERGENIC
10753	CakSNP10753	*Kabuli*	Ca_Kabuli_Chr06	27192557	[C/T]	Ca_14627	RNA polymerase Rpb1, domain 5	INTRON
10754	CakSNP10754	*Kabuli*	Ca_Kabuli_Chr06	27310342	[A/T]	Ca_14618	Pathogenesis-related transcriptional factor/ERF, DNA-binding	INTRON
10755	CakSNP10755	*Kabuli*	Ca_Kabuli_Chr06	27310341	[G/T]	Ca_14618	Pathogenesis-related transcriptional factor/ERF, DNA-binding	INTRON
10756	CakSNP10756	*Kabuli*	Ca_Kabuli_Chr06	27310336	[A/T]	Ca_14618	Pathogenesis-related transcriptional factor/ERF, DNA-binding	INTRON
10757	CakSNP10757	*Kabuli*	Ca_Kabuli_Chr06	27310333	[C/T]	Ca_14618	Pathogenesis-related transcriptional factor/ERF, DNA-binding	INTRON
10758	CakSNP10758	*Kabuli*	Ca_Kabuli_Chr06	27310328	[T/A]	Ca_14618	Pathogenesis-related transcriptional factor/ERF, DNA-binding	INTRON
10759	CakSNP10759	*Kabuli*	Ca_Kabuli_Chr06	27310327	[A/T]	Ca_14618	Pathogenesis-related transcriptional factor/ERF, DNA-binding	INTRON
10760	CakSNP10760	*Kabuli*	Ca_Kabuli_Chr06	27310325	[G/T]	Ca_14618	Pathogenesis-related transcriptional factor/ERF, DNA-binding	INTRON
10761	CakSNP10761	*Kabuli*	Ca_Kabuli_Chr06	27310315	[C/T]	Ca_14618	Pathogenesis-related transcriptional factor/ERF, DNA-binding	INTRON

S.N.	SNP IDs	Chickpea cultivars	Chromosomes	Physical positions (bp)	SNPs	Gene accession IDs	Putative functions	Sequence components of genome
10762	CakSNP10762	*Kabuli*	Ca_Kabuli_Chr06	27326420	[G/A]		--	INTERGENIC
10763	CakSNP10763	*Kabuli*	Ca_Kabuli_Chr06	27326978	[C/T]	Ca_14617	RNA recognition motif domain	NON_SYNONYMOUS_CODING
10764	CakSNP10764	*Kabuli*	Ca_Kabuli_Chr06	27330059	[T/C]	Ca_14617	RNA recognition motif domain	SYNONYMOUS_CODING
10765	CakSNP10765	*Kabuli*	Ca_Kabuli_Chr06	27330159	[C/T]	Ca_14617	RNA recognition motif domain	NON_SYNONYMOUS_CODING
10766	CakSNP10766	*Kabuli*	Ca_Kabuli_Chr06	27331165	[T/C]	Ca_14617	RNA recognition motif domain	NON_SYNONYMOUS_CODING
10767	CakSNP10767	*Kabuli*	Ca_Kabuli_Chr06	27331318	[A/C]	Ca_14617	RNA recognition motif domain	INTRON
10768	CakSNP10768	*Kabuli*	Ca_Kabuli_Chr06	27364381	[A/G]	Ca_14616	Haem peroxidase, plant/fungal/bacterial	INTRON
10769	CakSNP10769	*Kabuli*	Ca_Kabuli_Chr06	27364395	[C/T]	Ca_14616	Haem peroxidase, plant/fungal/bacterial	INTRON
10770	CakSNP10770	*Kabuli*	Ca_Kabuli_Chr06	27364402	[T/A]	Ca_14616	Haem peroxidase, plant/fungal/bacterial	INTRON
10771	CakSNP10771	*Kabuli*	Ca_Kabuli_Chr06	27364428	[G/T]	Ca_14616	Haem peroxidase, plant/fungal/bacterial	INTRON

S.N.	SNP IDs	Chickpea cultivars	Chromosomes	Physical positions (bp)	SNPs	Gene accession IDs	Putative functions	Sequence components of genome
10772	CakSNP10772	*Kabuli*	Ca_Kabuli_Chr06	27364452	[A/T]	Ca_14616	Haem peroxidase, plant/fungal/bacterial	INTRON
10773	CakSNP10773	*Kabuli*	Ca_Kabuli_Chr06	27383746	[A/T]	Ca_14614	--	SYNONYMOUS_CODING
10774	CakSNP10774	*Kabuli*	Ca_Kabuli_Chr06	27383745	[G/T]	Ca_14614	--	NON_SYNONYMOUS_CODING
10775	CakSNP10775	*Kabuli*	Ca_Kabuli_Chr06	27383744	[G/T]	Ca_14614	--	NON_SYNONYMOUS_CODING
10776	CakSNP10776	*Kabuli*	Ca_Kabuli_Chr06	27383718	[C/T]	Ca_14614	--	NON_SYNONYMOUS_CODING
10777	CakSNP10777	*Kabuli*	Ca_Kabuli_Chr06	27515184	[C/T]	Ca_14606	Tetraspanin, subgroup	SYNONYMOUS_CODING
10778	CakSNP10778	*Kabuli*	Ca_Kabuli_Chr06	27515293	[G/A]	Ca_14606	Tetraspanin, subgroup	SYNONYMOUS_CODING
10779	CakSNP10779	*Kabuli*	Ca_Kabuli_Chr06	27537433	[A/G]	Ca_14604	Zinc finger, RING-type	SYNONYMOUS_CODING
10780	CakSNP10780	*Kabuli*	Ca_Kabuli_Chr06	27558154	[A/G]	Ca_14601	Alpha/beta hydrolase fold-1	INTRON
10781	CakSNP10781	*Kabuli*	Ca_Kabuli_Chr06	27578968	[A/G]	Ca_14599	--	SYNONYMOUS_CODING

S.N.	SNP IDs	Chickpea cultivars	Chromosomes	Physical positions (bp)	SNPs	Gene accession IDs	Putative functions	Sequence components of genome
10782	CakSNP10782	*Kabuli*	Ca_Kabuli_Chr06	27592117	[G/T]	Ca_14598	Defects-in-morphology protein 1-like, mitochondrial	INTRON
10783	CakSNP10783	*Kabuli*	Ca_Kabuli_Chr06	27592070	[A/G]	Ca_14598	Defects-in-morphology protein 1-like, mitochondrial	SYNONYMOUS_CODING
10784	CakSNP10784	*Kabuli*	Ca_Kabuli_Chr06	27606193	[A/C]	Ca_14597	Cupin 1	INTRON
10785	CakSNP10785	*Kabuli*	Ca_Kabuli_Chr06	27606210	[C/T]	Ca_14597	Cupin 1	INTRON
10786	CakSNP10786	*Kabuli*	Ca_Kabuli_Chr06	27622037	[A/C]		--	INTERGENIC
10787	CakSNP10787	*Kabuli*	Ca_Kabuli_Chr06	27622045	[A/G]		--	INTERGENIC
10788	CakSNP10788	*Kabuli*	Ca_Kabuli_Chr06	27622053	[A/G]		--	INTERGENIC
10789	CakSNP10789	*Kabuli*	Ca_Kabuli_Chr06	27636256	[A/G]	Ca_14594	Heat shock protein Hsp20	NON_SYNONYMOUS_CODING
10790	CakSNP10790	*Kabuli*	Ca_Kabuli_Chr06	27655605	[A/G]	Ca_14592	Cytochrome B561-related	NON_SYNONYMOUS_CODING
10791	CakSNP10791	*Kabuli*	Ca_Kabuli_Chr06	27660980	[C/T]	Ca_14591	Acyl-CoA dehydrogenase, conserved site	SYNONYMOUS_CODING

S.N.	SNP IDs	Chickpea cultivars	Chromosomes	Physical positions (bp)	SNPs	Gene accession IDs	Putative functions	Sequence components of genome
10792	CakSNP10792	*Kabuli*	Ca_Kabuli_Chr06	27660971	[T/C]	Ca_14591	Acyl-CoA dehydrogenase, conserved site	SYNONYMOUS_CODING
10793	CakSNP10793	*Kabuli*	Ca_Kabuli_Chr06	27661951	[C/T]	Ca_14591	Acyl-CoA dehydrogenase, conserved site	INTRON
10794	CakSNP10794	*Kabuli*	Ca_Kabuli_Chr06	27662026	[C/T]	Ca_14591	Acyl-CoA dehydrogenase, conserved site	INTRON
10795	CakSNP10795	*Kabuli*	Ca_Kabuli_Chr06	27710531	[T/G]	Ca_14588	Preprotein translocase Sec, Sec61-beta subunit	SYNONYMOUS_CODING
10796	CakSNP10796	*Kabuli*	Ca_Kabuli_Chr06	27710547	[A/T]	Ca_14588	Preprotein translocase Sec, Sec61-beta subunit	NON_SYNONYMOUS_CODING
10797	CakSNP10797	*Kabuli*	Ca_Kabuli_Chr06	27842297	[G/A]	Ca_14577	D-galactoside/L-rhamnose binding SUEL lectin domain	NON_SYNONYMOUS_CODING
10798	CakSNP10798	*Kabuli*	Ca_Kabuli_Chr06	27865048	[C/T]	Ca_14574	RNA recognition motif domain	NON_SYNONYMOUS_CODING
10799	CakSNP10799	*Kabuli*	Ca_Kabuli_Chr06	27865046	[G/A]	Ca_14574	RNA recognition motif domain	SYNONYMOUS_CODING
10800	CakSNP10800	*Kabuli*	Ca_Kabuli_Chr06	27864997	[C/A]	Ca_14574	RNA recognition motif domain	INTRON
10801	CakSNP10801	*Kabuli*	Ca_Kabuli_Chr06	27986719	[A/G]	Ca_14567	Ribosomal protein L11	NON_SYNONYMOUS_CODING

S.N.	SNP IDs	Chickpea cultivars	Chromosomes	Physical positions (bp)	SNPs	Gene accession IDs	Putative functions	Sequence components of genome
10802	CakSNP10802	*Kabuli*	Ca_Kabuli_Chr06	28021899	[C/T]	Ca_14563	DNA-directed DNA polymerase, family B, exonuclease domain	NON_SYNONYMOUS_CODING
10803	CakSNP10803	*Kabuli*	Ca_Kabuli_Chr06	28044830	[C/A]	Ca_14563	DNA-directed DNA polymerase, family B, exonuclease domain	NON_SYNONYMOUS_CODING
10804	CakSNP10804	*Kabuli*	Ca_Kabuli_Chr06	28052463	[T/G]	Ca_14562	Vacuolar fusion protein MON1	SYNONYMOUS_CODING
10805	CakSNP10805	*Kabuli*	Ca_Kabuli_Chr06	28074393	[T/C]	Ca_14561	Double-stranded RNA-binding	SYNONYMOUS_CODING
10806	CakSNP10806	*Kabuli*	Ca_Kabuli_Chr06	28076323	[T/A]	Ca_14561	Double-stranded RNA-binding	INTRON
10807	CakSNP10807	*Kabuli*	Ca_Kabuli_Chr06	28076364	[T/G]	Ca_14561	Double-stranded RNA-binding	INTRON
10808	CakSNP10808	*Kabuli*	Ca_Kabuli_Chr06	28076436	[A/T]	Ca_14561	Double-stranded RNA-binding	INTRON
10809	CakSNP10809	*Kabuli*	Ca_Kabuli_Chr06	28177397	[A/C]	Ca_26122	SIT4 phosphatase-associated protein family	INTRON
10810	CakSNP10810	*Kabuli*	Ca_Kabuli_Chr06	28325392	[C/T]	Ca_25968	Ribonuclease T2	NON_SYNONYMOUS_CODING
10811	CakSNP10811	*Kabuli*	Ca_Kabuli_Chr06	28372130	[C/T]		--	INTERGENIC

S.N.	SNP IDs	Chickpea cultivars	Chromosomes	Physical positions (bp)	SNPs	Gene accession IDs	Putative functions	Sequence components of genome
10812	CakSNP10812	*Kabuli*	Ca_Kabuli_Chr06	28574699	[T/C]	Ca_17492	Exoribonuclease, phosphorolytic domain 1	INTRON
10813	CakSNP10813	*Kabuli*	Ca_Kabuli_Chr06	28575509	[T/C]	Ca_17492	Exoribonuclease, phosphorolytic domain 1	INTRON
10814	CakSNP10814	*Kabuli*	Ca_Kabuli_Chr06	28575491	[G/A]	Ca_17492	Exoribonuclease, phosphorolytic domain 1	SYNONYMOUS_CODING
10815	CakSNP10815	*Kabuli*	Ca_Kabuli_Chr06	28615404	[G/T]		--	INTERGENIC
10816	CakSNP10816	*Kabuli*	Ca_Kabuli_Chr06	28615475	[A/G]		--	INTERGENIC
10817	CakSNP10817	*Kabuli*	Ca_Kabuli_Chr06	28650056	[A/G]		--	INTERGENIC
10818	CakSNP10818	*Kabuli*	Ca_Kabuli_Chr06	28727478	[T/C]	Ca_17478	Chaperonin Cpn60	SYNONYMOUS_CODING
10819	CakSNP10819	*Kabuli*	Ca_Kabuli_Chr06	28728293	[A/C]	Ca_17478	Chaperonin Cpn60	NON_SYNONYMOUS_CODING
10820	CakSNP10820	*Kabuli*	Ca_Kabuli_Chr06	28755298	[T/A]	Ca_17477	--	INTRON
10821	CakSNP10821	*Kabuli*	Ca_Kabuli_Chr06	28757208	[C/A]	Ca_17477	--	INTRON

S.N.	SNP IDs	Chickpea cultivars	Chromosomes	Physical positions (bp)	SNPs	Gene accession IDs	Putative functions	Sequence components of genome
10822	CakSNP10822	*Kabuli*	Ca_Kabuli_Chr06	28757204	[G/A]	Ca_17477	--	INTRON
10823	CakSNP10823	*Kabuli*	Ca_Kabuli_Chr06	28757139	[C/A]	Ca_17477	--	INTRON
10824	CakSNP10824	*Kabuli*	Ca_Kabuli_Chr06	28887176	[G/A]		--	INTERGENIC
10825	CakSNP10825	*Kabuli*	Ca_Kabuli_Chr06	28887188	[G/T]		--	INTERGENIC
10826	CakSNP10826	*Kabuli*	Ca_Kabuli_Chr06	28887201	[A/G]		--	INTERGENIC
10827	CakSNP10827	*Kabuli*	Ca_Kabuli_Chr06	28887212	[C/T]		--	INTERGENIC
10828	CakSNP10828	*Kabuli*	Ca_Kabuli_Chr06	28892743	[G/C]	Ca_17474	Major facilitator superfamily MFS-1	SYNONYMOUS_CODING
10829	CakSNP10829	*Kabuli*	Ca_Kabuli_Chr06	28893411	[C/T]	Ca_17474	Major facilitator superfamily MFS-1	SYNONYMOUS_CODING
10830	CakSNP10830	*Kabuli*	Ca_Kabuli_Chr06	28917705	[T/A]	Ca_17473	ABC transporter-like	INTRON
10831	CakSNP10831	*Kabuli*	Ca_Kabuli_Chr06	28947529	[A/C]	Ca_17471	Glyoxalase/bleomycin resistance protein/dioxygenase	NON_SYNONYMOUS_CODING

S.N.	SNP IDs	Chickpea cultivars	Chromosomes	Physical positions (bp)	SNPs	Gene accession IDs	Putative functions	Sequence components of genome
10832	CakSNP10832	*Kabuli*	Ca_Kabuli_Chr06	28990518	[A/G]	Ca_17469	Nicotinamide N-methyltransferase, putative	NON_SYNONYMOUS_CODING
10833	CakSNP10833	*Kabuli*	Ca_Kabuli_Chr06	29085558	[T/C]		--	INTERGENIC
10834	CakSNP10834	*Kabuli*	Ca_Kabuli_Chr06	29163667	[C/A]	Ca_17460	Shugoshin, C-terminal	INTRON
10835	CakSNP10835	*Kabuli*	Ca_Kabuli_Chr06	29163714	[G/C]	Ca_17460	Shugoshin, C-terminal	INTRON
10836	CakSNP10836	*Kabuli*	Ca_Kabuli_Chr06	29163699	[T/A]	Ca_17460	Shugoshin, C-terminal	INTRON
10837	CakSNP10837	*Kabuli*	Ca_Kabuli_Chr06	29163642	[G/A]	Ca_17460	Shugoshin, C-terminal	SYNONYMOUS_CODING
10838	CakSNP10838	*Kabuli*	Ca_Kabuli_Chr06	29163640	[A/C]	Ca_17460	Shugoshin, C-terminal	SYNONYMOUS_CODING
10839	CakSNP10839	*Kabuli*	Ca_Kabuli_Chr06	29203129	[C/G]	Ca_17457	DNA ligase, ATP-dependent, N-terminal	INTRON
10840	CakSNP10840	*Kabuli*	Ca_Kabuli_Chr06	29203201	[G/C]	Ca_17457	DNA ligase, ATP-dependent, N-terminal	INTRON
10841	CakSNP10841	*Kabuli*	Ca_Kabuli_Chr06	29203210	[T/C]	Ca_17457	DNA ligase, ATP-dependent, N-terminal	INTRON

S.N.	SNP IDs	Chickpea cultivars	Chromosomes	Physical positions (bp)	SNPs	Gene accession IDs	Putative functions	Sequence components of genome
10842	CakSNP10842	*Kabuli*	Ca_Kabuli_Chr06	29205260	[A/G]	Ca_17457	DNA ligase, ATP-dependent, N-terminal	INTRON
10843	CakSNP10843	*Kabuli*	Ca_Kabuli_Chr06	29205940	[A/G]	Ca_17457	DNA ligase, ATP-dependent, N-terminal	NON_SYNONYMOUS_CODING
10844	CakSNP10844	*Kabuli*	Ca_Kabuli_Chr06	29206008	[A/G]	Ca_17457	DNA ligase, ATP-dependent, N-terminal	SYNONYMOUS_CODING
10845	CakSNP10845	*Kabuli*	Ca_Kabuli_Chr06	29307254	[A/G]	Ca_17448	Argonaute/Dicer protein, PAZ	SYNONYMOUS_CODING
10846	CakSNP10846	*Kabuli*	Ca_Kabuli_Chr06	29495856	[C/T]	Ca_17443	--	SYNONYMOUS_CODING
10847	CakSNP10847	*Kabuli*	Ca_Kabuli_Chr06	29934610	[A/T]	Ca_24058	Cyclin-dependent kinase, regulatory subunit	SYNONYMOUS_CODING
10848	CakSNP10848	*Kabuli*	Ca_Kabuli_Chr06	30127756	[T/G]	Ca_24048	Domain of unknown function DUF296	SYNONYMOUS_CODING
10849	CakSNP10849	*Kabuli*	Ca_Kabuli_Chr06	30257217	[C/T]	Ca_16442	Peptidase C48, SUMO/Sentrin/Ubl1	INTRON
10850	CakSNP10850	*Kabuli*	Ca_Kabuli_Chr06	30257284	[A/C]	Ca_16442	Peptidase C48, SUMO/Sentrin/Ubl1	INTRON
10851	CakSNP10851	*Kabuli*	Ca_Kabuli_Chr06	30567073	[G/A]		--	INTERGENIC

S.N.	SNP IDs	Chickpea cultivars	Chromosomes	Physical positions (bp)	SNPs	Gene accession IDs	Putative functions	Sequence components of genome
10852	CakSNP10852	*Kabuli*	Ca_Kabuli_Chr06	30676031	[G/T]	Ca_16465	Translation elongation factor EFG/EF2, C-terminal	SYNONYMOUS_CODING
10853	CakSNP10853	*Kabuli*	Ca_Kabuli_Chr06	30676320	[T/C]	Ca_16465	Translation elongation factor EFG/EF2, C-terminal	SYNONYMOUS_CODING
10854	CakSNP10854	*Kabuli*	Ca_Kabuli_Chr06	30676248	[C/T]	Ca_16465	Translation elongation factor EFG/EF2, C-terminal	SYNONYMOUS_CODING
10855	CakSNP10855	*Kabuli*	Ca_Kabuli_Chr06	30761620	[C/T]	Ca_16470	Peptidase S8/S53, subtilisin/kexin/sedolisin	INTRON
10856	CakSNP10856	*Kabuli*	Ca_Kabuli_Chr06	30761641	[G/T]	Ca_16470	Peptidase S8/S53, subtilisin/kexin/sedolisin	INTRON
10857	CakSNP10857	*Kabuli*	Ca_Kabuli_Chr06	30913932	[T/C]	Ca_16484	--	INTRON
10858	CakSNP10858	*Kabuli*	Ca_Kabuli_Chr06	30926084	[G/A]	Ca_16485	--	NON_SYNONYMOUS_CODING
10859	CakSNP10859	*Kabuli*	Ca_Kabuli_Chr06	30927221	[C/G]	Ca_16485	--	NON_SYNONYMOUS_CODING
10860	CakSNP10860	*Kabuli*	Ca_Kabuli_Chr06	31011743	[A/G]	Ca_16492	RNA recognition motif domain	INTRON
10861	CakSNP10861	*Kabuli*	Ca_Kabuli_Chr06	31024728	[A/T]	Ca_16493	Zinc finger, Sec23/Sec24-type	SYNONYMOUS_CODING

S.N.	SNP IDs	Chickpea cultivars	Chromosomes	Physical positions (bp)	SNPs	Gene accession IDs	Putative functions	Sequence components of genome
10862	CakSNP10862	*Kabuli*	Ca_Kabuli_Chr06	31025560	[C/G]	Ca_16493	Zinc finger, Sec23/Sec24-type	INTRON
10863	CakSNP10863	*Kabuli*	Ca_Kabuli_Chr06	31038808	[C/T]		--	INTERGENIC
10864	CakSNP10864	*Kabuli*	Ca_Kabuli_Chr06	31048949	[T/C]	Ca_16497	Late embryogenesis abundant protein, group 2	SYNONYMOUS_CODING
10865	CakSNP10865	*Kabuli*	Ca_Kabuli_Chr06	31057806	[C/T]		--	INTERGENIC
10866	CakSNP10866	*Kabuli*	Ca_Kabuli_Chr06	31068678	[G/A]		--	INTERGENIC
10867	CakSNP10867	*Kabuli*	Ca_Kabuli_Chr06	31324962	[A/C]		--	INTERGENIC
10868	CakSNP10868	*Kabuli*	Ca_Kabuli_Chr06	31324976	[A/G]		--	INTERGENIC
10869	CakSNP10869	*Kabuli*	Ca_Kabuli_Chr06	31408895	[G/T]	Ca_15173	--	SYNONYMOUS_CODING
10870	CakSNP10870	*Kabuli*	Ca_Kabuli_Chr06	31408969	[C/T]	Ca_15173	--	INTRON
10871	CakSNP10871	*Kabuli*	Ca_Kabuli_Chr06	31495897	[C/A]	Ca_15179	Putative 5-3 exonuclease	INTRON

S.N.	SNP IDs	Chickpea cultivars	Chromosomes	Physical positions (bp)	SNPs	Gene accession IDs	Putative functions	Sequence components of genome
10872	CakSNP10872	*Kabuli*	Ca_Kabuli_Chr06	31495903	[A/G]	Ca_15179	Putative 5-3 exonuclease	INTRON
10873	CakSNP10873	*Kabuli*	Ca_Kabuli_Chr06	31592707	[A/C]	Ca_15186	Protein kinase, catalytic domain	SYNONYMOUS_CODING
10874	CakSNP10874	*Kabuli*	Ca_Kabuli_Chr06	31595142	[A/C]	Ca_15186	Protein kinase, catalytic domain	SYNONYMOUS_CODING
10875	CakSNP10875	*Kabuli*	Ca_Kabuli_Chr06	31595136	[A/T]	Ca_15186	Protein kinase, catalytic domain	NON_SYNONYMOUS_CODING
10876	CakSNP10876	*Kabuli*	Ca_Kabuli_Chr06	31895214	[G/C]	Ca_15200	Metallo-dependent phosphatase	INTRON
10877	CakSNP10877	*Kabuli*	Ca_Kabuli_Chr06	31895264	[C/T]	Ca_15200	Metallo-dependent phosphatase	INTRON
10878	CakSNP10878	*Kabuli*	Ca_Kabuli_Chr06	31900781	[T/G]	Ca_15200	Metallo-dependent phosphatase	INTRON
10879	CakSNP10879	*Kabuli*	Ca_Kabuli_Chr06	31900785	[T/G]	Ca_15200	Metallo-dependent phosphatase	INTRON
10880	CakSNP10880	*Kabuli*	Ca_Kabuli_Chr06	31900790	[A/C]	Ca_15200	Metallo-dependent phosphatase	INTRON
10881	CakSNP10881	*Kabuli*	Ca_Kabuli_Chr06	31966982	[C/T]	Ca_15203	Lipase, class 3	SYNONYMOUS_CODING

S.N.	SNP IDs	Chickpea cultivars	Chromosomes	Physical positions (bp)	SNPs	Gene accession IDs	Putative functions	Sequence components of genome
10882	CakSNP10882	*Kabuli*	Ca_Kabuli_Chr06	31967089	[T/G]	Ca_15203	Lipase, class 3	INTRON
10883	CakSNP10883	*Kabuli*	Ca_Kabuli_Chr06	31967106	[G/A]	Ca_15203	Lipase, class 3	INTRON
10884	CakSNP10884	*Kabuli*	Ca_Kabuli_Chr06	31967109	[T/C]	Ca_15203	Lipase, class 3	INTRON
10885	CakSNP10885	*Kabuli*	Ca_Kabuli_Chr06	31967144	[T/C]	Ca_15203	Lipase, class 3	INTRON
10886	CakSNP10886	*Kabuli*	Ca_Kabuli_Chr06	31967201	[C/T]	Ca_15203	Lipase, class 3	INTRON
10887	CakSNP10887	*Kabuli*	Ca_Kabuli_Chr06	31967195	[G/C]	Ca_15203	Lipase, class 3	INTRON
10888	CakSNP10888	*Kabuli*	Ca_Kabuli_Chr06	31992907	[A/G]	Ca_15204	--	INTRON
10889	CakSNP10889	*Kabuli*	Ca_Kabuli_Chr06	32035882	[C/T]	Ca_15208	MD-2-related lipid-recognition	INTRON
10890	CakSNP10890	*Kabuli*	Ca_Kabuli_Chr06	32036033	[G/A]	Ca_15208	MD-2-related lipid-recognition	INTRON
10891	CakSNP10891	*Kabuli*	Ca_Kabuli_Chr06	32035976	[T/C]	Ca_15208	MD-2-related lipid-recognition	INTRON

S.N.	SNP IDs	Chickpea cultivars	Chromosomes	Physical positions (bp)	SNPs	Gene accession IDs	Putative functions	Sequence components of genome
10892	CakSNP10892	*Kabuli*	Ca_Kabuli_Chr06	32140428	[C/A]	Ca_15214	BRCT	NON_SYNONYMOUS_CODING
10893	CakSNP10893	*Kabuli*	Ca_Kabuli_Chr06	32140429	[G/T]	Ca_15214	BRCT	SYNONYMOUS_CODING
10894	CakSNP10894	*Kabuli*	Ca_Kabuli_Chr06	32262807	[T/C]		--	INTERGENIC
10895	CakSNP10895	*Kabuli*	Ca_Kabuli_Chr06	32262855	[C/T]		--	INTERGENIC
10896	CakSNP10896	*Kabuli*	Ca_Kabuli_Chr06	32328468	[T/C]	Ca_15224	DNA-binding WRKY	NON_SYNONYMOUS_CODING
10897	CakSNP10897	*Kabuli*	Ca_Kabuli_Chr06	32717450	[G/A]	Ca_22219	Protein of unknown function DUF936, plant	INTRON
10898	CakSNP10898	*Kabuli*	Ca_Kabuli_Chr06	32717665	[C/A]	Ca_22219	Protein of unknown function DUF936, plant	INTRON
10899	CakSNP10899	*Kabuli*	Ca_Kabuli_Chr06	32825567	[C/A]		--	INTERGENIC
10900	CakSNP10900	*Kabuli*	Ca_Kabuli_Chr06	32825561	[C/T]		--	INTERGENIC
10901	CakSNP10901	*Kabuli*	Ca_Kabuli_Chr06	32825547	[A/G]		--	INTERGENIC

S.N.	SNP IDs	Chickpea cultivars	Chromosomes	Physical positions (bp)	SNPs	Gene accession IDs	Putative functions	Sequence components of genome
10902	CakSNP10902	*Kabuli*	Ca_Kabuli_Chr06	32825579	[C/T]		--	INTERGENIC
10903	CakSNP10903	*Kabuli*	Ca_Kabuli_Chr06	32825587	[G/A]		--	INTERGENIC
10904	CakSNP10904	*Kabuli*	Ca_Kabuli_Chr06	32825612	[G/A]		--	INTERGENIC
10905	CakSNP10905	*Kabuli*	Ca_Kabuli_Chr06	32825619	[T/C]		--	INTERGENIC
10906	CakSNP10906	*Kabuli*	Ca_Kabuli_Chr06	32825631	[C/A]		--	INTERGENIC
10907	CakSNP10907	*Kabuli*	Ca_Kabuli_Chr06	32914927	[C/G]	Ca_22229	Peptidase M48	INTRON
10908	CakSNP10908	*Kabuli*	Ca_Kabuli_Chr06	33502994	[C/A]		--	INTERGENIC
10909	CakSNP10909	*Kabuli*	Ca_Kabuli_Chr06	33502997	[T/A]		--	INTERGENIC
10910	CakSNP10910	*Kabuli*	Ca_Kabuli_Chr06	33503007	[T/C]		--	INTERGENIC
10911	CakSNP10911	*Kabuli*	Ca_Kabuli_Chr06	33503014	[G/T]		--	INTERGENIC

S.N.	SNP IDs	Chickpea cultivars	Chromosomes	Physical positions (bp)	SNPs	Gene accession IDs	Putative functions	Sequence components of genome
10912	CakSNP10912	*Kabuli*	Ca_Kabuli_Chr06	33503020	[G/A]		--	INTERGENIC
10913	CakSNP10913	*Kabuli*	Ca_Kabuli_Chr06	33503032	[A/C]		--	INTERGENIC
10914	CakSNP10914	*Kabuli*	Ca_Kabuli_Chr06	33503037	[A/T]		--	INTERGENIC
10915	CakSNP10915	*Kabuli*	Ca_Kabuli_Chr06	33503051	[G/C]		--	INTERGENIC
10916	CakSNP10916	*Kabuli*	Ca_Kabuli_Chr06	33503063	[C/G]		--	INTERGENIC
10917	CakSNP10917	*Kabuli*	Ca_Kabuli_Chr06	33503065	[C/A]		--	INTERGENIC
10918	CakSNP10918	*Kabuli*	Ca_Kabuli_Chr06	33503071	[A/G]		--	INTERGENIC
10919	CakSNP10919	*Kabuli*	Ca_Kabuli_Chr06	33598926	[G/A]	Ca_22069	--	INTRON
10920	CakSNP10920	*Kabuli*	Ca_Kabuli_Chr06	33704792	[C/T]		--	INTERGENIC
10921	CakSNP10921	*Kabuli*	Ca_Kabuli_Chr06	33704807	[C/A]		--	INTERGENIC

S.N.	SNP IDs	Chickpea cultivars	Chromosomes	Physical positions (bp)	SNPs	Gene accession IDs	Putative functions	Sequence components of genome
10922	CakSNP10922	*Kabuli*	Ca_Kabuli_Chr06	33704839	[C/T]		--	INTERGENIC
10923	CakSNP10923	*Kabuli*	Ca_Kabuli_Chr06	33704782	[C/T]		--	INTERGENIC
10924	CakSNP10924	*Kabuli*	Ca_Kabuli_Chr06	33733259	[A/G]	Ca_22063	Zinc finger, RING-type	SYNONYMOUS_CODING
10925	CakSNP10925	*Kabuli*	Ca_Kabuli_Chr06	34235650	[T/C]		--	INTERGENIC
10926	CakSNP10926	*Kabuli*	Ca_Kabuli_Chr06	34407309	[T/C]		--	INTERGENIC
10927	CakSNP10927	*Kabuli*	Ca_Kabuli_Chr06	34481266	[A/G]	Ca_15818	--	INTRON
10928	CakSNP10928	*Kabuli*	Ca_Kabuli_Chr06	34481256	[C/T]	Ca_15818	--	INTRON
10929	CakSNP10929	*Kabuli*	Ca_Kabuli_Chr06	34535695	[T/C]	Ca_15817	Major facilitator superfamily MFS-1	SYNONYMOUS_CODING
10930	CakSNP10930	*Kabuli*	Ca_Kabuli_Chr06	34535983	[T/C]	Ca_15817	Major facilitator superfamily MFS-1	SYNONYMOUS_CODING
10931	CakSNP10931	*Kabuli*	Ca_Kabuli_Chr06	34596792	[A/G]		--	INTERGENIC

S.N.	SNP IDs	Chickpea cultivars	Chromosomes	Physical positions (bp)	SNPs	Gene accession IDs	Putative functions	Sequence components of genome
10932	CakSNP10932	*Kabuli*	Ca_Kabuli_Chr06	34634028	[T/A]	Ca_15813	Conserved oligomeric Golgi complex, subunit 7	INTRON
10933	CakSNP10933	*Kabuli*	Ca_Kabuli_Chr06	34744433	[A/G]		--	INTERGENIC
10934	CakSNP10934	*Kabuli*	Ca_Kabuli_Chr06	34744438	[T/G]		--	INTERGENIC
10935	CakSNP10935	*Kabuli*	Ca_Kabuli_Chr06	34744681	[T/G]		--	INTERGENIC
10936	CakSNP10936	*Kabuli*	Ca_Kabuli_Chr06	34744665	[G/A]		--	INTERGENIC
10937	CakSNP10937	*Kabuli*	Ca_Kabuli_Chr06	34773476	[T/A]	Ca_15807	--	INTRON
10938	CakSNP10938	*Kabuli*	Ca_Kabuli_Chr06	34793312	[T/G]	Ca_15805	Major intrinsic protein	SYNONYMOUS_CODING
10939	CakSNP10939	*Kabuli*	Ca_Kabuli_Chr06	34974503	[C/G]	Ca_15797	Peptidase C19, ubiquitin carboxyl-terminal hydrolase 2	INTRON
10940	CakSNP10940	*Kabuli*	Ca_Kabuli_Chr06	35126227	[C/T]		--	INTERGENIC
10941	CakSNP10941	*Kabuli*	Ca_Kabuli_Chr06	35126273	[T/C]		--	INTERGENIC

S.N.	SNP IDs	Chickpea cultivars	Chromosomes	Physical positions (bp)	SNPs	Gene accession IDs	Putative functions	Sequence components of genome
10942	CakSNP10942	*Kabuli*	Ca_Kabuli_Chr06	35126389	[G/T]		--	INTERGENIC
10943	CakSNP10943	*Kabuli*	Ca_Kabuli_Chr06	35129588	[G/A]	Ca_15788	Zinc finger, PHD-type	SYNONYMOUS_CODING
10944	CakSNP10944	*Kabuli*	Ca_Kabuli_Chr06	35596037	[C/A]		--	INTERGENIC
10945	CakSNP10945	*Kabuli*	Ca_Kabuli_Chr06	35596048	[G/A]		--	INTERGENIC
10946	CakSNP10946	*Kabuli*	Ca_Kabuli_Chr06	35596064	[T/C]		--	INTERGENIC
10947	CakSNP10947	*Kabuli*	Ca_Kabuli_Chr06	35596070	[G/A]		--	INTERGENIC
10948	CakSNP10948	*Kabuli*	Ca_Kabuli_Chr06	35596161	[C/T]		--	INTERGENIC
10949	CakSNP10949	*Kabuli*	Ca_Kabuli_Chr06	35596174	[G/A]		--	INTERGENIC
10950	CakSNP10950	*Kabuli*	Ca_Kabuli_Chr06	35596184	[A/G]		--	INTERGENIC
10951	CakSNP10951	*Kabuli*	Ca_Kabuli_Chr06	35596195	[G/C]		--	INTERGENIC

S.N.	SNP IDs	Chickpea cultivars	Chromosomes	Physical positions (bp)	SNPs	Gene accession IDs	Putative functions	Sequence components of genome
10952	CakSNP10952	*Kabuli*	Ca_Kabuli_Chr06	35596197	[G/A]		--	INTERGENIC
10953	CakSNP10953	*Kabuli*	Ca_Kabuli_Chr06	35596207	[G/T]		--	INTERGENIC
10954	CakSNP10954	*Kabuli*	Ca_Kabuli_Chr06	35596218	[T/C]		--	INTERGENIC
10955	CakSNP10955	*Kabuli*	Ca_Kabuli_Chr06	35596220	[G/A]		--	INTERGENIC
10956	CakSNP10956	*Kabuli*	Ca_Kabuli_Chr06	35596230	[T/A]		--	INTERGENIC
10957	CakSNP10957	*Kabuli*	Ca_Kabuli_Chr06	35596300	[G/A]		--	INTERGENIC
10958	CakSNP10958	*Kabuli*	Ca_Kabuli_Chr06	35628131	[T/C]	Ca_15775	SNF2-related	INTRON
10959	CakSNP10959	*Kabuli*	Ca_Kabuli_Chr06	35719930	[A/C]	Ca_15773	--	INTRON
10960	CakSNP10960	*Kabuli*	Ca_Kabuli_Chr06	35743792	[A/G]	Ca_15772	--	SYNONYMOUS_CODING
10961	CakSNP10961	*Kabuli*	Ca_Kabuli_Chr06	35962309	[C/T]		--	INTERGENIC

S.N.	SNP IDs	Chickpea cultivars	Chromosomes	Physical positions (bp)	SNPs	Gene accession IDs	Putative functions	Sequence components of genome
10962	CakSNP10962	*Kabuli*	Ca_Kabuli_Chr06	35962320	[T/C]		--	INTERGENIC
10963	CakSNP10963	*Kabuli*	Ca_Kabuli_Chr06	35962360	[T/C]		--	INTERGENIC
10964	CakSNP10964	*Kabuli*	Ca_Kabuli_Chr06	35962364	[T/G]		--	INTERGENIC
10965	CakSNP10965	*Kabuli*	Ca_Kabuli_Chr06	35962365	[T/C]		--	INTERGENIC
10966	CakSNP10966	*Kabuli*	Ca_Kabuli_Chr06	35962366	[G/A]		--	INTERGENIC
10967	CakSNP10967	*Kabuli*	Ca_Kabuli_Chr06	36094855	[T/G]	Ca_26434	B302/SPRY domain	NON_SYNONYMOUS_CODING
10968	CakSNP10968	*Kabuli*	Ca_Kabuli_Chr06	36371775	[A/G]		--	INTERGENIC
10969	CakSNP10969	*Kabuli*	Ca_Kabuli_Chr06	36374561	[C/T]	Ca_23965	UvrABC complex, subunit B	INTRON
10970	CakSNP10970	*Kabuli*	Ca_Kabuli_Chr06	36574836	[A/G]		--	INTERGENIC
10971	CakSNP10971	*Kabuli*	Ca_Kabuli_Chr06	36790467	[G/A]	Ca_19812	--	NON_SYNONYMOUS_CODING

S.N.	SNP IDs	Chickpea cultivars	Chromosomes	Physical positions (bp)	SNPs	Gene accession IDs	Putative functions	Sequence components of genome
10972	CakSNP10972	*Kabuli*	Ca_Kabuli_Chr06	36790458	[G/A]	Ca_19812	--	NON_SYNONYMOUS_CODING
10973	CakSNP10973	*Kabuli*	Ca_Kabuli_Chr06	36790428	[G/A]	Ca_19812	--	NON_SYNONYMOUS_CODING
10974	CakSNP10974	*Kabuli*	Ca_Kabuli_Chr06	36790455	[C/T]	Ca_19812	--	NON_SYNONYMOUS_CODING
10975	CakSNP10975	*Kabuli*	Ca_Kabuli_Chr06	36790460	[A/G]	Ca_19812	--	SYNONYMOUS_CODING
10976	CakSNP10976	*Kabuli*	Ca_Kabuli_Chr06	37139425	[C/T]	Ca_19803	Peptidase C48, SUMO/Sentrin/Ubl1	SYNONYMOUS_CODING
10977	CakSNP10977	*Kabuli*	Ca_Kabuli_Chr06	37139466	[G/C]	Ca_19803	Peptidase C48, SUMO/Sentrin/Ubl1	SYNONYMOUS_CODING
10978	CakSNP10978	*Kabuli*	Ca_Kabuli_Chr06	37139484	[T/C]	Ca_19803	Peptidase C48, SUMO/Sentrin/Ubl1	NON_SYNONYMOUS_CODING
10979	CakSNP10979	*Kabuli*	Ca_Kabuli_Chr06	37139486	[G/A]	Ca_19803	Peptidase C48, SUMO/Sentrin/Ubl1	NON_SYNONYMOUS_CODING
10980	CakSNP10980	*Kabuli*	Ca_Kabuli_Chr06	37139533	[T/A]	Ca_19803	Peptidase C48, SUMO/Sentrin/Ubl1	SYNONYMOUS_CODING
10981	CakSNP10981	*Kabuli*	Ca_Kabuli_Chr06	37139514	[C/T]	Ca_19803	Peptidase C48, SUMO/Sentrin/Ubl1	NON_SYNONYMOUS_CODING

S.N.	SNP IDs	Chickpea cultivars	Chromosomes	Physical positions (bp)	SNPs	Gene accession IDs	Putative functions	Sequence components of genome
10982	CakSNP10982	*Kabuli*	Ca_Kabuli_Chr06	37139499	[C/G]	Ca_19803	Peptidase C48, SUMO/Sentrin/Ubl1	NON_SYNONYMOUS_CODING
10983	CakSNP10983	*Kabuli*	Ca_Kabuli_Chr06	37139610	[T/G]	Ca_19803	Peptidase C48, SUMO/Sentrin/Ubl1	NON_SYNONYMOUS_CODING
10984	CakSNP10984	*Kabuli*	Ca_Kabuli_Chr06	37139604	[G/T]	Ca_19803	Peptidase C48, SUMO/Sentrin/Ubl1	NON_SYNONYMOUS_CODING
10985	CakSNP10985	*Kabuli*	Ca_Kabuli_Chr06	37321783	[A/T]		--	INTERGENIC
10986	CakSNP10986	*Kabuli*	Ca_Kabuli_Chr06	37476426	[C/T]		--	INTERGENIC
10987	CakSNP10987	*Kabuli*	Ca_Kabuli_Chr06	37476437	[A/C]		--	INTERGENIC
10988	CakSNP10988	*Kabuli*	Ca_Kabuli_Chr06	37476516	[G/T]		--	INTERGENIC
10989	CakSNP10989	*Kabuli*	Ca_Kabuli_Chr06	37476465	[G/A]		--	INTERGENIC
10990	CakSNP10990	*Kabuli*	Ca_Kabuli_Chr06	37484467	[C/T]		--	INTERGENIC
10991	CakSNP10991	*Kabuli*	Ca_Kabuli_Chr06	37484468	[G/T]		--	INTERGENIC

S.N.	SNP IDs	Chickpea cultivars	Chromosomes	Physical positions (bp)	SNPs	Gene accession IDs	Putative functions	Sequence components of genome
10992	CakSNP10992	*Kabuli*	Ca_Kabuli_Chr06	37484470	[C/A]		--	INTERGENIC
10993	CakSNP10993	*Kabuli*	Ca_Kabuli_Chr06	37484489	[C/G]		--	INTERGENIC
10994	CakSNP10994	*Kabuli*	Ca_Kabuli_Chr06	37484513	[T/C]		--	INTERGENIC
10995	CakSNP10995	*Kabuli*	Ca_Kabuli_Chr06	38816345	[C/T]	Ca_15898	Transcription factor GRAS	SYNONYMOUS_CODING
10996	CakSNP10996	*Kabuli*	Ca_Kabuli_Chr06	38838320	[A/G]	Ca_15899	Calcium-binding EF-hand	NON_SYNONYMOUS_CODING
10997	CakSNP10997	*Kabuli*	Ca_Kabuli_Chr06	38840279	[G/A]		--	INTERGENIC
10998	CakSNP10998	*Kabuli*	Ca_Kabuli_Chr06	38840315	[G/A]		--	INTERGENIC
10999	CakSNP10999	*Kabuli*	Ca_Kabuli_Chr06	38840273	[G/A]		--	INTERGENIC
11000	CakSNP11000	*Kabuli*	Ca_Kabuli_Chr06	38881909	[C/A]		--	INTERGENIC
11001	CakSNP11001	*Kabuli*	Ca_Kabuli_Chr06	38886735	[C/A]		--	INTERGENIC

S.N.	SNP IDs	Chickpea cultivars	Chromosomes	Physical positions (bp)	SNPs	Gene accession IDs	Putative functions	Sequence components of genome
11002	CakSNP11002	*Kabuli*	Ca_Kabuli_Chr06	38994879	[A/G]	Ca_15903	--	NON_SYNONYMOUS_CODING
11003	CakSNP11003	*Kabuli*	Ca_Kabuli_Chr06	38994925	[G/T]	Ca_15903	--	NON_SYNONYMOUS_CODING
11004	CakSNP11004	*Kabuli*	Ca_Kabuli_Chr06	38994954	[A/G]	Ca_15903	--	NON_SYNONYMOUS_CODING
11005	CakSNP11005	*Kabuli*	Ca_Kabuli_Chr06	38994962	[G/A]	Ca_15903	--	SYNONYMOUS_CODING
11006	CakSNP11006	*Kabuli*	Ca_Kabuli_Chr06	39028564	[T/G]		--	INTERGENIC
11007	CakSNP11007	*Kabuli*	Ca_Kabuli_Chr06	39107187	[C/T]		--	INTERGENIC
11008	CakSNP11008	*Kabuli*	Ca_Kabuli_Chr06	39107149	[A/C]		--	INTERGENIC
11009	CakSNP11009	*Kabuli*	Ca_Kabuli_Chr06	39107188	[A/C]		--	INTERGENIC
11010	CakSNP11010	*Kabuli*	Ca_Kabuli_Chr06	39317016	[T/G]	Ca_15914	PAP/25A-associated	INTRON
11011	CakSNP11011	*Kabuli*	Ca_Kabuli_Chr06	39380625	[A/C]	Ca_15918	Resolvase, holliday junction-type, YqgF-like	INTRON

S.N.	SNP IDs	Chickpea cultivars	Chromosomes	Physical positions (bp)	SNPs	Gene accession IDs	Putative functions	Sequence components of genome
11012	CakSNP11012	*Kabuli*	Ca_Kabuli_Chr06	39908314	[T/G]	Ca_15931	Ku70/Ku80 C-terminal arm	NON_SYNONYMOUS_CODING
11013	CakSNP11013	*Kabuli*	Ca_Kabuli_Chr06	40047823	[A/G]	Ca_15938	Chromo domain	NON_SYNONYMOUS_CODING
11014	CakSNP11014	*Kabuli*	Ca_Kabuli_Chr06	40128644	[T/C]	Ca_15941	Metal-dependent phosphohydrolase, HD domain	INTRON
11015	CakSNP11015	*Kabuli*	Ca_Kabuli_Chr06	40468356	[T/A]	Ca_21602	--	INTRON
11016	CakSNP11016	*Kabuli*	Ca_Kabuli_Chr06	40477285	[G/A]	Ca_21600	Nitroreductase-like	NON_SYNONYMOUS_CODING
11017	CakSNP11017	*Kabuli*	Ca_Kabuli_Chr06	40657695	[A/C]	Ca_21596	Peptidase M22, glycoprotease	SYNONYMOUS_CODING
11018	CakSNP11018	*Kabuli*	Ca_Kabuli_Chr06	40735186	[C/A]	Ca_21593	Pentatricopeptide repeat	NON_SYNONYMOUS_CODING
11019	CakSNP11019	*Kabuli*	Ca_Kabuli_Chr06	40767566	[A/G]		--	INTERGENIC
11020	CakSNP11020	*Kabuli*	Ca_Kabuli_Chr06	40821354	[C/T]	Ca_21586	Transketolase, N-terminal	INTRON
11021	CakSNP11021	*Kabuli*	Ca_Kabuli_Chr06	40935441	[A/T]	Ca_21581	Exocyst complex component Sec6	INTRON

S.N.	SNP IDs	Chickpea cultivars	Chromosomes	Physical positions (bp)	SNPs	Gene accession IDs	Putative functions	Sequence components of genome
11022	CakSNP11022	*Kabuli*	Ca_Kabuli_Chr06	41121337	[C/T]	Ca_23143	--	NON_SYNONYMOUS_CODING
11023	CakSNP11023	*Kabuli*	Ca_Kabuli_Chr06	41121330	[G/A]	Ca_23143	--	NON_SYNONYMOUS_CODING
11024	CakSNP11024	*Kabuli*	Ca_Kabuli_Chr06	41363546	[G/A]		--	INTERGENIC
11025	CakSNP11025	*Kabuli*	Ca_Kabuli_Chr06	41420918	[T/A]		--	INTERGENIC
11026	CakSNP11026	*Kabuli*	Ca_Kabuli_Chr06	41420917	[A/G]		--	INTERGENIC
11027	CakSNP11027	*Kabuli*	Ca_Kabuli_Chr06	41449028	[C/T]		--	INTERGENIC
11028	CakSNP11028	*Kabuli*	Ca_Kabuli_Chr06	41682983	[T/C]		--	INTERGENIC
11029	CakSNP11029	*Kabuli*	Ca_Kabuli_Chr06	41682966	[C/T]		--	INTERGENIC
11030	CakSNP11030	*Kabuli*	Ca_Kabuli_Chr06	41682943	[T/C]		--	INTERGENIC
11031	CakSNP11031	*Kabuli*	Ca_Kabuli_Chr06	41697812	[T/G]	Ca_21940	Ribonuclease II/R	INTRON

S.N.	SNP IDs	Chickpea cultivars	Chromosomes	Physical positions (bp)	SNPs	Gene accession IDs	Putative functions	Sequence components of genome
11032	CakSNP11032	*Kabuli*	Ca_Kabuli_Chr06	41708309	[G/A]		--	INTERGENIC
11033	CakSNP11033	*Kabuli*	Ca_Kabuli_Chr06	41708306	[G/A]		--	INTERGENIC
11034	CakSNP11034	*Kabuli*	Ca_Kabuli_Chr06	41708523	[T/G]		--	INTERGENIC
11035	CakSNP11035	*Kabuli*	Ca_Kabuli_Chr06	41708474	[G/A]		--	INTERGENIC
11036	CakSNP11036	*Kabuli*	Ca_Kabuli_Chr06	41708530	[C/A]		--	INTERGENIC
11037	CakSNP11037	*Kabuli*	Ca_Kabuli_Chr06	41907459	[C/G]		--	INTERGENIC
11038	CakSNP11038	*Kabuli*	Ca_Kabuli_Chr06	42004851	[A/G]	Ca_21951	TGS	NON_SYNONYMOUS_CODING
11039	CakSNP11039	*Kabuli*	Ca_Kabuli_Chr06	42004829	[T/G]	Ca_21951	TGS	SYNONYMOUS_CODING
11040	CakSNP11040	*Kabuli*	Ca_Kabuli_Chr06	42004818	[C/G]	Ca_21951	TGS	SYNONYMOUS_CODING
11041	CakSNP11041	*Kabuli*	Ca_Kabuli_Chr06	42004813	[C/T]	Ca_21951	TGS	SYNONYMOUS_CODING

S.N.	SNP IDs	Chickpea cultivars	Chromosomes	Physical positions (bp)	SNPs	Gene accession IDs	Putative functions	Sequence components of genome
11042	CakSNP11042	*Kabuli*	Ca_Kabuli_Chr06	42161851	[A/G]		--	INTERGENIC
11043	CakSNP11043	*Kabuli*	Ca_Kabuli_Chr06	42161857	[C/G]		--	INTERGENIC
11044	CakSNP11044	*Kabuli*	Ca_Kabuli_Chr06	42161866	[T/G]		--	INTERGENIC
11045	CakSNP11045	*Kabuli*	Ca_Kabuli_Chr06	42521162	[G/T]	Ca_23238	UDP-glucuronosyl/UDP-glucosyltransferase	INTRON
11046	CakSNP11046	*Kabuli*	Ca_Kabuli_Chr06	42855091	[T/G]	Ca_23252	BTB/POZ-like	NON_SYNONYMOUS_CODING
11047	CakSNP11047	*Kabuli*	Ca_Kabuli_Chr06	42904998	[C/G]	Ca_23254	Peptidase C48, SUMO/Sentrin/Ubl1	NON_SYNONYMOUS_CODING
11048	CakSNP11048	*Kabuli*	Ca_Kabuli_Chr06	42905012	[G/A]	Ca_23254	Peptidase C48, SUMO/Sentrin/Ubl1	SYNONYMOUS_CODING
11049	CakSNP11049	*Kabuli*	Ca_Kabuli_Chr06	42905039	[C/T]	Ca_23254	Peptidase C48, SUMO/Sentrin/Ubl1	NON_SYNONYMOUS_CODING
11050	CakSNP11050	*Kabuli*	Ca_Kabuli_Chr06	42905035	[T/A]	Ca_23254	Peptidase C48, SUMO/Sentrin/Ubl1	NON_SYNONYMOUS_CODING
11051	CakSNP11051	*Kabuli*	Ca_Kabuli_Chr06	42905111	[T/C]	Ca_23254	Peptidase C48, SUMO/Sentrin/Ubl1	NON_SYNONYMOUS_CODING

S.N.	SNP IDs	Chickpea cultivars	Chromosomes	Physical positions (bp)	SNPs	Gene accession IDs	Putative functions	Sequence components of genome
11052	CakSNP11052	*Kabuli*	Ca_Kabuli_Chr06	42905094	[C/T]	Ca_23254	Peptidase C48, SUMO/Sentrin/Ubl1	NON_SYNONYMOUS_CODING
11053	CakSNP11053	*Kabuli*	Ca_Kabuli_Chr06	42905090	[G/A]	Ca_23254	Peptidase C48, SUMO/Sentrin/Ubl1	NON_SYNONYMOUS_CODING
11054	CakSNP11054	*Kabuli*	Ca_Kabuli_Chr06	42905086	[T/C]	Ca_23254	Peptidase C48, SUMO/Sentrin/Ubl1	SYNONYMOUS_CODING
11055	CakSNP11055	*Kabuli*	Ca_Kabuli_Chr06	42905058	[C/G]	Ca_23254	Peptidase C48, SUMO/Sentrin/Ubl1	NON_SYNONYMOUS_CODING
11056	CakSNP11056	*Kabuli*	Ca_Kabuli_Chr06	42905059	[A/G]	Ca_23254	Peptidase C48, SUMO/Sentrin/Ubl1	SYNONYMOUS_CODING
11057	CakSNP11057	*Kabuli*	Ca_Kabuli_Chr06	42905072	[T/C]	Ca_23254	Peptidase C48, SUMO/Sentrin/Ubl1	NON_SYNONYMOUS_CODING
11058	CakSNP11058	*Kabuli*	Ca_Kabuli_Chr06	42905115	[G/T]	Ca_23254	Peptidase C48, SUMO/Sentrin/Ubl1	NON_SYNONYMOUS_CODING
11059	CakSNP11059	*Kabuli*	Ca_Kabuli_Chr06	42905116	[A/T]	Ca_23254	Peptidase C48, SUMO/Sentrin/Ubl1	SYNONYMOUS_CODING
11060	CakSNP11060	*Kabuli*	Ca_Kabuli_Chr06	42919899	[G/A]	Ca_23255	Zinc finger, RING-type	INTRON
11061	CakSNP11061	*Kabuli*	Ca_Kabuli_Chr06	42923969	[A/C]	Ca_23256	--	NON_SYNONYMOUS_CODING

S.N.	SNP IDs	Chickpea cultivars	Chromosomes	Physical positions (bp)	SNPs	Gene accession IDs	Putative functions	Sequence components of genome
11062	CakSNP11062	*Kabuli*	Ca_Kabuli_Chr06	42925974	[C/T]		--	INTERGENIC
11063	CakSNP11063	*Kabuli*	Ca_Kabuli_Chr06	43148829	[C/T]		--	INTERGENIC
11064	CakSNP11064	*Kabuli*	Ca_Kabuli_Chr06	43148824	[C/A]		--	INTERGENIC
11065	CakSNP11065	*Kabuli*	Ca_Kabuli_Chr06	43148784	[C/G]		--	INTERGENIC
11066	CakSNP11066	*Kabuli*	Ca_Kabuli_Chr06	43905509	[A/T]		--	INTERGENIC
11067	CakSNP11067	*Kabuli*	Ca_Kabuli_Chr06	43907445	[T/C]		--	INTERGENIC
11068	CakSNP11068	*Kabuli*	Ca_Kabuli_Chr06	44530888	[C/A]	Ca_24241	Aspartate/glutamate/uridylate kinase	NON_SYNONYMOUS_CODING
11069	CakSNP11069	*Kabuli*	Ca_Kabuli_Chr06	44530889	[A/G]	Ca_24241	Aspartate/glutamate/uridylate kinase	SYNONYMOUS_CODING
11070	CakSNP11070	*Kabuli*	Ca_Kabuli_Chr06	44707313	[T/A]		--	INTERGENIC
11071	CakSNP11071	*Kabuli*	Ca_Kabuli_Chr06	44711166	[A/G]		--	INTERGENIC

S.N.	SNP IDs	Chickpea cultivars	Chromosomes	Physical positions (bp)	SNPs	Gene accession IDs	Putative functions	Sequence components of genome
11072	CakSNP11072	*Kabuli*	Ca_Kabuli_Chr06	44792535	[C/T]	Ca_13807	--	NON_SYNONYMOUS_CODING
11073	CakSNP11073	*Kabuli*	Ca_Kabuli_Chr06	44792529	[G/C]	Ca_13807	--	NON_SYNONYMOUS_CODING
11074	CakSNP11074	*Kabuli*	Ca_Kabuli_Chr06	44792493	[C/T]	Ca_13807	--	NON_SYNONYMOUS_CODING
11075	CakSNP11075	*Kabuli*	Ca_Kabuli_Chr06	44792486	[G/A]	Ca_13807	--	NON_SYNONYMOUS_CODING
11076	CakSNP11076	*Kabuli*	Ca_Kabuli_Chr06	44937666	[C/T]	Ca_13812	GDP-fucose protein O-fucosyltransferase	SYNONYMOUS_CODING
11077	CakSNP11077	*Kabuli*	Ca_Kabuli_Chr06	44944917	[G/A]	Ca_13813	Carbohydrate/puine kinase, PfkB, conserved site	NON_SYNONYMOUS_CODING
11078	CakSNP11078	*Kabuli*	Ca_Kabuli_Chr06	45053093	[A/G]		--	INTERGENIC
11079	CakSNP11079	*Kabuli*	Ca_Kabuli_Chr06	45053214	[C/T]		--	INTERGENIC
11080	CakSNP11080	*Kabuli*	Ca_Kabuli_Chr06	45131295	[C/T]	Ca_13822	--	INTRON
11081	CakSNP11081	*Kabuli*	Ca_Kabuli_Chr06	45132545	[A/G]		--	INTERGENIC

S.N.	SNP IDs	Chickpea cultivars	Chromosomes	Physical positions (bp)	SNPs	Gene accession IDs	Putative functions	Sequence components of genome
11082	CakSNP11082	*Kabuli*	Ca_Kabuli_Chr06	45178060	[G/A]		--	INTERGENIC
11083	CakSNP11083	*Kabuli*	Ca_Kabuli_Chr06	45178056	[A/G]		--	INTERGENIC
11084	CakSNP11084	*Kabuli*	Ca_Kabuli_Chr06	45178045	[G/T]		--	INTERGENIC
11085	CakSNP11085	*Kabuli*	Ca_Kabuli_Chr06	45178044	[G/A]		--	INTERGENIC
11086	CakSNP11086	*Kabuli*	Ca_Kabuli_Chr06	45178033	[C/A]		--	INTERGENIC
11087	CakSNP11087	*Kabuli*	Ca_Kabuli_Chr06	45178061	[C/T]		--	INTERGENIC
11088	CakSNP11088	*Kabuli*	Ca_Kabuli_Chr06	45201783	[C/T]		--	INTERGENIC
11089	CakSNP11089	*Kabuli*	Ca_Kabuli_Chr06	45293271	[T/C]		--	INTERGENIC
11090	CakSNP11090	*Kabuli*	Ca_Kabuli_Chr06	45500961	[T/A]	Ca_13841	Ankyrin repeat	NON_SYNONYMOUS_CODING
11091	CakSNP11091	*Kabuli*	Ca_Kabuli_Chr06	45501099	[C/A]	Ca_13841	Ankyrin repeat	NON_SYNONYMOUS_CODING

S.N.	SNP IDs	Chickpea cultivars	Chromosomes	Physical positions (bp)	SNPs	Gene accession IDs	Putative functions	Sequence components of genome
11092	CakSNP11092	*Kabuli*	Ca_Kabuli_Chr06	45505942	[A/G]	Ca_13841	Ankyrin repeat	NON_SYNONYMOUS_CODING
11093	CakSNP11093	*Kabuli*	Ca_Kabuli_Chr06	45550031	[C/A]		--	INTERGENIC
11094	CakSNP11094	*Kabuli*	Ca_Kabuli_Chr06	45554040	[G/A]	Ca_13848	--	SYNONYMOUS_CODING
11095	CakSNP11095	*Kabuli*	Ca_Kabuli_Chr06	45557656	[T/C]	Ca_13848	--	SYNONYMOUS_CODING
11096	CakSNP11096	*Kabuli*	Ca_Kabuli_Chr06	45614017	[T/C]	Ca_13849	RNA recognition motif domain	SYNONYMOUS_CODING
11097	CakSNP11097	*Kabuli*	Ca_Kabuli_Chr06	45637271	[T/G]	Ca_13850	--	NON_SYNONYMOUS_CODING
11098	CakSNP11098	*Kabuli*	Ca_Kabuli_Chr06	45637292	[C/A]	Ca_13850	--	STOP_GAINED
11099	CakSNP11099	*Kabuli*	Ca_Kabuli_Chr06	45637293	[T/C]	Ca_13850	--	SYNONYMOUS_CODING
11100	CakSNP11100	*Kabuli*	Ca_Kabuli_Chr06	45637322	[C/A]	Ca_13850	--	NON_SYNONYMOUS_CODING
11101	CakSNP11101	*Kabuli*	Ca_Kabuli_Chr06	45637309	[G/A]	Ca_13850	--	NON_SYNONYMOUS_CODING

S.N.	SNP IDs	Chickpea cultivars	Chromosomes	Physical positions (bp)	SNPs	Gene accession IDs	Putative functions	Sequence components of genome
11102	CakSNP11102	*Kabuli*	Ca_Kabuli_Chr06	45637308	[G/T]	Ca_13850	--	SYNONYMOUS_CODING
11103	CakSNP11103	*Kabuli*	Ca_Kabuli_Chr06	45693275	[G/A]		--	INTERGENIC
11104	CakSNP11104	*Kabuli*	Ca_Kabuli_Chr06	45695051	[T/G]		--	INTERGENIC
11105	CakSNP11105	*Kabuli*	Ca_Kabuli_Chr06	45695043	[C/T]		--	INTERGENIC
11106	CakSNP11106	*Kabuli*	Ca_Kabuli_Chr06	45695035	[G/A]		--	INTERGENIC
11107	CakSNP11107	*Kabuli*	Ca_Kabuli_Chr06	45709318	[T/C]	Ca_13854	DNA-binding WRKY	SYNONYMOUS_CODING
11108	CakSNP11108	*Kabuli*	Ca_Kabuli_Chr06	45773318	[C/T]	Ca_13859	Disease resistance protein	NON_SYNONYMOUS_CODING
11109	CakSNP11109	*Kabuli*	Ca_Kabuli_Chr06	45773309	[A/G]	Ca_13859	Disease resistance protein	NON_SYNONYMOUS_CODING
11110	CakSNP11110	*Kabuli*	Ca_Kabuli_Chr06	45773298	[C/G]	Ca_13859	Disease resistance protein	NON_SYNONYMOUS_CODING
11111	CakSNP11111	*Kabuli*	Ca_Kabuli_Chr06	45773409	[T/A]	Ca_13859	Disease resistance protein	SYNONYMOUS_CODING

S.N.	SNP IDs	Chickpea cultivars	Chromosomes	Physical positions (bp)	SNPs	Gene accession IDs	Putative functions	Sequence components of genome
11112	CakSNP11112	*Kabuli*	Ca_Kabuli_Chr06	45773408	[T/A]	Ca_13859	Disease resistance protein	NON_SYNONYMOUS_CODING
11113	CakSNP11113	*Kabuli*	Ca_Kabuli_Chr06	45773373	[G/T]	Ca_13859	Disease resistance protein	NON_SYNONYMOUS_CODING
11114	CakSNP11114	*Kabuli*	Ca_Kabuli_Chr06	45773367	[G/T]	Ca_13859	Disease resistance protein	SYNONYMOUS_CODING
11115	CakSNP11115	*Kabuli*	Ca_Kabuli_Chr06	45773349	[C/T]	Ca_13859	Disease resistance protein	SYNONYMOUS_CODING
11116	CakSNP11116	*Kabuli*	Ca_Kabuli_Chr06	45812152	[G/A]	Ca_13861	ABC transporter, transmembrane domain	NON_SYNONYMOUS_CODING
11117	CakSNP11117	*Kabuli*	Ca_Kabuli_Chr06	45976962	[G/A]		--	INTERGENIC
11118	CakSNP11118	*Kabuli*	Ca_Kabuli_Chr06	45978206	[T/C]		--	INTERGENIC
11119	CakSNP11119	*Kabuli*	Ca_Kabuli_Chr06	45978263	[G/A]		--	INTERGENIC
11120	CakSNP11120	*Kabuli*	Ca_Kabuli_Chr06	46158211	[C/T]	Ca_13881	Aminoacyl-tRNA synthetase, class I, conserved site	SYNONYMOUS_CODING
11121	CakSNP11121	*Kabuli*	Ca_Kabuli_Chr06	46734706	[C/G]	Ca_17739	--	INTRON

S.N.	SNP IDs	Chickpea cultivars	Chromosomes	Physical positions (bp)	SNPs	Gene accession IDs	Putative functions	Sequence components of genome
11122	CakSNP11122	*Kabuli*	Ca_Kabuli_Chr06	46734725	[G/A]	Ca_17739	--	INTRON
11123	CakSNP11123	*Kabuli*	Ca_Kabuli_Chr06	46734726	[T/C]	Ca_17739	--	INTRON
11124	CakSNP11124	*Kabuli*	Ca_Kabuli_Chr06	46765659	[G/A]	Ca_17737	Peptidase C48, SUMO/Sentrin/Ubl1	NON_SYNONYMOUS_CODING
11125	CakSNP11125	*Kabuli*	Ca_Kabuli_Chr06	46765661	[A/T]	Ca_17737	Peptidase C48, SUMO/Sentrin/Ubl1	SYNONYMOUS_CODING
11126	CakSNP11126	*Kabuli*	Ca_Kabuli_Chr06	46765673	[G/A]	Ca_17737	Peptidase C48, SUMO/Sentrin/Ubl1	SYNONYMOUS_CODING
11127	CakSNP11127	*Kabuli*	Ca_Kabuli_Chr06	46765688	[A/C]	Ca_17737	Peptidase C48, SUMO/Sentrin/Ubl1	SYNONYMOUS_CODING
11128	CakSNP11128	*Kabuli*	Ca_Kabuli_Chr06	46765725	[C/T]	Ca_17737	Peptidase C48, SUMO/Sentrin/Ubl1	NON_SYNONYMOUS_CODING
11129	CakSNP11129	*Kabuli*	Ca_Kabuli_Chr06	46765778	[C/A]	Ca_17737	Peptidase C48, SUMO/Sentrin/Ubl1	SYNONYMOUS_CODING
11130	CakSNP11130	*Kabuli*	Ca_Kabuli_Chr06	46765696	[C/T]	Ca_17737	Peptidase C48, SUMO/Sentrin/Ubl1	NON_SYNONYMOUS_CODING
11131	CakSNP11131	*Kabuli*	Ca_Kabuli_Chr06	46830497	[A/T]		--	INTERGENIC

S.N.	SNP IDs	Chickpea cultivars	Chromosomes	Physical positions (bp)	SNPs	Gene accession IDs	Putative functions	Sequence components of genome
11132	CakSNP11132	*Kabuli*	Ca_Kabuli_Chr06	46896903	[C/T]		--	INTERGENIC
11133	CakSNP11133	*Kabuli*	Ca_Kabuli_Chr06	46896910	[A/C]		--	INTERGENIC
11134	CakSNP11134	*Kabuli*	Ca_Kabuli_Chr06	46898225	[A/C]		--	INTERGENIC
11135	CakSNP11135	*Kabuli*	Ca_Kabuli_Chr06	46898305	[T/G]		--	INTERGENIC
11136	CakSNP11136	*Kabuli*	Ca_Kabuli_Chr06	46898316	[T/G]		--	INTERGENIC
11137	CakSNP11137	*Kabuli*	Ca_Kabuli_Chr06	46898317	[C/T]		--	INTERGENIC
11138	CakSNP11138	*Kabuli*	Ca_Kabuli_Chr06	46898363	[C/T]		--	INTERGENIC
11139	CakSNP11139	*Kabuli*	Ca_Kabuli_Chr06	46898329	[T/A]		--	INTERGENIC
11140	CakSNP11140	*Kabuli*	Ca_Kabuli_Chr06	46898747	[G/A]		--	INTERGENIC
11141	CakSNP11141	*Kabuli*	Ca_Kabuli_Chr06	46898743	[A/G]		--	INTERGENIC

S.N.	SNP IDs	Chickpea cultivars	Chromosomes	Physical positions (bp)	SNPs	Gene accession IDs	Putative functions	Sequence components of genome
11142	CakSNP11142	*Kabuli*	Ca_Kabuli_Chr06	46899229	[G/A]		--	INTERGENIC
11143	CakSNP11143	*Kabuli*	Ca_Kabuli_Chr06	46979240	[C/T]		--	INTERGENIC
11144	CakSNP11144	*Kabuli*	Ca_Kabuli_Chr06	46979267	[C/A]		--	INTERGENIC
11145	CakSNP11145	*Kabuli*	Ca_Kabuli_Chr06	46998948	[G/A]		--	INTERGENIC
11146	CakSNP11146	*Kabuli*	Ca_Kabuli_Chr06	46999012	[C/T]		--	INTERGENIC
11147	CakSNP11147	*Kabuli*	Ca_Kabuli_Chr06	47104478	[T/G]		--	INTERGENIC
11148	CakSNP11148	*Kabuli*	Ca_Kabuli_Chr06	47104505	[C/T]	Ca_17723	Mitochondrial carrier protein	NON_SYNONYMOUS_CODING
11149	CakSNP11149	*Kabuli*	Ca_Kabuli_Chr06	47106174	[A/G]	Ca_17723	Mitochondrial carrier protein	SYNONYMOUS_CODING
11150	CakSNP11150	*Kabuli*	Ca_Kabuli_Chr06	47106238	[C/A]	Ca_17723	Mitochondrial carrier protein	INTRON
11151	CakSNP11151	*Kabuli*	Ca_Kabuli_Chr06	47232630	[C/T]	Ca_17716	Nicotinamide N-methyltransferase, putative	INTRON

S.N.	SNP IDs	Chickpea cultivars	Chromosomes	Physical positions (bp)	SNPs	Gene accession IDs	Putative functions	Sequence components of genome
11152	CakSNP11152	*Kabuli*	Ca_Kabuli_Chr06	47232631	[C/G]	Ca_17716	Nicotinamide N-methyltransferase, putative	INTRON
11153	CakSNP11153	*Kabuli*	Ca_Kabuli_Chr06	47232685	[T/A]		--	INTERGENIC
11154	CakSNP11154	*Kabuli*	Ca_Kabuli_Chr06	47440468	[A/T]	Ca_17707	Zinc finger, PHD-type	INTRON
11155	CakSNP11155	*Kabuli*	Ca_Kabuli_Chr06	47575655	[G/T]		--	INTERGENIC
11156	CakSNP11156	*Kabuli*	Ca_Kabuli_Chr06	47575659	[C/G]		--	INTERGENIC
11157	CakSNP11157	*Kabuli*	Ca_Kabuli_Chr06	47906683	[G/A]	Ca_23445	Zinc finger, CCHC-type	INTRON
11158	CakSNP11158	*Kabuli*	Ca_Kabuli_Chr06	47906680	[C/T]	Ca_23445	Zinc finger, CCHC-type	INTRON
11159	CakSNP11159	*Kabuli*	Ca_Kabuli_Chr06	47906629	[T/G]	Ca_23445	Zinc finger, CCHC-type	INTRON
11160	CakSNP11160	*Kabuli*	Ca_Kabuli_Chr06	48088550	[G/A]		--	INTERGENIC
11161	CakSNP11161	*Kabuli*	Ca_Kabuli_Chr06	48088592	[C/T]		--	INTERGENIC

S.N.	SNP IDs	Chickpea cultivars	Chromosomes	Physical positions (bp)	SNPs	Gene accession IDs	Putative functions	Sequence components of genome
11162	CakSNP11162	*Kabuli*	Ca_Kabuli_Chr06	48255392	[G/A]		--	INTERGENIC
11163	CakSNP11163	*Kabuli*	Ca_Kabuli_Chr06	48255538	[A/G]		--	INTERGENIC
11164	CakSNP11164	*Kabuli*	Ca_Kabuli_Chr06	48255558	[C/T]		--	INTERGENIC
11165	CakSNP11165	*Kabuli*	Ca_Kabuli_Chr06	48255559	[A/G]		--	INTERGENIC
11166	CakSNP11166	*Kabuli*	Ca_Kabuli_Chr06	48255568	[A/G]		--	INTERGENIC
11167	CakSNP11167	*Kabuli*	Ca_Kabuli_Chr06	48255599	[C/A]		--	INTERGENIC
11168	CakSNP11168	*Kabuli*	Ca_Kabuli_Chr06	48314052	[T/A]	Ca_24606	--	SYNONYMOUS_CODING
11169	CakSNP11169	*Kabuli*	Ca_Kabuli_Chr06	48314788	[T/C]		--	INTERGENIC
11170	CakSNP11170	*Kabuli*	Ca_Kabuli_Chr06	48315194	[C/A]		--	INTERGENIC
11171	CakSNP11171	*Kabuli*	Ca_Kabuli_Chr06	48315340	[C/A]		--	INTERGENIC

S.N.	SNP IDs	Chickpea cultivars	Chromosomes	Physical positions (bp)	SNPs	Gene accession IDs	Putative functions	Sequence components of genome
11172	CakSNP11172	*Kabuli*	Ca_Kabuli_Chr06	48315343	[G/T]		--	INTERGENIC
11173	CakSNP11173	*Kabuli*	Ca_Kabuli_Chr06	48810611	[A/T]	Ca_20703	--	INTRON
11174	CakSNP11174	*Kabuli*	Ca_Kabuli_Chr06	48810608	[C/T]	Ca_20703	--	INTRON
11175	CakSNP11175	*Kabuli*	Ca_Kabuli_Chr06	48864622	[T/C]		--	INTERGENIC
11176	CakSNP11176	*Kabuli*	Ca_Kabuli_Chr06	48864625	[G/A]		--	INTERGENIC
11177	CakSNP11177	*Kabuli*	Ca_Kabuli_Chr06	48864639	[A/C]		--	INTERGENIC
11178	CakSNP11178	*Kabuli*	Ca_Kabuli_Chr06	48864699	[T/C]		--	INTERGENIC
11179	CakSNP11179	*Kabuli*	Ca_Kabuli_Chr06	48869462	[C/T]		--	INTERGENIC
11180	CakSNP11180	*Kabuli*	Ca_Kabuli_Chr06	48869473	[C/A]		--	INTERGENIC
11181	CakSNP11181	*Kabuli*	Ca_Kabuli_Chr06	48869484	[G/A]		--	INTERGENIC

S.N.	SNP IDs	Chickpea cultivars	Chromosomes	Physical positions (bp)	SNPs	Gene accession IDs	Putative functions	Sequence components of genome
11182	CakSNP11182	*Kabuli*	Ca_Kabuli_Chr06	48869577	[C/T]		--	INTERGENIC
11183	CakSNP11183	*Kabuli*	Ca_Kabuli_Chr06	48869539	[A/G]		--	INTERGENIC
11184	CakSNP11184	*Kabuli*	Ca_Kabuli_Chr06	48869529	[T/C]		--	INTERGENIC
11185	CakSNP11185	*Kabuli*	Ca_Kabuli_Chr06	48869512	[G/C]		--	INTERGENIC
11186	CakSNP11186	*Kabuli*	Ca_Kabuli_Chr06	48940386	[A/C]	Ca_20710	Clathrin adaptor complex, small chain	SYNONYMOUS_CODING
11187	CakSNP11187	*Kabuli*	Ca_Kabuli_Chr06	49044772	[A/C]		--	INTERGENIC
11188	CakSNP11188	*Kabuli*	Ca_Kabuli_Chr06	49044785	[C/T]		--	INTERGENIC
11189	CakSNP11189	*Kabuli*	Ca_Kabuli_Chr06	49044794	[T/A]		--	INTERGENIC
11190	CakSNP11190	*Kabuli*	Ca_Kabuli_Chr06	49044802	[T/C]		--	INTERGENIC
11191	CakSNP11191	*Kabuli*	Ca_Kabuli_Chr06	49044805	[A/G]		--	INTERGENIC

S.N.	SNP IDs	Chickpea cultivars	Chromosomes	Physical positions (bp)	SNPs	Gene accession IDs	Putative functions	Sequence components of genome
11192	CakSNP11192	*Kabuli*	Ca_Kabuli_Chr06	49044814	[G/T]		--	INTERGENIC
11193	CakSNP11193	*Kabuli*	Ca_Kabuli_Chr06	49044824	[C/T]		--	INTERGENIC
11194	CakSNP11194	*Kabuli*	Ca_Kabuli_Chr06	49044839	[T/A]		--	INTERGENIC
11195	CakSNP11195	*Kabuli*	Ca_Kabuli_Chr06	49044850	[A/C]		--	INTERGENIC
11196	CakSNP11196	*Kabuli*	Ca_Kabuli_Chr06	49044847	[G/A]		--	INTERGENIC
11197	CakSNP11197	*Kabuli*	Ca_Kabuli_Chr06	49044842	[G/A]		--	INTERGENIC
11198	CakSNP11198	*Kabuli*	Ca_Kabuli_Chr06	49044838	[C/A]		--	INTERGENIC
11199	CakSNP11199	*Kabuli*	Ca_Kabuli_Chr06	49044835	[C/A]		--	INTERGENIC
11200	CakSNP11200	*Kabuli*	Ca_Kabuli_Chr06	49044826	[G/A]		--	INTERGENIC
11201	CakSNP11201	*Kabuli*	Ca_Kabuli_Chr06	49044821	[C/G]		--	INTERGENIC

S.N.	SNP IDs	Chickpea cultivars	Chromosomes	Physical positions (bp)	SNPs	Gene accession IDs	Putative functions	Sequence components of genome
11202	CakSNP11202	*Kabuli*	Ca_Kabuli_Chr06	49044810	[G/A]		--	INTERGENIC
11203	CakSNP11203	*Kabuli*	Ca_Kabuli_Chr06	49044808	[C/T]		--	INTERGENIC
11204	CakSNP11204	*Kabuli*	Ca_Kabuli_Chr06	49044788	[T/A]		--	INTERGENIC
11205	CakSNP11205	*Kabuli*	Ca_Kabuli_Chr06	49044780	[G/A]		--	INTERGENIC
11206	CakSNP11206	*Kabuli*	Ca_Kabuli_Chr06	49044915	[A/G]		--	INTERGENIC
11207	CakSNP11207	*Kabuli*	Ca_Kabuli_Chr06	49044928	[C/A]		--	INTERGENIC
11208	CakSNP11208	*Kabuli*	Ca_Kabuli_Chr06	49044936	[C/T]		--	INTERGENIC
11209	CakSNP11209	*Kabuli*	Ca_Kabuli_Chr06	49044937	[A/G]		--	INTERGENIC
11210	CakSNP11210	*Kabuli*	Ca_Kabuli_Chr06	49044944	[A/G]		--	INTERGENIC
11211	CakSNP11211	*Kabuli*	Ca_Kabuli_Chr06	49101829	[G/C]		--	INTERGENIC

S.N.	SNP IDs	Chickpea cultivars	Chromosomes	Physical positions (bp)	SNPs	Gene accession IDs	Putative functions	Sequence components of genome
11212	CakSNP11212	*Kabuli*	Ca_Kabuli_Chr06	49167341	[T/G]	Ca_20722	Macrophage migration inhibitory factor	SYNONYMOUS_CODING
11213	CakSNP11213	*Kabuli*	Ca_Kabuli_Chr06	49222291	[G/A]		--	INTERGENIC
11214	CakSNP11214	*Kabuli*	Ca_Kabuli_Chr06	49222290	[C/T]		--	INTERGENIC
11215	CakSNP11215	*Kabuli*	Ca_Kabuli_Chr06	49222275	[A/C]		--	INTERGENIC
11216	CakSNP11216	*Kabuli*	Ca_Kabuli_Chr06	49222268	[T/A]		--	INTERGENIC
11217	CakSNP11217	*Kabuli*	Ca_Kabuli_Chr06	49222251	[G/A]		--	INTERGENIC
11218	CakSNP11218	*Kabuli*	Ca_Kabuli_Chr06	49222235	[G/C]		--	INTERGENIC
11219	CakSNP11219	*Kabuli*	Ca_Kabuli_Chr06	49377275	[G/A]		--	INTERGENIC
11220	CakSNP11220	*Kabuli*	Ca_Kabuli_Chr06	49403430	[G/C]		--	INTERGENIC
11221	CakSNP11221	*Kabuli*	Ca_Kabuli_Chr06	49406604	[G/A]	Ca_13521	Ferroporti-1	INTRON

S.N.	SNP IDs	Chickpea cultivars	Chromosomes	Physical positions (bp)	SNPs	Gene accession IDs	Putative functions	Sequence components of genome
11222	CakSNP11222	*Kabuli*	Ca_Kabuli_Chr06	49406599	[C/A]	Ca_13521	Ferroporti-1	INTRON
11223	CakSNP11223	*Kabuli*	Ca_Kabuli_Chr06	49466066	[A/G]	Ca_13518	--	INTRON
11224	CakSNP11224	*Kabuli*	Ca_Kabuli_Chr06	49475716	[G/A]	Ca_13517	AMP-dependent synthetase/ligase	INTRON
11225	CakSNP11225	*Kabuli*	Ca_Kabuli_Chr06	49475715	[A/G]	Ca_13517	AMP-dependent synthetase/ligase	INTRON
11226	CakSNP11226	*Kabuli*	Ca_Kabuli_Chr06	49524179	[A/T]	Ca_13512	--	INTRON
11227	CakSNP11227	*Kabuli*	Ca_Kabuli_Chr06	49565934	[C/T]	Ca_13509	Ribosomal protein L37e	START_LOST
11228	CakSNP11228	*Kabuli*	Ca_Kabuli_Chr06	49664132	[C/G]	Ca_13499	Domain of unknown function DUF632	SYNONYMOUS_CODING
11229	CakSNP11229	*Kabuli*	Ca_Kabuli_Chr06	49724448	[C/T]	Ca_13495	Peptidase C48, SUMO/Sentrin/Ubl1	SYNONYMOUS_CODING
11230	CakSNP11230	*Kabuli*	Ca_Kabuli_Chr06	49724515	[C/T]	Ca_13495	Peptidase C48, SUMO/Sentrin/Ubl1	NON_SYNONYMOUS_CODING
11231	CakSNP11231	*Kabuli*	Ca_Kabuli_Chr06	49726734	[C/G]	Ca_13494	--	NON_SYNONYMOUS_CODING

S.N.	SNP IDs	Chickpea cultivars	Chromosomes	Physical positions (bp)	SNPs	Gene accession IDs	Putative functions	Sequence components of genome
11232	CakSNP11232	*Kabuli*	Ca_Kabuli_Chr06	49726732	[T/C]	Ca_13494	--	NON_SYNONYMOUS_CODING
11233	CakSNP11233	*Kabuli*	Ca_Kabuli_Chr06	49726729	[G/A]	Ca_13494	--	SYNONYMOUS_CODING
11234	CakSNP11234	*Kabuli*	Ca_Kabuli_Chr06	49726711	[C/T]	Ca_13494	--	NON_SYNONYMOUS_CODING
11235	CakSNP11235	*Kabuli*	Ca_Kabuli_Chr06	49726723	[G/A]	Ca_13494	--	NON_SYNONYMOUS_CODING
11236	CakSNP11236	*Kabuli*	Ca_Kabuli_Chr06	49830299	[A/G]	Ca_13490	Heat shock protein Hsp70	NON_SYNONYMOUS_CODING
11237	CakSNP11237	*Kabuli*	Ca_Kabuli_Chr06	49830384	[G/A]	Ca_13490	Heat shock protein Hsp70	SYNONYMOUS_CODING
11238	CakSNP11238	*Kabuli*	Ca_Kabuli_Chr06	49833934	[C/T]	Ca_13490	Heat shock protein Hsp70	NON_SYNONYMOUS_CODING
11239	CakSNP11239	*Kabuli*	Ca_Kabuli_Chr06	49843667	[G/A]	Ca_13488	Heat shock protein Hsp70	SYNONYMOUS_CODING
11240	CakSNP11240	*Kabuli*	Ca_Kabuli_Chr06	49846257	[A/T]	Ca_13488	Heat shock protein Hsp70	NON_SYNONYMOUS_CODING
11241	CakSNP11241	*Kabuli*	Ca_Kabuli_Chr06	49846303	[T/G]	Ca_13488	Heat shock protein Hsp70	SYNONYMOUS_CODING

S.N.	SNP IDs	Chickpea cultivars	Chromosomes	Physical positions (bp)	SNPs	Gene accession IDs	Putative functions	Sequence components of genome
11242	CakSNP11242	*Kabuli*	Ca_Kabuli_Chr06	49931821	[C/T]	Ca_13486	WD40 repeat	INTRON
11243	CakSNP11243	*Kabuli*	Ca_Kabuli_Chr06	49931879	[T/C]	Ca_13486	WD40 repeat	INTRON
11244	CakSNP11244	*Kabuli*	Ca_Kabuli_Chr06	49942397	[A/G]		--	INTERGENIC
11245	CakSNP11245	*Kabuli*	Ca_Kabuli_Chr06	50086781	[A/G]		--	INTERGENIC
11246	CakSNP11246	*Kabuli*	Ca_Kabuli_Chr06	50152053	[C/T]		--	INTERGENIC
11247	CakSNP11247	*Kabuli*	Ca_Kabuli_Chr06	50152129	[C/T]		--	INTERGENIC
11248	CakSNP11248	*Kabuli*	Ca_Kabuli_Chr06	50152124	[C/T]		--	INTERGENIC
11249	CakSNP11249	*Kabuli*	Ca_Kabuli_Chr06	50152119	[T/C]		--	INTERGENIC
11250	CakSNP11250	*Kabuli*	Ca_Kabuli_Chr06	50152083	[C/T]		--	INTERGENIC
11251	CakSNP11251	*Kabuli*	Ca_Kabuli_Chr06	50217734	[G/A]		--	INTERGENIC

S.N.	SNP IDs	Chickpea cultivars	Chromosomes	Physical positions (bp)	SNPs	Gene accession IDs	Putative functions	Sequence components of genome
11252	CakSNP11252	*Kabuli*	Ca_Kabuli_Chr06	50356239	[A/G]		--	INTERGENIC
11253	CakSNP11253	*Kabuli*	Ca_Kabuli_Chr06	50356229	[C/T]		--	INTERGENIC
11254	CakSNP11254	*Kabuli*	Ca_Kabuli_Chr06	50356222	[C/A]		--	INTERGENIC
11255	CakSNP11255	*Kabuli*	Ca_Kabuli_Chr06	50356217	[T/C]		--	INTERGENIC
11256	CakSNP11256	*Kabuli*	Ca_Kabuli_Chr06	50356206	[G/T]		--	INTERGENIC
11257	CakSNP11257	*Kabuli*	Ca_Kabuli_Chr06	50356201	[T/C]		--	INTERGENIC
11258	CakSNP11258	*Kabuli*	Ca_Kabuli_Chr06	50382452	[C/T]	Ca_13465	ABC transporter-like	SYNONYMOUS_CODING
11259	CakSNP11259	*Kabuli*	Ca_Kabuli_Chr06	50384101	[A/C]	Ca_13465	ABC transporter-like	START_LOST
11260	CakSNP11260	*Kabuli*	Ca_Kabuli_Chr06	50390752	[A/G]	Ca_13464	ABC transporter-like	INTRON
11261	CakSNP11261	*Kabuli*	Ca_Kabuli_Chr06	50476721	[T/C]	Ca_13459	--	SYNONYMOUS_CODING

S.N.	SNP IDs	Chickpea cultivars	Chromosomes	Physical positions (bp)	SNPs	Gene accession IDs	Putative functions	Sequence components of genome
11262	CakSNP11262	*Kabuli*	Ca_Kabuli_Chr06	50476775	[G/A]	Ca_13459	--	SYNONYMOUS_CODING
11263	CakSNP11263	*Kabuli*	Ca_Kabuli_Chr06	50477672	[A/C]	Ca_13459	--	SYNONYMOUS_CODING
11264	CakSNP11264	*Kabuli*	Ca_Kabuli_Chr06	50581295	[T/G]		--	INTERGENIC
11265	CakSNP11265	*Kabuli*	Ca_Kabuli_Chr06	50617926	[G/C]	Ca_13450	--	SYNONYMOUS_CODING
11266	CakSNP11266	*Kabuli*	Ca_Kabuli_Chr06	50617932	[G/A]	Ca_13450	--	SYNONYMOUS_CODING
11267	CakSNP11267	*Kabuli*	Ca_Kabuli_Chr06	50723595	[T/C]	Ca_13438	GDP-fucose protein O-fucosyltransferase	SYNONYMOUS_CODING
11268	CakSNP11268	*Kabuli*	Ca_Kabuli_Chr06	50723630	[C/T]	Ca_13438	GDP-fucose protein O-fucosyltransferase	NON_SYNONYMOUS_CODING
11269	CakSNP11269	*Kabuli*	Ca_Kabuli_Chr06	50723916	[T/C]	Ca_13438	GDP-fucose protein O-fucosyltransferase	SYNONYMOUS_CODING
11270	CakSNP11270	*Kabuli*	Ca_Kabuli_Chr06	50723950	[A/G]	Ca_13438	GDP-fucose protein O-fucosyltransferase	NON_SYNONYMOUS_CODING
11271	CakSNP11271	*Kabuli*	Ca_Kabuli_Chr06	50723980	[A/G]	Ca_13438	GDP-fucose protein O-fucosyltransferase	NON_SYNONYMOUS_CODING

S.N.	SNP IDs	Chickpea cultivars	Chromosomes	Physical positions (bp)	SNPs	Gene accession IDs	Putative functions	Sequence components of genome
11272	CakSNP11272	*Kabuli*	Ca_Kabuli_Chr06	50723988	[C/A]	Ca_13438	GDP-fucose protein O-fucosyltransferase	SYNONYMOUS_CODING
11273	CakSNP11273	*Kabuli*	Ca_Kabuli_Chr06	51157961	[C/T]	Ca_19183	--	NON_SYNONYMOUS_CODING
11274	CakSNP11274	*Kabuli*	Ca_Kabuli_Chr06	51157939	[A/C]	Ca_19183	--	NON_SYNONYMOUS_CODING
11275	CakSNP11275	*Kabuli*	Ca_Kabuli_Chr06	51162065	[T/C]		--	INTERGENIC
11276	CakSNP11276	*Kabuli*	Ca_Kabuli_Chr06	51281235	[G/A]		--	INTERGENIC
11277	CakSNP11277	*Kabuli*	Ca_Kabuli_Chr06	51281178	[C/G]		--	INTERGENIC
11278	CakSNP11278	*Kabuli*	Ca_Kabuli_Chr06	51345460	[G/C]	Ca_19171	Tetratricopeptide repeat-containing	NON_SYNONYMOUS_CODING
11279	CakSNP11279	*Kabuli*	Ca_Kabuli_Chr06	51392609	[T/C]		--	INTERGENIC
11280	CakSNP11280	*Kabuli*	Ca_Kabuli_Chr06	51471573	[T/C]	Ca_19165	--	NON_SYNONYMOUS_CODING
11281	CakSNP11281	*Kabuli*	Ca_Kabuli_Chr06	52007211	[T/C]		--	INTERGENIC

S.N.	SNP IDs	Chickpea cultivars	Chromosomes	Physical positions (bp)	SNPs	Gene accession IDs	Putative functions	Sequence components of genome
11282	CakSNP11282	*Kabuli*	Ca_Kabuli_Chr06	52007217	[G/A]		--	INTERGENIC
11283	CakSNP11283	*Kabuli*	Ca_Kabuli_Chr06	52007471	[C/T]		--	INTERGENIC
11284	CakSNP11284	*Kabuli*	Ca_Kabuli_Chr06	52007464	[C/G]		--	INTERGENIC
11285	CakSNP11285	*Kabuli*	Ca_Kabuli_Chr06	52007440	[C/T]		--	INTERGENIC
11286	CakSNP11286	*Kabuli*	Ca_Kabuli_Chr06	52033279	[G/A]		--	INTERGENIC
11287	CakSNP11287	*Kabuli*	Ca_Kabuli_Chr06	52033244	[A/T]		--	INTERGENIC
11288	CakSNP11288	*Kabuli*	Ca_Kabuli_Chr06	52059750	[A/G]	Ca_17511	Auxin responsive SAUR protein	SYNONYMOUS_CODING
11289	CakSNP11289	*Kabuli*	Ca_Kabuli_Chr06	52065393	[C/A]		--	INTERGENIC
11290	CakSNP11290	*Kabuli*	Ca_Kabuli_Chr06	52065527	[T/A]		--	INTERGENIC
11291	CakSNP11291	*Kabuli*	Ca_Kabuli_Chr06	52164571	[C/A]	Ca_17518	ATPase, P-type, K/Mg/Cd/Cu/Zn/Na/Ca/Na/H-transporter	INTRON

S.N.	SNP IDs	Chickpea cultivars	Chromosomes	Physical positions (bp)	SNPs	Gene accession IDs	Putative functions	Sequence components of genome
11292	CakSNP11292	*Kabuli*	Ca_Kabuli_Chr06	52227277	[T/C]	Ca_17522	Zinc finger, GATA-type	NON_SYNONYMOUS_CODING
11293	CakSNP11293	*Kabuli*	Ca_Kabuli_Chr06	52304094	[C/T]	Ca_17529	--	SYNONYMOUS_CODING
11294	CakSNP11294	*Kabuli*	Ca_Kabuli_Chr06	52304112	[A/G]	Ca_17529	--	SYNONYMOUS_CODING
11295	CakSNP11295	*Kabuli*	Ca_Kabuli_Chr06	52363121	[C/T]	Ca_17532	Pathogenesis-related transcriptional factor/ERF, DNA-binding	INTRON
11296	CakSNP11296	*Kabuli*	Ca_Kabuli_Chr06	52416366	[T/A]		--	INTERGENIC
11297	CakSNP11297	*Kabuli*	Ca_Kabuli_Chr06	52417684	[C/T]		--	INTERGENIC
11298	CakSNP11298	*Kabuli*	Ca_Kabuli_Chr06	52417717	[T/G]		--	INTERGENIC
11299	CakSNP11299	*Kabuli*	Ca_Kabuli_Chr06	52417720	[A/T]		--	INTERGENIC
11300	CakSNP11300	*Kabuli*	Ca_Kabuli_Chr06	52417736	[T/A]		--	INTERGENIC
11301	CakSNP11301	*Kabuli*	Ca_Kabuli_Chr06	52417747	[G/T]		--	INTERGENIC

S.N.	SNP IDs	Chickpea cultivars	Chromosomes	Physical positions (bp)	SNPs	Gene accession IDs	Putative functions	Sequence components of genome
11302	CakSNP11302	*Kabuli*	Ca_Kabuli_Chr06	52567474	[A/C]	Ca_17540	Transferase	NON_SYNONYMOUS_CODING
11303	CakSNP11303	*Kabuli*	Ca_Kabuli_Chr06	52596602	[T/A]	Ca_17541	Protein phosphatase 2C, manganese/magnesium aspartate binding site	INTRON
11304	CakSNP11304	*Kabuli*	Ca_Kabuli_Chr06	52596601	[A/T]	Ca_17541	Protein phosphatase 2C, manganese/magnesium aspartate binding site	INTRON
11305	CakSNP11305	*Kabuli*	Ca_Kabuli_Chr06	52858359	[A/G]		--	INTERGENIC
11306	CakSNP11306	*Kabuli*	Ca_Kabuli_Chr06	52993231	[A/G]		--	INTERGENIC
11307	CakSNP11307	*Kabuli*	Ca_Kabuli_Chr06	53068176	[C/T]		--	INTERGENIC
11308	CakSNP11308	*Kabuli*	Ca_Kabuli_Chr06	53068201	[G/T]		--	INTERGENIC
11309	CakSNP11309	*Kabuli*	Ca_Kabuli_Chr06	53097833	[G/C]		--	INTERGENIC
11310	CakSNP11310	*Kabuli*	Ca_Kabuli_Chr06	53097803	[A/G]		--	INTERGENIC
11311	CakSNP11311	*Kabuli*	Ca_Kabuli_Chr06	53097766	[G/A]	Ca_22909	IQ motif, EF-hand binding site	NON_SYNONYMOUS_CODING

S.N.	SNP IDs	Chickpea cultivars	Chromosomes	Physical positions (bp)	SNPs	Gene accession IDs	Putative functions	Sequence components of genome
11312	CakSNP11312	*Kabuli*	Ca_Kabuli_Chr06	53124182	[G/T]	Ca_22912	3-Oxoacyl-[acyl-carrier-protein (ACP)] synthase III C-terminal	NON_SYNONYMOUS_CODING
11313	CakSNP11313	*Kabuli*	Ca_Kabuli_Chr06	53168715	[T/G]	Ca_22916	Methyladenine glycosylase	INTRON
11314	CakSNP11314	*Kabuli*	Ca_Kabuli_Chr06	53348993	[C/A]	Ca_18877	Sodium/sulphate symporter	INTRON
11315	CakSNP11315	*Kabuli*	Ca_Kabuli_Chr06	53349034	[G/A]	Ca_18877	Sodium/sulphate symporter	INTRON
11316	CakSNP11316	*Kabuli*	Ca_Kabuli_Chr06	53390065	[A/G]	Ca_18882	--	NON_SYNONYMOUS_CODING
11317	CakSNP11317	*Kabuli*	Ca_Kabuli_Chr06	53461929	[C/A]		--	INTERGENIC
11318	CakSNP11318	*Kabuli*	Ca_Kabuli_Chr06	53461883	[A/G]		--	INTERGENIC
11319	CakSNP11319	*Kabuli*	Ca_Kabuli_Chr06	53541031	[G/A]	Ca_18890	Ctr copper transporter	SYNONYMOUS_CODING
11320	CakSNP11320	*Kabuli*	Ca_Kabuli_Chr06	53541094	[C/G]		--	INTERGENIC
11321	CakSNP11321	*Kabuli*	Ca_Kabuli_Chr06	53541111	[T/G]		--	INTERGENIC

S.N.	SNP IDs	Chickpea cultivars	Chromosomes	Physical positions (bp)	SNPs	Gene accession IDs	Putative functions	Sequence components of genome
11322	CakSNP11322	*Kabuli*	Ca_Kabuli_Chr06	53551878	[C/A]		--	INTERGENIC
11323	CakSNP11323	*Kabuli*	Ca_Kabuli_Chr06	53558430	[A/C]		--	INTERGENIC
11324	CakSNP11324	*Kabuli*	Ca_Kabuli_Chr06	53566496	[G/A]	Ca_18894	Pathogenesis-related transcriptional factor/ERF, DNA-binding	NON_SYNONYMOUS_CODING
11325	CakSNP11325	*Kabuli*	Ca_Kabuli_Chr06	53566506	[C/T]	Ca_18894	Pathogenesis-related transcriptional factor/ERF, DNA-binding	SYNONYMOUS_CODING
11326	CakSNP11326	*Kabuli*	Ca_Kabuli_Chr06	53625264	[A/C]	Ca_18898	Leucine-rich repeat	SYNONYMOUS_CODING
11327	CakSNP11327	*Kabuli*	Ca_Kabuli_Chr06	53683753	[T/C]	Ca_18901	--	INTRON
11328	CakSNP11328	*Kabuli*	Ca_Kabuli_Chr06	53683891	[C/A]	Ca_18901	--	INTRON
11329	CakSNP11329	*Kabuli*	Ca_Kabuli_Chr06	53683885	[C/T]	Ca_18901	--	INTRON
11330	CakSNP11330	*Kabuli*	Ca_Kabuli_Chr06	53683879	[C/T]	Ca_18901	--	INTRON
11331	CakSNP11331	*Kabuli*	Ca_Kabuli_Chr06	53683876	[C/T]	Ca_18901	--	INTRON

S.N.	SNP IDs	Chickpea cultivars	Chromosomes	Physical positions (bp)	SNPs	Gene accession IDs	Putative functions	Sequence components of genome
11332	CakSNP11332	*Kabuli*	Ca_Kabuli_Chr06	53692784	[G/A]	Ca_18902	--	NON_SYNONYMOUS_CODING
11333	CakSNP11333	*Kabuli*	Ca_Kabuli_Chr06	53769044	[A/C]	Ca_18909	Glycoside hydrolase, family 1	INTRON
11334	CakSNP11334	*Kabuli*	Ca_Kabuli_Chr06	53769160	[C/T]	Ca_18909	Glycoside hydrolase, family 1	INTRON
11335	CakSNP11335	*Kabuli*	Ca_Kabuli_Chr06	53769240	[T/A]	Ca_18909	Glycoside hydrolase, family 1	INTRON
11336	CakSNP11336	*Kabuli*	Ca_Kabuli_Chr06	53769241	[A/T]	Ca_18909	Glycoside hydrolase, family 1	INTRON
11337	CakSNP11337	*Kabuli*	Ca_Kabuli_Chr06	54091997	[A/C]	Ca_16263	Protein of unknown function DUF3527	NON_SYNONYMOUS_CODING
11338	CakSNP11338	*Kabuli*	Ca_Kabuli_Chr06	54151902	[T/G]		--	INTERGENIC
11339	CakSNP11339	*Kabuli*	Ca_Kabuli_Chr06	54151966	[G/C]		--	INTERGENIC
11340	CakSNP11340	*Kabuli*	Ca_Kabuli_Chr06	54151969	[T/G]		--	INTERGENIC
11341	CakSNP11341	*Kabuli*	Ca_Kabuli_Chr06	54203356	[G/A]	Ca_16276	NmrA-like	NON_SYNONYMOUS_CODING

S.N.	SNP IDs	Chickpea cultivars	Chromosomes	Physical positions (bp)	SNPs	Gene accession IDs	Putative functions	Sequence components of genome
11342	CakSNP11342	*Kabuli*	Ca_Kabuli_Chr06	54203400	[C/T]	Ca_16276	NmrA-like	SYNONYMOUS_CODING
11343	CakSNP11343	*Kabuli*	Ca_Kabuli_Chr06	54226451	[A/T]	Ca_16278	Sugar/inositol transporter	INTRON
11344	CakSNP11344	*Kabuli*	Ca_Kabuli_Chr06	54226432	[A/C]	Ca_16278	Sugar/inositol transporter	INTRON
11345	CakSNP11345	*Kabuli*	Ca_Kabuli_Chr06	54229449	[G/T]	Ca_16278	Sugar/inositol transporter	INTRON
11346	CakSNP11346	*Kabuli*	Ca_Kabuli_Chr06	54237764	[A/C]	Ca_16280	Bacterial surface antigen (D15)	SYNONYMOUS_CODING
11347	CakSNP11347	*Kabuli*	Ca_Kabuli_Chr06	54237815	[C/T]	Ca_16280	Bacterial surface antigen (D15)	SYNONYMOUS_CODING
11348	CakSNP11348	*Kabuli*	Ca_Kabuli_Chr06	54250702	[C/T]	Ca_16283	Pumilio RNA-binding repeat	NON_SYNONYMOUS_CODING
11349	CakSNP11349	*Kabuli*	Ca_Kabuli_Chr06	54368267	[G/A]		--	INTERGENIC
11350	CakSNP11350	*Kabuli*	Ca_Kabuli_Chr06	54869636	[C/T]	Ca_21207	Peptidase C48, SUMO/Sentrin/Ubl1	NON_SYNONYMOUS_CODING
11351	CakSNP11351	*Kabuli*	Ca_Kabuli_Chr06	54869666	[G/A]	Ca_21207	Peptidase C48, SUMO/Sentrin/Ubl1	NON_SYNONYMOUS_CODING

S.N.	SNP IDs	Chickpea cultivars	Chromosomes	Physical positions (bp)	SNPs	Gene accession IDs	Putative functions	Sequence components of genome
11352	CakSNP11352	*Kabuli*	Ca_Kabuli_Chr06	54997337	[A/T]	Ca_21212	Oligopeptide transporter	NON_SYNONYMOUS_CODING
11353	CakSNP11353	*Kabuli*	Ca_Kabuli_Chr06	55024071	[C/T]	Ca_21213	--	NON_SYNONYMOUS_CODING
11354	CakSNP11354	*Kabuli*	Ca_Kabuli_Chr06	55024091	[G/A]	Ca_21213	--	NON_SYNONYMOUS_CODING
11355	CakSNP11355	*Kabuli*	Ca_Kabuli_Chr06	55024146	[C/T]	Ca_21213	--	NON_SYNONYMOUS_CODING
11356	CakSNP11356	*Kabuli*	Ca_Kabuli_Chr06	55024140	[G/A]	Ca_21213	--	NON_SYNONYMOUS_CODING
11357	CakSNP11357	*Kabuli*	Ca_Kabuli_Chr06	55024130	[T/A]	Ca_21213	--	NON_SYNONYMOUS_CODING
11358	CakSNP11358	*Kabuli*	Ca_Kabuli_Chr06	55024112	[C/G]	Ca_21213	--	NON_SYNONYMOUS_CODING
11359	CakSNP11359	*Kabuli*	Ca_Kabuli_Chr06	55024093	[G/C]	Ca_21213	--	NON_SYNONYMOUS_CODING
11360	CakSNP11360	*Kabuli*	Ca_Kabuli_Chr06	55024108	[C/T]	Ca_21213	--	SYNONYMOUS_CODING
11361	CakSNP11361	*Kabuli*	Ca_Kabuli_Chr06	55024136	[C/T]	Ca_21213	--	NON_SYNONYMOUS_CODING

S.N.	SNP IDs	Chickpea cultivars	Chromosomes	Physical positions (bp)	SNPs	Gene accession IDs	Putative functions	Sequence components of genome
11362	CakSNP11362	*Kabuli*	Ca_Kabuli_Chr06	55024159	[T/A]	Ca_21213	--	SYNONYMOUS_CODING
11363	CakSNP11363	*Kabuli*	Ca_Kabuli_Chr06	55024198	[T/G]	Ca_21213	--	SYNONYMOUS_CODING
11364	CakSNP11364	*Kabuli*	Ca_Kabuli_Chr06	55028980	[G/A]		--	INTERGENIC
11365	CakSNP11365	*Kabuli*	Ca_Kabuli_Chr06	55074979	[T/A]		--	INTERGENIC
11366	CakSNP11366	*Kabuli*	Ca_Kabuli_Chr06	55083311	[T/A]	Ca_21214	Oligopeptide transporter	INTRON
11367	CakSNP11367	*Kabuli*	Ca_Kabuli_Chr06	55083721	[T/A]	Ca_21214	Oligopeptide transporter	SYNONYMOUS_CODING
11368	CakSNP11368	*Kabuli*	Ca_Kabuli_Chr06	55180211	[A/G]	Ca_21215	Oligopeptide transporter	NON_SYNONYMOUS_CODING
11369	CakSNP11369	*Kabuli*	Ca_Kabuli_Chr06	55332515	[G/A]	Ca_21219	Homeobox	SYNONYMOUS_CODING
11370	CakSNP11370	*Kabuli*	Ca_Kabuli_Chr06	55914455	[T/C]	Ca_19022	Pyridine nucleotide-disulphide oxidoreductase, NAD-binding region	INTRON
11371	CakSNP11371	*Kabuli*	Ca_Kabuli_Chr06	55914451	[A/G]	Ca_19022	Pyridine nucleotide-disulphide oxidoreductase, NAD-binding region	INTRON

S.N.	SNP IDs	Chickpea cultivars	Chromosomes	Physical positions (bp)	SNPs	Gene accession IDs	Putative functions	Sequence components of genome
11372	CakSNP11372	*Kabuli*	Ca_Kabuli_Chr06	55914443	[T/C]	Ca_19022	Pyridine nucleotide-disulphide oxidoreductase, NAD-binding region	INTRON
11373	CakSNP11373	*Kabuli*	Ca_Kabuli_Chr06	55914434	[C/T]	Ca_19022	Pyridine nucleotide-disulphide oxidoreductase, NAD-binding region	INTRON
11374	CakSNP11374	*Kabuli*	Ca_Kabuli_Chr06	55914431	[G/A]	Ca_19022	Pyridine nucleotide-disulphide oxidoreductase, NAD-binding region	INTRON
11375	CakSNP11375	*Kabuli*	Ca_Kabuli_Chr06	55922843	[T/A]	Ca_19023	Protein of unknown function wound-induced	NON_SYNONYMOUS_CODING
11376	CakSNP11376	*Kabuli*	Ca_Kabuli_Chr06	55922850	[C/T]	Ca_19023	Protein of unknown function wound-induced	NON_SYNONYMOUS_CODING
11377	CakSNP11377	*Kabuli*	Ca_Kabuli_Chr06	55942105	[A/G]		--	INTERGENIC
11378	CakSNP11378	*Kabuli*	Ca_Kabuli_Chr06	55953320	[T/A]	Ca_19026	Protein of unknown function wound-induced	NON_SYNONYMOUS_CODING
11379	CakSNP11379	*Kabuli*	Ca_Kabuli_Chr06	55953327	[C/T]	Ca_19026	Protein of unknown function wound-induced	NON_SYNONYMOUS_CODING
11380	CakSNP11380	*Kabuli*	Ca_Kabuli_Chr06	56025902	[A/G]	Ca_19029	Photosystem II Psp29, biogenesis	INTRON
11381	CakSNP11381	*Kabuli*	Ca_Kabuli_Chr06	56025888	[G/A]	Ca_19029	Photosystem II Psp29, biogenesis	INTRON

S.N.	SNP IDs	Chickpea cultivars	Chromosomes	Physical positions (bp)	SNPs	Gene accession IDs	Putative functions	Sequence components of genome
11382	CakSNP11382	*Kabuli*	Ca_Kabuli_Chr06	56025884	[A/G]	Ca_19029	Photosystem II Psp29, biogenesis	INTRON
11383	CakSNP11383	*Kabuli*	Ca_Kabuli_Chr06	56029491	[T/A]		--	INTERGENIC
11384	CakSNP11384	*Kabuli*	Ca_Kabuli_Chr06	56029544	[A/T]		--	INTERGENIC
11385	CakSNP11385	*Kabuli*	Ca_Kabuli_Chr06	56029549	[C/T]		--	INTERGENIC
11386	CakSNP11386	*Kabuli*	Ca_Kabuli_Chr06	56137878	[T/C]		--	INTERGENIC
11387	CakSNP11387	*Kabuli*	Ca_Kabuli_Chr06	56553930	[G/A]	Ca_17393	Tetratricopeptide TPR-1	NON_SYNONYMOUS_CODING
11388	CakSNP11388	*Kabuli*	Ca_Kabuli_Chr06	56564249	[G/C]	Ca_17394	Pyridoxal phosphate-dependent enzyme, beta subunit	INTRON
11389	CakSNP11389	*Kabuli*	Ca_Kabuli_Chr06	56587094	[C/G]		--	INTERGENIC
11390	CakSNP11390	*Kabuli*	Ca_Kabuli_Chr06	56593148	[A/G]	Ca_17397	Helicase, C-terminal	NON_SYNONYMOUS_CODING
11391	CakSNP11391	*Kabuli*	Ca_Kabuli_Chr06	56593121	[T/G]	Ca_17397	Helicase, C-terminal	SYNONYMOUS_CODING

S.N.	SNP IDs	Chickpea cultivars	Chromosomes	Physical positions (bp)	SNPs	Gene accession IDs	Putative functions	Sequence components of genome
11392	CakSNP11392	*Kabuli*	Ca_Kabuli_Chr06	56624316	[A/T]	Ca_17398	Proline dehydrogenase	INTRON
11393	CakSNP11393	*Kabuli*	Ca_Kabuli_Chr06	56624674	[T/C]	Ca_17398	Proline dehydrogenase	INTRON
11394	CakSNP11394	*Kabuli*	Ca_Kabuli_Chr06	56624718	[C/A]	Ca_17398	Proline dehydrogenase	INTRON
11395	CakSNP11395	*Kabuli*	Ca_Kabuli_Chr06	56694898	[T/C]	Ca_17408	ATPase, AAA+ type, core	NON_SYNONYMOUS_CODING
11396	CakSNP11396	*Kabuli*	Ca_Kabuli_Chr06	56765524	[C/A]	Ca_17415	Glycosyl transferase, family 8	SYNONYMOUS_CODING
11397	CakSNP11397	*Kabuli*	Ca_Kabuli_Chr06	56793077	[G/A]		--	INTERGENIC
11398	CakSNP11398	*Kabuli*	Ca_Kabuli_Chr06	56793037	[A/T]		--	INTERGENIC
11399	CakSNP11399	*Kabuli*	Ca_Kabuli_Chr06	56793021	[G/T]		--	INTERGENIC
11400	CakSNP11400	*Kabuli*	Ca_Kabuli_Chr06	56793122	[C/T]		--	INTERGENIC
11401	CakSNP11401	*Kabuli*	Ca_Kabuli_Chr06	56822084	[G/T]	Ca_17420	Domain of unknown function DUF231, plant	INTRON

S.N.	SNP IDs	Chickpea cultivars	Chromosomes	Physical positions (bp)	SNPs	Gene accession IDs	Putative functions	Sequence components of genome
11402	CakSNP11402	*Kabuli*	Ca_Kabuli_Chr06	56822060	[G/A]	Ca_17420	Domain of unknown function DUF231, plant	INTRON
11403	CakSNP11403	*Kabuli*	Ca_Kabuli_Chr06	56840030	[A/C]		--	INTERGENIC
11404	CakSNP11404	*Kabuli*	Ca_Kabuli_Chr06	56840084	[A/T]		--	INTERGENIC
11405	CakSNP11405	*Kabuli*	Ca_Kabuli_Chr06	56840063	[T/A]		--	INTERGENIC
11406	CakSNP11406	*Kabuli*	Ca_Kabuli_Chr06	56840038	[A/T]		--	INTERGENIC
11407	CakSNP11407	*Kabuli*	Ca_Kabuli_Chr06	56854753	[T/C]	Ca_17422	--	SYNONYMOUS_CODING
11408	CakSNP11408	*Kabuli*	Ca_Kabuli_Chr06	56946415	[A/G]	Ca_17427	Protein kinase, catalytic domain	INTRON
11409	CakSNP11409	*Kabuli*	Ca_Kabuli_Chr06	56946431	[T/C]	Ca_17427	Protein kinase, catalytic domain	INTRON
11410	CakSNP11410	*Kabuli*	Ca_Kabuli_Chr06	56950487	[G/T]		--	INTERGENIC
11411	CakSNP11411	*Kabuli*	Ca_Kabuli_Chr06	56980313	[T/C]	Ca_17429	GDP-fucose protein O-fucosyltransferase	INTRON

S.N.	SNP IDs	Chickpea cultivars	Chromosomes	Physical positions (bp)	SNPs	Gene accession IDs	Putative functions	Sequence components of genome
11412	CakSNP11412	*Kabuli*	Ca_Kabuli_Chr06	56980286	[T/C]	Ca_17429	GDP-fucose protein O-fucosyltransferase	INTRON
11413	CakSNP11413	*Kabuli*	Ca_Kabuli_Chr06	56980321	[T/C]	Ca_17429	GDP-fucose protein O-fucosyltransferase	INTRON
11414	CakSNP11414	*Kabuli*	Ca_Kabuli_Chr06	57147008	[A/G]	Ca_17439	Glycosyl-phosphatidyl inositol-anchored, plant	INTRON
11415	CakSNP11415	*Kabuli*	Ca_Kabuli_Chr06	57198837	[G/A]	Ca_17442	--	INTRON
11416	CakSNP11416	*Kabuli*	Ca_Kabuli_Chr06	57259982	[C/A]	Ca_13710	Glycoside hydrolase, family 1	INTRON
11417	CakSNP11417	*Kabuli*	Ca_Kabuli_Chr06	57260020	[A/G]	Ca_13710	Glycoside hydrolase, family 1	INTRON
11418	CakSNP11418	*Kabuli*	Ca_Kabuli_Chr06	57260022	[T/C]	Ca_13710	Glycoside hydrolase, family 1	INTRON
11419	CakSNP11419	*Kabuli*	Ca_Kabuli_Chr06	57272604	[A/G]		--	INTERGENIC
11420	CakSNP11420	*Kabuli*	Ca_Kabuli_Chr06	57272607	[G/A]		--	INTERGENIC
11421	CakSNP11421	*Kabuli*	Ca_Kabuli_Chr06	57272608	[C/A]		--	INTERGENIC

S.N.	SNP IDs	Chickpea cultivars	Chromosomes	Physical positions (bp)	SNPs	Gene accession IDs	Putative functions	Sequence components of genome
11422	CakSNP11422	*Kabuli*	Ca_Kabuli_Chr06	57277430	[A/G]		--	INTERGENIC
11423	CakSNP11423	*Kabuli*	Ca_Kabuli_Chr06	57277440	[C/T]		--	INTERGENIC
11424	CakSNP11424	*Kabuli*	Ca_Kabuli_Chr06	57344963	[G/T]	Ca_13706	Glycoside hydrolase, family 1	SYNONYMOUS_CODING
11425	CakSNP11425	*Kabuli*	Ca_Kabuli_Chr06	57382220	[G/A]	Ca_13705	Zinc finger, RING-type	NON_SYNONYMOUS_CODING
11426	CakSNP11426	*Kabuli*	Ca_Kabuli_Chr06	57433674	[T/A]		--	INTERGENIC
11427	CakSNP11427	*Kabuli*	Ca_Kabuli_Chr06	57436112	[A/T]	Ca_13700	Bromodomain	INTRON
11428	CakSNP11428	*Kabuli*	Ca_Kabuli_Chr06	57436119	[T/C]	Ca_13700	Bromodomain	INTRON
11429	CakSNP11429	*Kabuli*	Ca_Kabuli_Chr06	57436149	[T/A]	Ca_13700	Bromodomain	INTRON
11430	CakSNP11430	*Kabuli*	Ca_Kabuli_Chr06	57436320	[T/G]	Ca_13700	Bromodomain	NON_SYNONYMOUS_CODING
11431	CakSNP11431	*Kabuli*	Ca_Kabuli_Chr06	57437904	[A/G]	Ca_13700	Bromodomain	SYNONYMOUS_CODING

S.N.	SNP IDs	Chickpea cultivars	Chromosomes	Physical positions (bp)	SNPs	Gene accession IDs	Putative functions	Sequence components of genome
11432	CakSNP11432	*Kabuli*	Ca_Kabuli_Chr06	57449892	[A/G]	Ca_13699	--	NON_SYNONYMOUS_CODING
11433	CakSNP11433	*Kabuli*	Ca_Kabuli_Chr06	57475853	[G/T]		--	INTERGENIC
11434	CakSNP11434	*Kabuli*	Ca_Kabuli_Chr06	57475892	[A/G]		--	INTERGENIC
11435	CakSNP11435	*Kabuli*	Ca_Kabuli_Chr06	57475982	[A/G]		--	INTERGENIC
11436	CakSNP11436	*Kabuli*	Ca_Kabuli_Chr06	57533087	[G/A]		--	INTERGENIC
11437	CakSNP11437	*Kabuli*	Ca_Kabuli_Chr06	57579770	[G/T]	Ca_13686	RNA recognition motif domain	SYNONYMOUS_CODING
11438	CakSNP11438	*Kabuli*	Ca_Kabuli_Chr06	57579768	[C/T]	Ca_13686	RNA recognition motif domain	SYNONYMOUS_CODING
11439	CakSNP11439	*Kabuli*	Ca_Kabuli_Chr06	57580569	[C/T]	Ca_13686	RNA recognition motif domain	SYNONYMOUS_CODING
11440	CakSNP11440	*Kabuli*	Ca_Kabuli_Chr06	57603257	[T/A]	Ca_13685	3-beta hydroxysteroid dehydrogenase/isomerase	INTRON
11441	CakSNP11441	*Kabuli*	Ca_Kabuli_Chr06	57625134	[G/A]	Ca_13683	Calreticulin/calnexin	INTRON

S.N.	SNP IDs	Chickpea cultivars	Chromosomes	Physical positions (bp)	SNPs	Gene accession IDs	Putative functions	Sequence components of genome
11442	CakSNP11442	*Kabuli*	Ca_Kabuli_Chr06	57625320	[G/A]	Ca_13683	Calreticulin/calnexin	SYNONYMOUS_CODING
11443	CakSNP11443	*Kabuli*	Ca_Kabuli_Chr06	57632486	[C/T]	Ca_13682	Protein of unknown function DUF936, plant	SYNONYMOUS_CODING
11444	CakSNP11444	*Kabuli*	Ca_Kabuli_Chr06	57634387	[G/T]	Ca_13682	Protein of unknown function DUF936, plant	NON_SYNONYMOUS_CODING
11445	CakSNP11445	*Kabuli*	Ca_Kabuli_Chr06	57634677	[G/A]	Ca_13682	Protein of unknown function DUF936, plant	SYNONYMOUS_CODING
11446	CakSNP11446	*Kabuli*	Ca_Kabuli_Chr06	57637091	[G/A]		--	INTERGENIC
11447	CakSNP11447	*Kabuli*	Ca_Kabuli_Chr06	57637083	[T/G]		--	INTERGENIC
11448	CakSNP11448	*Kabuli*	Ca_Kabuli_Chr06	57637075	[C/T]		--	INTERGENIC
11449	CakSNP11449	*Kabuli*	Ca_Kabuli_Chr06	57662858	[T/A]	Ca_13679	Phox/Bem1p	INTRON
11450	CakSNP11450	*Kabuli*	Ca_Kabuli_Chr06	57667515	[A/T]	Ca_13678	Protein synthesis factor, GTP-binding	INTRON
11451	CakSNP11451	*Kabuli*	Ca_Kabuli_Chr06	57688937	[C/T]	Ca_13676	Peptidyl-prolyl cis-trans isomerase, FKBP-type, domain	NON_SYNONYMOUS_CODING

S.N.	SNP IDs	Chickpea cultivars	Chromosomes	Physical positions (bp)	SNPs	Gene accession IDs	Putative functions	Sequence components of genome
11452	CakSNP11452	*Kabuli*	Ca_Kabuli_Chr06	57704057	[C/T]	Ca_13674	Orn/Lys/Arg decarboxylase, major domain	SYNONYMOUS_CODING
11453	CakSNP11453	*Kabuli*	Ca_Kabuli_Chr06	57704802	[T/C]	Ca_13674	Orn/Lys/Arg decarboxylase, major domain	SYNONYMOUS_CODING
11454	CakSNP11454	*Kabuli*	Ca_Kabuli_Chr06	57705011	[G/A]	Ca_13674	Orn/Lys/Arg decarboxylase, major domain	NON_SYNONYMOUS_CODING
11455	CakSNP11455	*Kabuli*	Ca_Kabuli_Chr06	57707716	[C/T]	Ca_13673	Trigger factor, ribosome-binding, bacterial	INTRON
11456	CakSNP11456	*Kabuli*	Ca_Kabuli_Chr06	57707724	[A/C]	Ca_13673	Trigger factor, ribosome-binding, bacterial	INTRON
11457	CakSNP11457	*Kabuli*	Ca_Kabuli_Chr06	57707727	[T/G]	Ca_13673	Trigger factor, ribosome-binding, bacterial	INTRON
11458	CakSNP11458	*Kabuli*	Ca_Kabuli_Chr06	57707848	[A/G]	Ca_13673	Trigger factor, ribosome-binding, bacterial	INTRON
11459	CakSNP11459	*Kabuli*	Ca_Kabuli_Chr06	57707846	[G/A]	Ca_13673	Trigger factor, ribosome-binding, bacterial	INTRON
11460	CakSNP11460	*Kabuli*	Ca_Kabuli_Chr06	57707912	[G/C]	Ca_13673	Trigger factor, ribosome-binding, bacterial	NON_SYNONYMOUS_CODING
11461	CakSNP11461	*Kabuli*	Ca_Kabuli_Chr06	57708019	[A/G]	Ca_13673	Trigger factor, ribosome-binding, bacterial	INTRON

S.N.	SNP IDs	Chickpea cultivars	Chromosomes	Physical positions (bp)	SNPs	Gene accession IDs	Putative functions	Sequence components of genome
11462	CakSNP11462	*Kabuli*	Ca_Kabuli_Chr06	57708130	[T/C]	Ca_13673	Trigger factor, ribosome-binding, bacterial	INTRON
11463	CakSNP11463	*Kabuli*	Ca_Kabuli_Chr06	57708101	[T/G]	Ca_13673	Trigger factor, ribosome-binding, bacterial	INTRON
11464	CakSNP11464	*Kabuli*	Ca_Kabuli_Chr06	57708198	[C/T]	Ca_13673	Trigger factor, ribosome-binding, bacterial	INTRON
11465	CakSNP11465	*Kabuli*	Ca_Kabuli_Chr06	57710133	[C/G]	Ca_13672	--	INTRON
11466	CakSNP11466	*Kabuli*	Ca_Kabuli_Chr06	57720446	[T/C]	Ca_13671	Transcriptional factor B3	SYNONYMOUS_CODING
11467	CakSNP11467	*Kabuli*	Ca_Kabuli_Chr06	57723992	[A/C]	Ca_13670	Ribosomal protein L11	SYNONYMOUS_CODING
11468	CakSNP11468	*Kabuli*	Ca_Kabuli_Chr06	57725372	[A/C]	Ca_13669	Ribosomal protein L11	SYNONYMOUS_CODING
11469	CakSNP11469	*Kabuli*	Ca_Kabuli_Chr06	57746989	[C/T]	Ca_13666	--	NON_SYNONYMOUS_CODING
11470	CakSNP11470	*Kabuli*	Ca_Kabuli_Chr06	57747101	[C/T]	Ca_13666	--	SYNONYMOUS_CODING
11471	CakSNP11471	*Kabuli*	Ca_Kabuli_Chr06	57748565	[A/G]	Ca_13666	--	SYNONYMOUS_CODING

S.N.	SNP IDs	Chickpea cultivars	Chromosomes	Physical positions (bp)	SNPs	Gene accession IDs	Putative functions	Sequence components of genome
11472	CakSNP11472	*Kabuli*	Ca_Kabuli_Chr06	57748602	[A/T]	Ca_13666	--	NON_SYNONYMOUS_CODING
11473	CakSNP11473	*Kabuli*	Ca_Kabuli_Chr06	57753076	[T/C]		--	INTERGENIC
11474	CakSNP11474	*Kabuli*	Ca_Kabuli_Chr06	57756699	[G/A]	Ca_13665	Phosphatidylinositol 3-/4-kinase, catalytic	INTRON
11475	CakSNP11475	*Kabuli*	Ca_Kabuli_Chr06	57760109	[T/G]	Ca_13665	Phosphatidylinositol 3-/4-kinase, catalytic	INTRON
11476	CakSNP11476	*Kabuli*	Ca_Kabuli_Chr06	57760176	[C/T]	Ca_13665	Phosphatidylinositol 3-/4-kinase, catalytic	INTRON
11477	CakSNP11477	*Kabuli*	Ca_Kabuli_Chr06	57760227	[C/T]	Ca_13665	Phosphatidylinositol 3-/4-kinase, catalytic	SYNONYMOUS_CODING
11478	CakSNP11478	*Kabuli*	Ca_Kabuli_Chr06	57760290	[A/T]	Ca_13665	Phosphatidylinositol 3-/4-kinase, catalytic	NON_SYNONYMOUS_CODING
11479	CakSNP11479	*Kabuli*	Ca_Kabuli_Chr06	57760291	[G/T]	Ca_13665	Phosphatidylinositol 3-/4-kinase, catalytic	NON_SYNONYMOUS_CODING
11480	CakSNP11480	*Kabuli*	Ca_Kabuli_Chr06	57760360	[A/G]	Ca_13665	Phosphatidylinositol 3-/4-kinase, catalytic	INTRON
11481	CakSNP11481	*Kabuli*	Ca_Kabuli_Chr06	57761758	[G/A]	Ca_13665	Phosphatidylinositol 3-/4-kinase, catalytic	NON_SYNONYMOUS_CODING

S.N.	SNP IDs	Chickpea cultivars	Chromosomes	Physical positions (bp)	SNPs	Gene accession IDs	Putative functions	Sequence components of genome
11482	CakSNP11482	*Kabuli*	Ca_Kabuli_Chr06	57761722	[T/C]	Ca_13665	Phosphatidylinositol 3-/4-kinase, catalytic	INTRON
11483	CakSNP11483	*Kabuli*	Ca_Kabuli_Chr06	57766444	[C/T]	Ca_13664	Pentatricopeptide repeat	INTRON
11484	CakSNP11484	*Kabuli*	Ca_Kabuli_Chr06	57766484	[T/A]	Ca_13664	Pentatricopeptide repeat	NON_SYNONYMOUS_CODING
11485	CakSNP11485	*Kabuli*	Ca_Kabuli_Chr06	57766483	[C/A]	Ca_13664	Pentatricopeptide repeat	NON_SYNONYMOUS_CODING
11486	CakSNP11486	*Kabuli*	Ca_Kabuli_Chr06	57766570	[C/T]	Ca_13664	Pentatricopeptide repeat	NON_SYNONYMOUS_CODING
11487	CakSNP11487	*Kabuli*	Ca_Kabuli_Chr06	57766588	[G/A]	Ca_13664	Pentatricopeptide repeat	INTRON
11488	CakSNP11488	*Kabuli*	Ca_Kabuli_Chr06	57766598	[G/A]	Ca_13664	Pentatricopeptide repeat	INTRON
11489	CakSNP11489	*Kabuli*	Ca_Kabuli_Chr06	57768314	[T/C]	Ca_13664	Pentatricopeptide repeat	INTRON
11490	CakSNP11490	*Kabuli*	Ca_Kabuli_Chr06	57801985	[A/C]	Ca_13661	Proteasome assembly chaperone 2	INTRON
11491	CakSNP11491	*Kabuli*	Ca_Kabuli_Chr06	57831050	[G/T]	Ca_13657	Cold acclimation WCOR413	NON_SYNONYMOUS_CODING

S.N.	SNP IDs	Chickpea cultivars	Chromosomes	Physical positions (bp)	SNPs	Gene accession IDs	Putative functions	Sequence components of genome
11492	CakSNP11492	*Kabuli*	Ca_Kabuli_Chr06	57831049	[C/T]	Ca_13657	Cold acclimation WCOR413	SYNONYMOUS_CODING
11493	CakSNP11493	*Kabuli*	Ca_Kabuli_Chr06	57831040	[T/C]	Ca_13657	Cold acclimation WCOR413	SYNONYMOUS_CODING
11494	CakSNP11494	*Kabuli*	Ca_Kabuli_Chr06	57835093	[T/A]	Ca_13656	AMP-dependent synthetase/ligase	INTRON
11495	CakSNP11495	*Kabuli*	Ca_Kabuli_Chr06	57835120	[G/A]	Ca_13656	AMP-dependent synthetase/ligase	INTRON
11496	CakSNP11496	*Kabuli*	Ca_Kabuli_Chr06	57835917	[G/C]	Ca_13656	AMP-dependent synthetase/ligase	NON_SYNONYMOUS_CODING
11497	CakSNP11497	*Kabuli*	Ca_Kabuli_Chr06	57835899	[C/T]	Ca_13656	AMP-dependent synthetase/ligase	INTRON
11498	CakSNP11498	*Kabuli*	Ca_Kabuli_Chr06	57836024	[G/A]	Ca_13656	AMP-dependent synthetase/ligase	INTRON
11499	CakSNP11499	*Kabuli*	Ca_Kabuli_Chr06	57836237	[A/G]	Ca_13656	AMP-dependent synthetase/ligase	INTRON
11500	CakSNP11500	*Kabuli*	Ca_Kabuli_Chr06	57836284	[G/A]	Ca_13656	AMP-dependent synthetase/ligase	INTRON
11501	CakSNP11501	*Kabuli*	Ca_Kabuli_Chr06	57836314	[T/A]	Ca_13656	AMP-dependent synthetase/ligase	INTRON

S.N.	SNP IDs	Chickpea cultivars	Chromosomes	Physical positions (bp)	SNPs	Gene accession IDs	Putative functions	Sequence components of genome
11502	CakSNP11502	*Kabuli*	Ca_Kabuli_Chr06	57836316	[G/A]	Ca_13656	AMP-dependent synthetase/ligase	INTRON
11503	CakSNP11503	*Kabuli*	Ca_Kabuli_Chr06	57870893	[C/A]		--	INTERGENIC
11504	CakSNP11504	*Kabuli*	Ca_Kabuli_Chr06	57877446	[C/A]	Ca_13654	Uncharacterised protein family UPF0497, trans-membrane plant	SYNONYMOUS_CODING
11505	CakSNP11505	*Kabuli*	Ca_Kabuli_Chr06	57882364	[C/T]	Ca_13652	DC1	SYNONYMOUS_CODING
11506	CakSNP11506	*Kabuli*	Ca_Kabuli_Chr06	57894377	[A/G]		--	INTERGENIC
11507	CakSNP11507	*Kabuli*	Ca_Kabuli_Chr06	57894495	[C/G]	Ca_13650	--	SYNONYMOUS_CODING
11508	CakSNP11508	*Kabuli*	Ca_Kabuli_Chr06	57894590	[G/C]	Ca_13650	--	NON_SYNONYMOUS_CODING
11509	CakSNP11509	*Kabuli*	Ca_Kabuli_Chr06	57894585	[T/G]	Ca_13650	--	NON_SYNONYMOUS_CODING
11510	CakSNP11510	*Kabuli*	Ca_Kabuli_Chr06	57904599	[T/C]		--	INTERGENIC
11511	CakSNP11511	*Kabuli*	Ca_Kabuli_Chr06	57921373	[C/T]	Ca_13646	Methyltransferase type 11	INTRON

S.N.	SNP IDs	Chickpea cultivars	Chromosomes	Physical positions (bp)	SNPs	Gene accession IDs	Putative functions	Sequence components of genome
11512	CakSNP11512	*Kabuli*	Ca_Kabuli_Chr06	57943585	[A/T]		--	INTERGENIC
11513	CakSNP11513	*Kabuli*	Ca_Kabuli_Chr06	57943626	[G/A]		--	INTERGENIC
11514	CakSNP11514	*Kabuli*	Ca_Kabuli_Chr06	57943628	[A/C]		--	INTERGENIC
11515	CakSNP11515	*Kabuli*	Ca_Kabuli_Chr06	57944439	[C/T]		--	INTERGENIC
11516	CakSNP11516	*Kabuli*	Ca_Kabuli_Chr06	57968924	[A/T]	Ca_13640	WD40 repeat	INTRON
11517	CakSNP11517	*Kabuli*	Ca_Kabuli_Chr06	58036471	[C/T]		--	INTERGENIC
11518	CakSNP11518	*Kabuli*	Ca_Kabuli_Chr06	58174702	[T/C]	Ca_13623	Glycoside hydrolase, family 17	INTRON
11519	CakSNP11519	*Kabuli*	Ca_Kabuli_Chr06	58187822	[T/G]	Ca_13622	Adenylate cyclase-associated CAP	INTRON
11520	CakSNP11520	*Kabuli*	Ca_Kabuli_Chr06	58187859	[T/A]	Ca_13622	Adenylate cyclase-associated CAP	INTRON
11521	CakSNP11521	*Kabuli*	Ca_Kabuli_Chr06	58187957	[A/G]	Ca_13622	Adenylate cyclase-associated CAP	SYNONYMOUS_CODING

S.N.	SNP IDs	Chickpea cultivars	Chromosomes	Physical positions (bp)	SNPs	Gene accession IDs	Putative functions	Sequence components of genome
11522	CakSNP11522	*Kabuli*	Ca_Kabuli_Chr06	58191050	[T/C]	Ca_13622	Adenylate cyclase-associated CAP	NON_SYNONYMOUS_CODING
11523	CakSNP11523	*Kabuli*	Ca_Kabuli_Chr06	58224023	[G/T]	Ca_15364	--	SYNONYMOUS_CODING
11524	CakSNP11524	*Kabuli*	Ca_Kabuli_Chr06	58226908	[T/G]	Ca_15365	--	INTRON
11525	CakSNP11525	*Kabuli*	Ca_Kabuli_Chr06	58227006	[T/C]	Ca_15365	--	INTRON
11526	CakSNP11526	*Kabuli*	Ca_Kabuli_Chr06	58237503	[A/T]	Ca_15367	Phenylalanyl-tRNA synthetase	INTRON
11527	CakSNP11527	*Kabuli*	Ca_Kabuli_Chr06	58238371	[T/C]	Ca_15367	Phenylalanyl-tRNA synthetase	SYNONYMOUS_CODING
11528	CakSNP11528	*Kabuli*	Ca_Kabuli_Chr06	58238397	[A/G]	Ca_15367	Phenylalanyl-tRNA synthetase	INTRON
11529	CakSNP11529	*Kabuli*	Ca_Kabuli_Chr06	58243505	[G/A]	Ca_15368	Mitochodrial transcription termination factor-related	SYNONYMOUS_CODING
11530	CakSNP11530	*Kabuli*	Ca_Kabuli_Chr06	58326344	[A/T]		--	INTERGENIC
11531	CakSNP11531	*Kabuli*	Ca_Kabuli_Chr06	58380367	[T/C]	Ca_15376	Ribosomal RNA methyltransferase RrmJ/FtsJ	SYNONYMOUS_CODING

S.N.	SNP IDs	Chickpea cultivars	Chromosomes	Physical positions (bp)	SNPs	Gene accession IDs	Putative functions	Sequence components of genome
11532	CakSNP11532	*Kabuli*	Ca_Kabuli_Chr06	58380394	[G/A]	Ca_15376	Ribosomal RNA methyltransferase RrmJ/FtsJ	SYNONYMOUS_CODING
11533	CakSNP11533	*Kabuli*	Ca_Kabuli_Chr06	58380563	[G/A]	Ca_15376	Ribosomal RNA methyltransferase RrmJ/FtsJ	NON_SYNONYMOUS_CODING
11534	CakSNP11534	*Kabuli*	Ca_Kabuli_Chr06	58381203	[G/C]	Ca_15376	Ribosomal RNA methyltransferase RrmJ/FtsJ	SYNONYMOUS_CODING
11535	CakSNP11535	*Kabuli*	Ca_Kabuli_Chr06	58381259	[A/G]	Ca_15376	Ribosomal RNA methyltransferase RrmJ/FtsJ	INTRON
11536	CakSNP11536	*Kabuli*	Ca_Kabuli_Chr06	58434217	[C/G]	Ca_15379	--	NON_SYNONYMOUS_CODING
11537	CakSNP11537	*Kabuli*	Ca_Kabuli_Chr06	58452843	[A/C]		--	INTERGENIC
11538	CakSNP11538	*Kabuli*	Ca_Kabuli_Chr06	58569576	[G/T]	Ca_15398	Pre-mRNA processing ribonucleoprotein, snoRNA-binding domain	NON_SYNONYMOUS_CODING
11539	CakSNP11539	*Kabuli*	Ca_Kabuli_Chr06	58569569	[T/C]	Ca_15398	Pre-mRNA processing ribonucleoprotein, snoRNA-binding domain	SYNONYMOUS_CODING
11540	CakSNP11540	*Kabuli*	Ca_Kabuli_Chr06	58571660	[T/C]	Ca_15398	Pre-mRNA processing ribonucleoprotein, snoRNA-binding domain	SYNONYMOUS_CODING
11541	CakSNP11541	*Kabuli*	Ca_Kabuli_Chr06	58596821	[T/G]	Ca_15402	Pectinesterase inhibitor	NON_SYNONYMOUS_CODING

S.N.	SNP IDs	Chickpea cultivars	Chromosomes	Physical positions (bp)	SNPs	Gene accession IDs	Putative functions	Sequence components of genome
11542	CakSNP11542	*Kabuli*	Ca_Kabuli_Chr06	58596878	[C/A]	Ca_15402	Pectinesterase inhibitor	SYNONYMOUS_CODING
11543	CakSNP11543	*Kabuli*	Ca_Kabuli_Chr06	58596968	[T/C]		--	INTERGENIC
11544	CakSNP11544	*Kabuli*	Ca_Kabuli_Chr06	58596918	[A/G]	Ca_15402	Pectinesterase inhibitor	NON_SYNONYMOUS_CODING
11545	CakSNP11545	*Kabuli*	Ca_Kabuli_Chr06	58600930	[C/A]	Ca_15403	Calmodulin-binding protein, plant	SYNONYMOUS_CODING
11546	CakSNP11546	*Kabuli*	Ca_Kabuli_Chr06	58600952	[A/G]	Ca_15403	Calmodulin-binding protein, plant	NON_SYNONYMOUS_CODING
11547	CakSNP11547	*Kabuli*	Ca_Kabuli_Chr06	58600974	[A/G]	Ca_15403	Calmodulin-binding protein, plant	SYNONYMOUS_CODING
11548	CakSNP11548	*Kabuli*	Ca_Kabuli_Chr06	58623095	[A/G]	Ca_15407	ATPase, V0/A0 complex, 116kDa subunit	INTRON
11549	CakSNP11549	*Kabuli*	Ca_Kabuli_Chr06	58623096	[A/T]	Ca_15407	ATPase, V0/A0 complex, 116kDa subunit	INTRON
11550	CakSNP11550	*Kabuli*	Ca_Kabuli_Chr06	58623220	[T/C]	Ca_15407	ATPase, V0/A0 complex, 116kDa subunit	SYNONYMOUS_CODING
11551	CakSNP11551	*Kabuli*	Ca_Kabuli_Chr06	58624183	[G/A]	Ca_15407	ATPase, V0/A0 complex, 116kDa subunit	INTRON

S.N.	SNP IDs	Chickpea cultivars	Chromosomes	Physical positions (bp)	SNPs	Gene accession IDs	Putative functions	Sequence components of genome
11552	CakSNP11552	*Kabuli*	Ca_Kabuli_Chr06	58624233	[C/A]	Ca_15407	ATPase, V0/A0 complex, 116kDa subunit	INTRON
11553	CakSNP11553	*Kabuli*	Ca_Kabuli_Chr06	58624299	[A/G]	Ca_15407	ATPase, V0/A0 complex, 116kDa subunit	INTRON
11554	CakSNP11554	*Kabuli*	Ca_Kabuli_Chr06	58624281	[A/G]	Ca_15407	ATPase, V0/A0 complex, 116kDa subunit	INTRON
11555	CakSNP11555	*Kabuli*	Ca_Kabuli_Chr06	58624251	[C/T]	Ca_15407	ATPase, V0/A0 complex, 116kDa subunit	INTRON
11556	CakSNP11556	*Kabuli*	Ca_Kabuli_Chr06	58650354	[A/G]	Ca_15410	WD40 repeat	SYNONYMOUS_CODING
11557	CakSNP11557	*Kabuli*	Ca_Kabuli_Chr06	58650330	[G/A]	Ca_15410	WD40 repeat	SYNONYMOUS_CODING
11558	CakSNP11558	*Kabuli*	Ca_Kabuli_Chr06	58650318	[A/C]	Ca_15410	WD40 repeat	SYNONYMOUS_CODING
11559	CakSNP11559	*Kabuli*	Ca_Kabuli_Chr06	58650459	[A/T]	Ca_15410	WD40 repeat	NON_SYNONYMOUS_CODING
11560	CakSNP11560	*Kabuli*	Ca_Kabuli_Chr06	58650491	[A/G]	Ca_15410	WD40 repeat	NON_SYNONYMOUS_CODING
11561	CakSNP11561	*Kabuli*	Ca_Kabuli_Chr06	58652881	[A/G]	Ca_15410	WD40 repeat	SYNONYMOUS_CODING

S.N.	SNP IDs	Chickpea cultivars	Chromosomes	Physical positions (bp)	SNPs	Gene accession IDs	Putative functions	Sequence components of genome
11562	CakSNP11562	*Kabuli*	Ca_Kabuli_Chr06	58672317	[A/G]	Ca_15412	Zinc finger, B-box	NON_SYNONYMOUS_CODING
11563	CakSNP11563	*Kabuli*	Ca_Kabuli_Chr06	58675788	[T/C]	Ca_15413	Kinesin, motor domain	INTRON
11564	CakSNP11564	*Kabuli*	Ca_Kabuli_Chr06	58675749	[T/C]	Ca_15413	Kinesin, motor domain	SYNONYMOUS_CODING
11565	CakSNP11565	*Kabuli*	Ca_Kabuli_Chr06	58676507	[A/G]	Ca_15413	Kinesin, motor domain	SYNONYMOUS_CODING
11566	CakSNP11566	*Kabuli*	Ca_Kabuli_Chr06	58676517	[A/C]	Ca_15413	Kinesin, motor domain	NON_SYNONYMOUS_CODING
11567	CakSNP11567	*Kabuli*	Ca_Kabuli_Chr06	58682427	[G/T]	Ca_15413	Kinesin, motor domain	SYNONYMOUS_CODING
11568	CakSNP11568	*Kabuli*	Ca_Kabuli_Chr06	58682397	[G/A]	Ca_15413	Kinesin, motor domain	INTRON
11569	CakSNP11569	*Kabuli*	Ca_Kabuli_Chr06	58682387	[T/A]	Ca_15413	Kinesin, motor domain	INTRON
11570	CakSNP11570	*Kabuli*	Ca_Kabuli_Chr06	58730925	[T/C]	Ca_15417	Multi antimicrobial extrusion protein	INTRON
11571	CakSNP11571	*Kabuli*	Ca_Kabuli_Chr06	58730969	[C/A]	Ca_15417	Multi antimicrobial extrusion protein	INTRON

S.N.	SNP IDs	Chickpea cultivars	Chromosomes	Physical positions (bp)	SNPs	Gene accession IDs	Putative functions	Sequence components of genome
11572	CakSNP11572	*Kabuli*	Ca_Kabuli_Chr06	58730967	[T/G]	Ca_15417	Multi antimicrobial extrusion protein	INTRON
11573	CakSNP11573	*Kabuli*	Ca_Kabuli_Chr06	58732424	[G/T]	Ca_15417	Multi antimicrobial extrusion protein	INTRON
11574	CakSNP11574	*Kabuli*	Ca_Kabuli_Chr06	58732423	[C/T]	Ca_15417	Multi antimicrobial extrusion protein	INTRON
11575	CakSNP11575	*Kabuli*	Ca_Kabuli_Chr06	58732514	[G/A]	Ca_15417	Multi antimicrobial extrusion protein	SYNONYMOUS_CODING
11576	CakSNP11576	*Kabuli*	Ca_Kabuli_Chr06	58732451	[T/C]	Ca_15417	Multi antimicrobial extrusion protein	SYNONYMOUS_CODING
11577	CakSNP11577	*Kabuli*	Ca_Kabuli_Chr06	58732475	[G/A]	Ca_15417	Multi antimicrobial extrusion protein	SYNONYMOUS_CODING
11578	CakSNP11578	*Kabuli*	Ca_Kabuli_Chr06	58745356	[A/G]	Ca_15419	Multi antimicrobial extrusion protein	SYNONYMOUS_CODING
11579	CakSNP11579	*Kabuli*	Ca_Kabuli_Chr06	58745473	[A/G]	Ca_15419	Multi antimicrobial extrusion protein	SYNONYMOUS_CODING
11580	CakSNP11580	*Kabuli*	Ca_Kabuli_Chr06	58745426	[T/C]	Ca_15419	Multi antimicrobial extrusion protein	INTRON
11581	CakSNP11581	*Kabuli*	Ca_Kabuli_Chr06	58745583	[C/T]	Ca_15419	Multi antimicrobial extrusion protein	NON_SYNONYMOUS_CODING

S.N.	SNP IDs	Chickpea cultivars	Chromosomes	Physical positions (bp)	SNPs	Gene accession IDs	Putative functions	Sequence components of genome
11582	CakSNP11582	*Kabuli*	Ca_Kabuli_Chr06	58761879	[G/A]		--	INTERGENIC
11583	CakSNP11583	*Kabuli*	Ca_Kabuli_Chr06	58779344	[A/G]	Ca_15421	Src homology-3 domain	INTRON
11584	CakSNP11584	*Kabuli*	Ca_Kabuli_Chr06	58779389	[T/G]	Ca_15421	Src homology-3 domain	SYNONYMOUS_CODING
11585	CakSNP11585	*Kabuli*	Ca_Kabuli_Chr06	58820632	[A/C]		--	INTERGENIC
11586	CakSNP11586	*Kabuli*	Ca_Kabuli_Chr06	58844717	[T/C]		--	INTERGENIC
11587	CakSNP11587	*Kabuli*	Ca_Kabuli_Chr06	58844744	[A/T]		--	INTERGENIC
11588	CakSNP11588	*Kabuli*	Ca_Kabuli_Chr06	58844776	[C/G]		--	INTERGENIC
11589	CakSNP11589	*Kabuli*	Ca_Kabuli_Chr06	58861614	[G/A]	Ca_15427	Kinesin, motor domain	SYNONYMOUS_CODING
11590	CakSNP11590	*Kabuli*	Ca_Kabuli_Chr06	58878604	[C/G]	Ca_15428	Ornithine/DAP/Arg decarboxylase	SYNONYMOUS_CODING
11591	CakSNP11591	*Kabuli*	Ca_Kabuli_Chr06	58879570	[T/C]	Ca_15428	Ornithine/DAP/Arg decarboxylase	SYNONYMOUS_CODING

S.N.	SNP IDs	Chickpea cultivars	Chromosomes	Physical positions (bp)	SNPs	Gene accession IDs	Putative functions	Sequence components of genome
11592	CakSNP11592	*Kabuli*	Ca_Kabuli_Chr06	58903346	[G/A]	Ca_15431	Aldo/keto reductase	INTRON
11593	CakSNP11593	*Kabuli*	Ca_Kabuli_Chr06	58903381	[G/A]	Ca_15431	Aldo/keto reductase	INTRON
11594	CakSNP11594	*Kabuli*	Ca_Kabuli_Chr06	58903395	[A/G]	Ca_15431	Aldo/keto reductase	INTRON
11595	CakSNP11595	*Kabuli*	Ca_Kabuli_Chr06	58903416	[T/G]	Ca_15431	Aldo/keto reductase	INTRON
11596	CakSNP11596	*Kabuli*	Ca_Kabuli_Chr06	58903497	[G/C]	Ca_15431	Aldo/keto reductase	INTRON
11597	CakSNP11597	*Kabuli*	Ca_Kabuli_Chr06	58903538	[G/A]	Ca_15431	Aldo/keto reductase	INTRON
11598	CakSNP11598	*Kabuli*	Ca_Kabuli_Chr06	58908024	[G/A]	Ca_15431	Aldo/keto reductase	SYNONYMOUS_CODING
11599	CakSNP11599	*Kabuli*	Ca_Kabuli_Chr06	58908045	[A/G]	Ca_15431	Aldo/keto reductase	SYNONYMOUS_CODING
11600	CakSNP11600	*Kabuli*	Ca_Kabuli_Chr06	58908069	[G/A]	Ca_15431	Aldo/keto reductase	SYNONYMOUS_CODING
11601	CakSNP11601	*Kabuli*	Ca_Kabuli_Chr06	58911548	[T/C]	Ca_15432	3-oxo-5-alpha-steroid 4-dehydrogenase, C-terminal	NON_SYNONYMOUS_CODING

S.N.	SNP IDs	Chickpea cultivars	Chromosomes	Physical positions (bp)	SNPs	Gene accession IDs	Putative functions	Sequence components of genome
11602	CakSNP11602	*Kabuli*	Ca_Kabuli_Chr06	58951079	[T/C]	Ca_18874	Clathrin/coatomer adaptor, adaptin-like, N-terminal	SYNONYMOUS_CODING
11603	CakSNP11603	*Kabuli*	Ca_Kabuli_Chr06	58951298	[A/G]	Ca_18874	Clathrin/coatomer adaptor, adaptin-like, N-terminal	SYNONYMOUS_CODING
11604	CakSNP11604	*Kabuli*	Ca_Kabuli_Chr06	58951583	[C/A]	Ca_18874	Clathrin/coatomer adaptor, adaptin-like, N-terminal	SYNONYMOUS_CODING
11605	CakSNP11605	*Kabuli*	Ca_Kabuli_Chr06	58953219	[A/G]	Ca_18874	Clathrin/coatomer adaptor, adaptin-like, N-terminal	INTRON
11606	CakSNP11606	*Kabuli*	Ca_Kabuli_Chr06	58994014	[C/T]	Ca_18872	Vps51/Vps68	SYNONYMOUS_CODING
11607	CakSNP11607	*Kabuli*	Ca_Kabuli_Chr06	58994083	[A/G]	Ca_18872	Vps51/Vps69	SYNONYMOUS_CODING
11608	CakSNP11608	*Kabuli*	Ca_Kabuli_Chr06	58994281	[G/A]	Ca_18872	Vps51/Vps70	SYNONYMOUS_CODING
11609	CakSNP11609	*Kabuli*	Ca_Kabuli_Chr06	59001443	[C/T]		--	INTERGENIC
11610	CakSNP11610	*Kabuli*	Ca_Kabuli_Chr06	59003792	[A/C]	Ca_18871	RNA recognition motif domain	INTRON
11611	CakSNP11611	*Kabuli*	Ca_Kabuli_Chr06	59064798	[G/T]		--	INTERGENIC

S.N.	SNP IDs	Chickpea cultivars	Chromosomes	Physical positions (bp)	SNPs	Gene accession IDs	Putative functions	Sequence components of genome
11612	CakSNP11612	*Kabuli*	Ca_Kabuli_Chr06	59064815	[C/T]		--	INTERGENIC
11613	CakSNP11613	*Kabuli*	Ca_Kabuli_Chr06	59081169	[C/T]	Ca_18866	Glycosyl transferase, family 2	SYNONYMOUS_CODING
11614	CakSNP11614	*Kabuli*	Ca_Kabuli_Chr06	59107027	[A/T]	Ca_18863	RNA-binding, CRM domain	NON_SYNONYMOUS_CODING
11615	CakSNP11615	*Kabuli*	Ca_Kabuli_Chr06	59107007	[G/T]	Ca_18863	RNA-binding, CRM domain	NON_SYNONYMOUS_CODING
11616	CakSNP11616	*Kabuli*	Ca_Kabuli_Chr06	59135944	[A/C]	Ca_18861	Protein kinase, catalytic domain	SYNONYMOUS_CODING
11617	CakSNP11617	*Kabuli*	Ca_Kabuli_Chr06	59194015	[C/G]	Ca_18855	--	SYNONYMOUS_CODING
11618	CakSNP11618	*Kabuli*	Ca_Kabuli_Chr06	59193994	[G/T]	Ca_18855	--	SYNONYMOUS_CODING
11619	CakSNP11619	*Kabuli*	Ca_Kabuli_Chr06	59194081	[T/A]	Ca_18855	--	SYNONYMOUS_CODING
11620	CakSNP11620	*Kabuli*	Ca_Kabuli_Chr06	59202825	[T/G]		--	INTERGENIC
11621	CakSNP11621	*Kabuli*	Ca_Kabuli_Chr06	59219099	[T/C]	Ca_18853	Ergosterol biosynthesis ERG4/ERG24	SYNONYMOUS_CODING

S.N.	SNP IDs	Chickpea cultivars	Chromosomes	Physical positions (bp)	SNPs	Gene accession IDs	Putative functions	Sequence components of genome
11622	CakSNP11622	*Kabuli*	Ca_Kabuli_Chr06	59222848	[C/T]	Ca_18852	Protein of unknown function DUF1754, eukaryotic	INTRON
11623	CakSNP11623	*Kabuli*	Ca_Kabuli_Chr06	59296368	[C/T]	Ca_18847	YTH domain	NON_SYNONYMOUS_CODING
11624	CakSNP11624	*Kabuli*	Ca_Kabuli_Chr06	59296460	[G/A]	Ca_18847	YTH domain	SYNONYMOUS_CODING
11625	CakSNP11625	*Kabuli*	Ca_Kabuli_Chr06	59407761	[G/A]	Ca_18841	Mitochondrial carrier protein	NON_SYNONYMOUS_CODING
11626	CakSNP11626	*Kabuli*	Ca_Kabuli_Chr06	59417984	[G/A]	Ca_18838	Ribosomal protein L34e	INTRON
11627	CakSNP11627	*Kabuli*	Ca_Kabuli_Chr07	16760	[T/G]	Ca_20246	RNA helicase, ATP-dependent, DEAD-box, conserved site	SYNONYMOUS_CODING
11628	CakSNP11628	*Kabuli*	Ca_Kabuli_Chr07	16850	[C/T]	Ca_20246	RNA helicase, ATP-dependent, DEAD-box, conserved site	SYNONYMOUS_CODING
11629	CakSNP11629	*Kabuli*	Ca_Kabuli_Chr07	16880	[G/A]	Ca_20246	RNA helicase, ATP-dependent, DEAD-box, conserved site	SYNONYMOUS_CODING
11630	CakSNP11630	*Kabuli*	Ca_Kabuli_Chr07	16881	[A/G]	Ca_20246	RNA helicase, ATP-dependent, DEAD-box, conserved site	NON_SYNONYMOUS_CODING
11631	CakSNP11631	*Kabuli*	Ca_Kabuli_Chr07	41841	[G/A]	Ca_20241	Thiolase	INTRON

S.N.	SNP IDs	Chickpea cultivars	Chromosomes	Physical positions (bp)	SNPs	Gene accession IDs	Putative functions	Sequence components of genome
11632	CakSNP11632	*Kabuli*	Ca_Kabuli_Chr07	55131	[T/C]	Ca_20240	--	SYNONYMOUS_CODING
11633	CakSNP11633	*Kabuli*	Ca_Kabuli_Chr07	208824	[A/G]	Ca_20224	X8	SYNONYMOUS_CODING
11634	CakSNP11634	*Kabuli*	Ca_Kabuli_Chr07	218346	[C/A]	Ca_20223	Transcription factor GRAS	NON_SYNONYMOUS_CODING
11635	CakSNP11635	*Kabuli*	Ca_Kabuli_Chr07	218341	[C/G]	Ca_20223	Transcription factor GRAS	NON_SYNONYMOUS_CODING
11636	CakSNP11636	*Kabuli*	Ca_Kabuli_Chr07	419301	[C/T]		--	INTERGENIC
11637	CakSNP11637	*Kabuli*	Ca_Kabuli_Chr07	419316	[A/G]		--	INTERGENIC
11638	CakSNP11638	*Kabuli*	Ca_Kabuli_Chr07	419334	[A/C]		--	INTERGENIC
11639	CakSNP11639	*Kabuli*	Ca_Kabuli_Chr07	419385	[T/G]		--	INTERGENIC
11640	CakSNP11640	*Kabuli*	Ca_Kabuli_Chr07	419328	[C/T]		--	INTERGENIC
11641	CakSNP11641	*Kabuli*	Ca_Kabuli_Chr07	419679	[C/T]		--	INTERGENIC

S.N.	SNP IDs	Chickpea cultivars	Chromosomes	Physical positions (bp)	SNPs	Gene accession IDs	Putative functions	Sequence components of genome
11642	CakSNP11642	*Kabuli*	Ca_Kabuli_Chr07	419638	[C/T]		--	INTERGENIC
11643	CakSNP11643	*Kabuli*	Ca_Kabuli_Chr07	419628	[A/C]		--	INTERGENIC
11644	CakSNP11644	*Kabuli*	Ca_Kabuli_Chr07	528521	[G/A]		--	INTERGENIC
11645	CakSNP11645	*Kabuli*	Ca_Kabuli_Chr07	601563	[T/A]		--	INTERGENIC
11646	CakSNP11646	*Kabuli*	Ca_Kabuli_Chr07	601551	[T/C]		--	INTERGENIC
11647	CakSNP11647	*Kabuli*	Ca_Kabuli_Chr07	601545	[C/T]		--	INTERGENIC
11648	CakSNP11648	*Kabuli*	Ca_Kabuli_Chr07	601538	[C/A]		--	INTERGENIC
11649	CakSNP11649	*Kabuli*	Ca_Kabuli_Chr07	601521	[G/A]		--	INTERGENIC
11650	CakSNP11650	*Kabuli*	Ca_Kabuli_Chr07	601516	[G/A]		--	INTERGENIC
11651	CakSNP11651	*Kabuli*	Ca_Kabuli_Chr07	601513	[C/G]		--	INTERGENIC

S.N.	SNP IDs	Chickpea cultivars	Chromosomes	Physical positions (bp)	SNPs	Gene accession IDs	Putative functions	Sequence components of genome
11652	CakSNP11652	*Kabuli*	Ca_Kabuli_Chr07	601502	[G/T]		--	INTERGENIC
11653	CakSNP11653	*Kabuli*	Ca_Kabuli_Chr07	601496	[C/T]		--	INTERGENIC
11654	CakSNP11654	*Kabuli*	Ca_Kabuli_Chr07	601586	[C/T]		--	INTERGENIC
11655	CakSNP11655	*Kabuli*	Ca_Kabuli_Chr07	601601	[C/T]		--	INTERGENIC
11656	CakSNP11656	*Kabuli*	Ca_Kabuli_Chr07	601612	[A/G]		--	INTERGENIC
11657	CakSNP11657	*Kabuli*	Ca_Kabuli_Chr07	601643	[G/T]		--	INTERGENIC
11658	CakSNP11658	*Kabuli*	Ca_Kabuli_Chr07	601622	[G/C]		--	INTERGENIC
11659	CakSNP11659	*Kabuli*	Ca_Kabuli_Chr07	601657	[C/T]		--	INTERGENIC
11660	CakSNP11660	*Kabuli*	Ca_Kabuli_Chr07	601719	[G/T]		--	INTERGENIC
11661	CakSNP11661	*Kabuli*	Ca_Kabuli_Chr07	601718	[G/C]		--	INTERGENIC

S.N.	SNP IDs	Chickpea cultivars	Chromosomes	Physical positions (bp)	SNPs	Gene accession IDs	Putative functions	Sequence components of genome
11662	CakSNP11662	*Kabuli*	Ca_Kabuli_Chr07	601714	[A/T]		--	INTERGENIC
11663	CakSNP11663	*Kabuli*	Ca_Kabuli_Chr07	601709	[A/C]		--	INTERGENIC
11664	CakSNP11664	*Kabuli*	Ca_Kabuli_Chr07	601683	[G/A]		--	INTERGENIC
11665	CakSNP11665	*Kabuli*	Ca_Kabuli_Chr07	601647	[C/T]		--	INTERGENIC
11666	CakSNP11666	*Kabuli*	Ca_Kabuli_Chr07	610737	[C/A]	Ca_24417	Transcription factor GRAS	NON_SYNONYMOUS_CODING
11667	CakSNP11667	*Kabuli*	Ca_Kabuli_Chr07	610723	[G/T]	Ca_24417	Transcription factor GRAS	NON_SYNONYMOUS_CODING
11668	CakSNP11668	*Kabuli*	Ca_Kabuli_Chr07	667597	[C/T]		--	INTERGENIC
11669	CakSNP11669	*Kabuli*	Ca_Kabuli_Chr07	674791	[A/T]	Ca_03417	HECT	INTRON
11670	CakSNP11670	*Kabuli*	Ca_Kabuli_Chr07	676785	[G/T]	Ca_03416	--	NON_SYNONYMOUS_CODING
11671	CakSNP11671	*Kabuli*	Ca_Kabuli_Chr07	676786	[G/A]	Ca_03416	--	NON_SYNONYMOUS_CODING

S.N.	SNP IDs	Chickpea cultivars	Chromosomes	Physical positions (bp)	SNPs	Gene accession IDs	Putative functions	Sequence components of genome
11672	CakSNP11672	*Kabuli*	Ca_Kabuli_Chr07	676790	[T/C]	Ca_03416	--	SYNONYMOUS_CODING
11673	CakSNP11673	*Kabuli*	Ca_Kabuli_Chr07	683245	[T/C]	Ca_03415	Nucleolar, Nop52	SYNONYMOUS_CODING
11674	CakSNP11674	*Kabuli*	Ca_Kabuli_Chr07	761710	[A/G]	Ca_03403	Short-chain dehydrogenase/reductase SDR	INTRON
11675	CakSNP11675	*Kabuli*	Ca_Kabuli_Chr07	767708	[G/A]	Ca_03402	EPS15 homology (EH)	STOP_GAINED
11676	CakSNP11676	*Kabuli*	Ca_Kabuli_Chr07	781377	[G/T]	Ca_03400	Phox/Bem1p	NON_SYNONYMOUS_CODING
11677	CakSNP11677	*Kabuli*	Ca_Kabuli_Chr07	781409	[A/G]	Ca_03400	Phox/Bem1p	NON_SYNONYMOUS_CODING
11678	CakSNP11678	*Kabuli*	Ca_Kabuli_Chr07	807095	[A/T]	Ca_03397	Phosphofructokinase domain	INTRON
11679	CakSNP11679	*Kabuli*	Ca_Kabuli_Chr07	807110	[T/C]	Ca_03397	Phosphofructokinase domain	INTRON
11680	CakSNP11680	*Kabuli*	Ca_Kabuli_Chr07	807133	[T/A]	Ca_03397	Phosphofructokinase domain	INTRON
11681	CakSNP11681	*Kabuli*	Ca_Kabuli_Chr07	815477	[A/T]	Ca_03396	--	SYNONYMOUS_CODING

S.N.	SNP IDs	Chickpea cultivars	Chromosomes	Physical positions (bp)	SNPs	Gene accession IDs	Putative functions	Sequence components of genome
11682	CakSNP11682	*Kabuli*	Ca_Kabuli_Chr07	815526	[C/A]	Ca_03396	--	NON_SYNONYMOUS_CODING
11683	CakSNP11683	*Kabuli*	Ca_Kabuli_Chr07	838581	[C/A]		--	INTERGENIC
11684	CakSNP11684	*Kabuli*	Ca_Kabuli_Chr07	838651	[A/C]		--	INTERGENIC
11685	CakSNP11685	*Kabuli*	Ca_Kabuli_Chr07	841988	[T/C]	Ca_03393	Domain of unknown function DUF221	INTRON
11686	CakSNP11686	*Kabuli*	Ca_Kabuli_Chr07	844248	[G/A]	Ca_03393	Domain of unknown function DUF221	INTRON
11687	CakSNP11687	*Kabuli*	Ca_Kabuli_Chr07	845488	[A/G]	Ca_03393	Domain of unknown function DUF221	SYNONYMOUS_CODING
11688	CakSNP11688	*Kabuli*	Ca_Kabuli_Chr07	865007	[C/T]	Ca_03392	Aminoacyl-tRNA synthetase, class Ia	INTRON
11689	CakSNP11689	*Kabuli*	Ca_Kabuli_Chr07	865170	[C/T]	Ca_03392	Aminoacyl-tRNA synthetase, class Ia	INTRON
11690	CakSNP11690	*Kabuli*	Ca_Kabuli_Chr07	873207	[A/G]	Ca_03391	Plant peroxidase	SYNONYMOUS_CODING
11691	CakSNP11691	*Kabuli*	Ca_Kabuli_Chr07	888900	[G/A]	Ca_03388	DNA-binding WRKY	SYNONYMOUS_CODING

S.N.	SNP IDs	Chickpea cultivars	Chromosomes	Physical positions (bp)	SNPs	Gene accession IDs	Putative functions	Sequence components of genome
11692	CakSNP11692	*Kabuli*	Ca_Kabuli_Chr07	888987	[A/G]	Ca_03388	DNA-binding WRKY	SYNONYMOUS_CODING
11693	CakSNP11693	*Kabuli*	Ca_Kabuli_Chr07	975236	[T/A]	Ca_03381	Multicopper oxidase, type 1	INTRON
11694	CakSNP11694	*Kabuli*	Ca_Kabuli_Chr07	993031	[G/T]	Ca_03378	Prephenate dehydratase	NON_SYNONYMOUS_CODING
11695	CakSNP11695	*Kabuli*	Ca_Kabuli_Chr07	994976	[G/A]	Ca_03378	Prephenate dehydratase	SYNONYMOUS_CODING
11696	CakSNP11696	*Kabuli*	Ca_Kabuli_Chr07	995691	[C/T]	Ca_03378	Prephenate dehydratase	NON_SYNONYMOUS_CODING
11697	CakSNP11697	*Kabuli*	Ca_Kabuli_Chr07	995674	[T/C]	Ca_03378	Prephenate dehydratase	SYNONYMOUS_CODING
11698	CakSNP11698	*Kabuli*	Ca_Kabuli_Chr07	1022946	[C/T]	Ca_03375	--	NON_SYNONYMOUS_CODING
11699	CakSNP11699	*Kabuli*	Ca_Kabuli_Chr07	1045113	[A/C]	Ca_03372	--	INTRON
11700	CakSNP11700	*Kabuli*	Ca_Kabuli_Chr07	1045127	[T/A]	Ca_03372	--	INTRON
11701	CakSNP11701	*Kabuli*	Ca_Kabuli_Chr07	1096379	[C/T]	Ca_03366	IQ motif, EF-hand binding site	SYNONYMOUS_CODING

S.N.	SNP IDs	Chickpea cultivars	Chromosomes	Physical positions (bp)	SNPs	Gene accession IDs	Putative functions	Sequence components of genome
11702	CakSNP11702	*Kabuli*	Ca_Kabuli_Chr07	1153267	[A/C]	Ca_03359	Glycoside hydrolase, family 1	SYNONYMOUS_CODING
11703	CakSNP11703	*Kabuli*	Ca_Kabuli_Chr07	1174188	[C/T]	Ca_03356	Helicase, C-terminal	INTRON
11704	CakSNP11704	*Kabuli*	Ca_Kabuli_Chr07	1293601	[C/G]	Ca_03339	Protein kinase, catalytic domain	SYNONYMOUS_CODING
11705	CakSNP11705	*Kabuli*	Ca_Kabuli_Chr07	1350605	[C/T]	Ca_03334	Peptidase C19, ubiquitin carboxyl-terminal hydrolase 2	NON_SYNONYMOUS_CODING
11706	CakSNP11706	*Kabuli*	Ca_Kabuli_Chr07	1350856	[G/T]		--	INTERGENIC
11707	CakSNP11707	*Kabuli*	Ca_Kabuli_Chr07	1350846	[A/T]		--	INTERGENIC
11708	CakSNP11708	*Kabuli*	Ca_Kabuli_Chr07	1350947	[T/G]		--	INTERGENIC
11709	CakSNP11709	*Kabuli*	Ca_Kabuli_Chr07	1414206	[C/A]		--	INTERGENIC
11710	CakSNP11710	*Kabuli*	Ca_Kabuli_Chr07	1505646	[G/A]	Ca_03315	Pentatricopeptide repeat	INTRON
11711	CakSNP11711	*Kabuli*	Ca_Kabuli_Chr07	1509156	[G/A]	Ca_03315	Pentatricopeptide repeat	INTRON

S.N.	SNP IDs	Chickpea cultivars	Chromosomes	Physical positions (bp)	SNPs	Gene accession IDs	Putative functions	Sequence components of genome
11712	CakSNP11712	*Kabuli*	Ca_Kabuli_Chr07	1511252	[C/T]	Ca_03315	Pentatricopeptide repeat	INTRON
11713	CakSNP11713	*Kabuli*	Ca_Kabuli_Chr07	1585622	[T/C]	Ca_03308	--	NON_SYNONYMOUS_CODING
11714	CakSNP11714	*Kabuli*	Ca_Kabuli_Chr07	1635519	[G/A]	Ca_03304	WD40 repeat	INTRON
11715	CakSNP11715	*Kabuli*	Ca_Kabuli_Chr07	1635471	[G/T]	Ca_03304	WD40 repeat	INTRON
11716	CakSNP11716	*Kabuli*	Ca_Kabuli_Chr07	1702033	[G/T]		--	INTERGENIC
11717	CakSNP11717	*Kabuli*	Ca_Kabuli_Chr07	1702063	[A/G]		--	INTERGENIC
11718	CakSNP11718	*Kabuli*	Ca_Kabuli_Chr07	1702077	[G/C]		--	INTERGENIC
11719	CakSNP11719	*Kabuli*	Ca_Kabuli_Chr07	1704780	[T/G]		--	INTERGENIC
11720	CakSNP11720	*Kabuli*	Ca_Kabuli_Chr07	1704802	[A/G]		--	INTERGENIC
11721	CakSNP11721	*Kabuli*	Ca_Kabuli_Chr07	1704892	[A/G]		--	INTERGENIC

S.N.	SNP IDs	Chickpea cultivars	Chromosomes	Physical positions (bp)	SNPs	Gene accession IDs	Putative functions	Sequence components of genome
11722	CakSNP11722	*Kabuli*	Ca_Kabuli_Chr07	1704947	[C/T]		--	INTERGENIC
11723	CakSNP11723	*Kabuli*	Ca_Kabuli_Chr07	1705197	[A/C]	Ca_03296	Cytochrome c oxidase, subunit VIb	NON_SYNONYMOUS_CODING
11724	CakSNP11724	*Kabuli*	Ca_Kabuli_Chr07	1707283	[G/T]	Ca_03296	Cytochrome c oxidase, subunit VIb	NON_SYNONYMOUS_CODING
11725	CakSNP11725	*Kabuli*	Ca_Kabuli_Chr07	1707375	[C/G]	Ca_03296	Cytochrome c oxidase, subunit VIb	SYNONYMOUS_CODING
11726	CakSNP11726	*Kabuli*	Ca_Kabuli_Chr07	1739369	[C/A]		--	INTERGENIC
11727	CakSNP11727	*Kabuli*	Ca_Kabuli_Chr07	1787308	[T/A]	Ca_03291	Domain of unknown function DUF828	NON_SYNONYMOUS_CODING
11728	CakSNP11728	*Kabuli*	Ca_Kabuli_Chr07	1787417	[G/A]	Ca_03291	Domain of unknown function DUF828	NON_SYNONYMOUS_CODING
11729	CakSNP11729	*Kabuli*	Ca_Kabuli_Chr07	1946020	[G/A]	Ca_03279	Diacylglycerol kinase, accessory domain	INTRON
11730	CakSNP11730	*Kabuli*	Ca_Kabuli_Chr07	1963659	[T/A]		--	INTERGENIC
11731	CakSNP11731	*Kabuli*	Ca_Kabuli_Chr07	1963757	[A/G]		--	INTERGENIC

S.N.	SNP IDs	Chickpea cultivars	Chromosomes	Physical positions (bp)	SNPs	Gene accession IDs	Putative functions	Sequence components of genome
11732	CakSNP11732	*Kabuli*	Ca_Kabuli_Chr07	1991642	[G/T]	Ca_03275	Zinc finger, B-box	SYNONYMOUS_CODING
11733	CakSNP11733	*Kabuli*	Ca_Kabuli_Chr07	2012854	[T/A]	Ca_03273	Xylose isomerase	INTRON
11734	CakSNP11734	*Kabuli*	Ca_Kabuli_Chr07	2103626	[C/T]	Ca_03262	Calmodulin binding protein-like	INTRON
11735	CakSNP11735	*Kabuli*	Ca_Kabuli_Chr07	2106876	[C/A]	Ca_03262	Calmodulin binding protein-like	NON_SYNONYMOUS_CODING
11736	CakSNP11736	*Kabuli*	Ca_Kabuli_Chr07	2106917	[A/T]	Ca_03262	Calmodulin binding protein-like	NON_SYNONYMOUS_CODING
11737	CakSNP11737	*Kabuli*	Ca_Kabuli_Chr07	2146554	[G/A]		--	INTERGENIC
11738	CakSNP11738	*Kabuli*	Ca_Kabuli_Chr07	2167517	[A/C]		--	INTERGENIC
11739	CakSNP11739	*Kabuli*	Ca_Kabuli_Chr07	2167540	[G/C]		--	INTERGENIC
11740	CakSNP11740	*Kabuli*	Ca_Kabuli_Chr07	2211897	[G/A]	Ca_03250	SET domain	INTRON
11741	CakSNP11741	*Kabuli*	Ca_Kabuli_Chr07	2219764	[C/T]	Ca_03249	Syntaxin 6, N-terminal	INTRON

S.N.	SNP IDs	Chickpea cultivars	Chromosomes	Physical positions (bp)	SNPs	Gene accession IDs	Putative functions	Sequence components of genome
11742	CakSNP11742	*Kabuli*	Ca_Kabuli_Chr07	2230434	[A/G]		--	INTERGENIC
11743	CakSNP11743	*Kabuli*	Ca_Kabuli_Chr07	2262749	[C/G]	Ca_03245	Haloacid dehalogenase-like hydrolase	NON_SYNONYMOUS_CODING
11744	CakSNP11744	*Kabuli*	Ca_Kabuli_Chr07	2262865	[A/G]	Ca_03245	Haloacid dehalogenase-like hydrolase	INTRON
11745	CakSNP11745	*Kabuli*	Ca_Kabuli_Chr07	2262843	[T/A]	Ca_03245	Haloacid dehalogenase-like hydrolase	INTRON
11746	CakSNP11746	*Kabuli*	Ca_Kabuli_Chr07	2361064	[T/G]	Ca_03238	Fibronectin, type III	NON_SYNONYMOUS_CODING
11747	CakSNP11747	*Kabuli*	Ca_Kabuli_Chr07	2387109	[C/T]		--	INTERGENIC
11748	CakSNP11748	*Kabuli*	Ca_Kabuli_Chr07	2391392	[G/A]	Ca_03236	WD40 repeat	INTRON
11749	CakSNP11749	*Kabuli*	Ca_Kabuli_Chr07	2401235	[T/C]		--	INTERGENIC
11750	CakSNP11750	*Kabuli*	Ca_Kabuli_Chr07	2493033	[A/G]	Ca_03229	Methyltransferase type 11	INTRON
11751	CakSNP11751	*Kabuli*	Ca_Kabuli_Chr07	2538167	[C/G]	Ca_03225	Glycosyl transferase, family 14	INTRON

S.N.	SNP IDs	Chickpea cultivars	Chromosomes	Physical positions (bp)	SNPs	Gene accession IDs	Putative functions	Sequence components of genome
11752	CakSNP11752	*Kabuli*	Ca_Kabuli_Chr07	2541501	[A/G]		--	INTERGENIC
11753	CakSNP11753	*Kabuli*	Ca_Kabuli_Chr07	2541551	[G/A]		--	INTERGENIC
11754	CakSNP11754	*Kabuli*	Ca_Kabuli_Chr07	2541653	[C/G]		--	INTERGENIC
11755	CakSNP11755	*Kabuli*	Ca_Kabuli_Chr07	2545613	[T/C]		--	INTERGENIC
11756	CakSNP11756	*Kabuli*	Ca_Kabuli_Chr07	2552454	[G/T]	Ca_03223	Peptidase S8/S53, subtilisin/kexin/sedolisin	NON_SYNONYMOUS_CODING
11757	CakSNP11757	*Kabuli*	Ca_Kabuli_Chr07	2603595	[C/T]	Ca_03218	K Homology	INTRON
11758	CakSNP11758	*Kabuli*	Ca_Kabuli_Chr07	2603633	[C/T]	Ca_03218	K Homology	INTRON
11759	CakSNP11759	*Kabuli*	Ca_Kabuli_Chr07	2603649	[T/C]	Ca_03218	K Homology	INTRON
11760	CakSNP11760	*Kabuli*	Ca_Kabuli_Chr07	2609831	[C/A]	Ca_03217	Macrophage migration inhibitory factor	NON_SYNONYMOUS_CODING
11761	CakSNP11761	*Kabuli*	Ca_Kabuli_Chr07	2618478	[C/T]	Ca_03215	Glycoside hydrolase, family 47	SYNONYMOUS_CODING

S.N.	SNP IDs	Chickpea cultivars	Chromosomes	Physical positions (bp)	SNPs	Gene accession IDs	Putative functions	Sequence components of genome
11762	CakSNP11762	*Kabuli*	Ca_Kabuli_Chr07	2654442	[T/G]	Ca_03212	Uncharacterised protein family FPL	INTRON
11763	CakSNP11763	*Kabuli*	Ca_Kabuli_Chr07	2654478	[C/T]	Ca_03212	Uncharacterised protein family FPL	INTRON
11764	CakSNP11764	*Kabuli*	Ca_Kabuli_Chr07	2684213	[A/G]	Ca_03210	LisH dimerisation motif	INTRON
11765	CakSNP11765	*Kabuli*	Ca_Kabuli_Chr07	2734680	[A/G]	Ca_03205	Protein kinase, catalytic domain	INTRON
11766	CakSNP11766	*Kabuli*	Ca_Kabuli_Chr07	2734625	[C/T]	Ca_03205	Protein kinase, catalytic domain	INTRON
11767	CakSNP11767	*Kabuli*	Ca_Kabuli_Chr07	2745665	[A/C]		--	INTERGENIC
11768	CakSNP11768	*Kabuli*	Ca_Kabuli_Chr07	2745656	[A/C]		--	INTERGENIC
11769	CakSNP11769	*Kabuli*	Ca_Kabuli_Chr07	2757260	[C/T]	Ca_03202	Transcription factor jumonji/aspartyl beta-hydroxylase	SYNONYMOUS_CODING
11770	CakSNP11770	*Kabuli*	Ca_Kabuli_Chr07	2757255	[G/C]	Ca_03202	Transcription factor jumonji/aspartyl beta-hydroxylase	NON_SYNONYMOUS_CODING
11771	CakSNP11771	*Kabuli*	Ca_Kabuli_Chr07	2757253	[T/G]	Ca_03202	Transcription factor jumonji/aspartyl beta-hydroxylase	NON_SYNONYMOUS_CODING

S.N.	SNP IDs	Chickpea cultivars	Chromosomes	Physical positions (bp)	SNPs	Gene accession IDs	Putative functions	Sequence components of genome
11772	CakSNP11772	*Kabuli*	Ca_Kabuli_Chr07	2762396	[G/A]		--	INTERGENIC
11773	CakSNP11773	*Kabuli*	Ca_Kabuli_Chr07	2762406	[C/T]		--	INTERGENIC
11774	CakSNP11774	*Kabuli*	Ca_Kabuli_Chr07	2762423	[G/A]		--	INTERGENIC
11775	CakSNP11775	*Kabuli*	Ca_Kabuli_Chr07	2832369	[C/T]	Ca_03193	Zinc finger, GATA-type	SYNONYMOUS_CODING
11776	CakSNP11776	*Kabuli*	Ca_Kabuli_Chr07	2857630	[T/G]	Ca_03190	Protein kinase, catalytic domain	INTRON
11777	CakSNP11777	*Kabuli*	Ca_Kabuli_Chr07	2857818	[T/A]	Ca_03190	Protein kinase, catalytic domain	INTRON
11778	CakSNP11778	*Kabuli*	Ca_Kabuli_Chr07	2857844	[C/T]	Ca_03190	Protein kinase, catalytic domain	INTRON
11779	CakSNP11779	*Kabuli*	Ca_Kabuli_Chr07	2909641	[A/G]		--	INTERGENIC
11780	CakSNP11780	*Kabuli*	Ca_Kabuli_Chr07	2915375	[C/G]		--	INTERGENIC
11781	CakSNP11781	*Kabuli*	Ca_Kabuli_Chr07	2932309	[C/G]	Ca_03182	IQ motif, EF-hand binding site	NON_SYNONYMOUS_CODING

S.N.	SNP IDs	Chickpea cultivars	Chromosomes	Physical positions (bp)	SNPs	Gene accession IDs	Putative functions	Sequence components of genome
11782	CakSNP11782	*Kabuli*	Ca_Kabuli_Chr07	2932301	[C/A]	Ca_03182	IQ motif, EF-hand binding site	NON_SYNONYMOUS_CODING
11783	CakSNP11783	*Kabuli*	Ca_Kabuli_Chr07	2932377	[C/T]	Ca_03182	IQ motif, EF-hand binding site	SYNONYMOUS_CODING
11784	CakSNP11784	*Kabuli*	Ca_Kabuli_Chr07	2956665	[C/T]	Ca_03178	Membrane bound O-acyl transferase, MBOAT	INTRON
11785	CakSNP11785	*Kabuli*	Ca_Kabuli_Chr07	2956767	[A/G]	Ca_03178	Membrane bound O-acyl transferase, MBOAT	INTRON
11786	CakSNP11786	*Kabuli*	Ca_Kabuli_Chr07	2969653	[A/G]	Ca_03177	RNA helicase, ATP-dependent, DEAD-box, conserved site	INTRON
11787	CakSNP11787	*Kabuli*	Ca_Kabuli_Chr07	2997261	[A/G]		--	INTERGENIC
11788	CakSNP11788	*Kabuli*	Ca_Kabuli_Chr07	3023182	[T/C]	Ca_03170	--	INTRON
11789	CakSNP11789	*Kabuli*	Ca_Kabuli_Chr07	3023154	[T/C]	Ca_03170	--	INTRON
11790	CakSNP11790	*Kabuli*	Ca_Kabuli_Chr07	3070531	[A/C]	Ca_03164	Serine/threonine dehydratase, pyridoxal-phosphate-binding site	NON_SYNONYMOUS_CODING
11791	CakSNP11791	*Kabuli*	Ca_Kabuli_Chr07	3090898	[G/A]	Ca_03163	IQ motif, EF-hand binding site	NON_SYNONYMOUS_CODING

S.N.	SNP IDs	Chickpea cultivars	Chromosomes	Physical positions (bp)	SNPs	Gene accession IDs	Putative functions	Sequence components of genome
11792	CakSNP11792	*Kabuli*	Ca_Kabuli_Chr07	3093884	[T/A]	Ca_03163	IQ motif, EF-hand binding site	INTRON
11793	CakSNP11793	*Kabuli*	Ca_Kabuli_Chr07	3093953	[T/G]	Ca_03163	IQ motif, EF-hand binding site	NON_SYNONYMOUS_CODING
11794	CakSNP11794	*Kabuli*	Ca_Kabuli_Chr07	3094090	[G/A]	Ca_03163	IQ motif, EF-hand binding site	SYNONYMOUS_CODING
11795	CakSNP11795	*Kabuli*	Ca_Kabuli_Chr07	3094087	[A/G]	Ca_03163	IQ motif, EF-hand binding site	SYNONYMOUS_CODING
11796	CakSNP11796	*Kabuli*	Ca_Kabuli_Chr07	3107197	[C/T]	Ca_03162	Peptidoglycan-binding Lysin subgroup	SYNONYMOUS_CODING
11797	CakSNP11797	*Kabuli*	Ca_Kabuli_Chr07	3107239	[T/A]	Ca_03162	Peptidoglycan-binding Lysin subgroup	NON_SYNONYMOUS_CODING
11798	CakSNP11798	*Kabuli*	Ca_Kabuli_Chr07	3109250	[T/C]	Ca_03161	Peptidase S9, prolyl oligopeptidase, catalytic domain	NON_SYNONYMOUS_CODING
11799	CakSNP11799	*Kabuli*	Ca_Kabuli_Chr07	3109352	[T/C]	Ca_03161	Peptidase S9, prolyl oligopeptidase, catalytic domain	NON_SYNONYMOUS_CODING
11800	CakSNP11800	*Kabuli*	Ca_Kabuli_Chr07	3110316	[A/G]	Ca_03161	Peptidase S9, prolyl oligopeptidase, catalytic domain	SYNONYMOUS_CODING
11801	CakSNP11801	*Kabuli*	Ca_Kabuli_Chr07	3130070	[A/G]	Ca_03158	Photosystem II PsbX	NON_SYNONYMOUS_CODING

S.N.	SNP IDs	Chickpea cultivars	Chromosomes	Physical positions (bp)	SNPs	Gene accession IDs	Putative functions	Sequence components of genome
11802	CakSNP11802	*Kabuli*	Ca_Kabuli_Chr07	3130069	[A/G]	Ca_03158	Photosystem II PsbX	NON_SYNONYMOUS_CODING
11803	CakSNP11803	*Kabuli*	Ca_Kabuli_Chr07	3143409	[C/T]	Ca_03156	Stress responsive alpha-beta barrel	NON_SYNONYMOUS_CODING
11804	CakSNP11804	*Kabuli*	Ca_Kabuli_Chr07	3186341	[C/T]	Ca_03151	NADH:flavin oxidoreductase/NADH oxidase, N-terminal	NON_SYNONYMOUS_CODING
11805	CakSNP11805	*Kabuli*	Ca_Kabuli_Chr07	3186262	[T/G]	Ca_03151	NADH:flavin oxidoreductase/NADH oxidase, N-terminal	SYNONYMOUS_CODING
11806	CakSNP11806	*Kabuli*	Ca_Kabuli_Chr07	3198197	[C/A]		--	INTERGENIC
11807	CakSNP11807	*Kabuli*	Ca_Kabuli_Chr07	3198263	[T/C]		--	INTERGENIC
11808	CakSNP11808	*Kabuli*	Ca_Kabuli_Chr07	3217916	[T/G]	Ca_03145	--	SYNONYMOUS_CODING
11809	CakSNP11809	*Kabuli*	Ca_Kabuli_Chr07	3236150	[A/C]		--	INTERGENIC
11810	CakSNP11810	*Kabuli*	Ca_Kabuli_Chr07	3265823	[A/G]	Ca_03142	Peptidase S8/S53, subtilisin/kexin/sedolisin	NON_SYNONYMOUS_CODING
11811	CakSNP11811	*Kabuli*	Ca_Kabuli_Chr07	3297560	[G/A]	Ca_03139	Domain of unknown function DUF231, plant	SYNONYMOUS_CODING

S.N.	SNP IDs	Chickpea cultivars	Chromosomes	Physical positions (bp)	SNPs	Gene accession IDs	Putative functions	Sequence components of genome
11812	CakSNP11812	*Kabuli*	Ca_Kabuli_Chr07	3297677	[G/T]	Ca_03139	Domain of unknown function DUF231, plant	NON_SYNONYMOUS_CODING
11813	CakSNP11813	*Kabuli*	Ca_Kabuli_Chr07	3315157	[A/G]	Ca_03135	Protein kinase, catalytic domain	SYNONYMOUS_CODING
11814	CakSNP11814	*Kabuli*	Ca_Kabuli_Chr07	3351037	[G/T]	Ca_03129	Chaperonin Cpn10, conserved site	INTRON
11815	CakSNP11815	*Kabuli*	Ca_Kabuli_Chr07	3353095	[C/T]		--	INTERGENIC
11816	CakSNP11816	*Kabuli*	Ca_Kabuli_Chr07	3355627	[A/G]	Ca_03128	AUX/IAA protein	NON_SYNONYMOUS_CODING
11817	CakSNP11817	*Kabuli*	Ca_Kabuli_Chr07	3355763	[A/G]	Ca_03128	AUX/IAA protein	SYNONYMOUS_CODING
11818	CakSNP11818	*Kabuli*	Ca_Kabuli_Chr07	3356399	[G/A]	Ca_03128	AUX/IAA protein	INTRON
11819	CakSNP11819	*Kabuli*	Ca_Kabuli_Chr07	3357452	[G/A]	Ca_03128	AUX/IAA protein	NON_SYNONYMOUS_CODING
11820	CakSNP11820	*Kabuli*	Ca_Kabuli_Chr07	3430492	[A/T]		--	INTERGENIC
11821	CakSNP11821	*Kabuli*	Ca_Kabuli_Chr07	3430479	[G/A]		--	INTERGENIC

S.N.	SNP IDs	Chickpea cultivars	Chromosomes	Physical positions (bp)	SNPs	Gene accession IDs	Putative functions	Sequence components of genome
11822	CakSNP11822	*Kabuli*	Ca_Kabuli_Chr07	3445031	[T/C]		--	INTERGENIC
11823	CakSNP11823	*Kabuli*	Ca_Kabuli_Chr07	3476546	[T/C]	Ca_03119	--	SYNONYMOUS_CODING
11824	CakSNP11824	*Kabuli*	Ca_Kabuli_Chr07	3504900	[G/A]		--	INTERGENIC
11825	CakSNP11825	*Kabuli*	Ca_Kabuli_Chr07	3505012	[A/C]		--	INTERGENIC
11826	CakSNP11826	*Kabuli*	Ca_Kabuli_Chr07	3518048	[G/A]	Ca_03116	Synaptojanin, N-terminal	SYNONYMOUS_CODING
11827	CakSNP11827	*Kabuli*	Ca_Kabuli_Chr07	3519510	[A/G]	Ca_03116	Synaptojanin, N-terminal	INTRON
11828	CakSNP11828	*Kabuli*	Ca_Kabuli_Chr07	3519701	[A/C]	Ca_03116	Synaptojanin, N-terminal	INTRON
11829	CakSNP11829	*Kabuli*	Ca_Kabuli_Chr07	3521150	[A/G]	Ca_03116	Synaptojanin, N-terminal	INTRON
11830	CakSNP11830	*Kabuli*	Ca_Kabuli_Chr07	3521257	[G/A]	Ca_03116	Synaptojanin, N-terminal	INTRON
11831	CakSNP11831	*Kabuli*	Ca_Kabuli_Chr07	3521192	[C/A]	Ca_03116	Synaptojanin, N-terminal	INTRON

S.N.	SNP IDs	Chickpea cultivars	Chromosomes	Physical positions (bp)	SNPs	Gene accession IDs	Putative functions	Sequence components of genome
11832	CakSNP11832	*Kabuli*	Ca_Kabuli_Chr07	3536194	[C/T]		--	INTERGENIC
11833	CakSNP11833	*Kabuli*	Ca_Kabuli_Chr07	3536233	[T/C]	Ca_03115	Zinc finger, FYVE-type	SYNONYMOUS_CODING
11834	CakSNP11834	*Kabuli*	Ca_Kabuli_Chr07	3543765	[A/G]	Ca_03114	Zinc finger, FYVE-type	SYNONYMOUS_CODING
11835	CakSNP11835	*Kabuli*	Ca_Kabuli_Chr07	3543768	[T/C]	Ca_03114	Zinc finger, FYVE-type	SYNONYMOUS_CODING
11836	CakSNP11836	*Kabuli*	Ca_Kabuli_Chr07	3557570	[C/T]	Ca_03113	Zinc finger, FYVE-type	INTRON
11837	CakSNP11837	*Kabuli*	Ca_Kabuli_Chr07	3560182	[G/A]	Ca_03113	Zinc finger, FYVE-type	NON_SYNONYMOUS_CODING
11838	CakSNP11838	*Kabuli*	Ca_Kabuli_Chr07	3560250	[C/T]	Ca_03113	Zinc finger, FYVE-type	SYNONYMOUS_CODING
11839	CakSNP11839	*Kabuli*	Ca_Kabuli_Chr07	3572963	[T/C]	Ca_03112	Sec34-like protein	INTRON
11840	CakSNP11840	*Kabuli*	Ca_Kabuli_Chr07	3581123	[G/A]	Ca_03112	Sec34-like protein	INTRON
11841	CakSNP11841	*Kabuli*	Ca_Kabuli_Chr07	3600826	[C/T]	Ca_03108	Pectinesterase, catalytic	NON_SYNONYMOUS_CODING

S.N.	SNP IDs	Chickpea cultivars	Chromosomes	Physical positions (bp)	SNPs	Gene accession IDs	Putative functions	Sequence components of genome
11842	CakSNP11842	*Kabuli*	Ca_Kabuli_Chr07	3600833	[T/G]	Ca_03108	Pectinesterase, catalytic	SYNONYMOUS_CODING
11843	CakSNP11843	*Kabuli*	Ca_Kabuli_Chr07	3600866	[T/C]	Ca_03108	Pectinesterase, catalytic	NON_SYNONYMOUS_CODING
11844	CakSNP11844	*Kabuli*	Ca_Kabuli_Chr07	3600867	[C/T]	Ca_03108	Pectinesterase, catalytic	SYNONYMOUS_CODING
11845	CakSNP11845	*Kabuli*	Ca_Kabuli_Chr07	3600987	[A/C]		--	INTERGENIC
11846	CakSNP11846	*Kabuli*	Ca_Kabuli_Chr07	3628086	[G/A]	Ca_03106	Saccharopine dehydrogenase / Homospermidine synthase	INTRON
11847	CakSNP11847	*Kabuli*	Ca_Kabuli_Chr07	3635307	[G/A]	Ca_03106	Saccharopine dehydrogenase / Homospermidine synthase	INTRON
11848	CakSNP11848	*Kabuli*	Ca_Kabuli_Chr07	3635309	[C/T]	Ca_03106	Saccharopine dehydrogenase / Homospermidine synthase	INTRON
11849	CakSNP11849	*Kabuli*	Ca_Kabuli_Chr07	3635318	[T/A]	Ca_03106	Saccharopine dehydrogenase / Homospermidine synthase	INTRON
11850	CakSNP11850	*Kabuli*	Ca_Kabuli_Chr07	3635325	[T/C]	Ca_03106	Saccharopine dehydrogenase / Homospermidine synthase	INTRON
11851	CakSNP11851	*Kabuli*	Ca_Kabuli_Chr07	3635369	[G/T]	Ca_03106	Saccharopine dehydrogenase / Homospermidine synthase	INTRON

S.N.	SNP IDs	Chickpea cultivars	Chromosomes	Physical positions (bp)	SNPs	Gene accession IDs	Putative functions	Sequence components of genome
11852	CakSNP11852	*Kabuli*	Ca_Kabuli_Chr07	3651051	[T/C]	Ca_03103	SEC7-like	INTRON
11853	CakSNP11853	*Kabuli*	Ca_Kabuli_Chr07	3655451	[G/T]	Ca_03103	SEC7-like	INTRON
11854	CakSNP11854	*Kabuli*	Ca_Kabuli_Chr07	3665651	[G/C]	Ca_03103	SEC7-like	SYNONYMOUS_CODING
11855	CakSNP11855	*Kabuli*	Ca_Kabuli_Chr07	3672192	[T/C]	Ca_03102	Zinc finger, FYVE-type	INTRON
11856	CakSNP11856	*Kabuli*	Ca_Kabuli_Chr07	3672236	[G/A]	Ca_03102	Zinc finger, FYVE-type	INTRON
11857	CakSNP11857	*Kabuli*	Ca_Kabuli_Chr07	3734030	[T/A]	Ca_03095	Oligopeptide transporter	SYNONYMOUS_CODING
11858	CakSNP11858	*Kabuli*	Ca_Kabuli_Chr07	3738769	[T/C]		--	INTERGENIC
11859	CakSNP11859	*Kabuli*	Ca_Kabuli_Chr07	3754778	[G/A]	Ca_03092	SNF5/SMARCB1/INI1	INTRON
11860	CakSNP11860	*Kabuli*	Ca_Kabuli_Chr07	3754743	[G/C]	Ca_03092	SNF5/SMARCB1/INI1	INTRON
11861	CakSNP11861	*Kabuli*	Ca_Kabuli_Chr07	3795825	[T/C]	Ca_03085	SNF2-related	INTRON

S.N.	SNP IDs	Chickpea cultivars	Chromosomes	Physical positions (bp)	SNPs	Gene accession IDs	Putative functions	Sequence components of genome
11862	CakSNP11862	*Kabuli*	Ca_Kabuli_Chr07	3795798	[G/A]	Ca_03085	SNF2-related	INTRON
11863	CakSNP11863	*Kabuli*	Ca_Kabuli_Chr07	3795776	[C/A]	Ca_03085	SNF2-related	INTRON
11864	CakSNP11864	*Kabuli*	Ca_Kabuli_Chr07	3796826	[A/G]	Ca_03085	SNF2-related	INTRON
11865	CakSNP11865	*Kabuli*	Ca_Kabuli_Chr07	3796894	[A/G]	Ca_03085	SNF2-related	INTRON
11866	CakSNP11866	*Kabuli*	Ca_Kabuli_Chr07	3802188	[T/A]	Ca_03083	--	SYNONYMOUS_CODING
11867	CakSNP11867	*Kabuli*	Ca_Kabuli_Chr07	3814791	[A/G]		--	INTERGENIC
11868	CakSNP11868	*Kabuli*	Ca_Kabuli_Chr07	3817087	[C/T]	Ca_03082	ABC transporter, transmembrane domain	NON_SYNONYMOUS_CODING
11869	CakSNP11869	*Kabuli*	Ca_Kabuli_Chr07	3817733	[T/G]	Ca_03082	ABC transporter, transmembrane domain	NON_SYNONYMOUS_CODING
11870	CakSNP11870	*Kabuli*	Ca_Kabuli_Chr07	3817740	[G/T]	Ca_03082	ABC transporter, transmembrane domain	NON_SYNONYMOUS_CODING
11871	CakSNP11871	*Kabuli*	Ca_Kabuli_Chr07	3829115	[C/T]	Ca_03081	ABC transporter, transmembrane domain	NON_SYNONYMOUS_CODING

S.N.	SNP IDs	Chickpea cultivars	Chromosomes	Physical positions (bp)	SNPs	Gene accession IDs	Putative functions	Sequence components of genome
11872	CakSNP11872	*Kabuli*	Ca_Kabuli_Chr07	3843168	[A/T]	Ca_03080	ABC transporter, transmembrane domain	SYNONYMOUS_CODING
11873	CakSNP11873	*Kabuli*	Ca_Kabuli_Chr07	3845953	[A/C]	Ca_03080	ABC transporter, transmembrane domain	INTRON
11874	CakSNP11874	*Kabuli*	Ca_Kabuli_Chr07	3845958	[G/A]	Ca_03080	ABC transporter, transmembrane domain	INTRON
11875	CakSNP11875	*Kabuli*	Ca_Kabuli_Chr07	3845996	[G/A]	Ca_03080	ABC transporter, transmembrane domain	INTRON
11876	CakSNP11876	*Kabuli*	Ca_Kabuli_Chr07	3856308	[C/T]	Ca_03079	HAD-superfamily hydrolase, subfamily IG, 5'-nucleotidase	INTRON
11877	CakSNP11877	*Kabuli*	Ca_Kabuli_Chr07	3859451	[G/A]	Ca_03078	Pseudouridine synthase I, TruA	SYNONYMOUS_CODING
11878	CakSNP11878	*Kabuli*	Ca_Kabuli_Chr07	3916849	[A/C]	Ca_03070	Pentatricopeptide repeat	SYNONYMOUS_CODING
11879	CakSNP11879	*Kabuli*	Ca_Kabuli_Chr07	3924920	[T/C]	Ca_03069	ABC transporter-like	INTRON
11880	CakSNP11880	*Kabuli*	Ca_Kabuli_Chr07	3946712	[G/A]	Ca_03066	Kinesin, motor domain	INTRON
11881	CakSNP11881	*Kabuli*	Ca_Kabuli_Chr07	3982635	[T/G]	Ca_03063	YTH domain	INTRON

S.N.	SNP IDs	Chickpea cultivars	Chromosomes	Physical positions (bp)	SNPs	Gene accession IDs	Putative functions	Sequence components of genome
11882	CakSNP11882	*Kabuli*	Ca_Kabuli_Chr07	3982622	[G/A]	Ca_03063	YTH domain	INTRON
11883	CakSNP11883	*Kabuli*	Ca_Kabuli_Chr07	4017780	[C/T]	Ca_03061	--	INTRON
11884	CakSNP11884	*Kabuli*	Ca_Kabuli_Chr07	4017851	[T/C]	Ca_03061	--	INTRON
11885	CakSNP11885	*Kabuli*	Ca_Kabuli_Chr07	4039347	[C/A]	Ca_03059	Ferlin/Peroxisome membrane	INTRON
11886	CakSNP11886	*Kabuli*	Ca_Kabuli_Chr07	4039300	[T/C]	Ca_03059	Ferlin/Peroxisome membrane	INTRON
11887	CakSNP11887	*Kabuli*	Ca_Kabuli_Chr07	4039290	[A/G]	Ca_03059	Ferlin/Peroxisome membrane	INTRON
11888	CakSNP11888	*Kabuli*	Ca_Kabuli_Chr07	4042123	[T/G]	Ca_03059	Ferlin/Peroxisome membrane	INTRON
11889	CakSNP11889	*Kabuli*	Ca_Kabuli_Chr07	4095293	[A/G]	Ca_03057	Galectin, carbohydrate recognition domain	NON_SYNONYMOUS_CODING
11890	CakSNP11890	*Kabuli*	Ca_Kabuli_Chr07	4168933	[G/T]		--	INTERGENIC
11891	CakSNP11891	*Kabuli*	Ca_Kabuli_Chr07	4194809	[T/A]	Ca_03044	Pectinesterase inhibitor	NON_SYNONYMOUS_CODING

S.N.	SNP IDs	Chickpea cultivars	Chromosomes	Physical positions (bp)	SNPs	Gene accession IDs	Putative functions	Sequence components of genome
11892	CakSNP11892	*Kabuli*	Ca_Kabuli_Chr07	4194890	[C/A]	Ca_03044	Pectinesterase inhibitor	NON_SYNONYMOUS_CODING
11893	CakSNP11893	*Kabuli*	Ca_Kabuli_Chr07	4194891	[C/G]	Ca_03044	Pectinesterase inhibitor	SYNONYMOUS_CODING
11894	CakSNP11894	*Kabuli*	Ca_Kabuli_Chr07	4198812	[A/C]		--	INTERGENIC
11895	CakSNP11895	*Kabuli*	Ca_Kabuli_Chr07	4209425	[C/T]	Ca_03041	Pectinesterase inhibitor	NON_SYNONYMOUS_CODING
11896	CakSNP11896	*Kabuli*	Ca_Kabuli_Chr07	4209465	[A/C]	Ca_03041	Pectinesterase inhibitor	SYNONYMOUS_CODING
11897	CakSNP11897	*Kabuli*	Ca_Kabuli_Chr07	4225314	[T/C]	Ca_03037	Pectinesterase inhibitor	NON_SYNONYMOUS_CODING
11898	CakSNP11898	*Kabuli*	Ca_Kabuli_Chr07	4256529	[G/A]	Ca_03034	Pentatricopeptide repeat	INTRON
11899	CakSNP11899	*Kabuli*	Ca_Kabuli_Chr07	4256703	[C/G]	Ca_03034	Pentatricopeptide repeat	NON_SYNONYMOUS_CODING
11900	CakSNP11900	*Kabuli*	Ca_Kabuli_Chr07	4257726	[A/T]	Ca_03034	Pentatricopeptide repeat	NON_SYNONYMOUS_CODING
11901	CakSNP11901	*Kabuli*	Ca_Kabuli_Chr07	4257727	[T/A]	Ca_03034	Pentatricopeptide repeat	NON_SYNONYMOUS_CODING

S.N.	SNP IDs	Chickpea cultivars	Chromosomes	Physical positions (bp)	SNPs	Gene accession IDs	Putative functions	Sequence components of genome
11902	CakSNP11902	*Kabuli*	Ca_Kabuli_Chr07	4257838	[C/T]		--	INTERGENIC
11903	CakSNP11903	*Kabuli*	Ca_Kabuli_Chr07	4257830	[G/T]		--	INTERGENIC
11904	CakSNP11904	*Kabuli*	Ca_Kabuli_Chr07	4257799	[G/A]	Ca_03034	Pentatricopeptide repeat	SYNONYMOUS_CODING
11905	CakSNP11905	*Kabuli*	Ca_Kabuli_Chr07	4289975	[G/A]	Ca_03031	RNA recognition motif domain	SYNONYMOUS_CODING
11906	CakSNP11906	*Kabuli*	Ca_Kabuli_Chr07	4300525	[C/T]		--	INTERGENIC
11907	CakSNP11907	*Kabuli*	Ca_Kabuli_Chr07	4300650	[T/C]		--	INTERGENIC
11908	CakSNP11908	*Kabuli*	Ca_Kabuli_Chr07	4300645	[C/T]		--	INTERGENIC
11909	CakSNP11909	*Kabuli*	Ca_Kabuli_Chr07	4300592	[G/A]		--	INTERGENIC
11910	CakSNP11910	*Kabuli*	Ca_Kabuli_Chr07	4300584	[C/T]		--	INTERGENIC
11911	CakSNP11911	*Kabuli*	Ca_Kabuli_Chr07	4305424	[G/T]		--	INTERGENIC

S.N.	SNP IDs	Chickpea cultivars	Chromosomes	Physical positions (bp)	SNPs	Gene accession IDs	Putative functions	Sequence components of genome
11912	CakSNP11912	*Kabuli*	Ca_Kabuli_Chr07	4431138	[A/G]	Ca_03015	--	NON_SYNONYMOUS_CODING
11913	CakSNP11913	*Kabuli*	Ca_Kabuli_Chr07	4431253	[T/C]	Ca_03015	--	INTRON
11914	CakSNP11914	*Kabuli*	Ca_Kabuli_Chr07	4438929	[A/G]	Ca_03014	RNA-processing protein, HAT helix	INTRON
11915	CakSNP11915	*Kabuli*	Ca_Kabuli_Chr07	4455045	[T/C]	Ca_03012	--	INTRON
11916	CakSNP11916	*Kabuli*	Ca_Kabuli_Chr07	4461990	[A/C]		--	INTERGENIC
11917	CakSNP11917	*Kabuli*	Ca_Kabuli_Chr07	4470285	[C/G]	Ca_03010	Ubiquitin-conjugating enzyme, E2	NON_SYNONYMOUS_CODING
11918	CakSNP11918	*Kabuli*	Ca_Kabuli_Chr07	4490514	[G/A]	Ca_03004	--	SYNONYMOUS_CODING
11919	CakSNP11919	*Kabuli*	Ca_Kabuli_Chr07	4490964	[G/T]		--	INTERGENIC
11920	CakSNP11920	*Kabuli*	Ca_Kabuli_Chr07	4490966	[A/G]		--	INTERGENIC
11921	CakSNP11921	*Kabuli*	Ca_Kabuli_Chr07	4497784	[C/T]	Ca_03002	Domain of unknown function DUF625	NON_SYNONYMOUS_CODING

S.N.	SNP IDs	Chickpea cultivars	Chromosomes	Physical positions (bp)	SNPs	Gene accession IDs	Putative functions	Sequence components of genome
11922	CakSNP11922	*Kabuli*	Ca_Kabuli_Chr07	4520606	[T/C]	Ca_03001	Pyridine nucleotide-disulphide oxidoreductase, NAD-binding region	SYNONYMOUS_CODING
11923	CakSNP11923	*Kabuli*	Ca_Kabuli_Chr07	4525645	[A/G]	Ca_03001	Pyridine nucleotide-disulphide oxidoreductase, NAD-binding region	INTRON
11924	CakSNP11924	*Kabuli*	Ca_Kabuli_Chr07	4552790	[T/C]	Ca_02998	IQ motif, EF-hand binding site	NON_SYNONYMOUS_CODING
11925	CakSNP11925	*Kabuli*	Ca_Kabuli_Chr07	4573236	[G/A]	Ca_02995	--	NON_SYNONYMOUS_CODING
11926	CakSNP11926	*Kabuli*	Ca_Kabuli_Chr07	4625723	[C/T]	Ca_02989	Signal transduction response regulator, receiver domain	SYNONYMOUS_CODING
11927	CakSNP11927	*Kabuli*	Ca_Kabuli_Chr07	4648569	[A/C]		--	INTERGENIC
11928	CakSNP11928	*Kabuli*	Ca_Kabuli_Chr07	4648697	[T/A]		--	INTERGENIC
11929	CakSNP11929	*Kabuli*	Ca_Kabuli_Chr07	4648677	[A/T]		--	INTERGENIC
11930	CakSNP11930	*Kabuli*	Ca_Kabuli_Chr07	4648662	[G/A]		--	INTERGENIC
11931	CakSNP11931	*Kabuli*	Ca_Kabuli_Chr07	4649287	[A/G]		--	INTERGENIC

S.N.	SNP IDs	Chickpea cultivars	Chromosomes	Physical positions (bp)	SNPs	Gene accession IDs	Putative functions	Sequence components of genome
11932	CakSNP11932	*Kabuli*	Ca_Kabuli_Chr07	4651931	[T/C]	Ca_02988	Protein of unknown function DUF789	INTRON
11933	CakSNP11933	*Kabuli*	Ca_Kabuli_Chr07	4652083	[G/A]	Ca_02988	Protein of unknown function DUF789	INTRON
11934	CakSNP11934	*Kabuli*	Ca_Kabuli_Chr07	4653235	[A/C]		--	INTERGENIC
11935	CakSNP11935	*Kabuli*	Ca_Kabuli_Chr07	4700570	[C/A]	Ca_02979	WD40 repeat	INTRON
11936	CakSNP11936	*Kabuli*	Ca_Kabuli_Chr07	4749212	[T/G]	Ca_02973	Tetratricopeptide repeat-containing	SYNONYMOUS_CODING
11937	CakSNP11937	*Kabuli*	Ca_Kabuli_Chr07	4752954	[G/C]		--	INTERGENIC
11938	CakSNP11938	*Kabuli*	Ca_Kabuli_Chr07	4752887	[T/G]		--	INTERGENIC
11939	CakSNP11939	*Kabuli*	Ca_Kabuli_Chr07	4756338	[G/A]	Ca_02971	Glycoside hydrolase, family 28	SYNONYMOUS_CODING
11940	CakSNP11940	*Kabuli*	Ca_Kabuli_Chr07	4832993	[A/G]	Ca_02961	Late embryogenesis abundant protein, group 3	NON_SYNONYMOUS_CODING
11941	CakSNP11941	*Kabuli*	Ca_Kabuli_Chr07	4834177	[G/T]		--	INTERGENIC

S.N.	SNP IDs	Chickpea cultivars	Chromosomes	Physical positions (bp)	SNPs	Gene accession IDs	Putative functions	Sequence components of genome
11942	CakSNP11942	*Kabuli*	Ca_Kabuli_Chr07	4849553	[T/G]	Ca_02958	Kinesin, motor domain	INTRON
11943	CakSNP11943	*Kabuli*	Ca_Kabuli_Chr07	4849573	[T/C]	Ca_02958	Kinesin, motor domain	INTRON
11944	CakSNP11944	*Kabuli*	Ca_Kabuli_Chr07	4907315	[C/T]	Ca_02953	Rapid ALkalinization Factor	SYNONYMOUS_CODING
11945	CakSNP11945	*Kabuli*	Ca_Kabuli_Chr07	4926759	[A/T]		--	INTERGENIC
11946	CakSNP11946	*Kabuli*	Ca_Kabuli_Chr07	4942847	[A/G]		--	INTERGENIC
11947	CakSNP11947	*Kabuli*	Ca_Kabuli_Chr07	4942902	[G/C]		--	INTERGENIC
11948	CakSNP11948	*Kabuli*	Ca_Kabuli_Chr07	4945935	[C/G]		--	INTERGENIC
11949	CakSNP11949	*Kabuli*	Ca_Kabuli_Chr07	4981846	[T/C]		--	INTERGENIC
11950	CakSNP11950	*Kabuli*	Ca_Kabuli_Chr07	5122536	[T/G]	Ca_06853	Manganese/iron superoxide dismutase	SYNONYMOUS_CODING
11951	CakSNP11951	*Kabuli*	Ca_Kabuli_Chr07	5251897	[G/C]	Ca_06846	Mitochondrial carrier protein	NON_SYNONYMOUS_CODING

S.N.	SNP IDs	Chickpea cultivars	Chromosomes	Physical positions (bp)	SNPs	Gene accession IDs	Putative functions	Sequence components of genome
11952	CakSNP11952	*Kabuli*	Ca_Kabuli_Chr07	5361558	[T/C]	Ca_06835	Pathogenesis-related transcriptional factor/ERF, DNA-binding	SYNONYMOUS_CODING
11953	CakSNP11953	*Kabuli*	Ca_Kabuli_Chr07	5383564	[G/C]		--	INTERGENIC
11954	CakSNP11954	*Kabuli*	Ca_Kabuli_Chr07	5383566	[A/T]		--	INTERGENIC
11955	CakSNP11955	*Kabuli*	Ca_Kabuli_Chr07	5383671	[G/T]		--	INTERGENIC
11956	CakSNP11956	*Kabuli*	Ca_Kabuli_Chr07	5383695	[T/G]		--	INTERGENIC
11957	CakSNP11957	*Kabuli*	Ca_Kabuli_Chr07	5383767	[G/A]		--	INTERGENIC
11958	CakSNP11958	*Kabuli*	Ca_Kabuli_Chr07	5384447	[G/C]		--	INTERGENIC
11959	CakSNP11959	*Kabuli*	Ca_Kabuli_Chr07	5384467	[C/T]		--	INTERGENIC
11960	CakSNP11960	*Kabuli*	Ca_Kabuli_Chr07	5406524	[T/G]		--	INTERGENIC
11961	CakSNP11961	*Kabuli*	Ca_Kabuli_Chr07	5416862	[C/G]		--	INTERGENIC

S.N.	SNP IDs	Chickpea cultivars	Chromosomes	Physical positions (bp)	SNPs	Gene accession IDs	Putative functions	Sequence components of genome
11962	CakSNP11962	*Kabuli*	Ca_Kabuli_Chr07	5416865	[A/G]		--	INTERGENIC
11963	CakSNP11963	*Kabuli*	Ca_Kabuli_Chr07	5416874	[C/T]		--	INTERGENIC
11964	CakSNP11964	*Kabuli*	Ca_Kabuli_Chr07	5464941	[G/A]	Ca_06821	Uncharacterised protein family UPF0497, trans-membrane plant	NON_SYNONYMOUS_CODING
11965	CakSNP11965	*Kabuli*	Ca_Kabuli_Chr07	5464942	[A/G]	Ca_06821	Uncharacterised protein family UPF0497, trans-membrane plant	NON_SYNONYMOUS_CODING
11966	CakSNP11966	*Kabuli*	Ca_Kabuli_Chr07	5467744	[G/C]	Ca_06820	F-box domain, cyclin-like	NON_SYNONYMOUS_CODING
11967	CakSNP11967	*Kabuli*	Ca_Kabuli_Chr07	5500318	[C/A]	Ca_06816	SNF2-related	NON_SYNONYMOUS_CODING
11968	CakSNP11968	*Kabuli*	Ca_Kabuli_Chr07	5508162	[A/C]	Ca_06816	SNF2-related	SYNONYMOUS_CODING
11969	CakSNP11969	*Kabuli*	Ca_Kabuli_Chr07	5520720	[T/G]	Ca_06814	Heat shock protein DnaJ, N-terminal	NON_SYNONYMOUS_CODING
11970	CakSNP11970	*Kabuli*	Ca_Kabuli_Chr07	5520934	[T/C]	Ca_06814	Heat shock protein DnaJ, N-terminal	NON_SYNONYMOUS_CODING
11971	CakSNP11971	*Kabuli*	Ca_Kabuli_Chr07	5521596	[T/C]	Ca_06814	Heat shock protein DnaJ, N-terminal	NON_SYNONYMOUS_CODING

S.N.	SNP IDs	Chickpea cultivars	Chromosomes	Physical positions (bp)	SNPs	Gene accession IDs	Putative functions	Sequence components of genome
11972	CakSNP11972	*Kabuli*	Ca_Kabuli_Chr07	5521659	[G/T]	Ca_06814	Heat shock protein DnaJ, N-terminal	NON_SYNONYMOUS_CODING
11973	CakSNP11973	*Kabuli*	Ca_Kabuli_Chr07	5529577	[T/G]	Ca_06812	Dehydrogenase, E1 component	SYNONYMOUS_CODING
11974	CakSNP11974	*Kabuli*	Ca_Kabuli_Chr07	5529729	[A/G]	Ca_06812	Dehydrogenase, E1 component	INTRON
11975	CakSNP11975	*Kabuli*	Ca_Kabuli_Chr07	5529888	[A/G]	Ca_06812	Dehydrogenase, E1 component	INTRON
11976	CakSNP11976	*Kabuli*	Ca_Kabuli_Chr07	5529844	[C/A]	Ca_06812	Dehydrogenase, E1 component	INTRON
11977	CakSNP11977	*Kabuli*	Ca_Kabuli_Chr07	5537851	[G/C]	Ca_06811	Protamine P1	INTRON
11978	CakSNP11978	*Kabuli*	Ca_Kabuli_Chr07	5537880	[G/T]	Ca_06811	Protamine P1	INTRON
11979	CakSNP11979	*Kabuli*	Ca_Kabuli_Chr07	5561607	[G/T]	Ca_06808	Inosine/uridine-preferring nucleoside hydrolase domain	INTRON
11980	CakSNP11980	*Kabuli*	Ca_Kabuli_Chr07	5568114	[C/A]	Ca_06806	--	INTRON
11981	CakSNP11981	*Kabuli*	Ca_Kabuli_Chr07	5577467	[G/T]	Ca_06805	--	SYNONYMOUS_CODING

S.N.	SNP IDs	Chickpea cultivars	Chromosomes	Physical positions (bp)	SNPs	Gene accession IDs	Putative functions	Sequence components of genome
11982	CakSNP11982	*Kabuli*	Ca_Kabuli_Chr07	5577439	[A/G]	Ca_06805	--	NON_SYNONYMOUS_CODING
11983	CakSNP11983	*Kabuli*	Ca_Kabuli_Chr07	5581968	[G/C]		--	INTERGENIC
11984	CakSNP11984	*Kabuli*	Ca_Kabuli_Chr07	5602106	[T/C]		--	INTERGENIC
11985	CakSNP11985	*Kabuli*	Ca_Kabuli_Chr07	5602144	[G/T]		--	INTERGENIC
11986	CakSNP11986	*Kabuli*	Ca_Kabuli_Chr07	5605118	[G/A]		--	INTERGENIC
11987	CakSNP11987	*Kabuli*	Ca_Kabuli_Chr07	5605106	[G/A]		--	INTERGENIC
11988	CakSNP11988	*Kabuli*	Ca_Kabuli_Chr07	5658445	[G/T]		--	INTERGENIC
11989	CakSNP11989	*Kabuli*	Ca_Kabuli_Chr07	5671902	[G/T]		--	INTERGENIC
11990	CakSNP11990	*Kabuli*	Ca_Kabuli_Chr07	5672009	[G/C]		--	INTERGENIC
11991	CakSNP11991	*Kabuli*	Ca_Kabuli_Chr07	5672156	[T/C]		--	INTERGENIC

S.N.	SNP IDs	Chickpea cultivars	Chromosomes	Physical positions (bp)	SNPs	Gene accession IDs	Putative functions	Sequence components of genome
11992	CakSNP11992	*Kabuli*	Ca_Kabuli_Chr07	5683412	[T/A]	Ca_06789	RNA polymerase I specific transcription initiation factor RRN4	INTRON
11993	CakSNP11993	*Kabuli*	Ca_Kabuli_Chr07	5713977	[T/C]	Ca_06786	AWPM-19-like	SYNONYMOUS_CODING
11994	CakSNP11994	*Kabuli*	Ca_Kabuli_Chr07	5735293	[T/A]	Ca_06783	Protein kinase, catalytic domain	SYNONYMOUS_CODING
11995	CakSNP11995	*Kabuli*	Ca_Kabuli_Chr07	5735448	[A/T]	Ca_06783	Protein kinase, catalytic domain	NON_SYNONYMOUS_CODING
11996	CakSNP11996	*Kabuli*	Ca_Kabuli_Chr07	5737341	[C/T]	Ca_06783	Protein kinase, catalytic domain	SYNONYMOUS_CODING
11997	CakSNP11997	*Kabuli*	Ca_Kabuli_Chr07	5737305	[G/A]	Ca_06783	Protein kinase, catalytic domain	SYNONYMOUS_CODING
11998	CakSNP11998	*Kabuli*	Ca_Kabuli_Chr07	5756269	[C/T]		--	INTERGENIC
11999	CakSNP11999	*Kabuli*	Ca_Kabuli_Chr07	5756832	[A/C]		--	INTERGENIC
12000	CakSNP12000	*Kabuli*	Ca_Kabuli_Chr07	5774218	[T/A]	Ca_06781	Dienelactone hydrolase	INTRON
12001	CakSNP12001	*Kabuli*	Ca_Kabuli_Chr07	5774219	[T/A]	Ca_06781	Dienelactone hydrolase	INTRON

S.N.	SNP IDs	Chickpea cultivars	Chromosomes	Physical positions (bp)	SNPs	Gene accession IDs	Putative functions	Sequence components of genome
12002	CakSNP12002	*Kabuli*	Ca_Kabuli_Chr07	5774220	[A/T]	Ca_06781	Dienelactone hydrolase	INTRON
12003	CakSNP12003	*Kabuli*	Ca_Kabuli_Chr07	5774221	[A/T]	Ca_06781	Dienelactone hydrolase	INTRON
12004	CakSNP12004	*Kabuli*	Ca_Kabuli_Chr07	5774230	[G/A]	Ca_06781	Dienelactone hydrolase	INTRON
12005	CakSNP12005	*Kabuli*	Ca_Kabuli_Chr07	5774257	[C/T]	Ca_06781	Dienelactone hydrolase	INTRON
12006	CakSNP12006	*Kabuli*	Ca_Kabuli_Chr07	5774310	[A/G]	Ca_06781	Dienelactone hydrolase	INTRON
12007	CakSNP12007	*Kabuli*	Ca_Kabuli_Chr07	5811097	[G/A]		--	INTERGENIC
12008	CakSNP12008	*Kabuli*	Ca_Kabuli_Chr07	5811060	[T/C]		--	INTERGENIC
12009	CakSNP12009	*Kabuli*	Ca_Kabuli_Chr07	5811044	[T/A]		--	INTERGENIC
12010	CakSNP12010	*Kabuli*	Ca_Kabuli_Chr07	5836421	[A/T]		--	INTERGENIC
12011	CakSNP12011	*Kabuli*	Ca_Kabuli_Chr07	5846477	[A/G]	Ca_06774	SNARE associated Golgi protein	INTRON

S.N.	SNP IDs	Chickpea cultivars	Chromosomes	Physical positions (bp)	SNPs	Gene accession IDs	Putative functions	Sequence components of genome
12012	CakSNP12012	*Kabuli*	Ca_Kabuli_Chr07	5849936	[C/T]	Ca_06773	--	SYNONYMOUS_CODING
12013	CakSNP12013	*Kabuli*	Ca_Kabuli_Chr07	5849904	[C/A]	Ca_06773	--	SYNONYMOUS_CODING
12014	CakSNP12014	*Kabuli*	Ca_Kabuli_Chr07	5850058	[T/C]	Ca_06773	--	NON_SYNONYMOUS_CODING
12015	CakSNP12015	*Kabuli*	Ca_Kabuli_Chr07	5854598	[C/T]	Ca_06772	G10 protein	INTRON
12016	CakSNP12016	*Kabuli*	Ca_Kabuli_Chr07	5929009	[C/G]	Ca_06764	ATPase, P-type, H+ transporting proton pump	INTRON
12017	CakSNP12017	*Kabuli*	Ca_Kabuli_Chr07	5946113	[G/A]	Ca_06762	DNA-dependent ATPase MCM	NON_SYNONYMOUS_CODING
12018	CakSNP12018	*Kabuli*	Ca_Kabuli_Chr07	5950352	[T/A]	Ca_06762	DNA-dependent ATPase MCM	INTRON
12019	CakSNP12019	*Kabuli*	Ca_Kabuli_Chr07	5950492	[T/C]	Ca_06762	DNA-dependent ATPase MCM	SYNONYMOUS_CODING
12020	CakSNP12020	*Kabuli*	Ca_Kabuli_Chr07	5957696	[T/C]	Ca_06761	Ribonuclease III	INTRON
12021	CakSNP12021	*Kabuli*	Ca_Kabuli_Chr07	5963611	[C/G]	Ca_06761	Ribonuclease III	INTRON

S.N.	SNP IDs	Chickpea cultivars	Chromosomes	Physical positions (bp)	SNPs	Gene accession IDs	Putative functions	Sequence components of genome
12022	CakSNP12022	*Kabuli*	Ca_Kabuli_Chr07	5963609	[C/A]	Ca_06761	Ribonuclease III	INTRON
12023	CakSNP12023	*Kabuli*	Ca_Kabuli_Chr07	6012561	[A/C]	Ca_06758	Sugar/inositol transporter	SYNONYMOUS_CODING
12024	CakSNP12024	*Kabuli*	Ca_Kabuli_Chr07	6012611	[A/C]	Ca_06758	Sugar/inositol transporter	SYNONYMOUS_CODING
12025	CakSNP12025	*Kabuli*	Ca_Kabuli_Chr07	6029991	[G/A]	Ca_06756	Nonaspanin (TM9SF)	SYNONYMOUS_CODING
12026	CakSNP12026	*Kabuli*	Ca_Kabuli_Chr07	6029990	[C/G]	Ca_06756	Nonaspanin (TM9SF)	NON_SYNONYMOUS_CODING
12027	CakSNP12027	*Kabuli*	Ca_Kabuli_Chr07	6066322	[G/A]		--	INTERGENIC
12028	CakSNP12028	*Kabuli*	Ca_Kabuli_Chr07	6074408	[T/A]	Ca_06752	Phox/Bem1p	SYNONYMOUS_CODING
12029	CakSNP12029	*Kabuli*	Ca_Kabuli_Chr07	6075124	[T/G]	Ca_06752	Phox/Bem1p	NON_SYNONYMOUS_CODING
12030	CakSNP12030	*Kabuli*	Ca_Kabuli_Chr07	6075218	[C/G]	Ca_06752	Phox/Bem1p	START_LOST
12031	CakSNP12031	*Kabuli*	Ca_Kabuli_Chr07	6096238	[C/A]	Ca_06749	--	NON_SYNONYMOUS_CODING

S.N.	SNP IDs	Chickpea cultivars	Chromosomes	Physical positions (bp)	SNPs	Gene accession IDs	Putative functions	Sequence components of genome
12032	CakSNP12032	*Kabuli*	Ca_Kabuli_Chr07	6096237	[C/G]	Ca_06749	--	NON_SYNONYMOUS_CODING
12033	CakSNP12033	*Kabuli*	Ca_Kabuli_Chr07	6110019	[T/C]	Ca_06746	Translation elongation factor EFG/EF2, C-terminal	SYNONYMOUS_CODING
12034	CakSNP12034	*Kabuli*	Ca_Kabuli_Chr07	6132021	[G/A]	Ca_06744	Ubiquitin	INTRON
12035	CakSNP12035	*Kabuli*	Ca_Kabuli_Chr07	6232777	[T/C]		--	INTERGENIC
12036	CakSNP12036	*Kabuli*	Ca_Kabuli_Chr07	6282979	[C/A]	Ca_06730	Major facilitator superfamily MFS-1	INTRON
12037	CakSNP12037	*Kabuli*	Ca_Kabuli_Chr07	6282974	[T/A]	Ca_06730	Major facilitator superfamily MFS-1	INTRON
12038	CakSNP12038	*Kabuli*	Ca_Kabuli_Chr07	6282949	[T/A]	Ca_06730	Major facilitator superfamily MFS-1	INTRON
12039	CakSNP12039	*Kabuli*	Ca_Kabuli_Chr07	6299248	[A/G]		--	INTERGENIC
12040	CakSNP12040	*Kabuli*	Ca_Kabuli_Chr07	6299249	[C/G]		--	INTERGENIC
12041	CakSNP12041	*Kabuli*	Ca_Kabuli_Chr07	6330237	[A/T]	Ca_06724	Glycosyl transferase, family 28, C-terminal	INTRON

S.N.	SNP IDs	Chickpea cultivars	Chromosomes	Physical positions (bp)	SNPs	Gene accession IDs	Putative functions	Sequence components of genome
12042	CakSNP12042	*Kabuli*	Ca_Kabuli_Chr07	6340051	[A/G]		--	INTERGENIC
12043	CakSNP12043	*Kabuli*	Ca_Kabuli_Chr07	6340064	[C/G]		--	INTERGENIC
12044	CakSNP12044	*Kabuli*	Ca_Kabuli_Chr07	6340111	[C/G]		--	INTERGENIC
12045	CakSNP12045	*Kabuli*	Ca_Kabuli_Chr07	6340113	[G/A]		--	INTERGENIC
12046	CakSNP12046	*Kabuli*	Ca_Kabuli_Chr07	6340167	[T/A]		--	INTERGENIC
12047	CakSNP12047	*Kabuli*	Ca_Kabuli_Chr07	6340216	[T/C]		--	INTERGENIC
12048	CakSNP12048	*Kabuli*	Ca_Kabuli_Chr07	6372037	[A/G]		--	INTERGENIC
12049	CakSNP12049	*Kabuli*	Ca_Kabuli_Chr07	6393366	[G/A]	Ca_06712	Metallo-dependent phosphatase	INTRON
12050	CakSNP12050	*Kabuli*	Ca_Kabuli_Chr07	6430396	[T/A]	Ca_06708	Sucrose synthase	SYNONYMOUS_CODING
12051	CakSNP12051	*Kabuli*	Ca_Kabuli_Chr07	6433480	[C/G]		--	INTERGENIC

S.N.	SNP IDs	Chickpea cultivars	Chromosomes	Physical positions (bp)	SNPs	Gene accession IDs	Putative functions	Sequence components of genome
12052	CakSNP12052	*Kabuli*	Ca_Kabuli_Chr07	6467903	[C/T]	Ca_06705	Mak10 subunit, NatC N(alpha)-terminal acetyltransferase	SYNONYMOUS_CODING
12053	CakSNP12053	*Kabuli*	Ca_Kabuli_Chr07	6540624	[A/G]	Ca_06700	Rap1-interacting factor 1 N-terminal	INTRON
12054	CakSNP12054	*Kabuli*	Ca_Kabuli_Chr07	6540602	[G/A]	Ca_06700	Rap1-interacting factor 1 N-terminal	INTRON
12055	CakSNP12055	*Kabuli*	Ca_Kabuli_Chr07	6540601	[G/C]	Ca_06700	Rap1-interacting factor 1 N-terminal	INTRON
12056	CakSNP12056	*Kabuli*	Ca_Kabuli_Chr07	6566322	[T/C]		--	INTERGENIC
12057	CakSNP12057	*Kabuli*	Ca_Kabuli_Chr07	6654718	[C/T]	Ca_06694	Rab-GAP/TBC domain	INTRON
12058	CakSNP12058	*Kabuli*	Ca_Kabuli_Chr07	6681050	[C/T]	Ca_06692	Glycosyl transferase, family 17	SYNONYMOUS_CODING
12059	CakSNP12059	*Kabuli*	Ca_Kabuli_Chr07	6682456	[T/A]		--	INTERGENIC
12060	CakSNP12060	*Kabuli*	Ca_Kabuli_Chr07	6682454	[A/G]		--	INTERGENIC
12061	CakSNP12061	*Kabuli*	Ca_Kabuli_Chr07	6682453	[G/T]		--	INTERGENIC

S.N.	SNP IDs	Chickpea cultivars	Chromosomes	Physical positions (bp)	SNPs	Gene accession IDs	Putative functions	Sequence components of genome
12062	CakSNP12062	*Kabuli*	Ca_Kabuli_Chr07	6682448	[T/C]		--	INTERGENIC
12063	CakSNP12063	*Kabuli*	Ca_Kabuli_Chr07	6682443	[C/T]		--	INTERGENIC
12064	CakSNP12064	*Kabuli*	Ca_Kabuli_Chr07	6682439	[T/A]		--	INTERGENIC
12065	CakSNP12065	*Kabuli*	Ca_Kabuli_Chr07	6682455	[G/T]		--	INTERGENIC
12066	CakSNP12066	*Kabuli*	Ca_Kabuli_Chr07	6703759	[C/T]	Ca_06691	WD40 repeat	INTRON
12067	CakSNP12067	*Kabuli*	Ca_Kabuli_Chr07	6703849	[G/T]	Ca_06691	WD40 repeat	NON_SYNONYMOUS_CODING
12068	CakSNP12068	*Kabuli*	Ca_Kabuli_Chr07	6767204	[G/T]		--	INTERGENIC
12069	CakSNP12069	*Kabuli*	Ca_Kabuli_Chr07	6769694	[T/G]	Ca_06686	Blue (type 1) copper domain	NON_SYNONYMOUS_CODING
12070	CakSNP12070	*Kabuli*	Ca_Kabuli_Chr07	6792352	[T/C]	Ca_06682	Methyl-CpG DNA binding	NON_SYNONYMOUS_CODING
12071	CakSNP12071	*Kabuli*	Ca_Kabuli_Chr07	6792606	[T/G]	Ca_06682	Methyl-CpG DNA binding	NON_SYNONYMOUS_CODING

S.N.	SNP IDs	Chickpea cultivars	Chromosomes	Physical positions (bp)	SNPs	Gene accession IDs	Putative functions	Sequence components of genome
12072	CakSNP12072	*Kabuli*	Ca_Kabuli_Chr07	6807817	[A/C]		--	INTERGENIC
12073	CakSNP12073	*Kabuli*	Ca_Kabuli_Chr07	6814954	[G/T]	Ca_06681	YABBY protein	NON_SYNONYMOUS_CODING
12074	CakSNP12074	*Kabuli*	Ca_Kabuli_Chr07	6815151	[G/T]	Ca_06681	YABBY protein	SYNONYMOUS_CODING
12075	CakSNP12075	*Kabuli*	Ca_Kabuli_Chr07	6815113	[T/C]	Ca_06681	YABBY protein	INTRON
12076	CakSNP12076	*Kabuli*	Ca_Kabuli_Chr07	6859511	[C/G]		--	INTERGENIC
12077	CakSNP12077	*Kabuli*	Ca_Kabuli_Chr07	6904686	[G/C]		--	INTERGENIC
12078	CakSNP12078	*Kabuli*	Ca_Kabuli_Chr07	7026448	[C/T]	Ca_06659	H/ACA ribonucleoprotein complex, subunit Nhp2, eukaryote	INTRON
12079	CakSNP12079	*Kabuli*	Ca_Kabuli_Chr07	7026516	[T/C]	Ca_06659	H/ACA ribonucleoprotein complex, subunit Nhp2, eukaryote	INTRON
12080	CakSNP12080	*Kabuli*	Ca_Kabuli_Chr07	7026500	[T/A]	Ca_06659	H/ACA ribonucleoprotein complex, subunit Nhp2, eukaryote	INTRON
12081	CakSNP12081	*Kabuli*	Ca_Kabuli_Chr07	7072853	[G/A]		--	INTERGENIC

S.N.	SNP IDs	Chickpea cultivars	Chromosomes	Physical positions (bp)	SNPs	Gene accession IDs	Putative functions	Sequence components of genome
12082	CakSNP12082	*Kabuli*	Ca_Kabuli_Chr07	7126974	[T/C]	Ca_06645	Protein kinase, catalytic domain	INTRON
12083	CakSNP12083	*Kabuli*	Ca_Kabuli_Chr07	7126979	[C/G]	Ca_06645	Protein kinase, catalytic domain	INTRON
12084	CakSNP12084	*Kabuli*	Ca_Kabuli_Chr07	7127030	[G/A]	Ca_06645	Protein kinase, catalytic domain	INTRON
12085	CakSNP12085	*Kabuli*	Ca_Kabuli_Chr07	7146159	[C/G]		--	INTERGENIC
12086	CakSNP12086	*Kabuli*	Ca_Kabuli_Chr07	7147557	[A/G]	Ca_06642	Protein of unknown function DUF1644	SYNONYMOUS_CODING
12087	CakSNP12087	*Kabuli*	Ca_Kabuli_Chr07	7234422	[T/G]	Ca_06636	--	SYNONYMOUS_CODING
12088	CakSNP12088	*Kabuli*	Ca_Kabuli_Chr07	7234507	[T/C]	Ca_06636	--	SYNONYMOUS_CODING
12089	CakSNP12089	*Kabuli*	Ca_Kabuli_Chr07	7234484	[G/A]	Ca_06636	--	NON_SYNONYMOUS_CODING
12090	CakSNP12090	*Kabuli*	Ca_Kabuli_Chr07	7234430	[C/T]	Ca_06636	--	NON_SYNONYMOUS_CODING
12091	CakSNP12091	*Kabuli*	Ca_Kabuli_Chr07	7261971	[C/T]	Ca_06635	Cyclic nucleotide-binding domain	INTRON

S.N.	SNP IDs	Chickpea cultivars	Chromosomes	Physical positions (bp)	SNPs	Gene accession IDs	Putative functions	Sequence components of genome
12092	CakSNP12092	*Kabuli*	Ca_Kabuli_Chr07	7261941	[T/C]	Ca_06635	Cyclic nucleotide-binding domain	INTRON
12093	CakSNP12093	*Kabuli*	Ca_Kabuli_Chr07	7265386	[T/A]		--	INTERGENIC
12094	CakSNP12094	*Kabuli*	Ca_Kabuli_Chr07	7277353	[G/A]	Ca_06633	Cytochrome P450	SYNONYMOUS_CODING
12095	CakSNP12095	*Kabuli*	Ca_Kabuli_Chr07	7304614	[T/G]	Ca_06627	Myb, DNA-binding	SYNONYMOUS_CODING
12096	CakSNP12096	*Kabuli*	Ca_Kabuli_Chr07	7304617	[T/G]	Ca_06627	Myb, DNA-binding	SYNONYMOUS_CODING
12097	CakSNP12097	*Kabuli*	Ca_Kabuli_Chr07	7423419	[G/A]	Ca_06617	Heat shock protein DnaJ, N-terminal	NON_SYNONYMOUS_CODING
12098	CakSNP12098	*Kabuli*	Ca_Kabuli_Chr07	7433827	[A/T]	Ca_06617	Heat shock protein DnaJ, N-terminal	SYNONYMOUS_CODING
12099	CakSNP12099	*Kabuli*	Ca_Kabuli_Chr07	7449489	[T/C]		--	INTERGENIC
12100	CakSNP12100	*Kabuli*	Ca_Kabuli_Chr07	7451931	[G/C]	Ca_06614	Protein of unknown function Met10	INTRON
12101	CakSNP12101	*Kabuli*	Ca_Kabuli_Chr07	7491960	[G/A]	Ca_06610	DNA mismatch repair protein MutS, C-terminal domain	INTRON

S.N.	SNP IDs	Chickpea cultivars	Chromosomes	Physical positions (bp)	SNPs	Gene accession IDs	Putative functions	Sequence components of genome
12102	CakSNP12102	*Kabuli*	Ca_Kabuli_Chr07	7498598	[C/T]	Ca_06610	DNA mismatch repair protein MutS, C-terminal domain	SYNONYMOUS_CODING
12103	CakSNP12103	*Kabuli*	Ca_Kabuli_Chr07	7544142	[A/G]	Ca_06607	Transcription factor TFIIE, alpha subunit	INTRON
12104	CakSNP12104	*Kabuli*	Ca_Kabuli_Chr07	7573889	[C/G]	Ca_06604	Oleosin	NON_SYNONYMOUS_CODING
12105	CakSNP12105	*Kabuli*	Ca_Kabuli_Chr07	7576668	[A/C]		--	INTERGENIC
12106	CakSNP12106	*Kabuli*	Ca_Kabuli_Chr07	7580158	[T/C]		--	INTERGENIC
12107	CakSNP12107	*Kabuli*	Ca_Kabuli_Chr07	7580147	[C/A]		--	INTERGENIC
12108	CakSNP12108	*Kabuli*	Ca_Kabuli_Chr07	7580118	[G/A]		--	INTERGENIC
12109	CakSNP12109	*Kabuli*	Ca_Kabuli_Chr07	7612046	[A/T]	Ca_06599	PBS lyase HEAT-like repeat	NON_SYNONYMOUS_CODING
12110	CakSNP12110	*Kabuli*	Ca_Kabuli_Chr07	7624697	[G/A]	Ca_06598	Kinesin, motor domain	SYNONYMOUS_CODING
12111	CakSNP12111	*Kabuli*	Ca_Kabuli_Chr07	7625226	[G/T]	Ca_06598	Kinesin, motor domain	NON_SYNONYMOUS_CODING

S.N.	SNP IDs	Chickpea cultivars	Chromosomes	Physical positions (bp)	SNPs	Gene accession IDs	Putative functions	Sequence components of genome
12112	CakSNP12112	*Kabuli*	Ca_Kabuli_Chr07	7625277	[A/T]	Ca_06598	Kinesin, motor domain	NON_SYNONYMOUS_CODING
12113	CakSNP12113	*Kabuli*	Ca_Kabuli_Chr07	7625901	[A/C]		--	INTERGENIC
12114	CakSNP12114	*Kabuli*	Ca_Kabuli_Chr07	7625943	[C/G]		--	INTERGENIC
12115	CakSNP12115	*Kabuli*	Ca_Kabuli_Chr07	7663564	[A/G]	Ca_06594	ATPase, P-type, K/Mg/Cd/Cu/Zn/Na/Ca/Na/H-transporter	SYNONYMOUS_CODING
12116	CakSNP12116	*Kabuli*	Ca_Kabuli_Chr07	7706816	[A/G]	Ca_06587	Tetraspanin	NON_SYNONYMOUS_CODING
12117	CakSNP12117	*Kabuli*	Ca_Kabuli_Chr07	7756455	[C/T]	Ca_13243	SNF2-related	NON_SYNONYMOUS_CODING
12118	CakSNP12118	*Kabuli*	Ca_Kabuli_Chr07	7756482	[G/A]	Ca_13243	SNF2-related	NON_SYNONYMOUS_CODING
12119	CakSNP12119	*Kabuli*	Ca_Kabuli_Chr07	7756566	[G/A]	Ca_13243	SNF2-related	INTRON
12120	CakSNP12120	*Kabuli*	Ca_Kabuli_Chr07	7809179	[T/G]	Ca_13237	Peptidase S49	INTRON
12121	CakSNP12121	*Kabuli*	Ca_Kabuli_Chr07	7809192	[A/C]	Ca_13237	Peptidase S49	INTRON

S.N.	SNP IDs	Chickpea cultivars	Chromosomes	Physical positions (bp)	SNPs	Gene accession IDs	Putative functions	Sequence components of genome
12122	CakSNP12122	*Kabuli*	Ca_Kabuli_Chr07	7875189	[C/A]	Ca_13230	GDP-fucose protein O-fucosyltransferase	NON_SYNONYMOUS_CODING
12123	CakSNP12123	*Kabuli*	Ca_Kabuli_Chr07	7876554	[G/A]	Ca_13230	GDP-fucose protein O-fucosyltransferase	INTRON
12124	CakSNP12124	*Kabuli*	Ca_Kabuli_Chr07	7876561	[C/T]	Ca_13230	GDP-fucose protein O-fucosyltransferase	INTRON
12125	CakSNP12125	*Kabuli*	Ca_Kabuli_Chr07	7876570	[C/A]	Ca_13230	GDP-fucose protein O-fucosyltransferase	INTRON
12126	CakSNP12126	*Kabuli*	Ca_Kabuli_Chr07	7928398	[C/T]	Ca_13226	--	NON_SYNONYMOUS_CODING
12127	CakSNP12127	*Kabuli*	Ca_Kabuli_Chr07	7982650	[G/T]	Ca_13220	ABC transporter, transmembrane domain	SYNONYMOUS_CODING
12128	CakSNP12128	*Kabuli*	Ca_Kabuli_Chr07	7982871	[T/C]	Ca_13220	ABC transporter, transmembrane domain	INTRON
12129	CakSNP12129	*Kabuli*	Ca_Kabuli_Chr07	7982890	[T/A]	Ca_13220	ABC transporter, transmembrane domain	INTRON
12130	CakSNP12130	*Kabuli*	Ca_Kabuli_Chr07	7983519	[C/T]	Ca_13220	ABC transporter, transmembrane domain	SYNONYMOUS_CODING
12131	CakSNP12131	*Kabuli*	Ca_Kabuli_Chr07	8041465	[T/G]		--	INTERGENIC

S.N.	SNP IDs	Chickpea cultivars	Chromosomes	Physical positions (bp)	SNPs	Gene accession IDs	Putative functions	Sequence components of genome
12132	CakSNP12132	*Kabuli*	Ca_Kabuli_Chr07	8054489	[G/A]	Ca_13211	Survival motor neuron interacting protein 1	SYNONYMOUS_CODING
12133	CakSNP12133	*Kabuli*	Ca_Kabuli_Chr07	8061808	[A/G]	Ca_13209	Nucleic acid binding, OB-fold, tRNA/helicase-type	SYNONYMOUS_CODING
12134	CakSNP12134	*Kabuli*	Ca_Kabuli_Chr07	8111735	[T/C]	Ca_13205	RNA recognition motif domain	SYNONYMOUS_CODING
12135	CakSNP12135	*Kabuli*	Ca_Kabuli_Chr07	8111754	[A/C]	Ca_13205	RNA recognition motif domain	NON_SYNONYMOUS_CODING
12136	CakSNP12136	*Kabuli*	Ca_Kabuli_Chr07	8111759	[G/T]	Ca_13205	RNA recognition motif domain	NON_SYNONYMOUS_CODING
12137	CakSNP12137	*Kabuli*	Ca_Kabuli_Chr07	8169857	[A/G]	Ca_13197	Uncharacterised protein family, transmembrane-40	INTRON
12138	CakSNP12138	*Kabuli*	Ca_Kabuli_Chr07	8169866	[A/G]	Ca_13197	Uncharacterised protein family, transmembrane-40	INTRON
12139	CakSNP12139	*Kabuli*	Ca_Kabuli_Chr07	8169960	[A/T]	Ca_13197	Uncharacterised protein family, transmembrane-40	INTRON
12140	CakSNP12140	*Kabuli*	Ca_Kabuli_Chr07	8169947	[T/C]	Ca_13197	Uncharacterised protein family, transmembrane-40	INTRON
12141	CakSNP12141	*Kabuli*	Ca_Kabuli_Chr07	8187181	[G/A]	Ca_13195	Chaperonin ClpA/B	SYNONYMOUS_CODING

S.N.	SNP IDs	Chickpea cultivars	Chromosomes	Physical positions (bp)	SNPs	Gene accession IDs	Putative functions	Sequence components of genome
12142	CakSNP12142	*Kabuli*	Ca_Kabuli_Chr07	8188004	[C/G]	Ca_13195	Chaperonin ClpA/B	NON_SYNONYMOUS_CODING
12143	CakSNP12143	*Kabuli*	Ca_Kabuli_Chr07	8210963	[G/A]	Ca_13194	--	INTRON
12144	CakSNP12144	*Kabuli*	Ca_Kabuli_Chr07	8261062	[C/A]	Ca_13188	Alcohol dehydrogenase superfamily, zinc-containing	STOP_GAINED
12145	CakSNP12145	*Kabuli*	Ca_Kabuli_Chr07	8286115	[A/T]	Ca_13185	--	INTRON
12146	CakSNP12146	*Kabuli*	Ca_Kabuli_Chr07	8515092	[C/T]	Ca_13153	Lactate/malate dehydrogenase, N-terminal	SYNONYMOUS_CODING
12147	CakSNP12147	*Kabuli*	Ca_Kabuli_Chr07	8535189	[C/T]	Ca_13149	ZF-HD homeobox protein, Cys/His-rich dimerisation domain	SYNONYMOUS_CODING
12148	CakSNP12148	*Kabuli*	Ca_Kabuli_Chr07	8553114	[C/T]	Ca_15363	ZF-HD homeobox protein, Cys/His-rich dimerisation domain	SYNONYMOUS_CODING
12149	CakSNP12149	*Kabuli*	Ca_Kabuli_Chr07	8569632	[A/G]	Ca_15361	Pentatricopeptide repeat	NON_SYNONYMOUS_CODING
12150	CakSNP12150	*Kabuli*	Ca_Kabuli_Chr07	8583890	[T/C]	Ca_15359	Zinc finger, C2HC5-type	SYNONYMOUS_CODING
12151	CakSNP12151	*Kabuli*	Ca_Kabuli_Chr07	8609831	[A/T]		--	INTERGENIC

S.N.	SNP IDs	Chickpea cultivars	Chromosomes	Physical positions (bp)	SNPs	Gene accession IDs	Putative functions	Sequence components of genome
12152	CakSNP12152	*Kabuli*	Ca_Kabuli_Chr07	8626840	[A/C]	Ca_15356	--	NON_SYNONYMOUS_CODING
12153	CakSNP12153	*Kabuli*	Ca_Kabuli_Chr07	8626875	[A/T]	Ca_15356	--	NON_SYNONYMOUS_CODING
12154	CakSNP12154	*Kabuli*	Ca_Kabuli_Chr07	8626916	[T/C]	Ca_15356	--	SYNONYMOUS_CODING
12155	CakSNP12155	*Kabuli*	Ca_Kabuli_Chr07	8741098	[T/C]	Ca_15342	Aldehyde dehydrogenase domain	INTRON
12156	CakSNP12156	*Kabuli*	Ca_Kabuli_Chr07	8777670	[A/G]	Ca_15336	Band 7 protein	SYNONYMOUS_CODING
12157	CakSNP12157	*Kabuli*	Ca_Kabuli_Chr07	8777673	[A/G]	Ca_15336	Band 7 protein	SYNONYMOUS_CODING
12158	CakSNP12158	*Kabuli*	Ca_Kabuli_Chr07	8777690	[G/A]	Ca_15336	Band 7 protein	NON_SYNONYMOUS_CODING
12159	CakSNP12159	*Kabuli*	Ca_Kabuli_Chr07	8824586	[A/G]	Ca_15330	Oligopeptide transporter	NON_SYNONYMOUS_CODING
12160	CakSNP12160	*Kabuli*	Ca_Kabuli_Chr07	8824591	[C/T]	Ca_15330	Oligopeptide transporter	SYNONYMOUS_CODING
12161	CakSNP12161	*Kabuli*	Ca_Kabuli_Chr07	8830772	[A/C]	Ca_15329	Oligopeptide transporter	SYNONYMOUS_CODING

S.N.	SNP IDs	Chickpea cultivars	Chromosomes	Physical positions (bp)	SNPs	Gene accession IDs	Putative functions	Sequence components of genome
12162	CakSNP12162	*Kabuli*	Ca_Kabuli_Chr07	8830985	[A/G]	Ca_15329	Oligopeptide transporter	SYNONYMOUS_CODING
12163	CakSNP12163	*Kabuli*	Ca_Kabuli_Chr07	8831024	[C/T]	Ca_15329	Oligopeptide transporter	SYNONYMOUS_CODING
12164	CakSNP12164	*Kabuli*	Ca_Kabuli_Chr07	8831096	[G/A]		--	INTERGENIC
12165	CakSNP12165	*Kabuli*	Ca_Kabuli_Chr07	8835601	[G/A]	Ca_15328	Oligopeptide transporter	SYNONYMOUS_CODING
12166	CakSNP12166	*Kabuli*	Ca_Kabuli_Chr07	8835602	[A/G]	Ca_15328	Oligopeptide transporter	NON_SYNONYMOUS_CODING
12167	CakSNP12167	*Kabuli*	Ca_Kabuli_Chr07	8835610	[G/A]	Ca_15328	Oligopeptide transporter	SYNONYMOUS_CODING
12168	CakSNP12168	*Kabuli*	Ca_Kabuli_Chr07	8835659	[G/T]	Ca_15328	Oligopeptide transporter	STOP_GAINED
12169	CakSNP12169	*Kabuli*	Ca_Kabuli_Chr07	8835757	[T/C]	Ca_15328	Oligopeptide transporter	SYNONYMOUS_CODING
12170	CakSNP12170	*Kabuli*	Ca_Kabuli_Chr07	8835718	[T/G]	Ca_15328	Oligopeptide transporter	SYNONYMOUS_CODING
12171	CakSNP12171	*Kabuli*	Ca_Kabuli_Chr07	8835705	[T/A]	Ca_15328	Oligopeptide transporter	NON_SYNONYMOUS_CODING

S.N.	SNP IDs	Chickpea cultivars	Chromosomes	Physical positions (bp)	SNPs	Gene accession IDs	Putative functions	Sequence components of genome
12172	CakSNP12172	*Kabuli*	Ca_Kabuli_Chr07	8844902	[C/T]	Ca_15327	ATPase, P-type, H+ transporting proton pump	INTRON
12173	CakSNP12173	*Kabuli*	Ca_Kabuli_Chr07	8894362	[A/T]	Ca_15325	Domain of unknown function DUF629	NON_SYNONYMOUS_CODING
12174	CakSNP12174	*Kabuli*	Ca_Kabuli_Chr07	8895087	[T/C]	Ca_15325	Domain of unknown function DUF629	SYNONYMOUS_CODING
12175	CakSNP12175	*Kabuli*	Ca_Kabuli_Chr07	8913092	[C/G]	Ca_15322	Zinc finger, RING-type	INTRON
12176	CakSNP12176	*Kabuli*	Ca_Kabuli_Chr07	8937391	[A/G]	Ca_15321	WW domain binding protein 11	SYNONYMOUS_CODING
12177	CakSNP12177	*Kabuli*	Ca_Kabuli_Chr07	8970387	[A/G]	Ca_15317	Galectin, carbohydrate recognition domain	SYNONYMOUS_CODING
12178	CakSNP12178	*Kabuli*	Ca_Kabuli_Chr07	9128044	[A/G]		--	INTERGENIC
12179	CakSNP12179	*Kabuli*	Ca_Kabuli_Chr07	9128050	[C/A]		--	INTERGENIC
12180	CakSNP12180	*Kabuli*	Ca_Kabuli_Chr07	9147367	[A/T]	Ca_15301	Transcription factor E2F/dimerisation partner (TDP)	INTRON
12181	CakSNP12181	*Kabuli*	Ca_Kabuli_Chr07	9147366	[T/G]	Ca_15301	Transcription factor E2F/dimerisation partner (TDP)	INTRON

S.N.	SNP IDs	Chickpea cultivars	Chromosomes	Physical positions (bp)	SNPs	Gene accession IDs	Putative functions	Sequence components of genome
12182	CakSNP12182	*Kabuli*	Ca_Kabuli_Chr07	9147502	[C/T]	Ca_15301	Transcription factor E2F/dimerisation partner (TDP)	INTRON
12183	CakSNP12183	*Kabuli*	Ca_Kabuli_Chr07	9151709	[T/G]	Ca_15300	Aldehyde dehydrogenase domain	INTRON
12184	CakSNP12184	*Kabuli*	Ca_Kabuli_Chr07	9151755	[C/T]	Ca_15300	Aldehyde dehydrogenase domain	INTRON
12185	CakSNP12185	*Kabuli*	Ca_Kabuli_Chr07	9153104	[C/A]	Ca_15300	Aldehyde dehydrogenase domain	SYNONYMOUS_CODING
12186	CakSNP12186	*Kabuli*	Ca_Kabuli_Chr07	9153062	[G/A]	Ca_15300	Aldehyde dehydrogenase domain	SYNONYMOUS_CODING
12187	CakSNP12187	*Kabuli*	Ca_Kabuli_Chr07	9214457	[T/A]		--	INTERGENIC
12188	CakSNP12188	*Kabuli*	Ca_Kabuli_Chr07	9214422	[C/T]		--	INTERGENIC
12189	CakSNP12189	*Kabuli*	Ca_Kabuli_Chr07	9301825	[G/C]	Ca_17330	rRNA small subunit methyltransferase G	NON_SYNONYMOUS_CODING
12190	CakSNP12190	*Kabuli*	Ca_Kabuli_Chr07	9301881	[C/T]		--	INTERGENIC
12191	CakSNP12191	*Kabuli*	Ca_Kabuli_Chr07	9327709	[C/T]	Ca_17327	6-phosphogluconate dehydrogenase, NAD-binding	NON_SYNONYMOUS_CODING

S.N.	SNP IDs	Chickpea cultivars	Chromosomes	Physical positions (bp)	SNPs	Gene accession IDs	Putative functions	Sequence components of genome
12192	CakSNP12192	*Kabuli*	Ca_Kabuli_Chr07	9327687	[G/A]	Ca_17327	6-phosphogluconate dehydrogenase, NAD-binding	NON_SYNONYMOUS_CODING
12193	CakSNP12193	*Kabuli*	Ca_Kabuli_Chr07	9348281	[T/C]	Ca_17325	Glycoside hydrolase, family 47	SYNONYMOUS_CODING
12194	CakSNP12194	*Kabuli*	Ca_Kabuli_Chr07	9350506	[T/G]	Ca_17325	Glycoside hydrolase, family 47	INTRON
12195	CakSNP12195	*Kabuli*	Ca_Kabuli_Chr07	9350535	[A/C]	Ca_17325	Glycoside hydrolase, family 47	INTRON
12196	CakSNP12196	*Kabuli*	Ca_Kabuli_Chr07	9351783	[C/G]	Ca_17325	Glycoside hydrolase, family 47	INTRON
12197	CakSNP12197	*Kabuli*	Ca_Kabuli_Chr07	9351863	[G/A]	Ca_17325	Glycoside hydrolase, family 47	NON_SYNONYMOUS_CODING
12198	CakSNP12198	*Kabuli*	Ca_Kabuli_Chr07	9422842	[T/C]	Ca_17317	Hypoxia induced protein, domain	INTRON
12199	CakSNP12199	*Kabuli*	Ca_Kabuli_Chr07	9422859	[T/C]	Ca_17317	Hypoxia induced protein, domain	INTRON
12200	CakSNP12200	*Kabuli*	Ca_Kabuli_Chr07	9422876	[T/C]	Ca_17317	Hypoxia induced protein, domain	INTRON
12201	CakSNP12201	*Kabuli*	Ca_Kabuli_Chr07	9422946	[G/C]	Ca_17317	Hypoxia induced protein, domain	INTRON

S.N.	SNP IDs	Chickpea cultivars	Chromosomes	Physical positions (bp)	SNPs	Gene accession IDs	Putative functions	Sequence components of genome
12202	CakSNP12202	*Kabuli*	Ca_Kabuli_Chr07	9442836	[A/C]	Ca_17314	RNA recognition motif domain	SYNONYMOUS_CODING
12203	CakSNP12203	*Kabuli*	Ca_Kabuli_Chr07	9475275	[T/G]	Ca_17307	Protein kinase, catalytic domain	SYNONYMOUS_CODING
12204	CakSNP12204	*Kabuli*	Ca_Kabuli_Chr07	9475817	[C/A]	Ca_17307	Protein kinase, catalytic domain	INTRON
12205	CakSNP12205	*Kabuli*	Ca_Kabuli_Chr07	9475922	[A/G]	Ca_17307	Protein kinase, catalytic domain	INTRON
12206	CakSNP12206	*Kabuli*	Ca_Kabuli_Chr07	9475874	[C/T]	Ca_17307	Protein kinase, catalytic domain	INTRON
12207	CakSNP12207	*Kabuli*	Ca_Kabuli_Chr07	9523902	[C/T]	Ca_17302	Protein kinase, catalytic domain	INTRON
12208	CakSNP12208	*Kabuli*	Ca_Kabuli_Chr07	9528324	[A/G]	Ca_17301	Tetratricopeptide repeat-containing	SYNONYMOUS_CODING
12209	CakSNP12209	*Kabuli*	Ca_Kabuli_Chr07	9536012	[C/T]	Ca_17300	Peroxisome membrane anchor protein Pex14p, N-terminal	NON_SYNONYMOUS_CODING
12210	CakSNP12210	*Kabuli*	Ca_Kabuli_Chr07	9550284	[G/C]		--	INTERGENIC
12211	CakSNP12211	*Kabuli*	Ca_Kabuli_Chr07	9550230	[C/T]	Ca_17299	1-deoxy-D-xylulose 5-phosphate reductoisomerase, N-terminal	SYNONYMOUS_CODING

S.N.	SNP IDs	Chickpea cultivars	Chromosomes	Physical positions (bp)	SNPs	Gene accession IDs	Putative functions	Sequence components of genome
12212	CakSNP12212	*Kabuli*	Ca_Kabuli_Chr07	9555319	[C/G]	Ca_17298	Protein of unknown function DUF1645	SYNONYMOUS_CODING
12213	CakSNP12213	*Kabuli*	Ca_Kabuli_Chr07	9577441	[G/A]		--	INTERGENIC
12214	CakSNP12214	*Kabuli*	Ca_Kabuli_Chr07	9577424	[G/C]		--	INTERGENIC
12215	CakSNP12215	*Kabuli*	Ca_Kabuli_Chr07	9593677	[C/T]		--	INTERGENIC
12216	CakSNP12216	*Kabuli*	Ca_Kabuli_Chr07	9612558	[A/C]	Ca_17294	Drug/metabolite transporter	INTRON
12217	CakSNP12217	*Kabuli*	Ca_Kabuli_Chr07	9616335	[G/A]	Ca_17294	Drug/metabolite transporter	INTRON
12218	CakSNP12218	*Kabuli*	Ca_Kabuli_Chr07	9616347	[C/G]	Ca_17294	Drug/metabolite transporter	INTRON
12219	CakSNP12219	*Kabuli*	Ca_Kabuli_Chr07	9616357	[C/T]	Ca_17294	Drug/metabolite transporter	INTRON
12220	CakSNP12220	*Kabuli*	Ca_Kabuli_Chr07	9616363	[A/C]	Ca_17294	Drug/metabolite transporter	INTRON
12221	CakSNP12221	*Kabuli*	Ca_Kabuli_Chr07	9616367	[C/G]	Ca_17294	Drug/metabolite transporter	INTRON

S.N.	SNP IDs	Chickpea cultivars	Chromosomes	Physical positions (bp)	SNPs	Gene accession IDs	Putative functions	Sequence components of genome
12222	CakSNP12222	*Kabuli*	Ca_Kabuli_Chr07	9616378	[G/A]	Ca_17294	Drug/metabolite transporter	INTRON
12223	CakSNP12223	*Kabuli*	Ca_Kabuli_Chr07	9637456	[C/T]	Ca_17292	Mpp10 protein	INTRON
12224	CakSNP12224	*Kabuli*	Ca_Kabuli_Chr07	9639993	[C/A]		--	INTERGENIC
12225	CakSNP12225	*Kabuli*	Ca_Kabuli_Chr07	9661050	[A/C]	Ca_17290	Protein kinase, catalytic domain	INTRON
12226	CakSNP12226	*Kabuli*	Ca_Kabuli_Chr07	9671210	[T/C]	Ca_17289	Leucine-rich repeat	NON_SYNONYMOUS_CODING
12227	CakSNP12227	*Kabuli*	Ca_Kabuli_Chr07	9696620	[A/G]		--	INTERGENIC
12228	CakSNP12228	*Kabuli*	Ca_Kabuli_Chr07	9738801	[T/C]	Ca_17283	Protein kinase, catalytic domain	NON_SYNONYMOUS_CODING
12229	CakSNP12229	*Kabuli*	Ca_Kabuli_Chr07	9744436	[G/T]		--	INTERGENIC
12230	CakSNP12230	*Kabuli*	Ca_Kabuli_Chr07	9890779	[A/C]	Ca_12755	GDP-fucose protein O-fucosyltransferase	INTRON
12231	CakSNP12231	*Kabuli*	Ca_Kabuli_Chr07	9890777	[A/G]	Ca_12755	GDP-fucose protein O-fucosyltransferase	INTRON

S.N.	SNP IDs	Chickpea cultivars	Chromosomes	Physical positions (bp)	SNPs	Gene accession IDs	Putative functions	Sequence components of genome
12232	CakSNP12232	*Kabuli*	Ca_Kabuli_Chr07	9893688	[A/G]	Ca_12755	GDP-fucose protein O-fucosyltransferase	SYNONYMOUS_CODING
12233	CakSNP12233	*Kabuli*	Ca_Kabuli_Chr07	9897698	[T/G]	Ca_12756	Agenet	SYNONYMOUS_CODING
12234	CakSNP12234	*Kabuli*	Ca_Kabuli_Chr07	9898718	[A/T]	Ca_12756	Agenet	NON_SYNONYMOUS_CODING
12235	CakSNP12235	*Kabuli*	Ca_Kabuli_Chr07	9899348	[C/T]	Ca_12756	Agenet	NON_SYNONYMOUS_CODING
12236	CakSNP12236	*Kabuli*	Ca_Kabuli_Chr07	9914751	[G/A]		--	INTERGENIC
12237	CakSNP12237	*Kabuli*	Ca_Kabuli_Chr07	9914764	[G/A]		--	INTERGENIC
12238	CakSNP12238	*Kabuli*	Ca_Kabuli_Chr07	9914770	[T/C]		--	INTERGENIC
12239	CakSNP12239	*Kabuli*	Ca_Kabuli_Chr07	9914785	[T/C]		--	INTERGENIC
12240	CakSNP12240	*Kabuli*	Ca_Kabuli_Chr07	9914794	[G/A]		--	INTERGENIC
12241	CakSNP12241	*Kabuli*	Ca_Kabuli_Chr07	9914737	[C/G]		--	INTERGENIC

S.N.	SNP IDs	Chickpea cultivars	Chromosomes	Physical positions (bp)	SNPs	Gene accession IDs	Putative functions	Sequence components of genome
12242	CakSNP12242	*Kabuli*	Ca_Kabuli_Chr07	9914745	[T/C]		--	INTERGENIC
12243	CakSNP12243	*Kabuli*	Ca_Kabuli_Chr07	9914856	[G/A]		--	INTERGENIC
12244	CakSNP12244	*Kabuli*	Ca_Kabuli_Chr07	9914845	[A/C]		--	INTERGENIC
12245	CakSNP12245	*Kabuli*	Ca_Kabuli_Chr07	9914832	[C/T]		--	INTERGENIC
12246	CakSNP12246	*Kabuli*	Ca_Kabuli_Chr07	9914802	[T/G]		--	INTERGENIC
12247	CakSNP12247	*Kabuli*	Ca_Kabuli_Chr07	9932369	[A/G]	Ca_12759	DWNN domain	SYNONYMOUS_CODING
12248	CakSNP12248	*Kabuli*	Ca_Kabuli_Chr07	9936661	[C/T]	Ca_12759	DWNN domain	SYNONYMOUS_CODING
12249	CakSNP12249	*Kabuli*	Ca_Kabuli_Chr07	9936854	[A/G]	Ca_12759	DWNN domain	INTRON
12250	CakSNP12250	*Kabuli*	Ca_Kabuli_Chr07	9938937	[T/C]	Ca_12759	DWNN domain	INTRON
12251	CakSNP12251	*Kabuli*	Ca_Kabuli_Chr07	10050020	[A/C]		--	INTERGENIC

S.N.	SNP IDs	Chickpea cultivars	Chromosomes	Physical positions (bp)	SNPs	Gene accession IDs	Putative functions	Sequence components of genome
12252	CakSNP12252	*Kabuli*	Ca_Kabuli_Chr07	10049998	[T/A]		--	INTERGENIC
12253	CakSNP12253	*Kabuli*	Ca_Kabuli_Chr07	10049996	[A/G]		--	INTERGENIC
12254	CakSNP12254	*Kabuli*	Ca_Kabuli_Chr07	10089428	[T/C]	Ca_12771	Dual specificity phosphatase, catalytic domain	SYNONYMOUS_CODING
12255	CakSNP12255	*Kabuli*	Ca_Kabuli_Chr07	10089539	[T/A]	Ca_12771	Dual specificity phosphatase, catalytic domain	SYNONYMOUS_CODING
12256	CakSNP12256	*Kabuli*	Ca_Kabuli_Chr07	10106084	[C/G]		--	INTERGENIC
12257	CakSNP12257	*Kabuli*	Ca_Kabuli_Chr07	10106065	[A/G]		--	INTERGENIC
12258	CakSNP12258	*Kabuli*	Ca_Kabuli_Chr07	10106054	[G/C]		--	INTERGENIC
12259	CakSNP12259	*Kabuli*	Ca_Kabuli_Chr07	10137842	[A/G]	Ca_12775	Pathogenesis-related transcriptional factor/ERF, DNA-binding	SYNONYMOUS_CODING
12260	CakSNP12260	*Kabuli*	Ca_Kabuli_Chr07	10137890	[A/G]	Ca_12775	Pathogenesis-related transcriptional factor/ERF, DNA-binding	SYNONYMOUS_CODING
12261	CakSNP12261	*Kabuli*	Ca_Kabuli_Chr07	10181630	[G/T]		--	INTERGENIC

S.N.	SNP IDs	Chickpea cultivars	Chromosomes	Physical positions (bp)	SNPs	Gene accession IDs	Putative functions	Sequence components of genome
12262	CakSNP12262	*Kabuli*	Ca_Kabuli_Chr07	10208029	[T/C]	Ca_12781	Pentatricopeptide repeat	INTRON
12263	CakSNP12263	*Kabuli*	Ca_Kabuli_Chr07	10216813	[G/A]		--	INTERGENIC
12264	CakSNP12264	*Kabuli*	Ca_Kabuli_Chr07	10223046	[C/T]		--	INTERGENIC
12265	CakSNP12265	*Kabuli*	Ca_Kabuli_Chr07	10223068	[G/T]		--	INTERGENIC
12266	CakSNP12266	*Kabuli*	Ca_Kabuli_Chr07	10223069	[G/T]		--	INTERGENIC
12267	CakSNP12267	*Kabuli*	Ca_Kabuli_Chr07	10223071	[T/A]		--	INTERGENIC
12268	CakSNP12268	*Kabuli*	Ca_Kabuli_Chr07	10223074	[G/T]		--	INTERGENIC
12269	CakSNP12269	*Kabuli*	Ca_Kabuli_Chr07	10223075	[C/T]		--	INTERGENIC
12270	CakSNP12270	*Kabuli*	Ca_Kabuli_Chr07	10322726	[T/C]	Ca_12794	tRNA/rRNA methyltransferase, SpoU	SYNONYMOUS_CODING
12271	CakSNP12271	*Kabuli*	Ca_Kabuli_Chr07	10340199	[T/C]		--	INTERGENIC

S.N.	SNP IDs	Chickpea cultivars	Chromosomes	Physical positions (bp)	SNPs	Gene accession IDs	Putative functions	Sequence components of genome
12272	CakSNP12272	*Kabuli*	Ca_Kabuli_Chr07	10435983	[C/A]	Ca_12803	Casein kinase II, regulatory subunit	NON_SYNONYMOUS_CODING
12273	CakSNP12273	*Kabuli*	Ca_Kabuli_Chr07	10465140	[G/A]	Ca_12808	Glycoside hydrolase, family 14	SYNONYMOUS_CODING
12274	CakSNP12274	*Kabuli*	Ca_Kabuli_Chr07	10480401	[A/C]	Ca_12810	Protein of unknown function DUF702	INTRON
12275	CakSNP12275	*Kabuli*	Ca_Kabuli_Chr07	10480462	[A/G]	Ca_12810	Protein of unknown function DUF702	INTRON
12276	CakSNP12276	*Kabuli*	Ca_Kabuli_Chr07	10484200	[G/A]	Ca_12810	Protein of unknown function DUF702	INTRON
12277	CakSNP12277	*Kabuli*	Ca_Kabuli_Chr07	10484225	[C/A]	Ca_12810	Protein of unknown function DUF702	INTRON
12278	CakSNP12278	*Kabuli*	Ca_Kabuli_Chr07	10484196	[C/T]	Ca_12810	Protein of unknown function DUF702	INTRON
12279	CakSNP12279	*Kabuli*	Ca_Kabuli_Chr07	10485414	[C/T]	Ca_12810	Protein of unknown function DUF702	NON_SYNONYMOUS_CODING
12280	CakSNP12280	*Kabuli*	Ca_Kabuli_Chr07	10521423	[G/T]	Ca_12811	Zinc finger, RING-type	SYNONYMOUS_CODING
12281	CakSNP12281	*Kabuli*	Ca_Kabuli_Chr07	10521449	[C/A]	Ca_12811	Zinc finger, RING-type	NON_SYNONYMOUS_CODING

S.N.	SNP IDs	Chickpea cultivars	Chromosomes	Physical positions (bp)	SNPs	Gene accession IDs	Putative functions	Sequence components of genome
12282	CakSNP12282	*Kabuli*	Ca_Kabuli_Chr07	10614159	[G/A]	Ca_12815	Peptidase S16, Lon protease, C-terminal	NON_SYNONYMOUS_CODING
12283	CakSNP12283	*Kabuli*	Ca_Kabuli_Chr07	10658367	[A/T]		--	INTERGENIC
12284	CakSNP12284	*Kabuli*	Ca_Kabuli_Chr07	10658446	[G/T]		--	INTERGENIC
12285	CakSNP12285	*Kabuli*	Ca_Kabuli_Chr07	10688189	[T/G]	Ca_12825	Signal transduction response regulator, receiver domain	INTRON
12286	CakSNP12286	*Kabuli*	Ca_Kabuli_Chr07	10688401	[A/G]	Ca_12825	Signal transduction response regulator, receiver domain	INTRON
12287	CakSNP12287	*Kabuli*	Ca_Kabuli_Chr07	10688464	[G/A]	Ca_12825	Signal transduction response regulator, receiver domain	INTRON
12288	CakSNP12288	*Kabuli*	Ca_Kabuli_Chr07	10688434	[T/C]	Ca_12825	Signal transduction response regulator, receiver domain	INTRON
12289	CakSNP12289	*Kabuli*	Ca_Kabuli_Chr07	10688499	[T/C]	Ca_12825	Signal transduction response regulator, receiver domain	INTRON
12290	CakSNP12290	*Kabuli*	Ca_Kabuli_Chr07	10703569	[A/T]		--	INTERGENIC
12291	CakSNP12291	*Kabuli*	Ca_Kabuli_Chr07	10703612	[A/C]		--	INTERGENIC

S.N.	SNP IDs	Chickpea cultivars	Chromosomes	Physical positions (bp)	SNPs	Gene accession IDs	Putative functions	Sequence components of genome
12292	CakSNP12292	*Kabuli*	Ca_Kabuli_Chr07	10703704	[G/A]		--	INTERGENIC
12293	CakSNP12293	*Kabuli*	Ca_Kabuli_Chr07	10703679	[T/C]		--	INTERGENIC
12294	CakSNP12294	*Kabuli*	Ca_Kabuli_Chr07	10712202	[T/C]		--	INTERGENIC
12295	CakSNP12295	*Kabuli*	Ca_Kabuli_Chr07	10712231	[G/A]		--	INTERGENIC
12296	CakSNP12296	*Kabuli*	Ca_Kabuli_Chr07	10758973	[A/T]	Ca_12830	Oligopeptide transporter	INTRON
12297	CakSNP12297	*Kabuli*	Ca_Kabuli_Chr07	10759142	[T/G]	Ca_12830	Oligopeptide transporter	INTRON
12298	CakSNP12298	*Kabuli*	Ca_Kabuli_Chr07	10775881	[C/T]		--	INTERGENIC
12299	CakSNP12299	*Kabuli*	Ca_Kabuli_Chr07	10775875	[A/G]		--	INTERGENIC
12300	CakSNP12300	*Kabuli*	Ca_Kabuli_Chr07	10775851	[C/T]		--	INTERGENIC
12301	CakSNP12301	*Kabuli*	Ca_Kabuli_Chr07	10775860	[A/G]		--	INTERGENIC

S.N.	SNP IDs	Chickpea cultivars	Chromosomes	Physical positions (bp)	SNPs	Gene accession IDs	Putative functions	Sequence components of genome
12302	CakSNP12302	*Kabuli*	Ca_Kabuli_Chr07	10780680	[C/G]		--	INTERGENIC
12303	CakSNP12303	*Kabuli*	Ca_Kabuli_Chr07	10780682	[G/A]		--	INTERGENIC
12304	CakSNP12304	*Kabuli*	Ca_Kabuli_Chr07	10780686	[T/C]		--	INTERGENIC
12305	CakSNP12305	*Kabuli*	Ca_Kabuli_Chr07	10797204	[T/C]	Ca_12832	Protein kinase, catalytic domain	INTRON
12306	CakSNP12306	*Kabuli*	Ca_Kabuli_Chr07	10812617	[G/T]	Ca_12834	Smr protein/MutS2 C-terminal	SYNONYMOUS_CODING
12307	CakSNP12307	*Kabuli*	Ca_Kabuli_Chr07	10816523	[T/G]	Ca_12835	Protein phosphatase 2C-like	INTRON
12308	CakSNP12308	*Kabuli*	Ca_Kabuli_Chr07	10816674	[C/T]	Ca_12835	Protein phosphatase 2C-like	INTRON
12309	CakSNP12309	*Kabuli*	Ca_Kabuli_Chr07	10816662	[T/A]	Ca_12835	Protein phosphatase 2C-like	INTRON
12310	CakSNP12310	*Kabuli*	Ca_Kabuli_Chr07	10859907	[G/A]	Ca_12840	Zinc finger, RING-type	SYNONYMOUS_CODING
12311	CakSNP12311	*Kabuli*	Ca_Kabuli_Chr07	10859842	[G/T]	Ca_12840	Zinc finger, RING-type	SYNONYMOUS_CODING

S.N.	SNP IDs	Chickpea cultivars	Chromosomes	Physical positions (bp)	SNPs	Gene accession IDs	Putative functions	Sequence components of genome
12312	CakSNP12312	*Kabuli*	Ca_Kabuli_Chr07	10900910	[T/C]	Ca_12846	Zinc finger, LSD1-type	INTRON
12313	CakSNP12313	*Kabuli*	Ca_Kabuli_Chr07	11086047	[G/A]	Ca_09377	--	SYNONYMOUS_CODING
12314	CakSNP12314	*Kabuli*	Ca_Kabuli_Chr07	11086071	[A/T]	Ca_09377	--	SYNONYMOUS_CODING
12315	CakSNP12315	*Kabuli*	Ca_Kabuli_Chr07	11105847	[C/G]	Ca_09376	Hexokinase	NON_SYNONYMOUS_CODING
12316	CakSNP12316	*Kabuli*	Ca_Kabuli_Chr07	11105961	[A/C]	Ca_09376	Hexokinase	INTRON
12317	CakSNP12317	*Kabuli*	Ca_Kabuli_Chr07	11192667	[G/A]	Ca_09371	Homeobox	SYNONYMOUS_CODING
12318	CakSNP12318	*Kabuli*	Ca_Kabuli_Chr07	11192712	[C/T]	Ca_09371	Homeobox	SYNONYMOUS_CODING
12319	CakSNP12319	*Kabuli*	Ca_Kabuli_Chr07	11192703	[A/C]	Ca_09371	Homeobox	SYNONYMOUS_CODING
12320	CakSNP12320	*Kabuli*	Ca_Kabuli_Chr07	11261924	[G/A]		--	INTERGENIC
12321	CakSNP12321	*Kabuli*	Ca_Kabuli_Chr07	11339511	[C/T]		--	INTERGENIC

S.N.	SNP IDs	Chickpea cultivars	Chromosomes	Physical positions (bp)	SNPs	Gene accession IDs	Putative functions	Sequence components of genome
12322	CakSNP12322	*Kabuli*	Ca_Kabuli_Chr07	11393795	[A/G]		--	INTERGENIC
12323	CakSNP12323	*Kabuli*	Ca_Kabuli_Chr07	11393986	[A/G]		--	INTERGENIC
12324	CakSNP12324	*Kabuli*	Ca_Kabuli_Chr07	11473766	[A/G]	Ca_09347	Actin-binding FH2/DRF autoregulatory	INTRON
12325	CakSNP12325	*Kabuli*	Ca_Kabuli_Chr07	11521694	[G/A]		--	INTERGENIC
12326	CakSNP12326	*Kabuli*	Ca_Kabuli_Chr07	11527428	[G/A]		--	INTERGENIC
12327	CakSNP12327	*Kabuli*	Ca_Kabuli_Chr07	11545846	[A/G]	Ca_09342	Lateral organ boundaries, LOB	NON_SYNONYMOUS_CODING
12328	CakSNP12328	*Kabuli*	Ca_Kabuli_Chr07	11568963	[T/A]		--	INTERGENIC
12329	CakSNP12329	*Kabuli*	Ca_Kabuli_Chr07	11568945	[G/T]		--	INTERGENIC
12330	CakSNP12330	*Kabuli*	Ca_Kabuli_Chr07	11590987	[A/C]	Ca_09339	Plant peroxidase	INTRON
12331	CakSNP12331	*Kabuli*	Ca_Kabuli_Chr07	11630509	[A/G]		--	INTERGENIC

S.N.	SNP IDs	Chickpea cultivars	Chromosomes	Physical positions (bp)	SNPs	Gene accession IDs	Putative functions	Sequence components of genome
12332	CakSNP12332	*Kabuli*	Ca_Kabuli_Chr07	11630511	[C/T]		--	INTERGENIC
12333	CakSNP12333	*Kabuli*	Ca_Kabuli_Chr07	11638885	[C/A]	Ca_09335	BTB/POZ-like	NON_SYNONYMOUS_CODING
12334	CakSNP12334	*Kabuli*	Ca_Kabuli_Chr07	11639117	[C/G]	Ca_09335	BTB/POZ-like	INTRON
12335	CakSNP12335	*Kabuli*	Ca_Kabuli_Chr07	11685818	[G/T]	Ca_09331	Peptidase S8/S53, subtilisin/kexin/sedolisin	SYNONYMOUS_CODING
12336	CakSNP12336	*Kabuli*	Ca_Kabuli_Chr07	11685899	[A/G]	Ca_09331	Peptidase S8/S53, subtilisin/kexin/sedolisin	SYNONYMOUS_CODING
12337	CakSNP12337	*Kabuli*	Ca_Kabuli_Chr07	11686238	[C/T]	Ca_09331	Peptidase S8/S53, subtilisin/kexin/sedolisin	SYNONYMOUS_CODING
12338	CakSNP12338	*Kabuli*	Ca_Kabuli_Chr07	11725655	[G/A]	Ca_09324	Peptidase A1	SYNONYMOUS_CODING
12339	CakSNP12339	*Kabuli*	Ca_Kabuli_Chr07	11730926	[T/C]	Ca_09324	Peptidase A1	NON_SYNONYMOUS_CODING
12340	CakSNP12340	*Kabuli*	Ca_Kabuli_Chr07	11730946	[A/C]		--	INTERGENIC
12341	CakSNP12341	*Kabuli*	Ca_Kabuli_Chr07	11735177	[T/C]	Ca_09323	Frigida-like	SYNONYMOUS_CODING

S.N.	SNP IDs	Chickpea cultivars	Chromosomes	Physical positions (bp)	SNPs	Gene accession IDs	Putative functions	Sequence components of genome
12342	CakSNP12342	*Kabuli*	Ca_Kabuli_Chr07	11735381	[T/C]	Ca_09323	Frigida-like	SYNONYMOUS_CODING
12343	CakSNP12343	*Kabuli*	Ca_Kabuli_Chr07	11735368	[G/C]	Ca_09323	Frigida-like	NON_SYNONYMOUS_CODING
12344	CakSNP12344	*Kabuli*	Ca_Kabuli_Chr07	11736006	[C/A]		--	INTERGENIC
12345	CakSNP12345	*Kabuli*	Ca_Kabuli_Chr07	11792042	[G/A]	Ca_09316	Protein kinase, catalytic domain	NON_SYNONYMOUS_CODING
12346	CakSNP12346	*Kabuli*	Ca_Kabuli_Chr07	11792126	[T/A]		--	INTERGENIC
12347	CakSNP12347	*Kabuli*	Ca_Kabuli_Chr07	11792136	[G/A]		--	INTERGENIC
12348	CakSNP12348	*Kabuli*	Ca_Kabuli_Chr07	11803091	[T/G]		--	INTERGENIC
12349	CakSNP12349	*Kabuli*	Ca_Kabuli_Chr07	11812410	[G/A]		--	INTERGENIC
12350	CakSNP12350	*Kabuli*	Ca_Kabuli_Chr07	11812441	[A/C]		--	INTERGENIC
12351	CakSNP12351	*Kabuli*	Ca_Kabuli_Chr07	11858425	[C/T]	Ca_09311	MRG	INTRON

S.N.	SNP IDs	Chickpea cultivars	Chromosomes	Physical positions (bp)	SNPs	Gene accession IDs	Putative functions	Sequence components of genome
12352	CakSNP12352	*Kabuli*	Ca_Kabuli_Chr07	11886954	[A/G]		--	INTERGENIC
12353	CakSNP12353	*Kabuli*	Ca_Kabuli_Chr07	11902897	[A/G]		--	INTERGENIC
12354	CakSNP12354	*Kabuli*	Ca_Kabuli_Chr07	11902985	[G/T]		--	INTERGENIC
12355	CakSNP12355	*Kabuli*	Ca_Kabuli_Chr07	12010432	[G/A]		--	INTERGENIC
12356	CakSNP12356	*Kabuli*	Ca_Kabuli_Chr07	12010471	[C/T]		--	INTERGENIC
12357	CakSNP12357	*Kabuli*	Ca_Kabuli_Chr07	12010504	[G/A]		--	INTERGENIC
12358	CakSNP12358	*Kabuli*	Ca_Kabuli_Chr07	12010499	[C/T]		--	INTERGENIC
12359	CakSNP12359	*Kabuli*	Ca_Kabuli_Chr07	12010496	[G/A]		--	INTERGENIC
12360	CakSNP12360	*Kabuli*	Ca_Kabuli_Chr07	12010494	[G/A]		--	INTERGENIC
12361	CakSNP12361	*Kabuli*	Ca_Kabuli_Chr07	12010491	[G/T]		--	INTERGENIC

S.N.	SNP IDs	Chickpea cultivars	Chromosomes	Physical positions (bp)	SNPs	Gene accession IDs	Putative functions	Sequence components of genome
12362	CakSNP12362	*Kabuli*	Ca_Kabuli_Chr07	12010483	[C/T]		--	INTERGENIC
12363	CakSNP12363	*Kabuli*	Ca_Kabuli_Chr07	12010457	[C/T]		--	INTERGENIC
12364	CakSNP12364	*Kabuli*	Ca_Kabuli_Chr07	12010544	[G/A]		--	INTERGENIC
12365	CakSNP12365	*Kabuli*	Ca_Kabuli_Chr07	12010489	[C/T]		--	INTERGENIC
12366	CakSNP12366	*Kabuli*	Ca_Kabuli_Chr07	12030080	[A/C]	Ca_09301	tRNA-dihydrouridine synthase	SYNONYMOUS_CODING
12367	CakSNP12367	*Kabuli*	Ca_Kabuli_Chr07	12071764	[T/C]	Ca_09297	Kinesin, motor domain	INTRON
12368	CakSNP12368	*Kabuli*	Ca_Kabuli_Chr07	12071773	[A/T]	Ca_09297	Kinesin, motor domain	INTRON
12369	CakSNP12369	*Kabuli*	Ca_Kabuli_Chr07	12203726	[T/G]		--	INTERGENIC
12370	CakSNP12370	*Kabuli*	Ca_Kabuli_Chr07	12203722	[T/C]		--	INTERGENIC
12371	CakSNP12371	*Kabuli*	Ca_Kabuli_Chr07	12203706	[T/C]		--	INTERGENIC

S.N.	SNP IDs	Chickpea cultivars	Chromosomes	Physical positions (bp)	SNPs	Gene accession IDs	Putative functions	Sequence components of genome
12372	CakSNP12372	*Kabuli*	Ca_Kabuli_Chr07	12203690	[C/T]		--	INTERGENIC
12373	CakSNP12373	*Kabuli*	Ca_Kabuli_Chr07	12244234	[C/T]	Ca_09285	Nicotinate phosphoribosyltransferase-related	INTRON
12374	CakSNP12374	*Kabuli*	Ca_Kabuli_Chr07	12244259	[G/A]	Ca_09285	Nicotinate phosphoribosyltransferase-related	INTRON
12375	CakSNP12375	*Kabuli*	Ca_Kabuli_Chr07	12284289	[T/A]		--	INTERGENIC
12376	CakSNP12376	*Kabuli*	Ca_Kabuli_Chr07	12284304	[A/G]		--	INTERGENIC
12377	CakSNP12377	*Kabuli*	Ca_Kabuli_Chr07	12385785	[C/T]	Ca_09275	--	SYNONYMOUS_CODING
12378	CakSNP12378	*Kabuli*	Ca_Kabuli_Chr07	12390774	[G/A]	Ca_09275	--	SYNONYMOUS_CODING
12379	CakSNP12379	*Kabuli*	Ca_Kabuli_Chr07	12481556	[G/C]	Ca_09268	Pentatricopeptide repeat	NON_SYNONYMOUS_CODING
12380	CakSNP12380	*Kabuli*	Ca_Kabuli_Chr07	12481627	[A/C]	Ca_09268	Pentatricopeptide repeat	SYNONYMOUS_CODING
12381	CakSNP12381	*Kabuli*	Ca_Kabuli_Chr07	12481715	[A/G]	Ca_09268	Pentatricopeptide repeat	NON_SYNONYMOUS_CODING

S.N.	SNP IDs	Chickpea cultivars	Chromosomes	Physical positions (bp)	SNPs	Gene accession IDs	Putative functions	Sequence components of genome
12382	CakSNP12382	*Kabuli*	Ca_Kabuli_Chr07	12481835	[C/T]		--	INTERGENIC
12383	CakSNP12383	*Kabuli*	Ca_Kabuli_Chr07	12482851	[G/A]		--	INTERGENIC
12384	CakSNP12384	*Kabuli*	Ca_Kabuli_Chr07	12482822	[A/G]		--	INTERGENIC
12385	CakSNP12385	*Kabuli*	Ca_Kabuli_Chr07	12482953	[A/C]		--	INTERGENIC
12386	CakSNP12386	*Kabuli*	Ca_Kabuli_Chr07	12483290	[A/C]		--	INTERGENIC
12387	CakSNP12387	*Kabuli*	Ca_Kabuli_Chr07	12492075	[G/A]	Ca_09267	--	NON_SYNONYMOUS_CODING
12388	CakSNP12388	*Kabuli*	Ca_Kabuli_Chr07	12492052	[C/T]	Ca_09267	--	NON_SYNONYMOUS_CODING
12389	CakSNP12389	*Kabuli*	Ca_Kabuli_Chr07	12492046	[A/T]	Ca_09267	--	NON_SYNONYMOUS_CODING
12390	CakSNP12390	*Kabuli*	Ca_Kabuli_Chr07	12616871	[T/C]	Ca_09259	--	INTRON
12391	CakSNP12391	*Kabuli*	Ca_Kabuli_Chr07	12616870	[G/A]	Ca_09259	--	INTRON

S.N.	SNP IDs	Chickpea cultivars	Chromosomes	Physical positions (bp)	SNPs	Gene accession IDs	Putative functions	Sequence components of genome
12392	CakSNP12392	*Kabuli*	Ca_Kabuli_Chr07	12616957	[C/T]	Ca_09259	--	INTRON
12393	CakSNP12393	*Kabuli*	Ca_Kabuli_Chr07	12616937	[C/T]	Ca_09259	--	INTRON
12394	CakSNP12394	*Kabuli*	Ca_Kabuli_Chr07	12623129	[T/C]	Ca_09259	--	INTRON
12395	CakSNP12395	*Kabuli*	Ca_Kabuli_Chr07	12623128	[G/A]	Ca_09259	--	INTRON
12396	CakSNP12396	*Kabuli*	Ca_Kabuli_Chr07	12623215	[C/T]	Ca_09259	--	INTRON
12397	CakSNP12397	*Kabuli*	Ca_Kabuli_Chr07	12623195	[C/T]	Ca_09259	--	INTRON
12398	CakSNP12398	*Kabuli*	Ca_Kabuli_Chr07	12628148	[A/G]	Ca_09259	--	INTRON
12399	CakSNP12399	*Kabuli*	Ca_Kabuli_Chr07	12628211	[A/G]	Ca_09259	--	INTRON
12400	CakSNP12400	*Kabuli*	Ca_Kabuli_Chr07	12628265	[T/A]	Ca_09259	--	INTRON
12401	CakSNP12401	*Kabuli*	Ca_Kabuli_Chr07	12629986	[A/G]	Ca_09259	--	SYNONYMOUS_CODING

S.N.	SNP IDs	Chickpea cultivars	Chromosomes	Physical positions (bp)	SNPs	Gene accession IDs	Putative functions	Sequence components of genome
12402	CakSNP12402	*Kabuli*	Ca_Kabuli_Chr07	12633590	[A/C]		--	INTERGENIC
12403	CakSNP12403	*Kabuli*	Ca_Kabuli_Chr07	12633642	[T/G]		--	INTERGENIC
12404	CakSNP12404	*Kabuli*	Ca_Kabuli_Chr07	12633643	[A/T]		--	INTERGENIC
12405	CakSNP12405	*Kabuli*	Ca_Kabuli_Chr07	12681440	[T/C]		--	INTERGENIC
12406	CakSNP12406	*Kabuli*	Ca_Kabuli_Chr07	12718820	[C/G]		--	INTERGENIC
12407	CakSNP12407	*Kabuli*	Ca_Kabuli_Chr07	12718853	[G/A]		--	INTERGENIC
12408	CakSNP12408	*Kabuli*	Ca_Kabuli_Chr07	12730600	[C/A]	Ca_09247	Lateral organ boundaries, LOB	STOP_GAINED
12409	CakSNP12409	*Kabuli*	Ca_Kabuli_Chr07	12737516	[T/C]		--	INTERGENIC
12410	CakSNP12410	*Kabuli*	Ca_Kabuli_Chr07	12893655	[C/T]	Ca_09231	--	SYNONYMOUS_CODING
12411	CakSNP12411	*Kabuli*	Ca_Kabuli_Chr07	12895182	[A/G]	Ca_09231	--	SYNONYMOUS_CODING

S.N.	SNP IDs	Chickpea cultivars	Chromosomes	Physical positions (bp)	SNPs	Gene accession IDs	Putative functions	Sequence components of genome
12412	CakSNP12412	*Kabuli*	Ca_Kabuli_Chr07	12898640	[A/C]	Ca_09230	Peptidase S9, prolyl oligopeptidase, catalytic domain	INTRON
12413	CakSNP12413	*Kabuli*	Ca_Kabuli_Chr07	12898709	[T/A]	Ca_09230	Peptidase S9, prolyl oligopeptidase, catalytic domain	INTRON
12414	CakSNP12414	*Kabuli*	Ca_Kabuli_Chr07	12898707	[G/A]	Ca_09230	Peptidase S9, prolyl oligopeptidase, catalytic domain	INTRON
12415	CakSNP12415	*Kabuli*	Ca_Kabuli_Chr07	12899398	[T/C]	Ca_09230	Peptidase S9, prolyl oligopeptidase, catalytic domain	INTRON
12416	CakSNP12416	*Kabuli*	Ca_Kabuli_Chr07	12913811	[T/A]	Ca_09229	--	SYNONYMOUS_CODING
12417	CakSNP12417	*Kabuli*	Ca_Kabuli_Chr07	13020788	[C/T]	Ca_09225	Vacuolar protein sorting-associated protein	INTRON
12418	CakSNP12418	*Kabuli*	Ca_Kabuli_Chr07	13020772	[A/G]	Ca_09225	Vacuolar protein sorting-associated protein	INTRON
12419	CakSNP12419	*Kabuli*	Ca_Kabuli_Chr07	13021047	[A/G]	Ca_09225	Vacuolar protein sorting-associated protein	INTRON
12420	CakSNP12420	*Kabuli*	Ca_Kabuli_Chr07	13021030	[A/G]	Ca_09225	Vacuolar protein sorting-associated protein	INTRON
12421	CakSNP12421	*Kabuli*	Ca_Kabuli_Chr07	13059710	[C/A]	Ca_09222	BTB/POZ-like	NON_SYNONYMOUS_CODING

S.N.	SNP IDs	Chickpea cultivars	Chromosomes	Physical positions (bp)	SNPs	Gene accession IDs	Putative functions	Sequence components of genome
12422	CakSNP12422	*Kabuli*	Ca_Kabuli_Chr07	13059712	[C/A]	Ca_09222	BTB/POZ-like	NON_SYNONYMOUS_CODING
12423	CakSNP12423	*Kabuli*	Ca_Kabuli_Chr07	13059717	[T/A]	Ca_09222	BTB/POZ-like	NON_SYNONYMOUS_CODING
12424	CakSNP12424	*Kabuli*	Ca_Kabuli_Chr07	13059852	[G/A]	Ca_09222	BTB/POZ-like	SYNONYMOUS_CODING
12425	CakSNP12425	*Kabuli*	Ca_Kabuli_Chr07	13172542	[A/G]		--	INTERGENIC
12426	CakSNP12426	*Kabuli*	Ca_Kabuli_Chr07	13220439	[G/A]	Ca_16023	Domain of unknown function DUF547	INTRON
12427	CakSNP12427	*Kabuli*	Ca_Kabuli_Chr07	13220418	[A/G]	Ca_16023	Domain of unknown function DUF547	INTRON
12428	CakSNP12428	*Kabuli*	Ca_Kabuli_Chr07	13262430	[C/T]	Ca_16024	SET domain	SYNONYMOUS_CODING
12429	CakSNP12429	*Kabuli*	Ca_Kabuli_Chr07	13281255	[T/C]		--	INTERGENIC
12430	CakSNP12430	*Kabuli*	Ca_Kabuli_Chr07	13281290	[A/G]		--	INTERGENIC
12431	CakSNP12431	*Kabuli*	Ca_Kabuli_Chr07	13281297	[C/G]		--	INTERGENIC

S.N.	SNP IDs	Chickpea cultivars	Chromosomes	Physical positions (bp)	SNPs	Gene accession IDs	Putative functions	Sequence components of genome
12432	CakSNP12432	*Kabuli*	Ca_Kabuli_Chr07	13281307	[C/T]		--	INTERGENIC
12433	CakSNP12433	*Kabuli*	Ca_Kabuli_Chr07	13281313	[T/C]		--	INTERGENIC
12434	CakSNP12434	*Kabuli*	Ca_Kabuli_Chr07	13281314	[G/A]		--	INTERGENIC
12435	CakSNP12435	*Kabuli*	Ca_Kabuli_Chr07	13281374	[G/T]		--	INTERGENIC
12436	CakSNP12436	*Kabuli*	Ca_Kabuli_Chr07	13281351	[T/A]		--	INTERGENIC
12437	CakSNP12437	*Kabuli*	Ca_Kabuli_Chr07	13288828	[G/A]	Ca_16025	--	SYNONYMOUS_CODING
12438	CakSNP12438	*Kabuli*	Ca_Kabuli_Chr07	13288813	[G/A]	Ca_16025	--	SYNONYMOUS_CODING
12439	CakSNP12439	*Kabuli*	Ca_Kabuli_Chr07	13288753	[A/C]	Ca_16025	--	SYNONYMOUS_CODING
12440	CakSNP12440	*Kabuli*	Ca_Kabuli_Chr07	13319871	[A/G]	Ca_16026	Homeobox	SYNONYMOUS_CODING
12441	CakSNP12441	*Kabuli*	Ca_Kabuli_Chr07	13514074	[C/T]	Ca_16037	Protein of unknown function DUF677	SYNONYMOUS_CODING

S.N.	SNP IDs	Chickpea cultivars	Chromosomes	Physical positions (bp)	SNPs	Gene accession IDs	Putative functions	Sequence components of genome
12442	CakSNP12442	*Kabuli*	Ca_Kabuli_Chr07	13605200	[A/C]	Ca_16044	Zinc finger, C2H2-type	SYNONYMOUS_CODING
12443	CakSNP12443	*Kabuli*	Ca_Kabuli_Chr07	13605191	[A/C]	Ca_16044	Zinc finger, C2H2-type	SYNONYMOUS_CODING
12444	CakSNP12444	*Kabuli*	Ca_Kabuli_Chr07	13605188	[A/C]	Ca_16044	Zinc finger, C2H2-type	SYNONYMOUS_CODING
12445	CakSNP12445	*Kabuli*	Ca_Kabuli_Chr07	13605578	[C/G]	Ca_16044	Zinc finger, C2H2-type	SYNONYMOUS_CODING
12446	CakSNP12446	*Kabuli*	Ca_Kabuli_Chr07	13667102	[C/T]		--	INTERGENIC
12447	CakSNP12447	*Kabuli*	Ca_Kabuli_Chr07	13667103	[T/G]		--	INTERGENIC
12448	CakSNP12448	*Kabuli*	Ca_Kabuli_Chr07	13730350	[A/C]	Ca_16051	Fumarate reductase/succinate dehydrogenase, FAD-binding site	INTRON
12449	CakSNP12449	*Kabuli*	Ca_Kabuli_Chr07	13757159	[A/C]	Ca_16053	Citron-like	SYNONYMOUS_CODING
12450	CakSNP12450	*Kabuli*	Ca_Kabuli_Chr07	13776110	[A/C]	Ca_16055	FAD dependent oxidoreductase	NON_SYNONYMOUS_CODING
12451	CakSNP12451	*Kabuli*	Ca_Kabuli_Chr07	13781815	[T/C]		--	INTERGENIC

S.N.	SNP IDs	Chickpea cultivars	Chromosomes	Physical positions (bp)	SNPs	Gene accession IDs	Putative functions	Sequence components of genome
12452	CakSNP12452	*Kabuli*	Ca_Kabuli_Chr07	13819387	[C/T]	Ca_16058	Alpha/beta hydrolase fold-1	SYNONYMOUS_CODING
12453	CakSNP12453	*Kabuli*	Ca_Kabuli_Chr07	13881348	[G/A]		--	INTERGENIC
12454	CakSNP12454	*Kabuli*	Ca_Kabuli_Chr07	13893199	[T/C]		--	INTERGENIC
12455	CakSNP12455	*Kabuli*	Ca_Kabuli_Chr07	13893344	[G/C]		--	INTERGENIC
12456	CakSNP12456	*Kabuli*	Ca_Kabuli_Chr07	13893382	[G/T]		--	INTERGENIC
12457	CakSNP12457	*Kabuli*	Ca_Kabuli_Chr07	13893383	[C/T]		--	INTERGENIC
12458	CakSNP12458	*Kabuli*	Ca_Kabuli_Chr07	13903980	[T/G]	Ca_16064	Tubby, C-terminal	NON_SYNONYMOUS_CODING
12459	CakSNP12459	*Kabuli*	Ca_Kabuli_Chr07	13904163	[T/C]	Ca_16064	Tubby, C-terminal	SYNONYMOUS_CODING
12460	CakSNP12460	*Kabuli*	Ca_Kabuli_Chr07	13913735	[A/G]	Ca_16065	Alpha/beta hydrolase fold-1	INTRON
12461	CakSNP12461	*Kabuli*	Ca_Kabuli_Chr07	13921214	[C/T]	Ca_16066	Inorganic pyrophosphatase	INTRON

S.N.	SNP IDs	Chickpea cultivars	Chromosomes	Physical positions (bp)	SNPs	Gene accession IDs	Putative functions	Sequence components of genome
12462	CakSNP12462	*Kabuli*	Ca_Kabuli_Chr07	13921289	[T/C]	Ca_16066	Inorganic pyrophosphatase	INTRON
12463	CakSNP12463	*Kabuli*	Ca_Kabuli_Chr07	13949259	[A/G]	Ca_16067	Heat shock protein DnaJ, N-terminal	NON_SYNONYMOUS_CODING
12464	CakSNP12464	*Kabuli*	Ca_Kabuli_Chr07	13949314	[G/A]	Ca_16067	Heat shock protein DnaJ, N-terminal	SYNONYMOUS_CODING
12465	CakSNP12465	*Kabuli*	Ca_Kabuli_Chr07	13951874	[T/C]	Ca_16067	Heat shock protein DnaJ, N-terminal	SYNONYMOUS_CODING
12466	CakSNP12466	*Kabuli*	Ca_Kabuli_Chr07	13951856	[T/C]	Ca_16067	Heat shock protein DnaJ, N-terminal	SYNONYMOUS_CODING
12467	CakSNP12467	*Kabuli*	Ca_Kabuli_Chr07	14030136	[C/T]	Ca_16074	--	NON_SYNONYMOUS_CODING
12468	CakSNP12468	*Kabuli*	Ca_Kabuli_Chr07	14051146	[G/T]	Ca_16074	--	INTRON
12469	CakSNP12469	*Kabuli*	Ca_Kabuli_Chr07	14179629	[T/G]	Ca_23033	Ubiquitin-conjugating enzyme E2C-binding protein	SYNONYMOUS_CODING
12470	CakSNP12470	*Kabuli*	Ca_Kabuli_Chr07	14179733	[C/A]	Ca_23033	Ubiquitin-conjugating enzyme E2C-binding protein	NON_SYNONYMOUS_CODING
12471	CakSNP12471	*Kabuli*	Ca_Kabuli_Chr07	14212570	[C/A]	Ca_23038	Mitochondrial substrate/solute carrier	INTRON

S.N.	SNP IDs	Chickpea cultivars	Chromosomes	Physical positions (bp)	SNPs	Gene accession IDs	Putative functions	Sequence components of genome
12472	CakSNP12472	*Kabuli*	Ca_Kabuli_Chr07	14212597	[C/G]	Ca_23038	Mitochondrial substrate/solute carrier	INTRON
12473	CakSNP12473	*Kabuli*	Ca_Kabuli_Chr07	14233343	[T/G]	Ca_23041	Protein of unknown function DUF248, methyltransferase putative	NON_SYNONYMOUS_CODING
12474	CakSNP12474	*Kabuli*	Ca_Kabuli_Chr07	14233368	[T/C]	Ca_23041	Protein of unknown function DUF248, methyltransferase putative	SYNONYMOUS_CODING
12475	CakSNP12475	*Kabuli*	Ca_Kabuli_Chr07	14281993	[G/T]	Ca_23042	Protein of unknown function DUF702	NON_SYNONYMOUS_CODING
12476	CakSNP12476	*Kabuli*	Ca_Kabuli_Chr07	14282088	[T/C]	Ca_23042	Protein of unknown function DUF702	SYNONYMOUS_CODING
12477	CakSNP12477	*Kabuli*	Ca_Kabuli_Chr07	14320216	[T/C]		--	INTERGENIC
12478	CakSNP12478	*Kabuli*	Ca_Kabuli_Chr07	14328655	[T/C]		--	INTERGENIC
12479	CakSNP12479	*Kabuli*	Ca_Kabuli_Chr07	14328649	[T/C]		--	INTERGENIC
12480	CakSNP12480	*Kabuli*	Ca_Kabuli_Chr07	14329126	[C/T]		--	INTERGENIC
12481	CakSNP12481	*Kabuli*	Ca_Kabuli_Chr07	14329175	[G/A]		--	INTERGENIC

S.N.	SNP IDs	Chickpea cultivars	Chromosomes	Physical positions (bp)	SNPs	Gene accession IDs	Putative functions	Sequence components of genome
12482	CakSNP12482	*Kabuli*	Ca_Kabuli_Chr07	14329196	[G/A]		--	INTERGENIC
12483	CakSNP12483	*Kabuli*	Ca_Kabuli_Chr07	14329217	[C/T]		--	INTERGENIC
12484	CakSNP12484	*Kabuli*	Ca_Kabuli_Chr07	14329167	[A/T]		--	INTERGENIC
12485	CakSNP12485	*Kabuli*	Ca_Kabuli_Chr07	14345827	[T/C]	Ca_23045	D-galactoside/L-rhamnose binding SUEL lectin domain	SYNONYMOUS_CODING
12486	CakSNP12486	*Kabuli*	Ca_Kabuli_Chr07	14345799	[T/A]	Ca_23045	D-galactoside/L-rhamnose binding SUEL lectin domain	NON_SYNONYMOUS_CODING
12487	CakSNP12487	*Kabuli*	Ca_Kabuli_Chr07	14470549	[C/G]	Ca_09992	Forkhead-associated (FHA) domain	NON_SYNONYMOUS_CODING
12488	CakSNP12488	*Kabuli*	Ca_Kabuli_Chr07	14518292	[A/T]		--	INTERGENIC
12489	CakSNP12489	*Kabuli*	Ca_Kabuli_Chr07	14526546	[A/G]	Ca_09990	Cytochrome P450	SYNONYMOUS_CODING
12490	CakSNP12490	*Kabuli*	Ca_Kabuli_Chr07	14526495	[T/C]	Ca_09990	Cytochrome P450	SYNONYMOUS_CODING
12491	CakSNP12491	*Kabuli*	Ca_Kabuli_Chr07	14526493	[C/T]	Ca_09990	Cytochrome P450	NON_SYNONYMOUS_CODING

S.N.	SNP IDs	Chickpea cultivars	Chromosomes	Physical positions (bp)	SNPs	Gene accession IDs	Putative functions	Sequence components of genome
12492	CakSNP12492	*Kabuli*	Ca_Kabuli_Chr07	14526480	[T/C]	Ca_09990	Cytochrome P450	SYNONYMOUS_CODING
12493	CakSNP12493	*Kabuli*	Ca_Kabuli_Chr07	14526478	[G/C]	Ca_09990	Cytochrome P450	NON_SYNONYMOUS_CODING
12494	CakSNP12494	*Kabuli*	Ca_Kabuli_Chr07	14558741	[C/A]	Ca_09987	Protein of unknown function DUF726	INTRON
12495	CakSNP12495	*Kabuli*	Ca_Kabuli_Chr07	14558785	[C/T]	Ca_09987	Protein of unknown function DUF726	INTRON
12496	CakSNP12496	*Kabuli*	Ca_Kabuli_Chr07	14559033	[G/A]	Ca_09987	Protein of unknown function DUF726	INTRON
12497	CakSNP12497	*Kabuli*	Ca_Kabuli_Chr07	14666535	[T/A]	Ca_09978	Peptide methionine sulphoxide reductase MsrA	INTRON
12498	CakSNP12498	*Kabuli*	Ca_Kabuli_Chr07	14668939	[G/A]		--	INTERGENIC
12499	CakSNP12499	*Kabuli*	Ca_Kabuli_Chr07	14668922	[A/C]		--	INTERGENIC
12500	CakSNP12500	*Kabuli*	Ca_Kabuli_Chr07	14685011	[G/C]	Ca_09975	--	NON_SYNONYMOUS_CODING
12501	CakSNP12501	*Kabuli*	Ca_Kabuli_Chr07	14685017	[C/T]	Ca_09975	--	NON_SYNONYMOUS_CODING

S.N.	SNP IDs	Chickpea cultivars	Chromosomes	Physical positions (bp)	SNPs	Gene accession IDs	Putative functions	Sequence components of genome
12502	CakSNP12502	*Kabuli*	Ca_Kabuli_Chr07	14685024	[A/G]	Ca_09975	--	NON_SYNONYMOUS_CODING
12503	CakSNP12503	*Kabuli*	Ca_Kabuli_Chr07	14685034	[T/G]	Ca_09975	--	SYNONYMOUS_CODING
12504	CakSNP12504	*Kabuli*	Ca_Kabuli_Chr07	14712518	[C/A]		--	INTERGENIC
12505	CakSNP12505	*Kabuli*	Ca_Kabuli_Chr07	14712568	[C/A]		--	INTERGENIC
12506	CakSNP12506	*Kabuli*	Ca_Kabuli_Chr07	14712596	[T/C]		--	INTERGENIC
12507	CakSNP12507	*Kabuli*	Ca_Kabuli_Chr07	14712601	[C/T]		--	INTERGENIC
12508	CakSNP12508	*Kabuli*	Ca_Kabuli_Chr07	14748257	[T/A]		--	INTERGENIC
12509	CakSNP12509	*Kabuli*	Ca_Kabuli_Chr07	14775135	[C/G]	Ca_09969	Mediator complex, subunit Med4	SYNONYMOUS_CODING
12510	CakSNP12510	*Kabuli*	Ca_Kabuli_Chr07	14782453	[A/G]	Ca_09968	Heat shock protein DnaJ, N-terminal	SYNONYMOUS_CODING
12511	CakSNP12511	*Kabuli*	Ca_Kabuli_Chr07	14899028	[C/A]	Ca_09960	von Willebrand factor, type A	INTRON

S.N.	SNP IDs	Chickpea cultivars	Chromosomes	Physical positions (bp)	SNPs	Gene accession IDs	Putative functions	Sequence components of genome
12512	CakSNP12512	*Kabuli*	Ca_Kabuli_Chr07	14899101	[T/C]	Ca_09960	von Willebrand factor, type A	INTRON
12513	CakSNP12513	*Kabuli*	Ca_Kabuli_Chr07	14933294	[T/C]	Ca_09959	Glycoside hydrolase, family 38, core	SYNONYMOUS_CODING
12514	CakSNP12514	*Kabuli*	Ca_Kabuli_Chr07	14933286	[G/A]	Ca_09959	Glycoside hydrolase, family 38, core	NON_SYNONYMOUS_CODING
12515	CakSNP12515	*Kabuli*	Ca_Kabuli_Chr07	14983364	[T/G]		--	INTERGENIC
12516	CakSNP12516	*Kabuli*	Ca_Kabuli_Chr07	14997794	[G/A]	Ca_09954	RNA helicase, ATP-dependent, DEAD-box, conserved site	NON_SYNONYMOUS_CODING
12517	CakSNP12517	*Kabuli*	Ca_Kabuli_Chr07	14997883	[T/C]	Ca_09954	RNA helicase, ATP-dependent, DEAD-box, conserved site	INTRON
12518	CakSNP12518	*Kabuli*	Ca_Kabuli_Chr07	15028182	[T/G]	Ca_09951	Ras GTPase	INTRON
12519	CakSNP12519	*Kabuli*	Ca_Kabuli_Chr07	15039606	[T/A]		--	INTERGENIC
12520	CakSNP12520	*Kabuli*	Ca_Kabuli_Chr07	15069817	[G/A]	Ca_09947	Uncharacterised protein family Cys-rich	INTRON
12521	CakSNP12521	*Kabuli*	Ca_Kabuli_Chr07	15070091	[T/A]	Ca_09947	Uncharacterised protein family Cys-rich	INTRON

S.N.	SNP IDs	Chickpea cultivars	Chromosomes	Physical positions (bp)	SNPs	Gene accession IDs	Putative functions	Sequence components of genome
12522	CakSNP12522	*Kabuli*	Ca_Kabuli_Chr07	15119205	[T/C]	Ca_09943	Plant peroxidase	SYNONYMOUS_CODING
12523	CakSNP12523	*Kabuli*	Ca_Kabuli_Chr07	15125638	[G/C]	Ca_09942	Peptidase A1	NON_SYNONYMOUS_CODING
12524	CakSNP12524	*Kabuli*	Ca_Kabuli_Chr07	15125619	[T/G]	Ca_09942	Peptidase A1	NON_SYNONYMOUS_CODING
12525	CakSNP12525	*Kabuli*	Ca_Kabuli_Chr07	15185603	[A/G]	Ca_09937	Pentatricopeptide repeat	SYNONYMOUS_CODING
12526	CakSNP12526	*Kabuli*	Ca_Kabuli_Chr07	15201600	[A/G]	Ca_09934	Ammonium transporter	SYNONYMOUS_CODING
12527	CakSNP12527	*Kabuli*	Ca_Kabuli_Chr07	15399421	[T/C]	Ca_09920	Glycoside hydrolase, family 19, catalytic	NON_SYNONYMOUS_CODING
12528	CakSNP12528	*Kabuli*	Ca_Kabuli_Chr07	15441316	[A/T]		--	INTERGENIC
12529	CakSNP12529	*Kabuli*	Ca_Kabuli_Chr07	15463183	[G/T]	Ca_09916	AMP-dependent synthetase/ligase	NON_SYNONYMOUS_CODING
12530	CakSNP12530	*Kabuli*	Ca_Kabuli_Chr07	15463170	[G/A]	Ca_09916	AMP-dependent synthetase/ligase	SYNONYMOUS_CODING
12531	CakSNP12531	*Kabuli*	Ca_Kabuli_Chr07	15463169	[G/A]	Ca_09916	AMP-dependent synthetase/ligase	SYNONYMOUS_CODING

S.N.	SNP IDs	Chickpea cultivars	Chromosomes	Physical positions (bp)	SNPs	Gene accession IDs	Putative functions	Sequence components of genome
12532	CakSNP12532	*Kabuli*	Ca_Kabuli_Chr07	15530329	[G/T]	Ca_09913	AMP-dependent synthetase/ligase	NON_SYNONYMOUS_CODING
12533	CakSNP12533	*Kabuli*	Ca_Kabuli_Chr07	15530316	[G/A]	Ca_09913	AMP-dependent synthetase/ligase	SYNONYMOUS_CODING
12534	CakSNP12534	*Kabuli*	Ca_Kabuli_Chr07	15530315	[G/A]	Ca_09913	AMP-dependent synthetase/ligase	SYNONYMOUS_CODING
12535	CakSNP12535	*Kabuli*	Ca_Kabuli_Chr07	15568060	[G/T]	Ca_09912	AMP-dependent synthetase/ligase	NON_SYNONYMOUS_CODING
12536	CakSNP12536	*Kabuli*	Ca_Kabuli_Chr07	15568047	[G/A]	Ca_09912	AMP-dependent synthetase/ligase	SYNONYMOUS_CODING
12537	CakSNP12537	*Kabuli*	Ca_Kabuli_Chr07	15568046	[G/A]	Ca_09912	AMP-dependent synthetase/ligase	SYNONYMOUS_CODING
12538	CakSNP12538	*Kabuli*	Ca_Kabuli_Chr07	15622247	[G/A]	Ca_09907	Ubiquitin-associated/translation elongation factor EF1B, N-terminal	INTRON
12539	CakSNP12539	*Kabuli*	Ca_Kabuli_Chr07	15622245	[T/A]	Ca_09907	Ubiquitin-associated/translation elongation factor EF1B, N-terminal	INTRON
12540	CakSNP12540	*Kabuli*	Ca_Kabuli_Chr07	15762068	[A/T]	Ca_09895	WD40 repeat	INTRON
12541	CakSNP12541	*Kabuli*	Ca_Kabuli_Chr07	15826588	[A/C]	Ca_09889	Lipid-binding START	SYNONYMOUS_CODING

S.N.	SNP IDs	Chickpea cultivars	Chromosomes	Physical positions (bp)	SNPs	Gene accession IDs	Putative functions	Sequence components of genome
12542	CakSNP12542	*Kabuli*	Ca_Kabuli_Chr07	15856789	[A/G]	Ca_09887	Ionotropic glutamate receptor	NON_SYNONYMOUS_CODING
12543	CakSNP12543	*Kabuli*	Ca_Kabuli_Chr07	15856955	[T/G]	Ca_09887	Ionotropic glutamate receptor	INTRON
12544	CakSNP12544	*Kabuli*	Ca_Kabuli_Chr07	15860298	[T/C]	Ca_09887	Ionotropic glutamate receptor	SYNONYMOUS_CODING
12545	CakSNP12545	*Kabuli*	Ca_Kabuli_Chr07	15887790	[T/C]	Ca_09885	Ribonuclease II/R	INTRON
12546	CakSNP12546	*Kabuli*	Ca_Kabuli_Chr07	15966680	[A/G]	Ca_09880	Sugar/inositol transporter	SYNONYMOUS_CODING
12547	CakSNP12547	*Kabuli*	Ca_Kabuli_Chr07	15966714	[G/A]	Ca_09880	Sugar/inositol transporter	NON_SYNONYMOUS_CODING
12548	CakSNP12548	*Kabuli*	Ca_Kabuli_Chr07	16008746	[T/C]	Ca_09875	RecF/RecN/SMC	INTRON
12549	CakSNP12549	*Kabuli*	Ca_Kabuli_Chr07	16008733	[A/G]	Ca_09875	RecF/RecN/SMC	INTRON
12550	CakSNP12550	*Kabuli*	Ca_Kabuli_Chr07	16289811	[C/T]	Ca_09857	Tafazzin	SYNONYMOUS_CODING
12551	CakSNP12551	*Kabuli*	Ca_Kabuli_Chr07	16289802	[C/T]	Ca_09857	Tafazzin	SYNONYMOUS_CODING

S.N.	SNP IDs	Chickpea cultivars	Chromosomes	Physical positions (bp)	SNPs	Gene accession IDs	Putative functions	Sequence components of genome
12552	CakSNP12552	*Kabuli*	Ca_Kabuli_Chr07	16289794	[G/T]	Ca_09857	Tafazzin	NON_SYNONYMOUS_CODING
12553	CakSNP12553	*Kabuli*	Ca_Kabuli_Chr07	16324985	[T/C]	Ca_09853	Drug/metabolite transporter	INTRON
12554	CakSNP12554	*Kabuli*	Ca_Kabuli_Chr07	16331943	[A/G]	Ca_09852	--	SYNONYMOUS_CODING
12555	CakSNP12555	*Kabuli*	Ca_Kabuli_Chr07	16381854	[T/C]	Ca_09849	Clathrin/coatomer adaptor, adaptin-like, N-terminal	INTRON
12556	CakSNP12556	*Kabuli*	Ca_Kabuli_Chr07	16381891	[T/G]	Ca_09849	Clathrin/coatomer adaptor, adaptin-like, N-terminal	INTRON
12557	CakSNP12557	*Kabuli*	Ca_Kabuli_Chr07	16381934	[T/C]	Ca_09849	Clathrin/coatomer adaptor, adaptin-like, N-terminal	INTRON
12558	CakSNP12558	*Kabuli*	Ca_Kabuli_Chr07	16466619	[T/C]		--	INTERGENIC
12559	CakSNP12559	*Kabuli*	Ca_Kabuli_Chr07	16581695	[C/G]	Ca_20559	Pectinacetylesterase	INTRON
12560	CakSNP12560	*Kabuli*	Ca_Kabuli_Chr07	16626047	[T/G]	Ca_20562	Nodulin-like	SYNONYMOUS_CODING
12561	CakSNP12561	*Kabuli*	Ca_Kabuli_Chr07	16626703	[A/G]		--	INTERGENIC

S.N.	SNP IDs	Chickpea cultivars	Chromosomes	Physical positions (bp)	SNPs	Gene accession IDs	Putative functions	Sequence components of genome
12562	CakSNP12562	*Kabuli*	Ca_Kabuli_Chr07	16665577	[A/G]	Ca_20568	Tetratricopeptide repeat-containing	SYNONYMOUS_CODING
12563	CakSNP12563	*Kabuli*	Ca_Kabuli_Chr07	16735437	[G/A]		--	INTERGENIC
12564	CakSNP12564	*Kabuli*	Ca_Kabuli_Chr07	16735427	[A/G]		--	INTERGENIC
12565	CakSNP12565	*Kabuli*	Ca_Kabuli_Chr07	16735423	[G/C]		--	INTERGENIC
12566	CakSNP12566	*Kabuli*	Ca_Kabuli_Chr07	16816296	[G/C]		--	INTERGENIC
12567	CakSNP12567	*Kabuli*	Ca_Kabuli_Chr07	16821274	[A/C]	Ca_20581	--	NON_SYNONYMOUS_CODING
12568	CakSNP12568	*Kabuli*	Ca_Kabuli_Chr07	16829081	[C/A]	Ca_20582	Transglutaminase-like	INTRON
12569	CakSNP12569	*Kabuli*	Ca_Kabuli_Chr07	16897329	[A/C]		--	INTERGENIC
12570	CakSNP12570	*Kabuli*	Ca_Kabuli_Chr07	16897285	[T/C]		--	INTERGENIC
12571	CakSNP12571	*Kabuli*	Ca_Kabuli_Chr07	16936123	[C/G]	Ca_15885	H/ACA ribonucleoprotein complex, subunit Nop10	NON_SYNONYMOUS_CODING

S.N.	SNP IDs	Chickpea cultivars	Chromosomes	Physical positions (bp)	SNPs	Gene accession IDs	Putative functions	Sequence components of genome
12572	CakSNP12572	*Kabuli*	Ca_Kabuli_Chr07	17017466	[C/G]	Ca_15881	Domain of unknown function DUF231, plant	INTRON
12573	CakSNP12573	*Kabuli*	Ca_Kabuli_Chr07	17017453	[A/G]	Ca_15881	Domain of unknown function DUF231, plant	INTRON
12574	CakSNP12574	*Kabuli*	Ca_Kabuli_Chr07	17017447	[A/G]	Ca_15881	Domain of unknown function DUF231, plant	INTRON
12575	CakSNP12575	*Kabuli*	Ca_Kabuli_Chr07	17017438	[A/T]	Ca_15881	Domain of unknown function DUF231, plant	INTRON
12576	CakSNP12576	*Kabuli*	Ca_Kabuli_Chr07	17017421	[G/A]	Ca_15881	Domain of unknown function DUF231, plant	INTRON
12577	CakSNP12577	*Kabuli*	Ca_Kabuli_Chr07	17017418	[T/G]	Ca_15881	Domain of unknown function DUF231, plant	INTRON
12578	CakSNP12578	*Kabuli*	Ca_Kabuli_Chr07	17038317	[G/A]	Ca_15879	RNA-binding, CRM domain	SYNONYMOUS_CODING
12579	CakSNP12579	*Kabuli*	Ca_Kabuli_Chr07	17064968	[T/C]	Ca_15876	Alpha/beta hydrolase fold-1	SYNONYMOUS_CODING
12580	CakSNP12580	*Kabuli*	Ca_Kabuli_Chr07	17088603	[A/G]	Ca_15873	Thiamine pyrophosphate enzyme, N-terminal TPP-binding domain	INTRON
12581	CakSNP12581	*Kabuli*	Ca_Kabuli_Chr07	17140664	[C/T]		--	INTERGENIC

S.N.	SNP IDs	Chickpea cultivars	Chromosomes	Physical positions (bp)	SNPs	Gene accession IDs	Putative functions	Sequence components of genome
12582	CakSNP12582	*Kabuli*	Ca_Kabuli_Chr07	17178847	[A/C]	Ca_15868	ATPase, P-type, K/Mg/Cd/Cu/Zn/Na/Ca/Na/H-transporter	INTRON
12583	CakSNP12583	*Kabuli*	Ca_Kabuli_Chr07	17181160	[G/C]	Ca_15868	ATPase, P-type, K/Mg/Cd/Cu/Zn/Na/Ca/Na/H-transporter	INTRON
12584	CakSNP12584	*Kabuli*	Ca_Kabuli_Chr07	17228904	[T/G]	Ca_15865	SPX, N-terminal	SYNONYMOUS_CODING
12585	CakSNP12585	*Kabuli*	Ca_Kabuli_Chr07	17318174	[G/T]	Ca_15859	SET domain	NON_SYNONYMOUS_CODING
12586	CakSNP12586	*Kabuli*	Ca_Kabuli_Chr07	17539110	[G/A]	Ca_15853	UBX	INTRON
12587	CakSNP12587	*Kabuli*	Ca_Kabuli_Chr07	17539136	[C/A]	Ca_15853	UBX	INTRON
12588	CakSNP12588	*Kabuli*	Ca_Kabuli_Chr07	17539207	[G/A]	Ca_15853	UBX	INTRON
12589	CakSNP12589	*Kabuli*	Ca_Kabuli_Chr07	17586154	[C/T]	Ca_15850	Bromodomain	INTRON
12590	CakSNP12590	*Kabuli*	Ca_Kabuli_Chr07	17591798	[T/A]	Ca_15850	Bromodomain	INTRON
12591	CakSNP12591	*Kabuli*	Ca_Kabuli_Chr07	17591781	[G/A]	Ca_15850	Bromodomain	INTRON

S.N.	SNP IDs	Chickpea cultivars	Chromosomes	Physical positions (bp)	SNPs	Gene accession IDs	Putative functions	Sequence components of genome
12592	CakSNP12592	*Kabuli*	Ca_Kabuli_Chr07	17591843	[G/T]	Ca_15850	Bromodomain	INTRON
12593	CakSNP12593	*Kabuli*	Ca_Kabuli_Chr07	17591974	[A/G]	Ca_15850	Bromodomain	INTRON
12594	CakSNP12594	*Kabuli*	Ca_Kabuli_Chr07	17591971	[A/G]	Ca_15850	Bromodomain	INTRON
12595	CakSNP12595	*Kabuli*	Ca_Kabuli_Chr07	17591924	[T/C]	Ca_15850	Bromodomain	INTRON
12596	CakSNP12596	*Kabuli*	Ca_Kabuli_Chr07	17592701	[C/A]	Ca_15850	Bromodomain	INTRON
12597	CakSNP12597	*Kabuli*	Ca_Kabuli_Chr07	17597348	[T/C]	Ca_15849	Protein of unknown function DUF1639	NON_SYNONYMOUS_CODING
12598	CakSNP12598	*Kabuli*	Ca_Kabuli_Chr07	17597421	[A/G]	Ca_15849	Protein of unknown function DUF1639	SYNONYMOUS_CODING
12599	CakSNP12599	*Kabuli*	Ca_Kabuli_Chr07	17597420	[C/T]	Ca_15849	Protein of unknown function DUF1639	NON_SYNONYMOUS_CODING
12600	CakSNP12600	*Kabuli*	Ca_Kabuli_Chr07	17624143	[G/A]		--	INTERGENIC
12601	CakSNP12601	*Kabuli*	Ca_Kabuli_Chr07	17624225	[T/C]		--	INTERGENIC

S.N.	SNP IDs	Chickpea cultivars	Chromosomes	Physical positions (bp)	SNPs	Gene accession IDs	Putative functions	Sequence components of genome
12602	CakSNP12602	*Kabuli*	Ca_Kabuli_Chr07	17766520	[T/C]		--	INTERGENIC
12603	CakSNP12603	*Kabuli*	Ca_Kabuli_Chr07	17766952	[T/A]		--	INTERGENIC
12604	CakSNP12604	*Kabuli*	Ca_Kabuli_Chr07	17766914	[C/A]		--	INTERGENIC
12605	CakSNP12605	*Kabuli*	Ca_Kabuli_Chr07	17779414	[A/G]	Ca_15839	Basic-leucine zipper (bZIP) transcription factor	NON_SYNONYMOUS_CODING
12606	CakSNP12606	*Kabuli*	Ca_Kabuli_Chr07	17779594	[T/C]	Ca_15839	Basic-leucine zipper (bZIP) transcription factor	NON_SYNONYMOUS_CODING
12607	CakSNP12607	*Kabuli*	Ca_Kabuli_Chr07	17779700	[A/T]	Ca_15839	Basic-leucine zipper (bZIP) transcription factor	INTRON
12608	CakSNP12608	*Kabuli*	Ca_Kabuli_Chr07	17782558	[C/T]	Ca_15838	LrgB-like protein	INTRON
12609	CakSNP12609	*Kabuli*	Ca_Kabuli_Chr07	18011604	[G/C]	Ca_15826	Protein kinase, catalytic domain	NON_SYNONYMOUS_CODING
12610	CakSNP12610	*Kabuli*	Ca_Kabuli_Chr07	18090289	[T/A]		--	INTERGENIC
12611	CakSNP12611	*Kabuli*	Ca_Kabuli_Chr07	18265803	[A/C]		--	INTERGENIC

S.N.	SNP IDs	Chickpea cultivars	Chromosomes	Physical positions (bp)	SNPs	Gene accession IDs	Putative functions	Sequence components of genome
12612	CakSNP12612	*Kabuli*	Ca_Kabuli_Chr07	18265791	[G/C]		--	INTERGENIC
12613	CakSNP12613	*Kabuli*	Ca_Kabuli_Chr07	18265782	[G/C]		--	INTERGENIC
12614	CakSNP12614	*Kabuli*	Ca_Kabuli_Chr07	18319049	[T/G]		--	INTERGENIC
12615	CakSNP12615	*Kabuli*	Ca_Kabuli_Chr07	18319073	[T/G]		--	INTERGENIC
12616	CakSNP12616	*Kabuli*	Ca_Kabuli_Chr07	18365218	[G/A]		--	INTERGENIC
12617	CakSNP12617	*Kabuli*	Ca_Kabuli_Chr07	18430468	[C/T]	Ca_22129	Clathrin adaptor, mu subunit	INTRON
12618	CakSNP12618	*Kabuli*	Ca_Kabuli_Chr07	18469372	[G/A]	Ca_22127	--	SYNONYMOUS_CODING
12619	CakSNP12619	*Kabuli*	Ca_Kabuli_Chr07	18469422	[C/T]	Ca_22127	--	NON_SYNONYMOUS_CODING
12620	CakSNP12620	*Kabuli*	Ca_Kabuli_Chr07	18638565	[A/G]	Ca_12302	--	NON_SYNONYMOUS_CODING
12621	CakSNP12621	*Kabuli*	Ca_Kabuli_Chr07	18642538	[A/G]	Ca_12302	--	NON_SYNONYMOUS_CODING

S.N.	SNP IDs	Chickpea cultivars	Chromosomes	Physical positions (bp)	SNPs	Gene accession IDs	Putative functions	Sequence components of genome
12622	CakSNP12622	*Kabuli*	Ca_Kabuli_Chr07	18658563	[C/G]	Ca_12303	Phosphate transporter	SYNONYMOUS_CODING
12623	CakSNP12623	*Kabuli*	Ca_Kabuli_Chr07	18696645	[G/A]		--	INTERGENIC
12624	CakSNP12624	*Kabuli*	Ca_Kabuli_Chr07	18796748	[T/C]	Ca_12308	ZF-HD homeobox protein, Cys/His-rich dimerisation domain	NON_SYNONYMOUS_CODING
12625	CakSNP12625	*Kabuli*	Ca_Kabuli_Chr07	18796768	[A/G]	Ca_12308	ZF-HD homeobox protein, Cys/His-rich dimerisation domain	SYNONYMOUS_CODING
12626	CakSNP12626	*Kabuli*	Ca_Kabuli_Chr07	18910468	[A/G]	Ca_12311	Manganese/iron superoxide dismutase	SYNONYMOUS_CODING
12627	CakSNP12627	*Kabuli*	Ca_Kabuli_Chr07	19042183	[C/T]	Ca_12316	Kinesin, motor domain	INTRON
12628	CakSNP12628	*Kabuli*	Ca_Kabuli_Chr07	19189737	[A/C]		--	INTERGENIC
12629	CakSNP12629	*Kabuli*	Ca_Kabuli_Chr07	19217501	[A/C]	Ca_12328	Pathogenesis-related transcriptional factor/ERF, DNA-binding	NON_SYNONYMOUS_CODING
12630	CakSNP12630	*Kabuli*	Ca_Kabuli_Chr07	19217598	[G/C]	Ca_12328	Pathogenesis-related transcriptional factor/ERF, DNA-binding	NON_SYNONYMOUS_CODING
12631	CakSNP12631	*Kabuli*	Ca_Kabuli_Chr07	19232818	[T/A]	Ca_12330	Protein kinase, catalytic domain	INTRON

S.N.	SNP IDs	Chickpea cultivars	Chromosomes	Physical positions (bp)	SNPs	Gene accession IDs	Putative functions	Sequence components of genome
12632	CakSNP12632	*Kabuli*	Ca_Kabuli_Chr07	19290521	[G/A]		--	INTERGENIC
12633	CakSNP12633	*Kabuli*	Ca_Kabuli_Chr07	19470527	[T/C]	Ca_12344	AUX/IAA protein	SYNONYMOUS_CODING
12634	CakSNP12634	*Kabuli*	Ca_Kabuli_Chr07	19472719	[G/A]		--	INTERGENIC
12635	CakSNP12635	*Kabuli*	Ca_Kabuli_Chr07	19472689	[T/A]		--	INTERGENIC
12636	CakSNP12636	*Kabuli*	Ca_Kabuli_Chr07	19542901	[A/G]	Ca_12349	--	INTRON
12637	CakSNP12637	*Kabuli*	Ca_Kabuli_Chr07	19549636	[C/T]	Ca_12350	Mitochondrial substrate/solute carrier	SYNONYMOUS_CODING
12638	CakSNP12638	*Kabuli*	Ca_Kabuli_Chr07	19707812	[T/A]		--	INTERGENIC
12639	CakSNP12639	*Kabuli*	Ca_Kabuli_Chr07	19707842	[G/A]		--	INTERGENIC
12640	CakSNP12640	*Kabuli*	Ca_Kabuli_Chr07	19818789	[C/A]		--	INTERGENIC
12641	CakSNP12641	*Kabuli*	Ca_Kabuli_Chr07	19818770	[T/C]		--	INTERGENIC

S.N.	SNP IDs	Chickpea cultivars	Chromosomes	Physical positions (bp)	SNPs	Gene accession IDs	Putative functions	Sequence components of genome
12642	CakSNP12642	*Kabuli*	Ca_Kabuli_Chr07	19818759	[G/C]		--	INTERGENIC
12643	CakSNP12643	*Kabuli*	Ca_Kabuli_Chr07	19818757	[G/C]		--	INTERGENIC
12644	CakSNP12644	*Kabuli*	Ca_Kabuli_Chr07	20031805	[T/C]		--	INTERGENIC
12645	CakSNP12645	*Kabuli*	Ca_Kabuli_Chr07	20032205	[A/C]	Ca_12371	General substrate transporter	SYNONYMOUS_CODING
12646	CakSNP12646	*Kabuli*	Ca_Kabuli_Chr07	20082842	[T/C]	Ca_12374	RNA recognition motif domain	SYNONYMOUS_CODING
12647	CakSNP12647	*Kabuli*	Ca_Kabuli_Chr07	20083040	[A/T]	Ca_12374	RNA recognition motif domain	SYNONYMOUS_CODING
12648	CakSNP12648	*Kabuli*	Ca_Kabuli_Chr07	20099384	[C/T]		--	INTERGENIC
12649	CakSNP12649	*Kabuli*	Ca_Kabuli_Chr07	20111019	[T/C]	Ca_12376	PIG-X/PBN1	NON_SYNONYMOUS_CODING
12650	CakSNP12650	*Kabuli*	Ca_Kabuli_Chr07	20111123	[T/C]	Ca_12376	PIG-X/PBN1	NON_SYNONYMOUS_CODING
12651	CakSNP12651	*Kabuli*	Ca_Kabuli_Chr07	20112897	[G/A]		--	INTERGENIC

S.N.	SNP IDs	Chickpea cultivars	Chromosomes	Physical positions (bp)	SNPs	Gene accession IDs	Putative functions	Sequence components of genome
12652	CakSNP12652	*Kabuli*	Ca_Kabuli_Chr07	20135647	[C/G]	Ca_12378	FY-rich, C-terminal	INTRON
12653	CakSNP12653	*Kabuli*	Ca_Kabuli_Chr07	20168870	[T/A]	Ca_12379	YABBY protein	INTRON
12654	CakSNP12654	*Kabuli*	Ca_Kabuli_Chr07	20252133	[T/C]		--	INTERGENIC
12655	CakSNP12655	*Kabuli*	Ca_Kabuli_Chr07	20252134	[G/A]		--	INTERGENIC
12656	CakSNP12656	*Kabuli*	Ca_Kabuli_Chr07	20252135	[A/G]		--	INTERGENIC
12657	CakSNP12657	*Kabuli*	Ca_Kabuli_Chr07	20252144	[G/A]		--	INTERGENIC
12658	CakSNP12658	*Kabuli*	Ca_Kabuli_Chr07	20252145	[A/G]		--	INTERGENIC
12659	CakSNP12659	*Kabuli*	Ca_Kabuli_Chr07	20252181	[C/T]		--	INTERGENIC
12660	CakSNP12660	*Kabuli*	Ca_Kabuli_Chr07	20252192	[G/A]		--	INTERGENIC
12661	CakSNP12661	*Kabuli*	Ca_Kabuli_Chr07	20252201	[C/A]		--	INTERGENIC

S.N.	SNP IDs	Chickpea cultivars	Chromosomes	Physical positions (bp)	SNPs	Gene accession IDs	Putative functions	Sequence components of genome
12662	CakSNP12662	*Kabuli*	Ca_Kabuli_Chr07	20252212	[C/T]		--	INTERGENIC
12663	CakSNP12663	*Kabuli*	Ca_Kabuli_Chr07	20252214	[C/G]		--	INTERGENIC
12664	CakSNP12664	*Kabuli*	Ca_Kabuli_Chr07	20252236	[G/A]		--	INTERGENIC
12665	CakSNP12665	*Kabuli*	Ca_Kabuli_Chr07	20252234	[A/C]		--	INTERGENIC
12666	CakSNP12666	*Kabuli*	Ca_Kabuli_Chr07	20252233	[C/T]		--	INTERGENIC
12667	CakSNP12667	*Kabuli*	Ca_Kabuli_Chr07	20252221	[T/C]		--	INTERGENIC
12668	CakSNP12668	*Kabuli*	Ca_Kabuli_Chr07	20252209	[G/A]		--	INTERGENIC
12669	CakSNP12669	*Kabuli*	Ca_Kabuli_Chr07	20252309	[C/T]		--	INTERGENIC
12670	CakSNP12670	*Kabuli*	Ca_Kabuli_Chr07	20252334	[A/T]	Ca_12384	--	SYNONYMOUS_STOP
12671	CakSNP12671	*Kabuli*	Ca_Kabuli_Chr07	20252340	[A/G]	Ca_12384	--	SYNONYMOUS_CODING

S.N.	SNP IDs	Chickpea cultivars	Chromosomes	Physical positions (bp)	SNPs	Gene accession IDs	Putative functions	Sequence components of genome
12672	CakSNP12672	*Kabuli*	Ca_Kabuli_Chr07	20362883	[G/A]		--	INTERGENIC
12673	CakSNP12673	*Kabuli*	Ca_Kabuli_Chr07	20362879	[C/T]		--	INTERGENIC
12674	CakSNP12674	*Kabuli*	Ca_Kabuli_Chr07	20362876	[G/A]		--	INTERGENIC
12675	CakSNP12675	*Kabuli*	Ca_Kabuli_Chr07	20362838	[T/C]		--	INTERGENIC
12676	CakSNP12676	*Kabuli*	Ca_Kabuli_Chr07	20414653	[G/A]	Ca_12393	Amino acid transporter, transmembrane	SYNONYMOUS_CODING
12677	CakSNP12677	*Kabuli*	Ca_Kabuli_Chr07	20414703	[C/A]	Ca_12393	Amino acid transporter, transmembrane	NON_SYNONYMOUS_CODING
12678	CakSNP12678	*Kabuli*	Ca_Kabuli_Chr07	20486247	[T/G]		--	INTERGENIC
12679	CakSNP12679	*Kabuli*	Ca_Kabuli_Chr07	20486280	[G/C]		--	INTERGENIC
12680	CakSNP12680	*Kabuli*	Ca_Kabuli_Chr07	20496378	[G/A]	Ca_12400	Pathogenesis-related transcriptional factor/ERF, DNA-binding	SYNONYMOUS_CODING
12681	CakSNP12681	*Kabuli*	Ca_Kabuli_Chr07	20496303	[C/T]	Ca_12400	Pathogenesis-related transcriptional factor/ERF, DNA-binding	SYNONYMOUS_CODING

S.N.	SNP IDs	Chickpea cultivars	Chromosomes	Physical positions (bp)	SNPs	Gene accession IDs	Putative functions	Sequence components of genome
12682	CakSNP12682	*Kabuli*	Ca_Kabuli_Chr07	20496438	[C/T]	Ca_12400	Pathogenesis-related transcriptional factor/ERF, DNA-binding	SYNONYMOUS_CODING
12683	CakSNP12683	*Kabuli*	Ca_Kabuli_Chr07	20498552	[T/C]	Ca_12400	Pathogenesis-related transcriptional factor/ERF, DNA-binding	INTRON
12684	CakSNP12684	*Kabuli*	Ca_Kabuli_Chr07	20498626	[T/G]	Ca_12400	Pathogenesis-related transcriptional factor/ERF, DNA-binding	INTRON
12685	CakSNP12685	*Kabuli*	Ca_Kabuli_Chr07	20540780	[C/T]	Ca_12404	Xanthine/uracil/vitamin C permease	NON_SYNONYMOUS_CODING
12686	CakSNP12686	*Kabuli*	Ca_Kabuli_Chr07	20540791	[G/A]	Ca_12404	Xanthine/uracil/vitamin C permease	SYNONYMOUS_CODING
12687	CakSNP12687	*Kabuli*	Ca_Kabuli_Chr07	20543119	[T/C]	Ca_12404	Xanthine/uracil/vitamin C permease	INTRON
12688	CakSNP12688	*Kabuli*	Ca_Kabuli_Chr07	20550563	[G/A]		--	INTERGENIC
12689	CakSNP12689	*Kabuli*	Ca_Kabuli_Chr07	20550575	[C/G]		--	INTERGENIC
12690	CakSNP12690	*Kabuli*	Ca_Kabuli_Chr07	20552041	[G/A]	Ca_12405	SGT1	SYNONYMOUS_CODING
12691	CakSNP12691	*Kabuli*	Ca_Kabuli_Chr07	20552053	[C/G]	Ca_12405	SGT1	SYNONYMOUS_CODING

S.N.	SNP IDs	Chickpea cultivars	Chromosomes	Physical positions (bp)	SNPs	Gene accession IDs	Putative functions	Sequence components of genome
12692	CakSNP12692	*Kabuli*	Ca_Kabuli_Chr07	20556466	[G/C]		--	INTERGENIC
12693	CakSNP12693	*Kabuli*	Ca_Kabuli_Chr07	20556576	[C/T]		--	INTERGENIC
12694	CakSNP12694	*Kabuli*	Ca_Kabuli_Chr07	20563931	[G/A]	Ca_12406	KIP1-like	SYNONYMOUS_CODING
12695	CakSNP12695	*Kabuli*	Ca_Kabuli_Chr07	20564024	[G/A]	Ca_12406	KIP1-like	SYNONYMOUS_CODING
12696	CakSNP12696	*Kabuli*	Ca_Kabuli_Chr07	20564266	[G/A]	Ca_12406	KIP1-like	NON_SYNONYMOUS_CODING
12697	CakSNP12697	*Kabuli*	Ca_Kabuli_Chr07	20564269	[C/T]	Ca_12406	KIP1-like	NON_SYNONYMOUS_CODING
12698	CakSNP12698	*Kabuli*	Ca_Kabuli_Chr07	20564297	[A/G]	Ca_12406	KIP1-like	SYNONYMOUS_CODING
12699	CakSNP12699	*Kabuli*	Ca_Kabuli_Chr07	20579963	[G/T]		--	INTERGENIC
12700	CakSNP12700	*Kabuli*	Ca_Kabuli_Chr07	20579974	[A/T]		--	INTERGENIC
12701	CakSNP12701	*Kabuli*	Ca_Kabuli_Chr07	20585997	[T/G]		--	INTERGENIC

S.N.	SNP IDs	Chickpea cultivars	Chromosomes	Physical positions (bp)	SNPs	Gene accession IDs	Putative functions	Sequence components of genome
12702	CakSNP12702	*Kabuli*	Ca_Kabuli_Chr07	20605389	[G/A]	Ca_12412	--	SYNONYMOUS_CODING
12703	CakSNP12703	*Kabuli*	Ca_Kabuli_Chr07	20605384	[C/G]	Ca_12412	--	NON_SYNONYMOUS_CODING
12704	CakSNP12704	*Kabuli*	Ca_Kabuli_Chr07	20605373	[C/A]	Ca_12412	--	NON_SYNONYMOUS_CODING
12705	CakSNP12705	*Kabuli*	Ca_Kabuli_Chr07	20633040	[A/G]		--	INTERGENIC
12706	CakSNP12706	*Kabuli*	Ca_Kabuli_Chr07	20637152	[G/A]	Ca_12415	Protein synthesis factor, GTP-binding	SYNONYMOUS_CODING
12707	CakSNP12707	*Kabuli*	Ca_Kabuli_Chr07	20917747	[A/G]	Ca_14483	Protein kinase, catalytic domain	NON_SYNONYMOUS_CODING
12708	CakSNP12708	*Kabuli*	Ca_Kabuli_Chr07	20917743	[T/G]	Ca_14483	Protein kinase, catalytic domain	NON_SYNONYMOUS_CODING
12709	CakSNP12709	*Kabuli*	Ca_Kabuli_Chr07	20917740	[A/G]	Ca_14483	Protein kinase, catalytic domain	INTRON
12710	CakSNP12710	*Kabuli*	Ca_Kabuli_Chr07	21073385	[A/C]	Ca_14490	Alcohol dehydrogenase superfamily, zinc-containing	NON_SYNONYMOUS_CODING
12711	CakSNP12711	*Kabuli*	Ca_Kabuli_Chr07	21081969	[A/G]		--	INTERGENIC

S.N.	SNP IDs	Chickpea cultivars	Chromosomes	Physical positions (bp)	SNPs	Gene accession IDs	Putative functions	Sequence components of genome
12712	CakSNP12712	*Kabuli*	Ca_Kabuli_Chr07	21099782	[G/T]		--	INTERGENIC
12713	CakSNP12713	*Kabuli*	Ca_Kabuli_Chr07	21402075	[A/G]	Ca_14503	Aldehyde dehydrogenase domain	SYNONYMOUS_CODING
12714	CakSNP12714	*Kabuli*	Ca_Kabuli_Chr07	21402117	[A/C]	Ca_14503	Aldehyde dehydrogenase domain	INTRON
12715	CakSNP12715	*Kabuli*	Ca_Kabuli_Chr07	21595525	[C/T]	Ca_14511	--	NON_SYNONYMOUS_CODING
12716	CakSNP12716	*Kabuli*	Ca_Kabuli_Chr07	21595527	[T/G]	Ca_14511	--	NON_SYNONYMOUS_CODING
12717	CakSNP12717	*Kabuli*	Ca_Kabuli_Chr07	21616285	[A/G]	Ca_14512	Clathrin/coatomer adaptor, adaptin-like, N-terminal	INTRON
12718	CakSNP12718	*Kabuli*	Ca_Kabuli_Chr07	21616287	[T/G]	Ca_14512	Clathrin/coatomer adaptor, adaptin-like, N-terminal	INTRON
12719	CakSNP12719	*Kabuli*	Ca_Kabuli_Chr07	21616294	[T/G]	Ca_14512	Clathrin/coatomer adaptor, adaptin-like, N-terminal	INTRON
12720	CakSNP12720	*Kabuli*	Ca_Kabuli_Chr07	21626752	[T/C]		--	INTERGENIC
12721	CakSNP12721	*Kabuli*	Ca_Kabuli_Chr07	21633103	[C/G]	Ca_14514	BRCT	INTRON

S.N.	SNP IDs	Chickpea cultivars	Chromosomes	Physical positions (bp)	SNPs	Gene accession IDs	Putative functions	Sequence components of genome
12722	CakSNP12722	*Kabuli*	Ca_Kabuli_Chr07	21639234	[G/A]	Ca_14515	Clathrin/coatomer adaptor, adaptin-like, N-terminal	INTRON
12723	CakSNP12723	*Kabuli*	Ca_Kabuli_Chr07	21639238	[G/A]	Ca_14515	Clathrin/coatomer adaptor, adaptin-like, N-terminal	INTRON
12724	CakSNP12724	*Kabuli*	Ca_Kabuli_Chr07	21639244	[T/G]	Ca_14515	Clathrin/coatomer adaptor, adaptin-like, N-terminal	INTRON
12725	CakSNP12725	*Kabuli*	Ca_Kabuli_Chr07	21639251	[T/G]	Ca_14515	Clathrin/coatomer adaptor, adaptin-like, N-terminal	INTRON
12726	CakSNP12726	*Kabuli*	Ca_Kabuli_Chr07	21639254	[T/G]	Ca_14515	Clathrin/coatomer adaptor, adaptin-like, N-terminal	INTRON
12727	CakSNP12727	*Kabuli*	Ca_Kabuli_Chr07	21639375	[T/G]	Ca_14515	Clathrin/coatomer adaptor, adaptin-like, N-terminal	INTRON
12728	CakSNP12728	*Kabuli*	Ca_Kabuli_Chr07	21644279	[C/T]		--	INTERGENIC
12729	CakSNP12729	*Kabuli*	Ca_Kabuli_Chr07	21644342	[G/A]		--	INTERGENIC
12730	CakSNP12730	*Kabuli*	Ca_Kabuli_Chr07	21713147	[T/G]	Ca_14519	--	INTRON
12731	CakSNP12731	*Kabuli*	Ca_Kabuli_Chr07	21744514	[T/C]	Ca_14522	SANT domain, DNA binding	NON_SYNONYMOUS_CODING

S.N.	SNP IDs	Chickpea cultivars	Chromosomes	Physical positions (bp)	SNPs	Gene accession IDs	Putative functions	Sequence components of genome
12732	CakSNP12732	*Kabuli*	Ca_Kabuli_Chr07	21918052	[G/C]		--	INTERGENIC
12733	CakSNP12733	*Kabuli*	Ca_Kabuli_Chr07	22707297	[T/C]		--	INTERGENIC
12734	CakSNP12734	*Kabuli*	Ca_Kabuli_Chr07	22721356	[A/C]	Ca_19530	Haloacid dehalogenase-like hydrolase	SYNONYMOUS_CODING
12735	CakSNP12735	*Kabuli*	Ca_Kabuli_Chr07	22738152	[C/G]	Ca_19530	Haloacid dehalogenase-like hydrolase	INTRON
12736	CakSNP12736	*Kabuli*	Ca_Kabuli_Chr07	22738307	[G/C]	Ca_19530	Haloacid dehalogenase-like hydrolase	INTRON
12737	CakSNP12737	*Kabuli*	Ca_Kabuli_Chr07	22738482	[A/G]	Ca_19530	Haloacid dehalogenase-like hydrolase	INTRON
12738	CakSNP12738	*Kabuli*	Ca_Kabuli_Chr07	22808696	[A/G]		--	INTERGENIC
12739	CakSNP12739	*Kabuli*	Ca_Kabuli_Chr07	22808695	[C/T]		--	INTERGENIC
12740	CakSNP12740	*Kabuli*	Ca_Kabuli_Chr07	22808687	[C/T]		--	INTERGENIC
12741	CakSNP12741	*Kabuli*	Ca_Kabuli_Chr07	22808717	[C/T]		--	INTERGENIC

S.N.	SNP IDs	Chickpea cultivars	Chromosomes	Physical positions (bp)	SNPs	Gene accession IDs	Putative functions	Sequence components of genome
12742	CakSNP12742	*Kabuli*	Ca_Kabuli_Chr07	22808679	[A/C]		--	INTERGENIC
12743	CakSNP12743	*Kabuli*	Ca_Kabuli_Chr07	22839776	[G/A]		--	INTERGENIC
12744	CakSNP12744	*Kabuli*	Ca_Kabuli_Chr07	22875652	[A/G]	Ca_19526	UbiA prenyltransferase family	INTRON
12745	CakSNP12745	*Kabuli*	Ca_Kabuli_Chr07	23087696	[T/C]		--	INTERGENIC
12746	CakSNP12746	*Kabuli*	Ca_Kabuli_Chr07	23087732	[T/C]		--	INTERGENIC
12747	CakSNP12747	*Kabuli*	Ca_Kabuli_Chr07	23439214	[A/G]	Ca_26248	--	INTRON
12748	CakSNP12748	*Kabuli*	Ca_Kabuli_Chr07	23439225	[C/A]	Ca_26248	--	INTRON
12749	CakSNP12749	*Kabuli*	Ca_Kabuli_Chr07	23574823	[C/T]	Ca_26017	Phosphatidylinositol 3-/4-kinase, catalytic	NON_SYNONYMOUS_CODING
12750	CakSNP12750	*Kabuli*	Ca_Kabuli_Chr07	23575629	[G/A]	Ca_26017	Phosphatidylinositol 3-/4-kinase, catalytic	NON_SYNONYMOUS_CODING
12751	CakSNP12751	*Kabuli*	Ca_Kabuli_Chr07	23616050	[A/G]	Ca_26014	Translin	INTRON

S.N.	SNP IDs	Chickpea cultivars	Chromosomes	Physical positions (bp)	SNPs	Gene accession IDs	Putative functions	Sequence components of genome
12752	CakSNP12752	*Kabuli*	Ca_Kabuli_Chr07	24021690	[T/A]	Ca_19916	ZF-HD homeobox protein, Cys/His-rich dimerisation domain	NON_SYNONYMOUS_CODING
12753	CakSNP12753	*Kabuli*	Ca_Kabuli_Chr07	24181321	[G/A]	Ca_19912	Zinc finger, RING-CH-type	NON_SYNONYMOUS_CODING
12754	CakSNP12754	*Kabuli*	Ca_Kabuli_Chr07	24190633	[G/A]		--	INTERGENIC
12755	CakSNP12755	*Kabuli*	Ca_Kabuli_Chr07	24190636	[G/A]		--	INTERGENIC
12756	CakSNP12756	*Kabuli*	Ca_Kabuli_Chr07	24190645	[A/T]		--	INTERGENIC
12757	CakSNP12757	*Kabuli*	Ca_Kabuli_Chr07	24190650	[G/A]		--	INTERGENIC
12758	CakSNP12758	*Kabuli*	Ca_Kabuli_Chr07	24190671	[C/T]		--	INTERGENIC
12759	CakSNP12759	*Kabuli*	Ca_Kabuli_Chr07	24245096	[C/T]		--	INTERGENIC
12760	CakSNP12760	*Kabuli*	Ca_Kabuli_Chr07	24415819	[T/A]	Ca_19906	Disease resistance protein	NON_SYNONYMOUS_CODING
12761	CakSNP12761	*Kabuli*	Ca_Kabuli_Chr07	25218407	[C/T]		--	INTERGENIC

S.N.	SNP IDs	Chickpea cultivars	Chromosomes	Physical positions (bp)	SNPs	Gene accession IDs	Putative functions	Sequence components of genome
12762	CakSNP12762	*Kabuli*	Ca_Kabuli_Chr07	25362272	[T/C]		--	INTERGENIC
12763	CakSNP12763	*Kabuli*	Ca_Kabuli_Chr07	25428518	[T/C]		--	INTERGENIC
12764	CakSNP12764	*Kabuli*	Ca_Kabuli_Chr07	25428525	[C/G]		--	INTERGENIC
12765	CakSNP12765	*Kabuli*	Ca_Kabuli_Chr07	25428548	[G/T]		--	INTERGENIC
12766	CakSNP12766	*Kabuli*	Ca_Kabuli_Chr07	25428566	[T/C]		--	INTERGENIC
12767	CakSNP12767	*Kabuli*	Ca_Kabuli_Chr07	25428660	[G/A]		--	INTERGENIC
12768	CakSNP12768	*Kabuli*	Ca_Kabuli_Chr07	25569665	[T/G]	Ca_23609	Zinc finger, PHD-type	INTRON
12769	CakSNP12769	*Kabuli*	Ca_Kabuli_Chr07	25569657	[T/C]	Ca_23609	Zinc finger, PHD-type	INTRON
12770	CakSNP12770	*Kabuli*	Ca_Kabuli_Chr07	25740866	[G/T]		--	INTERGENIC
12771	CakSNP12771	*Kabuli*	Ca_Kabuli_Chr07	26070513	[G/T]		--	INTERGENIC

S.N.	SNP IDs	Chickpea cultivars	Chromosomes	Physical positions (bp)	SNPs	Gene accession IDs	Putative functions	Sequence components of genome
12772	CakSNP12772	*Kabuli*	Ca_Kabuli_Chr07	26070515	[A/G]		--	INTERGENIC
12773	CakSNP12773	*Kabuli*	Ca_Kabuli_Chr07	26094578	[T/C]		--	INTERGENIC
12774	CakSNP12774	*Kabuli*	Ca_Kabuli_Chr07	26094572	[C/T]		--	INTERGENIC
12775	CakSNP12775	*Kabuli*	Ca_Kabuli_Chr07	26094562	[G/T]		--	INTERGENIC
12776	CakSNP12776	*Kabuli*	Ca_Kabuli_Chr07	26094567	[G/A]		--	INTERGENIC
12777	CakSNP12777	*Kabuli*	Ca_Kabuli_Chr07	26094609	[C/T]		--	INTERGENIC
12778	CakSNP12778	*Kabuli*	Ca_Kabuli_Chr07	26137315	[T/C]	Ca_23640	IQ motif, EF-hand binding site	SYNONYMOUS_CODING
12779	CakSNP12779	*Kabuli*	Ca_Kabuli_Chr07	26146779	[C/T]	Ca_23640	IQ motif, EF-hand binding site	INTRON
12780	CakSNP12780	*Kabuli*	Ca_Kabuli_Chr07	26226146	[G/C]		--	INTERGENIC
12781	CakSNP12781	*Kabuli*	Ca_Kabuli_Chr07	26487440	[A/C]	Ca_16258	26S proteasome non-ATPase regulatory subunit 5	NON_SYNONYMOUS_CODING

S.N.	SNP IDs	Chickpea cultivars	Chromosomes	Physical positions (bp)	SNPs	Gene accession IDs	Putative functions	Sequence components of genome
12782	CakSNP12782	*Kabuli*	Ca_Kabuli_Chr07	26692131	[T/G]		--	INTERGENIC
12783	CakSNP12783	*Kabuli*	Ca_Kabuli_Chr07	26692130	[C/T]		--	INTERGENIC
12784	CakSNP12784	*Kabuli*	Ca_Kabuli_Chr07	26692128	[A/C]		--	INTERGENIC
12785	CakSNP12785	*Kabuli*	Ca_Kabuli_Chr07	26692194	[C/A]		--	INTERGENIC
12786	CakSNP12786	*Kabuli*	Ca_Kabuli_Chr07	26692195	[G/T]		--	INTERGENIC
12787	CakSNP12787	*Kabuli*	Ca_Kabuli_Chr07	26694365	[G/A]	Ca_16250	--	SYNONYMOUS_CODING
12788	CakSNP12788	*Kabuli*	Ca_Kabuli_Chr07	26694440	[C/A]	Ca_16250	--	SYNONYMOUS_CODING
12789	CakSNP12789	*Kabuli*	Ca_Kabuli_Chr07	26694441	[G/A]	Ca_16250	--	NON_SYNONYMOUS_CODING
12790	CakSNP12790	*Kabuli*	Ca_Kabuli_Chr07	26897878	[A/G]		--	INTERGENIC
12791	CakSNP12791	*Kabuli*	Ca_Kabuli_Chr07	26897910	[A/G]		--	INTERGENIC

S.N.	SNP IDs	Chickpea cultivars	Chromosomes	Physical positions (bp)	SNPs	Gene accession IDs	Putative functions	Sequence components of genome
12792	CakSNP12792	*Kabuli*	Ca_Kabuli_Chr07	26897920	[A/G]		--	INTERGENIC
12793	CakSNP12793	*Kabuli*	Ca_Kabuli_Chr07	26897923	[G/A]		--	INTERGENIC
12794	CakSNP12794	*Kabuli*	Ca_Kabuli_Chr07	26897929	[T/C]		--	INTERGENIC
12795	CakSNP12795	*Kabuli*	Ca_Kabuli_Chr07	26897940	[A/G]		--	INTERGENIC
12796	CakSNP12796	*Kabuli*	Ca_Kabuli_Chr07	26897948	[A/T]		--	INTERGENIC
12797	CakSNP12797	*Kabuli*	Ca_Kabuli_Chr07	26940917	[G/A]	Ca_16240	Plastocyanin-like	NON_SYNONYMOUS_CODING
12798	CakSNP12798	*Kabuli*	Ca_Kabuli_Chr07	26940967	[A/G]	Ca_16240	Plastocyanin-like	SYNONYMOUS_CODING
12799	CakSNP12799	*Kabuli*	Ca_Kabuli_Chr07	26940979	[A/G]	Ca_16240	Plastocyanin-like	SYNONYMOUS_CODING
12800	CakSNP12800	*Kabuli*	Ca_Kabuli_Chr07	26940985	[T/G]	Ca_16240	Plastocyanin-like	SYNONYMOUS_CODING
12801	CakSNP12801	*Kabuli*	Ca_Kabuli_Chr07	27050259	[C/G]	Ca_16234	Dynamin, GTPase domain	NON_SYNONYMOUS_CODING

S.N.	SNP IDs	Chickpea cultivars	Chromosomes	Physical positions (bp)	SNPs	Gene accession IDs	Putative functions	Sequence components of genome
12802	CakSNP12802	*Kabuli*	Ca_Kabuli_Chr07	27176481	[C/A]	Ca_16229	Lactate/malate dehydrogenase, N-terminal	NON_SYNONYMOUS_CODING
12803	CakSNP12803	*Kabuli*	Ca_Kabuli_Chr07	27210919	[C/T]		--	INTERGENIC
12804	CakSNP12804	*Kabuli*	Ca_Kabuli_Chr07	27210881	[C/T]		--	INTERGENIC
12805	CakSNP12805	*Kabuli*	Ca_Kabuli_Chr07	27210871	[C/A]		--	INTERGENIC
12806	CakSNP12806	*Kabuli*	Ca_Kabuli_Chr07	27210865	[T/C]		--	INTERGENIC
12807	CakSNP12807	*Kabuli*	Ca_Kabuli_Chr07	27210858	[C/T]		--	INTERGENIC
12808	CakSNP12808	*Kabuli*	Ca_Kabuli_Chr07	27210853	[A/G]		--	INTERGENIC
12809	CakSNP12809	*Kabuli*	Ca_Kabuli_Chr07	27210848	[A/T]		--	INTERGENIC
12810	CakSNP12810	*Kabuli*	Ca_Kabuli_Chr07	27210951	[T/C]		--	INTERGENIC
12811	CakSNP12811	*Kabuli*	Ca_Kabuli_Chr07	27210945	[C/T]		--	INTERGENIC

S.N.	SNP IDs	Chickpea cultivars	Chromosomes	Physical positions (bp)	SNPs	Gene accession IDs	Putative functions	Sequence components of genome
12812	CakSNP12812	*Kabuli*	Ca_Kabuli_Chr07	27210988	[C/T]		--	INTERGENIC
12813	CakSNP12813	*Kabuli*	Ca_Kabuli_Chr07	27210989	[C/T]		--	INTERGENIC
12814	CakSNP12814	*Kabuli*	Ca_Kabuli_Chr07	27210992	[C/A]		--	INTERGENIC
12815	CakSNP12815	*Kabuli*	Ca_Kabuli_Chr07	27211004	[T/C]		--	INTERGENIC
12816	CakSNP12816	*Kabuli*	Ca_Kabuli_Chr07	27211036	[G/A]		--	INTERGENIC
12817	CakSNP12817	*Kabuli*	Ca_Kabuli_Chr07	27211051	[C/A]		--	INTERGENIC
12818	CakSNP12818	*Kabuli*	Ca_Kabuli_Chr07	27211084	[G/T]		--	INTERGENIC
12819	CakSNP12819	*Kabuli*	Ca_Kabuli_Chr07	27211073	[C/T]		--	INTERGENIC
12820	CakSNP12820	*Kabuli*	Ca_Kabuli_Chr07	27211060	[C/T]		--	INTERGENIC
12821	CakSNP12821	*Kabuli*	Ca_Kabuli_Chr07	27633960	[C/A]		--	INTERGENIC

S.N.	SNP IDs	Chickpea cultivars	Chromosomes	Physical positions (bp)	SNPs	Gene accession IDs	Putative functions	Sequence components of genome
12822	CakSNP12822	*Kabuli*	Ca_Kabuli_Chr07	27735234	[A/G]	Ca_16212	Cyclin, C-terminal	INTRON
12823	CakSNP12823	*Kabuli*	Ca_Kabuli_Chr07	27899782	[T/C]		--	INTERGENIC
12824	CakSNP12824	*Kabuli*	Ca_Kabuli_Chr07	27899743	[G/A]		--	INTERGENIC
12825	CakSNP12825	*Kabuli*	Ca_Kabuli_Chr07	27971186	[T/G]		--	INTERGENIC
12826	CakSNP12826	*Kabuli*	Ca_Kabuli_Chr07	28676139	[T/C]	Ca_18955	Cytochrome P450	NON_SYNONYMOUS_CODING
12827	CakSNP12827	*Kabuli*	Ca_Kabuli_Chr07	28931212	[A/C]	Ca_11808	--	NON_SYNONYMOUS_CODING
12828	CakSNP12828	*Kabuli*	Ca_Kabuli_Chr07	28931223	[C/A]	Ca_11808	--	NON_SYNONYMOUS_CODING
12829	CakSNP12829	*Kabuli*	Ca_Kabuli_Chr07	29150927	[A/C]	Ca_11791	--	INTRON
12830	CakSNP12830	*Kabuli*	Ca_Kabuli_Chr07	29153994	[T/C]	Ca_11791	--	SYNONYMOUS_CODING
12831	CakSNP12831	*Kabuli*	Ca_Kabuli_Chr07	29154220	[C/A]	Ca_11791	--	NON_SYNONYMOUS_CODING

S.N.	SNP IDs	Chickpea cultivars	Chromosomes	Physical positions (bp)	SNPs	Gene accession IDs	Putative functions	Sequence components of genome
12832	CakSNP12832	*Kabuli*	Ca_Kabuli_Chr07	29157286	[A/G]	Ca_11791	--	SYNONYMOUS_CODING
12833	CakSNP12833	*Kabuli*	Ca_Kabuli_Chr07	29168982	[G/A]		--	INTERGENIC
12834	CakSNP12834	*Kabuli*	Ca_Kabuli_Chr07	29168980	[C/T]		--	INTERGENIC
12835	CakSNP12835	*Kabuli*	Ca_Kabuli_Chr07	29190659	[T/G]	Ca_11786	Glutamyl/glutaminyl-tRNA synthetase, class Ic	NON_SYNONYMOUS_CODING
12836	CakSNP12836	*Kabuli*	Ca_Kabuli_Chr07	29225385	[T/G]	Ca_11783	NADH:ubiquinone oxidoreductase, 17.2kDa subunit	INTRON
12837	CakSNP12837	*Kabuli*	Ca_Kabuli_Chr07	29225351	[T/G]	Ca_11783	NADH:ubiquinone oxidoreductase, 17.2kDa subunit	INTRON
12838	CakSNP12838	*Kabuli*	Ca_Kabuli_Chr07	29225348	[G/C]	Ca_11783	NADH:ubiquinone oxidoreductase, 17.2kDa subunit	INTRON
12839	CakSNP12839	*Kabuli*	Ca_Kabuli_Chr07	29229965	[A/G]	Ca_11782	Zinc finger, C2H2-type matrin	INTRON
12840	CakSNP12840	*Kabuli*	Ca_Kabuli_Chr07	29313114	[G/T]		--	INTERGENIC
12841	CakSNP12841	*Kabuli*	Ca_Kabuli_Chr07	29313080	[T/C]		--	INTERGENIC

S.N.	SNP IDs	Chickpea cultivars	Chromosomes	Physical positions (bp)	SNPs	Gene accession IDs	Putative functions	Sequence components of genome
12842	CakSNP12842	*Kabuli*	Ca_Kabuli_Chr07	29313067	[G/T]		--	INTERGENIC
12843	CakSNP12843	*Kabuli*	Ca_Kabuli_Chr07	29313059	[G/A]		--	INTERGENIC
12844	CakSNP12844	*Kabuli*	Ca_Kabuli_Chr07	29313058	[T/C]		--	INTERGENIC
12845	CakSNP12845	*Kabuli*	Ca_Kabuli_Chr07	29313051	[T/C]		--	INTERGENIC
12846	CakSNP12846	*Kabuli*	Ca_Kabuli_Chr07	29313257	[G/A]		--	INTERGENIC
12847	CakSNP12847	*Kabuli*	Ca_Kabuli_Chr07	29434818	[T/C]		--	INTERGENIC
12848	CakSNP12848	*Kabuli*	Ca_Kabuli_Chr07	29482556	[G/A]	Ca_11769	Protein kinase, catalytic domain	NON_SYNONYMOUS_CODING
12849	CakSNP12849	*Kabuli*	Ca_Kabuli_Chr07	29484304	[G/A]		--	INTERGENIC
12850	CakSNP12850	*Kabuli*	Ca_Kabuli_Chr07	29607943	[C/T]		--	INTERGENIC
12851	CakSNP12851	*Kabuli*	Ca_Kabuli_Chr07	29613699	[C/T]	Ca_11764	F-box domain, cyclin-like	SYNONYMOUS_CODING

S.N.	SNP IDs	Chickpea cultivars	Chromosomes	Physical positions (bp)	SNPs	Gene accession IDs	Putative functions	Sequence components of genome
12852	CakSNP12852	*Kabuli*	Ca_Kabuli_Chr07	29625884	[T/C]		--	INTERGENIC
12853	CakSNP12853	*Kabuli*	Ca_Kabuli_Chr07	29705531	[T/G]	Ca_11752	--	NON_SYNONYMOUS_CODING
12854	CakSNP12854	*Kabuli*	Ca_Kabuli_Chr07	29705499	[T/C]	Ca_11752	--	NON_SYNONYMOUS_CODING
12855	CakSNP12855	*Kabuli*	Ca_Kabuli_Chr07	29796564	[G/T]	Ca_11746	Spc97/Spc98	NON_SYNONYMOUS_CODING
12856	CakSNP12856	*Kabuli*	Ca_Kabuli_Chr07	29801148	[A/G]	Ca_11746	Spc97/Spc98	NON_SYNONYMOUS_CODING
12857	CakSNP12857	*Kabuli*	Ca_Kabuli_Chr07	29801154	[T/C]	Ca_11746	Spc97/Spc98	NON_SYNONYMOUS_CODING
12858	CakSNP12858	*Kabuli*	Ca_Kabuli_Chr07	29837035	[G/A]	Ca_11740	Aminoacyl-tRNA synthetase, class II (D/K/N)	SYNONYMOUS_CODING
12859	CakSNP12859	*Kabuli*	Ca_Kabuli_Chr07	29838073	[C/T]	Ca_11740	Aminoacyl-tRNA synthetase, class II (D/K/N)	NON_SYNONYMOUS_CODING
12860	CakSNP12860	*Kabuli*	Ca_Kabuli_Chr07	29848248	[T/C]	Ca_11739	Lipase, class 3	INTRON
12861	CakSNP12861	*Kabuli*	Ca_Kabuli_Chr07	29848433	[C/T]	Ca_11739	Lipase, class 3	SYNONYMOUS_CODING

S.N.	SNP IDs	Chickpea cultivars	Chromosomes	Physical positions (bp)	SNPs	Gene accession IDs	Putative functions	Sequence components of genome
12862	CakSNP12862	*Kabuli*	Ca_Kabuli_Chr07	29849509	[C/T]	Ca_11739	Lipase, class 3	NON_SYNONYMOUS_CODING
12863	CakSNP12863	*Kabuli*	Ca_Kabuli_Chr07	29849544	[A/G]	Ca_11739	Lipase, class 3	SYNONYMOUS_CODING
12864	CakSNP12864	*Kabuli*	Ca_Kabuli_Chr07	29884719	[G/T]	Ca_11736	Tetratricopeptide TPR-1	NON_SYNONYMOUS_CODING
12865	CakSNP12865	*Kabuli*	Ca_Kabuli_Chr07	29884718	[C/A]	Ca_11736	Tetratricopeptide TPR-1	SYNONYMOUS_CODING
12866	CakSNP12866	*Kabuli*	Ca_Kabuli_Chr07	29925008	[G/A]	Ca_11732	Disease resistance protein	NON_SYNONYMOUS_CODING
12867	CakSNP12867	*Kabuli*	Ca_Kabuli_Chr07	30004767	[C/T]		--	INTERGENIC
12868	CakSNP12868	*Kabuli*	Ca_Kabuli_Chr07	30004934	[A/C]		--	INTERGENIC
12869	CakSNP12869	*Kabuli*	Ca_Kabuli_Chr07	30026039	[T/G]	Ca_11727	Glycosyl transferase, family 2	INTRON
12870	CakSNP12870	*Kabuli*	Ca_Kabuli_Chr07	30026017	[C/T]	Ca_11727	Glycosyl transferase, family 2	INTRON
12871	CakSNP12871	*Kabuli*	Ca_Kabuli_Chr07	30029793	[A/C]	Ca_11726	Ribosomal protein S8e, conserved site	INTRON

S.N.	SNP IDs	Chickpea cultivars	Chromosomes	Physical positions (bp)	SNPs	Gene accession IDs	Putative functions	Sequence components of genome
12872	CakSNP12872	*Kabuli*	Ca_Kabuli_Chr07	30029820	[T/C]	Ca_11726	Ribosomal protein S8e, conserved site	SYNONYMOUS_CODING
12873	CakSNP12873	*Kabuli*	Ca_Kabuli_Chr07	30060299	[C/A]	Ca_11722	ATPase, P-type, K/Mg/Cd/Cu/Zn/Na/Ca/Na/H-transporter	SYNONYMOUS_CODING
12874	CakSNP12874	*Kabuli*	Ca_Kabuli_Chr07	30085847	[C/T]	Ca_11718	Domain of unknown function DUF828	NON_SYNONYMOUS_CODING
12875	CakSNP12875	*Kabuli*	Ca_Kabuli_Chr07	30085883	[T/G]	Ca_11718	Domain of unknown function DUF828	NON_SYNONYMOUS_CODING
12876	CakSNP12876	*Kabuli*	Ca_Kabuli_Chr07	30085906	[C/T]		--	INTERGENIC
12877	CakSNP12877	*Kabuli*	Ca_Kabuli_Chr07	30085910	[T/A]		--	INTERGENIC
12878	CakSNP12878	*Kabuli*	Ca_Kabuli_Chr07	30099003	[C/T]		--	INTERGENIC
12879	CakSNP12879	*Kabuli*	Ca_Kabuli_Chr07	30107340	[A/G]		--	INTERGENIC
12880	CakSNP12880	*Kabuli*	Ca_Kabuli_Chr07	30398855	[A/C]		--	INTERGENIC
12881	CakSNP12881	*Kabuli*	Ca_Kabuli_Chr07	30403615	[A/G]		--	INTERGENIC

S.N.	SNP IDs	Chickpea cultivars	Chromosomes	Physical positions (bp)	SNPs	Gene accession IDs	Putative functions	Sequence components of genome
12882	CakSNP12882	*Kabuli*	Ca_Kabuli_Chr07	30403618	[G/A]		--	INTERGENIC
12883	CakSNP12883	*Kabuli*	Ca_Kabuli_Chr07	30403700	[C/T]		--	INTERGENIC
12884	CakSNP12884	*Kabuli*	Ca_Kabuli_Chr07	30403692	[G/A]		--	INTERGENIC
12885	CakSNP12885	*Kabuli*	Ca_Kabuli_Chr07	30403621	[G/T]		--	INTERGENIC
12886	CakSNP12886	*Kabuli*	Ca_Kabuli_Chr07	30691324	[A/C]	Ca_23327	--	NON_SYNONYMOUS_CODING
12887	CakSNP12887	*Kabuli*	Ca_Kabuli_Chr07	30691318	[A/C]	Ca_23327	--	NON_SYNONYMOUS_CODING
12888	CakSNP12888	*Kabuli*	Ca_Kabuli_Chr07	30768315	[G/A]		--	INTERGENIC
12889	CakSNP12889	*Kabuli*	Ca_Kabuli_Chr07	30850950	[A/C]		--	INTERGENIC
12890	CakSNP12890	*Kabuli*	Ca_Kabuli_Chr07	31190059	[G/A]	Ca_10139	RNA polymerase II-associated, Paf1	SYNONYMOUS_CODING
12891	CakSNP12891	*Kabuli*	Ca_Kabuli_Chr07	31193786	[T/G]	Ca_10139	RNA polymerase II-associated, Paf1	SYNONYMOUS_CODING

S.N.	SNP IDs	Chickpea cultivars	Chromosomes	Physical positions (bp)	SNPs	Gene accession IDs	Putative functions	Sequence components of genome
12892	CakSNP12892	*Kabuli*	Ca_Kabuli_Chr07	31314421	[T/A]	Ca_10128	--	NON_SYNONYMOUS_CODING
12893	CakSNP12893	*Kabuli*	Ca_Kabuli_Chr07	31324279	[A/G]		--	INTERGENIC
12894	CakSNP12894	*Kabuli*	Ca_Kabuli_Chr07	31401807	[T/C]	Ca_10118	--	SYNONYMOUS_CODING
12895	CakSNP12895	*Kabuli*	Ca_Kabuli_Chr07	31442425	[A/C]	Ca_10114	PWWP	INTRON
12896	CakSNP12896	*Kabuli*	Ca_Kabuli_Chr07	31612697	[T/A]	Ca_10100	ABC transporter, transmembrane domain	NON_SYNONYMOUS_CODING
12897	CakSNP12897	*Kabuli*	Ca_Kabuli_Chr07	31639701	[C/T]	Ca_10099	Domain of unknown function DUF250	INTRON
12898	CakSNP12898	*Kabuli*	Ca_Kabuli_Chr07	31830238	[A/G]		--	INTERGENIC
12899	CakSNP12899	*Kabuli*	Ca_Kabuli_Chr07	31851096	[T/A]	Ca_10079	Prenyltransferase/squalene oxidase	NON_SYNONYMOUS_CODING
12900	CakSNP12900	*Kabuli*	Ca_Kabuli_Chr07	31873079	[G/T]		--	INTERGENIC
12901	CakSNP12901	*Kabuli*	Ca_Kabuli_Chr07	31996345	[A/C]		--	INTERGENIC

S.N.	SNP IDs	Chickpea cultivars	Chromosomes	Physical positions (bp)	SNPs	Gene accession IDs	Putative functions	Sequence components of genome
12902	CakSNP12902	*Kabuli*	Ca_Kabuli_Chr07	32017294	[G/A]		--	INTERGENIC
12903	CakSNP12903	*Kabuli*	Ca_Kabuli_Chr07	32017288	[G/T]		--	INTERGENIC
12904	CakSNP12904	*Kabuli*	Ca_Kabuli_Chr07	32017286	[T/G]		--	INTERGENIC
12905	CakSNP12905	*Kabuli*	Ca_Kabuli_Chr07	32017284	[C/T]		--	INTERGENIC
12906	CakSNP12906	*Kabuli*	Ca_Kabuli_Chr07	32017253	[G/A]		--	INTERGENIC
12907	CakSNP12907	*Kabuli*	Ca_Kabuli_Chr07	32017252	[G/A]		--	INTERGENIC
12908	CakSNP12908	*Kabuli*	Ca_Kabuli_Chr07	32017238	[C/T]		--	INTERGENIC
12909	CakSNP12909	*Kabuli*	Ca_Kabuli_Chr07	32017228	[G/A]		--	INTERGENIC
12910	CakSNP12910	*Kabuli*	Ca_Kabuli_Chr07	32017221	[C/T]		--	INTERGENIC
12911	CakSNP12911	*Kabuli*	Ca_Kabuli_Chr07	32017211	[C/A]		--	INTERGENIC

S.N.	SNP IDs	Chickpea cultivars	Chromosomes	Physical positions (bp)	SNPs	Gene accession IDs	Putative functions	Sequence components of genome
12912	CakSNP12912	*Kabuli*	Ca_Kabuli_Chr07	32127288	[T/A]	Ca_10057	Lipoxygenase	SYNONYMOUS_CODING
12913	CakSNP12913	*Kabuli*	Ca_Kabuli_Chr07	32283585	[T/G]	Ca_10045	von Willebrand factor, type A	INTRON
12914	CakSNP12914	*Kabuli*	Ca_Kabuli_Chr07	32309397	[A/T]	Ca_10045	von Willebrand factor, type A	INTRON
12915	CakSNP12915	*Kabuli*	Ca_Kabuli_Chr07	32309411	[G/T]	Ca_10045	von Willebrand factor, type A	INTRON
12916	CakSNP12916	*Kabuli*	Ca_Kabuli_Chr07	32309415	[C/T]	Ca_10045	von Willebrand factor, type A	INTRON
12917	CakSNP12917	*Kabuli*	Ca_Kabuli_Chr07	32309429	[C/T]	Ca_10045	von Willebrand factor, type A	INTRON
12918	CakSNP12918	*Kabuli*	Ca_Kabuli_Chr07	32309377	[C/T]	Ca_10045	von Willebrand factor, type A	INTRON
12919	CakSNP12919	*Kabuli*	Ca_Kabuli_Chr07	32309376	[C/G]	Ca_10045	von Willebrand factor, type A	INTRON
12920	CakSNP12920	*Kabuli*	Ca_Kabuli_Chr07	32399759	[C/T]	Ca_10038	LsmAD domain	SYNONYMOUS_CODING
12921	CakSNP12921	*Kabuli*	Ca_Kabuli_Chr07	32404932	[T/C]		--	INTERGENIC

S.N.	SNP IDs	Chickpea cultivars	Chromosomes	Physical positions (bp)	SNPs	Gene accession IDs	Putative functions	Sequence components of genome
12922	CakSNP12922	*Kabuli*	Ca_Kabuli_Chr07	32451377	[A/C]	Ca_10034	Protein of unknown function DUF1296	INTRON
12923	CakSNP12923	*Kabuli*	Ca_Kabuli_Chr07	32522201	[A/G]	Ca_10028	SANT domain, DNA binding	SYNONYMOUS_CODING
12924	CakSNP12924	*Kabuli*	Ca_Kabuli_Chr07	32522203	[A/G]	Ca_10028	SANT domain, DNA binding	NON_SYNONYMOUS_CODING
12925	CakSNP12925	*Kabuli*	Ca_Kabuli_Chr07	32522207	[T/G]	Ca_10028	SANT domain, DNA binding	SYNONYMOUS_CODING
12926	CakSNP12926	*Kabuli*	Ca_Kabuli_Chr07	32522213	[A/G]	Ca_10028	SANT domain, DNA binding	SYNONYMOUS_CODING
12927	CakSNP12927	*Kabuli*	Ca_Kabuli_Chr07	32522230	[T/G]	Ca_10028	SANT domain, DNA binding	NON_SYNONYMOUS_CODING
12928	CakSNP12928	*Kabuli*	Ca_Kabuli_Chr07	32557599	[G/A]		--	INTERGENIC
12929	CakSNP12929	*Kabuli*	Ca_Kabuli_Chr07	32669570	[A/G]		--	INTERGENIC
12930	CakSNP12930	*Kabuli*	Ca_Kabuli_Chr07	32754633	[T/G]	Ca_10017	--	INTRON
12931	CakSNP12931	*Kabuli*	Ca_Kabuli_Chr07	32755647	[A/C]	Ca_10017	--	SYNONYMOUS_CODING

S.N.	SNP IDs	Chickpea cultivars	Chromosomes	Physical positions (bp)	SNPs	Gene accession IDs	Putative functions	Sequence components of genome
12932	CakSNP12932	*Kabuli*	Ca_Kabuli_Chr07	32755662	[G/T]	Ca_10017	--	NON_SYNONYMOUS_CODING
12933	CakSNP12933	*Kabuli*	Ca_Kabuli_Chr07	32784903	[T/G]	Ca_10015	Transcription initiation factor TFIID	SYNONYMOUS_CODING
12934	CakSNP12934	*Kabuli*	Ca_Kabuli_Chr07	32799037	[A/G]		--	INTERGENIC
12935	CakSNP12935	*Kabuli*	Ca_Kabuli_Chr07	32807457	[T/A]		--	INTERGENIC
12936	CakSNP12936	*Kabuli*	Ca_Kabuli_Chr07	32807499	[A/G]		--	INTERGENIC
12937	CakSNP12937	*Kabuli*	Ca_Kabuli_Chr07	32874847	[C/T]		--	INTERGENIC
12938	CakSNP12938	*Kabuli*	Ca_Kabuli_Chr07	32874840	[C/T]		--	INTERGENIC
12939	CakSNP12939	*Kabuli*	Ca_Kabuli_Chr07	32875799	[T/A]	Ca_10011	Germin	SYNONYMOUS_CODING
12940	CakSNP12940	*Kabuli*	Ca_Kabuli_Chr07	32942199	[A/G]	Ca_10004	Transcription factor GRAS	NON_SYNONYMOUS_CODING
12941	CakSNP12941	*Kabuli*	Ca_Kabuli_Chr07	32942951	[T/G]	Ca_10004	Transcription factor GRAS	SYNONYMOUS_CODING

S.N.	SNP IDs	Chickpea cultivars	Chromosomes	Physical positions (bp)	SNPs	Gene accession IDs	Putative functions	Sequence components of genome
12942	CakSNP12942	*Kabuli*	Ca_Kabuli_Chr07	32989796	[T/A]	Ca_10000	Protein kinase, catalytic domain	SYNONYMOUS_CODING
12943	CakSNP12943	*Kabuli*	Ca_Kabuli_Chr07	32989838	[C/T]	Ca_10000	Protein kinase, catalytic domain	SYNONYMOUS_CODING
12944	CakSNP12944	*Kabuli*	Ca_Kabuli_Chr07	32989903	[A/G]	Ca_10000	Protein kinase, catalytic domain	INTRON
12945	CakSNP12945	*Kabuli*	Ca_Kabuli_Chr07	32989902	[C/A]	Ca_10000	Protein kinase, catalytic domain	INTRON
12946	CakSNP12946	*Kabuli*	Ca_Kabuli_Chr07	33065611	[G/T]		--	INTERGENIC
12947	CakSNP12947	*Kabuli*	Ca_Kabuli_Chr07	33121674	[G/C]	Ca_16143	Peptidase S8/S53, subtilisin/kexin/sedolisin	NON_SYNONYMOUS_CODING
12948	CakSNP12948	*Kabuli*	Ca_Kabuli_Chr07	33121640	[C/A]	Ca_16143	Peptidase S8/S53, subtilisin/kexin/sedolisin	SYNONYMOUS_CODING
12949	CakSNP12949	*Kabuli*	Ca_Kabuli_Chr07	33122473	[A/C]	Ca_16143	Peptidase S8/S53, subtilisin/kexin/sedolisin	SYNONYMOUS_CODING
12950	CakSNP12950	*Kabuli*	Ca_Kabuli_Chr07	33182932	[G/A]	Ca_16149	Lipase, class 3	SYNONYMOUS_CODING
12951	CakSNP12951	*Kabuli*	Ca_Kabuli_Chr07	33267485	[T/C]		--	INTERGENIC

S.N.	SNP IDs	Chickpea cultivars	Chromosomes	Physical positions (bp)	SNPs	Gene accession IDs	Putative functions	Sequence components of genome
12952	CakSNP12952	*Kabuli*	Ca_Kabuli_Chr07	33267496	[T/A]		--	INTERGENIC
12953	CakSNP12953	*Kabuli*	Ca_Kabuli_Chr07	33335978	[C/A]	Ca_16158	RNA recognition motif domain	NON_SYNONYMOUS_CODING
12954	CakSNP12954	*Kabuli*	Ca_Kabuli_Chr07	33364329	[C/T]	Ca_16162	--	INTRON
12955	CakSNP12955	*Kabuli*	Ca_Kabuli_Chr07	33364354	[G/A]	Ca_16162	--	INTRON
12956	CakSNP12956	*Kabuli*	Ca_Kabuli_Chr07	33378427	[C/T]	Ca_16163	Spt20 family	INTRON
12957	CakSNP12957	*Kabuli*	Ca_Kabuli_Chr07	33411746	[C/A]	Ca_16164	Zinc finger, CCCH-type	INTRON
12958	CakSNP12958	*Kabuli*	Ca_Kabuli_Chr07	33465828	[C/A]	Ca_16168	--	SYNONYMOUS_CODING
12959	CakSNP12959	*Kabuli*	Ca_Kabuli_Chr07	33465818	[G/A]	Ca_16168	--	SYNONYMOUS_CODING
12960	CakSNP12960	*Kabuli*	Ca_Kabuli_Chr07	33465805	[T/C]	Ca_16168	--	SYNONYMOUS_CODING
12961	CakSNP12961	*Kabuli*	Ca_Kabuli_Chr07	33465802	[C/A]	Ca_16168	--	SYNONYMOUS_CODING

S.N.	SNP IDs	Chickpea cultivars	Chromosomes	Physical positions (bp)	SNPs	Gene accession IDs	Putative functions	Sequence components of genome
12962	CakSNP12962	*Kabuli*	Ca_Kabuli_Chr07	33465800	[T/A]	Ca_16168	--	SYNONYMOUS_CODING
12963	CakSNP12963	*Kabuli*	Ca_Kabuli_Chr07	33465781	[G/T]	Ca_16168	--	NON_SYNONYMOUS_CODING
12964	CakSNP12964	*Kabuli*	Ca_Kabuli_Chr07	33465855	[A/C]	Ca_16168	--	SYNONYMOUS_CODING
12965	CakSNP12965	*Kabuli*	Ca_Kabuli_Chr07	33480940	[C/T]		--	INTERGENIC
12966	CakSNP12966	*Kabuli*	Ca_Kabuli_Chr07	33573605	[C/T]	Ca_16177	CCAAT-binding factor	SYNONYMOUS_CODING
12967	CakSNP12967	*Kabuli*	Ca_Kabuli_Chr07	33573655	[A/C]	Ca_16177	CCAAT-binding factor	NON_SYNONYMOUS_CODING
12968	CakSNP12968	*Kabuli*	Ca_Kabuli_Chr07	33619268	[G/C]		--	INTERGENIC
12969	CakSNP12969	*Kabuli*	Ca_Kabuli_Chr07	33641799	[G/T]	Ca_16180	Pathogenesis-related transcriptional factor/ERF, DNA-binding	SYNONYMOUS_CODING
12970	CakSNP12970	*Kabuli*	Ca_Kabuli_Chr07	33641882	[A/T]	Ca_16180	Pathogenesis-related transcriptional factor/ERF, DNA-binding	INTRON
12971	CakSNP12971	*Kabuli*	Ca_Kabuli_Chr07	33674454	[T/C]	Ca_16181	Peptidase S8/S53, subtilisin/kexin/sedolisin	SYNONYMOUS_CODING

S.N.	SNP IDs	Chickpea cultivars	Chromosomes	Physical positions (bp)	SNPs	Gene accession IDs	Putative functions	Sequence components of genome
12972	CakSNP12972	*Kabuli*	Ca_Kabuli_Chr07	33744521	[C/G]	Ca_16182	ARID/BRIGHT DNA-binding domain	INTRON
12973	CakSNP12973	*Kabuli*	Ca_Kabuli_Chr07	33753917	[G/A]	Ca_16183	Initiation factor 2B-related	SYNONYMOUS_CODING
12974	CakSNP12974	*Kabuli*	Ca_Kabuli_Chr07	33761944	[G/A]	Ca_16184	--	SYNONYMOUS_CODING
12975	CakSNP12975	*Kabuli*	Ca_Kabuli_Chr07	33762053	[T/G]	Ca_16184	--	NON_SYNONYMOUS_CODING
12976	CakSNP12976	*Kabuli*	Ca_Kabuli_Chr07	33821658	[C/T]	Ca_16190	1-aminocyclopropane-1-carboxylate synthase	INTRON
12977	CakSNP12977	*Kabuli*	Ca_Kabuli_Chr07	33865463	[T/C]	Ca_16193	Exocyst complex component Sec10	INTRON
12978	CakSNP12978	*Kabuli*	Ca_Kabuli_Chr07	33865475	[G/A]	Ca_16193	Exocyst complex component Sec10	INTRON
12979	CakSNP12979	*Kabuli*	Ca_Kabuli_Chr07	33867334	[C/T]	Ca_16193	Exocyst complex component Sec10	INTRON
12980	CakSNP12980	*Kabuli*	Ca_Kabuli_Chr07	33867491	[C/T]	Ca_16193	Exocyst complex component Sec10	INTRON
12981	CakSNP12981	*Kabuli*	Ca_Kabuli_Chr07	33867674	[C/T]	Ca_16193	Exocyst complex component Sec10	INTRON

S.N.	SNP IDs	Chickpea cultivars	Chromosomes	Physical positions (bp)	SNPs	Gene accession IDs	Putative functions	Sequence components of genome
12982	CakSNP12982	*Kabuli*	Ca_Kabuli_Chr07	33869367	[G/A]	Ca_16193	Exocyst complex component Sec10	SYNONYMOUS_CODING
12983	CakSNP12983	*Kabuli*	Ca_Kabuli_Chr07	33879559	[C/G]	Ca_16194	Ubiquitin-conjugating enzyme, E2	INTRON
12984	CakSNP12984	*Kabuli*	Ca_Kabuli_Chr07	33910324	[C/A]		--	INTERGENIC
12985	CakSNP12985	*Kabuli*	Ca_Kabuli_Chr07	33910870	[C/A]		--	INTERGENIC
12986	CakSNP12986	*Kabuli*	Ca_Kabuli_Chr07	33912719	[T/G]		--	INTERGENIC
12987	CakSNP12987	*Kabuli*	Ca_Kabuli_Chr07	33912766	[G/T]		--	INTERGENIC
12988	CakSNP12988	*Kabuli*	Ca_Kabuli_Chr07	33912866	[T/C]		--	INTERGENIC
12989	CakSNP12989	*Kabuli*	Ca_Kabuli_Chr07	33912899	[T/A]		--	INTERGENIC
12990	CakSNP12990	*Kabuli*	Ca_Kabuli_Chr07	33912902	[A/T]		--	INTERGENIC
12991	CakSNP12991	*Kabuli*	Ca_Kabuli_Chr07	33912913	[A/T]		--	INTERGENIC

S.N.	SNP IDs	Chickpea cultivars	Chromosomes	Physical positions (bp)	SNPs	Gene accession IDs	Putative functions	Sequence components of genome
12992	CakSNP12992	*Kabuli*	Ca_Kabuli_Chr07	33912916	[G/T]		--	INTERGENIC
12993	CakSNP12993	*Kabuli*	Ca_Kabuli_Chr07	33912921	[T/G]		--	INTERGENIC
12994	CakSNP12994	*Kabuli*	Ca_Kabuli_Chr07	33912950	[C/T]		--	INTERGENIC
12995	CakSNP12995	*Kabuli*	Ca_Kabuli_Chr07	33912966	[T/C]		--	INTERGENIC
12996	CakSNP12996	*Kabuli*	Ca_Kabuli_Chr07	33913094	[A/G]		--	INTERGENIC
12997	CakSNP12997	*Kabuli*	Ca_Kabuli_Chr07	33913047	[C/T]		--	INTERGENIC
12998	CakSNP12998	*Kabuli*	Ca_Kabuli_Chr07	33913045	[G/T]		--	INTERGENIC
12999	CakSNP12999	*Kabuli*	Ca_Kabuli_Chr07	34107603	[C/A]		--	INTERGENIC
13000	CakSNP13000	*Kabuli*	Ca_Kabuli_Chr07	34152124	[G/C]	Ca_21006	DNA-directed RNA polymerase, subunit 2, domain 6	NON_SYNONYMOUS_CODING
13001	CakSNP13001	*Kabuli*	Ca_Kabuli_Chr07	34271486	[A/G]	Ca_20992	Pentatricopeptide repeat	NON_SYNONYMOUS_CODING

S.N.	SNP IDs	Chickpea cultivars	Chromosomes	Physical positions (bp)	SNPs	Gene accession IDs	Putative functions	Sequence components of genome
13002	CakSNP13002	*Kabuli*	Ca_Kabuli_Chr07	34277014	[G/T]		--	INTERGENIC
13003	CakSNP13003	*Kabuli*	Ca_Kabuli_Chr07	34277010	[A/G]		--	INTERGENIC
13004	CakSNP13004	*Kabuli*	Ca_Kabuli_Chr07	34279673	[G/A]	Ca_20990	Brix domain	INTRON
13005	CakSNP13005	*Kabuli*	Ca_Kabuli_Chr07	34279725	[A/G]	Ca_20990	Brix domain	INTRON
13006	CakSNP13006	*Kabuli*	Ca_Kabuli_Chr07	34279715	[A/C]	Ca_20990	Brix domain	INTRON
13007	CakSNP13007	*Kabuli*	Ca_Kabuli_Chr07	34318860	[C/T]		--	INTERGENIC
13008	CakSNP13008	*Kabuli*	Ca_Kabuli_Chr07	34318862	[A/G]		--	INTERGENIC
13009	CakSNP13009	*Kabuli*	Ca_Kabuli_Chr07	34474614	[A/C]	Ca_17602	SSXT	NON_SYNONYMOUS_CODING
13010	CakSNP13010	*Kabuli*	Ca_Kabuli_Chr07	34801839	[A/T]	Ca_17614	--	SYNONYMOUS_CODING
13011	CakSNP13011	*Kabuli*	Ca_Kabuli_Chr07	34857693	[C/T]	Ca_17617	Armadillo	NON_SYNONYMOUS_CODING

S.N.	SNP IDs	Chickpea cultivars	Chromosomes	Physical positions (bp)	SNPs	Gene accession IDs	Putative functions	Sequence components of genome
13012	CakSNP13012	*Kabuli*	Ca_Kabuli_Chr07	34947743	[A/C]	Ca_17626	Phosphoribosyltransferase	INTRON
13013	CakSNP13013	*Kabuli*	Ca_Kabuli_Chr07	35016516	[G/C]	Ca_17629	Multicopper oxidase, type 1	NON_SYNONYMOUS_CODING
13014	CakSNP13014	*Kabuli*	Ca_Kabuli_Chr07	35072425	[A/T]		--	INTERGENIC
13015	CakSNP13015	*Kabuli*	Ca_Kabuli_Chr07	35072407	[G/A]		--	INTERGENIC
13016	CakSNP13016	*Kabuli*	Ca_Kabuli_Chr07	35072461	[A/T]		--	INTERGENIC
13017	CakSNP13017	*Kabuli*	Ca_Kabuli_Chr07	35074345	[T/A]		--	INTERGENIC
13018	CakSNP13018	*Kabuli*	Ca_Kabuli_Chr07	35074348	[T/C]		--	INTERGENIC
13019	CakSNP13019	*Kabuli*	Ca_Kabuli_Chr07	35074362	[T/C]		--	INTERGENIC
13020	CakSNP13020	*Kabuli*	Ca_Kabuli_Chr07	35112785	[T/C]	Ca_17637	Importin-beta, N-terminal	SYNONYMOUS_CODING
13021	CakSNP13021	*Kabuli*	Ca_Kabuli_Chr07	35114049	[C/T]	Ca_17637	Importin-beta, N-terminal	NON_SYNONYMOUS_CODING

S.N.	SNP IDs	Chickpea cultivars	Chromosomes	Physical positions (bp)	SNPs	Gene accession IDs	Putative functions	Sequence components of genome
13022	CakSNP13022	*Kabuli*	Ca_Kabuli_Chr07	35134809	[A/C]	Ca_17638	Pathogenesis-related transcriptional factor/ERF, DNA-binding	NON_SYNONYMOUS_CODING
13023	CakSNP13023	*Kabuli*	Ca_Kabuli_Chr07	35134863	[T/C]	Ca_17638	Pathogenesis-related transcriptional factor/ERF, DNA-binding	SYNONYMOUS_CODING
13024	CakSNP13024	*Kabuli*	Ca_Kabuli_Chr07	35181619	[T/G]	Ca_17639	Helix-loop-helix DNA-binding domain	NON_SYNONYMOUS_CODING
13025	CakSNP13025	*Kabuli*	Ca_Kabuli_Chr07	35181661	[T/C]	Ca_17639	Helix-loop-helix DNA-binding domain	SYNONYMOUS_CODING
13026	CakSNP13026	*Kabuli*	Ca_Kabuli_Chr07	35181660	[G/A]	Ca_17639	Helix-loop-helix DNA-binding domain	NON_SYNONYMOUS_CODING
13027	CakSNP13027	*Kabuli*	Ca_Kabuli_Chr07	35181635	[G/C]	Ca_17639	Helix-loop-helix DNA-binding domain	NON_SYNONYMOUS_CODING
13028	CakSNP13028	*Kabuli*	Ca_Kabuli_Chr07	35227794	[A/C]	Ca_17641	--	SYNONYMOUS_CODING
13029	CakSNP13029	*Kabuli*	Ca_Kabuli_Chr07	35258029	[T/G]	Ca_17644	Dynamin central domain	INTRON
13030	CakSNP13030	*Kabuli*	Ca_Kabuli_Chr07	35263711	[A/T]		--	INTERGENIC
13031	CakSNP13031	*Kabuli*	Ca_Kabuli_Chr07	35263712	[G/T]		--	INTERGENIC

S.N.	SNP IDs	Chickpea cultivars	Chromosomes	Physical positions (bp)	SNPs	Gene accession IDs	Putative functions	Sequence components of genome
13032	CakSNP13032	*Kabuli*	Ca_Kabuli_Chr07	35263736	[C/G]		--	INTERGENIC
13033	CakSNP13033	*Kabuli*	Ca_Kabuli_Chr07	35263758	[A/G]		--	INTERGENIC
13034	CakSNP13034	*Kabuli*	Ca_Kabuli_Chr07	35274368	[A/G]		--	INTERGENIC
13035	CakSNP13035	*Kabuli*	Ca_Kabuli_Chr07	35274859	[T/G]		--	INTERGENIC
13036	CakSNP13036	*Kabuli*	Ca_Kabuli_Chr07	35274858	[G/A]		--	INTERGENIC
13037	CakSNP13037	*Kabuli*	Ca_Kabuli_Chr07	35274828	[A/G]		--	INTERGENIC
13038	CakSNP13038	*Kabuli*	Ca_Kabuli_Chr07	35279675	[A/G]	Ca_17646	--	NON_SYNONYMOUS_CODING
13039	CakSNP13039	*Kabuli*	Ca_Kabuli_Chr07	35280189	[G/A]	Ca_17646	--	SYNONYMOUS_CODING
13040	CakSNP13040	*Kabuli*	Ca_Kabuli_Chr07	35280167	[A/G]	Ca_17646	--	SYNONYMOUS_CODING
13041	CakSNP13041	*Kabuli*	Ca_Kabuli_Chr07	35576427	[C/A]	Ca_17765	Reticulon	NON_SYNONYMOUS_CODING

S.N.	SNP IDs	Chickpea cultivars	Chromosomes	Physical positions (bp)	SNPs	Gene accession IDs	Putative functions	Sequence components of genome
13042	CakSNP13042	*Kabuli*	Ca_Kabuli_Chr07	35619530	[G/A]		--	INTERGENIC
13043	CakSNP13043	*Kabuli*	Ca_Kabuli_Chr07	35619487	[G/A]		--	INTERGENIC
13044	CakSNP13044	*Kabuli*	Ca_Kabuli_Chr07	35619518	[G/C]		--	INTERGENIC
13045	CakSNP13045	*Kabuli*	Ca_Kabuli_Chr07	35622168	[A/G]	Ca_17769	Ketopantoate hydroxymethyltransferase	NON_SYNONYMOUS_CODING
13046	CakSNP13046	*Kabuli*	Ca_Kabuli_Chr07	35637056	[T/C]	Ca_17772	--	INTRON
13047	CakSNP13047	*Kabuli*	Ca_Kabuli_Chr07	35680423	[A/C]		--	INTERGENIC
13048	CakSNP13048	*Kabuli*	Ca_Kabuli_Chr07	35799096	[A/C]	Ca_17781	BTB/POZ-like	NON_SYNONYMOUS_CODING
13049	CakSNP13049	*Kabuli*	Ca_Kabuli_Chr07	35799083	[T/A]	Ca_17781	BTB/POZ-like	SYNONYMOUS_CODING
13050	CakSNP13050	*Kabuli*	Ca_Kabuli_Chr07	35799074	[G/C]	Ca_17781	BTB/POZ-like	NON_SYNONYMOUS_CODING
13051	CakSNP13051	*Kabuli*	Ca_Kabuli_Chr07	35799073	[T/C]	Ca_17781	BTB/POZ-like	NON_SYNONYMOUS_CODING

S.N.	SNP IDs	Chickpea cultivars	Chromosomes	Physical positions (bp)	SNPs	Gene accession IDs	Putative functions	Sequence components of genome
13052	CakSNP13052	*Kabuli*	Ca_Kabuli_Chr07	35807000	[A/G]		--	INTERGENIC
13053	CakSNP13053	*Kabuli*	Ca_Kabuli_Chr07	35806999	[C/G]		--	INTERGENIC
13054	CakSNP13054	*Kabuli*	Ca_Kabuli_Chr07	35806990	[A/T]		--	INTERGENIC
13055	CakSNP13055	*Kabuli*	Ca_Kabuli_Chr07	35806977	[T/G]		--	INTERGENIC
13056	CakSNP13056	*Kabuli*	Ca_Kabuli_Chr07	35911620	[T/G]		--	INTERGENIC
13057	CakSNP13057	*Kabuli*	Ca_Kabuli_Chr07	35911617	[A/C]		--	INTERGENIC
13058	CakSNP13058	*Kabuli*	Ca_Kabuli_Chr07	35911615	[C/T]		--	INTERGENIC
13059	CakSNP13059	*Kabuli*	Ca_Kabuli_Chr07	35911612	[T/G]		--	INTERGENIC
13060	CakSNP13060	*Kabuli*	Ca_Kabuli_Chr07	35911606	[T/C]		--	INTERGENIC
13061	CakSNP13061	*Kabuli*	Ca_Kabuli_Chr07	35947529	[T/C]	Ca_17800	Transposase, Ptta/En/Spm, plant	NON_SYNONYMOUS_CODING

S.N.	SNP IDs	Chickpea cultivars	Chromosomes	Physical positions (bp)	SNPs	Gene accession IDs	Putative functions	Sequence components of genome
13062	CakSNP13062	*Kabuli*	Ca_Kabuli_Chr07	35947550	[C/T]	Ca_17800	Transposase, Ptta/En/Spm, plant	NON_SYNONYMOUS_CODING
13063	CakSNP13063	*Kabuli*	Ca_Kabuli_Chr07	35947551	[A/G]	Ca_17800	Transposase, Ptta/En/Spm, plant	SYNONYMOUS_CODING
13064	CakSNP13064	*Kabuli*	Ca_Kabuli_Chr07	35959805	[C/T]		--	INTERGENIC
13065	CakSNP13065	*Kabuli*	Ca_Kabuli_Chr07	35959825	[G/A]		--	INTERGENIC
13066	CakSNP13066	*Kabuli*	Ca_Kabuli_Chr07	35959878	[G/A]		--	INTERGENIC
13067	CakSNP13067	*Kabuli*	Ca_Kabuli_Chr07	35959917	[C/T]		--	INTERGENIC
13068	CakSNP13068	*Kabuli*	Ca_Kabuli_Chr07	35959865	[T/C]		--	INTERGENIC
13069	CakSNP13069	*Kabuli*	Ca_Kabuli_Chr07	36711954	[C/A]	Ca_13756	Mediator complex, subunit Med12	NON_SYNONYMOUS_CODING
13070	CakSNP13070	*Kabuli*	Ca_Kabuli_Chr07	36715249	[T/C]	Ca_13756	Mediator complex, subunit Med12	SYNONYMOUS_CODING
13071	CakSNP13071	*Kabuli*	Ca_Kabuli_Chr07	36745439	[T/G]		--	INTERGENIC

S.N.	SNP IDs	Chickpea cultivars	Chromosomes	Physical positions (bp)	SNPs	Gene accession IDs	Putative functions	Sequence components of genome
13072	CakSNP13072	*Kabuli*	Ca_Kabuli_Chr07	36916272	[T/G]		--	INTERGENIC
13073	CakSNP13073	*Kabuli*	Ca_Kabuli_Chr07	36916273	[T/A]		--	INTERGENIC
13074	CakSNP13074	*Kabuli*	Ca_Kabuli_Chr07	36998096	[T/C]	Ca_13784	Peptidase C48, SUMO/Sentrin/Ubl1	SYNONYMOUS_CODING
13075	CakSNP13075	*Kabuli*	Ca_Kabuli_Chr07	36998113	[C/T]	Ca_13784	Peptidase C48, SUMO/Sentrin/Ubl1	SYNONYMOUS_CODING
13076	CakSNP13076	*Kabuli*	Ca_Kabuli_Chr07	37058979	[C/T]	Ca_13790	Hydroxymethylglutaryl-CoA lyase, active site	NON_SYNONYMOUS_CODING
13077	CakSNP13077	*Kabuli*	Ca_Kabuli_Chr07	37187439	[A/G]	Ca_13800	Short-chain dehydrogenase/reductase SDR	SYNONYMOUS_CODING
13078	CakSNP13078	*Kabuli*	Ca_Kabuli_Chr07	37626729	[G/T]	Ca_22806	Transposase, Ptta/En/Spm, plant	NON_SYNONYMOUS_CODING
13079	CakSNP13079	*Kabuli*	Ca_Kabuli_Chr07	37626653	[A/C]	Ca_22806	Transposase, Ptta/En/Spm, plant	NON_SYNONYMOUS_CODING
13080	CakSNP13080	*Kabuli*	Ca_Kabuli_Chr07	37626687	[A/G]	Ca_22806	Transposase, Ptta/En/Spm, plant	NON_SYNONYMOUS_CODING
13081	CakSNP13081	*Kabuli*	Ca_Kabuli_Chr07	37626666	[C/T]	Ca_22806	Transposase, Ptta/En/Spm, plant	NON_SYNONYMOUS_CODING

S.N.	SNP IDs	Chickpea cultivars	Chromosomes	Physical positions (bp)	SNPs	Gene accession IDs	Putative functions	Sequence components of genome
13082	CakSNP13082	*Kabuli*	Ca_Kabuli_Chr07	37703967	[G/A]		--	INTERGENIC
13083	CakSNP13083	*Kabuli*	Ca_Kabuli_Chr07	37703966	[C/T]		--	INTERGENIC
13084	CakSNP13084	*Kabuli*	Ca_Kabuli_Chr07	37703965	[C/T]		--	INTERGENIC
13085	CakSNP13085	*Kabuli*	Ca_Kabuli_Chr07	37703921	[G/T]		--	INTERGENIC
13086	CakSNP13086	*Kabuli*	Ca_Kabuli_Chr07	37703912	[G/A]		--	INTERGENIC
13087	CakSNP13087	*Kabuli*	Ca_Kabuli_Chr07	37703913	[C/G]		--	INTERGENIC
13088	CakSNP13088	*Kabuli*	Ca_Kabuli_Chr07	37703936	[C/T]		--	INTERGENIC
13089	CakSNP13089	*Kabuli*	Ca_Kabuli_Chr07	37857936	[G/T]	Ca_22787	Peptidase C48, SUMO/Sentrin/Ubl1	NON_SYNONYMOUS_CODING
13090	CakSNP13090	*Kabuli*	Ca_Kabuli_Chr07	37857928	[A/T]	Ca_22787	Peptidase C48, SUMO/Sentrin/Ubl1	SYNONYMOUS_CODING
13091	CakSNP13091	*Kabuli*	Ca_Kabuli_Chr07	37857917	[G/A]	Ca_22787	Peptidase C48, SUMO/Sentrin/Ubl1	NON_SYNONYMOUS_CODING

S.N.	SNP IDs	Chickpea cultivars	Chromosomes	Physical positions (bp)	SNPs	Gene accession IDs	Putative functions	Sequence components of genome
13092	CakSNP13092	*Kabuli*	Ca_Kabuli_Chr07	37857885	[C/T]	Ca_22787	Peptidase C48, SUMO/Sentrin/Ubl1	NON_SYNONYMOUS_CODING
13093	CakSNP13093	*Kabuli*	Ca_Kabuli_Chr07	38047374	[A/G]		--	INTERGENIC
13094	CakSNP13094	*Kabuli*	Ca_Kabuli_Chr07	38047378	[C/A]		--	INTERGENIC
13095	CakSNP13095	*Kabuli*	Ca_Kabuli_Chr07	38143361	[A/G]	Ca_16396	RNA recognition motif domain	INTRON
13096	CakSNP13096	*Kabuli*	Ca_Kabuli_Chr07	38143524	[T/A]	Ca_16396	RNA recognition motif domain	INTRON
13097	CakSNP13097	*Kabuli*	Ca_Kabuli_Chr07	38143452	[G/A]	Ca_16396	RNA recognition motif domain	INTRON
13098	CakSNP13098	*Kabuli*	Ca_Kabuli_Chr07	38460464	[G/A]		--	INTERGENIC
13099	CakSNP13099	*Kabuli*	Ca_Kabuli_Chr07	38460452	[C/T]		--	INTERGENIC
13100	CakSNP13100	*Kabuli*	Ca_Kabuli_Chr07	38460425	[G/A]		--	INTERGENIC
13101	CakSNP13101	*Kabuli*	Ca_Kabuli_Chr07	38460394	[C/A]		--	INTERGENIC

S.N.	SNP IDs	Chickpea cultivars	Chromosomes	Physical positions (bp)	SNPs	Gene accession IDs	Putative functions	Sequence components of genome
13102	CakSNP13102	*Kabuli*	Ca_Kabuli_Chr07	39181880	[G/A]	Ca_19581	BEACH domain	NON_SYNONYMOUS_CODING
13103	CakSNP13103	*Kabuli*	Ca_Kabuli_Chr07	39182564	[A/T]	Ca_19581	BEACH domain	NON_SYNONYMOUS_CODING
13104	CakSNP13104	*Kabuli*	Ca_Kabuli_Chr07	39339510	[T/G]	Ca_19588	WRC	NON_SYNONYMOUS_CODING
13105	CakSNP13105	*Kabuli*	Ca_Kabuli_Chr07	39339509	[G/A]	Ca_19588	WRC	SYNONYMOUS_CODING
13106	CakSNP13106	*Kabuli*	Ca_Kabuli_Chr07	39339508	[G/A]	Ca_19588	WRC	NON_SYNONYMOUS_CODING
13107	CakSNP13107	*Kabuli*	Ca_Kabuli_Chr07	39339503	[C/A]	Ca_19588	WRC	SYNONYMOUS_CODING
13108	CakSNP13108	*Kabuli*	Ca_Kabuli_Chr07	39339502	[C/A]	Ca_19588	WRC	NON_SYNONYMOUS_CODING
13109	CakSNP13109	*Kabuli*	Ca_Kabuli_Chr07	39339501	[G/C]	Ca_19588	WRC	NON_SYNONYMOUS_CODING
13110	CakSNP13110	*Kabuli*	Ca_Kabuli_Chr07	39339500	[G/T]	Ca_19588	WRC	SYNONYMOUS_CODING
13111	CakSNP13111	*Kabuli*	Ca_Kabuli_Chr07	39339499	[C/T]	Ca_19588	WRC	NON_SYNONYMOUS_CODING

S.N.	SNP IDs	Chickpea cultivars	Chromosomes	Physical positions (bp)	SNPs	Gene accession IDs	Putative functions	Sequence components of genome
13112	CakSNP13112	*Kabuli*	Ca_Kabuli_Chr07	39339498	[G/T]	Ca_19588	WRC	NON_SYNONYMOUS_CODING
13113	CakSNP13113	*Kabuli*	Ca_Kabuli_Chr07	39339495	[G/T]	Ca_19588	WRC	NON_SYNONYMOUS_CODING
13114	CakSNP13114	*Kabuli*	Ca_Kabuli_Chr07	39399280	[T/C]	Ca_19592	Domain of unknown function DUF296	SYNONYMOUS_CODING
13115	CakSNP13115	*Kabuli*	Ca_Kabuli_Chr07	39680221	[A/C]	Ca_19607	Domain of unknown function DUF296	INTRON
13116	CakSNP13116	*Kabuli*	Ca_Kabuli_Chr07	39680222	[G/A]	Ca_19607	Domain of unknown function DUF296	INTRON
13117	CakSNP13117	*Kabuli*	Ca_Kabuli_Chr07	39768248	[T/C]	Ca_25098	Zinc finger, CCCH-type	SYNONYMOUS_CODING
13118	CakSNP13118	*Kabuli*	Ca_Kabuli_Chr07	39944444	[T/C]	Ca_25106	Mitochondrial carrier protein	SYNONYMOUS_CODING
13119	CakSNP13119	*Kabuli*	Ca_Kabuli_Chr07	39944937	[T/C]	Ca_25106	Mitochondrial carrier protein	SYNONYMOUS_CODING
13120	CakSNP13120	*Kabuli*	Ca_Kabuli_Chr07	39944889	[T/C]	Ca_25106	Mitochondrial carrier protein	SYNONYMOUS_CODING
13121	CakSNP13121	*Kabuli*	Ca_Kabuli_Chr07	40092198	[G/A]	Ca_24269	--	NON_SYNONYMOUS_CODING

S.N.	SNP IDs	Chickpea cultivars	Chromosomes	Physical positions (bp)	SNPs	Gene accession IDs	Putative functions	Sequence components of genome
13122	CakSNP13122	*Kabuli*	Ca_Kabuli_Chr07	40092181	[C/T]	Ca_24269	--	NON_SYNONYMOUS_CODING
13123	CakSNP13123	*Kabuli*	Ca_Kabuli_Chr07	40092218	[A/T]	Ca_24269	--	NON_SYNONYMOUS_CODING
13124	CakSNP13124	*Kabuli*	Ca_Kabuli_Chr07	40092213	[G/A]	Ca_24269	--	NON_SYNONYMOUS_CODING
13125	CakSNP13125	*Kabuli*	Ca_Kabuli_Chr07	40092163	[T/C]	Ca_24269	--	NON_SYNONYMOUS_CODING
13126	CakSNP13126	*Kabuli*	Ca_Kabuli_Chr07	40092155	[T/A]	Ca_24269	--	SYNONYMOUS_CODING
13127	CakSNP13127	*Kabuli*	Ca_Kabuli_Chr07	40092156	[C/A]	Ca_24269	--	NON_SYNONYMOUS_CODING
13128	CakSNP13128	*Kabuli*	Ca_Kabuli_Chr07	40092168	[G/A]	Ca_24269	--	NON_SYNONYMOUS_CODING
13129	CakSNP13129	*Kabuli*	Ca_Kabuli_Chr07	40092201	[G/A]	Ca_24269	--	NON_SYNONYMOUS_CODING
13130	CakSNP13130	*Kabuli*	Ca_Kabuli_Chr07	40092203	[T/G]	Ca_24269	--	SYNONYMOUS_CODING
13131	CakSNP13131	*Kabuli*	Ca_Kabuli_Chr07	40092211	[C/T]	Ca_24269	--	NON_SYNONYMOUS_CODING

S.N.	SNP IDs	Chickpea cultivars	Chromosomes	Physical positions (bp)	SNPs	Gene accession IDs	Putative functions	Sequence components of genome
13132	CakSNP13132	*Kabuli*	Ca_Kabuli_Chr07	40092214	[C/G]	Ca_24269	--	NON_SYNONYMOUS_CODING
13133	CakSNP13133	*Kabuli*	Ca_Kabuli_Chr07	40092229	[C/A]	Ca_24269	--	NON_SYNONYMOUS_CODING
13134	CakSNP13134	*Kabuli*	Ca_Kabuli_Chr07	40092237	[C/T]	Ca_24269	--	SYNONYMOUS_CODING
13135	CakSNP13135	*Kabuli*	Ca_Kabuli_Chr07	40092239	[G/A]	Ca_24269	--	SYNONYMOUS_CODING
13136	CakSNP13136	*Kabuli*	Ca_Kabuli_Chr07	40092250	[A/C]	Ca_24269	--	NON_SYNONYMOUS_CODING
13137	CakSNP13137	*Kabuli*	Ca_Kabuli_Chr07	40092253	[C/T]	Ca_24269	--	NON_SYNONYMOUS_CODING
13138	CakSNP13138	*Kabuli*	Ca_Kabuli_Chr07	40092256	[T/C]	Ca_24269	--	NON_SYNONYMOUS_CODING
13139	CakSNP13139	*Kabuli*	Ca_Kabuli_Chr07	40092267	[G/C]	Ca_24269	--	NON_SYNONYMOUS_CODING
13140	CakSNP13140	*Kabuli*	Ca_Kabuli_Chr07	40183449	[C/A]		--	INTERGENIC
13141	CakSNP13141	*Kabuli*	Ca_Kabuli_Chr07	40183452	[A/C]		--	INTERGENIC

S.N.	SNP IDs	Chickpea cultivars	Chromosomes	Physical positions (bp)	SNPs	Gene accession IDs	Putative functions	Sequence components of genome
13142	CakSNP13142	*Kabuli*	Ca_Kabuli_Chr07	40241674	[A/C]	Ca_24261	RNA recognition motif domain	NON_SYNONYMOUS_CODING
13143	CakSNP13143	*Kabuli*	Ca_Kabuli_Chr07	40242539	[A/G]	Ca_24261	RNA recognition motif domain	INTRON
13144	CakSNP13144	*Kabuli*	Ca_Kabuli_Chr07	40242765	[A/G]	Ca_24261	RNA recognition motif domain	INTRON
13145	CakSNP13145	*Kabuli*	Ca_Kabuli_Chr07	40243784	[G/A]	Ca_24261	RNA recognition motif domain	NON_SYNONYMOUS_CODING
13146	CakSNP13146	*Kabuli*	Ca_Kabuli_Chr07	40323213	[C/A]		--	INTERGENIC
13147	CakSNP13147	*Kabuli*	Ca_Kabuli_Chr07	40323251	[T/A]		--	INTERGENIC
13148	CakSNP13148	*Kabuli*	Ca_Kabuli_Chr07	40429548	[A/G]		--	INTERGENIC
13149	CakSNP13149	*Kabuli*	Ca_Kabuli_Chr07	40429552	[C/T]		--	INTERGENIC
13150	CakSNP13150	*Kabuli*	Ca_Kabuli_Chr07	40429564	[G/T]		--	INTERGENIC
13151	CakSNP13151	*Kabuli*	Ca_Kabuli_Chr07	40429573	[G/T]		--	INTERGENIC

S.N.	SNP IDs	Chickpea cultivars	Chromosomes	Physical positions (bp)	SNPs	Gene accession IDs	Putative functions	Sequence components of genome
13152	CakSNP13152	*Kabuli*	Ca_Kabuli_Chr07	40429629	[G/T]		--	INTERGENIC
13153	CakSNP13153	*Kabuli*	Ca_Kabuli_Chr07	40429621	[G/T]		--	INTERGENIC
13154	CakSNP13154	*Kabuli*	Ca_Kabuli_Chr07	40429604	[C/T]		--	INTERGENIC
13155	CakSNP13155	*Kabuli*	Ca_Kabuli_Chr07	40429600	[C/T]		--	INTERGENIC
13156	CakSNP13156	*Kabuli*	Ca_Kabuli_Chr07	40429596	[G/A]		--	INTERGENIC
13157	CakSNP13157	*Kabuli*	Ca_Kabuli_Chr07	40429567	[G/A]		--	INTERGENIC
13158	CakSNP13158	*Kabuli*	Ca_Kabuli_Chr07	40456574	[T/C]		--	INTERGENIC
13159	CakSNP13159	*Kabuli*	Ca_Kabuli_Chr07	40645278	[A/G]		--	INTERGENIC
13160	CakSNP13160	*Kabuli*	Ca_Kabuli_Chr07	40645276	[A/G]		--	INTERGENIC
13161	CakSNP13161	*Kabuli*	Ca_Kabuli_Chr07	40645208	[C/G]		--	INTERGENIC

S.N.	SNP IDs	Chickpea cultivars	Chromosomes	Physical positions (bp)	SNPs	Gene accession IDs	Putative functions	Sequence components of genome
13162	CakSNP13162	*Kabuli*	Ca_Kabuli_Chr07	40889596	[A/C]	Ca_26517	--	NON_SYNONYMOUS_CODING
13163	CakSNP13163	*Kabuli*	Ca_Kabuli_Chr07	40889598	[C/G]	Ca_26517	--	NON_SYNONYMOUS_CODING
13164	CakSNP13164	*Kabuli*	Ca_Kabuli_Chr07	40889623	[G/A]	Ca_26517	--	NON_SYNONYMOUS_CODING
13165	CakSNP13165	*Kabuli*	Ca_Kabuli_Chr07	40889632	[C/T]	Ca_26517	--	NON_SYNONYMOUS_CODING
13166	CakSNP13166	*Kabuli*	Ca_Kabuli_Chr07	40889633	[G/A]	Ca_26517	--	SYNONYMOUS_CODING
13167	CakSNP13167	*Kabuli*	Ca_Kabuli_Chr07	40889640	[G/A]	Ca_26517	--	NON_SYNONYMOUS_CODING
13168	CakSNP13168	*Kabuli*	Ca_Kabuli_Chr07	40889641	[C/T]	Ca_26517	--	NON_SYNONYMOUS_CODING
13169	CakSNP13169	*Kabuli*	Ca_Kabuli_Chr07	40889643	[G/A]	Ca_26517	--	NON_SYNONYMOUS_CODING
13170	CakSNP13170	*Kabuli*	Ca_Kabuli_Chr07	40889648	[T/A]	Ca_26517	--	SYNONYMOUS_CODING
13171	CakSNP13171	*Kabuli*	Ca_Kabuli_Chr07	40889649	[G/A]	Ca_26517	--	NON_SYNONYMOUS_CODING

S.N.	SNP IDs	Chickpea cultivars	Chromosomes	Physical positions (bp)	SNPs	Gene accession IDs	Putative functions	Sequence components of genome
13172	CakSNP13172	*Kabuli*	Ca_Kabuli_Chr07	40889673	[C/A]	Ca_26517	--	NON_SYNONYMOUS_CODING
13173	CakSNP13173	*Kabuli*	Ca_Kabuli_Chr07	40889670	[C/T]	Ca_26517	--	NON_SYNONYMOUS_CODING
13174	CakSNP13174	*Kabuli*	Ca_Kabuli_Chr07	40889723	[C/G]	Ca_26517	--	SYNONYMOUS_CODING
13175	CakSNP13175	*Kabuli*	Ca_Kabuli_Chr07	40889684	[C/T]	Ca_26517	--	SYNONYMOUS_CODING
13176	CakSNP13176	*Kabuli*	Ca_Kabuli_Chr07	41282883	[A/T]		--	INTERGENIC
13177	CakSNP13177	*Kabuli*	Ca_Kabuli_Chr07	41282961	[G/T]		--	INTERGENIC
13178	CakSNP13178	*Kabuli*	Ca_Kabuli_Chr07	41282960	[A/C]		--	INTERGENIC
13179	CakSNP13179	*Kabuli*	Ca_Kabuli_Chr07	41282957	[G/A]		--	INTERGENIC
13180	CakSNP13180	*Kabuli*	Ca_Kabuli_Chr07	41325886	[A/G]	Ca_18002	Orotidine 5'-phosphate decarboxylase domain	SYNONYMOUS_CODING
13181	CakSNP13181	*Kabuli*	Ca_Kabuli_Chr07	41339748	[C/A]	Ca_18001	C2 calcium-dependent membrane targeting	NON_SYNONYMOUS_CODING

S.N.	SNP IDs	Chickpea cultivars	Chromosomes	Physical positions (bp)	SNPs	Gene accession IDs	Putative functions	Sequence components of genome
13182	CakSNP13182	*Kabuli*	Ca_Kabuli_Chr07	41349604	[T/C]		--	INTERGENIC
13183	CakSNP13183	*Kabuli*	Ca_Kabuli_Chr07	41387509	[A/C]	Ca_17996	WD40 repeat	SYNONYMOUS_CODING
13184	CakSNP13184	*Kabuli*	Ca_Kabuli_Chr07	41387502	[A/C]	Ca_17996	WD40 repeat	NON_SYNONYMOUS_CODING
13185	CakSNP13185	*Kabuli*	Ca_Kabuli_Chr07	41387475	[A/C]	Ca_17996	WD40 repeat	NON_SYNONYMOUS_CODING
13186	CakSNP13186	*Kabuli*	Ca_Kabuli_Chr07	41657180	[G/T]	Ca_17988	RNA polymerase, alpha subunit	INTRON
13187	CakSNP13187	*Kabuli*	Ca_Kabuli_Chr07	41657241	[T/C]	Ca_17988	RNA polymerase, alpha subunit	NON_SYNONYMOUS_CODING
13188	CakSNP13188	*Kabuli*	Ca_Kabuli_Chr07	41821410	[T/C]		--	INTERGENIC
13189	CakSNP13189	*Kabuli*	Ca_Kabuli_Chr07	42166004	[C/T]	Ca_17969	Protein of unknown function DUF810	SYNONYMOUS_CODING
13190	CakSNP13190	*Kabuli*	Ca_Kabuli_Chr07	42268628	[A/C]	Ca_17965	Mannosyltransferase, DXD	SYNONYMOUS_CODING
13191	CakSNP13191	*Kabuli*	Ca_Kabuli_Chr07	42272939	[G/C]	Ca_17964	SOUL haem-binding protein	NON_SYNONYMOUS_CODING

S.N.	SNP IDs	Chickpea cultivars	Chromosomes	Physical positions (bp)	SNPs	Gene accession IDs	Putative functions	Sequence components of genome
13192	CakSNP13192	*Kabuli*	Ca_Kabuli_Chr07	42355002	[T/G]		--	INTERGENIC
13193	CakSNP13193	*Kabuli*	Ca_Kabuli_Chr07	42354958	[G/A]		--	INTERGENIC
13194	CakSNP13194	*Kabuli*	Ca_Kabuli_Chr07	42354938	[C/T]		--	INTERGENIC
13195	CakSNP13195	*Kabuli*	Ca_Kabuli_Chr07	42354999	[C/T]		--	INTERGENIC
13196	CakSNP13196	*Kabuli*	Ca_Kabuli_Chr07	42355011	[G/T]		--	INTERGENIC
13197	CakSNP13197	*Kabuli*	Ca_Kabuli_Chr07	42566717	[A/G]	Ca_20213	Cytochrome P450	NON_SYNONYMOUS_CODING
13198	CakSNP13198	*Kabuli*	Ca_Kabuli_Chr07	42846882	[C/T]	Ca_20198	--	NON_SYNONYMOUS_CODING
13199	CakSNP13199	*Kabuli*	Ca_Kabuli_Chr07	42851182	[T/C]		--	INTERGENIC
13200	CakSNP13200	*Kabuli*	Ca_Kabuli_Chr07	43080817	[T/C]	Ca_20188	--	NON_SYNONYMOUS_CODING
13201	CakSNP13201	*Kabuli*	Ca_Kabuli_Chr07	43118954	[A/C]	Ca_20187	Ubiquitin-associated/translation elongation factor EF1B, N-terminal	SYNONYMOUS_CODING

**S.N.**	**SNP IDs**	**Chickpea cultivars**	**Chromosomes**	**Physical positions (bp)**	**SNPs**	**Gene accession IDs**	**Putative functions**	**Sequence components of genome**
13202	CakSNP13202	*Kabuli*	Ca_Kabuli_Chr07	43119266	[C/T]	Ca_20187	Ubiquitin-associated/translation elongation factor EF1B, N-terminal	SYNONYMOUS_CODING
13203	CakSNP13203	*Kabuli*	Ca_Kabuli_Chr07	43210821	[A/T]		--	INTERGENIC
13204	CakSNP13204	*Kabuli*	Ca_Kabuli_Chr07	43646117	[G/A]	Ca_25031	Protein kinase, catalytic domain	SYNONYMOUS_CODING
13205	CakSNP13205	*Kabuli*	Ca_Kabuli_Chr07	44232239	[G/A]	Ca_21369	--	SYNONYMOUS_CODING
13206	CakSNP13206	*Kabuli*	Ca_Kabuli_Chr07	44232204	[T/A]	Ca_21369	--	SYNONYMOUS_CODING
13207	CakSNP13207	*Kabuli*	Ca_Kabuli_Chr07	44232221	[C/T]	Ca_21369	--	NON_SYNONYMOUS_CODING
13208	CakSNP13208	*Kabuli*	Ca_Kabuli_Chr07	44232266	[C/T]	Ca_21369	--	NON_SYNONYMOUS_CODING
13209	CakSNP13209	*Kabuli*	Ca_Kabuli_Chr07	44540553	[T/G]		--	INTERGENIC
13210	CakSNP13210	*Kabuli*	Ca_Kabuli_Chr07	44540595	[G/A]		--	INTERGENIC
13211	CakSNP13211	*Kabuli*	Ca_Kabuli_Chr07	44540596	[C/A]		--	INTERGENIC

S.N.	SNP IDs	Chickpea cultivars	Chromosomes	Physical positions (bp)	SNPs	Gene accession IDs	Putative functions	Sequence components of genome
13212	CakSNP13212	*Kabuli*	Ca_Kabuli_Chr07	44540629	[G/A]		--	INTERGENIC
13213	CakSNP13213	*Kabuli*	Ca_Kabuli_Chr07	44540616	[C/A]		--	INTERGENIC
13214	CakSNP13214	*Kabuli*	Ca_Kabuli_Chr07	44612651	[C/A]	Ca_15697	Ethylene insensitive 3	NON_SYNONYMOUS_CODING
13215	CakSNP13215	*Kabuli*	Ca_Kabuli_Chr07	44747668	[C/T]	Ca_15701	--	SYNONYMOUS_CODING
13216	CakSNP13216	*Kabuli*	Ca_Kabuli_Chr07	44765450	[C/T]	Ca_15703	Transposase, Ptta/En/Spm, plant	SYNONYMOUS_CODING
13217	CakSNP13217	*Kabuli*	Ca_Kabuli_Chr07	44765467	[G/T]	Ca_15703	Transposase, Ptta/En/Spm, plant	NON_SYNONYMOUS_CODING
13218	CakSNP13218	*Kabuli*	Ca_Kabuli_Chr07	44765522	[A/C]	Ca_15703	Transposase, Ptta/En/Spm, plant	SYNONYMOUS_CODING
13219	CakSNP13219	*Kabuli*	Ca_Kabuli_Chr07	44877324	[A/C]	Ca_15708	Zinc finger, C2H2-type	NON_SYNONYMOUS_CODING
13220	CakSNP13220	*Kabuli*	Ca_Kabuli_Chr07	44926768	[C/T]		--	INTERGENIC
13221	CakSNP13221	*Kabuli*	Ca_Kabuli_Chr07	44926783	[C/A]		--	INTERGENIC

S.N.	SNP IDs	Chickpea cultivars	Chromosomes	Physical positions (bp)	SNPs	Gene accession IDs	Putative functions	Sequence components of genome
13222	CakSNP13222	*Kabuli*	Ca_Kabuli_Chr07	44926815	[C/T]		--	INTERGENIC
13223	CakSNP13223	*Kabuli*	Ca_Kabuli_Chr07	45147550	[A/G]		--	INTERGENIC
13224	CakSNP13224	*Kabuli*	Ca_Kabuli_Chr07	45280659	[T/A]		--	INTERGENIC
13225	CakSNP13225	*Kabuli*	Ca_Kabuli_Chr07	45289633	[T/C]	Ca_15728	Short-chain dehydrogenase/reductase SDR	INTRON
13226	CakSNP13226	*Kabuli*	Ca_Kabuli_Chr07	45936581	[C/A]	Ca_15757	Dedicator of cytokinesis	NON_SYNONYMOUS_CODING
13227	CakSNP13227	*Kabuli*	Ca_Kabuli_Chr07	46053109	[G/A]	Ca_15758	Peptidase C48, SUMO/Sentrin/Ubl1	NON_SYNONYMOUS_CODING
13228	CakSNP13228	*Kabuli*	Ca_Kabuli_Chr07	46394656	[A/G]	Ca_22753	Pathogenesis-related transcriptional factor/ERF, DNA-binding	INTRON
13229	CakSNP13229	*Kabuli*	Ca_Kabuli_Chr07	46485934	[A/G]	Ca_22756	--	INTRON
13230	CakSNP13230	*Kabuli*	Ca_Kabuli_Chr07	46747255	[A/C]	Ca_19472	Glycoside hydrolase, family 18, catalytic domain	SYNONYMOUS_CODING
13231	CakSNP13231	*Kabuli*	Ca_Kabuli_Chr07	46747283	[T/C]	Ca_19472	Glycoside hydrolase, family 18, catalytic domain	NON_SYNONYMOUS_CODING

S.N.	SNP IDs	Chickpea cultivars	Chromosomes	Physical positions (bp)	SNPs	Gene accession IDs	Putative functions	Sequence components of genome
13232	CakSNP13232	*Kabuli*	Ca_Kabuli_Chr07	46747290	[G/T]	Ca_19472	Glycoside hydrolase, family 18, catalytic domain	NON_SYNONYMOUS_CODING
13233	CakSNP13233	*Kabuli*	Ca_Kabuli_Chr07	46747295	[G/C]	Ca_19472	Glycoside hydrolase, family 18, catalytic domain	NON_SYNONYMOUS_CODING
13234	CakSNP13234	*Kabuli*	Ca_Kabuli_Chr07	46747309	[C/T]	Ca_19472	Glycoside hydrolase, family 18, catalytic domain	SYNONYMOUS_CODING
13235	CakSNP13235	*Kabuli*	Ca_Kabuli_Chr07	46747330	[G/A]	Ca_19472	Glycoside hydrolase, family 18, catalytic domain	SYNONYMOUS_CODING
13236	CakSNP13236	*Kabuli*	Ca_Kabuli_Chr07	46823471	[T/G]		--	INTERGENIC
13237	CakSNP13237	*Kabuli*	Ca_Kabuli_Chr07	46823949	[G/T]		--	INTERGENIC
13238	CakSNP13238	*Kabuli*	Ca_Kabuli_Chr07	46826036	[T/A]	Ca_19475	Glycoside hydrolase, family 18, catalytic domain	SYNONYMOUS_CODING
13239	CakSNP13239	*Kabuli*	Ca_Kabuli_Chr07	46826030	[T/C]	Ca_19475	Glycoside hydrolase, family 18, catalytic domain	SYNONYMOUS_CODING
13240	CakSNP13240	*Kabuli*	Ca_Kabuli_Chr07	46826028	[C/A]	Ca_19475	Glycoside hydrolase, family 18, catalytic domain	NON_SYNONYMOUS_CODING
13241	CakSNP13241	*Kabuli*	Ca_Kabuli_Chr07	46826020	[G/A]	Ca_19475	Glycoside hydrolase, family 18, catalytic domain	NON_SYNONYMOUS_CODING

S.N.	SNP IDs	Chickpea cultivars	Chromosomes	Physical positions (bp)	SNPs	Gene accession IDs	Putative functions	Sequence components of genome
13242	CakSNP13242	*Kabuli*	Ca_Kabuli_Chr07	46826015	[G/A]	Ca_19475	Glycoside hydrolase, family 18, catalytic domain	SYNONYMOUS_CODING
13243	CakSNP13243	*Kabuli*	Ca_Kabuli_Chr07	46826008	[A/T]	Ca_19475	Glycoside hydrolase, family 18, catalytic domain	NON_SYNONYMOUS_CODING
13244	CakSNP13244	*Kabuli*	Ca_Kabuli_Chr07	46825997	[A/G]	Ca_19475	Glycoside hydrolase, family 18, catalytic domain	SYNONYMOUS_CODING
13245	CakSNP13245	*Kabuli*	Ca_Kabuli_Chr07	46825992	[T/G]	Ca_19475	Glycoside hydrolase, family 18, catalytic domain	NON_SYNONYMOUS_CODING
13246	CakSNP13246	*Kabuli*	Ca_Kabuli_Chr07	46825985	[T/G]	Ca_19475	Glycoside hydrolase, family 18, catalytic domain	SYNONYMOUS_CODING
13247	CakSNP13247	*Kabuli*	Ca_Kabuli_Chr07	46825957	[A/T]	Ca_19475	Glycoside hydrolase, family 18, catalytic domain	NON_SYNONYMOUS_CODING
13248	CakSNP13248	*Kabuli*	Ca_Kabuli_Chr07	46825952	[A/G]	Ca_19475	Glycoside hydrolase, family 18, catalytic domain	SYNONYMOUS_CODING
13249	CakSNP13249	*Kabuli*	Ca_Kabuli_Chr07	47127331	[T/G]		--	INTERGENIC
13250	CakSNP13250	*Kabuli*	Ca_Kabuli_Chr07	47127342	[T/G]		--	INTERGENIC
13251	CakSNP13251	*Kabuli*	Ca_Kabuli_Chr07	47127343	[C/T]		--	INTERGENIC

S.N.	SNP IDs	Chickpea cultivars	Chromosomes	Physical positions (bp)	SNPs	Gene accession IDs	Putative functions	Sequence components of genome
13252	CakSNP13252	*Kabuli*	Ca_Kabuli_Chr07	47159646	[A/C]		--	INTERGENIC
13253	CakSNP13253	*Kabuli*	Ca_Kabuli_Chr07	47335940	[G/A]		--	INTERGENIC
13254	CakSNP13254	*Kabuli*	Ca_Kabuli_Chr07	47335916	[C/G]		--	INTERGENIC
13255	CakSNP13255	*Kabuli*	Ca_Kabuli_Chr07	47524173	[A/C]	Ca_19499	Protein kinase, catalytic domain	NON_SYNONYMOUS_CODING
13256	CakSNP13256	*Kabuli*	Ca_Kabuli_Chr07	47554330	[C/G]	Ca_19500	Ubiquitin-conjugating enzyme, E2	INTRON
13257	CakSNP13257	*Kabuli*	Ca_Kabuli_Chr07	47694706	[A/C]	Ca_22538	Cytochrome c oxidase copper chaperone	SYNONYMOUS_CODING
13258	CakSNP13258	*Kabuli*	Ca_Kabuli_Chr07	47839934	[G/A]	Ca_22546	Ubiquitin-conjugating enzyme, E2	NON_SYNONYMOUS_CODING
13259	CakSNP13259	*Kabuli*	Ca_Kabuli_Chr07	47839952	[G/T]	Ca_22546	Ubiquitin-conjugating enzyme, E2	NON_SYNONYMOUS_CODING
13260	CakSNP13260	*Kabuli*	Ca_Kabuli_Chr07	47840034	[A/G]	Ca_22546	Ubiquitin-conjugating enzyme, E2	SYNONYMOUS_CODING
13261	CakSNP13261	*Kabuli*	Ca_Kabuli_Chr07	47840092	[A/C]	Ca_22546	Ubiquitin-conjugating enzyme, E2	SYNONYMOUS_CODING

S.N.	SNP IDs	Chickpea cultivars	Chromosomes	Physical positions (bp)	SNPs	Gene accession IDs	Putative functions	Sequence components of genome
13262	CakSNP13262	*Kabuli*	Ca_Kabuli_Chr07	48102586	[G/T]		--	INTERGENIC
13263	CakSNP13263	*Kabuli*	Ca_Kabuli_Chr07	48200865	[A/G]		--	INTERGENIC
13264	CakSNP13264	*Kabuli*	Ca_Kabuli_Chr07	48200852	[G/A]		--	INTERGENIC
13265	CakSNP13265	*Kabuli*	Ca_Kabuli_Chr07	48200822	[G/A]		--	INTERGENIC
13266	CakSNP13266	*Kabuli*	Ca_Kabuli_Chr07	48200820	[A/C]		--	INTERGENIC
13267	CakSNP13267	*Kabuli*	Ca_Kabuli_Chr07	48200823	[G/A]		--	INTERGENIC
13268	CakSNP13268	*Kabuli*	Ca_Kabuli_Chr07	48200848	[T/G]		--	INTERGENIC
13269	CakSNP13269	*Kabuli*	Ca_Kabuli_Chr07	48509506	[G/T]		--	INTERGENIC
13270	CakSNP13270	*Kabuli*	Ca_Kabuli_Chr08	54095	[A/C]	Ca_11935	Heat shock protein DnaJ, N-terminal	SYNONYMOUS_CODING
13271	CakSNP13271	*Kabuli*	Ca_Kabuli_Chr08	85693	[T/G]	Ca_11933	--	NON_SYNONYMOUS_CODING

S.N.	SNP IDs	Chickpea cultivars	Chromosomes	Physical positions (bp)	SNPs	Gene accession IDs	Putative functions	Sequence components of genome
13272	CakSNP13272	*Kabuli*	Ca_Kabuli_Chr08	85697	[G/A]	Ca_11933	--	NON_SYNONYMOUS_CODING
13273	CakSNP13273	*Kabuli*	Ca_Kabuli_Chr08	85707	[C/A]	Ca_11933	--	NON_SYNONYMOUS_CODING
13274	CakSNP13274	*Kabuli*	Ca_Kabuli_Chr08	85710	[C/A]	Ca_11933	--	NON_SYNONYMOUS_CODING
13275	CakSNP13275	*Kabuli*	Ca_Kabuli_Chr08	158986	[C/G]	Ca_11928	SNF2-related	INTRON
13276	CakSNP13276	*Kabuli*	Ca_Kabuli_Chr08	177457	[C/T]		--	INTERGENIC
13277	CakSNP13277	*Kabuli*	Ca_Kabuli_Chr08	177436	[A/G]		--	INTERGENIC
13278	CakSNP13278	*Kabuli*	Ca_Kabuli_Chr08	177405	[G/A]		--	INTERGENIC
13279	CakSNP13279	*Kabuli*	Ca_Kabuli_Chr08	184444	[G/A]	Ca_11925	Spc97/Spc98	INTRON
13280	CakSNP13280	*Kabuli*	Ca_Kabuli_Chr08	218813	[C/T]		--	INTERGENIC
13281	CakSNP13281	*Kabuli*	Ca_Kabuli_Chr08	218828	[T/G]		--	INTERGENIC

S.N.	SNP IDs	Chickpea cultivars	Chromosomes	Physical positions (bp)	SNPs	Gene accession IDs	Putative functions	Sequence components of genome
13282	CakSNP13282	*Kabuli*	Ca_Kabuli_Chr08	218957	[G/T]		--	INTERGENIC
13283	CakSNP13283	*Kabuli*	Ca_Kabuli_Chr08	218928	[A/G]		--	INTERGENIC
13284	CakSNP13284	*Kabuli*	Ca_Kabuli_Chr08	249654	[A/C]	Ca_11915	Pentatricopeptide repeat	NON_SYNONYMOUS_CODING
13285	CakSNP13285	*Kabuli*	Ca_Kabuli_Chr08	253786	[G/A]	Ca_11914	--	INTRON
13286	CakSNP13286	*Kabuli*	Ca_Kabuli_Chr08	281622	[G/A]	Ca_11909	Helix-loop-helix DNA-binding domain	SYNONYMOUS_CODING
13287	CakSNP13287	*Kabuli*	Ca_Kabuli_Chr08	281608	[T/A]	Ca_11909	Helix-loop-helix DNA-binding domain	NON_SYNONYMOUS_CODING
13288	CakSNP13288	*Kabuli*	Ca_Kabuli_Chr08	281630	[A/C]	Ca_11909	Helix-loop-helix DNA-binding domain	SYNONYMOUS_CODING
13289	CakSNP13289	*Kabuli*	Ca_Kabuli_Chr08	283147	[C/T]	Ca_11908	EGF-like calcium-binding	SYNONYMOUS_STOP
13290	CakSNP13290	*Kabuli*	Ca_Kabuli_Chr08	285779	[G/A]	Ca_11908	EGF-like calcium-binding	SYNONYMOUS_CODING
13291	CakSNP13291	*Kabuli*	Ca_Kabuli_Chr08	356295	[A/T]	Ca_11899	HEAT	INTRON

S.N.	SNP IDs	Chickpea cultivars	Chromosomes	Physical positions (bp)	SNPs	Gene accession IDs	Putative functions	Sequence components of genome
13292	CakSNP13292	*Kabuli*	Ca_Kabuli_Chr08	411967	[G/A]	Ca_11894	Protein kinase, catalytic domain	SYNONYMOUS_CODING
13293	CakSNP13293	*Kabuli*	Ca_Kabuli_Chr08	499819	[A/C]	Ca_11887	Protein phosphatase 2C, manganese/magnesium aspartate binding site	INTRON
13294	CakSNP13294	*Kabuli*	Ca_Kabuli_Chr08	499890	[T/C]	Ca_11887	Protein phosphatase 2C, manganese/magnesium aspartate binding site	INTRON
13295	CakSNP13295	*Kabuli*	Ca_Kabuli_Chr08	510824	[G/A]	Ca_11886	SNF2-related	SYNONYMOUS_CODING
13296	CakSNP13296	*Kabuli*	Ca_Kabuli_Chr08	560477	[C/T]	Ca_11882	Cystathionine beta-synthase, core	INTRON
13297	CakSNP13297	*Kabuli*	Ca_Kabuli_Chr08	560451	[C/T]	Ca_11882	Cystathionine beta-synthase, core	INTRON
13298	CakSNP13298	*Kabuli*	Ca_Kabuli_Chr08	560604	[T/G]	Ca_11882	Cystathionine beta-synthase, core	INTRON
13299	CakSNP13299	*Kabuli*	Ca_Kabuli_Chr08	560746	[A/G]	Ca_11882	Cystathionine beta-synthase, core	INTRON
13300	CakSNP13300	*Kabuli*	Ca_Kabuli_Chr08	566112	[A/G]	Ca_11881	Plastocyanin-like	INTRON
13301	CakSNP13301	*Kabuli*	Ca_Kabuli_Chr08	566107	[C/T]	Ca_11881	Plastocyanin-like	INTRON

S.N.	SNP IDs	Chickpea cultivars	Chromosomes	Physical positions (bp)	SNPs	Gene accession IDs	Putative functions	Sequence components of genome
13302	CakSNP13302	*Kabuli*	Ca_Kabuli_Chr08	566094	[A/G]	Ca_11881	Plastocyanin-like	INTRON
13303	CakSNP13303	*Kabuli*	Ca_Kabuli_Chr08	596171	[C/T]	Ca_11877	Photosystem I PsaD	NON_SYNONYMOUS_CODING
13304	CakSNP13304	*Kabuli*	Ca_Kabuli_Chr08	614324	[G/A]		--	INTERGENIC
13305	CakSNP13305	*Kabuli*	Ca_Kabuli_Chr08	614301	[C/A]		--	INTERGENIC
13306	CakSNP13306	*Kabuli*	Ca_Kabuli_Chr08	619605	[T/C]	Ca_11872	Ribosomal protein L18/L5	SYNONYMOUS_CODING
13307	CakSNP13307	*Kabuli*	Ca_Kabuli_Chr08	634862	[C/T]		--	INTERGENIC
13308	CakSNP13308	*Kabuli*	Ca_Kabuli_Chr08	634843	[G/A]		--	INTERGENIC
13309	CakSNP13309	*Kabuli*	Ca_Kabuli_Chr08	634818	[G/T]		--	INTERGENIC
13310	CakSNP13310	*Kabuli*	Ca_Kabuli_Chr08	634921	[C/G]		--	INTERGENIC
13311	CakSNP13311	*Kabuli*	Ca_Kabuli_Chr08	649491	[A/C]	Ca_11866	Protein of unknown function DUF1423, plant	SYNONYMOUS_CODING

S.N.	SNP IDs	Chickpea cultivars	Chromosomes	Physical positions (bp)	SNPs	Gene accession IDs	Putative functions	Sequence components of genome
13312	CakSNP13312	*Kabuli*	Ca_Kabuli_Chr08	660610	[T/G]		--	INTERGENIC
13313	CakSNP13313	*Kabuli*	Ca_Kabuli_Chr08	666294	[G/A]		--	INTERGENIC
13314	CakSNP13314	*Kabuli*	Ca_Kabuli_Chr08	666288	[A/T]		--	INTERGENIC
13315	CakSNP13315	*Kabuli*	Ca_Kabuli_Chr08	666284	[T/A]		--	INTERGENIC
13316	CakSNP13316	*Kabuli*	Ca_Kabuli_Chr08	666274	[T/G]		--	INTERGENIC
13317	CakSNP13317	*Kabuli*	Ca_Kabuli_Chr08	672962	[C/T]	Ca_11861	KIP1-like	NON_SYNONYMOUS_CODING
13318	CakSNP13318	*Kabuli*	Ca_Kabuli_Chr08	673820	[A/G]	Ca_11861	KIP1-like	NON_SYNONYMOUS_CODING
13319	CakSNP13319	*Kabuli*	Ca_Kabuli_Chr08	674902	[T/G]	Ca_11861	KIP1-like	NON_SYNONYMOUS_CODING
13320	CakSNP13320	*Kabuli*	Ca_Kabuli_Chr08	701803	[C/T]	Ca_11858	Protein kinase, catalytic domain	INTRON
13321	CakSNP13321	*Kabuli*	Ca_Kabuli_Chr08	701811	[T/G]	Ca_11858	Protein kinase, catalytic domain	INTRON

S.N.	SNP IDs	Chickpea cultivars	Chromosomes	Physical positions (bp)	SNPs	Gene accession IDs	Putative functions	Sequence components of genome
13322	CakSNP13322	*Kabuli*	Ca_Kabuli_Chr08	701837	[G/A]	Ca_11858	Protein kinase, catalytic domain	INTRON
13323	CakSNP13323	*Kabuli*	Ca_Kabuli_Chr08	706514	[T/C]	Ca_11857	Pentatricopeptide repeat	NON_SYNONYMOUS_CODING
13324	CakSNP13324	*Kabuli*	Ca_Kabuli_Chr08	706482	[G/C]	Ca_11857	Pentatricopeptide repeat	NON_SYNONYMOUS_CODING
13325	CakSNP13325	*Kabuli*	Ca_Kabuli_Chr08	706614	[C/T]	Ca_11857	Pentatricopeptide repeat	SYNONYMOUS_CODING
13326	CakSNP13326	*Kabuli*	Ca_Kabuli_Chr08	706793	[A/G]	Ca_11857	Pentatricopeptide repeat	NON_SYNONYMOUS_CODING
13327	CakSNP13327	*Kabuli*	Ca_Kabuli_Chr08	706800	[G/A]	Ca_11857	Pentatricopeptide repeat	SYNONYMOUS_CODING
13328	CakSNP13328	*Kabuli*	Ca_Kabuli_Chr08	709063	[T/C]	Ca_11856	Protein of unknown function DUF1950	NON_SYNONYMOUS_CODING
13329	CakSNP13329	*Kabuli*	Ca_Kabuli_Chr08	709094	[T/C]	Ca_11856	Protein of unknown function DUF1950	NON_SYNONYMOUS_CODING
13330	CakSNP13330	*Kabuli*	Ca_Kabuli_Chr08	709124	[A/T]	Ca_11856	Protein of unknown function DUF1950	SYNONYMOUS_CODING
13331	CakSNP13331	*Kabuli*	Ca_Kabuli_Chr08	714967	[A/G]	Ca_11855	Protein of unknown function DUF1950	NON_SYNONYMOUS_CODING

S.N.	SNP IDs	Chickpea cultivars	Chromosomes	Physical positions (bp)	SNPs	Gene accession IDs	Putative functions	Sequence components of genome
13332	CakSNP13332	*Kabuli*	Ca_Kabuli_Chr08	714974	[G/A]	Ca_11855	Protein of unknown function DUF1950	SYNONYMOUS_CODING
13333	CakSNP13333	*Kabuli*	Ca_Kabuli_Chr08	777760	[A/T]	Ca_11846	Plant lipid transfer protein/Par allergen	SYNONYMOUS_CODING
13334	CakSNP13334	*Kabuli*	Ca_Kabuli_Chr08	777772	[G/A]	Ca_11846	Plant lipid transfer protein/Par allergen	SYNONYMOUS_CODING
13335	CakSNP13335	*Kabuli*	Ca_Kabuli_Chr08	777776	[C/G]	Ca_11846	Plant lipid transfer protein/Par allergen	NON_SYNONYMOUS_CODING
13336	CakSNP13336	*Kabuli*	Ca_Kabuli_Chr08	779589	[C/G]	Ca_11845	Plant lipid transfer protein/Par allergen	SYNONYMOUS_CODING
13337	CakSNP13337	*Kabuli*	Ca_Kabuli_Chr08	779611	[T/C]	Ca_11845	Plant lipid transfer protein/Par allergen	SYNONYMOUS_CODING
13338	CakSNP13338	*Kabuli*	Ca_Kabuli_Chr08	779640	[T/C]	Ca_11845	Plant lipid transfer protein/Par allergen	SYNONYMOUS_CODING
13339	CakSNP13339	*Kabuli*	Ca_Kabuli_Chr08	817321	[T/G]	Ca_11839	Zinc finger, PHD-type	INTRON
13340	CakSNP13340	*Kabuli*	Ca_Kabuli_Chr08	822302	[A/G]	Ca_11839	Zinc finger, PHD-type	NON_SYNONYMOUS_CODING
13341	CakSNP13341	*Kabuli*	Ca_Kabuli_Chr08	822942	[T/G]	Ca_11839	Zinc finger, PHD-type	NON_SYNONYMOUS_CODING

S.N.	SNP IDs	Chickpea cultivars	Chromosomes	Physical positions (bp)	SNPs	Gene accession IDs	Putative functions	Sequence components of genome
13342	CakSNP13342	*Kabuli*	Ca_Kabuli_Chr08	822976	[C/T]	Ca_11839	Zinc finger, PHD-type	SYNONYMOUS_CODING
13343	CakSNP13343	*Kabuli*	Ca_Kabuli_Chr08	839924	[C/G]		--	INTERGENIC
13344	CakSNP13344	*Kabuli*	Ca_Kabuli_Chr08	855332	[G/A]	Ca_11834	Protein of unknown function DUF827, plant	NON_SYNONYMOUS_CODING
13345	CakSNP13345	*Kabuli*	Ca_Kabuli_Chr08	866741	[A/C]	Ca_11832	SANT domain, DNA binding	NON_SYNONYMOUS_CODING
13346	CakSNP13346	*Kabuli*	Ca_Kabuli_Chr08	871666	[C/G]		--	INTERGENIC
13347	CakSNP13347	*Kabuli*	Ca_Kabuli_Chr08	875236	[T/G]	Ca_11831	Peptidase M16, C-terminal	INTRON
13348	CakSNP13348	*Kabuli*	Ca_Kabuli_Chr08	894184	[C/A]	Ca_11829	--	INTRON
13349	CakSNP13349	*Kabuli*	Ca_Kabuli_Chr08	895883	[G/A]	Ca_11829	--	INTRON
13350	CakSNP13350	*Kabuli*	Ca_Kabuli_Chr08	895947	[C/T]	Ca_11829	--	INTRON
13351	CakSNP13351	*Kabuli*	Ca_Kabuli_Chr08	897417	[T/C]	Ca_11829	--	SYNONYMOUS_CODING

S.N.	SNP IDs	Chickpea cultivars	Chromosomes	Physical positions (bp)	SNPs	Gene accession IDs	Putative functions	Sequence components of genome
13352	CakSNP13352	*Kabuli*	Ca_Kabuli_Chr08	898089	[C/T]	Ca_11829	--	INTRON
13353	CakSNP13353	*Kabuli*	Ca_Kabuli_Chr08	898150	[C/A]	Ca_11829	--	INTRON
13354	CakSNP13354	*Kabuli*	Ca_Kabuli_Chr08	898152	[A/G]	Ca_11829	--	INTRON
13355	CakSNP13355	*Kabuli*	Ca_Kabuli_Chr08	898173	[G/T]	Ca_11829	--	NON_SYNONYMOUS_CODING
13356	CakSNP13356	*Kabuli*	Ca_Kabuli_Chr08	898164	[G/T]	Ca_11829	--	INTRON
13357	CakSNP13357	*Kabuli*	Ca_Kabuli_Chr08	898226	[C/G]	Ca_11829	--	NON_SYNONYMOUS_CODING
13358	CakSNP13358	*Kabuli*	Ca_Kabuli_Chr08	898251	[G/T]	Ca_11829	--	SYNONYMOUS_CODING
13359	CakSNP13359	*Kabuli*	Ca_Kabuli_Chr08	919690	[T/C]	Ca_11826	AMP-dependent synthetase/ligase	INTRON
13360	CakSNP13360	*Kabuli*	Ca_Kabuli_Chr08	923820	[T/C]	Ca_11826	AMP-dependent synthetase/ligase	INTRON
13361	CakSNP13361	*Kabuli*	Ca_Kabuli_Chr08	924720	[G/T]		--	INTERGENIC

S.N.	SNP IDs	Chickpea cultivars	Chromosomes	Physical positions (bp)	SNPs	Gene accession IDs	Putative functions	Sequence components of genome
13362	CakSNP13362	*Kabuli*	Ca_Kabuli_Chr08	924681	[C/T]		--	INTERGENIC
13363	CakSNP13363	*Kabuli*	Ca_Kabuli_Chr08	924715	[A/G]		--	INTERGENIC
13364	CakSNP13364	*Kabuli*	Ca_Kabuli_Chr08	924732	[G/T]		--	INTERGENIC
13365	CakSNP13365	*Kabuli*	Ca_Kabuli_Chr08	974170	[T/C]	Ca_11819	--	NON_SYNONYMOUS_CODING
13366	CakSNP13366	*Kabuli*	Ca_Kabuli_Chr08	974159	[T/G]	Ca_11819	--	NON_SYNONYMOUS_CODING
13367	CakSNP13367	*Kabuli*	Ca_Kabuli_Chr08	1011333	[C/T]	Ca_11813	Metal-dependent phosphohydrolase, HD domain	SYNONYMOUS_CODING
13368	CakSNP13368	*Kabuli*	Ca_Kabuli_Chr08	1011320	[G/T]	Ca_11813	Metal-dependent phosphohydrolase, HD domain	NON_SYNONYMOUS_CODING
13369	CakSNP13369	*Kabuli*	Ca_Kabuli_Chr08	1011327	[G/A]	Ca_11813	Metal-dependent phosphohydrolase, HD domain	SYNONYMOUS_CODING
13370	CakSNP13370	*Kabuli*	Ca_Kabuli_Chr08	1043905	[T/C]	Ca_15083	Ribonuclease P/MRP, subunit p29	INTRON
13371	CakSNP13371	*Kabuli*	Ca_Kabuli_Chr08	1070728	[T/C]	Ca_15078	WD40 repeat	NON_SYNONYMOUS_CODING

S.N.	SNP IDs	Chickpea cultivars	Chromosomes	Physical positions (bp)	SNPs	Gene accession IDs	Putative functions	Sequence components of genome
13372	CakSNP13372	*Kabuli*	Ca_Kabuli_Chr08	1077334	[C/T]	Ca_15077	WD40 repeat	SYNONYMOUS_CODING
13373	CakSNP13373	*Kabuli*	Ca_Kabuli_Chr08	1077430	[C/T]	Ca_15077	WD40 repeat	SYNONYMOUS_CODING
13374	CakSNP13374	*Kabuli*	Ca_Kabuli_Chr08	1119568	[T/C]		--	INTERGENIC
13375	CakSNP13375	*Kabuli*	Ca_Kabuli_Chr08	1119781	[A/G]		--	INTERGENIC
13376	CakSNP13376	*Kabuli*	Ca_Kabuli_Chr08	1119773	[T/G]		--	INTERGENIC
13377	CakSNP13377	*Kabuli*	Ca_Kabuli_Chr08	1119761	[G/T]		--	INTERGENIC
13378	CakSNP13378	*Kabuli*	Ca_Kabuli_Chr08	1138275	[G/A]	Ca_15067	K Homology	INTRON
13379	CakSNP13379	*Kabuli*	Ca_Kabuli_Chr08	1138394	[A/G]	Ca_15067	K Homology	INTRON
13380	CakSNP13380	*Kabuli*	Ca_Kabuli_Chr08	1155597	[C/T]	Ca_15064	Pathogenesis-related transcriptional factor/ERF, DNA-binding	SYNONYMOUS_CODING
13381	CakSNP13381	*Kabuli*	Ca_Kabuli_Chr08	1155636	[T/C]	Ca_15064	Pathogenesis-related transcriptional factor/ERF, DNA-binding	SYNONYMOUS_CODING

S.N.	SNP IDs	Chickpea cultivars	Chromosomes	Physical positions (bp)	SNPs	Gene accession IDs	Putative functions	Sequence components of genome
13382	CakSNP13382	*Kabuli*	Ca_Kabuli_Chr08	1155639	[T/A]	Ca_15064	Pathogenesis-related transcriptional factor/ERF, DNA-binding	NON_SYNONYMOUS_CODING
13383	CakSNP13383	*Kabuli*	Ca_Kabuli_Chr08	1155641	[C/G]	Ca_15064	Pathogenesis-related transcriptional factor/ERF, DNA-binding	NON_SYNONYMOUS_CODING
13384	CakSNP13384	*Kabuli*	Ca_Kabuli_Chr08	1174369	[A/G]		--	INTERGENIC
13385	CakSNP13385	*Kabuli*	Ca_Kabuli_Chr08	1193297	[C/T]	Ca_15060	Alpha/beta hydrolase fold-1	INTRON
13386	CakSNP13386	*Kabuli*	Ca_Kabuli_Chr08	1194886	[G/A]	Ca_15060	Alpha/beta hydrolase fold-1	INTRON
13387	CakSNP13387	*Kabuli*	Ca_Kabuli_Chr08	1194888	[A/G]	Ca_15060	Alpha/beta hydrolase fold-1	INTRON
13388	CakSNP13388	*Kabuli*	Ca_Kabuli_Chr08	1241739	[C/T]	Ca_15053	Pentatricopeptide repeat	NON_SYNONYMOUS_CODING
13389	CakSNP13389	*Kabuli*	Ca_Kabuli_Chr08	1262576	[T/C]	Ca_15049	Zinc finger, CCCH-type	SYNONYMOUS_CODING
13390	CakSNP13390	*Kabuli*	Ca_Kabuli_Chr08	1262561	[C/T]	Ca_15049	Zinc finger, CCCH-type	SYNONYMOUS_CODING
13391	CakSNP13391	*Kabuli*	Ca_Kabuli_Chr08	1262660	[C/A]	Ca_15049	Zinc finger, CCCH-type	INTRON

S.N.	SNP IDs	Chickpea cultivars	Chromosomes	Physical positions (bp)	SNPs	Gene accession IDs	Putative functions	Sequence components of genome
13392	CakSNP13392	*Kabuli*	Ca_Kabuli_Chr08	1262686	[T/G]	Ca_15049	Zinc finger, CCCH-type	INTRON
13393	CakSNP13393	*Kabuli*	Ca_Kabuli_Chr08	1265537	[G/A]	Ca_15049	Zinc finger, CCCH-type	NON_SYNONYMOUS_CODING
13394	CakSNP13394	*Kabuli*	Ca_Kabuli_Chr08	1265535	[A/G]	Ca_15049	Zinc finger, CCCH-type	NON_SYNONYMOUS_CODING
13395	CakSNP13395	*Kabuli*	Ca_Kabuli_Chr08	1265509	[T/C]	Ca_15049	Zinc finger, CCCH-type	SYNONYMOUS_CODING
13396	CakSNP13396	*Kabuli*	Ca_Kabuli_Chr08	1265595	[G/T]	Ca_15049	Zinc finger, CCCH-type	INTRON
13397	CakSNP13397	*Kabuli*	Ca_Kabuli_Chr08	1265579	[A/G]	Ca_15049	Zinc finger, CCCH-type	INTRON
13398	CakSNP13398	*Kabuli*	Ca_Kabuli_Chr08	1265577	[T/G]	Ca_15049	Zinc finger, CCCH-type	INTRON
13399	CakSNP13399	*Kabuli*	Ca_Kabuli_Chr08	1382266	[G/A]	Ca_15035	Zinc finger, PHD-type	NON_SYNONYMOUS_CODING
13400	CakSNP13400	*Kabuli*	Ca_Kabuli_Chr08	1382433	[G/T]	Ca_15035	Zinc finger, PHD-type	NON_SYNONYMOUS_CODING
13401	CakSNP13401	*Kabuli*	Ca_Kabuli_Chr08	1382454	[A/C]	Ca_15035	Zinc finger, PHD-type	SYNONYMOUS_CODING

S.N.	SNP IDs	Chickpea cultivars	Chromosomes	Physical positions (bp)	SNPs	Gene accession IDs	Putative functions	Sequence components of genome
13402	CakSNP13402	*Kabuli*	Ca_Kabuli_Chr08	1433436	[T/C]	Ca_15031	Pathogenesis-related transcriptional factor/ERF, DNA-binding	SYNONYMOUS_CODING
13403	CakSNP13403	*Kabuli*	Ca_Kabuli_Chr08	1477523	[T/C]	Ca_15024	WD40 repeat	SYNONYMOUS_CODING
13404	CakSNP13404	*Kabuli*	Ca_Kabuli_Chr08	1487202	[G/C]		--	INTERGENIC
13405	CakSNP13405	*Kabuli*	Ca_Kabuli_Chr08	1503621	[C/T]	Ca_15021	Protein kinase, catalytic domain	NON_SYNONYMOUS_CODING
13406	CakSNP13406	*Kabuli*	Ca_Kabuli_Chr08	1503595	[C/T]	Ca_15021	Protein kinase, catalytic domain	SYNONYMOUS_CODING
13407	CakSNP13407	*Kabuli*	Ca_Kabuli_Chr08	1503588	[T/C]	Ca_15021	Protein kinase, catalytic domain	NON_SYNONYMOUS_CODING
13408	CakSNP13408	*Kabuli*	Ca_Kabuli_Chr08	1504859	[A/G]	Ca_15021	Protein kinase, catalytic domain	SYNONYMOUS_CODING
13409	CakSNP13409	*Kabuli*	Ca_Kabuli_Chr08	1507709	[T/G]		--	INTERGENIC
13410	CakSNP13410	*Kabuli*	Ca_Kabuli_Chr08	1507782	[G/C]	Ca_15020	Aldose 1-epimerase	NON_SYNONYMOUS_CODING
13411	CakSNP13411	*Kabuli*	Ca_Kabuli_Chr08	1518285	[A/C]	Ca_15019	Glycosyl transferase, family 31	NON_SYNONYMOUS_CODING

S.N.	SNP IDs	Chickpea cultivars	Chromosomes	Physical positions (bp)	SNPs	Gene accession IDs	Putative functions	Sequence components of genome
13412	CakSNP13412	*Kabuli*	Ca_Kabuli_Chr08	1518297	[T/G]	Ca_15019	Glycosyl transferase, family 31	NON_SYNONYMOUS_CODING
13413	CakSNP13413	*Kabuli*	Ca_Kabuli_Chr08	1518355	[G/A]	Ca_15019	Glycosyl transferase, family 31	INTRON
13414	CakSNP13414	*Kabuli*	Ca_Kabuli_Chr08	1534461	[A/C]		--	INTERGENIC
13415	CakSNP13415	*Kabuli*	Ca_Kabuli_Chr08	1544916	[C/T]		--	INTERGENIC
13416	CakSNP13416	*Kabuli*	Ca_Kabuli_Chr08	1552082	[T/A]	Ca_15015	Cation/H+ exchanger	NON_SYNONYMOUS_CODING
13417	CakSNP13417	*Kabuli*	Ca_Kabuli_Chr08	1552143	[A/G]	Ca_15015	Cation/H+ exchanger	SYNONYMOUS_CODING
13418	CakSNP13418	*Kabuli*	Ca_Kabuli_Chr08	1554964	[T/C]	Ca_15014	Uncharacterised protein family UPF0016	INTRON
13419	CakSNP13419	*Kabuli*	Ca_Kabuli_Chr08	1555017	[C/A]	Ca_15014	Uncharacterised protein family UPF0016	NON_SYNONYMOUS_CODING
13420	CakSNP13420	*Kabuli*	Ca_Kabuli_Chr08	1555122	[A/G]	Ca_15014	Uncharacterised protein family UPF0016	NON_SYNONYMOUS_CODING
13421	CakSNP13421	*Kabuli*	Ca_Kabuli_Chr08	1557492	[C/T]	Ca_15014	Uncharacterised protein family UPF0016	INTRON

S.N.	SNP IDs	Chickpea cultivars	Chromosomes	Physical positions (bp)	SNPs	Gene accession IDs	Putative functions	Sequence components of genome
13422	CakSNP13422	*Kabuli*	Ca_Kabuli_Chr08	1573877	[G/T]	Ca_15012	ATPase, AAA+ type, core	NON_SYNONYMOUS_CODING
13423	CakSNP13423	*Kabuli*	Ca_Kabuli_Chr08	1612107	[G/A]		--	INTERGENIC
13424	CakSNP13424	*Kabuli*	Ca_Kabuli_Chr08	1612099	[A/G]		--	INTERGENIC
13425	CakSNP13425	*Kabuli*	Ca_Kabuli_Chr08	1612221	[C/G]		--	INTERGENIC
13426	CakSNP13426	*Kabuli*	Ca_Kabuli_Chr08	1667832	[T/C]		--	INTERGENIC
13427	CakSNP13427	*Kabuli*	Ca_Kabuli_Chr08	1675895	[C/T]	Ca_02440	Protein of unknown function DUF604	NON_SYNONYMOUS_CODING
13428	CakSNP13428	*Kabuli*	Ca_Kabuli_Chr08	1675888	[C/T]	Ca_02440	Protein of unknown function DUF604	NON_SYNONYMOUS_CODING
13429	CakSNP13429	*Kabuli*	Ca_Kabuli_Chr08	1708743	[C/T]	Ca_02436	--	SYNONYMOUS_CODING
13430	CakSNP13430	*Kabuli*	Ca_Kabuli_Chr08	1708860	[T/C]	Ca_02436	--	SYNONYMOUS_CODING
13431	CakSNP13431	*Kabuli*	Ca_Kabuli_Chr08	1718032	[C/T]	Ca_02435	Major intrinsic protein	SYNONYMOUS_CODING

S.N.	SNP IDs	Chickpea cultivars	Chromosomes	Physical positions (bp)	SNPs	Gene accession IDs	Putative functions	Sequence components of genome
13432	CakSNP13432	*Kabuli*	Ca_Kabuli_Chr08	1723593	[G/A]		--	INTERGENIC
13433	CakSNP13433	*Kabuli*	Ca_Kabuli_Chr08	1743097	[G/A]		--	INTERGENIC
13434	CakSNP13434	*Kabuli*	Ca_Kabuli_Chr08	1747523	[T/A]	Ca_02431	Zinc finger, RING-type	INTRON
13435	CakSNP13435	*Kabuli*	Ca_Kabuli_Chr08	1747522	[T/C]	Ca_02431	Zinc finger, RING-type	INTRON
13436	CakSNP13436	*Kabuli*	Ca_Kabuli_Chr08	1782034	[C/G]	Ca_02429	Domain of unknown function DUF296	INTRON
13437	CakSNP13437	*Kabuli*	Ca_Kabuli_Chr08	1794755	[C/A]	Ca_02426	ATPase, P-type, K/Mg/Cd/Cu/Zn/Na/Ca/Na/H-transporter	NON_SYNONYMOUS_CODING
13438	CakSNP13438	*Kabuli*	Ca_Kabuli_Chr08	1794756	[G/T]	Ca_02426	ATPase, P-type, K/Mg/Cd/Cu/Zn/Na/Ca/Na/H-transporter	SYNONYMOUS_CODING
13439	CakSNP13439	*Kabuli*	Ca_Kabuli_Chr08	1794759	[C/T]	Ca_02426	ATPase, P-type, K/Mg/Cd/Cu/Zn/Na/Ca/Na/H-transporter	SYNONYMOUS_CODING
13440	CakSNP13440	*Kabuli*	Ca_Kabuli_Chr08	1794810	[G/A]	Ca_02426	ATPase, P-type, K/Mg/Cd/Cu/Zn/Na/Ca/Na/H-transporter	SYNONYMOUS_CODING
13441	CakSNP13441	*Kabuli*	Ca_Kabuli_Chr08	1817388	[T/C]	Ca_02424	Protein kinase, catalytic domain	SYNONYMOUS_CODING

S.N.	SNP IDs	Chickpea cultivars	Chromosomes	Physical positions (bp)	SNPs	Gene accession IDs	Putative functions	Sequence components of genome
13442	CakSNP13442	*Kabuli*	Ca_Kabuli_Chr08	1830230	[A/T]	Ca_02423	--	NON_SYNONYMOUS_CODING
13443	CakSNP13443	*Kabuli*	Ca_Kabuli_Chr08	1830348	[T/C]	Ca_02423	--	NON_SYNONYMOUS_CODING
13444	CakSNP13444	*Kabuli*	Ca_Kabuli_Chr08	1846478	[A/G]	Ca_02421	Transcription factor TFIIE beta subunit, DNA-binding domain	INTRON
13445	CakSNP13445	*Kabuli*	Ca_Kabuli_Chr08	1893613	[T/C]	Ca_02413	--	INTRON
13446	CakSNP13446	*Kabuli*	Ca_Kabuli_Chr08	1932975	[T/C]	Ca_02408	Pathogenesis-related transcriptional factor/ERF, DNA-binding	SYNONYMOUS_CODING
13447	CakSNP13447	*Kabuli*	Ca_Kabuli_Chr08	1932981	[T/C]	Ca_02408	Pathogenesis-related transcriptional factor/ERF, DNA-binding	SYNONYMOUS_CODING
13448	CakSNP13448	*Kabuli*	Ca_Kabuli_Chr08	1932990	[A/C]	Ca_02408	Pathogenesis-related transcriptional factor/ERF, DNA-binding	SYNONYMOUS_CODING
13449	CakSNP13449	*Kabuli*	Ca_Kabuli_Chr08	1932993	[C/T]	Ca_02408	Pathogenesis-related transcriptional factor/ERF, DNA-binding	SYNONYMOUS_CODING
13450	CakSNP13450	*Kabuli*	Ca_Kabuli_Chr08	1932997	[G/T]	Ca_02408	Pathogenesis-related transcriptional factor/ERF, DNA-binding	NON_SYNONYMOUS_CODING
13451	CakSNP13451	*Kabuli*	Ca_Kabuli_Chr08	1933026	[G/A]	Ca_02408	Pathogenesis-related transcriptional factor/ERF, DNA-binding	SYNONYMOUS_CODING

S.N.	SNP IDs	Chickpea cultivars	Chromosomes	Physical positions (bp)	SNPs	Gene accession IDs	Putative functions	Sequence components of genome
13452	CakSNP13452	*Kabuli*	Ca_Kabuli_Chr08	1933064	[G/C]	Ca_02408	Pathogenesis-related transcriptional factor/ERF, DNA-binding	NON_SYNONYMOUS_CODING
13453	CakSNP13453	*Kabuli*	Ca_Kabuli_Chr08	1933053	[G/A]	Ca_02408	Pathogenesis-related transcriptional factor/ERF, DNA-binding	SYNONYMOUS_CODING
13454	CakSNP13454	*Kabuli*	Ca_Kabuli_Chr08	1933052	[T/C]	Ca_02408	Pathogenesis-related transcriptional factor/ERF, DNA-binding	NON_SYNONYMOUS_CODING
13455	CakSNP13455	*Kabuli*	Ca_Kabuli_Chr08	1933039	[G/T]	Ca_02408	Pathogenesis-related transcriptional factor/ERF, DNA-binding	NON_SYNONYMOUS_CODING
13456	CakSNP13456	*Kabuli*	Ca_Kabuli_Chr08	1933035	[T/A]	Ca_02408	Pathogenesis-related transcriptional factor/ERF, DNA-binding	SYNONYMOUS_CODING
13457	CakSNP13457	*Kabuli*	Ca_Kabuli_Chr08	1958433	[G/C]	Ca_02407	Pathogenesis-related transcriptional factor/ERF, DNA-binding	NON_SYNONYMOUS_CODING
13458	CakSNP13458	*Kabuli*	Ca_Kabuli_Chr08	1958415	[T/G]	Ca_02407	Pathogenesis-related transcriptional factor/ERF, DNA-binding	NON_SYNONYMOUS_CODING
13459	CakSNP13459	*Kabuli*	Ca_Kabuli_Chr08	1978964	[G/C]	Ca_02404	RNA polymerase, alpha subunit	SYNONYMOUS_CODING
13460	CakSNP13460	*Kabuli*	Ca_Kabuli_Chr08	1980189	[T/A]	Ca_02404	RNA polymerase, alpha subunit	NON_SYNONYMOUS_CODING
13461	CakSNP13461	*Kabuli*	Ca_Kabuli_Chr08	2004509	[A/G]	Ca_02402	Hydroxymethylglutaryl-coenzyme A synthase, active site	SYNONYMOUS_CODING

S.N.	SNP IDs	Chickpea cultivars	Chromosomes	Physical positions (bp)	SNPs	Gene accession IDs	Putative functions	Sequence components of genome
13462	CakSNP13462	*Kabuli*	Ca_Kabuli_Chr08	2004577	[G/C]	Ca_02402	Hydroxymethylglutaryl-coenzyme A synthase, active site	INTRON
13463	CakSNP13463	*Kabuli*	Ca_Kabuli_Chr08	2029405	[C/A]	Ca_02400	Protein of unknown function DUF544	SYNONYMOUS_CODING
13464	CakSNP13464	*Kabuli*	Ca_Kabuli_Chr08	2029384	[T/C]	Ca_02400	Protein of unknown function DUF544	SYNONYMOUS_CODING
13465	CakSNP13465	*Kabuli*	Ca_Kabuli_Chr08	2052263	[T/G]		--	INTERGENIC
13466	CakSNP13466	*Kabuli*	Ca_Kabuli_Chr08	2052286	[T/C]		--	INTERGENIC
13467	CakSNP13467	*Kabuli*	Ca_Kabuli_Chr08	2070694	[G/T]		--	INTERGENIC
13468	CakSNP13468	*Kabuli*	Ca_Kabuli_Chr08	2073807	[C/G]	Ca_02393	Peptidase S8/S53, subtilisin/kexin/sedolisin	NON_SYNONYMOUS_CODING
13469	CakSNP13469	*Kabuli*	Ca_Kabuli_Chr08	2074619	[T/G]	Ca_02393	Peptidase S8/S53, subtilisin/kexin/sedolisin	NON_SYNONYMOUS_CODING
13470	CakSNP13470	*Kabuli*	Ca_Kabuli_Chr08	2075296	[T/C]	Ca_02393	Peptidase S8/S53, subtilisin/kexin/sedolisin	NON_SYNONYMOUS_CODING
13471	CakSNP13471	*Kabuli*	Ca_Kabuli_Chr08	2075290	[G/T]	Ca_02393	Peptidase S8/S53, subtilisin/kexin/sedolisin	NON_SYNONYMOUS_CODING

S.N.	SNP IDs	Chickpea cultivars	Chromosomes	Physical positions (bp)	SNPs	Gene accession IDs	Putative functions	Sequence components of genome
13472	CakSNP13472	*Kabuli*	Ca_Kabuli_Chr08	2075278	[T/G]	Ca_02393	Peptidase S8/S53, subtilisin/kexin/sedolisin	NON_SYNONYMOUS_CODING
13473	CakSNP13473	*Kabuli*	Ca_Kabuli_Chr08	2079723	[A/G]	Ca_02392	Peptidase S8/S53, subtilisin/kexin/sedolisin	SYNONYMOUS_CODING
13474	CakSNP13474	*Kabuli*	Ca_Kabuli_Chr08	2081228	[C/T]	Ca_02392	Peptidase S8/S53, subtilisin/kexin/sedolisin	NON_SYNONYMOUS_CODING
13475	CakSNP13475	*Kabuli*	Ca_Kabuli_Chr08	2088903	[T/C]	Ca_02391	Cell cycle regulated microtubule associated protein	NON_SYNONYMOUS_CODING
13476	CakSNP13476	*Kabuli*	Ca_Kabuli_Chr08	2088892	[T/G]	Ca_02391	Cell cycle regulated microtubule associated protein	NON_SYNONYMOUS_CODING
13477	CakSNP13477	*Kabuli*	Ca_Kabuli_Chr08	2090852	[A/G]		--	INTERGENIC
13478	CakSNP13478	*Kabuli*	Ca_Kabuli_Chr08	2095607	[A/G]	Ca_02389	Protein kinase, catalytic domain	NON_SYNONYMOUS_CODING
13479	CakSNP13479	*Kabuli*	Ca_Kabuli_Chr08	2107370	[C/T]	Ca_02387	Bile acid:sodium symporter	SYNONYMOUS_CODING
13480	CakSNP13480	*Kabuli*	Ca_Kabuli_Chr08	2113823	[T/G]	Ca_02386	Bile acid:sodium symporter	NON_SYNONYMOUS_CODING
13481	CakSNP13481	*Kabuli*	Ca_Kabuli_Chr08	2113816	[T/C]	Ca_02386	Bile acid:sodium symporter	SYNONYMOUS_CODING

S.N.	SNP IDs	Chickpea cultivars	Chromosomes	Physical positions (bp)	SNPs	Gene accession IDs	Putative functions	Sequence components of genome
13482	CakSNP13482	*Kabuli*	Ca_Kabuli_Chr08	2128450	[A/G]	Ca_02385	Armadillo	SYNONYMOUS_CODING
13483	CakSNP13483	*Kabuli*	Ca_Kabuli_Chr08	2194927	[G/A]		--	INTERGENIC
13484	CakSNP13484	*Kabuli*	Ca_Kabuli_Chr08	2194898	[A/T]		--	INTERGENIC
13485	CakSNP13485	*Kabuli*	Ca_Kabuli_Chr08	2250195	[C/G]		--	INTERGENIC
13486	CakSNP13486	*Kabuli*	Ca_Kabuli_Chr08	2250148	[T/C]		--	INTERGENIC
13487	CakSNP13487	*Kabuli*	Ca_Kabuli_Chr08	2264108	[G/A]	Ca_02364	Pentapeptide repeat	SYNONYMOUS_CODING
13488	CakSNP13488	*Kabuli*	Ca_Kabuli_Chr08	2279413	[G/A]	Ca_02362	--	SYNONYMOUS_STOP
13489	CakSNP13489	*Kabuli*	Ca_Kabuli_Chr08	2309552	[C/G]	Ca_02358	Oligopeptide transporter	NON_SYNONYMOUS_CODING
13490	CakSNP13490	*Kabuli*	Ca_Kabuli_Chr08	2309549	[A/G]	Ca_02358	Oligopeptide transporter	SYNONYMOUS_CODING
13491	CakSNP13491	*Kabuli*	Ca_Kabuli_Chr08	2413731	[A/G]	Ca_02345	--	SYNONYMOUS_CODING

S.N.	SNP IDs	Chickpea cultivars	Chromosomes	Physical positions (bp)	SNPs	Gene accession IDs	Putative functions	Sequence components of genome
13492	CakSNP13492	*Kabuli*	Ca_Kabuli_Chr08	2456352	[T/C]	Ca_02341	Pentatricopeptide repeat	SYNONYMOUS_CODING
13493	CakSNP13493	*Kabuli*	Ca_Kabuli_Chr08	2482834	[A/G]	Ca_02338	Major intrinsic protein	SYNONYMOUS_CODING
13494	CakSNP13494	*Kabuli*	Ca_Kabuli_Chr08	2482849	[T/C]	Ca_02338	Major intrinsic protein	SYNONYMOUS_CODING
13495	CakSNP13495	*Kabuli*	Ca_Kabuli_Chr08	2483021	[G/A]	Ca_02338	Major intrinsic protein	INTRON
13496	CakSNP13496	*Kabuli*	Ca_Kabuli_Chr08	2488709	[A/G]	Ca_02336	Domain of unknown function DUF828	SYNONYMOUS_CODING
13497	CakSNP13497	*Kabuli*	Ca_Kabuli_Chr08	2488769	[C/G]	Ca_02336	Domain of unknown function DUF828	SYNONYMOUS_CODING
13498	CakSNP13498	*Kabuli*	Ca_Kabuli_Chr08	2488772	[A/G]	Ca_02336	Domain of unknown function DUF828	SYNONYMOUS_CODING
13499	CakSNP13499	*Kabuli*	Ca_Kabuli_Chr08	2515612	[A/G]	Ca_02333	DNA mismatch repair protein MutS, C-terminal domain	INTRON
13500	CakSNP13500	*Kabuli*	Ca_Kabuli_Chr08	2524188	[A/G]	Ca_02332	--	NON_SYNONYMOUS_CODING
13501	CakSNP13501	*Kabuli*	Ca_Kabuli_Chr08	2524148	[A/G]	Ca_02332	--	SYNONYMOUS_CODING

S.N.	SNP IDs	Chickpea cultivars	Chromosomes	Physical positions (bp)	SNPs	Gene accession IDs	Putative functions	Sequence components of genome
13502	CakSNP13502	*Kabuli*	Ca_Kabuli_Chr08	2539375	[G/A]		--	INTERGENIC
13503	CakSNP13503	*Kabuli*	Ca_Kabuli_Chr08	2540644	[G/A]	Ca_02330	--	SYNONYMOUS_CODING
13504	CakSNP13504	*Kabuli*	Ca_Kabuli_Chr08	2540704	[A/T]	Ca_02330	--	NON_SYNONYMOUS_CODING
13505	CakSNP13505	*Kabuli*	Ca_Kabuli_Chr08	2543156	[A/G]	Ca_02330	--	NON_SYNONYMOUS_CODING
13506	CakSNP13506	*Kabuli*	Ca_Kabuli_Chr08	2545070	[T/A]	Ca_02330	--	INTRON
13507	CakSNP13507	*Kabuli*	Ca_Kabuli_Chr08	2604759	[C/T]	Ca_02321	Polycomb protein, VEFS-Box	INTRON
13508	CakSNP13508	*Kabuli*	Ca_Kabuli_Chr08	2605423	[A/T]		--	INTERGENIC
13509	CakSNP13509	*Kabuli*	Ca_Kabuli_Chr08	2605460	[C/T]		--	INTERGENIC
13510	CakSNP13510	*Kabuli*	Ca_Kabuli_Chr08	2621132	[T/A]		--	INTERGENIC
13511	CakSNP13511	*Kabuli*	Ca_Kabuli_Chr08	2622009	[G/T]	Ca_02319	Monooxygenase, FAD-binding	NON_SYNONYMOUS_CODING

S.N.	SNP IDs	Chickpea cultivars	Chromosomes	Physical positions (bp)	SNPs	Gene accession IDs	Putative functions	Sequence components of genome
13512	CakSNP13512	*Kabuli*	Ca_Kabuli_Chr08	2621987	[C/G]	Ca_02319	Monooxygenase, FAD-binding	NON_SYNONYMOUS_CODING
13513	CakSNP13513	*Kabuli*	Ca_Kabuli_Chr08	2621973	[T/G]	Ca_02319	Monooxygenase, FAD-binding	SYNONYMOUS_CODING
13514	CakSNP13514	*Kabuli*	Ca_Kabuli_Chr08	2651439	[G/T]	Ca_02314	Inhibitor of apoptosis-promoting Bax1-related	INTRON
13515	CakSNP13515	*Kabuli*	Ca_Kabuli_Chr08	2651425	[C/T]	Ca_02314	Inhibitor of apoptosis-promoting Bax1-related	INTRON
13516	CakSNP13516	*Kabuli*	Ca_Kabuli_Chr08	2651422	[T/C]	Ca_02314	Inhibitor of apoptosis-promoting Bax1-related	INTRON
13517	CakSNP13517	*Kabuli*	Ca_Kabuli_Chr08	2651393	[G/T]	Ca_02314	Inhibitor of apoptosis-promoting Bax1-related	INTRON
13518	CakSNP13518	*Kabuli*	Ca_Kabuli_Chr08	2651373	[G/T]	Ca_02314	Inhibitor of apoptosis-promoting Bax1-related	INTRON
13519	CakSNP13519	*Kabuli*	Ca_Kabuli_Chr08	2651921	[C/T]	Ca_02314	Inhibitor of apoptosis-promoting Bax1-related	SYNONYMOUS_CODING
13520	CakSNP13520	*Kabuli*	Ca_Kabuli_Chr08	2664625	[T/C]	Ca_02313	Zinc finger, GATA-type	NON_SYNONYMOUS_CODING
13521	CakSNP13521	*Kabuli*	Ca_Kabuli_Chr08	2664711	[T/G]	Ca_02313	Zinc finger, GATA-type	INTRON

S.N.	SNP IDs	Chickpea cultivars	Chromosomes	Physical positions (bp)	SNPs	Gene accession IDs	Putative functions	Sequence components of genome
13522	CakSNP13522	*Kabuli*	Ca_Kabuli_Chr08	2722673	[C/A]	Ca_02304	RAI1-like	INTRON
13523	CakSNP13523	*Kabuli*	Ca_Kabuli_Chr08	2735849	[T/C]	Ca_02302	Ribosomal protein L37ae	INTRON
13524	CakSNP13524	*Kabuli*	Ca_Kabuli_Chr08	2743075	[T/C]		--	INTERGENIC
13525	CakSNP13525	*Kabuli*	Ca_Kabuli_Chr08	2743088	[A/G]		--	INTERGENIC
13526	CakSNP13526	*Kabuli*	Ca_Kabuli_Chr08	2743109	[G/C]		--	INTERGENIC
13527	CakSNP13527	*Kabuli*	Ca_Kabuli_Chr08	2743139	[A/T]		--	INTERGENIC
13528	CakSNP13528	*Kabuli*	Ca_Kabuli_Chr08	2783576	[T/G]		--	INTERGENIC
13529	CakSNP13529	*Kabuli*	Ca_Kabuli_Chr08	2783655	[G/C]		--	INTERGENIC
13530	CakSNP13530	*Kabuli*	Ca_Kabuli_Chr08	2827822	[C/T]	Ca_02297	Pleckstrin homology domain	INTRON
13531	CakSNP13531	*Kabuli*	Ca_Kabuli_Chr08	2827984	[T/C]	Ca_02297	Pleckstrin homology domain	INTRON

S.N.	SNP IDs	Chickpea cultivars	Chromosomes	Physical positions (bp)	SNPs	Gene accession IDs	Putative functions	Sequence components of genome
13532	CakSNP13532	*Kabuli*	Ca_Kabuli_Chr08	2838179	[T/A]	Ca_02297	Pleckstrin homology domain	INTRON
13533	CakSNP13533	*Kabuli*	Ca_Kabuli_Chr08	2871095	[G/A]	Ca_02297	Pleckstrin homology domain	INTRON
13534	CakSNP13534	*Kabuli*	Ca_Kabuli_Chr08	2871064	[G/A]	Ca_02297	Pleckstrin homology domain	INTRON
13535	CakSNP13535	*Kabuli*	Ca_Kabuli_Chr08	2888353	[G/T]	Ca_02296	Peptidase S16, Lon protease, C-terminal	INTRON
13536	CakSNP13536	*Kabuli*	Ca_Kabuli_Chr08	2888317	[G/A]	Ca_02296	Peptidase S16, Lon protease, C-terminal	INTRON
13537	CakSNP13537	*Kabuli*	Ca_Kabuli_Chr08	2943631	[G/T]	Ca_02290	Pentatricopeptide repeat	NON_SYNONYMOUS_CODING
13538	CakSNP13538	*Kabuli*	Ca_Kabuli_Chr08	2947498	[A/G]	Ca_02289	Alpha-D-phosphohexomutase, C-terminal	INTRON
13539	CakSNP13539	*Kabuli*	Ca_Kabuli_Chr08	2974759	[G/C]	Ca_02285	Signal transduction response regulator, receiver domain	INTRON
13540	CakSNP13540	*Kabuli*	Ca_Kabuli_Chr08	2976709	[C/T]	Ca_02285	Signal transduction response regulator, receiver domain	SYNONYMOUS_CODING
13541	CakSNP13541	*Kabuli*	Ca_Kabuli_Chr08	2977543	[T/C]	Ca_02285	Signal transduction response regulator, receiver domain	SYNONYMOUS_CODING

S.N.	SNP IDs	Chickpea cultivars	Chromosomes	Physical positions (bp)	SNPs	Gene accession IDs	Putative functions	Sequence components of genome
13542	CakSNP13542	*Kabuli*	Ca_Kabuli_Chr08	2977628	[C/T]	Ca_02285	Signal transduction response regulator, receiver domain	INTRON
13543	CakSNP13543	*Kabuli*	Ca_Kabuli_Chr08	2977603	[T/A]	Ca_02285	Signal transduction response regulator, receiver domain	INTRON
13544	CakSNP13544	*Kabuli*	Ca_Kabuli_Chr08	3013928	[G/C]	Ca_02280	Pyruvate kinase	INTRON
13545	CakSNP13545	*Kabuli*	Ca_Kabuli_Chr08	3013917	[G/A]	Ca_02280	Pyruvate kinase	INTRON
13546	CakSNP13546	*Kabuli*	Ca_Kabuli_Chr08	3041947	[A/G]	Ca_02278	Protein of unknown function DUF947	SYNONYMOUS_CODING
13547	CakSNP13547	*Kabuli*	Ca_Kabuli_Chr08	3041981	[A/G]	Ca_02278	Protein of unknown function DUF947	INTRON
13548	CakSNP13548	*Kabuli*	Ca_Kabuli_Chr08	3050452	[T/C]	Ca_02276	Glycine cleavage H-protein	SYNONYMOUS_CODING
13549	CakSNP13549	*Kabuli*	Ca_Kabuli_Chr08	3064477	[G/A]	Ca_02273	Malate transporter, aliminium toerance	NON_SYNONYMOUS_CODING
13550	CakSNP13550	*Kabuli*	Ca_Kabuli_Chr08	3141617	[C/T]	Ca_02265	--	NON_SYNONYMOUS_CODING
13551	CakSNP13551	*Kabuli*	Ca_Kabuli_Chr08	3171324	[C/T]		--	INTERGENIC

S.N.	SNP IDs	Chickpea cultivars	Chromosomes	Physical positions (bp)	SNPs	Gene accession IDs	Putative functions	Sequence components of genome
13552	CakSNP13552	*Kabuli*	Ca_Kabuli_Chr08	3210879	[C/A]	Ca_02260	Protein kinase, catalytic domain	NON_SYNONYMOUS_CODING
13553	CakSNP13553	*Kabuli*	Ca_Kabuli_Chr08	3248044	[A/G]	Ca_02254	Aminotransferase, class V/Cysteine desulfurase	SYNONYMOUS_CODING
13554	CakSNP13554	*Kabuli*	Ca_Kabuli_Chr08	3248104	[A/G]	Ca_02254	Aminotransferase, class V/Cysteine desulfurase	SYNONYMOUS_CODING
13555	CakSNP13555	*Kabuli*	Ca_Kabuli_Chr08	3250707	[C/T]		--	INTERGENIC
13556	CakSNP13556	*Kabuli*	Ca_Kabuli_Chr08	3263822	[T/C]		--	INTERGENIC
13557	CakSNP13557	*Kabuli*	Ca_Kabuli_Chr08	3269124	[G/A]	Ca_02251	Homeobox	SYNONYMOUS_CODING
13558	CakSNP13558	*Kabuli*	Ca_Kabuli_Chr08	3269118	[A/G]	Ca_02251	Homeobox	SYNONYMOUS_CODING
13559	CakSNP13559	*Kabuli*	Ca_Kabuli_Chr08	3269088	[A/C]	Ca_02251	Homeobox	SYNONYMOUS_CODING
13560	CakSNP13560	*Kabuli*	Ca_Kabuli_Chr08	3269085	[A/C]	Ca_02251	Homeobox	SYNONYMOUS_CODING
13561	CakSNP13561	*Kabuli*	Ca_Kabuli_Chr08	3297410	[T/C]	Ca_02249	SANT domain, DNA binding	NON_SYNONYMOUS_CODING

S.N.	SNP IDs	Chickpea cultivars	Chromosomes	Physical positions (bp)	SNPs	Gene accession IDs	Putative functions	Sequence components of genome
13562	CakSNP13562	*Kabuli*	Ca_Kabuli_Chr08	3386165	[C/A]		--	INTERGENIC
13563	CakSNP13563	*Kabuli*	Ca_Kabuli_Chr08	3386181	[C/A]	Ca_02242	--	NON_SYNONYMOUS_CODING
13564	CakSNP13564	*Kabuli*	Ca_Kabuli_Chr08	3392929	[A/C]	Ca_02241	Amino acid-binding ACT	SYNONYMOUS_CODING
13565	CakSNP13565	*Kabuli*	Ca_Kabuli_Chr08	3394796	[A/C]		--	INTERGENIC
13566	CakSNP13566	*Kabuli*	Ca_Kabuli_Chr08	3394833	[T/C]		--	INTERGENIC
13567	CakSNP13567	*Kabuli*	Ca_Kabuli_Chr08	3394931	[T/C]		--	INTERGENIC
13568	CakSNP13568	*Kabuli*	Ca_Kabuli_Chr08	3396820	[T/C]	Ca_02240	BSD	NON_SYNONYMOUS_CODING
13569	CakSNP13569	*Kabuli*	Ca_Kabuli_Chr08	3438274	[A/T]	Ca_02235	Hly-III-related	INTRON
13570	CakSNP13570	*Kabuli*	Ca_Kabuli_Chr08	3445568	[G/T]	Ca_02234	Transmembrane protein 194	INTRON
13571	CakSNP13571	*Kabuli*	Ca_Kabuli_Chr08	3448194	[C/T]	Ca_02234	Transmembrane protein 194	INTRON

S.N.	SNP IDs	Chickpea cultivars	Chromosomes	Physical positions (bp)	SNPs	Gene accession IDs	Putative functions	Sequence components of genome
13572	CakSNP13572	*Kabuli*	Ca_Kabuli_Chr08	3460627	[G/A]	Ca_02233	GCN5-related N-acetyltransferase (GNAT) domain	INTRON
13573	CakSNP13573	*Kabuli*	Ca_Kabuli_Chr08	3499010	[G/A]	Ca_02229	Ras GTPase	INTRON
13574	CakSNP13574	*Kabuli*	Ca_Kabuli_Chr08	3539690	[A/G]		--	INTERGENIC
13575	CakSNP13575	*Kabuli*	Ca_Kabuli_Chr08	3539723	[G/C]		--	INTERGENIC
13576	CakSNP13576	*Kabuli*	Ca_Kabuli_Chr08	3539745	[T/C]		--	INTERGENIC
13577	CakSNP13577	*Kabuli*	Ca_Kabuli_Chr08	3539851	[T/C]		--	INTERGENIC
13578	CakSNP13578	*Kabuli*	Ca_Kabuli_Chr08	3539838	[T/G]		--	INTERGENIC
13579	CakSNP13579	*Kabuli*	Ca_Kabuli_Chr08	3539832	[T/C]		--	INTERGENIC
13580	CakSNP13580	*Kabuli*	Ca_Kabuli_Chr08	3560271	[G/A]	Ca_02220	ATPase, P-type, H+ transporting proton pump	INTRON
13581	CakSNP13581	*Kabuli*	Ca_Kabuli_Chr08	3563692	[A/C]	Ca_02220	ATPase, P-type, H+ transporting proton pump	SYNONYMOUS_CODING

S.N.	SNP IDs	Chickpea cultivars	Chromosomes	Physical positions (bp)	SNPs	Gene accession IDs	Putative functions	Sequence components of genome
13582	CakSNP13582	*Kabuli*	Ca_Kabuli_Chr08	3563825	[G/T]		--	INTERGENIC
13583	CakSNP13583	*Kabuli*	Ca_Kabuli_Chr08	3563824	[T/A]		--	INTERGENIC
13584	CakSNP13584	*Kabuli*	Ca_Kabuli_Chr08	3599091	[G/C]		--	INTERGENIC
13585	CakSNP13585	*Kabuli*	Ca_Kabuli_Chr08	3601517	[T/C]	Ca_02217	Zinc finger, TAZ-type	SYNONYMOUS_CODING
13586	CakSNP13586	*Kabuli*	Ca_Kabuli_Chr08	3605513	[T/C]	Ca_02216	Actin-binding FH2/DRF autoregulatory	SYNONYMOUS_CODING
13587	CakSNP13587	*Kabuli*	Ca_Kabuli_Chr08	3617810	[T/C]		--	INTERGENIC
13588	CakSNP13588	*Kabuli*	Ca_Kabuli_Chr08	3617757	[G/C]		--	INTERGENIC
13589	CakSNP13589	*Kabuli*	Ca_Kabuli_Chr08	3723085	[C/T]	Ca_02205	Ribonuclease III	INTRON
13590	CakSNP13590	*Kabuli*	Ca_Kabuli_Chr08	3730369	[A/C]		--	INTERGENIC
13591	CakSNP13591	*Kabuli*	Ca_Kabuli_Chr08	3775945	[G/A]	Ca_02199	Peptidase S8/S53, subtilisin/kexin/sedolisin	SYNONYMOUS_CODING

S.N.	SNP IDs	Chickpea cultivars	Chromosomes	Physical positions (bp)	SNPs	Gene accession IDs	Putative functions	Sequence components of genome
13592	CakSNP13592	*Kabuli*	Ca_Kabuli_Chr08	3775960	[T/C]	Ca_02199	Peptidase S8/S53, subtilisin/kexin/sedolisin	SYNONYMOUS_CODING
13593	CakSNP13593	*Kabuli*	Ca_Kabuli_Chr08	3813978	[C/T]	Ca_02193	--	SYNONYMOUS_CODING
13594	CakSNP13594	*Kabuli*	Ca_Kabuli_Chr08	3829425	[C/G]		--	INTERGENIC
13595	CakSNP13595	*Kabuli*	Ca_Kabuli_Chr08	3829576	[A/G]	Ca_02191	Natural resistance-associated macrophage protein	SYNONYMOUS_CODING
13596	CakSNP13596	*Kabuli*	Ca_Kabuli_Chr08	3837237	[A/G]		--	INTERGENIC
13597	CakSNP13597	*Kabuli*	Ca_Kabuli_Chr08	3837306	[G/C]		--	INTERGENIC
13598	CakSNP13598	*Kabuli*	Ca_Kabuli_Chr08	3868511	[A/C]	Ca_02188	WD40 repeat	INTRON
13599	CakSNP13599	*Kabuli*	Ca_Kabuli_Chr08	3877130	[G/T]	Ca_02187	Polyprenyl synthetase	INTRON
13600	CakSNP13600	*Kabuli*	Ca_Kabuli_Chr08	3877176	[T/G]	Ca_02187	Polyprenyl synthetase	INTRON
13601	CakSNP13601	*Kabuli*	Ca_Kabuli_Chr08	3911930	[A/C]	Ca_02182	SANT domain, DNA binding	SYNONYMOUS_CODING

S.N.	SNP IDs	Chickpea cultivars	Chromosomes	Physical positions (bp)	SNPs	Gene accession IDs	Putative functions	Sequence components of genome
13602	CakSNP13602	*Kabuli*	Ca_Kabuli_Chr08	3911876	[G/C]	Ca_02182	SANT domain, DNA binding	SYNONYMOUS_CODING
13603	CakSNP13603	*Kabuli*	Ca_Kabuli_Chr08	3951767	[C/A]	Ca_02175	Ribosomal protein S2	NON_SYNONYMOUS_CODING
13604	CakSNP13604	*Kabuli*	Ca_Kabuli_Chr08	3951768	[A/G]	Ca_02175	Ribosomal protein S2	SYNONYMOUS_CODING
13605	CakSNP13605	*Kabuli*	Ca_Kabuli_Chr08	3954853	[T/A]		--	INTERGENIC
13606	CakSNP13606	*Kabuli*	Ca_Kabuli_Chr08	4042166	[A/C]	Ca_02167	Protein of unknown function DUF869, plant	SYNONYMOUS_CODING
13607	CakSNP13607	*Kabuli*	Ca_Kabuli_Chr08	4042233	[G/A]	Ca_02167	Protein of unknown function DUF869, plant	SYNONYMOUS_CODING
13608	CakSNP13608	*Kabuli*	Ca_Kabuli_Chr08	4042424	[G/A]	Ca_02167	Protein of unknown function DUF869, plant	NON_SYNONYMOUS_CODING
13609	CakSNP13609	*Kabuli*	Ca_Kabuli_Chr08	4052732	[C/T]	Ca_02166	SET domain	INTRON
13610	CakSNP13610	*Kabuli*	Ca_Kabuli_Chr08	4052702	[C/T]	Ca_02166	SET domain	SYNONYMOUS_CODING
13611	CakSNP13611	*Kabuli*	Ca_Kabuli_Chr08	4073528	[C/T]	Ca_02163	Zinc finger, RING-type	NON_SYNONYMOUS_CODING

S.N.	SNP IDs	Chickpea cultivars	Chromosomes	Physical positions (bp)	SNPs	Gene accession IDs	Putative functions	Sequence components of genome
13612	CakSNP13612	*Kabuli*	Ca_Kabuli_Chr08	4073603	[A/G]	Ca_02163	Zinc finger, RING-type	NON_SYNONYMOUS_CODING
13613	CakSNP13613	*Kabuli*	Ca_Kabuli_Chr08	4074135	[T/C]	Ca_02163	Zinc finger, RING-type	SYNONYMOUS_CODING
13614	CakSNP13614	*Kabuli*	Ca_Kabuli_Chr08	4091946	[T/C]	Ca_02160	DNA-directed DNA polymerase, family B, exonuclease domain	SYNONYMOUS_CODING
13615	CakSNP13615	*Kabuli*	Ca_Kabuli_Chr08	4092348	[T/A]	Ca_02160	DNA-directed DNA polymerase, family B, exonuclease domain	INTRON
13616	CakSNP13616	*Kabuli*	Ca_Kabuli_Chr08	4092350	[G/C]	Ca_02160	DNA-directed DNA polymerase, family B, exonuclease domain	INTRON
13617	CakSNP13617	*Kabuli*	Ca_Kabuli_Chr08	4095131	[A/C]	Ca_02160	DNA-directed DNA polymerase, family B, exonuclease domain	INTRON
13618	CakSNP13618	*Kabuli*	Ca_Kabuli_Chr08	4095119	[G/A]	Ca_02160	DNA-directed DNA polymerase, family B, exonuclease domain	INTRON
13619	CakSNP13619	*Kabuli*	Ca_Kabuli_Chr08	4106580	[T/C]	Ca_02159	Protein kinase, catalytic domain	INTRON
13620	CakSNP13620	*Kabuli*	Ca_Kabuli_Chr08	4106583	[T/C]	Ca_02159	Protein kinase, catalytic domain	INTRON
13621	CakSNP13621	*Kabuli*	Ca_Kabuli_Chr08	4106644	[C/T]	Ca_02159	Protein kinase, catalytic domain	INTRON

S.N.	SNP IDs	Chickpea cultivars	Chromosomes	Physical positions (bp)	SNPs	Gene accession IDs	Putative functions	Sequence components of genome
13622	CakSNP13622	*Kabuli*	Ca_Kabuli_Chr08	4172342	[A/G]	Ca_02150	Forkhead-associated (FHA) domain	NON_SYNONYMOUS_CODING
13623	CakSNP13623	*Kabuli*	Ca_Kabuli_Chr08	4172368	[T/C]	Ca_02150	Forkhead-associated (FHA) domain	SYNONYMOUS_CODING
13624	CakSNP13624	*Kabuli*	Ca_Kabuli_Chr08	4183744	[A/G]		--	INTERGENIC
13625	CakSNP13625	*Kabuli*	Ca_Kabuli_Chr08	4183733	[G/A]		--	INTERGENIC
13626	CakSNP13626	*Kabuli*	Ca_Kabuli_Chr08	4198214	[A/C]	Ca_02148	Splicing factor, suppressor of white apricot	NON_SYNONYMOUS_CODING
13627	CakSNP13627	*Kabuli*	Ca_Kabuli_Chr08	4201285	[A/G]	Ca_02148	Splicing factor, suppressor of white apricot	INTRON
13628	CakSNP13628	*Kabuli*	Ca_Kabuli_Chr08	4237620	[T/A]	Ca_02144	--	INTRON
13629	CakSNP13629	*Kabuli*	Ca_Kabuli_Chr08	4271620	[T/A]	Ca_02139	Peptidase M24, structural domain	INTRON
13630	CakSNP13630	*Kabuli*	Ca_Kabuli_Chr08	4290690	[T/A]	Ca_02137	Patatin/Phospholipase A2-related	NON_SYNONYMOUS_CODING
13631	CakSNP13631	*Kabuli*	Ca_Kabuli_Chr08	4322058	[A/G]	Ca_02135	Protein of unknown function DUF3651, TMEM131	SYNONYMOUS_CODING

S.N.	SNP IDs	Chickpea cultivars	Chromosomes	Physical positions (bp)	SNPs	Gene accession IDs	Putative functions	Sequence components of genome
13632	CakSNP13632	*Kabuli*	Ca_Kabuli_Chr08	4322202	[C/T]	Ca_02135	Protein of unknown function DUF3651, TMEM131	SYNONYMOUS_CODING
13633	CakSNP13633	*Kabuli*	Ca_Kabuli_Chr08	4344459	[C/A]	Ca_02132	--	NON_SYNONYMOUS_CODING
13634	CakSNP13634	*Kabuli*	Ca_Kabuli_Chr08	4346552	[A/G]		--	INTERGENIC
13635	CakSNP13635	*Kabuli*	Ca_Kabuli_Chr08	4346749	[A/G]		--	INTERGENIC
13636	CakSNP13636	*Kabuli*	Ca_Kabuli_Chr08	4346811	[C/T]		--	INTERGENIC
13637	CakSNP13637	*Kabuli*	Ca_Kabuli_Chr08	4346827	[A/C]		--	INTERGENIC
13638	CakSNP13638	*Kabuli*	Ca_Kabuli_Chr08	4347535	[A/C]		--	INTERGENIC
13639	CakSNP13639	*Kabuli*	Ca_Kabuli_Chr08	4351751	[G/A]		--	INTERGENIC
13640	CakSNP13640	*Kabuli*	Ca_Kabuli_Chr08	4353397	[A/C]	Ca_02130	WD40 repeat	INTRON
13641	CakSNP13641	*Kabuli*	Ca_Kabuli_Chr08	4355388	[G/T]	Ca_02130	WD40 repeat	INTRON

S.N.	SNP IDs	Chickpea cultivars	Chromosomes	Physical positions (bp)	SNPs	Gene accession IDs	Putative functions	Sequence components of genome
13642	CakSNP13642	*Kabuli*	Ca_Kabuli_Chr08	4355531	[A/C]	Ca_02130	WD40 repeat	INTRON
13643	CakSNP13643	*Kabuli*	Ca_Kabuli_Chr08	4368163	[T/C]	Ca_02128	Protein kinase, catalytic domain	SYNONYMOUS_CODING
13644	CakSNP13644	*Kabuli*	Ca_Kabuli_Chr08	4368184	[T/C]	Ca_02128	Protein kinase, catalytic domain	SYNONYMOUS_CODING
13645	CakSNP13645	*Kabuli*	Ca_Kabuli_Chr08	4368213	[T/C]	Ca_02128	Protein kinase, catalytic domain	NON_SYNONYMOUS_CODING
13646	CakSNP13646	*Kabuli*	Ca_Kabuli_Chr08	4368403	[G/A]	Ca_02128	Protein kinase, catalytic domain	SYNONYMOUS_CODING
13647	CakSNP13647	*Kabuli*	Ca_Kabuli_Chr08	4368463	[A/G]	Ca_02128	Protein kinase, catalytic domain	SYNONYMOUS_CODING
13648	CakSNP13648	*Kabuli*	Ca_Kabuli_Chr08	4392707	[T/C]	Ca_02126	Protein kinase, catalytic domain	INTRON
13649	CakSNP13649	*Kabuli*	Ca_Kabuli_Chr08	4392767	[T/G]	Ca_02126	Protein kinase, catalytic domain	INTRON
13650	CakSNP13650	*Kabuli*	Ca_Kabuli_Chr08	4392818	[C/A]	Ca_02126	Protein kinase, catalytic domain	INTRON
13651	CakSNP13651	*Kabuli*	Ca_Kabuli_Chr08	4406956	[C/G]	Ca_02125	Lateral organ boundaries, LOB	SYNONYMOUS_CODING

S.N.	SNP IDs	Chickpea cultivars	Chromosomes	Physical positions (bp)	SNPs	Gene accession IDs	Putative functions	Sequence components of genome
13652	CakSNP13652	*Kabuli*	Ca_Kabuli_Chr08	4421609	[A/G]	Ca_02124	Lateral organ boundaries, LOB	NON_SYNONYMOUS_CODING
13653	CakSNP13653	*Kabuli*	Ca_Kabuli_Chr08	4421629	[T/A]	Ca_02124	Lateral organ boundaries, LOB	SYNONYMOUS_CODING
13654	CakSNP13654	*Kabuli*	Ca_Kabuli_Chr08	4472423	[A/T]	Ca_02119	Disease resistance protein	SYNONYMOUS_CODING
13655	CakSNP13655	*Kabuli*	Ca_Kabuli_Chr08	4483130	[T/C]	Ca_02117	Zinc finger, LIM-type	NON_SYNONYMOUS_CODING
13656	CakSNP13656	*Kabuli*	Ca_Kabuli_Chr08	4483274	[T/C]	Ca_02117	Zinc finger, LIM-type	INTRON
13657	CakSNP13657	*Kabuli*	Ca_Kabuli_Chr08	4484606	[T/A]	Ca_02117	Zinc finger, LIM-type	INTRON
13658	CakSNP13658	*Kabuli*	Ca_Kabuli_Chr08	4507729	[G/T]		--	INTERGENIC
13659	CakSNP13659	*Kabuli*	Ca_Kabuli_Chr08	4510845	[C/T]		--	INTERGENIC
13660	CakSNP13660	*Kabuli*	Ca_Kabuli_Chr08	4510810	[A/G]		--	INTERGENIC
13661	CakSNP13661	*Kabuli*	Ca_Kabuli_Chr08	4512311	[T/C]	Ca_02113	Pentatricopeptide repeat	NON_SYNONYMOUS_CODING

S.N.	SNP IDs	Chickpea cultivars	Chromosomes	Physical positions (bp)	SNPs	Gene accession IDs	Putative functions	Sequence components of genome
13662	CakSNP13662	*Kabuli*	Ca_Kabuli_Chr08	4512436	[G/A]	Ca_02113	Pentatricopeptide repeat	NON_SYNONYMOUS_CODING
13663	CakSNP13663	*Kabuli*	Ca_Kabuli_Chr08	4512492	[T/G]	Ca_02113	Pentatricopeptide repeat	SYNONYMOUS_CODING
13664	CakSNP13664	*Kabuli*	Ca_Kabuli_Chr08	4524914	[C/T]		--	INTERGENIC
13665	CakSNP13665	*Kabuli*	Ca_Kabuli_Chr08	4557334	[A/G]		--	INTERGENIC
13666	CakSNP13666	*Kabuli*	Ca_Kabuli_Chr08	4557328	[T/C]		--	INTERGENIC
13667	CakSNP13667	*Kabuli*	Ca_Kabuli_Chr08	4557401	[A/C]		--	INTERGENIC
13668	CakSNP13668	*Kabuli*	Ca_Kabuli_Chr08	4557407	[A/C]		--	INTERGENIC
13669	CakSNP13669	*Kabuli*	Ca_Kabuli_Chr08	4557440	[A/G]		--	INTERGENIC
13670	CakSNP13670	*Kabuli*	Ca_Kabuli_Chr08	4557488	[C/T]		--	INTERGENIC
13671	CakSNP13671	*Kabuli*	Ca_Kabuli_Chr08	4580363	[C/A]	Ca_02100	--	NON_SYNONYMOUS_CODING

S.N.	SNP IDs	Chickpea cultivars	Chromosomes	Physical positions (bp)	SNPs	Gene accession IDs	Putative functions	Sequence components of genome
13672	CakSNP13672	*Kabuli*	Ca_Kabuli_Chr08	4588869	[T/C]		--	INTERGENIC
13673	CakSNP13673	*Kabuli*	Ca_Kabuli_Chr08	4588915	[A/C]		--	INTERGENIC
13674	CakSNP13674	*Kabuli*	Ca_Kabuli_Chr08	4611094	[C/T]	Ca_02096	SET domain	NON_SYNONYMOUS_CODING
13675	CakSNP13675	*Kabuli*	Ca_Kabuli_Chr08	4658502	[A/G]	Ca_02094	Zinc finger, LIM-type	INTRON
13676	CakSNP13676	*Kabuli*	Ca_Kabuli_Chr08	4714566	[G/A]	Ca_02087	Pentatricopeptide repeat	SYNONYMOUS_CODING
13677	CakSNP13677	*Kabuli*	Ca_Kabuli_Chr08	4744388	[C/T]	Ca_02083	Cystinosin/ERS1p repeat	SYNONYMOUS_CODING
13678	CakSNP13678	*Kabuli*	Ca_Kabuli_Chr08	4767567	[A/C]		--	INTERGENIC
13679	CakSNP13679	*Kabuli*	Ca_Kabuli_Chr08	4767586	[C/G]		--	INTERGENIC
13680	CakSNP13680	*Kabuli*	Ca_Kabuli_Chr08	4773205	[A/C]		--	INTERGENIC
13681	CakSNP13681	*Kabuli*	Ca_Kabuli_Chr08	4810954	[A/C]	Ca_02076	Major facilitator superfamily MFS-1	INTRON

S.N.	SNP IDs	Chickpea cultivars	Chromosomes	Physical positions (bp)	SNPs	Gene accession IDs	Putative functions	Sequence components of genome
13682	CakSNP13682	*Kabuli*	Ca_Kabuli_Chr08	4810968	[G/A]	Ca_02076	Major facilitator superfamily MFS-1	INTRON
13683	CakSNP13683	*Kabuli*	Ca_Kabuli_Chr08	4810986	[A/C]	Ca_02076	Major facilitator superfamily MFS-1	INTRON
13684	CakSNP13684	*Kabuli*	Ca_Kabuli_Chr08	4858262	[C/A]	Ca_02071	NADPH-dependent FMN reductase	NON_SYNONYMOUS_CODING
13685	CakSNP13685	*Kabuli*	Ca_Kabuli_Chr08	4899964	[T/G]	Ca_02066	Protein phosphatase 2C-like	NON_SYNONYMOUS_CODING
13686	CakSNP13686	*Kabuli*	Ca_Kabuli_Chr08	4899983	[G/A]	Ca_02066	Protein phosphatase 2C-like	SYNONYMOUS_CODING
13687	CakSNP13687	*Kabuli*	Ca_Kabuli_Chr08	4980949	[A/T]		--	INTERGENIC
13688	CakSNP13688	*Kabuli*	Ca_Kabuli_Chr08	4980916	[C/T]		--	INTERGENIC
13689	CakSNP13689	*Kabuli*	Ca_Kabuli_Chr08	5044456	[T/C]	Ca_02050	Fumarate reductase/succinate dehydrogenase, FAD-binding site	SYNONYMOUS_CODING
13690	CakSNP13690	*Kabuli*	Ca_Kabuli_Chr08	5044423	[T/A]	Ca_02050	Fumarate reductase/succinate dehydrogenase, FAD-binding site	SYNONYMOUS_CODING
13691	CakSNP13691	*Kabuli*	Ca_Kabuli_Chr08	5096194	[T/G]		--	INTERGENIC

S.N.	SNP IDs	Chickpea cultivars	Chromosomes	Physical positions (bp)	SNPs	Gene accession IDs	Putative functions	Sequence components of genome
13692	CakSNP13692	*Kabuli*	Ca_Kabuli_Chr08	5115451	[C/G]		--	INTERGENIC
13693	CakSNP13693	*Kabuli*	Ca_Kabuli_Chr08	5115498	[A/T]		--	INTERGENIC
13694	CakSNP13694	*Kabuli*	Ca_Kabuli_Chr08	5115512	[T/G]		--	INTERGENIC
13695	CakSNP13695	*Kabuli*	Ca_Kabuli_Chr08	5115522	[G/C]		--	INTERGENIC
13696	CakSNP13696	*Kabuli*	Ca_Kabuli_Chr08	5134706	[A/C]		--	INTERGENIC
13697	CakSNP13697	*Kabuli*	Ca_Kabuli_Chr08	5184227	[C/G]	Ca_02036	Sugar/inositol transporter	NON_SYNONYMOUS_CODING
13698	CakSNP13698	*Kabuli*	Ca_Kabuli_Chr08	5184276	[G/A]	Ca_02036	Sugar/inositol transporter	INTRON
13699	CakSNP13699	*Kabuli*	Ca_Kabuli_Chr08	5197004	[G/T]	Ca_02034	Homeobox	NON_SYNONYMOUS_CODING
13700	CakSNP13700	*Kabuli*	Ca_Kabuli_Chr08	5197254	[G/A]	Ca_02034	Homeobox	SYNONYMOUS_CODING
13701	CakSNP13701	*Kabuli*	Ca_Kabuli_Chr08	5204956	[A/G]	Ca_02032	ATPase, AAA+ type, core	SYNONYMOUS_CODING

S.N.	SNP IDs	Chickpea cultivars	Chromosomes	Physical positions (bp)	SNPs	Gene accession IDs	Putative functions	Sequence components of genome
13702	CakSNP13702	*Kabuli*	Ca_Kabuli_Chr08	5313792	[T/C]		--	INTERGENIC
13703	CakSNP13703	*Kabuli*	Ca_Kabuli_Chr08	5313782	[T/G]		--	INTERGENIC
13704	CakSNP13704	*Kabuli*	Ca_Kabuli_Chr08	5313774	[C/G]		--	INTERGENIC
13705	CakSNP13705	*Kabuli*	Ca_Kabuli_Chr08	5313770	[A/T]		--	INTERGENIC
13706	CakSNP13706	*Kabuli*	Ca_Kabuli_Chr08	5379556	[G/A]	Ca_02010	ATPase, AAA+ type, core	SYNONYMOUS_CODING
13707	CakSNP13707	*Kabuli*	Ca_Kabuli_Chr08	5390415	[C/T]	Ca_02008	Plant peroxidase	SYNONYMOUS_CODING
13708	CakSNP13708	*Kabuli*	Ca_Kabuli_Chr08	5390602	[T/C]	Ca_02008	Plant peroxidase	NON_SYNONYMOUS_CODING
13709	CakSNP13709	*Kabuli*	Ca_Kabuli_Chr08	5399503	[G/A]	Ca_02005	Protein of unknown function DUF248, methyltransferase putative	STOP_GAINED
13710	CakSNP13710	*Kabuli*	Ca_Kabuli_Chr08	5400188	[A/G]	Ca_02005	Protein of unknown function DUF248, methyltransferase putative	INTRON
13711	CakSNP13711	*Kabuli*	Ca_Kabuli_Chr08	5412501	[A/G]	Ca_02004	Protein of unknown function DUF702	SYNONYMOUS_CODING

S.N.	SNP IDs	Chickpea cultivars	Chromosomes	Physical positions (bp)	SNPs	Gene accession IDs	Putative functions	Sequence components of genome
13712	CakSNP13712	*Kabuli*	Ca_Kabuli_Chr08	5412489	[T/C]	Ca_02004	Protein of unknown function DUF702	SYNONYMOUS_CODING
13713	CakSNP13713	*Kabuli*	Ca_Kabuli_Chr08	5434223	[C/T]	Ca_02002	Drug/metabolite transporter	INTRON
13714	CakSNP13714	*Kabuli*	Ca_Kabuli_Chr08	5434225	[G/C]	Ca_02002	Drug/metabolite transporter	INTRON
13715	CakSNP13715	*Kabuli*	Ca_Kabuli_Chr08	5434232	[A/G]	Ca_02002	Drug/metabolite transporter	INTRON
13716	CakSNP13716	*Kabuli*	Ca_Kabuli_Chr08	5434238	[T/G]	Ca_02002	Drug/metabolite transporter	INTRON
13717	CakSNP13717	*Kabuli*	Ca_Kabuli_Chr08	5434242	[T/A]	Ca_02002	Drug/metabolite transporter	INTRON
13718	CakSNP13718	*Kabuli*	Ca_Kabuli_Chr08	5487769	[G/A]		--	INTERGENIC
13719	CakSNP13719	*Kabuli*	Ca_Kabuli_Chr08	5487830	[G/A]		--	INTERGENIC
13720	CakSNP13720	*Kabuli*	Ca_Kabuli_Chr08	5536418	[T/G]	Ca_01989	Protein of unknown function DUF726	INTRON
13721	CakSNP13721	*Kabuli*	Ca_Kabuli_Chr08	5538696	[A/C]	Ca_01989	Protein of unknown function DUF726	INTRON

S.N.	SNP IDs	Chickpea cultivars	Chromosomes	Physical positions (bp)	SNPs	Gene accession IDs	Putative functions	Sequence components of genome
13722	CakSNP13722	*Kabuli*	Ca_Kabuli_Chr08	5538695	[T/C]	Ca_01989	Protein of unknown function DUF726	INTRON
13723	CakSNP13723	*Kabuli*	Ca_Kabuli_Chr08	5538683	[T/C]	Ca_01989	Protein of unknown function DUF726	INTRON
13724	CakSNP13724	*Kabuli*	Ca_Kabuli_Chr08	5539044	[C/T]	Ca_01989	Protein of unknown function DUF726	NON_SYNONYMOUS_CODING
13725	CakSNP13725	*Kabuli*	Ca_Kabuli_Chr08	5586106	[G/A]	Ca_01986	Uncharacterised protein family UPF0183	INTRON
13726	CakSNP13726	*Kabuli*	Ca_Kabuli_Chr08	5591654	[T/C]		--	INTERGENIC
13727	CakSNP13727	*Kabuli*	Ca_Kabuli_Chr08	5591788	[G/C]		--	INTERGENIC
13728	CakSNP13728	*Kabuli*	Ca_Kabuli_Chr08	5650080	[C/G]		--	INTERGENIC
13729	CakSNP13729	*Kabuli*	Ca_Kabuli_Chr08	5712245	[T/A]	Ca_01977	Protein of unknown function DUF869, plant	INTRON
13730	CakSNP13730	*Kabuli*	Ca_Kabuli_Chr08	5811179	[G/C]	Ca_01971	Phosphatidylinositol 3-/4-kinase, catalytic	INTRON
13731	CakSNP13731	*Kabuli*	Ca_Kabuli_Chr08	5819008	[G/A]	Ca_01971	Phosphatidylinositol 3-/4-kinase, catalytic	INTRON

S.N.	SNP IDs	Chickpea cultivars	Chromosomes	Physical positions (bp)	SNPs	Gene accession IDs	Putative functions	Sequence components of genome
13732	CakSNP13732	*Kabuli*	Ca_Kabuli_Chr08	5840141	[G/A]	Ca_01967	Xanthine/uracil/vitamin C permease	INTRON
13733	CakSNP13733	*Kabuli*	Ca_Kabuli_Chr08	5870455	[A/G]	Ca_01965	Armadillo	SYNONYMOUS_CODING
13734	CakSNP13734	*Kabuli*	Ca_Kabuli_Chr08	5870664	[T/C]	Ca_01965	Armadillo	NON_SYNONYMOUS_CODING
13735	CakSNP13735	*Kabuli*	Ca_Kabuli_Chr08	5870749	[C/T]	Ca_01965	Armadillo	SYNONYMOUS_CODING
13736	CakSNP13736	*Kabuli*	Ca_Kabuli_Chr08	5871358	[G/C]	Ca_01965	Armadillo	SYNONYMOUS_CODING
13737	CakSNP13737	*Kabuli*	Ca_Kabuli_Chr08	5871361	[T/G]	Ca_01965	Armadillo	SYNONYMOUS_CODING
13738	CakSNP13738	*Kabuli*	Ca_Kabuli_Chr08	5871364	[T/C]	Ca_01965	Armadillo	SYNONYMOUS_CODING
13739	CakSNP13739	*Kabuli*	Ca_Kabuli_Chr08	5871367	[T/A]	Ca_01965	Armadillo	SYNONYMOUS_CODING
13740	CakSNP13740	*Kabuli*	Ca_Kabuli_Chr08	5871421	[A/G]	Ca_01965	Armadillo	SYNONYMOUS_CODING
13741	CakSNP13741	*Kabuli*	Ca_Kabuli_Chr08	5910629	[C/G]	Ca_01961	Nodulin-like	SYNONYMOUS_CODING

S.N.	SNP IDs	Chickpea cultivars	Chromosomes	Physical positions (bp)	SNPs	Gene accession IDs	Putative functions	Sequence components of genome
13742	CakSNP13742	*Kabuli*	Ca_Kabuli_Chr08	5927870	[G/T]	Ca_01958	von Willebrand factor, type A	INTRON
13743	CakSNP13743	*Kabuli*	Ca_Kabuli_Chr08	5935435	[A/C]	Ca_01957	Senescence/spartin-associated	SYNONYMOUS_CODING
13744	CakSNP13744	*Kabuli*	Ca_Kabuli_Chr08	5963855	[T/A]	Ca_01953	--	NON_SYNONYMOUS_CODING
13745	CakSNP13745	*Kabuli*	Ca_Kabuli_Chr08	5992761	[A/G]	Ca_01949	Vacuolar protein sorting-associated protein 35	NON_SYNONYMOUS_CODING
13746	CakSNP13746	*Kabuli*	Ca_Kabuli_Chr08	6084594	[G/A]	Ca_01938	--	SYNONYMOUS_CODING
13747	CakSNP13747	*Kabuli*	Ca_Kabuli_Chr08	6084591	[C/T]	Ca_01938	--	NON_SYNONYMOUS_CODING
13748	CakSNP13748	*Kabuli*	Ca_Kabuli_Chr08	6115961	[T/G]	Ca_01935	Plant peroxidase	SYNONYMOUS_CODING
13749	CakSNP13749	*Kabuli*	Ca_Kabuli_Chr08	6115965	[T/C]	Ca_01935	Plant peroxidase	SYNONYMOUS_CODING
13750	CakSNP13750	*Kabuli*	Ca_Kabuli_Chr08	6116096	[C/T]	Ca_01935	Plant peroxidase	INTRON
13751	CakSNP13751	*Kabuli*	Ca_Kabuli_Chr08	6116097	[C/A]	Ca_01935	Plant peroxidase	INTRON

S.N.	SNP IDs	Chickpea cultivars	Chromosomes	Physical positions (bp)	SNPs	Gene accession IDs	Putative functions	Sequence components of genome
13752	CakSNP13752	*Kabuli*	Ca_Kabuli_Chr08	6116101	[G/A]	Ca_01935	Plant peroxidase	INTRON
13753	CakSNP13753	*Kabuli*	Ca_Kabuli_Chr08	6145326	[G/A]	Ca_01932	Aconitase A/isopropylmalate dehydratase small subunit, swivel	INTRON
13754	CakSNP13754	*Kabuli*	Ca_Kabuli_Chr08	6149097	[A/C]	Ca_01932	Aconitase A/isopropylmalate dehydratase small subunit, swivel	NON_SYNONYMOUS_CODING
13755	CakSNP13755	*Kabuli*	Ca_Kabuli_Chr08	6192944	[T/G]	Ca_01927	Pentatricopeptide repeat	SYNONYMOUS_CODING
13756	CakSNP13756	*Kabuli*	Ca_Kabuli_Chr08	6192986	[C/T]	Ca_01927	Pentatricopeptide repeat	SYNONYMOUS_CODING
13757	CakSNP13757	*Kabuli*	Ca_Kabuli_Chr08	6193078	[G/A]	Ca_01927	Pentatricopeptide repeat	NON_SYNONYMOUS_CODING
13758	CakSNP13758	*Kabuli*	Ca_Kabuli_Chr08	6218250	[G/A]	Ca_01924	Ankyrin repeat	INTRON
13759	CakSNP13759	*Kabuli*	Ca_Kabuli_Chr08	6218275	[C/T]	Ca_01924	Ankyrin repeat	INTRON
13760	CakSNP13760	*Kabuli*	Ca_Kabuli_Chr08	6218298	[A/G]	Ca_01924	Ankyrin repeat	INTRON
13761	CakSNP13761	*Kabuli*	Ca_Kabuli_Chr08	6218299	[T/A]	Ca_01924	Ankyrin repeat	INTRON

S.N.	SNP IDs	Chickpea cultivars	Chromosomes	Physical positions (bp)	SNPs	Gene accession IDs	Putative functions	Sequence components of genome
13762	CakSNP13762	*Kabuli*	Ca_Kabuli_Chr08	6266432	[A/G]	Ca_23577	Protein kinase, catalytic domain	INTRON
13763	CakSNP13763	*Kabuli*	Ca_Kabuli_Chr08	6372236	[A/T]		--	INTERGENIC
13764	CakSNP13764	*Kabuli*	Ca_Kabuli_Chr08	6372230	[G/A]		--	INTERGENIC
13765	CakSNP13765	*Kabuli*	Ca_Kabuli_Chr08	6372223	[G/A]		--	INTERGENIC
13766	CakSNP13766	*Kabuli*	Ca_Kabuli_Chr08	6374794	[C/A]		--	INTERGENIC
13767	CakSNP13767	*Kabuli*	Ca_Kabuli_Chr08	6437758	[T/G]	Ca_23591	GRIP	INTRON
13768	CakSNP13768	*Kabuli*	Ca_Kabuli_Chr08	6503723	[T/C]	Ca_10738	Poly A polymerase, head domain	INTRON
13769	CakSNP13769	*Kabuli*	Ca_Kabuli_Chr08	6525633	[A/T]		--	INTERGENIC
13770	CakSNP13770	*Kabuli*	Ca_Kabuli_Chr08	6526612	[T/C]		--	INTERGENIC
13771	CakSNP13771	*Kabuli*	Ca_Kabuli_Chr08	6544248	[G/T]	Ca_10735	Zinc finger, C2H2-type	SYNONYMOUS_CODING

S.N.	SNP IDs	Chickpea cultivars	Chromosomes	Physical positions (bp)	SNPs	Gene accession IDs	Putative functions	Sequence components of genome
13772	CakSNP13772	*Kabuli*	Ca_Kabuli_Chr08	6544278	[C/A]		--	INTERGENIC
13773	CakSNP13773	*Kabuli*	Ca_Kabuli_Chr08	6624006	[C/T]	Ca_10724	Nucleoside triphosphate phosphohydrolase, predicted	INTRON
13774	CakSNP13774	*Kabuli*	Ca_Kabuli_Chr08	6630663	[C/T]		--	INTERGENIC
13775	CakSNP13775	*Kabuli*	Ca_Kabuli_Chr08	6630661	[T/C]		--	INTERGENIC
13776	CakSNP13776	*Kabuli*	Ca_Kabuli_Chr08	6664509	[T/A]		--	INTERGENIC
13777	CakSNP13777	*Kabuli*	Ca_Kabuli_Chr08	6673855	[C/T]	Ca_10719	DNA mismatch repair protein	INTRON
13778	CakSNP13778	*Kabuli*	Ca_Kabuli_Chr08	6673891	[A/G]	Ca_10719	DNA mismatch repair protein	NON_SYNONYMOUS_CODING
13779	CakSNP13779	*Kabuli*	Ca_Kabuli_Chr08	6674024	[G/A]	Ca_10719	DNA mismatch repair protein	NON_SYNONYMOUS_CODING
13780	CakSNP13780	*Kabuli*	Ca_Kabuli_Chr08	6673956	[G/A]	Ca_10719	DNA mismatch repair protein	SYNONYMOUS_CODING
13781	CakSNP13781	*Kabuli*	Ca_Kabuli_Chr08	6708869	[G/T]	Ca_10713	Protein kinase, catalytic domain	NON_SYNONYMOUS_CODING

S.N.	SNP IDs	Chickpea cultivars	Chromosomes	Physical positions (bp)	SNPs	Gene accession IDs	Putative functions	Sequence components of genome
13782	CakSNP13782	*Kabuli*	Ca_Kabuli_Chr08	6708945	[A/G]	Ca_10713	Protein kinase, catalytic domain	SYNONYMOUS_CODING
13783	CakSNP13783	*Kabuli*	Ca_Kabuli_Chr08	6747091	[T/C]	Ca_10708	Sugar/inositol transporter	INTRON
13784	CakSNP13784	*Kabuli*	Ca_Kabuli_Chr08	6747995	[A/G]	Ca_10708	Sugar/inositol transporter	SYNONYMOUS_CODING
13785	CakSNP13785	*Kabuli*	Ca_Kabuli_Chr08	6748136	[C/T]	Ca_10708	Sugar/inositol transporter	SYNONYMOUS_CODING
13786	CakSNP13786	*Kabuli*	Ca_Kabuli_Chr08	6748294	[T/C]	Ca_10708	Sugar/inositol transporter	NON_SYNONYMOUS_CODING
13787	CakSNP13787	*Kabuli*	Ca_Kabuli_Chr08	6780592	[T/G]	Ca_10705	ATPase-like, ATP-binding domain	NON_SYNONYMOUS_CODING
13788	CakSNP13788	*Kabuli*	Ca_Kabuli_Chr08	6940139	[A/G]	Ca_10698	ARID/BRIGHT DNA-binding domain	INTRON
13789	CakSNP13789	*Kabuli*	Ca_Kabuli_Chr08	6942651	[G/T]	Ca_10698	ARID/BRIGHT DNA-binding domain	INTRON
13790	CakSNP13790	*Kabuli*	Ca_Kabuli_Chr08	6943710	[A/G]	Ca_10698	ARID/BRIGHT DNA-binding domain	SYNONYMOUS_CODING
13791	CakSNP13791	*Kabuli*	Ca_Kabuli_Chr08	6968036	[A/G]		--	INTERGENIC

S.N.	SNP IDs	Chickpea cultivars	Chromosomes	Physical positions (bp)	SNPs	Gene accession IDs	Putative functions	Sequence components of genome
13792	CakSNP13792	*Kabuli*	Ca_Kabuli_Chr08	6986090	[G/A]	Ca_10694	Peptidase S8/S53, subtilisin/kexin/sedolisin	NON_SYNONYMOUS_CODING
13793	CakSNP13793	*Kabuli*	Ca_Kabuli_Chr08	6986130	[A/G]	Ca_10694	Peptidase S8/S53, subtilisin/kexin/sedolisin	SYNONYMOUS_CODING
13794	CakSNP13794	*Kabuli*	Ca_Kabuli_Chr08	6987609	[G/T]	Ca_10694	Peptidase S8/S53, subtilisin/kexin/sedolisin	NON_SYNONYMOUS_CODING
13795	CakSNP13795	*Kabuli*	Ca_Kabuli_Chr08	6987615	[A/C]	Ca_10694	Peptidase S8/S53, subtilisin/kexin/sedolisin	NON_SYNONYMOUS_CODING
13796	CakSNP13796	*Kabuli*	Ca_Kabuli_Chr08	6989342	[G/T]		--	INTERGENIC
13797	CakSNP13797	*Kabuli*	Ca_Kabuli_Chr08	6989433	[A/G]		--	INTERGENIC
13798	CakSNP13798	*Kabuli*	Ca_Kabuli_Chr08	7013049	[G/A]	Ca_10690	MyTH4 domain	INTRON
13799	CakSNP13799	*Kabuli*	Ca_Kabuli_Chr08	7037715	[G/A]	Ca_10687	Protein of unknown function DUF647	SYNONYMOUS_CODING
13800	CakSNP13800	*Kabuli*	Ca_Kabuli_Chr08	7041901	[A/C]	Ca_10686	Transcription factor jumonji/aspartyl beta-hydroxylase	NON_SYNONYMOUS_CODING
13801	CakSNP13801	*Kabuli*	Ca_Kabuli_Chr08	7047509	[T/G]		--	INTERGENIC

S.N.	SNP IDs	Chickpea cultivars	Chromosomes	Physical positions (bp)	SNPs	Gene accession IDs	Putative functions	Sequence components of genome
13802	CakSNP13802	*Kabuli*	Ca_Kabuli_Chr08	7047469	[T/A]		--	INTERGENIC
13803	CakSNP13803	*Kabuli*	Ca_Kabuli_Chr08	7099455	[C/T]	Ca_10679	Conserved oligomeric complex COG6	INTRON
13804	CakSNP13804	*Kabuli*	Ca_Kabuli_Chr08	7099569	[T/C]	Ca_10679	Conserved oligomeric complex COG6	INTRON
13805	CakSNP13805	*Kabuli*	Ca_Kabuli_Chr08	7099530	[C/A]	Ca_10679	Conserved oligomeric complex COG6	INTRON
13806	CakSNP13806	*Kabuli*	Ca_Kabuli_Chr08	7099756	[A/G]	Ca_10679	Conserved oligomeric complex COG6	SYNONYMOUS_CODING
13807	CakSNP13807	*Kabuli*	Ca_Kabuli_Chr08	7162826	[T/G]		--	INTERGENIC
13808	CakSNP13808	*Kabuli*	Ca_Kabuli_Chr08	7162976	[A/G]		--	INTERGENIC
13809	CakSNP13809	*Kabuli*	Ca_Kabuli_Chr08	7174066	[C/T]	Ca_10671	F-box domain, cyclin-like	SYNONYMOUS_CODING
13810	CakSNP13810	*Kabuli*	Ca_Kabuli_Chr08	7175086	[A/C]	Ca_10671	F-box domain, cyclin-like	INTRON
13811	CakSNP13811	*Kabuli*	Ca_Kabuli_Chr08	7191825	[A/G]	Ca_10668	Pentatricopeptide repeat	SYNONYMOUS_CODING

S.N.	SNP IDs	Chickpea cultivars	Chromosomes	Physical positions (bp)	SNPs	Gene accession IDs	Putative functions	Sequence components of genome
13812	CakSNP13812	*Kabuli*	Ca_Kabuli_Chr08	7193946	[C/T]	Ca_10668	Pentatricopeptide repeat	SYNONYMOUS_CODING
13813	CakSNP13813	*Kabuli*	Ca_Kabuli_Chr08	7193960	[A/T]	Ca_10668	Pentatricopeptide repeat	NON_SYNONYMOUS_CODING
13814	CakSNP13814	*Kabuli*	Ca_Kabuli_Chr08	7295151	[T/C]		--	INTERGENIC
13815	CakSNP13815	*Kabuli*	Ca_Kabuli_Chr08	7295206	[A/G]		--	INTERGENIC
13816	CakSNP13816	*Kabuli*	Ca_Kabuli_Chr08	7295177	[G/T]		--	INTERGENIC
13817	CakSNP13817	*Kabuli*	Ca_Kabuli_Chr08	7307737	[G/T]	Ca_10659	Uncharacterised protein family UPF0114	SYNONYMOUS_CODING
13818	CakSNP13818	*Kabuli*	Ca_Kabuli_Chr08	7307787	[A/T]	Ca_10659	Uncharacterised protein family UPF0114	NON_SYNONYMOUS_CODING
13819	CakSNP13819	*Kabuli*	Ca_Kabuli_Chr08	7357621	[A/G]		--	INTERGENIC
13820	CakSNP13820	*Kabuli*	Ca_Kabuli_Chr08	7357583	[T/C]		--	INTERGENIC
13821	CakSNP13821	*Kabuli*	Ca_Kabuli_Chr08	7503013	[T/A]	Ca_10639	Protein kinase, catalytic domain	INTRON

S.N.	SNP IDs	Chickpea cultivars	Chromosomes	Physical positions (bp)	SNPs	Gene accession IDs	Putative functions	Sequence components of genome
13822	CakSNP13822	*Kabuli*	Ca_Kabuli_Chr08	7503010	[T/C]	Ca_10639	Protein kinase, catalytic domain	INTRON
13823	CakSNP13823	*Kabuli*	Ca_Kabuli_Chr08	7506004	[A/G]	Ca_10639	Protein kinase, catalytic domain	INTRON
13824	CakSNP13824	*Kabuli*	Ca_Kabuli_Chr08	7519758	[C/A]		--	INTERGENIC
13825	CakSNP13825	*Kabuli*	Ca_Kabuli_Chr08	7519815	[G/A]		--	INTERGENIC
13826	CakSNP13826	*Kabuli*	Ca_Kabuli_Chr08	7526956	[A/G]	Ca_10635	Ankyrin repeat	SYNONYMOUS_CODING
13827	CakSNP13827	*Kabuli*	Ca_Kabuli_Chr08	7563385	[C/G]		--	INTERGENIC
13828	CakSNP13828	*Kabuli*	Ca_Kabuli_Chr08	7563370	[T/C]		--	INTERGENIC
13829	CakSNP13829	*Kabuli*	Ca_Kabuli_Chr08	7563373	[T/A]		--	INTERGENIC
13830	CakSNP13830	*Kabuli*	Ca_Kabuli_Chr08	7566548	[T/C]	Ca_10630	Glycoside hydrolase, family 31	SYNONYMOUS_CODING
13831	CakSNP13831	*Kabuli*	Ca_Kabuli_Chr08	7614202	[A/C]	Ca_10626	Ubiquitin	INTRON

S.N.	SNP IDs	Chickpea cultivars	Chromosomes	Physical positions (bp)	SNPs	Gene accession IDs	Putative functions	Sequence components of genome
13832	CakSNP13832	*Kabuli*	Ca_Kabuli_Chr08	7628690	[T/A]	Ca_10626	Ubiquitin	INTRON
13833	CakSNP13833	*Kabuli*	Ca_Kabuli_Chr08	7665291	[T/C]	Ca_10624	Acyl-CoA-binding protein, ACBP	INTRON
13834	CakSNP13834	*Kabuli*	Ca_Kabuli_Chr08	7665383	[G/T]	Ca_10624	Acyl-CoA-binding protein, ACBP	INTRON
13835	CakSNP13835	*Kabuli*	Ca_Kabuli_Chr08	7694747	[A/G]	Ca_10620	Ankyrin repeat	SYNONYMOUS_CODING
13836	CakSNP13836	*Kabuli*	Ca_Kabuli_Chr08	7695990	[A/T]		--	INTERGENIC
13837	CakSNP13837	*Kabuli*	Ca_Kabuli_Chr08	7695989	[T/A]		--	INTERGENIC
13838	CakSNP13838	*Kabuli*	Ca_Kabuli_Chr08	7697886	[G/C]	Ca_10619	Tetratricopeptide repeat-containing	SYNONYMOUS_CODING
13839	CakSNP13839	*Kabuli*	Ca_Kabuli_Chr08	7726921	[G/A]	Ca_10614	Zinc finger, CCHC-type	SYNONYMOUS_CODING
13840	CakSNP13840	*Kabuli*	Ca_Kabuli_Chr08	7726894	[G/T]	Ca_10614	Zinc finger, CCHC-type	SYNONYMOUS_CODING
13841	CakSNP13841	*Kabuli*	Ca_Kabuli_Chr08	7726879	[A/G]	Ca_10614	Zinc finger, CCHC-type	SYNONYMOUS_CODING

S.N.	SNP IDs	Chickpea cultivars	Chromosomes	Physical positions (bp)	SNPs	Gene accession IDs	Putative functions	Sequence components of genome
13842	CakSNP13842	*Kabuli*	Ca_Kabuli_Chr08	7728085	[T/G]	Ca_10614	Zinc finger, CCHC-type	SYNONYMOUS_CODING
13843	CakSNP13843	*Kabuli*	Ca_Kabuli_Chr08	7728076	[T/C]	Ca_10614	Zinc finger, CCHC-type	SYNONYMOUS_CODING
13844	CakSNP13844	*Kabuli*	Ca_Kabuli_Chr08	7729693	[T/C]	Ca_10613	Zinc finger, CCHC-type	SYNONYMOUS_CODING
13845	CakSNP13845	*Kabuli*	Ca_Kabuli_Chr08	7733508	[C/T]	Ca_10612	--	SYNONYMOUS_CODING
13846	CakSNP13846	*Kabuli*	Ca_Kabuli_Chr08	7733573	[A/C]	Ca_10612	--	SYNONYMOUS_CODING
13847	CakSNP13847	*Kabuli*	Ca_Kabuli_Chr08	7733574	[G/A]	Ca_10612	--	SYNONYMOUS_CODING
13848	CakSNP13848	*Kabuli*	Ca_Kabuli_Chr08	7733670	[C/T]	Ca_10612	--	SYNONYMOUS_CODING
13849	CakSNP13849	*Kabuli*	Ca_Kabuli_Chr08	7733647	[G/T]	Ca_10612	--	NON_SYNONYMOUS_CODING
13850	CakSNP13850	*Kabuli*	Ca_Kabuli_Chr08	7776466	[G/A]	Ca_10607	Kinesin, motor domain	SYNONYMOUS_CODING
13851	CakSNP13851	*Kabuli*	Ca_Kabuli_Chr08	7776592	[T/C]	Ca_10607	Kinesin, motor domain	SYNONYMOUS_CODING

S.N.	SNP IDs	Chickpea cultivars	Chromosomes	Physical positions (bp)	SNPs	Gene accession IDs	Putative functions	Sequence components of genome
13852	CakSNP13852	*Kabuli*	Ca_Kabuli_Chr08	7776897	[A/T]	Ca_10607	Kinesin, motor domain	INTRON
13853	CakSNP13853	*Kabuli*	Ca_Kabuli_Chr08	7776934	[G/T]	Ca_10607	Kinesin, motor domain	INTRON
13854	CakSNP13854	*Kabuli*	Ca_Kabuli_Chr08	7776970	[T/G]	Ca_10607	Kinesin, motor domain	SYNONYMOUS_CODING
13855	CakSNP13855	*Kabuli*	Ca_Kabuli_Chr08	7777023	[G/A]	Ca_10607	Kinesin, motor domain	INTRON
13856	CakSNP13856	*Kabuli*	Ca_Kabuli_Chr08	7807065	[T/C]	Ca_10604	SET domain	SYNONYMOUS_CODING
13857	CakSNP13857	*Kabuli*	Ca_Kabuli_Chr08	7978269	[C/T]	Ca_11434	--	SYNONYMOUS_CODING
13858	CakSNP13858	*Kabuli*	Ca_Kabuli_Chr08	8029283	[T/A]	Ca_11440	Phosphoribulokinase	SYNONYMOUS_CODING
13859	CakSNP13859	*Kabuli*	Ca_Kabuli_Chr08	8039078	[A/T]	Ca_11441	SCAMP	INTRON
13860	CakSNP13860	*Kabuli*	Ca_Kabuli_Chr08	8059732	[A/G]	Ca_11443	Disease resistance protein	SYNONYMOUS_CODING
13861	CakSNP13861	*Kabuli*	Ca_Kabuli_Chr08	8066006	[C/A]		--	INTERGENIC

S.N.	SNP IDs	Chickpea cultivars	Chromosomes	Physical positions (bp)	SNPs	Gene accession IDs	Putative functions	Sequence components of genome
13862	CakSNP13862	*Kabuli*	Ca_Kabuli_Chr08	8066005	[A/G]		--	INTERGENIC
13863	CakSNP13863	*Kabuli*	Ca_Kabuli_Chr08	8104804	[G/T]	Ca_11445	--	INTRON
13864	CakSNP13864	*Kabuli*	Ca_Kabuli_Chr08	8104801	[T/C]	Ca_11445	--	INTRON
13865	CakSNP13865	*Kabuli*	Ca_Kabuli_Chr08	8104884	[T/A]	Ca_11445	--	INTRON
13866	CakSNP13866	*Kabuli*	Ca_Kabuli_Chr08	8104892	[C/T]	Ca_11445	--	INTRON
13867	CakSNP13867	*Kabuli*	Ca_Kabuli_Chr08	8106804	[A/G]		--	INTERGENIC
13868	CakSNP13868	*Kabuli*	Ca_Kabuli_Chr08	8106851	[G/A]		--	INTERGENIC
13869	CakSNP13869	*Kabuli*	Ca_Kabuli_Chr08	8114316	[A/C]	Ca_11446	Proteasome component (PCI) domain	SYNONYMOUS_CODING
13870	CakSNP13870	*Kabuli*	Ca_Kabuli_Chr08	8194206	[C/T]	Ca_11454	Aspartyl/Asparaginyl-tRNA synthetase, class IIb	SYNONYMOUS_CODING
13871	CakSNP13871	*Kabuli*	Ca_Kabuli_Chr08	8194232	[C/A]	Ca_11454	Aspartyl/Asparaginyl-tRNA synthetase, class IIb	NON_SYNONYMOUS_CODING

S.N.	SNP IDs	Chickpea cultivars	Chromosomes	Physical positions (bp)	SNPs	Gene accession IDs	Putative functions	Sequence components of genome
13872	CakSNP13872	*Kabuli*	Ca_Kabuli_Chr08	8194234	[C/A]	Ca_11454	Aspartyl/Asparaginyl-tRNA synthetase, class IIb	NON_SYNONYMOUS_CODING
13873	CakSNP13873	*Kabuli*	Ca_Kabuli_Chr08	8194342	[T/C]	Ca_11454	Aspartyl/Asparaginyl-tRNA synthetase, class IIb	INTRON
13874	CakSNP13874	*Kabuli*	Ca_Kabuli_Chr08	8194464	[A/G]	Ca_11454	Aspartyl/Asparaginyl-tRNA synthetase, class IIb	SYNONYMOUS_CODING
13875	CakSNP13875	*Kabuli*	Ca_Kabuli_Chr08	8194436	[T/A]	Ca_11454	Aspartyl/Asparaginyl-tRNA synthetase, class IIb	NON_SYNONYMOUS_CODING
13876	CakSNP13876	*Kabuli*	Ca_Kabuli_Chr08	8202726	[G/A]	Ca_11455	Basic-leucine zipper (bZIP) transcription factor	INTRON
13877	CakSNP13877	*Kabuli*	Ca_Kabuli_Chr08	8270488	[T/C]	Ca_11460	Ribosomal protein L22/L17	SYNONYMOUS_CODING
13878	CakSNP13878	*Kabuli*	Ca_Kabuli_Chr08	8288041	[A/G]	Ca_11461	Ribosomal protein L22/L17	INTRON
13879	CakSNP13879	*Kabuli*	Ca_Kabuli_Chr08	8293716	[G/A]	Ca_11463	Phospholipase/carboxylesterase	SYNONYMOUS_CODING
13880	CakSNP13880	*Kabuli*	Ca_Kabuli_Chr08	8293704	[T/C]	Ca_11463	Phospholipase/carboxylesterase	SYNONYMOUS_CODING
13881	CakSNP13881	*Kabuli*	Ca_Kabuli_Chr08	8293693	[T/C]	Ca_11463	Phospholipase/carboxylesterase	NON_SYNONYMOUS_CODING

S.N.	SNP IDs	Chickpea cultivars	Chromosomes	Physical positions (bp)	SNPs	Gene accession IDs	Putative functions	Sequence components of genome
13882	CakSNP13882	*Kabuli*	Ca_Kabuli_Chr08	8318110	[A/G]	Ca_11464	Phospholipid/glycerol acyltransferase	INTRON
13883	CakSNP13883	*Kabuli*	Ca_Kabuli_Chr08	8389117	[C/T]	Ca_11471	Transcription factor jumonji/aspartyl beta-hydroxylase	NON_SYNONYMOUS_CODING
13884	CakSNP13884	*Kabuli*	Ca_Kabuli_Chr08	8458383	[G/A]	Ca_11477	Poly(ADP-ribose) polymerase, catalytic domain	SYNONYMOUS_CODING
13885	CakSNP13885	*Kabuli*	Ca_Kabuli_Chr08	8533479	[G/T]	Ca_11485	--	INTRON
13886	CakSNP13886	*Kabuli*	Ca_Kabuli_Chr08	8533978	[G/A]		--	INTERGENIC
13887	CakSNP13887	*Kabuli*	Ca_Kabuli_Chr08	8560330	[C/A]		--	INTERGENIC
13888	CakSNP13888	*Kabuli*	Ca_Kabuli_Chr08	8560331	[C/G]		--	INTERGENIC
13889	CakSNP13889	*Kabuli*	Ca_Kabuli_Chr08	8560332	[A/C]		--	INTERGENIC
13890	CakSNP13890	*Kabuli*	Ca_Kabuli_Chr08	8560326	[G/T]		--	INTERGENIC
13891	CakSNP13891	*Kabuli*	Ca_Kabuli_Chr08	8661327	[T/C]	Ca_11497	RNA recognition motif domain	NON_SYNONYMOUS_CODING

S.N.	SNP IDs	Chickpea cultivars	Chromosomes	Physical positions (bp)	SNPs	Gene accession IDs	Putative functions	Sequence components of genome
13892	CakSNP13892	*Kabuli*	Ca_Kabuli_Chr08	8670242	[T/G]	Ca_11498	Tetratricopeptide repeat-containing	NON_SYNONYMOUS_CODING
13893	CakSNP13893	*Kabuli*	Ca_Kabuli_Chr08	8772131	[C/G]		--	INTERGENIC
13894	CakSNP13894	*Kabuli*	Ca_Kabuli_Chr08	8772124	[G/A]		--	INTERGENIC
13895	CakSNP13895	*Kabuli*	Ca_Kabuli_Chr08	8853602	[G/A]	Ca_11515	--	SYNONYMOUS_CODING
13896	CakSNP13896	*Kabuli*	Ca_Kabuli_Chr08	8853664	[A/G]	Ca_11515	--	NON_SYNONYMOUS_CODING
13897	CakSNP13897	*Kabuli*	Ca_Kabuli_Chr08	8856046	[C/T]	Ca_11516	--	NON_SYNONYMOUS_CODING
13898	CakSNP13898	*Kabuli*	Ca_Kabuli_Chr08	8856047	[C/T]	Ca_11516	--	SYNONYMOUS_CODING
13899	CakSNP13899	*Kabuli*	Ca_Kabuli_Chr08	8856150	[A/G]	Ca_11516	--	NON_SYNONYMOUS_CODING
13900	CakSNP13900	*Kabuli*	Ca_Kabuli_Chr08	8856143	[G/A]	Ca_11516	--	SYNONYMOUS_CODING
13901	CakSNP13901	*Kabuli*	Ca_Kabuli_Chr08	8864079	[C/T]	Ca_11517	VQ	SYNONYMOUS_CODING

S.N.	SNP IDs	Chickpea cultivars	Chromosomes	Physical positions (bp)	SNPs	Gene accession IDs	Putative functions	Sequence components of genome
13902	CakSNP13902	*Kabuli*	Ca_Kabuli_Chr08	8864157	[G/A]	Ca_11517	VQ	SYNONYMOUS_CODING
13903	CakSNP13903	*Kabuli*	Ca_Kabuli_Chr08	8888412	[A/G]	Ca_11520	Mitochondrial substrate/solute carrier	INTRON
13904	CakSNP13904	*Kabuli*	Ca_Kabuli_Chr08	8888414	[G/T]	Ca_11520	Mitochondrial substrate/solute carrier	INTRON
13905	CakSNP13905	*Kabuli*	Ca_Kabuli_Chr08	8888426	[T/C]	Ca_11520	Mitochondrial substrate/solute carrier	INTRON
13906	CakSNP13906	*Kabuli*	Ca_Kabuli_Chr08	8888476	[A/C]	Ca_11520	Mitochondrial substrate/solute carrier	INTRON
13907	CakSNP13907	*Kabuli*	Ca_Kabuli_Chr08	8904346	[C/A]	Ca_11521	Major facilitator superfamily MFS-1	INTRON
13908	CakSNP13908	*Kabuli*	Ca_Kabuli_Chr08	8904366	[A/G]	Ca_11521	Major facilitator superfamily MFS-1	INTRON
13909	CakSNP13909	*Kabuli*	Ca_Kabuli_Chr08	8914018	[A/C]	Ca_11522	Starch synthase, catalytic domain	INTRON
13910	CakSNP13910	*Kabuli*	Ca_Kabuli_Chr08	9097585	[A/C]	Ca_11533	Glycoside hydrolase, family 3, N-terminal	SYNONYMOUS_CODING
13911	CakSNP13911	*Kabuli*	Ca_Kabuli_Chr08	9099798	[G/T]	Ca_11533	Glycoside hydrolase, family 3, N-terminal	INTRON

S.N.	SNP IDs	Chickpea cultivars	Chromosomes	Physical positions (bp)	SNPs	Gene accession IDs	Putative functions	Sequence components of genome
13912	CakSNP13912	*Kabuli*	Ca_Kabuli_Chr08	9100111	[C/T]	Ca_11533	Glycoside hydrolase, family 3, N-terminal	INTRON
13913	CakSNP13913	*Kabuli*	Ca_Kabuli_Chr08	9109896	[T/A]		--	INTERGENIC
13914	CakSNP13914	*Kabuli*	Ca_Kabuli_Chr08	9109907	[A/T]		--	INTERGENIC
13915	CakSNP13915	*Kabuli*	Ca_Kabuli_Chr08	9109920	[C/A]		--	INTERGENIC
13916	CakSNP13916	*Kabuli*	Ca_Kabuli_Chr08	9112178	[G/A]	Ca_11534	Protein of unknown function DUF1399	SYNONYMOUS_CODING
13917	CakSNP13917	*Kabuli*	Ca_Kabuli_Chr08	9112172	[A/C]	Ca_11534	Protein of unknown function DUF1399	SYNONYMOUS_CODING
13918	CakSNP13918	*Kabuli*	Ca_Kabuli_Chr08	9118582	[C/T]	Ca_11535	--	SYNONYMOUS_CODING
13919	CakSNP13919	*Kabuli*	Ca_Kabuli_Chr08	9118667	[A/G]	Ca_11535	--	INTRON
13920	CakSNP13920	*Kabuli*	Ca_Kabuli_Chr08	9143840	[C/A]	Ca_11537	Protein kinase, catalytic domain	INTRON
13921	CakSNP13921	*Kabuli*	Ca_Kabuli_Chr08	9151722	[G/T]	Ca_11539	Peptidase M3A/M3B, thimet/oligopeptidase F	SYNONYMOUS_CODING

S.N.	SNP IDs	Chickpea cultivars	Chromosomes	Physical positions (bp)	SNPs	Gene accession IDs	Putative functions	Sequence components of genome
13922	CakSNP13922	*Kabuli*	Ca_Kabuli_Chr08	9389347	[G/A]		--	INTERGENIC
13923	CakSNP13923	*Kabuli*	Ca_Kabuli_Chr08	10004481	[T/C]		--	INTERGENIC
13924	CakSNP13924	*Kabuli*	Ca_Kabuli_Chr08	10155981	[A/C]	Ca_18411	NADH:flavin oxidoreductase/NADH oxidase, N-terminal	SYNONYMOUS_CODING
13925	CakSNP13925	*Kabuli*	Ca_Kabuli_Chr08	10258892	[T/G]	Ca_18416	Zinc finger, PHD-type	SYNONYMOUS_CODING
13926	CakSNP13926	*Kabuli*	Ca_Kabuli_Chr08	10486601	[A/C]		--	INTERGENIC
13927	CakSNP13927	*Kabuli*	Ca_Kabuli_Chr08	10556124	[T/C]		--	INTERGENIC
13928	CakSNP13928	*Kabuli*	Ca_Kabuli_Chr08	10668422	[A/C]		--	INTERGENIC
13929	CakSNP13929	*Kabuli*	Ca_Kabuli_Chr08	10728369	[G/C]		--	INTERGENIC
13930	CakSNP13930	*Kabuli*	Ca_Kabuli_Chr08	10733404	[A/G]	Ca_19724	Ankyrin repeat	SYNONYMOUS_CODING
13931	CakSNP13931	*Kabuli*	Ca_Kabuli_Chr08	10733503	[C/T]	Ca_19724	Ankyrin repeat	SYNONYMOUS_CODING

S.N.	SNP IDs	Chickpea cultivars	Chromosomes	Physical positions (bp)	SNPs	Gene accession IDs	Putative functions	Sequence components of genome
13932	CakSNP13932	*Kabuli*	Ca_Kabuli_Chr08	10733661	[G/A]	Ca_19724	Ankyrin repeat	SYNONYMOUS_CODING
13933	CakSNP13933	*Kabuli*	Ca_Kabuli_Chr08	10733747	[C/T]	Ca_19724	Ankyrin repeat	NON_SYNONYMOUS_CODING
13934	CakSNP13934	*Kabuli*	Ca_Kabuli_Chr08	10759078	[G/A]		--	INTERGENIC
13935	CakSNP13935	*Kabuli*	Ca_Kabuli_Chr08	10759060	[G/A]		--	INTERGENIC
13936	CakSNP13936	*Kabuli*	Ca_Kabuli_Chr08	10759056	[C/T]		--	INTERGENIC
13937	CakSNP13937	*Kabuli*	Ca_Kabuli_Chr08	10759102	[C/T]		--	INTERGENIC
13938	CakSNP13938	*Kabuli*	Ca_Kabuli_Chr08	10763656	[A/T]	Ca_19727	AWPM-19-like	SYNONYMOUS_CODING
13939	CakSNP13939	*Kabuli*	Ca_Kabuli_Chr08	10953767	[C/T]		--	INTERGENIC
13940	CakSNP13940	*Kabuli*	Ca_Kabuli_Chr08	11015680	[G/T]		--	INTERGENIC
13941	CakSNP13941	*Kabuli*	Ca_Kabuli_Chr08	11015678	[C/G]		--	INTERGENIC

S.N.	SNP IDs	Chickpea cultivars	Chromosomes	Physical positions (bp)	SNPs	Gene accession IDs	Putative functions	Sequence components of genome
13942	CakSNP13942	*Kabuli*	Ca_Kabuli_Chr08	11015666	[G/C]		--	INTERGENIC
13943	CakSNP13943	*Kabuli*	Ca_Kabuli_Chr08	11066644	[C/T]	Ca_16843	Multi antimicrobial extrusion protein	SYNONYMOUS_CODING
13944	CakSNP13944	*Kabuli*	Ca_Kabuli_Chr08	11066728	[G/A]	Ca_16843	Multi antimicrobial extrusion protein	INTRON
13945	CakSNP13945	*Kabuli*	Ca_Kabuli_Chr08	11087143	[A/G]		--	INTERGENIC
13946	CakSNP13946	*Kabuli*	Ca_Kabuli_Chr08	11087169	[C/T]		--	INTERGENIC
13947	CakSNP13947	*Kabuli*	Ca_Kabuli_Chr08	11087302	[A/T]		--	INTERGENIC
13948	CakSNP13948	*Kabuli*	Ca_Kabuli_Chr08	11087250	[T/C]		--	INTERGENIC
13949	CakSNP13949	*Kabuli*	Ca_Kabuli_Chr08	11087247	[T/C]		--	INTERGENIC
13950	CakSNP13950	*Kabuli*	Ca_Kabuli_Chr08	11186919	[C/G]	Ca_16839	Zinc finger, RanBP2-type	NON_SYNONYMOUS_CODING
13951	CakSNP13951	*Kabuli*	Ca_Kabuli_Chr08	11187037	[T/C]	Ca_16839	Zinc finger, RanBP2-type	SYNONYMOUS_CODING

S.N.	SNP IDs	Chickpea cultivars	Chromosomes	Physical positions (bp)	SNPs	Gene accession IDs	Putative functions	Sequence components of genome
13952	CakSNP13952	*Kabuli*	Ca_Kabuli_Chr08	11186986	[A/C]	Ca_16839	Zinc finger, RanBP2-type	SYNONYMOUS_CODING
13953	CakSNP13953	*Kabuli*	Ca_Kabuli_Chr08	11186974	[A/C]	Ca_16839	Zinc finger, RanBP2-type	NON_SYNONYMOUS_CODING
13954	CakSNP13954	*Kabuli*	Ca_Kabuli_Chr08	11231812	[A/G]	Ca_16835	Protein kinase, catalytic domain	SYNONYMOUS_CODING
13955	CakSNP13955	*Kabuli*	Ca_Kabuli_Chr08	11240258	[C/A]	Ca_16834	Histone H1/H5	INTRON
13956	CakSNP13956	*Kabuli*	Ca_Kabuli_Chr08	11243263	[A/C]	Ca_16834	Histone H1/H5	NON_SYNONYMOUS_CODING
13957	CakSNP13957	*Kabuli*	Ca_Kabuli_Chr08	11243256	[A/C]	Ca_16834	Histone H1/H5	SYNONYMOUS_CODING
13958	CakSNP13958	*Kabuli*	Ca_Kabuli_Chr08	11266447	[G/A]		--	INTERGENIC
13959	CakSNP13959	*Kabuli*	Ca_Kabuli_Chr08	11310460	[G/A]		--	INTERGENIC
13960	CakSNP13960	*Kabuli*	Ca_Kabuli_Chr08	11310480	[T/A]		--	INTERGENIC
13961	CakSNP13961	*Kabuli*	Ca_Kabuli_Chr08	11332934	[C/T]		--	INTERGENIC

S.N.	SNP IDs	Chickpea cultivars	Chromosomes	Physical positions (bp)	SNPs	Gene accession IDs	Putative functions	Sequence components of genome
13962	CakSNP13962	*Kabuli*	Ca_Kabuli_Chr08	11332940	[T/C]		--	INTERGENIC
13963	CakSNP13963	*Kabuli*	Ca_Kabuli_Chr08	11333057	[C/G]		--	INTERGENIC
13964	CakSNP13964	*Kabuli*	Ca_Kabuli_Chr08	11334190	[A/T]		--	INTERGENIC
13965	CakSNP13965	*Kabuli*	Ca_Kabuli_Chr08	11334210	[C/T]		--	INTERGENIC
13966	CakSNP13966	*Kabuli*	Ca_Kabuli_Chr08	11349891	[T/A]	Ca_16822	Glycoside hydrolase, family 1	NON_SYNONYMOUS_CODING
13967	CakSNP13967	*Kabuli*	Ca_Kabuli_Chr08	11349995	[A/G]	Ca_16822	Glycoside hydrolase, family 1	INTRON
13968	CakSNP13968	*Kabuli*	Ca_Kabuli_Chr08	11403805	[G/A]	Ca_16820	SANT domain, DNA binding	SYNONYMOUS_CODING
13969	CakSNP13969	*Kabuli*	Ca_Kabuli_Chr08	11403754	[G/A]	Ca_16820	SANT domain, DNA binding	NON_SYNONYMOUS_CODING
13970	CakSNP13970	*Kabuli*	Ca_Kabuli_Chr08	11562814	[A/C]		--	INTERGENIC
13971	CakSNP13971	*Kabuli*	Ca_Kabuli_Chr08	11562833	[A/G]		--	INTERGENIC

S.N.	SNP IDs	Chickpea cultivars	Chromosomes	Physical positions (bp)	SNPs	Gene accession IDs	Putative functions	Sequence components of genome
13972	CakSNP13972	*Kabuli*	Ca_Kabuli_Chr08	11631836	[C/T]	Ca_16809	Ketose-bisphosphate aldolase, class-II	NON_SYNONYMOUS_CODING
13973	CakSNP13973	*Kabuli*	Ca_Kabuli_Chr08	11656700	[G/T]	Ca_16807	--	NON_SYNONYMOUS_CODING
13974	CakSNP13974	*Kabuli*	Ca_Kabuli_Chr08	11656687	[A/G]	Ca_16807	--	NON_SYNONYMOUS_CODING
13975	CakSNP13975	*Kabuli*	Ca_Kabuli_Chr08	11656675	[T/C]	Ca_16807	--	NON_SYNONYMOUS_CODING
13976	CakSNP13976	*Kabuli*	Ca_Kabuli_Chr08	11656670	[A/G]	Ca_16807	--	SYNONYMOUS_CODING
13977	CakSNP13977	*Kabuli*	Ca_Kabuli_Chr08	11696021	[G/C]	Ca_16803	Protein kinase, catalytic domain	SYNONYMOUS_CODING
13978	CakSNP13978	*Kabuli*	Ca_Kabuli_Chr08	11735882	[A/C]	Ca_16799	Heat shock protein DnaJ, cysteine-rich domain	SYNONYMOUS_CODING
13979	CakSNP13979	*Kabuli*	Ca_Kabuli_Chr08	11962280	[T/C]	Ca_23842	BRO1 domain	INTRON
13980	CakSNP13980	*Kabuli*	Ca_Kabuli_Chr08	12875217	[A/G]		--	INTERGENIC
13981	CakSNP13981	*Kabuli*	Ca_Kabuli_Chr08	13248268	[T/C]	Ca_20108	C2 calcium-dependent membrane targeting	SYNONYMOUS_CODING

S.N.	SNP IDs	Chickpea cultivars	Chromosomes	Physical positions (bp)	SNPs	Gene accession IDs	Putative functions	Sequence components of genome
13982	CakSNP13982	*Kabuli*	Ca_Kabuli_Chr08	13745335	[A/G]		--	INTERGENIC
13983	CakSNP13983	*Kabuli*	Ca_Kabuli_Chr08	13840865	[A/C]		--	INTERGENIC
13984	CakSNP13984	*Kabuli*	Ca_Kabuli_Chr08	13860922	[C/G]	Ca_22727	C2 calcium-dependent membrane targeting	INTRON
13985	CakSNP13985	*Kabuli*	Ca_Kabuli_Chr08	13864026	[T/G]	Ca_22728	--	SYNONYMOUS_CODING
13986	CakSNP13986	*Kabuli*	Ca_Kabuli_Chr08	13864077	[T/C]	Ca_22728	--	SYNONYMOUS_CODING
13987	CakSNP13987	*Kabuli*	Ca_Kabuli_Chr08	13997385	[C/T]	Ca_22740	--	INTRON
13988	CakSNP13988	*Kabuli*	Ca_Kabuli_Chr08	13998102	[A/G]		--	INTERGENIC
13989	CakSNP13989	*Kabuli*	Ca_Kabuli_Chr08	14006709	[G/A]	Ca_22742	--	INTRON
13990	CakSNP13990	*Kabuli*	Ca_Kabuli_Chr08	14006694	[C/T]	Ca_22742	--	INTRON
13991	CakSNP13991	*Kabuli*	Ca_Kabuli_Chr08	14007431	[A/G]	Ca_22742	--	INTRON

S.N.	SNP IDs	Chickpea cultivars	Chromosomes	Physical positions (bp)	SNPs	Gene accession IDs	Putative functions	Sequence components of genome
13992	CakSNP13992	*Kabuli*	Ca_Kabuli_Chr08	14124588	[C/A]		--	INTERGENIC
13993	CakSNP13993	*Kabuli*	Ca_Kabuli_Chr08	14129040	[G/A]	Ca_13047	Protein phosphatase 2C-like	NON_SYNONYMOUS_CODING
13994	CakSNP13994	*Kabuli*	Ca_Kabuli_Chr08	14325980	[C/A]	Ca_13032	Kinesin, motor domain	INTRON
13995	CakSNP13995	*Kabuli*	Ca_Kabuli_Chr08	14437006	[C/A]	Ca_13023	Ribosomal protein S7e	INTRON
13996	CakSNP13996	*Kabuli*	Ca_Kabuli_Chr08	14444424	[G/A]	Ca_13021	Mov34/MPN/PAD-1	SYNONYMOUS_CODING
13997	CakSNP13997	*Kabuli*	Ca_Kabuli_Chr08	14444438	[A/C]	Ca_13021	Mov34/MPN/PAD-1	SYNONYMOUS_CODING
13998	CakSNP13998	*Kabuli*	Ca_Kabuli_Chr08	14445205	[C/A]	Ca_13021	Mov34/MPN/PAD-1	INTRON
13999	CakSNP13999	*Kabuli*	Ca_Kabuli_Chr08	14460426	[T/C]		--	INTERGENIC
14000	CakSNP14000	*Kabuli*	Ca_Kabuli_Chr08	14528882	[T/G]	Ca_13014	RNA-binding, CRM domain	NON_SYNONYMOUS_CODING
14001	CakSNP14001	*Kabuli*	Ca_Kabuli_Chr08	14528966	[C/G]	Ca_13014	RNA-binding, CRM domain	NON_SYNONYMOUS_CODING

S.N.	SNP IDs	Chickpea cultivars	Chromosomes	Physical positions (bp)	SNPs	Gene accession IDs	Putative functions	Sequence components of genome
14002	CakSNP14002	*Kabuli*	Ca_Kabuli_Chr08	14613302	[G/A]	Ca_13006	Endonuclease/exonuclease/phosphatase	INTRON
14003	CakSNP14003	*Kabuli*	Ca_Kabuli_Chr08	14640967	[A/C]	Ca_13004	Toprim domain	INTRON
14004	CakSNP14004	*Kabuli*	Ca_Kabuli_Chr08	14715495	[T/G]	Ca_12997	HEAT	INTRON
14005	CakSNP14005	*Kabuli*	Ca_Kabuli_Chr08	14753763	[T/C]		--	INTERGENIC
14006	CakSNP14006	*Kabuli*	Ca_Kabuli_Chr08	14753750	[T/C]		--	INTERGENIC
14007	CakSNP14007	*Kabuli*	Ca_Kabuli_Chr08	14754849	[A/T]	Ca_12993	Proteasome, subunit alpha/beta	INTRON
14008	CakSNP14008	*Kabuli*	Ca_Kabuli_Chr08	14759784	[G/T]	Ca_12992	Ribosomal protein S7e	INTRON
14009	CakSNP14009	*Kabuli*	Ca_Kabuli_Chr08	14760479	[C/G]	Ca_12992	Ribosomal protein S7e	INTRON
14010	CakSNP14010	*Kabuli*	Ca_Kabuli_Chr08	14760497	[G/A]	Ca_12992	Ribosomal protein S7e	INTRON
14011	CakSNP14011	*Kabuli*	Ca_Kabuli_Chr08	14760503	[A/T]	Ca_12992	Ribosomal protein S7e	INTRON

S.N.	SNP IDs	Chickpea cultivars	Chromosomes	Physical positions (bp)	SNPs	Gene accession IDs	Putative functions	Sequence components of genome
14012	CakSNP14012	*Kabuli*	Ca_Kabuli_Chr08	14821416	[G/A]	Ca_12987	Mrp, conserved site	SYNONYMOUS_CODING
14013	CakSNP14013	*Kabuli*	Ca_Kabuli_Chr08	15000109	[A/G]	Ca_12972	Aldehyde dehydrogenase domain	NON_SYNONYMOUS_CODING
14014	CakSNP14014	*Kabuli*	Ca_Kabuli_Chr08	15034465	[T/C]		--	INTERGENIC
14015	CakSNP14015	*Kabuli*	Ca_Kabuli_Chr08	15131175	[G/A]	Ca_12965	Sugar/inositol transporter	SYNONYMOUS_CODING
14016	CakSNP14016	*Kabuli*	Ca_Kabuli_Chr08	15192125	[A/G]		--	INTERGENIC
14017	CakSNP14017	*Kabuli*	Ca_Kabuli_Chr08	15207944	[C/T]	Ca_12957	tRNA(Ile)-lysidine/2-thiocytidine synthase	INTRON
14018	CakSNP14018	*Kabuli*	Ca_Kabuli_Chr08	15207976	[A/G]	Ca_12957	tRNA(Ile)-lysidine/2-thiocytidine synthase	NON_SYNONYMOUS_CODING
14019	CakSNP14019	*Kabuli*	Ca_Kabuli_Chr08	15220986	[C/G]	Ca_12956	Tesmin/TSO1-like, CXC	SYNONYMOUS_CODING
14020	CakSNP14020	*Kabuli*	Ca_Kabuli_Chr08	15421764	[T/C]		--	INTERGENIC
14021	CakSNP14021	*Kabuli*	Ca_Kabuli_Chr08	15421744	[T/G]		--	INTERGENIC

S.N.	SNP IDs	Chickpea cultivars	Chromosomes	Physical positions (bp)	SNPs	Gene accession IDs	Putative functions	Sequence components of genome
14022	CakSNP14022	*Kabuli*	Ca_Kabuli_Chr08	15421719	[C/G]		--	INTERGENIC
14023	CakSNP14023	*Kabuli*	Ca_Kabuli_Chr08	15422163	[A/T]		--	INTERGENIC
14024	CakSNP14024	*Kabuli*	Ca_Kabuli_Chr08	15546669	[T/C]	Ca_17889	Glyoxalase/bleomycin resistance protein/dioxygenase	INTRON
14025	CakSNP14025	*Kabuli*	Ca_Kabuli_Chr08	15546671	[A/C]	Ca_17889	Glyoxalase/bleomycin resistance protein/dioxygenase	INTRON
14026	CakSNP14026	*Kabuli*	Ca_Kabuli_Chr08	15546696	[C/T]	Ca_17889	Glyoxalase/bleomycin resistance protein/dioxygenase	INTRON
14027	CakSNP14027	*Kabuli*	Ca_Kabuli_Chr08	15546710	[T/A]	Ca_17889	Glyoxalase/bleomycin resistance protein/dioxygenase	INTRON
14028	CakSNP14028	*Kabuli*	Ca_Kabuli_Chr08	15546758	[G/C]	Ca_17889	Glyoxalase/bleomycin resistance protein/dioxygenase	SYNONYMOUS_CODING
14029	CakSNP14029	*Kabuli*	Ca_Kabuli_Chr08	15553251	[A/G]		--	INTERGENIC
14030	CakSNP14030	*Kabuli*	Ca_Kabuli_Chr08	15555771	[A/C]	Ca_17888	Protein of unknown function DUF1675	SYNONYMOUS_CODING
14031	CakSNP14031	*Kabuli*	Ca_Kabuli_Chr08	15699220	[T/A]	Ca_17876	RNA-dependent RNA polymerase, eukaryotic-type	NON_SYNONYMOUS_CODING

S.N.	SNP IDs	Chickpea cultivars	Chromosomes	Physical positions (bp)	SNPs	Gene accession IDs	Putative functions	Sequence components of genome
14032	CakSNP14032	*Kabuli*	Ca_Kabuli_Chr08	15699180	[T/C]	Ca_17876	RNA-dependent RNA polymerase, eukaryotic-type	INTRON
14033	CakSNP14033	*Kabuli*	Ca_Kabuli_Chr08	15741863	[C/G]		--	INTERGENIC
14034	CakSNP14034	*Kabuli*	Ca_Kabuli_Chr08	15764739	[C/T]	Ca_17866	Chromo domain	INTRON
14035	CakSNP14035	*Kabuli*	Ca_Kabuli_Chr08	15764748	[G/A]	Ca_17866	Chromo domain	INTRON
14036	CakSNP14036	*Kabuli*	Ca_Kabuli_Chr08	15764823	[G/A]	Ca_17866	Chromo domain	INTRON
14037	CakSNP14037	*Kabuli*	Ca_Kabuli_Chr08	15764808	[C/A]	Ca_17866	Chromo domain	INTRON
14038	CakSNP14038	*Kabuli*	Ca_Kabuli_Chr08	15764874	[G/A]	Ca_17866	Chromo domain	SYNONYMOUS_CODING
14039	CakSNP14039	*Kabuli*	Ca_Kabuli_Chr08	15857176	[G/A]	Ca_17860	Multi antimicrobial extrusion protein	INTRON
14040	CakSNP14040	*Kabuli*	Ca_Kabuli_Chr08	15857192	[A/G]	Ca_17860	Multi antimicrobial extrusion protein	INTRON
14041	CakSNP14041	*Kabuli*	Ca_Kabuli_Chr08	15857359	[T/G]	Ca_17860	Multi antimicrobial extrusion protein	SYNONYMOUS_CODING

S.N.	SNP IDs	Chickpea cultivars	Chromosomes	Physical positions (bp)	SNPs	Gene accession IDs	Putative functions	Sequence components of genome
14042	CakSNP14042	*Kabuli*	Ca_Kabuli_Chr08	15863509	[T/C]	Ca_17858	WD40 repeat	INTRON
14043	CakSNP14043	*Kabuli*	Ca_Kabuli_Chr08	15873071	[A/C]	Ca_17857	HEAT	SYNONYMOUS_CODING
14044	CakSNP14044	*Kabuli*	Ca_Kabuli_Chr08	15873133	[A/G]	Ca_17857	HEAT	NON_SYNONYMOUS_CODING
14045	CakSNP14045	*Kabuli*	Ca_Kabuli_Chr08	16004976	[G/C]	Ca_15507	Glycoside hydrolase, family 17	NON_SYNONYMOUS_CODING
14046	CakSNP14046	*Kabuli*	Ca_Kabuli_Chr08	16005046	[C/T]	Ca_15507	Glycoside hydrolase, family 17	SYNONYMOUS_CODING
14047	CakSNP14047	*Kabuli*	Ca_Kabuli_Chr08	16005078	[A/G]		--	INTERGENIC
14048	CakSNP14048	*Kabuli*	Ca_Kabuli_Chr08	16005235	[A/G]		--	INTERGENIC
14049	CakSNP14049	*Kabuli*	Ca_Kabuli_Chr08	16005253	[G/A]		--	INTERGENIC
14050	CakSNP14050	*Kabuli*	Ca_Kabuli_Chr08	16020239	[C/A]	Ca_15510	Cullin, N-terminal	INTRON
14051	CakSNP14051	*Kabuli*	Ca_Kabuli_Chr08	16020277	[T/G]	Ca_15510	Cullin, N-terminal	INTRON

S.N.	SNP IDs	Chickpea cultivars	Chromosomes	Physical positions (bp)	SNPs	Gene accession IDs	Putative functions	Sequence components of genome
14052	CakSNP14052	*Kabuli*	Ca_Kabuli_Chr08	16020323	[G/T]	Ca_15510	Cullin, N-terminal	STOP_GAINED
14053	CakSNP14053	*Kabuli*	Ca_Kabuli_Chr08	16020305	[G/A]	Ca_15510	Cullin, N-terminal	NON_SYNONYMOUS_CODING
14054	CakSNP14054	*Kabuli*	Ca_Kabuli_Chr08	16022432	[C/T]	Ca_15511	Pentatricopeptide repeat	NON_SYNONYMOUS_CODING
14055	CakSNP14055	*Kabuli*	Ca_Kabuli_Chr08	16027974	[A/T]	Ca_15513	Leucine-rich repeat	NON_SYNONYMOUS_CODING
14056	CakSNP14056	*Kabuli*	Ca_Kabuli_Chr08	16028064	[G/T]		--	INTERGENIC
14057	CakSNP14057	*Kabuli*	Ca_Kabuli_Chr08	16028062	[G/A]		--	INTERGENIC
14058	CakSNP14058	*Kabuli*	Ca_Kabuli_Chr08	16028040	[A/G]		--	INTERGENIC
14059	CakSNP14059	*Kabuli*	Ca_Kabuli_Chr08	16033779	[T/G]		--	INTERGENIC
14060	CakSNP14060	*Kabuli*	Ca_Kabuli_Chr08	16076161	[C/G]	Ca_15517	Gamma-glutamyltranspeptidase	NON_SYNONYMOUS_CODING
14061	CakSNP14061	*Kabuli*	Ca_Kabuli_Chr08	16076168	[A/G]	Ca_15517	Gamma-glutamyltranspeptidase	SYNONYMOUS_CODING

S.N.	SNP IDs	Chickpea cultivars	Chromosomes	Physical positions (bp)	SNPs	Gene accession IDs	Putative functions	Sequence components of genome
14062	CakSNP14062	*Kabuli*	Ca_Kabuli_Chr08	16102413	[C/T]		--	INTERGENIC
14063	CakSNP14063	*Kabuli*	Ca_Kabuli_Chr08	16102395	[G/A]		--	INTERGENIC
14064	CakSNP14064	*Kabuli*	Ca_Kabuli_Chr08	16102363	[T/C]		--	INTERGENIC
14065	CakSNP14065	*Kabuli*	Ca_Kabuli_Chr08	16102362	[G/T]		--	INTERGENIC
14066	CakSNP14066	*Kabuli*	Ca_Kabuli_Chr08	16102348	[A/T]		--	INTERGENIC
14067	CakSNP14067	*Kabuli*	Ca_Kabuli_Chr08	16102343	[C/T]		--	INTERGENIC
14068	CakSNP14068	*Kabuli*	Ca_Kabuli_Chr08	16102335	[A/T]		--	INTERGENIC
14069	CakSNP14069	*Kabuli*	Ca_Kabuli_Chr08	16102414	[G/T]		--	INTERGENIC
14070	CakSNP14070	*Kabuli*	Ca_Kabuli_Chr08	16144279	[T/A]	Ca_15527	Domain of unknown function DUF292, eukaryotic	INTRON
14071	CakSNP14071	*Kabuli*	Ca_Kabuli_Chr08	16145275	[A/G]	Ca_15527	Domain of unknown function DUF292, eukaryotic	INTRON

S.N.	SNP IDs	Chickpea cultivars	Chromosomes	Physical positions (bp)	SNPs	Gene accession IDs	Putative functions	Sequence components of genome
14072	CakSNP14072	*Kabuli*	Ca_Kabuli_Chr08	16145419	[A/G]	Ca_15527	Domain of unknown function DUF292, eukaryotic	SYNONYMOUS_CODING
14073	CakSNP14073	*Kabuli*	Ca_Kabuli_Chr08	16145411	[C/G]	Ca_15527	Domain of unknown function DUF292, eukaryotic	NON_SYNONYMOUS_CODING
14074	CakSNP14074	*Kabuli*	Ca_Kabuli_Chr08	16145370	[C/A]	Ca_15527	Domain of unknown function DUF292, eukaryotic	INTRON
14075	CakSNP14075	*Kabuli*	Ca_Kabuli_Chr08	16157015	[C/T]	Ca_15529	Phospholipase A2, eukaryotic	INTRON
14076	CakSNP14076	*Kabuli*	Ca_Kabuli_Chr08	16157134	[A/C]	Ca_15529	Phospholipase A2, eukaryotic	NON_SYNONYMOUS_CODING
14077	CakSNP14077	*Kabuli*	Ca_Kabuli_Chr08	16158594	[A/C]	Ca_15530	--	NON_SYNONYMOUS_CODING
14078	CakSNP14078	*Kabuli*	Ca_Kabuli_Chr08	16264310	[G/T]	Ca_15543	CMP/dCMP deaminase, zinc-binding	NON_SYNONYMOUS_CODING
14079	CakSNP14079	*Kabuli*	Ca_Kabuli_Chr08	16268878	[T/C]	Ca_15545	Thioredoxin	INTRON
14080	CakSNP14080	*Kabuli*	Ca_Kabuli_Chr08	16278758	[T/C]	Ca_15548	Phospholipase A2, eukaryotic	SYNONYMOUS_CODING
14081	CakSNP14081	*Kabuli*	Ca_Kabuli_Chr08	16283660	[C/T]	Ca_15549	--	SYNONYMOUS_CODING

S.N.	SNP IDs	Chickpea cultivars	Chromosomes	Physical positions (bp)	SNPs	Gene accession IDs	Putative functions	Sequence components of genome
14082	CakSNP14082	*Kabuli*	Ca_Kabuli_Chr08	16283661	[A/G]	Ca_15549	--	NON_SYNONYMOUS_CODING
14083	CakSNP14083	*Kabuli*	Ca_Kabuli_Chr08	16283722	[A/G]	Ca_15549	--	INTRON
14084	CakSNP14084	*Kabuli*	Ca_Kabuli_Chr08	16283738	[G/C]	Ca_15549	--	INTRON
14085	CakSNP14085	*Kabuli*	Ca_Kabuli_Chr08	16283859	[T/C]	Ca_15549	--	NON_SYNONYMOUS_CODING
14086	CakSNP14086	*Kabuli*	Ca_Kabuli_Chr08	16284269	[G/A]	Ca_15549	--	INTRON
14087	CakSNP14087	*Kabuli*	Ca_Kabuli_Chr08	16284279	[A/C]	Ca_15549	--	INTRON
14088	CakSNP14088	*Kabuli*	Ca_Kabuli_Chr08	16284306	[C/T]	Ca_15549	--	INTRON
14089	CakSNP14089	*Kabuli*	Ca_Kabuli_Chr08	16309857	[G/T]		--	INTERGENIC
14090	CakSNP14090	*Kabuli*	Ca_Kabuli_Chr08	16321524	[A/G]		--	INTERGENIC
14091	CakSNP14091	*Kabuli*	Ca_Kabuli_Chr08	16321545	[G/T]		--	INTERGENIC

S.N.	SNP IDs	Chickpea cultivars	Chromosomes	Physical positions (bp)	SNPs	Gene accession IDs	Putative functions	Sequence components of genome
14092	CakSNP14092	*Kabuli*	Ca_Kabuli_Chr08	16366490	[G/A]	Ca_15558	Prenyltransferase/squalene oxidase	SYNONYMOUS_CODING
14093	CakSNP14093	*Kabuli*	Ca_Kabuli_Chr08	16381928	[T/G]	Ca_15559	Prenyltransferase/squalene oxidase	INTRON
14094	CakSNP14094	*Kabuli*	Ca_Kabuli_Chr08	16411550	[A/G]	Ca_15562	--	INTRON
14095	CakSNP14095	*Kabuli*	Ca_Kabuli_Chr08	16412672	[T/C]	Ca_15562	--	INTRON
14096	CakSNP14096	*Kabuli*	Ca_Kabuli_Chr08	16421407	[G/A]		--	INTERGENIC
14097	CakSNP14097	*Kabuli*	Ca_Kabuli_Chr08	16421441	[C/A]	Ca_15563	--	NON_SYNONYMOUS_CODING
14098	CakSNP14098	*Kabuli*	Ca_Kabuli_Chr08	16421442	[C/G]	Ca_15563	--	SYNONYMOUS_CODING
14099	CakSNP14099	*Kabuli*	Ca_Kabuli_Chr08	16421545	[G/T]	Ca_15563	--	NON_SYNONYMOUS_CODING
14100	CakSNP14100	*Kabuli*	Ca_Kabuli_Chr08	16421497	[C/T]	Ca_15563	--	NON_SYNONYMOUS_CODING
14101	CakSNP14101	*Kabuli*	Ca_Kabuli_Chr08	16421595	[A/C]	Ca_15563	--	SYNONYMOUS_CODING

S.N.	SNP IDs	Chickpea cultivars	Chromosomes	Physical positions (bp)	SNPs	Gene accession IDs	Putative functions	Sequence components of genome
14102	CakSNP14102	*Kabuli*	Ca_Kabuli_Chr08	16432907	[T/G]	Ca_15563	--	INTRON
14103	CakSNP14103	*Kabuli*	Ca_Kabuli_Chr08	16472919	[T/A]		--	INTERGENIC
14104	CakSNP14104	*Kabuli*	Ca_Kabuli_Chr08	16472932	[C/T]		--	INTERGENIC
14105	CakSNP14105	*Kabuli*	Ca_Kabuli_Chr08	16472933	[G/C]		--	INTERGENIC
14106	CakSNP14106	*Kabuli*	Ca_Kabuli_Chr08	16472999	[A/C]		--	INTERGENIC
14107	CakSNP14107	*Kabuli*	Ca_Kabuli_Chr08	16473047	[C/G]		--	INTERGENIC
14108	CakSNP14108	*Kabuli*	Ca_Kabuli_Chr08	16473139	[C/T]		--	INTERGENIC
14109	CakSNP14109	*Kabuli*	Ca_Kabuli_Chr08	16473154	[T/A]		--	INTERGENIC
14110	CakSNP14110	*Kabuli*	Ca_Kabuli_Chr08	16473157	[C/T]		--	INTERGENIC
14111	CakSNP14111	*Kabuli*	Ca_Kabuli_Chr08	16473311	[A/G]		--	INTERGENIC

S.N.	SNP IDs	Chickpea cultivars	Chromosomes	Physical positions (bp)	SNPs	Gene accession IDs	Putative functions	Sequence components of genome
14112	CakSNP14112	*Kabuli*	Ca_Kabuli_Chr08	16473290	[A/G]		--	INTERGENIC
14113	CakSNP14113	*Kabuli*	Ca_Kabuli_Chr08	16473920	[A/G]		--	INTERGENIC
14114	CakSNP14114	*Kabuli*	Ca_Kabuli_Chr08	16473939	[A/G]		--	INTERGENIC
14115	CakSNP14115	*Kabuli*	Ca_Kabuli_Chr08	16473987	[C/T]		--	INTERGENIC
14116	CakSNP14116	*Kabuli*	Ca_Kabuli_Scaffold_C1105808	341	[G/A]		--	INTERGENIC
14117	CakSNP14117	*Kabuli*	Ca_Kabuli_Scaffold_C1105827	143	[T/A]		--	INTERGENIC
14118	CakSNP14118	*Kabuli*	Ca_Kabuli_Scaffold_C1106233	412	[G/A]		--	INTERGENIC
14119	CakSNP14119	*Kabuli*	Ca_Kabuli_Scaffold_C1106243	381	[T/A]		--	INTERGENIC
14120	CakSNP14120	*Kabuli*	Ca_Kabuli_Scaffold_C1106254	677	[G/T]		--	INTERGENIC
14121	CakSNP14121	*Kabuli*	Ca_Kabuli_Scaffold_C1107670	989	[G/A]		--	INTERGENIC

S.N.	SNP IDs	Chickpea cultivars	Chromosomes	Physical positions (bp)	SNPs	Gene accession IDs	Putative functions	Sequence components of genome
14122	CakSNP14122	*Kabuli*	Ca_Kabuli_Scaffold_C110784	885	[G/A]		--	INTERGENIC
14123	CakSNP14123	*Kabuli*	Ca_Kabuli_Scaffold_C110797	1418	[A/C]		--	INTERGENIC
14124	CakSNP14124	*Kabuli*	Ca_Kabuli_Scaffold_C110797	1422	[G/C]		--	INTERGENIC
14125	CakSNP14125	*Kabuli*	Ca_Kabuli_Scaffold_C110797	1412	[A/T]		--	INTERGENIC
14126	CakSNP14126	*Kabuli*	Ca_Kabuli_Scaffold_C110807	1568	[A/C]		--	INTERGENIC
14127	CakSNP14127	*Kabuli*	Ca_Kabuli_Scaffold_C110846	656	[G/A]		--	INTERGENIC
14128	CakSNP14128	*Kabuli*	Ca_Kabuli_Scaffold_C110849	516	[A/G]		--	INTERGENIC
14129	CakSNP14129	*Kabuli*	Ca_Kabuli_Scaffold_C110849	537	[G/T]		--	INTERGENIC
14130	CakSNP14130	*Kabuli*	Ca_Kabuli_Scaffold_C110860	641	[A/C]		--	INTERGENIC
14131	CakSNP14131	*Kabuli*	Ca_Kabuli_Scaffold_C110860	624	[A/C]		--	INTERGENIC

S.N.	SNP IDs	Chickpea cultivars	Chromosomes	Physical positions (bp)	SNPs	Gene accession IDs	Putative functions	Sequence components of genome
14132	CakSNP14132	*Kabuli*	Ca_Kabuli_Scaffold_C1108869	514	[G/C]		--	INTERGENIC
14133	CakSNP14133	*Kabuli*	Ca_Kabuli_Scaffold_C1111949	682	[T/A]		--	INTERGENIC
14134	CakSNP14134	*Kabuli*	Ca_Kabuli_Scaffold_C1111949	686	[C/A]		--	INTERGENIC
14135	CakSNP14135	*Kabuli*	Ca_Kabuli_Scaffold_C1111949	689	[C/A]		--	INTERGENIC
14136	CakSNP14136	*Kabuli*	Ca_Kabuli_Scaffold_C1111949	693	[G/A]		--	INTERGENIC
14137	CakSNP14137	*Kabuli*	Ca_Kabuli_Scaffold_C1113276	2177	[G/A]		--	INTERGENIC
14138	CakSNP14138	*Kabuli*	Ca_Kabuli_Scaffold_C1114221	2902	[G/T]		--	INTERGENIC
14139	CakSNP14139	*Kabuli*	Ca_Kabuli_Scaffold_C1114225	3299	[A/C]	Ca_27871	Ribosomal protein S7	INTRON
14140	CakSNP14140	*Kabuli*	Ca_Kabuli_Scaffold_C1114360	1389	[A/T]	Ca_27926	DNA-directed RNA polymerase, subunit 2, domain 6	INTRON
14141	CakSNP14141	*Kabuli*	Ca_Kabuli_Scaffold_C1114763	3004	[C/A]	Ca_28253	Domain of unknown function DUF231, plant	INTRON

S.N.	SNP IDs	Chickpea cultivars	Chromosomes	Physical positions (bp)	SNPs	Gene accession IDs	Putative functions	Sequence components of genome
14142	CakSNP14142	*Kabuli*	Ca_Kabuli_Scaffold_C111476;	3028	[C/T]	Ca_28253	Domain of unknown function DUF231, plant	INTRON
14143	CakSNP14143	*Kabuli*	Ca_Kabuli_Scaffold_C111490!	2181	[A/G]		--	INTERGENIC
14144	CakSNP14144	*Kabuli*	Ca_Kabuli_Scaffold_C111490!	2281	[T/C]		--	INTERGENIC
14145	CakSNP14145	*Kabuli*	Ca_Kabuli_Scaffold_C111490!	2474	[T/A]		--	INTERGENIC
14146	CakSNP14146	*Kabuli*	Ca_Kabuli_Scaffold_C111490!	3190	[T/G]		--	INTERGENIC
14147	CakSNP14147	*Kabuli*	Ca_Kabuli_Scaffold_C111490!	3284	[C/G]		--	INTERGENIC
14148	CakSNP14148	*Kabuli*	Ca_Kabuli_Scaffold_C111536(	4275	[C/T]	Ca_28089	UDP-glucuronosyl/UDP-glucosyltransferase	INTRON
14149	CakSNP14149	*Kabuli*	Ca_Kabuli_Scaffold_C111573;	766	[C/G]	Ca_28268	GCN5-related N-acetyltransferase (GNAT) domain	INTRON
14150	CakSNP14150	*Kabuli*	Ca_Kabuli_Scaffold_C111573;	2169	[T/A]	Ca_28268	GCN5-related N-acetyltransferase (GNAT) domain	INTRON
14151	CakSNP14151	*Kabuli*	Ca_Kabuli_Scaffold_C111573;	2152	[G/C]	Ca_28268	GCN5-related N-acetyltransferase (GNAT) domain	INTRON

S.N.	SNP IDs	Chickpea cultivars	Chromosomes	Physical positions (bp)	SNPs	Gene accession IDs	Putative functions	Sequence components of genome
14152	CakSNP14152	*Kabuli*	Ca_Kabuli_Scaffold_C111580	2186	[A/T]	Ca_28046	HEAT	INTRON
14153	CakSNP14153	*Kabuli*	Ca_Kabuli_Scaffold_C111605	4522	[A/G]	Ca_28216	Cytochrome P450	NON_SYNONYMOUS_CODING
14154	CakSNP14154	*Kabuli*	Ca_Kabuli_Scaffold_C111620	3914	[C/T]		--	INTERGENIC
14155	CakSNP14155	*Kabuli*	Ca_Kabuli_Scaffold_C111620	3926	[T/G]		--	INTERGENIC
14156	CakSNP14156	*Kabuli*	Ca_Kabuli_Scaffold_C111620	5515	[G/A]	Ca_28006	Mov34/MPN/PAD-1	INTRON
14157	CakSNP14157	*Kabuli*	Ca_Kabuli_Scaffold_C111649	5778	[T/A]	Ca_28056	Protein of unknown function DUF2930	NON_SYNONYMOUS_CODING
14158	CakSNP14158	*Kabuli*	Ca_Kabuli_Scaffold_C111649	5784	[G/A]	Ca_28056	Protein of unknown function DUF2930	NON_SYNONYMOUS_CODING
14159	CakSNP14159	*Kabuli*	Ca_Kabuli_Scaffold_C111654	6285	[T/A]		--	INTERGENIC
14160	CakSNP14160	*Kabuli*	Ca_Kabuli_Scaffold_C111658	154	[C/G]		--	INTERGENIC
14161	CakSNP14161	*Kabuli*	Ca_Kabuli_Scaffold_C111747	8931	[C/G]	Ca_27829	Protein kinase, catalytic domain	SYNONYMOUS_CODING

S.N.	SNP IDs	Chickpea cultivars	Chromosomes	Physical positions (bp)	SNPs	Gene accession IDs	Putative functions	Sequence components of genome
14162	CakSNP14162	*Kabuli*	Ca_Kabuli_Scaffold_C111754	987	[A/G]	Ca_27726	Protein of unknown function DUF3743	SYNONYMOUS_CODING
14163	CakSNP14163	*Kabuli*	Ca_Kabuli_Scaffold_C111754	3929	[T/G]	Ca_27727	ABC transporter, transmembrane domain	NON_SYNONYMOUS_CODING
14164	CakSNP14164	*Kabuli*	Ca_Kabuli_Scaffold_C111754	3936	[G/T]	Ca_27727	ABC transporter, transmembrane domain	NON_SYNONYMOUS_CODING
14165	CakSNP14165	*Kabuli*	Ca_Kabuli_Scaffold_C111754	3952	[T/C]	Ca_27727	ABC transporter, transmembrane domain	NON_SYNONYMOUS_CODING
14166	CakSNP14166	*Kabuli*	Ca_Kabuli_Scaffold_C111766	4197	[G/C]	Ca_28151	Zinc finger, Sec23/Sec24-type	INTRON
14167	CakSNP14167	*Kabuli*	Ca_Kabuli_Scaffold_C111771	2529	[T/G]	Ca_28257	U3 small nucleolar RNA-associated protein 10	INTRON
14168	CakSNP14168	*Kabuli*	Ca_Kabuli_Scaffold_C111771	2515	[T/A]	Ca_28257	U3 small nucleolar RNA-associated protein 10	INTRON
14169	CakSNP14169	*Kabuli*	Ca_Kabuli_Scaffold_C111771	5324	[G/A]	Ca_28257	U3 small nucleolar RNA-associated protein 10	INTRON
14170	CakSNP14170	*Kabuli*	Ca_Kabuli_Scaffold_C111777	1264	[T/A]		--	INTERGENIC
14171	CakSNP14171	*Kabuli*	Ca_Kabuli_Scaffold_C111785	2107	[A/T]	Ca_27936	Protein kinase, catalytic domain	INTRON

S.N.	SNP IDs	Chickpea cultivars	Chromosomes	Physical positions (bp)	SNPs	Gene accession IDs	Putative functions	Sequence components of genome
14172	CakSNP14172	*Kabuli*	Ca_Kabuli_Scaffold_C1117853	3524	[A/G]	Ca_27936	Protein kinase, catalytic domain	SYNONYMOUS_CODING
14173	CakSNP14173	*Kabuli*	Ca_Kabuli_Scaffold_C1118178	6215	[A/G]	Ca_27691	Domain of unknown function DUF1084	SYNONYMOUS_CODING
14174	CakSNP14174	*Kabuli*	Ca_Kabuli_Scaffold_C1118184	19192	[A/G]	Ca_28165	Domain of unknown function DUF547	DRR
14175	CakSNP14175	*Kabuli*	Ca_Kabuli_Scaffold_C1118184	19202	[T/C]	Ca_28165	Domain of unknown function DUF547	DRR
14176	CakSNP14176	*Kabuli*	Ca_Kabuli_Scaffold_1006	40651	[T/A]		--	INTERGENIC
14177	CakSNP14177	*Kabuli*	Ca_Kabuli_Scaffold_1006	382579	[C/T]		--	INTERGENIC
14178	CakSNP14178	*Kabuli*	Ca_Kabuli_Scaffold_1006	436923	[T/C]	Ca_18033	--	INTRON
14179	CakSNP14179	*Kabuli*	Ca_Kabuli_Scaffold_1006	644893	[A/C]	Ca_18050	CoA-binding	NON_SYNONYMOUS_CODING
14180	CakSNP14180	*Kabuli*	Ca_Kabuli_Scaffold_1006	667620	[A/G]	Ca_18053	--	INTRON
14181	CakSNP14181	*Kabuli*	Ca_Kabuli_Scaffold_1006	667713	[G/A]	Ca_18053	--	INTRON

S.N.	SNP IDs	Chickpea cultivars	Chromosomes	Physical positions (bp)	SNPs	Gene accession IDs	Putative functions	Sequence components of genome
14182	CakSNP14182	*Kabuli*	Ca_Kabuli_Scaffold_1006	674495	[A/G]	Ca_18055	Oligopeptide transporter OPT superfamily	NON_SYNONYMOUS_CODING
14183	CakSNP14183	*Kabuli*	Ca_Kabuli_Scaffold_1006	674823	[G/A]	Ca_18055	Oligopeptide transporter OPT superfamily	SYNONYMOUS_CODING
14184	CakSNP14184	*Kabuli*	Ca_Kabuli_Scaffold_1006	674826	[C/T]	Ca_18055	Oligopeptide transporter OPT superfamily	SYNONYMOUS_CODING
14185	CakSNP14185	*Kabuli*	Ca_Kabuli_Scaffold_1006	674844	[T/C]	Ca_18055	Oligopeptide transporter OPT superfamily	SYNONYMOUS_CODING
14186	CakSNP14186	*Kabuli*	Ca_Kabuli_Scaffold_1006	674847	[T/G]	Ca_18055	Oligopeptide transporter OPT superfamily	SYNONYMOUS_CODING
14187	CakSNP14187	*Kabuli*	Ca_Kabuli_Scaffold_1006	674862	[G/A]	Ca_18055	Oligopeptide transporter OPT superfamily	SYNONYMOUS_CODING
14188	CakSNP14188	*Kabuli*	Ca_Kabuli_Scaffold_1006	674865	[T/C]	Ca_18055	Oligopeptide transporter OPT superfamily	SYNONYMOUS_CODING
14189	CakSNP14189	*Kabuli*	Ca_Kabuli_Scaffold_1006	674871	[A/G]	Ca_18055	Oligopeptide transporter OPT superfamily	SYNONYMOUS_CODING
14190	CakSNP14190	*Kabuli*	Ca_Kabuli_Scaffold_1006	674881	[G/A]	Ca_18055	Oligopeptide transporter OPT superfamily	NON_SYNONYMOUS_CODING
14191	CakSNP14191	*Kabuli*	Ca_Kabuli_Scaffold_1006	674981	[G/A]	Ca_18055	Oligopeptide transporter OPT superfamily	NON_SYNONYMOUS_CODING

S.N.	SNP IDs	Chickpea cultivars	Chromosomes	Physical positions (bp)	SNPs	Gene accession IDs	Putative functions	Sequence components of genome
14192	CakSNP14192	*Kabuli*	Ca_Kabuli_Scaffold_1006	674946	[G/A]	Ca_18055	Oligopeptide transporter OPT superfamily	SYNONYMOUS_CODING
14193	CakSNP14193	*Kabuli*	Ca_Kabuli_Scaffold_1010	90092	[G/A]		--	INTERGENIC
14194	CakSNP14194	*Kabuli*	Ca_Kabuli_Scaffold_1013	56140	[G/A]		--	INTERGENIC
14195	CakSNP14195	*Kabuli*	Ca_Kabuli_Scaffold_1013	56116	[C/T]		--	INTERGENIC
14196	CakSNP14196	*Kabuli*	Ca_Kabuli_Scaffold_1013	56114	[C/A]		--	INTERGENIC
14197	CakSNP14197	*Kabuli*	Ca_Kabuli_Scaffold_1013	56075	[T/A]		--	INTERGENIC
14198	CakSNP14198	*Kabuli*	Ca_Kabuli_Scaffold_1013	56074	[C/T]		--	INTERGENIC
14199	CakSNP14199	*Kabuli*	Ca_Kabuli_Scaffold_1013	56069	[C/T]		--	INTERGENIC
14200	CakSNP14200	*Kabuli*	Ca_Kabuli_Scaffold_10186	416	[C/T]		--	INTERGENIC
14201	CakSNP14201	*Kabuli*	Ca_Kabuli_Scaffold_10186	410	[A/C]		--	INTERGENIC

S.N.	SNP IDs	Chickpea cultivars	Chromosomes	Physical positions (bp)	SNPs	Gene accession IDs	Putative functions	Sequence components of genome
14202	CakSNP14202	*Kabuli*	Ca_Kabuli_Scaffold_10186	378	[A/T]		--	INTERGENIC
14203	CakSNP14203	*Kabuli*	Ca_Kabuli_Scaffold_10186	354	[T/A]		--	INTERGENIC
14204	CakSNP14204	*Kabuli*	Ca_Kabuli_Scaffold_10186	397	[G/A]		--	INTERGENIC
14205	CakSNP14205	*Kabuli*	Ca_Kabuli_Scaffold_10186	573	[T/G]		--	INTERGENIC
14206	CakSNP14206	*Kabuli*	Ca_Kabuli_Scaffold_102	82102	[T/C]		--	INTERGENIC
14207	CakSNP14207	*Kabuli*	Ca_Kabuli_Scaffold_102	82107	[C/A]		--	INTERGENIC
14208	CakSNP14208	*Kabuli*	Ca_Kabuli_Scaffold_1027	256612	[A/C]	Ca_25976	--	URR
14209	CakSNP14209	*Kabuli*	Ca_Kabuli_Scaffold_1034	73684	[G/A]	Ca_27683	--	STOP_GAINED
14210	CakSNP14210	*Kabuli*	Ca_Kabuli_Scaffold_1034	73695	[G/T]	Ca_27683	--	NON_SYNONYMOUS_CODING
14211	CakSNP14211	*Kabuli*	Ca_Kabuli_Scaffold_10420	19974	[A/G]	Ca_26535	ATPase, AAA+ type, core	NON_SYNONYMOUS_CODING

S.N.	SNP IDs	Chickpea cultivars	Chromosomes	Physical positions (bp)	SNPs	Gene accession IDs	Putative functions	Sequence components of genome
14212	CakSNP14212	*Kabuli*	Ca_Kabuli_Scaffold_10420	31513	[C/T]	Ca_26536	--	INTRON
14213	CakSNP14213	*Kabuli*	Ca_Kabuli_Scaffold_10420	31563	[A/G]	Ca_26536	--	INTRON
14214	CakSNP14214	*Kabuli*	Ca_Kabuli_Scaffold_10420	31817	[C/T]	Ca_26536	--	INTRON
14215	CakSNP14215	*Kabuli*	Ca_Kabuli_Scaffold_10420	40193	[G/T]	Ca_26538	--	SYNONYMOUS_CODING
14216	CakSNP14216	*Kabuli*	Ca_Kabuli_Scaffold_1043	16328	[A/T]		--	INTERGENIC
14217	CakSNP14217	*Kabuli*	Ca_Kabuli_Scaffold_1043	16331	[G/T]		--	INTERGENIC
14218	CakSNP14218	*Kabuli*	Ca_Kabuli_Scaffold_1043	16333	[A/T]		--	INTERGENIC
14219	CakSNP14219	*Kabuli*	Ca_Kabuli_Scaffold_1043	16313	[A/G]		--	INTERGENIC
14220	CakSNP14220	*Kabuli*	Ca_Kabuli_Scaffold_1047	235352	[T/C]	Ca_17194	Engulfment/cell motility, ELMO	INTRON
14221	CakSNP14221	*Kabuli*	Ca_Kabuli_Scaffold_1047	577874	[T/G]		--	INTERGENIC

S.N.	SNP IDs	Chickpea cultivars	Chromosomes	Physical positions (bp)	SNPs	Gene accession IDs	Putative functions	Sequence components of genome
14222	CakSNP14222	*Kabuli*	Ca_Kabuli_Scaffold_1050	3398	[G/A]	Ca_25913	Peptidase C48, SUMO/Sentrin/Ubl1	SYNONYMOUS_CODING
14223	CakSNP14223	*Kabuli*	Ca_Kabuli_Scaffold_1050	3415	[C/T]	Ca_25913	Peptidase C48, SUMO/Sentrin/Ubl1	NON_SYNONYMOUS_CODING
14224	CakSNP14224	*Kabuli*	Ca_Kabuli_Scaffold_1050	3463	[C/T]	Ca_25913	Peptidase C48, SUMO/Sentrin/Ubl1	NON_SYNONYMOUS_CODING
14225	CakSNP14225	*Kabuli*	Ca_Kabuli_Scaffold_1050	14057	[C/A]		--	INTERGENIC
14226	CakSNP14226	*Kabuli*	Ca_Kabuli_Scaffold_1050	126986	[T/G]	Ca_25916	Synaptojanin, N-terminal	INTRON
14227	CakSNP14227	*Kabuli*	Ca_Kabuli_Scaffold_1050	127022	[G/A]	Ca_25916	Synaptojanin, N-terminal	INTRON
14228	CakSNP14228	*Kabuli*	Ca_Kabuli_Scaffold_1050	127023	[G/T]	Ca_25916	Synaptojanin, N-terminal	INTRON
14229	CakSNP14229	*Kabuli*	Ca_Kabuli_Scaffold_1050	127054	[C/T]	Ca_25916	Synaptojanin, N-terminal	INTRON
14230	CakSNP14230	*Kabuli*	Ca_Kabuli_Scaffold_1050	128763	[A/G]	Ca_25916	Synaptojanin, N-terminal	INTRON
14231	CakSNP14231	*Kabuli*	Ca_Kabuli_Scaffold_1055	344459	[G/A]		--	INTERGENIC

S.N.	SNP IDs	Chickpea cultivars	Chromosomes	Physical positions (bp)	SNPs	Gene accession IDs	Putative functions	Sequence components of genome
14232	CakSNP14232	*Kabuli*	Ca_Kabuli_Scaffold_1055	344443	[G/A]		--	INTERGENIC
14233	CakSNP14233	*Kabuli*	Ca_Kabuli_Scaffold_1055	344399	[C/T]		--	INTERGENIC
14234	CakSNP14234	*Kabuli*	Ca_Kabuli_Scaffold_1060	3278	[A/C]	Ca_21773	Pathogenesis-related transcriptional factor/ERF, DNA-binding	INTRON
14235	CakSNP14235	*Kabuli*	Ca_Kabuli_Scaffold_1060	3310	[G/T]	Ca_21773	Pathogenesis-related transcriptional factor/ERF, DNA-binding	INTRON
14236	CakSNP14236	*Kabuli*	Ca_Kabuli_Scaffold_1060	394867	[C/T]	Ca_21793	Transposase, Ptta/En/Spm, plant	INTRON
14237	CakSNP14237	*Kabuli*	Ca_Kabuli_Scaffold_1060	395029	[C/T]	Ca_21793	Transposase, Ptta/En/Spm, plant	INTRON
14238	CakSNP14238	*Kabuli*	Ca_Kabuli_Scaffold_1060	395042	[A/G]	Ca_21793	Transposase, Ptta/En/Spm, plant	INTRON
14239	CakSNP14239	*Kabuli*	Ca_Kabuli_Scaffold_1061	63613	[A/G]		--	INTERGENIC
14240	CakSNP14240	*Kabuli*	Ca_Kabuli_Scaffold_1061	63620	[C/T]		--	INTERGENIC
14241	CakSNP14241	*Kabuli*	Ca_Kabuli_Scaffold_1065	142678	[A/C]		--	INTERGENIC

S.N.	SNP IDs	Chickpea cultivars	Chromosomes	Physical positions (bp)	SNPs	Gene accession IDs	Putative functions	Sequence components of genome
14242	CakSNP14242	*Kabuli*	Ca_Kabuli_Scaffold_1067	38623	[C/A]	Ca_24418	Mediator complex subunit Med27	INTRON
14243	CakSNP14243	*Kabuli*	Ca_Kabuli_Scaffold_1081	768	[A/C]		--	INTERGENIC
14244	CakSNP14244	*Kabuli*	Ca_Kabuli_Scaffold_1081	798	[T/C]		--	INTERGENIC
14245	CakSNP14245	*Kabuli*	Ca_Kabuli_Scaffold_1081	800	[T/C]		--	INTERGENIC
14246	CakSNP14246	*Kabuli*	Ca_Kabuli_Scaffold_1081	805	[A/C]		--	INTERGENIC
14247	CakSNP14247	*Kabuli*	Ca_Kabuli_Scaffold_1081	818	[A/C]		--	INTERGENIC
14248	CakSNP14248	*Kabuli*	Ca_Kabuli_Scaffold_1089	1154	[C/A]		--	INTERGENIC
14249	CakSNP14249	*Kabuli*	Ca_Kabuli_Scaffold_1089	1240	[T/G]	Ca_24272	--	NON_SYNONYMOUS_CODING
14250	CakSNP14250	*Kabuli*	Ca_Kabuli_Scaffold_1089	1281	[G/A]	Ca_24272	--	NON_SYNONYMOUS_CODING
14251	CakSNP14251	*Kabuli*	Ca_Kabuli_Scaffold_1089	1278	[T/C]	Ca_24272	--	NON_SYNONYMOUS_CODING

S.N.	SNP IDs	Chickpea cultivars	Chromosomes	Physical positions (bp)	SNPs	Gene accession IDs	Putative functions	Sequence components of genome
14252	CakSNP14252	*Kabuli*	Ca_Kabuli_Scaffold_1089	3160	[C/A]		--	INTERGENIC
14253	CakSNP14253	*Kabuli*	Ca_Kabuli_Scaffold_1089	3173	[G/T]		--	INTERGENIC
14254	CakSNP14254	*Kabuli*	Ca_Kabuli_Scaffold_1089	3204	[G/C]		--	INTERGENIC
14255	CakSNP14255	*Kabuli*	Ca_Kabuli_Scaffold_1089	3236	[T/G]		--	INTERGENIC
14256	CakSNP14256	*Kabuli*	Ca_Kabuli_Scaffold_1089	3249	[T/G]		--	INTERGENIC
14257	CakSNP14257	*Kabuli*	Ca_Kabuli_Scaffold_1089	3248	[C/T]		--	INTERGENIC
14258	CakSNP14258	*Kabuli*	Ca_Kabuli_Scaffold_1089	3240	[G/C]		--	INTERGENIC
14259	CakSNP14259	*Kabuli*	Ca_Kabuli_Scaffold_1089	3228	[C/T]		--	INTERGENIC
14260	CakSNP14260	*Kabuli*	Ca_Kabuli_Scaffold_1089	3191	[G/A]		--	INTERGENIC
14261	CakSNP14261	*Kabuli*	Ca_Kabuli_Scaffold_1089	3274	[T/C]		--	INTERGENIC

S.N.	SNP IDs	Chickpea cultivars	Chromosomes	Physical positions (bp)	SNPs	Gene accession IDs	Putative functions	Sequence components of genome
14262	CakSNP14262	*Kabuli*	Ca_Kabuli_Scaffold_1089	137675	[T/C]	Ca_24281	Glycoside hydrolase, family 18, catalytic domain	NON_SYNONYMOUS_CODING
14263	CakSNP14263	*Kabuli*	Ca_Kabuli_Scaffold_1089	137679	[G/T]	Ca_24281	Glycoside hydrolase, family 18, catalytic domain	NON_SYNONYMOUS_CODING
14264	CakSNP14264	*Kabuli*	Ca_Kabuli_Scaffold_109_1	156485	[C/T]	Ca_11572	Membrane bound O-acyl transferase, MBOAT	INTRON
14265	CakSNP14265	*Kabuli*	Ca_Kabuli_Scaffold_109_1	230701	[G/C]	Ca_11576	Lycopene cyclase-type, FAD-binding	INTRON
14266	CakSNP14266	*Kabuli*	Ca_Kabuli_Scaffold_109_1	244141	[A/C]	Ca_11577	--	SYNONYMOUS_CODING
14267	CakSNP14267	*Kabuli*	Ca_Kabuli_Scaffold_109_1	244150	[C/T]	Ca_11577	--	SYNONYMOUS_CODING
14268	CakSNP14268	*Kabuli*	Ca_Kabuli_Scaffold_109_1	244156	[A/C]	Ca_11577	--	SYNONYMOUS_CODING
14269	CakSNP14269	*Kabuli*	Ca_Kabuli_Scaffold_109_1	244176	[A/T]	Ca_11577	--	NON_SYNONYMOUS_CODING
14270	CakSNP14270	*Kabuli*	Ca_Kabuli_Scaffold_109_1	328757	[C/T]	Ca_11580	--	NON_SYNONYMOUS_CODING
14271	CakSNP14271	*Kabuli*	Ca_Kabuli_Scaffold_109_1	343415	[G/A]	Ca_11581	Monooxygenase, FAD-binding	INTRON

S.N.	SNP IDs	Chickpea cultivars	Chromosomes	Physical positions (bp)	SNPs	Gene accession IDs	Putative functions	Sequence components of genome
14272	CakSNP14272	*Kabuli*	Ca_Kabuli_Scaffold_109_1	343454	[G/A]	Ca_11581	Monooxygenase, FAD-binding	INTRON
14273	CakSNP14273	*Kabuli*	Ca_Kabuli_Scaffold_109_1	385548	[T/C]	Ca_11585	--	SYNONYMOUS_CODING
14274	CakSNP14274	*Kabuli*	Ca_Kabuli_Scaffold_109_1	385578	[C/G]	Ca_11585	--	SYNONYMOUS_CODING
14275	CakSNP14275	*Kabuli*	Ca_Kabuli_Scaffold_109_1	385602	[C/T]	Ca_11585	--	SYNONYMOUS_CODING
14276	CakSNP14276	*Kabuli*	Ca_Kabuli_Scaffold_109_1	385660	[G/A]	Ca_11585	--	NON_SYNONYMOUS_CODING
14277	CakSNP14277	*Kabuli*	Ca_Kabuli_Scaffold_109_1	385653	[C/T]	Ca_11585	--	SYNONYMOUS_CODING
14278	CakSNP14278	*Kabuli*	Ca_Kabuli_Scaffold_109_1	442149	[A/T]	Ca_11589	Peptidoglycan-binding Lysin subgroup	SYNONYMOUS_CODING
14279	CakSNP14279	*Kabuli*	Ca_Kabuli_Scaffold_109_1	535504	[G/C]	Ca_11595	Snf7	SYNONYMOUS_CODING
14280	CakSNP14280	*Kabuli*	Ca_Kabuli_Scaffold_109_1	586181	[A/C]	Ca_11598	Photosystem II PsbX	SYNONYMOUS_CODING
14281	CakSNP14281	*Kabuli*	Ca_Kabuli_Scaffold_109_1	586286	[C/T]	Ca_11598	Photosystem II PsbX	SYNONYMOUS_CODING

S.N.	SNP IDs	Chickpea cultivars	Chromosomes	Physical positions (bp)	SNPs	Gene accession IDs	Putative functions	Sequence components of genome
14282	CakSNP14282	*Kabuli*	Ca_Kabuli_Scaffold_109_1	696869	[A/C]	Ca_11606	Tetratricopeptide TPR-1	INTRON
14283	CakSNP14283	*Kabuli*	Ca_Kabuli_Scaffold_109_1	734137	[G/A]	Ca_11608	DENN	INTRON
14284	CakSNP14284	*Kabuli*	Ca_Kabuli_Scaffold_109_1	734141	[C/T]	Ca_11608	DENN	INTRON
14285	CakSNP14285	*Kabuli*	Ca_Kabuli_Scaffold_109_1	741607	[G/A]	Ca_11609	uDENN	SYNONYMOUS_CODING
14286	CakSNP14286	*Kabuli*	Ca_Kabuli_Scaffold_109_1	763160	[G/A]		--	INTERGENIC
14287	CakSNP14287	*Kabuli*	Ca_Kabuli_Scaffold_1109	150672	[G/A]	Ca_25534	--	NON_SYNONYMOUS_CODING
14288	CakSNP14288	*Kabuli*	Ca_Kabuli_Scaffold_1109	150720	[G/A]	Ca_25534	--	NON_SYNONYMOUS_CODING
14289	CakSNP14289	*Kabuli*	Ca_Kabuli_Scaffold_1109	150676	[C/T]	Ca_25534	--	NON_SYNONYMOUS_CODING
14290	CakSNP14290	*Kabuli*	Ca_Kabuli_Scaffold_1115	6234	[G/C]		--	INTERGENIC
14291	CakSNP14291	*Kabuli*	Ca_Kabuli_Scaffold_1115	131039	[G/A]	Ca_24642	Uncharacterised protein family UPF0406	INTRON

S.N.	SNP IDs	Chickpea cultivars	Chromosomes	Physical positions (bp)	SNPs	Gene accession IDs	Putative functions	Sequence components of genome
14292	CakSNP14292	*Kabuli*	Ca_Kabuli_Scaffold_1115	153349	[T/C]		--	INTERGENIC
14293	CakSNP14293	*Kabuli*	Ca_Kabuli_Scaffold_1118	2215	[T/C]		--	INTERGENIC
14294	CakSNP14294	*Kabuli*	Ca_Kabuli_Scaffold_1118	2224	[T/A]		--	INTERGENIC
14295	CakSNP14295	*Kabuli*	Ca_Kabuli_Scaffold_1118	2225	[C/T]		--	INTERGENIC
14296	CakSNP14296	*Kabuli*	Ca_Kabuli_Scaffold_1118	2226	[T/G]		--	INTERGENIC
14297	CakSNP14297	*Kabuli*	Ca_Kabuli_Scaffold_1118	2315	[T/C]		--	INTERGENIC
14298	CakSNP14298	*Kabuli*	Ca_Kabuli_Scaffold_1118	9568	[A/G]	Ca_27404	Inositol polyphosphate-related phosphatase	INTRON
14299	CakSNP14299	*Kabuli*	Ca_Kabuli_Scaffold_1118	9556	[C/T]	Ca_27404	Inositol polyphosphate-related phosphatase	INTRON
14300	CakSNP14300	*Kabuli*	Ca_Kabuli_Scaffold_1128	94196	[T/A]	Ca_23728	ABC transporter-like	INTRON
14301	CakSNP14301	*Kabuli*	Ca_Kabuli_Scaffold_1128	112702	[G/A]		--	INTERGENIC

S.N.	SNP IDs	Chickpea cultivars	Chromosomes	Physical positions (bp)	SNPs	Gene accession IDs	Putative functions	Sequence components of genome
14302	CakSNP14302	*Kabuli*	Ca_Kabuli_Scaffold_1128	341416	[T/A]		--	INTERGENIC
14303	CakSNP14303	*Kabuli*	Ca_Kabuli_Scaffold_1128	360567	[T/A]		--	INTERGENIC
14304	CakSNP14304	*Kabuli*	Ca_Kabuli_Scaffold_1128	360529	[A/C]		--	INTERGENIC
14305	CakSNP14305	*Kabuli*	Ca_Kabuli_Scaffold_1128	423553	[A/G]		--	INTERGENIC
14306	CakSNP14306	*Kabuli*	Ca_Kabuli_Scaffold_1132	26497	[C/T]		--	INTERGENIC
14307	CakSNP14307	*Kabuli*	Ca_Kabuli_Scaffold_1132	26492	[G/A]		--	INTERGENIC
14308	CakSNP14308	*Kabuli*	Ca_Kabuli_Scaffold_1134	50630	[T/C]	Ca_27657	--	INTRON
14309	CakSNP14309	*Kabuli*	Ca_Kabuli_Scaffold_1151	228312	[C/G]		--	INTERGENIC
14310	CakSNP14310	*Kabuli*	Ca_Kabuli_Scaffold_1151	228304	[A/T]		--	INTERGENIC
14311	CakSNP14311	*Kabuli*	Ca_Kabuli_Scaffold_1151	228291	[C/T]		--	INTERGENIC

S.N.	SNP IDs	Chickpea cultivars	Chromosomes	Physical positions (bp)	SNPs	Gene accession IDs	Putative functions	Sequence components of genome
14312	CakSNP14312	*Kabuli*	Ca_Kabuli_Scaffold_1152	9541	[A/G]		--	INTERGENIC
14313	CakSNP14313	*Kabuli*	Ca_Kabuli_Scaffold_1176_2	52186	[C/T]		--	INTERGENIC
14314	CakSNP14314	*Kabuli*	Ca_Kabuli_Scaffold_1176_2	52146	[T/C]		--	INTERGENIC
14315	CakSNP14315	*Kabuli*	Ca_Kabuli_Scaffold_1176_2	52142	[G/T]		--	INTERGENIC
14316	CakSNP14316	*Kabuli*	Ca_Kabuli_Scaffold_1176_2	52125	[C/T]		--	INTERGENIC
14317	CakSNP14317	*Kabuli*	Ca_Kabuli_Scaffold_1176_2	52124	[C/T]		--	INTERGENIC
14318	CakSNP14318	*Kabuli*	Ca_Kabuli_Scaffold_1176_2	330840	[C/A]		--	INTERGENIC
14319	CakSNP14319	*Kabuli*	Ca_Kabuli_Scaffold_118	206562	[A/G]	Ca_22950	--	NON_SYNONYMOUS_CODING
14320	CakSNP14320	*Kabuli*	Ca_Kabuli_Scaffold_118	303784	[A/C]	Ca_22952	Sulfatase	INTRON
14321	CakSNP14321	*Kabuli*	Ca_Kabuli_Scaffold_118	332673	[T/C]		--	INTERGENIC

S.N.	SNP IDs	Chickpea cultivars	Chromosomes	Physical positions (bp)	SNPs	Gene accession IDs	Putative functions	Sequence components of genome
14322	CakSNP14322	*Kabuli*	Ca_Kabuli_Scaffold_1180	114508	[C/A]	Ca_25739	Staphylococcal nuclease (SNase-like)	SYNONYMOUS_CODING
14323	CakSNP14323	*Kabuli*	Ca_Kabuli_Scaffold_1185	944	[G/A]		--	INTERGENIC
14324	CakSNP14324	*Kabuli*	Ca_Kabuli_Scaffold_1185	1110	[G/A]		--	INTERGENIC
14325	CakSNP14325	*Kabuli*	Ca_Kabuli_Scaffold_1185	1134	[T/A]		--	INTERGENIC
14326	CakSNP14326	*Kabuli*	Ca_Kabuli_Scaffold_1185	1098	[T/C]		--	INTERGENIC
14327	CakSNP14327	*Kabuli*	Ca_Kabuli_Scaffold_119	12259	[A/G]		--	INTERGENIC
14328	CakSNP14328	*Kabuli*	Ca_Kabuli_Scaffold_119	12263	[C/A]		--	INTERGENIC
14329	CakSNP14329	*Kabuli*	Ca_Kabuli_Scaffold_119	12267	[C/A]		--	INTERGENIC
14330	CakSNP14330	*Kabuli*	Ca_Kabuli_Scaffold_119	12279	[G/A]		--	INTERGENIC
14331	CakSNP14331	*Kabuli*	Ca_Kabuli_Scaffold_1196	70506	[G/A]		--	INTERGENIC

S.N.	SNP IDs	Chickpea cultivars	Chromosomes	Physical positions (bp)	SNPs	Gene accession IDs	Putative functions	Sequence components of genome
14332	CakSNP14332	*Kabuli*	Ca_Kabuli_Scaffold_1196	70552	[A/C]		--	INTERGENIC
14333	CakSNP14333	*Kabuli*	Ca_Kabuli_Scaffold_1196	70549	[G/A]		--	INTERGENIC
14334	CakSNP14334	*Kabuli*	Ca_Kabuli_Scaffold_1196	70547	[G/C]		--	INTERGENIC
14335	CakSNP14335	*Kabuli*	Ca_Kabuli_Scaffold_1196	70535	[G/A]		--	INTERGENIC
14336	CakSNP14336	*Kabuli*	Ca_Kabuli_Scaffold_1196	70530	[C/T]		--	INTERGENIC
14337	CakSNP14337	*Kabuli*	Ca_Kabuli_Scaffold_1196	70526	[T/C]		--	INTERGENIC
14338	CakSNP14338	*Kabuli*	Ca_Kabuli_Scaffold_1196	70520	[G/A]		--	INTERGENIC
14339	CakSNP14339	*Kabuli*	Ca_Kabuli_Scaffold_1196	70503	[C/T]		--	INTERGENIC
14340	CakSNP14340	*Kabuli*	Ca_Kabuli_Scaffold_1196	203001	[G/A]	Ca_26186	Peptidase C48, SUMO/Sentrin/Ubl1	NON_SYNONYMOUS_CODING
14341	CakSNP14341	*Kabuli*	Ca_Kabuli_Scaffold_1197	219637	[A/T]		--	INTERGENIC

S.N.	SNP IDs	Chickpea cultivars	Chromosomes	Physical positions (bp)	SNPs	Gene accession IDs	Putative functions	Sequence components of genome
14342	CakSNP14342	*Kabuli*	Ca_Kabuli_Scaffold_1197	344239	[A/C]		--	INTERGENIC
14343	CakSNP14343	*Kabuli*	Ca_Kabuli_Scaffold_1197	402506	[T/C]		--	INTERGENIC
14344	CakSNP14344	*Kabuli*	Ca_Kabuli_Scaffold_1197	407641	[G/A]	Ca_24073	TGS	SYNONYMOUS_CODING
14345	CakSNP14345	*Kabuli*	Ca_Kabuli_Scaffold_1197	407658	[C/G]	Ca_24073	TGS	STOP_GAINED
14346	CakSNP14346	*Kabuli*	Ca_Kabuli_Scaffold_1197	407691	[T/C]	Ca_24073	TGS	NON_SYNONYMOUS_CODING
14347	CakSNP14347	*Kabuli*	Ca_Kabuli_Scaffold_1197	407695	[T/C]	Ca_24073	TGS	SYNONYMOUS_CODING
14348	CakSNP14348	*Kabuli*	Ca_Kabuli_Scaffold_1197	447890	[C/T]	Ca_24074	--	SYNONYMOUS_CODING
14349	CakSNP14349	*Kabuli*	Ca_Kabuli_Scaffold_1197	520164	[T/A]		--	INTERGENIC
14350	CakSNP14350	*Kabuli*	Ca_Kabuli_Scaffold_1197	520153	[C/G]		--	INTERGENIC
14351	CakSNP14351	*Kabuli*	Ca_Kabuli_Scaffold_1197	520148	[A/T]		--	INTERGENIC

S.N.	SNP IDs	Chickpea cultivars	Chromosomes	Physical positions (bp)	SNPs	Gene accession IDs	Putative functions	Sequence components of genome
14352	CakSNP14352	*Kabuli*	Ca_Kabuli_Scaffold_12	107756	[G/A]	Ca_22972	Peptidase C48, SUMO/Sentrin/Ubl1	NON_SYNONYMOUS_CODING
14353	CakSNP14353	*Kabuli*	Ca_Kabuli_Scaffold_12	200573	[G/C]		--	INTERGENIC
14354	CakSNP14354	*Kabuli*	Ca_Kabuli_Scaffold_12	331877	[G/A]	Ca_22985	Aminotransferase-like, plant mobile domain	INTRON
14355	CakSNP14355	*Kabuli*	Ca_Kabuli_Scaffold_12	331890	[A/G]	Ca_22985	Aminotransferase-like, plant mobile domain	INTRON
14356	CakSNP14356	*Kabuli*	Ca_Kabuli_Scaffold_12	332909	[C/T]	Ca_22985	Aminotransferase-like, plant mobile domain	SYNONYMOUS_CODING
14357	CakSNP14357	*Kabuli*	Ca_Kabuli_Scaffold_12	332921	[G/A]	Ca_22985	Aminotransferase-like, plant mobile domain	NON_SYNONYMOUS_CODING
14358	CakSNP14358	*Kabuli*	Ca_Kabuli_Scaffold_12	332963	[C/T]	Ca_22985	Aminotransferase-like, plant mobile domain	NON_SYNONYMOUS_CODING
14359	CakSNP14359	*Kabuli*	Ca_Kabuli_Scaffold_1202	46904	[C/T]	Ca_24659	CDP-alcohol phosphatidyltransferase	INTRON
14360	CakSNP14360	*Kabuli*	Ca_Kabuli_Scaffold_1202	46945	[G/A]	Ca_24659	CDP-alcohol phosphatidyltransferase	INTRON
14361	CakSNP14361	*Kabuli*	Ca_Kabuli_Scaffold_1215	40741	[G/T]	Ca_28164	Peptidase S8/S53, subtilisin/kexin/sedolisin	SYNONYMOUS_CODING

S.N.	SNP IDs	Chickpea cultivars	Chromosomes	Physical positions (bp)	SNPs	Gene accession IDs	Putative functions	Sequence components of genome
14362	CakSNP14362	*Kabuli*	Ca_Kabuli_Scaffold_1215	40793	[A/G]	Ca_28164	Peptidase S8/S53, subtilisin/kexin/sedolisin	SYNONYMOUS_CODING
14363	CakSNP14363	*Kabuli*	Ca_Kabuli_Scaffold_1215	40797	[A/T]	Ca_28164	Peptidase S8/S53, subtilisin/kexin/sedolisin	NON_SYNONYMOUS_CODING
14364	CakSNP14364	*Kabuli*	Ca_Kabuli_Scaffold_1215	40817	[G/A]	Ca_28164	Peptidase S8/S53, subtilisin/kexin/sedolisin	NON_SYNONYMOUS_CODING
14365	CakSNP14365	*Kabuli*	Ca_Kabuli_Scaffold_1215	40814	[A/T]	Ca_28164	Peptidase S8/S53, subtilisin/kexin/sedolisin	NON_SYNONYMOUS_CODING
14366	CakSNP14366	*Kabuli*	Ca_Kabuli_Scaffold_1219	34791	[G/A]		--	INTERGENIC
14367	CakSNP14367	*Kabuli*	Ca_Kabuli_Scaffold_1219	85015	[A/C]	Ca_27098	Major facilitator superfamily MFS-1	NON_SYNONYMOUS_CODING
14368	CakSNP14368	*Kabuli*	Ca_Kabuli_Scaffold_1259	71428	[C/T]		--	INTERGENIC
14369	CakSNP14369	*Kabuli*	Ca_Kabuli_Scaffold_1259	71574	[A/T]		--	INTERGENIC
14370	CakSNP14370	*Kabuli*	Ca_Kabuli_Scaffold_1259	71649	[C/T]		--	INTERGENIC
14371	CakSNP14371	*Kabuli*	Ca_Kabuli_Scaffold_127	28809	[A/T]		--	INTERGENIC

S.N.	SNP IDs	Chickpea cultivars	Chromosomes	Physical positions (bp)	SNPs	Gene accession IDs	Putative functions	Sequence components of genome
14372	CakSNP14372	*Kabuli*	Ca_Kabuli_Scaffold_127	28780	[G/A]		--	INTERGENIC
14373	CakSNP14373	*Kabuli*	Ca_Kabuli_Scaffold_127	28778	[C/T]		--	INTERGENIC
14374	CakSNP14374	*Kabuli*	Ca_Kabuli_Scaffold_127	28776	[T/G]		--	INTERGENIC
14375	CakSNP14375	*Kabuli*	Ca_Kabuli_Scaffold_127	28772	[G/A]		--	INTERGENIC
14376	CakSNP14376	*Kabuli*	Ca_Kabuli_Scaffold_1272	57303	[C/A]	Ca_26853	Glycosyl transferase, family 20	INTRON
14377	CakSNP14377	*Kabuli*	Ca_Kabuli_Scaffold_1272	57356	[C/G]	Ca_26853	Glycosyl transferase, family 20	INTRON
14378	CakSNP14378	*Kabuli*	Ca_Kabuli_Scaffold_128	19256	[A/C]		--	INTERGENIC
14379	CakSNP14379	*Kabuli*	Ca_Kabuli_Scaffold_128	19315	[C/T]		--	INTERGENIC
14380	CakSNP14380	*Kabuli*	Ca_Kabuli_Scaffold_128	52881	[G/A]		--	INTERGENIC
14381	CakSNP14381	*Kabuli*	Ca_Kabuli_Scaffold_128	52855	[G/C]		--	INTERGENIC

S.N.	SNP IDs	Chickpea cultivars	Chromosomes	Physical positions (bp)	SNPs	Gene accession IDs	Putative functions	Sequence components of genome
14382	CakSNP14382	*Kabuli*	Ca_Kabuli_Scaffold_128	52844	[A/T]		--	INTERGENIC
14383	CakSNP14383	*Kabuli*	Ca_Kabuli_Scaffold_128	263590	[G/T]		--	INTERGENIC
14384	CakSNP14384	*Kabuli*	Ca_Kabuli_Scaffold_128	263586	[T/A]		--	INTERGENIC
14385	CakSNP14385	*Kabuli*	Ca_Kabuli_Scaffold_128	263572	[T/A]		--	INTERGENIC
14386	CakSNP14386	*Kabuli*	Ca_Kabuli_Scaffold_128	334180	[G/T]		--	INTERGENIC
14387	CakSNP14387	*Kabuli*	Ca_Kabuli_Scaffold_128	334129	[G/A]		--	INTERGENIC
14388	CakSNP14388	*Kabuli*	Ca_Kabuli_Scaffold_128	593782	[T/C]		--	INTERGENIC
14389	CakSNP14389	*Kabuli*	Ca_Kabuli_Scaffold_128	860975	[G/A]	Ca_21452	Helicase, C-terminal	INTRON
14390	CakSNP14390	*Kabuli*	Ca_Kabuli_Scaffold_128	860981	[T/A]	Ca_21452	Helicase, C-terminal	INTRON
14391	CakSNP14391	*Kabuli*	Ca_Kabuli_Scaffold_128	860983	[A/T]	Ca_21452	Helicase, C-terminal	INTRON

S.N.	SNP IDs	Chickpea cultivars	Chromosomes	Physical positions (bp)	SNPs	Gene accession IDs	Putative functions	Sequence components of genome
14392	CakSNP14392	*Kabuli*	Ca_Kabuli_Scaffold_128	860987	[C/T]	Ca_21452	Helicase, C-terminal	INTRON
14393	CakSNP14393	*Kabuli*	Ca_Kabuli_Scaffold_128	861003	[C/A]	Ca_21452	Helicase, C-terminal	INTRON
14394	CakSNP14394	*Kabuli*	Ca_Kabuli_Scaffold_128	861010	[C/T]	Ca_21452	Helicase, C-terminal	INTRON
14395	CakSNP14395	*Kabuli*	Ca_Kabuli_Scaffold_128	861022	[T/C]	Ca_21452	Helicase, C-terminal	INTRON
14396	CakSNP14396	*Kabuli*	Ca_Kabuli_Scaffold_128	861028	[T/C]	Ca_21452	Helicase, C-terminal	INTRON
14397	CakSNP14397	*Kabuli*	Ca_Kabuli_Scaffold_128	861072	[G/T]	Ca_21452	Helicase, C-terminal	INTRON
14398	CakSNP14398	*Kabuli*	Ca_Kabuli_Scaffold_128	861053	[G/A]	Ca_21452	Helicase, C-terminal	INTRON
14399	CakSNP14399	*Kabuli*	Ca_Kabuli_Scaffold_128	861036	[G/A]	Ca_21452	Helicase, C-terminal	INTRON
14400	CakSNP14400	*Kabuli*	Ca_Kabuli_Scaffold_128	861015	[C/T]	Ca_21452	Helicase, C-terminal	INTRON
14401	CakSNP14401	*Kabuli*	Ca_Kabuli_Scaffold_1281	211456	[A/T]	Ca_21514	RINT-1/TIP-1	NON_SYNONYMOUS_CODING

S.N.	SNP IDs	Chickpea cultivars	Chromosomes	Physical positions (bp)	SNPs	Gene accession IDs	Putative functions	Sequence components of genome
14402	CakSNP14402	*Kabuli*	Ca_Kabuli_Scaffold_1281	239742	[T/C]		--	INTERGENIC
14403	CakSNP14403	*Kabuli*	Ca_Kabuli_Scaffold_1281	244153	[A/C]	Ca_21517	Protein kinase, catalytic domain	SYNONYMOUS_CODING
14404	CakSNP14404	*Kabuli*	Ca_Kabuli_Scaffold_1281	258565	[G/A]	Ca_21518	Transcriptional factor B3	SYNONYMOUS_CODING
14405	CakSNP14405	*Kabuli*	Ca_Kabuli_Scaffold_1281	316037	[A/G]	Ca_21523	Zinc finger, RING-type	SYNONYMOUS_CODING
14406	CakSNP14406	*Kabuli*	Ca_Kabuli_Scaffold_1281	396888	[G/A]		--	INTERGENIC
14407	CakSNP14407	*Kabuli*	Ca_Kabuli_Scaffold_1281	396996	[C/T]		--	INTERGENIC
14408	CakSNP14408	*Kabuli*	Ca_Kabuli_Scaffold_1281	396992	[A/G]		--	INTERGENIC
14409	CakSNP14409	*Kabuli*	Ca_Kabuli_Scaffold_1281	398693	[G/A]	Ca_21525	Armadillo	NON_SYNONYMOUS_CODING
14410	CakSNP14410	*Kabuli*	Ca_Kabuli_Scaffold_1281	398729	[T/G]	Ca_21525	Armadillo	NON_SYNONYMOUS_CODING
14411	CakSNP14411	*Kabuli*	Ca_Kabuli_Scaffold_1281	398887	[G/A]	Ca_21525	Armadillo	SYNONYMOUS_CODING

S.N.	SNP IDs	Chickpea cultivars	Chromosomes	Physical positions (bp)	SNPs	Gene accession IDs	Putative functions	Sequence components of genome
14412	CakSNP14412	*Kabuli*	Ca_Kabuli_Scaffold_1281	408214	[C/T]		--	INTERGENIC
14413	CakSNP14413	*Kabuli*	Ca_Kabuli_Scaffold_1281	408170	[C/T]		--	INTERGENIC
14414	CakSNP14414	*Kabuli*	Ca_Kabuli_Scaffold_1281	526446	[C/T]		--	INTERGENIC
14415	CakSNP14415	*Kabuli*	Ca_Kabuli_Scaffold_1281	526442	[T/C]		--	INTERGENIC
14416	CakSNP14416	*Kabuli*	Ca_Kabuli_Scaffold_1281	527012	[C/T]		--	INTERGENIC
14417	CakSNP14417	*Kabuli*	Ca_Kabuli_Scaffold_1281	527008	[T/C]		--	INTERGENIC
14418	CakSNP14418	*Kabuli*	Ca_Kabuli_Scaffold_1285	68330	[G/A]	Ca_24805	Peptidase C48, SUMO/Sentrin/Ubl1	INTRON
14419	CakSNP14419	*Kabuli*	Ca_Kabuli_Scaffold_1285	68327	[G/T]	Ca_24805	Peptidase C48, SUMO/Sentrin/Ubl1	INTRON
14420	CakSNP14420	*Kabuli*	Ca_Kabuli_Scaffold_1285	68324	[T/C]	Ca_24805	Peptidase C48, SUMO/Sentrin/Ubl1	INTRON
14421	CakSNP14421	*Kabuli*	Ca_Kabuli_Scaffold_1285	68315	[T/C]	Ca_24805	Peptidase C48, SUMO/Sentrin/Ubl1	SYNONYMOUS_CODING

S.N.	SNP IDs	Chickpea cultivars	Chromosomes	Physical positions (bp)	SNPs	Gene accession IDs	Putative functions	Sequence components of genome
14422	CakSNP14422	*Kabuli*	Ca_Kabuli_Scaffold_1285	68311	[G/A]	Ca_24805	Peptidase C48, SUMO/Sentrin/Ubl1	SYNONYMOUS_CODING
14423	CakSNP14423	*Kabuli*	Ca_Kabuli_Scaffold_1285	96310	[A/C]		--	INTERGENIC
14424	CakSNP14424	*Kabuli*	Ca_Kabuli_Scaffold_1285	96358	[T/C]		--	INTERGENIC
14425	CakSNP14425	*Kabuli*	Ca_Kabuli_Scaffold_1285	96429	[C/T]		--	INTERGENIC
14426	CakSNP14426	*Kabuli*	Ca_Kabuli_Scaffold_1285	96428	[G/A]		--	INTERGENIC
14427	CakSNP14427	*Kabuli*	Ca_Kabuli_Scaffold_1285	96414	[C/T]		--	INTERGENIC
14428	CakSNP14428	*Kabuli*	Ca_Kabuli_Scaffold_1285	96411	[G/A]		--	INTERGENIC
14429	CakSNP14429	*Kabuli*	Ca_Kabuli_Scaffold_1285	96409	[C/T]		--	INTERGENIC
14430	CakSNP14430	*Kabuli*	Ca_Kabuli_Scaffold_1285	96399	[C/T]		--	INTERGENIC
14431	CakSNP14431	*Kabuli*	Ca_Kabuli_Scaffold_1285	105553	[T/C]		--	INTERGENIC

S.N.	SNP IDs	Chickpea cultivars	Chromosomes	Physical positions (bp)	SNPs	Gene accession IDs	Putative functions	Sequence components of genome
14432	CakSNP14432	*Kabuli*	Ca_Kabuli_Scaffold_1285	105507	[A/T]		--	INTERGENIC
14433	CakSNP14433	*Kabuli*	Ca_Kabuli_Scaffold_1285	105504	[G/A]		--	INTERGENIC
14434	CakSNP14434	*Kabuli*	Ca_Kabuli_Scaffold_1285	192068	[T/G]		--	INTERGENIC
14435	CakSNP14435	*Kabuli*	Ca_Kabuli_Scaffold_1285	240762	[C/T]	Ca_24811	--	INTRON
14436	CakSNP14436	*Kabuli*	Ca_Kabuli_Scaffold_1285	240735	[G/A]	Ca_24811	--	INTRON
14437	CakSNP14437	*Kabuli*	Ca_Kabuli_Scaffold_1285	240730	[T/C]	Ca_24811	--	INTRON
14438	CakSNP14438	*Kabuli*	Ca_Kabuli_Scaffold_1285	240723	[C/T]	Ca_24811	--	INTRON
14439	CakSNP14439	*Kabuli*	Ca_Kabuli_Scaffold_1301_1	43433	[T/C]	Ca_11165	--	SYNONYMOUS_CODING
14440	CakSNP14440	*Kabuli*	Ca_Kabuli_Scaffold_1301_1	45565	[A/C]	Ca_11165	--	SYNONYMOUS_CODING
14441	CakSNP14441	*Kabuli*	Ca_Kabuli_Scaffold_1301_1	45566	[G/A]	Ca_11165	--	SYNONYMOUS_CODING

S.N.	SNP IDs	Chickpea cultivars	Chromosomes	Physical positions (bp)	SNPs	Gene accession IDs	Putative functions	Sequence components of genome
14442	CakSNP14442	*Kabuli*	Ca_Kabuli_Scaffold_1301_1	58243	[A/G]		--	INTERGENIC
14443	CakSNP14443	*Kabuli*	Ca_Kabuli_Scaffold_1301_1	58337	[C/T]		--	INTERGENIC
14444	CakSNP14444	*Kabuli*	Ca_Kabuli_Scaffold_1301_1	58460	[A/T]		--	INTERGENIC
14445	CakSNP14445	*Kabuli*	Ca_Kabuli_Scaffold_1301_1	63148	[T/C]	Ca_11167	DEP domain	SYNONYMOUS_CODING
14446	CakSNP14446	*Kabuli*	Ca_Kabuli_Scaffold_1301_1	95495	[G/A]	Ca_11170	Crotonase, core	NON_SYNONYMOUS_CODING
14447	CakSNP14447	*Kabuli*	Ca_Kabuli_Scaffold_1301_1	95497	[G/A]	Ca_11170	Crotonase, core	NON_SYNONYMOUS_CODING
14448	CakSNP14448	*Kabuli*	Ca_Kabuli_Scaffold_1301_1	95602	[T/C]	Ca_11170	Crotonase, core	NON_SYNONYMOUS_CODING
14449	CakSNP14449	*Kabuli*	Ca_Kabuli_Scaffold_1301_1	95533	[C/G]	Ca_11170	Crotonase, core	NON_SYNONYMOUS_CODING
14450	CakSNP14450	*Kabuli*	Ca_Kabuli_Scaffold_1301_1	122496	[T/A]	Ca_11173	Serine hydroxymethyltransferase	INTRON
14451	CakSNP14451	*Kabuli*	Ca_Kabuli_Scaffold_1301_1	122563	[T/C]	Ca_11173	Serine hydroxymethyltransferase	INTRON

S.N.	SNP IDs	Chickpea cultivars	Chromosomes	Physical positions (bp)	SNPs	Gene accession IDs	Putative functions	Sequence components of genome
14452	CakSNP14452	*Kabuli*	Ca_Kabuli_Scaffold_1301_1	154342	[A/C]	Ca_11176	Glycosyl transferase, family 31	INTRON
14453	CakSNP14453	*Kabuli*	Ca_Kabuli_Scaffold_1301_1	157390	[A/G]	Ca_11177	Vacuolar sorting protein 9	INTRON
14454	CakSNP14454	*Kabuli*	Ca_Kabuli_Scaffold_1301_1	218538	[G/A]		--	INTERGENIC
14455	CakSNP14455	*Kabuli*	Ca_Kabuli_Scaffold_1308	44260	[A/G]		--	INTERGENIC
14456	CakSNP14456	*Kabuli*	Ca_Kabuli_Scaffold_1309	49259	[G/A]	Ca_26762	Transposase, Ptta/En/Spm, plant	SYNONYMOUS_CODING
14457	CakSNP14457	*Kabuli*	Ca_Kabuli_Scaffold_1312	98400	[T/C]		--	INTERGENIC
14458	CakSNP14458	*Kabuli*	Ca_Kabuli_Scaffold_1313	18173	[A/G]	Ca_25873	Homeobox	INTRON
14459	CakSNP14459	*Kabuli*	Ca_Kabuli_Scaffold_1313	18412	[G/A]	Ca_25873	Homeobox	NON_SYNONYMOUS_CODING
14460	CakSNP14460	*Kabuli*	Ca_Kabuli_Scaffold_1313	227732	[G/A]	Ca_25880	Peptidase C48, SUMO/Sentrin/Ubl1	NON_SYNONYMOUS_CODING
14461	CakSNP14461	*Kabuli*	Ca_Kabuli_Scaffold_1313	227744	[C/T]	Ca_25880	Peptidase C48, SUMO/Sentrin/Ubl1	NON_SYNONYMOUS_CODING

S.N.	SNP IDs	Chickpea cultivars	Chromosomes	Physical positions (bp)	SNPs	Gene accession IDs	Putative functions	Sequence components of genome
14462	CakSNP14462	*Kabuli*	Ca_Kabuli_Scaffold_1315	115480	[G/A]		--	INTERGENIC
14463	CakSNP14463	*Kabuli*	Ca_Kabuli_Scaffold_1315	115476	[A/C]		--	INTERGENIC
14464	CakSNP14464	*Kabuli*	Ca_Kabuli_Scaffold_1315	115471	[G/A]		--	INTERGENIC
14465	CakSNP14465	*Kabuli*	Ca_Kabuli_Scaffold_1315	115446	[C/A]		--	INTERGENIC
14466	CakSNP14466	*Kabuli*	Ca_Kabuli_Scaffold_1315	115481	[G/T]		--	INTERGENIC
14467	CakSNP14467	*Kabuli*	Ca_Kabuli_Scaffold_132	153364	[A/G]		--	INTERGENIC
14468	CakSNP14468	*Kabuli*	Ca_Kabuli_Scaffold_132	153361	[A/G]		--	INTERGENIC
14469	CakSNP14469	*Kabuli*	Ca_Kabuli_Scaffold_1324	228151	[G/A]	Ca_19765	--	SYNONYMOUS_CODING
14470	CakSNP14470	*Kabuli*	Ca_Kabuli_Scaffold_1324	228159	[A/C]	Ca_19765	--	NON_SYNONYMOUS_CODING
14471	CakSNP14471	*Kabuli*	Ca_Kabuli_Scaffold_1324	262208	[C/G]	Ca_19766	--	NON_SYNONYMOUS_CODING

S.N.	SNP IDs	Chickpea cultivars	Chromosomes	Physical positions (bp)	SNPs	Gene accession IDs	Putative functions	Sequence components of genome
14472	CakSNP14472	*Kabuli*	Ca_Kabuli_Scaffold_134	218290	[C/T]	Ca_22581	SET domain	SYNONYMOUS_CODING
14473	CakSNP14473	*Kabuli*	Ca_Kabuli_Scaffold_134	218352	[T/G]	Ca_22581	SET domain	NON_SYNONYMOUS_CODING
14474	CakSNP14474	*Kabuli*	Ca_Kabuli_Scaffold_134	218336	[T/G]	Ca_22581	SET domain	NON_SYNONYMOUS_CODING
14475	CakSNP14475	*Kabuli*	Ca_Kabuli_Scaffold_134	223318	[C/T]	Ca_22581	SET domain	INTRON
14476	CakSNP14476	*Kabuli*	Ca_Kabuli_Scaffold_134	223303	[T/C]	Ca_22581	SET domain	INTRON
14477	CakSNP14477	*Kabuli*	Ca_Kabuli_Scaffold_134	233007	[C/T]	Ca_22581	SET domain	INTRON
14478	CakSNP14478	*Kabuli*	Ca_Kabuli_Scaffold_134	275661	[T/A]	Ca_22585	Pathogenesis-related transcriptional factor/ERF, DNA-binding	NON_SYNONYMOUS_CODING
14479	CakSNP14479	*Kabuli*	Ca_Kabuli_Scaffold_1348_1	84644	[G/C]	Ca_08694	Peptidase C13, legumain	NON_SYNONYMOUS_CODING
14480	CakSNP14480	*Kabuli*	Ca_Kabuli_Scaffold_1348_1	134575	[T/C]		--	INTERGENIC
14481	CakSNP14481	*Kabuli*	Ca_Kabuli_Scaffold_1348_1	134572	[G/A]		--	INTERGENIC

S.N.	SNP IDs	Chickpea cultivars	Chromosomes	Physical positions (bp)	SNPs	Gene accession IDs	Putative functions	Sequence components of genome
14482	CakSNP14482	*Kabuli*	Ca_Kabuli_Scaffold_1348_1	294458	[A/G]	Ca_08713	Peptide chain release factor class I/class II	NON_SYNONYMOUS_CODING
14483	CakSNP14483	*Kabuli*	Ca_Kabuli_Scaffold_1348_1	294456	[A/G]	Ca_08713	Peptide chain release factor class I/class II	SYNONYMOUS_CODING
14484	CakSNP14484	*Kabuli*	Ca_Kabuli_Scaffold_1348_1	380156	[A/T]	Ca_08725	Peptidase S8/S53, subtilisin/kexin/sedolisin	NON_SYNONYMOUS_CODING
14485	CakSNP14485	*Kabuli*	Ca_Kabuli_Scaffold_1348_1	380225	[T/G]	Ca_08725	Peptidase S8/S53, subtilisin/kexin/sedolisin	SYNONYMOUS_CODING
14486	CakSNP14486	*Kabuli*	Ca_Kabuli_Scaffold_1348_1	380227	[C/G]	Ca_08725	Peptidase S8/S53, subtilisin/kexin/sedolisin	NON_SYNONYMOUS_CODING
14487	CakSNP14487	*Kabuli*	Ca_Kabuli_Scaffold_1348_1	380244	[C/G]	Ca_08725	Peptidase S8/S53, subtilisin/kexin/sedolisin	NON_SYNONYMOUS_CODING
14488	CakSNP14488	*Kabuli*	Ca_Kabuli_Scaffold_1348_1	380289	[C/T]	Ca_08725	Peptidase S8/S53, subtilisin/kexin/sedolisin	NON_SYNONYMOUS_CODING
14489	CakSNP14489	*Kabuli*	Ca_Kabuli_Scaffold_1348_1	380336	[G/C]	Ca_08725	Peptidase S8/S53, subtilisin/kexin/sedolisin	SYNONYMOUS_CODING
14490	CakSNP14490	*Kabuli*	Ca_Kabuli_Scaffold_1348_1	381026	[G/T]	Ca_08725	Peptidase S8/S53, subtilisin/kexin/sedolisin	SYNONYMOUS_CODING
14491	CakSNP14491	*Kabuli*	Ca_Kabuli_Scaffold_1348_1	452011	[G/T]	Ca_08730	Glucose-methanol-choline oxidoreductase, N-terminal	NON_SYNONYMOUS_CODING

S.N.	SNP IDs	Chickpea cultivars	Chromosomes	Physical positions (bp)	SNPs	Gene accession IDs	Putative functions	Sequence components of genome
14492	CakSNP14492	*Kabuli*	Ca_Kabuli_Scaffold_1348_1	454342	[G/T]		--	INTERGENIC
14493	CakSNP14493	*Kabuli*	Ca_Kabuli_Scaffold_1348_1	515602	[A/C]	Ca_08740	GPI transamidase subunit PIG-U	SYNONYMOUS_CODING
14494	CakSNP14494	*Kabuli*	Ca_Kabuli_Scaffold_1348_1	535984	[G/T]	Ca_08741	IQ motif, EF-hand binding site	NON_SYNONYMOUS_CODING
14495	CakSNP14495	*Kabuli*	Ca_Kabuli_Scaffold_1348_1	552192	[C/A]	Ca_08743	Zinc finger, RING-type	INTRON
14496	CakSNP14496	*Kabuli*	Ca_Kabuli_Scaffold_1348_1	552299	[T/C]	Ca_08743	Zinc finger, RING-type	INTRON
14497	CakSNP14497	*Kabuli*	Ca_Kabuli_Scaffold_1348_1	552342	[G/A]	Ca_08743	Zinc finger, RING-type	INTRON
14498	CakSNP14498	*Kabuli*	Ca_Kabuli_Scaffold_1348_1	552328	[A/G]	Ca_08743	Zinc finger, RING-type	INTRON
14499	CakSNP14499	*Kabuli*	Ca_Kabuli_Scaffold_1348_1	553863	[G/T]	Ca_08743	Zinc finger, RING-type	NON_SYNONYMOUS_CODING
14500	CakSNP14500	*Kabuli*	Ca_Kabuli_Scaffold_1348_1	553978	[C/T]		--	INTERGENIC
14501	CakSNP14501	*Kabuli*	Ca_Kabuli_Scaffold_1348_1	554003	[G/C]		--	INTERGENIC

S.N.	SNP IDs	Chickpea cultivars	Chromosomes	Physical positions (bp)	SNPs	Gene accession IDs	Putative functions	Sequence components of genome
14502	CakSNP14502	*Kabuli*	Ca_Kabuli_Scaffold_1348_1	554134	[A/G]		--	INTERGENIC
14503	CakSNP14503	*Kabuli*	Ca_Kabuli_Scaffold_1348_1	554215	[T/G]		--	INTERGENIC
14504	CakSNP14504	*Kabuli*	Ca_Kabuli_Scaffold_1348_1	651906	[A/T]	Ca_08753	Peptidase S8/S53, subtilisin/kexin/sedolisin	SYNONYMOUS_CODING
14505	CakSNP14505	*Kabuli*	Ca_Kabuli_Scaffold_1348_1	652067	[A/G]	Ca_08753	Peptidase S8/S53, subtilisin/kexin/sedolisin	NON_SYNONYMOUS_CODING
14506	CakSNP14506	*Kabuli*	Ca_Kabuli_Scaffold_1348_1	667541	[A/C]	Ca_08754	Peptidase S8/S53, subtilisin/kexin/sedolisin	SYNONYMOUS_CODING
14507	CakSNP14507	*Kabuli*	Ca_Kabuli_Scaffold_1348_1	667504	[C/T]	Ca_08754	Peptidase S8/S53, subtilisin/kexin/sedolisin	NON_SYNONYMOUS_CODING
14508	CakSNP14508	*Kabuli*	Ca_Kabuli_Scaffold_1348_1	679305	[T/C]	Ca_08755	Peptidase S8/S53, subtilisin/kexin/sedolisin	NON_SYNONYMOUS_CODING
14509	CakSNP14509	*Kabuli*	Ca_Kabuli_Scaffold_1348_1	680734	[G/T]	Ca_08755	Peptidase S8/S53, subtilisin/kexin/sedolisin	NON_SYNONYMOUS_CODING
14510	CakSNP14510	*Kabuli*	Ca_Kabuli_Scaffold_1348_1	680730	[C/T]	Ca_08755	Peptidase S8/S53, subtilisin/kexin/sedolisin	NON_SYNONYMOUS_CODING
14511	CakSNP14511	*Kabuli*	Ca_Kabuli_Scaffold_1348_1	680718	[A/T]	Ca_08755	Peptidase S8/S53, subtilisin/kexin/sedolisin	NON_SYNONYMOUS_CODING

S.N.	SNP IDs	Chickpea cultivars	Chromosomes	Physical positions (bp)	SNPs	Gene accession IDs	Putative functions	Sequence components of genome
14512	CakSNP14512	*Kabuli*	Ca_Kabuli_Scaffold_1348_1	708276	[G/A]	Ca_08758	Cystathionine beta-synthase, core	INTRON
14513	CakSNP14513	*Kabuli*	Ca_Kabuli_Scaffold_1348_1	708302	[G/T]	Ca_08758	Cystathionine beta-synthase, core	INTRON
14514	CakSNP14514	*Kabuli*	Ca_Kabuli_Scaffold_1348_1	708344	[G/A]	Ca_08758	Cystathionine beta-synthase, core	SYNONYMOUS_CODING
14515	CakSNP14515	*Kabuli*	Ca_Kabuli_Scaffold_1348_1	732450	[A/C]	Ca_08761	Forkhead-associated (FHA) domain	SYNONYMOUS_CODING
14516	CakSNP14516	*Kabuli*	Ca_Kabuli_Scaffold_1348_1	732454	[T/G]	Ca_08761	Forkhead-associated (FHA) domain	INTRON
14517	CakSNP14517	*Kabuli*	Ca_Kabuli_Scaffold_1348_1	732460	[T/A]	Ca_08761	Forkhead-associated (FHA) domain	INTRON
14518	CakSNP14518	*Kabuli*	Ca_Kabuli_Scaffold_1348_1	732496	[G/T]	Ca_08761	Forkhead-associated (FHA) domain	INTRON
14519	CakSNP14519	*Kabuli*	Ca_Kabuli_Scaffold_1348_1	735782	[C/T]	Ca_08761	Forkhead-associated (FHA) domain	SYNONYMOUS_CODING
14520	CakSNP14520	*Kabuli*	Ca_Kabuli_Scaffold_1348_1	735812	[G/A]	Ca_08761	Forkhead-associated (FHA) domain	SYNONYMOUS_CODING
14521	CakSNP14521	*Kabuli*	Ca_Kabuli_Scaffold_1348_1	750170	[C/A]	Ca_08762	COG complex component, COG2	INTRON

S.N.	SNP IDs	Chickpea cultivars	Chromosomes	Physical positions (bp)	SNPs	Gene accession IDs	Putative functions	Sequence components of genome
14522	CakSNP14522	*Kabuli*	Ca_Kabuli_Scaffold_1348_1	750162	[T/C]	Ca_08762	COG complex component, COG2	INTRON
14523	CakSNP14523	*Kabuli*	Ca_Kabuli_Scaffold_1348_1	750213	[C/A]	Ca_08762	COG complex component, COG2	INTRON
14524	CakSNP14524	*Kabuli*	Ca_Kabuli_Scaffold_1348_1	771029	[T/A]	Ca_08766	Argininosuccinate synthase	SYNONYMOUS_CODING
14525	CakSNP14525	*Kabuli*	Ca_Kabuli_Scaffold_1348_1	871724	[G/A]	Ca_08775	Glyceraldehyde 3-phosphate dehydrogenase, NAD(P) binding domain	INTRON
14526	CakSNP14526	*Kabuli*	Ca_Kabuli_Scaffold_1348_1	909909	[A/G]		--	INTERGENIC
14527	CakSNP14527	*Kabuli*	Ca_Kabuli_Scaffold_1348_1	909967	[C/G]		--	INTERGENIC
14528	CakSNP14528	*Kabuli*	Ca_Kabuli_Scaffold_1348_1	909965	[T/A]		--	INTERGENIC
14529	CakSNP14529	*Kabuli*	Ca_Kabuli_Scaffold_1348_1	909959	[A/C]		--	INTERGENIC
14530	CakSNP14530	*Kabuli*	Ca_Kabuli_Scaffold_1348_1	924164	[T/G]	Ca_08781	Proteasome component (PCI) domain	SYNONYMOUS_CODING
14531	CakSNP14531	*Kabuli*	Ca_Kabuli_Scaffold_1348_1	934720	[C/A]	Ca_08783	Zinc finger, C2H2-type	INTRON

S.N.	SNP IDs	Chickpea cultivars	Chromosomes	Physical positions (bp)	SNPs	Gene accession IDs	Putative functions	Sequence components of genome
14532	CakSNP14532	*Kabuli*	Ca_Kabuli_Scaffold_1348_1	934791	[C/T]	Ca_08783	Zinc finger, C2H2-type	INTRON
14533	CakSNP14533	*Kabuli*	Ca_Kabuli_Scaffold_1348_1	950995	[A/G]	Ca_08786	Zinc finger, CCCH-type	SYNONYMOUS_CODING
14534	CakSNP14534	*Kabuli*	Ca_Kabuli_Scaffold_1348_1	959608	[A/C]	Ca_08787	Transcription factor jumonji/aspartyl beta-hydroxylase	NON_SYNONYMOUS_CODING
14535	CakSNP14535	*Kabuli*	Ca_Kabuli_Scaffold_1348_1	959828	[G/A]	Ca_08787	Transcription factor jumonji/aspartyl beta-hydroxylase	SYNONYMOUS_CODING
14536	CakSNP14536	*Kabuli*	Ca_Kabuli_Scaffold_1348_1	959780	[G/A]	Ca_08787	Transcription factor jumonji/aspartyl beta-hydroxylase	SYNONYMOUS_CODING
14537	CakSNP14537	*Kabuli*	Ca_Kabuli_Scaffold_1348_1	970927	[A/G]	Ca_08788	Initiation factor eIF-4 gamma, MA3	SYNONYMOUS_CODING
14538	CakSNP14538	*Kabuli*	Ca_Kabuli_Scaffold_1348_1	981964	[T/C]	Ca_08789	ATPase, AAA+ type, core	NON_SYNONYMOUS_CODING
14539	CakSNP14539	*Kabuli*	Ca_Kabuli_Scaffold_1348_1	983101	[T/G]	Ca_08789	ATPase, AAA+ type, core	SYNONYMOUS_CODING
14540	CakSNP14540	*Kabuli*	Ca_Kabuli_Scaffold_1348_1	1020258	[C/T]		--	INTERGENIC
14541	CakSNP14541	*Kabuli*	Ca_Kabuli_Scaffold_1348_1	1023898	[G/A]		--	INTERGENIC

S.N.	SNP IDs	Chickpea cultivars	Chromosomes	Physical positions (bp)	SNPs	Gene accession IDs	Putative functions	Sequence components of genome
14542	CakSNP14542	*Kabuli*	Ca_Kabuli_Scaffold_1348_1	1023923	[G/A]		--	INTERGENIC
14543	CakSNP14543	*Kabuli*	Ca_Kabuli_Scaffold_1348_1	1023954	[G/C]		--	INTERGENIC
14544	CakSNP14544	*Kabuli*	Ca_Kabuli_Scaffold_1348_1	1066818	[A/C]	Ca_08793	Peptidase, cysteine peptidase active site	INTRON
14545	CakSNP14545	*Kabuli*	Ca_Kabuli_Scaffold_1348_1	1066783	[C/T]	Ca_08793	Peptidase, cysteine peptidase active site	INTRON
14546	CakSNP14546	*Kabuli*	Ca_Kabuli_Scaffold_1348_1	1066776	[T/C]	Ca_08793	Peptidase, cysteine peptidase active site	INTRON
14547	CakSNP14547	*Kabuli*	Ca_Kabuli_Scaffold_1348_1	1072750	[C/T]	Ca_08794	WD40 repeat	SYNONYMOUS_CODING
14548	CakSNP14548	*Kabuli*	Ca_Kabuli_Scaffold_1348_1	1145082	[A/C]		--	INTERGENIC
14549	CakSNP14549	*Kabuli*	Ca_Kabuli_Scaffold_1348_1	1145124	[T/C]		--	INTERGENIC
14550	CakSNP14550	*Kabuli*	Ca_Kabuli_Scaffold_1348_1	1207442	[G/A]	Ca_08810	Pentatricopeptide repeat	SYNONYMOUS_CODING
14551	CakSNP14551	*Kabuli*	Ca_Kabuli_Scaffold_1348_1	1236956	[A/G]	Ca_08813	Phox/Bem1p	SYNONYMOUS_CODING

S.N.	SNP IDs	Chickpea cultivars	Chromosomes	Physical positions (bp)	SNPs	Gene accession IDs	Putative functions	Sequence components of genome
14552	CakSNP14552	*Kabuli*	Ca_Kabuli_Scaffold_1348_1	1263560	[T/G]		--	INTERGENIC
14553	CakSNP14553	*Kabuli*	Ca_Kabuli_Scaffold_1348_1	1265627	[T/C]	Ca_08816	Protein kinase, catalytic domain	SYNONYMOUS_CODING
14554	CakSNP14554	*Kabuli*	Ca_Kabuli_Scaffold_1348_1	1265635	[T/G]	Ca_08816	Protein kinase, catalytic domain	NON_SYNONYMOUS_CODING
14555	CakSNP14555	*Kabuli*	Ca_Kabuli_Scaffold_1348_1	1273341	[G/A]	Ca_08818	Ferric reductase, NAD binding	INTRON
14556	CakSNP14556	*Kabuli*	Ca_Kabuli_Scaffold_1348_1	1273388	[G/A]	Ca_08818	Ferric reductase, NAD binding	INTRON
14557	CakSNP14557	*Kabuli*	Ca_Kabuli_Scaffold_1348_1	1288448	[A/G]	Ca_08820	RNA helicase, ATP-dependent, DEAD-box, conserved site	INTRON
14558	CakSNP14558	*Kabuli*	Ca_Kabuli_Scaffold_1348_1	1294675	[A/T]		--	INTERGENIC
14559	CakSNP14559	*Kabuli*	Ca_Kabuli_Scaffold_1348_1	1296453	[G/A]		--	INTERGENIC
14560	CakSNP14560	*Kabuli*	Ca_Kabuli_Scaffold_1348_1	1330256	[A/G]		--	INTERGENIC
14561	CakSNP14561	*Kabuli*	Ca_Kabuli_Scaffold_1348_1	1330288	[A/G]		--	INTERGENIC

S.N.	SNP IDs	Chickpea cultivars	Chromosomes	Physical positions (bp)	SNPs	Gene accession IDs	Putative functions	Sequence components of genome
14562	CakSNP14562	*Kabuli*	Ca_Kabuli_Scaffold_1348_1	1330400	[A/G]		--	INTERGENIC
14563	CakSNP14563	*Kabuli*	Ca_Kabuli_Scaffold_1348_1	1346914	[G/T]	Ca_08826	Allinase, C-terminal	INTRON
14564	CakSNP14564	*Kabuli*	Ca_Kabuli_Scaffold_1348_1	1346943	[G/A]	Ca_08826	Allinase, C-terminal	INTRON
14565	CakSNP14565	*Kabuli*	Ca_Kabuli_Scaffold_1348_1	1372535	[G/A]	Ca_08827	Raffinose synthase	SYNONYMOUS_CODING
14566	CakSNP14566	*Kabuli*	Ca_Kabuli_Scaffold_1348_1	1403452	[G/A]	Ca_08830	Kinetochore-Ndc80 complex, subunit Spc25	INTRON
14567	CakSNP14567	*Kabuli*	Ca_Kabuli_Scaffold_1348_1	1403480	[A/G]	Ca_08830	Kinetochore-Ndc80 complex, subunit Spc25	INTRON
14568	CakSNP14568	*Kabuli*	Ca_Kabuli_Scaffold_1351	172111	[G/A]	Ca_21995	Protein of unknown function DUF2419	SYNONYMOUS_CODING
14569	CakSNP14569	*Kabuli*	Ca_Kabuli_Scaffold_1351	172110	[T/C]	Ca_21995	Protein of unknown function DUF2419	NON_SYNONYMOUS_CODING
14570	CakSNP14570	*Kabuli*	Ca_Kabuli_Scaffold_1351	177159	[G/A]		--	INTERGENIC
14571	CakSNP14571	*Kabuli*	Ca_Kabuli_Scaffold_1351	177172	[G/A]		--	INTERGENIC

S.N.	SNP IDs	Chickpea cultivars	Chromosomes	Physical positions (bp)	SNPs	Gene accession IDs	Putative functions	Sequence components of genome
14572	CakSNP14572	*Kabuli*	Ca_Kabuli_Scaffold_1351	177178	[T/C]		--	INTERGENIC
14573	CakSNP14573	*Kabuli*	Ca_Kabuli_Scaffold_1351	177181	[A/G]		--	INTERGENIC
14574	CakSNP14574	*Kabuli*	Ca_Kabuli_Scaffold_1351	177194	[G/A]		--	INTERGENIC
14575	CakSNP14575	*Kabuli*	Ca_Kabuli_Scaffold_1351	177202	[G/A]		--	INTERGENIC
14576	CakSNP14576	*Kabuli*	Ca_Kabuli_Scaffold_1351	177171	[T/G]		--	INTERGENIC
14577	CakSNP14577	*Kabuli*	Ca_Kabuli_Scaffold_1351	177193	[T/C]		--	INTERGENIC
14578	CakSNP14578	*Kabuli*	Ca_Kabuli_Scaffold_1351	177256	[T/G]		--	INTERGENIC
14579	CakSNP14579	*Kabuli*	Ca_Kabuli_Scaffold_1351	177248	[G/T]		--	INTERGENIC
14580	CakSNP14580	*Kabuli*	Ca_Kabuli_Scaffold_1351	177247	[G/A]		--	INTERGENIC
14581	CakSNP14581	*Kabuli*	Ca_Kabuli_Scaffold_1351	177242	[G/A]		--	INTERGENIC

S.N.	SNP IDs	Chickpea cultivars	Chromosomes	Physical positions (bp)	SNPs	Gene accession IDs	Putative functions	Sequence components of genome
14582	CakSNP14582	*Kabuli*	Ca_Kabuli_Scaffold_1351	177241	[T/G]		--	INTERGENIC
14583	CakSNP14583	*Kabuli*	Ca_Kabuli_Scaffold_1351	177240	[C/A]		--	INTERGENIC
14584	CakSNP14584	*Kabuli*	Ca_Kabuli_Scaffold_1351	177235	[G/T]		--	INTERGENIC
14585	CakSNP14585	*Kabuli*	Ca_Kabuli_Scaffold_1351	177234	[A/C]		--	INTERGENIC
14586	CakSNP14586	*Kabuli*	Ca_Kabuli_Scaffold_1351	177210	[T/A]		--	INTERGENIC
14587	CakSNP14587	*Kabuli*	Ca_Kabuli_Scaffold_1351	177200	[T/G]		--	INTERGENIC
14588	CakSNP14588	*Kabuli*	Ca_Kabuli_Scaffold_1351	177191	[C/T]		--	INTERGENIC
14589	CakSNP14589	*Kabuli*	Ca_Kabuli_Scaffold_1351	177188	[G/T]		--	INTERGENIC
14590	CakSNP14590	*Kabuli*	Ca_Kabuli_Scaffold_1351	204656	[C/A]		--	INTERGENIC
14591	CakSNP14591	*Kabuli*	Ca_Kabuli_Scaffold_1351	335227	[T/C]	Ca_22005	--	SYNONYMOUS_CODING

S.N.	SNP IDs	Chickpea cultivars	Chromosomes	Physical positions (bp)	SNPs	Gene accession IDs	Putative functions	Sequence components of genome
14592	CakSNP14592	*Kabuli*	Ca_Kabuli_Scaffold_1351	335239	[G/T]	Ca_22005	--	NON_SYNONYMOUS_CODING
14593	CakSNP14593	*Kabuli*	Ca_Kabuli_Scaffold_1351	367576	[A/G]	Ca_22006	Aldehyde dehydrogenase NAD(P)-dependent	URR
14594	CakSNP14594	*Kabuli*	Ca_Kabuli_Scaffold_1351	367578	[G/A]	Ca_22006	Aldehyde dehydrogenase NAD(P)-dependent	URR
14595	CakSNP14595	*Kabuli*	Ca_Kabuli_Scaffold_1351	367745	[G/A]	Ca_22006	Aldehyde dehydrogenase NAD(P)-dependent	URR
14596	CakSNP14596	*Kabuli*	Ca_Kabuli_Scaffold_1369	8015	[T/A]		--	INTERGENIC
14597	CakSNP14597	*Kabuli*	Ca_Kabuli_Scaffold_1369	8115	[T/G]		--	INTERGENIC
14598	CakSNP14598	*Kabuli*	Ca_Kabuli_Scaffold_1369	8154	[T/G]		--	INTERGENIC
14599	CakSNP14599	*Kabuli*	Ca_Kabuli_Scaffold_1369	8221	[T/G]		--	INTERGENIC
14600	CakSNP14600	*Kabuli*	Ca_Kabuli_Scaffold_1369	8327	[G/A]		--	INTERGENIC
14601	CakSNP14601	*Kabuli*	Ca_Kabuli_Scaffold_1369	31000	[A/G]	Ca_25519	Oligopeptide transporter	SYNONYMOUS_CODING

S.N.	SNP IDs	Chickpea cultivars	Chromosomes	Physical positions (bp)	SNPs	Gene accession IDs	Putative functions	Sequence components of genome
14602	CakSNP14602	*Kabuli*	Ca_Kabuli_Scaffold_1390	98354	[A/G]		--	INTERGENIC
14603	CakSNP14603	*Kabuli*	Ca_Kabuli_Scaffold_1401	89077	[T/C]	Ca_26388	Peptidase S8/S53, subtilisin/kexin/sedolisin	NON_SYNONYMOUS_CODING
14604	CakSNP14604	*Kabuli*	Ca_Kabuli_Scaffold_1401	89074	[T/C]	Ca_26388	Peptidase S8/S53, subtilisin/kexin/sedolisin	NON_SYNONYMOUS_CODING
14605	CakSNP14605	*Kabuli*	Ca_Kabuli_Scaffold_1417	4863	[A/G]	Ca_25454	--	NON_SYNONYMOUS_CODING
14606	CakSNP14606	*Kabuli*	Ca_Kabuli_Scaffold_1417	4906	[G/A]	Ca_25454	--	STOP_GAINED
14607	CakSNP14607	*Kabuli*	Ca_Kabuli_Scaffold_1417	4917	[G/T]	Ca_25454	--	NON_SYNONYMOUS_CODING
14608	CakSNP14608	*Kabuli*	Ca_Kabuli_Scaffold_1417	76627	[A/C]		--	INTERGENIC
14609	CakSNP14609	*Kabuli*	Ca_Kabuli_Scaffold_1417	230210	[T/G]		--	INTERGENIC
14610	CakSNP14610	*Kabuli*	Ca_Kabuli_Scaffold_1419	24845	[C/T]		--	INTERGENIC
14611	CakSNP14611	*Kabuli*	Ca_Kabuli_Scaffold_143	18492	[A/G]		--	INTERGENIC

S.N.	SNP IDs	Chickpea cultivars	Chromosomes	Physical positions (bp)	SNPs	Gene accession IDs	Putative functions	Sequence components of genome
14612	CakSNP14612	*Kabuli*	Ca_Kabuli_Scaffold_1439	242972	[A/G]	Ca_23658	--	SYNONYMOUS_CODING
14613	CakSNP14613	*Kabuli*	Ca_Kabuli_Scaffold_1448	47306	[A/G]	Ca_26687	Importin-beta, N-terminal	INTRON
14614	CakSNP14614	*Kabuli*	Ca_Kabuli_Scaffold_1448	74436	[C/T]	Ca_26687	Importin-beta, N-terminal	INTRON
14615	CakSNP14615	*Kabuli*	Ca_Kabuli_Scaffold_1448	74434	[C/T]	Ca_26687	Importin-beta, N-terminal	INTRON
14616	CakSNP14616	*Kabuli*	Ca_Kabuli_Scaffold_1448	74529	[C/T]	Ca_26687	Importin-beta, N-terminal	INTRON
14617	CakSNP14617	*Kabuli*	Ca_Kabuli_Scaffold_1448	76809	[G/A]	Ca_26687	Importin-beta, N-terminal	INTRON
14618	CakSNP14618	*Kabuli*	Ca_Kabuli_Scaffold_1449	55474	[T/G]	Ca_28129	--	SYNONYMOUS_CODING
14619	CakSNP14619	*Kabuli*	Ca_Kabuli_Scaffold_1449	55529	[G/A]	Ca_28129	--	NON_SYNONYMOUS_CODING
14620	CakSNP14620	*Kabuli*	Ca_Kabuli_Scaffold_1449	55532	[G/A]	Ca_28129	--	NON_SYNONYMOUS_CODING
14621	CakSNP14621	*Kabuli*	Ca_Kabuli_Scaffold_1452	3501	[C/A]		--	INTERGENIC

S.N.	SNP IDs	Chickpea cultivars	Chromosomes	Physical positions (bp)	SNPs	Gene accession IDs	Putative functions	Sequence components of genome
14622	CakSNP14622	*Kabuli*	Ca_Kabuli_Scaffold_1452	3496	[A/T]		--	INTERGENIC
14623	CakSNP14623	*Kabuli*	Ca_Kabuli_Scaffold_1452	3495	[C/A]		--	INTERGENIC
14624	CakSNP14624	*Kabuli*	Ca_Kabuli_Scaffold_1452	3737	[C/A]		--	INTERGENIC
14625	CakSNP14625	*Kabuli*	Ca_Kabuli_Scaffold_1462	1556	[T/G]	Ca_28230	Protein kinase, catalytic domain	INTRON
14626	CakSNP14626	*Kabuli*	Ca_Kabuli_Scaffold_1466	90846	[C/A]		--	INTERGENIC
14627	CakSNP14627	*Kabuli*	Ca_Kabuli_Scaffold_1466	165472	[C/T]	Ca_24036	Ribosomal protein L13	INTRON
14628	CakSNP14628	*Kabuli*	Ca_Kabuli_Scaffold_1466	322287	[G/A]		--	INTERGENIC
14629	CakSNP14629	*Kabuli*	Ca_Kabuli_Scaffold_1466	322315	[G/T]		--	INTERGENIC
14630	CakSNP14630	*Kabuli*	Ca_Kabuli_Scaffold_1466	322450	[G/T]		--	INTERGENIC
14631	CakSNP14631	*Kabuli*	Ca_Kabuli_Scaffold_1466	322448	[T/G]		--	INTERGENIC

S.N.	SNP IDs	Chickpea cultivars	Chromosomes	Physical positions (bp)	SNPs	Gene accession IDs	Putative functions	Sequence components of genome
14632	CakSNP14632	*Kabuli*	Ca_Kabuli_Scaffold_1466	322421	[C/T]		--	INTERGENIC
14633	CakSNP14633	*Kabuli*	Ca_Kabuli_Scaffold_1466	322415	[G/A]		--	INTERGENIC
14634	CakSNP14634	*Kabuli*	Ca_Kabuli_Scaffold_1466	322414	[G/A]		--	INTERGENIC
14635	CakSNP14635	*Kabuli*	Ca_Kabuli_Scaffold_1466	322400	[C/T]		--	INTERGENIC
14636	CakSNP14636	*Kabuli*	Ca_Kabuli_Scaffold_1466	322390	[G/A]		--	INTERGENIC
14637	CakSNP14637	*Kabuli*	Ca_Kabuli_Scaffold_1466	322383	[C/T]		--	INTERGENIC
14638	CakSNP14638	*Kabuli*	Ca_Kabuli_Scaffold_1467	205779	[A/G]		--	INTERGENIC
14639	CakSNP14639	*Kabuli*	Ca_Kabuli_Scaffold_1467	205803	[C/T]		--	INTERGENIC
14640	CakSNP14640	*Kabuli*	Ca_Kabuli_Scaffold_1467	205833	[G/A]		--	INTERGENIC
14641	CakSNP14641	*Kabuli*	Ca_Kabuli_Scaffold_1467	205840	[G/A]		--	INTERGENIC

S.N.	SNP IDs	Chickpea cultivars	Chromosomes	Physical positions (bp)	SNPs	Gene accession IDs	Putative functions	Sequence components of genome
14642	CakSNP14642	*Kabuli*	Ca_Kabuli_Scaffold_1467	205845	[A/G]		--	INTERGENIC
14643	CakSNP14643	*Kabuli*	Ca_Kabuli_Scaffold_1467	206121	[G/A]		--	INTERGENIC
14644	CakSNP14644	*Kabuli*	Ca_Kabuli_Scaffold_1467	206118	[C/T]		--	INTERGENIC
14645	CakSNP14645	*Kabuli*	Ca_Kabuli_Scaffold_1467	206100	[G/A]		--	INTERGENIC
14646	CakSNP14646	*Kabuli*	Ca_Kabuli_Scaffold_1467	206094	[T/C]		--	INTERGENIC
14647	CakSNP14647	*Kabuli*	Ca_Kabuli_Scaffold_1467	206085	[T/C]		--	INTERGENIC
14648	CakSNP14648	*Kabuli*	Ca_Kabuli_Scaffold_1467	206068	[A/G]		--	INTERGENIC
14649	CakSNP14649	*Kabuli*	Ca_Kabuli_Scaffold_1467	206067	[T/G]		--	INTERGENIC
14650	CakSNP14650	*Kabuli*	Ca_Kabuli_Scaffold_1475	4820	[G/A]		--	INTERGENIC
14651	CakSNP14651	*Kabuli*	Ca_Kabuli_Scaffold_1475	4796	[C/T]		--	INTERGENIC

S.N.	SNP IDs	Chickpea cultivars	Chromosomes	Physical positions (bp)	SNPs	Gene accession IDs	Putative functions	Sequence components of genome
14652	CakSNP14652	*Kabuli*	Ca_Kabuli_Scaffold_1475	4794	[C/A]		--	INTERGENIC
14653	CakSNP14653	*Kabuli*	Ca_Kabuli_Scaffold_1475	4755	[T/A]		--	INTERGENIC
14654	CakSNP14654	*Kabuli*	Ca_Kabuli_Scaffold_1475	4754	[C/T]		--	INTERGENIC
14655	CakSNP14655	*Kabuli*	Ca_Kabuli_Scaffold_1475	4749	[C/T]		--	INTERGENIC
14656	CakSNP14656	*Kabuli*	Ca_Kabuli_Scaffold_1475	4873	[G/T]		--	INTERGENIC
14657	CakSNP14657	*Kabuli*	Ca_Kabuli_Scaffold_1475	4845	[A/T]		--	INTERGENIC
14658	CakSNP14658	*Kabuli*	Ca_Kabuli_Scaffold_1475	4833	[G/A]		--	INTERGENIC
14659	CakSNP14659	*Kabuli*	Ca_Kabuli_Scaffold_1475	4818	[C/T]		--	INTERGENIC
14660	CakSNP14660	*Kabuli*	Ca_Kabuli_Scaffold_1483	56686	[A/T]		--	INTERGENIC
14661	CakSNP14661	*Kabuli*	Ca_Kabuli_Scaffold_1483	56721	[G/C]		--	INTERGENIC

S.N.	SNP IDs	Chickpea cultivars	Chromosomes	Physical positions (bp)	SNPs	Gene accession IDs	Putative functions	Sequence components of genome
14662	CakSNP14662	*Kabuli*	Ca_Kabuli_Scaffold_1493	81740	[C/T]	Ca_26514	Peptidase C48, SUMO/Sentrin/Ubl1	INTRON
14663	CakSNP14663	*Kabuli*	Ca_Kabuli_Scaffold_1493	81784	[C/A]	Ca_26514	Peptidase C48, SUMO/Sentrin/Ubl1	INTRON
14664	CakSNP14664	*Kabuli*	Ca_Kabuli_Scaffold_1493	81789	[G/A]	Ca_26514	Peptidase C48, SUMO/Sentrin/Ubl1	INTRON
14665	CakSNP14665	*Kabuli*	Ca_Kabuli_Scaffold_1496	289997	[G/T]		--	INTERGENIC
14666	CakSNP14666	*Kabuli*	Ca_Kabuli_Scaffold_1496	290018	[C/A]		--	INTERGENIC
14667	CakSNP14667	*Kabuli*	Ca_Kabuli_Scaffold_1498	3474	[A/C]	Ca_28063	Multi antimicrobial extrusion protein	SYNONYMOUS_CODING
14668	CakSNP14668	*Kabuli*	Ca_Kabuli_Scaffold_1498	3657	[C/T]	Ca_28063	Multi antimicrobial extrusion protein	INTRON
14669	CakSNP14669	*Kabuli*	Ca_Kabuli_Scaffold_1498	3641	[T/C]	Ca_28063	Multi antimicrobial extrusion protein	INTRON
14670	CakSNP14670	*Kabuli*	Ca_Kabuli_Scaffold_1504	160623	[G/T]	Ca_24092	--	SYNONYMOUS_CODING
14671	CakSNP14671	*Kabuli*	Ca_Kabuli_Scaffold_1504	189700	[A/G]		--	INTERGENIC

S.N.	SNP IDs	Chickpea cultivars	Chromosomes	Physical positions (bp)	SNPs	Gene accession IDs	Putative functions	Sequence components of genome
14672	CakSNP14672	*Kabuli*	Ca_Kabuli_Scaffold_1504	189699	[C/T]		--	INTERGENIC
14673	CakSNP14673	*Kabuli*	Ca_Kabuli_Scaffold_1504	189681	[C/T]		--	INTERGENIC
14674	CakSNP14674	*Kabuli*	Ca_Kabuli_Scaffold_1504	189664	[G/A]		--	INTERGENIC
14675	CakSNP14675	*Kabuli*	Ca_Kabuli_Scaffold_1504	189663	[G/A]		--	INTERGENIC
14676	CakSNP14676	*Kabuli*	Ca_Kabuli_Scaffold_1504	197789	[G/A]		--	INTERGENIC
14677	CakSNP14677	*Kabuli*	Ca_Kabuli_Scaffold_1504	197790	[G/A]		--	INTERGENIC
14678	CakSNP14678	*Kabuli*	Ca_Kabuli_Scaffold_1504	197819	[T/G]		--	INTERGENIC
14679	CakSNP14679	*Kabuli*	Ca_Kabuli_Scaffold_1505	33297	[A/C]	Ca_28120	--	SYNONYMOUS_CODING
14680	CakSNP14680	*Kabuli*	Ca_Kabuli_Scaffold_151	267060	[T/G]	Ca_25631	LETM1-like	SYNONYMOUS_CODING
14681	CakSNP14681	*Kabuli*	Ca_Kabuli_Scaffold_151	275743	[T/A]	Ca_25631	LETM1-like	NON_SYNONYMOUS_CODING

S.N.	SNP IDs	Chickpea cultivars	Chromosomes	Physical positions (bp)	SNPs	Gene accession IDs	Putative functions	Sequence components of genome
14682	CakSNP14682	*Kabuli*	Ca_Kabuli_Scaffold_1521	20734	[C/A]	Ca_26979	Protein kinase, catalytic domain	DRR
14683	CakSNP14683	*Kabuli*	Ca_Kabuli_Scaffold_1521	20779	[T/C]	Ca_26979	Protein kinase, catalytic domain	DRR
14684	CakSNP14684	*Kabuli*	Ca_Kabuli_Scaffold_1521	20851	[C/T]	Ca_26979	Protein kinase, catalytic domain	DRR
14685	CakSNP14685	*Kabuli*	Ca_Kabuli_Scaffold_1545	111476	[G/A]	Ca_23856	Phosphoenolpyruvate carboxylase, active site	NON_SYNONYMOUS_CODING
14686	CakSNP14686	*Kabuli*	Ca_Kabuli_Scaffold_1545	113148	[T/C]	Ca_23856	Phosphoenolpyruvate carboxylase, active site	SYNONYMOUS_CODING
14687	CakSNP14687	*Kabuli*	Ca_Kabuli_Scaffold_1553	5078	[G/A]	Ca_27877	Peptidase C48, SUMO/Sentrin/Ubl1	NON_SYNONYMOUS_CODING
14688	CakSNP14688	*Kabuli*	Ca_Kabuli_Scaffold_1553	5115	[C/T]	Ca_27877	Peptidase C48, SUMO/Sentrin/Ubl1	NON_SYNONYMOUS_CODING
14689	CakSNP14689	*Kabuli*	Ca_Kabuli_Scaffold_1553	5125	[A/G]	Ca_27877	Peptidase C48, SUMO/Sentrin/Ubl1	SYNONYMOUS_CODING
14690	CakSNP14690	*Kabuli*	Ca_Kabuli_Scaffold_157	100228	[A/G]	Ca_21065	RNA recognition motif domain	SYNONYMOUS_CODING
14691	CakSNP14691	*Kabuli*	Ca_Kabuli_Scaffold_157	100529	[T/C]	Ca_21065	RNA recognition motif domain	NON_SYNONYMOUS_CODING

S.N.	SNP IDs	Chickpea cultivars	Chromosomes	Physical positions (bp)	SNPs	Gene accession IDs	Putative functions	Sequence components of genome
14692	CakSNP14692	*Kabuli*	Ca_Kabuli_Scaffold_157	102779	[C/T]	Ca_21065	RNA recognition motif domain	INTRON
14693	CakSNP14693	*Kabuli*	Ca_Kabuli_Scaffold_157	106331	[G/A]	Ca_21065	RNA recognition motif domain	INTRON
14694	CakSNP14694	*Kabuli*	Ca_Kabuli_Scaffold_157	106349	[C/T]	Ca_21065	RNA recognition motif domain	INTRON
14695	CakSNP14695	*Kabuli*	Ca_Kabuli_Scaffold_157	133602	[A/G]		--	INTERGENIC
14696	CakSNP14696	*Kabuli*	Ca_Kabuli_Scaffold_157	133583	[T/G]		--	INTERGENIC
14697	CakSNP14697	*Kabuli*	Ca_Kabuli_Scaffold_157	141880	[A/G]	Ca_21068	Ammonium transporter	SYNONYMOUS_CODING
14698	CakSNP14698	*Kabuli*	Ca_Kabuli_Scaffold_157	142444	[A/T]	Ca_21068	Ammonium transporter	INTRON
14699	CakSNP14699	*Kabuli*	Ca_Kabuli_Scaffold_157	142455	[T/C]	Ca_21068	Ammonium transporter	INTRON
14700	CakSNP14700	*Kabuli*	Ca_Kabuli_Scaffold_157	142470	[G/T]	Ca_21068	Ammonium transporter	INTRON
14701	CakSNP14701	*Kabuli*	Ca_Kabuli_Scaffold_157	151081	[G/A]	Ca_21070	Zinc finger, DHHC-type, palmitoyltransferase	NON_SYNONYMOUS_CODING

S.N.	SNP IDs	Chickpea cultivars	Chromosomes	Physical positions (bp)	SNPs	Gene accession IDs	Putative functions	Sequence components of genome
14702	CakSNP14702	*Kabuli*	Ca_Kabuli_Scaffold_157	151199	[T/A]	Ca_21070	Zinc finger, DHHC-type, palmitoyltransferase	INTRON
14703	CakSNP14703	*Kabuli*	Ca_Kabuli_Scaffold_157	151192	[T/C]	Ca_21070	Zinc finger, DHHC-type, palmitoyltransferase	INTRON
14704	CakSNP14704	*Kabuli*	Ca_Kabuli_Scaffold_157	151177	[A/G]	Ca_21070	Zinc finger, DHHC-type, palmitoyltransferase	INTRON
14705	CakSNP14705	*Kabuli*	Ca_Kabuli_Scaffold_157	151131	[T/A]	Ca_21070	Zinc finger, DHHC-type, palmitoyltransferase	SYNONYMOUS_CODING
14706	CakSNP14706	*Kabuli*	Ca_Kabuli_Scaffold_157	213380	[C/G]	Ca_21071	Mitochondrial substrate/solute carrier	NON_SYNONYMOUS_CODING
14707	CakSNP14707	*Kabuli*	Ca_Kabuli_Scaffold_157	213398	[T/G]	Ca_21071	Mitochondrial substrate/solute carrier	NON_SYNONYMOUS_CODING
14708	CakSNP14708	*Kabuli*	Ca_Kabuli_Scaffold_157	213404	[T/G]	Ca_21071	Mitochondrial substrate/solute carrier	SYNONYMOUS_CODING
14709	CakSNP14709	*Kabuli*	Ca_Kabuli_Scaffold_157	213518	[T/C]	Ca_21071	Mitochondrial substrate/solute carrier	INTRON
14710	CakSNP14710	*Kabuli*	Ca_Kabuli_Scaffold_157	213487	[G/C]	Ca_21071	Mitochondrial substrate/solute carrier	INTRON
14711	CakSNP14711	*Kabuli*	Ca_Kabuli_Scaffold_157	213477	[G/A]	Ca_21071	Mitochondrial substrate/solute carrier	INTRON

S.N.	SNP IDs	Chickpea cultivars	Chromosomes	Physical positions (bp)	SNPs	Gene accession IDs	Putative functions	Sequence components of genome
14712	CakSNP14712	*Kabuli*	Ca_Kabuli_Scaffold_157	229917	[G/A]		--	INTERGENIC
14713	CakSNP14713	*Kabuli*	Ca_Kabuli_Scaffold_157	229900	[G/A]		--	INTERGENIC
14714	CakSNP14714	*Kabuli*	Ca_Kabuli_Scaffold_157	321386	[C/T]		--	INTERGENIC
14715	CakSNP14715	*Kabuli*	Ca_Kabuli_Scaffold_157	326434	[A/G]	Ca_21078	Protein of unknown function DUF3755	INTRON
14716	CakSNP14716	*Kabuli*	Ca_Kabuli_Scaffold_157	326467	[C/T]	Ca_21078	Protein of unknown function DUF3755	INTRON
14717	CakSNP14717	*Kabuli*	Ca_Kabuli_Scaffold_157	326494	[A/G]	Ca_21078	Protein of unknown function DUF3755	INTRON
14718	CakSNP14718	*Kabuli*	Ca_Kabuli_Scaffold_157	326497	[A/G]	Ca_21078	Protein of unknown function DUF3755	INTRON
14719	CakSNP14719	*Kabuli*	Ca_Kabuli_Scaffold_157	352687	[C/T]	Ca_21079	DEP domain	INTRON
14720	CakSNP14720	*Kabuli*	Ca_Kabuli_Scaffold_157	352712	[G/A]	Ca_21079	DEP domain	INTRON
14721	CakSNP14721	*Kabuli*	Ca_Kabuli_Scaffold_157	352747	[T/C]	Ca_21079	DEP domain	INTRON

S.N.	SNP IDs	Chickpea cultivars	Chromosomes	Physical positions (bp)	SNPs	Gene accession IDs	Putative functions	Sequence components of genome
14722	CakSNP14722	*Kabuli*	Ca_Kabuli_Scaffold_157	355222	[G/A]	Ca_21079	DEP domain	INTRON
14723	CakSNP14723	*Kabuli*	Ca_Kabuli_Scaffold_157	469198	[A/C]		--	INTERGENIC
14724	CakSNP14724	*Kabuli*	Ca_Kabuli_Scaffold_157	535057	[T/G]		--	INTERGENIC
14725	CakSNP14725	*Kabuli*	Ca_Kabuli_Scaffold_1575	2230	[C/T]	Ca_27016	Peptidase C48, SUMO/Sentrin/Ubl1	NON_SYNONYMOUS_CODING
14726	CakSNP14726	*Kabuli*	Ca_Kabuli_Scaffold_1580	71861	[G/T]		--	INTERGENIC
14727	CakSNP14727	*Kabuli*	Ca_Kabuli_Scaffold_1580	119410	[T/C]	Ca_20791	NAD-dependent histone deacetylase, silent information regulator Sir3	INTRON
14728	CakSNP14728	*Kabuli*	Ca_Kabuli_Scaffold_1580	119678	[T/C]	Ca_20791	NAD-dependent histone deacetylase, silent information regulator Sir4	INTRON
14729	CakSNP14729	*Kabuli*	Ca_Kabuli_Scaffold_1580	163148	[A/T]	Ca_20794	AMP-dependent synthetase/ligase	NON_SYNONYMOUS_CODING
14730	CakSNP14730	*Kabuli*	Ca_Kabuli_Scaffold_1580	163194	[T/C]	Ca_20794	AMP-dependent synthetase/ligase	NON_SYNONYMOUS_CODING
14731	CakSNP14731	*Kabuli*	Ca_Kabuli_Scaffold_1580	380649	[A/T]		--	INTERGENIC

S.N.	SNP IDs	Chickpea cultivars	Chromosomes	Physical positions (bp)	SNPs	Gene accession IDs	Putative functions	Sequence components of genome
14732	CakSNP14732	*Kabuli*	Ca_Kabuli_Scaffold_1585	59010	[C/A]		--	INTERGENIC
14733	CakSNP14733	*Kabuli*	Ca_Kabuli_Scaffold_1603	11828	[C/G]		--	INTERGENIC
14734	CakSNP14734	*Kabuli*	Ca_Kabuli_Scaffold_1607	107222	[T/C]	Ca_27087	Mediator complex subunit Med28	INTRON
14735	CakSNP14735	*Kabuli*	Ca_Kabuli_Scaffold_1613	48917	[T/G]	Ca_27472	Protein kinase, catalytic domain	INTRON
14736	CakSNP14736	*Kabuli*	Ca_Kabuli_Scaffold_1613	107664	[T/A]	Ca_27474	Photosynthetic reaction centre, L/M	NON_SYNONYMOUS_CODING
14737	CakSNP14737	*Kabuli*	Ca_Kabuli_Scaffold_1613	107750	[T/A]	Ca_27474	Photosynthetic reaction centre, L/M	NON_SYNONYMOUS_CODING
14738	CakSNP14738	*Kabuli*	Ca_Kabuli_Scaffold_1631	202418	[A/G]		--	INTERGENIC
14739	CakSNP14739	*Kabuli*	Ca_Kabuli_Scaffold_1635	83227	[G/C]		--	INTERGENIC
14740	CakSNP14740	*Kabuli*	Ca_Kabuli_Scaffold_1635	83345	[A/T]		--	INTERGENIC
14741	CakSNP14741	*Kabuli*	Ca_Kabuli_Scaffold_1654	26432	[G/A]		--	INTERGENIC

S.N.	SNP IDs	Chickpea cultivars	Chromosomes	Physical positions (bp)	SNPs	Gene accession IDs	Putative functions	Sequence components of genome
14742	CakSNP14742	*Kabuli*	Ca_Kabuli_Scaffold_1659	303945	[T/G]		--	INTERGENIC
14743	CakSNP14743	*Kabuli*	Ca_Kabuli_Scaffold_1661	70434	[A/G]		--	INTERGENIC
14744	CakSNP14744	*Kabuli*	Ca_Kabuli_Scaffold_1664	94989	[G/A]		--	INTERGENIC
14745	CakSNP14745	*Kabuli*	Ca_Kabuli_Scaffold_1664	94984	[A/T]		--	INTERGENIC
14746	CakSNP14746	*Kabuli*	Ca_Kabuli_Scaffold_1664	94930	[G/C]		--	INTERGENIC
14747	CakSNP14747	*Kabuli*	Ca_Kabuli_Scaffold_167	11018	[T/A]		--	INTERGENIC
14748	CakSNP14748	*Kabuli*	Ca_Kabuli_Scaffold_167	11009	[A/T]		--	INTERGENIC
14749	CakSNP14749	*Kabuli*	Ca_Kabuli_Scaffold_167	137869	[C/T]		--	INTERGENIC
14750	CakSNP14750	*Kabuli*	Ca_Kabuli_Scaffold_167	137848	[A/G]		--	INTERGENIC
14751	CakSNP14751	*Kabuli*	Ca_Kabuli_Scaffold_167	239687	[G/A]		--	INTERGENIC

S.N.	SNP IDs	Chickpea cultivars	Chromosomes	Physical positions (bp)	SNPs	Gene accession IDs	Putative functions	Sequence components of genome
14752	CakSNP14752	*Kabuli*	Ca_Kabuli_Scaffold_167	327829	[T/C]		--	INTERGENIC
14753	CakSNP14753	*Kabuli*	Ca_Kabuli_Scaffold_1687	82935	[A/C]	Ca_26679	Plant lipid transfer protein/Par allergen	SYNONYMOUS_CODING
14754	CakSNP14754	*Kabuli*	Ca_Kabuli_Scaffold_1699	27556	[A/C]		--	INTERGENIC
14755	CakSNP14755	*Kabuli*	Ca_Kabuli_Scaffold_1699	27607	[T/G]		--	INTERGENIC
14756	CakSNP14756	*Kabuli*	Ca_Kabuli_Scaffold_1708	69664	[A/T]		--	INTERGENIC
14757	CakSNP14757	*Kabuli*	Ca_Kabuli_Scaffold_171	96763	[T/C]	Ca_25945	--	SYNONYMOUS_CODING
14758	CakSNP14758	*Kabuli*	Ca_Kabuli_Scaffold_171	183718	[T/C]	Ca_25948	Short-chain dehydrogenase/reductase SDR	INTRON
14759	CakSNP14759	*Kabuli*	Ca_Kabuli_Scaffold_171	183720	[C/T]	Ca_25948	Short-chain dehydrogenase/reductase SDR	INTRON
14760	CakSNP14760	*Kabuli*	Ca_Kabuli_Scaffold_171	183721	[G/A]	Ca_25948	Short-chain dehydrogenase/reductase SDR	INTRON
14761	CakSNP14761	*Kabuli*	Ca_Kabuli_Scaffold_171	183727	[C/T]	Ca_25948	Short-chain dehydrogenase/reductase SDR	INTRON

S.N.	SNP IDs	Chickpea cultivars	Chromosomes	Physical positions (bp)	SNPs	Gene accession IDs	Putative functions	Sequence components of genome
14762	CakSNP14762	*Kabuli*	Ca_Kabuli_Scaffold_171	183759	[T/C]	Ca_25948	Short-chain dehydrogenase/reductase SDR	INTRON
14763	CakSNP14763	*Kabuli*	Ca_Kabuli_Scaffold_171	186125	[C/A]	Ca_25948	Short-chain dehydrogenase/reductase SDR	NON_SYNONYMOUS_CODING
14764	CakSNP14764	*Kabuli*	Ca_Kabuli_Scaffold_171	208050	[A/G]		--	INTERGENIC
14765	CakSNP14765	*Kabuli*	Ca_Kabuli_Scaffold_171	209964	[G/T]		--	INTERGENIC
14766	CakSNP14766	*Kabuli*	Ca_Kabuli_Scaffold_1718	302	[G/A]		--	INTERGENIC
14767	CakSNP14767	*Kabuli*	Ca_Kabuli_Scaffold_1718	295	[G/A]		--	INTERGENIC
14768	CakSNP14768	*Kabuli*	Ca_Kabuli_Scaffold_1718	289	[C/T]		--	INTERGENIC
14769	CakSNP14769	*Kabuli*	Ca_Kabuli_Scaffold_1718	268	[C/G]		--	INTERGENIC
14770	CakSNP14770	*Kabuli*	Ca_Kabuli_Scaffold_1718	3898	[G/C]		--	INTERGENIC
14771	CakSNP14771	*Kabuli*	Ca_Kabuli_Scaffold_1718	21521	[A/G]	Ca_27100	Protein kinase, catalytic domain	INTRON

S.N.	SNP IDs	Chickpea cultivars	Chromosomes	Physical positions (bp)	SNPs	Gene accession IDs	Putative functions	Sequence components of genome
14772	CakSNP14772	*Kabuli*	Ca_Kabuli_Scaffold_1718	40669	[T/C]		--	INTERGENIC
14773	CakSNP14773	*Kabuli*	Ca_Kabuli_Scaffold_1722	65999	[C/A]	Ca_26630	Ribulose-phosphate 3-epimerase	INTRON
14774	CakSNP14774	*Kabuli*	Ca_Kabuli_Scaffold_1722	135589	[C/T]		--	INTERGENIC
14775	CakSNP14775	*Kabuli*	Ca_Kabuli_Scaffold_1728	33301	[A/C]		--	INTERGENIC
14776	CakSNP14776	*Kabuli*	Ca_Kabuli_Scaffold_174	17761	[A/C]		--	INTERGENIC
14777	CakSNP14777	*Kabuli*	Ca_Kabuli_Scaffold_174	32366	[T/C]	Ca_21702	Heat shock protein DnaJ, N-terminal	SYNONYMOUS_CODING
14778	CakSNP14778	*Kabuli*	Ca_Kabuli_Scaffold_174	44014	[T/G]		--	INTERGENIC
14779	CakSNP14779	*Kabuli*	Ca_Kabuli_Scaffold_174	61454	[T/C]	Ca_21706	Protein kinase, catalytic domain	NON_SYNONYMOUS_CODING
14780	CakSNP14780	*Kabuli*	Ca_Kabuli_Scaffold_174	263899	[G/A]	Ca_21715	RNA polymerase, alpha subunit	NON_SYNONYMOUS_CODING
14781	CakSNP14781	*Kabuli*	Ca_Kabuli_Scaffold_174	335077	[C/T]	Ca_21719	RNA polymerase, alpha subunit	INTRON

S.N.	SNP IDs	Chickpea cultivars	Chromosomes	Physical positions (bp)	SNPs	Gene accession IDs	Putative functions	Sequence components of genome
14782	CakSNP14782	*Kabuli*	Ca_Kabuli_Scaffold_174	341322	[C/T]	Ca_21719	RNA polymerase, alpha subunit	INTRON
14783	CakSNP14783	*Kabuli*	Ca_Kabuli_Scaffold_174	457627	[G/A]	Ca_21723	--	INTRON
14784	CakSNP14784	*Kabuli*	Ca_Kabuli_Scaffold_1750	15510	[C/T]	Ca_24921	UBA/THIF-type NAD/FAD binding fold	SYNONYMOUS_CODING
14785	CakSNP14785	*Kabuli*	Ca_Kabuli_Scaffold_1750	137106	[A/C]		--	INTERGENIC
14786	CakSNP14786	*Kabuli*	Ca_Kabuli_Scaffold_1750	204638	[G/A]	Ca_24928	WD40 repeat	INTRON
14787	CakSNP14787	*Kabuli*	Ca_Kabuli_Scaffold_1751	189367	[T/G]		--	INTERGENIC
14788	CakSNP14788	*Kabuli*	Ca_Kabuli_Scaffold_1751	192417	[C/A]		--	INTERGENIC
14789	CakSNP14789	*Kabuli*	Ca_Kabuli_Scaffold_1751	192713	[G/T]		--	INTERGENIC
14790	CakSNP14790	*Kabuli*	Ca_Kabuli_Scaffold_1751	193026	[T/A]		--	INTERGENIC
14791	CakSNP14791	*Kabuli*	Ca_Kabuli_Scaffold_1751	193013	[T/G]		--	INTERGENIC

S.N.	SNP IDs	Chickpea cultivars	Chromosomes	Physical positions (bp)	SNPs	Gene accession IDs	Putative functions	Sequence components of genome
14792	CakSNP14792	*Kabuli*	Ca_Kabuli_Scaffold_1751	266583	[G/A]		--	INTERGENIC
14793	CakSNP14793	*Kabuli*	Ca_Kabuli_Scaffold_1751	496626	[G/A]	Ca_23938	--	STOP_GAINED
14794	CakSNP14794	*Kabuli*	Ca_Kabuli_Scaffold_1751	528896	[A/C]		--	INTERGENIC
14795	CakSNP14795	*Kabuli*	Ca_Kabuli_Scaffold_1751	529005	[C/T]		--	INTERGENIC
14796	CakSNP14796	*Kabuli*	Ca_Kabuli_Scaffold_1751	528979	[T/C]		--	INTERGENIC
14797	CakSNP14797	*Kabuli*	Ca_Kabuli_Scaffold_1751	528973	[C/G]		--	INTERGENIC
14798	CakSNP14798	*Kabuli*	Ca_Kabuli_Scaffold_1751	625364	[G/A]		--	INTERGENIC
14799	CakSNP14799	*Kabuli*	Ca_Kabuli_Scaffold_1751	625362	[C/T]		--	INTERGENIC
14800	CakSNP14800	*Kabuli*	Ca_Kabuli_Scaffold_1751	625355	[C/T]		--	INTERGENIC
14801	CakSNP14801	*Kabuli*	Ca_Kabuli_Scaffold_1751	625342	[C/T]		--	INTERGENIC

S.N.	SNP IDs	Chickpea cultivars	Chromosomes	Physical positions (bp)	SNPs	Gene accession IDs	Putative functions	Sequence components of genome
14802	CakSNP14802	*Kabuli*	Ca_Kabuli_Scaffold_1751	625335	[G/A]		--	INTERGENIC
14803	CakSNP14803	*Kabuli*	Ca_Kabuli_Scaffold_1751	625395	[A/G]		--	INTERGENIC
14804	CakSNP14804	*Kabuli*	Ca_Kabuli_Scaffold_1751	625394	[C/T]		--	INTERGENIC
14805	CakSNP14805	*Kabuli*	Ca_Kabuli_Scaffold_1751	625389	[A/C]		--	INTERGENIC
14806	CakSNP14806	*Kabuli*	Ca_Kabuli_Scaffold_1751	625378	[G/A]		--	INTERGENIC
14807	CakSNP14807	*Kabuli*	Ca_Kabuli_Scaffold_1751	625375	[G/A]		--	INTERGENIC
14808	CakSNP14808	*Kabuli*	Ca_Kabuli_Scaffold_1751	625366	[T/C]		--	INTERGENIC
14809	CakSNP14809	*Kabuli*	Ca_Kabuli_Scaffold_1751	625365	[T/C]		--	INTERGENIC
14810	CakSNP14810	*Kabuli*	Ca_Kabuli_Scaffold_1751	625332	[A/C]		--	INTERGENIC
14811	CakSNP14811	*Kabuli*	Ca_Kabuli_Scaffold_1751	625396	[G/A]		--	INTERGENIC

S.N.	SNP IDs	Chickpea cultivars	Chromosomes	Physical positions (bp)	SNPs	Gene accession IDs	Putative functions	Sequence components of genome
14812	CakSNP14812	*Kabuli*	Ca_Kabuli_Scaffold_1776	740	[C/T]		--	INTERGENIC
14813	CakSNP14813	*Kabuli*	Ca_Kabuli_Scaffold_1777	164715	[G/A]		--	INTERGENIC
14814	CakSNP14814	*Kabuli*	Ca_Kabuli_Scaffold_1777	164722	[C/G]		--	INTERGENIC
14815	CakSNP14815	*Kabuli*	Ca_Kabuli_Scaffold_1777	164747	[G/A]		--	INTERGENIC
14816	CakSNP14816	*Kabuli*	Ca_Kabuli_Scaffold_1777	164791	[T/G]		--	INTERGENIC
14817	CakSNP14817	*Kabuli*	Ca_Kabuli_Scaffold_1777	164758	[C/T]		--	INTERGENIC
14818	CakSNP14818	*Kabuli*	Ca_Kabuli_Scaffold_1777	164753	[A/G]		--	INTERGENIC
14819	CakSNP14819	*Kabuli*	Ca_Kabuli_Scaffold_1777	164730	[T/G]		--	INTERGENIC
14820	CakSNP14820	*Kabuli*	Ca_Kabuli_Scaffold_1777	169037	[G/T]		--	INTERGENIC
14821	CakSNP14821	*Kabuli*	Ca_Kabuli_Scaffold_1777	343173	[C/T]		--	INTERGENIC

S.N.	SNP IDs	Chickpea cultivars	Chromosomes	Physical positions (bp)	SNPs	Gene accession IDs	Putative functions	Sequence components of genome
14822	CakSNP14822	*Kabuli*	Ca_Kabuli_Scaffold_1777	343185	[G/A]		--	INTERGENIC
14823	CakSNP14823	*Kabuli*	Ca_Kabuli_Scaffold_1777	448166	[A/G]		--	INTERGENIC
14824	CakSNP14824	*Kabuli*	Ca_Kabuli_Scaffold_1777	448176	[C/T]		--	INTERGENIC
14825	CakSNP14825	*Kabuli*	Ca_Kabuli_Scaffold_1777	448187	[G/T]		--	INTERGENIC
14826	CakSNP14826	*Kabuli*	Ca_Kabuli_Scaffold_1777	448216	[C/T]		--	INTERGENIC
14827	CakSNP14827	*Kabuli*	Ca_Kabuli_Scaffold_1779	957	[G/A]		--	INTERGENIC
14828	CakSNP14828	*Kabuli*	Ca_Kabuli_Scaffold_1779	896	[A/G]		--	INTERGENIC
14829	CakSNP14829	*Kabuli*	Ca_Kabuli_Scaffold_1779	890	[G/A]		--	INTERGENIC
14830	CakSNP14830	*Kabuli*	Ca_Kabuli_Scaffold_1802	17068	[A/G]		--	INTERGENIC
14831	CakSNP14831	*Kabuli*	Ca_Kabuli_Scaffold_1802	17033	[A/G]		--	INTERGENIC

S.N.	SNP IDs	Chickpea cultivars	Chromosomes	Physical positions (bp)	SNPs	Gene accession IDs	Putative functions	Sequence components of genome
14832	CakSNP14832	*Kabuli*	Ca_Kabuli_Scaffold_1802	17111	[C/T]		--	INTERGENIC
14833	CakSNP14833	*Kabuli*	Ca_Kabuli_Scaffold_1802	17117	[T/C]		--	INTERGENIC
14834	CakSNP14834	*Kabuli*	Ca_Kabuli_Scaffold_1802	17120	[C/T]		--	INTERGENIC
14835	CakSNP14835	*Kabuli*	Ca_Kabuli_Scaffold_1802	17123	[G/T]		--	INTERGENIC
14836	CakSNP14836	*Kabuli*	Ca_Kabuli_Scaffold_1802	17132	[C/T]		--	INTERGENIC
14837	CakSNP14837	*Kabuli*	Ca_Kabuli_Scaffold_1802	17150	[T/C]		--	INTERGENIC
14838	CakSNP14838	*Kabuli*	Ca_Kabuli_Scaffold_1802	17151	[T/C]		--	INTERGENIC
14839	CakSNP14839	*Kabuli*	Ca_Kabuli_Scaffold_1802	17168	[T/C]		--	INTERGENIC
14840	CakSNP14840	*Kabuli*	Ca_Kabuli_Scaffold_1802	17177	[T/A]		--	INTERGENIC
14841	CakSNP14841	*Kabuli*	Ca_Kabuli_Scaffold_1835	9398	[C/A]		--	INTERGENIC

S.N.	SNP IDs	Chickpea cultivars	Chromosomes	Physical positions (bp)	SNPs	Gene accession IDs	Putative functions	Sequence components of genome
14842	CakSNP14842	*Kabuli*	Ca_Kabuli_Scaffold_1835	10541	[C/G]		--	INTERGENIC
14843	CakSNP14843	*Kabuli*	Ca_Kabuli_Scaffold_1835	10563	[T/C]		--	INTERGENIC
14844	CakSNP14844	*Kabuli*	Ca_Kabuli_Scaffold_1844	41899	[C/A]	Ca_24135	Epsin domain, N-terminal	SYNONYMOUS_CODING
14845	CakSNP14845	*Kabuli*	Ca_Kabuli_Scaffold_1845	16292	[C/G]		--	INTERGENIC
14846	CakSNP14846	*Kabuli*	Ca_Kabuli_Scaffold_1845	16317	[C/G]		--	INTERGENIC
14847	CakSNP14847	*Kabuli*	Ca_Kabuli_Scaffold_1845	40886	[T/A]		--	INTERGENIC
14848	CakSNP14848	*Kabuli*	Ca_Kabuli_Scaffold_1845	40872	[T/G]		--	INTERGENIC
14849	CakSNP14849	*Kabuli*	Ca_Kabuli_Scaffold_1845	40871	[T/A]		--	INTERGENIC
14850	CakSNP14850	*Kabuli*	Ca_Kabuli_Scaffold_1845	40842	[T/G]		--	INTERGENIC
14851	CakSNP14851	*Kabuli*	Ca_Kabuli_Scaffold_1845	40821	[T/G]		--	INTERGENIC

S.N.	SNP IDs	Chickpea cultivars	Chromosomes	Physical positions (bp)	SNPs	Gene accession IDs	Putative functions	Sequence components of genome
14852	CakSNP14852	*Kabuli*	Ca_Kabuli_Scaffold_1848	583	[A/G]		--	INTERGENIC
14853	CakSNP14853	*Kabuli*	Ca_Kabuli_Scaffold_1848	565	[T/C]		--	INTERGENIC
14854	CakSNP14854	*Kabuli*	Ca_Kabuli_Scaffold_1848	562	[G/C]		--	INTERGENIC
14855	CakSNP14855	*Kabuli*	Ca_Kabuli_Scaffold_1848	532	[G/C]		--	INTERGENIC
14856	CakSNP14856	*Kabuli*	Ca_Kabuli_Scaffold_186	11965	[A/G]		--	INTERGENIC
14857	CakSNP14857	*Kabuli*	Ca_Kabuli_Scaffold_186	279942	[A/G]		--	INTERGENIC
14858	CakSNP14858	*Kabuli*	Ca_Kabuli_Scaffold_186	280062	[T/C]		--	INTERGENIC
14859	CakSNP14859	*Kabuli*	Ca_Kabuli_Scaffold_186	280047	[A/G]		--	INTERGENIC
14860	CakSNP14860	*Kabuli*	Ca_Kabuli_Scaffold_186	280023	[C/T]		--	INTERGENIC
14861	CakSNP14861	*Kabuli*	Ca_Kabuli_Scaffold_186	280015	[T/C]		--	INTERGENIC

S.N.	SNP IDs	Chickpea cultivars	Chromosomes	Physical positions (bp)	SNPs	Gene accession IDs	Putative functions	Sequence components of genome
14862	CakSNP14862	*Kabuli*	Ca_Kabuli_Scaffold_186	280009	[G/T]		--	INTERGENIC
14863	CakSNP14863	*Kabuli*	Ca_Kabuli_Scaffold_1866	106318	[T/A]	Ca_26044	Ankyrin repeat	NON_SYNONYMOUS_CODING
14864	CakSNP14864	*Kabuli*	Ca_Kabuli_Scaffold_1866	106330	[A/T]	Ca_26044	Ankyrin repeat	NON_SYNONYMOUS_CODING
14865	CakSNP14865	*Kabuli*	Ca_Kabuli_Scaffold_1866	106371	[C/A]	Ca_26044	Ankyrin repeat	STOP_GAINED
14866	CakSNP14866	*Kabuli*	Ca_Kabuli_Scaffold_1866	106374	[A/T]	Ca_26044	Ankyrin repeat	NON_SYNONYMOUS_CODING
14867	CakSNP14867	*Kabuli*	Ca_Kabuli_Scaffold_1866	106402	[C/T]	Ca_26044	Ankyrin repeat	SYNONYMOUS_CODING
14868	CakSNP14868	*Kabuli*	Ca_Kabuli_Scaffold_1866	110550	[A/G]	Ca_26045	Ankyrin repeat	SYNONYMOUS_CODING
14869	CakSNP14869	*Kabuli*	Ca_Kabuli_Scaffold_1866	110570	[G/A]	Ca_26045	Ankyrin repeat	INTRON
14870	CakSNP14870	*Kabuli*	Ca_Kabuli_Scaffold_1866	110578	[C/A]	Ca_26045	Ankyrin repeat	INTRON
14871	CakSNP14871	*Kabuli*	Ca_Kabuli_Scaffold_1899	148	[T/G]		--	INTERGENIC

S.N.	SNP IDs	Chickpea cultivars	Chromosomes	Physical positions (bp)	SNPs	Gene accession IDs	Putative functions	Sequence components of genome
14872	CakSNP14872	*Kabuli*	Ca_Kabuli_Scaffold_1899	187	[A/G]		--	INTERGENIC
14873	CakSNP14873	*Kabuli*	Ca_Kabuli_Scaffold_1899	267	[T/A]		--	INTERGENIC
14874	CakSNP14874	*Kabuli*	Ca_Kabuli_Scaffold_1899	200	[G/A]		--	INTERGENIC
14875	CakSNP14875	*Kabuli*	Ca_Kabuli_Scaffold_1918	216231	[T/G]		--	INTERGENIC
14876	CakSNP14876	*Kabuli*	Ca_Kabuli_Scaffold_1918	216213	[T/C]		--	INTERGENIC
14877	CakSNP14877	*Kabuli*	Ca_Kabuli_Scaffold_1918	216180	[C/A]		--	INTERGENIC
14878	CakSNP14878	*Kabuli*	Ca_Kabuli_Scaffold_1918	237787	[G/A]		--	INTERGENIC
14879	CakSNP14879	*Kabuli*	Ca_Kabuli_Scaffold_1918	245929	[T/C]	Ca_24715	Trehalose-phosphatase	SYNONYMOUS_CODING
14880	CakSNP14880	*Kabuli*	Ca_Kabuli_Scaffold_1928	103092	[A/G]		--	INTERGENIC
14881	CakSNP14881	*Kabuli*	Ca_Kabuli_Scaffold_1928	103114	[G/A]		--	INTERGENIC

S.N.	SNP IDs	Chickpea cultivars	Chromosomes	Physical positions (bp)	SNPs	Gene accession IDs	Putative functions	Sequence components of genome
14882	CakSNP14882	*Kabuli*	Ca_Kabuli_Scaffold_193	38279	[C/A]	Ca_25161	--	NON_SYNONYMOUS_CODING
14883	CakSNP14883	*Kabuli*	Ca_Kabuli_Scaffold_193	186538	[C/T]		--	INTERGENIC
[truncated: 326,383 more chars]
